# Managing Delayed or Missed Doses of Prolonged‐Release Tacrolimus in Transplant Recipients: Implications for Drug Exposure and Recovery Strategies

**DOI:** 10.1111/bcpt.70157

**Published:** 2025-12-04

**Authors:** S. Arraki Zava, F. Maizaud, H. Sayadi, Y. Fromage, P. Marquet, J. B. Woillard, C. Monchaud

**Affiliations:** ^1^ Pharmacology, Toxicology and Pharmacovigilance Department Limoges University Hospital Limoges France; ^2^ Pharmacology and Transplantation, INSERM U1248 University of Limoges Limoges France; ^3^ Hospital‐University Federation “Survival Optimization in Organ Transplantation” (FHU SUPORT) Limoges France

## Abstract

In lifelong immunosuppressive therapy, missed doses of prolonged‐release tacrolimus are almost inevitable and may reduce drug exposure, compromising transplant outcomes. Using pharmacokinetic models, we simulated delayed and missed doses in virtual kidney and liver transplant recipients. Our simulations showed that a missed dose significantly lowers exposure, with recovery taking 2–4 days. For delays shorter than 12 h, the full dose should be taken immediately; for delays between 12 and 24 h, half the dose should be administered; for a missed dose, 150% should be given at the next intake. These strategies may help maintain therapeutic exposure and preserve transplant outcomes.

## Summary

People who receive an organ transplant must take tacrolimus every day to prevent rejection. Sometimes, doses are missed or taken late, lowering drug levels in the blood and putting both the transplant and patient at risk. Using simulations, we studied how timing mistakes affect tacrolimus levels. We found that missing a dose can lower drug levels for several days. Our results suggest practical advice: If the delay is under 12 h, take the full dose; between 12 and 24 h, take half; if a dose is missed, take 150% next time to maintain the drug level needed to protect the transplant.

## Graphical abstract

This simulation–based analysis quantified the impact of delayed and missed doses of prolonged–release tacrolimus in kidney and liver transplant recipients. A single missed dose reduced AUC24h by up to 50% and required 2–4 days to return to steady state. Model–informed recommendations indicate full–dose intake for delays 〈12 h, half–dose for longer delays, and a 150% compensatory dose after a missed intake.

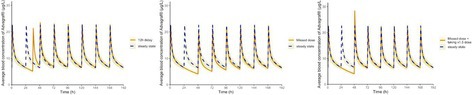

**Abbreviations**
AUC: area under the curve (area under the concentration–time curve)
AUC_12h_
: area under the curve over 12 h
AUC_24h_
: area under the curve over 24 h
AUC_ss_
: area under the curve at steady state
C_0_
: trough concentration
C_0ss_
: C_0_ at steady state
LCP‐tac: life‐cycle pharma tacrolimus
PopPK: population pharmacokinetic
PR‐tac: prolonged release tacrolimus
RD: relative difference
SPC: summary of product characteristics
ss: steady state
TDM: therapeutic drug monitoring
XR‐tac: extended‐release tacrolimus

## 1 Introduction

Calcineurin inhibitors, particularly tacrolimus, form the cornerstone of immunosuppressive therapy after solid organ transplantation. When combined with mycophenolate mofetil and corticosteroids, they help balance the risk of rejection and toxicity [1, 2].

However, tacrolimus use remains challenging due to its narrow therapeutic index—targeting trough concentrations (C_0_) of 5–8 μg/L in liver and 4–12 μg/L in kidney transplant recipients [3]—and wide inter‐ and intra‐individual pharmacokinetic variability. Tacrolimus pharmacokinetic variability is largely influenced by *CYP3A5* genetic polymorphisms (including donor genotype in liver transplantation) as well as by drug–drug interactions, liver function and therapeutic adherence [4, 5].

Therapeutic drug monitoring (TDM) is essential to maintain adequate exposure [3]. Although the monitoring of residual blood concentrations (C_0_) is routinely used, it is an imperfect surrogate of mean exposure, best estimated by the area under the curve (AUC). Therefore, the 12‐ or 24‐h AUC is the most reliable indicator of exposure to tacrolimus. However, full AUC measurement requires multiple blood samples, making routine implementation challenging. Instead, AUC can be estimated for immediate and prolonged‐release tacrolimus using limited sampling strategies combined with Bayesian a posteriori estimators based on PopPK models, as implemented in the Immunosuppressant Bayesian Dose Adjustment expert system (https://abis.chu‐limoges.fr) developed at Limoges University Hospital [6, 7].

One of the key barriers to maintaining optimal exposure is non‐adherence, often unintentional, due to lifestyle disruption, forgetfulness (especially for evening doses) or adverse events [8]. Such deviations increase variability and have been linked to late rejection episodes and graft loss, representing an additional cost in healthcare expenditure [9, 10, 11].

Non‐adherence has a profound impact on clinical outcomes: in kidney transplant recipients, it is associated with a 2.66‐fold increase in mortality risk, a 2.28‐fold increase in acute rejection risk and a 6.44‐fold increase in graft loss [12]. These findings highlight the critical importance of maintaining consistent adherence over the long term.

Several interventions have been proposed to enhance adherence, including digital reminders (telephone alarms, mobile applications and electronic monitoring of medication intake), pillboxes and once‐daily prolonged‐release tacrolimus formulations [13, 14]. Despite this, missed doses remain unavoidable over a lifetime of therapy.

Current summary of product characteristics (SPC) of immediate‐release and prolonged‐release tacrolimus formulations recommend taking a missed dose as soon as possible during the day while avoiding taking a double dose the following day. However, these recommendations are not based on pharmacokinetic data and do not quantify the impact of missed doses on tacrolimus exposure. Only one study has examined missed doses in the context of immediate‐release tacrolimus, highlighting substantial underexposure and proposing mitigation strategies [15].

To our knowledge, no study has investigated the impact of missed or delayed doses of prolonged‐release (either extended‐release [XR]‐tacrolimus or LCP‐tacrolimus), in kidney or liver transplant recipients, despite the availability of published PopPK models [16, 17, 18]. The aim of this study was to simulate and quantify the impact of non‐adherence scenarios—including delayed and missed doses on exposure to tacrolimus and to propose model‐informed recommendations to guide clinical management, based on the AUC_24h_.

## 2 Material and Methods

The study was conducted in accordance with the *Basic & Clinical Pharmacology & Toxicology* policy for experimental and clinical studies [19].

As this work was based exclusively on pharmacokinetic simulations and did not involve any patient data or biological samples, ethical approval and informed consent were not required. The R codes used for the simulations are present in the files SDC 1, SDC 2 and SDC 3.

### 2.1 Pharmacokinetic Models

Three PopPK models of tacrolimus were selected from the literature, based on the following criteria: (i) developed in adult, stable kidney or liver transplant recipients; (ii) describing prolonged‐release formulations (XR‐ or LCP‐tacrolimus); (iii) fully specified structural and statistical components, allowing implementation in the mrgsolve package in R; and (iv) inclusion of key clinical covariates, such as *CYP3A5* genotype or haematocrit, when available. These models allowed exploration of interindividual variability in exposure across transplant types, formulations and pharmacogenetic profiles. In all three source studies, patients were in the maintenance phase, at least 3 months post‐transplantation for liver recipients and ≥ 12 months post‐transplantation for kidney recipients, reflecting stable dosing conditions. These post‐transplant timeframes guided our selection of therapeutic target ranges (5–8 μg/L for liver and 4–12 μg/L for kidney), consistent with current maintenance‐phase recommendations [3].

The Woillard et al. model [16], developed on 41 stable kidney transplant recipients (more than 12 months post‐transplantation) treated with XR‐tac for more than 6 months, included haematocrit (median: 38.5%) and *CYP3A5* genotype (expressors: 12.2%, non‐expressors: 87.8%) as covariates.

The Moes et al. model [17], based on 49 adult stable liver transplant recipients (at least 3 months post‐transplantation) treated with XR‐tac, stratified patients into four groups according to recipient and donor *CYP3A5* status. In group C1 (65.3%), neither the recipient nor the donor carried the CYP3A5*1 allele. In groups C2 (16.3%) and C3 (8.2%), either the recipient or the donor carried it, whereas in group C4 (10.2%), both were carriers. Apparent tacrolimus clearance varied according to the combination of genotypes, ranging from ‘normal’ clearance in C1 (non‐expressors) to a 71% increase in C4 (donor and recipient both expressors).

Finally, the Martial et al. [18] model, derived from 55 stable adult liver transplant recipients (at least 6 months after transplantation) treated with LCP‐tac, did not include covariates.

All models were implemented in the *mrgsolve* package (RStudio) using the a priori PopPK parameters and covariate distributions (Table 1). For the Woillard model, the haematocrit covariate was fixed at 38.5%. But *CYP3A5* status was simulated according to the proportions reported in the source populations used to develop the models.

**TABLE 1:** A priori pharmacokinetic parameters used for PK modelling.

Models	XR‐tac, renal transplantation [16]	XR‐tac, hepatic transplantation [17]	LCP‐tac, hepatic transplantation [18]
Software	NONMEM	NONMEM	NONMEM
PopPK parameters			
F (%)	NA	0.23	0.23
Ntrans	NA	NA	1.58
MTT (h)	NA	NA	3.39
CL (L/h)	21.2^a^	4.21	3.27
Vc (L)	486^a^	88.3	94.9
Vp (L)	271^a^	145	500
Ka (h^−1^)	NA	3.76	2.97
Ktr (h^−1^)	3.34^a^	NA	NA
Q (L/h)	79^a^	14	9.62
Interindividual variability (Variance values)			
IIV_F_	NA	NA	0.1296
IIV_Cl_	0.0784	0.183	0.1156
IIV_Vc_	0.0961	0.744	1.9881
IIV_Q_	0.2916	NA	0.0576
IIV_Vp_	0.36	NA	NA
IIV_Ka_	NA	0.434	3.0276
IIV_Ktr_	0.0576	NA	NA
Residual error			
Original ε add (μg/L)	0.71	NA	NA
Original ε prop (%)	11.3	13.0	10.5
ε add used (μg/L)	0.000001^b^	NA	NA
ε prop used (%)	0.000001^b^	0.000001^b^	0.000001^b^

Abbreviations: ε add, additive residual error; ε prop, proportional residual error; CL, clearance; F, bioavailability; IIV, interindividual variability; Ka, absorption rate constant; Ktr, transfer rate constant; MTT, mean transit time; NA, not applicable; Ntrans, mean number of transit compartments; Q, inter‐compartmental clearance; Vc, central compartment volume; Vp, peripheral compartment volume.

^a^ For the Woillard et al. model, the population pharmacokinetic parameters (clearance, central and peripheral volumes of distribution, transit rate constant and intercompartmental clearance) are apparent values (not corrected for bioavailability).

^b^ Residual error (ε) was minimized for the purposes of the simulation [20]. To reduce the influence of residual variability and ensure smoother simulated concentration–time profiles, the additive and proportional error terms were set to near‐zero values.

### 2.2 Simulation Design

For each model, 12 000 virtual pharmacokinetic profiles were simulated under various scenarios, yielding a total of 36 000 profiles. Each simulation followed a three‐phase structure: (1) steady‐state reference dose administration; (2) dose delay scenarios, with or without catch‐up strategies; and (3) return to reference dosing for 10 consecutive days.

Doses ranged from 0.5 to 15 mg per day in 0.5 mg increments, across 30 groups of 400 simulated profiles. For each profile, the C_0_ was calculated and compared to target ranges: 5–8 μg/L for liver and 4–12 μg/L for kidney transplantation [3]. Only profiles with steady‐state C_0_ within the target range were retained for further analysis.

Dosing deviations were simulated by shifting the dosing time in 3‐h increments, and two categories were considered. A ‘delayed dose’ was defined as a late administration occurring within the same 24‐h interval (i.e., delay < 24 h), whereas a ‘missed dose’ was defined as a complete omission of a scheduled dose, that is, a 24‐h gap.

For delayed doses, the full dose was taken at the delayed timepoint. For delays of up to 12 h, strategies consisting in taking 50% and 150% of the reference dose were also tested. In scenarios where the delay exceeded 12 h, the alternative strategy of taking 50% of the regular dose was evaluated to account for the shortened interval before the next scheduled intake. For missed doses, two corrective strategies were tested at the next scheduled intake: (i) administering 150% of the regular dose (to partially compensate for the missed dose) and (ii) administering 200% (i.e., the full missed dose in addition to the scheduled dose).

### 2.3 Outcome Measures

**FIGURE 1:**
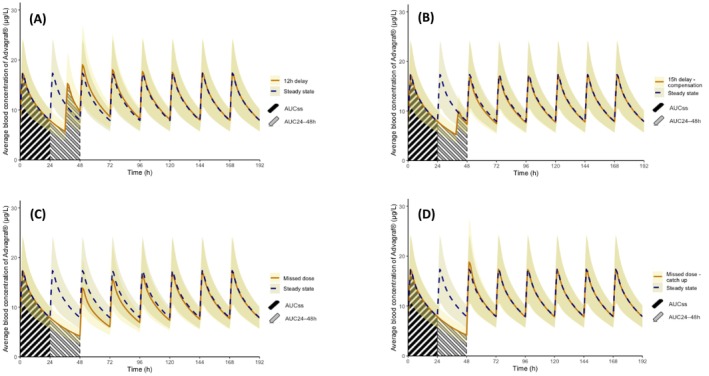
Mean whole blood concentration profiles of XR‐tac in CYP3A5 non‐expressors (renal transplantation, Woillard et al.) under different dosing scenarios. (A) Steady‐state versus 12‐h delay. (B) Steady‐state versus 15‐h delay using compensation strategy: half‐dose intake. (C) Steady‐state versus missed dose. (D) Steady‐state versus missed dose using catch‐up strategy: intake of one and a half‐dose.

The impact of dose deviations and the effectiveness of corrective strategies were evaluated using the following metrics: (i) the relative difference (RD) in C_0_ and AUC_24h_ from steady state (C_0ss_ and AUC_ss_), where AUC_ss_ refers to the 24‐h steady‐state AUC, and the post‐intervention AUC was calculated over the subsequent 24 h (AUC_24–48h_) (Figure 1), and (ii) the time required for these exposure biomarkers to return to steady state.

### 2.4 Acceptability Criteria for Evaluated Scenarios

Given the narrow therapeutic window of tacrolimus, the evaluation of delayed or missed doses was based on the extent and duration of deviation from steady‐state exposure rather than on fixed numerical thresholds. A 10% margin on the AUC_24h_ was pragmatically applied to define return to steady state, consistent with bioequivalence margins recommended for narrow therapeutic index drugs [21]. For delays and corrective strategies, interpretation focused on identifying the point at which the loss of exposure became clinically meaningful and justified corrective action, balancing the transient impact of non‐adherence with the need to minimize risks of underexposure and toxicity.

## 3 Results

### 3.1 Pharmacokinetic Profiles at Steady State

**FIGURE 2:**
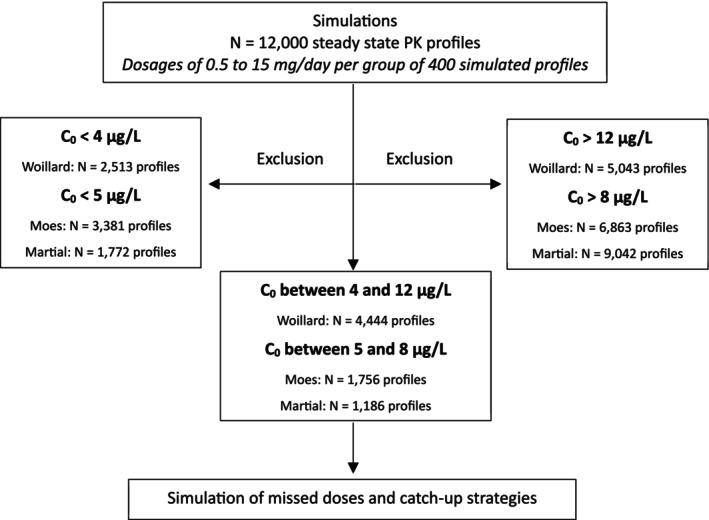
Selection of pharmacokinetic profiles according to target concentrations.

Among the 36 000 virtual PK profiles simulated across the three models, 7386 profiles were retained based on therapeutic trough concentrations at steady state (Figure 2). These included 4444 profiles for XR‐tac in kidney transplant recipients, 1756 for XR‐tac in liver transplant recipients and 1186 for LCP‐tac in liver transplant recipients.

For XR‐tac in renal transplantation, C_0ss_ averaged 6.8 ± 2.1 μg/L in expressors and 7.9 ± 2.2 μg/L in non‐expressors, corresponding to mean AUC_ss_ values of 293 ± 76.2 h.μg/L and 279 ± 87.1 h.μg/L, respectively. The mean daily doses required to reach target C_0ss_ were 10.5 ± 2.9 mg in expressors and 6.2 ± 2.9 mg in non‐expressors.

**FIGURE 3:**
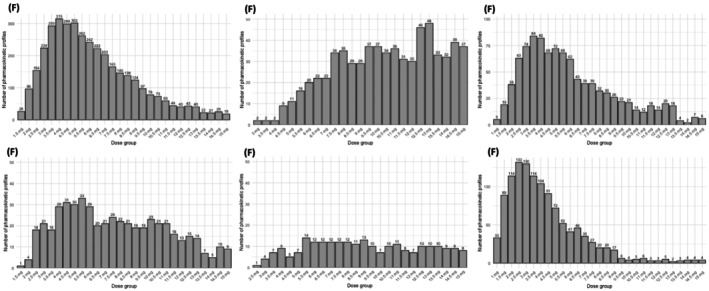
Distribution of doses received by pharmacokinetic profiles, at steady state, by model and CYP3A5 status. (A) Simulated profiles of CYP3A5 non‐expressors receiving XR‐tac in renal transplantation (Woillard et al.). (B) Simulated profiles of CYP3A5 expressors receiving XR‐tac in renal transplantation (Woillard et al.). (C) Simulated profiles of C1 group receiving XR‐tac in hepatic transplantation (Moes et al.). (D) Simulated profiles of C2/C3 group receiving XR‐tac in hepatic transplantation (Moes et al.). (E) Simulated profiles of C4 group receiving XR‐tac in hepatic transplantation (Moes et al.). (F) Simulated profiles receiving LCP‐tac in hepatic transplantation (Martial et al.).

In liver transplant recipients receiving XR‐tacrolimus, mean C_0ss_ ranged from 6.4 ± 0.9 μg/L to 6.5 ± 0.8 μg/L across *CYP3A5* expression groups, with corresponding AUC_ss_ values ranging from 231 ± 47.3 h.μg/L to 256 ± 47.4 h.μg/L. Required doses increased from 5.9 ± 3.1 mg in non‐expressors to 9.1 ± 3.4 mg in double expressors. For LCP‐tac, C_0ss_ averaged 6.5 ± 0.9 μg/L with an AUC_ss_ of 205 ± 43.2 h.μg/L and a mean daily dose of 4.3 ± 2.5 mg. The distribution of the required doses to reach the target C_0ss_, stratified by model and *CYP3A5* status, is shown in Figure 3. Furthermore, the interindividual variability (average CV) based on AUC ranged from 18.5% to 31.2% (and ranged from 12.3% to 30.8% based on C_0_) depending on *CYP3A5* expression status, transplant type or tacrolimus formulation.

### 3.2 Delayed Doses

Mean C_0_ and AUC_24h_ values, along with their mean relative differences from steady state, for a 12‐h delay in tacrolimus administration and compensatory strategies are shown in Table 2. Additional results for other delay durations and applied catch‐up strategies are provided in Supplemental Digital Content (SDC tables).

**TABLE 2:** Impact of delayed doses and catch‐up strategies on prolonged‐release tacrolimus exposure indices (C_0_, μg/L; AUC_24h_, h.μg/L), and relative differences (RD, %) compared to steady state. Results are presented as means ± standard deviations.

a. XR‐tac, renal transplantation (Woillard et al.)							
Days	Exposure biomarker	12‐h delay	12‐h delay 50% dose intake	12‐h delay 150% dose intake	Missed dose	Missed dose followed by 150% dose intake	Missed dose followed by 200% dose intake
**CYP3A5 non‐expressors (*N* = 3771), AUC**_**ss**_ **: 279 ± 87.1 h.μg/L, C**_**0ss**_ **: 6.8 ± 2.1 μg/L**							
Dgap	AUC_24h_	224 ± 66.7	182 ± 51.6	266 ± 85.0	140 ± 43.1	140 ± 43.1	140 ± 43.1
	*RD AUC*_24h_	*−19.1% ± 4.0*	*−33.7% ± 7.8*	*−4.6% ± 2.7*	*−48.2% ± 12.0*	*−48.2% ± 12.0*	*−48.2% ± 12.0*
	**C**_**0**_	9.8 ± 3.0	7.0 ± 2.0	12.6 ± 4.2	4.2 ± 1.6	4.2 ± 1.6	4.2 ± 1.6
	***RD C***_***0***_	*23.8% ± 17.1*	*−11.6% ± 4.2*	*59.1% ± 31.4*	*−46.9% ± 12.8*	*−46.9% ± 12.8*	*−46.9% ± 12.8*
**D + 1**	**AUC**_**24h**_	307 ± 101.7	261 ± 84.5	353 ± 119.3	215 ± 67.8	284 ± 98.5	354 ± 130.7
	***RD AUC***_***24h***_	*9.6% ± 4.2*	*−6.6% ± 2.1*	*25.8% ± 6.8*	*−22.8% ± 2.6*	*1.3% ± 6.0*	*25.4% ± 11.8*
	**C**_**0**_	8.7 ± 2.5	7.4 ± 2.1	9.9 ± 2.9	6.1 ± 1.7	7.9 ± 2.3	9.8 ± 2.9
	***RD C***_***0***_	*9.4% ± 4.2*	*−6.9% ± 2.2*	*25.7% ± 6.6*	*−23.2% ± 2.2*	*0.3% ± 6.3*	*23.8% ± 12.5*
**D + 2**	**AUC**_**24h**_	291 ± 91.9	269 ± 86.2	314 ± 97.8	246 ± 80.6	278 ± 90.3	310 ± 100.3
	***RD AUC***_***24h***_	*4.4% ± 1.1*	*−3.8% ± 1.6*	*12.6% ± 1.6*	*−12.0% ± 2.6*	*−0.6% ± 2.6*	*10.8% ± 3.2*
	**C**_**0**_	8.3 ± 2.3	7.6 ± 2.2	8.9 ± 2.5	6.9 ± 2.0	7.9 ± 2.2	8.8 ± 2.5
	***RD C***_***0***_	*4.4% ± 1.1*	*−3.9% ± 1.7*	*12.8% ± 1.4*	*−12.3% ± 2.7*	*−0.7% ± 2.7*	*10.9% ± 3.1*
**CYP3A5 expressors (*N* = 673), AUC**_**ss**_ **: 293 ± 76.2 h.μg/L, C**_**0ss**_ **: 7.9 ± 2.2 μg/L**							
**Dgap**	**AUC**_**24h**_	227 ± 59.1	167 ± 45.7	288 ± 76.6	107 ± 40.2	107 ± 40.2	107 ± 40.2
	***RD AUC***_***24h***_	*−22.2% ± 3.1*	*−42.6% ± 6.5*	*−1.7% ± 4.3*	*−63.0% ± 10.7*	*−63.0% ± 10.7*	*−63.0% ± 10.7*
	**C**_**0**_	9.9 ± 2.7	6.3 ± 1.8	13.5 ± 3.8	2.8 ± 1.4	2.8 ± 1.4	2.8 ± 1.4
	***RD C***_***0***_	*48.4% ± 28.6*	*−6.3% ± 9.6*	*103.2% ± 48.2*	*−61.1% ± 12.0*	*−61.1% ± 12.0*	*−61.1% ± 12.0*
**D + 1**	**AUC**_**24h**_	334 ± 89.3	282 ± 75.4	386 ± 103.7	230 ± 61.7	323 ± 90.2	416 ± 119.8
	***RD AUC***_***24h***_	*14% ± 4.0*	*−3.6% ± 3.0*	*31.7% ± 5.6*	*−21.3% ± 3.2*	*10.2% ± 7.9*	*41.7% ± 13.2*
	**C**_**0**_	7.8 ± 2.3	6.5 ± 1.9	9.0 ± 2.6	5.3 ± 1.5	7.3 ± 2.1	9.4 ± 2.6
	***RD C***_***0***_	*14.0% ± 3.9*	*−4.1% ± 3.2*	*32.1% ± 4.9*	*−22.2% ± 3.0*	*8.3% ± 8.6*	*38.9% ± 14.5*
**D + 2**	**AUC**_**24h**_	307 ± 79.8	287 ± 75.7	326 ± 84.5	268 ± 71.5	299 ± 78.8	330 ± 86.5
	***RD AUC***_***24h***_	*4.8% ± 0.8*	*−1.8% ± 1.5*	*11.5% ± 2.2*	*−8.5% ± 3.1*	*2.2% ± 2.2*	*12.8% ± 2.3*
	**C**_**0**_	7.2 ± 2.2	6.7 ± 2.0	7.7 ± 2.4	6.2 ± 1.8	7.0 ± 2.1	7.7 ± 2.3
	***RD C***_***0***_	*4.9% ± 0.7*	*−2.0% ± 1.7*	*11.9% ± 2.2*	*−9.0% ± 3.4*	*2.1% ± 2.3*	*13.2% ± 1.9*

b. XR‐tac, hepatic transplantation (Moes et al.)							
**CYP3A5 expression group C1 (*N* = 1002), AUC**_**ss**_ **: 231 ± 47.3 h.μg/L, C**_**0ss**_ **: 6.5 ± 0.8 μg/L**							
**Dgap**	**AUC**_**24h**_	198 ± 37.0	161 ± 22.6	235 ± 54.3	124 ± 19.4	124 ± 19.4	124 ± 19.4
	***RD AUC***_***24h***_	*−14.1% ± 2.6*	*−29.3% ± 7.7*	*1.0% ± 3.3*	*−44.4% ± 13.0*	*−44.4% ± 13.0*	*−44.4% ± 13.0*
	**C**_**0**_	7.4 ± 1.1	5.7 ± 0.8	9.0 ± 1.5	4.1 ± 0.9	4.1 ± 0.9	4.1 ± 0.9
	***RD C***_***0***_	*14.3% ± 7.7*	*−11.4% ± 2.2*	*39.9% ± 16.5*	*−37% ± 10.4*	*−37% ± 10.4*	*−37% ± 10.4*
**D + 1**	**AUC**_**24h**_	245 ± 54.0	216 ± 45.4	275 ± 62.7	187 ± 37.0	240 ± 60.0	294 ± 83.8
	***RD AUC***_***24h***_	*5.7% ± 2.2*	*−6.7% ± 0.9*	*18.1% ± 4.1*	*−19.2% ± 2.1*	*3.0% ± 5.4*	*25.2% ± 11.7*
	**C**_**0**_	6.9 ± 0.9	6.0 ± 0.8	7.8 ± 1.1	5.0 ± 0.7	6.2 ± 0.8	7.4 ± 1.1
	***RD C***_***0***_	*6.3% ± 3*	*−8.0% ±* 0.8	*20.5% ± 6.0*	*−22.1% ± 3.2*	*−3.6% ± 2.6*	*14.9% ± 7.6*
**D + 2**	**AUC**_**24h**_	239 ± 50.2	222 ± 47.2	256 ± 53.3	204 ± 44.3	226 ± 49.5	249 ± 54.7
	***RD AUC***_***24h***_	*3.1% ± 0.9*	*−4.4% ± 1.1*	*10.7% ± 1.3*	*−11.9% ± 1.5*	*−2.4% ± 1.7*	*7.2% ± 2.3*
	**C**_**0**_	6.7 ± 0.9	6.1 ± 0.8	7.3 ± 1.0	5.6 ± 0.7	6.3 ± 0.8	7.0 ± 0.9
	***RD C***_***0***_	*3.6% ± 1.3*	*−5.1% ± 0.9*	*12.3% ± 1.9*	*−13.7% ± 1.3*	*−2.6% ± 1.8*	*8.4% ± 3.2*
**CYP3A5 expression group C2/C3 (*N* = 514), AUC**_**ss**_ **: 248 ± 50.6 h.μg/L, C**_**0ss**_ **: 6.4 ± 0.8 μg/L**							
**Dgap**	**AUC**_**24h**_	210 ± 40.2	165 ± 23.6	255 ± 60.0	119 ± 19.4	119 ± 19.4	119 ± 19.4
	***RD AUC***_***24h***_	*−15.2% ± 2.3*	*−32.5% ± 7.6*	*2.1% ± 3.9*	*−49.8% ± 13.1*	*−49.8% ± 13.1*	*−49.8% ± 13.1*
	**C**_**0**_	7.6 ± 1.1	5.7 ± 0.8	9.6 ± 1.6	3.8 ± 0.9	3.8 ± 0.9	3.8 ± 0.9
	***RD C***_***0***_	*18.6% ± 8.7*	*−11.5% ± 2.0*	*48.8% ± 17.9*	*−41.7% ± 10.5*	*−41.7% ± 10.5*	*−41.7% ± 10.5*
**D + 1**	**AUC**_**24h**_	265 ± 57.8	233 ± 49.4	299 ± 66.9	199 ± 40.8	263 ± 66.1	328 ± 92.1
	***RD AUC***_***24h***_	*6.8% ± 2.2*	*−6.4% ± 1.0*	*19.9% ± 3.8*	*−19.5% ± 1.7*	*5.4% ± 6.1*	*30.3% ± 12.5*
	**C**_**0**_	6.9 ± 0.9	6.0 ± 0.8	7.9 ± 1.1	5.0 ± 0.7	6.3 ± 0.8	7.6 ± 1.1
	***RD C***_***0***_	*7.7% ± 3.2*	*−7.7% ±* 0.9	*23.1% ± 5.9*	*−23.1% ± 2.7*	*−2.3% ± 3.1*	*18.5% ± 8.2*
**D + 2**	**AUC**_**24h**_	257 ± 53.6	239 ± 51.2	275 ± 56.6	220 ± 48.4	245 ± 53.3	269 ± 58.4
	***RD AUC***_***24h***_	*3.5% ± 0.8*	*−3.9% ± 1.2*	*10.9% ± 1.1*	*−11.3% ± 1.8*	*−1.6% ± 1.8*	*8.2% ± 2.2*
	**C**_**0**_	6.7 ± 0.9	6.2 ± 0.8	7.3 ± 1.0	5.6 ± 0.7	6.3 ± 0.8	7.1 ± 0.9
	***RD C***_***0***_	*4.1% ± 1.2*	*−4.6% ± 1.1*	*12.9% ± 1.6*	*−13.3% ± 1.4*	*−1.8% ± 2.0*	*9.8% ± 3.2*
**CYP3A5 expression group C4 (*N* = 240), AUC**_**ss**_ **: 256 ± 47.4 h.μg/L, C**_**0ss**_ **: 6.4 ± 0.9 μg/L**							
**Dgap**	**AUC**_**24h**_	214 ± 38.5	164 ± 23.1	264 ± 56.5	115 ± 19.0	115 ± 19.0	115 ± 19.0
	***RD AUC***_***24h***_	*−16.1% ± 1.7*	*−34.9% ± 6.0*	*2.7% ± 4.2*	*−53.7% ± 10.7*	*−53.7% ± 10.7*	*−53.7% ± 10.7*
	**C**_**0**_	7.8 ± 1.1	5.6 ± 0.8	9.9 ± 1.6	3.5 ± 0.8	3.5 ± 0.8	3.5 ± 0.8
	***RD C***_***0***_	*22.2% ± 8.2*	*−11.5% ± 1.7*	*55.9% ± 16.2*	*−45.2% ± 8.5*	*−45.2% ± 8.5*	*−45.2% ± 8.5*
**D + 1**	**AUC**_**24h**_	276 ± 53.6	240 ± 46.4	311 ± 60.9	205 ± 39.3	275 ± 62.2	346 ± 85.6
	***RD AUC***_***24h***_	*7.7% ± 1.8*	*−6.1% ± 1.1*	*21.5% ± 2.8*	*−20% ± 1.4*	*6.9% ± 5.9*	*33.8% ± 11.2*
	**C**_**0**_	6.9 ± 0.9	5.9 ± 0.8	8.0 ± 1.1	4.9 ± 0.7	6.3 ± 0.8	7.7 ± 1.1
	***RD C***_***0***_	*8.7% ± 2.7*	*−7.6% ±* 0.9	*25.1% ± 4.8*	*−23.9% ± 1.6*	*−1.3% ± 3.1*	*21.3% ± 7.2*
**D + 2**	**AUC**_**24h**_	266 ± 49.9	247 ± 47.7	284 ± 52.0	228 ± 45.7	254 ± 49.7	279 ± 53.8
	***RD AUC***_***24h***_	*3.8% ± 0.6*	*−3.6% ± 1.1*	*11.1% ± 0.8*	*−11.0% ± 1.9*	*−1.0% ± 1.6*	*9.0% ± 1.6*
	**C**_**0**_	6.7 ± 0.9	6.1 ± 0.8	7.2 ± 1.0	5.5 ± 0.7	6.3 ± 0.8	7.1 ± 0.9
	***RD C***_***0***_	*4.5% ± 0.9*	*−4.3% ± 1.1*	*13.3% ± 1.0*	*−13.1% ± 1.4*	*−1.1% ± 1.8*	*10.8% ± 2.5*

2c. LCP‐tac, hepatic transplantation (Martial et al.)							
**AUC**_**ss**_ **: 205 ± 43.2 h.μg/L, C**_**0ss**_ **: 6.5 ± 0.9 μg/L**							
**Dgap**	**AUC**_**24h**_	191 ± 41.0	167 ± 27.5	215 ± 56.2	144 ± 19.8	144 ± 19.8	144 ± 19.8
	***RD AUC***_***24h***_	*−6.7% ± 2.7*	*−17.3% ± 6.3*	*3.9% ± 6.3*	*−27.9% ± 11.8*	*−27.9% ± 11.8*	*−27.9% ± 11.8*
	**C**_**0**_	7.3 ± 1.2	6.4 ± 0.9	8.1 ± 1.6	5.6 ± 0.8	5.6 ± 0.8	5.6 ± 0.8
	***RD C***_***0***_	*12.1% ± 10.8*	*−0.6% ± 4.5*	*24.9% ± 17.5*	*−13.4% ± 4.4*	*−13.4% ± 4.4*	*−13.4% ± 4.4*
**D + 1**	**AUC**_**24h**_	211 ± 44.4	199 ± 42.0	222 ± 46.9	188 ± 40.1	218 ± 56.5	249 ± 73.6
	***RD AUC***_***24h***_	*2.8% ± 2.2*	*−2.8% ± 1.0*	*8.4% ± 4.0*	*−8.4% ± 2.1*	*5.6% ± 5.9*	*19.5% ± 11.6*
	**C**_**0**_	6.6 ± 0.9	6.2 ± 0.8	6.9 ± 0.9	5.9 ± 0.8	6.3 ± 0.8	6.7 ± 0.9
	***RD C***_***0***_	*1.1% ± 0.9*	*−4.2% ± 1.1*	*6.5% ± 2.1*	*−9.6% ± 2.4*	*−2.9% ± 1.6*	*3.9% ± 3.1*
**D + 2**	**AUC**_**24h**_	206 ± 43.3	199 ± 41.7	214 ± 44.9	191 ± 40.2	200 ± 41.8	208 ± 43.4
	***RD AUC***_***24h***_	*0.6% ± 0.5*	*−3.0% ± 0.6*	*4.3% ± 1.1*	*−6.7% ± 1.2*	*−2.5% ± 0.6*	*1.7% ± 1.2*
	**C**_**0**_	6.5 ± 0.9	6.2 ± 0.8	6.8 ± 0.9	5.9 ± 0.8	6.3 ± 0.8	6.6 ± 0.9
	***RD C***_***0***_	*0.6% ± 0.3*	*−3.8% ± 0.9*	*4.9% ± 1.3*	*−8.2% ± 1.9*	*−3.4% ± 0.8*	*1.4% ± 0.7*

*Note:* ***Dgap*** refers to the day of the delayed or missed dose. ***D + x*** refers to the number of days following the delayed or missed dose. Bold is relevant for titles of the columns and to separate models and subgroups. Italics was intended to mark the difference between mean absolute values in each situation and mean relative differences.

Abbreviations: **AUC**_**24h**_, mean area under the curve over 24 h (h.μg/L); **C**_**0**_, mean trough concentration (μg/L); ***RD AUC***_***24h***_, mean relative difference in AUC_24h_ (%); ***RD C***_***0***_, mean relative difference in C_0_ (%).

Delays of up to 12 h without a catch‐up strategy resulted in a decrease in AUC_24h_ of less than 25% for XR‐tac in kidney transplantation and less than 20% for both XR‐tac and LCP‐tac in liver transplantation. Despite reduced AUC, delayed dosing led to an apparent increase in C_0_ values, with elevations reaching up to 48% for XR‐tac in *CYP3A5* expressors and over 20% for LCP‐tac or XR‐tac in liver. These fluctuations were generally transient: 2 days after the delayed dose, both C_0_ and AUC_24h_ returned to within ±5% of baseline values in the majority of profiles.

When a half‐dose catch‐up strategy was applied for delays beyond 12 h, the day‐of‐delay AUC_24h_ was markedly reduced—up to 58% for XR‐tac in kidney transplant recipients with *CYP3A5* expression—but generally returned close to baseline the following day. C_0_ deviations, however, were more variable, occasionally exceeding a 50% increase, particularly for long delays in expressors.

### 3.3 Missed Doses

The mean C_0_ and AUC_24h_ values after missed doses, including catch‐up strategies, and their relative difference from steady state are presented in Table 2.

Missed doses resulted in significant reductions in exposure across all models. For XR‐tac in kidney transplantation, the AUC_24h_ decreased by approximately 50%, with 82.3% of expressors and 49.4% of non‐expressors presenting a C_0_ below the therapeutic range (4–12 μg/L) on the day following the missed dose. In liver transplantation, missed doses of XR‐tac led to an average C_0_ decrease of 39% and an AUC_24h_ reduction of 47%, with more pronounced underexposure observed in dual *CYP3A5* expressors (C4 group), in whom 95% of profiles showed subtherapeutic C_0_ values (< 5 μg/L). LCP‐tac was associated with more moderate reductions (13% for C_0_, 28% for AUC_24h_), and only 26% of profiles dropped below the C_0_ therapeutic range (5–8 μg/L). The average time required to return to steady‐state AUC_24h_ was 2 days for LCP‐tac and 3–4 days for XR‐tac, depending on the transplanted organ and CYP3A5 status.

To mitigate the impact of a fully missed dose (i.e., 24‐h omission), two catch‐up strategies were evaluated. Administering 150% of the reference dose at the next scheduled time moderately increased AUC_24h_—typically within 10% of steady‐state values—and restored target C_0_ in the majority of profiles without excessive overexposure. Conversely, a 200% dose resulted in tacrolimus overexposure, particularly in simulated XR‐tac PK profiles for kidney transplant recipients expressing *CYP3A5*, with AUC_24h_ and C_0_ exceeding steady‐state values by up to 40%.

## 4 Discussion

This simulation‐based study, leveraging three published PopPK models of prolonged‐release tacrolimus in kidney or liver transplant recipients [16, 17, 18], provides a quantitative evaluation of the impact of delayed or missed doses on exposure. These models were sourced from the literature and not developed de novo. Minor differences in clearance values between formulations reflect inter‐study variability rather than formulation effects, as clearance estimates in liver transplant recipients were comparable. The slower‐release profile of the LCP formulation in our simulations is therefore mainly explained by a lower absorption rate constant, which avoids overestimating concentrations after a missed dose.

Our findings underscore a substantial and sustained reduction in tacrolimus exposure, as measured by AUC_24h_, following missed doses, with decreases often exceeding 50%. This reduction was associated with a proportion of profiles falling below the therapeutic threshold ranging between 26% and 90% and an average time to return to steady‐state AUC_24h_ ranging from 2 to 4 days, depending on the formulation, transplanted organ and *CYP3A5* status. These variations, especially in *CYP3A5* expressors, are likely to be clinically meaningful and merit specific mitigation strategies.

To date, no clinical data have established recommendations for the management of delayed or missed doses of prolonged‐release tacrolimus. Given the ethical limitations and practical difficulties of conducting prospective studies in this context, our study based on PopPK modelling and simulations provides useful guidance to clinicians. The study by Dai et al., using PopPK models and Monte Carlo simulations, demonstrated that prolonged‐release tacrolimus is less forgiving to missed doses than the immediate‐release formulation, despite generally higher adherence rates. Notably, their findings highlight the significant underexposure associated with a single missed dose of prolonged‐release tacrolimus [22]. In this context, ‘forgiveness’ refers to the capacity of a drug formulation to maintain therapeutic exposure despite occasional missed or delayed doses. These observations underscore the clinical importance of developing specific, model‐informed mitigation strategies for PR‐tacrolimus—a gap our study directly addresses. Consistently, with previous work on immediate‐release tacrolimus by Saint‐Marcoux et al. [15], who reported up to a 70% drop in AUC_12h_ following missed doses, our study extends these concerns to prolonged‐release formulations and supports the value of catch‐up strategies. Notably, our simulations offer an expanded framework by incorporating different formulations (XR‐tac and LCP‐tac), two organ types and pharmacogenetic variability, thereby broadening the applicability of our findings.

To reflect real‐world clinical practice, only virtual profiles achieving therapeutic C_0_ at steady state were retained, simulating patients under stable dosing after initial titration. This approach, which mirrors routine clinical practice where dosing is initiated based on body weight without prior knowledge of the *CYP3A5* genotype and subsequently adjusted to reach target C_0_ levels, resulted in 7386 of the 36 000 profiles being included for analysis. Mean daily doses in the retained profiles were slightly higher than in the original model cohorts, particularly in CYP3A5 expressors, likely due to our selection of profiles near the upper end of the target range. In the XR‐tac renal model, for example, expressors required 10.5 ± 2.9 mg/day compared to 4 mg [2–10] in the source population [16]. A similar trend was observed in the liver model. This difference reinforces the relevance of our simulations, which are anchored in therapeutic ranges representative of clinical targets.

Despite slightly elevated exposure at baseline, a substantial proportion of profiles still fell below the therapeutic range following a missed dose: 26% with LCP‐tac, 50% with XR‐tac in non‐expressors and over 80% in expressors in the renal model. In the liver model, over 90% of CYP3A5 expressors were similarly affected. These findings suggest that in actual practice, where some patients may be managed at lower target levels, the risk of underexposure due to non‐adherence could be even more important.

Despite the significant initial drop in exposure after a missed dose, recovery to steady‐state AUC_24h_ levels (within a 10% margin) was generally quick in most cases, occurring in 2 days for LCP‐tac and within 3–4 days for XR‐tac. LCP‐tac showed a lower reduction in AUC_24h_ (approximately 30%) and a rapid return to steady state, potentially due to its flatter PK profile. This apparent tolerance to missed doses may be attributed to the reduced peak‐to‐trough fluctuations characteristic of the formulation [23].

Interestingly, from the second day after a missed dose—regardless of the tacrolimus formulation, transplant type or *CYP3A5* genotype—the mean relative deviation in AUC_24h_ was consistently close to the lower boundary of the bioequivalence interval (90–111%), as defined for narrow therapeutic index drugs [21]. This finding raises concerns about the capacity of C_0_ alone to reliably detect non‐adherence, particularly when missed doses are occasional and partially compensated by physiological variability.

Even occasional, missed doses lead to prolonged underexposure, associated with a variation in exposure, which has been reported to increase the risk of rejection or even graft loss [24, 25]. To mitigate the consequences of a missed dose, we evaluated several catch‐up strategies. Taking 150% of the usual dose at the next scheduled intake performed well across all models, restoring C_0_ and AUC_24h_ values without excessive overshoot. In contrast, a full 200% catch‐up dose caused marked overexposure, particularly in kidney transplant recipients who were *CYP3A5* expressors treated with XR‐tac, where C_0_ and AUC_24h_ increased by over 40%. These data reinforce the current SPC recommendation against doubling the dose and support the need for tailored, pharmacologically informed guidance.

Our study also addressed the impact of dose delays. Delays of less than 12 h, when managed by immediate full‐dose intake, produced moderate changes in AUC_24h_ and transient increases in C_0_, particularly in *CYP3A5* expressors. These effects typically resolved within 2 days. Given the once‐daily morning dose of prolonged‐release tacrolimus, it is unlikely that patients will detect or report a delay exceeding 12 h, making this threshold clinically relevant. For longer delays, administering only 50% of the missed dose helped stabilize exposure while minimizing the risk of overcompensation. Other tested strategies led to higher variations of the exposure and were therefore discarded. Although LCP‐tac appeared more pharmacokinetically forgiving, reflected by a lower relative decrease in AUC_0–24h_ and faster return to steady‐state exposure, this formulation may even allow full‐dose intake when the delay approaches 18 h. However, this still falls short of a complete 24‐h omission. Our recommendation remains pragmatic: take the full dose if the delay is less than 12 h; take half the dose if more than 12 h have elapsed; and take 150% of the regular dose at the next scheduled administration in case of missed dose.

Importantly, our study also confirms that a delayed intake prior to sampling can cause high C_0_ values, primarily due to blood sampling occurring closer to drug intake. This ‘apparent’ overexposure may mislead clinicians, especially when they are unaware of the delay. Saint‐Marcoux et al. also observed this phenomenon [15], emphasizing that the ‘trough’ value is no longer a true 24‐h concentration in such cases. In clinical practice, a sudden elevation in C_0_ often prompts a search for pharmacokinetic interactions (e.g., CYP3A inhibition) or clinical events (e.g., diarrhoea), both known to increase tacrolimus exposure [26]. However, delayed drug intake prior to sampling should also be considered, as failing to do so may lead to inappropriate dose reductions and true underexposure in the subsequent days.

In addition, C_0_ levels in the days following a delayed or missed dose may be misleading for TDM, and dose adjustments based solely on trough concentrations should be avoided until a new steady‐state measurement is available. The extent of this effect is highest in patients with a high dose/C_0_ ratio, reflecting higher tacrolimus clearance, particularly in CYP3A5 expressors.

These observations further support the use of AUC rather than C_0_ alone for tacrolimus TDM. AUC provides a more robust estimate of total drug exposure and is less sensitive to the timing of blood sampling, although AUC estimation is not always feasible in routine clinical practice. Consistently, the IATDMCT recommends the use of AUC whenever feasible [3]. Van Gelder et al. also proposed AUC‐guided monitoring to improve precision in exposure assessment and clinical decision‐making [27]. These considerations justified our choice to employ AUC measurements in this study to quantify average exposure and to evaluate the pharmacokinetic impact of delayed or missed doses.

However, to date, no universally accepted AUC_24h_ target ranges have been established specifically for PR‐tacrolimus. The second international consensus report on tacrolimus TDM recommended a minimal AUC_12h_ of 150 μg·h/L for IR‐tacrolimus in renal transplantation but did not define equivalent targets for prolonged‐release formulations. AUC_24h_ ranges extrapolated from C_0_ values have been proposed: 150–275 μg·h/L for C_0_ 3–7 μg/L; 180–350 μg·h/L for 5–10 μg/L; 260–400 μg·h/L for 8–12 μg/L; and 310–475 μg·h/L for 10–15 μg/L. More recently, Van Gelder et al. suggested a target AUC_24h_ of 160 μg·h/L for PR‐tacrolimus. These proposed ranges are not stratified by days post‐transplant and should therefore be interpreted with caution.

In our simulations, mean AUC_24h_ values at steady state ranged from approximately 180 to 330 μg·h/L, depending on the formulation, organ type, and *CYP3A5* genotype. These values fall within or near the proposed target ranges of 150–275 μg·h/L and 180–350 μg·h/L for tacrolimus C_0_ levels between 3 and 10 μg/L, as cited above. This alignment supports the clinical plausibility of our simulated profiles and reinforces the relevance of our exposure‐based recommendations. However, we caution that these AUC targets remain extrapolated and may not fully account for interindividual variability.

Taken together, these findings reinforce the need for integrated therapeutic education strategies aimed not only at preventing non‐adherence but also at empowering patients with clear, practical guidance on how to respond appropriately when a dose is missed or delayed. The proposed mitigation strategies—taking the full dose if the delay is under 12 h, half the dose if the delay exceeds 12 h and administering 150% of the usual dose at the next scheduled intake after a missed dose—are simple, evidence based and specifically designed to minimize fluctuations in tacrolimus exposure that could otherwise compromise graft function. Incorporating these recommendations into structured patient education programmes would help ensure that transplant recipients are equipped to make informed decisions when deviations from prescribed dosing occur, thereby improving long‐term adherence and clinical outcomes. These messages could be supported by written tools, digital reminders or decision aids tailored to patient literacy levels. Importantly, such initiatives would complement conventional counselling focused on the importance of daily intake by also addressing how to manage errors safely and consistently.

Although our simulations were based on renal and hepatic transplant models, the underlying principles of exposure dynamics and adherence management are likely relevant across solid organ transplant populations. Pending prospective validation in external cohorts and clinical settings, these recommendations may also be extended to other transplant groups—such as heart, lung or pancreas recipients—thereby supporting a more unified and proactive approach to tacrolimus therapeutic drug management.

An additional point to consider is that the population pharmacokinetic models used in this study for PR‐tacrolimus were not developed by us but were directly taken from previously published studies, each conducted in different patient cohorts. This explains the slight differences observed in some pharmacokinetic parameters between models, which likely reflect inter‐study population variability rather than formulation effects. In particular, the clearance values reported for liver transplant recipients were very similar between the XR‐tacrolimus and LCP‐tacrolimus models (4.21 vs. 3.27 L/h equivalent to apparent CL of 18.3 and 14.2 L/h), indicating no clinically relevant difference in elimination between the two formulations. The higher clearance observed in the kidney transplant model (21.2 L/h) most probably reflects population differences rather than a formulation effect. In our simulations, the difference between the two formulations was therefore considered to lie mainly in the absorption process: The LCP‐tacrolimus model uses a lower absorption rate constant (ka) to reflect its slower release, whereas the elimination parameters (including clearance) were considered comparable to those of the XR‐tacrolimus model. This ensures that the differences observed after a missed dose are driven by the delayed absorption profile of the LCP formulation and not by artificial differences in drug elimination, thereby avoiding overestimation of drug concentrations.

This study has certain limitations. Although various factors may influence the variability of tacrolimus exposure, only the impact of *CYP3A5* polymorphism was analysed, as it was a covariate included in the PKPOP models used. Furthermore, although no direct correlation between tacrolimus exposure metrics and the occurrence of adverse events has been demonstrated to date [28], our study does not allow for an assessment of the potential risk of toxicity associated with a single overexposure induced by the administration of a catch‐up dose. However, given the significant risk of underexposure following a missed dose, the benefit–risk balance argues in favour of administering a compensatory dose.

## 5 Conclusion

This simulation‐based study, using PopPK models developed in kidney and liver transplant recipients, quantitatively assessed the impact of delayed and missed doses of two prolonged‐release tacrolimus formulations (XR‐tac and LCP‐tac). It extends the findings previously reported for immediate‐release tacrolimus by Saint‐Marcoux et al. and provides complementary evidence to guide clinical decision‐making across formulations.

Our results confirm that missed doses can lead to a substantial decrease in tacrolimus exposure—often around 50%—although both missed and delayed intakes introduce significant variability, which may increase the risk of rejection and graft loss. In this context, optimizing adherence and implementing well‐defined catch‐up strategies are critical to maintaining therapeutic exposure.

Based on our simulations, we propose the following pragmatic recommendations for all prolonged‐release tacrolimus formulations: If the delay is less than 12 h, the full dose should be taken as soon as possible; if the delay exceeds 12 h, 50% of the missed dose should be taken immediately; and if the dose is completely missed, 150% of the usual dose should be administered at the next scheduled intake. These strategies are simple and pharmacologically rational and can be readily incorporated into therapeutic education programmes to better support patients in managing dosing deviations. Although the AUC thresholds applied in this study align with bioequivalence margins for narrow therapeutic index drugs, slight deviations from typical clinical targets were considered to balance patient safety and therapeutic efficacy.

Although our models were derived from kidney and liver transplant populations, the underlying pharmacokinetic principles likely apply to other solid organ recipients. Prospective validation in heart, lung or pancreas transplantation would be a valuable next step to confirm the generalizability of these recommendations.

## Funding

The authors received no specific funding for this work.

## Conflicts of Interest

The authors declare no conflicts of interest.

## Supporting information


**Data S1:** Supporting Information.

**Data S2:** Supporting Information.

**Data S3:** Supporting Information.

**Data S4:** Supporting Information.
