# Supplementary material for: Managing Delayed or Missed Doses of Prolonged‐Release Tacrolimus in Transplant Recipients: Implications for Drug Exposure and Recovery Strategies
Source: Basic Clin Pharmacol Toxicol. 2025 Dec 4;138(1):e70157. doi: 10.1111/bcpt.70157 (PMC12676261; doi:10.1111/bcpt.70157)
Supplement: Supplementary file 3 — Data S3: Supporting Information. [file BCPT-138-0-s004.pdf]

Simulations of Woillard model

Arraki Zava Selim

2025-05-06

##Loading of the packages

```
library(mrgsolve)
```

```
##
## Attaching package: 'mrgsolve'
```

```
## The following object is masked from 'package:stats':
##
##     filter
```

```
library(stats)
library(tidyverse)
```

```
## ── Attaching core tidyverse packages ──────────────────────── tidyverse 2.0.0 ─
─
## ✔ dplyr     1.1.4     ✔ readr     2.1.5
## ✔ forcats   1.0.0     ✔ stringr   1.5.1
## ✔ ggplot2   3.5.1     ✔ tibble    3.2.1
## ✔ lubridate 1.9.4     ✔ tidyr     1.3.1
## ✔ purrr     1.0.2
```

```
## ── Conflicts ────────────────────────────────────────── tidyverse_conflicts() ─
─
## ✖ dplyr::filter() masks mrgsolve::filter(), stats::filter()
## ✖ dplyr::lag()    masks stats::lag()
## ℹ Use the conflicted package (<http://conflicted.r-lib.org/>) to force all conf
licts to become errors
```

```
library(truncnorm)
library(ggplot2)
library(conflicted)
library(PKNCA)
library(Pmetrics)
```

```
##
## CRITICAL: Execute PMbuild() in R to complete Pmetrics installation.
```

```
library(skimr)
library(stats)
library(tidyr)
library(pander)
library(DT)
library(purrr)
library(gridExtra)
library(patchwork)
library(stringr)
```

##Population pharmacokinetics model and bayesian estimator for two tacrolimus formulations - twice daily Prograf and once daily Advagraf, Woillard, 2011 (tacrolimus stable renal transplant) OK

```
code <-
"
[SET] end=100, delta=0.1

[PARAM] @annotated

TVKTR : 3.34 : Typical transfert rate constant (1/h)
TVCL  : 21.2 : Typical value of clearance (L/h)
TVVC  : 486 : Typical central volume of distribution (L)
TVQ   : 79 : Typical intercomp clearance 1 (L/h)
TVVP  : 271 : Typical peripheral volume of distribution 1 (L)
HTCL  : -1.14 : effect of HT on cl
CYPCL : 2.00 : effect of CYP on cl
STKTR : 1.53 : effect of study on ktr
STVC  : 0.29 : effect of study on vc
HT    : 38.5 : hematocrit (perc) paramètre fixé à 38.5


[PARAM] @annotated @covariates
ST    : 0 : Prograf(1) advagraf(0)
CYP   : 0 : expressor (1) non exp(0)


[OMEGA] @annotated
ETACL  : 0.0784 : ETA on clearance
ETAVC  : 0.0961 : ETA on VC
ETAQ   : 0.2916 : ETA on Q
ETAVP  : 0.3600 : ETA on VP
ETAKTR : 0.0576 : ETA on KTR


[CMT] @annotated
GUT : estomac [ADM]
TRANS1 : CPT TR 1 (μg)
TRANS2 : CPT TR 2 (μg)
TRANS3 : CPT TR 3 (μg)
```

```
CENT  : Central compartment [OBS]
PERIPH : peripheral compartment (µg)


[SIGMA]
0.000001 // proportionnal Residual unexplained variability
0.000001 // additive Residual unexplained variability ng/mL


[MAIN]

double KTR   = TVKTR * pow(STKTR,ST) * exp(ETAKTR);
double VC    = TVVC * pow(STVC,ST) * exp(ETAVC);
double Q     = TVQ * exp(ETAQ);
double VP    = TVVP * exp (ETAVP);
double CL    = (TVCL * pow((HT/35),HTCL) * pow(CYPCL,CYP)) * exp(ETACL);


[ODE]
dxdt_GUT    = -KTR*GUT;
dxdt_TRANS1 = KTR*GUT - KTR*TRANS1;
dxdt_TRANS2 = KTR*TRANS1 - KTR*TRANS2;
dxdt_TRANS3 = KTR*TRANS2 - KTR*TRANS3;
dxdt_CENT   = -CL*CENT/VC -Q*CENT/VC + Q*PERIPH/VP + KTR*TRANS3;
dxdt_PERIPH = Q*CENT/VC - Q*PERIPH/VP;

[TABLE]
double DV = ((CENT/VC)*1000) * (1 + EPS(1)) + EPS(2);
int i = 0;
while(DV<0 && i <100) {
simeps();
DV = ((CENT/VC)*1000) * (1 + EPS(1)) + EPS(2);
++i;

}


[CAPTURE] DV CL VC VP Q
"



my_model_tacro_Woillard <- mcode("TACRO_model_JB", code)
```

```
## Building TACRO_model_JB ... done.
```

Simulations 6000: simulation with different dose each 400 profiles (including CYP3A5

status variations)

```
set.seed(123456)

generate_CYPCL_woillard_6000 <- function(n = 400) {
  frequencies <- c(36/41, 5/41)
  values <- c(0, 1)
  # cette ligne
  sample(values, n, replace = TRUE, prob = frequencies)
  }

CYPCL_data_woillard_6000 <- tibble(ID = 1:6000) %>%
  group_by(group = (ID - 1) %/% 400) %>%
  mutate(CYP = generate_CYPCL_woillard_6000()) %>%
  ungroup() %>%
  select(-group)

# Scenarios simulation
Simulation_data_woillard_6000 <- as_tibble( seq(
  # Steady-state simulation
ev(ID = 1:400, amt = 1*1, ii= 24, ss= 1, addl=0),
ev(ID = 1:400, amt = 1*1, ii= 24, ss= 0, addl=0),
ev(ID = 1:400, amt = 1*1, ii= 24, ss= 0, addl=10),
#3h delay simulation
ev(ID = 1:400, amt = 1*1, ii= 24, ss= 1, addl=0),
ev(ID = 1:400, amt = 1*0, ii= 3, ss= 0, addl=0),
ev(ID = 1:400, amt = 1*1, ii= 21, ss= 0, addl=0),
ev(ID = 1:400, amt = 1*1, ii= 24, ss= 0, addl=10),
#6h delay simulation
ev(ID = 1:400, amt = 1*1, ii= 24, ss= 1, addl=0),
ev(ID = 1:400, amt = 1*0, ii= 6, ss= 0, addl=0),
ev(ID = 1:400, amt = 1*1, ii= 18, ss= 0, addl=0),
ev(ID = 1:400, amt = 1*1, ii= 24, ss= 0, addl=10),
#9h delay simulation
ev(ID = 1:400, amt = 1*1, ii= 24, ss= 1, addl=0),
ev(ID = 1:400, amt = 1*0, ii= 9, ss= 0, addl=0),
ev(ID = 1:400, amt = 1*1, ii= 15, ss= 0, addl=0),
ev(ID = 1:400, amt = 1*1, ii= 24, ss= 0, addl=10),
#12h delay simulation
ev(ID = 1:400, amt = 1*1, ii= 24, ss= 1, addl=0),
ev(ID = 1:400, amt = 1*0, ii= 12, ss= 0, addl=0),
ev(ID = 1:400, amt = 1*1, ii= 12, ss= 0, addl=0),
ev(ID = 1:400, amt = 1*1, ii= 24, ss= 0, addl=10),
#15h delay + 50% dose intake simulation
ev(ID = 1:400, amt = 1*1, ii= 24, ss= 1, addl=0),
ev(ID = 1:400, amt = 1*0, ii= 15, ss= 0, addl=0),
ev(ID = 1:400, amt = 1*0.5, ii= 9, ss= 0, addl=0),
ev(ID = 1:400, amt = 1*1, ii= 24, ss= 0, addl=10),
#15h delay simulation
```

```
ev(ID = 1:400, amt = 1*1, ii= 24, ss= 1, addl=0),
ev(ID = 1:400, amt = 1*0, ii= 15, ss= 0, addl=0),
ev(ID = 1:400, amt = 1*1, ii= 9, ss= 0, addl=0),
ev(ID = 1:400, amt = 1*1, ii= 24, ss= 0, addl=10),
#18h delay + 50% dose intake simulation
ev(ID = 1:400, amt = 1*1, ii= 24, ss= 1, addl=0),
ev(ID = 1:400, amt = 1*0, ii= 18, ss= 0, addl=0),
ev(ID = 1:400, amt = 1*0.5, ii= 6, ss= 0, addl=0),
ev(ID = 1:400, amt = 1*1, ii= 24, ss= 0, addl=10),
#18h delay simulation
ev(ID = 1:400, amt = 1*1, ii= 24, ss= 1, addl=0),
ev(ID = 1:400, amt = 1*0, ii= 18, ss= 0, addl=0),
ev(ID = 1:400, amt = 1*1, ii= 6, ss= 0, addl=0),
ev(ID = 1:400, amt = 1*1, ii= 24, ss= 0, addl=10),
#21h delay + 50% dose intake simulation
ev(ID = 1:400, amt = 1*1, ii= 24, ss= 1, addl=0),
ev(ID = 1:400, amt = 1*0, ii= 21, ss= 0, addl=0),
ev(ID = 1:400, amt = 1*0.5, ii= 3, ss= 0, addl=0),
ev(ID = 1:400, amt = 1*1, ii= 24, ss= 0, addl=10),
#21h delay simulation
ev(ID = 1:400, amt = 1*1, ii= 24, ss= 1, addl=0),
ev(ID = 1:400, amt = 1*0, ii= 21, ss= 0, addl=0),
ev(ID = 1:400, amt = 1*1, ii= 3, ss= 0, addl=0),
ev(ID = 1:400, amt = 1*1, ii= 24, ss= 0, addl=10),
#Missed dose simulation
ev(ID = 1:400, amt = 1*1, ii= 24, ss= 1, addl=0),
ev(ID = 1:400, amt = 1*0, ii= 24, ss= 0, addl=0),
ev(ID = 1:400, amt = 1*1, ii= 24, ss= 0, addl=0),
ev(ID = 1:400, amt = 1*1, ii= 24, ss= 0, addl=10),
#Missed dose + 150% dose intake simulation
ev(ID = 1:400, amt = 1*1, ii= 24, ss= 1, addl=0),
ev(ID = 1:400, amt = 1*0, ii= 24, ss= 0, addl=0),
ev(ID = 1:400, amt = 1*1.5, ii= 24, ss= 0, addl=0),
ev(ID = 1:400, amt = 1*1, ii= 24, ss= 0, addl=10),
#Missed dose + 200% dose intake simulation
ev(ID = 1:400, amt = 1*1, ii= 24, ss= 1, addl=0),
ev(ID = 1:400, amt = 1*0, ii= 24, ss= 0, addl=0),
ev(ID = 1:400, amt = 1*2, ii= 24, ss= 0, addl=0),
ev(ID = 1:400, amt = 1*1, ii= 24, ss= 0, addl=10))) %>%
             mutate(dose_group = "1 mg") %>%

  bind_rows(as_tibble( seq(
  #Steady-state simulation
ev(ID = 401:800, amt = 2*1, ii= 24, ss= 1, addl=0),
ev(ID = 401:800, amt = 2*1, ii= 24, ss= 0, addl=0),
ev(ID = 401:800, amt = 2*1, ii= 24, ss= 0, addl=10),
#3h delay simulation
ev(ID = 401:800, amt = 2*1, ii= 24, ss= 1, addl=0),
ev(ID = 401:800, amt = 2*0, ii= 3, ss= 0, addl=0),
ev(ID = 401:800, amt = 2*1, ii= 21, ss= 0, addl=0),
```

```
ev(ID = 401:800, amt = 2*1, ii= 24, ss= 0, addl=10),
#6h delay simulation
ev(ID = 401:800, amt = 2*1, ii= 24, ss= 1, addl=0),
ev(ID = 401:800, amt = 2*0, ii= 6, ss= 0, addl=0),
ev(ID = 401:800, amt = 2*1, ii= 18, ss= 0, addl=0),
ev(ID = 401:800, amt = 2*1, ii= 24, ss= 0, addl=10),
#9h delay simulation
ev(ID = 401:800, amt = 2*1, ii= 24, ss= 1, addl=0),
ev(ID = 401:800, amt = 2*0, ii= 9, ss= 0, addl=0),
ev(ID = 401:800, amt = 2*1, ii= 15, ss= 0, addl=0),
ev(ID = 401:800, amt = 2*1, ii= 24, ss= 0, addl=10),
#12h delay simulation
ev(ID = 401:800, amt = 2*1, ii= 24, ss= 1, addl=0),
ev(ID = 401:800, amt = 2*0, ii= 12, ss= 0, addl=0),
ev(ID = 401:800, amt = 2*1, ii= 12, ss= 0, addl=0),
ev(ID = 401:800, amt = 2*1, ii= 24, ss= 0, addl=10),
#15h delay + 50% dose intake simulation
ev(ID = 401:800, amt = 2*1, ii= 24, ss= 1, addl=0),
ev(ID = 401:800, amt = 2*0, ii= 15, ss= 0, addl=0),
ev(ID = 401:800, amt = 2*0.5, ii= 9, ss= 0, addl=0),
ev(ID = 401:800, amt = 2*1, ii= 24, ss= 0, addl=10),
#15h delay simulation
ev(ID = 401:800, amt = 2*1, ii= 24, ss= 1, addl=0),
ev(ID = 401:800, amt = 2*0, ii= 15, ss= 0, addl=0),
ev(ID = 401:800, amt = 2*1, ii= 9, ss= 0, addl=0),
ev(ID = 401:800, amt = 2*1, ii= 24, ss= 0, addl=10),
#18h delay + 50% dose intake simulation
ev(ID = 401:800, amt = 2*1, ii= 24, ss= 1, addl=0),
ev(ID = 401:800, amt = 2*0, ii= 18, ss= 0, addl=0),
ev(ID = 401:800, amt = 2*0.5, ii= 6, ss= 0, addl=0),
ev(ID = 401:800, amt = 2*1, ii= 24, ss= 0, addl=10),
#18h delay simulation
ev(ID = 401:800, amt = 2*1, ii= 24, ss= 1, addl=0),
ev(ID = 401:800, amt = 2*0, ii= 18, ss= 0, addl=0),
ev(ID = 401:800, amt = 2*1, ii= 6, ss= 0, addl=0),
ev(ID = 401:800, amt = 2*1, ii= 24, ss= 0, addl=10),
#21h delay + 50% dose intake simulation
ev(ID = 401:800, amt = 2*1, ii= 24, ss= 1, addl=0),
ev(ID = 401:800, amt = 2*0, ii= 21, ss= 0, addl=0),
ev(ID = 401:800, amt = 2*0.5, ii= 3, ss= 0, addl=0),
ev(ID = 401:800, amt = 2*1, ii= 24, ss= 0, addl=10),
#21h delay simulation
ev(ID = 401:800, amt = 2*1, ii= 24, ss= 1, addl=0),
ev(ID = 401:800, amt = 2*0, ii= 21, ss= 0, addl=0),
ev(ID = 401:800, amt = 2*1, ii= 3, ss= 0, addl=0),
ev(ID = 401:800, amt = 2*1, ii= 24, ss= 0, addl=10),
#Missed dose simulation
ev(ID = 401:800, amt = 2*1, ii= 24, ss= 1, addl=0),
ev(ID = 401:800, amt = 2*0, ii= 24, ss= 0, addl=0),
ev(ID = 401:800, amt = 2*1, ii= 24, ss= 0, addl=0),
```

```
ev(ID = 401:800, amt = 2*1, ii= 24, ss= 0, addl=10),
#Missed dose + 150% dose intake simulation
ev(ID = 401:800, amt = 2*1, ii= 24, ss= 1, addl=0),
ev(ID = 401:800, amt = 2*0, ii= 24, ss= 0, addl=0),
ev(ID = 401:800, amt = 2*1.5, ii= 24, ss= 0, addl=0),
ev(ID = 401:800, amt = 2*1, ii= 24, ss= 0, addl=10),
#Missed dose + 200% dose intake simulation
ev(ID = 401:800, amt = 2*1, ii= 24, ss= 1, addl=0),
ev(ID = 401:800, amt = 2*0, ii= 24, ss= 0, addl=0),
ev(ID = 401:800, amt = 2*2, ii= 24, ss= 0, addl=0),
ev(ID = 401:800, amt = 2*1, ii= 24, ss= 0, addl=10))))%>%
              mutate(dose_group = "2 mg") %>%

  bind_rows(as_tibble( seq(
  #Steady-state simulation
ev(ID = 801:1200, amt = 3*1, ii= 24, ss= 1, addl=0),
ev(ID = 801:1200, amt = 3*1, ii= 24, ss= 0, addl=0),
ev(ID = 801:1200, amt = 3*1, ii= 24, ss= 0, addl=10),
#3h delay simulation
ev(ID = 801:1200, amt = 3*1, ii= 24, ss= 1, addl=0),
ev(ID = 801:1200, amt = 3*0, ii= 3, ss= 0, addl=0),
ev(ID = 801:1200, amt = 3*1, ii= 21, ss= 0, addl=0),
ev(ID = 801:1200, amt = 3*1, ii= 24, ss= 0, addl=10),
#6h delay simulation
ev(ID = 801:1200, amt = 3*1, ii= 24, ss= 1, addl=0),
ev(ID = 801:1200, amt = 3*0, ii= 6, ss= 0, addl=0),
ev(ID = 801:1200, amt = 3*1, ii= 18, ss= 0, addl=0),
ev(ID = 801:1200, amt = 3*1, ii= 24, ss= 0, addl=10),
#9h delay simulation
ev(ID = 801:1200, amt = 3*1, ii= 24, ss= 1, addl=0),
ev(ID = 801:1200, amt = 3*0, ii= 9, ss= 0, addl=0),
ev(ID = 801:1200, amt = 3*1, ii= 15, ss= 0, addl=0),
ev(ID = 801:1200, amt = 3*1, ii= 24, ss= 0, addl=10),
#12h delay simulation
ev(ID = 801:1200, amt = 3*1, ii= 24, ss= 1, addl=0),
ev(ID = 801:1200, amt = 3*0, ii= 12, ss= 0, addl=0),
ev(ID = 801:1200, amt = 3*1, ii= 12, ss= 0, addl=0),
ev(ID = 801:1200, amt = 3*1, ii= 24, ss= 0, addl=10),
#15h delay + 50% dose intake simulation
ev(ID = 801:1200, amt = 3*1, ii= 24, ss= 1, addl=0),
ev(ID = 801:1200, amt = 3*0, ii= 15, ss= 0, addl=0),
ev(ID = 801:1200, amt = 3*0.5, ii= 9, ss= 0, addl=0),
ev(ID = 801:1200, amt = 3*1, ii= 24, ss= 0, addl=10),
#15h delay simulation
ev(ID = 801:1200, amt = 3*1, ii= 24, ss= 1, addl=0),
ev(ID = 801:1200, amt = 3*0, ii= 15, ss= 0, addl=0),
ev(ID = 801:1200, amt = 3*1, ii= 9, ss= 0, addl=0),
ev(ID = 801:1200, amt = 3*1, ii= 24, ss= 0, addl=10),
#18h delay + 50% dose intake simulation
ev(ID = 801:1200, amt = 3*1, ii= 24, ss= 1, addl=0),
```

```
ev(ID = 801:1200, amt = 3*0, ii= 18, ss= 0, addl=0),
ev(ID = 801:1200, amt = 3*0.5, ii= 6, ss= 0, addl=0),
ev(ID = 801:1200, amt = 3*1, ii= 24, ss= 0, addl=10),
#18h delay simulation
ev(ID = 801:1200, amt = 3*1, ii= 24, ss= 1, addl=0),
ev(ID = 801:1200, amt = 3*0, ii= 18, ss= 0, addl=0),
ev(ID = 801:1200, amt = 3*1, ii= 6, ss= 0, addl=0),
ev(ID = 801:1200, amt = 3*1, ii= 24, ss= 0, addl=10),
#21h delay + 50% dose intake simulation
ev(ID = 801:1200, amt = 3*1, ii= 24, ss= 1, addl=0),
ev(ID = 801:1200, amt = 3*0, ii= 21, ss= 0, addl=0),
ev(ID = 801:1200, amt = 3*0.5, ii= 3, ss= 0, addl=0),
ev(ID = 801:1200, amt = 3*1, ii= 24, ss= 0, addl=10),
#21h delay simulation
ev(ID = 801:1200, amt = 3*1, ii= 24, ss= 1, addl=0),
ev(ID = 801:1200, amt = 3*0, ii= 21, ss= 0, addl=0),
ev(ID = 801:1200, amt = 3*1, ii= 3, ss= 0, addl=0),
ev(ID = 801:1200, amt = 3*1, ii= 24, ss= 0, addl=10),
#Missed dose simulation
ev(ID = 801:1200, amt = 3*1, ii= 24, ss= 1, addl=0),
ev(ID = 801:1200, amt = 3*0, ii= 24, ss= 0, addl=0),
ev(ID = 801:1200, amt = 3*1, ii= 24, ss= 0, addl=0),
ev(ID = 801:1200, amt = 3*1, ii= 24, ss= 0, addl=10),
#Missed dose + 150% dose intake simulation
ev(ID = 801:1200, amt = 3*1, ii= 24, ss= 1, addl=0),
ev(ID = 801:1200, amt = 3*0, ii= 24, ss= 0, addl=0),
ev(ID = 801:1200, amt = 3*1.5, ii= 24, ss= 0, addl=0),
ev(ID = 801:1200, amt = 3*1, ii= 24, ss= 0, addl=10),
#Missed dose + 200% dose intake simulation
ev(ID = 801:1200, amt = 3*1, ii= 24, ss= 1, addl=0),
ev(ID = 801:1200, amt = 3*0, ii= 24, ss= 0, addl=0),
ev(ID = 801:1200, amt = 3*2, ii= 24, ss= 0, addl=0),
ev(ID = 801:1200, amt = 3*1, ii= 24, ss= 0, addl=10))))%>%
              mutate(dose_group = "3 mg") %>%

    bind_rows(as_tibble( seq(
  #Steady-state simulation
ev(ID = 1201:1600, amt = 4*1, ii= 24, ss= 1, addl=0),
ev(ID = 1201:1600, amt = 4*1, ii= 24, ss= 0, addl=0),
ev(ID = 1201:1600, amt = 4*1, ii= 24, ss= 0, addl=10),
#3h delay simulation
ev(ID = 1201:1600, amt = 4*1, ii= 24, ss= 1, addl=0),
ev(ID = 1201:1600, amt = 4*0, ii= 3, ss= 0, addl=0),
ev(ID = 1201:1600, amt = 4*1, ii= 21, ss= 0, addl=0),
ev(ID = 1201:1600, amt = 4*1, ii= 24, ss= 0, addl=10),
#6h delay simulation
ev(ID = 1201:1600, amt = 4*1, ii= 24, ss= 1, addl=0),
ev(ID = 1201:1600, amt = 4*0, ii= 6, ss= 0, addl=0),
ev(ID = 1201:1600, amt = 4*1, ii= 18, ss= 0, addl=0),
ev(ID = 1201:1600, amt = 4*1, ii= 24, ss= 0, addl=10),
```

```
#9h delay simulation
ev(ID = 1201:1600, amt = 4*1, ii= 24, ss= 1, addl=0),
ev(ID = 1201:1600, amt = 4*0, ii= 9, ss= 0, addl=0),
ev(ID = 1201:1600, amt = 4*1, ii= 15, ss= 0, addl=0),
ev(ID = 1201:1600, amt = 4*1, ii= 24, ss= 0, addl=10),
#12h delay simulation
ev(ID = 1201:1600, amt = 4*1, ii= 24, ss= 1, addl=0),
ev(ID = 1201:1600, amt = 4*0, ii= 12, ss= 0, addl=0),
ev(ID = 1201:1600, amt = 4*1, ii= 12, ss= 0, addl=0),
ev(ID = 1201:1600, amt = 4*1, ii= 24, ss= 0, addl=10),
#15h delay + 50% dose intake simulation
ev(ID = 1201:1600, amt = 4*1, ii= 24, ss= 1, addl=0),
ev(ID = 1201:1600, amt = 4*0, ii= 15, ss= 0, addl=0),
ev(ID = 1201:1600, amt = 4*0.5, ii= 9, ss= 0, addl=0),
ev(ID = 1201:1600, amt = 4*1, ii= 24, ss= 0, addl=10),
#15h delay simulation
ev(ID = 1201:1600, amt = 4*1, ii= 24, ss= 1, addl=0),
ev(ID = 1201:1600, amt = 4*0, ii= 15, ss= 0, addl=0),
ev(ID = 1201:1600, amt = 4*1, ii= 9, ss= 0, addl=0),
ev(ID = 1201:1600, amt = 4*1, ii= 24, ss= 0, addl=10),
#18h delay + 50% dose intake simulation
ev(ID = 1201:1600, amt = 4*1, ii= 24, ss= 1, addl=0),
ev(ID = 1201:1600, amt = 4*0, ii= 18, ss= 0, addl=0),
ev(ID = 1201:1600, amt = 4*0.5, ii= 6, ss= 0, addl=0),
ev(ID = 1201:1600, amt = 4*1, ii= 24, ss= 0, addl=10),
#18h delay simulation
ev(ID = 1201:1600, amt = 4*1, ii= 24, ss= 1, addl=0),
ev(ID = 1201:1600, amt = 4*0, ii= 18, ss= 0, addl=0),
ev(ID = 1201:1600, amt = 4*1, ii= 6, ss= 0, addl=0),
ev(ID = 1201:1600, amt = 4*1, ii= 24, ss= 0, addl=10),
#21h delay + 50% dose intake simulation
ev(ID = 1201:1600, amt = 4*1, ii= 24, ss= 1, addl=0),
ev(ID = 1201:1600, amt = 4*0, ii= 21, ss= 0, addl=0),
ev(ID = 1201:1600, amt = 4*0.5, ii= 3, ss= 0, addl=0),
ev(ID = 1201:1600, amt = 4*1, ii= 24, ss= 0, addl=10),
#21h delay simulation
ev(ID = 1201:1600, amt = 4*1, ii= 24, ss= 1, addl=0),
ev(ID = 1201:1600, amt = 4*0, ii= 21, ss= 0, addl=0),
ev(ID = 1201:1600, amt = 4*1, ii= 3, ss= 0, addl=0),
ev(ID = 1201:1600, amt = 4*1, ii= 24, ss= 0, addl=10),
#Missed dose simulation
ev(ID = 1201:1600, amt = 4*1, ii= 24, ss= 1, addl=0),
ev(ID = 1201:1600, amt = 4*0, ii= 24, ss= 0, addl=0),
ev(ID = 1201:1600, amt = 4*1, ii= 24, ss= 0, addl=0),
ev(ID = 1201:1600, amt = 4*1, ii= 24, ss= 0, addl=10),
#Missed dose + 150% dose intake simulation
ev(ID = 1201:1600, amt = 4*1, ii= 24, ss= 1, addl=0),
ev(ID = 1201:1600, amt = 4*0, ii= 24, ss= 0, addl=0),
ev(ID = 1201:1600, amt = 4*1.5, ii= 24, ss= 0, addl=0),
ev(ID = 1201:1600, amt = 4*1, ii= 24, ss= 0, addl=10),
```

```
#Missed dose + 200% dose intake simulation
ev(ID = 1201:1600, amt = 4*1, ii= 24, ss= 1, addl=0),
ev(ID = 1201:1600, amt = 4*0, ii= 24, ss= 0, addl=0),
ev(ID = 1201:1600, amt = 4*2, ii= 24, ss= 0, addl=0),
ev(ID = 1201:1600, amt = 4*1, ii= 24, ss= 0, addl=10))))%>%
              mutate(dose_group = "4 mg") %>%

    bind_rows(as_tibble( seq(
  #Steady-state simulation
ev(ID = 1601:2000, amt = 5*1, ii= 24, ss= 1, addl=0),
ev(ID = 1601:2000, amt = 5*1, ii= 24, ss= 0, addl=0),
ev(ID = 1601:2000, amt = 5*1, ii= 24, ss= 0, addl=10),
#3h delay simulation
ev(ID = 1601:2000, amt = 5*1, ii= 24, ss= 1, addl=0),
ev(ID = 1601:2000, amt = 5*0, ii= 3, ss= 0, addl=0),
ev(ID = 1601:2000, amt = 5*1, ii= 21, ss= 0, addl=0),
ev(ID = 1601:2000, amt = 5*1, ii= 24, ss= 0, addl=10),
#6h delay simulation
ev(ID = 1601:2000, amt = 5*1, ii= 24, ss= 1, addl=0),
ev(ID = 1601:2000, amt = 5*0, ii= 6, ss= 0, addl=0),
ev(ID = 1601:2000, amt = 5*1, ii= 18, ss= 0, addl=0),
ev(ID = 1601:2000, amt = 5*1, ii= 24, ss= 0, addl=10),
#9h delay simulation
ev(ID = 1601:2000, amt = 5*1, ii= 24, ss= 1, addl=0),
ev(ID = 1601:2000, amt = 5*0, ii= 9, ss= 0, addl=0),
ev(ID = 1601:2000, amt = 5*1, ii= 15, ss= 0, addl=0),
ev(ID = 1601:2000, amt = 5*1, ii= 24, ss= 0, addl=10),
#12h delay simulation
ev(ID = 1601:2000, amt = 5*1, ii= 24, ss= 1, addl=0),
ev(ID = 1601:2000, amt = 5*0, ii= 12, ss= 0, addl=0),
ev(ID = 1601:2000, amt = 5*1, ii= 12, ss= 0, addl=0),
ev(ID = 1601:2000, amt = 5*1, ii= 24, ss= 0, addl=10),
#15h delay + 50% dose intake simulation
ev(ID = 1601:2000, amt = 5*1, ii= 24, ss= 1, addl=0),
ev(ID = 1601:2000, amt = 5*0, ii= 15, ss= 0, addl=0),
ev(ID = 1601:2000, amt = 5*0.5, ii= 9, ss= 0, addl=0),
ev(ID = 1601:2000, amt = 5*1, ii= 24, ss= 0, addl=10),
#15h delay simulation
ev(ID = 1601:2000, amt = 5*1, ii= 24, ss= 1, addl=0),
ev(ID = 1601:2000, amt = 5*0, ii= 15, ss= 0, addl=0),
ev(ID = 1601:2000, amt = 5*1, ii= 9, ss= 0, addl=0),
ev(ID = 1601:2000, amt = 5*1, ii= 24, ss= 0, addl=10),
#18h delay + 50% dose intake simulation
ev(ID = 1601:2000, amt = 5*1, ii= 24, ss= 1, addl=0),
ev(ID = 1601:2000, amt = 5*0, ii= 18, ss= 0, addl=0),
ev(ID = 1601:2000, amt = 5*0.5, ii= 6, ss= 0, addl=0),
ev(ID = 1601:2000, amt = 5*1, ii= 24, ss= 0, addl=10),
#18h delay simulation
ev(ID = 1601:2000, amt = 5*1, ii= 24, ss= 1, addl=0),
ev(ID = 1601:2000, amt = 5*0, ii= 18, ss= 0, addl=0),
```

```
ev(ID = 1601:2000, amt = 5*1, ii= 6, ss= 0, addl=0),
ev(ID = 1601:2000, amt = 5*1, ii= 24, ss= 0, addl=10),
#21h delay + 50% dose intake simulation
ev(ID = 1601:2000, amt = 5*1, ii= 24, ss= 1, addl=0),
ev(ID = 1601:2000, amt = 5*0, ii= 21, ss= 0, addl=0),
ev(ID = 1601:2000, amt = 5*0.5, ii= 3, ss= 0, addl=0),
ev(ID = 1601:2000, amt = 5*1, ii= 24, ss= 0, addl=10),
#21h delay simulation
ev(ID = 1601:2000, amt = 5*1, ii= 24, ss= 1, addl=0),
ev(ID = 1601:2000, amt = 5*0, ii= 21, ss= 0, addl=0),
ev(ID = 1601:2000, amt = 5*1, ii= 3, ss= 0, addl=0),
ev(ID = 1601:2000, amt = 5*1, ii= 24, ss= 0, addl=10),
#Missed dose simulation
ev(ID = 1601:2000, amt = 5*1, ii= 24, ss= 1, addl=0),
ev(ID = 1601:2000, amt = 5*0, ii= 24, ss= 0, addl=0),
ev(ID = 1601:2000, amt = 5*1, ii= 24, ss= 0, addl=0),
ev(ID = 1601:2000, amt = 5*1, ii= 24, ss= 0, addl=10),
#Missed dose + 150% dose intake simulation
ev(ID = 1601:2000, amt = 5*1, ii= 24, ss= 1, addl=0),
ev(ID = 1601:2000, amt = 5*0, ii= 24, ss= 0, addl=0),
ev(ID = 1601:2000, amt = 5*1.5, ii= 24, ss= 0, addl=0),
ev(ID = 1601:2000, amt = 5*1, ii= 24, ss= 0, addl=10),
#Missed dose + 200% dose intake simulation
ev(ID = 1601:2000, amt = 5*1, ii= 24, ss= 1, addl=0),
ev(ID = 1601:2000, amt = 5*0, ii= 24, ss= 0, addl=0),
ev(ID = 1601:2000, amt = 5*2, ii= 24, ss= 0, addl=0),
ev(ID = 1601:2000, amt = 5*1, ii= 24, ss= 0, addl=10))))%>%
              mutate(dose_group = "5 mg") %>%

    bind_rows(as_tibble( seq(
  #Steady-state simulation
ev(ID = 2001:2400, amt = 6*1, ii= 24, ss= 1, addl=0),
ev(ID = 2001:2400, amt = 6*1, ii= 24, ss= 0, addl=0),
ev(ID = 2001:2400, amt = 6*1, ii= 24, ss= 0, addl=10),
#3h delay simulation
ev(ID = 2001:2400, amt = 6*1, ii= 24, ss= 1, addl=0),
ev(ID = 2001:2400, amt = 6*0, ii= 3, ss= 0, addl=0),
ev(ID = 2001:2400, amt = 6*1, ii= 21, ss= 0, addl=0),
ev(ID = 2001:2400, amt = 6*1, ii= 24, ss= 0, addl=10),
#6h delay simulation
ev(ID = 2001:2400, amt = 6*1, ii= 24, ss= 1, addl=0),
ev(ID = 2001:2400, amt = 6*0, ii= 6, ss= 0, addl=0),
ev(ID = 2001:2400, amt = 6*1, ii= 18, ss= 0, addl=0),
ev(ID = 2001:2400, amt = 6*1, ii= 24, ss= 0, addl=10),
#9h delay simulation
ev(ID = 2001:2400, amt = 6*1, ii= 24, ss= 1, addl=0),
ev(ID = 2001:2400, amt = 6*0, ii= 9, ss= 0, addl=0),
ev(ID = 2001:2400, amt = 6*1, ii= 15, ss= 0, addl=0),
ev(ID = 2001:2400, amt = 6*1, ii= 24, ss= 0, addl=10),
#12h delay simulation
```

```
ev(ID = 2001:2400, amt = 6*1, ii= 24, ss= 1, addl=0),
ev(ID = 2001:2400, amt = 6*0, ii= 12, ss= 0, addl=0),
ev(ID = 2001:2400, amt = 6*1, ii= 12, ss= 0, addl=0),
ev(ID = 2001:2400, amt = 6*1, ii= 24, ss= 0, addl=10),
#15h delay + 50% dose intake simulation
ev(ID = 2001:2400, amt = 6*1, ii= 24, ss= 1, addl=0),
ev(ID = 2001:2400, amt = 6*0, ii= 15, ss= 0, addl=0),
ev(ID = 2001:2400, amt = 6*0.5, ii= 9, ss= 0, addl=0),
ev(ID = 2001:2400, amt = 6*1, ii= 24, ss= 0, addl=10),
#15h delay simulation
ev(ID = 2001:2400, amt = 6*1, ii= 24, ss= 1, addl=0),
ev(ID = 2001:2400, amt = 6*0, ii= 15, ss= 0, addl=0),
ev(ID = 2001:2400, amt = 6*1, ii= 9, ss= 0, addl=0),
ev(ID = 2001:2400, amt = 6*1, ii= 24, ss= 0, addl=10),
#18h delay + 50% dose intake simulation
ev(ID = 2001:2400, amt = 6*1, ii= 24, ss= 1, addl=0),
ev(ID = 2001:2400, amt = 6*0, ii= 18, ss= 0, addl=0),
ev(ID = 2001:2400, amt = 6*0.5, ii= 6, ss= 0, addl=0),
ev(ID = 2001:2400, amt = 6*1, ii= 24, ss= 0, addl=10),
#18h delay simulation
ev(ID = 2001:2400, amt = 6*1, ii= 24, ss= 1, addl=0),
ev(ID = 2001:2400, amt = 6*0, ii= 18, ss= 0, addl=0),
ev(ID = 2001:2400, amt = 6*1, ii= 6, ss= 0, addl=0),
ev(ID = 2001:2400, amt = 6*1, ii= 24, ss= 0, addl=10),
#21h delay + 50% dose intake simulation
ev(ID = 2001:2400, amt = 6*1, ii= 24, ss= 1, addl=0),
ev(ID = 2001:2400, amt = 6*0, ii= 21, ss= 0, addl=0),
ev(ID = 2001:2400, amt = 6*0.5, ii= 3, ss= 0, addl=0),
ev(ID = 2001:2400, amt = 6*1, ii= 24, ss= 0, addl=10),
#21h delay simulation
ev(ID = 2001:2400, amt = 6*1, ii= 24, ss= 1, addl=0),
ev(ID = 2001:2400, amt = 6*0, ii= 21, ss= 0, addl=0),
ev(ID = 2001:2400, amt = 6*1, ii= 3, ss= 0, addl=0),
ev(ID = 2001:2400, amt = 6*1, ii= 24, ss= 0, addl=10),
#Missed dose simulation
ev(ID = 2001:2400, amt = 6*1, ii= 24, ss= 1, addl=0),
ev(ID = 2001:2400, amt = 6*0, ii= 24, ss= 0, addl=0),
ev(ID = 2001:2400, amt = 6*1, ii= 24, ss= 0, addl=0),
ev(ID = 2001:2400, amt = 6*1, ii= 24, ss= 0, addl=10),
#Missed dose + 150% dose intake simulation
ev(ID = 2001:2400, amt = 6*1, ii= 24, ss= 1, addl=0),
ev(ID = 2001:2400, amt = 6*0, ii= 24, ss= 0, addl=0),
ev(ID = 2001:2400, amt = 6*1.5, ii= 24, ss= 0, addl=0),
ev(ID = 2001:2400, amt = 6*1, ii= 24, ss= 0, addl=10),
#Missed dose + 200% dose intake simulation
ev(ID = 2001:2400, amt = 6*1, ii= 24, ss= 1, addl=0),
ev(ID = 2001:2400, amt = 6*0, ii= 24, ss= 0, addl=0),
ev(ID = 2001:2400, amt = 6*2, ii= 24, ss= 0, addl=0),
ev(ID = 2001:2400, amt = 6*1, ii= 24, ss= 0, addl=10))))%>%
              mutate(dose_group = "6 mg") %>%
```

```
    bind_rows(as_tibble( seq(
  #Steady-state simulation
ev(ID = 2401:2800, amt = 7*1, ii= 24, ss= 1, addl=0),
ev(ID = 2401:2800, amt = 7*1, ii= 24, ss= 0, addl=0),
ev(ID = 2401:2800, amt = 7*1, ii= 24, ss= 0, addl=10),
#3h delay simulation
ev(ID = 2401:2800, amt = 7*1, ii= 24, ss= 1, addl=0),
ev(ID = 2401:2800, amt = 7*0, ii= 3, ss= 0, addl=0),
ev(ID = 2401:2800, amt = 7*1, ii= 21, ss= 0, addl=0),
ev(ID = 2401:2800, amt = 7*1, ii= 24, ss= 0, addl=10),
#6h delay simulation
ev(ID = 2401:2800, amt = 7*1, ii= 24, ss= 1, addl=0),
ev(ID = 2401:2800, amt = 7*0, ii= 6, ss= 0, addl=0),
ev(ID = 2401:2800, amt = 7*1, ii= 18, ss= 0, addl=0),
ev(ID = 2401:2800, amt = 7*1, ii= 24, ss= 0, addl=10),
#9h delay simulation
ev(ID = 2401:2800, amt = 7*1, ii= 24, ss= 1, addl=0),
ev(ID = 2401:2800, amt = 7*0, ii= 9, ss= 0, addl=0),
ev(ID = 2401:2800, amt = 7*1, ii= 15, ss= 0, addl=0),
ev(ID = 2401:2800, amt = 7*1, ii= 24, ss= 0, addl=10),
#12h delay simulation
ev(ID = 2401:2800, amt = 7*1, ii= 24, ss= 1, addl=0),
ev(ID = 2401:2800, amt = 7*0, ii= 12, ss= 0, addl=0),
ev(ID = 2401:2800, amt = 7*1, ii= 12, ss= 0, addl=0),
ev(ID = 2401:2800, amt = 7*1, ii= 24, ss= 0, addl=10),
#15h delay + 50% dose intake simulation
ev(ID = 2401:2800, amt = 7*1, ii= 24, ss= 1, addl=0),
ev(ID = 2401:2800, amt = 7*0, ii= 15, ss= 0, addl=0),
ev(ID = 2401:2800, amt = 7*0.5, ii= 9, ss= 0, addl=0),
ev(ID = 2401:2800, amt = 7*1, ii= 24, ss= 0, addl=10),
#15h delay simulation
ev(ID = 2401:2800, amt = 7*1, ii= 24, ss= 1, addl=0),
ev(ID = 2401:2800, amt = 7*0, ii= 15, ss= 0, addl=0),
ev(ID = 2401:2800, amt = 7*1, ii= 9, ss= 0, addl=0),
ev(ID = 2401:2800, amt = 7*1, ii= 24, ss= 0, addl=10),
#18h delay + 50% dose intake simulation
ev(ID = 2401:2800, amt = 7*1, ii= 24, ss= 1, addl=0),
ev(ID = 2401:2800, amt = 7*0, ii= 18, ss= 0, addl=0),
ev(ID = 2401:2800, amt = 7*0.5, ii= 6, ss= 0, addl=0),
ev(ID = 2401:2800, amt = 7*1, ii= 24, ss= 0, addl=10),
#18h delay simulation
ev(ID = 2401:2800, amt = 7*1, ii= 24, ss= 1, addl=0),
ev(ID = 2401:2800, amt = 7*0, ii= 18, ss= 0, addl=0),
ev(ID = 2401:2800, amt = 7*1, ii= 6, ss= 0, addl=0),
ev(ID = 2401:2800, amt = 7*1, ii= 24, ss= 0, addl=10),
#21h delay + 50% dose intake simulation
ev(ID = 2401:2800, amt = 7*1, ii= 24, ss= 1, addl=0),
ev(ID = 2401:2800, amt = 7*0, ii= 21, ss= 0, addl=0),
ev(ID = 2401:2800, amt = 7*0.5, ii= 3, ss= 0, addl=0),
```

```
ev(ID = 2401:2800, amt = 7*1, ii= 24, ss= 0, addl=10),
#21h delay simulation
ev(ID = 2401:2800, amt = 7*1, ii= 24, ss= 1, addl=0),
ev(ID = 2401:2800, amt = 7*0, ii= 21, ss= 0, addl=0),
ev(ID = 2401:2800, amt = 7*1, ii= 3, ss= 0, addl=0),
ev(ID = 2401:2800, amt = 7*1, ii= 24, ss= 0, addl=10),
#Missed dose simulation
ev(ID = 2401:2800, amt = 7*1, ii= 24, ss= 1, addl=0),
ev(ID = 2401:2800, amt = 7*0, ii= 24, ss= 0, addl=0),
ev(ID = 2401:2800, amt = 7*1, ii= 24, ss= 0, addl=0),
ev(ID = 2401:2800, amt = 7*1, ii= 24, ss= 0, addl=10),
#Missed dose + 150% dose intake simulation
ev(ID = 2401:2800, amt = 7*1, ii= 24, ss= 1, addl=0),
ev(ID = 2401:2800, amt = 7*0, ii= 24, ss= 0, addl=0),
ev(ID = 2401:2800, amt = 7*1.5, ii= 24, ss= 0, addl=0),
ev(ID = 2401:2800, amt = 7*1, ii= 24, ss= 0, addl=10),
#Missed dose + 200% dose intake simulation
ev(ID = 2401:2800, amt = 7*1, ii= 24, ss= 1, addl=0),
ev(ID = 2401:2800, amt = 7*0, ii= 24, ss= 0, addl=0),
ev(ID = 2401:2800, amt = 7*2, ii= 24, ss= 0, addl=0),
ev(ID = 2401:2800, amt = 7*1, ii= 24, ss= 0, addl=10))))%>%
              mutate(dose_group = "7 mg") %>%

    bind_rows(as_tibble( seq(
  #Steady-state simulation
ev(ID = 2801:3200, amt = 8*1, ii= 24, ss= 1, addl=0),
ev(ID = 2801:3200, amt = 8*1, ii= 24, ss= 0, addl=0),
ev(ID = 2801:3200, amt = 8*1, ii= 24, ss= 0, addl=10),
#3h delay simulation
ev(ID = 2801:3200, amt = 8*1, ii= 24, ss= 1, addl=0),
ev(ID = 2801:3200, amt = 8*0, ii= 3, ss= 0, addl=0),
ev(ID = 2801:3200, amt = 8*1, ii= 21, ss= 0, addl=0),
ev(ID = 2801:3200, amt = 8*1, ii= 24, ss= 0, addl=10),
#6h delay simulation
ev(ID = 2801:3200, amt = 8*1, ii= 24, ss= 1, addl=0),
ev(ID = 2801:3200, amt = 8*0, ii= 6, ss= 0, addl=0),
ev(ID = 2801:3200, amt = 8*1, ii= 18, ss= 0, addl=0),
ev(ID = 2801:3200, amt = 8*1, ii= 24, ss= 0, addl=10),
#9h delay simulation
ev(ID = 2801:3200, amt = 8*1, ii= 24, ss= 1, addl=0),
ev(ID = 2801:3200, amt = 8*0, ii= 9, ss= 0, addl=0),
ev(ID = 2801:3200, amt = 8*1, ii= 15, ss= 0, addl=0),
ev(ID = 2801:3200, amt = 8*1, ii= 24, ss= 0, addl=10),
#12h delay simulation
ev(ID = 2801:3200, amt = 8*1, ii= 24, ss= 1, addl=0),
ev(ID = 2801:3200, amt = 8*0, ii= 12, ss= 0, addl=0),
ev(ID = 2801:3200, amt = 8*1, ii= 12, ss= 0, addl=0),
ev(ID = 2801:3200, amt = 8*1, ii= 24, ss= 0, addl=10),
#15h delay + 50% dose intake simulation
ev(ID = 2801:3200, amt = 8*1, ii= 24, ss= 1, addl=0),
```

```
ev(ID = 2801:3200, amt = 8*0, ii= 15, ss= 0, addl=0),
ev(ID = 2801:3200, amt = 8*0.5, ii= 9, ss= 0, addl=0),
ev(ID = 2801:3200, amt = 8*1, ii= 24, ss= 0, addl=10),
#15h delay simulation
ev(ID = 2801:3200, amt = 8*1, ii= 24, ss= 1, addl=0),
ev(ID = 2801:3200, amt = 8*0, ii= 15, ss= 0, addl=0),
ev(ID = 2801:3200, amt = 8*1, ii= 9, ss= 0, addl=0),
ev(ID = 2801:3200, amt = 8*1, ii= 24, ss= 0, addl=10),
#18h delay + 50% dose intake simulation
ev(ID = 2801:3200, amt = 8*1, ii= 24, ss= 1, addl=0),
ev(ID = 2801:3200, amt = 8*0, ii= 18, ss= 0, addl=0),
ev(ID = 2801:3200, amt = 8*0.5, ii= 6, ss= 0, addl=0),
ev(ID = 2801:3200, amt = 8*1, ii= 24, ss= 0, addl=10),
#18h delay simulation
ev(ID = 2801:3200, amt = 8*1, ii= 24, ss= 1, addl=0),
ev(ID = 2801:3200, amt = 8*0, ii= 18, ss= 0, addl=0),
ev(ID = 2801:3200, amt = 8*1, ii= 6, ss= 0, addl=0),
ev(ID = 2801:3200, amt = 8*1, ii= 24, ss= 0, addl=10),
#21h delay + 50% dose intake simulation
ev(ID = 2801:3200, amt = 8*1, ii= 24, ss= 1, addl=0),
ev(ID = 2801:3200, amt = 8*0, ii= 21, ss= 0, addl=0),
ev(ID = 2801:3200, amt = 8*0.5, ii= 3, ss= 0, addl=0),
ev(ID = 2801:3200, amt = 8*1, ii= 24, ss= 0, addl=10),
#21h delay simulation
ev(ID = 2801:3200, amt = 8*1, ii= 24, ss= 1, addl=0),
ev(ID = 2801:3200, amt = 8*0, ii= 21, ss= 0, addl=0),
ev(ID = 2801:3200, amt = 8*1, ii= 3, ss= 0, addl=0),
ev(ID = 2801:3200, amt = 8*1, ii= 24, ss= 0, addl=10),
#Missed dose simulation
ev(ID = 2801:3200, amt = 8*1, ii= 24, ss= 1, addl=0),
ev(ID = 2801:3200, amt = 8*0, ii= 24, ss= 0, addl=0),
ev(ID = 2801:3200, amt = 8*1, ii= 24, ss= 0, addl=0),
ev(ID = 2801:3200, amt = 8*1, ii= 24, ss= 0, addl=10),
#Missed dose + 150% dose intake simulation
ev(ID = 2801:3200, amt = 8*1, ii= 24, ss= 1, addl=0),
ev(ID = 2801:3200, amt = 8*0, ii= 24, ss= 0, addl=0),
ev(ID = 2801:3200, amt = 8*1.5, ii= 24, ss= 0, addl=0),
ev(ID = 2801:3200, amt = 8*1, ii= 24, ss= 0, addl=10),
#Missed dose + 200% dose intake simulation
ev(ID = 2801:3200, amt = 8*1, ii= 24, ss= 1, addl=0),
ev(ID = 2801:3200, amt = 8*0, ii= 24, ss= 0, addl=0),
ev(ID = 2801:3200, amt = 8*2, ii= 24, ss= 0, addl=0),
ev(ID = 2801:3200, amt = 8*1, ii= 24, ss= 0, addl=10))))%>%
              mutate(dose_group = "8 mg") %>%

  bind_rows(as_tibble( seq(
  #Steady-state simulation
ev(ID = 3201:3600, amt = 9*1, ii= 24, ss= 1, addl=0),
ev(ID = 3201:3600, amt = 9*1, ii= 24, ss= 0, addl=0),
ev(ID = 3201:3600, amt = 9*1, ii= 24, ss= 0, addl=10),
```

```
#3h delay simulation
ev(ID = 3201:3600, amt = 9*1, ii= 24, ss= 1, addl=0),
ev(ID = 3201:3600, amt = 9*0, ii= 3, ss= 0, addl=0),
ev(ID = 3201:3600, amt = 9*1, ii= 21, ss= 0, addl=0),
ev(ID = 3201:3600, amt = 9*1, ii= 24, ss= 0, addl=10),
#6h delay simulation
ev(ID = 3201:3600, amt = 9*1, ii= 24, ss= 1, addl=0),
ev(ID = 3201:3600, amt = 9*0, ii= 6, ss= 0, addl=0),
ev(ID = 3201:3600, amt = 9*1, ii= 18, ss= 0, addl=0),
ev(ID = 3201:3600, amt = 9*1, ii= 24, ss= 0, addl=10),
#9h delay simulation
ev(ID = 3201:3600, amt = 9*1, ii= 24, ss= 1, addl=0),
ev(ID = 3201:3600, amt = 9*0, ii= 9, ss= 0, addl=0),
ev(ID = 3201:3600, amt = 9*1, ii= 15, ss= 0, addl=0),
ev(ID = 3201:3600, amt = 9*1, ii= 24, ss= 0, addl=10),
#12h delay simulation
ev(ID = 3201:3600, amt = 9*1, ii= 24, ss= 1, addl=0),
ev(ID = 3201:3600, amt = 9*0, ii= 12, ss= 0, addl=0),
ev(ID = 3201:3600, amt = 9*1, ii= 12, ss= 0, addl=0),
ev(ID = 3201:3600, amt = 9*1, ii= 24, ss= 0, addl=10),
#15h delay + 50% dose intake simulation
ev(ID = 3201:3600, amt = 9*1, ii= 24, ss= 1, addl=0),
ev(ID = 3201:3600, amt = 9*0, ii= 15, ss= 0, addl=0),
ev(ID = 3201:3600, amt = 9*0.5, ii= 9, ss= 0, addl=0),
ev(ID = 3201:3600, amt = 9*1, ii= 24, ss= 0, addl=10),
#15h delay simulation
ev(ID = 3201:3600, amt = 9*1, ii= 24, ss= 1, addl=0),
ev(ID = 3201:3600, amt = 9*0, ii= 15, ss= 0, addl=0),
ev(ID = 3201:3600, amt = 9*1, ii= 9, ss= 0, addl=0),
ev(ID = 3201:3600, amt = 9*1, ii= 24, ss= 0, addl=10),
#18h delay + 50% dose intake simulation
ev(ID = 3201:3600, amt = 9*1, ii= 24, ss= 1, addl=0),
ev(ID = 3201:3600, amt = 9*0, ii= 18, ss= 0, addl=0),
ev(ID = 3201:3600, amt = 9*0.5, ii= 6, ss= 0, addl=0),
ev(ID = 3201:3600, amt = 9*1, ii= 24, ss= 0, addl=10),
#18h delay simulation
ev(ID = 3201:3600, amt = 9*1, ii= 24, ss= 1, addl=0),
ev(ID = 3201:3600, amt = 9*0, ii= 18, ss= 0, addl=0),
ev(ID = 3201:3600, amt = 9*1, ii= 6, ss= 0, addl=0),
ev(ID = 3201:3600, amt = 9*1, ii= 24, ss= 0, addl=10),
#21h delay + 50% dose intake simulation
ev(ID = 3201:3600, amt = 9*1, ii= 24, ss= 1, addl=0),
ev(ID = 3201:3600, amt = 9*0, ii= 21, ss= 0, addl=0),
ev(ID = 3201:3600, amt = 9*0.5, ii= 3, ss= 0, addl=0),
ev(ID = 3201:3600, amt = 9*1, ii= 24, ss= 0, addl=10),
#21h delay simulation
ev(ID = 3201:3600, amt = 9*1, ii= 24, ss= 1, addl=0),
ev(ID = 3201:3600, amt = 9*0, ii= 21, ss= 0, addl=0),
ev(ID = 3201:3600, amt = 9*1, ii= 3, ss= 0, addl=0),
ev(ID = 3201:3600, amt = 9*1, ii= 24, ss= 0, addl=10),
```

```
#Missed dose simulation
ev(ID = 3201:3600, amt = 9*1, ii= 24, ss= 1, addl=0),
ev(ID = 3201:3600, amt = 9*0, ii= 24, ss= 0, addl=0),
ev(ID = 3201:3600, amt = 9*1, ii= 24, ss= 0, addl=0),
ev(ID = 3201:3600, amt = 9*1, ii= 24, ss= 0, addl=10),
#Missed dose + 150% dose intake simulation
ev(ID = 3201:3600, amt = 9*1, ii= 24, ss= 1, addl=0),
ev(ID = 3201:3600, amt = 9*0, ii= 24, ss= 0, addl=0),
ev(ID = 3201:3600, amt = 9*1.5, ii= 24, ss= 0, addl=0),
ev(ID = 3201:3600, amt = 9*1, ii= 24, ss= 0, addl=10),
#Missed dose + 200% dose intake simulation
ev(ID = 3201:3600, amt = 9*1, ii= 24, ss= 1, addl=0),
ev(ID = 3201:3600, amt = 9*0, ii= 24, ss= 0, addl=0),
ev(ID = 3201:3600, amt = 9*2, ii= 24, ss= 0, addl=0),
ev(ID = 3201:3600, amt = 9*1, ii= 24, ss= 0, addl=10))))%>%
              mutate(dose_group = "9 mg") %>%

    bind_rows(as_tibble( seq(
  #Steady-state simulation
ev(ID = 3601:4000, amt = 10*1, ii= 24, ss= 1, addl=0),
ev(ID = 3601:4000, amt = 10*1, ii= 24, ss= 0, addl=0),
ev(ID = 3601:4000, amt = 10*1, ii= 24, ss= 0, addl=10),
#3h delay simulation
ev(ID = 3601:4000, amt = 10*1, ii= 24, ss= 1, addl=0),
ev(ID = 3601:4000, amt = 10*0, ii= 3, ss= 0, addl=0),
ev(ID = 3601:4000, amt = 10*1, ii= 21, ss= 0, addl=0),
ev(ID = 3601:4000, amt = 10*1, ii= 24, ss= 0, addl=10),
#6h delay simulation
ev(ID = 3601:4000, amt = 10*1, ii= 24, ss= 1, addl=0),
ev(ID = 3601:4000, amt = 10*0, ii= 6, ss= 0, addl=0),
ev(ID = 3601:4000, amt = 10*1, ii= 18, ss= 0, addl=0),
ev(ID = 3601:4000, amt = 10*1, ii= 24, ss= 0, addl=10),
#9h delay simulation
ev(ID = 3601:4000, amt = 10*1, ii= 24, ss= 1, addl=0),
ev(ID = 3601:4000, amt = 10*0, ii= 9, ss= 0, addl=0),
ev(ID = 3601:4000, amt = 10*1, ii= 15, ss= 0, addl=0),
ev(ID = 3601:4000, amt = 10*1, ii= 24, ss= 0, addl=10),
#12h delay simulation
ev(ID = 3601:4000, amt = 10*1, ii= 24, ss= 1, addl=0),
ev(ID = 3601:4000, amt = 10*0, ii= 12, ss= 0, addl=0),
ev(ID = 3601:4000, amt = 10*1, ii= 12, ss= 0, addl=0),
ev(ID = 3601:4000, amt = 10*1, ii= 24, ss= 0, addl=10),
#15h delay + 50% dose intake simulation
ev(ID = 3601:4000, amt = 10*1, ii= 24, ss= 1, addl=0),
ev(ID = 3601:4000, amt = 10*0, ii= 15, ss= 0, addl=0),
ev(ID = 3601:4000, amt = 10*0.5, ii= 9, ss= 0, addl=0),
ev(ID = 3601:4000, amt = 10*1, ii= 24, ss= 0, addl=10),
#15h delay simulation
ev(ID = 3601:4000, amt = 10*1, ii= 24, ss= 1, addl=0),
ev(ID = 3601:4000, amt = 10*0, ii= 15, ss= 0, addl=0),
```

```
ev(ID = 3601:4000, amt = 10*1, ii= 9, ss= 0, addl=0),
ev(ID = 3601:4000, amt = 10*1, ii= 24, ss= 0, addl=10),
#18h delay + 50% dose intake simulation
ev(ID = 3601:4000, amt = 10*1, ii= 24, ss= 1, addl=0),
ev(ID = 3601:4000, amt = 10*0, ii= 18, ss= 0, addl=0),
ev(ID = 3601:4000, amt = 10*0.5, ii= 6, ss= 0, addl=0),
ev(ID = 3601:4000, amt = 10*1, ii= 24, ss= 0, addl=10),
#18h delay simulation
ev(ID = 3601:4000, amt = 10*1, ii= 24, ss= 1, addl=0),
ev(ID = 3601:4000, amt = 10*0, ii= 18, ss= 0, addl=0),
ev(ID = 3601:4000, amt = 10*1, ii= 6, ss= 0, addl=0),
ev(ID = 3601:4000, amt = 10*1, ii= 24, ss= 0, addl=10),
#21h delay + 50% dose intake simulation
ev(ID = 3601:4000, amt = 10*1, ii= 24, ss= 1, addl=0),
ev(ID = 3601:4000, amt = 10*0, ii= 21, ss= 0, addl=0),
ev(ID = 3601:4000, amt = 10*0.5, ii= 3, ss= 0, addl=0),
ev(ID = 3601:4000, amt = 10*1, ii= 24, ss= 0, addl=10),
#21h delay simulation
ev(ID = 3601:4000, amt = 10*1, ii= 24, ss= 1, addl=0),
ev(ID = 3601:4000, amt = 10*0, ii= 21, ss= 0, addl=0),
ev(ID = 3601:4000, amt = 10*1, ii= 3, ss= 0, addl=0),
ev(ID = 3601:4000, amt = 10*1, ii= 24, ss= 0, addl=10),
#Missed dose simulation
ev(ID = 3601:4000, amt = 10*1, ii= 24, ss= 1, addl=0),
ev(ID = 3601:4000, amt = 10*0, ii= 24, ss= 0, addl=0),
ev(ID = 3601:4000, amt = 10*1, ii= 24, ss= 0, addl=0),
ev(ID = 3601:4000, amt = 10*1, ii= 24, ss= 0, addl=10),
#Missed dose + 150% dose intake simulation
ev(ID = 3601:4000, amt = 10*1, ii= 24, ss= 1, addl=0),
ev(ID = 3601:4000, amt = 10*0, ii= 24, ss= 0, addl=0),
ev(ID = 3601:4000, amt = 10*1.5, ii= 24, ss= 0, addl=0),
ev(ID = 3601:4000, amt = 10*1, ii= 24, ss= 0, addl=10),
#Missed dose + 200% dose intake simulation
ev(ID = 3601:4000, amt = 10*1, ii= 24, ss= 1, addl=0),
ev(ID = 3601:4000, amt = 10*0, ii= 24, ss= 0, addl=0),
ev(ID = 3601:4000, amt = 10*2, ii= 24, ss= 0, addl=0),
ev(ID = 3601:4000, amt = 10*1, ii= 24, ss= 0, addl=10))))%>%
              mutate(dose_group = "10 mg") %>%

    bind_rows(as_tibble( seq(
  #Steady-state simulation
ev(ID = 4001:4400, amt = 11*1, ii= 24, ss= 1, addl=0),
ev(ID = 4001:4400, amt = 11*1, ii= 24, ss= 0, addl=0),
ev(ID = 4001:4400, amt = 11*1, ii= 24, ss= 0, addl=10),
#3h delay simulation
ev(ID = 4001:4400, amt = 11*1, ii= 24, ss= 1, addl=0),
ev(ID = 4001:4400, amt = 11*0, ii= 3, ss= 0, addl=0),
ev(ID = 4001:4400, amt = 11*1, ii= 21, ss= 0, addl=0),
ev(ID = 4001:4400, amt = 11*1, ii= 24, ss= 0, addl=10),
#6h delay simulation
```

```
ev(ID = 4001:4400, amt = 11*1, ii= 24, ss= 1, addl=0),
ev(ID = 4001:4400, amt = 11*0, ii= 6, ss= 0, addl=0),
ev(ID = 4001:4400, amt = 11*1, ii= 18, ss= 0, addl=0),
ev(ID = 4001:4400, amt = 11*1, ii= 24, ss= 0, addl=10),
#9h delay simulation
ev(ID = 4001:4400, amt = 11*1, ii= 24, ss= 1, addl=0),
ev(ID = 4001:4400, amt = 11*0, ii= 9, ss= 0, addl=0),
ev(ID = 4001:4400, amt = 11*1, ii= 15, ss= 0, addl=0),
ev(ID = 4001:4400, amt = 11*1, ii= 24, ss= 0, addl=10),
#12h delay simulation
ev(ID = 4001:4400, amt = 11*1, ii= 24, ss= 1, addl=0),
ev(ID = 4001:4400, amt = 11*0, ii= 12, ss= 0, addl=0),
ev(ID = 4001:4400, amt = 11*1, ii= 12, ss= 0, addl=0),
ev(ID = 4001:4400, amt = 11*1, ii= 24, ss= 0, addl=10),
#15h delay + 50% dose intake simulation
ev(ID = 4001:4400, amt = 11*1, ii= 24, ss= 1, addl=0),
ev(ID = 4001:4400, amt = 11*0, ii= 15, ss= 0, addl=0),
ev(ID = 4001:4400, amt = 11*0.5, ii= 9, ss= 0, addl=0),
ev(ID = 4001:4400, amt = 11*1, ii= 24, ss= 0, addl=10),
#15h delay simulation
ev(ID = 4001:4400, amt = 11*1, ii= 24, ss= 1, addl=0),
ev(ID = 4001:4400, amt = 11*0, ii= 15, ss= 0, addl=0),
ev(ID = 4001:4400, amt = 11*1, ii= 9, ss= 0, addl=0),
ev(ID = 4001:4400, amt = 11*1, ii= 24, ss= 0, addl=10),
#18h delay + 50% dose intake simulation
ev(ID = 4001:4400, amt = 11*1, ii= 24, ss= 1, addl=0),
ev(ID = 4001:4400, amt = 11*0, ii= 18, ss= 0, addl=0),
ev(ID = 4001:4400, amt = 11*0.5, ii= 6, ss= 0, addl=0),
ev(ID = 4001:4400, amt = 11*1, ii= 24, ss= 0, addl=10),
#18h delay simulation
ev(ID = 4001:4400, amt = 11*1, ii= 24, ss= 1, addl=0),
ev(ID = 4001:4400, amt = 11*0, ii= 18, ss= 0, addl=0),
ev(ID = 4001:4400, amt = 11*1, ii= 6, ss= 0, addl=0),
ev(ID = 4001:4400, amt = 11*1, ii= 24, ss= 0, addl=10),
#21h delay + 50% dose intake simulation
ev(ID = 4001:4400, amt = 11*1, ii= 24, ss= 1, addl=0),
ev(ID = 4001:4400, amt = 11*0, ii= 21, ss= 0, addl=0),
ev(ID = 4001:4400, amt = 11*0.5, ii= 3, ss= 0, addl=0),
ev(ID = 4001:4400, amt = 11*1, ii= 24, ss= 0, addl=10),
#21h delay simulation
ev(ID = 4001:4400, amt = 11*1, ii= 24, ss= 1, addl=0),
ev(ID = 4001:4400, amt = 11*0, ii= 21, ss= 0, addl=0),
ev(ID = 4001:4400, amt = 11*1, ii= 3, ss= 0, addl=0),
ev(ID = 4001:4400, amt = 11*1, ii= 24, ss= 0, addl=10),
#Missed dose simulation
ev(ID = 4001:4400, amt = 11*1, ii= 24, ss= 1, addl=0),
ev(ID = 4001:4400, amt = 11*0, ii= 24, ss= 0, addl=0),
ev(ID = 4001:4400, amt = 11*1, ii= 24, ss= 0, addl=0),
ev(ID = 4001:4400, amt = 11*1, ii= 24, ss= 0, addl=10),
#Missed dose + 150% dose intake simulation
```

```
ev(ID = 4001:4400, amt = 11*1, ii= 24, ss= 1, addl=0),
ev(ID = 4001:4400, amt = 11*0, ii= 24, ss= 0, addl=0),
ev(ID = 4001:4400, amt = 11*1.5, ii= 24, ss= 0, addl=0),
ev(ID = 4001:4400, amt = 11*1, ii= 24, ss= 0, addl=10),
#Missed dose + 200% dose intake simulation
ev(ID = 4001:4400, amt = 11*1, ii= 24, ss= 1, addl=0),
ev(ID = 4001:4400, amt = 11*0, ii= 24, ss= 0, addl=0),
ev(ID = 4001:4400, amt = 11*2, ii= 24, ss= 0, addl=0),
ev(ID = 4001:4400, amt = 11*1, ii= 24, ss= 0, addl=10))))%>%
              mutate(dose_group = "11 mg") %>%

    bind_rows(as_tibble( seq(
  #Steady-state simulation
ev(ID = 4401:4800, amt = 12*1, ii= 24, ss= 1, addl=0),
ev(ID = 4401:4800, amt = 12*1, ii= 24, ss= 0, addl=0),
ev(ID = 4401:4800, amt = 12*1, ii= 24, ss= 0, addl=10),
#3h delay simulation
ev(ID = 4401:4800, amt = 12*1, ii= 24, ss= 1, addl=0),
ev(ID = 4401:4800, amt = 12*0, ii= 3, ss= 0, addl=0),
ev(ID = 4401:4800, amt = 12*1, ii= 21, ss= 0, addl=0),
ev(ID = 4401:4800, amt = 12*1, ii= 24, ss= 0, addl=10),
#6h delay simulation
ev(ID = 4401:4800, amt = 12*1, ii= 24, ss= 1, addl=0),
ev(ID = 4401:4800, amt = 12*0, ii= 6, ss= 0, addl=0),
ev(ID = 4401:4800, amt = 12*1, ii= 18, ss= 0, addl=0),
ev(ID = 4401:4800, amt = 12*1, ii= 24, ss= 0, addl=10),
#9h delay simulation
ev(ID = 4401:4800, amt = 12*1, ii= 24, ss= 1, addl=0),
ev(ID = 4401:4800, amt = 12*0, ii= 9, ss= 0, addl=0),
ev(ID = 4401:4800, amt = 12*1, ii= 15, ss= 0, addl=0),
ev(ID = 4401:4800, amt = 12*1, ii= 24, ss= 0, addl=10),
#12h delay simulation
ev(ID = 4401:4800, amt = 12*1, ii= 24, ss= 1, addl=0),
ev(ID = 4401:4800, amt = 12*0, ii= 12, ss= 0, addl=0),
ev(ID = 4401:4800, amt = 12*1, ii= 12, ss= 0, addl=0),
ev(ID = 4401:4800, amt = 12*1, ii= 24, ss= 0, addl=10),
#15h delay + 50% dose intake simulation
ev(ID = 4401:4800, amt = 12*1, ii= 24, ss= 1, addl=0),
ev(ID = 4401:4800, amt = 12*0, ii= 15, ss= 0, addl=0),
ev(ID = 4401:4800, amt = 12*0.5, ii= 9, ss= 0, addl=0),
ev(ID = 4401:4800, amt = 12*1, ii= 24, ss= 0, addl=10),
#15h delay simulation
ev(ID = 4401:4800, amt = 12*1, ii= 24, ss= 1, addl=0),
ev(ID = 4401:4800, amt = 12*0, ii= 15, ss= 0, addl=0),
ev(ID = 4401:4800, amt = 12*1, ii= 9, ss= 0, addl=0),
ev(ID = 4401:4800, amt = 12*1, ii= 24, ss= 0, addl=10),
#18h delay + 50% dose intake simulation
ev(ID = 4401:4800, amt = 12*1, ii= 24, ss= 1, addl=0),
ev(ID = 4401:4800, amt = 12*0, ii= 18, ss= 0, addl=0),
ev(ID = 4401:4800, amt = 12*0.5, ii= 6, ss= 0, addl=0),
```

```
ev(ID = 4401:4800, amt = 12*1, ii= 24, ss= 0, addl=10),
#18h delay simulation
ev(ID = 4401:4800, amt = 12*1, ii= 24, ss= 1, addl=0),
ev(ID = 4401:4800, amt = 12*0, ii= 18, ss= 0, addl=0),
ev(ID = 4401:4800, amt = 12*1, ii= 6, ss= 0, addl=0),
ev(ID = 4401:4800, amt = 12*1, ii= 24, ss= 0, addl=10),
#21h delay + 50% dose intake simulation
ev(ID = 4401:4800, amt = 12*1, ii= 24, ss= 1, addl=0),
ev(ID = 4401:4800, amt = 12*0, ii= 21, ss= 0, addl=0),
ev(ID = 4401:4800, amt = 12*0.5, ii= 3, ss= 0, addl=0),
ev(ID = 4401:4800, amt = 12*1, ii= 24, ss= 0, addl=10),
#21h delay simulation
ev(ID = 4401:4800, amt = 12*1, ii= 24, ss= 1, addl=0),
ev(ID = 4401:4800, amt = 12*0, ii= 21, ss= 0, addl=0),
ev(ID = 4401:4800, amt = 12*1, ii= 3, ss= 0, addl=0),
ev(ID = 4401:4800, amt = 12*1, ii= 24, ss= 0, addl=10),
#Missed dose simulation
ev(ID = 4401:4800, amt = 12*1, ii= 24, ss= 1, addl=0),
ev(ID = 4401:4800, amt = 12*0, ii= 24, ss= 0, addl=0),
ev(ID = 4401:4800, amt = 12*1, ii= 24, ss= 0, addl=0),
ev(ID = 4401:4800, amt = 12*1, ii= 24, ss= 0, addl=10),
#Missed dose + 150% dose intake simulation
ev(ID = 4401:4800, amt = 12*1, ii= 24, ss= 1, addl=0),
ev(ID = 4401:4800, amt = 12*0, ii= 24, ss= 0, addl=0),
ev(ID = 4401:4800, amt = 12*1.5, ii= 24, ss= 0, addl=0),
ev(ID = 4401:4800, amt = 12*1, ii= 24, ss= 0, addl=10),
#Missed dose + 200% dose intake simulation
ev(ID = 4401:4800, amt = 12*1, ii= 24, ss= 1, addl=0),
ev(ID = 4401:4800, amt = 12*0, ii= 24, ss= 0, addl=0),
ev(ID = 4401:4800, amt = 12*2, ii= 24, ss= 0, addl=0),
ev(ID = 4401:4800, amt = 12*1, ii= 24, ss= 0, addl=10))))%>%
              mutate(dose_group = "12 mg") %>%

    bind_rows(as_tibble( seq(
  #Steady-state simulation
ev(ID = 4801:5200, amt = 13*1, ii= 24, ss= 1, addl=0),
ev(ID = 4801:5200, amt = 13*1, ii= 24, ss= 0, addl=0),
ev(ID = 4801:5200, amt = 13*1, ii= 24, ss= 0, addl=10),
#3h delay simulation
ev(ID = 4801:5200, amt = 13*1, ii= 24, ss= 1, addl=0),
ev(ID = 4801:5200, amt = 13*0, ii= 3, ss= 0, addl=0),
ev(ID = 4801:5200, amt = 13*1, ii= 21, ss= 0, addl=0),
ev(ID = 4801:5200, amt = 13*1, ii= 24, ss= 0, addl=10),
#6h delay simulation
ev(ID = 4801:5200, amt = 13*1, ii= 24, ss= 1, addl=0),
ev(ID = 4801:5200, amt = 13*0, ii= 6, ss= 0, addl=0),
ev(ID = 4801:5200, amt = 13*1, ii= 18, ss= 0, addl=0),
ev(ID = 4801:5200, amt = 13*1, ii= 24, ss= 0, addl=10),
#9h delay simulation
ev(ID = 4801:5200, amt = 13*1, ii= 24, ss= 1, addl=0),
```

```
ev(ID = 4801:5200, amt = 13*0, ii= 9, ss= 0, addl=0),
ev(ID = 4801:5200, amt = 13*1, ii= 15, ss= 0, addl=0),
ev(ID = 4801:5200, amt = 13*1, ii= 24, ss= 0, addl=10),
#12h delay simulation
ev(ID = 4801:5200, amt = 13*1, ii= 24, ss= 1, addl=0),
ev(ID = 4801:5200, amt = 13*0, ii= 12, ss= 0, addl=0),
ev(ID = 4801:5200, amt = 13*1, ii= 12, ss= 0, addl=0),
ev(ID = 4801:5200, amt = 13*1, ii= 24, ss= 0, addl=10),
#15h delay + 50% dose intake simulation
ev(ID = 4801:5200, amt = 13*1, ii= 24, ss= 1, addl=0),
ev(ID = 4801:5200, amt = 13*0, ii= 15, ss= 0, addl=0),
ev(ID = 4801:5200, amt = 13*0.5, ii= 9, ss= 0, addl=0),
ev(ID = 4801:5200, amt = 13*1, ii= 24, ss= 0, addl=10),
#15h delay simulation
ev(ID = 4801:5200, amt = 13*1, ii= 24, ss= 1, addl=0),
ev(ID = 4801:5200, amt = 13*0, ii= 15, ss= 0, addl=0),
ev(ID = 4801:5200, amt = 13*1, ii= 9, ss= 0, addl=0),
ev(ID = 4801:5200, amt = 13*1, ii= 24, ss= 0, addl=10),
#18h delay + 50% dose intake simulation
ev(ID = 4801:5200, amt = 13*1, ii= 24, ss= 1, addl=0),
ev(ID = 4801:5200, amt = 13*0, ii= 18, ss= 0, addl=0),
ev(ID = 4801:5200, amt = 13*0.5, ii= 6, ss= 0, addl=0),
ev(ID = 4801:5200, amt = 13*1, ii= 24, ss= 0, addl=10),
#18h delay simulation
ev(ID = 4801:5200, amt = 13*1, ii= 24, ss= 1, addl=0),
ev(ID = 4801:5200, amt = 13*0, ii= 18, ss= 0, addl=0),
ev(ID = 4801:5200, amt = 13*1, ii= 6, ss= 0, addl=0),
ev(ID = 4801:5200, amt = 13*1, ii= 24, ss= 0, addl=10),
#21h delay + 50% dose intake simulation
ev(ID = 4801:5200, amt = 13*1, ii= 24, ss= 1, addl=0),
ev(ID = 4801:5200, amt = 13*0, ii= 21, ss= 0, addl=0),
ev(ID = 4801:5200, amt = 13*0.5, ii= 3, ss= 0, addl=0),
ev(ID = 4801:5200, amt = 13*1, ii= 24, ss= 0, addl=10),
#21h delay simulation
ev(ID = 4801:5200, amt = 13*1, ii= 24, ss= 1, addl=0),
ev(ID = 4801:5200, amt = 13*0, ii= 21, ss= 0, addl=0),
ev(ID = 4801:5200, amt = 13*1, ii= 3, ss= 0, addl=0),
ev(ID = 4801:5200, amt = 13*1, ii= 24, ss= 0, addl=10),
#Missed dose simulation
ev(ID = 4801:5200, amt = 13*1, ii= 24, ss= 1, addl=0),
ev(ID = 4801:5200, amt = 13*0, ii= 24, ss= 0, addl=0),
ev(ID = 4801:5200, amt = 13*1, ii= 24, ss= 0, addl=0),
ev(ID = 4801:5200, amt = 13*1, ii= 24, ss= 0, addl=10),
#Missed dose + 150% dose intake simulation
ev(ID = 4801:5200, amt = 13*1, ii= 24, ss= 1, addl=0),
ev(ID = 4801:5200, amt = 13*0, ii= 24, ss= 0, addl=0),
ev(ID = 4801:5200, amt = 13*1.5, ii= 24, ss= 0, addl=0),
ev(ID = 4801:5200, amt = 13*1, ii= 24, ss= 0, addl=10),
#Missed dose + 200% dose intake simulation
ev(ID = 4801:5200, amt = 13*1, ii= 24, ss= 1, addl=0),
```

```
ev(ID = 4801:5200, amt = 13*0, ii= 24, ss= 0, addl=0),
ev(ID = 4801:5200, amt = 13*2, ii= 24, ss= 0, addl=0),
ev(ID = 4801:5200, amt = 13*1, ii= 24, ss= 0, addl=10))))%>%
              mutate(dose_group = "13 mg") %>%

    bind_rows(as_tibble( seq(
  #Steady-state simulation
ev(ID = 5201:5600, amt = 14*1, ii= 24, ss= 1, addl=0),
ev(ID = 5201:5600, amt = 14*1, ii= 24, ss= 0, addl=0),
ev(ID = 5201:5600, amt = 14*1, ii= 24, ss= 0, addl=10),
#3h delay simulation
ev(ID = 5201:5600, amt = 14*1, ii= 24, ss= 1, addl=0),
ev(ID = 5201:5600, amt = 14*0, ii= 3, ss= 0, addl=0),
ev(ID = 5201:5600, amt = 14*1, ii= 21, ss= 0, addl=0),
ev(ID = 5201:5600, amt = 14*1, ii= 24, ss= 0, addl=10),
#6h delay simulation
ev(ID = 5201:5600, amt = 14*1, ii= 24, ss= 1, addl=0),
ev(ID = 5201:5600, amt = 14*0, ii= 6, ss= 0, addl=0),
ev(ID = 5201:5600, amt = 14*1, ii= 18, ss= 0, addl=0),
ev(ID = 5201:5600, amt = 14*1, ii= 24, ss= 0, addl=10),
#9h delay simulation
ev(ID = 5201:5600, amt = 14*1, ii= 24, ss= 1, addl=0),
ev(ID = 5201:5600, amt = 14*0, ii= 9, ss= 0, addl=0),
ev(ID = 5201:5600, amt = 14*1, ii= 15, ss= 0, addl=0),
ev(ID = 5201:5600, amt = 14*1, ii= 24, ss= 0, addl=10),
#12h delay simulation
ev(ID = 5201:5600, amt = 14*1, ii= 24, ss= 1, addl=0),
ev(ID = 5201:5600, amt = 14*0, ii= 12, ss= 0, addl=0),
ev(ID = 5201:5600, amt = 14*1, ii= 12, ss= 0, addl=0),
ev(ID = 5201:5600, amt = 14*1, ii= 24, ss= 0, addl=10),
#15h delay + 50% dose intake simulation
ev(ID = 5201:5600, amt = 14*1, ii= 24, ss= 1, addl=0),
ev(ID = 5201:5600, amt = 14*0, ii= 15, ss= 0, addl=0),
ev(ID = 5201:5600, amt = 14*0.5, ii= 9, ss= 0, addl=0),
ev(ID = 5201:5600, amt = 14*1, ii= 24, ss= 0, addl=10),
#15h delay simulation
ev(ID = 5201:5600, amt = 14*1, ii= 24, ss= 1, addl=0),
ev(ID = 5201:5600, amt = 14*0, ii= 15, ss= 0, addl=0),
ev(ID = 5201:5600, amt = 14*1, ii= 9, ss= 0, addl=0),
ev(ID = 5201:5600, amt = 14*1, ii= 24, ss= 0, addl=10),
#18h delay + 50% dose intake simulation
ev(ID = 5201:5600, amt = 14*1, ii= 24, ss= 1, addl=0),
ev(ID = 5201:5600, amt = 14*0, ii= 18, ss= 0, addl=0),
ev(ID = 5201:5600, amt = 14*0.5, ii= 6, ss= 0, addl=0),
ev(ID = 5201:5600, amt = 14*1, ii= 24, ss= 0, addl=10),
#18h delay simulation
ev(ID = 5201:5600, amt = 14*1, ii= 24, ss= 1, addl=0),
ev(ID = 5201:5600, amt = 14*0, ii= 18, ss= 0, addl=0),
ev(ID = 5201:5600, amt = 14*1, ii= 6, ss= 0, addl=0),
ev(ID = 5201:5600, amt = 14*1, ii= 24, ss= 0, addl=10),
```

```
#21h delay + 50% dose intake simulation
ev(ID = 5201:5600, amt = 14*1, ii= 24, ss= 1, addl=0),
ev(ID = 5201:5600, amt = 14*0, ii= 21, ss= 0, addl=0),
ev(ID = 5201:5600, amt = 14*0.5, ii= 3, ss= 0, addl=0),
ev(ID = 5201:5600, amt = 14*1, ii= 24, ss= 0, addl=10),
#21h delay simulation
ev(ID = 5201:5600, amt = 14*1, ii= 24, ss= 1, addl=0),
ev(ID = 5201:5600, amt = 14*0, ii= 21, ss= 0, addl=0),
ev(ID = 5201:5600, amt = 14*1, ii= 3, ss= 0, addl=0),
ev(ID = 5201:5600, amt = 14*1, ii= 24, ss= 0, addl=10),
#Missed dose simulation
ev(ID = 5201:5600, amt = 14*1, ii= 24, ss= 1, addl=0),
ev(ID = 5201:5600, amt = 14*0, ii= 24, ss= 0, addl=0),
ev(ID = 5201:5600, amt = 14*1, ii= 24, ss= 0, addl=0),
ev(ID = 5201:5600, amt = 14*1, ii= 24, ss= 0, addl=10),
#Missed dose + 150% dose intake simulation
ev(ID = 5201:5600, amt = 14*1, ii= 24, ss= 1, addl=0),
ev(ID = 5201:5600, amt = 14*0, ii= 24, ss= 0, addl=0),
ev(ID = 5201:5600, amt = 14*1.5, ii= 24, ss= 0, addl=0),
ev(ID = 5201:5600, amt = 14*1, ii= 24, ss= 0, addl=10),
#Missed dose + 200% dose intake simulation
ev(ID = 5201:5600, amt = 14*1, ii= 24, ss= 1, addl=0),
ev(ID = 5201:5600, amt = 14*0, ii= 24, ss= 0, addl=0),
ev(ID = 5201:5600, amt = 14*2, ii= 24, ss= 0, addl=0),
ev(ID = 5201:5600, amt = 14*1, ii= 24, ss= 0, addl=10))))%>%
              mutate(dose_group = "14 mg") %>%

    bind_rows(as_tibble( seq(
  #Steady-state simulation
ev(ID = 5601:6000, amt = 15*1, ii= 24, ss= 1, addl=0),
ev(ID = 5601:6000, amt = 15*1, ii= 24, ss= 0, addl=0),
ev(ID = 5601:6000, amt = 15*1, ii= 24, ss= 0, addl=10),
#3h delay simulation
ev(ID = 5601:6000, amt = 15*1, ii= 24, ss= 1, addl=0),
ev(ID = 5601:6000, amt = 15*0, ii= 3, ss= 0, addl=0),
ev(ID = 5601:6000, amt = 15*1, ii= 21, ss= 0, addl=0),
ev(ID = 5601:6000, amt = 15*1, ii= 24, ss= 0, addl=10),
#6h delay simulation
ev(ID = 5601:6000, amt = 15*1, ii= 24, ss= 1, addl=0),
ev(ID = 5601:6000, amt = 15*0, ii= 6, ss= 0, addl=0),
ev(ID = 5601:6000, amt = 15*1, ii= 18, ss= 0, addl=0),
ev(ID = 5601:6000, amt = 15*1, ii= 24, ss= 0, addl=10),
#9h delay simulation
ev(ID = 5601:6000, amt = 15*1, ii= 24, ss= 1, addl=0),
ev(ID = 5601:6000, amt = 15*0, ii= 9, ss= 0, addl=0),
ev(ID = 5601:6000, amt = 15*1, ii= 15, ss= 0, addl=0),
ev(ID = 5601:6000, amt = 15*1, ii= 24, ss= 0, addl=10),
#12h delay simulation
ev(ID = 5601:6000, amt = 15*1, ii= 24, ss= 1, addl=0),
ev(ID = 5601:6000, amt = 15*0, ii= 12, ss= 0, addl=0),
```

```
ev(ID = 5601:6000, amt = 15*1, ii= 12, ss= 0, addl=0),
ev(ID = 5601:6000, amt = 15*1, ii= 24, ss= 0, addl=10),
#15h delay + 50% dose intake simulation
ev(ID = 5601:6000, amt = 15*1, ii= 24, ss= 1, addl=0),
ev(ID = 5601:6000, amt = 15*0, ii= 15, ss= 0, addl=0),
ev(ID = 5601:6000, amt = 15*0.5, ii= 9, ss= 0, addl=0),
ev(ID = 5601:6000, amt = 15*1, ii= 24, ss= 0, addl=10),
#15h delay simulation
ev(ID = 5601:6000, amt = 15*1, ii= 24, ss= 1, addl=0),
ev(ID = 5601:6000, amt = 15*0, ii= 15, ss= 0, addl=0),
ev(ID = 5601:6000, amt = 15*1, ii= 9, ss= 0, addl=0),
ev(ID = 5601:6000, amt = 15*1, ii= 24, ss= 0, addl=10),
#18h delay + 50% dose intake simulation
ev(ID = 5601:6000, amt = 15*1, ii= 24, ss= 1, addl=0),
ev(ID = 5601:6000, amt = 15*0, ii= 18, ss= 0, addl=0),
ev(ID = 5601:6000, amt = 15*0.5, ii= 6, ss= 0, addl=0),
ev(ID = 5601:6000, amt = 15*1, ii= 24, ss= 0, addl=10),
#18h delay simulation
ev(ID = 5601:6000, amt = 15*1, ii= 24, ss= 1, addl=0),
ev(ID = 5601:6000, amt = 15*0, ii= 18, ss= 0, addl=0),
ev(ID = 5601:6000, amt = 15*1, ii= 6, ss= 0, addl=0),
ev(ID = 5601:6000, amt = 15*1, ii= 24, ss= 0, addl=10),
#21h delay + 50% dose intake simulation
ev(ID = 5601:6000, amt = 15*1, ii= 24, ss= 1, addl=0),
ev(ID = 5601:6000, amt = 15*0, ii= 21, ss= 0, addl=0),
ev(ID = 5601:6000, amt = 15*0.5, ii= 3, ss= 0, addl=0),
ev(ID = 5601:6000, amt = 15*1, ii= 24, ss= 0, addl=10),
#21h delay simulation
ev(ID = 5601:6000, amt = 15*1, ii= 24, ss= 1, addl=0),
ev(ID = 5601:6000, amt = 15*0, ii= 21, ss= 0, addl=0),
ev(ID = 5601:6000, amt = 15*1, ii= 3, ss= 0, addl=0),
ev(ID = 5601:6000, amt = 15*1, ii= 24, ss= 0, addl=10),
#Missed dose simulation
ev(ID = 5601:6000, amt = 15*1, ii= 24, ss= 1, addl=0),
ev(ID = 5601:6000, amt = 15*0, ii= 24, ss= 0, addl=0),
ev(ID = 5601:6000, amt = 15*1, ii= 24, ss= 0, addl=0),
ev(ID = 5601:6000, amt = 15*1, ii= 24, ss= 0, addl=10),
#Missed dose + 150% dose intake simulation
ev(ID = 5601:6000, amt = 15*1, ii= 24, ss= 1, addl=0),
ev(ID = 5601:6000, amt = 15*0, ii= 24, ss= 0, addl=0),
ev(ID = 5601:6000, amt = 15*1.5, ii= 24, ss= 0, addl=0),
ev(ID = 5601:6000, amt = 15*1, ii= 24, ss= 0, addl=10),
#Missed dose + 200% dose intake simulation
ev(ID = 5601:6000, amt = 15*1, ii= 24, ss= 1, addl=0),
ev(ID = 5601:6000, amt = 15*0, ii= 24, ss= 0, addl=0),
ev(ID = 5601:6000, amt = 15*2, ii= 24, ss= 0, addl=0),
ev(ID = 5601:6000, amt = 15*1, ii= 24, ss= 0, addl=10)))) %>%
              mutate(dose_group = "15 mg")
```

```
Dataset_woillard_6000 <- Simulation_data_woillard_6000 %>%
  arrange(ID) %>%
  left_join(CYPCL_data_woillard_6000, by = "ID")


## test simulation
set.seed(123456)
Sim_Test_woillard_6000 <- my_model_tacro_Woillard %>%
 data_set(Dataset_woillard_6000) %>%
 Req(DV) %>%
 mrgsim(end = 7000, delta = 1)
```

Suite 12000

```
set.seed(123456)

generate_CYPCL_woillard_12000 <- function(n = 400) {
  frequencies <- c(36/41, 5/41)
  values <- c(0, 1)
  sample(values, n, replace = TRUE, prob = frequencies)
  }

CYPCL_data_woillard_12000 <- tibble(ID = 6001:12000) %>%
  group_by(group = (ID - 1) %/% 400) %>%
  mutate(CYP = generate_CYPCL_woillard_12000()) %>%
  ungroup() %>%
  select(-group)

Simulation_data_12000_W <- bind_rows(as_tibble( seq(
  #Steady-state simulation
ev(ID = 6001:6400, amt = 0.5*1, ii= 24, ss= 1, addl=0),
ev(ID = 6001:6400, amt = 0.5*1, ii= 24, ss= 0, addl=0),
ev(ID = 6001:6400, amt = 0.5*1, ii= 24, ss= 0, addl=10),
#3h delay simulation
ev(ID = 6001:6400, amt = 0.5*1, ii= 24, ss= 1, addl=0),
ev(ID = 6001:6400, amt = 0.5*0, ii= 3, ss= 0, addl=0),
ev(ID = 6001:6400, amt = 0.5*1, ii= 21, ss= 0, addl=0),
ev(ID = 6001:6400, amt = 0.5*1, ii= 24, ss= 0, addl=10),
#6h delay simulation
ev(ID = 6001:6400, amt = 0.5*1, ii= 24, ss= 1, addl=0),
ev(ID = 6001:6400, amt = 0.5*0, ii= 6, ss= 0, addl=0),
ev(ID = 6001:6400, amt = 0.5*1, ii= 18, ss= 0, addl=0),
ev(ID = 6001:6400, amt = 0.5*1, ii= 24, ss= 0, addl=10),
#9h delay simulation
ev(ID = 6001:6400, amt = 0.5*1, ii= 24, ss= 1, addl=0),
ev(ID = 6001:6400, amt = 0.5*0, ii= 9, ss= 0, addl=0),
ev(ID = 6001:6400, amt = 0.5*1, ii= 15, ss= 0, addl=0),
ev(ID = 6001:6400, amt = 0.5*1, ii= 24, ss= 0, addl=10),
#12h delay simulation
```

```
ev(ID = 6001:6400, amt = 0.5*1, ii= 24, ss= 1, addl=0),
ev(ID = 6001:6400, amt = 0.5*0, ii= 12, ss= 0, addl=0),
ev(ID = 6001:6400, amt = 0.5*1, ii= 12, ss= 0, addl=0),
ev(ID = 6001:6400, amt = 0.5*1, ii= 24, ss= 0, addl=10),
#15h delay + 50% dose intake simulation
ev(ID = 6001:6400, amt = 0.5*1, ii= 24, ss= 1, addl=0),
ev(ID = 6001:6400, amt = 0.5*0, ii= 15, ss= 0, addl=0),
ev(ID = 6001:6400, amt = 0.5*0.5, ii= 9, ss= 0, addl=0),
ev(ID = 6001:6400, amt = 0.5*1, ii= 24, ss= 0, addl=10),
#15h delay simulation
ev(ID = 6001:6400, amt = 0.5*1, ii= 24, ss= 1, addl=0),
ev(ID = 6001:6400, amt = 0.5*0, ii= 15, ss= 0, addl=0),
ev(ID = 6001:6400, amt = 0.5*1, ii= 9, ss= 0, addl=0),
ev(ID = 6001:6400, amt = 0.5*1, ii= 24, ss= 0, addl=10),
#18h delay + 50% dose intake simulation
ev(ID = 6001:6400, amt = 0.5*1, ii= 24, ss= 1, addl=0),
ev(ID = 6001:6400, amt = 0.5*0, ii= 18, ss= 0, addl=0),
ev(ID = 6001:6400, amt = 0.5*0.5, ii= 6, ss= 0, addl=0),
ev(ID = 6001:6400, amt = 0.5*1, ii= 24, ss= 0, addl=10),
#18h delay simulation
ev(ID = 6001:6400, amt = 0.5*1, ii= 24, ss= 1, addl=0),
ev(ID = 6001:6400, amt = 0.5*0, ii= 18, ss= 0, addl=0),
ev(ID = 6001:6400, amt = 0.5*1, ii= 6, ss= 0, addl=0),
ev(ID = 6001:6400, amt = 0.5*1, ii= 24, ss= 0, addl=10),
#21h delay + 50% dose intake simulation
ev(ID = 6001:6400, amt = 0.5*1, ii= 24, ss= 1, addl=0),
ev(ID = 6001:6400, amt = 0.5*0, ii= 21, ss= 0, addl=0),
ev(ID = 6001:6400, amt = 0.5*0.5, ii= 3, ss= 0, addl=0),
ev(ID = 6001:6400, amt = 0.5*1, ii= 24, ss= 0, addl=10),
#21h delay simulation
ev(ID = 6001:6400, amt = 0.5*1, ii= 24, ss= 1, addl=0),
ev(ID = 6001:6400, amt = 0.5*0, ii= 21, ss= 0, addl=0),
ev(ID = 6001:6400, amt = 0.5*1, ii= 3, ss= 0, addl=0),
ev(ID = 6001:6400, amt = 0.5*1, ii= 24, ss= 0, addl=10),
#Missed dose simulation
ev(ID = 6001:6400, amt = 0.5*1, ii= 24, ss= 1, addl=0),
ev(ID = 6001:6400, amt = 0.5*0, ii= 24, ss= 0, addl=0),
ev(ID = 6001:6400, amt = 0.5*1, ii= 24, ss= 0, addl=0),
ev(ID = 6001:6400, amt = 0.5*1, ii= 24, ss= 0, addl=10),
#Missed dose + 150% dose intake simulation
ev(ID = 6001:6400, amt = 0.5*1, ii= 24, ss= 1, addl=0),
ev(ID = 6001:6400, amt = 0.5*0, ii= 24, ss= 0, addl=0),
ev(ID = 6001:6400, amt = 0.5*1.5, ii= 24, ss= 0, addl=0),
ev(ID = 6001:6400, amt = 0.5*1, ii= 24, ss= 0, addl=10),
#Missed dose + 200% dose intake simulation
ev(ID = 6001:6400, amt = 0.5*1, ii= 24, ss= 1, addl=0),
ev(ID = 6001:6400, amt = 0.5*0, ii= 24, ss= 0, addl=0),
ev(ID = 6001:6400, amt = 0.5*2, ii= 24, ss= 0, addl=0),
ev(ID = 6001:6400, amt = 0.5*1, ii= 24, ss= 0, addl=10)))) %>%
              mutate(dose_group = "0,5 mg") %>%
```

```
    bind_rows(as_tibble( seq(
  #Steady-state simulation
ev(ID = 6401:6800, amt = 1.5*1, ii= 24, ss= 1, addl=0),
ev(ID = 6401:6800, amt = 1.5*1, ii= 24, ss= 0, addl=0),
ev(ID = 6401:6800, amt = 1.5*1, ii= 24, ss= 0, addl=10),
#3h delay simulation
ev(ID = 6401:6800, amt = 1.5*1, ii= 24, ss= 1, addl=0),
ev(ID = 6401:6800, amt = 1.5*0, ii= 3, ss= 0, addl=0),
ev(ID = 6401:6800, amt = 1.5*1, ii= 21, ss= 0, addl=0),
ev(ID = 6401:6800, amt = 1.5*1, ii= 24, ss= 0, addl=10),
#6h delay simulation
ev(ID = 6401:6800, amt = 1.5*1, ii= 24, ss= 1, addl=0),
ev(ID = 6401:6800, amt = 1.5*0, ii= 6, ss= 0, addl=0),
ev(ID = 6401:6800, amt = 1.5*1, ii= 18, ss= 0, addl=0),
ev(ID = 6401:6800, amt = 1.5*1, ii= 24, ss= 0, addl=10),
#9h delay simulation
ev(ID = 6401:6800, amt = 1.5*1, ii= 24, ss= 1, addl=0),
ev(ID = 6401:6800, amt = 1.5*0, ii= 9, ss= 0, addl=0),
ev(ID = 6401:6800, amt = 1.5*1, ii= 15, ss= 0, addl=0),
ev(ID = 6401:6800, amt = 1.5*1, ii= 24, ss= 0, addl=10),
#12h delay simulation
ev(ID = 6401:6800, amt = 1.5*1, ii= 24, ss= 1, addl=0),
ev(ID = 6401:6800, amt = 1.5*0, ii= 12, ss= 0, addl=0),
ev(ID = 6401:6800, amt = 1.5*1, ii= 12, ss= 0, addl=0),
ev(ID = 6401:6800, amt = 1.5*1, ii= 24, ss= 0, addl=10),
#15h delay + 50% dose intake simulation
ev(ID = 6401:6800, amt = 1.5*1, ii= 24, ss= 1, addl=0),
ev(ID = 6401:6800, amt = 1.5*0, ii= 15, ss= 0, addl=0),
ev(ID = 6401:6800, amt = 1.5*0.5, ii= 9, ss= 0, addl=0),
ev(ID = 6401:6800, amt = 1.5*1, ii= 24, ss= 0, addl=10),
#15h delay simulation
ev(ID = 6401:6800, amt = 1.5*1, ii= 24, ss= 1, addl=0),
ev(ID = 6401:6800, amt = 1.5*0, ii= 15, ss= 0, addl=0),
ev(ID = 6401:6800, amt = 1.5*1, ii= 9, ss= 0, addl=0),
ev(ID = 6401:6800, amt = 1.5*1, ii= 24, ss= 0, addl=10),
#18h delay + 50% dose intake simulation
ev(ID = 6401:6800, amt = 1.5*1, ii= 24, ss= 1, addl=0),
ev(ID = 6401:6800, amt = 1.5*0, ii= 18, ss= 0, addl=0),
ev(ID = 6401:6800, amt = 1.5*0.5, ii= 6, ss= 0, addl=0),
ev(ID = 6401:6800, amt = 1.5*1, ii= 24, ss= 0, addl=10),
#18h delay simulation
ev(ID = 6401:6800, amt = 1.5*1, ii= 24, ss= 1, addl=0),
ev(ID = 6401:6800, amt = 1.5*0, ii= 18, ss= 0, addl=0),
ev(ID = 6401:6800, amt = 1.5*1, ii= 6, ss= 0, addl=0),
ev(ID = 6401:6800, amt = 1.5*1, ii= 24, ss= 0, addl=10),
#21h delay + 50% dose intake simulation
ev(ID = 6401:6800, amt = 1.5*1, ii= 24, ss= 1, addl=0),
ev(ID = 6401:6800, amt = 1.5*0, ii= 21, ss= 0, addl=0),
ev(ID = 6401:6800, amt = 1.5*0.5, ii= 3, ss= 0, addl=0),
```

```
ev(ID = 6401:6800, amt = 1.5*1, ii= 24, ss= 0, addl=10),
#21h delay simulation
ev(ID = 6401:6800, amt = 1.5*1, ii= 24, ss= 1, addl=0),
ev(ID = 6401:6800, amt = 1.5*0, ii= 21, ss= 0, addl=0),
ev(ID = 6401:6800, amt = 1.5*1, ii= 3, ss= 0, addl=0),
ev(ID = 6401:6800, amt = 1.5*1, ii= 24, ss= 0, addl=10),
#Missed dose simulation
ev(ID = 6401:6800, amt = 1.5*1, ii= 24, ss= 1, addl=0),
ev(ID = 6401:6800, amt = 1.5*0, ii= 24, ss= 0, addl=0),
ev(ID = 6401:6800, amt = 1.5*1, ii= 24, ss= 0, addl=0),
ev(ID = 6401:6800, amt = 1.5*1, ii= 24, ss= 0, addl=10),
#Missed dose + 150% dose intake simulation
ev(ID = 6401:6800, amt = 1.5*1, ii= 24, ss= 1, addl=0),
ev(ID = 6401:6800, amt = 1.5*0, ii= 24, ss= 0, addl=0),
ev(ID = 6401:6800, amt = 1.5*1.5, ii= 24, ss= 0, addl=0),
ev(ID = 6401:6800, amt = 1.5*1, ii= 24, ss= 0, addl=10),
#Missed dose + 200% dose intake simulation
ev(ID = 6401:6800, amt = 1.5*1, ii= 24, ss= 1, addl=0),
ev(ID = 6401:6800, amt = 1.5*0, ii= 24, ss= 0, addl=0),
ev(ID = 6401:6800, amt = 1.5*2, ii= 24, ss= 0, addl=0),
ev(ID = 6401:6800, amt = 1.5*1, ii= 24, ss= 0, addl=10)))) %>%
              mutate(dose_group = "1,5 mg") %>%

    bind_rows(as_tibble( seq(
  #Steady-state simulation
ev(ID = 6801:7200, amt = 2.5*1, ii= 24, ss= 1, addl=0),
ev(ID = 6801:7200, amt = 2.5*1, ii= 24, ss= 0, addl=0),
ev(ID = 6801:7200, amt = 2.5*1, ii= 24, ss= 0, addl=10),
#3h delay simulation
ev(ID = 6801:7200, amt = 2.5*1, ii= 24, ss= 1, addl=0),
ev(ID = 6801:7200, amt = 2.5*0, ii= 3, ss= 0, addl=0),
ev(ID = 6801:7200, amt = 2.5*1, ii= 21, ss= 0, addl=0),
ev(ID = 6801:7200, amt = 2.5*1, ii= 24, ss= 0, addl=10),
#6h delay simulation
ev(ID = 6801:7200, amt = 2.5*1, ii= 24, ss= 1, addl=0),
ev(ID = 6801:7200, amt = 2.5*0, ii= 6, ss= 0, addl=0),
ev(ID = 6801:7200, amt = 2.5*1, ii= 18, ss= 0, addl=0),
ev(ID = 6801:7200, amt = 2.5*1, ii= 24, ss= 0, addl=10),
#9h delay simulation
ev(ID = 6801:7200, amt = 2.5*1, ii= 24, ss= 1, addl=0),
ev(ID = 6801:7200, amt = 2.5*0, ii= 9, ss= 0, addl=0),
ev(ID = 6801:7200, amt = 2.5*1, ii= 15, ss= 0, addl=0),
ev(ID = 6801:7200, amt = 2.5*1, ii= 24, ss= 0, addl=10),
#12h delay simulation
ev(ID = 6801:7200, amt = 2.5*1, ii= 24, ss= 1, addl=0),
ev(ID = 6801:7200, amt = 2.5*0, ii= 12, ss= 0, addl=0),
ev(ID = 6801:7200, amt = 2.5*1, ii= 12, ss= 0, addl=0),
ev(ID = 6801:7200, amt = 2.5*1, ii= 24, ss= 0, addl=10),
#15h delay + 50% dose intake simulation
ev(ID = 6801:7200, amt = 2.5*1, ii= 24, ss= 1, addl=0),
```

```
ev(ID = 6801:7200, amt = 2.5*0, ii= 15, ss= 0, addl=0),
ev(ID = 6801:7200, amt = 2.5*0.5, ii= 9, ss= 0, addl=0),
ev(ID = 6801:7200, amt = 2.5*1, ii= 24, ss= 0, addl=10),
#15h delay simulation
ev(ID = 6801:7200, amt = 2.5*1, ii= 24, ss= 1, addl=0),
ev(ID = 6801:7200, amt = 2.5*0, ii= 15, ss= 0, addl=0),
ev(ID = 6801:7200, amt = 2.5*1, ii= 9, ss= 0, addl=0),
ev(ID = 6801:7200, amt = 2.5*1, ii= 24, ss= 0, addl=10),
#18h delay + 50% dose intake simulation
ev(ID = 6801:7200, amt = 2.5*1, ii= 24, ss= 1, addl=0),
ev(ID = 6801:7200, amt = 2.5*0, ii= 18, ss= 0, addl=0),
ev(ID = 6801:7200, amt = 2.5*0.5, ii= 6, ss= 0, addl=0),
ev(ID = 6801:7200, amt = 2.5*1, ii= 24, ss= 0, addl=10),
#18h delay simulation
ev(ID = 6801:7200, amt = 2.5*1, ii= 24, ss= 1, addl=0),
ev(ID = 6801:7200, amt = 2.5*0, ii= 18, ss= 0, addl=0),
ev(ID = 6801:7200, amt = 2.5*1, ii= 6, ss= 0, addl=0),
ev(ID = 6801:7200, amt = 2.5*1, ii= 24, ss= 0, addl=10),
#21h delay + 50% dose intake simulation
ev(ID = 6801:7200, amt = 2.5*1, ii= 24, ss= 1, addl=0),
ev(ID = 6801:7200, amt = 2.5*0, ii= 21, ss= 0, addl=0),
ev(ID = 6801:7200, amt = 2.5*0.5, ii= 3, ss= 0, addl=0),
ev(ID = 6801:7200, amt = 2.5*1, ii= 24, ss= 0, addl=10),
#21h delay simulation
ev(ID = 6801:7200, amt = 2.5*1, ii= 24, ss= 1, addl=0),
ev(ID = 6801:7200, amt = 2.5*0, ii= 21, ss= 0, addl=0),
ev(ID = 6801:7200, amt = 2.5*1, ii= 3, ss= 0, addl=0),
ev(ID = 6801:7200, amt = 2.5*1, ii= 24, ss= 0, addl=10),
#Missed dose simulation
ev(ID = 6801:7200, amt = 2.5*1, ii= 24, ss= 1, addl=0),
ev(ID = 6801:7200, amt = 2.5*0, ii= 24, ss= 0, addl=0),
ev(ID = 6801:7200, amt = 2.5*1, ii= 24, ss= 0, addl=0),
ev(ID = 6801:7200, amt = 2.5*1, ii= 24, ss= 0, addl=10),
#Missed dose + 150% dose intake simulation
ev(ID = 6801:7200, amt = 2.5*1, ii= 24, ss= 1, addl=0),
ev(ID = 6801:7200, amt = 2.5*0, ii= 24, ss= 0, addl=0),
ev(ID = 6801:7200, amt = 2.5*1.5, ii= 24, ss= 0, addl=0),
ev(ID = 6801:7200, amt = 2.5*1, ii= 24, ss= 0, addl=10),
#Missed dose + 200% dose intake simulation
ev(ID = 6801:7200, amt = 2.5*1, ii= 24, ss= 1, addl=0),
ev(ID = 6801:7200, amt = 2.5*0, ii= 24, ss= 0, addl=0),
ev(ID = 6801:7200, amt = 2.5*2, ii= 24, ss= 0, addl=0),
ev(ID = 6801:7200, amt = 2.5*1, ii= 24, ss= 0, addl=10)))) %>%
              mutate(dose_group = "2,5 mg") %>%

    bind_rows(as_tibble( seq(
  #Steady-state simulation
ev(ID = 7201:7600, amt = 3.5*1, ii= 24, ss= 1, addl=0),
ev(ID = 7201:7600, amt = 3.5*1, ii= 24, ss= 0, addl=0),
ev(ID = 7201:7600, amt = 3.5*1, ii= 24, ss= 0, addl=10),
```

```
#3h delay simulation
ev(ID = 7201:7600, amt = 3.5*1, ii= 24, ss= 1, addl=0),
ev(ID = 7201:7600, amt = 3.5*0, ii= 3, ss= 0, addl=0),
ev(ID = 7201:7600, amt = 3.5*1, ii= 21, ss= 0, addl=0),
ev(ID = 7201:7600, amt = 3.5*1, ii= 24, ss= 0, addl=10),
#6h delay simulation
ev(ID = 7201:7600, amt = 3.5*1, ii= 24, ss= 1, addl=0),
ev(ID = 7201:7600, amt = 3.5*0, ii= 6, ss= 0, addl=0),
ev(ID = 7201:7600, amt = 3.5*1, ii= 18, ss= 0, addl=0),
ev(ID = 7201:7600, amt = 3.5*1, ii= 24, ss= 0, addl=10),
#9h delay simulation
ev(ID = 7201:7600, amt = 3.5*1, ii= 24, ss= 1, addl=0),
ev(ID = 7201:7600, amt = 3.5*0, ii= 9, ss= 0, addl=0),
ev(ID = 7201:7600, amt = 3.5*1, ii= 15, ss= 0, addl=0),
ev(ID = 7201:7600, amt = 3.5*1, ii= 24, ss= 0, addl=10),
#12h delay simulation
ev(ID = 7201:7600, amt = 3.5*1, ii= 24, ss= 1, addl=0),
ev(ID = 7201:7600, amt = 3.5*0, ii= 12, ss= 0, addl=0),
ev(ID = 7201:7600, amt = 3.5*1, ii= 12, ss= 0, addl=0),
ev(ID = 7201:7600, amt = 3.5*1, ii= 24, ss= 0, addl=10),
#15h delay + 50% dose intake simulation
ev(ID = 7201:7600, amt = 3.5*1, ii= 24, ss= 1, addl=0),
ev(ID = 7201:7600, amt = 3.5*0, ii= 15, ss= 0, addl=0),
ev(ID = 7201:7600, amt = 3.5*0.5, ii= 9, ss= 0, addl=0),
ev(ID = 7201:7600, amt = 3.5*1, ii= 24, ss= 0, addl=10),
#15h delay simulation
ev(ID = 7201:7600, amt = 3.5*1, ii= 24, ss= 1, addl=0),
ev(ID = 7201:7600, amt = 3.5*0, ii= 15, ss= 0, addl=0),
ev(ID = 7201:7600, amt = 3.5*1, ii= 9, ss= 0, addl=0),
ev(ID = 7201:7600, amt = 3.5*1, ii= 24, ss= 0, addl=10),
#18h delay + 50% dose intake simulation
ev(ID = 7201:7600, amt = 3.5*1, ii= 24, ss= 1, addl=0),
ev(ID = 7201:7600, amt = 3.5*0, ii= 18, ss= 0, addl=0),
ev(ID = 7201:7600, amt = 3.5*0.5, ii= 6, ss= 0, addl=0),
ev(ID = 7201:7600, amt = 3.5*1, ii= 24, ss= 0, addl=10),
#18h delay simulation
ev(ID = 7201:7600, amt = 3.5*1, ii= 24, ss= 1, addl=0),
ev(ID = 7201:7600, amt = 3.5*0, ii= 18, ss= 0, addl=0),
ev(ID = 7201:7600, amt = 3.5*1, ii= 6, ss= 0, addl=0),
ev(ID = 7201:7600, amt = 3.5*1, ii= 24, ss= 0, addl=10),
#21h delay + 50% dose intake simulation
ev(ID = 7201:7600, amt = 3.5*1, ii= 24, ss= 1, addl=0),
ev(ID = 7201:7600, amt = 3.5*0, ii= 21, ss= 0, addl=0),
ev(ID = 7201:7600, amt = 3.5*0.5, ii= 3, ss= 0, addl=0),
ev(ID = 7201:7600, amt = 3.5*1, ii= 24, ss= 0, addl=10),
#21h delay simulation
ev(ID = 7201:7600, amt = 3.5*1, ii= 24, ss= 1, addl=0),
ev(ID = 7201:7600, amt = 3.5*0, ii= 21, ss= 0, addl=0),
ev(ID = 7201:7600, amt = 3.5*1, ii= 3, ss= 0, addl=0),
ev(ID = 7201:7600, amt = 3.5*1, ii= 24, ss= 0, addl=10),
```

```
#Missed dose simulation
ev(ID = 7201:7600, amt = 3.5*1, ii= 24, ss= 1, addl=0),
ev(ID = 7201:7600, amt = 3.5*0, ii= 24, ss= 0, addl=0),
ev(ID = 7201:7600, amt = 3.5*1, ii= 24, ss= 0, addl=0),
ev(ID = 7201:7600, amt = 3.5*1, ii= 24, ss= 0, addl=10),
#Missed dose + 150% dose intake simulation
ev(ID = 7201:7600, amt = 3.5*1, ii= 24, ss= 1, addl=0),
ev(ID = 7201:7600, amt = 3.5*0, ii= 24, ss= 0, addl=0),
ev(ID = 7201:7600, amt = 3.5*1.5, ii= 24, ss= 0, addl=0),
ev(ID = 7201:7600, amt = 3.5*1, ii= 24, ss= 0, addl=10),
#Missed dose + 200% dose intake simulation
ev(ID = 7201:7600, amt = 3.5*1, ii= 24, ss= 1, addl=0),
ev(ID = 7201:7600, amt = 3.5*0, ii= 24, ss= 0, addl=0),
ev(ID = 7201:7600, amt = 3.5*2, ii= 24, ss= 0, addl=0),
ev(ID = 7201:7600, amt = 3.5*1, ii= 24, ss= 0, addl=10)))) %>%
              mutate(dose_group = "3,5 mg") %>%

    bind_rows(as_tibble( seq(
  #Steady-state simulation
ev(ID = 7601:8000, amt = 4.5*1, ii= 24, ss= 1, addl=0),
ev(ID = 7601:8000, amt = 4.5*1, ii= 24, ss= 0, addl=0),
ev(ID = 7601:8000, amt = 4.5*1, ii= 24, ss= 0, addl=10),
#3h delay simulation
ev(ID = 7601:8000, amt = 4.5*1, ii= 24, ss= 1, addl=0),
ev(ID = 7601:8000, amt = 4.5*0, ii= 3, ss= 0, addl=0),
ev(ID = 7601:8000, amt = 4.5*1, ii= 21, ss= 0, addl=0),
ev(ID = 7601:8000, amt = 4.5*1, ii= 24, ss= 0, addl=10),
#6h delay simulation
ev(ID = 7601:8000, amt = 4.5*1, ii= 24, ss= 1, addl=0),
ev(ID = 7601:8000, amt = 4.5*0, ii= 6, ss= 0, addl=0),
ev(ID = 7601:8000, amt = 4.5*1, ii= 18, ss= 0, addl=0),
ev(ID = 7601:8000, amt = 4.5*1, ii= 24, ss= 0, addl=10),
#9h delay simulation
ev(ID = 7601:8000, amt = 4.5*1, ii= 24, ss= 1, addl=0),
ev(ID = 7601:8000, amt = 4.5*0, ii= 9, ss= 0, addl=0),
ev(ID = 7601:8000, amt = 4.5*1, ii= 15, ss= 0, addl=0),
ev(ID = 7601:8000, amt = 4.5*1, ii= 24, ss= 0, addl=10),
#12h delay simulation
ev(ID = 7601:8000, amt = 4.5*1, ii= 24, ss= 1, addl=0),
ev(ID = 7601:8000, amt = 4.5*0, ii= 12, ss= 0, addl=0),
ev(ID = 7601:8000, amt = 4.5*1, ii= 12, ss= 0, addl=0),
ev(ID = 7601:8000, amt = 4.5*1, ii= 24, ss= 0, addl=10),
#15h delay + 50% dose intake simulation
ev(ID = 7601:8000, amt = 4.5*1, ii= 24, ss= 1, addl=0),
ev(ID = 7601:8000, amt = 4.5*0, ii= 15, ss= 0, addl=0),
ev(ID = 7601:8000, amt = 4.5*0.5, ii= 9, ss= 0, addl=0),
ev(ID = 7601:8000, amt = 4.5*1, ii= 24, ss= 0, addl=10),
#15h delay simulation
ev(ID = 7601:8000, amt = 4.5*1, ii= 24, ss= 1, addl=0),
ev(ID = 7601:8000, amt = 4.5*0, ii= 15, ss= 0, addl=0),
```

```
ev(ID = 7601:8000, amt = 4.5*1, ii= 9, ss= 0, addl=0),
ev(ID = 7601:8000, amt = 4.5*1, ii= 24, ss= 0, addl=10),
#18h delay + 50% dose intake simulation
ev(ID = 7601:8000, amt = 4.5*1, ii= 24, ss= 1, addl=0),
ev(ID = 7601:8000, amt = 4.5*0, ii= 18, ss= 0, addl=0),
ev(ID = 7601:8000, amt = 4.5*0.5, ii= 6, ss= 0, addl=0),
ev(ID = 7601:8000, amt = 4.5*1, ii= 24, ss= 0, addl=10),
#18h delay simulation
ev(ID = 7601:8000, amt = 4.5*1, ii= 24, ss= 1, addl=0),
ev(ID = 7601:8000, amt = 4.5*0, ii= 18, ss= 0, addl=0),
ev(ID = 7601:8000, amt = 4.5*1, ii= 6, ss= 0, addl=0),
ev(ID = 7601:8000, amt = 4.5*1, ii= 24, ss= 0, addl=10),
#21h delay + 50% dose intake simulation
ev(ID = 7601:8000, amt = 4.5*1, ii= 24, ss= 1, addl=0),
ev(ID = 7601:8000, amt = 4.5*0, ii= 21, ss= 0, addl=0),
ev(ID = 7601:8000, amt = 4.5*0.5, ii= 3, ss= 0, addl=0),
ev(ID = 7601:8000, amt = 4.5*1, ii= 24, ss= 0, addl=10),
#21h delay simulation
ev(ID = 7601:8000, amt = 4.5*1, ii= 24, ss= 1, addl=0),
ev(ID = 7601:8000, amt = 4.5*0, ii= 21, ss= 0, addl=0),
ev(ID = 7601:8000, amt = 4.5*1, ii= 3, ss= 0, addl=0),
ev(ID = 7601:8000, amt = 4.5*1, ii= 24, ss= 0, addl=10),
#Missed dose simulation
ev(ID = 7601:8000, amt = 4.5*1, ii= 24, ss= 1, addl=0),
ev(ID = 7601:8000, amt = 4.5*0, ii= 24, ss= 0, addl=0),
ev(ID = 7601:8000, amt = 4.5*1, ii= 24, ss= 0, addl=0),
ev(ID = 7601:8000, amt = 4.5*1, ii= 24, ss= 0, addl=10),
#Missed dose + 150% dose intake simulation
ev(ID = 7601:8000, amt = 4.5*1, ii= 24, ss= 1, addl=0),
ev(ID = 7601:8000, amt = 4.5*0, ii= 24, ss= 0, addl=0),
ev(ID = 7601:8000, amt = 4.5*1.5, ii= 24, ss= 0, addl=0),
ev(ID = 7601:8000, amt = 4.5*1, ii= 24, ss= 0, addl=10),
#Missed dose + 200% dose intake simulation
ev(ID = 7601:8000, amt = 4.5*1, ii= 24, ss= 1, addl=0),
ev(ID = 7601:8000, amt = 4.5*0, ii= 24, ss= 0, addl=0),
ev(ID = 7601:8000, amt = 4.5*2, ii= 24, ss= 0, addl=0),
ev(ID = 7601:8000, amt = 4.5*1, ii= 24, ss= 0, addl=10)))) %>%
              mutate(dose_group = "4,5 mg") %>%

    bind_rows(as_tibble( seq(
  #Steady-state simulation
ev(ID = 8001:8400, amt = 5.5*1, ii= 24, ss= 1, addl=0),
ev(ID = 8001:8400, amt = 5.5*1, ii= 24, ss= 0, addl=0),
ev(ID = 8001:8400, amt = 5.5*1, ii= 24, ss= 0, addl=10),
#3h delay simulation
ev(ID = 8001:8400, amt = 5.5*1, ii= 24, ss= 1, addl=0),
ev(ID = 8001:8400, amt = 5.5*0, ii= 3, ss= 0, addl=0),
ev(ID = 8001:8400, amt = 5.5*1, ii= 21, ss= 0, addl=0),
ev(ID = 8001:8400, amt = 5.5*1, ii= 24, ss= 0, addl=10),
#6h delay simulation
```

```
ev(ID = 8001:8400, amt = 5.5*1, ii= 24, ss= 1, addl=0),
ev(ID = 8001:8400, amt = 5.5*0, ii= 6, ss= 0, addl=0),
ev(ID = 8001:8400, amt = 5.5*1, ii= 18, ss= 0, addl=0),
ev(ID = 8001:8400, amt = 5.5*1, ii= 24, ss= 0, addl=10),
#9h delay simulation
ev(ID = 8001:8400, amt = 5.5*1, ii= 24, ss= 1, addl=0),
ev(ID = 8001:8400, amt = 5.5*0, ii= 9, ss= 0, addl=0),
ev(ID = 8001:8400, amt = 5.5*1, ii= 15, ss= 0, addl=0),
ev(ID = 8001:8400, amt = 5.5*1, ii= 24, ss= 0, addl=10),
#12h delay simulation
ev(ID = 8001:8400, amt = 5.5*1, ii= 24, ss= 1, addl=0),
ev(ID = 8001:8400, amt = 5.5*0, ii= 12, ss= 0, addl=0),
ev(ID = 8001:8400, amt = 5.5*1, ii= 12, ss= 0, addl=0),
ev(ID = 8001:8400, amt = 5.5*1, ii= 24, ss= 0, addl=10),
#15h delay + 50% dose intake simulation
ev(ID = 8001:8400, amt = 5.5*1, ii= 24, ss= 1, addl=0),
ev(ID = 8001:8400, amt = 5.5*0, ii= 15, ss= 0, addl=0),
ev(ID = 8001:8400, amt = 5.5*0.5, ii= 9, ss= 0, addl=0),
ev(ID = 8001:8400, amt = 5.5*1, ii= 24, ss= 0, addl=10),
#15h delay simulation
ev(ID = 8001:8400, amt = 5.5*1, ii= 24, ss= 1, addl=0),
ev(ID = 8001:8400, amt = 5.5*0, ii= 15, ss= 0, addl=0),
ev(ID = 8001:8400, amt = 5.5*1, ii= 9, ss= 0, addl=0),
ev(ID = 8001:8400, amt = 5.5*1, ii= 24, ss= 0, addl=10),
#18h delay + 50% dose intake simulation
ev(ID = 8001:8400, amt = 5.5*1, ii= 24, ss= 1, addl=0),
ev(ID = 8001:8400, amt = 5.5*0, ii= 18, ss= 0, addl=0),
ev(ID = 8001:8400, amt = 5.5*0.5, ii= 6, ss= 0, addl=0),
ev(ID = 8001:8400, amt = 5.5*1, ii= 24, ss= 0, addl=10),
#18h delay simulation
ev(ID = 8001:8400, amt = 5.5*1, ii= 24, ss= 1, addl=0),
ev(ID = 8001:8400, amt = 5.5*0, ii= 18, ss= 0, addl=0),
ev(ID = 8001:8400, amt = 5.5*1, ii= 6, ss= 0, addl=0),
ev(ID = 8001:8400, amt = 5.5*1, ii= 24, ss= 0, addl=10),
#21h delay + 50% dose intake simulation
ev(ID = 8001:8400, amt = 5.5*1, ii= 24, ss= 1, addl=0),
ev(ID = 8001:8400, amt = 5.5*0, ii= 21, ss= 0, addl=0),
ev(ID = 8001:8400, amt = 5.5*0.5, ii= 3, ss= 0, addl=0),
ev(ID = 8001:8400, amt = 5.5*1, ii= 24, ss= 0, addl=10),
#21h delay simulation
ev(ID = 8001:8400, amt = 5.5*1, ii= 24, ss= 1, addl=0),
ev(ID = 8001:8400, amt = 5.5*0, ii= 21, ss= 0, addl=0),
ev(ID = 8001:8400, amt = 5.5*1, ii= 3, ss= 0, addl=0),
ev(ID = 8001:8400, amt = 5.5*1, ii= 24, ss= 0, addl=10),
#Missed dose simulation
ev(ID = 8001:8400, amt = 5.5*1, ii= 24, ss= 1, addl=0),
ev(ID = 8001:8400, amt = 5.5*0, ii= 24, ss= 0, addl=0),
ev(ID = 8001:8400, amt = 5.5*1, ii= 24, ss= 0, addl=0),
ev(ID = 8001:8400, amt = 5.5*1, ii= 24, ss= 0, addl=10),
#Missed dose + 150% dose intake simulation
```

```
ev(ID = 8001:8400, amt = 5.5*1, ii= 24, ss= 1, addl=0),
ev(ID = 8001:8400, amt = 5.5*0, ii= 24, ss= 0, addl=0),
ev(ID = 8001:8400, amt = 5.5*1.5, ii= 24, ss= 0, addl=0),
ev(ID = 8001:8400, amt = 5.5*1, ii= 24, ss= 0, addl=10),
#Missed dose + 200% dose intake simulation
ev(ID = 8001:8400, amt = 5.5*1, ii= 24, ss= 1, addl=0),
ev(ID = 8001:8400, amt = 5.5*0, ii= 24, ss= 0, addl=0),
ev(ID = 8001:8400, amt = 5.5*2, ii= 24, ss= 0, addl=0),
ev(ID = 8001:8400, amt = 5.5*1, ii= 24, ss= 0, addl=10)))) %>%
              mutate(dose_group = "5,5 mg") %>%

    bind_rows(as_tibble( seq(
  #Steady-state simulation
ev(ID = 8401:8800, amt = 6.5*1, ii= 24, ss= 1, addl=0),
ev(ID = 8401:8800, amt = 6.5*1, ii= 24, ss= 0, addl=0),
ev(ID = 8401:8800, amt = 6.5*1, ii= 24, ss= 0, addl=10),
#3h delay simulation
ev(ID = 8401:8800, amt = 6.5*1, ii= 24, ss= 1, addl=0),
ev(ID = 8401:8800, amt = 6.5*0, ii= 3, ss= 0, addl=0),
ev(ID = 8401:8800, amt = 6.5*1, ii= 21, ss= 0, addl=0),
ev(ID = 8401:8800, amt = 6.5*1, ii= 24, ss= 0, addl=10),
#6h delay simulation
ev(ID = 8401:8800, amt = 6.5*1, ii= 24, ss= 1, addl=0),
ev(ID = 8401:8800, amt = 6.5*0, ii= 6, ss= 0, addl=0),
ev(ID = 8401:8800, amt = 6.5*1, ii= 18, ss= 0, addl=0),
ev(ID = 8401:8800, amt = 6.5*1, ii= 24, ss= 0, addl=10),
#9h delay simulation
ev(ID = 8401:8800, amt = 6.5*1, ii= 24, ss= 1, addl=0),
ev(ID = 8401:8800, amt = 6.5*0, ii= 9, ss= 0, addl=0),
ev(ID = 8401:8800, amt = 6.5*1, ii= 15, ss= 0, addl=0),
ev(ID = 8401:8800, amt = 6.5*1, ii= 24, ss= 0, addl=10),
#12h delay simulation
ev(ID = 8401:8800, amt = 6.5*1, ii= 24, ss= 1, addl=0),
ev(ID = 8401:8800, amt = 6.5*0, ii= 12, ss= 0, addl=0),
ev(ID = 8401:8800, amt = 6.5*1, ii= 12, ss= 0, addl=0),
ev(ID = 8401:8800, amt = 6.5*1, ii= 24, ss= 0, addl=10),
#15h delay + 50% dose intake simulation
ev(ID = 8401:8800, amt = 6.5*1, ii= 24, ss= 1, addl=0),
ev(ID = 8401:8800, amt = 6.5*0, ii= 15, ss= 0, addl=0),
ev(ID = 8401:8800, amt = 6.5*0.5, ii= 9, ss= 0, addl=0),
ev(ID = 8401:8800, amt = 6.5*1, ii= 24, ss= 0, addl=10),
#15h delay simulation
ev(ID = 8401:8800, amt = 6.5*1, ii= 24, ss= 1, addl=0),
ev(ID = 8401:8800, amt = 6.5*0, ii= 15, ss= 0, addl=0),
ev(ID = 8401:8800, amt = 6.5*1, ii= 9, ss= 0, addl=0),
ev(ID = 8401:8800, amt = 6.5*1, ii= 24, ss= 0, addl=10),
#18h delay + 50% dose intake simulation
ev(ID = 8401:8800, amt = 6.5*1, ii= 24, ss= 1, addl=0),
ev(ID = 8401:8800, amt = 6.5*0, ii= 18, ss= 0, addl=0),
ev(ID = 8401:8800, amt = 6.5*0.5, ii= 6, ss= 0, addl=0),
```

```
ev(ID = 8401:8800, amt = 6.5*1, ii= 24, ss= 0, addl=10),
#18h delay simulation
ev(ID = 8401:8800, amt = 6.5*1, ii= 24, ss= 1, addl=0),
ev(ID = 8401:8800, amt = 6.5*0, ii= 18, ss= 0, addl=0),
ev(ID = 8401:8800, amt = 6.5*1, ii= 6, ss= 0, addl=0),
ev(ID = 8401:8800, amt = 6.5*1, ii= 24, ss= 0, addl=10),
#21h delay + 50% dose intake simulation
ev(ID = 8401:8800, amt = 6.5*1, ii= 24, ss= 1, addl=0),
ev(ID = 8401:8800, amt = 6.5*0, ii= 21, ss= 0, addl=0),
ev(ID = 8401:8800, amt = 6.5*0.5, ii= 3, ss= 0, addl=0),
ev(ID = 8401:8800, amt = 6.5*1, ii= 24, ss= 0, addl=10),
#21h delay simulation
ev(ID = 8401:8800, amt = 6.5*1, ii= 24, ss= 1, addl=0),
ev(ID = 8401:8800, amt = 6.5*0, ii= 21, ss= 0, addl=0),
ev(ID = 8401:8800, amt = 6.5*1, ii= 3, ss= 0, addl=0),
ev(ID = 8401:8800, amt = 6.5*1, ii= 24, ss= 0, addl=10),
#Missed dose simulation
ev(ID = 8401:8800, amt = 6.5*1, ii= 24, ss= 1, addl=0),
ev(ID = 8401:8800, amt = 6.5*0, ii= 24, ss= 0, addl=0),
ev(ID = 8401:8800, amt = 6.5*1, ii= 24, ss= 0, addl=0),
ev(ID = 8401:8800, amt = 6.5*1, ii= 24, ss= 0, addl=10),
#Missed dose + 150% dose intake simulation
ev(ID = 8401:8800, amt = 6.5*1, ii= 24, ss= 1, addl=0),
ev(ID = 8401:8800, amt = 6.5*0, ii= 24, ss= 0, addl=0),
ev(ID = 8401:8800, amt = 6.5*1.5, ii= 24, ss= 0, addl=0),
ev(ID = 8401:8800, amt = 6.5*1, ii= 24, ss= 0, addl=10),
#Missed dose + 200% dose intake simulation
ev(ID = 8401:8800, amt = 6.5*1, ii= 24, ss= 1, addl=0),
ev(ID = 8401:8800, amt = 6.5*0, ii= 24, ss= 0, addl=0),
ev(ID = 8401:8800, amt = 6.5*2, ii= 24, ss= 0, addl=0),
ev(ID = 8401:8800, amt = 6.5*1, ii= 24, ss= 0, addl=10)))) %>%
              mutate(dose_group = "6,5 mg") %>%

    bind_rows(as_tibble( seq(
  #Steady-state simulation
ev(ID = 8801:9200, amt = 7.5*1, ii= 24, ss= 1, addl=0),
ev(ID = 8801:9200, amt = 7.5*1, ii= 24, ss= 0, addl=0),
ev(ID = 8801:9200, amt = 7.5*1, ii= 24, ss= 0, addl=10),
#3h delay simulation
ev(ID = 8801:9200, amt = 7.5*1, ii= 24, ss= 1, addl=0),
ev(ID = 8801:9200, amt = 7.5*0, ii= 3, ss= 0, addl=0),
ev(ID = 8801:9200, amt = 7.5*1, ii= 21, ss= 0, addl=0),
ev(ID = 8801:9200, amt = 7.5*1, ii= 24, ss= 0, addl=10),
#6h delay simulation
ev(ID = 8801:9200, amt = 7.5*1, ii= 24, ss= 1, addl=0),
ev(ID = 8801:9200, amt = 7.5*0, ii= 6, ss= 0, addl=0),
ev(ID = 8801:9200, amt = 7.5*1, ii= 18, ss= 0, addl=0),
ev(ID = 8801:9200, amt = 7.5*1, ii= 24, ss= 0, addl=10),
#9h delay simulation
ev(ID = 8801:9200, amt = 7.5*1, ii= 24, ss= 1, addl=0),
```

```
ev(ID = 8801:9200, amt = 7.5*0, ii= 9, ss= 0, addl=0),
ev(ID = 8801:9200, amt = 7.5*1, ii= 15, ss= 0, addl=0),
ev(ID = 8801:9200, amt = 7.5*1, ii= 24, ss= 0, addl=10),
#12h delay simulation
ev(ID = 8801:9200, amt = 7.5*1, ii= 24, ss= 1, addl=0),
ev(ID = 8801:9200, amt = 7.5*0, ii= 12, ss= 0, addl=0),
ev(ID = 8801:9200, amt = 7.5*1, ii= 12, ss= 0, addl=0),
ev(ID = 8801:9200, amt = 7.5*1, ii= 24, ss= 0, addl=10),
#15h delay + 50% dose intake simulation
ev(ID = 8801:9200, amt = 7.5*1, ii= 24, ss= 1, addl=0),
ev(ID = 8801:9200, amt = 7.5*0, ii= 15, ss= 0, addl=0),
ev(ID = 8801:9200, amt = 7.5*0.5, ii= 9, ss= 0, addl=0),
ev(ID = 8801:9200, amt = 7.5*1, ii= 24, ss= 0, addl=10),
#15h delay simulation
ev(ID = 8801:9200, amt = 7.5*1, ii= 24, ss= 1, addl=0),
ev(ID = 8801:9200, amt = 7.5*0, ii= 15, ss= 0, addl=0),
ev(ID = 8801:9200, amt = 7.5*1, ii= 9, ss= 0, addl=0),
ev(ID = 8801:9200, amt = 7.5*1, ii= 24, ss= 0, addl=10),
#18h delay + 50% dose intake simulation
ev(ID = 8801:9200, amt = 7.5*1, ii= 24, ss= 1, addl=0),
ev(ID = 8801:9200, amt = 7.5*0, ii= 18, ss= 0, addl=0),
ev(ID = 8801:9200, amt = 7.5*0.5, ii= 6, ss= 0, addl=0),
ev(ID = 8801:9200, amt = 7.5*1, ii= 24, ss= 0, addl=10),
#18h delay simulation
ev(ID = 8801:9200, amt = 7.5*1, ii= 24, ss= 1, addl=0),
ev(ID = 8801:9200, amt = 7.5*0, ii= 18, ss= 0, addl=0),
ev(ID = 8801:9200, amt = 7.5*1, ii= 6, ss= 0, addl=0),
ev(ID = 8801:9200, amt = 7.5*1, ii= 24, ss= 0, addl=10),
#21h delay + 50% dose intake simulation
ev(ID = 8801:9200, amt = 7.5*1, ii= 24, ss= 1, addl=0),
ev(ID = 8801:9200, amt = 7.5*0, ii= 21, ss= 0, addl=0),
ev(ID = 8801:9200, amt = 7.5*0.5, ii= 3, ss= 0, addl=0),
ev(ID = 8801:9200, amt = 7.5*1, ii= 24, ss= 0, addl=10),
#21h delay simulation
ev(ID = 8801:9200, amt = 7.5*1, ii= 24, ss= 1, addl=0),
ev(ID = 8801:9200, amt = 7.5*0, ii= 21, ss= 0, addl=0),
ev(ID = 8801:9200, amt = 7.5*1, ii= 3, ss= 0, addl=0),
ev(ID = 8801:9200, amt = 7.5*1, ii= 24, ss= 0, addl=10),
#Missed dose simulation
ev(ID = 8801:9200, amt = 7.5*1, ii= 24, ss= 1, addl=0),
ev(ID = 8801:9200, amt = 7.5*0, ii= 24, ss= 0, addl=0),
ev(ID = 8801:9200, amt = 7.5*1, ii= 24, ss= 0, addl=0),
ev(ID = 8801:9200, amt = 7.5*1, ii= 24, ss= 0, addl=10),
#Missed dose + 150% dose intake simulation
ev(ID = 8801:9200, amt = 7.5*1, ii= 24, ss= 1, addl=0),
ev(ID = 8801:9200, amt = 7.5*0, ii= 24, ss= 0, addl=0),
ev(ID = 8801:9200, amt = 7.5*1.5, ii= 24, ss= 0, addl=0),
ev(ID = 8801:9200, amt = 7.5*1, ii= 24, ss= 0, addl=10),
#Missed dose + 200% dose intake simulation
ev(ID = 8801:9200, amt = 7.5*1, ii= 24, ss= 1, addl=0),
```

```
ev(ID = 8801:9200, amt = 7.5*0, ii= 24, ss= 0, addl=0),
ev(ID = 8801:9200, amt = 7.5*2, ii= 24, ss= 0, addl=0),
ev(ID = 8801:9200, amt = 7.5*1, ii= 24, ss= 0, addl=10)))) %>%
              mutate(dose_group = "7,5 mg") %>%

    bind_rows(as_tibble( seq(
  #Steady-state simulation
ev(ID = 9201:9600, amt = 8.5*1, ii= 24, ss= 1, addl=0),
ev(ID = 9201:9600, amt = 8.5*1, ii= 24, ss= 0, addl=0),
ev(ID = 9201:9600, amt = 8.5*1, ii= 24, ss= 0, addl=10),
#3h delay simulation
ev(ID = 9201:9600, amt = 8.5*1, ii= 24, ss= 1, addl=0),
ev(ID = 9201:9600, amt = 8.5*0, ii= 3, ss= 0, addl=0),
ev(ID = 9201:9600, amt = 8.5*1, ii= 21, ss= 0, addl=0),
ev(ID = 9201:9600, amt = 8.5*1, ii= 24, ss= 0, addl=10),
#6h delay simulation
ev(ID = 9201:9600, amt = 8.5*1, ii= 24, ss= 1, addl=0),
ev(ID = 9201:9600, amt = 8.5*0, ii= 6, ss= 0, addl=0),
ev(ID = 9201:9600, amt = 8.5*1, ii= 18, ss= 0, addl=0),
ev(ID = 9201:9600, amt = 8.5*1, ii= 24, ss= 0, addl=10),
#9h delay simulation
ev(ID = 9201:9600, amt = 8.5*1, ii= 24, ss= 1, addl=0),
ev(ID = 9201:9600, amt = 8.5*0, ii= 9, ss= 0, addl=0),
ev(ID = 9201:9600, amt = 8.5*1, ii= 15, ss= 0, addl=0),
ev(ID = 9201:9600, amt = 8.5*1, ii= 24, ss= 0, addl=10),
#12h delay simulation
ev(ID = 9201:9600, amt = 8.5*1, ii= 24, ss= 1, addl=0),
ev(ID = 9201:9600, amt = 8.5*0, ii= 12, ss= 0, addl=0),
ev(ID = 9201:9600, amt = 8.5*1, ii= 12, ss= 0, addl=0),
ev(ID = 9201:9600, amt = 8.5*1, ii= 24, ss= 0, addl=10),
#15h delay + 50% dose intake simulation
ev(ID = 9201:9600, amt = 8.5*1, ii= 24, ss= 1, addl=0),
ev(ID = 9201:9600, amt = 8.5*0, ii= 15, ss= 0, addl=0),
ev(ID = 9201:9600, amt = 8.5*0.5, ii= 9, ss= 0, addl=0),
ev(ID = 9201:9600, amt = 8.5*1, ii= 24, ss= 0, addl=10),
#15h delay simulation
ev(ID = 9201:9600, amt = 8.5*1, ii= 24, ss= 1, addl=0),
ev(ID = 9201:9600, amt = 8.5*0, ii= 15, ss= 0, addl=0),
ev(ID = 9201:9600, amt = 8.5*1, ii= 9, ss= 0, addl=0),
ev(ID = 9201:9600, amt = 8.5*1, ii= 24, ss= 0, addl=10),
#18h delay + 50% dose intake simulation
ev(ID = 9201:9600, amt = 8.5*1, ii= 24, ss= 1, addl=0),
ev(ID = 9201:9600, amt = 8.5*0, ii= 18, ss= 0, addl=0),
ev(ID = 9201:9600, amt = 8.5*0.5, ii= 6, ss= 0, addl=0),
ev(ID = 9201:9600, amt = 8.5*1, ii= 24, ss= 0, addl=10),
#18h delay simulation
ev(ID = 9201:9600, amt = 8.5*1, ii= 24, ss= 1, addl=0),
ev(ID = 9201:9600, amt = 8.5*0, ii= 18, ss= 0, addl=0),
ev(ID = 9201:9600, amt = 8.5*1, ii= 6, ss= 0, addl=0),
ev(ID = 9201:9600, amt = 8.5*1, ii= 24, ss= 0, addl=10),
```

```
#21h delay + 50% dose intake simulation
ev(ID = 9201:9600, amt = 8.5*1, ii= 24, ss= 1, addl=0),
ev(ID = 9201:9600, amt = 8.5*0, ii= 21, ss= 0, addl=0),
ev(ID = 9201:9600, amt = 8.5*0.5, ii= 3, ss= 0, addl=0),
ev(ID = 9201:9600, amt = 8.5*1, ii= 24, ss= 0, addl=10),
#21h delay simulation
ev(ID = 9201:9600, amt = 8.5*1, ii= 24, ss= 1, addl=0),
ev(ID = 9201:9600, amt = 8.5*0, ii= 21, ss= 0, addl=0),
ev(ID = 9201:9600, amt = 8.5*1, ii= 3, ss= 0, addl=0),
ev(ID = 9201:9600, amt = 8.5*1, ii= 24, ss= 0, addl=10),
#Missed dose simulation
ev(ID = 9201:9600, amt = 8.5*1, ii= 24, ss= 1, addl=0),
ev(ID = 9201:9600, amt = 8.5*0, ii= 24, ss= 0, addl=0),
ev(ID = 9201:9600, amt = 8.5*1, ii= 24, ss= 0, addl=0),
ev(ID = 9201:9600, amt = 8.5*1, ii= 24, ss= 0, addl=10),
#Missed dose + 150% dose intake simulation
ev(ID = 9201:9600, amt = 8.5*1, ii= 24, ss= 1, addl=0),
ev(ID = 9201:9600, amt = 8.5*0, ii= 24, ss= 0, addl=0),
ev(ID = 9201:9600, amt = 8.5*1.5, ii= 24, ss= 0, addl=0),
ev(ID = 9201:9600, amt = 8.5*1, ii= 24, ss= 0, addl=10),
#Missed dose + 200% dose intake simulation
ev(ID = 9201:9600, amt = 8.5*1, ii= 24, ss= 1, addl=0),
ev(ID = 9201:9600, amt = 8.5*0, ii= 24, ss= 0, addl=0),
ev(ID = 9201:9600, amt = 8.5*2, ii= 24, ss= 0, addl=0),
ev(ID = 9201:9600, amt = 8.5*1, ii= 24, ss= 0, addl=10)))) %>%
              mutate(dose_group = "8,5 mg") %>%

    bind_rows(as_tibble( seq(
  #Steady-state simulation
ev(ID = 9601:10000, amt = 9.5*1, ii= 24, ss= 1, addl=0),
ev(ID = 9601:10000, amt = 9.5*1, ii= 24, ss= 0, addl=0),
ev(ID = 9601:10000, amt = 9.5*1, ii= 24, ss= 0, addl=10),
#3h delay simulation
ev(ID = 9601:10000, amt = 9.5*1, ii= 24, ss= 1, addl=0),
ev(ID = 9601:10000, amt = 9.5*0, ii= 3, ss= 0, addl=0),
ev(ID = 9601:10000, amt = 9.5*1, ii= 21, ss= 0, addl=0),
ev(ID = 9601:10000, amt = 9.5*1, ii= 24, ss= 0, addl=10),
#6h delay simulation
ev(ID = 9601:10000, amt = 9.5*1, ii= 24, ss= 1, addl=0),
ev(ID = 9601:10000, amt = 9.5*0, ii= 6, ss= 0, addl=0),
ev(ID = 9601:10000, amt = 9.5*1, ii= 18, ss= 0, addl=0),
ev(ID = 9601:10000, amt = 9.5*1, ii= 24, ss= 0, addl=10),
#9h delay simulation
ev(ID = 9601:10000, amt = 9.5*1, ii= 24, ss= 1, addl=0),
ev(ID = 9601:10000, amt = 9.5*0, ii= 9, ss= 0, addl=0),
ev(ID = 9601:10000, amt = 9.5*1, ii= 15, ss= 0, addl=0),
ev(ID = 9601:10000, amt = 9.5*1, ii= 24, ss= 0, addl=10),
#12h delay simulation
ev(ID = 9601:10000, amt = 9.5*1, ii= 24, ss= 1, addl=0),
ev(ID = 9601:10000, amt = 9.5*0, ii= 12, ss= 0, addl=0),
```

```
ev(ID = 9601:10000, amt = 9.5*1, ii= 12, ss= 0, addl=0),
ev(ID = 9601:10000, amt = 9.5*1, ii= 24, ss= 0, addl=10),
#15h delay + 50% dose intake simulation
ev(ID = 9601:10000, amt = 9.5*1, ii= 24, ss= 1, addl=0),
ev(ID = 9601:10000, amt = 9.5*0, ii= 15, ss= 0, addl=0),
ev(ID = 9601:10000, amt = 9.5*0.5, ii= 9, ss= 0, addl=0),
ev(ID = 9601:10000, amt = 9.5*1, ii= 24, ss= 0, addl=10),
#15h delay simulation
ev(ID = 9601:10000, amt = 9.5*1, ii= 24, ss= 1, addl=0),
ev(ID = 9601:10000, amt = 9.5*0, ii= 15, ss= 0, addl=0),
ev(ID = 9601:10000, amt = 9.5*1, ii= 9, ss= 0, addl=0),
ev(ID = 9601:10000, amt = 9.5*1, ii= 24, ss= 0, addl=10),
#18h delay + 50% dose intake simulation
ev(ID = 9601:10000, amt = 9.5*1, ii= 24, ss= 1, addl=0),
ev(ID = 9601:10000, amt = 9.5*0, ii= 18, ss= 0, addl=0),
ev(ID = 9601:10000, amt = 9.5*0.5, ii= 6, ss= 0, addl=0),
ev(ID = 9601:10000, amt = 9.5*1, ii= 24, ss= 0, addl=10),
#18h delay simulation
ev(ID = 9601:10000, amt = 9.5*1, ii= 24, ss= 1, addl=0),
ev(ID = 9601:10000, amt = 9.5*0, ii= 18, ss= 0, addl=0),
ev(ID = 9601:10000, amt = 9.5*1, ii= 6, ss= 0, addl=0),
ev(ID = 9601:10000, amt = 9.5*1, ii= 24, ss= 0, addl=10),
#21h delay + 50% dose intake simulation
ev(ID = 9601:10000, amt = 9.5*1, ii= 24, ss= 1, addl=0),
ev(ID = 9601:10000, amt = 9.5*0, ii= 21, ss= 0, addl=0),
ev(ID = 9601:10000, amt = 9.5*0.5, ii= 3, ss= 0, addl=0),
ev(ID = 9601:10000, amt = 9.5*1, ii= 24, ss= 0, addl=10),
#21h delay simulation
ev(ID = 9601:10000, amt = 9.5*1, ii= 24, ss= 1, addl=0),
ev(ID = 9601:10000, amt = 9.5*0, ii= 21, ss= 0, addl=0),
ev(ID = 9601:10000, amt = 9.5*1, ii= 3, ss= 0, addl=0),
ev(ID = 9601:10000, amt = 9.5*1, ii= 24, ss= 0, addl=10),
#Missed dose simulation
ev(ID = 9601:10000, amt = 9.5*1, ii= 24, ss= 1, addl=0),
ev(ID = 9601:10000, amt = 9.5*0, ii= 24, ss= 0, addl=0),
ev(ID = 9601:10000, amt = 9.5*1, ii= 24, ss= 0, addl=0),
ev(ID = 9601:10000, amt = 9.5*1, ii= 24, ss= 0, addl=10),
#Missed dose + 150% dose intake simulation
ev(ID = 9601:10000, amt = 9.5*1, ii= 24, ss= 1, addl=0),
ev(ID = 9601:10000, amt = 9.5*0, ii= 24, ss= 0, addl=0),
ev(ID = 9601:10000, amt = 9.5*1.5, ii= 24, ss= 0, addl=0),
ev(ID = 9601:10000, amt = 9.5*1, ii= 24, ss= 0, addl=10),
#Missed dose + 200% dose intake simulation
ev(ID = 9601:10000, amt = 9.5*1, ii= 24, ss= 1, addl=0),
ev(ID = 9601:10000, amt = 9.5*0, ii= 24, ss= 0, addl=0),
ev(ID = 9601:10000, amt = 9.5*2, ii= 24, ss= 0, addl=0),
ev(ID = 9601:10000, amt = 9.5*1, ii= 24, ss= 0, addl=10)))) %>%
              mutate(dose_group = "9,5 mg") %>%

    bind_rows(as_tibble( seq(
```

```
  #Steady-state simulation
ev(ID = 10001:10400, amt = 10.5*1, ii= 24, ss= 1, addl=0),
ev(ID = 10001:10400, amt = 10.5*1, ii= 24, ss= 0, addl=0),
ev(ID = 10001:10400, amt = 10.5*1, ii= 24, ss= 0, addl=10),
#3h delay simulation
ev(ID = 10001:10400, amt = 10.5*1, ii= 24, ss= 1, addl=0),
ev(ID = 10001:10400, amt = 10.5*0, ii= 3, ss= 0, addl=0),
ev(ID = 10001:10400, amt = 10.5*1, ii= 21, ss= 0, addl=0),
ev(ID = 10001:10400, amt = 10.5*1, ii= 24, ss= 0, addl=10),
#6h delay simulation
ev(ID = 10001:10400, amt = 10.5*1, ii= 24, ss= 1, addl=0),
ev(ID = 10001:10400, amt = 10.5*0, ii= 6, ss= 0, addl=0),
ev(ID = 10001:10400, amt = 10.5*1, ii= 18, ss= 0, addl=0),
ev(ID = 10001:10400, amt = 10.5*1, ii= 24, ss= 0, addl=10),
#9h delay simulation
ev(ID = 10001:10400, amt = 10.5*1, ii= 24, ss= 1, addl=0),
ev(ID = 10001:10400, amt = 10.5*0, ii= 9, ss= 0, addl=0),
ev(ID = 10001:10400, amt = 10.5*1, ii= 15, ss= 0, addl=0),
ev(ID = 10001:10400, amt = 10.5*1, ii= 24, ss= 0, addl=10),
#12h delay simulation
ev(ID = 10001:10400, amt = 10.5*1, ii= 24, ss= 1, addl=0),
ev(ID = 10001:10400, amt = 10.5*0, ii= 12, ss= 0, addl=0),
ev(ID = 10001:10400, amt = 10.5*1, ii= 12, ss= 0, addl=0),
ev(ID = 10001:10400, amt = 10.5*1, ii= 24, ss= 0, addl=10),
#15h delay + 50% dose intake simulation
ev(ID = 10001:10400, amt = 10.5*1, ii= 24, ss= 1, addl=0),
ev(ID = 10001:10400, amt = 10.5*0, ii= 15, ss= 0, addl=0),
ev(ID = 10001:10400, amt = 10.5*0.5, ii= 9, ss= 0, addl=0),
ev(ID = 10001:10400, amt = 10.5*1, ii= 24, ss= 0, addl=10),
#15h delay simulation
ev(ID = 10001:10400, amt = 10.5*1, ii= 24, ss= 1, addl=0),
ev(ID = 10001:10400, amt = 10.5*0, ii= 15, ss= 0, addl=0),
ev(ID = 10001:10400, amt = 10.5*1, ii= 9, ss= 0, addl=0),
ev(ID = 10001:10400, amt = 10.5*1, ii= 24, ss= 0, addl=10),
#18h delay + 50% dose intake simulation
ev(ID = 10001:10400, amt = 10.5*1, ii= 24, ss= 1, addl=0),
ev(ID = 10001:10400, amt = 10.5*0, ii= 18, ss= 0, addl=0),
ev(ID = 10001:10400, amt = 10.5*0.5, ii= 6, ss= 0, addl=0),
ev(ID = 10001:10400, amt = 10.5*1, ii= 24, ss= 0, addl=10),
#18h delay simulation
ev(ID = 10001:10400, amt = 10.5*1, ii= 24, ss= 1, addl=0),
ev(ID = 10001:10400, amt = 10.5*0, ii= 18, ss= 0, addl=0),
ev(ID = 10001:10400, amt = 10.5*1, ii= 6, ss= 0, addl=0),
ev(ID = 10001:10400, amt = 10.5*1, ii= 24, ss= 0, addl=10),
#21h delay + 50% dose intake simulation
ev(ID = 10001:10400, amt = 10.5*1, ii= 24, ss= 1, addl=0),
ev(ID = 10001:10400, amt = 10.5*0, ii= 21, ss= 0, addl=0),
ev(ID = 10001:10400, amt = 10.5*0.5, ii= 3, ss= 0, addl=0),
ev(ID = 10001:10400, amt = 10.5*1, ii= 24, ss= 0, addl=10),
#21h delay simulation
```

```
ev(ID = 10001:10400, amt = 10.5*1, ii= 24, ss= 1, addl=0),
ev(ID = 10001:10400, amt = 10.5*0, ii= 21, ss= 0, addl=0),
ev(ID = 10001:10400, amt = 10.5*1, ii= 3, ss= 0, addl=0),
ev(ID = 10001:10400, amt = 10.5*1, ii= 24, ss= 0, addl=10),
#Missed dose simulation
ev(ID = 10001:10400, amt = 10.5*1, ii= 24, ss= 1, addl=0),
ev(ID = 10001:10400, amt = 10.5*0, ii= 24, ss= 0, addl=0),
ev(ID = 10001:10400, amt = 10.5*1, ii= 24, ss= 0, addl=0),
ev(ID = 10001:10400, amt = 10.5*1, ii= 24, ss= 0, addl=10),
#Missed dose + 150% dose intake simulation
ev(ID = 10001:10400, amt = 10.5*1, ii= 24, ss= 1, addl=0),
ev(ID = 10001:10400, amt = 10.5*0, ii= 24, ss= 0, addl=0),
ev(ID = 10001:10400, amt = 10.5*1.5, ii= 24, ss= 0, addl=0),
ev(ID = 10001:10400, amt = 10.5*1, ii= 24, ss= 0, addl=10),
#Missed dose + 200% dose intake simulation
ev(ID = 10001:10400, amt = 10.5*1, ii= 24, ss= 1, addl=0),
ev(ID = 10001:10400, amt = 10.5*0, ii= 24, ss= 0, addl=0),
ev(ID = 10001:10400, amt = 10.5*2, ii= 24, ss= 0, addl=0),
ev(ID = 10001:10400, amt = 10.5*1, ii= 24, ss= 0, addl=10)))) %>%
              mutate(dose_group = "10,5 mg") %>%

    bind_rows(as_tibble( seq(
  #Steady-state simulation
ev(ID = 10401:10800, amt = 11.5*1, ii= 24, ss= 1, addl=0),
ev(ID = 10401:10800, amt = 11.5*1, ii= 24, ss= 0, addl=0),
ev(ID = 10401:10800, amt = 11.5*1, ii= 24, ss= 0, addl=10),
#3h delay simulation
ev(ID = 10401:10800, amt = 11.5*1, ii= 24, ss= 1, addl=0),
ev(ID = 10401:10800, amt = 11.5*0, ii= 3, ss= 0, addl=0),
ev(ID = 10401:10800, amt = 11.5*1, ii= 21, ss= 0, addl=0),
ev(ID = 10401:10800, amt = 11.5*1, ii= 24, ss= 0, addl=10),
#6h delay simulation
ev(ID = 10401:10800, amt = 11.5*1, ii= 24, ss= 1, addl=0),
ev(ID = 10401:10800, amt = 11.5*0, ii= 6, ss= 0, addl=0),
ev(ID = 10401:10800, amt = 11.5*1, ii= 18, ss= 0, addl=0),
ev(ID = 10401:10800, amt = 11.5*1, ii= 24, ss= 0, addl=10),
#9h delay simulation
ev(ID = 10401:10800, amt = 11.5*1, ii= 24, ss= 1, addl=0),
ev(ID = 10401:10800, amt = 11.5*0, ii= 9, ss= 0, addl=0),
ev(ID = 10401:10800, amt = 11.5*1, ii= 15, ss= 0, addl=0),
ev(ID = 10401:10800, amt = 11.5*1, ii= 24, ss= 0, addl=10),
#12h delay simulation
ev(ID = 10401:10800, amt = 11.5*1, ii= 24, ss= 1, addl=0),
ev(ID = 10401:10800, amt = 11.5*0, ii= 12, ss= 0, addl=0),
ev(ID = 10401:10800, amt = 11.5*1, ii= 12, ss= 0, addl=0),
ev(ID = 10401:10800, amt = 11.5*1, ii= 24, ss= 0, addl=10),
#15h delay + 50% dose intake simulation
ev(ID = 10401:10800, amt = 11.5*1, ii= 24, ss= 1, addl=0),
ev(ID = 10401:10800, amt = 11.5*0, ii= 15, ss= 0, addl=0),
ev(ID = 10401:10800, amt = 11.5*0.5, ii= 9, ss= 0, addl=0),
```

```
ev(ID = 10401:10800, amt = 11.5*1, ii= 24, ss= 0, addl=10),
#15h delay simulation
ev(ID = 10401:10800, amt = 11.5*1, ii= 24, ss= 1, addl=0),
ev(ID = 10401:10800, amt = 11.5*0, ii= 15, ss= 0, addl=0),
ev(ID = 10401:10800, amt = 11.5*1, ii= 9, ss= 0, addl=0),
#18h delay + 50% dose intake simulation
ev(ID = 10401:10800, amt = 11.5*1, ii= 24, ss= 1, addl=0),
ev(ID = 10401:10800, amt = 11.5*0, ii= 18, ss= 0, addl=0),
ev(ID = 10401:10800, amt = 11.5*0.5, ii= 6, ss= 0, addl=0),
ev(ID = 10401:10800, amt = 11.5*1, ii= 24, ss= 0, addl=10),
#18h delay simulation
ev(ID = 10401:10800, amt = 11.5*1, ii= 24, ss= 1, addl=0),
ev(ID = 10401:10800, amt = 11.5*0, ii= 18, ss= 0, addl=0),
ev(ID = 10401:10800, amt = 11.5*1, ii= 6, ss= 0, addl=0),
ev(ID = 10401:10800, amt = 11.5*1, ii= 24, ss= 0, addl=10),
#21h delay + 50% dose intake simulation
ev(ID = 10401:10800, amt = 11.5*1, ii= 24, ss= 1, addl=0),
ev(ID = 10401:10800, amt = 11.5*0, ii= 21, ss= 0, addl=0),
ev(ID = 10401:10800, amt = 11.5*0.5, ii= 3, ss= 0, addl=0),
ev(ID = 10401:10800, amt = 11.5*1, ii= 24, ss= 0, addl=10),
#21h delay simulation
ev(ID = 10401:10800, amt = 11.5*1, ii= 24, ss= 1, addl=0),
ev(ID = 10401:10800, amt = 11.5*0, ii= 21, ss= 0, addl=0),
ev(ID = 10401:10800, amt = 11.5*1, ii= 3, ss= 0, addl=0),
ev(ID = 10401:10800, amt = 11.5*1, ii= 24, ss= 0, addl=10),
#Missed dose simulation
ev(ID = 10401:10800, amt = 11.5*1, ii= 24, ss= 1, addl=0),
ev(ID = 10401:10800, amt = 11.5*0, ii= 24, ss= 0, addl=0),
ev(ID = 10401:10800, amt = 11.5*1, ii= 24, ss= 0, addl=0),
ev(ID = 10401:10800, amt = 11.5*1, ii= 24, ss= 0, addl=10),
#Missed dose + 150% dose intake simulation
ev(ID = 10401:10800, amt = 11.5*1, ii= 24, ss= 1, addl=0),
ev(ID = 10401:10800, amt = 11.5*0, ii= 24, ss= 0, addl=0),
ev(ID = 10401:10800, amt = 11.5*1.5, ii= 24, ss= 0, addl=0),
ev(ID = 10401:10800, amt = 11.5*1, ii= 24, ss= 0, addl=10),
#Missed dose + 200% dose intake simulation
ev(ID = 10401:10800, amt = 11.5*1, ii= 24, ss= 1, addl=0),
ev(ID = 10401:10800, amt = 11.5*0, ii= 24, ss= 0, addl=0),
ev(ID = 10401:10800, amt = 11.5*2, ii= 24, ss= 0, addl=0),
ev(ID = 10401:10800, amt = 11.5*1, ii= 24, ss= 0, addl=10)))) %>%
              mutate(dose_group = "11,5 mg") %>%

    bind_rows(as_tibble( seq(
  #Steady-state simulation
ev(ID = 10801:11200, amt = 12.5*1, ii= 24, ss= 1, addl=0),
ev(ID = 10801:11200, amt = 12.5*1, ii= 24, ss= 0, addl=0),
ev(ID = 10801:11200, amt = 12.5*1, ii= 24, ss= 0, addl=10),
#3h delay simulation
ev(ID = 10801:11200, amt = 12.5*1, ii= 24, ss= 1, addl=0),
ev(ID = 10801:11200, amt = 12.5*0, ii= 3, ss= 0, addl=0),
```

```
ev(ID = 10801:11200, amt = 12.5*1, ii= 21, ss= 0, addl=0),
ev(ID = 10801:11200, amt = 12.5*1, ii= 24, ss= 0, addl=10),
#6h delay simulation
ev(ID = 10801:11200, amt = 12.5*1, ii= 24, ss= 1, addl=0),
ev(ID = 10801:11200, amt = 12.5*0, ii= 6, ss= 0, addl=0),
ev(ID = 10801:11200, amt = 12.5*1, ii= 18, ss= 0, addl=0),
ev(ID = 10801:11200, amt = 12.5*1, ii= 24, ss= 0, addl=10),
#9h delay simulation
ev(ID = 10801:11200, amt = 12.5*1, ii= 24, ss= 1, addl=0),
ev(ID = 10801:11200, amt = 12.5*0, ii= 9, ss= 0, addl=0),
ev(ID = 10801:11200, amt = 12.5*1, ii= 15, ss= 0, addl=0),
ev(ID = 10801:11200, amt = 12.5*1, ii= 24, ss= 0, addl=10),
#12h delay simulation
ev(ID = 10801:11200, amt = 12.5*1, ii= 24, ss= 1, addl=0),
ev(ID = 10801:11200, amt = 12.5*0, ii= 12, ss= 0, addl=0),
ev(ID = 10801:11200, amt = 12.5*1, ii= 12, ss= 0, addl=0),
ev(ID = 10801:11200, amt = 12.5*1, ii= 24, ss= 0, addl=10),
#15h delay + 50% dose intake simulation
ev(ID = 10801:11200, amt = 12.5*1, ii= 24, ss= 1, addl=0),
ev(ID = 10801:11200, amt = 12.5*0, ii= 15, ss= 0, addl=0),
ev(ID = 10801:11200, amt = 12.5*0.5, ii= 9, ss= 0, addl=0),
ev(ID = 10801:11200, amt = 12.5*1, ii= 24, ss= 0, addl=10),
#15h delay simulation
ev(ID = 10801:11200, amt = 12.5*1, ii= 24, ss= 1, addl=0),
ev(ID = 10801:11200, amt = 12.5*0, ii= 15, ss= 0, addl=0),
ev(ID = 10801:11200, amt = 12.5*1, ii= 9, ss= 0, addl=0),
ev(ID = 10801:11200, amt = 12.5*1, ii= 24, ss= 0, addl=10),
#18h delay + 50% dose intake simulation
ev(ID = 10801:11200, amt = 12.5*1, ii= 24, ss= 1, addl=0),
ev(ID = 10801:11200, amt = 12.5*0, ii= 18, ss= 0, addl=0),
ev(ID = 10801:11200, amt = 12.5*0.5, ii= 6, ss= 0, addl=0),
ev(ID = 10801:11200, amt = 12.5*1, ii= 24, ss= 0, addl=10),
#18h delay simulation
ev(ID = 10801:11200, amt = 12.5*1, ii= 24, ss= 1, addl=0),
ev(ID = 10801:11200, amt = 12.5*0, ii= 18, ss= 0, addl=0),
ev(ID = 10801:11200, amt = 12.5*1, ii= 6, ss= 0, addl=0),
ev(ID = 10801:11200, amt = 12.5*1, ii= 24, ss= 0, addl=10),
#21h delay + 50% dose intake simulation
ev(ID = 10801:11200, amt = 12.5*1, ii= 24, ss= 1, addl=0),
ev(ID = 10801:11200, amt = 12.5*0, ii= 21, ss= 0, addl=0),
ev(ID = 10801:11200, amt = 12.5*0.5, ii= 3, ss= 0, addl=0),
ev(ID = 10801:11200, amt = 12.5*1, ii= 24, ss= 0, addl=10),
#21h delay simulation
ev(ID = 10801:11200, amt = 12.5*1, ii= 24, ss= 1, addl=0),
ev(ID = 10801:11200, amt = 12.5*0, ii= 21, ss= 0, addl=0),
ev(ID = 10801:11200, amt = 12.5*1, ii= 3, ss= 0, addl=0),
ev(ID = 10801:11200, amt = 12.5*1, ii= 24, ss= 0, addl=10),
#Missed dose simulation
ev(ID = 10801:11200, amt = 12.5*1, ii= 24, ss= 1, addl=0),
ev(ID = 10801:11200, amt = 12.5*0, ii= 24, ss= 0, addl=0),
```

```
ev(ID = 10801:11200, amt = 12.5*1, ii= 24, ss= 0, addl=0),
ev(ID = 10801:11200, amt = 12.5*1, ii= 24, ss= 0, addl=10),
#Missed dose + 150% dose intake simulation
ev(ID = 10801:11200, amt = 12.5*1, ii= 24, ss= 1, addl=0),
ev(ID = 10801:11200, amt = 12.5*0, ii= 24, ss= 0, addl=0),
ev(ID = 10801:11200, amt = 12.5*1.5, ii= 24, ss= 0, addl=0),
ev(ID = 10801:11200, amt = 12.5*1, ii= 24, ss= 0, addl=10),
#Missed dose + 200% dose intake simulation
ev(ID = 10801:11200, amt = 12.5*1, ii= 24, ss= 1, addl=0),
ev(ID = 10801:11200, amt = 12.5*0, ii= 24, ss= 0, addl=0),
ev(ID = 10801:11200, amt = 12.5*2, ii= 24, ss= 0, addl=0),
ev(ID = 10801:11200, amt = 12.5*1, ii= 24, ss= 0, addl=10)))) %>%
              mutate(dose_group = "12,5 mg") %>%

    bind_rows(as_tibble( seq(
  #Steady-state simulation
ev(ID = 11201:11600, amt = 13.5*1, ii= 24, ss= 1, addl=0),
ev(ID = 11201:11600, amt = 13.5*1, ii= 24, ss= 0, addl=0),
ev(ID = 11201:11600, amt = 13.5*1, ii= 24, ss= 0, addl=10),
#3h delay simulation
ev(ID = 11201:11600, amt = 13.5*1, ii= 24, ss= 1, addl=0),
ev(ID = 11201:11600, amt = 13.5*0, ii= 3, ss= 0, addl=0),
ev(ID = 11201:11600, amt = 13.5*1, ii= 21, ss= 0, addl=0),
ev(ID = 11201:11600, amt = 13.5*1, ii= 24, ss= 0, addl=10),
#6h delay simulation
ev(ID = 11201:11600, amt = 13.5*1, ii= 24, ss= 1, addl=0),
ev(ID = 11201:11600, amt = 13.5*0, ii= 6, ss= 0, addl=0),
ev(ID = 11201:11600, amt = 13.5*1, ii= 18, ss= 0, addl=0),
ev(ID = 11201:11600, amt = 13.5*1, ii= 24, ss= 0, addl=10),
#9h delay simulation
ev(ID = 11201:11600, amt = 13.5*1, ii= 24, ss= 1, addl=0),
ev(ID = 11201:11600, amt = 13.5*0, ii= 9, ss= 0, addl=0),
ev(ID = 11201:11600, amt = 13.5*1, ii= 15, ss= 0, addl=0),
ev(ID = 11201:11600, amt = 13.5*1, ii= 24, ss= 0, addl=10),
#12h delay simulation
ev(ID = 11201:11600, amt = 13.5*1, ii= 24, ss= 1, addl=0),
ev(ID = 11201:11600, amt = 13.5*0, ii= 12, ss= 0, addl=0),
ev(ID = 11201:11600, amt = 13.5*1, ii= 12, ss= 0, addl=0),
ev(ID = 11201:11600, amt = 13.5*1, ii= 24, ss= 0, addl=10),
#15h delay + 50% dose intake simulation
ev(ID = 11201:11600, amt = 13.5*1, ii= 24, ss= 1, addl=0),
ev(ID = 11201:11600, amt = 13.5*0, ii= 15, ss= 0, addl=0),
ev(ID = 11201:11600, amt = 13.5*0.5, ii= 9, ss= 0, addl=0),
ev(ID = 11201:11600, amt = 13.5*1, ii= 24, ss= 0, addl=10),
#15h delay simulation
ev(ID = 11201:11600, amt = 13.5*1, ii= 24, ss= 1, addl=0),
ev(ID = 11201:11600, amt = 13.5*0, ii= 15, ss= 0, addl=0),
ev(ID = 11201:11600, amt = 13.5*1, ii= 9, ss= 0, addl=0),
ev(ID = 11201:11600, amt = 13.5*1, ii= 24, ss= 0, addl=10),
#18h delay + 50% dose intake simulation
```

```
ev(ID = 11201:11600, amt = 13.5*1, ii= 24, ss= 1, addl=0),
ev(ID = 11201:11600, amt = 13.5*0, ii= 18, ss= 0, addl=0),
ev(ID = 11201:11600, amt = 13.5*0.5, ii= 6, ss= 0, addl=0),
ev(ID = 11201:11600, amt = 13.5*1, ii= 24, ss= 0, addl=10),
#18h delay simulation
ev(ID = 11201:11600, amt = 13.5*1, ii= 24, ss= 1, addl=0),
ev(ID = 11201:11600, amt = 13.5*0, ii= 18, ss= 0, addl=0),
ev(ID = 11201:11600, amt = 13.5*1, ii= 6, ss= 0, addl=0),
ev(ID = 11201:11600, amt = 13.5*1, ii= 24, ss= 0, addl=10),
#21h delay + 50% dose intake simulation
ev(ID = 11201:11600, amt = 13.5*1, ii= 24, ss= 1, addl=0),
ev(ID = 11201:11600, amt = 13.5*0, ii= 21, ss= 0, addl=0),
ev(ID = 11201:11600, amt = 13.5*0.5, ii= 3, ss= 0, addl=0),
ev(ID = 11201:11600, amt = 13.5*1, ii= 24, ss= 0, addl=10),
#21h delay simulation
ev(ID = 11201:11600, amt = 13.5*1, ii= 24, ss= 1, addl=0),
ev(ID = 11201:11600, amt = 13.5*0, ii= 21, ss= 0, addl=0),
ev(ID = 11201:11600, amt = 13.5*1, ii= 3, ss= 0, addl=0),
ev(ID = 11201:11600, amt = 13.5*1, ii= 24, ss= 0, addl=10),
#Missed dose simulation
ev(ID = 11201:11600, amt = 13.5*1, ii= 24, ss= 1, addl=0),
ev(ID = 11201:11600, amt = 13.5*0, ii= 24, ss= 0, addl=0),
ev(ID = 11201:11600, amt = 13.5*1, ii= 24, ss= 0, addl=0),
ev(ID = 11201:11600, amt = 13.5*1, ii= 24, ss= 0, addl=10),
#Missed dose + 150% dose intake simulation
ev(ID = 11201:11600, amt = 13.5*1, ii= 24, ss= 1, addl=0),
ev(ID = 11201:11600, amt = 13.5*0, ii= 24, ss= 0, addl=0),
ev(ID = 11201:11600, amt = 13.5*1.5, ii= 24, ss= 0, addl=0),
ev(ID = 11201:11600, amt = 13.5*1, ii= 24, ss= 0, addl=10),
#Missed dose + 200% dose intake simulation
ev(ID = 11201:11600, amt = 13.5*1, ii= 24, ss= 1, addl=0),
ev(ID = 11201:11600, amt = 13.5*0, ii= 24, ss= 0, addl=0),
ev(ID = 11201:11600, amt = 13.5*2, ii= 24, ss= 0, addl=0),
ev(ID = 11201:11600, amt = 13.5*1, ii= 24, ss= 0, addl=10)))) %>%
              mutate(dose_group = "13,5 mg") %>%

    bind_rows(as_tibble( seq(
  #Steady-state simulation
ev(ID = 11601:12000, amt = 14.5*1, ii= 24, ss= 1, addl=0),
ev(ID = 11601:12000, amt = 14.5*1, ii= 24, ss= 0, addl=0),
ev(ID = 11601:12000, amt = 14.5*1, ii= 24, ss= 0, addl=10),
#3h delay simulation
ev(ID = 11601:12000, amt = 14.5*1, ii= 24, ss= 1, addl=0),
ev(ID = 11601:12000, amt = 14.5*0, ii= 3, ss= 0, addl=0),
ev(ID = 11601:12000, amt = 14.5*1, ii= 21, ss= 0, addl=0),
ev(ID = 11601:12000, amt = 14.5*1, ii= 24, ss= 0, addl=10),
#6h delay simulation
ev(ID = 11601:12000, amt = 14.5*1, ii= 24, ss= 1, addl=0),
ev(ID = 11601:12000, amt = 14.5*0, ii= 6, ss= 0, addl=0),
ev(ID = 11601:12000, amt = 14.5*1, ii= 18, ss= 0, addl=0),
```

```
ev(ID = 11601:12000, amt = 14.5*1, ii= 24, ss= 0, addl=10),
#9h delay simulation
ev(ID = 11601:12000, amt = 14.5*1, ii= 24, ss= 1, addl=0),
ev(ID = 11601:12000, amt = 14.5*0, ii= 9, ss= 0, addl=0),
ev(ID = 11601:12000, amt = 14.5*1, ii= 15, ss= 0, addl=0),
ev(ID = 11601:12000, amt = 14.5*1, ii= 24, ss= 0, addl=10),
#12h delay simulation
ev(ID = 11601:12000, amt = 14.5*1, ii= 24, ss= 1, addl=0),
ev(ID = 11601:12000, amt = 14.5*0, ii= 12, ss= 0, addl=0),
ev(ID = 11601:12000, amt = 14.5*1, ii= 12, ss= 0, addl=0),
ev(ID = 11601:12000, amt = 14.5*1, ii= 24, ss= 0, addl=10),
#15h delay + 50% dose intake simulation
ev(ID = 11601:12000, amt = 14.5*1, ii= 24, ss= 1, addl=0),
ev(ID = 11601:12000, amt = 14.5*0, ii= 15, ss= 0, addl=0),
ev(ID = 11601:12000, amt = 14.5*0.5, ii= 9, ss= 0, addl=0),
ev(ID = 11601:12000, amt = 14.5*1, ii= 24, ss= 0, addl=10),
#15h delay simulation
ev(ID = 11601:12000, amt = 14.5*1, ii= 24, ss= 1, addl=0),
ev(ID = 11601:12000, amt = 14.5*0, ii= 15, ss= 0, addl=0),
ev(ID = 11601:12000, amt = 14.5*1, ii= 9, ss= 0, addl=0),
ev(ID = 11601:12000, amt = 14.5*1, ii= 24, ss= 0, addl=10),
#18h delay + 50% dose intake simulation
ev(ID = 11601:12000, amt = 14.5*1, ii= 24, ss= 1, addl=0),
ev(ID = 11601:12000, amt = 14.5*0, ii= 18, ss= 0, addl=0),
ev(ID = 11601:12000, amt = 14.5*0.5, ii= 6, ss= 0, addl=0),
ev(ID = 11601:12000, amt = 14.5*1, ii= 24, ss= 0, addl=10),
#18h delay simulation
ev(ID = 11601:12000, amt = 14.5*1, ii= 24, ss= 1, addl=0),
ev(ID = 11601:12000, amt = 14.5*0, ii= 18, ss= 0, addl=0),
ev(ID = 11601:12000, amt = 14.5*1, ii= 6, ss= 0, addl=0),
ev(ID = 11601:12000, amt = 14.5*1, ii= 24, ss= 0, addl=10),
#21h delay + 50% dose intake simulation
ev(ID = 11601:12000, amt = 14.5*1, ii= 24, ss= 1, addl=0),
ev(ID = 11601:12000, amt = 14.5*0, ii= 21, ss= 0, addl=0),
ev(ID = 11601:12000, amt = 14.5*0.5, ii= 3, ss= 0, addl=0),
ev(ID = 11601:12000, amt = 14.5*1, ii= 24, ss= 0, addl=10),
#21h delay simulation
ev(ID = 11601:12000, amt = 14.5*1, ii= 24, ss= 1, addl=0),
ev(ID = 11601:12000, amt = 14.5*0, ii= 21, ss= 0, addl=0),
ev(ID = 11601:12000, amt = 14.5*1, ii= 3, ss= 0, addl=0),
ev(ID = 11601:12000, amt = 14.5*1, ii= 24, ss= 0, addl=10),
#Missed dose simulation
ev(ID = 11601:12000, amt = 14.5*1, ii= 24, ss= 1, addl=0),
ev(ID = 11601:12000, amt = 14.5*0, ii= 24, ss= 0, addl=0),
ev(ID = 11601:12000, amt = 14.5*1, ii= 24, ss= 0, addl=0),
ev(ID = 11601:12000, amt = 14.5*1, ii= 24, ss= 0, addl=10),
#Missed dose + 150% dose intake simulation
ev(ID = 11601:12000, amt = 14.5*1, ii= 24, ss= 1, addl=0),
ev(ID = 11601:12000, amt = 14.5*0, ii= 24, ss= 0, addl=0),
ev(ID = 11601:12000, amt = 14.5*1.5, ii= 24, ss= 0, addl=0),
```

```
ev(ID = 11601:12000, amt = 14.5*1, ii= 24, ss= 0, addl=10),
#Missed dose + 200% dose intake simulation
ev(ID = 11601:12000, amt = 14.5*1, ii= 24, ss= 1, addl=0),
ev(ID = 11601:12000, amt = 14.5*0, ii= 24, ss= 0, addl=0),
ev(ID = 11601:12000, amt = 14.5*2, ii= 24, ss= 0, addl=0),
ev(ID = 11601:12000, amt = 14.5*1, ii= 24, ss= 0, addl=10)))) %>%
              mutate(dose_group = "14,5 mg")


Dataset_woillard_12000 <- Simulation_data_12000_W %>%
  arrange(ID) %>%
  left_join(CYPCL_data_woillard_12000, by = "ID")


## test simulation
set.seed(123456)
Sim_Test_woillard_12000 <- my_model_tacro_Woillard %>%
 data_set(Dataset_woillard_12000) %>%
 Req(DV) %>%
 mrgsim(end = 7000, delta = 1)
```

Visualisation of C0 profiles at steady-state: <4, >12 or between 4 and 12 ug/L (6000 profiles)

```
ID_C0_Woillard_6000 <- as_tibble(Sim_Test_woillard_6000) %>%
  dplyr::filter(time == 24) %>%
  select(ID, DV, time) %>%
  group_by(ID, time) %>%
  dplyr::filter(DV == max(DV)) %>%
  ungroup() %>%
  mutate(Group = case_when(
    DV < 4  ~ "Below 4",
    DV > 12  ~ "Above 12",
    TRUE    ~ "Between 4 and 12"
  ))

print(ID_C0_Woillard_6000)
```

```
## # A tibble: 6,000 × 4
##       ID    DV  time Group
##    <dbl> <dbl> <dbl> <chr>
##  1     1 1.57     24 Below 4
##  2     2 1.27     24 Below 4
##  3     3 1.51     24 Below 4
##  4     4 1.18     24 Below 4
##  5     5 3.10     24 Below 4
##  6     6 1.88     24 Below 4
##  7     7 0.973    24 Below 4
##  8     8 1.78     24 Below 4
##  9     9 0.299    24 Below 4
## 10    10 1.66     24 Below 4
## # ℹ 5,990 more rows
```

Profiles couting (6000 profiles)

```
count_Below_4_W_6000 <- nrow(ID_C0_Woillard_6000 %>% dplyr::filter(Group == "Below
4"))
count_Above_12_W_6000 <- nrow(ID_C0_Woillard_6000 %>% dplyr::filter(Group == "Abov
e 12"))
count_Between_4_12_W_6000 <- nrow(ID_C0_Woillard_6000 %>% dplyr::filter(Group == "
Between 4 and 12"))

print(count_Below_4_W_6000)
```

```
## [1] 1156
```

```
print(count_Above_12_W_6000)
```

```
## [1] 2594
```

```
print(count_Between_4_12_W_6000)
```

```
## [1] 2250
```

#Selection of profiles with a C0 in the therapeutic target: 4-12 ug/L (6000 profiles)

```
selection_4_12_W_6000 <- ID_C0_Woillard_6000 %>%
  dplyr::filter(Group == "Between 4 and 12") %>%
  select(ID, DV, time)

Results_test_6000_W <- Sim_Test_woillard_6000 %>%
  dplyr::filter(ID %in%selection_4_12_W_6000$ID)

sim_data_filtered_6000_Woillard <- Results_test_6000_W %>%
  group_by(ID, time) %>%
  dplyr::filter(DV == max(DV)) %>%
  ungroup()

sim_data_filtered_6000_Woillard
```

```
## # A tibble: 15,752,250 × 3
##       ID  time    DV
##    <dbl> <dbl> <dbl>
##  1   404     0  5.37
##  2   404     1  9.48
##  3   404     2 10.8
##  4   404     3  9.99
##  5   404     4  9.37
##  6   404     5  8.97
##  7   404     6  8.67
##  8   404     7  8.40
##  9   404     8  8.17
## 10   404     9  7.94
## # ℹ 15,752,240 more rows
```

Visualisation of C0 profiles at steady-state: <4, >12 or between 4 and 12 ug/L (12000 profiles)

```
ID_C0_Woillard_12000 <- as_tibble(Sim_Test_woillard_12000) %>%
  dplyr::filter(time == 24) %>%
  select(ID, DV, time) %>%
  group_by(ID, time) %>%
  dplyr::filter(DV == max(DV)) %>%
  ungroup() %>%
  mutate(Group = case_when(
    DV < 4  ~ "Below 4",
    DV > 12  ~ "Above 12",
    TRUE    ~ "Between 4 and 12"
  ))

print(ID_C0_Woillard_12000)
```

```
## # A tibble: 6,000 × 4
##       ID    DV  time Group
##    <dbl> <dbl> <dbl> <chr>
##  1  6001 0.788    24 Below 4
##  2  6002 0.636    24 Below 4
##  3  6003 0.756    24 Below 4
##  4  6004 0.593    24 Below 4
##  5  6005 1.55     24 Below 4
##  6  6006 0.936    24 Below 4
##  7  6007 0.487    24 Below 4
##  8  6008 0.893    24 Below 4
##  9  6009 0.149    24 Below 4
## 10  6010 0.830    24 Below 4
## # ℹ 5,990 more rows
```

Profiles couting (12000 profiles)

```
count_Below_4_W_12000 <- nrow(ID_C0_Woillard_12000 %>% dplyr::filter(Group == "Bel
ow 4"))
count_Above_12_W_12000 <- nrow(ID_C0_Woillard_12000 %>% dplyr::filter(Group == "Ab
ove 12"))
count_Between_4_12_W_12000 <- nrow(ID_C0_Woillard_12000 %>% dplyr::filter(Group ==
"Between 4 and 12"))

# Affichage des résultats
print(count_Below_4_W_12000)
```

```
## [1] 1356
```

```
print(count_Above_12_W_12000)
```

```
## [1] 2447
```

```
print(count_Between_4_12_W_12000)
```

```
## [1] 2197
```

#Selection of profiles with a C0 in the therapeutic target: 4-12 ug/L (12000 profiles)

```
selection_4_12_W_12000 <- ID_C0_Woillard_12000 %>%
  dplyr::filter(Group == "Between 4 and 12") %>%
  select(ID, DV, time)

Results_test_12000_W <- Sim_Test_woillard_12000 %>%
  dplyr::filter(ID %in%selection_4_12_W_12000$ID)

sim_data_filtered_12000_Woillard <- Results_test_12000_W %>%
  group_by(ID, time) %>%
  dplyr::filter(DV == max(DV)) %>%
  ungroup()

sim_data_filtered_12000_Woillard
```

```
## # A tibble: 15,381,197 × 3
##       ID  time    DV
##    <dbl> <dbl> <dbl>
##  1  6404     0  4.03
##  2  6404     1  7.11
##  3  6404     2  8.07
##  4  6404     3  7.49
##  5  6404     4  7.03
##  6  6404     5  6.72
##  7  6404     6  6.50
##  8  6404     7  6.30
##  9  6404     8  6.13
## 10  6404     9  5.96
## # ℹ 15,381,187 more rows
```

Dose distribution of selected profiles

```
ids_to_keep_6000_W <- sim_data_filtered_6000_Woillard$ID

filtered_data_with_info_6000_W <- Dataset_woillard_6000 %>%
  dplyr::filter(ID %in% ids_to_keep_6000_W) %>%
  select(ID, CYP) %>%
  distinct(ID, .keep_all = TRUE) %>%
```

```
  mutate(
    Group_of_dose = case_when(
      ID >= 1 & ID <= 400 ~ "1 mg",
      ID >= 401 & ID <= 800 ~ "2 mg",
      ID >= 801 & ID <= 1200 ~ "3 mg",
      ID >= 1201 & ID <= 1600 ~ "4 mg",
      ID >= 1601 & ID <= 2000 ~ "5 mg",
      ID >= 2001 & ID <= 2400 ~ "6 mg",
      ID >= 2401 & ID <= 2800 ~ "7 mg",
      ID >= 2801 & ID <= 3200 ~ "8 mg",
      ID >= 3201 & ID <= 3600 ~ "9 mg",
      ID >= 3601 & ID <= 4000 ~ "10 mg",
      ID >= 4001 & ID <= 4400 ~ "11 mg",
      ID >= 4401 & ID <= 4800 ~ "12 mg",
      ID >= 4801 & ID <= 5200 ~ "13 mg",
      ID >= 5201 & ID <= 5600 ~ "14 mg",
      ID >= 5601 & ID <= 6000 ~ "15 mg",
      TRUE ~ NA_character_
    )
  )


ids_to_keep_12000_W <- sim_data_filtered_12000_Woillard$ID

filtered_data_with_info_12000_W <- Dataset_woillard_12000 %>%
  dplyr::filter(ID %in% ids_to_keep_12000_W) %>%
  select(ID, CYP) %>%
  distinct(ID, .keep_all = TRUE) %>%
  mutate(
    Group_of_dose = case_when(
      ID >= 6001 & ID <= 6400 ~ "0,5 mg",
      ID >= 6401 & ID <= 6800 ~ "1,5 mg",
      ID >= 6801 & ID <= 7200 ~ "2,5 mg",
      ID >= 7201 & ID <= 7600 ~ "3,5 mg",
      ID >= 7601 & ID <= 8000 ~ "4,5 mg",
      ID >= 8001 & ID <= 8400 ~ "5,5 mg",
      ID >= 8401 & ID <= 8800 ~ "6,5 mg",
      ID >= 8801 & ID <= 9200 ~ "7,5 mg",
      ID >= 9201 & ID <= 9600 ~ "8,5 mg",
      ID >= 9601 & ID <= 10000 ~ "9,5 mg",
      ID >= 10001 & ID <= 10400 ~ "10,5 mg",
      ID >= 10401 & ID <= 10800 ~ "11,5 mg",
      ID >= 10801 & ID <= 11200 ~ "12,5 mg",
      ID >= 11201 & ID <= 11600 ~ "13,5 mg",
      ID >= 11601 & ID <= 12000 ~ "14,5 mg",
      TRUE ~ NA_character_
    )
  )

filtered_data_with_CYP_W <- bind_rows(filtered_data_with_info_6000_W, filtered_dat
```

```
a_with_info_12000_W)

filtered_data_with_CYP_W
```

```
## # A tibble: 4,447 × 3
##       ID   CYP Group_of_dose
##    <int> <dbl> <chr>
##  1   404     0 2 mg
##  2   405     0 2 mg
##  3   417     0 2 mg
##  4   426     0 2 mg
##  5   427     0 2 mg
##  6   428     0 2 mg
##  7   437     0 2 mg
##  8   442     0 2 mg
##  9   444     0 2 mg
## 10   450     0 2 mg
## # ℹ 4,437 more rows
```

#Distribution of doses received by selected non-expressors :

```
filtered_non_expresseurs <- filtered_data_with_CYP_W %>%
  dplyr::filter(CYP == 0) %>%
    mutate(
    Group_of_dose = factor(
      Group_of_dose,
      levels = c(
        "0,5 mg", "1 mg", "1,5 mg", "2 mg", "2,5 mg", "3 mg",
        "3,5 mg", "4 mg", "4,5 mg", "5 mg", "5,5 mg", "6 mg",
        "6,5 mg", "7 mg", "7,5 mg", "8 mg", "8,5 mg", "9 mg",
        "9,5 mg", "10 mg", "10,5 mg", "11 mg", "11,5 mg", "12 mg",
        "12,5 mg", "13 mg", "13,5 mg", "14 mg", "14,5 mg", "15 mg")))

ggplot(filtered_non_expresseurs, aes(x = Group_of_dose)) +
  geom_bar(fill = "grey", color = "black") +
  geom_text(
    stat = "count",
    aes(label = ..count..),
    vjust = -0.5,
    size = 3
  ) +
  labs(
    title = "                               CYP3A5 non-expressors, Woillard mode
l",
    x = "Dose group",
    y = "Number of pharmacokinetic profiles"
  ) +
  theme_minimal() +
  theme(axis.text.x = element_text(angle = 45, hjust = 1))
```

```
## Warning: The dot-dot notation (`..count..`) was deprecated in ggplot2 3.4.0.
## ℹ Please use `after_stat(count)` instead.
## This warning is displayed once every 8 hours.
## Call `lifecycle::last_lifecycle_warnings()` to see where this warning was
## generated.
```

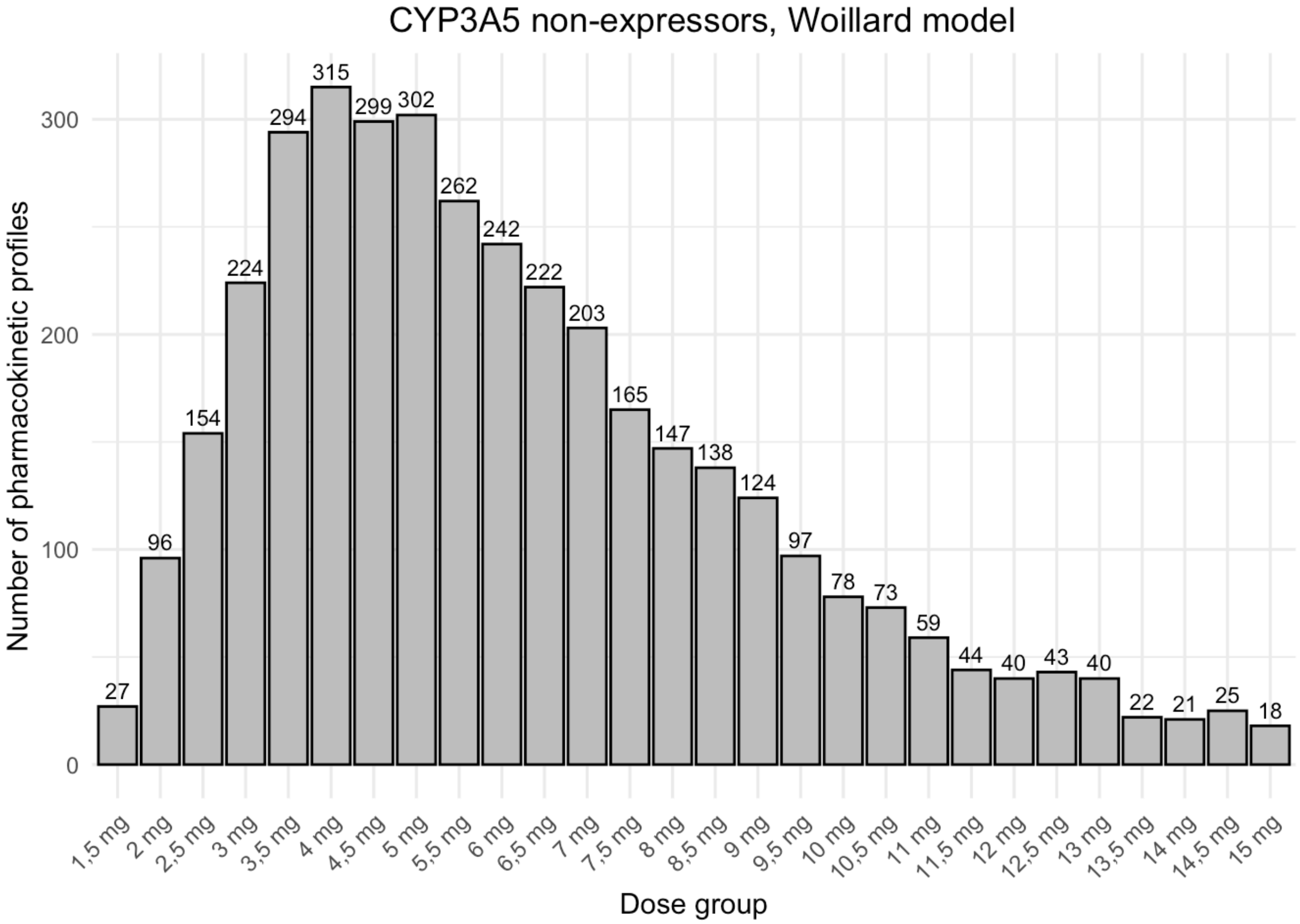

#Distribution of doses received by selected expressors :

```
filtered_expresseurs <- filtered_data_with_CYP_W %>%
  dplyr::filter(CYP == 1) %>%
    mutate(
    Group_of_dose = factor(
      Group_of_dose,
      levels = c(
        "0,5 mg", "1 mg", "1,5 mg", "2 mg", "2,5 mg", "3 mg",
        "3,5 mg", "4 mg", "4,5 mg", "5 mg", "5,5 mg", "6 mg",
        "6,5 mg", "7 mg", "7,5 mg", "8 mg", "8,5 mg", "9 mg",
        "9,5 mg", "10 mg", "10,5 mg", "11 mg", "11,5 mg", "12 mg",
        "12,5 mg", "13 mg", "13,5 mg", "14 mg", "14,5 mg", "15 mg")))

ggplot(filtered_expresseurs, aes(x = Group_of_dose)) +
  geom_bar(fill = "grey", color = "black") +
  geom_text(
    stat = "count",
    aes(label = ..count..),
    vjust = -0.5,
    size = 3
  ) +
  labs(
    title = "                               CYP3A5 expressors, Woillard model",
    x = "Dose group",
    y = "Number of pharmacokinetic profiles"
  ) +
  theme_minimal() +
  scale_y_continuous(limits = c(0, 50), expand = expansion(mult = c(0, 0.05))) +
  theme(axis.text.x = element_text(angle = 45, hjust = 1))
```

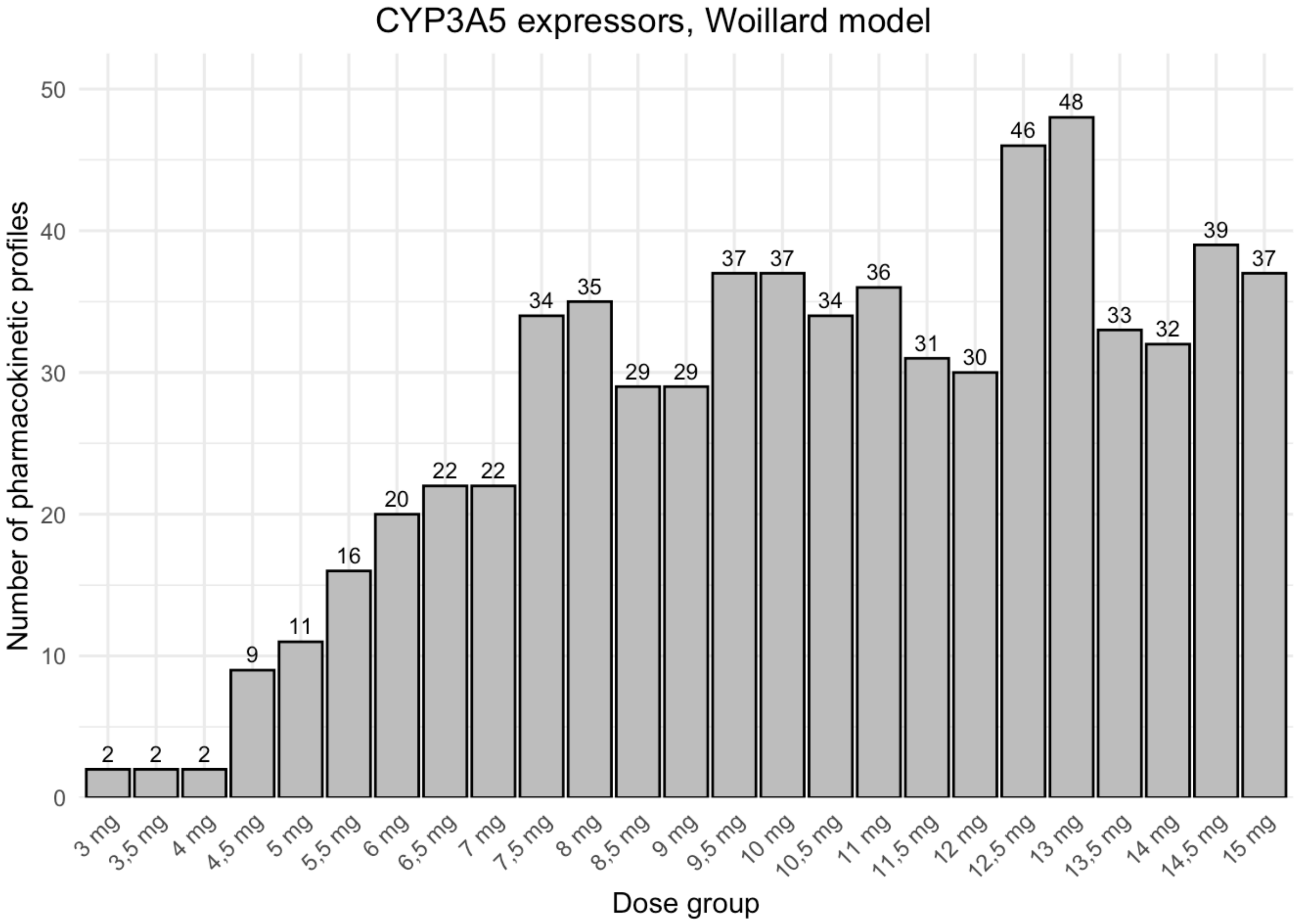

#Average dose received by non-expressors

```
filtered_non_expresseurs_W <- filtered_data_with_CYP_W %>%
  dplyr::filter(CYP == 0) %>%
  mutate(
    Dose_numeric = as.numeric(sub(" mg", "", gsub(",", ".", Group_of_dose)))
  )

dose_stats_non_expresseurs_W <- filtered_non_expresseurs_W %>%
  summarize(
    Mean_dose = mean(Dose_numeric, na.rm = TRUE),
    SD_dose = sd(Dose_numeric, na.rm = TRUE)
  )

dose_stats_non_expresseurs_W
```

```
## # A tibble: 1 × 2
##   Mean_dose SD_dose
##       <dbl>   <dbl>
## 1      6.17    2.87
```

#Average dose received by expressors

```
filtered_expresseurs_W <- filtered_data_with_CYP_W %>%
  dplyr::filter(CYP == 1) %>%
  mutate(
    Dose_numeric = as.numeric(sub(" mg", "", gsub(",", ".", Group_of_dose)))
  )

dose_stats_expresseurs_W <- filtered_expresseurs_W %>%
  summarize(
    Mean_dose = mean(Dose_numeric, na.rm = TRUE),
    SD_dose = sd(Dose_numeric, na.rm = TRUE)
  )

dose_stats_expresseurs_W
```

```
## # A tibble: 1 × 2
##   Mean_dose SD_dose
##       <dbl>   <dbl>
## 1      10.5    2.93
```

Time separation by simulated event (6000 profiles)

```
sim_data_by_scenario_6000_W <- sim_data_filtered_6000_Woillard %>%
  mutate(
    forget_event = case_when(
      time >= 0 & time < 312 ~ "no_forget",
            time >= 312 & time < 624 ~ "forget_3h_retake_x1dose",
      time >= 624 & time < 936 ~ "forget_6h_retake_x1dose",
            time >= 936 & time < 1248 ~ "forget_9h_retake_x1dose",
      time >= 1248 & time < 1560 ~ "forget_12h_retake_x1dose",
            time >= 1560 & time < 1872 ~ "forget_15h_retake_x0.5dose",
      time >= 1872 & time < 2184 ~ "forget_15h_retake_x1dose",
            time >= 2184 & time < 2496 ~ "forget_18h_retake_x0.5dose",
      time >= 2496 & time < 2808 ~ "forget_18h_retake_x1dose",
      time >= 2808 & time < 3120 ~ "forget_21h_retake_x0.5dose",
            time >= 3120 & time < 3432 ~ "forget_21h_retake_x1dose",
      time >= 3456 & time < 3768 ~ "forget_24h_retake_x1dose",
      time >= 3788 & time < 4104 ~ "forget_24h_retake_x1.5dose",
            time >= 4128 & time < 4440 ~ "forget_24h_retake_x2dose"))

sim_data_by_scenario_6000_W
```

```
## # A tibble: 15,752,250 × 4
##       ID  time    DV forget_event
##    <dbl> <dbl> <dbl> <chr>
##  1   404     0  5.37 no_forget
##  2   404     1  9.48 no_forget
##  3   404     2 10.8  no_forget
##  4   404     3  9.99 no_forget
##  5   404     4  9.37 no_forget
##  6   404     5  8.97 no_forget
##  7   404     6  8.67 no_forget
##  8   404     7  8.40 no_forget
##  9   404     8  8.17 no_forget
## 10   404     9  7.94 no_forget
## # ℹ 15,752,240 more rows
```

Time separation by simulated event (12000 profiles)

```
sim_data_by_scenario_12000_W <- sim_data_filtered_12000_Woillard %>%
  mutate(
    forget_event = case_when(
      time >= 0 & time < 312 ~ "no_forget",
            time >= 312 & time < 624 ~ "forget_3h_retake_x1dose",
      time >= 624 & time < 936 ~ "forget_6h_retake_x1dose",
            time >= 936 & time < 1248 ~ "forget_9h_retake_x1dose",
      time >= 1248 & time < 1560 ~ "forget_12h_retake_x1dose",
            time >= 1560 & time < 1872 ~ "forget_15h_retake_x0.5dose",
      time >= 1872 & time < 2184 ~ "forget_15h_retake_x1dose",
            time >= 2184 & time < 2496 ~ "forget_18h_retake_x0.5dose",
      time >= 2496 & time < 2808 ~ "forget_18h_retake_x1dose",
      time >= 2808 & time < 3120 ~ "forget_21h_retake_x0.5dose",
            time >= 3120 & time < 3432 ~ "forget_21h_retake_x1dose",
      time >= 3456 & time < 3768 ~ "forget_24h_retake_x1dose",
      time >= 3788 & time < 4104 ~ "forget_24h_retake_x1.5dose",
            time >= 4128 & time < 4440 ~ "forget_24h_retake_x2dose"))

sim_data_by_scenario_12000_W
```

```
## # A tibble: 15,381,197 × 4
##        ID  time    DV forget_event
##     <dbl> <dbl> <dbl> <chr>
##  1  6404     0  4.03 no_forget
##  2  6404     1  7.11 no_forget
##  3  6404     2  8.07 no_forget
##  4  6404     3  7.49 no_forget
##  5  6404     4  7.03 no_forget
##  6  6404     5  6.72 no_forget
##  7  6404     6  6.50 no_forget
##  8  6404     7  6.30 no_forget
##  9  6404     8  6.13 no_forget
## 10  6404     9  5.96 no_forget
## # ℹ 15,381,187 more rows
```

Fusion des dataframes

```
Final_data_combined_Woillard <- rbind(sim_data_by_scenario_6000_W, sim_data_by_sce
nario_12000_W)

Final_data_combined_Woillard
```

```
## # A tibble: 31,133,447 × 4
##        ID  time    DV forget_event
##     <dbl> <dbl> <dbl> <chr>
##  1   404     0  5.37 no_forget
##  2   404     1  9.48 no_forget
##  3   404     2 10.8  no_forget
##  4   404     3  9.99 no_forget
##  5   404     4  9.37 no_forget
##  6   404     5  8.97 no_forget
##  7   404     6  8.67 no_forget
##  8   404     7  8.40 no_forget
##  9   404     8  8.17 no_forget
## 10   404     9  7.94 no_forget
## # ℹ 31,133,437 more rows
```

#C0 measurements for each profile every 24 hours

```
time_intervals <- list(
  c(24, 312), c(336, 624), c(648, 936), c(960, 1248),
  c(1272, 1560), c(1584, 1872), c(1896, 2184), c(2208, 2496),
  c(2520, 2808), c(2832, 3120), c(3144, 3432), c(3456, 3768),
  c(3772, 4104), c(4128, 4440)
)

filter_by_interval <- function(forget_event_group, interval) {
  Final_data_combined_Woillard %>%
    dplyr::filter(forget_event == forget_event_group,
                  time >= interval[1],
                  time < interval[2],
                  time %% 24 == 0)
}

forget_events <- unique(Final_data_combined_Woillard$forget_event)

results_final_C0_scenario_W <- map_dfr(forget_events, function(event) {
  map_dfr(time_intervals, function(interval) {
    filter_by_interval(event, interval)
  })
})

results_final_C0_scenario_W <- results_final_C0_scenario_W %>%
  arrange(forget_event, time)

print(results_final_C0_scenario_W)
```

```
## # A tibble: 760,437 × 4
##       ID  time    DV forget_event
##    <dbl> <dbl> <dbl> <chr>
##  1   404  1272  5.39 forget_12h_retake_x1dose
##  2   405  1272  4.64 forget_12h_retake_x1dose
##  3   417  1272  4.78 forget_12h_retake_x1dose
##  4   426  1272  5.91 forget_12h_retake_x1dose
##  5   427  1272  4.06 forget_12h_retake_x1dose
##  6   428  1272  4.23 forget_12h_retake_x1dose
##  7   437  1272  6.43 forget_12h_retake_x1dose
##  8   442  1272  4.13 forget_12h_retake_x1dose
##  9   444  1272  6.65 forget_12h_retake_x1dose
## 10   450  1272  4.12 forget_12h_retake_x1dose
## # ℹ 760,427 more rows
```

Calcul des différences relatives de C0 pour chaque patient

```
C0_basale_by_scenario_W <- results_final_C0_scenario_W %>%
  group_by(ID, forget_event) %>%
  arrange(time) %>%
  mutate(C0_basale = first(DV)) %>%
  ungroup()

Relative_C0_change_by_scenario_W <- C0_basale_by_scenario_W %>%
  group_by(forget_event) %>%
  mutate(relative_C0 = (DV - C0_basale) / C0_basale * 100) %>%
  ungroup()

Relative_C0_change_by_scenario_W
```

```
## # A tibble: 760,437 × 6
##       ID  time    DV forget_event C0_basale relative_C0
##    <dbl> <dbl> <dbl> <chr>            <dbl>       <dbl>
##  1   404    24  5.38 no_forget         5.38           0
##  2   405    24  4.64 no_forget         4.64           0
##  3   417    24  4.77 no_forget         4.77           0
##  4   426    24  5.91 no_forget         5.91           0
##  5   427    24  4.06 no_forget         4.06           0
##  6   428    24  4.23 no_forget         4.23           0
##  7   437    24  6.43 no_forget         6.43           0
##  8   442    24  4.13 no_forget         4.13           0
##  9   444    24  6.66 no_forget         6.66           0
## 10   450    24  4.12 no_forget         4.12           0
## # ℹ 760,427 more rows
```

Rename time for each group of forget_event

```
Data_with_time_renamed_W <- Relative_C0_change_by_scenario_W %>%
  mutate(time_adjusted = case_when(
    time >= 24 & time < 312 ~ paste0((time - 24) / 24 * 24 + 24, "h"),
    time >= 336 & time < 624 ~ paste0((time - 336) / 24 * 24 + 24, "h"),
    time >= 648 & time < 936 ~ paste0((time - 648) / 24 * 24 + 24, "h"),
    time >= 960 & time < 1248 ~ paste0((time - 960) / 24 * 24 + 24, "h"),
    time >= 1272 & time < 1560 ~ paste0((time - 1272) / 24 * 24 + 24, "h"),
    time >= 1584 & time < 1872 ~ paste0((time - 1584) / 24 * 24 + 24, "h"),
    time >= 1896 & time < 2184 ~ paste0((time - 1896) / 24 * 24 + 24, "h"),
    time >= 2208 & time < 2496 ~ paste0((time - 2208) / 24 * 24 + 24, "h"),
    time >= 2520 & time < 2808 ~ paste0((time - 2520) / 24 * 24 + 24, "h"),
    time >= 2832 & time < 3120 ~ paste0((time - 2832) / 24 * 24 + 24, "h"),
    time >= 3144 & time < 3432 ~ paste0((time - 3144) / 24 * 24 + 24, "h"),

      time >= 3456 & time < 3768 ~ paste0((time - 3456) / 24 * 24 + 24, "h"),
    time >= 3792 & time < 4104 ~ paste0((time - 3792) / 24 * 24 + 24, "h"),
    time >= 4128 & time < 4440 ~ paste0((time - 4128) / 24 * 24 + 24, "h"),

    TRUE ~ paste0(time, "h")
  ))

print(Data_with_time_renamed_W)
```

```
## # A tibble: 760,437 × 7
##       ID  time    DV forget_event C0_basale relative_C0 time_adjusted
##    <dbl> <dbl> <dbl> <chr>            <dbl>       <dbl> <chr>
##  1   404    24  5.38 no_forget         5.38           0 24h
##  2   405    24  4.64 no_forget         4.64           0 24h
##  3   417    24  4.77 no_forget         4.77           0 24h
##  4   426    24  5.91 no_forget         5.91           0 24h
##  5   427    24  4.06 no_forget         4.06           0 24h
##  6   428    24  4.23 no_forget         4.23           0 24h
##  7   437    24  6.43 no_forget         6.43           0 24h
##  8   442    24  4.13 no_forget         4.13           0 24h
##  9   444    24  6.66 no_forget         6.66           0 24h
## 10   450    24  4.12 no_forget         4.12           0 24h
## # ℹ 760,427 more rows
```

#Average statistics of C0 and RD: all profiles

```
summary_stats_expanded_W <- Data_with_time_renamed_W %>%
  group_by(forget_event, time_adjusted) %>%
  summarize(
    min_DV = fivenum(DV)[1],        # Minimum
    Q1_DV = fivenum(DV)[2],         # First quartile (Q1)
    median_DV = fivenum(DV)[3],     # Median (Q2)
    Q3_DV = fivenum(DV)[4],         # Third quartile (Q3)
    max_DV = fivenum(DV)[5],        # Maximum
    mean_DV = mean(DV, na.rm = TRUE),  # Mean
    sd_DV = sd(DV, na.rm = TRUE),      # SD

    min_relative_C0 = fivenum(relative_C0)[1],        # Minimum
    Q1_relative_C0 = fivenum(relative_C0)[2],         # First quartile (Q1)
    median_relative_C0 = fivenum(relative_C0)[3],     # Median (Q2)
    Q3_relative_C0 = fivenum(relative_C0)[4],         # Third quartile (Q3)
    max_relative_C0 = fivenum(relative_C0)[5],        # Maximum
    mean_relative_C0 = mean(relative_C0, na.rm = TRUE),  # Mean
    sd_relative_C0 = sd(relative_C0, na.rm = TRUE)       # SD
  ) %>%
  ungroup() %>%
  arrange(forget_event, time_adjusted)
```

```
## `summarise()` has grouped output by 'forget_event'. You can override using the
## `.groups` argument.
```

```
summary_stats_expanded_W
```

```
## # A tibble: 171 × 16
##    forget_event  time_adjusted min_DV Q1_DV median_DV Q3_DV max_DV mean_DV sd_D
V
##    <chr>         <chr>          <dbl> <dbl>     <dbl> <dbl>  <dbl>   <dbl> <dbl
>
##  1 forget_12h_r… 120h            4.02  5.94      7.82  9.87   12.3    7.92  2.3
1
##  2 forget_12h_r… 144h            4.01  5.88      7.74  9.76   12.2    7.84  2.2
8
##  3 forget_12h_r… 168h            4.00  5.85      7.71  9.71   12.1    7.80  2.2
7
##  4 forget_12h_r… 192h            3.99  5.83      7.69  9.68   12.1    7.78  2.2
6
##  5 forget_12h_r… 216h            4.00  5.82      7.68  9.67   12.0    7.76  2.2
6
##  6 forget_12h_r… 240h            3.99  5.82      7.66  9.66   12.0    7.76  2.2
6
##  7 forget_12h_r… 24h             4.00  5.81      7.66  9.65   12.0    7.75  2.2
5
##  8 forget_12h_r… 264h            3.99  5.82      7.67  9.65   12.0    7.75  2.2
6
##  9 forget_12h_r… 288h            3.99  5.82      7.66  9.65   12.0    7.75  2.2
5
## 10 forget_12h_r… 48h             4.14  7.43      9.64 12.0    22.4    9.79  2.9
4
## # ℹ 161 more rows
## # ℹ 7 more variables: min_relative_C0 <dbl>, Q1_relative_C0 <dbl>,
## #   median_relative_C0 <dbl>, Q3_relative_C0 <dbl>, max_relative_C0 <dbl>,
## #   mean_relative_C0 <dbl>, sd_relative_C0 <dbl>
```

#Histograms of mean C0 and mean RD by simulated event

```
desired_order <- c("no_forget",
                   "forget_3h_retake_x1dose",
                   "forget_6h_retake_x1dose",
                   "forget_9h_retake_x1dose",
                   "forget_12h_retake_x1dose",
                   "forget_15h_retake_x0.5dose",
                   "forget_15h_retake_x1dose",
                   "forget_18h_retake_x0.5dose",
                   "forget_18h_retake_x1dose",
                   "forget_21h_retake_x0.5dose",
                   "forget_21h_retake_x1dose",
                   "forget_24h_retake_x1dose",
                   "forget_24h_retake_x1.5dose",
                   "forget_24h_retake_x2dose")

summary_stats_expanded_W$forget_event <- factor(summary_stats_expanded_W$forget_ev
ent, levels = desired_order)
```

```
time_adjusted_order <- c("24h", "48h", "72h", "96h", "120h", "144h", "168h", "192
h",
                         "216h", "240h", "264h", "288h", "312h")

summary_stats_expanded_W <- summary_stats_expanded_W %>%
  mutate(time_adjusted = factor(time_adjusted, levels = time_adjusted_order))

forget_events_W_C0 <- levels(summary_stats_expanded_W$forget_event)

for (event in forget_events_W_C0) {
  data_subset_W_C0 <- summary_stats_expanded_W %>%
    dplyr::filter(forget_event == event)

  p6 <- ggplot(data_subset_W_C0, aes(x = time_adjusted, y = mean_DV)) +
    geom_bar(stat = "identity", fill = "grey", color = "black") +
    geom_text(aes(label = round(mean_DV, 1)), vjust = -0.4, color = "black") +
    labs(title = paste("Mean residual concentration for :", event),
         x = "Time (h)", y = "Mean Residual Concentration") +
    theme_bw() +
    theme(plot.title = element_text(hjust = 0.5),
          axis.text.x = element_text(angle = 45, hjust = 1))

  print(p6)

  p7 <- ggplot(data_subset_W_C0, aes(x = time_adjusted, y = mean_relative_C0)) +
    geom_bar(stat = "identity", fill = "steelblue", color = "black") +
    geom_text(aes(label = paste0(round(mean_relative_C0, 1), "%")), vjust = -0.4,
color = "black") +
    labs(title = paste("Relative difference of mean through concentration for:", e
vent),
         x = "Time (h)", y = "Mean relative difference of through concentration")
+
    theme_bw() +
    theme(plot.title = element_text(hjust = 0.5),
          axis.text.x = element_text(angle = 45, hjust = 1)) +
    ylim(-100, 170) +
    geom_hline(yintercept = c(-10, 10), linetype = "dashed", color = "red")

  print(p7)

}
```

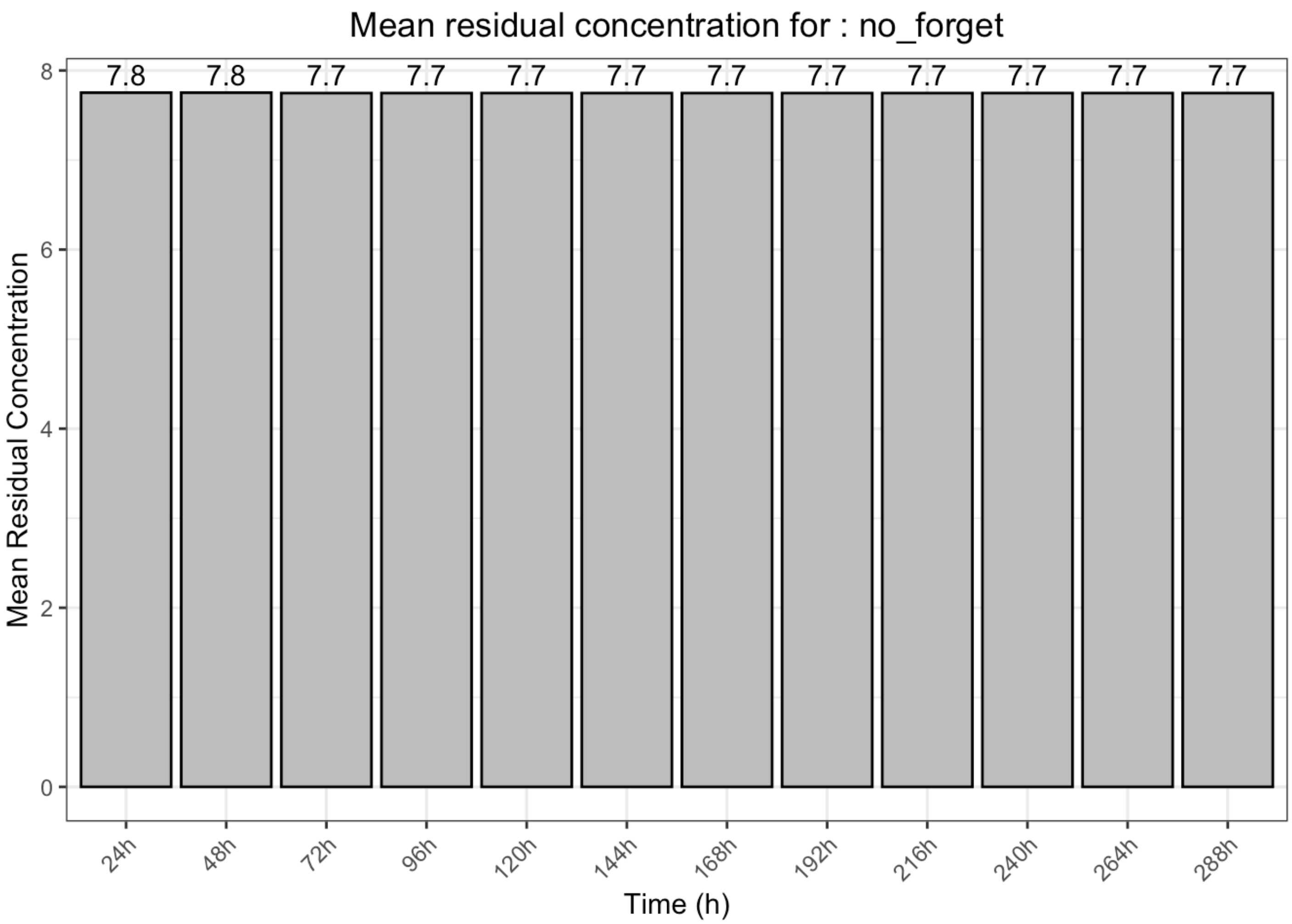
Mean residual concentration for : no_forget
Mean Residual Concentration
Time (h)
7.8 7.8 7.7 7.7 7.7 7.7 7.7 7.7 7.7 7.7 7.7 7.7
24h 48h 72h 96h 120h 144h 168h 192h 216h 240h 264h 288h

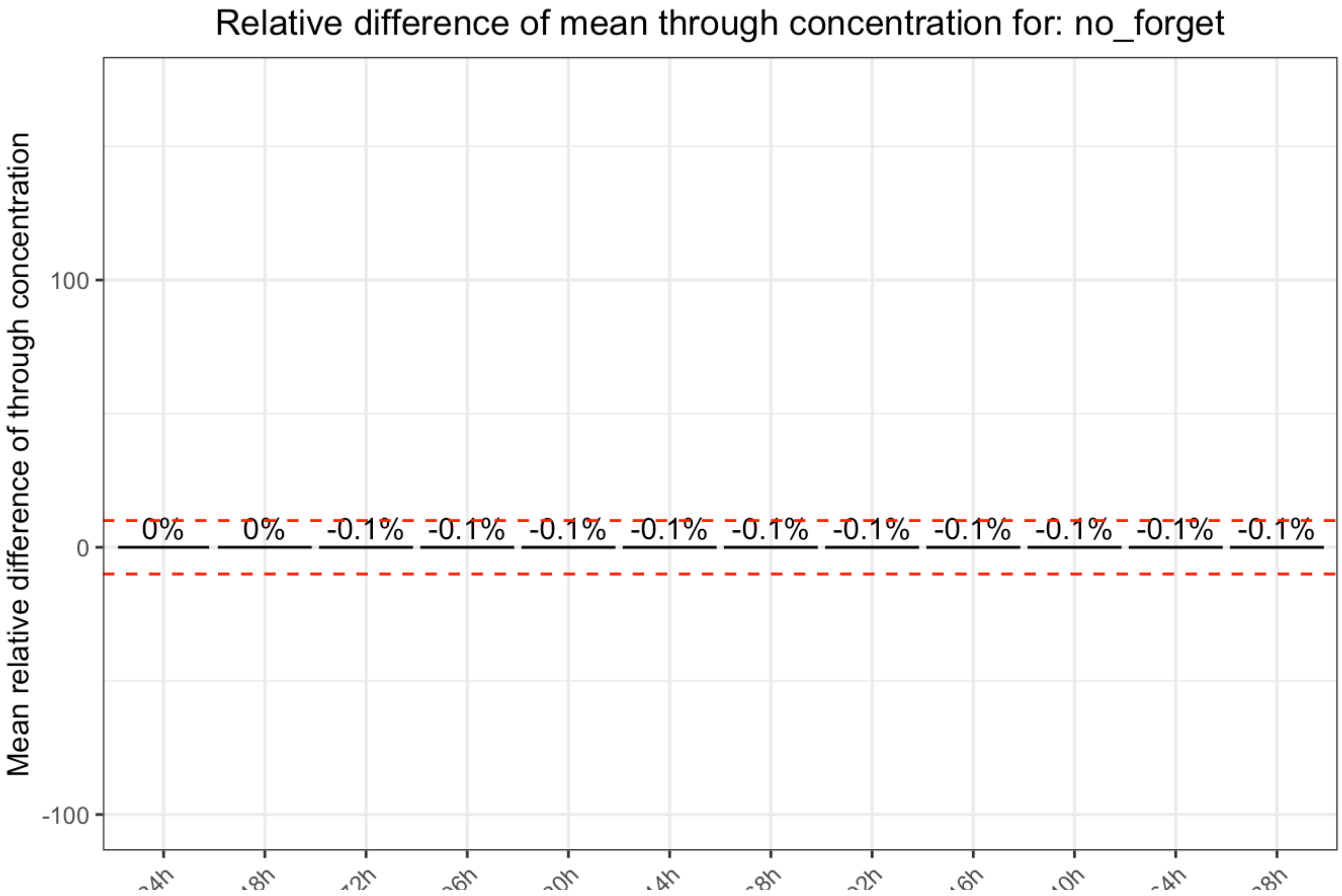
Relative difference of mean through concentration for: no_forget
Mean relative difference of through concentration
0% 0% -0.1% -0.1% -0.1% -0.1% -0.1% -0.1% -0.1% -0.1% -0.1% -0.1%

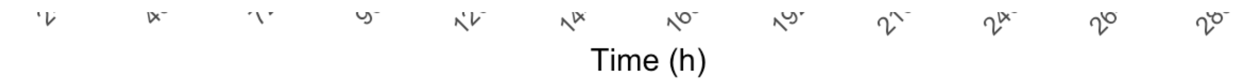
Time (h)

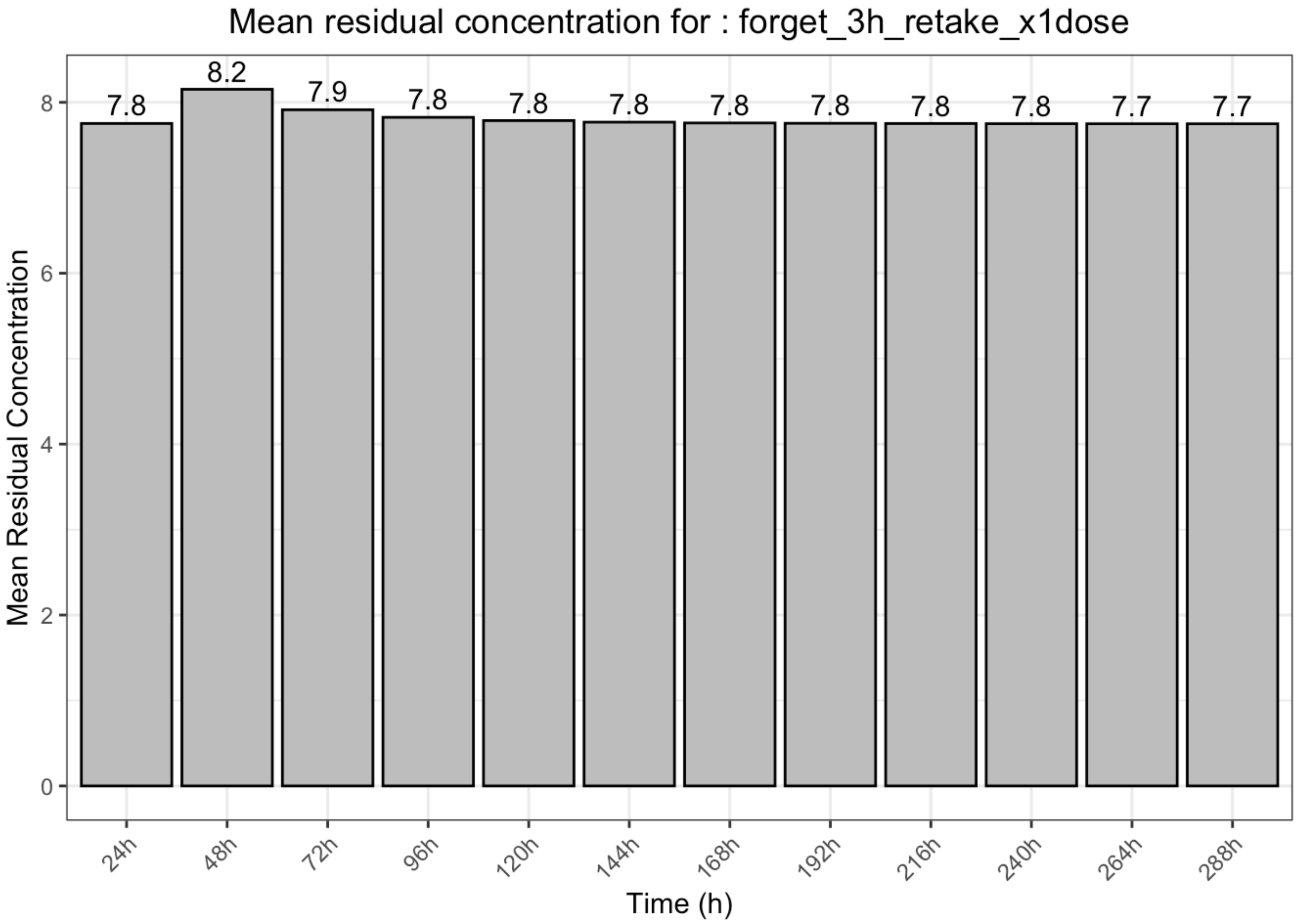
Mean residual concentration for : forget_3h_retake_x1dose
Mean Residual Concentration
Time (h)
0
2
4
6
8
7.8
8.2
7.9
7.8
7.8
7.8
7.8
7.8
7.8
7.8
7.7
7.7
24h
48h
72h
96h
120h
144h
168h
192h
216h
240h
264h
288h

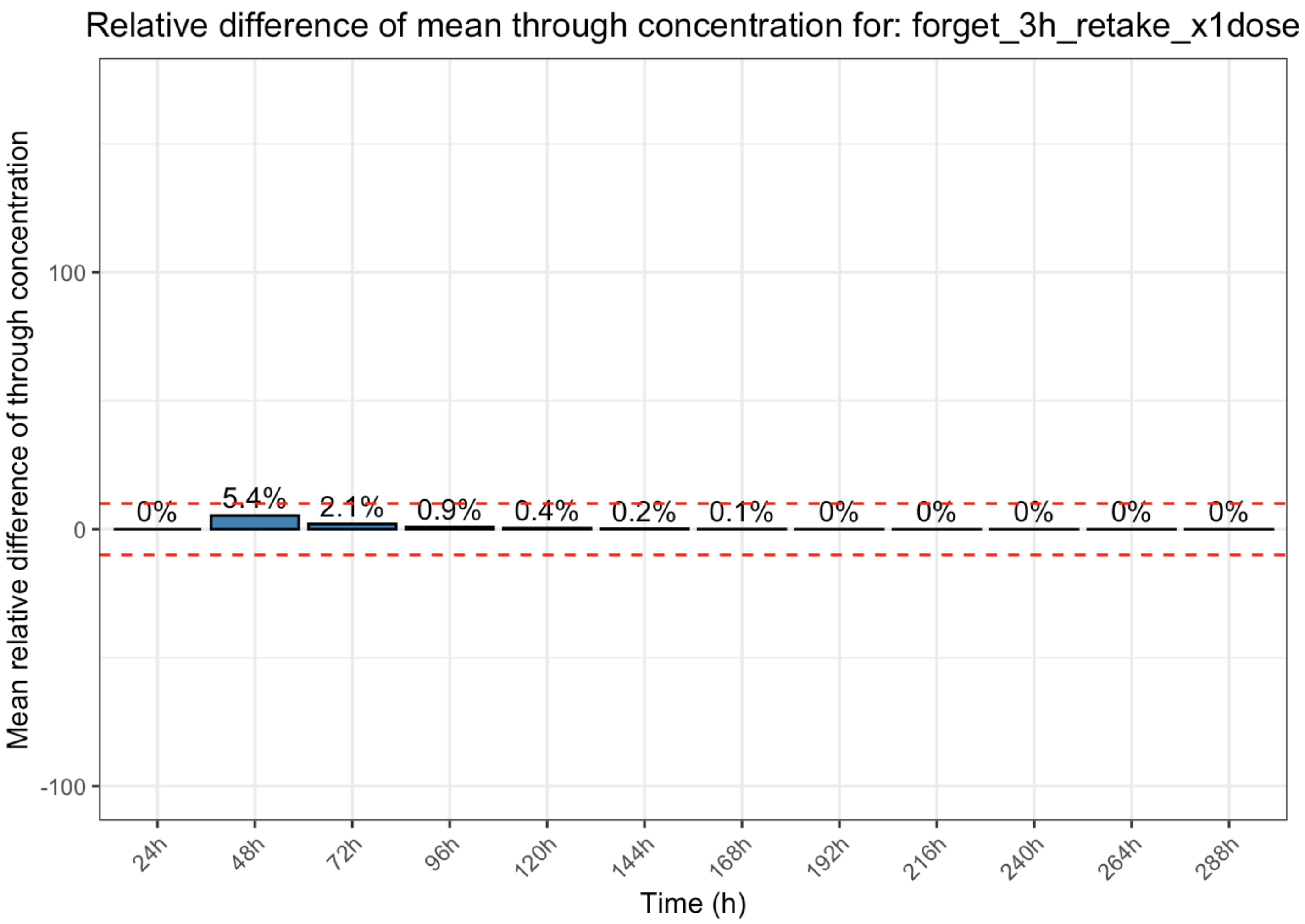
Relative difference of mean through concentration for: forget_3h_retake_x1dose
Mean relative difference of through concentration
100
0
-100
0%
5.4%
2.1%
0.9%
0.4%
0.2%
0.1%
0%
0%
0%
0%
0%
24h
48h
72h
96h
120h
144h
168h
192h
216h
240h
264h
288h
Time (h)

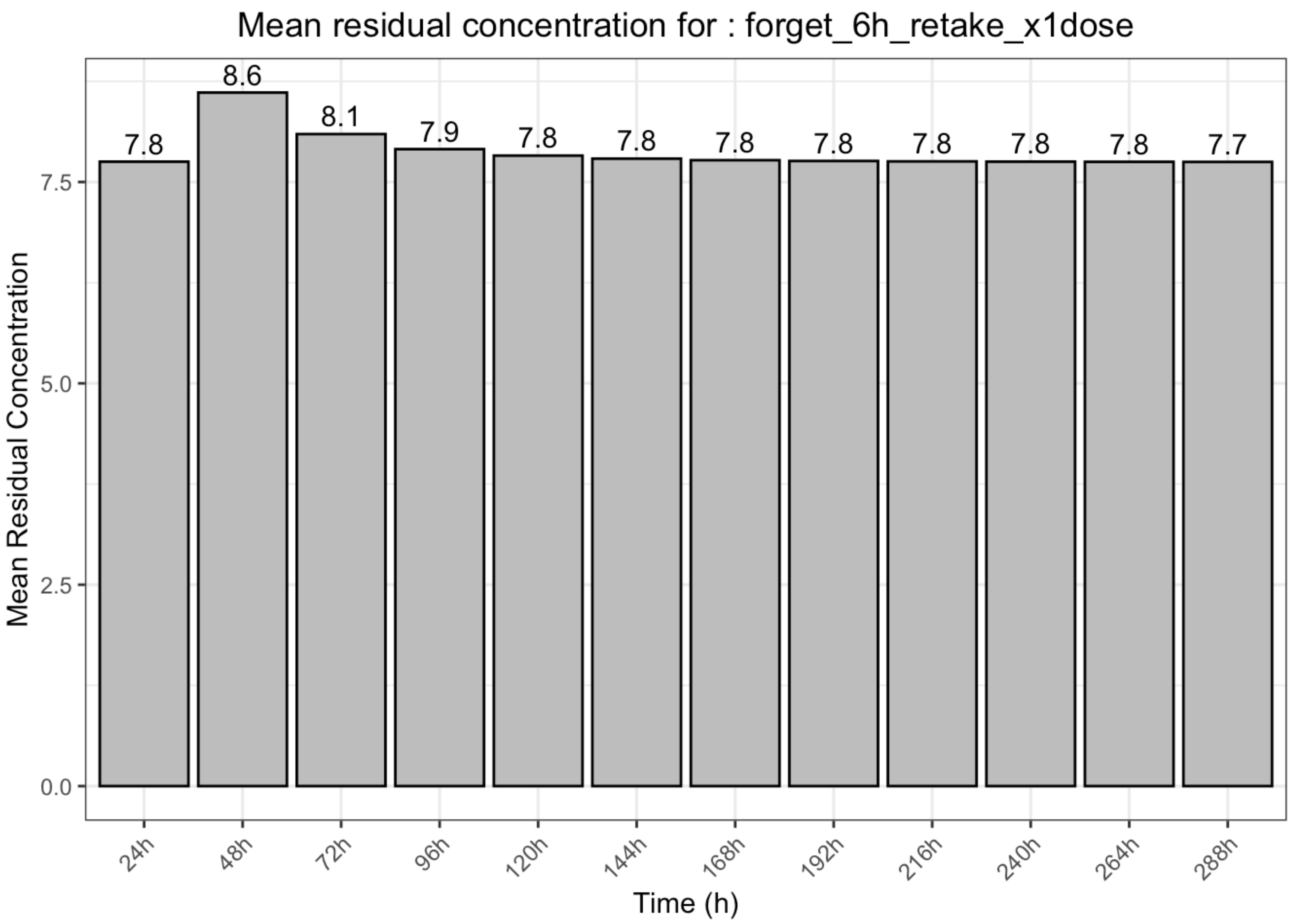
Mean residual concentration for : forget_6h_retake_x1dose
Mean Residual Concentration
Time (h)
7.8
8.6
8.1
7.9
7.8
7.8
7.8
7.8
7.8
7.8
7.8
7.7
0.0
2.5
5.0
7.5
24h
48h
72h
96h
120h
144h
168h
192h
216h
240h
264h
288h

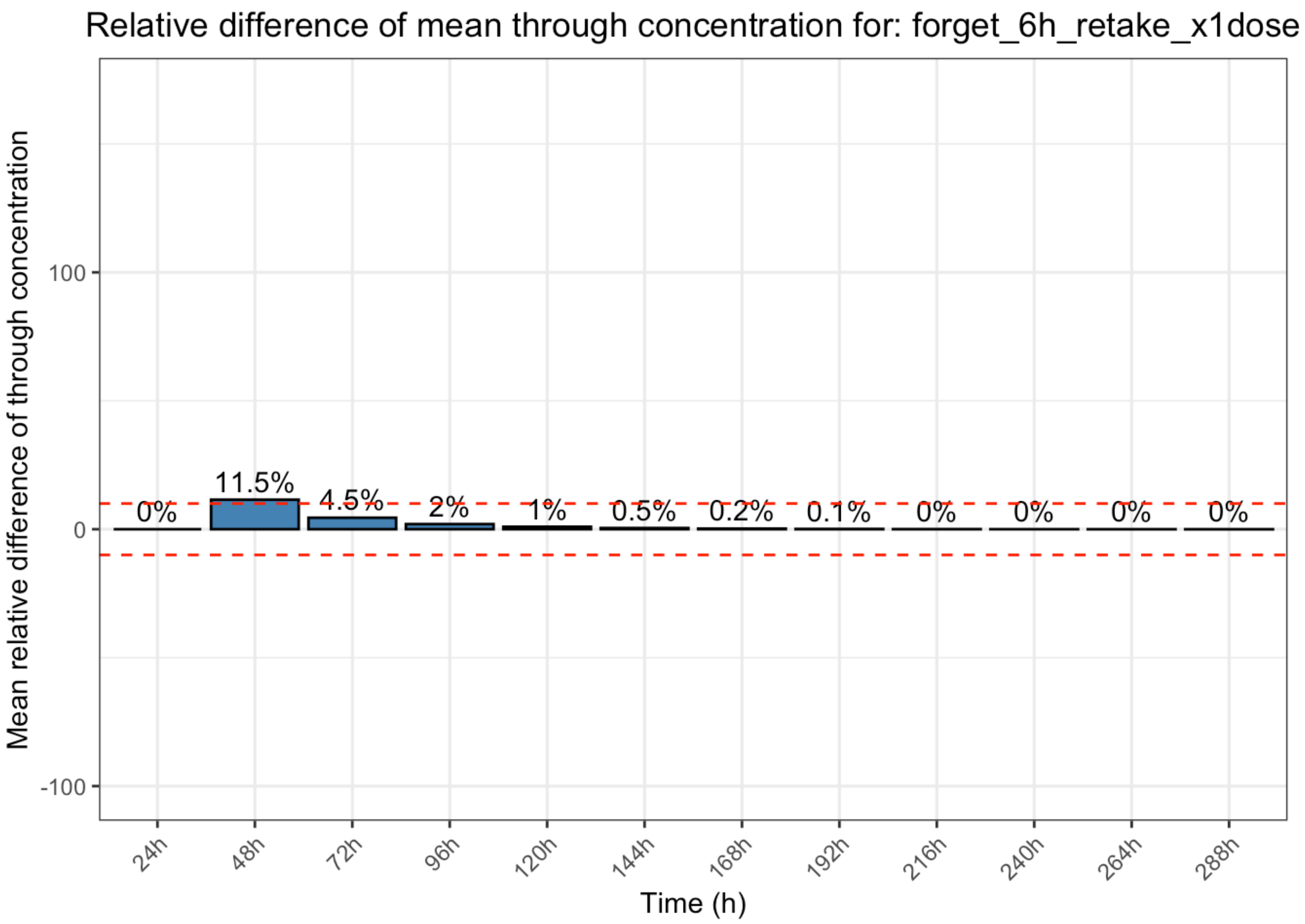
Relative difference of mean through concentration for: forget_6h_retake_x1dose
Mean relative difference of through concentration
100
0
-100
0%
11.5%
4.5%
2%
1%
0.5%
0.2%
0.1%
0%
0%
0%
0%
24h
48h
72h
96h
120h
144h
168h
192h
216h
240h
264h
288h
Time (h)

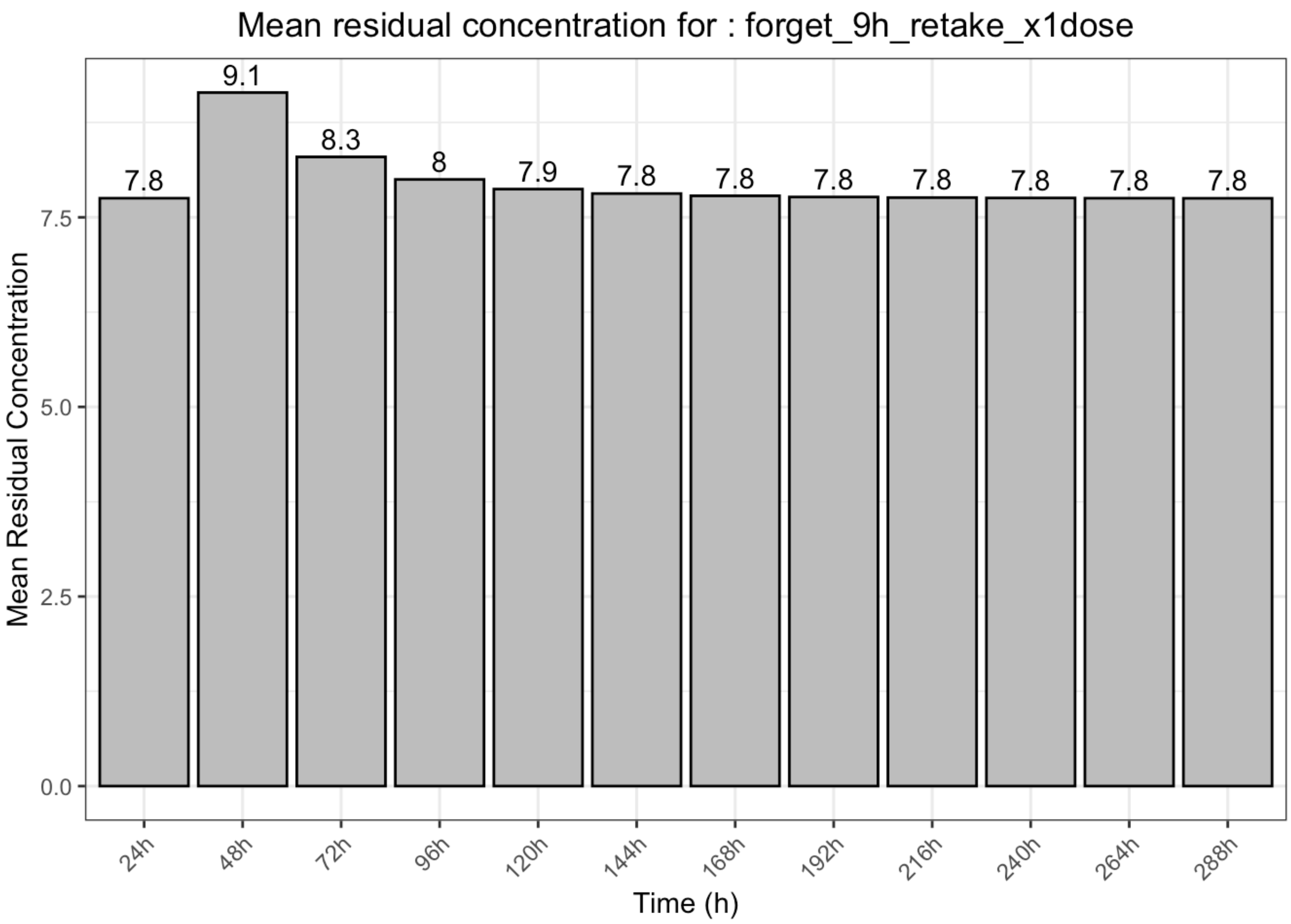
Mean residual concentration for : forget_9h_retake_x1dose
Mean Residual Concentration
Time (h)
0.0
2.5
5.0
7.5
7.8
9.1
8.3
8
7.9
7.8
7.8
7.8
7.8
7.8
7.8
7.8
24h
48h
72h
96h
120h
144h
168h
192h
216h
240h
264h
288h

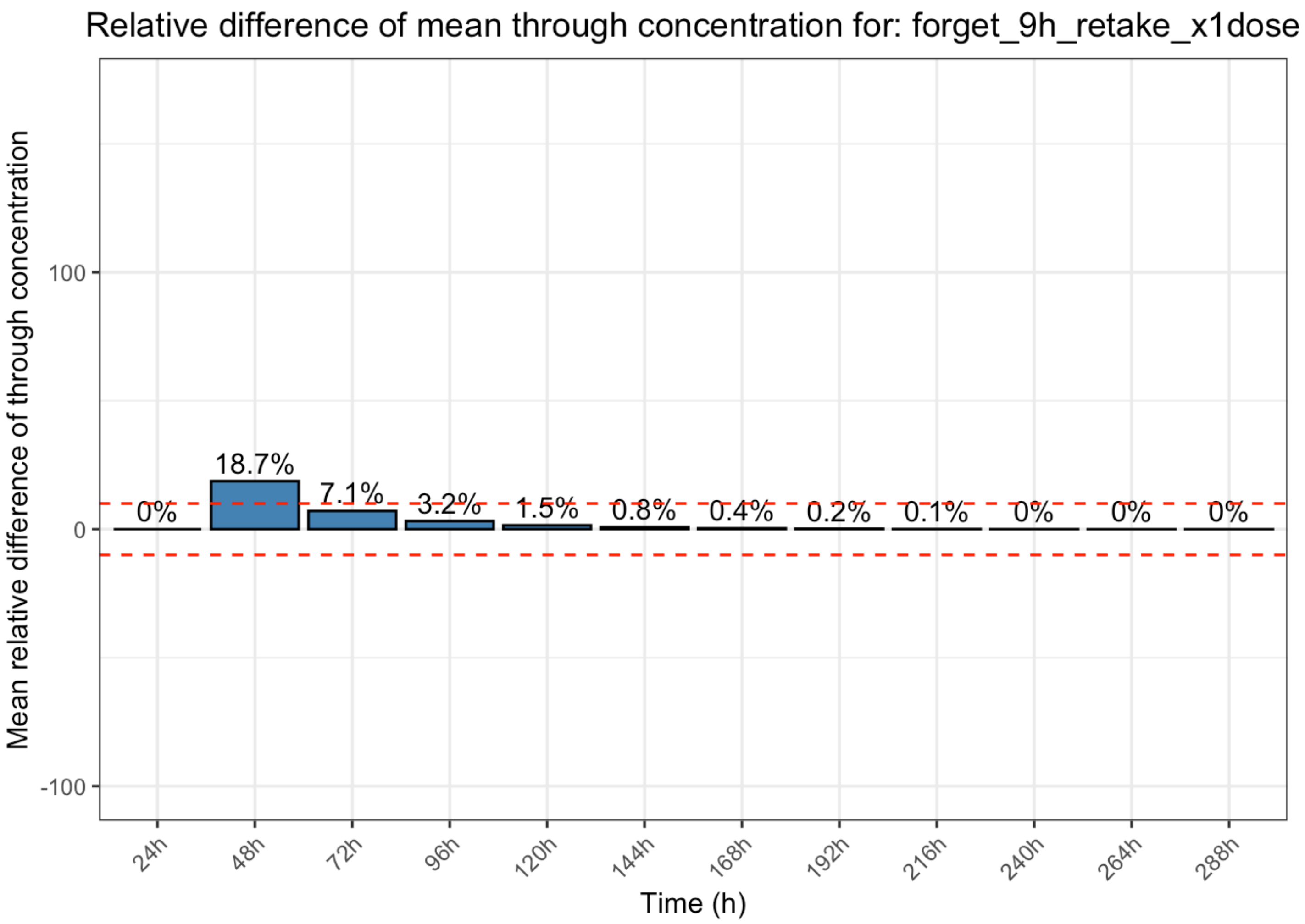
Relative difference of mean through concentration for: forget_9h_retake_x1dose
Mean relative difference of through concentration
100
0
-100
0%
18.7%
7.1%
3.2%
1.5%
0.8%
0.4%
0.2%
0.1%
0%
0%
0%
24h
48h
72h
96h
120h
144h
168h
192h
216h
240h
264h
288h
Time (h)

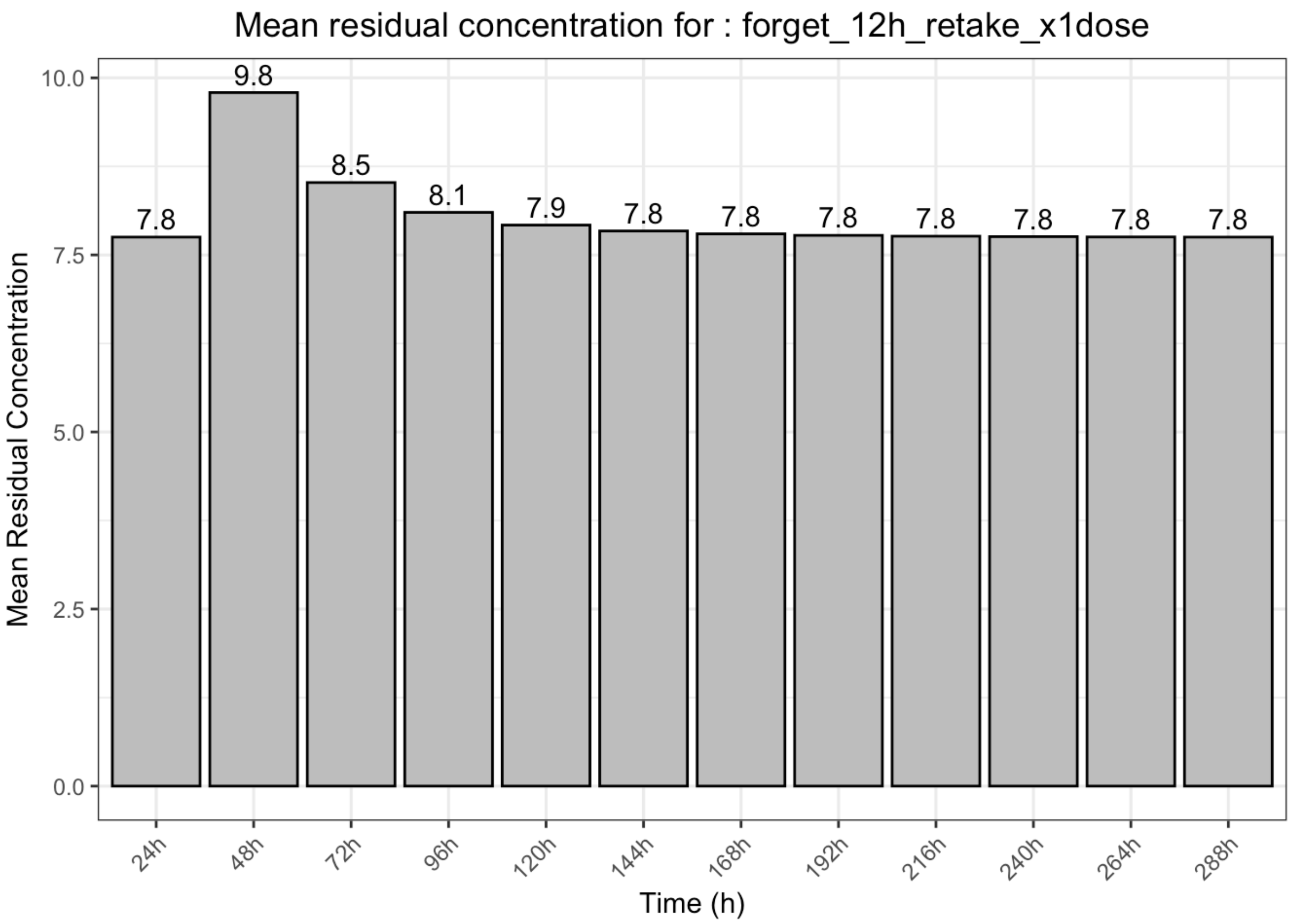
Mean residual concentration for : forget_12h_retake_x1dose
Mean Residual Concentration
10.0
7.5
5.0
2.5
0.0
7.8
9.8
8.5
8.1
7.9
7.8
7.8
7.8
7.8
7.8
7.8
7.8
24h
48h
72h
96h
120h
144h
168h
192h
216h
240h
264h
288h
Time (h)

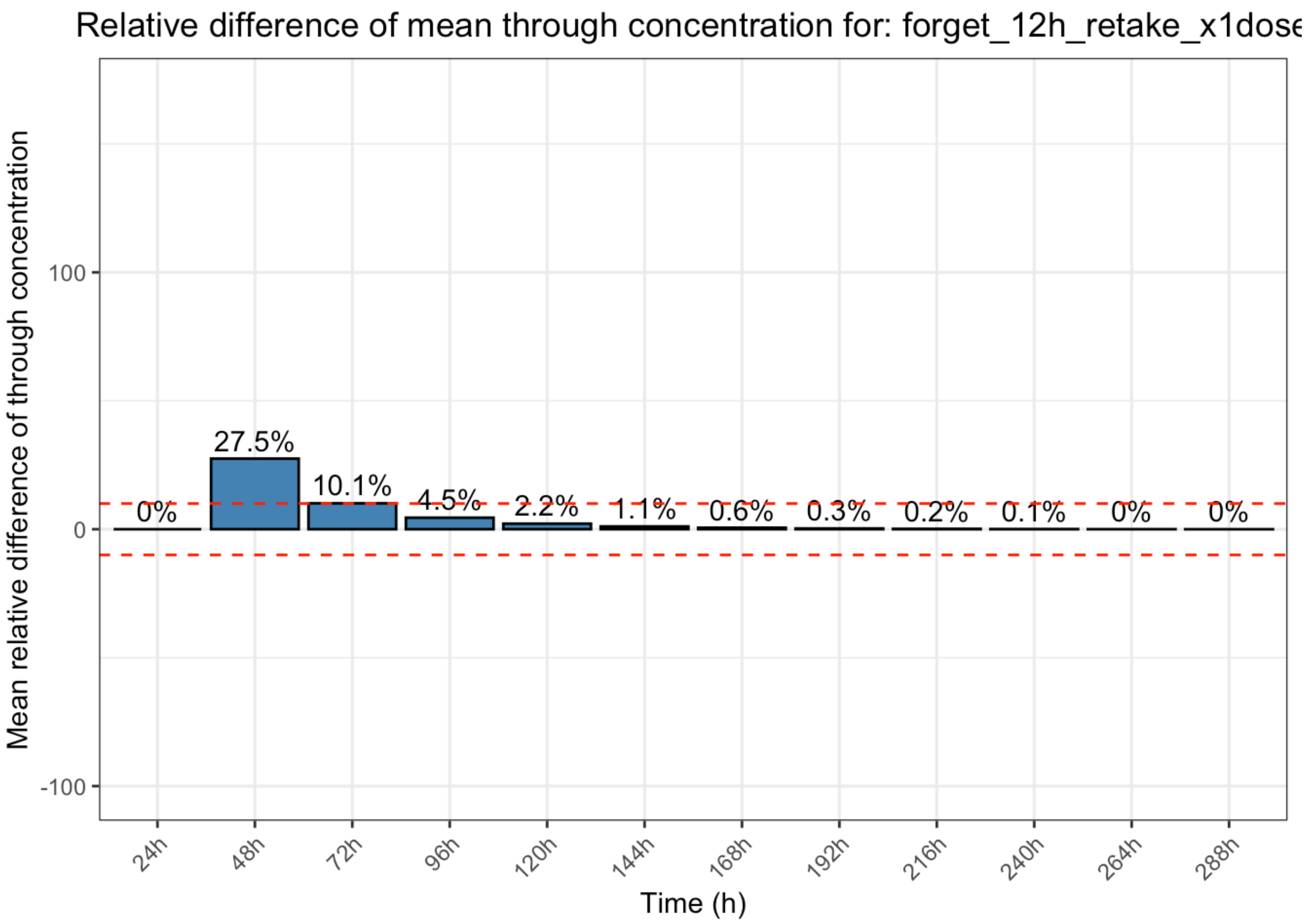
Relative difference of mean through concentration for: forget_12h_retake_x1dose
Mean relative difference of through concentration
100
0
-100
0%
27.5%
10.1%
4.5%
2.2%
1.1%
0.6%
0.3%
0.2%
0.1%
0%
0%
24h
48h
72h
96h
120h
144h
168h
192h
216h
240h
264h
288h
Time (h)

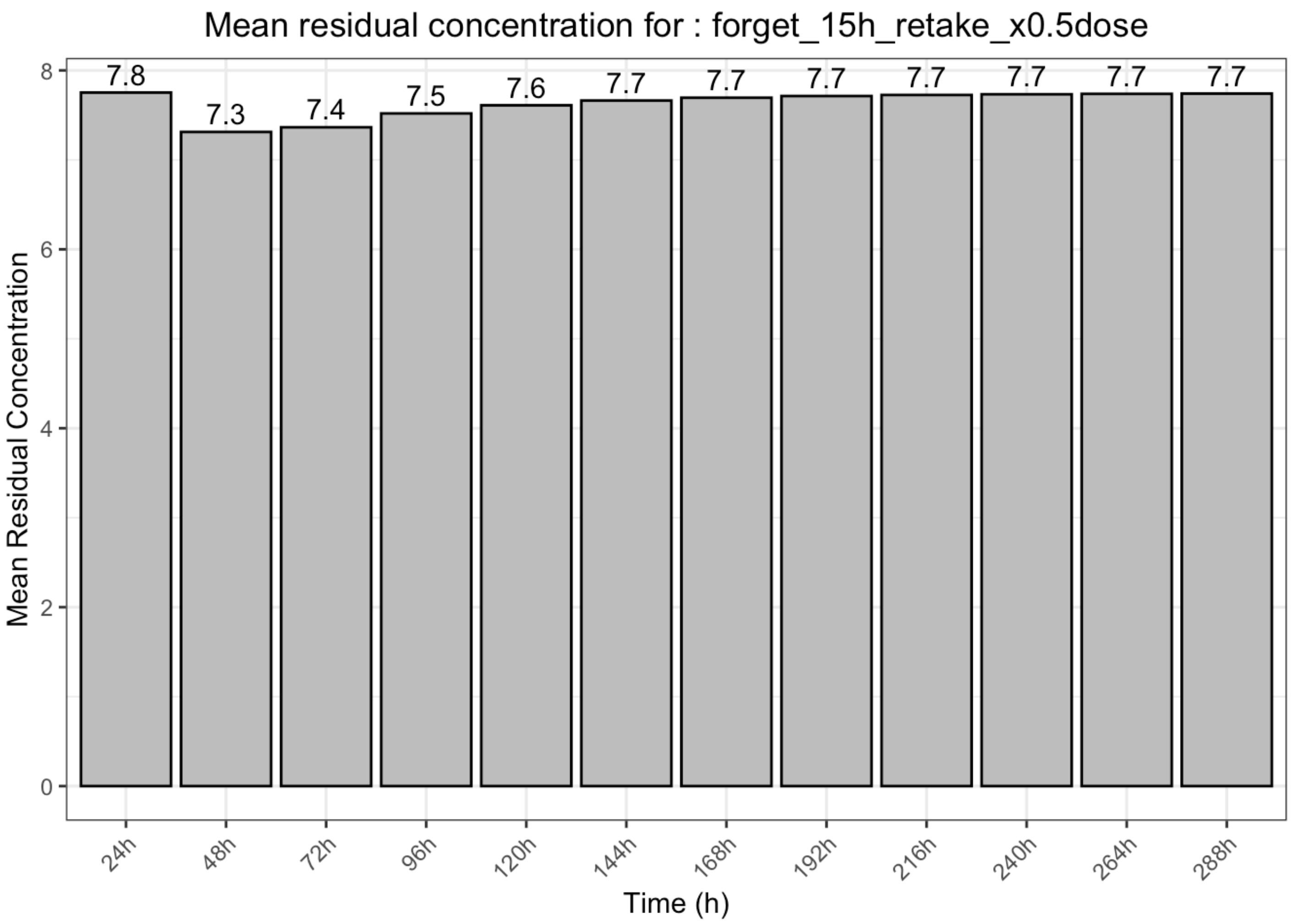
Mean residual concentration for : forget_15h_retake_x0.5dose
Mean Residual Concentration
Time (h)
0
2
4
6
8
7.8
7.3
7.4
7.5
7.6
7.7
7.7
7.7
7.7
7.7
7.7
7.7
24h
48h
72h
96h
120h
144h
168h
192h
216h
240h
264h
288h

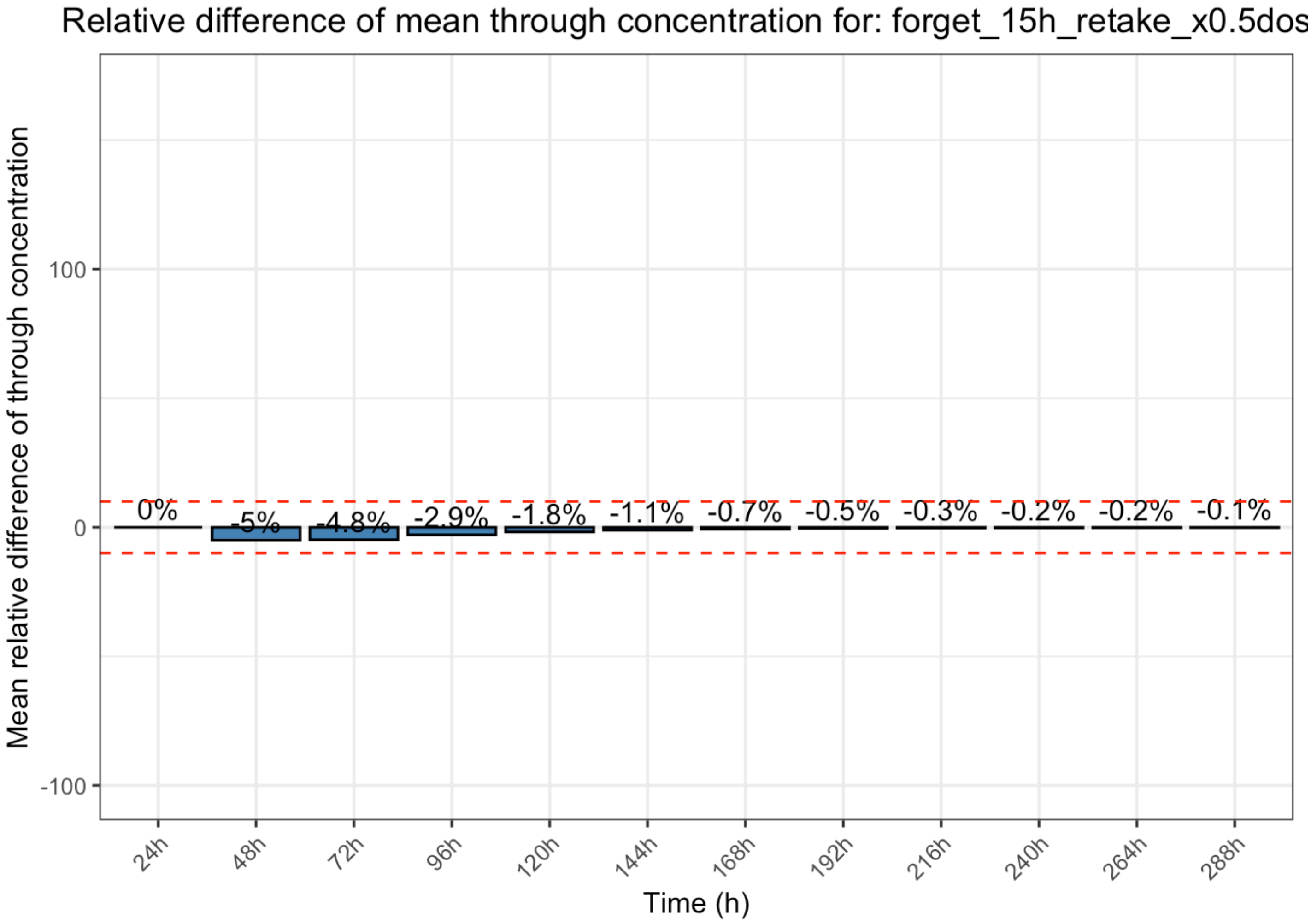
Relative difference of mean through concentration for: forget_15h_retake_x0.5dos
Mean relative difference of through concentration
100
0
-100
0%
-5%
-4.8%
-2.9%
-1.8%
-1.1%
-0.7%
-0.5%
-0.3%
-0.2%
-0.2%
-0.1%
24h
48h
72h
96h
120h
144h
168h
192h
216h
240h
264h
288h
Time (h)

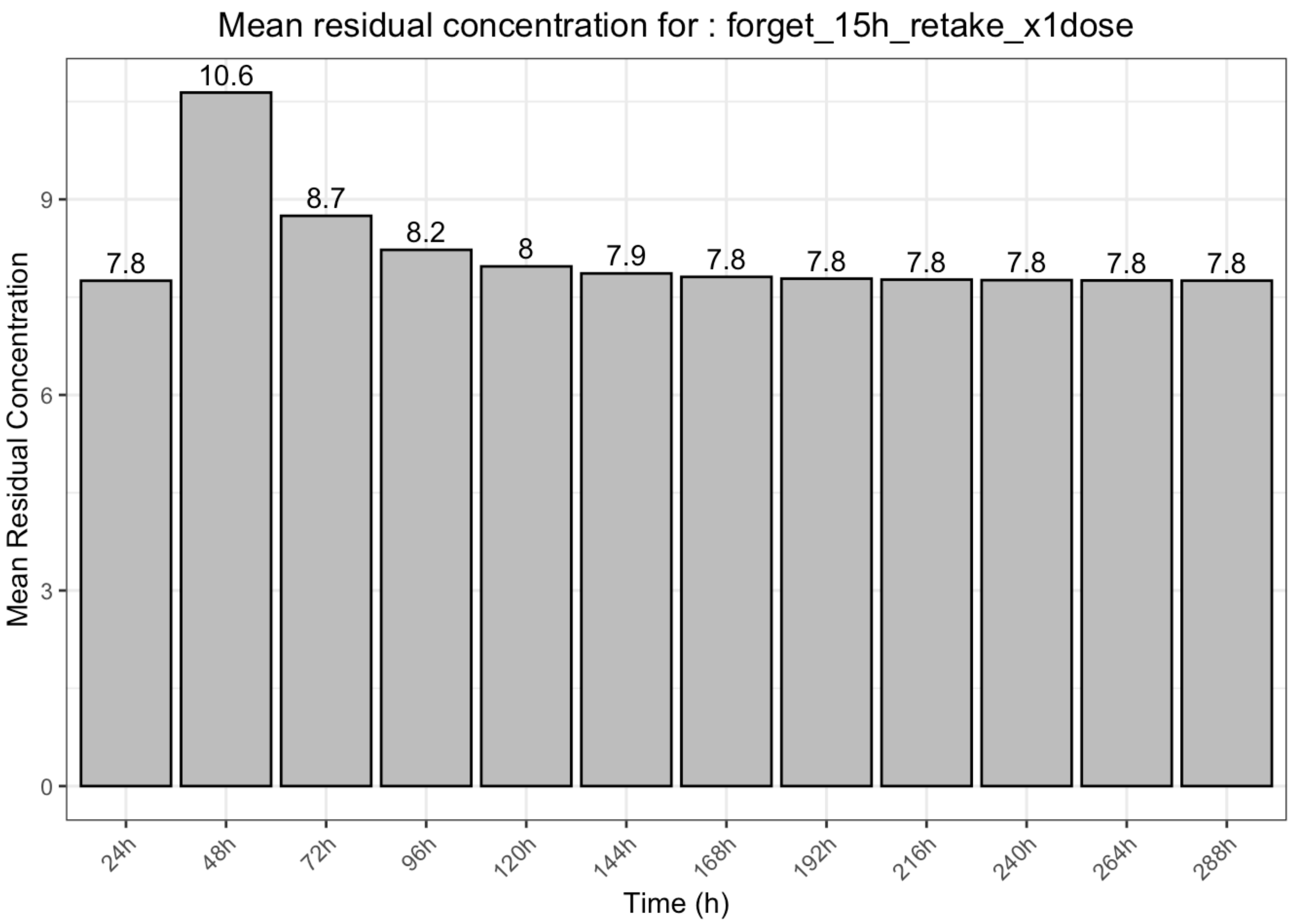
Mean residual concentration for : forget_15h_retake_x1dose
Mean Residual Concentration
Time (h)
0
3
6
9
7.8
10.6
8.7
8.2
8
7.9
7.8
7.8
7.8
7.8
7.8
7.8
24h
48h
72h
96h
120h
144h
168h
192h
216h
240h
264h
288h

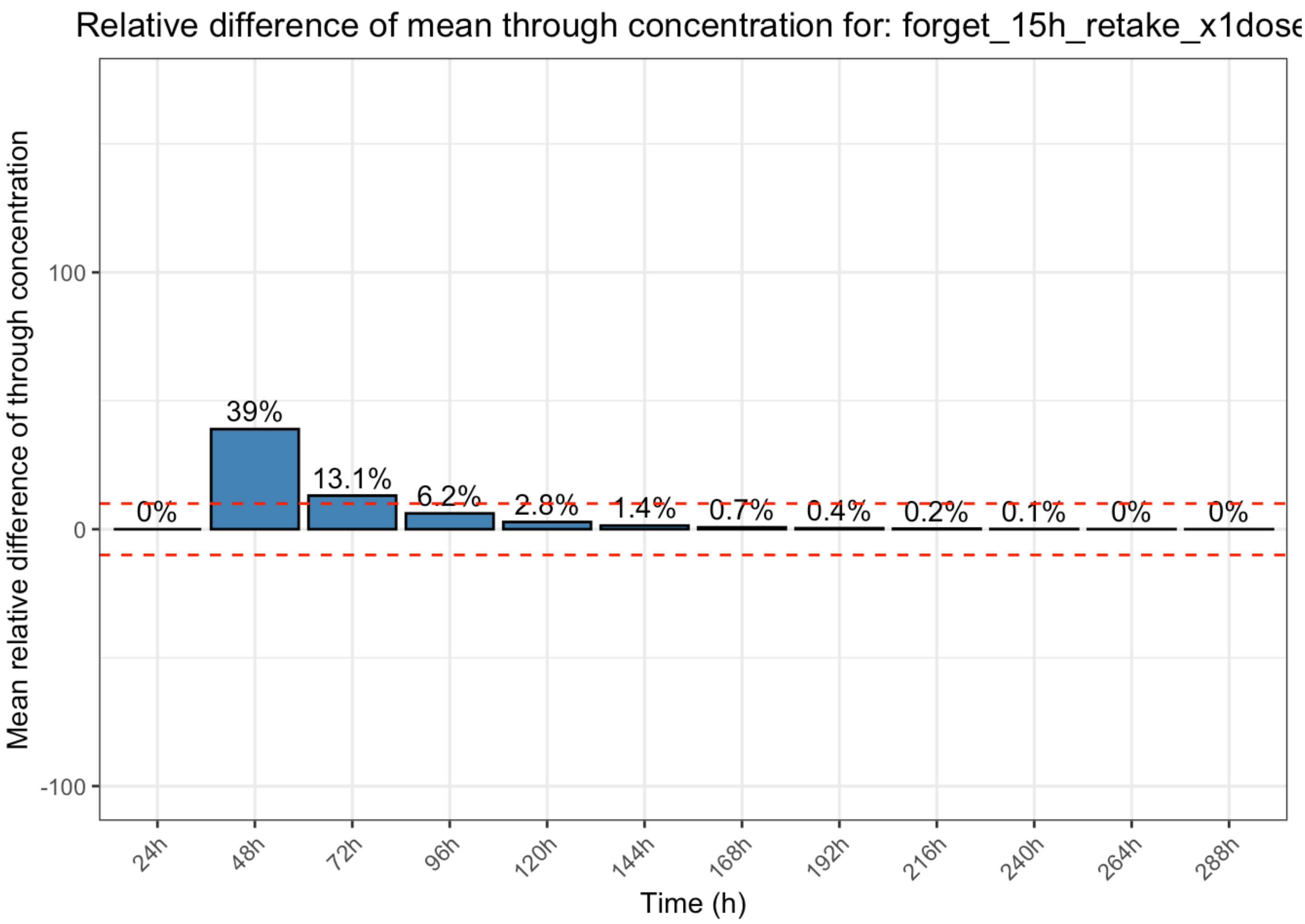
Relative difference of mean through concentration for: forget_15h_retake_x1dose
Mean relative difference of through concentration
Time (h)
100
0
-100
24h
48h
72h
96h
120h
144h
168h
192h
216h
240h
264h
288h
0%
39%
13.1%
6.2%
2.8%
1.4%
0.7%
0.4%
0.2%
0.1%
0%
0%

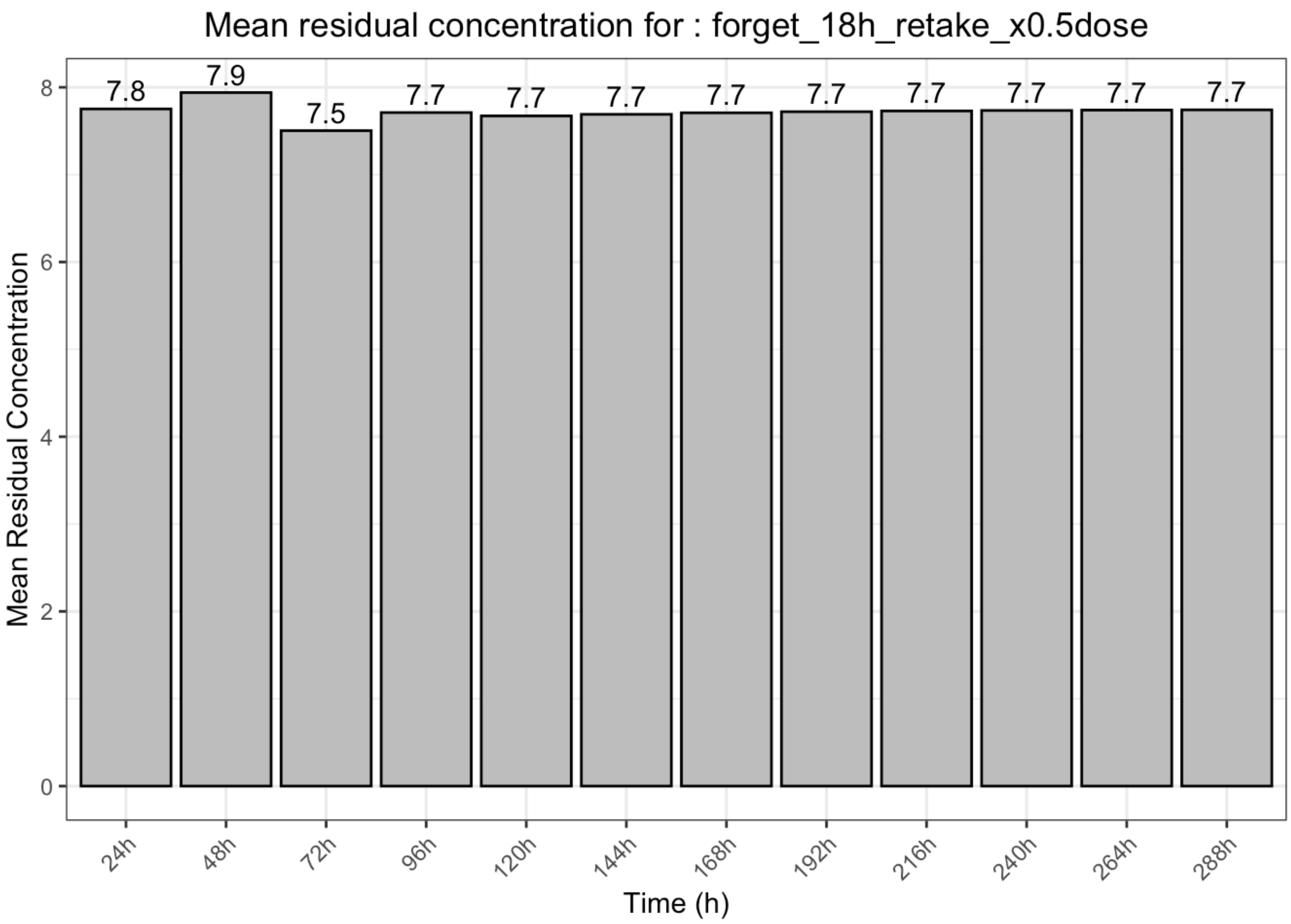
Mean residual concentration for : forget_18h_retake_x0.5dose
Mean Residual Concentration
0
2
4
6
8
7.8
7.9
7.5
7.7
7.7
7.7
7.7
7.7
7.7
7.7
7.7
7.7
24h
48h
72h
96h
120h
144h
168h
192h
216h
240h
264h
288h
Time (h)

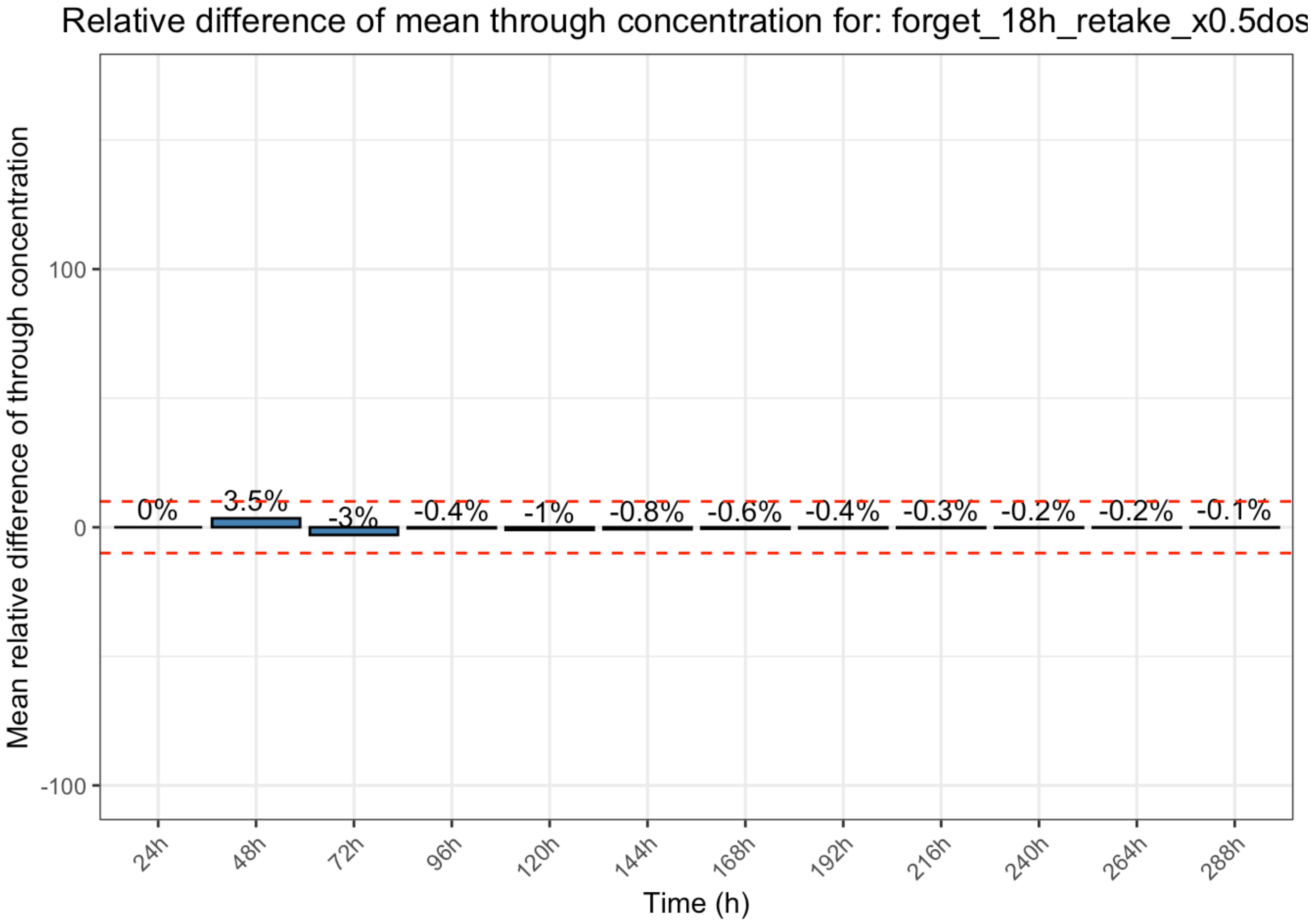
Relative difference of mean through concentration for: forget_18h_retake_x0.5dos
Mean relative difference of through concentration
100
0
-100
0%
3.5%
-3%
-0.4%
-1%
-0.8%
-0.6%
-0.4%
-0.3%
-0.2%
-0.2%
-0.1%
24h
48h
72h
96h
120h
144h
168h
192h
216h
240h
264h
288h
Time (h)

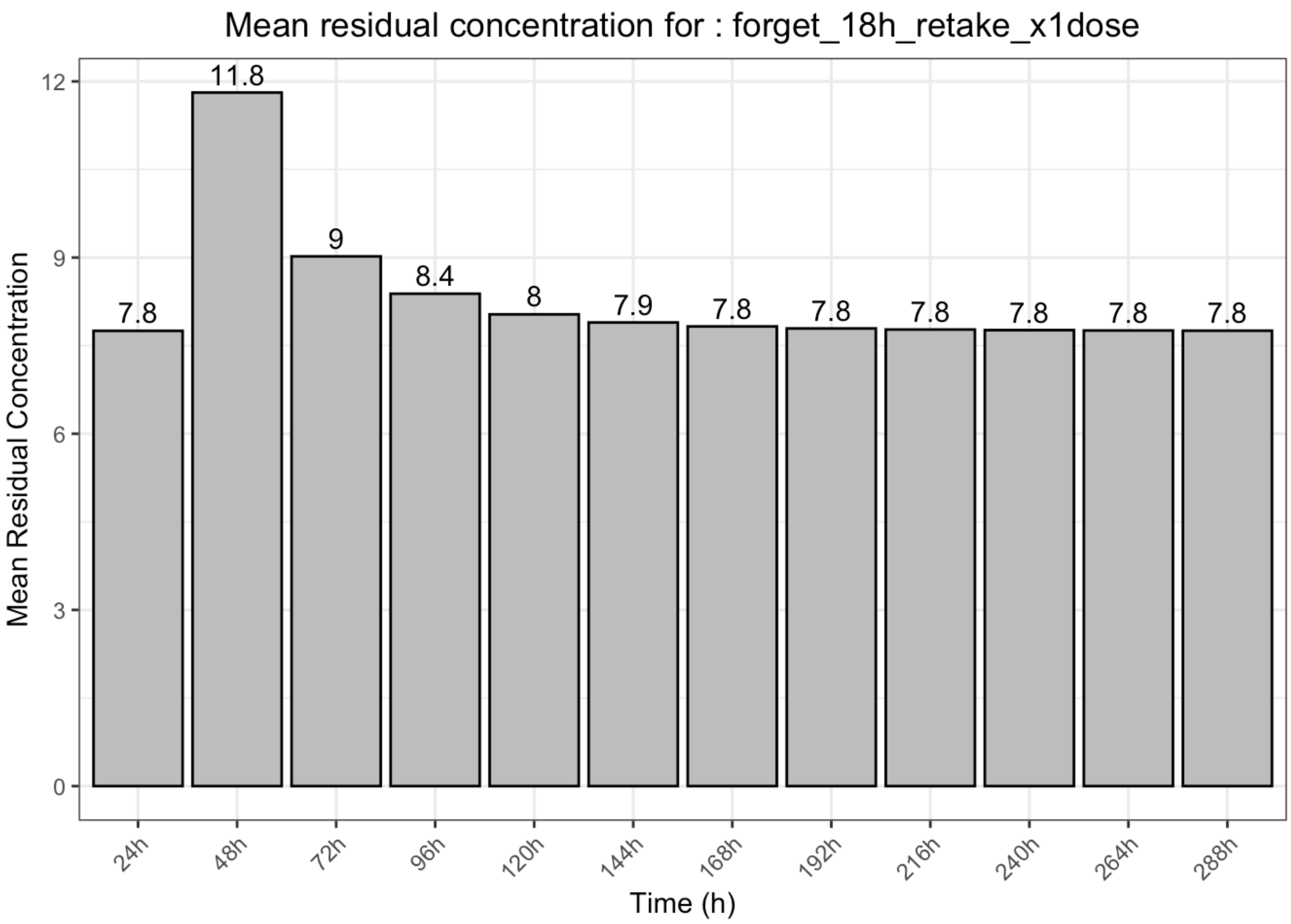
Mean residual concentration for : forget_18h_retake_x1dose
Mean Residual Concentration
Time (h)
0
3
6
9
12
7.8
11.8
9
8.4
8
7.9
7.8
7.8
7.8
7.8
7.8
7.8
24h
48h
72h
96h
120h
144h
168h
192h
216h
240h
264h
288h

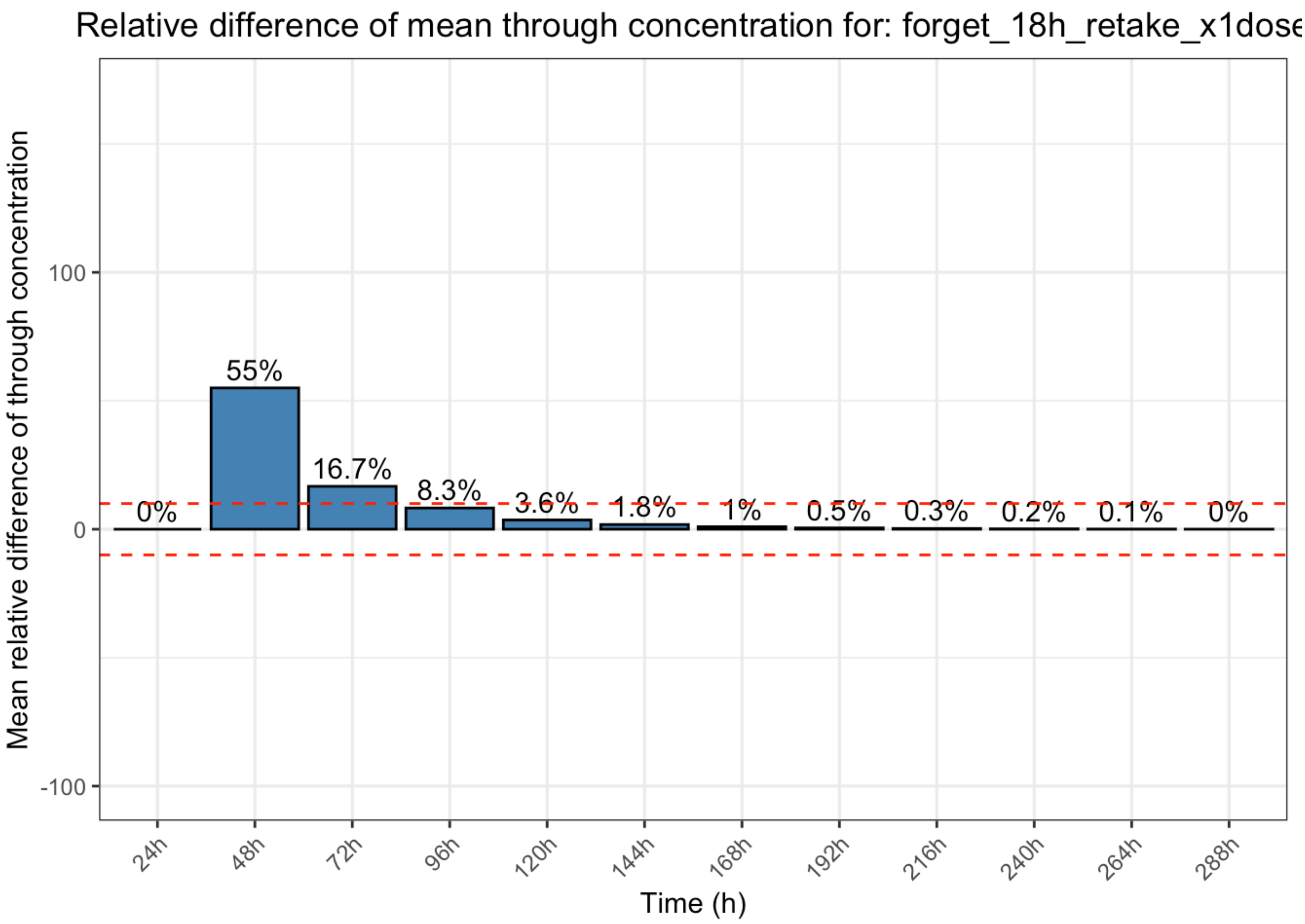
Relative difference of mean through concentration for: forget_18h_retake_x1dose
Mean relative difference of through concentration
Time (h)
100
0
-100
0%
55%
16.7%
8.3%
3.6%
1.8%
1%
0.5%
0.3%
0.2%
0.1%
0%
24h
48h
72h
96h
120h
144h
168h
192h
216h
240h
264h
288h

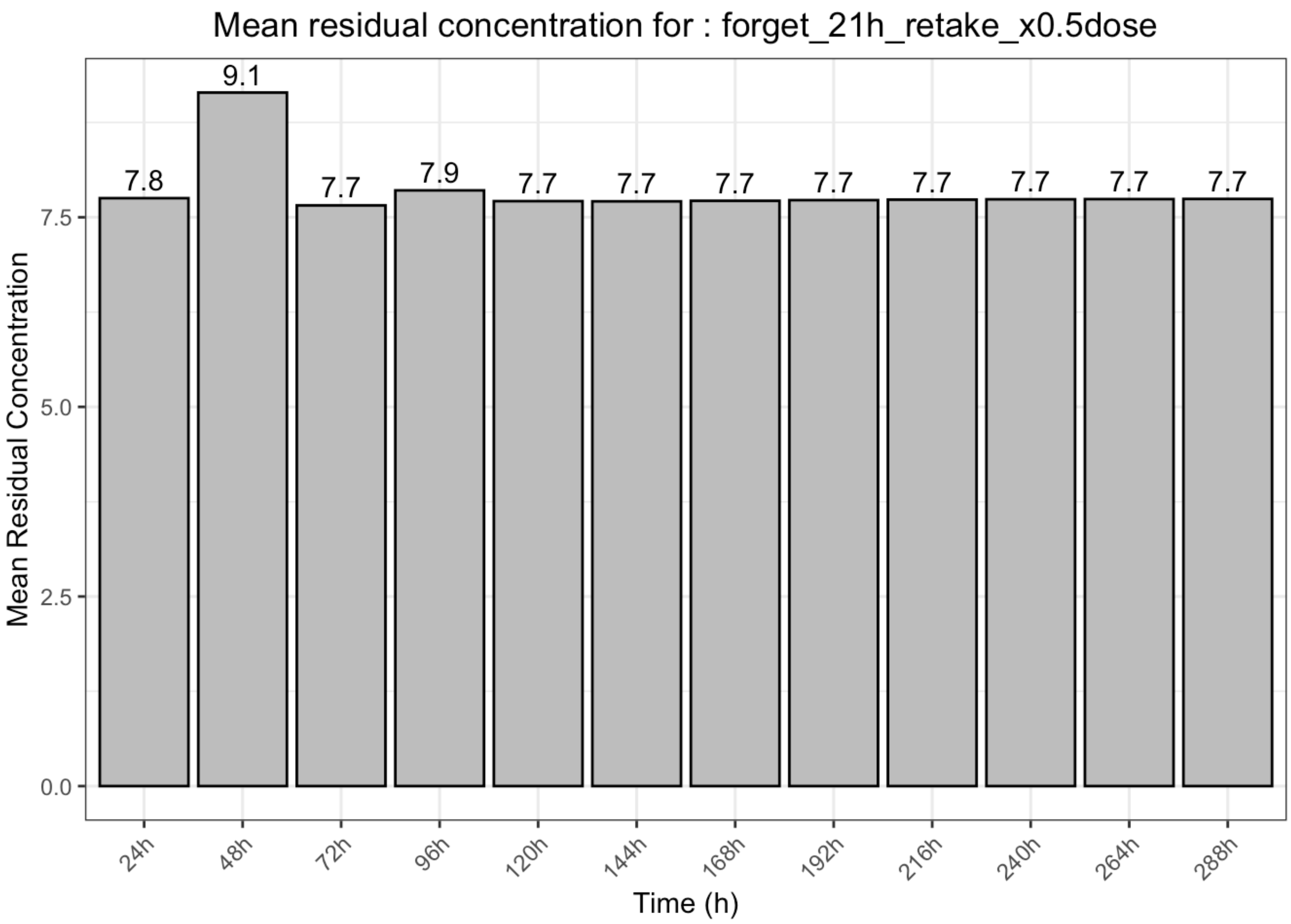
Mean residual concentration for : forget_21h_retake_x0.5dose
Mean Residual Concentration
Time (h)
0.0
2.5
5.0
7.5
7.8
9.1
7.7
7.9
7.7
7.7
7.7
7.7
7.7
7.7
7.7
7.7
24h
48h
72h
96h
120h
144h
168h
192h
216h
240h
264h
288h

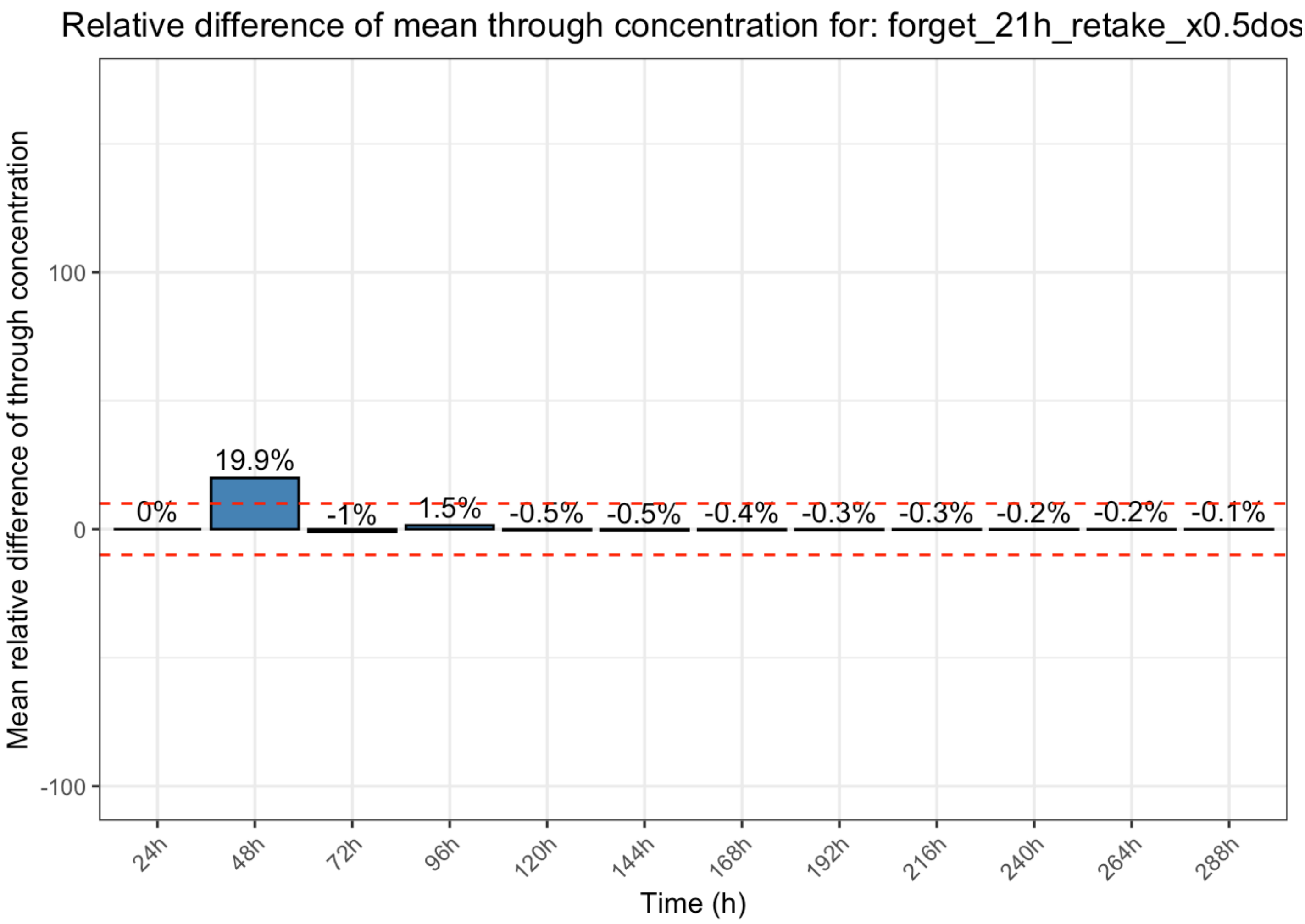
Relative difference of mean through concentration for: forget_21h_retake_x0.5dos
Mean relative difference of through concentration
100
0
-100
0%
19.9%
-1%
1.5%
-0.5%
-0.5%
-0.4%
-0.3%
-0.3%
-0.2%
-0.2%
-0.1%
24h
48h
72h
96h
120h
144h
168h
192h
216h
240h
264h
288h
Time (h)

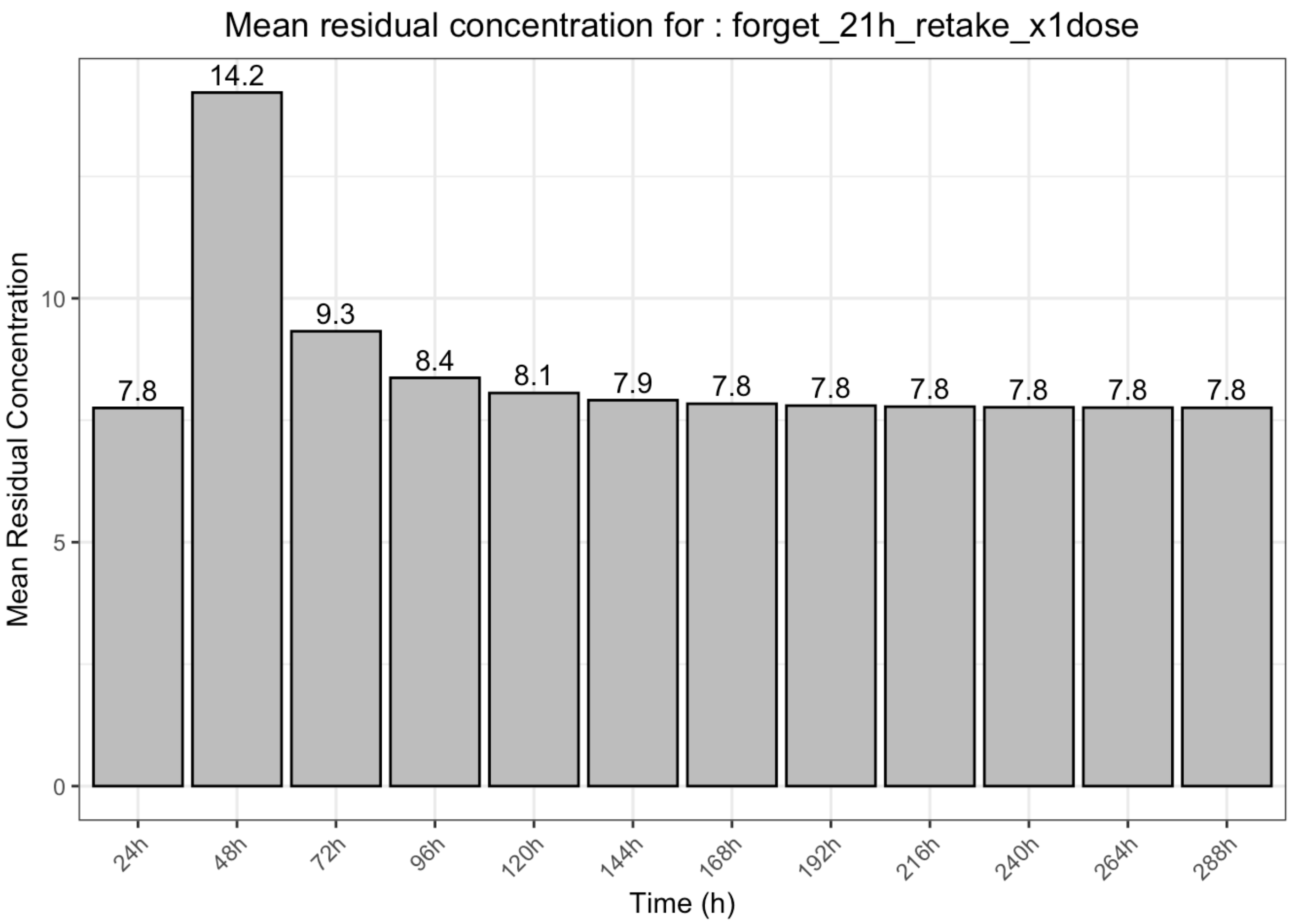
Mean residual concentration for : forget_21h_retake_x1dose
Mean Residual Concentration
Time (h)
0
5
10
7.8
14.2
9.3
8.4
8.1
7.9
7.8
7.8
7.8
7.8
7.8
7.8
24h
48h
72h
96h
120h
144h
168h
192h
216h
240h
264h
288h

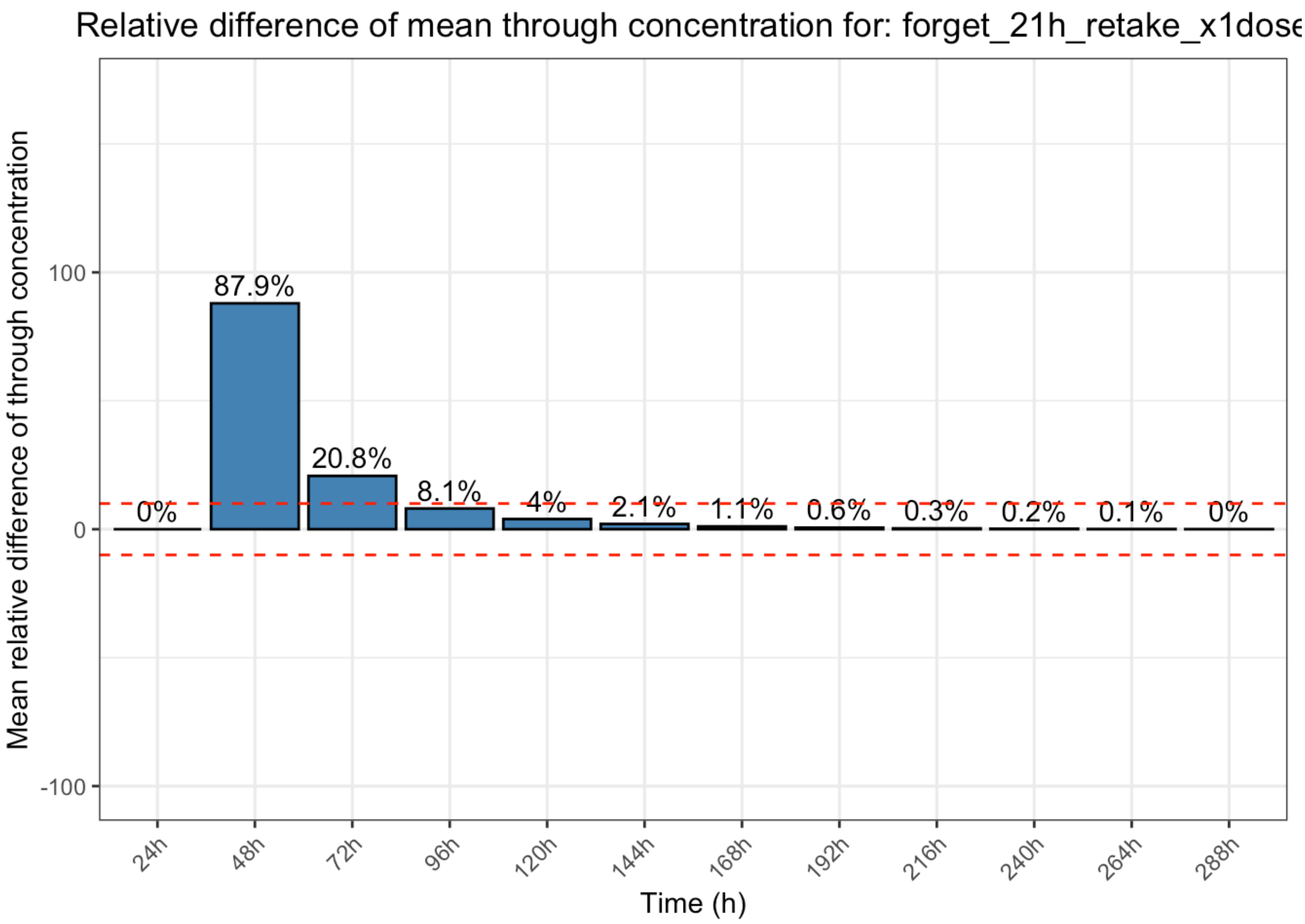
Relative difference of mean through concentration for: forget_21h_retake_x1dose
Mean relative difference of through concentration
100
0
-100
87.9%
20.8%
8.1%
4%
2.1%
1.1%
0.6%
0.3%
0.2%
0.1%
0%
0%
24h
48h
72h
96h
120h
144h
168h
192h
216h
240h
264h
288h
Time (h)

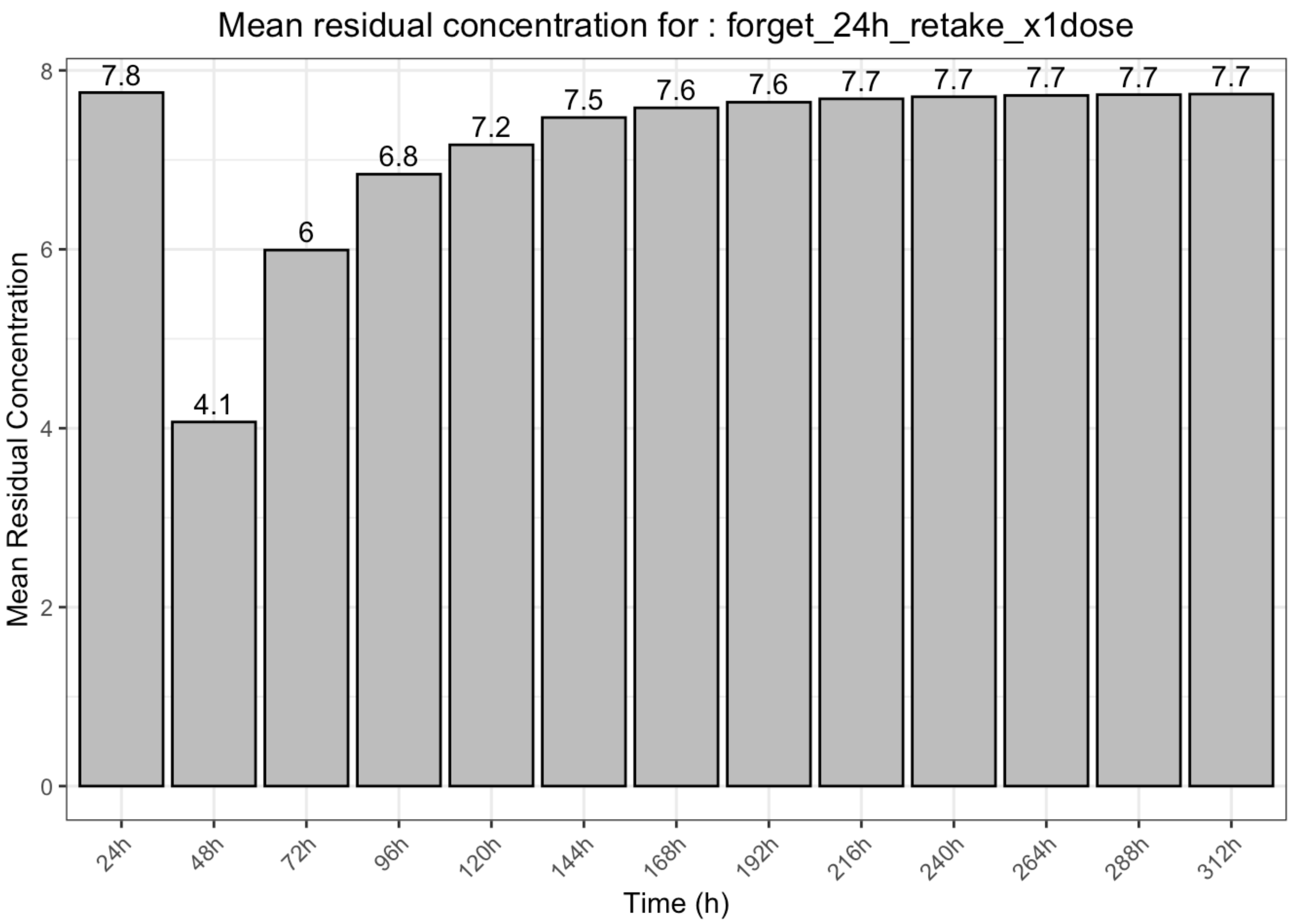
Mean residual concentration for : forget_24h_retake_x1dose
Mean Residual Concentration
Time (h)
0
2
4
6
8
7.8
4.1
6
6.8
7.2
7.5
7.6
7.6
7.7
7.7
7.7
7.7
7.7
24h
48h
72h
96h
120h
144h
168h
192h
216h
240h
264h
288h
312h

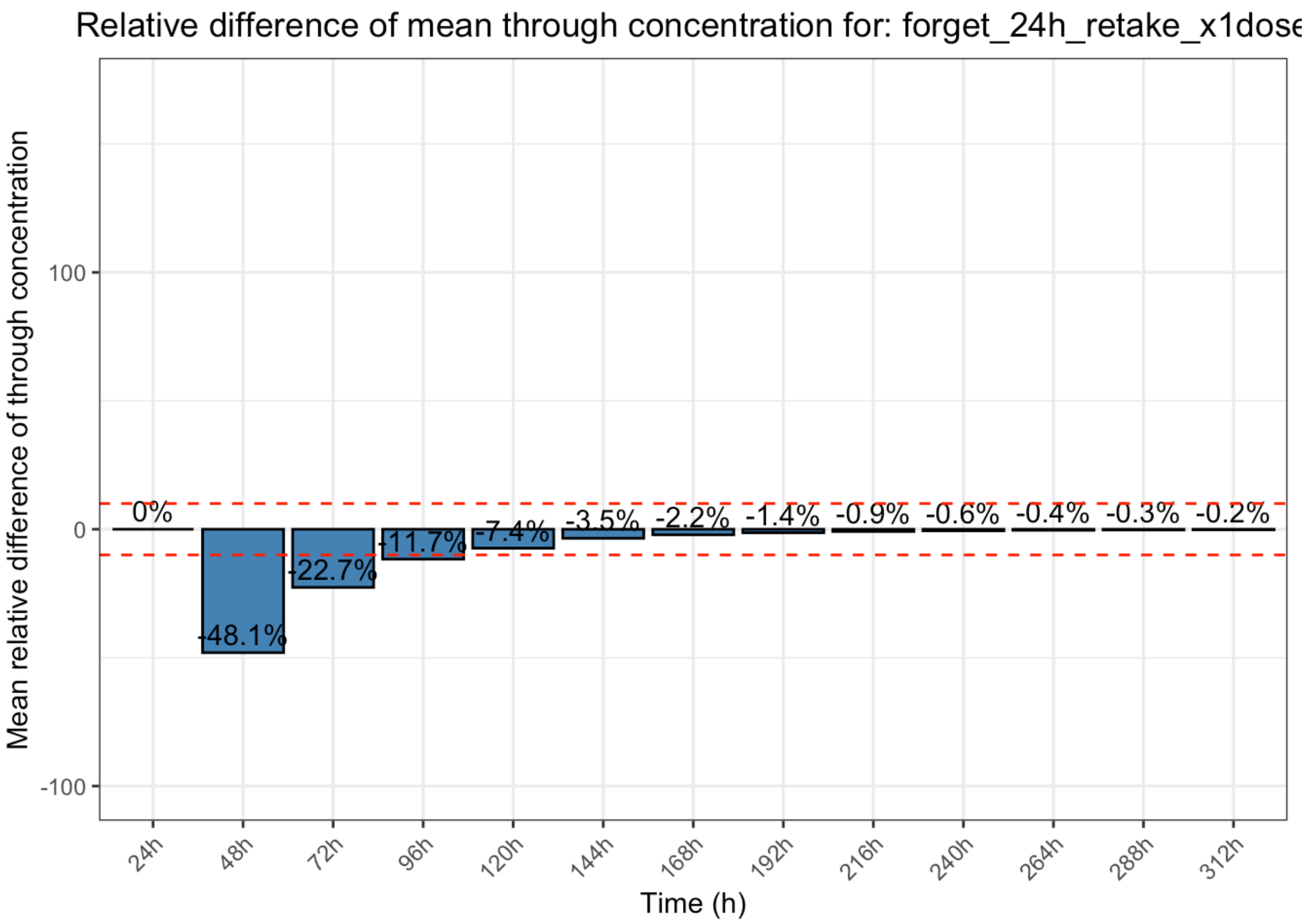
Relative difference of mean through concentration for: forget_24h_retake_x1dose
Mean relative difference of through concentration
100
0
-100
0%
-48.1%
-22.7%
-11.7%
-7.4%
-3.5%
-2.2%
-1.4%
-0.9%
-0.6%
-0.4%
-0.3%
-0.2%
24h
48h
72h
96h
120h
144h
168h
192h
216h
240h
264h
288h
312h
Time (h)

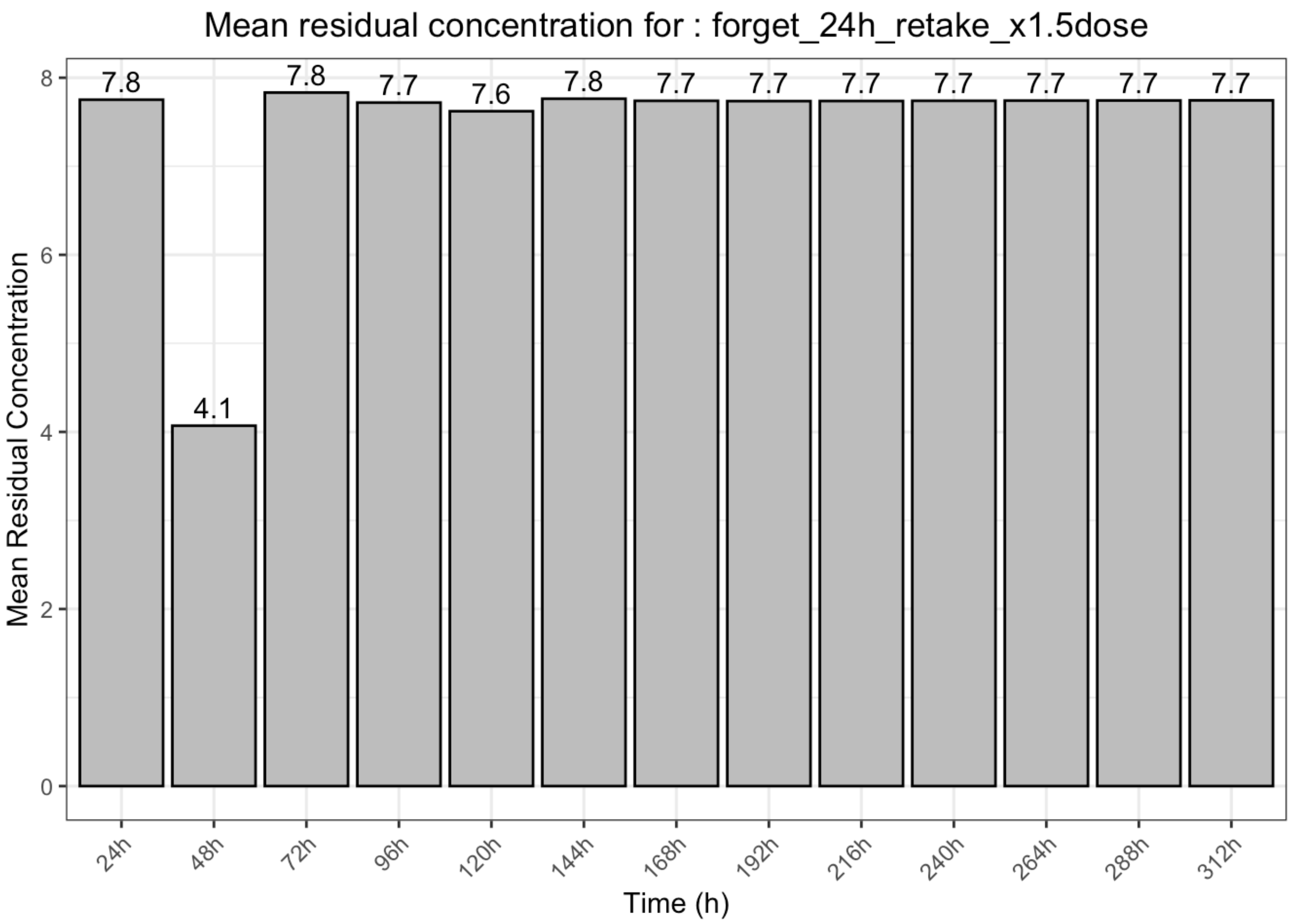
Mean residual concentration for : forget_24h_retake_x1.5dose
Mean Residual Concentration
Time (h)
0
2
4
6
8
7.8
4.1
7.8
7.7
7.6
7.8
7.7
7.7
7.7
7.7
7.7
7.7
7.7
24h
48h
72h
96h
120h
144h
168h
192h
216h
240h
264h
288h
312h

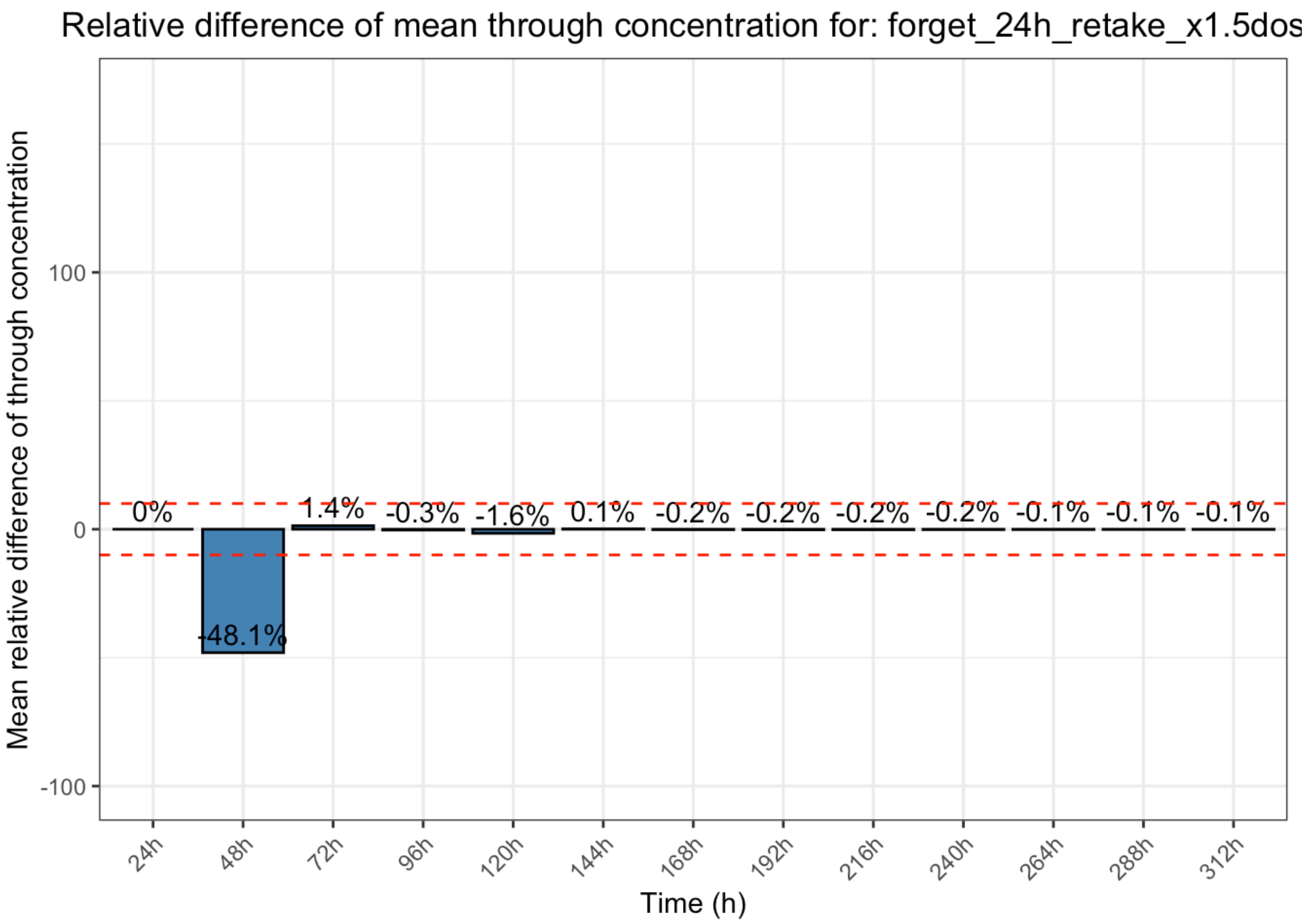
Relative difference of mean through concentration for: forget_24h_retake_x1.5dos
Mean relative difference of through concentration
100
0
-100
0%
-48.1%
1.4%
-0.3%
-1.6%
0.1%
-0.2%
-0.2%
-0.2%
-0.2%
-0.1%
-0.1%
-0.1%
24h
48h
72h
96h
120h
144h
168h
192h
216h
240h
264h
288h
312h
Time (h)

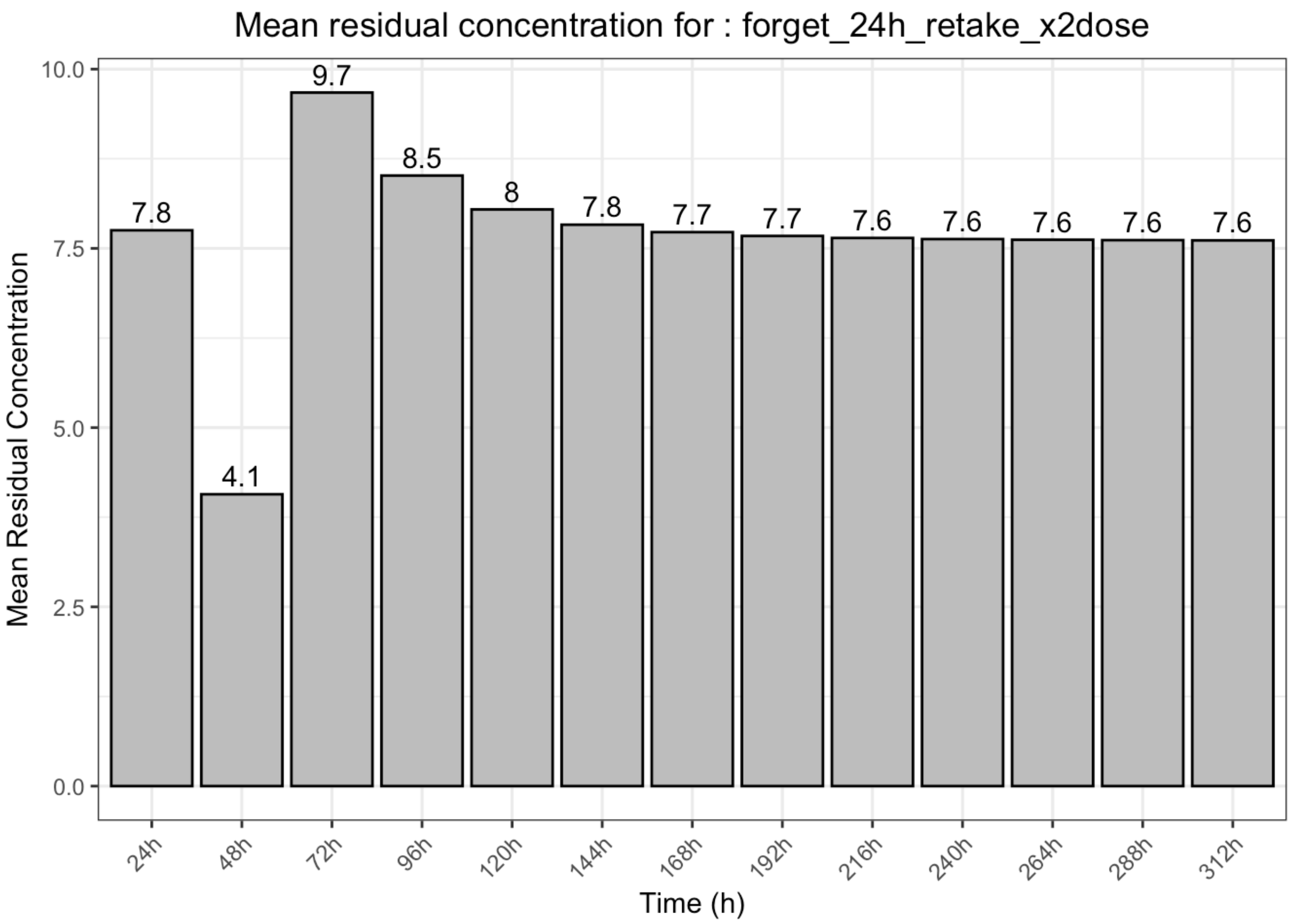
Mean residual concentration for : forget_24h_retake_x2dose
Mean Residual Concentration
10.0
7.5
5.0
2.5
0.0
7.8
4.1
9.7
8.5
8
7.8
7.7
7.7
7.6
7.6
7.6
7.6
7.6
24h
48h
72h
96h
120h
144h
168h
192h
216h
240h
264h
288h
312h
Time (h)

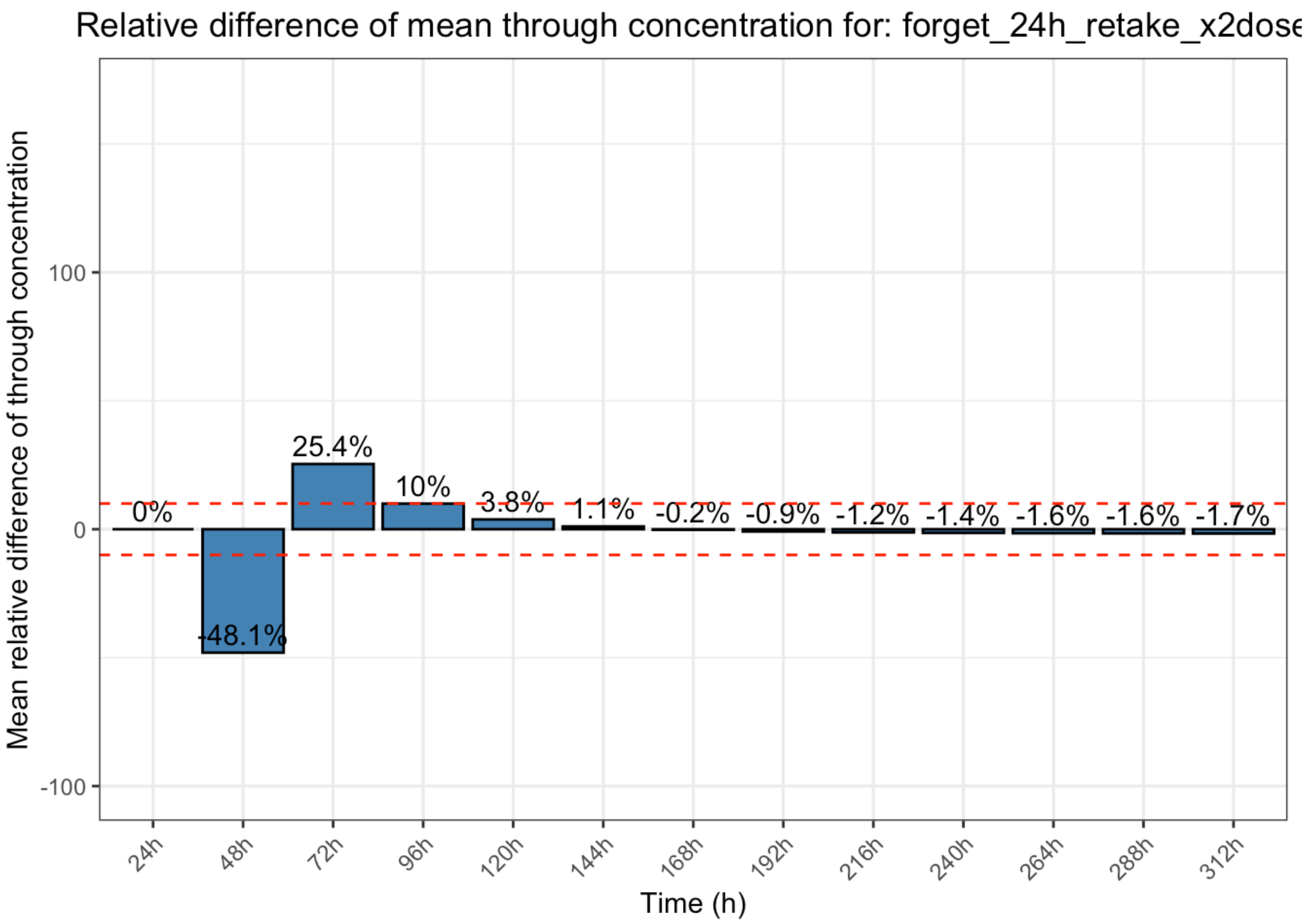

Add the CYP_W column to Data_with_time_renamed_W

```
Data_with_CYP_W <- Data_with_time_renamed_W %>%
  left_join(
    filtered_data_with_CYP_W %>% select(ID, CYP),
    by = "ID"
  )

Data_with_CYP_W
```

```
## # A tibble: 760,437 × 8
##       ID  time    DV forget_event C0_basale relative_C0 time_adjusted   CYP
##    <dbl> <dbl> <dbl> <chr>            <dbl>       <dbl> <chr>         <dbl>
##  1   404    24  5.38 no_forget         5.38           0 24h               0
##  2   405    24  4.64 no_forget         4.64           0 24h               0
##  3   417    24  4.77 no_forget         4.77           0 24h               0
##  4   426    24  5.91 no_forget         5.91           0 24h               0
##  5   427    24  4.06 no_forget         4.06           0 24h               0
##  6   428    24  4.23 no_forget         4.23           0 24h               0
##  7   437    24  6.43 no_forget         6.43           0 24h               0
##  8   442    24  4.13 no_forget         4.13           0 24h               0
##  9   444    24  6.66 no_forget         6.66           0 24h               0
## 10   450    24  4.12 no_forget         4.12           0 24h               0
## # ℹ 760,427 more rows
```

Stats for patients with CYP1: expressors

```
summary_stats_expanded_CYP_1_W <- Data_with_CYP_W %>%
  dplyr::filter(CYP == 1) %>%
  group_by(forget_event, time_adjusted) %>%
  summarize(
    min_DV = fivenum(DV)[1],        # Minimum
    Q1_DV = fivenum(DV)[2],         # First quartile (Q1)
    median_DV = fivenum(DV)[3],     # Median (Q2)
    Q3_DV = fivenum(DV)[4],         # Third quartile (Q3)
    max_DV = fivenum(DV)[5],        # Maximum
    mean_DV = mean(DV, na.rm = TRUE),  # Mean
    sd_DV = sd(DV, na.rm = TRUE),      # SD

    min_relative_C0 = fivenum(relative_C0)[1],        # Minimum
    Q1_relative_C0 = fivenum(relative_C0)[2],         # First quartile (Q1)
    median_relative_C0 = fivenum(relative_C0)[3],     # Median (Q2)
    Q3_relative_C0 = fivenum(relative_C0)[4],         # Third quartile (Q3)
    max_relative_C0 = fivenum(relative_C0)[5],        # Maximum
    mean_relative_C0 = mean(relative_C0, na.rm = TRUE),  # Mean
    sd_relative_C0 = sd(relative_C0, na.rm = TRUE)       # SD
  ) %>%
  ungroup() %>%
  arrange(forget_event, time_adjusted)
```

```
## `summarise()` has grouped output by 'forget_event'. You can override using the
## `.groups` argument.
```

```
summary_stats_expanded_CYP_1_W
```

```
## # A tibble: 171 × 16
##    forget_event  time_adjusted min_DV Q1_DV median_DV Q3_DV max_DV mean_DV sd_D
V
##    <chr>         <chr>          <dbl> <dbl>     <dbl> <dbl>  <dbl>   <dbl> <dbl
>
##  1 forget_12h_r… 120h            4.02  5.18      6.50  8.45   12.2    6.97  2.1
4
##  2 forget_12h_r… 144h            4.01  5.13      6.44  8.33   12.0    6.89  2.1
1
##  3 forget_12h_r… 168h            4.00  5.12      6.41  8.30   11.9    6.86  2.0
9
##  4 forget_12h_r… 192h            3.99  5.11      6.40  8.27   11.9    6.85  2.0
9
##  5 forget_12h_r… 216h            4.00  5.11      6.38  8.25   11.9    6.84  2.0
8
##  6 forget_12h_r… 240h            4.00  5.11      6.38  8.24   11.9    6.84  2.0
8
##  7 forget_12h_r… 24h             4.00  5.11      6.38  8.26   11.9    6.84  2.0
8
##  8 forget_12h_r… 264h            4.00  5.10      6.38  8.25   11.9    6.83  2.0
8
##  9 forget_12h_r… 288h            4.00  5.10      6.38  8.25   11.9    6.83  2.0
8
## 10 forget_12h_r… 48h             4.58  7.80      9.60 11.8    18.3    9.91  2.6
7
## # ℹ 161 more rows
## # ℹ 7 more variables: min_relative_C0 <dbl>, Q1_relative_C0 <dbl>,
## #   median_relative_C0 <dbl>, Q3_relative_C0 <dbl>, max_relative_C0 <dbl>,
## #   mean_relative_C0 <dbl>, sd_relative_C0 <dbl>
```

Stats for patients with CYP0: non-expressors

```
summary_stats_expanded_CYP_0_W <- Data_with_CYP_W %>%
  dplyr::filter(CYP == 0) %>%
  group_by(forget_event, time_adjusted) %>%
  summarize(
    min_DV = fivenum(DV)[1],        # Minimum
    Q1_DV = fivenum(DV)[2],         # First quartile (Q1)
    median_DV = fivenum(DV)[3],     # Median (Q2)
    Q3_DV = fivenum(DV)[4],         # Third quartile (Q3)
    max_DV = fivenum(DV)[5],        # Maximum
    mean_DV = mean(DV, na.rm = TRUE),  # Mean
    sd_DV = sd(DV, na.rm = TRUE),      # SD

    min_relative_C0 = fivenum(relative_C0)[1],        # Minimum
    Q1_relative_C0 = fivenum(relative_C0)[2],         # First quartile (Q1)
    median_relative_C0 = fivenum(relative_C0)[3],     # Median (Q2)
    Q3_relative_C0 = fivenum(relative_C0)[4],         # Third quartile (Q3)
    max_relative_C0 = fivenum(relative_C0)[5],        # Maximum
    mean_relative_C0 = mean(relative_C0, na.rm = TRUE),  # Mean
    sd_relative_C0 = sd(relative_C0, na.rm = TRUE)       # SD
  ) %>%
  ungroup() %>%
  arrange(forget_event, time_adjusted)
```

```
## `summarise()` has grouped output by 'forget_event'. You can override using the
## `.groups` argument.
```

```
summary_stats_expanded_CYP_0_W
```

```
## # A tibble: 171 × 16
##    forget_event  time_adjusted min_DV Q1_DV median_DV Q3_DV max_DV mean_DV sd_D
V
##    <chr>         <chr>          <dbl> <dbl>     <dbl> <dbl>  <dbl>   <dbl> <dbl
>
##  1 forget_12h_r… 120h            4.06  6.14      8.08 10.0    12.3    8.09  2.3
0
##  2 forget_12h_r… 144h            4.03  6.08      7.99  9.93   12.2    8.01  2.2
7
##  3 forget_12h_r… 168h            4.01  6.04      7.95  9.88   12.1    7.97  2.2
6
##  4 forget_12h_r… 192h            4.00  6.02      7.93  9.85   12.1    7.94  2.2
5
##  5 forget_12h_r… 216h            4.00  6.02      7.92  9.83   12.0    7.93  2.2
5
##  6 forget_12h_r… 240h            3.99  6.01      7.91  9.82   12.0    7.92  2.2
5
##  7 forget_12h_r… 24h             4.00  6.01      7.90  9.82   12.0    7.92  2.2
5
##  8 forget_12h_r… 264h            3.99  6.01      7.91  9.82   12.0    7.92  2.2
5
##  9 forget_12h_r… 288h            3.99  6.00      7.91  9.82   12.0    7.92  2.2
5
## 10 forget_12h_r… 48h             4.14  7.34      9.64 12.1    22.4    9.77  2.9
8
## # ℹ 161 more rows
## # ℹ 7 more variables: min_relative_C0 <dbl>, Q1_relative_C0 <dbl>,
## #   median_relative_C0 <dbl>, Q3_relative_C0 <dbl>, max_relative_C0 <dbl>,
## #   mean_relative_C0 <dbl>, sd_relative_C0 <dbl>
```

#AUC measurements, every 24h

```
start <- seq(0, 4416, by = 24)
end <- seq(24, 4440, by = 24)

iterate_auc <- function(start, end) {
  Final_data_combined_Woillard %>%
    group_by(ID) %>%
    rename(id = ID) %>%
    makeAUC(DV ~ time, start = start, end = end) %>%
    mutate(
      auc = round(tau, 0),
      intervalle = paste0(start, "_", end)
    ) %>%
    select(id, auc, intervalle)
}

auc_test_TEST_W <- map2_df(start, end, iterate_auc) %>%
  arrange(id, intervalle)
```

```
assign_forget_event <- function(start, end) {
  case_when(
    start >= 0 & end <= 312 ~ "no_forget",
    start >= 312 & end <= 624 ~ "forget_3h_retake_x1dose",
    start >= 624 & end <= 936 ~ "forget_6h_retake_x1dose",
    start >= 936 & end <= 1248 ~ "forget_9h_retake_x1dose",
    start >= 1248 & end <= 1560 ~ "forget_12h_retake_x1dose",
    start >= 1560 & end <= 1872 ~ "forget_15h_retake_x0.5dose",
    start >= 1872 & end <= 2184 ~ "forget_15h_retake_x1dose",
    start >= 2184 & end <= 2496 ~ "forget_18h_retake_x0.5dose",
    start >= 2496 & end <= 2808 ~ "forget_18h_retake_x1dose",
    start >= 2808 & end <= 3120 ~ "forget_21h_retake_x0.5dose",
    start >= 3120 & end <= 3432 ~ "forget_21h_retake_x1dose",
    start >= 3432 & end <= 3768 ~ "forget_24h_retake_x1dose",
    start >= 3768 & end <= 4104 ~ "forget_24h_retake_x1.5dose",
    start >= 4104 & end <= 4440 ~ "forget_24h_retake_x2dose",
    TRUE ~ NA_character_
  )
}

auc_test_TEST_by_scenario_W <- auc_test_TEST_W %>%
  mutate(
    start = as.numeric(str_extract(intervalle, "^[0-9]+")),
    end = as.numeric(str_extract(intervalle, "(?<=_)[0-9]+")),
    forget_event = assign_forget_event(start, end)  # Attribuer les groupes
  )

auc_test_TEST_by_scenario_W <- auc_test_TEST_by_scenario_W %>%
  select(-start, -end)

print(auc_test_TEST_by_scenario_W)
```

```
## # A tibble: 822,695 × 4
## # Groups:   id [4,447]
##       id   auc intervalle forget_event
##    <dbl> <dbl> <chr>      <chr>
##  1   404   179 0_24       no_forget
##  2   404   185 1008_1032  forget_9h_retake_x1dose
##  3   404   183 1032_1056  forget_9h_retake_x1dose
##  4   404   181 1056_1080  forget_9h_retake_x1dose
##  5   404   180 1080_1104  forget_9h_retake_x1dose
##  6   404   180 1104_1128  forget_9h_retake_x1dose
##  7   404   179 1128_1152  forget_9h_retake_x1dose
##  8   404   179 1152_1176  forget_9h_retake_x1dose
##  9   404   179 1176_1200  forget_9h_retake_x1dose
## 10   404   179 1200_1224  forget_9h_retake_x1dose
## # ℹ 822,685 more rows
```

Calculation of relative differences in AUC for each profile

```
AUC_basale_by_scenario_W <- auc_test_TEST_by_scenario_W %>%
  group_by(id, forget_event) %>%
  arrange(intervalle) %>%
  mutate(AUC_basale = first(auc)) %>%
  ungroup()

Relative_AUC_change_by_scenario_W <- AUC_basale_by_scenario_W %>%
  group_by(forget_event) %>%
  mutate(relative_AUC_by_scenario = (auc - AUC_basale) / AUC_basale * 100) %>%
  ungroup()

Relative_AUC_change_by_scenario_W
```

```
## # A tibble: 822,695 × 6
##       id   auc intervalle forget_event AUC_basale relative_AUC_by_scenario
##    <dbl> <dbl> <chr>      <chr>             <dbl>                    <dbl>
##  1   404   179 0_24       no_forget           179                        0
##  2   405   131 0_24       no_forget           131                        0
##  3   417   147 0_24       no_forget           147                        0
##  4   426   170 0_24       no_forget           170                        0
##  5   427   123 0_24       no_forget           123                        0
##  6   428   133 0_24       no_forget           133                        0
##  7   437   187 0_24       no_forget           187                        0
##  8   442   132 0_24       no_forget           132                        0
##  9   444   184 0_24       no_forget           184                        0
## 10   450   124 0_24       no_forget           124                        0
## # ℹ 822,685 more rows
```

#Renaming intervals

```
rename_interval <- function(intervalle) {
  start <- as.numeric(sub("_.*", "", intervalle))
  end <- as.numeric(sub(".*_", "", intervalle))

  if ((start >= 3432 && end <= 3768) || (start >= 3768 && end <= 4104) || (start >
= 4104 && end <= 4440)) {
    interval_index <- ((start - 3432) %% 336) %/% 24
    new_start <- interval_index * 24
    new_end <- new_start + 24
  } else {
    interval_index <- ((start - 312) %% 312) %/% 24
    new_start <- interval_index * 24
    new_end <- new_start + 24
  }

  return(paste0(new_start, "_", new_end))
}

Renamed_AUC_W <- Relative_AUC_change_by_scenario_W %>%
  mutate(intervalle_renamed = sapply(intervalle, rename_interval))

print(Renamed_AUC_W)
```

```
## # A tibble: 822,695 × 7
##       id   auc intervalle forget_event AUC_basale relative_AUC_by_scenario
##    <dbl> <dbl> <chr>      <chr>             <dbl>                    <dbl>
##  1   404   179 0_24       no_forget           179                        0
##  2   405   131 0_24       no_forget           131                        0
##  3   417   147 0_24       no_forget           147                        0
##  4   426   170 0_24       no_forget           170                        0
##  5   427   123 0_24       no_forget           123                        0
##  6   428   133 0_24       no_forget           133                        0
##  7   437   187 0_24       no_forget           187                        0
##  8   442   132 0_24       no_forget           132                        0
##  9   444   184 0_24       no_forget           184                        0
## 10   450   124 0_24       no_forget           124                        0
## # ℹ 822,685 more rows
## # ℹ 1 more variable: intervalle_renamed <chr>
```

#AUC and RD average statistics: all profiles

```
summary_stats_auc_W <- Renamed_AUC_W %>%
  group_by(forget_event, intervalle_renamed) %>%
  summarize(
    min_auc = fivenum(auc)[1],        # Minimum
    Q1_auc = fivenum(auc)[2],         # First quartile (Q1)
    median_auc = fivenum(auc)[3],     # Median (Q2)
    Q3_auc = fivenum(auc)[4],         # Third quartile (Q3)
    max_auc = fivenum(auc)[5],        # Maximum
    mean_auc = mean(auc, na.rm = TRUE),  # Mean
    sd_auc = sd(auc, na.rm = TRUE),      # SD

    min_relative_auc = fivenum(relative_AUC_by_scenario)[1],        # Minimum
    Q1_relative_auc = fivenum(relative_AUC_by_scenario)[2],         # First quarti
le (Q1)
    median_relative_auc = fivenum(relative_AUC_by_scenario)[3],     # Median (Q2)
    Q3_relative_auc = fivenum(relative_AUC_by_scenario)[4],         # Third quarti
le (Q3)
    max_relative_auc = fivenum(relative_AUC_by_scenario)[5],        # Maximum
    mean_relative_auc = mean(relative_AUC_by_scenario, na.rm = TRUE),  # Mean
    sd_relative_auc = sd(relative_AUC_by_scenario, na.rm = TRUE)       # SD
  ) %>%
  ungroup() %>%
  arrange(intervalle_renamed)
```

```
## `summarise()` has grouped output by 'forget_event'. You can override using the
## `.groups` argument.
```

```
summary_stats_auc_W
```

```
## # A tibble: 185 × 16
##    forget_event      intervalle_renamed min_auc Q1_auc median_auc Q3_auc max_au
c
##    <chr>             <chr>                <dbl>  <dbl>      <dbl>  <dbl>   <dbl
>
##  1 forget_12h_retak… 0_24                   112    214        276    343     61
8
##  2 forget_15h_retak… 0_24                   112    214        276    343     61
8
##  3 forget_15h_retak… 0_24                   112    214        276    343     61
8
##  4 forget_18h_retak… 0_24                   112    214        276    343     61
8
##  5 forget_18h_retak… 0_24                   111    214        276    343     61
8
##  6 forget_21h_retak… 0_24                   112    214        276    343     61
8
##  7 forget_21h_retak… 0_24                   112    214        276    343     61
8
##  8 forget_24h_retak… 0_24                   112    214        276    343     61
8
##  9 forget_24h_retak… 0_24                   111    214        276    343     61
8
## 10 forget_24h_retak… 0_24                   112    214        276    343     61
8
## # ℹ 175 more rows
## # ℹ 9 more variables: mean_auc <dbl>, sd_auc <dbl>, min_relative_auc <dbl>,
## #   Q1_relative_auc <dbl>, median_relative_auc <dbl>, Q3_relative_auc <dbl>,
## #   max_relative_auc <dbl>, mean_relative_auc <dbl>, sd_relative_auc <dbl>
```

#AUC and RD histograms: all profiles

```
desired_order <- c("no_forget",
                   "forget_3h_retake_x1dose",
                   "forget_6h_retake_x1dose",
                   "forget_9h_retake_x1dose",
                   "forget_12h_retake_x1dose",
                   "forget_15h_retake_x0.5dose",
                   "forget_15h_retake_x1dose",
                   "forget_18h_retake_x0.5dose",
                   "forget_18h_retake_x1dose",
                   "forget_21h_retake_x0.5dose",
                   "forget_21h_retake_x1dose",
                   "forget_24h_retake_x1dose",
                   "forget_24h_retake_x1.5dose",
                   "forget_24h_retake_x2dose")

summary_stats_auc_W <- summary_stats_auc_W %>%
  mutate(
```

```
    interval_start = as.numeric(str_extract(intervalle_renamed, "^[0-9]+"))
  )

summary_stats_auc_W <- summary_stats_auc_W %>%
  arrange(forget_event, interval_start)

summary_stats_auc_W$intervalle_renamed <- factor(summary_stats_auc_W$intervalle_re
named, levels = unique(summary_stats_auc_W$intervalle_renamed))

summary_stats_auc_W$forget_event <- factor(summary_stats_auc_W$forget_event, level
s = desired_order)

forget_events_W <- levels(summary_stats_auc_W$forget_event)

for (event in forget_events_W) {
  data_subset_W <- summary_stats_auc_W %>%
    dplyr::filter(forget_event == event)

  p7 <- ggplot(data_subset_W, aes(x = intervalle_renamed, y = mean_auc)) +
    geom_bar(stat = "identity", fill = "grey", color = "black") +
    geom_text(aes(label = round(mean_auc, 1)), vjust = -0.5, color = "black") +
    labs(title = paste("Mean AUC for Forget Event:", event),
         x = "Interval of time (h)", y = "Mean AUC") +
    theme_bw() +
    theme(plot.title = element_text(hjust = 0.5)) +
  theme(axis.text.x = element_text(angle = 45, hjust = 1))

  print(p7)

p8 <- ggplot(data_subset_W, aes(x = intervalle_renamed, y = mean_relative_auc)) +
  geom_bar(stat = "identity", fill = "steelblue", color = "black") +
  geom_text(aes(label = round(mean_relative_auc, 1)), vjust = -0.5, color = "blac
k") +
  labs(title = paste("Mean Relative Difference of AUC by Forget Event:", event),
       x = "Interval of time (h)", y = "Mean RD of AUC") +
  theme_bw() +
  theme(plot.title = element_text(hjust = 0.5)) +
  ylim(-100, 100) +
  geom_hline(yintercept = c(-5, 5), linetype = "dashed", color = "red") +
  theme(plot.title = element_text(hjust = 0.5)) +
  theme(axis.text.x = element_text(angle = 45, hjust = 1))

print(p8)
}
```

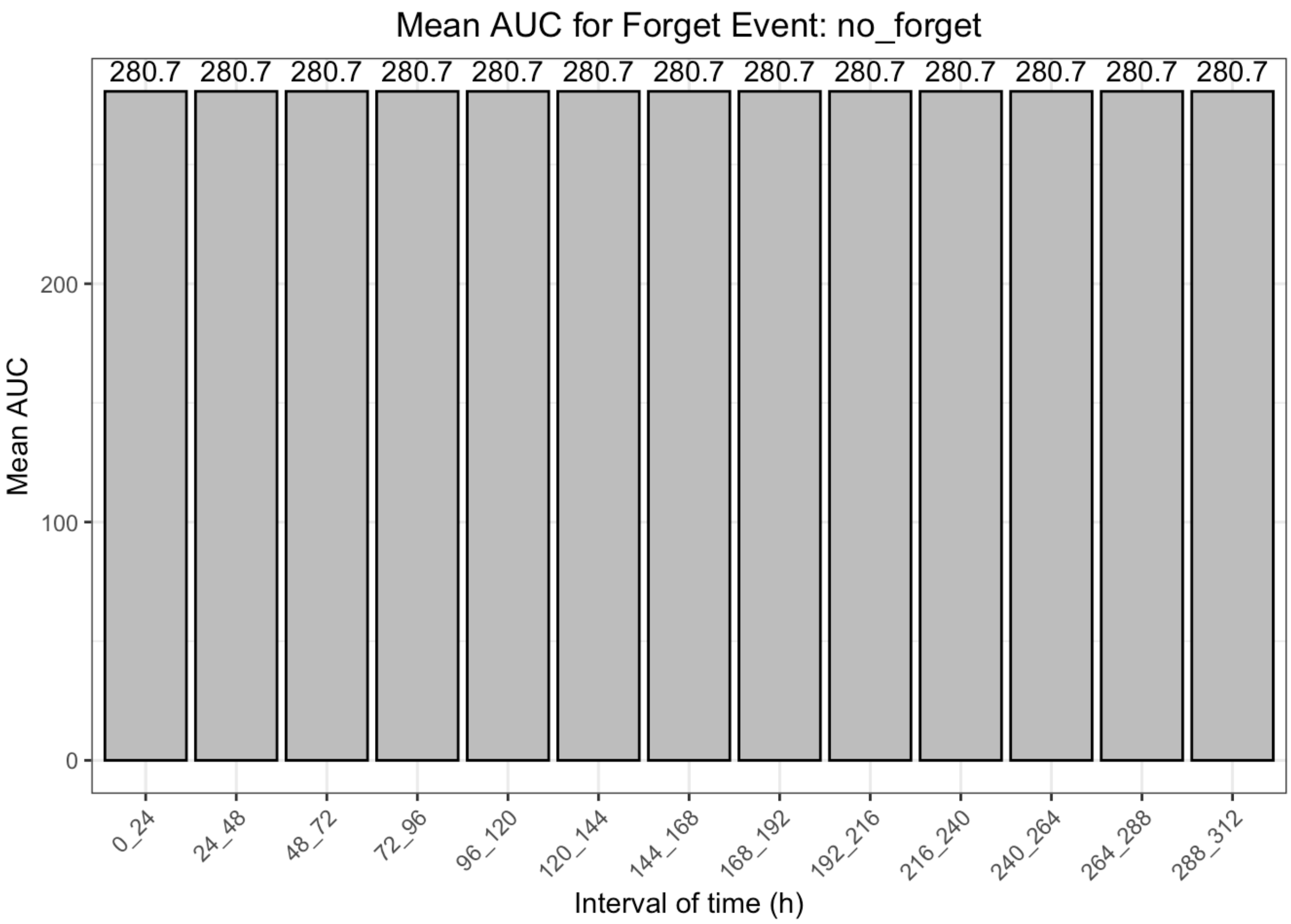
Mean AUC for Forget Event: no_forget
280.7 280.7 280.7 280.7 280.7 280.7 280.7 280.7 280.7 280.7 280.7 280.7 280.7
Mean AUC
0
100
200
0_24 24_48 48_72 72_96 96_120 120_144 144_168 168_192 192_216 216_240 240_264 264_288 288_312
Interval of time (h)

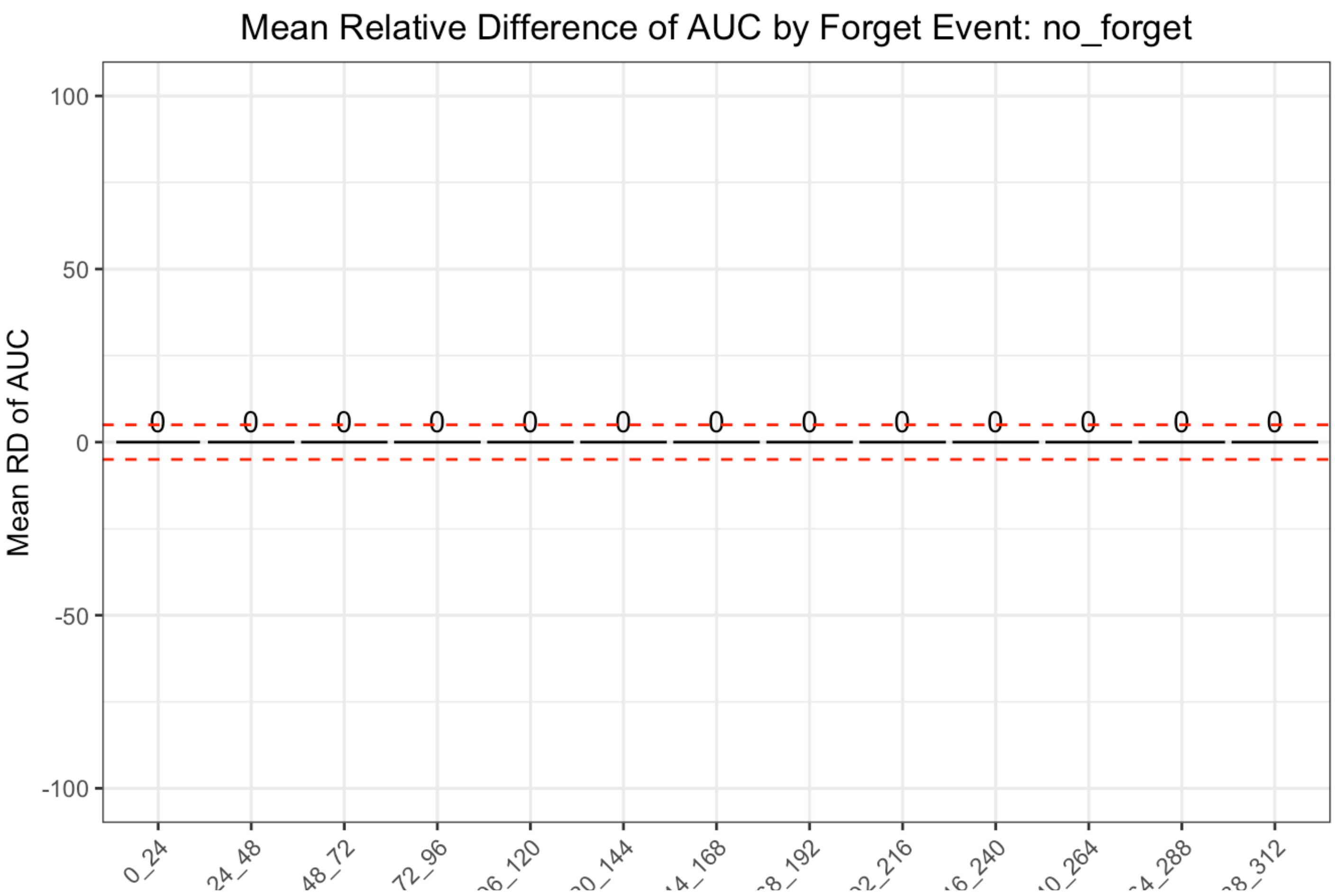
Mean Relative Difference of AUC by Forget Event: no_forget
Mean RD of AUC
100
50
0
-50
-100
0 0 0 0 0 0 0 0 0 0 0 0 0
0_24 24_48 48_72 72_96

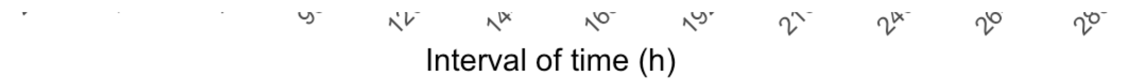
Interval of time (h)

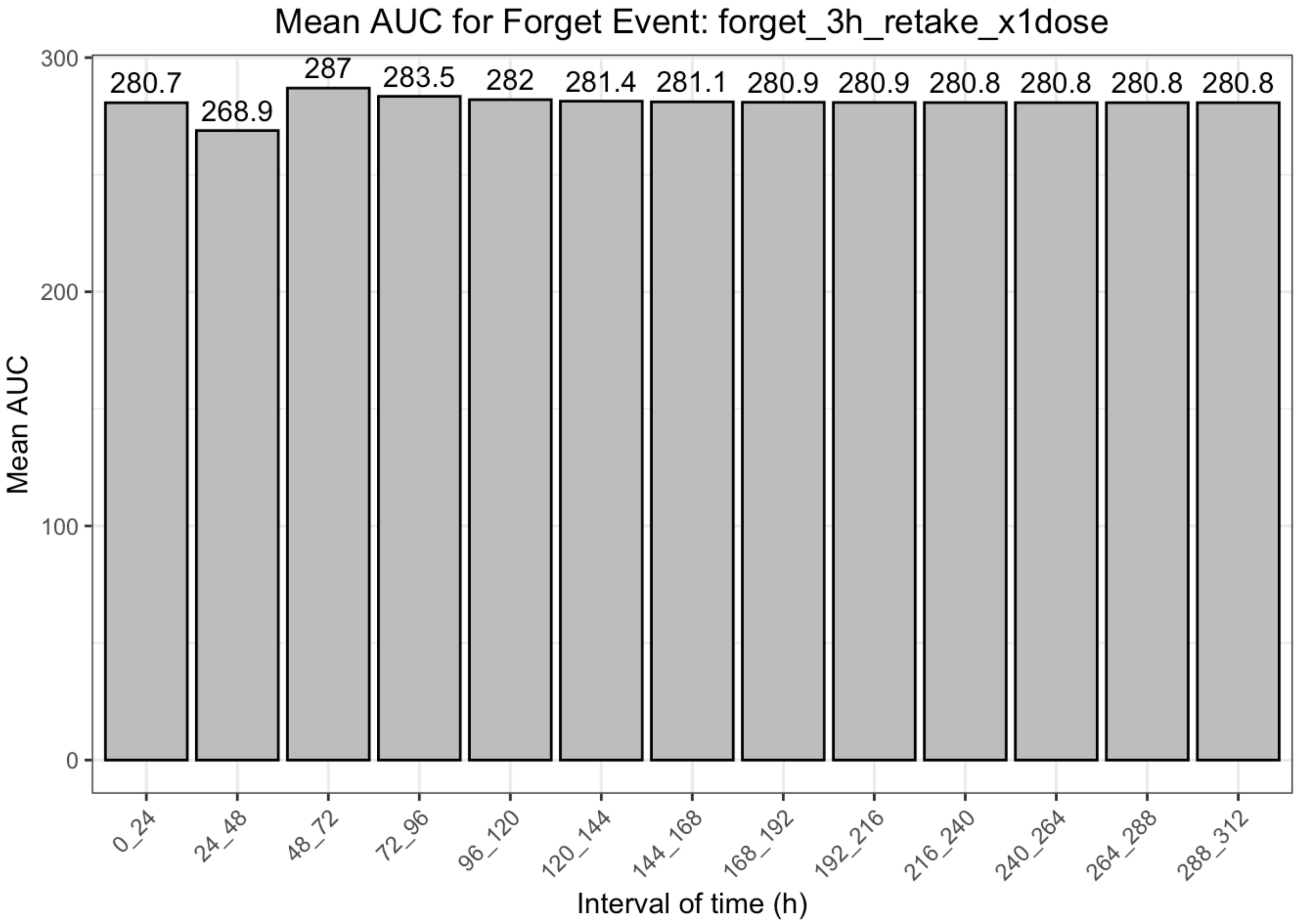
Mean AUC for Forget Event: forget_3h_retake_x1dose
Mean AUC
Interval of time (h)
0
100
200
300
280.7
268.9
287
283.5
282
281.4
281.1
280.9
280.9
280.8
280.8
280.8
280.8
0_24
24_48
48_72
72_96
96_120
120_144
144_168
168_192
192_216
216_240
240_264
264_288
288_312

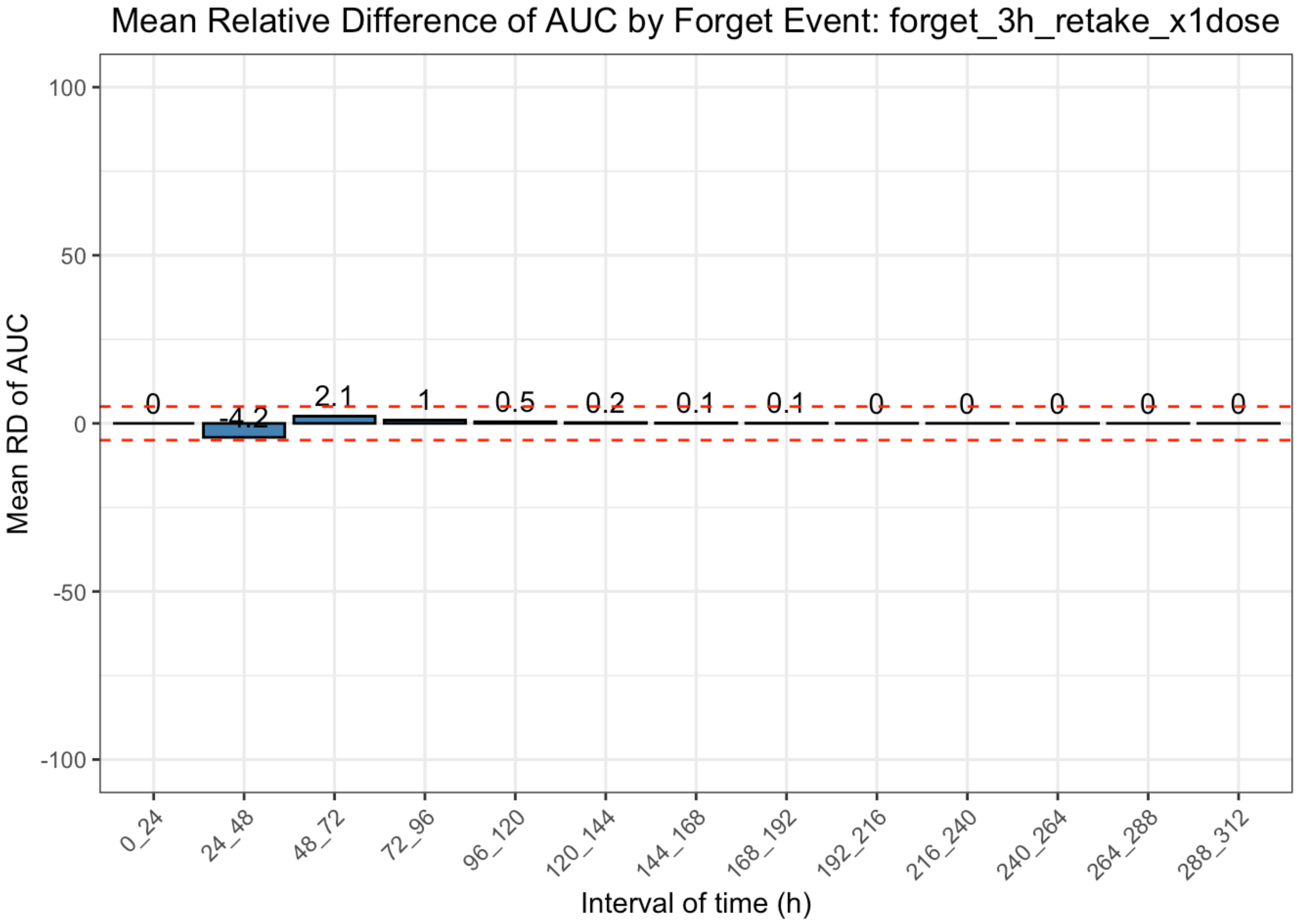
Mean Relative Difference of AUC by Forget Event: forget_3h_retake_x1dose
Mean RD of AUC
100
50
0
-50
-100
0
-4.2
2.1
1
0.5
0.2
0.1
0.1
0
0
0
0
0
0_24
24_48
48_72
72_96
96_120
120_144
144_168
168_192
192_216
216_240
240_264
264_288
288_312
Interval of time (h)

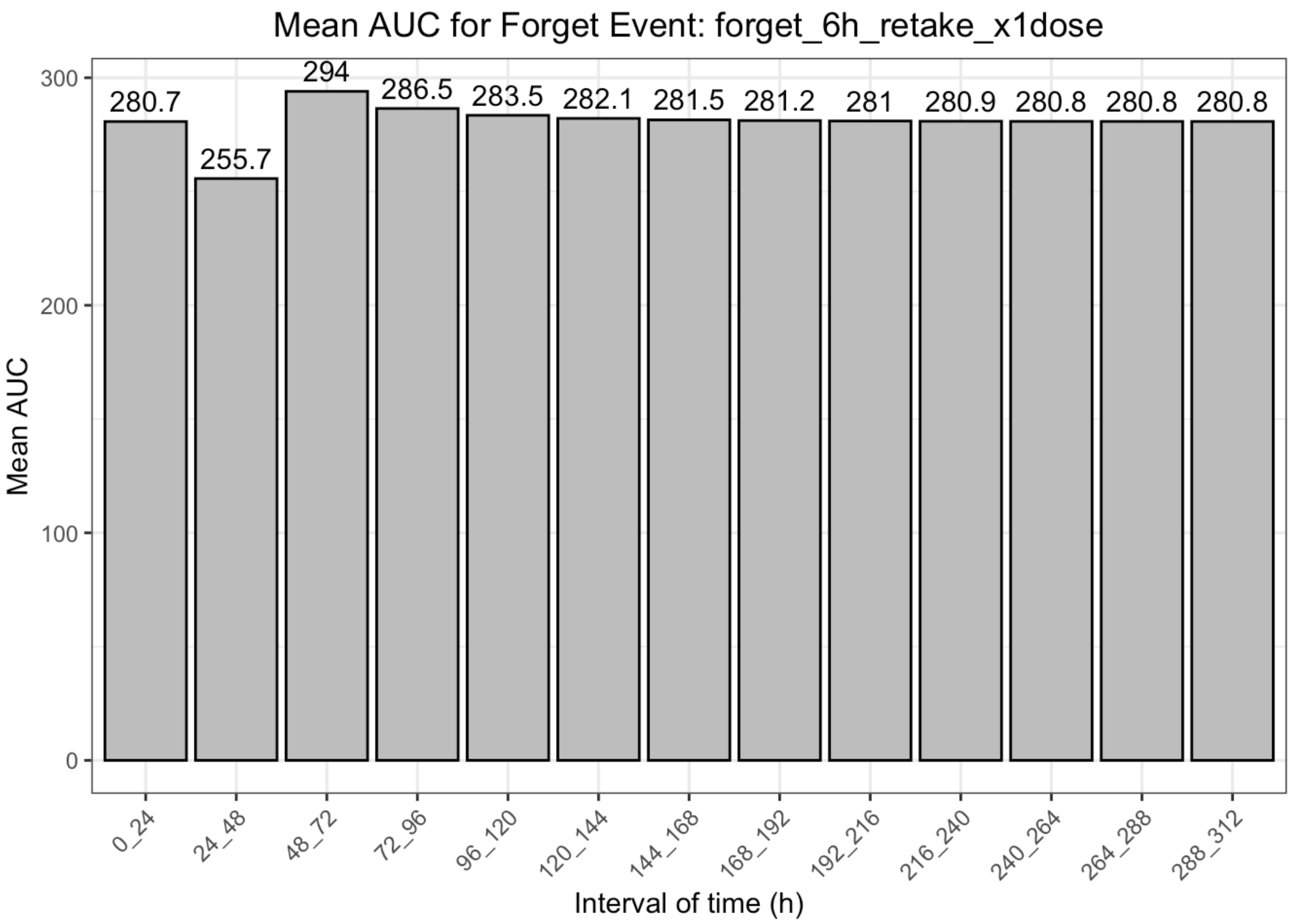
Mean AUC for Forget Event: forget_6h_retake_x1dose
Mean AUC
300
200
100
0
280.7
255.7
294
286.5
283.5
282.1
281.5
281.2
281
280.9
280.8
280.8
280.8
0_24
24_48
48_72
72_96
96_120
120_144
144_168
168_192
192_216
216_240
240_264
264_288
288_312
Interval of time (h)

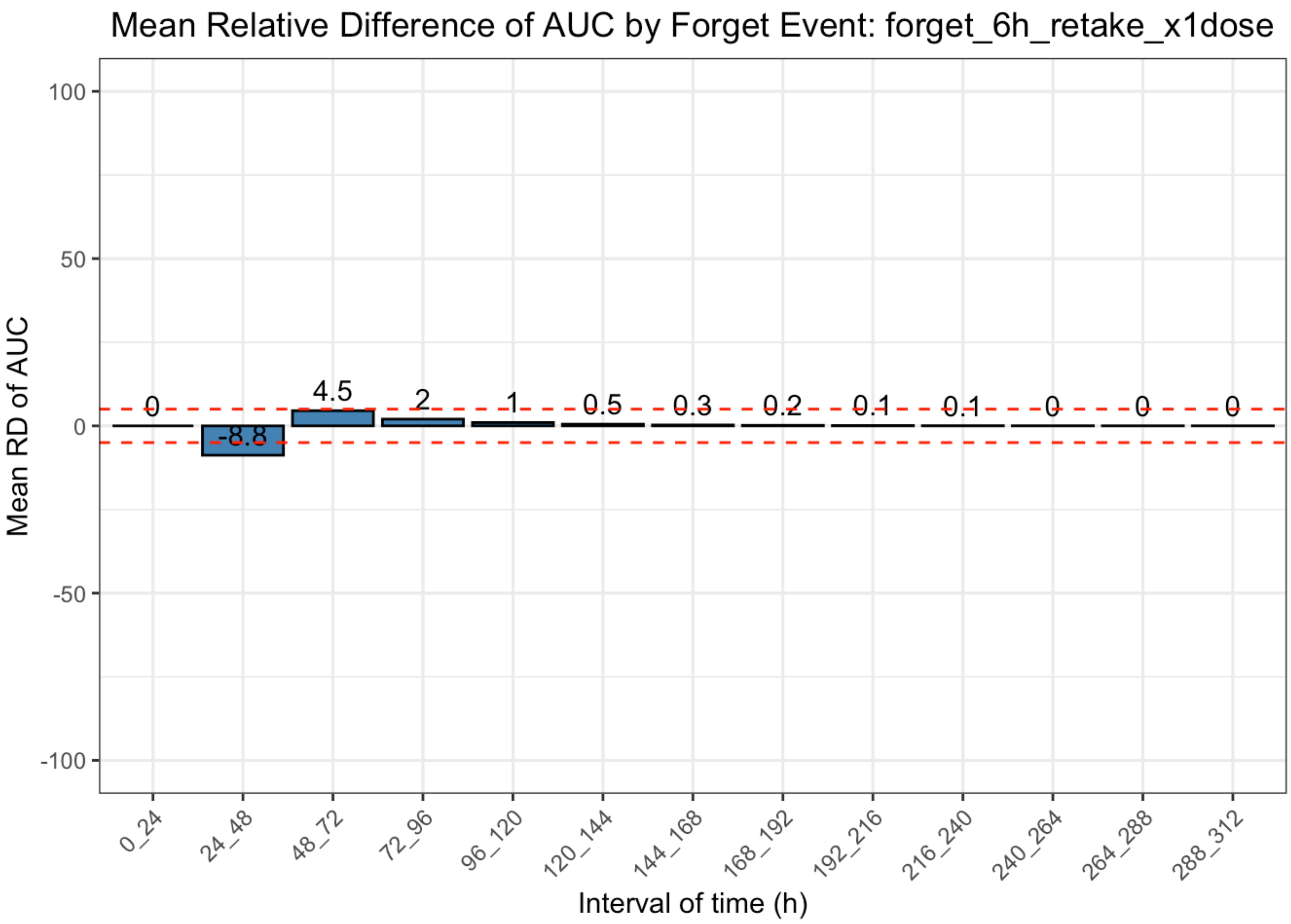
Mean Relative Difference of AUC by Forget Event: forget_6h_retake_x1dose
Mean RD of AUC
100
50
0
-50
-100
0
-8.8
4.5
2
1
0.5
0.3
0.2
0.1
0.1
0
0
0
0_24
24_48
48_72
72_96
96_120
120_144
144_168
168_192
192_216
216_240
240_264
264_288
288_312
Interval of time (h)

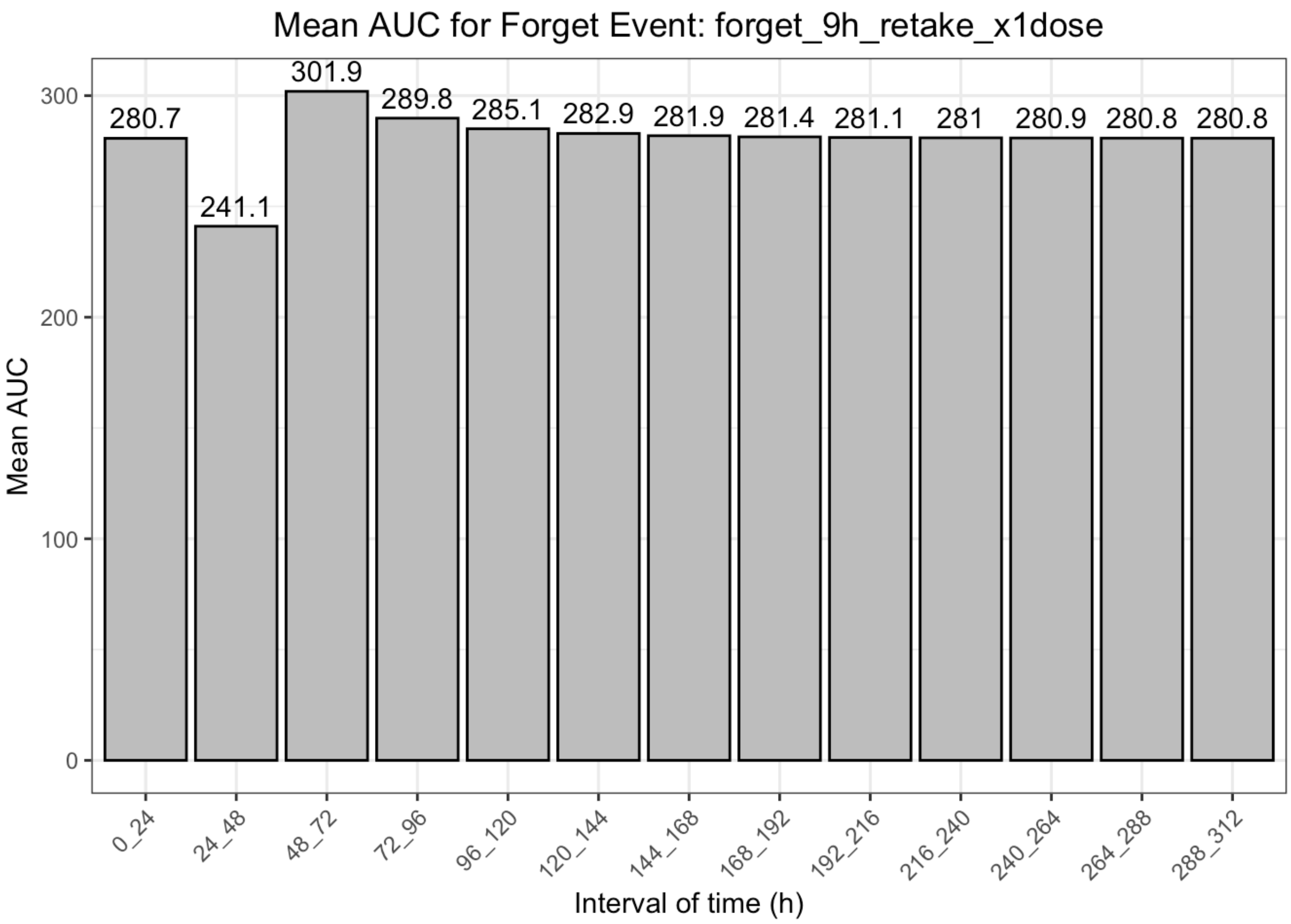
Mean AUC for Forget Event: forget_9h_retake_x1dose
Mean AUC
Interval of time (h)
280.7
241.1
301.9
289.8
285.1
282.9
281.9
281.4
281.1
281
280.9
280.8
280.8
0
100
200
300
0_24
24_48
48_72
72_96
96_120
120_144
144_168
168_192
192_216
216_240
240_264
264_288
288_312

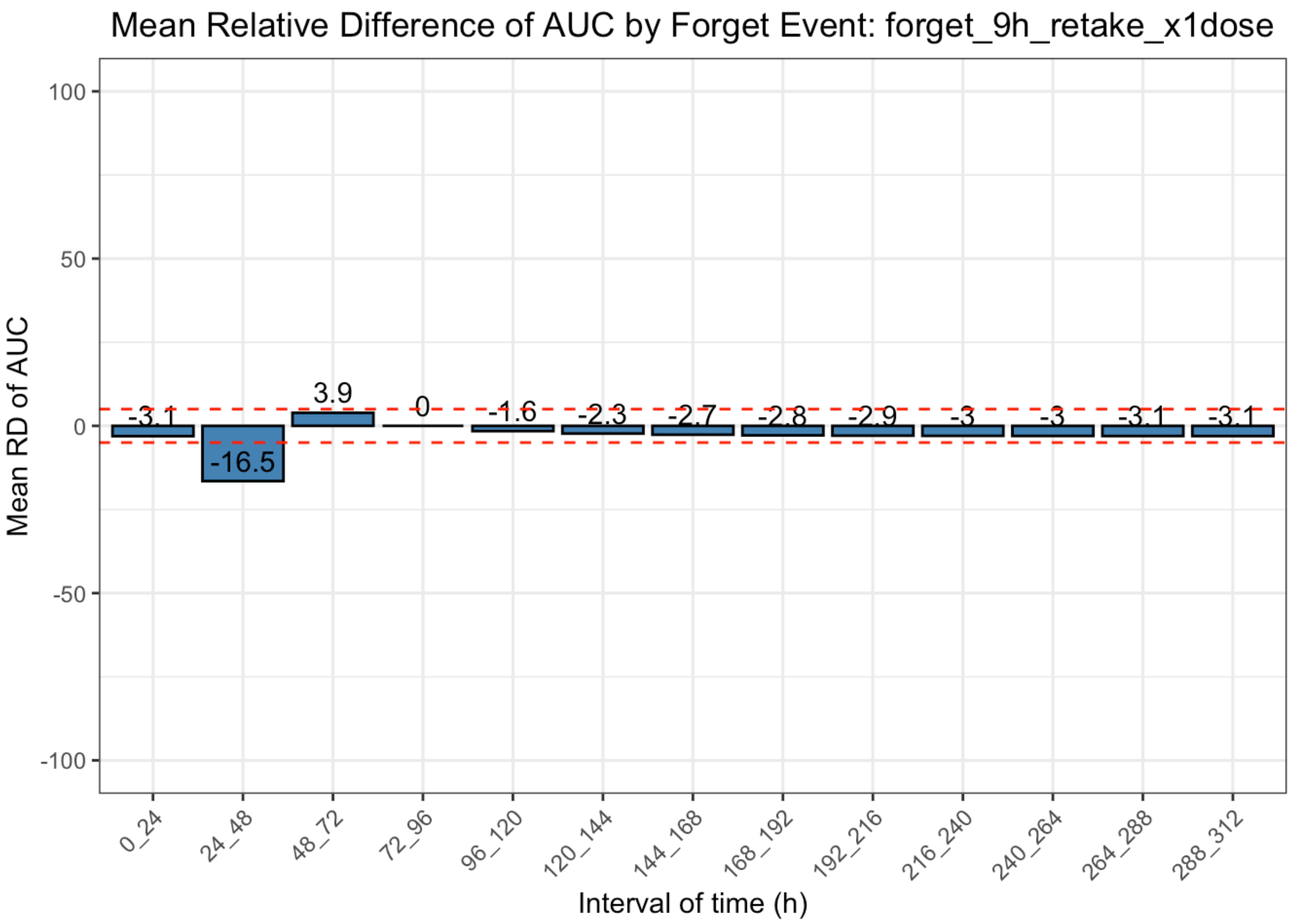
Mean Relative Difference of AUC by Forget Event: forget_9h_retake_x1dose
Mean RD of AUC
100
50
0
-50
-100
-3.1
-16.5
3.9
0
-1.6
-2.3
-2.7
-2.8
-2.9
-3
-3
-3.1
-3.1
0_24
24_48
48_72
72_96
96_120
120_144
144_168
168_192
192_216
216_240
240_264
264_288
288_312
Interval of time (h)

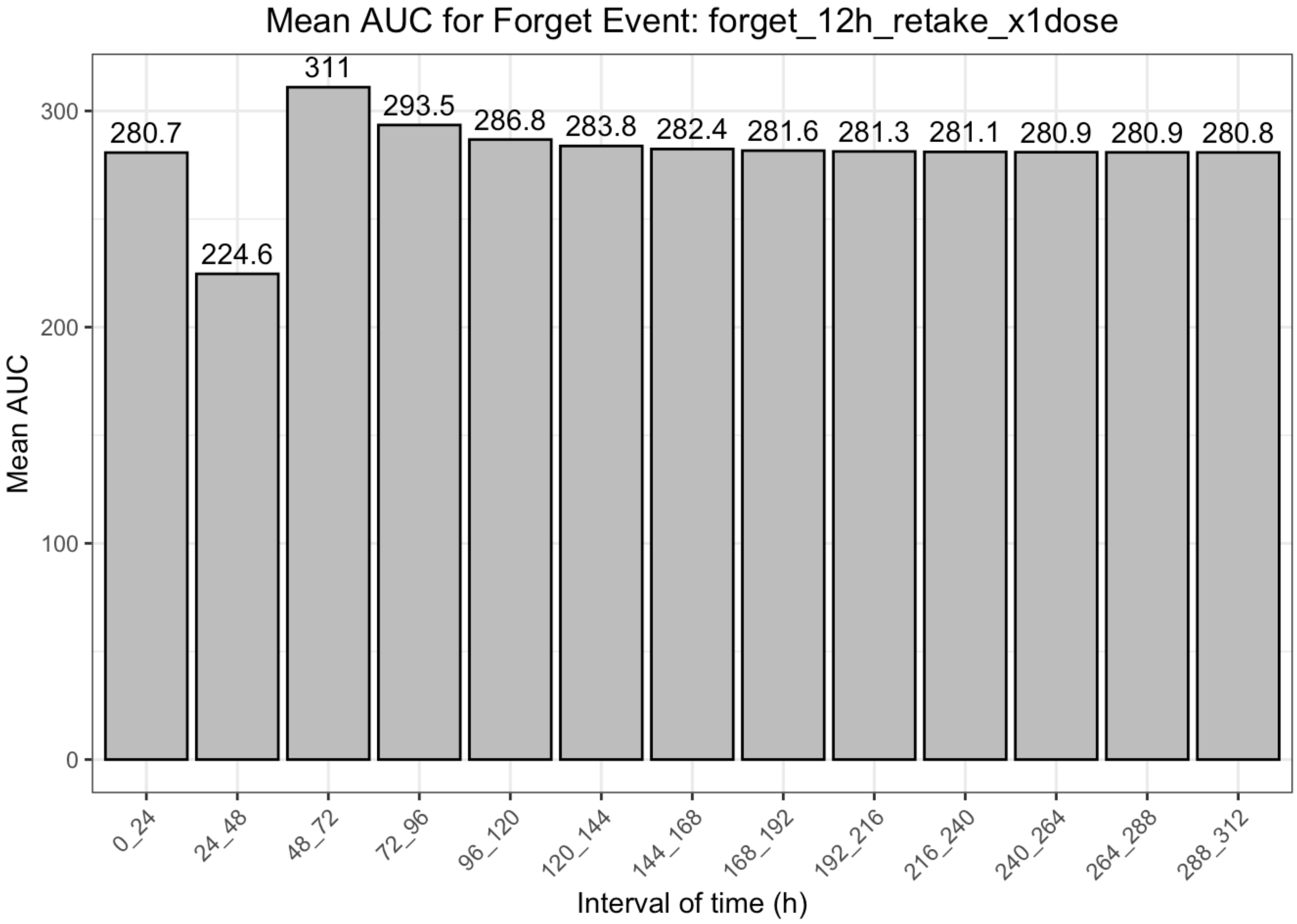
Mean AUC for Forget Event: forget_12h_retake_x1dose
Mean AUC
Interval of time (h)
0
100
200
300
280.7
224.6
311
293.5
286.8
283.8
282.4
281.6
281.3
281.1
280.9
280.9
280.8
0_24
24_48
48_72
72_96
96_120
120_144
144_168
168_192
192_216
216_240
240_264
264_288
288_312

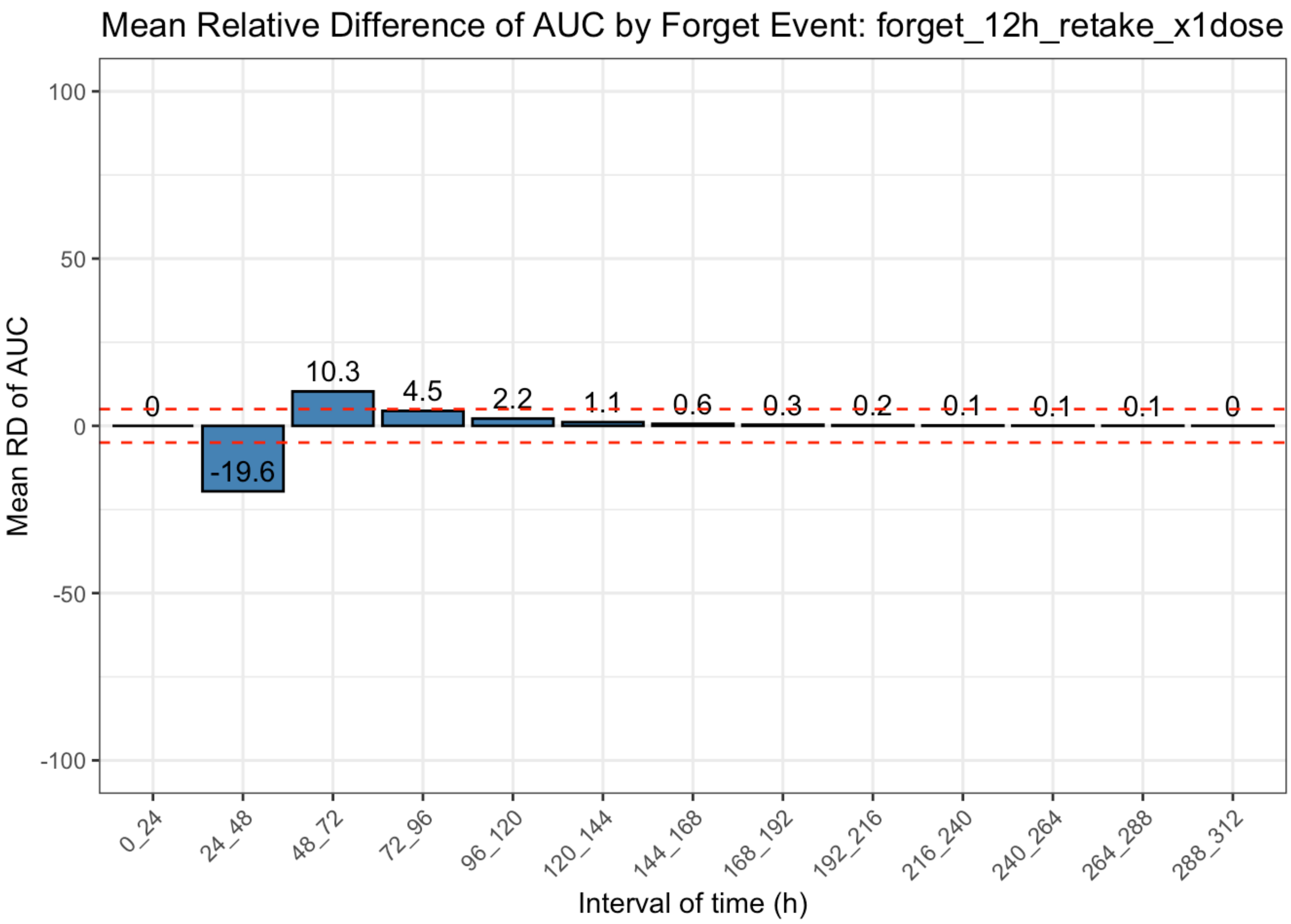
Mean Relative Difference of AUC by Forget Event: forget_12h_retake_x1dose
Mean RD of AUC
100
50
0
-50
-100
0
-19.6
10.3
4.5
2.2
1.1
0.6
0.3
0.2
0.1
0.1
0.1
0
0_24
24_48
48_72
72_96
96_120
120_144
144_168
168_192
192_216
216_240
240_264
264_288
288_312
Interval of time (h)

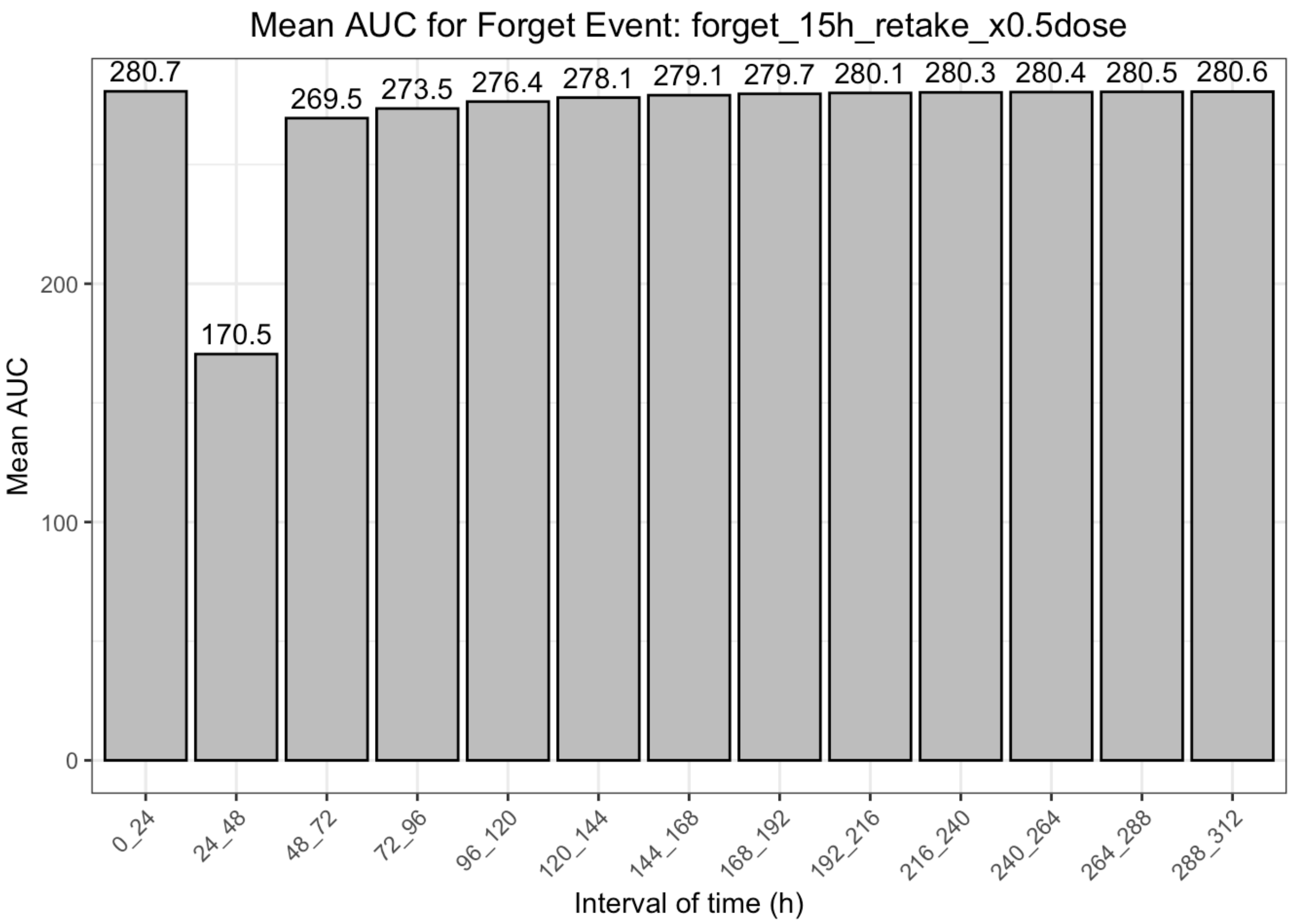
Mean AUC for Forget Event: forget_15h_retake_x0.5dose
280.7
170.5
269.5
273.5
276.4
278.1
279.1
279.7
280.1
280.3
280.4
280.5
280.6
Mean AUC
0
100
200
0_24
24_48
48_72
72_96
96_120
120_144
144_168
168_192
192_216
216_240
240_264
264_288
288_312
Interval of time (h)

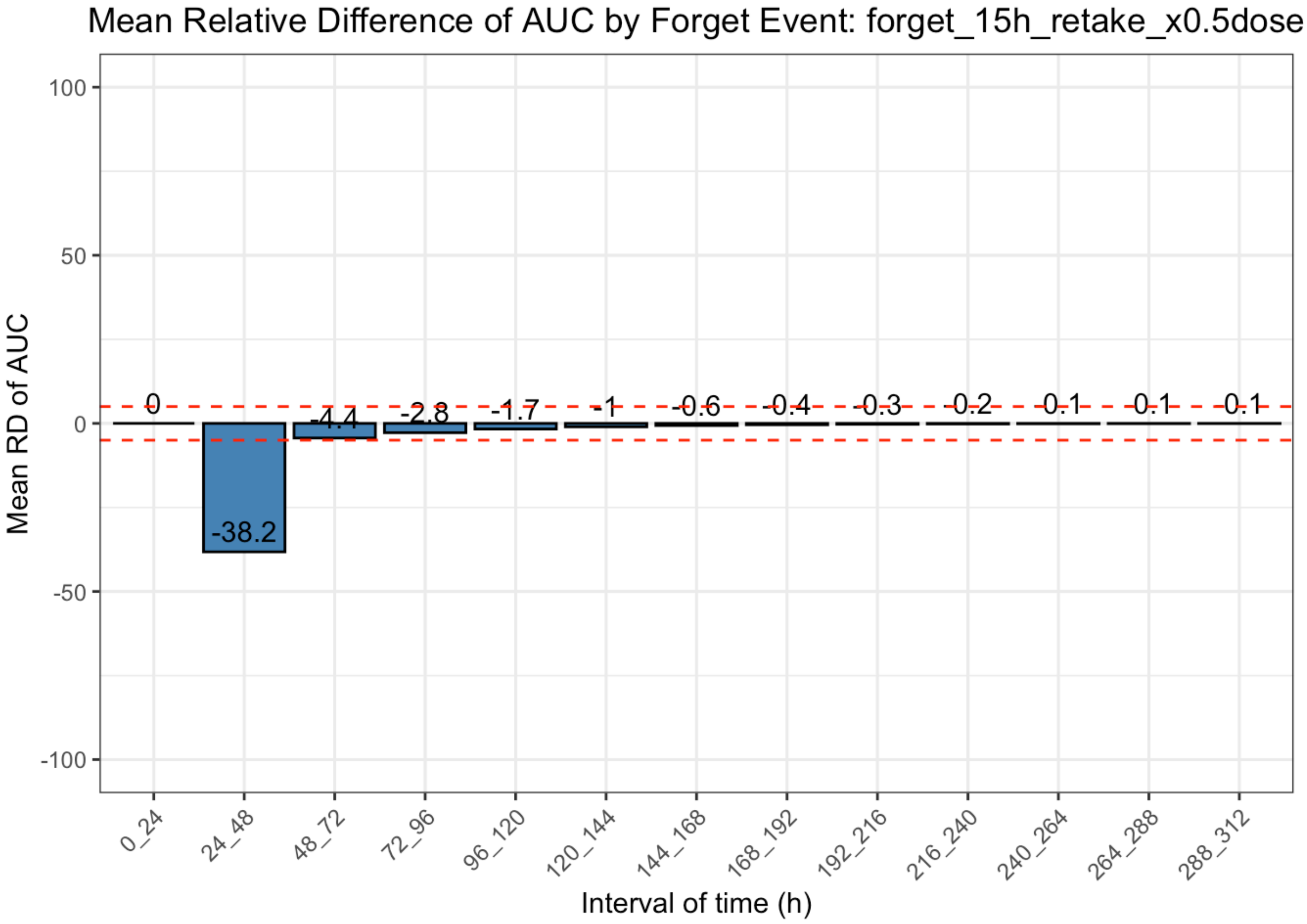
Mean Relative Difference of AUC by Forget Event: forget_15h_retake_x0.5dose
Mean RD of AUC
Interval of time (h)
100
50
0
-50
-100
0
-38.2
-4.4
-2.8
-1.7
-1
-0.6
-0.4
-0.3
-0.2
-0.1
-0.1
-0.1
0_24
24_48
48_72
72_96
96_120
120_144
144_168
168_192
192_216
216_240
240_264
264_288
288_312

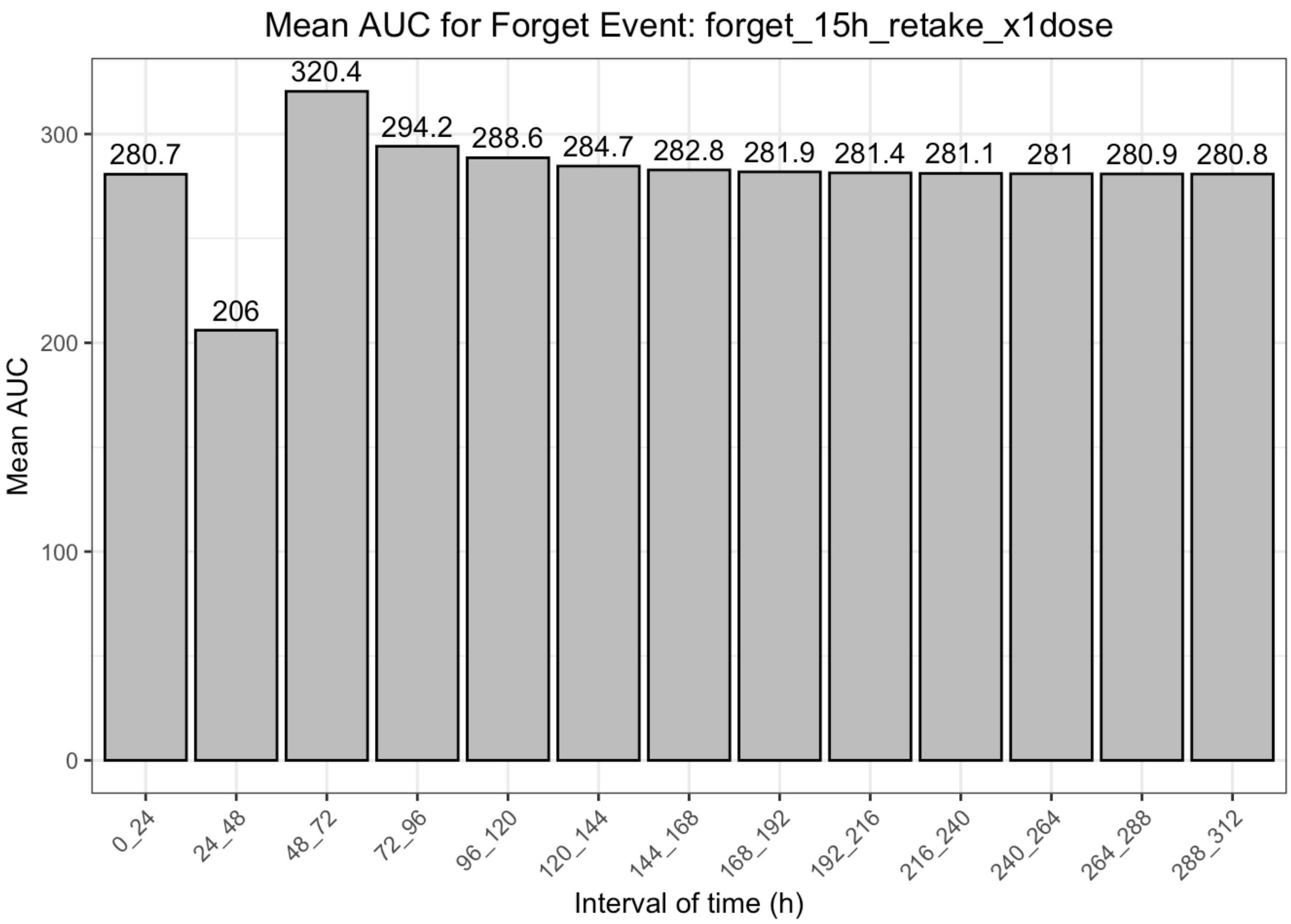
Mean AUC for Forget Event: forget_15h_retake_x1dose
Mean AUC
Interval of time (h)
0
100
200
300
280.7
206
320.4
294.2
288.6
284.7
282.8
281.9
281.4
281.1
281
280.9
280.8
0_24
24_48
48_72
72_96
96_120
120_144
144_168
168_192
192_216
216_240
240_264
264_288
288_312

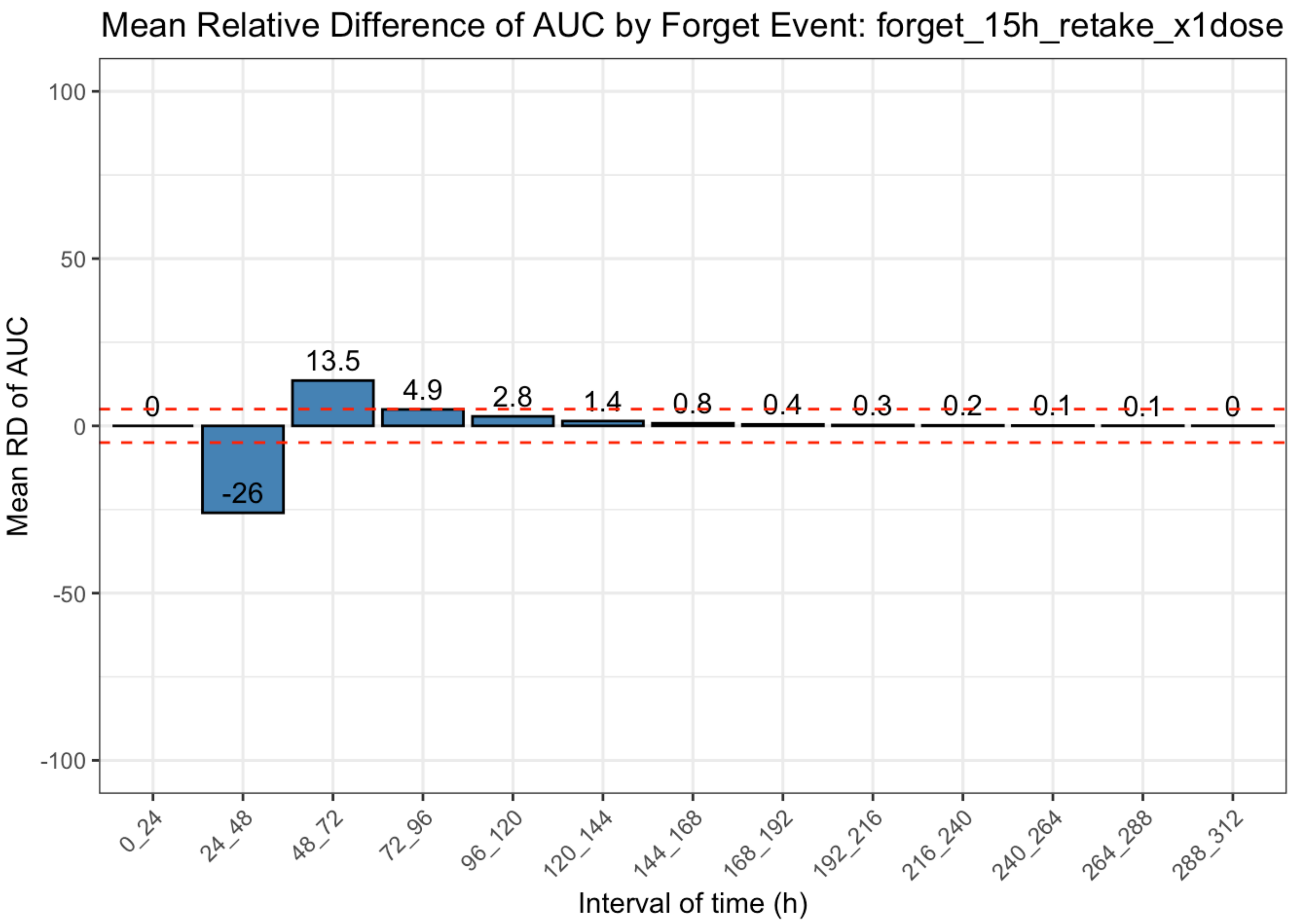
Mean Relative Difference of AUC by Forget Event: forget_15h_retake_x1dose
Mean RD of AUC
100
50
0
-50
-100
0
-26
13.5
4.9
2.8
1.4
0.8
0.4
0.3
0.2
0.1
0.1
0
0_24
24_48
48_72
72_96
96_120
120_144
144_168
168_192
192_216
216_240
240_264
264_288
288_312
Interval of time (h)

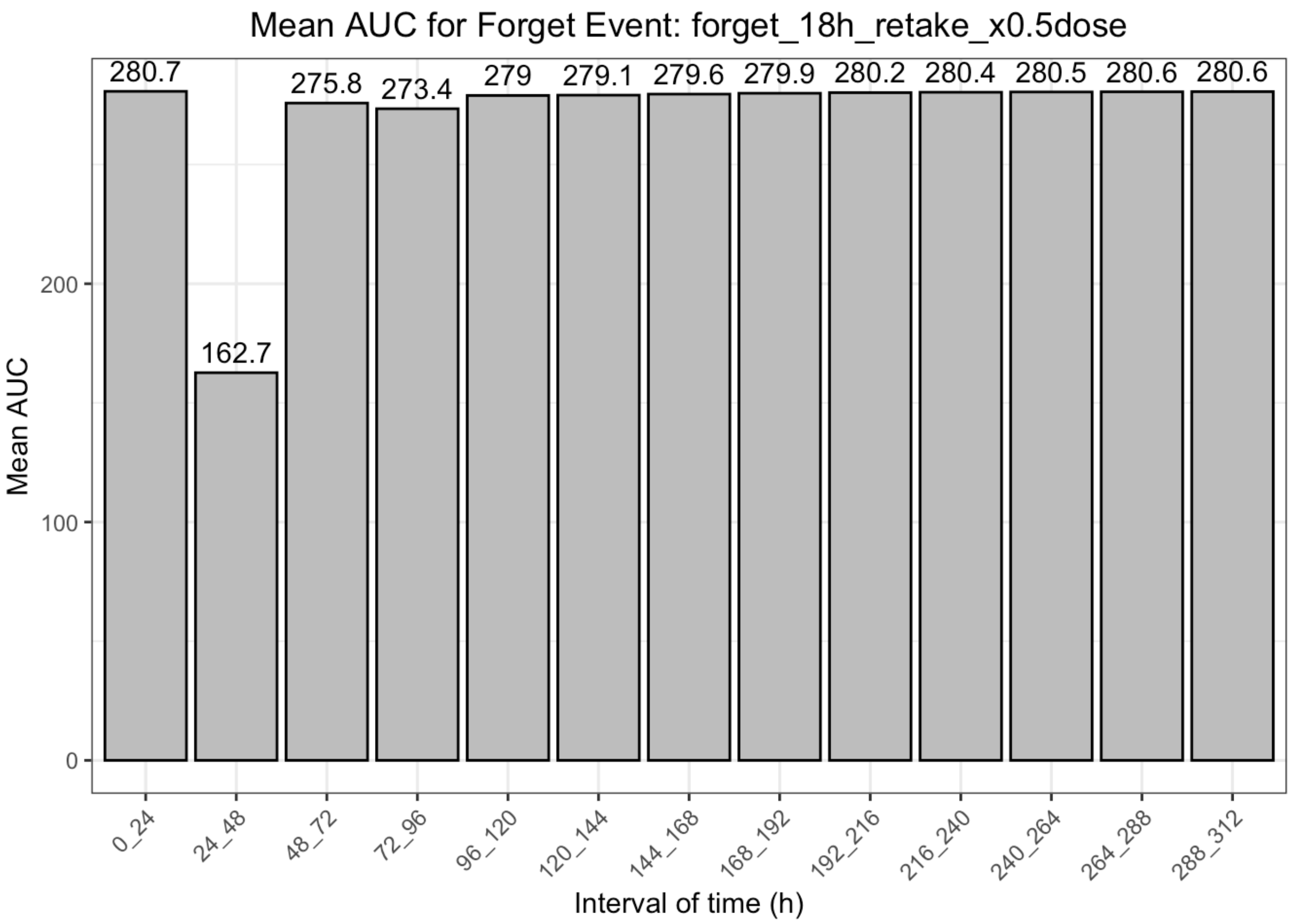
Mean AUC for Forget Event: forget_18h_retake_x0.5dose
280.7
162.7
275.8
273.4
279
279.1
279.6
279.9
280.2
280.4
280.5
280.6
280.6
Mean AUC
0
100
200
0_24
24_48
48_72
72_96
96_120
120_144
144_168
168_192
192_216
216_240
240_264
264_288
288_312
Interval of time (h)

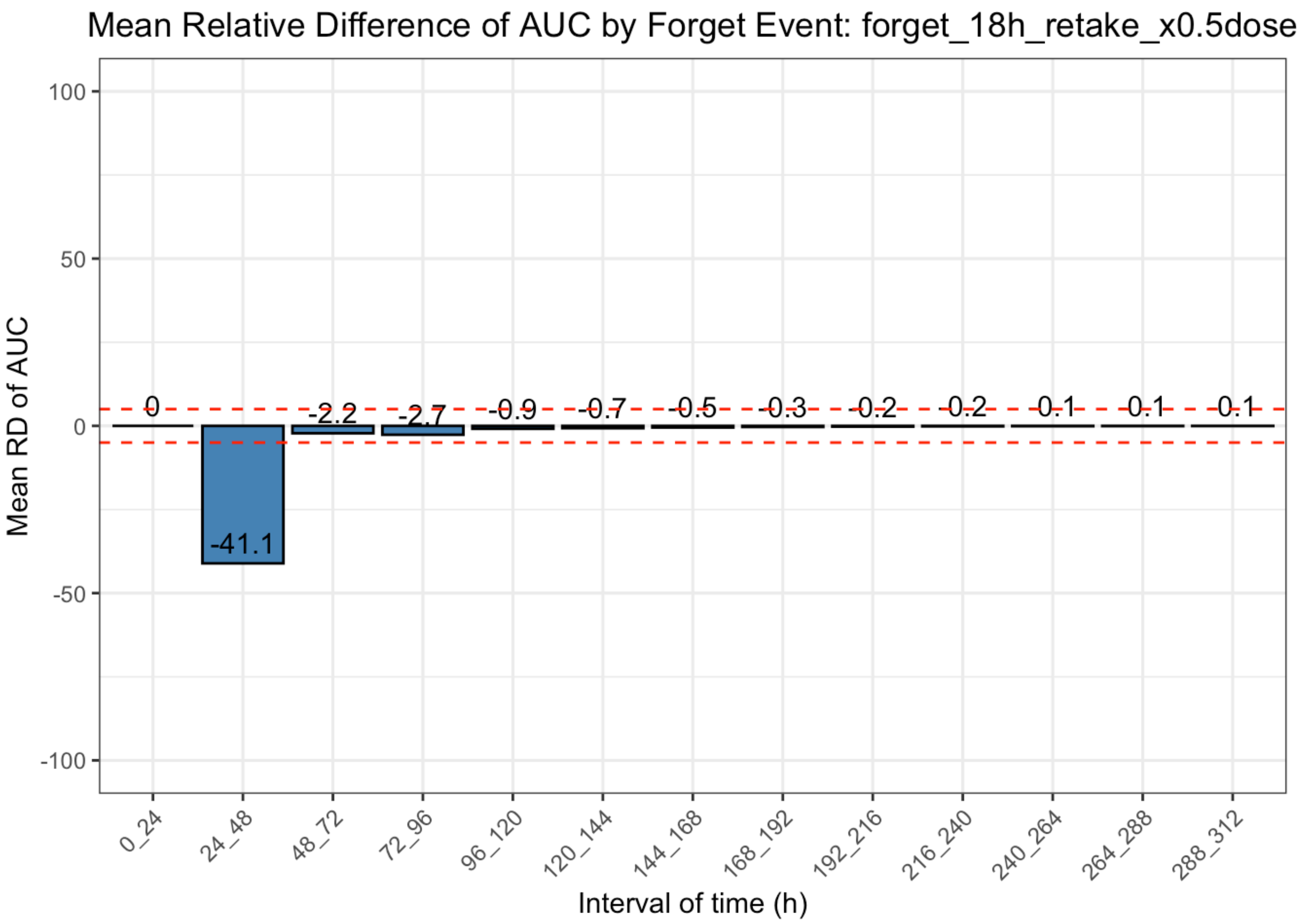
Mean Relative Difference of AUC by Forget Event: forget_18h_retake_x0.5dose
Mean RD of AUC
100
50
0
-50
-100
0
-41.1
-2.2
-2.7
-0.9
-0.7
-0.5
-0.3
-0.2
-0.2
-0.1
-0.1
-0.1
0_24
24_48
48_72
72_96
96_120
120_144
144_168
168_192
192_216
216_240
240_264
264_288
288_312
Interval of time (h)

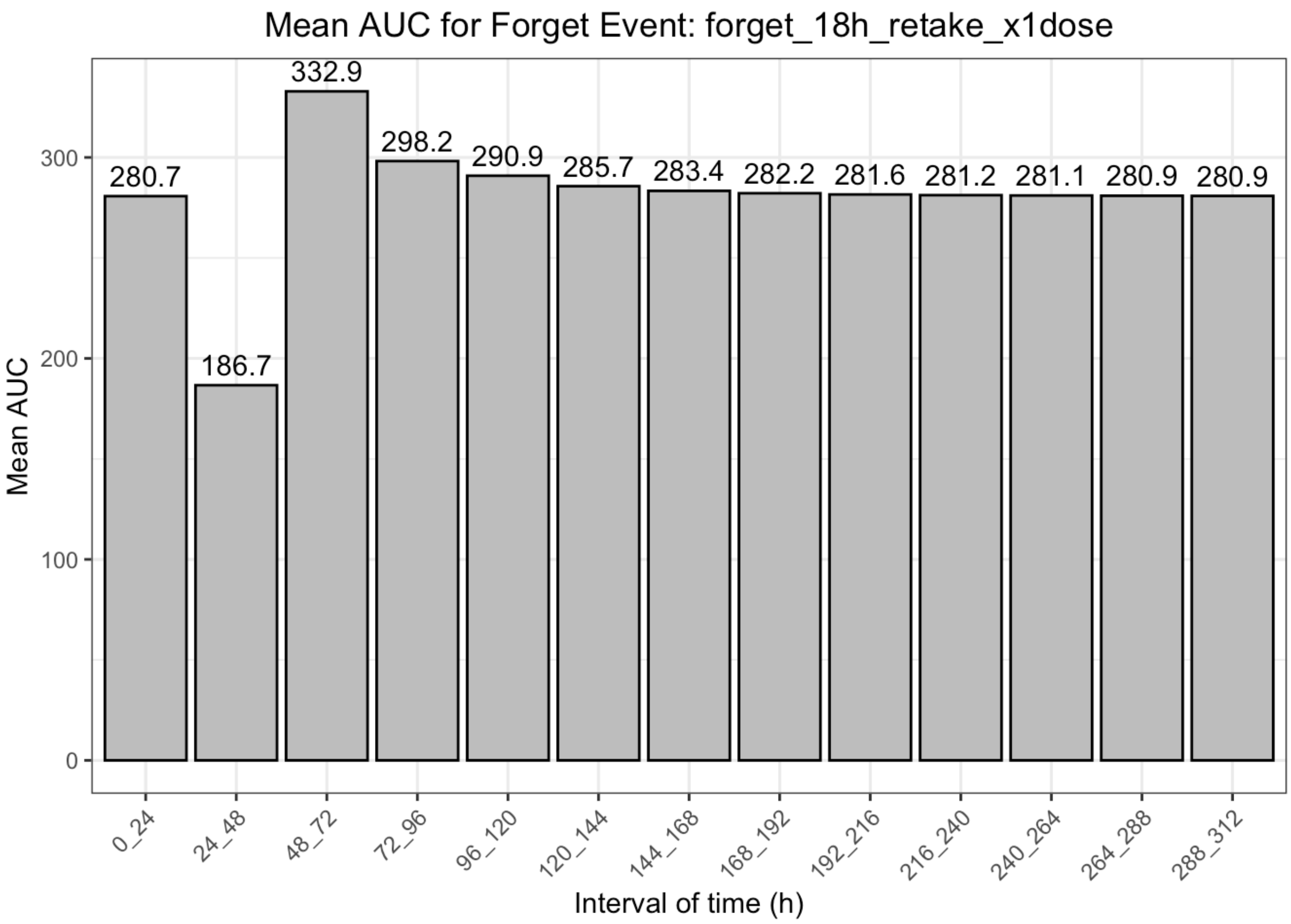
Mean AUC for Forget Event: forget_18h_retake_x1dose
Mean AUC
Interval of time (h)
0
100
200
300
0_24
24_48
48_72
72_96
96_120
120_144
144_168
168_192
192_216
216_240
240_264
264_288
288_312
280.7
186.7
332.9
298.2
290.9
285.7
283.4
282.2
281.6
281.2
281.1
280.9
280.9

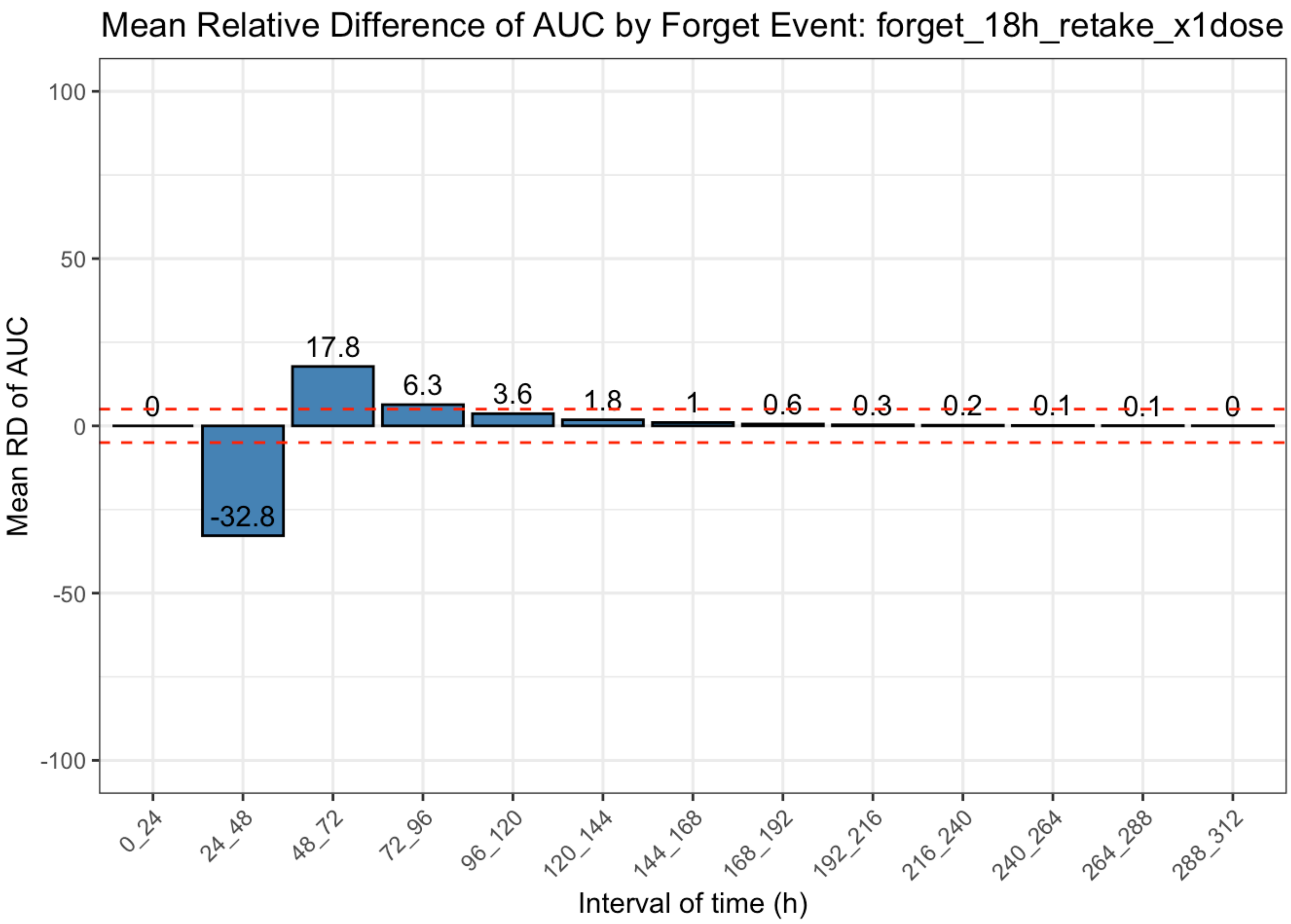
Mean Relative Difference of AUC by Forget Event: forget_18h_retake_x1dose
Mean RD of AUC
100
50
0
-50
-100
0
-32.8
17.8
6.3
3.6
1.8
1
0.6
0.3
0.2
0.1
0.1
0
0_24
24_48
48_72
72_96
96_120
120_144
144_168
168_192
192_216
216_240
240_264
264_288
288_312
Interval of time (h)

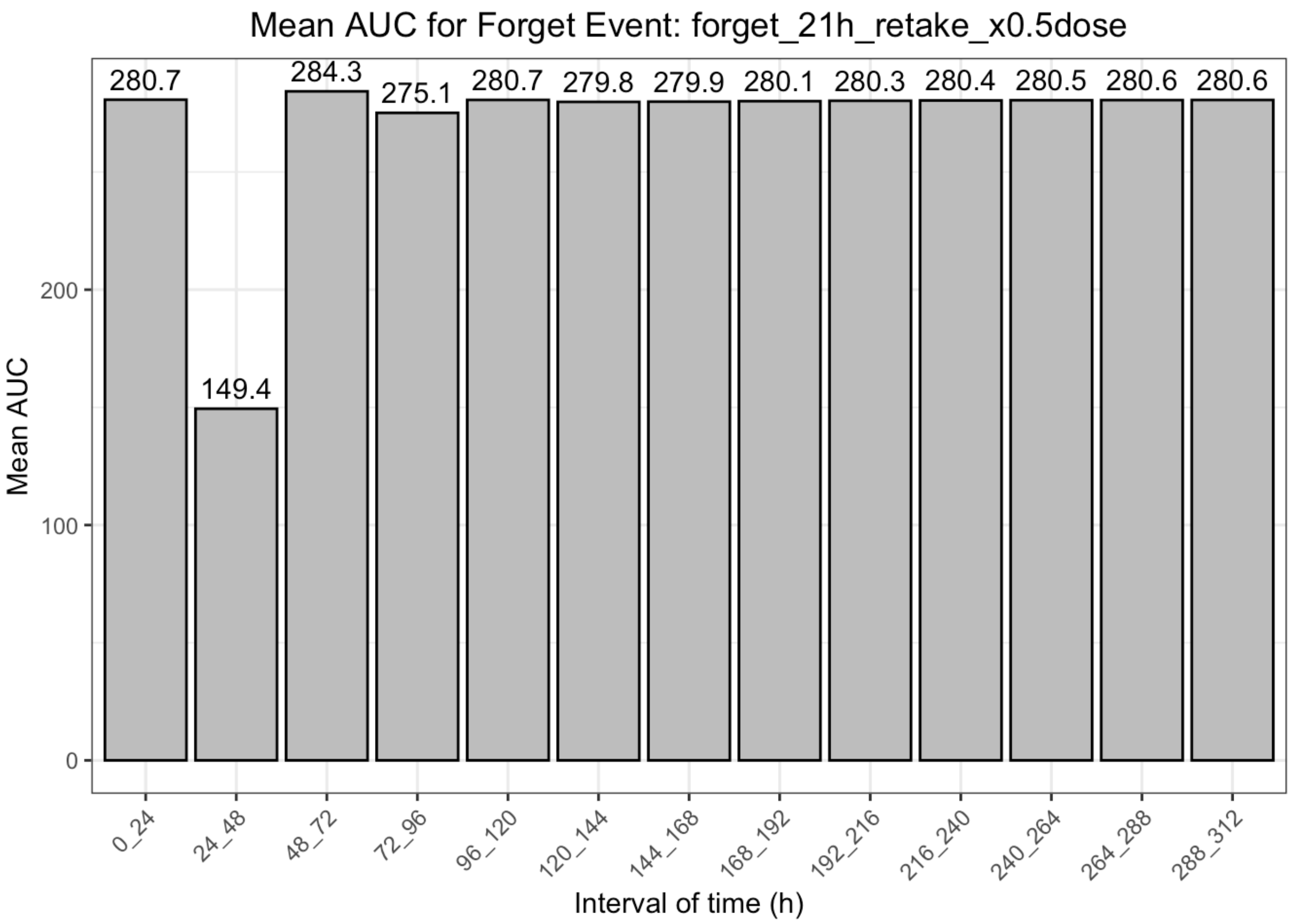
Mean AUC for Forget Event: forget_21h_retake_x0.5dose
280.7
149.4
284.3
275.1
280.7
279.8
279.9
280.1
280.3
280.4
280.5
280.6
280.6
Mean AUC
200
100
0
0_24
24_48
48_72
72_96
96_120
120_144
144_168
168_192
192_216
216_240
240_264
264_288
288_312
Interval of time (h)

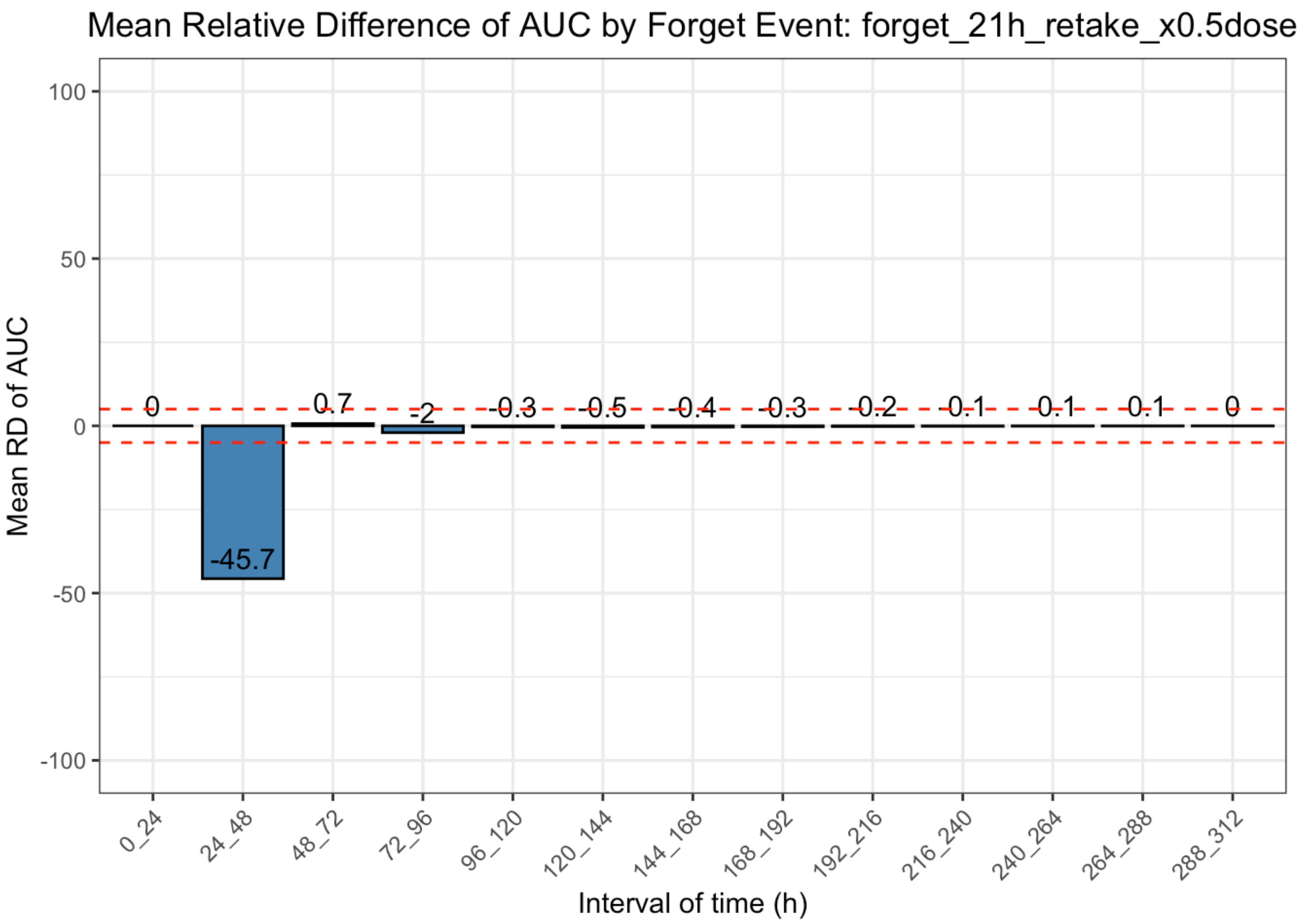
Mean Relative Difference of AUC by Forget Event: forget_21h_retake_x0.5dose
Mean RD of AUC
100
50
0
-50
-100
0
-45.7
0.7
-2
-0.3
-0.5
-0.4
-0.3
-0.2
-0.1
-0.1
-0.1
0
0_24
24_48
48_72
72_96
96_120
120_144
144_168
168_192
192_216
216_240
240_264
264_288
288_312
Interval of time (h)

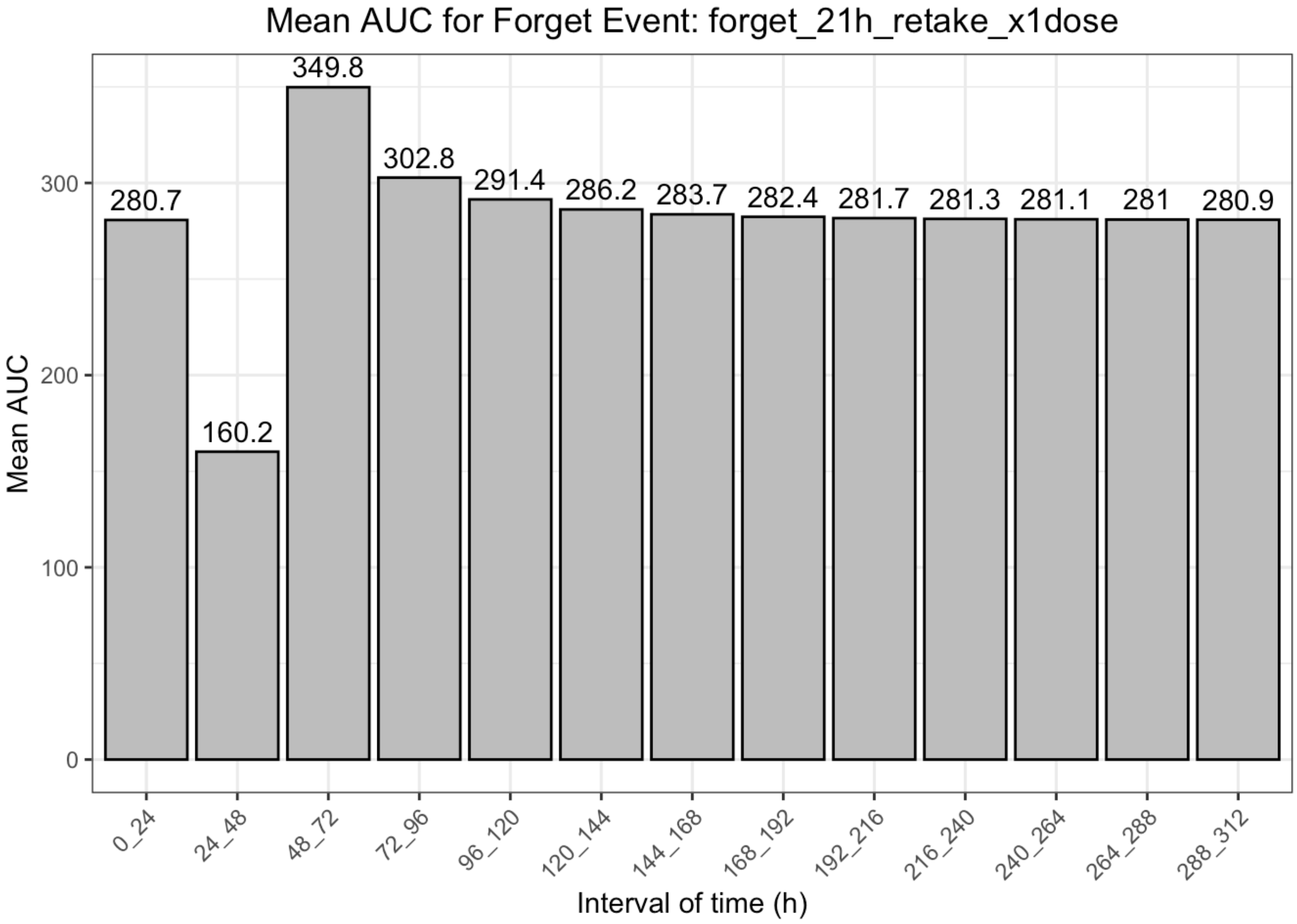
Mean AUC for Forget Event: forget_21h_retake_x1dose
Mean AUC
Interval of time (h)
0
100
200
300
280.7
160.2
349.8
302.8
291.4
286.2
283.7
282.4
281.7
281.3
281.1
281
280.9
0_24
24_48
48_72
72_96
96_120
120_144
144_168
168_192
192_216
216_240
240_264
264_288
288_312

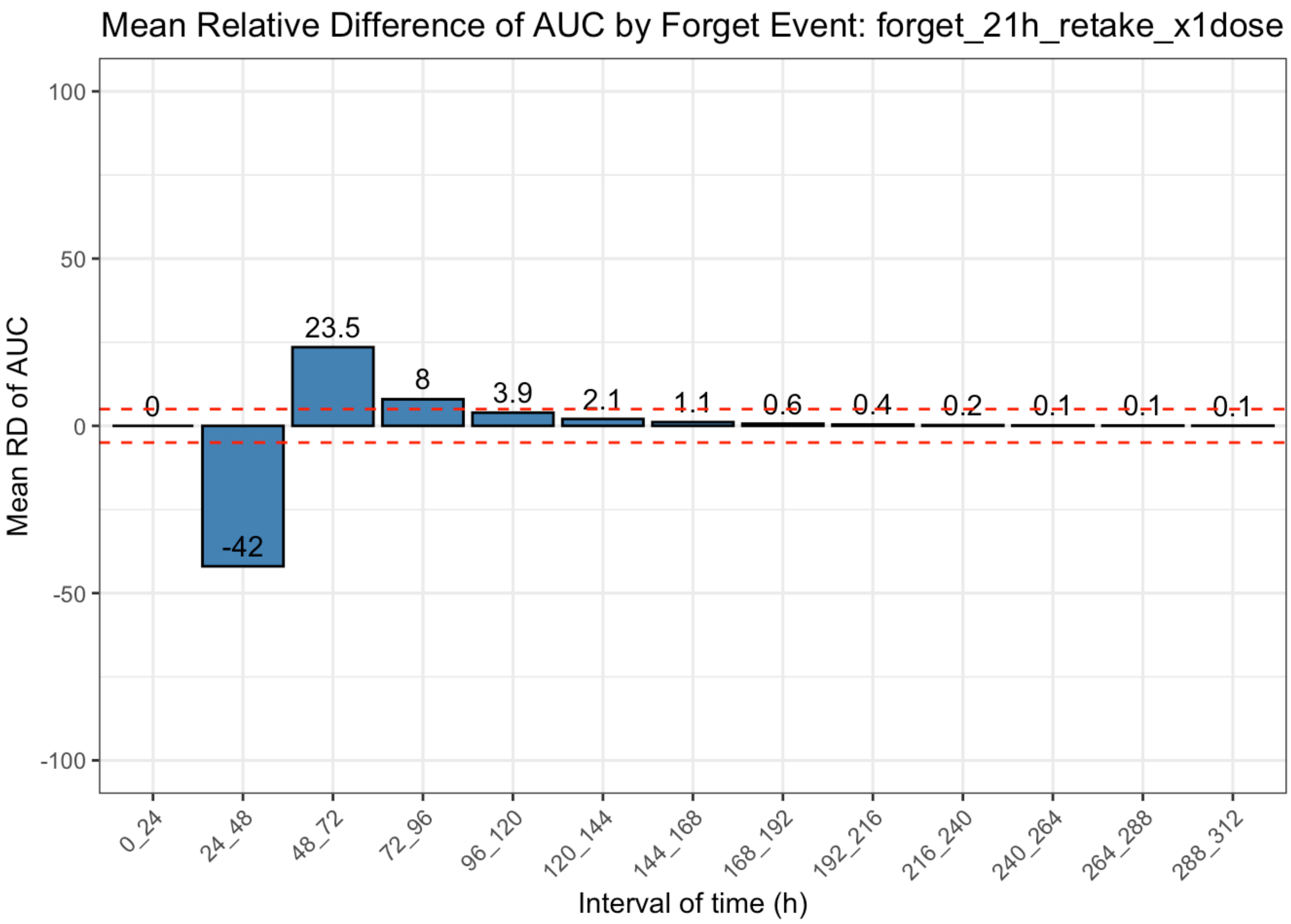
Mean Relative Difference of AUC by Forget Event: forget_21h_retake_x1dose
Mean RD of AUC
100
50
0
-50
-100
0
-42
23.5
8
3.9
2.1
1.1
0.6
0.4
0.2
0.1
0.1
0.1
0_24
24_48
48_72
72_96
96_120
120_144
144_168
168_192
192_216
216_240
240_264
264_288
288_312
Interval of time (h)

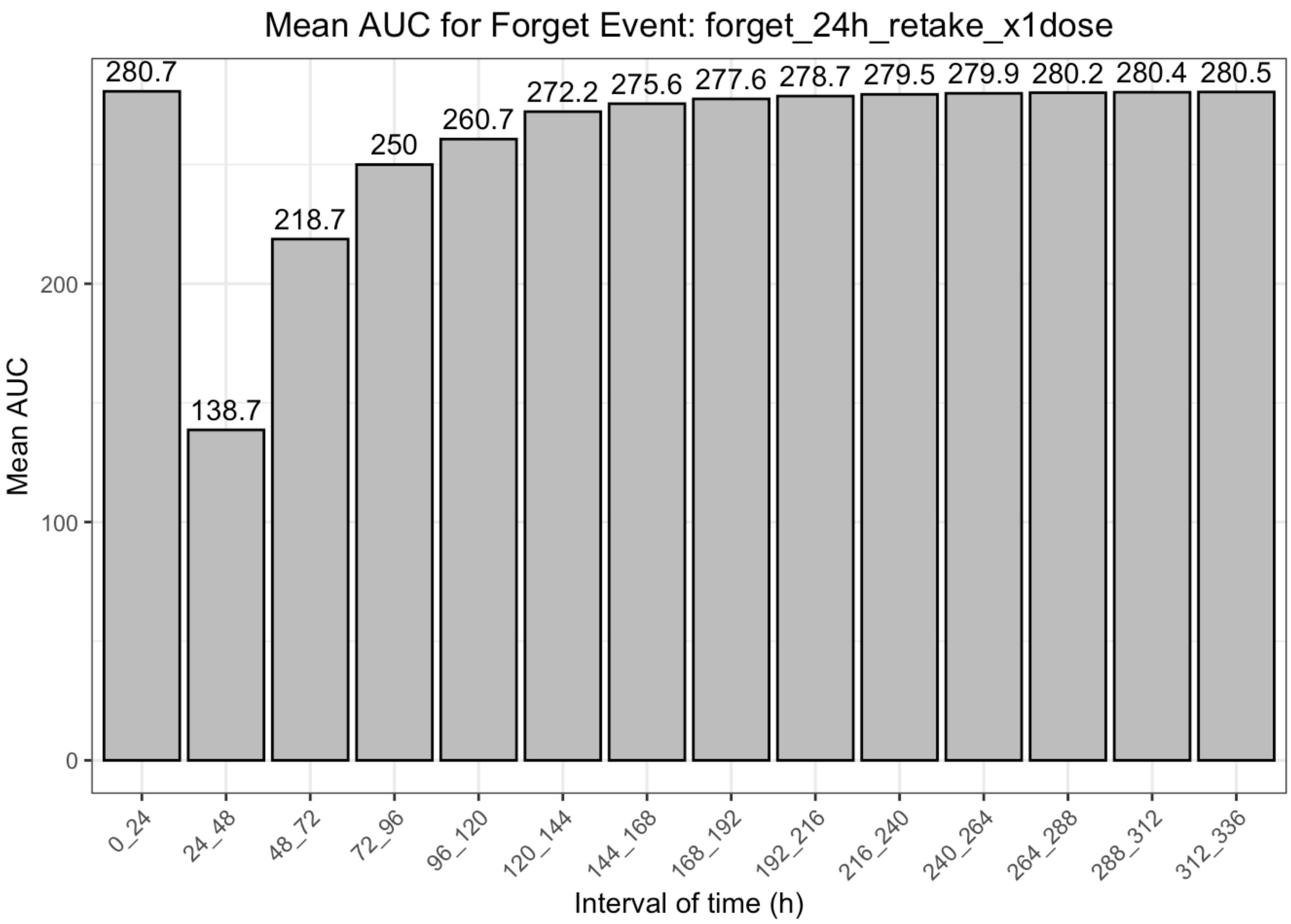
Mean AUC for Forget Event: forget_24h_retake_x1dose
Mean AUC
Interval of time (h)
280.7
138.7
218.7
250
260.7
272.2
275.6
277.6
278.7
279.5
279.9
280.2
280.4
280.5
0
100
200
0_24
24_48
48_72
72_96
96_120
120_144
144_168
168_192
192_216
216_240
240_264
264_288
288_312
312_336

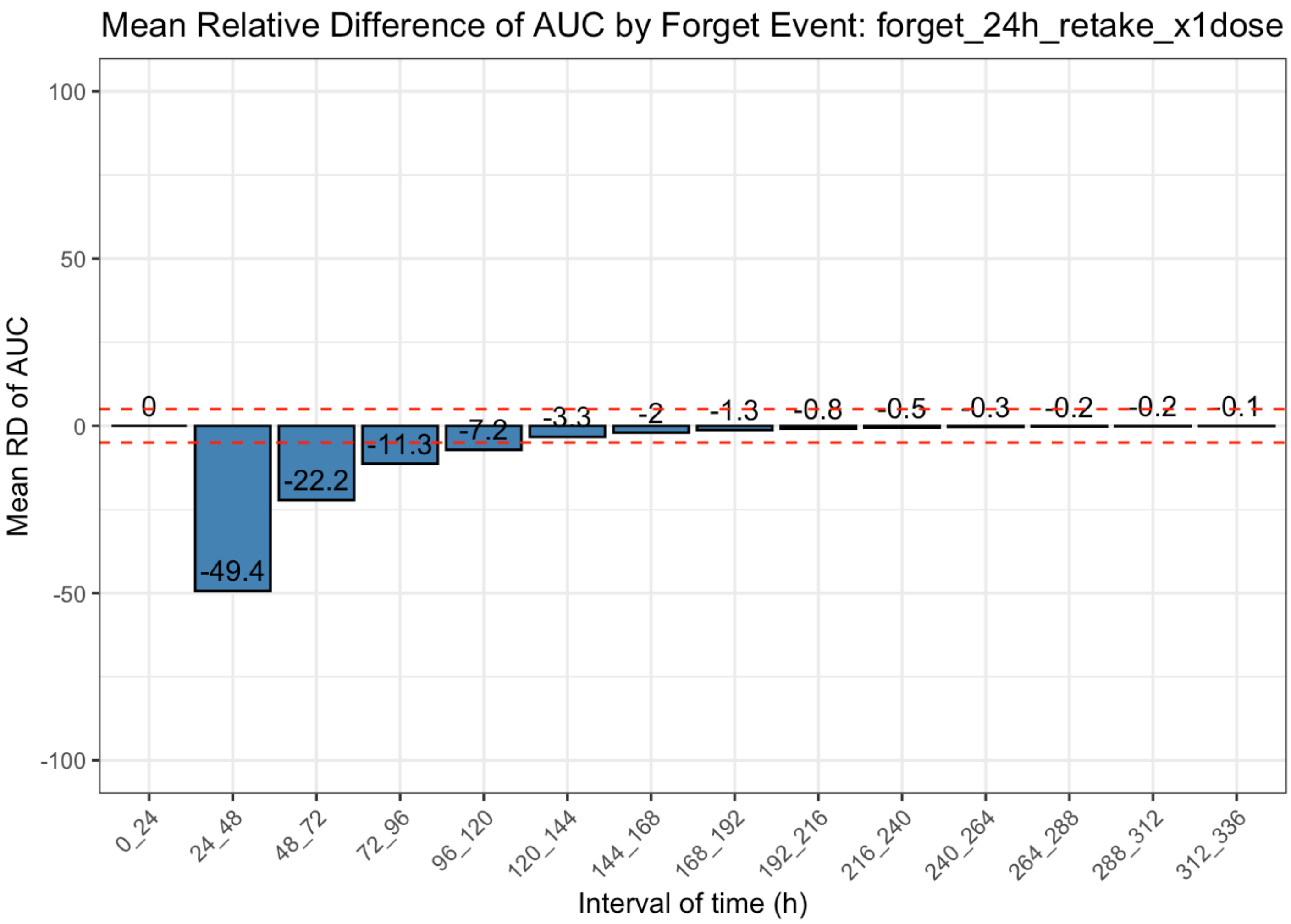
Mean Relative Difference of AUC by Forget Event: forget_24h_retake_x1dose
Mean RD of AUC
Interval of time (h)
100
50
0
-50
-100
0
-49.4
-22.2
-11.3
-7.2
-3.3
-2
-1.3
-0.8
-0.5
-0.3
-0.2
-0.2
-0.1
0_24
24_48
48_72
72_96
96_120
120_144
144_168
168_192
192_216
216_240
240_264
264_288
288_312
312_336

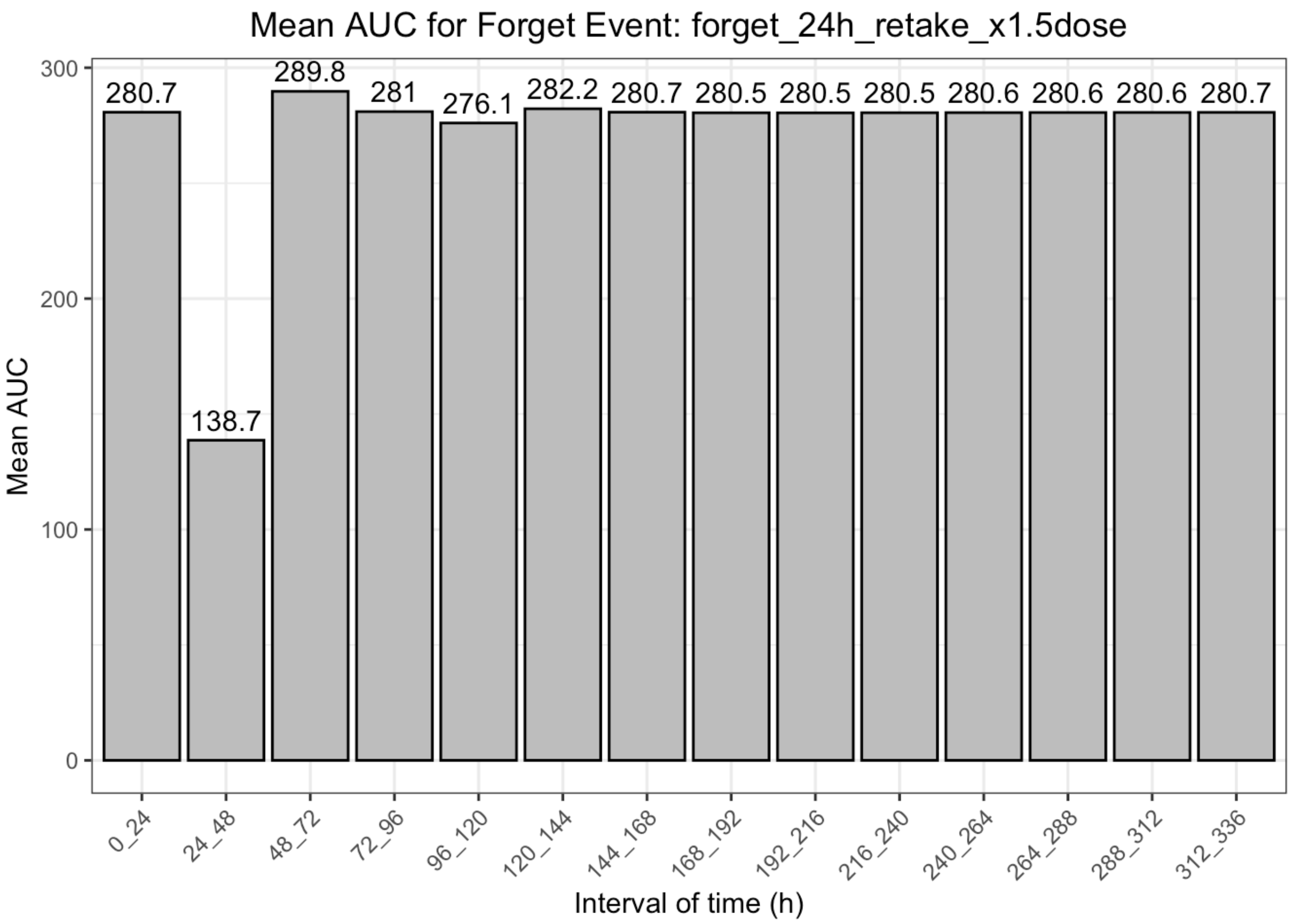

Mean AUC for Forget Event: forget_24h_retake_x1.5dose
Mean AUC
300
200
100
0
280.7
138.7
289.8
281
276.1
282.2
280.7
280.5
280.5
280.5
280.6
280.6
280.6
280.7
0_24
24_48
48_72
72_96
96_120
120_144
144_168
168_192
192_216
216_240
240_264
264_288
288_312
312_336
Interval of time (h)

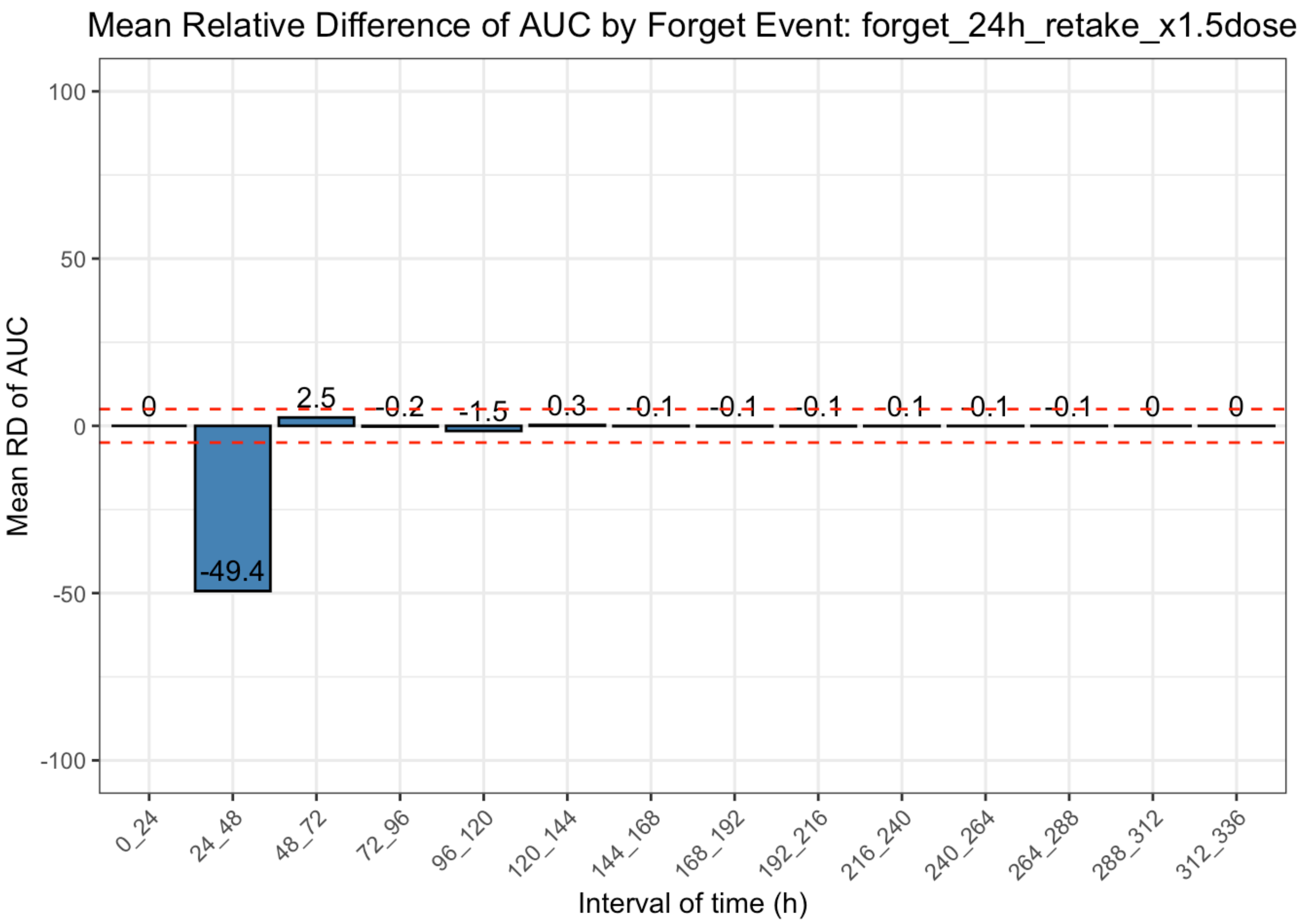
Mean Relative Difference of AUC by Forget Event: forget_24h_retake_x1.5dose
Mean RD of AUC
100
50
0
-50
-100
0
-49.4
2.5
-0.2
-1.5
0.3
-0.1
-0.1
-0.1
-0.1
-0.1
-0.1
0
0
0_24
24_48
48_72
72_96
96_120
120_144
144_168
168_192
192_216
216_240
240_264
264_288
288_312
312_336
Interval of time (h)

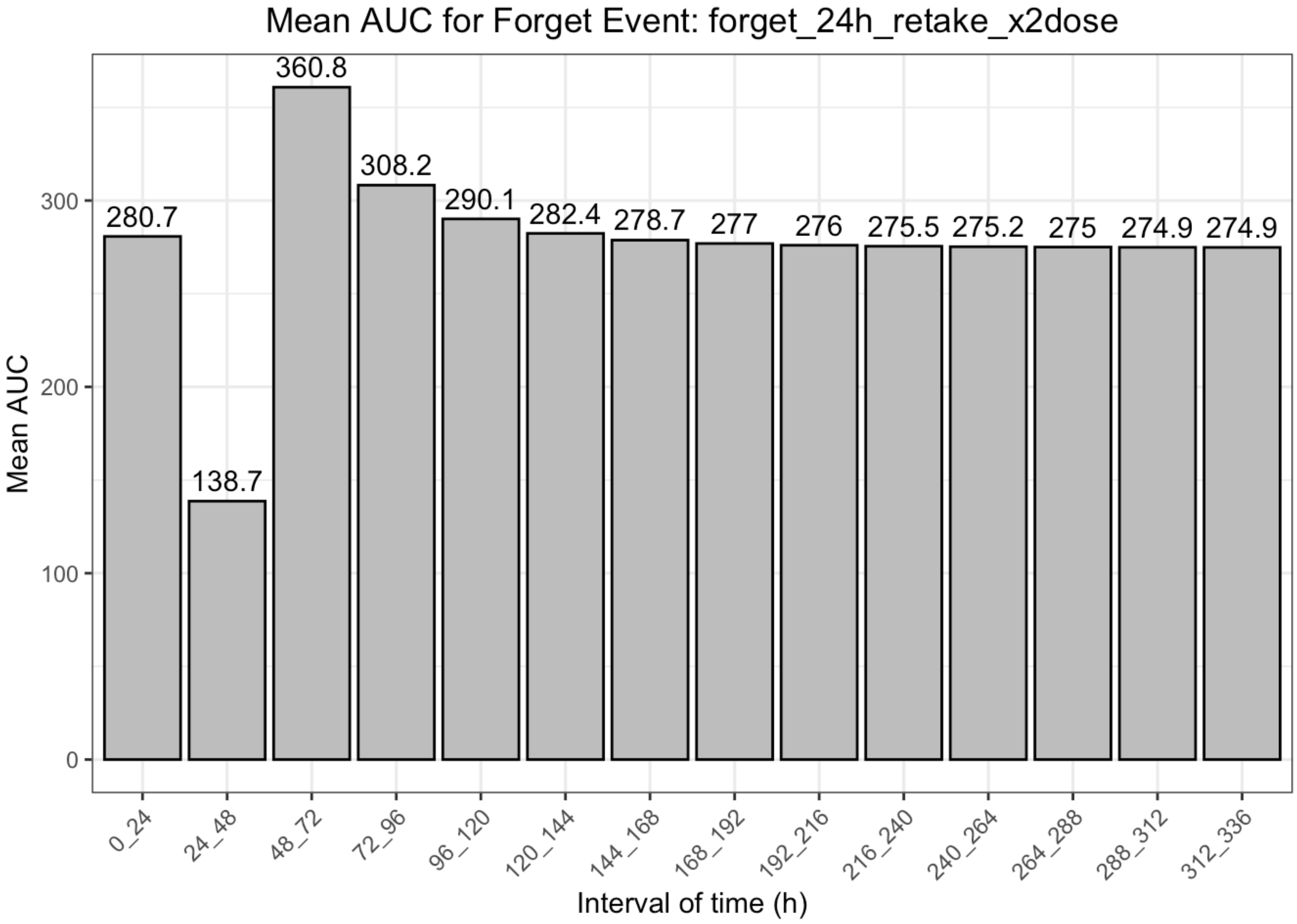
Mean AUC for Forget Event: forget_24h_retake_x2dose
Mean AUC
0
100
200
300
280.7
138.7
360.8
308.2
290.1
282.4
278.7
277
276
275.5
275.2
275
274.9
274.9
0_24
24_48
48_72
72_96
96_120
120_144
144_168
168_192
192_216
216_240
240_264
264_288
288_312
312_336
Interval of time (h)

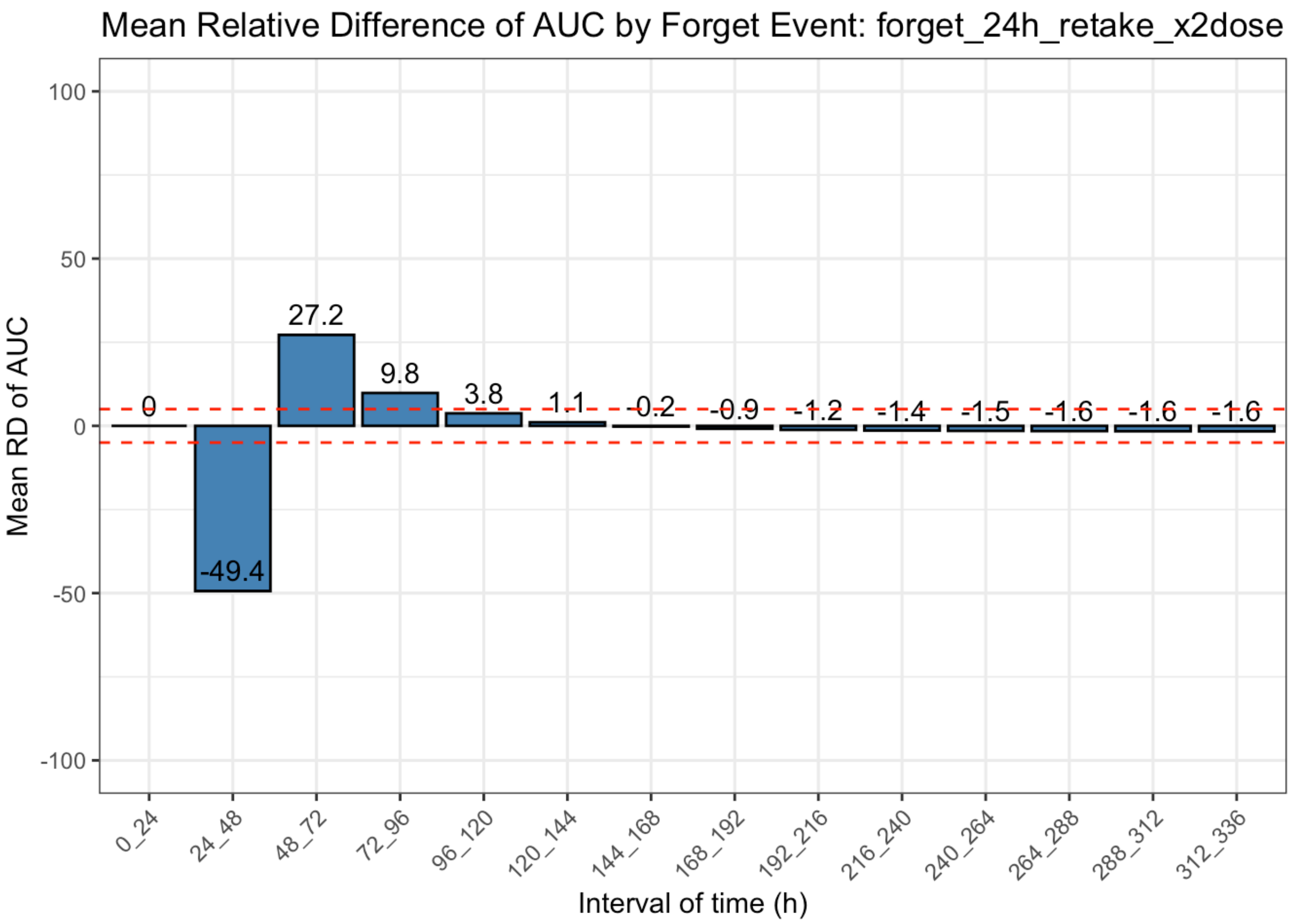

CYP_W column added to Renamed_AUC

```
AUC_with_CYP_W <- Renamed_AUC_W %>%
  left_join(
    filtered_data_with_CYP_W %>% select(ID, CYP),
    by = c("id" = "ID")
  )

AUC_with_CYP_W
```

```
## # A tibble: 822,695 × 8
##       id   auc intervalle forget_event AUC_basale relative_AUC_by_scenario
##    <dbl> <dbl> <chr>      <chr>             <dbl>                    <dbl>
##  1   404   179 0_24       no_forget           179                        0
##  2   405   131 0_24       no_forget           131                        0
##  3   417   147 0_24       no_forget           147                        0
##  4   426   170 0_24       no_forget           170                        0
##  5   427   123 0_24       no_forget           123                        0
##  6   428   133 0_24       no_forget           133                        0
##  7   437   187 0_24       no_forget           187                        0
##  8   442   132 0_24       no_forget           132                        0
##  9   444   184 0_24       no_forget           184                        0
## 10   450   124 0_24       no_forget           124                        0
## # ℹ 822,685 more rows
## # ℹ 2 more variables: intervalle_renamed <chr>, CYP <dbl>
```

AUC statistics for patients with CYP = 1: expressors

```
summary_stats_AUC_CYP_1 <- AUC_with_CYP_W %>%
  dplyr::filter(CYP == 1) %>%
  group_by(forget_event, intervalle_renamed) %>%
    summarize(
    min_auc = fivenum(auc)[1],        # Minimum
    Q1_auc = fivenum(auc)[2],         # First quartile (Q1)
    median_auc = fivenum(auc)[3],     # Median (Q2)
    Q3_auc = fivenum(auc)[4],         # Third quartile (Q3)
    max_auc = fivenum(auc)[5],        # Maximum
    mean_auc = mean(auc, na.rm = TRUE),  # Mean
    sd_auc = sd(auc, na.rm = TRUE),      # SD

    min_relative_auc = fivenum(relative_AUC_by_scenario)[1],        # Minimum
    Q1_relative_auc = fivenum(relative_AUC_by_scenario)[2],         # First quarti
le (Q1)
    median_relative_auc = fivenum(relative_AUC_by_scenario)[3],     # Median (Q2)
    Q3_relative_auc = fivenum(relative_AUC_by_scenario)[4],         # Third quarti
le (Q3)
    max_relative_auc = fivenum(relative_AUC_by_scenario)[5],        # Maximum
    mean_relative_auc = mean(relative_AUC_by_scenario, na.rm = TRUE),  # Mean
    sd_relative_auc = sd(relative_AUC_by_scenario, na.rm = TRUE)       # SD
  ) %>%
  ungroup() %>%
  arrange(forget_event, intervalle_renamed)
```

```
## `summarise()` has grouped output by 'forget_event'. You can override using the
## `.groups` argument.
```

```
summary_stats_AUC_CYP_1
```

```
## # A tibble: 185 × 16
##    forget_event      intervalle_renamed min_auc Q1_auc median_auc Q3_auc max_au
c
##    <chr>             <chr>                <dbl>  <dbl>      <dbl>  <dbl>   <dbl
>
##  1 forget_12h_retak… 0_24                   128    236        285    346     50
4
##  2 forget_12h_retak… 120_144                130    237        287    349     50
5
##  3 forget_12h_retak… 144_168                129    237        286    347     50
4
##  4 forget_12h_retak… 168_192                129    236        286    346     50
4
##  5 forget_12h_retak… 192_216                129    236        286    346     50
4
##  6 forget_12h_retak… 216_240                128    236        286    346     50
4
##  7 forget_12h_retak… 240_264                128    236        285    346     50
4
##  8 forget_12h_retak… 24_48                  105    182        222    270     39
1
##  9 forget_12h_retak… 264_288                128    236        285    346     50
4
## 10 forget_12h_retak… 288_312                128    236        286    346     50
4
## # ℹ 175 more rows
## # ℹ 9 more variables: mean_auc <dbl>, sd_auc <dbl>, min_relative_auc <dbl>,
## #   Q1_relative_auc <dbl>, median_relative_auc <dbl>, Q3_relative_auc <dbl>,
## #   max_relative_auc <dbl>, mean_relative_auc <dbl>, sd_relative_auc <dbl>
```

AUC statistics for patients with CYP = 0: non-expressors

```
summary_stats_AUC_CYP_0 <- AUC_with_CYP_W %>%
  dplyr::filter(CYP == 0) %>%
  group_by(forget_event, intervalle_renamed) %>%
    summarize(
    min_auc = fivenum(auc)[1],        # Minimum
    Q1_auc = fivenum(auc)[2],         # First quartile (Q1)
    median_auc = fivenum(auc)[3],     # Median (Q2)
    Q3_auc = fivenum(auc)[4],         # Third quartile (Q3)
    max_auc = fivenum(auc)[5],        # Maximum
    mean_auc = mean(auc, na.rm = TRUE),  # Mean
    sd_auc = sd(auc, na.rm = TRUE),      # SD

    min_relative_auc = fivenum(relative_AUC_by_scenario)[1],        # Minimum
    Q1_relative_auc = fivenum(relative_AUC_by_scenario)[2],         # First quarti
le (Q1)
    median_relative_auc = fivenum(relative_AUC_by_scenario)[3],     # Median (Q2)
    Q3_relative_auc = fivenum(relative_AUC_by_scenario)[4],         # Third quarti
le (Q3)
    max_relative_auc = fivenum(relative_AUC_by_scenario)[5],        # Maximum
    mean_relative_auc = mean(relative_AUC_by_scenario, na.rm = TRUE),  # Mean
    sd_relative_auc = sd(relative_AUC_by_scenario, na.rm = TRUE)       # SD
  ) %>%
  ungroup() %>%
  arrange(forget_event, intervalle_renamed)
```

```
## `summarise()` has grouped output by 'forget_event'. You can override using the
## `.groups` argument.
```

```
summary_stats_AUC_CYP_0
```

```
## # A tibble: 185 × 16
##    forget_event      intervalle_renamed min_auc Q1_auc median_auc Q3_auc max_au
c
##    <chr>             <chr>                <dbl>  <dbl>      <dbl>  <dbl>   <dbl
>
##  1 forget_12h_retak… 0_24                   112    208        273    342     61
8
##  2 forget_12h_retak… 120_144                113    211        277    346     61
9
##  3 forget_12h_retak… 144_168                112    210        275    344     61
8
##  4 forget_12h_retak… 168_192                112    209        274.   343     61
8
##  5 forget_12h_retak… 192_216                112    209        274    343     61
8
##  6 forget_12h_retak… 216_240                112    209        274    342     61
8
##  7 forget_12h_retak… 240_264                112    209        274.   342     61
8
##  8 forget_12h_retak… 24_48                   98    169        224    275     45
2
##  9 forget_12h_retak… 264_288                112    209        273    342     61
8
## 10 forget_12h_retak… 288_312                112    209        273    342     61
8
## # ℹ 175 more rows
## # ℹ 9 more variables: mean_auc <dbl>, sd_auc <dbl>, min_relative_auc <dbl>,
## #   Q1_relative_auc <dbl>, median_relative_auc <dbl>, Q3_relative_auc <dbl>,
## #   max_relative_auc <dbl>, mean_relative_auc <dbl>, sd_relative_auc <dbl>
```
