# Supplementary material for: Managing Delayed or Missed Doses of Prolonged‐Release Tacrolimus in Transplant Recipients: Implications for Drug Exposure and Recovery Strategies
Source: Basic Clin Pharmacol Toxicol. 2025 Dec 4;138(1):e70157. doi: 10.1111/bcpt.70157 (PMC12676261; doi:10.1111/bcpt.70157)
Supplement: Supplementary file 4 — Data S4: Supporting Information. [file BCPT-138-0-s001.pdf]

Table 1. Impact of delayed doses from 3h to 9h and catch-up strategies on prolonged-release tacrolimus exposure indices (C_0, µg/L; AUC_{24h}, h.µg/L), and *relative differences (RD, %)* compared to steady-state. Results are presented as means ± standard deviations.

a. XR-tac, renal transplantation (Woillard et al.)

Days	Exposure biomarker	3h delay	6h delay	9h delay	6h delay - 50% dose intake	6h delay - 150% dose intake	9h delay - 50% dose intake	9h delay - 150% dose intake
**CYP3A5 non-expressors (N = 3771), AUC_{ss}: 279 ± 87.1 h.µg/L, C_{0ss}: 6.8 ± 2.1 µg/L**								
**Dgap**	**AUC_{24h}**	267 ± 82.9	254 ± 78.1	240 ± 72.7	197 ± 56.1	311 ± 103.5	190 ± 53.9	290 ± 94.8
	RD AUC_{24h}	*-4.1 % ± 0.7*	*-10.4 % ± 2.0*	*-13.6 % ± 2.7*	*-28.4 % ± 6.7*	*11.2 % ± 4.2*	*-30.9 % ± 7.2*	*3.7 % ± 3.2*
	**C_0**	8.3 ± 2.3	8.7 ± 2.5	9.2 ± 2.7	6.5 ± 1.8	11.0 ± 3.3	6.7 ± 1.9	11.7 ± 3.7
	RD C_0	*4.7 % ± 3.2*	*10.1 % ± 7.0*	*16.3 % ± 11.5*	*-18.4 % ± 3.5*	*38.6 % ± 16.5*	*-15.3 % ± 2.7*	*47.9 % ± 23.2*
**D+1**	**AUC_{24h}**	285 ± 89.9	291 ± 93.2	299 ± 97.1	253 ± 80.4	329 ± 106.3	257 ± 82.3	340 ± 112.2
	RD AUC_{24h}	*2.0 % ± 0.8*	*2.2 % ± 1.4*	*6.8 % ± 2.9*	*-9.2 % ± 1.3*	*17.8 % ± 3.3*	*-8.0 % ± 1.6*	*21.6 % ± 4.9*
	**C_0**	8.1 ± 2.3	8.2 ± 2.3	8.4 ± 2.4	7.2 ± 2.0	9.3 ± 2.7	7.3 ± 2.1	9.6 ± 2.8
	RD C_0	*2.0 % ± 0.8*	*4.2 % ± 1.7*	*6.7 % ± 2.8*	*-9.5 % ± 1.2*	*17.9 % ± 3.1*	*-8.3 % ± 1.6*	*21.6 % ± 4.7*
**D+2**	**AUC_{24h}**	281 ± 88.1	284 ± 89.2	288 ± 90.5	265 ± 84.8	303 ± 93.7	267 ± 85.5	308 ± 95.6
	RD AUC_{24h}	*1.0 % ± 0.3*	*0.0 % ± 0.0*	*3.2 % ± 0.8*	*-5.0 % ± 1.4*	*9.0 % ± 1.3*	*-4.4 % ± 1.5*	*10.7 % ± 1.4*
	**C_0**	8.0 ± 2.3	8.1 ± 2.3	8.2 ± 2.3	7.5 ± 2.1	8.6 ± 2.5	7.6 ± 2.1	8.8 ± 2.5
	RD C_0	*0.9 % ± 0.2*	*2.0 % ± 0.5*	*3.1 % ± 0.7*	*-5.2 % ± 1.4*	*9.1 % ± 1.2*	*-4.6 % ± 1.5*	*10.9 % ± 1.3*
**CYP3A5 expressors (N = 673), AUC_{ss}: 293 ± 76.2 h.µg/L, C_{0ss}: 7.9 ± 2.2 µg/L**								
**Dgap**	**AUC_{24h}**	279 ± 72.7	264 ± 68.8	247 ± 64.2	186 ± 49.5	343 ± 92.2	177 ± 47.7	318 ± 85.0
	RD AUC_{24h}	*-4.5 % ± 0.6*	*-11.4 % ± 1.4*	*-15.4 % ± 2.1*	*-36.3 % ± 5.6*	*17.2 % ± 5.0*	*-39.2 % ± 6.0*	*8.4 % ± 4.6*
	**C_0**	7.4 ± 2.1	8.1 ± 2.2	8.9 ± 2.4	5.4 ± 1.7	10.8 ± 2.9	5.8 ± 1.7	12.0 ± 3.3
	RD C_0	*9 % ± 4.8*	*19.6 % ± 10.8*	*32.4 % ± 18.6*	*-20.8 % ± 2.4*	*59.9 % ± 21.9*	*-14.4 % ± 4.7*	*79.1 % ± 33.4*
**D+1**	**AUC_{24h}**	301 ± 78.6	310 ± 81.4	321 ± 84.9	270 ± 71.5	350 ± 91.8	276 ± 73.2	366 ± 97.1
	RD AUC_{24h}	*2.8 % ± 0.7*	*3.8 % ± 1.5*	*9.7 % ± 2.7*	*-7.7 % ± 1.9*	*19.6 % ± 2.5*	*-5.8 % ± 2.3*	*25.2 % ± 3.8*
	**C_0**	7.0 ± 2.1	7.2 ± 2.2	7.5 ± 2.2	6.3 ± 1.9	8.2 ± 2.5	6.4 ± 1.9	8.6 ± 2.6
	RD C_0	*2.8 % ± 0.6*	*6.0 % ± 1.5*	*9.7 % ± 2.5*	*-8.1 % ± 2.0*	*20.2 % ± 1.8*	*-6.3 % ± 2.5*	*25.8 % ± 3.0*
**D+2**	**AUC_{24h}**	295 ± 76.9	299 ± 77.8	302 ± 78.7	284 ± 74.7	314 ± 81.4	285 ± 75.1	320 ± 82.8
	RD AUC_{24h}	*1.0 % ± 0.2*	*0.0 % ± 0.0*	*3.4 % ± 0.6*	*-3.2 % ± 1.5*	*7.4 % ± 1.8*	*-2.5 % ± 1.5*	*9.3 % ± 2.0*
	**C_0**	6.9 ± 2.1	7.0 ± 2.1	7.1 ± 2.2	6.6 ± 2.0	7.4 ± 2.3	6.6 ± 2.0	7.5 ± 2.3
	RD C_0	*1.0 % ± 0.2*	*2.1 % ± 0.4*	*3.5 % ± 0.5*	*-3.4 % ± 1.6*	*7.7 % ± 2.0*	*-2.8 % ± 1.6*	*9.7 % ± 2.1*

b. XR-tac, hepatic transplantation (Moes et al.)

Days	Exposure biomarker	3h delay	6h delay	9h delay	6h delay - 50% dose intake	6h delay - 150% dose intake	9h delay - 50% dose intake	9h delay - 150% dose intake
**CYP3A5 expression group C1 (N = 1002), AUC_{ss}: 231 ± 47.3 h.µg/L, C_{0ss}: 6.5 ± 0.8 µg/L**								
**Dgap**	**AUC_{24h}**	224 ± 45.1	216 ± 42.7	207 ± 40.0	170 ± 24.8	262 ± 63.3	166 ± 23.7	249 ± 59.0
	RD AUC_{24h}	*-3.1 % ± 0.5*	*-7.9 % ± 1.5*	*-10.1 % ± 1.8*	*-25.5 % ± 7.0*	*12.4 % ± 5.0*	*-27.3 % ± 7.3*	*7.0 % ± 4.2*
	**C_0**	6.6 ± 0.9	6.8 ± 0.9	7.1 ± 1.0	5.5 ± 0.8	8.2 ± 1.2	5.6 ± 0.8	8.6 ± 1.4
	RD C_0	*2.7 % ± 1.5*	*5.7 % ± 3.1*	*9.4 % ± 5.1*	*-15.6 % ± 3.7*	*27.1 % ± 9.8*	*-13.8 % ± 2.9*	*32.5 % ± 12.7*
**D+1**	**AUC_{24h}**	234 ± 48.7	238 ± 50.3	241 ± 52.0	212 ± 43.5	263 ± 57.0	214 ± 44.4	268 ± 59.7
	RD AUC_{24h}	*1.2 % ± 0.5*	*1.1 % ± 0.6*	*4 % ± 1.6*	*-8.3 % ± 0.8*	*13.4 % ± 2.3*	*-7.6 % ± 0.8*	*15.6 % ± 3.1*
	**C_0**	6.6 ± 0.9	6.7 ± 0.9	6.8 ± 0.9	5.9 ± 0.8	7.5 ± 1.0	5.9 ± 0.8	7.6 ± 1.0
	RD C_0	*1.3 % ± 0.7*	*2.9 % ± 1.4*	*4.5 % ± 2.1*	*-9.6 % ± 1.1*	*15.4 % ± 3.5*	*-8.8 % ± 0.9*	*17.9 % ± 4.7*
**D+2**	**AUC_{24h}**	233 ± 47.9	235 ± 48.6	237 ± 49.3	220 ± 46.4	250 ± 50.8	221 ± 46.8	253 ± 52.0
	RD AUC_{24h}	*0.7 % ± 0.3*	*0.0 % ± 0.0*	*2.3 % ± 0.7*	*-5.2 % ± 0.9*	*8.2 % ± 0.8*	*-4.8 % ± 1.0*	*9.4 % ± 1.0*
	**C_0**	6.5 ± 0.9	6.6 ± 0.9	6.6 ± 0.9	6.1 ± 0.8	7.1 ± 0.9	6.1 ± 0.8	7.2 ± 0.9
	RD C_0	*0.8 % ± 0.3*	*1.7 % ± 0.6*	*2.6 % ± 0.9*	*-6.0 % ± 0.7*	*9.3 % ± 1.0*	*-5.5 % ±0.8*	*10.8 % ± 1.4*
**CYP3A5 expression group C2/C3 (N = 514), AUC_{ss}: 248 ± 50,6 h.µg/L, C_{0ss}: 6.4 ± 0.8 µg/L**								
**Dgap**	**AUC_{24h}**	239 ± 48.4	230 ± 46.0	220 ± 43.2	175 ± 25.8	286 ± 68.8	170 ± 24.7	271 ± 64.6
	RD AUC_{24h}	*-3.3 % ± 0.5*	*-8.4 % ± 1.3*	*-10.8 % ± 1.6*	*-28.3 % ± 7.0*	*14.5 % ± 5.4*	*-30.3 % ± 7.3*	*8.6 % ± 4.6*
	**C_0**	6.7 ± 0.9	6.9 ± 0.9	7.2 ± 1.0	5.4 ± 0.8	8.5 ± 1.2	5.5 ± 0.8	9.0 ± 1.4
	RD C_0	*3.4 % ± 1.6*	*7.4 % ± 3.5*	*12.2 % ± 5.7*	*-17.1 % ± 3.6*	*31.9 % ± 10.4*	*-14.7 % ± 2.7*	*39.1 % ± 13.6*
**D+1**	**AUC_{24h}**	251 ± 52.1	256 ± 53.8	260 ± 55.7	228 ± 47.4	284 ± 60.5	230 ± 48.4	291 ± 63.4
	RD AUC_{24h}	*1.4 % ± 0.5*	*1.4 % ± 0.7*	*4.8 % ± 1.6*	*-8.2 % ± 0.8*	*14.3 % ± 2.0*	*-7.4 % ± 0.9*	*16.9 % ± 2.8*
	**C_0**	6.6 ± 0.9	6.7 ± 0.9	6.8 ± 0.9	5.8 ± 0.8	7.5 ± 1.0	5.9 ± 0.8	7.7 ± 1.0
	RD C_0	*1.7 % ± 0.7*	*3.5 % ± 1.4*	*5.5 % ± 2.3*	*-9.8 % ± 0.9*	*16.8 % ± 3.3*	*-8.8 % ± 0.7*	*19.9 % ± 4.6*
**D+2**	**AUC_{24h}**	250 ± 51.3	252 ± 52.0	254 ± 52.8	236 ± 50.4	268 ± 53.9	238 ± 50.8	271 ± 55.0
	RD AUC_{24h}	*0.8 % ± 0.2*	*0.0 % ± 0.0*	*2.5 % ± 0.6*	*-4.9 % ± 1.0*	*8.1 % ± 0.8*	*-4.4 % ± 1.1*	*9.4 % ± 0.9*
	**C_0**	6.5 ± 0.9	6.6 ± 0.9	6.6 ± 0.9	6.1 ± 0.8	7.1 ± 0.9	6.1 ± 0.8	7.2 ± 0.9
	RD C_0	*0.9 % ± 0.3*	*1.9 % ± 0.5*	*2.9 % ± 0.9*	*-5.7 % ± 0.8*	*9.5 % ± 0.8*	*-5.2 % ± 0.9*	*11.1 % ± 1.2*
**CYP3A5 expression group C4 (N = 240), AUC_{ss}: 256 ± 47.4 h.µg/L, C_{0ss}: 6.4 ± 0.9 µg/L**								
**Dgap**	**AUC_{24h}**	247 ± 45.5	237 ± 43.5	226 ± 41.1	176 ± 25.3	298 ± 64.1	170 ± 24.2	282 ± 60.5
	RD AUC_{24h}	*-3.5 % ± 0.3*	*-8.9 % ± 0.9*	*-11.5 % ± 1.1*	*-30.5 % ± 5.6*	*15.9 % ± 4.8*	*-32.6 % ± 5.8*	*9.7 % ± 4.5*
	**C_0**	6.6 ± 0.9	6.9 ± 0.9	7.3 ± 1	5.2 ± 0.8	8.6 ± 1.2	5.4 ± 0.8	9.2 ± 1.3
	RD C_0	*4.0 % ± 1.5*	*8.7 % ± 3.2*	*14.4 % ± 5.3*	*-18.2 % ± 2.7*	*35.7 % ± 9.0*	*-15.4 % ± 1.9*	*44.3 % ± 12.0*
**D+1**	**AUC_{24h}**	260 ± 48.7	265 ± 50.1	270 ± 51.7	235 ± 44.7	294 ± 55.6	237 ± 45.5	302 ± 58.0
	RD AUC_{24h}	*1.6 % ± 0.4*	*1.6 % ± 0.6*	*5.4 % ± 1.3*	*-8.3 % ± 0.8*	*15.1 % ± 1.4*	*-7.3 % ± 0.9*	*18.1 % ± 2.0*
	**C_0**	6.5 ± 0.9	6.6 ± 0.9	6.8 ± 0.9	5.7 ± 0.8	7.5 ± 1.0	5.8 ± 0.8	7.7 ± 1.0
	RD C_0	*1.9 % ± 0.6*	*4 % ± 1.2*	*6.2 % ± 1.9*	*-10.0 % ± 0.5*	*17.9 % ± 2.5*	*-8.9 % ± 0.6*	*21.4 % ± 3.6*
**D+2**	**AUC_{24h}**	258 ± 47.9	260 ± 48.4	263 ± 49.1	244 ± 47.1	276 ± 50.0	245 ± 47.4	280 ± 50.9
	RD AUC_{24h}	*0.9 % ± 0.2*	*0.0 % ± 0.0*	*2.7 % ± 0.4*	*-4.6 % ± 1.0*	*8.1 % ± 0.8*	*-4.1 % ± 1.1*	*9.6 % ± 0.8*
	**C_0**	6.4 ± 0.9	6.5 ± 0.9	6.6 ± 0.9	6.0 ± 0.8	7.0 ± 1.0	6.1 ± 0.8	7.1 ± 1.0
	RD C_0	*0.9 % ± 0.2*	*2.1 % ± 0.4*	*3.2 % ± 0.6*	*-5.5 % ± 0.8*	*9.6 % ± 0.5*	*-4.9 % ± 1.0*	*11.4 % ± 0.7*

c. LCP-tac, hepatic transplantation (Martial et al.)

Days	Exposure biomarker	3h delay	6h delay	9h delay	6h delay - 50% dose intake	6h delay - 150% dose intake	9h delay - 50% dose intake	9h delay - 150% dose intake
**AUC_{ss}: 205 ± 43.2 h.µg/L, C_{0ss}: 6.5 ± 0.9 µg/L**								
**Dgap**	**AUC_{24h}**	202 ± 42.7	199 ± 42.3	196 ± 41.8	171 ± 28.1	227 ± 58.3	170 ± 27.9	221 ± 57.4
	RD AUC_{24h}	*-1.3 % ± 0.5*	*-3.1 % ± 1.2*	*-4.6 % ± 1.8*	*-15.3 % ± 6.0*	*9.7 % ± 5.9*	*-16.2 % ± 6.1*	*7.1 % ± 6.0*
	**C_0**	6.6 ± 0.9	6.7 ± 0.9	6.9 ± 1.0	6.1 ± 0.8	7.2 ± 1.0	6.2 ± 0.8	7.5 ± 1.2
	RD C_0	*1.3 % ± 1.2*	*3.3 % ± 3.0*	*6.5 % ± 5.8*	*-5.0 % ± 1.6*	*11.7 % ± 6.3*	*-3.5 % ± 2.2*	*16.4 % ± 10.3*
**D+1**	**AUC_{24h}**	206 ± 43.2	207 ± 43.3	208 ± 43.6	197 ± 41.6	216 ± 45.2	198 ± 41.7	219 ± 45.7
	RD AUC_{24h}	*0.4 % ± 0.4*	*0.7 % ± 0.7*	*1.7 % ± 1.4*	*-3.7 % ± 0.8*	*5.6 % ± 2.1*	*-3.3 % ± 0.8*	*6.8 % ± 2.9*
	**C_0**	6.5 ± 0.9	6.5 ± 0.9	6.5 ± 0.9	6.1 ± 0.8	6.8 ± 0.9	6.2 ± 0.8	6.9 ± 0.9
	RD C_0	*0.2 % ± 0.2*	*0.5 % ± 0.4*	*0.8 % ± 0.6*	*-4.6 % ± 1.1*	*5.5 % ± 1.5*	*-4.4 % ± 1.1*	*6.0 % ± 1.8*
**D+2**	**AUC_{24h}**	205 ± 43.2	205 ± 43.3	206 ± 43.3	198 ± 41.7	212 ± 44.8	198 ± 41.7	213 ± 44.9
	RD AUC_{24h}	*0.2 % ± 0.2*	*0.0 % ± 0.0*	*0.4 % ± 0.3*	*-3.2 % ± 0.6*	*3.8 % ± 0.8*	*-3.1 % ± 0.6*	*4.0 % ± 1.0*
	**C_0**	6.5 ± 0.9	6.5 ± 0.9	6.5 ± 0.9	6.2 ± 0.8	6.8 ± 0.9	6.2 ± 0.8	6.8 ± 0.9
	RD C_0	*0.1 % ± 0.1*	*0.2 % ± 0.2*	*0.4 % ± 0.2*	*-4.0 % ± 0.9*	*4.5 % ± 1.1*	*-3.9 % ± 0.9*	*4.7 % ± 1.2*

Notes:

- ***Dgap*** refers to the day of the delayed or missed dose
- ***D+x*** refers to the number of days following the delayed or missed dose
- **AUC_{24h}** = mean Area Under the Curve over 24 hours (h.µg/L)
- ***RD AUC_{24h}*** = mean Relative Difference in AUC_{24h} (%)
- **C_0** = mean trough concentration (µg/L)
- ***RD C_0*** = mean Relative Difference in C_0 (%)

Table 2. Impact of delayed doses from 15h to 21h and catch-up strategies on prolonged-release tacrolimus exposure indices (C_0, µg/L; AUC_{24h}, h.µg/L), and *relative differences (RD, %)* compared to steady-state. Results are presented as means ± standard deviations.

a. XR-tac, renal transplantation (Woillard et al.)

Days	Exposure biomarker	15h delay	18h delay	21h delay	15h delay - 50% dose intake	18h delay - 50% dose intake	21h delay - 50% dose intake
**CYP3A5 non-expressors (N = 3771), AUC_{ss}: 279 ± 87.1 h.µg/L, C_{0ss}: 6.8 ± 2.1 µg/L**							
**Dgap**	**AUC_{24h}**	206 ± 60.2	186 ± 53.2	160 ± 46.4	173 ± 49.1	163 ± 46.7	150 ± 44.3
	RD AUC_{24h}	*-25.3 % ± 5.6*	*-32.4 % ± 7.5*	*-41.2 % ± 9.9*	*-36.8 % ± 8.6*	*-40.3 % ± 9.6*	*-44.7 % ± 10.9*
	**C_0**	10.5 ± 3.4	11.7 ± 4.0	13.9 ± 5.4	7.4 ± 2.1	7.9 ± 2.4	9.0 ± 3.0
	RD C_0	*33.5 % ± 24.1*	*48.3 % ± 34.3*	*76.6 % ± 54.1*	*-6.7 % ± 7.5*	*0.7 % ± 12.8*	*14.8 % ± 23*
**D+1**	**AUC_{24h}**	317 ± 107.3	328 ± 114.2	344 ± 123.5	266 ± 87.2	272 ± 90.5	280 ± 95.0
	RD AUC_{24h}	*12.9 % ± 5.7*	*16.8 % ± 7.5*	*22.2 % ± 9.8*	*-4.9 % ± 2.9*	*-3.0 % ± 3.8*	*-0.3 % ± 5.0*
	**C_0**	8.9 ± 2.5	9.2 ± 2.6	9.5 ± 2.8	7.5 ± 2.1	7.6 ± 2.2	7.8 ± 2.2
	RD C_0	*12.4 % ± 5.7*	*15.8 % ± 7.6*	*19.5 % ± 9.8*	*-5.4 % ± 2.9*	*-3.7 % ± 3.8*	*-1.9 % ± 4.9*
**D+2**	**AUC_{24h}**	295 ± 93.6	299 ± 95.5	304 ± 97.7	271 ± 87.0	273 ± 88.0	275 ± 89.1
	RD AUC_{24h}	*5.8 % ± 1.5*	*7.3 % ± 2.0*	*9.0 % ± 2.6*	*-3.1 % ± 1.8*	*-2.3 % ± 2.0*	*-1.5 % ± 2.3*
	**C_0**	8.4 ± 2.4	8.5 ± 2.4	8.6 ± 2.5	7.7 ± 2.2	7.7 ± 2.2	7.8 ± 2.2
	RD C_0	*5.8 % ± 1.4*	*7.3 % ± 1.9*	*9.0 % ± 2.4*	*-3.2 % ± 1.8*	*-2.5 % ± 2.1*	*-1.6 % ± 2.3*
**CYP3A5 expressors (N = 673), AUC_{ss}: 293 ± 76.2 h.µg/L, C_{0ss}: 7.9 ± 2.2 µg/L**							
**Dgap**	**AUC_{24h}**	204 ± 53.3	176 ± 47.2	138 ± 41.5	155 ± 43.6	141 ± 41.7	122 ± 40.3
	RD AUC_{24h}	*-30.1 % ± 4.5*	*-39.7 % ± 6.2*	*-52.4 % ± 8.5*	*-46.6 % ± 7.2*	*-51.4 % ± 8.2*	*-57.7 % ± 9.5*
	**C_0**	11.3 ± 3.1	13.4 ± 3.9	17.5 ± 5.7	7.0 ± 1.9	8.1 ± 2.2	10.1 ± 3
	RD C_0	*70.1 % ± 42.4*	*104.5 % ± 64.4*	*170.6 % ±109.2*	*4.5 % ± 16.6*	*21.7 % ± 28.1*	*54.7 % ± 50.9*
**D+1**	**AUC_{24h}**	349 ± 94.8	369 ± 102.0	396 ± 112.0	290 ± 78	300 ± 81.5	313 ± 86.4
	RD AUC_{24h}	*19.2 % ± 5.7*	*25.8 % ± 7.8*	*35.1 % ± 10.6*	*-1.0 % ± 3.9*	*2.2 % ± 5.0*	*6.9 % ± 6.5*
	**C_0**	8.1 ± 2.3	8.5 ± 2.4	8.9 ± 2.5	6.7 ± 1.9	6.9 ± 2.0	7.1 ± 2.0
	RD C_0	*18.9 % ± 5.7*	*24.6 % ± 7.9*	*31.2 % ± 10.8*	*-1.7 % ± 4.1*	*1.1 % ± 5.2*	*4.4 % ± 6.7*
**D+2**	**AUC_{24h}**	311 ± 81.1	317 ± 82.6	323 ± 84.4	290 ± 76.1	292 ± 76.9	296 ± 77.8
	RD AUC_{24h}	*6.4 % ± 1.1*	*8.3 % ± 1.4*	*10.4 % ± 1.8*	*-1.0 % ± 1.6*	*-0.1 % ± 1.8*	*1.0 % ± 1.9*
	**C_0**	7.3 ± 2.2	7.4 ± 2.3	7.6 ± 2.3	6.7 ± 2.0	6.8 ± 2.0	6.9 ± 2.0
	RD C_0	*6.6 % ± 0.9*	*8.6 % ± 1.2*	*10.7 % ± 1.5*	*-1.2 % ± 1.7*	*-0.2 % ± 1.9*	*0.9 % ± 2.0*

b. XR-tac, hepatic transplantation (Moes et al.)

Days	Exposure biomarker	15h delay	18h delay	21h delay	15h delay - 50% dose intake	18h delay - 50% dose intake	21h delay - 50% dose intake
**CYP3A5 expression group C1 (N = 1002), AUC_{ss}: 231 ± 47.3 h.µg/L, C_{0ss}: 6.5 ± 0.8 µg/L**							
**Dgap**	**AUC_{24h}**	187 ± 33.7	175 ± 29.9	156 ± 24.5	156 ± 21.5	149 ± 20.3	140 ± 19.1
	RD AUC_{24h}	*-18.6 % ± 3.6*	*-23.9 % ± 5.0*	*-31.7 % ± 7.4*	*-31.5 % ± 8.1*	*-34.2 % ± 8.8*	*-38.1 % ± 10.0*
	**C_0**	7.9 ± 1.2	9.0 ± 1.8	12.4 ± 4.0	6.0 ± 0.8	6.5 ± 0.9	8.2 ± 1.9
	RD C_0	*22.1 % ± 12.1*	*38.8 % ± 22.5*	*92.3 % ± 60.5*	*-7.5 % ± 2.9*	*0.9 % ± 7.5*	*27.7 % ± 25.8*
**D+1**	**AUC_{24h}**	250 ± 56.3	257 ± 59.4	269 ± 65.7	218 ± 46.5	222 ± 48.0	228 ± 51.1
	RD AUC_{24h}	*7.7 % ± 3*	*10.5 % ± 4.1*	*15.5 % ± 6.3*	*-5.7 % ± 1.1*	*-4.3 % ± 1.6*	*-1.8 % ± 2.6*
	**C_0**	7.0 ± 0.9	7.1 ± 1.0	7.3 ± 1.0	6.0 ± 0.8	6.1 ± 0.8	6.2 ± 0.8
	RD C_0	*8.1 % ± 4*	*10.2 % ± 5.1*	*12.4 % ± 6.2*	*-7.0 % ± 1.0*	*-6.0 % ± 1.4*	*-4.9 % ± 2.0*
**D+2**	**AUC_{24h}**	241 ± 51.2	243 ± 52.2	246 ± 53.4	223 ± 47.7	224 ± 48.2	225 ± 48.8
	RD AUC_{24h}	*4.1 % ± 1.2*	*5.0 % ± 1.5*	*6.1 % ± 1.9*	*-4.0 % ± 1.2*	*-3.5 % ± 1.3*	*-2.9 % ± 1.5*
	**C_0**	6.8 ± 0.9	6.9 ± 0.9	6.9 ± 0.9	6.2 ± 0.8	6.2 ± 0.8	6.3 ± 0.8
	RD C_0	*4.7 % ± 1.7*	*5.8 % ± 2.1*	*7.1 % ± 2.6*	*-4.5 % ± 1.1*	*-3.9 % ± 1.3*	*-3.3 % ± 1.5*
**CYP3A5 expression group C2/C3 (N = 514), AUC_{ss}: 248 ± 50,6 h.µg/L, C_{0ss}: 6.4 ± 0.8 µg/L**							
**Dgap**	**AUC_{24h}**	197 ± 36.7	182 ± 32.7	159 ± 26.4	158 ± 22.2	151 ± 20.9	139 ± 19.3
	RD AUC_{24h}	*-20.1 % ± 3.3*	*-26.2 % ± 4.7*	*-35.1 % ± 7.2*	*-34.9 % ± 8*	*-38 % ± 8.6*	*-42.4 % ± 9.9*
	**C_0**	8.3 ± 1.3	9.7 ± 1.9	14.0 ± 4.6	6.0 ± 0.8	6.7 ± 1.0	8.9 ± 2.2
	RD C_0	*28.9 % ± 13.8*	*50.6 % ± 25.9*	*118.6 % ± 70*	*-6.4 % ± 3.4*	*4.5 % ± 9.0*	*38.5 % ± 30.5*
**D+1**	**AUC_{24h}**	272 ± 60.3	280 ± 63.8	296 ± 71.1	235 ± 50.4	240 ± 52.1	248 ± 55.7
	RD AUC_{24h}	*9.2 % ± 3.1*	*12.6 % ± 4.2*	*18.7 % ± 6.6*	*-5.1 % ± 1.4*	*-3.4 % ± 1.9*	*-0.4 % ± 3.0*
	**C_0**	7.1 ± 0.9	7.3 ± 1.0	7.4 ± 1.0	6.0 ± 0.8	6.1 ± 0.8	6.2 ± 0.8
	RD C_0	*10.0 % ± 4.2*	*12.6 % ± 5.4*	*15.4 % ± 6.7*	*-6.5 % ± 1.3*	*-5.3 % ± 1.8*	*-3.9 % ± 2.4*
**D+2**	**AUC_{24h}**	259 ± 54.6	262 ± 55.8	265 ± 57.0	240 ± 51.5	241 ± 52.0	243 ± 52.7
	RD AUC_{24h}	*4.5 % ± 1.1*	*5.7 % ± 1.4*	*6.9 % ± 1.8*	*-3.4 % ± 1.3*	*-2.8 % ± 1.5*	*-2.2 % ± 1.6*
	**C_0**	6.8 ± 0.9	6.9 ± 0.9	7.0 ± 0.9	6.2 ± 0.8	6.2 ± 0.8	6.3 ± 0.8
	RD C_0	*5.4 % ± 1.6*	*6.7 % ± 2.1*	*8.2 % ± 2.6*	*-4.0 % ± 1.3*	*-3.3 % ± 1.5*	*-2.5 % ± 1.7*
**CYP3A5 expression group C4 (N = 240), AUC_{ss}: 256 ± 47.4 h.µg/L, C_{0ss}: 6.4 ± 0.9 µg/L**							
**Dgap**	**AUC_{24h}**	200 ± 35.4	183 ± 31.3	157 ± 24.3	158 ± 22.0	149 ± 20.5	136 ± 18.4
	RD AUC_{24h}	*-21.5 % ± 2.5*	*-28.2 % ± 3.6*	*-38.2 % ± 5.7*	*-37.6 % ± 6.3*	*-41.0 % ± 6.8*	*-46.0 % ± 7.9*
	**C_0**	8.6 ± 1.4	10.2 ± 2.1	15.1 ± 4.8	6.0 ± 0.9	6.9 ± 1.1	9.3 ± 2.3
	RD C_0	*34.7 % ± 13.4*	*61.1 % ± 26.8*	*139.5 % ± 76.5*	*-5.3 % ± 3.8*	*7.9 % ± 10.1*	*47.2 % ± 34.6*
**D+1**	**AUC_{24h}**	283 ± 55.9	294 ± 59.6	312 ± 67.5	244 ± 47.5	249 ± 49.3	259 ± 53.1
	RD AUC_{24h}	*10.5 % ± 2.5*	*14.4 % ± 3.7*	*21.4 % ± 5.8*	*-4.7 % ± 1.4*	*-2.7 % ± 1.9*	*0.8 % ± 3.0*
	**C_0**	7.1 ± 0.9	7.3 ± 1.0	7.5 ± 1.0	6.0 ± 0.8	6.1 ± 0.8	6.2 ± 0.8
	RD C_0	*11.4 % ± 3.7*	*14.3 % ± 4.7*	*17.6 % ± 5.9*	*-6.3 % ± 1.3*	*-4.8 % ± 1.8*	*-3.2 % ± 2.4*
**D+2**	**AUC_{24h}**	268 ± 50.7	272 ± 51.6	275 ± 52.6	248 ± 48.1	250 ± 48.6	252 ± 49.2
	RD AUC_{24h}	*4.9 % ± 0.8*	*6.2 % ± 1.0*	*7.5 % ± 1.3*	*-3.0 % ± 1.2*	*-2.4 % ± 1.3*	*-1.7 % ± 1.5*
	**C_0**	6.8 ± 0.9	6.8 ± 0.9	6.9 ± 0.9	6.1 ± 0.8	6.2 ± 0.8	6.2 ± 0.8
	RD C_0	*5.9 % ± 1.2*	*7.4 % ± 1.6*	*9.0 % ± 2.0*	*-3.6 % ± 1.2*	*-2.8 % ± 1.4*	*-2.0 % ± 1.6*

c. LCP-tac, hepatic transplantation (Martial et al.)

Days	Exposure biomarker	15h delay	18h delay	21h delay	15h delay - 50% dose intake	18h delay - 50% dose intake	21h delay - 50% dose intake
**AUC_{ss}: 205 ± 43.2 h.µg/L, C_{0ss}: 6.5 ± 0.9 µg/L**							
**Dgap**	**AUC_{24h}**	185 ± 39.6	176 ± 36.6	161 ± 28.6	165 ± 27.0	160 ± 25.8	152 ± 22.8
	RD AUC_{24h}	*-9.5 % ± 3.9*	*-13.7 % ± 5.7*	*-20.7 % ± 8.2*	*-18.7 % ± 6.8*	*-20.8 % ± 7.7*	*-24.3 % ± 9.6*
	**C_0**	8.0 ± 1.7	9.5 ± 2.8	12.3 ± 6.0	6.8 ± 1.1	7.5 ± 1.6	9.0 ± 3.1
	RD C_0	*23.1 % ± 20.0*	*46.4 % ± 38.7*	*89.9 % ± 88.7*	*4.8 % ± 9.3*	*16.5 % ± 18.8*	*38.3 % ± 44.2*
**D+1**	**AUC_{24h}**	214 ± 46.1	221 ± 50.4	234 ± 60.4	201 ± 42.9	204 ± 44.9	211 ± 49.9
	RD AUC_{24h}	*4.6 % ± 3.3*	*7.7 % ± 5.1*	*13.6 % ± 7.8*	*-1.9 % ± 1.5*	*-0.3 % ± 2.4*	*2.6 % ± 3.9*
	**C_0**	6.6 ± 0.9	6.6 ± 0.9	6.7 ± 0.9	6.2 ± 0.8	6.2 ± 0.8	6.3 ± 0.8
	RD C_0	*1.6 % ± 1.2*	*2.1 % ± 1.7*	*2.8 % ± 2.3*	*-4.0 % ± 1.1*	*-3.7 % ± 1.2*	*-3.4 % ± 1.4*
**D+2**	**AUC_{24h}**	206 ± 43.3	207 ± 43.4	208 ± 43.4	199 ± 41.8	199 ± 41.8	199 ± 41.8
	RD AUC_{24h}	*0.9 % ± 0.6*	*1.1 % ± 0.8*	*1.4 % ± 1.0*	*-2.9 % ± 0.5*	*-2.8 % ± 0.6*	*-2.7 % ± 0.6*
	**C_0**	6.5 ± 0.9	6.5 ± 0.9	6.6 ± 0.9	6.2 ± 0.8	6.2 ± 0.8	6.2 ± 0.8
	RD C_0	*0.7 % ± 0.4*	*0.9 % ± 0.5*	*1.1 % ± 0.6*	*-3.7 % ± 0.8*	*-3.6 % ± 0.8*	*-3.5 % ± 0.8*

Notes:

- ***Dgap*** refers to the day of the delayed or missed dose
- ***D+x*** refers to the number of days following the delayed or missed dose
- **AUC_{24h}** = mean Area Under the Curve over 24 hours (h.µg/L)
- ***RD AUC_{24h}*** = mean Relative Difference in AUC_{24h} (%)
- **C_0** = mean trough concentration (µg/L)
- ***RD C_0*** = mean Relative Difference in C_0 (%)
