# Supplementary material for: Managing Delayed or Missed Doses of Prolonged‐Release Tacrolimus in Transplant Recipients: Implications for Drug Exposure and Recovery Strategies
Source: Basic Clin Pharmacol Toxicol. 2025 Dec 4;138(1):e70157. doi: 10.1111/bcpt.70157 (PMC12676261; doi:10.1111/bcpt.70157)
Supplement: Supplementary file 1 — Data S1: Supporting Information. [file BCPT-138-0-s003.pdf]

Moes simulation

Arraki Zava Selim

2025-05-06

##Loading of the packages

```r
library(mrgsolve)
```

```
##
## Attaching package: 'mrgsolve'
```

```
## The following object is masked from 'package:stats':
##
##     filter
```

```r
library(stats)
library(tidyverse)
```

```
## ── Attaching core tidyverse packages ──────────────────────── tidyverse 2.0.0 ──
## ✔ dplyr     1.1.4     ✔ readr     2.1.5
## ✔ forcats   1.0.0     ✔ stringr   1.5.1
## ✔ ggplot2   3.5.1     ✔ tibble    3.2.1
## ✔ lubridate 1.9.4     ✔ tidyr     1.3.1
## ✔ purrr     1.0.2
```

```
## ── Conflicts ────────────────────────────────────────── tidyverse_conflicts() ──
## ✖ dplyr::filter() masks mrgsolve::filter(), stats::filter()
## ✖ dplyr::lag()    masks stats::lag()
## ℹ Use the conflicted package (<http://conflicted.r-lib.org/>) to force all conflicts to become errors
```

```r
library(truncnorm)
library(ggplot2)
library(conflicted)
library(PKNCA)
library(Pmetrics)
```

```
##
## CRITICAL: Execute PMbuild() in R to complete Pmetrics installation.
```

```r
library(skimr)
library(stats)
library(tidyr)
library(pander)
library(DT)
library(purrr)
library(gridExtra)
library(patchwork)
library(stringr)
library(ggtext)
```

##Population pharmacokinetics and pharmacogenetics of once daily tacrolimus formulation in stable liver transplant recipients, Moes, 2015 (Stable liver transplant patients receiving once daily tacrolimus (N = 66) were included) OK

```r
code <-
"
[SET] end=100, delta=0.1

[PARAM] @annotated
TVKA : 3.76 : Typical value of intercomp absorbance (1/h)
TVVC : 88.3 : Typical value of central volume (L)
TVCL : 4.21 : Typical value of Clearance (L/h)
F    : 0.23 : value of biodiponibility (%)
Q    : 14 : value of intercomp clearance (L/h)
VP   : 145 : value of periph volume (L)


[PARAM] @annotated @covariates
CYPCL : 0 : effect of CYP on CL (0=C1, 0.33=C2, 0.33=C3, 0.71=C4)


[OMEGA] @annotated
ETACL : 0.183 : IIV on Clearance
ETAVC : 0.744 : IIV on central volume
ETAKA : 0.434 : IVV on KA absorbance


[SIGMA]
0.000001 // proportional 0.13


[CMT] @annotated
GUT : estomac [ADM]
CENT  : Central compartment [OBS]
PERIPH : peripheral compartment


[MAIN]
double CL = (TVCL* (1 + CYPCL)) * exp(ETACL);
double VC = TVVC * exp(ETAVC);
double KA = TVKA * exp(ETAKA);
F_GUT = F;


[ODE]
dxdt_GUT    = -KA*GUT;
dxdt_CENT   = -(CL+Q)*CENT/VC + Q*PERIPH/VP + KA*GUT;
dxdt_PERIPH = Q*CENT/VC - Q*PERIPH/VP;


[TABLE]
double DV = ((CENT/VC)*1000) * (1 + EPS(1));
int i = 0;
while(DV<0 && i <100) {
simeps();
DV = ((CENT/VC)*1000) * (1 + EPS(1));
++i;
}


[CAPTURE] DV CL VP VC Q
"


my_model_Moes <- mcode("tacro_model", code)
```

```
## Building tacro_model ... done.
```

Simulations 6000: simulation with different dose each 400 profiles (including CYP3A5 status variations)

```r
set.seed(123456)
# Frequency of CYPCL over 400 profiles
generate_CYPCL_6000 <- function(n = 400) {
  frequencies <- c(32/49, 12/49, 5/49)
  values <- c(0, 0.33, 0.71)
  # cette ligne
  sample(values, n, replace = TRUE, prob = frequencies)
  }

# Application of CYPCL over 6000 profiles
CYPCL_data_6000 <- tibble(ID = 1:6000) %>%
  group_by(group = (ID - 1) %/% 400) %>%
  mutate(CYPCL = generate_CYPCL_6000()) %>%
  ungroup() %>%
  select(-group)

# Scenarios simulation
Simulation_data_6000 <- as_tibble( seq(
  # Steady-state simulation
ev(ID = 1:400, amt = 1*1, ii= 24, ss= 1, addl=0),
ev(ID = 1:400, amt = 1*1, ii= 24, ss= 0, addl=0),
ev(ID = 1:400, amt = 1*1, ii= 24, ss= 0, addl=10),
#3h delay simulation
ev(ID = 1:400, amt = 1*1, ii= 24, ss= 1, addl=0),
ev(ID = 1:400, amt = 1*0, ii= 3, ss= 0, addl=0),
ev(ID = 1:400, amt = 1*1, ii= 21, ss= 0, addl=0),
ev(ID = 1:400, amt = 1*1, ii= 24, ss= 0, addl=10),
#6h delay simulation
ev(ID = 1:400, amt = 1*1, ii= 24, ss= 1, addl=0),
ev(ID = 1:400, amt = 1*0, ii= 6, ss= 0, addl=0),
ev(ID = 1:400, amt = 1*1, ii= 18, ss= 0, addl=0),
ev(ID = 1:400, amt = 1*1, ii= 24, ss= 0, addl=10),
#9h delay simulation
ev(ID = 1:400, amt = 1*1, ii= 24, ss= 1, addl=0),
ev(ID = 1:400, amt = 1*0, ii= 9, ss= 0, addl=0),
ev(ID = 1:400, amt = 1*1, ii= 15, ss= 0, addl=0),
ev(ID = 1:400, amt = 1*1, ii= 24, ss= 0, addl=10),
#12h delay simulation
ev(ID = 1:400, amt = 1*1, ii= 24, ss= 1, addl=0),
ev(ID = 1:400, amt = 1*0, ii= 12, ss= 0, addl=0),
ev(ID = 1:400, amt = 1*1, ii= 12, ss= 0, addl=0),
ev(ID = 1:400, amt = 1*1, ii= 24, ss= 0, addl=10),
#15h delay + 50% dose intake simulation
ev(ID = 1:400, amt = 1*1, ii= 24, ss= 1, addl=0),
ev(ID = 1:400, amt = 1*0, ii= 15, ss= 0, addl=0),
ev(ID = 1:400, amt = 1*0.5, ii= 9, ss= 0, addl=0),
ev(ID = 1:400, amt = 1*1, ii= 24, ss= 0, addl=10),
#15h delay simulation
ev(ID = 1:400, amt = 1*1, ii= 24, ss= 1, addl=0),
ev(ID = 1:400, amt = 1*0, ii= 15, ss= 0, addl=0),
ev(ID = 1:400, amt = 1*1, ii= 9, ss= 0, addl=0),
ev(ID = 1:400, amt = 1*1, ii= 24, ss= 0, addl=10),
#18h delay + 50% dose intake simulation
ev(ID = 1:400, amt = 1*1, ii= 24, ss= 1, addl=0),
ev(ID = 1:400, amt = 1*0, ii= 18, ss= 0, addl=0),
ev(ID = 1:400, amt = 1*0.5, ii= 6, ss= 0, addl=0),
ev(ID = 1:400, amt = 1*1, ii= 24, ss= 0, addl=10),
#18h delay simulation
ev(ID = 1:400, amt = 1*1, ii= 24, ss= 1, addl=0),
ev(ID = 1:400, amt = 1*0, ii= 18, ss= 0, addl=0),
ev(ID = 1:400, amt = 1*1, ii= 6, ss= 0, addl=0),
ev(ID = 1:400, amt = 1*1, ii= 24, ss= 0, addl=10),
#21h delay + 50% dose intake simulation
ev(ID = 1:400, amt = 1*1, ii= 24, ss= 1, addl=0),
ev(ID = 1:400, amt = 1*0, ii= 21, ss= 0, addl=0),
ev(ID = 1:400, amt = 1*0.5, ii= 3, ss= 0, addl=0),
ev(ID = 1:400, amt = 1*1, ii= 24, ss= 0, addl=10),
#21h delay simulation
ev(ID = 1:400, amt = 1*1, ii= 24, ss= 1, addl=0),
ev(ID = 1:400, amt = 1*0, ii= 21, ss= 0, addl=0),
ev(ID = 1:400, amt = 1*1, ii= 3, ss= 0, addl=0),
ev(ID = 1:400, amt = 1*1, ii= 24, ss= 0, addl=10),
#Missed dose simulation
ev(ID = 1:400, amt = 1*1, ii= 24, ss= 1, addl=0),
ev(ID = 1:400, amt = 1*0, ii= 24, ss= 0, addl=0),
ev(ID = 1:400, amt = 1*1, ii= 24, ss= 0, addl=0),
ev(ID = 1:400, amt = 1*1, ii= 24, ss= 0, addl=10),
#Missed dose + 150% dose intake simulation
ev(ID = 1:400, amt = 1*1, ii= 24, ss= 1, addl=0),
ev(ID = 1:400, amt = 1*0, ii= 24, ss= 0, addl=0),
ev(ID = 1:400, amt = 1*1.5, ii= 24, ss= 0, addl=0),
ev(ID = 1:400, amt = 1*1, ii= 24, ss= 0, addl=10),
#Missed dose + 200% dose intake simulation
ev(ID = 1:400, amt = 1*1, ii= 24, ss= 1, addl=0),
ev(ID = 1:400, amt = 1*0, ii= 24, ss= 0, addl=0),
ev(ID = 1:400, amt = 1*2, ii= 24, ss= 0, addl=0),
ev(ID = 1:400, amt = 1*1, ii= 24, ss= 0, addl=10))) %>%
              mutate(dose_group = "1 mg") %>%

  bind_rows(as_tibble( seq(
  #Steady-state simulation
ev(ID = 401:800, amt = 2*1, ii= 24, ss= 1, addl=0),
ev(ID = 401:800, amt = 2*1, ii= 24, ss= 0, addl=0),
ev(ID = 401:800, amt = 2*1, ii= 24, ss= 0, addl=10),
#3h delay simulation
ev(ID = 401:800, amt = 2*1, ii= 24, ss= 1, addl=0),
ev(ID = 401:800, amt = 2*0, ii= 3, ss= 0, addl=0),
ev(ID = 401:800, amt = 2*1, ii= 21, ss= 0, addl=0),
ev(ID = 401:800, amt = 2*1, ii= 24, ss= 0, addl=10),
#6h delay simulation
ev(ID = 401:800, amt = 2*1, ii= 24, ss= 1, addl=0),
ev(ID = 401:800, amt = 2*0, ii= 6, ss= 0, addl=0),
ev(ID = 401:800, amt = 2*1, ii= 18, ss= 0, addl=0),
ev(ID = 401:800, amt = 2*1, ii= 24, ss= 0, addl=10),
#9h delay simulation
ev(ID = 401:800, amt = 2*1, ii= 24, ss= 1, addl=0),
ev(ID = 401:800, amt = 2*0, ii= 9, ss= 0, addl=0),
ev(ID = 401:800, amt = 2*1, ii= 15, ss= 0, addl=0),
ev(ID = 401:800, amt = 2*1, ii= 24, ss= 0, addl=10),
#12h delay simulation
ev(ID = 401:800, amt = 2*1, ii= 24, ss= 1, addl=0),
ev(ID = 401:800, amt = 2*0, ii= 12, ss= 0, addl=0),
ev(ID = 401:800, amt = 2*1, ii= 12, ss= 0, addl=0),
ev(ID = 401:800, amt = 2*1, ii= 24, ss= 0, addl=10),
#15h delay + 50% dose intake simulation
ev(ID = 401:800, amt = 2*1, ii= 24, ss= 1, addl=0),
ev(ID = 401:800, amt = 2*0, ii= 15, ss= 0, addl=0),
ev(ID = 401:800, amt = 2*0.5, ii= 9, ss= 0, addl=0),
ev(ID = 401:800, amt = 2*1, ii= 24, ss= 0, addl=10),
#15h delay simulation
ev(ID = 401:800, amt = 2*1, ii= 24, ss= 1, addl=0),
ev(ID = 401:800, amt = 2*0, ii= 15, ss= 0, addl=0),
ev(ID = 401:800, amt = 2*1, ii= 9, ss= 0, addl=0),
ev(ID = 401:800, amt = 2*1, ii= 24, ss= 0, addl=10),
#18h delay + 50% dose intake simulation
ev(ID = 401:800, amt = 2*1, ii= 24, ss= 1, addl=0),
ev(ID = 401:800, amt = 2*0, ii= 18, ss= 0, addl=0),
ev(ID = 401:800, amt = 2*0.5, ii= 6, ss= 0, addl=0),
ev(ID = 401:800, amt = 2*1, ii= 24, ss= 0, addl=10),
#18h delay simulation
ev(ID = 401:800, amt = 2*1, ii= 24, ss= 1, addl=0),
ev(ID = 401:800, amt = 2*0, ii= 18, ss= 0, addl=0),
ev(ID = 401:800, amt = 2*1, ii= 6, ss= 0, addl=0),
ev(ID = 401:800, amt = 2*1, ii= 24, ss= 0, addl=10),
#21h delay + 50% dose intake simulation
ev(ID = 401:800, amt = 2*1, ii= 24, ss= 1, addl=0),
ev(ID = 401:800, amt = 2*0, ii= 21, ss= 0, addl=0),
ev(ID = 401:800, amt = 2*0.5, ii= 3, ss= 0, addl=0),
ev(ID = 401:800, amt = 2*1, ii= 24, ss= 0, addl=10),
#21h delay simulation
ev(ID = 401:800, amt = 2*1, ii= 24, ss= 1, addl=0),
ev(ID = 401:800, amt = 2*0, ii= 21, ss= 0, addl=0),
ev(ID = 401:800, amt = 2*1, ii= 3, ss= 0, addl=0),
ev(ID = 401:800, amt = 2*1, ii= 24, ss= 0, addl=10),
#Missed dose simulation
ev(ID = 401:800, amt = 2*1, ii= 24, ss= 1, addl=0),
ev(ID = 401:800, amt = 2*0, ii= 24, ss= 0, addl=0),
ev(ID = 401:800, amt = 2*1, ii= 24, ss= 0, addl=0),
ev(ID = 401:800, amt = 2*1, ii= 24, ss= 0, addl=10),
#Missed dose + 150% dose intake simulation
ev(ID = 401:800, amt = 2*1, ii= 24, ss= 1, addl=0),
ev(ID = 401:800, amt = 2*0, ii= 24, ss= 0, addl=0),
ev(ID = 401:800, amt = 2*1.5, ii= 24, ss= 0, addl=0),
ev(ID = 401:800, amt = 2*1, ii= 24, ss= 0, addl=10),
#Missed dose + 200% dose intake simulation
ev(ID = 401:800, amt = 2*1, ii= 24, ss= 1, addl=0),
ev(ID = 401:800, amt = 2*0, ii= 24, ss= 0, addl=0),
ev(ID = 401:800, amt = 2*2, ii= 24, ss= 0, addl=0),
ev(ID = 401:800, amt = 2*1, ii= 24, ss= 0, addl=10))))%>%
              mutate(dose_group = "2 mg") %>%

  bind_rows(as_tibble( seq(
  #Steady-state simulation
ev(ID = 801:1200, amt = 3*1, ii= 24, ss= 1, addl=0),
ev(ID = 801:1200, amt = 3*1, ii= 24, ss= 0, addl=0),
ev(ID = 801:1200, amt = 3*1, ii= 24, ss= 0, addl=10),
#3h delay simulation
ev(ID = 801:1200, amt = 3*1, ii= 24, ss= 1, addl=0),
ev(ID = 801:1200, amt = 3*0, ii= 3, ss= 0, addl=0),
ev(ID = 801:1200, amt = 3*1, ii= 21, ss= 0, addl=0),
ev(ID = 801:1200, amt = 3*1, ii= 24, ss= 0, addl=10),
#6h delay simulation
ev(ID = 801:1200, amt = 3*1, ii= 24, ss= 1, addl=0),
ev(ID = 801:1200, amt = 3*0, ii= 6, ss= 0, addl=0),
ev(ID = 801:1200, amt = 3*1, ii= 18, ss= 0, addl=0),
ev(ID = 801:1200, amt = 3*1, ii= 24, ss= 0, addl=10),
#9h delay simulation
ev(ID = 801:1200, amt = 3*1, ii= 24, ss= 1, addl=0),
ev(ID = 801:1200, amt = 3*0, ii= 9, ss= 0, addl=0),
ev(ID = 801:1200, amt = 3*1, ii= 15, ss= 0, addl=0),
ev(ID = 801:1200, amt = 3*1, ii= 24, ss= 0, addl=10),
#12h delay simulation
ev(ID = 801:1200, amt = 3*1, ii= 24, ss= 1, addl=0),
ev(ID = 801:1200, amt = 3*0, ii= 12, ss= 0, addl=0),
ev(ID = 801:1200, amt = 3*1, ii= 12, ss= 0, addl=0),
ev(ID = 801:1200, amt = 3*1, ii= 24, ss= 0, addl=10),
#15h delay + 50% dose intake simulation
ev(ID = 801:1200, amt = 3*1, ii= 24, ss= 1, addl=0),
ev(ID = 801:1200, amt = 3*0, ii= 15, ss= 0, addl=0),
ev(ID = 801:1200, amt = 3*0.5, ii= 9, ss= 0, addl=0),
ev(ID = 801:1200, amt = 3*1, ii= 24, ss= 0, addl=10),
#15h delay simulation
ev(ID = 801:1200, amt = 3*1, ii= 24, ss= 1, addl=0),
ev(ID = 801:1200, amt = 3*0, ii= 15, ss= 0, addl=0),
ev(ID = 801:1200, amt = 3*1, ii= 9, ss= 0, addl=0),
ev(ID = 801:1200, amt = 3*1, ii= 24, ss= 0, addl=10),
#18h delay + 50% dose intake simulation
ev(ID = 801:1200, amt = 3*1, ii= 24, ss= 1, addl=0),
ev(ID = 801:1200, amt = 3*0, ii= 18, ss= 0, addl=0),
ev(ID = 801:1200, amt = 3*0.5, ii= 6, ss= 0, addl=0),
ev(ID = 801:1200, amt = 3*1, ii= 24, ss= 0, addl=10),
#18h delay simulation
ev(ID = 801:1200, amt = 3*1, ii= 24, ss= 1, addl=0),
ev(ID = 801:1200, amt = 3*0, ii= 18, ss= 0, addl=0),
ev(ID = 801:1200, amt = 3*1, ii= 6, ss= 0, addl=0),
ev(ID = 801:1200, amt = 3*1, ii= 24, ss= 0, addl=10),
#21h delay + 50% dose intake simulation
ev(ID = 801:1200, amt = 3*1, ii= 24, ss= 1, addl=0),
ev(ID = 801:1200, amt = 3*0, ii= 21, ss= 0, addl=0),
ev(ID = 801:1200, amt = 3*0.5, ii= 3, ss= 0, addl=0),
ev(ID = 801:1200, amt = 3*1, ii= 24, ss= 0, addl=10),
#21h delay simulation
ev(ID = 801:1200, amt = 3*1, ii= 24, ss= 1, addl=0),
ev(ID = 801:1200, amt = 3*0, ii= 21, ss= 0, addl=0),
ev(ID = 801:1200, amt = 3*1, ii= 3, ss= 0, addl=0),
ev(ID = 801:1200, amt = 3*1, ii= 24, ss= 0, addl=10),
#Missed dose simulation
ev(ID = 801:1200, amt = 3*1, ii= 24, ss= 1, addl=0),
ev(ID = 801:1200, amt = 3*0, ii= 24, ss= 0, addl=0),
ev(ID = 801:1200, amt = 3*1, ii= 24, ss= 0, addl=0),
ev(ID = 801:1200, amt = 3*1, ii= 24, ss= 0, addl=10),
#Missed dose + 150% dose intake simulation
ev(ID = 801:1200, amt = 3*1, ii= 24, ss= 1, addl=0),
ev(ID = 801:1200, amt = 3*0, ii= 24, ss= 0, addl=0),
ev(ID = 801:1200, amt = 3*1.5, ii= 24, ss= 0, addl=0),
ev(ID = 801:1200, amt = 3*1, ii= 24, ss= 0, addl=10),
#Missed dose + 200% dose intake simulation
ev(ID = 801:1200, amt = 3*1, ii= 24, ss= 1, addl=0),
ev(ID = 801:1200, amt = 3*0, ii= 24, ss= 0, addl=0),
ev(ID = 801:1200, amt = 3*2, ii= 24, ss= 0, addl=0),
ev(ID = 801:1200, amt = 3*1, ii= 24, ss= 0, addl=10))))%>%
              mutate(dose_group = "3 mg") %>%

    bind_rows(as_tibble( seq(
  #Steady-state simulation
ev(ID = 1201:1600, amt = 4*1, ii= 24, ss= 1, addl=0),
ev(ID = 1201:1600, amt = 4*1, ii= 24, ss= 0, addl=0),
ev(ID = 1201:1600, amt = 4*1, ii= 24, ss= 0, addl=10),
#3h delay simulation
ev(ID = 1201:1600, amt = 4*1, ii= 24, ss= 1, addl=0),
ev(ID = 1201:1600, amt = 4*0, ii= 3, ss= 0, addl=0),
ev(ID = 1201:1600, amt = 4*1, ii= 21, ss= 0, addl=0),
ev(ID = 1201:1600, amt = 4*1, ii= 24, ss= 0, addl=10),
#6h delay simulation
ev(ID = 1201:1600, amt = 4*1, ii= 24, ss= 1, addl=0),
ev(ID = 1201:1600, amt = 4*0, ii= 6, ss= 0, addl=0),
ev(ID = 1201:1600, amt = 4*1, ii= 18, ss= 0, addl=0),
ev(ID = 1201:1600, amt = 4*1, ii= 24, ss= 0, addl=10),
#9h delay simulation
ev(ID = 1201:1600, amt = 4*1, ii= 24, ss= 1, addl=0),
ev(ID = 1201:1600, amt = 4*0, ii= 9, ss= 0, addl=0),
ev(ID = 1201:1600, amt = 4*1, ii= 15, ss= 0, addl=0),
ev(ID = 1201:1600, amt = 4*1, ii= 24, ss= 0, addl=10),
#12h delay simulation
ev(ID = 1201:1600, amt = 4*1, ii= 24, ss= 1, addl=0),
ev(ID = 1201:1600, amt = 4*0, ii= 12, ss= 0, addl=0),
ev(ID = 1201:1600, amt = 4*1, ii= 12, ss= 0, addl=0),
ev(ID = 1201:1600, amt = 4*1, ii= 24, ss= 0, addl=10),
#15h delay + 50% dose intake simulation
ev(ID = 1201:1600, amt = 4*1, ii= 24, ss= 1, addl=0),
ev(ID = 1201:1600, amt = 4*0, ii= 15, ss= 0, addl=0),
ev(ID = 1201:1600, amt = 4*0.5, ii= 9, ss= 0, addl=0),
ev(ID = 1201:1600, amt = 4*1, ii= 24, ss= 0, addl=10),
#15h delay simulation
ev(ID = 1201:1600, amt = 4*1, ii= 24, ss= 1, addl=0),
ev(ID = 1201:1600, amt = 4*0, ii= 15, ss= 0, addl=0),
ev(ID = 1201:1600, amt = 4*1, ii= 9, ss= 0, addl=0),
ev(ID = 1201:1600, amt = 4*1, ii= 24, ss= 0, addl=10),
#18h delay + 50% dose intake simulation
ev(ID = 1201:1600, amt = 4*1, ii= 24, ss= 1, addl=0),
ev(ID = 1201:1600, amt = 4*0, ii= 18, ss= 0, addl=0),
ev(ID = 1201:1600, amt = 4*0.5, ii= 6, ss= 0, addl=0),
ev(ID = 1201:1600, amt = 4*1, ii= 24, ss= 0, addl=10),
#18h delay simulation
ev(ID = 1201:1600, amt = 4*1, ii= 24, ss= 1, addl=0),
ev(ID = 1201:1600, amt = 4*0, ii= 18, ss= 0, addl=0),
ev(ID = 1201:1600, amt = 4*1, ii= 6, ss= 0, addl=0),
ev(ID = 1201:1600, amt = 4*1, ii= 24, ss= 0, addl=10),
#21h delay + 50% dose intake simulation
ev(ID = 1201:1600, amt = 4*1, ii= 24, ss= 1, addl=0),
ev(ID = 1201:1600, amt = 4*0, ii= 21, ss= 0, addl=0),
ev(ID = 1201:1600, amt = 4*0.5, ii= 3, ss= 0, addl=0),
ev(ID = 1201:1600, amt = 4*1, ii= 24, ss= 0, addl=10),
#21h delay simulation
ev(ID = 1201:1600, amt = 4*1, ii= 24, ss= 1, addl=0),
ev(ID = 1201:1600, amt = 4*0, ii= 21, ss= 0, addl=0),
ev(ID = 1201:1600, amt = 4*1, ii= 3, ss= 0, addl=0),
ev(ID = 1201:1600, amt = 4*1, ii= 24, ss= 0, addl=10),
#Missed dose simulation
ev(ID = 1201:1600, amt = 4*1, ii= 24, ss= 1, addl=0),
ev(ID = 1201:1600, amt = 4*0, ii= 24, ss= 0, addl=0),
ev(ID = 1201:1600, amt = 4*1, ii= 24, ss= 0, addl=0),
ev(ID = 1201:1600, amt = 4*1, ii= 24, ss= 0, addl=10),
#Missed dose + 150% dose intake simulation
ev(ID = 1201:1600, amt = 4*1, ii= 24, ss= 1, addl=0),
ev(ID = 1201:1600, amt = 4*0, ii= 24, ss= 0, addl=0),
ev(ID = 1201:1600, amt = 4*1.5, ii= 24, ss= 0, addl=0),
ev(ID = 1201:1600, amt = 4*1, ii= 24, ss= 0, addl=10),
#Missed dose + 200% dose intake simulation
ev(ID = 1201:1600, amt = 4*1, ii= 24, ss= 1, addl=0),
ev(ID = 1201:1600, amt = 4*0, ii= 24, ss= 0, addl=0),
ev(ID = 1201:1600, amt = 4*2, ii= 24, ss= 0, addl=0),
ev(ID = 1201:1600, amt = 4*1, ii= 24, ss= 0, addl=10))))%>%
              mutate(dose_group = "4 mg") %>%

    bind_rows(as_tibble( seq(
  #Steady-state simulation
ev(ID = 1601:2000, amt = 5*1, ii= 24, ss= 1, addl=0),
ev(ID = 1601:2000, amt = 5*1, ii= 24, ss= 0, addl=0),
ev(ID = 1601:2000, amt = 5*1, ii= 24, ss= 0, addl=10),
#3h delay simulation
ev(ID = 1601:2000, amt = 5*1, ii= 24, ss= 1, addl=0),
ev(ID = 1601:2000, amt = 5*0, ii= 3, ss= 0, addl=0),
ev(ID = 1601:2000, amt = 5*1, ii= 21, ss= 0, addl=0),
ev(ID = 1601:2000, amt = 5*1, ii= 24, ss= 0, addl=10),
#6h delay simulation
ev(ID = 1601:2000, amt = 5*1, ii= 24, ss= 1, addl=0),
ev(ID = 1601:2000, amt = 5*0, ii= 6, ss= 0, addl=0),
ev(ID = 1601:2000, amt = 5*1, ii= 18, ss= 0, addl=0),
ev(ID = 1601:2000, amt = 5*1, ii= 24, ss= 0, addl=10),
#9h delay simulation
ev(ID = 1601:2000, amt = 5*1, ii= 24, ss= 1, addl=0),
ev(ID = 1601:2000, amt = 5*0, ii= 9, ss= 0, addl=0),
ev(ID = 1601:2000, amt = 5*1, ii= 15, ss= 0, addl=0),
ev(ID = 1601:2000, amt = 5*1, ii= 24, ss= 0, addl=10),
#12h delay simulation
ev(ID = 1601:2000, amt = 5*1, ii= 24, ss= 1, addl=0),
ev(ID = 1601:2000, amt = 5*0, ii= 12, ss= 0, addl=0),
ev(ID = 1601:2000, amt = 5*1, ii= 12, ss= 0, addl=0),
ev(ID = 1601:2000, amt = 5*1, ii= 24, ss= 0, addl=10),
#15h delay + 50% dose intake simulation
ev(ID = 1601:2000, amt = 5*1, ii= 24, ss= 1, addl=0),
ev(ID = 1601:2000, amt = 5*0, ii= 15, ss= 0, addl=0),
ev(ID = 1601:2000, amt = 5*0.5, ii= 9, ss= 0, addl=0),
ev(ID = 1601:2000, amt = 5*1, ii= 24, ss= 0, addl=10),
#15h delay simulation
ev(ID = 1601:2000, amt = 5*1, ii= 24, ss= 1, addl=0),
ev(ID = 1601:2000, amt = 5*0, ii= 15, ss= 0, addl=0),
ev(ID = 1601:2000, amt = 5*1, ii= 9, ss= 0, addl=0),
ev(ID = 1601:2000, amt = 5*1, ii= 24, ss= 0, addl=10),
#18h delay + 50% dose intake simulation
ev(ID = 1601:2000, amt = 5*1, ii= 24, ss= 1, addl=0),
ev(ID = 1601:2000, amt = 5*0, ii= 18, ss= 0, addl=0),
ev(ID = 1601:2000, amt = 5*0.5, ii= 6, ss= 0, addl=0),
ev(ID = 1601:2000, amt = 5*1, ii= 24, ss= 0, addl=10),
#18h delay simulation
ev(ID = 1601:2000, amt = 5*1, ii= 24, ss= 1, addl=0),
ev(ID = 1601:2000, amt = 5*0, ii= 18, ss= 0, addl=0),
ev(ID = 1601:2000, amt = 5*1, ii= 6, ss= 0, addl=0),
ev(ID = 1601:2000, amt = 5*1, ii= 24, ss= 0, addl=10),
#21h delay + 50% dose intake simulation
ev(ID = 1601:2000, amt = 5*1, ii= 24, ss= 1, addl=0),
ev(ID = 1601:2000, amt = 5*0, ii= 21, ss= 0, addl=0),
ev(ID = 1601:2000, amt = 5*0.5, ii= 3, ss= 0, addl=0),
ev(ID = 1601:2000, amt = 5*1, ii= 24, ss= 0, addl=10),
#21h delay simulation
ev(ID = 1601:2000, amt = 5*1, ii= 24, ss= 1, addl=0),
ev(ID = 1601:2000, amt = 5*0, ii= 21, ss= 0, addl=0),
ev(ID = 1601:2000, amt = 5*1, ii= 3, ss= 0, addl=0),
ev(ID = 1601:2000, amt = 5*1, ii= 24, ss= 0, addl=10),
#Missed dose simulation
ev(ID = 1601:2000, amt = 5*1, ii= 24, ss= 1, addl=0),
ev(ID = 1601:2000, amt = 5*0, ii= 24, ss= 0, addl=0),
ev(ID = 1601:2000, amt = 5*1, ii= 24, ss= 0, addl=0),
ev(ID = 1601:2000, amt = 5*1, ii= 24, ss= 0, addl=10),
#Missed dose + 150% dose intake simulation
ev(ID = 1601:2000, amt = 5*1, ii= 24, ss= 1, addl=0),
ev(ID = 1601:2000, amt = 5*0, ii= 24, ss= 0, addl=0),
ev(ID = 1601:2000, amt = 5*1.5, ii= 24, ss= 0, addl=0),
ev(ID = 1601:2000, amt = 5*1, ii= 24, ss= 0, addl=10),
#Missed dose + 200% dose intake simulation
ev(ID = 1601:2000, amt = 5*1, ii= 24, ss= 1, addl=0),
ev(ID = 1601:2000, amt = 5*0, ii= 24, ss= 0, addl=0),
ev(ID = 1601:2000, amt = 5*2, ii= 24, ss= 0, addl=0),
ev(ID = 1601:2000, amt = 5*1, ii= 24, ss= 0, addl=10))))%>%
              mutate(dose_group = "5 mg") %>%

    bind_rows(as_tibble( seq(
  #Steady-state simulation
ev(ID = 2001:2400, amt = 6*1, ii= 24, ss= 1, addl=0),
ev(ID = 2001:2400, amt = 6*1, ii= 24, ss= 0, addl=0),
ev(ID = 2001:2400, amt = 6*1, ii= 24, ss= 0, addl=10),
#3h delay simulation
ev(ID = 2001:2400, amt = 6*1, ii= 24, ss= 1, addl=0),
ev(ID = 2001:2400, amt = 6*0, ii= 3, ss= 0, addl=0),
ev(ID = 2001:2400, amt = 6*1, ii= 21, ss= 0, addl=0),
ev(ID = 2001:2400, amt = 6*1, ii= 24, ss= 0, addl=10),
#6h delay simulation
ev(ID = 2001:2400, amt = 6*1, ii= 24, ss= 1, addl=0),
ev(ID = 2001:2400, amt = 6*0, ii= 6, ss= 0, addl=0),
ev(ID = 2001:2400, amt = 6*1, ii= 18, ss= 0, addl=0),
ev(ID = 2001:2400, amt = 6*1, ii= 24, ss= 0, addl=10),
#9h delay simulation
ev(ID = 2001:2400, amt = 6*1, ii= 24, ss= 1, addl=0),
ev(ID = 2001:2400, amt = 6*0, ii= 9, ss= 0, addl=0),
ev(ID = 2001:2400, amt = 6*1, ii= 15, ss= 0, addl=0),
ev(ID = 2001:2400, amt = 6*1, ii= 24, ss= 0, addl=10),
#12h delay simulation
ev(ID = 2001:2400, amt = 6*1, ii= 24, ss= 1, addl=0),
ev(ID = 2001:2400, amt = 6*0, ii= 12, ss= 0, addl=0),
ev(ID = 2001:2400, amt = 6*1, ii= 12, ss= 0, addl=0),
ev(ID = 2001:2400, amt = 6*1, ii= 24, ss= 0, addl=10),
#15h delay + 50% dose intake simulation
ev(ID = 2001:2400, amt = 6*1, ii= 24, ss= 1, addl=0),
ev(ID = 2001:2400, amt = 6*0, ii= 15, ss= 0, addl=0),
ev(ID = 2001:2400, amt = 6*0.5, ii= 9, ss= 0, addl=0),
ev(ID = 2001:2400, amt = 6*1, ii= 24, ss= 0, addl=10),
#15h delay simulation
ev(ID = 2001:2400, amt = 6*1, ii= 24, ss= 1, addl=0),
ev(ID = 2001:2400, amt = 6*0, ii= 15, ss= 0, addl=0),
ev(ID = 2001:2400, amt = 6*1, ii= 9, ss= 0, addl=0),
ev(ID = 2001:2400, amt = 6*1, ii= 24, ss= 0, addl=10),
#18h delay + 50% dose intake simulation
ev(ID = 2001:2400, amt = 6*1, ii= 24, ss= 1, addl=0),
ev(ID = 2001:2400, amt = 6*0, ii= 18, ss= 0, addl=0),
ev(ID = 2001:2400, amt = 6*0.5, ii= 6, ss= 0, addl=0),
ev(ID = 2001:2400, amt = 6*1, ii= 24, ss= 0, addl=10),
#18h delay simulation
ev(ID = 2001:2400, amt = 6*1, ii= 24, ss= 1, addl=0),
ev(ID = 2001:2400, amt = 6*0, ii= 18, ss= 0, addl=0),
ev(ID = 2001:2400, amt = 6*1, ii= 6, ss= 0, addl=0),
ev(ID = 2001:2400, amt = 6*1, ii= 24, ss= 0, addl=10),
#21h delay + 50% dose intake simulation
ev(ID = 2001:2400, amt = 6*1, ii= 24, ss= 1, addl=0),
ev(ID = 2001:2400, amt = 6*0, ii= 21, ss= 0, addl=0),
ev(ID = 2001:2400, amt = 6*0.5, ii= 3, ss= 0, addl=0),
ev(ID = 2001:2400, amt = 6*1, ii= 24, ss= 0, addl=10),
#21h delay simulation
ev(ID = 2001:2400, amt = 6*1, ii= 24, ss= 1, addl=0),
ev(ID = 2001:2400, amt = 6*0, ii= 21, ss= 0, addl=0),
ev(ID = 2001:2400, amt = 6*1, ii= 3, ss= 0, addl=0),
ev(ID = 2001:2400, amt = 6*1, ii= 24, ss= 0, addl=10),
#Missed dose simulation
ev(ID = 2001:2400, amt = 6*1, ii= 24, ss= 1, addl=0),
ev(ID = 2001:2400, amt = 6*0, ii= 24, ss= 0, addl=0),
ev(ID = 2001:2400, amt = 6*1, ii= 24, ss= 0, addl=0),
ev(ID = 2001:2400, amt = 6*1, ii= 24, ss= 0, addl=10),
#Missed dose + 150% dose intake simulation
ev(ID = 2001:2400, amt = 6*1, ii= 24, ss= 1, addl=0),
ev(ID = 2001:2400, amt = 6*0, ii= 24, ss= 0, addl=0),
ev(ID = 2001:2400, amt = 6*1.5, ii= 24, ss= 0, addl=0),
ev(ID = 2001:2400, amt = 6*1, ii= 24, ss= 0, addl=10),
#Missed dose + 200% dose intake simulation
ev(ID = 2001:2400, amt = 6*1, ii= 24, ss= 1, addl=0),
ev(ID = 2001:2400, amt = 6*0, ii= 24, ss= 0, addl=0),
ev(ID = 2001:2400, amt = 6*2, ii= 24, ss= 0, addl=0),
ev(ID = 2001:2400, amt = 6*1, ii= 24, ss= 0, addl=10))))%>%
              mutate(dose_group = "6 mg") %>%

    bind_rows(as_tibble( seq(
  #Steady-state simulation
ev(ID = 2401:2800, amt = 7*1, ii= 24, ss= 1, addl=0),
ev(ID = 2401:2800, amt = 7*1, ii= 24, ss= 0, addl=0),
ev(ID = 2401:2800, amt = 7*1, ii= 24, ss= 0, addl=10),
#3h delay simulation
ev(ID = 2401:2800, amt = 7*1, ii= 24, ss= 1, addl=0),
ev(ID = 2401:2800, amt = 7*0, ii= 3, ss= 0, addl=0),
ev(ID = 2401:2800, amt = 7*1, ii= 21, ss= 0, addl=0),
ev(ID = 2401:2800, amt = 7*1, ii= 24, ss= 0, addl=10),
#6h delay simulation
ev(ID = 2401:2800, amt = 7*1, ii= 24, ss= 1, addl=0),
ev(ID = 2401:2800, amt = 7*0, ii= 6, ss= 0, addl=0),
ev(ID = 2401:2800, amt = 7*1, ii= 18, ss= 0, addl=0),
ev(ID = 2401:2800, amt = 7*1, ii= 24, ss= 0, addl=10),
#9h delay simulation
ev(ID = 2401:2800, amt = 7*1, ii= 24, ss= 1, addl=0),
ev(ID = 2401:2800, amt = 7*0, ii= 9, ss= 0, addl=0),
ev(ID = 2401:2800, amt = 7*1, ii= 15, ss= 0, addl=0),
ev(ID = 2401:2800, amt = 7*1, ii= 24, ss= 0, addl=10),
#12h delay simulation
ev(ID = 2401:2800, amt = 7*1, ii= 24, ss= 1, addl=0),
ev(ID = 2401:2800, amt = 7*0, ii= 12, ss= 0, addl=0),
ev(ID = 2401:2800, amt = 7*1, ii= 12, ss= 0, addl=0),
ev(ID = 2401:2800, amt = 7*1, ii= 24, ss= 0, addl=10),
#15h delay + 50% dose intake simulation
ev(ID = 2401:2800, amt = 7*1, ii= 24, ss= 1, addl=0),
ev(ID = 2401:2800, amt = 7*0, ii= 15, ss= 0, addl=0),
ev(ID = 2401:2800, amt = 7*0.5, ii= 9, ss= 0, addl=0),
ev(ID = 2401:2800, amt = 7*1, ii= 24, ss= 0, addl=10),
#15h delay simulation
ev(ID = 2401:2800, amt = 7*1, ii= 24, ss= 1, addl=0),
ev(ID = 2401:2800, amt = 7*0, ii= 15, ss= 0, addl=0),
ev(ID = 2401:2800, amt = 7*1, ii= 9, ss= 0, addl=0),
ev(ID = 2401:2800, amt = 7*1, ii= 24, ss= 0, addl=10),
#18h delay + 50% dose intake simulation
ev(ID = 2401:2800, amt = 7*1, ii= 24, ss= 1, addl=0),
ev(ID = 2401:2800, amt = 7*0, ii= 18, ss= 0, addl=0),
ev(ID = 2401:2800, amt = 7*0.5, ii= 6, ss= 0, addl=0),
ev(ID = 2401:2800, amt = 7*1, ii= 24, ss= 0, addl=10),
#18h delay simulation
ev(ID = 2401:2800, amt = 7*1, ii= 24, ss= 1, addl=0),
ev(ID = 2401:2800, amt = 7*0, ii= 18, ss= 0, addl=0),
ev(ID = 2401:2800, amt = 7*1, ii= 6, ss= 0, addl=0),
ev(ID = 2401:2800, amt = 7*1, ii= 24, ss= 0, addl=10),
#21h delay + 50% dose intake simulation
ev(ID = 2401:2800, amt = 7*1, ii= 24, ss= 1, addl=0),
ev(ID = 2401:2800, amt = 7*0, ii= 21, ss= 0, addl=0),
ev(ID = 2401:2800, amt = 7*0.5, ii= 3, ss= 0, addl=0),
ev(ID = 2401:2800, amt = 7*1, ii= 24, ss= 0, addl=10),
#21h delay simulation
ev(ID = 2401:2800, amt = 7*1, ii= 24, ss= 1, addl=0),
ev(ID = 2401:2800, amt = 7*0, ii= 21, ss= 0, addl=0),
ev(ID = 2401:2800, amt = 7*1, ii= 3, ss= 0, addl=0),
ev(ID = 2401:2800, amt = 7*1, ii= 24, ss= 0, addl=10),
#Missed dose simulation
ev(ID = 2401:2800, amt = 7*1, ii= 24, ss= 1, addl=0),
ev(ID = 2401:2800, amt = 7*0, ii= 24, ss= 0, addl=0),
ev(ID = 2401:2800, amt = 7*1, ii= 24, ss= 0, addl=0),
ev(ID = 2401:2800, amt = 7*1, ii= 24, ss= 0, addl=10),
#Missed dose + 150% dose intake simulation
ev(ID = 2401:2800, amt = 7*1, ii= 24, ss= 1, addl=0),
ev(ID = 2401:2800, amt = 7*0, ii= 24, ss= 0, addl=0),
ev(ID = 2401:2800, amt = 7*1.5, ii= 24, ss= 0, addl=0),
ev(ID = 2401:2800, amt = 7*1, ii= 24, ss= 0, addl=10),
#Missed dose + 200% dose intake simulation
ev(ID = 2401:2800, amt = 7*1, ii= 24, ss= 1, addl=0),
ev(ID = 2401:2800, amt = 7*0, ii= 24, ss= 0, addl=0),
ev(ID = 2401:2800, amt = 7*2, ii= 24, ss= 0, addl=0),
ev(ID = 2401:2800, amt = 7*1, ii= 24, ss= 0, addl=10))))%>%
              mutate(dose_group = "7 mg") %>%

    bind_rows(as_tibble( seq(
  #Steady-state simulation
ev(ID = 2801:3200, amt = 8*1, ii= 24, ss= 1, addl=0),
ev(ID = 2801:3200, amt = 8*1, ii= 24, ss= 0, addl=0),
ev(ID = 2801:3200, amt = 8*1, ii= 24, ss= 0, addl=10),
#3h delay simulation
ev(ID = 2801:3200, amt = 8*1, ii= 24, ss= 1, addl=0),
ev(ID = 2801:3200, amt = 8*0, ii= 3, ss= 0, addl=0),
ev(ID = 2801:3200, amt = 8*1, ii= 21, ss= 0, addl=0),
ev(ID = 2801:3200, amt = 8*1, ii= 24, ss= 0, addl=10),
#6h delay simulation
ev(ID = 2801:3200, amt = 8*1, ii= 24, ss= 1, addl=0),
ev(ID = 2801:3200, amt = 8*0, ii= 6, ss= 0, addl=0),
ev(ID = 2801:3200, amt = 8*1, ii= 18, ss= 0, addl=0),
ev(ID = 2801:3200, amt = 8*1, ii= 24, ss= 0, addl=10),
#9h delay simulation
ev(ID = 2801:3200, amt = 8*1, ii= 24, ss= 1, addl=0),
ev(ID = 2801:3200, amt = 8*0, ii= 9, ss= 0, addl=0),
ev(ID = 2801:3200, amt = 8*1, ii= 15, ss= 0, addl=0),
ev(ID = 2801:3200, amt = 8*1, ii= 24, ss= 0, addl=10),
#12h delay simulation
ev(ID = 2801:3200, amt = 8*1, ii= 24, ss= 1, addl=0),
ev(ID = 2801:3200, amt = 8*0, ii= 12, ss= 0, addl=0),
ev(ID = 2801:3200, amt = 8*1, ii= 12, ss= 0, addl=0),
ev(ID = 2801:3200, amt = 8*1, ii= 24, ss= 0, addl=10),
#15h delay + 50% dose intake simulation
ev(ID = 2801:3200, amt = 8*1, ii= 24, ss= 1, addl=0),
ev(ID = 2801:3200, amt = 8*0, ii= 15, ss= 0, addl=0),
ev(ID = 2801:3200, amt = 8*0.5, ii= 9, ss= 0, addl=0),
ev(ID = 2801:3200, amt = 8*1, ii= 24, ss= 0, addl=10),
#15h delay simulation
ev(ID = 2801:3200, amt = 8*1, ii= 24, ss= 1, addl=0),
ev(ID = 2801:3200, amt = 8*0, ii= 15, ss= 0, addl=0),
ev(ID = 2801:3200, amt = 8*1, ii= 9, ss= 0, addl=0),
ev(ID = 2801:3200, amt = 8*1, ii= 24, ss= 0, addl=10),
#18h delay + 50% dose intake simulation
ev(ID = 2801:3200, amt = 8*1, ii= 24, ss= 1, addl=0),
ev(ID = 2801:3200, amt = 8*0, ii= 18, ss= 0, addl=0),
ev(ID = 2801:3200, amt = 8*0.5, ii= 6, ss= 0, addl=0),
ev(ID = 2801:3200, amt = 8*1, ii= 24, ss= 0, addl=10),
#18h delay simulation
ev(ID = 2801:3200, amt = 8*1, ii= 24, ss= 1, addl=0),
ev(ID = 2801:3200, amt = 8*0, ii= 18, ss= 0, addl=0),
ev(ID = 2801:3200, amt = 8*1, ii= 6, ss= 0, addl=0),
ev(ID = 2801:3200, amt = 8*1, ii= 24, ss= 0, addl=10),
#21h delay + 50% dose intake simulation
ev(ID = 2801:3200, amt = 8*1, ii= 24, ss= 1, addl=0),
ev(ID = 2801:3200, amt = 8*0, ii= 21, ss= 0, addl=0),
ev(ID = 2801:3200, amt = 8*0.5, ii= 3, ss= 0, addl=0),
ev(ID = 2801:3200, amt = 8*1, ii= 24, ss= 0, addl=10),
#21h delay simulation
ev(ID = 2801:3200, amt = 8*1, ii= 24, ss= 1, addl=0),
ev(ID = 2801:3200, amt = 8*0, ii= 21, ss= 0, addl=0),
ev(ID = 2801:3200, amt = 8*1, ii= 3, ss= 0, addl=0),
ev(ID = 2801:3200, amt = 8*1, ii= 24, ss= 0, addl=10),
#Missed dose simulation
ev(ID = 2801:3200, amt = 8*1, ii= 24, ss= 1, addl=0),
ev(ID = 2801:3200, amt = 8*0, ii= 24, ss= 0, addl=0),
ev(ID = 2801:3200, amt = 8*1, ii= 24, ss= 0, addl=0),
ev(ID = 2801:3200, amt = 8*1, ii= 24, ss= 0, addl=10),
#Missed dose + 150% dose intake simulation
ev(ID = 2801:3200, amt = 8*1, ii= 24, ss= 1, addl=0),
ev(ID = 2801:3200, amt = 8*0, ii= 24, ss= 0, addl=0),
ev(ID = 2801:3200, amt = 8*1.5, ii= 24, ss= 0, addl=0),
ev(ID = 2801:3200, amt = 8*1, ii= 24, ss= 0, addl=10),
#Missed dose + 200% dose intake simulation
ev(ID = 2801:3200, amt = 8*1, ii= 24, ss= 1, addl=0),
ev(ID = 2801:3200, amt = 8*0, ii= 24, ss= 0, addl=0),
ev(ID = 2801:3200, amt = 8*2, ii= 24, ss= 0, addl=0),
ev(ID = 2801:3200, amt = 8*1, ii= 24, ss= 0, addl=10))))%>%
              mutate(dose_group = "8 mg") %>%

  bind_rows(as_tibble( seq(
  #Steady-state simulation
ev(ID = 3201:3600, amt = 9*1, ii= 24, ss= 1, addl=0),
ev(ID = 3201:3600, amt = 9*1, ii= 24, ss= 0, addl=0),
ev(ID = 3201:3600, amt = 9*1, ii= 24, ss= 0, addl=10),
#3h delay simulation
ev(ID = 3201:3600, amt = 9*1, ii= 24, ss= 1, addl=0),
ev(ID = 3201:3600, amt = 9*0, ii= 3, ss= 0, addl=0),
ev(ID = 3201:3600, amt = 9*1, ii= 21, ss= 0, addl=0),
ev(ID = 3201:3600, amt = 9*1, ii= 24, ss= 0, addl=10),
#6h delay simulation
ev(ID = 3201:3600, amt = 9*1, ii= 24, ss= 1, addl=0),
ev(ID = 3201:3600, amt = 9*0, ii= 6, ss= 0, addl=0),
ev(ID = 3201:3600, amt = 9*1, ii= 18, ss= 0, addl=0),
ev(ID = 3201:3600, amt = 9*1, ii= 24, ss= 0, addl=10),
#9h delay simulation
ev(ID = 3201:3600, amt = 9*1, ii= 24, ss= 1, addl=0),
ev(ID = 3201:3600, amt = 9*0, ii= 9, ss= 0, addl=0),
ev(ID = 3201:3600, amt = 9*1, ii= 15, ss= 0, addl=0),
ev(ID = 3201:3600, amt = 9*1, ii= 24, ss= 0, addl=10),
#12h delay simulation
ev(ID = 3201:3600, amt = 9*1, ii= 24, ss= 1, addl=0),
ev(ID = 3201:3600, amt = 9*0, ii= 12, ss= 0, addl=0),
ev(ID = 3201:3600, amt = 9*1, ii= 12, ss= 0, addl=0),
ev(ID = 3201:3600, amt = 9*1, ii= 24, ss= 0, addl=10),
#15h delay + 50% dose intake simulation
ev(ID = 3201:3600, amt = 9*1, ii= 24, ss= 1, addl=0),
ev(ID = 3201:3600, amt = 9*0, ii= 15, ss= 0, addl=0),
ev(ID = 3201:3600, amt = 9*0.5, ii= 9, ss= 0, addl=0),
ev(ID = 3201:3600, amt = 9*1, ii= 24, ss= 0, addl=10),
#15h delay simulation
ev(ID = 3201:3600, amt = 9*1, ii= 24, ss= 1, addl=0),
ev(ID = 3201:3600, amt = 9*0, ii= 15, ss= 0, addl=0),
ev(ID = 3201:3600, amt = 9*1, ii= 9, ss= 0, addl=0),
ev(ID = 3201:3600, amt = 9*1, ii= 24, ss= 0, addl=10),
#18h delay + 50% dose intake simulation
ev(ID = 3201:3600, amt = 9*1, ii= 24, ss= 1, addl=0),
ev(ID = 3201:3600, amt = 9*0, ii= 18, ss= 0, addl=0),
ev(ID = 3201:3600, amt = 9*0.5, ii= 6, ss= 0, addl=0),
ev(ID = 3201:3600, amt = 9*1, ii= 24, ss= 0, addl=10),
#18h delay simulation
ev(ID = 3201:3600, amt = 9*1, ii= 24, ss= 1, addl=0),
ev(ID = 3201:3600, amt = 9*0, ii= 18, ss= 0, addl=0),
ev(ID = 3201:3600, amt = 9*1, ii= 6, ss= 0, addl=0),
ev(ID = 3201:3600, amt = 9*1, ii= 24, ss= 0, addl=10),
#21h delay + 50% dose intake simulation
ev(ID = 3201:3600, amt = 9*1, ii= 24, ss= 1, addl=0),
ev(ID = 3201:3600, amt = 9*0, ii= 21, ss= 0, addl=0),
ev(ID = 3201:3600, amt = 9*0.5, ii= 3, ss= 0, addl=0),
ev(ID = 3201:3600, amt = 9*1, ii= 24, ss= 0, addl=10),
#21h delay simulation
ev(ID = 3201:3600, amt = 9*1, ii= 24, ss= 1, addl=0),
ev(ID = 3201:3600, amt = 9*0, ii= 21, ss= 0, addl=0),
ev(ID = 3201:3600, amt = 9*1, ii= 3, ss= 0, addl=0),
ev(ID = 3201:3600, amt = 9*1, ii= 24, ss= 0, addl=10),
#Missed dose simulation
ev(ID = 3201:3600, amt = 9*1, ii= 24, ss= 1, addl=0),
ev(ID = 3201:3600, amt = 9*0, ii= 24, ss= 0, addl=0),
ev(ID = 3201:3600, amt = 9*1, ii= 24, ss= 0, addl=0),
ev(ID = 3201:3600, amt = 9*1, ii= 24, ss= 0, addl=10),
#Missed dose + 150% dose intake simulation
ev(ID = 3201:3600, amt = 9*1, ii= 24, ss= 1, addl=0),
ev(ID = 3201:3600, amt = 9*0, ii= 24, ss= 0, addl=0),
ev(ID = 3201:3600, amt = 9*1.5, ii= 24, ss= 0, addl=0),
ev(ID = 3201:3600, amt = 9*1, ii= 24, ss= 0, addl=10),
#Missed dose + 200% dose intake simulation
ev(ID = 3201:3600, amt = 9*1, ii= 24, ss= 1, addl=0),
ev(ID = 3201:3600, amt = 9*0, ii= 24, ss= 0, addl=0),
ev(ID = 3201:3600, amt = 9*2, ii= 24, ss= 0, addl=0),
ev(ID = 3201:3600, amt = 9*1, ii= 24, ss= 0, addl=10))))%>%
              mutate(dose_group = "9 mg") %>%

    bind_rows(as_tibble( seq(
  #Steady-state simulation
```

```
  #Steady-state simulation
ev(ID = 3601:4000, amt = 10*1, ii= 24, ss= 1, addl=0),
ev(ID = 3601:4000, amt = 10*1, ii= 24, ss= 0, addl=0),
ev(ID = 3601:4000, amt = 10*1, ii= 24, ss= 0, addl=10),
#3h delay simulation
ev(ID = 3601:4000, amt = 10*1, ii= 24, ss= 1, addl=0),
ev(ID = 3601:4000, amt = 10*0, ii= 3, ss= 0, addl=0),
ev(ID = 3601:4000, amt = 10*1, ii= 21, ss= 0, addl=0),
ev(ID = 3601:4000, amt = 10*1, ii= 24, ss= 0, addl=10),
#6h delay simulation
ev(ID = 3601:4000, amt = 10*1, ii= 24, ss= 1, addl=0),
ev(ID = 3601:4000, amt = 10*0, ii= 6, ss= 0, addl=0),
ev(ID = 3601:4000, amt = 10*1, ii= 18, ss= 0, addl=0),
ev(ID = 3601:4000, amt = 10*1, ii= 24, ss= 0, addl=10),
#9h delay simulation
ev(ID = 3601:4000, amt = 10*1, ii= 24, ss= 1, addl=0),
ev(ID = 3601:4000, amt = 10*0, ii= 9, ss= 0, addl=0),
ev(ID = 3601:4000, amt = 10*1, ii= 15, ss= 0, addl=0),
ev(ID = 3601:4000, amt = 10*1, ii= 24, ss= 0, addl=10),
#12h delay simulation
ev(ID = 3601:4000, amt = 10*1, ii= 24, ss= 1, addl=0),
ev(ID = 3601:4000, amt = 10*0, ii= 12, ss= 0, addl=0),
ev(ID = 3601:4000, amt = 10*1, ii= 12, ss= 0, addl=0),
ev(ID = 3601:4000, amt = 10*1, ii= 24, ss= 0, addl=10),
#15h delay + 50% dose intake simulation
ev(ID = 3601:4000, amt = 10*1, ii= 24, ss= 1, addl=0),
ev(ID = 3601:4000, amt = 10*0, ii= 15, ss= 0, addl=0),
ev(ID = 3601:4000, amt = 10*0.5, ii= 9, ss= 0, addl=0),
ev(ID = 3601:4000, amt = 10*1, ii= 24, ss= 0, addl=10),
#15h delay simulation
ev(ID = 3601:4000, amt = 10*1, ii= 24, ss= 1, addl=0),
ev(ID = 3601:4000, amt = 10*0, ii= 15, ss= 0, addl=0),
ev(ID = 3601:4000, amt = 10*1, ii= 9, ss= 0, addl=0),
ev(ID = 3601:4000, amt = 10*1, ii= 24, ss= 0, addl=10),
#18h delay + 50% dose intake simulation
ev(ID = 3601:4000, amt = 10*1, ii= 24, ss= 1, addl=0),
ev(ID = 3601:4000, amt = 10*0, ii= 18, ss= 0, addl=0),
ev(ID = 3601:4000, amt = 10*0.5, ii= 6, ss= 0, addl=0),
ev(ID = 3601:4000, amt = 10*1, ii= 24, ss= 0, addl=10),
#18h delay simulation
ev(ID = 3601:4000, amt = 10*1, ii= 24, ss= 1, addl=0),
ev(ID = 3601:4000, amt = 10*0, ii= 18, ss= 0, addl=0),
ev(ID = 3601:4000, amt = 10*1, ii= 6, ss= 0, addl=0),
ev(ID = 3601:4000, amt = 10*1, ii= 24, ss= 0, addl=10),
#21h delay + 50% dose intake simulation
ev(ID = 3601:4000, amt = 10*1, ii= 24, ss= 1, addl=0),
ev(ID = 3601:4000, amt = 10*0, ii= 21, ss= 0, addl=0),
ev(ID = 3601:4000, amt = 10*0.5, ii= 3, ss= 0, addl=0),
ev(ID = 3601:4000, amt = 10*1, ii= 24, ss= 0, addl=10),
#21h delay simulation
ev(ID = 3601:4000, amt = 10*1, ii= 24, ss= 1, addl=0),
ev(ID = 3601:4000, amt = 10*0, ii= 21, ss= 0, addl=0),
ev(ID = 3601:4000, amt = 10*1, ii= 3, ss= 0, addl=0),
ev(ID = 3601:4000, amt = 10*1, ii= 24, ss= 0, addl=10),
#Missed dose simulation
ev(ID = 3601:4000, amt = 10*1, ii= 24, ss= 1, addl=0),
ev(ID = 3601:4000, amt = 10*0, ii= 24, ss= 0, addl=0),
ev(ID = 3601:4000, amt = 10*1, ii= 24, ss= 0, addl=0),
ev(ID = 3601:4000, amt = 10*1, ii= 24, ss= 0, addl=10),
#Missed dose + 150% dose intake simulation
ev(ID = 3601:4000, amt = 10*1, ii= 24, ss= 1, addl=0),
ev(ID = 3601:4000, amt = 10*0, ii= 24, ss= 0, addl=0),
ev(ID = 3601:4000, amt = 10*1.5, ii= 24, ss= 0, addl=0),
ev(ID = 3601:4000, amt = 10*1, ii= 24, ss= 0, addl=10),
#Missed dose + 200% dose intake simulation
ev(ID = 3601:4000, amt = 10*1, ii= 24, ss= 1, addl=0),
ev(ID = 3601:4000, amt = 10*0, ii= 24, ss= 0, addl=0),
ev(ID = 3601:4000, amt = 10*2, ii= 24, ss= 0, addl=0),
ev(ID = 3601:4000, amt = 10*1, ii= 24, ss= 0, addl=10))))%>%
              mutate(dose_group = "10 mg") %>%

    bind_rows(as_tibble( seq(
  #Steady-state simulation
ev(ID = 4001:4400, amt = 11*1, ii= 24, ss= 1, addl=0),
ev(ID = 4001:4400, amt = 11*1, ii= 24, ss= 0, addl=0),
ev(ID = 4001:4400, amt = 11*1, ii= 24, ss= 0, addl=10),
#3h delay simulation
ev(ID = 4001:4400, amt = 11*1, ii= 24, ss= 1, addl=0),
ev(ID = 4001:4400, amt = 11*0, ii= 3, ss= 0, addl=0),
ev(ID = 4001:4400, amt = 11*1, ii= 21, ss= 0, addl=0),
ev(ID = 4001:4400, amt = 11*1, ii= 24, ss= 0, addl=10),
#6h delay simulation
ev(ID = 4001:4400, amt = 11*1, ii= 24, ss= 1, addl=0),
ev(ID = 4001:4400, amt = 11*0, ii= 6, ss= 0, addl=0),
ev(ID = 4001:4400, amt = 11*1, ii= 18, ss= 0, addl=0),
ev(ID = 4001:4400, amt = 11*1, ii= 24, ss= 0, addl=10),
#9h delay simulation
ev(ID = 4001:4400, amt = 11*1, ii= 24, ss= 1, addl=0),
ev(ID = 4001:4400, amt = 11*0, ii= 9, ss= 0, addl=0),
ev(ID = 4001:4400, amt = 11*1, ii= 15, ss= 0, addl=0),
ev(ID = 4001:4400, amt = 11*1, ii= 24, ss= 0, addl=10),
#12h delay simulation
ev(ID = 4001:4400, amt = 11*1, ii= 24, ss= 1, addl=0),
ev(ID = 4001:4400, amt = 11*0, ii= 12, ss= 0, addl=0),
ev(ID = 4001:4400, amt = 11*1, ii= 12, ss= 0, addl=0),
ev(ID = 4001:4400, amt = 11*1, ii= 24, ss= 0, addl=10),
#15h delay + 50% dose intake simulation
ev(ID = 4001:4400, amt = 11*1, ii= 24, ss= 1, addl=0),
ev(ID = 4001:4400, amt = 11*0, ii= 15, ss= 0, addl=0),
ev(ID = 4001:4400, amt = 11*0.5, ii= 9, ss= 0, addl=0),
ev(ID = 4001:4400, amt = 11*1, ii= 24, ss= 0, addl=10),
#15h delay simulation
ev(ID = 4001:4400, amt = 11*1, ii= 24, ss= 1, addl=0),
ev(ID = 4001:4400, amt = 11*0, ii= 15, ss= 0, addl=0),
ev(ID = 4001:4400, amt = 11*1, ii= 9, ss= 0, addl=0),
ev(ID = 4001:4400, amt = 11*1, ii= 24, ss= 0, addl=10),
#18h delay + 50% dose intake simulation
ev(ID = 4001:4400, amt = 11*1, ii= 24, ss= 1, addl=0),
ev(ID = 4001:4400, amt = 11*0, ii= 18, ss= 0, addl=0),
ev(ID = 4001:4400, amt = 11*0.5, ii= 6, ss= 0, addl=0),
ev(ID = 4001:4400, amt = 11*1, ii= 24, ss= 0, addl=10),
#18h delay simulation
ev(ID = 4001:4400, amt = 11*1, ii= 24, ss= 1, addl=0),
ev(ID = 4001:4400, amt = 11*0, ii= 18, ss= 0, addl=0),
ev(ID = 4001:4400, amt = 11*1, ii= 6, ss= 0, addl=0),
ev(ID = 4001:4400, amt = 11*1, ii= 24, ss= 0, addl=10),
#21h delay + 50% dose intake simulation
ev(ID = 4001:4400, amt = 11*1, ii= 24, ss= 1, addl=0),
ev(ID = 4001:4400, amt = 11*0, ii= 21, ss= 0, addl=0),
ev(ID = 4001:4400, amt = 11*0.5, ii= 3, ss= 0, addl=0),
ev(ID = 4001:4400, amt = 11*1, ii= 24, ss= 0, addl=10),
#21h delay simulation
ev(ID = 4001:4400, amt = 11*1, ii= 24, ss= 1, addl=0),
ev(ID = 4001:4400, amt = 11*0, ii= 21, ss= 0, addl=0),
ev(ID = 4001:4400, amt = 11*1, ii= 3, ss= 0, addl=0),
ev(ID = 4001:4400, amt = 11*1, ii= 24, ss= 0, addl=10),
#Missed dose simulation
ev(ID = 4001:4400, amt = 11*1, ii= 24, ss= 1, addl=0),
ev(ID = 4001:4400, amt = 11*0, ii= 24, ss= 0, addl=0),
ev(ID = 4001:4400, amt = 11*1, ii= 24, ss= 0, addl=0),
ev(ID = 4001:4400, amt = 11*1, ii= 24, ss= 0, addl=10),
#Missed dose + 150% dose intake simulation
ev(ID = 4001:4400, amt = 11*1, ii= 24, ss= 1, addl=0),
ev(ID = 4001:4400, amt = 11*0, ii= 24, ss= 0, addl=0),
ev(ID = 4001:4400, amt = 11*1.5, ii= 24, ss= 0, addl=0),
ev(ID = 4001:4400, amt = 11*1, ii= 24, ss= 0, addl=10),
#Missed dose + 200% dose intake simulation
ev(ID = 4001:4400, amt = 11*1, ii= 24, ss= 1, addl=0),
ev(ID = 4001:4400, amt = 11*0, ii= 24, ss= 0, addl=0),
ev(ID = 4001:4400, amt = 11*2, ii= 24, ss= 0, addl=0),
ev(ID = 4001:4400, amt = 11*1, ii= 24, ss= 0, addl=10))))%>%
              mutate(dose_group = "11 mg") %>%

    bind_rows(as_tibble( seq(
  #Steady-state simulation
ev(ID = 4401:4800, amt = 12*1, ii= 24, ss= 1, addl=0),
ev(ID = 4401:4800, amt = 12*1, ii= 24, ss= 0, addl=0),
ev(ID = 4401:4800, amt = 12*1, ii= 24, ss= 0, addl=10),
#3h delay simulation
ev(ID = 4401:4800, amt = 12*1, ii= 24, ss= 1, addl=0),
ev(ID = 4401:4800, amt = 12*0, ii= 3, ss= 0, addl=0),
ev(ID = 4401:4800, amt = 12*1, ii= 21, ss= 0, addl=0),
ev(ID = 4401:4800, amt = 12*1, ii= 24, ss= 0, addl=10),
#6h delay simulation
ev(ID = 4401:4800, amt = 12*1, ii= 24, ss= 1, addl=0),
ev(ID = 4401:4800, amt = 12*0, ii= 6, ss= 0, addl=0),
ev(ID = 4401:4800, amt = 12*1, ii= 18, ss= 0, addl=0),
ev(ID = 4401:4800, amt = 12*1, ii= 24, ss= 0, addl=10),
#9h delay simulation
ev(ID = 4401:4800, amt = 12*1, ii= 24, ss= 1, addl=0),
ev(ID = 4401:4800, amt = 12*0, ii= 9, ss= 0, addl=0),
ev(ID = 4401:4800, amt = 12*1, ii= 15, ss= 0, addl=0),
ev(ID = 4401:4800, amt = 12*1, ii= 24, ss= 0, addl=10),
#12h delay simulation
ev(ID = 4401:4800, amt = 12*1, ii= 24, ss= 1, addl=0),
ev(ID = 4401:4800, amt = 12*0, ii= 12, ss= 0, addl=0),
ev(ID = 4401:4800, amt = 12*1, ii= 12, ss= 0, addl=0),
ev(ID = 4401:4800, amt = 12*1, ii= 24, ss= 0, addl=10),
#15h delay + 50% dose intake simulation
ev(ID = 4401:4800, amt = 12*1, ii= 24, ss= 1, addl=0),
ev(ID = 4401:4800, amt = 12*0, ii= 15, ss= 0, addl=0),
ev(ID = 4401:4800, amt = 12*0.5, ii= 9, ss= 0, addl=0),
ev(ID = 4401:4800, amt = 12*1, ii= 24, ss= 0, addl=10),
#15h delay simulation
ev(ID = 4401:4800, amt = 12*1, ii= 24, ss= 1, addl=0),
ev(ID = 4401:4800, amt = 12*0, ii= 15, ss= 0, addl=0),
ev(ID = 4401:4800, amt = 12*1, ii= 9, ss= 0, addl=0),
ev(ID = 4401:4800, amt = 12*1, ii= 24, ss= 0, addl=10),
#18h delay + 50% dose intake simulation
ev(ID = 4401:4800, amt = 12*1, ii= 24, ss= 1, addl=0),
ev(ID = 4401:4800, amt = 12*0, ii= 18, ss= 0, addl=0),
ev(ID = 4401:4800, amt = 12*0.5, ii= 6, ss= 0, addl=0),
ev(ID = 4401:4800, amt = 12*1, ii= 24, ss= 0, addl=10),
#18h delay simulation
ev(ID = 4401:4800, amt = 12*1, ii= 24, ss= 1, addl=0),
ev(ID = 4401:4800, amt = 12*0, ii= 18, ss= 0, addl=0),
ev(ID = 4401:4800, amt = 12*1, ii= 6, ss= 0, addl=0),
ev(ID = 4401:4800, amt = 12*1, ii= 24, ss= 0, addl=10),
#21h delay + 50% dose intake simulation
ev(ID = 4401:4800, amt = 12*1, ii= 24, ss= 1, addl=0),
ev(ID = 4401:4800, amt = 12*0, ii= 21, ss= 0, addl=0),
ev(ID = 4401:4800, amt = 12*0.5, ii= 3, ss= 0, addl=0),
ev(ID = 4401:4800, amt = 12*1, ii= 24, ss= 0, addl=10),
#21h delay simulation
ev(ID = 4401:4800, amt = 12*1, ii= 24, ss= 1, addl=0),
ev(ID = 4401:4800, amt = 12*0, ii= 21, ss= 0, addl=0),
ev(ID = 4401:4800, amt = 12*1, ii= 3, ss= 0, addl=0),
ev(ID = 4401:4800, amt = 12*1, ii= 24, ss= 0, addl=10),
#Missed dose simulation
ev(ID = 4401:4800, amt = 12*1, ii= 24, ss= 1, addl=0),
ev(ID = 4401:4800, amt = 12*0, ii= 24, ss= 0, addl=0),
ev(ID = 4401:4800, amt = 12*1, ii= 24, ss= 0, addl=0),
ev(ID = 4401:4800, amt = 12*1, ii= 24, ss= 0, addl=10),
#Missed dose + 150% dose intake simulation
ev(ID = 4401:4800, amt = 12*1, ii= 24, ss= 1, addl=0),
ev(ID = 4401:4800, amt = 12*0, ii= 24, ss= 0, addl=0),
ev(ID = 4401:4800, amt = 12*1.5, ii= 24, ss= 0, addl=0),
ev(ID = 4401:4800, amt = 12*1, ii= 24, ss= 0, addl=10),
#Missed dose + 200% dose intake simulation
ev(ID = 4401:4800, amt = 12*1, ii= 24, ss= 1, addl=0),
ev(ID = 4401:4800, amt = 12*0, ii= 24, ss= 0, addl=0),
ev(ID = 4401:4800, amt = 12*2, ii= 24, ss= 0, addl=0),
ev(ID = 4401:4800, amt = 12*1, ii= 24, ss= 0, addl=10))))%>%
              mutate(dose_group = "12 mg") %>%

    bind_rows(as_tibble( seq(
  #Steady-state simulation
ev(ID = 4801:5200, amt = 13*1, ii= 24, ss= 1, addl=0),
ev(ID = 4801:5200, amt = 13*1, ii= 24, ss= 0, addl=0),
ev(ID = 4801:5200, amt = 13*1, ii= 24, ss= 0, addl=10),
#3h delay simulation
ev(ID = 4801:5200, amt = 13*1, ii= 24, ss= 1, addl=0),
ev(ID = 4801:5200, amt = 13*0, ii= 3, ss= 0, addl=0),
ev(ID = 4801:5200, amt = 13*1, ii= 21, ss= 0, addl=0),
ev(ID = 4801:5200, amt = 13*1, ii= 24, ss= 0, addl=10),
#6h delay simulation
ev(ID = 4801:5200, amt = 13*1, ii= 24, ss= 1, addl=0),
ev(ID = 4801:5200, amt = 13*0, ii= 6, ss= 0, addl=0),
ev(ID = 4801:5200, amt = 13*1, ii= 18, ss= 0, addl=0),
ev(ID = 4801:5200, amt = 13*1, ii= 24, ss= 0, addl=10),
#9h delay simulation
ev(ID = 4801:5200, amt = 13*1, ii= 24, ss= 1, addl=0),
ev(ID = 4801:5200, amt = 13*0, ii= 9, ss= 0, addl=0),
ev(ID = 4801:5200, amt = 13*1, ii= 15, ss= 0, addl=0),
ev(ID = 4801:5200, amt = 13*1, ii= 24, ss= 0, addl=10),
#12h delay simulation
ev(ID = 4801:5200, amt = 13*1, ii= 24, ss= 1, addl=0),
ev(ID = 4801:5200, amt = 13*0, ii= 12, ss= 0, addl=0),
ev(ID = 4801:5200, amt = 13*1, ii= 12, ss= 0, addl=0),
ev(ID = 4801:5200, amt = 13*1, ii= 24, ss= 0, addl=10),
#15h delay + 50% dose intake simulation
ev(ID = 4801:5200, amt = 13*1, ii= 24, ss= 1, addl=0),
ev(ID = 4801:5200, amt = 13*0, ii= 15, ss= 0, addl=0),
ev(ID = 4801:5200, amt = 13*0.5, ii= 9, ss= 0, addl=0),
ev(ID = 4801:5200, amt = 13*1, ii= 24, ss= 0, addl=10),
#15h delay simulation
ev(ID = 4801:5200, amt = 13*1, ii= 24, ss= 1, addl=0),
ev(ID = 4801:5200, amt = 13*0, ii= 15, ss= 0, addl=0),
ev(ID = 4801:5200, amt = 13*1, ii= 9, ss= 0, addl=0),
ev(ID = 4801:5200, amt = 13*1, ii= 24, ss= 0, addl=10),
#18h delay + 50% dose intake simulation
ev(ID = 4801:5200, amt = 13*1, ii= 24, ss= 1, addl=0),
ev(ID = 4801:5200, amt = 13*0, ii= 18, ss= 0, addl=0),
ev(ID = 4801:5200, amt = 13*0.5, ii= 6, ss= 0, addl=0),
ev(ID = 4801:5200, amt = 13*1, ii= 24, ss= 0, addl=10),
#18h delay simulation
ev(ID = 4801:5200, amt = 13*1, ii= 24, ss= 1, addl=0),
ev(ID = 4801:5200, amt = 13*0, ii= 18, ss= 0, addl=0),
ev(ID = 4801:5200, amt = 13*1, ii= 6, ss= 0, addl=0),
ev(ID = 4801:5200, amt = 13*1, ii= 24, ss= 0, addl=10),
#21h delay + 50% dose intake simulation
ev(ID = 4801:5200, amt = 13*1, ii= 24, ss= 1, addl=0),
ev(ID = 4801:5200, amt = 13*0, ii= 21, ss= 0, addl=0),
ev(ID = 4801:5200, amt = 13*0.5, ii= 3, ss= 0, addl=0),
ev(ID = 4801:5200, amt = 13*1, ii= 24, ss= 0, addl=10),
#21h delay simulation
ev(ID = 4801:5200, amt = 13*1, ii= 24, ss= 1, addl=0),
ev(ID = 4801:5200, amt = 13*0, ii= 21, ss= 0, addl=0),
ev(ID = 4801:5200, amt = 13*1, ii= 3, ss= 0, addl=0),
ev(ID = 4801:5200, amt = 13*1, ii= 24, ss= 0, addl=10),
#Missed dose simulation
ev(ID = 4801:5200, amt = 13*1, ii= 24, ss= 1, addl=0),
ev(ID = 4801:5200, amt = 13*0, ii= 24, ss= 0, addl=0),
ev(ID = 4801:5200, amt = 13*1, ii= 24, ss= 0, addl=0),
ev(ID = 4801:5200, amt = 13*1, ii= 24, ss= 0, addl=10),
#Missed dose + 150% dose intake simulation
ev(ID = 4801:5200, amt = 13*1, ii= 24, ss= 1, addl=0),
ev(ID = 4801:5200, amt = 13*0, ii= 24, ss= 0, addl=0),
ev(ID = 4801:5200, amt = 13*1.5, ii= 24, ss= 0, addl=0),
ev(ID = 4801:5200, amt = 13*1, ii= 24, ss= 0, addl=10),
#Missed dose + 200% dose intake simulation
ev(ID = 4801:5200, amt = 13*1, ii= 24, ss= 1, addl=0),
ev(ID = 4801:5200, amt = 13*0, ii= 24, ss= 0, addl=0),
ev(ID = 4801:5200, amt = 13*2, ii= 24, ss= 0, addl=0),
ev(ID = 4801:5200, amt = 13*1, ii= 24, ss= 0, addl=10))))%>%
              mutate(dose_group = "13 mg") %>%

    bind_rows(as_tibble( seq(
  #Steady-state simulation
ev(ID = 5201:5600, amt = 14*1, ii= 24, ss= 1, addl=0),
ev(ID = 5201:5600, amt = 14*1, ii= 24, ss= 0, addl=0),
ev(ID = 5201:5600, amt = 14*1, ii= 24, ss= 0, addl=10),
#3h delay simulation
ev(ID = 5201:5600, amt = 14*1, ii= 24, ss= 1, addl=0),
ev(ID = 5201:5600, amt = 14*0, ii= 3, ss= 0, addl=0),
ev(ID = 5201:5600, amt = 14*1, ii= 21, ss= 0, addl=0),
ev(ID = 5201:5600, amt = 14*1, ii= 24, ss= 0, addl=10),
#6h delay simulation
ev(ID = 5201:5600, amt = 14*1, ii= 24, ss= 1, addl=0),
ev(ID = 5201:5600, amt = 14*0, ii= 6, ss= 0, addl=0),
ev(ID = 5201:5600, amt = 14*1, ii= 18, ss= 0, addl=0),
ev(ID = 5201:5600, amt = 14*1, ii= 24, ss= 0, addl=10),
#9h delay simulation
ev(ID = 5201:5600, amt = 14*1, ii= 24, ss= 1, addl=0),
ev(ID = 5201:5600, amt = 14*0, ii= 9, ss= 0, addl=0),
ev(ID = 5201:5600, amt = 14*1, ii= 15, ss= 0, addl=0),
ev(ID = 5201:5600, amt = 14*1, ii= 24, ss= 0, addl=10),
#12h delay simulation
ev(ID = 5201:5600, amt = 14*1, ii= 24, ss= 1, addl=0),
ev(ID = 5201:5600, amt = 14*0, ii= 12, ss= 0, addl=0),
ev(ID = 5201:5600, amt = 14*1, ii= 12, ss= 0, addl=0),
ev(ID = 5201:5600, amt = 14*1, ii= 24, ss= 0, addl=10),
#15h delay + 50% dose intake simulation
ev(ID = 5201:5600, amt = 14*1, ii= 24, ss= 1, addl=0),
ev(ID = 5201:5600, amt = 14*0, ii= 15, ss= 0, addl=0),
ev(ID = 5201:5600, amt = 14*0.5, ii= 9, ss= 0, addl=0),
ev(ID = 5201:5600, amt = 14*1, ii= 24, ss= 0, addl=10),
#15h delay simulation
ev(ID = 5201:5600, amt = 14*1, ii= 24, ss= 1, addl=0),
ev(ID = 5201:5600, amt = 14*0, ii= 15, ss= 0, addl=0),
ev(ID = 5201:5600, amt = 14*1, ii= 9, ss= 0, addl=0),
ev(ID = 5201:5600, amt = 14*1, ii= 24, ss= 0, addl=10),
#18h delay + 50% dose intake simulation
ev(ID = 5201:5600, amt = 14*1, ii= 24, ss= 1, addl=0),
ev(ID = 5201:5600, amt = 14*0, ii= 18, ss= 0, addl=0),
ev(ID = 5201:5600, amt = 14*0.5, ii= 6, ss= 0, addl=0),
ev(ID = 5201:5600, amt = 14*1, ii= 24, ss= 0, addl=10),
#18h delay simulation
ev(ID = 5201:5600, amt = 14*1, ii= 24, ss= 1, addl=0),
ev(ID = 5201:5600, amt = 14*0, ii= 18, ss= 0, addl=0),
ev(ID = 5201:5600, amt = 14*1, ii= 6, ss= 0, addl=0),
ev(ID = 5201:5600, amt = 14*1, ii= 24, ss= 0, addl=10),
#21h delay + 50% dose intake simulation
ev(ID = 5201:5600, amt = 14*1, ii= 24, ss= 1, addl=0),
ev(ID = 5201:5600, amt = 14*0, ii= 21, ss= 0, addl=0),
ev(ID = 5201:5600, amt = 14*0.5, ii= 3, ss= 0, addl=0),
ev(ID = 5201:5600, amt = 14*1, ii= 24, ss= 0, addl=10),
#21h delay simulation
ev(ID = 5201:5600, amt = 14*1, ii= 24, ss= 1, addl=0),
ev(ID = 5201:5600, amt = 14*0, ii= 21, ss= 0, addl=0),
ev(ID = 5201:5600, amt = 14*1, ii= 3, ss= 0, addl=0),
ev(ID = 5201:5600, amt = 14*1, ii= 24, ss= 0, addl=10),
#Missed dose simulation
ev(ID = 5201:5600, amt = 14*1, ii= 24, ss= 1, addl=0),
ev(ID = 5201:5600, amt = 14*0, ii= 24, ss= 0, addl=0),
ev(ID = 5201:5600, amt = 14*1, ii= 24, ss= 0, addl=0),
ev(ID = 5201:5600, amt = 14*1, ii= 24, ss= 0, addl=10),
#Missed dose + 150% dose intake simulation
ev(ID = 5201:5600, amt = 14*1, ii= 24, ss= 1, addl=0),
ev(ID = 5201:5600, amt = 14*0, ii= 24, ss= 0, addl=0),
ev(ID = 5201:5600, amt = 14*1.5, ii= 24, ss= 0, addl=0),
ev(ID = 5201:5600, amt = 14*1, ii= 24, ss= 0, addl=10),
#Missed dose + 200% dose intake simulation
ev(ID = 5201:5600, amt = 14*1, ii= 24, ss= 1, addl=0),
ev(ID = 5201:5600, amt = 14*0, ii= 24, ss= 0, addl=0),
ev(ID = 5201:5600, amt = 14*2, ii= 24, ss= 0, addl=0),
ev(ID = 5201:5600, amt = 14*1, ii= 24, ss= 0, addl=10))))%>%
              mutate(dose_group = "14 mg") %>%

    bind_rows(as_tibble( seq(
  #Steady-state simulation
ev(ID = 5601:6000, amt = 15*1, ii= 24, ss= 1, addl=0),
ev(ID = 5601:6000, amt = 15*1, ii= 24, ss= 0, addl=0),
ev(ID = 5601:6000, amt = 15*1, ii= 24, ss= 0, addl=10),
#3h delay simulation
ev(ID = 5601:6000, amt = 15*1, ii= 24, ss= 1, addl=0),
ev(ID = 5601:6000, amt = 15*0, ii= 3, ss= 0, addl=0),
ev(ID = 5601:6000, amt = 15*1, ii= 21, ss= 0, addl=0),
ev(ID = 5601:6000, amt = 15*1, ii= 24, ss= 0, addl=10),
#6h delay simulation
ev(ID = 5601:6000, amt = 15*1, ii= 24, ss= 1, addl=0),
ev(ID = 5601:6000, amt = 15*0, ii= 6, ss= 0, addl=0),
ev(ID = 5601:6000, amt = 15*1, ii= 18, ss= 0, addl=0),
ev(ID = 5601:6000, amt = 15*1, ii= 24, ss= 0, addl=10),
#9h delay simulation
ev(ID = 5601:6000, amt = 15*1, ii= 24, ss= 1, addl=0),
ev(ID = 5601:6000, amt = 15*0, ii= 9, ss= 0, addl=0),
ev(ID = 5601:6000, amt = 15*1, ii= 15, ss= 0, addl=0),
ev(ID = 5601:6000, amt = 15*1, ii= 24, ss= 0, addl=10),
#12h delay simulation
ev(ID = 5601:6000, amt = 15*1, ii= 24, ss= 1, addl=0),
ev(ID = 5601:6000, amt = 15*0, ii= 12, ss= 0, addl=0),
ev(ID = 5601:6000, amt = 15*1, ii= 12, ss= 0, addl=0),
ev(ID = 5601:6000, amt = 15*1, ii= 24, ss= 0, addl=10),
#15h delay + 50% dose intake simulation
ev(ID = 5601:6000, amt = 15*1, ii= 24, ss= 1, addl=0),
ev(ID = 5601:6000, amt = 15*0, ii= 15, ss= 0, addl=0),
ev(ID = 5601:6000, amt = 15*0.5, ii= 9, ss= 0, addl=0),
ev(ID = 5601:6000, amt = 15*1, ii= 24, ss= 0, addl=10),
#15h delay simulation
ev(ID = 5601:6000, amt = 15*1, ii= 24, ss= 1, addl=0),
ev(ID = 5601:6000, amt = 15*0, ii= 15, ss= 0, addl=0),
ev(ID = 5601:6000, amt = 15*1, ii= 9, ss= 0, addl=0),
ev(ID = 5601:6000, amt = 15*1, ii= 24, ss= 0, addl=10),
#18h delay + 50% dose intake simulation
ev(ID = 5601:6000, amt = 15*1, ii= 24, ss= 1, addl=0),
ev(ID = 5601:6000, amt = 15*0, ii= 18, ss= 0, addl=0),
ev(ID = 5601:6000, amt = 15*0.5, ii= 6, ss= 0, addl=0),
ev(ID = 5601:6000, amt = 15*1, ii= 24, ss= 0, addl=10),
#18h delay simulation
ev(ID = 5601:6000, amt = 15*1, ii= 24, ss= 1, addl=0),
ev(ID = 5601:6000, amt = 15*0, ii= 18, ss= 0, addl=0),
ev(ID = 5601:6000, amt = 15*1, ii= 6, ss= 0, addl=0),
ev(ID = 5601:6000, amt = 15*1, ii= 24, ss= 0, addl=10),
#21h delay + 50% dose intake simulation
ev(ID = 5601:6000, amt = 15*1, ii= 24, ss= 1, addl=0),
ev(ID = 5601:6000, amt = 15*0, ii= 21, ss= 0, addl=0),
ev(ID = 5601:6000, amt = 15*0.5, ii= 3, ss= 0, addl=0),
ev(ID = 5601:6000, amt = 15*1, ii= 24, ss= 0, addl=10),
#21h delay simulation
ev(ID = 5601:6000, amt = 15*1, ii= 24, ss= 1, addl=0),
ev(ID = 5601:6000, amt = 15*0, ii= 21, ss= 0, addl=0),
ev(ID = 5601:6000, amt = 15*1, ii= 3, ss= 0, addl=0),
ev(ID = 5601:6000, amt = 15*1, ii= 24, ss= 0, addl=10),
#Missed dose simulation
ev(ID = 5601:6000, amt = 15*1, ii= 24, ss= 1, addl=0),
ev(ID = 5601:6000, amt = 15*0, ii= 24, ss= 0, addl=0),
ev(ID = 5601:6000, amt = 15*1, ii= 24, ss= 0, addl=0),
ev(ID = 5601:6000, amt = 15*1, ii= 24, ss= 0, addl=10),
#Missed dose + 150% dose intake simulation
ev(ID = 5601:6000, amt = 15*1, ii= 24, ss= 1, addl=0),
ev(ID = 5601:6000, amt = 15*0, ii= 24, ss= 0, addl=0),
ev(ID = 5601:6000, amt = 15*1.5, ii= 24, ss= 0, addl=0),
ev(ID = 5601:6000, amt = 15*1, ii= 24, ss= 0, addl=10),
#Missed dose + 200% dose intake simulation
ev(ID = 5601:6000, amt = 15*1, ii= 24, ss= 1, addl=0),
ev(ID = 5601:6000, amt = 15*0, ii= 24, ss= 0, addl=0),
ev(ID = 5601:6000, amt = 15*2, ii= 24, ss= 0, addl=0),
ev(ID = 5601:6000, amt = 15*1, ii= 24, ss= 0, addl=10)))) %>%
              mutate(dose_group = "15 mg")


Dataset_6000 <- Simulation_data_6000 %>%
  arrange(ID) %>%
  left_join(CYPCL_data_6000, by = "ID")


## test simulation
set.seed(123456)
Sim_Test_6000 <- my_model_Moes %>%
 data_set(Dataset_6000) %>%
 Req(DV) %>%
 mrgsim(end = 7000, delta = 1)
```

suite 6000

```
set.seed(123456)

generate_CYPCL_12000 <- function(n = 400) {
  frequencies <- c(32/49, 12/49, 5/49)
  values <- c(0, 0.33, 0.71)
  sample(values, n, replace = TRUE, prob = frequencies)
  }

CYPCL_data_12000 <- tibble(ID = 6001:12000) %>%
  group_by(group = (ID - 1) %/% 400) %>%
  mutate(CYPCL = generate_CYPCL_12000()) %>%
  ungroup() %>%
  select(-group)

Simulation_data_12000 <- bind_rows(as_tibble( seq(
  #Steady-state simulation
ev(ID = 6001:6400, amt = 0.5*1, ii= 24, ss= 1, addl=0),
ev(ID = 6001:6400, amt = 0.5*1, ii= 24, ss= 0, addl=0),
ev(ID = 6001:6400, amt = 0.5*1, ii= 24, ss= 0, addl=10),
#3h delay simulation
ev(ID = 6001:6400, amt = 0.5*1, ii= 24, ss= 1, addl=0),
ev(ID = 6001:6400, amt = 0.5*0, ii= 3, ss= 0, addl=0),
ev(ID = 6001:6400, amt = 0.5*1, ii= 21, ss= 0, addl=0),
ev(ID = 6001:6400, amt = 0.5*1, ii= 24, ss= 0, addl=10),
#6h delay simulation
ev(ID = 6001:6400, amt = 0.5*1, ii= 24, ss= 1, addl=0),
ev(ID = 6001:6400, amt = 0.5*0, ii= 6, ss= 0, addl=0),
ev(ID = 6001:6400, amt = 0.5*1, ii= 18, ss= 0, addl=0),
ev(ID = 6001:6400, amt = 0.5*1, ii= 24, ss= 0, addl=10),
#9h delay simulation
ev(ID = 6001:6400, amt = 0.5*1, ii= 24, ss= 1, addl=0),
ev(ID = 6001:6400, amt = 0.5*0, ii= 9, ss= 0, addl=0),
ev(ID = 6001:6400, amt = 0.5*1, ii= 15, ss= 0, addl=0),
ev(ID = 6001:6400, amt = 0.5*1, ii= 24, ss= 0, addl=10),
#12h delay simulation
ev(ID = 6001:6400, amt = 0.5*1, ii= 24, ss= 1, addl=0),
ev(ID = 6001:6400, amt = 0.5*0, ii= 12, ss= 0, addl=0),
ev(ID = 6001:6400, amt = 0.5*1, ii= 12, ss= 0, addl=0),
ev(ID = 6001:6400, amt = 0.5*1, ii= 24, ss= 0, addl=10),
#15h delay + 50% dose intake simulation
ev(ID = 6001:6400, amt = 0.5*1, ii= 24, ss= 1, addl=0),
ev(ID = 6001:6400, amt = 0.5*0, ii= 15, ss= 0, addl=0),
ev(ID = 6001:6400, amt = 0.5*0.5, ii= 9, ss= 0, addl=0),
ev(ID = 6001:6400, amt = 0.5*1, ii= 24, ss= 0, addl=10),
#15h delay simulation
ev(ID = 6001:6400, amt = 0.5*1, ii= 24, ss= 1, addl=0),
ev(ID = 6001:6400, amt = 0.5*0, ii= 15, ss= 0, addl=0),
ev(ID = 6001:6400, amt = 0.5*1, ii= 9, ss= 0, addl=0),
ev(ID = 6001:6400, amt = 0.5*1, ii= 24, ss= 0, addl=10),
#18h delay + 50% dose intake simulation
ev(ID = 6001:6400, amt = 0.5*1, ii= 24, ss= 1, addl=0),
ev(ID = 6001:6400, amt = 0.5*0, ii= 18, ss= 0, addl=0),
ev(ID = 6001:6400, amt = 0.5*0.5, ii= 6, ss= 0, addl=0),
ev(ID = 6001:6400, amt = 0.5*1, ii= 24, ss= 0, addl=10),
#18h delay simulation
ev(ID = 6001:6400, amt = 0.5*1, ii= 24, ss= 1, addl=0),
ev(ID = 6001:6400, amt = 0.5*0, ii= 18, ss= 0, addl=0),
ev(ID = 6001:6400, amt = 0.5*1, ii= 6, ss= 0, addl=0),
ev(ID = 6001:6400, amt = 0.5*1, ii= 24, ss= 0, addl=10),
#21h delay + 50% dose intake simulation
ev(ID = 6001:6400, amt = 0.5*1, ii= 24, ss= 1, addl=0),
ev(ID = 6001:6400, amt = 0.5*0, ii= 21, ss= 0, addl=0),
ev(ID = 6001:6400, amt = 0.5*0.5, ii= 3, ss= 0, addl=0),
ev(ID = 6001:6400, amt = 0.5*1, ii= 24, ss= 0, addl=10),
#21h delay simulation
ev(ID = 6001:6400, amt = 0.5*1, ii= 24, ss= 1, addl=0),
ev(ID = 6001:6400, amt = 0.5*0, ii= 21, ss= 0, addl=0),
ev(ID = 6001:6400, amt = 0.5*1, ii= 3, ss= 0, addl=0),
ev(ID = 6001:6400, amt = 0.5*1, ii= 24, ss= 0, addl=10),
#Missed dose simulation
ev(ID = 6001:6400, amt = 0.5*1, ii= 24, ss= 1, addl=0),
ev(ID = 6001:6400, amt = 0.5*0, ii= 24, ss= 0, addl=0),
ev(ID = 6001:6400, amt = 0.5*1, ii= 24, ss= 0, addl=0),
ev(ID = 6001:6400, amt = 0.5*1, ii= 24, ss= 0, addl=10),
#Missed dose + 150% dose intake simulation
ev(ID = 6001:6400, amt = 0.5*1, ii= 24, ss= 1, addl=0),
ev(ID = 6001:6400, amt = 0.5*0, ii= 24, ss= 0, addl=0),
ev(ID = 6001:6400, amt = 0.5*1.5, ii= 24, ss= 0, addl=0),
ev(ID = 6001:6400, amt = 0.5*1, ii= 24, ss= 0, addl=10),
#Missed dose + 200% dose intake simulation
ev(ID = 6001:6400, amt = 0.5*1, ii= 24, ss= 1, addl=0),
ev(ID = 6001:6400, amt = 0.5*0, ii= 24, ss= 0, addl=0),
ev(ID = 6001:6400, amt = 0.5*2, ii= 24, ss= 0, addl=0),
ev(ID = 6001:6400, amt = 0.5*1, ii= 24, ss= 0, addl=10)))) %>%
              mutate(dose_group = "0,5 mg") %>%

    bind_rows(as_tibble( seq(
  #Steady-state simulation
ev(ID = 6401:6800, amt = 1.5*1, ii= 24, ss= 1, addl=0),
ev(ID = 6401:6800, amt = 1.5*1, ii= 24, ss= 0, addl=0),
ev(ID = 6401:6800, amt = 1.5*1, ii= 24, ss= 0, addl=10),
#3h delay simulation
ev(ID = 6401:6800, amt = 1.5*1, ii= 24, ss= 1, addl=0),
ev(ID = 6401:6800, amt = 1.5*0, ii= 3, ss= 0, addl=0),
ev(ID = 6401:6800, amt = 1.5*1, ii= 21, ss= 0, addl=0),
ev(ID = 6401:6800, amt = 1.5*1, ii= 24, ss= 0, addl=10),
#6h delay simulation
ev(ID = 6401:6800, amt = 1.5*1, ii= 24, ss= 1, addl=0),
ev(ID = 6401:6800, amt = 1.5*0, ii= 6, ss= 0, addl=0),
ev(ID = 6401:6800, amt = 1.5*1, ii= 18, ss= 0, addl=0),
ev(ID = 6401:6800, amt = 1.5*1, ii= 24, ss= 0, addl=10),
#9h delay simulation
ev(ID = 6401:6800, amt = 1.5*1, ii= 24, ss= 1, addl=0),
ev(ID = 6401:6800, amt = 1.5*0, ii= 9, ss= 0, addl=0),
ev(ID = 6401:6800, amt = 1.5*1, ii= 15, ss= 0, addl=0),
ev(ID = 6401:6800, amt = 1.5*1, ii= 24, ss= 0, addl=10),
#12h delay simulation
ev(ID = 6401:6800, amt = 1.5*1, ii= 24, ss= 1, addl=0),
ev(ID = 6401:6800, amt = 1.5*0, ii= 12, ss= 0, addl=0),
ev(ID = 6401:6800, amt = 1.5*1, ii= 12, ss= 0, addl=0),
ev(ID = 6401:6800, amt = 1.5*1, ii= 24, ss= 0, addl=10),
#15h delay + 50% dose intake simulation
ev(ID = 6401:6800, amt = 1.5*1, ii= 24, ss= 1, addl=0),
ev(ID = 6401:6800, amt = 1.5*0, ii= 15, ss= 0, addl=0),
ev(ID = 6401:6800, amt = 1.5*0.5, ii= 9, ss= 0, addl=0),
ev(ID = 6401:6800, amt = 1.5*1, ii= 24, ss= 0, addl=10),
#15h delay simulation
ev(ID = 6401:6800, amt = 1.5*1, ii= 24, ss= 1, addl=0),
ev(ID = 6401:6800, amt = 1.5*0, ii= 15, ss= 0, addl=0),
ev(ID = 6401:6800, amt = 1.5*1, ii= 9, ss= 0, addl=0),
ev(ID = 6401:6800, amt = 1.5*1, ii= 24, ss= 0, addl=10),
#18h delay + 50% dose intake simulation
ev(ID = 6401:6800, amt = 1.5*1, ii= 24, ss= 1, addl=0),
ev(ID = 6401:6800, amt = 1.5*0, ii= 18, ss= 0, addl=0),
ev(ID = 6401:6800, amt = 1.5*0.5, ii= 6, ss= 0, addl=0),
ev(ID = 6401:6800, amt = 1.5*1, ii= 24, ss= 0, addl=10),
#18h delay simulation
ev(ID = 6401:6800, amt = 1.5*1, ii= 24, ss= 1, addl=0),
ev(ID = 6401:6800, amt = 1.5*0, ii= 18, ss= 0, addl=0),
ev(ID = 6401:6800, amt = 1.5*1, ii= 6, ss= 0, addl=0),
ev(ID = 6401:6800, amt = 1.5*1, ii= 24, ss= 0, addl=10),
#21h delay + 50% dose intake simulation
ev(ID = 6401:6800, amt = 1.5*1, ii= 24, ss= 1, addl=0),
ev(ID = 6401:6800, amt = 1.5*0, ii= 21, ss= 0, addl=0),
ev(ID = 6401:6800, amt = 1.5*0.5, ii= 3, ss= 0, addl=0),
ev(ID = 6401:6800, amt = 1.5*1, ii= 24, ss= 0, addl=10),
#21h delay simulation
ev(ID = 6401:6800, amt = 1.5*1, ii= 24, ss= 1, addl=0),
ev(ID = 6401:6800, amt = 1.5*0, ii= 21, ss= 0, addl=0),
ev(ID = 6401:6800, amt = 1.5*1, ii= 3, ss= 0, addl=0),
ev(ID = 6401:6800, amt = 1.5*1, ii= 24, ss= 0, addl=10),
#Missed dose simulation
ev(ID = 6401:6800, amt = 1.5*1, ii= 24, ss= 1, addl=0),
ev(ID = 6401:6800, amt = 1.5*0, ii= 24, ss= 0, addl=0),
ev(ID = 6401:6800, amt = 1.5*1, ii= 24, ss= 0, addl=0),
ev(ID = 6401:6800, amt = 1.5*1, ii= 24, ss= 0, addl=10),
#Missed dose + 150% dose intake simulation
ev(ID = 6401:6800, amt = 1.5*1, ii= 24, ss= 1, addl=0),
ev(ID = 6401:6800, amt = 1.5*0, ii= 24, ss= 0, addl=0),
ev(ID = 6401:6800, amt = 1.5*1.5, ii= 24, ss= 0, addl=0),
ev(ID = 6401:6800, amt = 1.5*1, ii= 24, ss= 0, addl=10),
#Missed dose + 200% dose intake simulation
ev(ID = 6401:6800, amt = 1.5*1, ii= 24, ss= 1, addl=0),
ev(ID = 6401:6800, amt = 1.5*0, ii= 24, ss= 0, addl=0),
ev(ID = 6401:6800, amt = 1.5*2, ii= 24, ss= 0, addl=0),
ev(ID = 6401:6800, amt = 1.5*1, ii= 24, ss= 0, addl=10)))) %>%
              mutate(dose_group = "1,5 mg") %>%

    bind_rows(as_tibble( seq(
  #Steady-state simulation
ev(ID = 6801:7200, amt = 2.5*1, ii= 24, ss= 1, addl=0),
ev(ID = 6801:7200, amt = 2.5*1, ii= 24, ss= 0, addl=0),
ev(ID = 6801:7200, amt = 2.5*1, ii= 24, ss= 0, addl=10),
#3h delay simulation
ev(ID = 6801:7200, amt = 2.5*1, ii= 24, ss= 1, addl=0),
ev(ID = 6801:7200, amt = 2.5*0, ii= 3, ss= 0, addl=0),
ev(ID = 6801:7200, amt = 2.5*1, ii= 21, ss= 0, addl=0),
ev(ID = 6801:7200, amt = 2.5*1, ii= 24, ss= 0, addl=10),
#6h delay simulation
ev(ID = 6801:7200, amt = 2.5*1, ii= 24, ss= 1, addl=0),
ev(ID = 6801:7200, amt = 2.5*0, ii= 6, ss= 0, addl=0),
ev(ID = 6801:7200, amt = 2.5*1, ii= 18, ss= 0, addl=0),
ev(ID = 6801:7200, amt = 2.5*1, ii= 24, ss= 0, addl=10),
#9h delay simulation
ev(ID = 6801:7200, amt = 2.5*1, ii= 24, ss= 1, addl=0),
ev(ID = 6801:7200, amt = 2.5*0, ii= 9, ss= 0, addl=0),
ev(ID = 6801:7200, amt = 2.5*1, ii= 15, ss= 0, addl=0),
ev(ID = 6801:7200, amt = 2.5*1, ii= 24, ss= 0, addl=10),
#12h delay simulation
ev(ID = 6801:7200, amt = 2.5*1, ii= 24, ss= 1, addl=0),
ev(ID = 6801:7200, amt = 2.5*0, ii= 12, ss= 0, addl=0),
ev(ID = 6801:7200, amt = 2.5*1, ii= 12, ss= 0, addl=0),
ev(ID = 6801:7200, amt = 2.5*1, ii= 24, ss= 0, addl=10),
#15h delay + 50% dose intake simulation
ev(ID = 6801:7200, amt = 2.5*1, ii= 24, ss= 1, addl=0),
ev(ID = 6801:7200, amt = 2.5*0, ii= 15, ss= 0, addl=0),
ev(ID = 6801:7200, amt = 2.5*0.5, ii= 9, ss= 0, addl=0),
ev(ID = 6801:7200, amt = 2.5*1, ii= 24, ss= 0, addl=10),
#15h delay simulation
ev(ID = 6801:7200, amt = 2.5*1, ii= 24, ss= 1, addl=0),
ev(ID = 6801:7200, amt = 2.5*0, ii= 15, ss= 0, addl=0),
ev(ID = 6801:7200, amt = 2.5*1, ii= 9, ss= 0, addl=0),
ev(ID = 6801:7200, amt = 2.5*1, ii= 24, ss= 0, addl=10),
#18h delay + 50% dose intake simulation
ev(ID = 6801:7200, amt = 2.5*1, ii= 24, ss= 1, addl=0),
ev(ID = 6801:7200, amt = 2.5*0, ii= 18, ss= 0, addl=0),
ev(ID = 6801:7200, amt = 2.5*0.5, ii= 6, ss= 0, addl=0),
ev(ID = 6801:7200, amt = 2.5*1, ii= 24, ss= 0, addl=10),
#18h delay simulation
ev(ID = 6801:7200, amt = 2.5*1, ii= 24, ss= 1, addl=0),
ev(ID = 6801:7200, amt = 2.5*0, ii= 18, ss= 0, addl=0),
ev(ID = 6801:7200, amt = 2.5*1, ii= 6, ss= 0, addl=0),
ev(ID = 6801:7200, amt = 2.5*1, ii= 24, ss= 0, addl=10),
#21h delay + 50% dose intake simulation
ev(ID = 6801:7200, amt = 2.5*1, ii= 24, ss= 1, addl=0),
ev(ID = 6801:7200, amt = 2.5*0, ii= 21, ss= 0, addl=0),
ev(ID = 6801:7200, amt = 2.5*0.5, ii= 3, ss= 0, addl=0),
ev(ID = 6801:7200, amt = 2.5*1, ii= 24, ss= 0, addl=10),
#21h delay simulation
ev(ID = 6801:7200, amt = 2.5*1, ii= 24, ss= 1, addl=0),
ev(ID = 6801:7200, amt = 2.5*0, ii= 21, ss= 0, addl=0),
ev(ID = 6801:7200, amt = 2.5*1, ii= 3, ss= 0, addl=0),
ev(ID = 6801:7200, amt = 2.5*1, ii= 24, ss= 0, addl=10),
#Missed dose simulation
ev(ID = 6801:7200, amt = 2.5*1, ii= 24, ss= 1, addl=0),
ev(ID = 6801:7200, amt = 2.5*0, ii= 24, ss= 0, addl=0),
ev(ID = 6801:7200, amt = 2.5*1, ii= 24, ss= 0, addl=0),
ev(ID = 6801:7200, amt = 2.5*1, ii= 24, ss= 0, addl=10),
#Missed dose + 150% dose intake simulation
ev(ID = 6801:7200, amt = 2.5*1, ii= 24, ss= 1, addl=0),
ev(ID = 6801:7200, amt = 2.5*0, ii= 24, ss= 0, addl=0),
ev(ID = 6801:7200, amt = 2.5*1.5, ii= 24, ss= 0, addl=0),
ev(ID = 6801:7200, amt = 2.5*1, ii= 24, ss= 0, addl=10),
#Missed dose + 200% dose intake simulation
ev(ID = 6801:7200, amt = 2.5*1, ii= 24, ss= 1, addl=0),
ev(ID = 6801:7200, amt = 2.5*0, ii= 24, ss= 0, addl=0),
ev(ID = 6801:7200, amt = 2.5*2, ii= 24, ss= 0, addl=0),
ev(ID = 6801:7200, amt = 2.5*1, ii= 24, ss= 0, addl=10)))) %>%
              mutate(dose_group = "2,5 mg") %>%

    bind_rows(as_tibble( seq(
  #Steady-state simulation
ev(ID = 7201:7600, amt = 3.5*1, ii= 24, ss= 1, addl=0),
ev(ID = 7201:7600, amt = 3.5*1, ii= 24, ss= 0, addl=0),
ev(ID = 7201:7600, amt = 3.5*1, ii= 24, ss= 0, addl=10),
#3h delay simulation
ev(ID = 7201:7600, amt = 3.5*1, ii= 24, ss= 1, addl=0),
ev(ID = 7201:7600, amt = 3.5*0, ii= 3, ss= 0, addl=0),
ev(ID = 7201:7600, amt = 3.5*1, ii= 21, ss= 0, addl=0),
ev(ID = 7201:7600, amt = 3.5*1, ii= 24, ss= 0, addl=10),
#6h delay simulation
ev(ID = 7201:7600, amt = 3.5*1, ii= 24, ss= 1, addl=0),
ev(ID = 7201:7600, amt = 3.5*0, ii= 6, ss= 0, addl=0),
ev(ID = 7201:7600, amt = 3.5*1, ii= 18, ss= 0, addl=0),
ev(ID = 7201:7600, amt = 3.5*1, ii= 24, ss= 0, addl=10),
#9h delay simulation
ev(ID = 7201:7600, amt = 3.5*1, ii= 24, ss= 1, addl=0),
ev(ID = 7201:7600, amt = 3.5*0, ii= 9, ss= 0, addl=0),
ev(ID = 7201:7600, amt = 3.5*1, ii= 15, ss= 0, addl=0),
ev(ID = 7201:7600, amt = 3.5*1, ii= 24, ss= 0, addl=10),
#12h delay simulation
ev(ID = 7201:7600, amt = 3.5*1, ii= 24, ss= 1, addl=0),
ev(ID = 7201:7600, amt = 3.5*0, ii= 12, ss= 0, addl=0),
ev(ID = 7201:7600, amt = 3.5*1, ii= 12, ss= 0, addl=0),
ev(ID = 7201:7600, amt = 3.5*1, ii= 24, ss= 0, addl=10),
#15h delay + 50% dose intake simulation
ev(ID = 7201:7600, amt = 3.5*1, ii= 24, ss= 1, addl=0),
ev(ID = 7201:7600, amt = 3.5*0, ii= 15, ss= 0, addl=0),
ev(ID = 7201:7600, amt = 3.5*0.5, ii= 9, ss= 0, addl=0),
ev(ID = 7201:7600, amt = 3.5*1, ii= 24, ss= 0, addl=10),
#15h delay simulation
ev(ID = 7201:7600, amt = 3.5*1, ii= 24, ss= 1, addl=0),
ev(ID = 7201:7600, amt = 3.5*0, ii= 15, ss= 0, addl=0),
ev(ID = 7201:7600, amt = 3.5*1, ii= 9, ss= 0, addl=0),
ev(ID = 7201:7600, amt = 3.5*1, ii= 24, ss= 0, addl=10),
#18h delay + 50% dose intake simulation
ev(ID = 7201:7600, amt = 3.5*1, ii= 24, ss= 1, addl=0),
ev(ID = 7201:7600, amt = 3.5*0, ii= 18, ss= 0, addl=0),
ev(ID = 7201:7600, amt = 3.5*0.5, ii= 6, ss= 0, addl=0),
ev(ID = 7201:7600, amt = 3.5*1, ii= 24, ss= 0, addl=10),
#18h delay simulation
ev(ID = 7201:7600, amt = 3.5*1, ii= 24, ss= 1, addl=0),
ev(ID = 7201:7600, amt = 3.5*0, ii= 18, ss= 0, addl=0),
ev(ID = 7201:7600, amt = 3.5*1, ii= 6, ss= 0, addl=0),
ev(ID = 7201:7600, amt = 3.5*1, ii= 24, ss= 0, addl=10),
#21h delay + 50% dose intake simulation
ev(ID = 7201:7600, amt = 3.5*1, ii= 24, ss= 1, addl=0),
ev(ID = 7201:7600, amt = 3.5*0, ii= 21, ss= 0, addl=0),
ev(ID = 7201:7600, amt = 3.5*0.5, ii= 3, ss= 0, addl=0),
ev(ID = 7201:7600, amt = 3.5*1, ii= 24, ss= 0, addl=10),
#21h delay simulation
ev(ID = 7201:7600, amt = 3.5*1, ii= 24, ss= 1, addl=0),
ev(ID = 7201:7600, amt = 3.5*0, ii= 21, ss= 0, addl=0),
ev(ID = 7201:7600, amt = 3.5*1, ii= 3, ss= 0, addl=0),
ev(ID = 7201:7600, amt = 3.5*1, ii= 24, ss= 0, addl=10),
#Missed dose simulation
ev(ID = 7201:7600, amt = 3.5*1, ii= 24, ss= 1, addl=0),
ev(ID = 7201:7600, amt = 3.5*0, ii= 24, ss= 0, addl=0),
ev(ID = 7201:7600, amt = 3.5*1, ii= 24, ss= 0, addl=0),
ev(ID = 7201:7600, amt = 3.5*1, ii= 24, ss= 0, addl=10),
#Missed dose + 150% dose intake simulation
ev(ID = 7201:7600, amt = 3.5*1, ii= 24, ss= 1, addl=0),
ev(ID = 7201:7600, amt = 3.5*0, ii= 24, ss= 0, addl=0),
ev(ID = 7201:7600, amt = 3.5*1.5, ii= 24, ss= 0, addl=0),
ev(ID = 7201:7600, amt = 3.5*1, ii= 24, ss= 0, addl=10),
#Missed dose + 200% dose intake simulation
ev(ID = 7201:7600, amt = 3.5*1, ii= 24, ss= 1, addl=0),
ev(ID = 7201:7600, amt = 3.5*0, ii= 24, ss= 0, addl=0),
ev(ID = 7201:7600, amt = 3.5*2, ii= 24, ss= 0, addl=0),
ev(ID = 7201:7600, amt = 3.5*1, ii= 24, ss= 0, addl=10)))) %>%
              mutate(dose_group = "3,5 mg") %>%

    bind_rows(as_tibble( seq(
  #Steady-state simulation
ev(ID = 7601:8000, amt = 4.5*1, ii= 24, ss= 1, addl=0),
ev(ID = 7601:8000, amt = 4.5*1, ii= 24, ss= 0, addl=0),
ev(ID = 7601:8000, amt = 4.5*1, ii= 24, ss= 0, addl=10),
#3h delay simulation
ev(ID = 7601:8000, amt = 4.5*1, ii= 24, ss= 1, addl=0),
ev(ID = 7601:8000, amt = 4.5*0, ii= 3, ss= 0, addl=0),
ev(ID = 7601:8000, amt = 4.5*1, ii= 21, ss= 0, addl=0),
ev(ID = 7601:8000, amt = 4.5*1, ii= 24, ss= 0, addl=10),
#6h delay simulation
ev(ID = 7601:8000, amt = 4.5*1, ii= 24, ss= 1, addl=0),
ev(ID = 7601:8000, amt = 4.5*0, ii= 6, ss= 0, addl=0),
ev(ID = 7601:8000, amt = 4.5*1, ii= 18, ss= 0, addl=0),
ev(ID = 7601:8000, amt = 4.5*1, ii= 24, ss= 0, addl=10),
#9h delay simulation
ev(ID = 7601:8000, amt = 4.5*1, ii= 24, ss= 1, addl=0),
ev(ID = 7601:8000, amt = 4.5*0, ii= 9, ss= 0, addl=0),
ev(ID = 7601:8000, amt = 4.5*1, ii= 15, ss= 0, addl=0),
ev(ID = 7601:8000, amt = 4.5*1, ii= 24, ss= 0, addl=10),
#12h delay simulation
ev(ID = 7601:8000, amt = 4.5*1, ii= 24, ss= 1, addl=0),
ev(ID = 7601:8000, amt = 4.5*0, ii= 12, ss= 0, addl=0),
ev(ID = 7601:8000, amt = 4.5*1, ii= 12, ss= 0, addl=0),
ev(ID = 7601:8000, amt = 4.5*1, ii= 24, ss= 0, addl=10),
#15h delay + 50% dose intake simulation
ev(ID = 7601:8000, amt = 4.5*1, ii= 24, ss= 1, addl=0),
ev(ID = 7601:8000, amt = 4.5*0, ii= 15, ss= 0, addl=0),
ev(ID = 7601:8000, amt = 4.5*0.5, ii= 9, ss= 0, addl=0),
ev(ID = 7601:8000, amt = 4.5*1, ii= 24, ss= 0, addl=10),
#15h delay simulation
ev(ID = 7601:8000, amt = 4.5*1, ii= 24, ss= 1, addl=0),
ev(ID = 7601:8000, amt = 4.5*0, ii= 15, ss= 0, addl=0),
ev(ID = 7601:8000, amt = 4.5*1, ii= 9, ss= 0, addl=0),
ev(ID = 7601:8000, amt = 4.5*1, ii= 24, ss= 0, addl=10),
#18h delay + 50% dose intake simulation
ev(ID = 7601:8000, amt = 4.5*1, ii= 24, ss= 1, addl=0),
ev(ID = 7601:8000, amt = 4.5*0, ii= 18, ss= 0, addl=0),
ev(ID = 7601:8000, amt = 4.5*0.5, ii= 6, ss= 0, addl=0),
ev(ID = 7601:8000, amt = 4.5*1, ii= 24, ss= 0, addl=10),
#18h delay simulation
ev(ID = 7601:8000, amt = 4.5*1, ii= 24, ss= 1, addl=0),
ev(ID = 7601:8000, amt = 4.5*0, ii= 18, ss= 0, addl=0),
ev(ID = 7601:8000, amt = 4.5*1, ii= 6, ss= 0, addl=0),
ev(ID = 7601:8000, amt = 4.5*1, ii= 24, ss= 0, addl=10),
#21h delay + 50% dose intake simulation
ev(ID = 7601:8000, amt = 4.5*1, ii= 24, ss= 1, addl=0),
ev(ID = 7601:8000, amt = 4.5*0, ii= 21, ss= 0, addl=0),
ev(ID = 7601:8000, amt = 4.5*0.5, ii= 3, ss= 0, addl=0),
ev(ID = 7601:8000, amt = 4.5*1, ii= 24, ss= 0, addl=10),
#21h delay simulation
```

```r
ev(ID = 7601:8000, amt = 4.5*1, ii= 24, ss= 1, addl=0),
ev(ID = 7601:8000, amt = 4.5*0, ii= 21, ss= 0, addl=0),
ev(ID = 7601:8000, amt = 4.5*1, ii= 3, ss= 0, addl=0),
ev(ID = 7601:8000, amt = 4.5*1, ii= 24, ss= 0, addl=10),
#Missed dose simulation
ev(ID = 7601:8000, amt = 4.5*1, ii= 24, ss= 1, addl=0),
ev(ID = 7601:8000, amt = 4.5*0, ii= 24, ss= 0, addl=0),
ev(ID = 7601:8000, amt = 4.5*1, ii= 24, ss= 0, addl=0),
ev(ID = 7601:8000, amt = 4.5*1, ii= 24, ss= 0, addl=10),
#Missed dose + 150% dose intake simulation
ev(ID = 7601:8000, amt = 4.5*1, ii= 24, ss= 1, addl=0),
ev(ID = 7601:8000, amt = 4.5*0, ii= 24, ss= 0, addl=0),
ev(ID = 7601:8000, amt = 4.5*1.5, ii= 24, ss= 0, addl=0),
ev(ID = 7601:8000, amt = 4.5*1, ii= 24, ss= 0, addl=10),
#Missed dose + 200% dose intake simulation
ev(ID = 7601:8000, amt = 4.5*1, ii= 24, ss= 1, addl=0),
ev(ID = 7601:8000, amt = 4.5*0, ii= 24, ss= 0, addl=0),
ev(ID = 7601:8000, amt = 4.5*2, ii= 24, ss= 0, addl=0),
ev(ID = 7601:8000, amt = 4.5*1, ii= 24, ss= 0, addl=10)))) %>%
             mutate(dose_group = "4,5 mg") %>%

    bind_rows(as_tibble( seq(
  #Steady-state simulation
ev(ID = 8001:8400, amt = 5.5*1, ii= 24, ss= 1, addl=0),
ev(ID = 8001:8400, amt = 5.5*1, ii= 24, ss= 0, addl=0),
ev(ID = 8001:8400, amt = 5.5*1, ii= 24, ss= 0, addl=10),
#3h delay simulation
ev(ID = 8001:8400, amt = 5.5*1, ii= 24, ss= 1, addl=0),
ev(ID = 8001:8400, amt = 5.5*0, ii= 3, ss= 0, addl=0),
ev(ID = 8001:8400, amt = 5.5*1, ii= 21, ss= 0, addl=0),
ev(ID = 8001:8400, amt = 5.5*1, ii= 24, ss= 0, addl=10),
#6h delay simulation
ev(ID = 8001:8400, amt = 5.5*1, ii= 24, ss= 1, addl=0),
ev(ID = 8001:8400, amt = 5.5*0, ii= 6, ss= 0, addl=0),
ev(ID = 8001:8400, amt = 5.5*1, ii= 18, ss= 0, addl=0),
ev(ID = 8001:8400, amt = 5.5*1, ii= 24, ss= 0, addl=10),
#9h delay simulation
ev(ID = 8001:8400, amt = 5.5*1, ii= 24, ss= 1, addl=0),
ev(ID = 8001:8400, amt = 5.5*0, ii= 9, ss= 0, addl=0),
ev(ID = 8001:8400, amt = 5.5*1, ii= 15, ss= 0, addl=0),
ev(ID = 8001:8400, amt = 5.5*1, ii= 24, ss= 0, addl=10),
#12h delay simulation
ev(ID = 8001:8400, amt = 5.5*1, ii= 24, ss= 1, addl=0),
ev(ID = 8001:8400, amt = 5.5*0, ii= 12, ss= 0, addl=0),
ev(ID = 8001:8400, amt = 5.5*1, ii= 12, ss= 0, addl=0),
ev(ID = 8001:8400, amt = 5.5*1, ii= 24, ss= 0, addl=10),
#15h delay + 50% dose intake simulation
ev(ID = 8001:8400, amt = 5.5*1, ii= 24, ss= 1, addl=0),
ev(ID = 8001:8400, amt = 5.5*0, ii= 15, ss= 0, addl=0),
ev(ID = 8001:8400, amt = 5.5*0.5, ii= 9, ss= 0, addl=0),
ev(ID = 8001:8400, amt = 5.5*1, ii= 24, ss= 0, addl=10),
#15h delay simulation
ev(ID = 8001:8400, amt = 5.5*1, ii= 24, ss= 1, addl=0),
ev(ID = 8001:8400, amt = 5.5*0, ii= 15, ss= 0, addl=0),
ev(ID = 8001:8400, amt = 5.5*1, ii= 9, ss= 0, addl=0),
ev(ID = 8001:8400, amt = 5.5*1, ii= 24, ss= 0, addl=10),
#18h delay + 50% dose intake simulation
ev(ID = 8001:8400, amt = 5.5*1, ii= 24, ss= 1, addl=0),
ev(ID = 8001:8400, amt = 5.5*0, ii= 18, ss= 0, addl=0),
ev(ID = 8001:8400, amt = 5.5*0.5, ii= 6, ss= 0, addl=0),
ev(ID = 8001:8400, amt = 5.5*1, ii= 24, ss= 0, addl=10),
#18h delay simulation
ev(ID = 8001:8400, amt = 5.5*1, ii= 24, ss= 1, addl=0),
ev(ID = 8001:8400, amt = 5.5*0, ii= 18, ss= 0, addl=0),
ev(ID = 8001:8400, amt = 5.5*1, ii= 6, ss= 0, addl=0),
ev(ID = 8001:8400, amt = 5.5*1, ii= 24, ss= 0, addl=10),
#21h delay + 50% dose intake simulation
ev(ID = 8001:8400, amt = 5.5*1, ii= 24, ss= 1, addl=0),
ev(ID = 8001:8400, amt = 5.5*0, ii= 21, ss= 0, addl=0),
ev(ID = 8001:8400, amt = 5.5*0.5, ii= 3, ss= 0, addl=0),
ev(ID = 8001:8400, amt = 5.5*1, ii= 24, ss= 0, addl=10),
#21h delay simulation
ev(ID = 8001:8400, amt = 5.5*1, ii= 24, ss= 1, addl=0),
ev(ID = 8001:8400, amt = 5.5*0, ii= 21, ss= 0, addl=0),
ev(ID = 8001:8400, amt = 5.5*1, ii= 3, ss= 0, addl=0),
ev(ID = 8001:8400, amt = 5.5*1, ii= 24, ss= 0, addl=10),
#Missed dose simulation
ev(ID = 8001:8400, amt = 5.5*1, ii= 24, ss= 1, addl=0),
ev(ID = 8001:8400, amt = 5.5*0, ii= 24, ss= 0, addl=0),
ev(ID = 8001:8400, amt = 5.5*1, ii= 24, ss= 0, addl=0),
ev(ID = 8001:8400, amt = 5.5*1, ii= 24, ss= 0, addl=10),
#Missed dose + 150% dose intake simulation
ev(ID = 8001:8400, amt = 5.5*1, ii= 24, ss= 1, addl=0),
ev(ID = 8001:8400, amt = 5.5*0, ii= 24, ss= 0, addl=0),
ev(ID = 8001:8400, amt = 5.5*1.5, ii= 24, ss= 0, addl=0),
ev(ID = 8001:8400, amt = 5.5*1, ii= 24, ss= 0, addl=10),
#Missed dose + 200% dose intake simulation
ev(ID = 8001:8400, amt = 5.5*1, ii= 24, ss= 1, addl=0),
ev(ID = 8001:8400, amt = 5.5*0, ii= 24, ss= 0, addl=0),
ev(ID = 8001:8400, amt = 5.5*2, ii= 24, ss= 0, addl=0),
ev(ID = 8001:8400, amt = 5.5*1, ii= 24, ss= 0, addl=10)))) %>%
             mutate(dose_group = "5,5 mg") %>%

    bind_rows(as_tibble( seq(
  #Steady-state simulation
ev(ID = 8401:8800, amt = 6.5*1, ii= 24, ss= 1, addl=0),
ev(ID = 8401:8800, amt = 6.5*1, ii= 24, ss= 0, addl=0),
ev(ID = 8401:8800, amt = 6.5*1, ii= 24, ss= 0, addl=10),
#3h delay simulation
ev(ID = 8401:8800, amt = 6.5*1, ii= 24, ss= 1, addl=0),
ev(ID = 8401:8800, amt = 6.5*0, ii= 3, ss= 0, addl=0),
ev(ID = 8401:8800, amt = 6.5*1, ii= 21, ss= 0, addl=0),
ev(ID = 8401:8800, amt = 6.5*1, ii= 24, ss= 0, addl=10),
#6h delay simulation
ev(ID = 8401:8800, amt = 6.5*1, ii= 24, ss= 1, addl=0),
ev(ID = 8401:8800, amt = 6.5*0, ii= 6, ss= 0, addl=0),
ev(ID = 8401:8800, amt = 6.5*1, ii= 18, ss= 0, addl=0),
ev(ID = 8401:8800, amt = 6.5*1, ii= 24, ss= 0, addl=10),
#9h delay simulation
ev(ID = 8401:8800, amt = 6.5*1, ii= 24, ss= 1, addl=0),
ev(ID = 8401:8800, amt = 6.5*0, ii= 9, ss= 0, addl=0),
ev(ID = 8401:8800, amt = 6.5*1, ii= 15, ss= 0, addl=0),
ev(ID = 8401:8800, amt = 6.5*1, ii= 24, ss= 0, addl=10),
#12h delay simulation
ev(ID = 8401:8800, amt = 6.5*1, ii= 24, ss= 1, addl=0),
ev(ID = 8401:8800, amt = 6.5*0, ii= 12, ss= 0, addl=0),
ev(ID = 8401:8800, amt = 6.5*1, ii= 12, ss= 0, addl=0),
ev(ID = 8401:8800, amt = 6.5*1, ii= 24, ss= 0, addl=10),
#15h delay + 50% dose intake simulation
ev(ID = 8401:8800, amt = 6.5*1, ii= 24, ss= 1, addl=0),
ev(ID = 8401:8800, amt = 6.5*0, ii= 15, ss= 0, addl=0),
ev(ID = 8401:8800, amt = 6.5*0.5, ii= 9, ss= 0, addl=0),
ev(ID = 8401:8800, amt = 6.5*1, ii= 24, ss= 0, addl=10),
#15h delay simulation
ev(ID = 8401:8800, amt = 6.5*1, ii= 24, ss= 1, addl=0),
ev(ID = 8401:8800, amt = 6.5*0, ii= 15, ss= 0, addl=0),
ev(ID = 8401:8800, amt = 6.5*1, ii= 9, ss= 0, addl=0),
ev(ID = 8401:8800, amt = 6.5*1, ii= 24, ss= 0, addl=10),
#18h delay + 50% dose intake simulation
ev(ID = 8401:8800, amt = 6.5*1, ii= 24, ss= 1, addl=0),
ev(ID = 8401:8800, amt = 6.5*0, ii= 18, ss= 0, addl=0),
ev(ID = 8401:8800, amt = 6.5*0.5, ii= 6, ss= 0, addl=0),
ev(ID = 8401:8800, amt = 6.5*1, ii= 24, ss= 0, addl=10),
#18h delay simulation
ev(ID = 8401:8800, amt = 6.5*1, ii= 24, ss= 1, addl=0),
ev(ID = 8401:8800, amt = 6.5*0, ii= 18, ss= 0, addl=0),
ev(ID = 8401:8800, amt = 6.5*1, ii= 6, ss= 0, addl=0),
ev(ID = 8401:8800, amt = 6.5*1, ii= 24, ss= 0, addl=10),
#21h delay + 50% dose intake simulation
ev(ID = 8401:8800, amt = 6.5*1, ii= 24, ss= 1, addl=0),
ev(ID = 8401:8800, amt = 6.5*0, ii= 21, ss= 0, addl=0),
ev(ID = 8401:8800, amt = 6.5*0.5, ii= 3, ss= 0, addl=0),
ev(ID = 8401:8800, amt = 6.5*1, ii= 24, ss= 0, addl=10),
#21h delay simulation
ev(ID = 8401:8800, amt = 6.5*1, ii= 24, ss= 1, addl=0),
ev(ID = 8401:8800, amt = 6.5*0, ii= 21, ss= 0, addl=0),
ev(ID = 8401:8800, amt = 6.5*1, ii= 3, ss= 0, addl=0),
ev(ID = 8401:8800, amt = 6.5*1, ii= 24, ss= 0, addl=10),
#Missed dose simulation
ev(ID = 8401:8800, amt = 6.5*1, ii= 24, ss= 1, addl=0),
ev(ID = 8401:8800, amt = 6.5*0, ii= 24, ss= 0, addl=0),
ev(ID = 8401:8800, amt = 6.5*1, ii= 24, ss= 0, addl=0),
ev(ID = 8401:8800, amt = 6.5*1, ii= 24, ss= 0, addl=10),
#Missed dose + 150% dose intake simulation
ev(ID = 8401:8800, amt = 6.5*1, ii= 24, ss= 1, addl=0),
ev(ID = 8401:8800, amt = 6.5*0, ii= 24, ss= 0, addl=0),
ev(ID = 8401:8800, amt = 6.5*1.5, ii= 24, ss= 0, addl=0),
ev(ID = 8401:8800, amt = 6.5*1, ii= 24, ss= 0, addl=10),
#Missed dose + 200% dose intake simulation
ev(ID = 8401:8800, amt = 6.5*1, ii= 24, ss= 1, addl=0),
ev(ID = 8401:8800, amt = 6.5*0, ii= 24, ss= 0, addl=0),
ev(ID = 8401:8800, amt = 6.5*2, ii= 24, ss= 0, addl=0),
ev(ID = 8401:8800, amt = 6.5*1, ii= 24, ss= 0, addl=10)))) %>%
             mutate(dose_group = "6,5 mg") %>%

    bind_rows(as_tibble( seq(
  #Steady-state simulation
ev(ID = 8801:9200, amt = 7.5*1, ii= 24, ss= 1, addl=0),
ev(ID = 8801:9200, amt = 7.5*1, ii= 24, ss= 0, addl=0),
ev(ID = 8801:9200, amt = 7.5*1, ii= 24, ss= 0, addl=10),
#3h delay simulation
ev(ID = 8801:9200, amt = 7.5*1, ii= 24, ss= 1, addl=0),
ev(ID = 8801:9200, amt = 7.5*0, ii= 3, ss= 0, addl=0),
ev(ID = 8801:9200, amt = 7.5*1, ii= 21, ss= 0, addl=0),
ev(ID = 8801:9200, amt = 7.5*1, ii= 24, ss= 0, addl=10),
#6h delay simulation
ev(ID = 8801:9200, amt = 7.5*1, ii= 24, ss= 1, addl=0),
ev(ID = 8801:9200, amt = 7.5*0, ii= 6, ss= 0, addl=0),
ev(ID = 8801:9200, amt = 7.5*1, ii= 18, ss= 0, addl=0),
ev(ID = 8801:9200, amt = 7.5*1, ii= 24, ss= 0, addl=10),
#9h delay simulation
ev(ID = 8801:9200, amt = 7.5*1, ii= 24, ss= 1, addl=0),
ev(ID = 8801:9200, amt = 7.5*0, ii= 9, ss= 0, addl=0),
ev(ID = 8801:9200, amt = 7.5*1, ii= 15, ss= 0, addl=0),
ev(ID = 8801:9200, amt = 7.5*1, ii= 24, ss= 0, addl=10),
#12h delay simulation
ev(ID = 8801:9200, amt = 7.5*1, ii= 24, ss= 1, addl=0),
ev(ID = 8801:9200, amt = 7.5*0, ii= 12, ss= 0, addl=0),
ev(ID = 8801:9200, amt = 7.5*1, ii= 12, ss= 0, addl=0),
ev(ID = 8801:9200, amt = 7.5*1, ii= 24, ss= 0, addl=10),
#15h delay + 50% dose intake simulation
ev(ID = 8801:9200, amt = 7.5*1, ii= 24, ss= 1, addl=0),
ev(ID = 8801:9200, amt = 7.5*0, ii= 15, ss= 0, addl=0),
ev(ID = 8801:9200, amt = 7.5*0.5, ii= 9, ss= 0, addl=0),
ev(ID = 8801:9200, amt = 7.5*1, ii= 24, ss= 0, addl=10),
#15h delay simulation
ev(ID = 8801:9200, amt = 7.5*1, ii= 24, ss= 1, addl=0),
ev(ID = 8801:9200, amt = 7.5*0, ii= 15, ss= 0, addl=0),
ev(ID = 8801:9200, amt = 7.5*1, ii= 9, ss= 0, addl=0),
ev(ID = 8801:9200, amt = 7.5*1, ii= 24, ss= 0, addl=10),
#18h delay + 50% dose intake simulation
ev(ID = 8801:9200, amt = 7.5*1, ii= 24, ss= 1, addl=0),
ev(ID = 8801:9200, amt = 7.5*0, ii= 18, ss= 0, addl=0),
ev(ID = 8801:9200, amt = 7.5*0.5, ii= 6, ss= 0, addl=0),
ev(ID = 8801:9200, amt = 7.5*1, ii= 24, ss= 0, addl=10),
#18h delay simulation
ev(ID = 8801:9200, amt = 7.5*1, ii= 24, ss= 1, addl=0),
ev(ID = 8801:9200, amt = 7.5*0, ii= 18, ss= 0, addl=0),
ev(ID = 8801:9200, amt = 7.5*1, ii= 6, ss= 0, addl=0),
ev(ID = 8801:9200, amt = 7.5*1, ii= 24, ss= 0, addl=10),
#21h delay + 50% dose intake simulation
ev(ID = 8801:9200, amt = 7.5*1, ii= 24, ss= 1, addl=0),
ev(ID = 8801:9200, amt = 7.5*0, ii= 21, ss= 0, addl=0),
ev(ID = 8801:9200, amt = 7.5*0.5, ii= 3, ss= 0, addl=0),
ev(ID = 8801:9200, amt = 7.5*1, ii= 24, ss= 0, addl=10),
#21h delay simulation
ev(ID = 8801:9200, amt = 7.5*1, ii= 24, ss= 1, addl=0),
ev(ID = 8801:9200, amt = 7.5*0, ii= 21, ss= 0, addl=0),
ev(ID = 8801:9200, amt = 7.5*1, ii= 3, ss= 0, addl=0),
ev(ID = 8801:9200, amt = 7.5*1, ii= 24, ss= 0, addl=10),
#Missed dose simulation
ev(ID = 8801:9200, amt = 7.5*1, ii= 24, ss= 1, addl=0),
ev(ID = 8801:9200, amt = 7.5*0, ii= 24, ss= 0, addl=0),
ev(ID = 8801:9200, amt = 7.5*1, ii= 24, ss= 0, addl=0),
ev(ID = 8801:9200, amt = 7.5*1, ii= 24, ss= 0, addl=10),
#Missed dose + 150% dose intake simulation
ev(ID = 8801:9200, amt = 7.5*1, ii= 24, ss= 1, addl=0),
ev(ID = 8801:9200, amt = 7.5*0, ii= 24, ss= 0, addl=0),
ev(ID = 8801:9200, amt = 7.5*1.5, ii= 24, ss= 0, addl=0),
ev(ID = 8801:9200, amt = 7.5*1, ii= 24, ss= 0, addl=10),
#Missed dose + 200% dose intake simulation
ev(ID = 8801:9200, amt = 7.5*1, ii= 24, ss= 1, addl=0),
ev(ID = 8801:9200, amt = 7.5*0, ii= 24, ss= 0, addl=0),
ev(ID = 8801:9200, amt = 7.5*2, ii= 24, ss= 0, addl=0),
ev(ID = 8801:9200, amt = 7.5*1, ii= 24, ss= 0, addl=10)))) %>%
             mutate(dose_group = "7,5 mg") %>%

    bind_rows(as_tibble( seq(
  #Steady-state simulation
ev(ID = 9201:9600, amt = 8.5*1, ii= 24, ss= 1, addl=0),
ev(ID = 9201:9600, amt = 8.5*1, ii= 24, ss= 0, addl=0),
ev(ID = 9201:9600, amt = 8.5*1, ii= 24, ss= 0, addl=10),
#3h delay simulation
ev(ID = 9201:9600, amt = 8.5*1, ii= 24, ss= 1, addl=0),
ev(ID = 9201:9600, amt = 8.5*0, ii= 3, ss= 0, addl=0),
ev(ID = 9201:9600, amt = 8.5*1, ii= 21, ss= 0, addl=0),
ev(ID = 9201:9600, amt = 8.5*1, ii= 24, ss= 0, addl=10),
#6h delay simulation
ev(ID = 9201:9600, amt = 8.5*1, ii= 24, ss= 1, addl=0),
ev(ID = 9201:9600, amt = 8.5*0, ii= 6, ss= 0, addl=0),
ev(ID = 9201:9600, amt = 8.5*1, ii= 18, ss= 0, addl=0),
ev(ID = 9201:9600, amt = 8.5*1, ii= 24, ss= 0, addl=10),
#9h delay simulation
ev(ID = 9201:9600, amt = 8.5*1, ii= 24, ss= 1, addl=0),
ev(ID = 9201:9600, amt = 8.5*0, ii= 9, ss= 0, addl=0),
ev(ID = 9201:9600, amt = 8.5*1, ii= 15, ss= 0, addl=0),
ev(ID = 9201:9600, amt = 8.5*1, ii= 24, ss= 0, addl=10),
#12h delay simulation
ev(ID = 9201:9600, amt = 8.5*1, ii= 24, ss= 1, addl=0),
ev(ID = 9201:9600, amt = 8.5*0, ii= 12, ss= 0, addl=0),
ev(ID = 9201:9600, amt = 8.5*1, ii= 12, ss= 0, addl=0),
ev(ID = 9201:9600, amt = 8.5*1, ii= 24, ss= 0, addl=10),
#15h delay + 50% dose intake simulation
ev(ID = 9201:9600, amt = 8.5*1, ii= 24, ss= 1, addl=0),
ev(ID = 9201:9600, amt = 8.5*0, ii= 15, ss= 0, addl=0),
ev(ID = 9201:9600, amt = 8.5*0.5, ii= 9, ss= 0, addl=0),
ev(ID = 9201:9600, amt = 8.5*1, ii= 24, ss= 0, addl=10),
#15h delay simulation
ev(ID = 9201:9600, amt = 8.5*1, ii= 24, ss= 1, addl=0),
ev(ID = 9201:9600, amt = 8.5*0, ii= 15, ss= 0, addl=0),
ev(ID = 9201:9600, amt = 8.5*1, ii= 9, ss= 0, addl=0),
ev(ID = 9201:9600, amt = 8.5*1, ii= 24, ss= 0, addl=10),
#18h delay + 50% dose intake simulation
ev(ID = 9201:9600, amt = 8.5*1, ii= 24, ss= 1, addl=0),
ev(ID = 9201:9600, amt = 8.5*0, ii= 18, ss= 0, addl=0),
ev(ID = 9201:9600, amt = 8.5*0.5, ii= 6, ss= 0, addl=0),
ev(ID = 9201:9600, amt = 8.5*1, ii= 24, ss= 0, addl=10),
#18h delay simulation
ev(ID = 9201:9600, amt = 8.5*1, ii= 24, ss= 1, addl=0),
ev(ID = 9201:9600, amt = 8.5*0, ii= 18, ss= 0, addl=0),
ev(ID = 9201:9600, amt = 8.5*1, ii= 6, ss= 0, addl=0),
ev(ID = 9201:9600, amt = 8.5*1, ii= 24, ss= 0, addl=10),
#21h delay + 50% dose intake simulation
ev(ID = 9201:9600, amt = 8.5*1, ii= 24, ss= 1, addl=0),
ev(ID = 9201:9600, amt = 8.5*0, ii= 21, ss= 0, addl=0),
ev(ID = 9201:9600, amt = 8.5*0.5, ii= 3, ss= 0, addl=0),
ev(ID = 9201:9600, amt = 8.5*1, ii= 24, ss= 0, addl=10),
#21h delay simulation
ev(ID = 9201:9600, amt = 8.5*1, ii= 24, ss= 1, addl=0),
ev(ID = 9201:9600, amt = 8.5*0, ii= 21, ss= 0, addl=0),
ev(ID = 9201:9600, amt = 8.5*1, ii= 3, ss= 0, addl=0),
ev(ID = 9201:9600, amt = 8.5*1, ii= 24, ss= 0, addl=10),
#Missed dose simulation
ev(ID = 9201:9600, amt = 8.5*1, ii= 24, ss= 1, addl=0),
ev(ID = 9201:9600, amt = 8.5*0, ii= 24, ss= 0, addl=0),
ev(ID = 9201:9600, amt = 8.5*1, ii= 24, ss= 0, addl=0),
ev(ID = 9201:9600, amt = 8.5*1, ii= 24, ss= 0, addl=10),
#Missed dose + 150% dose intake simulation
ev(ID = 9201:9600, amt = 8.5*1, ii= 24, ss= 1, addl=0),
ev(ID = 9201:9600, amt = 8.5*0, ii= 24, ss= 0, addl=0),
ev(ID = 9201:9600, amt = 8.5*1.5, ii= 24, ss= 0, addl=0),
ev(ID = 9201:9600, amt = 8.5*1, ii= 24, ss= 0, addl=10),
#Missed dose + 200% dose intake simulation
ev(ID = 9201:9600, amt = 8.5*1, ii= 24, ss= 1, addl=0),
ev(ID = 9201:9600, amt = 8.5*0, ii= 24, ss= 0, addl=0),
ev(ID = 9201:9600, amt = 8.5*2, ii= 24, ss= 0, addl=0),
ev(ID = 9201:9600, amt = 8.5*1, ii= 24, ss= 0, addl=10)))) %>%
             mutate(dose_group = "8,5 mg") %>%

    bind_rows(as_tibble( seq(
  #Steady-state simulation
ev(ID = 9601:10000, amt = 9.5*1, ii= 24, ss= 1, addl=0),
ev(ID = 9601:10000, amt = 9.5*1, ii= 24, ss= 0, addl=0),
ev(ID = 9601:10000, amt = 9.5*1, ii= 24, ss= 0, addl=10),
#3h delay simulation
ev(ID = 9601:10000, amt = 9.5*1, ii= 24, ss= 1, addl=0),
ev(ID = 9601:10000, amt = 9.5*0, ii= 3, ss= 0, addl=0),
ev(ID = 9601:10000, amt = 9.5*1, ii= 21, ss= 0, addl=0),
ev(ID = 9601:10000, amt = 9.5*1, ii= 24, ss= 0, addl=10),
#6h delay simulation
ev(ID = 9601:10000, amt = 9.5*1, ii= 24, ss= 1, addl=0),
ev(ID = 9601:10000, amt = 9.5*0, ii= 6, ss= 0, addl=0),
ev(ID = 9601:10000, amt = 9.5*1, ii= 18, ss= 0, addl=0),
ev(ID = 9601:10000, amt = 9.5*1, ii= 24, ss= 0, addl=10),
#9h delay simulation
ev(ID = 9601:10000, amt = 9.5*1, ii= 24, ss= 1, addl=0),
ev(ID = 9601:10000, amt = 9.5*0, ii= 9, ss= 0, addl=0),
ev(ID = 9601:10000, amt = 9.5*1, ii= 15, ss= 0, addl=0),
ev(ID = 9601:10000, amt = 9.5*1, ii= 24, ss= 0, addl=10),
#12h delay simulation
ev(ID = 9601:10000, amt = 9.5*1, ii= 24, ss= 1, addl=0),
ev(ID = 9601:10000, amt = 9.5*0, ii= 12, ss= 0, addl=0),
ev(ID = 9601:10000, amt = 9.5*1, ii= 12, ss= 0, addl=0),
ev(ID = 9601:10000, amt = 9.5*1, ii= 24, ss= 0, addl=10),
#15h delay + 50% dose intake simulation
ev(ID = 9601:10000, amt = 9.5*1, ii= 24, ss= 1, addl=0),
ev(ID = 9601:10000, amt = 9.5*0, ii= 15, ss= 0, addl=0),
ev(ID = 9601:10000, amt = 9.5*0.5, ii= 9, ss= 0, addl=0),
ev(ID = 9601:10000, amt = 9.5*1, ii= 24, ss= 0, addl=10),
#15h delay simulation
ev(ID = 9601:10000, amt = 9.5*1, ii= 24, ss= 1, addl=0),
ev(ID = 9601:10000, amt = 9.5*0, ii= 15, ss= 0, addl=0),
ev(ID = 9601:10000, amt = 9.5*1, ii= 9, ss= 0, addl=0),
ev(ID = 9601:10000, amt = 9.5*1, ii= 24, ss= 0, addl=10),
#18h delay + 50% dose intake simulation
ev(ID = 9601:10000, amt = 9.5*1, ii= 24, ss= 1, addl=0),
ev(ID = 9601:10000, amt = 9.5*0, ii= 18, ss= 0, addl=0),
ev(ID = 9601:10000, amt = 9.5*0.5, ii= 6, ss= 0, addl=0),
ev(ID = 9601:10000, amt = 9.5*1, ii= 24, ss= 0, addl=10),
#18h delay simulation
ev(ID = 9601:10000, amt = 9.5*1, ii= 24, ss= 1, addl=0),
ev(ID = 9601:10000, amt = 9.5*0, ii= 18, ss= 0, addl=0),
ev(ID = 9601:10000, amt = 9.5*1, ii= 6, ss= 0, addl=0),
ev(ID = 9601:10000, amt = 9.5*1, ii= 24, ss= 0, addl=10),
#21h delay + 50% dose intake simulation
ev(ID = 9601:10000, amt = 9.5*1, ii= 24, ss= 1, addl=0),
ev(ID = 9601:10000, amt = 9.5*0, ii= 21, ss= 0, addl=0),
ev(ID = 9601:10000, amt = 9.5*0.5, ii= 3, ss= 0, addl=0),
ev(ID = 9601:10000, amt = 9.5*1, ii= 24, ss= 0, addl=10),
#21h delay simulation
ev(ID = 9601:10000, amt = 9.5*1, ii= 24, ss= 1, addl=0),
ev(ID = 9601:10000, amt = 9.5*0, ii= 21, ss= 0, addl=0),
ev(ID = 9601:10000, amt = 9.5*1, ii= 3, ss= 0, addl=0),
ev(ID = 9601:10000, amt = 9.5*1, ii= 24, ss= 0, addl=10),
#Missed dose simulation
ev(ID = 9601:10000, amt = 9.5*1, ii= 24, ss= 1, addl=0),
ev(ID = 9601:10000, amt = 9.5*0, ii= 24, ss= 0, addl=0),
ev(ID = 9601:10000, amt = 9.5*1, ii= 24, ss= 0, addl=0),
ev(ID = 9601:10000, amt = 9.5*1, ii= 24, ss= 0, addl=10),
#Missed dose + 150% dose intake simulation
ev(ID = 9601:10000, amt = 9.5*1, ii= 24, ss= 1, addl=0),
ev(ID = 9601:10000, amt = 9.5*0, ii= 24, ss= 0, addl=0),
ev(ID = 9601:10000, amt = 9.5*1.5, ii= 24, ss= 0, addl=0),
ev(ID = 9601:10000, amt = 9.5*1, ii= 24, ss= 0, addl=10),
#Missed dose + 200% dose intake simulation
ev(ID = 9601:10000, amt = 9.5*1, ii= 24, ss= 1, addl=0),
ev(ID = 9601:10000, amt = 9.5*0, ii= 24, ss= 0, addl=0),
ev(ID = 9601:10000, amt = 9.5*2, ii= 24, ss= 0, addl=0),
ev(ID = 9601:10000, amt = 9.5*1, ii= 24, ss= 0, addl=10)))) %>%
             mutate(dose_group = "9,5 mg") %>%

    bind_rows(as_tibble( seq(
  #Steady-state simulation
ev(ID = 10001:10400, amt = 10.5*1, ii= 24, ss= 1, addl=0),
ev(ID = 10001:10400, amt = 10.5*1, ii= 24, ss= 0, addl=0),
ev(ID = 10001:10400, amt = 10.5*1, ii= 24, ss= 0, addl=10),
#3h delay simulation
ev(ID = 10001:10400, amt = 10.5*1, ii= 24, ss= 1, addl=0),
ev(ID = 10001:10400, amt = 10.5*0, ii= 3, ss= 0, addl=0),
ev(ID = 10001:10400, amt = 10.5*1, ii= 21, ss= 0, addl=0),
ev(ID = 10001:10400, amt = 10.5*1, ii= 24, ss= 0, addl=10),
#6h delay simulation
ev(ID = 10001:10400, amt = 10.5*1, ii= 24, ss= 1, addl=0),
ev(ID = 10001:10400, amt = 10.5*0, ii= 6, ss= 0, addl=0),
ev(ID = 10001:10400, amt = 10.5*1, ii= 18, ss= 0, addl=0),
ev(ID = 10001:10400, amt = 10.5*1, ii= 24, ss= 0, addl=10),
#9h delay simulation
ev(ID = 10001:10400, amt = 10.5*1, ii= 24, ss= 1, addl=0),
ev(ID = 10001:10400, amt = 10.5*0, ii= 9, ss= 0, addl=0),
ev(ID = 10001:10400, amt = 10.5*1, ii= 15, ss= 0, addl=0),
ev(ID = 10001:10400, amt = 10.5*1, ii= 24, ss= 0, addl=10),
#12h delay simulation
ev(ID = 10001:10400, amt = 10.5*1, ii= 24, ss= 1, addl=0),
ev(ID = 10001:10400, amt = 10.5*0, ii= 12, ss= 0, addl=0),
ev(ID = 10001:10400, amt = 10.5*1, ii= 12, ss= 0, addl=0),
ev(ID = 10001:10400, amt = 10.5*1, ii= 24, ss= 0, addl=10),
#15h delay + 50% dose intake simulation
ev(ID = 10001:10400, amt = 10.5*1, ii= 24, ss= 1, addl=0),
ev(ID = 10001:10400, amt = 10.5*0, ii= 15, ss= 0, addl=0),
ev(ID = 10001:10400, amt = 10.5*0.5, ii= 9, ss= 0, addl=0),
ev(ID = 10001:10400, amt = 10.5*1, ii= 24, ss= 0, addl=10),
#15h delay simulation
ev(ID = 10001:10400, amt = 10.5*1, ii= 24, ss= 1, addl=0),
ev(ID = 10001:10400, amt = 10.5*0, ii= 15, ss= 0, addl=0),
ev(ID = 10001:10400, amt = 10.5*1, ii= 9, ss= 0, addl=0),
ev(ID = 10001:10400, amt = 10.5*1, ii= 24, ss= 0, addl=10),
#18h delay + 50% dose intake simulation
ev(ID = 10001:10400, amt = 10.5*1, ii= 24, ss= 1, addl=0),
ev(ID = 10001:10400, amt = 10.5*0, ii= 18, ss= 0, addl=0),
ev(ID = 10001:10400, amt = 10.5*0.5, ii= 6, ss= 0, addl=0),
ev(ID = 10001:10400, amt = 10.5*1, ii= 24, ss= 0, addl=10),
#18h delay simulation
ev(ID = 10001:10400, amt = 10.5*1, ii= 24, ss= 1, addl=0),
ev(ID = 10001:10400, amt = 10.5*0, ii= 18, ss= 0, addl=0),
ev(ID = 10001:10400, amt = 10.5*1, ii= 6, ss= 0, addl=0),
ev(ID = 10001:10400, amt = 10.5*1, ii= 24, ss= 0, addl=10),
#21h delay + 50% dose intake simulation
ev(ID = 10001:10400, amt = 10.5*1, ii= 24, ss= 1, addl=0),
ev(ID = 10001:10400, amt = 10.5*0, ii= 21, ss= 0, addl=0),
ev(ID = 10001:10400, amt = 10.5*0.5, ii= 3, ss= 0, addl=0),
ev(ID = 10001:10400, amt = 10.5*1, ii= 24, ss= 0, addl=10),
#21h delay simulation
ev(ID = 10001:10400, amt = 10.5*1, ii= 24, ss= 1, addl=0),
ev(ID = 10001:10400, amt = 10.5*0, ii= 21, ss= 0, addl=0),
ev(ID = 10001:10400, amt = 10.5*1, ii= 3, ss= 0, addl=0),
ev(ID = 10001:10400, amt = 10.5*1, ii= 24, ss= 0, addl=10),
#Missed dose simulation
ev(ID = 10001:10400, amt = 10.5*1, ii= 24, ss= 1, addl=0),
ev(ID = 10001:10400, amt = 10.5*0, ii= 24, ss= 0, addl=0),
ev(ID = 10001:10400, amt = 10.5*1, ii= 24, ss= 0, addl=0),
ev(ID = 10001:10400, amt = 10.5*1, ii= 24, ss= 0, addl=10),
#Missed dose + 150% dose intake simulation
ev(ID = 10001:10400, amt = 10.5*1, ii= 24, ss= 1, addl=0),
ev(ID = 10001:10400, amt = 10.5*0, ii= 24, ss= 0, addl=0),
ev(ID = 10001:10400, amt = 10.5*1.5, ii= 24, ss= 0, addl=0),
ev(ID = 10001:10400, amt = 10.5*1, ii= 24, ss= 0, addl=10),
#Missed dose + 200% dose intake simulation
ev(ID = 10001:10400, amt = 10.5*1, ii= 24, ss= 1, addl=0),
ev(ID = 10001:10400, amt = 10.5*0, ii= 24, ss= 0, addl=0),
ev(ID = 10001:10400, amt = 10.5*2, ii= 24, ss= 0, addl=0),
ev(ID = 10001:10400, amt = 10.5*1, ii= 24, ss= 0, addl=10)))) %>%
             mutate(dose_group = "10,5 mg") %>%

    bind_rows(as_tibble( seq(
  #Steady-state simulation
ev(ID = 10401:10800, amt = 11.5*1, ii= 24, ss= 1, addl=0),
ev(ID = 10401:10800, amt = 11.5*1, ii= 24, ss= 0, addl=0),
ev(ID = 10401:10800, amt = 11.5*1, ii= 24, ss= 0, addl=10),
#3h delay simulation
ev(ID = 10401:10800, amt = 11.5*1, ii= 24, ss= 1, addl=0),
ev(ID = 10401:10800, amt = 11.5*0, ii= 3, ss= 0, addl=0),
ev(ID = 10401:10800, amt = 11.5*1, ii= 21, ss= 0, addl=0),
ev(ID = 10401:10800, amt = 11.5*1, ii= 24, ss= 0, addl=10),
#6h delay simulation
ev(ID = 10401:10800, amt = 11.5*1, ii= 24, ss= 1, addl=0),
ev(ID = 10401:10800, amt = 11.5*0, ii= 6, ss= 0, addl=0),
ev(ID = 10401:10800, amt = 11.5*1, ii= 18, ss= 0, addl=0),
ev(ID = 10401:10800, amt = 11.5*1, ii= 24, ss= 0, addl=10),
#9h delay simulation
ev(ID = 10401:10800, amt = 11.5*1, ii= 24, ss= 1, addl=0),
ev(ID = 10401:10800, amt = 11.5*0, ii= 9, ss= 0, addl=0),
ev(ID = 10401:10800, amt = 11.5*1, ii= 15, ss= 0, addl=0),
ev(ID = 10401:10800, amt = 11.5*1, ii= 24, ss= 0, addl=10),
#12h delay simulation
ev(ID = 10401:10800, amt = 11.5*1, ii= 24, ss= 1, addl=0),
ev(ID = 10401:10800, amt = 11.5*0, ii= 12, ss= 0, addl=0),
ev(ID = 10401:10800, amt = 11.5*1, ii= 12, ss= 0, addl=0),
ev(ID = 10401:10800, amt = 11.5*1, ii= 24, ss= 0, addl=10),
#15h delay + 50% dose intake simulation
ev(ID = 10401:10800, amt = 11.5*1, ii= 24, ss= 1, addl=0),
ev(ID = 10401:10800, amt = 11.5*0, ii= 15, ss= 0, addl=0),
ev(ID = 10401:10800, amt = 11.5*0.5, ii= 9, ss= 0, addl=0),
ev(ID = 10401:10800, amt = 11.5*1, ii= 24, ss= 0, addl=10),
#15h delay simulation
ev(ID = 10401:10800, amt = 11.5*1, ii= 24, ss= 1, addl=0),
ev(ID = 10401:10800, amt = 11.5*0, ii= 15, ss= 0, addl=0),
ev(ID = 10401:10800, amt = 11.5*1, ii= 9, ss= 0, addl=0),
#18h delay + 50% dose intake simulation
ev(ID = 10401:10800, amt = 11.5*1, ii= 24, ss= 1, addl=0),
ev(ID = 10401:10800, amt = 11.5*0, ii= 18, ss= 0, addl=0),
ev(ID = 10401:10800, amt = 11.5*0.5, ii= 6, ss= 0, addl=0),
ev(ID = 10401:10800, amt = 11.5*1, ii= 24, ss= 0, addl=10),
#18h delay simulation
ev(ID = 10401:10800, amt = 11.5*1, ii= 24, ss= 1, addl=0),
ev(ID = 10401:10800, amt = 11.5*0, ii= 18, ss= 0, addl=0),
ev(ID = 10401:10800, amt = 11.5*1, ii= 6, ss= 0, addl=0),
ev(ID = 10401:10800, amt = 11.5*1, ii= 24, ss= 0, addl=10),
#21h delay + 50% dose intake simulation
ev(ID = 10401:10800, amt = 11.5*1, ii= 24, ss= 1, addl=0),
ev(ID = 10401:10800, amt = 11.5*0, ii= 21, ss= 0, addl=0),
ev(ID = 10401:10800, amt = 11.5*0.5, ii= 3, ss= 0, addl=0),
ev(ID = 10401:10800, amt = 11.5*1, ii= 24, ss= 0, addl=10),
#21h delay simulation
ev(ID = 10401:10800, amt = 11.5*1, ii= 24, ss= 1, addl=0),
ev(ID = 10401:10800, amt = 11.5*0, ii= 21, ss= 0, addl=0),
ev(ID = 10401:10800, amt = 11.5*1, ii= 3, ss= 0, addl=0),
ev(ID = 10401:10800, amt = 11.5*1, ii= 24, ss= 0, addl=10),
#Missed dose simulation
ev(ID = 10401:10800, amt = 11.5*1, ii= 24, ss= 1, addl=0),
ev(ID = 10401:10800, amt = 11.5*0, ii= 24, ss= 0, addl=0),
ev(ID = 10401:10800, amt = 11.5*1, ii= 24, ss= 0, addl=0),
ev(ID = 10401:10800, amt = 11.5*1, ii= 24, ss= 0, addl=10),
#Missed dose + 150% dose intake simulation
ev(ID = 10401:10800, amt = 11.5*1, ii= 24, ss= 1, addl=0),
ev(ID = 10401:10800, amt = 11.5*0, ii= 24, ss= 0, addl=0),
ev(ID = 10401:10800, amt = 11.5*1.5, ii= 24, ss= 0, addl=0),
ev(ID = 10401:10800, amt = 11.5*1, ii= 24, ss= 0, addl=10),
#Missed dose + 200% dose intake simulation
ev(ID = 10401:10800, amt = 11.5*1, ii= 24, ss= 1, addl=0),
ev(ID = 10401:10800, amt = 11.5*0, ii= 24, ss= 0, addl=0),
ev(ID = 10401:10800, amt = 11.5*2, ii= 24, ss= 0, addl=0),
ev(ID = 10401:10800, amt = 11.5*1, ii= 24, ss= 0, addl=10)))) %>%
             mutate(dose_group = "11,5 mg") %>%

    bind_rows(as_tibble( seq(
  #Steady-state simulation
ev(ID = 10801:11200, amt = 12.5*1, ii= 24, ss= 1, addl=0),
ev(ID = 10801:11200, amt = 12.5*1, ii= 24, ss= 0, addl=0),
ev(ID = 10801:11200, amt = 12.5*1, ii= 24, ss= 0, addl=10),
#3h delay simulation
ev(ID = 10801:11200, amt = 12.5*1, ii= 24, ss= 1, addl=0),
ev(ID = 10801:11200, amt = 12.5*0, ii= 3, ss= 0, addl=0),
ev(ID = 10801:11200, amt = 12.5*1, ii= 21, ss= 0, addl=0),
ev(ID = 10801:11200, amt = 12.5*1, ii= 24, ss= 0, addl=10),
#6h delay simulation
ev(ID = 10801:11200, amt = 12.5*1, ii= 24, ss= 1, addl=0),
ev(ID = 10801:11200, amt = 12.5*0, ii= 6, ss= 0, addl=0),
ev(ID = 10801:11200, amt = 12.5*1, ii= 18, ss= 0, addl=0),
ev(ID = 10801:11200, amt = 12.5*1, ii= 24, ss= 0, addl=10),
#9h delay simulation
ev(ID = 10801:11200, amt = 12.5*1, ii= 24, ss= 1, addl=0),
ev(ID = 10801:11200, amt = 12.5*0, ii= 9, ss= 0, addl=0),
ev(ID = 10801:11200, amt = 12.5*1, ii= 15, ss= 0, addl=0),
ev(ID = 10801:11200, amt = 12.5*1, ii= 24, ss= 0, addl=10),
#12h delay simulation
ev(ID = 10801:11200, amt = 12.5*1, ii= 24, ss= 1, addl=0),
ev(ID = 10801:11200, amt = 12.5*0, ii= 12, ss= 0, addl=0),
ev(ID = 10801:11200, amt = 12.5*1, ii= 12, ss= 0, addl=0),
ev(ID = 10801:11200, amt = 12.5*1, ii= 24, ss= 0, addl=10),
#15h delay + 50% dose intake simulation
ev(ID = 10801:11200, amt = 12.5*1, ii= 24, ss= 1, addl=0),
ev(ID = 10801:11200, amt = 12.5*0, ii= 15, ss= 0, addl=0),
ev(ID = 10801:11200, amt = 12.5*0.5, ii= 9, ss= 0, addl=0),
ev(ID = 10801:11200, amt = 12.5*1, ii= 24, ss= 0, addl=10),
#15h delay simulation
ev(ID = 10801:11200, amt = 12.5*1, ii= 24, ss= 1, addl=0),
ev(ID = 10801:11200, amt = 12.5*0, ii= 15, ss= 0, addl=0),
ev(ID = 10801:11200, amt = 12.5*1, ii= 9, ss= 0, addl=0),
ev(ID = 10801:11200, amt = 12.5*1, ii= 24, ss= 0, addl=10),
#18h delay + 50% dose intake simulation
ev(ID = 10801:11200, amt = 12.5*1, ii= 24, ss= 1, addl=0),
ev(ID = 10801:11200, amt = 12.5*0, ii= 18, ss= 0, addl=0),
ev(ID = 10801:11200, amt = 12.5*0.5, ii= 6, ss= 0, addl=0),
ev(ID = 10801:11200, amt = 12.5*1, ii= 24, ss= 0, addl=10),
#18h delay simulation
ev(ID = 10801:11200, amt = 12.5*1, ii= 24, ss= 1, addl=0),
ev(ID = 10801:11200, amt = 12.5*0, ii= 18, ss= 0, addl=0),
ev(ID = 10801:11200, amt = 12.5*1, ii= 6, ss= 0, addl=0),
ev(ID = 10801:11200, amt = 12.5*1, ii= 24, ss= 0, addl=10),
#21h delay + 50% dose intake simulation
ev(ID = 10801:11200, amt = 12.5*1, ii= 24, ss= 1, addl=0),
ev(ID = 10801:11200, amt = 12.5*0, ii= 21, ss= 0, addl=0),
ev(ID = 10801:11200, amt = 12.5*0.5, ii= 3, ss= 0, addl=0),
ev(ID = 10801:11200, amt = 12.5*1, ii= 24, ss= 0, addl=10),
#21h delay simulation
ev(ID = 10801:11200, amt = 12.5*1, ii= 24, ss= 1, addl=0),
ev(ID = 10801:11200, amt = 12.5*0, ii= 21, ss= 0, addl=0),
ev(ID = 10801:11200, amt = 12.5*1, ii= 3, ss= 0, addl=0),
ev(ID = 10801:11200, amt = 12.5*1, ii= 24, ss= 0, addl=10),
#Missed dose simulation
ev(ID = 10801:11200, amt = 12.5*1, ii= 24, ss= 1, addl=0),
ev(ID = 10801:11200, amt = 12.5*0, ii= 24, ss= 0, addl=0),
ev(ID = 10801:11200, amt = 12.5*1, ii= 24, ss= 0, addl=0),
ev(ID = 10801:11200, amt = 12.5*1, ii= 24, ss= 0, addl=10),
#Missed dose + 150% dose intake simulation
ev(ID = 10801:11200, amt = 12.5*1, ii= 24, ss= 1, addl=0),
ev(ID = 10801:11200, amt = 12.5*0, ii= 24, ss= 0, addl=0),
ev(ID = 10801:11200, amt = 12.5*1.5, ii= 24, ss= 0, addl=0),
ev(ID = 10801:11200, amt = 12.5*1, ii= 24, ss= 0, addl=10),
#Missed dose + 200% dose intake simulation
ev(ID = 10801:11200, amt = 12.5*1, ii= 24, ss= 1, addl=0),
ev(ID = 10801:11200, amt = 12.5*0, ii= 24, ss= 0, addl=0),
ev(ID = 10801:11200, amt = 12.5*2, ii= 24, ss= 0, addl=0),
ev(ID = 10801:11200, amt = 12.5*1, ii= 24, ss= 0, addl=10)))) %>%
             mutate(dose_group = "12,5 mg") %>%

    bind_rows(as_tibble( seq(
  #Steady-state simulation
ev(ID = 11201:11600, amt = 13.5*1, ii= 24, ss= 1, addl=0),
ev(ID = 11201:11600, amt = 13.5*1, ii= 24, ss= 0, addl=0),
ev(ID = 11201:11600, amt = 13.5*1, ii= 24, ss= 0, addl=10),
#3h delay simulation
ev(ID = 11201:11600, amt = 13.5*1, ii= 24, ss= 1, addl=0),
ev(ID = 11201:11600, amt = 13.5*0, ii= 3, ss= 0, addl=0),
ev(ID = 11201:11600, amt = 13.5*1, ii= 21, ss= 0, addl=0),
ev(ID = 11201:11600, amt = 13.5*1, ii= 24, ss= 0, addl=10),
#6h delay simulation
ev(ID = 11201:11600, amt = 13.5*1, ii= 24, ss= 1, addl=0),
ev(ID = 11201:11600, amt = 13.5*0, ii= 6, ss= 0, addl=0),
ev(ID = 11201:11600, amt = 13.5*1, ii= 18, ss= 0, addl=0),
ev(ID = 11201:11600, amt = 13.5*1, ii= 24, ss= 0, addl=10),
#9h delay simulation
ev(ID = 11201:11600, amt = 13.5*1, ii= 24, ss= 1, addl=0),
ev(ID = 11201:11600, amt = 13.5*0, ii= 9, ss= 0, addl=0),
ev(ID = 11201:11600, amt = 13.5*1, ii= 15, ss= 0, addl=0),
ev(ID = 11201:11600, amt = 13.5*1, ii= 24, ss= 0, addl=10),
#12h delay simulation
ev(ID = 11201:11600, amt = 13.5*1, ii= 24, ss= 1, addl=0),
ev(ID = 11201:11600, amt = 13.5*0, ii= 12, ss= 0, addl=0),
ev(ID = 11201:11600, amt = 13.5*1, ii= 12, ss= 0, addl=0),
ev(ID = 11201:11600, amt = 13.5*1, ii= 24, ss= 0, addl=10),
#15h delay + 50% dose intake simulation
ev(ID = 11201:11600, amt = 13.5*1, ii= 24, ss= 1, addl=0),
ev(ID = 11201:11600, amt = 13.5*0, ii= 15, ss= 0, addl=0),
ev(ID = 11201:11600, amt = 13.5*0.5, ii= 9, ss= 0, addl=0),
ev(ID = 11201:11600, amt = 13.5*1, ii= 24, ss= 0, addl=10),
#15h delay simulation
ev(ID = 11201:11600, amt = 13.5*1, ii= 24, ss= 1, addl=0),
ev(ID = 11201:11600, amt = 13.5*0, ii= 15, ss= 0, addl=0),
ev(ID = 11201:11600, amt = 13.5*1, ii= 9, ss= 0, addl=0),
ev(ID = 11201:11600, amt = 13.5*1, ii= 24, ss= 0, addl=10),
#18h delay + 50% dose intake simulation
ev(ID = 11201:11600, amt = 13.5*1, ii= 24, ss= 1, addl=0),
ev(ID = 11201:11600, amt = 13.5*0, ii= 18, ss= 0, addl=0),
ev(ID = 11201:11600, amt = 13.5*0.5, ii= 6, ss= 0, addl=0),
ev(ID = 11201:11600, amt = 13.5*1, ii= 24, ss= 0, addl=10),
#18h delay simulation
ev(ID = 11201:11600, amt = 13.5*1, ii= 24, ss= 1, addl=0),
ev(ID = 11201:11600, amt = 13.5*0, ii= 18, ss= 0, addl=0),
ev(ID = 11201:11600, amt = 13.5*1, ii= 6, ss= 0, addl=0),
ev(ID = 11201:11600, amt = 13.5*1, ii= 24, ss= 0, addl=10),
#21h delay + 50% dose intake simulation
ev(ID = 11201:11600, amt = 13.5*1, ii= 24, ss= 1, addl=0),
ev(ID = 11201:11600, amt = 13.5*0, ii= 21, ss= 0, addl=0),
ev(ID = 11201:11600, amt = 13.5*0.5, ii= 3, ss= 0, addl=0),
ev(ID = 11201:11600, amt = 13.5*1, ii= 24, ss= 0, addl=10),
#21h delay simulation
ev(ID = 11201:11600, amt = 13.5*1, ii= 24, ss= 1, addl=0),
ev(ID = 11201:11600, amt = 13.5*0, ii= 21, ss= 0, addl=0),
ev(ID = 11201:11600, amt = 13.5*1, ii= 3, ss= 0, addl=0),
ev(ID = 11201:11600, amt = 13.5*1, ii= 24, ss= 0, addl=10),
#Missed dose simulation
ev(ID = 11201:11600, amt = 13.5*1, ii= 24, ss= 1, addl=0),
ev(ID = 11201:11600, amt = 13.5*0, ii= 24, ss= 0, addl=0),
ev(ID = 11201:11600, amt = 13.5*1, ii= 24, ss= 0, addl=0),
ev(ID = 11201:11600, amt = 13.5*1, ii= 24, ss= 0, addl=10),
#Missed dose + 150% dose intake simulation
ev(ID = 11201:11600, amt = 13.5*1, ii= 24, ss= 1, addl=0),
ev(ID = 11201:11600, amt = 13.5*0, ii= 24, ss= 0, addl=0),
ev(ID = 11201:11600, amt = 13.5*1.5, ii= 24, ss= 0, addl=0),
ev(ID = 11201:11600, amt = 13.5*1, ii= 24, ss= 0, addl=10),
#Missed dose + 200% dose intake simulation
ev(ID = 11201:11600, amt = 13.5*1, ii= 24, ss= 1, addl=0),
ev(ID = 11201:11600, amt = 13.5*0, ii= 24, ss= 0, addl=0),
ev(ID = 11201:11600, amt = 13.5*2, ii= 24, ss= 0, addl=0),
ev(ID = 11201:11600, amt = 13.5*1, ii= 24, ss= 0, addl=10)))) %>%
             mutate(dose_group = "13,5 mg") %>%

    bind_rows(as_tibble( seq(
  #Steady-state simulation
ev(ID = 11601:12000, amt = 14.5*1, ii= 24, ss= 1, addl=0),
ev(ID = 11601:12000, amt = 14.5*1, ii= 24, ss= 0, addl=0),
ev(ID = 11601:12000, amt = 14.5*1, ii= 24, ss= 0, addl=10),
#3h delay simulation
ev(ID = 11601:12000, amt = 14.5*1, ii= 24, ss= 1, addl=0),
ev(ID = 11601:12000, amt = 14.5*0, ii= 3, ss= 0, addl=0),
ev(ID = 11601:12000, amt = 14.5*1, ii= 21, ss= 0, addl=0),
ev(ID = 11601:12000, amt = 14.5*1, ii= 24, ss= 0, addl=10),
#6h delay simulation
ev(ID = 11601:12000, amt = 14.5*1, ii= 24, ss= 1, addl=0),
ev(ID = 11601:12000, amt = 14.5*0, ii= 6, ss= 0, addl=0),
ev(ID = 11601:12000, amt = 14.5*1, ii= 18, ss= 0, addl=0),
ev(ID = 11601:12000, amt = 14.5*1, ii= 24, ss= 0, addl=10),
#9h delay simulation
ev(ID = 11601:12000, amt = 14.5*1, ii= 24, ss= 1, addl=0),
ev(ID = 11601:12000, amt = 14.5*0, ii= 9, ss= 0, addl=0),
ev(ID = 11601:12000, amt = 14.5*1, ii= 15, ss= 0, addl=0),
ev(ID = 11601:12000, amt = 14.5*1, ii= 24, ss= 0, addl=10),
#12h delay simulation
ev(ID = 11601:12000, amt = 14.5*1, ii= 24, ss= 1, addl=0),
ev(ID = 11601:12000, amt = 14.5*0, ii= 12, ss= 0, addl=0),
ev(ID = 11601:12000, amt = 14.5*1, ii= 12, ss= 0, addl=0),
ev(ID = 11601:12000, amt = 14.5*1, ii= 24, ss= 0, addl=10),
#15h delay + 50% dose intake simulation
ev(ID = 11601:12000, amt = 14.5*1, ii= 24, ss= 1, addl=0),
ev(ID = 11601:12000, amt = 14.5*0, ii= 15, ss= 0, addl=0),
ev(ID = 11601:12000, amt = 14.5*0.5, ii= 9, ss= 0, addl=0),
ev(ID = 11601:12000, amt = 14.5*1, ii= 24, ss= 0, addl=10),
#15h delay simulation
ev(ID = 11601:12000, amt = 14.5*1, ii= 24, ss= 1, addl=0),
ev(ID = 11601:12000, amt = 14.5*0, ii= 15, ss= 0, addl=0),
ev(ID = 11601:12000, amt = 14.5*1, ii= 9, ss= 0, addl=0),
ev(ID = 11601:12000, amt = 14.5*1, ii= 24, ss= 0, addl=10),
#18h delay + 50% dose intake simulation
ev(ID = 11601:12000, amt = 14.5*1, ii= 24, ss= 1, addl=0),
ev(ID = 11601:12000, amt = 14.5*0, ii= 18, ss= 0, addl=0),
ev(ID = 11601:12000, amt = 14.5*0.5, ii= 6, ss= 0, addl=0),
ev(ID = 11601:12000, amt = 14.5*1, ii= 24, ss= 0, addl=10),
#18h delay simulation
ev(ID = 11601:12000, amt = 14.5*1, ii= 24, ss= 1, addl=0),
ev(ID = 11601:12000, amt = 14.5*0, ii= 18, ss= 0, addl=0),
ev(ID = 11601:12000, amt = 14.5*1, ii= 6, ss= 0, addl=0),
ev(ID = 11601:12000, amt = 14.5*1, ii= 24, ss= 0, addl=10),
#21h delay + 50% dose intake simulation
ev(ID = 11601:12000, amt = 14.5*1, ii= 24, ss= 1, addl=0),
ev(ID = 11601:12000, amt = 14.5*0, ii= 21, ss= 0, addl=0),
ev(ID = 11601:12000, amt = 14.5*0.5, ii= 3, ss= 0, addl=0),
ev(ID = 11601:12000, amt = 14.5*1, ii= 24, ss= 0, addl=10),
#21h delay simulation
ev(ID = 11601:12000, amt = 14.5*1, ii= 24, ss= 1, addl=0),
ev(ID = 11601:12000, amt = 14.5*0, ii= 21, ss= 0, addl=0),
ev(ID = 11601:12000, amt = 14.5*1, ii= 3, ss= 0, addl=0),
ev(ID = 11601:12000, amt = 14.5*1, ii= 24, ss= 0, addl=10),
#Missed dose simulation
ev(ID = 11601:12000, amt = 14.5*1, ii= 24, ss= 1, addl=0),
ev(ID = 11601:12000, amt = 14.5*0, ii= 24, ss= 0, addl=0),
ev(ID = 11601:12000, amt = 14.5*1, ii= 24, ss= 0, addl=0),
ev(ID = 11601:12000, amt = 14.5*1, ii= 24, ss= 0, addl=10),
#Missed dose + 150% dose intake simulation
ev(ID = 11601:12000, amt = 14.5*1, ii= 24, ss= 1, addl=0),
ev(ID = 11601:12000, amt = 14.5*0, ii= 24, ss= 0, addl=0),
ev(ID = 11601:12000, amt = 14.5*1.5, ii= 24, ss= 0, addl=0),
ev(ID = 11601:12000, amt = 14.5*1, ii= 24, ss= 0, addl=10),
#Missed dose + 200% dose intake simulation
ev(ID = 11601:12000, amt = 14.5*1, ii= 24, ss= 1, addl=0),
ev(ID = 11601:12000, amt = 14.5*0, ii= 24, ss= 0, addl=0),
ev(ID = 11601:12000, amt = 14.5*2, ii= 24, ss= 0, addl=0),
ev(ID = 11601:12000, amt = 14.5*1, ii= 24, ss= 0, addl=10)))) %>%
             mutate(dose_group = "14,5 mg")


Dataset_12000 <- Simulation_data_12000 %>%
  arrange(ID) %>%
  left_join(CYPCL_data_12000, by = "ID")

## test simulation
set.seed(123456)
Sim_Test_12000 <- my_model_Moes %>%
 data_set(Dataset_12000) %>%
 Req(DV) %>%
 mrgsim(end = 7000, delta = 1)
```

Fusion des dataframes

```r
Sim_Test_6000_df <- as.data.frame(Sim_Test_6000)
Sim_Test_12000_df <- as.data.frame(Sim_Test_12000)

Data_combined <- rbind(Sim_Test_6000_df, Sim_Test_12000_df)

Data_combined
```

```
##         ID time           DV
## 1        1    0 0.000000e+00
## 2        1    0 6.393983e-01
## 3        1    1 3.688770e+00
## 4        1    2 2.839635e+00
## 5        1    3 2.208022e+00
## 6        1    4 1.794712e+00
## 7        1    5 1.520401e+00
## 8        1    6 1.340514e+00
## 9        1    7 1.212370e+00
## 10       1    8 1.123766e+00
## 11       1    9 1.057123e+00
## 12       1   10 1.004392e+00
## 13       1   11 9.617093e-01
## 14       1   12 9.235940e-01
## 15       1   13 8.924323e-01
## 16       1   14 8.650522e-01
## 17       1   15 8.389556e-01
## 18       1   16 8.107720e-01
## 19       1   17 7.865124e-01
## 20       1   18 7.646043e-01
## 21       1   19 7.409990e-01
## 22       1   20 7.194918e-01
## 23       1   21 6.992719e-01
## 24       1   22 6.783634e-01
## 25       1   23 6.583545e-01
## 26       1   24 6.402097e-01
## 27       1   24 6.393476e-01
## 28       1   25 3.685825e+00
## 29       1   26 2.835588e+00
## 30       1   27 2.207882e+00
## 31       1   28 1.791425e+00
## 32       1   29 1.520375e+00
## 33       1   30 1.336863e+00
## 34       1   31 1.213870e+00
## 35       1   32 1.124909e+00
```

```
## 36    1   33 1.055368e+00
## 37    1   34 1.004589e+00
## 38    1   35 9.614541e-01
## 39    1   36 9.253704e-01
## 40    1   37 8.921946e-01
## 41    1   38 8.625982e-01
## 42    1   39 8.370826e-01
## 43    1   40 8.112926e-01
## 44    1   41 7.857345e-01
## 45    1   42 7.630301e-01
## 46    1   43 7.410497e-01
## 47    1   44 7.194239e-01
## 48    1   45 6.982655e-01
## 49    1   46 6.773897e-01
## 50    1   47 6.583973e-01
## 51    1   48 6.393287e-01
## 52    1   48 6.400526e-01
## 53    1   49 3.682578e+00
## 54    1   50 2.836052e+00
## 55    1   51 2.203238e+00
## 56    1   52 1.793052e+00
## 57    1   53 1.518309e+00
## 58    1   54 1.340552e+00
## 59    1   55 1.210917e+00
## 60    1   56 1.122839e+00
## 61    1   57 1.056237e+00
## 62    1   58 1.003609e+00
## 63    1   59 9.610469e-01
## 64    1   60 9.250660e-01
## 65    1   61 8.923767e-01
## 66    1   62 8.620727e-01
## 67    1   63 8.377422e-01
## 68    1   64 8.117826e-01
## 69    1   65 7.867382e-01
## 70    1   66 7.637247e-01
## 71    1   67 7.402710e-01
## 72    1   68 7.196391e-01
## 73    1   69 6.987783e-01
## 74    1   70 6.777203e-01
## 75    1   71 6.587856e-01
## 76    1   72 6.391723e-01
## 77    1   73 3.682168e+00
## 78    1   74 2.834965e+00
## 79    1   75 2.201233e+00
## 80    1   76 1.793605e+00
## 81    1   77 1.521543e+00
## 82    1   78 1.336965e+00
## 83    1   79 1.212715e+00
## 84    1   80 1.124795e+00
## 85    1   81 1.055803e+00
## 86    1   82 1.004722e+00
## 87    1   83 9.608746e-01
## 88    1   84 9.266752e-01
## 89    1   85 8.916122e-01
## 90    1   86 8.635748e-01
## 91    1   87 8.380044e-01
## 92    1   88 8.107311e-01
## 93    1   89 7.872477e-01
## 94    1   90 7.633559e-01
## 95    1   91 7.413226e-01
## 96    1   92 7.203032e-01
## 97    1   93 6.988798e-01
## 98    1   94 6.777316e-01
## 99    1   95 6.564156e-01
## 100   1   96 6.395968e-01
## 101   1   97 3.684537e+00
## 102   1   98 2.837089e+00
## 103   1   99 2.206047e+00
## 104   1  100 1.794762e+00
## 105   1  101 1.520405e+00
## 106   1  102 1.339156e+00
## 107   1  103 1.213411e+00
## 108   1  104 1.124399e+00
## 109   1  105 1.054737e+00
## 110   1  106 1.002690e+00
## 111   1  107 9.621585e-01
## 112   1  108 9.256379e-01
## 113   1  109 8.943716e-01
## 114   1  110 8.615588e-01
## 115   1  111 8.371231e-01
## 116   1  112 8.094063e-01
## 117   1  113 7.861606e-01
## 118   1  114 7.619800e-01
## 119   1  115 7.410400e-01
## 120   1  116 7.185469e-01
## 121   1  117 6.971954e-01
## 122   1  118 6.775062e-01
## 123   1  119 6.573996e-01
## 124   1  120 6.388001e-01
## 125   1  121 3.685471e+00
## 126   1  122 2.843204e+00
## 127   1  123 2.206388e+00
## 128   1  124 1.792359e+00
## 129   1  125 1.518024e+00
## 130   1  126 1.340759e+00
## 131   1  127 1.212422e+00
## 132   1  128 1.124696e+00
## 133   1  129 1.054476e+00
## 134   1  130 1.002978e+00
## 135   1  131 9.624017e-01
## 136   1  132 9.254170e-01
## 137   1  133 8.928423e-01
## 138   1  134 8.630033e-01
## 139   1  135 8.364654e-01
## 140   1  136 8.099565e-01
## 141   1  137 7.862212e-01
## 142   1  138 7.641126e-01
## 143   1  139 7.402824e-01
## 144   1  140 7.190572e-01
## 145   1  141 6.979065e-01
## 146   1  142 6.789144e-01
## 147   1  143 6.575338e-01
## 148   1  144 6.394445e-01
## 149   1  145 3.685632e+00
## 150   1  146 2.834051e+00
## 151   1  147 2.204682e+00
## 152   1  148 1.790330e+00
## 153   1  149 1.521747e+00
## 154   1  150 1.339060e+00
## 155   1  151 1.212734e+00
## 156   1  152 1.123788e+00
## 157   1  153 1.057317e+00
## 158   1  154 1.003613e+00
## 159   1  155 9.613438e-01
## 160   1  156 9.235591e-01
## 161   1  157 8.933205e-01
## 162   1  158 8.643746e-01
## 163   1  159 8.365733e-01
## 164   1  160 8.108222e-01
## 165   1  161 7.866978e-01
## 166   1  162 7.644504e-01
## 167   1  163 7.421704e-01
## 168   1  164 7.183966e-01
## 169   1  165 6.987197e-01
## 170   1  166 6.775704e-01
## 171   1  167 6.594501e-01
## 172   1  168 6.398750e-01
## 173   1  169 3.687181e+00
## 174   1  170 2.839118e+00
## 175   1  171 2.207211e+00
## 176   1  172 1.792594e+00
## 177   1  173 1.521199e+00
## 178   1  174 1.340621e+00
## 179   1  175 1.213139e+00
## 180   1  176 1.123772e+00
## 181   1  177 1.056762e+00
## 182   1  178 1.004677e+00
## 183   1  179 9.622478e-01
## 184   1  180 9.248912e-01
## 185   1  181 8.921397e-01
## 186   1  182 8.626712e-01
## 187   1  183 8.367532e-01
## 188   1  184 8.113658e-01
## 189   1  185 7.876429e-01
## 190   1  186 7.633006e-01
## 191   1  187 7.404780e-01
## 192   1  188 7.197836e-01
## 193   1  189 6.993527e-01
## 194   1  190 6.783420e-01
## 195   1  191 6.584998e-01
## 196   1  192 6.387647e-01
## 197   1  193 3.683723e+00
## 198   1  194 2.840297e+00
## 199   1  195 2.202575e+00
## 200   1  196 1.791502e+00
## 201   1  197 1.520343e+00
## 202   1  198 1.338369e+00
## 203   1  199 1.210025e+00
## 204   1  200 1.121587e+00
## 205   1  201 1.056266e+00
## 206   1  202 1.002238e+00
## 207   1  203 9.598116e-01
## 208   1  204 9.272378e-01
## 209   1  205 8.931351e-01
## 210   1  206 8.642614e-01
## 211   1  207 8.359414e-01
## 212   1  208 8.106344e-01
## 213   1  209 7.870773e-01
## 214   1  210 7.634442e-01
## 215   1  211 7.407783e-01
## 216   1  212 7.186106e-01
## 217   1  213 6.979301e-01
## 218   1  214 6.781743e-01
## 219   1  215 6.581364e-01
## 220   1  216 6.391393e-01
## 221   1  217 3.684292e+00
## 222   1  218 2.837594e+00
## 223   1  219 2.205026e+00
## 224   1  220 1.790498e+00
## 225   1  221 1.520563e+00
## 226   1  222 1.337752e+00
## 227   1  223 1.212059e+00
## 228   1  224 1.123332e+00
## 229   1  225 1.055108e+00
## 230   1  226 1.002248e+00
## 231   1  227 9.601407e-01
## 232   1  228 9.260108e-01
## 233   1  229 8.930470e-01
## 234   1  230 8.642273e-01
## 235   1  231 8.380520e-01
## 236   1  232 8.114435e-01
## 237   1  233 7.867759e-01
## 238   1  234 7.629094e-01
## 239   1  235 7.412629e-01
## 240   1  236 7.177724e-01
## 241   1  237 6.982787e-01
## 242   1  238 6.772181e-01
## 243   1  239 6.582792e-01
## 244   1  240 6.393268e-01
## 245   1  241 3.689379e+00
## 246   1  242 2.835047e+00
## 247   1  243 2.206882e+00
## 248   1  244 1.792333e+00
## 249   1  245 1.517183e+00
## 250   1  246 1.338217e+00
## 251   1  247 1.213918e+00
## 252   1  248 1.125870e+00
## 253   1  249 1.054970e+00
## 254   1  250 1.002553e+00
## 255   1  251 9.609659e-01
## 256   1  252 9.246092e-01
## 257   1  253 8.923932e-01
## 258   1  254 8.635193e-01
## 259   1  255 8.370308e-01
## 260   1  256 8.110746e-01
## 261   1  257 7.872354e-01
## 262   1  258 7.640629e-01
## 263   1  259 7.411023e-01
## 264   1  260 7.187672e-01
## 265   1  261 6.981703e-01
## 266   1  262 6.780836e-01
## 267   1  263 6.580096e-01
## 268   1  264 6.389961e-01
## 269   1  265 3.689595e+00
## 270   1  266 2.839078e+00
## 271   1  267 2.206660e+00
## 272   1  268 1.791670e+00
## 273   1  269 1.518537e+00
## 274   1  270 1.339921e+00
## 275   1  271 1.211805e+00
## 276   1  272 1.122887e+00
## 277   1  273 1.055124e+00
## 278   1  274 1.004492e+00
## 279   1  275 9.601607e-01
## 280   1  276 9.254861e-01
## 281   1  277 8.944650e-01
## 282   1  278 8.630746e-01
## 283   1  279 8.367248e-01
## 284   1  280 8.100218e-01
## 285   1  281 7.866486e-01
## 286   1  282 7.631548e-01
## 287   1  283 7.407354e-01
## 288   1  284 7.193503e-01
## 289   1  285 6.986866e-01
## 290   1  286 6.785293e-01
## 291   1  287 6.579497e-01
## 292   1  288 6.395117e-01
## 293   1  289 3.688567e+00
## 294   1  290 2.833429e+00
## 295   1  291 2.204197e+00
## 296   1  292 1.792650e+00
## 297   1  293 1.522472e+00
## 298   1  294 1.335329e+00
## 299   1  295 1.213053e+00
## 300   1  296 1.123585e+00
## 301   1  297 1.055457e+00
## 302   1  298 1.002215e+00
## 303   1  299 9.624401e-01
## 304   1  300 9.247622e-01
## 305   1  301 8.920399e-01
## 306   1  302 8.645334e-01
## 307   1  303 8.374095e-01
## 308   1  304 8.113316e-01
## 309   1  305 7.870501e-01
## 310   1  306 7.639365e-01
## 311   1  307 7.407174e-01
## 312   1  308 7.191903e-01
## 313   1  309 6.983573e-01
## 314   1  310 6.785077e-01
## 315   1  311 6.583710e-01
## 316   1  312 6.394261e-01
## 317   1  312 6.394918e-01
## 318   1  313 3.693018e+00
## 319   1  314 2.838335e+00
## 320   1  315 2.209657e+00
## 321   1  316 1.795254e+00
## 322   1  317 1.518391e+00
## 323   1  318 1.341145e+00
## 324   1  319 1.209500e+00
## 325   1  320 1.124086e+00
## 326   1  321 1.057477e+00
## 327   1  322 1.003000e+00
## 328   1  323 9.621007e-01
## 329   1  324 9.260706e-01
## 330   1  325 8.926761e-01
## 331   1  326 8.640622e-01
## 332   1  327 8.362072e-01
## 333   1  328 8.098845e-01
## 334   1  329 7.868145e-01
## 335   1  330 7.635739e-01
## 336   1  331 7.411368e-01
## 337   1  332 7.201548e-01
## 338   1  333 6.985227e-01
## 339   1  334 6.784567e-01
## 340   1  335 6.588171e-01
## 341   1  336 6.393112e-01
## 342   1  336 6.392057e-01
## 343   1  337 6.207619e-01
## 344   1  338 6.025348e-01
## 345   1  339 5.847180e-01
## 346   1  339 5.864586e-01
## 347   1  340 3.631032e+00
## 348   1  341 2.787296e+00
## 349   1  342 2.152024e+00
## 350   1  343 1.746194e+00
## 351   1  344 1.475138e+00
## 352   1  345 1.290741e+00
## 353   1  346 1.171196e+00
## 354   1  347 1.080944e+00
## 355   1  348 1.015659e+00
## 356   1  349 9.635976e-01
## 357   1  350 9.230870e-01
## 358   1  351 8.876774e-01
## 359   1  352 8.563552e-01
## 360   1  353 8.266804e-01
## 361   1  354 8.026193e-01
## 362   1  355 7.765200e-01
## 363   1  356 7.540068e-01
## 364   1  357 7.314521e-01
## 365   1  358 7.092008e-01
## 366   1  359 6.889469e-01
## 367   1  360 6.687622e-01
## 368   1  360 6.696113e-01
## 369   1  361 3.711205e+00
## 370   1  362 2.872465e+00
## 371   1  363 2.233135e+00
## 372   1  364 1.820242e+00
## 373   1  365 1.546107e+00
## 374   1  366 1.363205e+00
## 375   1  367 1.235650e+00
## 376   1  368 1.146362e+00
## 377   1  369 1.078121e+00
## 378   1  370 1.026266e+00
## 379   1  371 9.824851e-01
## 380   1  372 9.456803e-01
## 381   1  373 9.135373e-01
## 382   1  374 8.831211e-01
## 383   1  375 8.550829e-01
## 384   1  376 8.297407e-01
## 385   1  377 8.043418e-01
## 386   1  378 7.808886e-01
## 387   1  379 7.578198e-01
## 388   1  380 7.352372e-01
## 389   1  381 7.150313e-01
## 390   1  382 6.929511e-01
## 391   1  383 6.738975e-01
## 392   1  384 6.535594e-01
## 393   1  385 3.702101e+00
## 394   1  386 2.852091e+00
## 395   1  387 2.220072e+00
## 396   1  388 1.807798e+00
## 397   1  389 1.532726e+00
## 398   1  390 1.350388e+00
## 399   1  391 1.225813e+00
## 400   1  392 1.135116e+00
## 401   1  393 1.067404e+00
## 402   1  394 1.014318e+00
## 403   1  395 9.720419e-01
## 404   1  396 9.353429e-01
## 405   1  397 9.036574e-01
## 406   1  398 8.735353e-01
## 407   1  399 8.471021e-01
## 408   1  400 8.208316e-01
## 409   1  401 7.957438e-01
## 410   1  402 7.714226e-01
## 411   1  403 7.493350e-01
## 412   1  404 7.276603e-01
## 413   1  405 7.062458e-01
## 414   1  406 6.865586e-01
## 415   1  407 6.649153e-01
## 416   1  408 6.463950e-01
## 417   1  409 3.698580e+00
## 418   1  410 2.844163e+00
## 419   1  411 2.210493e+00
## 420   1  412 1.800293e+00
## 421   1  413 1.526111e+00
## 422   1  414 1.344722e+00
## 423   1  415 1.217777e+00
## 424   1  416 1.128547e+00
## 425   1  417 1.062701e+00
## 426   1  418 1.009943e+00
## 427   1  419 9.668683e-01
## 428   1  420 9.307733e-01
## 429   1  421 8.955473e-01
## 430   1  422 8.669127e-01
## 431   1  423 8.403099e-01
## 432   1  424 8.148491e-01
## 433   1  425 7.917222e-01
## 434   1  426 7.691106e-01
## 435   1  427 7.448663e-01
## 436   1  428 7.216775e-01
## 437   1  429 7.008431e-01
## 438   1  430 6.823262e-01
## 439   1  431 6.623993e-01
## 440   1  432 6.430126e-01
## 441   1  433 3.683590e+00
## 442   1  434 2.845732e+00
## 443   1  435 2.205281e+00
## 444   1  436 1.796711e+00
## 445   1  437 1.525136e+00
## 446   1  438 1.341142e+00
## 447   1  439 1.214285e+00
## 448   1  440 1.125826e+00
## 449   1  441 1.058814e+00
## 450   1  442 1.006104e+00
## 451   1  443 9.629423e-01
## 452   1  444 9.280767e-01
## 453   1  445 8.938163e-01
## 454   1  446 8.668842e-01
## 455   1  447 8.378887e-01
## 456   1  448 8.129378e-01
## 457   1  449 7.883859e-01
## 458   1  450 7.650889e-01
## 459   1  451 7.435157e-01
## 460   1  452 7.212824e-01
## 461   1  453 7.005330e-01
## 462   1  454 6.788324e-01
## 463   1  455 6.610825e-01
## 464   1  456 6.409737e-01
## 465   1  457 3.682400e+00
## 466   1  458 2.837560e+00
## 467   1  459 2.205023e+00
## 468   1  460 1.793647e+00
## 469   1  461 1.522361e+00
## 470   1  462 1.340156e+00
## 471   1  463 1.214450e+00
## 472   1  464 1.122294e+00
## 473   1  465 1.058544e+00
## 474   1  466 1.005431e+00
## 475   1  467 9.621265e-01
## 476   1  468 9.263603e-01
## 477   1  469 8.949777e-01
## 478   1  470 8.652773e-01
## 479   1  471 8.374388e-01
## 480   1  472 8.118285e-01
## 481   1  473 7.881875e-01
## 482   1  474 7.631152e-01
## 483   1  475 7.429167e-01
## 484   1  476 7.206615e-01
## 485   1  477 6.993241e-01
## 486   1  478 6.796445e-01
## 487   1  479 6.589957e-01
## 488   1  480 6.404436e-01
## 489   1  481 3.687892e+00
## 490   1  482 2.842343e+00
## 491   1  483 2.205408e+00
## 492   1  484 1.794075e+00
## 493   1  485 1.519602e+00
## 494   1  486 1.337229e+00
## 495   1  487 1.213511e+00
## 496   1  488 1.122994e+00
## 497   1  489 1.057354e+00
## 498   1  490 1.005415e+00
## 499   1  491 9.633444e-01
## 500   1  492 9.265920e-01
## 501   1  493 8.931299e-01
## 502   1  494 8.641538e-01
## 503   1  495 8.364817e-01
## 504   1  496 8.121039e-01
## 505   1  497 7.876156e-01
## 506   1  498 7.647619e-01
## 507   1  499 7.417538e-01
## 508   1  500 7.194024e-01
## 509   1  501 6.985210e-01
## 510   1  502 6.778531e-01
## 511   1  503 6.593418e-01
## 512   1  504 6.399681e-01
## 513   1  505 3.686315e+00
## 514   1  506 2.842402e+00
## 515   1  507 2.203569e+00
## 516   1  508 1.792601e+00
## 517   1  509 1.522515e+00
## 518   1  510 1.340182e+00
## 519   1  511 1.212779e+00
## 520   1  512 1.123611e+00
## 521   1  513 1.057201e+00
## 522   1  514 1.003872e+00
## 523   1  515 9.601999e-01
## 524   1  516 9.247148e-01
## 525   1  517 8.948276e-01
## 526   1  518 8.640564e-01
## 527   1  519 8.361823e-01
## 528   1  520 8.100572e-01
## 529   1  521 7.879255e-01
## 530   1  522 7.618279e-01
## 531   1  523 7.420902e-01
## 532   1  524 7.204753e-01
## 533   1  525 6.978801e-01
## 534   1  526 6.781877e-01
## 535   1  527 6.581368e-01
## 536   1  528 6.399032e-01
## 537   1  529 3.678966e+00
## 538   1  530 2.839332e+00
## 539   1  531 2.206121e+00
## 540   1  532 1.791756e+00
## 541   1  533 1.524265e+00
## 542   1  534 1.339526e+00
## 543   1  535 1.210967e+00
## 544   1  536 1.122859e+00
## 545   1  537 1.056096e+00
## 546   1  538 1.004257e+00
## 547   1  539 9.611500e-01
## 548   1  540 9.241366e-01
## 549   1  541 8.934300e-01
## 550   1  542 8.642039e-01
## 551   1  543 8.360405e-01
## 552   1  544 8.105056e-01
## 553   1  545 7.880538e-01
## 554   1  546 7.625775e-01
## 555   1  547 7.413710e-01
## 556   1  548 7.197911e-01
## 557   1  549 6.986355e-01
## 558   1  550 6.783062e-01
## 559   1  551 6.591868e-01
## 560   1  552 6.396111e-01
## 561   1  553 3.687448e+00
## 562   1  554 2.836388e+00
## 563   1  555 2.203935e+00
## 564   1  556 1.789136e+00
## 565   1  557 1.518778e+00
## 566   1  558 1.338184e+00
## 567   1  559 1.212053e+00
## 568   1  560 1.123296e+00
## 569   1  561 1.054805e+00
## 570   1  562 1.003890e+00
## 571   1  563 9.590002e-01
## 572   1  564 9.245411e-01
## 573   1  565 8.927533e-01
## 574   1  566 8.629485e-01
## 575   1  567 8.365248e-01
## 576   1  568 8.109872e-01
## 577   1  569 7.875459e-01
## 578   1  570 7.624586e-01
## 579   1  571 7.405516e-01
## 580   1  572 7.192506e-01
## 581   1  573 6.990087e-01
## 582   1  574 6.775312e-01
## 583   1  575 6.592241e-01
## 584   1  576 6.405506e-01
## 585   1  577 3.682806e+00
## 586   1  578 2.840918e+00
## 587   1  579 2.203760e+00
## 588   1  580 1.790450e+00
## 589   1  581 1.520454e+00
## 590   1  582 1.337393e+00
## 591   1  583 1.213841e+00
## 592   1  584 1.123180e+00
## 593   1  585 1.055970e+00
## 594   1  586 1.003838e+00
## 595   1  587 9.610113e-01
## 596   1  588 9.266153e-01
## 597   1  589 8.952458e-01
## 598   1  590 8.637879e-01
## 599   1  591 8.371190e-01
## 600   1  592 8.096556e-01
## 601   1  593 7.869169e-01
## 602   1  594 7.637756e-01
## 603   1  595 7.415702e-01
## 604   1  596 7.194714e-01
## 605   1  597 6.980992e-01
## 606   1  598 6.780075e-01
## 607   1  599 6.578614e-01
## 608   1  600 6.388816e-01
## 609   1  601 3.690318e+00
## 610   1  602 2.843263e+00
## 611   1  603 2.203739e+00
## 612   1  604 1.792600e+00
## 613   1  605 1.518832e+00
## 614   1  606 1.339261e+00
## 615   1  607 1.212774e+00
## 616   1  608 1.123283e+00
## 617   1  609 1.059121e+00
## 618   1  610 1.002434e+00
## 619   1  611 9.615048e-01
## 620   1  612 9.255725e-01
## 621   1  613 8.912204e-01
## 622   1  614 8.632477e-01
## 623   1  615 8.378533e-01
## 624   1  616 8.105803e-01
## 625   1  617 7.856289e-01
## 626   1  618 7.633981e-01
## 627   1  619 7.396095e-01
## 628   1  620 7.180400e-01
## 629   1  621 6.985547e-01
## 630   1  622 6.771318e-01
## 631   1  623 6.581033e-01
## 632   1  624 6.398467e-01
## 633   1  624 6.394146e-01
## 634   1  625 3.685050e+00
## 635   1  626 2.833518e+00
## 636   1  627 2.206283e+00
## 637   1  628 1.793598e+00
## 638   1  629 1.520730e+00
## 639   1  630 1.338211e+00
## 640   1  631 1.213972e+00
## 641   1  632 1.121601e+00
## 642   1  633 1.055969e+00
## 643   1  634 1.002585e+00
## 644   1  635 9.620808e-01
## 645   1  636 9.249182e-01
## 646   1  637 8.942632e-01
## 647   1  638 8.642202e-01
## 648   1  639 8.366718e-01
## 649   1  640 8.119113e-01
## 650   1  641 7.860408e-01
## 651   1  642 7.642396e-01
## 652   1  643 7.410015e-01
## 653   1  644 7.200048e-01
## 654   1  645 6.984040e-01
## 655   1  646 6.781367e-01
## 656   1  647 6.569706e-01
## 657   1  648 6.382813e-01
## 658   1  648 6.378384e-01
## 659   1  649 6.221376e-01
## 660   1  650 6.023730e-01
## 661   1  651 5.849440e-01
## 662   1  652 5.683349e-01
## 663   1  653 5.526815e-01
## 664   1  654 5.359145e-01
## 665   1  654 5.365000e-01
## 666   1  655 3.586638e+00
## 667   1  656 2.736458e+00
## 668   1  657 2.113450e+00
## 669   1  658 1.699213e+00
## 670   1  659 1.431390e+00
## 671   1  660 1.251115e+00
## 672   1  661 1.128284e+00
## 673   1  662 1.039732e+00
## 674   1  663 9.771750e-01
## 675   1  664 9.277134e-01
## 676   1  665 8.860960e-01
## 677   1  666 8.531697e-01
## 678   1  667 8.220297e-01
## 679   1  668 7.947055e-01
## 680   1  669 7.690923e-01
## 681   1  670 7.467786e-01
## 682   1  671 7.244488e-01
## 683   1  672 7.028886e-01
## 684   1  672 7.018973e-01
## 685   1  673 3.747193e+00
## 686   1  674 2.896165e+00
## 687   1  675 2.265362e+00
## 688   1  676 1.850993e+00
## 689   1  677 1.573838e+00
## 690   1  678 1.389519e+00
## 691   1  679 1.262530e+00
## 692   1  680 1.174022e+00
## 693   1  681 1.103954e+00
## 694   1  682 1.051459e+00
## 695   1  683 1.005666e+00
## 696   1  684 9.705803e-01
## 697   1  685 9.337990e-01
## 698   1  686 9.046137e-01
## 699   1  687 8.762210e-01
## 700   1  688 8.491666e-01
## 701   1  689 8.257555e-01
## 702   1  690 7.995108e-01
## 703   1  691 7.768310e-01
## 704   1  692 7.544190e-01
## 705   1  693 7.329631e-01
## 706   1  694 7.095576e-01
## 707   1  695 6.896813e-01
## 708   1  696 6.698490e-01
## 709   1  697 3.713378e+00
## 710   1  698 2.861535e+00
## 711   1  699 2.232642e+00
## 712   1  700 1.817730e+00
## 713   1  701 1.546081e+00
## 714   1  702 1.363768e+00
## 715   1  703 1.234971e+00
## 716   1  704 1.147409e+00
## 717   1  705 1.081682e+00
## 718   1  706 1.028219e+00
## 719   1  707 9.839206e-01
## 720   1  708 9.458688e-01
## 721   1  709 9.153236e-01
## 722   1  710 8.841040e-01
## 723   1  711 8.575748e-01
## 724   1  712 8.313172e-01
## 725   1  713 8.061223e-01
## 726   1  714 7.817660e-01
## 727   1  715 7.592338e-01
## 728   1  716 7.364117e-01
## 729   1  717 7.138996e-01
## 730   1  718 6.938354e-01
## 731   1  719 6.745758e-01
## 732   1  720 6.548777e-01
## 733   1  721 3.700059e+00
## 734   1  722 2.848432e+00
## 735   1  723 2.218656e+00
## 736   1  724 1.808817e+00
## 737   1  725 1.531805e+00
## 738   1  726 1.352157e+00
## 739   1  727 1.223674e+00
## 740   1  728 1.135752e+00
## 741   1  729 1.067442e+00
## 742   1  730 1.016320e+00
## 743   1  731 9.719217e-01
## 744   1  732 9.330177e-01
## 745   1  733 9.048932e-01
## 746   1  734 8.726344e-01
## 747   1  735 8.466107e-01
## 748   1  736 8.212840e-01
## 749   1  737 7.963239e-01
## 750   1  738 7.723461e-01
## 751   1  739 7.492154e-01
## 752   1  740 7.275876e-01
## 753   1  741 7.057011e-01
## 754   1  742 6.855744e-01
## 755   1  743 6.653800e-01
## 756   1  744 6.461653e-01
## 757   1  745 3.694540e+00
## 758   1  746 2.845729e+00
## 759   1  747 2.212871e+00
## 760   1  748 1.799268e+00
## 761   1  749 1.529082e+00
## 762   1  750 1.344943e+00
## 763   1  751 1.219262e+00
## 764   1  752 1.130548e+00
## 765   1  753 1.061470e+00
## 766   1  754 1.009207e+00
## 767   1  755 9.658946e-01
## 768   1  756 9.304033e-01
## 769   1  757 8.980746e-01
## 770   1  758 8.694351e-01
## 771   1  759 8.408993e-01
## 772   1  760 8.152570e-01
## 773   1  761 7.916483e-01
## 774   1  762 7.673048e-01
## 775   1  763 7.455801e-01
## 776   1  764 7.222622e-01
## 777   1  765 7.030736e-01
## 778   1  766 6.823909e-01
## 779   1  767 6.616797e-01
## 780   1  768 6.419470e-01
## 781   1  769 3.688490e+00
## 782   1  770 2.842834e+00
## 783   1  771 2.207960e+00
## 784   1  772 1.796514e+00
## 785   1  773 1.523223e+00
## 786   1  774 1.341247e+00
## 787   1  775 1.215931e+00
## 788   1  776 1.124517e+00
## 789   1  777 1.060325e+00
## 790   1  778 1.007741e+00
## 791   1  779 9.652255e-01
## 792   1  780 9.269564e-01
## 793   1  781 8.955146e-01
## 794   1  782 8.657148e-01
## 795   1  783 8.398373e-01
## 796   1  784 8.134378e-01
## 797   1  785 7.886840e-01
## 798   1  786 7.670192e-01
## 799   1  787 7.440203e-01
## 800   1  788 7.215323e-01
## 801   1  789 7.008895e-01
## 802   1  790 6.812295e-01
## 803   1  791 6.600437e-01
## 804   1  792 6.410710e-01
## 805   1  793 3.683082e+00
## 806   1  794 2.837827e+00
## 807   1  795 2.204972e+00
## 808   1  796 1.792225e+00
## 809   1  797 1.521286e+00
## 810   1  798 1.341166e+00
## 811   1  799 1.214561e+00
## 812   1  800 1.123658e+00
## 813   1  801 1.056302e+00
## 814   1  802 1.006221e+00
## 815   1  803 9.639435e-01
## 816   1  804 9.258197e-01
## 817   1  805 8.942614e-01
## 818   1  806 8.642227e-01
## 819   1  807 8.383880e-01
## 820   1  808 8.118241e-01
## 821   1  809 7.876718e-01
## 822   1  810 7.649784e-01
## 823   1  811 7.424564e-01
## 824   1  812 7.201770e-01
## 825   1  813 6.994566e-01
## 826   1  814 6.783791e-01
## 827   1  815 6.584888e-01
## 828   1  816 6.409079e-01
## 829   1  817 3.682409e+00
## 830   1  818 2.838817e+00
## 831   1  819 2.204971e+00
## 832   1  820 1.793930e+00
## 833   1  821 1.523700e+00
## 834   1  822 1.338680e+00
## 835   1  823 1.211957e+00
```

```
## 836     1  824  1.125301e+00
## 837     1  825  1.055408e+00
## 838     1  826  1.003647e+00
## 839     1  827  9.614019e-01
## 840     1  828  9.238227e-01
## 841     1  829  8.929318e-01
## 842     1  830  8.658088e-01
## 843     1  831  8.374595e-01
## 844     1  832  8.113759e-01
## 845     1  833  7.867321e-01
## 846     1  834  7.640231e-01
## 847     1  835  7.418927e-01
## 848     1  836  7.188198e-01
## 849     1  837  6.996196e-01
## 850     1  838  6.783182e-01
## 851     1  839  6.579610e-01
## 852     1  840  6.390958e-01
## 853     1  841  3.680553e+00
## 854     1  842  2.840404e+00
## 855     1  843  2.206615e+00
## 856     1  844  1.794862e+00
## 857     1  845  1.521474e+00
## 858     1  846  1.340809e+00
## 859     1  847  1.212670e+00
## 860     1  848  1.121838e+00
## 861     1  849  1.055634e+00
## 862     1  850  1.004731e+00
## 863     1  851  9.605784e-01
## 864     1  852  9.232656e-01
## 865     1  853  8.930046e-01
## 866     1  854  8.641963e-01
## 867     1  855  8.369208e-01
## 868     1  856  8.120859e-01
## 869     1  857  7.849936e-01
## 870     1  858  7.633121e-01
## 871     1  859  7.408381e-01
## 872     1  860  7.207317e-01
## 873     1  861  6.986622e-01
## 874     1  862  6.787559e-01
## 875     1  863  6.581229e-01
## 876     1  864  6.396648e-01
## 877     1  865  3.688637e+00
## 878     1  866  2.843223e+00
## 879     1  867  2.205322e+00
## 880     1  868  1.792025e+00
## 881     1  869  1.521672e+00
## 882     1  870  1.340413e+00
## 883     1  871  1.213954e+00
## 884     1  872  1.123345e+00
## 885     1  873  1.054483e+00
## 886     1  874  1.004245e+00
## 887     1  875  9.620994e-01
## 888     1  876  9.243307e-01
## 889     1  877  8.924426e-01
## 890     1  878  8.638823e-01
## 891     1  879  8.368480e-01
## 892     1  880  8.126504e-01
## 893     1  881  7.872913e-01
## 894     1  882  7.638532e-01
## 895     1  883  7.412948e-01
## 896     1  884  7.193917e-01
## 897     1  885  6.982325e-01
## 898     1  886  6.779427e-01
## 899     1  887  6.590322e-01
## 900     1  888  6.405128e-01
## 901     1  889  3.683453e+00
## 902     1  890  2.836099e+00
## 903     1  891  2.202738e+00
## 904     1  892  1.790213e+00
## 905     1  893  1.518915e+00
## 906     1  894  1.338360e+00
## 907     1  895  1.214112e+00
## 908     1  896  1.124728e+00
## 909     1  897  1.055843e+00
## 910     1  898  1.004104e+00
## 911     1  899  9.613491e-01
## 912     1  900  9.253753e-01
## 913     1  901  8.936300e-01
## 914     1  902  8.646801e-01
## 915     1  903  8.355419e-01
## 916     1  904  8.118930e-01
## 917     1  905  7.859979e-01
## 918     1  906  7.647125e-01
## 919     1  907  7.415453e-01
## 920     1  908  7.184362e-01
## 921     1  909  6.987460e-01
## 922     1  910  6.782850e-01
## 923     1  911  6.589082e-01
## 924     1  912  6.403467e-01
## 925     1  913  3.693230e+00
## 926     1  914  2.839352e+00
## 927     1  915  2.208303e+00
## 928     1  916  1.791433e+00
## 929     1  917  1.520040e+00
## 930     1  918  1.338126e+00
## 931     1  919  1.212772e+00
## 932     1  920  1.122196e+00
## 933     1  921  1.058228e+00
## 934     1  922  1.003742e+00
## 935     1  923  9.617471e-01
## 936     1  924  9.249852e-01
## 937     1  925  8.928743e-01
## 938     1  926  8.645994e-01
## 939     1  927  8.349936e-01
## 940     1  928  8.104663e-01
## 941     1  929  7.865069e-01
## 942     1  930  7.640738e-01
## 943     1  931  7.408752e-01
## 944     1  932  7.190971e-01
## 945     1  933  6.978068e-01
## 946     1  934  6.785672e-01
## 947     1  935  6.586213e-01
## 948     1  936  6.397984e-01
## 949     1  936  6.390207e-01
## 950     1  937  3.691259e+00
## 951     1  938  2.836837e+00
## 952     1  939  2.205554e+00
## 953     1  940  1.791739e+00
## 954     1  941  1.523199e+00
## 955     1  942  1.339100e+00
## 956     1  943  1.212106e+00
## 957     1  944  1.123604e+00
## 958     1  945  1.058395e+00
## 959     1  946  1.005450e+00
## 960     1  947  9.613261e-01
## 961     1  948  9.242309e-01
## 962     1  949  8.939049e-01
## 963     1  950  8.637816e-01
## 964     1  951  8.350963e-01
## 965     1  952  8.113322e-01
## 966     1  953  7.872538e-01
## 967     1  954  7.636137e-01
## 968     1  955  7.417298e-01
## 969     1  956  7.193412e-01
## 970     1  957  6.988264e-01
## 971     1  958  6.777677e-01
## 972     1  959  6.578795e-01
## 973     1  960  6.392045e-01
## 974     1  960  6.398408e-01
## 975     1  961  6.211767e-01
## 976     1  962  6.023590e-01
## 977     1  963  5.860786e-01
## 978     1  964  5.683468e-01
## 979     1  965  5.509920e-01
## 980     1  966  5.356581e-01
## 981     1  967  5.191124e-01
## 982     1  968  5.051901e-01
## 983     1  969  4.895905e-01
## 984     1  969  4.905633e-01
## 985     1  970  3.531993e+00
## 986     1  971  2.696792e+00
## 987     1  972  2.067326e+00
## 988     1  973  1.657388e+00
## 989     1  974  1.389544e+00
## 990     1  975  1.214221e+00
## 991     1  976  1.091088e+00
## 992     1  977  1.003758e+00
## 993     1  978  9.427093e-01
## 994     1  979  8.938107e-01
## 995     1  980  8.556441e-01
## 996     1  981  8.185224e-01
## 997     1  982  7.919787e-01
## 998     1  983  7.656701e-01
## 999     1  984  7.401855e-01
## 1000    1  984  7.398737e-01
## 1001    1  985  3.781936e+00
## 1002    1  986  2.932627e+00
## 1003    1  987  2.294654e+00
## 1004    1  988  1.879856e+00
## 1005    1  989  1.606017e+00
## 1006    1  990  1.420170e+00
## 1007    1  991  1.294725e+00
## 1008    1  992  1.200570e+00
## 1009    1  993  1.129966e+00
## 1010    1  994  1.076443e+00
## 1011    1  995  1.031454e+00
## 1012    1  996  9.931650e-01
## 1013    1  997  9.599601e-01
## 1014    1  998  9.285281e-01
## 1015    1  999  9.002885e-01
## 1016    1 1000  8.731801e-01
## 1017    1 1001  8.460237e-01
## 1018    1 1002  8.202010e-01
## 1019    1 1003  7.986777e-01
## 1020    1 1004  7.738731e-01
## 1021    1 1005  7.523991e-01
## 1022    1 1006  7.299324e-01
## 1023    1 1007  7.077046e-01
## 1024    1 1008  6.869500e-01
## 1025    1 1009  3.728556e+00
## 1026    1 1010  2.880778e+00
## 1027    1 1011  2.249107e+00
## 1028    1 1012  1.834029e+00
## 1029    1 1013  1.559655e+00
## 1030    1 1014  1.378892e+00
## 1031    1 1015  1.253731e+00
## 1032    1 1016  1.162599e+00
## 1033    1 1017  1.094619e+00
## 1034    1 1018  1.038892e+00
## 1035    1 1019  9.963949e-01
## 1036    1 1020  9.570268e-01
## 1037    1 1021  9.256031e-01
## 1038    1 1022  8.953056e-01
## 1039    1 1023  8.684637e-01
## 1040    1 1024  8.415051e-01
## 1041    1 1025  8.166928e-01
## 1042    1 1026  7.924660e-01
## 1043    1 1027  7.689723e-01
## 1044    1 1028  7.465923e-01
## 1045    1 1029  7.236068e-01
## 1046    1 1030  7.041575e-01
## 1047    1 1031  6.827792e-01
## 1048    1 1032  6.634577e-01
## 1049    1 1033  3.700732e+00
## 1050    1 1034  2.854480e+00
## 1051    1 1035  2.225200e+00
## 1052    1 1036  1.811711e+00
## 1053    1 1037  1.539246e+00
## 1054    1 1038  1.361670e+00
## 1055    1 1039  1.232488e+00
## 1056    1 1040  1.139978e+00
## 1057    1 1041  1.074036e+00
## 1058    1 1042  1.024068e+00
## 1059    1 1043  9.777232e-01
## 1060    1 1044  9.419581e-01
## 1061    1 1045  9.077745e-01
## 1062    1 1046  8.795965e-01
## 1063    1 1047  8.530900e-01
## 1064    1 1048  8.254593e-01
## 1065    1 1049  8.010472e-01
## 1066    1 1050  7.765550e-01
## 1067    1 1051  7.548946e-01
## 1068    1 1052  7.313927e-01
## 1069    1 1053  7.120955e-01
## 1070    1 1054  6.906386e-01
## 1071    1 1055  6.704278e-01
## 1072    1 1056  6.503485e-01
## 1073    1 1057  3.698430e+00
## 1074    1 1058  2.843935e+00
## 1075    1 1059  2.213716e+00
## 1076    1 1060  1.807399e+00
## 1077    1 1061  1.530597e+00
## 1078    1 1062  1.348353e+00
## 1079    1 1063  1.219777e+00
## 1080    1 1064  1.130489e+00
## 1081    1 1065  1.065562e+00
## 1082    1 1066  1.012039e+00
## 1083    1 1067  9.695079e-01
## 1084    1 1068  9.348257e-01
## 1085    1 1069  9.003976e-01
## 1086    1 1070  8.720488e-01
## 1087    1 1071  8.438881e-01
## 1088    1 1072  8.188066e-01
## 1089    1 1073  7.943347e-01
## 1090    1 1074  7.703390e-01
## 1091    1 1075  7.473818e-01
## 1092    1 1076  7.266157e-01
## 1093    1 1077  7.060079e-01
## 1094    1 1078  6.850408e-01
## 1095    1 1079  6.639972e-01
## 1096    1 1080  6.459608e-01
## 1097    1 1081  3.689047e+00
## 1098    1 1082  2.833011e+00
## 1099    1 1083  2.210298e+00
## 1100    1 1084  1.797693e+00
## 1101    1 1085  1.524895e+00
## 1102    1 1086  1.342796e+00
## 1103    1 1087  1.218245e+00
## 1104    1 1088  1.127795e+00
## 1105    1 1089  1.060797e+00
## 1106    1 1090  1.009357e+00
## 1107    1 1091  9.664074e-01
## 1108    1 1092  9.295340e-01
## 1109    1 1093  8.951960e-01
## 1110    1 1094  8.655767e-01
## 1111    1 1095  8.413508e-01
## 1112    1 1096  8.133145e-01
## 1113    1 1097  7.904791e-01
## 1114    1 1098  7.680002e-01
## 1115    1 1099  7.438545e-01
## 1116    1 1100  7.232390e-01
## 1117    1 1101  7.023766e-01
## 1118    1 1102  6.816652e-01
## 1119    1 1103  6.615040e-01
## 1120    1 1104  6.421720e-01
## 1121    1 1105  3.689072e+00
## 1122    1 1106  2.843036e+00
## 1123    1 1107  2.205607e+00
## 1124    1 1108  1.798954e+00
## 1125    1 1109  1.525712e+00
## 1126    1 1110  1.342206e+00
## 1127    1 1111  1.216079e+00
## 1128    1 1112  1.126147e+00
## 1129    1 1113  1.058611e+00
## 1130    1 1114  1.007246e+00
## 1131    1 1115  9.645952e-01
## 1132    1 1116  9.274193e-01
## 1133    1 1117  8.942833e-01
## 1134    1 1118  8.657953e-01
## 1135    1 1119  8.394430e-01
## 1136    1 1120  8.127572e-01
## 1137    1 1121  7.891480e-01
## 1138    1 1122  7.644711e-01
## 1139    1 1123  7.427960e-01
## 1140    1 1124  7.200288e-01
## 1141    1 1125  7.001132e-01
## 1142    1 1126  6.800036e-01
## 1143    1 1127  6.595107e-01
## 1144    1 1128  6.407456e-01
## 1145    1 1129  3.681910e+00
## 1146    1 1130  2.838521e+00
## 1147    1 1131  2.204888e+00
## 1148    1 1132  1.792312e+00
## 1149    1 1133  1.522532e+00
## 1150    1 1134  1.338413e+00
## 1151    1 1135  1.211333e+00
## 1152    1 1136  1.124138e+00
## 1153    1 1137  1.059295e+00
## 1154    1 1138  1.006014e+00
## 1155    1 1139  9.633391e-01
## 1156    1 1140  9.255092e-01
## 1157    1 1141  8.946603e-01
## 1158    1 1142  8.652168e-01
## 1159    1 1143  8.388718e-01
## 1160    1 1144  8.124351e-01
## 1161    1 1145  7.874208e-01
## 1162    1 1146  7.652230e-01
## 1163    1 1147  7.410366e-01
## 1164    1 1148  7.199281e-01
## 1165    1 1149  6.985430e-01
## 1166    1 1150  6.781091e-01
## 1167    1 1151  6.587422e-01
## 1168    1 1152  6.393053e-01
## 1169    1 1153  3.685322e+00
## 1170    1 1154  2.842166e+00
## 1171    1 1155  2.203047e+00
## 1172    1 1156  1.794384e+00
## 1173    1 1157  1.523190e+00
## 1174    1 1158  1.337995e+00
## 1175    1 1159  1.213261e+00
## 1176    1 1160  1.122493e+00
## 1177    1 1161  1.056559e+00
## 1178    1 1162  1.006497e+00
## 1179    1 1163  9.619645e-01
## 1180    1 1164  9.265441e-01
## 1181    1 1165  8.928754e-01
## 1182    1 1166  8.634986e-01
## 1183    1 1167  8.369945e-01
## 1184    1 1168  8.107957e-01
## 1185    1 1169  7.879965e-01
## 1186    1 1170  7.642231e-01
## 1187    1 1171  7.410727e-01
## 1188    1 1172  7.182430e-01
## 1189    1 1173  6.997814e-01
## 1190    1 1174  6.772366e-01
## 1191    1 1175  6.576216e-01
## 1192    1 1176  6.405663e-01
## 1193    1 1177  3.682386e+00
## 1194    1 1178  2.834504e+00
## 1195    1 1179  2.204152e+00
## 1196    1 1180  1.793467e+00
## 1197    1 1181  1.520317e+00
## 1198    1 1182  1.338833e+00
## 1199    1 1183  1.213415e+00
## 1200    1 1184  1.125413e+00
## 1201    1 1185  1.056521e+00
## 1202    1 1186  1.003201e+00
## 1203    1 1187  9.597707e-01
## 1204    1 1188  9.237583e-01
## 1205    1 1189  8.941700e-01
## 1206    1 1190  8.640232e-01
## 1207    1 1191  8.366348e-01
## 1208    1 1192  8.114797e-01
## 1209    1 1193  7.873767e-01
## 1210    1 1194  7.643296e-01
## 1211    1 1195  7.400297e-01
## 1212    1 1196  7.206749e-01
## 1213    1 1197  6.970185e-01
## 1214    1 1198  6.775000e-01
## 1215    1 1199  6.586890e-01
## 1216    1 1200  6.394920e-01
## 1217    1 1201  3.678915e+00
## 1218    1 1202  2.833670e+00
## 1219    1 1203  2.207598e+00
## 1220    1 1204  1.792555e+00
## 1221    1 1205  1.520883e+00
## 1222    1 1206  1.339711e+00
## 1223    1 1207  1.214388e+00
## 1224    1 1208  1.125165e+00
## 1225    1 1209  1.056645e+00
## 1226    1 1210  1.003743e+00
## 1227    1 1211  9.628616e-01
## 1228    1 1212  9.266991e-01
## 1229    1 1213  8.936353e-01
## 1230    1 1214  8.635897e-01
## 1231    1 1215  8.356055e-01
## 1232    1 1216  8.107431e-01
## 1233    1 1217  7.872287e-01
## 1234    1 1218  7.637639e-01
## 1235    1 1219  7.417532e-01
## 1236    1 1220  7.183369e-01
## 1237    1 1221  6.985287e-01
## 1238    1 1222  6.773711e-01
## 1239    1 1223  6.587335e-01
## 1240    1 1224  6.393715e-01
## 1241    1 1225  3.688271e+00
## 1242    1 1226  2.837664e+00
## 1243    1 1227  2.204507e+00
## 1244    1 1228  1.790370e+00
## 1245    1 1229  1.521013e+00
## 1246    1 1230  1.337874e+00
## 1247    1 1231  1.213798e+00
## 1248    1 1232  1.121746e+00
## 1249    1 1233  1.055642e+00
## 1250    1 1234  1.004318e+00
## 1251    1 1235  9.611968e-01
## 1252    1 1236  9.235579e-01
## 1253    1 1237  8.929067e-01
## 1254    1 1238  8.629131e-01
## 1255    1 1239  8.361336e-01
## 1256    1 1240  8.102713e-01
## 1257    1 1241  7.860126e-01
## 1258    1 1242  7.635363e-01
## 1259    1 1243  7.413946e-01
## 1260    1 1244  7.192652e-01
## 1261    1 1245  6.987897e-01
## 1262    1 1246  6.785484e-01
## 1263    1 1247  6.583163e-01
## 1264    1 1248  6.397891e-01
## 1265    1 1248  6.384147e-01
## 1266    1 1249  3.686639e+00
## 1267    1 1250  2.836142e+00
## 1268    1 1251  2.206956e+00
## 1269    1 1252  1.792370e+00
## 1270    1 1253  1.519324e+00
## 1271    1 1254  1.341440e+00
## 1272    1 1255  1.215749e+00
## 1273    1 1256  1.122052e+00
## 1274    1 1257  1.057256e+00
## 1275    1 1258  1.004678e+00
## 1276    1 1259  9.626891e-01
## 1277    1 1260  9.265176e-01
## 1278    1 1261  8.929022e-01
## 1279    1 1262  8.634082e-01
## 1280    1 1263  8.360769e-01
## 1281    1 1264  8.108355e-01
## 1282    1 1265  7.866409e-01
## 1283    1 1266  7.628572e-01
## 1284    1 1267  7.415460e-01
## 1285    1 1268  7.194876e-01
## 1286    1 1269  6.982769e-01
## 1287    1 1270  6.787546e-01
## 1288    1 1271  6.587558e-01
## 1289    1 1272  6.394158e-01
## 1290    1 1272  6.392272e-01
## 1291    1 1273  6.217765e-01
## 1292    1 1274  6.029824e-01
## 1293    1 1275  5.853759e-01
## 1294    1 1276  5.687568e-01
## 1295    1 1277  5.519710e-01
## 1296    1 1278  5.360730e-01
## 1297    1 1279  5.204445e-01
## 1298    1 1280  5.054818e-01
## 1299    1 1281  4.905393e-01
## 1300    1 1282  4.766759e-01
## 1301    1 1283  4.624535e-01
## 1302    1 1284  4.494216e-01
## 1303    1 1284  4.490625e-01
## 1304    1 1285  3.496691e+00
## 1305    1 1286  2.653578e+00
## 1306    1 1287  2.029538e+00
## 1307    1 1288  1.625972e+00
## 1308    1 1289  1.354868e+00
## 1309    1 1290  1.180857e+00
## 1310    1 1291  1.059509e+00
## 1311    1 1292  9.725669e-01
## 1312    1 1293  9.098984e-01
## 1313    1 1294  8.612287e-01
## 1314    1 1295  8.235184e-01
## 1315    1 1296  7.917491e-01
## 1316    1 1296  7.913419e-01
## 1317    1 1297  3.833346e+00
## 1318    1 1298  2.970895e+00
## 1319    1 1299  2.333417e+00
## 1320    1 1300  1.913838e+00
## 1321    1 1301  1.639673e+00
## 1322    1 1302  1.454345e+00
## 1323    1 1303  1.325167e+00
## 1324    1 1304  1.231075e+00
## 1325    1 1305  1.161280e+00
## 1326    1 1306  1.109001e+00
## 1327    1 1307  1.059994e+00
## 1328    1 1308  1.023492e+00
## 1329    1 1309  9.894961e-01
## 1330    1 1310  9.542683e-01
## 1331    1 1311  9.235741e-01
## 1332    1 1312  8.953990e-01
## 1333    1 1313  8.699601e-01
## 1334    1 1314  8.430702e-01
## 1335    1 1315  8.180579e-01
## 1336    1 1316  7.952117e-01
## 1337    1 1317  7.708232e-01
## 1338    1 1318  7.509442e-01
## 1339    1 1319  7.276459e-01
## 1340    1 1320  7.072696e-01
## 1341    1 1321  3.749319e+00
## 1342    1 1322  2.897976e+00
## 1343    1 1323  2.267274e+00
## 1344    1 1324  1.855860e+00
## 1345    1 1325  1.578386e+00
## 1346    1 1326  1.395978e+00
## 1347    1 1327  1.265994e+00
## 1348    1 1328  1.176205e+00
## 1349    1 1329  1.106962e+00
## 1350    1 1330  1.054426e+00
## 1351    1 1331  1.012061e+00
## 1352    1 1332  9.722167e-01
## 1353    1 1333  9.397556e-01
## 1354    1 1334  9.086051e-01
## 1355    1 1335  8.798071e-01
## 1356    1 1336  8.530410e-01
## 1357    1 1337  8.272283e-01
## 1358    1 1338  8.032195e-01
## 1359    1 1339  7.797722e-01
## 1360    1 1340  7.568404e-01
## 1361    1 1341  7.350310e-01
## 1362    1 1342  7.134222e-01
## 1363    1 1343  6.926333e-01
## 1364    1 1344  6.723644e-01
## 1365    1 1345  3.717057e+00
## 1366    1 1346  2.863343e+00
## 1367    1 1347  2.232789e+00
## 1368    1 1348  1.820018e+00
## 1369    1 1349  1.549004e+00
## 1370    1 1350  1.366551e+00
## 1371    1 1351  1.238906e+00
## 1372    1 1352  1.147368e+00
## 1373    1 1353  1.083391e+00
## 1374    1 1354  1.031414e+00
## 1375    1 1355  9.859311e-01
## 1376    1 1356  9.493010e-01
## 1377    1 1357  9.140482e-01
## 1378    1 1358  8.852270e-01
## 1379    1 1359  8.591323e-01
## 1380    1 1360  8.308595e-01
## 1381    1 1361  8.077366e-01
## 1382    1 1362  7.827641e-01
## 1383    1 1363  7.597746e-01
## 1384    1 1364  7.387514e-01
## 1385    1 1365  7.150936e-01
## 1386    1 1366  6.966750e-01
## 1387    1 1367  6.750476e-01
## 1388    1 1368  6.566416e-01
## 1389    1 1369  3.701505e+00
## 1390    1 1370  2.852331e+00
## 1391    1 1371  2.222852e+00
## 1392    1 1372  1.807346e+00
## 1393    1 1373  1.535931e+00
## 1394    1 1374  1.351272e+00
## 1395    1 1375  1.225705e+00
## 1396    1 1376  1.137052e+00
## 1397    1 1377  1.069506e+00
## 1398    1 1378  1.015188e+00
## 1399    1 1379  9.744239e-01
## 1400    1 1380  9.376428e-01
## 1401    1 1381  9.058466e-01
## 1402    1 1382  8.752977e-01
## 1403    1 1383  8.473111e-01
## 1404    1 1384  8.211514e-01
## 1405    1 1385  7.972141e-01
## 1406    1 1386  7.739721e-01
## 1407    1 1387  7.496379e-01
## 1408    1 1388  7.278330e-01
## 1409    1 1389  7.074074e-01
## 1410    1 1390  6.861676e-01
## 1411    1 1391  6.670514e-01
## 1412    1 1392  6.476576e-01
## 1413    1 1393  3.691191e+00
## 1414    1 1394  2.843697e+00
## 1415    1 1395  2.209026e+00
## 1416    1 1396  1.800896e+00
## 1417    1 1397  1.527120e+00
## 1418    1 1398  1.344813e+00
## 1419    1 1399  1.220516e+00
## 1420    1 1400  1.128797e+00
## 1421    1 1401  1.062600e+00
## 1422    1 1402  1.011256e+00
## 1423    1 1403  9.682287e-01
## 1424    1 1404  9.305607e-01
## 1425    1 1405  8.981742e-01
## 1426    1 1406  8.689551e-01
## 1427    1 1407  8.413994e-01
## 1428    1 1408  8.157793e-01
## 1429    1 1409  7.917897e-01
## 1430    1 1410  7.682252e-01
## 1431    1 1411  7.454662e-01
## 1432    1 1412  7.246845e-01
## 1433    1 1413  7.027132e-01
## 1434    1 1414  6.814999e-01
## 1435    1 1415  6.633301e-01
## 1436    1 1416  6.431085e-01
## 1437    1 1417  3.686421e+00
## 1438    1 1418  2.840342e+00
## 1439    1 1419  2.206977e+00
## 1440    1 1420  1.794811e+00
## 1441    1 1421  1.523017e+00
## 1442    1 1422  1.342174e+00
## 1443    1 1423  1.218249e+00
## 1444    1 1424  1.126750e+00
## 1445    1 1425  1.060025e+00
## 1446    1 1426  1.006874e+00
## 1447    1 1427  9.646405e-01
## 1448    1 1428  9.263798e-01
## 1449    1 1429  8.958193e-01
## 1450    1 1430  8.659920e-01
## 1451    1 1431  8.383093e-01
## 1452    1 1432  8.131686e-01
## 1453    1 1433  7.872363e-01
## 1454    1 1434  7.657338e-01
## 1455    1 1435  7.438105e-01
## 1456    1 1436  7.212307e-01
## 1457    1 1437  7.002888e-01
## 1458    1 1438  6.790271e-01
## 1459    1 1439  6.613573e-01
## 1460    1 1440  6.408976e-01
## 1461    1 1441  3.686595e+00
## 1462    1 1442  2.839952e+00
## 1463    1 1443  2.208138e+00
## 1464    1 1444  1.796679e+00
## 1465    1 1445  1.523738e+00
## 1466    1 1446  1.340905e+00
## 1467    1 1447  1.213516e+00
## 1468    1 1448  1.123912e+00
## 1469    1 1449  1.054469e+00
## 1470    1 1450  1.004679e+00
## 1471    1 1451  9.623135e-01
## 1472    1 1452  9.263372e-01
## 1473    1 1453  8.958411e-01
## 1474    1 1454  8.649047e-01
## 1475    1 1455  8.367184e-01
## 1476    1 1456  8.127027e-01
## 1477    1 1457  7.896060e-01
## 1478    1 1458  7.639538e-01
## 1479    1 1459  7.415476e-01
## 1480    1 1460  7.208402e-01
## 1481    1 1461  6.991282e-01
## 1482    1 1462  6.795782e-01
## 1483    1 1463  6.587851e-01
## 1484    1 1464  6.410840e-01
## 1485    1 1465  3.683087e+00
## 1486    1 1466  2.840448e+00
## 1487    1 1467  2.207696e+00
## 1488    1 1468  1.793364e+00
## 1489    1 1469  1.521476e+00
## 1490    1 1470  1.340893e+00
## 1491    1 1471  1.214210e+00
## 1492    1 1472  1.123295e+00
## 1493    1 1473  1.057307e+00
## 1494    1 1474  1.002702e+00
## 1495    1 1475  9.600521e-01
## 1496    1 1476  9.253939e-01
## 1497    1 1477  8.944778e-01
## 1498    1 1478  8.658919e-01
## 1499    1 1479  8.374764e-01
## 1500    1 1480  8.112388e-01
## 1501    1 1481  7.868629e-01
## 1502    1 1482  7.630589e-01
## 1503    1 1483  7.405981e-01
## 1504    1 1484  7.205068e-01
## 1505    1 1485  6.977287e-01
## 1506    1 1486  6.796214e-01
## 1507    1 1487  6.594668e-01
## 1508    1 1488  6.397333e-01
## 1509    1 1489  3.680595e+00
## 1510    1 1490  2.837531e+00
## 1511    1 1491  2.204473e+00
## 1512    1 1492  1.790344e+00
## 1513    1 1493  1.520322e+00
## 1514    1 1494  1.340637e+00
## 1515    1 1495  1.211354e+00
## 1516    1 1496  1.124230e+00
## 1517    1 1497  1.056156e+00
## 1518    1 1498  1.004335e+00
## 1519    1 1499  9.611739e-01
## 1520    1 1500  9.257284e-01
## 1521    1 1501  8.943497e-01
## 1522    1 1502  8.639211e-01
## 1523    1 1503  8.371301e-01
## 1524    1 1504  8.123498e-01
## 1525    1 1505  7.856161e-01
## 1526    1 1506  7.634523e-01
## 1527    1 1507  7.420712e-01
## 1528    1 1508  7.186739e-01
## 1529    1 1509  6.991674e-01
## 1530    1 1510  6.783598e-01
## 1531    1 1511  6.578581e-01
## 1532    1 1512  6.393514e-01
## 1533    1 1513  3.679658e+00
## 1534    1 1514  2.840671e+00
## 1535    1 1515  2.200692e+00
## 1536    1 1516  1.789669e+00
## 1537    1 1517  1.521145e+00
## 1538    1 1518  1.338691e+00
## 1539    1 1519  1.214044e+00
## 1540    1 1520  1.123026e+00
## 1541    1 1521  1.057545e+00
## 1542    1 1522  1.003257e+00
## 1543    1 1523  9.597291e-01
## 1544    1 1524  9.254817e-01
## 1545    1 1525  8.935927e-01
## 1546    1 1526  8.646150e-01
## 1547    1 1527  8.347976e-01
## 1548    1 1528  8.104186e-01
## 1549    1 1529  7.871785e-01
## 1550    1 1530  7.618467e-01
## 1551    1 1531  7.403311e-01
## 1552    1 1532  7.192307e-01
## 1553    1 1533  6.976184e-01
## 1554    1 1534  6.786278e-01
## 1555    1 1535  6.575098e-01
## 1556    1 1536  6.387229e-01
## 1557    1 1537  3.674035e+00
## 1558    1 1538  2.834546e+00
## 1559    1 1539  2.204648e+00
## 1560    1 1540  1.791503e+00
## 1561    1 1541  1.522156e+00
## 1562    1 1542  1.340848e+00
## 1563    1 1543  1.214051e+00
## 1564    1 1544  1.124459e+00
## 1565    1 1545  1.056434e+00
## 1566    1 1546  1.005312e+00
## 1567    1 1547  9.605157e-01
## 1568    1 1548  9.256833e-01
## 1569    1 1549  8.937316e-01
## 1570    1 1550  8.642064e-01
## 1571    1 1551  8.376806e-01
## 1572    1 1552  8.110334e-01
## 1573    1 1553  7.871863e-01
## 1574    1 1554  7.624688e-01
## 1575    1 1555  7.409965e-01
## 1576    1 1556  7.195751e-01
## 1577    1 1557  6.989660e-01
## 1578    1 1558  6.775283e-01
## 1579    1 1559  6.579912e-01
## 1580    1 1560  6.398525e-01
## 1581    1 1560  6.392010e-01
## 1582    1 1561  3.684619e+00
## 1583    1 1562  2.836086e+00
## 1584    1 1563  2.202564e+00
## 1585    1 1564  1.791325e+00
## 1586    1 1565  1.520643e+00
## 1587    1 1566  1.337613e+00
## 1588    1 1567  1.212850e+00
## 1589    1 1568  1.122757e+00
## 1590    1 1569  1.056722e+00
## 1591    1 1570  1.005632e+00
## 1592    1 1571  9.622828e-01
## 1593    1 1572  9.253337e-01
## 1594    1 1573  8.916494e-01
## 1595    1 1574  8.615726e-01
## 1596    1 1575  8.355858e-01
## 1597    1 1576  8.112733e-01
## 1598    1 1577  7.870219e-01
## 1599    1 1578  7.638994e-01
## 1600    1 1579  7.409730e-01
## 1601    1 1580  7.192567e-01
## 1602    1 1581  6.979544e-01
## 1603    1 1582  6.782347e-01
## 1604    1 1583  6.595216e-01
## 1605    1 1584  6.385911e-01
## 1606    1 1584  6.385653e-01
## 1607    1 1585  6.203312e-01
## 1608    1 1586  6.028979e-01
## 1609    1 1587  5.862485e-01
## 1610    1 1588  5.686766e-01
## 1611    1 1589  5.512872e-01
## 1612    1 1590  5.354254e-01
## 1613    1 1591  5.206250e-01
## 1614    1 1592  5.051636e-01
## 1615    1 1593  4.905563e-01
## 1616    1 1594  4.776306e-01
## 1617    1 1595  4.622425e-01
## 1618    1 1596  4.490793e-01
## 1619    1 1597  4.364937e-01
## 1620    1 1598  4.237279e-01
## 1621    1 1599  4.112232e-01
## 1622    1 1599  4.120597e-01
## 1623    1 1600  1.931201e+00
## 1624    1 1601  1.506152e+00
## 1625    1 1602  1.187096e+00
## 1626    1 1603  9.766705e-01
## 1627    1 1604  8.391244e-01
## 1628    1 1605  7.452750e-01
## 1629    1 1606  6.827986e-01
## 1630    1 1607  6.348955e-01
## 1631    1 1608  5.986960e-01
## 1632    1 1608  5.992783e-01
## 1633    1 1609  3.631976e+00
## 1634    1 1610  2.784966e+00
## 1635    1 1611  2.145188e+00
```

```
## 1636   1 1612  1.729042e+00
## 1637   1 1613  1.461717e+00
## 1638   1 1614  1.279215e+00
## 1639   1 1615  1.155430e+00
## 1640   1 1616  1.066856e+00
## 1641   1 1617  1.001210e+00
## 1642   1 1618  9.515272e-01
## 1643   1 1619  9.094931e-01
## 1644   1 1620  8.737540e-01
## 1645   1 1621  8.448088e-01
## 1646   1 1622  8.159561e-01
## 1647   1 1623  7.904060e-01
## 1648   1 1624  7.652623e-01
## 1649   1 1625  7.426112e-01
## 1650   1 1626  7.199521e-01
## 1651   1 1627  6.998546e-01
## 1652   1 1628  6.790184e-01
## 1653   1 1629  6.582027e-01
## 1654   1 1630  6.408409e-01
## 1655   1 1631  6.218751e-01
## 1656   1 1632  6.034751e-01
## 1657   1 1633  3.650919e+00
## 1658   1 1634  2.804214e+00
## 1659   1 1635  2.172727e+00
## 1660   1 1636  1.761211e+00
## 1661   1 1637  1.489801e+00
## 1662   1 1638  1.308363e+00
## 1663   1 1639  1.182406e+00
## 1664   1 1640  1.094718e+00
## 1665   1 1641  1.027448e+00
## 1666   1 1642  9.791875e-01
## 1667   1 1643  9.356868e-01
## 1668   1 1644  8.993716e-01
## 1669   1 1645  8.679808e-01
## 1670   1 1646  8.397782e-01
## 1671   1 1647  8.132358e-01
## 1672   1 1648  7.888600e-01
## 1673   1 1649  7.639075e-01
## 1674   1 1650  7.421479e-01
## 1675   1 1651  7.210517e-01
## 1676   1 1652  6.981496e-01
## 1677   1 1653  6.788947e-01
## 1678   1 1654  6.589676e-01
## 1679   1 1655  6.396599e-01
## 1680   1 1656  6.222010e-01
## 1681   1 1657  3.667908e+00
## 1682   1 1658  2.824066e+00
## 1683   1 1659  2.189246e+00
## 1684   1 1660  1.775374e+00
## 1685   1 1661  1.503815e+00
## 1686   1 1662  1.325764e+00
## 1687   1 1663  1.201429e+00
## 1688   1 1664  1.109094e+00
## 1689   1 1665  1.041383e+00
## 1690   1 1666  9.925856e-01
## 1691   1 1667  9.481694e-01
## 1692   1 1668  9.138059e-01
## 1693   1 1669  8.795000e-01
## 1694   1 1670  8.523105e-01
## 1695   1 1671  8.245952e-01
## 1696   1 1672  8.006061e-01
## 1697   1 1673  7.765119e-01
## 1698   1 1674  7.537830e-01
## 1699   1 1675  7.308442e-01
## 1700   1 1676  7.096089e-01
## 1701   1 1677  6.896489e-01
## 1702   1 1678  6.700764e-01
## 1703   1 1679  6.493999e-01
## 1704   1 1680  6.307077e-01
## 1705   1 1681  3.676847e+00
## 1706   1 1682  2.829700e+00
## 1707   1 1683  2.195047e+00
## 1708   1 1684  1.785184e+00
## 1709   1 1685  1.514231e+00
## 1710   1 1686  1.334135e+00
## 1711   1 1687  1.207736e+00
## 1712   1 1688  1.115216e+00
## 1713   1 1689  1.048383e+00
## 1714   1 1690  9.977820e-01
## 1715   1 1691  9.554490e-01
## 1716   1 1692  9.188645e-01
## 1717   1 1693  8.863335e-01
## 1718   1 1694  8.590851e-01
## 1719   1 1695  8.316355e-01
## 1720   1 1696  8.061309e-01
## 1721   1 1697  7.817553e-01
## 1722   1 1698  7.583763e-01
## 1723   1 1699  7.362028e-01
## 1724   1 1700  7.140786e-01
## 1725   1 1701  6.940962e-01
## 1726   1 1702  6.728828e-01
## 1727   1 1703  6.537892e-01
## 1728   1 1704  6.352078e-01
## 1729   1 1705  3.677888e+00
## 1730   1 1706  2.837133e+00
## 1731   1 1707  2.202691e+00
## 1732   1 1708  1.791391e+00
## 1733   1 1709  1.516629e+00
## 1734   1 1710  1.335130e+00
## 1735   1 1711  1.209131e+00
## 1736   1 1712  1.120642e+00
## 1737   1 1713  1.052552e+00
## 1738   1 1714  9.985395e-01
## 1739   1 1715  9.568725e-01
## 1740   1 1716  9.221219e-01
## 1741   1 1717  8.898979e-01
## 1742   1 1718  8.598528e-01
## 1743   1 1719  8.325993e-01
## 1744   1 1720  8.083734e-01
## 1745   1 1721  7.841140e-01
## 1746   1 1722  7.611870e-01
## 1747   1 1723  7.379119e-01
## 1748   1 1724  7.173339e-01
## 1749   1 1725  6.956437e-01
## 1750   1 1726  6.750833e-01
## 1751   1 1727  6.562523e-01
## 1752   1 1728  6.370329e-01
## 1753   1 1729  3.677633e+00
## 1754   1 1730  2.835853e+00
## 1755   1 1731  2.202514e+00
## 1756   1 1732  1.789503e+00
## 1757   1 1733  1.518987e+00
## 1758   1 1734  1.334955e+00
## 1759   1 1735  1.212846e+00
## 1760   1 1736  1.119853e+00
## 1761   1 1737  1.055865e+00
## 1762   1 1738  1.001354e+00
## 1763   1 1739  9.613944e-01
## 1764   1 1740  9.234622e-01
## 1765   1 1741  8.914314e-01
## 1766   1 1742  8.620305e-01
## 1767   1 1743  8.352414e-01
## 1768   1 1744  8.082292e-01
## 1769   1 1745  7.853774e-01
## 1770   1 1746  7.629489e-01
## 1771   1 1747  7.395997e-01
## 1772   1 1748  7.177144e-01
## 1773   1 1749  6.969222e-01
## 1774   1 1750  6.768568e-01
## 1775   1 1751  6.570588e-01
## 1776   1 1752  6.370747e-01
## 1777   1 1753  3.691832e+00
## 1778   1 1754  2.837231e+00
## 1779   1 1755  2.204208e+00
## 1780   1 1756  1.793575e+00
## 1781   1 1757  1.521665e+00
## 1782   1 1758  1.335834e+00
## 1783   1 1759  1.209794e+00
## 1784   1 1760  1.120636e+00
## 1785   1 1761  1.055279e+00
## 1786   1 1762  1.003854e+00
## 1787   1 1763  9.613026e-01
## 1788   1 1764  9.247065e-01
## 1789   1 1765  8.934951e-01
## 1790   1 1766  8.634907e-01
## 1791   1 1767  8.361360e-01
## 1792   1 1768  8.107064e-01
## 1793   1 1769  7.859048e-01
## 1794   1 1770  7.637925e-01
## 1795   1 1771  7.397619e-01
## 1796   1 1772  7.196350e-01
## 1797   1 1773  6.972770e-01
## 1798   1 1774  6.782419e-01
## 1799   1 1775  6.588156e-01
## 1800   1 1776  6.385520e-01
## 1801   1 1777  3.686117e+00
## 1802   1 1778  2.840728e+00
## 1803   1 1779  2.204275e+00
## 1804   1 1780  1.790990e+00
## 1805   1 1781  1.523720e+00
## 1806   1 1782  1.336665e+00
## 1807   1 1783  1.212188e+00
## 1808   1 1784  1.122776e+00
## 1809   1 1785  1.055618e+00
## 1810   1 1786  1.004098e+00
## 1811   1 1787  9.613359e-01
## 1812   1 1788  9.242072e-01
## 1813   1 1789  8.909815e-01
## 1814   1 1790  8.640070e-01
## 1815   1 1791  8.363944e-01
## 1816   1 1792  8.120374e-01
## 1817   1 1793  7.862586e-01
## 1818   1 1794  7.632241e-01
## 1819   1 1795  7.411317e-01
## 1820   1 1796  7.201534e-01
## 1821   1 1797  6.980317e-01
## 1822   1 1798  6.781651e-01
## 1823   1 1799  6.572201e-01
## 1824   1 1800  6.383062e-01
## 1825   1 1801  3.686729e+00
## 1826   1 1802  2.837977e+00
## 1827   1 1803  2.201774e+00
## 1828   1 1804  1.790640e+00
## 1829   1 1805  1.523228e+00
## 1830   1 1806  1.336246e+00
## 1831   1 1807  1.211045e+00
## 1832   1 1808  1.124728e+00
## 1833   1 1809  1.056175e+00
## 1834   1 1810  1.003990e+00
## 1835   1 1811  9.612849e-01
## 1836   1 1812  9.253495e-01
## 1837   1 1813  8.930281e-01
## 1838   1 1814  8.625738e-01
## 1839   1 1815  8.375758e-01
## 1840   1 1816  8.107924e-01
## 1841   1 1817  7.873751e-01
## 1842   1 1818  7.636550e-01
## 1843   1 1819  7.411405e-01
## 1844   1 1820  7.192910e-01
## 1845   1 1821  6.977524e-01
## 1846   1 1822  6.774739e-01
## 1847   1 1823  6.580837e-01
## 1848   1 1824  6.384982e-01
## 1849   1 1825  3.683952e+00
## 1850   1 1826  2.839952e+00
## 1851   1 1827  2.203106e+00
## 1852   1 1828  1.791784e+00
## 1853   1 1829  1.521296e+00
## 1854   1 1830  1.338700e+00
## 1855   1 1831  1.213650e+00
## 1856   1 1832  1.120863e+00
## 1857   1 1833  1.053199e+00
## 1858   1 1834  1.005076e+00
## 1859   1 1835  9.612835e-01
## 1860   1 1836  9.260922e-01
## 1861   1 1837  8.919829e-01
## 1862   1 1838  8.634629e-01
## 1863   1 1839  8.357770e-01
## 1864   1 1840  8.113284e-01
## 1865   1 1841  7.890554e-01
## 1866   1 1842  7.637531e-01
## 1867   1 1843  7.410867e-01
## 1868   1 1844  7.195236e-01
## 1869   1 1845  6.979036e-01
## 1870   1 1846  6.785695e-01
## 1871   1 1847  6.577625e-01
## 1872   1 1848  6.390818e-01
## 1873   1 1849  3.686593e+00
## 1874   1 1850  2.837192e+00
## 1875   1 1851  2.207063e+00
## 1876   1 1852  1.791125e+00
## 1877   1 1853  1.521141e+00
## 1878   1 1854  1.340020e+00
## 1879   1 1855  1.211652e+00
## 1880   1 1856  1.123954e+00
## 1881   1 1857  1.056034e+00
## 1882   1 1858  1.003082e+00
## 1883   1 1859  9.614774e-01
## 1884   1 1860  9.261099e-01
## 1885   1 1861  8.916591e-01
## 1886   1 1862  8.639415e-01
## 1887   1 1863  8.381206e-01
## 1888   1 1864  8.111864e-01
## 1889   1 1865  7.870146e-01
## 1890   1 1866  7.634976e-01
## 1891   1 1867  7.405933e-01
## 1892   1 1868  7.184374e-01
## 1893   1 1869  6.974404e-01
## 1894   1 1870  6.786042e-01
## 1895   1 1871  6.596425e-01
## 1896   1 1872  6.380589e-01
## 1897   1 1872  6.399038e-01
## 1898   1 1873  3.682886e+00
## 1899   1 1874  2.840214e+00
## 1900   1 1875  2.204458e+00
## 1901   1 1876  1.788598e+00
## 1902   1 1877  1.519193e+00
## 1903   1 1878  1.339640e+00
## 1904   1 1879  1.215136e+00
## 1905   1 1880  1.123545e+00
## 1906   1 1881  1.055160e+00
## 1907   1 1882  1.005694e+00
## 1908   1 1883  9.620183e-01
## 1909   1 1884  9.266776e-01
## 1910   1 1885  8.920600e-01
## 1911   1 1886  8.629384e-01
## 1912   1 1887  8.372360e-01
## 1913   1 1888  8.109211e-01
## 1914   1 1889  7.864125e-01
## 1915   1 1890  7.637668e-01
## 1916   1 1891  7.404855e-01
## 1917   1 1892  7.201370e-01
## 1918   1 1893  6.992835e-01
## 1919   1 1894  6.780967e-01
## 1920   1 1895  6.588866e-01
## 1921   1 1896  6.396214e-01
## 1922   1 1896  6.394658e-01
## 1923   1 1897  6.207678e-01
## 1924   1 1898  6.019145e-01
## 1925   1 1899  5.854232e-01
## 1926   1 1900  5.679048e-01
## 1927   1 1901  5.514627e-01
## 1928   1 1902  5.357380e-01
## 1929   1 1903  5.203678e-01
## 1930   1 1904  5.056805e-01
## 1931   1 1905  4.901337e-01
## 1932   1 1906  4.766889e-01
## 1933   1 1907  4.623390e-01
## 1934   1 1908  4.484042e-01
## 1935   1 1909  4.366878e-01
## 1936   1 1910  4.243939e-01
## 1937   1 1911  4.113791e-01
## 1938   1 1911  4.109973e-01
## 1939   1 1912  3.467370e+00
## 1940   1 1913  2.621634e+00
## 1941   1 1914  1.996342e+00
## 1942   1 1915  1.591151e+00
## 1943   1 1916  1.322793e+00
## 1944   1 1917  1.149106e+00
## 1945   1 1918  1.028056e+00
## 1946   1 1919  9.426559e-01
## 1947   1 1920  8.804215e-01
## 1948   1 1920  8.803947e-01
## 1949   1 1921  3.896294e+00
## 1950   1 1922  3.035482e+00
## 1951   1 1923  2.389200e+00
## 1952   1 1924  1.957708e+00
## 1953   1 1925  1.680758e+00
## 1954   1 1926  1.491996e+00
## 1955   1 1927  1.362689e+00
## 1956   1 1928  1.265319e+00
## 1957   1 1929  1.193291e+00
## 1958   1 1930  1.136547e+00
## 1959   1 1931  1.090017e+00
## 1960   1 1932  1.049745e+00
## 1961   1 1933  1.016601e+00
## 1962   1 1934  9.823381e-01
## 1963   1 1935  9.533027e-01
## 1964   1 1936  9.227825e-01
## 1965   1 1937  8.948497e-01
## 1966   1 1938  8.693259e-01
## 1967   1 1939  8.438152e-01
## 1968   1 1940  8.191396e-01
## 1969   1 1941  7.962736e-01
## 1970   1 1942  7.713426e-01
## 1971   1 1943  7.497109e-01
## 1972   1 1944  7.274982e-01
## 1973   1 1945  3.772075e+00
## 1974   1 1946  2.924742e+00
## 1975   1 1947  2.286116e+00
## 1976   1 1948  1.872244e+00
## 1977   1 1949  1.595728e+00
## 1978   1 1950  1.409413e+00
## 1979   1 1951  1.285078e+00
## 1980   1 1952  1.193160e+00
## 1981   1 1953  1.122382e+00
## 1982   1 1954  1.070341e+00
## 1983   1 1955  1.025725e+00
## 1984   1 1956  9.859490e-01
## 1985   1 1957  9.552961e-01
## 1986   1 1958  9.206613e-01
## 1987   1 1959  8.945351e-01
## 1988   1 1960  8.655091e-01
## 1989   1 1961  8.398656e-01
## 1990   1 1962  8.153114e-01
## 1991   1 1963  7.918088e-01
## 1992   1 1964  7.692070e-01
## 1993   1 1965  7.462669e-01
## 1994   1 1966  7.249138e-01
## 1995   1 1967  7.033714e-01
## 1996   1 1968  6.832998e-01
## 1997   1 1969  3.729361e+00
## 1998   1 1970  2.879961e+00
## 1999   1 1971  2.241657e+00
## 2000   1 1972  1.831099e+00
## 2001   1 1973  1.557320e+00
## 2002   1 1974  1.373187e+00
## 2003   1 1975  1.248629e+00
## 2004   1 1976  1.158496e+00
## 2005   1 1977  1.089969e+00
## 2006   1 1978  1.035588e+00
## 2007   1 1979  9.926253e-01
## 2008   1 1980  9.553352e-01
## 2009   1 1981  9.228021e-01
## 2010   1 1982  8.934121e-01
## 2011   1 1983  8.643341e-01
## 2012   1 1984  8.376309e-01
## 2013   1 1985  8.125491e-01
## 2014   1 1986  7.900416e-01
## 2015   1 1987  7.654974e-01
## 2016   1 1988  7.441488e-01
## 2017   1 1989  7.219791e-01
## 2018   1 1990  7.016848e-01
## 2019   1 1991  6.802335e-01
## 2020   1 1992  6.610945e-01
## 2021   1 1993  3.710478e+00
## 2022   1 1994  2.856190e+00
## 2023   1 1995  2.226981e+00
## 2024   1 1996  1.811191e+00
## 2025   1 1997  1.537454e+00
## 2026   1 1998  1.356773e+00
## 2027   1 1999  1.230192e+00
## 2028   1 2000  1.139387e+00
## 2029   1 2001  1.074088e+00
## 2030   1 2002  1.018944e+00
## 2031   1 2003  9.768183e-01
## 2032   1 2004  9.402351e-01
## 2033   1 2005  9.070336e-01
## 2034   1 2006  8.770918e-01
## 2035   1 2007  8.501411e-01
## 2036   1 2008  8.241366e-01
## 2037   1 2009  8.011256e-01
## 2038   1 2010  7.753142e-01
## 2039   1 2011  7.536494e-01
## 2040   1 2012  7.308369e-01
## 2041   1 2013  7.102372e-01
## 2042   1 2014  6.887735e-01
## 2043   1 2015  6.699024e-01
## 2044   1 2016  6.509636e-01
## 2045   1 2017  3.696421e+00
## 2046   1 2018  2.847911e+00
## 2047   1 2019  2.215970e+00
## 2048   1 2020  1.801107e+00
## 2049   1 2021  1.527874e+00
## 2050   1 2022  1.346818e+00
## 2051   1 2023  1.223160e+00
## 2052   1 2024  1.133012e+00
## 2053   1 2025  1.065146e+00
## 2054   1 2026  1.012858e+00
## 2055   1 2027  9.700382e-01
## 2056   1 2028  9.322190e-01
## 2057   1 2029  8.998253e-01
## 2058   1 2030  8.706570e-01
## 2059   1 2031  8.427402e-01
## 2060   1 2032  8.165420e-01
## 2061   1 2033  7.937948e-01
## 2062   1 2034  7.695245e-01
## 2063   1 2035  7.463861e-01
## 2064   1 2036  7.236324e-01
## 2065   1 2037  7.041320e-01
## 2066   1 2038  6.833243e-01
## 2067   1 2039  6.635213e-01
## 2068   1 2040  6.432514e-01
## 2069   1 2041  3.693872e+00
## 2070   1 2042  2.837827e+00
## 2071   1 2043  2.206979e+00
## 2072   1 2044  1.795522e+00
## 2073   1 2045  1.525771e+00
## 2074   1 2046  1.342436e+00
## 2075   1 2047  1.219597e+00
## 2076   1 2048  1.126537e+00
## 2077   1 2049  1.060879e+00
## 2078   1 2050  1.008790e+00
## 2079   1 2051  9.643012e-01
## 2080   1 2052  9.291783e-01
## 2081   1 2053  8.966277e-01
## 2082   1 2054  8.661752e-01
## 2083   1 2055  8.407403e-01
## 2084   1 2056  8.153724e-01
## 2085   1 2057  7.896807e-01
## 2086   1 2058  7.665441e-01
## 2087   1 2059  7.440111e-01
## 2088   1 2060  7.217955e-01
## 2089   1 2061  7.017732e-01
## 2090   1 2062  6.819225e-01
## 2091   1 2063  6.610862e-01
## 2092   1 2064  6.427757e-01
## 2093   1 2065  3.680280e+00
## 2094   1 2066  2.841871e+00
## 2095   1 2067  2.205195e+00
## 2096   1 2068  1.797605e+00
## 2097   1 2069  1.522275e+00
## 2098   1 2070  1.339487e+00
## 2099   1 2071  1.215214e+00
## 2100   1 2072  1.126635e+00
## 2101   1 2073  1.059766e+00
## 2102   1 2074  1.006973e+00
## 2103   1 2075  9.632055e-01
## 2104   1 2076  9.276448e-01
## 2105   1 2077  8.958201e-01
## 2106   1 2078  8.650903e-01
## 2107   1 2079  8.393399e-01
## 2108   1 2080  8.120749e-01
## 2109   1 2081  7.886464e-01
## 2110   1 2082  7.641651e-01
## 2111   1 2083  7.435608e-01
## 2112   1 2084  7.196880e-01
## 2113   1 2085  7.009485e-01
## 2114   1 2086  6.786542e-01
## 2115   1 2087  6.588840e-01
## 2116   1 2088  6.406440e-01
## 2117   1 2089  3.678857e+00
## 2118   1 2090  2.837343e+00
## 2119   1 2091  2.208063e+00
## 2120   1 2092  1.791751e+00
## 2121   1 2093  1.523325e+00
## 2122   1 2094  1.339156e+00
## 2123   1 2095  1.215209e+00
## 2124   1 2096  1.125413e+00
## 2125   1 2097  1.057452e+00
## 2126   1 2098  1.005601e+00
## 2127   1 2099  9.623771e-01
## 2128   1 2100  9.274356e-01
## 2129   1 2101  8.958123e-01
## 2130   1 2102  8.650702e-01
## 2131   1 2103  8.385259e-01
## 2132   1 2104  8.132701e-01
## 2133   1 2105  7.876603e-01
## 2134   1 2106  7.641923e-01
## 2135   1 2107  7.425779e-01
## 2136   1 2108  7.207361e-01
## 2137   1 2109  6.981386e-01
## 2138   1 2110  6.789916e-01
## 2139   1 2111  6.593753e-01
## 2140   1 2112  6.401162e-01
## 2141   1 2113  3.685345e+00
## 2142   1 2114  2.839075e+00
## 2143   1 2115  2.203775e+00
## 2144   1 2116  1.793239e+00
## 2145   1 2117  1.520411e+00
## 2146   1 2118  1.337428e+00
## 2147   1 2119  1.212854e+00
## 2148   1 2120  1.122947e+00
## 2149   1 2121  1.056742e+00
## 2150   1 2122  1.003708e+00
## 2151   1 2123  9.616485e-01
## 2152   1 2124  9.256664e-01
## 2153   1 2125  8.949432e-01
## 2154   1 2126  8.673440e-01
## 2155   1 2127  8.355000e-01
## 2156   1 2128  8.109262e-01
## 2157   1 2129  7.870896e-01
## 2158   1 2130  7.640485e-01
## 2159   1 2131  7.411986e-01
## 2160   1 2132  7.201701e-01
## 2161   1 2133  6.988549e-01
## 2162   1 2134  6.792233e-01
## 2163   1 2135  6.600056e-01
## 2164   1 2136  6.385601e-01
## 2165   1 2137  3.684100e+00
## 2166   1 2138  2.841447e+00
## 2167   1 2139  2.206125e+00
## 2168   1 2140  1.792899e+00
## 2169   1 2141  1.521713e+00
## 2170   1 2142  1.338213e+00
## 2171   1 2143  1.213864e+00
## 2172   1 2144  1.124574e+00
## 2173   1 2145  1.056418e+00
## 2174   1 2146  1.006231e+00
## 2175   1 2147  9.617087e-01
## 2176   1 2148  9.249621e-01
## 2177   1 2149  8.935576e-01
## 2178   1 2150  8.639257e-01
## 2179   1 2151  8.373670e-01
## 2180   1 2152  8.100818e-01
## 2181   1 2153  7.879524e-01
## 2182   1 2154  7.624595e-01
## 2183   1 2155  7.415683e-01
## 2184   1 2156  7.193958e-01
## 2185   1 2157  6.977266e-01
## 2186   1 2158  6.779250e-01
## 2187   1 2159  6.594321e-01
## 2188   1 2160  6.396016e-01
## 2189   1 2161  3.680656e+00
## 2190   1 2162  2.835849e+00
## 2191   1 2163  2.207103e+00
## 2192   1 2164  1.792791e+00
## 2193   1 2165  1.523524e+00
## 2194   1 2166  1.338065e+00
## 2195   1 2167  1.214770e+00
## 2196   1 2168  1.122551e+00
## 2197   1 2169  1.055846e+00
## 2198   1 2170  1.004674e+00
## 2199   1 2171  9.586404e-01
## 2200   1 2172  9.248744e-01
## 2201   1 2173  8.925206e-01
## 2202   1 2174  8.626689e-01
## 2203   1 2175  8.364833e-01
## 2204   1 2176  8.111215e-01
## 2205   1 2177  7.871578e-01
## 2206   1 2178  7.627177e-01
## 2207   1 2179  7.406836e-01
## 2208   1 2180  7.187512e-01
## 2209   1 2181  6.989129e-01
## 2210   1 2182  6.778527e-01
## 2211   1 2183  6.588568e-01
## 2212   1 2184  6.404661e-01
## 2213   1 2184  6.395849e-01
## 2214   1 2185  3.679565e+00
## 2215   1 2186  2.833922e+00
## 2216   1 2187  2.202919e+00
## 2217   1 2188  1.796036e+00
## 2218   1 2189  1.520946e+00
## 2219   1 2190  1.339551e+00
## 2220   1 2191  1.213888e+00
## 2221   1 2192  1.122631e+00
## 2222   1 2193  1.056664e+00
## 2223   1 2194  1.001762e+00
## 2224   1 2195  9.602145e-01
## 2225   1 2196  9.248884e-01
## 2226   1 2197  8.933703e-01
## 2227   1 2198  8.644066e-01
## 2228   1 2199  8.373714e-01
## 2229   1 2200  8.113189e-01
## 2230   1 2201  7.866139e-01
## 2231   1 2202  7.637216e-01
## 2232   1 2203  7.406695e-01
## 2233   1 2204  7.195804e-01
## 2234   1 2205  6.976568e-01
## 2235   1 2206  6.784306e-01
## 2236   1 2207  6.581830e-01
## 2237   1 2208  6.382506e-01
## 2238   1 2208  6.397998e-01
## 2239   1 2209  6.193224e-01
## 2240   1 2210  6.028152e-01
## 2241   1 2211  5.837149e-01
## 2242   1 2212  5.682834e-01
## 2243   1 2213  5.518109e-01
## 2244   1 2214  5.359559e-01
## 2245   1 2215  5.208924e-01
## 2246   1 2216  5.059320e-01
## 2247   1 2217  4.912883e-01
## 2248   1 2218  4.763346e-01
## 2249   1 2219  4.628986e-01
## 2250   1 2220  4.490740e-01
## 2251   1 2221  4.368039e-01
## 2252   1 2222  4.241202e-01
## 2253   1 2223  4.105960e-01
## 2254   1 2224  3.988775e-01
## 2255   1 2225  3.879571e-01
## 2256   1 2226  3.759488e-01
## 2257   1 2226  3.763282e-01
## 2258   1 2227  1.895178e+00
## 2259   1 2228  1.473509e+00
## 2260   1 2229  1.154165e+00
## 2261   1 2230  9.472957e-01
## 2262   1 2231  8.092685e-01
## 2263   1 2232  7.163825e-01
## 2264   1 2232  7.166395e-01
## 2265   1 2233  3.718799e+00
## 2266   1 2234  2.843521e+00
## 2267   1 2235  2.190567e+00
## 2268   1 2236  1.768822e+00
## 2269   1 2237  1.486104e+00
## 2270   1 2238  1.305110e+00
## 2271   1 2239  1.177044e+00
## 2272   1 2240  1.088893e+00
## 2273   1 2241  1.019702e+00
## 2274   1 2242  9.676404e-01
## 2275   1 2243  9.251290e-01
## 2276   1 2244  8.914892e-01
## 2277   1 2245  8.610655e-01
## 2278   1 2246  8.307621e-01
## 2279   1 2247  8.063403e-01
## 2280   1 2248  7.802545e-01
## 2281   1 2249  7.555927e-01
## 2282   1 2250  7.339073e-01
## 2283   1 2251  7.127315e-01
## 2284   1 2252  6.922569e-01
## 2285   1 2253  6.730333e-01
## 2286   1 2254  6.524999e-01
## 2287   1 2255  6.333403e-01
## 2288   1 2256  6.157743e-01
## 2289   1 2257  3.666665e+00
## 2290   1 2258  2.814063e+00
## 2291   1 2259  2.180925e+00
## 2292   1 2260  1.774500e+00
## 2293   1 2261  1.498814e+00
## 2294   1 2262  1.319378e+00
## 2295   1 2263  1.192727e+00
## 2296   1 2264  1.104184e+00
## 2297   1 2265  1.037047e+00
## 2298   1 2266  9.879762e-01
## 2299   1 2267  9.436187e-01
## 2300   1 2268  9.085832e-01
## 2301   1 2269  8.757267e-01
## 2302   1 2270  8.481453e-01
## 2303   1 2271  8.219813e-01
## 2304   1 2272  7.964893e-01
## 2305   1 2273  7.714139e-01
## 2306   1 2274  7.493646e-01
## 2307   1 2275  7.270586e-01
## 2308   1 2276  7.050002e-01
## 2309   1 2277  6.847002e-01
## 2310   1 2278  6.654415e-01
## 2311   1 2279  6.463168e-01
## 2312   1 2280  6.287407e-01
## 2313   1 2281  3.677329e+00
## 2314   1 2282  2.826514e+00
## 2315   1 2283  2.191053e+00
## 2316   1 2284  1.780132e+00
## 2317   1 2285  1.510029e+00
## 2318   1 2286  1.329539e+00
## 2319   1 2287  1.202457e+00
## 2320   1 2288  1.113775e+00
## 2321   1 2289  1.045559e+00
## 2322   1 2290  9.946618e-01
## 2323   1 2291  9.528829e-01
## 2324   1 2292  9.160812e-01
## 2325   1 2293  8.849104e-01
## 2326   1 2294  8.557788e-01
## 2327   1 2295  8.284167e-01
## 2328   1 2296  8.048767e-01
## 2329   1 2297  7.788787e-01
## 2330   1 2298  7.562306e-01
## 2331   1 2299  7.331550e-01
## 2332   1 2300  7.116931e-01
## 2333   1 2301  6.930169e-01
## 2334   1 2302  6.710826e-01
## 2335   1 2303  6.524075e-01
## 2336   1 2304  6.334185e-01
## 2337   1 2305  3.678022e+00
## 2338   1 2306  2.833741e+00
## 2339   1 2307  2.198756e+00
## 2340   1 2308  1.785262e+00
## 2341   1 2309  1.514633e+00
## 2342   1 2310  1.332628e+00
## 2343   1 2311  1.206772e+00
## 2344   1 2312  1.119209e+00
## 2345   1 2313  1.051259e+00
## 2346   1 2314  1.001328e+00
## 2347   1 2315  9.570654e-01
## 2348   1 2316  9.189835e-01
## 2349   1 2317  8.896409e-01
## 2350   1 2318  8.601797e-01
## 2351   1 2319  8.340011e-01
## 2352   1 2320  8.066471e-01
## 2353   1 2321  7.825982e-01
## 2354   1 2322  7.607240e-01
## 2355   1 2323  7.379314e-01
## 2356   1 2324  7.149886e-01
## 2357   1 2325  6.951949e-01
## 2358   1 2326  6.752334e-01
## 2359   1 2327  6.555009e-01
## 2360   1 2328  6.358746e-01
## 2361   1 2329  3.683005e+00
## 2362   1 2330  2.836298e+00
## 2363   1 2331  2.200511e+00
## 2364   1 2332  1.791195e+00
## 2365   1 2333  1.515437e+00
## 2366   1 2334  1.335652e+00
## 2367   1 2335  1.211230e+00
## 2368   1 2336  1.120760e+00
## 2369   1 2337  1.056070e+00
## 2370   1 2338  1.002228e+00
## 2371   1 2339  9.604840e-01
## 2372   1 2340  9.228660e-01
## 2373   1 2341  8.908316e-01
## 2374   1 2342  8.624076e-01
## 2375   1 2343  8.356264e-01
## 2376   1 2344  8.082387e-01
## 2377   1 2345  7.849927e-01
## 2378   1 2346  7.617391e-01
## 2379   1 2347  7.393910e-01
## 2380   1 2348  7.175076e-01
## 2381   1 2349  6.967063e-01
## 2382   1 2350  6.753882e-01
## 2383   1 2351  6.573509e-01
## 2384   1 2352  6.386395e-01
## 2385   1 2353  3.681062e+00
## 2386   1 2354  2.835102e+00
## 2387   1 2355  2.199900e+00
## 2388   1 2356  1.790669e+00
## 2389   1 2357  1.517914e+00
## 2390   1 2358  1.338220e+00
## 2391   1 2359  1.212305e+00
## 2392   1 2360  1.123878e+00
## 2393   1 2361  1.055282e+00
## 2394   1 2362  1.003914e+00
## 2395   1 2363  9.621934e-01
## 2396   1 2364  9.238733e-01
## 2397   1 2365  8.919634e-01
## 2398   1 2366  8.633526e-01
## 2399   1 2367  8.340586e-01
## 2400   1 2368  8.105010e-01
## 2401   1 2369  7.867569e-01
## 2402   1 2370  7.624447e-01
## 2403   1 2371  7.404108e-01
## 2404   1 2372  7.178856e-01
## 2405   1 2373  6.985137e-01
## 2406   1 2374  6.765997e-01
## 2407   1 2375  6.570825e-01
## 2408   1 2376  6.383527e-01
## 2409   1 2377  3.679517e+00
## 2410   1 2378  2.838417e+00
## 2411   1 2379  2.205877e+00
## 2412   1 2380  1.789934e+00
## 2413   1 2381  1.520898e+00
## 2414   1 2382  1.336325e+00
## 2415   1 2383  1.212599e+00
## 2416   1 2384  1.122278e+00
## 2417   1 2385  1.055677e+00
## 2418   1 2386  1.004292e+00
## 2419   1 2387  9.604908e-01
## 2420   1 2388  9.256520e-01
## 2421   1 2389  8.923121e-01
## 2422   1 2390  8.651050e-01
## 2423   1 2391  8.352251e-01
## 2424   1 2392  8.115163e-01
## 2425   1 2393  7.861689e-01
## 2426   1 2394  7.631548e-01
## 2427   1 2395  7.401366e-01
## 2428   1 2396  7.197279e-01
## 2429   1 2397  6.988434e-01
## 2430   1 2398  6.785448e-01
## 2431   1 2399  6.587476e-01
## 2432   1 2400  6.388350e-01
## 2433   1 2401  3.685390e+00
## 2434   1 2402  2.832552e+00
## 2435   1 2403  2.207249e+00
```

```
## 2436    1 2404 1.788465e+00
## 2437    1 2405 1.518115e+00
## 2438    1 2406 1.339621e+00
## 2439    1 2407 1.213199e+00
## 2440    1 2408 1.123603e+00
## 2441    1 2409 1.056031e+00
## 2442    1 2410 1.002408e+00
## 2443    1 2411 9.629680e-01
## 2444    1 2412 9.253831e-01
## 2445    1 2413 8.939159e-01
## 2446    1 2414 8.621138e-01
## 2447    1 2415 8.375709e-01
## 2448    1 2416 8.102857e-01
## 2449    1 2417 7.878113e-01
## 2450    1 2418 7.632096e-01
## 2451    1 2419 7.415282e-01
## 2452    1 2420 7.197923e-01
## 2453    1 2421 6.987218e-01
## 2454    1 2422 6.784895e-01
## 2455    1 2423 6.577009e-01
## 2456    1 2424 6.388932e-01
## 2457    1 2425 3.680671e+00
## 2458    1 2426 2.842730e+00
## 2459    1 2427 2.207305e+00
## 2460    1 2428 1.791361e+00
## 2461    1 2429 1.519288e+00
## 2462    1 2430 1.337752e+00
## 2463    1 2431 1.211762e+00
## 2464    1 2432 1.122993e+00
## 2465    1 2433 1.054759e+00
## 2466    1 2434 1.003145e+00
## 2467    1 2435 9.608731e-01
## 2468    1 2436 9.240491e-01
## 2469    1 2437 8.913570e-01
## 2470    1 2438 8.620830e-01
## 2471    1 2439 8.365317e-01
## 2472    1 2440 8.106874e-01
## 2473    1 2441 7.859914e-01
## 2474    1 2442 7.625610e-01
## 2475    1 2443 7.419375e-01
## 2476    1 2444 7.181299e-01
## 2477    1 2445 6.987552e-01
## 2478    1 2446 6.785747e-01
## 2479    1 2447 6.589852e-01
## 2480    1 2448 6.396278e-01
## 2481    1 2449 3.682649e+00
## 2482    1 2450 2.837735e+00
## 2483    1 2451 2.204555e+00
## 2484    1 2452 1.791678e+00
## 2485    1 2453 1.519978e+00
## 2486    1 2454 1.340081e+00
## 2487    1 2455 1.212389e+00
## 2488    1 2456 1.123345e+00
## 2489    1 2457 1.055420e+00
## 2490    1 2458 1.004699e+00
## 2491    1 2459 9.613936e-01
## 2492    1 2460 9.233557e-01
## 2493    1 2461 8.921658e-01
## 2494    1 2462 8.638446e-01
## 2495    1 2463 8.362694e-01
## 2496    1 2464 8.094429e-01
## 2497    1 2465 7.866398e-01
## 2498    1 2466 7.653051e-01
## 2499    1 2467 7.407379e-01
## 2500    1 2468 7.190142e-01
## 2501    1 2469 6.972121e-01
## 2502    1 2470 6.787535e-01
## 2503    1 2471 6.584132e-01
## 2504    1 2472 6.385838e-01
## 2505    1 2473 3.685920e+00
## 2506    1 2474 2.832365e+00
## 2507    1 2475 2.209307e+00
## 2508    1 2476 1.788787e+00
## 2509    1 2477 1.520371e+00
## 2510    1 2478 1.338996e+00
## 2511    1 2479 1.213687e+00
## 2512    1 2480 1.122927e+00
## 2513    1 2481 1.054775e+00
## 2514    1 2482 1.003980e+00
## 2515    1 2483 9.610941e-01
## 2516    1 2484 9.238350e-01
## 2517    1 2485 8.944519e-01
## 2518    1 2486 8.650369e-01
## 2519    1 2487 8.374763e-01
## 2520    1 2488 8.112466e-01
## 2521    1 2489 7.868321e-01
## 2522    1 2490 7.640414e-01
## 2523    1 2491 7.418668e-01
## 2524    1 2492 7.206793e-01
## 2525    1 2493 6.989761e-01
## 2526    1 2494 6.781773e-01
## 2527    1 2495 6.575139e-01
## 2528    1 2496 6.396270e-01
## 2529    1 2496 6.393332e-01
## 2530    1 2497 3.686593e+00
## 2531    1 2498 2.839450e+00
## 2532    1 2499 2.209453e+00
## 2533    1 2500 1.793829e+00
## 2534    1 2501 1.520353e+00
## 2535    1 2502 1.336995e+00
## 2536    1 2503 1.214915e+00
## 2537    1 2504 1.123125e+00
## 2538    1 2505 1.056466e+00
## 2539    1 2506 1.004411e+00
## 2540    1 2507 9.609154e-01
## 2541    1 2508 9.254172e-01
## 2542    1 2509 8.915052e-01
## 2543    1 2510 8.646104e-01
## 2544    1 2511 8.343658e-01
## 2545    1 2512 8.094209e-01
## 2546    1 2513 7.874863e-01
## 2547    1 2514 7.637647e-01
## 2548    1 2515 7.415110e-01
## 2549    1 2516 7.192201e-01
## 2550    1 2517 6.990826e-01
## 2551    1 2518 6.776949e-01
## 2552    1 2519 6.588405e-01
## 2553    1 2520 6.382750e-01
## 2554    1 2520 6.387297e-01
## 2555    1 2521 6.205419e-01
## 2556    1 2522 6.027531e-01
## 2557    1 2523 5.850246e-01
## 2558    1 2524 5.685426e-01
## 2559    1 2525 5.532783e-01
## 2560    1 2526 5.345468e-01
## 2561    1 2527 5.200566e-01
## 2562    1 2528 5.055377e-01
## 2563    1 2529 4.905586e-01
## 2564    1 2530 4.764556e-01
## 2565    1 2531 4.638640e-01
## 2566    1 2532 4.492699e-01
## 2567    1 2533 4.357561e-01
## 2568    1 2534 4.234519e-01
## 2569    1 2535 4.119719e-01
## 2570    1 2536 3.996979e-01
## 2571    1 2537 3.881130e-01
## 2572    1 2538 3.765800e-01
## 2573    1 2538 3.768291e-01
## 2574    1 2539 3.433643e+00
## 2575    1 2540 2.584054e+00
## 2576    1 2541 1.965377e+00
## 2577    1 2542 1.560769e+00
## 2578    1 2543 1.294480e+00
## 2579    1 2544 1.118755e+00
## 2580    1 2544 1.118426e+00
## 2581    1 2545 4.065255e+00
## 2582    1 2546 3.150850e+00
## 2583    1 2547 2.477697e+00
## 2584    1 2548 2.029619e+00
## 2585    1 2549 1.740967e+00
## 2586    1 2550 1.542850e+00
## 2587    1 2551 1.405927e+00
## 2588    1 2552 1.308667e+00
## 2589    1 2553 1.234257e+00
## 2590    1 2554 1.173843e+00
## 2591    1 2555 1.125600e+00
## 2592    1 2556 1.086248e+00
## 2593    1 2557 1.046825e+00
## 2594    1 2558 1.014328e+00
## 2595    1 2559 9.831767e-01
## 2596    1 2560 9.518688e-01
## 2597    1 2561 9.222776e-01
## 2598    1 2562 8.975778e-01
## 2599    1 2563 8.714092e-01
## 2600    1 2564 8.451523e-01
## 2601    1 2565 8.203920e-01
## 2602    1 2566 7.964634e-01
## 2603    1 2567 7.715756e-01
## 2604    1 2568 7.510635e-01
## 2605    1 2569 3.796107e+00
## 2606    1 2570 2.945990e+00
## 2607    1 2571 2.308175e+00
## 2608    1 2572 1.890317e+00
## 2609    1 2573 1.616087e+00
## 2610    1 2574 1.433444e+00
## 2611    1 2575 1.303205e+00
## 2612    1 2576 1.212087e+00
## 2613    1 2577 1.140266e+00
## 2614    1 2578 1.085903e+00
## 2615    1 2579 1.041661e+00
## 2616    1 2580 1.004038e+00
## 2617    1 2581 9.706054e-01
## 2618    1 2582 9.370961e-01
## 2619    1 2583 9.084775e-01
## 2620    1 2584 8.801989e-01
## 2621    1 2585 8.543500e-01
## 2622    1 2586 8.290626e-01
## 2623    1 2587 8.045678e-01
## 2624    1 2588 7.814864e-01
## 2625    1 2589 7.582126e-01
## 2626    1 2590 7.359590e-01
## 2627    1 2591 7.146337e-01
## 2628    1 2592 6.940030e-01
## 2629    1 2593 3.736263e+00
## 2630    1 2594 2.889406e+00
## 2631    1 2595 2.256692e+00
## 2632    1 2596 1.838994e+00
## 2633    1 2597 1.564930e+00
## 2634    1 2598 1.382551e+00
## 2635    1 2599 1.257707e+00
## 2636    1 2600 1.166240e+00
## 2637    1 2601 1.098103e+00
## 2638    1 2602 1.046303e+00
## 2639    1 2603 1.001898e+00
## 2640    1 2604 9.627398e-01
## 2641    1 2605 9.314711e-01
## 2642    1 2606 9.011672e-01
## 2643    1 2607 8.716158e-01
## 2644    1 2608 8.438082e-01
## 2645    1 2609 8.187236e-01
## 2646    1 2610 7.957066e-01
## 2647    1 2611 7.729435e-01
## 2648    1 2612 7.498461e-01
## 2649    1 2613 7.275166e-01
## 2650    1 2614 7.073917e-01
## 2651    1 2615 6.859840e-01
## 2652    1 2616 6.656215e-01
## 2653    1 2617 3.707628e+00
## 2654    1 2618 2.865463e+00
## 2655    1 2619 2.227992e+00
## 2656    1 2620 1.812735e+00
## 2657    1 2621 1.542168e+00
## 2658    1 2622 1.359040e+00
## 2659    1 2623 1.236041e+00
## 2660    1 2624 1.144446e+00
## 2661    1 2625 1.075772e+00
## 2662    1 2626 1.024658e+00
## 2663    1 2627 9.807709e-01
## 2664    1 2628 9.447319e-01
## 2665    1 2629 9.104095e-01
## 2666    1 2630 8.810138e-01
## 2667    1 2631 8.540646e-01
## 2668    1 2632 8.289093e-01
## 2669    1 2633 8.029079e-01
## 2670    1 2634 7.785301e-01
## 2671    1 2635 7.562599e-01
## 2672    1 2636 7.341824e-01
## 2673    1 2637 7.131093e-01
## 2674    1 2638 6.926745e-01
## 2675    1 2639 6.719961e-01
## 2676    1 2640 6.520061e-01
## 2677    1 2641 3.698653e+00
## 2678    1 2642 2.854460e+00
## 2679    1 2643 2.219248e+00
## 2680    1 2644 1.804105e+00
## 2681    1 2645 1.530311e+00
## 2682    1 2646 1.349312e+00
## 2683    1 2647 1.224629e+00
## 2684    1 2648 1.131922e+00
## 2685    1 2649 1.067010e+00
## 2686    1 2650 1.014634e+00
## 2687    1 2651 9.710449e-01
## 2688    1 2652 9.353761e-01
## 2689    1 2653 9.017042e-01
## 2690    1 2654 8.716696e-01
## 2691    1 2655 8.450766e-01
## 2692    1 2656 8.198327e-01
## 2693    1 2657 7.957111e-01
## 2694    1 2658 7.717284e-01
## 2695    1 2659 7.504344e-01
## 2696    1 2660 7.268112e-01
## 2697    1 2661 7.062958e-01
## 2698    1 2662 6.859905e-01
## 2699    1 2663 6.642887e-01
## 2700    1 2664 6.442763e-01
## 2701    1 2665 3.692737e+00
## 2702    1 2666 2.842198e+00
## 2703    1 2667 2.208505e+00
## 2704    1 2668 1.796378e+00
## 2705    1 2669 1.525640e+00
## 2706    1 2670 1.344881e+00
## 2707    1 2671 1.219344e+00
## 2708    1 2672 1.128953e+00
## 2709    1 2673 1.064294e+00
## 2710    1 2674 1.007883e+00
## 2711    1 2675 9.663101e-01
## 2712    1 2676 9.297190e-01
## 2713    1 2677 8.966072e-01
## 2714    1 2678 8.684162e-01
## 2715    1 2679 8.410388e-01
## 2716    1 2680 8.145177e-01
## 2717    1 2681 7.900006e-01
## 2718    1 2682 7.677409e-01
## 2719    1 2683 7.452710e-01
## 2720    1 2684 7.221925e-01
## 2721    1 2685 7.030154e-01
## 2722    1 2686 6.820536e-01
## 2723    1 2687 6.622484e-01
## 2724    1 2688 6.421633e-01
## 2725    1 2689 3.689938e+00
## 2726    1 2690 2.841790e+00
## 2727    1 2691 2.206667e+00
## 2728    1 2692 1.795051e+00
## 2729    1 2693 1.521936e+00
## 2730    1 2694 1.339122e+00
## 2731    1 2695 1.216826e+00
## 2732    1 2696 1.126264e+00
## 2733    1 2697 1.058327e+00
## 2734    1 2698 1.006257e+00
## 2735    1 2699 9.635355e-01
## 2736    1 2700 9.285906e-01
## 2737    1 2701 8.968247e-01
## 2738    1 2702 8.646607e-01
## 2739    1 2703 8.397138e-01
## 2740    1 2704 8.142157e-01
## 2741    1 2705 7.880814e-01
## 2742    1 2706 7.654949e-01
## 2743    1 2707 7.438415e-01
## 2744    1 2708 7.211129e-01
## 2745    1 2709 7.005095e-01
## 2746    1 2710 6.804601e-01
## 2747    1 2711 6.604112e-01
## 2748    1 2712 6.412024e-01
## 2749    1 2713 3.681135e+00
## 2750    1 2714 2.843179e+00
## 2751    1 2715 2.204976e+00
## 2752    1 2716 1.792573e+00
## 2753    1 2717 1.524197e+00
## 2754    1 2718 1.339958e+00
## 2755    1 2719 1.213573e+00
## 2756    1 2720 1.123682e+00
## 2757    1 2721 1.057608e+00
## 2758    1 2722 1.005060e+00
## 2759    1 2723 9.624224e-01
## 2760    1 2724 9.253417e-01
## 2761    1 2725 8.942127e-01
## 2762    1 2726 8.644800e-01
## 2763    1 2727 8.360490e-01
## 2764    1 2728 8.121025e-01
## 2765    1 2729 7.881259e-01
## 2766    1 2730 7.645356e-01
## 2767    1 2731 7.429111e-01
## 2768    1 2732 7.202006e-01
## 2769    1 2733 6.993750e-01
## 2770    1 2734 6.786240e-01
## 2771    1 2735 6.583183e-01
## 2772    1 2736 6.395552e-01
## 2773    1 2737 3.677562e+00
## 2774    1 2738 2.838255e+00
## 2775    1 2739 2.204468e+00
## 2776    1 2740 1.792669e+00
## 2777    1 2741 1.521580e+00
## 2778    1 2742 1.337836e+00
## 2779    1 2743 1.214120e+00
## 2780    1 2744 1.126295e+00
## 2781    1 2745 1.056536e+00
## 2782    1 2746 1.005397e+00
## 2783    1 2747 9.624527e-01
## 2784    1 2748 9.257316e-01
## 2785    1 2749 8.943630e-01
## 2786    1 2750 8.634164e-01
## 2787    1 2751 8.366542e-01
## 2788    1 2752 8.106095e-01
## 2789    1 2753 7.863779e-01
## 2790    1 2754 7.629070e-01
## 2791    1 2755 7.411656e-01
## 2792    1 2756 7.204124e-01
## 2793    1 2757 6.987572e-01
## 2794    1 2758 6.787351e-01
## 2795    1 2759 6.583401e-01
## 2796    1 2760 6.398104e-01
## 2797    1 2761 3.687706e+00
## 2798    1 2762 2.838909e+00
## 2799    1 2763 2.205691e+00
## 2800    1 2764 1.791095e+00
## 2801    1 2765 1.519752e+00
## 2802    1 2766 1.337723e+00
## 2803    1 2767 1.213486e+00
## 2804    1 2768 1.122496e+00
## 2805    1 2769 1.055714e+00
## 2806    1 2770 1.005783e+00
## 2807    1 2771 9.615221e-01
## 2808    1 2772 9.252356e-01
## 2809    1 2773 8.938956e-01
## 2810    1 2774 8.634320e-01
## 2811    1 2775 8.360263e-01
## 2812    1 2776 8.112337e-01
## 2813    1 2777 7.868315e-01
## 2814    1 2778 7.635964e-01
## 2815    1 2779 7.430045e-01
## 2816    1 2780 7.180435e-01
## 2817    1 2781 6.980011e-01
## 2818    1 2782 6.795207e-01
## 2819    1 2783 6.593082e-01
## 2820    1 2784 6.397951e-01
## 2821    1 2785 3.685161e+00
## 2822    1 2786 2.836031e+00
## 2823    1 2787 2.207030e+00
## 2824    1 2788 1.792801e+00
## 2825    1 2789 1.522231e+00
## 2826    1 2790 1.338173e+00
## 2827    1 2791 1.210980e+00
## 2828    1 2792 1.121959e+00
## 2829    1 2793 1.058290e+00
## 2830    1 2794 1.005521e+00
## 2831    1 2795 9.640041e-01
## 2832    1 2796 9.249586e-01
## 2833    1 2797 8.928847e-01
## 2834    1 2798 8.638269e-01
## 2835    1 2799 8.352155e-01
## 2836    1 2800 8.126013e-01
## 2837    1 2801 7.864216e-01
## 2838    1 2802 7.632796e-01
## 2839    1 2803 7.403723e-01
## 2840    1 2804 7.189665e-01
## 2841    1 2805 6.977882e-01
## 2842    1 2806 6.779595e-01
## 2843    1 2807 6.582122e-01
## 2844    1 2808 6.392166e-01
## 2845    1 2808 6.390437e-01
## 2846    1 2809 3.687715e+00
## 2847    1 2810 2.835041e+00
## 2848    1 2811 2.204487e+00
## 2849    1 2812 1.788879e+00
## 2850    1 2813 1.520552e+00
## 2851    1 2814 1.335652e+00
## 2852    1 2815 1.213821e+00
## 2853    1 2816 1.123267e+00
## 2854    1 2817 1.054510e+00
## 2855    1 2818 1.003376e+00
## 2856    1 2819 9.615955e-01
## 2857    1 2820 9.247015e-01
## 2858    1 2821 8.926969e-01
## 2859    1 2822 8.630961e-01
## 2860    1 2823 8.351171e-01
## 2861    1 2824 8.100042e-01
## 2862    1 2825 7.869670e-01
## 2863    1 2826 7.633566e-01
## 2864    1 2827 7.400494e-01
## 2865    1 2828 7.185604e-01
## 2866    1 2829 6.983964e-01
## 2867    1 2830 6.787367e-01
## 2868    1 2831 6.574716e-01
## 2869    1 2832 6.393416e-01
## 2870    1 2832 6.407283e-01
## 2871    1 2833 6.206918e-01
## 2872    1 2834 6.030923e-01
## 2873    1 2835 5.854849e-01
## 2874    1 2836 5.689031e-01
## 2875    1 2837 5.520150e-01
## 2876    1 2838 5.358865e-01
## 2877    1 2839 5.196260e-01
## 2878    1 2840 5.051852e-01
## 2879    1 2841 4.911488e-01
## 2880    1 2842 4.763936e-01
## 2881    1 2843 4.626946e-01
## 2882    1 2844 4.495409e-01
## 2883    1 2845 4.359957e-01
## 2884    1 2846 4.233973e-01
## 2885    1 2847 4.121054e-01
## 2886    1 2848 3.994342e-01
## 2887    1 2849 3.871445e-01
## 2888    1 2850 3.763135e-01
## 2889    1 2851 3.654428e-01
## 2890    1 2852 3.550214e-01
## 2891    1 2853 3.446991e-01
## 2892    1 2853 3.448034e-01
## 2893    1 2854 1.868495e+00
## 2894    1 2855 1.442458e+00
## 2895    1 2856 1.123734e+00
## 2896    1 2856 1.124948e+00
## 2897    1 2857 3.989653e+00
## 2898    1 2858 3.015236e+00
## 2899    1 2859 2.304676e+00
## 2900    1 2860 1.849634e+00
## 2901    1 2861 1.550255e+00
## 2902    1 2862 1.350610e+00
## 2903    1 2863 1.212297e+00
## 2904    1 2864 1.118046e+00
## 2905    1 2865 1.046678e+00
## 2906    1 2866 9.944805e-01
## 2907    1 2867 9.479914e-01
## 2908    1 2868 9.106668e-01
## 2909    1 2869 8.778472e-01
## 2910    1 2870 8.485327e-01
## 2911    1 2871 8.222745e-01
## 2912    1 2872 7.954245e-01
## 2913    1 2873 7.712449e-01
## 2914    1 2874 7.500708e-01
## 2915    1 2875 7.278394e-01
## 2916    1 2876 7.063396e-01
## 2917    1 2877 6.850081e-01
## 2918    1 2878 6.658003e-01
## 2919    1 2879 6.463698e-01
## 2920    1 2880 6.271754e-01
## 2921    1 2881 3.672710e+00
## 2922    1 2882 2.824077e+00
## 2923    1 2883 2.196854e+00
## 2924    1 2884 1.783902e+00
## 2925    1 2885 1.509182e+00
## 2926    1 2886 1.330301e+00
## 2927    1 2887 1.204007e+00
## 2928    1 2888 1.112668e+00
## 2929    1 2889 1.047856e+00
## 2930    1 2890 9.947605e-01
## 2931    1 2891 9.531942e-01
## 2932    1 2892 9.170733e-01
## 2933    1 2893 8.858846e-01
## 2934    1 2894 8.541620e-01
## 2935    1 2895 8.288651e-01
## 2936    1 2896 8.042728e-01
## 2937    1 2897 7.806432e-01
## 2938    1 2898 7.573364e-01
## 2939    1 2899 7.345908e-01
## 2940    1 2900 7.124797e-01
## 2941    1 2901 6.923955e-01
## 2942    1 2902 6.718951e-01
## 2943    1 2903 6.524941e-01
## 2944    1 2904 6.339177e-01
## 2945    1 2905 3.678763e+00
## 2946    1 2906 2.829023e+00
## 2947    1 2907 2.196868e+00
## 2948    1 2908 1.785981e+00
## 2949    1 2909 1.514797e+00
## 2950    1 2910 1.334115e+00
## 2951    1 2911 1.207453e+00
## 2952    1 2912 1.117495e+00
## 2953    1 2913 1.051963e+00
## 2954    1 2914 9.976818e-01
## 2955    1 2915 9.568518e-01
## 2956    1 2916 9.228126e-01
## 2957    1 2917 8.886447e-01
## 2958    1 2918 8.597184e-01
## 2959    1 2919 8.334091e-01
## 2960    1 2920 8.071452e-01
## 2961    1 2921 7.829800e-01
## 2962    1 2922 7.600872e-01
## 2963    1 2923 7.369317e-01
## 2964    1 2924 7.160314e-01
## 2965    1 2925 6.953849e-01
## 2966    1 2926 6.739671e-01
## 2967    1 2927 6.556805e-01
## 2968    1 2928 6.357373e-01
## 2969    1 2929 3.680368e+00
## 2970    1 2930 2.834697e+00
## 2971    1 2931 2.200428e+00
## 2972    1 2932 1.789871e+00
## 2973    1 2933 1.520335e+00
## 2974    1 2934 1.337525e+00
## 2975    1 2935 1.209384e+00
## 2976    1 2936 1.122481e+00
## 2977    1 2937 1.053817e+00
## 2978    1 2938 1.002443e+00
## 2979    1 2939 9.590085e-01
## 2980    1 2940 9.239734e-01
## 2981    1 2941 8.918721e-01
## 2982    1 2942 8.626672e-01
## 2983    1 2943 8.345690e-01
## 2984    1 2944 8.100916e-01
## 2985    1 2945 7.858711e-01
## 2986    1 2946 7.616349e-01
## 2987    1 2947 7.396374e-01
## 2988    1 2948 7.174214e-01
## 2989    1 2949 6.989477e-01
## 2990    1 2950 6.766249e-01
## 2991    1 2951 6.568749e-01
## 2992    1 2952 6.377312e-01
## 2993    1 2953 3.685869e+00
## 2994    1 2954 2.837771e+00
## 2995    1 2955 2.204686e+00
## 2996    1 2956 1.787181e+00
## 2997    1 2957 1.519177e+00
## 2998    1 2958 1.336992e+00
## 2999    1 2959 1.212575e+00
## 3000    1 2960 1.121958e+00
## 3001    1 2961 1.054189e+00
## 3002    1 2962 1.003578e+00
## 3003    1 2963 9.609660e-01
## 3004    1 2964 9.231565e-01
## 3005    1 2965 8.922793e-01
## 3006    1 2966 8.616910e-01
## 3007    1 2967 8.362564e-01
## 3008    1 2968 8.102171e-01
## 3009    1 2969 7.843083e-01
## 3010    1 2970 7.621526e-01
## 3011    1 2971 7.408110e-01
## 3012    1 2972 7.181892e-01
## 3013    1 2973 6.986619e-01
## 3014    1 2974 6.760559e-01
## 3015    1 2975 6.571198e-01
## 3016    1 2976 6.386505e-01
## 3017    1 2977 3.682742e+00
## 3018    1 2978 2.843382e+00
## 3019    1 2979 2.205354e+00
## 3020    1 2980 1.789051e+00
## 3021    1 2981 1.519390e+00
## 3022    1 2982 1.338199e+00
## 3023    1 2983 1.210430e+00
## 3024    1 2984 1.122265e+00
## 3025    1 2985 1.052095e+00
## 3026    1 2986 1.003479e+00
## 3027    1 2987 9.640584e-01
## 3028    1 2988 9.245740e-01
## 3029    1 2989 8.939266e-01
## 3030    1 2990 8.618464e-01
## 3031    1 2991 8.380794e-01
## 3032    1 2992 8.103797e-01
## 3033    1 2993 7.861829e-01
## 3034    1 2994 7.637631e-01
## 3035    1 2995 7.406685e-01
## 3036    1 2996 7.182025e-01
## 3037    1 2997 6.990799e-01
## 3038    1 2998 6.765338e-01
## 3039    1 2999 6.587251e-01
## 3040    1 3000 6.396271e-01
## 3041    1 3001 3.682906e+00
## 3042    1 3002 2.836864e+00
## 3043    1 3003 2.206358e+00
## 3044    1 3004 1.791846e+00
## 3045    1 3005 1.518733e+00
## 3046    1 3006 1.337806e+00
## 3047    1 3007 1.212383e+00
## 3048    1 3008 1.123246e+00
## 3049    1 3009 1.055233e+00
## 3050    1 3010 1.002815e+00
## 3051    1 3011 9.624994e-01
## 3052    1 3012 9.255634e-01
## 3053    1 3013 8.930493e-01
## 3054    1 3014 8.623975e-01
## 3055    1 3015 8.346718e-01
## 3056    1 3016 8.096554e-01
## 3057    1 3017 7.868848e-01
## 3058    1 3018 7.636202e-01
## 3059    1 3019 7.404295e-01
## 3060    1 3020 7.186475e-01
## 3061    1 3021 6.988992e-01
## 3062    1 3022 6.770203e-01
## 3063    1 3023 6.581020e-01
## 3064    1 3024 6.397555e-01
## 3065    1 3025 3.678037e+00
## 3066    1 3026 2.834717e+00
## 3067    1 3027 2.205421e+00
## 3068    1 3028 1.793797e+00
## 3069    1 3029 1.520638e+00
## 3070    1 3030 1.337900e+00
## 3071    1 3031 1.213290e+00
## 3072    1 3032 1.123032e+00
## 3073    1 3033 1.056812e+00
## 3074    1 3034 1.004844e+00
## 3075    1 3035 9.618784e-01
## 3076    1 3036 9.255123e-01
## 3077    1 3037 8.933218e-01
## 3078    1 3038 8.637855e-01
## 3079    1 3039 8.353821e-01
## 3080    1 3040 8.104949e-01
## 3081    1 3041 7.863168e-01
## 3082    1 3042 7.626847e-01
## 3083    1 3043 7.396447e-01
## 3084    1 3044 7.205126e-01
## 3085    1 3045 6.990222e-01
## 3086    1 3046 6.775726e-01
## 3087    1 3047 6.575761e-01
## 3088    1 3048 6.384036e-01
## 3089    1 3049 3.684330e+00
## 3090    1 3050 2.834584e+00
## 3091    1 3051 2.199732e+00
## 3092    1 3052 1.790584e+00
## 3093    1 3053 1.520442e+00
## 3094    1 3054 1.336463e+00
## 3095    1 3055 1.211696e+00
## 3096    1 3056 1.123202e+00
## 3097    1 3057 1.056989e+00
## 3098    1 3058 1.004466e+00
## 3099    1 3059 9.619801e-01
## 3100    1 3060 9.249566e-01
## 3101    1 3061 8.912715e-01
## 3102    1 3062 8.624738e-01
## 3103    1 3063 8.373163e-01
## 3104    1 3064 8.112684e-01
## 3105    1 3065 7.872191e-01
## 3106    1 3066 7.649708e-01
## 3107    1 3067 7.421066e-01
## 3108    1 3068 7.191165e-01
## 3109    1 3069 6.978778e-01
## 3110    1 3070 6.769298e-01
## 3111    1 3071 6.588119e-01
## 3112    1 3072 6.393970e-01
## 3113    1 3073 3.685490e+00
## 3114    1 3074 2.831132e+00
## 3115    1 3075 2.207170e+00
## 3116    1 3076 1.791795e+00
## 3117    1 3077 1.524582e+00
## 3118    1 3078 1.337822e+00
## 3119    1 3079 1.212159e+00
## 3120    1 3080 1.122082e+00
## 3121    1 3081 1.054308e+00
## 3122    1 3082 1.005151e+00
## 3123    1 3083 9.621577e-01
## 3124    1 3084 9.263148e-01
## 3125    1 3085 8.930432e-01
## 3126    1 3086 8.637640e-01
## 3127    1 3087 8.393154e-01
## 3128    1 3088 8.102206e-01
## 3129    1 3089 7.869428e-01
## 3130    1 3090 7.636580e-01
## 3131    1 3091 7.406343e-01
## 3132    1 3092 7.186966e-01
## 3133    1 3093 6.973229e-01
## 3134    1 3094 6.781103e-01
## 3135    1 3095 6.578447e-01
## 3136    1 3096 6.385387e-01
## 3137    1 3097 3.684951e+00
## 3138    1 3098 2.837644e+00
## 3139    1 3099 2.205257e+00
## 3140    1 3100 1.791819e+00
## 3141    1 3101 1.522600e+00
## 3142    1 3102 1.339690e+00
## 3143    1 3103 1.212255e+00
## 3144    1 3104 1.122024e+00
## 3145    1 3105 1.056202e+00
## 3146    1 3106 1.003257e+00
## 3147    1 3107 9.625577e-01
## 3148    1 3108 9.265218e-01
## 3149    1 3109 8.937761e-01
## 3150    1 3110 8.629522e-01
## 3151    1 3111 8.360181e-01
## 3152    1 3112 8.127390e-01
## 3153    1 3113 7.878753e-01
## 3154    1 3114 7.635577e-01
## 3155    1 3115 7.406706e-01
## 3156    1 3116 7.184480e-01
## 3157    1 3117 6.974186e-01
## 3158    1 3118 6.777492e-01
## 3159    1 3119 6.582742e-01
## 3160    1 3120 6.387459e-01
## 3161    1 3120 6.393724e-01
## 3162    1 3121 3.679980e+00
## 3163    1 3122 2.840498e+00
## 3164    1 3123 2.205539e+00
## 3165    1 3124 1.792701e+00
## 3166    1 3125 1.519262e+00
## 3167    1 3126 1.338290e+00
## 3168    1 3127 1.213734e+00
## 3169    1 3128 1.122758e+00
## 3170    1 3129 1.056684e+00
## 3171    1 3130 1.003897e+00
## 3172    1 3131 9.615307e-01
## 3173    1 3132 9.255447e-01
## 3174    1 3133 8.922372e-01
## 3175    1 3134 8.633277e-01
## 3176    1 3135 8.351400e-01
## 3177    1 3136 8.114841e-01
## 3178    1 3137 7.862343e-01
## 3179    1 3138 7.633061e-01
## 3180    1 3139 7.416870e-01
## 3181    1 3140 7.196647e-01
## 3182    1 3141 6.978602e-01
## 3183    1 3142 6.777779e-01
## 3184    1 3143 6.581797e-01
## 3185    1 3144 6.400877e-01
## 3186    1 3144 6.388782e-01
## 3187    1 3145 6.194490e-01
## 3188    1 3146 6.030387e-01
## 3189    1 3147 5.853732e-01
## 3190    1 3148 5.681088e-01
## 3191    1 3149 5.519593e-01
## 3192    1 3150 5.345854e-01
## 3193    1 3151 5.206433e-01
## 3194    1 3152 5.041644e-01
## 3195    1 3153 4.895113e-01
## 3196    1 3154 4.766918e-01
## 3197    1 3155 4.622798e-01
## 3198    1 3156 4.494791e-01
## 3199    1 3157 4.364627e-01
## 3200    1 3158 4.233932e-01
## 3201    1 3159 4.110536e-01
## 3202    1 3160 3.994033e-01
## 3203    1 3161 3.877967e-01
## 3204    1 3162 3.766071e-01
## 3205    1 3163 3.659950e-01
## 3206    1 3164 3.549628e-01
## 3207    1 3165 3.448459e-01
## 3208    1 3165 3.443594e-01
## 3209    1 3166 3.395187e+00
## 3210    1 3167 2.562365e+00
## 3211    1 3168 1.934353e+00
## 3212    1 3168 1.935740e+00
## 3213    1 3169 4.592207e+00
## 3214    1 3170 3.496459e+00
## 3215    1 3171 2.710326e+00
## 3216    1 3172 2.196180e+00
## 3217    1 3173 1.859240e+00
## 3218    1 3174 1.633709e+00
## 3219    1 3175 1.476399e+00
## 3220    1 3176 1.366566e+00
## 3221    1 3177 1.283367e+00
## 3222    1 3178 1.221002e+00
## 3223    1 3179 1.166238e+00
## 3224    1 3180 1.124695e+00
## 3225    1 3181 1.085694e+00
## 3226    1 3182 1.047006e+00
## 3227    1 3183 1.016133e+00
## 3228    1 3184 9.852910e-01
## 3229    1 3185 9.541146e-01
## 3230    1 3186 9.246327e-01
## 3231    1 3187 8.995292e-01
## 3232    1 3188 8.725490e-01
## 3233    1 3189 8.482176e-01
## 3234    1 3190 8.233062e-01
## 3235    1 3191 7.991082e-01
```

```
## 3236   1 3192  7.753396e-01
## 3237   1 3193  3.811274e+00
## 3238   1 3194  2.966649e+00
## 3239   1 3195  2.325599e+00
## 3240   1 3196  1.911830e+00
## 3241   1 3197  1.638103e+00
## 3242   1 3198  1.455847e+00
## 3243   1 3199  1.323267e+00
## 3244   1 3200  1.231590e+00
## 3245   1 3201  1.162597e+00
## 3246   1 3202  1.105164e+00
## 3247   1 3203  1.061766e+00
## 3248   1 3204  1.020114e+00
## 3249   1 3205  9.878605e-01
## 3250   1 3206  9.546543e-01
## 3251   1 3207  9.242112e-01
## 3252   1 3208  8.977542e-01
## 3253   1 3209  8.703529e-01
## 3254   1 3210  8.451423e-01
## 3255   1 3211  8.190329e-01
## 3256   1 3212  7.963788e-01
## 3257   1 3213  7.715246e-01
## 3258   1 3214  7.501443e-01
## 3259   1 3215  7.286084e-01
## 3260   1 3216  7.066245e-01
## 3261   1 3217  3.750549e+00
## 3262   1 3218  2.900088e+00
## 3263   1 3219  2.266882e+00
## 3264   1 3220  1.856243e+00
## 3265   1 3221  1.578017e+00
## 3266   1 3222  1.394536e+00
## 3267   1 3223  1.267593e+00
## 3268   1 3224  1.177255e+00
## 3269   1 3225  1.107165e+00
## 3270   1 3226  1.054669e+00
## 3271   1 3227  1.011252e+00
## 3272   1 3228  9.733954e-01
## 3273   1 3229  9.374897e-01
## 3274   1 3230  9.093171e-01
## 3275   1 3231  8.806788e-01
## 3276   1 3232  8.531761e-01
## 3277   1 3233  8.276724e-01
## 3278   1 3234  8.033862e-01
## 3279   1 3235  7.795566e-01
## 3280   1 3236  7.554734e-01
## 3281   1 3237  7.346929e-01
## 3282   1 3238  7.134375e-01
## 3283   1 3239  6.933866e-01
## 3284   1 3240  6.726073e-01
## 3285   1 3241  3.713993e+00
## 3286   1 3242  2.874750e+00
## 3287   1 3243  2.231219e+00
## 3288   1 3244  1.821419e+00
## 3289   1 3245  1.551653e+00
## 3290   1 3246  1.364365e+00
## 3291   1 3247  1.239909e+00
## 3292   1 3248  1.150056e+00
## 3293   1 3249  1.082389e+00
## 3294   1 3250  1.030826e+00
## 3295   1 3251  9.847963e-01
## 3296   1 3252  9.472307e-01
## 3297   1 3253  9.169971e-01
## 3298   1 3254  8.859009e-01
## 3299   1 3255  8.560474e-01
## 3300   1 3256  8.319064e-01
## 3301   1 3257  8.071399e-01
## 3302   1 3258  7.826504e-01
## 3303   1 3259  7.603724e-01
## 3304   1 3260  7.373020e-01
## 3305   1 3261  7.164767e-01
## 3306   1 3262  6.965416e-01
## 3307   1 3263  6.750450e-01
## 3308   1 3264  6.559863e-01
## 3309   1 3265  3.698313e+00
## 3310   1 3266  2.855940e+00
## 3311   1 3267  2.219929e+00
## 3312   1 3268  1.807425e+00
## 3313   1 3269  1.534591e+00
## 3314   1 3270  1.352052e+00
## 3315   1 3271  1.223408e+00
## 3316   1 3272  1.135693e+00
## 3317   1 3273  1.069226e+00
## 3318   1 3274  1.018062e+00
## 3319   1 3275  9.747153e-01
## 3320   1 3276  9.366619e-01
## 3321   1 3277  9.043492e-01
## 3322   1 3278  8.750643e-01
## 3323   1 3279  8.477675e-01
## 3324   1 3280  8.214783e-01
## 3325   1 3281  7.974304e-01
## 3326   1 3282  7.731492e-01
## 3327   1 3283  7.495552e-01
## 3328   1 3284  7.276217e-01
## 3329   1 3285  7.075789e-01
## 3330   1 3286  6.868843e-01
## 3331   1 3287  6.659601e-01
## 3332   1 3288  6.472764e-01
## 3333   1 3289  3.693297e+00
## 3334   1 3290  2.846477e+00
## 3335   1 3291  2.213441e+00
## 3336   1 3292  1.800247e+00
## 3337   1 3293  1.530489e+00
## 3338   1 3294  1.344893e+00
## 3339   1 3295  1.220754e+00
## 3340   1 3296  1.128731e+00
## 3341   1 3297  1.063366e+00
## 3342   1 3298  1.010062e+00
## 3343   1 3299  9.669228e-01
## 3344   1 3300  9.308399e-01
## 3345   1 3301  8.969696e-01
## 3346   1 3302  8.694007e-01
## 3347   1 3303  8.420118e-01
## 3348   1 3304  8.159803e-01
## 3349   1 3305  7.915291e-01
## 3350   1 3306  7.656534e-01
## 3351   1 3307  7.448423e-01
## 3352   1 3308  7.244471e-01
## 3353   1 3309  7.014006e-01
## 3354   1 3310  6.831740e-01
## 3355   1 3311  6.633920e-01
## 3356   1 3312  6.427567e-01
## 3357   1 3313  3.685197e+00
## 3358   1 3314  2.841217e+00
## 3359   1 3315  2.212288e+00
## 3360   1 3316  1.794995e+00
## 3361   1 3317  1.524225e+00
## 3362   1 3318  1.342869e+00
## 3363   1 3319  1.215727e+00
## 3364   1 3320  1.125043e+00
## 3365   1 3321  1.060704e+00
## 3366   1 3322  1.005196e+00
## 3367   1 3323  9.651771e-01
## 3368   1 3324  9.257164e-01
## 3369   1 3325  8.953480e-01
## 3370   1 3326  8.656073e-01
## 3371   1 3327  8.391113e-01
## 3372   1 3328  8.138418e-01
## 3373   1 3329  7.888538e-01
## 3374   1 3330  7.654931e-01
## 3375   1 3331  7.429427e-01
## 3376   1 3332  7.212275e-01
## 3377   1 3333  7.007945e-01
## 3378   1 3334  6.810714e-01
## 3379   1 3335  6.600574e-01
## 3380   1 3336  6.418376e-01
## 3381   1 3337  3.686689e+00
## 3382   1 3338  2.838924e+00
## 3383   1 3339  2.209265e+00
## 3384   1 3340  1.792419e+00
## 3385   1 3341  1.521513e+00
## 3386   1 3342  1.339117e+00
## 3387   1 3343  1.215124e+00
## 3388   1 3344  1.123307e+00
## 3389   1 3345  1.058148e+00
## 3390   1 3346  1.005762e+00
## 3391   1 3347  9.624670e-01
## 3392   1 3348  9.268597e-01
## 3393   1 3349  8.951939e-01
## 3394   1 3350  8.656245e-01
## 3395   1 3351  8.365260e-01
## 3396   1 3352  8.137046e-01
## 3397   1 3353  7.880447e-01
## 3398   1 3354  7.644636e-01
## 3399   1 3355  7.425363e-01
## 3400   1 3356  7.204905e-01
## 3401   1 3357  6.996625e-01
## 3402   1 3358  6.794432e-01
## 3403   1 3359  6.583193e-01
## 3404   1 3360  6.407165e-01
## 3405   1 3361  3.689554e+00
## 3406   1 3362  2.838490e+00
## 3407   1 3363  2.204787e+00
## 3408   1 3364  1.794935e+00
## 3409   1 3365  1.523303e+00
## 3410   1 3366  1.337277e+00
## 3411   1 3367  1.213366e+00
## 3412   1 3368  1.124629e+00
## 3413   1 3369  1.057710e+00
## 3414   1 3370  1.002224e+00
## 3415   1 3371  9.618702e-01
## 3416   1 3372  9.242035e-01
## 3417   1 3373  8.952940e-01
## 3418   1 3374  8.637394e-01
## 3419   1 3375  8.363184e-01
## 3420   1 3376  8.109838e-01
## 3421   1 3377  7.886354e-01
## 3422   1 3378  7.645095e-01
## 3423   1 3379  7.428760e-01
## 3424   1 3380  7.212202e-01
## 3425   1 3381  6.980088e-01
## 3426   1 3382  6.783833e-01
## 3427   1 3383  6.593256e-01
## 3428   1 3384  6.400754e-01
## 3429   1 3385  3.687195e+00
## 3430   1 3386  2.838650e+00
## 3431   1 3387  2.207513e+00
## 3432   1 3388  1.793118e+00
## 3433   1 3389  1.519711e+00
## 3434   1 3390  1.338147e+00
## 3435   1 3391  1.213642e+00
## 3436   1 3392  1.123347e+00
## 3437   1 3393  1.055799e+00
## 3438   1 3394  1.004261e+00
## 3439   1 3395  9.631758e-01
## 3440   1 3396  9.270079e-01
## 3441   1 3397  8.951688e-01
## 3442   1 3398  8.645184e-01
## 3443   1 3399  8.371357e-01
## 3444   1 3400  8.114690e-01
## 3445   1 3401  7.870095e-01
## 3446   1 3402  7.633689e-01
## 3447   1 3403  7.416569e-01
## 3448   1 3404  7.213474e-01
## 3449   1 3405  6.987498e-01
## 3450   1 3406  6.784957e-01
## 3451   1 3407  6.579981e-01
## 3452   1 3408  6.391934e-01
## 3453   1 3409  3.690112e+00
## 3454   1 3410  2.841715e+00
## 3455   1 3411  2.204165e+00
## 3456   1 3412  1.794569e+00
## 3457   1 3413  1.520643e+00
## 3458   1 3414  1.339177e+00
## 3459   1 3415  1.214633e+00
## 3460   1 3416  1.122420e+00
## 3461   1 3417  1.056113e+00
## 3462   1 3418  1.003620e+00
## 3463   1 3419  9.605628e-01
## 3464   1 3420  9.254684e-01
## 3465   1 3421  8.928061e-01
## 3466   1 3422  8.635267e-01
## 3467   1 3423  8.354116e-01
## 3468   1 3424  8.106553e-01
## 3469   1 3425  7.872346e-01
## 3470   1 3426  7.625662e-01
## 3471   1 3427  7.402854e-01
## 3472   1 3428  7.198484e-01
## 3473   1 3429  6.995874e-01
## 3474   1 3430  6.784239e-01
## 3475   1 3431  6.585397e-01
## 3476   1 3432  6.398389e-01
## 3477   1 3432  6.392523e-01
## 3478   1 3433  3.683128e+00
## 3479   1 3434  2.837868e+00
## 3480   1 3435  2.203989e+00
## 3481   1 3436  1.793952e+00
## 3482   1 3437  1.519464e+00
## 3483   1 3438  1.338881e+00
## 3484   1 3439  1.212248e+00
## 3485   1 3440  1.122660e+00
## 3486   1 3441  1.056280e+00
## 3487   1 3442  1.004359e+00
## 3488   1 3443  9.618746e-01
## 3489   1 3444  9.235229e-01
## 3490   1 3445  8.932869e-01
## 3491   1 3446  8.632669e-01
## 3492   1 3447  8.355611e-01
## 3493   1 3448  8.117326e-01
## 3494   1 3449  7.868719e-01
## 3495   1 3450  7.632818e-01
## 3496   1 3451  7.411504e-01
## 3497   1 3452  7.187477e-01
## 3498   1 3453  6.966871e-01
## 3499   1 3454  6.766220e-01
## 3500   1 3455  6.582016e-01
## 3501   1 3456  6.401786e-01
## 3502   1 3456  6.399261e-01
## 3503   1 3457  6.200010e-01
## 3504   1 3458  6.037005e-01
## 3505   1 3459  5.848429e-01
## 3506   1 3460  5.676158e-01
## 3507   1 3461  5.518042e-01
## 3508   1 3462  5.365317e-01
## 3509   1 3463  5.205219e-01
## 3510   1 3464  5.047008e-01
## 3511   1 3465  4.903119e-01
## 3512   1 3466  4.766032e-01
## 3513   1 3467  4.624982e-01
## 3514   1 3468  4.503689e-01
## 3515   1 3469  4.366463e-01
## 3516   1 3470  4.232462e-01
## 3517   1 3471  4.108367e-01
## 3518   1 3472  3.996088e-01
## 3519   1 3473  3.881386e-01
## 3520   1 3474  3.767382e-01
## 3521   1 3475  3.655985e-01
## 3522   1 3476  3.556524e-01
## 3523   1 3477  3.451211e-01
## 3524   1 3478  3.351008e-01
## 3525   1 3479  3.250983e-01
## 3526   1 3480  3.153865e-01
## 3527   1 3480  3.158363e-01
## 3528   1 3481  3.376064e+00
## 3529   1 3482  2.530560e+00
## 3530   1 3483  1.905869e+00
## 3531   1 3484  1.504966e+00
## 3532   1 3485  1.238662e+00
## 3533   1 3486  1.067712e+00
## 3534   1 3487  9.483456e-01
## 3535   1 3488  8.680331e-01
## 3536   1 3489  8.081228e-01
## 3537   1 3490  7.623027e-01
## 3538   1 3491  7.264221e-01
## 3539   1 3492  6.971381e-01
## 3540   1 3493  6.716207e-01
## 3541   1 3494  6.497077e-01
## 3542   1 3495  6.287317e-01
## 3543   1 3496  6.096448e-01
## 3544   1 3497  5.903692e-01
## 3545   1 3498  5.733156e-01
## 3546   1 3499  5.557856e-01
## 3547   1 3500  5.402723e-01
## 3548   1 3501  5.235595e-01
## 3549   1 3502  5.089422e-01
## 3550   1 3503  4.937712e-01
## 3551   1 3504  4.793707e-01
## 3552   1 3504  4.802830e-01
## 3553   1 3505  3.528960e+00
## 3554   1 3506  2.687976e+00
## 3555   1 3507  2.059598e+00
## 3556   1 3508  1.647122e+00
## 3557   1 3509  1.381007e+00
## 3558   1 3510  1.204230e+00
## 3559   1 3511  1.083590e+00
## 3560   1 3512  9.959930e-01
## 3561   1 3513  9.335474e-01
## 3562   1 3514  8.855926e-01
## 3563   1 3515  8.457025e-01
## 3564   1 3516  8.122508e-01
## 3565   1 3517  7.837166e-01
## 3566   1 3518  7.584167e-01
## 3567   1 3519  7.335343e-01
## 3568   1 3520  7.118809e-01
## 3569   1 3521  6.907800e-01
## 3570   1 3522  6.691717e-01
## 3571   1 3523  6.478654e-01
## 3572   1 3524  6.316203e-01
## 3573   1 3525  6.117598e-01
## 3574   1 3526  5.946716e-01
## 3575   1 3527  5.781565e-01
## 3576   1 3528  5.600595e-01
## 3577   1 3529  3.610942e+00
## 3578   1 3530  2.767516e+00
## 3579   1 3531  2.137022e+00
## 3580   1 3532  1.721437e+00
## 3581   1 3533  1.452150e+00
## 3582   1 3534  1.272565e+00
## 3583   1 3535  1.147690e+00
## 3584   1 3536  1.059425e+00
## 3585   1 3537  9.955744e-01
## 3586   1 3538  9.432075e-01
## 3587   1 3539  9.033938e-01
## 3588   1 3540  8.684592e-01
## 3589   1 3541  8.402087e-01
## 3590   1 3542  8.114451e-01
## 3591   1 3543  7.867572e-01
## 3592   1 3544  7.613991e-01
## 3593   1 3545  7.389412e-01
## 3594   1 3546  7.172835e-01
## 3595   1 3547  6.962341e-01
## 3596   1 3548  6.762740e-01
## 3597   1 3549  6.565295e-01
## 3598   1 3550  6.366252e-01
## 3599   1 3551  6.193248e-01
## 3600   1 3552  6.003422e-01
## 3601   1 3553  3.651018e+00
## 3602   1 3554  2.804527e+00
## 3603   1 3555  2.166369e+00
## 3604   1 3556  1.758208e+00
## 3605   1 3557  1.485956e+00
## 3606   1 3558  1.303982e+00
## 3607   1 3559  1.180386e+00
## 3608   1 3560  1.092064e+00
## 3609   1 3561  1.026784e+00
## 3610   1 3562  9.756612e-01
## 3611   1 3563  9.322864e-01
## 3612   1 3564  8.975094e-01
## 3613   1 3565  8.642816e-01
## 3614   1 3566  8.389984e-01
## 3615   1 3567  8.120568e-01
## 3616   1 3568  7.858127e-01
## 3617   1 3569  7.624107e-01
## 3618   1 3570  7.400792e-01
## 3619   1 3571  7.189929e-01
## 3620   1 3572  6.962483e-01
## 3621   1 3573  6.774049e-01
## 3622   1 3574  6.578601e-01
## 3623   1 3575  6.381301e-01
## 3624   1 3576  6.202155e-01
## 3625   1 3577  3.661926e+00
## 3626   1 3578  2.815426e+00
## 3627   1 3579  2.186453e+00
## 3628   1 3580  1.774857e+00
## 3629   1 3581  1.504596e+00
## 3630   1 3582  1.322379e+00
## 3631   1 3583  1.197810e+00
## 3632   1 3584  1.107784e+00
## 3633   1 3585  1.040552e+00
## 3634   1 3586  9.894035e-01
## 3635   1 3587  9.481550e-01
## 3636   1 3588  9.123560e-01
## 3637   1 3589  8.806544e-01
## 3638   1 3590  8.519475e-01
## 3639   1 3591  8.249208e-01
## 3640   1 3592  7.998393e-01
## 3641   1 3593  7.752881e-01
## 3642   1 3594  7.507791e-01
## 3643   1 3595  7.300163e-01
## 3644   1 3596  7.083480e-01
## 3645   1 3597  6.871229e-01
## 3646   1 3598  6.683868e-01
## 3647   1 3599  6.487325e-01
## 3648   1 3600  6.291006e-01
## 3649   1 3601  3.672272e+00
## 3650   1 3602  2.824642e+00
## 3651   1 3603  2.196968e+00
## 3652   1 3604  1.781927e+00
## 3653   1 3605  1.512606e+00
## 3654   1 3606  1.329488e+00
## 3655   1 3607  1.206165e+00
## 3656   1 3608  1.115218e+00
## 3657   1 3609  1.050227e+00
## 3658   1 3610  9.983864e-01
## 3659   1 3611  9.544807e-01
## 3660   1 3612  9.187331e-01
## 3661   1 3613  8.871044e-01
## 3662   1 3614  8.564992e-01
## 3663   1 3615  8.300629e-01
## 3664   1 3616  8.054315e-01
## 3665   1 3617  7.805864e-01
## 3666   1 3618  7.564747e-01
## 3667   1 3619  7.347182e-01
## 3668   1 3620  7.141463e-01
## 3669   1 3621  6.939490e-01
## 3670   1 3622  6.733053e-01
## 3671   1 3623  6.520636e-01
## 3672   1 3624  6.345420e-01
## 3673   1 3625  3.680289e+00
## 3674   1 3626  2.833071e+00
## 3675   1 3627  2.197999e+00
## 3676   1 3628  1.785537e+00
## 3677   1 3629  1.516330e+00
## 3678   1 3630  1.334057e+00
## 3679   1 3631  1.206082e+00
## 3680   1 3632  1.118624e+00
## 3681   1 3633  1.050949e+00
## 3682   1 3634  9.995831e-01
## 3683   1 3635  9.578304e-01
## 3684   1 3636  9.203199e-01
## 3685   1 3637  8.904174e-01
## 3686   1 3638  8.605240e-01
## 3687   1 3639  8.343766e-01
## 3688   1 3640  8.094940e-01
## 3689   1 3641  7.838952e-01
## 3690   1 3642  7.599911e-01
## 3691   1 3643  7.370789e-01
## 3692   1 3644  7.167199e-01
## 3693   1 3645  6.954667e-01
## 3694   1 3646  6.744196e-01
## 3695   1 3647  6.552261e-01
## 3696   1 3648  6.377794e-01
## 3697   1 3649  3.677716e+00
## 3698   1 3650  2.836186e+00
## 3699   1 3651  2.204364e+00
## 3700   1 3652  1.791114e+00
## 3701   1 3653  1.520193e+00
## 3702   1 3654  1.335304e+00
## 3703   1 3655  1.209792e+00
## 3704   1 3656  1.120046e+00
## 3705   1 3657  1.053878e+00
## 3706   1 3658  1.003570e+00
## 3707   1 3659  9.609231e-01
## 3708   1 3660  9.246428e-01
## 3709   1 3661  8.914216e-01
## 3710   1 3662  8.621195e-01
## 3711   1 3663  8.350189e-01
## 3712   1 3664  8.090952e-01
## 3713   1 3665  7.864145e-01
## 3714   1 3666  7.612801e-01
## 3715   1 3667  7.379563e-01
## 3716   1 3668  7.186363e-01
## 3717   1 3669  6.968306e-01
## 3718   1 3670  6.777122e-01
## 3719   1 3671  6.578091e-01
## 3720   1 3672  6.382027e-01
## 3721   1 3673  3.681679e+00
## 3722   1 3674  2.837155e+00
## 3723   1 3675  2.205721e+00
## 3724   1 3676  1.790108e+00
## 3725   1 3677  1.522491e+00
## 3726   1 3678  1.338343e+00
## 3727   1 3679  1.211704e+00
## 3728   1 3680  1.123233e+00
## 3729   1 3681  1.055957e+00
## 3730   1 3682  1.003623e+00
## 3731   1 3683  9.610585e-01
## 3732   1 3684  9.229154e-01
## 3733   1 3685  8.922819e-01
## 3734   1 3686  8.638316e-01
## 3735   1 3687  8.362227e-01
## 3736   1 3688  8.108415e-01
## 3737   1 3689  7.860407e-01
## 3738   1 3690  7.620505e-01
## 3739   1 3691  7.392805e-01
## 3740   1 3692  7.191512e-01
## 3741   1 3693  6.978874e-01
## 3742   1 3694  6.757804e-01
## 3743   1 3695  6.579111e-01
## 3744   1 3696  6.393219e-01
## 3745   1 3697  3.682204e+00
## 3746   1 3698  2.843459e+00
## 3747   1 3699  2.207207e+00
## 3748   1 3700  1.791100e+00
## 3749   1 3701  1.522379e+00
## 3750   1 3702  1.337526e+00
## 3751   1 3703  1.211725e+00
## 3752   1 3704  1.124315e+00
## 3753   1 3705  1.055652e+00
## 3754   1 3706  1.003182e+00
## 3755   1 3707  9.618547e-01
## 3756   1 3708  9.239614e-01
## 3757   1 3709  8.918244e-01
## 3758   1 3710  8.636946e-01
## 3759   1 3711  8.371098e-01
## 3760   1 3712  8.110757e-01
## 3761   1 3713  7.863320e-01
## 3762   1 3714  7.631287e-01
## 3763   1 3715  7.400920e-01
## 3764   1 3716  7.184793e-01
## 3765   1 3717  6.978673e-01
## 3766   1 3718  6.775486e-01
## 3767   1 3719  6.583320e-01
## 3768   1 3720  6.388112e-01
## 3769   1 3721  3.687837e+00
## 3770   1 3722  2.839196e+00
## 3771   1 3723  2.205690e+00
## 3772   1 3724  1.791511e+00
## 3773   1 3725  1.520753e+00
## 3774   1 3726  1.337803e+00
## 3775   1 3727  1.211608e+00
## 3776   1 3728  1.121914e+00
## 3777   1 3729  1.056610e+00
## 3778   1 3730  1.003079e+00
## 3779   1 3731  9.608128e-01
## 3780   1 3732  9.250707e-01
## 3781   1 3733  8.928584e-01
## 3782   1 3734  8.630815e-01
## 3783   1 3735  8.369809e-01
## 3784   1 3736  8.119498e-01
## 3785   1 3737  7.880125e-01
## 3786   1 3738  7.628457e-01
## 3787   1 3739  7.394589e-01
## 3788   1 3740  7.191250e-01
## 3789   1 3741  6.985912e-01
## 3790   1 3742  6.766565e-01
## 3791   1 3743  6.582618e-01
## 3792   1 3744  6.391198e-01
## 3793   1 3745  3.683951e+00
## 3794   1 3746  2.836174e+00
## 3795   1 3747  2.202777e+00
## 3796   1 3748  1.792496e+00
## 3797   1 3749  1.517303e+00
## 3798   1 3750  1.338781e+00
## 3799   1 3751  1.212899e+00
## 3800   1 3752  1.121250e+00
## 3801   1 3753  1.056319e+00
## 3802   1 3754  1.003298e+00
## 3803   1 3755  9.606172e-01
## 3804   1 3756  9.249325e-01
## 3805   1 3757  8.926836e-01
## 3806   1 3758  8.631220e-01
## 3807   1 3759  8.348737e-01
## 3808   1 3760  8.101356e-01
## 3809   1 3761  7.867999e-01
## 3810   1 3762  7.637504e-01
## 3811   1 3763  7.406777e-01
## 3812   1 3764  7.195423e-01
## 3813   1 3765  6.976644e-01
## 3814   1 3766  6.772388e-01
## 3815   1 3767  6.576615e-01
## 3816   1 3768  6.388510e-01
## 3817   1 3768  6.389720e-01
## 3818   1 3769  3.685720e+00
## 3819   1 3770  2.838377e+00
## 3820   1 3771  2.204135e+00
## 3821   1 3772  1.787970e+00
## 3822   1 3773  1.521144e+00
## 3823   1 3774  1.336444e+00
## 3824   1 3775  1.211917e+00
## 3825   1 3776  1.122623e+00
## 3826   1 3777  1.055605e+00
## 3827   1 3778  1.004631e+00
## 3828   1 3779  9.609269e-01
## 3829   1 3780  9.252679e-01
## 3830   1 3781  8.925713e-01
## 3831   1 3782  8.649564e-01
## 3832   1 3783  8.361471e-01
## 3833   1 3784  8.113419e-01
## 3834   1 3785  7.881240e-01
## 3835   1 3786  7.632912e-01
## 3836   1 3787  7.393347e-01
## 3837   1 3788  7.192132e-01
## 3838   1 3789  6.979898e-01
## 3839   1 3790  6.762077e-01
## 3840   1 3791  6.587693e-01
## 3841   1 3792  6.391593e-01
## 3842   1 3792  6.398496e-01
## 3843   1 3793  6.210564e-01
## 3844   1 3794  6.021170e-01
## 3845   1 3795  5.843544e-01
## 3846   1 3796  5.677061e-01
## 3847   1 3797  5.521022e-01
## 3848   1 3798  5.369479e-01
## 3849   1 3799  5.205027e-01
## 3850   1 3800  5.044840e-01
## 3851   1 3801  4.908246e-01
## 3852   1 3802  4.767573e-01
## 3853   1 3803  4.632496e-01
## 3854   1 3804  4.501078e-01
## 3855   1 3805  4.361822e-01
## 3856   1 3806  4.237768e-01
## 3857   1 3807  4.110840e-01
## 3858   1 3808  3.992389e-01
## 3859   1 3809  3.880346e-01
## 3860   1 3810  3.765847e-01
## 3861   1 3811  3.661035e-01
## 3862   1 3812  3.553335e-01
## 3863   1 3813  3.444835e-01
## 3864   1 3814  3.349331e-01
## 3865   1 3815  3.253340e-01
## 3866   1 3816  3.159052e-01
## 3867   1 3816  3.157663e-01
## 3868   1 3817  4.895611e+00
## 3869   1 3818  3.650586e+00
## 3870   1 3819  2.721274e+00
## 3871   1 3820  2.115483e+00
## 3872   1 3821  1.726383e+00
## 3873   1 3822  1.471294e+00
## 3874   1 3823  1.295441e+00
## 3875   1 3824  1.179702e+00
## 3876   1 3825  1.091475e+00
## 3877   1 3826  1.024602e+00
## 3878   1 3827  9.769062e-01
## 3879   1 3828  9.356020e-01
## 3880   1 3829  9.003794e-01
## 3881   1 3830  8.682472e-01
## 3882   1 3831  8.421736e-01
## 3883   1 3832  8.145319e-01
## 3884   1 3833  7.903688e-01
## 3885   1 3834  7.667559e-01
## 3886   1 3835  7.431550e-01
## 3887   1 3836  7.214699e-01
## 3888   1 3837  7.003823e-01
## 3889   1 3838  6.805413e-01
## 3890   1 3839  6.603474e-01
## 3891   1 3840  6.420047e-01
## 3892   1 3840  6.404148e-01
## 3893   1 3841  3.683243e+00
## 3894   1 3842  2.838687e+00
## 3895   1 3843  2.203076e+00
## 3896   1 3844  1.794519e+00
## 3897   1 3845  1.522848e+00
## 3898   1 3846  1.338537e+00
## 3899   1 3847  1.214700e+00
## 3900   1 3848  1.124724e+00
## 3901   1 3849  1.057877e+00
## 3902   1 3850  1.005124e+00
## 3903   1 3851  9.632075e-01
## 3904   1 3852  9.257514e-01
## 3905   1 3853  8.952038e-01
## 3906   1 3854  8.639150e-01
## 3907   1 3855  8.372584e-01
## 3908   1 3856  8.122050e-01
## 3909   1 3857  7.872952e-01
## 3910   1 3858  7.639027e-01
## 3911   1 3859  7.421110e-01
## 3912   1 3860  7.208120e-01
## 3913   1 3861  6.995845e-01
## 3914   1 3862  6.801390e-01
## 3915   1 3863  6.595826e-01
## 3916   1 3864  6.400070e-01
## 3917   1 3865  3.686333e+00
## 3918   1 3866  2.834411e+00
## 3919   1 3867  2.208989e+00
## 3920   1 3868  1.792224e+00
## 3921   1 3869  1.519018e+00
## 3922   1 3870  1.337853e+00
## 3923   1 3871  1.212792e+00
## 3924   1 3872  1.124368e+00
## 3925   1 3873  1.057596e+00
## 3926   1 3874  1.005362e+00
## 3927   1 3875  9.618594e-01
## 3928   1 3876  9.249636e-01
## 3929   1 3877  8.934795e-01
## 3930   1 3878  8.660887e-01
## 3931   1 3879  8.368448e-01
## 3932   1 3880  8.110039e-01
## 3933   1 3881  7.870075e-01
## 3934   1 3882  7.634919e-01
## 3935   1 3883  7.402648e-01
## 3936   1 3884  7.203775e-01
## 3937   1 3885  6.997068e-01
## 3938   1 3886  6.785820e-01
## 3939   1 3887  6.591470e-01
## 3940   1 3888  6.391405e-01
## 3941   1 3889  3.684872e+00
## 3942   1 3890  2.842455e+00
## 3943   1 3891  2.203161e+00
## 3944   1 3892  1.792769e+00
## 3945   1 3893  1.523075e+00
## 3946   1 3894  1.337611e+00
## 3947   1 3895  1.212085e+00
## 3948   1 3896  1.125878e+00
## 3949   1 3897  1.057728e+00
## 3950   1 3898  1.006064e+00
## 3951   1 3899  9.615355e-01
## 3952   1 3900  9.238523e-01
## 3953   1 3901  8.897623e-01
## 3954   1 3902  8.628920e-01
## 3955   1 3903  8.373035e-01
## 3956   1 3904  8.115651e-01
## 3957   1 3905  7.867575e-01
## 3958   1 3906  7.630058e-01
## 3959   1 3907  7.383990e-01
## 3960   1 3908  7.185708e-01
## 3961   1 3909  6.991439e-01
## 3962   1 3910  6.779222e-01
## 3963   1 3911  6.579695e-01
## 3964   1 3912  6.393943e-01
## 3965   1 3913  3.686787e+00
## 3966   1 3914  2.832557e+00
## 3967   1 3915  2.202920e+00
## 3968   1 3916  1.794361e+00
## 3969   1 3917  1.520597e+00
## 3970   1 3918  1.339416e+00
## 3971   1 3919  1.212222e+00
## 3972   1 3920  1.124176e+00
## 3973   1 3921  1.056228e+00
## 3974   1 3922  1.004010e+00
## 3975   1 3923  9.618709e-01
## 3976   1 3924  9.255557e-01
## 3977   1 3925  8.929815e-01
## 3978   1 3926  8.642331e-01
## 3979   1 3927  8.376332e-01
## 3980   1 3928  8.123055e-01
## 3981   1 3929  7.878099e-01
## 3982   1 3930  7.643525e-01
## 3983   1 3931  7.405684e-01
## 3984   1 3932  7.204961e-01
## 3985   1 3933  6.992938e-01
## 3986   1 3934  6.782244e-01
## 3987   1 3935  6.579841e-01
## 3988   1 3936  6.402136e-01
## 3989   1 3937  3.685492e+00
## 3990   1 3938  2.836339e+00
## 3991   1 3939  2.203580e+00
## 3992   1 3940  1.790032e+00
## 3993   1 3941  1.520952e+00
## 3994   1 3942  1.339598e+00
## 3995   1 3943  1.212543e+00
## 3996   1 3944  1.123680e+00
## 3997   1 3945  1.057632e+00
## 3998   1 3946  1.003995e+00
## 3999   1 3947  9.598639e-01
## 4000   1 3948  9.247651e-01
## 4001   1 3949  8.937074e-01
## 4002   1 3950  8.644065e-01
## 4003   1 3951  8.370573e-01
## 4004   1 3952  8.097380e-01
## 4005   1 3953  7.861692e-01
## 4006   1 3954  7.639959e-01
## 4007   1 3955  7.426644e-01
## 4008   1 3956  7.202903e-01
## 4009   1 3957  6.984925e-01
## 4010   1 3958  6.782166e-01
## 4011   1 3959  6.585105e-01
## 4012   1 3960  6.398431e-01
## 4013   1 3961  3.682069e+00
## 4014   1 3962  2.839038e+00
## 4015   1 3963  2.204990e+00
## 4016   1 3964  1.793403e+00
## 4017   1 3965  1.519273e+00
## 4018   1 3966  1.340492e+00
## 4019   1 3967  1.213363e+00
## 4020   1 3968  1.124050e+00
## 4021   1 3969  1.055756e+00
## 4022   1 3970  1.004103e+00
## 4023   1 3971  9.638812e-01
## 4024   1 3972  9.239799e-01
## 4025   1 3973  8.927240e-01
## 4026   1 3974  8.639704e-01
## 4027   1 3975  8.367930e-01
## 4028   1 3976  8.106002e-01
## 4029   1 3977  7.873614e-01
## 4030   1 3978  7.633334e-01
## 4031   1 3979  7.417257e-01
## 4032   1 3980  7.188730e-01
## 4033   1 3981  6.981681e-01
## 4034   1 3982  6.781684e-01
## 4035   1 3983  6.583252e-01
```

```
## 4036   1 3984  6.386839e-01
## 4037   1 3985  3.686147e+00
## 4038   1 3986  2.836245e+00
## 4039   1 3987  2.204016e+00
## 4040   1 3988  1.791686e+00
## 4041   1 3989  1.522321e+00
## 4042   1 3990  1.339845e+00
## 4043   1 3991  1.210616e+00
## 4044   1 3992  1.123328e+00
## 4045   1 3993  1.055588e+00
## 4046   1 3994  1.004991e+00
## 4047   1 3995  9.609699e-01
## 4048   1 3996  9.243183e-01
## 4049   1 3997  8.936322e-01
## 4050   1 3998  8.628996e-01
## 4051   1 3999  8.351809e-01
## 4052   1 4000  8.113069e-01
## 4053   1 4001  7.889346e-01
## 4054   1 4002  7.648689e-01
## 4055   1 4003  7.417947e-01
## 4056   1 4004  7.200827e-01
## 4057   1 4005  6.978412e-01
## 4058   1 4006  6.769948e-01
## 4059   1 4007  6.577973e-01
## 4060   1 4008  6.388097e-01
## 4061   1 4009  3.694713e+00
## 4062   1 4010  2.841507e+00
## 4063   1 4011  2.206588e+00
## 4064   1 4012  1.791002e+00
## 4065   1 4013  1.521462e+00
## 4066   1 4014  1.336889e+00
## 4067   1 4015  1.213536e+00
## 4068   1 4016  1.122463e+00
## 4069   1 4017  1.055754e+00
## 4070   1 4018  1.004358e+00
## 4071   1 4019  9.612031e-01
## 4072   1 4020  9.263100e-01
## 4073   1 4021  8.935294e-01
## 4074   1 4022  8.635523e-01
## 4075   1 4023  8.349140e-01
## 4076   1 4024  8.105682e-01
## 4077   1 4025  7.864990e-01
## 4078   1 4026  7.650190e-01
## 4079   1 4027  7.402470e-01
## 4080   1 4028  7.194740e-01
## 4081   1 4029  6.983934e-01
## 4082   1 4030  6.780250e-01
## 4083   1 4031  6.585298e-01
## 4084   1 4032  6.395901e-01
## 4085   1 4033  3.680498e+00
## 4086   1 4034  2.836348e+00
## 4087   1 4035  2.208126e+00
## 4088   1 4036  1.791205e+00
## 4089   1 4037  1.518896e+00
## 4090   1 4038  1.339246e+00
## 4091   1 4039  1.211956e+00
## 4092   1 4040  1.122459e+00
## 4093   1 4041  1.057325e+00
## 4094   1 4042  1.004052e+00
## 4095   1 4043  9.618418e-01
## 4096   1 4044  9.243871e-01
## 4097   1 4045  8.930981e-01
## 4098   1 4046  8.630959e-01
## 4099   1 4047  8.356221e-01
## 4100   1 4048  8.118701e-01
## 4101   1 4049  7.865352e-01
## 4102   1 4050  7.639419e-01
## 4103   1 4051  7.411935e-01
## 4104   1 4052  7.188388e-01
## 4105   1 4053  6.986993e-01
## 4106   1 4054  6.770481e-01
## 4107   1 4055  6.591094e-01
## 4108   1 4056  6.392599e-01
## 4109   1 4057  3.687733e+00
## 4110   1 4058  2.839639e+00
## 4111   1 4059  2.209777e+00
## 4112   1 4060  1.791846e+00
## 4113   1 4061  1.519305e+00
## 4114   1 4062  1.338367e+00
## 4115   1 4063  1.211908e+00
## 4116   1 4064  1.123022e+00
## 4117   1 4065  1.054807e+00
## 4118   1 4066  1.003811e+00
## 4119   1 4067  9.610710e-01
## 4120   1 4068  9.243443e-01
## 4121   1 4069  8.929860e-01
## 4122   1 4070  8.646761e-01
## 4123   1 4071  8.356419e-01
## 4124   1 4072  8.105896e-01
## 4125   1 4073  7.867440e-01
## 4126   1 4074  7.629833e-01
## 4127   1 4075  7.408454e-01
## 4128   1 4076  7.178659e-01
## 4129   1 4077  6.978470e-01
## 4130   1 4078  6.774194e-01
## 4131   1 4079  6.573017e-01
## 4132   1 4080  6.389130e-01
## 4133   1 4081  3.683221e+00
## 4134   1 4082  2.837949e+00
## 4135   1 4083  2.206444e+00
## 4136   1 4084  1.789210e+00
## 4137   1 4085  1.520051e+00
## 4138   1 4086  1.339672e+00
## 4139   1 4087  1.213954e+00
## 4140   1 4088  1.122466e+00
## 4141   1 4089  1.056270e+00
## 4142   1 4090  1.004489e+00
## 4143   1 4091  9.608323e-01
## 4144   1 4092  9.256031e-01
## 4145   1 4093  8.944070e-01
## 4146   1 4094  8.643948e-01
## 4147   1 4095  8.359989e-01
## 4148   1 4096  8.101654e-01
## 4149   1 4097  7.856792e-01
## 4150   1 4098  7.625990e-01
## 4151   1 4099  7.407286e-01
## 4152   1 4100  7.198349e-01
## 4153   1 4101  6.980224e-01
## 4154   1 4102  6.782654e-01
## 4155   1 4103  6.573675e-01
## 4156   1 4104  6.400272e-01
## 4157   1 4104  6.401119e-01
## 4158   1 4105  3.682165e+00
## 4159   1 4106  2.837419e+00
## 4160   1 4107  2.207717e+00
## 4161   1 4108  1.792357e+00
## 4162   1 4109  1.519656e+00
## 4163   1 4110  1.338279e+00
## 4164   1 4111  1.213006e+00
## 4165   1 4112  1.121923e+00
## 4166   1 4113  1.056017e+00
## 4167   1 4114  1.003130e+00
## 4168   1 4115  9.616396e-01
## 4169   1 4116  9.240740e-01
## 4170   1 4117  8.936959e-01
## 4171   1 4118  8.632303e-01
## 4172   1 4119  8.363162e-01
## 4173   1 4120  8.120752e-01
## 4174   1 4121  7.858332e-01
## 4175   1 4122  7.638637e-01
## 4176   1 4123  7.414715e-01
## 4177   1 4124  7.198429e-01
## 4178   1 4125  6.987682e-01
## 4179   1 4126  6.784912e-01
## 4180   1 4127  6.586031e-01
## 4181   1 4128  6.397700e-01
## 4182   1 4128  6.394463e-01
## 4183   1 4129  6.199352e-01
## 4184   1 4130  6.026281e-01
## 4185   1 4131  5.839854e-01
## 4186   1 4132  5.675278e-01
## 4187   1 4133  5.516250e-01
## 4188   1 4134  5.360343e-01
## 4189   1 4135  5.201005e-01
## 4190   1 4136  5.050990e-01
## 4191   1 4137  4.900053e-01
## 4192   1 4138  4.763806e-01
## 4193   1 4139  4.627582e-01
## 4194   1 4140  4.488476e-01
## 4195   1 4141  4.360245e-01
## 4196   1 4142  4.240337e-01
## 4197   1 4143  4.112995e-01
## 4198   1 4144  3.997876e-01
## 4199   1 4145  3.881060e-01
## 4200   1 4146  3.770843e-01
## 4201   1 4147  3.663709e-01
## 4202   1 4148  3.552350e-01
## 4203   1 4149  3.445106e-01
## 4204   1 4150  3.347058e-01
## 4205   1 4151  3.252126e-01
## 4206   1 4152  3.157427e-01
## 4207   1 4152  3.158348e-01
## 4208   1 4153  6.427678e+00
## 4209   1 4154  4.768507e+00
## 4210   1 4155  3.527082e+00
## 4211   1 4156  2.727909e+00
## 4212   1 4157  2.211202e+00
## 4213   1 4158  1.871162e+00
## 4214   1 4159  1.646935e+00
## 4215   1 4160  1.484494e+00
## 4216   1 4161  1.373326e+00
## 4217   1 4162  1.293710e+00
## 4218   1 4163  1.225464e+00
## 4219   1 4164  1.175052e+00
## 4220   1 4165  1.128767e+00
## 4221   1 4166  1.087387e+00
## 4222   1 4167  1.051208e+00
## 4223   1 4168  1.020723e+00
## 4224   1 4169  9.888649e-01
## 4225   1 4170  9.604671e-01
## 4226   1 4171  9.321840e-01
## 4227   1 4172  9.028241e-01
## 4228   1 4173  8.778642e-01
## 4229   1 4174  8.517540e-01
## 4230   1 4175  8.271489e-01
## 4231   1 4176  8.024087e-01
## 4232   1 4176  8.031985e-01
## 4233   1 4177  3.840425e+00
## 4234   1 4178  2.990545e+00
## 4235   1 4179  2.352968e+00
## 4236   1 4180  1.935734e+00
## 4237   1 4181  1.661339e+00
## 4238   1 4182  1.475037e+00
## 4239   1 4183  1.346144e+00
## 4240   1 4184  1.254701e+00
## 4241   1 4185  1.180704e+00
## 4242   1 4186  1.125943e+00
## 4243   1 4187  1.080018e+00
## 4244   1 4188  1.041652e+00
## 4245   1 4189  1.003093e+00
## 4246   1 4190  9.732598e-01
## 4247   1 4191  9.413243e-01
## 4248   1 4192  9.120879e-01
## 4249   1 4193  8.863505e-01
## 4250   1 4194  8.586283e-01
## 4251   1 4195  8.333870e-01
## 4252   1 4196  8.104458e-01
## 4253   1 4197  7.859524e-01
## 4254   1 4198  7.635986e-01
## 4255   1 4199  7.422545e-01
## 4256   1 4200  7.211179e-01
## 4257   1 4201  3.756552e+00
## 4258   1 4202  2.916196e+00
## 4259   1 4203  2.279200e+00
## 4260   1 4204  1.863691e+00
## 4261   1 4205  1.591747e+00
## 4262   1 4206  1.406421e+00
## 4263   1 4207  1.281918e+00
## 4264   1 4208  1.188117e+00
## 4265   1 4209  1.117537e+00
## 4266   1 4210  1.064103e+00
## 4267   1 4211  1.022208e+00
## 4268   1 4212  9.808931e-01
## 4269   1 4213  9.464380e-01
## 4270   1 4214  9.164860e-01
## 4271   1 4215  8.891629e-01
## 4272   1 4216  8.630054e-01
## 4273   1 4217  8.357666e-01
## 4274   1 4218  8.112411e-01
## 4275   1 4219  7.874495e-01
## 4276   1 4220  7.633024e-01
## 4277   1 4221  7.412034e-01
## 4278   1 4222  7.193658e-01
## 4279   1 4223  6.997598e-01
## 4280   1 4224  6.791260e-01
## 4281   1 4225  3.724514e+00
## 4282   1 4226  2.877334e+00
## 4283   1 4227  2.237897e+00
## 4284   1 4228  1.829299e+00
## 4285   1 4229  1.555547e+00
## 4286   1 4230  1.370531e+00
## 4287   1 4231  1.244732e+00
## 4288   1 4232  1.154899e+00
## 4289   1 4233  1.088243e+00
## 4290   1 4234  1.032816e+00
## 4291   1 4235  9.899608e-01
## 4292   1 4236  9.524976e-01
## 4293   1 4237  9.194871e-01
## 4294   1 4238  8.914867e-01
## 4295   1 4239  8.632799e-01
## 4296   1 4240  8.366411e-01
## 4297   1 4241  8.112647e-01
## 4298   1 4242  7.862560e-01
## 4299   1 4243  7.637069e-01
## 4300   1 4244  7.413475e-01
## 4301   1 4245  7.196661e-01
## 4302   1 4246  6.996362e-01
## 4303   1 4247  6.782003e-01
## 4304   1 4248  6.585524e-01
## 4305   1 4249  3.707664e+00
## 4306   1 4250  2.854225e+00
## 4307   1 4251  2.222530e+00
## 4308   1 4252  1.809569e+00
## 4309   1 4253  1.534499e+00
## 4310   1 4254  1.353927e+00
## 4311   1 4255  1.226932e+00
## 4312   1 4256  1.139248e+00
## 4313   1 4257  1.073093e+00
## 4314   1 4258  1.017060e+00
## 4315   1 4259  9.766569e-01
## 4316   1 4260  9.386872e-01
## 4317   1 4261  9.062385e-01
## 4318   1 4262  8.767417e-01
## 4319   1 4263  8.498787e-01
## 4320   1 4264  8.237976e-01
## 4321   1 4265  8.001496e-01
## 4322   1 4266  7.749304e-01
## 4323   1 4267  7.514468e-01
## 4324   1 4268  7.295996e-01
## 4325   1 4269  7.078962e-01
## 4326   1 4270  6.890073e-01
## 4327   1 4271  6.689190e-01
## 4328   1 4272  6.475976e-01
## 4329   1 4273  3.695100e+00
## 4330   1 4274  2.843882e+00
## 4331   1 4275  2.213159e+00
## 4332   1 4276  1.798793e+00
## 4333   1 4277  1.526866e+00
## 4334   1 4278  1.344843e+00
## 4335   1 4279  1.220114e+00
## 4336   1 4280  1.130373e+00
## 4337   1 4281  1.063770e+00
## 4338   1 4282  1.012052e+00
## 4339   1 4283  9.688259e-01
## 4340   1 4284  9.318017e-01
## 4341   1 4285  9.006196e-01
## 4342   1 4286  8.710407e-01
## 4343   1 4287  8.424687e-01
## 4344   1 4288  8.173246e-01
## 4345   1 4289  7.930741e-01
## 4346   1 4290  7.699599e-01
## 4347   1 4291  7.472710e-01
## 4348   1 4292  7.250177e-01
## 4349   1 4293  7.036918e-01
## 4350   1 4294  6.834935e-01
## 4351   1 4295  6.640688e-01
## 4352   1 4296  6.447796e-01
## 4353   1 4297  3.693040e+00
## 4354   1 4298  2.841238e+00
## 4355   1 4299  2.207859e+00
## 4356   1 4300  1.796564e+00
## 4357   1 4301  1.522274e+00
## 4358   1 4302  1.342659e+00
## 4359   1 4303  1.217317e+00
## 4360   1 4304  1.126147e+00
## 4361   1 4305  1.060084e+00
## 4362   1 4306  1.008193e+00
## 4363   1 4307  9.658620e-01
## 4364   1 4308  9.286735e-01
## 4365   1 4309  8.963778e-01
## 4366   1 4310  8.668335e-01
## 4367   1 4311  8.386345e-01
## 4368   1 4312  8.148777e-01
## 4369   1 4313  7.895205e-01
## 4370   1 4314  7.667000e-01
## 4371   1 4315  7.433863e-01
## 4372   1 4316  7.214530e-01
## 4373   1 4317  6.995629e-01
## 4374   1 4318  6.815737e-01
## 4375   1 4319  6.606947e-01
## 4376   1 4320  6.423382e-01
## 4377   1 4321  3.694115e+00
## 4378   1 4322  2.838662e+00
## 4379   1 4323  2.208582e+00
## 4380   1 4324  1.794279e+00
## 4381   1 4325  1.523089e+00
## 4382   1 4326  1.338741e+00
## 4383   1 4327  1.214443e+00
## 4384   1 4328  1.125009e+00
## 4385   1 4329  1.057833e+00
## 4386   1 4330  1.005221e+00
## 4387   1 4331  9.632941e-01
## 4388   1 4332  9.272782e-01
## 4389   1 4333  8.943501e-01
## 4390   1 4334  8.668854e-01
## 4391   1 4335  8.378371e-01
## 4392   1 4336  8.128148e-01
## 4393   1 4337  7.888951e-01
## 4394   1 4338  7.646528e-01
## 4395   1 4339  7.417226e-01
## 4396   1 4340  7.211456e-01
## 4397   1 4341  6.992366e-01
## 4398   1 4342  6.803003e-01
## 4399   1 4343  6.599145e-01
## 4400   1 4344  6.403702e-01
## 4401   1 4345  3.685497e+00
## 4402   1 4346  2.844403e+00
## 4403   1 4347  2.205308e+00
## 4404   1 4348  1.794146e+00
## 4405   1 4349  1.524607e+00
## 4406   1 4350  1.338326e+00
## 4407   1 4351  1.212145e+00
## 4408   1 4352  1.125584e+00
## 4409   1 4353  1.055702e+00
## 4410   1 4354  1.005301e+00
## 4411   1 4355  9.620718e-01
## 4412   1 4356  9.271931e-01
## 4413   1 4357  8.942701e-01
## 4414   1 4358  8.648093e-01
## 4415   1 4359  8.366814e-01
## 4416   1 4360  8.125372e-01
## 4417   1 4361  7.880896e-01
## 4418   1 4362  7.645947e-01
## 4419   1 4363  7.409648e-01
## 4420   1 4364  7.198211e-01
## 4421   1 4365  6.990287e-01
## 4422   1 4366  6.786562e-01
## 4423   1 4367  6.586717e-01
## 4424   1 4368  6.401358e-01
## 4425   1 4369  3.683149e+00
## 4426   1 4370  2.839369e+00
## 4427   1 4371  2.203784e+00
## 4428   1 4372  1.793251e+00
## 4429   1 4373  1.523247e+00
## 4430   1 4374  1.339643e+00
## 4431   1 4375  1.211123e+00
## 4432   1 4376  1.124115e+00
## 4433   1 4377  1.057312e+00
## 4434   1 4378  1.003767e+00
## 4435   1 4379  9.624360e-01
## 4436   1 4380  9.263262e-01
## 4437   1 4381  8.926704e-01
## 4438   1 4382  8.626182e-01
## 4439   1 4383  8.365320e-01
## 4440   1 4384  8.120164e-01
## 4441   1 4385  7.871776e-01
## 4442   1 4386  7.639314e-01
## 4443   1 4387  7.417182e-01
## 4444   1 4388  7.190467e-01
## 4445   1 4389  6.985536e-01
## 4446   1 4390  6.775980e-01
## 4447   1 4391  6.586701e-01
## 4448   1 4392  6.391552e-01
## 4449   1 4393  3.686099e+00
## 4450   1 4394  2.839546e+00
## 4451   1 4395  2.206881e+00
## 4452   1 4396  1.788204e+00
## 4453   1 4397  1.520535e+00
## 4454   1 4398  1.338291e+00
## 4455   1 4399  1.212705e+00
## 4456   1 4400  1.122530e+00
## 4457   1 4401  1.056241e+00
## 4458   1 4402  1.002072e+00
## 4459   1 4403  9.615368e-01
## 4460   1 4404  9.263409e-01
## 4461   1 4405  8.917278e-01
## 4462   1 4406  8.620409e-01
## 4463   1 4407  8.367708e-01
## 4464   1 4408  8.116660e-01
## 4465   1 4409  7.861625e-01
## 4466   1 4410  7.647748e-01
## 4467   1 4411  7.409236e-01
## 4468   1 4412  7.181703e-01
## 4469   1 4413  6.988200e-01
## 4470   1 4414  6.779144e-01
## 4471   1 4415  6.583408e-01
## 4472   1 4416  6.390455e-01
## 4473   1 4417  3.683429e+00
## 4474   1 4418  2.838318e+00
## 4475   1 4419  2.203645e+00
## 4476   1 4420  1.789484e+00
## 4477   1 4421  1.518806e+00
## 4478   1 4422  1.338334e+00
## 4479   1 4423  1.211677e+00
## 4480   1 4424  1.125126e+00
## 4481   1 4425  1.059012e+00
## 4482   1 4426  1.003320e+00
## 4483   1 4427  9.582759e-01
## 4484   1 4428  9.266536e-01
## 4485   1 4429  8.934773e-01
## 4486   1 4430  8.644051e-01
## 4487   1 4431  8.380819e-01
## 4488   1 4432  8.109244e-01
## 4489   1 4433  7.878963e-01
## 4490   1 4434  7.627878e-01
## 4491   1 4435  7.417860e-01
## 4492   1 4436  7.180323e-01
## 4493   1 4437  6.985411e-01
## 4494   1 4438  6.778468e-01
## 4495   1 4439  6.571556e-01
## 4496   1 4440  6.391041e-01
## 4497   1 4441  6.214157e-01
## 4498   1 4442  6.026720e-01
## 4499   1 4443  5.857301e-01
## 4500   1 4444  5.683220e-01
## 4501   1 4445  5.518252e-01
## 4502   1 4446  5.359454e-01
## 4503   1 4447  5.203088e-01
## 4504   1 4448  5.055969e-01
## 4505   1 4449  4.914065e-01
## 4506   1 4450  4.765240e-01
## 4507   1 4451  4.629496e-01
## 4508   1 4452  4.499512e-01
## 4509   1 4453  4.362052e-01
## 4510   1 4454  4.242126e-01
## 4511   1 4455  4.122434e-01
## 4512   1 4456  3.995573e-01
## 4513   1 4457  3.875403e-01
## 4514   1 4458  3.759745e-01
## 4515   1 4459  3.656107e-01
## 4516   1 4460  3.547254e-01
## 4517   1 4461  3.447568e-01
## 4518   1 4462  3.348258e-01
## 4519   1 4463  3.247733e-01
## 4520   1 4464  3.157274e-01
## 4521   1 4465  3.067407e-01
## 4522   1 4466  2.978683e-01
## 4523   1 4467  2.891116e-01
## 4524   1 4468  2.808752e-01
## 4525   1 4469  2.726768e-01
## 4526   1 4470  2.647347e-01
## 4527   1 4471  2.573966e-01
## 4528   1 4472  2.501083e-01
## 4529   1 4473  2.422444e-01
## 4530   1 4474  2.358516e-01
## 4531   1 4475  2.287401e-01
## 4532   1 4476  2.218359e-01
## 4533   1 4477  2.155608e-01
## 4534   1 4478  2.093035e-01
## 4535   1 4479  2.031470e-01
## 4536   1 4480  1.973613e-01
## 4537   1 4481  1.915629e-01
## 4538   1 4482  1.861346e-01
## 4539   1 4483  1.809246e-01
## 4540   1 4484  1.756247e-01
## 4541   1 4485  1.704868e-01
## 4542   1 4486  1.653575e-01
## 4543   1 4487  1.603735e-01
## 4544   1 4488  1.560187e-01
## 4545   1 4489  1.514096e-01
## 4546   1 4490  1.469857e-01
## 4547   1 4491  1.428992e-01
## 4548   1 4492  1.387502e-01
## 4549   1 4493  1.347198e-01
## 4550   1 4494  1.308132e-01
## 4551   1 4495  1.270551e-01
## 4552   1 4496  1.232472e-01
## 4553   1 4497  1.197776e-01
## 4554   1 4498  1.163974e-01
## 4555   1 4499  1.130097e-01
## 4556   1 4500  1.097743e-01
## 4557   1 4501  1.065337e-01
## 4558   1 4502  1.034348e-01
## 4559   1 4503  1.006974e-01
## 4560   1 4504  9.763497e-02
## 4561   1 4505  9.461040e-02
## 4562   1 4506  9.195039e-02
## 4563   1 4507  8.918320e-02
## 4564   1 4508  8.673600e-02
## 4565   1 4509  8.429569e-02
## 4566   1 4510  8.171150e-02
## 4567   1 4511  7.938501e-02
## 4568   1 4512  7.708503e-02
## 4569   1 4513  7.474779e-02
## 4570   1 4514  7.255255e-02
## 4571   1 4515  7.051747e-02
## 4572   1 4516  6.851960e-02
## 4573   1 4517  6.645849e-02
## 4574   1 4518  6.471714e-02
## 4575   1 4519  6.272139e-02
## 4576   1 4520  6.097502e-02
## 4577   1 4521  5.912671e-02
## 4578   1 4522  5.740234e-02
## 4579   1 4523  5.578398e-02
## 4580   1 4524  5.412698e-02
## 4581   1 4525  5.261514e-02
## 4582   1 4526  5.119259e-02
## 4583   1 4527  4.966238e-02
## 4584   1 4528  4.819440e-02
## 4585   1 4529  4.677173e-02
## 4586   1 4530  4.540157e-02
## 4587   1 4531  4.416138e-02
## 4588   1 4532  4.283821e-02
## 4589   1 4533  4.158780e-02
## 4590   1 4534  4.041323e-02
## 4591   1 4535  3.910748e-02
## 4592   1 4536  3.814124e-02
## 4593   1 4537  3.700002e-02
## 4594   1 4538  3.586124e-02
## 4595   1 4539  3.483598e-02
## 4596   1 4540  3.387183e-02
## 4597   1 4541  3.290568e-02
## 4598   1 4542  3.187449e-02
## 4599   1 4543  3.100243e-02
## 4600   1 4544  3.011055e-02
## 4601   1 4545  2.924324e-02
## 4602   1 4546  2.837163e-02
## 4603   1 4547  2.753786e-02
## 4604   1 4548  2.678476e-02
## 4605   1 4549  2.601054e-02
## 4606   1 4550  2.522733e-02
## 4607   1 4551  2.446403e-02
## 4608   1 4552  2.378742e-02
## 4609   1 4553  2.307400e-02
## 4610   1 4554  2.246269e-02
## 4611   1 4555  2.180171e-02
## 4612   1 4556  2.118258e-02
## 4613   1 4557  2.057034e-02
## 4614   1 4558  1.998185e-02
## 4615   1 4559  1.937667e-02
## 4616   1 4560  1.881460e-02
## 4617   1 4561  1.827443e-02
## 4618   1 4562  1.777609e-02
## 4619   1 4563  1.722626e-02
## 4620   1 4564  1.671904e-02
## 4621   1 4565  1.623981e-02
## 4622   1 4566  1.577950e-02
## 4623   1 4567  1.531584e-02
## 4624   1 4568  1.483732e-02
## 4625   1 4569  1.445186e-02
## 4626   1 4570  1.402176e-02
## 4627   1 4571  1.363315e-02
## 4628   1 4572  1.323175e-02
## 4629   1 4573  1.282817e-02
## 4630   1 4574  1.247524e-02
## 4631   1 4575  1.212999e-02
## 4632   1 4576  1.175110e-02
## 4633   1 4577  1.139769e-02
## 4634   1 4578  1.106892e-02
## 4635   1 4579  1.075433e-02
## 4636   1 4580  1.043254e-02
## 4637   1 4581  1.014642e-02
## 4638   1 4582  9.868310e-03
## 4639   1 4583  9.566516e-03
## 4640   1 4584  9.291000e-03
## 4641   1 4585  9.008888e-03
## 4642   1 4586  8.763865e-03
## 4643   1 4587  8.513251e-03
## 4644   1 4588  8.257710e-03
## 4645   1 4589  8.024349e-03
## 4646   1 4590  7.778542e-03
## 4647   1 4591  7.571519e-03
## 4648   1 4592  7.355727e-03
## 4649   1 4593  7.143967e-03
## 4650   1 4594  6.923019e-03
## 4651   1 4595  6.729121e-03
## 4652   1 4596  6.539798e-03
## 4653   1 4597  6.350743e-03
## 4654   1 4598  6.163352e-03
## 4655   1 4599  5.980142e-03
## 4656   1 4600  5.811479e-03
## 4657   1 4601  5.634417e-03
## 4658   1 4602  5.480930e-03
## 4659   1 4603  5.314205e-03
## 4660   1 4604  5.154114e-03
## 4661   1 4605  5.011655e-03
## 4662   1 4606  4.868001e-03
## 4663   1 4607  4.721701e-03
## 4664   1 4608  4.591314e-03
## 4665   1 4609  4.453907e-03
## 4666   1 4610  4.323680e-03
## 4667   1 4611  4.198458e-03
## 4668   1 4612  4.084122e-03
## 4669   1 4613  3.965545e-03
## 4670   1 4614  3.848817e-03
## 4671   1 4615  3.738682e-03
## 4672   1 4616  3.628812e-03
## 4673   1 4617  3.528902e-03
## 4674   1 4618  3.426454e-03
## 4675   1 4619  3.324496e-03
## 4676   1 4620  3.230348e-03
## 4677   1 4621  3.131013e-03
## 4678   1 4622  3.041935e-03
## 4679   1 4623  2.956115e-03
## 4680   1 4624  2.868483e-03
## 4681   1 4625  2.785402e-03
## 4682   1 4626  2.707126e-03
## 4683   1 4627  2.626151e-03
## 4684   1 4628  2.550599e-03
## 4685   1 4629  2.476760e-03
## 4686   1 4630  2.407247e-03
## 4687   1 4631  2.337163e-03
## 4688   1 4632  2.264858e-03
## 4689   1 4633  2.203140e-03
## 4690   1 4634  2.137696e-03
## 4691   1 4635  2.079827e-03
## 4692   1 4636  2.015176e-03
## 4693   1 4637  1.956696e-03
## 4694   1 4638  1.901876e-03
## 4695   1 4639  1.844846e-03
## 4696   1 4640  1.793024e-03
## 4697   1 4641  1.742318e-03
## 4698   1 4642  1.690350e-03
## 4699   1 4643  1.639397e-03
## 4700   1 4644  1.595357e-03
## 4701   1 4645  1.547430e-03
## 4702   1 4646  1.500195e-03
## 4703   1 4647  1.459454e-03
## 4704   1 4648  1.416354e-03
## 4705   1 4649  1.375021e-03
## 4706   1 4650  1.336695e-03
## 4707   1 4651  1.296309e-03
## 4708   1 4652  1.260037e-03
## 4709   1 4653  1.225528e-03
## 4710   1 4654  1.188297e-03
## 4711   1 4655  1.154849e-03
## 4712   1 4656  1.120136e-03
## 4713   1 4657  1.088843e-03
## 4714   1 4658  1.057099e-03
## 4715   1 4659  1.025052e-03
## 4716   1 4660  9.974063e-04
## 4717   1 4661  9.687808e-04
## 4718   1 4662  9.410003e-04
## 4719   1 4663  9.125909e-04
## 4720   1 4664  8.849409e-04
## 4721   1 4665  8.597926e-04
## 4722   1 4666  8.354141e-04
## 4723   1 4667  8.110531e-04
## 4724   1 4668  7.888869e-04
## 4725   1 4669  7.646054e-04
## 4726   1 4670  7.423215e-04
## 4727   1 4671  7.214576e-04
## 4728   1 4672  7.020673e-04
## 4729   1 4673  6.799414e-04
## 4730   1 4674  6.591145e-04
## 4731   1 4675  6.404200e-04
## 4732   1 4676  6.238018e-04
## 4733   1 4677  6.044836e-04
## 4734   1 4678  5.866225e-04
## 4735   1 4679  5.704838e-04
## 4736   1 4680  5.534956e-04
## 4737   1 4681  5.372142e-04
## 4738   1 4682  5.223859e-04
## 4739   1 4683  5.063802e-04
## 4740   1 4684  4.924452e-04
## 4741   1 4685  4.785622e-04
## 4742   1 4686  4.636009e-04
## 4743   1 4687  4.507513e-04
## 4744   1 4688  4.386353e-04
## 4745   1 4689  4.250168e-04
## 4746   1 4690  4.133703e-04
## 4747   1 4691  4.000274e-04
## 4748   1 4692  3.884329e-04
## 4749   1 4693  3.780667e-04
## 4750   1 4694  3.664762e-04
## 4751   1 4695  3.562417e-04
## 4752   1 4696  3.454491e-04
## 4753   1 4697  3.359808e-04
## 4754   1 4698  3.263218e-04
## 4755   1 4699  3.167597e-04
## 4756   1 4700  3.073239e-04
## 4757   1 4701  2.986437e-04
## 4758   1 4702  2.905649e-04
## 4759   1 4703  2.813773e-04
## 4760   1 4704  2.731948e-04
## 4761   1 4705  2.653298e-04
## 4762   1 4706  2.576361e-04
## 4763   1 4707  2.504609e-04
## 4764   1 4708  2.434413e-04
## 4765   1 4709  2.361239e-04
## 4766   1 4710  2.293192e-04
## 4767   1 4711  2.227583e-04
## 4768   1 4712  2.166103e-04
## 4769   1 4713  2.100601e-04
## 4770   1 4714  2.037060e-04
## 4771   1 4715  1.982782e-04
## 4772   1 4716  1.922547e-04
## 4773   1 4717  1.867530e-04
## 4774   1 4718  1.811227e-04
## 4775   1 4719  1.760717e-04
## 4776   1 4720  1.708357e-04
## 4777   1 4721  1.662742e-04
## 4778   1 4722  1.616149e-04
## 4779   1 4723  1.565381e-04
## 4780   1 4724  1.521557e-04
## 4781   1 4725  1.474651e-04
## 4782   1 4726  1.434475e-04
## 4783   1 4727  1.393090e-04
## 4784   1 4728  1.351042e-04
## 4785   1 4729  1.310855e-04
## 4786   1 4730  1.275575e-04
## 4787   1 4731  1.238721e-04
## 4788   1 4732  1.200612e-04
## 4789   1 4733  1.164289e-04
## 4790   1 4734  1.130604e-04
## 4791   1 4735  1.101113e-04
## 4792   1 4736  1.068458e-04
## 4793   1 4737  1.038230e-04
## 4794   1 4738  1.007806e-04
## 4795   1 4739  9.798357e-05
## 4796   1 4740  9.483394e-05
## 4797   1 4741  9.219509e-05
## 4798   1 4742  8.959563e-05
## 4799   1 4743  8.695112e-05
## 4800   1 4744  8.449426e-05
## 4801   1 4745  8.196533e-05
## 4802   1 4746  7.979169e-05
## 4803   1 4747  7.733580e-05
## 4804   1 4748  7.498939e-05
## 4805   1 4749  7.302755e-05
## 4806   1 4750  7.071793e-05
## 4807   1 4751  6.874680e-05
## 4808   1 4752  6.677573e-05
## 4809   1 4753  6.487099e-05
## 4810   1 4754  6.308368e-05
## 4811   1 4755  6.113938e-05
## 4812   1 4756  5.943525e-05
## 4813   1 4757  5.775468e-05
## 4814   1 4758  5.599854e-05
## 4815   1 4759  5.439924e-05
## 4816   1 4760  5.282924e-05
## 4817   1 4761  5.126149e-05
## 4818   1 4762  4.987568e-05
## 4819   1 4763  4.835447e-05
## 4820   1 4764  4.692103e-05
## 4821   1 4765  4.560184e-05
## 4822   1 4766  4.425444e-05
## 4823   1 4767  4.299696e-05
## 4824   1 4768  4.176832e-05
## 4825   1 4769  4.057373e-05
## 4826   1 4770  3.933459e-05
## 4827   1 4771  3.819841e-05
## 4828   1 4772  3.714679e-05
## 4829   1 4773  3.604631e-05
## 4830   1 4774  3.499403e-05
## 4831   1 4775  3.403296e-05
## 4832   1 4776  3.298548e-05
## 4833   1 4777  3.203764e-05
## 4834   1 4778  3.116776e-05
## 4835   1 4779  3.026765e-05
```

```
## 4836     1 4780  2.935524e-05
## 4837     1 4781  2.851275e-05
## 4838     1 4782  2.765813e-05
## 4839     1 4783  2.689524e-05
## 4840     1 4784  2.612902e-05
## 4841     1 4785  2.535001e-05
## 4842     1 4786  2.466417e-05
## 4843     1 4787  2.391873e-05
## 4844     1 4788  2.325550e-05
## 4845     1 4789  2.254943e-05
## 4846     1 4790  2.190421e-05
## 4847     1 4791  2.127152e-05
## 4848     1 4792  2.064383e-05
## 4849     1 4793  2.005080e-05
## 4850     1 4794  1.946599e-05
## 4851     1 4795  1.889236e-05
## 4852     1 4796  1.836401e-05
## 4853     1 4797  1.782554e-05
## 4854     1 4798  1.731095e-05
## 4855     1 4799  1.681759e-05
## 4856     1 4800  1.631662e-05
## 4857     1 4801  1.586009e-05
## 4858     1 4802  1.537509e-05
## 4859     1 4803  1.493443e-05
## 4860     1 4804  1.450636e-05
## 4861     1 4805  1.408386e-05
## 4862     1 4806  1.368772e-05
## 4863     1 4807  1.330416e-05
## 4864     1 4808  1.291059e-05
## 4865     1 4809  1.251330e-05
## 4866     1 4810  1.217431e-05
## 4867     1 4811  1.180734e-05
## 4868     1 4812  1.145972e-05
## 4869     1 4813  1.114609e-05
## 4870     1 4814  1.081814e-05
## 4871     1 4815  1.050836e-05
## 4872     1 4816  1.021941e-05
## 4873     1 4817  9.905929e-06
## 4874     1 4818  9.622969e-06
## 4875     1 4819  9.347688e-06
## 4876     1 4820  9.078505e-06
## 4877     1 4821  8.819545e-06
## 4878     1 4822  8.549793e-06
## 4879     1 4823  8.320331e-06
## 4880     1 4824  8.083275e-06
## 4881     1 4825  7.850093e-06
## 4882     1 4826  7.624132e-06
## 4883     1 4827  7.394263e-06
## 4884     1 4828  7.199749e-06
## 4885     1 4829  6.969712e-06
## 4886     1 4830  6.781186e-06
## 4887     1 4831  6.606154e-06
## 4888     1 4832  6.398024e-06
## 4889     1 4833  6.216603e-06
## 4890     1 4834  6.033739e-06
## 4891     1 4835  5.859761e-06
## 4892     1 4836  5.699429e-06
## 4893     1 4837  5.520053e-06
## 4894     1 4838  5.360395e-06
## 4895     1 4839  5.206535e-06
## 4896     1 4840  5.072175e-06
## 4897     1 4841  4.915042e-06
## 4898     1 4842  4.765993e-06
## 4899     1 4843  4.623626e-06
## 4900     1 4844  4.497268e-06
## 4901     1 4845  4.366735e-06
## 4902     1 4846  4.244642e-06
## 4903     1 4847  4.119295e-06
## 4904     1 4848  4.006425e-06
## 4905     1 4849  3.896228e-06
## 4906     1 4850  3.773423e-06
## 4907     1 4851  3.676313e-06
## 4908     1 4852  3.571525e-06
## 4909     1 4853  3.479852e-06
## 4910     1 4854  3.395080e-06
## 4911     1 4855  3.305483e-06
## 4912     1 4856  3.209457e-06
## 4913     1 4857  3.129710e-06
## 4914     1 4858  3.039535e-06
## 4915     1 4859  2.966904e-06
## 4916     1 4860  2.882842e-06
## 4917     1 4861  2.799185e-06
## 4918     1 4862  2.722924e-06
## 4919     1 4863  2.650972e-06
## 4920     1 4864  2.574162e-06
## 4921     1 4865  2.495016e-06
## 4922     1 4866  2.422713e-06
## 4923     1 4867  2.350857e-06
## 4924     1 4868  2.275681e-06
## 4925     1 4869  2.203116e-06
## 4926     1 4870  2.135638e-06
## 4927     1 4871  2.069064e-06
## 4928     1 4872  2.007853e-06
## 4929     1 4873  1.944758e-06
## 4930     1 4874  1.883766e-06
## 4931     1 4875  1.827191e-06
## 4932     1 4876  1.767749e-06
## 4933     1 4877  1.708863e-06
## 4934     1 4878  1.650014e-06
## 4935     1 4879  1.597308e-06
## 4936     1 4880  1.548325e-06
## 4937     1 4881  1.496013e-06
## 4938     1 4882  1.446355e-06
## 4939     1 4883  1.396180e-06
## 4940     1 4884  1.352968e-06
## 4941     1 4885  1.306341e-06
## 4942     1 4886  1.265304e-06
## 4943     1 4887  1.220784e-06
## 4944     1 4888  1.182130e-06
## 4945     1 4889  1.144941e-06
## 4946     1 4890  1.113740e-06
## 4947     1 4891  1.079560e-06
## 4948     1 4892  1.050465e-06
## 4949     1 4893  1.026397e-06
## 4950     1 4894  1.001084e-06
## 4951     1 4895  9.817749e-07
## 4952     1 4896  9.620076e-07
## 4953     1 4897  9.355460e-07
## 4954     1 4898  9.100655e-07
## 4955     1 4899  8.892948e-07
## 4956     1 4900  8.663505e-07
## 4957     1 4901  8.454729e-07
## 4958     1 4902  8.237017e-07
## 4959     1 4903  8.033184e-07
## 4960     1 4904  7.841109e-07
## 4961     1 4905  7.657841e-07
## 4962     1 4906  7.453716e-07
## 4963     1 4907  7.263999e-07
## 4964     1 4908  7.077636e-07
## 4965     1 4909  6.881823e-07
## 4966     1 4910  6.670366e-07
## 4967     1 4911  6.458634e-07
## 4968     1 4912  6.244895e-07
## 4969     1 4913  6.040506e-07
## 4970     1 4914  5.875777e-07
## 4971     1 4915  5.717863e-07
## 4972     1 4916  5.569019e-07
## 4973     1 4917  5.417032e-07
## 4974     1 4918  5.268393e-07
## 4975     1 4919  5.141030e-07
## 4976     1 4920  4.996909e-07
## 4977     1 4921  4.860249e-07
## 4978     1 4922  4.728686e-07
## 4979     1 4923  4.592717e-07
## 4980     1 4924  4.457768e-07
## 4981     1 4925  4.337700e-07
## 4982     1 4926  4.198810e-07
## 4983     1 4927  4.069445e-07
## 4984     1 4928  3.948502e-07
## 4985     1 4929  3.828601e-07
## 4986     1 4930  3.696362e-07
## 4987     1 4931  3.585582e-07
## 4988     1 4932  3.470721e-07
## 4989     1 4933  3.356960e-07
## 4990     1 4934  3.250490e-07
## 4991     1 4935  3.146553e-07
## 4992     1 4936  3.045004e-07
## 4993     1 4937  2.945044e-07
## 4994     1 4938  2.851816e-07
## 4995     1 4939  2.768899e-07
## 4996     1 4940  2.682505e-07
## 4997     1 4941  2.600186e-07
## 4998     1 4942  2.529986e-07
## 4999     1 4943  2.462391e-07
## 5000     1 4944  2.403271e-07
## 5001     1 4945  2.348001e-07
## 5002     1 4946  2.302693e-07
## 5003     1 4947  2.255721e-07
## 5004     1 4948  2.200722e-07
## 5005     1 4949  2.141075e-07
## 5006     1 4950  2.086175e-07
## 5007     1 4951  2.032789e-07
## 5008     1 4952  1.979909e-07
## 5009     1 4953  1.927257e-07
## 5010     1 4954  1.875073e-07
## 5011     1 4955  1.825177e-07
## 5012     1 4956  1.779725e-07
## 5013     1 4957  1.727278e-07
## 5014     1 4958  1.680281e-07
## 5015     1 4959  1.634618e-07
## 5016     1 4960  1.585683e-07
## 5017     1 4961  1.533310e-07
## 5018     1 4962  1.482721e-07
## 5019     1 4963  1.432978e-07
## 5020     1 4964  1.377893e-07
## 5021     1 4965  1.340888e-07
## 5022     1 4966  1.302235e-07
## 5023     1 4967  1.265276e-07
## 5024     1 4968  1.230282e-07
## 5025     1 4969  1.196181e-07
## 5026     1 4970  1.158418e-07
## 5027     1 4971  1.125067e-07
## 5028     1 4972  1.094356e-07
## 5029     1 4973  1.060208e-07
## 5030     1 4974  1.028883e-07
## 5031     1 4975  1.000760e-07
## 5032     1 4976  9.708337e-08
## 5033     1 4977  9.414681e-08
## 5034     1 4978  9.144573e-08
## 5035     1 4979  8.853190e-08
## 5036     1 4980  8.611233e-08
## 5037     1 4981  8.355522e-08
## 5038     1 4982  8.101446e-08
## 5039     1 4983  7.851331e-08
## 5040     1 4984  7.624856e-08
## 5041     1 4985  7.384150e-08
## 5042     1 4986  7.181923e-08
## 5043     1 4987  6.973459e-08
## 5044     1 4988  6.747062e-08
## 5045     1 4989  6.554294e-08
## 5046     1 4990  6.367974e-08
## 5047     1 4991  6.188332e-08
## 5048     1 4992  6.016438e-08
## 5049     1 4993  5.848784e-08
## 5050     1 4994  5.686201e-08
## 5051     1 4995  5.534727e-08
## 5052     1 4996  5.394692e-08
## 5053     1 4997  5.255288e-08
## 5054     1 4998  5.126651e-08
## 5055     1 4999  4.981770e-08
## 5056     1 5000  4.825991e-08
## 5057     1 5001  4.693981e-08
## 5058     1 5002  4.538504e-08
## 5059     1 5003  4.412261e-08
## 5060     1 5004  4.277503e-08
## 5061     1 5005  4.154697e-08
## 5062     1 5006  4.035123e-08
## 5063     1 5007  3.913403e-08
## 5064     1 5008  3.802369e-08
## 5065     1 5009  3.692311e-08
## 5066     1 5010  3.586785e-08
## 5067     1 5011  3.489349e-08
## 5068     1 5012  3.401530e-08
## 5069     1 5013  3.311818e-08
## 5070     1 5014  3.228452e-08
## 5071     1 5015  3.157349e-08
## 5072     1 5016  3.083344e-08
## 5073     1 5017  3.013266e-08
## 5074     1 5018  2.966542e-08
## 5075     1 5019  2.908902e-08
## 5076     1 5020  2.867415e-08
## 5077     1 5021  2.831903e-08
## 5078     1 5022  2.801541e-08
## 5079     1 5023  2.776483e-08
## 5080     1 5024  2.761941e-08
## 5081     1 5025  2.758907e-08
## 5082     1 5026  2.759931e-08
## 5083     1 5027  2.771543e-08
## 5084     1 5028  2.757336e-08
## 5085     1 5029  2.724921e-08
## 5086     1 5030  2.691009e-08
## 5087     1 5031  2.665753e-08
## 5088     1 5032  2.641439e-08
## 5089     1 5033  2.620355e-08
## 5090     1 5034  2.593494e-08
## 5091     1 5035  2.574307e-08
## 5092     1 5036  2.555811e-08
## 5093     1 5037  2.531873e-08
## 5094     1 5038  2.509769e-08
## 5095     1 5039  2.492102e-08
## 5096     1 5040  2.470932e-08
## 5097     1 5041  2.450237e-08
## 5098     1 5042  2.438903e-08
## 5099     1 5043  2.414267e-08
## 5100     1 5044  2.391611e-08
## 5101     1 5045  2.367945e-08
## 5102     1 5046  2.344627e-08
## 5103     1 5047  2.315457e-08
## 5104     1 5048  2.292779e-08
## 5105     1 5049  2.258081e-08
## 5106     1 5050  2.222662e-08
## 5107     1 5051  2.189232e-08
## 5108     1 5052  2.152027e-08
## 5109     1 5053  2.107642e-08
## 5110     1 5054  2.058949e-08
## 5111     1 5055  2.007733e-08
## 5112     1 5056  1.951387e-08
## 5113     1 5057  1.895199e-08
## 5114     1 5058  1.874093e-08
## 5115     1 5059  1.843375e-08
## 5116     1 5060  1.817341e-08
## 5117     1 5061  1.793937e-08
## 5118     1 5062  1.766215e-08
## 5119     1 5063  1.739073e-08
## 5120     1 5064  1.716947e-08
## 5121     1 5065  1.686512e-08
## 5122     1 5066  1.660833e-08
## 5123     1 5067  1.632740e-08
## 5124     1 5068  1.605018e-08
## 5125     1 5069  1.579880e-08
## 5126     1 5070  1.546618e-08
## 5127     1 5071  1.519304e-08
## 5128     1 5072  1.488671e-08
## 5129     1 5073  1.460782e-08
## 5130     1 5074  1.433133e-08
## 5131     1 5075  1.401227e-08
## 5132     1 5076  1.369185e-08
## 5133     1 5077  1.340835e-08
## 5134     1 5078  1.309169e-08
## 5135     1 5079  1.280946e-08
## 5136     1 5080  1.248422e-08
## 5137     1 5081  1.216589e-08
## 5138     1 5082  1.187744e-08
## 5139     1 5083  1.153951e-08
## 5140     1 5084  1.122078e-08
## 5141     1 5085  1.090640e-08
## 5142     1 5086  1.059490e-08
## 5143     1 5087  1.027191e-08
## 5144     1 5088  9.934384e-09
## 5145     1 5089  9.650052e-09
## 5146     1 5090  9.332688e-09
## 5147     1 5091  9.001952e-09
## 5148     1 5092  8.715429e-09
## 5149     1 5093  8.409297e-09
## 5150     1 5094  8.103349e-09
## 5151     1 5095  7.798221e-09
## 5152     1 5096  7.494184e-09
## 5153     1 5097  7.214406e-09
## 5154     1 5098  6.923644e-09
## 5155     1 5099  6.643749e-09
## 5156     1 5100  6.368538e-09
## 5157     1 5101  6.099317e-09
## 5158     1 5102  5.840584e-09
## 5159     1 5103  5.574639e-09
## 5160     1 5104  5.333801e-09
## 5161     1 5105  5.097278e-09
## 5162     1 5106  4.870300e-09
## 5163     1 5107  4.652975e-09
## 5164     1 5108  4.466522e-09
## 5165     1 5109  4.272542e-09
## 5166     1 5110  4.098910e-09
## 5167     1 5111  3.933605e-09
## 5168     1 5112  3.790539e-09
## 5169     1 5113  3.656808e-09
## 5170     1 5114  3.545219e-09
## 5171     1 5115  3.451747e-09
## 5172     1 5116  3.351673e-09
## 5173     1 5117  3.190996e-09
## 5174     1 5118  3.043427e-09
## 5175     1 5119  2.894273e-09
## 5176     1 5120  2.752597e-09
## 5177     1 5121  2.627293e-09
## 5178     1 5122  2.497629e-09
## 5179     1 5123  2.377019e-09
## 5180     1 5124  2.270358e-09
## 5181     1 5125  2.160321e-09
## 5182     1 5126  2.056905e-09
## 5183     1 5127  1.963446e-09
## 5184     1 5128  1.876579e-09
## 5185     1 5129  1.792430e-09
## 5186     1 5130  1.717083e-09
## 5187     1 5131  1.643497e-09
## 5188     1 5132  1.576383e-09
## 5189     1 5133  1.511460e-09
## 5190     1 5134  1.456509e-09
## 5191     1 5135  1.400254e-09
## 5192     1 5136  1.349908e-09
## 5193     1 5137  1.300690e-09
## 5194     1 5138  1.257359e-09
## 5195     1 5139  1.214174e-09
## 5196     1 5140  1.171077e-09
## 5197     1 5141  1.133877e-09
## 5198     1 5142  1.094859e-09
## 5199     1 5143  1.056467e-09
## 5200     1 5144  1.019198e-09
## 5201     1 5145  9.780115e-10
## 5202     1 5146  9.708155e-10
## 5203     1 5147  9.810581e-10
## 5204     1 5148  9.938619e-10
## 5205     1 5149  1.013848e-09
## 5206     1 5150  1.033548e-09
## 5207     1 5151  1.059926e-09
## 5208     1 5152  1.086428e-09
## 5209     1 5153  1.116717e-09
## 5210     1 5154  1.149343e-09
## 5211     1 5155  1.182117e-09
## 5212     1 5156  1.219796e-09
## 5213     1 5157  1.255750e-09
## 5214     1 5158  1.290456e-09
## 5215     1 5159  1.333512e-09
## 5216     1 5160  1.367940e-09
## 5217     1 5161  1.401759e-09
## 5218     1 5162  1.436698e-09
## 5219     1 5163  1.469357e-09
## 5220     1 5164  1.501606e-09
## 5221     1 5165  1.529737e-09
## 5222     1 5166  1.554025e-09
## 5223     1 5167  1.569452e-09
## 5224     1 5168  1.586044e-09
## 5225     1 5169  1.592974e-09
## 5226     1 5170  1.594247e-09
## 5227     1 5171  1.592624e-09
## 5228     1 5172  1.579179e-09
## 5229     1 5173  1.558829e-09
## 5230     1 5174  1.529915e-09
## 5231     1 5175  1.528933e-09
## 5232     1 5176  1.660751e-09
## 5233     1 5177  1.797944e-09
## 5234     1 5178  1.940146e-09
## 5235     1 5179  2.089334e-09
## 5236     1 5180  2.232537e-09
## 5237     1 5181  2.389574e-09
## 5238     1 5182  2.541314e-09
## 5239     1 5183  2.697521e-09
## 5240     1 5184  2.851660e-09
## 5241     1 5185  3.009595e-09
## 5242     1 5186  3.171904e-09
## 5243     1 5187  3.323327e-09
## 5244     1 5188  3.486351e-09
## 5245     1 5189  3.648285e-09
## 5246     1 5190  3.819788e-09
## 5247     1 5191  3.982627e-09
## 5248     1 5192  4.141439e-09
## 5249     1 5193  4.301982e-09
## 5250     1 5194  4.451538e-09
## 5251     1 5195  4.614193e-09
## 5252     1 5196  4.771064e-09
## 5253     1 5197  4.913457e-09
## 5254     1 5198  5.070692e-09
## 5255     1 5199  5.203411e-09
## 5256     1 5200  5.344630e-09
## 5257     1 5201  5.481967e-09
## 5258     1 5202  5.604174e-09
## 5259     1 5203  5.724278e-09
## 5260     1 5204  5.843109e-09
## 5261     1 5205  5.948277e-09
## 5262     1 5206  6.051789e-09
## 5263     1 5207  6.143974e-09
## 5264     1 5208  6.215174e-09
## 5265     1 5209  6.285129e-09
## 5266     1 5210  6.358665e-09
## 5267     1 5211  6.398367e-09
## 5268     1 5212  6.436218e-09
## 5269     1 5213  6.464589e-09
## 5270     1 5214  6.470932e-09
## 5271     1 5215  6.483358e-09
## 5272     1 5216  6.457069e-09
## 5273     1 5217  6.440477e-09
## 5274     1 5218  6.377645e-09
## 5275     1 5219  6.324705e-09
## 5276     1 5220  6.250641e-09
## 5277     1 5221  6.131090e-09
## 5278     1 5222  6.013292e-09
## 5279     1 5223  5.870914e-09
## 5280     1 5224  5.830608e-09
## 5281     1 5225  5.902233e-09
## 5282     1 5226  5.959536e-09
## 5283     1 5227  6.034301e-09
## 5284     1 5228  6.071551e-09
## 5285     1 5229  6.140736e-09
## 5286     1 5230  6.172735e-09
## 5287     1 5231  6.222378e-09
## 5288     1 5232  6.266529e-09
## 5289     1 5233  6.299914e-09
## 5290     1 5234  6.347915e-09
## 5291     1 5235  6.372897e-09
## 5292     1 5236  6.409452e-09
## 5293     1 5237  6.422275e-09
## 5294     1 5238  6.439934e-09
## 5295     1 5239  6.452304e-09
## 5296     1 5240  6.470810e-09
## 5297     1 5241  6.478818e-09
## 5298     1 5242  6.490962e-09
## 5299     1 5243  6.499151e-09
## 5300     1 5244  6.483424e-09
## 5301     1 5245  6.473712e-09
## 5302     1 5246  6.471326e-09
## 5303     1 5247  6.469288e-09
## 5304     1 5248  6.451821e-09
## 5305     1 5249  6.405725e-09
## 5306     1 5250  6.385215e-09
## 5307     1 5251  6.351769e-09
## 5308     1 5252  6.328363e-09
## 5309     1 5253  6.295819e-09
## 5310     1 5254  6.242364e-09
## 5311     1 5255  6.190402e-09
## 5312     1 5256  6.135086e-09
## 5313     1 5257  6.089093e-09
## 5314     1 5258  6.010143e-09
## 5315     1 5259  5.946657e-09
## 5316     1 5260  5.869618e-09
## 5317     1 5261  5.795638e-09
## 5318     1 5262  5.721135e-09
## 5319     1 5263  5.624948e-09
## 5320     1 5264  5.544512e-09
## 5321     1 5265  5.439151e-09
## 5322     1 5266  5.334000e-09
## 5323     1 5267  5.233971e-09
## 5324     1 5268  5.123839e-09
## 5325     1 5269  5.008081e-09
## 5326     1 5270  4.886788e-09
## 5327     1 5271  4.752712e-09
## 5328     1 5272  4.620152e-09
## 5329     1 5273  4.516711e-09
## 5330     1 5274  4.411618e-09
## 5331     1 5275  4.303254e-09
## 5332     1 5276  4.179576e-09
## 5333     1 5277  4.070555e-09
## 5334     1 5278  3.949315e-09
## 5335     1 5279  3.828258e-09
## 5336     1 5280  3.707631e-09
## 5337     1 5281  3.579853e-09
## 5338     1 5282  3.456592e-09
## 5339     1 5283  3.335929e-09
## 5340     1 5284  3.207830e-09
## 5341     1 5285  3.080405e-09
## 5342     1 5286  2.956101e-09
## 5343     1 5287  2.822532e-09
## 5344     1 5288  2.695642e-09
## 5345     1 5289  2.564581e-09
## 5346     1 5290  2.436157e-09
## 5347     1 5291  2.310039e-09
## 5348     1 5292  2.178685e-09
## 5349     1 5293  2.051754e-09
## 5350     1 5294  1.923704e-09
## 5351     1 5295  1.799672e-09
## 5352     1 5296  1.671215e-09
## 5353     1 5297  1.550512e-09
## 5354     1 5298  1.428410e-09
## 5355     1 5299  1.310783e-09
## 5356     1 5300  1.189481e-09
## 5357     1 5301  1.077430e-09
## 5358     1 5302  9.668904e-10
## 5359     1 5303  8.584019e-10
## 5360     1 5304  7.529160e-10
## 5361     1 5305  6.504838e-10
## 5362     1 5306  5.534587e-10
## 5363     1 5307  4.613474e-10
## 5364     1 5308  3.724669e-10
## 5365     1 5309  2.886921e-10
## 5366     1 5310  2.104883e-10
## 5367     1 5311  1.379867e-10
## 5368     1 5312  7.121097e-11
## 5369     1 5313  1.086003e-11
## 5370     1 5314 -4.283863e-11
## 5371     1 5315 -8.947964e-11
## 5372     1 5316 -1.285316e-10
## 5373     1 5317 -1.600215e-10
## 5374     1 5318 -1.833165e-10
## 5375     1 5319 -1.980763e-10
## 5376     1 5320 -2.038946e-10
## 5377     1 5321 -2.289954e-10
## 5378     1 5322 -3.186122e-10
## 5379     1 5323 -4.049474e-10
## 5380     1 5324 -4.910995e-10
## 5381     1 5325 -5.757442e-10
## 5382     1 5326 -6.568184e-10
## 5383     1 5327 -7.386551e-10
## 5384     1 5328 -8.168493e-10
## 5385     1 5329 -8.945885e-10
## 5386     1 5330 -9.685365e-10
## 5387     1 5331 -1.042108e-09
## 5388     1 5332 -1.112687e-09
## 5389     1 5333 -1.179348e-09
## 5390     1 5334 -1.245010e-09
## 5391     1 5335 -1.309353e-09
## 5392     1 5336 -1.369987e-09
## 5393     1 5337 -1.426038e-09
## 5394     1 5338 -1.482246e-09
## 5395     1 5339 -1.537077e-09
## 5396     1 5340 -1.590170e-09
## 5397     1 5341 -1.636621e-09
## 5398     1 5342 -1.681226e-09
## 5399     1 5343 -1.727047e-09
## 5400     1 5344 -1.766331e-09
## 5401     1 5345 -1.800723e-09
## 5402     1 5346 -1.834305e-09
## 5403     1 5347 -1.863121e-09
## 5404     1 5348 -1.893544e-09
## 5405     1 5349 -1.921848e-09
## 5406     1 5350 -1.940348e-09
## 5407     1 5351 -1.960714e-09
## 5408     1 5352 -1.973081e-09
## 5409     1 5353 -1.985306e-09
## 5410     1 5354 -1.997590e-09
## 5411     1 5355 -2.003606e-09
## 5412     1 5356 -2.002913e-09
## 5413     1 5357 -1.998918e-09
## 5414     1 5358 -1.992353e-09
## 5415     1 5359 -1.983603e-09
## 5416     1 5360 -1.975952e-09
## 5417     1 5361 -1.955664e-09
## 5418     1 5362 -1.938312e-09
## 5419     1 5363 -1.914107e-09
## 5420     1 5364 -1.884041e-09
## 5421     1 5365 -1.857786e-09
## 5422     1 5366 -1.820410e-09
## 5423     1 5367 -1.783233e-09
## 5424     1 5368 -1.740420e-09
## 5425     1 5369 -1.692087e-09
## 5426     1 5370 -1.656501e-09
## 5427     1 5371 -1.625589e-09
## 5428     1 5372 -1.592641e-09
## 5429     1 5373 -1.557427e-09
## 5430     1 5374 -1.523463e-09
## 5431     1 5375 -1.483013e-09
## 5432     1 5376 -1.442048e-09
## 5433     1 5377 -1.397538e-09
## 5434     1 5378 -1.356477e-09
## 5435     1 5379 -1.308743e-09
## 5436     1 5380 -1.265965e-09
## 5437     1 5381 -1.216125e-09
## 5438     1 5382 -1.167489e-09
## 5439     1 5383 -1.115742e-09
## 5440     1 5384 -1.065394e-09
## 5441     1 5385 -1.012147e-09
## 5442     1 5386 -9.579576e-10
## 5443     1 5387 -9.048801e-10
## 5444     1 5388 -8.499465e-10
## 5445     1 5389 -7.943706e-10
## 5446     1 5390 -7.390972e-10
## 5447     1 5391 -6.835294e-10
## 5448     1 5392 -6.267063e-10
## 5449     1 5393 -5.730862e-10
## 5450     1 5394 -5.169810e-10
## 5451     1 5395 -4.625052e-10
## 5452     1 5396 -4.077559e-10
## 5453     1 5397 -3.545227e-10
## 5454     1 5398 -3.008974e-10
## 5455     1 5399 -2.494348e-10
## 5456     1 5400 -1.986395e-10
## 5457     1 5401 -1.493506e-10
## 5458     1 5402 -1.016100e-10
## 5459     1 5403 -5.546493e-11
## 5460     1 5404 -1.146205e-11
## 5461     1 5405  3.051085e-11
## 5462     1 5406  7.008781e-11
## 5463     1 5407  1.073764e-10
## 5464     1 5408  1.418639e-10
## 5465     1 5409  1.729196e-10
## 5466     1 5410  2.009189e-10
## 5467     1 5411  2.262825e-10
## 5468     1 5412  2.471449e-10
## 5469     1 5413  2.648231e-10
## 5470     1 5414  2.780481e-10
## 5471     1 5415  2.871785e-10
## 5472     1 5416  2.912140e-10
## 5473     1 5417  2.901033e-10
## 5474     1 5418  3.042946e-10
## 5475     1 5419  3.522750e-10
## 5476     1 5420  4.011066e-10
## 5477     1 5421  4.483481e-10
## 5478     1 5422  4.954657e-10
## 5479     1 5423  5.425749e-10
## 5480     1 5424  5.890472e-10
## 5481     1 5425  6.354047e-10
## 5482     1 5426  6.796249e-10
## 5483     1 5427  7.252871e-10
## 5484     1 5428  7.696580e-10
## 5485     1 5429  8.121109e-10
## 5486     1 5430  8.546239e-10
## 5487     1 5431  8.965236e-10
## 5488     1 5432  9.355958e-10
## 5489     1 5433  9.758173e-10
## 5490     1 5434  1.011874e-09
## 5491     1 5435  1.049683e-09
## 5492     1 5436  1.083633e-09
## 5493     1 5437  1.117288e-09
## 5494     1 5438  1.149118e-09
## 5495     1 5439  1.184643e-09
## 5496     1 5440  1.211847e-09
## 5497     1 5441  1.240999e-09
## 5498     1 5442  1.268483e-09
## 5499     1 5443  1.290700e-09
## 5500     1 5444  1.312554e-09
## 5501     1 5445  1.334658e-09
## 5502     1 5446  1.354258e-09
## 5503     1 5447  1.370162e-09
## 5504     1 5448  1.385853e-09
## 5505     1 5449  1.398288e-09
## 5506     1 5450  1.409882e-09
## 5507     1 5451  1.421037e-09
## 5508     1 5452  1.426527e-09
## 5509     1 5453  1.431480e-09
## 5510     1 5454  1.431270e-09
## 5511     1 5455  1.430797e-09
## 5512     1 5456  1.429591e-09
## 5513     1 5457  1.422391e-09
## 5514     1 5458  1.415091e-09
## 5515     1 5459  1.402569e-09
## 5516     1 5460  1.386553e-09
## 5517     1 5461  1.370728e-09
## 5518     1 5462  1.353352e-09
## 5519     1 5463  1.327723e-09
## 5520     1 5464  1.302562e-09
## 5521     1 5465  1.272408e-09
## 5522     1 5466  1.240514e-09
## 5523     1 5467  1.232258e-09
## 5524     1 5468  1.267390e-09
## 5525     1 5469  1.299879e-09
## 5526     1 5470  1.330730e-09
## 5527     1 5471  1.364454e-09
## 5528     1 5472  1.399231e-09
## 5529     1 5473  1.428404e-09
## 5530     1 5474  1.458537e-09
## 5531     1 5475  1.491416e-09
## 5532     1 5476  1.520017e-09
## 5533     1 5477  1.550155e-09
## 5534     1 5478  1.578909e-09
## 5535     1 5479  1.608078e-09
## 5536     1 5480  1.633302e-09
## 5537     1 5481  1.660774e-09
## 5538     1 5482  1.682585e-09
## 5539     1 5483  1.709605e-09
## 5540     1 5484  1.736150e-09
## 5541     1 5485  1.753284e-09
## 5542     1 5486  1.777166e-09
## 5543     1 5487  1.799629e-09
## 5544     1 5488  1.824441e-09
## 5545     1 5489  1.839800e-09
## 5546     1 5490  1.859046e-09
## 5547     1 5491  1.882131e-09
## 5548     1 5492  1.896432e-09
## 5549     1 5493  1.913573e-09
## 5550     1 5494  1.933271e-09
## 5551     1 5495  1.948206e-09
## 5552     1 5496  1.962519e-09
## 5553     1 5497  1.977146e-09
## 5554     1 5498  1.990512e-09
## 5555     1 5499  1.998433e-09
## 5556     1 5500  2.015642e-09
## 5557     1 5501  2.024521e-09
## 5558     1 5502  2.035260e-09
## 5559     1 5503  2.042778e-09
## 5560     1 5504  2.050735e-09
## 5561     1 5505  2.056560e-09
## 5562     1 5506  2.062343e-09
## 5563     1 5507  2.072037e-09
## 5564     1 5508  2.071299e-09
## 5565     1 5509  2.077454e-09
## 5566     1 5510  2.077934e-09
## 5567     1 5511  2.079476e-09
## 5568     1 5512  2.081599e-09
## 5569     1 5513  2.080114e-09
## 5570     1 5514  2.084594e-09
## 5571     1 5515  2.078625e-09
## 5572     1 5516  2.077094e-09
## 5573     1 5517  2.074870e-09
## 5574     1 5518  2.075062e-09
## 5575     1 5519  2.067341e-09
## 5576     1 5520  2.062970e-09
## 5577     1 5521  2.051124e-09
## 5578     1 5522  2.049703e-09
## 5579     1 5523  2.037702e-09
## 5580     1 5524  2.032164e-09
## 5581     1 5525  2.018717e-09
## 5582     1 5526  2.009346e-09
## 5583     1 5527  1.999888e-09
## 5584     1 5528  1.984542e-09
## 5585     1 5529  1.967203e-09
## 5586     1 5530  1.955118e-09
## 5587     1 5531  1.942634e-09
## 5588     1 5532  1.926360e-09
## 5589     1 5533  1.907551e-09
## 5590     1 5534  1.891978e-09
## 5591     1 5535  1.875229e-09
## 5592     1 5536  1.852761e-09
## 5593     1 5537  1.833229e-09
## 5594     1 5538  1.814174e-09
## 5595     1 5539  1.794830e-09
## 5596     1 5540  1.773364e-09
## 5597     1 5541  1.746359e-09
## 5598     1 5542  1.727571e-09
## 5599     1 5543  1.705014e-09
## 5600     1 5544  1.679052e-09
## 5601     1 5545  1.652931e-09
## 5602     1 5546  1.629516e-09
## 5603     1 5547  1.599367e-09
## 5604     1 5548  1.575170e-09
## 5605     1 5549  1.544541e-09
## 5606     1 5550  1.517072e-09
## 5607     1 5551  1.490098e-09
## 5608     1 5552  1.460182e-09
## 5609     1 5553  1.430900e-09
## 5610     1 5554  1.397648e-09
## 5611     1 5555  1.368095e-09
## 5612     1 5556  1.336662e-09
## 5613     1 5557  1.306339e-09
## 5614     1 5558  1.272454e-09
## 5615     1 5559  1.237857e-09
## 5616     1 5560  1.205418e-09
## 5617     1 5561  1.171709e-09
## 5618     1 5562  1.133520e-09
## 5619     1 5563  1.096851e-09
## 5620     1 5564  1.059578e-09
## 5621     1 5565  1.022799e-09
## 5622     1 5566  9.814218e-10
## 5623     1 5567  9.421181e-10
## 5624     1 5568  9.043992e-10
## 5625     1 5569  8.616112e-10
## 5626     1 5570  8.218062e-10
## 5627     1 5571  7.815708e-10
## 5628     1 5572  7.402535e-10
## 5629     1 5573  6.995073e-10
## 5630     1 5574  6.578219e-10
## 5631     1 5575  6.168612e-10
## 5632     1 5576  5.733652e-10
## 5633     1 5577  5.317326e-10
## 5634     1 5578  4.887915e-10
## 5635     1 5579  4.455659e-10
```

```
## 5636   1 5580  4.023023e-10
## 5637   1 5581  3.596311e-10
## 5638   1 5582  3.164145e-10
## 5639   1 5583  2.731981e-10
## 5640   1 5584  2.293041e-10
## 5641   1 5585  1.858244e-10
## 5642   1 5586  1.421084e-10
## 5643   1 5587  9.880695e-11
## 5644   1 5588  5.509740e-11
## 5645   1 5589  1.159679e-11
## 5646   1 5590 -3.184879e-11
## 5647   1 5591 -7.527382e-11
## 5648   1 5592 -1.181825e-10
## 5649   1 5593 -1.614053e-10
## 5650   1 5594 -2.047741e-10
## 5651   1 5595 -2.477333e-10
## 5652   1 5596 -2.894876e-10
## 5653   1 5597 -3.316861e-10
## 5654   1 5598 -3.736533e-10
## 5655   1 5599 -4.148068e-10
## 5656   1 5600 -4.565894e-10
## 5657   1 5601 -4.977438e-10
## 5658   1 5602 -5.381645e-10
## 5659   1 5603 -5.785576e-10
## 5660   1 5604 -6.176134e-10
## 5661   1 5605 -6.563098e-10
## 5662   1 5606 -6.963131e-10
## 5663   1 5607 -7.341376e-10
## 5664   1 5608 -7.710402e-10
## 5665   1 5609 -8.092738e-10
## 5666   1 5610 -8.445887e-10
## 5667   1 5611 -8.799529e-10
## 5668   1 5612 -9.127595e-10
## 5669   1 5613 -9.499522e-10
## 5670   1 5614 -9.817636e-10
## 5671   1 5615 -1.013010e-09
## 5672   1 5616 -1.046267e-09
## 5673   1 5617 -1.078191e-09
## 5674   1 5618 -1.105470e-09
## 5675   1 5619 -1.132749e-09
## 5676   1 5620 -1.163950e-09
## 5677   1 5621 -1.191875e-09
## 5678   1 5622 -1.216192e-09
## 5679   1 5623 -1.242119e-09
## 5680   1 5624 -1.262257e-09
## 5681   1 5625 -1.285753e-09
## 5682   1 5626 -1.306454e-09
## 5683   1 5627 -1.329489e-09
## 5684   1 5628 -1.345602e-09
## 5685   1 5629 -1.362049e-09
## 5686   1 5630 -1.380571e-09
## 5687   1 5631 -1.397626e-09
## 5688   1 5632 -1.409871e-09
## 5689   1 5633 -1.419952e-09
## 5690   1 5634 -1.431299e-09
## 5691   1 5635 -1.437157e-09
## 5692   1 5636 -1.445823e-09
## 5693   1 5637 -1.452136e-09
## 5694   1 5638 -1.451760e-09
## 5695   1 5639 -1.458239e-09
## 5696   1 5640 -1.454366e-09
## 5697   1 5641 -1.454506e-09
## 5698   1 5642 -1.452491e-09
## 5699   1 5643 -1.445829e-09
## 5700   1 5644 -1.441067e-09
## 5701   1 5645 -1.427912e-09
## 5702   1 5646 -1.417074e-09
## 5703   1 5647 -1.401423e-09
## 5704   1 5648 -1.389122e-09
## 5705   1 5649 -1.370199e-09
## 5706   1 5650 -1.351041e-09
## 5707   1 5651 -1.330167e-09
## 5708   1 5652 -1.303114e-09
## 5709   1 5653 -1.274206e-09
## 5710   1 5654 -1.248311e-09
## 5711   1 5655 -1.216680e-09
## 5712   1 5656 -1.193806e-09
## 5713   1 5657 -1.219652e-09
## 5714   1 5658 -1.245674e-09
## 5715   1 5659 -1.271922e-09
## 5716   1 5660 -1.292202e-09
## 5717   1 5661 -1.313999e-09
## 5718   1 5662 -1.339201e-09
## 5719   1 5663 -1.365548e-09
## 5720   1 5664 -1.388498e-09
## 5721   1 5665 -1.408798e-09
## 5722   1 5666 -1.432150e-09
## 5723   1 5667 -1.454028e-09
## 5724   1 5668 -1.478544e-09
## 5725   1 5669 -1.497306e-09
## 5726   1 5670 -1.519579e-09
## 5727   1 5671 -1.541811e-09
## 5728   1 5672 -1.563526e-09
## 5729   1 5673 -1.579041e-09
## 5730   1 5674 -1.603264e-09
## 5731   1 5675 -1.621209e-09
## 5732   1 5676 -1.638381e-09
## 5733   1 5677 -1.660199e-09
## 5734   1 5678 -1.678184e-09
## 5735   1 5679 -1.697579e-09
## 5736   1 5680 -1.715016e-09
## 5737   1 5681 -1.731712e-09
## 5738   1 5682 -1.747486e-09
## 5739   1 5683 -1.765936e-09
## 5740   1 5684 -1.779601e-09
## 5741   1 5685 -1.796129e-09
## 5742   1 5686 -1.813750e-09
## 5743   1 5687 -1.825043e-09
## 5744   1 5688 -1.839000e-09
## 5745   1 5689 -1.853505e-09
## 5746   1 5690 -1.864975e-09
## 5747   1 5691 -1.879813e-09
## 5748   1 5692 -1.890199e-09
## 5749   1 5693 -1.898200e-09
## 5750   1 5694 -1.911326e-09
## 5751   1 5695 -1.924094e-09
## 5752   1 5696 -1.931503e-09
## 5753   1 5697 -1.940410e-09
## 5754   1 5698 -1.948939e-09
## 5755   1 5699 -1.962581e-09
## 5756   1 5700 -1.966208e-09
## 5757   1 5701 -1.975455e-09
## 5758   1 5702 -1.976546e-09
## 5759   1 5703 -1.982918e-09
## 5760   1 5704 -1.990630e-09
## 5761   1 5705 -1.995571e-09
## 5762   1 5706 -1.997376e-09
## 5763   1 5707 -1.999616e-09
## 5764   1 5708 -1.998944e-09
## 5765   1 5709 -2.002203e-09
## 5766   1 5710 -2.006748e-09
## 5767   1 5711 -2.005215e-09
## 5768   1 5712 -2.006099e-09
## 5769   1 5713 -2.003435e-09
## 5770   1 5714 -1.997784e-09
## 5771   1 5715 -2.001599e-09
## 5772   1 5716 -1.993834e-09
## 5773   1 5717 -1.991348e-09
## 5774   1 5718 -1.988012e-09
## 5775   1 5719 -1.978068e-09
## 5776   1 5720 -1.971484e-09
## 5777   1 5721 -1.959494e-09
## 5778   1 5722 -1.954563e-09
## 5779   1 5723 -1.943327e-09
## 5780   1 5724 -1.936475e-09
## 5781   1 5725 -1.924192e-09
## 5782   1 5726 -1.912647e-09
## 5783   1 5727 -1.902183e-09
## 5784   1 5728 -1.887636e-09
## 5785   1 5729 -1.868921e-09
## 5786   1 5730 -1.855054e-09
## 5787   1 5731 -1.839523e-09
## 5788   1 5732 -1.817650e-09
## 5789   1 5733 -1.799485e-09
## 5790   1 5734 -1.779822e-09
## 5791   1 5735 -1.759581e-09
## 5792   1 5736 -1.738787e-09
## 5793   1 5737 -1.716395e-09
## 5794   1 5738 -1.692565e-09
## 5795   1 5739 -1.665484e-09
## 5796   1 5740 -1.639692e-09
## 5797   1 5741 -1.616461e-09
## 5798   1 5742 -1.585090e-09
## 5799   1 5743 -1.556487e-09
## 5800   1 5744 -1.522999e-09
## 5801   1 5745 -1.495998e-09
## 5802   1 5746 -1.461324e-09
## 5803   1 5747 -1.430280e-09
## 5804   1 5748 -1.392845e-09
## 5805   1 5749 -1.355900e-09
## 5806   1 5750 -1.320733e-09
## 5807   1 5751 -1.298932e-09
## 5808   1 5752 -1.284587e-09
## 5809   1 5753 -1.272211e-09
## 5810   1 5754 -1.260157e-09
## 5811   1 5755 -1.246605e-09
## 5812   1 5756 -1.234258e-09
## 5813   1 5757 -1.220877e-09
## 5814   1 5758 -1.207723e-09
## 5815   1 5759 -1.192284e-09
## 5816   1 5760 -1.179687e-09
## 5817   1 5761 -1.166420e-09
## 5818   1 5762 -1.153112e-09
## 5819   1 5763 -1.135402e-09
## 5820   1 5764 -1.123373e-09
## 5821   1 5765 -1.107749e-09
## 5822   1 5766 -1.093518e-09
## 5823   1 5767 -1.080548e-09
## 5824   1 5768 -1.064520e-09
## 5825   1 5769 -1.049022e-09
## 5826   1 5770 -1.034274e-09
## 5827   1 5771 -1.017329e-09
## 5828   1 5772 -1.003633e-09
## 5829   1 5773 -9.874871e-10
## 5830   1 5774 -9.707853e-10
## 5831   1 5775 -9.558392e-10
## 5832   1 5776 -9.392982e-10
## 5833   1 5777 -9.251518e-10
## 5834   1 5778 -9.100051e-10
## 5835   1 5779 -8.918743e-10
## 5836   1 5780 -8.759692e-10
## 5837   1 5781 -8.615025e-10
## 5838   1 5782 -8.449658e-10
## 5839   1 5783 -8.288356e-10
## 5840   1 5784 -8.124067e-10
## 5841   1 5785 -7.971714e-10
## 5842   1 5786 -7.812250e-10
## 5843   1 5787 -7.650006e-10
## 5844   1 5788 -7.480436e-10
## 5845   1 5789 -7.323254e-10
## 5846   1 5790 -7.164039e-10
## 5847   1 5791 -7.010360e-10
## 5848   1 5792 -6.836002e-10
## 5849   1 5793 -6.675565e-10
## 5850   1 5794 -6.530292e-10
## 5851   1 5795 -6.363076e-10
## 5852   1 5796 -6.196280e-10
## 5853   1 5797 -6.034217e-10
## 5854   1 5798 -5.885634e-10
## 5855   1 5799 -5.730205e-10
## 5856   1 5800 -5.578022e-10
## 5857   1 5801 -5.421929e-10
## 5858   1 5802 -5.260007e-10
## 5859   1 5803 -5.103744e-10
## 5860   1 5804 -4.964379e-10
## 5861   1 5805 -4.814275e-10
## 5862   1 5806 -4.664072e-10
## 5863   1 5807 -4.512901e-10
## 5864   1 5808 -4.365793e-10
## 5865   1 5809 -4.220911e-10
## 5866   1 5810 -4.075806e-10
## 5867   1 5811 -3.945796e-10
## 5868   1 5812 -3.802038e-10
## 5869   1 5813 -3.662097e-10
## 5870   1 5814 -3.535652e-10
## 5871   1 5815 -3.396761e-10
## 5872   1 5816 -3.268190e-10
## 5873   1 5817 -3.138067e-10
## 5874   1 5818 -3.012110e-10
## 5875   1 5819 -2.888661e-10
## 5876   1 5820 -2.772043e-10
## 5877   1 5821 -2.648743e-10
## 5878   1 5822 -2.533672e-10
## 5879   1 5823 -2.420965e-10
## 5880   1 5824 -2.312835e-10
## 5881   1 5825 -2.201081e-10
## 5882   1 5826 -2.096793e-10
## 5883   1 5827 -1.999493e-10
## 5884   1 5828 -1.902369e-10
## 5885   1 5829 -1.809436e-10
## 5886   1 5830 -1.717249e-10
## 5887   1 5831 -1.630687e-10
## 5888   1 5832 -1.547246e-10
## 5889   1 5833 -1.469553e-10
## 5890   1 5834 -1.392689e-10
## 5891   1 5835 -1.317141e-10
## 5892   1 5836 -1.250962e-10
## 5893   1 5837 -1.187392e-10
## 5894   1 5838 -1.128030e-10
## 5895   1 5839 -1.074006e-10
## 5896   1 5840 -1.022028e-10
## 5897   1 5841 -9.770729e-11
## 5898   1 5842 -9.355291e-11
## 5899   1 5843 -8.981249e-11
## 5900   1 5844 -8.670074e-11
## 5901   1 5845 -3.216038e-11
## 5902   1 5846 -2.071389e-11
## 5903   1 5847 -9.310282e-12
## 5904   1 5848  2.067463e-12
## 5905   1 5849  1.344829e-11
## 5906   1 5850  2.478545e-11
## 5907   1 5851  3.609447e-11
## 5908   1 5852  4.740606e-11
## 5909   1 5853  5.853894e-11
## 5910   1 5854  6.987965e-11
## 5911   1 5855  8.085058e-11
## 5912   1 5856  9.209578e-11
## 5913   1 5857  1.029472e-10
## 5914   1 5858  1.138584e-10
## 5915   1 5859  1.248627e-10
## 5916   1 5860  1.357052e-10
## 5917   1 5861  1.463411e-10
## 5918   1 5862  1.570030e-10
## 5919   1 5863  1.674907e-10
## 5920   1 5864  1.779774e-10
## 5921   1 5865  1.884478e-10
## 5922   1 5866  1.987979e-10
## 5923   1 5867  2.091570e-10
## 5924   1 5868  2.188597e-10
## 5925   1 5869  2.286162e-10
## 5926   1 5870  2.388329e-10
## 5927   1 5871  2.484649e-10
## 5928   1 5872  2.579741e-10
## 5929   1 5873  2.671539e-10
## 5930   1 5874  2.768797e-10
## 5931   1 5875  2.857054e-10
## 5932   1 5876  2.950246e-10
## 5933   1 5877  3.035910e-10
## 5934   1 5878  3.123113e-10
## 5935   1 5879  3.204955e-10
## 5936   1 5880  3.290454e-10
## 5937   1 5881  3.367016e-10
## 5938   1 5882  3.448510e-10
## 5939   1 5883  3.530900e-10
## 5940   1 5884  3.609223e-10
## 5941   1 5885  3.681824e-10
## 5942   1 5886  3.756290e-10
## 5943   1 5887  3.828803e-10
## 5944   1 5888  3.892959e-10
## 5945   1 5889  3.961051e-10
## 5946   1 5890  4.024099e-10
## 5947   1 5891  4.080677e-10
## 5948   1 5892  4.137687e-10
## 5949   1 5893  4.199670e-10
## 5950   1 5894  4.261751e-10
## 5951   1 5895  4.316496e-10
## 5952   1 5896  4.359982e-10
## 5953   1 5897  4.400414e-10
## 5954   1 5898  4.447279e-10
## 5955   1 5899  4.494964e-10
## 5956   1 5900  4.539188e-10
## 5957   1 5901  4.570561e-10
## 5958   1 5902  4.608397e-10
## 5959   1 5903  4.641066e-10
## 5960   1 5904  4.669603e-10
## 5961   1 5905  4.690086e-10
## 5962   1 5906  4.718933e-10
## 5963   1 5907  4.745587e-10
## 5964   1 5908  4.747515e-10
## 5965   1 5909  4.767307e-10
## 5966   1 5910  4.783580e-10
## 5967   1 5911  4.793898e-10
## 5968   1 5912  4.797740e-10
## 5969   1 5913  4.791226e-10
## 5970   1 5914  4.797374e-10
## 5971   1 5915  4.788995e-10
## 5972   1 5916  4.791134e-10
## 5973   1 5917  4.774024e-10
## 5974   1 5918  4.759382e-10
## 5975   1 5919  4.740553e-10
## 5976   1 5920  4.713266e-10
## 5977   1 5921  4.688982e-10
## 5978   1 5922  4.650211e-10
## 5979   1 5923  4.624153e-10
## 5980   1 5924  4.592136e-10
## 5981   1 5925  4.550514e-10
## 5982   1 5926  4.496235e-10
## 5983   1 5927  4.466863e-10
## 5984   1 5928  4.407086e-10
## 5985   1 5929  4.349383e-10
## 5986   1 5930  4.290841e-10
## 5987   1 5931  4.229625e-10
## 5988   1 5932  4.154110e-10
## 5989   1 5933  4.090686e-10
## 5990   1 5934  4.008836e-10
## 5991   1 5935  3.929504e-10
## 5992   1 5936  3.840207e-10
## 5993   1 5937  3.751710e-10
## 5994   1 5938  3.657666e-10
## 5995   1 5939  3.561409e-10
## 5996   1 5940  3.662381e-10
## 5997   1 5941  3.978744e-10
## 5998   1 5942  4.296033e-10
## 5999   1 5943  4.617346e-10
## 6000   1 5944  4.933373e-10
## 6001   1 5945  5.243422e-10
## 6002   1 5946  5.574155e-10
## 6003   1 5947  5.882087e-10
## 6004   1 5948  6.199614e-10
## 6005   1 5949  6.521493e-10
## 6006   1 5950  6.834282e-10
## 6007   1 5951  7.149226e-10
## 6008   1 5952  7.463903e-10
## 6009   1 5953  7.786405e-10
## 6010   1 5954  8.102771e-10
## 6011   1 5955  8.430812e-10
## 6012   1 5956  8.741426e-10
## 6013   1 5957  9.046261e-10
## 6014   1 5958  9.367708e-10
## 6015   1 5959  9.699901e-10
## 6016   1 5960  1.000486e-09
## 6017   1 5961  1.031236e-09
## 6018   1 5962  1.063841e-09
## 6019   1 5963  1.095401e-09
## 6020   1 5964  1.127987e-09
## 6021   1 5965  1.158012e-09
## 6022   1 5966  1.190705e-09
## 6023   1 5967  1.219971e-09
## 6024   1 5968  1.254266e-09
## 6025   1 5969  1.287633e-09
## 6026   1 5970  1.317624e-09
## 6027   1 5971  1.347844e-09
## 6028   1 5972  1.378440e-09
## 6029   1 5973  1.410672e-09
## 6030   1 5974  1.442013e-09
## 6031   1 5975  1.469901e-09
## 6032   1 5976  1.505665e-09
## 6033   1 5977  1.536411e-09
## 6034   1 5978  1.562345e-09
## 6035   1 5979  1.598497e-09
## 6036   1 5980  1.625623e-09
## 6037   1 5981  1.658497e-09
## 6038   1 5982  1.688318e-09
## 6039   1 5983  1.719080e-09
## 6040   1 5984  1.747763e-09
## 6041   1 5985  1.775452e-09
## 6042   1 5986  1.809634e-09
## 6043   1 5987  1.840676e-09
## 6044   1 5988  1.871813e-09
## 6045   1 5989  1.907166e-09
## 6046   1 5990  1.928244e-09
## 6047   1 5991  1.959260e-09
## 6048   1 5992  1.992793e-09
## 6049   1 5993  2.024605e-09
## 6050   1 5994  2.048676e-09
## 6051   1 5995  2.079254e-09
## 6052   1 5996  2.107230e-09
## 6053   1 5997  2.136214e-09
## 6054   1 5998  2.168799e-09
## 6055   1 5999  2.196444e-09
## 6056   1 6000  2.218287e-09
## 6057   1 6001  2.250265e-09
## 6058   1 6002  2.278170e-09
## 6059   1 6003  2.312637e-09
## 6060   1 6004  2.336569e-09
## 6061   1 6005  2.365645e-09
## 6062   1 6006  2.392079e-09
## 6063   1 6007  2.421314e-09
## 6064   1 6008  2.448824e-09
## 6065   1 6009  2.476059e-09
## 6066   1 6010  2.500944e-09
## 6067   1 6011  2.531150e-09
## 6068   1 6012  2.556485e-09
## 6069   1 6013  2.579647e-09
## 6070   1 6014  2.611820e-09
## 6071   1 6015  2.633209e-09
## 6072   1 6016  2.662727e-09
## 6073   1 6017  2.690534e-09
## 6074   1 6018  2.714814e-09
## 6075   1 6019  2.742854e-09
## 6076   1 6020  2.767746e-09
## 6077   1 6021  2.796034e-09
## 6078   1 6022  2.821434e-09
## 6079   1 6023  2.842957e-09
## 6080   1 6024  2.868462e-09
## 6081   1 6025  2.892690e-09
## 6082   1 6026  2.914816e-09
## 6083   1 6027  2.939045e-09
## 6084   1 6028  2.963898e-09
## 6085   1 6029  2.982920e-09
## 6086   1 6030  3.010658e-09
## 6087   1 6031  3.025080e-09
## 6088   1 6032  3.055975e-09
## 6089   1 6033  3.078895e-09
## 6090   1 6034  3.106129e-09
## 6091   1 6035  3.124067e-09
## 6092   1 6036  3.144731e-09
## 6093   1 6037  3.173979e-09
## 6094   1 6038  3.191162e-09
## 6095   1 6039  3.213738e-09
## 6096   1 6040  3.238344e-09
## 6097   1 6041  3.254735e-09
## 6098   1 6042  3.271098e-09
## 6099   1 6043  3.293415e-09
## 6100   1 6044  3.313319e-09
## 6101   1 6045  3.337228e-09
## 6102   1 6046  3.347772e-09
## 6103   1 6047  3.376005e-09
## 6104   1 6048  3.398029e-09
## 6105   1 6049  3.409635e-09
## 6106   1 6050  3.429265e-09
## 6107   1 6051  3.453808e-09
## 6108   1 6052  3.470995e-09
## 6109   1 6053  3.491105e-09
## 6110   1 6054  3.501486e-09
## 6111   1 6055  3.516750e-09
## 6112   1 6056  3.542877e-09
## 6113   1 6057  3.556249e-09
## 6114   1 6058  3.575374e-09
## 6115   1 6059  3.584375e-09
## 6116   1 6060  3.606052e-09
## 6117   1 6061  3.623594e-09
## 6118   1 6062  3.629630e-09
## 6119   1 6063  3.658927e-09
## 6120   1 6064  3.659288e-09
## 6121   1 6065  3.679495e-09
## 6122   1 6066  3.688176e-09
## 6123   1 6067  3.711970e-09
## 6124   1 6068  3.715215e-09
## 6125   1 6069  3.730903e-09
## 6126   1 6070  3.743246e-09
## 6127   1 6071  3.751896e-09
## 6128   1 6072  3.768578e-09
## 6129   1 6073  3.781432e-09
## 6130   1 6074  3.790483e-09
## 6131   1 6075  3.799007e-09
## 6132   1 6076  3.813582e-09
## 6133   1 6077  3.829250e-09
## 6134   1 6078  3.836464e-09
## 6135   1 6079  3.844871e-09
## 6136   1 6080  3.850055e-09
## 6137   1 6081  3.860820e-09
## 6138   1 6082  3.870178e-09
## 6139   1 6083  3.878202e-09
## 6140   1 6084  3.887870e-09
## 6141   1 6085  3.901910e-09
## 6142   1 6086  3.901403e-09
## 6143   1 6087  3.905239e-09
## 6144   1 6088  3.915772e-09
## 6145   1 6089  3.927294e-09
## 6146   1 6090  3.926535e-09
## 6147   1 6091  3.937540e-09
## 6148   1 6092  3.934947e-09
## 6149   1 6093  3.937939e-09
## 6150   1 6094  3.938968e-09
## 6151   1 6095  3.943945e-09
## 6152   1 6096  3.954014e-09
## 6153   1 6097  3.953099e-09
## 6154   1 6098  3.961176e-09
## 6155   1 6099  3.967896e-09
## 6156   1 6100  3.958826e-09
## 6157   1 6101  3.953268e-09
## 6158   1 6102  3.964665e-09
## 6159   1 6103  3.955844e-09
## 6160   1 6104  3.970274e-09
## 6161   1 6105  3.961028e-09
## 6162   1 6106  3.965584e-09
## 6163   1 6107  3.959762e-09
## 6164   1 6108  3.959375e-09
## 6165   1 6109  3.957375e-09
## 6166   1 6110  3.951978e-09
## 6167   1 6111  3.955012e-09
## 6168   1 6112  3.938684e-09
## 6169   1 6113  3.948891e-09
## 6170   1 6114  3.945484e-09
## 6171   1 6115  3.933408e-09
## 6172   1 6116  3.922355e-09
## 6173   1 6117  3.925884e-09
## 6174   1 6118  3.914668e-09
## 6175   1 6119  3.912220e-09
## 6176   1 6120  3.902991e-09
## 6177   1 6121  3.892590e-09
## 6178   1 6122  3.880533e-09
## 6179   1 6123  3.882002e-09
## 6180   1 6124  3.866334e-09
## 6181   1 6125  3.856220e-09
## 6182   1 6126  3.848966e-09
## 6183   1 6127  3.841968e-09
## 6184   1 6128  3.826582e-09
## 6185   1 6129  3.818136e-09
## 6186   1 6130  3.807409e-09
## 6187   1 6131  3.786802e-09
## 6188   1 6132  3.784633e-09
## 6189   1 6133  3.758360e-09
## 6190   1 6134  3.751334e-09
## 6191   1 6135  3.739265e-09
## 6192   1 6136  3.722741e-09
## 6193   1 6137  3.708919e-09
## 6194   1 6138  3.693651e-09
## 6195   1 6139  3.676662e-09
## 6196   1 6140  3.666240e-09
## 6197   1 6141  3.642468e-09
## 6198   1 6142  3.632228e-09
## 6199   1 6143  3.603058e-09
## 6200   1 6144  3.581594e-09
## 6201   1 6145  3.560250e-09
## 6202   1 6146  3.539370e-09
## 6203   1 6147  3.522832e-09
## 6204   1 6148  3.503789e-09
## 6205   1 6149  3.477683e-09
## 6206   1 6150  3.469003e-09
## 6207   1 6151  3.439439e-09
## 6208   1 6152  3.412479e-09
## 6209   1 6153  3.391029e-09
## 6210   1 6154  3.362800e-09
## 6211   1 6155  3.341165e-09
## 6212   1 6156  3.314254e-09
## 6213   1 6157  3.290340e-09
## 6214   1 6158  3.252853e-09
## 6215   1 6159  3.231560e-09
## 6216   1 6160  3.205640e-09
## 6217   1 6161  3.172827e-09
## 6218   1 6162  3.150855e-09
## 6219   1 6163  3.115136e-09
## 6220   1 6164  3.086394e-09
## 6221   1 6165  3.053560e-09
## 6222   1 6166  3.020093e-09
## 6223   1 6167  2.989723e-09
## 6224   1 6168  2.962253e-09
## 6225   1 6169  2.926179e-09
## 6226   1 6170  2.891523e-09
## 6227   1 6171  2.866127e-09
## 6228   1 6172  2.819954e-09
## 6229   1 6173  2.787461e-09
## 6230   1 6174  2.752300e-09
## 6231   1 6175  2.717482e-09
## 6232   1 6176  2.669266e-09
## 6233   1 6177  2.635864e-09
## 6234   1 6178  2.597648e-09
## 6235   1 6179  2.557152e-09
## 6236   1 6180  2.517558e-09
## 6237   1 6181  2.475673e-09
## 6238   1 6182  2.435221e-09
## 6239   1 6183  2.395667e-09
## 6240   1 6184  2.351034e-09
## 6241   1 6185  2.308557e-09
## 6242   1 6186  2.266836e-09
## 6243   1 6187  2.221284e-09
## 6244   1 6188  2.176950e-09
## 6245   1 6189  2.127866e-09
## 6246   1 6190  2.087865e-09
## 6247   1 6191  2.036837e-09
## 6248   1 6192  1.986133e-09
## 6249   1 6193  1.940042e-09
## 6250   1 6194  1.888040e-09
## 6251   1 6195  1.841535e-09
## 6252   1 6196  1.789345e-09
## 6253   1 6197  1.753682e-09
## 6254   1 6198  1.754403e-09
## 6255   1 6199  1.760801e-09
## 6256   1 6200  1.760731e-09
## 6257   1 6201  1.765403e-09
## 6258   1 6202  1.771001e-09
## 6259   1 6203  1.774185e-09
## 6260   1 6204  1.771566e-09
## 6261   1 6205  1.786218e-09
## 6262   1 6206  1.782661e-09
## 6263   1 6207  1.785683e-09
## 6264   1 6208  1.790348e-09
## 6265   1 6209  1.792826e-09
## 6266   1 6210  1.795470e-09
## 6267   1 6211  1.797631e-09
## 6268   1 6212  1.801002e-09
## 6269   1 6213  1.806624e-09
## 6270   1 6214  1.806487e-09
## 6271   1 6215  1.810854e-09
## 6272   1 6216  1.812087e-09
## 6273   1 6217  1.816694e-09
## 6274   1 6218  1.816703e-09
## 6275   1 6219  1.820071e-09
## 6276   1 6220  1.823823e-09
## 6277   1 6221  1.824798e-09
## 6278   1 6222  1.829609e-09
## 6279   1 6223  1.833895e-09
## 6280   1 6224  1.832998e-09
## 6281   1 6225  1.838592e-09
## 6282   1 6226  1.838019e-09
## 6283   1 6227  1.842561e-09
## 6284   1 6228  1.845591e-09
## 6285   1 6229  1.848198e-09
## 6286   1 6230  1.847840e-09
## 6287   1 6231  1.850546e-09
## 6288   1 6232  1.853630e-09
## 6289   1 6233  1.857067e-09
## 6290   1 6234  1.859419e-09
## 6291   1 6235  1.860551e-09
## 6292   1 6236  1.860654e-09
## 6293   1 6237  1.863503e-09
## 6294   1 6238  1.863461e-09
## 6295   1 6239  1.867631e-09
## 6296   1 6240  1.869968e-09
## 6297   1 6241  1.871809e-09
## 6298   1 6242  1.872381e-09
## 6299   1 6243  1.870407e-09
## 6300   1 6244  1.873641e-09
## 6301   1 6245  1.878708e-09
## 6302   1 6246  1.874357e-09
## 6303   1 6247  1.878189e-09
## 6304   1 6248  1.878503e-09
## 6305   1 6249  1.878962e-09
## 6306   1 6250  1.884745e-09
## 6307   1 6251  1.883265e-09
## 6308   1 6252  1.884326e-09
## 6309   1 6253  1.884893e-09
## 6310   1 6254  1.887966e-09
## 6311   1 6255  1.884559e-09
## 6312   1 6256  1.889084e-09
## 6313   1 6257  1.887686e-09
## 6314   1 6258  1.888474e-09
## 6315   1 6259  1.889633e-09
## 6316   1 6260  1.886556e-09
## 6317   1 6261  1.893121e-09
## 6318   1 6262  1.893883e-09
## 6319   1 6263  1.892877e-09
## 6320   1 6264  1.892584e-09
## 6321   1 6265  1.892700e-09
## 6322   1 6266  1.892161e-09
## 6323   1 6267  1.892796e-09
## 6324   1 6268  1.896111e-09
## 6325   1 6269  1.894979e-09
## 6326   1 6270  1.894193e-09
## 6327   1 6271  1.896221e-09
## 6328   1 6272  1.889689e-09
## 6329   1 6273  1.893297e-09
## 6330   1 6274  1.894106e-09
## 6331   1 6275  1.891526e-09
## 6332   1 6276  1.891908e-09
## 6333   1 6277  1.893278e-09
## 6334   1 6278  1.889879e-09
## 6335   1 6279  1.892193e-09
## 6336   1 6280  1.887659e-09
## 6337   1 6281  1.891153e-09
## 6338   1 6282  1.888616e-09
## 6339   1 6283  1.886553e-09
## 6340   1 6284  1.890186e-09
## 6341   1 6285  1.884667e-09
## 6342   1 6286  1.884806e-09
## 6343   1 6287  1.881152e-09
## 6344   1 6288  1.883203e-09
## 6345   1 6289  1.878468e-09
## 6346   1 6290  1.881492e-09
## 6347   1 6291  1.882240e-09
## 6348   1 6292  1.877272e-09
## 6349   1 6293  1.875154e-09
## 6350   1 6294  1.873858e-09
## 6351   1 6295  1.870408e-09
## 6352   1 6296  1.871784e-09
## 6353   1 6297  1.870859e-09
## 6354   1 6298  1.869228e-09
## 6355   1 6299  1.864852e-09
## 6356   1 6300  1.863336e-09
## 6357   1 6301  1.863421e-09
## 6358   1 6302  1.859689e-09
## 6359   1 6303  1.859007e-09
## 6360   1 6304  1.854313e-09
## 6361   1 6305  1.852863e-09
## 6362   1 6306  1.851560e-09
## 6363   1 6307  1.847974e-09
## 6364   1 6308  1.845207e-09
## 6365   1 6309  1.844284e-09
## 6366   1 6310  1.843373e-09
## 6367   1 6311  1.838760e-09
## 6368   1 6312  1.833263e-09
## 6369   1 6313  1.833114e-09
## 6370   1 6314  1.830263e-09
## 6371   1 6315  1.828126e-09
## 6372   1 6316  1.823444e-09
## 6373   1 6317  1.819838e-09
## 6374   1 6318  1.816522e-09
## 6375   1 6319  1.814192e-09
## 6376   1 6320  1.810265e-09
## 6377   1 6321  1.806504e-09
## 6378   1 6322  1.802311e-09
## 6379   1 6323  1.799205e-09
## 6380   1 6324  1.797554e-09
## 6381   1 6325  1.791123e-09
## 6382   1 6326  1.786574e-09
## 6383   1 6327  1.782441e-09
## 6384   1 6328  1.778459e-09
## 6385   1 6329  1.777422e-09
## 6386   1 6330  1.771496e-09
## 6387   1 6331  1.767943e-09
## 6388   1 6332  1.759223e-09
## 6389   1 6333  1.761005e-09
## 6390   1 6334  1.753247e-09
## 6391   1 6335  1.749018e-09
## 6392   1 6336  1.745688e-09
## 6393   1 6337  1.741093e-09
## 6394   1 6338  1.737228e-09
## 6395   1 6339  1.727463e-09
## 6396   1 6340  1.724214e-09
## 6397   1 6341  1.720821e-09
## 6398   1 6342  1.712850e-09
## 6399   1 6343  1.712606e-09
## 6400   1 6344  1.705116e-09
## 6401   1 6345  1.701323e-09
## 6402   1 6346  1.693394e-09
## 6403   1 6347  1.686617e-09
## 6404   1 6348  1.681251e-09
## 6405   1 6349  1.677521e-09
## 6406   1 6350  1.672634e-09
## 6407   1 6351  1.666426e-09
## 6408   1 6352  1.661355e-09
## 6409   1 6353  1.651782e-09
## 6410   1 6354  1.649624e-09
## 6411   1 6355  1.642979e-09
## 6412   1 6356  1.634615e-09
## 6413   1 6357  1.625743e-09
## 6414   1 6358  1.623867e-09
## 6415   1 6359  1.613451e-09
## 6416   1 6360  1.610295e-09
## 6417   1 6361  1.599954e-09
## 6418   1 6362  1.594827e-09
## 6419   1 6363  1.589234e-09
## 6420   1 6364  1.581159e-09
## 6421   1 6365  1.574207e-09
## 6422   1 6366  1.566265e-09
## 6423   1 6367  1.561093e-09
## 6424   1 6368  1.551827e-09
## 6425   1 6369  1.544407e-09
## 6426   1 6370  1.537929e-09
## 6427   1 6371  1.529000e-09
## 6428   1 6372  1.522273e-09
## 6429   1 6373  1.513748e-09
## 6430   1 6374  1.505789e-09
## 6431   1 6375  1.494246e-09
## 6432   1 6376  1.492493e-09
## 6433   1 6377  1.482342e-09
## 6434   1 6378  1.471476e-09
## 6435   1 6379  1.462780e-09
```

```
## 6436   1 6380  1.456504e-09
## 6437   1 6381  1.449350e-09
## 6438   1 6382  1.439398e-09
## 6439   1 6383  1.429943e-09
## 6440   1 6384  1.422194e-09
## 6441   1 6385  1.410885e-09
## 6442   1 6386  1.403555e-09
## 6443   1 6387  1.394048e-09
## 6444   1 6388  1.386492e-09
## 6445   1 6389  1.375589e-09
## 6446   1 6390  1.366717e-09
## 6447   1 6391  1.357444e-09
## 6448   1 6392  1.346559e-09
## 6449   1 6393  1.339068e-09
## 6450   1 6394  1.329582e-09
## 6451   1 6395  1.318215e-09
## 6452   1 6396  1.309621e-09
## 6453   1 6397  1.296760e-09
## 6454   1 6398  1.287823e-09
## 6455   1 6399  1.277388e-09
## 6456   1 6400  1.268244e-09
## 6457   1 6401  1.255771e-09
## 6458   1 6402  1.247072e-09
## 6459   1 6403  1.235523e-09
## 6460   1 6404  1.226813e-09
## 6461   1 6405  1.212974e-09
## 6462   1 6406  1.202791e-09
## 6463   1 6407  1.191497e-09
## 6464   1 6408  1.180511e-09
## 6465   1 6409  1.167869e-09
## 6466   1 6410  1.158073e-09
## 6467   1 6411  1.145738e-09
## 6468   1 6412  1.133557e-09
## 6469   1 6413  1.122544e-09
## 6470   1 6414  1.110996e-09
## 6471   1 6415  1.096799e-09
## 6472   1 6416  1.086143e-09
## 6473   1 6417  1.075071e-09
## 6474   1 6418  1.062392e-09
## 6475   1 6419  1.048455e-09
## 6476   1 6420  1.038189e-09
## 6477   1 6421  1.027164e-09
## 6478   1 6422  1.012220e-09
## 6479   1 6423  1.000837e-09
## 6480   1 6424  9.879786e-10
## 6481   1 6425  9.735163e-10
## 6482   1 6426  9.610585e-10
## 6483   1 6427  9.474315e-10
## 6484   1 6428  9.334213e-10
## 6485   1 6429  9.222441e-10
## 6486   1 6430  9.070679e-10
## 6487   1 6431  8.951502e-10
## 6488   1 6432  8.815437e-10
## 6489   1 6433  8.672245e-10
## 6490   1 6434  8.532038e-10
## 6491   1 6435  8.387473e-10
## 6492   1 6436  8.238165e-10
## 6493   1 6437  8.107592e-10
## 6494   1 6438  7.960333e-10
## 6495   1 6439  7.813417e-10
## 6496   1 6440  7.687303e-10
## 6497   1 6441  7.535705e-10
## 6498   1 6442  7.375307e-10
## 6499   1 6443  7.238481e-10
## 6500   1 6444  7.070589e-10
## 6501   1 6445  6.911708e-10
## 6502   1 6446  6.774257e-10
## 6503   1 6447  6.614784e-10
## 6504   1 6448  6.446508e-10
## 6505   1 6449  6.315281e-10
## 6506   1 6450  6.154018e-10
## 6507   1 6451  6.001305e-10
## 6508   1 6452  5.836752e-10
## 6509   1 6453  5.676032e-10
## 6510   1 6454  5.515712e-10
## 6511   1 6455  5.458986e-10
## 6512   1 6456  5.404709e-10
## 6513   1 6457  5.359429e-10
## 6514   1 6458  5.296593e-10
## 6515   1 6459  5.229589e-10
## 6516   1 6460  5.188345e-10
## 6517   1 6461  5.127197e-10
## 6518   1 6462  5.072421e-10
## 6519   1 6463  5.017114e-10
## 6520   1 6464  4.957783e-10
## 6521   1 6465  4.906669e-10
## 6522   1 6466  4.841639e-10
## 6523   1 6467  4.798593e-10
## 6524   1 6468  4.743792e-10
## 6525   1 6469  4.685931e-10
## 6526   1 6470  4.624466e-10
## 6527   1 6471  4.579946e-10
## 6528   1 6472  4.519548e-10
## 6529   1 6473  4.471041e-10
## 6530   1 6474  4.403783e-10
## 6531   1 6475  4.354398e-10
## 6532   1 6476  4.309195e-10
## 6533   1 6477  4.251773e-10
## 6534   1 6478  4.202843e-10
## 6535   1 6479  4.133709e-10
## 6536   1 6480  4.084272e-10
## 6537   1 6481  4.032525e-10
## 6538   1 6482  3.976364e-10
## 6539   1 6483  3.928132e-10
## 6540   1 6484  3.877523e-10
## 6541   1 6485  3.824912e-10
## 6542   1 6486  3.770379e-10
## 6543   1 6487  3.712897e-10
## 6544   1 6488  3.669360e-10
## 6545   1 6489  3.608275e-10
## 6546   1 6490  3.561137e-10
## 6547   1 6491  3.510315e-10
## 6548   1 6492  3.455159e-10
## 6549   1 6493  3.414931e-10
## 6550   1 6494  3.347093e-10
## 6551   1 6495  3.302623e-10
## 6552   1 6496  3.251070e-10
## 6553   1 6497  3.202902e-10
## 6554   1 6498  3.150623e-10
## 6555   1 6499  3.096899e-10
## 6556   1 6500  3.050509e-10
## 6557   1 6501  2.998616e-10
## 6558   1 6502  2.945636e-10
## 6559   1 6503  2.899812e-10
## 6560   1 6504  2.852861e-10
## 6561   1 6505  2.801890e-10
## 6562   1 6506  2.748870e-10
## 6563   1 6507  2.699626e-10
## 6564   1 6508  2.652445e-10
## 6565   1 6509  2.600611e-10
## 6566   1 6510  2.551097e-10
## 6567   1 6511  2.504082e-10
## 6568   1 6512  2.456050e-10
## 6569   1 6513  2.408886e-10
## 6570   1 6514  2.362043e-10
## 6571   1 6515  2.311059e-10
## 6572   1 6516  2.265686e-10
## 6573   1 6517  2.216025e-10
## 6574   1 6518  2.167894e-10
## 6575   1 6519  2.121355e-10
## 6576   1 6520  2.077980e-10
## 6577   1 6521  2.028143e-10
## 6578   1 6522  1.985773e-10
## 6579   1 6523  1.936624e-10
## 6580   1 6524  1.896032e-10
## 6581   1 6525  1.844312e-10
## 6582   1 6526  1.800400e-10
## 6583   1 6527  1.754910e-10
## 6584   1 6528  1.710572e-10
## 6585   1 6529  1.663756e-10
## 6586   1 6530  1.619725e-10
## 6587   1 6531  1.575659e-10
## 6588   1 6532  1.532721e-10
## 6589   1 6533  1.487550e-10
## 6590   1 6534  1.444451e-10
## 6591   1 6535  1.400262e-10
## 6592   1 6536  1.357442e-10
## 6593   1 6537  1.314934e-10
## 6594   1 6538  1.272970e-10
## 6595   1 6539  1.230102e-10
## 6596   1 6540  1.188138e-10
## 6597   1 6541  1.146287e-10
## 6598   1 6542  1.103016e-10
## 6599   1 6543  1.061291e-10
## 6600   1 6544  1.018960e-10
## 6601   1 6545  9.788624e-11
## 6602   1 6546  9.370020e-11
## 6603   1 6547  8.970400e-11
## 6604   1 6548  8.587541e-11
## 6605   1 6549  8.179050e-11
## 6606   1 6550  7.766946e-11
## 6607   1 6551  7.374087e-11
## 6608   1 6552  6.988954e-11
## 6609   1 6553  6.614745e-11
## 6610   1 6554  6.206954e-11
## 6611   1 6555  5.820842e-11
## 6612   1 6556  5.451364e-11
## 6613   1 6557  5.065311e-11
## 6614   1 6558  4.696369e-11
## 6615   1 6559  4.318132e-11
## 6616   1 6560  3.949169e-11
## 6617   1 6561  3.581606e-11
## 6618   1 6562  3.216334e-11
## 6619   1 6563  2.852533e-11
## 6620   1 6564  2.498530e-11
## 6621   1 6565  2.136879e-11
## 6622   1 6566  1.790179e-11
## 6623   1 6567  1.436121e-11
## 6624   1 6568  1.088279e-11
## 6625   1 6569  7.443961e-12
## 6626   1 6570  4.030220e-12
## 6627   1 6571  6.369046e-13
## 6628   1 6572 -2.719001e-12
## 6629   1 6573 -6.043665e-12
## 6630   1 6574 -9.328126e-12
## 6631   1 6575 -1.261326e-11
## 6632   1 6576 -1.582782e-11
## 6633   1 6577 -1.904247e-11
## 6634   1 6578 -2.222068e-11
## 6635   1 6579 -2.533179e-11
## 6636   1 6580 -2.846654e-11
## 6637   1 6581 -3.149184e-11
## 6638   1 6582 -3.456082e-11
## 6639   1 6583 -3.761750e-11
## 6640   1 6584 -4.061742e-11
## 6641   1 6585 -4.351583e-11
## 6642   1 6586 -4.640221e-11
## 6643   1 6587 -4.937977e-11
## 6644   1 6588 -5.219810e-11
## 6645   1 6589 -5.498257e-11
## 6646   1 6590 -5.784917e-11
## 6647   1 6591 -6.052775e-11
## 6648   1 6592 -6.335812e-11
## 6649   1 6593 -6.594257e-11
## 6650   1 6594 -6.856876e-11
## 6651   1 6595 -7.116113e-11
## 6652   1 6596 -7.366663e-11
## 6653   1 6597 -7.629565e-11
## 6654   1 6598 -7.892528e-11
## 6655   1 6599 -8.131890e-11
## 6656   1 6600 -8.379034e-11
## 6657   1 6601 -8.597825e-11
## 6658   1 6602 -8.849984e-11
## 6659   1 6603 -9.078611e-11
## 6660   1 6604 -9.328451e-11
## 6661   1 6605 -9.514958e-11
## 6662   1 6606 -9.766523e-11
## 6663   1 6607 -9.968337e-11
## 6664   1 6608 -1.018083e-10
## 6665   1 6609 -1.041910e-10
## 6666   1 6610 -1.063591e-10
## 6667   1 6611 -1.081557e-10
## 6668   1 6612 -1.099750e-10
## 6669   1 6613 -1.121343e-10
## 6670   1 6614 -1.140532e-10
## 6671   1 6615 -1.159544e-10
## 6672   1 6616 -1.177931e-10
## 6673   1 6617 -1.196744e-10
## 6674   1 6618 -1.213538e-10
## 6675   1 6619 -1.234114e-10
## 6676   1 6620 -1.247037e-10
## 6677   1 6621 -1.262959e-10
## 6678   1 6622 -1.280210e-10
## 6679   1 6623 -1.293603e-10
## 6680   1 6624 -1.311397e-10
## 6681   1 6625 -1.325303e-10
## 6682   1 6626 -1.339688e-10
## 6683   1 6627 -1.356538e-10
## 6684   1 6628 -1.367643e-10
## 6685   1 6629 -1.382949e-10
## 6686   1 6630 -1.392348e-10
## 6687   1 6631 -1.408552e-10
## 6688   1 6632 -1.421545e-10
## 6689   1 6633 -1.431397e-10
## 6690   1 6634 -1.444033e-10
## 6691   1 6635 -1.455291e-10
## 6692   1 6636 -1.463053e-10
## 6693   1 6637 -1.473632e-10
## 6694   1 6638 -1.481715e-10
## 6695   1 6639 -1.493534e-10
## 6696   1 6640 -1.500200e-10
## 6697   1 6641 -1.510325e-10
## 6698   1 6642 -1.515107e-10
## 6699   1 6643 -1.525481e-10
## 6700   1 6644 -1.531046e-10
## 6701   1 6645 -1.539313e-10
## 6702   1 6646 -1.546059e-10
## 6703   1 6647 -1.549908e-10
## 6704   1 6648 -1.552071e-10
## 6705   1 6649 -1.559331e-10
## 6706   1 6650 -1.563727e-10
## 6707   1 6651 -1.567411e-10
## 6708   1 6652 -1.573112e-10
## 6709   1 6653 -1.572604e-10
## 6710   1 6654 -1.575326e-10
## 6711   1 6655 -1.579542e-10
## 6712   1 6656 -1.584791e-10
## 6713   1 6657 -1.586000e-10
## 6714   1 6658 -1.584306e-10
## 6715   1 6659 -1.585779e-10
## 6716   1 6660 -1.582460e-10
## 6717   1 6661 -1.581694e-10
## 6718   1 6662 -1.582545e-10
## 6719   1 6663 -1.580184e-10
## 6720   1 6664 -1.577530e-10
## 6721   1 6665 -1.575409e-10
## 6722   1 6666 -1.570843e-10
## 6723   1 6667 -1.568213e-10
## 6724   1 6668 -1.565684e-10
## 6725   1 6669 -1.561250e-10
## 6726   1 6670 -1.556902e-10
## 6727   1 6671 -1.551769e-10
## 6728   1 6672 -1.543831e-10
## 6729   1 6673 -1.537008e-10
## 6730   1 6674 -1.529884e-10
## 6731   1 6675 -1.521092e-10
## 6732   1 6676 -1.515080e-10
## 6733   1 6677 -1.507745e-10
## 6734   1 6678 -1.496607e-10
## 6735   1 6679 -1.490157e-10
## 6736   1 6680 -1.478016e-10
## 6737   1 6681 -1.468109e-10
## 6738   1 6682 -1.458321e-10
## 6739   1 6683 -1.446183e-10
## 6740   1 6684 -1.436120e-10
## 6741   1 6685 -1.424637e-10
## 6742   1 6686 -1.407513e-10
## 6743   1 6687 -1.396321e-10
## 6744   1 6688 -1.379916e-10
## 6745   1 6689 -1.366774e-10
## 6746   1 6690 -1.351691e-10
## 6747   1 6691 -1.337459e-10
## 6748   1 6692 -1.320363e-10
## 6749   1 6693 -1.303371e-10
## 6750   1 6694 -1.286405e-10
## 6751   1 6695 -1.269264e-10
## 6752   1 6696 -1.251872e-10
## 6753   1 6697 -1.232469e-10
## 6754   1 6698 -1.214303e-10
## 6755   1 6699 -1.192788e-10
## 6756   1 6700 -1.173489e-10
## 6757   1 6701 -1.152390e-10
## 6758   1 6702 -1.128408e-10
## 6759   1 6703 -1.110261e-10
## 6760   1 6704 -1.087278e-10
## 6761   1 6705 -1.064846e-10
## 6762   1 6706 -1.040806e-10
## 6763   1 6707 -1.016348e-10
## 6764   1 6708 -9.899352e-11
## 6765   1 6709 -9.668332e-11
## 6766   1 6710 -9.408261e-11
## 6767   1 6711 -9.131133e-11
## 6768   1 6712 -7.175402e-11
## 6769   1 6713 -7.326170e-11
## 6770   1 6714 -7.488511e-11
## 6771   1 6715 -7.603916e-11
## 6772   1 6716 -7.769863e-11
## 6773   1 6717 -7.906025e-11
## 6774   1 6718 -8.058562e-11
## 6775   1 6719 -8.198163e-11
## 6776   1 6720 -8.326823e-11
## 6777   1 6721 -8.469159e-11
## 6778   1 6722 -8.595450e-11
## 6779   1 6723 -8.744031e-11
## 6780   1 6724 -8.869152e-11
## 6781   1 6725 -9.019319e-11
## 6782   1 6726 -9.141469e-11
## 6783   1 6727 -9.285903e-11
## 6784   1 6728 -9.391554e-11
## 6785   1 6729 -9.524582e-11
## 6786   1 6730 -9.650662e-11
## 6787   1 6731 -9.765133e-11
## 6788   1 6732 -9.899906e-11
## 6789   1 6733 -1.002718e-10
## 6790   1 6734 -1.014821e-10
## 6791   1 6735 -1.026983e-10
## 6792   1 6736 -1.039004e-10
## 6793   1 6737 -1.051077e-10
## 6794   1 6738 -1.064374e-10
## 6795   1 6739 -1.076317e-10
## 6796   1 6740 -1.084155e-10
## 6797   1 6741 -1.097470e-10
## 6798   1 6742 -1.109332e-10
## 6799   1 6743 -1.120301e-10
## 6800   1 6744 -1.129309e-10
## 6801   1 6745 -1.144638e-10
## 6802   1 6746 -1.152483e-10
## 6803   1 6747 -1.165392e-10
## 6804   1 6748 -1.175267e-10
## 6805   1 6749 -1.185336e-10
## 6806   1 6750 -1.196507e-10
## 6807   1 6751 -1.205544e-10
## 6808   1 6752 -1.217454e-10
## 6809   1 6753 -1.226311e-10
## 6810   1 6754 -1.236196e-10
## 6811   1 6755 -1.246721e-10
## 6812   1 6756 -1.255429e-10
## 6813   1 6757 -1.264598e-10
## 6814   1 6758 -1.277195e-10
## 6815   1 6759 -1.282796e-10
## 6816   1 6760 -1.292224e-10
## 6817   1 6761 -1.303113e-10
## 6818   1 6762 -1.310919e-10
## 6819   1 6763 -1.320699e-10
## 6820   1 6764 -1.325969e-10
## 6821   1 6765 -1.335254e-10
## 6822   1 6766 -1.344886e-10
## 6823   1 6767 -1.352343e-10
## 6824   1 6768 -1.358420e-10
## 6825   1 6769 -1.369563e-10
## 6826   1 6770 -1.375417e-10
## 6827   1 6771 -1.384442e-10
## 6828   1 6772 -1.391525e-10
## 6829   1 6773 -1.398339e-10
## 6830   1 6774 -1.408506e-10
## 6831   1 6775 -1.412912e-10
## 6832   1 6776 -1.421598e-10
## 6833   1 6777 -1.427800e-10
## 6834   1 6778 -1.437227e-10
## 6835   1 6779 -1.447354e-10
## 6836   1 6780 -1.450959e-10
## 6837   1 6781 -1.455838e-10
## 6838   1 6782 -1.462130e-10
## 6839   1 6783 -1.467173e-10
## 6840   1 6784 -1.474884e-10
## 6841   1 6785 -1.481350e-10
## 6842   1 6786 -1.487714e-10
## 6843   1 6787 -1.493107e-10
## 6844   1 6788 -1.498778e-10
## 6845   1 6789 -1.504708e-10
## 6846   1 6790 -1.511111e-10
## 6847   1 6791 -1.514350e-10
## 6848   1 6792 -1.521368e-10
## 6849   1 6793 -1.524500e-10
## 6850   1 6794 -1.525965e-10
## 6851   1 6795 -1.534089e-10
## 6852   1 6796 -1.542758e-10
## 6853   1 6797 -1.544611e-10
## 6854   1 6798 -1.551990e-10
## 6855   1 6799 -1.552772e-10
## 6856   1 6800 -1.558476e-10
## 6857   1 6801 -1.563162e-10
## 6858   1 6802 -1.567039e-10
## 6859   1 6803 -1.570957e-10
## 6860   1 6804 -1.572317e-10
## 6861   1 6805 -1.578393e-10
## 6862   1 6806 -1.579668e-10
## 6863   1 6807 -1.582777e-10
## 6864   1 6808 -1.588280e-10
## 6865   1 6809 -1.593312e-10
## 6866   1 6810 -1.592971e-10
## 6867   1 6811 -1.594754e-10
## 6868   1 6812 -1.602197e-10
## 6869   1 6813 -1.604735e-10
## 6870   1 6814 -1.602403e-10
## 6871   1 6815 -1.607423e-10
## 6872   1 6816 -1.609384e-10
## 6873   1 6817 -1.610488e-10
## 6874   1 6818 -1.614509e-10
## 6875   1 6819 -1.616870e-10
## 6876   1 6820 -1.617587e-10
## 6877   1 6821 -1.618806e-10
## 6878   1 6822 -1.623122e-10
## 6879   1 6823 -1.620564e-10
## 6880   1 6824 -1.623266e-10
## 6881   1 6825 -1.626751e-10
## 6882   1 6826 -1.622689e-10
## 6883   1 6827 -1.627490e-10
## 6884   1 6828 -1.628602e-10
## 6885   1 6829 -1.627199e-10
## 6886   1 6830 -1.627862e-10
## 6887   1 6831 -1.629658e-10
## 6888   1 6832 -1.630502e-10
## 6889   1 6833 -1.631970e-10
## 6890   1 6834 -1.632919e-10
## 6891   1 6835 -1.631164e-10
## 6892   1 6836 -1.630036e-10
## 6893   1 6837 -1.632438e-10
## 6894   1 6838 -1.630578e-10
## 6895   1 6839 -1.629117e-10
## 6896   1 6840 -1.630691e-10
## 6897   1 6841 -1.628538e-10
## 6898   1 6842 -1.630163e-10
## 6899   1 6843 -1.626809e-10
## 6900   1 6844 -1.625882e-10
## 6901   1 6845 -1.629524e-10
## 6902   1 6846 -1.623821e-10
## 6903   1 6847 -1.619297e-10
## 6904   1 6848 -1.621263e-10
## 6905   1 6849 -1.620716e-10
## 6906   1 6850 -1.615694e-10
## 6907   1 6851 -1.616118e-10
## 6908   1 6852 -1.615306e-10
## 6909   1 6853 -1.615412e-10
## 6910   1 6854 -1.607299e-10
## 6911   1 6855 -1.604931e-10
## 6912   1 6856 -1.605072e-10
## 6913   1 6857 -1.600810e-10
## 6914   1 6858 -1.599239e-10
## 6915   1 6859 -1.595368e-10
## 6916   1 6860 -1.592488e-10
## 6917   1 6861 -1.589322e-10
## 6918   1 6862 -1.586827e-10
## 6919   1 6863 -1.582748e-10
## 6920   1 6864 -1.578747e-10
## 6921   1 6865 -1.574573e-10
## 6922   1 6866 -1.573307e-10
## 6923   1 6867 -1.568966e-10
## 6924   1 6868 -1.563585e-10
## 6925   1 6869 -1.560413e-10
## 6926   1 6870 -1.554854e-10
## 6927   1 6871 -1.551120e-10
## 6928   1 6872 -1.543883e-10
## 6929   1 6873 -1.542043e-10
## 6930   1 6874 -1.539481e-10
## 6931   1 6875 -1.534886e-10
## 6932   1 6876 -1.524866e-10
## 6933   1 6877 -1.525356e-10
## 6934   1 6878 -1.518798e-10
## 6935   1 6879 -1.512251e-10
## 6936   1 6880 -1.506415e-10
## 6937   1 6881 -1.503528e-10
## 6938   1 6882 -1.498797e-10
## 6939   1 6883 -1.488492e-10
## 6940   1 6884 -1.486244e-10
## 6941   1 6885 -1.480833e-10
## 6942   1 6886 -1.473143e-10
## 6943   1 6887 -1.465277e-10
## 6944   1 6888 -1.462384e-10
## 6945   1 6889 -1.453391e-10
## 6946   1 6890 -1.444882e-10
## 6947   1 6891 -1.439059e-10
## 6948   1 6892 -1.433250e-10
## 6949   1 6893 -1.427684e-10
## 6950   1 6894 -1.420798e-10
## 6951   1 6895 -1.411010e-10
## 6952   1 6896 -1.406670e-10
## 6953   1 6897 -1.398804e-10
## 6954   1 6898 -1.389107e-10
## 6955   1 6899 -1.381995e-10
## 6956   1 6900 -1.374320e-10
## 6957   1 6901 -1.367090e-10
## 6958   1 6902 -1.358121e-10
## 6959   1 6903 -1.351324e-10
## 6960   1 6904 -1.341851e-10
## 6961   1 6905 -1.336328e-10
## 6962   1 6906 -1.326217e-10
## 6963   1 6907 -1.313762e-10
## 6964   1 6908 -1.305799e-10
## 6965   1 6909 -1.298316e-10
## 6966   1 6910 -1.288241e-10
## 6967   1 6911 -1.279340e-10
## 6968   1 6912 -1.271294e-10
## 6969   1 6913 -1.261518e-10
## 6970   1 6914 -1.251214e-10
## 6971   1 6915 -1.240995e-10
## 6972   1 6916 -1.231864e-10
## 6973   1 6917 -1.219395e-10
## 6974   1 6918 -1.212628e-10
## 6975   1 6919 -1.202580e-10
## 6976   1 6920 -1.191226e-10
## 6977   1 6921 -1.181010e-10
## 6978   1 6922 -1.172212e-10
## 6979   1 6923 -1.161219e-10
## 6980   1 6924 -1.149578e-10
## 6981   1 6925 -1.138468e-10
## 6982   1 6926 -1.125821e-10
## 6983   1 6927 -1.117901e-10
## 6984   1 6928 -1.105322e-10
## 6985   1 6929 -1.095648e-10
## 6986   1 6930 -1.082376e-10
## 6987   1 6931 -1.070367e-10
## 6988   1 6932 -1.059389e-10
## 6989   1 6933 -1.048686e-10
## 6990   1 6934 -1.036520e-10
## 6991   1 6935 -1.019982e-10
## 6992   1 6936 -1.011693e-10
## 6993   1 6937 -9.985030e-11
## 6994   1 6938 -9.863096e-11
## 6995   1 6939 -9.743734e-11
## 6996   1 6940 -9.625188e-11
## 6997   1 6941 -9.484222e-11
## 6998   1 6942 -9.352097e-11
## 6999   1 6943 -9.226973e-11
## 7000   1 6944 -9.096247e-11
## 7001   1 6945 -8.965653e-11
## 7002   1 6946 -8.827231e-11
## 7003   1 6947 -8.693703e-11
## 7004   1 6948 -8.563390e-11
## 7005   1 6949 -8.412904e-11
## 7006   1 6950 -8.280297e-11
## 7007   1 6951 -8.147354e-11
## 7008   1 6952 -7.993803e-11
## 7009   1 6953 -7.868839e-11
## 7010   1 6954 -7.707587e-11
## 7011   1 6955 -7.556521e-11
## 7012   1 6956 -7.408786e-11
## 7013   1 6957 -7.268374e-11
## 7014   1 6958 -7.115098e-11
## 7015   1 6959 -6.982669e-11
## 7016   1 6960 -6.823295e-11
## 7017   1 6961 -6.671173e-11
## 7018   1 6962 -6.519936e-11
## 7019   1 6963 -6.364032e-11
## 7020   1 6964 -6.213657e-11
## 7021   1 6965 -6.049304e-11
## 7022   1 6966 -5.885491e-11
## 7023   1 6967 -5.717466e-11
## 7024   1 6968 -5.572991e-11
## 7025   1 6969 -5.831909e-11
## 7026   1 6970 -6.403022e-11
## 7027   1 6971 -6.938090e-11
## 7028   1 6972 -7.494857e-11
## 7029   1 6973 -8.047878e-11
## 7030   1 6974 -8.607453e-11
## 7031   1 6975 -9.166125e-11
## 7032   1 6976 -9.695034e-11
## 7033   1 6977 -1.024305e-10
## 7034   1 6978 -1.078678e-10
## 7035   1 6979 -1.135420e-10
## 7036   1 6980 -1.191902e-10
## 7037   1 6981 -1.245842e-10
## 7038   1 6982 -1.297488e-10
## 7039   1 6983 -1.354793e-10
## 7040   1 6984 -1.408490e-10
## 7041   1 6985 -1.461130e-10
## 7042   1 6986 -1.517635e-10
## 7043   1 6987 -1.571194e-10
## 7044   1 6988 -1.628728e-10
## 7045   1 6989 -1.681937e-10
## 7046   1 6990 -1.734061e-10
## 7047   1 6991 -1.789869e-10
## 7048   1 6992 -1.842024e-10
## 7049   1 6993 -1.899252e-10
## 7050   1 6994 -1.953040e-10
## 7051   1 6995 -2.009677e-10
## 7052   1 6996 -2.057315e-10
## 7053   1 6997 -2.114400e-10
## 7054   1 6998 -2.162116e-10
## 7055   1 6999 -2.222186e-10
## 7056   1 7000 -2.277758e-10
## 7057   2    0  0.000000e+00
## 7058   2    0  9.151588e-01
## 7059   2    1  1.988222e+00
## 7060   2    2  1.865837e+00
## 7061   2    3  1.758285e+00
## 7062   2    4  1.661976e+00
## 7063   2    5  1.581484e+00
## 7064   2    6  1.511822e+00
## 7065   2    7  1.447727e+00
## 7066   2    8  1.392494e+00
## 7067   2    9  1.344830e+00
## 7068   2   10  1.297706e+00
## 7069   2   11  1.257939e+00
## 7070   2   12  1.220153e+00
## 7071   2   13  1.182577e+00
## 7072   2   14  1.152148e+00
## 7073   2   15  1.124472e+00
## 7074   2   16  1.094796e+00
## 7075   2   17  1.069001e+00
## 7076   2   18  1.043979e+00
## 7077   2   19  1.019307e+00
## 7078   2   20  9.970165e-01
## 7079   2   21  9.760275e-01
## 7080   2   22  9.532035e-01
## 7081   2   23  9.336528e-01
## 7082   2   24  9.147985e-01
## 7083   2   24  9.146283e-01
## 7084   2   25  1.986181e+00
## 7085   2   26  1.865797e+00
## 7086   2   27  1.754521e+00
## 7087   2   28  1.661667e+00
## 7088   2   29  1.580918e+00
## 7089   2   30  1.511761e+00
## 7090   2   31  1.448927e+00
## 7091   2   32  1.392755e+00
## 7092   2   33  1.342132e+00
## 7093   2   34  1.297144e+00
## 7094   2   35  1.257338e+00
## 7095   2   36  1.219761e+00
## 7096   2   37  1.183991e+00
## 7097   2   38  1.154433e+00
## 7098   2   39  1.123426e+00
## 7099   2   40  1.094355e+00
## 7100   2   41  1.068471e+00
## 7101   2   42  1.044029e+00
## 7102   2   43  1.020137e+00
## 7103   2   44  9.963836e-01
## 7104   2   45  9.746697e-01
## 7105   2   46  9.549108e-01
## 7106   2   47  9.333137e-01
## 7107   2   48  9.145197e-01
## 7108   2   48  9.145356e-01
## 7109   2   49  1.987006e+00
## 7110   2   50  1.861770e+00
## 7111   2   51  1.756639e+00
## 7112   2   52  1.664344e+00
## 7113   2   53  1.584461e+00
## 7114   2   54  1.512094e+00
## 7115   2   55  1.448721e+00
## 7116   2   56  1.392879e+00
## 7117   2   57  1.344747e+00
## 7118   2   58  1.298045e+00
## 7119   2   59  1.255747e+00
## 7120   2   60  1.220374e+00
## 7121   2   61  1.185442e+00
## 7122   2   62  1.152930e+00
## 7123   2   63  1.123623e+00
## 7124   2   64  1.093711e+00
## 7125   2   65  1.068625e+00
## 7126   2   66  1.042394e+00
## 7127   2   67  1.021023e+00
## 7128   2   68  9.968008e-01
## 7129   2   69  9.761548e-01
## 7130   2   70  9.536815e-01
## 7131   2   71  9.350309e-01
## 7132   2   72  9.137827e-01
## 7133   2   73  1.987318e+00
## 7134   2   74  1.863992e+00
## 7135   2   75  1.756580e+00
## 7136   2   76  1.665303e+00
## 7137   2   77  1.582807e+00
## 7138   2   78  1.511884e+00
## 7139   2   79  1.448963e+00
## 7140   2   80  1.393832e+00
## 7141   2   81  1.340149e+00
## 7142   2   82  1.297067e+00
## 7143   2   83  1.255268e+00
## 7144   2   84  1.218651e+00
## 7145   2   85  1.188318e+00
## 7146   2   86  1.154994e+00
## 7147   2   87  1.123819e+00
## 7148   2   88  1.092289e+00
## 7149   2   89  1.068512e+00
## 7150   2   90  1.041090e+00
## 7151   2   91  1.019158e+00
## 7152   2   92  9.972172e-01
## 7153   2   93  9.729873e-01
## 7154   2   94  9.534011e-01
## 7155   2   95  9.334384e-01
## 7156   2   96  9.124392e-01
## 7157   2   97  1.988874e+00
## 7158   2   98  1.863368e+00
## 7159   2   99  1.757901e+00
## 7160   2  100  1.663939e+00
## 7161   2  101  1.581305e+00
## 7162   2  102  1.512626e+00
## 7163   2  103  1.449282e+00
## 7164   2  104  1.392196e+00
## 7165   2  105  1.344435e+00
## 7166   2  106  1.298018e+00
## 7167   2  107  1.256579e+00
## 7168   2  108  1.218485e+00
## 7169   2  109  1.181755e+00
## 7170   2  110  1.152885e+00
## 7171   2  111  1.122124e+00
## 7172   2  112  1.094228e+00
## 7173   2  113  1.069842e+00
## 7174   2  114  1.044859e+00
## 7175   2  115  1.018914e+00
## 7176   2  116  9.963887e-01
## 7177   2  117  9.761006e-01
## 7178   2  118  9.533501e-01
## 7179   2  119  9.337502e-01
## 7180   2  120  9.134364e-01
## 7181   2  121  1.989566e+00
## 7182   2  122  1.862538e+00
## 7183   2  123  1.757465e+00
## 7184   2  124  1.664187e+00
## 7185   2  125  1.580788e+00
## 7186   2  126  1.511269e+00
## 7187   2  127  1.448993e+00
## 7188   2  128  1.391684e+00
## 7189   2  129  1.345014e+00
## 7190   2  130  1.296530e+00
## 7191   2  131  1.257482e+00
## 7192   2  132  1.219934e+00
## 7193   2  133  1.183232e+00
## 7194   2  134  1.152447e+00
## 7195   2  135  1.121673e+00
## 7196   2  136  1.093445e+00
## 7197   2  137  1.067688e+00
## 7198   2  138  1.043061e+00
## 7199   2  139  1.021232e+00
## 7200   2  140  9.967346e-01
## 7201   2  141  9.741699e-01
## 7202   2  142  9.531242e-01
## 7203   2  143  9.329740e-01
## 7204   2  144  9.148616e-01
## 7205   2  145  1.988025e+00
## 7206   2  146  1.859361e+00
## 7207   2  147  1.758630e+00
## 7208   2  148  1.662777e+00
## 7209   2  149  1.582173e+00
## 7210   2  150  1.511256e+00
## 7211   2  151  1.446847e+00
## 7212   2  152  1.392645e+00
## 7213   2  153  1.341755e+00
## 7214   2  154  1.296564e+00
## 7215   2  155  1.256246e+00
## 7216   2  156  1.218574e+00
## 7217   2  157  1.185477e+00
## 7218   2  158  1.150964e+00
## 7219   2  159  1.123078e+00
## 7220   2  160  1.095953e+00
## 7221   2  161  1.067791e+00
## 7222   2  162  1.042495e+00
## 7223   2  163  1.019178e+00
## 7224   2  164  9.959273e-01
## 7225   2  165  9.745673e-01
## 7226   2  166  9.530320e-01
## 7227   2  167  9.332513e-01
## 7228   2  168  9.136999e-01
## 7229   2  169  1.983432e+00
## 7230   2  170  1.862105e+00
## 7231   2  171  1.755302e+00
## 7232   2  172  1.661322e+00
## 7233   2  173  1.580226e+00
## 7234   2  174  1.510313e+00
## 7235   2  175  1.448954e+00
```

```
## 7236   2  176  1.393477e+00
## 7237   2  177  1.342507e+00
## 7238   2  178  1.299816e+00
## 7239   2  179  1.255879e+00
## 7240   2  180  1.220113e+00
## 7241   2  181  1.183276e+00
## 7242   2  182  1.153340e+00
## 7243   2  183  1.121170e+00
## 7244   2  184  1.094955e+00
## 7245   2  185  1.067816e+00
## 7246   2  186  1.043111e+00
## 7247   2  187  1.019876e+00
## 7248   2  188  9.980255e-01
## 7249   2  189  9.749083e-01
## 7250   2  190  9.540213e-01
## 7251   2  191  9.321264e-01
## 7252   2  192  9.140135e-01
## 7253   2  193  1.987001e+00
## 7254   2  194  1.865769e+00
## 7255   2  195  1.754938e+00
## 7256   2  196  1.662735e+00
## 7257   2  197  1.581365e+00
## 7258   2  198  1.510850e+00
## 7259   2  199  1.451027e+00
## 7260   2  200  1.390528e+00
## 7261   2  201  1.343918e+00
## 7262   2  202  1.300085e+00
## 7263   2  203  1.257369e+00
## 7264   2  204  1.217621e+00
## 7265   2  205  1.184145e+00
## 7266   2  206  1.152976e+00
## 7267   2  207  1.122920e+00
## 7268   2  208  1.096350e+00
## 7269   2  209  1.069282e+00
## 7270   2  210  1.043267e+00
## 7271   2  211  1.019450e+00
## 7272   2  212  9.968152e-01
## 7273   2  213  9.751033e-01
## 7274   2  214  9.538898e-01
## 7275   2  215  9.321729e-01
## 7276   2  216  9.143961e-01
## 7277   2  217  1.983425e+00
## 7278   2  218  1.863604e+00
## 7279   2  219  1.758835e+00
## 7280   2  220  1.663361e+00
## 7281   2  221  1.580603e+00
## 7282   2  222  1.510197e+00
## 7283   2  223  1.450397e+00
## 7284   2  224  1.389704e+00
## 7285   2  225  1.343992e+00
## 7286   2  226  1.298683e+00
## 7287   2  227  1.256762e+00
## 7288   2  228  1.218582e+00
## 7289   2  229  1.187295e+00
## 7290   2  230  1.153076e+00
## 7291   2  231  1.121023e+00
## 7292   2  232  1.094294e+00
## 7293   2  233  1.068691e+00
## 7294   2  234  1.043473e+00
## 7295   2  235  1.020297e+00
## 7296   2  236  9.941744e-01
## 7297   2  237  9.742623e-01
## 7298   2  238  9.530635e-01
## 7299   2  239  9.329943e-01
## 7300   2  240  9.136793e-01
## 7301   2  241  1.982070e+00
## 7302   2  242  1.861125e+00
## 7303   2  243  1.755075e+00
## 7304   2  244  1.662849e+00
## 7305   2  245  1.581204e+00
## 7306   2  246  1.510994e+00
## 7307   2  247  1.446453e+00
## 7308   2  248  1.393750e+00
## 7309   2  249  1.341957e+00
## 7310   2  250  1.296054e+00
## 7311   2  251  1.255815e+00
## 7312   2  252  1.218192e+00
## 7313   2  253  1.183974e+00
## 7314   2  254  1.154844e+00
## 7315   2  255  1.123152e+00
## 7316   2  256  1.094981e+00
## 7317   2  257  1.068429e+00
## 7318   2  258  1.043127e+00
## 7319   2  259  1.018797e+00
## 7320   2  260  9.960744e-01
## 7321   2  261  9.766157e-01
## 7322   2  262  9.542988e-01
## 7323   2  263  9.328893e-01
## 7324   2  264  9.156969e-01
## 7325   2  265  1.986916e+00
## 7326   2  266  1.863334e+00
## 7327   2  267  1.756065e+00
## 7328   2  268  1.663157e+00
## 7329   2  269  1.581988e+00
## 7330   2  270  1.512674e+00
## 7331   2  271  1.446258e+00
## 7332   2  272  1.394969e+00
## 7333   2  273  1.343768e+00
## 7334   2  274  1.299402e+00
## 7335   2  275  1.257661e+00
## 7336   2  276  1.220290e+00
## 7337   2  277  1.185554e+00
## 7338   2  278  1.153168e+00
## 7339   2  279  1.120789e+00
## 7340   2  280  1.092005e+00
## 7341   2  281  1.069417e+00
## 7342   2  282  1.043109e+00
## 7343   2  283  1.018918e+00
## 7344   2  284  9.960858e-01
## 7345   2  285  9.738656e-01
## 7346   2  286  9.537615e-01
## 7347   2  287  9.335282e-01
## 7348   2  288  9.143084e-01
## 7349   2  289  1.985467e+00
## 7350   2  290  1.864864e+00
## 7351   2  291  1.757822e+00
## 7352   2  292  1.664054e+00
## 7353   2  293  1.583227e+00
## 7354   2  294  1.512880e+00
## 7355   2  295  1.448326e+00
## 7356   2  296  1.394812e+00
## 7357   2  297  1.341087e+00
## 7358   2  298  1.296257e+00
## 7359   2  299  1.256049e+00
## 7360   2  300  1.220873e+00
## 7361   2  301  1.185256e+00
## 7362   2  302  1.151994e+00
## 7363   2  303  1.120590e+00
## 7364   2  304  1.094616e+00
## 7365   2  305  1.069321e+00
## 7366   2  306  1.042384e+00
## 7367   2  307  1.019486e+00
## 7368   2  308  9.960913e-01
## 7369   2  309  9.732605e-01
## 7370   2  310  9.539561e-01
## 7371   2  311  9.329883e-01
## 7372   2  312  9.122778e-01
## 7373   2  312  9.132317e-01
## 7374   2  313  1.983898e+00
## 7375   2  314  1.863537e+00
## 7376   2  315  1.754258e+00
## 7377   2  316  1.662874e+00
## 7378   2  317  1.584234e+00
## 7379   2  318  1.509306e+00
## 7380   2  319  1.450341e+00
## 7381   2  320  1.392925e+00
## 7382   2  321  1.344393e+00
## 7383   2  322  1.295963e+00
## 7384   2  323  1.257330e+00
## 7385   2  324  1.220587e+00
## 7386   2  325  1.184949e+00
## 7387   2  326  1.152452e+00
## 7388   2  327  1.122516e+00
## 7389   2  328  1.094142e+00
## 7390   2  329  1.068592e+00
## 7391   2  330  1.042930e+00
## 7392   2  331  1.019977e+00
## 7393   2  332  9.956972e-01
## 7394   2  333  9.750878e-01
## 7395   2  334  9.547124e-01
## 7396   2  335  9.324874e-01
## 7397   2  336  9.145956e-01
## 7398   2  336  9.133686e-01
## 7399   2  337  8.953387e-01
## 7400   2  338  8.761847e-01
## 7401   2  339  8.578936e-01
## 7402   2  339  8.575083e-01
## 7403   2  340  1.935513e+00
## 7404   2  341  1.812344e+00
## 7405   2  342  1.707392e+00
## 7406   2  343  1.613254e+00
## 7407   2  344  1.531701e+00
## 7408   2  345  1.464614e+00
## 7409   2  346  1.401130e+00
## 7410   2  347  1.345665e+00
## 7411   2  348  1.299281e+00
## 7412   2  349  1.255128e+00
## 7413   2  350  1.213770e+00
## 7414   2  351  1.177643e+00
## 7415   2  352  1.143917e+00
## 7416   2  353  1.113274e+00
## 7417   2  354  1.083654e+00
## 7418   2  355  1.056895e+00
## 7419   2  356  1.030035e+00
## 7420   2  357  1.006253e+00
## 7421   2  358  9.829649e-01
## 7422   2  359  9.642331e-01
## 7423   2  360  9.410621e-01
## 7424   2  360  9.409540e-01
## 7425   2  361  2.012387e+00
## 7426   2  362  1.884963e+00
## 7427   2  363  1.781627e+00
## 7428   2  364  1.687591e+00
## 7429   2  365  1.604494e+00
## 7430   2  366  1.527460e+00
## 7431   2  367  1.465452e+00
## 7432   2  368  1.412427e+00
## 7433   2  369  1.363548e+00
## 7434   2  370  1.313890e+00
## 7435   2  371  1.275874e+00
## 7436   2  372  1.235793e+00
## 7437   2  373  1.202352e+00
## 7438   2  374  1.167848e+00
## 7439   2  375  1.138751e+00
## 7440   2  376  1.109062e+00
## 7441   2  377  1.082668e+00
## 7442   2  378  1.057669e+00
## 7443   2  379  1.033708e+00
## 7444   2  380  1.010006e+00
## 7445   2  381  9.871710e-01
## 7446   2  382  9.667810e-01
## 7447   2  383  9.472689e-01
## 7448   2  384  9.274739e-01
## 7449   2  385  2.000945e+00
## 7450   2  386  1.874168e+00
## 7451   2  387  1.772362e+00
## 7452   2  388  1.672781e+00
## 7453   2  389  1.594735e+00
## 7454   2  390  1.523037e+00
## 7455   2  391  1.460950e+00
## 7456   2  392  1.401450e+00
## 7457   2  393  1.353242e+00
## 7458   2  394  1.307229e+00
## 7459   2  395  1.267245e+00
## 7460   2  396  1.229559e+00
## 7461   2  397  1.193838e+00
## 7462   2  398  1.161533e+00
## 7463   2  399  1.132395e+00
## 7464   2  400  1.104000e+00
## 7465   2  401  1.075168e+00
## 7466   2  402  1.052758e+00
## 7467   2  403  1.027469e+00
## 7468   2  404  1.004549e+00
## 7469   2  405  9.831849e-01
## 7470   2  406  9.625860e-01
## 7471   2  407  9.425204e-01
## 7472   2  408  9.225983e-01
## 7473   2  409  1.995916e+00
## 7474   2  410  1.871036e+00
## 7475   2  411  1.762342e+00
## 7476   2  412  1.669198e+00
## 7477   2  413  1.589785e+00
## 7478   2  414  1.521816e+00
## 7479   2  415  1.455780e+00
## 7480   2  416  1.400319e+00
## 7481   2  417  1.348914e+00
## 7482   2  418  1.303857e+00
## 7483   2  419  1.263105e+00
## 7484   2  420  1.224678e+00
## 7485   2  421  1.191976e+00
## 7486   2  422  1.160438e+00
## 7487   2  423  1.129328e+00
## 7488   2  424  1.101074e+00
## 7489   2  425  1.072803e+00
## 7490   2  426  1.049309e+00
## 7491   2  427  1.027020e+00
## 7492   2  428  1.001256e+00
## 7493   2  429  9.820735e-01
## 7494   2  430  9.597529e-01
## 7495   2  431  9.398019e-01
## 7496   2  432  9.184546e-01
## 7497   2  433  1.991212e+00
## 7498   2  434  1.866408e+00
## 7499   2  435  1.755693e+00
## 7500   2  436  1.669423e+00
## 7501   2  437  1.587108e+00
## 7502   2  438  1.515380e+00
## 7503   2  439  1.454990e+00
## 7504   2  440  1.396976e+00
## 7505   2  441  1.347269e+00
## 7506   2  442  1.303861e+00
## 7507   2  443  1.259610e+00
## 7508   2  444  1.222021e+00
## 7509   2  445  1.188093e+00
## 7510   2  446  1.156365e+00
## 7511   2  447  1.128250e+00
## 7512   2  448  1.099658e+00
## 7513   2  449  1.071737e+00
## 7514   2  450  1.046005e+00
## 7515   2  451  1.024017e+00
## 7516   2  452  9.986961e-01
## 7517   2  453  9.780395e-01
## 7518   2  454  9.572334e-01
## 7519   2  455  9.376096e-01
## 7520   2  456  9.172632e-01
## 7521   2  457  1.990048e+00
## 7522   2  458  1.866284e+00
## 7523   2  459  1.757515e+00
## 7524   2  460  1.667420e+00
## 7525   2  461  1.583708e+00
## 7526   2  462  1.513602e+00
## 7527   2  463  1.449867e+00
## 7528   2  464  1.394053e+00
## 7529   2  465  1.347464e+00
## 7530   2  466  1.300856e+00
## 7531   2  467  1.261055e+00
## 7532   2  468  1.221826e+00
## 7533   2  469  1.186766e+00
## 7534   2  470  1.155706e+00
## 7535   2  471  1.125021e+00
## 7536   2  472  1.096520e+00
## 7537   2  473  1.071293e+00
## 7538   2  474  1.045213e+00
## 7539   2  475  1.020090e+00
## 7540   2  476  1.000093e+00
## 7541   2  477  9.764098e-01
## 7542   2  478  9.546389e-01
## 7543   2  479  9.361633e-01
## 7544   2  480  9.147277e-01
## 7545   2  481  1.989298e+00
## 7546   2  482  1.865244e+00
## 7547   2  483  1.755165e+00
## 7548   2  484  1.664870e+00
## 7549   2  485  1.584395e+00
## 7550   2  486  1.513696e+00
## 7551   2  487  1.449420e+00
## 7552   2  488  1.394004e+00
## 7553   2  489  1.346439e+00
## 7554   2  490  1.301121e+00
## 7555   2  491  1.260711e+00
## 7556   2  492  1.221043e+00
## 7557   2  493  1.185449e+00
## 7558   2  494  1.154133e+00
## 7559   2  495  1.123813e+00
## 7560   2  496  1.096687e+00
## 7561   2  497  1.065944e+00
## 7562   2  498  1.044829e+00
## 7563   2  499  1.018864e+00
## 7564   2  500  9.961805e-01
## 7565   2  501  9.760554e-01
## 7566   2  502  9.550463e-01
## 7567   2  503  9.338749e-01
## 7568   2  504  9.146808e-01
## 7569   2  505  1.985781e+00
## 7570   2  506  1.863610e+00
## 7571   2  507  1.754998e+00
## 7572   2  508  1.664255e+00
## 7573   2  509  1.585916e+00
## 7574   2  510  1.512769e+00
## 7575   2  511  1.447824e+00
## 7576   2  512  1.391600e+00
## 7577   2  513  1.343863e+00
## 7578   2  514  1.298203e+00
## 7579   2  515  1.256501e+00
## 7580   2  516  1.218571e+00
## 7581   2  517  1.186188e+00
## 7582   2  518  1.154299e+00
## 7583   2  519  1.123104e+00
## 7584   2  520  1.095328e+00
## 7585   2  521  1.068827e+00
## 7586   2  522  1.044709e+00
## 7587   2  523  1.019845e+00
## 7588   2  524  9.967297e-01
## 7589   2  525  9.743798e-01
## 7590   2  526  9.543893e-01
## 7591   2  527  9.330450e-01
## 7592   2  528  9.148071e-01
## 7593   2  529  1.987869e+00
## 7594   2  530  1.863305e+00
## 7595   2  531  1.756717e+00
## 7596   2  532  1.660867e+00
## 7597   2  533  1.584342e+00
## 7598   2  534  1.513267e+00
## 7599   2  535  1.449137e+00
## 7600   2  536  1.390192e+00
## 7601   2  537  1.342427e+00
## 7602   2  538  1.299688e+00
## 7603   2  539  1.257187e+00
## 7604   2  540  1.219060e+00
## 7605   2  541  1.184605e+00
## 7606   2  542  1.152404e+00
## 7607   2  543  1.122794e+00
## 7608   2  544  1.094464e+00
## 7609   2  545  1.065308e+00
## 7610   2  546  1.046547e+00
## 7611   2  547  1.020970e+00
## 7612   2  548  9.972385e-01
## 7613   2  549  9.754814e-01
## 7614   2  550  9.532245e-01
## 7615   2  551  9.348784e-01
## 7616   2  552  9.143443e-01
## 7617   2  553  1.987165e+00
## 7618   2  554  1.864926e+00
## 7619   2  555  1.756775e+00
## 7620   2  556  1.665924e+00
## 7621   2  557  1.584243e+00
## 7622   2  558  1.512004e+00
## 7623   2  559  1.451002e+00
## 7624   2  560  1.394654e+00
## 7625   2  561  1.343419e+00
## 7626   2  562  1.296786e+00
## 7627   2  563  1.256254e+00
## 7628   2  564  1.220230e+00
## 7629   2  565  1.185153e+00
## 7630   2  566  1.151146e+00
## 7631   2  567  1.123351e+00
## 7632   2  568  1.094979e+00
## 7633   2  569  1.068264e+00
## 7634   2  570  1.044041e+00
## 7635   2  571  1.018511e+00
## 7636   2  572  9.960729e-01
## 7637   2  573  9.736742e-01
## 7638   2  574  9.541015e-01
## 7639   2  575  9.353438e-01
## 7640   2  576  9.138943e-01
## 7641   2  577  1.985456e+00
## 7642   2  578  1.861780e+00
## 7643   2  579  1.756462e+00
## 7644   2  580  1.663401e+00
## 7645   2  581  1.583811e+00
## 7646   2  582  1.513270e+00
## 7647   2  583  1.447502e+00
## 7648   2  584  1.390421e+00
## 7649   2  585  1.343818e+00
## 7650   2  586  1.296696e+00
## 7651   2  587  1.256780e+00
## 7652   2  588  1.219620e+00
## 7653   2  589  1.186647e+00
## 7654   2  590  1.152865e+00
## 7655   2  591  1.122390e+00
## 7656   2  592  1.096911e+00
## 7657   2  593  1.067834e+00
## 7658   2  594  1.042279e+00
## 7659   2  595  1.019994e+00
## 7660   2  596  9.960501e-01
## 7661   2  597  9.750289e-01
## 7662   2  598  9.528718e-01
## 7663   2  599  9.326302e-01
## 7664   2  600  9.141332e-01
## 7665   2  601  1.983978e+00
## 7666   2  602  1.865475e+00
## 7667   2  603  1.756731e+00
## 7668   2  604  1.664241e+00
## 7669   2  605  1.581188e+00
## 7670   2  606  1.512050e+00
## 7671   2  607  1.448571e+00
## 7672   2  608  1.393214e+00
## 7673   2  609  1.340329e+00
## 7674   2  610  1.297110e+00
## 7675   2  611  1.256435e+00
## 7676   2  612  1.219766e+00
## 7677   2  613  1.186188e+00
## 7678   2  614  1.152889e+00
## 7679   2  615  1.122540e+00
## 7680   2  616  1.096213e+00
## 7681   2  617  1.068068e+00
## 7682   2  618  1.042531e+00
## 7683   2  619  1.018467e+00
## 7684   2  620  9.967617e-01
## 7685   2  621  9.737666e-01
## 7686   2  622  9.541214e-01
## 7687   2  623  9.336510e-01
## 7688   2  624  9.113323e-01
## 7689   2  624  9.143769e-01
## 7690   2  625  1.987704e+00
## 7691   2  626  1.861915e+00
## 7692   2  627  1.756979e+00
## 7693   2  628  1.661779e+00
## 7694   2  629  1.582292e+00
## 7695   2  630  1.509669e+00
## 7696   2  631  1.449937e+00
## 7697   2  632  1.390810e+00
## 7698   2  633  1.342017e+00
## 7699   2  634  1.296187e+00
## 7700   2  635  1.254977e+00
## 7701   2  636  1.218118e+00
## 7702   2  637  1.185520e+00
## 7703   2  638  1.153314e+00
## 7704   2  639  1.122482e+00
## 7705   2  640  1.094123e+00
## 7706   2  641  1.068231e+00
## 7707   2  642  1.042577e+00
## 7708   2  643  1.017967e+00
## 7709   2  644  9.948707e-01
## 7710   2  645  9.752318e-01
## 7711   2  646  9.514430e-01
## 7712   2  647  9.342878e-01
## 7713   2  648  9.130925e-01
## 7714   2  648  9.141058e-01
## 7715   2  649  8.952298e-01
## 7716   2  650  8.782928e-01
## 7717   2  651  8.579794e-01
## 7718   2  652  8.411276e-01
## 7719   2  653  8.236528e-01
## 7720   2  654  8.080201e-01
## 7721   2  654  8.067694e-01
## 7722   2  655  1.885666e+00
## 7723   2  656  1.760811e+00
## 7724   2  657  1.655975e+00
## 7725   2  658  1.567337e+00
## 7726   2  659  1.487494e+00
## 7727   2  660  1.423212e+00
## 7728   2  661  1.358318e+00
## 7729   2  662  1.306566e+00
## 7730   2  663  1.255810e+00
## 7731   2  664  1.213292e+00
## 7732   2  665  1.173525e+00
## 7733   2  666  1.137720e+00
## 7734   2  667  1.105755e+00
## 7735   2  668  1.075778e+00
## 7736   2  669  1.046797e+00
## 7737   2  670  1.018669e+00
## 7738   2  671  9.967349e-01
## 7739   2  672  9.722668e-01
## 7740   2  672  9.719516e-01
## 7741   2  673  2.038875e+00
## 7742   2  674  1.915129e+00
## 7743   2  675  1.805294e+00
## 7744   2  676  1.708664e+00
## 7745   2  677  1.628526e+00
## 7746   2  678  1.554495e+00
## 7747   2  679  1.493722e+00
## 7748   2  680  1.432072e+00
## 7749   2  681  1.382050e+00
## 7750   2  682  1.334935e+00
## 7751   2  683  1.292500e+00
## 7752   2  684  1.255365e+00
## 7753   2  685  1.217049e+00
## 7754   2  686  1.183086e+00
## 7755   2  687  1.156900e+00
## 7756   2  688  1.125401e+00
## 7757   2  689  1.099016e+00
## 7758   2  690  1.074244e+00
## 7759   2  691  1.049423e+00
## 7760   2  692  1.023537e+00
## 7761   2  693  1.003825e+00
## 7762   2  694  9.824842e-01
## 7763   2  695  9.595799e-01
## 7764   2  696  9.408554e-01
## 7765   2  697  2.014243e+00
## 7766   2  698  1.889685e+00
## 7767   2  699  1.780752e+00
## 7768   2  700  1.692199e+00
## 7769   2  701  1.604233e+00
## 7770   2  702  1.536635e+00
## 7771   2  703  1.471738e+00
## 7772   2  704  1.416994e+00
## 7773   2  705  1.364637e+00
## 7774   2  706  1.319700e+00
## 7775   2  707  1.279471e+00
## 7776   2  708  1.240466e+00
## 7777   2  709  1.205841e+00
## 7778   2  710  1.173346e+00
## 7779   2  711  1.143530e+00
## 7780   2  712  1.115312e+00
## 7781   2  713  1.085512e+00
## 7782   2  714  1.062026e+00
## 7783   2  715  1.036847e+00
## 7784   2  716  1.013932e+00
## 7785   2  717  9.927090e-01
## 7786   2  718  9.720241e-01
## 7787   2  719  9.484306e-01
## 7788   2  720  9.308595e-01
## 7789   2  721  2.000247e+00
## 7790   2  722  1.877086e+00
## 7791   2  723  1.771489e+00
## 7792   2  724  1.681552e+00
## 7793   2  725  1.600227e+00
## 7794   2  726  1.526398e+00
## 7795   2  727  1.463894e+00
## 7796   2  728  1.406174e+00
## 7797   2  729  1.356962e+00
## 7798   2  730  1.308651e+00
## 7799   2  731  1.270181e+00
## 7800   2  732  1.232347e+00
## 7801   2  733  1.196925e+00
## 7802   2  734  1.164270e+00
## 7803   2  735  1.134762e+00
## 7804   2  736  1.106098e+00
## 7805   2  737  1.078899e+00
## 7806   2  738  1.053492e+00
## 7807   2  739  1.030291e+00
## 7808   2  740  1.007110e+00
## 7809   2  741  9.847466e-01
## 7810   2  742  9.640948e-01
## 7811   2  743  9.422464e-01
## 7812   2  744  9.250348e-01
## 7813   2  745  1.996769e+00
## 7814   2  746  1.875887e+00
## 7815   2  747  1.763041e+00
## 7816   2  748  1.674459e+00
## 7817   2  749  1.592628e+00
## 7818   2  750  1.521256e+00
## 7819   2  751  1.459303e+00
## 7820   2  752  1.403666e+00
## 7821   2  753  1.350128e+00
## 7822   2  754  1.304973e+00
## 7823   2  755  1.264860e+00
## 7824   2  756  1.226558e+00
## 7825   2  757  1.191298e+00
## 7826   2  758  1.157945e+00
## 7827   2  759  1.129703e+00
## 7828   2  760  1.102658e+00
## 7829   2  761  1.075982e+00
## 7830   2  762  1.049751e+00
## 7831   2  763  1.026976e+00
## 7832   2  764  1.002922e+00
## 7833   2  765  9.817704e-01
## 7834   2  766  9.600839e-01
## 7835   2  767  9.405331e-01
## 7836   2  768  9.210427e-01
## 7837   2  769  1.989812e+00
## 7838   2  770  1.871872e+00
## 7839   2  771  1.762802e+00
## 7840   2  772  1.672238e+00
## 7841   2  773  1.590797e+00
## 7842   2  774  1.517229e+00
## 7843   2  775  1.454626e+00
## 7844   2  776  1.398012e+00
## 7845   2  777  1.348160e+00
## 7846   2  778  1.302856e+00
## 7847   2  779  1.263525e+00
## 7848   2  780  1.225578e+00
## 7849   2  781  1.187826e+00
## 7850   2  782  1.157160e+00
## 7851   2  783  1.128346e+00
## 7852   2  784  1.099419e+00
## 7853   2  785  1.072038e+00
## 7854   2  786  1.046135e+00
## 7855   2  787  1.022219e+00
## 7856   2  788  1.000244e+00
## 7857   2  789  9.813064e-01
## 7858   2  790  9.566329e-01
## 7859   2  791  9.377314e-01
## 7860   2  792  9.167411e-01
## 7861   2  793  1.992208e+00
## 7862   2  794  1.867975e+00
## 7863   2  795  1.756612e+00
## 7864   2  796  1.668298e+00
## 7865   2  797  1.585497e+00
## 7866   2  798  1.515025e+00
## 7867   2  799  1.451296e+00
## 7868   2  800  1.395016e+00
## 7869   2  801  1.345549e+00
## 7870   2  802  1.300319e+00
## 7871   2  803  1.259017e+00
## 7872   2  804  1.221605e+00
## 7873   2  805  1.188596e+00
## 7874   2  806  1.155234e+00
## 7875   2  807  1.125089e+00
## 7876   2  808  1.097059e+00
## 7877   2  809  1.070536e+00
## 7878   2  810  1.046172e+00
## 7879   2  811  1.020975e+00
## 7880   2  812  9.983487e-01
## 7881   2  813  9.778360e-01
## 7882   2  814  9.580687e-01
## 7883   2  815  9.353886e-01
## 7884   2  816  9.160430e-01
## 7885   2  817  1.988434e+00
## 7886   2  818  1.866354e+00
## 7887   2  819  1.757211e+00
## 7888   2  820  1.665342e+00
## 7889   2  821  1.583848e+00
## 7890   2  822  1.513060e+00
## 7891   2  823  1.449943e+00
## 7892   2  824  1.395051e+00
## 7893   2  825  1.347068e+00
## 7894   2  826  1.299639e+00
## 7895   2  827  1.257384e+00
## 7896   2  828  1.222354e+00
## 7897   2  829  1.186493e+00
## 7898   2  830  1.154846e+00
## 7899   2  831  1.125437e+00
## 7900   2  832  1.095688e+00
## 7901   2  833  1.068314e+00
## 7902   2  834  1.044239e+00
## 7903   2  835  1.020348e+00
## 7904   2  836  9.985543e-01
## 7905   2  837  9.745605e-01
## 7906   2  838  9.562332e-01
## 7907   2  839  9.340358e-01
## 7908   2  840  9.144658e-01
## 7909   2  841  1.986470e+00
## 7910   2  842  1.862761e+00
## 7911   2  843  1.758481e+00
## 7912   2  844  1.661281e+00
## 7913   2  845  1.584515e+00
## 7914   2  846  1.510820e+00
## 7915   2  847  1.449184e+00
## 7916   2  848  1.392366e+00
## 7917   2  849  1.344844e+00
## 7918   2  850  1.298566e+00
## 7919   2  851  1.257784e+00
## 7920   2  852  1.220803e+00
## 7921   2  853  1.186713e+00
## 7922   2  854  1.151429e+00
## 7923   2  855  1.123173e+00
## 7924   2  856  1.093570e+00
## 7925   2  857  1.069107e+00
## 7926   2  858  1.043000e+00
## 7927   2  859  1.020507e+00
## 7928   2  860  9.963280e-01
## 7929   2  861  9.756517e-01
## 7930   2  862  9.555726e-01
## 7931   2  863  9.350982e-01
## 7932   2  864  9.153821e-01
## 7933   2  865  1.987602e+00
## 7934   2  866  1.863409e+00
## 7935   2  867  1.756145e+00
## 7936   2  868  1.663294e+00
## 7937   2  869  1.579640e+00
## 7938   2  870  1.513217e+00
## 7939   2  871  1.449760e+00
## 7940   2  872  1.393352e+00
## 7941   2  873  1.345310e+00
## 7942   2  874  1.296936e+00
## 7943   2  875  1.258927e+00
## 7944   2  876  1.219505e+00
## 7945   2  877  1.185069e+00
## 7946   2  878  1.154178e+00
## 7947   2  879  1.121169e+00
## 7948   2  880  1.096603e+00
## 7949   2  881  1.067311e+00
## 7950   2  882  1.043142e+00
## 7951   2  883  1.018304e+00
## 7952   2  884  9.978450e-01
## 7953   2  885  9.775326e-01
## 7954   2  886  9.550259e-01
## 7955   2  887  9.335877e-01
## 7956   2  888  9.147133e-01
## 7957   2  889  1.990634e+00
## 7958   2  890  1.864916e+00
## 7959   2  891  1.759060e+00
## 7960   2  892  1.665143e+00
## 7961   2  893  1.581613e+00
## 7962   2  894  1.510970e+00
## 7963   2  895  1.448623e+00
## 7964   2  896  1.391845e+00
## 7965   2  897  1.344952e+00
## 7966   2  898  1.297605e+00
## 7967   2  899  1.256227e+00
## 7968   2  900  1.219606e+00
## 7969   2  901  1.185034e+00
## 7970   2  902  1.151739e+00
## 7971   2  903  1.120284e+00
## 7972   2  904  1.094438e+00
## 7973   2  905  1.068169e+00
## 7974   2  906  1.043110e+00
## 7975   2  907  1.019631e+00
## 7976   2  908  9.984701e-01
## 7977   2  909  9.737834e-01
## 7978   2  910  9.533790e-01
## 7979   2  911  9.331488e-01
## 7980   2  912  9.124751e-01
## 7981   2  913  1.988041e+00
## 7982   2  914  1.864126e+00
## 7983   2  915  1.758299e+00
## 7984   2  916  1.660319e+00
## 7985   2  917  1.584531e+00
## 7986   2  918  1.512052e+00
## 7987   2  919  1.450345e+00
## 7988   2  920  1.395305e+00
## 7989   2  921  1.345346e+00
## 7990   2  922  1.297976e+00
## 7991   2  923  1.257157e+00
## 7992   2  924  1.220227e+00
## 7993   2  925  1.184718e+00
## 7994   2  926  1.152027e+00
## 7995   2  927  1.122890e+00
## 7996   2  928  1.094368e+00
## 7997   2  929  1.067232e+00
## 7998   2  930  1.042540e+00
## 7999   2  931  1.020644e+00
## 8000   2  932  9.957570e-01
## 8001   2  933  9.757549e-01
## 8002   2  934  9.545145e-01
## 8003   2  935  9.339195e-01
## 8004   2  936  9.141355e-01
## 8005   2  936  9.149194e-01
## 8006   2  937  1.985177e+00
## 8007   2  938  1.862678e+00
## 8008   2  939  1.753074e+00
## 8009   2  940  1.661202e+00
## 8010   2  941  1.581349e+00
## 8011   2  942  1.510717e+00
## 8012   2  943  1.447655e+00
## 8013   2  944  1.393697e+00
## 8014   2  945  1.342683e+00
## 8015   2  946  1.296594e+00
## 8016   2  947  1.256540e+00
## 8017   2  948  1.222158e+00
## 8018   2  949  1.184629e+00
## 8019   2  950  1.152804e+00
## 8020   2  951  1.124310e+00
## 8021   2  952  1.095116e+00
## 8022   2  953  1.067026e+00
## 8023   2  954  1.043854e+00
## 8024   2  955  1.020882e+00
## 8025   2  956  9.962443e-01
## 8026   2  957  9.744667e-01
## 8027   2  958  9.520594e-01
## 8028   2  959  9.331128e-01
## 8029   2  960  9.161913e-01
## 8030   2  960  9.150924e-01
## 8031   2  961  8.957624e-01
## 8032   2  962  8.748641e-01
## 8033   2  963  8.586506e-01
## 8034   2  964  8.396083e-01
## 8035   2  965  8.241644e-01
```

```
## 8036   2  966 8.065561e-01
## 8037   2  967 7.911601e-01
## 8038   2  968 7.753092e-01
## 8039   2  969 7.607925e-01
## 8040   2  969 7.606687e-01
## 8041   2  970 1.840169e+00
## 8042   2  971 1.718700e+00
## 8043   2  972 1.613795e+00
## 8044   2  973 1.523201e+00
## 8045   2  974 1.445156e+00
## 8046   2  975 1.376864e+00
## 8047   2  976 1.319408e+00
## 8048   2  977 1.266423e+00
## 8049   2  978 1.219109e+00
## 8050   2  979 1.176606e+00
## 8051   2  980 1.136261e+00
## 8052   2  981 1.102380e+00
## 8053   2  982 1.071826e+00
## 8054   2  983 1.040492e+00
## 8055   2  984 1.015117e+00
## 8056   2  984 1.013759e+00
## 8057   2  985 2.080082e+00
## 8058   2  986 1.947664e+00
## 8059   2  987 1.835295e+00
## 8060   2  988 1.740490e+00
## 8061   2  989 1.656113e+00
## 8062   2  990 1.579829e+00
## 8063   2  991 1.517779e+00
## 8064   2  992 1.454531e+00
## 8065   2  993 1.403439e+00
## 8066   2  994 1.358029e+00
## 8067   2  995 1.314879e+00
## 8068   2  996 1.275789e+00
## 8069   2  997 1.238494e+00
## 8070   2  998 1.204980e+00
## 8071   2  999 1.174298e+00
## 8072   2 1000 1.144570e+00
## 8073   2 1001 1.118031e+00
## 8074   2 1002 1.091272e+00
## 8075   2 1003 1.067316e+00
## 8076   2 1004 1.041427e+00
## 8077   2 1005 1.018605e+00
## 8078   2 1006 9.970179e-01
## 8079   2 1007 9.760061e-01
## 8080   2 1008 9.564249e-01
## 8081   2 1009 2.027158e+00
## 8082   2 1010 1.904248e+00
## 8083   2 1011 1.794723e+00
## 8084   2 1012 1.703097e+00
## 8085   2 1013 1.623839e+00
## 8086   2 1014 1.549184e+00
## 8087   2 1015 1.486780e+00
## 8088   2 1016 1.426987e+00
## 8089   2 1017 1.377489e+00
## 8090   2 1018 1.331050e+00
## 8091   2 1019 1.291990e+00
## 8092   2 1020 1.251640e+00
## 8093   2 1021 1.216744e+00
## 8094   2 1022 1.184342e+00
## 8095   2 1023 1.152833e+00
## 8096   2 1024 1.124273e+00
## 8097   2 1025 1.097390e+00
## 8098   2 1026 1.072815e+00
## 8099   2 1027 1.049782e+00
## 8100   2 1028 1.025793e+00
## 8101   2 1029 1.002077e+00
## 8102   2 1030 9.800347e-01
## 8103   2 1031 9.607465e-01
## 8104   2 1032 9.403091e-01
## 8105   2 1033 2.010715e+00
## 8106   2 1034 1.885737e+00
## 8107   2 1035 1.778112e+00
## 8108   2 1036 1.684318e+00
## 8109   2 1037 1.607588e+00
## 8110   2 1038 1.534707e+00
## 8111   2 1039 1.471730e+00
## 8112   2 1040 1.413491e+00
## 8113   2 1041 1.363499e+00
## 8114   2 1042 1.317483e+00
## 8115   2 1043 1.274972e+00
## 8116   2 1044 1.237824e+00
## 8117   2 1045 1.205247e+00
## 8118   2 1046 1.171316e+00
## 8119   2 1047 1.141317e+00
## 8120   2 1048 1.112972e+00
## 8121   2 1049 1.085859e+00
## 8122   2 1050 1.062757e+00
## 8123   2 1051 1.037745e+00
## 8124   2 1052 1.012924e+00
## 8125   2 1053 9.922548e-01
## 8126   2 1054 9.695813e-01
## 8127   2 1055 9.506015e-01
## 8128   2 1056 9.281696e-01
## 8129   2 1057 2.005092e+00
## 8130   2 1058 1.878021e+00
## 8131   2 1059 1.769807e+00
## 8132   2 1060 1.676712e+00
## 8133   2 1061 1.597575e+00
## 8134   2 1062 1.525739e+00
## 8135   2 1063 1.464007e+00
## 8136   2 1064 1.407343e+00
## 8137   2 1065 1.354787e+00
## 8138   2 1066 1.309733e+00
## 8139   2 1067 1.269932e+00
## 8140   2 1068 1.230507e+00
## 8141   2 1069 1.196467e+00
## 8142   2 1070 1.164608e+00
## 8143   2 1071 1.134818e+00
## 8144   2 1072 1.106750e+00
## 8145   2 1073 1.079555e+00
## 8146   2 1074 1.052828e+00
## 8147   2 1075 1.029559e+00
## 8148   2 1076 1.005116e+00
## 8149   2 1077 9.859713e-01
## 8150   2 1078 9.621345e-01
## 8151   2 1079 9.435530e-01
## 8152   2 1080 9.229191e-01
## 8153   2 1081 1.994808e+00
## 8154   2 1082 1.871578e+00
## 8155   2 1083 1.765040e+00
## 8156   2 1084 1.673943e+00
## 8157   2 1085 1.591530e+00
## 8158   2 1086 1.521198e+00
## 8159   2 1087 1.456058e+00
## 8160   2 1088 1.398674e+00
## 8161   2 1089 1.350150e+00
## 8162   2 1090 1.306507e+00
## 8163   2 1091 1.265405e+00
## 8164   2 1092 1.226346e+00
## 8165   2 1093 1.192006e+00
## 8166   2 1094 1.161449e+00
## 8167   2 1095 1.131787e+00
## 8168   2 1096 1.102711e+00
## 8169   2 1097 1.075993e+00
## 8170   2 1098 1.049920e+00
## 8171   2 1099 1.024988e+00
## 8172   2 1100 1.005460e+00
## 8173   2 1101 9.824325e-01
## 8174   2 1102 9.597892e-01
## 8175   2 1103 9.400563e-01
## 8176   2 1104 9.195669e-01
## 8177   2 1105 1.993552e+00
## 8178   2 1106 1.865589e+00
## 8179   2 1107 1.763780e+00
## 8180   2 1108 1.670424e+00
## 8181   2 1109 1.586996e+00
## 8182   2 1110 1.518093e+00
## 8183   2 1111 1.452685e+00
## 8184   2 1112 1.398807e+00
## 8185   2 1113 1.348643e+00
## 8186   2 1114 1.303693e+00
## 8187   2 1115 1.261200e+00
## 8188   2 1116 1.222277e+00
## 8189   2 1117 1.189246e+00
## 8190   2 1118 1.157591e+00
## 8191   2 1119 1.125287e+00
## 8192   2 1120 1.098762e+00
## 8193   2 1121 1.070729e+00
## 8194   2 1122 1.047433e+00
## 8195   2 1123 1.025127e+00
## 8196   2 1124 9.988788e-01
## 8197   2 1125 9.772006e-01
## 8198   2 1126 9.574315e-01
## 8199   2 1127 9.375012e-01
## 8200   2 1128 9.177556e-01
## 8201   2 1129 1.991002e+00
## 8202   2 1130 1.866886e+00
## 8203   2 1131 1.756656e+00
## 8204   2 1132 1.668779e+00
## 8205   2 1133 1.585567e+00
## 8206   2 1134 1.514365e+00
## 8207   2 1135 1.451538e+00
## 8208   2 1136 1.397532e+00
## 8209   2 1137 1.345422e+00
## 8210   2 1138 1.299517e+00
## 8211   2 1139 1.259866e+00
## 8212   2 1140 1.218755e+00
## 8213   2 1141 1.188304e+00
## 8214   2 1142 1.155204e+00
## 8215   2 1143 1.125132e+00
## 8216   2 1144 1.097799e+00
## 8217   2 1145 1.070861e+00
## 8218   2 1146 1.043815e+00
## 8219   2 1147 1.023220e+00
## 8220   2 1148 1.000061e+00
## 8221   2 1149 9.776599e-01
## 8222   2 1150 9.546504e-01
## 8223   2 1151 9.355887e-01
## 8224   2 1152 9.184661e-01
## 8225   2 1153 1.992727e+00
## 8226   2 1154 1.866012e+00
## 8227   2 1155 1.759977e+00
## 8228   2 1156 1.666922e+00
## 8229   2 1157 1.586448e+00
## 8230   2 1158 1.513402e+00
## 8231   2 1159 1.449112e+00
## 8232   2 1160 1.394394e+00
## 8233   2 1161 1.345144e+00
## 8234   2 1162 1.299369e+00
## 8235   2 1163 1.259400e+00
## 8236   2 1164 1.220904e+00
## 8237   2 1165 1.185908e+00
## 8238   2 1166 1.153855e+00
## 8239   2 1167 1.125713e+00
## 8240   2 1168 1.096319e+00
## 8241   2 1169 1.069674e+00
## 8242   2 1170 1.043640e+00
## 8243   2 1171 1.020420e+00
## 8244   2 1172 9.965830e-01
## 8245   2 1173 9.764134e-01
## 8246   2 1174 9.541220e-01
## 8247   2 1175 9.351072e-01
## 8248   2 1176 9.147042e-01
## 8249   2 1177 1.990373e+00
## 8250   2 1178 1.864613e+00
## 8251   2 1179 1.755744e+00
## 8252   2 1180 1.667600e+00
## 8253   2 1181 1.581358e+00
## 8254   2 1182 1.512351e+00
## 8255   2 1183 1.452825e+00
## 8256   2 1184 1.394420e+00
## 8257   2 1185 1.340638e+00
## 8258   2 1186 1.296051e+00
## 8259   2 1187 1.260061e+00
## 8260   2 1188 1.218206e+00
## 8261   2 1189 1.184714e+00
## 8262   2 1190 1.153366e+00
## 8263   2 1191 1.123234e+00
## 8264   2 1192 1.096481e+00
## 8265   2 1193 1.068138e+00
## 8266   2 1194 1.043792e+00
## 8267   2 1195 1.019199e+00
## 8268   2 1196 9.983558e-01
## 8269   2 1197 9.751884e-01
## 8270   2 1198 9.530644e-01
## 8271   2 1199 9.336619e-01
## 8272   2 1200 9.130640e-01
## 8273   2 1201 1.982657e+00
## 8274   2 1202 1.860350e+00
## 8275   2 1203 1.758284e+00
## 8276   2 1204 1.663032e+00
## 8277   2 1205 1.583757e+00
## 8278   2 1206 1.511183e+00
## 8279   2 1207 1.450958e+00
## 8280   2 1208 1.395656e+00
## 8281   2 1209 1.343226e+00
## 8282   2 1210 1.299714e+00
## 8283   2 1211 1.257771e+00
## 8284   2 1212 1.219772e+00
## 8285   2 1213 1.183603e+00
## 8286   2 1214 1.152997e+00
## 8287   2 1215 1.122372e+00
## 8288   2 1216 1.095637e+00
## 8289   2 1217 1.069027e+00
## 8290   2 1218 1.043261e+00
## 8291   2 1219 1.019925e+00
## 8292   2 1220 9.978045e-01
## 8293   2 1221 9.744172e-01
## 8294   2 1222 9.529564e-01
## 8295   2 1223 9.335808e-01
## 8296   2 1224 9.151642e-01
## 8297   2 1225 1.989325e+00
## 8298   2 1226 1.863252e+00
## 8299   2 1227 1.758414e+00
## 8300   2 1228 1.664412e+00
## 8301   2 1229 1.581565e+00
## 8302   2 1230 1.511669e+00
## 8303   2 1231 1.448689e+00
## 8304   2 1232 1.392187e+00
## 8305   2 1233 1.342410e+00
## 8306   2 1234 1.296515e+00
## 8307   2 1235 1.256914e+00
## 8308   2 1236 1.219413e+00
## 8309   2 1237 1.182908e+00
## 8310   2 1238 1.151804e+00
## 8311   2 1239 1.123853e+00
## 8312   2 1240 1.095294e+00
## 8313   2 1241 1.069290e+00
## 8314   2 1242 1.042503e+00
## 8315   2 1243 1.018358e+00
## 8316   2 1244 9.961575e-01
## 8317   2 1245 9.740193e-01
## 8318   2 1246 9.550810e-01
## 8319   2 1247 9.338316e-01
## 8320   2 1248 9.138683e-01
## 8321   2 1248 9.137081e-01
## 8322   2 1249 1.985400e+00
## 8323   2 1250 1.864282e+00
## 8324   2 1251 1.754856e+00
## 8325   2 1252 1.664847e+00
## 8326   2 1253 1.581272e+00
## 8327   2 1254 1.512235e+00
## 8328   2 1255 1.449182e+00
## 8329   2 1256 1.391096e+00
## 8330   2 1257 1.345661e+00
## 8331   2 1258 1.295099e+00
## 8332   2 1259 1.256812e+00
## 8333   2 1260 1.219169e+00
## 8334   2 1261 1.184665e+00
## 8335   2 1262 1.151883e+00
## 8336   2 1263 1.121904e+00
## 8337   2 1264 1.093655e+00
## 8338   2 1265 1.067025e+00
## 8339   2 1266 1.044735e+00
## 8340   2 1267 1.018729e+00
## 8341   2 1268 9.959736e-01
## 8342   2 1269 9.758852e-01
## 8343   2 1270 9.549160e-01
## 8344   2 1271 9.333271e-01
## 8345   2 1272 9.139431e-01
## 8346   2 1272 9.149010e-01
## 8347   2 1273 8.936344e-01
## 8348   2 1274 8.772742e-01
## 8349   2 1275 8.568608e-01
## 8350   2 1276 8.406915e-01
## 8351   2 1277 8.253691e-01
## 8352   2 1278 8.091510e-01
## 8353   2 1279 7.926436e-01
## 8354   2 1280 7.756241e-01
## 8355   2 1281 7.598714e-01
## 8356   2 1282 7.460186e-01
## 8357   2 1283 7.303062e-01
## 8358   2 1284 7.156831e-01
## 8359   2 1284 7.161879e-01
## 8360   2 1285 1.795426e+00
## 8361   2 1286 1.676239e+00
## 8362   2 1287 1.571049e+00
## 8363   2 1288 1.486655e+00
## 8364   2 1289 1.404629e+00
## 8365   2 1290 1.339625e+00
## 8366   2 1291 1.280034e+00
## 8367   2 1292 1.229106e+00
## 8368   2 1293 1.181328e+00
## 8369   2 1294 1.139537e+00
## 8370   2 1295 1.103841e+00
## 8371   2 1296 1.066005e+00
## 8372   2 1296 1.067110e+00
## 8373   2 1297 2.129997e+00
## 8374   2 1298 1.994557e+00
## 8375   2 1299 1.879280e+00
## 8376   2 1300 1.774317e+00
## 8377   2 1301 1.689414e+00
## 8378   2 1302 1.615319e+00
## 8379   2 1303 1.541157e+00
## 8380   2 1304 1.486623e+00
## 8381   2 1305 1.431236e+00
## 8382   2 1306 1.383067e+00
## 8383   2 1307 1.337429e+00
## 8384   2 1308 1.297704e+00
## 8385   2 1309 1.259694e+00
## 8386   2 1310 1.228192e+00
## 8387   2 1311 1.196774e+00
## 8388   2 1312 1.162855e+00
## 8389   2 1313 1.136436e+00
## 8390   2 1314 1.109523e+00
## 8391   2 1315 1.084441e+00
## 8392   2 1316 1.059659e+00
## 8393   2 1317 1.037158e+00
## 8394   2 1318 1.013456e+00
## 8395   2 1319 9.914413e-01
## 8396   2 1320 9.718580e-01
## 8397   2 1321 2.042593e+00
## 8398   2 1322 1.921334e+00
## 8399   2 1323 1.811868e+00
## 8400   2 1324 1.718856e+00
## 8401   2 1325 1.633533e+00
## 8402   2 1326 1.565584e+00
## 8403   2 1327 1.497949e+00
## 8404   2 1328 1.439824e+00
## 8405   2 1329 1.391015e+00
## 8406   2 1330 1.343503e+00
## 8407   2 1331 1.303840e+00
## 8408   2 1332 1.265458e+00
## 8409   2 1333 1.230427e+00
## 8410   2 1334 1.195873e+00
## 8411   2 1335 1.163309e+00
## 8412   2 1336 1.136466e+00
## 8413   2 1337 1.108520e+00
## 8414   2 1338 1.084083e+00
## 8415   2 1339 1.058262e+00
## 8416   2 1340 1.034076e+00
## 8417   2 1341 1.010931e+00
## 8418   2 1342 9.902299e-01
## 8419   2 1343 9.703191e-01
## 8420   2 1344 9.484960e-01
## 8421   2 1345 2.021693e+00
## 8422   2 1346 1.895070e+00
## 8423   2 1347 1.786769e+00
## 8424   2 1348 1.698260e+00
## 8425   2 1349 1.616408e+00
## 8426   2 1350 1.544403e+00
## 8427   2 1351 1.479817e+00
## 8428   2 1352 1.424971e+00
## 8429   2 1353 1.371403e+00
## 8430   2 1354 1.325336e+00
## 8431   2 1355 1.284826e+00
## 8432   2 1356 1.246213e+00
## 8433   2 1357 1.213790e+00
## 8434   2 1358 1.179696e+00
## 8435   2 1359 1.145957e+00
## 8436   2 1360 1.120097e+00
## 8437   2 1361 1.093361e+00
## 8438   2 1362 1.066336e+00
## 8439   2 1363 1.044785e+00
## 8440   2 1364 1.021169e+00
## 8441   2 1365 9.979022e-01
## 8442   2 1366 9.760605e-01
## 8443   2 1367 9.550811e-01
## 8444   2 1368 9.361524e-01
## 8445   2 1369 2.008316e+00
## 8446   2 1370 1.882907e+00
## 8447   2 1371 1.776885e+00
## 8448   2 1372 1.684514e+00
## 8449   2 1373 1.602139e+00
## 8450   2 1374 1.532009e+00
## 8451   2 1375 1.470386e+00
## 8452   2 1376 1.411135e+00
## 8453   2 1377 1.363508e+00
## 8454   2 1378 1.316247e+00
## 8455   2 1379 1.275800e+00
## 8456   2 1380 1.235810e+00
## 8457   2 1381 1.200338e+00
## 8458   2 1382 1.170160e+00
## 8459   2 1383 1.139261e+00
## 8460   2 1384 1.110089e+00
## 8461   2 1385 1.082863e+00
## 8462   2 1386 1.059885e+00
## 8463   2 1387 1.033411e+00
## 8464   2 1388 1.011969e+00
## 8465   2 1389 9.880606e-01
## 8466   2 1390 9.681250e-01
## 8467   2 1391 9.470811e-01
## 8468   2 1392 9.286133e-01
## 8469   2 1393 2.002165e+00
## 8470   2 1394 1.873474e+00
## 8471   2 1395 1.772560e+00
## 8472   2 1396 1.674735e+00
## 8473   2 1397 1.596029e+00
## 8474   2 1398 1.523828e+00
## 8475   2 1399 1.461184e+00
## 8476   2 1400 1.403119e+00
## 8477   2 1401 1.354855e+00
## 8478   2 1402 1.309021e+00
## 8479   2 1403 1.266461e+00
## 8480   2 1404 1.230397e+00
## 8481   2 1405 1.196395e+00
## 8482   2 1406 1.162581e+00
## 8483   2 1407 1.132398e+00
## 8484   2 1408 1.105758e+00
## 8485   2 1409 1.077138e+00
## 8486   2 1410 1.052367e+00
## 8487   2 1411 1.028742e+00
## 8488   2 1412 1.006099e+00
## 8489   2 1413 9.838671e-01
## 8490   2 1414 9.623542e-01
## 8491   2 1415 9.425782e-01
## 8492   2 1416 9.226206e-01
## 8493   2 1417 1.996539e+00
## 8494   2 1418 1.871405e+00
## 8495   2 1419 1.763485e+00
## 8496   2 1420 1.672144e+00
## 8497   2 1421 1.588564e+00
## 8498   2 1422 1.517647e+00
## 8499   2 1423 1.456731e+00
## 8500   2 1424 1.401068e+00
## 8501   2 1425 1.347685e+00
## 8502   2 1426 1.304064e+00
## 8503   2 1427 1.264857e+00
## 8504   2 1428 1.223058e+00
## 8505   2 1429 1.191209e+00
## 8506   2 1430 1.157950e+00
## 8507   2 1431 1.130555e+00
## 8508   2 1432 1.099829e+00
## 8509   2 1433 1.075260e+00
## 8510   2 1434 1.048730e+00
## 8511   2 1435 1.026169e+00
## 8512   2 1436 1.001133e+00
## 8513   2 1437 9.807152e-01
## 8514   2 1438 9.598208e-01
## 8515   2 1439 9.389541e-01
## 8516   2 1440 9.185198e-01
## 8517   2 1441 1.988542e+00
## 8518   2 1442 1.869186e+00
## 8519   2 1443 1.760542e+00
## 8520   2 1444 1.669356e+00
## 8521   2 1445 1.588745e+00
## 8522   2 1446 1.514805e+00
## 8523   2 1447 1.451538e+00
## 8524   2 1448 1.398317e+00
## 8525   2 1449 1.348660e+00
## 8526   2 1450 1.303248e+00
## 8527   2 1451 1.261058e+00
## 8528   2 1452 1.224112e+00
## 8529   2 1453 1.188316e+00
## 8530   2 1454 1.157578e+00
## 8531   2 1455 1.124975e+00
## 8532   2 1456 1.097992e+00
## 8533   2 1457 1.071878e+00
## 8534   2 1458 1.048087e+00
## 8535   2 1459 1.023804e+00
## 8536   2 1460 9.998635e-01
## 8537   2 1461 9.794069e-01
## 8538   2 1462 9.564251e-01
## 8539   2 1463 9.379710e-01
## 8540   2 1464 9.181604e-01
## 8541   2 1465 1.992421e+00
## 8542   2 1466 1.862079e+00
## 8543   2 1467 1.757566e+00
## 8544   2 1468 1.666128e+00
## 8545   2 1469 1.584425e+00
## 8546   2 1470 1.515519e+00
## 8547   2 1471 1.451012e+00
## 8548   2 1472 1.395067e+00
## 8549   2 1473 1.344489e+00
## 8550   2 1474 1.298664e+00
## 8551   2 1475 1.257028e+00
## 8552   2 1476 1.221135e+00
## 8553   2 1477 1.187043e+00
## 8554   2 1478 1.154494e+00
## 8555   2 1479 1.125225e+00
## 8556   2 1480 1.097405e+00
## 8557   2 1481 1.070209e+00
## 8558   2 1482 1.045476e+00
## 8559   2 1483 1.021108e+00
## 8560   2 1484 9.998090e-01
## 8561   2 1485 9.787535e-01
## 8562   2 1486 9.551873e-01
## 8563   2 1487 9.358799e-01
## 8564   2 1488 9.148246e-01
## 8565   2 1489 1.988866e+00
## 8566   2 1490 1.867248e+00
## 8567   2 1491 1.758705e+00
## 8568   2 1492 1.664275e+00
## 8569   2 1493 1.587014e+00
## 8570   2 1494 1.514929e+00
## 8571   2 1495 1.449908e+00
## 8572   2 1496 1.397417e+00
## 8573   2 1497 1.342492e+00
## 8574   2 1498 1.299105e+00
## 8575   2 1499 1.259629e+00
## 8576   2 1500 1.220460e+00
## 8577   2 1501 1.187056e+00
## 8578   2 1502 1.154529e+00
## 8579   2 1503 1.122472e+00
## 8580   2 1504 1.095473e+00
## 8581   2 1505 1.069186e+00
## 8582   2 1506 1.044190e+00
## 8583   2 1507 1.020341e+00
## 8584   2 1508 9.975555e-01
## 8585   2 1509 9.758523e-01
## 8586   2 1510 9.543846e-01
## 8587   2 1511 9.345404e-01
## 8588   2 1512 9.153421e-01
## 8589   2 1513 1.990510e+00
## 8590   2 1514 1.863318e+00
## 8591   2 1515 1.757737e+00
## 8592   2 1516 1.665833e+00
## 8593   2 1517 1.585511e+00
## 8594   2 1518 1.510153e+00
## 8595   2 1519 1.450158e+00
## 8596   2 1520 1.393747e+00
## 8597   2 1521 1.342573e+00
## 8598   2 1522 1.297981e+00
## 8599   2 1523 1.259042e+00
## 8600   2 1524 1.221356e+00
## 8601   2 1525 1.185720e+00
## 8602   2 1526 1.152399e+00
## 8603   2 1527 1.124722e+00
## 8604   2 1528 1.095476e+00
## 8605   2 1529 1.069682e+00
## 8606   2 1530 1.043584e+00
## 8607   2 1531 1.019590e+00
## 8608   2 1532 9.966490e-01
## 8609   2 1533 9.765477e-01
## 8610   2 1534 9.540548e-01
## 8611   2 1535 9.356869e-01
## 8612   2 1536 9.149882e-01
## 8613   2 1537 1.982950e+00
## 8614   2 1538 1.863584e+00
## 8615   2 1539 1.757724e+00
## 8616   2 1540 1.664382e+00
## 8617   2 1541 1.584452e+00
## 8618   2 1542 1.510686e+00
## 8619   2 1543 1.450301e+00
## 8620   2 1544 1.393419e+00
## 8621   2 1545 1.345110e+00
## 8622   2 1546 1.297249e+00
## 8623   2 1547 1.256402e+00
## 8624   2 1548 1.219465e+00
## 8625   2 1549 1.186159e+00
## 8626   2 1550 1.151207e+00
## 8627   2 1551 1.120220e+00
## 8628   2 1552 1.094197e+00
## 8629   2 1553 1.069467e+00
## 8630   2 1554 1.045355e+00
## 8631   2 1555 1.019068e+00
## 8632   2 1556 9.963449e-01
## 8633   2 1557 9.748314e-01
## 8634   2 1558 9.537639e-01
## 8635   2 1559 9.341922e-01
## 8636   2 1560 9.134081e-01
## 8637   2 1560 9.147111e-01
## 8638   2 1561 1.985750e+00
## 8639   2 1562 1.865205e+00
## 8640   2 1563 1.754215e+00
## 8641   2 1564 1.663707e+00
## 8642   2 1565 1.580932e+00
## 8643   2 1566 1.510796e+00
## 8644   2 1567 1.448525e+00
## 8645   2 1568 1.392583e+00
## 8646   2 1569 1.343644e+00
## 8647   2 1570 1.298528e+00
## 8648   2 1571 1.252879e+00
## 8649   2 1572 1.220718e+00
## 8650   2 1573 1.186963e+00
## 8651   2 1574 1.152643e+00
## 8652   2 1575 1.120513e+00
## 8653   2 1576 1.094111e+00
## 8654   2 1577 1.067914e+00
## 8655   2 1578 1.041778e+00
## 8656   2 1579 1.019079e+00
## 8657   2 1580 9.967599e-01
## 8658   2 1581 9.753595e-01
## 8659   2 1582 9.531807e-01
## 8660   2 1583 9.328860e-01
## 8661   2 1584 9.136687e-01
## 8662   2 1584 9.142758e-01
## 8663   2 1585 8.936841e-01
## 8664   2 1586 8.751649e-01
## 8665   2 1587 8.592288e-01
## 8666   2 1588 8.401074e-01
## 8667   2 1589 8.226244e-01
## 8668   2 1590 8.069800e-01
## 8669   2 1591 7.914654e-01
## 8670   2 1592 7.750255e-01
## 8671   2 1593 7.610307e-01
## 8672   2 1594 7.454370e-01
## 8673   2 1595 7.298348e-01
## 8674   2 1596 7.152380e-01
## 8675   2 1597 7.026060e-01
## 8676   2 1598 6.886918e-01
## 8677   2 1599 6.737653e-01
## 8678   2 1599 6.745591e-01
## 8679   2 1600 1.205261e+00
## 8680   2 1601 1.143317e+00
## 8681   2 1602 1.083742e+00
## 8682   2 1603 1.034002e+00
## 8683   2 1604 9.903826e-01
## 8684   2 1605 9.516215e-01
## 8685   2 1606 9.174245e-01
## 8686   2 1607 8.845627e-01
## 8687   2 1608 8.568721e-01
## 8688   2 1608 8.566605e-01
## 8689   2 1609 1.921953e+00
## 8690   2 1610 1.791676e+00
## 8691   2 1611 1.682267e+00
## 8692   2 1612 1.585640e+00
## 8693   2 1613 1.501619e+00
## 8694   2 1614 1.426686e+00
## 8695   2 1615 1.367047e+00
## 8696   2 1616 1.308738e+00
## 8697   2 1617 1.258449e+00
## 8698   2 1618 1.214623e+00
## 8699   2 1619 1.172292e+00
## 8700   2 1620 1.135258e+00
## 8701   2 1621 1.102580e+00
## 8702   2 1622 1.069759e+00
## 8703   2 1623 1.040216e+00
## 8704   2 1624 1.015135e+00
## 8705   2 1625 9.881734e-01
## 8706   2 1626 9.659106e-01
## 8707   2 1627 9.441904e-01
## 8708   2 1628 9.242735e-01
## 8709   2 1629 9.030665e-01
## 8710   2 1630 8.821908e-01
## 8711   2 1631 8.650874e-01
## 8712   2 1632 8.434885e-01
## 8713   2 1633 1.917926e+00
## 8714   2 1634 1.794153e+00
## 8715   2 1635 1.690055e+00
## 8716   2 1636 1.598789e+00
## 8717   2 1637 1.519867e+00
## 8718   2 1638 1.450745e+00
## 8719   2 1639 1.387318e+00
## 8720   2 1640 1.332769e+00
## 8721   2 1641 1.287761e+00
## 8722   2 1642 1.242231e+00
## 8723   2 1643 1.198825e+00
## 8724   2 1644 1.164287e+00
## 8725   2 1645 1.129813e+00
## 8726   2 1646 1.100056e+00
## 8727   2 1647 1.071073e+00
## 8728   2 1648 1.043295e+00
## 8729   2 1649 1.019481e+00
## 8730   2 1650 9.931639e-01
## 8731   2 1651 9.702069e-01
## 8732   2 1652 9.492845e-01
## 8733   2 1653 9.264466e-01
## 8734   2 1654 9.098079e-01
## 8735   2 1655 8.888641e-01
## 8736   2 1656 8.703947e-01
## 8737   2 1657 1.942120e+00
## 8738   2 1658 1.818452e+00
## 8739   2 1659 1.713899e+00
## 8740   2 1660 1.621043e+00
## 8741   2 1661 1.543056e+00
## 8742   2 1662 1.474284e+00
## 8743   2 1663 1.406602e+00
## 8744   2 1664 1.355868e+00
## 8745   2 1665 1.304992e+00
## 8746   2 1666 1.263839e+00
## 8747   2 1667 1.219725e+00
## 8748   2 1668 1.185297e+00
## 8749   2 1669 1.151006e+00
## 8750   2 1670 1.118276e+00
## 8751   2 1671 1.089914e+00
## 8752   2 1672 1.063063e+00
## 8753   2 1673 1.035964e+00
## 8754   2 1674 1.011690e+00
## 8755   2 1675 9.911514e-01
## 8756   2 1676 9.670179e-01
## 8757   2 1677 9.451406e-01
## 8758   2 1678 9.257330e-01
## 8759   2 1679 9.064271e-01
## 8760   2 1680 8.864695e-01
## 8761   2 1681 1.959527e+00
## 8762   2 1682 1.841182e+00
## 8763   2 1683 1.731634e+00
## 8764   2 1684 1.637826e+00
## 8765   2 1685 1.555584e+00
## 8766   2 1686 1.486309e+00
## 8767   2 1687 1.427726e+00
## 8768   2 1688 1.370647e+00
## 8769   2 1689 1.319534e+00
## 8770   2 1690 1.275452e+00
## 8771   2 1691 1.234850e+00
## 8772   2 1692 1.196498e+00
## 8773   2 1693 1.163782e+00
## 8774   2 1694 1.131586e+00
## 8775   2 1695 1.103289e+00
## 8776   2 1696 1.073932e+00
## 8777   2 1697 1.048411e+00
## 8778   2 1698 1.024320e+00
## 8779   2 1699 1.002480e+00
## 8780   2 1700 9.781189e-01
## 8781   2 1701 9.567452e-01
## 8782   2 1702 9.363890e-01
## 8783   2 1703 9.151806e-01
## 8784   2 1704 8.958854e-01
## 8785   2 1705 1.970205e+00
## 8786   2 1706 1.850405e+00
## 8787   2 1707 1.737608e+00
## 8788   2 1708 1.647210e+00
## 8789   2 1709 1.566272e+00
## 8790   2 1710 1.496043e+00
## 8791   2 1711 1.433563e+00
## 8792   2 1712 1.378779e+00
## 8793   2 1713 1.328081e+00
## 8794   2 1714 1.284503e+00
## 8795   2 1715 1.241430e+00
## 8796   2 1716 1.205815e+00
## 8797   2 1717 1.173088e+00
## 8798   2 1718 1.139210e+00
## 8799   2 1719 1.109668e+00
## 8800   2 1720 1.082664e+00
## 8801   2 1721 1.056731e+00
## 8802   2 1722 1.030241e+00
## 8803   2 1723 1.006380e+00
## 8804   2 1724 9.865529e-01
## 8805   2 1725 9.642204e-01
## 8806   2 1726 9.418679e-01
## 8807   2 1727 9.216900e-01
## 8808   2 1728 9.031094e-01
## 8809   2 1729 1.975268e+00
## 8810   2 1730 1.854711e+00
## 8811   2 1731 1.743206e+00
## 8812   2 1732 1.653977e+00
## 8813   2 1733 1.572089e+00
## 8814   2 1734 1.502258e+00
## 8815   2 1735 1.436457e+00
## 8816   2 1736 1.384618e+00
## 8817   2 1737 1.334793e+00
## 8818   2 1738 1.290430e+00
## 8819   2 1739 1.249657e+00
## 8820   2 1740 1.210781e+00
## 8821   2 1741 1.176446e+00
## 8822   2 1742 1.143841e+00
## 8823   2 1743 1.114316e+00
## 8824   2 1744 1.088007e+00
## 8825   2 1745 1.059498e+00
## 8826   2 1746 1.036585e+00
## 8827   2 1747 1.012307e+00
## 8828   2 1748 9.887994e-01
## 8829   2 1749 9.674180e-01
## 8830   2 1750 9.480862e-01
## 8831   2 1751 9.276275e-01
## 8832   2 1752 9.074038e-01
## 8833   2 1753 1.978255e+00
## 8834   2 1754 1.856011e+00
## 8835   2 1755 1.751227e+00
```

```
## 8836   2 1756  1.654811e+00
## 8837   2 1757  1.575694e+00
## 8838   2 1758  1.504361e+00
## 8839   2 1759  1.444070e+00
## 8840   2 1760  1.388854e+00
## 8841   2 1761  1.337451e+00
## 8842   2 1762  1.292124e+00
## 8843   2 1763  1.249682e+00
## 8844   2 1764  1.213365e+00
## 8845   2 1765  1.178226e+00
## 8846   2 1766  1.146013e+00
## 8847   2 1767  1.119068e+00
## 8848   2 1768  1.088908e+00
## 8849   2 1769  1.062783e+00
## 8850   2 1770  1.040107e+00
## 8851   2 1771  1.016475e+00
## 8852   2 1772  9.921565e-01
## 8853   2 1773  9.699042e-01
## 8854   2 1774  9.506825e-01
## 8855   2 1775  9.297123e-01
## 8856   2 1776  9.102837e-01
## 8857   2 1777  1.983014e+00
## 8858   2 1778  1.857765e+00
## 8859   2 1779  1.752723e+00
## 8860   2 1780  1.658336e+00
## 8861   2 1781  1.579606e+00
## 8862   2 1782  1.507422e+00
## 8863   2 1783  1.443420e+00
## 8864   2 1784  1.386992e+00
## 8865   2 1785  1.340641e+00
## 8866   2 1786  1.294964e+00
## 8867   2 1787  1.252375e+00
## 8868   2 1788  1.214379e+00
## 8869   2 1789  1.180112e+00
## 8870   2 1790  1.148708e+00
## 8871   2 1791  1.118452e+00
## 8872   2 1792  1.091081e+00
## 8873   2 1793  1.063868e+00
## 8874   2 1794  1.039677e+00
## 8875   2 1795  1.015813e+00
## 8876   2 1796  9.923879e-01
## 8877   2 1797  9.732675e-01
## 8878   2 1798  9.517579e-01
## 8879   2 1799  9.326798e-01
## 8880   2 1800  9.101976e-01
## 8881   2 1801  1.987468e+00
## 8882   2 1802  1.857331e+00
## 8883   2 1803  1.758112e+00
## 8884   2 1804  1.660957e+00
## 8885   2 1805  1.579804e+00
## 8886   2 1806  1.509313e+00
## 8887   2 1807  1.446966e+00
## 8888   2 1808  1.392318e+00
## 8889   2 1809  1.342112e+00
## 8890   2 1810  1.294127e+00
## 8891   2 1811  1.252979e+00
## 8892   2 1812  1.215087e+00
## 8893   2 1813  1.181830e+00
## 8894   2 1814  1.150619e+00
## 8895   2 1815  1.119289e+00
## 8896   2 1816  1.092159e+00
## 8897   2 1817  1.064289e+00
## 8898   2 1818  1.041108e+00
## 8899   2 1819  1.016377e+00
## 8900   2 1820  9.954688e-01
## 8901   2 1821  9.716938e-01
## 8902   2 1822  9.517402e-01
## 8903   2 1823  9.309420e-01
## 8904   2 1824  9.114619e-01
## 8905   2 1825  1.987069e+00
## 8906   2 1826  1.861030e+00
## 8907   2 1827  1.751859e+00
## 8908   2 1828  1.663325e+00
## 8909   2 1829  1.581399e+00
## 8910   2 1830  1.512434e+00
## 8911   2 1831  1.446341e+00
## 8912   2 1832  1.391894e+00
## 8913   2 1833  1.341677e+00
## 8914   2 1834  1.295838e+00
## 8915   2 1835  1.256189e+00
## 8916   2 1836  1.219658e+00
## 8917   2 1837  1.183453e+00
## 8918   2 1838  1.151045e+00
## 8919   2 1839  1.122447e+00
## 8920   2 1840  1.093279e+00
## 8921   2 1841  1.067257e+00
## 8922   2 1842  1.039954e+00
## 8923   2 1843  1.016481e+00
## 8924   2 1844  9.973397e-01
## 8925   2 1845  9.748858e-01
## 8926   2 1846  9.533052e-01
## 8927   2 1847  9.313634e-01
## 8928   2 1848  9.138532e-01
## 8929   2 1849  1.985030e+00
## 8930   2 1850  1.865458e+00
## 8931   2 1851  1.757410e+00
## 8932   2 1852  1.659968e+00
## 8933   2 1853  1.583796e+00
## 8934   2 1854  1.512149e+00
## 8935   2 1855  1.447183e+00
## 8936   2 1856  1.391140e+00
## 8937   2 1857  1.340198e+00
## 8938   2 1858  1.297812e+00
## 8939   2 1859  1.255295e+00
## 8940   2 1860  1.216959e+00
## 8941   2 1861  1.183266e+00
## 8942   2 1862  1.152018e+00
## 8943   2 1863  1.124555e+00
## 8944   2 1864  1.092233e+00
## 8945   2 1865  1.065743e+00
## 8946   2 1866  1.041054e+00
## 8947   2 1867  1.018159e+00
## 8948   2 1868  9.936281e-01
## 8949   2 1869  9.723650e-01
## 8950   2 1870  9.534416e-01
## 8951   2 1871  9.321835e-01
## 8952   2 1872  9.127074e-01
## 8953   2 1872  9.156952e-01
## 8954   2 1873  1.986640e+00
## 8955   2 1874  1.864337e+00
## 8956   2 1875  1.759077e+00
## 8957   2 1876  1.664005e+00
## 8958   2 1877  1.582107e+00
## 8959   2 1878  1.511637e+00
## 8960   2 1879  1.449030e+00
## 8961   2 1880  1.390394e+00
## 8962   2 1881  1.342704e+00
## 8963   2 1882  1.296409e+00
## 8964   2 1883  1.256997e+00
## 8965   2 1884  1.219647e+00
## 8966   2 1885  1.185849e+00
## 8967   2 1886  1.152450e+00
## 8968   2 1887  1.123297e+00
## 8969   2 1888  1.095450e+00
## 8970   2 1889  1.068546e+00
## 8971   2 1890  1.043548e+00
## 8972   2 1891  1.019809e+00
## 8973   2 1892  9.961436e-01
## 8974   2 1893  9.741569e-01
## 8975   2 1894  9.540637e-01
## 8976   2 1895  9.343450e-01
## 8977   2 1896  9.133842e-01
## 8978   2 1896  9.133964e-01
## 8979   2 1897  8.949712e-01
## 8980   2 1898  8.759860e-01
## 8981   2 1899  8.597637e-01
## 8982   2 1900  8.411646e-01
## 8983   2 1901  8.243771e-01
## 8984   2 1902  8.078013e-01
## 8985   2 1903  7.925400e-01
## 8986   2 1904  7.744903e-01
## 8987   2 1905  7.607415e-01
## 8988   2 1906  7.445135e-01
## 8989   2 1907  7.307670e-01
## 8990   2 1908  7.162867e-01
## 8991   2 1909  7.021948e-01
## 8992   2 1910  6.900005e-01
## 8993   2 1911  6.740580e-01
## 8994   2 1911  6.753403e-01
## 8995   2 1912  1.750666e+00
## 8996   2 1913  1.635731e+00
## 8997   2 1914  1.532461e+00
## 8998   2 1915  1.445596e+00
## 8999   2 1916  1.369355e+00
## 9000   2 1917  1.300382e+00
## 9001   2 1918  1.243158e+00
## 9002   2 1919  1.194923e+00
## 9003   2 1920  1.148067e+00
## 9004   2 1920  1.146620e+00
## 9005   2 1921  2.198318e+00
## 9006   2 1922  2.056948e+00
## 9007   2 1923  1.931671e+00
## 9008   2 1924  1.823839e+00
## 9009   2 1925  1.738104e+00
## 9010   2 1926  1.650032e+00
## 9011   2 1927  1.582377e+00
## 9012   2 1928  1.518634e+00
## 9013   2 1929  1.462405e+00
## 9014   2 1930  1.412742e+00
## 9015   2 1931  1.366087e+00
## 9016   2 1932  1.322250e+00
## 9017   2 1933  1.285464e+00
## 9018   2 1934  1.250640e+00
## 9019   2 1935  1.215792e+00
## 9020   2 1936  1.185896e+00
## 9021   2 1937  1.155878e+00
## 9022   2 1938  1.130556e+00
## 9023   2 1939  1.103769e+00
## 9024   2 1940  1.078454e+00
## 9025   2 1941  1.055886e+00
## 9026   2 1942  1.032281e+00
## 9027   2 1943  1.010140e+00
## 9028   2 1944  9.885260e-01
## 9029   2 1945  2.061316e+00
## 9030   2 1946  1.931878e+00
## 9031   2 1947  1.828321e+00
## 9032   2 1948  1.733097e+00
## 9033   2 1949  1.649158e+00
## 9034   2 1950  1.577309e+00
## 9035   2 1951  1.512911e+00
## 9036   2 1952  1.456964e+00
## 9037   2 1953  1.404244e+00
## 9038   2 1954  1.357835e+00
## 9039   2 1955  1.316225e+00
## 9040   2 1956  1.278590e+00
## 9041   2 1957  1.244038e+00
## 9042   2 1958  1.208910e+00
## 9043   2 1959  1.175530e+00
## 9044   2 1960  1.147825e+00
## 9045   2 1961  1.119313e+00
## 9046   2 1962  1.094546e+00
## 9047   2 1963  1.070040e+00
## 9048   2 1964  1.044000e+00
## 9049   2 1965  1.020825e+00
## 9050   2 1966  9.997144e-01
## 9051   2 1967  9.794267e-01
## 9052   2 1968  9.588470e-01
## 9053   2 1969  2.034892e+00
## 9054   2 1970  1.901566e+00
## 9055   2 1971  1.798714e+00
## 9056   2 1972  1.705328e+00
## 9057   2 1973  1.622764e+00
## 9058   2 1974  1.549316e+00
## 9059   2 1975  1.487282e+00
## 9060   2 1976  1.431668e+00
## 9061   2 1977  1.379342e+00
## 9062   2 1978  1.335502e+00
## 9063   2 1979  1.294586e+00
## 9064   2 1980  1.255892e+00
## 9065   2 1981  1.221562e+00
## 9066   2 1982  1.186032e+00
## 9067   2 1983  1.157318e+00
## 9068   2 1984  1.127830e+00
## 9069   2 1985  1.100888e+00
## 9070   2 1986  1.076188e+00
## 9071   2 1987  1.049113e+00
## 9072   2 1988  1.027771e+00
## 9073   2 1989  1.005087e+00
## 9074   2 1990  9.842619e-01
## 9075   2 1991  9.607464e-01
## 9076   2 1992  9.429372e-01
## 9077   2 1993  2.018368e+00
## 9078   2 1994  1.892865e+00
## 9079   2 1995  1.782603e+00
## 9080   2 1996  1.689553e+00
## 9081   2 1997  1.610037e+00
## 9082   2 1998  1.538386e+00
## 9083   2 1999  1.472985e+00
## 9084   2 2000  1.416206e+00
## 9085   2 2001  1.366330e+00
## 9086   2 2002  1.322191e+00
## 9087   2 2003  1.278980e+00
## 9088   2 2004  1.241967e+00
## 9089   2 2005  1.205207e+00
## 9090   2 2006  1.173933e+00
## 9091   2 2007  1.143572e+00
## 9092   2 2008  1.117206e+00
## 9093   2 2009  1.092149e+00
## 9094   2 2010  1.062175e+00
## 9095   2 2011  1.039521e+00
## 9096   2 2012  1.014350e+00
## 9097   2 2013  9.939070e-01
## 9098   2 2014  9.718183e-01
## 9099   2 2015  9.520157e-01
## 9100   2 2016  9.327039e-01
## 9101   2 2017  2.005531e+00
## 9102   2 2018  1.878037e+00
## 9103   2 2019  1.774781e+00
## 9104   2 2020  1.678060e+00
## 9105   2 2021  1.597680e+00
## 9106   2 2022  1.528154e+00
## 9107   2 2023  1.463777e+00
## 9108   2 2024  1.406832e+00
## 9109   2 2025  1.356538e+00
## 9110   2 2026  1.311378e+00
## 9111   2 2027  1.271158e+00
## 9112   2 2028  1.234889e+00
## 9113   2 2029  1.199734e+00
## 9114   2 2030  1.168573e+00
## 9115   2 2031  1.134624e+00
## 9116   2 2032  1.107110e+00
## 9117   2 2033  1.079313e+00
## 9118   2 2034  1.055141e+00
## 9119   2 2035  1.031808e+00
## 9120   2 2036  1.008738e+00
## 9121   2 2037  9.862623e-01
## 9122   2 2038  9.653906e-01
## 9123   2 2039  9.438602e-01
## 9124   2 2040  9.235883e-01
## 9125   2 2041  1.997079e+00
## 9126   2 2042  1.874483e+00
## 9127   2 2043  1.768910e+00
## 9128   2 2044  1.669967e+00
## 9129   2 2045  1.590021e+00
## 9130   2 2046  1.521194e+00
## 9131   2 2047  1.459463e+00
## 9132   2 2048  1.401121e+00
## 9133   2 2049  1.350903e+00
## 9134   2 2050  1.307023e+00
## 9135   2 2051  1.265170e+00
## 9136   2 2052  1.228504e+00
## 9137   2 2053  1.194573e+00
## 9138   2 2054  1.161239e+00
## 9139   2 2055  1.129826e+00
## 9140   2 2056  1.102077e+00
## 9141   2 2057  1.075652e+00
## 9142   2 2058  1.050226e+00
## 9143   2 2059  1.026499e+00
## 9144   2 2060  1.005022e+00
## 9145   2 2061  9.804004e-01
## 9146   2 2062  9.595614e-01
## 9147   2 2063  9.413571e-01
## 9148   2 2064  9.193241e-01
## 9149   2 2065  1.995959e+00
## 9150   2 2066  1.870814e+00
## 9151   2 2067  1.763455e+00
## 9152   2 2068  1.672884e+00
## 9153   2 2069  1.588979e+00
## 9154   2 2070  1.515262e+00
## 9155   2 2071  1.453957e+00
## 9156   2 2072  1.398769e+00
## 9157   2 2073  1.346486e+00
## 9158   2 2074  1.303274e+00
## 9159   2 2075  1.262798e+00
## 9160   2 2076  1.223470e+00
## 9161   2 2077  1.189338e+00
## 9162   2 2078  1.157576e+00
## 9163   2 2079  1.128783e+00
## 9164   2 2080  1.100099e+00
## 9165   2 2081  1.071456e+00
## 9166   2 2082  1.050032e+00
## 9167   2 2083  1.023399e+00
## 9168   2 2084  1.002771e+00
## 9169   2 2085  9.780008e-01
## 9170   2 2086  9.576663e-01
## 9171   2 2087  9.381246e-01
## 9172   2 2088  9.160781e-01
## 9173   2 2089  1.987902e+00
## 9174   2 2090  1.865953e+00
## 9175   2 2091  1.760493e+00
## 9176   2 2092  1.666258e+00
## 9177   2 2093  1.582640e+00
## 9178   2 2094  1.515753e+00
## 9179   2 2095  1.452712e+00
## 9180   2 2096  1.397777e+00
## 9181   2 2097  1.345802e+00
## 9182   2 2098  1.300483e+00
## 9183   2 2099  1.260569e+00
## 9184   2 2100  1.221976e+00
## 9185   2 2101  1.186514e+00
## 9186   2 2102  1.156233e+00
## 9187   2 2103  1.123460e+00
## 9188   2 2104  1.095930e+00
## 9189   2 2105  1.070069e+00
## 9190   2 2106  1.046356e+00
## 9191   2 2107  1.023805e+00
## 9192   2 2108  9.994116e-01
## 9193   2 2109  9.781972e-01
## 9194   2 2110  9.573677e-01
## 9195   2 2111  9.352532e-01
## 9196   2 2112  9.175658e-01
## 9197   2 2113  1.989026e+00
## 9198   2 2114  1.865617e+00
## 9199   2 2115  1.756995e+00
## 9200   2 2116  1.665248e+00
## 9201   2 2117  1.583486e+00
## 9202   2 2118  1.513492e+00
## 9203   2 2119  1.449391e+00
## 9204   2 2120  1.393052e+00
## 9205   2 2121  1.345334e+00
## 9206   2 2122  1.300905e+00
## 9207   2 2123  1.259743e+00
## 9208   2 2124  1.221177e+00
## 9209   2 2125  1.184070e+00
## 9210   2 2126  1.152274e+00
## 9211   2 2127  1.125666e+00
## 9212   2 2128  1.097876e+00
## 9213   2 2129  1.070231e+00
## 9214   2 2130  1.047626e+00
## 9215   2 2131  1.020058e+00
## 9216   2 2132  9.993273e-01
## 9217   2 2133  9.770912e-01
## 9218   2 2134  9.544485e-01
## 9219   2 2135  9.352779e-01
## 9220   2 2136  9.161504e-01
## 9221   2 2137  1.989477e+00
## 9222   2 2138  1.866263e+00
## 9223   2 2139  1.759141e+00
## 9224   2 2140  1.663472e+00
## 9225   2 2141  1.582787e+00
## 9226   2 2142  1.511378e+00
## 9227   2 2143  1.448028e+00
## 9228   2 2144  1.395118e+00
## 9229   2 2145  1.344820e+00
## 9230   2 2146  1.300290e+00
## 9231   2 2147  1.257699e+00
## 9232   2 2148  1.220904e+00
## 9233   2 2149  1.185072e+00
## 9234   2 2150  1.154646e+00
## 9235   2 2151  1.123013e+00
## 9236   2 2152  1.094795e+00
## 9237   2 2153  1.069453e+00
## 9238   2 2154  1.044886e+00
## 9239   2 2155  1.021222e+00
## 9240   2 2156  9.976634e-01
## 9241   2 2157  9.799731e-01
## 9242   2 2158  9.539963e-01
## 9243   2 2159  9.356976e-01
## 9244   2 2160  9.139391e-01
## 9245   2 2161  1.989289e+00
## 9246   2 2162  1.863868e+00
## 9247   2 2163  1.761042e+00
## 9248   2 2164  1.666645e+00
## 9249   2 2165  1.583054e+00
## 9250   2 2166  1.514445e+00
## 9251   2 2167  1.449434e+00
## 9252   2 2168  1.392286e+00
## 9253   2 2169  1.346540e+00
## 9254   2 2170  1.298303e+00
## 9255   2 2171  1.257935e+00
## 9256   2 2172  1.221814e+00
## 9257   2 2173  1.186517e+00
## 9258   2 2174  1.153924e+00
## 9259   2 2175  1.122786e+00
## 9260   2 2176  1.093155e+00
## 9261   2 2177  1.068039e+00
## 9262   2 2178  1.043840e+00
## 9263   2 2179  1.020832e+00
## 9264   2 2180  9.993284e-01
## 9265   2 2181  9.757305e-01
## 9266   2 2182  9.546980e-01
## 9267   2 2183  9.338711e-01
## 9268   2 2184  9.135561e-01
## 9269   2 2184  9.140579e-01
## 9270   2 2185  1.984713e+00
## 9271   2 2186  1.865008e+00
## 9272   2 2187  1.757420e+00
## 9273   2 2188  1.660517e+00
## 9274   2 2189  1.583757e+00
## 9275   2 2190  1.512119e+00
## 9276   2 2191  1.449482e+00
## 9277   2 2192  1.394943e+00
## 9278   2 2193  1.342338e+00
## 9279   2 2194  1.298005e+00
## 9280   2 2195  1.256017e+00
## 9281   2 2196  1.218349e+00
## 9282   2 2197  1.182895e+00
## 9283   2 2198  1.151730e+00
## 9284   2 2199  1.121909e+00
## 9285   2 2200  1.092433e+00
## 9286   2 2201  1.069739e+00
## 9287   2 2202  1.042166e+00
## 9288   2 2203  1.021218e+00
## 9289   2 2204  9.954262e-01
## 9290   2 2205  9.728325e-01
## 9291   2 2206  9.536445e-01
## 9292   2 2207  9.321332e-01
## 9293   2 2208  9.129213e-01
## 9294   2 2208  9.131712e-01
## 9295   2 2209  8.948260e-01
## 9296   2 2210  8.780364e-01
## 9297   2 2211  8.580404e-01
## 9298   2 2212  8.409288e-01
## 9299   2 2213  8.246552e-01
## 9300   2 2214  8.070090e-01
## 9301   2 2215  7.928433e-01
## 9302   2 2216  7.756073e-01
## 9303   2 2217  7.608649e-01
## 9304   2 2218  7.462490e-01
## 9305   2 2219  7.311921e-01
## 9306   2 2220  7.160166e-01
## 9307   2 2221  7.011673e-01
## 9308   2 2222  6.878790e-01
## 9309   2 2223  6.735808e-01
## 9310   2 2224  6.610353e-01
## 9311   2 2225  6.491444e-01
## 9312   2 2226  6.362247e-01
## 9313   2 2226  6.358680e-01
## 9314   2 2227  1.168895e+00
## 9315   2 2228  1.103350e+00
## 9316   2 2229  1.048857e+00
## 9317   2 2230  9.983517e-01
## 9318   2 2231  9.551916e-01
## 9319   2 2232  9.166310e-01
## 9320   2 2232  9.159753e-01
## 9321   2 2233  1.972790e+00
## 9322   2 2234  1.840349e+00
## 9323   2 2235  1.721146e+00
## 9324   2 2236  1.616298e+00
## 9325   2 2237  1.533724e+00
## 9326   2 2238  1.455233e+00
## 9327   2 2239  1.387609e+00
## 9328   2 2240  1.332353e+00
## 9329   2 2241  1.279923e+00
## 9330   2 2242  1.231409e+00
## 9331   2 2243  1.190781e+00
## 9332   2 2244  1.149855e+00
## 9333   2 2245  1.117356e+00
## 9334   2 2246  1.086007e+00
## 9335   2 2247  1.056629e+00
## 9336   2 2248  1.026024e+00
## 9337   2 2249  1.002245e+00
## 9338   2 2250  9.789917e-01
## 9339   2 2251  9.555243e-01
## 9340   2 2252  9.329053e-01
## 9341   2 2253  9.125363e-01
## 9342   2 2254  8.923487e-01
## 9343   2 2255  8.714053e-01
## 9344   2 2256  8.536086e-01
## 9345   2 2257  1.928157e+00
## 9346   2 2258  1.806192e+00
## 9347   2 2259  1.699068e+00
## 9348   2 2260  1.605623e+00
## 9349   2 2261  1.527490e+00
## 9350   2 2262  1.458115e+00
## 9351   2 2263  1.395075e+00
## 9352   2 2264  1.341714e+00
## 9353   2 2265  1.290466e+00
## 9354   2 2266  1.249931e+00
## 9355   2 2267  1.208194e+00
## 9356   2 2268  1.171005e+00
## 9357   2 2269  1.137814e+00
## 9358   2 2270  1.105084e+00
## 9359   2 2271  1.076231e+00
## 9360   2 2272  1.049819e+00
## 9361   2 2273  1.024896e+00
## 9362   2 2274  9.978487e-01
## 9363   2 2275  9.764882e-01
## 9364   2 2276  9.552830e-01
## 9365   2 2277  9.356256e-01
## 9366   2 2278  9.131748e-01
## 9367   2 2279  8.945942e-01
## 9368   2 2280  8.752644e-01
## 9369   2 2281  1.949384e+00
## 9370   2 2282  1.825597e+00
## 9371   2 2283  1.719596e+00
## 9372   2 2284  1.625870e+00
## 9373   2 2285  1.548839e+00
## 9374   2 2286  1.475206e+00
## 9375   2 2287  1.417297e+00
## 9376   2 2288  1.357766e+00
## 9377   2 2289  1.311684e+00
## 9378   2 2290  1.265016e+00
## 9379   2 2291  1.226745e+00
## 9380   2 2292  1.188568e+00
## 9381   2 2293  1.153645e+00
## 9382   2 2294  1.125004e+00
## 9383   2 2295  1.095222e+00
## 9384   2 2296  1.067045e+00
## 9385   2 2297  1.041584e+00
## 9386   2 2298  1.017205e+00
## 9387   2 2299  9.934389e-01
## 9388   2 2300  9.707100e-01
## 9389   2 2301  9.498919e-01
## 9390   2 2302  9.282206e-01
## 9391   2 2303  9.085295e-01
## 9392   2 2304  8.891142e-01
## 9393   2 2305  1.965641e+00
## 9394   2 2306  1.839303e+00
## 9395   2 2307  1.734798e+00
## 9396   2 2308  1.641688e+00
## 9397   2 2309  1.558991e+00
## 9398   2 2310  1.490387e+00
## 9399   2 2311  1.428438e+00
## 9400   2 2312  1.371494e+00
## 9401   2 2313  1.320893e+00
## 9402   2 2314  1.279523e+00
## 9403   2 2315  1.236635e+00
## 9404   2 2316  1.200286e+00
## 9405   2 2317  1.166116e+00
## 9406   2 2318  1.134703e+00
## 9407   2 2319  1.103950e+00
## 9408   2 2320  1.076900e+00
## 9409   2 2321  1.052356e+00
## 9410   2 2322  1.027109e+00
## 9411   2 2323  1.002879e+00
## 9412   2 2324  9.821549e-01
## 9413   2 2325  9.607316e-01
## 9414   2 2326  9.393041e-01
## 9415   2 2327  9.181562e-01
## 9416   2 2328  8.981084e-01
## 9417   2 2329  1.969051e+00
## 9418   2 2330  1.848736e+00
## 9419   2 2331  1.741568e+00
## 9420   2 2332  1.648476e+00
## 9421   2 2333  1.570238e+00
## 9422   2 2334  1.498363e+00
## 9423   2 2335  1.436361e+00
## 9424   2 2336  1.377648e+00
## 9425   2 2337  1.331120e+00
## 9426   2 2338  1.286625e+00
## 9427   2 2339  1.244785e+00
## 9428   2 2340  1.207026e+00
## 9429   2 2341  1.175882e+00
## 9430   2 2342  1.141034e+00
## 9431   2 2343  1.109699e+00
## 9432   2 2344  1.083527e+00
## 9433   2 2345  1.056126e+00
## 9434   2 2346  1.031929e+00
## 9435   2 2347  1.010615e+00
## 9436   2 2348  9.860406e-01
## 9437   2 2349  9.647629e-01
## 9438   2 2350  9.438459e-01
## 9439   2 2351  9.238855e-01
## 9440   2 2352  9.051112e-01
## 9441   2 2353  1.978707e+00
## 9442   2 2354  1.854530e+00
## 9443   2 2355  1.748037e+00
## 9444   2 2356  1.651884e+00
## 9445   2 2357  1.571380e+00
## 9446   2 2358  1.505774e+00
## 9447   2 2359  1.442191e+00
## 9448   2 2360  1.385440e+00
## 9449   2 2361  1.333963e+00
## 9450   2 2362  1.290307e+00
## 9451   2 2363  1.248962e+00
## 9452   2 2364  1.210068e+00
## 9453   2 2365  1.176511e+00
## 9454   2 2366  1.145698e+00
## 9455   2 2367  1.115417e+00
## 9456   2 2368  1.090410e+00
## 9457   2 2369  1.062458e+00
## 9458   2 2370  1.035796e+00
## 9459   2 2371  1.012321e+00
## 9460   2 2372  9.911678e-01
## 9461   2 2373  9.697258e-01
## 9462   2 2374  9.469192e-01
## 9463   2 2375  9.269477e-01
## 9464   2 2376  9.083073e-01
## 9465   2 2377  1.982178e+00
## 9466   2 2378  1.857274e+00
## 9467   2 2379  1.749085e+00
## 9468   2 2380  1.658283e+00
## 9469   2 2381  1.578009e+00
## 9470   2 2382  1.505120e+00
## 9471   2 2383  1.443677e+00
## 9472   2 2384  1.388404e+00
## 9473   2 2385  1.338975e+00
## 9474   2 2386  1.293409e+00
## 9475   2 2387  1.252303e+00
## 9476   2 2388  1.216254e+00
## 9477   2 2389  1.179266e+00
## 9478   2 2390  1.147137e+00
## 9479   2 2391  1.119040e+00
## 9480   2 2392  1.089851e+00
## 9481   2 2393  1.062881e+00
## 9482   2 2394  1.039246e+00
## 9483   2 2395  1.013497e+00
## 9484   2 2396  9.919787e-01
## 9485   2 2397  9.714976e-01
## 9486   2 2398  9.496536e-01
## 9487   2 2399  9.297610e-01
## 9488   2 2400  9.100375e-01
## 9489   2 2401  1.987134e+00
## 9490   2 2402  1.858166e+00
## 9491   2 2403  1.749357e+00
## 9492   2 2404  1.660279e+00
## 9493   2 2405  1.579323e+00
## 9494   2 2406  1.508277e+00
## 9495   2 2407  1.446438e+00
## 9496   2 2408  1.388434e+00
## 9497   2 2409  1.340629e+00
## 9498   2 2410  1.294826e+00
## 9499   2 2411  1.251730e+00
## 9500   2 2412  1.218420e+00
## 9501   2 2413  1.181777e+00
## 9502   2 2414  1.149406e+00
## 9503   2 2415  1.120156e+00
## 9504   2 2416  1.091159e+00
## 9505   2 2417  1.064636e+00
## 9506   2 2418  1.040762e+00
## 9507   2 2419  1.016693e+00
## 9508   2 2420  9.945169e-01
## 9509   2 2421  9.717856e-01
## 9510   2 2422  9.506702e-01
## 9511   2 2423  9.304566e-01
## 9512   2 2424  9.116617e-01
## 9513   2 2425  1.984669e+00
## 9514   2 2426  1.863216e+00
## 9515   2 2427  1.754262e+00
## 9516   2 2428  1.657572e+00
## 9517   2 2429  1.580703e+00
## 9518   2 2430  1.511079e+00
## 9519   2 2431  1.446953e+00
## 9520   2 2432  1.391044e+00
## 9521   2 2433  1.340620e+00
## 9522   2 2434  1.294408e+00
## 9523   2 2435  1.255782e+00
## 9524   2 2436  1.215892e+00
## 9525   2 2437  1.181387e+00
## 9526   2 2438  1.148557e+00
## 9527   2 2439  1.121636e+00
## 9528   2 2440  1.092259e+00
## 9529   2 2441  1.068752e+00
## 9530   2 2442  1.040999e+00
## 9531   2 2443  1.016848e+00
## 9532   2 2444  9.940994e-01
## 9533   2 2445  9.742314e-01
## 9534   2 2446  9.522707e-01
## 9535   2 2447  9.329642e-01
## 9536   2 2448  9.136490e-01
## 9537   2 2449  1.982792e+00
## 9538   2 2450  1.861177e+00
## 9539   2 2451  1.755284e+00
## 9540   2 2452  1.661731e+00
## 9541   2 2453  1.580835e+00
## 9542   2 2454  1.509442e+00
## 9543   2 2455  1.447291e+00
## 9544   2 2456  1.395016e+00
## 9545   2 2457  1.343144e+00
## 9546   2 2458  1.296670e+00
## 9547   2 2459  1.254941e+00
## 9548   2 2460  1.219341e+00
## 9549   2 2461  1.183430e+00
## 9550   2 2462  1.149414e+00
## 9551   2 2463  1.122505e+00
## 9552   2 2464  1.092695e+00
## 9553   2 2465  1.065495e+00
## 9554   2 2466  1.041936e+00
## 9555   2 2467  1.018344e+00
## 9556   2 2468  9.964602e-01
## 9557   2 2469  9.750596e-01
## 9558   2 2470  9.523233e-01
## 9559   2 2471  9.324466e-01
## 9560   2 2472  9.142994e-01
## 9561   2 2473  1.986642e+00
## 9562   2 2474  1.862626e+00
## 9563   2 2475  1.755162e+00
## 9564   2 2476  1.662766e+00
## 9565   2 2477  1.579809e+00
## 9566   2 2478  1.508578e+00
## 9567   2 2479  1.449396e+00
## 9568   2 2480  1.392815e+00
## 9569   2 2481  1.341166e+00
## 9570   2 2482  1.297660e+00
## 9571   2 2483  1.256413e+00
## 9572   2 2484  1.218836e+00
## 9573   2 2485  1.184396e+00
## 9574   2 2486  1.151695e+00
## 9575   2 2487  1.121610e+00
## 9576   2 2488  1.093106e+00
## 9577   2 2489  1.066343e+00
## 9578   2 2490  1.040427e+00
## 9579   2 2491  1.018675e+00
## 9580   2 2492  9.962282e-01
## 9581   2 2493  9.737140e-01
## 9582   2 2494  9.533255e-01
## 9583   2 2495  9.335196e-01
## 9584   2 2496  9.115762e-01
## 9585   2 2496  9.132330e-01
## 9586   2 2497  1.987132e+00
## 9587   2 2498  1.864088e+00
## 9588   2 2499  1.757712e+00
## 9589   2 2500  1.665113e+00
## 9590   2 2501  1.583591e+00
## 9591   2 2502  1.512523e+00
## 9592   2 2503  1.447412e+00
## 9593   2 2504  1.393543e+00
## 9594   2 2505  1.341395e+00
## 9595   2 2506  1.297807e+00
## 9596   2 2507  1.255999e+00
## 9597   2 2508  1.219811e+00
## 9598   2 2509  1.181718e+00
## 9599   2 2510  1.152605e+00
## 9600   2 2511  1.121936e+00
## 9601   2 2512  1.094881e+00
## 9602   2 2513  1.068587e+00
## 9603   2 2514  1.043288e+00
## 9604   2 2515  1.021067e+00
## 9605   2 2516  9.945320e-01
## 9606   2 2517  9.741957e-01
## 9607   2 2518  9.527965e-01
## 9608   2 2519  9.336143e-01
## 9609   2 2520  9.141181e-01
## 9610   2 2520  9.135963e-01
## 9611   2 2521  8.950962e-01
## 9612   2 2522  8.764013e-01
## 9613   2 2523  8.602714e-01
## 9614   2 2524  8.408948e-01
## 9615   2 2525  8.234217e-01
## 9616   2 2526  8.075684e-01
## 9617   2 2527  7.903155e-01
## 9618   2 2528  7.766578e-01
## 9619   2 2529  7.610638e-01
## 9620   2 2530  7.449360e-01
## 9621   2 2531  7.301100e-01
## 9622   2 2532  7.162136e-01
## 9623   2 2533  7.019573e-01
## 9624   2 2534  6.880852e-01
## 9625   2 2535  6.754519e-01
## 9626   2 2536  6.618750e-01
## 9627   2 2537  6.486573e-01
## 9628   2 2538  6.365135e-01
## 9629   2 2538  6.370728e-01
## 9630   2 2539  1.717909e+00
## 9631   2 2540  1.597804e+00
## 9632   2 2541  1.494674e+00
## 9633   2 2542  1.408793e+00
## 9634   2 2543  1.332329e+00
## 9635   2 2544  1.270358e+00
```

```
## 9636   2 2544  1.270549e+00
## 9637   2 2545  2.303942e+00
## 9638   2 2546  2.149203e+00
## 9639   2 2547  2.013290e+00
## 9640   2 2548  1.893998e+00
## 9641   2 2549  1.792941e+00
## 9642   2 2550  1.707366e+00
## 9643   2 2551  1.631553e+00
## 9644   2 2552  1.563022e+00
## 9645   2 2553  1.501043e+00
## 9646   2 2554  1.447048e+00
## 9647   2 2555  1.402535e+00
## 9648   2 2556  1.355733e+00
## 9649   2 2557  1.314984e+00
## 9650   2 2558  1.278594e+00
## 9651   2 2559  1.244365e+00
## 9652   2 2560  1.209253e+00
## 9653   2 2561  1.182693e+00
## 9654   2 2562  1.152753e+00
## 9655   2 2563  1.126848e+00
## 9656   2 2564  1.099089e+00
## 9657   2 2565  1.075532e+00
## 9658   2 2566  1.051061e+00
## 9659   2 2567  1.028869e+00
## 9660   2 2568  1.008305e+00
## 9661   2 2569  2.078230e+00
## 9662   2 2570  1.954442e+00
## 9663   2 2571  1.846812e+00
## 9664   2 2572  1.747004e+00
## 9665   2 2573  1.666467e+00
## 9666   2 2574  1.589338e+00
## 9667   2 2575  1.529801e+00
## 9668   2 2576  1.472105e+00
## 9669   2 2577  1.418801e+00
## 9670   2 2578  1.372411e+00
## 9671   2 2579  1.329182e+00
## 9672   2 2580  1.291367e+00
## 9673   2 2581  1.255620e+00
## 9674   2 2582  1.221940e+00
## 9675   2 2583  1.189839e+00
## 9676   2 2584  1.160943e+00
## 9677   2 2585  1.134525e+00
## 9678   2 2586  1.106097e+00
## 9679   2 2587  1.081561e+00
## 9680   2 2588  1.058396e+00
## 9681   2 2589  1.034380e+00
## 9682   2 2590  1.012200e+00
## 9683   2 2591  9.924854e-01
## 9684   2 2592  9.698018e-01
## 9685   2 2593  2.041318e+00
## 9686   2 2594  1.917236e+00
## 9687   2 2595  1.811275e+00
## 9688   2 2596  1.715773e+00
## 9689   2 2597  1.633027e+00
## 9690   2 2598  1.558728e+00
## 9691   2 2599  1.496469e+00
## 9692   2 2600  1.441500e+00
## 9693   2 2601  1.392157e+00
## 9694   2 2602  1.343442e+00
## 9695   2 2603  1.303046e+00
## 9696   2 2604  1.264247e+00
## 9697   2 2605  1.229850e+00
## 9698   2 2606  1.195598e+00
## 9699   2 2607  1.164362e+00
## 9700   2 2608  1.136850e+00
## 9701   2 2609  1.108605e+00
## 9702   2 2610  1.081654e+00
## 9703   2 2611  1.058283e+00
## 9704   2 2612  1.033920e+00
## 9705   2 2613  1.011530e+00
## 9706   2 2614  9.904195e-01
## 9707   2 2615  9.699099e-01
## 9708   2 2616  9.500747e-01
## 9709   2 2617  2.022377e+00
## 9710   2 2618  1.898418e+00
## 9711   2 2619  1.787438e+00
## 9712   2 2620  1.695441e+00
## 9713   2 2621  1.613481e+00
## 9714   2 2622  1.541549e+00
## 9715   2 2623  1.477546e+00
## 9716   2 2624  1.421688e+00
## 9717   2 2625  1.374058e+00
## 9718   2 2626  1.326174e+00
## 9719   2 2627  1.285743e+00
## 9720   2 2628  1.248784e+00
## 9721   2 2629  1.211355e+00
## 9722   2 2630  1.178995e+00
## 9723   2 2631  1.149077e+00
## 9724   2 2632  1.119429e+00
## 9725   2 2633  1.094172e+00
## 9726   2 2634  1.068476e+00
## 9727   2 2635  1.042452e+00
## 9728   2 2636  1.019988e+00
## 9729   2 2637  9.976429e-01
## 9730   2 2638  9.752345e-01
## 9731   2 2639  9.578154e-01
## 9732   2 2640  9.344930e-01
## 9733   2 2641  2.007002e+00
## 9734   2 2642  1.883049e+00
## 9735   2 2643  1.776319e+00
## 9736   2 2644  1.684715e+00
## 9737   2 2645  1.602506e+00
## 9738   2 2646  1.532582e+00
## 9739   2 2647  1.466704e+00
## 9740   2 2648  1.409715e+00
## 9741   2 2649  1.360760e+00
## 9742   2 2650  1.315073e+00
## 9743   2 2651  1.273275e+00
## 9744   2 2652  1.235256e+00
## 9745   2 2653  1.199990e+00
## 9746   2 2654  1.167544e+00
## 9747   2 2655  1.138181e+00
## 9748   2 2656  1.110414e+00
## 9749   2 2657  1.084299e+00
## 9750   2 2658  1.057755e+00
## 9751   2 2659  1.033920e+00
## 9752   2 2660  1.012931e+00
## 9753   2 2661  9.909912e-01
## 9754   2 2662  9.670682e-01
## 9755   2 2663  9.462589e-01
## 9756   2 2664  9.254427e-01
## 9757   2 2665  2.002127e+00
## 9758   2 2666  1.878445e+00
## 9759   2 2667  1.768173e+00
## 9760   2 2668  1.677051e+00
## 9761   2 2669  1.592162e+00
## 9762   2 2670  1.523711e+00
## 9763   2 2671  1.459627e+00
## 9764   2 2672  1.403412e+00
## 9765   2 2673  1.355079e+00
## 9766   2 2674  1.309743e+00
## 9767   2 2675  1.266687e+00
## 9768   2 2676  1.228534e+00
## 9769   2 2677  1.196308e+00
## 9770   2 2678  1.163015e+00
## 9771   2 2679  1.132078e+00
## 9772   2 2680  1.102765e+00
## 9773   2 2681  1.078314e+00
## 9774   2 2682  1.054504e+00
## 9775   2 2683  1.026547e+00
## 9776   2 2684  1.006595e+00
## 9777   2 2685  9.822560e-01
## 9778   2 2686  9.630938e-01
## 9779   2 2687  9.420415e-01
## 9780   2 2688  9.207411e-01
## 9781   2 2689  1.993995e+00
## 9782   2 2690  1.872438e+00
## 9783   2 2691  1.763205e+00
## 9784   2 2692  1.671657e+00
## 9785   2 2693  1.589323e+00
## 9786   2 2694  1.517136e+00
## 9787   2 2695  1.454141e+00
## 9788   2 2696  1.399061e+00
## 9789   2 2697  1.354206e+00
## 9790   2 2698  1.303306e+00
## 9791   2 2699  1.262591e+00
## 9792   2 2700  1.226128e+00
## 9793   2 2701  1.193025e+00
## 9794   2 2702  1.159394e+00
## 9795   2 2703  1.128855e+00
## 9796   2 2704  1.101595e+00
## 9797   2 2705  1.072870e+00
## 9798   2 2706  1.048165e+00
## 9799   2 2707  1.025647e+00
## 9800   2 2708  1.002633e+00
## 9801   2 2709  9.797801e-01
## 9802   2 2710  9.599544e-01
## 9803   2 2711  9.401100e-01
## 9804   2 2712  9.201631e-01
## 9805   2 2713  1.989550e+00
## 9806   2 2714  1.870620e+00
## 9807   2 2715  1.760222e+00
## 9808   2 2716  1.667077e+00
## 9809   2 2717  1.588341e+00
## 9810   2 2718  1.515648e+00
## 9811   2 2719  1.455538e+00
## 9812   2 2720  1.395819e+00
## 9813   2 2721  1.346624e+00
## 9814   2 2722  1.298838e+00
## 9815   2 2723  1.262055e+00
## 9816   2 2724  1.224239e+00
## 9817   2 2725  1.189582e+00
## 9818   2 2726  1.158599e+00
## 9819   2 2727  1.128437e+00
## 9820   2 2728  1.097743e+00
## 9821   2 2729  1.073812e+00
## 9822   2 2730  1.046918e+00
## 9823   2 2731  1.023748e+00
## 9824   2 2732  9.995002e-01
## 9825   2 2733  9.770460e-01
## 9826   2 2734  9.565464e-01
## 9827   2 2735  9.368218e-01
## 9828   2 2736  9.165086e-01
## 9829   2 2737  1.986859e+00
## 9830   2 2738  1.867544e+00
## 9831   2 2739  1.759002e+00
## 9832   2 2740  1.666903e+00
## 9833   2 2741  1.583428e+00
## 9834   2 2742  1.514048e+00
## 9835   2 2743  1.454742e+00
## 9836   2 2744  1.396264e+00
## 9837   2 2745  1.344661e+00
## 9838   2 2746  1.301789e+00
## 9839   2 2747  1.259708e+00
## 9840   2 2748  1.221478e+00
## 9841   2 2749  1.185211e+00
## 9842   2 2750  1.154241e+00
## 9843   2 2751  1.127673e+00
## 9844   2 2752  1.097019e+00
## 9845   2 2753  1.070064e+00
## 9846   2 2754  1.044917e+00
## 9847   2 2755  1.020722e+00
## 9848   2 2756  9.983287e-01
## 9849   2 2757  9.767943e-01
## 9850   2 2758  9.551781e-01
## 9851   2 2759  9.353838e-01
## 9852   2 2760  9.158536e-01
## 9853   2 2761  1.986617e+00
## 9854   2 2762  1.864066e+00
## 9855   2 2763  1.758066e+00
## 9856   2 2764  1.664791e+00
## 9857   2 2765  1.582950e+00
## 9858   2 2766  1.511713e+00
## 9859   2 2767  1.450065e+00
## 9860   2 2768  1.394838e+00
## 9861   2 2769  1.345053e+00
## 9862   2 2770  1.298062e+00
## 9863   2 2771  1.257803e+00
## 9864   2 2772  1.221059e+00
## 9865   2 2773  1.186432e+00
## 9866   2 2774  1.152784e+00
## 9867   2 2775  1.124851e+00
## 9868   2 2776  1.095920e+00
## 9869   2 2777  1.069201e+00
## 9870   2 2778  1.045624e+00
## 9871   2 2779  1.020691e+00
## 9872   2 2780  9.982803e-01
## 9873   2 2781  9.753034e-01
## 9874   2 2782  9.569853e-01
## 9875   2 2783  9.346184e-01
## 9876   2 2784  9.150002e-01
## 9877   2 2785  1.982887e+00
## 9878   2 2786  1.863900e+00
## 9879   2 2787  1.755370e+00
## 9880   2 2788  1.665100e+00
## 9881   2 2789  1.582664e+00
## 9882   2 2790  1.511639e+00
## 9883   2 2791  1.449621e+00
## 9884   2 2792  1.393694e+00
## 9885   2 2793  1.340308e+00
## 9886   2 2794  1.300333e+00
## 9887   2 2795  1.259587e+00
## 9888   2 2796  1.220758e+00
## 9889   2 2797  1.187106e+00
## 9890   2 2798  1.153312e+00
## 9891   2 2799  1.123618e+00
## 9892   2 2800  1.095967e+00
## 9893   2 2801  1.069048e+00
## 9894   2 2802  1.043360e+00
## 9895   2 2803  1.020181e+00
## 9896   2 2804  9.984253e-01
## 9897   2 2805  9.765496e-01
## 9898   2 2806  9.551531e-01
## 9899   2 2807  9.339363e-01
## 9900   2 2808  9.152072e-01
## 9901   2 2808  9.128973e-01
## 9902   2 2809  1.984062e+00
## 9903   2 2810  1.862373e+00
## 9904   2 2811  1.756955e+00
## 9905   2 2812  1.660974e+00
## 9906   2 2813  1.580390e+00
## 9907   2 2814  1.513389e+00
## 9908   2 2815  1.446495e+00
## 9909   2 2816  1.392432e+00
## 9910   2 2817  1.341271e+00
## 9911   2 2818  1.299835e+00
## 9912   2 2819  1.256744e+00
## 9913   2 2820  1.218653e+00
## 9914   2 2821  1.184897e+00
## 9915   2 2822  1.152393e+00
## 9916   2 2823  1.122890e+00
## 9917   2 2824  1.093772e+00
## 9918   2 2825  1.068934e+00
## 9919   2 2826  1.040927e+00
## 9920   2 2827  1.019685e+00
## 9921   2 2828  9.969953e-01
## 9922   2 2829  9.746431e-01
## 9923   2 2830  9.549505e-01
## 9924   2 2831  9.329926e-01
## 9925   2 2832  9.131021e-01
## 9926   2 2832  9.151462e-01
## 9927   2 2833  8.951234e-01
## 9928   2 2834  8.770954e-01
## 9929   2 2835  8.588103e-01
## 9930   2 2836  8.414512e-01
## 9931   2 2837  8.248211e-01
## 9932   2 2838  8.063351e-01
## 9933   2 2839  7.913949e-01
## 9934   2 2840  7.758453e-01
## 9935   2 2841  7.598430e-01
## 9936   2 2842  7.447559e-01
## 9937   2 2843  7.316100e-01
## 9938   2 2844  7.166056e-01
## 9939   2 2845  7.023359e-01
## 9940   2 2846  6.897503e-01
## 9941   2 2847  6.750363e-01
## 9942   2 2848  6.607846e-01
## 9943   2 2849  6.492103e-01
## 9944   2 2850  6.365587e-01
## 9945   2 2851  6.246423e-01
## 9946   2 2852  6.109077e-01
## 9947   2 2853  5.988230e-01
## 9948   2 2853  5.987333e-01
## 9949   2 2854  1.134636e+00
## 9950   2 2855  1.068380e+00
## 9951   2 2856  1.013091e+00
## 9952   2 2856  1.014618e+00
## 9953   2 2857  2.056964e+00
## 9954   2 2858  1.908545e+00
## 9955   2 2859  1.781285e+00
## 9956   2 2860  1.672974e+00
## 9957   2 2861  1.576820e+00
## 9958   2 2862  1.495914e+00
## 9959   2 2863  1.426638e+00
## 9960   2 2864  1.360511e+00
## 9961   2 2865  1.308199e+00
## 9962   2 2866  1.256357e+00
## 9963   2 2867  1.215426e+00
## 9964   2 2868  1.172862e+00
## 9965   2 2869  1.137410e+00
## 9966   2 2870  1.102471e+00
## 9967   2 2871  1.071134e+00
## 9968   2 2872  1.043990e+00
## 9969   2 2873  1.015966e+00
## 9970   2 2874  9.912650e-01
## 9971   2 2875  9.667586e-01
## 9972   2 2876  9.460826e-01
## 9973   2 2877  9.217539e-01
## 9974   2 2878  9.028993e-01
## 9975   2 2879  8.826480e-01
## 9976   2 2880  8.633199e-01
## 9977   2 2881  1.939078e+00
## 9978   2 2882  1.818543e+00
## 9979   2 2883  1.708834e+00
## 9980   2 2884  1.619123e+00
## 9981   2 2885  1.535512e+00
## 9982   2 2886  1.466989e+00
## 9983   2 2887  1.403836e+00
## 9984   2 2888  1.349279e+00
## 9985   2 2889  1.299295e+00
## 9986   2 2890  1.256521e+00
## 9987   2 2891  1.213618e+00
## 9988   2 2892  1.177059e+00
## 9989   2 2893  1.146172e+00
## 9990   2 2894  1.115119e+00
## 9991   2 2895  1.085121e+00
## 9992   2 2896  1.056562e+00
## 9993   2 2897  1.030606e+00
## 9994   2 2898  1.006342e+00
## 9995   2 2899  9.855474e-01
## 9996   2 2900  9.630718e-01
## 9997   2 2901  9.425463e-01
## 9998   2 2902  9.191695e-01
## 9999   2 2903  8.988690e-01
## 10000  2 2904  8.818217e-01
## 10001  2 2905  1.953958e+00
## 10002  2 2906  1.832313e+00
## 10003  2 2907  1.723768e+00
## 10004  2 2908  1.635255e+00
## 10005  2 2909  1.553431e+00
## 10006  2 2910  1.484163e+00
## 10007  2 2911  1.417964e+00
## 10008  2 2912  1.366236e+00
## 10009  2 2913  1.315662e+00
## 10010  2 2914  1.271689e+00
## 10011  2 2915  1.232105e+00
## 10012  2 2916  1.195931e+00
## 10013  2 2917  1.159029e+00
## 10014  2 2918  1.126845e+00
## 10015  2 2919  1.098242e+00
## 10016  2 2920  1.069817e+00
## 10017  2 2921  1.042968e+00
## 10018  2 2922  1.019272e+00
## 10019  2 2923  9.963108e-01
## 10020  2 2924  9.745706e-01
## 10021  2 2925  9.532332e-01
## 10022  2 2926  9.338862e-01
## 10023  2 2927  9.127347e-01
## 10024  2 2928  8.933115e-01
## 10025  2 2929  1.966928e+00
## 10026  2 2930  1.845973e+00
## 10027  2 2931  1.737360e+00
## 10028  2 2932  1.645868e+00
## 10029  2 2933  1.565227e+00
## 10030  2 2934  1.495247e+00
## 10031  2 2935  1.432513e+00
## 10032  2 2936  1.376965e+00
## 10033  2 2937  1.325698e+00
## 10034  2 2938  1.283060e+00
## 10035  2 2939  1.237642e+00
## 10036  2 2940  1.204587e+00
## 10037  2 2941  1.167665e+00
## 10038  2 2942  1.138810e+00
## 10039  2 2943  1.109399e+00
## 10040  2 2944  1.080782e+00
## 10041  2 2945  1.052554e+00
## 10042  2 2946  1.030181e+00
## 10043  2 2947  1.005904e+00
## 10044  2 2948  9.829696e-01
## 10045  2 2949  9.618624e-01
## 10046  2 2950  9.407860e-01
## 10047  2 2951  9.220300e-01
## 10048  2 2952  9.009449e-01
## 10049  2 2953  1.974110e+00
## 10050  2 2954  1.853239e+00
## 10051  2 2955  1.744741e+00
## 10052  2 2956  1.648672e+00
## 10053  2 2957  1.570580e+00
## 10054  2 2958  1.499043e+00
## 10055  2 2959  1.438923e+00
## 10056  2 2960  1.381618e+00
## 10057  2 2961  1.333707e+00
## 10058  2 2962  1.288706e+00
## 10059  2 2963  1.245506e+00
## 10060  2 2964  1.207090e+00
## 10061  2 2965  1.173917e+00
## 10062  2 2966  1.140867e+00
## 10063  2 2967  1.109721e+00
## 10064  2 2968  1.085957e+00
## 10065  2 2969  1.059109e+00
## 10066  2 2970  1.032824e+00
## 10067  2 2971  1.008264e+00
## 10068  2 2972  9.881486e-01
## 10069  2 2973  9.672474e-01
## 10070  2 2974  9.466310e-01
## 10071  2 2975  9.258429e-01
## 10072  2 2976  9.049450e-01
## 10073  2 2977  1.979970e+00
## 10074  2 2978  1.858580e+00
## 10075  2 2979  1.746182e+00
## 10076  2 2980  1.654775e+00
## 10077  2 2981  1.575130e+00
## 10078  2 2982  1.504806e+00
## 10079  2 2983  1.443970e+00
## 10080  2 2984  1.386328e+00
## 10081  2 2985  1.334638e+00
## 10082  2 2986  1.292094e+00
## 10083  2 2987  1.249816e+00
## 10084  2 2988  1.214566e+00
## 10085  2 2989  1.177404e+00
## 10086  2 2990  1.146926e+00
## 10087  2 2991  1.116645e+00
## 10088  2 2992  1.090390e+00
## 10089  2 2993  1.060532e+00
## 10090  2 2994  1.039172e+00
## 10091  2 2995  1.013391e+00
## 10092  2 2996  9.909696e-01
## 10093  2 2997  9.700609e-01
## 10094  2 2998  9.492182e-01
## 10095  2 2999  9.294345e-01
## 10096  2 3000  9.071392e-01
## 10097  2 3001  1.978588e+00
## 10098  2 3002  1.860604e+00
## 10099  2 3003  1.751088e+00
## 10100  2 3004  1.659394e+00
## 10101  2 3005  1.579211e+00
## 10102  2 3006  1.504830e+00
## 10103  2 3007  1.445361e+00
## 10104  2 3008  1.387273e+00
## 10105  2 3009  1.337940e+00
## 10106  2 3010  1.292093e+00
## 10107  2 3011  1.252410e+00
## 10108  2 3012  1.215819e+00
## 10109  2 3013  1.179888e+00
## 10110  2 3014  1.148327e+00
## 10111  2 3015  1.118312e+00
## 10112  2 3016  1.092075e+00
## 10113  2 3017  1.064475e+00
## 10114  2 3018  1.040272e+00
## 10115  2 3019  1.014700e+00
## 10116  2 3020  9.930193e-01
## 10117  2 3021  9.717088e-01
## 10118  2 3022  9.499862e-01
## 10119  2 3023  9.298309e-01
## 10120  2 3024  9.092778e-01
## 10121  2 3025  1.987161e+00
## 10122  2 3026  1.860896e+00
## 10123  2 3027  1.753154e+00
## 10124  2 3028  1.661190e+00
## 10125  2 3029  1.579801e+00
## 10126  2 3030  1.511074e+00
## 10127  2 3031  1.445264e+00
## 10128  2 3032  1.389066e+00
## 10129  2 3033  1.341099e+00
## 10130  2 3034  1.297188e+00
## 10131  2 3035  1.254586e+00
## 10132  2 3036  1.213596e+00
## 10133  2 3037  1.181001e+00
## 10134  2 3038  1.148046e+00
## 10135  2 3039  1.119127e+00
## 10136  2 3040  1.090549e+00
## 10137  2 3041  1.067064e+00
## 10138  2 3042  1.040732e+00
## 10139  2 3043  1.018684e+00
## 10140  2 3044  9.955391e-01
## 10141  2 3045  9.737416e-01
## 10142  2 3046  9.520350e-01
## 10143  2 3047  9.312696e-01
## 10144  2 3048  9.127837e-01
## 10145  2 3049  1.985305e+00
## 10146  2 3050  1.862993e+00
## 10147  2 3051  1.754602e+00
## 10148  2 3052  1.660686e+00
## 10149  2 3053  1.580602e+00
## 10150  2 3054  1.508242e+00
## 10151  2 3055  1.446897e+00
## 10152  2 3056  1.390949e+00
## 10153  2 3057  1.340387e+00
## 10154  2 3058  1.295753e+00
## 10155  2 3059  1.255627e+00
## 10156  2 3060  1.218109e+00
## 10157  2 3061  1.184688e+00
## 10158  2 3062  1.150113e+00
## 10159  2 3063  1.119955e+00
## 10160  2 3064  1.093120e+00
## 10161  2 3065  1.066023e+00
## 10162  2 3066  1.042004e+00
## 10163  2 3067  1.019081e+00
## 10164  2 3068  9.962942e-01
## 10165  2 3069  9.749600e-01
## 10166  2 3070  9.529623e-01
## 10167  2 3071  9.290214e-01
## 10168  2 3072  9.131576e-01
## 10169  2 3073  1.985066e+00
## 10170  2 3074  1.860621e+00
## 10171  2 3075  1.757169e+00
## 10172  2 3076  1.661928e+00
## 10173  2 3077  1.581901e+00
## 10174  2 3078  1.510091e+00
## 10175  2 3079  1.445030e+00
## 10176  2 3080  1.389479e+00
## 10177  2 3081  1.342292e+00
## 10178  2 3082  1.296735e+00
## 10179  2 3083  1.257365e+00
## 10180  2 3084  1.218625e+00
## 10181  2 3085  1.182911e+00
## 10182  2 3086  1.148843e+00
## 10183  2 3087  1.120781e+00
## 10184  2 3088  1.095212e+00
## 10185  2 3089  1.067213e+00
## 10186  2 3090  1.042419e+00
## 10187  2 3091  1.019822e+00
## 10188  2 3092  9.968457e-01
## 10189  2 3093  9.745567e-01
## 10190  2 3094  9.529426e-01
## 10191  2 3095  9.310962e-01
## 10192  2 3096  9.137534e-01
## 10193  2 3097  1.989008e+00
## 10194  2 3098  1.862791e+00
## 10195  2 3099  1.753794e+00
## 10196  2 3100  1.667526e+00
## 10197  2 3101  1.582427e+00
## 10198  2 3102  1.510605e+00
## 10199  2 3103  1.447044e+00
## 10200  2 3104  1.392933e+00
## 10201  2 3105  1.341328e+00
## 10202  2 3106  1.299858e+00
## 10203  2 3107  1.258162e+00
## 10204  2 3108  1.218288e+00
## 10205  2 3109  1.184614e+00
## 10206  2 3110  1.152309e+00
## 10207  2 3111  1.121805e+00
## 10208  2 3112  1.094282e+00
## 10209  2 3113  1.066340e+00
## 10210  2 3114  1.043466e+00
## 10211  2 3115  1.019347e+00
## 10212  2 3116  9.947365e-01
## 10213  2 3117  9.743606e-01
## 10214  2 3118  9.524514e-01
## 10215  2 3119  9.341004e-01
## 10216  2 3120  9.115058e-01
## 10217  2 3120  9.141168e-01
## 10218  2 3121  1.984542e+00
## 10219  2 3122  1.860355e+00
## 10220  2 3123  1.759123e+00
## 10221  2 3124  1.664861e+00
## 10222  2 3125  1.585124e+00
## 10223  2 3126  1.512409e+00
## 10224  2 3127  1.450717e+00
## 10225  2 3128  1.392345e+00
## 10226  2 3129  1.342703e+00
## 10227  2 3130  1.296299e+00
## 10228  2 3131  1.257845e+00
## 10229  2 3132  1.216742e+00
## 10230  2 3133  1.183336e+00
## 10231  2 3134  1.152990e+00
## 10232  2 3135  1.122043e+00
## 10233  2 3136  1.093363e+00
## 10234  2 3137  1.068358e+00
## 10235  2 3138  1.041980e+00
## 10236  2 3139  1.020337e+00
## 10237  2 3140  9.966430e-01
## 10238  2 3141  9.760818e-01
## 10239  2 3142  9.540615e-01
## 10240  2 3143  9.329797e-01
## 10241  2 3144  9.142694e-01
## 10242  2 3144  9.152181e-01
## 10243  2 3145  8.964411e-01
## 10244  2 3146  8.768721e-01
## 10245  2 3147  8.597978e-01
## 10246  2 3148  8.423036e-01
## 10247  2 3149  8.240953e-01
## 10248  2 3150  8.067730e-01
## 10249  2 3151  7.913804e-01
## 10250  2 3152  7.744419e-01
## 10251  2 3153  7.611549e-01
## 10252  2 3154  7.449346e-01
## 10253  2 3155  7.319727e-01
## 10254  2 3156  7.163009e-01
## 10255  2 3157  7.008120e-01
## 10256  2 3158  6.875592e-01
## 10257  2 3159  6.744660e-01
## 10258  2 3160  6.613868e-01
## 10259  2 3161  6.486742e-01
## 10260  2 3162  6.364428e-01
## 10261  2 3163  6.240269e-01
## 10262  2 3164  6.108975e-01
## 10263  2 3165  5.994886e-01
## 10264  2 3165  5.985548e-01
## 10265  2 3166  1.676134e+00
## 10266  2 3167  1.564279e+00
## 10267  2 3168  1.462874e+00
## 10268  2 3168  1.461565e+00
## 10269  2 3169  2.471664e+00
## 10270  2 3170  2.290597e+00
## 10271  2 3171  2.135197e+00
## 10272  2 3172  2.003340e+00
## 10273  2 3173  1.887699e+00
## 10274  2 3174  1.790770e+00
## 10275  2 3175  1.705445e+00
## 10276  2 3176  1.625453e+00
## 10277  2 3177  1.561170e+00
## 10278  2 3178  1.497054e+00
## 10279  2 3179  1.443778e+00
## 10280  2 3180  1.395377e+00
## 10281  2 3181  1.351752e+00
## 10282  2 3182  1.313241e+00
## 10283  2 3183  1.273796e+00
## 10284  2 3184  1.241333e+00
## 10285  2 3185  1.208798e+00
## 10286  2 3186  1.178084e+00
## 10287  2 3187  1.148491e+00
## 10288  2 3188  1.124588e+00
## 10289  2 3189  1.097260e+00
## 10290  2 3190  1.072678e+00
## 10291  2 3191  1.051761e+00
## 10292  2 3192  1.026710e+00
## 10293  2 3193  2.095646e+00
## 10294  2 3194  1.972321e+00
## 10295  2 3195  1.861592e+00
## 10296  2 3196  1.763575e+00
## 10297  2 3197  1.681092e+00
## 10298  2 3198  1.609061e+00
## 10299  2 3199  1.545394e+00
## 10300  2 3200  1.485781e+00
## 10301  2 3201  1.435168e+00
## 10302  2 3202  1.389334e+00
## 10303  2 3203  1.344813e+00
## 10304  2 3204  1.304826e+00
## 10305  2 3205  1.270370e+00
## 10306  2 3206  1.236050e+00
## 10307  2 3207  1.204814e+00
## 10308  2 3208  1.177265e+00
## 10309  2 3209  1.144767e+00
## 10310  2 3210  1.120703e+00
## 10311  2 3211  1.093421e+00
## 10312  2 3212  1.070273e+00
## 10313  2 3213  1.046649e+00
## 10314  2 3214  1.022846e+00
## 10315  2 3215  1.001811e+00
## 10316  2 3216  9.822003e-01
## 10317  2 3217  2.054350e+00
## 10318  2 3218  1.926030e+00
## 10319  2 3219  1.820783e+00
## 10320  2 3220  1.725303e+00
## 10321  2 3221  1.646726e+00
## 10322  2 3222  1.573986e+00
## 10323  2 3223  1.507909e+00
## 10324  2 3224  1.450589e+00
## 10325  2 3225  1.399556e+00
## 10326  2 3226  1.354423e+00
## 10327  2 3227  1.310403e+00
## 10328  2 3228  1.272893e+00
## 10329  2 3229  1.236830e+00
## 10330  2 3230  1.202934e+00
## 10331  2 3231  1.173757e+00
## 10332  2 3232  1.146071e+00
## 10333  2 3233  1.115781e+00
## 10334  2 3234  1.092320e+00
## 10335  2 3235  1.065604e+00
## 10336  2 3236  1.043422e+00
## 10337  2 3237  1.019406e+00
## 10338  2 3238  9.978234e-01
## 10339  2 3239  9.750888e-01
## 10340  2 3240  9.554360e-01
## 10341  2 3241  2.030956e+00
## 10342  2 3242  1.903004e+00
## 10343  2 3243  1.792215e+00
## 10344  2 3244  1.701865e+00
## 10345  2 3245  1.621156e+00
## 10346  2 3246  1.547464e+00
## 10347  2 3247  1.484939e+00
## 10348  2 3248  1.428583e+00
## 10349  2 3249  1.378506e+00
## 10350  2 3250  1.332596e+00
## 10351  2 3251  1.290308e+00
## 10352  2 3252  1.251107e+00
## 10353  2 3253  1.217087e+00
## 10354  2 3254  1.183073e+00
## 10355  2 3255  1.153048e+00
## 10356  2 3256  1.124720e+00
## 10357  2 3257  1.099357e+00
## 10358  2 3258  1.071986e+00
## 10359  2 3259  1.048994e+00
## 10360  2 3260  1.024414e+00
## 10361  2 3261  1.001251e+00
## 10362  2 3262  9.828441e-01
## 10363  2 3263  9.604784e-01
## 10364  2 3264  9.401361e-01
## 10365  2 3265  2.008683e+00
## 10366  2 3266  1.887475e+00
## 10367  2 3267  1.782077e+00
## 10368  2 3268  1.688034e+00
## 10369  2 3269  1.605074e+00
## 10370  2 3270  1.534781e+00
## 10371  2 3271  1.470411e+00
## 10372  2 3272  1.416705e+00
## 10373  2 3273  1.365707e+00
## 10374  2 3274  1.317891e+00
## 10375  2 3275  1.275835e+00
## 10376  2 3276  1.239735e+00
## 10377  2 3277  1.206032e+00
## 10378  2 3278  1.173457e+00
## 10379  2 3279  1.143530e+00
## 10380  2 3280  1.115781e+00
## 10381  2 3281  1.087555e+00
## 10382  2 3282  1.061511e+00
## 10383  2 3283  1.037671e+00
## 10384  2 3284  1.015694e+00
## 10385  2 3285  9.909678e-01
## 10386  2 3286  9.731065e-01
## 10387  2 3287  9.500889e-01
## 10388  2 3288  9.304755e-01
## 10389  2 3289  2.000048e+00
## 10390  2 3290  1.878087e+00
## 10391  2 3291  1.773645e+00
## 10392  2 3292  1.679878e+00
## 10393  2 3293  1.597716e+00
## 10394  2 3294  1.523844e+00
## 10395  2 3295  1.460728e+00
## 10396  2 3296  1.406790e+00
## 10397  2 3297  1.356363e+00
## 10398  2 3298  1.310441e+00
## 10399  2 3299  1.269972e+00
## 10400  2 3300  1.229333e+00
## 10401  2 3301  1.197486e+00
## 10402  2 3302  1.165799e+00
## 10403  2 3303  1.135679e+00
## 10404  2 3304  1.107116e+00
## 10405  2 3305  1.079297e+00
## 10406  2 3306  1.054675e+00
## 10407  2 3307  1.030351e+00
## 10408  2 3308  1.009121e+00
## 10409  2 3309  9.851131e-01
## 10410  2 3310  9.632011e-01
## 10411  2 3311  9.431774e-01
## 10412  2 3312  9.252217e-01
## 10413  2 3313  1.995450e+00
## 10414  2 3314  1.872143e+00
## 10415  2 3315  1.766105e+00
## 10416  2 3316  1.670251e+00
## 10417  2 3317  1.593297e+00
## 10418  2 3318  1.519955e+00
## 10419  2 3319  1.456900e+00
## 10420  2 3320  1.403453e+00
## 10421  2 3321  1.350593e+00
## 10422  2 3322  1.306963e+00
## 10423  2 3323  1.263936e+00
## 10424  2 3324  1.224630e+00
## 10425  2 3325  1.191705e+00
## 10426  2 3326  1.162970e+00
## 10427  2 3327  1.129243e+00
## 10428  2 3328  1.101962e+00
## 10429  2 3329  1.074100e+00
## 10430  2 3330  1.050742e+00
## 10431  2 3331  1.026571e+00
## 10432  2 3332  1.003362e+00
## 10433  2 3333  9.803677e-01
## 10434  2 3334  9.604434e-01
## 10435  2 3335  9.403013e-01
```

```
## 10436  2 3336  9.196762e-01
## 10437  2 3337  1.994737e+00
## 10438  2 3338  1.865829e+00
## 10439  2 3339  1.766239e+00
## 10440  2 3340  1.667875e+00
## 10441  2 3341  1.589337e+00
## 10442  2 3342  1.515820e+00
## 10443  2 3343  1.453696e+00
## 10444  2 3344  1.398917e+00
## 10445  2 3345  1.349860e+00
## 10446  2 3346  1.303135e+00
## 10447  2 3347  1.261255e+00
## 10448  2 3348  1.222992e+00
## 10449  2 3349  1.188756e+00
## 10450  2 3350  1.157911e+00
## 10451  2 3351  1.127843e+00
## 10452  2 3352  1.099232e+00
## 10453  2 3353  1.073671e+00
## 10454  2 3354  1.046646e+00
## 10455  2 3355  1.023443e+00
## 10456  2 3356  1.001113e+00
## 10457  2 3357  9.796280e-01
## 10458  2 3358  9.588693e-01
## 10459  2 3359  9.369036e-01
## 10460  2 3360  9.179069e-01
## 10461  2 3361  1.992382e+00
## 10462  2 3362  1.866318e+00
## 10463  2 3363  1.760908e+00
## 10464  2 3364  1.668253e+00
## 10465  2 3365  1.589050e+00
## 10466  2 3366  1.515890e+00
## 10467  2 3367  1.450992e+00
## 10468  2 3368  1.395923e+00
## 10469  2 3369  1.346668e+00
## 10470  2 3370  1.303687e+00
## 10471  2 3371  1.258178e+00
## 10472  2 3372  1.221265e+00
## 10473  2 3373  1.185059e+00
## 10474  2 3374  1.155917e+00
## 10475  2 3375  1.124919e+00
## 10476  2 3376  1.097372e+00
## 10477  2 3377  1.071383e+00
## 10478  2 3378  1.045752e+00
## 10479  2 3379  1.020267e+00
## 10480  2 3380  9.993061e-01
## 10481  2 3381  9.771652e-01
## 10482  2 3382  9.562444e-01
## 10483  2 3383  9.353373e-01
## 10484  2 3384  9.156551e-01
## 10485  2 3385  1.988762e+00
## 10486  2 3386  1.865526e+00
## 10487  2 3387  1.757598e+00
## 10488  2 3388  1.664444e+00
## 10489  2 3389  1.583001e+00
## 10490  2 3390  1.513977e+00
## 10491  2 3391  1.451324e+00
## 10492  2 3392  1.396294e+00
## 10493  2 3393  1.344154e+00
## 10494  2 3394  1.297750e+00
## 10495  2 3395  1.259087e+00
## 10496  2 3396  1.221788e+00
## 10497  2 3397  1.186017e+00
## 10498  2 3398  1.156934e+00
## 10499  2 3399  1.123711e+00
## 10500  2 3400  1.097536e+00
## 10501  2 3401  1.070540e+00
## 10502  2 3402  1.045327e+00
## 10503  2 3403  1.020789e+00
## 10504  2 3404  9.989510e-01
## 10505  2 3405  9.760761e-01
## 10506  2 3406  9.528467e-01
## 10507  2 3407  9.363380e-01
## 10508  2 3408  9.158991e-01
## 10509  2 3409  1.987697e+00
## 10510  2 3410  1.861122e+00
## 10511  2 3411  1.760317e+00
## 10512  2 3412  1.666118e+00
## 10513  2 3413  1.584341e+00
## 10514  2 3414  1.511751e+00
## 10515  2 3415  1.449375e+00
## 10516  2 3416  1.392934e+00
## 10517  2 3417  1.343664e+00
## 10518  2 3418  1.299913e+00
## 10519  2 3419  1.258520e+00
## 10520  2 3420  1.220660e+00
## 10521  2 3421  1.185384e+00
## 10522  2 3422  1.153051e+00
## 10523  2 3423  1.124100e+00
## 10524  2 3424  1.096899e+00
## 10525  2 3425  1.071816e+00
## 10526  2 3426  1.045227e+00
## 10527  2 3427  1.020033e+00
## 10528  2 3428  9.969777e-01
## 10529  2 3429  9.766410e-01
## 10530  2 3430  9.564797e-01
## 10531  2 3431  9.352334e-01
## 10532  2 3432  9.150986e-01
## 10533  2 3432  9.146444e-01
## 10534  2 3433  1.985828e+00
## 10535  2 3434  1.861653e+00
## 10536  2 3435  1.757445e+00
## 10537  2 3436  1.662778e+00
## 10538  2 3437  1.581956e+00
## 10539  2 3438  1.509335e+00
## 10540  2 3439  1.450180e+00
## 10541  2 3440  1.392496e+00
## 10542  2 3441  1.342271e+00
## 10543  2 3442  1.296249e+00
## 10544  2 3443  1.256237e+00
## 10545  2 3444  1.219078e+00
## 10546  2 3445  1.186448e+00
## 10547  2 3446  1.153824e+00
## 10548  2 3447  1.121161e+00
## 10549  2 3448  1.095251e+00
## 10550  2 3449  1.069344e+00
## 10551  2 3450  1.041463e+00
## 10552  2 3451  1.019968e+00
## 10553  2 3452  9.967657e-01
## 10554  2 3453  9.765313e-01
## 10555  2 3454  9.554212e-01
## 10556  2 3455  9.341913e-01
## 10557  2 3456  9.147179e-01
## 10558  2 3456  9.137332e-01
## 10559  2 3457  8.959225e-01
## 10560  2 3458  8.771598e-01
## 10561  2 3459  8.583082e-01
## 10562  2 3460  8.409334e-01
## 10563  2 3461  8.259065e-01
## 10564  2 3462  8.079763e-01
## 10565  2 3463  7.920494e-01
## 10566  2 3464  7.771583e-01
## 10567  2 3465  7.607330e-01
## 10568  2 3466  7.452293e-01
## 10569  2 3467  7.311203e-01
## 10570  2 3468  7.167255e-01
## 10571  2 3469  7.023594e-01
## 10572  2 3470  6.896851e-01
## 10573  2 3471  6.745057e-01
## 10574  2 3472  6.617614e-01
## 10575  2 3473  6.493260e-01
## 10576  2 3474  6.362884e-01
## 10577  2 3475  6.236476e-01
## 10578  2 3476  6.100512e-01
## 10579  2 3477  5.989088e-01
## 10580  2 3478  5.878317e-01
## 10581  2 3479  5.763063e-01
## 10582  2 3480  5.644138e-01
## 10583  2 3480  5.648964e-01
## 10584  2 3481  1.644747e+00
## 10585  2 3482  1.530357e+00
## 10586  2 3483  1.429936e+00
## 10587  2 3484  1.343022e+00
## 10588  2 3485  1.269101e+00
## 10589  2 3486  1.205993e+00
## 10590  2 3487  1.149701e+00
## 10591  2 3488  1.098581e+00
## 10592  2 3489  1.054232e+00
## 10593  2 3490  1.014728e+00
## 10594  2 3491  9.799601e-01
## 10595  2 3492  9.493377e-01
## 10596  2 3493  9.193852e-01
## 10597  2 3494  8.930432e-01
## 10598  2 3495  8.675984e-01
## 10599  2 3496  8.444038e-01
## 10600  2 3497  8.238005e-01
## 10601  2 3498  8.021759e-01
## 10602  2 3499  7.842345e-01
## 10603  2 3500  7.660892e-01
## 10604  2 3501  7.470189e-01
## 10605  2 3502  7.324936e-01
## 10606  2 3503  7.164389e-01
## 10607  2 3504  7.014558e-01
## 10608  2 3504  7.004298e-01
## 10609  2 3505  1.777150e+00
## 10610  2 3506  1.659599e+00
## 10611  2 3507  1.553723e+00
## 10612  2 3508  1.465120e+00
## 10613  2 3509  1.388204e+00
## 10614  2 3510  1.322875e+00
## 10615  2 3511  1.260820e+00
## 10616  2 3512  1.208829e+00
## 10617  2 3513  1.164399e+00
## 10618  2 3514  1.122628e+00
## 10619  2 3515  1.084511e+00
## 10620  2 3516  1.049887e+00
## 10621  2 3517  1.018589e+00
## 10622  2 3518  9.913016e-01
## 10623  2 3519  9.659622e-01
## 10624  2 3520  9.393965e-01
## 10625  2 3521  9.160617e-01
## 10626  2 3522  8.940991e-01
## 10627  2 3523  8.728544e-01
## 10628  2 3524  8.537600e-01
## 10629  2 3525  8.345585e-01
## 10630  2 3526  8.154945e-01
## 10631  2 3527  7.998575e-01
## 10632  2 3528  7.808433e-01
## 10633  2 3529  1.857826e+00
## 10634  2 3530  1.733212e+00
## 10635  2 3531  1.633128e+00
## 10636  2 3532  1.537659e+00
## 10637  2 3533  1.463142e+00
## 10638  2 3534  1.394439e+00
## 10639  2 3535  1.332479e+00
## 10640  2 3536  1.279341e+00
## 10641  2 3537  1.229520e+00
## 10642  2 3538  1.191097e+00
## 10643  2 3539  1.147934e+00
## 10644  2 3540  1.113387e+00
## 10645  2 3541  1.080343e+00
## 10646  2 3542  1.051350e+00
## 10647  2 3543  1.024825e+00
## 10648  2 3544  9.978468e-01
## 10649  2 3545  9.727655e-01
## 10650  2 3546  9.495135e-01
## 10651  2 3547  9.288924e-01
## 10652  2 3548  9.070731e-01
## 10653  2 3549  8.847089e-01
## 10654  2 3550  8.673691e-01
## 10655  2 3551  8.485726e-01
## 10656  2 3552  8.303361e-01
## 10657  2 3553  1.905976e+00
## 10658  2 3554  1.786353e+00
## 10659  2 3555  1.677641e+00
## 10660  2 3556  1.585638e+00
## 10661  2 3557  1.510046e+00
## 10662  2 3558  1.439257e+00
## 10663  2 3559  1.379424e+00
## 10664  2 3560  1.325552e+00
## 10665  2 3561  1.274052e+00
## 10666  2 3562  1.229413e+00
## 10667  2 3563  1.190305e+00
## 10668  2 3564  1.152988e+00
## 10669  2 3565  1.121417e+00
## 10670  2 3566  1.090519e+00
## 10671  2 3567  1.061823e+00
## 10672  2 3568  1.033670e+00
## 10673  2 3569  1.009333e+00
## 10674  2 3570  9.858458e-01
## 10675  2 3571  9.629887e-01
## 10676  2 3572  9.409442e-01
## 10677  2 3573  9.199193e-01
## 10678  2 3574  8.989953e-01
## 10679  2 3575  8.822805e-01
## 10680  2 3576  8.628575e-01
## 10681  2 3577  1.935962e+00
## 10682  2 3578  1.813175e+00
## 10683  2 3579  1.708118e+00
## 10684  2 3580  1.615162e+00
## 10685  2 3581  1.536261e+00
## 10686  2 3582  1.466100e+00
## 10687  2 3583  1.403214e+00
## 10688  2 3584  1.349363e+00
## 10689  2 3585  1.301155e+00
## 10690  2 3586  1.256197e+00
## 10691  2 3587  1.214045e+00
## 10692  2 3588  1.178703e+00
## 10693  2 3589  1.145903e+00
## 10694  2 3590  1.114411e+00
## 10695  2 3591  1.083423e+00
## 10696  2 3592  1.057764e+00
## 10697  2 3593  1.031472e+00
## 10698  2 3594  1.007636e+00
## 10699  2 3595  9.835419e-01
## 10700  2 3596  9.611451e-01
## 10701  2 3597  9.401948e-01
## 10702  2 3598  9.216153e-01
## 10703  2 3599  8.985367e-01
## 10704  2 3600  8.832143e-01
## 10705  2 3601  1.955294e+00
## 10706  2 3602  1.829029e+00
## 10707  2 3603  1.726997e+00
## 10708  2 3604  1.633100e+00
## 10709  2 3605  1.554169e+00
## 10710  2 3606  1.483513e+00
## 10711  2 3607  1.421072e+00
## 10712  2 3608  1.364568e+00
## 10713  2 3609  1.313704e+00
## 10714  2 3610  1.272434e+00
## 10715  2 3611  1.229109e+00
## 10716  2 3612  1.194886e+00
## 10717  2 3613  1.159182e+00
## 10718  2 3614  1.128073e+00
## 10719  2 3615  1.098315e+00
## 10720  2 3616  1.070953e+00
## 10721  2 3617  1.046395e+00
## 10722  2 3618  1.022728e+00
## 10723  2 3619  9.980555e-01
## 10724  2 3620  9.773778e-01
## 10725  2 3621  9.530227e-01
## 10726  2 3622  9.328442e-01
## 10727  2 3623  9.131014e-01
## 10728  2 3624  8.920990e-01
## 10729  2 3625  1.967464e+00
## 10730  2 3626  1.845500e+00
## 10731  2 3627  1.736107e+00
## 10732  2 3628  1.645498e+00
## 10733  2 3629  1.561858e+00
## 10734  2 3630  1.494506e+00
## 10735  2 3631  1.431790e+00
## 10736  2 3632  1.376270e+00
## 10737  2 3633  1.326386e+00
## 10738  2 3634  1.279603e+00
## 10739  2 3635  1.239261e+00
## 10740  2 3636  1.201724e+00
## 10741  2 3637  1.167602e+00
## 10742  2 3638  1.135733e+00
## 10743  2 3639  1.108623e+00
## 10744  2 3640  1.079939e+00
## 10745  2 3641  1.054568e+00
## 10746  2 3642  1.027984e+00
## 10747  2 3643  1.006448e+00
## 10748  2 3644  9.833470e-01
## 10749  2 3645  9.640838e-01
## 10750  2 3646  9.405410e-01
## 10751  2 3647  9.206930e-01
## 10752  2 3648  9.013775e-01
## 10753  2 3649  1.971759e+00
## 10754  2 3650  1.846821e+00
## 10755  2 3651  1.742711e+00
## 10756  2 3652  1.649407e+00
## 10757  2 3653  1.571076e+00
## 10758  2 3654  1.497550e+00
## 10759  2 3655  1.438846e+00
## 10760  2 3656  1.379390e+00
## 10761  2 3657  1.334270e+00
## 10762  2 3658  1.289359e+00
## 10763  2 3659  1.247415e+00
## 10764  2 3660  1.210683e+00
## 10765  2 3661  1.175859e+00
## 10766  2 3662  1.143379e+00
## 10767  2 3663  1.113044e+00
## 10768  2 3664  1.083343e+00
## 10769  2 3665  1.059376e+00
## 10770  2 3666  1.035371e+00
## 10771  2 3667  1.009715e+00
## 10772  2 3668  9.901961e-01
## 10773  2 3669  9.678906e-01
## 10774  2 3670  9.451822e-01
## 10775  2 3671  9.255428e-01
## 10776  2 3672  9.055417e-01
## 10777  2 3673  1.976947e+00
## 10778  2 3674  1.855483e+00
## 10779  2 3675  1.749809e+00
## 10780  2 3676  1.656355e+00
## 10781  2 3677  1.573085e+00
## 10782  2 3678  1.504358e+00
## 10783  2 3679  1.440490e+00
## 10784  2 3680  1.384979e+00
## 10785  2 3681  1.335196e+00
## 10786  2 3682  1.291689e+00
## 10787  2 3683  1.251612e+00
## 10788  2 3684  1.213358e+00
## 10789  2 3685  1.177566e+00
## 10790  2 3686  1.145084e+00
## 10791  2 3687  1.116938e+00
## 10792  2 3688  1.088054e+00
## 10793  2 3689  1.065224e+00
## 10794  2 3690  1.037531e+00
## 10795  2 3691  1.014730e+00
## 10796  2 3692  9.911672e-01
## 10797  2 3693  9.694641e-01
## 10798  2 3694  9.486478e-01
## 10799  2 3695  9.288063e-01
## 10800  2 3696  9.094728e-01
## 10801  2 3697  1.981807e+00
## 10802  2 3698  1.859322e+00
## 10803  2 3699  1.748696e+00
## 10804  2 3700  1.655288e+00
## 10805  2 3701  1.578048e+00
## 10806  2 3702  1.506080e+00
## 10807  2 3703  1.444480e+00
## 10808  2 3704  1.388534e+00
## 10809  2 3705  1.338752e+00
## 10810  2 3706  1.292145e+00
## 10811  2 3707  1.252781e+00
## 10812  2 3708  1.215709e+00
## 10813  2 3709  1.181092e+00
## 10814  2 3710  1.148862e+00
## 10815  2 3711  1.117875e+00
## 10816  2 3712  1.090299e+00
## 10817  2 3713  1.063402e+00
## 10818  2 3714  1.039486e+00
## 10819  2 3715  1.015224e+00
## 10820  2 3716  9.945884e-01
## 10821  2 3717  9.713139e-01
## 10822  2 3718  9.498560e-01
## 10823  2 3719  9.293434e-01
## 10824  2 3720  9.119188e-01
## 10825  2 3721  1.983404e+00
## 10826  2 3722  1.863487e+00
## 10827  2 3723  1.753946e+00
## 10828  2 3724  1.659083e+00
## 10829  2 3725  1.577208e+00
## 10830  2 3726  1.508178e+00
## 10831  2 3727  1.446591e+00
## 10832  2 3728  1.389051e+00
## 10833  2 3729  1.338481e+00
## 10834  2 3730  1.294961e+00
## 10835  2 3731  1.253860e+00
## 10836  2 3732  1.215867e+00
## 10837  2 3733  1.181886e+00
## 10838  2 3734  1.150824e+00
## 10839  2 3735  1.119605e+00
## 10840  2 3736  1.093898e+00
## 10841  2 3737  1.066532e+00
## 10842  2 3738  1.040849e+00
## 10843  2 3739  1.017415e+00
## 10844  2 3740  9.937228e-01
## 10845  2 3741  9.723087e-01
## 10846  2 3742  9.507106e-01
## 10847  2 3743  9.314734e-01
## 10848  2 3744  9.117569e-01
## 10849  2 3745  1.984882e+00
## 10850  2 3746  1.861628e+00
## 10851  2 3747  1.753832e+00
## 10852  2 3748  1.661837e+00
## 10853  2 3749  1.579897e+00
## 10854  2 3750  1.510705e+00
## 10855  2 3751  1.446916e+00
## 10856  2 3752  1.391739e+00
## 10857  2 3753  1.341722e+00
## 10858  2 3754  1.296344e+00
## 10859  2 3755  1.255912e+00
## 10860  2 3756  1.217389e+00
## 10861  2 3757  1.183133e+00
## 10862  2 3758  1.151217e+00
## 10863  2 3759  1.118761e+00
## 10864  2 3760  1.091985e+00
## 10865  2 3761  1.065700e+00
## 10866  2 3762  1.040624e+00
## 10867  2 3763  1.016355e+00
## 10868  2 3764  9.952483e-01
## 10869  2 3765  9.737365e-01
## 10870  2 3766  9.530294e-01
## 10871  2 3767  9.321399e-01
## 10872  2 3768  9.129864e-01
## 10873  2 3768  9.140106e-01
## 10874  2 3769  1.986930e+00
## 10875  2 3770  1.863100e+00
## 10876  2 3771  1.754036e+00
## 10877  2 3772  1.661960e+00
## 10878  2 3773  1.582333e+00
## 10879  2 3774  1.511646e+00
## 10880  2 3775  1.447324e+00
## 10881  2 3776  1.392580e+00
## 10882  2 3777  1.341169e+00
## 10883  2 3778  1.297342e+00
## 10884  2 3779  1.255455e+00
## 10885  2 3780  1.217997e+00
## 10886  2 3781  1.182758e+00
## 10887  2 3782  1.153903e+00
## 10888  2 3783  1.125078e+00
## 10889  2 3784  1.094851e+00
## 10890  2 3785  1.067424e+00
## 10891  2 3786  1.042778e+00
## 10892  2 3787  1.018532e+00
## 10893  2 3788  9.953337e-01
## 10894  2 3789  9.744453e-01
## 10895  2 3790  9.525380e-01
## 10896  2 3791  9.340371e-01
## 10897  2 3792  9.139459e-01
## 10898  2 3792  9.139286e-01
## 10899  2 3793  8.960096e-01
## 10900  2 3794  8.776168e-01
## 10901  2 3795  8.578029e-01
## 10902  2 3796  8.425369e-01
## 10903  2 3797  8.236650e-01
## 10904  2 3798  8.077447e-01
## 10905  2 3799  7.920867e-01
## 10906  2 3800  7.761466e-01
## 10907  2 3801  7.616641e-01
## 10908  2 3802  7.455914e-01
## 10909  2 3803  7.303009e-01
## 10910  2 3804  7.172009e-01
## 10911  2 3805  7.018058e-01
## 10912  2 3806  6.888401e-01
## 10913  2 3807  6.750268e-01
## 10914  2 3808  6.608800e-01
## 10915  2 3809  6.488852e-01
## 10916  2 3810  6.359076e-01
## 10917  2 3811  6.233987e-01
## 10918  2 3812  6.103982e-01
## 10919  2 3813  5.992706e-01
## 10920  2 3814  5.873806e-01
## 10921  2 3815  5.759597e-01
## 10922  2 3816  5.656044e-01
## 10923  2 3816  5.644579e-01
## 10924  2 3817  2.187155e+00
## 10925  2 3818  2.026067e+00
## 10926  2 3819  1.880788e+00
## 10927  2 3820  1.754352e+00
## 10928  2 3821  1.647694e+00
## 10929  2 3822  1.558380e+00
## 10930  2 3823  1.476475e+00
## 10931  2 3824  1.406894e+00
## 10932  2 3825  1.347431e+00
## 10933  2 3826  1.290798e+00
## 10934  2 3827  1.243131e+00
## 10935  2 3828  1.199736e+00
## 10936  2 3829  1.158958e+00
## 10937  2 3830  1.127300e+00
## 10938  2 3831  1.091449e+00
## 10939  2 3832  1.061768e+00
## 10940  2 3833  1.032194e+00
## 10941  2 3834  1.005335e+00
## 10942  2 3835  9.805928e-01
## 10943  2 3836  9.607712e-01
## 10944  2 3837  9.371980e-01
## 10945  2 3838  9.154350e-01
## 10946  2 3839  8.935423e-01
## 10947  2 3840  8.762783e-01
## 10948  2 3840  8.755010e-01
## 10949  2 3841  1.947476e+00
## 10950  2 3842  1.827580e+00
## 10951  2 3843  1.718532e+00
## 10952  2 3844  1.629344e+00
## 10953  2 3845  1.545508e+00
## 10954  2 3846  1.476097e+00
## 10955  2 3847  1.413657e+00
## 10956  2 3848  1.356904e+00
## 10957  2 3849  1.307586e+00
## 10958  2 3850  1.261934e+00
## 10959  2 3851  1.224604e+00
## 10960  2 3852  1.183545e+00
## 10961  2 3853  1.153962e+00
## 10962  2 3854  1.121014e+00
## 10963  2 3855  1.090821e+00
## 10964  2 3856  1.063227e+00
## 10965  2 3857  1.037988e+00
## 10966  2 3858  1.014414e+00
## 10967  2 3859  9.903118e-01
## 10968  2 3860  9.668495e-01
## 10969  2 3861  9.453941e-01
## 10970  2 3862  9.265776e-01
## 10971  2 3863  9.058271e-01
## 10972  2 3864  8.873168e-01
## 10973  2 3865  1.957901e+00
## 10974  2 3866  1.838808e+00
## 10975  2 3867  1.729223e+00
## 10976  2 3868  1.638813e+00
## 10977  2 3869  1.559376e+00
## 10978  2 3870  1.488000e+00
## 10979  2 3871  1.425134e+00
## 10980  2 3872  1.372080e+00
## 10981  2 3873  1.318707e+00
## 10982  2 3874  1.277464e+00
## 10983  2 3875  1.234520e+00
## 10984  2 3876  1.197832e+00
## 10985  2 3877  1.164390e+00
## 10986  2 3878  1.130716e+00
## 10987  2 3879  1.102321e+00
## 10988  2 3880  1.075318e+00
## 10989  2 3881  1.050541e+00
## 10990  2 3882  1.023663e+00
## 10991  2 3883  1.001129e+00
## 10992  2 3884  9.797534e-01
## 10993  2 3885  9.580494e-01
## 10994  2 3886  9.348009e-01
## 10995  2 3887  9.175751e-01
## 10996  2 3888  8.969022e-01
## 10997  2 3889  1.973992e+00
## 10998  2 3890  1.847589e+00
## 10999  2 3891  1.736334e+00
## 11000  2 3892  1.646866e+00
## 11001  2 3893  1.568854e+00
## 11002  2 3894  1.497408e+00
## 11003  2 3895  1.430481e+00
## 11004  2 3896  1.378951e+00
## 11005  2 3897  1.326711e+00
## 11006  2 3898  1.282356e+00
## 11007  2 3899  1.243773e+00
## 11008  2 3900  1.207195e+00
## 11009  2 3901  1.173856e+00
## 11010  2 3902  1.140991e+00
## 11011  2 3903  1.106781e+00
## 11012  2 3904  1.083652e+00
## 11013  2 3905  1.055840e+00
## 11014  2 3906  1.031050e+00
## 11015  2 3907  1.009435e+00
## 11016  2 3908  9.849794e-01
## 11017  2 3909  9.644433e-01
## 11018  2 3910  9.436652e-01
## 11019  2 3911  9.227755e-01
## 11020  2 3912  9.035940e-01
## 11021  2 3913  1.975759e+00
## 11022  2 3914  1.857826e+00
## 11023  2 3915  1.745922e+00
## 11024  2 3916  1.651589e+00
## 11025  2 3917  1.574135e+00
## 11026  2 3918  1.501646e+00
## 11027  2 3919  1.438970e+00
## 11028  2 3920  1.382088e+00
## 11029  2 3921  1.336357e+00
## 11030  2 3922  1.289972e+00
## 11031  2 3923  1.247174e+00
## 11032  2 3924  1.210641e+00
## 11033  2 3925  1.174799e+00
## 11034  2 3926  1.142411e+00
## 11035  2 3927  1.115294e+00
## 11036  2 3928  1.088851e+00
## 11037  2 3929  1.060418e+00
## 11038  2 3930  1.036767e+00
## 11039  2 3931  1.012013e+00
## 11040  2 3932  9.889076e-01
## 11041  2 3933  9.678675e-01
## 11042  2 3934  9.469587e-01
## 11043  2 3935  9.262239e-01
## 11044  2 3936  9.077456e-01
## 11045  2 3937  1.982259e+00
## 11046  2 3938  1.856897e+00
## 11047  2 3939  1.748763e+00
## 11048  2 3940  1.659153e+00
## 11049  2 3941  1.578239e+00
## 11050  2 3942  1.506534e+00
## 11051  2 3943  1.443891e+00
## 11052  2 3944  1.387650e+00
## 11053  2 3945  1.338006e+00
## 11054  2 3946  1.293598e+00
## 11055  2 3947  1.250952e+00
## 11056  2 3948  1.214483e+00
## 11057  2 3949  1.179874e+00
## 11058  2 3950  1.146619e+00
## 11059  2 3951  1.117379e+00
## 11060  2 3952  1.089918e+00
## 11061  2 3953  1.063567e+00
## 11062  2 3954  1.041884e+00
## 11063  2 3955  1.016066e+00
## 11064  2 3956  9.905080e-01
## 11065  2 3957  9.724224e-01
## 11066  2 3958  9.501462e-01
## 11067  2 3959  9.295145e-01
## 11068  2 3960  9.111781e-01
## 11069  2 3961  1.986650e+00
## 11070  2 3962  1.855388e+00
## 11071  2 3963  1.751052e+00
## 11072  2 3964  1.659939e+00
## 11073  2 3965  1.579543e+00
## 11074  2 3966  1.505996e+00
## 11075  2 3967  1.445861e+00
## 11076  2 3968  1.390460e+00
## 11077  2 3969  1.338526e+00
## 11078  2 3970  1.294995e+00
## 11079  2 3971  1.254075e+00
## 11080  2 3972  1.214746e+00
## 11081  2 3973  1.179651e+00
## 11082  2 3974  1.152523e+00
## 11083  2 3975  1.120596e+00
## 11084  2 3976  1.092775e+00
## 11085  2 3977  1.066409e+00
## 11086  2 3978  1.039827e+00
## 11087  2 3979  1.016992e+00
## 11088  2 3980  9.912749e-01
## 11089  2 3981  9.723315e-01
## 11090  2 3982  9.513620e-01
## 11091  2 3983  9.320045e-01
## 11092  2 3984  9.106173e-01
## 11093  2 3985  1.983759e+00
## 11094  2 3986  1.862975e+00
## 11095  2 3987  1.751875e+00
## 11096  2 3988  1.658163e+00
## 11097  2 3989  1.579700e+00
## 11098  2 3990  1.510988e+00
## 11099  2 3991  1.446226e+00
## 11100  2 3992  1.391886e+00
## 11101  2 3993  1.339825e+00
## 11102  2 3994  1.294871e+00
## 11103  2 3995  1.255124e+00
## 11104  2 3996  1.216189e+00
## 11105  2 3997  1.182487e+00
## 11106  2 3998  1.150686e+00
## 11107  2 3999  1.121303e+00
## 11108  2 4000  1.090939e+00
## 11109  2 4001  1.065336e+00
## 11110  2 4002  1.041259e+00
## 11111  2 4003  1.016373e+00
## 11112  2 4004  9.947887e-01
## 11113  2 4005  9.737083e-01
## 11114  2 4006  9.520976e-01
## 11115  2 4007  9.324575e-01
## 11116  2 4008  9.141053e-01
## 11117  2 4009  1.984540e+00
## 11118  2 4010  1.859785e+00
## 11119  2 4011  1.756965e+00
## 11120  2 4012  1.661629e+00
## 11121  2 4013  1.579883e+00
## 11122  2 4014  1.508761e+00
## 11123  2 4015  1.445786e+00
## 11124  2 4016  1.392587e+00
## 11125  2 4017  1.339762e+00
## 11126  2 4018  1.294871e+00
## 11127  2 4019  1.252726e+00
## 11128  2 4020  1.217396e+00
## 11129  2 4021  1.183460e+00
## 11130  2 4022  1.152167e+00
## 11131  2 4023  1.120416e+00
## 11132  2 4024  1.093055e+00
## 11133  2 4025  1.065992e+00
## 11134  2 4026  1.040928e+00
## 11135  2 4027  1.016568e+00
## 11136  2 4028  9.961008e-01
## 11137  2 4029  9.729051e-01
## 11138  2 4030  9.519885e-01
## 11139  2 4031  9.338048e-01
## 11140  2 4032  9.119070e-01
## 11141  2 4033  1.986099e+00
## 11142  2 4034  1.861791e+00
## 11143  2 4035  1.753971e+00
## 11144  2 4036  1.660205e+00
## 11145  2 4037  1.582895e+00
## 11146  2 4038  1.509871e+00
## 11147  2 4039  1.449299e+00
## 11148  2 4040  1.395948e+00
## 11149  2 4041  1.339610e+00
## 11150  2 4042  1.297966e+00
## 11151  2 4043  1.254086e+00
## 11152  2 4044  1.220811e+00
## 11153  2 4045  1.183677e+00
## 11154  2 4046  1.151874e+00
## 11155  2 4047  1.119813e+00
## 11156  2 4048  1.094519e+00
## 11157  2 4049  1.067495e+00
## 11158  2 4050  1.042148e+00
## 11159  2 4051  1.019000e+00
## 11160  2 4052  9.952928e-01
## 11161  2 4053  9.754141e-01
## 11162  2 4054  9.527433e-01
## 11163  2 4055  9.336298e-01
## 11164  2 4056  9.136302e-01
## 11165  2 4057  1.987114e+00
## 11166  2 4058  1.863144e+00
## 11167  2 4059  1.756171e+00
## 11168  2 4060  1.664132e+00
## 11169  2 4061  1.584624e+00
## 11170  2 4062  1.511992e+00
## 11171  2 4063  1.447665e+00
## 11172  2 4064  1.393609e+00
## 11173  2 4065  1.341005e+00
## 11174  2 4066  1.295021e+00
## 11175  2 4067  1.257431e+00
## 11176  2 4068  1.220666e+00
## 11177  2 4069  1.182331e+00
## 11178  2 4070  1.151116e+00
## 11179  2 4071  1.120384e+00
## 11180  2 4072  1.094299e+00
## 11181  2 4073  1.067894e+00
## 11182  2 4074  1.042875e+00
## 11183  2 4075  1.019548e+00
## 11184  2 4076  9.953676e-01
## 11185  2 4077  9.747196e-01
## 11186  2 4078  9.525671e-01
## 11187  2 4079  9.319466e-01
## 11188  2 4080  9.139729e-01
## 11189  2 4081  1.986067e+00
## 11190  2 4082  1.864446e+00
## 11191  2 4083  1.756635e+00
## 11192  2 4084  1.662910e+00
## 11193  2 4085  1.583747e+00
## 11194  2 4086  1.510609e+00
## 11195  2 4087  1.449032e+00
## 11196  2 4088  1.391917e+00
## 11197  2 4089  1.342737e+00
## 11198  2 4090  1.295296e+00
## 11199  2 4091  1.256203e+00
## 11200  2 4092  1.220283e+00
## 11201  2 4093  1.182989e+00
## 11202  2 4094  1.151274e+00
## 11203  2 4095  1.123515e+00
## 11204  2 4096  1.095653e+00
## 11205  2 4097  1.066327e+00
## 11206  2 4098  1.042201e+00
## 11207  2 4099  1.017890e+00
## 11208  2 4100  9.965938e-01
## 11209  2 4101  9.748045e-01
## 11210  2 4102  9.531468e-01
## 11211  2 4103  9.341360e-01
## 11212  2 4104  9.135715e-01
## 11213  2 4104  9.145668e-01
## 11214  2 4105  1.984501e+00
## 11215  2 4106  1.862649e+00
## 11216  2 4107  1.753835e+00
## 11217  2 4108  1.663351e+00
## 11218  2 4109  1.582213e+00
## 11219  2 4110  1.510161e+00
## 11220  2 4111  1.447409e+00
## 11221  2 4112  1.390802e+00
## 11222  2 4113  1.341666e+00
## 11223  2 4114  1.297274e+00
## 11224  2 4115  1.255926e+00
## 11225  2 4116  1.218086e+00
## 11226  2 4117  1.183842e+00
## 11227  2 4118  1.153277e+00
## 11228  2 4119  1.121657e+00
## 11229  2 4120  1.093807e+00
## 11230  2 4121  1.068434e+00
## 11231  2 4122  1.042379e+00
## 11232  2 4123  1.018836e+00
## 11233  2 4124  9.966746e-01
## 11234  2 4125  9.758276e-01
## 11235  2 4126  9.522003e-01
```

```
## 11236  2 4127  9.342695e-01
## 11237  2 4128  9.148095e-01
## 11238  2 4128  9.133045e-01
## 11239  2 4129  8.944785e-01
## 11240  2 4130  8.754443e-01
## 11241  2 4131  8.582089e-01
## 11242  2 4132  8.388314e-01
## 11243  2 4133  8.257695e-01
## 11244  2 4134  8.069417e-01
## 11245  2 4135  7.929455e-01
## 11246  2 4136  7.762855e-01
## 11247  2 4137  7.615078e-01
## 11248  2 4138  7.446202e-01
## 11249  2 4139  7.302046e-01
## 11250  2 4140  7.164352e-01
## 11251  2 4141  7.021940e-01
## 11252  2 4142  6.875245e-01
## 11253  2 4143  6.750129e-01
## 11254  2 4144  6.611661e-01
## 11255  2 4145  6.496535e-01
## 11256  2 4146  6.367247e-01
## 11257  2 4147  6.233282e-01
## 11258  2 4148  6.111384e-01
## 11259  2 4149  6.001584e-01
## 11260  2 4150  5.868512e-01
## 11261  2 4151  5.754519e-01
## 11262  2 4152  5.650960e-01
## 11263  2 4152  5.652157e-01
## 11264  2 4153  2.735185e+00
## 11265  2 4154  2.515435e+00
## 11266  2 4155  2.328241e+00
## 11267  2 4156  2.168505e+00
## 11268  2 4157  2.026818e+00
## 11269  2 4158  1.910381e+00
## 11270  2 4159  1.805107e+00
## 11271  2 4160  1.715710e+00
## 11272  2 4161  1.637156e+00
## 11273  2 4162  1.570897e+00
## 11274  2 4163  1.506642e+00
## 11275  2 4164  1.452990e+00
## 11276  2 4165  1.400064e+00
## 11277  2 4166  1.356522e+00
## 11278  2 4167  1.315688e+00
## 11279  2 4168  1.278770e+00
## 11280  2 4169  1.242627e+00
## 11281  2 4170  1.210895e+00
## 11282  2 4171  1.179630e+00
## 11283  2 4172  1.150992e+00
## 11284  2 4173  1.123204e+00
## 11285  2 4174  1.098369e+00
## 11286  2 4175  1.071976e+00
## 11287  2 4176  1.050929e+00
## 11288  2 4176  1.050177e+00
## 11289  2 4177  2.118581e+00
## 11290  2 4178  1.988261e+00
## 11291  2 4179  1.881470e+00
## 11292  2 4180  1.783991e+00
## 11293  2 4181  1.703522e+00
## 11294  2 4182  1.628160e+00
## 11295  2 4183  1.565461e+00
## 11296  2 4184  1.506111e+00
## 11297  2 4185  1.451454e+00
## 11298  2 4186  1.404045e+00
## 11299  2 4187  1.361094e+00
## 11300  2 4188  1.320827e+00
## 11301  2 4189  1.285007e+00
## 11302  2 4190  1.250063e+00
## 11303  2 4191  1.219746e+00
## 11304  2 4192  1.188477e+00
## 11305  2 4193  1.159524e+00
## 11306  2 4194  1.135166e+00
## 11307  2 4195  1.109123e+00
## 11308  2 4196  1.083640e+00
## 11309  2 4197  1.060637e+00
## 11310  2 4198  1.037663e+00
## 11311  2 4199  1.014501e+00
## 11312  2 4200  9.944065e-01
## 11313  2 4201  2.066293e+00
## 11314  2 4202  1.936323e+00
## 11315  2 4203  1.834134e+00
## 11316  2 4204  1.738267e+00
## 11317  2 4205  1.656620e+00
## 11318  2 4206  1.582022e+00
## 11319  2 4207  1.519401e+00
## 11320  2 4208  1.462079e+00
## 11321  2 4209  1.409046e+00
## 11322  2 4210  1.362793e+00
## 11323  2 4211  1.321013e+00
## 11324  2 4212  1.281899e+00
## 11325  2 4213  1.246586e+00
## 11326  2 4214  1.212498e+00
## 11327  2 4215  1.181051e+00
## 11328  2 4216  1.152794e+00
## 11329  2 4217  1.124903e+00
## 11330  2 4218  1.099075e+00
## 11331  2 4219  1.072414e+00
## 11332  2 4220  1.049395e+00
## 11333  2 4221  1.028359e+00
## 11334  2 4222  1.006279e+00
## 11335  2 4223  9.838374e-01
## 11336  2 4224  9.632944e-01
## 11337  2 4225  2.037732e+00
## 11338  2 4226  1.908984e+00
## 11339  2 4227  1.802124e+00
## 11340  2 4228  1.708963e+00
## 11341  2 4229  1.629464e+00
## 11342  2 4230  1.553589e+00
## 11343  2 4231  1.491348e+00
## 11344  2 4232  1.436877e+00
## 11345  2 4233  1.383080e+00
## 11346  2 4234  1.336896e+00
## 11347  2 4235  1.295890e+00
## 11348  2 4236  1.260410e+00
## 11349  2 4237  1.224735e+00
## 11350  2 4238  1.189985e+00
## 11351  2 4239  1.160665e+00
## 11352  2 4240  1.130722e+00
## 11353  2 4241  1.103089e+00
## 11354  2 4242  1.078520e+00
## 11355  2 4243  1.054168e+00
## 11356  2 4244  1.028939e+00
## 11357  2 4245  1.009766e+00
## 11358  2 4246  9.871982e-01
## 11359  2 4247  9.657273e-01
## 11360  2 4248  9.449373e-01
## 11361  2 4249  2.014982e+00
## 11362  2 4250  1.892498e+00
## 11363  2 4251  1.785924e+00
## 11364  2 4252  1.692123e+00
## 11365  2 4253  1.612047e+00
## 11366  2 4254  1.537100e+00
## 11367  2 4255  1.476405e+00
## 11368  2 4256  1.419682e+00
## 11369  2 4257  1.368171e+00
## 11370  2 4258  1.322597e+00
## 11371  2 4259  1.282630e+00
## 11372  2 4260  1.242760e+00
## 11373  2 4261  1.208844e+00
## 11374  2 4262  1.175110e+00
## 11375  2 4263  1.144719e+00
## 11376  2 4264  1.117000e+00
## 11377  2 4265  1.092543e+00
## 11378  2 4266  1.063896e+00
## 11379  2 4267  1.040872e+00
## 11380  2 4268  1.017422e+00
## 11381  2 4269  9.957638e-01
## 11382  2 4270  9.727911e-01
## 11383  2 4271  9.536268e-01
## 11384  2 4272  9.322979e-01
## 11385  2 4273  2.007181e+00
## 11386  2 4274  1.880380e+00
## 11387  2 4275  1.774399e+00
## 11388  2 4276  1.682230e+00
## 11389  2 4277  1.597794e+00
## 11390  2 4278  1.530860e+00
## 11391  2 4279  1.466053e+00
## 11392  2 4280  1.406335e+00
## 11393  2 4281  1.357156e+00
## 11394  2 4282  1.313556e+00
## 11395  2 4283  1.271552e+00
## 11396  2 4284  1.232700e+00
## 11397  2 4285  1.199940e+00
## 11398  2 4286  1.165195e+00
## 11399  2 4287  1.137207e+00
## 11400  2 4288  1.109887e+00
## 11401  2 4289  1.081457e+00
## 11402  2 4290  1.056998e+00
## 11403  2 4291  1.031343e+00
## 11404  2 4292  1.008467e+00
## 11405  2 4293  9.873229e-01
## 11406  2 4294  9.640687e-01
## 11407  2 4295  9.459798e-01
## 11408  2 4296  9.256525e-01
## 11409  2 4297  1.998878e+00
## 11410  2 4298  1.874447e+00
## 11411  2 4299  1.768127e+00
## 11412  2 4300  1.678133e+00
## 11413  2 4301  1.591807e+00
## 11414  2 4302  1.523368e+00
## 11415  2 4303  1.461175e+00
## 11416  2 4304  1.402862e+00
## 11417  2 4305  1.351944e+00
## 11418  2 4306  1.307421e+00
## 11419  2 4307  1.267519e+00
## 11420  2 4308  1.228061e+00
## 11421  2 4309  1.193486e+00
## 11422  2 4310  1.162929e+00
## 11423  2 4311  1.132713e+00
## 11424  2 4312  1.104432e+00
## 11425  2 4313  1.076773e+00
## 11426  2 4314  1.050971e+00
## 11427  2 4315  1.027270e+00
## 11428  2 4316  1.003957e+00
## 11429  2 4317  9.836831e-01
## 11430  2 4318  9.631618e-01
## 11431  2 4319  9.410188e-01
## 11432  2 4320  9.215135e-01
## 11433  2 4321  1.989377e+00
## 11434  2 4322  1.869230e+00
## 11435  2 4323  1.763367e+00
## 11436  2 4324  1.669707e+00
## 11437  2 4325  1.587776e+00
## 11438  2 4326  1.516641e+00
## 11439  2 4327  1.454156e+00
## 11440  2 4328  1.398260e+00
## 11441  2 4329  1.345974e+00
## 11442  2 4330  1.301414e+00
## 11443  2 4331  1.263894e+00
## 11444  2 4332  1.225810e+00
## 11445  2 4333  1.188228e+00
## 11446  2 4334  1.157050e+00
## 11447  2 4335  1.128171e+00
## 11448  2 4336  1.100767e+00
## 11449  2 4337  1.074164e+00
## 11450  2 4338  1.048189e+00
## 11451  2 4339  1.024494e+00
## 11452  2 4340  1.001390e+00
## 11453  2 4341  9.801109e-01
## 11454  2 4342  9.575915e-01
## 11455  2 4343  9.371865e-01
## 11456  2 4344  9.179105e-01
## 11457  2 4345  1.992509e+00
## 11458  2 4346  1.869920e+00
## 11459  2 4347  1.758310e+00
## 11460  2 4348  1.668289e+00
## 11461  2 4349  1.585444e+00
## 11462  2 4350  1.513055e+00
## 11463  2 4351  1.451741e+00
## 11464  2 4352  1.396710e+00
## 11465  2 4353  1.345758e+00
## 11466  2 4354  1.299123e+00
## 11467  2 4355  1.260581e+00
## 11468  2 4356  1.222580e+00
## 11469  2 4357  1.188094e+00
## 11470  2 4358  1.156400e+00
## 11471  2 4359  1.125824e+00
## 11472  2 4360  1.098389e+00
## 11473  2 4361  1.072146e+00
## 11474  2 4362  1.046826e+00
## 11475  2 4363  1.023826e+00
## 11476  2 4364  9.986249e-01
## 11477  2 4365  9.786567e-01
## 11478  2 4366  9.564771e-01
## 11479  2 4367  9.367024e-01
## 11480  2 4368  9.169177e-01
## 11481  2 4369  1.988258e+00
## 11482  2 4370  1.867789e+00
## 11483  2 4371  1.758346e+00
## 11484  2 4372  1.665333e+00
## 11485  2 4373  1.586705e+00
## 11486  2 4374  1.512607e+00
## 11487  2 4375  1.450751e+00
## 11488  2 4376  1.395197e+00
## 11489  2 4377  1.346952e+00
## 11490  2 4378  1.298138e+00
## 11491  2 4379  1.260006e+00
## 11492  2 4380  1.221292e+00
## 11493  2 4381  1.189129e+00
## 11494  2 4382  1.154323e+00
## 11495  2 4383  1.124679e+00
## 11496  2 4384  1.096064e+00
## 11497  2 4385  1.071364e+00
## 11498  2 4386  1.044868e+00
## 11499  2 4387  1.022942e+00
## 11500  2 4388  9.979508e-01
## 11501  2 4389  9.755202e-01
## 11502  2 4390  9.561305e-01
## 11503  2 4391  9.355004e-01
## 11504  2 4392  9.153959e-01
## 11505  2 4393  1.986639e+00
## 11506  2 4394  1.866732e+00
## 11507  2 4395  1.756134e+00
## 11508  2 4396  1.667750e+00
## 11509  2 4397  1.580342e+00
## 11510  2 4398  1.516606e+00
## 11511  2 4399  1.448814e+00
## 11512  2 4400  1.392410e+00
## 11513  2 4401  1.343423e+00
## 11514  2 4402  1.297723e+00
## 11515  2 4403  1.258867e+00
## 11516  2 4404  1.221468e+00
## 11517  2 4405  1.188255e+00
## 11518  2 4406  1.154381e+00
## 11519  2 4407  1.124633e+00
## 11520  2 4408  1.095804e+00
## 11521  2 4409  1.068427e+00
## 11522  2 4410  1.044611e+00
## 11523  2 4411  1.020904e+00
## 11524  2 4412  9.981175e-01
## 11525  2 4413  9.769072e-01
## 11526  2 4414  9.544601e-01
## 11527  2 4415  9.347385e-01
## 11528  2 4416  9.150921e-01
## 11529  2 4417  1.988171e+00
## 11530  2 4418  1.860620e+00
## 11531  2 4419  1.757116e+00
## 11532  2 4420  1.661358e+00
## 11533  2 4421  1.582440e+00
## 11534  2 4422  1.513246e+00
## 11535  2 4423  1.451877e+00
## 11536  2 4424  1.394125e+00
## 11537  2 4425  1.343253e+00
## 11538  2 4426  1.295641e+00
## 11539  2 4427  1.257700e+00
## 11540  2 4428  1.218992e+00
## 11541  2 4429  1.187048e+00
## 11542  2 4430  1.152737e+00
## 11543  2 4431  1.122415e+00
## 11544  2 4432  1.095432e+00
## 11545  2 4433  1.067249e+00
## 11546  2 4434  1.043167e+00
## 11547  2 4435  1.020236e+00
## 11548  2 4436  9.961442e-01
## 11549  2 4437  9.763579e-01
## 11550  2 4438  9.539958e-01
## 11551  2 4439  9.350832e-01
## 11552  2 4440  9.136249e-01
## 11553  2 4441  8.958214e-01
## 11554  2 4442  8.769321e-01
## 11555  2 4443  8.594679e-01
## 11556  2 4444  8.414002e-01
## 11557  2 4445  8.240115e-01
## 11558  2 4446  8.096674e-01
## 11559  2 4447  7.921509e-01
## 11560  2 4448  7.771531e-01
## 11561  2 4449  7.626111e-01
## 11562  2 4450  7.459900e-01
## 11563  2 4451  7.316657e-01
## 11564  2 4452  7.165791e-01
## 11565  2 4453  7.038161e-01
## 11566  2 4454  6.885917e-01
## 11567  2 4455  6.753270e-01
## 11568  2 4456  6.624383e-01
## 11569  2 4457  6.489979e-01
## 11570  2 4458  6.364738e-01
## 11571  2 4459  6.239661e-01
## 11572  2 4460  6.125345e-01
## 11573  2 4461  5.987581e-01
## 11574  2 4462  5.871422e-01
## 11575  2 4463  5.762626e-01
## 11576  2 4464  5.646438e-01
## 11577  2 4465  5.544680e-01
## 11578  2 4466  5.437528e-01
## 11579  2 4467  5.320576e-01
## 11580  2 4468  5.213694e-01
## 11581  2 4469  5.116873e-01
## 11582  2 4470  5.031607e-01
## 11583  2 4471  4.924787e-01
## 11584  2 4472  4.829143e-01
## 11585  2 4473  4.730146e-01
## 11586  2 4474  4.635673e-01
## 11587  2 4475  4.553646e-01
## 11588  2 4476  4.455302e-01
## 11589  2 4477  4.382128e-01
## 11590  2 4478  4.286891e-01
## 11591  2 4479  4.204775e-01
## 11592  2 4480  4.125439e-01
## 11593  2 4481  4.045979e-01
## 11594  2 4482  3.972223e-01
## 11595  2 4483  3.887368e-01
## 11596  2 4484  3.811946e-01
## 11597  2 4485  3.743281e-01
## 11598  2 4486  3.667379e-01
## 11599  2 4487  3.590503e-01
## 11600  2 4488  3.525052e-01
## 11601  2 4489  3.451702e-01
## 11602  2 4490  3.378397e-01
## 11603  2 4491  3.321161e-01
## 11604  2 4492  3.254081e-01
## 11605  2 4493  3.186330e-01
## 11606  2 4494  3.132720e-01
## 11607  2 4495  3.068684e-01
## 11608  2 4496  3.007501e-01
## 11609  2 4497  2.948438e-01
## 11610  2 4498  2.893044e-01
## 11611  2 4499  2.836650e-01
## 11612  2 4500  2.783347e-01
## 11613  2 4501  2.725449e-01
## 11614  2 4502  2.674105e-01
## 11615  2 4503  2.620338e-01
## 11616  2 4504  2.572349e-01
## 11617  2 4505  2.516238e-01
## 11618  2 4506  2.474968e-01
## 11619  2 4507  2.424176e-01
## 11620  2 4508  2.375670e-01
## 11621  2 4509  2.329491e-01
## 11622  2 4510  2.285674e-01
## 11623  2 4511  2.239640e-01
## 11624  2 4512  2.191899e-01
## 11625  2 4513  2.151155e-01
## 11626  2 4514  2.111841e-01
## 11627  2 4515  2.068587e-01
## 11628  2 4516  2.029397e-01
## 11629  2 4517  1.988256e-01
## 11630  2 4518  1.951938e-01
## 11631  2 4519  1.915941e-01
## 11632  2 4520  1.880066e-01
## 11633  2 4521  1.836787e-01
## 11634  2 4522  1.801520e-01
## 11635  2 4523  1.767592e-01
## 11636  2 4524  1.732217e-01
## 11637  2 4525  1.698437e-01
## 11638  2 4526  1.666235e-01
## 11639  2 4527  1.633218e-01
## 11640  2 4528  1.599734e-01
## 11641  2 4529  1.571687e-01
## 11642  2 4530  1.539318e-01
## 11643  2 4531  1.510698e-01
## 11644  2 4532  1.480350e-01
## 11645  2 4533  1.451552e-01
## 11646  2 4534  1.421812e-01
## 11647  2 4535  1.394544e-01
## 11648  2 4536  1.368649e-01
## 11649  2 4537  1.337057e-01
## 11650  2 4538  1.311964e-01
## 11651  2 4539  1.289162e-01
## 11652  2 4540  1.265126e-01
## 11653  2 4541  1.238421e-01
## 11654  2 4542  1.212788e-01
## 11655  2 4543  1.191845e-01
## 11656  2 4544  1.169182e-01
## 11657  2 4545  1.145926e-01
## 11658  2 4546  1.120309e-01
## 11659  2 4547  1.101145e-01
## 11660  2 4548  1.078552e-01
## 11661  2 4549  1.058518e-01
## 11662  2 4550  1.038546e-01
## 11663  2 4551  1.019132e-01
## 11664  2 4552  9.961977e-02
## 11665  2 4553  9.766633e-02
## 11666  2 4554  9.605498e-02
## 11667  2 4555  9.390265e-02
## 11668  2 4556  9.234449e-02
## 11669  2 4557  9.047053e-02
## 11670  2 4558  8.862439e-02
## 11671  2 4559  8.691349e-02
## 11672  2 4560  8.533474e-02
## 11673  2 4561  8.371707e-02
## 11674  2 4562  8.207521e-02
## 11675  2 4563  8.015494e-02
## 11676  2 4564  7.879321e-02
## 11677  2 4565  7.728788e-02
## 11678  2 4566  7.575684e-02
## 11679  2 4567  7.424804e-02
## 11680  2 4568  7.275981e-02
## 11681  2 4569  7.134432e-02
## 11682  2 4570  6.998030e-02
## 11683  2 4571  6.875382e-02
## 11684  2 4572  6.736257e-02
## 11685  2 4573  6.599538e-02
## 11686  2 4574  6.466052e-02
## 11687  2 4575  6.336141e-02
## 11688  2 4576  6.219023e-02
## 11689  2 4577  6.100919e-02
## 11690  2 4578  5.971380e-02
## 11691  2 4579  5.874454e-02
## 11692  2 4580  5.744984e-02
## 11693  2 4581  5.629709e-02
## 11694  2 4582  5.520527e-02
## 11695  2 4583  5.423928e-02
## 11696  2 4584  5.309366e-02
## 11697  2 4585  5.202072e-02
## 11698  2 4586  5.110918e-02
## 11699  2 4587  5.009319e-02
## 11700  2 4588  4.904212e-02
## 11701  2 4589  4.812700e-02
## 11702  2 4590  4.720674e-02
## 11703  2 4591  4.626494e-02
## 11704  2 4592  4.531834e-02
## 11705  2 4593  4.448752e-02
## 11706  2 4594  4.359941e-02
## 11707  2 4595  4.277901e-02
## 11708  2 4596  4.196773e-02
## 11709  2 4597  4.107302e-02
## 11710  2 4598  4.035061e-02
## 11711  2 4599  3.953729e-02
## 11712  2 4600  3.877916e-02
## 11713  2 4601  3.791693e-02
## 11714  2 4602  3.724983e-02
## 11715  2 4603  3.647226e-02
## 11716  2 4604  3.574808e-02
## 11717  2 4605  3.517596e-02
## 11718  2 4606  3.443186e-02
## 11719  2 4607  3.377895e-02
## 11720  2 4608  3.305939e-02
## 11721  2 4609  3.245255e-02
## 11722  2 4610  3.180312e-02
## 11723  2 4611  3.122803e-02
## 11724  2 4612  3.054584e-02
## 11725  2 4613  3.002538e-02
## 11726  2 4614  2.945074e-02
## 11727  2 4615  2.882039e-02
## 11728  2 4616  2.823626e-02
## 11729  2 4617  2.767850e-02
## 11730  2 4618  2.715518e-02
## 11731  2 4619  2.669581e-02
## 11732  2 4620  2.616211e-02
## 11733  2 4621  2.563240e-02
## 11734  2 4622  2.513453e-02
## 11735  2 4623  2.466910e-02
## 11736  2 4624  2.416826e-02
## 11737  2 4625  2.370048e-02
## 11738  2 4626  2.319681e-02
## 11739  2 4627  2.276741e-02
## 11740  2 4628  2.234811e-02
## 11741  2 4629  2.190465e-02
## 11742  2 4630  2.146379e-02
## 11743  2 4631  2.100329e-02
## 11744  2 4632  2.062682e-02
## 11745  2 4633  2.023825e-02
## 11746  2 4634  1.980808e-02
## 11747  2 4635  1.943691e-02
## 11748  2 4636  1.906715e-02
## 11749  2 4637  1.869497e-02
## 11750  2 4638  1.832369e-02
## 11751  2 4639  1.796696e-02
## 11752  2 4640  1.762702e-02
## 11753  2 4641  1.728921e-02
## 11754  2 4642  1.695183e-02
## 11755  2 4643  1.660591e-02
## 11756  2 4644  1.627791e-02
## 11757  2 4645  1.594991e-02
## 11758  2 4646  1.567619e-02
## 11759  2 4647  1.536993e-02
## 11760  2 4648  1.506155e-02
## 11761  2 4649  1.477250e-02
## 11762  2 4650  1.445820e-02
## 11763  2 4651  1.417837e-02
## 11764  2 4652  1.390693e-02
## 11765  2 4653  1.365385e-02
## 11766  2 4654  1.336147e-02
## 11767  2 4655  1.310179e-02
## 11768  2 4656  1.283748e-02
## 11769  2 4657  1.259581e-02
## 11770  2 4658  1.234948e-02
## 11771  2 4659  1.212917e-02
## 11772  2 4660  1.188254e-02
## 11773  2 4661  1.164170e-02
## 11774  2 4662  1.142437e-02
## 11775  2 4663  1.118609e-02
## 11776  2 4664  1.096705e-02
## 11777  2 4665  1.076541e-02
## 11778  2 4666  1.055486e-02
## 11779  2 4667  1.034473e-02
## 11780  2 4668  1.012681e-02
## 11781  2 4669  9.950700e-03
## 11782  2 4670  9.742421e-03
## 11783  2 4671  9.556044e-03
## 11784  2 4672  9.372841e-03
## 11785  2 4673  9.196677e-03
## 11786  2 4674  9.002329e-03
## 11787  2 4675  8.845213e-03
## 11788  2 4676  8.668934e-03
## 11789  2 4677  8.481300e-03
## 11790  2 4678  8.336766e-03
## 11791  2 4679  8.181461e-03
## 11792  2 4680  7.999352e-03
## 11793  2 4681  7.865903e-03
## 11794  2 4682  7.697997e-03
## 11795  2 4683  7.551966e-03
## 11796  2 4684  7.390582e-03
## 11797  2 4685  7.251202e-03
## 11798  2 4686  7.116916e-03
## 11799  2 4687  6.966766e-03
## 11800  2 4688  6.854531e-03
## 11801  2 4689  6.709361e-03
## 11802  2 4690  6.574062e-03
## 11803  2 4691  6.453665e-03
## 11804  2 4692  6.322394e-03
## 11805  2 4693  6.198394e-03
## 11806  2 4694  6.083079e-03
## 11807  2 4695  5.956727e-03
## 11808  2 4696  5.850943e-03
## 11809  2 4697  5.721499e-03
## 11810  2 4698  5.618079e-03
## 11811  2 4699  5.501966e-03
## 11812  2 4700  5.409078e-03
## 11813  2 4701  5.297344e-03
## 11814  2 4702  5.194542e-03
## 11815  2 4703  5.088706e-03
## 11816  2 4704  4.978432e-03
## 11817  2 4705  4.886506e-03
## 11818  2 4706  4.801213e-03
## 11819  2 4707  4.697465e-03
## 11820  2 4708  4.615074e-03
## 11821  2 4709  4.522855e-03
## 11822  2 4710  4.434361e-03
## 11823  2 4711  4.345441e-03
## 11824  2 4712  4.265249e-03
## 11825  2 4713  4.177098e-03
## 11826  2 4714  4.089635e-03
## 11827  2 4715  4.018623e-03
## 11828  2 4716  3.940247e-03
## 11829  2 4717  3.870864e-03
## 11830  2 4718  3.792855e-03
## 11831  2 4719  3.711933e-03
## 11832  2 4720  3.636499e-03
## 11833  2 4721  3.568761e-03
## 11834  2 4722  3.507259e-03
## 11835  2 4723  3.435076e-03
## 11836  2 4724  3.363099e-03
## 11837  2 4725  3.300914e-03
## 11838  2 4726  3.231925e-03
## 11839  2 4727  3.173304e-03
## 11840  2 4728  3.108614e-03
## 11841  2 4729  3.051531e-03
## 11842  2 4730  2.995970e-03
## 11843  2 4731  2.935544e-03
## 11844  2 4732  2.874712e-03
## 11845  2 4733  2.818699e-03
## 11846  2 4734  2.761174e-03
## 11847  2 4735  2.707646e-03
## 11848  2 4736  2.659341e-03
## 11849  2 4737  2.607701e-03
## 11850  2 4738  2.553524e-03
## 11851  2 4739  2.503416e-03
## 11852  2 4740  2.457236e-03
## 11853  2 4741  2.412851e-03
## 11854  2 4742  2.360247e-03
## 11855  2 4743  2.317523e-03
## 11856  2 4744  2.269863e-03
## 11857  2 4745  2.231148e-03
## 11858  2 4746  2.185998e-03
## 11859  2 4747  2.141346e-03
## 11860  2 4748  2.092697e-03
## 11861  2 4749  2.056416e-03
## 11862  2 4750  2.017541e-03
## 11863  2 4751  1.978874e-03
## 11864  2 4752  1.935743e-03
## 11865  2 4753  1.900048e-03
## 11866  2 4754  1.864290e-03
## 11867  2 4755  1.829190e-03
## 11868  2 4756  1.789886e-03
## 11869  2 4757  1.759376e-03
## 11870  2 4758  1.723850e-03
## 11871  2 4759  1.688784e-03
## 11872  2 4760  1.654759e-03
## 11873  2 4761  1.624882e-03
## 11874  2 4762  1.591724e-03
## 11875  2 4763  1.560176e-03
## 11876  2 4764  1.528307e-03
## 11877  2 4765  1.500393e-03
## 11878  2 4766  1.469672e-03
## 11879  2 4767  1.440486e-03
## 11880  2 4768  1.413986e-03
## 11881  2 4769  1.387759e-03
## 11882  2 4770  1.358899e-03
## 11883  2 4771  1.333918e-03
## 11884  2 4772  1.306743e-03
## 11885  2 4773  1.280224e-03
## 11886  2 4774  1.255134e-03
## 11887  2 4775  1.230290e-03
## 11888  2 4776  1.207936e-03
## 11889  2 4777  1.184313e-03
## 11890  2 4778  1.160963e-03
## 11891  2 4779  1.136911e-03
## 11892  2 4780  1.116611e-03
## 11893  2 4781  1.093427e-03
## 11894  2 4782  1.074172e-03
## 11895  2 4783  1.052636e-03
## 11896  2 4784  1.027625e-03
## 11897  2 4785  1.010510e-03
## 11898  2 4786  9.904514e-04
## 11899  2 4787  9.725406e-04
## 11900  2 4788  9.536520e-04
## 11901  2 4789  9.347766e-04
## 11902  2 4790  9.154662e-04
## 11903  2 4791  8.985935e-04
## 11904  2 4792  8.805313e-04
## 11905  2 4793  8.629042e-04
## 11906  2 4794  8.471701e-04
## 11907  2 4795  8.287593e-04
## 11908  2 4796  8.141293e-04
## 11909  2 4797  7.991345e-04
## 11910  2 4798  7.823574e-04
## 11911  2 4799  7.672049e-04
## 11912  2 4800  7.519318e-04
## 11913  2 4801  7.361759e-04
## 11914  2 4802  7.240330e-04
## 11915  2 4803  7.103025e-04
## 11916  2 4804  6.960478e-04
## 11917  2 4805  6.827119e-04
## 11918  2 4806  6.682418e-04
## 11919  2 4807  6.563645e-04
## 11920  2 4808  6.428595e-04
## 11921  2 4809  6.306967e-04
## 11922  2 4810  6.177503e-04
## 11923  2 4811  6.055478e-04
## 11924  2 4812  5.943717e-04
## 11925  2 4813  5.825453e-04
## 11926  2 4814  5.707319e-04
## 11927  2 4815  5.600102e-04
## 11928  2 4816  5.491988e-04
## 11929  2 4817  5.373898e-04
## 11930  2 4818  5.275284e-04
## 11931  2 4819  5.165020e-04
## 11932  2 4820  5.072603e-04
## 11933  2 4821  4.971581e-04
## 11934  2 4822  4.886814e-04
## 11935  2 4823  4.789420e-04
## 11936  2 4824  4.688619e-04
## 11937  2 4825  4.592722e-04
## 11938  2 4826  4.516716e-04
## 11939  2 4827  4.422049e-04
## 11940  2 4828  4.337301e-04
## 11941  2 4829  4.252797e-04
## 11942  2 4830  4.166483e-04
## 11943  2 4831  4.077812e-04
## 11944  2 4832  4.005920e-04
## 11945  2 4833  3.930420e-04
## 11946  2 4834  3.844380e-04
## 11947  2 4835  3.778205e-04
## 11948  2 4836  3.696352e-04
## 11949  2 4837  3.632053e-04
## 11950  2 4838  3.559057e-04
## 11951  2 4839  3.487886e-04
## 11952  2 4840  3.417329e-04
## 11953  2 4841  3.357919e-04
## 11954  2 4842  3.293601e-04
## 11955  2 4843  3.223106e-04
## 11956  2 4844  3.156009e-04
## 11957  2 4845  3.097840e-04
## 11958  2 4846  3.036158e-04
## 11959  2 4847  2.977284e-04
## 11960  2 4848  2.920986e-04
## 11961  2 4849  2.869412e-04
## 11962  2 4850  2.806328e-04
## 11963  2 4851  2.755967e-04
## 11964  2 4852  2.703268e-04
## 11965  2 4853  2.646352e-04
## 11966  2 4854  2.594403e-04
## 11967  2 4855  2.548779e-04
## 11968  2 4856  2.499323e-04
## 11969  2 4857  2.447037e-04
## 11970  2 4858  2.400068e-04
## 11971  2 4859  2.350998e-04
## 11972  2 4860  2.307682e-04
## 11973  2 4861  2.260736e-04
## 11974  2 4862  2.216830e-04
## 11975  2 4863  2.174218e-04
## 11976  2 4864  2.133270e-04
## 11977  2 4865  2.092950e-04
## 11978  2 4866  2.049973e-04
## 11979  2 4867  2.008629e-04
## 11980  2 4868  1.971101e-04
## 11981  2 4869  1.935612e-04
## 11982  2 4870  1.894155e-04
## 11983  2 4871  1.857376e-04
## 11984  2 4872  1.819989e-04
## 11985  2 4873  1.787700e-04
## 11986  2 4874  1.750873e-04
## 11987  2 4875  1.716750e-04
## 11988  2 4876  1.682930e-04
## 11989  2 4877  1.649189e-04
## 11990  2 4878  1.618817e-04
## 11991  2 4879  1.584929e-04
## 11992  2 4880  1.557410e-04
## 11993  2 4881  1.523772e-04
## 11994  2 4882  1.495731e-04
## 11995  2 4883  1.464395e-04
## 11996  2 4884  1.433620e-04
## 11997  2 4885  1.408035e-04
## 11998  2 4886  1.382160e-04
## 11999  2 4887  1.357435e-04
## 12000  2 4888  1.327888e-04
## 12001  2 4889  1.304956e-04
## 12002  2 4890  1.278654e-04
## 12003  2 4891  1.252585e-04
## 12004  2 4892  1.227801e-04
## 12005  2 4893  1.202995e-04
## 12006  2 4894  1.179873e-04
## 12007  2 4895  1.158573e-04
## 12008  2 4896  1.134465e-04
## 12009  2 4897  1.112298e-04
## 12010  2 4898  1.090679e-04
## 12011  2 4899  1.070647e-04
## 12012  2 4900  1.048599e-04
## 12013  2 4901  1.029109e-04
## 12014  2 4902  1.006261e-04
## 12015  2 4903  9.871338e-05
## 12016  2 4904  9.693117e-05
## 12017  2 4905  9.499889e-05
## 12018  2 4906  9.337315e-05
## 12019  2 4907  9.154859e-05
## 12020  2 4908  8.970604e-05
## 12021  2 4909  8.782092e-05
## 12022  2 4910  8.629044e-05
## 12023  2 4911  8.454835e-05
## 12024  2 4912  8.284443e-05
## 12025  2 4913  8.125363e-05
## 12026  2 4914  7.957934e-05
## 12027  2 4915  7.799553e-05
## 12028  2 4916  7.646243e-05
## 12029  2 4917  7.522958e-05
## 12030  2 4918  7.366311e-05
## 12031  2 4919  7.216691e-05
## 12032  2 4920  7.062434e-05
## 12033  2 4921  6.924360e-05
## 12034  2 4922  6.813296e-05
## 12035  2 4923  6.667494e-05
```

```
## 12036  2 4924  6.541425e-05
## 12037  2 4925  6.401835e-05
## 12038  2 4926  6.285208e-05
## 12039  2 4927  6.155882e-05
## 12040  2 4928  6.044438e-05
## 12041  2 4929  5.919877e-05
## 12042  2 4930  5.800430e-05
## 12043  2 4931  5.691725e-05
## 12044  2 4932  5.578326e-05
## 12045  2 4933  5.470252e-05
## 12046  2 4934  5.369722e-05
## 12047  2 4935  5.258465e-05
## 12048  2 4936  5.161791e-05
## 12049  2 4937  5.065313e-05
## 12050  2 4938  4.954910e-05
## 12051  2 4939  4.865815e-05
## 12052  2 4940  4.768664e-05
## 12053  2 4941  4.676890e-05
## 12054  2 4942  4.580786e-05
## 12055  2 4943  4.502514e-05
## 12056  2 4944  4.408553e-05
## 12057  2 4945  4.312748e-05
## 12058  2 4946  4.228667e-05
## 12059  2 4947  4.146901e-05
## 12060  2 4948  4.071687e-05
## 12061  2 4949  3.991177e-05
## 12062  2 4950  3.913365e-05
## 12063  2 4951  3.845012e-05
## 12064  2 4952  3.759328e-05
## 12065  2 4953  3.692399e-05
## 12066  2 4954  3.622742e-05
## 12067  2 4955  3.546918e-05
## 12068  2 4956  3.479691e-05
## 12069  2 4957  3.409342e-05
## 12070  2 4958  3.338484e-05
## 12071  2 4959  3.276053e-05
## 12072  2 4960  3.212341e-05
## 12073  2 4961  3.151430e-05
## 12074  2 4962  3.097446e-05
## 12075  2 4963  3.026912e-05
## 12076  2 4964  2.973360e-05
## 12077  2 4965  2.910333e-05
## 12078  2 4966  2.860662e-05
## 12079  2 4967  2.795188e-05
## 12080  2 4968  2.741819e-05
## 12081  2 4969  2.693236e-05
## 12082  2 4970  2.634298e-05
## 12083  2 4971  2.586024e-05
## 12084  2 4972  2.538294e-05
## 12085  2 4973  2.487410e-05
## 12086  2 4974  2.440390e-05
## 12087  2 4975  2.385694e-05
## 12088  2 4976  2.342496e-05
## 12089  2 4977  2.297906e-05
## 12090  2 4978  2.254644e-05
## 12091  2 4979  2.209392e-05
## 12092  2 4980  2.170216e-05
## 12093  2 4981  2.124779e-05
## 12094  2 4982  2.081598e-05
## 12095  2 4983  2.041825e-05
## 12096  2 4984  2.004578e-05
## 12097  2 4985  1.964950e-05
## 12098  2 4986  1.927733e-05
## 12099  2 4987  1.887986e-05
## 12100  2 4988  1.848668e-05
## 12101  2 4989  1.814701e-05
## 12102  2 4990  1.778504e-05
## 12103  2 4991  1.745921e-05
## 12104  2 4992  1.709121e-05
## 12105  2 4993  1.676244e-05
## 12106  2 4994  1.643554e-05
## 12107  2 4995  1.612146e-05
## 12108  2 4996  1.579737e-05
## 12109  2 4997  1.550792e-05
## 12110  2 4998  1.519637e-05
## 12111  2 4999  1.491929e-05
## 12112  2 5000  1.461284e-05
## 12113  2 5001  1.430577e-05
## 12114  2 5002  1.404398e-05
## 12115  2 5003  1.376453e-05
## 12116  2 5004  1.350504e-05
## 12117  2 5005  1.323107e-05
## 12118  2 5006  1.296624e-05
## 12119  2 5007  1.272320e-05
## 12120  2 5008  1.249245e-05
## 12121  2 5009  1.221996e-05
## 12122  2 5010  1.196795e-05
## 12123  2 5011  1.176342e-05
## 12124  2 5012  1.151548e-05
## 12125  2 5013  1.130176e-05
## 12126  2 5014  1.107339e-05
## 12127  2 5015  1.084903e-05
## 12128  2 5016  1.063809e-05
## 12129  2 5017  1.043808e-05
## 12130  2 5018  1.024721e-05
## 12131  2 5019  1.003651e-05
## 12132  2 5020  9.844416e-06
## 12133  2 5021  9.666999e-06
## 12134  2 5022  9.466680e-06
## 12135  2 5023  9.265881e-06
## 12136  2 5024  9.080319e-06
## 12137  2 5025  8.916446e-06
## 12138  2 5026  8.750034e-06
## 12139  2 5027  8.570753e-06
## 12140  2 5028  8.413660e-06
## 12141  2 5029  8.242305e-06
## 12142  2 5030  8.089596e-06
## 12143  2 5031  7.941929e-06
## 12144  2 5032  7.776231e-06
## 12145  2 5033  7.614864e-06
## 12146  2 5034  7.474150e-06
## 12147  2 5035  7.334531e-06
## 12148  2 5036  7.183952e-06
## 12149  2 5037  7.041703e-06
## 12150  2 5038  6.918398e-06
## 12151  2 5039  6.779554e-06
## 12152  2 5040  6.651669e-06
## 12153  2 5041  6.519778e-06
## 12154  2 5042  6.381367e-06
## 12155  2 5043  6.282651e-06
## 12156  2 5044  6.147191e-06
## 12157  2 5045  6.033605e-06
## 12158  2 5046  5.919154e-06
## 12159  2 5047  5.800065e-06
## 12160  2 5048  5.695022e-06
## 12161  2 5049  5.586448e-06
## 12162  2 5050  5.474180e-06
## 12163  2 5051  5.370235e-06
## 12164  2 5052  5.262181e-06
## 12165  2 5053  5.167196e-06
## 12166  2 5054  5.066059e-06
## 12167  2 5055  4.963376e-06
## 12168  2 5056  4.868925e-06
## 12169  2 5057  4.777462e-06
## 12170  2 5058  4.696005e-06
## 12171  2 5059  4.601416e-06
## 12172  2 5060  4.499780e-06
## 12173  2 5061  4.416215e-06
## 12174  2 5062  4.336769e-06
## 12175  2 5063  4.246997e-06
## 12176  2 5064  4.170221e-06
## 12177  2 5065  4.096864e-06
## 12178  2 5066  4.017257e-06
## 12179  2 5067  3.933402e-06
## 12180  2 5068  3.864223e-06
## 12181  2 5069  3.784869e-06
## 12182  2 5070  3.724112e-06
## 12183  2 5071  3.644187e-06
## 12184  2 5072  3.577646e-06
## 12185  2 5073  3.512740e-06
## 12186  2 5074  3.439098e-06
## 12187  2 5075  3.376124e-06
## 12188  2 5076  3.310608e-06
## 12189  2 5077  3.247463e-06
## 12190  2 5078  3.181962e-06
## 12191  2 5079  3.130809e-06
## 12192  2 5080  3.058726e-06
## 12193  2 5081  3.001214e-06
## 12194  2 5082  2.949470e-06
## 12195  2 5083  2.888980e-06
## 12196  2 5084  2.827354e-06
## 12197  2 5085  2.776165e-06
## 12198  2 5086  2.726052e-06
## 12199  2 5087  2.671277e-06
## 12200  2 5088  2.618736e-06
## 12201  2 5089  2.570357e-06
## 12202  2 5090  2.519234e-06
## 12203  2 5091  2.466757e-06
## 12204  2 5092  2.420620e-06
## 12205  2 5093  2.380425e-06
## 12206  2 5094  2.330440e-06
## 12207  2 5095  2.288916e-06
## 12208  2 5096  2.241973e-06
## 12209  2 5097  2.195170e-06
## 12210  2 5098  2.153820e-06
## 12211  2 5099  2.112003e-06
## 12212  2 5100  2.069309e-06
## 12213  2 5101  2.030853e-06
## 12214  2 5102  1.990264e-06
## 12215  2 5103  1.952040e-06
## 12216  2 5104  1.910535e-06
## 12217  2 5105  1.878706e-06
## 12218  2 5106  1.842431e-06
## 12219  2 5107  1.806403e-06
## 12220  2 5108  1.767820e-06
## 12221  2 5109  1.730537e-06
## 12222  2 5110  1.698660e-06
## 12223  2 5111  1.665061e-06
## 12224  2 5112  1.630573e-06
## 12225  2 5113  1.599928e-06
## 12226  2 5114  1.567505e-06
## 12227  2 5115  1.538169e-06
## 12228  2 5116  1.506943e-06
## 12229  2 5117  1.479429e-06
## 12230  2 5118  1.451457e-06
## 12231  2 5119  1.421492e-06
## 12232  2 5120  1.394255e-06
## 12233  2 5121  1.365865e-06
## 12234  2 5122  1.340709e-06
## 12235  2 5123  1.312400e-06
## 12236  2 5124  1.287830e-06
## 12237  2 5125  1.260961e-06
## 12238  2 5126  1.234763e-06
## 12239  2 5127  1.212684e-06
## 12240  2 5128  1.188332e-06
## 12241  2 5129  1.165637e-06
## 12242  2 5130  1.144299e-06
## 12243  2 5131  1.120912e-06
## 12244  2 5132  1.099983e-06
## 12245  2 5133  1.077129e-06
## 12246  2 5134  1.055162e-06
## 12247  2 5135  1.034986e-06
## 12248  2 5136  1.013945e-06
## 12249  2 5137  9.931210e-07
## 12250  2 5138  9.754484e-07
## 12251  2 5139  9.559954e-07
## 12252  2 5140  9.370163e-07
## 12253  2 5141  9.204223e-07
## 12254  2 5142  9.013719e-07
## 12255  2 5143  8.836467e-07
## 12256  2 5144  8.671317e-07
## 12257  2 5145  8.481751e-07
## 12258  2 5146  8.333015e-07
## 12259  2 5147  8.171970e-07
## 12260  2 5148  8.028913e-07
## 12261  2 5149  7.865789e-07
## 12262  2 5150  7.711882e-07
## 12263  2 5151  7.562761e-07
## 12264  2 5152  7.427371e-07
## 12265  2 5153  7.274153e-07
## 12266  2 5154  7.130014e-07
## 12267  2 5155  6.992375e-07
## 12268  2 5156  6.868741e-07
## 12269  2 5157  6.737011e-07
## 12270  2 5158  6.614766e-07
## 12271  2 5159  6.489217e-07
## 12272  2 5160  6.376909e-07
## 12273  2 5161  6.249586e-07
## 12274  2 5162  6.146652e-07
## 12275  2 5163  6.031728e-07
## 12276  2 5164  5.908186e-07
## 12277  2 5165  5.804886e-07
## 12278  2 5166  5.703018e-07
## 12279  2 5167  5.594029e-07
## 12280  2 5168  5.485578e-07
## 12281  2 5169  5.399565e-07
## 12282  2 5170  5.308273e-07
## 12283  2 5171  5.208593e-07
## 12284  2 5172  5.112871e-07
## 12285  2 5173  5.025481e-07
## 12286  2 5174  4.920075e-07
## 12287  2 5175  4.830818e-07
## 12288  2 5176  4.752569e-07
## 12289  2 5177  4.661709e-07
## 12290  2 5178  4.573490e-07
## 12291  2 5179  4.489694e-07
## 12292  2 5180  4.399662e-07
## 12293  2 5181  4.326416e-07
## 12294  2 5182  4.233639e-07
## 12295  2 5183  4.149631e-07
## 12296  2 5184  4.088738e-07
## 12297  2 5185  4.010380e-07
## 12298  2 5186  3.940901e-07
## 12299  2 5187  3.875524e-07
## 12300  2 5188  3.802406e-07
## 12301  2 5189  3.736538e-07
## 12302  2 5190  3.678630e-07
## 12303  2 5191  3.610377e-07
## 12304  2 5192  3.544814e-07
## 12305  2 5193  3.486946e-07
## 12306  2 5194  3.422271e-07
## 12307  2 5195  3.363759e-07
## 12308  2 5196  3.301207e-07
## 12309  2 5197  3.245038e-07
## 12310  2 5198  3.190011e-07
## 12311  2 5199  3.135112e-07
## 12312  2 5200  3.075566e-07
## 12313  2 5201  3.025074e-07
## 12314  2 5202  2.972234e-07
## 12315  2 5203  2.918934e-07
## 12316  2 5204  2.869604e-07
## 12317  2 5205  2.815261e-07
## 12318  2 5206  2.757433e-07
## 12319  2 5207  2.709151e-07
## 12320  2 5208  2.656859e-07
## 12321  2 5209  2.611979e-07
## 12322  2 5210  2.560204e-07
## 12323  2 5211  2.507554e-07
## 12324  2 5212  2.467306e-07
## 12325  2 5213  2.424606e-07
## 12326  2 5214  2.376851e-07
## 12327  2 5215  2.330122e-07
## 12328  2 5216  2.287191e-07
## 12329  2 5217  2.248283e-07
## 12330  2 5218  2.208064e-07
## 12331  2 5219  2.164891e-07
## 12332  2 5220  2.125505e-07
## 12333  2 5221  2.080444e-07
## 12334  2 5222  2.042761e-07
## 12335  2 5223  2.005145e-07
## 12336  2 5224  1.964431e-07
## 12337  2 5225  1.930059e-07
## 12338  2 5226  1.888912e-07
## 12339  2 5227  1.854115e-07
## 12340  2 5228  1.822306e-07
## 12341  2 5229  1.786193e-07
## 12342  2 5230  1.750372e-07
## 12343  2 5231  1.714006e-07
## 12344  2 5232  1.679362e-07
## 12345  2 5233  1.648997e-07
## 12346  2 5234  1.617279e-07
## 12347  2 5235  1.581865e-07
## 12348  2 5236  1.553043e-07
## 12349  2 5237  1.523188e-07
## 12350  2 5238  1.493733e-07
## 12351  2 5239  1.464348e-07
## 12352  2 5240  1.430913e-07
## 12353  2 5241  1.409229e-07
## 12354  2 5242  1.376719e-07
## 12355  2 5243  1.347176e-07
## 12356  2 5244  1.321082e-07
## 12357  2 5245  1.294530e-07
## 12358  2 5246  1.266997e-07
## 12359  2 5247  1.240624e-07
## 12360  2 5248  1.213903e-07
## 12361  2 5249  1.187848e-07
## 12362  2 5250  1.160980e-07
## 12363  2 5251  1.138670e-07
## 12364  2 5252  1.114345e-07
## 12365  2 5253  1.090402e-07
## 12366  2 5254  1.067186e-07
## 12367  2 5255  1.044828e-07
## 12368  2 5256  1.023405e-07
## 12369  2 5257  1.001598e-07
## 12370  2 5258  9.797418e-08
## 12371  2 5259  9.585753e-08
## 12372  2 5260  9.371410e-08
## 12373  2 5261  9.190777e-08
## 12374  2 5262  9.007940e-08
## 12375  2 5263  8.810157e-08
## 12376  2 5264  8.639631e-08
## 12377  2 5265  8.456788e-08
## 12378  2 5266  8.287769e-08
## 12379  2 5267  8.116087e-08
## 12380  2 5268  7.968681e-08
## 12381  2 5269  7.806252e-08
## 12382  2 5270  7.639388e-08
## 12383  2 5271  7.474628e-08
## 12384  2 5272  7.330990e-08
## 12385  2 5273  7.164146e-08
## 12386  2 5274  7.034256e-08
## 12387  2 5275  6.879471e-08
## 12388  2 5276  6.749149e-08
## 12389  2 5277  6.604987e-08
## 12390  2 5278  6.474333e-08
## 12391  2 5279  6.342993e-08
## 12392  2 5280  6.223073e-08
## 12393  2 5281  6.107246e-08
## 12394  2 5282  5.968183e-08
## 12395  2 5283  5.873487e-08
## 12396  2 5284  5.747577e-08
## 12397  2 5285  5.634557e-08
## 12398  2 5286  5.524310e-08
## 12399  2 5287  5.423553e-08
## 12400  2 5288  5.324242e-08
## 12401  2 5289  5.214387e-08
## 12402  2 5290  5.113543e-08
## 12403  2 5291  5.018282e-08
## 12404  2 5292  4.927742e-08
## 12405  2 5293  4.835054e-08
## 12406  2 5294  4.746859e-08
## 12407  2 5295  4.647278e-08
## 12408  2 5296  4.562404e-08
## 12409  2 5297  4.480089e-08
## 12410  2 5298  4.407496e-08
## 12411  2 5299  4.343964e-08
## 12412  2 5300  4.285215e-08
## 12413  2 5301  4.229693e-08
## 12414  2 5302  4.175494e-08
## 12415  2 5303  4.123717e-08
## 12416  2 5304  4.072193e-08
## 12417  2 5305  4.022652e-08
## 12418  2 5306  3.977373e-08
## 12419  2 5307  3.935968e-08
## 12420  2 5308  3.897709e-08
## 12421  2 5309  3.855401e-08
## 12422  2 5310  3.820317e-08
## 12423  2 5311  3.783872e-08
## 12424  2 5312  3.749735e-08
## 12425  2 5313  3.720004e-08
## 12426  2 5314  3.686051e-08
## 12427  2 5315  3.659046e-08
## 12428  2 5316  3.623610e-08
## 12429  2 5317  3.601071e-08
## 12430  2 5318  3.563489e-08
## 12431  2 5319  3.542702e-08
## 12432  2 5320  3.514135e-08
## 12433  2 5321  3.491693e-08
## 12434  2 5322  3.464035e-08
## 12435  2 5323  3.430233e-08
## 12436  2 5324  3.401530e-08
## 12437  2 5325  3.374843e-08
## 12438  2 5326  3.344058e-08
## 12439  2 5327  3.310507e-08
## 12440  2 5328  3.276147e-08
## 12441  2 5329  3.240010e-08
## 12442  2 5330  3.203527e-08
## 12443  2 5331  3.160457e-08
## 12444  2 5332  3.120947e-08
## 12445  2 5333  3.075193e-08
## 12446  2 5334  3.027754e-08
## 12447  2 5335  2.971688e-08
## 12448  2 5336  2.923595e-08
## 12449  2 5337  2.907024e-08
## 12450  2 5338  2.894730e-08
## 12451  2 5339  2.879496e-08
## 12452  2 5340  2.868899e-08
## 12453  2 5341  2.862469e-08
## 12454  2 5342  2.853956e-08
## 12455  2 5343  2.839012e-08
## 12456  2 5344  2.834124e-08
## 12457  2 5345  2.825066e-08
## 12458  2 5346  2.809805e-08
## 12459  2 5347  2.805230e-08
## 12460  2 5348  2.798883e-08
## 12461  2 5349  2.785871e-08
## 12462  2 5350  2.770981e-08
## 12463  2 5351  2.766014e-08
## 12464  2 5352  2.755113e-08
## 12465  2 5353  2.750058e-08
## 12466  2 5354  2.742979e-08
## 12467  2 5355  2.726436e-08
## 12468  2 5356  2.716281e-08
## 12469  2 5357  2.703384e-08
## 12470  2 5358  2.692454e-08
## 12471  2 5359  2.676409e-08
## 12472  2 5360  2.658121e-08
## 12473  2 5361  2.643279e-08
## 12474  2 5362  2.624749e-08
## 12475  2 5363  2.605978e-08
## 12476  2 5364  2.584017e-08
## 12477  2 5365  2.566124e-08
## 12478  2 5366  2.547193e-08
## 12479  2 5367  2.512058e-08
## 12480  2 5368  2.486630e-08
## 12481  2 5369  2.459133e-08
## 12482  2 5370  2.427828e-08
## 12483  2 5371  2.395920e-08
## 12484  2 5372  2.363366e-08
## 12485  2 5373  2.327753e-08
## 12486  2 5374  2.289559e-08
## 12487  2 5375  2.250719e-08
## 12488  2 5376  2.209519e-08
## 12489  2 5377  2.189532e-08
## 12490  2 5378  2.166559e-08
## 12491  2 5379  2.139447e-08
## 12492  2 5380  2.117066e-08
## 12493  2 5381  2.093527e-08
## 12494  2 5382  2.063883e-08
## 12495  2 5383  2.040368e-08
## 12496  2 5384  2.013564e-08
## 12497  2 5385  1.982439e-08
## 12498  2 5386  1.956289e-08
## 12499  2 5387  1.930211e-08
## 12500  2 5388  1.895627e-08
## 12501  2 5389  1.869240e-08
## 12502  2 5390  1.840653e-08
## 12503  2 5391  1.811331e-08
## 12504  2 5392  1.780105e-08
## 12505  2 5393  1.752503e-08
## 12506  2 5394  1.718806e-08
## 12507  2 5395  1.689885e-08
## 12508  2 5396  1.661070e-08
## 12509  2 5397  1.631457e-08
## 12510  2 5398  1.604146e-08
## 12511  2 5399  1.572573e-08
## 12512  2 5400  1.544881e-08
## 12513  2 5401  1.511362e-08
## 12514  2 5402  1.480679e-08
## 12515  2 5403  1.451649e-08
## 12516  2 5404  1.420579e-08
## 12517  2 5405  1.392445e-08
## 12518  2 5406  1.363374e-08
## 12519  2 5407  1.335992e-08
## 12520  2 5408  1.304640e-08
## 12521  2 5409  1.280514e-08
## 12522  2 5410  1.252485e-08
## 12523  2 5411  1.227795e-08
## 12524  2 5412  1.202129e-08
## 12525  2 5413  1.179119e-08
## 12526  2 5414  1.154269e-08
## 12527  2 5415  1.131934e-08
## 12528  2 5416  1.093512e-08
## 12529  2 5417  1.065706e-08
## 12530  2 5418  1.034082e-08
## 12531  2 5419  1.000890e-08
## 12532  2 5420  9.688114e-09
## 12533  2 5421  9.372597e-09
## 12534  2 5422  9.055787e-09
## 12535  2 5423  8.749401e-09
## 12536  2 5424  8.436085e-09
## 12537  2 5425  8.116623e-09
## 12538  2 5426  7.820623e-09
## 12539  2 5427  7.513430e-09
## 12540  2 5428  7.219834e-09
## 12541  2 5429  6.916090e-09
## 12542  2 5430  6.632528e-09
## 12543  2 5431  6.344010e-09
## 12544  2 5432  6.061284e-09
## 12545  2 5433  5.782294e-09
## 12546  2 5434  5.523345e-09
## 12547  2 5435  5.261037e-09
## 12548  2 5436  4.993041e-09
## 12549  2 5437  4.753321e-09
## 12550  2 5438  4.513973e-09
## 12551  2 5439  4.277853e-09
## 12552  2 5440  4.055563e-09
## 12553  2 5441  3.840934e-09
## 12554  2 5442  3.639113e-09
## 12555  2 5443  3.440502e-09
## 12556  2 5444  3.254245e-09
## 12557  2 5445  3.074745e-09
## 12558  2 5446  2.916972e-09
## 12559  2 5447  2.763160e-09
## 12560  2 5448  2.618495e-09
## 12561  2 5449  2.498798e-09
## 12562  2 5450  2.381908e-09
## 12563  2 5451  2.287837e-09
## 12564  2 5452  2.201208e-09
## 12565  2 5453  2.133873e-09
## 12566  2 5454  2.079419e-09
## 12567  2 5455  1.964406e-09
## 12568  2 5456  1.828162e-09
## 12569  2 5457  1.698405e-09
## 12570  2 5458  1.575751e-09
## 12571  2 5459  1.460144e-09
## 12572  2 5460  1.344874e-09
## 12573  2 5461  1.238580e-09
## 12574  2 5462  1.139152e-09
## 12575  2 5463  1.041249e-09
## 12576  2 5464  9.534825e-10
## 12577  2 5465  8.675810e-10
## 12578  2 5466  7.901351e-10
## 12579  2 5467  7.154283e-10
## 12580  2 5468  6.463478e-10
## 12581  2 5469  5.848767e-10
## 12582  2 5470  5.265752e-10
## 12583  2 5471  4.728890e-10
## 12584  2 5472  4.244692e-10
## 12585  2 5473  3.807521e-10
## 12586  2 5474  3.415161e-10
## 12587  2 5475  3.063364e-10
## 12588  2 5476  2.748040e-10
## 12589  2 5477  2.478047e-10
## 12590  2 5478  2.245632e-10
## 12591  2 5479  2.043028e-10
## 12592  2 5480  1.876565e-10
## 12593  2 5481  1.742342e-10
## 12594  2 5482  1.629804e-10
## 12595  2 5483  1.549342e-10
## 12596  2 5484  1.487782e-10
## 12597  2 5485  1.443290e-10
## 12598  2 5486  1.417580e-10
## 12599  2 5487  1.402491e-10
## 12600  2 5488  1.400005e-10
## 12601  2 5489  1.401799e-10
## 12602  2 5490  1.406709e-10
## 12603  2 5491  1.409367e-10
## 12604  2 5492  1.410982e-10
## 12605  2 5493  1.396937e-10
## 12606  2 5494  8.024388e-11
## 12607  2 5495  1.373669e-10
## 12608  2 5496  1.967087e-10
## 12609  2 5497  2.584912e-10
## 12610  2 5498  3.223470e-10
## 12611  2 5499  3.882334e-10
## 12612  2 5500  4.547430e-10
## 12613  2 5501  5.238881e-10
## 12614  2 5502  5.939020e-10
## 12615  2 5503  6.655255e-10
## 12616  2 5504  7.375005e-10
## 12617  2 5505  8.115727e-10
## 12618  2 5506  8.851855e-10
## 12619  2 5507  9.604453e-10
## 12620  2 5508  1.034784e-09
## 12621  2 5509  1.109267e-09
## 12622  2 5510  1.185988e-09
## 12623  2 5511  1.255304e-09
## 12624  2 5512  1.328328e-09
## 12625  2 5513  1.403422e-09
## 12626  2 5514  1.474391e-09
## 12627  2 5515  1.538864e-09
## 12628  2 5516  1.608190e-09
## 12629  2 5517  1.669259e-09
## 12630  2 5518  1.728765e-09
## 12631  2 5519  1.785748e-09
## 12632  2 5520  1.840289e-09
## 12633  2 5521  1.887497e-09
## 12634  2 5522  1.933641e-09
## 12635  2 5523  1.971296e-09
## 12636  2 5524  2.010381e-09
## 12637  2 5525  2.035181e-09
## 12638  2 5526  2.062867e-09
## 12639  2 5527  2.074988e-09
## 12640  2 5528  2.083198e-09
## 12641  2 5529  2.085311e-09
## 12642  2 5530  2.083214e-09
## 12643  2 5531  2.069202e-09
## 12644  2 5532  2.038817e-09
## 12645  2 5533  2.007676e-09
## 12646  2 5534  2.178442e-09
## 12647  2 5535  2.362632e-09
## 12648  2 5536  2.545251e-09
## 12649  2 5537  2.729382e-09
## 12650  2 5538  2.922063e-09
## 12651  2 5539  3.117124e-09
## 12652  2 5540  3.321035e-09
## 12653  2 5541  3.515568e-09
## 12654  2 5542  3.715670e-09
## 12655  2 5543  3.919091e-09
## 12656  2 5544  4.116053e-09
## 12657  2 5545  4.334155e-09
## 12658  2 5546  4.527913e-09
## 12659  2 5547  4.749438e-09
## 12660  2 5548  4.934223e-09
## 12661  2 5549  5.146405e-09
## 12662  2 5550  5.355966e-09
## 12663  2 5551  5.554809e-09
## 12664  2 5552  5.776927e-09
## 12665  2 5553  5.977266e-09
## 12666  2 5554  6.163997e-09
## 12667  2 5555  6.362337e-09
## 12668  2 5556  6.558567e-09
## 12669  2 5557  6.759445e-09
## 12670  2 5558  6.959534e-09
## 12671  2 5559  7.132310e-09
## 12672  2 5560  7.315716e-09
## 12673  2 5561  7.505582e-09
## 12674  2 5562  7.685721e-09
## 12675  2 5563  7.866854e-09
## 12676  2 5564  8.038977e-09
## 12677  2 5565  8.193812e-09
## 12678  2 5566  8.345344e-09
## 12679  2 5567  8.505619e-09
## 12680  2 5568  8.632374e-09
## 12681  2 5569  8.762577e-09
## 12682  2 5570  8.902105e-09
## 12683  2 5571  9.020636e-09
## 12684  2 5572  9.120745e-09
## 12685  2 5573  9.221981e-09
## 12686  2 5574  9.326704e-09
## 12687  2 5575  9.412311e-09
## 12688  2 5576  9.482836e-09
## 12689  2 5577  9.551310e-09
## 12690  2 5578  9.603292e-09
## 12691  2 5579  9.643862e-09
## 12692  2 5580  9.648668e-09
## 12693  2 5581  9.696475e-09
## 12694  2 5582  9.688986e-09
## 12695  2 5583  9.676789e-09
## 12696  2 5584  9.645577e-09
## 12697  2 5585  9.603977e-09
## 12698  2 5586  9.550336e-09
## 12699  2 5587  9.479535e-09
## 12700  2 5588  9.394294e-09
## 12701  2 5589  9.302546e-09
## 12702  2 5590  9.160321e-09
## 12703  2 5591  9.019733e-09
## 12704  2 5592  8.854551e-09
## 12705  2 5593  8.907805e-09
## 12706  2 5594  9.036486e-09
## 12707  2 5595  9.133149e-09
## 12708  2 5596  9.261208e-09
## 12709  2 5597  9.375255e-09
## 12710  2 5598  9.489527e-09
## 12711  2 5599  9.610813e-09
## 12712  2 5600  9.709496e-09
## 12713  2 5601  9.815551e-09
## 12714  2 5602  9.900401e-09
## 12715  2 5603  1.001171e-08
## 12716  2 5604  1.009838e-08
## 12717  2 5605  1.020018e-08
## 12718  2 5606  1.030294e-08
## 12719  2 5607  1.037459e-08
## 12720  2 5608  1.048069e-08
## 12721  2 5609  1.054173e-08
## 12722  2 5610  1.061664e-08
## 12723  2 5611  1.070632e-08
## 12724  2 5612  1.079103e-08
## 12725  2 5613  1.085989e-08
## 12726  2 5614  1.092010e-08
## 12727  2 5615  1.097888e-08
## 12728  2 5616  1.102834e-08
## 12729  2 5617  1.109268e-08
## 12730  2 5618  1.115720e-08
## 12731  2 5619  1.117413e-08
## 12732  2 5620  1.123656e-08
## 12733  2 5621  1.127009e-08
## 12734  2 5622  1.128529e-08
## 12735  2 5623  1.132885e-08
## 12736  2 5624  1.134533e-08
## 12737  2 5625  1.137409e-08
## 12738  2 5626  1.138009e-08
## 12739  2 5627  1.139097e-08
## 12740  2 5628  1.139625e-08
## 12741  2 5629  1.137256e-08
## 12742  2 5630  1.136358e-08
## 12743  2 5631  1.133556e-08
## 12744  2 5632  1.133435e-08
## 12745  2 5633  1.130420e-08
## 12746  2 5634  1.125147e-08
## 12747  2 5635  1.121733e-08
## 12748  2 5636  1.116225e-08
## 12749  2 5637  1.110869e-08
## 12750  2 5638  1.103218e-08
## 12751  2 5639  1.093816e-08
## 12752  2 5640  1.087647e-08
## 12753  2 5641  1.078890e-08
## 12754  2 5642  1.068680e-08
## 12755  2 5643  1.057928e-08
## 12756  2 5644  1.047762e-08
## 12757  2 5645  1.037079e-08
## 12758  2 5646  1.023110e-08
## 12759  2 5647  1.007723e-08
## 12760  2 5648  9.969062e-09
## 12761  2 5649  9.794823e-09
## 12762  2 5650  9.612268e-09
## 12763  2 5651  9.427166e-09
## 12764  2 5652  9.339509e-09
## 12765  2 5653  9.278972e-09
## 12766  2 5654  9.211101e-09
## 12767  2 5655  9.151638e-09
## 12768  2 5656  9.081431e-09
## 12769  2 5657  9.021534e-09
## 12770  2 5658  8.947929e-09
## 12771  2 5659  8.876773e-09
## 12772  2 5660  8.837613e-09
## 12773  2 5661  8.747969e-09
## 12774  2 5662  8.688838e-09
## 12775  2 5663  8.603124e-09
## 12776  2 5664  8.526091e-09
## 12777  2 5665  8.448508e-09
## 12778  2 5666  8.364560e-09
## 12779  2 5667  8.302734e-09
## 12780  2 5668  8.210973e-09
## 12781  2 5669  8.114958e-09
## 12782  2 5670  8.044480e-09
## 12783  2 5671  7.951639e-09
## 12784  2 5672  7.869028e-09
## 12785  2 5673  7.788945e-09
## 12786  2 5674  7.694579e-09
## 12787  2 5675  7.597158e-09
## 12788  2 5676  7.508998e-09
## 12789  2 5677  7.410087e-09
## 12790  2 5678  7.326317e-09
## 12791  2 5679  7.237171e-09
## 12792  2 5680  7.148550e-09
## 12793  2 5681  7.049774e-09
## 12794  2 5682  6.949026e-09
## 12795  2 5683  6.861147e-09
## 12796  2 5684  6.751288e-09
## 12797  2 5685  6.670097e-09
## 12798  2 5686  6.558221e-09
## 12799  2 5687  6.469544e-09
## 12800  2 5688  6.359421e-09
## 12801  2 5689  6.263575e-09
## 12802  2 5690  6.182168e-09
## 12803  2 5691  6.071663e-09
## 12804  2 5692  5.980080e-09
## 12805  2 5693  5.880180e-09
## 12806  2 5694  5.784018e-09
## 12807  2 5695  5.681783e-09
## 12808  2 5696  5.588903e-09
## 12809  2 5697  5.486625e-09
## 12810  2 5698  5.399781e-09
## 12811  2 5699  5.302846e-09
## 12812  2 5700  5.193504e-09
## 12813  2 5701  5.115227e-09
## 12814  2 5702  5.010229e-09
## 12815  2 5703  4.924363e-09
## 12816  2 5704  4.836904e-09
## 12817  2 5705  4.735772e-09
## 12818  2 5706  4.652028e-09
## 12819  2 5707  4.558569e-09
## 12820  2 5708  4.474724e-09
## 12821  2 5709  4.382624e-09
## 12822  2 5710  4.303227e-09
## 12823  2 5711  4.199991e-09
## 12824  2 5712  4.099881e-09
## 12825  2 5713  3.989676e-09
## 12826  2 5714  3.883943e-09
## 12827  2 5715  3.780993e-09
## 12828  2 5716  3.677830e-09
## 12829  2 5717  3.572412e-09
## 12830  2 5718  3.465774e-09
## 12831  2 5719  3.364142e-09
## 12832  2 5720  3.260572e-09
## 12833  2 5721  3.162150e-09
## 12834  2 5722  3.059512e-09
## 12835  2 5723  2.964094e-09
```

```
## 12836  2 5724  2.859562e-09
## 12837  2 5725  2.753399e-09
## 12838  2 5726  2.660070e-09
## 12839  2 5727  2.559356e-09
## 12840  2 5728  2.464727e-09
## 12841  2 5729  2.376306e-09
## 12842  2 5730  2.276065e-09
## 12843  2 5731  2.181887e-09
## 12844  2 5732  2.092438e-09
## 12845  2 5733  2.000030e-09
## 12846  2 5734  1.907300e-09
## 12847  2 5735  1.820233e-09
## 12848  2 5736  1.731859e-09
## 12849  2 5737  1.647878e-09
## 12850  2 5738  1.565556e-09
## 12851  2 5739  1.478848e-09
## 12852  2 5740  1.399507e-09
## 12853  2 5741  1.322416e-09
## 12854  2 5742  1.243174e-09
## 12855  2 5743  1.168934e-09
## 12856  2 5744  1.092151e-09
## 12857  2 5745  1.021385e-09
## 12858  2 5746  9.526509e-10
## 12859  2 5747  8.833679e-10
## 12860  2 5748  8.186236e-10
## 12861  2 5749  7.556457e-10
## 12862  2 5750  6.928534e-10
## 12863  2 5751  6.350398e-10
## 12864  2 5752  5.781384e-10
## 12865  2 5753  5.245777e-10
## 12866  2 5754  4.733470e-10
## 12867  2 5755  4.242900e-10
## 12868  2 5756  3.770739e-10
## 12869  2 5757  3.343398e-10
## 12870  2 5758  2.929713e-10
## 12871  2 5759  2.554223e-10
## 12872  2 5760  2.205552e-10
## 12873  2 5761  1.880491e-10
## 12874  2 5762  1.590630e-10
## 12875  2 5763  1.335821e-10
## 12876  2 5764  1.110883e-10
## 12877  2 5765  9.205892e-11
## 12878  2 5766  7.589398e-11
## 12879  2 5767  6.348735e-11
## 12880  2 5768  5.452152e-11
## 12881  2 5769  4.911581e-11
## 12882  2 5770  2.003969e-10
## 12883  2 5771  1.466349e-10
## 12884  2 5772  9.498206e-11
## 12885  2 5773  4.481667e-11
## 12886  2 5774 -3.640456e-12
## 12887  2 5775 -5.055502e-11
## 12888  2 5776 -9.603734e-11
## 12889  2 5777 -1.396677e-10
## 12890  2 5778 -1.822593e-10
## 12891  2 5779 -2.226131e-10
## 12892  2 5780 -2.617290e-10
## 12893  2 5781 -2.992268e-10
## 12894  2 5782 -3.362955e-10
## 12895  2 5783 -3.702670e-10
## 12896  2 5784 -4.043818e-10
## 12897  2 5785 -4.360762e-10
## 12898  2 5786 -4.670881e-10
## 12899  2 5787 -4.968213e-10
## 12900  2 5788 -5.245135e-10
## 12901  2 5789 -5.503037e-10
## 12902  2 5790 -5.760614e-10
## 12903  2 5791 -5.996576e-10
## 12904  2 5792 -6.218492e-10
## 12905  2 5793 -6.437701e-10
## 12906  2 5794 -6.614756e-10
## 12907  2 5795 -6.820269e-10
## 12908  2 5796 -6.989319e-10
## 12909  2 5797 -7.151976e-10
## 12910  2 5798 -7.314241e-10
## 12911  2 5799 -7.437325e-10
## 12912  2 5800 -7.580500e-10
## 12913  2 5801 -7.690355e-10
## 12914  2 5802 -7.802898e-10
## 12915  2 5803 -7.894450e-10
## 12916  2 5804 -7.984648e-10
## 12917  2 5805 -8.033207e-10
## 12918  2 5806 -8.101100e-10
## 12919  2 5807 -8.162269e-10
## 12920  2 5808 -8.185302e-10
## 12921  2 5809 -8.215990e-10
## 12922  2 5810 -8.241370e-10
## 12923  2 5811 -8.261066e-10
## 12924  2 5812 -8.266100e-10
## 12925  2 5813 -8.260298e-10
## 12926  2 5814 -8.226715e-10
## 12927  2 5815 -8.211014e-10
## 12928  2 5816 -8.165703e-10
## 12929  2 5817 -8.114659e-10
## 12930  2 5818 -8.061647e-10
## 12931  2 5819 -8.013206e-10
## 12932  2 5820 -7.947313e-10
## 12933  2 5821 -7.869494e-10
## 12934  2 5822 -7.778747e-10
## 12935  2 5823 -7.684558e-10
## 12936  2 5824 -7.591310e-10
## 12937  2 5825 -7.487409e-10
## 12938  2 5826 -7.358956e-10
## 12939  2 5827 -7.253876e-10
## 12940  2 5828 -7.120750e-10
## 12941  2 5829 -6.987116e-10
## 12942  2 5830 -6.812998e-10
## 12943  2 5831 -6.647902e-10
## 12944  2 5832 -6.481797e-10
## 12945  2 5833 -6.307431e-10
## 12946  2 5834 -6.125369e-10
## 12947  2 5835 -5.931551e-10
## 12948  2 5836 -5.741441e-10
## 12949  2 5837 -5.538326e-10
## 12950  2 5838 -5.328780e-10
## 12951  2 5839 -5.113460e-10
## 12952  2 5840 -4.905649e-10
## 12953  2 5841 -4.684265e-10
## 12954  2 5842 -4.454143e-10
## 12955  2 5843 -4.227238e-10
## 12956  2 5844 -3.982403e-10
## 12957  2 5845 -3.748266e-10
## 12958  2 5846 -3.495087e-10
## 12959  2 5847 -3.249367e-10
## 12960  2 5848 -2.992733e-10
## 12961  2 5849 -2.740415e-10
## 12962  2 5850 -2.482176e-10
## 12963  2 5851 -2.212672e-10
## 12964  2 5852 -1.953723e-10
## 12965  2 5853 -1.679108e-10
## 12966  2 5854 -1.407179e-10
## 12967  2 5855 -1.132080e-10
## 12968  2 5856 -8.524690e-11
## 12969  2 5857 -5.724034e-11
## 12970  2 5858 -2.902345e-11
## 12971  2 5859 -5.925914e-13
## 12972  2 5860  2.807301e-11
## 12973  2 5861  5.690974e-11
## 12974  2 5862  8.592439e-11
## 12975  2 5863  1.148321e-10
## 12976  2 5864  1.442912e-10
## 12977  2 5865  1.735990e-10
## 12978  2 5866  2.031468e-10
## 12979  2 5867  2.322161e-10
## 12980  2 5868  2.618860e-10
## 12981  2 5869  2.915404e-10
## 12982  2 5870  3.214548e-10
## 12983  2 5871  3.509334e-10
## 12984  2 5872  3.804217e-10
## 12985  2 5873  4.094780e-10
## 12986  2 5874  4.402775e-10
## 12987  2 5875  4.687346e-10
## 12988  2 5876  4.985557e-10
## 12989  2 5877  5.276471e-10
## 12990  2 5878  5.565996e-10
## 12991  2 5879  5.861080e-10
## 12992  2 5880  6.144475e-10
## 12993  2 5881  6.436741e-10
## 12994  2 5882  6.718148e-10
## 12995  2 5883  6.995094e-10
## 12996  2 5884  7.294701e-10
## 12997  2 5885  7.565448e-10
## 12998  2 5886  7.842692e-10
## 12999  2 5887  8.104310e-10
## 13000  2 5888  8.382304e-10
## 13001  2 5889  8.647783e-10
## 13002  2 5890  8.918856e-10
## 13003  2 5891  9.178272e-10
## 13004  2 5892  9.440230e-10
## 13005  2 5893  9.693935e-10
## 13006  2 5894  9.948536e-10
## 13007  2 5895  1.016072e-09
## 13008  2 5896  1.043792e-09
## 13009  2 5897  1.064268e-09
## 13010  2 5898  1.092360e-09
## 13011  2 5899  1.113715e-09
## 13012  2 5900  1.136145e-09
## 13013  2 5901  1.156656e-09
## 13014  2 5902  1.179456e-09
## 13015  2 5903  1.197504e-09
## 13016  2 5904  1.221942e-09
## 13017  2 5905  1.241135e-09
## 13018  2 5906  1.261302e-09
## 13019  2 5907  1.279370e-09
## 13020  2 5908  1.295927e-09
## 13021  2 5909  1.314870e-09
## 13022  2 5910  1.328851e-09
## 13023  2 5911  1.347607e-09
## 13024  2 5912  1.360828e-09
## 13025  2 5913  1.376796e-09
## 13026  2 5914  1.390317e-09
## 13027  2 5915  1.403773e-09
## 13028  2 5916  1.415662e-09
## 13029  2 5917  1.427272e-09
## 13030  2 5918  1.436862e-09
## 13031  2 5919  1.451733e-09
## 13032  2 5920  1.454805e-09
## 13033  2 5921  1.466938e-09
## 13034  2 5922  1.474065e-09
## 13035  2 5923  1.480639e-09
## 13036  2 5924  1.486601e-09
## 13037  2 5925  1.496307e-09
## 13038  2 5926  1.495381e-09
## 13039  2 5927  1.496540e-09
## 13040  2 5928  1.499748e-09
## 13041  2 5929  1.502178e-09
## 13042  2 5930  1.501709e-09
## 13043  2 5931  1.497963e-09
## 13044  2 5932  1.497298e-09
## 13045  2 5933  1.494191e-09
## 13046  2 5934  1.493449e-09
## 13047  2 5935  1.488305e-09
## 13048  2 5936  1.482358e-09
## 13049  2 5937  1.475390e-09
## 13050  2 5938  1.466457e-09
## 13051  2 5939  1.457901e-09
## 13052  2 5940  1.449697e-09
## 13053  2 5941  1.435169e-09
## 13054  2 5942  1.422303e-09
## 13055  2 5943  1.406414e-09
## 13056  2 5944  1.392579e-09
## 13057  2 5945  1.375542e-09
## 13058  2 5946  1.359679e-09
## 13059  2 5947  1.340591e-09
## 13060  2 5948  1.318742e-09
## 13061  2 5949  1.296793e-09
## 13062  2 5950  1.275808e-09
## 13063  2 5951  1.261099e-09
## 13064  2 5952  1.285512e-09
## 13065  2 5953  1.312239e-09
## 13066  2 5954  1.335955e-09
## 13067  2 5955  1.363573e-09
## 13068  2 5956  1.387513e-09
## 13069  2 5957  1.414021e-09
## 13070  2 5958  1.437884e-09
## 13071  2 5959  1.464365e-09
## 13072  2 5960  1.486318e-09
## 13073  2 5961  1.512975e-09
## 13074  2 5962  1.538493e-09
## 13075  2 5963  1.561203e-09
## 13076  2 5964  1.586545e-09
## 13077  2 5965  1.610429e-09
## 13078  2 5966  1.636129e-09
## 13079  2 5967  1.659692e-09
## 13080  2 5968  1.682853e-09
## 13081  2 5969  1.706243e-09
## 13082  2 5970  1.727940e-09
## 13083  2 5971  1.750916e-09
## 13084  2 5972  1.774664e-09
## 13085  2 5973  1.796619e-09
## 13086  2 5974  1.819423e-09
## 13087  2 5975  1.841861e-09
## 13088  2 5976  1.861811e-09
## 13089  2 5977  1.885138e-09
## 13090  2 5978  1.908102e-09
## 13091  2 5979  1.924665e-09
## 13092  2 5980  1.948193e-09
## 13093  2 5981  1.966170e-09
## 13094  2 5982  1.987186e-09
## 13095  2 5983  2.006899e-09
## 13096  2 5984  2.026221e-09
## 13097  2 5985  2.045024e-09
## 13098  2 5986  2.066196e-09
## 13099  2 5987  2.078134e-09
## 13100  2 5988  2.097273e-09
## 13101  2 5989  2.117419e-09
## 13102  2 5990  2.134968e-09
## 13103  2 5991  2.153503e-09
## 13104  2 5992  2.170010e-09
## 13105  2 5993  2.182668e-09
## 13106  2 5994  2.201926e-09
## 13107  2 5995  2.215569e-09
## 13108  2 5996  2.228916e-09
## 13109  2 5997  2.248759e-09
## 13110  2 5998  2.260017e-09
## 13111  2 5999  2.272734e-09
## 13112  2 6000  2.288045e-09
## 13113  2 6001  2.301011e-09
## 13114  2 6002  2.311118e-09
## 13115  2 6003  2.323257e-09
## 13116  2 6004  2.338038e-09
## 13117  2 6005  2.346219e-09
## 13118  2 6006  2.358384e-09
## 13119  2 6007  2.368929e-09
## 13120  2 6008  2.383886e-09
## 13121  2 6009  2.388539e-09
## 13122  2 6010  2.401925e-09
## 13123  2 6011  2.406313e-09
## 13124  2 6012  2.417329e-09
## 13125  2 6013  2.425891e-09
## 13126  2 6014  2.430596e-09
## 13127  2 6015  2.435772e-09
## 13128  2 6016  2.441569e-09
## 13129  2 6017  2.448214e-09
## 13130  2 6018  2.455919e-09
## 13131  2 6019  2.461029e-09
## 13132  2 6020  2.465579e-09
## 13133  2 6021  2.467670e-09
## 13134  2 6022  2.472132e-09
## 13135  2 6023  2.467544e-09
## 13136  2 6024  2.473273e-09
## 13137  2 6025  2.475418e-09
## 13138  2 6026  2.474863e-09
## 13139  2 6027  2.475927e-09
## 13140  2 6028  2.474637e-09
## 13141  2 6029  2.474099e-09
## 13142  2 6030  2.470289e-09
## 13143  2 6031  2.462796e-09
## 13144  2 6032  2.469354e-09
## 13145  2 6033  2.463461e-09
## 13146  2 6034  2.455967e-09
## 13147  2 6035  2.448684e-09
## 13148  2 6036  2.445291e-09
## 13149  2 6037  2.441548e-09
## 13150  2 6038  2.430044e-09
## 13151  2 6039  2.428253e-09
## 13152  2 6040  2.418271e-09
## 13153  2 6041  2.409334e-09
## 13154  2 6042  2.400254e-09
## 13155  2 6043  2.383880e-09
## 13156  2 6044  2.378814e-09
## 13157  2 6045  2.369520e-09
## 13158  2 6046  2.358984e-09
## 13159  2 6047  2.341869e-09
## 13160  2 6048  2.326864e-09
## 13161  2 6049  2.314479e-09
## 13162  2 6050  2.295205e-09
## 13163  2 6051  2.279652e-09
## 13164  2 6052  2.263365e-09
## 13165  2 6053  2.247579e-09
## 13166  2 6054  2.227968e-09
## 13167  2 6055  2.205657e-09
## 13168  2 6056  2.192527e-09
## 13169  2 6057  2.172482e-09
## 13170  2 6058  2.143891e-09
## 13171  2 6059  2.125018e-09
## 13172  2 6060  2.105181e-09
## 13173  2 6061  2.076155e-09
## 13174  2 6062  2.052599e-09
## 13175  2 6063  2.030116e-09
## 13176  2 6064  2.000371e-09
## 13177  2 6065  1.976058e-09
## 13178  2 6066  1.948139e-09
## 13179  2 6067  1.918904e-09
## 13180  2 6068  1.890960e-09
## 13181  2 6069  1.859848e-09
## 13182  2 6070  1.822522e-09
## 13183  2 6071  1.793330e-09
## 13184  2 6072  1.760991e-09
## 13185  2 6073  1.736742e-09
## 13186  2 6074  1.733435e-09
## 13187  2 6075  1.733970e-09
## 13188  2 6076  1.731361e-09
## 13189  2 6077  1.722999e-09
## 13190  2 6078  1.725995e-09
## 13191  2 6079  1.719266e-09
## 13192  2 6080  1.716751e-09
## 13193  2 6081  1.714925e-09
## 13194  2 6082  1.710138e-09
## 13195  2 6083  1.709864e-09
## 13196  2 6084  1.706922e-09
## 13197  2 6085  1.700159e-09
## 13198  2 6086  1.698611e-09
## 13199  2 6087  1.694247e-09
## 13200  2 6088  1.690368e-09
## 13201  2 6089  1.690896e-09
## 13202  2 6090  1.684105e-09
## 13203  2 6091  1.680283e-09
## 13204  2 6092  1.675460e-09
## 13205  2 6093  1.674714e-09
## 13206  2 6094  1.668249e-09
## 13207  2 6095  1.665206e-09
## 13208  2 6096  1.660713e-09
## 13209  2 6097  1.656292e-09
## 13210  2 6098  1.651385e-09
## 13211  2 6099  1.645658e-09
## 13212  2 6100  1.643745e-09
## 13213  2 6101  1.635933e-09
## 13214  2 6102  1.632812e-09
## 13215  2 6103  1.626044e-09
## 13216  2 6104  1.622114e-09
## 13217  2 6105  1.615634e-09
## 13218  2 6106  1.612242e-09
## 13219  2 6107  1.607030e-09
## 13220  2 6108  1.597679e-09
## 13221  2 6109  1.592495e-09
## 13222  2 6110  1.588758e-09
## 13223  2 6111  1.580233e-09
## 13224  2 6112  1.576086e-09
## 13225  2 6113  1.567577e-09
## 13226  2 6114  1.563674e-09
## 13227  2 6115  1.557873e-09
## 13228  2 6116  1.550576e-09
## 13229  2 6117  1.547246e-09
## 13230  2 6118  1.538239e-09
## 13231  2 6119  1.530774e-09
## 13232  2 6120  1.524079e-09
## 13233  2 6121  1.521878e-09
## 13234  2 6122  1.510221e-09
## 13235  2 6123  1.503337e-09
## 13236  2 6124  1.494520e-09
## 13237  2 6125  1.486210e-09
## 13238  2 6126  1.479265e-09
## 13239  2 6127  1.473258e-09
## 13240  2 6128  1.466080e-09
## 13241  2 6129  1.459186e-09
## 13242  2 6130  1.450721e-09
## 13243  2 6131  1.441581e-09
## 13244  2 6132  1.432840e-09
## 13245  2 6133  1.423572e-09
## 13246  2 6134  1.417885e-09
## 13247  2 6135  1.408851e-09
## 13248  2 6136  1.401270e-09
## 13249  2 6137  1.392966e-09
## 13250  2 6138  1.385987e-09
## 13251  2 6139  1.375256e-09
## 13252  2 6140  1.365961e-09
## 13253  2 6141  1.358064e-09
## 13254  2 6142  1.348269e-09
## 13255  2 6143  1.340276e-09
## 13256  2 6144  1.332986e-09
## 13257  2 6145  1.321371e-09
## 13258  2 6146  1.312839e-09
## 13259  2 6147  1.302335e-09
## 13260  2 6148  1.291982e-09
## 13261  2 6149  1.280843e-09
## 13262  2 6150  1.271167e-09
## 13263  2 6151  1.263155e-09
## 13264  2 6152  1.253297e-09
## 13265  2 6153  1.243395e-09
## 13266  2 6154  1.232828e-09
## 13267  2 6155  1.220976e-09
## 13268  2 6156  1.212281e-09
## 13269  2 6157  1.202284e-09
## 13270  2 6158  1.188886e-09
## 13271  2 6159  1.182136e-09
## 13272  2 6160  1.169331e-09
## 13273  2 6161  1.156829e-09
## 13274  2 6162  1.147082e-09
## 13275  2 6163  1.135406e-09
## 13276  2 6164  1.124440e-09
## 13277  2 6165  1.112513e-09
## 13278  2 6166  1.103774e-09
## 13279  2 6167  1.089180e-09
## 13280  2 6168  1.078952e-09
## 13281  2 6169  1.067265e-09
## 13282  2 6170  1.055171e-09
## 13283  2 6171  1.041046e-09
## 13284  2 6172  1.030267e-09
## 13285  2 6173  1.017172e-09
## 13286  2 6174  1.006403e-09
## 13287  2 6175  9.930733e-10
## 13288  2 6176  9.804494e-10
## 13289  2 6177  9.687798e-10
## 13290  2 6178  9.563861e-10
## 13291  2 6179  9.431679e-10
## 13292  2 6180  9.300720e-10
## 13293  2 6181  9.182017e-10
## 13294  2 6182  9.046231e-10
## 13295  2 6183  8.908030e-10
## 13296  2 6184  8.772422e-10
## 13297  2 6185  8.635058e-10
## 13298  2 6186  8.510260e-10
## 13299  2 6187  8.378337e-10
## 13300  2 6188  8.252544e-10
## 13301  2 6189  8.120310e-10
## 13302  2 6190  7.976499e-10
## 13303  2 6191  7.823606e-10
## 13304  2 6192  7.686401e-10
## 13305  2 6193  7.540729e-10
## 13306  2 6194  7.403593e-10
## 13307  2 6195  1.020185e-09
## 13308  2 6196  1.003071e-09
## 13309  2 6197  9.863364e-10
## 13310  2 6198  9.698107e-10
## 13311  2 6199  9.551718e-10
## 13312  2 6200  9.377459e-10
## 13313  2 6201  9.205955e-10
## 13314  2 6202  9.068261e-10
## 13315  2 6203  8.893870e-10
## 13316  2 6204  8.744300e-10
## 13317  2 6205  8.582685e-10
## 13318  2 6206  8.438458e-10
## 13319  2 6207  8.283634e-10
## 13320  2 6208  8.139555e-10
## 13321  2 6209  7.992248e-10
## 13322  2 6210  7.837752e-10
## 13323  2 6211  7.697516e-10
## 13324  2 6212  7.547019e-10
## 13325  2 6213  7.407483e-10
## 13326  2 6214  7.258697e-10
## 13327  2 6215  7.114088e-10
## 13328  2 6216  6.990627e-10
## 13329  2 6217  6.848690e-10
## 13330  2 6218  6.711827e-10
## 13331  2 6219  6.576643e-10
## 13332  2 6220  6.442497e-10
## 13333  2 6221  6.303910e-10
## 13334  2 6222  6.173432e-10
## 13335  2 6223  6.043237e-10
## 13336  2 6224  5.916469e-10
## 13337  2 6225  5.796221e-10
## 13338  2 6226  5.669291e-10
## 13339  2 6227  5.557267e-10
## 13340  2 6228  5.431997e-10
## 13341  2 6229  5.297738e-10
## 13342  2 6230  5.176368e-10
## 13343  2 6231  5.071419e-10
## 13344  2 6232  4.950252e-10
## 13345  2 6233  4.832052e-10
## 13346  2 6234  4.713765e-10
## 13347  2 6235  4.607518e-10
## 13348  2 6236  4.491958e-10
## 13349  2 6237  4.384437e-10
## 13350  2 6238  4.277208e-10
## 13351  2 6239  4.163490e-10
## 13352  2 6240  4.062586e-10
## 13353  2 6241  3.952560e-10
## 13354  2 6242  3.855303e-10
## 13355  2 6243  3.747768e-10
## 13356  2 6244  3.646261e-10
## 13357  2 6245  3.549238e-10
## 13358  2 6246  3.447470e-10
## 13359  2 6247  3.355779e-10
## 13360  2 6248  3.260766e-10
## 13361  2 6249  3.165534e-10
## 13362  2 6250  3.070834e-10
## 13363  2 6251  2.979808e-10
## 13364  2 6252  2.894396e-10
## 13365  2 6253  2.804713e-10
## 13366  2 6254  2.718819e-10
## 13367  2 6255  2.633451e-10
## 13368  2 6256  2.540834e-10
## 13369  2 6257  2.463329e-10
## 13370  2 6258  2.380709e-10
## 13371  2 6259  2.303320e-10
## 13372  2 6260  2.227561e-10
## 13373  2 6261  2.149249e-10
## 13374  2 6262  2.076082e-10
## 13375  2 6263  1.998975e-10
## 13376  2 6264  1.922905e-10
## 13377  2 6265  1.853104e-10
## 13378  2 6266  1.779411e-10
## 13379  2 6267  1.714747e-10
## 13380  2 6268  1.645859e-10
## 13381  2 6269  1.578355e-10
## 13382  2 6270  1.515190e-10
## 13383  2 6271  1.451280e-10
## 13384  2 6272  1.388108e-10
## 13385  2 6273  1.327343e-10
## 13386  2 6274  1.266267e-10
## 13387  2 6275  1.209127e-10
## 13388  2 6276  1.153497e-10
## 13389  2 6277  1.098617e-10
## 13390  2 6278  1.042952e-10
## 13391  2 6279  9.912107e-11
## 13392  2 6280  9.416985e-11
## 13393  2 6281  8.903237e-11
## 13394  2 6282  8.420981e-11
## 13395  2 6283  7.942508e-11
## 13396  2 6284  7.510390e-11
## 13397  2 6285  7.049928e-11
## 13398  2 6286  6.612055e-11
## 13399  2 6287  6.198119e-11
## 13400  2 6288  5.802637e-11
## 13401  2 6289  5.420341e-11
## 13402  2 6290  5.052506e-11
## 13403  2 6291  4.673812e-11
## 13404  2 6292  4.332661e-11
## 13405  2 6293  3.991993e-11
## 13406  2 6294  3.671008e-11
## 13407  2 6295  3.361486e-11
## 13408  2 6296  3.078574e-11
## 13409  2 6297  2.798236e-11
## 13410  2 6298  2.537045e-11
## 13411  2 6299  2.283319e-11
## 13412  2 6300  2.045375e-11
## 13413  2 6301  1.819334e-11
## 13414  2 6302  1.613803e-11
## 13415  2 6303  1.412693e-11
## 13416  2 6304  1.232921e-11
## 13417  2 6305  1.064781e-11
## 13418  2 6306  9.105047e-12
## 13419  2 6307  7.675296e-12
## 13420  2 6308  6.401043e-12
## 13421  2 6309  5.273458e-12
## 13422  2 6310  4.265475e-12
## 13423  2 6311  3.402101e-12
## 13424  2 6312  2.670749e-12
## 13425  2 6313  2.076419e-12
## 13426  2 6314  1.627383e-12
## 13427  2 6315  1.312895e-12
## 13428  2 6316  1.135192e-12
## 13429  2 6317 -4.372628e-12
## 13430  2 6318 -1.942878e-11
## 13431  2 6319 -3.440465e-11
## 13432  2 6320 -4.933812e-11
## 13433  2 6321 -6.407973e-11
## 13434  2 6322 -7.891891e-11
## 13435  2 6323 -9.361714e-11
## 13436  2 6324 -1.081375e-10
## 13437  2 6325 -1.227868e-10
## 13438  2 6326 -1.373948e-10
## 13439  2 6327 -1.518240e-10
## 13440  2 6328 -1.663495e-10
## 13441  2 6329 -1.805154e-10
## 13442  2 6330 -1.945794e-10
## 13443  2 6331 -2.092430e-10
## 13444  2 6332 -2.230891e-10
## 13445  2 6333 -2.372555e-10
## 13446  2 6334 -2.518246e-10
## 13447  2 6335 -2.652054e-10
## 13448  2 6336 -2.790536e-10
## 13449  2 6337 -2.923577e-10
## 13450  2 6338 -3.064302e-10
## 13451  2 6339 -3.204895e-10
## 13452  2 6340 -3.341786e-10
## 13453  2 6341 -3.472792e-10
## 13454  2 6342 -3.605956e-10
## 13455  2 6343 -3.741365e-10
## 13456  2 6344 -3.877306e-10
## 13457  2 6345 -4.009160e-10
## 13458  2 6346 -4.138971e-10
## 13459  2 6347 -4.272557e-10
## 13460  2 6348 -4.405268e-10
## 13461  2 6349 -4.531097e-10
## 13462  2 6350 -4.669044e-10
## 13463  2 6351 -4.790790e-10
## 13464  2 6352 -4.914554e-10
## 13465  2 6353 -5.052655e-10
## 13466  2 6354 -5.174567e-10
## 13467  2 6355 -5.300116e-10
## 13468  2 6356 -5.425952e-10
## 13469  2 6357 -5.552989e-10
## 13470  2 6358 -5.684251e-10
## 13471  2 6359 -5.803363e-10
## 13472  2 6360 -5.937226e-10
## 13473  2 6361 -6.051179e-10
## 13474  2 6362 -6.164693e-10
## 13475  2 6363 -6.289748e-10
## 13476  2 6364 -6.410094e-10
## 13477  2 6365 -6.536389e-10
## 13478  2 6366 -6.644428e-10
## 13479  2 6367 -6.770500e-10
## 13480  2 6368 -6.872134e-10
## 13481  2 6369 -6.996919e-10
## 13482  2 6370 -7.112979e-10
## 13483  2 6371 -7.227532e-10
## 13484  2 6372 -7.358687e-10
## 13485  2 6373 -7.472049e-10
## 13486  2 6374 -7.571487e-10
## 13487  2 6375 -7.694461e-10
## 13488  2 6376 -7.788181e-10
## 13489  2 6377 -7.907899e-10
## 13490  2 6378 -8.036565e-10
## 13491  2 6379 -8.130067e-10
## 13492  2 6380 -8.253347e-10
## 13493  2 6381 -8.370023e-10
## 13494  2 6382 -8.479462e-10
## 13495  2 6383 -8.577471e-10
## 13496  2 6384 -8.669134e-10
## 13497  2 6385 -8.781173e-10
## 13498  2 6386 -8.896797e-10
## 13499  2 6387 -9.000851e-10
## 13500  2 6388 -9.105593e-10
## 13501  2 6389 -9.211541e-10
## 13502  2 6390 -9.308739e-10
## 13503  2 6391 -9.412504e-10
## 13504  2 6392 -9.539564e-10
## 13505  2 6393 -9.620342e-10
## 13506  2 6394 -9.707946e-10
## 13507  2 6395 -9.812103e-10
## 13508  2 6396 -9.925204e-10
## 13509  2 6397 -1.001494e-09
## 13510  2 6398 -1.014027e-09
## 13511  2 6399 -1.023753e-09
## 13512  2 6400 -1.028856e-09
## 13513  2 6401 -1.041434e-09
## 13514  2 6402 -1.049515e-09
## 13515  2 6403 -1.060526e-09
## 13516  2 6404 -1.070962e-09
## 13517  2 6405 -1.079527e-09
## 13518  2 6406 -1.090569e-09
## 13519  2 6407 -1.097505e-09
## 13520  2 6408 -1.107305e-09
## 13521  2 6409 -1.115105e-09
## 13522  2 6410 -1.126448e-09
## 13523  2 6411 -1.136502e-09
## 13524  2 6412 -1.143518e-09
## 13525  2 6413 -1.148266e-09
## 13526  2 6414 -1.159754e-09
## 13527  2 6415 -1.170982e-09
## 13528  2 6416 -1.177846e-09
## 13529  2 6417 -1.186678e-09
## 13530  2 6418 -1.194480e-09
## 13531  2 6419 -1.203244e-09
## 13532  2 6420 -1.210669e-09
## 13533  2 6421 -1.220442e-09
## 13534  2 6422 -1.227645e-09
## 13535  2 6423 -1.237184e-09
## 13536  2 6424 -1.245017e-09
## 13537  2 6425 -1.252940e-09
## 13538  2 6426 -1.259996e-09
## 13539  2 6427 -1.271267e-09
## 13540  2 6428 -1.274769e-09
## 13541  2 6429 -1.284486e-09
## 13542  2 6430 -1.292330e-09
## 13543  2 6431 -1.299062e-09
## 13544  2 6432 -1.309954e-09
## 13545  2 6433 -1.316836e-09
## 13546  2 6434 -1.322171e-09
## 13547  2 6435 -1.330662e-09
## 13548  2 6436 -1.336408e-09
## 13549  2 6437 -1.345725e-09
## 13550  2 6438 -1.352725e-09
## 13551  2 6439 -1.358742e-09
## 13552  2 6440 -1.364903e-09
## 13553  2 6441 -1.371882e-09
## 13554  2 6442 -1.376813e-09
## 13555  2 6443 -1.386791e-09
## 13556  2 6444 -1.395852e-09
## 13557  2 6445 -1.401064e-09
## 13558  2 6446 -1.406144e-09
## 13559  2 6447 -1.412600e-09
## 13560  2 6448 -1.421912e-09
## 13561  2 6449 -1.425491e-09
## 13562  2 6450 -1.433447e-09
## 13563  2 6451 -1.439081e-09
## 13564  2 6452 -1.447307e-09
## 13565  2 6453 -1.454379e-09
## 13566  2 6454 -1.462375e-09
## 13567  2 6455 -1.467010e-09
## 13568  2 6456 -1.470680e-09
## 13569  2 6457 -1.473438e-09
## 13570  2 6458 -1.480355e-09
## 13571  2 6459 -1.486552e-09
## 13572  2 6460 -1.493369e-09
## 13573  2 6461 -1.500070e-09
## 13574  2 6462 -1.504883e-09
## 13575  2 6463 -1.511631e-09
## 13576  2 6464 -1.519498e-09
## 13577  2 6465 -1.524369e-09
## 13578  2 6466 -1.526805e-09
## 13579  2 6467 -1.532411e-09
## 13580  2 6468 -1.541510e-09
## 13581  2 6469 -1.542415e-09
## 13582  2 6470 -1.550508e-09
## 13583  2 6471 -1.553184e-09
## 13584  2 6472 -1.560093e-09
## 13585  2 6473 -1.561012e-09
## 13586  2 6474 -1.568057e-09
## 13587  2 6475 -1.575150e-09
## 13588  2 6476 -1.577239e-09
## 13589  2 6477 -1.582093e-09
## 13590  2 6478 -1.586690e-09
## 13591  2 6479 -1.592931e-09
## 13592  2 6480 -1.597234e-09
## 13593  2 6481 -1.602393e-09
## 13594  2 6482 -1.603844e-09
## 13595  2 6483 -1.607830e-09
## 13596  2 6484 -1.614786e-09
## 13597  2 6485 -1.619461e-09
## 13598  2 6486 -1.622856e-09
## 13599  2 6487 -1.624138e-09
## 13600  2 6488 -1.629539e-09
## 13601  2 6489 -1.630514e-09
## 13602  2 6490 -1.636764e-09
## 13603  2 6491 -1.640680e-09
## 13604  2 6492 -1.643626e-09
## 13605  2 6493 -1.647012e-09
## 13606  2 6494 -1.654031e-09
## 13607  2 6495 -1.657164e-09
## 13608  2 6496 -1.658657e-09
## 13609  2 6497 -1.665280e-09
## 13610  2 6498 -1.664647e-09
## 13611  2 6499 -1.672846e-09
## 13612  2 6500 -1.669806e-09
## 13613  2 6501 -1.674270e-09
## 13614  2 6502 -1.678760e-09
## 13615  2 6503 -1.681530e-09
## 13616  2 6504 -1.683445e-09
## 13617  2 6505 -1.688409e-09
## 13618  2 6506 -1.690006e-09
## 13619  2 6507 -1.691632e-09
## 13620  2 6508 -1.694433e-09
## 13621  2 6509 -1.700427e-09
## 13622  2 6510 -1.700905e-09
## 13623  2 6511 -1.704541e-09
## 13624  2 6512 -1.708862e-09
## 13625  2 6513 -1.707787e-09
## 13626  2 6514 -1.711378e-09
## 13627  2 6515 -1.711128e-09
## 13628  2 6516 -1.718046e-09
## 13629  2 6517 -1.716440e-09
## 13630  2 6518 -1.721617e-09
## 13631  2 6519 -1.720325e-09
## 13632  2 6520 -1.723365e-09
## 13633  2 6521 -1.719680e-09
## 13634  2 6522 -1.726116e-09
## 13635  2 6523 -1.725475e-09
```

```
# 13636  2 6524 -1.729819e-09
# 13637  2 6525 -1.731815e-09
# 13638  2 6526 -1.729900e-09
# 13639  2 6527 -1.734178e-09
# 13640  2 6528 -1.736430e-09
# 13641  2 6529 -1.735780e-09
# 13642  2 6530 -1.736884e-09
# 13643  2 6531 -1.738332e-09
# 13644  2 6532 -1.736100e-09
# 13645  2 6533 -1.744042e-09
# 13646  2 6534 -1.740769e-09
# 13647  2 6535 -1.742168e-09
# 13648  2 6536 -1.742566e-09
# 13649  2 6537 -1.745735e-09
# 13650  2 6538 -1.746354e-09
# 13651  2 6539 -1.746749e-09
# 13652  2 6540 -1.746345e-09
# 13653  2 6541 -1.749340e-09
# 13654  2 6542 -1.748918e-09
# 13655  2 6543 -1.745415e-09
# 13656  2 6544 -1.751544e-09
# 13657  2 6545 -1.749478e-09
# 13658  2 6546 -1.750895e-09
# 13659  2 6547 -1.751869e-09
# 13660  2 6548 -1.749509e-09
# 13661  2 6549 -1.748972e-09
# 13662  2 6550 -1.749763e-09
# 13663  2 6551 -1.750804e-09
# 13664  2 6552 -1.752960e-09
# 13665  2 6553 -1.749181e-09
# 13666  2 6554 -1.747866e-09
# 13667  2 6555 -1.749936e-09
# 13668  2 6556 -1.745749e-09
# 13669  2 6557 -1.749057e-09
# 13670  2 6558 -1.747508e-09
# 13671  2 6559 -1.745076e-09
# 13672  2 6560 -1.742873e-09
# 13673  2 6561 -1.738546e-09
# 13674  2 6562 -1.742519e-09
# 13675  2 6563 -1.738564e-09
# 13676  2 6564 -1.741510e-09
# 13677  2 6565 -1.737662e-09
# 13678  2 6566 -1.740021e-09
# 13679  2 6567 -1.738067e-09
# 13680  2 6568 -1.735821e-09
# 13681  2 6569 -1.733976e-09
# 13682  2 6570 -1.733096e-09
# 13683  2 6571 -1.732931e-09
# 13684  2 6572 -1.730420e-09
# 13685  2 6573 -1.730002e-09
# 13686  2 6574 -1.726778e-09
# 13687  2 6575 -1.726254e-09
# 13688  2 6576 -1.721125e-09
# 13689  2 6577 -1.722596e-09
# 13690  2 6578 -1.719364e-09
# 13691  2 6579 -1.716531e-09
# 13692  2 6580 -1.717395e-09
# 13693  2 6581 -1.711629e-09
# 13694  2 6582 -1.710250e-09
# 13695  2 6583 -1.710593e-09
# 13696  2 6584 -1.706145e-09
# 13697  2 6585 -1.701429e-09
# 13698  2 6586 -1.702792e-09
# 13699  2 6587 -1.699040e-09
# 13700  2 6588 -1.699358e-09
# 13701  2 6589 -1.697163e-09
# 13702  2 6590 -1.687495e-09
# 13703  2 6591 -1.691474e-09
# 13704  2 6592 -1.686719e-09
# 13705  2 6593 -1.684113e-09
# 13706  2 6594 -1.680299e-09
# 13707  2 6595 -1.677117e-09
# 13708  2 6596 -1.674646e-09
# 13709  2 6597 -1.669269e-09
# 13710  2 6598 -1.670632e-09
# 13711  2 6599 -1.664701e-09
# 13712  2 6600 -1.664623e-09
# 13713  2 6601 -1.655541e-09
# 13714  2 6602 -1.656263e-09
# 13715  2 6603 -1.652155e-09
# 13716  2 6604 -1.647841e-09
# 13717  2 6605 -1.643994e-09
# 13718  2 6606 -1.641305e-09
# 13719  2 6607 -1.634782e-09
# 13720  2 6608 -1.634048e-09
# 13721  2 6609 -1.627718e-09
# 13722  2 6610 -1.626476e-09
# 13723  2 6611 -1.621749e-09
# 13724  2 6612 -1.615720e-09
# 13725  2 6613 -1.615841e-09
# 13726  2 6614 -1.607998e-09
# 13727  2 6615 -1.604019e-09
# 13728  2 6616 -1.602160e-09
# 13729  2 6617 -1.598348e-09
# 13730  2 6618 -1.590842e-09
# 13731  2 6619 -1.586270e-09
# 13732  2 6620 -1.579234e-09
# 13733  2 6621 -1.575847e-09
# 13734  2 6622 -1.572101e-09
# 13735  2 6623 -1.568336e-09
# 13736  2 6624 -1.565914e-09
# 13737  2 6625 -1.557273e-09
# 13738  2 6626 -1.551716e-09
# 13739  2 6627 -1.549693e-09
# 13740  2 6628 -1.543425e-09
# 13741  2 6629 -1.538855e-09
# 13742  2 6630 -1.529800e-09
# 13743  2 6631 -1.528110e-09
# 13744  2 6632 -1.522735e-09
# 13745  2 6633 -1.516306e-09
# 13746  2 6634 -1.506453e-09
# 13747  2 6635 -1.502695e-09
# 13748  2 6636 -1.497157e-09
# 13749  2 6637 -1.493081e-09
# 13750  2 6638 -1.488545e-09
# 13751  2 6639 -1.483419e-09
# 13752  2 6640 -1.475800e-09
# 13753  2 6641 -1.467433e-09
# 13754  2 6642 -1.462185e-09
# 13755  2 6643 -1.457973e-09
# 13756  2 6644 -1.451384e-09
# 13757  2 6645 -1.444326e-09
# 13758  2 6646 -1.438028e-09
# 13759  2 6647 -1.430661e-09
# 13760  2 6648 -1.427105e-09
# 13761  2 6649 -1.417935e-09
# 13762  2 6650 -1.413985e-09
# 13763  2 6651 -1.403400e-09
# 13764  2 6652 -1.398293e-09
# 13765  2 6653 -1.393364e-09
# 13766  2 6654 -1.386046e-09
# 13767  2 6655 -1.376800e-09
# 13768  2 6656 -1.372502e-09
# 13769  2 6657 -1.363903e-09
# 13770  2 6658 -1.356022e-09
# 13771  2 6659 -1.350268e-09
# 13772  2 6660 -1.346979e-09
# 13773  2 6661 -1.336371e-09
# 13774  2 6662 -1.327965e-09
# 13775  2 6663 -1.320005e-09
# 13776  2 6664 -1.312963e-09
# 13777  2 6665 -1.306052e-09
# 13778  2 6666 -1.299153e-09
# 13779  2 6667 -1.288482e-09
# 13780  2 6668 -1.280018e-09
# 13781  2 6669 -1.275328e-09
# 13782  2 6670 -1.266265e-09
# 13783  2 6671 -1.258902e-09
# 13784  2 6672 -1.250898e-09
# 13785  2 6673 -1.243821e-09
# 13786  2 6674 -1.235639e-09
# 13787  2 6675 -1.225915e-09
# 13788  2 6676 -1.216746e-09
# 13789  2 6677 -1.209855e-09
# 13790  2 6678 -1.198676e-09
# 13791  2 6679 -1.191852e-09
# 13792  2 6680 -1.183913e-09
# 13793  2 6681 -1.175065e-09
# 13794  2 6682 -1.167104e-09
# 13795  2 6683 -1.158046e-09
# 13796  2 6684 -1.150764e-09
# 13797  2 6685 -1.143642e-09
# 13798  2 6686 -1.132463e-09
# 13799  2 6687 -1.122458e-09
# 13800  2 6688 -1.112428e-09
# 13801  2 6689 -1.106653e-09
# 13802  2 6690 -1.094564e-09
# 13803  2 6691 -1.085734e-09
# 13804  2 6692 -1.079343e-09
# 13805  2 6693 -1.069787e-09
# 13806  2 6694 -1.058161e-09
# 13807  2 6695 -1.048206e-09
# 13808  2 6696 -1.038455e-09
# 13809  2 6697 -1.029816e-09
# 13810  2 6698 -1.019462e-09
# 13811  2 6699 -1.009729e-09
# 13812  2 6700 -1.001313e-09
# 13813  2 6701 -9.932774e-10
# 13814  2 6702 -9.802690e-10
# 13815  2 6703 -9.709156e-10
# 13816  2 6704 -9.593296e-10
# 13817  2 6705 -9.508339e-10
# 13818  2 6706 -9.403704e-10
# 13819  2 6707 -9.283717e-10
# 13820  2 6708 -9.180448e-10
# 13821  2 6709 -9.091196e-10
# 13822  2 6710 -8.975622e-10
# 13823  2 6711 -8.885685e-10
# 13824  2 6712 -8.749321e-10
# 13825  2 6713 -8.660806e-10
# 13826  2 6714 -8.540397e-10
# 13827  2 6715 -8.441709e-10
# 13828  2 6716 -8.338162e-10
# 13829  2 6717 -8.226720e-10
# 13830  2 6718 -8.115512e-10
# 13831  2 6719 -8.013855e-10
# 13832  2 6720 -7.896925e-10
# 13833  2 6721 -7.787552e-10
# 13834  2 6722 -7.684291e-10
# 13835  2 6723 -7.557647e-10
# 13836  2 6724 -7.447394e-10
# 13837  2 6725 -7.323002e-10
# 13838  2 6726 -7.204148e-10
# 13839  2 6727 -7.102454e-10
# 13840  2 6728 -6.979794e-10
# 13841  2 6729 -6.866866e-10
# 13842  2 6730 -6.751908e-10
# 13843  2 6731 -6.625666e-10
# 13844  2 6732 -6.517877e-10
# 13845  2 6733 -6.396355e-10
# 13846  2 6734 -6.276850e-10
# 13847  2 6735 -6.163025e-10
# 13848  2 6736 -6.167540e-10
# 13849  2 6737 -6.163993e-10
# 13850  2 6738 -6.161440e-10
# 13851  2 6739 -6.158478e-10
# 13852  2 6740 -6.172258e-10
# 13853  2 6741 -6.167149e-10
# 13854  2 6742 -6.181198e-10
# 13855  2 6743 -6.176111e-10
# 13856  2 6744 -6.169036e-10
# 13857  2 6745 -6.186689e-10
# 13858  2 6746 -6.191557e-10
# 13859  2 6747 -6.182401e-10
# 13860  2 6748 -6.183809e-10
# 13861  2 6749 -6.174746e-10
# 13862  2 6750 -6.183364e-10
# 13863  2 6751 -6.188429e-10
# 13864  2 6752 -6.184286e-10
# 13865  2 6753 -6.189699e-10
# 13866  2 6754 -6.178431e-10
# 13867  2 6755 -6.185829e-10
# 13868  2 6756 -6.188857e-10
# 13869  2 6757 -6.185184e-10
# 13870  2 6758 -6.187895e-10
# 13871  2 6759 -6.188084e-10
# 13872  2 6760 -6.174257e-10
# 13873  2 6761 -6.186111e-10
# 13874  2 6762 -6.187569e-10
# 13875  2 6763 -6.176293e-10
# 13876  2 6764 -6.175474e-10
# 13877  2 6765 -6.177174e-10
# 13878  2 6766 -6.179563e-10
# 13879  2 6767 -6.180233e-10
# 13880  2 6768 -6.176891e-10
# 13881  2 6769 -6.182492e-10
# 13882  2 6770 -6.179920e-10
# 13883  2 6771 -6.164831e-10
# 13884  2 6772 -6.167232e-10
# 13885  2 6773 -6.174786e-10
# 13886  2 6774 -6.170477e-10
# 13887  2 6775 -6.178742e-10
# 13888  2 6776 -6.172200e-10
# 13889  2 6777 -6.176545e-10
# 13890  2 6778 -6.169064e-10
# 13891  2 6779 -6.175186e-10
# 13892  2 6780 -6.165562e-10
# 13893  2 6781 -6.166109e-10
# 13894  2 6782 -6.167733e-10
# 13895  2 6783 -6.161423e-10
# 13896  2 6784 -6.159166e-10
# 13897  2 6785 -6.154409e-10
# 13898  2 6786 -6.166257e-10
# 13899  2 6787 -6.149197e-10
# 13900  2 6788 -6.155813e-10
# 13901  2 6789 -6.157423e-10
# 13902  2 6790 -6.144683e-10
# 13903  2 6791 -6.153791e-10
# 13904  2 6792 -6.155981e-10
# 13905  2 6793 -6.147548e-10
# 13906  2 6794 -6.134448e-10
# 13907  2 6795 -6.127555e-10
# 13908  2 6796 -6.146725e-10
# 13909  2 6797 -6.128684e-10
# 13910  2 6798 -6.133758e-10
# 13911  2 6799 -6.133731e-10
# 13912  2 6800 -6.129747e-10
# 13913  2 6801 -6.132350e-10
# 13914  2 6802 -6.123427e-10
# 13915  2 6803 -6.134240e-10
# 13916  2 6804 -6.118939e-10
# 13917  2 6805 -6.113386e-10
# 13918  2 6806 -6.112781e-10
# 13919  2 6807 -6.103965e-10
# 13920  2 6808 -6.116097e-10
# 13921  2 6809 -6.101736e-10
# 13922  2 6810 -6.108930e-10
# 13923  2 6811 -6.097153e-10
# 13924  2 6812 -6.084388e-10
# 13925  2 6813 -6.088660e-10
# 13926  2 6814 -6.093775e-10
# 13927  2 6815 -6.085936e-10
# 13928  2 6816 -6.081566e-10
# 13929  2 6817 -6.073515e-10
# 13930  2 6818 -6.064095e-10
# 13931  2 6819 -6.066797e-10
# 13932  2 6820 -6.052092e-10
# 13933  2 6821 -6.052737e-10
# 13934  2 6822 -6.055040e-10
# 13935  2 6823 -6.061630e-10
# 13936  2 6824 -6.045573e-10
# 13937  2 6825 -6.046678e-10
# 13938  2 6826 -6.039834e-10
# 13939  2 6827 -6.036488e-10
# 13940  2 6828 -6.022050e-10
# 13941  2 6829 -6.028012e-10
# 13942  2 6830 -6.019345e-10
# 13943  2 6831 -6.018809e-10
# 13944  2 6832 -6.011284e-10
# 13945  2 6833 -6.005994e-10
# 13946  2 6834 -6.003029e-10
# 13947  2 6835 -6.008619e-10
# 13948  2 6836 -6.005317e-10
# 13949  2 6837 -5.979099e-10
# 13950  2 6838 -5.984747e-10
# 13951  2 6839 -5.984995e-10
# 13952  2 6840 -5.982331e-10
# 13953  2 6841 -5.978414e-10
# 13954  2 6842 -5.964990e-10
# 13955  2 6843 -5.952948e-10
# 13956  2 6844 -5.951759e-10
# 13957  2 6845 -5.942236e-10
# 13958  2 6846 -5.943776e-10
# 13959  2 6847 -5.938942e-10
# 13960  2 6848 -5.929748e-10
# 13961  2 6849 -5.928975e-10
# 13962  2 6850 -5.926464e-10
# 13963  2 6851 -5.928196e-10
# 13964  2 6852 -5.912194e-10
# 13965  2 6853 -5.902689e-10
# 13966  2 6854 -5.902578e-10
# 13967  2 6855 -5.891314e-10
# 13968  2 6856 -5.887830e-10
# 13969  2 6857 -5.878991e-10
# 13970  2 6858 -5.866917e-10
# 13971  2 6859 -5.864115e-10
# 13972  2 6860 -5.863797e-10
# 13973  2 6861 -5.849197e-10
# 13974  2 6862 -5.848903e-10
# 13975  2 6863 -5.845260e-10
# 13976  2 6864 -5.831913e-10
# 13977  2 6865 -5.833945e-10
# 13978  2 6866 -5.816660e-10
# 13979  2 6867 -5.798242e-10
# 13980  2 6868 -5.805492e-10
# 13981  2 6869 -5.796610e-10
# 13982  2 6870 -5.794986e-10
# 13983  2 6871 -5.780613e-10
# 13984  2 6872 -5.787420e-10
# 13985  2 6873 -5.766544e-10
# 13986  2 6874 -5.769652e-10
# 13987  2 6875 -5.767531e-10
# 13988  2 6876 -5.757013e-10
# 13989  2 6877 -5.739805e-10
# 13990  2 6878 -5.733730e-10
# 13991  2 6879 -5.725266e-10
# 13992  2 6880 -5.729279e-10
# 13993  2 6881 -5.708162e-10
# 13994  2 6882 -5.712915e-10
# 13995  2 6883 -5.700209e-10
# 13996  2 6884 -5.688241e-10
# 13997  2 6885 -5.677252e-10
# 13998  2 6886 -5.672791e-10
# 13999  2 6887 -5.665707e-10
# 14000  2 6888 -5.657638e-10
# 14001  2 6889 -5.649643e-10
# 14002  2 6890 -5.636014e-10
# 14003  2 6891 -5.628454e-10
# 14004  2 6892 -5.613658e-10
# 14005  2 6893 -5.606964e-10
# 14006  2 6894 -5.607043e-10
# 14007  2 6895 -5.595287e-10
# 14008  2 6896 -5.578842e-10
# 14009  2 6897 -5.584917e-10
# 14010  2 6898 -5.575320e-10
# 14011  2 6899 -5.562629e-10
# 14012  2 6900 -5.565052e-10
# 14013  2 6901 -5.540881e-10
# 14014  2 6902 -5.543494e-10
# 14015  2 6903 -5.522596e-10
# 14016  2 6904 -5.524140e-10
# 14017  2 6905 -5.505365e-10
# 14018  2 6906 -5.495153e-10
# 14019  2 6907 -5.488226e-10
# 14020  2 6908 -5.482613e-10
# 14021  2 6909 -5.479331e-10
# 14022  2 6910 -5.467603e-10
# 14023  2 6911 -5.449414e-10
# 14024  2 6912 -5.448007e-10
# 14025  2 6913 -5.434009e-10
# 14026  2 6914 -5.414775e-10
# 14027  2 6915 -5.417712e-10
# 14028  2 6916 -5.404935e-10
# 14029  2 6917 -5.385556e-10
# 14030  2 6918 -5.385200e-10
# 14031  2 6919 -5.377528e-10
# 14032  2 6920 -5.375215e-10
# 14033  2 6921 -5.351984e-10
# 14034  2 6922 -5.333915e-10
# 14035  2 6923 -5.331786e-10
# 14036  2 6924 -5.320634e-10
# 14037  2 6925 -5.313302e-10
# 14038  2 6926 -5.289279e-10
# 14039  2 6927 -5.292152e-10
# 14040  2 6928 -5.278310e-10
# 14041  2 6929 -5.266298e-10
# 14042  2 6930 -5.258091e-10
# 14043  2 6931 -5.242139e-10
# 14044  2 6932 -5.231106e-10
# 14045  2 6933 -5.219740e-10
# 14046  2 6934 -5.214205e-10
# 14047  2 6935 -5.204828e-10
# 14048  2 6936 -5.188353e-10
# 14049  2 6937 -5.184412e-10
# 14050  2 6938 -5.174502e-10
# 14051  2 6939 -5.153412e-10
# 14052  2 6940 -5.148162e-10
# 14053  2 6941 -5.130153e-10
# 14054  2 6942 -5.126234e-10
# 14055  2 6943 -5.111283e-10
# 14056  2 6944 -5.099148e-10
# 14057  2 6945 -5.083676e-10
# 14058  2 6946 -5.080771e-10
# 14059  2 6947 -5.060928e-10
# 14060  2 6948 -5.046353e-10
# 14061  2 6949 -5.041152e-10
# 14062  2 6950 -5.028444e-10
# 14063  2 6951 -5.008478e-10
# 14064  2 6952 -5.008052e-10
# 14065  2 6953 -4.992344e-10
# 14066  2 6954 -4.974135e-10
# 14067  2 6955 -4.966609e-10
# 14068  2 6956 -4.952535e-10
# 14069  2 6957 -4.948898e-10
# 14070  2 6958 -4.925660e-10
# 14071  2 6959 -4.908588e-10
# 14072  2 6960 -4.902675e-10
# 14073  2 6961 -4.895936e-10
# 14074  2 6962 -4.870793e-10
# 14075  2 6963 -4.858096e-10
# 14076  2 6964 -4.851399e-10
# 14077  2 6965 -4.832921e-10
# 14078  2 6966 -4.820947e-10
# 14079  2 6967 -4.816198e-10
# 14080  2 6968 -4.804672e-10
# 14081  2 6969 -4.789676e-10
# 14082  2 6970 -4.770309e-10
# 14083  2 6971 -4.760911e-10
# 14084  2 6972 -4.737338e-10
# 14085  2 6973 -4.732911e-10
# 14086  2 6974 -4.727297e-10
# 14087  2 6975 -4.712286e-10
# 14088  2 6976 -4.694835e-10
# 14089  2 6977 -4.685172e-10
# 14090  2 6978 -4.662544e-10
# 14091  2 6979 -4.649805e-10
# 14092  2 6980 -4.636153e-10
# 14093  2 6981 -4.630592e-10
# 14094  2 6982 -4.603558e-10
# 14095  2 6983 -4.596332e-10
# 14096  2 6984 -4.577158e-10
# 14097  2 6985 -4.565879e-10
# 14098  2 6986 -4.549206e-10
# 14099  2 6987 -4.526004e-10
# 14100  2 6988 -4.522347e-10
# 14101  2 6989 -4.505718e-10
# 14102  2 6990 -4.488924e-10
# 14103  2 6991 -4.479287e-10
# 14104  2 6992 -4.464050e-10
# 14105  2 6993 -4.439604e-10
# 14106  2 6994 -4.438893e-10
# 14107  2 6995 -4.413887e-10
# 14108  2 6996 -4.403431e-10
# 14109  2 6997 -4.390499e-10
# 14110  2 6998 -4.379113e-10
# 14111  2 6999 -4.352656e-10
# 14112  2 7000 -4.337048e-10
# 14113  3    0  0.000000e+00
# 14114  3    0  2.790807e+00
# 14115  3    1  6.986381e+00
# 14116  3    2  5.707124e+00
# 14117  3    3  4.867668e+00
# 14118  3    4  4.323843e+00
# 14119  3    5  4.003883e+00
# 14120  3    6  3.773474e+00
# 14121  3    7  3.615448e+00
# 14122  3    8  3.516618e+00
# 14123  3    9  3.434901e+00
# 14124  3   10  3.374194e+00
# 14125  3   11  3.315128e+00
# 14126  3   12  3.263946e+00
# 14127  3   13  3.216696e+00
# 14128  3   14  3.172819e+00
# 14129  3   15  3.135480e+00
# 14130  3   16  3.088155e+00
# 14131  3   17  3.053169e+00
# 14132  3   18  3.010821e+00
# 14133  3   19  2.977153e+00
# 14134  3   20  2.937879e+00
# 14135  3   21  2.900564e+00
# 14136  3   22  2.861596e+00
# 14137  3   23  2.831247e+00
# 14138  3   24  2.793512e+00
# 14139  3   24  2.793406e+00
# 14140  3   25  6.986900e+00
# 14141  3   26  5.719558e+00
# 14142  3   27  4.867996e+00
# 14143  3   28  4.337187e+00
# 14144  3   29  3.988515e+00
# 14145  3   30  3.775380e+00
# 14146  3   31  3.624535e+00
# 14147  3   32  3.520150e+00
# 14148  3   33  3.434337e+00
# 14149  3   34  3.373428e+00
# 14150  3   35  3.316151e+00
# 14151  3   36  3.273901e+00
# 14152  3   37  3.219969e+00
# 14153  3   38  3.175171e+00
# 14154  3   39  3.135404e+00
# 14155  3   40  3.088686e+00
# 14156  3   41  3.053786e+00
# 14157  3   42  3.009188e+00
# 14158  3   43  2.971232e+00
# 14159  3   44  2.935368e+00
# 14160  3   45  2.904061e+00
# 14161  3   46  2.869723e+00
# 14162  3   47  2.830419e+00
# 14163  3   48  2.792059e+00
# 14164  3   48  2.791528e+00
# 14165  3   49  6.991299e+00
# 14166  3   50  5.711554e+00
# 14167  3   51  4.863978e+00
# 14168  3   52  4.336231e+00
# 14169  3   53  3.998215e+00
# 14170  3   54  3.768396e+00
# 14171  3   55  3.619291e+00
# 14172  3   56  3.515316e+00
# 14173  3   57  3.434105e+00
# 14174  3   58  3.368009e+00
# 14175  3   59  3.318498e+00
# 14176  3   60  3.270783e+00
# 14177  3   61  3.221342e+00
# 14178  3   62  3.172971e+00
# 14179  3   63  3.132344e+00
# 14180  3   64  3.092453e+00
# 14181  3   65  3.058455e+00
# 14182  3   66  3.010793e+00
# 14183  3   67  2.974584e+00
# 14184  3   68  2.935427e+00
# 14185  3   69  2.905791e+00
# 14186  3   70  2.867962e+00
# 14187  3   71  2.825973e+00
# 14188  3   72  2.791658e+00
# 14189  3   73  6.990972e+00
# 14190  3   74  5.718401e+00
# 14191  3   75  4.867580e+00
# 14192  3   76  4.335747e+00
# 14193  3   77  3.998242e+00
# 14194  3   78  3.780258e+00
# 14195  3   79  3.624584e+00
# 14196  3   80  3.513312e+00
# 14197  3   81  3.433305e+00
# 14198  3   82  3.365917e+00
# 14199  3   83  3.311431e+00
# 14200  3   84  3.266190e+00
# 14201  3   85  3.217184e+00
# 14202  3   86  3.173193e+00
# 14203  3   87  3.132175e+00
# 14204  3   88  3.094000e+00
# 14205  3   89  3.051606e+00
# 14206  3   90  3.014580e+00
# 14207  3   91  2.974728e+00
# 14208  3   92  2.937967e+00
# 14209  3   93  2.906588e+00
# 14210  3   94  2.869536e+00
# 14211  3   95  2.826560e+00
# 14212  3   96  2.788458e+00
# 14213  3   97  6.988696e+00
# 14214  3   98  5.715468e+00
# 14215  3   99  4.867918e+00
# 14216  3  100  4.334610e+00
# 14217  3  101  3.994277e+00
# 14218  3  102  3.776180e+00
# 14219  3  103  3.627002e+00
# 14220  3  104  3.519276e+00
# 14221  3  105  3.440693e+00
# 14222  3  106  3.370391e+00
# 14223  3  107  3.314764e+00
# 14224  3  108  3.269917e+00
# 14225  3  109  3.214700e+00
# 14226  3  110  3.182547e+00
# 14227  3  111  3.131803e+00
# 14228  3  112  3.089949e+00
# 14229  3  113  3.052877e+00
# 14230  3  114  3.020481e+00
# 14231  3  115  2.975355e+00
# 14232  3  116  2.934628e+00
# 14233  3  117  2.899021e+00
# 14234  3  118  2.864574e+00
# 14235  3  119  2.827156e+00
# 14236  3  120  2.788923e+00
# 14237  3  121  6.986716e+00
# 14238  3  122  5.707861e+00
# 14239  3  123  4.860464e+00
# 14240  3  124  4.332170e+00
# 14241  3  125  3.995385e+00
# 14242  3  126  3.776011e+00
# 14243  3  127  3.631880e+00
# 14244  3  128  3.515141e+00
# 14245  3  129  3.436347e+00
# 14246  3  130  3.364104e+00
# 14247  3  131  3.316331e+00
# 14248  3  132  3.264951e+00
# 14249  3  133  3.222037e+00
# 14250  3  134  3.179649e+00
# 14251  3  135  3.135948e+00
# 14252  3  136  3.093816e+00
# 14253  3  137  3.056658e+00
# 14254  3  138  3.010102e+00
# 14255  3  139  2.975222e+00
# 14256  3  140  2.936395e+00
# 14257  3  141  2.902901e+00
# 14258  3  142  2.860505e+00
# 14259  3  143  2.825591e+00
# 14260  3  144  2.789984e+00
# 14261  3  145  6.986251e+00
# 14262  3  146  5.717032e+00
# 14263  3  147  4.870241e+00
# 14264  3  148  4.335422e+00
# 14265  3  149  3.996750e+00
# 14266  3  150  3.769067e+00
# 14267  3  151  3.627416e+00
# 14268  3  152  3.516720e+00
# 14269  3  153  3.443426e+00
# 14270  3  154  3.368975e+00
# 14271  3  155  3.316242e+00
# 14272  3  156  3.263055e+00
# 14273  3  157  3.222797e+00
# 14274  3  158  3.177606e+00
# 14275  3  159  3.135019e+00
# 14276  3  160  3.092397e+00
# 14277  3  161  3.049604e+00
# 14278  3  162  3.013442e+00
# 14279  3  163  2.977410e+00
# 14280  3  164  2.937279e+00
# 14281  3  165  2.898085e+00
# 14282  3  166  2.862441e+00
# 14283  3  167  2.823563e+00
# 14284  3  168  2.796408e+00
# 14285  3  169  6.988023e+00
# 14286  3  170  5.707747e+00
# 14287  3  171  4.859328e+00
# 14288  3  172  4.328619e+00
# 14289  3  173  3.990225e+00
# 14290  3  174  3.772953e+00
# 14291  3  175  3.624551e+00
# 14292  3  176  3.517774e+00
# 14293  3  177  3.437639e+00
# 14294  3  178  3.366334e+00
# 14295  3  179  3.318710e+00
# 14296  3  180  3.266670e+00
# 14297  3  181  3.218635e+00
# 14298  3  182  3.172524e+00
# 14299  3  183  3.137384e+00
# 14300  3  184  3.089422e+00
# 14301  3  185  3.051675e+00
# 14302  3  186  3.012376e+00
# 14303  3  187  2.981898e+00
# 14304  3  188  2.938828e+00
# 14305  3  189  2.897795e+00
# 14306  3  190  2.866456e+00
# 14307  3  191  2.825972e+00
# 14308  3  192  2.787356e+00
# 14309  3  193  6.973066e+00
# 14310  3  194  5.714652e+00
# 14311  3  195  4.860679e+00
# 14312  3  196  4.336147e+00
# 14313  3  197  3.995967e+00
# 14314  3  198  3.770562e+00
# 14315  3  199  3.630705e+00
# 14316  3  200  3.516228e+00
# 14317  3  201  3.431684e+00
# 14318  3  202  3.367493e+00
# 14319  3  203  3.311750e+00
# 14320  3  204  3.272953e+00
# 14321  3  205  3.214841e+00
# 14322  3  206  3.171134e+00
# 14323  3  207  3.135041e+00
# 14324  3  208  3.092932e+00
# 14325  3  209  3.048359e+00
# 14326  3  210  3.015819e+00
# 14327  3  211  2.975986e+00
# 14328  3  212  2.938369e+00
# 14329  3  213  2.898071e+00
# 14330  3  214  2.860536e+00
# 14331  3  215  2.828842e+00
# 14332  3  216  2.788666e+00
# 14333  3  217  6.975921e+00
# 14334  3  218  5.720159e+00
# 14335  3  219  4.861101e+00
# 14336  3  220  4.332784e+00
# 14337  3  221  3.999146e+00
# 14338  3  222  3.770244e+00
# 14339  3  223  3.628674e+00
# 14340  3  224  3.520189e+00
# 14341  3  225  3.432440e+00
# 14342  3  226  3.372329e+00
# 14343  3  227  3.308466e+00
# 14344  3  228  3.270057e+00
# 14345  3  229  3.219369e+00
# 14346  3  230  3.173079e+00
# 14347  3  231  3.138353e+00
# 14348  3  232  3.090493e+00
# 14349  3  233  3.052476e+00
# 14350  3  234  3.013857e+00
# 14351  3  235  2.977154e+00
# 14352  3  236  2.939999e+00
# 14353  3  237  2.903472e+00
# 14354  3  238  2.864749e+00
# 14355  3  239  2.825931e+00
# 14356  3  240  2.795474e+00
# 14357  3  241  6.978920e+00
# 14358  3  242  5.714020e+00
# 14359  3  243  4.860779e+00
# 14360  3  244  4.328338e+00
# 14361  3  245  3.997441e+00
# 14362  3  246  3.773462e+00
# 14363  3  247  3.619174e+00
# 14364  3  248  3.521685e+00
# 14365  3  249  3.441761e+00
# 14366  3  250  3.362805e+00
# 14367  3  251  3.316486e+00
# 14368  3  252  3.266059e+00
# 14369  3  253  3.215239e+00
# 14370  3  254  3.175621e+00
# 14371  3  255  3.127275e+00
# 14372  3  256  3.092550e+00
# 14373  3  257  3.055149e+00
# 14374  3  258  3.017498e+00
# 14375  3  259  2.969434e+00
# 14376  3  260  2.935526e+00
# 14377  3  261  2.896565e+00
# 14378  3  262  2.865597e+00
# 14379  3  263  2.833097e+00
# 14380  3  264  2.791076e+00
# 14381  3  265  6.987253e+00
# 14382  3  266  5.713391e+00
# 14383  3  267  4.871968e+00
# 14384  3  268  4.337491e+00
# 14385  3  269  3.992342e+00
# 14386  3  270  3.774225e+00
# 14387  3  271  3.622476e+00
# 14388  3  272  3.515884e+00
# 14389  3  273  3.434435e+00
# 14390  3  274  3.372463e+00
# 14391  3  275  3.312421e+00
# 14392  3  276  3.263341e+00
# 14393  3  277  3.218439e+00
# 14394  3  278  3.172917e+00
# 14395  3  279  3.133348e+00
# 14396  3  280  3.102290e+00
# 14397  3  281  3.048293e+00
# 14398  3  282  3.016740e+00
# 14399  3  283  2.973219e+00
# 14400  3  284  2.937661e+00
# 14401  3  285  2.902106e+00
# 14402  3  286  2.860873e+00
# 14403  3  287  2.827668e+00
# 14404  3  288  2.792161e+00
# 14405  3  289  6.987357e+00
# 14406  3  290  5.714626e+00
# 14407  3  291  4.871307e+00
# 14408  3  292  4.331259e+00
# 14409  3  293  3.992916e+00
# 14410  3  294  3.775549e+00
# 14411  3  295  3.626564e+00
# 14412  3  296  3.516902e+00
# 14413  3  297  3.432453e+00
# 14414  3  298  3.365147e+00
# 14415  3  299  3.317623e+00
# 14416  3  300  3.267547e+00
# 14417  3  301  3.214566e+00
# 14418  3  302  3.170186e+00
# 14419  3  303  3.134692e+00
# 14420  3  304  3.091143e+00
# 14421  3  305  3.053460e+00
# 14422  3  306  3.013042e+00
# 14423  3  307  2.969662e+00
# 14424  3  308  2.935705e+00
# 14425  3  309  2.903927e+00
# 14426  3  310  2.865474e+00
# 14427  3  311  2.827340e+00
# 14428  3  312  2.794288e+00
# 14429  3  312  2.791809e+00
# 14430  3  313  6.976954e+00
# 14431  3  314  5.720698e+00
# 14432  3  315  4.852996e+00
# 14433  3  316  4.331479e+00
# 14434  3  317  3.998009e+00
# 14435  3  318  3.774869e+00
```

```
## 14436  3  319  3.627373e+00
## 14437  3  320  3.520596e+00
## 14438  3  321  3.433822e+00
## 14439  3  322  3.375769e+00
## 14440  3  323  3.313421e+00
## 14441  3  324  3.265532e+00
## 14442  3  325  3.220481e+00
## 14443  3  326  3.172900e+00
## 14444  3  327  3.133366e+00
## 14445  3  328  3.098100e+00
## 14446  3  329  3.052163e+00
## 14447  3  330  3.018266e+00
## 14448  3  331  2.972321e+00
## 14449  3  332  2.929772e+00
## 14450  3  333  2.897477e+00
## 14451  3  334  2.864170e+00
## 14452  3  335  2.826575e+00
## 14453  3  336  2.798208e+00
## 14454  3  336  2.789468e+00
## 14455  3  337  2.754542e+00
## 14456  3  338  2.718616e+00
## 14457  3  339  2.687351e+00
## 14458  3  339  2.688768e+00
## 14459  3  340  6.875456e+00
## 14460  3  341  5.615898e+00
## 14461  3  342  4.771293e+00
## 14462  3  343  4.231373e+00
## 14463  3  344  3.903427e+00
## 14464  3  345  3.673869e+00
## 14465  3  346  3.534305e+00
## 14466  3  347  3.421614e+00
## 14467  3  348  3.344596e+00
## 14468  3  349  3.275568e+00
## 14469  3  350  3.223357e+00
## 14470  3  351  3.173532e+00
## 14471  3  352  3.131944e+00
## 14472  3  353  3.094572e+00
## 14473  3  354  3.046362e+00
## 14474  3  355  3.003020e+00
## 14475  3  356  2.972098e+00
## 14476  3  357  2.933291e+00
## 14477  3  358  2.894166e+00
## 14478  3  359  2.859374e+00
## 14479  3  360  2.819356e+00
## 14480  3  360  2.820528e+00
## 14481  3  361  7.016012e+00
## 14482  3  362  5.751917e+00
## 14483  3  363  4.890969e+00
## 14484  3  364  4.360550e+00
## 14485  3  365  4.015742e+00
## 14486  3  366  3.801931e+00
## 14487  3  367  3.653633e+00
## 14488  3  368  3.546039e+00
## 14489  3  369  3.468506e+00
## 14490  3  370  3.392805e+00
## 14491  3  371  3.342533e+00
## 14492  3  372  3.291077e+00
## 14493  3  373  3.243626e+00
## 14494  3  374  3.200082e+00
## 14495  3  375  3.156000e+00
## 14496  3  376  3.117895e+00
## 14497  3  377  3.075078e+00
## 14498  3  378  3.035894e+00
## 14499  3  379  3.002061e+00
## 14500  3  380  2.960895e+00
## 14501  3  381  2.920673e+00
## 14502  3  382  2.883196e+00
## 14503  3  383  2.856463e+00
## 14504  3  384  2.812066e+00
## 14505  3  385  7.000614e+00
## 14506  3  386  5.732117e+00
## 14507  3  387  4.884119e+00
## 14508  3  388  4.346156e+00
## 14509  3  389  4.020239e+00
## 14510  3  390  3.800644e+00
## 14511  3  391  3.651449e+00
## 14512  3  392  3.537972e+00
## 14513  3  393  3.456729e+00
## 14514  3  394  3.390638e+00
## 14515  3  395  3.329261e+00
## 14516  3  396  3.283571e+00
## 14517  3  397  3.234935e+00
## 14518  3  398  3.191697e+00
## 14519  3  399  3.153342e+00
## 14520  3  400  3.111334e+00
## 14521  3  401  3.069069e+00
## 14522  3  402  3.028355e+00
## 14523  3  403  2.984949e+00
## 14524  3  404  2.959528e+00
## 14525  3  405  2.919797e+00
## 14526  3  406  2.879583e+00
## 14527  3  407  2.843273e+00
## 14528  3  408  2.807290e+00
## 14529  3  409  7.008115e+00
## 14530  3  410  5.734207e+00
## 14531  3  411  4.872425e+00
## 14532  3  412  4.346558e+00
## 14533  3  413  4.015834e+00
## 14534  3  414  3.791964e+00
## 14535  3  415  3.638010e+00
## 14536  3  416  3.537275e+00
## 14537  3  417  3.448741e+00
## 14538  3  418  3.384081e+00
## 14539  3  419  3.322903e+00
## 14540  3  420  3.280448e+00
## 14541  3  421  3.235755e+00
## 14542  3  422  3.183268e+00
## 14543  3  423  3.150273e+00
## 14544  3  424  3.102888e+00
## 14545  3  425  3.066383e+00
## 14546  3  426  3.025769e+00
## 14547  3  427  2.980016e+00
## 14548  3  428  2.946413e+00
## 14549  3  429  2.913621e+00
## 14550  3  430  2.876171e+00
## 14551  3  431  2.834551e+00
## 14552  3  432  2.801747e+00
## 14553  3  433  6.994033e+00
## 14554  3  434  5.727786e+00
## 14555  3  435  4.876035e+00
## 14556  3  436  4.334449e+00
## 14557  3  437  4.004691e+00
## 14558  3  438  3.783795e+00
## 14559  3  439  3.634841e+00
## 14560  3  440  3.534970e+00
## 14561  3  441  3.447449e+00
## 14562  3  442  3.381537e+00
## 14563  3  443  3.328918e+00
## 14564  3  444  3.273936e+00
## 14565  3  445  3.227495e+00
## 14566  3  446  3.181310e+00
## 14567  3  447  3.145466e+00
## 14568  3  448  3.098994e+00
## 14569  3  449  3.063264e+00
## 14570  3  450  3.022958e+00
## 14571  3  451  2.986742e+00
## 14572  3  452  2.944925e+00
## 14573  3  453  2.909992e+00
## 14574  3  454  2.874488e+00
## 14575  3  455  2.836456e+00
## 14576  3  456  2.802808e+00
## 14577  3  457  6.988679e+00
## 14578  3  458  5.720569e+00
## 14579  3  459  4.875684e+00
## 14580  3  460  4.344888e+00
## 14581  3  461  4.002740e+00
## 14582  3  462  3.787384e+00
## 14583  3  463  3.628434e+00
## 14584  3  464  3.523613e+00
## 14585  3  465  3.442280e+00
## 14586  3  466  3.375265e+00
## 14587  3  467  3.321618e+00
## 14588  3  468  3.280249e+00
## 14589  3  469  3.233416e+00
## 14590  3  470  3.180520e+00
## 14591  3  471  3.135774e+00
## 14592  3  472  3.100095e+00
## 14593  3  473  3.058829e+00
## 14594  3  474  3.024928e+00
## 14595  3  475  2.978410e+00
## 14596  3  476  2.943678e+00
## 14597  3  477  2.908098e+00
## 14598  3  478  2.870983e+00
## 14599  3  479  2.838580e+00
## 14600  3  480  2.801975e+00
## 14601  3  481  6.982445e+00
## 14602  3  482  5.722727e+00
## 14603  3  483  4.871798e+00
## 14604  3  484  4.330894e+00
## 14605  3  485  3.999690e+00
## 14606  3  486  3.777167e+00
## 14607  3  487  3.626753e+00
## 14608  3  488  3.526039e+00
## 14609  3  489  3.435555e+00
## 14610  3  490  3.378105e+00
## 14611  3  491  3.323926e+00
## 14612  3  492  3.272576e+00
## 14613  3  493  3.224203e+00
## 14614  3  494  3.186932e+00
## 14615  3  495  3.136913e+00
## 14616  3  496  3.098906e+00
## 14617  3  497  3.057263e+00
## 14618  3  498  3.018710e+00
## 14619  3  499  2.978349e+00
## 14620  3  500  2.947793e+00
## 14621  3  501  2.901502e+00
## 14622  3  502  2.870281e+00
## 14623  3  503  2.833840e+00
## 14624  3  504  2.796840e+00
## 14625  3  505  6.984361e+00
## 14626  3  506  5.723221e+00
## 14627  3  507  4.863951e+00
## 14628  3  508  4.338608e+00
## 14629  3  509  4.003254e+00
## 14630  3  510  3.778376e+00
## 14631  3  511  3.629125e+00
## 14632  3  512  3.529186e+00
## 14633  3  513  3.436939e+00
## 14634  3  514  3.374024e+00
## 14635  3  515  3.315738e+00
## 14636  3  516  3.265401e+00
## 14637  3  517  3.225208e+00
## 14638  3  518  3.184662e+00
## 14639  3  519  3.136381e+00
## 14640  3  520  3.096880e+00
## 14641  3  521  3.056367e+00
## 14642  3  522  3.013633e+00
## 14643  3  523  2.978383e+00
## 14644  3  524  2.938889e+00
## 14645  3  525  2.901763e+00
## 14646  3  526  2.868784e+00
## 14647  3  527  2.833727e+00
## 14648  3  528  2.800852e+00
## 14649  3  529  6.996461e+00
## 14650  3  530  5.716665e+00
## 14651  3  531  4.870314e+00
## 14652  3  532  4.328176e+00
## 14653  3  533  3.995017e+00
## 14654  3  534  3.781862e+00
## 14655  3  535  3.624674e+00
## 14656  3  536  3.515350e+00
## 14657  3  537  3.435595e+00
## 14658  3  538  3.377778e+00
## 14659  3  539  3.320181e+00
## 14660  3  540  3.268843e+00
## 14661  3  541  3.218966e+00
## 14662  3  542  3.172600e+00
## 14663  3  543  3.134530e+00
## 14664  3  544  3.096504e+00
## 14665  3  545  3.058833e+00
## 14666  3  546  3.017219e+00
## 14667  3  547  2.980760e+00
## 14668  3  548  2.944645e+00
## 14669  3  549  2.905577e+00
## 14670  3  550  2.868304e+00
## 14671  3  551  2.830503e+00
## 14672  3  552  2.801458e+00
## 14673  3  553  6.995206e+00
## 14674  3  554  5.715175e+00
## 14675  3  555  4.877957e+00
## 14676  3  556  4.327416e+00
## 14677  3  557  3.990453e+00
## 14678  3  558  3.772810e+00
## 14679  3  559  3.629401e+00
## 14680  3  560  3.516054e+00
## 14681  3  561  3.442576e+00
## 14682  3  562  3.370535e+00
## 14683  3  563  3.314086e+00
## 14684  3  564  3.265972e+00
## 14685  3  565  3.220160e+00
## 14686  3  566  3.178099e+00
## 14687  3  567  3.132539e+00
## 14688  3  568  3.099173e+00
## 14689  3  569  3.052117e+00
## 14690  3  570  3.010327e+00
## 14691  3  571  2.979027e+00
## 14692  3  572  2.940849e+00
## 14693  3  573  2.899532e+00
## 14694  3  574  2.871545e+00
## 14695  3  575  2.831740e+00
## 14696  3  576  2.794533e+00
## 14697  3  577  6.984015e+00
## 14698  3  578  5.709813e+00
## 14699  3  579  4.867119e+00
## 14700  3  580  4.328810e+00
## 14701  3  581  3.994310e+00
## 14702  3  582  3.778532e+00
## 14703  3  583  3.630158e+00
## 14704  3  584  3.517134e+00
## 14705  3  585  3.441628e+00
## 14706  3  586  3.369992e+00
## 14707  3  587  3.317190e+00
## 14708  3  588  3.263283e+00
## 14709  3  589  3.223044e+00
## 14710  3  590  3.178506e+00
## 14711  3  591  3.137259e+00
## 14712  3  592  3.088057e+00
## 14713  3  593  3.057510e+00
## 14714  3  594  3.009182e+00
## 14715  3  595  2.978175e+00
## 14716  3  596  2.942835e+00
## 14717  3  597  2.901374e+00
## 14718  3  598  2.860553e+00
## 14719  3  599  2.825367e+00
## 14720  3  600  2.786874e+00
## 14721  3  601  6.984303e+00
## 14722  3  602  5.714462e+00
## 14723  3  603  4.859883e+00
## 14724  3  604  4.336466e+00
## 14725  3  605  4.002942e+00
## 14726  3  606  3.778495e+00
## 14727  3  607  3.619284e+00
## 14728  3  608  3.519672e+00
## 14729  3  609  3.438909e+00
## 14730  3  610  3.367931e+00
## 14731  3  611  3.316641e+00
## 14732  3  612  3.268221e+00
## 14733  3  613  3.215705e+00
## 14734  3  614  3.176324e+00
## 14735  3  615  3.140442e+00
## 14736  3  616  3.093745e+00
## 14737  3  617  3.056999e+00
## 14738  3  618  3.011724e+00
## 14739  3  619  2.980688e+00
## 14740  3  620  2.940637e+00
## 14741  3  621  2.894697e+00
## 14742  3  622  2.868709e+00
## 14743  3  623  2.831454e+00
## 14744  3  624  2.791947e+00
## 14745  3  624  2.792760e+00
## 14746  3  625  6.981066e+00
## 14747  3  626  5.721924e+00
## 14748  3  627  4.869824e+00
## 14749  3  628  4.325386e+00
## 14750  3  629  3.991439e+00
## 14751  3  630  3.774597e+00
## 14752  3  631  3.616812e+00
## 14753  3  632  3.517248e+00
## 14754  3  633  3.432516e+00
## 14755  3  634  3.367687e+00
## 14756  3  635  3.316554e+00
## 14757  3  636  3.263598e+00
## 14758  3  637  3.222459e+00
## 14759  3  638  3.172786e+00
## 14760  3  639  3.132738e+00
## 14761  3  640  3.096611e+00
## 14762  3  641  3.052264e+00
## 14763  3  642  3.016937e+00
## 14764  3  643  2.973801e+00
## 14765  3  644  2.936378e+00
## 14766  3  645  2.902998e+00
## 14767  3  646  2.867235e+00
## 14768  3  647  2.835572e+00
## 14769  3  648  2.788220e+00
## 14770  3  648  2.793873e+00
## 14771  3  649  2.756339e+00
## 14772  3  650  2.727451e+00
## 14773  3  651  2.692746e+00
## 14774  3  652  2.655429e+00
## 14775  3  653  2.622065e+00
## 14776  3  654  2.588231e+00
## 14777  3  654  2.587529e+00
## 14778  3  655  6.773503e+00
## 14779  3  656  5.520055e+00
## 14780  3  657  4.665638e+00
## 14781  3  658  4.135724e+00
## 14782  3  659  3.801537e+00
## 14783  3  660  3.583453e+00
## 14784  3  661  3.433620e+00
## 14785  3  662  3.328196e+00
## 14786  3  663  3.250088e+00
## 14787  3  664  3.192260e+00
## 14788  3  665  3.135435e+00
## 14789  3  666  3.090751e+00
## 14790  3  667  3.050748e+00
## 14791  3  668  3.004055e+00
## 14792  3  669  2.962679e+00
## 14793  3  670  2.918039e+00
## 14794  3  671  2.889300e+00
## 14795  3  672  2.853790e+00
## 14796  3  672  2.849952e+00
## 14797  3  673  7.038421e+00
## 14798  3  674  5.772635e+00
## 14799  3  675  4.921693e+00
## 14800  3  676  4.383807e+00
## 14801  3  677  4.050260e+00
## 14802  3  678  3.831837e+00
## 14803  3  679  3.679347e+00
## 14804  3  680  3.573193e+00
## 14805  3  681  3.486214e+00
## 14806  3  682  3.423613e+00
## 14807  3  683  3.363210e+00
## 14808  3  684  3.310505e+00
## 14809  3  685  3.261800e+00
## 14810  3  686  3.220577e+00
## 14811  3  687  3.186078e+00
## 14812  3  688  3.142821e+00
## 14813  3  689  3.100885e+00
## 14814  3  690  3.064494e+00
## 14815  3  691  3.023087e+00
## 14816  3  692  2.986014e+00
## 14817  3  693  2.940438e+00
## 14818  3  694  2.908846e+00
## 14819  3  695  2.869699e+00
## 14820  3  696  2.835175e+00
## 14821  3  697  7.032875e+00
## 14822  3  698  5.754977e+00
## 14823  3  699  4.903125e+00
## 14824  3  700  4.381857e+00
## 14825  3  701  4.027517e+00
## 14826  3  702  3.810642e+00
## 14827  3  703  3.663377e+00
## 14828  3  704  3.558381e+00
## 14829  3  705  3.475351e+00
## 14830  3  706  3.408909e+00
## 14831  3  707  3.346615e+00
## 14832  3  708  3.302297e+00
## 14833  3  709  3.255644e+00
## 14834  3  710  3.207704e+00
## 14835  3  711  3.167119e+00
## 14836  3  712  3.134424e+00
## 14837  3  713  3.091920e+00
## 14838  3  714  3.051032e+00
## 14839  3  715  3.005648e+00
## 14840  3  716  2.973537e+00
## 14841  3  717  2.934245e+00
## 14842  3  718  2.895580e+00
## 14843  3  719  2.862314e+00
## 14844  3  720  2.821584e+00
## 14845  3  721  7.013257e+00
## 14846  3  722  5.734806e+00
## 14847  3  723  4.888562e+00
## 14848  3  724  4.365324e+00
## 14849  3  725  4.022703e+00
## 14850  3  726  3.804379e+00
## 14851  3  727  3.656410e+00
## 14852  3  728  3.543116e+00
## 14853  3  729  3.465743e+00
## 14854  3  730  3.398452e+00
## 14855  3  731  3.344457e+00
## 14856  3  732  3.294679e+00
## 14857  3  733  3.247511e+00
## 14858  3  734  3.204516e+00
## 14859  3  735  3.160210e+00
## 14860  3  736  3.115328e+00
## 14861  3  737  3.075577e+00
## 14862  3  738  3.036662e+00
## 14863  3  739  3.004296e+00
## 14864  3  740  2.961200e+00
## 14865  3  741  2.922160e+00
## 14866  3  742  2.888933e+00
## 14867  3  743  2.852463e+00
## 14868  3  744  2.814370e+00
## 14869  3  745  7.020767e+00
## 14870  3  746  5.737980e+00
## 14871  3  747  4.887503e+00
## 14872  3  748  4.357294e+00
## 14873  3  749  4.022107e+00
## 14874  3  750  3.797185e+00
## 14875  3  751  3.645015e+00
## 14876  3  752  3.541915e+00
## 14877  3  753  3.460787e+00
## 14878  3  754  3.394425e+00
## 14879  3  755  3.334342e+00
## 14880  3  756  3.279078e+00
## 14881  3  757  3.241971e+00
## 14882  3  758  3.196182e+00
## 14883  3  759  3.149495e+00
## 14884  3  760  3.113087e+00
## 14885  3  761  3.074039e+00
## 14886  3  762  3.036592e+00
## 14887  3  763  2.995782e+00
## 14888  3  764  2.957815e+00
## 14889  3  765  2.918242e+00
## 14890  3  766  2.876614e+00
## 14891  3  767  2.846631e+00
## 14892  3  768  2.813200e+00
## 14893  3  769  7.005049e+00
## 14894  3  770  5.730464e+00
## 14895  3  771  4.875214e+00
## 14896  3  772  4.354860e+00
## 14897  3  773  4.008850e+00
## 14898  3  774  3.790197e+00
## 14899  3  775  3.637408e+00
## 14900  3  776  3.529304e+00
## 14901  3  777  3.454074e+00
## 14902  3  778  3.381806e+00
## 14903  3  779  3.329487e+00
## 14904  3  780  3.282968e+00
## 14905  3  781  3.235736e+00
## 14906  3  782  3.193136e+00
## 14907  3  783  3.143530e+00
## 14908  3  784  3.106691e+00
## 14909  3  785  3.071022e+00
## 14910  3  786  3.030564e+00
## 14911  3  787  2.989932e+00
## 14912  3  788  2.950179e+00
## 14913  3  789  2.916825e+00
## 14914  3  790  2.878545e+00
## 14915  3  791  2.840294e+00
## 14916  3  792  2.799381e+00
## 14917  3  793  6.990820e+00
## 14918  3  794  5.733223e+00
## 14919  3  795  4.886237e+00
## 14920  3  796  4.344721e+00
## 14921  3  797  4.007818e+00
## 14922  3  798  3.788355e+00
## 14923  3  799  3.637646e+00
## 14924  3  800  3.524773e+00
## 14925  3  801  3.446526e+00
## 14926  3  802  3.390179e+00
## 14927  3  803  3.325824e+00
## 14928  3  804  3.271677e+00
## 14929  3  805  3.228234e+00
## 14930  3  806  3.183834e+00
## 14931  3  807  3.153160e+00
## 14932  3  808  3.106422e+00
## 14933  3  809  3.068856e+00
## 14934  3  810  3.025493e+00
## 14935  3  811  2.991183e+00
## 14936  3  812  2.947723e+00
## 14937  3  813  2.909460e+00
## 14938  3  814  2.872452e+00
## 14939  3  815  2.840926e+00
## 14940  3  816  2.798888e+00
## 14941  3  817  6.991913e+00
## 14942  3  818  5.730013e+00
## 14943  3  819  4.868968e+00
## 14944  3  820  4.346502e+00
## 14945  3  821  4.005096e+00
## 14946  3  822  3.781308e+00
## 14947  3  823  3.635088e+00
## 14948  3  824  3.526424e+00
## 14949  3  825  3.447924e+00
## 14950  3  826  3.372785e+00
## 14951  3  827  3.323241e+00
## 14952  3  828  3.266534e+00
## 14953  3  829  3.225594e+00
## 14954  3  830  3.187125e+00
## 14955  3  831  3.135821e+00
## 14956  3  832  3.100853e+00
## 14957  3  833  3.061448e+00
## 14958  3  834  3.021150e+00
## 14959  3  835  2.977299e+00
## 14960  3  836  2.944455e+00
## 14961  3  837  2.905835e+00
## 14962  3  838  2.870349e+00
## 14963  3  839  2.834210e+00
## 14964  3  840  2.795414e+00
## 14965  3  841  6.987162e+00
## 14966  3  842  5.712627e+00
## 14967  3  843  4.866353e+00
## 14968  3  844  4.337775e+00
## 14969  3  845  4.005168e+00
## 14970  3  846  3.782506e+00
## 14971  3  847  3.631266e+00
## 14972  3  848  3.524519e+00
## 14973  3  849  3.438949e+00
## 14974  3  850  3.376115e+00
## 14975  3  851  3.322526e+00
## 14976  3  852  3.271918e+00
## 14977  3  853  3.226378e+00
## 14978  3  854  3.184748e+00
## 14979  3  855  3.133876e+00
## 14980  3  856  3.095562e+00
## 14981  3  857  3.058322e+00
## 14982  3  858  3.018079e+00
## 14983  3  859  2.977901e+00
## 14984  3  860  2.946466e+00
## 14985  3  861  2.906298e+00
## 14986  3  862  2.861801e+00
## 14987  3  863  2.833769e+00
## 14988  3  864  2.798699e+00
## 14989  3  865  6.991164e+00
## 14990  3  866  5.713027e+00
## 14991  3  867  4.857366e+00
## 14992  3  868  4.334163e+00
## 14993  3  869  4.000545e+00
## 14994  3  870  3.778777e+00
## 14995  3  871  3.627745e+00
## 14996  3  872  3.525581e+00
## 14997  3  873  3.437862e+00
## 14998  3  874  3.371671e+00
## 14999  3  875  3.309645e+00
## 15000  3  876  3.274976e+00
## 15001  3  877  3.226414e+00
## 15002  3  878  3.171038e+00
## 15003  3  879  3.138910e+00
## 15004  3  880  3.096678e+00
## 15005  3  881  3.052101e+00
## 15006  3  882  3.012509e+00
## 15007  3  883  2.981732e+00
## 15008  3  884  2.933100e+00
## 15009  3  885  2.902882e+00
## 15010  3  886  2.867633e+00
## 15011  3  887  2.837209e+00
## 15012  3  888  2.794721e+00
## 15013  3  889  6.989490e+00
## 15014  3  890  5.723227e+00
## 15015  3  891  4.862767e+00
## 15016  3  892  4.337992e+00
## 15017  3  893  4.000554e+00
## 15018  3  894  3.778794e+00
## 15019  3  895  3.626332e+00
## 15020  3  896  3.522260e+00
## 15021  3  897  3.442130e+00
## 15022  3  898  3.367685e+00
## 15023  3  899  3.314505e+00
## 15024  3  900  3.269061e+00
## 15025  3  901  3.216676e+00
## 15026  3  902  3.176419e+00
## 15027  3  903  3.137199e+00
## 15028  3  904  3.092694e+00
## 15029  3  905  3.062339e+00
## 15030  3  906  3.014959e+00
## 15031  3  907  2.977198e+00
## 15032  3  908  2.941861e+00
## 15033  3  909  2.906115e+00
## 15034  3  910  2.863378e+00
## 15035  3  911  2.829031e+00
## 15036  3  912  2.794062e+00
## 15037  3  913  6.986767e+00
## 15038  3  914  5.709458e+00
## 15039  3  915  4.879297e+00
## 15040  3  916  4.333437e+00
## 15041  3  917  4.000942e+00
## 15042  3  918  3.774260e+00
## 15043  3  919  3.628855e+00
## 15044  3  920  3.517923e+00
## 15045  3  921  3.439686e+00
## 15046  3  922  3.379240e+00
## 15047  3  923  3.320510e+00
## 15048  3  924  3.264889e+00
## 15049  3  925  3.222768e+00
## 15050  3  926  3.172677e+00
## 15051  3  927  3.134413e+00
## 15052  3  928  3.096290e+00
## 15053  3  929  3.056058e+00
## 15054  3  930  3.017240e+00
## 15055  3  931  2.976296e+00
## 15056  3  932  2.943945e+00
## 15057  3  933  2.904031e+00
## 15058  3  934  2.860333e+00
## 15059  3  935  2.825858e+00
## 15060  3  936  2.791993e+00
## 15061  3  936  2.790288e+00
## 15062  3  937  6.977827e+00
## 15063  3  938  5.716372e+00
## 15064  3  939  4.860079e+00
## 15065  3  940  4.329263e+00
## 15066  3  941  3.995415e+00
## 15067  3  942  3.772037e+00
## 15068  3  943  3.627917e+00
## 15069  3  944  3.520845e+00
## 15070  3  945  3.436871e+00
## 15071  3  946  3.370364e+00
## 15072  3  947  3.312590e+00
## 15073  3  948  3.264042e+00
## 15074  3  949  3.212482e+00
## 15075  3  950  3.170580e+00
## 15076  3  951  3.132058e+00
## 15077  3  952  3.092433e+00
## 15078  3  953  3.051387e+00
## 15079  3  954  3.016159e+00
## 15080  3  955  2.976646e+00
## 15081  3  956  2.941719e+00
## 15082  3  957  2.900807e+00
## 15083  3  958  2.865187e+00
## 15084  3  959  2.832768e+00
## 15085  3  960  2.795835e+00
## 15086  3  960  2.788428e+00
## 15087  3  961  2.754470e+00
## 15088  3  962  2.722824e+00
## 15089  3  963  2.685820e+00
## 15090  3  964  2.649301e+00
## 15091  3  965  2.623124e+00
## 15092  3  966  2.587028e+00
## 15093  3  967  2.552009e+00
## 15094  3  968  2.520975e+00
## 15095  3  969  2.491978e+00
## 15096  3  969  2.489390e+00
## 15097  3  970  6.688763e+00
## 15098  3  971  5.409675e+00
## 15099  3  972  4.572594e+00
## 15100  3  973  4.045508e+00
## 15101  3  974  3.714331e+00
## 15102  3  975  3.494081e+00
## 15103  3  976  3.350362e+00
## 15104  3  977  3.240978e+00
## 15105  3  978  3.163658e+00
## 15106  3  979  3.104282e+00
## 15107  3  980  3.049581e+00
## 15108  3  981  3.005216e+00
## 15109  3  982  2.962016e+00
## 15110  3  983  2.924869e+00
## 15111  3  984  2.882467e+00
## 15112  3  984  2.882010e+00
## 15113  3  985  7.081159e+00
## 15114  3  986  5.807480e+00
## 15115  3  987  4.952703e+00
## 15116  3  988  4.418506e+00
## 15117  3  989  4.076686e+00
## 15118  3  990  3.855030e+00
## 15119  3  991  3.701493e+00
## 15120  3  992  3.605146e+00
## 15121  3  993  3.515800e+00
## 15122  3  994  3.442149e+00
## 15123  3  995  3.388111e+00
## 15124  3  996  3.341936e+00
## 15125  3  997  3.291557e+00
## 15126  3  998  3.243732e+00
## 15127  3  999  3.206533e+00
## 15128  3 1000  3.161553e+00
## 15129  3 1001  3.122234e+00
## 15130  3 1002  3.077713e+00
## 15131  3 1003  3.046012e+00
## 15132  3 1004  3.006620e+00
## 15133  3 1005  2.967550e+00
## 15134  3 1006  2.930723e+00
## 15135  3 1007  2.892834e+00
## 15136  3 1008  2.857897e+00
## 15137  3 1009  7.049589e+00
## 15138  3 1010  5.781118e+00
## 15139  3 1011  4.925154e+00
## 15140  3 1012  4.387303e+00
## 15141  3 1013  4.062283e+00
## 15142  3 1014  3.832931e+00
## 15143  3 1015  3.689835e+00
## 15144  3 1016  3.579460e+00
## 15145  3 1017  3.489687e+00
## 15146  3 1018  3.427334e+00
## 15147  3 1019  3.378315e+00
## 15148  3 1020  3.314647e+00
## 15149  3 1021  3.275836e+00
## 15150  3 1022  3.221222e+00
## 15151  3 1023  3.183975e+00
## 15152  3 1024  3.145660e+00
## 15153  3 1025  3.103994e+00
## 15154  3 1026  3.067232e+00
## 15155  3 1027  3.027155e+00
## 15156  3 1028  2.990959e+00
## 15157  3 1029  2.946515e+00
## 15158  3 1030  2.910961e+00
## 15159  3 1031  2.874220e+00
## 15160  3 1032  2.837394e+00
## 15161  3 1033  7.029119e+00
## 15162  3 1034  5.775434e+00
## 15163  3 1035  4.914600e+00
## 15164  3 1036  4.369246e+00
## 15165  3 1037  4.039220e+00
## 15166  3 1038  3.821462e+00
## 15167  3 1039  3.672173e+00
## 15168  3 1040  3.560508e+00
## 15169  3 1041  3.477390e+00
## 15170  3 1042  3.413957e+00
## 15171  3 1043  3.353199e+00
## 15172  3 1044  3.307354e+00
## 15173  3 1045  3.258826e+00
## 15174  3 1046  3.218795e+00
## 15175  3 1047  3.175411e+00
## 15176  3 1048  3.139231e+00
## 15177  3 1049  3.089478e+00
## 15178  3 1050  3.054446e+00
## 15179  3 1051  3.020847e+00
## 15180  3 1052  2.976100e+00
## 15181  3 1053  2.935344e+00
## 15182  3 1054  2.897025e+00
## 15183  3 1055  2.858654e+00
## 15184  3 1056  2.826258e+00
## 15185  3 1057  7.013864e+00
## 15186  3 1058  5.755167e+00
## 15187  3 1059  4.896725e+00
## 15188  3 1060  4.363390e+00
## 15189  3 1061  4.033204e+00
## 15190  3 1062  3.805949e+00
## 15191  3 1063  3.659983e+00
## 15192  3 1064  3.550216e+00
## 15193  3 1065  3.470886e+00
## 15194  3 1066  3.402629e+00
## 15195  3 1067  3.351668e+00
## 15196  3 1068  3.292863e+00
## 15197  3 1069  3.254459e+00
## 15198  3 1070  3.205795e+00
## 15199  3 1071  3.165092e+00
## 15200  3 1072  3.122423e+00
## 15201  3 1073  3.080253e+00
## 15202  3 1074  3.042226e+00
## 15203  3 1075  3.001969e+00
## 15204  3 1076  2.965668e+00
## 15205  3 1077  2.933089e+00
## 15206  3 1078  2.886973e+00
## 15207  3 1079  2.858738e+00
## 15208  3 1080  2.813733e+00
## 15209  3 1081  7.017541e+00
## 15210  3 1082  5.740550e+00
## 15211  3 1083  4.890772e+00
## 15212  3 1084  4.355224e+00
## 15213  3 1085  4.014521e+00
## 15214  3 1086  3.795771e+00
## 15215  3 1087  3.650599e+00
## 15216  3 1088  3.539283e+00
## 15217  3 1089  3.459665e+00
## 15218  3 1090  3.392844e+00
## 15219  3 1091  3.336599e+00
## 15220  3 1092  3.286853e+00
## 15221  3 1093  3.237435e+00
## 15222  3 1094  3.198593e+00
## 15223  3 1095  3.152397e+00
## 15224  3 1096  3.112914e+00
## 15225  3 1097  3.072060e+00
## 15226  3 1098  3.031253e+00
## 15227  3 1099  2.996098e+00
## 15228  3 1100  2.955347e+00
## 15229  3 1101  2.918997e+00
## 15230  3 1102  2.880872e+00
## 15231  3 1103  2.846999e+00
## 15232  3 1104  2.813066e+00
## 15233  3 1105  7.001180e+00
## 15234  3 1106  5.737101e+00
## 15235  3 1107  4.887955e+00
```

```
## 15236  3 1108  4.344221e+00
## 15237  3 1109  4.010386e+00
## 15238  3 1110  3.786997e+00
## 15239  3 1111  3.646043e+00
## 15240  3 1112  3.536898e+00
## 15241  3 1113  3.458492e+00
## 15242  3 1114  3.388409e+00
## 15243  3 1115  3.334816e+00
## 15244  3 1116  3.279430e+00
## 15245  3 1117  3.230846e+00
## 15246  3 1118  3.187440e+00
## 15247  3 1119  3.150674e+00
## 15248  3 1120  3.113229e+00
## 15249  3 1121  3.068995e+00
## 15250  3 1122  3.030319e+00
## 15251  3 1123  2.986846e+00
## 15252  3 1124  2.956981e+00
## 15253  3 1125  2.913726e+00
## 15254  3 1126  2.875607e+00
## 15255  3 1127  2.842621e+00
## 15256  3 1128  2.805282e+00
## 15257  3 1129  7.005587e+00
## 15258  3 1130  5.730264e+00
## 15259  3 1131  4.871939e+00
## 15260  3 1132  4.347098e+00
## 15261  3 1133  4.010598e+00
## 15262  3 1134  3.791102e+00
## 15263  3 1135  3.638376e+00
## 15264  3 1136  3.535498e+00
## 15265  3 1137  3.449579e+00
## 15266  3 1138  3.383914e+00
## 15267  3 1139  3.326802e+00
## 15268  3 1140  3.275356e+00
## 15269  3 1141  3.232391e+00
## 15270  3 1142  3.189656e+00
## 15271  3 1143  3.146335e+00
## 15272  3 1144  3.101856e+00
## 15273  3 1145  3.070004e+00
## 15274  3 1146  3.026812e+00
## 15275  3 1147  2.984568e+00
## 15276  3 1148  2.944636e+00
## 15277  3 1149  2.911141e+00
## 15278  3 1150  2.871827e+00
## 15279  3 1151  2.837999e+00
## 15280  3 1152  2.802435e+00
## 15281  3 1153  6.992194e+00
## 15282  3 1154  5.718202e+00
## 15283  3 1155  4.871106e+00
## 15284  3 1156  4.346696e+00
## 15285  3 1157  4.005343e+00
## 15286  3 1158  3.781750e+00
## 15287  3 1159  3.630865e+00
## 15288  3 1160  3.524274e+00
## 15289  3 1161  3.454478e+00
## 15290  3 1162  3.382086e+00
## 15291  3 1163  3.320408e+00
## 15292  3 1164  3.273621e+00
## 15293  3 1165  3.223553e+00
## 15294  3 1166  3.181751e+00
## 15295  3 1167  3.140055e+00
## 15296  3 1168  3.103363e+00
## 15297  3 1169  3.067497e+00
## 15298  3 1170  3.019705e+00
## 15299  3 1171  2.986391e+00
## 15300  3 1172  2.942829e+00
## 15301  3 1173  2.913282e+00
## 15302  3 1174  2.871315e+00
## 15303  3 1175  2.830820e+00
## 15304  3 1176  2.801625e+00
## 15305  3 1177  6.996636e+00
## 15306  3 1178  5.714648e+00
## 15307  3 1179  4.871238e+00
## 15308  3 1180  4.344225e+00
## 15309  3 1181  4.012175e+00
## 15310  3 1182  3.782967e+00
## 15311  3 1183  3.623303e+00
## 15312  3 1184  3.523765e+00
## 15313  3 1185  3.445870e+00
## 15314  3 1186  3.379749e+00
## 15315  3 1187  3.323236e+00
## 15316  3 1188  3.269094e+00
## 15317  3 1189  3.225651e+00
## 15318  3 1190  3.188779e+00
## 15319  3 1191  3.139096e+00
## 15320  3 1192  3.100211e+00
## 15321  3 1193  3.061001e+00
## 15322  3 1194  3.020678e+00
## 15323  3 1195  2.986732e+00
## 15324  3 1196  2.943918e+00
## 15325  3 1197  2.905916e+00
## 15326  3 1198  2.868121e+00
## 15327  3 1199  2.826521e+00
## 15328  3 1200  2.793179e+00
## 15329  3 1201  6.983200e+00
## 15330  3 1202  5.712331e+00
## 15331  3 1203  4.869081e+00
## 15332  3 1204  4.340529e+00
## 15333  3 1205  4.002354e+00
## 15334  3 1206  3.777226e+00
## 15335  3 1207  3.631432e+00
## 15336  3 1208  3.520108e+00
## 15337  3 1209  3.441778e+00
## 15338  3 1210  3.373458e+00
## 15339  3 1211  3.318553e+00
## 15340  3 1212  3.268101e+00
## 15341  3 1213  3.216610e+00
## 15342  3 1214  3.177427e+00
## 15343  3 1215  3.144038e+00
## 15344  3 1216  3.096589e+00
## 15345  3 1217  3.054954e+00
## 15346  3 1218  3.020067e+00
## 15347  3 1219  2.975270e+00
## 15348  3 1220  2.943804e+00
## 15349  3 1221  2.906641e+00
## 15350  3 1222  2.869492e+00
## 15351  3 1223  2.830201e+00
## 15352  3 1224  2.794164e+00
## 15353  3 1225  6.991393e+00
## 15354  3 1226  5.716345e+00
## 15355  3 1227  4.869420e+00
## 15356  3 1228  4.339629e+00
## 15357  3 1229  4.001419e+00
## 15358  3 1230  3.779874e+00
## 15359  3 1231  3.624111e+00
## 15360  3 1232  3.522640e+00
## 15361  3 1233  3.442785e+00
## 15362  3 1234  3.373392e+00
## 15363  3 1235  3.318166e+00
## 15364  3 1236  3.268670e+00
## 15365  3 1237  3.219635e+00
## 15366  3 1238  3.179656e+00
## 15367  3 1239  3.139647e+00
## 15368  3 1240  3.095165e+00
## 15369  3 1241  3.057570e+00
## 15370  3 1242  3.018801e+00
## 15371  3 1243  2.972160e+00
## 15372  3 1244  2.950471e+00
## 15373  3 1245  2.908026e+00
## 15374  3 1246  2.860608e+00
## 15375  3 1247  2.828870e+00
## 15376  3 1248  2.795195e+00
## 15377  3 1248  2.788616e+00
## 15378  3 1249  6.989839e+00
## 15379  3 1250  5.715446e+00
## 15380  3 1251  4.862264e+00
## 15381  3 1252  4.333456e+00
## 15382  3 1253  3.993957e+00
## 15383  3 1254  3.777054e+00
## 15384  3 1255  3.631398e+00
## 15385  3 1256  3.516210e+00
## 15386  3 1257  3.439292e+00
## 15387  3 1258  3.374811e+00
## 15388  3 1259  3.317300e+00
## 15389  3 1260  3.262133e+00
## 15390  3 1261  3.219928e+00
## 15391  3 1262  3.174048e+00
## 15392  3 1263  3.131024e+00
## 15393  3 1264  3.093846e+00
## 15394  3 1265  3.052738e+00
## 15395  3 1266  3.014220e+00
## 15396  3 1267  2.974110e+00
## 15397  3 1268  2.938644e+00
## 15398  3 1269  2.897932e+00
## 15399  3 1270  2.863851e+00
## 15400  3 1271  2.828869e+00
## 15401  3 1272  2.794392e+00
## 15402  3 1272  2.793190e+00
## 15403  3 1273  2.757972e+00
## 15404  3 1274  2.722053e+00
## 15405  3 1275  2.687855e+00
## 15406  3 1276  2.656740e+00
## 15407  3 1277  2.621746e+00
## 15408  3 1278  2.582329e+00
## 15409  3 1279  2.555151e+00
## 15410  3 1280  2.522097e+00
## 15411  3 1281  2.488834e+00
## 15412  3 1282  2.458358e+00
## 15413  3 1283  2.428115e+00
## 15414  3 1284  2.400043e+00
## 15415  3 1284  2.398515e+00
## 15416  3 1285  6.611296e+00
## 15417  3 1286  5.325534e+00
## 15418  3 1287  4.481349e+00
## 15419  3 1288  3.956801e+00
## 15420  3 1289  3.623802e+00
## 15421  3 1290  3.410177e+00
## 15422  3 1291  3.259248e+00
## 15423  3 1292  3.161227e+00
## 15424  3 1293  3.077232e+00
## 15425  3 1294  3.019244e+00
## 15426  3 1295  2.971325e+00
## 15427  3 1296  2.921990e+00
## 15428  3 1296  2.922865e+00
## 15429  3 1297  7.108397e+00
## 15430  3 1298  5.839202e+00
## 15431  3 1299  4.983792e+00
## 15432  3 1300  4.456993e+00
## 15433  3 1301  4.106801e+00
## 15434  3 1302  3.880910e+00
## 15435  3 1303  3.741017e+00
## 15436  3 1304  3.625956e+00
## 15437  3 1305  3.545102e+00
## 15438  3 1306  3.478015e+00
## 15439  3 1307  3.416188e+00
## 15440  3 1308  3.365546e+00
## 15441  3 1309  3.322298e+00
## 15442  3 1310  3.276467e+00
## 15443  3 1311  3.236893e+00
## 15444  3 1312  3.192068e+00
## 15445  3 1313  3.151847e+00
## 15446  3 1314  3.116902e+00
## 15447  3 1315  3.070169e+00
## 15448  3 1316  3.032653e+00
## 15449  3 1317  2.988899e+00
## 15450  3 1318  2.951215e+00
## 15451  3 1319  2.922974e+00
## 15452  3 1320  2.882835e+00
## 15453  3 1321  7.071366e+00
## 15454  3 1322  5.796146e+00
## 15455  3 1323  4.944199e+00
## 15456  3 1324  4.411136e+00
## 15457  3 1325  4.082199e+00
## 15458  3 1326  3.858902e+00
## 15459  3 1327  3.708966e+00
## 15460  3 1328  3.595907e+00
## 15461  3 1329  3.515848e+00
## 15462  3 1330  3.455127e+00
## 15463  3 1331  3.393882e+00
## 15464  3 1332  3.341024e+00
## 15465  3 1333  3.295941e+00
## 15466  3 1334  3.248354e+00
## 15467  3 1335  3.206412e+00
## 15468  3 1336  3.162805e+00
## 15469  3 1337  3.125162e+00
## 15470  3 1338  3.085358e+00
## 15471  3 1339  3.043352e+00
## 15472  3 1340  3.005241e+00
## 15473  3 1341  2.964535e+00
## 15474  3 1342  2.926660e+00
## 15475  3 1343  2.890440e+00
## 15476  3 1344  2.853029e+00
## 15477  3 1345  7.050396e+00
## 15478  3 1346  5.775095e+00
## 15479  3 1347  4.928794e+00
## 15480  3 1348  4.388275e+00
## 15481  3 1349  4.050542e+00
## 15482  3 1350  3.837008e+00
## 15483  3 1351  3.683643e+00
## 15484  3 1352  3.577778e+00
## 15485  3 1353  3.491422e+00
## 15486  3 1354  3.426314e+00
## 15487  3 1355  3.363874e+00
## 15488  3 1356  3.320567e+00
## 15489  3 1357  3.272123e+00
## 15490  3 1358  3.228508e+00
## 15491  3 1359  3.186464e+00
## 15492  3 1360  3.143332e+00
## 15493  3 1361  3.104621e+00
## 15494  3 1362  3.065223e+00
## 15495  3 1363  3.028083e+00
## 15496  3 1364  2.990296e+00
## 15497  3 1365  2.949715e+00
## 15498  3 1366  2.907569e+00
## 15499  3 1367  2.877997e+00
## 15500  3 1368  2.839323e+00
## 15501  3 1369  7.028105e+00
## 15502  3 1370  5.776620e+00
## 15503  3 1371  4.910050e+00
## 15504  3 1372  4.369669e+00
## 15505  3 1373  4.040166e+00
## 15506  3 1374  3.823283e+00
## 15507  3 1375  3.663932e+00
## 15508  3 1376  3.563454e+00
## 15509  3 1377  3.481842e+00
## 15510  3 1378  3.412206e+00
## 15511  3 1379  3.356436e+00
## 15512  3 1380  3.300247e+00
## 15513  3 1381  3.258022e+00
## 15514  3 1382  3.214003e+00
## 15515  3 1383  3.170287e+00
## 15516  3 1384  3.135786e+00
## 15517  3 1385  3.092120e+00
## 15518  3 1386  3.055896e+00
## 15519  3 1387  3.017861e+00
## 15520  3 1388  2.976166e+00
## 15521  3 1389  2.933671e+00
## 15522  3 1390  2.899287e+00
## 15523  3 1391  2.860942e+00
## 15524  3 1392  2.835108e+00
## 15525  3 1393  7.021193e+00
## 15526  3 1394  5.751342e+00
## 15527  3 1395  4.895309e+00
## 15528  3 1396  4.366825e+00
## 15529  3 1397  4.030856e+00
## 15530  3 1398  3.808240e+00
## 15531  3 1399  3.655271e+00
## 15532  3 1400  3.544743e+00
## 15533  3 1401  3.461441e+00
## 15534  3 1402  3.403489e+00
## 15535  3 1403  3.347482e+00
## 15536  3 1404  3.297178e+00
## 15537  3 1405  3.248960e+00
## 15538  3 1406  3.205977e+00
## 15539  3 1407  3.163896e+00
## 15540  3 1408  3.119254e+00
## 15541  3 1409  3.082571e+00
## 15542  3 1410  3.045265e+00
## 15543  3 1411  3.014109e+00
## 15544  3 1412  2.968132e+00
## 15545  3 1413  2.922562e+00
## 15546  3 1414  2.889621e+00
## 15547  3 1415  2.847676e+00
## 15548  3 1416  2.821426e+00
## 15549  3 1417  7.016177e+00
## 15550  3 1418  5.743392e+00
## 15551  3 1419  4.888678e+00
## 15552  3 1420  4.356813e+00
## 15553  3 1421  4.025209e+00
## 15554  3 1422  3.802131e+00
## 15555  3 1423  3.647964e+00
## 15556  3 1424  3.542321e+00
## 15557  3 1425  3.461767e+00
## 15558  3 1426  3.392588e+00
## 15559  3 1427  3.332570e+00
## 15560  3 1428  3.293193e+00
## 15561  3 1429  3.245115e+00
## 15562  3 1430  3.196188e+00
## 15563  3 1431  3.153577e+00
## 15564  3 1432  3.114641e+00
## 15565  3 1433  3.079627e+00
## 15566  3 1434  3.034083e+00
## 15567  3 1435  2.996031e+00
## 15568  3 1436  2.952550e+00
## 15569  3 1437  2.916625e+00
## 15570  3 1438  2.882476e+00
## 15571  3 1439  2.846686e+00
## 15572  3 1440  2.813827e+00
## 15573  3 1441  7.003900e+00
## 15574  3 1442  5.737228e+00
## 15575  3 1443  4.879784e+00
## 15576  3 1444  4.354919e+00
## 15577  3 1445  4.016989e+00
## 15578  3 1446  3.796679e+00
## 15579  3 1447  3.641232e+00
## 15580  3 1448  3.539624e+00
## 15581  3 1449  3.449443e+00
## 15582  3 1450  3.380514e+00
## 15583  3 1451  3.335787e+00
## 15584  3 1452  3.288228e+00
## 15585  3 1453  3.232251e+00
## 15586  3 1454  3.189783e+00
## 15587  3 1455  3.149924e+00
## 15588  3 1456  3.108284e+00
## 15589  3 1457  3.065895e+00
## 15590  3 1458  3.030791e+00
## 15591  3 1459  2.993275e+00
## 15592  3 1460  2.947151e+00
## 15593  3 1461  2.913730e+00
## 15594  3 1462  2.884584e+00
## 15595  3 1463  2.844631e+00
## 15596  3 1464  2.806116e+00
## 15597  3 1465  7.011813e+00
## 15598  3 1466  5.733247e+00
## 15599  3 1467  4.874550e+00
## 15600  3 1468  4.343602e+00
## 15601  3 1469  4.011934e+00
## 15602  3 1470  3.790777e+00
## 15603  3 1471  3.636099e+00
## 15604  3 1472  3.530189e+00
## 15605  3 1473  3.447750e+00
## 15606  3 1474  3.393591e+00
## 15607  3 1475  3.327167e+00
## 15608  3 1476  3.283262e+00
## 15609  3 1477  3.234126e+00
## 15610  3 1478  3.184802e+00
## 15611  3 1479  3.145131e+00
## 15612  3 1480  3.102248e+00
## 15613  3 1481  3.061493e+00
## 15614  3 1482  3.024455e+00
## 15615  3 1483  2.988320e+00
## 15616  3 1484  2.948972e+00
## 15617  3 1485  2.909795e+00
## 15618  3 1486  2.875542e+00
## 15619  3 1487  2.837647e+00
## 15620  3 1488  2.799917e+00
## 15621  3 1489  7.000473e+00
## 15622  3 1490  5.725843e+00
## 15623  3 1491  4.875953e+00
## 15624  3 1492  4.345142e+00
## 15625  3 1493  4.007366e+00
## 15626  3 1494  3.788315e+00
## 15627  3 1495  3.632767e+00
## 15628  3 1496  3.526579e+00
## 15629  3 1497  3.450883e+00
## 15630  3 1498  3.378717e+00
## 15631  3 1499  3.322595e+00
## 15632  3 1500  3.273815e+00
## 15633  3 1501  3.226620e+00
## 15634  3 1502  3.188811e+00
## 15635  3 1503  3.138545e+00
## 15636  3 1504  3.099283e+00
## 15637  3 1505  3.059245e+00
## 15638  3 1506  3.023186e+00
## 15639  3 1507  2.986032e+00
## 15640  3 1508  2.949696e+00
## 15641  3 1509  2.907481e+00
## 15642  3 1510  2.872197e+00
## 15643  3 1511  2.836072e+00
## 15644  3 1512  2.802062e+00
## 15645  3 1513  6.991554e+00
## 15646  3 1514  5.726298e+00
## 15647  3 1515  4.880400e+00
## 15648  3 1516  4.346776e+00
## 15649  3 1517  4.008207e+00
## 15650  3 1518  3.786222e+00
## 15651  3 1519  3.632920e+00
## 15652  3 1520  3.519531e+00
## 15653  3 1521  3.449180e+00
## 15654  3 1522  3.378574e+00
## 15655  3 1523  3.316135e+00
## 15656  3 1524  3.272240e+00
## 15657  3 1525  3.226420e+00
## 15658  3 1526  3.183615e+00
## 15659  3 1527  3.137276e+00
## 15660  3 1528  3.104338e+00
## 15661  3 1529  3.058309e+00
## 15662  3 1530  3.019628e+00
## 15663  3 1531  2.981891e+00
## 15664  3 1532  2.948113e+00
## 15665  3 1533  2.910556e+00
## 15666  3 1534  2.872054e+00
## 15667  3 1535  2.833856e+00
## 15668  3 1536  2.795066e+00
## 15669  3 1537  6.989369e+00
## 15670  3 1538  5.724010e+00
## 15671  3 1539  4.873048e+00
## 15672  3 1540  4.341164e+00
## 15673  3 1541  4.003698e+00
## 15674  3 1542  3.778499e+00
## 15675  3 1543  3.625402e+00
## 15676  3 1544  3.523609e+00
## 15677  3 1545  3.439715e+00
## 15678  3 1546  3.380140e+00
## 15679  3 1547  3.317066e+00
## 15680  3 1548  3.272709e+00
## 15681  3 1549  3.221879e+00
## 15682  3 1550  3.176604e+00
## 15683  3 1551  3.140584e+00
## 15684  3 1552  3.097860e+00
## 15685  3 1553  3.056323e+00
## 15686  3 1554  3.021567e+00
## 15687  3 1555  2.980013e+00
## 15688  3 1556  2.940468e+00
## 15689  3 1557  2.903918e+00
## 15690  3 1558  2.865807e+00
## 15691  3 1559  2.832574e+00
## 15692  3 1560  2.795715e+00
## 15693  3 1560  2.792569e+00
## 15694  3 1561  6.986630e+00
## 15695  3 1562  5.722276e+00
## 15696  3 1563  4.857736e+00
## 15697  3 1564  4.333275e+00
## 15698  3 1565  3.996885e+00
## 15699  3 1566  3.770173e+00
## 15700  3 1567  3.629108e+00
## 15701  3 1568  3.523947e+00
## 15702  3 1569  3.434452e+00
## 15703  3 1570  3.369853e+00
## 15704  3 1571  3.314706e+00
## 15705  3 1572  3.265418e+00
## 15706  3 1573  3.219554e+00
## 15707  3 1574  3.179483e+00
## 15708  3 1575  3.132926e+00
## 15709  3 1576  3.089723e+00
## 15710  3 1577  3.047075e+00
## 15711  3 1578  3.014379e+00
## 15712  3 1579  2.980723e+00
## 15713  3 1580  2.945310e+00
## 15714  3 1581  2.903119e+00
## 15715  3 1582  2.855637e+00
## 15716  3 1583  2.827508e+00
## 15717  3 1584  2.793320e+00
## 15718  3 1584  2.790722e+00
## 15719  3 1585  2.756250e+00
## 15720  3 1586  2.721571e+00
## 15721  3 1587  2.684189e+00
## 15722  3 1588  2.649906e+00
## 15723  3 1589  2.618948e+00
## 15724  3 1590  2.588885e+00
## 15725  3 1591  2.553460e+00
## 15726  3 1592  2.523820e+00
## 15727  3 1593  2.487202e+00
## 15728  3 1594  2.456518e+00
## 15729  3 1595  2.423311e+00
## 15730  3 1596  2.397800e+00
## 15731  3 1597  2.366484e+00
## 15732  3 1598  2.338003e+00
## 15733  3 1599  2.306814e+00
## 15734  3 1599  2.307578e+00
## 15735  3 1600  4.389229e+00
## 15736  3 1601  3.740829e+00
## 15737  3 1602  3.312014e+00
## 15738  3 1603  3.030328e+00
## 15739  3 1604  2.847575e+00
## 15740  3 1605  2.734134e+00
## 15741  3 1606  2.643630e+00
## 15742  3 1607  2.580383e+00
## 15743  3 1608  2.530806e+00
## 15744  3 1608  2.525753e+00
## 15745  3 1609  6.718554e+00
## 15746  3 1610  5.444969e+00
## 15747  3 1611  4.598361e+00
## 15748  3 1612  4.063050e+00
## 15749  3 1613  3.728723e+00
## 15750  3 1614  3.508997e+00
## 15751  3 1615  3.361859e+00
## 15752  3 1616  3.248617e+00
## 15753  3 1617  3.178434e+00
## 15754  3 1618  3.108843e+00
## 15755  3 1619  3.058034e+00
## 15756  3 1620  3.020196e+00
## 15757  3 1621  2.972800e+00
## 15758  3 1622  2.929411e+00
## 15759  3 1623  2.889408e+00
## 15760  3 1624  2.858022e+00
## 15761  3 1625  2.818126e+00
## 15762  3 1626  2.783686e+00
## 15763  3 1627  2.743527e+00
## 15764  3 1628  2.713553e+00
## 15765  3 1629  2.677178e+00
## 15766  3 1630  2.644083e+00
## 15767  3 1631  2.612442e+00
## 15768  3 1632  2.577580e+00
## 15769  3 1633  6.772699e+00
## 15770  3 1634  5.506535e+00
## 15771  3 1635  4.663711e+00
## 15772  3 1636  4.128877e+00
## 15773  3 1637  3.796911e+00
## 15774  3 1638  3.578137e+00
## 15775  3 1639  3.427988e+00
## 15776  3 1640  3.324909e+00
## 15777  3 1641  3.247752e+00
## 15778  3 1642  3.188257e+00
## 15779  3 1643  3.126481e+00
## 15780  3 1644  3.082436e+00
## 15781  3 1645  3.033941e+00
## 15782  3 1646  2.995027e+00
## 15783  3 1647  2.957044e+00
## 15784  3 1648  2.920172e+00
## 15785  3 1649  2.880624e+00
## 15786  3 1650  2.842850e+00
## 15787  3 1651  2.806866e+00
## 15788  3 1652  2.772510e+00
## 15789  3 1653  2.736100e+00
## 15790  3 1654  2.702008e+00
## 15791  3 1655  2.672444e+00
## 15792  3 1656  2.630659e+00
## 15793  3 1657  6.826752e+00
## 15794  3 1658  5.564022e+00
## 15795  3 1659  4.703578e+00
## 15796  3 1660  4.181044e+00
## 15797  3 1661  3.849338e+00
## 15798  3 1662  3.622794e+00
## 15799  3 1663  3.484903e+00
## 15800  3 1664  3.373750e+00
## 15801  3 1665  3.291763e+00
## 15802  3 1666  3.228916e+00
## 15803  3 1667  3.180485e+00
## 15804  3 1668  3.127172e+00
## 15805  3 1669  3.082628e+00
## 15806  3 1670  3.038261e+00
## 15807  3 1671  3.003861e+00
## 15808  3 1672  2.962002e+00
## 15809  3 1673  2.928365e+00
## 15810  3 1674  2.882991e+00
## 15811  3 1675  2.851381e+00
## 15812  3 1676  2.812761e+00
## 15813  3 1677  2.779984e+00
## 15814  3 1678  2.746159e+00
## 15815  3 1679  2.708206e+00
## 15816  3 1680  2.676854e+00
## 15817  3 1681  6.868356e+00
## 15818  3 1682  5.583755e+00
## 15819  3 1683  4.744895e+00
## 15820  3 1684  4.230968e+00
## 15821  3 1685  3.892772e+00
## 15822  3 1686  3.668994e+00
## 15823  3 1687  3.522939e+00
## 15824  3 1688  3.411502e+00
## 15825  3 1689  3.331191e+00
## 15826  3 1690  3.270320e+00
## 15827  3 1691  3.212431e+00
## 15828  3 1692  3.166924e+00
## 15829  3 1693  3.119819e+00
## 15830  3 1694  3.081272e+00
## 15831  3 1695  3.040952e+00
## 15832  3 1696  2.993035e+00
## 15833  3 1697  2.957452e+00
## 15834  3 1698  2.921797e+00
## 15835  3 1699  2.883522e+00
## 15836  3 1700  2.846900e+00
## 15837  3 1701  2.813624e+00
## 15838  3 1702  2.772800e+00
## 15839  3 1703  2.737238e+00
## 15840  3 1704  2.706090e+00
## 15841  3 1705  6.904657e+00
## 15842  3 1706  5.626776e+00
## 15843  3 1707  4.777917e+00
## 15844  3 1708  4.253934e+00
## 15845  3 1709  3.915413e+00
## 15846  3 1710  3.699419e+00
## 15847  3 1711  3.548724e+00
## 15848  3 1712  3.442868e+00
## 15849  3 1713  3.364226e+00
## 15850  3 1714  3.292038e+00
## 15851  3 1715  3.238472e+00
## 15852  3 1716  3.193821e+00
## 15853  3 1717  3.147917e+00
## 15854  3 1718  3.105059e+00
## 15855  3 1719  3.064660e+00
## 15856  3 1720  3.016728e+00
## 15857  3 1721  2.991198e+00
## 15858  3 1722  2.946484e+00
## 15859  3 1723  2.902155e+00
## 15860  3 1724  2.876061e+00
## 15861  3 1725  2.836468e+00
## 15862  3 1726  2.798890e+00
## 15863  3 1727  2.759551e+00
## 15864  3 1728  2.734057e+00
## 15865  3 1729  6.915470e+00
## 15866  3 1730  5.657490e+00
## 15867  3 1731  4.799165e+00
## 15868  3 1732  4.277878e+00
## 15869  3 1733  3.930805e+00
## 15870  3 1734  3.715073e+00
## 15871  3 1735  3.569277e+00
## 15872  3 1736  3.458574e+00
## 15873  3 1737  3.381419e+00
## 15874  3 1738  3.318150e+00
## 15875  3 1739  3.263652e+00
## 15876  3 1740  3.203456e+00
## 15877  3 1741  3.159835e+00
## 15878  3 1742  3.123749e+00
## 15879  3 1743  3.078373e+00
## 15880  3 1744  3.042692e+00
## 15881  3 1745  3.004025e+00
## 15882  3 1746  2.961205e+00
## 15883  3 1747  2.928790e+00
## 15884  3 1748  2.887224e+00
## 15885  3 1749  2.851578e+00
## 15886  3 1750  2.820453e+00
## 15887  3 1751  2.781417e+00
## 15888  3 1752  2.746817e+00
## 15889  3 1753  6.932045e+00
## 15890  3 1754  5.671332e+00
## 15891  3 1755  4.819163e+00
## 15892  3 1756  4.293814e+00
## 15893  3 1757  3.948864e+00
## 15894  3 1758  3.729220e+00
## 15895  3 1759  3.580297e+00
## 15896  3 1760  3.483387e+00
## 15897  3 1761  3.396100e+00
## 15898  3 1762  3.330062e+00
## 15899  3 1763  3.268611e+00
## 15900  3 1764  3.225617e+00
## 15901  3 1765  3.178960e+00
## 15902  3 1766  3.139982e+00
## 15903  3 1767  3.092027e+00
## 15904  3 1768  3.057122e+00
## 15905  3 1769  3.015180e+00
## 15906  3 1770  2.977235e+00
## 15907  3 1771  2.937649e+00
## 15908  3 1772  2.902865e+00
## 15909  3 1773  2.867028e+00
## 15910  3 1774  2.829719e+00
## 15911  3 1775  2.794615e+00
## 15912  3 1776  2.755039e+00
## 15913  3 1777  6.937177e+00
## 15914  3 1778  5.680354e+00
## 15915  3 1779  4.825545e+00
## 15916  3 1780  4.293305e+00
## 15917  3 1781  3.961096e+00
## 15918  3 1782  3.749979e+00
## 15919  3 1783  3.595438e+00
## 15920  3 1784  3.483873e+00
## 15921  3 1785  3.402962e+00
## 15922  3 1786  3.347877e+00
## 15923  3 1787  3.288836e+00
## 15924  3 1788  3.235287e+00
## 15925  3 1789  3.189963e+00
## 15926  3 1790  3.144383e+00
## 15927  3 1791  3.103563e+00
## 15928  3 1792  3.066294e+00
## 15929  3 1793  3.029567e+00
## 15930  3 1794  2.985263e+00
## 15931  3 1795  2.944250e+00
## 15932  3 1796  2.911129e+00
## 15933  3 1797  2.873272e+00
## 15934  3 1798  2.838502e+00
## 15935  3 1799  2.802005e+00
## 15936  3 1800  2.768179e+00
## 15937  3 1801  6.965266e+00
## 15938  3 1802  5.684614e+00
## 15939  3 1803  4.848915e+00
## 15940  3 1804  4.307564e+00
## 15941  3 1805  3.969816e+00
## 15942  3 1806  3.752889e+00
## 15943  3 1807  3.604142e+00
## 15944  3 1808  3.496585e+00
## 15945  3 1809  3.414193e+00
## 15946  3 1810  3.355628e+00
## 15947  3 1811  3.295414e+00
## 15948  3 1812  3.243772e+00
## 15949  3 1813  3.196308e+00
## 15950  3 1814  3.153015e+00
## 15951  3 1815  3.110858e+00
## 15952  3 1816  3.071832e+00
## 15953  3 1817  3.033923e+00
## 15954  3 1818  2.992639e+00
## 15955  3 1819  2.952445e+00
## 15956  3 1820  2.919285e+00
## 15957  3 1821  2.878508e+00
## 15958  3 1822  2.840119e+00
## 15959  3 1823  2.809305e+00
## 15960  3 1824  2.774610e+00
## 15961  3 1825  6.954399e+00
## 15962  3 1826  5.689438e+00
## 15963  3 1827  4.841972e+00
## 15964  3 1828  4.313819e+00
## 15965  3 1829  3.971864e+00
## 15966  3 1830  3.763697e+00
## 15967  3 1831  3.607625e+00
## 15968  3 1832  3.498446e+00
## 15969  3 1833  3.414987e+00
## 15970  3 1834  3.356357e+00
## 15971  3 1835  3.297911e+00
## 15972  3 1836  3.250616e+00
## 15973  3 1837  3.200748e+00
## 15974  3 1838  3.156358e+00
## 15975  3 1839  3.116844e+00
## 15976  3 1840  3.079333e+00
## 15977  3 1841  3.036619e+00
## 15978  3 1842  3.001113e+00
## 15979  3 1843  2.959953e+00
## 15980  3 1844  2.926749e+00
## 15981  3 1845  2.885129e+00
## 15982  3 1846  2.847072e+00
## 15983  3 1847  2.814234e+00
## 15984  3 1848  2.776107e+00
## 15985  3 1849  6.967945e+00
## 15986  3 1850  5.701338e+00
## 15987  3 1851  4.848608e+00
## 15988  3 1852  4.325186e+00
## 15989  3 1853  3.979767e+00
## 15990  3 1854  3.759845e+00
## 15991  3 1855  3.614757e+00
## 15992  3 1856  3.502752e+00
## 15993  3 1857  3.422275e+00
## 15994  3 1858  3.359463e+00
## 15995  3 1859  3.306665e+00
## 15996  3 1860  3.257575e+00
## 15997  3 1861  3.206003e+00
## 15998  3 1862  3.170626e+00
## 15999  3 1863  3.125451e+00
## 16000  3 1864  3.081909e+00
## 16001  3 1865  3.041396e+00
## 16002  3 1866  3.006842e+00
## 16003  3 1867  2.959439e+00
## 16004  3 1868  2.928071e+00
## 16005  3 1869  2.889598e+00
## 16006  3 1870  2.855945e+00
## 16007  3 1871  2.822709e+00
## 16008  3 1872  2.780514e+00
## 16009  3 1872  2.791041e+00
## 16010  3 1873  6.986931e+00
## 16011  3 1874  5.718199e+00
## 16012  3 1875  4.863603e+00
## 16013  3 1876  4.332668e+00
## 16014  3 1877  3.993321e+00
## 16015  3 1878  3.765291e+00
## 16016  3 1879  3.632685e+00
## 16017  3 1880  3.516819e+00
## 16018  3 1881  3.435902e+00
## 16019  3 1882  3.369011e+00
## 16020  3 1883  3.309459e+00
## 16021  3 1884  3.266719e+00
## 16022  3 1885  3.212268e+00
## 16023  3 1886  3.173182e+00
## 16024  3 1887  3.126939e+00
## 16025  3 1888  3.089444e+00
## 16026  3 1889  3.051917e+00
## 16027  3 1890  3.018344e+00
## 16028  3 1891  2.978432e+00
## 16029  3 1892  2.934491e+00
## 16030  3 1893  2.900130e+00
## 16031  3 1894  2.859646e+00
## 16032  3 1895  2.829413e+00
## 16033  3 1896  2.792612e+00
## 16034  3 1896  2.798201e+00
## 16035  3 1897  2.754052e+00
```

```
## 16036  3 1898  2.724567e+00
## 16037  3 1899  2.690434e+00
## 16038  3 1900  2.652007e+00
## 16039  3 1901  2.619442e+00
## 16040  3 1902  2.588204e+00
## 16041  3 1903  2.548463e+00
## 16042  3 1904  2.524982e+00
## 16043  3 1905  2.488418e+00
## 16044  3 1906  2.455033e+00
## 16045  3 1907  2.427061e+00
## 16046  3 1908  2.391266e+00
## 16047  3 1909  2.368487e+00
## 16048  3 1910  2.336403e+00
## 16049  3 1911  2.306362e+00
## 16050  3 1911  2.304373e+00
## 16051  3 1912  6.511321e+00
## 16052  3 1913  5.236911e+00
## 16053  3 1914  4.395092e+00
## 16054  3 1915  3.875430e+00
## 16055  3 1916  3.538350e+00
## 16056  3 1917  3.325243e+00
## 16057  3 1918  3.172674e+00
## 16058  3 1919  3.081546e+00
## 16059  3 1920  3.002924e+00
## 16060  3 1920  3.000176e+00
## 16061  3 1921  7.172736e+00
## 16062  3 1922  5.893524e+00
## 16063  3 1923  5.025913e+00
## 16064  3 1924  4.487996e+00
## 16065  3 1925  4.140752e+00
## 16066  3 1926  3.920765e+00
## 16067  3 1927  3.770152e+00
## 16068  3 1928  3.657156e+00
## 16069  3 1929  3.580411e+00
## 16070  3 1930  3.510069e+00
## 16071  3 1931  3.450015e+00
## 16072  3 1932  3.388913e+00
## 16073  3 1933  3.350765e+00
## 16074  3 1934  3.303606e+00
## 16075  3 1935  3.264877e+00
## 16076  3 1936  3.220191e+00
## 16077  3 1937  3.177108e+00
## 16078  3 1938  3.140450e+00
## 16079  3 1939  3.102184e+00
## 16080  3 1940  3.057052e+00
## 16081  3 1941  3.019452e+00
## 16082  3 1942  2.982368e+00
## 16083  3 1943  2.949135e+00
## 16084  3 1944  2.908362e+00
## 16085  3 1945  7.088587e+00
## 16086  3 1946  5.826895e+00
## 16087  3 1947  4.979017e+00
## 16088  3 1948  4.437417e+00
## 16089  3 1949  4.104318e+00
## 16090  3 1950  3.877177e+00
## 16091  3 1951  3.732149e+00
## 16092  3 1952  3.624339e+00
## 16093  3 1953  3.541552e+00
## 16094  3 1954  3.476433e+00
## 16095  3 1955  3.419001e+00
## 16096  3 1956  3.366206e+00
## 16097  3 1957  3.310467e+00
## 16098  3 1958  3.267293e+00
## 16099  3 1959  3.231213e+00
## 16100  3 1960  3.178526e+00
## 16101  3 1961  3.140667e+00
## 16102  3 1962  3.104402e+00
## 16103  3 1963  3.068232e+00
## 16104  3 1964  3.027414e+00
## 16105  3 1965  2.985572e+00
## 16106  3 1966  2.940487e+00
## 16107  3 1967  2.913081e+00
## 16108  3 1968  2.876743e+00
## 16109  3 1969  7.068738e+00
## 16110  3 1970  5.802487e+00
## 16111  3 1971  4.941653e+00
## 16112  3 1972  4.417626e+00
## 16113  3 1973  4.074360e+00
## 16114  3 1974  3.853200e+00
## 16115  3 1975  3.699784e+00
## 16116  3 1976  3.593521e+00
## 16117  3 1977  3.507609e+00
## 16118  3 1978  3.444881e+00
## 16119  3 1979  3.384816e+00
## 16120  3 1980  3.340113e+00
## 16121  3 1981  3.292427e+00
## 16122  3 1982  3.242811e+00
## 16123  3 1983  3.207612e+00
## 16124  3 1984  3.160259e+00
## 16125  3 1985  3.119163e+00
## 16126  3 1986  3.082204e+00
## 16127  3 1987  3.042191e+00
## 16128  3 1988  3.006132e+00
## 16129  3 1989  2.962781e+00
## 16130  3 1990  2.922925e+00
## 16131  3 1991  2.888606e+00
## 16132  3 1992  2.856543e+00
## 16133  3 1993  7.040817e+00
## 16134  3 1994  5.772711e+00
## 16135  3 1995  4.925228e+00
## 16136  3 1996  4.389488e+00
## 16137  3 1997  4.055388e+00
## 16138  3 1998  3.835994e+00
## 16139  3 1999  3.674360e+00
## 16140  3 2000  3.575073e+00
## 16141  3 2001  3.496110e+00
## 16142  3 2002  3.424220e+00
## 16143  3 2003  3.368295e+00
## 16144  3 2004  3.320725e+00
## 16145  3 2005  3.262097e+00
## 16146  3 2006  3.229383e+00
## 16147  3 2007  3.186674e+00
## 16148  3 2008  3.144813e+00
## 16149  3 2009  3.101152e+00
## 16150  3 2010  3.062970e+00
## 16151  3 2011  3.019725e+00
## 16152  3 2012  2.985532e+00
## 16153  3 2013  2.948155e+00
## 16154  3 2014  2.909776e+00
## 16155  3 2015  2.873213e+00
## 16156  3 2016  2.841698e+00
## 16157  3 2017  7.024759e+00
## 16158  3 2018  5.766038e+00
## 16159  3 2019  4.903148e+00
## 16160  3 2020  4.373515e+00
## 16161  3 2021  4.037504e+00
## 16162  3 2022  3.817632e+00
## 16163  3 2023  3.664885e+00
## 16164  3 2024  3.559223e+00
## 16165  3 2025  3.473545e+00
## 16166  3 2026  3.413502e+00
## 16167  3 2027  3.351968e+00
## 16168  3 2028  3.306558e+00
## 16169  3 2029  3.254688e+00
## 16170  3 2030  3.213688e+00
## 16171  3 2031  3.172188e+00
## 16172  3 2032  3.129366e+00
## 16173  3 2033  3.089796e+00
## 16174  3 2034  3.049974e+00
## 16175  3 2035  3.011790e+00
## 16176  3 2036  2.970330e+00
## 16177  3 2037  2.937781e+00
## 16178  3 2038  2.895527e+00
## 16179  3 2039  2.867117e+00
## 16180  3 2040  2.827225e+00
## 16181  3 2041  7.022360e+00
## 16182  3 2042  5.744910e+00
## 16183  3 2043  4.891888e+00
## 16184  3 2044  4.356267e+00
## 16185  3 2045  4.026847e+00
## 16186  3 2046  3.811316e+00
## 16187  3 2047  3.655622e+00
## 16188  3 2048  3.553210e+00
## 16189  3 2049  3.468458e+00
## 16190  3 2050  3.400765e+00
## 16191  3 2051  3.346604e+00
## 16192  3 2052  3.294366e+00
## 16193  3 2053  3.250721e+00
## 16194  3 2054  3.201827e+00
## 16195  3 2055  3.164337e+00
## 16196  3 2056  3.119185e+00
## 16197  3 2057  3.074544e+00
## 16198  3 2058  3.040764e+00
## 16199  3 2059  2.997544e+00
## 16200  3 2060  2.963270e+00
## 16201  3 2061  2.926629e+00
## 16202  3 2062  2.889908e+00
## 16203  3 2063  2.851737e+00
## 16204  3 2064  2.814677e+00
## 16205  3 2065  7.001093e+00
## 16206  3 2066  5.739814e+00
## 16207  3 2067  4.886811e+00
## 16208  3 2068  4.362992e+00
## 16209  3 2069  4.016655e+00
## 16210  3 2070  3.800989e+00
## 16211  3 2071  3.650743e+00
## 16212  3 2072  3.541734e+00
## 16213  3 2073  3.455799e+00
## 16214  3 2074  3.403390e+00
## 16215  3 2075  3.330789e+00
## 16216  3 2076  3.281482e+00
## 16217  3 2077  3.246260e+00
## 16218  3 2078  3.192561e+00
## 16219  3 2079  3.154223e+00
## 16220  3 2080  3.108422e+00
## 16221  3 2081  3.072333e+00
## 16222  3 2082  3.036023e+00
## 16223  3 2083  2.994383e+00
## 16224  3 2084  2.957064e+00
## 16225  3 2085  2.921379e+00
## 16226  3 2086  2.882267e+00
## 16227  3 2087  2.852363e+00
## 16228  3 2088  2.808231e+00
## 16229  3 2089  6.995324e+00
## 16230  3 2090  5.728155e+00
## 16231  3 2091  4.882000e+00
## 16232  3 2092  4.348966e+00
## 16233  3 2093  4.014738e+00
## 16234  3 2094  3.789249e+00
## 16235  3 2095  3.643344e+00
## 16236  3 2096  3.536034e+00
## 16237  3 2097  3.452693e+00
## 16238  3 2098  3.392840e+00
## 16239  3 2099  3.331779e+00
## 16240  3 2100  3.278626e+00
## 16241  3 2101  3.232571e+00
## 16242  3 2102  3.189397e+00
## 16243  3 2103  3.146114e+00
## 16244  3 2104  3.110459e+00
## 16245  3 2105  3.070847e+00
## 16246  3 2106  3.033021e+00
## 16247  3 2107  2.983567e+00
## 16248  3 2108  2.951338e+00
## 16249  3 2109  2.915105e+00
## 16250  3 2110  2.876199e+00
## 16251  3 2111  2.841073e+00
## 16252  3 2112  2.804833e+00
## 16253  3 2113  6.994354e+00
## 16254  3 2114  5.730135e+00
## 16255  3 2115  4.872096e+00
## 16256  3 2116  4.347653e+00
## 16257  3 2117  4.007720e+00
## 16258  3 2118  3.789357e+00
## 16259  3 2119  3.640263e+00
## 16260  3 2120  3.528280e+00
## 16261  3 2121  3.449129e+00
## 16262  3 2122  3.382028e+00
## 16263  3 2123  3.321963e+00
## 16264  3 2124  3.273656e+00
## 16265  3 2125  3.228859e+00
## 16266  3 2126  3.186662e+00
## 16267  3 2127  3.149043e+00
## 16268  3 2128  3.102191e+00
## 16269  3 2129  3.068073e+00
## 16270  3 2130  3.026240e+00
## 16271  3 2131  2.982079e+00
## 16272  3 2132  2.946880e+00
## 16273  3 2133  2.914300e+00
## 16274  3 2134  2.875196e+00
## 16275  3 2135  2.839118e+00
## 16276  3 2136  2.798555e+00
## 16277  3 2137  6.979462e+00
## 16278  3 2138  5.721637e+00
## 16279  3 2139  4.881479e+00
## 16280  3 2140  4.345254e+00
## 16281  3 2141  4.014394e+00
## 16282  3 2142  3.778824e+00
## 16283  3 2143  3.635201e+00
## 16284  3 2144  3.526982e+00
## 16285  3 2145  3.446891e+00
## 16286  3 2146  3.378246e+00
## 16287  3 2147  3.321762e+00
## 16288  3 2148  3.267622e+00
## 16289  3 2149  3.224758e+00
## 16290  3 2150  3.181366e+00
## 16291  3 2151  3.138302e+00
## 16292  3 2152  3.103121e+00
## 16293  3 2153  3.060396e+00
## 16294  3 2154  3.021601e+00
## 16295  3 2155  2.984377e+00
## 16296  3 2156  2.942536e+00
## 16297  3 2157  2.905049e+00
## 16298  3 2158  2.874528e+00
## 16299  3 2159  2.828356e+00
## 16300  3 2160  2.797761e+00
## 16301  3 2161  6.982066e+00
## 16302  3 2162  5.714847e+00
## 16303  3 2163  4.865631e+00
## 16304  3 2164  4.338578e+00
## 16305  3 2165  4.003997e+00
## 16306  3 2166  3.778441e+00
## 16307  3 2167  3.629136e+00
## 16308  3 2168  3.525004e+00
## 16309  3 2169  3.446538e+00
## 16310  3 2170  3.376222e+00
## 16311  3 2171  3.321858e+00
## 16312  3 2172  3.272707e+00
## 16313  3 2173  3.225032e+00
## 16314  3 2174  3.178833e+00
## 16315  3 2175  3.141563e+00
## 16316  3 2176  3.098658e+00
## 16317  3 2177  3.058698e+00
## 16318  3 2178  3.015092e+00
## 16319  3 2179  2.984262e+00
## 16320  3 2180  2.940504e+00
## 16321  3 2181  2.907777e+00
## 16322  3 2182  2.870544e+00
## 16323  3 2183  2.829562e+00
## 16324  3 2184  2.800286e+00
## 16325  3 2184  2.791422e+00
## 16326  3 2185  6.980992e+00
## 16327  3 2186  5.712378e+00
## 16328  3 2187  4.860630e+00
## 16329  3 2188  4.340595e+00
## 16330  3 2189  3.989373e+00
## 16331  3 2190  3.773615e+00
## 16332  3 2191  3.623324e+00
## 16333  3 2192  3.520275e+00
## 16334  3 2193  3.431519e+00
## 16335  3 2194  3.375315e+00
## 16336  3 2195  3.314598e+00
## 16337  3 2196  3.270427e+00
## 16338  3 2197  3.224771e+00
## 16339  3 2198  3.172775e+00
## 16340  3 2199  3.135972e+00
## 16341  3 2200  3.089101e+00
## 16342  3 2201  3.055707e+00
## 16343  3 2202  3.017876e+00
## 16344  3 2203  2.975237e+00
## 16345  3 2204  2.937129e+00
## 16346  3 2205  2.898873e+00
## 16347  3 2206  2.867804e+00
## 16348  3 2207  2.827792e+00
## 16349  3 2208  2.791655e+00
## 16350  3 2208  2.795102e+00
## 16351  3 2209  2.758117e+00
## 16352  3 2210  2.721630e+00
## 16353  3 2211  2.684794e+00
## 16354  3 2212  2.653172e+00
## 16355  3 2213  2.625764e+00
## 16356  3 2214  2.584724e+00
## 16357  3 2215  2.555381e+00
## 16358  3 2216  2.520433e+00
## 16359  3 2217  2.487172e+00
## 16360  3 2218  2.461021e+00
## 16361  3 2219  2.426508e+00
## 16362  3 2220  2.397489e+00
## 16363  3 2221  2.369562e+00
## 16364  3 2222  2.333694e+00
## 16365  3 2223  2.310411e+00
## 16366  3 2224  2.278319e+00
## 16367  3 2225  2.249716e+00
## 16368  3 2226  2.222659e+00
## 16369  3 2226  2.221750e+00
## 16370  3 2227  4.295824e+00
## 16371  3 2228  3.652933e+00
## 16372  3 2229  3.227052e+00
## 16373  3 2230  2.951400e+00
## 16374  3 2231  2.771400e+00
## 16375  3 2232  2.654935e+00
## 16376  3 2232  2.649360e+00
## 16377  3 2233  6.786922e+00
## 16378  3 2234  5.500565e+00
## 16379  3 2235  4.637771e+00
## 16380  3 2236  4.093829e+00
## 16381  3 2237  3.750147e+00
## 16382  3 2238  3.522866e+00
## 16383  3 2239  3.380739e+00
## 16384  3 2240  3.271997e+00
## 16385  3 2241  3.190975e+00
## 16386  3 2242  3.128946e+00
## 16387  3 2243  3.084586e+00
## 16388  3 2244  3.036532e+00
## 16389  3 2245  2.992291e+00
## 16390  3 2246  2.953685e+00
## 16391  3 2247  2.908285e+00
## 16392  3 2248  2.863942e+00
## 16393  3 2249  2.831395e+00
## 16394  3 2250  2.797297e+00
## 16395  3 2251  2.760195e+00
## 16396  3 2252  2.728251e+00
## 16397  3 2253  2.690138e+00
## 16398  3 2254  2.656095e+00
## 16399  3 2255  2.629713e+00
## 16400  3 2256  2.587249e+00
## 16401  3 2257  6.783232e+00
## 16402  3 2258  5.524090e+00
## 16403  3 2259  4.669593e+00
## 16404  3 2260  4.145682e+00
## 16405  3 2261  3.806134e+00
## 16406  3 2262  3.587220e+00
## 16407  3 2263  3.437957e+00
## 16408  3 2264  3.334086e+00
## 16409  3 2265  3.254973e+00
## 16410  3 2266  3.195367e+00
## 16411  3 2267  3.139166e+00
## 16412  3 2268  3.091858e+00
## 16413  3 2269  3.052030e+00
## 16414  3 2270  3.002149e+00
## 16415  3 2271  2.965288e+00
## 16416  3 2272  2.928532e+00
## 16417  3 2273  2.885882e+00
## 16418  3 2274  2.852983e+00
## 16419  3 2275  2.813964e+00
## 16420  3 2276  2.780242e+00
## 16421  3 2277  2.748102e+00
## 16422  3 2278  2.712880e+00
## 16423  3 2279  2.679505e+00
## 16424  3 2280  2.645873e+00
## 16425  3 2281  6.837355e+00
## 16426  3 2282  5.575722e+00
## 16427  3 2283  4.723732e+00
## 16428  3 2284  4.184016e+00
## 16429  3 2285  3.854481e+00
## 16430  3 2286  3.638808e+00
## 16431  3 2287  3.495799e+00
## 16432  3 2288  3.382151e+00
## 16433  3 2289  3.305754e+00
## 16434  3 2290  3.241013e+00
## 16435  3 2291  3.185148e+00
## 16436  3 2292  3.137042e+00
## 16437  3 2293  3.090113e+00
## 16438  3 2294  3.052573e+00
## 16439  3 2295  3.011704e+00
## 16440  3 2296  2.970171e+00
## 16441  3 2297  2.933462e+00
## 16442  3 2298  2.898350e+00
## 16443  3 2299  2.857530e+00
## 16444  3 2300  2.820994e+00
## 16445  3 2301  2.795479e+00
## 16446  3 2302  2.754093e+00
## 16447  3 2303  2.721041e+00
## 16448  3 2304  2.683753e+00
## 16449  3 2305  6.866530e+00
## 16450  3 2306  5.594983e+00
## 16451  3 2307  4.759650e+00
## 16452  3 2308  4.224820e+00
## 16453  3 2309  3.895338e+00
## 16454  3 2310  3.673069e+00
## 16455  3 2311  3.525076e+00
## 16456  3 2312  3.419070e+00
## 16457  3 2313  3.333557e+00
## 16458  3 2314  3.271840e+00
## 16459  3 2315  3.217376e+00
## 16460  3 2316  3.170314e+00
## 16461  3 2317  3.126776e+00
## 16462  3 2318  3.088747e+00
## 16463  3 2319  3.043706e+00
## 16464  3 2320  3.000099e+00
## 16465  3 2321  2.966390e+00
## 16466  3 2322  2.933108e+00
## 16467  3 2323  2.886273e+00
## 16468  3 2324  2.858695e+00
## 16469  3 2325  2.822420e+00
## 16470  3 2326  2.778249e+00
## 16471  3 2327  2.745452e+00
## 16472  3 2328  2.717766e+00
## 16473  3 2329  6.890986e+00
## 16474  3 2330  5.631119e+00
## 16475  3 2331  4.788738e+00
## 16476  3 2332  4.261719e+00
## 16477  3 2333  3.918456e+00
## 16478  3 2334  3.703539e+00
## 16479  3 2335  3.548863e+00
## 16480  3 2336  3.446339e+00
## 16481  3 2337  3.372893e+00
## 16482  3 2338  3.300633e+00
## 16483  3 2339  3.243007e+00
## 16484  3 2340  3.196338e+00
## 16485  3 2341  3.153797e+00
## 16486  3 2342  3.110647e+00
## 16487  3 2343  3.075116e+00
## 16488  3 2344  3.024263e+00
## 16489  3 2345  2.989846e+00
## 16490  3 2346  2.944131e+00
## 16491  3 2347  2.911385e+00
## 16492  3 2348  2.881138e+00
## 16493  3 2349  2.839716e+00
## 16494  3 2350  2.803046e+00
## 16495  3 2351  2.761844e+00
## 16496  3 2352  2.732732e+00
## 16497  3 2353  6.919683e+00
## 16498  3 2354  5.660813e+00
## 16499  3 2355  4.802905e+00
## 16500  3 2356  4.283268e+00
## 16501  3 2357  3.937348e+00
## 16502  3 2358  3.729858e+00
## 16503  3 2359  3.574112e+00
## 16504  3 2360  3.469056e+00
## 16505  3 2361  3.381515e+00
## 16506  3 2362  3.316324e+00
## 16507  3 2363  3.265114e+00
## 16508  3 2364  3.212774e+00
## 16509  3 2365  3.169632e+00
## 16510  3 2366  3.127667e+00
## 16511  3 2367  3.085910e+00
## 16512  3 2368  3.046502e+00
## 16513  3 2369  3.000891e+00
## 16514  3 2370  2.969192e+00
## 16515  3 2371  2.923972e+00
## 16516  3 2372  2.890274e+00
## 16517  3 2373  2.851973e+00
## 16518  3 2374  2.816491e+00
## 16519  3 2375  2.787916e+00
## 16520  3 2376  2.751143e+00
## 16521  3 2377  6.950782e+00
## 16522  3 2378  5.667066e+00
## 16523  3 2379  4.821918e+00
## 16524  3 2380  4.284927e+00
## 16525  3 2381  3.956549e+00
## 16526  3 2382  3.731309e+00
## 16527  3 2383  3.586166e+00
## 16528  3 2384  3.475973e+00
## 16529  3 2385  3.397817e+00
## 16530  3 2386  3.331820e+00
## 16531  3 2387  3.274573e+00
## 16532  3 2388  3.229213e+00
## 16533  3 2389  3.181352e+00
## 16534  3 2390  3.136710e+00
## 16535  3 2391  3.092709e+00
## 16536  3 2392  3.058981e+00
## 16537  3 2393  3.022225e+00
## 16538  3 2394  2.983047e+00
## 16539  3 2395  2.938534e+00
## 16540  3 2396  2.901414e+00
## 16541  3 2397  2.869746e+00
## 16542  3 2398  2.827028e+00
## 16543  3 2399  2.795667e+00
## 16544  3 2400  2.763875e+00
## 16545  3 2401  6.959046e+00
## 16546  3 2402  5.686396e+00
## 16547  3 2403  4.836777e+00
## 16548  3 2404  4.294837e+00
## 16549  3 2405  3.967840e+00
## 16550  3 2406  3.739515e+00
## 16551  3 2407  3.596777e+00
## 16552  3 2408  3.493773e+00
## 16553  3 2409  3.405946e+00
## 16554  3 2410  3.341854e+00
## 16555  3 2411  3.288809e+00
## 16556  3 2412  3.245171e+00
## 16557  3 2413  3.190012e+00
## 16558  3 2414  3.148226e+00
## 16559  3 2415  3.107877e+00
## 16560  3 2416  3.062485e+00
## 16561  3 2417  3.029493e+00
## 16562  3 2418  2.987049e+00
## 16563  3 2419  2.948889e+00
## 16564  3 2420  2.912734e+00
## 16565  3 2421  2.874182e+00
## 16566  3 2422  2.843582e+00
## 16567  3 2423  2.807956e+00
## 16568  3 2424  2.773725e+00
## 16569  3 2425  6.959476e+00
## 16570  3 2426  5.690635e+00
## 16571  3 2427  4.830725e+00
## 16572  3 2428  4.296929e+00
## 16573  3 2429  3.969830e+00
## 16574  3 2430  3.757258e+00
## 16575  3 2431  3.606506e+00
## 16576  3 2432  3.493964e+00
## 16577  3 2433  3.415541e+00
## 16578  3 2434  3.347183e+00
## 16579  3 2435  3.286604e+00
## 16580  3 2436  3.242554e+00
## 16581  3 2437  3.196304e+00
## 16582  3 2438  3.155682e+00
## 16583  3 2439  3.111372e+00
## 16584  3 2440  3.067540e+00
## 16585  3 2441  3.031458e+00
## 16586  3 2442  2.997326e+00
## 16587  3 2443  2.961544e+00
## 16588  3 2444  2.919281e+00
## 16589  3 2445  2.880638e+00
## 16590  3 2446  2.848610e+00
## 16591  3 2447  2.810963e+00
## 16592  3 2448  2.775076e+00
## 16593  3 2449  6.963660e+00
## 16594  3 2450  5.702285e+00
## 16595  3 2451  4.846242e+00
## 16596  3 2452  4.317661e+00
## 16597  3 2453  3.977895e+00
## 16598  3 2454  3.757067e+00
## 16599  3 2455  3.608146e+00
## 16600  3 2456  3.499851e+00
## 16601  3 2457  3.424690e+00
## 16602  3 2458  3.354635e+00
## 16603  3 2459  3.297700e+00
## 16604  3 2460  3.250624e+00
## 16605  3 2461  3.203649e+00
## 16606  3 2462  3.159277e+00
## 16607  3 2463  3.119463e+00
## 16608  3 2464  3.078030e+00
## 16609  3 2465  3.044801e+00
## 16610  3 2466  2.999779e+00
## 16611  3 2467  2.961529e+00
## 16612  3 2468  2.925199e+00
## 16613  3 2469  2.884570e+00
## 16614  3 2470  2.847364e+00
## 16615  3 2471  2.811696e+00
## 16616  3 2472  2.779572e+00
## 16617  3 2473  6.961157e+00
## 16618  3 2474  5.690438e+00
## 16619  3 2475  4.847565e+00
## 16620  3 2476  4.325171e+00
## 16621  3 2477  3.986139e+00
## 16622  3 2478  3.764095e+00
## 16623  3 2479  3.611127e+00
## 16624  3 2480  3.503730e+00
## 16625  3 2481  3.419860e+00
## 16626  3 2482  3.359413e+00
## 16627  3 2483  3.299769e+00
## 16628  3 2484  3.251464e+00
## 16629  3 2485  3.207539e+00
## 16630  3 2486  3.163767e+00
## 16631  3 2487  3.127203e+00
## 16632  3 2488  3.077682e+00
## 16633  3 2489  3.046108e+00
## 16634  3 2490  3.001363e+00
## 16635  3 2491  2.966611e+00
## 16636  3 2492  2.925024e+00
## 16637  3 2493  2.892256e+00
## 16638  3 2494  2.851411e+00
## 16639  3 2495  2.819201e+00
## 16640  3 2496  2.785020e+00
## 16641  3 2496  2.791384e+00
## 16642  3 2497  6.978269e+00
## 16643  3 2498  5.708686e+00
## 16644  3 2499  4.862503e+00
## 16645  3 2500  4.329944e+00
## 16646  3 2501  3.995415e+00
## 16647  3 2502  3.773888e+00
## 16648  3 2503  3.627448e+00
## 16649  3 2504  3.514690e+00
## 16650  3 2505  3.433062e+00
## 16651  3 2506  3.372356e+00
## 16652  3 2507  3.315176e+00
## 16653  3 2508  3.262676e+00
## 16654  3 2509  3.219117e+00
## 16655  3 2510  3.173505e+00
## 16656  3 2511  3.138416e+00
## 16657  3 2512  3.092648e+00
## 16658  3 2513  3.051470e+00
## 16659  3 2514  3.012550e+00
## 16660  3 2515  2.968261e+00
## 16661  3 2516  2.933253e+00
## 16662  3 2517  2.898797e+00
## 16663  3 2518  2.866971e+00
## 16664  3 2519  2.820717e+00
## 16665  3 2520  2.791950e+00
## 16666  3 2520  2.789441e+00
## 16667  3 2521  2.756487e+00
## 16668  3 2522  2.721852e+00
## 16669  3 2523  2.687547e+00
## 16670  3 2524  2.652922e+00
## 16671  3 2525  2.623371e+00
## 16672  3 2526  2.586334e+00
## 16673  3 2527  2.554967e+00
## 16674  3 2528  2.521969e+00
## 16675  3 2529  2.489344e+00
## 16676  3 2530  2.456956e+00
## 16677  3 2531  2.423775e+00
## 16678  3 2532  2.396471e+00
## 16679  3 2533  2.364627e+00
## 16680  3 2534  2.337031e+00
## 16681  3 2535  2.307179e+00
## 16682  3 2536  2.276332e+00
## 16683  3 2537  2.247853e+00
## 16684  3 2538  2.221286e+00
## 16685  3 2538  2.221560e+00
## 16686  3 2539  6.419771e+00
## 16687  3 2540  5.159163e+00
## 16688  3 2541  4.318201e+00
## 16689  3 2542  3.788830e+00
## 16690  3 2543  3.458790e+00
## 16691  3 2544  3.246459e+00
## 16692  3 2544  3.244259e+00
## 16693  3 2545  7.328931e+00
## 16694  3 2546  5.986901e+00
## 16695  3 2547  5.101821e+00
## 16696  3 2548  4.547692e+00
## 16697  3 2549  4.194081e+00
## 16698  3 2550  3.965521e+00
## 16699  3 2551  3.806349e+00
## 16700  3 2552  3.689950e+00
## 16701  3 2553  3.605855e+00
## 16702  3 2554  3.542377e+00
## 16703  3 2555  3.483474e+00
## 16704  3 2556  3.420743e+00
## 16705  3 2557  3.375744e+00
## 16706  3 2558  3.337221e+00
## 16707  3 2559  3.293886e+00
## 16708  3 2560  3.247707e+00
## 16709  3 2561  3.205346e+00
## 16710  3 2562  3.164610e+00
## 16711  3 2563  3.122771e+00
## 16712  3 2564  3.080748e+00
## 16713  3 2565  3.047927e+00
## 16714  3 2566  3.005347e+00
## 16715  3 2567  2.965824e+00
## 16716  3 2568  2.931251e+00
## 16717  3 2569  7.111027e+00
## 16718  3 2570  5.843380e+00
## 16719  3 2571  4.997543e+00
## 16720  3 2572  4.471604e+00
## 16721  3 2573  4.120968e+00
## 16722  3 2574  3.903882e+00
## 16723  3 2575  3.762406e+00
## 16724  3 2576  3.641895e+00
## 16725  3 2577  3.555148e+00
## 16726  3 2578  3.492140e+00
## 16727  3 2579  3.442903e+00
## 16728  3 2580  3.383785e+00
## 16729  3 2581  3.338573e+00
## 16730  3 2582  3.286572e+00
## 16731  3 2583  3.243761e+00
## 16732  3 2584  3.200027e+00
## 16733  3 2585  3.161133e+00
## 16734  3 2586  3.128800e+00
## 16735  3 2587  3.083276e+00
## 16736  3 2588  3.043661e+00
## 16737  3 2589  3.007440e+00
## 16738  3 2590  2.970701e+00
## 16739  3 2591  2.926065e+00
## 16740  3 2592  2.895880e+00
## 16741  3 2593  7.095943e+00
## 16742  3 2594  5.814523e+00
## 16743  3 2595  4.965421e+00
## 16744  3 2596  4.438256e+00
## 16745  3 2597  4.091160e+00
## 16746  3 2598  3.866255e+00
## 16747  3 2599  3.715189e+00
## 16748  3 2600  3.611197e+00
## 16749  3 2601  3.527486e+00
## 16750  3 2602  3.463546e+00
## 16751  3 2603  3.400972e+00
## 16752  3 2604  3.356842e+00
## 16753  3 2605  3.312050e+00
## 16754  3 2606  3.263215e+00
## 16755  3 2607  3.218135e+00
## 16756  3 2608  3.174769e+00
## 16757  3 2609  3.136322e+00
## 16758  3 2610  3.092661e+00
## 16759  3 2611  3.054918e+00
## 16760  3 2612  3.012796e+00
## 16761  3 2613  2.977339e+00
## 16762  3 2614  2.940628e+00
## 16763  3 2615  2.904908e+00
## 16764  3 2616  2.865217e+00
## 16765  3 2617  7.042844e+00
## 16766  3 2618  5.786302e+00
## 16767  3 2619  4.943422e+00
## 16768  3 2620  4.403678e+00
## 16769  3 2621  4.065269e+00
## 16770  3 2622  3.840305e+00
## 16771  3 2623  3.694815e+00
## 16772  3 2624  3.588124e+00
## 16773  3 2625  3.506030e+00
## 16774  3 2626  3.438171e+00
## 16775  3 2627  3.381108e+00
## 16776  3 2628  3.333240e+00
## 16777  3 2629  3.284704e+00
## 16778  3 2630  3.242416e+00
## 16779  3 2631  3.194155e+00
## 16780  3 2632  3.147981e+00
## 16781  3 2633  3.117392e+00
## 16782  3 2634  3.072414e+00
## 16783  3 2635  3.033754e+00
## 16784  3 2636  2.992246e+00
## 16785  3 2637  2.956682e+00
## 16786  3 2638  2.921591e+00
## 16787  3 2639  2.885067e+00
## 16788  3 2640  2.846500e+00
## 16789  3 2641  7.036118e+00
## 16790  3 2642  5.766246e+00
## 16791  3 2643  4.918266e+00
## 16792  3 2644  4.380790e+00
## 16793  3 2645  4.046831e+00
## 16794  3 2646  3.831219e+00
## 16795  3 2647  3.677343e+00
## 16796  3 2648  3.570635e+00
## 16797  3 2649  3.484765e+00
## 16798  3 2650  3.423944e+00
## 16799  3 2651  3.356872e+00
## 16800  3 2652  3.314244e+00
## 16801  3 2653  3.266321e+00
## 16802  3 2654  3.219739e+00
## 16803  3 2655  3.178921e+00
## 16804  3 2656  3.140356e+00
## 16805  3 2657  3.100878e+00
## 16806  3 2658  3.053306e+00
## 16807  3 2659  3.016400e+00
## 16808  3 2660  2.981642e+00
## 16809  3 2661  2.945260e+00
## 16810  3 2662  2.908686e+00
## 16811  3 2663  2.869860e+00
## 16812  3 2664  2.834899e+00
## 16813  3 2665  7.023535e+00
## 16814  3 2666  5.749408e+00
## 16815  3 2667  4.906663e+00
## 16816  3 2668  4.372888e+00
## 16817  3 2669  4.029399e+00
## 16818  3 2670  3.810312e+00
## 16819  3 2671  3.662385e+00
## 16820  3 2672  3.552990e+00
## 16821  3 2673  3.467697e+00
## 16822  3 2674  3.398151e+00
## 16823  3 2675  3.351345e+00
## 16824  3 2676  3.304406e+00
## 16825  3 2677  3.258349e+00
## 16826  3 2678  3.205564e+00
## 16827  3 2679  3.167219e+00
## 16828  3 2680  3.121544e+00
## 16829  3 2681  3.085340e+00
## 16830  3 2682  3.040966e+00
## 16831  3 2683  3.007951e+00
## 16832  3 2684  2.966171e+00
## 16833  3 2685  2.931026e+00
## 16834  3 2686  2.896341e+00
## 16835  3 2687  2.859035e+00
```

```
## 16836  3 2688  2.826966e+00
## 16837  3 2689  7.012428e+00
## 16838  3 2690  5.753784e+00
## 16839  3 2691  4.890270e+00
## 16840  3 2692  4.360519e+00
## 16841  3 2693  4.022557e+00
## 16842  3 2694  3.810639e+00
## 16843  3 2695  3.650959e+00
## 16844  3 2696  3.542209e+00
## 16845  3 2697  3.456240e+00
## 16846  3 2698  3.398579e+00
## 16847  3 2699  3.340588e+00
## 16848  3 2700  3.286356e+00
## 16849  3 2701  3.244005e+00
## 16850  3 2702  3.208259e+00
## 16851  3 2703  3.160824e+00
## 16852  3 2704  3.116031e+00
## 16853  3 2705  3.073444e+00
## 16854  3 2706  3.035295e+00
## 16855  3 2707  3.001035e+00
## 16856  3 2708  2.959916e+00
## 16857  3 2709  2.925891e+00
## 16858  3 2710  2.886476e+00
## 16859  3 2711  2.848111e+00
## 16860  3 2712  2.811800e+00
## 16861  3 2713  7.018646e+00
## 16862  3 2714  5.744507e+00
## 16863  3 2715  4.886286e+00
## 16864  3 2716  4.355317e+00
## 16865  3 2717  4.011747e+00
## 16866  3 2718  3.792759e+00
## 16867  3 2719  3.645581e+00
## 16868  3 2720  3.536679e+00
## 16869  3 2721  3.450245e+00
## 16870  3 2722  3.388243e+00
## 16871  3 2723  3.333823e+00
## 16872  3 2724  3.279799e+00
## 16873  3 2725  3.239544e+00
## 16874  3 2726  3.186876e+00
## 16875  3 2727  3.155478e+00
## 16876  3 2728  3.112817e+00
## 16877  3 2729  3.065385e+00
## 16878  3 2730  3.033368e+00
## 16879  3 2731  2.993874e+00
## 16880  3 2732  2.952433e+00
## 16881  3 2733  2.922131e+00
## 16882  3 2734  2.880980e+00
## 16883  3 2735  2.844099e+00
## 16884  3 2736  2.814066e+00
## 16885  3 2737  6.999798e+00
## 16886  3 2738  5.727484e+00
## 16887  3 2739  4.876825e+00
## 16888  3 2740  4.348876e+00
## 16889  3 2741  4.003681e+00
## 16890  3 2742  3.789427e+00
## 16891  3 2743  3.633590e+00
## 16892  3 2744  3.529826e+00
## 16893  3 2745  3.455047e+00
## 16894  3 2746  3.384693e+00
## 16895  3 2747  3.327537e+00
## 16896  3 2748  3.282665e+00
## 16897  3 2749  3.230184e+00
## 16898  3 2750  3.189915e+00
## 16899  3 2751  3.143508e+00
## 16900  3 2752  3.109731e+00
## 16901  3 2753  3.069395e+00
## 16902  3 2754  3.027105e+00
## 16903  3 2755  2.988635e+00
## 16904  3 2756  2.949102e+00
## 16905  3 2757  2.912441e+00
## 16906  3 2758  2.875876e+00
## 16907  3 2759  2.839050e+00
## 16908  3 2760  2.802617e+00
## 16909  3 2761  7.002410e+00
## 16910  3 2762  5.733312e+00
## 16911  3 2763  4.880949e+00
## 16912  3 2764  4.342101e+00
## 16913  3 2765  4.000233e+00
## 16914  3 2766  3.788781e+00
## 16915  3 2767  3.633282e+00
## 16916  3 2768  3.530767e+00
## 16917  3 2769  3.443410e+00
## 16918  3 2770  3.380993e+00
## 16919  3 2771  3.325119e+00
## 16920  3 2772  3.274004e+00
## 16921  3 2773  3.236636e+00
## 16922  3 2774  3.189548e+00
## 16923  3 2775  3.141172e+00
## 16924  3 2776  3.102474e+00
## 16925  3 2777  3.059970e+00
## 16926  3 2778  3.023732e+00
## 16927  3 2779  2.987126e+00
## 16928  3 2780  2.948514e+00
## 16929  3 2781  2.902485e+00
## 16930  3 2782  2.872779e+00
## 16931  3 2783  2.837273e+00
## 16932  3 2784  2.800801e+00
## 16933  3 2785  6.991523e+00
## 16934  3 2786  5.717553e+00
## 16935  3 2787  4.863276e+00
## 16936  3 2788  4.342138e+00
## 16937  3 2789  4.007342e+00
## 16938  3 2790  3.777605e+00
## 16939  3 2791  3.634846e+00
## 16940  3 2792  3.527536e+00
## 16941  3 2793  3.443781e+00
## 16942  3 2794  3.377031e+00
## 16943  3 2795  3.322856e+00
## 16944  3 2796  3.273882e+00
## 16945  3 2797  3.228860e+00
## 16946  3 2798  3.187708e+00
## 16947  3 2799  3.143753e+00
## 16948  3 2800  3.095767e+00
## 16949  3 2801  3.060884e+00
## 16950  3 2802  3.024284e+00
## 16951  3 2803  2.982471e+00
## 16952  3 2804  2.942599e+00
## 16953  3 2805  2.909901e+00
## 16954  3 2806  2.866249e+00
## 16955  3 2807  2.836211e+00
## 16956  3 2808  2.797002e+00
## 16957  3 2808  2.792487e+00
## 16958  3 2809  6.982893e+00
## 16959  3 2810  5.715122e+00
## 16960  3 2811  4.871418e+00
## 16961  3 2812  4.334126e+00
## 16962  3 2813  3.997685e+00
## 16963  3 2814  3.770923e+00
## 16964  3 2815  3.631228e+00
## 16965  3 2816  3.519887e+00
## 16966  3 2817  3.435558e+00
## 16967  3 2818  3.371545e+00
## 16968  3 2819  3.310009e+00
## 16969  3 2820  3.267015e+00
## 16970  3 2821  3.217759e+00
## 16971  3 2822  3.181673e+00
## 16972  3 2823  3.134725e+00
## 16973  3 2824  3.092435e+00
## 16974  3 2825  3.049532e+00
## 16975  3 2826  3.008521e+00
## 16976  3 2827  2.975340e+00
## 16977  3 2828  2.941016e+00
## 16978  3 2829  2.903338e+00
## 16979  3 2830  2.862526e+00
## 16980  3 2831  2.824665e+00
## 16981  3 2832  2.802402e+00
## 16982  3 2832  2.794552e+00
## 16983  3 2833  2.759038e+00
## 16984  3 2834  2.717531e+00
## 16985  3 2835  2.690207e+00
## 16986  3 2836  2.649947e+00
## 16987  3 2837  2.622894e+00
## 16988  3 2838  2.588930e+00
## 16989  3 2839  2.552635e+00
## 16990  3 2840  2.520902e+00
## 16991  3 2841  2.488948e+00
## 16992  3 2842  2.460102e+00
## 16993  3 2843  2.427635e+00
## 16994  3 2844  2.395621e+00
## 16995  3 2845  2.368457e+00
## 16996  3 2846  2.342479e+00
## 16997  3 2847  2.308383e+00
## 16998  3 2848  2.276262e+00
## 16999  3 2849  2.252173e+00
## 17000  3 2850  2.222139e+00
## 17001  3 2851  2.196719e+00
## 17002  3 2852  2.167308e+00
## 17003  3 2853  2.138840e+00
## 17004  3 2853  2.140000e+00
## 17005  3 2854  4.223619e+00
## 17006  3 2855  3.579403e+00
## 17007  3 2856  3.144787e+00
## 17008  3 2856  3.148546e+00
## 17009  3 2857  7.104483e+00
## 17010  3 2858  5.684548e+00
## 17011  3 2859  4.751804e+00
## 17012  3 2860  4.167931e+00
## 17013  3 2861  3.801892e+00
## 17014  3 2862  3.561618e+00
## 17015  3 2863  3.410604e+00
## 17016  3 2864  3.299918e+00
## 17017  3 2865  3.217031e+00
## 17018  3 2866  3.149343e+00
## 17019  3 2867  3.094380e+00
## 17020  3 2868  3.056071e+00
## 17021  3 2869  3.005039e+00
## 17022  3 2870  2.962596e+00
## 17023  3 2871  2.927204e+00
## 17024  3 2872  2.890046e+00
## 17025  3 2873  2.844632e+00
## 17026  3 2874  2.809659e+00
## 17027  3 2875  2.776891e+00
## 17028  3 2876  2.747848e+00
## 17029  3 2877  2.704090e+00
## 17030  3 2878  2.671856e+00
## 17031  3 2879  2.638051e+00
## 17032  3 2880  2.601452e+00
## 17033  3 2881  6.805296e+00
## 17034  3 2882  5.534121e+00
## 17035  3 2883  4.683663e+00
## 17036  3 2884  4.157108e+00
## 17037  3 2885  3.823282e+00
## 17038  3 2886  3.601383e+00
## 17039  3 2887  3.451685e+00
## 17040  3 2888  3.348103e+00
## 17041  3 2889  3.272312e+00
## 17042  3 2890  3.209237e+00
## 17043  3 2891  3.146780e+00
## 17044  3 2892  3.106725e+00
## 17045  3 2893  3.064746e+00
## 17046  3 2894  3.024360e+00
## 17047  3 2895  2.975631e+00
## 17048  3 2896  2.936397e+00
## 17049  3 2897  2.902966e+00
## 17050  3 2898  2.866741e+00
## 17051  3 2899  2.832072e+00
## 17052  3 2900  2.788904e+00
## 17053  3 2901  2.756408e+00
## 17054  3 2902  2.719799e+00
## 17055  3 2903  2.681999e+00
## 17056  3 2904  2.653349e+00
## 17057  3 2905  6.852387e+00
## 17058  3 2906  5.584873e+00
## 17059  3 2907  4.735755e+00
## 17060  3 2908  4.198873e+00
## 17061  3 2909  3.869918e+00
## 17062  3 2910  3.648383e+00
## 17063  3 2911  3.503828e+00
## 17064  3 2912  3.391248e+00
## 17065  3 2913  3.310248e+00
## 17066  3 2914  3.252887e+00
## 17067  3 2915  3.192580e+00
## 17068  3 2916  3.146515e+00
## 17069  3 2917  3.096670e+00
## 17070  3 2918  3.056835e+00
## 17071  3 2919  3.021023e+00
## 17072  3 2920  2.975959e+00
## 17073  3 2921  2.944530e+00
## 17074  3 2922  2.905564e+00
## 17075  3 2923  2.864311e+00
## 17076  3 2924  2.826940e+00
## 17077  3 2925  2.793670e+00
## 17078  3 2926  2.758440e+00
## 17079  3 2927  2.728006e+00
## 17080  3 2928  2.686549e+00
## 17081  3 2929  6.894324e+00
## 17082  3 2930  5.612008e+00
## 17083  3 2931  4.761378e+00
## 17084  3 2932  4.233239e+00
## 17085  3 2933  3.902262e+00
## 17086  3 2934  3.684838e+00
## 17087  3 2935  3.527953e+00
## 17088  3 2936  3.427739e+00
## 17089  3 2937  3.343725e+00
## 17090  3 2938  3.282611e+00
## 17091  3 2939  3.224645e+00
## 17092  3 2940  3.180096e+00
## 17093  3 2941  3.130072e+00
## 17094  3 2942  3.089228e+00
## 17095  3 2943  3.052220e+00
## 17096  3 2944  3.010347e+00
## 17097  3 2945  2.968037e+00
## 17098  3 2946  2.938067e+00
## 17099  3 2947  2.898950e+00
## 17100  3 2948  2.857847e+00
## 17101  3 2949  2.824138e+00
## 17102  3 2950  2.782853e+00
## 17103  3 2951  2.751006e+00
## 17104  3 2952  2.716834e+00
## 17105  3 2953  6.906844e+00
## 17106  3 2954  5.644781e+00
## 17107  3 2955  4.788948e+00
## 17108  3 2956  4.260746e+00
## 17109  3 2957  3.917631e+00
## 17110  3 2958  3.708621e+00
## 17111  3 2959  3.552858e+00
## 17112  3 2960  3.444664e+00
## 17113  3 2961  3.369164e+00
## 17114  3 2962  3.302627e+00
## 17115  3 2963  3.246781e+00
## 17116  3 2964  3.200566e+00
## 17117  3 2965  3.155369e+00
## 17118  3 2966  3.107149e+00
## 17119  3 2967  3.069544e+00
## 17120  3 2968  3.029508e+00
## 17121  3 2969  2.987266e+00
## 17122  3 2970  2.952155e+00
## 17123  3 2971  2.915769e+00
## 17124  3 2972  2.878634e+00
## 17125  3 2973  2.844747e+00
## 17126  3 2974  2.806333e+00
## 17127  3 2975  2.772847e+00
## 17128  3 2976  2.734613e+00
## 17129  3 2977  6.923530e+00
## 17130  3 2978  5.664281e+00
## 17131  3 2979  4.801580e+00
## 17132  3 2980  4.276098e+00
## 17133  3 2981  3.933685e+00
## 17134  3 2982  3.723607e+00
## 17135  3 2983  3.568980e+00
## 17136  3 2984  3.474383e+00
## 17137  3 2985  3.385322e+00
## 17138  3 2986  3.319524e+00
## 17139  3 2987  3.264199e+00
## 17140  3 2988  3.220495e+00
## 17141  3 2989  3.171229e+00
## 17142  3 2990  3.128908e+00
## 17143  3 2991  3.090368e+00
## 17144  3 2992  3.047430e+00
## 17145  3 2993  3.012689e+00
## 17146  3 2994  2.964821e+00
## 17147  3 2995  2.930064e+00
## 17148  3 2996  2.894947e+00
## 17149  3 2997  2.860994e+00
## 17150  3 2998  2.819908e+00
## 17151  3 2999  2.782948e+00
## 17152  3 3000  2.748725e+00
## 17153  3 3001  6.934567e+00
## 17154  3 3002  5.674707e+00
## 17155  3 3003  4.821438e+00
## 17156  3 3004  4.294129e+00
## 17157  3 3005  3.956819e+00
## 17158  3 3006  3.737385e+00
## 17159  3 3007  3.586822e+00
## 17160  3 3008  3.478550e+00
## 17161  3 3009  3.399733e+00
## 17162  3 3010  3.328535e+00
## 17163  3 3011  3.275164e+00
## 17164  3 3012  3.229944e+00
## 17165  3 3013  3.190101e+00
## 17166  3 3014  3.139551e+00
## 17167  3 3015  3.099258e+00
## 17168  3 3016  3.061889e+00
## 17169  3 3017  3.020025e+00
## 17170  3 3018  2.980539e+00
## 17171  3 3019  2.942766e+00
## 17172  3 3020  2.905245e+00
## 17173  3 3021  2.867893e+00
## 17174  3 3022  2.833886e+00
## 17175  3 3023  2.800387e+00
## 17176  3 3024  2.766337e+00
## 17177  3 3025  6.951637e+00
## 17178  3 3026  5.681379e+00
## 17179  3 3027  4.841071e+00
## 17180  3 3028  4.304707e+00
## 17181  3 3029  3.964521e+00
## 17182  3 3030  3.746106e+00
## 17183  3 3031  3.602057e+00
## 17184  3 3032  3.492564e+00
## 17185  3 3033  3.407120e+00
## 17186  3 3034  3.346151e+00
## 17187  3 3035  3.292699e+00
## 17188  3 3036  3.237950e+00
## 17189  3 3037  3.194160e+00
## 17190  3 3038  3.147649e+00
## 17191  3 3039  3.109887e+00
## 17192  3 3040  3.066529e+00
## 17193  3 3041  3.028395e+00
## 17194  3 3042  2.986691e+00
## 17195  3 3043  2.950473e+00
## 17196  3 3044  2.907703e+00
## 17197  3 3045  2.874618e+00
## 17198  3 3046  2.843671e+00
## 17199  3 3047  2.808484e+00
## 17200  3 3048  2.772594e+00
## 17201  3 3049  6.963184e+00
## 17202  3 3050  5.691859e+00
## 17203  3 3051  4.844190e+00
## 17204  3 3052  4.309840e+00
## 17205  3 3053  3.972135e+00
## 17206  3 3054  3.761257e+00
## 17207  3 3055  3.603179e+00
## 17208  3 3056  3.496804e+00
## 17209  3 3057  3.418808e+00
## 17210  3 3058  3.354537e+00
## 17211  3 3059  3.292175e+00
## 17212  3 3060  3.252294e+00
## 17213  3 3061  3.199145e+00
## 17214  3 3062  3.157977e+00
## 17215  3 3063  3.116189e+00
## 17216  3 3064  3.070535e+00
## 17217  3 3065  3.036300e+00
## 17218  3 3066  2.995437e+00
## 17219  3 3067  2.959406e+00
## 17220  3 3068  2.918819e+00
## 17221  3 3069  2.881883e+00
## 17222  3 3070  2.849762e+00
## 17223  3 3071  2.814149e+00
## 17224  3 3072  2.774629e+00
## 17225  3 3073  6.975448e+00
## 17226  3 3074  5.693353e+00
## 17227  3 3075  4.847561e+00
## 17228  3 3076  4.314366e+00
## 17229  3 3077  3.979801e+00
## 17230  3 3078  3.758125e+00
## 17231  3 3079  3.614614e+00
## 17232  3 3080  3.501070e+00
## 17233  3 3081  3.425964e+00
## 17234  3 3082  3.353201e+00
## 17235  3 3083  3.300806e+00
## 17236  3 3084  3.250949e+00
## 17237  3 3085  3.205160e+00
## 17238  3 3086  3.159693e+00
## 17239  3 3087  3.120298e+00
## 17240  3 3088  3.079434e+00
## 17241  3 3089  3.044711e+00
## 17242  3 3090  3.000886e+00
## 17243  3 3091  2.962079e+00
## 17244  3 3092  2.929157e+00
## 17245  3 3093  2.886822e+00
## 17246  3 3094  2.849439e+00
## 17247  3 3095  2.817266e+00
## 17248  3 3096  2.775606e+00
## 17249  3 3097  6.969962e+00
## 17250  3 3098  5.699869e+00
## 17251  3 3099  4.847553e+00
## 17252  3 3100  4.320664e+00
## 17253  3 3101  3.983740e+00
## 17254  3 3102  3.760081e+00
## 17255  3 3103  3.612453e+00
## 17256  3 3104  3.511093e+00
## 17257  3 3105  3.423342e+00
## 17258  3 3106  3.355841e+00
## 17259  3 3107  3.300925e+00
## 17260  3 3108  3.259520e+00
## 17261  3 3109  3.205699e+00
## 17262  3 3110  3.161460e+00
## 17263  3 3111  3.119705e+00
## 17264  3 3112  3.084603e+00
## 17265  3 3113  3.043050e+00
## 17266  3 3114  3.001549e+00
## 17267  3 3115  2.966701e+00
## 17268  3 3116  2.929599e+00
## 17269  3 3117  2.887595e+00
## 17270  3 3118  2.850003e+00
## 17271  3 3119  2.817832e+00
## 17272  3 3120  2.777775e+00
## 17273  3 3120  2.792726e+00
## 17274  3 3121  7.003141e+00
## 17275  3 3122  5.708048e+00
## 17276  3 3123  4.858066e+00
## 17277  3 3124  4.335300e+00
## 17278  3 3125  3.990826e+00
## 17279  3 3126  3.777189e+00
## 17280  3 3127  3.627737e+00
## 17281  3 3128  3.518333e+00
## 17282  3 3129  3.432287e+00
## 17283  3 3130  3.366903e+00
## 17284  3 3131  3.312736e+00
## 17285  3 3132  3.265150e+00
## 17286  3 3133  3.224276e+00
## 17287  3 3134  3.177247e+00
## 17288  3 3135  3.133507e+00
## 17289  3 3136  3.099334e+00
## 17290  3 3137  3.050951e+00
## 17291  3 3138  3.012484e+00
## 17292  3 3139  2.973083e+00
## 17293  3 3140  2.933717e+00
## 17294  3 3141  2.900545e+00
## 17295  3 3142  2.865973e+00
## 17296  3 3143  2.824831e+00
## 17297  3 3144  2.791252e+00
## 17298  3 3144  2.792736e+00
## 17299  3 3145  2.754191e+00
## 17300  3 3146  2.726391e+00
## 17301  3 3147  2.684063e+00
## 17302  3 3148  2.652546e+00
## 17303  3 3149  2.624718e+00
## 17304  3 3150  2.585952e+00
## 17305  3 3151  2.555831e+00
## 17306  3 3152  2.523920e+00
## 17307  3 3153  2.491452e+00
## 17308  3 3154  2.458318e+00
## 17309  3 3155  2.430224e+00
## 17310  3 3156  2.398694e+00
## 17311  3 3157  2.365418e+00
## 17312  3 3158  2.336809e+00
## 17313  3 3159  2.307401e+00
## 17314  3 3160  2.279971e+00
## 17315  3 3161  2.251480e+00
## 17316  3 3162  2.222892e+00
## 17317  3 3163  2.196586e+00
## 17318  3 3164  2.162788e+00
## 17319  3 3165  2.133859e+00
## 17320  3 3165  2.137529e+00
## 17321  3 3166  6.336829e+00
## 17322  3 3167  5.078131e+00
## 17323  3 3168  4.227423e+00
## 17324  3 3168  4.229515e+00
## 17325  3 3169  7.952175e+00
## 17326  3 3170  6.377322e+00
## 17327  3 3171  5.331445e+00
## 17328  3 3172  4.710000e+00
## 17329  3 3173  4.298033e+00
## 17330  3 3174  4.049537e+00
## 17331  3 3175  3.858651e+00
## 17332  3 3176  3.737572e+00
## 17333  3 3177  3.652729e+00
## 17334  3 3178  3.580210e+00
## 17335  3 3179  3.513815e+00
## 17336  3 3180  3.462782e+00
## 17337  3 3181  3.408551e+00
## 17338  3 3182  3.367309e+00
## 17339  3 3183  3.314142e+00
## 17340  3 3184  3.280245e+00
## 17341  3 3185  3.232616e+00
## 17342  3 3186  3.192962e+00
## 17343  3 3187  3.151595e+00
## 17344  3 3188  3.115548e+00
## 17345  3 3189  3.072414e+00
## 17346  3 3190  3.036472e+00
## 17347  3 3191  2.990874e+00
## 17348  3 3192  2.955307e+00
## 17349  3 3193  7.155293e+00
## 17350  3 3194  5.887978e+00
## 17351  3 3195  5.018897e+00
## 17352  3 3196  4.494406e+00
## 17353  3 3197  4.148937e+00
## 17354  3 3198  3.929795e+00
## 17355  3 3199  3.771945e+00
## 17356  3 3200  3.670065e+00
## 17357  3 3201  3.580099e+00
## 17358  3 3202  3.516840e+00
## 17359  3 3203  3.460538e+00
## 17360  3 3204  3.406381e+00
## 17361  3 3205  3.361390e+00
## 17362  3 3206  3.311009e+00
## 17363  3 3207  3.272881e+00
## 17364  3 3208  3.228740e+00
## 17365  3 3209  3.188944e+00
## 17366  3 3210  3.147149e+00
## 17367  3 3211  3.106433e+00
## 17368  3 3212  3.065520e+00
## 17369  3 3213  3.027824e+00
## 17370  3 3214  2.994170e+00
## 17371  3 3215  2.951850e+00
## 17372  3 3216  2.914593e+00
## 17373  3 3217  7.100431e+00
## 17374  3 3218  5.827047e+00
## 17375  3 3219  4.980845e+00
## 17376  3 3220  4.449696e+00
## 17377  3 3221  4.100068e+00
## 17378  3 3222  3.889463e+00
## 17379  3 3223  3.739782e+00
## 17380  3 3224  3.633334e+00
## 17381  3 3225  3.542303e+00
## 17382  3 3226  3.479668e+00
## 17383  3 3227  3.413348e+00
## 17384  3 3228  3.370242e+00
## 17385  3 3229  3.321700e+00
## 17386  3 3230  3.274892e+00
## 17387  3 3231  3.230730e+00
## 17388  3 3232  3.193696e+00
## 17389  3 3233  3.153590e+00
## 17390  3 3234  3.109346e+00
## 17391  3 3235  3.070037e+00
## 17392  3 3236  3.033205e+00
## 17393  3 3237  2.998759e+00
## 17394  3 3238  2.956475e+00
## 17395  3 3239  2.921228e+00
## 17396  3 3240  2.876321e+00
## 17397  3 3241  7.077690e+00
## 17398  3 3242  5.801016e+00
## 17399  3 3243  4.949923e+00
## 17400  3 3244  4.409693e+00
## 17401  3 3245  4.078636e+00
## 17402  3 3246  3.852716e+00
## 17403  3 3247  3.706037e+00
## 17404  3 3248  3.602827e+00
## 17405  3 3249  3.513747e+00
## 17406  3 3250  3.444472e+00
## 17407  3 3251  3.399433e+00
## 17408  3 3252  3.344203e+00
## 17409  3 3253  3.294336e+00
## 17410  3 3254  3.247566e+00
## 17411  3 3255  3.210616e+00
## 17412  3 3256  3.165415e+00
## 17413  3 3257  3.123594e+00
## 17414  3 3258  3.086886e+00
## 17415  3 3259  3.045639e+00
## 17416  3 3260  3.002071e+00
## 17417  3 3261  2.959658e+00
## 17418  3 3262  2.933618e+00
## 17419  3 3263  2.898018e+00
## 17420  3 3264  2.855229e+00
## 17421  3 3265  7.052519e+00
## 17422  3 3266  5.776642e+00
## 17423  3 3267  4.931685e+00
## 17424  3 3268  4.395945e+00
## 17425  3 3269  4.060023e+00
## 17426  3 3270  3.842914e+00
## 17427  3 3271  3.694239e+00
## 17428  3 3272  3.584764e+00
## 17429  3 3273  3.496482e+00
## 17430  3 3274  3.431119e+00
## 17431  3 3275  3.377461e+00
## 17432  3 3276  3.322530e+00
## 17433  3 3277  3.272384e+00
## 17434  3 3278  3.227574e+00
## 17435  3 3279  3.186733e+00
## 17436  3 3280  3.155848e+00
## 17437  3 3281  3.107065e+00
## 17438  3 3282  3.075223e+00
## 17439  3 3283  3.022716e+00
## 17440  3 3284  2.984697e+00
## 17441  3 3285  2.945102e+00
## 17442  3 3286  2.915191e+00
## 17443  3 3287  2.875646e+00
## 17444  3 3288  2.839010e+00
## 17445  3 3289  7.018841e+00
## 17446  3 3290  5.758631e+00
## 17447  3 3291  4.908137e+00
## 17448  3 3292  4.375842e+00
## 17449  3 3293  4.040243e+00
## 17450  3 3294  3.817821e+00
## 17451  3 3295  3.665262e+00
## 17452  3 3296  3.561337e+00
## 17453  3 3297  3.481097e+00
## 17454  3 3298  3.409758e+00
## 17455  3 3299  3.355570e+00
## 17456  3 3300  3.309448e+00
## 17457  3 3301  3.256381e+00
## 17458  3 3302  3.215001e+00
## 17459  3 3303  3.178201e+00
## 17460  3 3304  3.130245e+00
## 17461  3 3305  3.095044e+00
## 17462  3 3306  3.050951e+00
## 17463  3 3307  3.011303e+00
## 17464  3 3308  2.977481e+00
## 17465  3 3309  2.944879e+00
## 17466  3 3310  2.901253e+00
## 17467  3 3311  2.864491e+00
## 17468  3 3312  2.832338e+00
## 17469  3 3313  7.028181e+00
## 17470  3 3314  5.751219e+00
## 17471  3 3315  4.890232e+00
## 17472  3 3316  4.368577e+00
## 17473  3 3317  4.022515e+00
## 17474  3 3318  3.816231e+00
## 17475  3 3319  3.656548e+00
## 17476  3 3320  3.549708e+00
## 17477  3 3321  3.467130e+00
## 17478  3 3322  3.398860e+00
## 17479  3 3323  3.348885e+00
## 17480  3 3324  3.295173e+00
## 17481  3 3325  3.244793e+00
## 17482  3 3326  3.202544e+00
## 17483  3 3327  3.164666e+00
## 17484  3 3328  3.119235e+00
## 17485  3 3329  3.077833e+00
## 17486  3 3330  3.040933e+00
## 17487  3 3331  3.002096e+00
## 17488  3 3332  2.967334e+00
## 17489  3 3333  2.934472e+00
## 17490  3 3334  2.885291e+00
## 17491  3 3335  2.854475e+00
## 17492  3 3336  2.815714e+00
## 17493  3 3337  7.019660e+00
## 17494  3 3338  5.740909e+00
## 17495  3 3339  4.878313e+00
## 17496  3 3340  4.361050e+00
## 17497  3 3341  4.017429e+00
## 17498  3 3342  3.808430e+00
## 17499  3 3343  3.656676e+00
## 17500  3 3344  3.542079e+00
## 17501  3 3345  3.458775e+00
## 17502  3 3346  3.395282e+00
## 17503  3 3347  3.336473e+00
## 17504  3 3348  3.290419e+00
## 17505  3 3349  3.239809e+00
## 17506  3 3350  3.201082e+00
## 17507  3 3351  3.158061e+00
## 17508  3 3352  3.116606e+00
## 17509  3 3353  3.075169e+00
## 17510  3 3354  3.033347e+00
## 17511  3 3355  2.998443e+00
## 17512  3 3356  2.951718e+00
## 17513  3 3357  2.925129e+00
## 17514  3 3358  2.883971e+00
## 17515  3 3359  2.850400e+00
## 17516  3 3360  2.811731e+00
## 17517  3 3361  7.005346e+00
## 17518  3 3362  5.729620e+00
## 17519  3 3363  4.880540e+00
## 17520  3 3364  4.345355e+00
## 17521  3 3365  4.015057e+00
## 17522  3 3366  3.792356e+00
## 17523  3 3367  3.645000e+00
## 17524  3 3368  3.529693e+00
## 17525  3 3369  3.455954e+00
## 17526  3 3370  3.382286e+00
## 17527  3 3371  3.338024e+00
## 17528  3 3372  3.279590e+00
## 17529  3 3373  3.235896e+00
## 17530  3 3374  3.186606e+00
## 17531  3 3375  3.142209e+00
## 17532  3 3376  3.100223e+00
## 17533  3 3377  3.066228e+00
## 17534  3 3378  3.030604e+00
## 17535  3 3379  2.993142e+00
## 17536  3 3380  2.950673e+00
## 17537  3 3381  2.919772e+00
## 17538  3 3382  2.879259e+00
## 17539  3 3383  2.843005e+00
## 17540  3 3384  2.806217e+00
## 17541  3 3385  7.010376e+00
## 17542  3 3386  5.731256e+00
## 17543  3 3387  4.874447e+00
## 17544  3 3388  4.347037e+00
## 17545  3 3389  4.015106e+00
## 17546  3 3390  3.795567e+00
## 17547  3 3391  3.638484e+00
## 17548  3 3392  3.532547e+00
## 17549  3 3393  3.448658e+00
## 17550  3 3394  3.383242e+00
## 17551  3 3395  3.326636e+00
## 17552  3 3396  3.276488e+00
## 17553  3 3397  3.224148e+00
## 17554  3 3398  3.193017e+00
## 17555  3 3399  3.145507e+00
## 17556  3 3400  3.102219e+00
## 17557  3 3401  3.065617e+00
## 17558  3 3402  3.027313e+00
## 17559  3 3403  2.985976e+00
## 17560  3 3404  2.947583e+00
## 17561  3 3405  2.910063e+00
## 17562  3 3406  2.872204e+00
## 17563  3 3407  2.837448e+00
## 17564  3 3408  2.799494e+00
## 17565  3 3409  7.003446e+00
## 17566  3 3410  5.721398e+00
## 17567  3 3411  4.875792e+00
## 17568  3 3412  4.346504e+00
## 17569  3 3413  4.002228e+00
## 17570  3 3414  3.783930e+00
## 17571  3 3415  3.637280e+00
## 17572  3 3416  3.527753e+00
## 17573  3 3417  3.445741e+00
## 17574  3 3418  3.383582e+00
## 17575  3 3419  3.320295e+00
## 17576  3 3420  3.275565e+00
## 17577  3 3421  3.225984e+00
## 17578  3 3422  3.183294e+00
## 17579  3 3423  3.144187e+00
## 17580  3 3424  3.100750e+00
## 17581  3 3425  3.062018e+00
## 17582  3 3426  3.023982e+00
## 17583  3 3427  2.986770e+00
## 17584  3 3428  2.941114e+00
## 17585  3 3429  2.906240e+00
## 17586  3 3430  2.873561e+00
## 17587  3 3431  2.837050e+00
## 17588  3 3432  2.799652e+00
## 17589  3 3432  2.787274e+00
## 17590  3 3433  6.978848e+00
## 17591  3 3434  5.713554e+00
## 17592  3 3435  4.865278e+00
## 17593  3 3436  4.332025e+00
## 17594  3 3437  3.997741e+00
## 17595  3 3438  3.776128e+00
## 17596  3 3439  3.623903e+00
## 17597  3 3440  3.517147e+00
## 17598  3 3441  3.428006e+00
## 17599  3 3442  3.370832e+00
## 17600  3 3443  3.316100e+00
## 17601  3 3444  3.261297e+00
## 17602  3 3445  3.220324e+00
## 17603  3 3446  3.177500e+00
## 17604  3 3447  3.127013e+00
## 17605  3 3448  3.093364e+00
## 17606  3 3449  3.053697e+00
## 17607  3 3450  3.020294e+00
## 17608  3 3451  2.975907e+00
## 17609  3 3452  2.931319e+00
## 17610  3 3453  2.895463e+00
## 17611  3 3454  2.867656e+00
## 17612  3 3455  2.826145e+00
## 17613  3 3456  2.794042e+00
## 17614  3 3456  2.791920e+00
## 17615  3 3457  2.759491e+00
## 17616  3 3458  2.719045e+00
## 17617  3 3459  2.684163e+00
## 17618  3 3460  2.656943e+00
## 17619  3 3461  2.616774e+00
## 17620  3 3462  2.585761e+00
## 17621  3 3463  2.553606e+00
## 17622  3 3464  2.522209e+00
## 17623  3 3465  2.493986e+00
## 17624  3 3466  2.459707e+00
## 17625  3 3467  2.425124e+00
## 17626  3 3468  2.396259e+00
## 17627  3 3469  2.363455e+00
## 17628  3 3470  2.338049e+00
## 17629  3 3471  2.305488e+00
## 17630  3 3472  2.276441e+00
## 17631  3 3473  2.246405e+00
## 17632  3 3474  2.222910e+00
## 17633  3 3475  2.197681e+00
## 17634  3 3476  2.163775e+00
## 17635  3 3477  2.141137e+00
```

```
## 17636  3 3478  2.111096e+00
## 17637  3 3479  2.085199e+00
## 17638  3 3480  2.055173e+00
## 17639  3 3480  2.055394e+00
## 17640  3 3481  6.271358e+00
## 17641  3 3482  5.000497e+00
## 17642  3 3483  4.152345e+00
## 17643  3 3484  3.632517e+00
## 17644  3 3485  3.304527e+00
## 17645  3 3486  3.092326e+00
## 17646  3 3487  2.953327e+00
## 17647  3 3488  2.858575e+00
## 17648  3 3489  2.778275e+00
## 17649  3 3490  2.725040e+00
## 17650  3 3491  2.676983e+00
## 17651  3 3492  2.633793e+00
## 17652  3 3493  2.596049e+00
## 17653  3 3494  2.559350e+00
## 17654  3 3495  2.527628e+00
## 17655  3 3496  2.490714e+00
## 17656  3 3497  2.460335e+00
## 17657  3 3498  2.431679e+00
## 17658  3 3499  2.398653e+00
## 17659  3 3500  2.367802e+00
## 17660  3 3501  2.340404e+00
## 17661  3 3502  2.309460e+00
## 17662  3 3503  2.277525e+00
## 17663  3 3504  2.248359e+00
## 17664  3 3504  2.246090e+00
## 17665  3 3505  6.446677e+00
## 17666  3 3506  5.186858e+00
## 17667  3 3507  4.347459e+00
## 17668  3 3508  3.818397e+00
## 17669  3 3509  3.489453e+00
## 17670  3 3510  3.276118e+00
## 17671  3 3511  3.127715e+00
## 17672  3 3512  3.036074e+00
## 17673  3 3513  2.949491e+00
## 17674  3 3514  2.895895e+00
## 17675  3 3515  2.847736e+00
## 17676  3 3516  2.796380e+00
## 17677  3 3517  2.760750e+00
## 17678  3 3518  2.721290e+00
## 17679  3 3519  2.688053e+00
## 17680  3 3520  2.653709e+00
## 17681  3 3521  2.618404e+00
## 17682  3 3522  2.582275e+00
## 17683  3 3523  2.547698e+00
## 17684  3 3524  2.524189e+00
## 17685  3 3525  2.487010e+00
## 17686  3 3526  2.453404e+00
## 17687  3 3527  2.423899e+00
## 17688  3 3528  2.393341e+00
## 17689  3 3529  6.588636e+00
## 17690  3 3530  5.332995e+00
## 17691  3 3531  4.474317e+00
## 17692  3 3532  3.948106e+00
## 17693  3 3533  3.622003e+00
## 17694  3 3534  3.402745e+00
## 17695  3 3535  3.263064e+00
## 17696  3 3536  3.156341e+00
## 17697  3 3537  3.081263e+00
## 17698  3 3538  3.018459e+00
## 17699  3 3539  2.967096e+00
## 17700  3 3540  2.921673e+00
## 17701  3 3541  2.882734e+00
## 17702  3 3542  2.838084e+00
## 17703  3 3543  2.810073e+00
## 17704  3 3544  2.767176e+00
## 17705  3 3545  2.729764e+00
## 17706  3 3546  2.692395e+00
## 17707  3 3547  2.656650e+00
## 17708  3 3548  2.626166e+00
## 17709  3 3549  2.594431e+00
## 17710  3 3550  2.562670e+00
## 17711  3 3551  2.531473e+00
## 17712  3 3552  2.493241e+00
## 17713  3 3553  6.696641e+00
## 17714  3 3554  5.428056e+00
## 17715  3 3555  4.584800e+00
## 17716  3 3556  4.054687e+00
## 17717  3 3557  3.722594e+00
## 17718  3 3558  3.507167e+00
## 17719  3 3559  3.358035e+00
## 17720  3 3560  3.252256e+00
## 17721  3 3561  3.174269e+00
## 17722  3 3562  3.108288e+00
## 17723  3 3563  3.056210e+00
## 17724  3 3564  3.013744e+00
## 17725  3 3565  2.969260e+00
## 17726  3 3566  2.928702e+00
## 17727  3 3567  2.886815e+00
## 17728  3 3568  2.854857e+00
## 17729  3 3569  2.817286e+00
## 17730  3 3570  2.780555e+00
## 17731  3 3571  2.740166e+00
## 17732  3 3572  2.707738e+00
## 17733  3 3573  2.667566e+00
## 17734  3 3574  2.644309e+00
## 17735  3 3575  2.609137e+00
## 17736  3 3576  2.576865e+00
## 17737  3 3577  6.761005e+00
## 17738  3 3578  5.503583e+00
## 17739  3 3579  4.656034e+00
## 17740  3 3580  4.133310e+00
## 17741  3 3581  3.791730e+00
## 17742  3 3582  3.573959e+00
## 17743  3 3583  3.423325e+00
## 17744  3 3584  3.328566e+00
## 17745  3 3585  3.243085e+00
## 17746  3 3586  3.178361e+00
## 17747  3 3587  3.127847e+00
## 17748  3 3588  3.080601e+00
## 17749  3 3589  3.029944e+00
## 17750  3 3590  2.993829e+00
## 17751  3 3591  2.953476e+00
## 17752  3 3592  2.919260e+00
## 17753  3 3593  2.878213e+00
## 17754  3 3594  2.839174e+00
## 17755  3 3595  2.809546e+00
## 17756  3 3596  2.767151e+00
## 17757  3 3597  2.737636e+00
## 17758  3 3598  2.701113e+00
## 17759  3 3599  2.666384e+00
## 17760  3 3600  2.635137e+00
## 17761  3 3601  6.820028e+00
## 17762  3 3602  5.558192e+00
## 17763  3 3603  4.706612e+00
## 17764  3 3604  4.179805e+00
## 17765  3 3605  3.844460e+00
## 17766  3 3606  3.630825e+00
## 17767  3 3607  3.476701e+00
## 17768  3 3608  3.373375e+00
## 17769  3 3609  3.295364e+00
## 17770  3 3610  3.233186e+00
## 17771  3 3611  3.177266e+00
## 17772  3 3612  3.132598e+00
## 17773  3 3613  3.084818e+00
## 17774  3 3614  3.047645e+00
## 17775  3 3615  3.000549e+00
## 17776  3 3616  2.963008e+00
## 17777  3 3617  2.924990e+00
## 17778  3 3618  2.887879e+00
## 17779  3 3619  2.851147e+00
## 17780  3 3620  2.811090e+00
## 17781  3 3621  2.785064e+00
## 17782  3 3622  2.744876e+00
## 17783  3 3623  2.711173e+00
## 17784  3 3624  2.676008e+00
## 17785  3 3625  6.870205e+00
## 17786  3 3626  5.594869e+00
## 17787  3 3627  4.750494e+00
## 17788  3 3628  4.223019e+00
## 17789  3 3629  3.886587e+00
## 17790  3 3630  3.666237e+00
## 17791  3 3631  3.521471e+00
## 17792  3 3632  3.413739e+00
## 17793  3 3633  3.330106e+00
## 17794  3 3634  3.267233e+00
## 17795  3 3635  3.215977e+00
## 17796  3 3636  3.164900e+00
## 17797  3 3637  3.119270e+00
## 17798  3 3638  3.075916e+00
## 17799  3 3639  3.034084e+00
## 17800  3 3640  2.992044e+00
## 17801  3 3641  2.960697e+00
## 17802  3 3642  2.916903e+00
## 17803  3 3643  2.881368e+00
## 17804  3 3644  2.846314e+00
## 17805  3 3645  2.812550e+00
## 17806  3 3646  2.779050e+00
## 17807  3 3647  2.741380e+00
## 17808  3 3648  2.707214e+00
## 17809  3 3649  6.899234e+00
## 17810  3 3650  5.642974e+00
## 17811  3 3651  4.782115e+00
## 17812  3 3652  4.252033e+00
## 17813  3 3653  3.914763e+00
## 17814  3 3654  3.695549e+00
## 17815  3 3655  3.540261e+00
## 17816  3 3656  3.439054e+00
## 17817  3 3657  3.362678e+00
## 17818  3 3658  3.292102e+00
## 17819  3 3659  3.237906e+00
## 17820  3 3660  3.194012e+00
## 17821  3 3661  3.146433e+00
## 17822  3 3662  3.099511e+00
## 17823  3 3663  3.062159e+00
## 17824  3 3664  3.018934e+00
## 17825  3 3665  2.980083e+00
## 17826  3 3666  2.941021e+00
## 17827  3 3667  2.899451e+00
## 17828  3 3668  2.873841e+00
## 17829  3 3669  2.837737e+00
## 17830  3 3670  2.803288e+00
## 17831  3 3671  2.763033e+00
## 17832  3 3672  2.731751e+00
## 17833  3 3673  6.916209e+00
## 17834  3 3674  5.654341e+00
## 17835  3 3675  4.800727e+00
## 17836  3 3676  4.273381e+00
## 17837  3 3677  3.931608e+00
## 17838  3 3678  3.712148e+00
## 17839  3 3679  3.572062e+00
## 17840  3 3680  3.460792e+00
## 17841  3 3681  3.375884e+00
## 17842  3 3682  3.308304e+00
## 17843  3 3683  3.256209e+00
## 17844  3 3684  3.209145e+00
## 17845  3 3685  3.165292e+00
## 17846  3 3686  3.118385e+00
## 17847  3 3687  3.078034e+00
## 17848  3 3688  3.038061e+00
## 17849  3 3689  2.999738e+00
## 17850  3 3690  2.960833e+00
## 17851  3 3691  2.925779e+00
## 17852  3 3692  2.890299e+00
## 17853  3 3693  2.850737e+00
## 17854  3 3694  2.814995e+00
## 17855  3 3695  2.784251e+00
## 17856  3 3696  2.743118e+00
## 17857  3 3697  6.934943e+00
## 17858  3 3698  5.666407e+00
## 17859  3 3699  4.817393e+00
## 17860  3 3700  4.285968e+00
## 17861  3 3701  3.950468e+00
## 17862  3 3702  3.734291e+00
## 17863  3 3703  3.579623e+00
## 17864  3 3704  3.478261e+00
## 17865  3 3705  3.399637e+00
## 17866  3 3706  3.330170e+00
## 17867  3 3707  3.271364e+00
## 17868  3 3708  3.223752e+00
## 17869  3 3709  3.178007e+00
## 17870  3 3710  3.135139e+00
## 17871  3 3711  3.091628e+00
## 17872  3 3712  3.053468e+00
## 17873  3 3713  3.011261e+00
## 17874  3 3714  2.978051e+00
## 17875  3 3715  2.935199e+00
## 17876  3 3716  2.897498e+00
## 17877  3 3717  2.859130e+00
## 17878  3 3718  2.827942e+00
## 17879  3 3719  2.791645e+00
## 17880  3 3720  2.756561e+00
## 17881  3 3721  6.941636e+00
## 17882  3 3722  5.682309e+00
## 17883  3 3723  4.843068e+00
## 17884  3 3724  4.299414e+00
## 17885  3 3725  3.970195e+00
## 17886  3 3726  3.744324e+00
## 17887  3 3727  3.593316e+00
## 17888  3 3728  3.489053e+00
## 17889  3 3729  3.399139e+00
## 17890  3 3730  3.343279e+00
## 17891  3 3731  3.283105e+00
## 17892  3 3732  3.236180e+00
## 17893  3 3733  3.182468e+00
## 17894  3 3734  3.146591e+00
## 17895  3 3735  3.102775e+00
## 17896  3 3736  3.060650e+00
## 17897  3 3737  3.018107e+00
## 17898  3 3738  2.983633e+00
## 17899  3 3739  2.950239e+00
## 17900  3 3740  2.911232e+00
## 17901  3 3741  2.873164e+00
## 17902  3 3742  2.835871e+00
## 17903  3 3743  2.799139e+00
## 17904  3 3744  2.760647e+00
## 17905  3 3745  6.964452e+00
## 17906  3 3746  5.682462e+00
## 17907  3 3747  4.842027e+00
## 17908  3 3748  4.312160e+00
## 17909  3 3749  3.971733e+00
## 17910  3 3750  3.750580e+00
## 17911  3 3751  3.601521e+00
## 17912  3 3752  3.498964e+00
## 17913  3 3753  3.415025e+00
## 17914  3 3754  3.344947e+00
## 17915  3 3755  3.292417e+00
## 17916  3 3756  3.245045e+00
## 17917  3 3757  3.197970e+00
## 17918  3 3758  3.157807e+00
## 17919  3 3759  3.107912e+00
## 17920  3 3760  3.072538e+00
## 17921  3 3761  3.037176e+00
## 17922  3 3762  2.993294e+00
## 17923  3 3763  2.958917e+00
## 17924  3 3764  2.917805e+00
## 17925  3 3765  2.882512e+00
## 17926  3 3766  2.848318e+00
## 17927  3 3767  2.807231e+00
## 17928  3 3768  2.771891e+00
## 17929  3 3768  2.785498e+00
## 17930  3 3769  6.981375e+00
## 17931  3 3770  5.711434e+00
## 17932  3 3771  4.857946e+00
## 17933  3 3772  4.333586e+00
## 17934  3 3773  4.006112e+00
## 17935  3 3774  3.780808e+00
## 17936  3 3775  3.627308e+00
## 17937  3 3776  3.517960e+00
## 17938  3 3777  3.437959e+00
## 17939  3 3778  3.368406e+00
## 17940  3 3779  3.315405e+00
## 17941  3 3780  3.264917e+00
## 17942  3 3781  3.215107e+00
## 17943  3 3782  3.176106e+00
## 17944  3 3783  3.132265e+00
## 17945  3 3784  3.096773e+00
## 17946  3 3785  3.053527e+00
## 17947  3 3786  3.011571e+00
## 17948  3 3787  2.974149e+00
## 17949  3 3788  2.935319e+00
## 17950  3 3789  2.898505e+00
## 17951  3 3790  2.867326e+00
## 17952  3 3791  2.820125e+00
## 17953  3 3792  2.796359e+00
## 17954  3 3792  2.792754e+00
## 17955  3 3793  2.751242e+00
## 17956  3 3794  2.721354e+00
## 17957  3 3795  2.681803e+00
## 17958  3 3796  2.656435e+00
## 17959  3 3797  2.618851e+00
## 17960  3 3798  2.587786e+00
## 17961  3 3799  2.553564e+00
## 17962  3 3800  2.521763e+00
## 17963  3 3801  2.491551e+00
## 17964  3 3802  2.455546e+00
## 17965  3 3803  2.426081e+00
## 17966  3 3804  2.399092e+00
## 17967  3 3805  2.368351e+00
## 17968  3 3806  2.333814e+00
## 17969  3 3807  2.310344e+00
## 17970  3 3808  2.278949e+00
## 17971  3 3809  2.248125e+00
## 17972  3 3810  2.218423e+00
## 17973  3 3811  2.192920e+00
## 17974  3 3812  2.162216e+00
## 17975  3 3813  2.139560e+00
## 17976  3 3814  2.111344e+00
## 17977  3 3815  2.082136e+00
## 17978  3 3816  2.055332e+00
## 17979  3 3816  2.056296e+00
## 17980  3 3817  8.366659e+00
## 17981  3 3818  6.490620e+00
## 17982  3 3819  5.235939e+00
## 17983  3 3820  4.468317e+00
## 17984  3 3821  3.986261e+00
## 17985  3 3822  3.689182e+00
## 17986  3 3823  3.490939e+00
## 17987  3 3824  3.359116e+00
## 17988  3 3825  3.249771e+00
## 17989  3 3826  3.182046e+00
## 17990  3 3827  3.117657e+00
## 17991  3 3828  3.069167e+00
## 17992  3 3829  3.023806e+00
## 17993  3 3830  2.978901e+00
## 17994  3 3831  2.934204e+00
## 17995  3 3832  2.899346e+00
## 17996  3 3833  2.857854e+00
## 17997  3 3834  2.824882e+00
## 17998  3 3835  2.788678e+00
## 17999  3 3836  2.751916e+00
## 18000  3 3837  2.718398e+00
## 18001  3 3838  2.684696e+00
## 18002  3 3839  2.654744e+00
## 18003  3 3840  2.615960e+00
## 18004  3 3840  2.616546e+00
## 18005  3 3841  6.809365e+00
## 18006  3 3842  5.539874e+00
## 18007  3 3843  4.695668e+00
## 18008  3 3844  4.162266e+00
## 18009  3 3845  3.828857e+00
## 18010  3 3846  3.621610e+00
## 18011  3 3847  3.463819e+00
## 18012  3 3848  3.355749e+00
## 18013  3 3849  3.283382e+00
## 18014  3 3850  3.215302e+00
## 18015  3 3851  3.158358e+00
## 18016  3 3852  3.114804e+00
## 18017  3 3853  3.065707e+00
## 18018  3 3854  3.031703e+00
## 18019  3 3855  2.989676e+00
## 18020  3 3856  2.948031e+00
## 18021  3 3857  2.911049e+00
## 18022  3 3858  2.874268e+00
## 18023  3 3859  2.837046e+00
## 18024  3 3860  2.802438e+00
## 18025  3 3861  2.768085e+00
## 18026  3 3862  2.731883e+00
## 18027  3 3863  2.694963e+00
## 18028  3 3864  2.665298e+00
## 18029  3 3865  6.852077e+00
## 18030  3 3866  5.595830e+00
## 18031  3 3867  4.735251e+00
## 18032  3 3868  4.220659e+00
## 18033  3 3869  3.876449e+00
## 18034  3 3870  3.657087e+00
## 18035  3 3871  3.509378e+00
## 18036  3 3872  3.400343e+00
## 18037  3 3873  3.324465e+00
## 18038  3 3874  3.255090e+00
## 18039  3 3875  3.200574e+00
## 18040  3 3876  3.154023e+00
## 18041  3 3877  3.106608e+00
## 18042  3 3878  3.071959e+00
## 18043  3 3879  3.032424e+00
## 18044  3 3880  2.985008e+00
## 18045  3 3881  2.944287e+00
## 18046  3 3882  2.911487e+00
## 18047  3 3883  2.875654e+00
## 18048  3 3884  2.834349e+00
## 18049  3 3885  2.802097e+00
## 18050  3 3886  2.768622e+00
## 18051  3 3887  2.728484e+00
## 18052  3 3888  2.697982e+00
## 18053  3 3889  6.892828e+00
## 18054  3 3890  5.630447e+00
## 18055  3 3891  4.779634e+00
## 18056  3 3892  4.245132e+00
## 18057  3 3893  3.906690e+00
## 18058  3 3894  3.685934e+00
## 18059  3 3895  3.536235e+00
## 18060  3 3896  3.437741e+00
## 18061  3 3897  3.349498e+00
## 18062  3 3898  3.283283e+00
## 18063  3 3899  3.231582e+00
## 18064  3 3900  3.183676e+00
## 18065  3 3901  3.144065e+00
## 18066  3 3902  3.098457e+00
## 18067  3 3903  3.057277e+00
## 18068  3 3904  3.012816e+00
## 18069  3 3905  2.977462e+00
## 18070  3 3906  2.936386e+00
## 18071  3 3907  2.903692e+00
## 18072  3 3908  2.866890e+00
## 18073  3 3909  2.824553e+00
## 18074  3 3910  2.791701e+00
## 18075  3 3911  2.754275e+00
## 18076  3 3912  2.720857e+00
## 18077  3 3913  6.905187e+00
## 18078  3 3914  5.653769e+00
## 18079  3 3915  4.792576e+00
## 18080  3 3916  4.266108e+00
## 18081  3 3917  3.928676e+00
## 18082  3 3918  3.705585e+00
## 18083  3 3919  3.561462e+00
## 18084  3 3920  3.454159e+00
## 18085  3 3921  3.375601e+00
## 18086  3 3922  3.304950e+00
## 18087  3 3923  3.253885e+00
## 18088  3 3924  3.204820e+00
## 18089  3 3925  3.155964e+00
## 18090  3 3926  3.114627e+00
## 18091  3 3927  3.081037e+00
## 18092  3 3928  3.037346e+00
## 18093  3 3929  3.003839e+00
## 18094  3 3930  2.959012e+00
## 18095  3 3931  2.920914e+00
## 18096  3 3932  2.880165e+00
## 18097  3 3933  2.843853e+00
## 18098  3 3934  2.811519e+00
## 18099  3 3935  2.774577e+00
## 18100  3 3936  2.734877e+00
## 18101  3 3937  6.930366e+00
## 18102  3 3938  5.669832e+00
## 18103  3 3939  4.806944e+00
## 18104  3 3940  4.290500e+00
## 18105  3 3941  3.947240e+00
## 18106  3 3942  3.726217e+00
## 18107  3 3943  3.575732e+00
## 18108  3 3944  3.472437e+00
## 18109  3 3945  3.388512e+00
## 18110  3 3946  3.324706e+00
## 18111  3 3947  3.264661e+00
## 18112  3 3948  3.220812e+00
## 18113  3 3949  3.171867e+00
## 18114  3 3950  3.133447e+00
## 18115  3 3951  3.089001e+00
## 18116  3 3952  3.047200e+00
## 18117  3 3953  3.013757e+00
## 18118  3 3954  2.971621e+00
## 18119  3 3955  2.930075e+00
## 18120  3 3956  2.895390e+00
## 18121  3 3957  2.864352e+00
## 18122  3 3958  2.826564e+00
## 18123  3 3959  2.788073e+00
## 18124  3 3960  2.750551e+00
## 18125  3 3961  6.935009e+00
## 18126  3 3962  5.672403e+00
## 18127  3 3963  4.824549e+00
## 18128  3 3964  4.289256e+00
## 18129  3 3965  3.953454e+00
## 18130  3 3966  3.742173e+00
## 18131  3 3967  3.592646e+00
## 18132  3 3968  3.485209e+00
## 18133  3 3969  3.403992e+00
## 18134  3 3970  3.338588e+00
## 18135  3 3971  3.283484e+00
## 18136  3 3972  3.225536e+00
## 18137  3 3973  3.184941e+00
## 18138  3 3974  3.149197e+00
## 18139  3 3975  3.102263e+00
## 18140  3 3976  3.063719e+00
## 18141  3 3977  3.022536e+00
## 18142  3 3978  2.986367e+00
## 18143  3 3979  2.945964e+00
## 18144  3 3980  2.904363e+00
## 18145  3 3981  2.870415e+00
## 18146  3 3982  2.835030e+00
## 18147  3 3983  2.801044e+00
## 18148  3 3984  2.769632e+00
## 18149  3 3985  6.958415e+00
## 18150  3 3986  5.691611e+00
## 18151  3 3987  4.846281e+00
## 18152  3 3988  4.304601e+00
## 18153  3 3989  3.964889e+00
## 18154  3 3990  3.754491e+00
## 18155  3 3991  3.600841e+00
## 18156  3 3992  3.491739e+00
## 18157  3 3993  3.415366e+00
## 18158  3 3994  3.349729e+00
## 18159  3 3995  3.294763e+00
## 18160  3 3996  3.236025e+00
## 18161  3 3997  3.194724e+00
## 18162  3 3998  3.152739e+00
## 18163  3 3999  3.105164e+00
## 18164  3 4000  3.072350e+00
## 18165  3 4001  3.031312e+00
## 18166  3 4002  2.989678e+00
## 18167  3 4003  2.951602e+00
## 18168  3 4004  2.917965e+00
## 18169  3 4005  2.875061e+00
## 18170  3 4006  2.841487e+00
## 18171  3 4007  2.808483e+00
## 18172  3 4008  2.774821e+00
## 18173  3 4009  6.960695e+00
## 18174  3 4010  5.699561e+00
## 18175  3 4011  4.841949e+00
## 18176  3 4012  4.310528e+00
## 18177  3 4013  3.977602e+00
## 18178  3 4014  3.750453e+00
## 18179  3 4015  3.605139e+00
## 18180  3 4016  3.498227e+00
## 18181  3 4017  3.419793e+00
## 18182  3 4018  3.353561e+00
## 18183  3 4019  3.296050e+00
## 18184  3 4020  3.248215e+00
## 18185  3 4021  3.197979e+00
## 18186  3 4022  3.154350e+00
## 18187  3 4023  3.121011e+00
## 18188  3 4024  3.072285e+00
## 18189  3 4025  3.028502e+00
## 18190  3 4026  3.002773e+00
## 18191  3 4027  2.958617e+00
## 18192  3 4028  2.916635e+00
## 18193  3 4029  2.885610e+00
## 18194  3 4030  2.854186e+00
## 18195  3 4031  2.812388e+00
## 18196  3 4032  2.777530e+00
## 18197  3 4033  6.966843e+00
## 18198  3 4034  5.698126e+00
## 18199  3 4035  4.851378e+00
## 18200  3 4036  4.314609e+00
## 18201  3 4037  3.973808e+00
## 18202  3 4038  3.760578e+00
## 18203  3 4039  3.612883e+00
## 18204  3 4040  3.498161e+00
## 18205  3 4041  3.423316e+00
## 18206  3 4042  3.357420e+00
## 18207  3 4043  3.302356e+00
## 18208  3 4044  3.256031e+00
## 18209  3 4045  3.206387e+00
## 18210  3 4046  3.165123e+00
## 18211  3 4047  3.123216e+00
## 18212  3 4048  3.083625e+00
## 18213  3 4049  3.042811e+00
## 18214  3 4050  2.999891e+00
## 18215  3 4051  2.969651e+00
## 18216  3 4052  2.926914e+00
## 18217  3 4053  2.889410e+00
## 18218  3 4054  2.850617e+00
## 18219  3 4055  2.817144e+00
## 18220  3 4056  2.781174e+00
## 18221  3 4057  6.980583e+00
## 18222  3 4058  5.709792e+00
## 18223  3 4059  4.848827e+00
## 18224  3 4060  4.329921e+00
## 18225  3 4061  3.989384e+00
## 18226  3 4062  3.760400e+00
## 18227  3 4063  3.620457e+00
## 18228  3 4064  3.508181e+00
## 18229  3 4065  3.427730e+00
## 18230  3 4066  3.361131e+00
## 18231  3 4067  3.304219e+00
## 18232  3 4068  3.254336e+00
## 18233  3 4069  3.207771e+00
## 18234  3 4070  3.161527e+00
## 18235  3 4071  3.128570e+00
## 18236  3 4072  3.084344e+00
## 18237  3 4073  3.044641e+00
## 18238  3 4074  3.001374e+00
## 18239  3 4075  2.964792e+00
## 18240  3 4076  2.930578e+00
## 18241  3 4077  2.890742e+00
## 18242  3 4078  2.855833e+00
## 18243  3 4079  2.822761e+00
## 18244  3 4080  2.782894e+00
## 18245  3 4081  6.974231e+00
## 18246  3 4082  5.702209e+00
## 18247  3 4083  4.856489e+00
## 18248  3 4084  4.330589e+00
## 18249  3 4085  3.982238e+00
## 18250  3 4086  3.768170e+00
## 18251  3 4087  3.622235e+00
## 18252  3 4088  3.502894e+00
## 18253  3 4089  3.429422e+00
## 18254  3 4090  3.366851e+00
## 18255  3 4091  3.302558e+00
## 18256  3 4092  3.256646e+00
## 18257  3 4093  3.212599e+00
## 18258  3 4094  3.166049e+00
## 18259  3 4095  3.121089e+00
## 18260  3 4096  3.086098e+00
## 18261  3 4097  3.042907e+00
## 18262  3 4098  3.005103e+00
## 18263  3 4099  2.967877e+00
## 18264  3 4100  2.925439e+00
## 18265  3 4101  2.895480e+00
## 18266  3 4102  2.860996e+00
## 18267  3 4103  2.820065e+00
## 18268  3 4104  2.784387e+00
## 18269  3 4104  2.798305e+00
## 18270  3 4105  6.983391e+00
## 18271  3 4106  5.717809e+00
## 18272  3 4107  4.867398e+00
## 18273  3 4108  4.340070e+00
## 18274  3 4109  3.997664e+00
## 18275  3 4110  3.774706e+00
## 18276  3 4111  3.624718e+00
## 18277  3 4112  3.512982e+00
## 18278  3 4113  3.434035e+00
## 18279  3 4114  3.366222e+00
## 18280  3 4115  3.314940e+00
## 18281  3 4116  3.265295e+00
## 18282  3 4117  3.222941e+00
## 18283  3 4118  3.176357e+00
## 18284  3 4119  3.134047e+00
## 18285  3 4120  3.089013e+00
## 18286  3 4121  3.050572e+00
## 18287  3 4122  3.017050e+00
## 18288  3 4123  2.973997e+00
## 18289  3 4124  2.936992e+00
## 18290  3 4125  2.897936e+00
## 18291  3 4126  2.859411e+00
## 18292  3 4127  2.830226e+00
## 18293  3 4128  2.790723e+00
## 18294  3 4128  2.789770e+00
## 18295  3 4129  2.758342e+00
## 18296  3 4130  2.719412e+00
## 18297  3 4131  2.687131e+00
## 18298  3 4132  2.655389e+00
## 18299  3 4133  2.616076e+00
## 18300  3 4134  2.587121e+00
## 18301  3 4135  2.550987e+00
## 18302  3 4136  2.520981e+00
## 18303  3 4137  2.491328e+00
## 18304  3 4138  2.454836e+00
## 18305  3 4139  2.428172e+00
## 18306  3 4140  2.394284e+00
## 18307  3 4141  2.366376e+00
## 18308  3 4142  2.337185e+00
## 18309  3 4143  2.306715e+00
## 18310  3 4144  2.276168e+00
## 18311  3 4145  2.247578e+00
## 18312  3 4146  2.220358e+00
## 18313  3 4147  2.191321e+00
## 18314  3 4148  2.168167e+00
## 18315  3 4149  2.139106e+00
## 18316  3 4150  2.116085e+00
## 18317  3 4151  2.081829e+00
## 18318  3 4152  2.055552e+00
## 18319  3 4152  2.056284e+00
## 18320  3 4153  1.048862e+01
## 18321  3 4154  7.999894e+00
## 18322  3 4155  6.336875e+00
## 18323  3 4156  5.317974e+00
## 18324  3 4157  4.679732e+00
## 18325  3 4158  4.286820e+00
## 18326  3 4159  4.019884e+00
## 18327  3 4160  3.845574e+00
## 18328  3 4161  3.728544e+00
## 18329  3 4162  3.630495e+00
## 18330  3 4163  3.564792e+00
## 18331  3 4164  3.499090e+00
## 18332  3 4165  3.449207e+00
## 18333  3 4166  3.402921e+00
## 18334  3 4167  3.351159e+00
## 18335  3 4168  3.307568e+00
## 18336  3 4169  3.269194e+00
## 18337  3 4170  3.226783e+00
## 18338  3 4171  3.182520e+00
## 18339  3 4172  3.144949e+00
## 18340  3 4173  3.103899e+00
## 18341  3 4174  3.057558e+00
## 18342  3 4175  3.022786e+00
## 18343  3 4176  2.983496e+00
## 18344  3 4176  2.989701e+00
## 18345  3 4177  7.168452e+00
## 18346  3 4178  5.900963e+00
## 18347  3 4179  5.060269e+00
## 18348  3 4180  4.513791e+00
## 18349  3 4181  4.169341e+00
## 18350  3 4182  3.953908e+00
## 18351  3 4183  3.799886e+00
## 18352  3 4184  3.691020e+00
## 18353  3 4185  3.605036e+00
## 18354  3 4186  3.538514e+00
## 18355  3 4187  3.485542e+00
## 18356  3 4188  3.440517e+00
## 18357  3 4189  3.380466e+00
## 18358  3 4190  3.338689e+00
## 18359  3 4191  3.294906e+00
## 18360  3 4192  3.245733e+00
## 18361  3 4193  3.201311e+00
## 18362  3 4194  3.166722e+00
## 18363  3 4195  3.125256e+00
## 18364  3 4196  3.091322e+00
## 18365  3 4197  3.049864e+00
## 18366  3 4198  3.008505e+00
## 18367  3 4199  2.979106e+00
## 18368  3 4200  2.934605e+00
## 18369  3 4201  7.131707e+00
## 18370  3 4202  5.860441e+00
## 18371  3 4203  4.994150e+00
## 18372  3 4204  4.468095e+00
## 18373  3 4205  4.129026e+00
## 18374  3 4206  3.904846e+00
## 18375  3 4207  3.762466e+00
## 18376  3 4208  3.648182e+00
## 18377  3 4209  3.563132e+00
## 18378  3 4210  3.495790e+00
## 18379  3 4211  3.436828e+00
## 18380  3 4212  3.391742e+00
## 18381  3 4213  3.337818e+00
## 18382  3 4214  3.295314e+00
## 18383  3 4215  3.249108e+00
## 18384  3 4216  3.214381e+00
## 18385  3 4217  3.167652e+00
## 18386  3 4218  3.130793e+00
## 18387  3 4219  3.082966e+00
## 18388  3 4220  3.047906e+00
## 18389  3 4221  3.010243e+00
## 18390  3 4222  2.973863e+00
## 18391  3 4223  2.929230e+00
## 18392  3 4224  2.898308e+00
## 18393  3 4225  7.085819e+00
## 18394  3 4226  5.822938e+00
## 18395  3 4227  4.964499e+00
## 18396  3 4228  4.440014e+00
## 18397  3 4229  4.097770e+00
## 18398  3 4230  3.871655e+00
## 18399  3 4231  3.719150e+00
## 18400  3 4232  3.614280e+00
## 18401  3 4233  3.531709e+00
## 18402  3 4234  3.464924e+00
## 18403  3 4235  3.404530e+00
## 18404  3 4236  3.355147e+00
## 18405  3 4237  3.309023e+00
## 18406  3 4238  3.265531e+00
## 18407  3 4239  3.219873e+00
## 18408  3 4240  3.179385e+00
## 18409  3 4241  3.138233e+00
## 18410  3 4242  3.098636e+00
## 18411  3 4243  3.051130e+00
## 18412  3 4244  3.017901e+00
## 18413  3 4245  2.979656e+00
## 18414  3 4246  2.942055e+00
## 18415  3 4247  2.906277e+00
## 18416  3 4248  2.870735e+00
## 18417  3 4249  7.060029e+00
## 18418  3 4250  5.799610e+00
## 18419  3 4251  4.938622e+00
## 18420  3 4252  4.407902e+00
## 18421  3 4253  4.066059e+00
## 18422  3 4254  3.848341e+00
## 18423  3 4255  3.698742e+00
## 18424  3 4256  3.590810e+00
## 18425  3 4257  3.506133e+00
## 18426  3 4258  3.439023e+00
## 18427  3 4259  3.376622e+00
## 18428  3 4260  3.330785e+00
## 18429  3 4261  3.285546e+00
## 18430  3 4262  3.235440e+00
## 18431  3 4263  3.202118e+00
## 18432  3 4264  3.157693e+00
## 18433  3 4265  3.115147e+00
## 18434  3 4266  3.077947e+00
## 18435  3 4267  3.040000e+00
```

```
## 18436  3 4268  2.995175e+00
## 18437  3 4269  2.967110e+00
## 18438  3 4270  2.921507e+00
## 18439  3 4271  2.886460e+00
## 18440  3 4272  2.847853e+00
## 18441  3 4273  7.042429e+00
## 18442  3 4274  5.764580e+00
## 18443  3 4275  4.921291e+00
## 18444  3 4276  4.384309e+00
## 18445  3 4277  4.051826e+00
## 18446  3 4278  3.831098e+00
## 18447  3 4279  3.685291e+00
## 18448  3 4280  3.571097e+00
## 18449  3 4281  3.489829e+00
## 18450  3 4282  3.420785e+00
## 18451  3 4283  3.362867e+00
## 18452  3 4284  3.312352e+00
## 18453  3 4285  3.263412e+00
## 18454  3 4286  3.220918e+00
## 18455  3 4287  3.183033e+00
## 18456  3 4288  3.140599e+00
## 18457  3 4289  3.098361e+00
## 18458  3 4290  3.063423e+00
## 18459  3 4291  3.020132e+00
## 18460  3 4292  2.985229e+00
## 18461  3 4293  2.940613e+00
## 18462  3 4294  2.911895e+00
## 18463  3 4295  2.874153e+00
## 18464  3 4296  2.831498e+00
## 18465  3 4297  7.019834e+00
## 18466  3 4298  5.744312e+00
## 18467  3 4299  4.910938e+00
## 18468  3 4300  4.369941e+00
## 18469  3 4301  4.037700e+00
## 18470  3 4302  3.818313e+00
## 18471  3 4303  3.662557e+00
## 18472  3 4304  3.562539e+00
## 18473  3 4305  3.464670e+00
## 18474  3 4306  3.408454e+00
## 18475  3 4307  3.346000e+00
## 18476  3 4308  3.297927e+00
## 18477  3 4309  3.251484e+00
## 18478  3 4310  3.206760e+00
## 18479  3 4311  3.171300e+00
## 18480  3 4312  3.123805e+00
## 18481  3 4313  3.086970e+00
## 18482  3 4314  3.050145e+00
## 18483  3 4315  3.007174e+00
## 18484  3 4316  2.967746e+00
## 18485  3 4317  2.936876e+00
## 18486  3 4318  2.894763e+00
## 18487  3 4319  2.859068e+00
## 18488  3 4320  2.821794e+00
## 18489  3 4321  7.019669e+00
## 18490  3 4322  5.755533e+00
## 18491  3 4323  4.892231e+00
## 18492  3 4324  4.368983e+00
## 18493  3 4325  4.029082e+00
## 18494  3 4326  3.799545e+00
## 18495  3 4327  3.654013e+00
## 18496  3 4328  3.545094e+00
## 18497  3 4329  3.464949e+00
## 18498  3 4330  3.392676e+00
## 18499  3 4331  3.344242e+00
## 18500  3 4332  3.287988e+00
## 18501  3 4333  3.243840e+00
## 18502  3 4334  3.200971e+00
## 18503  3 4335  3.158325e+00
## 18504  3 4336  3.122781e+00
## 18505  3 4337  3.073800e+00
## 18506  3 4338  3.040023e+00
## 18507  3 4339  2.999859e+00
## 18508  3 4340  2.959456e+00
## 18509  3 4341  2.930364e+00
## 18510  3 4342  2.885046e+00
## 18511  3 4343  2.851344e+00
## 18512  3 4344  2.817943e+00
## 18513  3 4345  7.014307e+00
## 18514  3 4346  5.739756e+00
## 18515  3 4347  4.879206e+00
## 18516  3 4348  4.350513e+00
## 18517  3 4349  4.016679e+00
## 18518  3 4350  3.799322e+00
## 18519  3 4351  3.647009e+00
## 18520  3 4352  3.541207e+00
## 18521  3 4353  3.454319e+00
## 18522  3 4354  3.383488e+00
## 18523  3 4355  3.334889e+00
## 18524  3 4356  3.285997e+00
## 18525  3 4357  3.237787e+00
## 18526  3 4358  3.188414e+00
## 18527  3 4359  3.150557e+00
## 18528  3 4360  3.111652e+00
## 18529  3 4361  3.071493e+00
## 18530  3 4362  3.033443e+00
## 18531  3 4363  2.998075e+00
## 18532  3 4364  2.955491e+00
## 18533  3 4365  2.916659e+00
## 18534  3 4366  2.876945e+00
## 18535  3 4367  2.841789e+00
## 18536  3 4368  2.808359e+00
## 18537  3 4369  6.990148e+00
## 18538  3 4370  5.730616e+00
## 18539  3 4371  4.877606e+00
## 18540  3 4372  4.344322e+00
## 18541  3 4373  4.006707e+00
## 18542  3 4374  3.792088e+00
## 18543  3 4375  3.643336e+00
## 18544  3 4376  3.526061e+00
## 18545  3 4377  3.448040e+00
## 18546  3 4378  3.383834e+00
## 18547  3 4379  3.329372e+00
## 18548  3 4380  3.275968e+00
## 18549  3 4381  3.236550e+00
## 18550  3 4382  3.188522e+00
## 18551  3 4383  3.148968e+00
## 18552  3 4384  3.109744e+00
## 18553  3 4385  3.060560e+00
## 18554  3 4386  3.026877e+00
## 18555  3 4387  2.995189e+00
## 18556  3 4388  2.953421e+00
## 18557  3 4389  2.913519e+00
## 18558  3 4390  2.873965e+00
## 18559  3 4391  2.838256e+00
## 18560  3 4392  2.805912e+00
## 18561  3 4393  7.005648e+00
## 18562  3 4394  5.726376e+00
## 18563  3 4395  4.879383e+00
## 18564  3 4396  4.351570e+00
## 18565  3 4397  4.000747e+00
## 18566  3 4398  3.779696e+00
## 18567  3 4399  3.642362e+00
## 18568  3 4400  3.530093e+00
## 18569  3 4401  3.451441e+00
## 18570  3 4402  3.374679e+00
## 18571  3 4403  3.325430e+00
## 18572  3 4404  3.275733e+00
## 18573  3 4405  3.226940e+00
## 18574  3 4406  3.185102e+00
## 18575  3 4407  3.137027e+00
## 18576  3 4408  3.102795e+00
## 18577  3 4409  3.057421e+00
## 18578  3 4410  3.022874e+00
## 18579  3 4411  2.987354e+00
## 18580  3 4412  2.945776e+00
## 18581  3 4413  2.910782e+00
## 18582  3 4414  2.872576e+00
## 18583  3 4415  2.839333e+00
## 18584  3 4416  2.799878e+00
## 18585  3 4417  6.984297e+00
## 18586  3 4418  5.719903e+00
## 18587  3 4419  4.870284e+00
## 18588  3 4420  4.337703e+00
## 18589  3 4421  4.000900e+00
## 18590  3 4422  3.787994e+00
## 18591  3 4423  3.635555e+00
## 18592  3 4424  3.523261e+00
## 18593  3 4425  3.441374e+00
## 18594  3 4426  3.376036e+00
## 18595  3 4427  3.325136e+00
## 18596  3 4428  3.269995e+00
## 18597  3 4429  3.228898e+00
## 18598  3 4430  3.184166e+00
## 18599  3 4431  3.140623e+00
## 18600  3 4432  3.096726e+00
## 18601  3 4433  3.058113e+00
## 18602  3 4434  3.018673e+00
## 18603  3 4435  2.986015e+00
## 18604  3 4436  2.943061e+00
## 18605  3 4437  2.907772e+00
## 18606  3 4438  2.872908e+00
## 18607  3 4439  2.833096e+00
## 18608  3 4440  2.795822e+00
## 18609  3 4441  2.765573e+00
## 18610  3 4442  2.728835e+00
## 18611  3 4443  2.691515e+00
## 18612  3 4444  2.660442e+00
## 18613  3 4445  2.623668e+00
## 18614  3 4446  2.591812e+00
## 18615  3 4447  2.560417e+00
## 18616  3 4448  2.524999e+00
## 18617  3 4449  2.497254e+00
## 18618  3 4450  2.465369e+00
## 18619  3 4451  2.433513e+00
## 18620  3 4452  2.399639e+00
## 18621  3 4453  2.373027e+00
## 18622  3 4454  2.342212e+00
## 18623  3 4455  2.314903e+00
## 18624  3 4456  2.284623e+00
## 18625  3 4457  2.251928e+00
## 18626  3 4458  2.224721e+00
## 18627  3 4459  2.199536e+00
## 18628  3 4460  2.170576e+00
## 18629  3 4461  2.143053e+00
## 18630  3 4462  2.118190e+00
## 18631  3 4463  2.089855e+00
## 18632  3 4464  2.062918e+00
## 18633  3 4465  2.034829e+00
## 18634  3 4466  2.011180e+00
## 18635  3 4467  1.984920e+00
## 18636  3 4468  1.958483e+00
## 18637  3 4469  1.936180e+00
## 18638  3 4470  1.912128e+00
## 18639  3 4471  1.883955e+00
## 18640  3 4472  1.861872e+00
## 18641  3 4473  1.836049e+00
## 18642  3 4474  1.818316e+00
## 18643  3 4475  1.794910e+00
## 18644  3 4476  1.772843e+00
## 18645  3 4477  1.745908e+00
## 18646  3 4478  1.724440e+00
## 18647  3 4479  1.704171e+00
## 18648  3 4480  1.682807e+00
## 18649  3 4481  1.660583e+00
## 18650  3 4482  1.638385e+00
## 18651  3 4483  1.621820e+00
## 18652  3 4484  1.597132e+00
## 18653  3 4485  1.577804e+00
## 18654  3 4486  1.558794e+00
## 18655  3 4487  1.540366e+00
## 18656  3 4488  1.518277e+00
## 18657  3 4489  1.500052e+00
## 18658  3 4490  1.479469e+00
## 18659  3 4491  1.463181e+00
## 18660  3 4492  1.443927e+00
## 18661  3 4493  1.425955e+00
## 18662  3 4494  1.408374e+00
## 18663  3 4495  1.390055e+00
## 18664  3 4496  1.371345e+00
## 18665  3 4497  1.356189e+00
## 18666  3 4498  1.337096e+00
## 18667  3 4499  1.322065e+00
## 18668  3 4500  1.304213e+00
## 18669  3 4501  1.287994e+00
## 18670  3 4502  1.271486e+00
## 18671  3 4503  1.255891e+00
## 18672  3 4504  1.240098e+00
## 18673  3 4505  1.223941e+00
## 18674  3 4506  1.207977e+00
## 18675  3 4507  1.190146e+00
## 18676  3 4508  1.178775e+00
## 18677  3 4509  1.164431e+00
## 18678  3 4510  1.146970e+00
## 18679  3 4511  1.132769e+00
## 18680  3 4512  1.119450e+00
## 18681  3 4513  1.107399e+00
## 18682  3 4514  1.092659e+00
## 18683  3 4515  1.077561e+00
## 18684  3 4516  1.063628e+00
## 18685  3 4517  1.050800e+00
## 18686  3 4518  1.037527e+00
## 18687  3 4519  1.023867e+00
## 18688  3 4520  1.011054e+00
## 18689  3 4521  9.983915e-01
## 18690  3 4522  9.841691e-01
## 18691  3 4523  9.746953e-01
## 18692  3 4524  9.601276e-01
## 18693  3 4525  9.501050e-01
## 18694  3 4526  9.384422e-01
## 18695  3 4527  9.252024e-01
## 18696  3 4528  9.142864e-01
## 18697  3 4529  9.019420e-01
## 18698  3 4530  8.919621e-01
## 18699  3 4531  8.787527e-01
## 18700  3 4532  8.707980e-01
## 18701  3 4533  8.576982e-01
## 18702  3 4534  8.467218e-01
## 18703  3 4535  8.358573e-01
## 18704  3 4536  8.252783e-01
## 18705  3 4537  8.145346e-01
## 18706  3 4538  8.036061e-01
## 18707  3 4539  7.951526e-01
## 18708  3 4540  7.867597e-01
## 18709  3 4541  7.739816e-01
## 18710  3 4542  7.649102e-01
## 18711  3 4543  7.545644e-01
## 18712  3 4544  7.453692e-01
## 18713  3 4545  7.357044e-01
## 18714  3 4546  7.266578e-01
## 18715  3 4547  7.176644e-01
## 18716  3 4548  7.082962e-01
## 18717  3 4549  6.991742e-01
## 18718  3 4550  6.899144e-01
## 18719  3 4551  6.822048e-01
## 18720  3 4552  6.731080e-01
## 18721  3 4553  6.640711e-01
## 18722  3 4554  6.563861e-01
## 18723  3 4555  6.469753e-01
## 18724  3 4556  6.395696e-01
## 18725  3 4557  6.316176e-01
## 18726  3 4558  6.239289e-01
## 18727  3 4559  6.157077e-01
## 18728  3 4560  6.086910e-01
## 18729  3 4561  6.010663e-01
## 18730  3 4562  5.939637e-01
## 18731  3 4563  5.852754e-01
## 18732  3 4564  5.771655e-01
## 18733  3 4565  5.710807e-01
## 18734  3 4566  5.637164e-01
## 18735  3 4567  5.558502e-01
## 18736  3 4568  5.483131e-01
## 18737  3 4569  5.428106e-01
## 18738  3 4570  5.354841e-01
## 18739  3 4571  5.289394e-01
## 18740  3 4572  5.215223e-01
## 18741  3 4573  5.159570e-01
## 18742  3 4574  5.092464e-01
## 18743  3 4575  5.022818e-01
## 18744  3 4576  4.969852e-01
## 18745  3 4577  4.895965e-01
## 18746  3 4578  4.842011e-01
## 18747  3 4579  4.776379e-01
## 18748  3 4580  4.714760e-01
## 18749  3 4581  4.654423e-01
## 18750  3 4582  4.592737e-01
## 18751  3 4583  4.529206e-01
## 18752  3 4584  4.475722e-01
## 18753  3 4585  4.418173e-01
## 18754  3 4586  4.368985e-01
## 18755  3 4587  4.314847e-01
## 18756  3 4588  4.264807e-01
## 18757  3 4589  4.198612e-01
## 18758  3 4590  4.145615e-01
## 18759  3 4591  4.106549e-01
## 18760  3 4592  4.042503e-01
## 18761  3 4593  3.999726e-01
## 18762  3 4594  3.942642e-01
## 18763  3 4595  3.902097e-01
## 18764  3 4596  3.844649e-01
## 18765  3 4597  3.797153e-01
## 18766  3 4598  3.747674e-01
## 18767  3 4599  3.697375e-01
## 18768  3 4600  3.655949e-01
## 18769  3 4601  3.606061e-01
## 18770  3 4602  3.562554e-01
## 18771  3 4603  3.520548e-01
## 18772  3 4604  3.476675e-01
## 18773  3 4605  3.433572e-01
## 18774  3 4606  3.383577e-01
## 18775  3 4607  3.346444e-01
## 18776  3 4608  3.299850e-01
## 18777  3 4609  3.261904e-01
## 18778  3 4610  3.220211e-01
## 18779  3 4611  3.180650e-01
## 18780  3 4612  3.141150e-01
## 18781  3 4613  3.097833e-01
## 18782  3 4614  3.063896e-01
## 18783  3 4615  3.014511e-01
## 18784  3 4616  2.986259e-01
## 18785  3 4617  2.948191e-01
## 18786  3 4618  2.906259e-01
## 18787  3 4619  2.870420e-01
## 18788  3 4620  2.827784e-01
## 18789  3 4621  2.800899e-01
## 18790  3 4622  2.760290e-01
## 18791  3 4623  2.728002e-01
## 18792  3 4624  2.696162e-01
## 18793  3 4625  2.660949e-01
## 18794  3 4626  2.629181e-01
## 18795  3 4627  2.591389e-01
## 18796  3 4628  2.560967e-01
## 18797  3 4629  2.531522e-01
## 18798  3 4630  2.495980e-01
## 18799  3 4631  2.463675e-01
## 18800  3 4632  2.436467e-01
## 18801  3 4633  2.399021e-01
## 18802  3 4634  2.368918e-01
## 18803  3 4635  2.340532e-01
## 18804  3 4636  2.312719e-01
## 18805  3 4637  2.283413e-01
## 18806  3 4638  2.256895e-01
## 18807  3 4639  2.224073e-01
## 18808  3 4640  2.202347e-01
## 18809  3 4641  2.168604e-01
## 18810  3 4642  2.141789e-01
## 18811  3 4643  2.113805e-01
## 18812  3 4644  2.089873e-01
## 18813  3 4645  2.063087e-01
## 18814  3 4646  2.035091e-01
## 18815  3 4647  2.006957e-01
## 18816  3 4648  1.984946e-01
## 18817  3 4649  1.960696e-01
## 18818  3 4650  1.933713e-01
## 18819  3 4651  1.911021e-01
## 18820  3 4652  1.886572e-01
## 18821  3 4653  1.860116e-01
## 18822  3 4654  1.836524e-01
## 18823  3 4655  1.814111e-01
## 18824  3 4656  1.791984e-01
## 18825  3 4657  1.770044e-01
## 18826  3 4658  1.746410e-01
## 18827  3 4659  1.723582e-01
## 18828  3 4660  1.703894e-01
## 18829  3 4661  1.683731e-01
## 18830  3 4662  1.661709e-01
## 18831  3 4663  1.640053e-01
## 18832  3 4664  1.621838e-01
## 18833  3 4665  1.598565e-01
## 18834  3 4666  1.580571e-01
## 18835  3 4667  1.558807e-01
## 18836  3 4668  1.538911e-01
## 18837  3 4669  1.519294e-01
## 18838  3 4670  1.502154e-01
## 18839  3 4671  1.478833e-01
## 18840  3 4672  1.466641e-01
## 18841  3 4673  1.445752e-01
## 18842  3 4674  1.428276e-01
## 18843  3 4675  1.405875e-01
## 18844  3 4676  1.391521e-01
## 18845  3 4677  1.373273e-01
## 18846  3 4678  1.355765e-01
## 18847  3 4679  1.337209e-01
## 18848  3 4680  1.322336e-01
## 18849  3 4681  1.301895e-01
## 18850  3 4682  1.288671e-01
## 18851  3 4683  1.272625e-01
## 18852  3 4684  1.257085e-01
## 18853  3 4685  1.239764e-01
## 18854  3 4686  1.223876e-01
## 18855  3 4687  1.208719e-01
## 18856  3 4688  1.191228e-01
## 18857  3 4689  1.178468e-01
## 18858  3 4690  1.162939e-01
## 18859  3 4691  1.149612e-01
## 18860  3 4692  1.134610e-01
## 18861  3 4693  1.119037e-01
## 18862  3 4694  1.107181e-01
## 18863  3 4695  1.093070e-01
## 18864  3 4696  1.076888e-01
## 18865  3 4697  1.065036e-01
## 18866  3 4698  1.052006e-01
## 18867  3 4699  1.039647e-01
## 18868  3 4700  1.023709e-01
## 18869  3 4701  1.011837e-01
## 18870  3 4702  9.992931e-02
## 18871  3 4703  9.843361e-02
## 18872  3 4704  9.757916e-02
## 18873  3 4705  9.602798e-02
## 18874  3 4706  9.491744e-02
## 18875  3 4707  9.382245e-02
## 18876  3 4708  9.255452e-02
## 18877  3 4709  9.126358e-02
## 18878  3 4710  9.038594e-02
## 18879  3 4711  8.907200e-02
## 18880  3 4712  8.784799e-02
## 18881  3 4713  8.679999e-02
## 18882  3 4714  8.584758e-02
## 18883  3 4715  8.473707e-02
## 18884  3 4716  8.353996e-02
## 18885  3 4717  8.260554e-02
## 18886  3 4718  8.159285e-02
## 18887  3 4719  8.043092e-02
## 18888  3 4720  7.947064e-02
## 18889  3 4721  7.846497e-02
## 18890  3 4722  7.740067e-02
## 18891  3 4723  7.644541e-02
## 18892  3 4724  7.565200e-02
## 18893  3 4725  7.454603e-02
## 18894  3 4726  7.362204e-02
## 18895  3 4727  7.270227e-02
## 18896  3 4728  7.172388e-02
## 18897  3 4729  7.092322e-02
## 18898  3 4730  7.003512e-02
## 18899  3 4731  6.906741e-02
## 18900  3 4732  6.818248e-02
## 18901  3 4733  6.734548e-02
## 18902  3 4734  6.633746e-02
## 18903  3 4735  6.568531e-02
## 18904  3 4736  6.480318e-02
## 18905  3 4737  6.397870e-02
## 18906  3 4738  6.329764e-02
## 18907  3 4739  6.232241e-02
## 18908  3 4740  6.157149e-02
## 18909  3 4741  6.080933e-02
## 18910  3 4742  5.986352e-02
## 18911  3 4743  5.925881e-02
## 18912  3 4744  5.843367e-02
## 18913  3 4745  5.783153e-02
## 18914  3 4746  5.708648e-02
## 18915  3 4747  5.636610e-02
## 18916  3 4748  5.573311e-02
## 18917  3 4749  5.497606e-02
## 18918  3 4750  5.425044e-02
## 18919  3 4751  5.355930e-02
## 18920  3 4752  5.288841e-02
## 18921  3 4753  5.225264e-02
## 18922  3 4754  5.160616e-02
## 18923  3 4755  5.086400e-02
## 18924  3 4756  5.022827e-02
## 18925  3 4757  4.958180e-02
## 18926  3 4758  4.902845e-02
## 18927  3 4759  4.836278e-02
## 18928  3 4760  4.773640e-02
## 18929  3 4761  4.710203e-02
## 18930  3 4762  4.663735e-02
## 18931  3 4763  4.601335e-02
## 18932  3 4764  4.537618e-02
## 18933  3 4765  4.488332e-02
## 18934  3 4766  4.421597e-02
## 18935  3 4767  4.372401e-02
## 18936  3 4768  4.323492e-02
## 18937  3 4769  4.262291e-02
## 18938  3 4770  4.206129e-02
## 18939  3 4771  4.160661e-02
## 18940  3 4772  4.095528e-02
## 18941  3 4773  4.043434e-02
## 18942  3 4774  3.994821e-02
## 18943  3 4775  3.942055e-02
## 18944  3 4776  3.902705e-02
## 18945  3 4777  3.850948e-02
## 18946  3 4778  3.797971e-02
## 18947  3 4779  3.753814e-02
## 18948  3 4780  3.704218e-02
## 18949  3 4781  3.661486e-02
## 18950  3 4782  3.608308e-02
## 18951  3 4783  3.569260e-02
## 18952  3 4784  3.518463e-02
## 18953  3 4785  3.477817e-02
## 18954  3 4786  3.430405e-02
## 18955  3 4787  3.385728e-02
## 18956  3 4788  3.343316e-02
## 18957  3 4789  3.303884e-02
## 18958  3 4790  3.258510e-02
## 18959  3 4791  3.224438e-02
## 18960  3 4792  3.180960e-02
## 18961  3 4793  3.141954e-02
## 18962  3 4794  3.095945e-02
## 18963  3 4795  3.066218e-02
## 18964  3 4796  3.019270e-02
## 18965  3 4797  2.983019e-02
## 18966  3 4798  2.939515e-02
## 18967  3 4799  2.909307e-02
## 18968  3 4800  2.876484e-02
## 18969  3 4801  2.837597e-02
## 18970  3 4802  2.800547e-02
## 18971  3 4803  2.762219e-02
## 18972  3 4804  2.735589e-02
## 18973  3 4805  2.695088e-02
## 18974  3 4806  2.657748e-02
## 18975  3 4807  2.627978e-02
## 18976  3 4808  2.595178e-02
## 18977  3 4809  2.555987e-02
## 18978  3 4810  2.527028e-02
## 18979  3 4811  2.496978e-02
## 18980  3 4812  2.464134e-02
## 18981  3 4813  2.435350e-02
## 18982  3 4814  2.399379e-02
## 18983  3 4815  2.373629e-02
## 18984  3 4816  2.340052e-02
## 18985  3 4817  2.312666e-02
## 18986  3 4818  2.285701e-02
## 18987  3 4819  2.255524e-02
## 18988  3 4820  2.225429e-02
## 18989  3 4821  2.196994e-02
## 18990  3 4822  2.172264e-02
## 18991  3 4823  2.142153e-02
## 18992  3 4824  2.118430e-02
## 18993  3 4825  2.089047e-02
## 18994  3 4826  2.064296e-02
## 18995  3 4827  2.037458e-02
## 18996  3 4828  2.012537e-02
## 18997  3 4829  1.986813e-02
## 18998  3 4830  1.962695e-02
## 18999  3 4831  1.935838e-02
## 19000  3 4832  1.910356e-02
## 19001  3 4833  1.886607e-02
## 19002  3 4834  1.863473e-02
## 19003  3 4835  1.839742e-02
## 19004  3 4836  1.815676e-02
## 19005  3 4837  1.792525e-02
## 19006  3 4838  1.771535e-02
## 19007  3 4839  1.746673e-02
## 19008  3 4840  1.723737e-02
## 19009  3 4841  1.705702e-02
## 19010  3 4842  1.682587e-02
## 19011  3 4843  1.661579e-02
## 19012  3 4844  1.639084e-02
## 19013  3 4845  1.620032e-02
## 19014  3 4846  1.597523e-02
## 19015  3 4847  1.579085e-02
## 19016  3 4848  1.558284e-02
## 19017  3 4849  1.540993e-02
## 19018  3 4850  1.518890e-02
## 19019  3 4851  1.502893e-02
## 19020  3 4852  1.483021e-02
## 19021  3 4853  1.463459e-02
## 19022  3 4854  1.445901e-02
## 19023  3 4855  1.425924e-02
## 19024  3 4856  1.407041e-02
## 19025  3 4857  1.392388e-02
## 19026  3 4858  1.374563e-02
## 19027  3 4859  1.355590e-02
## 19028  3 4860  1.337764e-02
## 19029  3 4861  1.323798e-02
## 19030  3 4862  1.306437e-02
## 19031  3 4863  1.290374e-02
## 19032  3 4864  1.270594e-02
## 19033  3 4865  1.254561e-02
## 19034  3 4866  1.239038e-02
## 19035  3 4867  1.225837e-02
## 19036  3 4868  1.208352e-02
## 19037  3 4869  1.192174e-02
## 19038  3 4870  1.179534e-02
## 19039  3 4871  1.164498e-02
## 19040  3 4872  1.147383e-02
## 19041  3 4873  1.133355e-02
## 19042  3 4874  1.119666e-02
## 19043  3 4875  1.106593e-02
## 19044  3 4876  1.092589e-02
## 19045  3 4877  1.077676e-02
## 19046  3 4878  1.064858e-02
## 19047  3 4879  1.050611e-02
## 19048  3 4880  1.037778e-02
## 19049  3 4881  1.023865e-02
## 19050  3 4882  1.010367e-02
## 19051  3 4883  1.000469e-02
## 19052  3 4884  9.865105e-03
## 19053  3 4885  9.746930e-03
## 19054  3 4886  9.613304e-03
## 19055  3 4887  9.478749e-03
## 19056  3 4888  9.376297e-03
## 19057  3 4889  9.247141e-03
## 19058  3 4890  9.135846e-03
## 19059  3 4891  9.043305e-03
## 19060  3 4892  8.895382e-03
## 19061  3 4893  8.809921e-03
## 19062  3 4894  8.687167e-03
## 19063  3 4895  8.567817e-03
## 19064  3 4896  8.474348e-03
## 19065  3 4897  8.357134e-03
## 19066  3 4898  8.262083e-03
## 19067  3 4899  8.143235e-03
## 19068  3 4900  8.048275e-03
## 19069  3 4901  7.955051e-03
## 19070  3 4902  7.857591e-03
## 19071  3 4903  7.742532e-03
## 19072  3 4904  7.663730e-03
## 19073  3 4905  7.538286e-03
## 19074  3 4906  7.449476e-03
## 19075  3 4907  7.354236e-03
## 19076  3 4908  7.273375e-03
## 19077  3 4909  7.163669e-03
## 19078  3 4910  7.090393e-03
## 19079  3 4911  7.003118e-03
## 19080  3 4912  6.904995e-03
## 19081  3 4913  6.815220e-03
## 19082  3 4914  6.729820e-03
## 19083  3 4915  6.654288e-03
## 19084  3 4916  6.573794e-03
## 19085  3 4917  6.484687e-03
## 19086  3 4918  6.395783e-03
## 19087  3 4919  6.315183e-03
## 19088  3 4920  6.238767e-03
## 19089  3 4921  6.156201e-03
## 19090  3 4922  6.076049e-03
## 19091  3 4923  5.997628e-03
## 19092  3 4924  5.927930e-03
## 19093  3 4925  5.862278e-03
## 19094  3 4926  5.781642e-03
## 19095  3 4927  5.702781e-03
## 19096  3 4928  5.638834e-03
## 19097  3 4929  5.553311e-03
## 19098  3 4930  5.485407e-03
## 19099  3 4931  5.426219e-03
## 19100  3 4932  5.367465e-03
## 19101  3 4933  5.277791e-03
## 19102  3 4934  5.216357e-03
## 19103  3 4935  5.164984e-03
## 19104  3 4936  5.091555e-03
## 19105  3 4937  5.019585e-03
## 19106  3 4938  4.963970e-03
## 19107  3 4939  4.895687e-03
## 19108  3 4940  4.838089e-03
## 19109  3 4941  4.773068e-03
## 19110  3 4942  4.717231e-03
## 19111  3 4943  4.665154e-03
## 19112  3 4944  4.610319e-03
## 19113  3 4945  4.538472e-03
## 19114  3 4946  4.488943e-03
## 19115  3 4947  4.420607e-03
## 19116  3 4948  4.369020e-03
## 19117  3 4949  4.322756e-03
## 19118  3 4950  4.266116e-03
## 19119  3 4951  4.195196e-03
## 19120  3 4952  4.152823e-03
## 19121  3 4953  4.096614e-03
## 19122  3 4954  4.051847e-03
## 19123  3 4955  4.001522e-03
## 19124  3 4956  3.946027e-03
## 19125  3 4957  3.895750e-03
## 19126  3 4958  3.841867e-03
## 19127  3 4959  3.793692e-03
## 19128  3 4960  3.747501e-03
## 19129  3 4961  3.708306e-03
## 19130  3 4962  3.650482e-03
## 19131  3 4963  3.607293e-03
## 19132  3 4964  3.564810e-03
## 19133  3 4965  3.514460e-03
## 19134  3 4966  3.471158e-03
## 19135  3 4967  3.434726e-03
## 19136  3 4968  3.389192e-03
## 19137  3 4969  3.341227e-03
## 19138  3 4970  3.301839e-03
## 19139  3 4971  3.262150e-03
## 19140  3 4972  3.217963e-03
## 19141  3 4973  3.181515e-03
## 19142  3 4974  3.136174e-03
## 19143  3 4975  3.097033e-03
## 19144  3 4976  3.061287e-03
## 19145  3 4977  3.022619e-03
## 19146  3 4978  2.982746e-03
## 19147  3 4979  2.942467e-03
## 19148  3 4980  2.912124e-03
## 19149  3 4981  2.871728e-03
## 19150  3 4982  2.830355e-03
## 19151  3 4983  2.801680e-03
## 19152  3 4984  2.767428e-03
## 19153  3 4985  2.725569e-03
## 19154  3 4986  2.689762e-03
## 19155  3 4987  2.659560e-03
## 19156  3 4988  2.625896e-03
## 19157  3 4989  2.598764e-03
## 19158  3 4990  2.566242e-03
## 19159  3 4991  2.529751e-03
## 19160  3 4992  2.498847e-03
## 19161  3 4993  2.467677e-03
## 19162  3 4994  2.431955e-03
## 19163  3 4995  2.402978e-03
## 19164  3 4996  2.372690e-03
## 19165  3 4997  2.339356e-03
## 19166  3 4998  2.311040e-03
## 19167  3 4999  2.283858e-03
## 19168  3 5000  2.255300e-03
## 19169  3 5001  2.228525e-03
## 19170  3 5002  2.198812e-03
## 19171  3 5003  2.171111e-03
## 19172  3 5004  2.145188e-03
## 19173  3 5005  2.114518e-03
## 19174  3 5006  2.089885e-03
## 19175  3 5007  2.066479e-03
## 19176  3 5008  2.036528e-03
## 19177  3 5009  2.016133e-03
## 19178  3 5010  1.985336e-03
## 19179  3 5011  1.961616e-03
## 19180  3 5012  1.931942e-03
## 19181  3 5013  1.911632e-03
## 19182  3 5014  1.889128e-03
## 19183  3 5015  1.861339e-03
## 19184  3 5016  1.839094e-03
## 19185  3 5017  1.817413e-03
## 19186  3 5018  1.793195e-03
## 19187  3 5019  1.769632e-03
## 19188  3 5020  1.747597e-03
## 19189  3 5021  1.725414e-03
## 19190  3 5022  1.702158e-03
## 19191  3 5023  1.684034e-03
## 19192  3 5024  1.658893e-03
## 19193  3 5025  1.641119e-03
## 19194  3 5026  1.621244e-03
## 19195  3 5027  1.598630e-03
## 19196  3 5028  1.579725e-03
## 19197  3 5029  1.558285e-03
## 19198  3 5030  1.536149e-03
## 19199  3 5031  1.520060e-03
## 19200  3 5032  1.499946e-03
## 19201  3 5033  1.480501e-03
## 19202  3 5034  1.463603e-03
## 19203  3 5035  1.445345e-03
## 19204  3 5036  1.427167e-03
## 19205  3 5037  1.408898e-03
## 19206  3 5038  1.391275e-03
## 19207  3 5039  1.372173e-03
## 19208  3 5040  1.357158e-03
## 19209  3 5041  1.338386e-03
## 19210  3 5042  1.322882e-03
## 19211  3 5043  1.305049e-03
## 19212  3 5044  1.290082e-03
## 19213  3 5045  1.272369e-03
## 19214  3 5046  1.255808e-03
## 19215  3 5047  1.240882e-03
## 19216  3 5048  1.223231e-03
## 19217  3 5049  1.210199e-03
## 19218  3 5050  1.192946e-03
## 19219  3 5051  1.178079e-03
## 19220  3 5052  1.163771e-03
## 19221  3 5053  1.148999e-03
## 19222  3 5054  1.134931e-03
## 19223  3 5055  1.119216e-03
## 19224  3 5056  1.105874e-03
## 19225  3 5057  1.091179e-03
## 19226  3 5058  1.078146e-03
## 19227  3 5059  1.063959e-03
## 19228  3 5060  1.052096e-03
## 19229  3 5061  1.037559e-03
## 19230  3 5062  1.023624e-03
## 19231  3 5063  1.011542e-03
## 19232  3 5064  9.976792e-04
## 19233  3 5065  9.860072e-04
## 19234  3 5066  9.741973e-04
## 19235  3 5067  9.609715e-04
```

```
## 19236  3 5068  9.493445e-04
## 19237  3 5069  9.381209e-04
## 19238  3 5070  9.244909e-04
## 19239  3 5071  9.121750e-04
## 19240  3 5072  9.032599e-04
## 19241  3 5073  8.911344e-04
## 19242  3 5074  8.801673e-04
## 19243  3 5075  8.676380e-04
## 19244  3 5076  8.583468e-04
## 19245  3 5077  8.489725e-04
## 19246  3 5078  8.360864e-04
## 19247  3 5079  8.258092e-04
## 19248  3 5080  8.161227e-04
## 19249  3 5081  8.035045e-04
## 19250  3 5082  7.952072e-04
## 19251  3 5083  7.852026e-04
## 19252  3 5084  7.754284e-04
## 19253  3 5085  7.650150e-04
## 19254  3 5086  7.556518e-04
## 19255  3 5087  7.453589e-04
## 19256  3 5088  7.370814e-04
## 19257  3 5089  7.269220e-04
## 19258  3 5090  7.186079e-04
## 19259  3 5091  7.093288e-04
## 19260  3 5092  6.996974e-04
## 19261  3 5093  6.895192e-04
## 19262  3 5094  6.829147e-04
## 19263  3 5095  6.731706e-04
## 19264  3 5096  6.654570e-04
## 19265  3 5097  6.558684e-04
## 19266  3 5098  6.484709e-04
## 19267  3 5099  6.404481e-04
## 19268  3 5100  6.320858e-04
## 19269  3 5101  6.245606e-04
## 19270  3 5102  6.146097e-04
## 19271  3 5103  6.093055e-04
## 19272  3 5104  5.988902e-04
## 19273  3 5105  5.919190e-04
## 19274  3 5106  5.862893e-04
## 19275  3 5107  5.784276e-04
## 19276  3 5108  5.710640e-04
## 19277  3 5109  5.628295e-04
## 19278  3 5110  5.573084e-04
## 19279  3 5111  5.496453e-04
## 19280  3 5112  5.414772e-04
## 19281  3 5113  5.355605e-04
## 19282  3 5114  5.286825e-04
## 19283  3 5115  5.216025e-04
## 19284  3 5116  5.145885e-04
## 19285  3 5117  5.091808e-04
## 19286  3 5118  5.026918e-04
## 19287  3 5119  4.969450e-04
## 19288  3 5120  4.899053e-04
## 19289  3 5121  4.843355e-04
## 19290  3 5122  4.777030e-04
## 19291  3 5123  4.717554e-04
## 19292  3 5124  4.658859e-04
## 19293  3 5125  4.598926e-04
## 19294  3 5126  4.540672e-04
## 19295  3 5127  4.479981e-04
## 19296  3 5128  4.429251e-04
## 19297  3 5129  4.364398e-04
## 19298  3 5130  4.326197e-04
## 19299  3 5131  4.254816e-04
## 19300  3 5132  4.201384e-04
## 19301  3 5133  4.153512e-04
## 19302  3 5134  4.103327e-04
## 19303  3 5135  4.052783e-04
## 19304  3 5136  3.997091e-04
## 19305  3 5137  3.949306e-04
## 19306  3 5138  3.898206e-04
## 19307  3 5139  3.844843e-04
## 19308  3 5140  3.799378e-04
## 19309  3 5141  3.747159e-04
## 19310  3 5142  3.704535e-04
## 19311  3 5143  3.659212e-04
## 19312  3 5144  3.611687e-04
## 19313  3 5145  3.566000e-04
## 19314  3 5146  3.516411e-04
## 19315  3 5147  3.478048e-04
## 19316  3 5148  3.431307e-04
## 19317  3 5149  3.390595e-04
## 19318  3 5150  3.346653e-04
## 19319  3 5151  3.303431e-04
## 19320  3 5152  3.262449e-04
## 19321  3 5153  3.219808e-04
## 19322  3 5154  3.182572e-04
## 19323  3 5155  3.139063e-04
## 19324  3 5156  3.109164e-04
## 19325  3 5157  3.053642e-04
## 19326  3 5158  3.025540e-04
## 19327  3 5159  2.988403e-04
## 19328  3 5160  2.947642e-04
## 19329  3 5161  2.906718e-04
## 19330  3 5162  2.872842e-04
## 19331  3 5163  2.839393e-04
## 19332  3 5164  2.797120e-04
## 19333  3 5165  2.763548e-04
## 19334  3 5166  2.726319e-04
## 19335  3 5167  2.694290e-04
## 19336  3 5168  2.659071e-04
## 19337  3 5169  2.632832e-04
## 19338  3 5170  2.595360e-04
## 19339  3 5171  2.562357e-04
## 19340  3 5172  2.529172e-04
## 19341  3 5173  2.497295e-04
## 19342  3 5174  2.467752e-04
## 19343  3 5175  2.431332e-04
## 19344  3 5176  2.402022e-04
## 19345  3 5177  2.376827e-04
## 19346  3 5178  2.341837e-04
## 19347  3 5179  2.315208e-04
## 19348  3 5180  2.285261e-04
## 19349  3 5181  2.259837e-04
## 19350  3 5182  2.226177e-04
## 19351  3 5183  2.199611e-04
## 19352  3 5184  2.172450e-04
## 19353  3 5185  2.144322e-04
## 19354  3 5186  2.119747e-04
## 19355  3 5187  2.085901e-04
## 19356  3 5188  2.063612e-04
## 19357  3 5189  2.036363e-04
## 19358  3 5190  2.013941e-04
## 19359  3 5191  1.987180e-04
## 19360  3 5192  1.961616e-04
## 19361  3 5193  1.936517e-04
## 19362  3 5194  1.912562e-04
## 19363  3 5195  1.886850e-04
## 19364  3 5196  1.864918e-04
## 19365  3 5197  1.838815e-04
## 19366  3 5198  1.818308e-04
## 19367  3 5199  1.791073e-04
## 19368  3 5200  1.769975e-04
## 19369  3 5201  1.749837e-04
## 19370  3 5202  1.727592e-04
## 19371  3 5203  1.702925e-04
## 19372  3 5204  1.685349e-04
## 19373  3 5205  1.663321e-04
## 19374  3 5206  1.642877e-04
## 19375  3 5207  1.621914e-04
## 19376  3 5208  1.598601e-04
## 19377  3 5209  1.581670e-04
## 19378  3 5210  1.560293e-04
## 19379  3 5211  1.537123e-04
## 19380  3 5212  1.521256e-04
## 19381  3 5213  1.500650e-04
## 19382  3 5214  1.483281e-04
## 19383  3 5215  1.464804e-04
## 19384  3 5216  1.445820e-04
## 19385  3 5217  1.426333e-04
## 19386  3 5218  1.408627e-04
## 19387  3 5219  1.393710e-04
## 19388  3 5220  1.372846e-04
## 19389  3 5221  1.355849e-04
## 19390  3 5222  1.336967e-04
## 19391  3 5223  1.321347e-04
## 19392  3 5224  1.304159e-04
## 19393  3 5225  1.290356e-04
## 19394  3 5226  1.270119e-04
## 19395  3 5227  1.258509e-04
## 19396  3 5228  1.242556e-04
## 19397  3 5229  1.224660e-04
## 19398  3 5230  1.207790e-04
## 19399  3 5231  1.194186e-04
## 19400  3 5232  1.179160e-04
## 19401  3 5233  1.163775e-04
## 19402  3 5234  1.149925e-04
## 19403  3 5235  1.135597e-04
## 19404  3 5236  1.120389e-04
## 19405  3 5237  1.106780e-04
## 19406  3 5238  1.092249e-04
## 19407  3 5239  1.079046e-04
## 19408  3 5240  1.064668e-04
## 19409  3 5241  1.050940e-04
## 19410  3 5242  1.038500e-04
## 19411  3 5243  1.024356e-04
## 19412  3 5244  1.013878e-04
## 19413  3 5245  1.001171e-04
## 19414  3 5246  9.845790e-05
## 19415  3 5247  9.760288e-05
## 19416  3 5248  9.611892e-05
## 19417  3 5249  9.499038e-05
## 19418  3 5250  9.383530e-05
## 19419  3 5251  9.262353e-05
## 19420  3 5252  9.136881e-05
## 19421  3 5253  9.034122e-05
## 19422  3 5254  8.911285e-05
## 19423  3 5255  8.808417e-05
## 19424  3 5256  8.690460e-05
## 19425  3 5257  8.592987e-05
## 19426  3 5258  8.461324e-05
## 19427  3 5259  8.368402e-05
## 19428  3 5260  8.250590e-05
## 19429  3 5261  8.166703e-05
## 19430  3 5262  8.056666e-05
## 19431  3 5263  7.953374e-05
## 19432  3 5264  7.853331e-05
## 19433  3 5265  7.753558e-05
## 19434  3 5266  7.656539e-05
## 19435  3 5267  7.545906e-05
## 19436  3 5268  7.460287e-05
## 19437  3 5269  7.364681e-05
## 19438  3 5270  7.265504e-05
## 19439  3 5271  7.182849e-05
## 19440  3 5272  7.106881e-05
## 19441  3 5273  7.008272e-05
## 19442  3 5274  6.916930e-05
## 19443  3 5275  6.825863e-05
## 19444  3 5276  6.738748e-05
## 19445  3 5277  6.660660e-05
## 19446  3 5278  6.555265e-05
## 19447  3 5279  6.483284e-05
## 19448  3 5280  6.408463e-05
## 19449  3 5281  6.321578e-05
## 19450  3 5282  6.244970e-05
## 19451  3 5283  6.165301e-05
## 19452  3 5284  6.091281e-05
## 19453  3 5285  6.008372e-05
## 19454  3 5286  5.936585e-05
## 19455  3 5287  5.858436e-05
## 19456  3 5288  5.786147e-05
## 19457  3 5289  5.722437e-05
## 19458  3 5290  5.647076e-05
## 19459  3 5291  5.572774e-05
## 19460  3 5292  5.491617e-05
## 19461  3 5293  5.417145e-05
## 19462  3 5294  5.357970e-05
## 19463  3 5295  5.287586e-05
## 19464  3 5296  5.233441e-05
## 19465  3 5297  5.162802e-05
## 19466  3 5298  5.105362e-05
## 19467  3 5299  5.026871e-05
## 19468  3 5300  4.985270e-05
## 19469  3 5301  4.893946e-05
## 19470  3 5302  4.844134e-05
## 19471  3 5303  4.777082e-05
## 19472  3 5304  4.721734e-05
## 19473  3 5305  4.653453e-05
## 19474  3 5306  4.600709e-05
## 19475  3 5307  4.542843e-05
## 19476  3 5308  4.480126e-05
## 19477  3 5309  4.425795e-05
## 19478  3 5310  4.372388e-05
## 19479  3 5311  4.326689e-05
## 19480  3 5312  4.267846e-05
## 19481  3 5313  4.208679e-05
## 19482  3 5314  4.155361e-05
## 19483  3 5315  4.099908e-05
## 19484  3 5316  4.050965e-05
## 19485  3 5317  4.002034e-05
## 19486  3 5318  3.953444e-05
## 19487  3 5319  3.905701e-05
## 19488  3 5320  3.850988e-05
## 19489  3 5321  3.803331e-05
## 19490  3 5322  3.759515e-05
## 19491  3 5323  3.711865e-05
## 19492  3 5324  3.660607e-05
## 19493  3 5325  3.615247e-05
## 19494  3 5326  3.571911e-05
## 19495  3 5327  3.521947e-05
## 19496  3 5328  3.479653e-05
## 19497  3 5329  3.433798e-05
## 19498  3 5330  3.390441e-05
## 19499  3 5331  3.354326e-05
## 19500  3 5332  3.305096e-05
## 19501  3 5333  3.272561e-05
## 19502  3 5334  3.225860e-05
## 19503  3 5335  3.181331e-05
## 19504  3 5336  3.143956e-05
## 19505  3 5337  3.103613e-05
## 19506  3 5338  3.064065e-05
## 19507  3 5339  3.027069e-05
## 19508  3 5340  2.988281e-05
## 19509  3 5341  2.950184e-05
## 19510  3 5342  2.912008e-05
## 19511  3 5343  2.880181e-05
## 19512  3 5344  2.839855e-05
## 19513  3 5345  2.809442e-05
## 19514  3 5346  2.766891e-05
## 19515  3 5347  2.733844e-05
## 19516  3 5348  2.697623e-05
## 19517  3 5349  2.662846e-05
## 19518  3 5350  2.630158e-05
## 19519  3 5351  2.602705e-05
## 19520  3 5352  2.566237e-05
## 19521  3 5353  2.529749e-05
## 19522  3 5354  2.503210e-05
## 19523  3 5355  2.469343e-05
## 19524  3 5356  2.435488e-05
## 19525  3 5357  2.405390e-05
## 19526  3 5358  2.374227e-05
## 19527  3 5359  2.348342e-05
## 19528  3 5360  2.314320e-05
## 19529  3 5361  2.283111e-05
## 19530  3 5362  2.260402e-05
## 19531  3 5363  2.234131e-05
## 19532  3 5364  2.202730e-05
## 19533  3 5365  2.175081e-05
## 19534  3 5366  2.150149e-05
## 19535  3 5367  2.122939e-05
## 19536  3 5368  2.093184e-05
## 19537  3 5369  2.064080e-05
## 19538  3 5370  2.042256e-05
## 19539  3 5371  2.014255e-05
## 19540  3 5372  1.991584e-05
## 19541  3 5373  1.966322e-05
## 19542  3 5374  1.938705e-05
## 19543  3 5375  1.916843e-05
## 19544  3 5376  1.893089e-05
## 19545  3 5377  1.867836e-05
## 19546  3 5378  1.846295e-05
## 19547  3 5379  1.825581e-05
## 19548  3 5380  1.798454e-05
## 19549  3 5381  1.774978e-05
## 19550  3 5382  1.753106e-05
## 19551  3 5383  1.729118e-05
## 19552  3 5384  1.713473e-05
## 19553  3 5385  1.688968e-05
## 19554  3 5386  1.670107e-05
## 19555  3 5387  1.645205e-05
## 19556  3 5388  1.624686e-05
## 19557  3 5389  1.604487e-05
## 19558  3 5390  1.585256e-05
## 19559  3 5391  1.565648e-05
## 19560  3 5392  1.545003e-05
## 19561  3 5393  1.525475e-05
## 19562  3 5394  1.504879e-05
## 19563  3 5395  1.487566e-05
## 19564  3 5396  1.471794e-05
## 19565  3 5397  1.449121e-05
## 19566  3 5398  1.430756e-05
## 19567  3 5399  1.411883e-05
## 19568  3 5400  1.393729e-05
## 19569  3 5401  1.377773e-05
## 19570  3 5402  1.360436e-05
## 19571  3 5403  1.342002e-05
## 19572  3 5404  1.328398e-05
## 19573  3 5405  1.308202e-05
## 19574  3 5406  1.293099e-05
## 19575  3 5407  1.276182e-05
## 19576  3 5408  1.264183e-05
## 19577  3 5409  1.244005e-05
## 19578  3 5410  1.226660e-05
## 19579  3 5411  1.213588e-05
## 19580  3 5412  1.198005e-05
## 19581  3 5413  1.183635e-05
## 19582  3 5414  1.168818e-05
## 19583  3 5415  1.152257e-05
## 19584  3 5416  1.140195e-05
## 19585  3 5417  1.126791e-05
## 19586  3 5418  1.112239e-05
## 19587  3 5419  1.097731e-05
## 19588  3 5420  1.082392e-05
## 19589  3 5421  1.070255e-05
## 19590  3 5422  1.057634e-05
## 19591  3 5423  1.044741e-05
## 19592  3 5424  1.032485e-05
## 19593  3 5425  1.018490e-05
## 19594  3 5426  1.007360e-05
## 19595  3 5427  9.937998e-06
## 19596  3 5428  9.777671e-06
## 19597  3 5429  9.696346e-06
## 19598  3 5430  9.562636e-06
## 19599  3 5431  9.454094e-06
## 19600  3 5432  9.342942e-06
## 19601  3 5433  9.201871e-06
## 19602  3 5434  9.097844e-06
## 19603  3 5435  8.953170e-06
## 19604  3 5436  8.859113e-06
## 19605  3 5437  8.761542e-06
## 19606  3 5438  8.630402e-06
## 19607  3 5439  8.530543e-06
## 19608  3 5440  8.414733e-06
## 19609  3 5441  8.286012e-06
## 19610  3 5442  8.189441e-06
## 19611  3 5443  8.075690e-06
## 19612  3 5444  7.986762e-06
## 19613  3 5445  7.861816e-06
## 19614  3 5446  7.765178e-06
## 19615  3 5447  7.655983e-06
## 19616  3 5448  7.548178e-06
## 19617  3 5449  7.446874e-06
## 19618  3 5450  7.333612e-06
## 19619  3 5451  7.228230e-06
## 19620  3 5452  7.128258e-06
## 19621  3 5453  7.035507e-06
## 19622  3 5454  6.944214e-06
## 19623  3 5455  6.858082e-06
## 19624  3 5456  6.759159e-06
## 19625  3 5457  6.672757e-06
## 19626  3 5458  6.583095e-06
## 19627  3 5459  6.500482e-06
## 19628  3 5460  6.400367e-06
## 19629  3 5461  6.330425e-06
## 19630  3 5462  6.231702e-06
## 19631  3 5463  6.163115e-06
## 19632  3 5464  6.077918e-06
## 19633  3 5465  6.010462e-06
## 19634  3 5466  5.925465e-06
## 19635  3 5467  5.846387e-06
## 19636  3 5468  5.789163e-06
## 19637  3 5469  5.711014e-06
## 19638  3 5470  5.647947e-06
## 19639  3 5471  5.587743e-06
## 19640  3 5472  5.504084e-06
## 19641  3 5473  5.463394e-06
## 19642  3 5474  5.400390e-06
## 19643  3 5475  5.346316e-06
## 19644  3 5476  5.294341e-06
## 19645  3 5477  5.240952e-06
## 19646  3 5478  5.186865e-06
## 19647  3 5479  5.151036e-06
## 19648  3 5480  5.094675e-06
## 19649  3 5481  5.016921e-06
## 19650  3 5482  4.951564e-06
## 19651  3 5483  4.872849e-06
## 19652  3 5484  4.785466e-06
## 19653  3 5485  4.733316e-06
## 19654  3 5486  4.669627e-06
## 19655  3 5487  4.612304e-06
## 19656  3 5488  4.567393e-06
## 19657  3 5489  4.510551e-06
## 19658  3 5490  4.445679e-06
## 19659  3 5491  4.392306e-06
## 19660  3 5492  4.342235e-06
## 19661  3 5493  4.286715e-06
## 19662  3 5494  4.229484e-06
## 19663  3 5495  4.179444e-06
## 19664  3 5496  4.124855e-06
## 19665  3 5497  4.074305e-06
## 19666  3 5498  4.020804e-06
## 19667  3 5499  3.970266e-06
## 19668  3 5500  3.917208e-06
## 19669  3 5501  3.872846e-06
## 19670  3 5502  3.824454e-06
## 19671  3 5503  3.772238e-06
## 19672  3 5504  3.714595e-06
## 19673  3 5505  3.669261e-06
## 19674  3 5506  3.617780e-06
## 19675  3 5507  3.581355e-06
## 19676  3 5508  3.538276e-06
## 19677  3 5509  3.489519e-06
## 19678  3 5510  3.441179e-06
## 19679  3 5511  3.408487e-06
## 19680  3 5512  3.359757e-06
## 19681  3 5513  3.321718e-06
## 19682  3 5514  3.272924e-06
## 19683  3 5515  3.228252e-06
## 19684  3 5516  3.191827e-06
## 19685  3 5517  3.155973e-06
## 19686  3 5518  3.108906e-06
## 19687  3 5519  3.075337e-06
## 19688  3 5520  3.037112e-06
## 19689  3 5521  3.002600e-06
## 19690  3 5522  2.963283e-06
## 19691  3 5523  2.922771e-06
## 19692  3 5524  2.885648e-06
## 19693  3 5525  2.847097e-06
## 19694  3 5526  2.813469e-06
## 19695  3 5527  2.777760e-06
## 19696  3 5528  2.747514e-06
## 19697  3 5529  2.711630e-06
## 19698  3 5530  2.675511e-06
## 19699  3 5531  2.640474e-06
## 19700  3 5532  2.610605e-06
## 19701  3 5533  2.574398e-06
## 19702  3 5534  2.542294e-06
## 19703  3 5535  2.507798e-06
## 19704  3 5536  2.477912e-06
## 19705  3 5537  2.446649e-06
## 19706  3 5538  2.417777e-06
## 19707  3 5539  2.385766e-06
## 19708  3 5540  2.355937e-06
## 19709  3 5541  2.323491e-06
## 19710  3 5542  2.301305e-06
## 19711  3 5543  2.268420e-06
## 19712  3 5544  2.239798e-06
## 19713  3 5545  2.211385e-06
## 19714  3 5546  2.184985e-06
## 19715  3 5547  2.155586e-06
## 19716  3 5548  2.126340e-06
## 19717  3 5549  2.109053e-06
## 19718  3 5550  2.077125e-06
## 19719  3 5551  2.052833e-06
## 19720  3 5552  2.024359e-06
## 19721  3 5553  1.996195e-06
## 19722  3 5554  1.974932e-06
## 19723  3 5555  1.951829e-06
## 19724  3 5556  1.925377e-06
## 19725  3 5557  1.903379e-06
## 19726  3 5558  1.877151e-06
## 19727  3 5559  1.853287e-06
## 19728  3 5560  1.832321e-06
## 19729  3 5561  1.806292e-06
## 19730  3 5562  1.781364e-06
## 19731  3 5563  1.762333e-06
## 19732  3 5564  1.740106e-06
## 19733  3 5565  1.720071e-06
## 19734  3 5566  1.699277e-06
## 19735  3 5567  1.677985e-06
## 19736  3 5568  1.652301e-06
## 19737  3 5569  1.634497e-06
## 19738  3 5570  1.613311e-06
## 19739  3 5571  1.593935e-06
## 19740  3 5572  1.575425e-06
## 19741  3 5573  1.554712e-06
## 19742  3 5574  1.533895e-06
## 19743  3 5575  1.514800e-06
## 19744  3 5576  1.497001e-06
## 19745  3 5577  1.476205e-06
## 19746  3 5578  1.457991e-06
## 19747  3 5579  1.441938e-06
## 19748  3 5580  1.424640e-06
## 19749  3 5581  1.408177e-06
## 19750  3 5582  1.390502e-06
## 19751  3 5583  1.368967e-06
## 19752  3 5584  1.355448e-06
## 19753  3 5585  1.337275e-06
## 19754  3 5586  1.321541e-06
## 19755  3 5587  1.303346e-06
## 19756  3 5588  1.287665e-06
## 19757  3 5589  1.269680e-06
## 19758  3 5590  1.258781e-06
## 19759  3 5591  1.244234e-06
## 19760  3 5592  1.224553e-06
## 19761  3 5593  1.209799e-06
## 19762  3 5594  1.197097e-06
## 19763  3 5595  1.179758e-06
## 19764  3 5596  1.165155e-06
## 19765  3 5597  1.152622e-06
## 19766  3 5598  1.134773e-06
## 19767  3 5599  1.121901e-06
## 19768  3 5600  1.108711e-06
## 19769  3 5601  1.093440e-06
## 19770  3 5602  1.081517e-06
## 19771  3 5603  1.067433e-06
## 19772  3 5604  1.055761e-06
## 19773  3 5605  1.043413e-06
## 19774  3 5606  1.031110e-06
## 19775  3 5607  1.015719e-06
## 19776  3 5608  1.003783e-06
## 19777  3 5609  9.922549e-07
## 19778  3 5610  9.789112e-07
## 19779  3 5611  9.668661e-07
## 19780  3 5612  9.572773e-07
## 19781  3 5613  9.427707e-07
## 19782  3 5614  9.329947e-07
## 19783  3 5615  9.206877e-07
## 19784  3 5616  9.099930e-07
## 19785  3 5617  8.980903e-07
## 19786  3 5618  8.865295e-07
## 19787  3 5619  8.760597e-07
## 19788  3 5620  8.635026e-07
## 19789  3 5621  8.550660e-07
## 19790  3 5622  8.435781e-07
## 19791  3 5623  8.336490e-07
## 19792  3 5624  8.234544e-07
## 19793  3 5625  8.126531e-07
## 19794  3 5626  8.024314e-07
## 19795  3 5627  7.936166e-07
## 19796  3 5628  7.838271e-07
## 19797  3 5629  7.719861e-07
## 19798  3 5630  7.646393e-07
## 19799  3 5631  7.552643e-07
## 19800  3 5632  7.441689e-07
## 19801  3 5633  7.356419e-07
## 19802  3 5634  7.265082e-07
## 19803  3 5635  7.185638e-07
## 19804  3 5636  7.095353e-07
## 19805  3 5637  7.015727e-07
## 19806  3 5638  6.922362e-07
## 19807  3 5639  6.834469e-07
## 19808  3 5640  6.745697e-07
## 19809  3 5641  6.669732e-07
## 19810  3 5642  6.588803e-07
## 19811  3 5643  6.500922e-07
## 19812  3 5644  6.421078e-07
## 19813  3 5645  6.333061e-07
## 19814  3 5646  6.257210e-07
## 19815  3 5647  6.201591e-07
## 19816  3 5648  6.106012e-07
## 19817  3 5649  6.036857e-07
## 19818  3 5650  5.960590e-07
## 19819  3 5651  5.884173e-07
## 19820  3 5652  5.808672e-07
## 19821  3 5653  5.733804e-07
## 19822  3 5654  5.675640e-07
## 19823  3 5655  5.600675e-07
## 19824  3 5656  5.523521e-07
## 19825  3 5657  5.455725e-07
## 19826  3 5658  5.387268e-07
## 19827  3 5659  5.325646e-07
## 19828  3 5660  5.254355e-07
## 19829  3 5661  5.188497e-07
## 19830  3 5662  5.123722e-07
## 19831  3 5663  5.063557e-07
## 19832  3 5664  4.999498e-07
## 19833  3 5665  4.936125e-07
## 19834  3 5666  4.890153e-07
## 19835  3 5667  4.818764e-07
## 19836  3 5668  4.763949e-07
## 19837  3 5669  4.690455e-07
## 19838  3 5670  4.649215e-07
## 19839  3 5671  4.588888e-07
## 19840  3 5672  4.529388e-07
## 19841  3 5673  4.462978e-07
## 19842  3 5674  4.410369e-07
## 19843  3 5675  4.355917e-07
## 19844  3 5676  4.304818e-07
## 19845  3 5677  4.243282e-07
## 19846  3 5678  4.197348e-07
## 19847  3 5679  4.140824e-07
## 19848  3 5680  4.085947e-07
## 19849  3 5681  4.040426e-07
## 19850  3 5682  3.990527e-07
## 19851  3 5683  3.933110e-07
## 19852  3 5684  3.890195e-07
## 19853  3 5685  3.843808e-07
## 19854  3 5686  3.787468e-07
## 19855  3 5687  3.742124e-07
## 19856  3 5688  3.696551e-07
## 19857  3 5689  3.645327e-07
## 19858  3 5690  3.603398e-07
## 19859  3 5691  3.554615e-07
## 19860  3 5692  3.505582e-07
## 19861  3 5693  3.467712e-07
## 19862  3 5694  3.416634e-07
## 19863  3 5695  3.376477e-07
## 19864  3 5696  3.333835e-07
## 19865  3 5697  3.293468e-07
## 19866  3 5698  3.243588e-07
## 19867  3 5699  3.209589e-07
## 19868  3 5700  3.167458e-07
## 19869  3 5701  3.131519e-07
## 19870  3 5702  3.088019e-07
## 19871  3 5703  3.046691e-07
## 19872  3 5704  3.002043e-07
## 19873  3 5705  2.959789e-07
## 19874  3 5706  2.920255e-07
## 19875  3 5707  2.881269e-07
## 19876  3 5708  2.846107e-07
## 19877  3 5709  2.808416e-07
## 19878  3 5710  2.764689e-07
## 19879  3 5711  2.727909e-07
## 19880  3 5712  2.686750e-07
## 19881  3 5713  2.653372e-07
## 19882  3 5714  2.615885e-07
## 19883  3 5715  2.582397e-07
## 19884  3 5716  2.539650e-07
## 19885  3 5717  2.503778e-07
## 19886  3 5718  2.468659e-07
## 19887  3 5719  2.436889e-07
## 19888  3 5720  2.398634e-07
## 19889  3 5721  2.369070e-07
## 19890  3 5722  2.333771e-07
## 19891  3 5723  2.298741e-07
## 19892  3 5724  2.267325e-07
## 19893  3 5725  2.233809e-07
## 19894  3 5726  2.202498e-07
## 19895  3 5727  2.174646e-07
## 19896  3 5728  2.138993e-07
## 19897  3 5729  2.110663e-07
## 19898  3 5730  2.076141e-07
## 19899  3 5731  2.050169e-07
## 19900  3 5732  2.017274e-07
## 19901  3 5733  1.988076e-07
## 19902  3 5734  1.964422e-07
## 19903  3 5735  1.932939e-07
## 19904  3 5736  1.911375e-07
## 19905  3 5737  1.882118e-07
## 19906  3 5738  1.855562e-07
## 19907  3 5739  1.832904e-07
## 19908  3 5740  1.810244e-07
## 19909  3 5741  1.781970e-07
## 19910  3 5742  1.756111e-07
## 19911  3 5743  1.734158e-07
## 19912  3 5744  1.708984e-07
## 19913  3 5745  1.688354e-07
## 19914  3 5746  1.664642e-07
## 19915  3 5747  1.639686e-07
## 19916  3 5748  1.615536e-07
## 19917  3 5749  1.591625e-07
## 19918  3 5750  1.565973e-07
## 19919  3 5751  1.544973e-07
## 19920  3 5752  1.517187e-07
## 19921  3 5753  1.496505e-07
## 19922  3 5754  1.472916e-07
## 19923  3 5755  1.452956e-07
## 19924  3 5756  1.426559e-07
## 19925  3 5757  1.405324e-07
## 19926  3 5758  1.384545e-07
## 19927  3 5759  1.361423e-07
## 19928  3 5760  1.338857e-07
## 19929  3 5761  1.319677e-07
## 19930  3 5762  1.298150e-07
## 19931  3 5763  1.276523e-07
## 19932  3 5764  1.256754e-07
## 19933  3 5765  1.235520e-07
## 19934  3 5766  1.217405e-07
## 19935  3 5767  1.197112e-07
## 19936  3 5768  1.179624e-07
## 19937  3 5769  1.161801e-07
## 19938  3 5770  1.142772e-07
## 19939  3 5771  1.125235e-07
## 19940  3 5772  1.107151e-07
## 19941  3 5773  1.086369e-07
## 19942  3 5774  1.070885e-07
## 19943  3 5775  1.055621e-07
## 19944  3 5776  1.038566e-07
## 19945  3 5777  1.022188e-07
## 19946  3 5778  1.006649e-07
## 19947  3 5779  9.916344e-08
## 19948  3 5780  9.775118e-08
## 19949  3 5781  9.631890e-08
## 19950  3 5782  9.480074e-08
## 19951  3 5783  9.342235e-08
## 19952  3 5784  9.210129e-08
## 19953  3 5785  9.072802e-08
## 19954  3 5786  8.957849e-08
## 19955  3 5787  8.831133e-08
## 19956  3 5788  8.704688e-08
## 19957  3 5789  8.584770e-08
## 19958  3 5790  8.483661e-08
## 19959  3 5791  8.362242e-08
## 19960  3 5792  8.224161e-08
## 19961  3 5793  8.097021e-08
## 19962  3 5794  7.976910e-08
## 19963  3 5795  7.849287e-08
## 19964  3 5796  7.718298e-08
## 19965  3 5797  7.600491e-08
## 19966  3 5798  7.481695e-08
## 19967  3 5799  7.373047e-08
## 19968  3 5800  7.252322e-08
## 19969  3 5801  7.145464e-08
## 19970  3 5802  7.022341e-08
## 19971  3 5803  6.924515e-08
## 19972  3 5804  6.816640e-08
## 19973  3 5805  6.713654e-08
## 19974  3 5806  6.597403e-08
## 19975  3 5807  6.509136e-08
## 19976  3 5808  6.412453e-08
## 19977  3 5809  6.311610e-08
## 19978  3 5810  6.197468e-08
## 19979  3 5811  6.113746e-08
## 19980  3 5812  6.015021e-08
## 19981  3 5813  5.926488e-08
## 19982  3 5814  5.832481e-08
## 19983  3 5815  5.749903e-08
## 19984  3 5816  5.660899e-08
## 19985  3 5817  5.577148e-08
## 19986  3 5818  5.502694e-08
## 19987  3 5819  5.410768e-08
## 19988  3 5820  5.334859e-08
## 19989  3 5821  5.261384e-08
## 19990  3 5822  5.178740e-08
## 19991  3 5823  5.107830e-08
## 19992  3 5824  5.029465e-08
## 19993  3 5825  4.956989e-08
## 19994  3 5826  4.889269e-08
## 19995  3 5827  4.823091e-08
## 19996  3 5828  4.753255e-08
## 19997  3 5829  4.694606e-08
## 19998  3 5830  4.632428e-08
## 19999  3 5831  4.553724e-08
## 20000  3 5832  4.502524e-08
## 20001  3 5833  4.441214e-08
## 20002  3 5834  4.384239e-08
## 20003  3 5835  4.333440e-08
## 20004  3 5836  4.274825e-08
## 20005  3 5837  4.209072e-08
## 20006  3 5838  4.155596e-08
## 20007  3 5839  4.100821e-08
## 20008  3 5840  4.046751e-08
## 20009  3 5841  3.984219e-08
## 20010  3 5842  3.934789e-08
## 20011  3 5843  3.884497e-08
## 20012  3 5844  3.834782e-08
## 20013  3 5845  3.786881e-08
## 20014  3 5846  3.734310e-08
## 20015  3 5847  3.683618e-08
## 20016  3 5848  3.635142e-08
## 20017  3 5849  3.585866e-08
## 20018  3 5850  3.546058e-08
## 20019  3 5851  3.500316e-08
## 20020  3 5852  3.456455e-08
## 20021  3 5853  3.406436e-08
## 20022  3 5854  3.367029e-08
## 20023  3 5855  3.328314e-08
## 20024  3 5856  3.280025e-08
## 20025  3 5857  3.244970e-08
## 20026  3 5858  3.201391e-08
## 20027  3 5859  3.163897e-08
## 20028  3 5860  3.122196e-08
## 20029  3 5861  3.088866e-08
## 20030  3 5862  3.053762e-08
## 20031  3 5863  3.014980e-08
## 20032  3 5864  2.976077e-08
## 20033  3 5865  2.938280e-08
## 20034  3 5866  2.898839e-08
## 20035  3 5867  2.864311e-08
```

```
## 20036  3 5868  2.834907e-08
## 20037  3 5869  2.797040e-08
## 20038  3 5870  2.761942e-08
## 20039  3 5871  2.730235e-08
## 20040  3 5872  2.695881e-08
## 20041  3 5873  2.662065e-08
## 20042  3 5874  2.628788e-08
## 20043  3 5875  2.594631e-08
## 20044  3 5876  2.571075e-08
## 20045  3 5877  2.533723e-08
## 20046  3 5878  2.502239e-08
## 20047  3 5879  2.476615e-08
## 20048  3 5880  2.440836e-08
## 20049  3 5881  2.428031e-08
## 20050  3 5882  2.424257e-08
## 20051  3 5883  2.428134e-08
## 20052  3 5884  2.428364e-08
## 20053  3 5885  2.428930e-08
## 20054  3 5886  2.431651e-08
## 20055  3 5887  2.430244e-08
## 20056  3 5888  2.432117e-08
## 20057  3 5889  2.437019e-08
## 20058  3 5890  2.439657e-08
## 20059  3 5891  2.444554e-08
## 20060  3 5892  2.452451e-08
## 20061  3 5893  2.459624e-08
## 20062  3 5894  2.462253e-08
## 20063  3 5895  2.470065e-08
## 20064  3 5896  2.481864e-08
## 20065  3 5897  2.486022e-08
## 20066  3 5898  2.497050e-08
## 20067  3 5899  2.514207e-08
## 20068  3 5900  2.518502e-08
## 20069  3 5901  2.530754e-08
## 20070  3 5902  2.537568e-08
## 20071  3 5903  2.552216e-08
## 20072  3 5904  2.562051e-08
## 20073  3 5905  2.574831e-08
## 20074  3 5906  2.590298e-08
## 20075  3 5907  2.600468e-08
## 20076  3 5908  2.611742e-08
## 20077  3 5909  2.628363e-08
## 20078  3 5910  2.643751e-08
## 20079  3 5911  2.654063e-08
## 20080  3 5912  2.676722e-08
## 20081  3 5913  2.686818e-08
## 20082  3 5914  2.703564e-08
## 20083  3 5915  2.722135e-08
## 20084  3 5916  2.734089e-08
## 20085  3 5917  2.752421e-08
## 20086  3 5918  2.767464e-08
## 20087  3 5919  2.783123e-08
## 20088  3 5920  2.803708e-08
## 20089  3 5921  2.816573e-08
## 20090  3 5922  2.838441e-08
## 20091  3 5923  2.853474e-08
## 20092  3 5924  2.875285e-08
## 20093  3 5925  2.889262e-08
## 20094  3 5926  2.908202e-08
## 20095  3 5927  2.931134e-08
## 20096  3 5928  2.944393e-08
## 20097  3 5929  2.965819e-08
## 20098  3 5930  2.985714e-08
## 20099  3 5931  3.003312e-08
## 20100  3 5932  3.023155e-08
## 20101  3 5933  3.031968e-08
## 20102  3 5934  3.060735e-08
## 20103  3 5935  3.069882e-08
## 20104  3 5936  3.094159e-08
## 20105  3 5937  3.107151e-08
## 20106  3 5938  3.124295e-08
## 20107  3 5939  3.145742e-08
## 20108  3 5940  3.163052e-08
## 20109  3 5941  3.178521e-08
## 20110  3 5942  3.197199e-08
## 20111  3 5943  3.217099e-08
## 20112  3 5944  3.230740e-08
## 20113  3 5945  3.251938e-08
## 20114  3 5946  3.265253e-08
## 20115  3 5947  3.285778e-08
## 20116  3 5948  3.299593e-08
## 20117  3 5949  3.321371e-08
## 20118  3 5950  3.336437e-08
## 20119  3 5951  3.347446e-08
## 20120  3 5952  3.359251e-08
## 20121  3 5953  3.375971e-08
## 20122  3 5954  3.392709e-08
## 20123  3 5955  3.406013e-08
## 20124  3 5956  3.422125e-08
## 20125  3 5957  3.432295e-08
## 20126  3 5958  3.444112e-08
## 20127  3 5959  3.460833e-08
## 20128  3 5960  3.471489e-08
## 20129  3 5961  3.478114e-08
## 20130  3 5962  3.494612e-08
## 20131  3 5963  3.509449e-08
## 20132  3 5964  3.521399e-08
## 20133  3 5965  3.524092e-08
## 20134  3 5966  3.538291e-08
## 20135  3 5967  3.550901e-08
## 20136  3 5968  3.555285e-08
## 20137  3 5969  3.563486e-08
## 20138  3 5970  3.562928e-08
## 20139  3 5971  3.575098e-08
## 20140  3 5972  3.589145e-08
## 20141  3 5973  3.597249e-08
## 20142  3 5974  3.593397e-08
## 20143  3 5975  3.592877e-08
## 20144  3 5976  3.596479e-08
## 20145  3 5977  3.601656e-08
## 20146  3 5978  3.601731e-08
## 20147  3 5979  3.605178e-08
## 20148  3 5980  3.600514e-08
## 20149  3 5981  3.602368e-08
## 20150  3 5982  3.602946e-08
## 20151  3 5983  3.598816e-08
## 20152  3 5984  3.601170e-08
## 20153  3 5985  3.592830e-08
## 20154  3 5986  3.600677e-08
## 20155  3 5987  3.583677e-08
## 20156  3 5988  3.582764e-08
## 20157  3 5989  3.573647e-08
## 20158  3 5990  3.563197e-08
## 20159  3 5991  3.553135e-08
## 20160  3 5992  3.545707e-08
## 20161  3 5993  3.538007e-08
## 20162  3 5994  3.527848e-08
## 20163  3 5995  3.514132e-08
## 20164  3 5996  3.498071e-08
## 20165  3 5997  3.485545e-08
## 20166  3 5998  3.470604e-08
## 20167  3 5999  3.452716e-08
## 20168  3 6000  3.436656e-08
## 20169  3 6001  3.417307e-08
## 20170  3 6002  3.398656e-08
## 20171  3 6003  3.377864e-08
## 20172  3 6004  3.352230e-08
## 20173  3 6005  3.332231e-08
## 20174  3 6006  3.307354e-08
## 20175  3 6007  3.281940e-08
## 20176  3 6008  3.258086e-08
## 20177  3 6009  3.230540e-08
## 20178  3 6010  3.203493e-08
## 20179  3 6011  3.169355e-08
## 20180  3 6012  3.136621e-08
## 20181  3 6013  3.101706e-08
## 20182  3 6014  3.074097e-08
## 20183  3 6015  3.035061e-08
## 20184  3 6016  3.000027e-08
## 20185  3 6017  2.976548e-08
## 20186  3 6018  2.972309e-08
## 20187  3 6019  2.985394e-08
## 20188  3 6020  3.002091e-08
## 20189  3 6021  2.997875e-08
## 20190  3 6022  2.999787e-08
## 20191  3 6023  3.001030e-08
## 20192  3 6024  3.007781e-08
## 20193  3 6025  3.011530e-08
## 20194  3 6026  3.012124e-08
## 20195  3 6027  3.018833e-08
## 20196  3 6028  3.022189e-08
## 20197  3 6029  3.024119e-08
## 20198  3 6030  3.026668e-08
## 20199  3 6031  3.021049e-08
## 20200  3 6032  3.028737e-08
## 20201  3 6033  3.038734e-08
## 20202  3 6034  3.033591e-08
## 20203  3 6035  3.036745e-08
## 20204  3 6036  3.036935e-08
## 20205  3 6037  3.041669e-08
## 20206  3 6038  3.034927e-08
## 20207  3 6039  3.038961e-08
## 20208  3 6040  3.032659e-08
## 20209  3 6041  3.035726e-08
## 20210  3 6042  3.039610e-08
## 20211  3 6043  3.031824e-08
## 20212  3 6044  3.031968e-08
## 20213  3 6045  3.024159e-08
## 20214  3 6046  3.031322e-08
## 20215  3 6047  3.021196e-08
## 20216  3 6048  3.016319e-08
## 20217  3 6049  3.012173e-08
## 20218  3 6050  3.019115e-08
## 20219  3 6051  3.008282e-08
## 20220  3 6052  3.009441e-08
## 20221  3 6053  2.997342e-08
## 20222  3 6054  2.998331e-08
## 20223  3 6055  2.993771e-08
## 20224  3 6056  2.984551e-08
## 20225  3 6057  2.980162e-08
## 20226  3 6058  2.969054e-08
## 20227  3 6059  2.962783e-08
## 20228  3 6060  2.952195e-08
## 20229  3 6061  2.949577e-08
## 20230  3 6062  2.940613e-08
## 20231  3 6063  2.925698e-08
## 20232  3 6064  2.921668e-08
## 20233  3 6065  2.905905e-08
## 20234  3 6066  2.900548e-08
## 20235  3 6067  2.889146e-08
## 20236  3 6068  2.879672e-08
## 20237  3 6069  2.862018e-08
## 20238  3 6070  2.854790e-08
## 20239  3 6071  2.845338e-08
## 20240  3 6072  2.831744e-08
## 20241  3 6073  2.814585e-08
## 20242  3 6074  2.797089e-08
## 20243  3 6075  2.788183e-08
## 20244  3 6076  2.777419e-08
## 20245  3 6077  2.758238e-08
## 20246  3 6078  2.740684e-08
## 20247  3 6079  2.719399e-08
## 20248  3 6080  2.704136e-08
## 20249  3 6081  2.687950e-08
## 20250  3 6082  2.671909e-08
## 20251  3 6083  2.652810e-08
## 20252  3 6084  2.631631e-08
## 20253  3 6085  2.610633e-08
## 20254  3 6086  2.589660e-08
## 20255  3 6087  2.566124e-08
## 20256  3 6088  2.550953e-08
## 20257  3 6089  2.525686e-08
## 20258  3 6090  2.503936e-08
## 20259  3 6091  2.483891e-08
## 20260  3 6092  2.455373e-08
## 20261  3 6093  2.433486e-08
## 20262  3 6094  2.408103e-08
## 20263  3 6095  2.381259e-08
## 20264  3 6096  2.354962e-08
## 20265  3 6097  2.326989e-08
## 20266  3 6098  2.299270e-08
## 20267  3 6099  2.273412e-08
## 20268  3 6100  2.243755e-08
## 20269  3 6101  2.213162e-08
## 20270  3 6102  2.183546e-08
## 20271  3 6103  2.153447e-08
## 20272  3 6104  2.123568e-08
## 20273  3 6105  2.091471e-08
## 20274  3 6106  2.056094e-08
## 20275  3 6107  2.021557e-08
## 20276  3 6108  1.988688e-08
## 20277  3 6109  1.952236e-08
## 20278  3 6110  1.916442e-08
## 20279  3 6111  1.879532e-08
## 20280  3 6112  1.845778e-08
## 20281  3 6113  1.808077e-08
## 20282  3 6114  1.764686e-08
## 20283  3 6115  1.727627e-08
## 20284  3 6116  1.690273e-08
## 20285  3 6117  1.646521e-08
## 20286  3 6118  1.609651e-08
## 20287  3 6119  1.565316e-08
## 20288  3 6120  1.524279e-08
## 20289  3 6121  1.483100e-08
## 20290  3 6122  1.434713e-08
## 20291  3 6123  1.388639e-08
## 20292  3 6124  1.343988e-08
## 20293  3 6125  1.297653e-08
## 20294  3 6126  1.248499e-08
## 20295  3 6127  1.200587e-08
## 20296  3 6128  1.154195e-08
## 20297  3 6129  1.102008e-08
## 20298  3 6130  1.050491e-08
## 20299  3 6131  1.030898e-08
## 20300  3 6132  1.007250e-08
## 20301  3 6133  9.900300e-09
## 20302  3 6134  9.654267e-09
## 20303  3 6135  9.441940e-09
## 20304  3 6136  9.248169e-09
## 20305  3 6137  9.036625e-09
## 20306  3 6138  8.815976e-09
## 20307  3 6139  8.617826e-09
## 20308  3 6140  8.389708e-09
## 20309  3 6141  8.198525e-09
## 20310  3 6142  7.970626e-09
## 20311  3 6143  7.775561e-09
## 20312  3 6144  7.551264e-09
## 20313  3 6145  7.353565e-09
## 20314  3 6146  7.139181e-09
## 20315  3 6147  6.935058e-09
## 20316  3 6148  6.718337e-09
## 20317  3 6149  6.507355e-09
## 20318  3 6150  6.311019e-09
## 20319  3 6151  6.090921e-09
## 20320  3 6152  5.893421e-09
## 20321  3 6153  5.676230e-09
## 20322  3 6154  5.469209e-09
## 20323  3 6155  5.257352e-09
## 20324  3 6156  5.057415e-09
## 20325  3 6157  4.845592e-09
## 20326  3 6158  4.638452e-09
## 20327  3 6159  4.430846e-09
## 20328  3 6160  4.227567e-09
## 20329  3 6161  4.018370e-09
## 20330  3 6162  3.819574e-09
## 20331  3 6163  3.605852e-09
## 20332  3 6164  3.405707e-09
## 20333  3 6165  3.195504e-09
## 20334  3 6166  2.990316e-09
## 20335  3 6167  2.791723e-09
## 20336  3 6168  2.583379e-09
## 20337  3 6169  2.383167e-09
## 20338  3 6170  2.176352e-09
## 20339  3 6171  1.974856e-09
## 20340  3 6172  1.774695e-09
## 20341  3 6173  1.571987e-09
## 20342  3 6174  1.368928e-09
## 20343  3 6175  1.168329e-09
## 20344  3 6176  9.674831e-10
## 20345  3 6177  7.705712e-10
## 20346  3 6178  5.714200e-10
## 20347  3 6179  3.721408e-10
## 20348  3 6180  1.750253e-10
## 20349  3 6181 -2.214557e-11
## 20350  3 6182 -2.182205e-10
## 20351  3 6183 -4.142893e-10
## 20352  3 6184 -6.096770e-10
## 20353  3 6185 -8.047781e-10
## 20354  3 6186 -9.972437e-10
## 20355  3 6187 -1.191487e-09
## 20356  3 6188 -1.385726e-09
## 20357  3 6189 -1.574376e-09
## 20358  3 6190 -1.765943e-09
## 20359  3 6191 -1.956422e-09
## 20360  3 6192 -2.145823e-09
## 20361  3 6193 -2.335783e-09
## 20362  3 6194 -2.524817e-09
## 20363  3 6195 -2.711314e-09
## 20364  3 6196 -2.892012e-09
## 20365  3 6197 -3.077367e-09
## 20366  3 6198 -3.267937e-09
## 20367  3 6199 -3.450299e-09
## 20368  3 6200 -3.628239e-09
## 20369  3 6201 -3.810844e-09
## 20370  3 6202 -3.991908e-09
## 20371  3 6203 -4.171893e-09
## 20372  3 6204 -4.347522e-09
## 20373  3 6205 -4.533186e-09
## 20374  3 6206 -4.705028e-09
## 20375  3 6207 -4.882931e-09
## 20376  3 6208 -5.057039e-09
## 20377  3 6209 -5.222451e-09
## 20378  3 6210 -5.403936e-09
## 20379  3 6211 -5.576758e-09
## 20380  3 6212 -5.742993e-09
## 20381  3 6213 -5.928472e-09
## 20382  3 6214 -6.074079e-09
## 20383  3 6215 -6.242129e-09
## 20384  3 6216 -6.407310e-09
## 20385  3 6217 -6.569344e-09
## 20386  3 6218 -6.750583e-09
## 20387  3 6219 -6.919102e-09
## 20388  3 6220 -7.077125e-09
## 20389  3 6221 -7.234047e-09
## 20390  3 6222 -7.391995e-09
## 20391  3 6223 -7.551055e-09
## 20392  3 6224 -7.685336e-09
## 20393  3 6225 -7.873155e-09
## 20394  3 6226 -8.015232e-09
## 20395  3 6227 -8.173788e-09
## 20396  3 6228 -8.319169e-09
## 20397  3 6229 -8.465013e-09
## 20398  3 6230 -8.604126e-09
## 20399  3 6231 -8.767575e-09
## 20400  3 6232 -8.893902e-09
## 20401  3 6233 -9.056612e-09
## 20402  3 6234 -9.217660e-09
## 20403  3 6235 -9.356941e-09
## 20404  3 6236 -9.477266e-09
## 20405  3 6237 -9.627724e-09
## 20406  3 6238 -9.747135e-09
## 20407  3 6239 -9.889355e-09
## 20408  3 6240 -1.002626e-08
## 20409  3 6241 -1.016836e-08
## 20410  3 6242 -1.029349e-08
## 20411  3 6243 -1.041769e-08
## 20412  3 6244 -1.054792e-08
## 20413  3 6245 -1.067109e-08
## 20414  3 6246 -1.080549e-08
## 20415  3 6247 -1.091701e-08
## 20416  3 6248 -1.104803e-08
## 20417  3 6249 -1.114131e-08
## 20418  3 6250 -1.130268e-08
## 20419  3 6251 -1.138677e-08
## 20420  3 6252 -1.150239e-08
## 20421  3 6253 -1.164020e-08
## 20422  3 6254 -1.173567e-08
## 20423  3 6255 -1.184020e-08
## 20424  3 6256 -1.195422e-08
## 20425  3 6257 -1.207289e-08
## 20426  3 6258 -1.216959e-08
## 20427  3 6259 -1.228231e-08
## 20428  3 6260 -1.237436e-08
## 20429  3 6261 -1.246665e-08
## 20430  3 6262 -1.257601e-08
## 20431  3 6263 -1.266904e-08
## 20432  3 6264 -1.276608e-08
## 20433  3 6265 -1.283101e-08
## 20434  3 6266 -1.293060e-08
## 20435  3 6267 -1.303963e-08
## 20436  3 6268 -1.310489e-08
## 20437  3 6269 -1.321219e-08
## 20438  3 6270 -1.328677e-08
## 20439  3 6271 -1.338288e-08
## 20440  3 6272 -1.346526e-08
## 20441  3 6273 -1.350624e-08
## 20442  3 6274 -1.358649e-08
## 20443  3 6275 -1.364973e-08
## 20444  3 6276 -1.375518e-08
## 20445  3 6277 -1.382653e-08
## 20446  3 6278 -1.387340e-08
## 20447  3 6279 -1.395010e-08
## 20448  3 6280 -1.398725e-08
## 20449  3 6281 -1.406500e-08
## 20450  3 6282 -1.414212e-08
## 20451  3 6283 -1.415441e-08
## 20452  3 6284 -1.423721e-08
## 20453  3 6285 -1.430349e-08
## 20454  3 6286 -1.434022e-08
## 20455  3 6287 -1.439063e-08
## 20456  3 6288 -1.441648e-08
## 20457  3 6289 -1.445324e-08
## 20458  3 6290 -1.451757e-08
## 20459  3 6291 -1.455163e-08
## 20460  3 6292 -1.458336e-08
## 20461  3 6293 -1.461521e-08
## 20462  3 6294 -1.468085e-08
## 20463  3 6295 -1.467729e-08
## 20464  3 6296 -1.471334e-08
## 20465  3 6297 -1.471468e-08
## 20466  3 6298 -1.477281e-08
## 20467  3 6299 -1.477593e-08
## 20468  3 6300 -1.479006e-08
## 20469  3 6301 -1.480350e-08
## 20470  3 6302 -1.481580e-08
## 20471  3 6303 -1.482663e-08
## 20472  3 6304 -1.481231e-08
## 20473  3 6305 -1.483892e-08
## 20474  3 6306 -1.483559e-08
## 20475  3 6307 -1.481022e-08
## 20476  3 6308 -1.485899e-08
## 20477  3 6309 -1.482793e-08
## 20478  3 6310 -1.483898e-08
## 20479  3 6311 -1.483823e-08
## 20480  3 6312 -1.479108e-08
## 20481  3 6313 -1.479464e-08
## 20482  3 6314 -1.473909e-08
## 20483  3 6315 -1.474431e-08
## 20484  3 6316 -1.472178e-08
## 20485  3 6317 -1.469998e-08
## 20486  3 6318 -1.466613e-08
## 20487  3 6319 -1.465025e-08
## 20488  3 6320 -1.462301e-08
## 20489  3 6321 -1.457042e-08
## 20490  3 6322 -1.452500e-08
## 20491  3 6323 -1.449336e-08
## 20492  3 6324 -1.441976e-08
## 20493  3 6325 -1.439536e-08
## 20494  3 6326 -1.432263e-08
## 20495  3 6327 -1.427095e-08
## 20496  3 6328 -1.424189e-08
## 20497  3 6329 -1.417713e-08
## 20498  3 6330 -1.409089e-08
## 20499  3 6331 -1.405510e-08
## 20500  3 6332 -1.397559e-08
## 20501  3 6333 -1.392193e-08
## 20502  3 6334 -1.383863e-08
## 20503  3 6335 -1.378812e-08
## 20504  3 6336 -1.369400e-08
## 20505  3 6337 -1.359716e-08
## 20506  3 6338 -1.354896e-08
## 20507  3 6339 -1.341212e-08
## 20508  3 6340 -1.334989e-08
## 20509  3 6341 -1.325524e-08
## 20510  3 6342 -1.317299e-08
## 20511  3 6343 -1.304061e-08
## 20512  3 6344 -1.294754e-08
## 20513  3 6345 -1.285412e-08
## 20514  3 6346 -1.272607e-08
## 20515  3 6347 -1.262006e-08
## 20516  3 6348 -1.249545e-08
## 20517  3 6349 -1.240078e-08
## 20518  3 6350 -1.225507e-08
## 20519  3 6351 -1.215316e-08
## 20520  3 6352 -1.201082e-08
## 20521  3 6353 -1.188420e-08
## 20522  3 6354 -1.174001e-08
## 20523  3 6355 -1.160333e-08
## 20524  3 6356 -1.149409e-08
## 20525  3 6357 -1.293377e-08
## 20526  3 6358 -1.285933e-08
## 20527  3 6359 -1.276207e-08
## 20528  3 6360 -1.264033e-08
## 20529  3 6361 -1.253872e-08
## 20530  3 6362 -1.246407e-08
## 20531  3 6363 -1.238392e-08
## 20532  3 6364 -1.227084e-08
## 20533  3 6365 -1.219147e-08
## 20534  3 6366 -1.206141e-08
## 20535  3 6367 -1.198735e-08
## 20536  3 6368 -1.188477e-08
## 20537  3 6369 -1.180649e-08
## 20538  3 6370 -1.171263e-08
## 20539  3 6371 -1.161267e-08
## 20540  3 6372 -1.152239e-08
## 20541  3 6373 -1.140287e-08
## 20542  3 6374 -1.132786e-08
## 20543  3 6375 -1.122097e-08
## 20544  3 6376 -1.112484e-08
## 20545  3 6377 -1.103444e-08
## 20546  3 6378 -1.093957e-08
## 20547  3 6379 -1.082208e-08
## 20548  3 6380 -1.074323e-08
## 20549  3 6381 -1.062414e-08
## 20550  3 6382 -1.054439e-08
## 20551  3 6383 -1.043351e-08
## 20552  3 6384 -1.036141e-08
## 20553  3 6385 -1.024141e-08
## 20554  3 6386 -1.016759e-08
## 20555  3 6387 -1.006654e-08
## 20556  3 6388 -9.968166e-09
## 20557  3 6389 -9.864492e-09
## 20558  3 6390 -9.772397e-09
## 20559  3 6391 -9.677757e-09
## 20560  3 6392 -9.577573e-09
## 20561  3 6393 -9.483030e-09
## 20562  3 6394 -9.373431e-09
## 20563  3 6395 -9.283328e-09
## 20564  3 6396 -9.172381e-09
## 20565  3 6397 -9.112549e-09
## 20566  3 6398 -8.982834e-09
## 20567  3 6399 -8.907518e-09
## 20568  3 6400 -8.803135e-09
## 20569  3 6401 -8.705401e-09
## 20570  3 6402 -8.602192e-09
## 20571  3 6403 -8.489341e-09
## 20572  3 6404 -8.387912e-09
## 20573  3 6405 -8.296871e-09
## 20574  3 6406 -8.209701e-09
## 20575  3 6407 -8.121420e-09
## 20576  3 6408 -8.007758e-09
## 20577  3 6409 -7.925440e-09
## 20578  3 6410 -7.818549e-09
## 20579  3 6411 -7.708045e-09
## 20580  3 6412 -7.609679e-09
## 20581  3 6413 -7.515581e-09
## 20582  3 6414 -7.414873e-09
## 20583  3 6415 -7.330007e-09
## 20584  3 6416 -7.230424e-09
## 20585  3 6417 -7.120564e-09
## 20586  3 6418 -7.018080e-09
## 20587  3 6419 -6.925676e-09
## 20588  3 6420 -6.823153e-09
## 20589  3 6421 -6.727902e-09
## 20590  3 6422 -6.636846e-09
## 20591  3 6423 -6.527521e-09
## 20592  3 6424 -6.425691e-09
## 20593  3 6425 -6.327959e-09
## 20594  3 6426 -6.235778e-09
## 20595  3 6427 -6.126926e-09
## 20596  3 6428 -6.036040e-09
## 20597  3 6429 -5.931456e-09
## 20598  3 6430 -5.830537e-09
## 20599  3 6431 -5.728573e-09
## 20600  3 6432 -5.634525e-09
## 20601  3 6433 -5.536238e-09
## 20602  3 6434 -5.425168e-09
## 20603  3 6435 -5.334942e-09
## 20604  3 6436 -5.234602e-09
## 20605  3 6437 -5.134396e-09
## 20606  3 6438 -5.027703e-09
## 20607  3 6439 -4.943011e-09
## 20608  3 6440 -4.828368e-09
## 20609  3 6441 -4.720665e-09
## 20610  3 6442 -4.626584e-09
## 20611  3 6443 -4.524541e-09
## 20612  3 6444 -4.427846e-09
## 20613  3 6445 -4.330577e-09
## 20614  3 6446 -4.224961e-09
## 20615  3 6447 -4.124116e-09
## 20616  3 6448 -4.023002e-09
## 20617  3 6449 -3.928697e-09
## 20618  3 6450 -3.823490e-09
## 20619  3 6451 -3.720871e-09
## 20620  3 6452 -3.620586e-09
## 20621  3 6453 -3.522465e-09
## 20622  3 6454 -3.412575e-09
## 20623  3 6455 -3.320138e-09
## 20624  3 6456 -3.215979e-09
## 20625  3 6457 -3.119012e-09
## 20626  3 6458 -3.013262e-09
## 20627  3 6459 -2.908785e-09
## 20628  3 6460 -2.809846e-09
## 20629  3 6461 -2.713220e-09
## 20630  3 6462 -2.609354e-09
## 20631  3 6463 -2.507279e-09
## 20632  3 6464 -2.404806e-09
## 20633  3 6465 -2.306462e-09
## 20634  3 6466 -2.201384e-09
## 20635  3 6467 -2.099671e-09
## 20636  3 6468 -1.994850e-09
## 20637  3 6469 -1.895247e-09
## 20638  3 6470 -1.794720e-09
## 20639  3 6471 -1.726287e-09
## 20640  3 6472 -1.672703e-09
## 20641  3 6473 -1.618285e-09
## 20642  3 6474 -1.564278e-09
## 20643  3 6475 -1.507978e-09
## 20644  3 6476 -1.456680e-09
## 20645  3 6477 -1.403148e-09
## 20646  3 6478 -1.345591e-09
## 20647  3 6479 -1.296360e-09
## 20648  3 6480 -1.245558e-09
## 20649  3 6481 -1.190533e-09
## 20650  3 6482 -1.138274e-09
## 20651  3 6483 -1.090599e-09
## 20652  3 6484 -1.038418e-09
## 20653  3 6485 -9.877037e-10
## 20654  3 6486 -9.375367e-10
## 20655  3 6487 -8.884243e-10
## 20656  3 6488 -8.393985e-10
## 20657  3 6489 -7.913789e-10
## 20658  3 6490 -7.396154e-10
## 20659  3 6491 -6.926452e-10
## 20660  3 6492 -6.445334e-10
## 20661  3 6493 -5.966609e-10
## 20662  3 6494 -5.495556e-10
## 20663  3 6495 -5.026388e-10
## 20664  3 6496 -4.569354e-10
## 20665  3 6497 -4.102168e-10
## 20666  3 6498 -3.637563e-10
## 20667  3 6499 -3.183543e-10
## 20668  3 6500 -2.729195e-10
## 20669  3 6501 -2.281766e-10
## 20670  3 6502 -1.836243e-10
## 20671  3 6503 -1.394740e-10
## 20672  3 6504 -9.536913e-11
## 20673  3 6505 -5.192550e-11
## 20674  3 6506 -8.806616e-12
## 20675  3 6507  3.408199e-11
## 20676  3 6508  7.658809e-11
## 20677  3 6509  1.185774e-10
## 20678  3 6510  1.602403e-10
## 20679  3 6511  2.018051e-10
## 20680  3 6512  2.426536e-10
## 20681  3 6513  2.833011e-10
## 20682  3 6514  3.232924e-10
## 20683  3 6515  3.641706e-10
## 20684  3 6516  4.041020e-10
## 20685  3 6517  4.427178e-10
## 20686  3 6518  4.820034e-10
## 20687  3 6519  5.203324e-10
## 20688  3 6520  5.586496e-10
## 20689  3 6521  5.957343e-10
## 20690  3 6522  6.338334e-10
## 20691  3 6523  6.722737e-10
## 20692  3 6524  7.098123e-10
## 20693  3 6525  7.445733e-10
## 20694  3 6526  7.799544e-10
## 20695  3 6527  8.163768e-10
## 20696  3 6528  8.520995e-10
## 20697  3 6529  8.849847e-10
## 20698  3 6530  9.226350e-10
## 20699  3 6531  9.568522e-10
## 20700  3 6532  9.893427e-10
## 20701  3 6533  1.024211e-09
## 20702  3 6534  1.057334e-09
## 20703  3 6535  1.090915e-09
## 20704  3 6536  1.121682e-09
## 20705  3 6537  1.154597e-09
## 20706  3 6538  1.188231e-09
## 20707  3 6539  1.219934e-09
## 20708  3 6540  1.249984e-09
## 20709  3 6541  1.279389e-09
## 20710  3 6542  1.311243e-09
## 20711  3 6543  1.340960e-09
## 20712  3 6544  1.371701e-09
## 20713  3 6545  1.400955e-09
## 20714  3 6546  1.432613e-09
## 20715  3 6547  1.459532e-09
## 20716  3 6548  1.486825e-09
## 20717  3 6549  1.517132e-09
## 20718  3 6550  1.540934e-09
## 20719  3 6551  1.570505e-09
## 20720  3 6552  1.601154e-09
## 20721  3 6553  1.622424e-09
## 20722  3 6554  1.653693e-09
## 20723  3 6555  1.677829e-09
## 20724  3 6556  1.702227e-09
## 20725  3 6557  1.728688e-09
## 20726  3 6558  1.754789e-09
## 20727  3 6559  1.776725e-09
## 20728  3 6560  1.802111e-09
## 20729  3 6561  1.826774e-09
## 20730  3 6562  1.849440e-09
## 20731  3 6563  1.873047e-09
## 20732  3 6564  1.899189e-09
## 20733  3 6565  1.914889e-09
## 20734  3 6566  1.940644e-09
## 20735  3 6567  1.960422e-09
## 20736  3 6568  1.982558e-09
## 20737  3 6569  2.004915e-09
## 20738  3 6570  2.025493e-09
## 20739  3 6571  2.045597e-09
## 20740  3 6572  2.069378e-09
## 20741  3 6573  2.084337e-09
## 20742  3 6574  2.106131e-09
## 20743  3 6575  2.121695e-09
## 20744  3 6576  2.143896e-09
## 20745  3 6577  2.159914e-09
## 20746  3 6578  2.178171e-09
## 20747  3 6579  2.198062e-09
## 20748  3 6580  2.212770e-09
## 20749  3 6581  2.231277e-09
## 20750  3 6582  2.244321e-09
## 20751  3 6583  2.262604e-09
## 20752  3 6584  2.280876e-09
## 20753  3 6585  2.299934e-09
## 20754  3 6586  2.309573e-09
## 20755  3 6587  2.327049e-09
## 20756  3 6588  2.339758e-09
## 20757  3 6589  2.353931e-09
## 20758  3 6590  2.367707e-09
## 20759  3 6591  2.379800e-09
## 20760  3 6592  2.392145e-09
## 20761  3 6593  2.407318e-09
## 20762  3 6594  2.418007e-09
## 20763  3 6595  2.432614e-09
## 20764  3 6596  2.441681e-09
## 20765  3 6597  2.454406e-09
## 20766  3 6598  2.465807e-09
## 20767  3 6599  2.479416e-09
## 20768  3 6600  2.488007e-09
## 20769  3 6601  2.493811e-09
## 20770  3 6602  2.505656e-09
## 20771  3 6603  2.515042e-09
## 20772  3 6604  2.523054e-09
## 20773  3 6605  2.532378e-09
## 20774  3 6606  2.537190e-09
## 20775  3 6607  2.549810e-09
## 20776  3 6608  2.556961e-09
## 20777  3 6609  2.561404e-09
## 20778  3 6610  2.568801e-09
## 20779  3 6611  2.578565e-09
## 20780  3 6612  2.582625e-09
## 20781  3 6613  2.587183e-09
## 20782  3 6614  2.591542e-09
## 20783  3 6615  2.600813e-09
## 20784  3 6616  2.601744e-09
## 20785  3 6617  2.611046e-09
## 20786  3 6618  2.616070e-09
## 20787  3 6619  2.615626e-09
## 20788  3 6620  2.619649e-09
## 20789  3 6621  2.618111e-09
## 20790  3 6622  2.624724e-09
## 20791  3 6623  2.627860e-09
## 20792  3 6624  2.628454e-09
## 20793  3 6625  2.630299e-09
## 20794  3 6626  2.630458e-09
## 20795  3 6627  2.627354e-09
## 20796  3 6628  2.628450e-09
## 20797  3 6629  2.635390e-09
## 20798  3 6630  2.630510e-09
## 20799  3 6631  2.634021e-09
## 20800  3 6632  2.632601e-09
## 20801  3 6633  2.627159e-09
## 20802  3 6634  2.627237e-09
## 20803  3 6635  2.630001e-09
## 20804  3 6636  2.621810e-09
## 20805  3 6637  2.621815e-09
## 20806  3 6638  2.614680e-09
## 20807  3 6639  2.617880e-09
## 20808  3 6640  2.607181e-09
## 20809  3 6641  2.607522e-09
## 20810  3 6642  2.602568e-09
## 20811  3 6643  2.601027e-09
## 20812  3 6644  2.593854e-09
## 20813  3 6645  2.584438e-09
## 20814  3 6646  2.578455e-09
## 20815  3 6647  2.576005e-09
## 20816  3 6648  2.565587e-09
## 20817  3 6649  2.562639e-09
## 20818  3 6650  2.553646e-09
## 20819  3 6651  2.548794e-09
## 20820  3 6652  2.541411e-09
## 20821  3 6653  2.528607e-09
## 20822  3 6654  2.518221e-09
## 20823  3 6655  2.510740e-09
## 20824  3 6656  2.505433e-09
## 20825  3 6657  2.495842e-09
## 20826  3 6658  2.485992e-09
## 20827  3 6659  2.476622e-09
## 20828  3 6660  2.461349e-09
## 20829  3 6661  2.456359e-09
## 20830  3 6662  2.442315e-09
## 20831  3 6663  2.431160e-09
## 20832  3 6664  2.416342e-09
## 20833  3 6665  2.402412e-09
## 20834  3 6666  2.392483e-09
## 20835  3 6667  2.380722e-09
```

```
## 20836  3 6668  2.362858e-09
## 20837  3 6669  2.350769e-09
## 20838  3 6670  2.336685e-09
## 20839  3 6671  2.324023e-09
## 20840  3 6672  2.306981e-09
## 20841  3 6673  2.289707e-09
## 20842  3 6674  2.274899e-09
## 20843  3 6675  2.259837e-09
## 20844  3 6676  2.246020e-09
## 20845  3 6677  2.231284e-09
## 20846  3 6678  2.214674e-09
## 20847  3 6679  2.195131e-09
## 20848  3 6680  2.173377e-09
## 20849  3 6681  2.159473e-09
## 20850  3 6682  2.142035e-09
## 20851  3 6683  2.118676e-09
## 20852  3 6684  2.098579e-09
## 20853  3 6685  2.082255e-09
## 20854  3 6686  2.063837e-09
## 20855  3 6687  2.044237e-09
## 20856  3 6688  2.016323e-09
## 20857  3 6689  2.001217e-09
## 20858  3 6690  1.978640e-09
## 20859  3 6691  1.956674e-09
## 20860  3 6692  1.937885e-09
## 20861  3 6693  1.915662e-09
## 20862  3 6694  1.892721e-09
## 20863  3 6695  1.870333e-09
## 20864  3 6696  1.844070e-09
## 20865  3 6697  1.823606e-09
## 20866  3 6698  1.825531e-09
## 20867  3 6699  1.834364e-09
## 20868  3 6700  1.838604e-09
## 20869  3 6701  1.842931e-09
## 20870  3 6702  1.850509e-09
## 20871  3 6703  1.856438e-09
## 20872  3 6704  1.865485e-09
## 20873  3 6705  1.867557e-09
## 20874  3 6706  1.871623e-09
## 20875  3 6707  1.880858e-09
## 20876  3 6708  1.885357e-09
## 20877  3 6709  1.888924e-09
## 20878  3 6710  1.894674e-09
## 20879  3 6711  1.898424e-09
## 20880  3 6712  1.904214e-09
## 20881  3 6713  1.906655e-09
## 20882  3 6714  1.912929e-09
## 20883  3 6715  1.915128e-09
## 20884  3 6716  1.923874e-09
## 20885  3 6717  1.923966e-09
## 20886  3 6718  1.932434e-09
## 20887  3 6719  1.938193e-09
## 20888  3 6720  1.939778e-09
## 20889  3 6721  1.944719e-09
## 20890  3 6722  1.950100e-09
## 20891  3 6723  1.951235e-09
## 20892  3 6724  1.956505e-09
## 20893  3 6725  1.959338e-09
## 20894  3 6726  1.964430e-09
## 20895  3 6727  1.972088e-09
## 20896  3 6728  1.970596e-09
## 20897  3 6729  1.975885e-09
## 20898  3 6730  1.977504e-09
## 20899  3 6731  1.982445e-09
## 20900  3 6732  1.985263e-09
## 20901  3 6733  1.986189e-09
## 20902  3 6734  1.991315e-09
## 20903  3 6735  1.994283e-09
## 20904  3 6736  1.993269e-09
## 20905  3 6737  2.002925e-09
## 20906  3 6738  2.001306e-09
## 20907  3 6739  2.003741e-09
## 20908  3 6740  2.009787e-09
## 20909  3 6741  2.011929e-09
## 20910  3 6742  2.010991e-09
## 20911  3 6743  2.017663e-09
## 20912  3 6744  2.014690e-09
## 20913  3 6745  2.019443e-09
## 20914  3 6746  2.022678e-09
## 20915  3 6747  2.022916e-09
## 20916  3 6748  2.022488e-09
## 20917  3 6749  2.026211e-09
## 20918  3 6750  2.029132e-09
## 20919  3 6751  2.030700e-09
## 20920  3 6752  2.031144e-09
## 20921  3 6753  2.028735e-09
## 20922  3 6754  2.033045e-09
## 20923  3 6755  2.037754e-09
## 20924  3 6756  2.039557e-09
## 20925  3 6757  2.035134e-09
## 20926  3 6758  2.039258e-09
## 20927  3 6759  2.038796e-09
## 20928  3 6760  2.039287e-09
## 20929  3 6761  2.043858e-09
## 20930  3 6762  2.039188e-09
## 20931  3 6763  2.042356e-09
## 20932  3 6764  2.044370e-09
## 20933  3 6765  2.043671e-09
## 20934  3 6766  2.043378e-09
## 20935  3 6767  2.044956e-09
## 20936  3 6768  2.043443e-09
## 20937  3 6769  2.045079e-09
## 20938  3 6770  2.045210e-09
## 20939  3 6771  2.046478e-09
## 20940  3 6772  2.041928e-09
## 20941  3 6773  2.045552e-09
## 20942  3 6774  2.044847e-09
## 20943  3 6775  2.043804e-09
## 20944  3 6776  2.042461e-09
## 20945  3 6777  2.043835e-09
## 20946  3 6778  2.041257e-09
## 20947  3 6779  2.036984e-09
## 20948  3 6780  2.040766e-09
## 20949  3 6781  2.035152e-09
## 20950  3 6782  2.037010e-09
## 20951  3 6783  2.038113e-09
## 20952  3 6784  2.039724e-09
## 20953  3 6785  2.032088e-09
## 20954  3 6786  2.037546e-09
## 20955  3 6787  2.029624e-09
## 20956  3 6788  2.033969e-09
## 20957  3 6789  2.029131e-09
## 20958  3 6790  2.028934e-09
## 20959  3 6791  2.025209e-09
## 20960  3 6792  2.024120e-09
## 20961  3 6793  2.023344e-09
## 20962  3 6794  2.019927e-09
## 20963  3 6795  2.019216e-09
## 20964  3 6796  2.015441e-09
## 20965  3 6797  2.013014e-09
## 20966  3 6798  2.011634e-09
## 20967  3 6799  2.006030e-09
## 20968  3 6800  2.006525e-09
## 20969  3 6801  2.003025e-09
## 20970  3 6802  2.004015e-09
## 20971  3 6803  2.001982e-09
## 20972  3 6804  1.994087e-09
## 20973  3 6805  1.993352e-09
## 20974  3 6806  1.989623e-09
## 20975  3 6807  1.983919e-09
## 20976  3 6808  1.982087e-09
## 20977  3 6809  1.979320e-09
## 20978  3 6810  1.973282e-09
## 20979  3 6811  1.971224e-09
## 20980  3 6812  1.968356e-09
## 20981  3 6813  1.966194e-09
## 20982  3 6814  1.962837e-09
## 20983  3 6815  1.957945e-09
## 20984  3 6816  1.951707e-09
## 20985  3 6817  1.948274e-09
## 20986  3 6818  1.949030e-09
## 20987  3 6819  1.939893e-09
## 20988  3 6820  1.940757e-09
## 20989  3 6821  1.933522e-09
## 20990  3 6822  1.927078e-09
## 20991  3 6823  1.922973e-09
## 20992  3 6824  1.920627e-09
## 20993  3 6825  1.912825e-09
## 20994  3 6826  1.912320e-09
## 20995  3 6827  1.905411e-09
## 20996  3 6828  1.902685e-09
## 20997  3 6829  1.894890e-09
## 20998  3 6830  1.889144e-09
## 20999  3 6831  1.883664e-09
## 21000  3 6832  1.879987e-09
## 21001  3 6833  1.874796e-09
## 21002  3 6834  1.869419e-09
## 21003  3 6835  1.861898e-09
## 21004  3 6836  1.857304e-09
## 21005  3 6837  1.852846e-09
## 21006  3 6838  1.847203e-09
## 21007  3 6839  1.843579e-09
## 21008  3 6840  1.833882e-09
## 21009  3 6841  1.823567e-09
## 21010  3 6842  1.824461e-09
## 21011  3 6843  1.819196e-09
## 21012  3 6844  1.812695e-09
## 21013  3 6845  1.800690e-09
## 21014  3 6846  1.797431e-09
## 21015  3 6847  1.790942e-09
## 21016  3 6848  1.784174e-09
## 21017  3 6849  1.776821e-09
## 21018  3 6850  1.770524e-09
## 21019  3 6851  1.763463e-09
## 21020  3 6852  1.757076e-09
## 21021  3 6853  1.746479e-09
## 21022  3 6854  1.741049e-09
## 21023  3 6855  1.737623e-09
## 21024  3 6856  1.729130e-09
## 21025  3 6857  1.722582e-09
## 21026  3 6858  1.713239e-09
## 21027  3 6859  1.703702e-09
## 21028  3 6860  1.695924e-09
## 21029  3 6861  1.688460e-09
## 21030  3 6862  1.681611e-09
## 21031  3 6863  1.677188e-09
## 21032  3 6864  1.665293e-09
## 21033  3 6865  1.655962e-09
## 21034  3 6866  1.650304e-09
## 21035  3 6867  1.644256e-09
## 21036  3 6868  1.635564e-09
## 21037  3 6869  1.624539e-09
## 21038  3 6870  1.618862e-09
## 21039  3 6871  1.608279e-09
## 21040  3 6872  1.597383e-09
## 21041  3 6873  1.591444e-09
## 21042  3 6874  1.581339e-09
## 21043  3 6875  1.571854e-09
## 21044  3 6876  1.562529e-09
## 21045  3 6877  1.557791e-09
## 21046  3 6878  1.545695e-09
## 21047  3 6879  1.534840e-09
## 21048  3 6880  1.527160e-09
## 21049  3 6881  1.514519e-09
## 21050  3 6882  1.508665e-09
## 21051  3 6883  1.497239e-09
## 21052  3 6884  1.487799e-09
## 21053  3 6885  1.479312e-09
## 21054  3 6886  1.466482e-09
## 21055  3 6887  1.458589e-09
## 21056  3 6888  1.451952e-09
## 21057  3 6889  1.439614e-09
## 21058  3 6890  1.426973e-09
## 21059  3 6891  1.417457e-09
## 21060  3 6892  1.406977e-09
## 21061  3 6893  1.396771e-09
## 21062  3 6894  1.387214e-09
## 21063  3 6895  1.375506e-09
## 21064  3 6896  1.365127e-09
## 21065  3 6897  1.353915e-09
## 21066  3 6898  1.345296e-09
## 21067  3 6899  1.333767e-09
## 21068  3 6900  1.322525e-09
## 21069  3 6901  1.310207e-09
## 21070  3 6902  1.299075e-09
## 21071  3 6903  1.290159e-09
## 21072  3 6904  1.276305e-09
## 21073  3 6905  1.264190e-09
## 21074  3 6906  1.255496e-09
## 21075  3 6907  1.243790e-09
## 21076  3 6908  1.229962e-09
## 21077  3 6909  1.217815e-09
## 21078  3 6910  1.209655e-09
## 21079  3 6911  1.195762e-09
## 21080  3 6912  1.185690e-09
## 21081  3 6913  1.173288e-09
## 21082  3 6914  1.160745e-09
## 21083  3 6915  1.146353e-09
## 21084  3 6916  1.135201e-09
## 21085  3 6917  1.121874e-09
## 21086  3 6918  1.108723e-09
## 21087  3 6919  1.095356e-09
## 21088  3 6920  1.084536e-09
## 21089  3 6921  1.071803e-09
## 21090  3 6922  1.055800e-09
## 21091  3 6923  1.043729e-09
## 21092  3 6924  1.025456e-09
## 21093  3 6925  1.022301e-09
## 21094  3 6926  1.018627e-09
## 21095  3 6927  1.014073e-09
## 21096  3 6928  1.010453e-09
## 21097  3 6929  1.008374e-09
## 21098  3 6930  1.005383e-09
## 21099  3 6931  1.002204e-09
## 21100  3 6932  9.978967e-10
## 21101  3 6933  9.959431e-10
## 21102  3 6934  9.909537e-10
## 21103  3 6935  9.872398e-10
## 21104  3 6936  9.857091e-10
## 21105  3 6937  9.806359e-10
## 21106  3 6938  9.763475e-10
## 21107  3 6939  9.741623e-10
## 21108  3 6940  9.712417e-10
## 21109  3 6941  9.674332e-10
## 21110  3 6942  9.651043e-10
## 21111  3 6943  9.591739e-10
## 21112  3 6944  9.575487e-10
## 21113  3 6945  9.559098e-10
## 21114  3 6946  9.521718e-10
## 21115  3 6947  9.475326e-10
## 21116  3 6948  9.416713e-10
## 21117  3 6949  9.406415e-10
## 21118  3 6950  9.378816e-10
## 21119  3 6951  9.343742e-10
## 21120  3 6952  9.316529e-10
## 21121  3 6953  9.258667e-10
## 21122  3 6954  9.254665e-10
## 21123  3 6955  9.205482e-10
## 21124  3 6956  9.167512e-10
## 21125  3 6957  9.150240e-10
## 21126  3 6958  9.110237e-10
## 21127  3 6959  9.066834e-10
## 21128  3 6960  9.042761e-10
## 21129  3 6961  9.004587e-10
## 21130  3 6962  8.976990e-10
## 21131  3 6963  8.940380e-10
## 21132  3 6964  8.902018e-10
## 21133  3 6965  8.858265e-10
## 21134  3 6966  8.843959e-10
## 21135  3 6967  8.809870e-10
## 21136  3 6968  8.771046e-10
## 21137  3 6969  8.729299e-10
## 21138  3 6970  8.698343e-10
## 21139  3 6971  8.679932e-10
## 21140  3 6972  8.634933e-10
## 21141  3 6973  8.597981e-10
## 21142  3 6974  8.573577e-10
## 21143  3 6975  8.525329e-10
## 21144  3 6976  8.496299e-10
## 21145  3 6977  8.461203e-10
## 21146  3 6978  8.416021e-10
## 21147  3 6979  8.389630e-10
## 21148  3 6980  8.369030e-10
## 21149  3 6981  8.326233e-10
## 21150  3 6982  8.296510e-10
## 21151  3 6983  8.256925e-10
## 21152  3 6984  8.243258e-10
## 21153  3 6985  8.220487e-10
## 21154  3 6986  8.181931e-10
## 21155  3 6987  8.124172e-10
## 21156  3 6988  8.113333e-10
## 21157  3 6989  8.077583e-10
## 21158  3 6990  8.025528e-10
## 21159  3 6991  7.997308e-10
## 21160  3 6992  7.973024e-10
## 21161  3 6993  7.953925e-10
## 21162  3 6994  7.904020e-10
## 21163  3 6995  7.851271e-10
## 21164  3 6996  7.831416e-10
## 21165  3 6997  7.791379e-10
## 21166  3 6998  7.767791e-10
## 21167  3 6999  7.737032e-10
## 21168  3 7000  7.698245e-10
## 21169  4    0  0.000000e+00
## 21170  4    0  3.879012e+00
## 21171  4    1  5.933213e+00
## 21172  4    2  5.612533e+00
## 21173  4    3  5.357767e+00
## 21174  4    4  5.157420e+00
## 21175  4    5  4.986489e+00
## 21176  4    6  4.850239e+00
## 21177  4    7  4.732668e+00
## 21178  4    8  4.625641e+00
## 21179  4    9  4.563343e+00
## 21180  4   10  4.476486e+00
## 21181  4   11  4.410785e+00
## 21182  4   12  4.358573e+00
## 21183  4   13  4.321983e+00
## 21184  4   14  4.265431e+00
## 21185  4   15  4.211489e+00
## 21186  4   16  4.170501e+00
## 21187  4   17  4.128325e+00
## 21188  4   18  4.089879e+00
## 21189  4   19  4.052534e+00
## 21190  4   20  4.018463e+00
## 21191  4   21  3.974185e+00
## 21192  4   22  3.951386e+00
## 21193  4   23  3.909925e+00
## 21194  4   24  3.881515e+00
## 21195  4   24  3.876560e+00
## 21196  4   25  5.942454e+00
## 21197  4   26  5.610764e+00
## 21198  4   27  5.368001e+00
## 21199  4   28  5.154365e+00
## 21200  4   29  4.975422e+00
## 21201  4   30  4.847518e+00
## 21202  4   31  4.740030e+00
## 21203  4   32  4.638638e+00
## 21204  4   33  4.554377e+00
## 21205  4   34  4.482906e+00
## 21206  4   35  4.423225e+00
## 21207  4   36  4.356519e+00
## 21208  4   37  4.306496e+00
## 21209  4   38  4.254005e+00
## 21210  4   39  4.213191e+00
## 21211  4   40  4.176411e+00
## 21212  4   41  4.127309e+00
## 21213  4   42  4.096469e+00
## 21214  4   43  4.055194e+00
## 21215  4   44  4.014494e+00
## 21216  4   45  3.985502e+00
## 21217  4   46  3.944979e+00
## 21218  4   47  3.907713e+00
## 21219  4   48  3.876522e+00
## 21220  4   48  3.882750e+00
## 21221  4   49  5.933706e+00
## 21222  4   50  5.617520e+00
## 21223  4   51  5.362426e+00
## 21224  4   52  5.149154e+00
## 21225  4   53  4.991697e+00
## 21226  4   54  4.841277e+00
## 21227  4   55  4.730360e+00
## 21228  4   56  4.640012e+00
## 21229  4   57  4.554078e+00
## 21230  4   58  4.476450e+00
## 21231  4   59  4.421101e+00
## 21232  4   60  4.360751e+00
## 21233  4   61  4.304274e+00
## 21234  4   62  4.264456e+00
## 21235  4   63  4.212772e+00
## 21236  4   64  4.176118e+00
## 21237  4   65  4.130792e+00
## 21238  4   66  4.093869e+00
## 21239  4   67  4.055958e+00
## 21240  4   68  4.025369e+00
## 21241  4   69  3.979401e+00
## 21242  4   70  3.954305e+00
## 21243  4   71  3.905783e+00
## 21244  4   72  3.877336e+00
## 21245  4   73  5.929227e+00
## 21246  4   74  5.615423e+00
## 21247  4   75  5.366583e+00
## 21248  4   76  5.163771e+00
## 21249  4   77  4.983511e+00
## 21250  4   78  4.847060e+00
## 21251  4   79  4.733117e+00
## 21252  4   80  4.634050e+00
## 21253  4   81  4.557932e+00
## 21254  4   82  4.475698e+00
## 21255  4   83  4.411422e+00
## 21256  4   84  4.358707e+00
## 21257  4   85  4.306058e+00
## 21258  4   86  4.257781e+00
## 21259  4   87  4.201503e+00
## 21260  4   88  4.168547e+00
## 21261  4   89  4.133178e+00
## 21262  4   90  4.100689e+00
## 21263  4   91  4.060260e+00
## 21264  4   92  4.018652e+00
## 21265  4   93  3.981179e+00
## 21266  4   94  3.947256e+00
## 21267  4   95  3.914314e+00
## 21268  4   96  3.880582e+00
## 21269  4   97  5.930161e+00
## 21270  4   98  5.618958e+00
## 21271  4   99  5.365119e+00
## 21272  4  100  5.157902e+00
## 21273  4  101  4.988199e+00
## 21274  4  102  4.849304e+00
## 21275  4  103  4.727622e+00
## 21276  4  104  4.641566e+00
## 21277  4  105  4.550025e+00
## 21278  4  106  4.477119e+00
## 21279  4  107  4.421969e+00
## 21280  4  108  4.356365e+00
## 21281  4  109  4.306872e+00
## 21282  4  110  4.263979e+00
## 21283  4  111  4.214428e+00
## 21284  4  112  4.177776e+00
## 21285  4  113  4.134832e+00
## 21286  4  114  4.098398e+00
## 21287  4  115  4.060692e+00
## 21288  4  116  4.016276e+00
## 21289  4  117  3.982365e+00
## 21290  4  118  3.943758e+00
## 21291  4  119  3.916862e+00
## 21292  4  120  3.880214e+00
## 21293  4  121  5.925996e+00
## 21294  4  122  5.615327e+00
## 21295  4  123  5.354607e+00
## 21296  4  124  5.152612e+00
## 21297  4  125  4.981833e+00
## 21298  4  126  4.844349e+00
## 21299  4  127  4.735737e+00
## 21300  4  128  4.642741e+00
## 21301  4  129  4.553321e+00
## 21302  4  130  4.485698e+00
## 21303  4  131  4.415772e+00
## 21304  4  132  4.355196e+00
## 21305  4  133  4.305117e+00
## 21306  4  134  4.262992e+00
## 21307  4  135  4.209724e+00
## 21308  4  136  4.172906e+00
## 21309  4  137  4.128102e+00
## 21310  4  138  4.085967e+00
## 21311  4  139  4.058769e+00
## 21312  4  140  4.018483e+00
## 21313  4  141  3.990909e+00
## 21314  4  142  3.947297e+00
## 21315  4  143  3.922560e+00
## 21316  4  144  3.878727e+00
## 21317  4  145  5.943774e+00
## 21318  4  146  5.621261e+00
## 21319  4  147  5.358088e+00
## 21320  4  148  5.146428e+00
## 21321  4  149  4.987236e+00
## 21322  4  150  4.849868e+00
## 21323  4  151  4.740010e+00
## 21324  4  152  4.636010e+00
## 21325  4  153  4.565600e+00
## 21326  4  154  4.482019e+00
## 21327  4  155  4.412158e+00
## 21328  4  156  4.353613e+00
## 21329  4  157  4.311449e+00
## 21330  4  158  4.258355e+00
## 21331  4  159  4.205767e+00
## 21332  4  160  4.167587e+00
## 21333  4  161  4.123518e+00
## 21334  4  162  4.086099e+00
## 21335  4  163  4.056553e+00
## 21336  4  164  4.014413e+00
## 21337  4  165  3.995780e+00
## 21338  4  166  3.950601e+00
## 21339  4  167  3.913644e+00
## 21340  4  168  3.882133e+00
## 21341  4  169  5.930620e+00
## 21342  4  170  5.617066e+00
## 21343  4  171  5.357540e+00
## 21344  4  172  5.145850e+00
## 21345  4  173  4.992418e+00
## 21346  4  174  4.852391e+00
## 21347  4  175  4.731805e+00
## 21348  4  176  4.642509e+00
## 21349  4  177  4.554140e+00
## 21350  4  178  4.479112e+00
## 21351  4  179  4.421758e+00
## 21352  4  180  4.356195e+00
## 21353  4  181  4.306444e+00
## 21354  4  182  4.262436e+00
## 21355  4  183  4.214008e+00
## 21356  4  184  4.172728e+00
## 21357  4  185  4.124038e+00
## 21358  4  186  4.089621e+00
## 21359  4  187  4.061674e+00
## 21360  4  188  4.030437e+00
## 21361  4  189  3.977197e+00
## 21362  4  190  3.950195e+00
## 21363  4  191  3.917603e+00
## 21364  4  192  3.880070e+00
## 21365  4  193  5.930768e+00
## 21366  4  194  5.619729e+00
## 21367  4  195  5.358600e+00
## 21368  4  196  5.149057e+00
## 21369  4  197  4.983959e+00
## 21370  4  198  4.843337e+00
## 21371  4  199  4.730401e+00
## 21372  4  200  4.643637e+00
## 21373  4  201  4.556722e+00
## 21374  4  202  4.479108e+00
## 21375  4  203  4.418545e+00
## 21376  4  204  4.355671e+00
## 21377  4  205  4.306621e+00
## 21378  4  206  4.254513e+00
## 21379  4  207  4.214883e+00
## 21380  4  208  4.170374e+00
## 21381  4  209  4.134577e+00
## 21382  4  210  4.090213e+00
## 21383  4  211  4.055537e+00
## 21384  4  212  4.013455e+00
## 21385  4  213  3.984262e+00
## 21386  4  214  3.950048e+00
## 21387  4  215  3.910718e+00
## 21388  4  216  3.878873e+00
## 21389  4  217  5.927991e+00
## 21390  4  218  5.622830e+00
## 21391  4  219  5.359739e+00
## 21392  4  220  5.154705e+00
## 21393  4  221  4.985711e+00
## 21394  4  222  4.843305e+00
## 21395  4  223  4.731577e+00
## 21396  4  224  4.632894e+00
## 21397  4  225  4.546822e+00
## 21398  4  226  4.486362e+00
## 21399  4  227  4.413376e+00
## 21400  4  228  4.354072e+00
## 21401  4  229  4.299921e+00
## 21402  4  230  4.260391e+00
## 21403  4  231  4.210507e+00
## 21404  4  232  4.171890e+00
## 21405  4  233  4.131915e+00
## 21406  4  234  4.091417e+00
## 21407  4  235  4.058799e+00
## 21408  4  236  4.013956e+00
## 21409  4  237  3.979579e+00
## 21410  4  238  3.951338e+00
## 21411  4  239  3.914509e+00
## 21412  4  240  3.878462e+00
## 21413  4  241  5.945422e+00
## 21414  4  242  5.623707e+00
## 21415  4  243  5.360499e+00
## 21416  4  244  5.168543e+00
## 21417  4  245  4.991394e+00
## 21418  4  246  4.853874e+00
## 21419  4  247  4.732280e+00
## 21420  4  248  4.645729e+00
## 21421  4  249  4.547945e+00
## 21422  4  250  4.471753e+00
## 21423  4  251  4.413136e+00
## 21424  4  252  4.353667e+00
## 21425  4  253  4.307280e+00
## 21426  4  254  4.258200e+00
## 21427  4  255  4.213534e+00
## 21428  4  256  4.177770e+00
## 21429  4  257  4.134790e+00
## 21430  4  258  4.081863e+00
## 21431  4  259  4.053801e+00
## 21432  4  260  4.016956e+00
## 21433  4  261  3.987841e+00
## 21434  4  262  3.946605e+00
## 21435  4  263  3.918446e+00
## 21436  4  264  3.881642e+00
## 21437  4  265  5.931113e+00
## 21438  4  266  5.617313e+00
## 21439  4  267  5.360006e+00
## 21440  4  268  5.151929e+00
## 21441  4  269  4.988834e+00
## 21442  4  270  4.853864e+00
## 21443  4  271  4.731963e+00
## 21444  4  272  4.631828e+00
## 21445  4  273  4.540416e+00
## 21446  4  274  4.481496e+00
## 21447  4  275  4.422350e+00
## 21448  4  276  4.358490e+00
## 21449  4  277  4.305228e+00
## 21450  4  278  4.253840e+00
## 21451  4  279  4.214735e+00
## 21452  4  280  4.169780e+00
## 21453  4  281  4.134172e+00
## 21454  4  282  4.093591e+00
## 21455  4  283  4.052025e+00
## 21456  4  284  4.021714e+00
## 21457  4  285  3.981327e+00
## 21458  4  286  3.942151e+00
## 21459  4  287  3.916301e+00
## 21460  4  288  3.879295e+00
## 21461  4  289  5.938320e+00
## 21462  4  290  5.612187e+00
## 21463  4  291  5.361690e+00
## 21464  4  292  5.149376e+00
## 21465  4  293  4.989008e+00
## 21466  4  294  4.850904e+00
## 21467  4  295  4.740472e+00
## 21468  4  296  4.637171e+00
## 21469  4  297  4.564303e+00
## 21470  4  298  4.479004e+00
## 21471  4  299  4.413860e+00
## 21472  4  300  4.356669e+00
## 21473  4  301  4.312141e+00
## 21474  4  302  4.249958e+00
## 21475  4  303  4.214851e+00
## 21476  4  304  4.175467e+00
## 21477  4  305  4.133962e+00
## 21478  4  306  4.093481e+00
## 21479  4  307  4.063225e+00
## 21480  4  308  4.016944e+00
## 21481  4  309  3.976080e+00
## 21482  4  310  3.948638e+00
## 21483  4  311  3.914404e+00
## 21484  4  312  3.879539e+00
## 21485  4  312  3.882087e+00
## 21486  4  313  5.945183e+00
## 21487  4  314  5.626279e+00
## 21488  4  315  5.355682e+00
## 21489  4  316  5.154263e+00
## 21490  4  317  4.992577e+00
## 21491  4  318  4.849424e+00
## 21492  4  319  4.731937e+00
## 21493  4  320  4.634754e+00
## 21494  4  321  4.551000e+00
## 21495  4  322  4.475411e+00
## 21496  4  323  4.411556e+00
## 21497  4  324  4.365606e+00
## 21498  4  325  4.311446e+00
## 21499  4  326  4.254589e+00
## 21500  4  327  4.211745e+00
## 21501  4  328  4.174951e+00
## 21502  4  329  4.126485e+00
## 21503  4  330  4.091509e+00
## 21504  4  331  4.052032e+00
## 21505  4  332  4.025245e+00
## 21506  4  333  3.987621e+00
## 21507  4  334  3.953870e+00
## 21508  4  335  3.924646e+00
## 21509  4  336  3.878506e+00
## 21510  4  336  3.882065e+00
## 21511  4  337  3.857552e+00
## 21512  4  338  3.815033e+00
## 21513  4  339  3.783330e+00
## 21514  4  339  3.781586e+00
## 21515  4  340  5.841273e+00
## 21516  4  341  5.521087e+00
## 21517  4  342  5.268107e+00
## 21518  4  343  5.059327e+00
## 21519  4  344  4.899111e+00
## 21520  4  345  4.752572e+00
## 21521  4  346  4.638398e+00
## 21522  4  347  4.550301e+00
## 21523  4  348  4.466705e+00
## 21524  4  349  4.398657e+00
## 21525  4  350  4.325126e+00
## 21526  4  351  4.271907e+00
## 21527  4  352  4.219610e+00
## 21528  4  353  4.165549e+00
## 21529  4  354  4.123686e+00
## 21530  4  355  4.087653e+00
## 21531  4  356  4.049113e+00
## 21532  4  357  4.012387e+00
## 21533  4  358  3.972110e+00
## 21534  4  359  3.938630e+00
## 21535  4  360  3.900166e+00
## 21536  4  360  3.897839e+00
## 21537  4  361  5.951337e+00
## 21538  4  362  5.641874e+00
## 21539  4  363  5.381243e+00
## 21540  4  364  5.169566e+00
## 21541  4  365  5.008551e+00
## 21542  4  366  4.870551e+00
## 21543  4  367  4.755708e+00
## 21544  4  368  4.648541e+00
## 21545  4  369  4.570517e+00
## 21546  4  370  4.498725e+00
## 21547  4  371  4.432675e+00
## 21548  4  372  4.370511e+00
## 21549  4  373  4.326840e+00
## 21550  4  374  4.274737e+00
## 21551  4  375  4.223386e+00
## 21552  4  376  4.193029e+00
## 21553  4  377  4.148617e+00
## 21554  4  378  4.106978e+00
## 21555  4  379  4.071435e+00
## 21556  4  380  4.032889e+00
## 21557  4  381  4.001496e+00
## 21558  4  382  3.971217e+00
## 21559  4  383  3.928170e+00
## 21560  4  384  3.889044e+00
## 21561  4  385  5.954787e+00
## 21562  4  386  5.633007e+00
## 21563  4  387  5.384315e+00
## 21564  4  388  5.172569e+00
## 21565  4  389  4.995939e+00
## 21566  4  390  4.858139e+00
## 21567  4  391  4.742572e+00
## 21568  4  392  4.649902e+00
## 21569  4  393  4.563912e+00
## 21570  4  394  4.495359e+00
## 21571  4  395  4.435400e+00
## 21572  4  396  4.386840e+00
## 21573  4  397  4.329522e+00
## 21574  4  398  4.273428e+00
## 21575  4  399  4.232376e+00
## 21576  4  400  4.184950e+00
## 21577  4  401  4.135656e+00
## 21578  4  402  4.108900e+00
## 21579  4  403  4.060921e+00
## 21580  4  404  4.029812e+00
## 21581  4  405  3.998668e+00
## 21582  4  406  3.962905e+00
## 21583  4  407  3.927311e+00
## 21584  4  408  3.895759e+00
## 21585  4  409  5.952140e+00
## 21586  4  410  5.633320e+00
## 21587  4  411  5.365143e+00
## 21588  4  412  5.168720e+00
## 21589  4  413  4.998597e+00
## 21590  4  414  4.861149e+00
## 21591  4  415  4.747269e+00
## 21592  4  416  4.635950e+00
## 21593  4  417  4.562534e+00
## 21594  4  418  4.489563e+00
## 21595  4  419  4.430122e+00
## 21596  4  420  4.368666e+00
## 21597  4  421  4.317393e+00
## 21598  4  422  4.272706e+00
## 21599  4  423  4.224778e+00
## 21600  4  424  4.180076e+00
## 21601  4  425  4.138614e+00
## 21602  4  426  4.097190e+00
## 21603  4  427  4.060021e+00
## 21604  4  428  4.033516e+00
## 21605  4  429  3.991058e+00
## 21606  4  430  3.953601e+00
## 21607  4  431  3.923442e+00
## 21608  4  432  3.887154e+00
## 21609  4  433  5.931845e+00
## 21610  4  434  5.627149e+00
## 21611  4  435  5.371070e+00
## 21612  4  436  5.164067e+00
## 21613  4  437  5.001273e+00
## 21614  4  438  4.860458e+00
## 21615  4  439  4.748906e+00
## 21616  4  440  4.652170e+00
## 21617  4  441  4.568238e+00
## 21618  4  442  4.490603e+00
## 21619  4  443  4.433860e+00
## 21620  4  444  4.363581e+00
## 21621  4  445  4.318633e+00
## 21622  4  446  4.264428e+00
## 21623  4  447  4.221350e+00
## 21624  4  448  4.180468e+00
## 21625  4  449  4.141866e+00
## 21626  4  450  4.097742e+00
## 21627  4  451  4.061595e+00
## 21628  4  452  4.029690e+00
## 21629  4  453  3.990749e+00
## 21630  4  454  3.954895e+00
## 21631  4  455  3.925979e+00
## 21632  4  456  3.886684e+00
## 21633  4  457  5.943045e+00
## 21634  4  458  5.624929e+00
## 21635  4  459  5.375180e+00
```

```
## 21636 4  460 5.166754e+00
## 21637 4  461 4.992021e+00
## 21638 4  462 4.854761e+00
## 21639 4  463 4.741253e+00
## 21640 4  464 4.646951e+00
## 21641 4  465 4.552017e+00
## 21642 4  466 4.490637e+00
## 21643 4  467 4.423139e+00
## 21644 4  468 4.360951e+00
## 21645 4  469 4.318462e+00
## 21646 4  470 4.270425e+00
## 21647 4  471 4.218600e+00
## 21648 4  472 4.179993e+00
## 21649 4  473 4.143635e+00
## 21650 4  474 4.098341e+00
## 21651 4  475 4.063184e+00
## 21652 4  476 4.027675e+00
## 21653 4  477 3.991305e+00
## 21654 4  478 3.953486e+00
## 21655 4  479 3.917341e+00
## 21656 4  480 3.889801e+00
## 21657 4  481 5.944367e+00
## 21658 4  482 5.628323e+00
## 21659 4  483 5.369563e+00
## 21660 4  484 5.163277e+00
## 21661 4  485 4.996046e+00
## 21662 4  486 4.857137e+00
## 21663 4  487 4.735170e+00
## 21664 4  488 4.642861e+00
## 21665 4  489 4.559178e+00
## 21666 4  490 4.478788e+00
## 21667 4  491 4.423897e+00
## 21668 4  492 4.362890e+00
## 21669 4  493 4.307955e+00
## 21670 4  494 4.263939e+00
## 21671 4  495 4.214948e+00
## 21672 4  496 4.174730e+00
## 21673 4  497 4.140350e+00
## 21674 4  498 4.094030e+00
## 21675 4  499 4.062722e+00
## 21676 4  500 4.024559e+00
## 21677 4  501 3.985142e+00
## 21678 4  502 3.955201e+00
## 21679 4  503 3.918513e+00
## 21680 4  504 3.885854e+00
## 21681 4  505 5.934042e+00
## 21682 4  506 5.627084e+00
## 21683 4  507 5.372673e+00
## 21684 4  508 5.161144e+00
## 21685 4  509 5.001103e+00
## 21686 4  510 4.858414e+00
## 21687 4  511 4.730433e+00
## 21688 4  512 4.639253e+00
## 21689 4  513 4.552269e+00
## 21690 4  514 4.485607e+00
## 21691 4  515 4.415794e+00
## 21692 4  516 4.355140e+00
## 21693 4  517 4.308826e+00
## 21694 4  518 4.254377e+00
## 21695 4  519 4.222965e+00
## 21696 4  520 4.180195e+00
## 21697 4  521 4.136603e+00
## 21698 4  522 4.099045e+00
## 21699 4  523 4.054822e+00
## 21700 4  524 4.017196e+00
## 21701 4  525 3.989551e+00
## 21702 4  526 3.957238e+00
## 21703 4  527 3.917531e+00
## 21704 4  528 3.888795e+00
## 21705 4  529 5.945423e+00
## 21706 4  530 5.611228e+00
## 21707 4  531 5.369935e+00
## 21708 4  532 5.172022e+00
## 21709 4  533 4.998616e+00
## 21710 4  534 4.844250e+00
## 21711 4  535 4.734258e+00
## 21712 4  536 4.637493e+00
## 21713 4  537 4.562610e+00
## 21714 4  538 4.481290e+00
## 21715 4  539 4.424838e+00
## 21716 4  540 4.362598e+00
## 21717 4  541 4.313076e+00
## 21718 4  542 4.265788e+00
## 21719 4  543 4.212812e+00
## 21720 4  544 4.180620e+00
## 21721 4  545 4.131674e+00
## 21722 4  546 4.097022e+00
## 21723 4  547 4.059730e+00
## 21724 4  548 4.024347e+00
## 21725 4  549 3.982241e+00
## 21726 4  550 3.952255e+00
## 21727 4  551 3.912110e+00
## 21728 4  552 3.887780e+00
## 21729 4  553 5.935110e+00
## 21730 4  554 5.618473e+00
## 21731 4  555 5.371819e+00
## 21732 4  556 5.163139e+00
## 21733 4  557 4.988227e+00
## 21734 4  558 4.851737e+00
## 21735 4  559 4.736488e+00
## 21736 4  560 4.643617e+00
## 21737 4  561 4.560686e+00
## 21738 4  562 4.479480e+00
## 21739 4  563 4.414219e+00
## 21740 4  564 4.366068e+00
## 21741 4  565 4.314512e+00
## 21742 4  566 4.259259e+00
## 21743 4  567 4.218780e+00
## 21744 4  568 4.178299e+00
## 21745 4  569 4.131874e+00
## 21746 4  570 4.099066e+00
## 21747 4  571 4.051748e+00
## 21748 4  572 4.032043e+00
## 21749 4  573 3.980542e+00
## 21750 4  574 3.946065e+00
## 21751 4  575 3.913534e+00
## 21752 4  576 3.887646e+00
## 21753 4  577 5.935941e+00
## 21754 4  578 5.626733e+00
## 21755 4  579 5.364680e+00
## 21756 4  580 5.163466e+00
## 21757 4  581 4.988769e+00
## 21758 4  582 4.854137e+00
## 21759 4  583 4.737288e+00
## 21760 4  584 4.630044e+00
## 21761 4  585 4.555545e+00
## 21762 4  586 4.480330e+00
## 21763 4  587 4.412717e+00
## 21764 4  588 4.361771e+00
## 21765 4  589 4.312414e+00
## 21766 4  590 4.262708e+00
## 21767 4  591 4.222973e+00
## 21768 4  592 4.169673e+00
## 21769 4  593 4.135662e+00
## 21770 4  594 4.103432e+00
## 21771 4  595 4.062950e+00
## 21772 4  596 4.022015e+00
## 21773 4  597 3.984730e+00
## 21774 4  598 3.956493e+00
## 21775 4  599 3.914148e+00
## 21776 4  600 3.889852e+00
## 21777 4  601 5.943826e+00
## 21778 4  602 5.634625e+00
## 21779 4  603 5.357696e+00
## 21780 4  604 5.162005e+00
## 21781 4  605 4.987373e+00
## 21782 4  606 4.849402e+00
## 21783 4  607 4.729889e+00
## 21784 4  608 4.631336e+00
## 21785 4  609 4.561575e+00
## 21786 4  610 4.475479e+00
## 21787 4  611 4.426619e+00
## 21788 4  612 4.357711e+00
## 21789 4  613 4.312417e+00
## 21790 4  614 4.263634e+00
## 21791 4  615 4.215480e+00
## 21792 4  616 4.167100e+00
## 21793 4  617 4.144709e+00
## 21794 4  618 4.093636e+00
## 21795 4  619 4.055561e+00
## 21796 4  620 4.013888e+00
## 21797 4  621 3.991543e+00
## 21798 4  622 3.950451e+00
## 21799 4  623 3.917862e+00
## 21800 4  624 3.889773e+00
## 21801 4  624 3.882762e+00
## 21802 4  625 5.919505e+00
## 21803 4  626 5.609843e+00
## 21804 4  627 5.370354e+00
## 21805 4  628 5.147328e+00
## 21806 4  629 4.994370e+00
## 21807 4  630 4.847740e+00
## 21808 4  631 4.735511e+00
## 21809 4  632 4.645992e+00
## 21810 4  633 4.555813e+00
## 21811 4  634 4.474804e+00
## 21812 4  635 4.421400e+00
## 21813 4  636 4.359933e+00
## 21814 4  637 4.296072e+00
## 21815 4  638 4.259729e+00
## 21816 4  639 4.213045e+00
## 21817 4  640 4.171146e+00
## 21818 4  641 4.137866e+00
## 21819 4  642 4.095250e+00
## 21820 4  643 4.053732e+00
## 21821 4  644 4.018673e+00
## 21822 4  645 3.981632e+00
## 21823 4  646 3.947828e+00
## 21824 4  647 3.918841e+00
## 21825 4  648 3.880260e+00
## 21826 4  648 3.880882e+00
## 21827 4  649 3.846479e+00
## 21828 4  650 3.821819e+00
## 21829 4  651 3.781362e+00
## 21830 4  652 3.753431e+00
## 21831 4  653 3.723337e+00
## 21832 4  654 3.682576e+00
## 21833 4  654 3.692090e+00
## 21834 4  655 5.748562e+00
## 21835 4  656 5.433399e+00
## 21836 4  657 5.174143e+00
## 21837 4  658 4.968363e+00
## 21838 4  659 4.803597e+00
## 21839 4  660 4.667253e+00
## 21840 4  661 4.556085e+00
## 21841 4  662 4.460816e+00
## 21842 4  663 4.375376e+00
## 21843 4  664 4.312335e+00
## 21844 4  665 4.246512e+00
## 21845 4  666 4.185932e+00
## 21846 4  667 4.141956e+00
## 21847 4  668 4.083522e+00
## 21848 4  669 4.041687e+00
## 21849 4  670 4.012464e+00
## 21850 4  671 3.963011e+00
## 21851 4  672 3.927200e+00
## 21852 4  672 3.932335e+00
## 21853 4  673 5.975918e+00
## 21854 4  674 5.665521e+00
## 21855 4  675 5.401097e+00
## 21856 4  676 5.208655e+00
## 21857 4  677 5.025683e+00
## 21858 4  678 4.881235e+00
## 21859 4  679 4.771144e+00
## 21860 4  680 4.676475e+00
## 21861 4  681 4.577262e+00
## 21862 4  682 4.508659e+00
## 21863 4  683 4.449000e+00
## 21864 4  684 4.395165e+00
## 21865 4  685 4.337541e+00
## 21866 4  686 4.288723e+00
## 21867 4  687 4.243266e+00
## 21868 4  688 4.209893e+00
## 21869 4  689 4.153973e+00
## 21870 4  690 4.127475e+00
## 21871 4  691 4.080190e+00
## 21872 4  692 4.045289e+00
## 21873 4  693 4.015293e+00
## 21874 4  694 3.982900e+00
## 21875 4  695 3.943006e+00
## 21876 4  696 3.915182e+00
## 21877 4  697 5.962077e+00
## 21878 4  698 5.650454e+00
## 21879 4  699 5.397346e+00
## 21880 4  700 5.186318e+00
## 21881 4  701 5.023929e+00
## 21882 4  702 4.875813e+00
## 21883 4  703 4.754702e+00
## 21884 4  704 4.662243e+00
## 21885 4  705 4.581298e+00
## 21886 4  706 4.505849e+00
## 21887 4  707 4.434724e+00
## 21888 4  708 4.388480e+00
## 21889 4  709 4.334864e+00
## 21890 4  710 4.289525e+00
## 21891 4  711 4.245416e+00
## 21892 4  712 4.201667e+00
## 21893 4  713 4.155609e+00
## 21894 4  714 4.111685e+00
## 21895 4  715 4.074182e+00
## 21896 4  716 4.042349e+00
## 21897 4  717 4.007377e+00
## 21898 4  718 3.970958e+00
## 21899 4  719 3.940364e+00
## 21900 4  720 3.910959e+00
## 21901 4  721 5.963672e+00
## 21902 4  722 5.645386e+00
## 21903 4  723 5.382325e+00
## 21904 4  724 5.181205e+00
## 21905 4  725 5.004298e+00
## 21906 4  726 4.877652e+00
## 21907 4  727 4.756557e+00
## 21908 4  728 4.661381e+00
## 21909 4  729 4.576473e+00
## 21910 4  730 4.502816e+00
## 21911 4  731 4.433600e+00
## 21912 4  732 4.385536e+00
## 21913 4  733 4.327185e+00
## 21914 4  734 4.287866e+00
## 21915 4  735 4.238088e+00
## 21916 4  736 4.190148e+00
## 21917 4  737 4.148150e+00
## 21918 4  738 4.116808e+00
## 21919 4  739 4.067618e+00
## 21920 4  740 4.030959e+00
## 21921 4  741 3.993993e+00
## 21922 4  742 3.966317e+00
## 21923 4  743 3.932849e+00
## 21924 4  744 3.893891e+00
## 21925 4  745 5.954920e+00
## 21926 4  746 5.639467e+00
## 21927 4  747 5.388614e+00
## 21928 4  748 5.174084e+00
## 21929 4  749 5.011620e+00
## 21930 4  750 4.862766e+00
## 21931 4  751 4.758596e+00
## 21932 4  752 4.656479e+00
## 21933 4  753 4.566560e+00
## 21934 4  754 4.503218e+00
## 21935 4  755 4.439204e+00
## 21936 4  756 4.373810e+00
## 21937 4  757 4.326051e+00
## 21938 4  758 4.270736e+00
## 21939 4  759 4.223782e+00
## 21940 4  760 4.189907e+00
## 21941 4  761 4.146404e+00
## 21942 4  762 4.112523e+00
## 21943 4  763 4.073926e+00
## 21944 4  764 4.037880e+00
## 21945 4  765 4.002366e+00
## 21946 4  766 3.969560e+00
## 21947 4  767 3.930274e+00
## 21948 4  768 3.903379e+00
## 21949 4  769 5.954761e+00
## 21950 4  770 5.631907e+00
## 21951 4  771 5.375361e+00
## 21952 4  772 5.172248e+00
## 21953 4  773 5.007269e+00
## 21954 4  774 4.866661e+00
## 21955 4  775 4.743022e+00
## 21956 4  776 4.659009e+00
## 21957 4  777 4.568654e+00
## 21958 4  778 4.494079e+00
## 21959 4  779 4.438150e+00
## 21960 4  780 4.371423e+00
## 21961 4  781 4.318626e+00
## 21962 4  782 4.270354e+00
## 21963 4  783 4.232062e+00
## 21964 4  784 4.184583e+00
## 21965 4  785 4.147198e+00
## 21966 4  786 4.106940e+00
## 21967 4  787 4.074935e+00
## 21968 4  788 4.034225e+00
## 21969 4  789 3.992247e+00
## 21970 4  790 3.962625e+00
## 21971 4  791 3.928998e+00
## 21972 4  792 3.900140e+00
## 21973 4  793 5.940517e+00
## 21974 4  794 5.632460e+00
## 21975 4  795 5.378463e+00
## 21976 4  796 5.178336e+00
## 21977 4  797 5.009496e+00
## 21978 4  798 4.859469e+00
## 21979 4  799 4.745539e+00
## 21980 4  800 4.646032e+00
## 21981 4  801 4.564363e+00
## 21982 4  802 4.496462e+00
## 21983 4  803 4.427220e+00
## 21984 4  804 4.373050e+00
## 21985 4  805 4.310033e+00
## 21986 4  806 4.279562e+00
## 21987 4  807 4.218440e+00
## 21988 4  808 4.187126e+00
## 21989 4  809 4.144926e+00
## 21990 4  810 4.096262e+00
## 21991 4  811 4.061877e+00
## 21992 4  812 4.031617e+00
## 21993 4  813 3.992494e+00
## 21994 4  814 3.962954e+00
## 21995 4  815 3.925607e+00
## 21996 4  816 3.889420e+00
## 21997 4  817 5.947484e+00
## 21998 4  818 5.627298e+00
## 21999 4  819 5.375884e+00
## 22000 4  820 5.159946e+00
## 22001 4  821 4.997631e+00
## 22002 4  822 4.868998e+00
## 22003 4  823 4.741202e+00
## 22004 4  824 4.646937e+00
## 22005 4  825 4.558389e+00
## 22006 4  826 4.484535e+00
## 22007 4  827 4.427095e+00
## 22008 4  828 4.367061e+00
## 22009 4  829 4.314849e+00
## 22010 4  830 4.262609e+00
## 22011 4  831 4.226688e+00
## 22012 4  832 4.178114e+00
## 22013 4  833 4.142480e+00
## 22014 4  834 4.105271e+00
## 22015 4  835 4.060795e+00
## 22016 4  836 4.028330e+00
## 22017 4  837 3.989745e+00
## 22018 4  838 3.951896e+00
## 22019 4  839 3.930003e+00
## 22020 4  840 3.896491e+00
## 22021 4  841 5.948488e+00
## 22022 4  842 5.626489e+00
## 22023 4  843 5.368450e+00
## 22024 4  844 5.166053e+00
## 22025 4  845 4.996645e+00
## 22026 4  846 4.856579e+00
## 22027 4  847 4.732396e+00
## 22028 4  848 4.650375e+00
## 22029 4  849 4.566897e+00
## 22030 4  850 4.488540e+00
## 22031 4  851 4.426089e+00
## 22032 4  852 4.370309e+00
## 22033 4  853 4.315584e+00
## 22034 4  854 4.274861e+00
## 22035 4  855 4.217830e+00
## 22036 4  856 4.171352e+00
## 22037 4  857 4.133066e+00
## 22038 4  858 4.099606e+00
## 22039 4  859 4.060405e+00
## 22040 4  860 4.033073e+00
## 22041 4  861 3.989990e+00
## 22042 4  862 3.955409e+00
## 22043 4  863 3.919727e+00
## 22044 4  864 3.890819e+00
## 22045 4  865 5.946662e+00
## 22046 4  866 5.626705e+00
## 22047 4  867 5.363307e+00
## 22048 4  868 5.161334e+00
## 22049 4  869 4.996055e+00
## 22050 4  870 4.854889e+00
## 22051 4  871 4.735666e+00
## 22052 4  872 4.646924e+00
## 22053 4  873 4.557608e+00
## 22054 4  874 4.487022e+00
## 22055 4  875 4.430849e+00
## 22056 4  876 4.372074e+00
## 22057 4  877 4.315856e+00
## 22058 4  878 4.264134e+00
## 22059 4  879 4.223148e+00
## 22060 4  880 4.173813e+00
## 22061 4  881 4.130516e+00
## 22062 4  882 4.096114e+00
## 22063 4  883 4.059995e+00
## 22064 4  884 4.026121e+00
## 22065 4  885 3.997032e+00
## 22066 4  886 3.952944e+00
## 22067 4  887 3.923177e+00
## 22068 4  888 3.888056e+00
## 22069 4  889 5.929113e+00
## 22070 4  890 5.634658e+00
## 22071 4  891 5.373539e+00
## 22072 4  892 5.164953e+00
## 22073 4  893 4.991981e+00
## 22074 4  894 4.853337e+00
## 22075 4  895 4.735823e+00
## 22076 4  896 4.644917e+00
## 22077 4  897 4.555744e+00
## 22078 4  898 4.482320e+00
## 22079 4  899 4.430107e+00
## 22080 4  900 4.361738e+00
## 22081 4  901 4.303999e+00
## 22082 4  902 4.259488e+00
## 22083 4  903 4.219351e+00
## 22084 4  904 4.177929e+00
## 22085 4  905 4.140816e+00
## 22086 4  906 4.098711e+00
## 22087 4  907 4.060870e+00
## 22088 4  908 4.020506e+00
## 22089 4  909 3.992204e+00
## 22090 4  910 3.956911e+00
## 22091 4  911 3.913433e+00
## 22092 4  912 3.883779e+00
## 22093 4  913 5.939195e+00
## 22094 4  914 5.620356e+00
## 22095 4  915 5.366888e+00
## 22096 4  916 5.158188e+00
## 22097 4  917 4.990974e+00
## 22098 4  918 4.858619e+00
## 22099 4  919 4.737266e+00
## 22100 4  920 4.637857e+00
## 22101 4  921 4.557348e+00
## 22102 4  922 4.487502e+00
## 22103 4  923 4.420316e+00
## 22104 4  924 4.355947e+00
## 22105 4  925 4.309536e+00
## 22106 4  926 4.264676e+00
## 22107 4  927 4.215906e+00
## 22108 4  928 4.178322e+00
## 22109 4  929 4.135284e+00
## 22110 4  930 4.099037e+00
## 22111 4  931 4.061190e+00
## 22112 4  932 4.024570e+00
## 22113 4  933 3.989310e+00
## 22114 4  934 3.959368e+00
## 22115 4  935 3.920500e+00
## 22116 4  936 3.886164e+00
## 22117 4  936 3.881059e+00
## 22118 4  937 5.942524e+00
## 22119 4  938 5.618241e+00
## 22120 4  939 5.369243e+00
## 22121 4  940 5.153955e+00
## 22122 4  941 4.979171e+00
## 22123 4  942 4.844834e+00
## 22124 4  943 4.740257e+00
## 22125 4  944 4.634291e+00
## 22126 4  945 4.558174e+00
## 22127 4  946 4.474230e+00
## 22128 4  947 4.412368e+00
## 22129 4  948 4.359585e+00
## 22130 4  949 4.304314e+00
## 22131 4  950 4.256725e+00
## 22132 4  951 4.212873e+00
## 22133 4  952 4.167500e+00
## 22134 4  953 4.134508e+00
## 22135 4  954 4.092290e+00
## 22136 4  955 4.047916e+00
## 22137 4  956 4.013154e+00
## 22138 4  957 3.983958e+00
## 22139 4  958 3.943281e+00
## 22140 4  959 3.919639e+00
## 22141 4  960 3.886207e+00
## 22142 4  960 3.881011e+00
## 22143 4  961 3.853135e+00
## 22144 4  962 3.812803e+00
## 22145 4  963 3.787131e+00
## 22146 4  964 3.749587e+00
## 22147 4  965 3.723282e+00
## 22148 4  966 3.689640e+00
## 22149 4  967 3.664969e+00
## 22150 4  968 3.627899e+00
## 22151 4  969 3.594123e+00
## 22152 4  969 3.605447e+00
## 22153 4  970 5.661561e+00
## 22154 4  971 5.348299e+00
## 22155 4  972 5.093547e+00
## 22156 4  973 4.882726e+00
## 22157 4  974 4.721905e+00
## 22158 4  975 4.579393e+00
## 22159 4  976 4.472788e+00
## 22160 4  977 4.370847e+00
## 22161 4  978 4.283050e+00
## 22162 4  979 4.229432e+00
## 22163 4  980 4.166351e+00
## 22164 4  981 4.104847e+00
## 22165 4  982 4.061534e+00
## 22166 4  983 4.008399e+00
## 22167 4  984 3.961040e+00
## 22168 4  984 3.963463e+00
## 22169 4  985 6.007760e+00
## 22170 4  986 5.693313e+00
## 22171 4  987 5.434885e+00
## 22172 4  988 5.220660e+00
## 22173 4  989 5.050246e+00
## 22174 4  990 4.908804e+00
## 22175 4  991 4.795367e+00
## 22176 4  992 4.684287e+00
## 22177 4  993 4.609518e+00
## 22178 4  994 4.531310e+00
## 22179 4  995 4.468016e+00
## 22180 4  996 4.409471e+00
## 22181 4  997 4.356515e+00
## 22182 4  998 4.301579e+00
## 22183 4  999 4.255910e+00
## 22184 4 1000 4.215559e+00
## 22185 4 1001 4.175757e+00
## 22186 4 1002 4.139662e+00
## 22187 4 1003 4.104574e+00
## 22188 4 1004 4.067116e+00
## 22189 4 1005 4.033381e+00
## 22190 4 1006 4.001910e+00
## 22191 4 1007 3.960053e+00
## 22192 4 1008 3.927629e+00
## 22193 4 1009 5.978905e+00
## 22194 4 1010 5.663503e+00
## 22195 4 1011 5.405445e+00
## 22196 4 1012 5.195027e+00
## 22197 4 1013 5.031560e+00
## 22198 4 1014 4.895754e+00
## 22199 4 1015 4.767804e+00
## 22200 4 1016 4.679720e+00
## 22201 4 1017 4.602811e+00
## 22202 4 1018 4.510501e+00
## 22203 4 1019 4.456745e+00
## 22204 4 1020 4.399738e+00
## 22205 4 1021 4.348431e+00
## 22206 4 1022 4.296475e+00
## 22207 4 1023 4.244474e+00
## 22208 4 1024 4.213194e+00
## 22209 4 1025 4.164745e+00
## 22210 4 1026 4.130900e+00
## 22211 4 1027 4.092506e+00
## 22212 4 1028 4.055627e+00
## 22213 4 1029 4.018091e+00
## 22214 4 1030 3.990570e+00
## 22215 4 1031 3.955964e+00
## 22216 4 1032 3.921359e+00
## 22217 4 1033 5.977795e+00
## 22218 4 1034 5.656325e+00
## 22219 4 1035 5.388330e+00
## 22220 4 1036 5.194201e+00
## 22221 4 1037 5.027675e+00
## 22222 4 1038 4.880871e+00
## 22223 4 1039 4.767059e+00
## 22224 4 1040 4.662329e+00
## 22225 4 1041 4.590770e+00
## 22226 4 1042 4.511484e+00
## 22227 4 1043 4.449970e+00
## 22228 4 1044 4.394553e+00
## 22229 4 1045 4.329346e+00
## 22230 4 1046 4.292784e+00
## 22231 4 1047 4.244784e+00
## 22232 4 1048 4.208879e+00
## 22233 4 1049 4.172234e+00
## 22234 4 1050 4.127719e+00
## 22235 4 1051 4.084608e+00
## 22236 4 1052 4.051336e+00
## 22237 4 1053 4.009614e+00
## 22238 4 1054 3.982134e+00
## 22239 4 1055 3.936329e+00
## 22240 4 1056 3.900587e+00
## 22241 4 1057 5.971974e+00
## 22242 4 1058 5.648322e+00
## 22243 4 1059 5.393944e+00
## 22244 4 1060 5.176335e+00
## 22245 4 1061 5.020333e+00
## 22246 4 1062 4.878149e+00
## 22247 4 1063 4.764561e+00
## 22248 4 1064 4.668173e+00
## 22249 4 1065 4.586332e+00
## 22250 4 1066 4.509984e+00
## 22251 4 1067 4.442846e+00
## 22252 4 1068 4.379165e+00
## 22253 4 1069 4.330520e+00
## 22254 4 1070 4.282772e+00
## 22255 4 1071 4.243419e+00
## 22256 4 1072 4.192755e+00
## 22257 4 1073 4.155597e+00
## 22258 4 1074 4.111150e+00
## 22259 4 1075 4.078480e+00
## 22260 4 1076 4.044863e+00
## 22261 4 1077 4.002666e+00
## 22262 4 1078 3.972380e+00
## 22263 4 1079 3.934097e+00
## 22264 4 1080 3.900879e+00
## 22265 4 1081 5.954591e+00
## 22266 4 1082 5.644053e+00
## 22267 4 1083 5.385001e+00
## 22268 4 1084 5.174620e+00
## 22269 4 1085 5.007734e+00
## 22270 4 1086 4.873348e+00
## 22271 4 1087 4.766780e+00
## 22272 4 1088 4.655413e+00
## 22273 4 1089 4.577798e+00
## 22274 4 1090 4.505709e+00
## 22275 4 1091 4.441854e+00
## 22276 4 1092 4.378183e+00
## 22277 4 1093 4.325672e+00
## 22278 4 1094 4.275604e+00
## 22279 4 1095 4.228839e+00
## 22280 4 1096 4.185177e+00
## 22281 4 1097 4.157913e+00
## 22282 4 1098 4.107404e+00
## 22283 4 1099 4.078412e+00
## 22284 4 1100 4.031035e+00
## 22285 4 1101 4.004571e+00
## 22286 4 1102 3.975759e+00
## 22287 4 1103 3.940055e+00
## 22288 4 1104 3.896872e+00
## 22289 4 1105 5.956702e+00
## 22290 4 1106 5.638700e+00
## 22291 4 1107 5.386418e+00
## 22292 4 1108 5.173675e+00
## 22293 4 1109 5.012831e+00
## 22294 4 1110 4.871127e+00
## 22295 4 1111 4.751105e+00
## 22296 4 1112 4.648290e+00
## 22297 4 1113 4.577804e+00
## 22298 4 1114 4.509640e+00
## 22299 4 1115 4.435104e+00
## 22300 4 1116 4.377608e+00
## 22301 4 1117 4.328281e+00
## 22302 4 1118 4.270760e+00
## 22303 4 1119 4.233874e+00
## 22304 4 1120 4.195904e+00
## 22305 4 1121 4.150444e+00
## 22306 4 1122 4.102022e+00
## 22307 4 1123 4.073016e+00
## 22308 4 1124 4.038146e+00
## 22309 4 1125 4.002407e+00
## 22310 4 1126 3.970886e+00
## 22311 4 1127 3.925295e+00
## 22312 4 1128 3.891587e+00
## 22313 4 1129 5.941083e+00
## 22314 4 1130 5.632766e+00
## 22315 4 1131 5.371453e+00
## 22316 4 1132 5.179307e+00
## 22317 4 1133 4.994271e+00
## 22318 4 1134 4.854311e+00
## 22319 4 1135 4.754437e+00
## 22320 4 1136 4.654657e+00
## 22321 4 1137 4.572201e+00
## 22322 4 1138 4.489404e+00
## 22323 4 1139 4.423152e+00
## 22324 4 1140 4.378488e+00
## 22325 4 1141 4.320787e+00
## 22326 4 1142 4.268568e+00
## 22327 4 1143 4.228911e+00
## 22328 4 1144 4.186149e+00
## 22329 4 1145 4.146875e+00
## 22330 4 1146 4.102078e+00
## 22331 4 1147 4.059409e+00
## 22332 4 1148 4.036829e+00
## 22333 4 1149 4.002215e+00
## 22334 4 1150 3.967797e+00
## 22335 4 1151 3.928223e+00
## 22336 4 1152 3.894299e+00
## 22337 4 1153 5.945655e+00
## 22338 4 1154 5.623534e+00
## 22339 4 1155 5.381849e+00
## 22340 4 1156 5.169569e+00
## 22341 4 1157 5.008800e+00
## 22342 4 1158 4.862909e+00
## 22343 4 1159 4.741842e+00
## 22344 4 1160 4.647612e+00
## 22345 4 1161 4.566024e+00
## 22346 4 1162 4.494983e+00
## 22347 4 1163 4.431989e+00
## 22348 4 1164 4.369595e+00
## 22349 4 1165 4.318763e+00
## 22350 4 1166 4.264188e+00
## 22351 4 1167 4.230103e+00
## 22352 4 1168 4.179826e+00
## 22353 4 1169 4.141213e+00
## 22354 4 1170 4.098532e+00
## 22355 4 1171 4.064933e+00
## 22356 4 1172 4.020951e+00
## 22357 4 1173 3.995465e+00
## 22358 4 1174 3.955912e+00
## 22359 4 1175 3.922650e+00
## 22360 4 1176 3.888703e+00
## 22361 4 1177 5.950964e+00
## 22362 4 1178 5.623041e+00
## 22363 4 1179 5.373556e+00
## 22364 4 1180 5.165919e+00
## 22365 4 1181 5.001579e+00
## 22366 4 1182 4.861496e+00
## 22367 4 1183 4.750342e+00
## 22368 4 1184 4.645779e+00
## 22369 4 1185 4.553544e+00
## 22370 4 1186 4.488243e+00
## 22371 4 1187 4.427398e+00
## 22372 4 1188 4.373048e+00
## 22373 4 1189 4.320817e+00
## 22374 4 1190 4.266986e+00
## 22375 4 1191 4.231549e+00
## 22376 4 1192 4.176776e+00
## 22377 4 1193 4.133589e+00
## 22378 4 1194 4.102416e+00
## 22379 4 1195 4.066352e+00
## 22380 4 1196 4.037502e+00
## 22381 4 1197 3.988800e+00
## 22382 4 1198 3.962199e+00
## 22383 4 1199 3.925019e+00
## 22384 4 1200 3.887682e+00
## 22385 4 1201 5.945090e+00
## 22386 4 1202 5.635526e+00
## 22387 4 1203 5.367423e+00
## 22388 4 1204 5.163955e+00
## 22389 4 1205 5.003243e+00
## 22390 4 1206 4.850582e+00
## 22391 4 1207 4.744334e+00
## 22392 4 1208 4.646705e+00
## 22393 4 1209 4.566826e+00
## 22394 4 1210 4.491982e+00
## 22395 4 1211 4.423627e+00
## 22396 4 1212 4.362567e+00
## 22397 4 1213 4.317828e+00
## 22398 4 1214 4.271103e+00
## 22399 4 1215 4.229255e+00
## 22400 4 1216 4.177298e+00
## 22401 4 1217 4.140809e+00
## 22402 4 1218 4.092732e+00
## 22403 4 1219 4.063363e+00
## 22404 4 1220 4.022578e+00
## 22405 4 1221 4.000948e+00
## 22406 4 1222 3.962486e+00
## 22407 4 1223 3.922557e+00
## 22408 4 1224 3.887931e+00
## 22409 4 1225 5.944180e+00
## 22410 4 1226 5.619092e+00
## 22411 4 1227 5.364281e+00
## 22412 4 1228 5.163214e+00
## 22413 4 1229 4.999111e+00
## 22414 4 1230 4.867083e+00
## 22415 4 1231 4.731830e+00
## 22416 4 1232 4.634168e+00
## 22417 4 1233 4.564166e+00
## 22418 4 1234 4.484136e+00
## 22419 4 1235 4.427582e+00
## 22420 4 1236 4.368929e+00
## 22421 4 1237 4.316642e+00
## 22422 4 1238 4.260031e+00
## 22423 4 1239 4.216602e+00
## 22424 4 1240 4.187110e+00
## 22425 4 1241 4.133085e+00
## 22426 4 1242 4.095844e+00
## 22427 4 1243 4.061928e+00
## 22428 4 1244 4.026757e+00
## 22429 4 1245 3.987855e+00
## 22430 4 1246 3.948436e+00
## 22431 4 1247 3.917852e+00
## 22432 4 1248 3.883512e+00
## 22433 4 1248 3.884006e+00
## 22434 4 1249 5.934129e+00
## 22435 4 1250 5.622146e+00
```

```
## 22436  4 1251  5.366559e+00
## 22437  4 1252  5.154231e+00
## 22438  4 1253  4.988607e+00
## 22439  4 1254  4.848202e+00
## 22440  4 1255  4.732817e+00
## 22441  4 1256  4.635368e+00
## 22442  4 1257  4.552127e+00
## 22443  4 1258  4.478529e+00
## 22444  4 1259  4.408142e+00
## 22445  4 1260  4.367051e+00
## 22446  4 1261  4.309973e+00
## 22447  4 1262  4.257111e+00
## 22448  4 1263  4.211710e+00
## 22449  4 1264  4.177719e+00
## 22450  4 1265  4.137052e+00
## 22451  4 1266  4.086184e+00
## 22452  4 1267  4.055255e+00
## 22453  4 1268  4.019711e+00
## 22454  4 1269  3.982607e+00
## 22455  4 1270  3.947751e+00
## 22456  4 1271  3.913488e+00
## 22457  4 1272  3.881970e+00
## 22458  4 1272  3.882014e+00
## 22459  4 1273  3.853432e+00
## 22460  4 1274  3.814749e+00
## 22461  4 1275  3.790834e+00
## 22462  4 1276  3.752161e+00
## 22463  4 1277  3.714545e+00
## 22464  4 1278  3.686170e+00
## 22465  4 1279  3.659927e+00
## 22466  4 1280  3.623540e+00
## 22467  4 1281  3.600765e+00
## 22468  4 1282  3.566300e+00
## 22469  4 1283  3.539134e+00
## 22470  4 1284  3.508811e+00
## 22471  4 1284  3.514274e+00
## 22472  4 1285  5.570023e+00
## 22473  4 1286  5.246494e+00
## 22474  4 1287  5.002189e+00
## 22475  4 1288  4.798774e+00
## 22476  4 1289  4.633238e+00
## 22477  4 1290  4.501244e+00
## 22478  4 1291  4.386394e+00
## 22479  4 1292  4.292365e+00
## 22480  4 1293  4.208390e+00
## 22481  4 1294  4.136518e+00
## 22482  4 1295  4.077217e+00
## 22483  4 1296  4.022144e+00
## 22484  4 1296  4.029731e+00
## 22485  4 1297  6.061668e+00
## 22486  4 1298  5.744914e+00
## 22487  4 1299  5.466233e+00
## 22488  4 1300  5.258596e+00
## 22489  4 1301  5.076855e+00
## 22490  4 1302  4.938489e+00
## 22491  4 1303  4.811082e+00
## 22492  4 1304  4.705145e+00
## 22493  4 1305  4.630792e+00
## 22494  4 1306  4.546411e+00
## 22495  4 1307  4.483220e+00
## 22496  4 1308  4.426126e+00
## 22497  4 1309  4.376815e+00
## 22498  4 1310  4.323660e+00
## 22499  4 1311  4.277868e+00
## 22500  4 1312  4.235605e+00
## 22501  4 1313  4.188779e+00
## 22502  4 1314  4.152902e+00
## 22503  4 1315  4.116236e+00
## 22504  4 1316  4.080564e+00
## 22505  4 1317  4.033643e+00
## 22506  4 1318  4.011786e+00
## 22507  4 1319  3.983039e+00
## 22508  4 1320  3.939256e+00
## 22509  4 1321  5.991856e+00
## 22510  4 1322  5.676579e+00
## 22511  4 1323  5.415726e+00
## 22512  4 1324  5.213255e+00
## 22513  4 1325  5.042084e+00
## 22514  4 1326  4.905156e+00
## 22515  4 1327  4.790355e+00
## 22516  4 1328  4.686287e+00
## 22517  4 1329  4.606143e+00
## 22518  4 1330  4.537920e+00
## 22519  4 1331  4.472526e+00
## 22520  4 1332  4.409928e+00
## 22521  4 1333  4.359356e+00
## 22522  4 1334  4.311955e+00
## 22523  4 1335  4.272299e+00
## 22524  4 1336  4.225079e+00
## 22525  4 1337  4.175207e+00
## 22526  4 1338  4.142217e+00
## 22527  4 1339  4.108126e+00
## 22528  4 1340  4.068131e+00
## 22529  4 1341  4.035270e+00
## 22530  4 1342  4.001769e+00
## 22531  4 1343  3.957962e+00
## 22532  4 1344  3.933046e+00
## 22533  4 1345  5.987178e+00
## 22534  4 1346  5.669502e+00
## 22535  4 1347  5.413946e+00
## 22536  4 1348  5.210364e+00
## 22537  4 1349  5.024976e+00
## 22538  4 1350  4.889757e+00
## 22539  4 1351  4.782403e+00
## 22540  4 1352  4.674514e+00
## 22541  4 1353  4.592132e+00
## 22542  4 1354  4.527405e+00
## 22543  4 1355  4.464269e+00
## 22544  4 1356  4.408553e+00
## 22545  4 1357  4.350608e+00
## 22546  4 1358  4.304816e+00
## 22547  4 1359  4.254614e+00
## 22548  4 1360  4.216301e+00
## 22549  4 1361  4.183135e+00
## 22550  4 1362  4.132292e+00
## 22551  4 1363  4.092437e+00
## 22552  4 1364  4.060754e+00
## 22553  4 1365  4.034663e+00
## 22554  4 1366  3.983411e+00
## 22555  4 1367  3.963830e+00
## 22556  4 1368  3.925693e+00
## 22557  4 1369  5.970123e+00
## 22558  4 1370  5.662140e+00
## 22559  4 1371  5.396325e+00
## 22560  4 1372  5.186682e+00
## 22561  4 1373  5.026586e+00
## 22562  4 1374  4.887656e+00
## 22563  4 1375  4.768448e+00
## 22564  4 1376  4.670176e+00
## 22565  4 1377  4.590848e+00
## 22566  4 1378  4.511336e+00
## 22567  4 1379  4.457666e+00
## 22568  4 1380  4.394690e+00
## 22569  4 1381  4.350511e+00
## 22570  4 1382  4.298316e+00
## 22571  4 1383  4.247727e+00
## 22572  4 1384  4.206674e+00
## 22573  4 1385  4.166247e+00
## 22574  4 1386  4.120569e+00
## 22575  4 1387  4.090108e+00
## 22576  4 1388  4.053540e+00
## 22577  4 1389  4.017634e+00
## 22578  4 1390  3.985932e+00
## 22579  4 1391  3.949608e+00
## 22580  4 1392  3.909075e+00
## 22581  4 1393  5.973870e+00
## 22582  4 1394  5.662482e+00
## 22583  4 1395  5.392252e+00
## 22584  4 1396  5.185365e+00
## 22585  4 1397  5.025356e+00
## 22586  4 1398  4.874315e+00
## 22587  4 1399  4.767129e+00
## 22588  4 1400  4.666735e+00
## 22589  4 1401  4.581612e+00
## 22590  4 1402  4.506376e+00
## 22591  4 1403  4.443605e+00
## 22592  4 1404  4.382089e+00
## 22593  4 1405  4.331467e+00
## 22594  4 1406  4.293707e+00
## 22595  4 1407  4.237084e+00
## 22596  4 1408  4.202417e+00
## 22597  4 1409  4.158002e+00
## 22598  4 1410  4.113765e+00
## 22599  4 1411  4.088156e+00
## 22600  4 1412  4.043675e+00
## 22601  4 1413  4.009164e+00
## 22602  4 1414  3.977180e+00
## 22603  4 1415  3.939790e+00
## 22604  4 1416  3.905228e+00
## 22605  4 1417  5.957844e+00
## 22606  4 1418  5.657655e+00
## 22607  4 1419  5.390710e+00
## 22608  4 1420  5.183671e+00
## 22609  4 1421  5.016699e+00
## 22610  4 1422  4.868491e+00
## 22611  4 1423  4.752583e+00
## 22612  4 1424  4.655340e+00
## 22613  4 1425  4.578899e+00
## 22614  4 1426  4.500044e+00
## 22615  4 1427  4.442164e+00
## 22616  4 1428  4.382385e+00
## 22617  4 1429  4.333739e+00
## 22618  4 1430  4.277159e+00
## 22619  4 1431  4.242832e+00
## 22620  4 1432  4.196363e+00
## 22621  4 1433  4.155439e+00
## 22622  4 1434  4.111295e+00
## 22623  4 1435  4.076829e+00
## 22624  4 1436  4.042624e+00
## 22625  4 1437  4.002797e+00
## 22626  4 1438  3.972227e+00
## 22627  4 1439  3.937723e+00
## 22628  4 1440  3.906941e+00
## 22629  4 1441  5.946096e+00
## 22630  4 1442  5.640181e+00
## 22631  4 1443  5.380344e+00
## 22632  4 1444  5.177363e+00
## 22633  4 1445  5.013000e+00
## 22634  4 1446  4.868520e+00
## 22635  4 1447  4.757869e+00
## 22636  4 1448  4.656108e+00
## 22637  4 1449  4.570571e+00
## 22638  4 1450  4.495662e+00
## 22639  4 1451  4.431447e+00
## 22640  4 1452  4.379189e+00
## 22641  4 1453  4.318948e+00
## 22642  4 1454  4.281274e+00
## 22643  4 1455  4.237203e+00
## 22644  4 1456  4.194908e+00
## 22645  4 1457  4.149166e+00
## 22646  4 1458  4.117346e+00
## 22647  4 1459  4.075132e+00
## 22648  4 1460  4.042839e+00
## 22649  4 1461  4.004822e+00
## 22650  4 1462  3.959675e+00
## 22651  4 1463  3.929426e+00
## 22652  4 1464  3.904194e+00
## 22653  4 1465  5.956655e+00
## 22654  4 1466  5.632247e+00
## 22655  4 1467  5.383518e+00
## 22656  4 1468  5.182280e+00
## 22657  4 1469  5.001663e+00
## 22658  4 1470  4.868425e+00
## 22659  4 1471  4.750169e+00
## 22660  4 1472  4.653338e+00
## 22661  4 1473  4.564371e+00
## 22662  4 1474  4.496443e+00
## 22663  4 1475  4.438433e+00
## 22664  4 1476  4.377325e+00
## 22665  4 1477  4.316130e+00
## 22666  4 1478  4.280602e+00
## 22667  4 1479  4.238700e+00
## 22668  4 1480  4.189648e+00
## 22669  4 1481  4.150328e+00
## 22670  4 1482  4.107552e+00
## 22671  4 1483  4.063328e+00
## 22672  4 1484  4.033933e+00
## 22673  4 1485  4.006259e+00
## 22674  4 1486  3.960563e+00
## 22675  4 1487  3.929019e+00
## 22676  4 1488  3.897788e+00
## 22677  4 1489  5.948426e+00
## 22678  4 1490  5.627928e+00
## 22679  4 1491  5.372914e+00
## 22680  4 1492  5.174179e+00
## 22681  4 1493  4.998251e+00
## 22682  4 1494  4.866030e+00
## 22683  4 1495  4.749238e+00
## 22684  4 1496  4.656813e+00
## 22685  4 1497  4.560483e+00
## 22686  4 1498  4.497479e+00
## 22687  4 1499  4.434727e+00
## 22688  4 1500  4.373185e+00
## 22689  4 1501  4.313839e+00
## 22690  4 1502  4.277836e+00
## 22691  4 1503  4.226050e+00
## 22692  4 1504  4.180680e+00
## 22693  4 1505  4.145322e+00
## 22694  4 1506  4.107829e+00
## 22695  4 1507  4.066224e+00
## 22696  4 1508  4.038088e+00
## 22697  4 1509  3.987072e+00
## 22698  4 1510  3.956640e+00
## 22699  4 1511  3.933174e+00
## 22700  4 1512  3.902749e+00
## 22701  4 1513  5.944909e+00
## 22702  4 1514  5.626707e+00
## 22703  4 1515  5.377909e+00
## 22704  4 1516  5.170859e+00
## 22705  4 1517  5.001961e+00
## 22706  4 1518  4.861821e+00
## 22707  4 1519  4.736376e+00
## 22708  4 1520  4.646700e+00
## 22709  4 1521  4.556024e+00
## 22710  4 1522  4.489143e+00
## 22711  4 1523  4.423429e+00
## 22712  4 1524  4.372033e+00
## 22713  4 1525  4.322386e+00
## 22714  4 1526  4.268429e+00
## 22715  4 1527  4.226550e+00
## 22716  4 1528  4.182060e+00
## 22717  4 1529  4.149884e+00
## 22718  4 1530  4.095138e+00
## 22719  4 1531  4.061657e+00
## 22720  4 1532  4.033961e+00
## 22721  4 1533  3.991254e+00
## 22722  4 1534  3.965080e+00
## 22723  4 1535  3.920514e+00
## 22724  4 1536  3.889771e+00
## 22725  4 1537  5.943401e+00
## 22726  4 1538  5.641921e+00
## 22727  4 1539  5.369718e+00
## 22728  4 1540  5.168852e+00
## 22729  4 1541  5.001683e+00
## 22730  4 1542  4.857369e+00
## 22731  4 1543  4.744791e+00
## 22732  4 1544  4.644387e+00
## 22733  4 1545  4.561507e+00
## 22734  4 1546  4.487925e+00
## 22735  4 1547  4.428892e+00
## 22736  4 1548  4.358491e+00
## 22737  4 1549  4.309196e+00
## 22738  4 1550  4.269793e+00
## 22739  4 1551  4.223051e+00
## 22740  4 1552  4.172405e+00
## 22741  4 1553  4.140723e+00
## 22742  4 1554  4.102435e+00
## 22743  4 1555  4.064502e+00
## 22744  4 1556  4.031360e+00
## 22745  4 1557  3.990920e+00
## 22746  4 1558  3.956320e+00
## 22747  4 1559  3.923267e+00
## 22748  4 1560  3.888880e+00
## 22749  4 1560  3.882301e+00
## 22750  4 1561  5.931049e+00
## 22751  4 1562  5.611309e+00
## 22752  4 1563  5.364283e+00
## 22753  4 1564  5.153474e+00
## 22754  4 1565  4.979220e+00
## 22755  4 1566  4.858764e+00
## 22756  4 1567  4.726566e+00
## 22757  4 1568  4.641397e+00
## 22758  4 1569  4.553592e+00
## 22759  4 1570  4.484649e+00
## 22760  4 1571  4.414432e+00
## 22761  4 1572  4.356970e+00
## 22762  4 1573  4.309140e+00
## 22763  4 1574  4.254985e+00
## 22764  4 1575  4.209510e+00
## 22765  4 1576  4.170653e+00
## 22766  4 1577  4.133567e+00
## 22767  4 1578  4.087210e+00
## 22768  4 1579  4.060733e+00
## 22769  4 1580  4.013896e+00
## 22770  4 1581  3.984205e+00
## 22771  4 1582  3.948563e+00
## 22772  4 1583  3.916653e+00
## 22773  4 1584  3.881212e+00
## 22774  4 1584  3.877458e+00
## 22775  4 1585  3.841781e+00
## 22776  4 1586  3.825254e+00
## 22777  4 1587  3.787896e+00
## 22778  4 1588  3.753635e+00
## 22779  4 1589  3.726067e+00
## 22780  4 1590  3.688840e+00
## 22781  4 1591  3.659881e+00
## 22782  4 1592  3.629753e+00
## 22783  4 1593  3.600313e+00
## 22784  4 1594  3.571244e+00
## 22785  4 1595  3.537246e+00
## 22786  4 1596  3.510525e+00
## 22787  4 1597  3.478009e+00
## 22788  4 1598  3.449630e+00
## 22789  4 1599  3.422498e+00
## 22790  4 1599  3.422636e+00
## 22791  4 1600  4.440747e+00
## 22792  4 1601  4.264382e+00
## 22793  4 1602  4.119087e+00
## 22794  4 1603  4.013592e+00
## 22795  4 1604  3.918988e+00
## 22796  4 1605  3.831015e+00
## 22797  4 1606  3.763861e+00
## 22798  4 1607  3.709545e+00
## 22799  4 1608  3.656122e+00
## 22800  4 1608  3.657339e+00
## 22801  4 1609  5.704640e+00
## 22802  4 1610  5.364043e+00
## 22803  4 1611  5.102077e+00
## 22804  4 1612  4.888302e+00
## 22805  4 1613  4.725220e+00
## 22806  4 1614  4.577491e+00
## 22807  4 1615  4.461988e+00
## 22808  4 1616  4.360266e+00
## 22809  4 1617  4.272011e+00
## 22810  4 1618  4.205710e+00
## 22811  4 1619  4.143083e+00
## 22812  4 1620  4.083803e+00
## 22813  4 1621  4.036555e+00
## 22814  4 1622  3.986774e+00
## 22815  4 1623  3.951450e+00
## 22816  4 1624  3.903844e+00
## 22817  4 1625  3.871828e+00
## 22818  4 1626  3.837040e+00
## 22819  4 1627  3.798848e+00
## 22820  4 1628  3.762810e+00
## 22821  4 1629  3.730378e+00
## 22822  4 1630  3.696805e+00
## 22823  4 1631  3.663279e+00
## 22824  4 1632  3.631097e+00
## 22825  4 1633  5.698218e+00
## 22826  4 1634  5.374442e+00
## 22827  4 1635  5.118238e+00
## 22828  4 1636  4.907180e+00
## 22829  4 1637  4.738635e+00
## 22830  4 1638  4.606650e+00
## 22831  4 1639  4.496844e+00
## 22832  4 1640  4.395808e+00
## 22833  4 1641  4.321335e+00
## 22834  4 1642  4.250405e+00
## 22835  4 1643  4.192641e+00
## 22836  4 1644  4.136061e+00
## 22837  4 1645  4.084147e+00
## 22838  4 1646  4.037954e+00
## 22839  4 1647  3.993310e+00
## 22840  4 1648  3.948807e+00
## 22841  4 1649  3.912614e+00
## 22842  4 1650  3.875642e+00
## 22843  4 1651  3.842918e+00
## 22844  4 1652  3.806299e+00
## 22845  4 1653  3.778263e+00
## 22846  4 1654  3.741544e+00
## 22847  4 1655  3.707943e+00
## 22848  4 1656  3.684860e+00
## 22849  4 1657  5.742186e+00
## 22850  4 1658  5.416807e+00
## 22851  4 1659  5.167810e+00
## 22852  4 1660  4.953553e+00
## 22853  4 1661  4.801871e+00
## 22854  4 1662  4.655894e+00
## 22855  4 1663  4.536885e+00
## 22856  4 1664  4.448186e+00
## 22857  4 1665  4.365354e+00
## 22858  4 1666  4.291575e+00
## 22859  4 1667  4.230424e+00
## 22860  4 1668  4.167746e+00
## 22861  4 1669  4.120768e+00
## 22862  4 1670  4.068274e+00
## 22863  4 1671  4.032271e+00
## 22864  4 1672  3.992545e+00
## 22865  4 1673  3.954924e+00
## 22866  4 1674  3.915962e+00
## 22867  4 1675  3.884920e+00
## 22868  4 1676  3.843351e+00
## 22869  4 1677  3.810247e+00
## 22870  4 1678  3.782339e+00
## 22871  4 1679  3.740573e+00
## 22872  4 1680  3.718060e+00
## 22873  4 1681  5.774042e+00
## 22874  4 1682  5.454753e+00
## 22875  4 1683  5.196869e+00
## 22876  4 1684  4.988124e+00
## 22877  4 1685  4.822321e+00
## 22878  4 1686  4.694759e+00
## 22879  4 1687  4.576357e+00
## 22880  4 1688  4.485217e+00
## 22881  4 1689  4.402164e+00
## 22882  4 1690  4.330348e+00
## 22883  4 1691  4.263790e+00
## 22884  4 1692  4.208448e+00
## 22885  4 1693  4.165365e+00
## 22886  4 1694  4.109743e+00
## 22887  4 1695  4.073276e+00
## 22888  4 1696  4.022948e+00
## 22889  4 1697  3.981600e+00
## 22890  4 1698  3.946259e+00
## 22891  4 1699  3.911343e+00
## 22892  4 1700  3.875971e+00
## 22893  4 1701  3.842463e+00
## 22894  4 1702  3.804962e+00
## 22895  4 1703  3.778916e+00
## 22896  4 1704  3.744932e+00
## 22897  4 1705  5.801532e+00
## 22898  4 1706  5.486139e+00
## 22899  4 1707  5.228804e+00
## 22900  4 1708  5.026059e+00
## 22901  4 1709  4.856752e+00
## 22902  4 1710  4.718610e+00
## 22903  4 1711  4.607312e+00
## 22904  4 1712  4.503028e+00
## 22905  4 1713  4.429081e+00
## 22906  4 1714  4.356349e+00
## 22907  4 1715  4.286932e+00
## 22908  4 1716  4.236647e+00
## 22909  4 1717  4.177528e+00
## 22910  4 1718  4.135339e+00
## 22911  4 1719  4.093713e+00
## 22912  4 1720  4.042851e+00
## 22913  4 1721  4.015077e+00
## 22914  4 1722  3.973034e+00
## 22915  4 1723  3.941645e+00
## 22916  4 1724  3.905174e+00
## 22917  4 1725  3.865104e+00
## 22918  4 1726  3.836545e+00
## 22919  4 1727  3.798674e+00
## 22920  4 1728  3.775634e+00
## 22921  4 1729  5.824732e+00
## 22922  4 1730  5.512181e+00
## 22923  4 1731  5.250514e+00
## 22924  4 1732  5.039226e+00
## 22925  4 1733  4.885786e+00
## 22926  4 1734  4.737955e+00
## 22927  4 1735  4.628952e+00
## 22928  4 1736  4.532397e+00
## 22929  4 1737  4.439865e+00
## 22930  4 1738  4.378199e+00
## 22931  4 1739  4.314668e+00
## 22932  4 1740  4.259144e+00
## 22933  4 1741  4.205486e+00
## 22934  4 1742  4.150542e+00
## 22935  4 1743  4.114994e+00
## 22936  4 1744  4.070287e+00
## 22937  4 1745  4.036634e+00
## 22938  4 1746  3.996250e+00
## 22939  4 1747  3.964328e+00
## 22940  4 1748  3.920710e+00
## 22941  4 1749  3.892830e+00
## 22942  4 1750  3.853187e+00
## 22943  4 1751  3.823695e+00
## 22944  4 1752  3.785786e+00
## 22945  4 1753  5.847669e+00
## 22946  4 1754  5.529972e+00
## 22947  4 1755  5.273879e+00
## 22948  4 1756  5.069050e+00
## 22949  4 1757  4.900618e+00
## 22950  4 1758  4.759776e+00
## 22951  4 1759  4.645960e+00
## 22952  4 1760  4.547398e+00
## 22953  4 1761  4.462359e+00
## 22954  4 1762  4.407830e+00
## 22955  4 1763  4.332547e+00
## 22956  4 1764  4.268478e+00
## 22957  4 1765  4.219874e+00
## 22958  4 1766  4.179277e+00
## 22959  4 1767  4.131098e+00
## 22960  4 1768  4.094635e+00
## 22961  4 1769  4.048318e+00
## 22962  4 1770  4.009184e+00
## 22963  4 1771  3.983670e+00
## 22964  4 1772  3.936072e+00
## 22965  4 1773  3.901252e+00
## 22966  4 1774  3.870282e+00
## 22967  4 1775  3.841796e+00
## 22968  4 1776  3.801216e+00
## 22969  4 1777  5.855579e+00
## 22970  4 1778  5.545087e+00
## 22971  4 1779  5.285862e+00
## 22972  4 1780  5.084499e+00
## 22973  4 1781  4.916569e+00
## 22974  4 1782  4.774623e+00
## 22975  4 1783  4.662966e+00
## 22976  4 1784  4.564956e+00
## 22977  4 1785  4.478491e+00
## 22978  4 1786  4.407084e+00
## 22979  4 1787  4.341466e+00
## 22980  4 1788  4.297227e+00
## 22981  4 1789  4.234845e+00
## 22982  4 1790  4.187652e+00
## 22983  4 1791  4.142587e+00
## 22984  4 1792  4.103945e+00
## 22985  4 1793  4.066010e+00
## 22986  4 1794  4.029195e+00
## 22987  4 1795  3.990762e+00
## 22988  4 1796  3.954365e+00
## 22989  4 1797  3.916252e+00
## 22990  4 1798  3.884244e+00
## 22991  4 1799  3.845761e+00
## 22992  4 1800  3.826553e+00
## 22993  4 1801  5.874382e+00
## 22994  4 1802  5.557462e+00
## 22995  4 1803  5.297614e+00
## 22996  4 1804  5.094520e+00
## 22997  4 1805  4.926938e+00
## 22998  4 1806  4.788859e+00
## 22999  4 1807  4.669480e+00
## 23000  4 1808  4.587423e+00
## 23001  4 1809  4.501348e+00
## 23002  4 1810  4.432963e+00
## 23003  4 1811  4.357484e+00
## 23004  4 1812  4.296183e+00
## 23005  4 1813  4.246127e+00
## 23006  4 1814  4.203191e+00
## 23007  4 1815  4.155572e+00
## 23008  4 1816  4.122012e+00
## 23009  4 1817  4.072962e+00
## 23010  4 1818  4.041108e+00
## 23011  4 1819  3.992962e+00
## 23012  4 1820  3.968846e+00
## 23013  4 1821  3.936124e+00
## 23014  4 1822  3.896077e+00
## 23015  4 1823  3.864731e+00
## 23016  4 1824  3.828959e+00
## 23017  4 1825  5.896298e+00
## 23018  4 1826  5.558278e+00
## 23019  4 1827  5.314653e+00
## 23020  4 1828  5.110303e+00
## 23021  4 1829  4.942128e+00
## 23022  4 1830  4.798299e+00
## 23023  4 1831  4.682292e+00
## 23024  4 1832  4.592265e+00
## 23025  4 1833  4.509053e+00
## 23026  4 1834  4.430726e+00
## 23027  4 1835  4.373080e+00
## 23028  4 1836  4.319260e+00
## 23029  4 1837  4.260621e+00
## 23030  4 1838  4.210559e+00
## 23031  4 1839  4.170411e+00
## 23032  4 1840  4.125022e+00
## 23033  4 1841  4.083755e+00
## 23034  4 1842  4.052589e+00
## 23035  4 1843  4.012352e+00
## 23036  4 1844  3.978580e+00
## 23037  4 1845  3.939706e+00
## 23038  4 1846  3.902822e+00
## 23039  4 1847  3.873419e+00
## 23040  4 1848  3.843090e+00
## 23041  4 1849  5.890647e+00
## 23042  4 1850  5.565257e+00
## 23043  4 1851  5.321361e+00
## 23044  4 1852  5.121890e+00
## 23045  4 1853  4.948851e+00
## 23046  4 1854  4.804894e+00
## 23047  4 1855  4.690897e+00
## 23048  4 1856  4.590761e+00
## 23049  4 1857  4.518394e+00
## 23050  4 1858  4.440142e+00
## 23051  4 1859  4.383794e+00
## 23052  4 1860  4.323409e+00
## 23053  4 1861  4.272938e+00
## 23054  4 1862  4.224986e+00
## 23055  4 1863  4.184210e+00
## 23056  4 1864  4.134425e+00
## 23057  4 1865  4.098279e+00
## 23058  4 1866  4.051469e+00
## 23059  4 1867  4.017715e+00
## 23060  4 1868  3.986194e+00
## 23061  4 1869  3.946968e+00
## 23062  4 1870  3.919502e+00
## 23063  4 1871  3.876971e+00
## 23064  4 1872  3.850898e+00
## 23065  4 1872  3.877338e+00
## 23066  4 1873  5.934178e+00
## 23067  4 1874  5.619397e+00
## 23068  4 1875  5.362178e+00
## 23069  4 1876  5.164678e+00
## 23070  4 1877  4.985445e+00
## 23071  4 1878  4.851047e+00
## 23072  4 1879  4.734783e+00
## 23073  4 1880  4.634041e+00
## 23074  4 1881  4.558506e+00
## 23075  4 1882  4.475773e+00
## 23076  4 1883  4.414780e+00
## 23077  4 1884  4.360454e+00
## 23078  4 1885  4.311899e+00
## 23079  4 1886  4.262010e+00
## 23080  4 1887  4.206870e+00
## 23081  4 1888  4.163689e+00
## 23082  4 1889  4.136833e+00
## 23083  4 1890  4.080173e+00
## 23084  4 1891  4.056976e+00
## 23085  4 1892  4.017251e+00
## 23086  4 1893  3.977590e+00
## 23087  4 1894  3.948237e+00
## 23088  4 1895  3.913795e+00
## 23089  4 1896  3.878311e+00
## 23090  4 1896  3.880329e+00
## 23091  4 1897  3.846981e+00
## 23092  4 1898  3.816077e+00
## 23093  4 1899  3.785263e+00
## 23094  4 1900  3.756859e+00
## 23095  4 1901  3.722364e+00
## 23096  4 1902  3.691628e+00
## 23097  4 1903  3.655784e+00
## 23098  4 1904  3.628604e+00
## 23099  4 1905  3.599689e+00
## 23100  4 1906  3.573047e+00
## 23101  4 1907  3.540727e+00
## 23102  4 1908  3.505692e+00
## 23103  4 1909  3.479943e+00
## 23104  4 1910  3.449452e+00
## 23105  4 1911  3.427955e+00
## 23106  4 1911  3.420172e+00
## 23107  4 1912  5.482122e+00
## 23108  4 1913  5.173632e+00
## 23109  4 1914  4.916902e+00
## 23110  4 1915  4.718519e+00
## 23111  4 1916  4.546471e+00
## 23112  4 1917  4.423176e+00
## 23113  4 1918  4.303712e+00
## 23114  4 1919  4.215397e+00
## 23115  4 1920  4.136210e+00
## 23116  4 1920  4.136897e+00
## 23117  4 1921  6.151785e+00
## 23118  4 1922  5.799886e+00
## 23119  4 1923  5.525455e+00
## 23120  4 1924  5.309588e+00
## 23121  4 1925  5.113422e+00
## 23122  4 1926  4.962203e+00
## 23123  4 1927  4.843589e+00
## 23124  4 1928  4.743173e+00
## 23125  4 1929  4.660391e+00
## 23126  4 1930  4.576142e+00
## 23127  4 1931  4.508211e+00
## 23128  4 1932  4.446932e+00
## 23129  4 1933  4.401300e+00
## 23130  4 1934  4.353235e+00
## 23131  4 1935  4.306837e+00
## 23132  4 1936  4.256842e+00
## 23133  4 1937  4.217482e+00
## 23134  4 1938  4.170571e+00
## 23135  4 1939  4.129262e+00
## 23136  4 1940  4.095538e+00
## 23137  4 1941  4.059651e+00
## 23138  4 1942  4.031080e+00
## 23139  4 1943  4.002899e+00
## 23140  4 1944  3.962736e+00
## 23141  4 1945  6.004401e+00
## 23142  4 1946  5.693774e+00
## 23143  4 1947  5.426046e+00
## 23144  4 1948  5.226891e+00
## 23145  4 1949  5.054334e+00
## 23146  4 1950  4.922087e+00
## 23147  4 1951  4.799073e+00
## 23148  4 1952  4.708740e+00
## 23149  4 1953  4.619881e+00
## 23150  4 1954  4.553904e+00
## 23151  4 1955  4.480957e+00
## 23152  4 1956  4.435900e+00
## 23153  4 1957  4.376737e+00
## 23154  4 1958  4.328402e+00
## 23155  4 1959  4.281389e+00
## 23156  4 1960  4.236643e+00
## 23157  4 1961  4.200979e+00
## 23158  4 1962  4.164110e+00
## 23159  4 1963  4.123554e+00
## 23160  4 1964  4.077590e+00
## 23161  4 1965  4.047410e+00
## 23162  4 1966  4.012015e+00
## 23163  4 1967  3.981036e+00
## 23164  4 1968  3.944587e+00
## 23165  4 1969  5.997987e+00
## 23166  4 1970  5.689400e+00
## 23167  4 1971  5.421652e+00
## 23168  4 1972  5.218628e+00
## 23169  4 1973  5.046217e+00
## 23170  4 1974  4.911069e+00
## 23171  4 1975  4.787099e+00
## 23172  4 1976  4.700701e+00
## 23173  4 1977  4.607128e+00
## 23174  4 1978  4.534723e+00
## 23175  4 1979  4.476284e+00
## 23176  4 1980  4.420519e+00
## 23177  4 1981  4.359022e+00
## 23178  4 1982  4.316801e+00
## 23179  4 1983  4.273779e+00
## 23180  4 1984  4.231470e+00
## 23181  4 1985  4.185492e+00
## 23182  4 1986  4.139333e+00
## 23183  4 1987  4.107351e+00
## 23184  4 1988  4.066987e+00
## 23185  4 1989  4.034835e+00
## 23186  4 1990  4.004221e+00
## 23187  4 1991  3.969895e+00
## 23188  4 1992  3.931122e+00
## 23189  4 1993  5.986575e+00
## 23190  4 1994  5.661616e+00
## 23191  4 1995  5.411830e+00
## 23192  4 1996  5.207071e+00
## 23193  4 1997  5.035203e+00
## 23194  4 1998  4.893994e+00
## 23195  4 1999  4.781218e+00
## 23196  4 2000  4.681003e+00
## 23197  4 2001  4.602860e+00
## 23198  4 2002  4.521538e+00
## 23199  4 2003  4.466566e+00
## 23200  4 2004  4.407881e+00
## 23201  4 2005  4.356319e+00
## 23202  4 2006  4.300021e+00
## 23203  4 2007  4.260027e+00
## 23204  4 2008  4.212471e+00
## 23205  4 2009  4.171786e+00
## 23206  4 2010  4.137810e+00
## 23207  4 2011  4.096237e+00
## 23208  4 2012  4.063334e+00
## 23209  4 2013  4.031408e+00
## 23210  4 2014  3.992320e+00
## 23211  4 2015  3.966266e+00
## 23212  4 2016  3.925602e+00
## 23213  4 2017  5.982532e+00
## 23214  4 2018  5.662928e+00
## 23215  4 2019  5.404422e+00
## 23216  4 2020  5.202308e+00
## 23217  4 2021  5.017531e+00
## 23218  4 2022  4.887968e+00
## 23219  4 2023  4.762471e+00
## 23220  4 2024  4.664814e+00
## 23221  4 2025  4.583960e+00
## 23222  4 2026  4.522408e+00
## 23223  4 2027  4.450670e+00
## 23224  4 2028  4.398166e+00
## 23225  4 2029  4.339532e+00
## 23226  4 2030  4.286918e+00
## 23227  4 2031  4.253112e+00
## 23228  4 2032  4.206935e+00
## 23229  4 2033  4.163918e+00
## 23230  4 2034  4.129854e+00
## 23231  4 2035  4.091006e+00
## 23232  4 2036  4.054274e+00
## 23233  4 2037  4.020126e+00
## 23234  4 2038  3.984932e+00
## 23235  4 2039  3.951928e+00
```

```
## 23236   4 2040  3.918432e+00
## 23237   4 2041  5.965415e+00
## 23238   4 2042  5.654249e+00
## 23239   4 2043  5.390073e+00
## 23240   4 2044  5.192626e+00
## 23241   4 2045  5.030154e+00
## 23242   4 2046  4.884289e+00
## 23243   4 2047  4.762085e+00
## 23244   4 2048  4.665750e+00
## 23245   4 2049  4.583926e+00
## 23246   4 2050  4.512038e+00
## 23247   4 2051  4.449703e+00
## 23248   4 2052  4.387666e+00
## 23249   4 2053  4.337326e+00
## 23250   4 2054  4.290143e+00
## 23251   4 2055  4.242332e+00
## 23252   4 2056  4.201875e+00
## 23253   4 2057  4.163015e+00
## 23254   4 2058  4.130811e+00
## 23255   4 2059  4.080919e+00
## 23256   4 2060  4.053795e+00
## 23257   4 2061  4.015544e+00
## 23258   4 2062  3.977665e+00
## 23259   4 2063  3.942284e+00
## 23260   4 2064  3.915575e+00
## 23261   4 2065  5.958226e+00
## 23262   4 2066  5.637088e+00
## 23263   4 2067  5.390855e+00
## 23264   4 2068  5.182656e+00
## 23265   4 2069  5.011260e+00
## 23266   4 2070  4.878557e+00
## 23267   4 2071  4.758507e+00
## 23268   4 2072  4.657752e+00
## 23269   4 2073  4.578415e+00
## 23270   4 2074  4.506040e+00
## 23271   4 2075  4.445229e+00
## 23272   4 2076  4.377835e+00
## 23273   4 2077  4.341809e+00
## 23274   4 2078  4.280343e+00
## 23275   4 2079  4.241926e+00
## 23276   4 2080  4.184123e+00
## 23277   4 2081  4.158016e+00
## 23278   4 2082  4.114482e+00
## 23279   4 2083  4.072918e+00
## 23280   4 2084  4.038834e+00
## 23281   4 2085  3.993880e+00
## 23282   4 2086  3.978247e+00
## 23283   4 2087  3.947167e+00
## 23284   4 2088  3.899929e+00
## 23285   4 2089  5.959810e+00
## 23286   4 2090  5.643410e+00
## 23287   4 2091  5.380723e+00
## 23288   4 2092  5.184157e+00
## 23289   4 2093  5.008828e+00
## 23290   4 2094  4.879756e+00
## 23291   4 2095  4.763114e+00
## 23292   4 2096  4.650190e+00
## 23293   4 2097  4.576538e+00
## 23294   4 2098  4.496015e+00
## 23295   4 2099  4.440505e+00
## 23296   4 2100  4.386150e+00
## 23297   4 2101  4.327359e+00
## 23298   4 2102  4.275812e+00
## 23299   4 2103  4.239119e+00
## 23300   4 2104  4.192246e+00
## 23301   4 2105  4.145988e+00
## 23302   4 2106  4.114602e+00
## 23303   4 2107  4.070602e+00
## 23304   4 2108  4.034356e+00
## 23305   4 2109  3.997418e+00
## 23306   4 2110  3.968478e+00
## 23307   4 2111  3.940477e+00
## 23308   4 2112  3.901073e+00
## 23309   4 2113  5.960095e+00
## 23310   4 2114  5.640317e+00
## 23311   4 2115  5.388637e+00
## 23312   4 2116  5.183399e+00
## 23313   4 2117  4.995782e+00
## 23314   4 2118  4.867808e+00
## 23315   4 2119  4.752311e+00
## 23316   4 2120  4.657280e+00
## 23317   4 2121  4.573816e+00
## 23318   4 2122  4.495014e+00
## 23319   4 2123  4.432138e+00
## 23320   4 2124  4.366630e+00
## 23321   4 2125  4.318683e+00
## 23322   4 2126  4.274234e+00
## 23323   4 2127  4.226700e+00
## 23324   4 2128  4.195208e+00
## 23325   4 2129  4.150243e+00
## 23326   4 2130  4.109725e+00
## 23327   4 2131  4.063424e+00
## 23328   4 2132  4.034189e+00
## 23329   4 2133  3.996650e+00
## 23330   4 2134  3.964110e+00
## 23331   4 2135  3.930541e+00
## 23332   4 2136  3.899375e+00
## 23333   4 2137  5.958133e+00
## 23334   4 2138  5.628828e+00
## 23335   4 2139  5.378238e+00
## 23336   4 2140  5.164426e+00
## 23337   4 2141  5.000875e+00
## 23338   4 2142  4.865472e+00
## 23339   4 2143  4.747228e+00
## 23340   4 2144  4.655206e+00
## 23341   4 2145  4.568851e+00
## 23342   4 2146  4.494555e+00
## 23343   4 2147  4.434760e+00
## 23344   4 2148  4.385252e+00
## 23345   4 2149  4.323932e+00
## 23346   4 2150  4.264635e+00
## 23347   4 2151  4.224471e+00
## 23348   4 2152  4.179762e+00
## 23349   4 2153  4.142093e+00
## 23350   4 2154  4.101016e+00
## 23351   4 2155  4.067236e+00
## 23352   4 2156  4.020095e+00
## 23353   4 2157  4.004718e+00
## 23354   4 2158  3.963399e+00
## 23355   4 2159  3.928127e+00
## 23356   4 2160  3.895223e+00
## 23357   4 2161  5.940472e+00
## 23358   4 2162  5.631662e+00
## 23359   4 2163  5.376310e+00
## 23360   4 2164  5.174712e+00
## 23361   4 2165  5.000253e+00
## 23362   4 2166  4.863553e+00
## 23363   4 2167  4.744654e+00
## 23364   4 2168  4.652246e+00
## 23365   4 2169  4.561940e+00
## 23366   4 2170  4.487218e+00
## 23367   4 2171  4.426936e+00
## 23368   4 2172  4.375366e+00
## 23369   4 2173  4.320095e+00
## 23370   4 2174  4.268699e+00
## 23371   4 2175  4.225708e+00
## 23372   4 2176  4.179816e+00
## 23373   4 2177  4.144939e+00
## 23374   4 2178  4.105659e+00
## 23375   4 2179  4.062328e+00
## 23376   4 2180  4.022931e+00
## 23377   4 2181  3.998714e+00
## 23378   4 2182  3.961845e+00
## 23379   4 2183  3.925371e+00
## 23380   4 2184  3.889634e+00
## 23381   4 2184  3.880692e+00
## 23382   4 2185  5.945984e+00
## 23383   4 2186  5.620954e+00
## 23384   4 2187  5.359649e+00
## 23385   4 2188  5.149321e+00
## 23386   4 2189  4.986063e+00
## 23387   4 2190  4.855859e+00
## 23388   4 2191  4.730279e+00
## 23389   4 2192  4.634824e+00
## 23390   4 2193  4.544616e+00
## 23391   4 2194  4.481813e+00
## 23392   4 2195  4.413878e+00
## 23393   4 2196  4.360222e+00
## 23394   4 2197  4.311227e+00
## 23395   4 2198  4.252699e+00
## 23396   4 2199  4.216917e+00
## 23397   4 2200  4.174084e+00
## 23398   4 2201  4.136443e+00
## 23399   4 2202  4.094315e+00
## 23400   4 2203  4.054326e+00
## 23401   4 2204  4.024891e+00
## 23402   4 2205  3.989713e+00
## 23403   4 2206  3.941581e+00
## 23404   4 2207  3.911025e+00
## 23405   4 2208  3.885999e+00
## 23406   4 2208  3.883493e+00
## 23407   4 2209  3.845653e+00
## 23408   4 2210  3.814808e+00
## 23409   4 2211  3.779006e+00
## 23410   4 2212  3.748844e+00
## 23411   4 2213  3.720715e+00
## 23412   4 2214  3.686268e+00
## 23413   4 2215  3.662177e+00
## 23414   4 2216  3.636252e+00
## 23415   4 2217  3.598144e+00
## 23416   4 2218  3.567693e+00
## 23417   4 2219  3.536794e+00
## 23418   4 2220  3.508699e+00
## 23419   4 2221  3.477090e+00
## 23420   4 2222  3.451654e+00
## 23421   4 2223  3.427707e+00
## 23422   4 2224  3.390589e+00
## 23423   4 2225  3.368607e+00
## 23424   4 2226  3.338742e+00
## 23425   4 2226  3.337111e+00
## 23426   4 2227  4.360238e+00
## 23427   4 2228  4.185789e+00
## 23428   4 2229  4.049064e+00
## 23429   4 2230  3.938354e+00
## 23430   4 2231  3.837258e+00
## 23431   4 2232  3.749957e+00
## 23432   4 2232  3.754597e+00
## 23433   4 2233  5.775177e+00
## 23434   4 2234  5.426430e+00
## 23435   4 2235  5.157637e+00
## 23436   4 2236  4.930081e+00
## 23437   4 2237  4.755361e+00
## 23438   4 2238  4.610205e+00
## 23439   4 2239  4.478240e+00
## 23440   4 2240  4.390566e+00
## 23441   4 2241  4.301507e+00
## 23442   4 2242  4.216500e+00
## 23443   4 2243  4.156942e+00
## 23444   4 2244  4.099167e+00
## 23445   4 2245  4.043019e+00
## 23446   4 2246  3.999366e+00
## 23447   4 2247  3.960009e+00
## 23448   4 2248  3.917606e+00
## 23449   4 2249  3.879100e+00
## 23450   4 2250  3.846791e+00
## 23451   4 2251  3.796917e+00
## 23452   4 2252  3.770807e+00
## 23453   4 2253  3.736997e+00
## 23454   4 2254  3.700334e+00
## 23455   4 2255  3.669357e+00
## 23456   4 2256  3.637766e+00
## 23457   4 2257  5.681920e+00
## 23458   4 2258  5.388863e+00
## 23459   4 2259  5.126862e+00
## 23460   4 2260  4.925733e+00
## 23461   4 2261  4.764185e+00
## 23462   4 2262  4.616265e+00
## 23463   4 2263  4.506957e+00
## 23464   4 2264  4.414139e+00
## 23465   4 2265  4.332009e+00
## 23466   4 2266  4.260704e+00
## 23467   4 2267  4.194825e+00
## 23468   4 2268  4.135734e+00
## 23469   4 2269  4.089413e+00
## 23470   4 2270  4.048641e+00
## 23471   4 2271  4.004845e+00
## 23472   4 2272  3.963277e+00
## 23473   4 2273  3.920102e+00
## 23474   4 2274  3.883542e+00
## 23475   4 2275  3.851823e+00
## 23476   4 2276  3.815080e+00
## 23477   4 2277  3.786878e+00
## 23478   4 2278  3.749476e+00
## 23479   4 2279  3.715731e+00
## 23480   4 2280  3.686499e+00
## 23481   4 2281  5.740486e+00
## 23482   4 2282  5.421938e+00
## 23483   4 2283  5.170747e+00
## 23484   4 2284  4.972078e+00
## 23485   4 2285  4.793969e+00
## 23486   4 2286  4.667621e+00
## 23487   4 2287  4.538531e+00
## 23488   4 2288  4.446726e+00
## 23489   4 2289  4.371206e+00
## 23490   4 2290  4.293870e+00
## 23491   4 2291  4.230733e+00
## 23492   4 2292  4.176933e+00
## 23493   4 2293  4.135437e+00
## 23494   4 2294  4.081656e+00
## 23495   4 2295  4.035847e+00
## 23496   4 2296  4.002607e+00
## 23497   4 2297  3.960478e+00
## 23498   4 2298  3.925926e+00
## 23499   4 2299  3.883696e+00
## 23500   4 2300  3.849490e+00
## 23501   4 2301  3.818038e+00
## 23502   4 2302  3.777324e+00
## 23503   4 2303  3.749026e+00
## 23504   4 2304  3.724596e+00
## 23505   4 2305  5.778564e+00
## 23506   4 2306  5.460167e+00
## 23507   4 2307  5.203850e+00
## 23508   4 2308  4.992670e+00
## 23509   4 2309  4.827950e+00
## 23510   4 2310  4.693972e+00
## 23511   4 2311  4.581973e+00
## 23512   4 2312  4.479149e+00
## 23513   4 2313  4.397571e+00
## 23514   4 2314  4.335696e+00
## 23515   4 2315  4.257808e+00
## 23516   4 2316  4.210215e+00
## 23517   4 2317  4.158608e+00
## 23518   4 2318  4.118727e+00
## 23519   4 2319  4.076980e+00
## 23520   4 2320  4.030518e+00
## 23521   4 2321  3.989266e+00
## 23522   4 2322  3.957101e+00
## 23523   4 2323  3.913296e+00
## 23524   4 2324  3.876831e+00
## 23525   4 2325  3.850185e+00
## 23526   4 2326  3.819614e+00
## 23527   4 2327  3.781250e+00
## 23528   4 2328  3.751084e+00
## 23529   4 2329  5.805698e+00
## 23530   4 2330  5.489572e+00
## 23531   4 2331  5.228813e+00
## 23532   4 2332  5.029700e+00
## 23533   4 2333  4.867878e+00
## 23534   4 2334  4.727276e+00
## 23535   4 2335  4.606529e+00
## 23536   4 2336  4.506330e+00
## 23537   4 2337  4.427013e+00
## 23538   4 2338  4.357075e+00
## 23539   4 2339  4.301276e+00
## 23540   4 2340  4.242296e+00
## 23541   4 2341  4.187201e+00
## 23542   4 2342  4.144013e+00
## 23543   4 2343  4.093485e+00
## 23544   4 2344  4.058977e+00
## 23545   4 2345  4.016997e+00
## 23546   4 2346  3.979103e+00
## 23547   4 2347  3.943626e+00
## 23548   4 2348  3.906284e+00
## 23549   4 2349  3.872163e+00
## 23550   4 2350  3.839273e+00
## 23551   4 2351  3.808920e+00
## 23552   4 2352  3.773533e+00
## 23553   4 2353  5.823693e+00
## 23554   4 2354  5.513490e+00
## 23555   4 2355  5.265087e+00
## 23556   4 2356  5.046647e+00
## 23557   4 2357  4.876437e+00
## 23558   4 2358  4.748692e+00
## 23559   4 2359  4.630540e+00
## 23560   4 2360  4.536783e+00
## 23561   4 2361  4.454243e+00
## 23562   4 2362  4.374390e+00
## 23563   4 2363  4.325633e+00
## 23564   4 2364  4.266857e+00
## 23565   4 2365  4.208881e+00
## 23566   4 2366  4.165720e+00
## 23567   4 2367  4.113710e+00
## 23568   4 2368  4.081242e+00
## 23569   4 2369  4.038281e+00
## 23570   4 2370  3.999762e+00
## 23571   4 2371  3.964562e+00
## 23572   4 2372  3.923917e+00
## 23573   4 2373  3.894247e+00
## 23574   4 2374  3.856783e+00
## 23575   4 2375  3.819033e+00
## 23576   4 2376  3.798456e+00
## 23577   4 2377  5.850545e+00
## 23578   4 2378  5.538143e+00
## 23579   4 2379  5.277348e+00
## 23580   4 2380  5.070004e+00
## 23581   4 2381  4.908039e+00
## 23582   4 2382  4.762874e+00
## 23583   4 2383  4.651644e+00
## 23584   4 2384  4.554620e+00
## 23585   4 2385  4.464218e+00
## 23586   4 2386  4.389719e+00
## 23587   4 2387  4.334444e+00
## 23588   4 2388  4.276695e+00
## 23589   4 2389  4.227335e+00
## 23590   4 2390  4.175579e+00
## 23591   4 2391  4.134675e+00
## 23592   4 2392  4.097979e+00
## 23593   4 2393  4.055085e+00
## 23594   4 2394  4.022227e+00
## 23595   4 2395  3.980044e+00
## 23596   4 2396  3.946607e+00
## 23597   4 2397  3.910083e+00
## 23598   4 2398  3.865075e+00
## 23599   4 2399  3.840667e+00
## 23600   4 2400  3.803524e+00
## 23601   4 2401  5.866579e+00
## 23602   4 2402  5.538977e+00
## 23603   4 2403  5.294981e+00
## 23604   4 2404  5.081537e+00
## 23605   4 2405  4.919886e+00
## 23606   4 2406  4.783025e+00
## 23607   4 2407  4.660770e+00
## 23608   4 2408  4.561833e+00
## 23609   4 2409  4.480916e+00
## 23610   4 2410  4.412652e+00
## 23611   4 2411  4.351105e+00
## 23612   4 2412  4.293776e+00
## 23613   4 2413  4.242849e+00
## 23614   4 2414  4.192114e+00
## 23615   4 2415  4.144424e+00
## 23616   4 2416  4.105201e+00
## 23617   4 2417  4.062395e+00
## 23618   4 2418  4.026176e+00
## 23619   4 2419  3.994162e+00
## 23620   4 2420  3.955108e+00
## 23621   4 2421  3.924222e+00
## 23622   4 2422  3.884959e+00
## 23623   4 2423  3.856213e+00
## 23624   4 2424  3.821697e+00
## 23625   4 2425  5.867864e+00
## 23626   4 2426  5.568614e+00
## 23627   4 2427  5.309246e+00
## 23628   4 2428  5.095954e+00
## 23629   4 2429  4.933428e+00
## 23630   4 2430  4.792418e+00
## 23631   4 2431  4.681386e+00
## 23632   4 2432  4.576847e+00
## 23633   4 2433  4.494059e+00
## 23634   4 2434  4.429033e+00
## 23635   4 2435  4.363527e+00
## 23636   4 2436  4.299037e+00
## 23637   4 2437  4.251718e+00
## 23638   4 2438  4.204032e+00
## 23639   4 2439  4.159068e+00
## 23640   4 2440  4.113949e+00
## 23641   4 2441  4.080405e+00
## 23642   4 2442  4.039706e+00
## 23643   4 2443  4.004847e+00
## 23644   4 2444  3.969031e+00
## 23645   4 2445  3.933652e+00
## 23646   4 2446  3.903319e+00
## 23647   4 2447  3.866486e+00
## 23648   4 2448  3.825783e+00
## 23649   4 2449  5.880960e+00
## 23650   4 2450  5.570098e+00
## 23651   4 2451  5.313990e+00
## 23652   4 2452  5.112831e+00
## 23653   4 2453  4.943664e+00
## 23654   4 2454  4.801385e+00
## 23655   4 2455  4.689529e+00
## 23656   4 2456  4.591169e+00
## 23657   4 2457  4.513023e+00
## 23658   4 2458  4.434045e+00
## 23659   4 2459  4.376044e+00
## 23660   4 2460  4.311647e+00
## 23661   4 2461  4.270188e+00
## 23662   4 2462  4.222143e+00
## 23663   4 2463  4.176930e+00
## 23664   4 2464  4.124358e+00
## 23665   4 2465  4.091877e+00
## 23666   4 2466  4.048521e+00
## 23667   4 2467  4.016586e+00
## 23668   4 2468  3.981270e+00
## 23669   4 2469  3.938827e+00
## 23670   4 2470  3.903874e+00
## 23671   4 2471  3.873979e+00
## 23672   4 2472  3.847222e+00
## 23673   4 2473  5.889451e+00
## 23674   4 2474  5.589099e+00
## 23675   4 2475  5.331431e+00
## 23676   4 2476  5.119278e+00
## 23677   4 2477  4.957258e+00
## 23678   4 2478  4.812502e+00
## 23679   4 2479  4.692694e+00
## 23680   4 2480  4.584910e+00
## 23681   4 2481  4.513679e+00
## 23682   4 2482  4.451139e+00
## 23683   4 2483  4.379297e+00
## 23684   4 2484  4.320976e+00
## 23685   4 2485  4.276282e+00
## 23686   4 2486  4.218590e+00
## 23687   4 2487  4.177282e+00
## 23688   4 2488  4.132480e+00
## 23689   4 2489  4.092743e+00
## 23690   4 2490  4.052504e+00
## 23691   4 2491  4.025708e+00
## 23692   4 2492  3.983726e+00
## 23693   4 2493  3.949950e+00
## 23694   4 2494  3.915009e+00
## 23695   4 2495  3.884608e+00
## 23696   4 2496  3.852980e+00
## 23697   4 2496  3.883716e+00
## 23698   4 2497  5.942117e+00
## 23699   4 2498  5.626529e+00
## 23700   4 2499  5.362365e+00
## 23701   4 2500  5.155342e+00
## 23702   4 2501  4.989605e+00
## 23703   4 2502  4.851119e+00
## 23704   4 2503  4.737852e+00
## 23705   4 2504  4.637527e+00
## 23706   4 2505  4.545896e+00
## 23707   4 2506  4.482940e+00
## 23708   4 2507  4.417204e+00
## 23709   4 2508  4.365964e+00
## 23710   4 2509  4.299757e+00
## 23711   4 2510  4.247978e+00
## 23712   4 2511  4.221296e+00
## 23713   4 2512  4.173117e+00
## 23714   4 2513  4.128536e+00
## 23715   4 2514  4.089537e+00
## 23716   4 2515  4.052562e+00
## 23717   4 2516  4.017879e+00
## 23718   4 2517  3.980987e+00
## 23719   4 2518  3.948566e+00
## 23720   4 2519  3.916583e+00
## 23721   4 2520  3.892582e+00
## 23722   4 2520  3.879419e+00
## 23723   4 2521  3.848479e+00
## 23724   4 2522  3.817594e+00
## 23725   4 2523  3.781791e+00
## 23726   4 2524  3.759941e+00
## 23727   4 2525  3.724314e+00
## 23728   4 2526  3.684039e+00
## 23729   4 2527  3.658792e+00
## 23730   4 2528  3.633930e+00
## 23731   4 2529  3.596856e+00
## 23732   4 2530  3.574508e+00
## 23733   4 2531  3.539831e+00
## 23734   4 2532  3.509472e+00
## 23735   4 2533  3.485591e+00
## 23736   4 2534  3.449724e+00
## 23737   4 2535  3.424636e+00
## 23738   4 2536  3.393692e+00
## 23739   4 2537  3.366447e+00
## 23740   4 2538  3.341783e+00
## 23741   4 2538  3.338021e+00
## 23742   4 2539  5.401265e+00
## 23743   4 2540  5.087134e+00
## 23744   4 2541  4.830404e+00
## 23745   4 2542  4.628043e+00
## 23746   4 2543  4.469036e+00
## 23747   4 2544  4.344598e+00
## 23748   4 2544  4.331555e+00
## 23749   4 2545  6.305872e+00
## 23750   4 2546  5.935518e+00
## 23751   4 2547  5.624844e+00
## 23752   4 2548  5.389641e+00
## 23753   4 2549  5.193270e+00
## 23754   4 2550  5.026683e+00
## 23755   4 2551  4.892249e+00
## 23756   4 2552  4.786929e+00
## 23757   4 2553  4.693159e+00
## 23758   4 2554  4.610852e+00
## 23759   4 2555  4.552048e+00
## 23760   4 2556  4.475056e+00
## 23761   4 2557  4.421538e+00
## 23762   4 2558  4.358657e+00
## 23763   4 2559  4.320716e+00
## 23764   4 2560  4.280361e+00
## 23765   4 2561  4.233718e+00
## 23766   4 2562  4.195803e+00
## 23767   4 2563  4.159214e+00
## 23768   4 2564  4.106467e+00
## 23769   4 2565  4.075669e+00
## 23770   4 2566  4.044402e+00
## 23771   4 2567  4.009666e+00
## 23772   4 2568  3.964777e+00
## 23773   4 2569  6.019136e+00
## 23774   4 2570  5.699901e+00
## 23775   4 2571  5.453937e+00
## 23776   4 2572  5.246065e+00
## 23777   4 2573  5.083008e+00
## 23778   4 2574  4.940393e+00
## 23779   4 2575  4.831720e+00
## 23780   4 2576  4.721733e+00
## 23781   4 2577  4.643135e+00
## 23782   4 2578  4.565851e+00
## 23783   4 2579  4.497540e+00
## 23784   4 2580  4.433862e+00
## 23785   4 2581  4.389601e+00
## 23786   4 2582  4.347628e+00
## 23787   4 2583  4.298638e+00
## 23788   4 2584  4.252858e+00
## 23789   4 2585  4.211858e+00
## 23790   4 2586  4.165334e+00
## 23791   4 2587  4.132548e+00
## 23792   4 2588  4.094162e+00
## 23793   4 2589  4.063903e+00
## 23794   4 2590  4.037314e+00
## 23795   4 2591  3.996816e+00
## 23796   4 2592  3.949553e+00
## 23797   4 2593  6.012588e+00
## 23798   4 2594  5.695823e+00
## 23799   4 2595  5.437895e+00
## 23800   4 2596  5.231062e+00
## 23801   4 2597  5.055860e+00
## 23802   4 2598  4.919223e+00
## 23803   4 2599  4.807801e+00
## 23804   4 2600  4.705440e+00
## 23805   4 2601  4.620434e+00
## 23806   4 2602  4.544947e+00
## 23807   4 2603  4.478361e+00
## 23808   4 2604  4.427814e+00
## 23809   4 2605  4.376683e+00
## 23810   4 2606  4.336217e+00
## 23811   4 2607  4.280909e+00
## 23812   4 2608  4.232433e+00
## 23813   4 2609  4.196353e+00
## 23814   4 2610  4.157191e+00
## 23815   4 2611  4.120588e+00
## 23816   4 2612  4.080417e+00
## 23817   4 2613  4.051920e+00
## 23818   4 2614  4.013333e+00
## 23819   4 2615  3.979332e+00
## 23820   4 2616  3.944187e+00
## 23821   4 2617  5.995203e+00
## 23822   4 2618  5.679806e+00
## 23823   4 2619  5.418771e+00
## 23824   4 2620  5.216373e+00
## 23825   4 2621  5.038472e+00
## 23826   4 2622  4.913105e+00
## 23827   4 2623  4.796138e+00
## 23828   4 2624  4.689374e+00
## 23829   4 2625  4.605506e+00
## 23830   4 2626  4.530675e+00
## 23831   4 2627  4.476643e+00
## 23832   4 2628  4.415615e+00
## 23833   4 2629  4.360848e+00
## 23834   4 2630  4.312281e+00
## 23835   4 2631  4.263936e+00
## 23836   4 2632  4.225182e+00
## 23837   4 2633  4.187126e+00
## 23838   4 2634  4.149698e+00
## 23839   4 2635  4.113160e+00
## 23840   4 2636  4.068709e+00
## 23841   4 2637  4.033388e+00
## 23842   4 2638  4.001725e+00
## 23843   4 2639  3.963255e+00
## 23844   4 2640  3.935771e+00
## 23845   4 2641  5.984227e+00
## 23846   4 2642  5.664617e+00
## 23847   4 2643  5.409989e+00
## 23848   4 2644  5.208961e+00
## 23849   4 2645  5.039787e+00
## 23850   4 2646  4.890210e+00
## 23851   4 2647  4.784495e+00
## 23852   4 2648  4.683709e+00
## 23853   4 2649  4.596885e+00
## 23854   4 2650  4.524285e+00
## 23855   4 2651  4.461217e+00
## 23856   4 2652  4.397588e+00
## 23857   4 2653  4.353868e+00
## 23858   4 2654  4.301774e+00
## 23859   4 2655  4.258388e+00
## 23860   4 2656  4.221390e+00
## 23861   4 2657  4.171360e+00
## 23862   4 2658  4.139811e+00
## 23863   4 2659  4.096227e+00
## 23864   4 2660  4.056736e+00
## 23865   4 2661  4.025169e+00
## 23866   4 2662  4.001694e+00
## 23867   4 2663  3.950966e+00
## 23868   4 2664  3.929708e+00
## 23869   4 2665  5.980186e+00
## 23870   4 2666  5.653930e+00
## 23871   4 2667  5.409985e+00
## 23872   4 2668  5.193682e+00
## 23873   4 2669  5.025780e+00
## 23874   4 2670  4.891014e+00
## 23875   4 2671  4.777423e+00
## 23876   4 2672  4.673978e+00
## 23877   4 2673  4.590174e+00
## 23878   4 2674  4.513575e+00
## 23879   4 2675  4.452963e+00
## 23880   4 2676  4.404210e+00
## 23881   4 2677  4.343687e+00
## 23882   4 2678  4.304856e+00
## 23883   4 2679  4.246482e+00
## 23884   4 2680  4.198774e+00
## 23885   4 2681  4.165555e+00
## 23886   4 2682  4.124783e+00
## 23887   4 2683  4.090022e+00
## 23888   4 2684  4.052279e+00
## 23889   4 2685  4.015220e+00
## 23890   4 2686  3.973482e+00
## 23891   4 2687  3.944992e+00
## 23892   4 2688  3.920362e+00
## 23893   4 2689  5.972611e+00
## 23894   4 2690  5.649372e+00
## 23895   4 2691  5.390866e+00
## 23896   4 2692  5.194576e+00
## 23897   4 2693  5.008639e+00
## 23898   4 2694  4.880178e+00
## 23899   4 2695  4.764828e+00
## 23900   4 2696  4.666126e+00
## 23901   4 2697  4.589681e+00
## 23902   4 2698  4.505952e+00
## 23903   4 2699  4.443948e+00
## 23904   4 2700  4.387603e+00
## 23905   4 2701  4.336288e+00
## 23906   4 2702  4.290728e+00
## 23907   4 2703  4.238516e+00
## 23908   4 2704  4.204313e+00
## 23909   4 2705  4.158278e+00
## 23910   4 2706  4.123257e+00
## 23911   4 2707  4.081746e+00
## 23912   4 2708  4.046321e+00
## 23913   4 2709  4.006567e+00
## 23914   4 2710  3.975989e+00
## 23915   4 2711  3.941198e+00
## 23916   4 2712  3.905159e+00
## 23917   4 2713  5.955652e+00
## 23918   4 2714  5.651976e+00
## 23919   4 2715  5.394798e+00
## 23920   4 2716  5.179842e+00
## 23921   4 2717  5.012264e+00
## 23922   4 2718  4.875409e+00
## 23923   4 2719  4.760875e+00
## 23924   4 2720  4.664507e+00
## 23925   4 2721  4.581916e+00
## 23926   4 2722  4.502852e+00
## 23927   4 2723  4.440510e+00
## 23928   4 2724  4.383404e+00
## 23929   4 2725  4.322676e+00
## 23930   4 2726  4.284237e+00
## 23931   4 2727  4.241684e+00
## 23932   4 2728  4.197049e+00
## 23933   4 2729  4.153315e+00
## 23934   4 2730  4.112052e+00
## 23935   4 2731  4.085902e+00
## 23936   4 2732  4.044046e+00
## 23937   4 2733  4.010461e+00
## 23938   4 2734  3.975307e+00
## 23939   4 2735  3.929558e+00
## 23940   4 2736  3.903311e+00
## 23941   4 2737  5.962905e+00
## 23942   4 2738  5.644143e+00
## 23943   4 2739  5.386029e+00
## 23944   4 2740  5.187025e+00
## 23945   4 2741  5.005943e+00
## 23946   4 2742  4.876479e+00
## 23947   4 2743  4.756092e+00
## 23948   4 2744  4.655205e+00
## 23949   4 2745  4.574746e+00
## 23950   4 2746  4.511427e+00
## 23951   4 2747  4.438990e+00
## 23952   4 2748  4.381770e+00
## 23953   4 2749  4.318092e+00
## 23954   4 2750  4.275753e+00
## 23955   4 2751  4.242743e+00
## 23956   4 2752  4.188764e+00
## 23957   4 2753  4.153113e+00
## 23958   4 2754  4.112284e+00
## 23959   4 2755  4.075792e+00
## 23960   4 2756  4.036264e+00
## 23961   4 2757  3.998770e+00
## 23962   4 2758  3.973486e+00
## 23963   4 2759  3.930123e+00
## 23964   4 2760  3.903160e+00
## 23965   4 2761  5.951792e+00
## 23966   4 2762  5.643998e+00
## 23967   4 2763  5.372506e+00
## 23968   4 2764  5.171605e+00
## 23969   4 2765  5.007022e+00
## 23970   4 2766  4.863236e+00
## 23971   4 2767  4.743429e+00
## 23972   4 2768  4.646392e+00
## 23973   4 2769  4.568419e+00
## 23974   4 2770  4.494130e+00
## 23975   4 2771  4.436245e+00
## 23976   4 2772  4.370819e+00
## 23977   4 2773  4.317312e+00
## 23978   4 2774  4.282734e+00
## 23979   4 2775  4.226430e+00
## 23980   4 2776  4.181360e+00
## 23981   4 2777  4.153756e+00
## 23982   4 2778  4.102836e+00
## 23983   4 2779  4.073967e+00
## 23984   4 2780  4.037173e+00
## 23985   4 2781  3.996724e+00
## 23986   4 2782  3.963079e+00
## 23987   4 2783  3.930774e+00
## 23988   4 2784  3.898654e+00
## 23989   4 2785  5.950750e+00
## 23990   4 2786  5.634927e+00
## 23991   4 2787  5.375659e+00
## 23992   4 2788  5.169086e+00
## 23993   4 2789  4.996458e+00
## 23994   4 2790  4.864975e+00
## 23995   4 2791  4.750087e+00
## 23996   4 2792  4.652047e+00
## 23997   4 2793  4.567068e+00
## 23998   4 2794  4.492504e+00
## 23999   4 2795  4.433395e+00
## 24000   4 2796  4.372006e+00
## 24001   4 2797  4.322618e+00
## 24002   4 2798  4.270308e+00
## 24003   4 2799  4.225341e+00
## 24004   4 2800  4.184285e+00
## 24005   4 2801  4.150755e+00
## 24006   4 2802  4.107585e+00
## 24007   4 2803  4.067642e+00
## 24008   4 2804  4.029672e+00
## 24009   4 2805  3.993645e+00
## 24010   4 2806  3.957210e+00
## 24011   4 2807  3.931301e+00
## 24012   4 2808  3.897008e+00
## 24013   4 2808  3.879536e+00
## 24014   4 2809  5.934352e+00
## 24015   4 2810  5.623383e+00
## 24016   4 2811  5.358775e+00
## 24017   4 2812  5.158884e+00
## 24018   4 2813  4.994530e+00
## 24019   4 2814  4.853850e+00
## 24020   4 2815  4.745862e+00
## 24021   4 2816  4.643042e+00
## 24022   4 2817  4.548137e+00
## 24023   4 2818  4.478058e+00
## 24024   4 2819  4.421635e+00
## 24025   4 2820  4.362628e+00
## 24026   4 2821  4.300191e+00
## 24027   4 2822  4.260499e+00
## 24028   4 2823  4.216904e+00
## 24029   4 2824  4.172924e+00
## 24030   4 2825  4.131299e+00
## 24031   4 2826  4.089019e+00
## 24032   4 2827  4.053946e+00
## 24033   4 2828  4.017265e+00
## 24034   4 2829  3.980668e+00
## 24035   4 2830  3.950176e+00
```

```
# 24036  4 2831  3.921040e+00
# 24037  4 2832  3.881910e+00
# 24038  4 2832  3.881218e+00
# 24039  4 2833  3.847412e+00
# 24040  4 2834  3.819348e+00
# 24041  4 2835  3.796509e+00
# 24042  4 2836  3.760306e+00
# 24043  4 2837  3.720789e+00
# 24044  4 2838  3.698914e+00
# 24045  4 2839  3.656533e+00
# 24046  4 2840  3.631122e+00
# 24047  4 2841  3.598043e+00
# 24048  4 2842  3.570845e+00
# 24049  4 2843  3.542235e+00
# 24050  4 2844  3.508139e+00
# 24051  4 2845  3.484186e+00
# 24052  4 2846  3.453727e+00
# 24053  4 2847  3.428843e+00
# 24054  4 2848  3.395132e+00
# 24055  4 2849  3.364714e+00
# 24056  4 2850  3.338787e+00
# 24057  4 2851  3.308321e+00
# 24058  4 2852  3.288054e+00
# 24059  4 2853  3.258665e+00
# 24060  4 2853  3.261044e+00
# 24061  4 2854  4.277389e+00
# 24062  4 2855  4.107610e+00
# 24063  4 2856  3.968737e+00
# 24064  4 2856  3.962404e+00
# 24065  4 2857  5.931941e+00
# 24066  4 2858  5.557426e+00
# 24067  4 2859  5.256119e+00
# 24068  4 2860  5.010527e+00
# 24069  4 2861  4.815230e+00
# 24070  4 2862  4.662086e+00
# 24071  4 2863  4.526607e+00
# 24072  4 2864  4.418413e+00
# 24073  4 2865  4.320317e+00
# 24074  4 2866  4.248880e+00
# 24075  4 2867  4.176565e+00
# 24076  4 2868  4.117100e+00
# 24077  4 2869  4.057922e+00
# 24078  4 2870  4.015689e+00
# 24079  4 2871  3.982658e+00
# 24080  4 2872  3.932607e+00
# 24081  4 2873  3.887862e+00
# 24082  4 2874  3.853767e+00
# 24083  4 2875  3.811499e+00
# 24084  4 2876  3.779554e+00
# 24085  4 2877  3.746092e+00
# 24086  4 2878  3.709629e+00
# 24087  4 2879  3.680046e+00
# 24088  4 2880  3.658216e+00
# 24089  4 2881  5.704277e+00
# 24090  4 2882  5.389381e+00
# 24091  4 2883  5.126475e+00
# 24092  4 2884  4.932510e+00
# 24093  4 2885  4.759763e+00
# 24094  4 2886  4.622022e+00
# 24095  4 2887  4.516276e+00
# 24096  4 2888  4.423185e+00
# 24097  4 2889  4.335151e+00
# 24098  4 2890  4.266253e+00
# 24099  4 2891  4.195657e+00
# 24100  4 2892  4.146719e+00
# 24101  4 2893  4.093805e+00
# 24102  4 2894  4.051996e+00
# 24103  4 2895  4.008563e+00
# 24104  4 2896  3.962117e+00
# 24105  4 2897  3.923249e+00
# 24106  4 2898  3.891376e+00
# 24107  4 2899  3.866274e+00
# 24108  4 2900  3.830372e+00
# 24109  4 2901  3.786927e+00
# 24110  4 2902  3.758800e+00
# 24111  4 2903  3.726233e+00
# 24112  4 2904  3.690586e+00
# 24113  4 2905  5.740794e+00
# 24114  4 2906  5.436688e+00
# 24115  4 2907  5.166890e+00
# 24116  4 2908  4.979706e+00
# 24117  4 2909  4.797324e+00
# 24118  4 2910  4.669855e+00
# 24119  4 2911  4.560871e+00
# 24120  4 2912  4.463890e+00
# 24121  4 2913  4.365899e+00
# 24122  4 2914  4.301189e+00
# 24123  4 2915  4.244318e+00
# 24124  4 2916  4.182197e+00
# 24125  4 2917  4.134744e+00
# 24126  4 2918  4.083748e+00
# 24127  4 2919  4.049792e+00
# 24128  4 2920  4.003222e+00
# 24129  4 2921  3.962472e+00
# 24130  4 2922  3.930887e+00
# 24131  4 2923  3.889620e+00
# 24132  4 2924  3.859240e+00
# 24133  4 2925  3.827697e+00
# 24134  4 2926  3.786859e+00
# 24135  4 2927  3.758768e+00
# 24136  4 2928  3.725383e+00
# 24137  4 2929  5.785894e+00
# 24138  4 2930  5.462728e+00
# 24139  4 2931  5.205169e+00
# 24140  4 2932  5.008319e+00
# 24141  4 2933  4.840849e+00
# 24142  4 2934  4.695837e+00
# 24143  4 2935  4.576776e+00
# 24144  4 2936  4.492787e+00
# 24145  4 2937  4.405352e+00
# 24146  4 2938  4.331859e+00
# 24147  4 2939  4.281138e+00
# 24148  4 2940  4.216983e+00
# 24149  4 2941  4.166489e+00
# 24150  4 2942  4.122086e+00
# 24151  4 2943  4.079052e+00
# 24152  4 2944  4.032461e+00
# 24153  4 2945  3.998475e+00
# 24154  4 2946  3.961517e+00
# 24155  4 2947  3.925002e+00
# 24156  4 2948  3.876425e+00
# 24157  4 2949  3.852446e+00
# 24158  4 2950  3.815296e+00
# 24159  4 2951  3.787645e+00
# 24160  4 2952  3.747080e+00
# 24161  4 2953  5.811841e+00
# 24162  4 2954  5.490469e+00
# 24163  4 2955  5.238361e+00
# 24164  4 2956  5.033118e+00
# 24165  4 2957  4.863209e+00
# 24166  4 2958  4.714237e+00
# 24167  4 2959  4.608145e+00
# 24168  4 2960  4.517267e+00
# 24169  4 2961  4.433399e+00
# 24170  4 2962  4.361095e+00
# 24171  4 2963  4.299231e+00
# 24172  4 2964  4.241350e+00
# 24173  4 2965  4.191966e+00
# 24174  4 2966  4.141295e+00
# 24175  4 2967  4.094312e+00
# 24176  4 2968  4.057890e+00
# 24177  4 2969  4.023552e+00
# 24178  4 2970  3.983825e+00
# 24179  4 2971  3.943157e+00
# 24180  4 2972  3.912843e+00
# 24181  4 2973  3.871320e+00
# 24182  4 2974  3.841491e+00
# 24183  4 2975  3.805339e+00
# 24184  4 2976  3.779707e+00
# 24185  4 2977  5.832026e+00
# 24186  4 2978  5.519685e+00
# 24187  4 2979  5.261415e+00
# 24188  4 2980  5.060724e+00
# 24189  4 2981  4.885886e+00
# 24190  4 2982  4.747598e+00
# 24191  4 2983  4.628488e+00
# 24192  4 2984  4.538440e+00
# 24193  4 2985  4.450401e+00
# 24194  4 2986  4.385913e+00
# 24195  4 2987  4.318234e+00
# 24196  4 2988  4.271920e+00
# 24197  4 2989  4.220314e+00
# 24198  4 2990  4.163649e+00
# 24199  4 2991  4.123317e+00
# 24200  4 2992  4.079156e+00
# 24201  4 2993  4.041272e+00
# 24202  4 2994  3.998985e+00
# 24203  4 2995  3.970058e+00
# 24204  4 2996  3.929328e+00
# 24205  4 2997  3.892045e+00
# 24206  4 2998  3.854718e+00
# 24207  4 2999  3.828204e+00
# 24208  4 3000  3.798505e+00
# 24209  4 3001  5.853819e+00
# 24210  4 3002  5.537167e+00
# 24211  4 3003  5.287336e+00
# 24212  4 3004  5.068431e+00
# 24213  4 3005  4.908285e+00
# 24214  4 3006  4.769357e+00
# 24215  4 3007  4.653905e+00
# 24216  4 3008  4.554375e+00
# 24217  4 3009  4.475018e+00
# 24218  4 3010  4.393643e+00
# 24219  4 3011  4.339110e+00
# 24220  4 3012  4.281768e+00
# 24221  4 3013  4.233733e+00
# 24222  4 3014  4.185629e+00
# 24223  4 3015  4.139328e+00
# 24224  4 3016  4.098753e+00
# 24225  4 3017  4.054226e+00
# 24226  4 3018  4.019459e+00
# 24227  4 3019  3.983240e+00
# 24228  4 3020  3.947221e+00
# 24229  4 3021  3.915941e+00
# 24230  4 3022  3.879174e+00
# 24231  4 3023  3.852818e+00
# 24232  4 3024  3.813773e+00
# 24233  4 3025  5.861582e+00
# 24234  4 3026  5.559429e+00
# 24235  4 3027  5.291885e+00
# 24236  4 3028  5.091656e+00
# 24237  4 3029  4.919523e+00
# 24238  4 3030  4.780559e+00
# 24239  4 3031  4.668794e+00
# 24240  4 3032  4.568590e+00
# 24241  4 3033  4.491627e+00
# 24242  4 3034  4.424552e+00
# 24243  4 3035  4.340038e+00
# 24244  4 3036  4.295731e+00
# 24245  4 3037  4.240171e+00
# 24246  4 3038  4.192854e+00
# 24247  4 3039  4.151360e+00
# 24248  4 3040  4.107367e+00
# 24249  4 3041  4.075438e+00
# 24250  4 3042  4.036607e+00
# 24251  4 3043  3.994640e+00
# 24252  4 3044  3.961443e+00
# 24253  4 3045  3.922187e+00
# 24254  4 3046  3.895027e+00
# 24255  4 3047  3.857682e+00
# 24256  4 3048  3.820673e+00
# 24257  4 3049  5.874180e+00
# 24258  4 3050  5.564011e+00
# 24259  4 3051  5.307088e+00
# 24260  4 3052  5.106246e+00
# 24261  4 3053  4.932800e+00
# 24262  4 3054  4.792332e+00
# 24263  4 3055  4.677093e+00
# 24264  4 3056  4.579274e+00
# 24265  4 3057  4.500700e+00
# 24266  4 3058  4.432400e+00
# 24267  4 3059  4.366831e+00
# 24268  4 3060  4.307650e+00
# 24269  4 3061  4.261084e+00
# 24270  4 3062  4.213763e+00
# 24271  4 3063  4.158891e+00
# 24272  4 3064  4.119381e+00
# 24273  4 3065  4.078541e+00
# 24274  4 3066  4.043335e+00
# 24275  4 3067  4.015085e+00
# 24276  4 3068  3.969920e+00
# 24277  4 3069  3.938345e+00
# 24278  4 3070  3.899219e+00
# 24279  4 3071  3.865548e+00
# 24280  4 3072  3.831309e+00
# 24281  4 3073  5.890368e+00
# 24282  4 3074  5.566746e+00
# 24283  4 3075  5.309105e+00
# 24284  4 3076  5.106718e+00
# 24285  4 3077  4.941285e+00
# 24286  4 3078  4.801400e+00
# 24287  4 3079  4.681646e+00
# 24288  4 3080  4.593854e+00
# 24289  4 3081  4.504610e+00
# 24290  4 3082  4.426291e+00
# 24291  4 3083  4.375795e+00
# 24292  4 3084  4.315344e+00
# 24293  4 3085  4.263600e+00
# 24294  4 3086  4.217488e+00
# 24295  4 3087  4.165247e+00
# 24296  4 3088  4.129060e+00
# 24297  4 3089  4.088665e+00
# 24298  4 3090  4.049465e+00
# 24299  4 3091  4.017242e+00
# 24300  4 3092  3.975025e+00
# 24301  4 3093  3.945213e+00
# 24302  4 3094  3.906153e+00
# 24303  4 3095  3.877633e+00
# 24304  4 3096  3.845621e+00
# 24305  4 3097  5.894819e+00
# 24306  4 3098  5.578451e+00
# 24307  4 3099  5.334891e+00
# 24308  4 3100  5.122691e+00
# 24309  4 3101  4.941484e+00
# 24310  4 3102  4.817690e+00
# 24311  4 3103  4.688788e+00
# 24312  4 3104  4.598763e+00
# 24313  4 3105  4.520150e+00
# 24314  4 3106  4.436794e+00
# 24315  4 3107  4.386134e+00
# 24316  4 3108  4.323427e+00
# 24317  4 3109  4.275824e+00
# 24318  4 3110  4.229106e+00
# 24319  4 3111  4.179671e+00
# 24320  4 3112  4.142069e+00
# 24321  4 3113  4.104070e+00
# 24322  4 3114  4.055787e+00
# 24323  4 3115  4.019521e+00
# 24324  4 3116  3.982062e+00
# 24325  4 3117  3.956648e+00
# 24326  4 3118  3.915149e+00
# 24327  4 3119  3.883080e+00
# 24328  4 3120  3.843966e+00
# 24329  4 3120  3.878750e+00
# 24330  4 3121  5.950438e+00
# 24331  4 3122  5.621664e+00
# 24332  4 3123  5.356372e+00
# 24333  4 3124  5.149750e+00
# 24334  4 3125  4.992686e+00
# 24335  4 3126  4.846368e+00
# 24336  4 3127  4.728648e+00
# 24337  4 3128  4.636014e+00
# 24338  4 3129  4.551018e+00
# 24339  4 3130  4.477932e+00
# 24340  4 3131  4.411878e+00
# 24341  4 3132  4.360612e+00
# 24342  4 3133  4.301799e+00
# 24343  4 3134  4.259650e+00
# 24344  4 3135  4.212671e+00
# 24345  4 3136  4.175869e+00
# 24346  4 3137  4.143298e+00
# 24347  4 3138  4.091281e+00
# 24348  4 3139  4.059733e+00
# 24349  4 3140  4.015899e+00
# 24350  4 3141  3.980741e+00
# 24351  4 3142  3.951738e+00
# 24352  4 3143  3.914315e+00
# 24353  4 3144  3.881261e+00
# 24354  4 3144  3.879943e+00
# 24355  4 3145  3.841924e+00
# 24356  4 3146  3.811983e+00
# 24357  4 3147  3.780084e+00
# 24358  4 3148  3.751323e+00
# 24359  4 3149  3.725942e+00
# 24360  4 3150  3.685594e+00
# 24361  4 3151  3.661912e+00
# 24362  4 3152  3.634380e+00
# 24363  4 3153  3.604231e+00
# 24364  4 3154  3.571759e+00
# 24365  4 3155  3.542124e+00
# 24366  4 3156  3.511128e+00
# 24367  4 3157  3.482296e+00
# 24368  4 3158  3.451060e+00
# 24369  4 3159  3.423679e+00
# 24370  4 3160  3.393089e+00
# 24371  4 3161  3.366286e+00
# 24372  4 3162  3.340102e+00
# 24373  4 3163  3.320373e+00
# 24374  4 3164  3.284097e+00
# 24375  4 3165  3.256433e+00
# 24376  4 3165  3.256513e+00
# 24377  4 3166  5.313081e+00
# 24378  4 3167  5.003813e+00
# 24379  4 3168  4.757792e+00
# 24380  4 3168  4.753982e+00
# 24381  4 3169  6.636833e+00
# 24382  4 3170  6.196953e+00
# 24383  4 3171  5.833309e+00
# 24384  4 3172  5.543615e+00
# 24385  4 3173  5.326397e+00
# 24386  4 3174  5.139556e+00
# 24387  4 3175  4.986979e+00
# 24388  4 3176  4.852773e+00
# 24389  4 3177  4.746380e+00
# 24390  4 3178  4.662964e+00
# 24391  4 3179  4.580503e+00
# 24392  4 3180  4.514435e+00
# 24393  4 3181  4.458109e+00
# 24394  4 3182  4.398147e+00
# 24395  4 3183  4.346316e+00
# 24396  4 3184  4.308564e+00
# 24397  4 3185  4.259339e+00
# 24398  4 3186  4.211764e+00
# 24399  4 3187  4.180002e+00
# 24400  4 3188  4.133069e+00
# 24401  4 3189  4.094317e+00
# 24402  4 3190  4.060984e+00
# 24403  4 3191  4.031632e+00
# 24404  4 3192  3.995922e+00
# 24405  4 3193  6.045296e+00
# 24406  4 3194  5.722024e+00
# 24407  4 3195  5.464647e+00
# 24408  4 3196  5.260211e+00
# 24409  4 3197  5.090947e+00
# 24410  4 3198  4.951453e+00
# 24411  4 3199  4.843032e+00
# 24412  4 3200  4.739771e+00
# 24413  4 3201  4.646219e+00
# 24414  4 3202  4.576961e+00
# 24415  4 3203  4.512015e+00
# 24416  4 3204  4.458117e+00
# 24417  4 3205  4.392974e+00
# 24418  4 3206  4.361056e+00
# 24419  4 3207  4.315039e+00
# 24420  4 3208  4.258916e+00
# 24421  4 3209  4.222705e+00
# 24422  4 3210  4.187104e+00
# 24423  4 3211  4.153037e+00
# 24424  4 3212  4.108998e+00
# 24425  4 3213  4.071761e+00
# 24426  4 3214  4.032268e+00
# 24427  4 3215  4.007632e+00
# 24428  4 3216  3.969907e+00
# 24429  4 3217  6.037381e+00
# 24430  4 3218  5.708330e+00
# 24431  4 3219  5.452813e+00
# 24432  4 3220  5.248444e+00
# 24433  4 3221  5.073357e+00
# 24434  4 3222  4.937835e+00
# 24435  4 3223  4.814469e+00
# 24436  4 3224  4.722989e+00
# 24437  4 3225  4.635570e+00
# 24438  4 3226  4.562440e+00
# 24439  4 3227  4.502094e+00
# 24440  4 3228  4.441317e+00
# 24441  4 3229  4.380793e+00
# 24442  4 3230  4.337961e+00
# 24443  4 3231  4.289374e+00
# 24444  4 3232  4.255680e+00
# 24445  4 3233  4.205471e+00
# 24446  4 3234  4.176733e+00
# 24447  4 3235  4.131069e+00
# 24448  4 3236  4.089681e+00
# 24449  4 3237  4.056830e+00
# 24450  4 3238  4.007612e+00
# 24451  4 3239  3.989775e+00
# 24452  4 3240  3.953044e+00
# 24453  4 3241  6.013825e+00
# 24454  4 3242  5.688895e+00
# 24455  4 3243  5.432272e+00
# 24456  4 3244  5.225992e+00
# 24457  4 3245  5.048577e+00
# 24458  4 3246  4.920675e+00
# 24459  4 3247  4.808576e+00
# 24460  4 3248  4.701365e+00
# 24461  4 3249  4.626177e+00
# 24462  4 3250  4.548908e+00
# 24463  4 3251  4.486995e+00
# 24464  4 3252  4.421017e+00
# 24465  4 3253  4.377118e+00
# 24466  4 3254  4.322880e+00
# 24467  4 3255  4.276935e+00
# 24468  4 3256  4.227954e+00
# 24469  4 3257  4.191662e+00
# 24470  4 3258  4.156129e+00
# 24471  4 3259  4.111480e+00
# 24472  4 3260  4.078221e+00
# 24473  4 3261  4.044836e+00
# 24474  4 3262  4.015686e+00
# 24475  4 3263  3.975431e+00
# 24476  4 3264  3.949157e+00
# 24477  4 3265  5.996817e+00
# 24478  4 3266  5.678209e+00
# 24479  4 3267  5.421880e+00
# 24480  4 3268  5.216989e+00
# 24481  4 3269  5.040096e+00
# 24482  4 3270  4.906432e+00
# 24483  4 3271  4.783687e+00
# 24484  4 3272  4.692948e+00
# 24485  4 3273  4.609780e+00
# 24486  4 3274  4.537593e+00
# 24487  4 3275  4.472361e+00
# 24488  4 3276  4.411770e+00
# 24489  4 3277  4.358419e+00
# 24490  4 3278  4.313341e+00
# 24491  4 3279  4.267717e+00
# 24492  4 3280  4.232296e+00
# 24493  4 3281  4.184626e+00
# 24494  4 3282  4.138183e+00
# 24495  4 3283  4.105584e+00
# 24496  4 3284  4.074626e+00
# 24497  4 3285  4.031150e+00
# 24498  4 3286  3.998089e+00
# 24499  4 3287  3.960722e+00
# 24500  4 3288  3.932985e+00
# 24501  4 3289  5.981365e+00
# 24502  4 3290  5.665157e+00
# 24503  4 3291  5.412262e+00
# 24504  4 3292  5.206053e+00
# 24505  4 3293  5.031951e+00
# 24506  4 3294  4.899020e+00
# 24507  4 3295  4.772642e+00
# 24508  4 3296  4.682040e+00
# 24509  4 3297  4.595966e+00
# 24510  4 3298  4.524125e+00
# 24511  4 3299  4.464662e+00
# 24512  4 3300  4.402066e+00
# 24513  4 3301  4.351093e+00
# 24514  4 3302  4.298548e+00
# 24515  4 3303  4.261103e+00
# 24516  4 3304  4.211529e+00
# 24517  4 3305  4.164870e+00
# 24518  4 3306  4.125937e+00
# 24519  4 3307  4.097625e+00
# 24520  4 3308  4.054483e+00
# 24521  4 3309  4.024192e+00
# 24522  4 3310  3.988771e+00
# 24523  4 3311  3.956567e+00
# 24524  4 3312  3.929920e+00
# 24525  4 3313  5.980391e+00
# 24526  4 3314  5.659278e+00
# 24527  4 3315  5.401662e+00
# 24528  4 3316  5.191814e+00
# 24529  4 3317  5.028132e+00
# 24530  4 3318  4.880742e+00
# 24531  4 3319  4.776727e+00
# 24532  4 3320  4.680676e+00
# 24533  4 3321  4.597535e+00
# 24534  4 3322  4.520070e+00
# 24535  4 3323  4.450279e+00
# 24536  4 3324  4.388349e+00
# 24537  4 3325  4.338291e+00
# 24538  4 3326  4.293779e+00
# 24539  4 3327  4.253888e+00
# 24540  4 3328  4.211128e+00
# 24541  4 3329  4.169177e+00
# 24542  4 3330  4.127129e+00
# 24543  4 3331  4.079319e+00
# 24544  4 3332  4.050690e+00
# 24545  4 3333  4.014072e+00
# 24546  4 3334  3.985191e+00
# 24547  4 3335  3.951091e+00
# 24548  4 3336  3.922521e+00
# 24549  4 3337  5.968268e+00
# 24550  4 3338  5.642603e+00
# 24551  4 3339  5.392877e+00
# 24552  4 3340  5.177251e+00
# 24553  4 3341  5.020730e+00
# 24554  4 3342  4.884368e+00
# 24555  4 3343  4.763641e+00
# 24556  4 3344  4.663598e+00
# 24557  4 3345  4.583262e+00
# 24558  4 3346  4.515839e+00
# 24559  4 3347  4.444577e+00
# 24560  4 3348  4.395205e+00
# 24561  4 3349  4.331066e+00
# 24562  4 3350  4.286640e+00
# 24563  4 3351  4.242475e+00
# 24564  4 3352  4.197263e+00
# 24565  4 3353  4.162425e+00
# 24566  4 3354  4.115515e+00
# 24567  4 3355  4.080898e+00
# 24568  4 3356  4.051073e+00
# 24569  4 3357  4.010142e+00
# 24570  4 3358  3.968132e+00
# 24571  4 3359  3.937756e+00
# 24572  4 3360  3.913470e+00
# 24573  4 3361  5.969227e+00
# 24574  4 3362  5.638876e+00
# 24575  4 3363  5.386823e+00
# 24576  4 3364  5.182742e+00
# 24577  4 3365  5.011464e+00
# 24578  4 3366  4.875242e+00
# 24579  4 3367  4.758693e+00
# 24580  4 3368  4.661886e+00
# 24581  4 3369  4.576759e+00
# 24582  4 3370  4.506382e+00
# 24583  4 3371  4.436615e+00
# 24584  4 3372  4.382411e+00
# 24585  4 3373  4.331333e+00
# 24586  4 3374  4.282665e+00
# 24587  4 3375  4.231967e+00
# 24588  4 3376  4.201263e+00
# 24589  4 3377  4.149559e+00
# 24590  4 3378  4.115729e+00
# 24591  4 3379  4.078488e+00
# 24592  4 3380  4.039470e+00
# 24593  4 3381  4.009002e+00
# 24594  4 3382  3.970832e+00
# 24595  4 3383  3.942052e+00
# 24596  4 3384  3.903254e+00
# 24597  4 3385  5.955333e+00
# 24598  4 3386  5.639392e+00
# 24599  4 3387  5.371965e+00
# 24600  4 3388  5.180347e+00
# 24601  4 3389  5.008644e+00
# 24602  4 3390  4.869468e+00
# 24603  4 3391  4.750868e+00
# 24604  4 3392  4.661187e+00
# 24605  4 3393  4.577135e+00
# 24606  4 3394  4.498566e+00
# 24607  4 3395  4.433121e+00
# 24608  4 3396  4.383775e+00
# 24609  4 3397  4.321044e+00
# 24610  4 3398  4.277867e+00
# 24611  4 3399  4.232956e+00
# 24612  4 3400  4.193325e+00
# 24613  4 3401  4.154176e+00
# 24614  4 3402  4.116171e+00
# 24615  4 3403  4.076812e+00
# 24616  4 3404  4.038526e+00
# 24617  4 3405  3.998418e+00
# 24618  4 3406  3.965697e+00
# 24619  4 3407  3.938376e+00
# 24620  4 3408  3.894799e+00
# 24621  4 3409  5.946528e+00
# 24622  4 3410  5.631422e+00
# 24623  4 3411  5.381368e+00
# 24624  4 3412  5.171213e+00
# 24625  4 3413  5.004603e+00
# 24626  4 3414  4.873191e+00
# 24627  4 3415  4.751499e+00
# 24628  4 3416  4.658398e+00
# 24629  4 3417  4.561366e+00
# 24630  4 3418  4.488332e+00
# 24631  4 3419  4.429910e+00
# 24632  4 3420  4.382888e+00
# 24633  4 3421  4.318757e+00
# 24634  4 3422  4.279726e+00
# 24635  4 3423  4.230050e+00
# 24636  4 3424  4.191943e+00
# 24637  4 3425  4.148950e+00
# 24638  4 3426  4.114164e+00
# 24639  4 3427  4.070466e+00
# 24640  4 3428  4.031754e+00
# 24641  4 3429  3.994341e+00
# 24642  4 3430  3.966864e+00
# 24643  4 3431  3.932338e+00
# 24644  4 3432  3.897986e+00
# 24645  4 3432  3.876133e+00
# 24646  4 3433  5.940631e+00
# 24647  4 3434  5.619875e+00
# 24648  4 3435  5.357223e+00
# 24649  4 3436  5.153511e+00
# 24650  4 3437  4.986284e+00
# 24651  4 3438  4.850624e+00
# 24652  4 3439  4.726926e+00
# 24653  4 3440  4.632426e+00
# 24654  4 3441  4.556239e+00
# 24655  4 3442  4.477446e+00
# 24656  4 3443  4.420114e+00
# 24657  4 3444  4.360356e+00
# 24658  4 3445  4.301790e+00
# 24659  4 3446  4.262366e+00
# 24660  4 3447  4.215129e+00
# 24661  4 3448  4.169562e+00
# 24662  4 3449  4.124063e+00
# 24663  4 3450  4.091902e+00
# 24664  4 3451  4.056432e+00
# 24665  4 3452  4.011066e+00
# 24666  4 3453  3.980142e+00
# 24667  4 3454  3.949844e+00
# 24668  4 3455  3.918125e+00
# 24669  4 3456  3.876976e+00
# 24670  4 3456  3.879371e+00
# 24671  4 3457  3.845301e+00
# 24672  4 3458  3.819041e+00
# 24673  4 3459  3.782321e+00
# 24674  4 3460  3.746858e+00
# 24675  4 3461  3.716757e+00
# 24676  4 3462  3.685490e+00
# 24677  4 3463  3.662938e+00
# 24678  4 3464  3.625409e+00
# 24679  4 3465  3.596998e+00
# 24680  4 3466  3.564763e+00
# 24681  4 3467  3.536985e+00
# 24682  4 3468  3.509306e+00
# 24683  4 3469  3.482686e+00
# 24684  4 3470  3.450701e+00
# 24685  4 3471  3.423862e+00
# 24686  4 3472  3.396244e+00
# 24687  4 3473  3.365135e+00
# 24688  4 3474  3.336440e+00
# 24689  4 3475  3.309372e+00
# 24690  4 3476  3.282986e+00
# 24691  4 3477  3.258088e+00
# 24692  4 3478  3.230784e+00
# 24693  4 3479  3.203891e+00
# 24694  4 3480  3.178257e+00
# 24695  4 3480  3.182894e+00
# 24696  4 3481  5.238787e+00
# 24697  4 3482  4.930012e+00
# 24698  4 3483  4.671514e+00
# 24699  4 3484  4.475357e+00
# 24700  4 3485  4.314606e+00
# 24701  4 3486  4.180640e+00
# 24702  4 3487  4.071022e+00
# 24703  4 3488  3.980721e+00
# 24704  4 3489  3.896095e+00
# 24705  4 3490  3.826983e+00
# 24706  4 3491  3.778158e+00
# 24707  4 3492  3.727736e+00
# 24708  4 3493  3.677237e+00
# 24709  4 3494  3.633144e+00
# 24710  4 3495  3.595226e+00
# 24711  4 3496  3.557413e+00
# 24712  4 3497  3.525121e+00
# 24713  4 3498  3.495587e+00
# 24714  4 3499  3.454819e+00
# 24715  4 3500  3.426509e+00
# 24716  4 3501  3.392667e+00
# 24717  4 3502  3.360221e+00
# 24718  4 3503  3.332064e+00
# 24719  4 3504  3.309221e+00
# 24720  4 3504  3.303591e+00
# 24721  4 3505  5.366005e+00
# 24722  4 3506  5.054008e+00
# 24723  4 3507  4.804708e+00
# 24724  4 3508  4.594898e+00
# 24725  4 3509  4.434254e+00
# 24726  4 3510  4.302800e+00
# 24727  4 3511  4.189377e+00
# 24728  4 3512  4.092641e+00
# 24729  4 3513  4.012572e+00
# 24730  4 3514  3.947222e+00
# 24731  4 3515  3.898013e+00
# 24732  4 3516  3.837383e+00
# 24733  4 3517  3.793438e+00
# 24734  4 3518  3.747310e+00
# 24735  4 3519  3.706568e+00
# 24736  4 3520  3.668475e+00
# 24737  4 3521  3.631147e+00
# 24738  4 3522  3.602165e+00
# 24739  4 3523  3.559895e+00
# 24740  4 3524  3.529800e+00
# 24741  4 3525  3.498701e+00
# 24742  4 3526  3.471224e+00
# 24743  4 3527  3.437533e+00
# 24744  4 3528  3.413065e+00
# 24745  4 3529  5.470869e+00
# 24746  4 3530  5.151656e+00
# 24747  4 3531  4.898791e+00
# 24748  4 3532  4.706732e+00
# 24749  4 3533  4.531250e+00
# 24750  4 3534  4.407277e+00
# 24751  4 3535  4.291112e+00
# 24752  4 3536  4.193129e+00
# 24753  4 3537  4.124057e+00
# 24754  4 3538  4.044072e+00
# 24755  4 3539  3.990093e+00
# 24756  4 3540  3.929580e+00
# 24757  4 3541  3.881892e+00
# 24758  4 3542  3.835526e+00
# 24759  4 3543  3.797862e+00
# 24760  4 3544  3.758307e+00
# 24761  4 3545  3.721470e+00
# 24762  4 3546  3.687123e+00
# 24763  4 3547  3.647246e+00
# 24764  4 3548  3.622859e+00
# 24765  4 3549  3.591305e+00
# 24766  4 3550  3.557186e+00
# 24767  4 3551  3.524622e+00
# 24768  4 3552  3.490703e+00
# 24769  4 3553  5.553745e+00
# 24770  4 3554  5.243782e+00
# 24771  4 3555  4.987777e+00
# 24772  4 3556  4.783679e+00
# 24773  4 3557  4.612888e+00
# 24774  4 3558  4.488978e+00
# 24775  4 3559  4.366679e+00
# 24776  4 3560  4.272484e+00
# 24777  4 3561  4.193435e+00
# 24778  4 3562  4.125730e+00
# 24779  4 3563  4.063460e+00
# 24780  4 3564  4.009890e+00
# 24781  4 3565  3.962405e+00
# 24782  4 3566  3.914667e+00
# 24783  4 3567  3.880326e+00
# 24784  4 3568  3.831123e+00
# 24785  4 3569  3.794874e+00
# 24786  4 3570  3.757880e+00
# 24787  4 3571  3.722764e+00
# 24788  4 3572  3.688378e+00
# 24789  4 3573  3.659227e+00
# 24790  4 3574  3.627568e+00
# 24791  4 3575  3.593875e+00
# 24792  4 3576  3.562325e+00
# 24793  4 3577  5.614628e+00
# 24794  4 3578  5.305527e+00
# 24795  4 3579  5.050775e+00
# 24796  4 3580  4.841937e+00
# 24797  4 3581  4.685927e+00
# 24798  4 3582  4.551485e+00
# 24799  4 3583  4.433799e+00
# 24800  4 3584  4.337568e+00
# 24801  4 3585  4.260768e+00
# 24802  4 3586  4.185663e+00
# 24803  4 3587  4.118215e+00
# 24804  4 3588  4.077337e+00
# 24805  4 3589  4.024496e+00
# 24806  4 3590  3.976832e+00
# 24807  4 3591  3.931290e+00
# 24808  4 3592  3.896560e+00
# 24809  4 3593  3.853514e+00
# 24810  4 3594  3.817139e+00
# 24811  4 3595  3.785800e+00
# 24812  4 3596  3.752718e+00
# 24813  4 3597  3.718337e+00
# 24814  4 3598  3.693675e+00
# 24815  4 3599  3.655923e+00
# 24816  4 3600  3.622997e+00
# 24817  4 3601  5.677560e+00
# 24818  4 3602  5.364219e+00
# 24819  4 3603  5.104148e+00
# 24820  4 3604  4.904131e+00
# 24821  4 3605  4.734417e+00
# 24822  4 3606  4.599899e+00
# 24823  4 3607  4.490122e+00
# 24824  4 3608  4.390724e+00
# 24825  4 3609  4.314149e+00
# 24826  4 3610  4.248846e+00
# 24827  4 3611  4.182064e+00
# 24828  4 3612  4.126742e+00
# 24829  4 3613  4.066298e+00
# 24830  4 3614  4.031472e+00
# 24831  4 3615  3.978975e+00
# 24832  4 3616  3.948025e+00
# 24833  4 3617  3.904023e+00
# 24834  4 3618  3.879740e+00
# 24835  4 3619  3.831198e+00
```

```
## 24836  4 3620  3.801019e+00
## 24837  4 3621  3.761649e+00
## 24838  4 3622  3.729534e+00
## 24839  4 3623  3.701164e+00
## 24840  4 3624  3.674338e+00
## 24841  4 3625  5.719071e+00
## 24842  4 3626  5.409696e+00
## 24843  4 3627  5.156503e+00
## 24844  4 3628  4.954052e+00
## 24845  4 3629  4.785891e+00
## 24846  4 3630  4.648378e+00
## 24847  4 3631  4.537217e+00
## 24848  4 3632  4.438801e+00
## 24849  4 3633  4.353642e+00
## 24850  4 3634  4.283061e+00
## 24851  4 3635  4.223874e+00
## 24852  4 3636  4.166008e+00
## 24853  4 3637  4.113882e+00
## 24854  4 3638  4.071040e+00
## 24855  4 3639  4.028486e+00
## 24856  4 3640  3.981928e+00
## 24857  4 3641  3.947108e+00
## 24858  4 3642  3.912784e+00
## 24859  4 3643  3.871505e+00
## 24860  4 3644  3.841170e+00
## 24861  4 3645  3.810201e+00
## 24862  4 3646  3.771516e+00
## 24863  4 3647  3.735721e+00
## 24864  4 3648  3.708750e+00
## 24865  4 3649  5.757030e+00
## 24866  4 3650  5.449830e+00
## 24867  4 3651  5.195489e+00
## 24868  4 3652  4.987851e+00
## 24869  4 3653  4.819384e+00
## 24870  4 3654  4.675909e+00
## 24871  4 3655  4.571423e+00
## 24872  4 3656  4.475770e+00
## 24873  4 3657  4.394107e+00
## 24874  4 3658  4.321860e+00
## 24875  4 3659  4.256019e+00
## 24876  4 3660  4.205895e+00
## 24877  4 3661  4.158911e+00
## 24878  4 3662  4.102961e+00
## 24879  4 3663  4.061867e+00
## 24880  4 3664  4.016969e+00
## 24881  4 3665  3.979481e+00
## 24882  4 3666  3.943828e+00
## 24883  4 3667  3.905537e+00
## 24884  4 3668  3.876710e+00
## 24885  4 3669  3.838673e+00
## 24886  4 3670  3.797334e+00
## 24887  4 3671  3.770949e+00
## 24888  4 3672  3.744833e+00
## 24889  4 3673  5.801528e+00
## 24890  4 3674  5.483656e+00
## 24891  4 3675  5.232128e+00
## 24892  4 3676  5.013552e+00
## 24893  4 3677  4.850372e+00
## 24894  4 3678  4.717636e+00
## 24895  4 3679  4.594620e+00
## 24896  4 3680  4.508979e+00
## 24897  4 3681  4.421785e+00
## 24898  4 3682  4.343794e+00
## 24899  4 3683  4.285556e+00
## 24900  4 3684  4.228774e+00
## 24901  4 3685  4.180822e+00
## 24902  4 3686  4.137819e+00
## 24903  4 3687  4.082430e+00
## 24904  4 3688  4.046438e+00
## 24905  4 3689  4.004797e+00
## 24906  4 3690  3.969703e+00
## 24907  4 3691  3.936740e+00
## 24908  4 3692  3.892145e+00
## 24909  4 3693  3.861919e+00
## 24910  4 3694  3.831569e+00
## 24911  4 3695  3.791660e+00
## 24912  4 3696  3.763904e+00
## 24913  4 3697  5.823771e+00
## 24914  4 3698  5.503543e+00
## 24915  4 3699  5.249318e+00
## 24916  4 3700  5.043325e+00
## 24917  4 3701  4.876600e+00
## 24918  4 3702  4.732332e+00
## 24919  4 3703  4.613616e+00
## 24920  4 3704  4.533374e+00
## 24921  4 3705  4.443762e+00
## 24922  4 3706  4.366728e+00
## 24923  4 3707  4.309199e+00
## 24924  4 3708  4.247695e+00
## 24925  4 3709  4.208000e+00
## 24926  4 3710  4.150517e+00
## 24927  4 3711  4.109549e+00
## 24928  4 3712  4.069565e+00
## 24929  4 3713  4.018461e+00
## 24930  4 3714  3.997354e+00
## 24931  4 3715  3.958582e+00
## 24932  4 3716  3.925293e+00
## 24933  4 3717  3.887362e+00
## 24934  4 3718  3.845682e+00
## 24935  4 3719  3.814947e+00
## 24936  4 3720  3.790703e+00
## 24937  4 3721  5.843231e+00
## 24938  4 3722  5.533469e+00
## 24939  4 3723  5.274891e+00
## 24940  4 3724  5.059300e+00
## 24941  4 3725  4.889428e+00
## 24942  4 3726  4.758996e+00
## 24943  4 3727  4.638113e+00
## 24944  4 3728  4.550498e+00
## 24945  4 3729  4.462255e+00
## 24946  4 3730  4.401570e+00
## 24947  4 3731  4.329450e+00
## 24948  4 3732  4.268743e+00
## 24949  4 3733  4.229547e+00
## 24950  4 3734  4.169617e+00
## 24951  4 3735  4.129699e+00
## 24952  4 3736  4.082980e+00
## 24953  4 3737  4.046688e+00
## 24954  4 3738  4.012427e+00
## 24955  4 3739  3.978843e+00
## 24956  4 3740  3.931949e+00
## 24957  4 3741  3.897783e+00
## 24958  4 3742  3.871441e+00
## 24959  4 3743  3.838246e+00
## 24960  4 3744  3.804344e+00
## 24961  4 3745  5.864124e+00
## 24962  4 3746  5.533878e+00
## 24963  4 3747  5.292853e+00
## 24964  4 3748  5.083012e+00
## 24965  4 3749  4.914226e+00
## 24966  4 3750  4.778080e+00
## 24967  4 3751  4.656308e+00
## 24968  4 3752  4.556569e+00
## 24969  4 3753  4.475969e+00
## 24970  4 3754  4.398709e+00
## 24971  4 3755  4.334013e+00
## 24972  4 3756  4.287236e+00
## 24973  4 3757  4.241458e+00
## 24974  4 3758  4.188913e+00
## 24975  4 3759  4.146561e+00
## 24976  4 3760  4.107322e+00
## 24977  4 3761  4.063523e+00
## 24978  4 3762  4.019120e+00
## 24979  4 3763  3.990355e+00
## 24980  4 3764  3.946498e+00
## 24981  4 3765  3.914653e+00
## 24982  4 3766  3.885422e+00
## 24983  4 3767  3.854875e+00
## 24984  4 3768  3.818191e+00
## 24985  4 3768  3.883660e+00
## 24986  4 3769  5.935146e+00
## 24987  4 3770  5.615098e+00
## 24988  4 3771  5.364866e+00
## 24989  4 3772  5.164141e+00
## 24990  4 3773  4.992109e+00
## 24991  4 3774  4.853003e+00
## 24992  4 3775  4.733044e+00
## 24993  4 3776  4.640521e+00
## 24994  4 3777  4.560144e+00
## 24995  4 3778  4.487280e+00
## 24996  4 3779  4.407243e+00
## 24997  4 3780  4.357917e+00
## 24998  4 3781  4.302465e+00
## 24999  4 3782  4.261113e+00
## 25000  4 3783  4.209261e+00
## 25001  4 3784  4.173437e+00
## 25002  4 3785  4.132202e+00
## 25003  4 3786  4.092733e+00
## 25004  4 3787  4.054180e+00
## 25005  4 3788  4.022994e+00
## 25006  4 3789  3.985275e+00
## 25007  4 3790  3.948981e+00
## 25008  4 3791  3.912299e+00
## 25009  4 3792  3.887852e+00
## 25010  4 3792  3.887102e+00
## 25011  4 3793  3.859865e+00
## 25012  4 3794  3.814881e+00
## 25013  4 3795  3.782669e+00
## 25014  4 3796  3.750172e+00
## 25015  4 3797  3.722467e+00
## 25016  4 3798  3.688282e+00
## 25017  4 3799  3.659017e+00
## 25018  4 3800  3.627237e+00
## 25019  4 3801  3.603869e+00
## 25020  4 3802  3.575808e+00
## 25021  4 3803  3.539606e+00
## 25022  4 3804  3.513451e+00
## 25023  4 3805  3.484175e+00
## 25024  4 3806  3.452850e+00
## 25025  4 3807  3.425700e+00
## 25026  4 3808  3.395639e+00
## 25027  4 3809  3.360831e+00
## 25028  4 3810  3.347126e+00
## 25029  4 3811  3.314265e+00
## 25030  4 3812  3.285736e+00
## 25031  4 3813  3.259540e+00
## 25032  4 3814  3.228663e+00
## 25033  4 3815  3.201714e+00
## 25034  4 3816  3.171982e+00
## 25035  4 3816  3.181241e+00
## 25036  4 3817  6.289542e+00
## 25037  4 3818  5.823392e+00
## 25038  4 3819  5.458868e+00
## 25039  4 3820  5.179927e+00
## 25040  4 3821  4.954870e+00
## 25041  4 3822  4.762470e+00
## 25042  4 3823  4.601449e+00
## 25043  4 3824  4.483456e+00
## 25044  4 3825  4.377106e+00
## 25045  4 3826  4.290186e+00
## 25046  4 3827  4.215162e+00
## 25047  4 3828  4.147464e+00
## 25048  4 3829  4.086600e+00
## 25049  4 3830  4.037938e+00
## 25050  4 3831  3.986616e+00
## 25051  4 3832  3.943370e+00
## 25052  4 3833  3.904968e+00
## 25053  4 3834  3.860002e+00
## 25054  4 3835  3.832111e+00
## 25055  4 3836  3.789471e+00
## 25056  4 3837  3.757921e+00
## 25057  4 3838  3.726798e+00
## 25058  4 3839  3.687046e+00
## 25059  4 3840  3.661866e+00
## 25060  4 3840  3.656817e+00
## 25061  4 3841  5.720653e+00
## 25062  4 3842  5.397967e+00
## 25063  4 3843  5.147151e+00
## 25064  4 3844  4.939199e+00
## 25065  4 3845  4.771760e+00
## 25066  4 3846  4.635782e+00
## 25067  4 3847  4.529935e+00
## 25068  4 3848  4.425598e+00
## 25069  4 3849  4.347816e+00
## 25070  4 3850  4.278856e+00
## 25071  4 3851  4.205765e+00
## 25072  4 3852  4.151081e+00
## 25073  4 3853  4.109271e+00
## 25074  4 3854  4.061965e+00
## 25075  4 3855  4.012483e+00
## 25076  4 3856  3.973298e+00
## 25077  4 3857  3.938327e+00
## 25078  4 3858  3.903177e+00
## 25079  4 3859  3.862378e+00
## 25080  4 3860  3.833972e+00
## 25081  4 3861  3.781828e+00
## 25082  4 3862  3.765857e+00
## 25083  4 3863  3.730342e+00
## 25084  4 3864  3.701416e+00
## 25085  4 3865  5.759980e+00
## 25086  4 3866  5.448448e+00
## 25087  4 3867  5.189088e+00
## 25088  4 3868  4.976003e+00
## 25089  4 3869  4.813895e+00
## 25090  4 3870  4.671310e+00
## 25091  4 3871  4.559161e+00
## 25092  4 3872  4.467099e+00
## 25093  4 3873  4.382067e+00
## 25094  4 3874  4.309928e+00
## 25095  4 3875  4.242326e+00
## 25096  4 3876  4.194369e+00
## 25097  4 3877  4.140723e+00
## 25098  4 3878  4.094849e+00
## 25099  4 3879  4.050008e+00
## 25100  4 3880  4.008237e+00
## 25101  4 3881  3.966020e+00
## 25102  4 3882  3.931572e+00
## 25103  4 3883  3.903209e+00
## 25104  4 3884  3.864443e+00
## 25105  4 3885  3.826993e+00
## 25106  4 3886  3.791345e+00
## 25107  4 3887  3.758939e+00
## 25108  4 3888  3.732617e+00
## 25109  4 3889  5.782310e+00
## 25110  4 3890  5.470446e+00
## 25111  4 3891  5.216754e+00
## 25112  4 3892  5.014308e+00
## 25113  4 3893  4.841305e+00
## 25114  4 3894  4.705633e+00
## 25115  4 3895  4.586477e+00
## 25116  4 3896  4.494669e+00
## 25117  4 3897  4.415597e+00
## 25118  4 3898  4.331612e+00
## 25119  4 3899  4.276035e+00
## 25120  4 3900  4.220177e+00
## 25121  4 3901  4.173511e+00
## 25122  4 3902  4.128156e+00
## 25123  4 3903  4.084245e+00
## 25124  4 3904  4.042843e+00
## 25125  4 3905  4.003878e+00
## 25126  4 3906  3.963052e+00
## 25127  4 3907  3.920992e+00
## 25128  4 3908  3.898112e+00
## 25129  4 3909  3.860067e+00
## 25130  4 3910  3.828444e+00
## 25131  4 3911  3.794586e+00
## 25132  4 3912  3.754537e+00
## 25133  4 3913  5.815037e+00
## 25134  4 3914  5.482031e+00
## 25135  4 3915  5.242207e+00
## 25136  4 3916  5.042726e+00
## 25137  4 3917  4.859306e+00
## 25138  4 3918  4.732392e+00
## 25139  4 3919  4.615925e+00
## 25140  4 3920  4.512551e+00
## 25141  4 3921  4.445518e+00
## 25142  4 3922  4.361165e+00
## 25143  4 3923  4.300432e+00
## 25144  4 3924  4.250569e+00
## 25145  4 3925  4.184370e+00
## 25146  4 3926  4.154580e+00
## 25147  4 3927  4.099969e+00
## 25148  4 3928  4.061713e+00
## 25149  4 3929  4.026802e+00
## 25150  4 3930  3.988048e+00
## 25151  4 3931  3.953754e+00
## 25152  4 3932  3.919692e+00
## 25153  4 3933  3.873040e+00
## 25154  4 3934  3.849404e+00
## 25155  4 3935  3.809637e+00
## 25156  4 3936  3.780001e+00
## 25157  4 3937  5.845030e+00
## 25158  4 3938  5.508895e+00
## 25159  4 3939  5.266882e+00
## 25160  4 3940  5.050610e+00
## 25161  4 3941  4.891046e+00
## 25162  4 3942  4.760522e+00
## 25163  4 3943  4.628391e+00
## 25164  4 3944  4.546101e+00
## 25165  4 3945  4.463327e+00
## 25166  4 3946  4.384700e+00
## 25167  4 3947  4.322966e+00
## 25168  4 3948  4.269025e+00
## 25169  4 3949  4.212454e+00
## 25170  4 3950  4.168968e+00
## 25171  4 3951  4.124556e+00
## 25172  4 3952  4.080309e+00
## 25173  4 3953  4.041438e+00
## 25174  4 3954  4.003831e+00
## 25175  4 3955  3.967682e+00
## 25176  4 3956  3.927773e+00
## 25177  4 3957  3.895742e+00
## 25178  4 3958  3.867015e+00
## 25179  4 3959  3.830124e+00
## 25180  4 3960  3.802646e+00
## 25181  4 3961  5.865133e+00
## 25182  4 3962  5.546096e+00
## 25183  4 3963  5.283975e+00
## 25184  4 3964  5.070970e+00
## 25185  4 3965  4.903424e+00
## 25186  4 3966  4.769616e+00
## 25187  4 3967  4.655531e+00
## 25188  4 3968  4.562436e+00
## 25189  4 3969  4.466226e+00
## 25190  4 3970  4.405646e+00
## 25191  4 3971  4.336767e+00
## 25192  4 3972  4.288441e+00
## 25193  4 3973  4.239607e+00
## 25194  4 3974  4.179931e+00
## 25195  4 3975  4.134795e+00
## 25196  4 3976  4.099371e+00
## 25197  4 3977  4.063427e+00
## 25198  4 3978  4.023547e+00
## 25199  4 3979  3.985574e+00
## 25200  4 3980  3.953261e+00
## 25201  4 3981  3.919854e+00
## 25202  4 3982  3.879136e+00
## 25203  4 3983  3.847272e+00
## 25204  4 3984  3.814546e+00
## 25205  4 3985  5.871541e+00
## 25206  4 3986  5.546581e+00
## 25207  4 3987  5.301682e+00
## 25208  4 3988  5.097677e+00
## 25209  4 3989  4.928036e+00
## 25210  4 3990  4.775589e+00
## 25211  4 3991  4.665855e+00
## 25212  4 3992  4.572999e+00
## 25213  4 3993  4.494623e+00
## 25214  4 3994  4.418333e+00
## 25215  4 3995  4.350273e+00
## 25216  4 3996  4.294865e+00
## 25217  4 3997  4.246397e+00
## 25218  4 3998  4.200863e+00
## 25219  4 3999  4.153746e+00
## 25220  4 4000  4.107045e+00
## 25221  4 4001  4.072965e+00
## 25222  4 4002  4.029877e+00
## 25223  4 4003  4.000094e+00
## 25224  4 4004  3.955829e+00
## 25225  4 4005  3.924773e+00
## 25226  4 4006  3.890722e+00
## 25227  4 4007  3.859519e+00
## 25228  4 4008  3.828488e+00
## 25229  4 4009  5.884030e+00
## 25230  4 4010  5.559475e+00
## 25231  4 4011  5.308213e+00
## 25232  4 4012  5.096350e+00
## 25233  4 4013  4.939854e+00
## 25234  4 4014  4.792972e+00
## 25235  4 4015  4.689929e+00
## 25236  4 4016  4.580941e+00
## 25237  4 4017  4.499417e+00
## 25238  4 4018  4.428780e+00
## 25239  4 4019  4.362762e+00
## 25240  4 4020  4.313134e+00
## 25241  4 4021  4.253011e+00
## 25242  4 4022  4.201777e+00
## 25243  4 4023  4.168253e+00
## 25244  4 4024  4.124963e+00
## 25245  4 4025  4.078168e+00
## 25246  4 4026  4.052092e+00
## 25247  4 4027  4.010152e+00
## 25248  4 4028  3.972116e+00
## 25249  4 4029  3.936764e+00
## 25250  4 4030  3.905273e+00
## 25251  4 4031  3.866152e+00
## 25252  4 4032  3.839656e+00
## 25253  4 4033  5.893710e+00
## 25254  4 4034  5.571589e+00
## 25255  4 4035  5.319534e+00
## 25256  4 4036  5.112675e+00
## 25257  4 4037  4.948330e+00
## 25258  4 4038  4.805627e+00
## 25259  4 4039  4.692759e+00
## 25260  4 4040  4.597164e+00
## 25261  4 4041  4.519049e+00
## 25262  4 4042  4.432600e+00
## 25263  4 4043  4.368101e+00
## 25264  4 4044  4.322983e+00
## 25265  4 4045  4.264662e+00
## 25266  4 4046  4.214519e+00
## 25267  4 4047  4.178368e+00
## 25268  4 4048  4.131163e+00
## 25269  4 4049  4.091310e+00
## 25270  4 4050  4.050773e+00
## 25271  4 4051  4.021591e+00
## 25272  4 4052  3.979711e+00
## 25273  4 4053  3.950483e+00
## 25274  4 4054  3.907938e+00
## 25275  4 4055  3.879066e+00
## 25276  4 4056  3.842035e+00
## 25277  4 4057  5.895591e+00
## 25278  4 4058  5.586250e+00
## 25279  4 4059  5.315676e+00
## 25280  4 4060  5.118570e+00
## 25281  4 4061  4.950189e+00
## 25282  4 4062  4.814983e+00
## 25283  4 4063  4.698069e+00
## 25284  4 4064  4.605193e+00
## 25285  4 4065  4.525155e+00
## 25286  4 4066  4.444321e+00
## 25287  4 4067  4.386636e+00
## 25288  4 4068  4.323020e+00
## 25289  4 4069  4.279958e+00
## 25290  4 4070  4.226120e+00
## 25291  4 4071  4.185911e+00
## 25292  4 4072  4.139993e+00
## 25293  4 4073  4.099742e+00
## 25294  4 4074  4.061485e+00
## 25295  4 4075  4.015517e+00
## 25296  4 4076  3.985116e+00
## 25297  4 4077  3.952992e+00
## 25298  4 4078  3.917818e+00
## 25299  4 4079  3.889344e+00
## 25300  4 4080  3.854686e+00
## 25301  4 4081  5.887461e+00
## 25302  4 4082  5.583963e+00
## 25303  4 4083  5.328342e+00
## 25304  4 4084  5.120054e+00
## 25305  4 4085  4.955676e+00
## 25306  4 4086  4.823801e+00
## 25307  4 4087  4.707592e+00
## 25308  4 4088  4.607007e+00
## 25309  4 4089  4.524620e+00
## 25310  4 4090  4.447913e+00
## 25311  4 4091  4.392498e+00
## 25312  4 4092  4.331296e+00
## 25313  4 4093  4.276920e+00
## 25314  4 4094  4.230756e+00
## 25315  4 4095  4.186068e+00
## 25316  4 4096  4.138855e+00
## 25317  4 4097  4.107452e+00
## 25318  4 4098  4.067997e+00
## 25319  4 4099  4.033081e+00
## 25320  4 4100  3.985430e+00
## 25321  4 4101  3.958042e+00
## 25322  4 4102  3.918943e+00
## 25323  4 4103  3.883015e+00
## 25324  4 4104  3.851969e+00
## 25325  4 4104  3.880176e+00
## 25326  4 4105  5.934314e+00
## 25327  4 4106  5.616081e+00
## 25328  4 4107  5.365050e+00
## 25329  4 4108  5.151687e+00
## 25330  4 4109  4.982689e+00
## 25331  4 4110  4.840929e+00
## 25332  4 4111  4.731688e+00
## 25333  4 4112  4.638462e+00
## 25334  4 4113  4.549399e+00
## 25335  4 4114  4.479690e+00
## 25336  4 4115  4.418080e+00
## 25337  4 4116  4.358562e+00
## 25338  4 4117  4.301604e+00
## 25339  4 4118  4.260133e+00
## 25340  4 4119  4.213401e+00
## 25341  4 4120  4.167298e+00
## 25342  4 4121  4.136334e+00
## 25343  4 4122  4.088346e+00
## 25344  4 4123  4.049754e+00
## 25345  4 4124  4.013450e+00
## 25346  4 4125  3.980568e+00
## 25347  4 4126  3.949501e+00
## 25348  4 4127  3.914124e+00
## 25349  4 4128  3.877797e+00
## 25350  4 4128  3.883619e+00
## 25351  4 4129  3.852672e+00
## 25352  4 4130  3.816407e+00
## 25353  4 4131  3.786299e+00
## 25354  4 4132  3.750826e+00
## 25355  4 4133  3.717580e+00
## 25356  4 4134  3.689437e+00
## 25357  4 4135  3.662130e+00
## 25358  4 4136  3.624727e+00
## 25359  4 4137  3.600247e+00
## 25360  4 4138  3.566981e+00
## 25361  4 4139  3.547084e+00
## 25362  4 4140  3.511454e+00
## 25363  4 4141  3.485985e+00
## 25364  4 4142  3.455425e+00
## 25365  4 4143  3.422286e+00
## 25366  4 4144  3.398771e+00
## 25367  4 4145  3.364226e+00
## 25368  4 4146  3.338548e+00
## 25369  4 4147  3.307853e+00
## 25370  4 4148  3.288063e+00
## 25371  4 4149  3.262693e+00
## 25372  4 4150  3.231685e+00
## 25373  4 4151  3.206448e+00
## 25374  4 4152  3.180186e+00
## 25375  4 4152  3.173747e+00
## 25376  4 4153  7.322246e+00
## 25377  4 4154  6.727335e+00
## 25378  4 4155  6.264515e+00
## 25379  4 4156  5.878090e+00
## 25380  4 4157  5.585950e+00
## 25381  4 4158  5.341726e+00
## 25382  4 4159  5.147957e+00
## 25383  4 4160  4.985999e+00
## 25384  4 4161  4.845207e+00
## 25385  4 4162  4.738562e+00
## 25386  4 4163  4.655119e+00
## 25387  4 4164  4.564036e+00
## 25388  4 4165  4.503707e+00
## 25389  4 4166  4.439402e+00
## 25390  4 4167  4.388942e+00
## 25391  4 4168  4.332433e+00
## 25392  4 4169  4.278375e+00
## 25393  4 4170  4.235639e+00
## 25394  4 4171  4.202098e+00
## 25395  4 4172  4.159726e+00
## 25396  4 4173  4.120506e+00
## 25397  4 4174  4.077749e+00
## 25398  4 4175  4.050628e+00
## 25399  4 4176  4.013521e+00
## 25400  4 4176  4.009910e+00
## 25401  4 4177  6.068741e+00
## 25402  4 4178  5.745774e+00
## 25403  4 4179  5.491435e+00
## 25404  4 4180  5.270904e+00
## 25405  4 4181  5.113105e+00
## 25406  4 4182  4.980662e+00
## 25407  4 4183  4.847275e+00
## 25408  4 4184  4.760785e+00
## 25409  4 4185  4.664480e+00
## 25410  4 4186  4.586407e+00
## 25411  4 4187  4.534237e+00
## 25412  4 4188  4.480239e+00
## 25413  4 4189  4.418779e+00
## 25414  4 4190  4.373137e+00
## 25415  4 4191  4.323514e+00
## 25416  4 4192  4.281575e+00
## 25417  4 4193  4.240930e+00
## 25418  4 4194  4.204536e+00
## 25419  4 4195  4.163931e+00
## 25420  4 4196  4.119825e+00
## 25421  4 4197  4.097455e+00
## 25422  4 4198  4.055798e+00
## 25423  4 4199  4.012172e+00
## 25424  4 4200  3.979800e+00
## 25425  4 4201  6.037699e+00
## 25426  4 4202  5.722502e+00
## 25427  4 4203  5.459967e+00
## 25428  4 4204  5.256094e+00
## 25429  4 4205  5.088999e+00
## 25430  4 4206  4.940607e+00
## 25431  4 4207  4.826942e+00
## 25432  4 4208  4.726506e+00
## 25433  4 4209  4.645269e+00
## 25434  4 4210  4.568609e+00
## 25435  4 4211  4.501380e+00
## 25436  4 4212  4.454176e+00
## 25437  4 4213  4.403668e+00
## 25438  4 4214  4.356154e+00
## 25439  4 4215  4.309429e+00
## 25440  4 4216  4.259574e+00
## 25441  4 4217  4.222290e+00
## 25442  4 4218  4.180862e+00
## 25443  4 4219  4.142366e+00
## 25444  4 4220  4.102559e+00
## 25445  4 4221  4.075066e+00
## 25446  4 4222  4.026785e+00
## 25447  4 4223  4.001756e+00
## 25448  4 4224  3.967517e+00
## 25449  4 4225  6.031270e+00
## 25450  4 4226  5.704822e+00
## 25451  4 4227  5.440885e+00
## 25452  4 4228  5.238626e+00
## 25453  4 4229  5.062517e+00
## 25454  4 4230  4.926603e+00
## 25455  4 4231  4.805895e+00
## 25456  4 4232  4.715862e+00
## 25457  4 4233  4.627086e+00
## 25458  4 4234  4.554830e+00
## 25459  4 4235  4.498752e+00
## 25460  4 4236  4.437406e+00
## 25461  4 4237  4.382804e+00
## 25462  4 4238  4.339367e+00
## 25463  4 4239  4.281980e+00
## 25464  4 4240  4.241751e+00
## 25465  4 4241  4.195643e+00
## 25466  4 4242  4.163950e+00
## 25467  4 4243  4.138425e+00
## 25468  4 4244  4.087430e+00
## 25469  4 4245  4.048596e+00
## 25470  4 4246  4.015768e+00
## 25471  4 4247  3.982725e+00
## 25472  4 4248  3.945873e+00
## 25473  4 4249  6.002998e+00
## 25474  4 4250  5.683417e+00
## 25475  4 4251  5.425666e+00
## 25476  4 4252  5.220492e+00
## 25477  4 4253  5.060976e+00
## 25478  4 4254  4.908682e+00
## 25479  4 4255  4.805236e+00
## 25480  4 4256  4.693853e+00
## 25481  4 4257  4.617486e+00
## 25482  4 4258  4.544975e+00
## 25483  4 4259  4.477676e+00
## 25484  4 4260  4.413656e+00
## 25485  4 4261  4.371008e+00
## 25486  4 4262  4.328722e+00
## 25487  4 4263  4.275148e+00
## 25488  4 4264  4.235244e+00
## 25489  4 4265  4.196393e+00
## 25490  4 4266  4.142889e+00
## 25491  4 4267  4.109182e+00
## 25492  4 4268  4.080384e+00
## 25493  4 4269  4.040832e+00
## 25494  4 4270  4.001578e+00
## 25495  4 4271  3.971651e+00
## 25496  4 4272  3.942958e+00
## 25497  4 4273  5.994791e+00
## 25498  4 4274  5.675843e+00
## 25499  4 4275  5.419399e+00
## 25500  4 4276  5.218372e+00
## 25501  4 4277  5.029289e+00
## 25502  4 4278  4.901661e+00
## 25503  4 4279  4.789409e+00
## 25504  4 4280  4.694241e+00
## 25505  4 4281  4.599689e+00
## 25506  4 4282  4.526912e+00
## 25507  4 4283  4.473410e+00
## 25508  4 4284  4.406845e+00
## 25509  4 4285  4.364267e+00
## 25510  4 4286  4.312187e+00
## 25511  4 4287  4.264705e+00
## 25512  4 4288  4.215472e+00
## 25513  4 4289  4.180006e+00
## 25514  4 4290  4.148616e+00
## 25515  4 4291  4.096485e+00
## 25516  4 4292  4.068246e+00
## 25517  4 4293  4.029310e+00
## 25518  4 4294  3.999343e+00
## 25519  4 4295  3.963515e+00
## 25520  4 4296  3.931050e+00
## 25521  4 4297  5.986610e+00
## 25522  4 4298  5.664340e+00
## 25523  4 4299  5.418370e+00
## 25524  4 4300  5.204452e+00
## 25525  4 4301  5.032891e+00
## 25526  4 4302  4.901924e+00
## 25527  4 4303  4.773753e+00
## 25528  4 4304  4.681006e+00
## 25529  4 4305  4.601488e+00
## 25530  4 4306  4.524901e+00
## 25531  4 4307  4.457045e+00
## 25532  4 4308  4.402838e+00
## 25533  4 4309  4.344569e+00
## 25534  4 4310  4.299540e+00
## 25535  4 4311  4.250832e+00
## 25536  4 4312  4.214802e+00
## 25537  4 4313  4.172789e+00
## 25538  4 4314  4.129499e+00
## 25539  4 4315  4.100045e+00
## 25540  4 4316  4.063541e+00
## 25541  4 4317  4.026540e+00
## 25542  4 4318  3.983657e+00
## 25543  4 4319  3.951471e+00
## 25544  4 4320  3.913360e+00
## 25545  4 4321  5.969488e+00
## 25546  4 4322  5.647724e+00
## 25547  4 4323  5.404108e+00
## 25548  4 4324  5.192154e+00
## 25549  4 4325  5.018783e+00
## 25550  4 4326  4.880328e+00
## 25551  4 4327  4.762085e+00
## 25552  4 4328  4.664482e+00
## 25553  4 4329  4.588137e+00
## 25554  4 4330  4.517829e+00
## 25555  4 4331  4.449112e+00
## 25556  4 4332  4.393435e+00
## 25557  4 4333  4.333830e+00
## 25558  4 4334  4.295677e+00
## 25559  4 4335  4.252246e+00
## 25560  4 4336  4.202744e+00
## 25561  4 4337  4.159171e+00
## 25562  4 4338  4.126289e+00
## 25563  4 4339  4.087419e+00
## 25564  4 4340  4.049100e+00
## 25565  4 4341  4.016977e+00
## 25566  4 4342  3.979448e+00
## 25567  4 4343  3.943774e+00
## 25568  4 4344  3.912901e+00
## 25569  4 4345  5.974072e+00
## 25570  4 4346  5.657977e+00
## 25571  4 4347  5.392720e+00
## 25572  4 4348  5.183064e+00
## 25573  4 4349  5.017045e+00
## 25574  4 4350  4.881085e+00
## 25575  4 4351  4.766828e+00
## 25576  4 4352  4.667793e+00
## 25577  4 4353  4.578986e+00
## 25578  4 4354  4.513248e+00
## 25579  4 4355  4.438427e+00
## 25580  4 4356  4.384988e+00
## 25581  4 4357  4.337735e+00
## 25582  4 4358  4.281225e+00
## 25583  4 4359  4.242372e+00
## 25584  4 4360  4.197956e+00
## 25585  4 4361  4.158550e+00
## 25586  4 4362  4.122381e+00
## 25587  4 4363  4.078870e+00
## 25588  4 4364  4.043722e+00
## 25589  4 4365  4.014594e+00
## 25590  4 4366  3.971220e+00
## 25591  4 4367  3.936598e+00
## 25592  4 4368  3.908877e+00
## 25593  4 4369  5.956485e+00
## 25594  4 4370  5.641216e+00
## 25595  4 4371  5.384967e+00
## 25596  4 4372  5.175997e+00
## 25597  4 4373  5.015355e+00
## 25598  4 4374  4.872317e+00
## 25599  4 4375  4.748751e+00
## 25600  4 4376  4.657730e+00
## 25601  4 4377  4.580494e+00
## 25602  4 4378  4.495878e+00
## 25603  4 4379  4.440095e+00
## 25604  4 4380  4.383106e+00
## 25605  4 4381  4.326378e+00
## 25606  4 4382  4.290249e+00
## 25607  4 4383  4.243180e+00
## 25608  4 4384  4.188086e+00
## 25609  4 4385  4.156509e+00
## 25610  4 4386  4.119829e+00
## 25611  4 4387  4.073490e+00
## 25612  4 4388  4.042354e+00
## 25613  4 4389  4.005871e+00
## 25614  4 4390  3.967183e+00
## 25615  4 4391  3.940060e+00
## 25616  4 4392  3.911286e+00
## 25617  4 4393  5.962972e+00
## 25618  4 4394  5.643463e+00
## 25619  4 4395  5.384961e+00
## 25620  4 4396  5.172731e+00
## 25621  4 4397  5.008412e+00
## 25622  4 4398  4.868771e+00
## 25623  4 4399  4.748397e+00
## 25624  4 4400  4.657922e+00
## 25625  4 4401  4.573274e+00
## 25626  4 4402  4.506364e+00
## 25627  4 4403  4.437770e+00
## 25628  4 4404  4.380913e+00
## 25629  4 4405  4.321538e+00
## 25630  4 4406  4.274040e+00
## 25631  4 4407  4.231210e+00
## 25632  4 4408  4.196460e+00
## 25633  4 4409  4.148844e+00
## 25634  4 4410  4.108979e+00
## 25635  4 4411  4.082273e+00
```

```
## 25636  4 4412  4.041669e+00
## 25637  4 4413  3.998334e+00
## 25638  4 4414  3.962715e+00
## 25639  4 4415  3.935965e+00
## 25640  4 4416  3.901791e+00
## 25641  4 4417  5.955921e+00
## 25642  4 4418  5.629861e+00
## 25643  4 4419  5.374323e+00
## 25644  4 4420  5.167759e+00
## 25645  4 4421  5.000711e+00
## 25646  4 4422  4.865656e+00
## 25647  4 4423  4.741920e+00
## 25648  4 4424  4.646147e+00
## 25649  4 4425  4.575001e+00
## 25650  4 4426  4.493278e+00
## 25651  4 4427  4.433847e+00
## 25652  4 4428  4.374573e+00
## 25653  4 4429  4.322674e+00
## 25654  4 4430  4.276114e+00
## 25655  4 4431  4.228191e+00
## 25656  4 4432  4.179843e+00
## 25657  4 4433  4.144776e+00
## 25658  4 4434  4.106608e+00
## 25659  4 4435  4.074571e+00
## 25660  4 4436  4.031490e+00
## 25661  4 4437  3.992866e+00
## 25662  4 4438  3.973028e+00
## 25663  4 4439  3.934225e+00
## 25664  4 4440  3.899990e+00
## 25665  4 4441  3.863135e+00
## 25666  4 4442  3.831936e+00
## 25667  4 4443  3.798367e+00
## 25668  4 4444  3.771985e+00
## 25669  4 4445  3.737039e+00
## 25670  4 4446  3.706969e+00
## 25671  4 4447  3.675028e+00
## 25672  4 4448  3.638892e+00
## 25673  4 4449  3.605091e+00
## 25674  4 4450  3.581363e+00
## 25675  4 4451  3.552631e+00
## 25676  4 4452  3.517563e+00
## 25677  4 4453  3.494391e+00
## 25678  4 4454  3.463968e+00
## 25679  4 4455  3.439058e+00
## 25680  4 4456  3.414547e+00
## 25681  4 4457  3.386265e+00
## 25682  4 4458  3.353411e+00
## 25683  4 4459  3.326477e+00
## 25684  4 4460  3.299886e+00
## 25685  4 4461  3.272655e+00
## 25686  4 4462  3.247473e+00
## 25687  4 4463  3.215056e+00
## 25688  4 4464  3.189441e+00
## 25689  4 4465  3.167112e+00
## 25690  4 4466  3.140420e+00
## 25691  4 4467  3.111593e+00
## 25692  4 4468  3.081687e+00
## 25693  4 4469  3.057994e+00
## 25694  4 4470  3.031749e+00
## 25695  4 4471  3.015481e+00
## 25696  4 4472  2.987596e+00
## 25697  4 4473  2.957913e+00
## 25698  4 4474  2.938014e+00
## 25699  4 4475  2.914590e+00
## 25700  4 4476  2.884756e+00
## 25701  4 4477  2.863002e+00
## 25702  4 4478  2.841900e+00
## 25703  4 4479  2.814847e+00
## 25704  4 4480  2.789965e+00
## 25705  4 4481  2.770825e+00
## 25706  4 4482  2.747080e+00
## 25707  4 4483  2.722066e+00
## 25708  4 4484  2.702057e+00
## 25709  4 4485  2.677639e+00
## 25710  4 4486  2.659151e+00
## 25711  4 4487  2.638019e+00
## 25712  4 4488  2.614630e+00
## 25713  4 4489  2.589657e+00
## 25714  4 4490  2.568984e+00
## 25715  4 4491  2.548464e+00
## 25716  4 4492  2.531771e+00
## 25717  4 4493  2.508970e+00
## 25718  4 4494  2.488946e+00
## 25719  4 4495  2.465933e+00
## 25720  4 4496  2.446376e+00
## 25721  4 4497  2.426881e+00
## 25722  4 4498  2.399750e+00
## 25723  4 4499  2.392220e+00
## 25724  4 4500  2.365322e+00
## 25725  4 4501  2.348171e+00
## 25726  4 4502  2.326422e+00
## 25727  4 4503  2.308412e+00
## 25728  4 4504  2.286518e+00
## 25729  4 4505  2.268122e+00
## 25730  4 4506  2.246189e+00
## 25731  4 4507  2.236666e+00
## 25732  4 4508  2.212082e+00
## 25733  4 4509  2.193448e+00
## 25734  4 4510  2.179551e+00
## 25735  4 4511  2.154390e+00
## 25736  4 4512  2.140412e+00
## 25737  4 4513  2.124643e+00
## 25738  4 4514  2.105493e+00
## 25739  4 4515  2.089797e+00
## 25740  4 4516  2.072316e+00
## 25741  4 4517  2.057544e+00
## 25742  4 4518  2.037892e+00
## 25743  4 4519  2.019794e+00
## 25744  4 4520  2.001536e+00
## 25745  4 4521  1.989576e+00
## 25746  4 4522  1.972765e+00
## 25747  4 4523  1.952415e+00
## 25748  4 4524  1.937710e+00
## 25749  4 4525  1.922241e+00
## 25750  4 4526  1.905310e+00
## 25751  4 4527  1.889510e+00
## 25752  4 4528  1.876894e+00
## 25753  4 4529  1.858129e+00
## 25754  4 4530  1.846924e+00
## 25755  4 4531  1.828704e+00
## 25756  4 4532  1.814102e+00
## 25757  4 4533  1.802084e+00
## 25758  4 4534  1.783916e+00
## 25759  4 4535  1.768720e+00
## 25760  4 4536  1.754905e+00
## 25761  4 4537  1.738716e+00
## 25762  4 4538  1.729791e+00
## 25763  4 4539  1.711996e+00
## 25764  4 4540  1.697758e+00
## 25765  4 4541  1.683482e+00
## 25766  4 4542  1.666343e+00
## 25767  4 4543  1.657834e+00
## 25768  4 4544  1.642754e+00
## 25769  4 4545  1.629040e+00
## 25770  4 4546  1.614691e+00
## 25771  4 4547  1.599844e+00
## 25772  4 4548  1.590532e+00
## 25773  4 4549  1.574346e+00
## 25774  4 4550  1.561369e+00
## 25775  4 4551  1.549504e+00
## 25776  4 4552  1.534599e+00
## 25777  4 4553  1.523964e+00
## 25778  4 4554  1.513818e+00
## 25779  4 4555  1.499329e+00
## 25780  4 4556  1.486625e+00
## 25781  4 4557  1.474885e+00
## 25782  4 4558  1.460331e+00
## 25783  4 4559  1.448148e+00
## 25784  4 4560  1.436537e+00
## 25785  4 4561  1.425376e+00
## 25786  4 4562  1.416118e+00
## 25787  4 4563  1.402602e+00
## 25788  4 4564  1.392291e+00
## 25789  4 4565  1.377751e+00
## 25790  4 4566  1.366239e+00
## 25791  4 4567  1.355612e+00
## 25792  4 4568  1.344561e+00
## 25793  4 4569  1.334760e+00
## 25794  4 4570  1.322943e+00
## 25795  4 4571  1.311893e+00
## 25796  4 4572  1.301875e+00
## 25797  4 4573  1.291958e+00
## 25798  4 4574  1.280370e+00
## 25799  4 4575  1.270802e+00
## 25800  4 4576  1.257282e+00
## 25801  4 4577  1.249672e+00
## 25802  4 4578  1.238716e+00
## 25803  4 4579  1.227967e+00
## 25804  4 4580  1.217824e+00
## 25805  4 4581  1.206878e+00
## 25806  4 4582  1.195777e+00
## 25807  4 4583  1.189339e+00
## 25808  4 4584  1.178708e+00
## 25809  4 4585  1.167945e+00
## 25810  4 4586  1.158846e+00
## 25811  4 4587  1.149875e+00
## 25812  4 4588  1.140363e+00
## 25813  4 4589  1.130792e+00
## 25814  4 4590  1.119356e+00
## 25815  4 4591  1.110951e+00
## 25816  4 4592  1.102509e+00
## 25817  4 4593  1.094010e+00
## 25818  4 4594  1.084174e+00
## 25819  4 4595  1.075573e+00
## 25820  4 4596  1.065945e+00
## 25821  4 4597  1.057493e+00
## 25822  4 4598  1.048102e+00
## 25823  4 4599  1.038325e+00
## 25824  4 4600  1.030724e+00
## 25825  4 4601  1.023017e+00
## 25826  4 4602  1.014452e+00
## 25827  4 4603  1.007845e+00
## 25828  4 4604  9.970974e-01
## 25829  4 4605  9.875753e-01
## 25830  4 4606  9.803588e-01
## 25831  4 4607  9.704912e-01
## 25832  4 4608  9.646237e-01
## 25833  4 4609  9.560081e-01
## 25834  4 4610  9.499509e-01
## 25835  4 4611  9.418701e-01
## 25836  4 4612  9.346607e-01
## 25837  4 4613  9.260070e-01
## 25838  4 4614  9.204185e-01
## 25839  4 4615  9.112120e-01
## 25840  4 4616  9.035169e-01
## 25841  4 4617  8.947578e-01
## 25842  4 4618  8.881513e-01
## 25843  4 4619  8.803413e-01
## 25844  4 4620  8.738698e-01
## 25845  4 4621  8.678427e-01
## 25846  4 4622  8.596166e-01
## 25847  4 4623  8.519784e-01
## 25848  4 4624  8.453533e-01
## 25849  4 4625  8.381953e-01
## 25850  4 4626  8.318106e-01
## 25851  4 4627  8.256653e-01
## 25852  4 4628  8.174603e-01
## 25853  4 4629  8.113042e-01
## 25854  4 4630  8.028745e-01
## 25855  4 4631  7.966044e-01
## 25856  4 4632  7.902290e-01
## 25857  4 4633  7.844707e-01
## 25858  4 4634  7.775161e-01
## 25859  4 4635  7.708326e-01
## 25860  4 4636  7.666170e-01
## 25861  4 4637  7.582982e-01
## 25862  4 4638  7.518004e-01
## 25863  4 4639  7.478138e-01
## 25864  4 4640  7.408602e-01
## 25865  4 4641  7.361946e-01
## 25866  4 4642  7.285713e-01
## 25867  4 4643  7.221064e-01
## 25868  4 4644  7.160859e-01
## 25869  4 4645  7.112940e-01
## 25870  4 4646  7.030042e-01
## 25871  4 4647  6.997377e-01
## 25872  4 4648  6.928078e-01
## 25873  4 4649  6.867981e-01
## 25874  4 4650  6.808910e-01
## 25875  4 4651  6.756744e-01
## 25876  4 4652  6.708577e-01
## 25877  4 4653  6.649373e-01
## 25878  4 4654  6.595517e-01
## 25879  4 4655  6.534630e-01
## 25880  4 4656  6.478984e-01
## 25881  4 4657  6.413713e-01
## 25882  4 4658  6.364671e-01
## 25883  4 4659  6.316023e-01
## 25884  4 4660  6.274062e-01
## 25885  4 4661  6.220320e-01
## 25886  4 4662  6.162598e-01
## 25887  4 4663  6.106726e-01
## 25888  4 4664  6.077481e-01
## 25889  4 4665  6.018772e-01
## 25890  4 4666  5.963910e-01
## 25891  4 4667  5.912763e-01
## 25892  4 4668  5.870303e-01
## 25893  4 4669  5.815960e-01
## 25894  4 4670  5.781973e-01
## 25895  4 4671  5.727823e-01
## 25896  4 4672  5.668621e-01
## 25897  4 4673  5.618173e-01
## 25898  4 4674  5.581387e-01
## 25899  4 4675  5.535071e-01
## 25900  4 4676  5.491637e-01
## 25901  4 4677  5.452238e-01
## 25902  4 4678  5.395053e-01
## 25903  4 4679  5.355698e-01
## 25904  4 4680  5.307476e-01
## 25905  4 4681  5.268767e-01
## 25906  4 4682  5.221662e-01
## 25907  4 4683  5.179310e-01
## 25908  4 4684  5.137329e-01
## 25909  4 4685  5.091933e-01
## 25910  4 4686  5.049746e-01
## 25911  4 4687  5.007971e-01
## 25912  4 4688  4.978288e-01
## 25913  4 4689  4.928036e-01
## 25914  4 4690  4.882797e-01
## 25915  4 4691  4.854206e-01
## 25916  4 4692  4.808516e-01
## 25917  4 4693  4.770617e-01
## 25918  4 4694  4.731093e-01
## 25919  4 4695  4.693638e-01
## 25920  4 4696  4.652221e-01
## 25921  4 4697  4.607738e-01
## 25922  4 4698  4.569576e-01
## 25923  4 4699  4.537990e-01
## 25924  4 4700  4.494349e-01
## 25925  4 4701  4.461312e-01
## 25926  4 4702  4.425176e-01
## 25927  4 4703  4.398448e-01
## 25928  4 4704  4.354629e-01
## 25929  4 4705  4.308812e-01
## 25930  4 4706  4.275519e-01
## 25931  4 4707  4.252294e-01
## 25932  4 4708  4.207835e-01
## 25933  4 4709  4.174671e-01
## 25934  4 4710  4.142200e-01
## 25935  4 4711  4.098682e-01
## 25936  4 4712  4.075474e-01
## 25937  4 4713  4.035903e-01
## 25938  4 4714  4.010700e-01
## 25939  4 4715  3.976917e-01
## 25940  4 4716  3.933139e-01
## 25941  4 4717  3.904265e-01
## 25942  4 4718  3.879328e-01
## 25943  4 4719  3.843951e-01
## 25944  4 4720  3.807676e-01
## 25945  4 4721  3.775220e-01
## 25946  4 4722  3.754204e-01
## 25947  4 4723  3.717134e-01
## 25948  4 4724  3.683285e-01
## 25949  4 4725  3.657317e-01
## 25950  4 4726  3.620922e-01
## 25951  4 4727  3.590986e-01
## 25952  4 4728  3.565461e-01
## 25953  4 4729  3.532261e-01
## 25954  4 4730  3.511648e-01
## 25955  4 4731  3.479953e-01
## 25956  4 4732  3.447865e-01
## 25957  4 4733  3.422584e-01
## 25958  4 4734  3.400063e-01
## 25959  4 4735  3.363156e-01
## 25960  4 4736  3.333022e-01
## 25961  4 4737  3.310409e-01
## 25962  4 4738  3.282429e-01
## 25963  4 4739  3.249318e-01
## 25964  4 4740  3.233316e-01
## 25965  4 4741  3.198419e-01
## 25966  4 4742  3.174619e-01
## 25967  4 4743  3.144762e-01
## 25968  4 4744  3.121905e-01
## 25969  4 4745  3.098374e-01
## 25970  4 4746  3.075964e-01
## 25971  4 4747  3.050244e-01
## 25972  4 4748  3.023225e-01
## 25973  4 4749  3.000350e-01
## 25974  4 4750  2.970978e-01
## 25975  4 4751  2.948117e-01
## 25976  4 4752  2.921185e-01
## 25977  4 4753  2.895773e-01
## 25978  4 4754  2.871169e-01
## 25979  4 4755  2.851752e-01
## 25980  4 4756  2.827583e-01
## 25981  4 4757  2.807676e-01
## 25982  4 4758  2.780003e-01
## 25983  4 4759  2.757389e-01
## 25984  4 4760  2.741334e-01
## 25985  4 4761  2.712097e-01
## 25986  4 4762  2.685865e-01
## 25987  4 4763  2.670846e-01
## 25988  4 4764  2.646086e-01
## 25989  4 4765  2.624555e-01
## 25990  4 4766  2.597499e-01
## 25991  4 4767  2.584411e-01
## 25992  4 4768  2.554119e-01
## 25993  4 4769  2.537256e-01
## 25994  4 4770  2.518513e-01
## 25995  4 4771  2.495453e-01
## 25996  4 4772  2.477490e-01
## 25997  4 4773  2.456594e-01
## 25998  4 4774  2.434875e-01
## 25999  4 4775  2.414572e-01
## 26000  4 4776  2.394155e-01
## 26001  4 4777  2.376969e-01
## 26002  4 4778  2.358144e-01
## 26003  4 4779  2.337324e-01
## 26004  4 4780  2.315236e-01
## 26005  4 4781  2.297015e-01
## 26006  4 4782  2.279172e-01
## 26007  4 4783  2.261168e-01
## 26008  4 4784  2.242370e-01
## 26009  4 4785  2.223015e-01
## 26010  4 4786  2.208438e-01
## 26011  4 4787  2.184006e-01
## 26012  4 4788  2.172563e-01
## 26013  4 4789  2.147399e-01
## 26014  4 4790  2.131964e-01
## 26015  4 4791  2.111283e-01
## 26016  4 4792  2.097392e-01
## 26017  4 4793  2.083129e-01
## 26018  4 4794  2.058525e-01
## 26019  4 4795  2.041922e-01
## 26020  4 4796  2.025210e-01
## 26021  4 4797  2.009214e-01
## 26022  4 4798  1.994582e-01
## 26023  4 4799  1.981901e-01
## 26024  4 4800  1.959952e-01
## 26025  4 4801  1.943125e-01
## 26026  4 4802  1.931172e-01
## 26027  4 4803  1.914787e-01
## 26028  4 4804  1.896964e-01
## 26029  4 4805  1.881549e-01
## 26030  4 4806  1.867035e-01
## 26031  4 4807  1.852667e-01
## 26032  4 4808  1.835429e-01
## 26033  4 4809  1.822039e-01
## 26034  4 4810  1.807487e-01
## 26035  4 4811  1.791702e-01
## 26036  4 4812  1.775078e-01
## 26037  4 4813  1.761360e-01
## 26038  4 4814  1.746922e-01
## 26039  4 4815  1.730674e-01
## 26040  4 4816  1.719043e-01
## 26041  4 4817  1.704307e-01
## 26042  4 4818  1.692397e-01
## 26043  4 4819  1.675521e-01
## 26044  4 4820  1.663776e-01
## 26045  4 4821  1.650965e-01
## 26046  4 4822  1.637942e-01
## 26047  4 4823  1.622799e-01
## 26048  4 4824  1.608500e-01
## 26049  4 4825  1.592735e-01
## 26050  4 4826  1.580667e-01
## 26051  4 4827  1.568325e-01
## 26052  4 4828  1.552164e-01
## 26053  4 4829  1.542055e-01
## 26054  4 4830  1.529830e-01
## 26055  4 4831  1.516573e-01
## 26056  4 4832  1.503594e-01
## 26057  4 4833  1.493938e-01
## 26058  4 4834  1.480169e-01
## 26059  4 4835  1.467481e-01
## 26060  4 4836  1.453952e-01
## 26061  4 4837  1.442516e-01
## 26062  4 4838  1.430198e-01
## 26063  4 4839  1.419096e-01
## 26064  4 4840  1.409300e-01
## 26065  4 4841  1.397240e-01
## 26066  4 4842  1.386286e-01
## 26067  4 4843  1.375273e-01
## 26068  4 4844  1.362797e-01
## 26069  4 4845  1.351175e-01
## 26070  4 4846  1.339686e-01
## 26071  4 4847  1.328101e-01
## 26072  4 4848  1.317221e-01
## 26073  4 4849  1.307895e-01
## 26074  4 4850  1.295644e-01
## 26075  4 4851  1.286098e-01
## 26076  4 4852  1.274406e-01
## 26077  4 4853  1.263622e-01
## 26078  4 4854  1.252665e-01
## 26079  4 4855  1.243784e-01
## 26080  4 4856  1.232693e-01
## 26081  4 4857  1.222855e-01
## 26082  4 4858  1.211941e-01
## 26083  4 4859  1.204520e-01
## 26084  4 4860  1.192213e-01
## 26085  4 4861  1.183678e-01
## 26086  4 4862  1.172539e-01
## 26087  4 4863  1.163293e-01
## 26088  4 4864  1.153319e-01
## 26089  4 4865  1.145576e-01
## 26090  4 4866  1.134372e-01
## 26091  4 4867  1.125266e-01
## 26092  4 4868  1.116287e-01
## 26093  4 4869  1.107082e-01
## 26094  4 4870  1.098147e-01
## 26095  4 4871  1.089420e-01
## 26096  4 4872  1.076779e-01
## 26097  4 4873  1.070780e-01
## 26098  4 4874  1.060303e-01
## 26099  4 4875  1.053373e-01
## 26100  4 4876  1.042432e-01
## 26101  4 4877  1.038060e-01
## 26102  4 4878  1.026453e-01
## 26103  4 4879  1.019754e-01
## 26104  4 4880  1.007804e-01
## 26105  4 4881  1.002899e-01
## 26106  4 4882  9.953803e-02
## 26107  4 4883  9.859866e-02
## 26108  4 4884  9.786461e-02
## 26109  4 4885  9.697320e-02
## 26110  4 4886  9.607199e-02
## 26111  4 4887  9.529282e-02
## 26112  4 4888  9.443626e-02
## 26113  4 4889  9.377105e-02
## 26114  4 4890  9.288702e-02
## 26115  4 4891  9.203220e-02
## 26116  4 4892  9.142931e-02
## 26117  4 4893  9.050061e-02
## 26118  4 4894  8.995195e-02
## 26119  4 4895  8.925297e-02
## 26120  4 4896  8.860980e-02
## 26121  4 4897  8.764942e-02
## 26122  4 4898  8.696872e-02
## 26123  4 4899  8.614501e-02
## 26124  4 4900  8.577163e-02
## 26125  4 4901  8.479803e-02
## 26126  4 4902  8.419334e-02
## 26127  4 4903  8.352242e-02
## 26128  4 4904  8.274094e-02
## 26129  4 4905  8.208449e-02
## 26130  4 4906  8.135412e-02
## 26131  4 4907  8.072875e-02
## 26132  4 4908  8.012686e-02
## 26133  4 4909  7.945515e-02
## 26134  4 4910  7.886048e-02
## 26135  4 4911  7.806395e-02
## 26136  4 4912  7.753187e-02
## 26137  4 4913  7.679119e-02
## 26138  4 4914  7.617600e-02
## 26139  4 4915  7.552368e-02
## 26140  4 4916  7.497513e-02
## 26141  4 4917  7.432786e-02
## 26142  4 4918  7.359256e-02
## 26143  4 4919  7.322383e-02
## 26144  4 4920  7.249468e-02
## 26145  4 4921  7.199652e-02
## 26146  4 4922  7.122254e-02
## 26147  4 4923  7.062824e-02
## 26148  4 4924  7.018803e-02
## 26149  4 4925  6.953187e-02
## 26150  4 4926  6.891232e-02
## 26151  4 4927  6.841877e-02
## 26152  4 4928  6.784292e-02
## 26153  4 4929  6.722533e-02
## 26154  4 4930  6.675089e-02
## 26155  4 4931  6.606590e-02
## 26156  4 4932  6.564170e-02
## 26157  4 4933  6.509812e-02
## 26158  4 4934  6.455589e-02
## 26159  4 4935  6.389074e-02
## 26160  4 4936  6.345491e-02
## 26161  4 4937  6.297840e-02
## 26162  4 4938  6.237059e-02
## 26163  4 4939  6.175928e-02
## 26164  4 4940  6.152814e-02
## 26165  4 4941  6.082479e-02
## 26166  4 4942  6.036822e-02
## 26167  4 4943  5.989528e-02
## 26168  4 4944  5.947951e-02
## 26169  4 4945  5.888002e-02
## 26170  4 4946  5.838584e-02
## 26171  4 4947  5.785825e-02
## 26172  4 4948  5.746980e-02
## 26173  4 4949  5.705967e-02
## 26174  4 4950  5.643125e-02
## 26175  4 4951  5.607527e-02
## 26176  4 4952  5.561191e-02
## 26177  4 4953  5.523205e-02
## 26178  4 4954  5.465715e-02
## 26179  4 4955  5.424842e-02
## 26180  4 4956  5.381818e-02
## 26181  4 4957  5.327626e-02
## 26182  4 4958  5.275533e-02
## 26183  4 4959  5.251445e-02
## 26184  4 4960  5.200622e-02
## 26185  4 4961  5.153491e-02
## 26186  4 4962  5.115800e-02
## 26187  4 4963  5.066807e-02
## 26188  4 4964  5.028926e-02
## 26189  4 4965  4.998503e-02
## 26190  4 4966  4.942839e-02
## 26191  4 4967  4.900861e-02
## 26192  4 4968  4.866606e-02
## 26193  4 4969  4.823628e-02
## 26194  4 4970  4.785435e-02
## 26195  4 4971  4.754630e-02
## 26196  4 4972  4.699375e-02
## 26197  4 4973  4.670894e-02
## 26198  4 4974  4.625076e-02
## 26199  4 4975  4.597669e-02
## 26200  4 4976  4.551823e-02
## 26201  4 4977  4.514537e-02
## 26202  4 4978  4.480073e-02
## 26203  4 4979  4.448560e-02
## 26204  4 4980  4.404762e-02
## 26205  4 4981  4.361936e-02
## 26206  4 4982  4.328246e-02
## 26207  4 4983  4.302953e-02
## 26208  4 4984  4.263857e-02
## 26209  4 4985  4.217757e-02
## 26210  4 4986  4.185350e-02
## 26211  4 4987  4.155387e-02
## 26212  4 4988  4.126411e-02
## 26213  4 4989  4.089342e-02
## 26214  4 4990  4.058161e-02
## 26215  4 4991  4.020093e-02
## 26216  4 4992  3.990439e-02
## 26217  4 4993  3.957728e-02
## 26218  4 4994  3.924133e-02
## 26219  4 4995  3.895420e-02
## 26220  4 4996  3.863282e-02
## 26221  4 4997  3.827602e-02
## 26222  4 4998  3.785774e-02
## 26223  4 4999  3.758332e-02
## 26224  4 5000  3.728087e-02
## 26225  4 5001  3.695227e-02
## 26226  4 5002  3.674933e-02
## 26227  4 5003  3.640199e-02
## 26228  4 5004  3.613624e-02
## 26229  4 5005  3.578707e-02
## 26230  4 5006  3.548701e-02
## 26231  4 5007  3.519242e-02
## 26232  4 5008  3.495069e-02
## 26233  4 5009  3.470357e-02
## 26234  4 5010  3.440134e-02
## 26235  4 5011  3.407535e-02
## 26236  4 5012  3.380842e-02
## 26237  4 5013  3.352859e-02
## 26238  4 5014  3.321737e-02
## 26239  4 5015  3.288419e-02
## 26240  4 5016  3.264587e-02
## 26241  4 5017  3.242384e-02
## 26242  4 5018  3.214583e-02
## 26243  4 5019  3.180536e-02
## 26244  4 5020  3.161697e-02
## 26245  4 5021  3.134368e-02
## 26246  4 5022  3.106529e-02
## 26247  4 5023  3.083621e-02
## 26248  4 5024  3.051635e-02
## 26249  4 5025  3.030867e-02
## 26250  4 5026  3.005512e-02
## 26251  4 5027  2.987015e-02
## 26252  4 5028  2.962671e-02
## 26253  4 5029  2.933493e-02
## 26254  4 5030  2.913927e-02
## 26255  4 5031  2.881648e-02
## 26256  4 5032  2.866427e-02
## 26257  4 5033  2.837578e-02
## 26258  4 5034  2.810040e-02
## 26259  4 5035  2.787336e-02
## 26260  4 5036  2.768743e-02
## 26261  4 5037  2.741294e-02
## 26262  4 5038  2.722130e-02
## 26263  4 5039  2.702251e-02
## 26264  4 5040  2.676541e-02
## 26265  4 5041  2.660399e-02
## 26266  4 5042  2.636874e-02
## 26267  4 5043  2.611972e-02
## 26268  4 5044  2.591148e-02
## 26269  4 5045  2.569677e-02
## 26270  4 5046  2.549774e-02
## 26271  4 5047  2.526669e-02
## 26272  4 5048  2.504068e-02
## 26273  4 5049  2.481628e-02
## 26274  4 5050  2.463618e-02
## 26275  4 5051  2.444364e-02
## 26276  4 5052  2.429969e-02
## 26277  4 5053  2.404819e-02
## 26278  4 5054  2.385893e-02
## 26279  4 5055  2.362313e-02
## 26280  4 5056  2.342763e-02
## 26281  4 5057  2.323401e-02
## 26282  4 5058  2.308835e-02
## 26283  4 5059  2.291214e-02
## 26284  4 5060  2.269621e-02
## 26285  4 5061  2.252035e-02
## 26286  4 5062  2.233540e-02
## 26287  4 5063  2.209226e-02
## 26288  4 5064  2.192847e-02
## 26289  4 5065  2.177153e-02
## 26290  4 5066  2.160083e-02
## 26291  4 5067  2.140221e-02
## 26292  4 5068  2.120100e-02
## 26293  4 5069  2.102431e-02
## 26294  4 5070  2.087415e-02
## 26295  4 5071  2.068769e-02
## 26296  4 5072  2.054034e-02
## 26297  4 5073  2.038135e-02
## 26298  4 5074  2.017281e-02
## 26299  4 5075  2.003565e-02
## 26300  4 5076  1.983438e-02
## 26301  4 5077  1.969810e-02
## 26302  4 5078  1.953398e-02
## 26303  4 5079  1.935741e-02
## 26304  4 5080  1.919597e-02
## 26305  4 5081  1.902920e-02
## 26306  4 5082  1.889995e-02
## 26307  4 5083  1.869339e-02
## 26308  4 5084  1.857488e-02
## 26309  4 5085  1.844281e-02
## 26310  4 5086  1.826355e-02
## 26311  4 5087  1.813825e-02
## 26312  4 5088  1.798988e-02
## 26313  4 5089  1.782878e-02
## 26314  4 5090  1.770694e-02
## 26315  4 5091  1.752043e-02
## 26316  4 5092  1.738735e-02
## 26317  4 5093  1.720356e-02
## 26318  4 5094  1.713161e-02
## 26319  4 5095  1.697206e-02
## 26320  4 5096  1.685535e-02
## 26321  4 5097  1.666479e-02
## 26322  4 5098  1.656248e-02
## 26323  4 5099  1.642919e-02
## 26324  4 5100  1.627887e-02
## 26325  4 5101  1.612529e-02
## 26326  4 5102  1.600158e-02
## 26327  4 5103  1.585374e-02
## 26328  4 5104  1.575464e-02
## 26329  4 5105  1.560001e-02
## 26330  4 5106  1.550427e-02
## 26331  4 5107  1.535381e-02
## 26332  4 5108  1.522431e-02
## 26333  4 5109  1.507723e-02
## 26334  4 5110  1.497140e-02
## 26335  4 5111  1.484676e-02
## 26336  4 5112  1.472814e-02
## 26337  4 5113  1.461405e-02
## 26338  4 5114  1.449554e-02
## 26339  4 5115  1.438299e-02
## 26340  4 5116  1.425782e-02
## 26341  4 5117  1.411086e-02
## 26342  4 5118  1.403142e-02
## 26343  4 5119  1.388826e-02
## 26344  4 5120  1.376006e-02
## 26345  4 5121  1.366640e-02
## 26346  4 5122  1.356435e-02
## 26347  4 5123  1.343017e-02
## 26348  4 5124  1.331310e-02
## 26349  4 5125  1.321959e-02
## 26350  4 5126  1.309673e-02
## 26351  4 5127  1.301638e-02
## 26352  4 5128  1.289755e-02
## 26353  4 5129  1.278877e-02
## 26354  4 5130  1.270706e-02
## 26355  4 5131  1.260485e-02
## 26356  4 5132  1.248210e-02
## 26357  4 5133  1.239540e-02
## 26358  4 5134  1.227122e-02
## 26359  4 5135  1.218614e-02
## 26360  4 5136  1.208779e-02
## 26361  4 5137  1.197717e-02
## 26362  4 5138  1.187461e-02
## 26363  4 5139  1.176881e-02
## 26364  4 5140  1.168656e-02
## 26365  4 5141  1.158992e-02
## 26366  4 5142  1.147668e-02
## 26367  4 5143  1.139327e-02
## 26368  4 5144  1.128888e-02
## 26369  4 5145  1.119349e-02
## 26370  4 5146  1.109922e-02
## 26371  4 5147  1.101920e-02
## 26372  4 5148  1.092837e-02
## 26373  4 5149  1.083434e-02
## 26374  4 5150  1.075460e-02
## 26375  4 5151  1.064232e-02
## 26376  4 5152  1.057862e-02
## 26377  4 5153  1.048090e-02
## 26378  4 5154  1.040149e-02
## 26379  4 5155  1.031147e-02
## 26380  4 5156  1.022179e-02
## 26381  4 5157  1.012534e-02
## 26382  4 5158  1.005536e-02
## 26383  4 5159  9.953913e-03
## 26384  4 5160  9.879360e-03
## 26385  4 5161  9.804168e-03
## 26386  4 5162  9.721312e-03
## 26387  4 5163  9.653002e-03
## 26388  4 5164  9.574828e-03
## 26389  4 5165  9.493590e-03
## 26390  4 5166  9.402052e-03
## 26391  4 5167  9.329014e-03
## 26392  4 5168  9.273965e-03
## 26393  4 5169  9.179754e-03
## 26394  4 5170  9.111188e-03
## 26395  4 5171  9.012663e-03
## 26396  4 5172  8.959574e-03
## 26397  4 5173  8.883375e-03
## 26398  4 5174  8.810126e-03
## 26399  4 5175  8.726081e-03
## 26400  4 5176  8.663466e-03
## 26401  4 5177  8.589820e-03
## 26402  4 5178  8.514502e-03
## 26403  4 5179  8.453503e-03
## 26404  4 5180  8.363281e-03
## 26405  4 5181  8.311770e-03
## 26406  4 5182  8.245575e-03
## 26407  4 5183  8.168405e-03
## 26408  4 5184  8.115203e-03
## 26409  4 5185  8.037893e-03
## 26410  4 5186  7.975733e-03
## 26411  4 5187  7.891664e-03
## 26412  4 5188  7.844427e-03
## 26413  4 5189  7.782696e-03
## 26414  4 5190  7.712938e-03
## 26415  4 5191  7.644339e-03
## 26416  4 5192  7.601253e-03
## 26417  4 5193  7.525939e-03
## 26418  4 5194  7.465571e-03
## 26419  4 5195  7.388133e-03
## 26420  4 5196  7.326766e-03
## 26421  4 5197  7.282697e-03
## 26422  4 5198  7.224918e-03
## 26423  4 5199  7.155069e-03
## 26424  4 5200  7.102232e-03
## 26425  4 5201  7.052809e-03
## 26426  4 5202  6.980759e-03
## 26427  4 5203  6.932148e-03
## 26428  4 5204  6.875813e-03
## 26429  4 5205  6.804555e-03
## 26430  4 5206  6.759348e-03
## 26431  4 5207  6.692508e-03
## 26432  4 5208  6.632865e-03
## 26433  4 5209  6.594061e-03
## 26434  4 5210  6.535636e-03
## 26435  4 5211  6.475471e-03
```

```
## 26436  4 5212  6.433247e-03
## 26437  4 5213  6.372206e-03
## 26438  4 5214  6.307817e-03
## 26439  4 5215  6.268446e-03
## 26440  4 5216  6.221768e-03
## 26441  4 5217  6.157998e-03
## 26442  4 5218  6.117261e-03
## 26443  4 5219  6.072103e-03
## 26444  4 5220  6.010303e-03
## 26445  4 5221  5.965483e-03
## 26446  4 5222  5.913817e-03
## 26447  4 5223  5.855401e-03
## 26448  4 5224  5.815386e-03
## 26449  4 5225  5.772161e-03
## 26450  4 5226  5.722514e-03
## 26451  4 5227  5.683325e-03
## 26452  4 5228  5.623360e-03
## 26453  4 5229  5.570926e-03
## 26454  4 5230  5.551162e-03
## 26455  4 5231  5.486099e-03
## 26456  4 5232  5.447130e-03
## 26457  4 5233  5.405299e-03
## 26458  4 5234  5.348644e-03
## 26459  4 5235  5.312137e-03
## 26460  4 5236  5.265670e-03
## 26461  4 5237  5.211384e-03
## 26462  4 5238  5.175675e-03
## 26463  4 5239  5.140008e-03
## 26464  4 5240  5.094503e-03
## 26465  4 5241  5.054714e-03
## 26466  4 5242  5.010443e-03
## 26467  4 5243  4.967573e-03
## 26468  4 5244  4.930919e-03
## 26469  4 5245  4.885954e-03
## 26470  4 5246  4.844757e-03
## 26471  4 5247  4.808297e-03
## 26472  4 5248  4.766940e-03
## 26473  4 5249  4.731802e-03
## 26474  4 5250  4.689478e-03
## 26475  4 5251  4.638410e-03
## 26476  4 5252  4.596061e-03
## 26477  4 5253  4.570370e-03
## 26478  4 5254  4.531330e-03
## 26479  4 5255  4.491563e-03
## 26480  4 5256  4.453373e-03
## 26481  4 5257  4.424900e-03
## 26482  4 5258  4.381047e-03
## 26483  4 5259  4.353199e-03
## 26484  4 5260  4.318530e-03
## 26485  4 5261  4.279265e-03
## 26486  4 5262  4.243852e-03
## 26487  4 5263  4.205075e-03
## 26488  4 5264  4.172335e-03
## 26489  4 5265  4.141087e-03
## 26490  4 5266  4.107223e-03
## 26491  4 5267  4.069983e-03
## 26492  4 5268  4.038206e-03
## 26493  4 5269  3.996593e-03
## 26494  4 5270  3.965804e-03
## 26495  4 5271  3.944047e-03
## 26496  4 5272  3.905531e-03
## 26497  4 5273  3.874110e-03
## 26498  4 5274  3.843489e-03
## 26499  4 5275  3.811877e-03
## 26500  4 5276  3.777103e-03
## 26501  4 5277  3.752803e-03
## 26502  4 5278  3.716561e-03
## 26503  4 5279  3.681092e-03
## 26504  4 5280  3.662127e-03
## 26505  4 5281  3.621967e-03
## 26506  4 5282  3.591991e-03
## 26507  4 5283  3.560002e-03
## 26508  4 5284  3.540406e-03
## 26509  4 5285  3.499562e-03
## 26510  4 5286  3.473221e-03
## 26511  4 5287  3.447894e-03
## 26512  4 5288  3.417554e-03
## 26513  4 5289  3.391819e-03
## 26514  4 5290  3.355377e-03
## 26515  4 5291  3.332009e-03
## 26516  4 5292  3.306597e-03
## 26517  4 5293  3.276731e-03
## 26518  4 5294  3.256283e-03
## 26519  4 5295  3.231037e-03
## 26520  4 5296  3.207437e-03
## 26521  4 5297  3.175583e-03
## 26522  4 5298  3.144274e-03
## 26523  4 5299  3.119666e-03
## 26524  4 5300  3.098623e-03
## 26525  4 5301  3.071209e-03
## 26526  4 5302  3.048266e-03
## 26527  4 5303  3.021530e-03
## 26528  4 5304  2.997783e-03
## 26529  4 5305  2.964584e-03
## 26530  4 5306  2.950743e-03
## 26531  4 5307  2.922511e-03
## 26532  4 5308  2.902162e-03
## 26533  4 5309  2.873134e-03
## 26534  4 5310  2.852151e-03
## 26535  4 5311  2.820219e-03
## 26536  4 5312  2.801140e-03
## 26537  4 5313  2.776371e-03
## 26538  4 5314  2.753207e-03
## 26539  4 5315  2.731987e-03
## 26540  4 5316  2.711084e-03
## 26541  4 5317  2.685655e-03
## 26542  4 5318  2.666153e-03
## 26543  4 5319  2.642475e-03
## 26544  4 5320  2.622232e-03
## 26545  4 5321  2.601265e-03
## 26546  4 5322  2.582827e-03
## 26547  4 5323  2.557830e-03
## 26548  4 5324  2.533986e-03
## 26549  4 5325  2.514598e-03
## 26550  4 5326  2.495951e-03
## 26551  4 5327  2.472547e-03
## 26552  4 5328  2.455946e-03
## 26553  4 5329  2.436165e-03
## 26554  4 5330  2.410404e-03
## 26555  4 5331  2.392177e-03
## 26556  4 5332  2.375247e-03
## 26557  4 5333  2.355601e-03
## 26558  4 5334  2.331661e-03
## 26559  4 5335  2.312352e-03
## 26560  4 5336  2.295136e-03
## 26561  4 5337  2.279035e-03
## 26562  4 5338  2.261453e-03
## 26563  4 5339  2.239056e-03
## 26564  4 5340  2.219091e-03
## 26565  4 5341  2.204310e-03
## 26566  4 5342  2.186962e-03
## 26567  4 5343  2.168975e-03
## 26568  4 5344  2.147503e-03
## 26569  4 5345  2.128387e-03
## 26570  4 5346  2.111611e-03
## 26571  4 5347  2.091915e-03
## 26572  4 5348  2.078961e-03
## 26573  4 5349  2.059595e-03
## 26574  4 5350  2.045478e-03
## 26575  4 5351  2.027953e-03
## 26576  4 5352  2.009843e-03
## 26577  4 5353  1.993709e-03
## 26578  4 5354  1.974691e-03
## 26579  4 5355  1.962234e-03
## 26580  4 5356  1.947834e-03
## 26581  4 5357  1.927509e-03
## 26582  4 5358  1.912773e-03
## 26583  4 5359  1.896882e-03
## 26584  4 5360  1.884336e-03
## 26585  4 5361  1.862354e-03
## 26586  4 5362  1.852507e-03
## 26587  4 5363  1.832623e-03
## 26588  4 5364  1.818358e-03
## 26589  4 5365  1.805615e-03
## 26590  4 5366  1.793623e-03
## 26591  4 5367  1.776171e-03
## 26592  4 5368  1.763258e-03
## 26593  4 5369  1.746131e-03
## 26594  4 5370  1.731560e-03
## 26595  4 5371  1.718048e-03
## 26596  4 5372  1.703004e-03
## 26597  4 5373  1.688807e-03
## 26598  4 5374  1.676629e-03
## 26599  4 5375  1.659430e-03
## 26600  4 5376  1.647941e-03
## 26601  4 5377  1.632693e-03
## 26602  4 5378  1.620340e-03
## 26603  4 5379  1.606931e-03
## 26604  4 5380  1.592209e-03
## 26605  4 5381  1.583680e-03
## 26606  4 5382  1.567077e-03
## 26607  4 5383  1.555503e-03
## 26608  4 5384  1.541428e-03
## 26609  4 5385  1.527489e-03
## 26610  4 5386  1.517248e-03
## 26611  4 5387  1.503217e-03
## 26612  4 5388  1.492297e-03
## 26613  4 5389  1.480473e-03
## 26614  4 5390  1.466897e-03
## 26615  4 5391  1.453554e-03
## 26616  4 5392  1.443270e-03
## 26617  4 5393  1.432216e-03
## 26618  4 5394  1.417729e-03
## 26619  4 5395  1.406320e-03
## 26620  4 5396  1.395342e-03
## 26621  4 5397  1.386029e-03
## 26622  4 5398  1.372376e-03
## 26623  4 5399  1.363025e-03
## 26624  4 5400  1.350672e-03
## 26625  4 5401  1.341365e-03
## 26626  4 5402  1.326343e-03
## 26627  4 5403  1.314782e-03
## 26628  4 5404  1.306264e-03
## 26629  4 5405  1.296661e-03
## 26630  4 5406  1.283702e-03
## 26631  4 5407  1.273151e-03
## 26632  4 5408  1.262542e-03
## 26633  4 5409  1.253624e-03
## 26634  4 5410  1.242695e-03
## 26635  4 5411  1.234465e-03
## 26636  4 5412  1.219931e-03
## 26637  4 5413  1.212387e-03
## 26638  4 5414  1.201595e-03
## 26639  4 5415  1.193527e-03
## 26640  4 5416  1.181609e-03
## 26641  4 5417  1.171163e-03
## 26642  4 5418  1.163000e-03
## 26643  4 5419  1.151756e-03
## 26644  4 5420  1.145197e-03
## 26645  4 5421  1.135366e-03
## 26646  4 5422  1.124705e-03
## 26647  4 5423  1.114585e-03
## 26648  4 5424  1.106796e-03
## 26649  4 5425  1.096868e-03
## 26650  4 5426  1.086383e-03
## 26651  4 5427  1.079591e-03
## 26652  4 5428  1.069349e-03
## 26653  4 5429  1.061241e-03
## 26654  4 5430  1.053113e-03
## 26655  4 5431  1.044242e-03
## 26656  4 5432  1.035702e-03
## 26657  4 5433  1.027287e-03
## 26658  4 5434  1.019111e-03
## 26659  4 5435  1.009413e-03
## 26660  4 5436  1.002156e-03
## 26661  4 5437  9.937350e-04
## 26662  4 5438  9.842246e-04
## 26663  4 5439  9.782363e-04
## 26664  4 5440  9.688795e-04
## 26665  4 5441  9.595740e-04
## 26666  4 5442  9.538389e-04
## 26667  4 5443  9.437777e-04
## 26668  4 5444  9.377194e-04
## 26669  4 5445  9.308172e-04
## 26670  4 5446  9.218154e-04
## 26671  4 5447  9.141283e-04
## 26672  4 5448  9.048061e-04
## 26673  4 5449  8.991421e-04
## 26674  4 5450  8.919736e-04
## 26675  4 5451  8.830326e-04
## 26676  4 5452  8.776822e-04
## 26677  4 5453  8.682430e-04
## 26678  4 5454  8.635119e-04
## 26679  4 5455  8.543125e-04
## 26680  4 5456  8.498733e-04
## 26681  4 5457  8.403764e-04
## 26682  4 5458  8.331044e-04
## 26683  4 5459  8.273238e-04
## 26684  4 5460  8.202733e-04
## 26685  4 5461  8.141306e-04
## 26686  4 5462  8.080858e-04
## 26687  4 5463  7.991274e-04
## 26688  4 5464  7.942359e-04
## 26689  4 5465  7.880771e-04
## 26690  4 5466  7.812909e-04
## 26691  4 5467  7.739170e-04
## 26692  4 5468  7.687121e-04
## 26693  4 5469  7.614368e-04
## 26694  4 5470  7.548975e-04
## 26695  4 5471  7.488773e-04
## 26696  4 5472  7.431202e-04
## 26697  4 5473  7.370881e-04
## 26698  4 5474  7.301088e-04
## 26699  4 5475  7.253324e-04
## 26700  4 5476  7.184203e-04
## 26701  4 5477  7.127108e-04
## 26702  4 5478  7.065339e-04
## 26703  4 5479  7.015825e-04
## 26704  4 5480  6.951942e-04
## 26705  4 5481  6.895084e-04
## 26706  4 5482  6.832351e-04
## 26707  4 5483  6.782850e-04
## 26708  4 5484  6.721585e-04
## 26709  4 5485  6.669880e-04
## 26710  4 5486  6.607563e-04
## 26711  4 5487  6.562914e-04
## 26712  4 5488  6.498969e-04
## 26713  4 5489  6.441693e-04
## 26714  4 5490  6.409822e-04
## 26715  4 5491  6.343384e-04
## 26716  4 5492  6.295647e-04
## 26717  4 5493  6.227342e-04
## 26718  4 5494  6.190186e-04
## 26719  4 5495  6.136221e-04
## 26720  4 5496  6.084228e-04
## 26721  4 5497  6.042353e-04
## 26722  4 5498  5.982590e-04
## 26723  4 5499  5.929087e-04
## 26724  4 5500  5.886430e-04
## 26725  4 5501  5.840470e-04
## 26726  4 5502  5.798221e-04
## 26727  4 5503  5.741134e-04
## 26728  4 5504  5.701633e-04
## 26729  4 5505  5.653912e-04
## 26730  4 5506  5.606708e-04
## 26731  4 5507  5.544170e-04
## 26732  4 5508  5.502741e-04
## 26733  4 5509  5.465139e-04
## 26734  4 5510  5.426466e-04
## 26735  4 5511  5.375768e-04
## 26736  4 5512  5.327476e-04
## 26737  4 5513  5.283885e-04
## 26738  4 5514  5.237988e-04
## 26739  4 5515  5.202753e-04
## 26740  4 5516  5.157875e-04
## 26741  4 5517  5.118240e-04
## 26742  4 5518  5.076752e-04
## 26743  4 5519  5.027872e-04
## 26744  4 5520  4.990027e-04
## 26745  4 5521  4.950995e-04
## 26746  4 5522  4.902710e-04
## 26747  4 5523  4.857773e-04
## 26748  4 5524  4.815498e-04
## 26749  4 5525  4.779600e-04
## 26750  4 5526  4.748936e-04
## 26751  4 5527  4.707564e-04
## 26752  4 5528  4.662907e-04
## 26753  4 5529  4.634092e-04
## 26754  4 5530  4.578694e-04
## 26755  4 5531  4.553752e-04
## 26756  4 5532  4.511555e-04
## 26757  4 5533  4.474422e-04
## 26758  4 5534  4.439324e-04
## 26759  4 5535  4.408945e-04
## 26760  4 5536  4.368354e-04
## 26761  4 5537  4.333915e-04
## 26762  4 5538  4.292993e-04
## 26763  4 5539  4.257785e-04
## 26764  4 5540  4.221338e-04
## 26765  4 5541  4.184870e-04
## 26766  4 5542  4.154077e-04
## 26767  4 5543  4.125384e-04
## 26768  4 5544  4.078097e-04
## 26769  4 5545  4.055066e-04
## 26770  4 5546  4.024697e-04
## 26771  4 5547  3.983868e-04
## 26772  4 5548  3.954025e-04
## 26773  4 5549  3.926353e-04
## 26774  4 5550  3.885753e-04
## 26775  4 5551  3.850483e-04
## 26776  4 5552  3.821897e-04
## 26777  4 5553  3.794277e-04
## 26778  4 5554  3.765141e-04
## 26779  4 5555  3.724659e-04
## 26780  4 5556  3.704900e-04
## 26781  4 5557  3.665627e-04
## 26782  4 5558  3.633265e-04
## 26783  4 5559  3.604545e-04
## 26784  4 5560  3.575017e-04
## 26785  4 5561  3.551343e-04
## 26786  4 5562  3.520831e-04
## 26787  4 5563  3.490951e-04
## 26788  4 5564  3.457002e-04
## 26789  4 5565  3.439427e-04
## 26790  4 5566  3.405547e-04
## 26791  4 5567  3.370455e-04
## 26792  4 5568  3.348782e-04
## 26793  4 5569  3.316129e-04
## 26794  4 5570  3.293367e-04
## 26795  4 5571  3.272416e-04
## 26796  4 5572  3.241167e-04
## 26797  4 5573  3.211865e-04
## 26798  4 5574  3.180903e-04
## 26799  4 5575  3.154290e-04
## 26800  4 5576  3.135074e-04
## 26801  4 5577  3.113128e-04
## 26802  4 5578  3.076634e-04
## 26803  4 5579  3.059168e-04
## 26804  4 5580  3.033888e-04
## 26805  4 5581  3.007165e-04
## 26806  4 5582  2.980823e-04
## 26807  4 5583  2.958894e-04
## 26808  4 5584  2.934420e-04
## 26809  4 5585  2.905429e-04
## 26810  4 5586  2.883681e-04
## 26811  4 5587  2.860524e-04
## 26812  4 5588  2.840101e-04
## 26813  4 5589  2.813285e-04
## 26814  4 5590  2.788568e-04
## 26815  4 5591  2.763912e-04
## 26816  4 5592  2.742211e-04
## 26817  4 5593  2.724085e-04
## 26818  4 5594  2.699803e-04
## 26819  4 5595  2.673658e-04
## 26820  4 5596  2.654524e-04
## 26821  4 5597  2.631138e-04
## 26822  4 5598  2.608203e-04
## 26823  4 5599  2.588295e-04
## 26824  4 5600  2.570536e-04
## 26825  4 5601  2.550074e-04
## 26826  4 5602  2.523835e-04
## 26827  4 5603  2.503944e-04
## 26828  4 5604  2.481533e-04
## 26829  4 5605  2.467526e-04
## 26830  4 5606  2.443918e-04
## 26831  4 5607  2.423759e-04
## 26832  4 5608  2.403318e-04
## 26833  4 5609  2.387004e-04
## 26834  4 5610  2.362548e-04
## 26835  4 5611  2.346452e-04
## 26836  4 5612  2.325541e-04
## 26837  4 5613  2.302785e-04
## 26838  4 5614  2.284269e-04
## 26839  4 5615  2.269656e-04
## 26840  4 5616  2.246869e-04
## 26841  4 5617  2.226236e-04
## 26842  4 5618  2.207802e-04
## 26843  4 5619  2.192987e-04
## 26844  4 5620  2.175395e-04
## 26845  4 5621  2.157606e-04
## 26846  4 5622  2.141552e-04
## 26847  4 5623  2.122144e-04
## 26848  4 5624  2.100724e-04
## 26849  4 5625  2.087975e-04
## 26850  4 5626  2.066687e-04
## 26851  4 5627  2.053534e-04
## 26852  4 5628  2.035137e-04
## 26853  4 5629  2.018130e-04
## 26854  4 5630  2.000164e-04
## 26855  4 5631  1.987978e-04
## 26856  4 5632  1.972253e-04
## 26857  4 5633  1.954524e-04
## 26858  4 5634  1.938100e-04
## 26859  4 5635  1.921001e-04
## 26860  4 5636  1.906672e-04
## 26861  4 5637  1.886326e-04
## 26862  4 5638  1.874724e-04
## 26863  4 5639  1.855905e-04
## 26864  4 5640  1.844495e-04
## 26865  4 5641  1.827723e-04
## 26866  4 5642  1.814031e-04
## 26867  4 5643  1.796877e-04
## 26868  4 5644  1.782174e-04
## 26869  4 5645  1.765051e-04
## 26870  4 5646  1.753367e-04
## 26871  4 5647  1.736856e-04
## 26872  4 5648  1.724781e-04
## 26873  4 5649  1.712734e-04
## 26874  4 5650  1.696016e-04
## 26875  4 5651  1.683082e-04
## 26876  4 5652  1.666593e-04
## 26877  4 5653  1.654477e-04
## 26878  4 5654  1.641362e-04
## 26879  4 5655  1.626608e-04
## 26880  4 5656  1.614169e-04
## 26881  4 5657  1.599295e-04
## 26882  4 5658  1.587035e-04
## 26883  4 5659  1.577381e-04
## 26884  4 5660  1.559090e-04
## 26885  4 5661  1.546436e-04
## 26886  4 5662  1.536875e-04
## 26887  4 5663  1.524173e-04
## 26888  4 5664  1.510377e-04
## 26889  4 5665  1.499779e-04
## 26890  4 5666  1.484755e-04
## 26891  4 5667  1.473929e-04
## 26892  4 5668  1.459119e-04
## 26893  4 5669  1.449061e-04
## 26894  4 5670  1.435560e-04
## 26895  4 5671  1.425027e-04
## 26896  4 5672  1.413559e-04
## 26897  4 5673  1.400929e-04
## 26898  4 5674  1.390637e-04
## 26899  4 5675  1.375676e-04
## 26900  4 5676  1.368219e-04
## 26901  4 5677  1.356050e-04
## 26902  4 5678  1.343901e-04
## 26903  4 5679  1.335366e-04
## 26904  4 5680  1.323239e-04
## 26905  4 5681  1.311894e-04
## 26906  4 5682  1.302464e-04
## 26907  4 5683  1.288918e-04
## 26908  4 5684  1.278765e-04
## 26909  4 5685  1.269208e-04
## 26910  4 5686  1.260139e-04
## 26911  4 5687  1.249314e-04
## 26912  4 5688  1.238868e-04
## 26913  4 5689  1.227244e-04
## 26914  4 5690  1.216154e-04
## 26915  4 5691  1.206383e-04
## 26916  4 5692  1.200237e-04
## 26917  4 5693  1.187057e-04
## 26918  4 5694  1.177037e-04
## 26919  4 5695  1.167892e-04
## 26920  4 5696  1.156651e-04
## 26921  4 5697  1.150326e-04
## 26922  4 5698  1.137653e-04
## 26923  4 5699  1.128210e-04
## 26924  4 5700  1.121114e-04
## 26925  4 5701  1.110322e-04
## 26926  4 5702  1.100250e-04
## 26927  4 5703  1.090294e-04
## 26928  4 5704  1.083507e-04
## 26929  4 5705  1.075845e-04
## 26930  4 5706  1.064421e-04
## 26931  4 5707  1.057378e-04
## 26932  4 5708  1.047105e-04
## 26933  4 5709  1.038785e-04
## 26934  4 5710  1.030433e-04
## 26935  4 5711  1.021780e-04
## 26936  4 5712  1.015632e-04
## 26937  4 5713  1.005804e-04
## 26938  4 5714  9.972831e-05
## 26939  4 5715  9.905428e-05
## 26940  4 5716  9.813629e-05
## 26941  4 5717  9.722729e-05
## 26942  4 5718  9.649188e-05
## 26943  4 5719  9.570877e-05
## 26944  4 5720  9.475471e-05
## 26945  4 5721  9.410247e-05
## 26946  4 5722  9.350439e-05
## 26947  4 5723  9.243095e-05
## 26948  4 5724  9.172775e-05
## 26949  4 5725  9.110500e-05
## 26950  4 5726  9.035443e-05
## 26951  4 5727  8.951184e-05
## 26952  4 5728  8.884856e-05
## 26953  4 5729  8.807574e-05
## 26954  4 5730  8.745099e-05
## 26955  4 5731  8.677173e-05
## 26956  4 5732  8.592421e-05
## 26957  4 5733  8.528424e-05
## 26958  4 5734  8.443958e-05
## 26959  4 5735  8.362971e-05
## 26960  4 5736  8.310263e-05
## 26961  4 5737  8.245450e-05
## 26962  4 5738  8.166179e-05
## 26963  4 5739  8.111239e-05
## 26964  4 5740  8.035716e-05
## 26965  4 5741  7.976886e-05
## 26966  4 5742  7.897416e-05
## 26967  4 5743  7.847681e-05
## 26968  4 5744  7.768632e-05
## 26969  4 5745  7.713754e-05
## 26970  4 5746  7.646549e-05
## 26971  4 5747  7.600545e-05
## 26972  4 5748  7.523164e-05
## 26973  4 5749  7.478814e-05
## 26974  4 5750  7.383742e-05
## 26975  4 5751  7.325867e-05
## 26976  4 5752  7.288952e-05
## 26977  4 5753  7.213616e-05
## 26978  4 5754  7.151839e-05
## 26979  4 5755  7.098045e-05
## 26980  4 5756  7.052745e-05
## 26981  4 5757  6.985684e-05
## 26982  4 5758  6.925954e-05
## 26983  4 5759  6.859567e-05
## 26984  4 5760  6.813095e-05
## 26985  4 5761  6.755896e-05
## 26986  4 5762  6.718022e-05
## 26987  4 5763  6.642057e-05
## 26988  4 5764  6.587706e-05
## 26989  4 5765  6.547167e-05
## 26990  4 5766  6.478179e-05
## 26991  4 5767  6.423332e-05
## 26992  4 5768  6.368482e-05
## 26993  4 5769  6.325985e-05
## 26994  4 5770  6.272061e-05
## 26995  4 5771  6.218748e-05
## 26996  4 5772  6.160025e-05
## 26997  4 5773  6.111760e-05
## 26998  4 5774  6.058284e-05
## 26999  4 5775  6.019092e-05
## 27000  4 5776  5.969551e-05
## 27001  4 5777  5.921504e-05
## 27002  4 5778  5.854356e-05
## 27003  4 5779  5.817505e-05
## 27004  4 5780  5.768129e-05
## 27005  4 5781  5.721552e-05
## 27006  4 5782  5.682610e-05
## 27007  4 5783  5.619510e-05
## 27008  4 5784  5.591328e-05
## 27009  4 5785  5.534687e-05
## 27010  4 5786  5.486324e-05
## 27011  4 5787  5.441897e-05
## 27012  4 5788  5.408302e-05
## 27013  4 5789  5.351987e-05
## 27014  4 5790  5.306509e-05
## 27015  4 5791  5.268900e-05
## 27016  4 5792  5.222709e-05
## 27017  4 5793  5.180463e-05
## 27018  4 5794  5.146872e-05
## 27019  4 5795  5.096331e-05
## 27020  4 5796  5.042314e-05
## 27021  4 5797  5.009729e-05
## 27022  4 5798  4.964149e-05
## 27023  4 5799  4.926660e-05
## 27024  4 5800  4.889143e-05
## 27025  4 5801  4.847550e-05
## 27026  4 5802  4.803442e-05
## 27027  4 5803  4.765780e-05
## 27028  4 5804  4.729673e-05
## 27029  4 5805  4.685797e-05
## 27030  4 5806  4.650569e-05
## 27031  4 5807  4.610236e-05
## 27032  4 5808  4.578678e-05
## 27033  4 5809  4.537841e-05
## 27034  4 5810  4.506424e-05
## 27035  4 5811  4.460156e-05
## 27036  4 5812  4.425050e-05
## 27037  4 5813  4.380388e-05
## 27038  4 5814  4.351933e-05
## 27039  4 5815  4.316352e-05
## 27040  4 5816  4.277697e-05
## 27041  4 5817  4.242661e-05
## 27042  4 5818  4.211367e-05
## 27043  4 5819  4.172383e-05
## 27044  4 5820  4.141822e-05
## 27045  4 5821  4.104601e-05
## 27046  4 5822  4.074735e-05
## 27047  4 5823  4.039351e-05
## 27048  4 5824  4.005886e-05
## 27049  4 5825  3.972543e-05
## 27050  4 5826  3.943103e-05
## 27051  4 5827  3.905731e-05
## 27052  4 5828  3.876906e-05
## 27053  4 5829  3.847255e-05
## 27054  4 5830  3.811945e-05
## 27055  4 5831  3.779881e-05
## 27056  4 5832  3.748647e-05
## 27057  4 5833  3.716528e-05
## 27058  4 5834  3.685333e-05
## 27059  4 5835  3.651024e-05
## 27060  4 5836  3.628546e-05
## 27061  4 5837  3.602547e-05
## 27062  4 5838  3.571379e-05
## 27063  4 5839  3.535484e-05
## 27064  4 5840  3.509200e-05
## 27065  4 5841  3.480668e-05
## 27066  4 5842  3.449901e-05
## 27067  4 5843  3.419451e-05
## 27068  4 5844  3.393266e-05
## 27069  4 5845  3.362560e-05
## 27070  4 5846  3.340509e-05
## 27071  4 5847  3.312533e-05
## 27072  4 5848  3.281087e-05
## 27073  4 5849  3.254296e-05
## 27074  4 5850  3.231952e-05
## 27075  4 5851  3.199635e-05
## 27076  4 5852  3.172181e-05
## 27077  4 5853  3.150962e-05
## 27078  4 5854  3.120335e-05
## 27079  4 5855  3.095151e-05
## 27080  4 5856  3.068368e-05
## 27081  4 5857  3.052577e-05
## 27082  4 5858  3.020785e-05
## 27083  4 5859  2.999891e-05
## 27084  4 5860  2.970677e-05
## 27085  4 5861  2.941812e-05
## 27086  4 5862  2.926188e-05
## 27087  4 5863  2.898835e-05
## 27088  4 5864  2.873571e-05
## 27089  4 5865  2.853606e-05
## 27090  4 5866  2.829398e-05
## 27091  4 5867  2.803496e-05
## 27092  4 5868  2.779105e-05
## 27093  4 5869  2.758948e-05
## 27094  4 5870  2.734722e-05
## 27095  4 5871  2.713496e-05
## 27096  4 5872  2.686103e-05
## 27097  4 5873  2.669123e-05
## 27098  4 5874  2.641085e-05
## 27099  4 5875  2.625326e-05
## 27100  4 5876  2.605588e-05
## 27101  4 5877  2.584167e-05
## 27102  4 5878  2.562914e-05
## 27103  4 5879  2.538462e-05
## 27104  4 5880  2.519249e-05
## 27105  4 5881  2.501229e-05
## 27106  4 5882  2.473303e-05
## 27107  4 5883  2.451935e-05
## 27108  4 5884  2.433369e-05
## 27109  4 5885  2.412661e-05
## 27110  4 5886  2.394496e-05
## 27111  4 5887  2.375986e-05
## 27112  4 5888  2.356171e-05
## 27113  4 5889  2.337862e-05
## 27114  4 5890  2.319523e-05
## 27115  4 5891  2.299497e-05
## 27116  4 5892  2.278933e-05
## 27117  4 5893  2.262432e-05
## 27118  4 5894  2.244860e-05
## 27119  4 5895  2.220976e-05
## 27120  4 5896  2.203088e-05
## 27121  4 5897  2.187659e-05
## 27122  4 5898  2.166544e-05
## 27123  4 5899  2.144618e-05
## 27124  4 5900  2.131979e-05
## 27125  4 5901  2.110985e-05
## 27126  4 5902  2.096138e-05
## 27127  4 5903  2.079972e-05
## 27128  4 5904  2.061873e-05
## 27129  4 5905  2.048214e-05
## 27130  4 5906  2.032040e-05
## 27131  4 5907  2.014078e-05
## 27132  4 5908  1.993931e-05
## 27133  4 5909  1.980542e-05
## 27134  4 5910  1.965517e-05
## 27135  4 5911  1.949491e-05
## 27136  4 5912  1.930121e-05
## 27137  4 5913  1.917005e-05
## 27138  4 5914  1.896266e-05
## 27139  4 5915  1.882243e-05
## 27140  4 5916  1.863111e-05
## 27141  4 5917  1.852949e-05
## 27142  4 5918  1.834625e-05
## 27143  4 5919  1.823409e-05
## 27144  4 5920  1.804197e-05
## 27145  4 5921  1.794384e-05
## 27146  4 5922  1.778102e-05
## 27147  4 5923  1.760192e-05
## 27148  4 5924  1.745380e-05
## 27149  4 5925  1.734671e-05
## 27150  4 5926  1.718225e-05
## 27151  4 5927  1.704367e-05
## 27152  4 5928  1.692388e-05
## 27153  4 5929  1.678965e-05
## 27154  4 5930  1.664325e-05
## 27155  4 5931  1.650101e-05
## 27156  4 5932  1.633130e-05
## 27157  4 5933  1.620769e-05
## 27158  4 5934  1.612895e-05
## 27159  4 5935  1.596933e-05
## 27160  4 5936  1.583734e-05
## 27161  4 5937  1.571271e-05
## 27162  4 5938  1.557375e-05
## 27163  4 5939  1.542008e-05
## 27164  4 5940  1.531476e-05
## 27165  4 5941  1.519479e-05
## 27166  4 5942  1.508805e-05
## 27167  4 5943  1.493773e-05
## 27168  4 5944  1.480933e-05
## 27169  4 5945  1.470725e-05
## 27170  4 5946  1.455496e-05
## 27171  4 5947  1.447463e-05
## 27172  4 5948  1.433461e-05
## 27173  4 5949  1.423250e-05
## 27174  4 5950  1.409943e-05
## 27175  4 5951  1.399452e-05
## 27176  4 5952  1.387315e-05
## 27177  4 5953  1.375340e-05
## 27178  4 5954  1.361820e-05
## 27179  4 5955  1.350844e-05
## 27180  4 5956  1.343826e-05
## 27181  4 5957  1.329270e-05
## 27182  4 5958  1.320159e-05
## 27183  4 5959  1.309211e-05
## 27184  4 5960  1.298812e-05
## 27185  4 5961  1.288499e-05
## 27186  4 5962  1.276608e-05
## 27187  4 5963  1.267135e-05
## 27188  4 5964  1.254438e-05
## 27189  4 5965  1.245050e-05
## 27190  4 5966  1.232551e-05
## 27191  4 5967  1.224497e-05
## 27192  4 5968  1.214119e-05
## 27193  4 5969  1.206270e-05
## 27194  4 5970  1.195060e-05
## 27195  4 5971  1.185276e-05
## 27196  4 5972  1.174916e-05
## 27197  4 5973  1.166748e-05
## 27198  4 5974  1.156300e-05
## 27199  4 5975  1.144893e-05
## 27200  4 5976  1.136943e-05
## 27201  4 5977  1.126533e-05
## 27202  4 5978  1.117656e-05
## 27203  4 5979  1.108748e-05
## 27204  4 5980  1.099200e-05
## 27205  4 5981  1.090058e-05
## 27206  4 5982  1.082683e-05
## 27207  4 5983  1.074496e-05
## 27208  4 5984  1.065062e-05
## 27209  4 5985  1.056157e-05
## 27210  4 5986  1.045977e-05
## 27211  4 5987  1.039778e-05
## 27212  4 5988  1.029659e-05
## 27213  4 5989  1.022645e-05
## 27214  4 5990  1.012625e-05
## 27215  4 5991  1.004776e-05
## 27216  4 5992  9.959294e-06
## 27217  4 5993  9.874457e-06
## 27218  4 5994  9.810688e-06
## 27219  4 5995  9.732614e-06
## 27220  4 5996  9.640721e-06
## 27221  4 5997  9.563973e-06
## 27222  4 5998  9.472793e-06
## 27223  4 5999  9.397904e-06
## 27224  4 6000  9.349994e-06
## 27225  4 6001  9.260803e-06
## 27226  4 6002  9.174928e-06
## 27227  4 6003  9.099455e-06
## 27228  4 6004  9.022930e-06
## 27229  4 6005  8.975790e-06
## 27230  4 6006  8.869044e-06
## 27231  4 6007  8.818202e-06
## 27232  4 6008  8.739025e-06
## 27233  4 6009  8.668820e-06
## 27234  4 6010  8.622083e-06
## 27235  4 6011  8.532559e-06
```

```
## 27236  4 6012  8.455806e-06
## 27237  4 6013  8.388995e-06
## 27238  4 6014  8.326503e-06
## 27239  4 6015  8.246548e-06
## 27240  4 6016  8.183714e-06
## 27241  4 6017  8.098671e-06
## 27242  4 6018  8.050323e-06
## 27243  4 6019  7.980548e-06
## 27244  4 6020  7.914193e-06
## 27245  4 6021  7.850547e-06
## 27246  4 6022  7.778001e-06
## 27247  4 6023  7.704367e-06
## 27248  4 6024  7.649158e-06
## 27249  4 6025  7.590166e-06
## 27250  4 6026  7.532100e-06
## 27251  4 6027  7.468293e-06
## 27252  4 6028  7.399940e-06
## 27253  4 6029  7.339141e-06
## 27254  4 6030  7.283379e-06
## 27255  4 6031  7.217413e-06
## 27256  4 6032  7.163746e-06
## 27257  4 6033  7.099268e-06
## 27258  4 6034  7.045992e-06
## 27259  4 6035  6.984319e-06
## 27260  4 6036  6.924920e-06
## 27261  4 6037  6.881697e-06
## 27262  4 6038  6.818155e-06
## 27263  4 6039  6.752916e-06
## 27264  4 6040  6.710352e-06
## 27265  4 6041  6.657021e-06
## 27266  4 6042  6.580256e-06
## 27267  4 6043  6.547376e-06
## 27268  4 6044  6.489239e-06
## 27269  4 6045  6.433659e-06
## 27270  4 6046  6.389593e-06
## 27271  4 6047  6.328085e-06
## 27272  4 6048  6.274167e-06
## 27273  4 6049  6.230992e-06
## 27274  4 6050  6.178234e-06
## 27275  4 6051  6.121984e-06
## 27276  4 6052  6.077616e-06
## 27277  4 6053  6.010820e-06
## 27278  4 6054  5.972682e-06
## 27279  4 6055  5.918337e-06
## 27280  4 6056  5.878127e-06
## 27281  4 6057  5.824327e-06
## 27282  4 6058  5.785850e-06
## 27283  4 6059  5.732603e-06
## 27284  4 6060  5.695819e-06
## 27285  4 6061  5.634395e-06
## 27286  4 6062  5.598395e-06
## 27287  4 6063  5.545322e-06
## 27288  4 6064  5.507662e-06
## 27289  4 6065  5.459045e-06
## 27290  4 6066  5.407278e-06
## 27291  4 6067  5.367793e-06
## 27292  4 6068  5.321295e-06
## 27293  4 6069  5.268104e-06
## 27294  4 6070  5.229942e-06
## 27295  4 6071  5.203127e-06
## 27296  4 6072  5.160831e-06
## 27297  4 6073  5.113724e-06
## 27298  4 6074  5.066786e-06
## 27299  4 6075  5.029812e-06
## 27300  4 6076  4.980794e-06
## 27301  4 6077  4.947833e-06
## 27302  4 6078  4.910420e-06
## 27303  4 6079  4.862276e-06
## 27304  4 6080  4.823058e-06
## 27305  4 6081  4.782466e-06
## 27306  4 6082  4.750047e-06
## 27307  4 6083  4.711767e-06
## 27308  4 6084  4.666814e-06
## 27309  4 6085  4.630876e-06
## 27310  4 6086  4.589654e-06
## 27311  4 6087  4.560971e-06
## 27312  4 6088  4.515636e-06
## 27313  4 6089  4.481950e-06
## 27314  4 6090  4.437291e-06
## 27315  4 6091  4.420540e-06
## 27316  4 6092  4.372180e-06
## 27317  4 6093  4.331115e-06
## 27318  4 6094  4.301042e-06
## 27319  4 6095  4.268778e-06
## 27320  4 6096  4.229249e-06
## 27321  4 6097  4.188242e-06
## 27322  4 6098  4.165455e-06
## 27323  4 6099  4.116496e-06
## 27324  4 6100  4.091695e-06
## 27325  4 6101  4.056558e-06
## 27326  4 6102  4.028475e-06
## 27327  4 6103  3.995668e-06
## 27328  4 6104  3.953285e-06
## 27329  4 6105  3.925654e-06
## 27330  4 6106  3.898003e-06
## 27331  4 6107  3.863763e-06
## 27332  4 6108  3.823086e-06
## 27333  4 6109  3.789410e-06
## 27334  4 6110  3.758430e-06
## 27335  4 6111  3.730063e-06
## 27336  4 6112  3.695106e-06
## 27337  4 6113  3.676221e-06
## 27338  4 6114  3.635004e-06
## 27339  4 6115  3.607850e-06
## 27340  4 6116  3.577801e-06
## 27341  4 6117  3.545161e-06
## 27342  4 6118  3.517099e-06
## 27343  4 6119  3.486421e-06
## 27344  4 6120  3.462622e-06
## 27345  4 6121  3.434481e-06
## 27346  4 6122  3.395890e-06
## 27347  4 6123  3.367566e-06
## 27348  4 6124  3.341969e-06
## 27349  4 6125  3.310422e-06
## 27350  4 6126  3.281143e-06
## 27351  4 6127  3.253693e-06
## 27352  4 6128  3.228520e-06
## 27353  4 6129  3.198544e-06
## 27354  4 6130  3.172454e-06
## 27355  4 6131  3.146057e-06
## 27356  4 6132  3.118714e-06
## 27357  4 6133  3.088886e-06
## 27358  4 6134  3.061749e-06
## 27359  4 6135  3.037912e-06
## 27360  4 6136  3.011393e-06
## 27361  4 6137  2.986851e-06
## 27362  4 6138  2.961858e-06
## 27363  4 6139  2.933797e-06
## 27364  4 6140  2.905663e-06
## 27365  4 6141  2.886704e-06
## 27366  4 6142  2.860490e-06
## 27367  4 6143  2.835183e-06
## 27368  4 6144  2.810381e-06
## 27369  4 6145  2.790011e-06
## 27370  4 6146  2.763466e-06
## 27371  4 6147  2.737118e-06
## 27372  4 6148  2.714779e-06
## 27373  4 6149  2.693768e-06
## 27374  4 6150  2.672678e-06
## 27375  4 6151  2.646279e-06
## 27376  4 6152  2.625060e-06
## 27377  4 6153  2.603414e-06
## 27378  4 6154  2.579311e-06
## 27379  4 6155  2.557742e-06
## 27380  4 6156  2.541135e-06
## 27381  4 6157  2.519332e-06
## 27382  4 6158  2.493026e-06
## 27383  4 6159  2.477444e-06
## 27384  4 6160  2.457932e-06
## 27385  4 6161  2.437149e-06
## 27386  4 6162  2.410463e-06
## 27387  4 6163  2.395119e-06
## 27388  4 6164  2.378360e-06
## 27389  4 6165  2.357103e-06
## 27390  4 6166  2.340696e-06
## 27391  4 6167  2.318769e-06
## 27392  4 6168  2.302010e-06
## 27393  4 6169  2.281222e-06
## 27394  4 6170  2.264078e-06
## 27395  4 6171  2.251170e-06
## 27396  4 6172  2.232321e-06
## 27397  4 6173  2.215846e-06
## 27398  4 6174  2.196179e-06
## 27399  4 6175  2.177034e-06
## 27400  4 6176  2.160330e-06
## 27401  4 6177  2.144208e-06
## 27402  4 6178  2.127701e-06
## 27403  4 6179  2.107537e-06
## 27404  4 6180  2.089312e-06
## 27405  4 6181  2.080186e-06
## 27406  4 6182  2.056723e-06
## 27407  4 6183  2.040192e-06
## 27408  4 6184  2.026623e-06
## 27409  4 6185  2.005700e-06
## 27410  4 6186  1.991582e-06
## 27411  4 6187  1.975813e-06
## 27412  4 6188  1.963317e-06
## 27413  4 6189  1.948393e-06
## 27414  4 6190  1.930780e-06
## 27415  4 6191  1.912876e-06
## 27416  4 6192  1.897097e-06
## 27417  4 6193  1.885636e-06
## 27418  4 6194  1.869019e-06
## 27419  4 6195  1.856695e-06
## 27420  4 6196  1.838012e-06
## 27421  4 6197  1.824972e-06
## 27422  4 6198  1.810824e-06
## 27423  4 6199  1.794156e-06
## 27424  4 6200  1.780711e-06
## 27425  4 6201  1.766566e-06
## 27426  4 6202  1.750411e-06
## 27427  4 6203  1.736245e-06
## 27428  4 6204  1.724442e-06
## 27429  4 6205  1.712133e-06
## 27430  4 6206  1.695790e-06
## 27431  4 6207  1.681453e-06
## 27432  4 6208  1.669692e-06
## 27433  4 6209  1.654107e-06
## 27434  4 6210  1.638734e-06
## 27435  4 6211  1.627574e-06
## 27436  4 6212  1.614325e-06
## 27437  4 6213  1.600218e-06
## 27438  4 6214  1.588015e-06
## 27439  4 6215  1.576195e-06
## 27440  4 6216  1.561073e-06
## 27441  4 6217  1.546445e-06
## 27442  4 6218  1.536433e-06
## 27443  4 6219  1.519253e-06
## 27444  4 6220  1.505715e-06
## 27445  4 6221  1.494942e-06
## 27446  4 6222  1.482476e-06
## 27447  4 6223  1.472171e-06
## 27448  4 6224  1.458384e-06
## 27449  4 6225  1.450260e-06
## 27450  4 6226  1.435297e-06
## 27451  4 6227  1.423661e-06
## 27452  4 6228  1.411348e-06
## 27453  4 6229  1.399617e-06
## 27454  4 6230  1.389216e-06
## 27455  4 6231  1.375605e-06
## 27456  4 6232  1.365164e-06
## 27457  4 6233  1.354634e-06
## 27458  4 6234  1.344066e-06
## 27459  4 6235  1.331337e-06
## 27460  4 6236  1.321676e-06
## 27461  4 6237  1.310870e-06
## 27462  4 6238  1.299519e-06
## 27463  4 6239  1.288920e-06
## 27464  4 6240  1.279806e-06
## 27465  4 6241  1.269352e-06
## 27466  4 6242  1.256274e-06
## 27467  4 6243  1.245795e-06
## 27468  4 6244  1.235810e-06
## 27469  4 6245  1.224860e-06
## 27470  4 6246  1.217041e-06
## 27471  4 6247  1.204572e-06
## 27472  4 6248  1.193799e-06
## 27473  4 6249  1.183325e-06
## 27474  4 6250  1.173864e-06
## 27475  4 6251  1.164053e-06
## 27476  4 6252  1.155265e-06
## 27477  4 6253  1.143463e-06
## 27478  4 6254  1.134401e-06
## 27479  4 6255  1.124646e-06
## 27480  4 6256  1.115872e-06
## 27481  4 6257  1.103033e-06
## 27482  4 6258  1.094773e-06
## 27483  4 6259  1.084991e-06
## 27484  4 6260  1.076649e-06
## 27485  4 6261  1.065783e-06
## 27486  4 6262  1.056519e-06
## 27487  4 6263  1.047257e-06
## 27488  4 6264  1.036205e-06
## 27489  4 6265  1.025555e-06
## 27490  4 6266  1.018000e-06
## 27491  4 6267  1.010073e-06
## 27492  4 6268  1.000869e-06
## 27493  4 6269  9.921626e-07
## 27494  4 6270  9.863087e-07
## 27495  4 6271  9.764521e-07
## 27496  4 6272  9.669106e-07
## 27497  4 6273  9.587396e-07
## 27498  4 6274  9.529268e-07
## 27499  4 6275  9.428405e-07
## 27500  4 6276  9.359811e-07
## 27501  4 6277  9.275988e-07
## 27502  4 6278  9.195318e-07
## 27503  4 6279  9.120884e-07
## 27504  4 6280  9.025865e-07
## 27505  4 6281  8.984385e-07
## 27506  4 6282  8.910604e-07
## 27507  4 6283  8.840269e-07
## 27508  4 6284  8.755593e-07
## 27509  4 6285  8.665723e-07
## 27510  4 6286  8.622942e-07
## 27511  4 6287  8.551765e-07
## 27512  4 6288  8.483587e-07
## 27513  4 6289  8.403779e-07
## 27514  4 6290  8.353206e-07
## 27515  4 6291  8.281588e-07
## 27516  4 6292  8.229504e-07
## 27517  4 6293  8.183686e-07
## 27518  4 6294  8.113505e-07
## 27519  4 6295  8.049612e-07
## 27520  4 6296  7.984965e-07
## 27521  4 6297  7.937160e-07
## 27522  4 6298  7.886509e-07
## 27523  4 6299  7.837377e-07
## 27524  4 6300  7.764961e-07
## 27525  4 6301  7.747636e-07
## 27526  4 6302  7.695634e-07
## 27527  4 6303  7.655845e-07
## 27528  4 6304  7.600034e-07
## 27529  4 6305  7.562769e-07
## 27530  4 6306  7.525819e-07
## 27531  4 6307  7.484619e-07
## 27532  4 6308  7.441408e-07
## 27533  4 6309  7.414599e-07
## 27534  4 6310  7.392699e-07
## 27535  4 6311  7.359569e-07
## 27536  4 6312  7.299449e-07
## 27537  4 6313  7.256251e-07
## 27538  4 6314  7.215954e-07
## 27539  4 6315  7.177328e-07
## 27540  4 6316  7.129016e-07
## 27541  4 6317  7.078719e-07
## 27542  4 6318  7.023209e-07
## 27543  4 6319  6.988019e-07
## 27544  4 6320  6.924886e-07
## 27545  4 6321  6.873745e-07
## 27546  4 6322  6.853873e-07
## 27547  4 6323  6.786190e-07
## 27548  4 6324  6.746670e-07
## 27549  4 6325  6.709380e-07
## 27550  4 6326  6.660265e-07
## 27551  4 6327  6.622650e-07
## 27552  4 6328  6.567978e-07
## 27553  4 6329  6.532482e-07
## 27554  4 6330  6.477905e-07
## 27555  4 6331  6.417313e-07
## 27556  4 6332  6.383682e-07
## 27557  4 6333  6.335075e-07
## 27558  4 6334  6.292843e-07
## 27559  4 6335  6.240887e-07
## 27560  4 6336  6.200966e-07
## 27561  4 6337  6.167475e-07
## 27562  4 6338  6.105489e-07
## 27563  4 6339  6.046824e-07
## 27564  4 6340  6.000220e-07
## 27565  4 6341  5.948843e-07
## 27566  4 6342  5.893741e-07
## 27567  4 6343  5.858132e-07
## 27568  4 6344  5.793034e-07
## 27569  4 6345  5.752943e-07
## 27570  4 6346  5.683217e-07
## 27571  4 6347  5.636545e-07
## 27572  4 6348  5.581834e-07
## 27573  4 6349  5.534649e-07
## 27574  4 6350  5.468226e-07
## 27575  4 6351  5.414578e-07
## 27576  4 6352  5.351662e-07
## 27577  4 6353  5.284382e-07
## 27578  4 6354  5.225716e-07
## 27579  4 6355  5.173799e-07
## 27580  4 6356  5.111333e-07
## 27581  4 6357  5.032006e-07
## 27582  4 6358  4.966201e-07
## 27583  4 6359  4.906317e-07
## 27584  4 6360  4.834371e-07
## 27585  4 6361  4.763658e-07
## 27586  4 6362  4.680127e-07
## 27587  4 6363  4.643878e-07
## 27588  4 6364  4.584908e-07
## 27589  4 6365  4.543602e-07
## 27590  4 6366  4.492015e-07
## 27591  4 6367  4.444919e-07
## 27592  4 6368  4.388317e-07
## 27593  4 6369  4.347326e-07
## 27594  4 6370  4.292805e-07
## 27595  4 6371  4.252453e-07
## 27596  4 6372  4.203798e-07
## 27597  4 6373  4.170964e-07
## 27598  4 6374  4.123931e-07
## 27599  4 6375  4.067500e-07
## 27600  4 6376  4.023179e-07
## 27601  4 6377  3.978553e-07
## 27602  4 6378  3.940999e-07
## 27603  4 6379  3.898365e-07
## 27604  4 6380  3.860457e-07
## 27605  4 6381  3.815708e-07
## 27606  4 6382  3.770786e-07
## 27607  4 6383  3.734513e-07
## 27608  4 6384  3.687717e-07
## 27609  4 6385  3.655868e-07
## 27610  4 6386  3.618492e-07
## 27611  4 6387  3.571263e-07
## 27612  4 6388  3.535710e-07
## 27613  4 6389  3.496470e-07
## 27614  4 6390  3.462166e-07
## 27615  4 6391  3.424169e-07
## 27616  4 6392  3.391017e-07
## 27617  4 6393  3.358537e-07
## 27618  4 6394  3.321826e-07
## 27619  4 6395  3.295528e-07
## 27620  4 6396  3.257603e-07
## 27621  4 6397  3.230621e-07
## 27622  4 6398  3.199045e-07
## 27623  4 6399  3.170923e-07
## 27624  4 6400  3.137416e-07
## 27625  4 6401  3.112687e-07
## 27626  4 6402  3.087252e-07
## 27627  4 6403  3.053553e-07
## 27628  4 6404  3.032238e-07
## 27629  4 6405  3.005902e-07
## 27630  4 6406  2.983980e-07
## 27631  4 6407  2.959574e-07
## 27632  4 6408  2.923265e-07
## 27633  4 6409  2.908007e-07
## 27634  4 6410  2.875263e-07
## 27635  4 6411  2.853675e-07
## 27636  4 6412  2.826631e-07
## 27637  4 6413  2.804159e-07
## 27638  4 6414  2.780841e-07
## 27639  4 6415  2.758771e-07
## 27640  4 6416  2.734913e-07
## 27641  4 6417  2.713796e-07
## 27642  4 6418  2.687339e-07
## 27643  4 6419  2.671617e-07
## 27644  4 6420  2.652408e-07
## 27645  4 6421  2.627740e-07
## 27646  4 6422  2.611345e-07
## 27647  4 6423  2.592001e-07
## 27648  4 6424  2.572624e-07
## 27649  4 6425  2.557787e-07
## 27650  4 6426  2.543589e-07
## 27651  4 6427  2.518854e-07
## 27652  4 6428  2.509797e-07
## 27653  4 6429  2.491218e-07
## 27654  4 6430  2.477935e-07
## 27655  4 6431  2.470316e-07
## 27656  4 6432  2.452945e-07
## 27657  4 6433  2.442927e-07
## 27658  4 6434  2.434629e-07
## 27659  4 6435  2.414388e-07
## 27660  4 6436  2.409290e-07
## 27661  4 6437  2.396133e-07
## 27662  4 6438  2.389261e-07
## 27663  4 6439  2.377640e-07
## 27664  4 6440  2.372954e-07
## 27665  4 6441  2.364263e-07
## 27666  4 6442  2.355718e-07
## 27667  4 6443  2.353697e-07
## 27668  4 6444  2.346038e-07
## 27669  4 6445  2.342382e-07
## 27670  4 6446  2.332802e-07
## 27671  4 6447  2.331711e-07
## 27672  4 6448  2.323846e-07
## 27673  4 6449  2.320642e-07
## 27674  4 6450  2.317205e-07
## 27675  4 6451  2.307695e-07
## 27676  4 6452  2.302795e-07
## 27677  4 6453  2.291683e-07
## 27678  4 6454  2.281079e-07
## 27679  4 6455  2.272472e-07
## 27680  4 6456  2.261202e-07
## 27681  4 6457  2.252630e-07
## 27682  4 6458  2.246127e-07
## 27683  4 6459  2.237398e-07
## 27684  4 6460  2.226399e-07
## 27685  4 6461  2.220900e-07
## 27686  4 6462  2.209451e-07
## 27687  4 6463  2.195496e-07
## 27688  4 6464  2.189585e-07
## 27689  4 6465  2.179643e-07
## 27690  4 6466  2.164611e-07
## 27691  4 6467  2.161285e-07
## 27692  4 6468  2.145401e-07
## 27693  4 6469  2.132338e-07
## 27694  4 6470  2.122127e-07
## 27695  4 6471  2.111479e-07
## 27696  4 6472  2.096238e-07
## 27697  4 6473  2.079701e-07
## 27698  4 6474  2.067939e-07
## 27699  4 6475  2.051112e-07
## 27700  4 6476  2.036222e-07
## 27701  4 6477  2.021139e-07
## 27702  4 6478  2.004743e-07
## 27703  4 6479  1.983580e-07
## 27704  4 6480  1.968932e-07
## 27705  4 6481  1.952982e-07
## 27706  4 6482  1.926825e-07
## 27707  4 6483  1.909791e-07
## 27708  4 6484  1.888474e-07
## 27709  4 6485  1.866862e-07
## 27710  4 6486  1.843950e-07
## 27711  4 6487  1.819877e-07
## 27712  4 6488  1.794835e-07
## 27713  4 6489  1.768139e-07
## 27714  4 6490  1.740187e-07
## 27715  4 6491  1.711493e-07
## 27716  4 6492  1.680605e-07
## 27717  4 6493  1.651408e-07
## 27718  4 6494  1.634292e-07
## 27719  4 6495  1.617670e-07
## 27720  4 6496  1.598178e-07
## 27721  4 6497  1.581936e-07
## 27722  4 6498  1.561309e-07
## 27723  4 6499  1.547183e-07
## 27724  4 6500  1.529465e-07
## 27725  4 6501  1.509764e-07
## 27726  4 6502  1.492225e-07
## 27727  4 6503  1.475280e-07
## 27728  4 6504  1.458439e-07
## 27729  4 6505  1.441243e-07
## 27730  4 6506  1.422380e-07
## 27731  4 6507  1.402508e-07
## 27732  4 6508  1.386985e-07
## 27733  4 6509  1.369013e-07
## 27734  4 6510  1.354655e-07
## 27735  4 6511  1.332341e-07
## 27736  4 6512  1.318522e-07
## 27737  4 6513  1.297776e-07
## 27738  4 6514  1.282691e-07
## 27739  4 6515  1.263688e-07
## 27740  4 6516  1.246516e-07
## 27741  4 6517  1.228630e-07
## 27742  4 6518  1.213580e-07
## 27743  4 6519  1.195651e-07
## 27744  4 6520  1.182007e-07
## 27745  4 6521  1.166781e-07
## 27746  4 6522  1.148236e-07
## 27747  4 6523  1.131436e-07
## 27748  4 6524  1.117091e-07
## 27749  4 6525  1.104067e-07
## 27750  4 6526  1.088024e-07
## 27751  4 6527  1.072524e-07
## 27752  4 6528  1.057290e-07
## 27753  4 6529  1.044660e-07
## 27754  4 6530  1.031672e-07
## 27755  4 6531  1.015661e-07
## 27756  4 6532  1.002886e-07
## 27757  4 6533  9.913770e-08
## 27758  4 6534  9.786692e-08
## 27759  4 6535  9.681532e-08
## 27760  4 6536  9.555499e-08
## 27761  4 6537  9.439913e-08
## 27762  4 6538  9.323980e-08
## 27763  4 6539  9.181704e-08
## 27764  4 6540  9.051821e-08
## 27765  4 6541  8.906459e-08
## 27766  4 6542  8.798222e-08
## 27767  4 6543  8.698808e-08
## 27768  4 6544  8.574025e-08
## 27769  4 6545  8.478226e-08
## 27770  4 6546  8.356826e-08
## 27771  4 6547  8.253116e-08
## 27772  4 6548  8.153173e-08
## 27773  4 6549  8.057321e-08
## 27774  4 6550  7.984777e-08
## 27775  4 6551  7.864470e-08
## 27776  4 6552  7.800935e-08
## 27777  4 6553  7.725438e-08
## 27778  4 6554  7.642348e-08
## 27779  4 6555  7.582554e-08
## 27780  4 6556  7.513675e-08
## 27781  4 6557  7.465904e-08
## 27782  4 6558  7.399196e-08
## 27783  4 6559  7.354217e-08
## 27784  4 6560  7.300895e-08
## 27785  4 6561  7.268230e-08
## 27786  4 6562  7.243910e-08
## 27787  4 6563  7.200796e-08
## 27788  4 6564  7.186411e-08
## 27789  4 6565  7.169410e-08
## 27790  4 6566  7.164461e-08
## 27791  4 6567  7.167186e-08
## 27792  4 6568  7.157636e-08
## 27793  4 6569  7.174410e-08
## 27794  4 6570  7.186873e-08
## 27795  4 6571  7.219568e-08
## 27796  4 6572  7.244260e-08
## 27797  4 6573  7.285390e-08
## 27798  4 6574  7.324829e-08
## 27799  4 6575  7.388326e-08
## 27800  4 6576  7.452093e-08
## 27801  4 6577  7.522061e-08
## 27802  4 6578  7.594774e-08
## 27803  4 6579  7.689597e-08
## 27804  4 6580  7.771486e-08
## 27805  4 6581  7.809907e-08
## 27806  4 6582  7.754982e-08
## 27807  4 6583  7.715629e-08
## 27808  4 6584  7.692618e-08
## 27809  4 6585  7.645386e-08
## 27810  4 6586  7.620509e-08
## 27811  4 6587  7.615507e-08
## 27812  4 6588  7.556925e-08
## 27813  4 6589  7.528943e-08
## 27814  4 6590  7.502177e-08
## 27815  4 6591  7.467996e-08
## 27816  4 6592  7.429153e-08
## 27817  4 6593  7.393720e-08
## 27818  4 6594  7.352847e-08
## 27819  4 6595  7.332106e-08
## 27820  4 6596  7.279735e-08
## 27821  4 6597  7.254500e-08
## 27822  4 6598  7.221697e-08
## 27823  4 6599  7.182855e-08
## 27824  4 6600  7.145646e-08
## 27825  4 6601  7.107140e-08
## 27826  4 6602  7.079861e-08
## 27827  4 6603  7.047086e-08
## 27828  4 6604  6.995124e-08
## 27829  4 6605  6.967115e-08
## 27830  4 6606  6.939004e-08
## 27831  4 6607  6.889119e-08
## 27832  4 6608  6.845838e-08
## 27833  4 6609  6.815961e-08
## 27834  4 6610  6.755526e-08
## 27835  4 6611  6.733599e-08
## 27836  4 6612  6.688906e-08
## 27837  4 6613  6.635019e-08
## 27838  4 6614  6.598027e-08
## 27839  4 6615  6.545739e-08
## 27840  4 6616  6.502881e-08
## 27841  4 6617  6.448327e-08
## 27842  4 6618  6.393276e-08
## 27843  4 6619  6.337242e-08
## 27844  4 6620  6.288304e-08
## 27845  4 6621  6.233668e-08
## 27846  4 6622  6.175589e-08
## 27847  4 6623  6.124314e-08
## 27848  4 6624  6.055239e-08
## 27849  4 6625  5.994194e-08
## 27850  4 6626  5.921552e-08
## 27851  4 6627  5.859993e-08
## 27852  4 6628  5.796795e-08
## 27853  4 6629  5.718449e-08
## 27854  4 6630  5.646028e-08
## 27855  4 6631  5.563408e-08
## 27856  4 6632  5.483266e-08
## 27857  4 6633  5.402019e-08
## 27858  4 6634  5.313773e-08
## 27859  4 6635  5.231862e-08
## 27860  4 6636  5.141819e-08
## 27861  4 6637  5.050703e-08
## 27862  4 6638  4.956203e-08
## 27863  4 6639  4.863238e-08
## 27864  4 6640  4.748261e-08
## 27865  4 6641  4.641874e-08
## 27866  4 6642  4.550523e-08
## 27867  4 6643  4.500481e-08
## 27868  4 6644  4.453447e-08
## 27869  4 6645  4.403138e-08
## 27870  4 6646  4.353590e-08
## 27871  4 6647  4.302752e-08
## 27872  4 6648  4.242874e-08
## 27873  4 6649  4.203558e-08
## 27874  4 6650  4.159948e-08
## 27875  4 6651  4.108940e-08
## 27876  4 6652  4.069918e-08
## 27877  4 6653  4.020610e-08
## 27878  4 6654  3.973071e-08
## 27879  4 6655  3.931790e-08
## 27880  4 6656  3.885126e-08
## 27881  4 6657  3.841839e-08
## 27882  4 6658  3.800319e-08
## 27883  4 6659  3.755413e-08
## 27884  4 6660  3.711022e-08
## 27885  4 6661  3.671023e-08
## 27886  4 6662  3.628357e-08
## 27887  4 6663  3.587061e-08
## 27888  4 6664  3.549418e-08
## 27889  4 6665  3.510076e-08
## 27890  4 6666  3.469487e-08
## 27891  4 6667  3.431049e-08
## 27892  4 6668  3.397657e-08
## 27893  4 6669  3.359218e-08
## 27894  4 6670  3.323453e-08
## 27895  4 6671  3.285133e-08
## 27896  4 6672  3.251032e-08
## 27897  4 6673  3.219893e-08
## 27898  4 6674  3.186387e-08
## 27899  4 6675  3.154521e-08
## 27900  4 6676  3.114105e-08
## 27901  4 6677  3.085190e-08
## 27902  4 6678  3.059021e-08
## 27903  4 6679  3.023569e-08
## 27904  4 6680  3.002631e-08
## 27905  4 6681  2.972636e-08
## 27906  4 6682  2.947350e-08
## 27907  4 6683  2.921108e-08
## 27908  4 6684  2.902209e-08
## 27909  4 6685  2.878876e-08
## 27910  4 6686  2.854350e-08
## 27911  4 6687  2.832457e-08
## 27912  4 6688  2.812905e-08
## 27913  4 6689  2.794202e-08
## 27914  4 6690  2.779893e-08
## 27915  4 6691  2.761760e-08
## 27916  4 6692  2.747829e-08
## 27917  4 6693  2.733368e-08
## 27918  4 6694  2.717404e-08
## 27919  4 6695  2.708966e-08
## 27920  4 6696  2.698104e-08
## 27921  4 6697  2.686536e-08
## 27922  4 6698  2.683797e-08
## 27923  4 6699  2.676659e-08
## 27924  4 6700  2.679043e-08
## 27925  4 6701  2.675570e-08
## 27926  4 6702  2.671574e-08
## 27927  4 6703  2.671786e-08
## 27928  4 6704  2.667871e-08
## 27929  4 6705  2.644070e-08
## 27930  4 6706  2.626810e-08
## 27931  4 6707  2.602476e-08
## 27932  4 6708  2.582584e-08
## 27933  4 6709  2.559426e-08
## 27934  4 6710  2.546963e-08
## 27935  4 6711  2.525120e-08
## 27936  4 6712  2.504766e-08
## 27937  4 6713  2.483354e-08
## 27938  4 6714  2.465552e-08
## 27939  4 6715  2.450805e-08
## 27940  4 6716  2.428176e-08
## 27941  4 6717  2.410535e-08
## 27942  4 6718  2.394559e-08
## 27943  4 6719  2.375887e-08
## 27944  4 6720  2.356493e-08
## 27945  4 6721  2.340250e-08
## 27946  4 6722  2.323120e-08
## 27947  4 6723  2.303387e-08
## 27948  4 6724  2.288648e-08
## 27949  4 6725  2.264192e-08
## 27950  4 6726  2.250971e-08
## 27951  4 6727  2.233703e-08
## 27952  4 6728  2.210774e-08
## 27953  4 6729  2.200988e-08
## 27954  4 6730  2.180996e-08
## 27955  4 6731  2.166483e-08
## 27956  4 6732  2.148338e-08
## 27957  4 6733  2.128567e-08
## 27958  4 6734  2.112641e-08
## 27959  4 6735  2.095207e-08
## 27960  4 6736  2.079213e-08
## 27961  4 6737  2.063641e-08
## 27962  4 6738  2.049907e-08
## 27963  4 6739  2.028189e-08
## 27964  4 6740  2.015203e-08
## 27965  4 6741  1.996625e-08
## 27966  4 6742  1.981607e-08
## 27967  4 6743  1.962199e-08
## 27968  4 6744  1.946685e-08
## 27969  4 6745  1.932735e-08
## 27970  4 6746  1.912854e-08
## 27971  4 6747  1.896829e-08
## 27972  4 6748  1.877077e-08
## 27973  4 6749  1.862186e-08
## 27974  4 6750  1.845185e-08
## 27975  4 6751  1.831229e-08
## 27976  4 6752  1.815404e-08
## 27977  4 6753  1.797043e-08
## 27978  4 6754  1.779969e-08
## 27979  4 6755  1.759607e-08
## 27980  4 6756  1.747997e-08
## 27981  4 6757  1.724232e-08
## 27982  4 6758  1.710291e-08
## 27983  4 6759  1.692997e-08
## 27984  4 6760  1.674477e-08
## 27985  4 6761  1.654303e-08
## 27986  4 6762  1.636358e-08
## 27987  4 6763  1.615146e-08
## 27988  4 6764  1.598474e-08
## 27989  4 6765  1.581704e-08
## 27990  4 6766  1.564717e-08
## 27991  4 6767  1.553203e-08
## 27992  4 6768  1.538959e-08
## 27993  4 6769  1.528895e-08
## 27994  4 6770  1.510870e-08
## 27995  4 6771  1.496981e-08
## 27996  4 6772  1.486224e-08
## 27997  4 6773  1.473811e-08
## 27998  4 6774  1.458518e-08
## 27999  4 6775  1.449197e-08
## 28000  4 6776  1.434462e-08
## 28001  4 6777  1.418964e-08
## 28002  4 6778  1.408175e-08
## 28003  4 6779  1.397088e-08
## 28004  4 6780  1.384354e-08
## 28005  4 6781  1.372352e-08
## 28006  4 6782  1.360132e-08
## 28007  4 6783  1.348039e-08
## 28008  4 6784  1.336530e-08
## 28009  4 6785  1.325680e-08
## 28010  4 6786  1.313655e-08
## 28011  4 6787  1.302467e-08
## 28012  4 6788  1.289532e-08
## 28013  4 6789  1.280564e-08
## 28014  4 6790  1.265011e-08
## 28015  4 6791  1.258477e-08
## 28016  4 6792  1.244434e-08
## 28017  4 6793  1.233374e-08
## 28018  4 6794  1.224780e-08
## 28019  4 6795  1.213044e-08
## 28020  4 6796  1.204987e-08
## 28021  4 6797  1.191400e-08
## 28022  4 6798  1.182329e-08
## 28023  4 6799  1.171827e-08
## 28024  4 6800  1.161282e-08
## 28025  4 6801  1.150931e-08
## 28026  4 6802  1.142425e-08
## 28027  4 6803  1.132346e-08
## 28028  4 6804  1.121538e-08
## 28029  4 6805  1.111867e-08
## 28030  4 6806  1.103334e-08
## 28031  4 6807  1.091361e-08
## 28032  4 6808  1.082352e-08
## 28033  4 6809  1.074088e-08
## 28034  4 6810  1.063606e-08
## 28035  4 6811  1.055413e-08
```

```
## 28036  4 6812  1.046281e-08
## 28037  4 6813  1.039988e-08
## 28038  4 6814  1.029662e-08
## 28039  4 6815  1.021079e-08
## 28040  4 6816  1.012213e-08
## 28041  4 6817  1.004227e-08
## 28042  4 6818  9.948999e-09
## 28043  4 6819  9.866475e-09
## 28044  4 6820  9.803940e-09
## 28045  4 6821  9.710950e-09
## 28046  4 6822  9.613437e-09
## 28047  4 6823  9.552694e-09
## 28048  4 6824  9.488890e-09
## 28049  4 6825  9.396414e-09
## 28050  4 6826  9.329981e-09
## 28051  4 6827  9.187759e-09
## 28052  4 6828  9.102994e-09
## 28053  4 6829  9.029009e-09
## 28054  4 6830  8.952753e-09
## 28055  4 6831  8.881285e-09
## 28056  4 6832  8.815208e-09
## 28057  4 6833  8.733907e-09
## 28058  4 6834  8.658406e-09
## 28059  4 6835  8.584097e-09
## 28060  4 6836  8.513931e-09
## 28061  4 6837  8.470146e-09
## 28062  4 6838  8.378097e-09
## 28063  4 6839  8.304286e-09
## 28064  4 6840  8.245029e-09
## 28065  4 6841  8.165848e-09
## 28066  4 6842  8.097879e-09
## 28067  4 6843  8.014003e-09
## 28068  4 6844  7.970201e-09
## 28069  4 6845  7.873274e-09
## 28070  4 6846  7.837161e-09
## 28071  4 6847  7.753536e-09
## 28072  4 6848  7.690509e-09
## 28073  4 6849  7.636312e-09
## 28074  4 6850  7.564111e-09
## 28075  4 6851  7.504116e-09
## 28076  4 6852  7.427390e-09
## 28077  4 6853  7.360990e-09
## 28078  4 6854  7.302667e-09
## 28079  4 6855  7.236257e-09
## 28080  4 6856  7.164543e-09
## 28081  4 6857  7.124156e-09
## 28082  4 6858  7.061976e-09
## 28083  4 6859  6.999521e-09
## 28084  4 6860  6.943630e-09
## 28085  4 6861  6.888005e-09
## 28086  4 6862  6.832232e-09
## 28087  4 6863  6.759584e-09
## 28088  4 6864  6.695245e-09
## 28089  4 6865  6.654838e-09
## 28090  4 6866  6.582450e-09
## 28091  4 6867  6.536475e-09
## 28092  4 6868  6.460886e-09
## 28093  4 6869  6.424258e-09
## 28094  4 6870  6.369272e-09
## 28095  4 6871  6.319161e-09
## 28096  4 6872  6.263042e-09
## 28097  4 6873  6.202086e-09
## 28098  4 6874  6.163924e-09
## 28099  4 6875  6.104715e-09
## 28100  4 6876  6.048913e-09
## 28101  4 6877  5.999582e-09
## 28102  4 6878  5.960511e-09
## 28103  4 6879  5.895355e-09
## 28104  4 6880  5.854198e-09
## 28105  4 6881  5.813644e-09
## 28106  4 6882  5.760685e-09
## 28107  4 6883  5.709584e-09
## 28108  4 6884  5.664201e-09
## 28109  4 6885  5.618621e-09
## 28110  4 6886  5.564890e-09
## 28111  4 6887  5.520450e-09
## 28112  4 6888  5.480141e-09
## 28113  4 6889  5.441647e-09
## 28114  4 6890  5.379574e-09
## 28115  4 6891  5.326437e-09
## 28116  4 6892  5.253536e-09
## 28117  4 6893  5.204082e-09
## 28118  4 6894  5.150590e-09
## 28119  4 6895  5.094154e-09
## 28120  4 6896  5.045506e-09
## 28121  4 6897  4.982478e-09
## 28122  4 6898  4.925002e-09
## 28123  4 6899  4.871487e-09
## 28124  4 6900  4.822273e-09
## 28125  4 6901  4.764606e-09
## 28126  4 6902  4.715016e-09
## 28127  4 6903  4.666641e-09
## 28128  4 6904  4.606390e-09
## 28129  4 6905  4.557171e-09
## 28130  4 6906  4.502552e-09
## 28131  4 6907  4.455109e-09
## 28132  4 6908  4.395705e-09
## 28133  4 6909  4.349506e-09
## 28134  4 6910  4.301255e-09
## 28135  4 6911  4.249487e-09
## 28136  4 6912  4.200190e-09
## 28137  4 6913  4.152271e-09
## 28138  4 6914  4.100405e-09
## 28139  4 6915  4.046795e-09
## 28140  4 6916  4.008960e-09
## 28141  4 6917  3.952329e-09
## 28142  4 6918  3.905300e-09
## 28143  4 6919  3.864337e-09
## 28144  4 6920  3.816493e-09
## 28145  4 6921  3.764492e-09
## 28146  4 6922  3.718146e-09
## 28147  4 6923  3.676276e-09
## 28148  4 6924  3.624515e-09
## 28149  4 6925  3.583715e-09
## 28150  4 6926  3.541774e-09
## 28151  4 6927  3.493247e-09
## 28152  4 6928  3.447691e-09
## 28153  4 6929  3.405296e-09
## 28154  4 6930  3.359028e-09
## 28155  4 6931  3.320419e-09
## 28156  4 6932  3.273666e-09
## 28157  4 6933  3.235995e-09
## 28158  4 6934  3.188150e-09
## 28159  4 6935  3.149056e-09
## 28160  4 6936  3.103060e-09
## 28161  4 6937  3.061709e-09
## 28162  4 6938  3.026203e-09
## 28163  4 6939  2.985881e-09
## 28164  4 6940  2.947729e-09
## 28165  4 6941  2.903849e-09
## 28166  4 6942  2.861844e-09
## 28167  4 6943  2.831689e-09
## 28168  4 6944  2.785109e-09
## 28169  4 6945  2.748236e-09
## 28170  4 6946  2.707432e-09
## 28171  4 6947  2.670266e-09
## 28172  4 6948  2.634458e-09
## 28173  4 6949  2.593921e-09
## 28174  4 6950  2.564297e-09
## 28175  4 6951  2.528391e-09
## 28176  4 6952  2.487929e-09
## 28177  4 6953  2.459424e-09
## 28178  4 6954  2.417340e-09
## 28179  4 6955  2.383996e-09
## 28180  4 6956  2.348467e-09
## 28181  4 6957  2.315212e-09
## 28182  4 6958  2.282699e-09
## 28183  4 6959  2.249152e-09
## 28184  4 6960  2.218423e-09
## 28185  4 6961  2.182357e-09
## 28186  4 6962  2.150230e-09
## 28187  4 6963  2.119512e-09
## 28188  4 6964  2.088495e-09
## 28189  4 6965  2.058025e-09
## 28190  4 6966  2.022353e-09
## 28191  4 6967  1.993480e-09
## 28192  4 6968  1.963180e-09
## 28193  4 6969  1.932084e-09
## 28194  4 6970  1.904996e-09
## 28195  4 6971  1.876002e-09
## 28196  4 6972  1.849399e-09
## 28197  4 6973  1.819461e-09
## 28198  4 6974  1.788105e-09
## 28199  4 6975  1.763628e-09
## 28200  4 6976  1.736709e-09
## 28201  4 6977  1.708070e-09
## 28202  4 6978  1.680356e-09
## 28203  4 6979  1.653528e-09
## 28204  4 6980  1.628281e-09
## 28205  4 6981  1.601967e-09
## 28206  4 6982  1.576083e-09
## 28207  4 6983  1.555565e-09
## 28208  4 6984  1.527716e-09
## 28209  4 6985  1.506924e-09
## 28210  4 6986  1.482103e-09
## 28211  4 6987  1.459793e-09
## 28212  4 6988  1.434784e-09
## 28213  4 6989  1.410603e-09
## 28214  4 6990  1.389004e-09
## 28215  4 6991  1.368393e-09
## 28216  4 6992  1.347763e-09
## 28217  4 6993  1.328680e-09
## 28218  4 6994  1.306160e-09
## 28219  4 6995  1.286326e-09
## 28220  4 6996  1.262963e-09
## 28221  4 6997  1.240788e-09
## 28222  4 6998  1.224888e-09
## 28223  4 6999  1.205154e-09
## 28224  4 7000  1.186637e-09
## 28225  5    0  0.000000e+00
## 28226  5    0  2.400888e+00
## 28227  5    1  3.318405e+00
## 28228  5    2  3.488647e+00
## 28229  5    3  3.446476e+00
## 28230  5    4  3.356694e+00
## 28231  5    5  3.262554e+00
## 28232  5    6  3.172098e+00
## 28233  5    7  3.093895e+00
## 28234  5    8  3.018076e+00
## 28235  5    9  2.955118e+00
## 28236  5   10  2.898477e+00
## 28237  5   11  2.856386e+00
## 28238  5   12  2.808482e+00
## 28239  5   13  2.759368e+00
## 28240  5   14  2.724442e+00
## 28241  5   15  2.679398e+00
## 28242  5   16  2.645623e+00
## 28243  5   17  2.613840e+00
## 28244  5   18  2.582645e+00
## 28245  5   19  2.551709e+00
## 28246  5   20  2.517774e+00
## 28247  5   21  2.490060e+00
## 28248  5   22  2.459762e+00
## 28249  5   23  2.435141e+00
## 28250  5   24  2.402123e+00
## 28251  5   24  2.407408e+00
## 28252  5   25  3.320569e+00
## 28253  5   26  3.482751e+00
## 28254  5   27  3.443275e+00
## 28255  5   28  3.350623e+00
## 28256  5   29  3.257761e+00
## 28257  5   30  3.170306e+00
## 28258  5   31  3.089475e+00
## 28259  5   32  3.019613e+00
## 28260  5   33  2.964477e+00
## 28261  5   34  2.905060e+00
## 28262  5   35  2.848968e+00
## 28263  5   36  2.805725e+00
## 28264  5   37  2.762042e+00
## 28265  5   38  2.722567e+00
## 28266  5   39  2.678299e+00
## 28267  5   40  2.649401e+00
## 28268  5   41  2.610878e+00
## 28269  5   42  2.575612e+00
## 28270  5   43  2.546684e+00
## 28271  5   44  2.523407e+00
## 28272  5   45  2.493563e+00
## 28273  5   46  2.466888e+00
## 28274  5   47  2.435772e+00
## 28275  5   48  2.408875e+00
## 28276  5   48  2.406109e+00
## 28277  5   49  3.319490e+00
## 28278  5   50  3.476187e+00
## 28279  5   51  3.436403e+00
## 28280  5   52  3.352359e+00
## 28281  5   53  3.258839e+00
## 28282  5   54  3.169231e+00
## 28283  5   55  3.093234e+00
## 28284  5   56  3.024049e+00
## 28285  5   57  2.964352e+00
## 28286  5   58  2.903089e+00
## 28287  5   59  2.851108e+00
## 28288  5   60  2.805776e+00
## 28289  5   61  2.759420e+00
## 28290  5   62  2.718768e+00
## 28291  5   63  2.680423e+00
## 28292  5   64  2.647722e+00
## 28293  5   65  2.615810e+00
## 28294  5   66  2.586284e+00
## 28295  5   67  2.548367e+00
## 28296  5   68  2.520508e+00
## 28297  5   69  2.488535e+00
## 28298  5   70  2.464273e+00
## 28299  5   71  2.432606e+00
## 28300  5   72  2.407997e+00
## 28301  5   73  3.314137e+00
## 28302  5   74  3.484752e+00
## 28303  5   75  3.441040e+00
## 28304  5   76  3.358617e+00
## 28305  5   77  3.258896e+00
## 28306  5   78  3.169265e+00
## 28307  5   79  3.090726e+00
## 28308  5   80  3.029078e+00
## 28309  5   81  2.958357e+00
## 28310  5   82  2.900251e+00
## 28311  5   83  2.855611e+00
## 28312  5   84  2.806758e+00
## 28313  5   85  2.760220e+00
## 28314  5   86  2.721295e+00
## 28315  5   87  2.681126e+00
## 28316  5   88  2.646996e+00
## 28317  5   89  2.609928e+00
## 28318  5   90  2.579448e+00
## 28319  5   91  2.547962e+00
## 28320  5   92  2.518168e+00
## 28321  5   93  2.486187e+00
## 28322  5   94  2.457293e+00
## 28323  5   95  2.432190e+00
## 28324  5   96  2.409053e+00
## 28325  5   97  3.319867e+00
## 28326  5   98  3.483388e+00
## 28327  5   99  3.438828e+00
## 28328  5  100  3.352477e+00
## 28329  5  101  3.252265e+00
## 28330  5  102  3.172497e+00
## 28331  5  103  3.098365e+00
## 28332  5  104  3.023432e+00
## 28333  5  105  2.964708e+00
## 28334  5  106  2.900507e+00
## 28335  5  107  2.850676e+00
## 28336  5  108  2.802632e+00
## 28337  5  109  2.766010e+00
## 28338  5  110  2.715994e+00
## 28339  5  111  2.681831e+00
## 28340  5  112  2.648622e+00
## 28341  5  113  2.615122e+00
## 28342  5  114  2.580892e+00
## 28343  5  115  2.550437e+00
## 28344  5  116  2.520961e+00
## 28345  5  117  2.494045e+00
## 28346  5  118  2.462198e+00
## 28347  5  119  2.434569e+00
## 28348  5  120  2.414438e+00
## 28349  5  121  3.318957e+00
## 28350  5  122  3.482577e+00
## 28351  5  123  3.446965e+00
## 28352  5  124  3.355582e+00
## 28353  5  125  3.256122e+00
## 28354  5  126  3.175316e+00
## 28355  5  127  3.092886e+00
## 28356  5  128  3.024104e+00
## 28357  5  129  2.956896e+00
## 28358  5  130  2.905732e+00
## 28359  5  131  2.850108e+00
## 28360  5  132  2.804784e+00
## 28361  5  133  2.760797e+00
## 28362  5  134  2.720695e+00
## 28363  5  135  2.683051e+00
## 28364  5  136  2.648697e+00
## 28365  5  137  2.611272e+00
## 28366  5  138  2.582109e+00
## 28367  5  139  2.550764e+00
## 28368  5  140  2.519398e+00
## 28369  5  141  2.484537e+00
## 28370  5  142  2.461174e+00
## 28371  5  143  2.430425e+00
## 28372  5  144  2.409850e+00
## 28373  5  145  3.322120e+00
## 28374  5  146  3.483982e+00
## 28375  5  147  3.443183e+00
## 28376  5  148  3.353656e+00
## 28377  5  149  3.258544e+00
## 28378  5  150  3.176679e+00
## 28379  5  151  3.092233e+00
## 28380  5  152  3.017886e+00
## 28381  5  153  2.962456e+00
## 28382  5  154  2.898767e+00
## 28383  5  155  2.854265e+00
## 28384  5  156  2.800887e+00
## 28385  5  157  2.761252e+00
## 28386  5  158  2.722962e+00
## 28387  5  159  2.683170e+00
## 28388  5  160  2.647872e+00
## 28389  5  161  2.613693e+00
## 28390  5  162  2.580762e+00
## 28391  5  163  2.551463e+00
## 28392  5  164  2.518424e+00
## 28393  5  165  2.491245e+00
## 28394  5  166  2.467180e+00
## 28395  5  167  2.437371e+00
## 28396  5  168  2.409533e+00
## 28397  5  169  3.319774e+00
## 28398  5  170  3.478734e+00
## 28399  5  171  3.441385e+00
## 28400  5  172  3.355017e+00
## 28401  5  173  3.261815e+00
## 28402  5  174  3.173010e+00
## 28403  5  175  3.097109e+00
## 28404  5  176  3.021393e+00
## 28405  5  177  2.953621e+00
## 28406  5  178  2.898262e+00
## 28407  5  179  2.848443e+00
## 28408  5  180  2.810688e+00
## 28409  5  181  2.757409e+00
## 28410  5  182  2.720883e+00
## 28411  5  183  2.679017e+00
## 28412  5  184  2.646740e+00
## 28413  5  185  2.612752e+00
## 28414  5  186  2.583393e+00
## 28415  5  187  2.550946e+00
## 28416  5  188  2.520380e+00
## 28417  5  189  2.496466e+00
## 28418  5  190  2.458293e+00
## 28419  5  191  2.434509e+00
## 28420  5  192  2.409643e+00
## 28421  5  193  3.318207e+00
## 28422  5  194  3.476388e+00
## 28423  5  195  3.444572e+00
## 28424  5  196  3.351657e+00
## 28425  5  197  3.256436e+00
## 28426  5  198  3.173179e+00
## 28427  5  199  3.092897e+00
## 28428  5  200  3.022804e+00
## 28429  5  201  2.960545e+00
## 28430  5  202  2.902868e+00
## 28431  5  203  2.851800e+00
## 28432  5  204  2.799992e+00
## 28433  5  205  2.761733e+00
## 28434  5  206  2.722488e+00
## 28435  5  207  2.682912e+00
## 28436  5  208  2.648141e+00
## 28437  5  209  2.613764e+00
## 28438  5  210  2.577133e+00
## 28439  5  211  2.552483e+00
## 28440  5  212  2.517768e+00
## 28441  5  213  2.484119e+00
## 28442  5  214  2.464078e+00
## 28443  5  215  2.435440e+00
## 28444  5  216  2.408662e+00
## 28445  5  217  3.317327e+00
## 28446  5  218  3.481533e+00
## 28447  5  219  3.438534e+00
## 28448  5  220  3.357202e+00
## 28449  5  221  3.261364e+00
## 28450  5  222  3.176454e+00
## 28451  5  223  3.093331e+00
## 28452  5  224  3.018268e+00
## 28453  5  225  2.963909e+00
## 28454  5  226  2.902700e+00
## 28455  5  227  2.852350e+00
## 28456  5  228  2.802254e+00
## 28457  5  229  2.765086e+00
## 28458  5  230  2.721403e+00
## 28459  5  231  2.681872e+00
## 28460  5  232  2.644363e+00
## 28461  5  233  2.607395e+00
## 28462  5  234  2.578757e+00
## 28463  5  235  2.551781e+00
## 28464  5  236  2.517734e+00
## 28465  5  237  2.492119e+00
## 28466  5  238  2.458801e+00
## 28467  5  239  2.435648e+00
## 28468  5  240  2.405754e+00
## 28469  5  241  3.314731e+00
## 28470  5  242  3.488737e+00
## 28471  5  243  3.445431e+00
## 28472  5  244  3.352117e+00
## 28473  5  245  3.261866e+00
## 28474  5  246  3.171216e+00
## 28475  5  247  3.088209e+00
## 28476  5  248  3.025612e+00
## 28477  5  249  2.957500e+00
## 28478  5  250  2.902099e+00
## 28479  5  251  2.853481e+00
## 28480  5  252  2.806865e+00
## 28481  5  253  2.759957e+00
## 28482  5  254  2.716883e+00
## 28483  5  255  2.684328e+00
## 28484  5  256  2.642778e+00
## 28485  5  257  2.609686e+00
## 28486  5  258  2.580995e+00
## 28487  5  259  2.544196e+00
## 28488  5  260  2.521774e+00
## 28489  5  261  2.487755e+00
## 28490  5  262  2.462509e+00
## 28491  5  263  2.433363e+00
## 28492  5  264  2.409363e+00
## 28493  5  265  3.317039e+00
## 28494  5  266  3.482999e+00
## 28495  5  267  3.436882e+00
## 28496  5  268  3.351046e+00
## 28497  5  269  3.260633e+00
## 28498  5  270  3.171596e+00
## 28499  5  271  3.089697e+00
## 28500  5  272  3.023818e+00
## 28501  5  273  2.960434e+00
## 28502  5  274  2.903202e+00
## 28503  5  275  2.849319e+00
## 28504  5  276  2.799247e+00
## 28505  5  277  2.762571e+00
## 28506  5  278  2.721484e+00
## 28507  5  279  2.675188e+00
## 28508  5  280  2.647161e+00
## 28509  5  281  2.608062e+00
## 28510  5  282  2.581946e+00
## 28511  5  283  2.549811e+00
## 28512  5  284  2.516110e+00
## 28513  5  285  2.491346e+00
## 28514  5  286  2.460139e+00
## 28515  5  287  2.437573e+00
## 28516  5  288  2.410260e+00
## 28517  5  289  3.324243e+00
## 28518  5  290  3.485630e+00
## 28519  5  291  3.446751e+00
## 28520  5  292  3.347966e+00
## 28521  5  293  3.257849e+00
## 28522  5  294  3.168104e+00
## 28523  5  295  3.088093e+00
## 28524  5  296  3.025081e+00
## 28525  5  297  2.961200e+00
## 28526  5  298  2.898026e+00
## 28527  5  299  2.854652e+00
## 28528  5  300  2.804621e+00
## 28529  5  301  2.763696e+00
## 28530  5  302  2.721308e+00
## 28531  5  303  2.685806e+00
## 28532  5  304  2.649757e+00
## 28533  5  305  2.614659e+00
## 28534  5  306  2.582434e+00
## 28535  5  307  2.554370e+00
## 28536  5  308  2.512489e+00
## 28537  5  309  2.493996e+00
## 28538  5  310  2.460936e+00
## 28539  5  311  2.437814e+00
## 28540  5  312  2.407766e+00
## 28541  5  312  2.409756e+00
## 28542  5  313  3.321263e+00
## 28543  5  314  3.485172e+00
## 28544  5  315  3.444397e+00
## 28545  5  316  3.350103e+00
## 28546  5  317  3.265243e+00
## 28547  5  318  3.169239e+00
## 28548  5  319  3.085894e+00
## 28549  5  320  3.023360e+00
## 28550  5  321  2.957735e+00
## 28551  5  322  2.908469e+00
## 28552  5  323  2.854361e+00
## 28553  5  324  2.801187e+00
## 28554  5  325  2.756693e+00
## 28555  5  326  2.721402e+00
## 28556  5  327  2.680033e+00
## 28557  5  328  2.648945e+00
## 28558  5  329  2.610075e+00
## 28559  5  330  2.576685e+00
## 28560  5  331  2.554714e+00
## 28561  5  332  2.518269e+00
## 28562  5  333  2.486244e+00
## 28563  5  334  2.459846e+00
## 28564  5  335  2.433822e+00
## 28565  5  336  2.409404e+00
## 28566  5  336  2.411390e+00
## 28567  5  337  2.380953e+00
## 28568  5  338  2.357078e+00
## 28569  5  339  2.334186e+00
## 28570  5  339  2.330903e+00
## 28571  5  340  3.245724e+00
## 28572  5  341  3.402151e+00
## 28573  5  342  3.373914e+00
## 28574  5  343  3.281862e+00
## 28575  5  344  3.187469e+00
## 28576  5  345  3.100751e+00
## 28577  5  346  3.023628e+00
## 28578  5  347  2.956447e+00
## 28579  5  348  2.891937e+00
## 28580  5  349  2.839771e+00
## 28581  5  350  2.785535e+00
## 28582  5  351  2.736308e+00
## 28583  5  352  2.692902e+00
## 28584  5  353  2.660284e+00
## 28585  5  354  2.619791e+00
## 28586  5  355  2.585074e+00
## 28587  5  356  2.552861e+00
## 28588  5  357  2.518082e+00
## 28589  5  358  2.490018e+00
## 28590  5  359  2.456361e+00
## 28591  5  360  2.428872e+00
## 28592  5  360  2.435730e+00
## 28593  5  361  3.332854e+00
## 28594  5  362  3.509033e+00
## 28595  5  363  3.467251e+00
## 28596  5  364  3.375022e+00
## 28597  5  365  3.283422e+00
## 28598  5  366  3.184891e+00
## 28599  5  367  3.107718e+00
## 28600  5  368  3.040377e+00
## 28601  5  369  2.978895e+00
## 28602  5  370  2.919947e+00
## 28603  5  371  2.863703e+00
## 28604  5  372  2.821153e+00
## 28605  5  373  2.776763e+00
## 28606  5  374  2.730582e+00
## 28607  5  375  2.698682e+00
## 28608  5  376  2.663417e+00
## 28609  5  377  2.628240e+00
## 28610  5  378  2.593802e+00
## 28611  5  379  2.562223e+00
## 28612  5  380  2.535398e+00
## 28613  5  381  2.503179e+00
## 28614  5  382  2.478861e+00
## 28615  5  383  2.447740e+00
## 28616  5  384  2.422641e+00
## 28617  5  385  3.328767e+00
## 28618  5  386  3.493936e+00
## 28619  5  387  3.459818e+00
## 28620  5  388  3.364192e+00
## 28621  5  389  3.268954e+00
## 28622  5  390  3.182042e+00
## 28623  5  391  3.101968e+00
## 28624  5  392  3.028191e+00
## 28625  5  393  2.969432e+00
## 28626  5  394  2.915011e+00
## 28627  5  395  2.863261e+00
## 28628  5  396  2.820555e+00
## 28629  5  397  2.776323e+00
## 28630  5  398  2.731807e+00
## 28631  5  399  2.694046e+00
## 28632  5  400  2.660979e+00
## 28633  5  401  2.622464e+00
## 28634  5  402  2.588845e+00
## 28635  5  403  2.559817e+00
## 28636  5  404  2.529055e+00
## 28637  5  405  2.502178e+00
## 28638  5  406  2.476105e+00
## 28639  5  407  2.444129e+00
## 28640  5  408  2.418282e+00
## 28641  5  409  3.331588e+00
## 28642  5  410  3.493419e+00
## 28643  5  411  3.455404e+00
## 28644  5  412  3.361025e+00
## 28645  5  413  3.270151e+00
## 28646  5  414  3.182649e+00
## 28647  5  415  3.101463e+00
## 28648  5  416  3.031952e+00
## 28649  5  417  2.965279e+00
## 28650  5  418  2.911083e+00
## 28651  5  419  2.860043e+00
## 28652  5  420  2.814630e+00
## 28653  5  421  2.768548e+00
## 28654  5  422  2.730688e+00
## 28655  5  423  2.689885e+00
## 28656  5  424  2.658345e+00
## 28657  5  425  2.622544e+00
## 28658  5  426  2.591073e+00
## 28659  5  427  2.558133e+00
## 28660  5  428  2.527417e+00
## 28661  5  429  2.501566e+00
## 28662  5  430  2.468153e+00
## 28663  5  431  2.439919e+00
## 28664  5  432  2.412748e+00
## 28665  5  433  3.324726e+00
## 28666  5  434  3.494592e+00
## 28667  5  435  3.453046e+00
## 28668  5  436  3.365432e+00
## 28669  5  437  3.262012e+00
## 28670  5  438  3.177889e+00
## 28671  5  439  3.102272e+00
## 28672  5  440  3.029766e+00
## 28673  5  441  2.965677e+00
## 28674  5  442  2.909014e+00
## 28675  5  443  2.860267e+00
## 28676  5  444  2.806895e+00
## 28677  5  445  2.770560e+00
## 28678  5  446  2.731238e+00
## 28679  5  447  2.691675e+00
## 28680  5  448  2.656874e+00
## 28681  5  449  2.616232e+00
## 28682  5  450  2.586515e+00
## 28683  5  451  2.554585e+00
## 28684  5  452  2.525605e+00
## 28685  5  453  2.495105e+00
## 28686  5  454  2.466955e+00
## 28687  5  455  2.438460e+00
## 28688  5  456  2.408875e+00
## 28689  5  457  3.323476e+00
## 28690  5  458  3.488856e+00
## 28691  5  459  3.444096e+00
## 28692  5  460  3.360153e+00
## 28693  5  461  3.265337e+00
## 28694  5  462  3.177523e+00
## 28695  5  463  3.095676e+00
## 28696  5  464  3.027841e+00
## 28697  5  465  2.969434e+00
## 28698  5  466  2.912766e+00
## 28699  5  467  2.858013e+00
## 28700  5  468  2.812160e+00
## 28701  5  469  2.766164e+00
## 28702  5  470  2.723448e+00
## 28703  5  471  2.692585e+00
## 28704  5  472  2.649483e+00
## 28705  5  473  2.621088e+00
## 28706  5  474  2.584510e+00
## 28707  5  475  2.558186e+00
## 28708  5  476  2.524229e+00
## 28709  5  477  2.495304e+00
## 28710  5  478  2.466541e+00
## 28711  5  479  2.442166e+00
## 28712  5  480  2.416613e+00
## 28713  5  481  3.321469e+00
## 28714  5  482  3.487150e+00
## 28715  5  483  3.450052e+00
## 28716  5  484  3.362389e+00
## 28717  5  485  3.262388e+00
## 28718  5  486  3.176845e+00
## 28719  5  487  3.095137e+00
## 28720  5  488  3.028295e+00
## 28721  5  489  2.962526e+00
## 28722  5  490  2.907818e+00
## 28723  5  491  2.854732e+00
## 28724  5  492  2.810172e+00
## 28725  5  493  2.769260e+00
## 28726  5  494  2.721728e+00
## 28727  5  495  2.687723e+00
## 28728  5  496  2.651014e+00
## 28729  5  497  2.619228e+00
## 28730  5  498  2.583915e+00
## 28731  5  499  2.554284e+00
## 28732  5  500  2.521468e+00
## 28733  5  501  2.494956e+00
## 28734  5  502  2.468970e+00
## 28735  5  503  2.435094e+00
## 28736  5  504  2.411950e+00
## 28737  5  505  3.323056e+00
## 28738  5  506  3.487503e+00
## 28739  5  507  3.444538e+00
## 28740  5  508  3.357618e+00
## 28741  5  509  3.264749e+00
## 28742  5  510  3.170994e+00
## 28743  5  511  3.088738e+00
## 28744  5  512  3.023952e+00
## 28745  5  513  2.960210e+00
## 28746  5  514  2.904231e+00
## 28747  5  515  2.853543e+00
## 28748  5  516  2.808267e+00
## 28749  5  517  2.761338e+00
## 28750  5  518  2.723463e+00
## 28751  5  519  2.685970e+00
## 28752  5  520  2.652356e+00
## 28753  5  521  2.616965e+00
## 28754  5  522  2.583405e+00
## 28755  5  523  2.555810e+00
## 28756  5  524  2.520396e+00
## 28757  5  525  2.491815e+00
## 28758  5  526  2.463533e+00
## 28759  5  527  2.435388e+00
## 28760  5  528  2.409955e+00
## 28761  5  529  3.319572e+00
## 28762  5  530  3.485050e+00
## 28763  5  531  3.444921e+00
## 28764  5  532  3.360706e+00
## 28765  5  533  3.266216e+00
## 28766  5  534  3.170435e+00
## 28767  5  535  3.091409e+00
## 28768  5  536  3.022990e+00
## 28769  5  537  2.966221e+00
## 28770  5  538  2.907193e+00
## 28771  5  539  2.854641e+00
## 28772  5  540  2.804847e+00
## 28773  5  541  2.763613e+00
## 28774  5  542  2.720769e+00
## 28775  5  543  2.688418e+00
## 28776  5  544  2.649682e+00
## 28777  5  545  2.611953e+00
## 28778  5  546  2.583365e+00
## 28779  5  547  2.548344e+00
## 28780  5  548  2.520800e+00
## 28781  5  549  2.492786e+00
## 28782  5  550  2.463345e+00
## 28783  5  551  2.439598e+00
## 28784  5  552  2.408913e+00
## 28785  5  553  3.320344e+00
## 28786  5  554  3.498642e+00
## 28787  5  555  3.446974e+00
## 28788  5  556  3.360594e+00
## 28789  5  557  3.263522e+00
## 28790  5  558  3.179270e+00
## 28791  5  559  3.090075e+00
## 28792  5  560  3.026409e+00
## 28793  5  561  2.954705e+00
## 28794  5  562  2.908186e+00
## 28795  5  563  2.850955e+00
## 28796  5  564  2.807677e+00
## 28797  5  565  2.758938e+00
## 28798  5  566  2.718628e+00
## 28799  5  567  2.685135e+00
## 28800  5  568  2.650506e+00
## 28801  5  569  2.615350e+00
## 28802  5  570  2.583505e+00
## 28803  5  571  2.554463e+00
## 28804  5  572  2.517240e+00
## 28805  5  573  2.488020e+00
## 28806  5  574  2.459583e+00
## 28807  5  575  2.437491e+00
## 28808  5  576  2.407750e+00
## 28809  5  577  3.317883e+00
## 28810  5  578  3.490075e+00
## 28811  5  579  3.445719e+00
## 28812  5  580  3.353034e+00
## 28813  5  581  3.261797e+00
## 28814  5  582  3.171680e+00
## 28815  5  583  3.089169e+00
## 28816  5  584  3.021576e+00
## 28817  5  585  2.962626e+00
## 28818  5  586  2.906782e+00
## 28819  5  587  2.853235e+00
## 28820  5  588  2.807650e+00
## 28821  5  589  2.761237e+00
## 28822  5  590  2.720830e+00
## 28823  5  591  2.686097e+00
## 28824  5  592  2.648493e+00
## 28825  5  593  2.610978e+00
## 28826  5  594  2.576092e+00
## 28827  5  595  2.549999e+00
## 28828  5  596  2.520364e+00
## 28829  5  597  2.492058e+00
## 28830  5  598  2.463376e+00
## 28831  5  599  2.436796e+00
## 28832  5  600  2.408387e+00
## 28833  5  601  3.319358e+00
## 28834  5  602  3.489785e+00
## 28835  5  603  3.443725e+00
```

```
## 28836  5  604  3.353813e+00
## 28837  5  605  3.262895e+00
## 28838  5  606  3.175599e+00
## 28839  5  607  3.095345e+00
## 28840  5  608  3.020235e+00
## 28841  5  609  2.959491e+00
## 28842  5  610  2.903007e+00
## 28843  5  611  2.855140e+00
## 28844  5  612  2.807703e+00
## 28845  5  613  2.766369e+00
## 28846  5  614  2.719975e+00
## 28847  5  615  2.684046e+00
## 28848  5  616  2.647173e+00
## 28849  5  617  2.611165e+00
## 28850  5  618  2.585081e+00
## 28851  5  619  2.548833e+00
## 28852  5  620  2.519539e+00
## 28853  5  621  2.493915e+00
## 28854  5  622  2.462998e+00
## 28855  5  623  2.434715e+00
## 28856  5  624  2.411397e+00
## 28857  5  624  2.407997e+00
## 28858  5  625  3.321314e+00
## 28859  5  626  3.486336e+00
## 28860  5  627  3.439462e+00
## 28861  5  628  3.353992e+00
## 28862  5  629  3.252785e+00
## 28863  5  630  3.164843e+00
## 28864  5  631  3.093543e+00
## 28865  5  632  3.018327e+00
## 28866  5  633  2.954443e+00
## 28867  5  634  2.899207e+00
## 28868  5  635  2.852219e+00
## 28869  5  636  2.802041e+00
## 28870  5  637  2.756723e+00
## 28871  5  638  2.721896e+00
## 28872  5  639  2.685303e+00
## 28873  5  640  2.645559e+00
## 28874  5  641  2.613703e+00
## 28875  5  642  2.579031e+00
## 28876  5  643  2.548798e+00
## 28877  5  644  2.519485e+00
## 28878  5  645  2.492765e+00
## 28879  5  646  2.461283e+00
## 28880  5  647  2.432519e+00
## 28881  5  648  2.402739e+00
## 28882  5  648  2.403734e+00
## 28883  5  649  2.381124e+00
## 28884  5  650  2.353061e+00
## 28885  5  651  2.329870e+00
## 28886  5  652  2.307845e+00
## 28887  5  653  2.281921e+00
## 28888  5  654  2.257475e+00
## 28889  5  654  2.258321e+00
## 28890  5  655  3.168811e+00
## 28891  5  656  3.335895e+00
## 28892  5  657  3.302370e+00
## 28893  5  658  3.215489e+00
## 28894  5  659  3.113548e+00
## 28895  5  660  3.034012e+00
## 28896  5  661  2.957083e+00
## 28897  5  662  2.880590e+00
## 28898  5  663  2.823648e+00
## 28899  5  664  2.774404e+00
## 28900  5  665  2.719992e+00
## 28901  5  666  2.679387e+00
## 28902  5  667  2.636142e+00
## 28903  5  668  2.595384e+00
## 28904  5  669  2.565118e+00
## 28905  5  670  2.523167e+00
## 28906  5  671  2.492577e+00
## 28907  5  672  2.466277e+00
## 28908  5  672  2.458890e+00
## 28909  5  673  3.371767e+00
## 28910  5  674  3.527071e+00
## 28911  5  675  3.489861e+00
## 28912  5  676  3.394437e+00
## 28913  5  677  3.296252e+00
## 28914  5  678  3.211427e+00
## 28915  5  679  3.131643e+00
## 28916  5  680  3.056386e+00
## 28917  5  681  2.998194e+00
## 28918  5  682  2.936142e+00
## 28919  5  683  2.884806e+00
## 28920  5  684  2.830201e+00
## 28921  5  685  2.792610e+00
## 28922  5  686  2.752189e+00
## 28923  5  687  2.713558e+00
## 28924  5  688  2.674600e+00
## 28925  5  689  2.639947e+00
## 28926  5  690  2.610538e+00
## 28927  5  691  2.574622e+00
## 28928  5  692  2.545489e+00
## 28929  5  693  2.517607e+00
## 28930  5  694  2.494296e+00
## 28931  5  695  2.457809e+00
## 28932  5  696  2.434490e+00
## 28933  5  697  3.348901e+00
## 28934  5  698  3.512101e+00
## 28935  5  699  3.472024e+00
## 28936  5  700  3.376638e+00
## 28937  5  701  3.282842e+00
## 28938  5  702  3.192818e+00
## 28939  5  703  3.120942e+00
## 28940  5  704  3.052102e+00
## 28941  5  705  2.983224e+00
## 28942  5  706  2.924749e+00
## 28943  5  707  2.873352e+00
## 28944  5  708  2.825937e+00
## 28945  5  709  2.784494e+00
## 28946  5  710  2.742279e+00
## 28947  5  711  2.704349e+00
## 28948  5  712  2.667124e+00
## 28949  5  713  2.636770e+00
## 28950  5  714  2.599249e+00
## 28951  5  715  2.571958e+00
## 28952  5  716  2.542395e+00
## 28953  5  717  2.508601e+00
## 28954  5  718  2.480294e+00
## 28955  5  719  2.457821e+00
## 28956  5  720  2.432642e+00
## 28957  5  721  3.338745e+00
## 28958  5  722  3.503450e+00
## 28959  5  723  3.462541e+00
## 28960  5  724  3.373201e+00
## 28961  5  725  3.279501e+00
## 28962  5  726  3.190308e+00
## 28963  5  727  3.108112e+00
## 28964  5  728  3.041104e+00
## 28965  5  729  2.976644e+00
## 28966  5  730  2.924493e+00
## 28967  5  731  2.872479e+00
## 28968  5  732  2.822697e+00
## 28969  5  733  2.784092e+00
## 28970  5  734  2.737341e+00
## 28971  5  735  2.698059e+00
## 28972  5  736  2.667069e+00
## 28973  5  737  2.636578e+00
## 28974  5  738  2.600076e+00
## 28975  5  739  2.566335e+00
## 28976  5  740  2.536847e+00
## 28977  5  741  2.508684e+00
## 28978  5  742  2.471794e+00
## 28979  5  743  2.449564e+00
## 28980  5  744  2.420698e+00
## 28981  5  745  3.338044e+00
## 28982  5  746  3.503226e+00
## 28983  5  747  3.453857e+00
## 28984  5  748  3.365023e+00
## 28985  5  749  3.277983e+00
## 28986  5  750  3.186025e+00
## 28987  5  751  3.104358e+00
## 28988  5  752  3.038457e+00
## 28989  5  753  2.974906e+00
## 28990  5  754  2.917979e+00
## 28991  5  755  2.868110e+00
## 28992  5  756  2.821202e+00
## 28993  5  757  2.775118e+00
## 28994  5  758  2.729279e+00
## 28995  5  759  2.695119e+00
## 28996  5  760  2.658248e+00
## 28997  5  761  2.625626e+00
## 28998  5  762  2.596392e+00
## 28999  5  763  2.563146e+00
## 29000  5  764  2.534453e+00
## 29001  5  765  2.504781e+00
## 29002  5  766  2.478778e+00
## 29003  5  767  2.446630e+00
## 29004  5  768  2.421596e+00
## 29005  5  769  3.332893e+00
## 29006  5  770  3.492633e+00
## 29007  5  771  3.457107e+00
## 29008  5  772  3.363039e+00
## 29009  5  773  3.270693e+00
## 29010  5  774  3.183008e+00
## 29011  5  775  3.104906e+00
## 29012  5  776  3.032604e+00
## 29013  5  777  2.969928e+00
## 29014  5  778  2.912334e+00
## 29015  5  779  2.865291e+00
## 29016  5  780  2.817500e+00
## 29017  5  781  2.771080e+00
## 29018  5  782  2.726435e+00
## 29019  5  783  2.693546e+00
## 29020  5  784  2.657507e+00
## 29021  5  785  2.627753e+00
## 29022  5  786  2.584741e+00
## 29023  5  787  2.560666e+00
## 29024  5  788  2.533604e+00
## 29025  5  789  2.500131e+00
## 29026  5  790  2.467350e+00
## 29027  5  791  2.447442e+00
## 29028  5  792  2.415942e+00
## 29029  5  793  3.332262e+00
## 29030  5  794  3.491091e+00
## 29031  5  795  3.449526e+00
## 29032  5  796  3.365420e+00
## 29033  5  797  3.265022e+00
## 29034  5  798  3.175740e+00
## 29035  5  799  3.103504e+00
## 29036  5  800  3.029640e+00
## 29037  5  801  2.967324e+00
## 29038  5  802  2.907491e+00
## 29039  5  803  2.861251e+00
## 29040  5  804  2.814332e+00
## 29041  5  805  2.768788e+00
## 29042  5  806  2.729205e+00
## 29043  5  807  2.693477e+00
## 29044  5  808  2.651228e+00
## 29045  5  809  2.616209e+00
## 29046  5  810  2.591209e+00
## 29047  5  811  2.558185e+00
## 29048  5  812  2.524871e+00
## 29049  5  813  2.496765e+00
## 29050  5  814  2.471376e+00
## 29051  5  815  2.441264e+00
## 29052  5  816  2.418395e+00
## 29053  5  817  3.323915e+00
## 29054  5  818  3.493125e+00
## 29055  5  819  3.460449e+00
## 29056  5  820  3.362029e+00
## 29057  5  821  3.264230e+00
## 29058  5  822  3.177460e+00
## 29059  5  823  3.096668e+00
## 29060  5  824  3.029395e+00
## 29061  5  825  2.971334e+00
## 29062  5  826  2.906618e+00
## 29063  5  827  2.859766e+00
## 29064  5  828  2.810812e+00
## 29065  5  829  2.765978e+00
## 29066  5  830  2.726914e+00
## 29067  5  831  2.691206e+00
## 29068  5  832  2.655411e+00
## 29069  5  833  2.616822e+00
## 29070  5  834  2.584150e+00
## 29071  5  835  2.557551e+00
## 29072  5  836  2.524816e+00
## 29073  5  837  2.498496e+00
## 29074  5  838  2.470457e+00
## 29075  5  839  2.439070e+00
## 29076  5  840  2.411313e+00
## 29077  5  841  3.325084e+00
## 29078  5  842  3.492192e+00
## 29079  5  843  3.452390e+00
## 29080  5  844  3.361834e+00
## 29081  5  845  3.266964e+00
## 29082  5  846  3.179748e+00
## 29083  5  847  3.096065e+00
## 29084  5  848  3.023686e+00
## 29085  5  849  2.963898e+00
## 29086  5  850  2.911140e+00
## 29087  5  851  2.856581e+00
## 29088  5  852  2.809739e+00
## 29089  5  853  2.766585e+00
## 29090  5  854  2.724803e+00
## 29091  5  855  2.689870e+00
## 29092  5  856  2.652272e+00
## 29093  5  857  2.617071e+00
## 29094  5  858  2.585381e+00
## 29095  5  859  2.554314e+00
## 29096  5  860  2.525571e+00
## 29097  5  861  2.489082e+00
## 29098  5  862  2.470497e+00
## 29099  5  863  2.436248e+00
## 29100  5  864  2.412825e+00
## 29101  5  865  3.328887e+00
## 29102  5  866  3.491127e+00
## 29103  5  867  3.445877e+00
## 29104  5  868  3.357097e+00
## 29105  5  869  3.266777e+00
## 29106  5  870  3.171172e+00
## 29107  5  871  3.095104e+00
## 29108  5  872  3.031349e+00
## 29109  5  873  2.959950e+00
## 29110  5  874  2.906146e+00
## 29111  5  875  2.852902e+00
## 29112  5  876  2.807057e+00
## 29113  5  877  2.766823e+00
## 29114  5  878  2.728244e+00
## 29115  5  879  2.688599e+00
## 29116  5  880  2.650172e+00
## 29117  5  881  2.614387e+00
## 29118  5  882  2.585391e+00
## 29119  5  883  2.549923e+00
## 29120  5  884  2.524106e+00
## 29121  5  885  2.492743e+00
## 29122  5  886  2.465219e+00
## 29123  5  887  2.439132e+00
## 29124  5  888  2.415703e+00
## 29125  5  889  3.321951e+00
## 29126  5  890  3.484616e+00
## 29127  5  891  3.451984e+00
## 29128  5  892  3.356842e+00
## 29129  5  893  3.253944e+00
## 29130  5  894  3.169175e+00
## 29131  5  895  3.089981e+00
## 29132  5  896  3.026420e+00
## 29133  5  897  2.961331e+00
## 29134  5  898  2.912284e+00
## 29135  5  899  2.854901e+00
## 29136  5  900  2.807858e+00
## 29137  5  901  2.766518e+00
## 29138  5  902  2.722643e+00
## 29139  5  903  2.681263e+00
## 29140  5  904  2.648242e+00
## 29141  5  905  2.615124e+00
## 29142  5  906  2.584324e+00
## 29143  5  907  2.548375e+00
## 29144  5  908  2.518082e+00
## 29145  5  909  2.492448e+00
## 29146  5  910  2.465737e+00
## 29147  5  911  2.440509e+00
## 29148  5  912  2.411532e+00
## 29149  5  913  3.322101e+00
## 29150  5  914  3.490676e+00
## 29151  5  915  3.446491e+00
## 29152  5  916  3.360762e+00
## 29153  5  917  3.266320e+00
## 29154  5  918  3.176101e+00
## 29155  5  919  3.094675e+00
## 29156  5  920  3.023622e+00
## 29157  5  921  2.958637e+00
## 29158  5  922  2.905661e+00
## 29159  5  923  2.853085e+00
## 29160  5  924  2.807505e+00
## 29161  5  925  2.764464e+00
## 29162  5  926  2.724655e+00
## 29163  5  927  2.682382e+00
## 29164  5  928  2.646699e+00
## 29165  5  929  2.617922e+00
## 29166  5  930  2.583843e+00
## 29167  5  931  2.553294e+00
## 29168  5  932  2.520132e+00
## 29169  5  933  2.492701e+00
## 29170  5  934  2.464978e+00
## 29171  5  935  2.440300e+00
## 29172  5  936  2.409796e+00
## 29173  5  936  2.404582e+00
## 29174  5  937  3.313615e+00
## 29175  5  938  3.487179e+00
## 29176  5  939  3.448238e+00
## 29177  5  940  3.353234e+00
## 29178  5  941  3.262374e+00
## 29179  5  942  3.174855e+00
## 29180  5  943  3.092334e+00
## 29181  5  944  3.027516e+00
## 29182  5  945  2.959022e+00
## 29183  5  946  2.904619e+00
## 29184  5  947  2.850074e+00
## 29185  5  948  2.801384e+00
## 29186  5  949  2.757157e+00
## 29187  5  950  2.728974e+00
## 29188  5  951  2.680164e+00
## 29189  5  952  2.646478e+00
## 29190  5  953  2.610105e+00
## 29191  5  954  2.581686e+00
## 29192  5  955  2.554118e+00
## 29193  5  956  2.522161e+00
## 29194  5  957  2.488187e+00
## 29195  5  958  2.462833e+00
## 29196  5  959  2.436310e+00
## 29197  5  960  2.409015e+00
## 29198  5  960  2.408819e+00
## 29199  5  961  2.385114e+00
## 29200  5  962  2.356104e+00
## 29201  5  963  2.328665e+00
## 29202  5  964  2.303982e+00
## 29203  5  965  2.280557e+00
## 29204  5  966  2.257867e+00
## 29205  5  967  2.237159e+00
## 29206  5  968  2.210413e+00
## 29207  5  969  2.185869e+00
## 29208  5  969  2.187974e+00
## 29209  5  970  3.098821e+00
## 29210  5  971  3.269489e+00
## 29211  5  972  3.231802e+00
## 29212  5  973  3.146537e+00
## 29213  5  974  3.054253e+00
## 29214  5  975  2.964026e+00
## 29215  5  976  2.896396e+00
## 29216  5  977  2.825570e+00
## 29217  5  978  2.760520e+00
## 29218  5  979  2.707802e+00
## 29219  5  980  2.661881e+00
## 29220  5  981  2.614529e+00
## 29221  5  982  2.568974e+00
## 29222  5  983  2.533863e+00
## 29223  5  984  2.495565e+00
## 29224  5  984  2.499622e+00
## 29225  5  985  3.398992e+00
## 29226  5  986  3.561262e+00
## 29227  5  987  3.513515e+00
## 29228  5  988  3.421140e+00
## 29229  5  989  3.322817e+00
## 29230  5  990  3.228207e+00
## 29231  5  991  3.151019e+00
## 29232  5  992  3.082401e+00
## 29233  5  993  3.012883e+00
## 29234  5  994  2.948506e+00
## 29235  5  995  2.902664e+00
## 29236  5  996  2.852907e+00
## 29237  5  997  2.813754e+00
## 29238  5  998  2.767624e+00
## 29239  5  999  2.729812e+00
## 29240  5 1000  2.693136e+00
## 29241  5 1001  2.655678e+00
## 29242  5 1002  2.620892e+00
## 29243  5 1003  2.591143e+00
## 29244  5 1004  2.557127e+00
## 29245  5 1005  2.531300e+00
## 29246  5 1006  2.505593e+00
## 29247  5 1007  2.477075e+00
## 29248  5 1008  2.448394e+00
## 29249  5 1009  3.358072e+00
## 29250  5 1010  3.522184e+00
## 29251  5 1011  3.483084e+00
## 29252  5 1012  3.391595e+00
## 29253  5 1013  3.294889e+00
## 29254  5 1014  3.205475e+00
## 29255  5 1015  3.126873e+00
## 29256  5 1016  3.054762e+00
## 29257  5 1017  3.000098e+00
## 29258  5 1018  2.942648e+00
## 29259  5 1019  2.891487e+00
## 29260  5 1020  2.839374e+00
## 29261  5 1021  2.797519e+00
## 29262  5 1022  2.748393e+00
## 29263  5 1023  2.719253e+00
## 29264  5 1024  2.678512e+00
## 29265  5 1025  2.643700e+00
## 29266  5 1026  2.611137e+00
## 29267  5 1027  2.578694e+00
## 29268  5 1028  2.545852e+00
## 29269  5 1029  2.519410e+00
## 29270  5 1030  2.493467e+00
## 29271  5 1031  2.465543e+00
## 29272  5 1032  2.442689e+00
## 29273  5 1033  3.352881e+00
## 29274  5 1034  3.517440e+00
## 29275  5 1035  3.471841e+00
## 29276  5 1036  3.384969e+00
## 29277  5 1037  3.285354e+00
## 29278  5 1038  3.197870e+00
## 29279  5 1039  3.121295e+00
## 29280  5 1040  3.051160e+00
## 29281  5 1041  2.991479e+00
## 29282  5 1042  2.928098e+00
## 29283  5 1043  2.883849e+00
## 29284  5 1044  2.830753e+00
## 29285  5 1045  2.787890e+00
## 29286  5 1046  2.749724e+00
## 29287  5 1047  2.710517e+00
## 29288  5 1048  2.672931e+00
## 29289  5 1049  2.642964e+00
## 29290  5 1050  2.604021e+00
## 29291  5 1051  2.574184e+00
## 29292  5 1052  2.545059e+00
## 29293  5 1053  2.514374e+00
## 29294  5 1054  2.492584e+00
## 29295  5 1055  2.459842e+00
## 29296  5 1056  2.432730e+00
## 29297  5 1057  3.349974e+00
## 29298  5 1058  3.510870e+00
## 29299  5 1059  3.469017e+00
## 29300  5 1060  3.376122e+00
## 29301  5 1061  3.284125e+00
## 29302  5 1062  3.192716e+00
## 29303  5 1063  3.114053e+00
## 29304  5 1064  3.048693e+00
## 29305  5 1065  2.976221e+00
## 29306  5 1066  2.922071e+00
## 29307  5 1067  2.872335e+00
## 29308  5 1068  2.827301e+00
## 29309  5 1069  2.780235e+00
## 29310  5 1070  2.739480e+00
## 29311  5 1071  2.699904e+00
## 29312  5 1072  2.667363e+00
## 29313  5 1073  2.636728e+00
## 29314  5 1074  2.601465e+00
## 29315  5 1075  2.572540e+00
## 29316  5 1076  2.540244e+00
## 29317  5 1077  2.509111e+00
## 29318  5 1078  2.484877e+00
## 29319  5 1079  2.452590e+00
## 29320  5 1080  2.427084e+00
## 29321  5 1081  3.336982e+00
## 29322  5 1082  3.512564e+00
## 29323  5 1083  3.465068e+00
## 29324  5 1084  3.376356e+00
## 29325  5 1085  3.281254e+00
## 29326  5 1086  3.187954e+00
## 29327  5 1087  3.106950e+00
## 29328  5 1088  3.035763e+00
## 29329  5 1089  2.978812e+00
## 29330  5 1090  2.920571e+00
## 29331  5 1091  2.868863e+00
## 29332  5 1092  2.820908e+00
## 29333  5 1093  2.778786e+00
## 29334  5 1094  2.739486e+00
## 29335  5 1095  2.704051e+00
## 29336  5 1096  2.660333e+00
## 29337  5 1097  2.630426e+00
## 29338  5 1098  2.595294e+00
## 29339  5 1099  2.564471e+00
## 29340  5 1100  2.538412e+00
## 29341  5 1101  2.507036e+00
## 29342  5 1102  2.477492e+00
## 29343  5 1103  2.446421e+00
## 29344  5 1104  2.423711e+00
## 29345  5 1105  3.336917e+00
## 29346  5 1106  3.501586e+00
## 29347  5 1107  3.460975e+00
## 29348  5 1108  3.369896e+00
## 29349  5 1109  3.272916e+00
## 29350  5 1110  3.182361e+00
## 29351  5 1111  3.106671e+00
## 29352  5 1112  3.032897e+00
## 29353  5 1113  2.975193e+00
## 29354  5 1114  2.918349e+00
## 29355  5 1115  2.866418e+00
## 29356  5 1116  2.819461e+00
## 29357  5 1117  2.773343e+00
## 29358  5 1118  2.733401e+00
## 29359  5 1119  2.697872e+00
## 29360  5 1120  2.659952e+00
## 29361  5 1121  2.627297e+00
## 29362  5 1122  2.595121e+00
## 29363  5 1123  2.562668e+00
## 29364  5 1124  2.532669e+00
## 29365  5 1125  2.502951e+00
## 29366  5 1126  2.472706e+00
## 29367  5 1127  2.448569e+00
## 29368  5 1128  2.418698e+00
## 29369  5 1129  3.332788e+00
## 29370  5 1130  3.495249e+00
## 29371  5 1131  3.455536e+00
## 29372  5 1132  3.361227e+00
## 29373  5 1133  3.266053e+00
## 29374  5 1134  3.183307e+00
## 29375  5 1135  3.101338e+00
## 29376  5 1136  3.034864e+00
## 29377  5 1137  2.973349e+00
## 29378  5 1138  2.909495e+00
## 29379  5 1139  2.868625e+00
## 29380  5 1140  2.811596e+00
## 29381  5 1141  2.768165e+00
## 29382  5 1142  2.731442e+00
## 29383  5 1143  2.685970e+00
## 29384  5 1144  2.655003e+00
## 29385  5 1145  2.624506e+00
## 29386  5 1146  2.586643e+00
## 29387  5 1147  2.562343e+00
## 29388  5 1148  2.528516e+00
## 29389  5 1149  2.493819e+00
## 29390  5 1150  2.471059e+00
## 29391  5 1151  2.442497e+00
## 29392  5 1152  2.417084e+00
## 29393  5 1153  3.330328e+00
## 29394  5 1154  3.493233e+00
## 29395  5 1155  3.453651e+00
## 29396  5 1156  3.359687e+00
## 29397  5 1157  3.262661e+00
## 29398  5 1158  3.179165e+00
## 29399  5 1159  3.098854e+00
## 29400  5 1160  3.031882e+00
## 29401  5 1161  2.967314e+00
## 29402  5 1162  2.911204e+00
## 29403  5 1163  2.862186e+00
## 29404  5 1164  2.812098e+00
## 29405  5 1165  2.769756e+00
## 29406  5 1166  2.726634e+00
## 29407  5 1167  2.689412e+00
## 29408  5 1168  2.654756e+00
## 29409  5 1169  2.618873e+00
## 29410  5 1170  2.585971e+00
## 29411  5 1171  2.557380e+00
## 29412  5 1172  2.526054e+00
## 29413  5 1173  2.499929e+00
## 29414  5 1174  2.469290e+00
## 29415  5 1175  2.442175e+00
## 29416  5 1176  2.413065e+00
## 29417  5 1177  3.324624e+00
## 29418  5 1178  3.497601e+00
## 29419  5 1179  3.449856e+00
## 29420  5 1180  3.361465e+00
## 29421  5 1181  3.261026e+00
## 29422  5 1182  3.169792e+00
## 29423  5 1183  3.100463e+00
## 29424  5 1184  3.029844e+00
## 29425  5 1185  2.965525e+00
## 29426  5 1186  2.908433e+00
## 29427  5 1187  2.856966e+00
## 29428  5 1188  2.807534e+00
## 29429  5 1189  2.764341e+00
## 29430  5 1190  2.727956e+00
## 29431  5 1191  2.689684e+00
## 29432  5 1192  2.653172e+00
## 29433  5 1193  2.619308e+00
## 29434  5 1194  2.585225e+00
## 29435  5 1195  2.554730e+00
## 29436  5 1196  2.527834e+00
## 29437  5 1197  2.495205e+00
## 29438  5 1198  2.468809e+00
## 29439  5 1199  2.440346e+00
## 29440  5 1200  2.414078e+00
## 29441  5 1201  3.327825e+00
## 29442  5 1202  3.495481e+00
## 29443  5 1203  3.453381e+00
## 29444  5 1204  3.355500e+00
## 29445  5 1205  3.264802e+00
## 29446  5 1206  3.177699e+00
## 29447  5 1207  3.098905e+00
## 29448  5 1208  3.028079e+00
## 29449  5 1209  2.966239e+00
## 29450  5 1210  2.901552e+00
## 29451  5 1211  2.857116e+00
## 29452  5 1212  2.811827e+00
## 29453  5 1213  2.761339e+00
## 29454  5 1214  2.731065e+00
## 29455  5 1215  2.688386e+00
## 29456  5 1216  2.650276e+00
## 29457  5 1217  2.621563e+00
## 29458  5 1218  2.584519e+00
## 29459  5 1219  2.554243e+00
## 29460  5 1220  2.520667e+00
## 29461  5 1221  2.497211e+00
## 29462  5 1222  2.466410e+00
## 29463  5 1223  2.441798e+00
## 29464  5 1224  2.411944e+00
## 29465  5 1225  3.316830e+00
## 29466  5 1226  3.494678e+00
## 29467  5 1227  3.443075e+00
## 29468  5 1228  3.354132e+00
## 29469  5 1229  3.261702e+00
## 29470  5 1230  3.167805e+00
## 29471  5 1231  3.102850e+00
## 29472  5 1232  3.029423e+00
## 29473  5 1233  2.963647e+00
## 29474  5 1234  2.903689e+00
## 29475  5 1235  2.851013e+00
## 29476  5 1236  2.807990e+00
## 29477  5 1237  2.762440e+00
## 29478  5 1238  2.721784e+00
## 29479  5 1239  2.681112e+00
## 29480  5 1240  2.653781e+00
## 29481  5 1241  2.620660e+00
## 29482  5 1242  2.581929e+00
## 29483  5 1243  2.555523e+00
## 29484  5 1244  2.519825e+00
## 29485  5 1245  2.494808e+00
## 29486  5 1246  2.466927e+00
## 29487  5 1247  2.443061e+00
## 29488  5 1248  2.409565e+00
## 29489  5 1248  2.409142e+00
## 29490  5 1249  3.319968e+00
## 29491  5 1250  3.487117e+00
## 29492  5 1251  3.442689e+00
## 29493  5 1252  3.358615e+00
## 29494  5 1253  3.256226e+00
## 29495  5 1254  3.172652e+00
## 29496  5 1255  3.087664e+00
## 29497  5 1256  3.019514e+00
## 29498  5 1257  2.958144e+00
## 29499  5 1258  2.897645e+00
## 29500  5 1259  2.855450e+00
## 29501  5 1260  2.805906e+00
## 29502  5 1261  2.761265e+00
## 29503  5 1262  2.719525e+00
## 29504  5 1263  2.680672e+00
## 29505  5 1264  2.644150e+00
## 29506  5 1265  2.612887e+00
## 29507  5 1266  2.582984e+00
## 29508  5 1267  2.546338e+00
## 29509  5 1268  2.519503e+00
## 29510  5 1269  2.489513e+00
## 29511  5 1270  2.461679e+00
## 29512  5 1271  2.434116e+00
## 29513  5 1272  2.407897e+00
## 29514  5 1272  2.410396e+00
## 29515  5 1273  2.384890e+00
## 29516  5 1274  2.353060e+00
## 29517  5 1275  2.331323e+00
## 29518  5 1276  2.301933e+00
## 29519  5 1277  2.280755e+00
## 29520  5 1278  2.253722e+00
## 29521  5 1279  2.228948e+00
## 29522  5 1280  2.210329e+00
## 29523  5 1281  2.188715e+00
## 29524  5 1282  2.161404e+00
## 29525  5 1283  2.143081e+00
## 29526  5 1284  2.116909e+00
## 29527  5 1284  2.121190e+00
## 29528  5 1285  3.036927e+00
## 29529  5 1286  3.200400e+00
## 29530  5 1287  3.167591e+00
## 29531  5 1288  3.082359e+00
## 29532  5 1289  2.992092e+00
## 29533  5 1290  2.905960e+00
## 29534  5 1291  2.832071e+00
## 29535  5 1292  2.764950e+00
## 29536  5 1293  2.703984e+00
## 29537  5 1294  2.647640e+00
## 29538  5 1295  2.596787e+00
## 29539  5 1296  2.556179e+00
## 29540  5 1296  2.553951e+00
## 29541  5 1297  3.453264e+00
## 29542  5 1298  3.603493e+00
## 29543  5 1299  3.555803e+00
## 29544  5 1300  3.453278e+00
## 29545  5 1301  3.355829e+00
## 29546  5 1302  3.265253e+00
## 29547  5 1303  3.182661e+00
## 29548  5 1304  3.101469e+00
## 29549  5 1305  3.036420e+00
## 29550  5 1306  2.978367e+00
## 29551  5 1307  2.922042e+00
## 29552  5 1308  2.872054e+00
## 29553  5 1309  2.827212e+00
## 29554  5 1310  2.788686e+00
## 29555  5 1311  2.745678e+00
## 29556  5 1312  2.706790e+00
## 29557  5 1313  2.675034e+00
## 29558  5 1314  2.643270e+00
## 29559  5 1315  2.610474e+00
## 29560  5 1316  2.574152e+00
## 29561  5 1317  2.550280e+00
## 29562  5 1318  2.519890e+00
## 29563  5 1319  2.488312e+00
## 29564  5 1320  2.461639e+00
## 29565  5 1321  3.372263e+00
## 29566  5 1322  3.533778e+00
## 29567  5 1323  3.496583e+00
## 29568  5 1324  3.407278e+00
## 29569  5 1325  3.311954e+00
## 29570  5 1326  3.220508e+00
## 29571  5 1327  3.146808e+00
## 29572  5 1328  3.074399e+00
## 29573  5 1329  3.011040e+00
## 29574  5 1330  2.953502e+00
## 29575  5 1331  2.898691e+00
## 29576  5 1332  2.849300e+00
## 29577  5 1333  2.803207e+00
## 29578  5 1334  2.767701e+00
## 29579  5 1335  2.729453e+00
## 29580  5 1336  2.687393e+00
## 29581  5 1337  2.657204e+00
## 29582  5 1338  2.623730e+00
## 29583  5 1339  2.596544e+00
## 29584  5 1340  2.563666e+00
## 29585  5 1341  2.536779e+00
## 29586  5 1342  2.502230e+00
## 29587  5 1343  2.476082e+00
## 29588  5 1344  2.446700e+00
## 29589  5 1345  3.359471e+00
## 29590  5 1346  3.518614e+00
## 29591  5 1347  3.492346e+00
## 29592  5 1348  3.391361e+00
## 29593  5 1349  3.300906e+00
## 29594  5 1350  3.213222e+00
## 29595  5 1351  3.130541e+00
## 29596  5 1352  3.057427e+00
## 29597  5 1353  2.999592e+00
## 29598  5 1354  2.940474e+00
## 29599  5 1355  2.883453e+00
## 29600  5 1356  2.836348e+00
## 29601  5 1357  2.796442e+00
## 29602  5 1358  2.757309e+00
## 29603  5 1359  2.718135e+00
## 29604  5 1360  2.680510e+00
## 29605  5 1361  2.649049e+00
## 29606  5 1362  2.614584e+00
## 29607  5 1363  2.582493e+00
## 29608  5 1364  2.546904e+00
## 29609  5 1365  2.522906e+00
## 29610  5 1366  2.494924e+00
## 29611  5 1367  2.468602e+00
## 29612  5 1368  2.443093e+00
## 29613  5 1369  3.348200e+00
## 29614  5 1370  3.514148e+00
## 29615  5 1371  3.480982e+00
## 29616  5 1372  3.377221e+00
## 29617  5 1373  3.291799e+00
## 29618  5 1374  3.196357e+00
## 29619  5 1375  3.128734e+00
## 29620  5 1376  3.051566e+00
## 29621  5 1377  2.992471e+00
## 29622  5 1378  2.936307e+00
## 29623  5 1379  2.880205e+00
## 29624  5 1380  2.831057e+00
## 29625  5 1381  2.790277e+00
## 29626  5 1382  2.751687e+00
## 29627  5 1383  2.713562e+00
## 29628  5 1384  2.674851e+00
## 29629  5 1385  2.641592e+00
## 29630  5 1386  2.610853e+00
## 29631  5 1387  2.573630e+00
## 29632  5 1388  2.551089e+00
## 29633  5 1389  2.520057e+00
## 29634  5 1390  2.495046e+00
## 29635  5 1391  2.461256e+00
```

```
## 29636  5 1392  2.431091e+00
## 29637  5 1393  3.347283e+00
## 29638  5 1394  3.517966e+00
## 29639  5 1395  3.460328e+00
## 29640  5 1396  3.374793e+00
## 29641  5 1397  3.281440e+00
## 29642  5 1398  3.193208e+00
## 29643  5 1399  3.113189e+00
## 29644  5 1400  3.041930e+00
## 29645  5 1401  2.976995e+00
## 29646  5 1402  2.927831e+00
## 29647  5 1403  2.871544e+00
## 29648  5 1404  2.825228e+00
## 29649  5 1405  2.787395e+00
## 29650  5 1406  2.742192e+00
## 29651  5 1407  2.704592e+00
## 29652  5 1408  2.662250e+00
## 29653  5 1409  2.632561e+00
## 29654  5 1410  2.603815e+00
## 29655  5 1411  2.571921e+00
## 29656  5 1412  2.536441e+00
## 29657  5 1413  2.511424e+00
## 29658  5 1414  2.480074e+00
## 29659  5 1415  2.455194e+00
## 29660  5 1416  2.419246e+00
## 29661  5 1417  3.338905e+00
## 29662  5 1418  3.510408e+00
## 29663  5 1419  3.462076e+00
## 29664  5 1420  3.374483e+00
## 29665  5 1421  3.278560e+00
## 29666  5 1422  3.188229e+00
## 29667  5 1423  3.112573e+00
## 29668  5 1424  3.041875e+00
## 29669  5 1425  2.982594e+00
## 29670  5 1426  2.921052e+00
## 29671  5 1427  2.866926e+00
## 29672  5 1428  2.821017e+00
## 29673  5 1429  2.778962e+00
## 29674  5 1430  2.735692e+00
## 29675  5 1431  2.702392e+00
## 29676  5 1432  2.664531e+00
## 29677  5 1433  2.629822e+00
## 29678  5 1434  2.599910e+00
## 29679  5 1435  2.570122e+00
## 29680  5 1436  2.532447e+00
## 29681  5 1437  2.506144e+00
## 29682  5 1438  2.478380e+00
## 29683  5 1439  2.448032e+00
## 29684  5 1440  2.421415e+00
## 29685  5 1441  3.339584e+00
## 29686  5 1442  3.499720e+00
## 29687  5 1443  3.462267e+00
## 29688  5 1444  3.373288e+00
## 29689  5 1445  3.271916e+00
## 29690  5 1446  3.186951e+00
## 29691  5 1447  3.100935e+00
## 29692  5 1448  3.036147e+00
## 29693  5 1449  2.978850e+00
## 29694  5 1450  2.911641e+00
## 29695  5 1451  2.861471e+00
## 29696  5 1452  2.821231e+00
## 29697  5 1453  2.773119e+00
## 29698  5 1454  2.736870e+00
## 29699  5 1455  2.694610e+00
## 29700  5 1456  2.662175e+00
## 29701  5 1457  2.623963e+00
## 29702  5 1458  2.590953e+00
## 29703  5 1459  2.562979e+00
## 29704  5 1460  2.534627e+00
## 29705  5 1461  2.501622e+00
## 29706  5 1462  2.477157e+00
## 29707  5 1463  2.448291e+00
## 29708  5 1464  2.419170e+00
## 29709  5 1465  3.332135e+00
## 29710  5 1466  3.500174e+00
## 29711  5 1467  3.460605e+00
## 29712  5 1468  3.362596e+00
## 29713  5 1469  3.270961e+00
## 29714  5 1470  3.178142e+00
## 29715  5 1471  3.100830e+00
## 29716  5 1472  3.033073e+00
## 29717  5 1473  2.971038e+00
## 29718  5 1474  2.912985e+00
## 29719  5 1475  2.854407e+00
## 29720  5 1476  2.816548e+00
## 29721  5 1477  2.771857e+00
## 29722  5 1478  2.728444e+00
## 29723  5 1479  2.697310e+00
## 29724  5 1480  2.659873e+00
## 29725  5 1481  2.619039e+00
## 29726  5 1482  2.595325e+00
## 29727  5 1483  2.561908e+00
## 29728  5 1484  2.527302e+00
## 29729  5 1485  2.499608e+00
## 29730  5 1486  2.472607e+00
## 29731  5 1487  2.445710e+00
## 29732  5 1488  2.417652e+00
## 29733  5 1489  3.327021e+00
## 29734  5 1490  3.495742e+00
## 29735  5 1491  3.455926e+00
## 29736  5 1492  3.361950e+00
## 29737  5 1493  3.271062e+00
## 29738  5 1494  3.172876e+00
## 29739  5 1495  3.100561e+00
## 29740  5 1496  3.028540e+00
## 29741  5 1497  2.964917e+00
## 29742  5 1498  2.908935e+00
## 29743  5 1499  2.856906e+00
## 29744  5 1500  2.812615e+00
## 29745  5 1501  2.769214e+00
## 29746  5 1502  2.732499e+00
## 29747  5 1503  2.687955e+00
## 29748  5 1504  2.653988e+00
## 29749  5 1505  2.617674e+00
## 29750  5 1506  2.584834e+00
## 29751  5 1507  2.555092e+00
## 29752  5 1508  2.527211e+00
## 29753  5 1509  2.499588e+00
## 29754  5 1510  2.469878e+00
## 29755  5 1511  2.440702e+00
## 29756  5 1512  2.414899e+00
## 29757  5 1513  3.324909e+00
## 29758  5 1514  3.497647e+00
## 29759  5 1515  3.453984e+00
## 29760  5 1516  3.357719e+00
## 29761  5 1517  3.266521e+00
## 29762  5 1518  3.173022e+00
## 29763  5 1519  3.099889e+00
## 29764  5 1520  3.031028e+00
## 29765  5 1521  2.966702e+00
## 29766  5 1522  2.914533e+00
## 29767  5 1523  2.854046e+00
## 29768  5 1524  2.810091e+00
## 29769  5 1525  2.769925e+00
## 29770  5 1526  2.728820e+00
## 29771  5 1527  2.690600e+00
## 29772  5 1528  2.652933e+00
## 29773  5 1529  2.620453e+00
## 29774  5 1530  2.586595e+00
## 29775  5 1531  2.554976e+00
## 29776  5 1532  2.528162e+00
## 29777  5 1533  2.496902e+00
## 29778  5 1534  2.467640e+00
## 29779  5 1535  2.437664e+00
## 29780  5 1536  2.416488e+00
## 29781  5 1537  3.324741e+00
## 29782  5 1538  3.486203e+00
## 29783  5 1539  3.443850e+00
## 29784  5 1540  3.355848e+00
## 29785  5 1541  3.262145e+00
## 29786  5 1542  3.175912e+00
## 29787  5 1543  3.096004e+00
## 29788  5 1544  3.031043e+00
## 29789  5 1545  2.964973e+00
## 29790  5 1546  2.904057e+00
## 29791  5 1547  2.856931e+00
## 29792  5 1548  2.810063e+00
## 29793  5 1549  2.763861e+00
## 29794  5 1550  2.722286e+00
## 29795  5 1551  2.686791e+00
## 29796  5 1552  2.655680e+00
## 29797  5 1553  2.620043e+00
## 29798  5 1554  2.586986e+00
## 29799  5 1555  2.554614e+00
## 29800  5 1556  2.525893e+00
## 29801  5 1557  2.494367e+00
## 29802  5 1558  2.468405e+00
## 29803  5 1559  2.439069e+00
## 29804  5 1560  2.412893e+00
## 29805  5 1560  2.411700e+00
## 29806  5 1561  3.314961e+00
## 29807  5 1562  3.482021e+00
## 29808  5 1563  3.445559e+00
## 29809  5 1564  3.354466e+00
## 29810  5 1565  3.254971e+00
## 29811  5 1566  3.170323e+00
## 29812  5 1567  3.089627e+00
## 29813  5 1568  3.023029e+00
## 29814  5 1569  2.949657e+00
## 29815  5 1570  2.898281e+00
## 29816  5 1571  2.851090e+00
## 29817  5 1572  2.804722e+00
## 29818  5 1573  2.762441e+00
## 29819  5 1574  2.720659e+00
## 29820  5 1575  2.680478e+00
## 29821  5 1576  2.651099e+00
## 29822  5 1577  2.613022e+00
## 29823  5 1578  2.581503e+00
## 29824  5 1579  2.544031e+00
## 29825  5 1580  2.517609e+00
## 29826  5 1581  2.491349e+00
## 29827  5 1582  2.459601e+00
## 29828  5 1583  2.433881e+00
## 29829  5 1584  2.409896e+00
## 29830  5 1584  2.407661e+00
## 29831  5 1585  2.381654e+00
## 29832  5 1586  2.358108e+00
## 29833  5 1587  2.333338e+00
## 29834  5 1588  2.305269e+00
## 29835  5 1589  2.284817e+00
## 29836  5 1590  2.257059e+00
## 29837  5 1591  2.231856e+00
## 29838  5 1592  2.207816e+00
## 29839  5 1593  2.184726e+00
## 29840  5 1594  2.166061e+00
## 29841  5 1595  2.142493e+00
## 29842  5 1596  2.118847e+00
## 29843  5 1597  2.100434e+00
## 29844  5 1598  2.077452e+00
## 29845  5 1599  2.054633e+00
## 29846  5 1599  2.051512e+00
## 29847  5 1600  2.504761e+00
## 29848  5 1601  2.580047e+00
## 29849  5 1602  2.546373e+00
## 29850  5 1603  2.498413e+00
## 29851  5 1604  2.439336e+00
## 29852  5 1605  2.391822e+00
## 29853  5 1606  2.340086e+00
## 29854  5 1607  2.300550e+00
## 29855  5 1608  2.259344e+00
## 29856  5 1608  2.259164e+00
## 29857  5 1609  3.156403e+00
## 29858  5 1610  3.316466e+00
## 29859  5 1611  3.270440e+00
## 29860  5 1612  3.172271e+00
## 29861  5 1613  3.080473e+00
## 29862  5 1614  2.992105e+00
## 29863  5 1615  2.904768e+00
## 29864  5 1616  2.835185e+00
## 29865  5 1617  2.773388e+00
## 29866  5 1618  2.714493e+00
## 29867  5 1619  2.665355e+00
## 29868  5 1620  2.618069e+00
## 29869  5 1621  2.573750e+00
## 29870  5 1622  2.534831e+00
## 29871  5 1623  2.500118e+00
## 29872  5 1624  2.462063e+00
## 29873  5 1625  2.428564e+00
## 29874  5 1626  2.397796e+00
## 29875  5 1627  2.374245e+00
## 29876  5 1628  2.341010e+00
## 29877  5 1629  2.312252e+00
## 29878  5 1630  2.289085e+00
## 29879  5 1631  2.265453e+00
## 29880  5 1632  2.239109e+00
## 29881  5 1633  3.156109e+00
## 29882  5 1634  3.323497e+00
## 29883  5 1635  3.276259e+00
## 29884  5 1636  3.186877e+00
## 29885  5 1637  3.097882e+00
## 29886  5 1638  3.013056e+00
## 29887  5 1639  2.936844e+00
## 29888  5 1640  2.861207e+00
## 29889  5 1641  2.800935e+00
## 29890  5 1642  2.746280e+00
## 29891  5 1643  2.699299e+00
## 29892  5 1644  2.656320e+00
## 29893  5 1645  2.608475e+00
## 29894  5 1646  2.570953e+00
## 29895  5 1647  2.536433e+00
## 29896  5 1648  2.502981e+00
## 29897  5 1649  2.470592e+00
## 29898  5 1650  2.436595e+00
## 29899  5 1651  2.408109e+00
## 29900  5 1652  2.384802e+00
## 29901  5 1653  2.351243e+00
## 29902  5 1654  2.325683e+00
## 29903  5 1655  2.302979e+00
## 29904  5 1656  2.273096e+00
## 29905  5 1657  3.188233e+00
## 29906  5 1658  3.350786e+00
## 29907  5 1659  3.316428e+00
## 29908  5 1660  3.231689e+00
## 29909  5 1661  3.129014e+00
## 29910  5 1662  3.041944e+00
## 29911  5 1663  2.968850e+00
## 29912  5 1664  2.899718e+00
## 29913  5 1665  2.834663e+00
## 29914  5 1666  2.782631e+00
## 29915  5 1667  2.733504e+00
## 29916  5 1668  2.690817e+00
## 29917  5 1669  2.646784e+00
## 29918  5 1670  2.609769e+00
## 29919  5 1671  2.568473e+00
## 29920  5 1672  2.533646e+00
## 29921  5 1673  2.504325e+00
## 29922  5 1674  2.470712e+00
## 29923  5 1675  2.442541e+00
## 29924  5 1676  2.412824e+00
## 29925  5 1677  2.381816e+00
## 29926  5 1678  2.352995e+00
## 29927  5 1679  2.326317e+00
## 29928  5 1680  2.303220e+00
## 29929  5 1681  3.212937e+00
## 29930  5 1682  3.382746e+00
## 29931  5 1683  3.345592e+00
## 29932  5 1684  3.261265e+00
## 29933  5 1685  3.158205e+00
## 29934  5 1686  3.069283e+00
## 29935  5 1687  2.994160e+00
## 29936  5 1688  2.934275e+00
## 29937  5 1689  2.862067e+00
## 29938  5 1690  2.808015e+00
## 29939  5 1691  2.751393e+00
## 29940  5 1692  2.710661e+00
## 29941  5 1693  2.666628e+00
## 29942  5 1694  2.633121e+00
## 29943  5 1695  2.591913e+00
## 29944  5 1696  2.556060e+00
## 29945  5 1697  2.523608e+00
## 29946  5 1698  2.489572e+00
## 29947  5 1699  2.465989e+00
## 29948  5 1700  2.436462e+00
## 29949  5 1701  2.402342e+00
## 29950  5 1702  2.376704e+00
## 29951  5 1703  2.356439e+00
## 29952  5 1704  2.324324e+00
## 29953  5 1705  3.243666e+00
## 29954  5 1706  3.409089e+00
## 29955  5 1707  3.362670e+00
## 29956  5 1708  3.276913e+00
## 29957  5 1709  3.181261e+00
## 29958  5 1710  3.092090e+00
## 29959  5 1711  3.020796e+00
## 29960  5 1712  2.949218e+00
## 29961  5 1713  2.885310e+00
## 29962  5 1714  2.829798e+00
## 29963  5 1715  2.777816e+00
## 29964  5 1716  2.731578e+00
## 29965  5 1717  2.685830e+00
## 29966  5 1718  2.654708e+00
## 29967  5 1719  2.610244e+00
## 29968  5 1720  2.578097e+00
## 29969  5 1721  2.541821e+00
## 29970  5 1722  2.513388e+00
## 29971  5 1723  2.481918e+00
## 29972  5 1724  2.455090e+00
## 29973  5 1725  2.423974e+00
## 29974  5 1726  2.400312e+00
## 29975  5 1727  2.367309e+00
## 29976  5 1728  2.343327e+00
## 29977  5 1729  3.257579e+00
## 29978  5 1730  3.422440e+00
## 29979  5 1731  3.381499e+00
## 29980  5 1732  3.290169e+00
## 29981  5 1733  3.193928e+00
## 29982  5 1734  3.114061e+00
## 29983  5 1735  3.031700e+00
## 29984  5 1736  2.965756e+00
## 29985  5 1737  2.906351e+00
## 29986  5 1738  2.850519e+00
## 29987  5 1739  2.791423e+00
## 29988  5 1740  2.753340e+00
## 29989  5 1741  2.707645e+00
## 29990  5 1742  2.663882e+00
## 29991  5 1743  2.626586e+00
## 29992  5 1744  2.595672e+00
## 29993  5 1745  2.558367e+00
## 29994  5 1746  2.527467e+00
## 29995  5 1747  2.497111e+00
## 29996  5 1748  2.468456e+00
## 29997  5 1749  2.443726e+00
## 29998  5 1750  2.413489e+00
## 29999  5 1751  2.386815e+00
## 30000  5 1752  2.359511e+00
## 30001  5 1753  3.265498e+00
## 30002  5 1754  3.440838e+00
## 30003  5 1755  3.393786e+00
## 30004  5 1756  3.306412e+00
## 30005  5 1757  3.212093e+00
## 30006  5 1758  3.117116e+00
## 30007  5 1759  3.050193e+00
## 30008  5 1760  2.974105e+00
## 30009  5 1761  2.913132e+00
## 30010  5 1762  2.862483e+00
## 30011  5 1763  2.806495e+00
## 30012  5 1764  2.760029e+00
## 30013  5 1765  2.721100e+00
## 30014  5 1766  2.680277e+00
## 30015  5 1767  2.639642e+00
## 30016  5 1768  2.603297e+00
## 30017  5 1769  2.572073e+00
## 30018  5 1770  2.543142e+00
## 30019  5 1771  2.508837e+00
## 30020  5 1772  2.480264e+00
## 30021  5 1773  2.446678e+00
## 30022  5 1774  2.421168e+00
## 30023  5 1775  2.399303e+00
## 30024  5 1776  2.367487e+00
## 30025  5 1777  3.277417e+00
## 30026  5 1778  3.449863e+00
## 30027  5 1779  3.411011e+00
## 30028  5 1780  3.319775e+00
## 30029  5 1781  3.223333e+00
## 30030  5 1782  3.135976e+00
## 30031  5 1783  3.053700e+00
## 30032  5 1784  2.991711e+00
## 30033  5 1785  2.920038e+00
## 30034  5 1786  2.869190e+00
## 30035  5 1787  2.812358e+00
## 30036  5 1788  2.771397e+00
## 30037  5 1789  2.727589e+00
## 30038  5 1790  2.688363e+00
## 30039  5 1791  2.651520e+00
## 30040  5 1792  2.613799e+00
## 30041  5 1793  2.584751e+00
## 30042  5 1794  2.549362e+00
## 30043  5 1795  2.513733e+00
## 30044  5 1796  2.489632e+00
## 30045  5 1797  2.461808e+00
## 30046  5 1798  2.429126e+00
## 30047  5 1799  2.405959e+00
## 30048  5 1800  2.378783e+00
## 30049  5 1801  3.291825e+00
## 30050  5 1802  3.456250e+00
## 30051  5 1803  3.414188e+00
## 30052  5 1804  3.327465e+00
## 30053  5 1805  3.226986e+00
## 30054  5 1806  3.140499e+00
## 30055  5 1807  3.062839e+00
## 30056  5 1808  2.994990e+00
## 30057  5 1809  2.932862e+00
## 30058  5 1810  2.878166e+00
## 30059  5 1811  2.819379e+00
## 30060  5 1812  2.776381e+00
## 30061  5 1813  2.733446e+00
## 30062  5 1814  2.696768e+00
## 30063  5 1815  2.661403e+00
## 30064  5 1816  2.619728e+00
## 30065  5 1817  2.586336e+00
## 30066  5 1818  2.552249e+00
## 30067  5 1819  2.525063e+00
## 30068  5 1820  2.491084e+00
## 30069  5 1821  2.466019e+00
## 30070  5 1822  2.440760e+00
## 30071  5 1823  2.413211e+00
## 30072  5 1824  2.385837e+00
## 30073  5 1825  3.297821e+00
## 30074  5 1826  3.469280e+00
## 30075  5 1827  3.417021e+00
## 30076  5 1828  3.336167e+00
## 30077  5 1829  3.237982e+00
## 30078  5 1830  3.146313e+00
## 30079  5 1831  3.065511e+00
## 30080  5 1832  2.997392e+00
## 30081  5 1833  2.936094e+00
## 30082  5 1834  2.882855e+00
## 30083  5 1835  2.828180e+00
## 30084  5 1836  2.789270e+00
## 30085  5 1837  2.742039e+00
## 30086  5 1838  2.700486e+00
## 30087  5 1839  2.662131e+00
## 30088  5 1840  2.620184e+00
## 30089  5 1841  2.589725e+00
## 30090  5 1842  2.560194e+00
## 30091  5 1843  2.532012e+00
## 30092  5 1844  2.501461e+00
## 30093  5 1845  2.473059e+00
## 30094  5 1846  2.445327e+00
## 30095  5 1847  2.422192e+00
## 30096  5 1848  2.392572e+00
## 30097  5 1849  3.300225e+00
## 30098  5 1850  3.461794e+00
## 30099  5 1851  3.426308e+00
## 30100  5 1852  3.331720e+00
## 30101  5 1853  3.238382e+00
## 30102  5 1854  3.157374e+00
## 30103  5 1855  3.073004e+00
## 30104  5 1856  3.004509e+00
## 30105  5 1857  2.942916e+00
## 30106  5 1858  2.882273e+00
## 30107  5 1859  2.834533e+00
## 30108  5 1860  2.786500e+00
## 30109  5 1861  2.743039e+00
## 30110  5 1862  2.704657e+00
## 30111  5 1863  2.670124e+00
## 30112  5 1864  2.630773e+00
## 30113  5 1865  2.595806e+00
## 30114  5 1866  2.566121e+00
## 30115  5 1867  2.535161e+00
## 30116  5 1868  2.500250e+00
## 30117  5 1869  2.475347e+00
## 30118  5 1870  2.450913e+00
## 30119  5 1871  2.423214e+00
## 30120  5 1872  2.395115e+00
## 30121  5 1872  2.412358e+00
## 30122  5 1873  3.322068e+00
## 30123  5 1874  3.481535e+00
## 30124  5 1875  3.443832e+00
## 30125  5 1876  3.346451e+00
## 30126  5 1877  3.257879e+00
## 30127  5 1878  3.174017e+00
## 30128  5 1879  3.089880e+00
## 30129  5 1880  3.024111e+00
## 30130  5 1881  2.958694e+00
## 30131  5 1882  2.900566e+00
## 30132  5 1883  2.852432e+00
## 30133  5 1884  2.802442e+00
## 30134  5 1885  2.757658e+00
## 30135  5 1886  2.718866e+00
## 30136  5 1887  2.683668e+00
## 30137  5 1888  2.644812e+00
## 30138  5 1889  2.615526e+00
## 30139  5 1890  2.583327e+00
## 30140  5 1891  2.550536e+00
## 30141  5 1892  2.518228e+00
## 30142  5 1893  2.489472e+00
## 30143  5 1894  2.464543e+00
## 30144  5 1895  2.431664e+00
## 30145  5 1896  2.407316e+00
## 30146  5 1896  2.409103e+00
## 30147  5 1897  2.385468e+00
## 30148  5 1898  2.360096e+00
## 30149  5 1899  2.332277e+00
## 30150  5 1900  2.305867e+00
## 30151  5 1901  2.287435e+00
## 30152  5 1902  2.261245e+00
## 30153  5 1903  2.234155e+00
## 30154  5 1904  2.210913e+00
## 30155  5 1905  2.183669e+00
## 30156  5 1906  2.166727e+00
## 30157  5 1907  2.146640e+00
## 30158  5 1908  2.122587e+00
## 30159  5 1909  2.098393e+00
## 30160  5 1910  2.075841e+00
## 30161  5 1911  2.056415e+00
## 30162  5 1911  2.057561e+00
## 30163  5 1912  2.967268e+00
## 30164  5 1913  3.140315e+00
## 30165  5 1914  3.106260e+00
## 30166  5 1915  3.023926e+00
## 30167  5 1916  2.932882e+00
## 30168  5 1917  2.836774e+00
## 30169  5 1918  2.774298e+00
## 30170  5 1919  2.702845e+00
## 30171  5 1920  2.640202e+00
## 30172  5 1920  2.645545e+00
## 30173  5 1921  3.529322e+00
## 30174  5 1922  3.676928e+00
## 30175  5 1923  3.616717e+00
## 30176  5 1924  3.506128e+00
## 30177  5 1925  3.398900e+00
## 30178  5 1926  3.297797e+00
## 30179  5 1927  3.213843e+00
## 30180  5 1928  3.135047e+00
## 30181  5 1929  3.068397e+00
## 30182  5 1930  3.010625e+00
## 30183  5 1931  2.946140e+00
## 30184  5 1932  2.891792e+00
## 30185  5 1933  2.848615e+00
## 30186  5 1934  2.803587e+00
## 30187  5 1935  2.764205e+00
## 30188  5 1936  2.727665e+00
## 30189  5 1937  2.687571e+00
## 30190  5 1938  2.659774e+00
## 30191  5 1939  2.629936e+00
## 30192  5 1940  2.595683e+00
## 30193  5 1941  2.562855e+00
## 30194  5 1942  2.532167e+00
## 30195  5 1943  2.508357e+00
## 30196  5 1944  2.476277e+00
## 30197  5 1945  3.390072e+00
## 30198  5 1946  3.554868e+00
## 30199  5 1947  3.512054e+00
## 30200  5 1948  3.421791e+00
## 30201  5 1949  3.327400e+00
## 30202  5 1950  3.235776e+00
## 30203  5 1951  3.160442e+00
## 30204  5 1952  3.089268e+00
## 30205  5 1953  3.017168e+00
## 30206  5 1954  2.962475e+00
## 30207  5 1955  2.912735e+00
## 30208  5 1956  2.867990e+00
## 30209  5 1957  2.825774e+00
## 30210  5 1958  2.781360e+00
## 30211  5 1959  2.739407e+00
## 30212  5 1960  2.706743e+00
## 30213  5 1961  2.673606e+00
## 30214  5 1962  2.636365e+00
## 30215  5 1963  2.604523e+00
## 30216  5 1964  2.574096e+00
## 30217  5 1965  2.540763e+00
## 30218  5 1966  2.512923e+00
## 30219  5 1967  2.489392e+00
## 30220  5 1968  2.459638e+00
## 30221  5 1969  3.369689e+00
## 30222  5 1970  3.538202e+00
## 30223  5 1971  3.497338e+00
## 30224  5 1972  3.398817e+00
## 30225  5 1973  3.311481e+00
## 30226  5 1974  3.226212e+00
## 30227  5 1975  3.143902e+00
## 30228  5 1976  3.080443e+00
## 30229  5 1977  3.012900e+00
## 30230  5 1978  2.949775e+00
## 30231  5 1979  2.897862e+00
## 30232  5 1980  2.847968e+00
## 30233  5 1981  2.814510e+00
## 30234  5 1982  2.761914e+00
## 30235  5 1983  2.724057e+00
## 30236  5 1984  2.687650e+00
## 30237  5 1985  2.660093e+00
## 30238  5 1986  2.624126e+00
## 30239  5 1987  2.592406e+00
## 30240  5 1988  2.565319e+00
## 30241  5 1989  2.534119e+00
## 30242  5 1990  2.507137e+00
## 30243  5 1991  2.474977e+00
## 30244  5 1992  2.450002e+00
## 30245  5 1993  3.358927e+00
## 30246  5 1994  3.528058e+00
## 30247  5 1995  3.477577e+00
## 30248  5 1996  3.394034e+00
## 30249  5 1997  3.300652e+00
## 30250  5 1998  3.210212e+00
## 30251  5 1999  3.132507e+00
## 30252  5 2000  3.060801e+00
## 30253  5 2001  2.993216e+00
## 30254  5 2002  2.942217e+00
## 30255  5 2003  2.892212e+00
## 30256  5 2004  2.843228e+00
## 30257  5 2005  2.795390e+00
## 30258  5 2006  2.758039e+00
## 30259  5 2007  2.716663e+00
## 30260  5 2008  2.682715e+00
## 30261  5 2009  2.646679e+00
## 30262  5 2010  2.614032e+00
## 30263  5 2011  2.583192e+00
## 30264  5 2012  2.554797e+00
## 30265  5 2013  2.522720e+00
## 30266  5 2014  2.497448e+00
## 30267  5 2015  2.468568e+00
## 30268  5 2016  2.440557e+00
## 30269  5 2017  3.354488e+00
## 30270  5 2018  3.520824e+00
## 30271  5 2019  3.474121e+00
## 30272  5 2020  3.389570e+00
## 30273  5 2021  3.292403e+00
## 30274  5 2022  3.206786e+00
## 30275  5 2023  3.119013e+00
## 30276  5 2024  3.046584e+00
## 30277  5 2025  2.985390e+00
## 30278  5 2026  2.930269e+00
## 30279  5 2027  2.877592e+00
## 30280  5 2028  2.831086e+00
## 30281  5 2029  2.788275e+00
## 30282  5 2030  2.746925e+00
## 30283  5 2031  2.714363e+00
## 30284  5 2032  2.677849e+00
## 30285  5 2033  2.642127e+00
## 30286  5 2034  2.603227e+00
## 30287  5 2035  2.575324e+00
## 30288  5 2036  2.545443e+00
## 30289  5 2037  2.515845e+00
## 30290  5 2038  2.487811e+00
## 30291  5 2039  2.459382e+00
## 30292  5 2040  2.433010e+00
## 30293  5 2041  3.345594e+00
## 30294  5 2042  3.503741e+00
## 30295  5 2043  3.470112e+00
## 30296  5 2044  3.379994e+00
## 30297  5 2045  3.276677e+00
## 30298  5 2046  3.195604e+00
## 30299  5 2047  3.120379e+00
## 30300  5 2048  3.044904e+00
## 30301  5 2049  2.982455e+00
## 30302  5 2050  2.922581e+00
## 30303  5 2051  2.873591e+00
## 30304  5 2052  2.823158e+00
## 30305  5 2053  2.783486e+00
## 30306  5 2054  2.738414e+00
## 30307  5 2055  2.700120e+00
## 30308  5 2056  2.669714e+00
## 30309  5 2057  2.636576e+00
## 30310  5 2058  2.601846e+00
## 30311  5 2059  2.572422e+00
## 30312  5 2060  2.539110e+00
## 30313  5 2061  2.509223e+00
## 30314  5 2062  2.480454e+00
## 30315  5 2063  2.454021e+00
## 30316  5 2064  2.429638e+00
## 30317  5 2065  3.334199e+00
## 30318  5 2066  3.497952e+00
## 30319  5 2067  3.458201e+00
## 30320  5 2068  3.374914e+00
## 30321  5 2069  3.277313e+00
## 30322  5 2070  3.194665e+00
## 30323  5 2071  3.106198e+00
## 30324  5 2072  3.041398e+00
## 30325  5 2073  2.980193e+00
## 30326  5 2074  2.920800e+00
## 30327  5 2075  2.867718e+00
## 30328  5 2076  2.823902e+00
## 30329  5 2077  2.776143e+00
## 30330  5 2078  2.737174e+00
## 30331  5 2079  2.697510e+00
## 30332  5 2080  2.667560e+00
## 30333  5 2081  2.629356e+00
## 30334  5 2082  2.598355e+00
## 30335  5 2083  2.565075e+00
## 30336  5 2084  2.537088e+00
## 30337  5 2085  2.508551e+00
## 30338  5 2086  2.476302e+00
## 30339  5 2087  2.452145e+00
## 30340  5 2088  2.422792e+00
## 30341  5 2089  3.330677e+00
## 30342  5 2090  3.494563e+00
## 30343  5 2091  3.465899e+00
## 30344  5 2092  3.363673e+00
## 30345  5 2093  3.276207e+00
## 30346  5 2094  3.188866e+00
## 30347  5 2095  3.108581e+00
## 30348  5 2096  3.034972e+00
## 30349  5 2097  2.970549e+00
## 30350  5 2098  2.916111e+00
## 30351  5 2099  2.863035e+00
## 30352  5 2100  2.815892e+00
## 30353  5 2101  2.778654e+00
## 30354  5 2102  2.736456e+00
## 30355  5 2103  2.696415e+00
## 30356  5 2104  2.653747e+00
## 30357  5 2105  2.625322e+00
## 30358  5 2106  2.590861e+00
## 30359  5 2107  2.563121e+00
## 30360  5 2108  2.526454e+00
## 30361  5 2109  2.505563e+00
## 30362  5 2110  2.472879e+00
## 30363  5 2111  2.443110e+00
## 30364  5 2112  2.420386e+00
## 30365  5 2113  3.329497e+00
## 30366  5 2114  3.497995e+00
## 30367  5 2115  3.452262e+00
## 30368  5 2116  3.365489e+00
## 30369  5 2117  3.275654e+00
## 30370  5 2118  3.184992e+00
## 30371  5 2119  3.104053e+00
## 30372  5 2120  3.030385e+00
## 30373  5 2121  2.966502e+00
## 30374  5 2122  2.913874e+00
## 30375  5 2123  2.860765e+00
## 30376  5 2124  2.811949e+00
## 30377  5 2125  2.772022e+00
## 30378  5 2126  2.731263e+00
## 30379  5 2127  2.690629e+00
## 30380  5 2128  2.657915e+00
## 30381  5 2129  2.619332e+00
## 30382  5 2130  2.590078e+00
## 30383  5 2131  2.560670e+00
## 30384  5 2132  2.530831e+00
## 30385  5 2133  2.502048e+00
## 30386  5 2134  2.472823e+00
## 30387  5 2135  2.442868e+00
## 30388  5 2136  2.416177e+00
## 30389  5 2137  3.327068e+00
## 30390  5 2138  3.496756e+00
## 30391  5 2139  3.447860e+00
## 30392  5 2140  3.357878e+00
## 30393  5 2141  3.265930e+00
## 30394  5 2142  3.175201e+00
## 30395  5 2143  3.104260e+00
## 30396  5 2144  3.032381e+00
## 30397  5 2145  2.969827e+00
## 30398  5 2146  2.912158e+00
## 30399  5 2147  2.860485e+00
## 30400  5 2148  2.812307e+00
## 30401  5 2149  2.771099e+00
## 30402  5 2150  2.724160e+00
## 30403  5 2151  2.693680e+00
## 30404  5 2152  2.652225e+00
## 30405  5 2153  2.621391e+00
## 30406  5 2154  2.587539e+00
## 30407  5 2155  2.555880e+00
## 30408  5 2156  2.530728e+00
## 30409  5 2157  2.497093e+00
## 30410  5 2158  2.466419e+00
## 30411  5 2159  2.440104e+00
## 30412  5 2160  2.417097e+00
## 30413  5 2161  3.324866e+00
## 30414  5 2162  3.492094e+00
## 30415  5 2163  3.450456e+00
## 30416  5 2164  3.354399e+00
## 30417  5 2165  3.263181e+00
## 30418  5 2166  3.174741e+00
## 30419  5 2167  3.099237e+00
## 30420  5 2168  3.030409e+00
## 30421  5 2169  2.964204e+00
## 30422  5 2170  2.908845e+00
## 30423  5 2171  2.859712e+00
## 30424  5 2172  2.808668e+00
## 30425  5 2173  2.770240e+00
## 30426  5 2174  2.724035e+00
## 30427  5 2175  2.685294e+00
## 30428  5 2176  2.654542e+00
## 30429  5 2177  2.619219e+00
## 30430  5 2178  2.585906e+00
## 30431  5 2179  2.554561e+00
## 30432  5 2180  2.523528e+00
## 30433  5 2181  2.491817e+00
## 30434  5 2182  2.466924e+00
## 30435  5 2183  2.439900e+00
```

```
## 30436  5 2184  2.415710e+00
## 30437  5 2184  2.411785e+00
## 30438  5 2185  3.320174e+00
## 30439  5 2186  3.488618e+00
## 30440  5 2187  3.443332e+00
## 30441  5 2188  3.354610e+00
## 30442  5 2189  3.258256e+00
## 30443  5 2190  3.169688e+00
## 30444  5 2191  3.090534e+00
## 30445  5 2192  3.023101e+00
## 30446  5 2193  2.958295e+00
## 30447  5 2194  2.905537e+00
## 30448  5 2195  2.853217e+00
## 30449  5 2196  2.803446e+00
## 30450  5 2197  2.759109e+00
## 30451  5 2198  2.720439e+00
## 30452  5 2199  2.683738e+00
## 30453  5 2200  2.645281e+00
## 30454  5 2201  2.615180e+00
## 30455  5 2202  2.580014e+00
## 30456  5 2203  2.549182e+00
## 30457  5 2204  2.519673e+00
## 30458  5 2205  2.494631e+00
## 30459  5 2206  2.459970e+00
## 30460  5 2207  2.434251e+00
## 30461  5 2208  2.406984e+00
## 30462  5 2208  2.409775e+00
## 30463  5 2209  2.379036e+00
## 30464  5 2210  2.355750e+00
## 30465  5 2211  2.331534e+00
## 30466  5 2212  2.301416e+00
## 30467  5 2213  2.284887e+00
## 30468  5 2214  2.257938e+00
## 30469  5 2215  2.234970e+00
## 30470  5 2216  2.212053e+00
## 30471  5 2217  2.189763e+00
## 30472  5 2218  2.163601e+00
## 30473  5 2219  2.143508e+00
## 30474  5 2220  2.117154e+00
## 30475  5 2221  2.100406e+00
## 30476  5 2222  2.075795e+00
## 30477  5 2223  2.055539e+00
## 30478  5 2224  2.038040e+00
## 30479  5 2225  2.013530e+00
## 30480  5 2226  1.991012e+00
## 30481  5 2226  1.991769e+00
## 30482  5 2227  2.437991e+00
## 30483  5 2228  2.519697e+00
## 30484  5 2229  2.488962e+00
## 30485  5 2230  2.437470e+00
## 30486  5 2231  2.380310e+00
## 30487  5 2232  2.327971e+00
## 30488  5 2232  2.330630e+00
## 30489  5 2233  3.221991e+00
## 30490  5 2234  3.367344e+00
## 30491  5 2235  3.312447e+00
## 30492  5 2236  3.210094e+00
## 30493  5 2237  3.109048e+00
## 30494  5 2238  3.017288e+00
## 30495  5 2239  2.930317e+00
## 30496  5 2240  2.861320e+00
## 30497  5 2241  2.792681e+00
## 30498  5 2242  2.735811e+00
## 30499  5 2243  2.677325e+00
## 30500  5 2244  2.628708e+00
## 30501  5 2245  2.592448e+00
## 30502  5 2246  2.550993e+00
## 30503  5 2247  2.510407e+00
## 30504  5 2248  2.472550e+00
## 30505  5 2249  2.444115e+00
## 30506  5 2250  2.408431e+00
## 30507  5 2251  2.382388e+00
## 30508  5 2252  2.352953e+00
## 30509  5 2253  2.326986e+00
## 30510  5 2254  2.297686e+00
## 30511  5 2255  2.266733e+00
## 30512  5 2256  2.246826e+00
## 30513  5 2257  3.164279e+00
## 30514  5 2258  3.322730e+00
## 30515  5 2259  3.290293e+00
## 30516  5 2260  3.203933e+00
## 30517  5 2261  3.108189e+00
## 30518  5 2262  3.015516e+00
## 30519  5 2263  2.939111e+00
## 30520  5 2264  2.876178e+00
## 30521  5 2265  2.808957e+00
## 30522  5 2266  2.754317e+00
## 30523  5 2267  2.706512e+00
## 30524  5 2268  2.664726e+00
## 30525  5 2269  2.616665e+00
## 30526  5 2270  2.575112e+00
## 30527  5 2271  2.543044e+00
## 30528  5 2272  2.506913e+00
## 30529  5 2273  2.474262e+00
## 30530  5 2274  2.444278e+00
## 30531  5 2275  2.410906e+00
## 30532  5 2276  2.383996e+00
## 30533  5 2277  2.362248e+00
## 30534  5 2278  2.329234e+00
## 30535  5 2279  2.305455e+00
## 30536  5 2280  2.277319e+00
## 30537  5 2281  3.191550e+00
## 30538  5 2282  3.358651e+00
## 30539  5 2283  3.324736e+00
## 30540  5 2284  3.229396e+00
## 30541  5 2285  3.131060e+00
## 30542  5 2286  3.054484e+00
## 30543  5 2287  2.970140e+00
## 30544  5 2288  2.904887e+00
## 30545  5 2289  2.847361e+00
## 30546  5 2290  2.784279e+00
## 30547  5 2291  2.731719e+00
## 30548  5 2292  2.690710e+00
## 30549  5 2293  2.646277e+00
## 30550  5 2294  2.611753e+00
## 30551  5 2295  2.572268e+00
## 30552  5 2296  2.537772e+00
## 30553  5 2297  2.505376e+00
## 30554  5 2298  2.470208e+00
## 30555  5 2299  2.451401e+00
## 30556  5 2300  2.417328e+00
## 30557  5 2301  2.387388e+00
## 30558  5 2302  2.358369e+00
## 30559  5 2303  2.337023e+00
## 30560  5 2304  2.308020e+00
## 30561  5 2305  3.225671e+00
## 30562  5 2306  3.390527e+00
## 30563  5 2307  3.343140e+00
## 30564  5 2308  3.255812e+00
## 30565  5 2309  3.165679e+00
## 30566  5 2310  3.078947e+00
## 30567  5 2311  3.001951e+00
## 30568  5 2312  2.928815e+00
## 30569  5 2313  2.869411e+00
## 30570  5 2314  2.808225e+00
## 30571  5 2315  2.764029e+00
## 30572  5 2316  2.712683e+00
## 30573  5 2317  2.670176e+00
## 30574  5 2318  2.635261e+00
## 30575  5 2319  2.597428e+00
## 30576  5 2320  2.565369e+00
## 30577  5 2321  2.525554e+00
## 30578  5 2322  2.497876e+00
## 30579  5 2323  2.469593e+00
## 30580  5 2324  2.435017e+00
## 30581  5 2325  2.410502e+00
## 30582  5 2326  2.382977e+00
## 30583  5 2327  2.356080e+00
## 30584  5 2328  2.329243e+00
## 30585  5 2329  3.243114e+00
## 30586  5 2330  3.413627e+00
## 30587  5 2331  3.368187e+00
## 30588  5 2332  3.276608e+00
## 30589  5 2333  3.184195e+00
## 30590  5 2334  3.101152e+00
## 30591  5 2335  3.013207e+00
## 30592  5 2336  2.955478e+00
## 30593  5 2337  2.886927e+00
## 30594  5 2338  2.839245e+00
## 30595  5 2339  2.780034e+00
## 30596  5 2340  2.737649e+00
## 30597  5 2341  2.688995e+00
## 30598  5 2342  2.655512e+00
## 30599  5 2343  2.617513e+00
## 30600  5 2344  2.584652e+00
## 30601  5 2345  2.546415e+00
## 30602  5 2346  2.515199e+00
## 30603  5 2347  2.487450e+00
## 30604  5 2348  2.458712e+00
## 30605  5 2349  2.426665e+00
## 30606  5 2350  2.402374e+00
## 30607  5 2351  2.373632e+00
## 30608  5 2352  2.345247e+00
## 30609  5 2353  3.253525e+00
## 30610  5 2354  3.424877e+00
## 30611  5 2355  3.385928e+00
## 30612  5 2356  3.298853e+00
## 30613  5 2357  3.200929e+00
## 30614  5 2358  3.114700e+00
## 30615  5 2359  3.040327e+00
## 30616  5 2360  2.968766e+00
## 30617  5 2361  2.909091e+00
## 30618  5 2362  2.851489e+00
## 30619  5 2363  2.793895e+00
## 30620  5 2364  2.750776e+00
## 30621  5 2365  2.712067e+00
## 30622  5 2366  2.665971e+00
## 30623  5 2367  2.631782e+00
## 30624  5 2368  2.599255e+00
## 30625  5 2369  2.563475e+00
## 30626  5 2370  2.527618e+00
## 30627  5 2371  2.502350e+00
## 30628  5 2372  2.465579e+00
## 30629  5 2373  2.441564e+00
## 30630  5 2374  2.411183e+00
## 30631  5 2375  2.388397e+00
## 30632  5 2376  2.362431e+00
## 30633  5 2377  3.275453e+00
## 30634  5 2378  3.438014e+00
## 30635  5 2379  3.401785e+00
## 30636  5 2380  3.308495e+00
## 30637  5 2381  3.209969e+00
## 30638  5 2382  3.122452e+00
## 30639  5 2383  3.051924e+00
## 30640  5 2384  2.978449e+00
## 30641  5 2385  2.913509e+00
## 30642  5 2386  2.862144e+00
## 30643  5 2387  2.810070e+00
## 30644  5 2388  2.759186e+00
## 30645  5 2389  2.724111e+00
## 30646  5 2390  2.676783e+00
## 30647  5 2391  2.643775e+00
## 30648  5 2392  2.605757e+00
## 30649  5 2393  2.570010e+00
## 30650  5 2394  2.542338e+00
## 30651  5 2395  2.509480e+00
## 30652  5 2396  2.481636e+00
## 30653  5 2397  2.454860e+00
## 30654  5 2398  2.424110e+00
## 30655  5 2399  2.403332e+00
## 30656  5 2400  2.372064e+00
## 30657  5 2401  3.287226e+00
## 30658  5 2402  3.447045e+00
## 30659  5 2403  3.412748e+00
## 30660  5 2404  3.318029e+00
## 30661  5 2405  3.220339e+00
## 30662  5 2406  3.131032e+00
## 30663  5 2407  3.055333e+00
## 30664  5 2408  2.985921e+00
## 30665  5 2409  2.931488e+00
## 30666  5 2410  2.869244e+00
## 30667  5 2411  2.817867e+00
## 30668  5 2412  2.770804e+00
## 30669  5 2413  2.727167e+00
## 30670  5 2414  2.694309e+00
## 30671  5 2415  2.654254e+00
## 30672  5 2416  2.615592e+00
## 30673  5 2417  2.586743e+00
## 30674  5 2418  2.550955e+00
## 30675  5 2419  2.519398e+00
## 30676  5 2420  2.491057e+00
## 30677  5 2421  2.461904e+00
## 30678  5 2422  2.432152e+00
## 30679  5 2423  2.409636e+00
## 30680  5 2424  2.382544e+00
## 30681  5 2425  3.293887e+00
## 30682  5 2426  3.458906e+00
## 30683  5 2427  3.415916e+00
## 30684  5 2428  3.324763e+00
## 30685  5 2429  3.232852e+00
## 30686  5 2430  3.144688e+00
## 30687  5 2431  3.064882e+00
## 30688  5 2432  2.999097e+00
## 30689  5 2433  2.928060e+00
## 30690  5 2434  2.878747e+00
## 30691  5 2435  2.824100e+00
## 30692  5 2436  2.779712e+00
## 30693  5 2437  2.735972e+00
## 30694  5 2438  2.693445e+00
## 30695  5 2439  2.660028e+00
## 30696  5 2440  2.627269e+00
## 30697  5 2441  2.588442e+00
## 30698  5 2442  2.557857e+00
## 30699  5 2443  2.523003e+00
## 30700  5 2444  2.494848e+00
## 30701  5 2445  2.467674e+00
## 30702  5 2446  2.438219e+00
## 30703  5 2447  2.414325e+00
## 30704  5 2448  2.386744e+00
## 30705  5 2449  3.293070e+00
## 30706  5 2450  3.463409e+00
## 30707  5 2451  3.423544e+00
## 30708  5 2452  3.329612e+00
## 30709  5 2453  3.236043e+00
## 30710  5 2454  3.153266e+00
## 30711  5 2455  3.077303e+00
## 30712  5 2456  2.997388e+00
## 30713  5 2457  2.938773e+00
## 30714  5 2458  2.876169e+00
## 30715  5 2459  2.831167e+00
## 30716  5 2460  2.784626e+00
## 30717  5 2461  2.745046e+00
## 30718  5 2462  2.697591e+00
## 30719  5 2463  2.662043e+00
## 30720  5 2464  2.631046e+00
## 30721  5 2465  2.592951e+00
## 30722  5 2466  2.562340e+00
## 30723  5 2467  2.526426e+00
## 30724  5 2468  2.497149e+00
## 30725  5 2469  2.473836e+00
## 30726  5 2470  2.443165e+00
## 30727  5 2471  2.417828e+00
## 30728  5 2472  2.389048e+00
## 30729  5 2473  3.304306e+00
## 30730  5 2474  3.461072e+00
## 30731  5 2475  3.423788e+00
## 30732  5 2476  3.336637e+00
## 30733  5 2477  3.243915e+00
## 30734  5 2478  3.159675e+00
## 30735  5 2479  3.076476e+00
## 30736  5 2480  3.008290e+00
## 30737  5 2481  2.943320e+00
## 30738  5 2482  2.882376e+00
## 30739  5 2483  2.833406e+00
## 30740  5 2484  2.787432e+00
## 30741  5 2485  2.751354e+00
## 30742  5 2486  2.707395e+00
## 30743  5 2487  2.671548e+00
## 30744  5 2488  2.629751e+00
## 30745  5 2489  2.600711e+00
## 30746  5 2490  2.565130e+00
## 30747  5 2491  2.532845e+00
## 30748  5 2492  2.508451e+00
## 30749  5 2493  2.470274e+00
## 30750  5 2494  2.445809e+00
## 30751  5 2495  2.420786e+00
## 30752  5 2496  2.396285e+00
## 30753  5 2496  2.408891e+00
## 30754  5 2497  3.320201e+00
## 30755  5 2498  3.488406e+00
## 30756  5 2499  3.439808e+00
## 30757  5 2500  3.357302e+00
## 30758  5 2501  3.262724e+00
## 30759  5 2502  3.172669e+00
## 30760  5 2503  3.089423e+00
## 30761  5 2504  3.021928e+00
## 30762  5 2505  2.963548e+00
## 30763  5 2506  2.904598e+00
## 30764  5 2507  2.848469e+00
## 30765  5 2508  2.803248e+00
## 30766  5 2509  2.760718e+00
## 30767  5 2510  2.720664e+00
## 30768  5 2511  2.685358e+00
## 30769  5 2512  2.647049e+00
## 30770  5 2513  2.615232e+00
## 30771  5 2514  2.577989e+00
## 30772  5 2515  2.551105e+00
## 30773  5 2516  2.516113e+00
## 30774  5 2517  2.489755e+00
## 30775  5 2518  2.463023e+00
## 30776  5 2519  2.436205e+00
## 30777  5 2520  2.401051e+00
## 30778  5 2520  2.406974e+00
## 30779  5 2521  2.385379e+00
## 30780  5 2522  2.357001e+00
## 30781  5 2523  2.332831e+00
## 30782  5 2524  2.306698e+00
## 30783  5 2525  2.279728e+00
## 30784  5 2526  2.259465e+00
## 30785  5 2527  2.236482e+00
## 30786  5 2528  2.208977e+00
## 30787  5 2529  2.189772e+00
## 30788  5 2530  2.164291e+00
## 30789  5 2531  2.141842e+00
## 30790  5 2532  2.120114e+00
## 30791  5 2533  2.096371e+00
## 30792  5 2534  2.075161e+00
## 30793  5 2535  2.057406e+00
## 30794  5 2536  2.034993e+00
## 30795  5 2537  2.014549e+00
## 30796  5 2538  1.993206e+00
## 30797  5 2538  1.994731e+00
## 30798  5 2539  2.909069e+00
## 30799  5 2540  3.074699e+00
## 30800  5 2541  3.044648e+00
## 30801  5 2542  2.960900e+00
## 30802  5 2543  2.869805e+00
## 30803  5 2544  2.786342e+00
## 30804  5 2544  2.789600e+00
## 30805  5 2545  3.652384e+00
## 30806  5 2546  3.778174e+00
## 30807  5 2547  3.696486e+00
## 30808  5 2548  3.575963e+00
## 30809  5 2549  3.462639e+00
## 30810  5 2550  3.361792e+00
## 30811  5 2551  3.260218e+00
## 30812  5 2552  3.177461e+00
## 30813  5 2553  3.107293e+00
## 30814  5 2554  3.037988e+00
## 30815  5 2555  2.983992e+00
## 30816  5 2556  2.923216e+00
## 30817  5 2557  2.875999e+00
## 30818  5 2558  2.832296e+00
## 30819  5 2559  2.792178e+00
## 30820  5 2560  2.751430e+00
## 30821  5 2561  2.715919e+00
## 30822  5 2562  2.680592e+00
## 30823  5 2563  2.643763e+00
## 30824  5 2564  2.608318e+00
## 30825  5 2565  2.580115e+00
## 30826  5 2566  2.553035e+00
## 30827  5 2567  2.520559e+00
## 30828  5 2568  2.496883e+00
## 30829  5 2569  3.405511e+00
## 30830  5 2570  3.565389e+00
## 30831  5 2571  3.525516e+00
## 30832  5 2572  3.437509e+00
## 30833  5 2573  3.338162e+00
## 30834  5 2574  3.248620e+00
## 30835  5 2575  3.171012e+00
## 30836  5 2576  3.100756e+00
## 30837  5 2577  3.036074e+00
## 30838  5 2578  2.978708e+00
## 30839  5 2579  2.927215e+00
## 30840  5 2580  2.878901e+00
## 30841  5 2581  2.836278e+00
## 30842  5 2582  2.789286e+00
## 30843  5 2583  2.751312e+00
## 30844  5 2584  2.720618e+00
## 30845  5 2585  2.682487e+00
## 30846  5 2586  2.653425e+00
## 30847  5 2587  2.613952e+00
## 30848  5 2588  2.583115e+00
## 30849  5 2589  2.558575e+00
## 30850  5 2590  2.530980e+00
## 30851  5 2591  2.505909e+00
## 30852  5 2592  2.472213e+00
## 30853  5 2593  3.387998e+00
## 30854  5 2594  3.547036e+00
## 30855  5 2595  3.512894e+00
## 30856  5 2596  3.417334e+00
## 30857  5 2597  3.326040e+00
## 30858  5 2598  3.230084e+00
## 30859  5 2599  3.155117e+00
## 30860  5 2600  3.078650e+00
## 30861  5 2601  3.019973e+00
## 30862  5 2602  2.964474e+00
## 30863  5 2603  2.904192e+00
## 30864  5 2604  2.865687e+00
## 30865  5 2605  2.817668e+00
## 30866  5 2606  2.774645e+00
## 30867  5 2607  2.741008e+00
## 30868  5 2608  2.703341e+00
## 30869  5 2609  2.671781e+00
## 30870  5 2610  2.634373e+00
## 30871  5 2611  2.599894e+00
## 30872  5 2612  2.573505e+00
## 30873  5 2613  2.543167e+00
## 30874  5 2614  2.514051e+00
## 30875  5 2615  2.486900e+00
## 30876  5 2616  2.461522e+00
## 30877  5 2617  3.364923e+00
## 30878  5 2618  3.531973e+00
## 30879  5 2619  3.495004e+00
## 30880  5 2620  3.397747e+00
## 30881  5 2621  3.303833e+00
## 30882  5 2622  3.218238e+00
## 30883  5 2623  3.138005e+00
## 30884  5 2624  3.073075e+00
## 30885  5 2625  3.006134e+00
## 30886  5 2626  2.948418e+00
## 30887  5 2627  2.892755e+00
## 30888  5 2628  2.849690e+00
## 30889  5 2629  2.808220e+00
## 30890  5 2630  2.763024e+00
## 30891  5 2631  2.728940e+00
## 30892  5 2632  2.684849e+00
## 30893  5 2633  2.657023e+00
## 30894  5 2634  2.622490e+00
## 30895  5 2635  2.592568e+00
## 30896  5 2636  2.559944e+00
## 30897  5 2637  2.535712e+00
## 30898  5 2638  2.504506e+00
## 30899  5 2639  2.476152e+00
## 30900  5 2640  2.451119e+00
## 30901  5 2641  3.361563e+00
## 30902  5 2642  3.522545e+00
## 30903  5 2643  3.482370e+00
## 30904  5 2644  3.391901e+00
## 30905  5 2645  3.296889e+00
## 30906  5 2646  3.209620e+00
## 30907  5 2647  3.132312e+00
## 30908  5 2648  3.054558e+00
## 30909  5 2649  2.994599e+00
## 30910  5 2650  2.937663e+00
## 30911  5 2651  2.883202e+00
## 30912  5 2652  2.839790e+00
## 30913  5 2653  2.792447e+00
## 30914  5 2654  2.756457e+00
## 30915  5 2655  2.717366e+00
## 30916  5 2656  2.677829e+00
## 30917  5 2657  2.642555e+00
## 30918  5 2658  2.613744e+00
## 30919  5 2659  2.580611e+00
## 30920  5 2660  2.555817e+00
## 30921  5 2661  2.525928e+00
## 30922  5 2662  2.493918e+00
## 30923  5 2663  2.465482e+00
## 30924  5 2664  2.436747e+00
## 30925  5 2665  3.348826e+00
## 30926  5 2666  3.517397e+00
## 30927  5 2667  3.471786e+00
## 30928  5 2668  3.386353e+00
## 30929  5 2669  3.286008e+00
## 30930  5 2670  3.199501e+00
## 30931  5 2671  3.120296e+00
## 30932  5 2672  3.046544e+00
## 30933  5 2673  2.985842e+00
## 30934  5 2674  2.929980e+00
## 30935  5 2675  2.880205e+00
## 30936  5 2676  2.832114e+00
## 30937  5 2677  2.783762e+00
## 30938  5 2678  2.748797e+00
## 30939  5 2679  2.708029e+00
## 30940  5 2680  2.677758e+00
## 30941  5 2681  2.639462e+00
## 30942  5 2682  2.606347e+00
## 30943  5 2683  2.574182e+00
## 30944  5 2684  2.551654e+00
## 30945  5 2685  2.517301e+00
## 30946  5 2686  2.490505e+00
## 30947  5 2687  2.457047e+00
## 30948  5 2688  2.431421e+00
## 30949  5 2689  3.346902e+00
## 30950  5 2690  3.509191e+00
## 30951  5 2691  3.467429e+00
## 30952  5 2692  3.373353e+00
## 30953  5 2693  3.272355e+00
## 30954  5 2694  3.187508e+00
## 30955  5 2695  3.117444e+00
## 30956  5 2696  3.041706e+00
## 30957  5 2697  2.982239e+00
## 30958  5 2698  2.923250e+00
## 30959  5 2699  2.872945e+00
## 30960  5 2700  2.831259e+00
## 30961  5 2701  2.784640e+00
## 30962  5 2702  2.741387e+00
## 30963  5 2703  2.705659e+00
## 30964  5 2704  2.669138e+00
## 30965  5 2705  2.633082e+00
## 30966  5 2706  2.597981e+00
## 30967  5 2707  2.564635e+00
## 30968  5 2708  2.538070e+00
## 30969  5 2709  2.508379e+00
## 30970  5 2710  2.478977e+00
## 30971  5 2711  2.453077e+00
## 30972  5 2712  2.428419e+00
## 30973  5 2713  3.339911e+00
## 30974  5 2714  3.501098e+00
## 30975  5 2715  3.459715e+00
## 30976  5 2716  3.372021e+00
## 30977  5 2717  3.279254e+00
## 30978  5 2718  3.194547e+00
## 30979  5 2719  3.113612e+00
## 30980  5 2720  3.039215e+00
## 30981  5 2721  2.973779e+00
## 30982  5 2722  2.918836e+00
## 30983  5 2723  2.871029e+00
## 30984  5 2724  2.818599e+00
## 30985  5 2725  2.774603e+00
## 30986  5 2726  2.740968e+00
## 30987  5 2727  2.696725e+00
## 30988  5 2728  2.666482e+00
## 30989  5 2729  2.627303e+00
## 30990  5 2730  2.597934e+00
## 30991  5 2731  2.560644e+00
## 30992  5 2732  2.534484e+00
## 30993  5 2733  2.506238e+00
## 30994  5 2734  2.479145e+00
## 30995  5 2735  2.452324e+00
## 30996  5 2736  2.425484e+00
## 30997  5 2737  3.335299e+00
## 30998  5 2738  3.499879e+00
## 30999  5 2739  3.457788e+00
## 31000  5 2740  3.373365e+00
## 31001  5 2741  3.272695e+00
## 31002  5 2742  3.189772e+00
## 31003  5 2743  3.110293e+00
## 31004  5 2744  3.034889e+00
## 31005  5 2745  2.972253e+00
## 31006  5 2746  2.916091e+00
## 31007  5 2747  2.866039e+00
## 31008  5 2748  2.815562e+00
## 31009  5 2749  2.772226e+00
## 31010  5 2750  2.736550e+00
## 31011  5 2751  2.690781e+00
## 31012  5 2752  2.661706e+00
## 31013  5 2753  2.622519e+00
## 31014  5 2754  2.597085e+00
## 31015  5 2755  2.564885e+00
## 31016  5 2756  2.534201e+00
## 31017  5 2757  2.497875e+00
## 31018  5 2758  2.473462e+00
## 31019  5 2759  2.445369e+00
## 31020  5 2760  2.420266e+00
## 31021  5 2761  3.325742e+00
## 31022  5 2762  3.494527e+00
## 31023  5 2763  3.458805e+00
## 31024  5 2764  3.365189e+00
## 31025  5 2765  3.269384e+00
## 31026  5 2766  3.186535e+00
## 31027  5 2767  3.105527e+00
## 31028  5 2768  3.031456e+00
## 31029  5 2769  2.969251e+00
## 31030  5 2770  2.916123e+00
## 31031  5 2771  2.860152e+00
## 31032  5 2772  2.812893e+00
## 31033  5 2773  2.766955e+00
## 31034  5 2774  2.735140e+00
## 31035  5 2775  2.692345e+00
## 31036  5 2776  2.656123e+00
## 31037  5 2777  2.622893e+00
## 31038  5 2778  2.587254e+00
## 31039  5 2779  2.556620e+00
## 31040  5 2780  2.522861e+00
## 31041  5 2781  2.503379e+00
## 31042  5 2782  2.471107e+00
## 31043  5 2783  2.444794e+00
## 31044  5 2784  2.421157e+00
## 31045  5 2785  3.332354e+00
## 31046  5 2786  3.490017e+00
## 31047  5 2787  3.453511e+00
## 31048  5 2788  3.362361e+00
## 31049  5 2789  3.265232e+00
## 31050  5 2790  3.175919e+00
## 31051  5 2791  3.100506e+00
## 31052  5 2792  3.033070e+00
## 31053  5 2793  2.974255e+00
## 31054  5 2794  2.908613e+00
## 31055  5 2795  2.854943e+00
## 31056  5 2796  2.810773e+00
## 31057  5 2797  2.765551e+00
## 31058  5 2798  2.729480e+00
## 31059  5 2799  2.691802e+00
## 31060  5 2800  2.653952e+00
## 31061  5 2801  2.620595e+00
## 31062  5 2802  2.594557e+00
## 31063  5 2803  2.555704e+00
## 31064  5 2804  2.530252e+00
## 31065  5 2805  2.495979e+00
## 31066  5 2806  2.462924e+00
## 31067  5 2807  2.446256e+00
## 31068  5 2808  2.418291e+00
## 31069  5 2808  2.407725e+00
## 31070  5 2809  3.323920e+00
## 31071  5 2810  3.490193e+00
## 31072  5 2811  3.437242e+00
## 31073  5 2812  3.352403e+00
## 31074  5 2813  3.258274e+00
## 31075  5 2814  3.170307e+00
## 31076  5 2815  3.090419e+00
## 31077  5 2816  3.016931e+00
## 31078  5 2817  2.963072e+00
## 31079  5 2818  2.904004e+00
## 31080  5 2819  2.852559e+00
## 31081  5 2820  2.804506e+00
## 31082  5 2821  2.760459e+00
## 31083  5 2822  2.717533e+00
## 31084  5 2823  2.685063e+00
## 31085  5 2824  2.645570e+00
## 31086  5 2825  2.615069e+00
## 31087  5 2826  2.576682e+00
## 31088  5 2827  2.551224e+00
## 31089  5 2828  2.518868e+00
## 31090  5 2829  2.489527e+00
## 31091  5 2830  2.463182e+00
## 31092  5 2831  2.435520e+00
## 31093  5 2832  2.405490e+00
## 31094  5 2832  2.410291e+00
## 31095  5 2833  2.382378e+00
## 31096  5 2834  2.359720e+00
## 31097  5 2835  2.331530e+00
## 31098  5 2836  2.305868e+00
## 31099  5 2837  2.284514e+00
## 31100  5 2838  2.259979e+00
## 31101  5 2839  2.231737e+00
## 31102  5 2840  2.212240e+00
## 31103  5 2841  2.188772e+00
## 31104  5 2842  2.164700e+00
## 31105  5 2843  2.143082e+00
## 31106  5 2844  2.119524e+00
## 31107  5 2845  2.100701e+00
## 31108  5 2846  2.075289e+00
## 31109  5 2847  2.053594e+00
## 31110  5 2848  2.032526e+00
## 31111  5 2849  2.012190e+00
## 31112  5 2850  1.991141e+00
## 31113  5 2851  1.972182e+00
## 31114  5 2852  1.954989e+00
## 31115  5 2853  1.934596e+00
## 31116  5 2853  1.932904e+00
## 31117  5 2854  2.380913e+00
## 31118  5 2855  2.456266e+00
## 31119  5 2856  2.428023e+00
## 31120  5 2856  2.426060e+00
## 31121  5 2857  3.311473e+00
## 31122  5 2858  3.445426e+00
## 31123  5 2859  3.384328e+00
## 31124  5 2860  3.270648e+00
## 31125  5 2861  3.161914e+00
## 31126  5 2862  3.063663e+00
## 31127  5 2863  2.969577e+00
## 31128  5 2864  2.892930e+00
## 31129  5 2865  2.818665e+00
## 31130  5 2866  2.761980e+00
## 31131  5 2867  2.704389e+00
## 31132  5 2868  2.653568e+00
## 31133  5 2869  2.606149e+00
## 31134  5 2870  2.563652e+00
## 31135  5 2871  2.528887e+00
## 31136  5 2872  2.488999e+00
## 31137  5 2873  2.458205e+00
## 31138  5 2874  2.418686e+00
## 31139  5 2875  2.390488e+00
## 31140  5 2876  2.359391e+00
## 31141  5 2877  2.331089e+00
## 31142  5 2878  2.308584e+00
## 31143  5 2879  2.280810e+00
## 31144  5 2880  2.253813e+00
## 31145  5 2881  3.157578e+00
## 31146  5 2882  3.336576e+00
## 31147  5 2883  3.295028e+00
## 31148  5 2884  3.202080e+00
## 31149  5 2885  3.113922e+00
## 31150  5 2886  3.024401e+00
## 31151  5 2887  2.946399e+00
## 31152  5 2888  2.875725e+00
## 31153  5 2889  2.820668e+00
## 31154  5 2890  2.764889e+00
## 31155  5 2891  2.708484e+00
## 31156  5 2892  2.667598e+00
## 31157  5 2893  2.626656e+00
## 31158  5 2894  2.587735e+00
## 31159  5 2895  2.551767e+00
## 31160  5 2896  2.515687e+00
## 31161  5 2897  2.480190e+00
## 31162  5 2898  2.449049e+00
## 31163  5 2899  2.420255e+00
## 31164  5 2900  2.393230e+00
## 31165  5 2901  2.364479e+00
## 31166  5 2902  2.337148e+00
## 31167  5 2903  2.309905e+00
## 31168  5 2904  2.284077e+00
## 31169  5 2905  3.199440e+00
## 31170  5 2906  3.366220e+00
## 31171  5 2907  3.324021e+00
## 31172  5 2908  3.231230e+00
## 31173  5 2909  3.146070e+00
## 31174  5 2910  3.054573e+00
## 31175  5 2911  2.979606e+00
## 31176  5 2912  2.913723e+00
## 31177  5 2913  2.848559e+00
## 31178  5 2914  2.795393e+00
## 31179  5 2915  2.738234e+00
## 31180  5 2916  2.697665e+00
## 31181  5 2917  2.654323e+00
## 31182  5 2918  2.616901e+00
## 31183  5 2919  2.578375e+00
## 31184  5 2920  2.541171e+00
## 31185  5 2921  2.509866e+00
## 31186  5 2922  2.476459e+00
## 31187  5 2923  2.449256e+00
## 31188  5 2924  2.424963e+00
## 31189  5 2925  2.396553e+00
## 31190  5 2926  2.360151e+00
## 31191  5 2927  2.334472e+00
## 31192  5 2928  2.311210e+00
## 31193  5 2929  3.224119e+00
## 31194  5 2930  3.391661e+00
## 31195  5 2931  3.341520e+00
## 31196  5 2932  3.260354e+00
## 31197  5 2933  3.166333e+00
## 31198  5 2934  3.081408e+00
## 31199  5 2935  3.007417e+00
## 31200  5 2936  2.933720e+00
## 31201  5 2937  2.871340e+00
## 31202  5 2938  2.818704e+00
## 31203  5 2939  2.768953e+00
## 31204  5 2940  2.718030e+00
## 31205  5 2941  2.678257e+00
## 31206  5 2942  2.639203e+00
## 31207  5 2943  2.596298e+00
## 31208  5 2944  2.563806e+00
## 31209  5 2945  2.535306e+00
## 31210  5 2946  2.505109e+00
## 31211  5 2947  2.471303e+00
## 31212  5 2948  2.442641e+00
## 31213  5 2949  2.414968e+00
## 31214  5 2950  2.385611e+00
## 31215  5 2951  2.361070e+00
## 31216  5 2952  2.335439e+00
## 31217  5 2953  3.248108e+00
## 31218  5 2954  3.411217e+00
## 31219  5 2955  3.366223e+00
## 31220  5 2956  3.287037e+00
## 31221  5 2957  3.188237e+00
## 31222  5 2958  3.103168e+00
## 31223  5 2959  3.027437e+00
## 31224  5 2960  2.959370e+00
## 31225  5 2961  2.894371e+00
## 31226  5 2962  2.838422e+00
## 31227  5 2963  2.786133e+00
## 31228  5 2964  2.736348e+00
## 31229  5 2965  2.694723e+00
## 31230  5 2966  2.658643e+00
## 31231  5 2967  2.620867e+00
## 31232  5 2968  2.585055e+00
## 31233  5 2969  2.548871e+00
## 31234  5 2970  2.517441e+00
## 31235  5 2971  2.491118e+00
```

```
## 31236  5 2972  2.458516e+00
## 31237  5 2973  2.429482e+00
## 31238  5 2974  2.399841e+00
## 31239  5 2975  2.375331e+00
## 31240  5 2976  2.348326e+00
## 31241  5 2977  3.261365e+00
## 31242  5 2978  3.428731e+00
## 31243  5 2979  3.390268e+00
## 31244  5 2980  3.293189e+00
## 31245  5 2981  3.196731e+00
## 31246  5 2982  3.119913e+00
## 31247  5 2983  3.041124e+00
## 31248  5 2984  2.966926e+00
## 31249  5 2985  2.911190e+00
## 31250  5 2986  2.849960e+00
## 31251  5 2987  2.797018e+00
## 31252  5 2988  2.753649e+00
## 31253  5 2989  2.708407e+00
## 31254  5 2990  2.672346e+00
## 31255  5 2991  2.633634e+00
## 31256  5 2992  2.593276e+00
## 31257  5 2993  2.564851e+00
## 31258  5 2994  2.533930e+00
## 31259  5 2995  2.503900e+00
## 31260  5 2996  2.477382e+00
## 31261  5 2997  2.444699e+00
## 31262  5 2998  2.417046e+00
## 31263  5 2999  2.389404e+00
## 31264  5 3000  2.368446e+00
## 31265  5 3001  3.273077e+00
## 31266  5 3002  3.435830e+00
## 31267  5 3003  3.402753e+00
## 31268  5 3004  3.308254e+00
## 31269  5 3005  3.215140e+00
## 31270  5 3006  3.125262e+00
## 31271  5 3007  3.050793e+00
## 31272  5 3008  2.984730e+00
## 31273  5 3009  2.922418e+00
## 31274  5 3010  2.864064e+00
## 31275  5 3011  2.810125e+00
## 31276  5 3012  2.762410e+00
## 31277  5 3013  2.718216e+00
## 31278  5 3014  2.686544e+00
## 31279  5 3015  2.645535e+00
## 31280  5 3016  2.608118e+00
## 31281  5 3017  2.572407e+00
## 31282  5 3018  2.541948e+00
## 31283  5 3019  2.509782e+00
## 31284  5 3020  2.484308e+00
## 31285  5 3021  2.454469e+00
## 31286  5 3022  2.425418e+00
## 31287  5 3023  2.393735e+00
## 31288  5 3024  2.376049e+00
## 31289  5 3025  3.282365e+00
## 31290  5 3026  3.446506e+00
## 31291  5 3027  3.415565e+00
## 31292  5 3028  3.316976e+00
## 31293  5 3029  3.226048e+00
## 31294  5 3030  3.133009e+00
## 31295  5 3031  3.059936e+00
## 31296  5 3032  2.987155e+00
## 31297  5 3033  2.935229e+00
## 31298  5 3034  2.877886e+00
## 31299  5 3035  2.819819e+00
## 31300  5 3036  2.777393e+00
## 31301  5 3037  2.736196e+00
## 31302  5 3038  2.691087e+00
## 31303  5 3039  2.654034e+00
## 31304  5 3040  2.614324e+00
## 31305  5 3041  2.584951e+00
## 31306  5 3042  2.552539e+00
## 31307  5 3043  2.517745e+00
## 31308  5 3044  2.488080e+00
## 31309  5 3045  2.458039e+00
## 31310  5 3046  2.431855e+00
## 31311  5 3047  2.405336e+00
## 31312  5 3048  2.379534e+00
## 31313  5 3049  3.294375e+00
## 31314  5 3050  3.461720e+00
## 31315  5 3051  3.417808e+00
## 31316  5 3052  3.328174e+00
## 31317  5 3053  3.232409e+00
## 31318  5 3054  3.144690e+00
## 31319  5 3055  3.064385e+00
## 31320  5 3056  2.995809e+00
## 31321  5 3057  2.928182e+00
## 31322  5 3058  2.878217e+00
## 31323  5 3059  2.826640e+00
## 31324  5 3060  2.783113e+00
## 31325  5 3061  2.733348e+00
## 31326  5 3062  2.698459e+00
## 31327  5 3063  2.657339e+00
## 31328  5 3064  2.623875e+00
## 31329  5 3065  2.592475e+00
## 31330  5 3066  2.559533e+00
## 31331  5 3067  2.526135e+00
## 31332  5 3068  2.495051e+00
## 31333  5 3069  2.466475e+00
## 31334  5 3070  2.441287e+00
## 31335  5 3071  2.412569e+00
## 31336  5 3072  2.385219e+00
## 31337  5 3073  3.299524e+00
## 31338  5 3074  3.464776e+00
## 31339  5 3075  3.426941e+00
## 31340  5 3076  3.330385e+00
## 31341  5 3077  3.239770e+00
## 31342  5 3078  3.153499e+00
## 31343  5 3079  3.075670e+00
## 31344  5 3080  3.003664e+00
## 31345  5 3081  2.943292e+00
## 31346  5 3082  2.884716e+00
## 31347  5 3083  2.835600e+00
## 31348  5 3084  2.786512e+00
## 31349  5 3085  2.741778e+00
## 31350  5 3086  2.705335e+00
## 31351  5 3087  2.663449e+00
## 31352  5 3088  2.628874e+00
## 31353  5 3089  2.597846e+00
## 31354  5 3090  2.564432e+00
## 31355  5 3091  2.536281e+00
## 31356  5 3092  2.501628e+00
## 31357  5 3093  2.470912e+00
## 31358  5 3094  2.448344e+00
## 31359  5 3095  2.414263e+00
## 31360  5 3096  2.396100e+00
## 31361  5 3097  3.305818e+00
## 31362  5 3098  3.469902e+00
## 31363  5 3099  3.431084e+00
## 31364  5 3100  3.335620e+00
## 31365  5 3101  3.247556e+00
## 31366  5 3102  3.151958e+00
## 31367  5 3103  3.075753e+00
## 31368  5 3104  3.007635e+00
## 31369  5 3105  2.947864e+00
## 31370  5 3106  2.887177e+00
## 31371  5 3107  2.843968e+00
## 31372  5 3108  2.788512e+00
## 31373  5 3109  2.743228e+00
## 31374  5 3110  2.710942e+00
## 31375  5 3111  2.664988e+00
## 31376  5 3112  2.637506e+00
## 31377  5 3113  2.600175e+00
## 31378  5 3114  2.568066e+00
## 31379  5 3115  2.539060e+00
## 31380  5 3116  2.507094e+00
## 31381  5 3117  2.478618e+00
## 31382  5 3118  2.453111e+00
## 31383  5 3119  2.420710e+00
## 31384  5 3120  2.395050e+00
## 31385  5 3120  2.407950e+00
## 31386  5 3121  3.318512e+00
## 31387  5 3122  3.489087e+00
## 31388  5 3123  3.440314e+00
## 31389  5 3124  3.349922e+00
## 31390  5 3125  3.260979e+00
## 31391  5 3126  3.164461e+00
## 31392  5 3127  3.089126e+00
## 31393  5 3128  3.023444e+00
## 31394  5 3129  2.962541e+00
## 31395  5 3130  2.900285e+00
## 31396  5 3131  2.848371e+00
## 31397  5 3132  2.809370e+00
## 31398  5 3133  2.760648e+00
## 31399  5 3134  2.718978e+00
## 31400  5 3135  2.684814e+00
## 31401  5 3136  2.649164e+00
## 31402  5 3137  2.612140e+00
## 31403  5 3138  2.580484e+00
## 31404  5 3139  2.552060e+00
## 31405  5 3140  2.518812e+00
## 31406  5 3141  2.489921e+00
## 31407  5 3142  2.465348e+00
## 31408  5 3143  2.430541e+00
## 31409  5 3144  2.407848e+00
## 31410  5 3144  2.408680e+00
## 31411  5 3145  2.378060e+00
## 31412  5 3146  2.355604e+00
## 31413  5 3147  2.334846e+00
## 31414  5 3148  2.304957e+00
## 31415  5 3149  2.284285e+00
## 31416  5 3150  2.259804e+00
## 31417  5 3151  2.231918e+00
## 31418  5 3152  2.211308e+00
## 31419  5 3153  2.187055e+00
## 31420  5 3154  2.165781e+00
## 31421  5 3155  2.143839e+00
## 31422  5 3156  2.123522e+00
## 31423  5 3157  2.099400e+00
## 31424  5 3158  2.076724e+00
## 31425  5 3159  2.052506e+00
## 31426  5 3160  2.034305e+00
## 31427  5 3161  2.012232e+00
## 31428  5 3162  1.992694e+00
## 31429  5 3163  1.970214e+00
## 31430  5 3164  1.953455e+00
## 31431  5 3165  1.929681e+00
## 31432  5 3165  1.930648e+00
## 31433  5 3166  2.850536e+00
## 31434  5 3167  3.025348e+00
## 31435  5 3168  2.985431e+00
## 31436  5 3168  2.982998e+00
## 31437  5 3169  3.845942e+00
## 31438  5 3170  3.939861e+00
## 31439  5 3171  3.840917e+00
## 31440  5 3172  3.704009e+00
## 31441  5 3173  3.563238e+00
## 31442  5 3174  3.450518e+00
## 31443  5 3175  3.334874e+00
## 31444  5 3176  3.248596e+00
## 31445  5 3177  3.162170e+00
## 31446  5 3178  3.093773e+00
## 31447  5 3179  3.027330e+00
## 31448  5 3180  2.966315e+00
## 31449  5 3181  2.912111e+00
## 31450  5 3182  2.858682e+00
## 31451  5 3183  2.818605e+00
## 31452  5 3184  2.776862e+00
## 31453  5 3185  2.740009e+00
## 31454  5 3186  2.702247e+00
## 31455  5 3187  2.666349e+00
## 31456  5 3188  2.631298e+00
## 31457  5 3189  2.604492e+00
## 31458  5 3190  2.570763e+00
## 31459  5 3191  2.547561e+00
## 31460  5 3192  2.512235e+00
## 31461  5 3193  3.418953e+00
## 31462  5 3194  3.587576e+00
## 31463  5 3195  3.542584e+00
## 31464  5 3196  3.447586e+00
## 31465  5 3197  3.357295e+00
## 31466  5 3198  3.265901e+00
## 31467  5 3199  3.184116e+00
## 31468  5 3200  3.111831e+00
## 31469  5 3201  3.046860e+00
## 31470  5 3202  2.993733e+00
## 31471  5 3203  2.938072e+00
## 31472  5 3204  2.894379e+00
## 31473  5 3205  2.852465e+00
## 31474  5 3206  2.806139e+00
## 31475  5 3207  2.765614e+00
## 31476  5 3208  2.733447e+00
## 31477  5 3209  2.698824e+00
## 31478  5 3210  2.658121e+00
## 31479  5 3211  2.629108e+00
## 31480  5 3212  2.602885e+00
## 31481  5 3213  2.571423e+00
## 31482  5 3214  2.539259e+00
## 31483  5 3215  2.517063e+00
## 31484  5 3216  2.485747e+00
## 31485  5 3217  3.395038e+00
## 31486  5 3218  3.562223e+00
## 31487  5 3219  3.520693e+00
## 31488  5 3220  3.430518e+00
## 31489  5 3221  3.332756e+00
## 31490  5 3222  3.240582e+00
## 31491  5 3223  3.168271e+00
## 31492  5 3224  3.087663e+00
## 31493  5 3225  3.030402e+00
## 31494  5 3226  2.971046e+00
## 31495  5 3227  2.916355e+00
## 31496  5 3228  2.872730e+00
## 31497  5 3229  2.828314e+00
## 31498  5 3230  2.784011e+00
## 31499  5 3231  2.749626e+00
## 31500  5 3232  2.713622e+00
## 31501  5 3233  2.678618e+00
## 31502  5 3234  2.642408e+00
## 31503  5 3235  2.613199e+00
## 31504  5 3236  2.583195e+00
## 31505  5 3237  2.553280e+00
## 31506  5 3238  2.521257e+00
## 31507  5 3239  2.495927e+00
## 31508  5 3240  2.469391e+00
## 31509  5 3241  3.381420e+00
## 31510  5 3242  3.536534e+00
## 31511  5 3243  3.502705e+00
## 31512  5 3244  3.412550e+00
## 31513  5 3245  3.312008e+00
## 31514  5 3246  3.229260e+00
## 31515  5 3247  3.143012e+00
## 31516  5 3248  3.084461e+00
## 31517  5 3249  3.011613e+00
## 31518  5 3250  2.957528e+00
## 31519  5 3251  2.907136e+00
## 31520  5 3252  2.859142e+00
## 31521  5 3253  2.810821e+00
## 31522  5 3254  2.774076e+00
## 31523  5 3255  2.734186e+00
## 31524  5 3256  2.696724e+00
## 31525  5 3257  2.664092e+00
## 31526  5 3258  2.634535e+00
## 31527  5 3259  2.599848e+00
## 31528  5 3260  2.567973e+00
## 31529  5 3261  2.539562e+00
## 31530  5 3262  2.515115e+00
## 31531  5 3263  2.482688e+00
## 31532  5 3264  2.457703e+00
## 31533  5 3265  3.366037e+00
## 31534  5 3266  3.530200e+00
## 31535  5 3267  3.492306e+00
## 31536  5 3268  3.393545e+00
## 31537  5 3269  3.304719e+00
## 31538  5 3270  3.213980e+00
## 31539  5 3271  3.134865e+00
## 31540  5 3272  3.073085e+00
## 31541  5 3273  3.006235e+00
## 31542  5 3274  2.948260e+00
## 31543  5 3275  2.896598e+00
## 31544  5 3276  2.845482e+00
## 31545  5 3277  2.801422e+00
## 31546  5 3278  2.758618e+00
## 31547  5 3279  2.725008e+00
## 31548  5 3280  2.684915e+00
## 31549  5 3281  2.650197e+00
## 31550  5 3282  2.623971e+00
## 31551  5 3283  2.592256e+00
## 31552  5 3284  2.554339e+00
## 31553  5 3285  2.526839e+00
## 31554  5 3286  2.499330e+00
## 31555  5 3287  2.469959e+00
## 31556  5 3288  2.445731e+00
## 31557  5 3289  3.358586e+00
## 31558  5 3290  3.520671e+00
## 31559  5 3291  3.479239e+00
## 31560  5 3292  3.389892e+00
## 31561  5 3293  3.288904e+00
## 31562  5 3294  3.205806e+00
## 31563  5 3295  3.131866e+00
## 31564  5 3296  3.051835e+00
## 31565  5 3297  2.993966e+00
## 31566  5 3298  2.933797e+00
## 31567  5 3299  2.884962e+00
## 31568  5 3300  2.841606e+00
## 31569  5 3301  2.791126e+00
## 31570  5 3302  2.750739e+00
## 31571  5 3303  2.711628e+00
## 31572  5 3304  2.680053e+00
## 31573  5 3305  2.645571e+00
## 31574  5 3306  2.612214e+00
## 31575  5 3307  2.585800e+00
## 31576  5 3308  2.553823e+00
## 31577  5 3309  2.521928e+00
## 31578  5 3310  2.495119e+00
## 31579  5 3311  2.463328e+00
## 31580  5 3312  2.437790e+00
## 31581  5 3313  3.347612e+00
## 31582  5 3314  3.512366e+00
## 31583  5 3315  3.472975e+00
## 31584  5 3316  3.376531e+00
## 31585  5 3317  3.283583e+00
## 31586  5 3318  3.204087e+00
## 31587  5 3319  3.123149e+00
## 31588  5 3320  3.049672e+00
## 31589  5 3321  2.987692e+00
## 31590  5 3322  2.928827e+00
## 31591  5 3323  2.878324e+00
## 31592  5 3324  2.832391e+00
## 31593  5 3325  2.787004e+00
## 31594  5 3326  2.748933e+00
## 31595  5 3327  2.710485e+00
## 31596  5 3328  2.672685e+00
## 31597  5 3329  2.634595e+00
## 31598  5 3330  2.604407e+00
## 31599  5 3331  2.572578e+00
## 31600  5 3332  2.541258e+00
## 31601  5 3333  2.513184e+00
## 31602  5 3334  2.481488e+00
## 31603  5 3335  2.457335e+00
## 31604  5 3336  2.429720e+00
## 31605  5 3337  3.339420e+00
## 31606  5 3338  3.502058e+00
## 31607  5 3339  3.458945e+00
## 31608  5 3340  3.379297e+00
## 31609  5 3341  3.279752e+00
## 31610  5 3342  3.191319e+00
## 31611  5 3343  3.112891e+00
## 31612  5 3344  3.042662e+00
## 31613  5 3345  2.975575e+00
## 31614  5 3346  2.919035e+00
## 31615  5 3347  2.874589e+00
## 31616  5 3348  2.825304e+00
## 31617  5 3349  2.786484e+00
## 31618  5 3350  2.736000e+00
## 31619  5 3351  2.701921e+00
## 31620  5 3352  2.669576e+00
## 31621  5 3353  2.632158e+00
## 31622  5 3354  2.600841e+00
## 31623  5 3355  2.570161e+00
## 31624  5 3356  2.538217e+00
## 31625  5 3357  2.504553e+00
## 31626  5 3358  2.476572e+00
## 31627  5 3359  2.456224e+00
## 31628  5 3360  2.421869e+00
## 31629  5 3361  3.344696e+00
## 31630  5 3362  3.505862e+00
## 31631  5 3363  3.457528e+00
## 31632  5 3364  3.368701e+00
## 31633  5 3365  3.271983e+00
## 31634  5 3366  3.189181e+00
## 31635  5 3367  3.112665e+00
## 31636  5 3368  3.037488e+00
## 31637  5 3369  2.980950e+00
## 31638  5 3370  2.924333e+00
## 31639  5 3371  2.869568e+00
## 31640  5 3372  2.819804e+00
## 31641  5 3373  2.784076e+00
## 31642  5 3374  2.738996e+00
## 31643  5 3375  2.697163e+00
## 31644  5 3376  2.662495e+00
## 31645  5 3377  2.628189e+00
## 31646  5 3378  2.595898e+00
## 31647  5 3379  2.562335e+00
## 31648  5 3380  2.528754e+00
## 31649  5 3381  2.502967e+00
## 31650  5 3382  2.473483e+00
## 31651  5 3383  2.448325e+00
## 31652  5 3384  2.419500e+00
## 31653  5 3385  3.329232e+00
## 31654  5 3386  3.499594e+00
## 31655  5 3387  3.459322e+00
## 31656  5 3388  3.368753e+00
## 31657  5 3389  3.274676e+00
## 31658  5 3390  3.187311e+00
## 31659  5 3391  3.109131e+00
## 31660  5 3392  3.032544e+00
## 31661  5 3393  2.971304e+00
## 31662  5 3394  2.920646e+00
## 31663  5 3395  2.861607e+00
## 31664  5 3396  2.818414e+00
## 31665  5 3397  2.772501e+00
## 31666  5 3398  2.729109e+00
## 31667  5 3399  2.694922e+00
## 31668  5 3400  2.663705e+00
## 31669  5 3401  2.622770e+00
## 31670  5 3402  2.593521e+00
## 31671  5 3403  2.559774e+00
## 31672  5 3404  2.528371e+00
## 31673  5 3405  2.503269e+00
## 31674  5 3406  2.474839e+00
## 31675  5 3407  2.448005e+00
## 31676  5 3408  2.417393e+00
## 31677  5 3409  3.329386e+00
## 31678  5 3410  3.495891e+00
## 31679  5 3411  3.452546e+00
## 31680  5 3412  3.358203e+00
## 31681  5 3413  3.271957e+00
## 31682  5 3414  3.184718e+00
## 31683  5 3415  3.102658e+00
## 31684  5 3416  3.034453e+00
## 31685  5 3417  2.973452e+00
## 31686  5 3418  2.914350e+00
## 31687  5 3419  2.862310e+00
## 31688  5 3420  2.816724e+00
## 31689  5 3421  2.769201e+00
## 31690  5 3422  2.732033e+00
## 31691  5 3423  2.690367e+00
## 31692  5 3424  2.655353e+00
## 31693  5 3425  2.618980e+00
## 31694  5 3426  2.589484e+00
## 31695  5 3427  2.554015e+00
## 31696  5 3428  2.522638e+00
## 31697  5 3429  2.493561e+00
## 31698  5 3430  2.467250e+00
## 31699  5 3431  2.443709e+00
## 31700  5 3432  2.416148e+00
## 31701  5 3432  2.407904e+00
## 31702  5 3433  3.317938e+00
## 31703  5 3434  3.482332e+00
## 31704  5 3435  3.448716e+00
## 31705  5 3436  3.353329e+00
## 31706  5 3437  3.255740e+00
## 31707  5 3438  3.169349e+00
## 31708  5 3439  3.091074e+00
## 31709  5 3440  3.020464e+00
## 31710  5 3441  2.958771e+00
## 31711  5 3442  2.904427e+00
## 31712  5 3443  2.851729e+00
## 31713  5 3444  2.805890e+00
## 31714  5 3445  2.757789e+00
## 31715  5 3446  2.721120e+00
## 31716  5 3447  2.687466e+00
## 31717  5 3448  2.648690e+00
## 31718  5 3449  2.615297e+00
## 31719  5 3450  2.574206e+00
## 31720  5 3451  2.548706e+00
## 31721  5 3452  2.519744e+00
## 31722  5 3453  2.491885e+00
## 31723  5 3454  2.462964e+00
## 31724  5 3455  2.438312e+00
## 31725  5 3456  2.410490e+00
## 31726  5 3456  2.408416e+00
## 31727  5 3457  2.379745e+00
## 31728  5 3458  2.355464e+00
## 31729  5 3459  2.332066e+00
## 31730  5 3460  2.299781e+00
## 31731  5 3461  2.279970e+00
## 31732  5 3462  2.256533e+00
## 31733  5 3463  2.234988e+00
## 31734  5 3464  2.212778e+00
## 31735  5 3465  2.190002e+00
## 31736  5 3466  2.162459e+00
## 31737  5 3467  2.142364e+00
## 31738  5 3468  2.122634e+00
## 31739  5 3469  2.097865e+00
## 31740  5 3470  2.074128e+00
## 31741  5 3471  2.050616e+00
## 31742  5 3472  2.036005e+00
## 31743  5 3473  2.012355e+00
## 31744  5 3474  1.994371e+00
## 31745  5 3475  1.973447e+00
## 31746  5 3476  1.951509e+00
## 31747  5 3477  1.933174e+00
## 31748  5 3478  1.910507e+00
## 31749  5 3479  1.890794e+00
## 31750  5 3480  1.873599e+00
## 31751  5 3480  1.871785e+00
## 31752  5 3481  2.792024e+00
## 31753  5 3482  2.964848e+00
## 31754  5 3483  2.931719e+00
## 31755  5 3484  2.846230e+00
## 31756  5 3485  2.754396e+00
## 31757  5 3486  2.671230e+00
## 31758  5 3487  2.601399e+00
## 31759  5 3488  2.535841e+00
## 31760  5 3489  2.475790e+00
## 31761  5 3490  2.428131e+00
## 31762  5 3491  2.380913e+00
## 31763  5 3492  2.333970e+00
## 31764  5 3493  2.298560e+00
## 31765  5 3494  2.265911e+00
## 31766  5 3495  2.233100e+00
## 31767  5 3496  2.196995e+00
## 31768  5 3497  2.172378e+00
## 31769  5 3498  2.140926e+00
## 31770  5 3499  2.113649e+00
## 31771  5 3500  2.088379e+00
## 31772  5 3501  2.062986e+00
## 31773  5 3502  2.041119e+00
## 31774  5 3503  2.022294e+00
## 31775  5 3504  1.995097e+00
## 31776  5 3504  1.999266e+00
## 31777  5 3505  2.909013e+00
## 31778  5 3506  3.082192e+00
## 31779  5 3507  3.043482e+00
## 31780  5 3508  2.958433e+00
## 31781  5 3509  2.865832e+00
## 31782  5 3510  2.783185e+00
## 31783  5 3511  2.708777e+00
## 31784  5 3512  2.647710e+00
## 31785  5 3513  2.580989e+00
## 31786  5 3514  2.531428e+00
## 31787  5 3515  2.483281e+00
## 31788  5 3516  2.441052e+00
## 31789  5 3517  2.399542e+00
## 31790  5 3518  2.364762e+00
## 31791  5 3519  2.328794e+00
## 31792  5 3520  2.300640e+00
## 31793  5 3521  2.268864e+00
## 31794  5 3522  2.239509e+00
## 31795  5 3523  2.207904e+00
## 31796  5 3524  2.183820e+00
## 31797  5 3525  2.158408e+00
## 31798  5 3526  2.133724e+00
## 31799  5 3527  2.110951e+00
## 31800  5 3528  2.088866e+00
## 31801  5 3529  3.004340e+00
## 31802  5 3530  3.166445e+00
## 31803  5 3531  3.138206e+00
## 31804  5 3532  3.047146e+00
## 31805  5 3533  2.951821e+00
## 31806  5 3534  2.871853e+00
## 31807  5 3535  2.797052e+00
## 31808  5 3536  2.729193e+00
## 31809  5 3537  2.668983e+00
## 31810  5 3538  2.614652e+00
## 31811  5 3539  2.565924e+00
## 31812  5 3540  2.520375e+00
## 31813  5 3541  2.478214e+00
## 31814  5 3542  2.443149e+00
## 31815  5 3543  2.407023e+00
## 31816  5 3544  2.373911e+00
## 31817  5 3545  2.344585e+00
## 31818  5 3546  2.314399e+00
## 31819  5 3547  2.286572e+00
## 31820  5 3548  2.257045e+00
## 31821  5 3549  2.231251e+00
## 31822  5 3550  2.204315e+00
## 31823  5 3551  2.182326e+00
## 31824  5 3552  2.157140e+00
## 31825  5 3553  3.072700e+00
## 31826  5 3554  3.233551e+00
## 31827  5 3555  3.204200e+00
## 31828  5 3556  3.110599e+00
## 31829  5 3557  3.023331e+00
## 31830  5 3558  2.936685e+00
## 31831  5 3559  2.858548e+00
## 31832  5 3560  2.795214e+00
## 31833  5 3561  2.731851e+00
## 31834  5 3562  2.676875e+00
## 31835  5 3563  2.630743e+00
## 31836  5 3564  2.581064e+00
## 31837  5 3565  2.538585e+00
## 31838  5 3566  2.499892e+00
## 31839  5 3567  2.471936e+00
## 31840  5 3568  2.436155e+00
## 31841  5 3569  2.401432e+00
## 31842  5 3570  2.374766e+00
## 31843  5 3571  2.341223e+00
## 31844  5 3572  2.316466e+00
## 31845  5 3573  2.288943e+00
## 31846  5 3574  2.261947e+00
## 31847  5 3575  2.237784e+00
## 31848  5 3576  2.214441e+00
## 31849  5 3577  3.129116e+00
## 31850  5 3578  3.298613e+00
## 31851  5 3579  3.254864e+00
## 31852  5 3580  3.164770e+00
## 31853  5 3581  3.068171e+00
## 31854  5 3582  2.986074e+00
## 31855  5 3583  2.909818e+00
## 31856  5 3584  2.835801e+00
## 31857  5 3585  2.785904e+00
## 31858  5 3586  2.726899e+00
## 31859  5 3587  2.675678e+00
## 31860  5 3588  2.629600e+00
## 31861  5 3589  2.588691e+00
## 31862  5 3590  2.555057e+00
## 31863  5 3591  2.517319e+00
## 31864  5 3592  2.480407e+00
## 31865  5 3593  2.449474e+00
## 31866  5 3594  2.417309e+00
## 31867  5 3595  2.383253e+00
## 31868  5 3596  2.362068e+00
## 31869  5 3597  2.335854e+00
## 31870  5 3598  2.305950e+00
## 31871  5 3599  2.282884e+00
## 31872  5 3600  2.252918e+00
## 31873  5 3601  3.170770e+00
## 31874  5 3602  3.333827e+00
## 31875  5 3603  3.294409e+00
## 31876  5 3604  3.210470e+00
## 31877  5 3605  3.113119e+00
## 31878  5 3606  3.027147e+00
## 31879  5 3607  2.949565e+00
## 31880  5 3608  2.881779e+00
## 31881  5 3609  2.819849e+00
## 31882  5 3610  2.763530e+00
## 31883  5 3611  2.715170e+00
## 31884  5 3612  2.667240e+00
## 31885  5 3613  2.623331e+00
## 31886  5 3614  2.585444e+00
## 31887  5 3615  2.544589e+00
## 31888  5 3616  2.517003e+00
## 31889  5 3617  2.489060e+00
## 31890  5 3618  2.451080e+00
## 31891  5 3619  2.419057e+00
## 31892  5 3620  2.393000e+00
## 31893  5 3621  2.366925e+00
## 31894  5 3622  2.343266e+00
## 31895  5 3623  2.317357e+00
## 31896  5 3624  2.288970e+00
## 31897  5 3625  3.196562e+00
## 31898  5 3626  3.365827e+00
## 31899  5 3627  3.322955e+00
## 31900  5 3628  3.242250e+00
## 31901  5 3629  3.148363e+00
## 31902  5 3630  3.057008e+00
## 31903  5 3631  2.985549e+00
## 31904  5 3632  2.909317e+00
## 31905  5 3633  2.850374e+00
## 31906  5 3634  2.795643e+00
## 31907  5 3635  2.743619e+00
## 31908  5 3636  2.699085e+00
## 31909  5 3637  2.656959e+00
## 31910  5 3638  2.621898e+00
## 31911  5 3639  2.580992e+00
## 31912  5 3640  2.541545e+00
## 31913  5 3641  2.509102e+00
## 31914  5 3642  2.484110e+00
## 31915  5 3643  2.449516e+00
## 31916  5 3644  2.424854e+00
## 31917  5 3645  2.393592e+00
## 31918  5 3646  2.370323e+00
## 31919  5 3647  2.340513e+00
## 31920  5 3648  2.315265e+00
## 31921  5 3649  3.227204e+00
## 31922  5 3650  3.390100e+00
## 31923  5 3651  3.354721e+00
## 31924  5 3652  3.260611e+00
## 31925  5 3653  3.170923e+00
## 31926  5 3654  3.088755e+00
## 31927  5 3655  3.010581e+00
## 31928  5 3656  2.934158e+00
## 31929  5 3657  2.871229e+00
## 31930  5 3658  2.816541e+00
## 31931  5 3659  2.770384e+00
## 31932  5 3660  2.722883e+00
## 31933  5 3661  2.681088e+00
## 31934  5 3662  2.640169e+00
## 31935  5 3663  2.602368e+00
## 31936  5 3664  2.565456e+00
## 31937  5 3665  2.537521e+00
## 31938  5 3666  2.504514e+00
## 31939  5 3667  2.478415e+00
## 31940  5 3668  2.443253e+00
## 31941  5 3669  2.412210e+00
## 31942  5 3670  2.387525e+00
## 31943  5 3671  2.359346e+00
## 31944  5 3672  2.336553e+00
## 31945  5 3673  3.246103e+00
## 31946  5 3674  3.410173e+00
## 31947  5 3675  3.375429e+00
## 31948  5 3676  3.279319e+00
## 31949  5 3677  3.195076e+00
## 31950  5 3678  3.102273e+00
## 31951  5 3679  3.019147e+00
## 31952  5 3680  2.959646e+00
## 31953  5 3681  2.891893e+00
## 31954  5 3682  2.833758e+00
## 31955  5 3683  2.783367e+00
## 31956  5 3684  2.738718e+00
## 31957  5 3685  2.701783e+00
## 31958  5 3686  2.657022e+00
## 31959  5 3687  2.619408e+00
## 31960  5 3688  2.583609e+00
## 31961  5 3689  2.552765e+00
## 31962  5 3690  2.519280e+00
## 31963  5 3691  2.486193e+00
## 31964  5 3692  2.456038e+00
## 31965  5 3693  2.429306e+00
## 31966  5 3694  2.406320e+00
## 31967  5 3695  2.378345e+00
## 31968  5 3696  2.351578e+00
## 31969  5 3697  3.269371e+00
## 31970  5 3698  3.432700e+00
## 31971  5 3699  3.387072e+00
## 31972  5 3700  3.298232e+00
## 31973  5 3701  3.198987e+00
## 31974  5 3702  3.117405e+00
## 31975  5 3703  3.038676e+00
## 31976  5 3704  2.968369e+00
## 31977  5 3705  2.911376e+00
## 31978  5 3706  2.850574e+00
## 31979  5 3707  2.799141e+00
## 31980  5 3708  2.756878e+00
## 31981  5 3709  2.714067e+00
## 31982  5 3710  2.673264e+00
## 31983  5 3711  2.637426e+00
## 31984  5 3712  2.598999e+00
## 31985  5 3713  2.560728e+00
## 31986  5 3714  2.536428e+00
## 31987  5 3715  2.502135e+00
## 31988  5 3716  2.473597e+00
## 31989  5 3717  2.448087e+00
## 31990  5 3718  2.417770e+00
## 31991  5 3719  2.392974e+00
## 31992  5 3720  2.364536e+00
## 31993  5 3721  3.278796e+00
## 31994  5 3722  3.438848e+00
## 31995  5 3723  3.400719e+00
## 31996  5 3724  3.315097e+00
## 31997  5 3725  3.214645e+00
## 31998  5 3726  3.128064e+00
## 31999  5 3727  3.049629e+00
## 32000  5 3728  2.976053e+00
## 32001  5 3729  2.914632e+00
## 32002  5 3730  2.860885e+00
## 32003  5 3731  2.810436e+00
## 32004  5 3732  2.764524e+00
## 32005  5 3733  2.722776e+00
## 32006  5 3734  2.682118e+00
## 32007  5 3735  2.645527e+00
## 32008  5 3736  2.612419e+00
## 32009  5 3737  2.571577e+00
## 32010  5 3738  2.544014e+00
## 32011  5 3739  2.514157e+00
## 32012  5 3740  2.480688e+00
## 32013  5 3741  2.456341e+00
## 32014  5 3742  2.426522e+00
## 32015  5 3743  2.398517e+00
## 32016  5 3744  2.373750e+00
## 32017  5 3745  3.288441e+00
## 32018  5 3746  3.452216e+00
## 32019  5 3747  3.413485e+00
## 32020  5 3748  3.321369e+00
## 32021  5 3749  3.226484e+00
## 32022  5 3750  3.139078e+00
## 32023  5 3751  3.060013e+00
## 32024  5 3752  2.996610e+00
## 32025  5 3753  2.931712e+00
## 32026  5 3754  2.871293e+00
## 32027  5 3755  2.820553e+00
## 32028  5 3756  2.771892e+00
## 32029  5 3757  2.731057e+00
## 32030  5 3758  2.694420e+00
## 32031  5 3759  2.660350e+00
## 32032  5 3760  2.620265e+00
## 32033  5 3761  2.586857e+00
## 32034  5 3762  2.552536e+00
## 32035  5 3763  2.520588e+00
```

```
## 32036  5 3764  2.493206e+00
## 32037  5 3765  2.461609e+00
## 32038  5 3766  2.436522e+00
## 32039  5 3767  2.406905e+00
## 32040  5 3768  2.382263e+00
## 32041  5 3768  2.408069e+00
## 32042  5 3769  3.315939e+00
## 32043  5 3770  3.486669e+00
## 32044  5 3771  3.446570e+00
## 32045  5 3772  3.348690e+00
## 32046  5 3773  3.254134e+00
## 32047  5 3774  3.168073e+00
## 32048  5 3775  3.093670e+00
## 32049  5 3776  3.019497e+00
## 32050  5 3777  2.956335e+00
## 32051  5 3778  2.897514e+00
## 32052  5 3779  2.851994e+00
## 32053  5 3780  2.805473e+00
## 32054  5 3781  2.762212e+00
## 32055  5 3782  2.718538e+00
## 32056  5 3783  2.678903e+00
## 32057  5 3784  2.645186e+00
## 32058  5 3785  2.616963e+00
## 32059  5 3786  2.580921e+00
## 32060  5 3787  2.548940e+00
## 32061  5 3788  2.518225e+00
## 32062  5 3789  2.490094e+00
## 32063  5 3790  2.463702e+00
## 32064  5 3791  2.437326e+00
## 32065  5 3792  2.406625e+00
## 32066  5 3792  2.406532e+00
## 32067  5 3793  2.380118e+00
## 32068  5 3794  2.355762e+00
## 32069  5 3795  2.330276e+00
## 32070  5 3796  2.308867e+00
## 32071  5 3797  2.277755e+00
## 32072  5 3798  2.257607e+00
## 32073  5 3799  2.231385e+00
## 32074  5 3800  2.203558e+00
## 32075  5 3801  2.188399e+00
## 32076  5 3802  2.164216e+00
## 32077  5 3803  2.139516e+00
## 32078  5 3804  2.118389e+00
## 32079  5 3805  2.097028e+00
## 32080  5 3806  2.078993e+00
## 32081  5 3807  2.057764e+00
## 32082  5 3808  2.034582e+00
## 32083  5 3809  2.015424e+00
## 32084  5 3810  1.995013e+00
## 32085  5 3811  1.970874e+00
## 32086  5 3812  1.946217e+00
## 32087  5 3813  1.930189e+00
## 32088  5 3814  1.913316e+00
## 32089  5 3815  1.892097e+00
## 32090  5 3816  1.872330e+00
## 32091  5 3816  1.871325e+00
## 32092  5 3817  3.251484e+00
## 32093  5 3818  3.524472e+00
## 32094  5 3819  3.484272e+00
## 32095  5 3820  3.367427e+00
## 32096  5 3821  3.242088e+00
## 32097  5 3822  3.129283e+00
## 32098  5 3823  3.029849e+00
## 32099  5 3824  2.938448e+00
## 32100  5 3825  2.865069e+00
## 32101  5 3826  2.797629e+00
## 32102  5 3827  2.741277e+00
## 32103  5 3828  2.680200e+00
## 32104  5 3829  2.630788e+00
## 32105  5 3830  2.584130e+00
## 32106  5 3831  2.542723e+00
## 32107  5 3832  2.504504e+00
## 32108  5 3833  2.475445e+00
## 32109  5 3834  2.444951e+00
## 32110  5 3835  2.406740e+00
## 32111  5 3836  2.371597e+00
## 32112  5 3837  2.342844e+00
## 32113  5 3838  2.312418e+00
## 32114  5 3839  2.291476e+00
## 32115  5 3840  2.264486e+00
## 32116  5 3840  2.262538e+00
## 32117  5 3841  3.177103e+00
## 32118  5 3842  3.338359e+00
## 32119  5 3843  3.310609e+00
## 32120  5 3844  3.214296e+00
## 32121  5 3845  3.116621e+00
## 32122  5 3846  3.032918e+00
## 32123  5 3847  2.954927e+00
## 32124  5 3848  2.887766e+00
## 32125  5 3849  2.826992e+00
## 32126  5 3850  2.774058e+00
## 32127  5 3851  2.723198e+00
## 32128  5 3852  2.675615e+00
## 32129  5 3853  2.626965e+00
## 32130  5 3854  2.586536e+00
## 32131  5 3855  2.555097e+00
## 32132  5 3856  2.519689e+00
## 32133  5 3857  2.489622e+00
## 32134  5 3858  2.456560e+00
## 32135  5 3859  2.428376e+00
## 32136  5 3860  2.404283e+00
## 32137  5 3861  2.370373e+00
## 32138  5 3862  2.341324e+00
## 32139  5 3863  2.316243e+00
## 32140  5 3864  2.291775e+00
## 32141  5 3865  3.201786e+00
## 32142  5 3866  3.365517e+00
## 32143  5 3867  3.333694e+00
## 32144  5 3868  3.245340e+00
## 32145  5 3869  3.149152e+00
## 32146  5 3870  3.067549e+00
## 32147  5 3871  2.982269e+00
## 32148  5 3872  2.914128e+00
## 32149  5 3873  2.854427e+00
## 32150  5 3874  2.801358e+00
## 32151  5 3875  2.748880e+00
## 32152  5 3876  2.701735e+00
## 32153  5 3877  2.661450e+00
## 32154  5 3878  2.618987e+00
## 32155  5 3879  2.581921e+00
## 32156  5 3880  2.544515e+00
## 32157  5 3881  2.516349e+00
## 32158  5 3882  2.492680e+00
## 32159  5 3883  2.457279e+00
## 32160  5 3884  2.430092e+00
## 32161  5 3885  2.399464e+00
## 32162  5 3886  2.368992e+00
## 32163  5 3887  2.344105e+00
## 32164  5 3888  2.320201e+00
## 32165  5 3889  3.227106e+00
## 32166  5 3890  3.395676e+00
## 32167  5 3891  3.356542e+00
## 32168  5 3892  3.269378e+00
## 32169  5 3893  3.168995e+00
## 32170  5 3894  3.083689e+00
## 32171  5 3895  3.013414e+00
## 32172  5 3896  2.938695e+00
## 32173  5 3897  2.876404e+00
## 32174  5 3898  2.818531e+00
## 32175  5 3899  2.772093e+00
## 32176  5 3900  2.726967e+00
## 32177  5 3901  2.676944e+00
## 32178  5 3902  2.643362e+00
## 32179  5 3903  2.601114e+00
## 32180  5 3904  2.572816e+00
## 32181  5 3905  2.539556e+00
## 32182  5 3906  2.503170e+00
## 32183  5 3907  2.475481e+00
## 32184  5 3908  2.447739e+00
## 32185  5 3909  2.418956e+00
## 32186  5 3910  2.390621e+00
## 32187  5 3911  2.364675e+00
## 32188  5 3912  2.339226e+00
## 32189  5 3913  3.249960e+00
## 32190  5 3914  3.413392e+00
## 32191  5 3915  3.377060e+00
## 32192  5 3916  3.290165e+00
## 32193  5 3917  3.186074e+00
## 32194  5 3918  3.105789e+00
## 32195  5 3919  3.026181e+00
## 32196  5 3920  2.953532e+00
## 32197  5 3921  2.890657e+00
## 32198  5 3922  2.841834e+00
## 32199  5 3923  2.789932e+00
## 32200  5 3924  2.739722e+00
## 32201  5 3925  2.703210e+00
## 32202  5 3926  2.661282e+00
## 32203  5 3927  2.624608e+00
## 32204  5 3928  2.589626e+00
## 32205  5 3929  2.555530e+00
## 32206  5 3930  2.525466e+00
## 32207  5 3931  2.492779e+00
## 32208  5 3932  2.463381e+00
## 32209  5 3933  2.432258e+00
## 32210  5 3934  2.404513e+00
## 32211  5 3935  2.381932e+00
## 32212  5 3936  2.356906e+00
## 32213  5 3937  3.265230e+00
## 32214  5 3938  3.433390e+00
## 32215  5 3939  3.389371e+00
## 32216  5 3940  3.301854e+00
## 32217  5 3941  3.198226e+00
## 32218  5 3942  3.120530e+00
## 32219  5 3943  3.044755e+00
## 32220  5 3944  2.972293e+00
## 32221  5 3945  2.908902e+00
## 32222  5 3946  2.854514e+00
## 32223  5 3947  2.805497e+00
## 32224  5 3948  2.749264e+00
## 32225  5 3949  2.712283e+00
## 32226  5 3950  2.670665e+00
## 32227  5 3951  2.636111e+00
## 32228  5 3952  2.597644e+00
## 32229  5 3953  2.566218e+00
## 32230  5 3954  2.535299e+00
## 32231  5 3955  2.504798e+00
## 32232  5 3956  2.473884e+00
## 32233  5 3957  2.444934e+00
## 32234  5 3958  2.416274e+00
## 32235  5 3959  2.393018e+00
## 32236  5 3960  2.364366e+00
## 32237  5 3961  3.280578e+00
## 32238  5 3962  3.448638e+00
## 32239  5 3963  3.405743e+00
## 32240  5 3964  3.310805e+00
## 32241  5 3965  3.214942e+00
## 32242  5 3966  3.126491e+00
## 32243  5 3967  3.056751e+00
## 32244  5 3968  2.987361e+00
## 32245  5 3969  2.914435e+00
## 32246  5 3970  2.864294e+00
## 32247  5 3971  2.815262e+00
## 32248  5 3972  2.767074e+00
## 32249  5 3973  2.725574e+00
## 32250  5 3974  2.679386e+00
## 32251  5 3975  2.649961e+00
## 32252  5 3976  2.611453e+00
## 32253  5 3977  2.572678e+00
## 32254  5 3978  2.545276e+00
## 32255  5 3979  2.510443e+00
## 32256  5 3980  2.485239e+00
## 32257  5 3981  2.450363e+00
## 32258  5 3982  2.430460e+00
## 32259  5 3983  2.398251e+00
## 32260  5 3984  2.378051e+00
## 32261  5 3985  3.287418e+00
## 32262  5 3986  3.455018e+00
## 32263  5 3987  3.416809e+00
## 32264  5 3988  3.322278e+00
## 32265  5 3989  3.228196e+00
## 32266  5 3990  3.140935e+00
## 32267  5 3991  3.055502e+00
## 32268  5 3992  2.991233e+00
## 32269  5 3993  2.935693e+00
## 32270  5 3994  2.875331e+00
## 32271  5 3995  2.817004e+00
## 32272  5 3996  2.769804e+00
## 32273  5 3997  2.728952e+00
## 32274  5 3998  2.694981e+00
## 32275  5 3999  2.655405e+00
## 32276  5 4000  2.620407e+00
## 32277  5 4001  2.587594e+00
## 32278  5 4002  2.550820e+00
## 32279  5 4003  2.518699e+00
## 32280  5 4004  2.491895e+00
## 32281  5 4005  2.460144e+00
## 32282  5 4006  2.434260e+00
## 32283  5 4007  2.407877e+00
## 32284  5 4008  2.384492e+00
## 32285  5 4009  3.286690e+00
## 32286  5 4010  3.457403e+00
## 32287  5 4011  3.417904e+00
## 32288  5 4012  3.330580e+00
## 32289  5 4013  3.235551e+00
## 32290  5 4014  3.148618e+00
## 32291  5 4015  3.063525e+00
## 32292  5 4016  2.997656e+00
## 32293  5 4017  2.939894e+00
## 32294  5 4018  2.881197e+00
## 32295  5 4019  2.830211e+00
## 32296  5 4020  2.783429e+00
## 32297  5 4021  2.737196e+00
## 32298  5 4022  2.700799e+00
## 32299  5 4023  2.661906e+00
## 32300  5 4024  2.620580e+00
## 32301  5 4025  2.595165e+00
## 32302  5 4026  2.558886e+00
## 32303  5 4027  2.530950e+00
## 32304  5 4028  2.493310e+00
## 32305  5 4029  2.468998e+00
## 32306  5 4030  2.444312e+00
## 32307  5 4031  2.415727e+00
## 32308  5 4032  2.389238e+00
## 32309  5 4033  3.293409e+00
## 32310  5 4034  3.470436e+00
## 32311  5 4035  3.423920e+00
## 32312  5 4036  3.331836e+00
## 32313  5 4037  3.237421e+00
## 32314  5 4038  3.153418e+00
## 32315  5 4039  3.072000e+00
## 32316  5 4040  3.010716e+00
## 32317  5 4041  2.941003e+00
## 32318  5 4042  2.885065e+00
## 32319  5 4043  2.832122e+00
## 32320  5 4044  2.787389e+00
## 32321  5 4045  2.743631e+00
## 32322  5 4046  2.694937e+00
## 32323  5 4047  2.662803e+00
## 32324  5 4048  2.629116e+00
## 32325  5 4049  2.594519e+00
## 32326  5 4050  2.558430e+00
## 32327  5 4051  2.534470e+00
## 32328  5 4052  2.506675e+00
## 32329  5 4053  2.475870e+00
## 32330  5 4054  2.447300e+00
## 32331  5 4055  2.425308e+00
## 32332  5 4056  2.389521e+00
## 32333  5 4057  3.308468e+00
## 32334  5 4058  3.467228e+00
## 32335  5 4059  3.426230e+00
## 32336  5 4060  3.336055e+00
## 32337  5 4061  3.241280e+00
## 32338  5 4062  3.154202e+00
## 32339  5 4063  3.079134e+00
## 32340  5 4064  3.009302e+00
## 32341  5 4065  2.939416e+00
## 32342  5 4066  2.887514e+00
## 32343  5 4067  2.834617e+00
## 32344  5 4068  2.791492e+00
## 32345  5 4069  2.747076e+00
## 32346  5 4070  2.706344e+00
## 32347  5 4071  2.667300e+00
## 32348  5 4072  2.635905e+00
## 32349  5 4073  2.598473e+00
## 32350  5 4074  2.564043e+00
## 32351  5 4075  2.540513e+00
## 32352  5 4076  2.505070e+00
## 32353  5 4077  2.477865e+00
## 32354  5 4078  2.448832e+00
## 32355  5 4079  2.424212e+00
## 32356  5 4080  2.398653e+00
## 32357  5 4081  3.305183e+00
## 32358  5 4082  3.477526e+00
## 32359  5 4083  3.433297e+00
## 32360  5 4084  3.341573e+00
## 32361  5 4085  3.240917e+00
## 32362  5 4086  3.160056e+00
## 32363  5 4087  3.083507e+00
## 32364  5 4088  3.018189e+00
## 32365  5 4089  2.950318e+00
## 32366  5 4090  2.886304e+00
## 32367  5 4091  2.842398e+00
## 32368  5 4092  2.789476e+00
## 32369  5 4093  2.746466e+00
## 32370  5 4094  2.711112e+00
## 32371  5 4095  2.673636e+00
## 32372  5 4096  2.635981e+00
## 32373  5 4097  2.599265e+00
## 32374  5 4098  2.570348e+00
## 32375  5 4099  2.539050e+00
## 32376  5 4100  2.507949e+00
## 32377  5 4101  2.481923e+00
## 32378  5 4102  2.450514e+00
## 32379  5 4103  2.426704e+00
## 32380  5 4104  2.397689e+00
## 32381  5 4104  2.406704e+00
## 32382  5 4105  3.323574e+00
## 32383  5 4106  3.485240e+00
## 32384  5 4107  3.443549e+00
## 32385  5 4108  3.361642e+00
## 32386  5 4109  3.258982e+00
## 32387  5 4110  3.170259e+00
## 32388  5 4111  3.090148e+00
## 32389  5 4112  3.019036e+00
## 32390  5 4113  2.954735e+00
## 32391  5 4114  2.903175e+00
## 32392  5 4115  2.852816e+00
## 32393  5 4116  2.807425e+00
## 32394  5 4117  2.760926e+00
## 32395  5 4118  2.722230e+00
## 32396  5 4119  2.682456e+00
## 32397  5 4120  2.643996e+00
## 32398  5 4121  2.608983e+00
## 32399  5 4122  2.586901e+00
## 32400  5 4123  2.544795e+00
## 32401  5 4124  2.522603e+00
## 32402  5 4125  2.484973e+00
## 32403  5 4126  2.463283e+00
## 32404  5 4127  2.437190e+00
## 32405  5 4128  2.410630e+00
## 32406  5 4128  2.407297e+00
## 32407  5 4129  2.385259e+00
## 32408  5 4130  2.360042e+00
## 32409  5 4131  2.328343e+00
## 32410  5 4132  2.304665e+00
## 32411  5 4133  2.284681e+00
## 32412  5 4134  2.256050e+00
## 32413  5 4135  2.232684e+00
## 32414  5 4136  2.211297e+00
## 32415  5 4137  2.188421e+00
## 32416  5 4138  2.164342e+00
## 32417  5 4139  2.146449e+00
## 32418  5 4140  2.123548e+00
## 32419  5 4141  2.096428e+00
## 32420  5 4142  2.078699e+00
## 32421  5 4143  2.054424e+00
## 32422  5 4144  2.034585e+00
## 32423  5 4145  2.010860e+00
## 32424  5 4146  1.989494e+00
## 32425  5 4147  1.971167e+00
## 32426  5 4148  1.951965e+00
## 32427  5 4149  1.934582e+00
## 32428  5 4150  1.910347e+00
## 32429  5 4151  1.892030e+00
## 32430  5 4152  1.874047e+00
## 32431  5 4152  1.872102e+00
## 32432  5 4153  3.726057e+00
## 32433  5 4154  4.094324e+00
## 32434  5 4155  4.044813e+00
## 32435  5 4156  3.898144e+00
## 32436  5 4157  3.737551e+00
## 32437  5 4158  3.595906e+00
## 32438  5 4159  3.457745e+00
## 32439  5 4160  3.344846e+00
## 32440  5 4161  3.248238e+00
## 32441  5 4162  3.161098e+00
## 32442  5 4163  3.090781e+00
## 32443  5 4164  3.021090e+00
## 32444  5 4165  2.966872e+00
## 32445  5 4166  2.911466e+00
## 32446  5 4167  2.860993e+00
## 32447  5 4168  2.810229e+00
## 32448  5 4169  2.770113e+00
## 32449  5 4170  2.729515e+00
## 32450  5 4171  2.691720e+00
## 32451  5 4172  2.662264e+00
## 32452  5 4173  2.626954e+00
## 32453  5 4174  2.591400e+00
## 32454  5 4175  2.564072e+00
## 32455  5 4176  2.528686e+00
## 32456  5 4176  2.535612e+00
## 32457  5 4177  3.440962e+00
## 32458  5 4178  3.607611e+00
## 32459  5 4179  3.555879e+00
## 32460  5 4180  3.464545e+00
## 32461  5 4181  3.369723e+00
## 32462  5 4182  3.282482e+00
## 32463  5 4183  3.204189e+00
## 32464  5 4184  3.129606e+00
## 32465  5 4185  3.070091e+00
## 32466  5 4186  3.007046e+00
## 32467  5 4187  2.958915e+00
## 32468  5 4188  2.907112e+00
## 32469  5 4189  2.859638e+00
## 32470  5 4190  2.815135e+00
## 32471  5 4191  2.782416e+00
## 32472  5 4192  2.745234e+00
## 32473  5 4193  2.709886e+00
## 32474  5 4194  2.670942e+00
## 32475  5 4195  2.639595e+00
## 32476  5 4196  2.613556e+00
## 32477  5 4197  2.585444e+00
## 32478  5 4198  2.555711e+00
## 32479  5 4199  2.528958e+00
## 32480  5 4200  2.495523e+00
## 32481  5 4201  3.407985e+00
## 32482  5 4202  3.577080e+00
## 32483  5 4203  3.538457e+00
## 32484  5 4204  3.440766e+00
## 32485  5 4205  3.350003e+00
## 32486  5 4206  3.258494e+00
## 32487  5 4207  3.173153e+00
## 32488  5 4208  3.102147e+00
## 32489  5 4209  3.039428e+00
## 32490  5 4210  2.987924e+00
## 32491  5 4211  2.935961e+00
## 32492  5 4212  2.886791e+00
## 32493  5 4213  2.839834e+00
## 32494  5 4214  2.802586e+00
## 32495  5 4215  2.759131e+00
## 32496  5 4216  2.726859e+00
## 32497  5 4217  2.688521e+00
## 32498  5 4218  2.659489e+00
## 32499  5 4219  2.624623e+00
## 32500  5 4220  2.589125e+00
## 32501  5 4221  2.561865e+00
## 32502  5 4222  2.529335e+00
## 32503  5 4223  2.506148e+00
## 32504  5 4224  2.476704e+00
## 32505  5 4225  3.390022e+00
## 32506  5 4226  3.562060e+00
## 32507  5 4227  3.508584e+00
## 32508  5 4228  3.422354e+00
## 32509  5 4229  3.325150e+00
## 32510  5 4230  3.236671e+00
## 32511  5 4231  3.165459e+00
## 32512  5 4232  3.092541e+00
## 32513  5 4233  3.023948e+00
## 32514  5 4234  2.964535e+00
## 32515  5 4235  2.918353e+00
## 32516  5 4236  2.870074e+00
## 32517  5 4237  2.825136e+00
## 32518  5 4238  2.784811e+00
## 32519  5 4239  2.741141e+00
## 32520  5 4240  2.701576e+00
## 32521  5 4241  2.674668e+00
## 32522  5 4242  2.638091e+00
## 32523  5 4243  2.613502e+00
## 32524  5 4244  2.573804e+00
## 32525  5 4245  2.549172e+00
## 32526  5 4246  2.520655e+00
## 32527  5 4247  2.493686e+00
## 32528  5 4248  2.463365e+00
## 32529  5 4249  3.376325e+00
## 32530  5 4250  3.545174e+00
## 32531  5 4251  3.497268e+00
## 32532  5 4252  3.409763e+00
## 32533  5 4253  3.313572e+00
## 32534  5 4254  3.224780e+00
## 32535  5 4255  3.145063e+00
## 32536  5 4256  3.065544e+00
## 32537  5 4257  3.008810e+00
## 32538  5 4258  2.957221e+00
## 32539  5 4259  2.901830e+00
## 32540  5 4260  2.849758e+00
## 32541  5 4261  2.806700e+00
## 32542  5 4262  2.772322e+00
## 32543  5 4263  2.733119e+00
## 32544  5 4264  2.694797e+00
## 32545  5 4265  2.659728e+00
## 32546  5 4266  2.626351e+00
## 32547  5 4267  2.596896e+00
## 32548  5 4268  2.564145e+00
## 32549  5 4269  2.538788e+00
## 32550  5 4270  2.508730e+00
## 32551  5 4271  2.481669e+00
## 32552  5 4272  2.451446e+00
## 32553  5 4273  3.366392e+00
## 32554  5 4274  3.528526e+00
## 32555  5 4275  3.486771e+00
## 32556  5 4276  3.396435e+00
## 32557  5 4277  3.294819e+00
## 32558  5 4278  3.210792e+00
## 32559  5 4279  3.132112e+00
## 32560  5 4280  3.062925e+00
## 32561  5 4281  2.998520e+00
## 32562  5 4282  2.937667e+00
## 32563  5 4283  2.893567e+00
## 32564  5 4284  2.845224e+00
## 32565  5 4285  2.796947e+00
## 32566  5 4286  2.762936e+00
## 32567  5 4287  2.717896e+00
## 32568  5 4288  2.681240e+00
## 32569  5 4289  2.651560e+00
## 32570  5 4290  2.618330e+00
## 32571  5 4291  2.579890e+00
## 32572  5 4292  2.555714e+00
## 32573  5 4293  2.521856e+00
## 32574  5 4294  2.494074e+00
## 32575  5 4295  2.467858e+00
## 32576  5 4296  2.439295e+00
## 32577  5 4297  3.348359e+00
## 32578  5 4298  3.518247e+00
## 32579  5 4299  3.476482e+00
## 32580  5 4300  3.387125e+00
## 32581  5 4301  3.297600e+00
## 32582  5 4302  3.204779e+00
## 32583  5 4303  3.119450e+00
## 32584  5 4304  3.054178e+00
## 32585  5 4305  2.991188e+00
## 32586  5 4306  2.931873e+00
## 32587  5 4307  2.885164e+00
## 32588  5 4308  2.837010e+00
## 32589  5 4309  2.791731e+00
## 32590  5 4310  2.751978e+00
## 32591  5 4311  2.713185e+00
## 32592  5 4312  2.676998e+00
## 32593  5 4313  2.639588e+00
## 32594  5 4314  2.610719e+00
## 32595  5 4315  2.579865e+00
## 32596  5 4316  2.548037e+00
## 32597  5 4317  2.519180e+00
## 32598  5 4318  2.489602e+00
## 32599  5 4319  2.455886e+00
## 32600  5 4320  2.434545e+00
## 32601  5 4321  3.347886e+00
## 32602  5 4322  3.508771e+00
## 32603  5 4323  3.467408e+00
## 32604  5 4324  3.375119e+00
## 32605  5 4325  3.280084e+00
## 32606  5 4326  3.194828e+00
## 32607  5 4327  3.117334e+00
## 32608  5 4328  3.041523e+00
## 32609  5 4329  2.984779e+00
## 32610  5 4330  2.924052e+00
## 32611  5 4331  2.872306e+00
## 32612  5 4332  2.827953e+00
## 32613  5 4333  2.785232e+00
## 32614  5 4334  2.741248e+00
## 32615  5 4335  2.704581e+00
## 32616  5 4336  2.670085e+00
## 32617  5 4337  2.632033e+00
## 32618  5 4338  2.602123e+00
## 32619  5 4339  2.568855e+00
## 32620  5 4340  2.537841e+00
## 32621  5 4341  2.512358e+00
## 32622  5 4342  2.481987e+00
## 32623  5 4343  2.457903e+00
## 32624  5 4344  2.429723e+00
## 32625  5 4345  3.331613e+00
## 32626  5 4346  3.503713e+00
## 32627  5 4347  3.464199e+00
## 32628  5 4348  3.375518e+00
## 32629  5 4349  3.276159e+00
## 32630  5 4350  3.190838e+00
## 32631  5 4351  3.110397e+00
## 32632  5 4352  3.047308e+00
## 32633  5 4353  2.977708e+00
## 32634  5 4354  2.920444e+00
## 32635  5 4355  2.865798e+00
## 32636  5 4356  2.823207e+00
## 32637  5 4357  2.780208e+00
## 32638  5 4358  2.739635e+00
## 32639  5 4359  2.697754e+00
## 32640  5 4360  2.663017e+00
## 32641  5 4361  2.630898e+00
## 32642  5 4362  2.597759e+00
## 32643  5 4363  2.568563e+00
## 32644  5 4364  2.536967e+00
## 32645  5 4365  2.505444e+00
## 32646  5 4366  2.476304e+00
## 32647  5 4367  2.450843e+00
## 32648  5 4368  2.420249e+00
## 32649  5 4369  3.333318e+00
## 32650  5 4370  3.507843e+00
## 32651  5 4371  3.463811e+00
## 32652  5 4372  3.371276e+00
## 32653  5 4373  3.274325e+00
## 32654  5 4374  3.189641e+00
## 32655  5 4375  3.100541e+00
## 32656  5 4376  3.038063e+00
## 32657  5 4377  2.973134e+00
## 32658  5 4378  2.917551e+00
## 32659  5 4379  2.864184e+00
## 32660  5 4380  2.822205e+00
## 32661  5 4381  2.775802e+00
## 32662  5 4382  2.732822e+00
## 32663  5 4383  2.695779e+00
## 32664  5 4384  2.664579e+00
## 32665  5 4385  2.629122e+00
## 32666  5 4386  2.597198e+00
## 32667  5 4387  2.561178e+00
## 32668  5 4388  2.527801e+00
## 32669  5 4389  2.499281e+00
## 32670  5 4390  2.475407e+00
## 32671  5 4391  2.446363e+00
## 32672  5 4392  2.416052e+00
## 32673  5 4393  3.325679e+00
## 32674  5 4394  3.490347e+00
## 32675  5 4395  3.451423e+00
## 32676  5 4396  3.359661e+00
## 32677  5 4397  3.278679e+00
## 32678  5 4398  3.189418e+00
## 32679  5 4399  3.106313e+00
## 32680  5 4400  3.032742e+00
## 32681  5 4401  2.971444e+00
## 32682  5 4402  2.915974e+00
## 32683  5 4403  2.862440e+00
## 32684  5 4404  2.817329e+00
## 32685  5 4405  2.771426e+00
## 32686  5 4406  2.729786e+00
## 32687  5 4407  2.696303e+00
## 32688  5 4408  2.662087e+00
## 32689  5 4409  2.622889e+00
## 32690  5 4410  2.586828e+00
## 32691  5 4411  2.565866e+00
## 32692  5 4412  2.528555e+00
## 32693  5 4413  2.498645e+00
## 32694  5 4414  2.472711e+00
## 32695  5 4415  2.444978e+00
## 32696  5 4416  2.418201e+00
## 32697  5 4417  3.330242e+00
## 32698  5 4418  3.495532e+00
## 32699  5 4419  3.456757e+00
## 32700  5 4420  3.365376e+00
## 32701  5 4421  3.265583e+00
## 32702  5 4422  3.172924e+00
## 32703  5 4423  3.100759e+00
## 32704  5 4424  3.024113e+00
## 32705  5 4425  2.961877e+00
## 32706  5 4426  2.910945e+00
## 32707  5 4427  2.862564e+00
## 32708  5 4428  2.812035e+00
## 32709  5 4429  2.770742e+00
## 32710  5 4430  2.733572e+00
## 32711  5 4431  2.688567e+00
## 32712  5 4432  2.655532e+00
## 32713  5 4433  2.622656e+00
## 32714  5 4434  2.592188e+00
## 32715  5 4435  2.560020e+00
## 32716  5 4436  2.523721e+00
## 32717  5 4437  2.495437e+00
## 32718  5 4438  2.467536e+00
## 32719  5 4439  2.443186e+00
## 32720  5 4440  2.420022e+00
## 32721  5 4441  2.387201e+00
## 32722  5 4442  2.366822e+00
## 32723  5 4443  2.338052e+00
## 32724  5 4444  2.312605e+00
## 32725  5 4445  2.291087e+00
## 32726  5 4446  2.267039e+00
## 32727  5 4447  2.244368e+00
## 32728  5 4448  2.219097e+00
## 32729  5 4449  2.194074e+00
## 32730  5 4450  2.172509e+00
## 32731  5 4451  2.152840e+00
## 32732  5 4452  2.127931e+00
## 32733  5 4453  2.105391e+00
## 32734  5 4454  2.081574e+00
## 32735  5 4455  2.063021e+00
## 32736  5 4456  2.043657e+00
## 32737  5 4457  2.017437e+00
## 32738  5 4458  1.997625e+00
## 32739  5 4459  1.977776e+00
## 32740  5 4460  1.954861e+00
## 32741  5 4461  1.938089e+00
## 32742  5 4462  1.918811e+00
## 32743  5 4463  1.895130e+00
## 32744  5 4464  1.876513e+00
## 32745  5 4465  1.859280e+00
## 32746  5 4466  1.842288e+00
## 32747  5 4467  1.819148e+00
## 32748  5 4468  1.800891e+00
## 32749  5 4469  1.782623e+00
## 32750  5 4470  1.762820e+00
## 32751  5 4471  1.751664e+00
## 32752  5 4472  1.730446e+00
## 32753  5 4473  1.711788e+00
## 32754  5 4474  1.693505e+00
## 32755  5 4475  1.677150e+00
## 32756  5 4476  1.658913e+00
## 32757  5 4477  1.642687e+00
## 32758  5 4478  1.628317e+00
## 32759  5 4479  1.606869e+00
## 32760  5 4480  1.592495e+00
## 32761  5 4481  1.578218e+00
## 32762  5 4482  1.562490e+00
## 32763  5 4483  1.544501e+00
## 32764  5 4484  1.529686e+00
## 32765  5 4485  1.512232e+00
## 32766  5 4486  1.498762e+00
## 32767  5 4487  1.481754e+00
## 32768  5 4488  1.465647e+00
## 32769  5 4489  1.453823e+00
## 32770  5 4490  1.436178e+00
## 32771  5 4491  1.421691e+00
## 32772  5 4492  1.406432e+00
## 32773  5 4493  1.389620e+00
## 32774  5 4494  1.375830e+00
## 32775  5 4495  1.363240e+00
## 32776  5 4496  1.352238e+00
## 32777  5 4497  1.333597e+00
## 32778  5 4498  1.322551e+00
## 32779  5 4499  1.306877e+00
## 32780  5 4500  1.295757e+00
## 32781  5 4501  1.283365e+00
## 32782  5 4502  1.268851e+00
## 32783  5 4503  1.255294e+00
## 32784  5 4504  1.244463e+00
## 32785  5 4505  1.228415e+00
## 32786  5 4506  1.217173e+00
## 32787  5 4507  1.205222e+00
## 32788  5 4508  1.191779e+00
## 32789  5 4509  1.177601e+00
## 32790  5 4510  1.170058e+00
## 32791  5 4511  1.155684e+00
## 32792  5 4512  1.143534e+00
## 32793  5 4513  1.132526e+00
## 32794  5 4514  1.119983e+00
## 32795  5 4515  1.109069e+00
## 32796  5 4516  1.098760e+00
## 32797  5 4517  1.085900e+00
## 32798  5 4518  1.074604e+00
## 32799  5 4519  1.065151e+00
## 32800  5 4520  1.052273e+00
## 32801  5 4521  1.043171e+00
## 32802  5 4522  1.032933e+00
## 32803  5 4523  1.023459e+00
## 32804  5 4524  1.010661e+00
## 32805  5 4525  1.000628e+00
## 32806  5 4526  9.886062e-01
## 32807  5 4527  9.803690e-01
## 32808  5 4528  9.701397e-01
## 32809  5 4529  9.615773e-01
## 32810  5 4530  9.509886e-01
## 32811  5 4531  9.415101e-01
## 32812  5 4532  9.315179e-01
## 32813  5 4533  9.197869e-01
## 32814  5 4534  9.117462e-01
## 32815  5 4535  9.035920e-01
## 32816  5 4536  8.929423e-01
## 32817  5 4537  8.846458e-01
## 32818  5 4538  8.754362e-01
## 32819  5 4539  8.656898e-01
## 32820  5 4540  8.568138e-01
## 32821  5 4541  8.476517e-01
## 32822  5 4542  8.384983e-01
## 32823  5 4543  8.303914e-01
## 32824  5 4544  8.222791e-01
## 32825  5 4545  8.128894e-01
## 32826  5 4546  8.066995e-01
## 32827  5 4547  7.973698e-01
## 32828  5 4548  7.876128e-01
## 32829  5 4549  7.817830e-01
## 32830  5 4550  7.725805e-01
## 32831  5 4551  7.665567e-01
## 32832  5 4552  7.581019e-01
## 32833  5 4553  7.513383e-01
## 32834  5 4554  7.407037e-01
## 32835  5 4555  7.341978e-01
```

```
## 32836  5 4556  7.264840e-01
## 32837  5 4557  7.182085e-01
## 32838  5 4558  7.111565e-01
## 32839  5 4559  7.039727e-01
## 32840  5 4560  6.973533e-01
## 32841  5 4561  6.914487e-01
## 32842  5 4562  6.825498e-01
## 32843  5 4563  6.752057e-01
## 32844  5 4564  6.690109e-01
## 32845  5 4565  6.617408e-01
## 32846  5 4566  6.558084e-01
## 32847  5 4567  6.485829e-01
## 32848  5 4568  6.415981e-01
## 32849  5 4569  6.355250e-01
## 32850  5 4570  6.285138e-01
## 32851  5 4571  6.218276e-01
## 32852  5 4572  6.156436e-01
## 32853  5 4573  6.095374e-01
## 32854  5 4574  6.033814e-01
## 32855  5 4575  5.961481e-01
## 32856  5 4576  5.918454e-01
## 32857  5 4577  5.846549e-01
## 32858  5 4578  5.786888e-01
## 32859  5 4579  5.735784e-01
## 32860  5 4580  5.675618e-01
## 32861  5 4581  5.595628e-01
## 32862  5 4582  5.562094e-01
## 32863  5 4583  5.502355e-01
## 32864  5 4584  5.445853e-01
## 32865  5 4585  5.377066e-01
## 32866  5 4586  5.324878e-01
## 32867  5 4587  5.270610e-01
## 32868  5 4588  5.219841e-01
## 32869  5 4589  5.165372e-01
## 32870  5 4590  5.109867e-01
## 32871  5 4591  5.069732e-01
## 32872  5 4592  5.011089e-01
## 32873  5 4593  4.956685e-01
## 32874  5 4594  4.907790e-01
## 32875  5 4595  4.865783e-01
## 32876  5 4596  4.816055e-01
## 32877  5 4597  4.757690e-01
## 32878  5 4598  4.710015e-01
## 32879  5 4599  4.661059e-01
## 32880  5 4600  4.619059e-01
## 32881  5 4601  4.572209e-01
## 32882  5 4602  4.518367e-01
## 32883  5 4603  4.473611e-01
## 32884  5 4604  4.420294e-01
## 32885  5 4605  4.379156e-01
## 32886  5 4606  4.331624e-01
## 32887  5 4607  4.298057e-01
## 32888  5 4608  4.242546e-01
## 32889  5 4609  4.201796e-01
## 32890  5 4610  4.169630e-01
## 32891  5 4611  4.126026e-01
## 32892  5 4612  4.072580e-01
## 32893  5 4613  4.029839e-01
## 32894  5 4614  3.988482e-01
## 32895  5 4615  3.954817e-01
## 32896  5 4616  3.907524e-01
## 32897  5 4617  3.871359e-01
## 32898  5 4618  3.825982e-01
## 32899  5 4619  3.790121e-01
## 32900  5 4620  3.750918e-01
## 32901  5 4621  3.711662e-01
## 32902  5 4622  3.681112e-01
## 32903  5 4623  3.637374e-01
## 32904  5 4624  3.601651e-01
## 32905  5 4625  3.562012e-01
## 32906  5 4626  3.522981e-01
## 32907  5 4627  3.490375e-01
## 32908  5 4628  3.452152e-01
## 32909  5 4629  3.415522e-01
## 32910  5 4630  3.389072e-01
## 32911  5 4631  3.347028e-01
## 32912  5 4632  3.309157e-01
## 32913  5 4633  3.278990e-01
## 32914  5 4634  3.246787e-01
## 32915  5 4635  3.210096e-01
## 32916  5 4636  3.178346e-01
## 32917  5 4637  3.150447e-01
## 32918  5 4638  3.114694e-01
## 32919  5 4639  3.085226e-01
## 32920  5 4640  3.059019e-01
## 32921  5 4641  3.021848e-01
## 32922  5 4642  2.989148e-01
## 32923  5 4643  2.956924e-01
## 32924  5 4644  2.928780e-01
## 32925  5 4645  2.897300e-01
## 32926  5 4646  2.868731e-01
## 32927  5 4647  2.842731e-01
## 32928  5 4648  2.805642e-01
## 32929  5 4649  2.782957e-01
## 32930  5 4650  2.753669e-01
## 32931  5 4651  2.726164e-01
## 32932  5 4652  2.698878e-01
## 32933  5 4653  2.666997e-01
## 32934  5 4654  2.640314e-01
## 32935  5 4655  2.611048e-01
## 32936  5 4656  2.587662e-01
## 32937  5 4657  2.557458e-01
## 32938  5 4658  2.534458e-01
## 32939  5 4659  2.512588e-01
## 32940  5 4660  2.482555e-01
## 32941  5 4661  2.461894e-01
## 32942  5 4662  2.432229e-01
## 32943  5 4663  2.405963e-01
## 32944  5 4664  2.380531e-01
## 32945  5 4665  2.356371e-01
## 32946  5 4666  2.334509e-01
## 32947  5 4667  2.310611e-01
## 32948  5 4668  2.284446e-01
## 32949  5 4669  2.262089e-01
## 32950  5 4670  2.238924e-01
## 32951  5 4671  2.216127e-01
## 32952  5 4672  2.192254e-01
## 32953  5 4673  2.171700e-01
## 32954  5 4674  2.150343e-01
## 32955  5 4675  2.129092e-01
## 32956  5 4676  2.105447e-01
## 32957  5 4677  2.083278e-01
## 32958  5 4678  2.061429e-01
## 32959  5 4679  2.039461e-01
## 32960  5 4680  2.018283e-01
## 32961  5 4681  1.998357e-01
## 32962  5 4682  1.979331e-01
## 32963  5 4683  1.956752e-01
## 32964  5 4684  1.941482e-01
## 32965  5 4685  1.917881e-01
## 32966  5 4686  1.896561e-01
## 32967  5 4687  1.878359e-01
## 32968  5 4688  1.859985e-01
## 32969  5 4689  1.840403e-01
## 32970  5 4690  1.819191e-01
## 32971  5 4691  1.803069e-01
## 32972  5 4692  1.783587e-01
## 32973  5 4693  1.763731e-01
## 32974  5 4694  1.747136e-01
## 32975  5 4695  1.731363e-01
## 32976  5 4696  1.713528e-01
## 32977  5 4697  1.695374e-01
## 32978  5 4698  1.675712e-01
## 32979  5 4699  1.658489e-01
## 32980  5 4700  1.645504e-01
## 32981  5 4701  1.624294e-01
## 32982  5 4702  1.605702e-01
## 32983  5 4703  1.593272e-01
## 32984  5 4704  1.576475e-01
## 32985  5 4705  1.559913e-01
## 32986  5 4706  1.543496e-01
## 32987  5 4707  1.527113e-01
## 32988  5 4708  1.513434e-01
## 32989  5 4709  1.496299e-01
## 32990  5 4710  1.482275e-01
## 32991  5 4711  1.465798e-01
## 32992  5 4712  1.451340e-01
## 32993  5 4713  1.436039e-01
## 32994  5 4714  1.420990e-01
## 32995  5 4715  1.407631e-01
## 32996  5 4716  1.392508e-01
## 32997  5 4717  1.378567e-01
## 32998  5 4718  1.363457e-01
## 32999  5 4719  1.351647e-01
## 33000  5 4720  1.335620e-01
## 33001  5 4721  1.324233e-01
## 33002  5 4722  1.310327e-01
## 33003  5 4723  1.294402e-01
## 33004  5 4724  1.282484e-01
## 33005  5 4725  1.269074e-01
## 33006  5 4726  1.258241e-01
## 33007  5 4727  1.242351e-01
## 33008  5 4728  1.228457e-01
## 33009  5 4729  1.218381e-01
## 33010  5 4730  1.203452e-01
## 33011  5 4731  1.191100e-01
## 33012  5 4732  1.178847e-01
## 33013  5 4733  1.169337e-01
## 33014  5 4734  1.156231e-01
## 33015  5 4735  1.145235e-01
## 33016  5 4736  1.131652e-01
## 33017  5 4737  1.123036e-01
## 33018  5 4738  1.109047e-01
## 33019  5 4739  1.097411e-01
## 33020  5 4740  1.085279e-01
## 33021  5 4741  1.076083e-01
## 33022  5 4742  1.064816e-01
## 33023  5 4743  1.054054e-01
## 33024  5 4744  1.041667e-01
## 33025  5 4745  1.031618e-01
## 33026  5 4746  1.023313e-01
## 33027  5 4747  1.011102e-01
## 33028  5 4748  1.000603e-01
## 33029  5 4749  9.903627e-02
## 33030  5 4750  9.813290e-02
## 33031  5 4751  9.713326e-02
## 33032  5 4752  9.613830e-02
## 33033  5 4753  9.501773e-02
## 33034  5 4754  9.390905e-02
## 33035  5 4755  9.307874e-02
## 33036  5 4756  9.211391e-02
## 33037  5 4757  9.133007e-02
## 33038  5 4758  9.015758e-02
## 33039  5 4759  8.929052e-02
## 33040  5 4760  8.829679e-02
## 33041  5 4761  8.741629e-02
## 33042  5 4762  8.660760e-02
## 33043  5 4763  8.556352e-02
## 33044  5 4764  8.482134e-02
## 33045  5 4765  8.415531e-02
## 33046  5 4766  8.320368e-02
## 33047  5 4767  8.223140e-02
## 33048  5 4768  8.123610e-02
## 33049  5 4769  8.062194e-02
## 33050  5 4770  7.969872e-02
## 33051  5 4771  7.897508e-02
## 33052  5 4772  7.800223e-02
## 33053  5 4773  7.734269e-02
## 33054  5 4774  7.655779e-02
## 33055  5 4775  7.568157e-02
## 33056  5 4776  7.494596e-02
## 33057  5 4777  7.418385e-02
## 33058  5 4778  7.342929e-02
## 33059  5 4779  7.264391e-02
## 33060  5 4780  7.190934e-02
## 33061  5 4781  7.115828e-02
## 33062  5 4782  7.044414e-02
## 33063  5 4783  6.956204e-02
## 33064  5 4784  6.895482e-02
## 33065  5 4785  6.835530e-02
## 33066  5 4786  6.757813e-02
## 33067  5 4787  6.688478e-02
## 33068  5 4788  6.621511e-02
## 33069  5 4789  6.550375e-02
## 33070  5 4790  6.489597e-02
## 33071  5 4791  6.423929e-02
## 33072  5 4792  6.341401e-02
## 33073  5 4793  6.298105e-02
## 33074  5 4794  6.218414e-02
## 33075  5 4795  6.150757e-02
## 33076  5 4796  6.090522e-02
## 33077  5 4797  6.024022e-02
## 33078  5 4798  5.964365e-02
## 33079  5 4799  5.905350e-02
## 33080  5 4800  5.851625e-02
## 33081  5 4801  5.781161e-02
## 33082  5 4802  5.730862e-02
## 33083  5 4803  5.673766e-02
## 33084  5 4804  5.608292e-02
## 33085  5 4805  5.551451e-02
## 33086  5 4806  5.497955e-02
## 33087  5 4807  5.439071e-02
## 33088  5 4808  5.385875e-02
## 33089  5 4809  5.335458e-02
## 33090  5 4810  5.275786e-02
## 33091  5 4811  5.219241e-02
## 33092  5 4812  5.164018e-02
## 33093  5 4813  5.116036e-02
## 33094  5 4814  5.056331e-02
## 33095  5 4815  5.007219e-02
## 33096  5 4816  4.966244e-02
## 33097  5 4817  4.911247e-02
## 33098  5 4818  4.852779e-02
## 33099  5 4819  4.803293e-02
## 33100  5 4820  4.763823e-02
## 33101  5 4821  4.708785e-02
## 33102  5 4822  4.662929e-02
## 33103  5 4823  4.610568e-02
## 33104  5 4824  4.568186e-02
## 33105  5 4825  4.511017e-02
## 33106  5 4826  4.480131e-02
## 33107  5 4827  4.429394e-02
## 33108  5 4828  4.388941e-02
## 33109  5 4829  4.342735e-02
## 33110  5 4830  4.292723e-02
## 33111  5 4831  4.258325e-02
## 33112  5 4832  4.201879e-02
## 33113  5 4833  4.158653e-02
## 33114  5 4834  4.125629e-02
## 33115  5 4835  4.078814e-02
## 33116  5 4836  4.037284e-02
## 33117  5 4837  3.993629e-02
## 33118  5 4838  3.950919e-02
## 33119  5 4839  3.911496e-02
## 33120  5 4840  3.871619e-02
## 33121  5 4841  3.831111e-02
## 33122  5 4842  3.788303e-02
## 33123  5 4843  3.754756e-02
## 33124  5 4844  3.707801e-02
## 33125  5 4845  3.679432e-02
## 33126  5 4846  3.639251e-02
## 33127  5 4847  3.598522e-02
## 33128  5 4848  3.559980e-02
## 33129  5 4849  3.536480e-02
## 33130  5 4850  3.497212e-02
## 33131  5 4851  3.455683e-02
## 33132  5 4852  3.423091e-02
## 33133  5 4853  3.383090e-02
## 33134  5 4854  3.345775e-02
## 33135  5 4855  3.316959e-02
## 33136  5 4856  3.274998e-02
## 33137  5 4857  3.248570e-02
## 33138  5 4858  3.214129e-02
## 33139  5 4859  3.188206e-02
## 33140  5 4860  3.146870e-02
## 33141  5 4861  3.115845e-02
## 33142  5 4862  3.084859e-02
## 33143  5 4863  3.053885e-02
## 33144  5 4864  3.021515e-02
## 33145  5 4865  2.987325e-02
## 33146  5 4866  2.965941e-02
## 33147  5 4867  2.930294e-02
## 33148  5 4868  2.900064e-02
## 33149  5 4869  2.867669e-02
## 33150  5 4870  2.835533e-02
## 33151  5 4871  2.808474e-02
## 33152  5 4872  2.780644e-02
## 33153  5 4873  2.750074e-02
## 33154  5 4874  2.727665e-02
## 33155  5 4875  2.698714e-02
## 33156  5 4876  2.661640e-02
## 33157  5 4877  2.642175e-02
## 33158  5 4878  2.612448e-02
## 33159  5 4879  2.586639e-02
## 33160  5 4880  2.562165e-02
## 33161  5 4881  2.533794e-02
## 33162  5 4882  2.508808e-02
## 33163  5 4883  2.482041e-02
## 33164  5 4884  2.455931e-02
## 33165  5 4885  2.434183e-02
## 33166  5 4886  2.407720e-02
## 33167  5 4887  2.384205e-02
## 33168  5 4888  2.362902e-02
## 33169  5 4889  2.335617e-02
## 33170  5 4890  2.310005e-02
## 33171  5 4891  2.284481e-02
## 33172  5 4892  2.266808e-02
## 33173  5 4893  2.240669e-02
## 33174  5 4894  2.216927e-02
## 33175  5 4895  2.193343e-02
## 33176  5 4896  2.173529e-02
## 33177  5 4897  2.148566e-02
## 33178  5 4898  2.123191e-02
## 33179  5 4899  2.106658e-02
## 33180  5 4900  2.082594e-02
## 33181  5 4901  2.062481e-02
## 33182  5 4902  2.041451e-02
## 33183  5 4903  2.019098e-02
## 33184  5 4904  1.998441e-02
## 33185  5 4905  1.976687e-02
## 33186  5 4906  1.956076e-02
## 33187  5 4907  1.937404e-02
## 33188  5 4908  1.917332e-02
## 33189  5 4909  1.899976e-02
## 33190  5 4910  1.878764e-02
## 33191  5 4911  1.858061e-02
## 33192  5 4912  1.842899e-02
## 33193  5 4913  1.820296e-02
## 33194  5 4914  1.803878e-02
## 33195  5 4915  1.784832e-02
## 33196  5 4916  1.763848e-02
## 33197  5 4917  1.748332e-02
## 33198  5 4918  1.729869e-02
## 33199  5 4919  1.707498e-02
## 33200  5 4920  1.695227e-02
## 33201  5 4921  1.677513e-02
## 33202  5 4922  1.662005e-02
## 33203  5 4923  1.641955e-02
## 33204  5 4924  1.627648e-02
## 33205  5 4925  1.610196e-02
## 33206  5 4926  1.592375e-02
## 33207  5 4927  1.577114e-02
## 33208  5 4928  1.559380e-02
## 33209  5 4929  1.545304e-02
## 33210  5 4930  1.526806e-02
## 33211  5 4931  1.510243e-02
## 33212  5 4932  1.497669e-02
## 33213  5 4933  1.484238e-02
## 33214  5 4934  1.466942e-02
## 33215  5 4935  1.449954e-02
## 33216  5 4936  1.435972e-02
## 33217  5 4937  1.421587e-02
## 33218  5 4938  1.406675e-02
## 33219  5 4939  1.391662e-02
## 33220  5 4940  1.378602e-02
## 33221  5 4941  1.363528e-02
## 33222  5 4942  1.348176e-02
## 33223  5 4943  1.335489e-02
## 33224  5 4944  1.323455e-02
## 33225  5 4945  1.308540e-02
## 33226  5 4946  1.295501e-02
## 33227  5 4947  1.284219e-02
## 33228  5 4948  1.268744e-02
## 33229  5 4949  1.256288e-02
## 33230  5 4950  1.243728e-02
## 33231  5 4951  1.230134e-02
## 33232  5 4952  1.216569e-02
## 33233  5 4953  1.204662e-02
## 33234  5 4954  1.193101e-02
## 33235  5 4955  1.181557e-02
## 33236  5 4956  1.167197e-02
## 33237  5 4957  1.154689e-02
## 33238  5 4958  1.144944e-02
## 33239  5 4959  1.132162e-02
## 33240  5 4960  1.121024e-02
## 33241  5 4961  1.111646e-02
## 33242  5 4962  1.097765e-02
## 33243  5 4963  1.087926e-02
## 33244  5 4964  1.073814e-02
## 33245  5 4965  1.066505e-02
## 33246  5 4966  1.056578e-02
## 33247  5 4967  1.041303e-02
## 33248  5 4968  1.032697e-02
## 33249  5 4969  1.022657e-02
## 33250  5 4970  1.012403e-02
## 33251  5 4971  1.001632e-02
## 33252  5 4972  9.916379e-03
## 33253  5 4973  9.824587e-03
## 33254  5 4974  9.688585e-03
## 33255  5 4975  9.600230e-03
## 33256  5 4976  9.525749e-03
## 33257  5 4977  9.396432e-03
## 33258  5 4978  9.320653e-03
## 33259  5 4979  9.206175e-03
## 33260  5 4980  9.110698e-03
## 33261  5 4981  9.031740e-03
## 33262  5 4982  8.929236e-03
## 33263  5 4983  8.836074e-03
## 33264  5 4984  8.753610e-03
## 33265  5 4985  8.653964e-03
## 33266  5 4986  8.570609e-03
## 33267  5 4987  8.481629e-03
## 33268  5 4988  8.395040e-03
## 33269  5 4989  8.289383e-03
## 33270  5 4990  8.215064e-03
## 33271  5 4991  8.133276e-03
## 33272  5 4992  8.060859e-03
## 33273  5 4993  7.971719e-03
## 33274  5 4994  7.884548e-03
## 33275  5 4995  7.810593e-03
## 33276  5 4996  7.736685e-03
## 33277  5 4997  7.651101e-03
## 33278  5 4998  7.564707e-03
## 33279  5 4999  7.505846e-03
## 33280  5 5000  7.419746e-03
## 33281  5 5001  7.349925e-03
## 33282  5 5002  7.270410e-03
## 33283  5 5003  7.202030e-03
## 33284  5 5004  7.109501e-03
## 33285  5 5005  7.052139e-03
## 33286  5 5006  6.982041e-03
## 33287  5 5007  6.914761e-03
## 33288  5 5008  6.829193e-03
## 33289  5 5009  6.770230e-03
## 33290  5 5010  6.696191e-03
## 33291  5 5011  6.632968e-03
## 33292  5 5012  6.552397e-03
## 33293  5 5013  6.489457e-03
## 33294  5 5014  6.427119e-03
## 33295  5 5015  6.337681e-03
## 33296  5 5016  6.283508e-03
## 33297  5 5017  6.227523e-03
## 33298  5 5018  6.160425e-03
## 33299  5 5019  6.102059e-03
## 33300  5 5020  6.038695e-03
## 33301  5 5021  5.971165e-03
## 33302  5 5022  5.908313e-03
## 33303  5 5023  5.848573e-03
## 33304  5 5024  5.794064e-03
## 33305  5 5025  5.732360e-03
## 33306  5 5026  5.655870e-03
## 33307  5 5027  5.605683e-03
## 33308  5 5028  5.552453e-03
## 33309  5 5029  5.508405e-03
## 33310  5 5030  5.447133e-03
## 33311  5 5031  5.386832e-03
## 33312  5 5032  5.332961e-03
## 33313  5 5033  5.265327e-03
## 33314  5 5034  5.217856e-03
## 33315  5 5035  5.162842e-03
## 33316  5 5036  5.122640e-03
## 33317  5 5037  5.060784e-03
## 33318  5 5038  5.006916e-03
## 33319  5 5039  4.954471e-03
## 33320  5 5040  4.903168e-03
## 33321  5 5041  4.850931e-03
## 33322  5 5042  4.805751e-03
## 33323  5 5043  4.758569e-03
## 33324  5 5044  4.708968e-03
## 33325  5 5045  4.663715e-03
## 33326  5 5046  4.615917e-03
## 33327  5 5047  4.572524e-03
## 33328  5 5048  4.521361e-03
## 33329  5 5049  4.477106e-03
## 33330  5 5050  4.426025e-03
## 33331  5 5051  4.388618e-03
## 33332  5 5052  4.337951e-03
## 33333  5 5053  4.291415e-03
##  [ reached 'max' / getOption("max.print") -- omitted 84638267 rows ]
```

Visualisation of C0 profiles at steady-state: <5, >8 or between 5 and 8 ug/L

```
ID_C0_Moes <- as_tibble(Data_combined) %>%
  dplyr::filter(time == 24) %>%
  select(ID, DV, time) %>%
  group_by(ID, time) %>%
  dplyr::filter(DV == max(DV)) %>%
  ungroup() %>%
  mutate(Group = case_when(
    DV < 5  ~ "Below 5",
    DV > 8  ~ "Above 8",
    TRUE    ~ "Between 5 and 8"
  ))

print(ID_C0_Moes)
```

```
## # A tibble: 12,000 × 4
##       ID    DV  time Group
##    <dbl> <dbl> <dbl> <chr>
##  1     1 0.640    24 Below 5
##  2     2 0.915    24 Below 5
##  3     3 2.79     24 Below 5
##  4     4 3.88     24 Below 5
##  5     5 2.41     24 Below 5
##  6     6 3.23     24 Below 5
##  7     7 1.74     24 Below 5
##  8     8 2.62     24 Below 5
##  9     9 0.321    24 Below 5
## 10    10 1.23     24 Below 5
## # ℹ 11,990 more rows
```

Profiles counting

```
count_Below_5 <- nrow(ID_C0_Moes %>% dplyr::filter(Group == "Below 5"))
count_Above_8 <- nrow(ID_C0_Moes %>% dplyr::filter(Group == "Above 8"))
count_Between_5_8 <- nrow(ID_C0_Moes %>% dplyr::filter(Group == "Between 5 and 8"))

# Affichage des résultats
print(count_Below_5)
```

```
## [1] 3380
```

```
print(count_Above_8)
```

```
## [1] 6862
```

```
print(count_Between_5_8)
```

```
## [1] 1758
```

#Selection of profiles with a C0 in the therapeutic target: 5-8 ug/L

```
selection_5_8 <- ID_C0_Moes %>%
  dplyr::filter(Group == "Between 5 and 8") %>%
  select(ID, DV, time)

Results_test_5_8 <- Data_combined %>%
  dplyr::filter(ID %in%selection_5_8$ID)

sim_data_filtered_12000 <- Results_test_5_8 %>%
  group_by(ID, time) %>%
  dplyr::filter(DV == max(DV)) %>%
  ungroup()

sim_data_filtered_12000
```

```
## # A tibble: 12,307,758 × 3
##       ID  time    DV
##    <dbl> <dbl> <dbl>
##  1   104     0  6.53
##  2   104     1  9.76
##  3   104     2  9.40
##  4   104     3  8.79
##  5   104     4  8.32
##  6   104     5  7.96
##  7   104     6  7.71
##  8   104     7  7.54
##  9   104     8  7.40
## 10   104     9  7.30
## # ℹ 12,307,748 more rows
```

Dose distribution of selected profiles

```
# For IDs de 1 à 6000
ids_to_keep_12000 <- sim_data_filtered_12000$ID

filtered_data_with_info_6000 <- Dataset_6000 %>%
  dplyr::filter(ID %in% ids_to_keep_12000) %>%
  select(ID, CYPCL) %>%
  distinct(ID, .keep_all = TRUE) %>%
  mutate(
    Group_of_dose = case_when(
      ID >= 1 & ID <= 400 ~ "1 mg",
      ID >= 401 & ID <= 800 ~ "2 mg",
      ID >= 801 & ID <= 1200 ~ "3 mg",
      ID >= 1201 & ID <= 1600 ~ "4 mg",
      ID >= 1601 & ID <= 2000 ~ "5 mg",
      ID >= 2001 & ID <= 2400 ~ "6 mg",
      ID >= 2401 & ID <= 2800 ~ "7 mg",
      ID >= 2801 & ID <= 3200 ~ "8 mg",
      ID >= 3201 & ID <= 3600 ~ "9 mg",
      ID >= 3601 & ID <= 4000 ~ "10 mg",
      ID >= 4001 & ID <= 4400 ~ "11 mg",
      ID >= 4401 & ID <= 4800 ~ "12 mg",
      ID >= 4801 & ID <= 5200 ~ "13 mg",
      ID >= 5201 & ID <= 5600 ~ "14 mg",
      ID >= 5601 & ID <= 6000 ~ "15 mg",
      TRUE ~ NA_character_))

# Pour les IDs de 6001 à 12000
ids_to_keep_12000 <- sim_data_filtered_12000$ID

filtered_data_with_info_12000 <- Dataset_12000 %>%
  dplyr::filter(ID %in% ids_to_keep_12000) %>%
  select(ID, CYPCL) %>%
  distinct(ID, .keep_all = TRUE) %>%
  mutate(
    Group_of_dose = case_when(
      ID >= 6001 & ID <= 6400 ~ "0,5 mg",
      ID >= 6401 & ID <= 6800 ~ "1,5 mg",
      ID >= 6801 & ID <= 7200 ~ "2,5 mg",
      ID >= 7201 & ID <= 7600 ~ "3,5 mg",
      ID >= 7601 & ID <= 8000 ~ "4,5 mg",
      ID >= 8001 & ID <= 8400 ~ "5,5 mg",
      ID >= 8401 & ID <= 8800 ~ "6,5 mg",
      ID >= 8801 & ID <= 9200 ~ "7,5 mg",
      ID >= 9201 & ID <= 9600 ~ "8,5 mg",
      ID >= 9601 & ID <= 10000 ~ "9,5 mg",
      ID >= 10001 & ID <= 10400 ~ "10,5 mg",
      ID >= 10401 & ID <= 10800 ~ "11,5 mg",
      ID >= 10801 & ID <= 11200 ~ "12,5 mg",
      ID >= 11201 & ID <= 11600 ~ "13,5 mg",
      ID >= 11601 & ID <= 12000 ~ "14,5 mg",
      TRUE ~ NA_character_))

# Combinaison des deux data frames
filtered_data_with_info <- bind_rows(filtered_data_with_info_6000, filtered_data_with_info_12000)

filtered_data_with_info
```

```
## # A tibble: 1,758 × 3
##       ID CYPCL Group_of_dose
##    <int> <dbl> <chr>
##  1   104  0     1 mg
##  2   221  0     1 mg
##  3   375  0     1 mg
##  4   392  0     1 mg
##  5   400  0     1 mg
##  6   403  0     2 mg
##  7   413  0     2 mg
##  8   416  0     2 mg
##  9   418  0     2 mg
## 10   423  0.33  2 mg
## # ℹ 1,748 more rows
```

#Distribution of doses received by group C1 :

```
filtered_group_C1 <- filtered_data_with_info %>%
  dplyr::filter(CYPCL == 0.00) %>%
  mutate(
    Group_of_dose = factor(
      Group_of_dose,
      levels = c(
        "0,5 mg", "1 mg", "1,5 mg", "2 mg", "2,5 mg", "3 mg",
        "3,5 mg", "4 mg", "4,5 mg", "5 mg", "5,5 mg", "6 mg",
        "6,5 mg", "7 mg", "7,5 mg", "8 mg", "8,5 mg", "9 mg",
        "9,5 mg", "10 mg", "10,5 mg", "11 mg", "11,5 mg", "12 mg",
        "12,5 mg", "13 mg", "13,5 mg", "14 mg", "14,5 mg", "15 mg")))  %>%
  mutate(
    Group_of_dose = gsub(",", ".", Group_of_dose) %>% factor(),
    numeric_dose = as.numeric(gsub(" mg", "", Group_of_dose)),
    Group_of_dose = factor(Group_of_dose, levels = unique(Group_of_dose[order(numeric_dose)]))
  )

plot_C1_doses <- ggplot(filtered_group_C1, aes(x = Group_of_dose)) +
  geom_bar(fill = "grey", color = "black") +
  geom_text(
    stat = "count",
    aes(label = ..count..),
    vjust = -0.5,
    size = 3
  ) +
  labs(
    title = "                                                    C1 group",
    x = "Dose group",
    y = "Number of pharmacokinetic profiles"
  ) +
  theme_minimal() +
  scale_y_continuous(limits = c(0, 100), expand = expansion(mult = c(0, 0.05))) +
  theme(axis.text.x = element_text(angle = 45, hjust = 1))

plot_C1_doses
```

```
## Warning: The dot-dot notation (`..count..`) was deprecated in ggplot2 3.4.0.
## ℹ Please use `after_stat(count)` instead.
## This warning is displayed once every 8 hours.
## Call `lifecycle::last_lifecycle_warnings()` to see where this warning was
## generated.
```

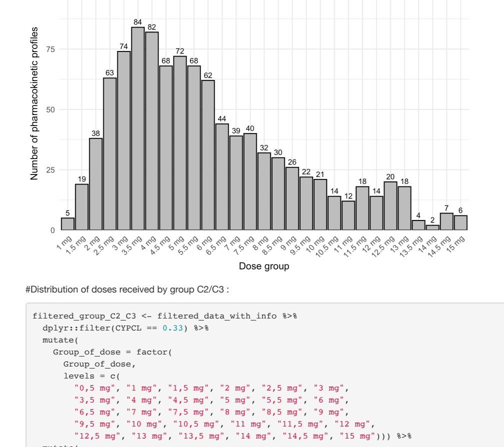

#Distribution of doses received by group C2/C3 :

```
filtered_group_C2_C3 <- filtered_data_with_info %>%
  dplyr::filter(CYPCL == 0.33) %>%
  mutate(
    Group_of_dose = factor(
      Group_of_dose,
      levels = c(
        "0,5 mg", "1 mg", "1,5 mg", "2 mg", "2,5 mg", "3 mg",
        "3,5 mg", "4 mg", "4,5 mg", "5 mg", "5,5 mg", "6 mg",
        "6,5 mg", "7 mg", "7,5 mg", "8 mg", "8,5 mg", "9 mg",
        "9,5 mg", "10 mg", "10,5 mg", "11 mg", "11,5 mg", "12 mg",
        "12,5 mg", "13 mg", "13,5 mg", "14 mg", "14,5 mg", "15 mg"))) %>%
  mutate(
    Group_of_dose = gsub(",", ".", Group_of_dose) %>% factor(),
    numeric_dose = as.numeric(gsub(" mg", "", Group_of_dose)),
    Group_of_dose = factor(Group_of_dose, levels = unique(Group_of_dose[order(numeric_dose)]))
  )

plot_C2_C3_doses <- ggplot(filtered_group_C2_C3, aes(x = Group_of_dose)) +
  geom_bar(fill = "grey", color = "black") +
  geom_text(
    stat = "count",
    aes(label = ..count..),
    vjust = -0.5,
    size = 3
  ) +
  labs(
    title = "                                                    C2/C3 group",
    x = "Dose group",
    y = "Number of pharmacokinetic profiles"
  ) +
  theme_minimal() +
  scale_y_continuous(limits = c(0, 50), expand = expansion(mult = c(0, 0.05))) +
  theme(axis.text.x = element_text(angle = 45, hjust = 1))

plot_C2_C3_doses
```

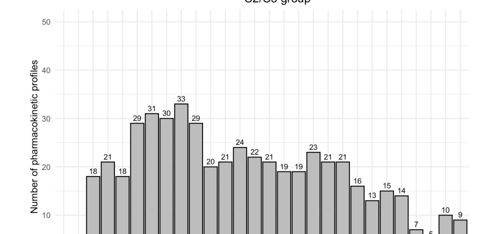

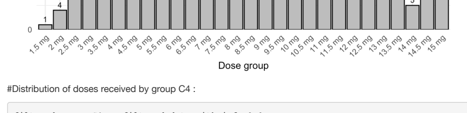

#Distribution of doses received by group C4 :

```
filtered_group_C4 <- filtered_data_with_info %>%
  dplyr::filter(CYPCL == 0.71) %>%
  mutate(
    Group_of_dose = factor(
      Group_of_dose,
      levels = c(
        "0,5 mg", "1 mg", "1,5 mg", "2 mg", "2,5 mg", "3 mg",
        "3,5 mg", "4 mg", "4,5 mg", "5 mg", "5,5 mg", "6 mg",
        "6,5 mg", "7 mg", "7,5 mg", "8 mg", "8,5 mg", "9 mg",
        "9,5 mg", "10 mg", "10,5 mg", "11 mg", "11,5 mg", "12 mg",
        "12,5 mg", "13 mg", "13,5 mg", "14 mg", "14,5 mg", "15 mg"))) %>%
  mutate(
    Group_of_dose = gsub(",", ".", Group_of_dose) %>% factor(),
    numeric_dose = as.numeric(gsub(" mg", "", Group_of_dose)),
    Group_of_dose = factor(Group_of_dose, levels = unique(Group_of_dose[order(numeric_dose)]))
  )

plot_C4_doses <- ggplot(filtered_group_C4, aes(x = Group_of_dose)) +
  geom_bar(fill = "grey", color = "black") +
  geom_text(
    stat = "count",
    aes(label = ..count..),
    vjust = -0.5,
    size = 3
  ) +
  labs(
    title = "                                                          C4 group",
    x = "Dose group",
    y = "Number of pharmacokinetic profiles"
  ) +
  theme_minimal() +
  scale_y_continuous(limits = c(0, 50), expand = expansion(mult = c(0, 0.05))) +
  theme(axis.text.x = element_text(angle = 45, hjust = 1))

plot_C4_doses
```

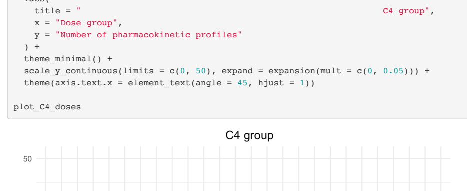

#Average dose received by all PK profiles

```
filtered_data_combined <- filtered_data_with_info %>%
  mutate(
    Dose_numeric = as.numeric(sub(" mg", "", gsub(",", ".", Group_of_dose)))
  )

dose_stats <- filtered_data_combined %>%
  summarize(
    Mean_dose = mean(Dose_numeric, na.rm = TRUE),
    SD_dose = sd(Dose_numeric, na.rm = TRUE)
  )

dose_stats
```

```
## # A tibble: 1 × 2
##   Mean_dose SD_dose
##       <dbl>   <dbl>
## 1      6.87    3.42
```

#Average dose received by C1

```
filtered_C1_Moes <- filtered_data_with_info %>%
  dplyr::filter(CYPCL == 0.00) %>%
  mutate(
    Dose_numeric = as.numeric(sub(" mg", "", gsub(",", ".", Group_of_dose)))
  )

dose_stats_C1 <- filtered_C1_Moes %>%
  summarize(
    Mean_dose = mean(Dose_numeric, na.rm = TRUE),
    SD_dose = sd(Dose_numeric, na.rm = TRUE)
  )

dose_stats_C1
```

```
## # A tibble: 1 × 2
##   Mean_dose SD_dose
##       <dbl>   <dbl>
## 1      5.93    3.10
```

#Average dose received by C2/C3

```
filtered_C2_C3_Moes <- filtered_data_with_info %>%
  dplyr::filter(CYPCL == 0.33) %>%
  mutate(
    Dose_numeric = as.numeric(sub(" mg", "", gsub(",", ".", Group_of_dose)))
  )

dose_stats_C2_C3 <- filtered_C2_C3_Moes %>%
  summarize(
    Mean_dose = mean(Dose_numeric, na.rm = TRUE),
    SD_dose = sd(Dose_numeric, na.rm = TRUE)
  )

dose_stats_C2_C3
```

```
## # A tibble: 1 × 2
##   Mean_dose SD_dose
##       <dbl>   <dbl>
## 1      7.67    3.38
```

#Average dose received by C4

```
filtered_C4_Moes <- filtered_data_with_info %>%
  dplyr::filter(CYPCL == 0.71) %>%
  mutate(
    Dose_numeric = as.numeric(sub(" mg", "", gsub(",", ".", Group_of_dose)))
  )

dose_stats_C4 <- filtered_C4_Moes %>%
  summarize(
    Mean_dose = mean(Dose_numeric, na.rm = TRUE),
    SD_dose = sd(Dose_numeric, na.rm = TRUE)
  )

dose_stats_C4
```

```
## # A tibble: 1 × 2
##   Mean_dose SD_dose
##       <dbl>   <dbl>
## 1      9.06    3.39
```

Time separation by simulated event

```
sim_data_by_scenario <- sim_data_filtered_12000 %>%
  mutate(
    forget_event = case_when(
      time >= 0 & time < 312 ~ "no_forget",
            time >= 312 & time < 624 ~ "forget_3h_retake_x1dose",
      time >= 624 & time < 936 ~ "forget_6h_retake_x1dose",
            time >= 936 & time < 1248 ~ "forget_9h_retake_x1dose",
      time >= 1248 & time < 1560 ~ "forget_12h_retake_x1dose",
            time >= 1560 & time < 1872 ~ "forget_15h_retake_x0.5dose",
      time >= 1872 & time < 2184 ~ "forget_15h_retake_x1dose",
            time >= 2184 & time < 2496 ~ "forget_18h_retake_x0.5dose",
      time >= 2496 & time < 2808 ~ "forget_18h_retake_x1dose",
      time >= 2808 & time < 3120 ~ "forget_21h_retake_x0.5dose",
            time >= 3120 & time < 3432 ~ "forget_21h_retake_x1dose",
      time >= 3456 & time < 3768 ~ "forget_24h_retake_x1dose",
      time >= 3788 & time < 4104 ~ "forget_24h_retake_x1.5dose",
            time >= 4128 & time < 4440 ~ "forget_24h_retake_x2dose"))

sim_data_by_scenario
```

```
## # A tibble: 12,307,758 × 4
##       ID  time    DV forget_event
##    <dbl> <dbl> <dbl> <chr>
##  1   104     0  6.53 no_forget
##  2   104     1  9.76 no_forget
##  3   104     2  9.40 no_forget
##  4   104     3  8.79 no_forget
##  5   104     4  8.32 no_forget
##  6   104     5  7.96 no_forget
##  7   104     6  7.71 no_forget
##  8   104     7  7.54 no_forget
##  9   104     8  7.40 no_forget
## 10   104     9  7.30 no_forget
## # ℹ 12,307,748 more rows
```

#C0 measurements for each profile every 24 hours

```
# Intervals of time
time_intervals <- list(
  c(24, 312), c(336, 624), c(648, 936), c(960, 1248),
  c(1272, 1560), c(1584, 1872), c(1896, 2184), c(2208, 2496),
  c(2520, 2808), c(2832, 3120), c(3144, 3432), c(3456, 3768),
  c(3772, 4104), c(4128, 4440)
)

filter_by_interval <- function(forget_event_group, interval) {
  sim_data_by_scenario %>%
    dplyr::filter(forget_event == forget_event_group,
                  time >= interval[1],
                  time < interval[2],
                  time %% 24 == 0)
}

forget_events <- unique(sim_data_by_scenario$forget_event)

# C0 calculation by forget_event and interval of time
results_final_C0_scenario <- map_dfr(forget_events, function(event) {
  map_dfr(time_intervals, function(interval) {
    filter_by_interval(event, interval)
  })
})

results_final_C0_scenario <- results_final_C0_scenario %>%
  arrange(forget_event, time)

print(results_final_C0_scenario)
```

```
## # A tibble: 300,618 × 4
##       ID  time    DV forget_event
##    <dbl> <dbl> <dbl> <chr>
##  1   104  1272  6.53 forget_12h_retake_x1dose
##  2   221  1272  5.70 forget_12h_retake_x1dose
##  3   375  1272  6.61 forget_12h_retake_x1dose
##  4   392  1272  6.11 forget_12h_retake_x1dose
##  5   400  1272  5.80 forget_12h_retake_x1dose
##  6   403  1272  5.60 forget_12h_retake_x1dose
##  7   413  1272  7.49 forget_12h_retake_x1dose
##  8   416  1272  5.21 forget_12h_retake_x1dose
##  9   418  1272  6.61 forget_12h_retake_x1dose
## 10   423  1272  7.81 forget_12h_retake_x1dose
## # ℹ 300,608 more rows
```

Calculation of relative differences in C0 for each profile

```
C0_basale_by_scenario <- results_final_C0_scenario %>%
  group_by(ID, forget_event) %>%
  arrange(time) %>%
  mutate(C0_basale = first(DV)) %>%
  ungroup()

Relative_C0_change_by_scenario <- C0_basale_by_scenario %>%
  group_by(forget_event) %>%
  mutate(relative_C0 = (DV - C0_basale) / C0_basale * 100) %>%
  ungroup()

Relative_C0_change_by_scenario
```

```
## # A tibble: 300,618 × 6
##       ID  time    DV forget_event C0_basale relative_C0
##    <dbl> <dbl> <dbl> <chr>            <dbl>       <dbl>
##  1   104    24  6.53 no_forget         6.53           0
##  2   221    24  5.71 no_forget         5.71           0
##  3   375    24  6.62 no_forget         6.62           0
##  4   392    24  6.10 no_forget         6.10           0
##  5   400    24  5.79 no_forget         5.79           0
##  6   403    24  5.60 no_forget         5.60           0
##  7   413    24  7.47 no_forget         7.47           0
##  8   416    24  5.22 no_forget         5.22           0
##  9   418    24  6.61 no_forget         6.61           0
## 10   423    24  7.79 no_forget         7.79           0
## # ℹ 300,608 more rows
```

Rename time for each group of forget_event

```
Data_with_time_renamed <- Relative_C0_change_by_scenario %>%
  mutate(time_adjusted = case_when(
    time >= 24 & time < 312 ~ paste0((time - 24) / 24 * 24 + 24, "h"),
    time >= 336 & time < 624 ~ paste0((time - 336) / 24 * 24 + 24, "h"),
    time >= 648 & time < 936 ~ paste0((time - 648) / 24 * 24 + 24, "h"),
    time >= 960 & time < 1248 ~ paste0((time - 960) / 24 * 24 + 24, "h"),
    time >= 1272 & time < 1560 ~ paste0((time - 1272) / 24 * 24 + 24, "h"),
    time >= 1584 & time < 1872 ~ paste0((time - 1584) / 24 * 24 + 24, "h"),
    time >= 1896 & time < 2184 ~ paste0((time - 1896) / 24 * 24 + 24, "h"),
    time >= 2208 & time < 2496 ~ paste0((time - 2208) / 24 * 24 + 24, "h"),
    time >= 2520 & time < 2808 ~ paste0((time - 2520) / 24 * 24 + 24, "h"),
    time >= 2832 & time < 3120 ~ paste0((time - 2832) / 24 * 24 + 24, "h"),
    time >= 3144 & time < 3432 ~ paste0((time - 3144) / 24 * 24 + 24, "h"),

      time >= 3456 & time < 3768 ~ paste0((time - 3456) / 24 * 24 + 24, "h"),
    time >= 3792 & time < 4104 ~ paste0((time - 3792) / 24 * 24 + 24, "h"),
    time >= 4128 & time < 4440 ~ paste0((time - 4128) / 24 * 24 + 24, "h"),

    TRUE ~ paste0(time, "h")
  ))

print(Data_with_time_renamed)
```

```
## # A tibble: 300,618 × 7
##       ID  time    DV forget_event C0_basale relative_C0 time_adjusted
##    <dbl> <dbl> <dbl> <chr>            <dbl>       <dbl> <chr>
##  1   104    24  6.53 no_forget         6.53           0 24h
##  2   221    24  5.71 no_forget         5.71           0 24h
##  3   375    24  6.62 no_forget         6.62           0 24h
##  4   392    24  6.10 no_forget         6.10           0 24h
##  5   400    24  5.79 no_forget         5.79           0 24h
##  6   403    24  5.60 no_forget         5.60           0 24h
##  7   413    24  7.47 no_forget         7.47           0 24h
##  8   416    24  5.22 no_forget         5.22           0 24h
##  9   418    24  6.61 no_forget         6.61           0 24h
## 10   423    24  7.79 no_forget         7.79           0 24h
## # ℹ 300,608 more rows
```

#Average statistics of C0 and RD: all profiles

```
summary_stats_expanded <- Data_with_time_renamed %>%
  group_by(forget_event, time_adjusted) %>%
  summarize(

    min_DV = fivenum(DV)[1],        # Minimum
    Q1_DV = fivenum(DV)[2],         # First quartile (Q1)
    median_DV = fivenum(DV)[3],     # Median (Q2)
    Q3_DV = fivenum(DV)[4],         # Third quartile (Q3)
    max_DV = fivenum(DV)[5],        # Maximum
    mean_DV = mean(DV, na.rm = TRUE),  # Average
    sd_DV = sd(DV, na.rm = TRUE),      # Standard deviation

    min_relative_C0 = fivenum(relative_C0)[1],        # Minimum
    Q1_relative_C0 = fivenum(relative_C0)[2],         # First quartile (Q1)
    median_relative_C0 = fivenum(relative_C0)[3],     # Median (Q2)
    Q3_relative_C0 = fivenum(relative_C0)[4],         # Third quartile (Q3)
    max_relative_C0 = fivenum(relative_C0)[5],        # Maximum
    mean_relative_C0 = mean(relative_C0, na.rm = TRUE),  # Average
    sd_relative_C0 = sd(relative_C0, na.rm = TRUE)       # Standard deviation
  ) %>%
  ungroup() %>%
  arrange(forget_event, time_adjusted)
```

```
## `summarise()` has grouped output by 'forget_event'. You can override using the
## `.groups` argument.
```

```
summary_stats_expanded
```

```
## # A tibble: 171 × 16
##    forget_event  time_adjusted min_DV Q1_DV median_DV Q3_DV max_DV mean_DV sd_DV
##    <chr>         <chr>          <dbl> <dbl>     <dbl> <dbl>  <dbl>   <dbl> <dbl>
##  1 forget_12h_r… 120h            5.11  5.84      6.56  7.33   8.21    6.60 0.870
##  2 forget_12h_r… 144h            5.06  5.78      6.49  7.26   8.12    6.54 0.864
##  3 forget_12h_r… 168h            5.03  5.75      6.46  7.22   8.09    6.50 0.860
##  4 forget_12h_r… 192h            5.01  5.73      6.44  7.20   8.06    6.48 0.857
##  5 forget_12h_r… 216h            5.01  5.72      6.43  7.19   8.04    6.47 0.855
##  6 forget_12h_r… 240h            5.01  5.72      6.42  7.18   8.02    6.47 0.855
##  7 forget_12h_r… 24h             5.00  5.70      6.42  7.17   8.00    6.46 0.852
##  8 forget_12h_r… 264h            5.01  5.71      6.42  7.17   8.01    6.46 0.854
##  9 forget_12h_r… 288h            5.00  5.71      6.41  7.16   8.02    6.46 0.853
## 10 forget_12h_r… 48h             5.14  6.66      7.51  8.32  10.4     7.52 1.08
## # ℹ 161 more rows
## # ℹ 7 more variables: min_relative_C0 <dbl>, Q1_relative_C0 <dbl>,
## #   median_relative_C0 <dbl>, Q3_relative_C0 <dbl>, max_relative_C0 <dbl>,
## #   mean_relative_C0 <dbl>, sd_relative_C0 <dbl>
```

#Histograms of mean C0 and mean RD by simulated event

```
desired_order <- c("no_forget",
                   "forget_3h_retake_x1dose",
                   "forget_6h_retake_x1dose",
                   "forget_9h_retake_x1dose",
                   "forget_12h_retake_x1dose",
                   "forget_15h_retake_x0.5dose",
                   "forget_15h_retake_x1dose",
                   "forget_18h_retake_x0.5dose",
                   "forget_18h_retake_x1dose",
                   "forget_21h_retake_x0.5dose",
                   "forget_21h_retake_x1dose",
                   "forget_24h_retake_x1dose",
                   "forget_24h_retake_x1.5dose",
                   "forget_24h_retake_x2dose")

summary_stats_expanded$forget_event <- factor(summary_stats_expanded$forget_event, levels = desired_order)

time_adjusted_order <- c("24h", "48h", "72h", "96h", "120h", "144h", "168h", "192h",
                         "216h", "240h", "264h", "288h", "312h")

summary_stats_expanded <- summary_stats_expanded %>%
  mutate(time_adjusted = factor(time_adjusted, levels = time_adjusted_order))

forget_events <- levels(summary_stats_expanded$forget_event)

for (event in forget_events) {
  data_subset <- summary_stats_expanded %>%
    dplyr::filter(forget_event == event)

  p1 <- ggplot(data_subset, aes(x = time_adjusted, y = mean_DV)) +
    geom_bar(stat = "identity", fill = "grey", color = "black") +
    geom_text(aes(label = round(mean_DV, 1)), vjust = -0.4, color = "black") +
    labs(title = paste("Mean residual concentration for :", event),
         x = "Time (h)", y = "Mean Residual Concentration") +
    theme_bw() +
    theme(plot.title = element_text(hjust = 0.5),
          axis.text.x = element_text(angle = 45, hjust = 1))

  print(p1)

  p2 <- ggplot(data_subset, aes(x = time_adjusted, y = mean_relative_C0)) +
  geom_bar(stat = "identity", fill = "steelblue", color = "black") +
  geom_hline(yintercept = c(-10, 10), linetype = "dashed", color = "red") +
  geom_text(aes(label = paste0(round(mean_relative_C0, 1), "%")),
            vjust = ifelse(data_subset$mean_relative_C0 >= 0, -0.5, 1.5),
            color = "black",
            size = 4,
            fontface = "bold",
            nudge_x = 0.05,
            nudge_y = 0.2) +
  labs(
    title = paste0(
      "<span style='color: #0072B2;'>Mean relative difference of residual concentration</span><br>",
      "<span style='color: orange;'>", event, "</span>"
    ),
    x = "Time (h)", y = "Mean relative difference of through concentration"
  ) +
  theme_bw() +
  theme(
    plot.title = element_markdown(hjust = 0.5, face = "bold"),
    axis.text.x = element_text(angle = 45, hjust = 1, size = 12),
    axis.title = element_text(face = "bold"),
    panel.grid.major = element_line(size = 0.5, color = "grey90"),
    panel.grid.minor = element_blank(),
    axis.line = element_line(color = "black")
  ) +
  ylim(min(data_subset$mean_relative_C0) - 10, max(data_subset$mean_relative_C0) + 10)



  print(p2)
}
```

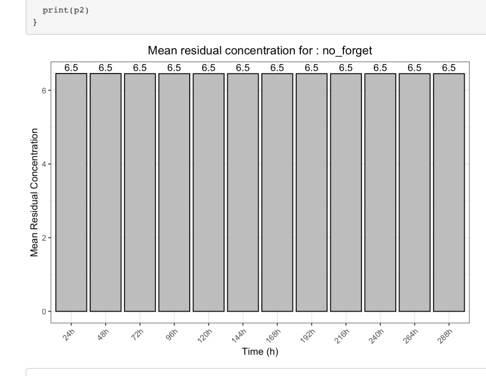


```
## Warning: The `size` argument of `element_line()` is deprecated as of ggplot2 3.4.0.
## ℹ Please use the `linewidth` argument instead.
## This warning is displayed once every 8 hours.
## Call `lifecycle::last_lifecycle_warnings()` to see where this warning was
## generated.
```

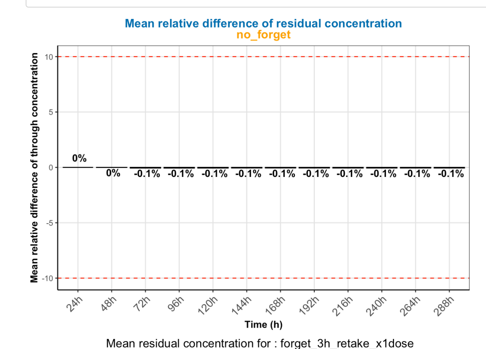

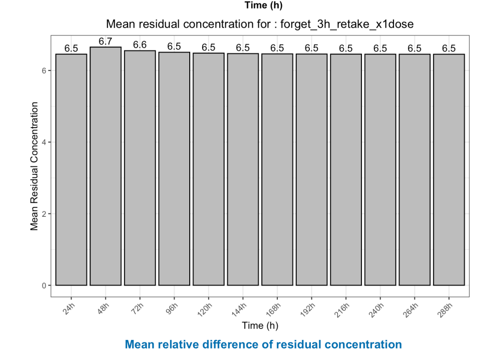

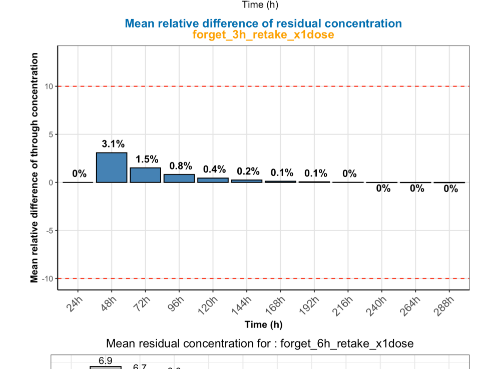

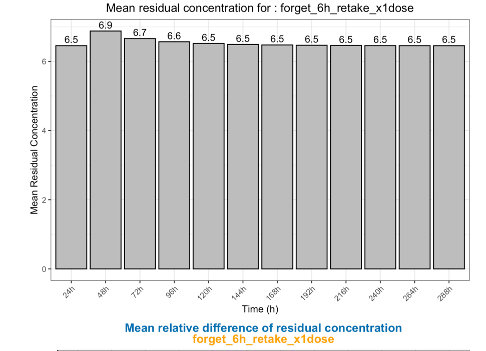

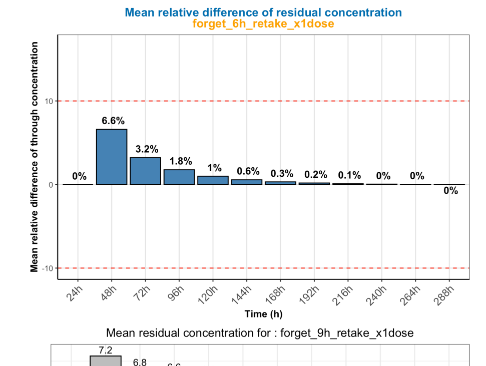

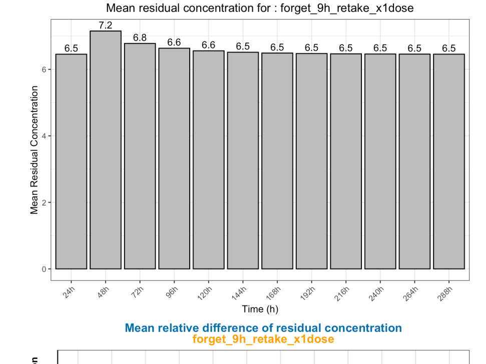

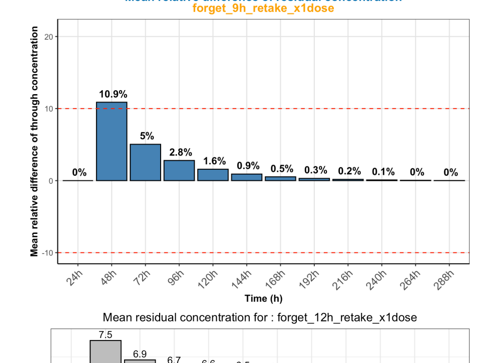

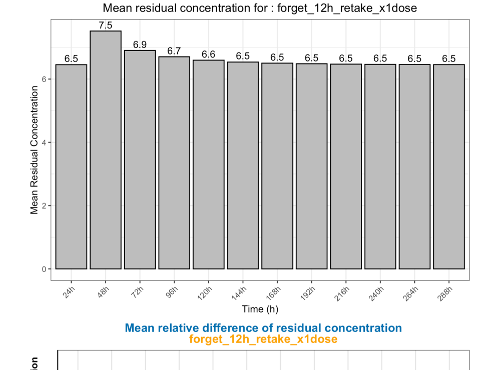

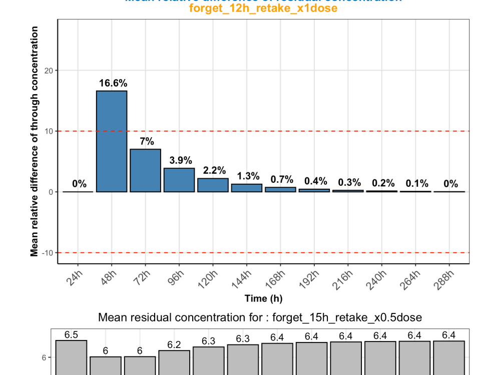

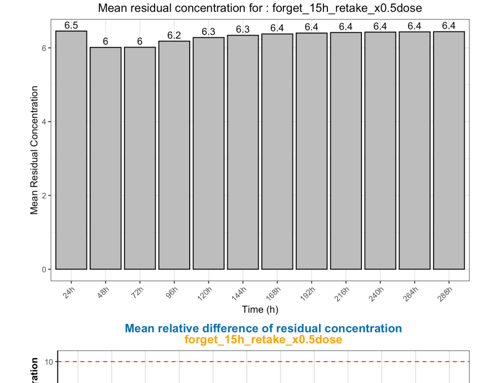

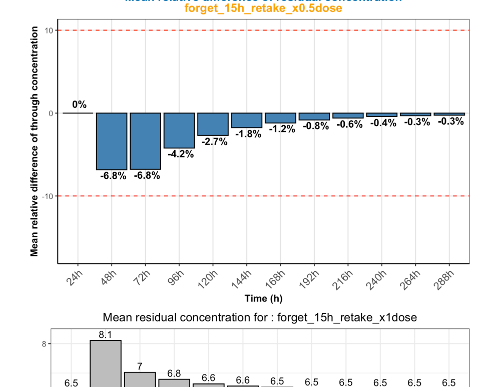

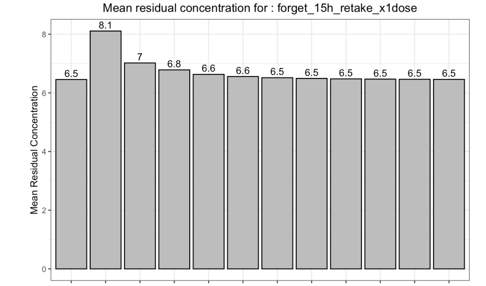

Time (h)

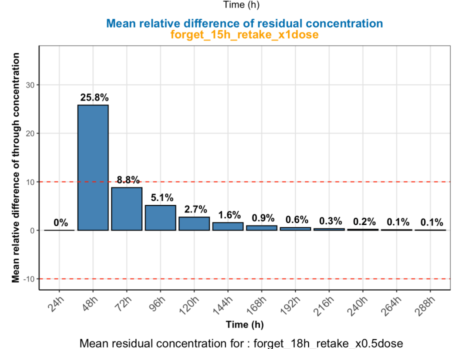

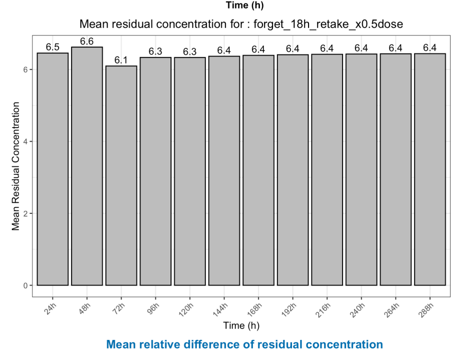

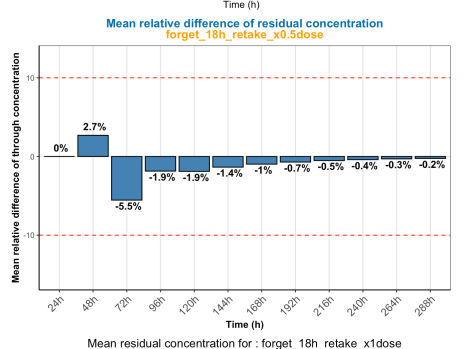

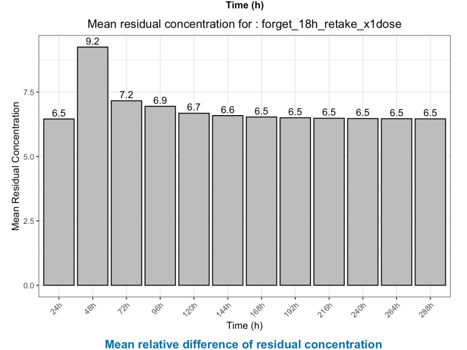

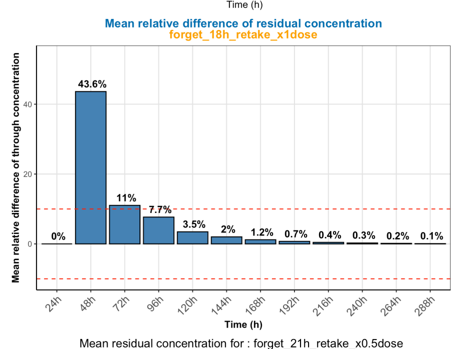

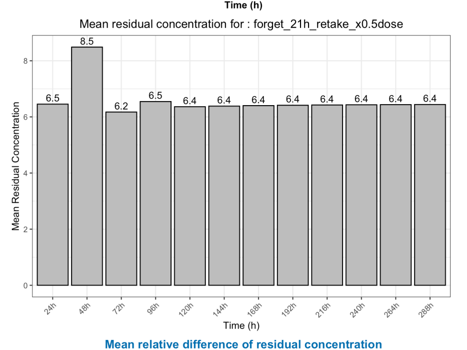

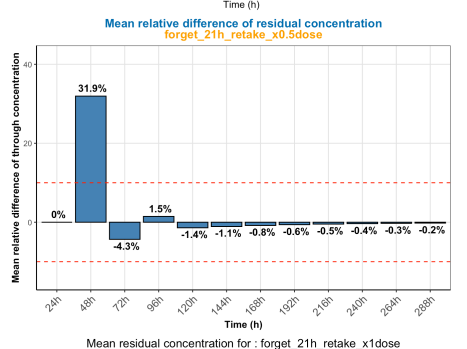

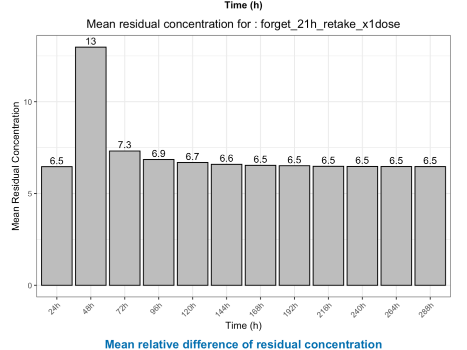

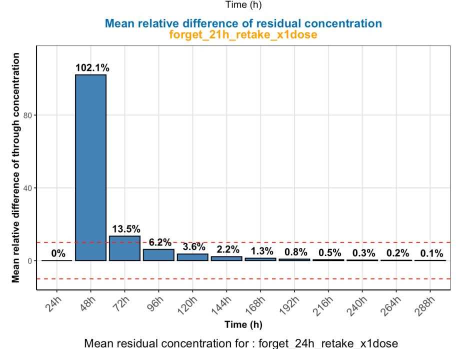

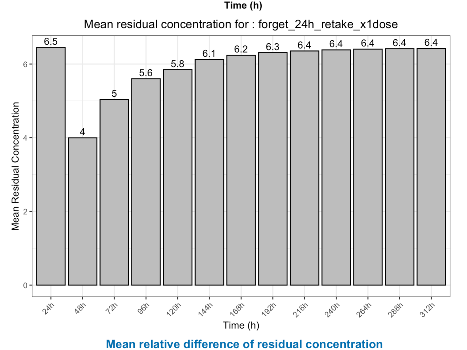

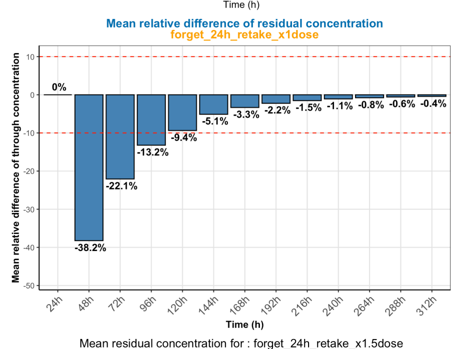

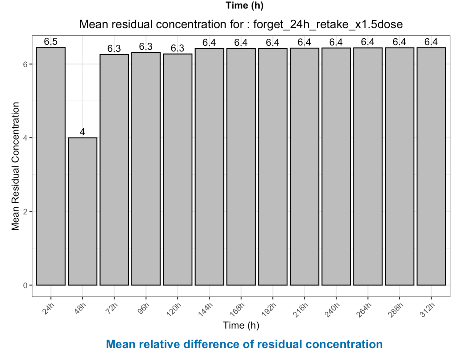

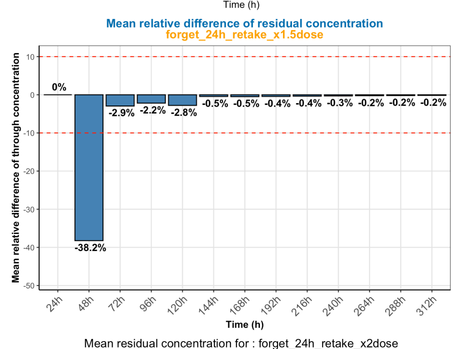

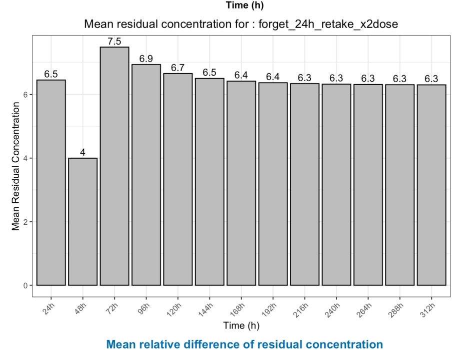

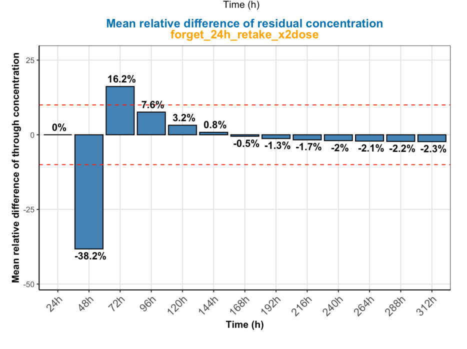

Attach CYP values to sim_data_filtered_6000_Woillard

```
sim_data_filtered_Moes_CYP <- Data_with_time_renamed %>%
  left_join(filtered_data_with_info, by = "ID")  # Jointure sur l'ID

sim_data_filtered_Moes_CYP
```

```
## # A tibble: 300,618 × 9
##       ID  time    DV forget_event C0_basale relative_C0 time_adjusted CYPCL
##    <dbl> <dbl> <dbl> <chr>            <dbl>       <dbl> <chr>         <dbl>
##  1   104    24  6.53 no_forget         6.53           0 24h            0
##  2   221    24  5.71 no_forget         5.71           0 24h            0
##  3   375    24  6.62 no_forget         6.62           0 24h            0
##  4   392    24  6.10 no_forget         6.10           0 24h            0
##  5   400    24  5.79 no_forget         5.79           0 24h            0
##  6   403    24  5.60 no_forget         5.60           0 24h            0
##  7   413    24  7.47 no_forget         7.47           0 24h            0
##  8   416    24  5.22 no_forget         5.22           0 24h            0
##  9   418    24  6.61 no_forget         6.61           0 24h            0
## 10   423    24  7.79 no_forget         7.79           0 24h            0.33
## # ℹ 300,608 more rows
## # ℹ 1 more variable: Group_of_dose <chr>
```

Stats for patients with CYPCL = 0

```
summary_stats_expanded_CYPCL_0 <- sim_data_filtered_Moes_CYP %>%
  dplyr::filter(CYPCL == 0) %>%
  group_by(forget_event, time_adjusted) %>%
  summarize(
    min_DV = fivenum(DV)[1],        # Minimum
    Q1_DV = fivenum(DV)[2],         # First quartile (Q1)
    median_DV = fivenum(DV)[3],     # Median (Q2)
    Q3_DV = fivenum(DV)[4],         # Third quartile (Q3)
    max_DV = fivenum(DV)[5],        # Maximum
    mean_DV = mean(DV, na.rm = TRUE),  # Mean
    sd_DV = sd(DV, na.rm = TRUE),      # SD

    min_relative_C0 = fivenum(relative_C0)[1],        # Minimum
    Q1_relative_C0 = fivenum(relative_C0)[2],         # First quartile (Q1)
    median_relative_C0 = fivenum(relative_C0)[3],     # Median (Q2)
    Q3_relative_C0 = fivenum(relative_C0)[4],         # Third quartile (Q3)
    max_relative_C0 = fivenum(relative_C0)[5],        # Maximum
    mean_relative_C0 = mean(relative_C0, na.rm = TRUE),  # Mean
    sd_relative_C0 = sd(relative_C0, na.rm = TRUE)       # SD
  ) %>%
  ungroup() %>%
  arrange(forget_event, time_adjusted)
```

```
## `summarise()` has grouped output by 'forget_event'. You can override using the
## `.groups` argument.
```

```
summary_stats_expanded_CYPCL_0
```

```
## # A tibble: 171 × 16
##    forget_event  time_adjusted min_DV Q1_DV median_DV Q3_DV max_DV mean_DV sd_DV
##    <chr>         <chr>          <dbl> <dbl>     <dbl> <dbl>  <dbl>   <dbl> <dbl>
##  1 forget_12h_r… 120h            5.11  5.85      6.58  7.35   8.20    6.61 0.869
##  2 forget_12h_r… 144h            5.06  5.82      6.53  7.29   8.11    6.56 0.862
##  3 forget_12h_r… 168h            5.04  5.79      6.50  7.25   8.09    6.52 0.859
##  4 forget_12h_r… 192h            5.02  5.77      6.47  7.22   8.06    6.51 0.856
##  5 forget_12h_r… 216h            5.01  5.77      6.46  7.22   8.04    6.49 0.855
##  6 forget_12h_r… 240h            5.01  5.76      6.46  7.21   8.02    6.49 0.854
##  7 forget_12h_r… 24h             5.00  5.74      6.44  7.20   8.00    6.47 0.851
##  8 forget_12h_r… 264h            5.01  5.75      6.45  7.20   8.01    6.48 0.853
##  9 forget_12h_r… 288h            5.00  5.76      6.45  7.20   8.00    6.48 0.852
## 10 forget_12h_r… 48h             5.36  6.53      7.38  8.22  10.3     7.39 1.06
## # ℹ 161 more rows
## # ℹ 7 more variables: min_relative_C0 <dbl>, Q1_relative_C0 <dbl>,
## #   median_relative_C0 <dbl>, Q3_relative_C0 <dbl>, max_relative_C0 <dbl>,
## #   mean_relative_C0 <dbl>, sd_relative_C0 <dbl>
```

Stats for patients with CYPCL = 0,33

```
summary_stats_expanded_CYPCL_33 <- sim_data_filtered_Moes_CYP %>%
  dplyr::filter(CYPCL == 0.33) %>%
  group_by(forget_event, time_adjusted) %>%
  summarize(
    min_DV = fivenum(DV)[1],        # Minimum
    Q1_DV = fivenum(DV)[2],         # First quartile (Q1)
    median_DV = fivenum(DV)[3],     # Median (Q2)
    Q3_DV = fivenum(DV)[4],         # Third quartile (Q3)
    max_DV = fivenum(DV)[5],        # Maximum
    mean_DV = mean(DV, na.rm = TRUE),  # Mean
    sd_DV = sd(DV, na.rm = TRUE),      # SD

    min_relative_C0 = fivenum(relative_C0)[1],        # Minimum
    Q1_relative_C0 = fivenum(relative_C0)[2],         # First quartile (Q1)
    median_relative_C0 = fivenum(relative_C0)[3],     # Median (Q2)
    Q3_relative_C0 = fivenum(relative_C0)[4],         # Third quartile (Q3)
    max_relative_C0 = fivenum(relative_C0)[5],        # Maximum
    mean_relative_C0 = mean(relative_C0, na.rm = TRUE),  # Mean
    sd_relative_C0 = sd(relative_C0, na.rm = TRUE)       # SD
  ) %>%
  ungroup() %>%
  arrange(forget_event, time_adjusted)
```

```
## `summarise()` has grouped output by 'forget_event'. You can override using the
## `.groups` argument.
```

```
summary_stats_expanded_CYPCL_33
```

```
## # A tibble: 171 × 16
##    forget_event  time_adjusted min_DV Q1_DV median_DV Q3_DV max_DV mean_DV sd_DV
##    <chr>         <chr>          <dbl> <dbl>     <dbl> <dbl>  <dbl>   <dbl> <dbl>
##  1 forget_12h_r… 120h            5.11  5.87      6.55  7.34   8.20    6.60 0.862
##  2 forget_12h_r… 144h            5.07  5.79      6.49  7.29   8.11    6.54 0.856
##  3 forget_12h_r… 168h            5.05  5.77      6.44  7.26   8.06    6.50 0.852
##  4 forget_12h_r… 192h            5.04  5.75      6.43  7.23   8.04    6.48 0.849
##  5 forget_12h_r… 216h            5.02  5.74      6.41  7.22   8.02    6.47 0.847
##  6 forget_12h_r… 240h            5.01  5.73      6.40  7.20   8.01    6.46 0.846
##  7 forget_12h_r… 24h             5.00  5.73      6.40  7.18   7.99    6.46 0.844
##  8 forget_12h_r… 264h            5.01  5.72      6.40  7.21   8.01    6.46 0.846
##  9 forget_12h_r… 288h            5.00  5.73      6.40  7.20   8.00    6.46 0.845
## 10 forget_12h_r… 48h             5.14  6.82      7.62  8.42  10.4     7.64 1.06
## # ℹ 161 more rows
## # ℹ 7 more variables: min_relative_C0 <dbl>, Q1_relative_C0 <dbl>,
## #   median_relative_C0 <dbl>, Q3_relative_C0 <dbl>, max_relative_C0 <dbl>,
## #   mean_relative_C0 <dbl>, sd_relative_C0 <dbl>
```

Stats for patients with CYPCL = 0,71

```
summary_stats_expanded_CYPCL_71 <- sim_data_filtered_Moes_CYP %>%
  dplyr::filter(CYPCL == 0.71) %>%
  group_by(forget_event, time_adjusted) %>%
  summarize(
    min_DV = fivenum(DV)[1],        # Minimum
    Q1_DV = fivenum(DV)[2],         # First quartile (Q1)
    median_DV = fivenum(DV)[3],     # Median (Q2)
    Q3_DV = fivenum(DV)[4],         # Third quartile (Q3)
    max_DV = fivenum(DV)[5],        # Maximum
    mean_DV = mean(DV, na.rm = TRUE),  # Mean
    sd_DV = sd(DV, na.rm = TRUE),      # SD

    min_relative_C0 = fivenum(relative_C0)[1],        # Minimum
    Q1_relative_C0 = fivenum(relative_C0)[2],         # First quartile (Q1)
    median_relative_C0 = fivenum(relative_C0)[3],     # Median (Q2)
    Q3_relative_C0 = fivenum(relative_C0)[4],         # Third quartile (Q3)
    max_relative_C0 = fivenum(relative_C0)[5],        # Maximum
    mean_relative_C0 = mean(relative_C0, na.rm = TRUE),  # Mean
    sd_relative_C0 = sd(relative_C0, na.rm = TRUE)       # SD
  ) %>%
  ungroup() %>%
  arrange(forget_event, time_adjusted)
```

```
## `summarise()` has grouped output by 'forget_event'. You can override using the
## `.groups` argument.
```

```
summary_stats_expanded_CYPCL_71
```

```
## # A tibble: 171 × 16
##    forget_event  time_adjusted min_DV Q1_DV median_DV Q3_DV max_DV mean_DV sd_DV
##    <chr>         <chr>          <dbl> <dbl>     <dbl> <dbl>  <dbl>   <dbl> <dbl>
##  1 forget_12h_r… 120h            5.12  5.73      6.51  7.18   8.21    6.53 0.892
##  2 forget_12h_r… 144h            5.06  5.66      6.45  7.10   8.12    6.46 0.884
##  3 forget_12h_r… 168h            5.03  5.63      6.41  7.07   8.07    6.42 0.879
##  4 forget_12h_r… 192h            5.01  5.62      6.38  7.05   8.02    6.40 0.876
##  5 forget_12h_r… 216h            5.01  5.60      6.37  7.03   8.03    6.39 0.874
##  6 forget_12h_r… 240h            5.01  5.60      6.37  7.02   8.02    6.38 0.874
##  7 forget_12h_r… 24h             5.02  5.59      6.36  7.01   8.00    6.37 0.871
##  8 forget_12h_r… 264h            5.02  5.59      6.37  7.02   8.01    6.38 0.872
##  9 forget_12h_r… 288h            5.00  5.59      6.36  7.01   8.02    6.37 0.870
## 10 forget_12h_r… 48h             5.62  6.89      7.78  8.59  10.4     7.78 1.11
## # ℹ 161 more rows
## # ℹ 7 more variables: min_relative_C0 <dbl>, Q1_relative_C0 <dbl>,
## #   median_relative_C0 <dbl>, Q3_relative_C0 <dbl>, max_relative_C0 <dbl>,
## #   mean_relative_C0 <dbl>, sd_relative_C0 <dbl>
```

#AUC measurements, every 24h

```
id_to_forget_event <- sim_data_by_scenario %>%
  select(ID, forget_event) %>%
  distinct()

start <- seq(0, 4416, by = 24)
end <- seq(24, 4440, by = 24)

iterate_auc <- function(start, end) {
  sim_data_by_scenario %>%
    group_by(ID) %>%
    rename(id = ID) %>%
    makeAUC(DV ~ time, start = start, end = end) %>%
    mutate(
      auc = round(tau, 0),
      intervalle = paste0(start, "_", end)
    ) %>%
    select(id, auc, intervalle)
}

auc_test_TEST <- map2_df(start, end, iterate_auc) %>%
  arrange(id, intervalle)


assign_forget_event <- function(start, end) {
  case_when(
    start >= 0 & end <= 312 ~ "no_forget",
    start >= 312 & end <= 624 ~ "forget_3h_retake_x1dose",
    start >= 624 & end <= 936 ~ "forget_6h_retake_x1dose",
    start >= 936 & end <= 1248 ~ "forget_9h_retake_x1dose",
    start >= 1248 & end <= 1560 ~ "forget_12h_retake_x1dose",
    start >= 1560 & end <= 1872 ~ "forget_15h_retake_x0.5dose",
    start >= 1872 & end <= 2184 ~ "forget_15h_retake_x1dose",
    start >= 2184 & end <= 2496 ~ "forget_18h_retake_x0.5dose",
    start >= 2496 & end <= 2808 ~ "forget_18h_retake_x1dose",
    start >= 2808 & end <= 3120 ~ "forget_21h_retake_x0.5dose",
    start >= 3120 & end <= 3432 ~ "forget_21h_retake_x1dose",
    start >= 3432 & end <= 3768 ~ "forget_24h_retake_x1dose",
    start >= 3768 & end <= 4104 ~ "forget_24h_retake_x1.5dose",
    start >= 4104 & end <= 4440 ~ "forget_24h_retake_x2dose",
    TRUE ~ NA_character_
  )
}

auc_test_TEST_by_scenario <- auc_test_TEST %>%
  mutate(
    start = as.numeric(str_extract(intervalle, "^[0-9]+")),
    end = as.numeric(str_extract(intervalle, "(?<=_)[0-9]+")),
    forget_event = assign_forget_event(start, end)
  )

auc_test_TEST_by_scenario <- auc_test_TEST_by_scenario %>%
  select(-start, -end)

print(auc_test_TEST_by_scenario)
```

```
## # A tibble: 325,230 × 4
## # Groups:   id [1,758]
##       id   auc intervalle forget_event
##    <dbl> <dbl> <chr>      <chr>
##  1   104   177 0_24       no_forget
##  2   104   178 1008_1032  forget_9h_retake_x1dose
##  3   104   178 1032_1056  forget_9h_retake_x1dose
##  4   104   177 1056_1080  forget_9h_retake_x1dose
##  5   104   177 1080_1104  forget_9h_retake_x1dose
##  6   104   177 1104_1128  forget_9h_retake_x1dose
##  7   104   177 1128_1152  forget_9h_retake_x1dose
##  8   104   177 1152_1176  forget_9h_retake_x1dose
##  9   104   177 1176_1200  forget_9h_retake_x1dose
## 10   104   177 1200_1224  forget_9h_retake_x1dose
## # ℹ 325,220 more rows
```

Calculation of relative differences in AUC for each profile

```
AUC_basale_by_scenario <- auc_test_TEST_by_scenario %>%
  group_by(id, forget_event) %>%
  arrange(intervalle) %>%
  mutate(AUC_basale = first(auc)) %>%
  ungroup()

Relative_AUC_change_by_scenario <- AUC_basale_by_scenario %>%
  group_by(forget_event) %>%
  mutate(relative_AUC_by_scenario = (auc - AUC_basale) / AUC_basale * 100) %>%
  ungroup()

Relative_AUC_change_by_scenario
```

```
## # A tibble: 325,230 × 6
##       id   auc intervalle forget_event AUC_basale relative_AUC_by_scenario
##    <dbl> <dbl> <chr>      <chr>             <dbl>                    <dbl>
##  1   104   177 0_24       no_forget           177                        0
##  2   221   152 0_24       no_forget           152                        0
##  3   375   180 0_24       no_forget           180                        0
##  4   392   162 0_24       no_forget           162                        0
##  5   400   153 0_24       no_forget           153                        0
##  6   403   172 0_24       no_forget           172                        0
##  7   413   217 0_24       no_forget           217                        0
##  8   416   154 0_24       no_forget           154                        0
##  9   418   172 0_24       no_forget           172                        0
## 10   423   206 0_24       no_forget           206                        0
## # ℹ 325,220 more rows
```

#Renaming intervals

```
rename_interval <- function(intervalle) {
  start <- as.numeric(sub("_.*", "", intervalle))
  end <- as.numeric(sub(".*_", "", intervalle))

  if ((start >= 3432 && end <= 3768) || (start >= 3768 && end <= 4104) || (start >= 4104 && end <= 4440)) {
    interval_index <- ((start - 3432) %% 336) %/% 24
    new_start <- interval_index * 24
    new_end <- new_start + 24
  } else {
    interval_index <- ((start - 312) %% 312) %/% 24
    new_start <- interval_index * 24
    new_end <- new_start + 24
  }

  return(paste0(new_start, "_", new_end))
}

Renamed_AUC <- Relative_AUC_change_by_scenario %>%
  mutate(intervalle_renamed = sapply(intervalle, rename_interval))

print(Renamed_AUC)
```

```
## # A tibble: 325,230 × 7
##       id   auc intervalle forget_event AUC_basale relative_AUC_by_scenario
##    <dbl> <dbl> <chr>      <chr>             <dbl>                    <dbl>
##  1   104   177 0_24       no_forget           177                        0
##  2   221   152 0_24       no_forget           152                        0
##  3   375   180 0_24       no_forget           180                        0
##  4   392   162 0_24       no_forget           162                        0
##  5   400   153 0_24       no_forget           153                        0
##  6   403   172 0_24       no_forget           172                        0
##  7   413   217 0_24       no_forget           217                        0
##  8   416   154 0_24       no_forget           154                        0
##  9   418   172 0_24       no_forget           172                        0
## 10   423   206 0_24       no_forget           206                        0
## # ℹ 325,220 more rows
## # ℹ 1 more variable: intervalle_renamed <chr>
```

#AUC and RD average statistics: all profiles

```
summary_stats_auc <- Renamed_AUC %>%
  group_by(forget_event, intervalle_renamed) %>%
  summarize(
    min_auc = fivenum(auc)[1],        # Minimum
    Q1_auc = fivenum(auc)[2],         # First quartile (Q1)
    median_auc = fivenum(auc)[3],     # Median (Q2)
    Q3_auc = fivenum(auc)[4],         # Third quartile (Q3)
    max_auc = fivenum(auc)[5],        # Maximum
    mean_auc = mean(auc, na.rm = TRUE),  # Mean
    sd_auc = sd(auc, na.rm = TRUE),      # SD

    min_relative_auc = fivenum(relative_AUC_by_scenario)[1],        # Minimum
    Q1_relative_auc = fivenum(relative_AUC_by_scenario)[2],         # First quartile (Q1)
    median_relative_auc = fivenum(relative_AUC_by_scenario)[3],     # Median (Q2)
    Q3_relative_auc = fivenum(relative_AUC_by_scenario)[4],         # Third quartile (Q3)
    max_relative_auc = fivenum(relative_AUC_by_scenario)[5],        # Maximum
    mean_relative_auc = mean(relative_AUC_by_scenario, na.rm = TRUE),  # Mean
    sd_relative_auc = sd(relative_AUC_by_scenario, na.rm = TRUE)       # SD
  ) %>%
  ungroup() %>%
  arrange(intervalle_renamed)
```

```
## `summarise()` has grouped output by 'forget_event'. You can override using the
## `.groups` argument.
```

```
summary_stats_auc
```

```
## # A tibble: 185 × 16
##    forget_event      intervalle_renamed min_auc Q1_auc median_auc Q3_auc max_auc
##    <chr>             <chr>                <dbl>  <dbl>      <dbl>  <dbl>   <dbl>
##  1 forget_12h_retak… 0_24                   130    202        236    272     388
##  2 forget_15h_retak… 0_24                   130    202        236    272     388
##  3 forget_15h_retak… 0_24                   130    202        236    272     388
##  4 forget_18h_retak… 0_24                   130    202        236    273     388
##  5 forget_18h_retak… 0_24                   130    202        236    272     388
##  6 forget_21h_retak… 0_24                   130    202        236    273     388
##  7 forget_21h_retak… 0_24                   130    202        236    272     388
##  8 forget_24h_retak… 0_24                   130    202        236    272     388
##  9 forget_24h_retak… 0_24                   130    202        236    272     388
## 10 forget_24h_retak… 0_24                   130    202        236    272     388
## # ℹ 175 more rows
## # ℹ 9 more variables: mean_auc <dbl>, sd_auc <dbl>, min_relative_auc <dbl>,
## #   Q1_relative_auc <dbl>, median_relative_auc <dbl>, Q3_relative_auc <dbl>,
## #   max_relative_auc <dbl>, mean_relative_auc <dbl>, sd_relative_auc <dbl>
```

#AUC and RD histograms: all profiles

```
desired_order <- c("no_forget",
                   "forget_3h_retake_x1dose",
                   "forget_6h_retake_x1dose",
                   "forget_9h_retake_x1dose",
                   "forget_12h_retake_x1dose",
                   "forget_15h_retake_x0.5dose",
                   "forget_15h_retake_x1dose",
                   "forget_18h_retake_x0.5dose",
                   "forget_18h_retake_x1dose",
                   "forget_21h_retake_x0.5dose",
                   "forget_21h_retake_x1dose",
                   "forget_24h_retake_x1dose",
                   "forget_24h_retake_x1.5dose",
                   "forget_24h_retake_x2dose")
```

```r
summary_stats_auc <- summary_stats_auc %>%
  mutate(
    interval_start = as.numeric(str_extract(intervalle_renamed, "^[0-9]+"))
  )

summary_stats_auc <- summary_stats_auc %>%
  arrange(forget_event, interval_start)

summary_stats_auc$intervalle_renamed <- factor(summary_stats_auc$intervalle_renamed, levels = unique(summary_stat
s_auc$intervalle_renamed))

summary_stats_auc$forget_event <- factor(summary_stats_auc$forget_event, levels = desired_order)

forget_events_Moes <- levels(summary_stats_auc$forget_event)

for (event in forget_events_Moes) {
  data_subset_moes <- summary_stats_auc %>%
    dplyr::filter(forget_event == event)

  p3 <- ggplot(data_subset_moes, aes(x = intervalle_renamed, y = mean_auc)) +
    geom_bar(stat = "identity", fill = "grey", color = "black") +
    geom_text(aes(label = round(mean_auc, 1)), vjust = -0.5, color = "black") +
    labs(title = paste("Mean AUC each interval of time:", event),
         x = "Interval of time (h)", y = "Mean AUC (µg·h/L)") +
    theme_bw() +
    theme(plot.title = element_text(hjust = 0.5)) +
    ylim(0, 360) +
    theme(axis.text.x = element_text(angle = 45, hjust = 1))

  print(p3)


p4 <- ggplot(data_subset_moes, aes(x = intervalle_renamed, y = mean_relative_auc)) +
  geom_bar(stat = "identity", fill = "steelblue", color = "black") +
  geom_hline(yintercept = c(-15, 15), linetype = "dashed", color = "red") +
  geom_text(aes(label = paste0(round(mean_relative_auc, 1), "%")),
            vjust = ifelse(data_subset_moes$mean_relative_auc >= 0, -0.5, 1.5),
            color = "black",
            size = 4,
            fontface = "bold",
            nudge_x = 0.05,
            nudge_y = 0.2) +
  labs(
    title = paste0(
      "<span style='color: #0072B2;'>Mean relative difference of AUC each interval of time</span><br>",
      "<span style='color: orange;'>", event, "</span>"
    ),
    x = "Time interval (h)", y = "Mean RD of AUC"
  ) +
  theme_bw() +
  theme(
    plot.title = element_markdown(hjust = 0.5, face = "bold"),
    axis.text.x = element_text(angle = 45, hjust = 1, size = 12),
    axis.title = element_text(face = "bold"),
    panel.grid.major = element_line(size = 0.5, color = "grey90"),
    panel.grid.minor = element_blank(),
    axis.line = element_line(color = "black")
  ) +
  ylim(min(data_subset_moes$mean_relative_auc) - 10, max(data_subset_moes$mean_relative_auc) + 10)


print(p4)
}
```

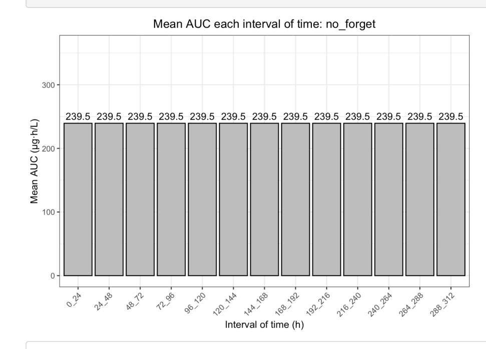


```
## Warning: Removed 2 rows containing missing values or values outside the scale range
## (`geom_hline()`).
```

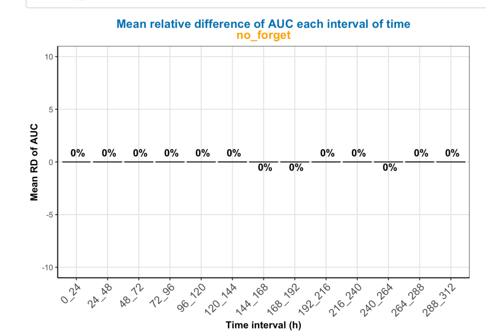

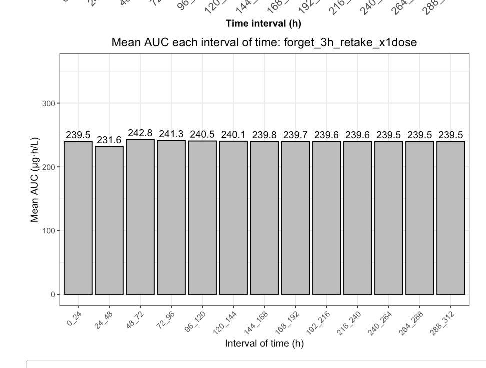


```
## Warning: Removed 2 rows containing missing values or values outside the scale range
## (`geom_hline()`).
```

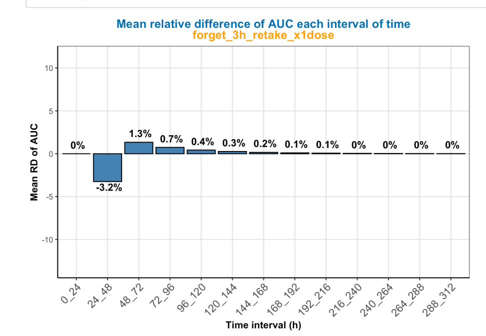

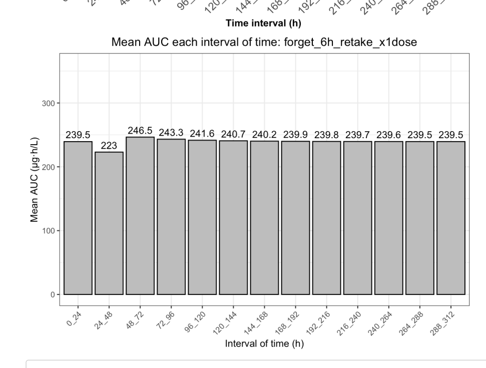


```
## Warning: Removed 1 row containing missing values or values outside the scale range
## (`geom_hline()`).
```

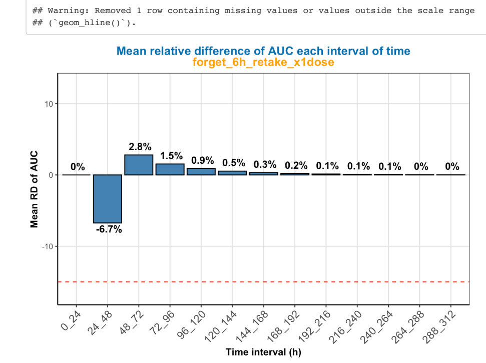

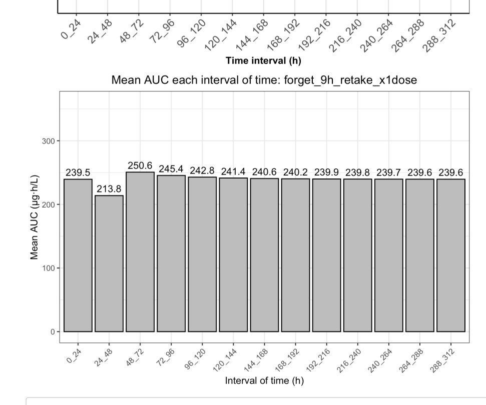


```
## Warning: Removed 1 row containing missing values or values outside the scale range
## (`geom_hline()`).
```

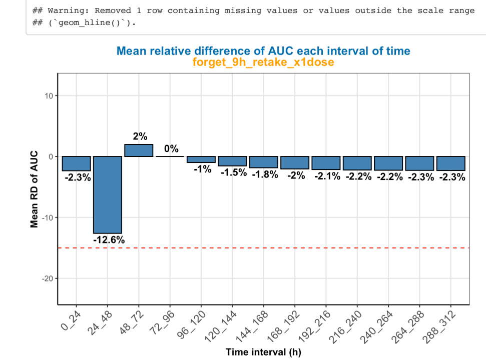

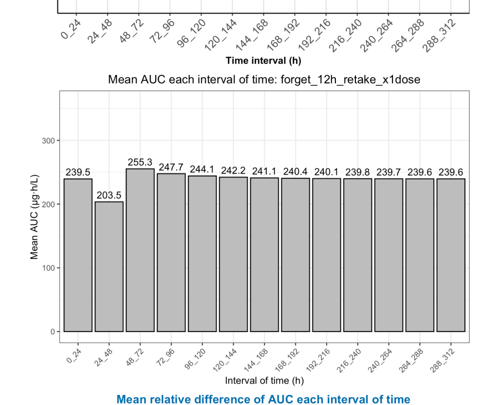

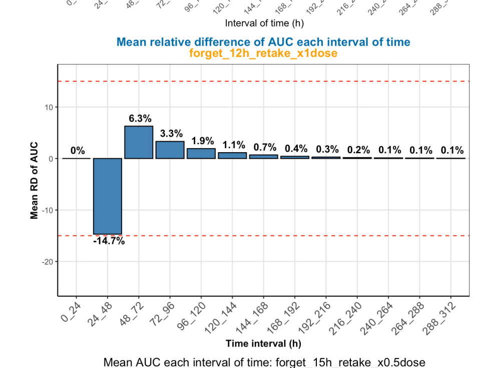

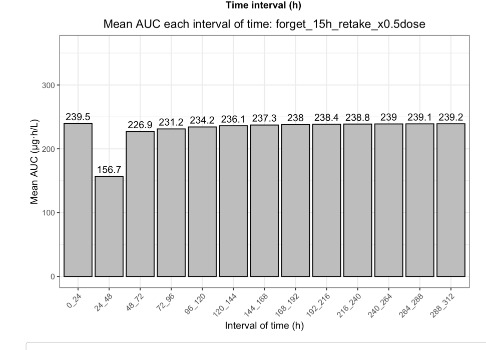


```
## Warning: Removed 1 row containing missing values or values outside the scale range
## (`geom_hline()`).
```

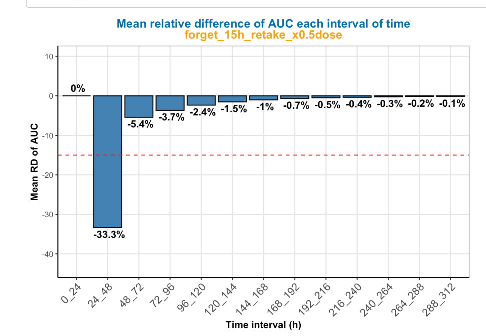

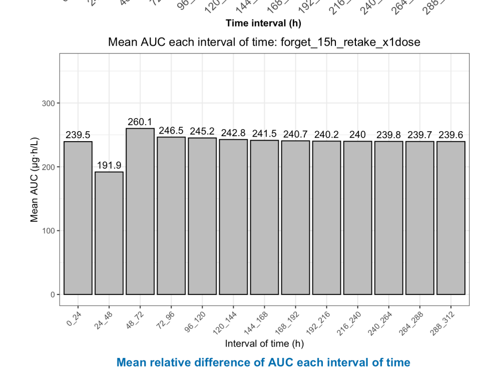

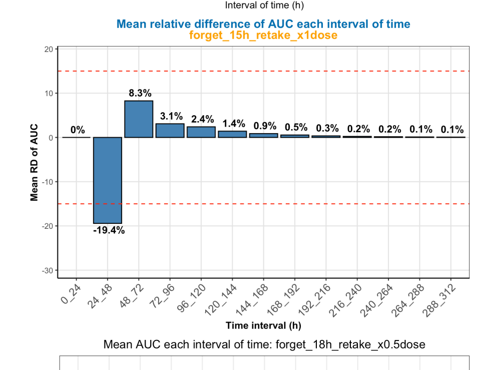

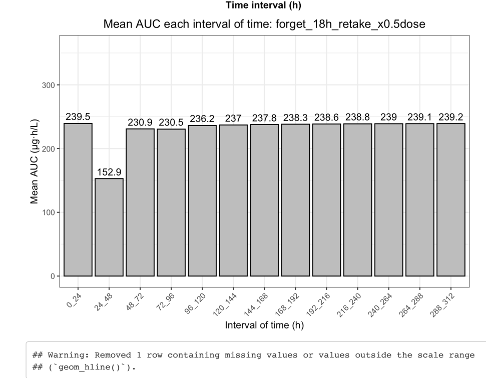


```
## Warning: Removed 1 row containing missing values or values outside the scale range
## (`geom_hline()`).
```

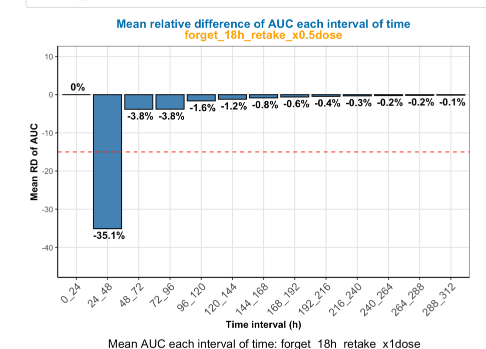

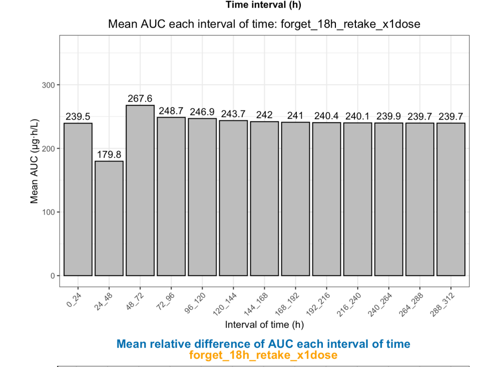

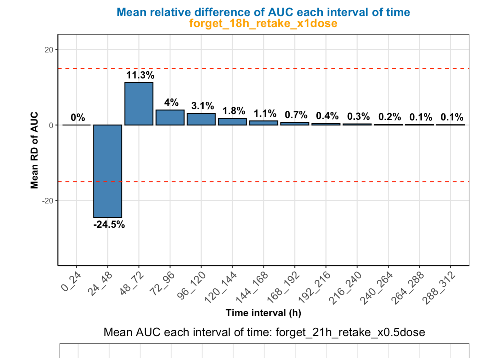

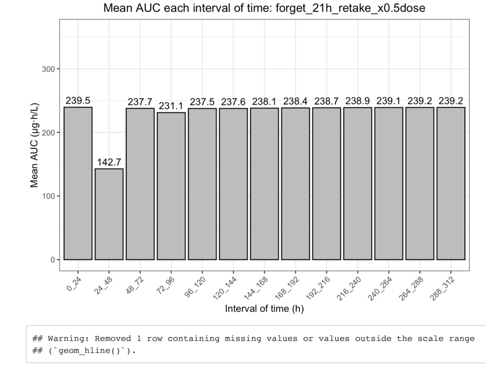


```
## Warning: Removed 1 row containing missing values or values outside the scale range
## (`geom_hline()`).
```

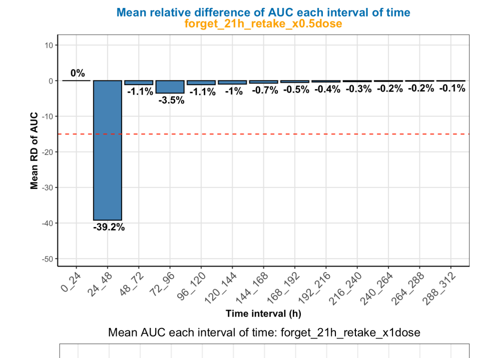

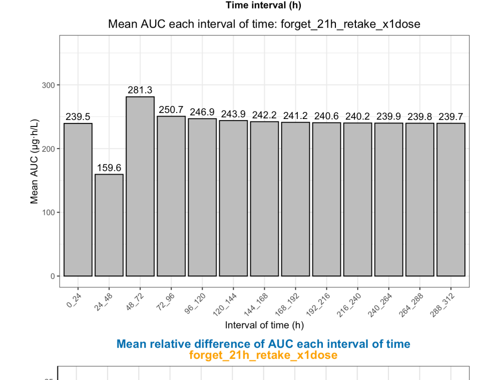

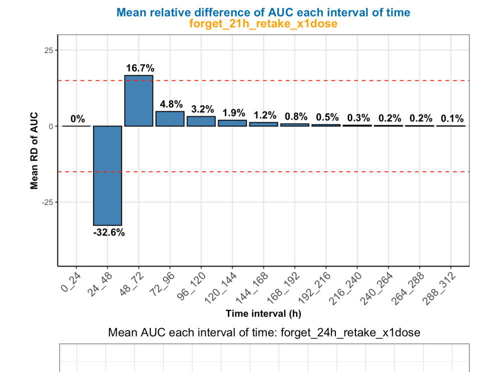

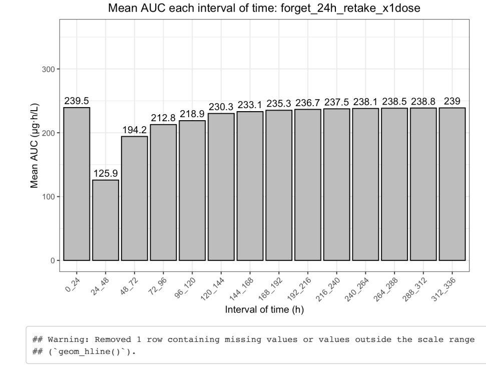


```
## Warning: Removed 1 row containing missing values or values outside the scale range
## (`geom_hline()`).
```

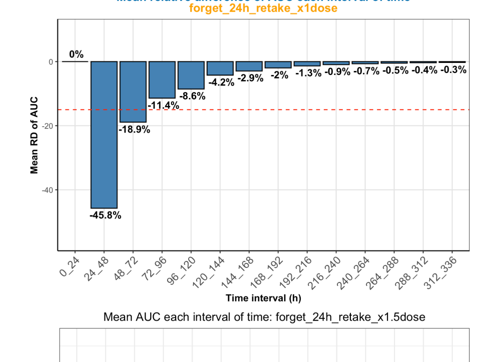

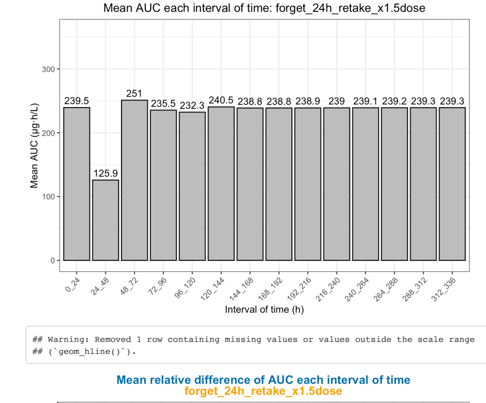


```
## Warning: Removed 1 row containing missing values or values outside the scale range
## (`geom_hline()`).
```

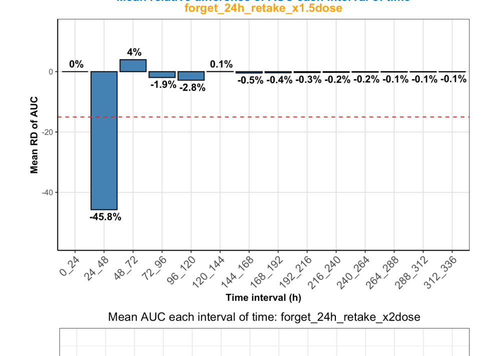

Mean AUC each interval of time: forget_24h_retake_x2dose

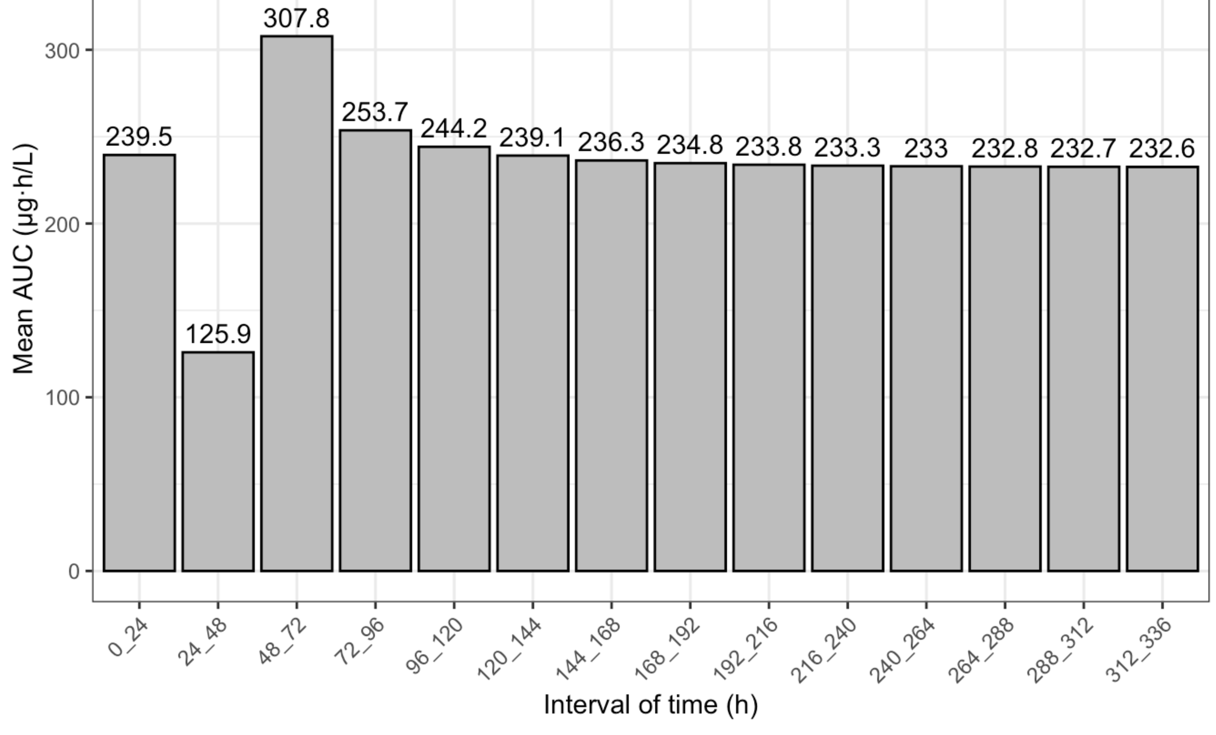

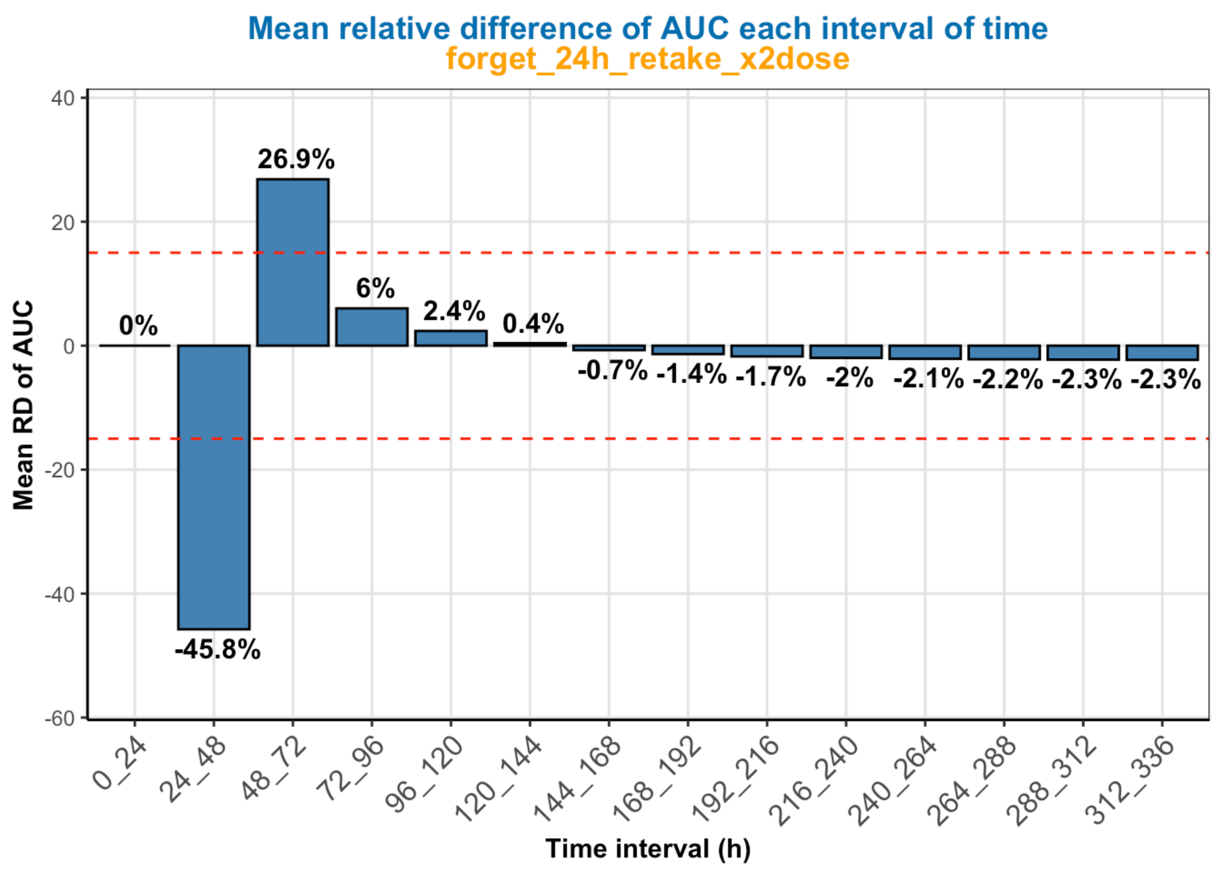

Add CYPCL column to Renamed_AUC

```
AUC_with_CYPCL <- Renamed_AUC %>%
  left_join(
    filtered_data_with_info %>% select(ID, CYPCL),
    by = c("id" = "ID")
  )

AUC_with_CYPCL
```

```
## # A tibble: 325,230 × 8
##       id   auc intervalle forget_event AUC_basale relative_AUC_by_scenario
##    <dbl> <dbl> <chr>      <chr>             <dbl>                    <dbl>
##  1   104   177 0_24       no_forget           177                        0
##  2   221   152 0_24       no_forget           152                        0
##  3   375   180 0_24       no_forget           180                        0
##  4   392   162 0_24       no_forget           162                        0
##  5   400   153 0_24       no_forget           153                        0
##  6   403   172 0_24       no_forget           172                        0
##  7   413   217 0_24       no_forget           217                        0
##  8   416   154 0_24       no_forget           154                        0
##  9   418   172 0_24       no_forget           172                        0
## 10   423   206 0_24       no_forget           206                        0
## # ℹ 325,220 more rows
## # ℹ 2 more variables: intervalle_renamed <chr>, CYPCL <dbl>
```

Stats AUC for patients with CYPCL = 0.00

```
summary_stats_AUC_CYPCL_0 <- AUC_with_CYPCL %>%
  dplyr::filter(CYPCL == 0.00) %>%
  group_by(forget_event, intervalle_renamed) %>%
    summarize(
    min_auc = fivenum(auc)[1],        # Minimum
    Q1_auc = fivenum(auc)[2],         # First quartile (Q1)
    median_auc = fivenum(auc)[3],     # Median (Q2)
    Q3_auc = fivenum(auc)[4],         # Third quartile (Q3)
    max_auc = fivenum(auc)[5],        # Maximum
    mean_auc = mean(auc, na.rm = TRUE),  # Mean
    sd_auc = sd(auc, na.rm = TRUE),      # SD

    min_relative_auc = fivenum(relative_AUC_by_scenario)[1],        # Minimum
    Q1_relative_auc = fivenum(relative_AUC_by_scenario)[2],         # First quartile (Q1)
    median_relative_auc = fivenum(relative_AUC_by_scenario)[3],     # Median (Q2)
    Q3_relative_auc = fivenum(relative_AUC_by_scenario)[4],         # Third quartile (Q3)
    max_relative_auc = fivenum(relative_AUC_by_scenario)[5],        # Maximum
    mean_relative_auc = mean(relative_AUC_by_scenario, na.rm = TRUE),  # Mean
    sd_relative_auc = sd(relative_AUC_by_scenario, na.rm = TRUE)       # SD
  ) %>%
  ungroup() %>%
  arrange(forget_event, intervalle_renamed)
```

```
## `summarise()` has grouped output by 'forget_event'. You can override using the
## `.groups` argument.
```

```
summary_stats_AUC_CYPCL_0
```

```
## # A tibble: 185 × 16
##    forget_event      intervalle_renamed min_auc Q1_auc median_auc Q3_auc max_auc
##    <chr>             <chr>                <dbl>  <dbl>      <dbl>  <dbl>   <dbl>
##  1 forget_12h_retak… 0_24                   140    195        225   262.     378
##  2 forget_12h_retak… 120_144                140    197        228   265      381
##  3 forget_12h_retak… 144_168                140    196        227   264.     380
##  4 forget_12h_retak… 168_192                140    196        227   262.     379
##  5 forget_12h_retak… 192_216                140    196        226   262      379
##  6 forget_12h_retak… 216_240                140    195        226   262      378
##  7 forget_12h_retak… 240_264                140    195        226   262      378
##  8 forget_12h_retak… 24_48                  129    170.       194   220      317
##  9 forget_12h_retak… 264_288                140    195        226.  262.     378
## 10 forget_12h_retak… 288_312                140    195        226.  262.     378
## # ℹ 175 more rows
## # ℹ 9 more variables: mean_auc <dbl>, sd_auc <dbl>, min_relative_auc <dbl>,
## #   Q1_relative_auc <dbl>, median_relative_auc <dbl>, Q3_relative_auc <dbl>,
## #   max_relative_auc <dbl>, mean_relative_auc <dbl>, sd_relative_auc <dbl>
```

Stats AUC for patients with CYPCL = 0,33

```
summary_stats_AUC_CYPCL_33 <- AUC_with_CYPCL %>%
  dplyr::filter(CYPCL == 0.33) %>%
  group_by(forget_event, intervalle_renamed) %>%
    summarize(
    min_auc = fivenum(auc)[1],        # Minimum
    Q1_auc = fivenum(auc)[2],         # First quartile (Q1)
    median_auc = fivenum(auc)[3],     # Median (Q2)
    Q3_auc = fivenum(auc)[4],         # Third quartile (Q3)
    max_auc = fivenum(auc)[5],        # Maximum
    mean_auc = mean(auc, na.rm = TRUE),  # Mean
    sd_auc = sd(auc, na.rm = TRUE),      # SD

    min_relative_auc = fivenum(relative_AUC_by_scenario)[1],        # Minimum
    Q1_relative_auc = fivenum(relative_AUC_by_scenario)[2],         # First quartile (Q1)
    median_relative_auc = fivenum(relative_AUC_by_scenario)[3],     # Median (Q2)
    Q3_relative_auc = fivenum(relative_AUC_by_scenario)[4],         # Third quartile (Q3)
    max_relative_auc = fivenum(relative_AUC_by_scenario)[5],        # Maximum
    mean_relative_auc = mean(relative_AUC_by_scenario, na.rm = TRUE),  # Mean
    sd_relative_auc = sd(relative_AUC_by_scenario, na.rm = TRUE)       # SD
  ) %>%
  ungroup() %>%
  arrange(forget_event, intervalle_renamed)
```

```
## `summarise()` has grouped output by 'forget_event'. You can override using the
## `.groups` argument.
```

```
summary_stats_AUC_CYPCL_33
```

```
## # A tibble: 185 × 16
##    forget_event      intervalle_renamed min_auc Q1_auc median_auc Q3_auc max_auc
##    <chr>             <chr>                <dbl>  <dbl>      <dbl>  <dbl>   <dbl>
##  1 forget_12h_retak… 0_24                   130    209       244.    280     388
##  2 forget_12h_retak… 120_144                131    212       248.    283     391
##  3 forget_12h_retak… 144_168                131    211       246.    282     389
##  4 forget_12h_retak… 168_192                131    210       246     281     389
##  5 forget_12h_retak… 192_216                130    210       246.    281     388
##  6 forget_12h_retak… 216_240                130    210       246.    280     388
##  7 forget_12h_retak… 240_264                130    210       244.    280     388
##  8 forget_12h_retak… 24_48                  123    181       208     235     324
##  9 forget_12h_retak… 264_288                130    210       244.    280     388
## 10 forget_12h_retak… 288_312                130    209       244.    280     388
## # ℹ 175 more rows
## # ℹ 9 more variables: mean_auc <dbl>, sd_auc <dbl>, min_relative_auc <dbl>,
## #   Q1_relative_auc <dbl>, median_relative_auc <dbl>, Q3_relative_auc <dbl>,
## #   max_relative_auc <dbl>, mean_relative_auc <dbl>, sd_relative_auc <dbl>
```

Stats AUC for patients with CYPCL = 0,71

```
summary_stats_AUC_CYPCL_71 <- AUC_with_CYPCL %>%
  dplyr::filter(CYPCL == 0.71) %>%
  group_by(forget_event, intervalle_renamed) %>%
    summarize(
    min_auc = fivenum(auc)[1],        # Minimum
    Q1_auc = fivenum(auc)[2],         # First quartile (Q1)
    median_auc = fivenum(auc)[3],     # Median (Q2)
    Q3_auc = fivenum(auc)[4],         # Third quartile (Q3)
    max_auc = fivenum(auc)[5],        # Maximum
    mean_auc = mean(auc, na.rm = TRUE),  # Mean
    sd_auc = sd(auc, na.rm = TRUE),      # SD

    min_relative_auc = fivenum(relative_AUC_by_scenario)[1],        # Minimum
    Q1_relative_auc = fivenum(relative_AUC_by_scenario)[2],         # First quartile (Q1)
    median_relative_auc = fivenum(relative_AUC_by_scenario)[3],     # Median (Q2)
    Q3_relative_auc = fivenum(relative_AUC_by_scenario)[4],         # Third quartile (Q3)
    max_relative_auc = fivenum(relative_AUC_by_scenario)[5],        # Maximum
    mean_relative_auc = mean(relative_AUC_by_scenario, na.rm = TRUE),  # Mean
    sd_relative_auc = sd(relative_AUC_by_scenario, na.rm = TRUE)       # SD
  ) %>%
  ungroup() %>%
  arrange(forget_event, intervalle_renamed)
```

```
## `summarise()` has grouped output by 'forget_event'. You can override using the
## `.groups` argument.
```

```
summary_stats_AUC_CYPCL_71
```

```
## # A tibble: 185 × 16
##    forget_event      intervalle_renamed min_auc Q1_auc median_auc Q3_auc max_auc
##    <chr>             <chr>                <dbl>  <dbl>      <dbl>  <dbl>   <dbl>
##  1 forget_12h_retak… 0_24                   148   221         257   288.     378
##  2 forget_12h_retak… 120_144                150   224.        260   291      381
##  3 forget_12h_retak… 144_168                150   222.        258   289      379
##  4 forget_12h_retak… 168_192                149   222.        258.  288.     378
##  5 forget_12h_retak… 192_216                149   222.        257   288      378
##  6 forget_12h_retak… 216_240                149   222.        257   288.     378
##  7 forget_12h_retak… 240_264                149   222.        257   288.     378
##  8 forget_12h_retak… 24_48                  133   188.        214   240.     315
##  9 forget_12h_retak… 264_288                149   222.        257   288.     377
## 10 forget_12h_retak… 288_312                149   222.        257   288.     378
## # ℹ 175 more rows
## # ℹ 9 more variables: mean_auc <dbl>, sd_auc <dbl>, min_relative_auc <dbl>,
## #   Q1_relative_auc <dbl>, median_relative_auc <dbl>, Q3_relative_auc <dbl>,
## #   max_relative_auc <dbl>, mean_relative_auc <dbl>, sd_relative_auc <dbl>
```
