# Supplementary material for: Managing Delayed or Missed Doses of Prolonged‐Release Tacrolimus in Transplant Recipients: Implications for Drug Exposure and Recovery Strategies
Source: Basic Clin Pharmacol Toxicol. 2025 Dec 4;138(1):e70157. doi: 10.1111/bcpt.70157 (PMC12676261; doi:10.1111/bcpt.70157)

Simulations of Martial model

Arraki Zava Selim

2025-05-06

##Loading of the packages

```
library(mrgsolve)
```

```
##
## Attaching package: 'mrgsolve'
```

```
## The following object is masked from 'package:stats':
##
##     filter
```

```
library(stats)
library(tidyverse)
```

```
## ── Attaching core tidyverse packages ──────────────────────── tidyverse 2.0.0 ──
## ✔ dplyr     1.1.4     ✔ readr     2.1.5
## ✔ forcats   1.0.0     ✔ stringr   1.5.1
## ✔ ggplot2   3.5.1     ✔ tibble    3.2.1
## ✔ lubridate 1.9.4     ✔ tidyr     1.3.1
## ✔ purrr     1.0.2
```

```
## ── Conflicts ────────────────────────────────────────── tidyverse_conflicts() ──
## ✖ dplyr::filter() masks mrgsolve::filter(), stats::filter()
## ✖ dplyr::lag()    masks stats::lag()
## ℹ Use the conflicted package (<http://conflicted.r-lib.org/>) to force all conflicts to become errors
```

```
library(truncnorm)
library(ggplot2)
library(conflicted)
library(PKNCA)
library(Pmetrics)
```

```
##
## CRITICAL: Execute PMbuild() in R to complete Pmetrics installation.
```

```
library(skimr)
library(openxlsx)
library(stats)
library(tidyr)
library(pander)
library(DT)
library(purrr)
library(gridExtra)
library(patchwork)
library(stringr)
library(ggtext)
```

##Population pharmacokinetics and genetics of oral meltdose tacrolimus (Envarsus) in stable adult liver transplant recipients, Martial, 2021

```
code <-
"
[SET] end=100, delta=0.1

[PARAM] @annotated

TVCL  : 3.27 : Typical value of clearance (L/h)
TVF   : 0.23 : bioavailability
Ntrans: 1.58 : number of transit compartment
MTT   : 3.39 : mean transit time (h)
TVVC  : 94.9 : Typical value of central volume of distribution (L)
TVQ   : 9.62 : Typical value of intercomp clearance 1 (L/h)
VP    : 500  : Typical value of peripheral volume of distribution 1 (L)
TVKA  : 2.97 : Typical value of absorption rate constant (1/h)


[OMEGA] @annotated
ETACL  : 0.1156 : ETA on clearance
ETAVC  : 1.9881 : ETA on VC
ETAQ   : 0.0576 : ETA on Q
ETAKA  : 3.0276 : ETA on KA
ETAF   : 0.1296 : ETA on bioavailability


[CMT] @annotated
GUT : estomac [ADM]
TRANS : compartments of transit
CENT  : Central compartment [OBS]
PERIPH : peripheral compartment (μg)


[SIGMA]
0.000001 // proportionnal Residual unexplained variability (0.011025)


[MAIN]
double KA    = TVKA * exp(ETAKA);
double F     = TVF * exp(ETAF);
double Q     = TVQ * exp(ETAQ);
double VC    = TVVC * exp (ETAVC);
double CL    = TVCL * exp(ETACL);
double KTR   = (Ntrans + 1)/MTT ;
F_GUT = F;


[ODE]
dxdt_GUT    = -KA*GUT;
dxdt_TRANS  = KA*GUT - KTR*TRANS;
dxdt_CENT   = -CL*CENT/VC -Q*CENT/VC + Q*PERIPH/VP + KTR*TRANS;
dxdt_PERIPH = Q*CENT/VC - Q*PERIPH/VP;

[TABLE]
double DV = ((CENT/VC)*1000) * (1 + EPS(1)) + EPS(2);
int i = 0;
while(DV<0 && i <100) {
simeps();
DV = ((CENT/VC)*1000) * (1 + EPS(1)) + EPS(2);
++i;

}


[CAPTURE] DV CL VC VP Q
"

my_model_envarsus <- mcode("Envarsus_Martial", code)
```

```
## Building Envarsus_Martial ... done.
```

Simulations 6000: simulation with different dose each 400 profiles (including CYP3A5 status variations)

```
# Scenarios simulation
Simulation_data_martial_6000 <- as_tibble( seq(
  # Steady-state simulation
ev(ID = 1:400, amt = 1*1, ii= 24, ss= 1, addl=0),
ev(ID = 1:400, amt = 1*1, ii= 24, ss= 0, addl=0),
ev(ID = 1:400, amt = 1*1, ii= 24, ss= 0, addl=10),
#3h delay simulation
ev(ID = 1:400, amt = 1*1, ii= 24, ss= 1, addl=0),
ev(ID = 1:400, amt = 1*0, ii= 3, ss= 0, addl=0),
ev(ID = 1:400, amt = 1*1, ii= 21, ss= 0, addl=0),
ev(ID = 1:400, amt = 1*1, ii= 24, ss= 0, addl=10),
#6h delay simulation
ev(ID = 1:400, amt = 1*1, ii= 24, ss= 1, addl=0),
ev(ID = 1:400, amt = 1*0, ii= 6, ss= 0, addl=0),
ev(ID = 1:400, amt = 1*1, ii= 18, ss= 0, addl=0),
ev(ID = 1:400, amt = 1*1, ii= 24, ss= 0, addl=10),
#9h delay simulation
ev(ID = 1:400, amt = 1*1, ii= 24, ss= 1, addl=0),
ev(ID = 1:400, amt = 1*0, ii= 9, ss= 0, addl=0),
ev(ID = 1:400, amt = 1*1, ii= 15, ss= 0, addl=0),
ev(ID = 1:400, amt = 1*1, ii= 24, ss= 0, addl=10),
#12h delay simulation
ev(ID = 1:400, amt = 1*1, ii= 24, ss= 1, addl=0),
ev(ID = 1:400, amt = 1*0, ii= 12, ss= 0, addl=0),
ev(ID = 1:400, amt = 1*1, ii= 12, ss= 0, addl=0),
ev(ID = 1:400, amt = 1*1, ii= 24, ss= 0, addl=10),
#15h delay + 50% dose intake simulation
ev(ID = 1:400, amt = 1*1, ii= 24, ss= 1, addl=0),
ev(ID = 1:400, amt = 1*0, ii= 15, ss= 0, addl=0),
ev(ID = 1:400, amt = 1*0.5, ii= 9, ss= 0, addl=0),
ev(ID = 1:400, amt = 1*1, ii= 24, ss= 0, addl=10),
#15h delay simulation
ev(ID = 1:400, amt = 1*1, ii= 24, ss= 1, addl=0),
ev(ID = 1:400, amt = 1*0, ii= 15, ss= 0, addl=0),
ev(ID = 1:400, amt = 1*1, ii= 9, ss= 0, addl=0),
ev(ID = 1:400, amt = 1*1, ii= 24, ss= 0, addl=10),
#18h delay + 50% dose intake simulation
ev(ID = 1:400, amt = 1*1, ii= 24, ss= 1, addl=0),
ev(ID = 1:400, amt = 1*0, ii= 18, ss= 0, addl=0),
ev(ID = 1:400, amt = 1*0.5, ii= 6, ss= 0, addl=0),
ev(ID = 1:400, amt = 1*1, ii= 24, ss= 0, addl=10),
#18h delay simulation
ev(ID = 1:400, amt = 1*1, ii= 24, ss= 1, addl=0),
ev(ID = 1:400, amt = 1*0, ii= 18, ss= 0, addl=0),
ev(ID = 1:400, amt = 1*1, ii= 6, ss= 0, addl=0),
ev(ID = 1:400, amt = 1*1, ii= 24, ss= 0, addl=10),
#21h delay + 50% dose intake simulation
ev(ID = 1:400, amt = 1*1, ii= 24, ss= 1, addl=0),
ev(ID = 1:400, amt = 1*0, ii= 21, ss= 0, addl=0),
ev(ID = 1:400, amt = 1*0.5, ii= 3, ss= 0, addl=0),
ev(ID = 1:400, amt = 1*1, ii= 24, ss= 0, addl=10),
#21h delay simulation
ev(ID = 1:400, amt = 1*1, ii= 24, ss= 1, addl=0),
ev(ID = 1:400, amt = 1*0, ii= 21, ss= 0, addl=0),
ev(ID = 1:400, amt = 1*1, ii= 3, ss= 0, addl=0),
ev(ID = 1:400, amt = 1*1, ii= 24, ss= 0, addl=10),
#Missed dose simulation
ev(ID = 1:400, amt = 1*1, ii= 24, ss= 1, addl=0),
ev(ID = 1:400, amt = 1*0, ii= 24, ss= 0, addl=0),
ev(ID = 1:400, amt = 1*1, ii= 24, ss= 0, addl=0),
ev(ID = 1:400, amt = 1*1, ii= 24, ss= 0, addl=10),
#Missed dose + 150% dose intake simulation
ev(ID = 1:400, amt = 1*1, ii= 24, ss= 1, addl=0),
ev(ID = 1:400, amt = 1*0, ii= 24, ss= 0, addl=0),
ev(ID = 1:400, amt = 1*1.5, ii= 24, ss= 0, addl=0),
ev(ID = 1:400, amt = 1*1, ii= 24, ss= 0, addl=10),
#Missed dose + 200% dose intake simulation
ev(ID = 1:400, amt = 1*1, ii= 24, ss= 1, addl=0),
ev(ID = 1:400, amt = 1*0, ii= 24, ss= 0, addl=0),
ev(ID = 1:400, amt = 1*2, ii= 24, ss= 0, addl=0),
ev(ID = 1:400, amt = 1*1, ii= 24, ss= 0, addl=10))) %>%
             mutate(dose_group = "1 mg") %>%

  bind_rows(as_tibble( seq(
  #Steady-state simulation
ev(ID = 401:800, amt = 2*1, ii= 24, ss= 1, addl=0),
ev(ID = 401:800, amt = 2*1, ii= 24, ss= 0, addl=0),
ev(ID = 401:800, amt = 2*1, ii= 24, ss= 0, addl=10),
#3h delay simulation
ev(ID = 401:800, amt = 2*1, ii= 24, ss= 1, addl=0),
ev(ID = 401:800, amt = 2*0, ii= 3, ss= 0, addl=0),
ev(ID = 401:800, amt = 2*1, ii= 21, ss= 0, addl=0),
ev(ID = 401:800, amt = 2*1, ii= 24, ss= 0, addl=10),
#6h delay simulation
ev(ID = 401:800, amt = 2*1, ii= 24, ss= 1, addl=0),
ev(ID = 401:800, amt = 2*0, ii= 6, ss= 0, addl=0),
ev(ID = 401:800, amt = 2*1, ii= 18, ss= 0, addl=0),
ev(ID = 401:800, amt = 2*1, ii= 24, ss= 0, addl=10),
#9h delay simulation
ev(ID = 401:800, amt = 2*1, ii= 24, ss= 1, addl=0),
ev(ID = 401:800, amt = 2*0, ii= 9, ss= 0, addl=0),
ev(ID = 401:800, amt = 2*1, ii= 15, ss= 0, addl=0),
ev(ID = 401:800, amt = 2*1, ii= 24, ss= 0, addl=10),
#12h delay simulation
ev(ID = 401:800, amt = 2*1, ii= 24, ss= 1, addl=0),
ev(ID = 401:800, amt = 2*0, ii= 12, ss= 0, addl=0),
ev(ID = 401:800, amt = 2*1, ii= 12, ss= 0, addl=0),
ev(ID = 401:800, amt = 2*1, ii= 24, ss= 0, addl=10),
#15h delay + 50% dose intake simulation
ev(ID = 401:800, amt = 2*1, ii= 24, ss= 1, addl=0),
ev(ID = 401:800, amt = 2*0, ii= 15, ss= 0, addl=0),
ev(ID = 401:800, amt = 2*0.5, ii= 9, ss= 0, addl=0),
ev(ID = 401:800, amt = 2*1, ii= 24, ss= 0, addl=10),
#15h delay simulation
ev(ID = 401:800, amt = 2*1, ii= 24, ss= 1, addl=0),
ev(ID = 401:800, amt = 2*0, ii= 15, ss= 0, addl=0),
ev(ID = 401:800, amt = 2*1, ii= 9, ss= 0, addl=0),
ev(ID = 401:800, amt = 2*1, ii= 24, ss= 0, addl=10),
#18h delay + 50% dose intake simulation
ev(ID = 401:800, amt = 2*1, ii= 24, ss= 1, addl=0),
ev(ID = 401:800, amt = 2*0, ii= 18, ss= 0, addl=0),
ev(ID = 401:800, amt = 2*0.5, ii= 6, ss= 0, addl=0),
ev(ID = 401:800, amt = 2*1, ii= 24, ss= 0, addl=10),
#18h delay simulation
ev(ID = 401:800, amt = 2*1, ii= 24, ss= 1, addl=0),
ev(ID = 401:800, amt = 2*0, ii= 18, ss= 0, addl=0),
ev(ID = 401:800, amt = 2*1, ii= 6, ss= 0, addl=0),
ev(ID = 401:800, amt = 2*1, ii= 24, ss= 0, addl=10),
#21h delay + 50% dose intake simulation
ev(ID = 401:800, amt = 2*1, ii= 24, ss= 1, addl=0),
ev(ID = 401:800, amt = 2*0, ii= 21, ss= 0, addl=0),
ev(ID = 401:800, amt = 2*0.5, ii= 3, ss= 0, addl=0),
ev(ID = 401:800, amt = 2*1, ii= 24, ss= 0, addl=10),
#21h delay simulation
ev(ID = 401:800, amt = 2*1, ii= 24, ss= 1, addl=0),
ev(ID = 401:800, amt = 2*0, ii= 21, ss= 0, addl=0),
ev(ID = 401:800, amt = 2*1, ii= 3, ss= 0, addl=0),
ev(ID = 401:800, amt = 2*1, ii= 24, ss= 0, addl=10),
#Missed dose simulation
ev(ID = 401:800, amt = 2*1, ii= 24, ss= 1, addl=0),
ev(ID = 401:800, amt = 2*0, ii= 24, ss= 0, addl=0),
ev(ID = 401:800, amt = 2*1, ii= 24, ss= 0, addl=0),
ev(ID = 401:800, amt = 2*1, ii= 24, ss= 0, addl=10),
#Missed dose + 150% dose intake simulation
ev(ID = 401:800, amt = 2*1, ii= 24, ss= 1, addl=0),
ev(ID = 401:800, amt = 2*0, ii= 24, ss= 0, addl=0),
ev(ID = 401:800, amt = 2*1.5, ii= 24, ss= 0, addl=0),
ev(ID = 401:800, amt = 2*1, ii= 24, ss= 0, addl=10),
#Missed dose + 200% dose intake simulation
ev(ID = 401:800, amt = 2*1, ii= 24, ss= 1, addl=0),
ev(ID = 401:800, amt = 2*0, ii= 24, ss= 0, addl=0),
ev(ID = 401:800, amt = 2*2, ii= 24, ss= 0, addl=0),
ev(ID = 401:800, amt = 2*1, ii= 24, ss= 0, addl=10))))%>%
             mutate(dose_group = "2 mg") %>%

  bind_rows(as_tibble( seq(
  #Steady-state simulation
ev(ID = 801:1200, amt = 3*1, ii= 24, ss= 1, addl=0),
ev(ID = 801:1200, amt = 3*1, ii= 24, ss= 0, addl=0),
ev(ID = 801:1200, amt = 3*1, ii= 24, ss= 0, addl=10),
#3h delay simulation
ev(ID = 801:1200, amt = 3*1, ii= 24, ss= 1, addl=0),
ev(ID = 801:1200, amt = 3*0, ii= 3, ss= 0, addl=0),
ev(ID = 801:1200, amt = 3*1, ii= 21, ss= 0, addl=0),
ev(ID = 801:1200, amt = 3*1, ii= 24, ss= 0, addl=10),
#6h delay simulation
ev(ID = 801:1200, amt = 3*1, ii= 24, ss= 1, addl=0),
ev(ID = 801:1200, amt = 3*0, ii= 6, ss= 0, addl=0),
ev(ID = 801:1200, amt = 3*1, ii= 18, ss= 0, addl=0),
ev(ID = 801:1200, amt = 3*1, ii= 24, ss= 0, addl=10),
#9h delay simulation
ev(ID = 801:1200, amt = 3*1, ii= 24, ss= 1, addl=0),
ev(ID = 801:1200, amt = 3*0, ii= 9, ss= 0, addl=0),
ev(ID = 801:1200, amt = 3*1, ii= 15, ss= 0, addl=0),
ev(ID = 801:1200, amt = 3*1, ii= 24, ss= 0, addl=10),
#12h delay simulation
ev(ID = 801:1200, amt = 3*1, ii= 24, ss= 1, addl=0),
ev(ID = 801:1200, amt = 3*0, ii= 12, ss= 0, addl=0),
ev(ID = 801:1200, amt = 3*1, ii= 12, ss= 0, addl=0),
ev(ID = 801:1200, amt = 3*1, ii= 24, ss= 0, addl=10),
#15h delay + 50% dose intake simulation
ev(ID = 801:1200, amt = 3*1, ii= 24, ss= 1, addl=0),
ev(ID = 801:1200, amt = 3*0, ii= 15, ss= 0, addl=0),
ev(ID = 801:1200, amt = 3*0.5, ii= 9, ss= 0, addl=0),
ev(ID = 801:1200, amt = 3*1, ii= 24, ss= 0, addl=10),
#15h delay simulation
ev(ID = 801:1200, amt = 3*1, ii= 24, ss= 1, addl=0),
ev(ID = 801:1200, amt = 3*0, ii= 15, ss= 0, addl=0),
ev(ID = 801:1200, amt = 3*1, ii= 9, ss= 0, addl=0),
ev(ID = 801:1200, amt = 3*1, ii= 24, ss= 0, addl=10),
#18h delay + 50% dose intake simulation
ev(ID = 801:1200, amt = 3*1, ii= 24, ss= 1, addl=0),
ev(ID = 801:1200, amt = 3*0, ii= 18, ss= 0, addl=0),
ev(ID = 801:1200, amt = 3*0.5, ii= 6, ss= 0, addl=0),
ev(ID = 801:1200, amt = 3*1, ii= 24, ss= 0, addl=10),
#18h delay simulation
ev(ID = 801:1200, amt = 3*1, ii= 24, ss= 1, addl=0),
ev(ID = 801:1200, amt = 3*0, ii= 18, ss= 0, addl=0),
ev(ID = 801:1200, amt = 3*1, ii= 6, ss= 0, addl=0),
ev(ID = 801:1200, amt = 3*1, ii= 24, ss= 0, addl=10),
#21h delay + 50% dose intake simulation
ev(ID = 801:1200, amt = 3*1, ii= 24, ss= 1, addl=0),
ev(ID = 801:1200, amt = 3*0, ii= 21, ss= 0, addl=0),
ev(ID = 801:1200, amt = 3*0.5, ii= 3, ss= 0, addl=0),
ev(ID = 801:1200, amt = 3*1, ii= 24, ss= 0, addl=10),
#21h delay simulation
ev(ID = 801:1200, amt = 3*1, ii= 24, ss= 1, addl=0),
ev(ID = 801:1200, amt = 3*0, ii= 21, ss= 0, addl=0),
ev(ID = 801:1200, amt = 3*1, ii= 3, ss= 0, addl=0),
ev(ID = 801:1200, amt = 3*1, ii= 24, ss= 0, addl=10),
#Missed dose simulation
ev(ID = 801:1200, amt = 3*1, ii= 24, ss= 1, addl=0),
ev(ID = 801:1200, amt = 3*0, ii= 24, ss= 0, addl=0),
ev(ID = 801:1200, amt = 3*1, ii= 24, ss= 0, addl=0),
ev(ID = 801:1200, amt = 3*1, ii= 24, ss= 0, addl=10),
#Missed dose + 150% dose intake simulation
ev(ID = 801:1200, amt = 3*1, ii= 24, ss= 1, addl=0),
ev(ID = 801:1200, amt = 3*0, ii= 24, ss= 0, addl=0),
ev(ID = 801:1200, amt = 3*1.5, ii= 24, ss= 0, addl=0),
ev(ID = 801:1200, amt = 3*1, ii= 24, ss= 0, addl=10),
#Missed dose + 200% dose intake simulation
ev(ID = 801:1200, amt = 3*1, ii= 24, ss= 1, addl=0),
ev(ID = 801:1200, amt = 3*0, ii= 24, ss= 0, addl=0),
ev(ID = 801:1200, amt = 3*2, ii= 24, ss= 0, addl=0),
ev(ID = 801:1200, amt = 3*1, ii= 24, ss= 0, addl=10))))%>%
             mutate(dose_group = "3 mg") %>%

    bind_rows(as_tibble( seq(
  #Steady-state simulation
ev(ID = 1201:1600, amt = 4*1, ii= 24, ss= 1, addl=0),
ev(ID = 1201:1600, amt = 4*1, ii= 24, ss= 0, addl=0),
ev(ID = 1201:1600, amt = 4*1, ii= 24, ss= 0, addl=10),
#3h delay simulation
ev(ID = 1201:1600, amt = 4*1, ii= 24, ss= 1, addl=0),
ev(ID = 1201:1600, amt = 4*0, ii= 3, ss= 0, addl=0),
ev(ID = 1201:1600, amt = 4*1, ii= 21, ss= 0, addl=0),
ev(ID = 1201:1600, amt = 4*1, ii= 24, ss= 0, addl=10),
#6h delay simulation
ev(ID = 1201:1600, amt = 4*1, ii= 24, ss= 1, addl=0),
ev(ID = 1201:1600, amt = 4*0, ii= 6, ss= 0, addl=0),
ev(ID = 1201:1600, amt = 4*1, ii= 18, ss= 0, addl=0),
ev(ID = 1201:1600, amt = 4*1, ii= 24, ss= 0, addl=10),
#9h delay simulation
ev(ID = 1201:1600, amt = 4*1, ii= 24, ss= 1, addl=0),
ev(ID = 1201:1600, amt = 4*0, ii= 9, ss= 0, addl=0),
ev(ID = 1201:1600, amt = 4*1, ii= 15, ss= 0, addl=0),
ev(ID = 1201:1600, amt = 4*1, ii= 24, ss= 0, addl=10),
#12h delay simulation
ev(ID = 1201:1600, amt = 4*1, ii= 24, ss= 1, addl=0),
ev(ID = 1201:1600, amt = 4*0, ii= 12, ss= 0, addl=0),
ev(ID = 1201:1600, amt = 4*1, ii= 12, ss= 0, addl=0),
ev(ID = 1201:1600, amt = 4*1, ii= 24, ss= 0, addl=10),
#15h delay + 50% dose intake simulation
ev(ID = 1201:1600, amt = 4*1, ii= 24, ss= 1, addl=0),
ev(ID = 1201:1600, amt = 4*0, ii= 15, ss= 0, addl=0),
ev(ID = 1201:1600, amt = 4*0.5, ii= 9, ss= 0, addl=0),
ev(ID = 1201:1600, amt = 4*1, ii= 24, ss= 0, addl=10),
#15h delay simulation
ev(ID = 1201:1600, amt = 4*1, ii= 24, ss= 1, addl=0),
ev(ID = 1201:1600, amt = 4*0, ii= 15, ss= 0, addl=0),
ev(ID = 1201:1600, amt = 4*1, ii= 9, ss= 0, addl=0),
ev(ID = 1201:1600, amt = 4*1, ii= 24, ss= 0, addl=10),
#18h delay + 50% dose intake simulation
ev(ID = 1201:1600, amt = 4*1, ii= 24, ss= 1, addl=0),
ev(ID = 1201:1600, amt = 4*0, ii= 18, ss= 0, addl=0),
ev(ID = 1201:1600, amt = 4*0.5, ii= 6, ss= 0, addl=0),
ev(ID = 1201:1600, amt = 4*1, ii= 24, ss= 0, addl=10),
#18h delay simulation
ev(ID = 1201:1600, amt = 4*1, ii= 24, ss= 1, addl=0),
ev(ID = 1201:1600, amt = 4*0, ii= 18, ss= 0, addl=0),
ev(ID = 1201:1600, amt = 4*1, ii= 6, ss= 0, addl=0),
ev(ID = 1201:1600, amt = 4*1, ii= 24, ss= 0, addl=10),
#21h delay + 50% dose intake simulation
ev(ID = 1201:1600, amt = 4*1, ii= 24, ss= 1, addl=0),
ev(ID = 1201:1600, amt = 4*0, ii= 21, ss= 0, addl=0),
ev(ID = 1201:1600, amt = 4*0.5, ii= 3, ss= 0, addl=0),
ev(ID = 1201:1600, amt = 4*1, ii= 24, ss= 0, addl=10),
#21h delay simulation
ev(ID = 1201:1600, amt = 4*1, ii= 24, ss= 1, addl=0),
ev(ID = 1201:1600, amt = 4*0, ii= 21, ss= 0, addl=0),
ev(ID = 1201:1600, amt = 4*1, ii= 3, ss= 0, addl=0),
ev(ID = 1201:1600, amt = 4*1, ii= 24, ss= 0, addl=10),
#Missed dose simulation
ev(ID = 1201:1600, amt = 4*1, ii= 24, ss= 1, addl=0),
ev(ID = 1201:1600, amt = 4*0, ii= 24, ss= 0, addl=0),
ev(ID = 1201:1600, amt = 4*1, ii= 24, ss= 0, addl=0),
ev(ID = 1201:1600, amt = 4*1, ii= 24, ss= 0, addl=10),
#Missed dose + 150% dose intake simulation
ev(ID = 1201:1600, amt = 4*1, ii= 24, ss= 1, addl=0),
ev(ID = 1201:1600, amt = 4*0, ii= 24, ss= 0, addl=0),
ev(ID = 1201:1600, amt = 4*1.5, ii= 24, ss= 0, addl=0),
ev(ID = 1201:1600, amt = 4*1, ii= 24, ss= 0, addl=10),
#Missed dose + 200% dose intake simulation
ev(ID = 1201:1600, amt = 4*1, ii= 24, ss= 1, addl=0),
ev(ID = 1201:1600, amt = 4*0, ii= 24, ss= 0, addl=0),
ev(ID = 1201:1600, amt = 4*2, ii= 24, ss= 0, addl=0),
ev(ID = 1201:1600, amt = 4*1, ii= 24, ss= 0, addl=10))))%>%
             mutate(dose_group = "4 mg") %>%

    bind_rows(as_tibble( seq(
  #Steady-state simulation
ev(ID = 1601:2000, amt = 5*1, ii= 24, ss= 1, addl=0),
ev(ID = 1601:2000, amt = 5*1, ii= 24, ss= 0, addl=0),
ev(ID = 1601:2000, amt = 5*1, ii= 24, ss= 0, addl=10),
#3h delay simulation
ev(ID = 1601:2000, amt = 5*1, ii= 24, ss= 1, addl=0),
ev(ID = 1601:2000, amt = 5*0, ii= 3, ss= 0, addl=0),
ev(ID = 1601:2000, amt = 5*1, ii= 21, ss= 0, addl=0),
ev(ID = 1601:2000, amt = 5*1, ii= 24, ss= 0, addl=10),
#6h delay simulation
ev(ID = 1601:2000, amt = 5*1, ii= 24, ss= 1, addl=0),
ev(ID = 1601:2000, amt = 5*0, ii= 6, ss= 0, addl=0),
ev(ID = 1601:2000, amt = 5*1, ii= 18, ss= 0, addl=0),
ev(ID = 1601:2000, amt = 5*1, ii= 24, ss= 0, addl=10),
#9h delay simulation
ev(ID = 1601:2000, amt = 5*1, ii= 24, ss= 1, addl=0),
ev(ID = 1601:2000, amt = 5*0, ii= 9, ss= 0, addl=0),
ev(ID = 1601:2000, amt = 5*1, ii= 15, ss= 0, addl=0),
ev(ID = 1601:2000, amt = 5*1, ii= 24, ss= 0, addl=10),
#12h delay simulation
ev(ID = 1601:2000, amt = 5*1, ii= 24, ss= 1, addl=0),
ev(ID = 1601:2000, amt = 5*0, ii= 12, ss= 0, addl=0),
ev(ID = 1601:2000, amt = 5*1, ii= 12, ss= 0, addl=0),
ev(ID = 1601:2000, amt = 5*1, ii= 24, ss= 0, addl=10),
#15h delay + 50% dose intake simulation
ev(ID = 1601:2000, amt = 5*1, ii= 24, ss= 1, addl=0),
ev(ID = 1601:2000, amt = 5*0, ii= 15, ss= 0, addl=0),
ev(ID = 1601:2000, amt = 5*0.5, ii= 9, ss= 0, addl=0),
ev(ID = 1601:2000, amt = 5*1, ii= 24, ss= 0, addl=10),
#15h delay simulation
ev(ID = 1601:2000, amt = 5*1, ii= 24, ss= 1, addl=0),
ev(ID = 1601:2000, amt = 5*0, ii= 15, ss= 0, addl=0),
ev(ID = 1601:2000, amt = 5*1, ii= 9, ss= 0, addl=0),
ev(ID = 1601:2000, amt = 5*1, ii= 24, ss= 0, addl=10),
#18h delay + 50% dose intake simulation
ev(ID = 1601:2000, amt = 5*1, ii= 24, ss= 1, addl=0),
ev(ID = 1601:2000, amt = 5*0, ii= 18, ss= 0, addl=0),
ev(ID = 1601:2000, amt = 5*0.5, ii= 6, ss= 0, addl=0),
ev(ID = 1601:2000, amt = 5*1, ii= 24, ss= 0, addl=10),
#18h delay simulation
ev(ID = 1601:2000, amt = 5*1, ii= 24, ss= 1, addl=0),
ev(ID = 1601:2000, amt = 5*0, ii= 18, ss= 0, addl=0),
ev(ID = 1601:2000, amt = 5*1, ii= 6, ss= 0, addl=0),
ev(ID = 1601:2000, amt = 5*1, ii= 24, ss= 0, addl=10),
#21h delay + 50% dose intake simulation
ev(ID = 1601:2000, amt = 5*1, ii= 24, ss= 1, addl=0),
ev(ID = 1601:2000, amt = 5*0, ii= 21, ss= 0, addl=0),
ev(ID = 1601:2000, amt = 5*0.5, ii= 3, ss= 0, addl=0),
ev(ID = 1601:2000, amt = 5*1, ii= 24, ss= 0, addl=10),
#21h delay simulation
ev(ID = 1601:2000, amt = 5*1, ii= 24, ss= 1, addl=0),
ev(ID = 1601:2000, amt = 5*0, ii= 21, ss= 0, addl=0),
ev(ID = 1601:2000, amt = 5*1, ii= 3, ss= 0, addl=0),
ev(ID = 1601:2000, amt = 5*1, ii= 24, ss= 0, addl=10),
#Missed dose simulation
ev(ID = 1601:2000, amt = 5*1, ii= 24, ss= 1, addl=0),
ev(ID = 1601:2000, amt = 5*0, ii= 24, ss= 0, addl=0),
ev(ID = 1601:2000, amt = 5*1, ii= 24, ss= 0, addl=0),
ev(ID = 1601:2000, amt = 5*1, ii= 24, ss= 0, addl=10),
#Missed dose + 150% dose intake simulation
ev(ID = 1601:2000, amt = 5*1, ii= 24, ss= 1, addl=0),
ev(ID = 1601:2000, amt = 5*0, ii= 24, ss= 0, addl=0),
ev(ID = 1601:2000, amt = 5*1.5, ii= 24, ss= 0, addl=0),
ev(ID = 1601:2000, amt = 5*1, ii= 24, ss= 0, addl=10),
#Missed dose + 200% dose intake simulation
ev(ID = 1601:2000, amt = 5*1, ii= 24, ss= 1, addl=0),
ev(ID = 1601:2000, amt = 5*0, ii= 24, ss= 0, addl=0),
ev(ID = 1601:2000, amt = 5*2, ii= 24, ss= 0, addl=0),
ev(ID = 1601:2000, amt = 5*1, ii= 24, ss= 0, addl=10))))%>%
             mutate(dose_group = "5 mg") %>%

    bind_rows(as_tibble( seq(
  #Steady-state simulation
ev(ID = 2001:2400, amt = 6*1, ii= 24, ss= 1, addl=0),
ev(ID = 2001:2400, amt = 6*1, ii= 24, ss= 0, addl=0),
ev(ID = 2001:2400, amt = 6*1, ii= 24, ss= 0, addl=10),
#3h delay simulation
ev(ID = 2001:2400, amt = 6*1, ii= 24, ss= 1, addl=0),
ev(ID = 2001:2400, amt = 6*0, ii= 3, ss= 0, addl=0),
ev(ID = 2001:2400, amt = 6*1, ii= 21, ss= 0, addl=0),
ev(ID = 2001:2400, amt = 6*1, ii= 24, ss= 0, addl=10),
#6h delay simulation
ev(ID = 2001:2400, amt = 6*1, ii= 24, ss= 1, addl=0),
ev(ID = 2001:2400, amt = 6*0, ii= 6, ss= 0, addl=0),
ev(ID = 2001:2400, amt = 6*1, ii= 18, ss= 0, addl=0),
ev(ID = 2001:2400, amt = 6*1, ii= 24, ss= 0, addl=10),
#9h delay simulation
ev(ID = 2001:2400, amt = 6*1, ii= 24, ss= 1, addl=0),
ev(ID = 2001:2400, amt = 6*0, ii= 9, ss= 0, addl=0),
ev(ID = 2001:2400, amt = 6*1, ii= 15, ss= 0, addl=0),
ev(ID = 2001:2400, amt = 6*1, ii= 24, ss= 0, addl=10),
#12h delay simulation
ev(ID = 2001:2400, amt = 6*1, ii= 24, ss= 1, addl=0),
ev(ID = 2001:2400, amt = 6*0, ii= 12, ss= 0, addl=0),
ev(ID = 2001:2400, amt = 6*1, ii= 12, ss= 0, addl=0),
ev(ID = 2001:2400, amt = 6*1, ii= 24, ss= 0, addl=10),
#15h delay + 50% dose intake simulation
ev(ID = 2001:2400, amt = 6*1, ii= 24, ss= 1, addl=0),
ev(ID = 2001:2400, amt = 6*0, ii= 15, ss= 0, addl=0),
ev(ID = 2001:2400, amt = 6*0.5, ii= 9, ss= 0, addl=0),
ev(ID = 2001:2400, amt = 6*1, ii= 24, ss= 0, addl=10),
#15h delay simulation
ev(ID = 2001:2400, amt = 6*1, ii= 24, ss= 1, addl=0),
ev(ID = 2001:2400, amt = 6*0, ii= 15, ss= 0, addl=0),
ev(ID = 2001:2400, amt = 6*1, ii= 9, ss= 0, addl=0),
ev(ID = 2001:2400, amt = 6*1, ii= 24, ss= 0, addl=10),
#18h delay + 50% dose intake simulation
ev(ID = 2001:2400, amt = 6*1, ii= 24, ss= 1, addl=0),
ev(ID = 2001:2400, amt = 6*0, ii= 18, ss= 0, addl=0),
ev(ID = 2001:2400, amt = 6*0.5, ii= 6, ss= 0, addl=0),
ev(ID = 2001:2400, amt = 6*1, ii= 24, ss= 0, addl=10),
#18h delay simulation
ev(ID = 2001:2400, amt = 6*1, ii= 24, ss= 1, addl=0),
ev(ID = 2001:2400, amt = 6*0, ii= 18, ss= 0, addl=0),
ev(ID = 2001:2400, amt = 6*1, ii= 6, ss= 0, addl=0),
ev(ID = 2001:2400, amt = 6*1, ii= 24, ss= 0, addl=10),
#21h delay + 50% dose intake simulation
ev(ID = 2001:2400, amt = 6*1, ii= 24, ss= 1, addl=0),
ev(ID = 2001:2400, amt = 6*0, ii= 21, ss= 0, addl=0),
ev(ID = 2001:2400, amt = 6*0.5, ii= 3, ss= 0, addl=0),
ev(ID = 2001:2400, amt = 6*1, ii= 24, ss= 0, addl=10),
#21h delay simulation
ev(ID = 2001:2400, amt = 6*1, ii= 24, ss= 1, addl=0),
ev(ID = 2001:2400, amt = 6*0, ii= 21, ss= 0, addl=0),
ev(ID = 2001:2400, amt = 6*1, ii= 3, ss= 0, addl=0),
ev(ID = 2001:2400, amt = 6*1, ii= 24, ss= 0, addl=10),
#Missed dose simulation
ev(ID = 2001:2400, amt = 6*1, ii= 24, ss= 1, addl=0),
ev(ID = 2001:2400, amt = 6*0, ii= 24, ss= 0, addl=0),
ev(ID = 2001:2400, amt = 6*1, ii= 24, ss= 0, addl=0),
ev(ID = 2001:2400, amt = 6*1, ii= 24, ss= 0, addl=10),
#Missed dose + 150% dose intake simulation
ev(ID = 2001:2400, amt = 6*1, ii= 24, ss= 1, addl=0),
ev(ID = 2001:2400, amt = 6*0, ii= 24, ss= 0, addl=0),
ev(ID = 2001:2400, amt = 6*1.5, ii= 24, ss= 0, addl=0),
ev(ID = 2001:2400, amt = 6*1, ii= 24, ss= 0, addl=10),
#Missed dose + 200% dose intake simulation
ev(ID = 2001:2400, amt = 6*1, ii= 24, ss= 1, addl=0),
ev(ID = 2001:2400, amt = 6*0, ii= 24, ss= 0, addl=0),
ev(ID = 2001:2400, amt = 6*2, ii= 24, ss= 0, addl=0),
ev(ID = 2001:2400, amt = 6*1, ii= 24, ss= 0, addl=10))))%>%
             mutate(dose_group = "6 mg") %>%

    bind_rows(as_tibble( seq(
  #Steady-state simulation
ev(ID = 2401:2800, amt = 7*1, ii= 24, ss= 1, addl=0),
ev(ID = 2401:2800, amt = 7*1, ii= 24, ss= 0, addl=0),
ev(ID = 2401:2800, amt = 7*1, ii= 24, ss= 0, addl=10),
#3h delay simulation
ev(ID = 2401:2800, amt = 7*1, ii= 24, ss= 1, addl=0),
ev(ID = 2401:2800, amt = 7*0, ii= 3, ss= 0, addl=0),
ev(ID = 2401:2800, amt = 7*1, ii= 21, ss= 0, addl=0),
ev(ID = 2401:2800, amt = 7*1, ii= 24, ss= 0, addl=10),
#6h delay simulation
ev(ID = 2401:2800, amt = 7*1, ii= 24, ss= 1, addl=0),
ev(ID = 2401:2800, amt = 7*0, ii= 6, ss= 0, addl=0),
ev(ID = 2401:2800, amt = 7*1, ii= 18, ss= 0, addl=0),
ev(ID = 2401:2800, amt = 7*1, ii= 24, ss= 0, addl=10),
#9h delay simulation
ev(ID = 2401:2800, amt = 7*1, ii= 24, ss= 1, addl=0),
ev(ID = 2401:2800, amt = 7*0, ii= 9, ss= 0, addl=0),
ev(ID = 2401:2800, amt = 7*1, ii= 15, ss= 0, addl=0),
ev(ID = 2401:2800, amt = 7*1, ii= 24, ss= 0, addl=10),
#12h delay simulation
ev(ID = 2401:2800, amt = 7*1, ii= 24, ss= 1, addl=0),
ev(ID = 2401:2800, amt = 7*0, ii= 12, ss= 0, addl=0),
ev(ID = 2401:2800, amt = 7*1, ii= 12, ss= 0, addl=0),
ev(ID = 2401:2800, amt = 7*1, ii= 24, ss= 0, addl=10),
#15h delay + 50% dose intake simulation
ev(ID = 2401:2800, amt = 7*1, ii= 24, ss= 1, addl=0),
ev(ID = 2401:2800, amt = 7*0, ii= 15, ss= 0, addl=0),
ev(ID = 2401:2800, amt = 7*0.5, ii= 9, ss= 0, addl=0),
ev(ID = 2401:2800, amt = 7*1, ii= 24, ss= 0, addl=10),
#15h delay simulation
ev(ID = 2401:2800, amt = 7*1, ii= 24, ss= 1, addl=0),
ev(ID = 2401:2800, amt = 7*0, ii= 15, ss= 0, addl=0),
ev(ID = 2401:2800, amt = 7*1, ii= 9, ss= 0, addl=0),
ev(ID = 2401:2800, amt = 7*1, ii= 24, ss= 0, addl=10),
#18h delay + 50% dose intake simulation
ev(ID = 2401:2800, amt = 7*1, ii= 24, ss= 1, addl=0),
ev(ID = 2401:2800, amt = 7*0, ii= 18, ss= 0, addl=0),
ev(ID = 2401:2800, amt = 7*0.5, ii= 6, ss= 0, addl=0),
ev(ID = 2401:2800, amt = 7*1, ii= 24, ss= 0, addl=10),
#18h delay simulation
ev(ID = 2401:2800, amt = 7*1, ii= 24, ss= 1, addl=0),
ev(ID = 2401:2800, amt = 7*0, ii= 18, ss= 0, addl=0),
ev(ID = 2401:2800, amt = 7*1, ii= 6, ss= 0, addl=0),
ev(ID = 2401:2800, amt = 7*1, ii= 24, ss= 0, addl=10),
#21h delay + 50% dose intake simulation
ev(ID = 2401:2800, amt = 7*1, ii= 24, ss= 1, addl=0),
ev(ID = 2401:2800, amt = 7*0, ii= 21, ss= 0, addl=0),
ev(ID = 2401:2800, amt = 7*0.5, ii= 3, ss= 0, addl=0),
ev(ID = 2401:2800, amt = 7*1, ii= 24, ss= 0, addl=10),
#21h delay simulation
ev(ID = 2401:2800, amt = 7*1, ii= 24, ss= 1, addl=0),
ev(ID = 2401:2800, amt = 7*0, ii= 21, ss= 0, addl=0),
ev(ID = 2401:2800, amt = 7*1, ii= 3, ss= 0, addl=0),
ev(ID = 2401:2800, amt = 7*1, ii= 24, ss= 0, addl=10),
#Missed dose simulation
ev(ID = 2401:2800, amt = 7*1, ii= 24, ss= 1, addl=0),
ev(ID = 2401:2800, amt = 7*0, ii= 24, ss= 0, addl=0),
ev(ID = 2401:2800, amt = 7*1, ii= 24, ss= 0, addl=0),
ev(ID = 2401:2800, amt = 7*1, ii= 24, ss= 0, addl=10),
#Missed dose + 150% dose intake simulation
ev(ID = 2401:2800, amt = 7*1, ii= 24, ss= 1, addl=0),
ev(ID = 2401:2800, amt = 7*0, ii= 24, ss= 0, addl=0),
ev(ID = 2401:2800, amt = 7*1.5, ii= 24, ss= 0, addl=0),
ev(ID = 2401:2800, amt = 7*1, ii= 24, ss= 0, addl=10),
#Missed dose + 200% dose intake simulation
ev(ID = 2401:2800, amt = 7*1, ii= 24, ss= 1, addl=0),
ev(ID = 2401:2800, amt = 7*0, ii= 24, ss= 0, addl=0),
ev(ID = 2401:2800, amt = 7*2, ii= 24, ss= 0, addl=0),
ev(ID = 2401:2800, amt = 7*1, ii= 24, ss= 0, addl=10))))%>%
             mutate(dose_group = "7 mg") %>%

    bind_rows(as_tibble( seq(
  #Steady-state simulation
ev(ID = 2801:3200, amt = 8*1, ii= 24, ss= 1, addl=0),
ev(ID = 2801:3200, amt = 8*1, ii= 24, ss= 0, addl=0),
ev(ID = 2801:3200, amt = 8*1, ii= 24, ss= 0, addl=10),
#3h delay simulation
ev(ID = 2801:3200, amt = 8*1, ii= 24, ss= 1, addl=0),
ev(ID = 2801:3200, amt = 8*0, ii= 3, ss= 0, addl=0),
ev(ID = 2801:3200, amt = 8*1, ii= 21, ss= 0, addl=0),
ev(ID = 2801:3200, amt = 8*1, ii= 24, ss= 0, addl=10),
#6h delay simulation
ev(ID = 2801:3200, amt = 8*1, ii= 24, ss= 1, addl=0),
ev(ID = 2801:3200, amt = 8*0, ii= 6, ss= 0, addl=0),
ev(ID = 2801:3200, amt = 8*1, ii= 18, ss= 0, addl=0),
ev(ID = 2801:3200, amt = 8*1, ii= 24, ss= 0, addl=10),
#9h delay simulation
ev(ID = 2801:3200, amt = 8*1, ii= 24, ss= 1, addl=0),
ev(ID = 2801:3200, amt = 8*0, ii= 9, ss= 0, addl=0),
ev(ID = 2801:3200, amt = 8*1, ii= 15, ss= 0, addl=0),
ev(ID = 2801:3200, amt = 8*1, ii= 24, ss= 0, addl=10),
#12h delay simulation
ev(ID = 2801:3200, amt = 8*1, ii= 24, ss= 1, addl=0),
ev(ID = 2801:3200, amt = 8*0, ii= 12, ss= 0, addl=0),
ev(ID = 2801:3200, amt = 8*1, ii= 12, ss= 0, addl=0),
ev(ID = 2801:3200, amt = 8*1, ii= 24, ss= 0, addl=10),
#15h delay + 50% dose intake simulation
ev(ID = 2801:3200, amt = 8*1, ii= 24, ss= 1, addl=0),
ev(ID = 2801:3200, amt = 8*0, ii= 15, ss= 0, addl=0),
ev(ID = 2801:3200, amt = 8*0.5, ii= 9, ss= 0, addl=0),
ev(ID = 2801:3200, amt = 8*1, ii= 24, ss= 0, addl=10),
#15h delay simulation
ev(ID = 2801:3200, amt = 8*1, ii= 24, ss= 1, addl=0),
ev(ID = 2801:3200, amt = 8*0, ii= 15, ss= 0, addl=0),
ev(ID = 2801:3200, amt = 8*1, ii= 9, ss= 0, addl=0),
ev(ID = 2801:3200, amt = 8*1, ii= 24, ss= 0, addl=10),
#18h delay + 50% dose intake simulation
ev(ID = 2801:3200, amt = 8*1, ii= 24, ss= 1, addl=0),
ev(ID = 2801:3200, amt = 8*0, ii= 18, ss= 0, addl=0),
ev(ID = 2801:3200, amt = 8*0.5, ii= 6, ss= 0, addl=0),
ev(ID = 2801:3200, amt = 8*1, ii= 24, ss= 0, addl=10),
#18h delay simulation
ev(ID = 2801:3200, amt = 8*1, ii= 24, ss= 1, addl=0),
ev(ID = 2801:3200, amt = 8*0, ii= 18, ss= 0, addl=0),
ev(ID = 2801:3200, amt = 8*1, ii= 6, ss= 0, addl=0),
ev(ID = 2801:3200, amt = 8*1, ii= 24, ss= 0, addl=10),
#21h delay + 50% dose intake simulation
ev(ID = 2801:3200, amt = 8*1, ii= 24, ss= 1, addl=0),
ev(ID = 2801:3200, amt = 8*0, ii= 21, ss= 0, addl=0),
ev(ID = 2801:3200, amt = 8*0.5, ii= 3, ss= 0, addl=0),
ev(ID = 2801:3200, amt = 8*1, ii= 24, ss= 0, addl=10),
#21h delay simulation
ev(ID = 2801:3200, amt = 8*1, ii= 24, ss= 1, addl=0),
ev(ID = 2801:3200, amt = 8*0, ii= 21, ss= 0, addl=0),
ev(ID = 2801:3200, amt = 8*1, ii= 3, ss= 0, addl=0),
ev(ID = 2801:3200, amt = 8*1, ii= 24, ss= 0, addl=10),
#Missed dose simulation
ev(ID = 2801:3200, amt = 8*1, ii= 24, ss= 1, addl=0),
ev(ID = 2801:3200, amt = 8*0, ii= 24, ss= 0, addl=0),
ev(ID = 2801:3200, amt = 8*1, ii= 24, ss= 0, addl=0),
ev(ID = 2801:3200, amt = 8*1, ii= 24, ss= 0, addl=10),
#Missed dose + 150% dose intake simulation
ev(ID = 2801:3200, amt = 8*1, ii= 24, ss= 1, addl=0),
ev(ID = 2801:3200, amt = 8*0, ii= 24, ss= 0, addl=0),
ev(ID = 2801:3200, amt = 8*1.5, ii= 24, ss= 0, addl=0),
ev(ID = 2801:3200, amt = 8*1, ii= 24, ss= 0, addl=10),
#Missed dose + 200% dose intake simulation
ev(ID = 2801:3200, amt = 8*1, ii= 24, ss= 1, addl=0),
ev(ID = 2801:3200, amt = 8*0, ii= 24, ss= 0, addl=0),
ev(ID = 2801:3200, amt = 8*2, ii= 24, ss= 0, addl=0),
ev(ID = 2801:3200, amt = 8*1, ii= 24, ss= 0, addl=10))))%>%
             mutate(dose_group = "8 mg") %>%

  bind_rows(as_tibble( seq(
  #Steady-state simulation
ev(ID = 3201:3600, amt = 9*1, ii= 24, ss= 1, addl=0),
ev(ID = 3201:3600, amt = 9*1, ii= 24, ss= 0, addl=0),
ev(ID = 3201:3600, amt = 9*1, ii= 24, ss= 0, addl=10),
#3h delay simulation
ev(ID = 3201:3600, amt = 9*1, ii= 24, ss= 1, addl=0),
ev(ID = 3201:3600, amt = 9*0, ii= 3, ss= 0, addl=0),
ev(ID = 3201:3600, amt = 9*1, ii= 21, ss= 0, addl=0),
ev(ID = 3201:3600, amt = 9*1, ii= 24, ss= 0, addl=10),
#6h delay simulation
ev(ID = 3201:3600, amt = 9*1, ii= 24, ss= 1, addl=0),
ev(ID = 3201:3600, amt = 9*0, ii= 6, ss= 0, addl=0),
ev(ID = 3201:3600, amt = 9*1, ii= 18, ss= 0, addl=0),
ev(ID = 3201:3600, amt = 9*1, ii= 24, ss= 0, addl=10),
#9h delay simulation
ev(ID = 3201:3600, amt = 9*1, ii= 24, ss= 1, addl=0),
ev(ID = 3201:3600, amt = 9*0, ii= 9, ss= 0, addl=0),
ev(ID = 3201:3600, amt = 9*1, ii= 15, ss= 0, addl=0),
ev(ID = 3201:3600, amt = 9*1, ii= 24, ss= 0, addl=10),
#12h delay simulation
ev(ID = 3201:3600, amt = 9*1, ii= 24, ss= 1, addl=0),
ev(ID = 3201:3600, amt = 9*0, ii= 12, ss= 0, addl=0),
ev(ID = 3201:3600, amt = 9*1, ii= 12, ss= 0, addl=0),
ev(ID = 3201:3600, amt = 9*1, ii= 24, ss= 0, addl=10),
#15h delay + 50% dose intake simulation
ev(ID = 3201:3600, amt = 9*1, ii= 24, ss= 1, addl=0),
ev(ID = 3201:3600, amt = 9*0, ii= 15, ss= 0, addl=0),
ev(ID = 3201:3600, amt = 9*0.5, ii= 9, ss= 0, addl=0),
ev(ID = 3201:3600, amt = 9*1, ii= 24, ss= 0, addl=10),
#15h delay simulation
ev(ID = 3201:3600, amt = 9*1, ii= 24, ss= 1, addl=0),
ev(ID = 3201:3600, amt = 9*0, ii= 15, ss= 0, addl=0),
ev(ID = 3201:3600, amt = 9*1, ii= 9, ss= 0, addl=0),
ev(ID = 3201:3600, amt = 9*1, ii= 24, ss= 0, addl=10),
#18h delay + 50% dose intake simulation
ev(ID = 3201:3600, amt = 9*1, ii= 24, ss= 1, addl=0),
ev(ID = 3201:3600, amt = 9*0, ii= 18, ss= 0, addl=0),
ev(ID = 3201:3600, amt = 9*0.5, ii= 6, ss= 0, addl=0),
ev(ID = 3201:3600, amt = 9*1, ii= 24, ss= 0, addl=10),
#18h delay simulation
ev(ID = 3201:3600, amt = 9*1, ii= 24, ss= 1, addl=0),
ev(ID = 3201:3600, amt = 9*0, ii= 18, ss= 0, addl=0),
ev(ID = 3201:3600, amt = 9*1, ii= 6, ss= 0, addl=0),
ev(ID = 3201:3600, amt = 9*1, ii= 24, ss= 0, addl=10),
#21h delay + 50% dose intake simulation
ev(ID = 3201:3600, amt = 9*1, ii= 24, ss= 1, addl=0),
ev(ID = 3201:3600, amt = 9*0, ii= 21, ss= 0, addl=0),
ev(ID = 3201:3600, amt = 9*0.5, ii= 3, ss= 0, addl=0),
ev(ID = 3201:3600, amt = 9*1, ii= 24, ss= 0, addl=10),
#21h delay simulation
ev(ID = 3201:3600, amt = 9*1, ii= 24, ss= 1, addl=0),
ev(ID = 3201:3600, amt = 9*0, ii= 21, ss= 0, addl=0),
ev(ID = 3201:3600, amt = 9*1, ii= 3, ss= 0, addl=0),
ev(ID = 3201:3600, amt = 9*1, ii= 24, ss= 0, addl=10),
#Missed dose simulation
ev(ID = 3201:3600, amt = 9*1, ii= 24, ss= 1, addl=0),
ev(ID = 3201:3600, amt = 9*0, ii= 24, ss= 0, addl=0),
ev(ID = 3201:3600, amt = 9*1, ii= 24, ss= 0, addl=0),
ev(ID = 3201:3600, amt = 9*1, ii= 24, ss= 0, addl=10),
#Missed dose + 150% dose intake simulation
ev(ID = 3201:3600, amt = 9*1, ii= 24, ss= 1, addl=0),
ev(ID = 3201:3600, amt = 9*0, ii= 24, ss= 0, addl=0),
ev(ID = 3201:3600, amt = 9*1.5, ii= 24, ss= 0, addl=0),
ev(ID = 3201:3600, amt = 9*1, ii= 24, ss= 0, addl=10),
#Missed dose + 200% dose intake simulation
ev(ID = 3201:3600, amt = 9*1, ii= 24, ss= 1, addl=0),
ev(ID = 3201:3600, amt = 9*0, ii= 24, ss= 0, addl=0),
ev(ID = 3201:3600, amt = 9*2, ii= 24, ss= 0, addl=0),
ev(ID = 3201:3600, amt = 9*1, ii= 24, ss= 0, addl=10))))%>%
             mutate(dose_group = "9 mg") %>%

    bind_rows(as_tibble( seq(
  #Steady-state simulation
ev(ID = 3601:4000, amt = 10*1, ii= 24, ss= 1, addl=0),
ev(ID = 3601:4000, amt = 10*1, ii= 24, ss= 0, addl=0),
ev(ID = 3601:4000, amt = 10*1, ii= 24, ss= 0, addl=10),
#3h delay simulation
ev(ID = 3601:4000, amt = 10*1, ii= 24, ss= 1, addl=0),
ev(ID = 3601:4000, amt = 10*0, ii= 3, ss= 0, addl=0),
ev(ID = 3601:4000, amt = 10*1, ii= 21, ss= 0, addl=0),
ev(ID = 3601:4000, amt = 10*1, ii= 24, ss= 0, addl=10),
#6h delay simulation
ev(ID = 3601:4000, amt = 10*1, ii= 24, ss= 1, addl=0),
ev(ID = 3601:4000, amt = 10*0, ii= 6, ss= 0, addl=0),
```

```
[illegible]
ev(ID = 3601:4000, amt = 10*1, ii= 18, ss= 0, addl=0),
ev(ID = 3601:4000, amt = 10*1, ii= 24, ss= 0, addl=10),
#9h delay simulation
ev(ID = 3601:4000, amt = 10*1, ii= 24, ss= 1, addl=0),
ev(ID = 3601:4000, amt = 10*0, ii= 9, ss= 0, addl=0),
ev(ID = 3601:4000, amt = 10*1, ii= 15, ss= 0, addl=0),
ev(ID = 3601:4000, amt = 10*1, ii= 24, ss= 0, addl=10),
#12h delay simulation
ev(ID = 3601:4000, amt = 10*1, ii= 24, ss= 1, addl=0),
ev(ID = 3601:4000, amt = 10*0, ii= 12, ss= 0, addl=0),
ev(ID = 3601:4000, amt = 10*1, ii= 12, ss= 0, addl=0),
ev(ID = 3601:4000, amt = 10*1, ii= 24, ss= 0, addl=10),
#15h delay + 50% dose intake simulation
ev(ID = 3601:4000, amt = 10*1, ii= 24, ss= 1, addl=0),
ev(ID = 3601:4000, amt = 10*0, ii= 15, ss= 0, addl=0),
ev(ID = 3601:4000, amt = 10*0.5, ii= 9, ss= 0, addl=0),
ev(ID = 3601:4000, amt = 10*1, ii= 24, ss= 0, addl=10),
#15h delay simulation
ev(ID = 3601:4000, amt = 10*1, ii= 24, ss= 1, addl=0),
ev(ID = 3601:4000, amt = 10*0, ii= 15, ss= 0, addl=0),
ev(ID = 3601:4000, amt = 10*1, ii= 9, ss= 0, addl=0),
ev(ID = 3601:4000, amt = 10*1, ii= 24, ss= 0, addl=10),
#18h delay + 50% dose intake simulation
ev(ID = 3601:4000, amt = 10*1, ii= 24, ss= 1, addl=0),
ev(ID = 3601:4000, amt = 10*0, ii= 18, ss= 0, addl=0),
ev(ID = 3601:4000, amt = 10*0.5, ii= 6, ss= 0, addl=0),
ev(ID = 3601:4000, amt = 10*1, ii= 24, ss= 0, addl=10),
#18h delay simulation
ev(ID = 3601:4000, amt = 10*1, ii= 24, ss= 1, addl=0),
ev(ID = 3601:4000, amt = 10*0, ii= 18, ss= 0, addl=0),
ev(ID = 3601:4000, amt = 10*1, ii= 6, ss= 0, addl=0),
ev(ID = 3601:4000, amt = 10*1, ii= 24, ss= 0, addl=10),
#21h delay + 50% dose intake simulation
ev(ID = 3601:4000, amt = 10*1, ii= 24, ss= 1, addl=0),
ev(ID = 3601:4000, amt = 10*0, ii= 21, ss= 0, addl=0),
ev(ID = 3601:4000, amt = 10*0.5, ii= 3, ss= 0, addl=0),
ev(ID = 3601:4000, amt = 10*1, ii= 24, ss= 0, addl=10),
#21h delay simulation
ev(ID = 3601:4000, amt = 10*1, ii= 24, ss= 1, addl=0),
ev(ID = 3601:4000, amt = 10*0, ii= 21, ss= 0, addl=0),
ev(ID = 3601:4000, amt = 10*1, ii= 3, ss= 0, addl=0),
ev(ID = 3601:4000, amt = 10*1, ii= 24, ss= 0, addl=10),
#Missed dose simulation
ev(ID = 3601:4000, amt = 10*1, ii= 24, ss= 1, addl=0),
ev(ID = 3601:4000, amt = 10*0, ii= 24, ss= 0, addl=0),
ev(ID = 3601:4000, amt = 10*1, ii= 24, ss= 0, addl=0),
ev(ID = 3601:4000, amt = 10*1, ii= 24, ss= 0, addl=10),
#Missed dose + 150% dose intake simulation
ev(ID = 3601:4000, amt = 10*1, ii= 24, ss= 1, addl=0),
ev(ID = 3601:4000, amt = 10*0, ii= 24, ss= 0, addl=0),
ev(ID = 3601:4000, amt = 10*1.5, ii= 24, ss= 0, addl=0),
ev(ID = 3601:4000, amt = 10*1, ii= 24, ss= 0, addl=10),
#Missed dose + 200% dose intake simulation
ev(ID = 3601:4000, amt = 10*1, ii= 24, ss= 1, addl=0),
ev(ID = 3601:4000, amt = 10*0, ii= 24, ss= 0, addl=0),
ev(ID = 3601:4000, amt = 10*2, ii= 24, ss= 0, addl=0),
ev(ID = 3601:4000, amt = 10*1, ii= 24, ss= 0, addl=10))))%>%
             mutate(dose_group = "10 mg") %>%

    bind_rows(as_tibble( seq(
  #Steady-state simulation
ev(ID = 4001:4400, amt = 11*1, ii= 24, ss= 1, addl=0),
ev(ID = 4001:4400, amt = 11*1, ii= 24, ss= 0, addl=0),
ev(ID = 4001:4400, amt = 11*1, ii= 24, ss= 0, addl=10),
#3h delay simulation
ev(ID = 4001:4400, amt = 11*1, ii= 24, ss= 1, addl=0),
ev(ID = 4001:4400, amt = 11*0, ii= 3, ss= 0, addl=0),
ev(ID = 4001:4400, amt = 11*1, ii= 21, ss= 0, addl=0),
ev(ID = 4001:4400, amt = 11*1, ii= 24, ss= 0, addl=10),
#6h delay simulation
ev(ID = 4001:4400, amt = 11*1, ii= 24, ss= 1, addl=0),
ev(ID = 4001:4400, amt = 11*0, ii= 6, ss= 0, addl=0),
ev(ID = 4001:4400, amt = 11*1, ii= 18, ss= 0, addl=0),
ev(ID = 4001:4400, amt = 11*1, ii= 24, ss= 0, addl=10),
#9h delay simulation
ev(ID = 4001:4400, amt = 11*1, ii= 24, ss= 1, addl=0),
ev(ID = 4001:4400, amt = 11*0, ii= 9, ss= 0, addl=0),
ev(ID = 4001:4400, amt = 11*1, ii= 15, ss= 0, addl=0),
ev(ID = 4001:4400, amt = 11*1, ii= 24, ss= 0, addl=10),
#12h delay simulation
ev(ID = 4001:4400, amt = 11*1, ii= 24, ss= 1, addl=0),
ev(ID = 4001:4400, amt = 11*0, ii= 12, ss= 0, addl=0),
ev(ID = 4001:4400, amt = 11*1, ii= 12, ss= 0, addl=0),
ev(ID = 4001:4400, amt = 11*1, ii= 24, ss= 0, addl=10),
#15h delay + 50% dose intake simulation
ev(ID = 4001:4400, amt = 11*1, ii= 24, ss= 1, addl=0),
ev(ID = 4001:4400, amt = 11*0, ii= 15, ss= 0, addl=0),
ev(ID = 4001:4400, amt = 11*0.5, ii= 9, ss= 0, addl=0),
ev(ID = 4001:4400, amt = 11*1, ii= 24, ss= 0, addl=10),
#15h delay simulation
ev(ID = 4001:4400, amt = 11*1, ii= 24, ss= 1, addl=0),
ev(ID = 4001:4400, amt = 11*0, ii= 15, ss= 0, addl=0),
ev(ID = 4001:4400, amt = 11*1, ii= 9, ss= 0, addl=0),
ev(ID = 4001:4400, amt = 11*1, ii= 24, ss= 0, addl=10),
#18h delay + 50% dose intake simulation
ev(ID = 4001:4400, amt = 11*1, ii= 24, ss= 1, addl=0),
ev(ID = 4001:4400, amt = 11*0, ii= 18, ss= 0, addl=0),
ev(ID = 4001:4400, amt = 11*0.5, ii= 6, ss= 0, addl=0),
ev(ID = 4001:4400, amt = 11*1, ii= 24, ss= 0, addl=10),
#18h delay simulation
ev(ID = 4001:4400, amt = 11*1, ii= 24, ss= 1, addl=0),
ev(ID = 4001:4400, amt = 11*0, ii= 18, ss= 0, addl=0),
ev(ID = 4001:4400, amt = 11*1, ii= 6, ss= 0, addl=0),
ev(ID = 4001:4400, amt = 11*1, ii= 24, ss= 0, addl=10),
#21h delay + 50% dose intake simulation
ev(ID = 4001:4400, amt = 11*1, ii= 24, ss= 1, addl=0),
ev(ID = 4001:4400, amt = 11*0, ii= 21, ss= 0, addl=0),
ev(ID = 4001:4400, amt = 11*0.5, ii= 3, ss= 0, addl=0),
ev(ID = 4001:4400, amt = 11*1, ii= 24, ss= 0, addl=10),
#21h delay simulation
ev(ID = 4001:4400, amt = 11*1, ii= 24, ss= 1, addl=0),
ev(ID = 4001:4400, amt = 11*0, ii= 21, ss= 0, addl=0),
ev(ID = 4001:4400, amt = 11*1, ii= 3, ss= 0, addl=0),
ev(ID = 4001:4400, amt = 11*1, ii= 24, ss= 0, addl=10),
#Missed dose simulation
ev(ID = 4001:4400, amt = 11*1, ii= 24, ss= 1, addl=0),
ev(ID = 4001:4400, amt = 11*0, ii= 24, ss= 0, addl=0),
ev(ID = 4001:4400, amt = 11*1, ii= 24, ss= 0, addl=0),
ev(ID = 4001:4400, amt = 11*1, ii= 24, ss= 0, addl=10),
#Missed dose + 150% dose intake simulation
ev(ID = 4001:4400, amt = 11*1, ii= 24, ss= 1, addl=0),
ev(ID = 4001:4400, amt = 11*0, ii= 24, ss= 0, addl=0),
ev(ID = 4001:4400, amt = 11*1.5, ii= 24, ss= 0, addl=0),
ev(ID = 4001:4400, amt = 11*1, ii= 24, ss= 0, addl=10),
#Missed dose + 200% dose intake simulation
ev(ID = 4001:4400, amt = 11*1, ii= 24, ss= 1, addl=0),
ev(ID = 4001:4400, amt = 11*0, ii= 24, ss= 0, addl=0),
ev(ID = 4001:4400, amt = 11*2, ii= 24, ss= 0, addl=0),
ev(ID = 4001:4400, amt = 11*1, ii= 24, ss= 0, addl=10))))%>%
             mutate(dose_group = "11 mg") %>%

    bind_rows(as_tibble( seq(
  #Steady-state simulation
ev(ID = 4401:4800, amt = 12*1, ii= 24, ss= 1, addl=0),
ev(ID = 4401:4800, amt = 12*1, ii= 24, ss= 0, addl=0),
ev(ID = 4401:4800, amt = 12*1, ii= 24, ss= 0, addl=10),
#3h delay simulation
ev(ID = 4401:4800, amt = 12*1, ii= 24, ss= 1, addl=0),
ev(ID = 4401:4800, amt = 12*0, ii= 3, ss= 0, addl=0),
ev(ID = 4401:4800, amt = 12*1, ii= 21, ss= 0, addl=0),
ev(ID = 4401:4800, amt = 12*1, ii= 24, ss= 0, addl=10),
#6h delay simulation
ev(ID = 4401:4800, amt = 12*1, ii= 24, ss= 1, addl=0),
ev(ID = 4401:4800, amt = 12*0, ii= 6, ss= 0, addl=0),
ev(ID = 4401:4800, amt = 12*1, ii= 18, ss= 0, addl=0),
ev(ID = 4401:4800, amt = 12*1, ii= 24, ss= 0, addl=10),
#9h delay simulation
ev(ID = 4401:4800, amt = 12*1, ii= 24, ss= 1, addl=0),
ev(ID = 4401:4800, amt = 12*0, ii= 9, ss= 0, addl=0),
ev(ID = 4401:4800, amt = 12*1, ii= 15, ss= 0, addl=0),
ev(ID = 4401:4800, amt = 12*1, ii= 24, ss= 0, addl=10),
#12h delay simulation
ev(ID = 4401:4800, amt = 12*1, ii= 24, ss= 1, addl=0),
ev(ID = 4401:4800, amt = 12*0, ii= 12, ss= 0, addl=0),
ev(ID = 4401:4800, amt = 12*1, ii= 12, ss= 0, addl=0),
ev(ID = 4401:4800, amt = 12*1, ii= 24, ss= 0, addl=10),
#15h delay + 50% dose intake simulation
ev(ID = 4401:4800, amt = 12*1, ii= 24, ss= 1, addl=0),
ev(ID = 4401:4800, amt = 12*0, ii= 15, ss= 0, addl=0),
ev(ID = 4401:4800, amt = 12*0.5, ii= 9, ss= 0, addl=0),
ev(ID = 4401:4800, amt = 12*1, ii= 24, ss= 0, addl=10),
#15h delay simulation
ev(ID = 4401:4800, amt = 12*1, ii= 24, ss= 1, addl=0),
ev(ID = 4401:4800, amt = 12*0, ii= 15, ss= 0, addl=0),
ev(ID = 4401:4800, amt = 12*1, ii= 9, ss= 0, addl=0),
ev(ID = 4401:4800, amt = 12*1, ii= 24, ss= 0, addl=10),
#18h delay + 50% dose intake simulation
ev(ID = 4401:4800, amt = 12*1, ii= 24, ss= 1, addl=0),
ev(ID = 4401:4800, amt = 12*0, ii= 18, ss= 0, addl=0),
ev(ID = 4401:4800, amt = 12*0.5, ii= 6, ss= 0, addl=0),
ev(ID = 4401:4800, amt = 12*1, ii= 24, ss= 0, addl=10),
#18h delay simulation
ev(ID = 4401:4800, amt = 12*1, ii= 24, ss= 1, addl=0),
ev(ID = 4401:4800, amt = 12*0, ii= 18, ss= 0, addl=0),
ev(ID = 4401:4800, amt = 12*1, ii= 6, ss= 0, addl=0),
ev(ID = 4401:4800, amt = 12*1, ii= 24, ss= 0, addl=10),
#21h delay + 50% dose intake simulation
ev(ID = 4401:4800, amt = 12*1, ii= 24, ss= 1, addl=0),
ev(ID = 4401:4800, amt = 12*0, ii= 21, ss= 0, addl=0),
ev(ID = 4401:4800, amt = 12*0.5, ii= 3, ss= 0, addl=0),
ev(ID = 4401:4800, amt = 12*1, ii= 24, ss= 0, addl=10),
#21h delay simulation
ev(ID = 4401:4800, amt = 12*1, ii= 24, ss= 1, addl=0),
ev(ID = 4401:4800, amt = 12*0, ii= 21, ss= 0, addl=0),
ev(ID = 4401:4800, amt = 12*1, ii= 3, ss= 0, addl=0),
ev(ID = 4401:4800, amt = 12*1, ii= 24, ss= 0, addl=10),
#Missed dose simulation
ev(ID = 4401:4800, amt = 12*1, ii= 24, ss= 1, addl=0),
ev(ID = 4401:4800, amt = 12*0, ii= 24, ss= 0, addl=0),
ev(ID = 4401:4800, amt = 12*1, ii= 24, ss= 0, addl=0),
ev(ID = 4401:4800, amt = 12*1, ii= 24, ss= 0, addl=10),
#Missed dose + 150% dose intake simulation
ev(ID = 4401:4800, amt = 12*1, ii= 24, ss= 1, addl=0),
ev(ID = 4401:4800, amt = 12*0, ii= 24, ss= 0, addl=0),
ev(ID = 4401:4800, amt = 12*1.5, ii= 24, ss= 0, addl=0),
ev(ID = 4401:4800, amt = 12*1, ii= 24, ss= 0, addl=10),
#Missed dose + 200% dose intake simulation
ev(ID = 4401:4800, amt = 12*1, ii= 24, ss= 1, addl=0),
ev(ID = 4401:4800, amt = 12*0, ii= 24, ss= 0, addl=0),
ev(ID = 4401:4800, amt = 12*2, ii= 24, ss= 0, addl=0),
ev(ID = 4401:4800, amt = 12*1, ii= 24, ss= 0, addl=10))))%>%
             mutate(dose_group = "12 mg") %>%

    bind_rows(as_tibble( seq(
  #Steady-state simulation
ev(ID = 4801:5200, amt = 13*1, ii= 24, ss= 1, addl=0),
ev(ID = 4801:5200, amt = 13*1, ii= 24, ss= 0, addl=0),
ev(ID = 4801:5200, amt = 13*1, ii= 24, ss= 0, addl=10),
#3h delay simulation
ev(ID = 4801:5200, amt = 13*1, ii= 24, ss= 1, addl=0),
ev(ID = 4801:5200, amt = 13*0, ii= 3, ss= 0, addl=0),
ev(ID = 4801:5200, amt = 13*1, ii= 21, ss= 0, addl=0),
ev(ID = 4801:5200, amt = 13*1, ii= 24, ss= 0, addl=10),
#6h delay simulation
ev(ID = 4801:5200, amt = 13*1, ii= 24, ss= 1, addl=0),
ev(ID = 4801:5200, amt = 13*0, ii= 6, ss= 0, addl=0),
ev(ID = 4801:5200, amt = 13*1, ii= 18, ss= 0, addl=0),
ev(ID = 4801:5200, amt = 13*1, ii= 24, ss= 0, addl=10),
#9h delay simulation
ev(ID = 4801:5200, amt = 13*1, ii= 24, ss= 1, addl=0),
ev(ID = 4801:5200, amt = 13*0, ii= 9, ss= 0, addl=0),
ev(ID = 4801:5200, amt = 13*1, ii= 15, ss= 0, addl=0),
ev(ID = 4801:5200, amt = 13*1, ii= 24, ss= 0, addl=10),
#12h delay simulation
ev(ID = 4801:5200, amt = 13*1, ii= 24, ss= 1, addl=0),
ev(ID = 4801:5200, amt = 13*0, ii= 12, ss= 0, addl=0),
ev(ID = 4801:5200, amt = 13*1, ii= 12, ss= 0, addl=0),
ev(ID = 4801:5200, amt = 13*1, ii= 24, ss= 0, addl=10),
#15h delay + 50% dose intake simulation
ev(ID = 4801:5200, amt = 13*1, ii= 24, ss= 1, addl=0),
ev(ID = 4801:5200, amt = 13*0, ii= 15, ss= 0, addl=0),
ev(ID = 4801:5200, amt = 13*0.5, ii= 9, ss= 0, addl=0),
ev(ID = 4801:5200, amt = 13*1, ii= 24, ss= 0, addl=10),
#15h delay simulation
ev(ID = 4801:5200, amt = 13*1, ii= 24, ss= 1, addl=0),
ev(ID = 4801:5200, amt = 13*0, ii= 15, ss= 0, addl=0),
ev(ID = 4801:5200, amt = 13*1, ii= 9, ss= 0, addl=0),
ev(ID = 4801:5200, amt = 13*1, ii= 24, ss= 0, addl=10),
#18h delay + 50% dose intake simulation
ev(ID = 4801:5200, amt = 13*1, ii= 24, ss= 1, addl=0),
ev(ID = 4801:5200, amt = 13*0, ii= 18, ss= 0, addl=0),
ev(ID = 4801:5200, amt = 13*0.5, ii= 6, ss= 0, addl=0),
ev(ID = 4801:5200, amt = 13*1, ii= 24, ss= 0, addl=10),
#18h delay simulation
ev(ID = 4801:5200, amt = 13*1, ii= 24, ss= 1, addl=0),
ev(ID = 4801:5200, amt = 13*0, ii= 18, ss= 0, addl=0),
ev(ID = 4801:5200, amt = 13*1, ii= 6, ss= 0, addl=0),
ev(ID = 4801:5200, amt = 13*1, ii= 24, ss= 0, addl=10),
#21h delay + 50% dose intake simulation
ev(ID = 4801:5200, amt = 13*1, ii= 24, ss= 1, addl=0),
ev(ID = 4801:5200, amt = 13*0, ii= 21, ss= 0, addl=0),
ev(ID = 4801:5200, amt = 13*0.5, ii= 3, ss= 0, addl=0),
ev(ID = 4801:5200, amt = 13*1, ii= 24, ss= 0, addl=10),
#21h delay simulation
ev(ID = 4801:5200, amt = 13*1, ii= 24, ss= 1, addl=0),
ev(ID = 4801:5200, amt = 13*0, ii= 21, ss= 0, addl=0),
ev(ID = 4801:5200, amt = 13*1, ii= 3, ss= 0, addl=0),
ev(ID = 4801:5200, amt = 13*1, ii= 24, ss= 0, addl=10),
#Missed dose simulation
ev(ID = 4801:5200, amt = 13*1, ii= 24, ss= 1, addl=0),
ev(ID = 4801:5200, amt = 13*0, ii= 24, ss= 0, addl=0),
ev(ID = 4801:5200, amt = 13*1, ii= 24, ss= 0, addl=0),
ev(ID = 4801:5200, amt = 13*1, ii= 24, ss= 0, addl=10),
#Missed dose + 150% dose intake simulation
ev(ID = 4801:5200, amt = 13*1, ii= 24, ss= 1, addl=0),
ev(ID = 4801:5200, amt = 13*0, ii= 24, ss= 0, addl=0),
ev(ID = 4801:5200, amt = 13*1.5, ii= 24, ss= 0, addl=0),
ev(ID = 4801:5200, amt = 13*1, ii= 24, ss= 0, addl=10),
#Missed dose + 200% dose intake simulation
ev(ID = 4801:5200, amt = 13*1, ii= 24, ss= 1, addl=0),
ev(ID = 4801:5200, amt = 13*0, ii= 24, ss= 0, addl=0),
ev(ID = 4801:5200, amt = 13*2, ii= 24, ss= 0, addl=0),
ev(ID = 4801:5200, amt = 13*1, ii= 24, ss= 0, addl=10))))%>%
             mutate(dose_group = "13 mg") %>%

    bind_rows(as_tibble( seq(
  #Steady-state simulation
ev(ID = 5201:5600, amt = 14*1, ii= 24, ss= 1, addl=0),
ev(ID = 5201:5600, amt = 14*1, ii= 24, ss= 0, addl=0),
ev(ID = 5201:5600, amt = 14*1, ii= 24, ss= 0, addl=10),
#3h delay simulation
ev(ID = 5201:5600, amt = 14*1, ii= 24, ss= 1, addl=0),
ev(ID = 5201:5600, amt = 14*0, ii= 3, ss= 0, addl=0),
ev(ID = 5201:5600, amt = 14*1, ii= 21, ss= 0, addl=0),
ev(ID = 5201:5600, amt = 14*1, ii= 24, ss= 0, addl=10),
#6h delay simulation
ev(ID = 5201:5600, amt = 14*1, ii= 24, ss= 1, addl=0),
ev(ID = 5201:5600, amt = 14*0, ii= 6, ss= 0, addl=0),
ev(ID = 5201:5600, amt = 14*1, ii= 18, ss= 0, addl=0),
ev(ID = 5201:5600, amt = 14*1, ii= 24, ss= 0, addl=10),
#9h delay simulation
ev(ID = 5201:5600, amt = 14*1, ii= 24, ss= 1, addl=0),
ev(ID = 5201:5600, amt = 14*0, ii= 9, ss= 0, addl=0),
ev(ID = 5201:5600, amt = 14*1, ii= 15, ss= 0, addl=0),
ev(ID = 5201:5600, amt = 14*1, ii= 24, ss= 0, addl=10),
#12h delay simulation
ev(ID = 5201:5600, amt = 14*1, ii= 24, ss= 1, addl=0),
ev(ID = 5201:5600, amt = 14*0, ii= 12, ss= 0, addl=0),
ev(ID = 5201:5600, amt = 14*1, ii= 12, ss= 0, addl=0),
ev(ID = 5201:5600, amt = 14*1, ii= 24, ss= 0, addl=10),
#15h delay + 50% dose intake simulation
ev(ID = 5201:5600, amt = 14*1, ii= 24, ss= 1, addl=0),
ev(ID = 5201:5600, amt = 14*0, ii= 15, ss= 0, addl=0),
ev(ID = 5201:5600, amt = 14*0.5, ii= 9, ss= 0, addl=0),
ev(ID = 5201:5600, amt = 14*1, ii= 24, ss= 0, addl=10),
#15h delay simulation
ev(ID = 5201:5600, amt = 14*1, ii= 24, ss= 1, addl=0),
ev(ID = 5201:5600, amt = 14*0, ii= 15, ss= 0, addl=0),
ev(ID = 5201:5600, amt = 14*1, ii= 9, ss= 0, addl=0),
ev(ID = 5201:5600, amt = 14*1, ii= 24, ss= 0, addl=10),
#18h delay + 50% dose intake simulation
ev(ID = 5201:5600, amt = 14*1, ii= 24, ss= 1, addl=0),
ev(ID = 5201:5600, amt = 14*0, ii= 18, ss= 0, addl=0),
ev(ID = 5201:5600, amt = 14*0.5, ii= 6, ss= 0, addl=0),
ev(ID = 5201:5600, amt = 14*1, ii= 24, ss= 0, addl=10),
#18h delay simulation
ev(ID = 5201:5600, amt = 14*1, ii= 24, ss= 1, addl=0),
ev(ID = 5201:5600, amt = 14*0, ii= 18, ss= 0, addl=0),
ev(ID = 5201:5600, amt = 14*1, ii= 6, ss= 0, addl=0),
ev(ID = 5201:5600, amt = 14*1, ii= 24, ss= 0, addl=10),
#21h delay + 50% dose intake simulation
ev(ID = 5201:5600, amt = 14*1, ii= 24, ss= 1, addl=0),
ev(ID = 5201:5600, amt = 14*0, ii= 21, ss= 0, addl=0),
ev(ID = 5201:5600, amt = 14*0.5, ii= 3, ss= 0, addl=0),
ev(ID = 5201:5600, amt = 14*1, ii= 24, ss= 0, addl=10),
#21h delay simulation
ev(ID = 5201:5600, amt = 14*1, ii= 24, ss= 1, addl=0),
ev(ID = 5201:5600, amt = 14*0, ii= 21, ss= 0, addl=0),
ev(ID = 5201:5600, amt = 14*1, ii= 3, ss= 0, addl=0),
ev(ID = 5201:5600, amt = 14*1, ii= 24, ss= 0, addl=10),
#Missed dose simulation
ev(ID = 5201:5600, amt = 14*1, ii= 24, ss= 1, addl=0),
ev(ID = 5201:5600, amt = 14*0, ii= 24, ss= 0, addl=0),
ev(ID = 5201:5600, amt = 14*1, ii= 24, ss= 0, addl=0),
ev(ID = 5201:5600, amt = 14*1, ii= 24, ss= 0, addl=10),
#Missed dose + 150% dose intake simulation
ev(ID = 5201:5600, amt = 14*1, ii= 24, ss= 1, addl=0),
ev(ID = 5201:5600, amt = 14*0, ii= 24, ss= 0, addl=0),
ev(ID = 5201:5600, amt = 14*1.5, ii= 24, ss= 0, addl=0),
ev(ID = 5201:5600, amt = 14*1, ii= 24, ss= 0, addl=10),
#Missed dose + 200% dose intake simulation
ev(ID = 5201:5600, amt = 14*1, ii= 24, ss= 1, addl=0),
ev(ID = 5201:5600, amt = 14*0, ii= 24, ss= 0, addl=0),
ev(ID = 5201:5600, amt = 14*2, ii= 24, ss= 0, addl=0),
ev(ID = 5201:5600, amt = 14*1, ii= 24, ss= 0, addl=10))))%>%
             mutate(dose_group = "14 mg") %>%

    bind_rows(as_tibble( seq(
  #Steady-state simulation
ev(ID = 5601:6000, amt = 15*1, ii= 24, ss= 1, addl=0),
ev(ID = 5601:6000, amt = 15*1, ii= 24, ss= 0, addl=0),
ev(ID = 5601:6000, amt = 15*1, ii= 24, ss= 0, addl=10),
#3h delay simulation
ev(ID = 5601:6000, amt = 15*1, ii= 24, ss= 1, addl=0),
ev(ID = 5601:6000, amt = 15*0, ii= 3, ss= 0, addl=0),
ev(ID = 5601:6000, amt = 15*1, ii= 21, ss= 0, addl=0),
ev(ID = 5601:6000, amt = 15*1, ii= 24, ss= 0, addl=10),
#6h delay simulation
ev(ID = 5601:6000, amt = 15*1, ii= 24, ss= 1, addl=0),
ev(ID = 5601:6000, amt = 15*0, ii= 6, ss= 0, addl=0),
ev(ID = 5601:6000, amt = 15*1, ii= 18, ss= 0, addl=0),
ev(ID = 5601:6000, amt = 15*1, ii= 24, ss= 0, addl=10),
#9h delay simulation
ev(ID = 5601:6000, amt = 15*1, ii= 24, ss= 1, addl=0),
ev(ID = 5601:6000, amt = 15*0, ii= 9, ss= 0, addl=0),
ev(ID = 5601:6000, amt = 15*1, ii= 15, ss= 0, addl=0),
ev(ID = 5601:6000, amt = 15*1, ii= 24, ss= 0, addl=10),
#12h delay simulation
ev(ID = 5601:6000, amt = 15*1, ii= 24, ss= 1, addl=0),
ev(ID = 5601:6000, amt = 15*0, ii= 12, ss= 0, addl=0),
ev(ID = 5601:6000, amt = 15*1, ii= 12, ss= 0, addl=0),
ev(ID = 5601:6000, amt = 15*1, ii= 24, ss= 0, addl=10),
#15h delay + 50% dose intake simulation
ev(ID = 5601:6000, amt = 15*1, ii= 24, ss= 1, addl=0),
ev(ID = 5601:6000, amt = 15*0, ii= 15, ss= 0, addl=0),
ev(ID = 5601:6000, amt = 15*0.5, ii= 9, ss= 0, addl=0),
ev(ID = 5601:6000, amt = 15*1, ii= 24, ss= 0, addl=10),
#15h delay simulation
ev(ID = 5601:6000, amt = 15*1, ii= 24, ss= 1, addl=0),
ev(ID = 5601:6000, amt = 15*0, ii= 15, ss= 0, addl=0),
ev(ID = 5601:6000, amt = 15*1, ii= 9, ss= 0, addl=0),
ev(ID = 5601:6000, amt = 15*1, ii= 24, ss= 0, addl=10),
#18h delay + 50% dose intake simulation
ev(ID = 5601:6000, amt = 15*1, ii= 24, ss= 1, addl=0),
ev(ID = 5601:6000, amt = 15*0, ii= 18, ss= 0, addl=0),
ev(ID = 5601:6000, amt = 15*0.5, ii= 6, ss= 0, addl=0),
ev(ID = 5601:6000, amt = 15*1, ii= 24, ss= 0, addl=10),
#18h delay simulation
ev(ID = 5601:6000, amt = 15*1, ii= 24, ss= 1, addl=0),
ev(ID = 5601:6000, amt = 15*0, ii= 18, ss= 0, addl=0),
ev(ID = 5601:6000, amt = 15*1, ii= 6, ss= 0, addl=0),
ev(ID = 5601:6000, amt = 15*1, ii= 24, ss= 0, addl=10),
#21h delay + 50% dose intake simulation
ev(ID = 5601:6000, amt = 15*1, ii= 24, ss= 1, addl=0),
ev(ID = 5601:6000, amt = 15*0, ii= 21, ss= 0, addl=0),
ev(ID = 5601:6000, amt = 15*0.5, ii= 3, ss= 0, addl=0),
ev(ID = 5601:6000, amt = 15*1, ii= 24, ss= 0, addl=10),
#21h delay simulation
ev(ID = 5601:6000, amt = 15*1, ii= 24, ss= 1, addl=0),
ev(ID = 5601:6000, amt = 15*0, ii= 21, ss= 0, addl=0),
ev(ID = 5601:6000, amt = 15*1, ii= 3, ss= 0, addl=0),
ev(ID = 5601:6000, amt = 15*1, ii= 24, ss= 0, addl=10),
#Missed dose simulation
ev(ID = 5601:6000, amt = 15*1, ii= 24, ss= 1, addl=0),
ev(ID = 5601:6000, amt = 15*0, ii= 24, ss= 0, addl=0),
ev(ID = 5601:6000, amt = 15*1, ii= 24, ss= 0, addl=0),
ev(ID = 5601:6000, amt = 15*1, ii= 24, ss= 0, addl=10),
#Missed dose + 150% dose intake simulation
ev(ID = 5601:6000, amt = 15*1, ii= 24, ss= 1, addl=0),
ev(ID = 5601:6000, amt = 15*0, ii= 24, ss= 0, addl=0),
ev(ID = 5601:6000, amt = 15*1.5, ii= 24, ss= 0, addl=0),
ev(ID = 5601:6000, amt = 15*1, ii= 24, ss= 0, addl=10),
#Missed dose + 200% dose intake simulation
ev(ID = 5601:6000, amt = 15*1, ii= 24, ss= 1, addl=0),
ev(ID = 5601:6000, amt = 15*0, ii= 24, ss= 0, addl=0),
ev(ID = 5601:6000, amt = 15*2, ii= 24, ss= 0, addl=0),
ev(ID = 5601:6000, amt = 15*1, ii= 24, ss= 0, addl=10)))) %>%
             mutate(dose_group = "15 mg")


Dataset_martial_6000 <- Simulation_data_martial_6000 %>%
  arrange(ID)

set.seed(123456)
Sim_MARTIAL_by_amt_6000 <- my_model_envarsus %>%
 data_set(Dataset_martial_6000) %>%
 Req(DV) %>%
 mrgsim(end = 7000, delta = 1)
```

```
## Warning in (function (x, data, idata = no_idata_set(), carry_out = carry.out, : [steady_bolus] ID 17 failed to
reach steady state
##   ss_n: 500, ss_rtol: 1e-08, ss_atol: 1e-08
```

```
## Warning in (function (x, data, idata = no_idata_set(), carry_out = carry.out, : [steady_bolus] ID 208 failed t
o reach steady state
##   ss_n: 500, ss_rtol: 1e-08, ss_atol: 1e-08
## Warning in (function (x, data, idata = no_idata_set(), carry_out = carry.out, : [steady_bolus] ID 208 failed t
o reach steady state
##   ss_n: 500, ss_rtol: 1e-08, ss_atol: 1e-08
## Warning in (function (x, data, idata = no_idata_set(), carry_out = carry.out, : [steady_bolus] ID 208 failed t
o reach steady state
##   ss_n: 500, ss_rtol: 1e-08, ss_atol: 1e-08
## Warning in (function (x, data, idata = no_idata_set(), carry_out = carry.out, : [steady_bolus] ID 208 failed t
o reach steady state
##   ss_n: 500, ss_rtol: 1e-08, ss_atol: 1e-08
## Warning in (function (x, data, idata = no_idata_set(), carry_out = carry.out, : [steady_bolus] ID 208 failed t
o reach steady state
##   ss_n: 500, ss_rtol: 1e-08, ss_atol: 1e-08
## Warning in (function (x, data, idata = no_idata_set(), carry_out = carry.out, : [steady_bolus] ID 208 failed t
o reach steady state
##   ss_n: 500, ss_rtol: 1e-08, ss_atol: 1e-08
```

```
## Warning in (function (x, data, idata = no_idata_set(), carry_out = carry.out, : [steady_bolus] ID 252 failed t
o reach steady state
##   ss_n: 500, ss_rtol: 1e-08, ss_atol: 1e-08
## Warning in (function (x, data, idata = no_idata_set(), carry_out = carry.out, : [steady_bolus] ID 252 failed t
o reach steady state
##   ss_n: 500, ss_rtol: 1e-08, ss_atol: 1e-08
```

```
## Warning in (function (x, data, idata = no_idata_set(), carry_out = carry.out, : [steady_bolus] ID 323 failed t
o reach steady state
##   ss_n: 500, ss_rtol: 1e-08, ss_atol: 1e-08
```

```
## Warning in (function (x, data, idata = no_idata_set(), carry_out = carry.out, : [steady_bolus] ID 370 failed t
o reach steady state
##   ss_n: 500, ss_rtol: 1e-08, ss_atol: 1e-08
## Warning in (function (x, data, idata = no_idata_set(), carry_out = carry.out, : [steady_bolus] ID 370 failed t
o reach steady state
##   ss_n: 500, ss_rtol: 1e-08, ss_atol: 1e-08
## Warning in (function (x, data, idata = no_idata_set(), carry_out = carry.out, : [steady_bolus] ID 370 failed t
o reach steady state
##   ss_n: 500, ss_rtol: 1e-08, ss_atol: 1e-08
## Warning in (function (x, data, idata = no_idata_set(), carry_out = carry.out, : [steady_bolus] ID 370 failed t
o reach steady state
##   ss_n: 500, ss_rtol: 1e-08, ss_atol: 1e-08
## Warning in (function (x, data, idata = no_idata_set(), carry_out = carry.out, : [steady_bolus] ID 370 failed t
o reach steady state
##   ss_n: 500, ss_rtol: 1e-08, ss_atol: 1e-08
## Warning in (function (x, data, idata = no_idata_set(), carry_out = carry.out, : [steady_bolus] ID 370 failed t
o reach steady state
##   ss_n: 500, ss_rtol: 1e-08, ss_atol: 1e-08
```

```
## Warning in (function (x, data, idata = no_idata_set(), carry_out = carry.out, : [steady_bolus] ID 417 failed t
o reach steady state
##   ss_n: 500, ss_rtol: 1e-08, ss_atol: 1e-08
```

```
## Warning in (function (x, data, idata = no_idata_set(), carry_out = carry.out, : [steady_bolus] ID 541 failed t
o reach steady state
##   ss_n: 500, ss_rtol: 1e-08, ss_atol: 1e-08
## Warning in (function (x, data, idata = no_idata_set(), carry_out = carry.out, : [steady_bolus] ID 541 failed t
o reach steady state
##   ss_n: 500, ss_rtol: 1e-08, ss_atol: 1e-08
```

```
## Warning in (function (x, data, idata = no_idata_set(), carry_out = carry.out, : [steady_bolus] ID 597 failed t
o reach steady state
##   ss_n: 500, ss_rtol: 1e-08, ss_atol: 1e-08
## Warning in (function (x, data, idata = no_idata_set(), carry_out = carry.out, : [steady_bolus] ID 597 failed t
o reach steady state
##   ss_n: 500, ss_rtol: 1e-08, ss_atol: 1e-08
## Warning in (function (x, data, idata = no_idata_set(), carry_out = carry.out, : [steady_bolus] ID 597 failed t
o reach steady state
##   ss_n: 500, ss_rtol: 1e-08, ss_atol: 1e-08
## Warning in (function (x, data, idata = no_idata_set(), carry_out = carry.out, : [steady_bolus] ID 597 failed t
o reach steady state
##   ss_n: 500, ss_rtol: 1e-08, ss_atol: 1e-08
## Warning in (function (x, data, idata = no_idata_set(), carry_out = carry.out, : [steady_bolus] ID 597 failed t
o reach steady state
##   ss_n: 500, ss_rtol: 1e-08, ss_atol: 1e-08
## Warning in (function (x, data, idata = no_idata_set(), carry_out = carry.out, : [steady_bolus] ID 597 failed t
o reach steady state
##   ss_n: 500, ss_rtol: 1e-08, ss_atol: 1e-08
## Warning in (function (x, data, idata = no_idata_set(), carry_out = carry.out, : [steady_bolus] ID 597 failed t
o reach steady state
##   ss_n: 500, ss_rtol: 1e-08, ss_atol: 1e-08
```

```
## Warning in (function (x, data, idata = no_idata_set(), carry_out = carry.out, : [steady_bolus] ID 617 failed t
o reach steady state
##   ss_n: 500, ss_rtol: 1e-08, ss_atol: 1e-08
## Warning in (function (x, data, idata = no_idata_set(), carry_out = carry.out, : [steady_bolus] ID 617 failed t
o reach steady state
##   ss_n: 500, ss_rtol: 1e-08, ss_atol: 1e-08
## Warning in (function (x, data, idata = no_idata_set(), carry_out = carry.out, : [steady_bolus] ID 617 failed t
o reach steady state
##   ss_n: 500, ss_rtol: 1e-08, ss_atol: 1e-08
## Warning in (function (x, data, idata = no_idata_set(), carry_out = carry.out, : [steady_bolus] ID 617 failed t
o reach steady state
##   ss_n: 500, ss_rtol: 1e-08, ss_atol: 1e-08
## Warning in (function (x, data, idata = no_idata_set(), carry_out = carry.out, : [steady_bolus] ID 617 failed t
o reach steady state
##   ss_n: 500, ss_rtol: 1e-08, ss_atol: 1e-08
## Warning in (function (x, data, idata = no_idata_set(), carry_out = carry.out, : [steady_bolus] ID 617 failed t
o reach steady state
##   ss_n: 500, ss_rtol: 1e-08, ss_atol: 1e-08
```

```
## Warning in (function (x, data, idata = no_idata_set(), carry_out = carry.out, : [steady_bolus] ID 659 failed t
o reach steady state
##   ss_n: 500, ss_rtol: 1e-08, ss_atol: 1e-08
## Warning in (function (x, data, idata = no_idata_set(), carry_out = carry.out, : [steady_bolus] ID 659 failed t
o reach steady state
##   ss_n: 500, ss_rtol: 1e-08, ss_atol: 1e-08
```

```
## Warning in (function (x, data, idata = no_idata_set(), carry_out = carry.out, : [steady_bolus] ID 892 failed t
o reach steady state
##   ss_n: 500, ss_rtol: 1e-08, ss_atol: 1e-08
```

```
## Warning in (function (x, data, idata = no_idata_set(), carry_out = carry.out, : [steady_bolus] ID 1054 failed
to reach steady state
##   ss_n: 500, ss_rtol: 1e-08, ss_atol: 1e-08
```

```
## Warning in (function (x, data, idata = no_idata_set(), carry_out = carry.out, : [steady_bolus] ID 1076 failed
to reach steady state
##   ss_n: 500, ss_rtol: 1e-08, ss_atol: 1e-08
## Warning in (function (x, data, idata = no_idata_set(), carry_out = carry.out, : [steady_bolus] ID 1076 failed
to reach steady state
##   ss_n: 500, ss_rtol: 1e-08, ss_atol: 1e-08
## Warning in (function (x, data, idata = no_idata_set(), carry_out = carry.out, : [steady_bolus] ID 1076 failed
to reach steady state
##   ss_n: 500, ss_rtol: 1e-08, ss_atol: 1e-08
## Warning in (function (x, data, idata = no_idata_set(), carry_out = carry.out, : [steady_bolus] ID 1076 failed
to reach steady state
##   ss_n: 500, ss_rtol: 1e-08, ss_atol: 1e-08
## Warning in (function (x, data, idata = no_idata_set(), carry_out = carry.out, : [steady_bolus] ID 1076 failed
to reach steady state
##   ss_n: 500, ss_rtol: 1e-08, ss_atol: 1e-08
## Warning in (function (x, data, idata = no_idata_set(), carry_out = carry.out, : [steady_bolus] ID 1076 failed
to reach steady state
##   ss_n: 500, ss_rtol: 1e-08, ss_atol: 1e-08
## Warning in (function (x, data, idata = no_idata_set(), carry_out = carry.out, : [steady_bolus] ID 1076 failed
to reach steady state
##   ss_n: 500, ss_rtol: 1e-08, ss_atol: 1e-08
```

```
## Warning in (function (x, data, idata = no_idata_set(), carry_out = carry.out, : [steady_bolus] ID 1114 failed
to reach steady state
##   ss_n: 500, ss_rtol: 1e-08, ss_atol: 1e-08
```

```
## Warning in (function (x, data, idata = no_idata_set(), carry_out = carry.out, : [steady_bolus] ID 1124 failed
to reach steady state
##   ss_n: 500, ss_rtol: 1e-08, ss_atol: 1e-08
## Warning in (function (x, data, idata = no_idata_set(), carry_out = carry.out, : [steady_bolus] ID 1124 failed
to reach steady state
##   ss_n: 500, ss_rtol: 1e-08, ss_atol: 1e-08
## Warning in (function (x, data, idata = no_idata_set(), carry_out = carry.out, : [steady_bolus] ID 1124 failed
to reach steady state
##   ss_n: 500, ss_rtol: 1e-08, ss_atol: 1e-08
## Warning in (function (x, data, idata = no_idata_set(), carry_out = carry.out, : [steady_bolus] ID 1124 failed
to reach steady state
##   ss_n: 500, ss_rtol: 1e-08, ss_atol: 1e-08
## Warning in (function (x, data, idata = no_idata_set(), carry_out = carry.out, : [steady_bolus] ID 1124 failed
to reach steady state
##   ss_n: 500, ss_rtol: 1e-08, ss_atol: 1e-08
## Warning in (function (x, data, idata = no_idata_set(), carry_out = carry.out, : [steady_bolus] ID 1124 failed
to reach steady state
##   ss_n: 500, ss_rtol: 1e-08, ss_atol: 1e-08
```

```
## Warning in (function (x, data, idata = no_idata_set(), carry_out = carry.out, : [steady_bolus] ID 1153 failed
to reach steady state
##   ss_n: 500, ss_rtol: 1e-08, ss_atol: 1e-08
## Warning in (function (x, data, idata = no_idata_set(), carry_out = carry.out, : [steady_bolus] ID 1153 failed
to reach steady state
##   ss_n: 500, ss_rtol: 1e-08, ss_atol: 1e-08
## Warning in (function (x, data, idata = no_idata_set(), carry_out = carry.out, : [steady_bolus] ID 1153 failed
to reach steady state
##   ss_n: 500, ss_rtol: 1e-08, ss_atol: 1e-08
## Warning in (function (x, data, idata = no_idata_set(), carry_out = carry.out, : [steady_bolus] ID 1153 failed
to reach steady state
##   ss_n: 500, ss_rtol: 1e-08, ss_atol: 1e-08
## Warning in (function (x, data, idata = no_idata_set(), carry_out = carry.out, : [steady_bolus] ID 1153 failed
to reach steady state
##   ss_n: 500, ss_rtol: 1e-08, ss_atol: 1e-08
## Warning in (function (x, data, idata = no_idata_set(), carry_out = carry.out, : [steady_bolus] ID 1153 failed
to reach steady state
##   ss_n: 500, ss_rtol: 1e-08, ss_atol: 1e-08
```

```
## Warning in (function (x, data, idata = no_idata_set(), carry_out = carry.out, : [steady_bolus] ID 1272 failed
to reach steady state
##   ss_n: 500, ss_rtol: 1e-08, ss_atol: 1e-08
## Warning in (function (x, data, idata = no_idata_set(), carry_out = carry.out, : [steady_bolus] ID 1272 failed
to reach steady state
##   ss_n: 500, ss_rtol: 1e-08, ss_atol: 1e-08
## Warning in (function (x, data, idata = no_idata_set(), carry_out = carry.out, : [steady_bolus] ID 1272 failed
to reach steady state
##   ss_n: 500, ss_rtol: 1e-08, ss_atol: 1e-08
## Warning in (function (x, data, idata = no_idata_set(), carry_out = carry.out, : [steady_bolus] ID 1272 failed
to reach steady state
##   ss_n: 500, ss_rtol: 1e-08, ss_atol: 1e-08
## Warning in (function (x, data, idata = no_idata_set(), carry_out = carry.out, : [steady_bolus] ID 1272 failed
to reach steady state
##   ss_n: 500, ss_rtol: 1e-08, ss_atol: 1e-08
## Warning in (function (x, data, idata = no_idata_set(), carry_out = carry.out, : [steady_bolus] ID 1272 failed
to reach steady state
##   ss_n: 500, ss_rtol: 1e-08, ss_atol: 1e-08
```

```
## Warning in (function (x, data, idata = no_idata_set(), carry_out = carry.out, : [steady_bolus] ID 1326 failed
to reach steady state
##   ss_n: 500, ss_rtol: 1e-08, ss_atol: 1e-08
## Warning in (function (x, data, idata = no_idata_set(), carry_out = carry.out, : [steady_bolus] ID 1326 failed
to reach steady state
##   ss_n: 500, ss_rtol: 1e-08, ss_atol: 1e-08
## Warning in (function (x, data, idata = no_idata_set(), carry_out = carry.out, : [steady_bolus] ID 1326 failed
to reach steady state
##   ss_n: 500, ss_rtol: 1e-08, ss_atol: 1e-08
## Warning in (function (x, data, idata = no_idata_set(), carry_out = carry.out, : [steady_bolus] ID 1326 failed
to reach steady state
##   ss_n: 500, ss_rtol: 1e-08, ss_atol: 1e-08
## Warning in (function (x, data, idata = no_idata_set(), carry_out = carry.out, : [steady_bolus] ID 1326 failed
to reach steady state
##   ss_n: 500, ss_rtol: 1e-08, ss_atol: 1e-08
## Warning in (function (x, data, idata = no_idata_set(), carry_out = carry.out, : [steady_bolus] ID 1326 failed
to reach steady state
##   ss_n: 500, ss_rtol: 1e-08, ss_atol: 1e-08
```

```
## Warning in (function (x, data, idata = no_idata_set(), carry_out = carry.out, : [steady_bolus] ID 1328 failed
to reach steady state
##   ss_n: 500, ss_rtol: 1e-08, ss_atol: 1e-08
```

```
## Warning in (function (x, data, idata = no_idata_set(), carry_out = carry.out, : [steady_bolus] ID 1329 failed
to reach steady state
##   ss_n: 500, ss_rtol: 1e-08, ss_atol: 1e-08
## Warning in (function (x, data, idata = no_idata_set(), carry_out = carry.out, : [steady_bolus] ID 1329 failed
to reach steady state
##   ss_n: 500, ss_rtol: 1e-08, ss_atol: 1e-08
## Warning in (function (x, data, idata = no_idata_set(), carry_out = carry.out, : [steady_bolus] ID 1329 failed
to reach steady state
##   ss_n: 500, ss_rtol: 1e-08, ss_atol: 1e-08
## Warning in (function (x, data, idata = no_idata_set(), carry_out = carry.out, : [steady_bolus] ID 1329 failed
to reach steady state
##   ss_n: 500, ss_rtol: 1e-08, ss_atol: 1e-08
## Warning in (function (x, data, idata = no_idata_set(), carry_out = carry.out, : [steady_bolus] ID 1329 failed
to reach steady state
##   ss_n: 500, ss_rtol: 1e-08, ss_atol: 1e-08
## Warning in (function (x, data, idata = no_idata_set(), carry_out = carry.out, : [steady_bolus] ID 1329 failed
to reach steady state
##   ss_n: 500, ss_rtol: 1e-08, ss_atol: 1e-08
## Warning in (function (x, data, idata = no_idata_set(), carry_out = carry.out, : [steady_bolus] ID 1329 failed
to reach steady state
##   ss_n: 500, ss_rtol: 1e-08, ss_atol: 1e-08
```

```
## Warning in (function (x, data, idata = no_idata_set(), carry_out = carry.out, : [steady_bolus] ID 1338 failed
to reach steady state
##   ss_n: 500, ss_rtol: 1e-08, ss_atol: 1e-08
```

```
## Warning in (function (x, data, idata = no_idata_set(), carry_out = carry.out, : [steady_bolus] ID 1374 failed
to reach steady state
##   ss_n: 500, ss_rtol: 1e-08, ss_atol: 1e-08
## Warning in (function (x, data, idata = no_idata_set(), carry_out = carry.out, : [steady_bolus] ID 1374 failed
to reach steady state
##   ss_n: 500, ss_rtol: 1e-08, ss_atol: 1e-08
## Warning in (function (x, data, idata = no_idata_set(), carry_out = carry.out, : [steady_bolus] ID 1374 failed
to reach steady state
##   ss_n: 500, ss_rtol: 1e-08, ss_atol: 1e-08
## Warning in (function (x, data, idata = no_idata_set(), carry_out = carry.out, : [steady_bolus] ID 1374 failed
to reach steady state
##   ss_n: 500, ss_rtol: 1e-08, ss_atol: 1e-08
## Warning in (function (x, data, idata = no_idata_set(), carry_out = carry.out, : [steady_bolus] ID 1374 failed
to reach steady state
##   ss_n: 500, ss_rtol: 1e-08, ss_atol: 1e-08
## Warning in (function (x, data, idata = no_idata_set(), carry_out = carry.out, : [steady_bolus] ID 1374 failed
to reach steady state
##   ss_n: 500, ss_rtol: 1e-08, ss_atol: 1e-08
```

```
## Warning in (function (x, data, idata = no_idata_set(), carry_out = carry.out, : [steady_bolus] ID 1629 failed
to reach steady state
##   ss_n: 500, ss_rtol: 1e-08, ss_atol: 1e-08
## Warning in (function (x, data, idata = no_idata_set(), carry_out = carry.out, : [steady_bolus] ID 1629 failed
to reach steady state
##   ss_n: 500, ss_rtol: 1e-08, ss_atol: 1e-08
## Warning in (function (x, data, idata = no_idata_set(), carry_out = carry.out, : [steady_bolus] ID 1629 failed
to reach steady state
##   ss_n: 500, ss_rtol: 1e-08, ss_atol: 1e-08
## Warning in (function (x, data, idata = no_idata_set(), carry_out = carry.out, : [steady_bolus] ID 1629 failed
to reach steady state
##   ss_n: 500, ss_rtol: 1e-08, ss_atol: 1e-08
## Warning in (function (x, data, idata = no_idata_set(), carry_out = carry.out, : [steady_bolus] ID 1629 failed
to reach steady state
##   ss_n: 500, ss_rtol: 1e-08, ss_atol: 1e-08
## Warning in (function (x, data, idata = no_idata_set(), carry_out = carry.out, : [steady_bolus] ID 1629 failed
to reach steady state
##   ss_n: 500, ss_rtol: 1e-08, ss_atol: 1e-08
## Warning in (function (x, data, idata = no_idata_set(), carry_out = carry.out, : [steady_bolus] ID 1629 failed
to reach steady state
##   ss_n: 500, ss_rtol: 1e-08, ss_atol: 1e-08
```

```
## Warning in (function (x, data, idata = no_idata_set(), carry_out = carry.out, : [steady_bolus] ID 1687 failed
to reach steady state
##   ss_n: 500, ss_rtol: 1e-08, ss_atol: 1e-08
```

```
## Warning in (function (x, data, idata = no_idata_set(), carry_out = carry.out, : [steady_bolus] ID 1805 failed
to reach steady state
##   ss_n: 500, ss_rtol: 1e-08, ss_atol: 1e-08
```

```
## Warning in (function (x, data, idata = no_idata_set(), carry_out = carry.out, : [steady_bolus] ID 1865 failed
to reach steady state
##   ss_n: 500, ss_rtol: 1e-08, ss_atol: 1e-08
## Warning in (function (x, data, idata = no_idata_set(), carry_out = carry.out, : [steady_bolus] ID 1865 failed
to reach steady state
##   ss_n: 500, ss_rtol: 1e-08, ss_atol: 1e-08
## Warning in (function (x, data, idata = no_idata_set(), carry_out = carry.out, : [steady_bolus] ID 1865 failed
to reach steady state
##   ss_n: 500, ss_rtol: 1e-08, ss_atol: 1e-08
## Warning in (function (x, data, idata = no_idata_set(), carry_out = carry.out, : [steady_bolus] ID 1865 failed
to reach steady state
##   ss_n: 500, ss_rtol: 1e-08, ss_atol: 1e-08
## Warning in (function (x, data, idata = no_idata_set(), carry_out = carry.out, : [steady_bolus] ID 1865 failed
to reach steady state
##   ss_n: 500, ss_rtol: 1e-08, ss_atol: 1e-08
## Warning in (function (x, data, idata = no_idata_set(), carry_out = carry.out, : [steady_bolus] ID 1865 failed
to reach steady state
##   ss_n: 500, ss_rtol: 1e-08, ss_atol: 1e-08
## Warning in (function (x, data, idata = no_idata_set(), carry_out = carry.out, : [steady_bolus] ID 1865 failed
to reach steady state
##   ss_n: 500, ss_rtol: 1e-08, ss_atol: 1e-08
```

```
## Warning in (function (x, data, idata = no_idata_set(), carry_out = carry.out, : [steady_bolus] ID 2075 failed
to reach steady state
##   ss_n: 500, ss_rtol: 1e-08, ss_atol: 1e-08
## Warning in (function (x, data, idata = no_idata_set(), carry_out = carry.out, : [steady_bolus] ID 2075 failed
to reach steady state
##   ss_n: 500, ss_rtol: 1e-08, ss_atol: 1e-08
```

```
## Warning in (function (x, data, idata = no_idata_set(), carry_out = carry.out, : [steady_bolus] ID 2129 failed
to reach steady state
##   ss_n: 500, ss_rtol: 1e-08, ss_atol: 1e-08
## Warning in (function (x, data, idata = no_idata_set(), carry_out = carry.out, : [steady_bolus] ID 2129 failed
to reach steady state
##   ss_n: 500, ss_rtol: 1e-08, ss_atol: 1e-08
```

```
## Warning in (function (x, data, idata = no_idata_set(), carry_out = carry.out, : [steady_bolus] ID 2154 failed
to reach steady state
##   ss_n: 500, ss_rtol: 1e-08, ss_atol: 1e-08
## Warning in (function (x, data, idata = no_idata_set(), carry_out = carry.out, : [steady_bolus] ID 2154 failed
to reach steady state
##   ss_n: 500, ss_rtol: 1e-08, ss_atol: 1e-08
## Warning in (function (x, data, idata = no_idata_set(), carry_out = carry.out, : [steady_bolus] ID 2154 failed
to reach steady state
##   ss_n: 500, ss_rtol: 1e-08, ss_atol: 1e-08
## Warning in (function (x, data, idata = no_idata_set(), carry_out = carry.out, : [steady_bolus] ID 2154 failed
```

```
to reach steady state
##   ss_n: 500, ss_rtol: 1e-08, ss_atol: 1e-08
## Warning in (function (x, data, idata = no_idata_set(), carry_out = carry.out, : [steady_bolus] ID 2154 failed
to reach steady state
##   ss_n: 500, ss_rtol: 1e-08, ss_atol: 1e-08
## Warning in (function (x, data, idata = no_idata_set(), carry_out = carry.out, : [steady_bolus] ID 2154 failed
to reach steady state
##   ss_n: 500, ss_rtol: 1e-08, ss_atol: 1e-08
## Warning in (function (x, data, idata = no_idata_set(), carry_out = carry.out, : [steady_bolus] ID 2154 failed
to reach steady state
##   ss_n: 500, ss_rtol: 1e-08, ss_atol: 1e-08
## Warning in (function (x, data, idata = no_idata_set(), carry_out = carry.out, : [steady_bolus] ID 2154 failed
to reach steady state
##   ss_n: 500, ss_rtol: 1e-08, ss_atol: 1e-08
## Warning in (function (x, data, idata = no_idata_set(), carry_out = carry.out, : [steady_bolus] ID 2154 failed
to reach steady state
##   ss_n: 500, ss_rtol: 1e-08, ss_atol: 1e-08
## Warning in (function (x, data, idata = no_idata_set(), carry_out = carry.out, : [steady_bolus] ID 2154 failed
to reach steady state
##   ss_n: 500, ss_rtol: 1e-08, ss_atol: 1e-08
## Warning in (function (x, data, idata = no_idata_set(), carry_out = carry.out, : [steady_bolus] ID 2154 failed
to reach steady state
##   ss_n: 500, ss_rtol: 1e-08, ss_atol: 1e-08
## Warning in (function (x, data, idata = no_idata_set(), carry_out = carry.out, : [steady_bolus] ID 2154 failed
to reach steady state
##   ss_n: 500, ss_rtol: 1e-08, ss_atol: 1e-08
```

```
## Warning in (function (x, data, idata = no_idata_set(), carry_out = carry.out, : [steady_bolus] ID 2187 failed
to reach steady state
##   ss_n: 500, ss_rtol: 1e-08, ss_atol: 1e-08
```

```
## Warning in (function (x, data, idata = no_idata_set(), carry_out = carry.out, : [steady_bolus] ID 2217 failed
to reach steady state
##   ss_n: 500, ss_rtol: 1e-08, ss_atol: 1e-08
```

```
## Warning in (function (x, data, idata = no_idata_set(), carry_out = carry.out, : [steady_bolus] ID 2292 failed
to reach steady state
##   ss_n: 500, ss_rtol: 1e-08, ss_atol: 1e-08
```

```
## Warning in (function (x, data, idata = no_idata_set(), carry_out = carry.out, : [steady_bolus] ID 2422 failed
to reach steady state
##   ss_n: 500, ss_rtol: 1e-08, ss_atol: 1e-08
```

```
## Warning in (function (x, data, idata = no_idata_set(), carry_out = carry.out, : [steady_bolus] ID 2498 failed
to reach steady state
##   ss_n: 500, ss_rtol: 1e-08, ss_atol: 1e-08
```

```
## Warning in (function (x, data, idata = no_idata_set(), carry_out = carry.out, : [steady_bolus] ID 2517 failed
to reach steady state
##   ss_n: 500, ss_rtol: 1e-08, ss_atol: 1e-08
```

```
## Warning in (function (x, data, idata = no_idata_set(), carry_out = carry.out, : [steady_bolus] ID 2559 failed
to reach steady state
##   ss_n: 500, ss_rtol: 1e-08, ss_atol: 1e-08
## Warning in (function (x, data, idata = no_idata_set(), carry_out = carry.out, : [steady_bolus] ID 2559 failed
to reach steady state
##   ss_n: 500, ss_rtol: 1e-08, ss_atol: 1e-08
## Warning in (function (x, data, idata = no_idata_set(), carry_out = carry.out, : [steady_bolus] ID 2559 failed
to reach steady state
##   ss_n: 500, ss_rtol: 1e-08, ss_atol: 1e-08
## Warning in (function (x, data, idata = no_idata_set(), carry_out = carry.out, : [steady_bolus] ID 2559 failed
to reach steady state
##   ss_n: 500, ss_rtol: 1e-08, ss_atol: 1e-08
## Warning in (function (x, data, idata = no_idata_set(), carry_out = carry.out, : [steady_bolus] ID 2559 failed
to reach steady state
##   ss_n: 500, ss_rtol: 1e-08, ss_atol: 1e-08
## Warning in (function (x, data, idata = no_idata_set(), carry_out = carry.out, : [steady_bolus] ID 2559 failed
to reach steady state
##   ss_n: 500, ss_rtol: 1e-08, ss_atol: 1e-08
## Warning in (function (x, data, idata = no_idata_set(), carry_out = carry.out, : [steady_bolus] ID 2559 failed
to reach steady state
##   ss_n: 500, ss_rtol: 1e-08, ss_atol: 1e-08
```

```
## Warning in (function (x, data, idata = no_idata_set(), carry_out = carry.out, : [steady_bolus] ID 2565 failed
to reach steady state
##   ss_n: 500, ss_rtol: 1e-08, ss_atol: 1e-08
```

```
## Warning in (function (x, data, idata = no_idata_set(), carry_out = carry.out, : [steady_bolus] ID 2695 failed
to reach steady state
##   ss_n: 500, ss_rtol: 1e-08, ss_atol: 1e-08
## Warning in (function (x, data, idata = no_idata_set(), carry_out = carry.out, : [steady_bolus] ID 2695 failed
to reach steady state
##   ss_n: 500, ss_rtol: 1e-08, ss_atol: 1e-08
## Warning in (function (x, data, idata = no_idata_set(), carry_out = carry.out, : [steady_bolus] ID 2695 failed
to reach steady state
##   ss_n: 500, ss_rtol: 1e-08, ss_atol: 1e-08
## Warning in (function (x, data, idata = no_idata_set(), carry_out = carry.out, : [steady_bolus] ID 2695 failed
to reach steady state
##   ss_n: 500, ss_rtol: 1e-08, ss_atol: 1e-08
## Warning in (function (x, data, idata = no_idata_set(), carry_out = carry.out, : [steady_bolus] ID 2695 failed
to reach steady state
##   ss_n: 500, ss_rtol: 1e-08, ss_atol: 1e-08
## Warning in (function (x, data, idata = no_idata_set(), carry_out = carry.out, : [steady_bolus] ID 2695 failed
to reach steady state
##   ss_n: 500, ss_rtol: 1e-08, ss_atol: 1e-08
```

```
## Warning in (function (x, data, idata = no_idata_set(), carry_out = carry.out, : [steady_bolus] ID 2709 failed
to reach steady state
##   ss_n: 500, ss_rtol: 1e-08, ss_atol: 1e-08
## Warning in (function (x, data, idata = no_idata_set(), carry_out = carry.out, : [steady_bolus] ID 2709 failed
to reach steady state
##   ss_n: 500, ss_rtol: 1e-08, ss_atol: 1e-08
## Warning in (function (x, data, idata = no_idata_set(), carry_out = carry.out, : [steady_bolus] ID 2709 failed
to reach steady state
##   ss_n: 500, ss_rtol: 1e-08, ss_atol: 1e-08
## Warning in (function (x, data, idata = no_idata_set(), carry_out = carry.out, : [steady_bolus] ID 2709 failed
to reach steady state
##   ss_n: 500, ss_rtol: 1e-08, ss_atol: 1e-08
## Warning in (function (x, data, idata = no_idata_set(), carry_out = carry.out, : [steady_bolus] ID 2709 failed
to reach steady state
##   ss_n: 500, ss_rtol: 1e-08, ss_atol: 1e-08
## Warning in (function (x, data, idata = no_idata_set(), carry_out = carry.out, : [steady_bolus] ID 2709 failed
to reach steady state
##   ss_n: 500, ss_rtol: 1e-08, ss_atol: 1e-08
## Warning in (function (x, data, idata = no_idata_set(), carry_out = carry.out, : [steady_bolus] ID 2709 failed
to reach steady state
##   ss_n: 500, ss_rtol: 1e-08, ss_atol: 1e-08
## Warning in (function (x, data, idata = no_idata_set(), carry_out = carry.out, : [steady_bolus] ID 2709 failed
to reach steady state
##   ss_n: 500, ss_rtol: 1e-08, ss_atol: 1e-08
```

```
## Warning in (function (x, data, idata = no_idata_set(), carry_out = carry.out, : [steady_bolus] ID 2780 failed
to reach steady state
##   ss_n: 500, ss_rtol: 1e-08, ss_atol: 1e-08
```

```
## Warning in (function (x, data, idata = no_idata_set(), carry_out = carry.out, : [steady_bolus] ID 2815 failed
to reach steady state
##   ss_n: 500, ss_rtol: 1e-08, ss_atol: 1e-08
```

```
## Warning in (function (x, data, idata = no_idata_set(), carry_out = carry.out, : [steady_bolus] ID 2831 failed
to reach steady state
##   ss_n: 500, ss_rtol: 1e-08, ss_atol: 1e-08
## Warning in (function (x, data, idata = no_idata_set(), carry_out = carry.out, : [steady_bolus] ID 2831 failed
to reach steady state
##   ss_n: 500, ss_rtol: 1e-08, ss_atol: 1e-08
```

```
## Warning in (function (x, data, idata = no_idata_set(), carry_out = carry.out, : [steady_bolus] ID 2870 failed
to reach steady state
##   ss_n: 500, ss_rtol: 1e-08, ss_atol: 1e-08
```

```
## Warning in (function (x, data, idata = no_idata_set(), carry_out = carry.out, : [steady_bolus] ID 2906 failed
to reach steady state
##   ss_n: 500, ss_rtol: 1e-08, ss_atol: 1e-08
## Warning in (function (x, data, idata = no_idata_set(), carry_out = carry.out, : [steady_bolus] ID 2906 failed
to reach steady state
##   ss_n: 500, ss_rtol: 1e-08, ss_atol: 1e-08
## Warning in (function (x, data, idata = no_idata_set(), carry_out = carry.out, : [steady_bolus] ID 2906 failed
to reach steady state
##   ss_n: 500, ss_rtol: 1e-08, ss_atol: 1e-08
## Warning in (function (x, data, idata = no_idata_set(), carry_out = carry.out, : [steady_bolus] ID 2906 failed
to reach steady state
##   ss_n: 500, ss_rtol: 1e-08, ss_atol: 1e-08
## Warning in (function (x, data, idata = no_idata_set(), carry_out = carry.out, : [steady_bolus] ID 2906 failed
to reach steady state
##   ss_n: 500, ss_rtol: 1e-08, ss_atol: 1e-08
## Warning in (function (x, data, idata = no_idata_set(), carry_out = carry.out, : [steady_bolus] ID 2906 failed
to reach steady state
##   ss_n: 500, ss_rtol: 1e-08, ss_atol: 1e-08
## Warning in (function (x, data, idata = no_idata_set(), carry_out = carry.out, : [steady_bolus] ID 2906 failed
to reach steady state
##   ss_n: 500, ss_rtol: 1e-08, ss_atol: 1e-08
## Warning in (function (x, data, idata = no_idata_set(), carry_out = carry.out, : [steady_bolus] ID 2906 failed
to reach steady state
##   ss_n: 500, ss_rtol: 1e-08, ss_atol: 1e-08
## Warning in (function (x, data, idata = no_idata_set(), carry_out = carry.out, : [steady_bolus] ID 2906 failed
to reach steady state
##   ss_n: 500, ss_rtol: 1e-08, ss_atol: 1e-08
```

```
## Warning in (function (x, data, idata = no_idata_set(), carry_out = carry.out, : [steady_bolus] ID 2989 failed
to reach steady state
##   ss_n: 500, ss_rtol: 1e-08, ss_atol: 1e-08
## Warning in (function (x, data, idata = no_idata_set(), carry_out = carry.out, : [steady_bolus] ID 2989 failed
to reach steady state
##   ss_n: 500, ss_rtol: 1e-08, ss_atol: 1e-08
## Warning in (function (x, data, idata = no_idata_set(), carry_out = carry.out, : [steady_bolus] ID 2989 failed
to reach steady state
##   ss_n: 500, ss_rtol: 1e-08, ss_atol: 1e-08
## Warning in (function (x, data, idata = no_idata_set(), carry_out = carry.out, : [steady_bolus] ID 2989 failed
to reach steady state
##   ss_n: 500, ss_rtol: 1e-08, ss_atol: 1e-08
## Warning in (function (x, data, idata = no_idata_set(), carry_out = carry.out, : [steady_bolus] ID 2989 failed
to reach steady state
##   ss_n: 500, ss_rtol: 1e-08, ss_atol: 1e-08
## Warning in (function (x, data, idata = no_idata_set(), carry_out = carry.out, : [steady_bolus] ID 2989 failed
to reach steady state
##   ss_n: 500, ss_rtol: 1e-08, ss_atol: 1e-08
```

```
## Warning in (function (x, data, idata = no_idata_set(), carry_out = carry.out, : [steady_bolus] ID 3018 failed
to reach steady state
##   ss_n: 500, ss_rtol: 1e-08, ss_atol: 1e-08
```

```
## Warning in (function (x, data, idata = no_idata_set(), carry_out = carry.out, : [steady_bolus] ID 3027 failed
to reach steady state
##   ss_n: 500, ss_rtol: 1e-08, ss_atol: 1e-08
## Warning in (function (x, data, idata = no_idata_set(), carry_out = carry.out, : [steady_bolus] ID 3027 failed
to reach steady state
##   ss_n: 500, ss_rtol: 1e-08, ss_atol: 1e-08
## Warning in (function (x, data, idata = no_idata_set(), carry_out = carry.out, : [steady_bolus] ID 3027 failed
to reach steady state
##   ss_n: 500, ss_rtol: 1e-08, ss_atol: 1e-08
## Warning in (function (x, data, idata = no_idata_set(), carry_out = carry.out, : [steady_bolus] ID 3027 failed
to reach steady state
##   ss_n: 500, ss_rtol: 1e-08, ss_atol: 1e-08
## Warning in (function (x, data, idata = no_idata_set(), carry_out = carry.out, : [steady_bolus] ID 3027 failed
to reach steady state
##   ss_n: 500, ss_rtol: 1e-08, ss_atol: 1e-08
## Warning in (function (x, data, idata = no_idata_set(), carry_out = carry.out, : [steady_bolus] ID 3027 failed
to reach steady state
##   ss_n: 500, ss_rtol: 1e-08, ss_atol: 1e-08
## Warning in (function (x, data, idata = no_idata_set(), carry_out = carry.out, : [steady_bolus] ID 3027 failed
to reach steady state
##   ss_n: 500, ss_rtol: 1e-08, ss_atol: 1e-08
```

```
## Warning in (function (x, data, idata = no_idata_set(), carry_out = carry.out, : [steady_bolus] ID 3031 failed
to reach steady state
##   ss_n: 500, ss_rtol: 1e-08, ss_atol: 1e-08
## Warning in (function (x, data, idata = no_idata_set(), carry_out = carry.out, : [steady_bolus] ID 3031 failed
to reach steady state
##   ss_n: 500, ss_rtol: 1e-08, ss_atol: 1e-08
```

```
## Warning in (function (x, data, idata = no_idata_set(), carry_out = carry.out, : [steady_bolus] ID 3117 failed
to reach steady state
##   ss_n: 500, ss_rtol: 1e-08, ss_atol: 1e-08
## Warning in (function (x, data, idata = no_idata_set(), carry_out = carry.out, : [steady_bolus] ID 3117 failed
to reach steady state
##   ss_n: 500, ss_rtol: 1e-08, ss_atol: 1e-08
## Warning in (function (x, data, idata = no_idata_set(), carry_out = carry.out, : [steady_bolus] ID 3117 failed
to reach steady state
##   ss_n: 500, ss_rtol: 1e-08, ss_atol: 1e-08
## Warning in (function (x, data, idata = no_idata_set(), carry_out = carry.out, : [steady_bolus] ID 3117 failed
to reach steady state
##   ss_n: 500, ss_rtol: 1e-08, ss_atol: 1e-08
## Warning in (function (x, data, idata = no_idata_set(), carry_out = carry.out, : [steady_bolus] ID 3117 failed
to reach steady state
##   ss_n: 500, ss_rtol: 1e-08, ss_atol: 1e-08
## Warning in (function (x, data, idata = no_idata_set(), carry_out = carry.out, : [steady_bolus] ID 3117 failed
to reach steady state
##   ss_n: 500, ss_rtol: 1e-08, ss_atol: 1e-08
## Warning in (function (x, data, idata = no_idata_set(), carry_out = carry.out, : [steady_bolus] ID 3117 failed
to reach steady state
##   ss_n: 500, ss_rtol: 1e-08, ss_atol: 1e-08
```

```
## Warning in (function (x, data, idata = no_idata_set(), carry_out = carry.out, : [steady_bolus] ID 3127 failed
to reach steady state
##   ss_n: 500, ss_rtol: 1e-08, ss_atol: 1e-08
## Warning in (function (x, data, idata = no_idata_set(), carry_out = carry.out, : [steady_bolus] ID 3127 failed
to reach steady state
##   ss_n: 500, ss_rtol: 1e-08, ss_atol: 1e-08
## Warning in (function (x, data, idata = no_idata_set(), carry_out = carry.out, : [steady_bolus] ID 3127 failed
to reach steady state
##   ss_n: 500, ss_rtol: 1e-08, ss_atol: 1e-08
## Warning in (function (x, data, idata = no_idata_set(), carry_out = carry.out, : [steady_bolus] ID 3127 failed
to reach steady state
##   ss_n: 500, ss_rtol: 1e-08, ss_atol: 1e-08
## Warning in (function (x, data, idata = no_idata_set(), carry_out = carry.out, : [steady_bolus] ID 3127 failed
to reach steady state
##   ss_n: 500, ss_rtol: 1e-08, ss_atol: 1e-08
## Warning in (function (x, data, idata = no_idata_set(), carry_out = carry.out, : [steady_bolus] ID 3127 failed
to reach steady state
##   ss_n: 500, ss_rtol: 1e-08, ss_atol: 1e-08
## Warning in (function (x, data, idata = no_idata_set(), carry_out = carry.out, : [steady_bolus] ID 3127 failed
to reach steady state
##   ss_n: 500, ss_rtol: 1e-08, ss_atol: 1e-08
## Warning in (function (x, data, idata = no_idata_set(), carry_out = carry.out, : [steady_bolus] ID 3127 failed
to reach steady state
##   ss_n: 500, ss_rtol: 1e-08, ss_atol: 1e-08
## Warning in (function (x, data, idata = no_idata_set(), carry_out = carry.out, : [steady_bolus] ID 3127 failed
to reach steady state
##   ss_n: 500, ss_rtol: 1e-08, ss_atol: 1e-08
## Warning in (function (x, data, idata = no_idata_set(), carry_out = carry.out, : [steady_bolus] ID 3127 failed
to reach steady state
##   ss_n: 500, ss_rtol: 1e-08, ss_atol: 1e-08
## Warning in (function (x, data, idata = no_idata_set(), carry_out = carry.out, : [steady_bolus] ID 3127 failed
to reach steady state
##   ss_n: 500, ss_rtol: 1e-08, ss_atol: 1e-08
```

```
## Warning in (function (x, data, idata = no_idata_set(), carry_out = carry.out, : [steady_bolus] ID 3152 failed
to reach steady state
##   ss_n: 500, ss_rtol: 1e-08, ss_atol: 1e-08
```

```
## Warning in (function (x, data, idata = no_idata_set(), carry_out = carry.out, : [steady_bolus] ID 3264 failed
to reach steady state
##   ss_n: 500, ss_rtol: 1e-08, ss_atol: 1e-08
## Warning in (function (x, data, idata = no_idata_set(), carry_out = carry.out, : [steady_bolus] ID 3264 failed
to reach steady state
##   ss_n: 500, ss_rtol: 1e-08, ss_atol: 1e-08
## Warning in (function (x, data, idata = no_idata_set(), carry_out = carry.out, : [steady_bolus] ID 3264 failed
to reach steady state
##   ss_n: 500, ss_rtol: 1e-08, ss_atol: 1e-08
## Warning in (function (x, data, idata = no_idata_set(), carry_out = carry.out, : [steady_bolus] ID 3264 failed
to reach steady state
##   ss_n: 500, ss_rtol: 1e-08, ss_atol: 1e-08
## Warning in (function (x, data, idata = no_idata_set(), carry_out = carry.out, : [steady_bolus] ID 3264 failed
to reach steady state
##   ss_n: 500, ss_rtol: 1e-08, ss_atol: 1e-08
## Warning in (function (x, data, idata = no_idata_set(), carry_out = carry.out, : [steady_bolus] ID 3264 failed
to reach steady state
##   ss_n: 500, ss_rtol: 1e-08, ss_atol: 1e-08
```

```
## Warning in (function (x, data, idata = no_idata_set(), carry_out = carry.out, : [steady_bolus] ID 3421 failed
to reach steady state
##   ss_n: 500, ss_rtol: 1e-08, ss_atol: 1e-08
```

```
## Warning in (function (x, data, idata = no_idata_set(), carry_out = carry.out, : [steady_bolus] ID 3441 failed
to reach steady state
##   ss_n: 500, ss_rtol: 1e-08, ss_atol: 1e-08
## Warning in (function (x, data, idata = no_idata_set(), carry_out = carry.out, : [steady_bolus] ID 3441 failed
to reach steady state
##   ss_n: 500, ss_rtol: 1e-08, ss_atol: 1e-08
## Warning in (function (x, data, idata = no_idata_set(), carry_out = carry.out, : [steady_bolus] ID 3441 failed
to reach steady state
##   ss_n: 500, ss_rtol: 1e-08, ss_atol: 1e-08
## Warning in (function (x, data, idata = no_idata_set(), carry_out = carry.out, : [steady_bolus] ID 3441 failed
to reach steady state
##   ss_n: 500, ss_rtol: 1e-08, ss_atol: 1e-08
## Warning in (function (x, data, idata = no_idata_set(), carry_out = carry.out, : [steady_bolus] ID 3441 failed
to reach steady state
##   ss_n: 500, ss_rtol: 1e-08, ss_atol: 1e-08
## Warning in (function (x, data, idata = no_idata_set(), carry_out = carry.out, : [steady_bolus] ID 3441 failed
to reach steady state
##   ss_n: 500, ss_rtol: 1e-08, ss_atol: 1e-08
```

```
## Warning in (function (x, data, idata = no_idata_set(), carry_out = carry.out, : [steady_bolus] ID 3448 failed
to reach steady state
##   ss_n: 500, ss_rtol: 1e-08, ss_atol: 1e-08
```

```
## Warning in (function (x, data, idata = no_idata_set(), carry_out = carry.out, : [steady_bolus] ID 3514 failed
to reach steady state
##   ss_n: 500, ss_rtol: 1e-08, ss_atol: 1e-08
## Warning in (function (x, data, idata = no_idata_set(), carry_out = carry.out, : [steady_bolus] ID 3514 failed
to reach steady state
##   ss_n: 500, ss_rtol: 1e-08, ss_atol: 1e-08
## Warning in (function (x, data, idata = no_idata_set(), carry_out = carry.out, : [steady_bolus] ID 3514 failed
to reach steady state
##   ss_n: 500, ss_rtol: 1e-08, ss_atol: 1e-08
## Warning in (function (x, data, idata = no_idata_set(), carry_out = carry.out, : [steady_bolus] ID 3514 failed
to reach steady state
##   ss_n: 500, ss_rtol: 1e-08, ss_atol: 1e-08
## Warning in (function (x, data, idata = no_idata_set(), carry_out = carry.out, : [steady_bolus] ID 3514 failed
to reach steady state
##   ss_n: 500, ss_rtol: 1e-08, ss_atol: 1e-08
## Warning in (function (x, data, idata = no_idata_set(), carry_out = carry.out, : [steady_bolus] ID 3514 failed
to reach steady state
##   ss_n: 500, ss_rtol: 1e-08, ss_atol: 1e-08
```

```
## Warning in (function (x, data, idata = no_idata_set(), carry_out = carry.out, : [steady_bolus] ID 3579 failed
to reach steady state
##   ss_n: 500, ss_rtol: 1e-08, ss_atol: 1e-08
```

```
## Warning in (function (x, data, idata = no_idata_set(), carry_out = carry.out, : [steady_bolus] ID 3614 failed
to reach steady state
##   ss_n: 500, ss_rtol: 1e-08, ss_atol: 1e-08
```

```
## Warning in (function (x, data, idata = no_idata_set(), carry_out = carry.out, : [steady_bolus] ID 3746 failed
to reach steady state
##   ss_n: 500, ss_rtol: 1e-08, ss_atol: 1e-08
```

```
## Warning in (function (x, data, idata = no_idata_set(), carry_out = carry.out, : [steady_bolus] ID 3873 failed
to reach steady state
##   ss_n: 500, ss_rtol: 1e-08, ss_atol: 1e-08
```

```
## Warning in (function (x, data, idata = no_idata_set(), carry_out = carry.out, : [steady_bolus] ID 3875 failed
to reach steady state
##   ss_n: 500, ss_rtol: 1e-08, ss_atol: 1e-08
## Warning in (function (x, data, idata = no_idata_set(), carry_out = carry.out, : [steady_bolus] ID 3875 failed
to reach steady state
##   ss_n: 500, ss_rtol: 1e-08, ss_atol: 1e-08
## Warning in (function (x, data, idata = no_idata_set(), carry_out = carry.out, : [steady_bolus] ID 3875 failed
to reach steady state
##   ss_n: 500, ss_rtol: 1e-08, ss_atol: 1e-08
## Warning in (function (x, data, idata = no_idata_set(), carry_out = carry.out, : [steady_bolus] ID 3875 failed
to reach steady state
##   ss_n: 500, ss_rtol: 1e-08, ss_atol: 1e-08
## Warning in (function (x, data, idata = no_idata_set(), carry_out = carry.out, : [steady_bolus] ID 3875 failed
to reach steady state
##   ss_n: 500, ss_rtol: 1e-08, ss_atol: 1e-08
## Warning in (function (x, data, idata = no_idata_set(), carry_out = carry.out, : [steady_bolus] ID 3875 failed
to reach steady state
##   ss_n: 500, ss_rtol: 1e-08, ss_atol: 1e-08
```

```
## Warning in (function (x, data, idata = no_idata_set(), carry_out = carry.out, : [steady_bolus] ID 3948 failed
to reach steady state
##   ss_n: 500, ss_rtol: 1e-08, ss_atol: 1e-08
```

```
## Warning in (function (x, data, idata = no_idata_set(), carry_out = carry.out, : [steady_bolus] ID 3950 failed
to reach steady state
##   ss_n: 500, ss_rtol: 1e-08, ss_atol: 1e-08
## Warning in (function (x, data, idata = no_idata_set(), carry_out = carry.out, : [steady_bolus] ID 3950 failed
to reach steady state
##   ss_n: 500, ss_rtol: 1e-08, ss_atol: 1e-08
```

```
## Warning in (function (x, data, idata = no_idata_set(), carry_out = carry.out, : [steady_bolus] ID 4007 failed
to reach steady state
##   ss_n: 500, ss_rtol: 1e-08, ss_atol: 1e-08
```

```
## Warning in (function (x, data, idata = no_idata_set(), carry_out = carry.out, : [steady_bolus] ID 4023 failed
to reach steady state
##   ss_n: 500, ss_rtol: 1e-08, ss_atol: 1e-08
## Warning in (function (x, data, idata = no_idata_set(), carry_out = carry.out, : [steady_bolus] ID 4023 failed
to reach steady state
##   ss_n: 500, ss_rtol: 1e-08, ss_atol: 1e-08
## Warning in (function (x, data, idata = no_idata_set(), carry_out = carry.out, : [steady_bolus] ID 4023 failed
to reach steady state
##   ss_n: 500, ss_rtol: 1e-08, ss_atol: 1e-08
## Warning in (function (x, data, idata = no_idata_set(), carry_out = carry.out, : [steady_bolus] ID 4023 failed
to reach steady state
##   ss_n: 500, ss_rtol: 1e-08, ss_atol: 1e-08
## Warning in (function (x, data, idata = no_idata_set(), carry_out = carry.out, : [steady_bolus] ID 4023 failed
to reach steady state
##   ss_n: 500, ss_rtol: 1e-08, ss_atol: 1e-08
## Warning in (function (x, data, idata = no_idata_set(), carry_out = carry.out, : [steady_bolus] ID 4023 failed
to reach steady state
##   ss_n: 500, ss_rtol: 1e-08, ss_atol: 1e-08
```

```
## Warning in (function (x, data, idata = no_idata_set(), carry_out = carry.out, : [steady_bolus] ID 4053 failed
to reach steady state
##   ss_n: 500, ss_rtol: 1e-08, ss_atol: 1e-08
## Warning in (function (x, data, idata = no_idata_set(), carry_out = carry.out, : [steady_bolus] ID 4053 failed
to reach steady state
##   ss_n: 500, ss_rtol: 1e-08, ss_atol: 1e-08
## Warning in (function (x, data, idata = no_idata_set(), carry_out = carry.out, : [steady_bolus] ID 4053 failed
to reach steady state
##   ss_n: 500, ss_rtol: 1e-08, ss_atol: 1e-08
## Warning in (function (x, data, idata = no_idata_set(), carry_out = carry.out, : [steady_bolus] ID 4053 failed
to reach steady state
##   ss_n: 500, ss_rtol: 1e-08, ss_atol: 1e-08
## Warning in (function (x, data, idata = no_idata_set(), carry_out = carry.out, : [steady_bolus] ID 4053 failed
to reach steady state
##   ss_n: 500, ss_rtol: 1e-08, ss_atol: 1e-08
## Warning in (function (x, data, idata = no_idata_set(), carry_out = carry.out, : [steady_bolus] ID 4053 failed
to reach steady state
##   ss_n: 500, ss_rtol: 1e-08, ss_atol: 1e-08
```

```
## Warning in (function (x, data, idata = no_idata_set(), carry_out = carry.out, : [steady_bolus] ID 4061 failed
to reach steady state
##   ss_n: 500, ss_rtol: 1e-08, ss_atol: 1e-08
## Warning in (function (x, data, idata = no_idata_set(), carry_out = carry.out, : [steady_bolus] ID 4061 failed
to reach steady state
##   ss_n: 500, ss_rtol: 1e-08, ss_atol: 1e-08
```

```
## Warning in (function (x, data, idata = no_idata_set(), carry_out = carry.out, : [steady_bolus] ID 4078 failed
to reach steady state
##   ss_n: 500, ss_rtol: 1e-08, ss_atol: 1e-08
```

```
## Warning in (function (x, data, idata = no_idata_set(), carry_out = carry.out, : [steady_bolus] ID 4214 failed
to reach steady state
##   ss_n: 500, ss_rtol: 1e-08, ss_atol: 1e-08
## Warning in (function (x, data, idata = no_idata_set(), carry_out = carry.out, : [steady_bolus] ID 4214 failed
to reach steady state
##   ss_n: 500, ss_rtol: 1e-08, ss_atol: 1e-08
## Warning in (function (x, data, idata = no_idata_set(), carry_out = carry.out, : [steady_bolus] ID 4214 failed
to reach steady state
##   ss_n: 500, ss_rtol: 1e-08, ss_atol: 1e-08
## Warning in (function (x, data, idata = no_idata_set(), carry_out = carry.out, : [steady_bolus] ID 4214 failed
to reach steady state
##   ss_n: 500, ss_rtol: 1e-08, ss_atol: 1e-08
## Warning in (function (x, data, idata = no_idata_set(), carry_out = carry.out, : [steady_bolus] ID 4214 failed
to reach steady state
##   ss_n: 500, ss_rtol: 1e-08, ss_atol: 1e-08
## Warning in (function (x, data, idata = no_idata_set(), carry_out = carry.out, : [steady_bolus] ID 4214 failed
to reach steady state
##   ss_n: 500, ss_rtol: 1e-08, ss_atol: 1e-08
## Warning in (function (x, data, idata = no_idata_set(), carry_out = carry.out, : [steady_bolus] ID 4214 failed
to reach steady state
##   ss_n: 500, ss_rtol: 1e-08, ss_atol: 1e-08
```

```
## Warning in (function (x, data, idata = no_idata_set(), carry_out = carry.out, : [steady_bolus] ID 4217 failed
to reach steady state
##   ss_n: 500, ss_rtol: 1e-08, ss_atol: 1e-08
```

```
## Warning in (function (x, data, idata = no_idata_set(), carry_out = carry.out, : [steady_bolus] ID 4263 failed
to reach steady state
##   ss_n: 500, ss_rtol: 1e-08, ss_atol: 1e-08
## Warning in (function (x, data, idata = no_idata_set(), carry_out = carry.out, : [steady_bolus] ID 4263 failed
to reach steady state
##   ss_n: 500, ss_rtol: 1e-08, ss_atol: 1e-08
## Warning in (function (x, data, idata = no_idata_set(), carry_out = carry.out, : [steady_bolus] ID 4263 failed
to reach steady state
##   ss_n: 500, ss_rtol: 1e-08, ss_atol: 1e-08
## Warning in (function (x, data, idata = no_idata_set(), carry_out = carry.out, : [steady_bolus] ID 4263 failed
to reach steady state
##   ss_n: 500, ss_rtol: 1e-08, ss_atol: 1e-08
## Warning in (function (x, data, idata = no_idata_set(), carry_out = carry.out, : [steady_bolus] ID 4263 failed
to reach steady state
##   ss_n: 500, ss_rtol: 1e-08, ss_atol: 1e-08
## Warning in (function (x, data, idata = no_idata_set(), carry_out = carry.out, : [steady_bolus] ID 4263 failed
to reach steady state
##   ss_n: 500, ss_rtol: 1e-08, ss_atol: 1e-08
```

```
## Warning in (function (x, data, idata = no_idata_set(), carry_out = carry.out, : [steady_bolus] ID 4402 failed
to reach steady state
##   ss_n: 500, ss_rtol: 1e-08, ss_atol: 1e-08
```

```
## Warning in (function (x, data, idata = no_idata_set(), carry_out = carry.out, : [steady_bolus] ID 4412 failed
to reach steady state
##   ss_n: 500, ss_rtol: 1e-08, ss_atol: 1e-08
```

```
## Warning in (function (x, data, idata = no_idata_set(), carry_out = carry.out, : [steady_bolus] ID 4481 failed
to reach steady state
##   ss_n: 500, ss_rtol: 1e-08, ss_atol: 1e-08
```

```
## Warning in (function (x, data, idata = no_idata_set(), carry_out = carry.out, : [steady_bolus] ID 4511 failed
to reach steady state
##   ss_n: 500, ss_rtol: 1e-08, ss_atol: 1e-08
```

```
## Warning in (function (x, data, idata = no_idata_set(), carry_out = carry.out, : [steady_bolus] ID 4559 failed
to reach steady state
##   ss_n: 500, ss_rtol: 1e-08, ss_atol: 1e-08
## Warning in (function (x, data, idata = no_idata_set(), carry_out = carry.out, : [steady_bolus] ID 4559 failed
to reach steady state
##   ss_n: 500, ss_rtol: 1e-08, ss_atol: 1e-08
## Warning in (function (x, data, idata = no_idata_set(), carry_out = carry.out, : [steady_bolus] ID 4559 failed
to reach steady state
##   ss_n: 500, ss_rtol: 1e-08, ss_atol: 1e-08
## Warning in (function (x, data, idata = no_idata_set(), carry_out = carry.out, : [steady_bolus] ID 4559 failed
to reach steady state
##   ss_n: 500, ss_rtol: 1e-08, ss_atol: 1e-08
## Warning in (function (x, data, idata = no_idata_set(), carry_out = carry.out, : [steady_bolus] ID 4559 failed
to reach steady state
##   ss_n: 500, ss_rtol: 1e-08, ss_atol: 1e-08
## Warning in (function (x, data, idata = no_idata_set(), carry_out = carry.out, : [steady_bolus] ID 4559 failed
to reach steady state
##   ss_n: 500, ss_rtol: 1e-08, ss_atol: 1e-08
## Warning in (function (x, data, idata = no_idata_set(), carry_out = carry.out, : [steady_bolus] ID 4559 failed
to reach steady state
##   ss_n: 500, ss_rtol: 1e-08, ss_atol: 1e-08
```

```
## Warning in (function (x, data, idata = no_idata_set(), carry_out = carry.out, : [steady_bolus] ID 4582 failed
to reach steady state
##   ss_n: 500, ss_rtol: 1e-08, ss_atol: 1e-08
```

```
## Warning in (function (x, data, idata = no_idata_set(), carry_out = carry.out, : [steady_bolus] ID 4618 failed
to reach steady state
##   ss_n: 500, ss_rtol: 1e-08, ss_atol: 1e-08
```

```
## Warning in (function (x, data, idata = no_idata_set(), carry_out = carry.out, : [steady_bolus] ID 4728 failed
to reach steady state
##   ss_n: 500, ss_rtol: 1e-08, ss_atol: 1e-08
## Warning in (function (x, data, idata = no_idata_set(), carry_out = carry.out, : [steady_bolus] ID 4728 failed
to reach steady state
##   ss_n: 500, ss_rtol: 1e-08, ss_atol: 1e-08
## Warning in (function (x, data, idata = no_idata_set(), carry_out = carry.out, : [steady_bolus] ID 4728 failed
to reach steady state
##   ss_n: 500, ss_rtol: 1e-08, ss_atol: 1e-08
## Warning in (function (x, data, idata = no_idata_set(), carry_out = carry.out, : [steady_bolus] ID 4728 failed
to reach steady state
##   ss_n: 500, ss_rtol: 1e-08, ss_atol: 1e-08
## Warning in (function (x, data, idata = no_idata_set(), carry_out = carry.out, : [steady_bolus] ID 4728 failed
to reach steady state
##   ss_n: 500, ss_rtol: 1e-08, ss_atol: 1e-08
## Warning in (function (x, data, idata = no_idata_set(), carry_out = carry.out, : [steady_bolus] ID 4728 failed
to reach steady state
##   ss_n: 500, ss_rtol: 1e-08, ss_atol: 1e-08
```

```
## Warning in (function (x, data, idata = no_idata_set(), carry_out = carry.out, : [steady_bolus] ID 4744 failed
to reach steady state
##   ss_n: 500, ss_rtol: 1e-08, ss_atol: 1e-08
## Warning in (function (x, data, idata = no_idata_set(), carry_out = carry.out, : [steady_bolus] ID 4744 failed
to reach steady state
##   ss_n: 500, ss_rtol: 1e-08, ss_atol: 1e-08
## Warning in (function (x, data, idata = no_idata_set(), carry_out = carry.out, : [steady_bolus] ID 4744 failed
to reach steady state
##   ss_n: 500, ss_rtol: 1e-08, ss_atol: 1e-08
## Warning in (function (x, data, idata = no_idata_set(), carry_out = carry.out, : [steady_bolus] ID 4744 failed
to reach steady state
##   ss_n: 500, ss_rtol: 1e-08, ss_atol: 1e-08
## Warning in (function (x, data, idata = no_idata_set(), carry_out = carry.out, : [steady_bolus] ID 4744 failed
to reach steady state
##   ss_n: 500, ss_rtol: 1e-08, ss_atol: 1e-08
## Warning in (function (x, data, idata = no_idata_set(), carry_out = carry.out, : [steady_bolus] ID 4744 failed
to reach steady state
##   ss_n: 500, ss_rtol: 1e-08, ss_atol: 1e-08
```

```
## Warning in (function (x, data, idata = no_idata_set(), carry_out = carry.out, : [steady_bolus] ID 4746 failed
to reach steady state
##   ss_n: 500, ss_rtol: 1e-08, ss_atol: 1e-08
```

```
## Warning in (function (x, data, idata = no_idata_set(), carry_out = carry.out, : [steady_bolus] ID 4778 failed
to reach steady state
##   ss_n: 500, ss_rtol: 1e-08, ss_atol: 1e-08
## Warning in (function (x, data, idata = no_idata_set(), carry_out = carry.out, : [steady_bolus] ID 4778 failed
to reach steady state
##   ss_n: 500, ss_rtol: 1e-08, ss_atol: 1e-08
## Warning in (function (x, data, idata = no_idata_set(), carry_out = carry.out, : [steady_bolus] ID 4778 failed
to reach steady state
##   ss_n: 500, ss_rtol: 1e-08, ss_atol: 1e-08
## Warning in (function (x, data, idata = no_idata_set(), carry_out = carry.out, : [steady_bolus] ID 4778 failed
to reach steady state
##   ss_n: 500, ss_rtol: 1e-08, ss_atol: 1e-08
## Warning in (function (x, data, idata = no_idata_set(), carry_out = carry.out, : [steady_bolus] ID 4778 failed
to reach steady state
##   ss_n: 500, ss_rtol: 1e-08, ss_atol: 1e-08
## Warning in (function (x, data, idata = no_idata_set(), carry_out = carry.out, : [steady_bolus] ID 4778 failed
to reach steady state
##   ss_n: 500, ss_rtol: 1e-08, ss_atol: 1e-08
## Warning in (function (x, data, idata = no_idata_set(), carry_out = carry.out, : [steady_bolus] ID 4778 failed
to reach steady state
##   ss_n: 500, ss_rtol: 1e-08, ss_atol: 1e-08
```

```
## Warning in (function (x, data, idata = no_idata_set(), carry_out = carry.out, : [steady_bolus] ID 4887 failed
to reach steady state
##   ss_n: 500, ss_rtol: 1e-08, ss_atol: 1e-08
```

```
## Warning in (function (x, data, idata = no_idata_set(), carry_out = carry.out, : [steady_bolus] ID 4896 failed
to reach steady state
##   ss_n: 500, ss_rtol: 1e-08, ss_atol: 1e-08
## Warning in (function (x, data, idata = no_idata_set(), carry_out = carry.out, : [steady_bolus] ID 4896 failed
to reach steady state
##   ss_n: 500, ss_rtol: 1e-08, ss_atol: 1e-08
## Warning in (function (x, data, idata = no_idata_set(), carry_out = carry.out, : [steady_bolus] ID 4896 failed
to reach steady state
##   ss_n: 500, ss_rtol: 1e-08, ss_atol: 1e-08
## Warning in (function (x, data, idata = no_idata_set(), carry_out = carry.out, : [steady_bolus] ID 4896 failed
to reach steady state
##   ss_n: 500, ss_rtol: 1e-08, ss_atol: 1e-08
## Warning in (function (x, data, idata = no_idata_set(), carry_out = carry.out, : [steady_bolus] ID 4896 failed
to reach steady state
##   ss_n: 500, ss_rtol: 1e-08, ss_atol: 1e-08
## Warning in (function (x, data, idata = no_idata_set(), carry_out = carry.out, : [steady_bolus] ID 4896 failed
to reach steady state
##   ss_n: 500, ss_rtol: 1e-08, ss_atol: 1e-08
```

```
## Warning in (function (x, data, idata = no_idata_set(), carry_out = carry.out, : [steady_bolus] ID 4915 failed
to reach steady state
##   ss_n: 500, ss_rtol: 1e-08, ss_atol: 1e-08
## Warning in (function (x, data, idata = no_idata_set(), carry_out = carry.out, : [steady_bolus] ID 4915 failed
to reach steady state
##   ss_n: 500, ss_rtol: 1e-08, ss_atol: 1e-08
## Warning in (function (x, data, idata = no_idata_set(), carry_out = carry.out, : [steady_bolus] ID 4915 failed
to reach steady state
##   ss_n: 500, ss_rtol: 1e-08, ss_atol: 1e-08
## Warning in (function (x, data, idata = no_idata_set(), carry_out = carry.out, : [steady_bolus] ID 4915 failed
to reach steady state
##   ss_n: 500, ss_rtol: 1e-08, ss_atol: 1e-08
## Warning in (function (x, data, idata = no_idata_set(), carry_out = carry.out, : [steady_bolus] ID 4915 failed
to reach steady state
##   ss_n: 500, ss_rtol: 1e-08, ss_atol: 1e-08
## Warning in (function (x, data, idata = no_idata_set(), carry_out = carry.out, : [steady_bolus] ID 4915 failed
to reach steady state
##   ss_n: 500, ss_rtol: 1e-08, ss_atol: 1e-08
## Warning in (function (x, data, idata = no_idata_set(), carry_out = carry.out, : [steady_bolus] ID 4915 failed
to reach steady state
##   ss_n: 500, ss_rtol: 1e-08, ss_atol: 1e-08
```

```
## Warning in (function (x, data, idata = no_idata_set(), carry_out = carry.out, : [steady_bolus] ID 4932 failed
to reach steady state
##   ss_n: 500, ss_rtol: 1e-08, ss_atol: 1e-08
## Warning in (function (x, data, idata = no_idata_set(), carry_out = carry.out, : [steady_bolus] ID 4932 failed
to reach steady state
##   ss_n: 500, ss_rtol: 1e-08, ss_atol: 1e-08
```

```
## Warning in (function (x, data, idata = no_idata_set(), carry_out = carry.out, : [steady_bolus] ID 4942 failed
to reach steady state
##   ss_n: 500, ss_rtol: 1e-08, ss_atol: 1e-08
```

```
## Warning in (function (x, data, idata = no_idata_set(), carry_out = carry.out, : [steady_bolus] ID 4949 failed
to reach steady state
##   ss_n: 500, ss_rtol: 1e-08, ss_atol: 1e-08
## Warning in (function (x, data, idata = no_idata_set(), carry_out = carry.out, : [steady_bolus] ID 4949 failed
to reach steady state
##   ss_n: 500, ss_rtol: 1e-08, ss_atol: 1e-08
```

```
## Warning in (function (x, data, idata = no_idata_set(), carry_out = carry.out, : [steady_bolus] ID 5025 failed
to reach steady state
##   ss_n: 500, ss_rtol: 1e-08, ss_atol: 1e-08
## Warning in (function (x, data, idata = no_idata_set(), carry_out = carry.out, : [steady_bolus] ID 5025 failed
to reach steady state
##   ss_n: 500, ss_rtol: 1e-08, ss_atol: 1e-08
## Warning in (function (x, data, idata = no_idata_set(), carry_out = carry.out, : [steady_bolus] ID 5025 failed
to reach steady state
##   ss_n: 500, ss_rtol: 1e-08, ss_atol: 1e-08
## Warning in (function (x, data, idata = no_idata_set(), carry_out = carry.out, : [steady_bolus] ID 5025 failed
to reach steady state
##   ss_n: 500, ss_rtol: 1e-08, ss_atol: 1e-08
## Warning in (function (x, data, idata = no_idata_set(), carry_out = carry.out, : [steady_bolus] ID 5025 failed
to reach steady state
##   ss_n: 500, ss_rtol: 1e-08, ss_atol: 1e-08
## Warning in (function (x, data, idata = no_idata_set(), carry_out = carry.out, : [steady_bolus] ID 5025 failed
to reach steady state
##   ss_n: 500, ss_rtol: 1e-08, ss_atol: 1e-08
```

```
## Warning in (function (x, data, idata = no_idata_set(), carry_out = carry.out, : [steady_bolus] ID 5125 failed
to reach steady state
##   ss_n: 500, ss_rtol: 1e-08, ss_atol: 1e-08
```

```
## Warning in (function (x, data, idata = no_idata_set(), carry_out = carry.out, : [steady_bolus] ID 5215 failed
to reach steady state
##   ss_n: 500, ss_rtol: 1e-08, ss_atol: 1e-08
## Warning in (function (x, data, idata = no_idata_set(), carry_out = carry.out, : [steady_bolus] ID 5215 failed
to reach steady state
##   ss_n: 500, ss_rtol: 1e-08, ss_atol: 1e-08
## Warning in (function (x, data, idata = no_idata_set(), carry_out = carry.out, : [steady_bolus] ID 5215 failed
to reach steady state
##   ss_n: 500, ss_rtol: 1e-08, ss_atol: 1e-08
## Warning in (function (x, data, idata = no_idata_set(), carry_out = carry.out, : [steady_bolus] ID 5215 failed
to reach steady state
##   ss_n: 500, ss_rtol: 1e-08, ss_atol: 1e-08
## Warning in (function (x, data, idata = no_idata_set(), carry_out = carry.out, : [steady_bolus] ID 5215 failed
to reach steady state
##   ss_n: 500, ss_rtol: 1e-08, ss_atol: 1e-08
## Warning in (function (x, data, idata = no_idata_set(), carry_out = carry.out, : [steady_bolus] ID 5215 failed
to reach steady state
##   ss_n: 500, ss_rtol: 1e-08, ss_atol: 1e-08
## Warning in (function (x, data, idata = no_idata_set(), carry_out = carry.out, : [steady_bolus] ID 5215 failed
to reach steady state
##   ss_n: 500, ss_rtol: 1e-08, ss_atol: 1e-08
```

```
## Warning in (function (x, data, idata = no_idata_set(), carry_out = carry.out, : [steady_bolus] ID 5237 failed
to reach steady state
##   ss_n: 500, ss_rtol: 1e-08, ss_atol: 1e-08
```

```
## Warning in (function (x, data, idata = no_idata_set(), carry_out = carry.out, : [steady_bolus] ID 5254 failed
to reach steady state
##   ss_n: 500, ss_rtol: 1e-08, ss_atol: 1e-08
```

```
## Warning in (function (x, data, idata = no_idata_set(), carry_out = carry.out, : [steady_bolus] ID 5276 failed
to reach steady state
##   ss_n: 500, ss_rtol: 1e-08, ss_atol: 1e-08
## Warning in (function (x, data, idata = no_idata_set(), carry_out = carry.out, : [steady_bolus] ID 5276 failed
to reach steady state
##   ss_n: 500, ss_rtol: 1e-08, ss_atol: 1e-08
## Warning in (function (x, data, idata = no_idata_set(), carry_out = carry.out, : [steady_bolus] ID 5276 failed
to reach steady state
##   ss_n: 500, ss_rtol: 1e-08, ss_atol: 1e-08
## Warning in (function (x, data, idata = no_idata_set(), carry_out = carry.out, : [steady_bolus] ID 5276 failed
to reach steady state
##   ss_n: 500, ss_rtol: 1e-08, ss_atol: 1e-08
## Warning in (function (x, data, idata = no_idata_set(), carry_out = carry.out, : [steady_bolus] ID 5276 failed
to reach steady state
##   ss_n: 500, ss_rtol: 1e-08, ss_atol: 1e-08
## Warning in (function (x, data, idata = no_idata_set(), carry_out = carry.out, : [steady_bolus] ID 5276 failed
to reach steady state
##   ss_n: 500, ss_rtol: 1e-08, ss_atol: 1e-08
```

```
## Warning in (function (x, data, idata = no_idata_set(), carry_out = carry.out, : [steady_bolus] ID 5290 failed
to reach steady state
##   ss_n: 500, ss_rtol: 1e-08, ss_atol: 1e-08
```

```
## Warning in (function (x, data, idata = no_idata_set(), carry_out = carry.out, : [steady_bolus] ID 5292 failed
to reach steady state
##   ss_n: 500, ss_rtol: 1e-08, ss_atol: 1e-08
```

```
## Warning in (function (x, data, idata = no_idata_set(), carry_out = carry.out, : [steady_bolus] ID 5304 failed
to reach steady state
##   ss_n: 500, ss_rtol: 1e-08, ss_atol: 1e-08
```

```
## Warning in (function (x, data, idata = no_idata_set(), carry_out = carry.out, : [steady_bolus] ID 5313 failed
to reach steady state
##   ss_n: 500, ss_rtol: 1e-08, ss_atol: 1e-08
```

```
## Warning in (function (x, data, idata = no_idata_set(), carry_out = carry.out, : [steady_bolus] ID 5418 failed
to reach steady state
##   ss_n: 500, ss_rtol: 1e-08, ss_atol: 1e-08
```

```
## Warning in (function (x, data, idata = no_idata_set(), carry_out = carry.out, : [steady_bolus] ID 5436 failed
to reach steady state
##   ss_n: 500, ss_rtol: 1e-08, ss_atol: 1e-08
## Warning in (function (x, data, idata = no_idata_set(), carry_out = carry.out, : [steady_bolus] ID 5436 failed
to reach steady state
##   ss_n: 500, ss_rtol: 1e-08, ss_atol: 1e-08
## Warning in (function (x, data, idata = no_idata_set(), carry_out = carry.out, : [steady_bolus] ID 5436 failed
to reach steady state
##   ss_n: 500, ss_rtol: 1e-08, ss_atol: 1e-08
## Warning in (function (x, data, idata = no_idata_set(), carry_out = carry.out, : [steady_bolus] ID 5436 failed
to reach steady state
##   ss_n: 500, ss_rtol: 1e-08, ss_atol: 1e-08
## Warning in (function (x, data, idata = no_idata_set(), carry_out = carry.out, : [steady_bolus] ID 5436 failed
to reach steady state
##   ss_n: 500, ss_rtol: 1e-08, ss_atol: 1e-08
## Warning in (function (x, data, idata = no_idata_set(), carry_out = carry.out, : [steady_bolus] ID 5436 failed
to reach steady state
##   ss_n: 500, ss_rtol: 1e-08, ss_atol: 1e-08
## Warning in (function (x, data, idata = no_idata_set(), carry_out = carry.out, : [steady_bolus] ID 5436 failed
to reach steady state
##   ss_n: 500, ss_rtol: 1e-08, ss_atol: 1e-08
## Warning in (function (x, data, idata = no_idata_set(), carry_out = carry.out, : [steady_bolus] ID 5436 failed
to reach steady state
##   ss_n: 500, ss_rtol: 1e-08, ss_atol: 1e-08
```

```
## Warning in (function (x, data, idata = no_idata_set(), carry_out = carry.out, : [steady_bolus] ID 5574 failed
to reach steady state
##   ss_n: 500, ss_rtol: 1e-08, ss_atol: 1e-08
```

```
## Warning in (function (x, data, idata = no_idata_set(), carry_out = carry.out, : [steady_bolus] ID 5653 failed
to reach steady state
##   ss_n: 500, ss_rtol: 1e-08, ss_atol: 1e-08
```

```
## Warning in (function (x, data, idata = no_idata_set(), carry_out = carry.out, : [steady_bolus] ID 5659 failed
to reach steady state
##   ss_n: 500, ss_rtol: 1e-08, ss_atol: 1e-08
```

```
## Warning in (function (x, data, idata = no_idata_set(), carry_out = carry.out, : [steady_bolus] ID 5695 failed
to reach steady state
##   ss_n: 500, ss_rtol: 1e-08, ss_atol: 1e-08
## Warning in (function (x, data, idata = no_idata_set(), carry_out = carry.out, : [steady_bolus] ID 5695 failed
to reach steady state
##   ss_n: 500, ss_rtol: 1e-08, ss_atol: 1e-08
## Warning in (function (x, data, idata = no_idata_set(), carry_out = carry.out, : [steady_bolus] ID 5695 failed
to reach steady state
##   ss_n: 500, ss_rtol: 1e-08, ss_atol: 1e-08
## Warning in (function (x, data, idata = no_idata_set(), carry_out = carry.out, : [steady_bolus] ID 5695 failed
to reach steady state
##   ss_n: 500, ss_rtol: 1e-08, ss_atol: 1e-08
## Warning in (function (x, data, idata = no_idata_set(), carry_out = carry.out, : [steady_bolus] ID 5695 failed
to reach steady state
##   ss_n: 500, ss_rtol: 1e-08, ss_atol: 1e-08
## Warning in (function (x, data, idata = no_idata_set(), carry_out = carry.out, : [steady_bolus] ID 5695 failed
to reach steady state
##   ss_n: 500, ss_rtol: 1e-08, ss_atol: 1e-08
## Warning in (function (x, data, idata = no_idata_set(), carry_out = carry.out, : [steady_bolus] ID 5695 failed
to reach steady state
##   ss_n: 500, ss_rtol: 1e-08, ss_atol: 1e-08
```

```
## Warning in (function (x, data, idata = no_idata_set(), carry_out = carry.out, : [steady_bolus] ID 5719 failed
to reach steady state
##   ss_n: 500, ss_rtol: 1e-08, ss_atol: 1e-08
## Warning in (function (x, data, idata = no_idata_set(), carry_out = carry.out, : [steady_bolus] ID 5719 failed
to reach steady state
##   ss_n: 500, ss_rtol: 1e-08, ss_atol: 1e-08
```

```
## Warning in (function (x, data, idata = no_idata_set(), carry_out = carry.out, : [steady_bolus] ID 5739 failed
to reach steady state
##   ss_n: 500, ss_rtol: 1e-08, ss_atol: 1e-08
## Warning in (function (x, data, idata = no_idata_set(), carry_out = carry.out, : [steady_bolus] ID 5739 failed
to reach steady state
##   ss_n: 500, ss_rtol: 1e-08, ss_atol: 1e-08
## Warning in (function (x, data, idata = no_idata_set(), carry_out = carry.out, : [steady_bolus] ID 5739 failed
to reach steady state
##   ss_n: 500, ss_rtol: 1e-08, ss_atol: 1e-08
## Warning in (function (x, data, idata = no_idata_set(), carry_out = carry.out, : [steady_bolus] ID 5739 failed
to reach steady state
##   ss_n: 500, ss_rtol: 1e-08, ss_atol: 1e-08
## Warning in (function (x, data, idata = no_idata_set(), carry_out = carry.out, : [steady_bolus] ID 5739 failed
to reach steady state
##   ss_n: 500, ss_rtol: 1e-08, ss_atol: 1e-08
## Warning in (function (x, data, idata = no_idata_set(), carry_out = carry.out, : [steady_bolus] ID 5739 failed
to reach steady state
##   ss_n: 500, ss_rtol: 1e-08, ss_atol: 1e-08
```

```
## Warning in (function (x, data, idata = no_idata_set(), carry_out = carry.out, : [steady_bolus] ID 5864 failed
to reach steady state
##   ss_n: 500, ss_rtol: 1e-08, ss_atol: 1e-08
## Warning in (function (x, data, idata = no_idata_set(), carry_out = carry.out, : [steady_bolus] ID 5864 failed
to reach steady state
##   ss_n: 500, ss_rtol: 1e-08, ss_atol: 1e-08
## Warning in (function (x, data, idata = no_idata_set(), carry_out = carry.out, : [steady_bolus] ID 5864 failed
to reach steady state
##   ss_n: 500, ss_rtol: 1e-08, ss_atol: 1e-08
## Warning in (function (x, data, idata = no_idata_set(), carry_out = carry.out, : [steady_bolus] ID 5864 failed
to reach steady state
##   ss_n: 500, ss_rtol: 1e-08, ss_atol: 1e-08
## Warning in (function (x, data, idata = no_idata_set(), carry_out = carry.out, : [steady_bolus] ID 5864 failed
to reach steady state
##   ss_n: 500, ss_rtol: 1e-08, ss_atol: 1e-08
## Warning in (function (x, data, idata = no_idata_set(), carry_out = carry.out, : [steady_bolus] ID 5864 failed
to reach steady state
##   ss_n: 500, ss_rtol: 1e-08, ss_atol: 1e-08
## Warning in (function (x, data, idata = no_idata_set(), carry_out = carry.out, : [steady_bolus] ID 5864 failed
to reach steady state
##   ss_n: 500, ss_rtol: 1e-08, ss_atol: 1e-08
```

```
## Warning in (function (x, data, idata = no_idata_set(), carry_out = carry.out, : [steady_bolus] ID 5875 failed
to reach steady state
##   ss_n: 500, ss_rtol: 1e-08, ss_atol: 1e-08
```

suite 6000

```
Simulation_data_martial_12000 <- bind_rows(as_tibble( seq(
  #Steady-state simulation
ev(ID = 6001:6400, amt = 0.5*1, ii= 24, ss= 1, addl=0),
ev(ID = 6001:6400, amt = 0.5*1, ii= 24, ss= 0, addl=0),
ev(ID = 6001:6400, amt = 0.5*1, ii= 24, ss= 0, addl=10),
#3h delay simulation
ev(ID = 6001:6400, amt = 0.5*1, ii= 24, ss= 1, addl=0),
ev(ID = 6001:6400, amt = 0.5*0, ii= 3, ss= 0, addl=0),
ev(ID = 6001:6400, amt = 0.5*1, ii= 21, ss= 0, addl=0),
ev(ID = 6001:6400, amt = 0.5*1, ii= 24, ss= 0, addl=10),
#6h delay simulation
ev(ID = 6001:6400, amt = 0.5*1, ii= 24, ss= 1, addl=0),
ev(ID = 6001:6400, amt = 0.5*0, ii= 6, ss= 0, addl=0),
ev(ID = 6001:6400, amt = 0.5*1, ii= 18, ss= 0, addl=0),
ev(ID = 6001:6400, amt = 0.5*1, ii= 24, ss= 0, addl=10),
#9h delay simulation
ev(ID = 6001:6400, amt = 0.5*1, ii= 24, ss= 1, addl=0),
ev(ID = 6001:6400, amt = 0.5*0, ii= 9, ss= 0, addl=0),
ev(ID = 6001:6400, amt = 0.5*1, ii= 15, ss= 0, addl=0),
ev(ID = 6001:6400, amt = 0.5*1, ii= 24, ss= 0, addl=10),
#12h delay simulation
ev(ID = 6001:6400, amt = 0.5*1, ii= 24, ss= 1, addl=0),
ev(ID = 6001:6400, amt = 0.5*0, ii= 12, ss= 0, addl=0),
ev(ID = 6001:6400, amt = 0.5*1, ii= 12, ss= 0, addl=0),
ev(ID = 6001:6400, amt = 0.5*1, ii= 24, ss= 0, addl=10),
#15h delay + 50% dose intake simulation
ev(ID = 6001:6400, amt = 0.5*1, ii= 24, ss= 1, addl=0),
ev(ID = 6001:6400, amt = 0.5*0, ii= 15, ss= 0, addl=0),
ev(ID = 6001:6400, amt = 0.5*0.5, ii= 9, ss= 0, addl=0),
ev(ID = 6001:6400, amt = 0.5*1, ii= 24, ss= 0, addl=10),
#15h delay simulation
ev(ID = 6001:6400, amt = 0.5*1, ii= 24, ss= 1, addl=0),
ev(ID = 6001:6400, amt = 0.5*0, ii= 15, ss= 0, addl=0),
ev(ID = 6001:6400, amt = 0.5*1, ii= 9, ss= 0, addl=0),
ev(ID = 6001:6400, amt = 0.5*1, ii= 24, ss= 0, addl=10),
#18h delay + 50% dose intake simulation
ev(ID = 6001:6400, amt = 0.5*1, ii= 24, ss= 1, addl=0),
ev(ID = 6001:6400, amt = 0.5*0, ii= 18, ss= 0, addl=0),
ev(ID = 6001:6400, amt = 0.5*0.5, ii= 6, ss= 0, addl=0),
ev(ID = 6001:6400, amt = 0.5*1, ii= 24, ss= 0, addl=10),
#18h delay simulation
ev(ID = 6001:6400, amt = 0.5*1, ii= 24, ss= 1, addl=0),
ev(ID = 6001:6400, amt = 0.5*0, ii= 18, ss= 0, addl=0),
ev(ID = 6001:6400, amt = 0.5*1, ii= 6, ss= 0, addl=0),
ev(ID = 6001:6400, amt = 0.5*1, ii= 24, ss= 0, addl=10),
#21h delay + 50% dose intake simulation
ev(ID = 6001:6400, amt = 0.5*1, ii= 24, ss= 1, addl=0),
ev(ID = 6001:6400, amt = 0.5*0, ii= 21, ss= 0, addl=0),
ev(ID = 6001:6400, amt = 0.5*0.5, ii= 3, ss= 0, addl=0),
ev(ID = 6001:6400, amt = 0.5*1, ii= 24, ss= 0, addl=10),
#21h delay simulation
ev(ID = 6001:6400, amt = 0.5*1, ii= 24, ss= 1, addl=0),
ev(ID = 6001:6400, amt = 0.5*0, ii= 21, ss= 0, addl=0),
ev(ID = 6001:6400, amt = 0.5*1, ii= 3, ss= 0, addl=0),
ev(ID = 6001:6400, amt = 0.5*1, ii= 24, ss= 0, addl=10),
#Missed dose simulation
ev(ID = 6001:6400, amt = 0.5*1, ii= 24, ss= 1, addl=0),
ev(ID = 6001:6400, amt = 0.5*0, ii= 24, ss= 0, addl=0),
ev(ID = 6001:6400, amt = 0.5*1, ii= 24, ss= 0, addl=0),
ev(ID = 6001:6400, amt = 0.5*1, ii= 24, ss= 0, addl=10),
#Missed dose + 150% dose intake simulation
ev(ID = 6001:6400, amt = 0.5*1, ii= 24, ss= 1, addl=0),
ev(ID = 6001:6400, amt = 0.5*0, ii= 24, ss= 0, addl=0),
ev(ID = 6001:6400, amt = 0.5*1.5, ii= 24, ss= 0, addl=0),
ev(ID = 6001:6400, amt = 0.5*1, ii= 24, ss= 0, addl=10),
#Missed dose + 200% dose intake simulation
ev(ID = 6001:6400, amt = 0.5*1, ii= 24, ss= 1, addl=0),
ev(ID = 6001:6400, amt = 0.5*0, ii= 24, ss= 0, addl=0),
ev(ID = 6001:6400, amt = 0.5*2, ii= 24, ss= 0, addl=0),
ev(ID = 6001:6400, amt = 0.5*1, ii= 24, ss= 0, addl=10)))) %>%
             mutate(dose_group = "0,5 mg") %>%

    bind_rows(as_tibble( seq(
  #Steady-state simulation
ev(ID = 6401:6800, amt = 1.5*1, ii= 24, ss= 1, addl=0),
ev(ID = 6401:6800, amt = 1.5*1, ii= 24, ss= 0, addl=0),
ev(ID = 6401:6800, amt = 1.5*1, ii= 24, ss= 0, addl=10),
#3h delay simulation
ev(ID = 6401:6800, amt = 1.5*1, ii= 24, ss= 1, addl=0),
ev(ID = 6401:6800, amt = 1.5*0, ii= 3, ss= 0, addl=0),
ev(ID = 6401:6800, amt = 1.5*1, ii= 21, ss= 0, addl=0),
ev(ID = 6401:6800, amt = 1.5*1, ii= 24, ss= 0, addl=10),
#6h delay simulation
ev(ID = 6401:6800, amt = 1.5*1, ii= 24, ss= 1, addl=0),
ev(ID = 6401:6800, amt = 1.5*0, ii= 6, ss= 0, addl=0),
ev(ID = 6401:6800, amt = 1.5*1, ii= 18, ss= 0, addl=0),
ev(ID = 6401:6800, amt = 1.5*1, ii= 24, ss= 0, addl=10),
#9h delay simulation
ev(ID = 6401:6800, amt = 1.5*1, ii= 24, ss= 1, addl=0),
ev(ID = 6401:6800, amt = 1.5*0, ii= 9, ss= 0, addl=0),
ev(ID = 6401:6800, amt = 1.5*1, ii= 15, ss= 0, addl=0),
ev(ID = 6401:6800, amt = 1.5*1, ii= 24, ss= 0, addl=10),
#12h delay simulation
ev(ID = 6401:6800, amt = 1.5*1, ii= 24, ss= 1, addl=0),
ev(ID = 6401:6800, amt = 1.5*0, ii= 12, ss= 0, addl=0),
ev(ID = 6401:6800, amt = 1.5*1, ii= 12, ss= 0, addl=0),
ev(ID = 6401:6800, amt = 1.5*1, ii= 24, ss= 0, addl=10),
#15h delay + 50% dose intake simulation
ev(ID = 6401:6800, amt = 1.5*1, ii= 24, ss= 1, addl=0),
ev(ID = 6401:6800, amt = 1.5*0, ii= 15, ss= 0, addl=0),
ev(ID = 6401:6800, amt = 1.5*0.5, ii= 9, ss= 0, addl=0),
ev(ID = 6401:6800, amt = 1.5*1, ii= 24, ss= 0, addl=10),
#15h delay simulation
ev(ID = 6401:6800, amt = 1.5*1, ii= 24, ss= 1, addl=0),
ev(ID = 6401:6800, amt = 1.5*0, ii= 15, ss= 0, addl=0),
ev(ID = 6401:6800, amt = 1.5*1, ii= 9, ss= 0, addl=0),
ev(ID = 6401:6800, amt = 1.5*1, ii= 24, ss= 0, addl=10),
#18h delay + 50% dose intake simulation
ev(ID = 6401:6800, amt = 1.5*1, ii= 24, ss= 1, addl=0),
ev(ID = 6401:6800, amt = 1.5*0, ii= 18, ss= 0, addl=0),
ev(ID = 6401:6800, amt = 1.5*0.5, ii= 6, ss= 0, addl=0),
ev(ID = 6401:6800, amt = 1.5*1, ii= 24, ss= 0, addl=10),
#18h delay simulation
ev(ID = 6401:6800, amt = 1.5*1, ii= 24, ss= 1, addl=0),
ev(ID = 6401:6800, amt = 1.5*0, ii= 18, ss= 0, addl=0),
ev(ID = 6401:6800, amt = 1.5*1, ii= 6, ss= 0, addl=0),
ev(ID = 6401:6800, amt = 1.5*1, ii= 24, ss= 0, addl=10),
#21h delay + 50% dose intake simulation
ev(ID = 6401:6800, amt = 1.5*1, ii= 24, ss= 1, addl=0),
ev(ID = 6401:6800, amt = 1.5*0, ii= 21, ss= 0, addl=0),
ev(ID = 6401:6800, amt = 1.5*0.5, ii= 3, ss= 0, addl=0),
ev(ID = 6401:6800, amt = 1.5*1, ii= 24, ss= 0, addl=10),
#21h delay simulation
ev(ID = 6401:6800, amt = 1.5*1, ii= 24, ss= 1, addl=0),
ev(ID = 6401:6800, amt = 1.5*0, ii= 21, ss= 0, addl=0),
ev(ID = 6401:6800, amt = 1.5*1, ii= 3, ss= 0, addl=0),
ev(ID = 6401:6800, amt = 1.5*1, ii= 24, ss= 0, addl=10),
#Missed dose simulation
ev(ID = 6401:6800, amt = 1.5*1, ii= 24, ss= 1, addl=0),
ev(ID = 6401:6800, amt = 1.5*0, ii= 24, ss= 0, addl=0),
ev(ID = 6401:6800, amt = 1.5*1, ii= 24, ss= 0, addl=0),
ev(ID = 6401:6800, amt = 1.5*1, ii= 24, ss= 0, addl=10),
#Missed dose + 150% dose intake simulation
ev(ID = 6401:6800, amt = 1.5*1, ii= 24, ss= 1, addl=0),
ev(ID = 6401:6800, amt = 1.5*0, ii= 24, ss= 0, addl=0),
ev(ID = 6401:6800, amt = 1.5*1.5, ii= 24, ss= 0, addl=0),
ev(ID = 6401:6800, amt = 1.5*1, ii= 24, ss= 0, addl=10),
#Missed dose + 200% dose intake simulation
ev(ID = 6401:6800, amt = 1.5*1, ii= 24, ss= 1, addl=0),
ev(ID = 6401:6800, amt = 1.5*0, ii= 24, ss= 0, addl=0),
ev(ID = 6401:6800, amt = 1.5*2, ii= 24, ss= 0, addl=0),
ev(ID = 6401:6800, amt = 1.5*1, ii= 24, ss= 0, addl=10)))) %>%
             mutate(dose_group = "1,5 mg") %>%

    bind_rows(as_tibble( seq(
  #Steady-state simulation
ev(ID = 6801:7200, amt = 2.5*1, ii= 24, ss= 1, addl=0),
ev(ID = 6801:7200, amt = 2.5*1, ii= 24, ss= 0, addl=0),
ev(ID = 6801:7200, amt = 2.5*1, ii= 24, ss= 0, addl=10),
#3h delay simulation
ev(ID = 6801:7200, amt = 2.5*1, ii= 24, ss= 1, addl=0),
ev(ID = 6801:7200, amt = 2.5*0, ii= 3, ss= 0, addl=0),
ev(ID = 6801:7200, amt = 2.5*1, ii= 21, ss= 0, addl=0),
ev(ID = 6801:7200, amt = 2.5*1, ii= 24, ss= 0, addl=10),
#6h delay simulation
ev(ID = 6801:7200, amt = 2.5*1, ii= 24, ss= 1, addl=0),
ev(ID = 6801:7200, amt = 2.5*0, ii= 6, ss= 0, addl=0),
ev(ID = 6801:7200, amt = 2.5*1, ii= 18, ss= 0, addl=0),
ev(ID = 6801:7200, amt = 2.5*1, ii= 24, ss= 0, addl=10),
#9h delay simulation
ev(ID = 6801:7200, amt = 2.5*1, ii= 24, ss= 1, addl=0),
ev(ID = 6801:7200, amt = 2.5*0, ii= 9, ss= 0, addl=0),
ev(ID = 6801:7200, amt = 2.5*1, ii= 15, ss= 0, addl=0),
ev(ID = 6801:7200, amt = 2.5*1, ii= 24, ss= 0, addl=10),
#12h delay simulation
ev(ID = 6801:7200, amt = 2.5*1, ii= 24, ss= 1, addl=0),
ev(ID = 6801:7200, amt = 2.5*0, ii= 12, ss= 0, addl=0),
ev(ID = 6801:7200, amt = 2.5*1, ii= 12, ss= 0, addl=0),
ev(ID = 6801:7200, amt = 2.5*1, ii= 24, ss= 0, addl=10),
#15h delay + 50% dose intake simulation
ev(ID = 6801:7200, amt = 2.5*1, ii= 24, ss= 1, addl=0),
ev(ID = 6801:7200, amt = 2.5*0, ii= 15, ss= 0, addl=0),
ev(ID = 6801:7200, amt = 2.5*0.5, ii= 9, ss= 0, addl=0),
ev(ID = 6801:7200, amt = 2.5*1, ii= 24, ss= 0, addl=10),
#15h delay simulation
ev(ID = 6801:7200, amt = 2.5*1, ii= 24, ss= 1, addl=0),
ev(ID = 6801:7200, amt = 2.5*0, ii= 15, ss= 0, addl=0),
ev(ID = 6801:7200, amt = 2.5*1, ii= 9, ss= 0, addl=0),
ev(ID = 6801:7200, amt = 2.5*1, ii= 24, ss= 0, addl=10),
#18h delay + 50% dose intake simulation
ev(ID = 6801:7200, amt = 2.5*1, ii= 24, ss= 1, addl=0),
ev(ID = 6801:7200, amt = 2.5*0, ii= 18, ss= 0, addl=0),
ev(ID = 6801:7200, amt = 2.5*0.5, ii= 6, ss= 0, addl=0),
ev(ID = 6801:7200, amt = 2.5*1, ii= 24, ss= 0, addl=10),
#18h delay simulation
ev(ID = 6801:7200, amt = 2.5*1, ii= 24, ss= 1, addl=0),
ev(ID = 6801:7200, amt = 2.5*0, ii= 18, ss= 0, addl=0),
ev(ID = 6801:7200, amt = 2.5*1, ii= 6, ss= 0, addl=0),
ev(ID = 6801:7200, amt = 2.5*1, ii= 24, ss= 0, addl=10),
#21h delay + 50% dose intake simulation
ev(ID = 6801:7200, amt = 2.5*1, ii= 24, ss= 1, addl=0),
ev(ID = 6801:7200, amt = 2.5*0, ii= 21, ss= 0, addl=0),
ev(ID = 6801:7200, amt = 2.5*0.5, ii= 3, ss= 0, addl=0),
ev(ID = 6801:7200, amt = 2.5*1, ii= 24, ss= 0, addl=10),
#21h delay simulation
ev(ID = 6801:7200, amt = 2.5*1, ii= 24, ss= 1, addl=0),
ev(ID = 6801:7200, amt = 2.5*0, ii= 21, ss= 0, addl=0),
ev(ID = 6801:7200, amt = 2.5*1, ii= 3, ss= 0, addl=0),
ev(ID = 6801:7200, amt = 2.5*1, ii= 24, ss= 0, addl=10),
#Missed dose simulation
ev(ID = 6801:7200, amt = 2.5*1, ii= 24, ss= 1, addl=0),
ev(ID = 6801:7200, amt = 2.5*0, ii= 24, ss= 0, addl=0),
ev(ID = 6801:7200, amt = 2.5*1, ii= 24, ss= 0, addl=0),
ev(ID = 6801:7200, amt = 2.5*1, ii= 24, ss= 0, addl=10),
#Missed dose + 150% dose intake simulation
ev(ID = 6801:7200, amt = 2.5*1, ii= 24, ss= 1, addl=0),
ev(ID = 6801:7200, amt = 2.5*0, ii= 24, ss= 0, addl=0),
ev(ID = 6801:7200, amt = 2.5*1.5, ii= 24, ss= 0, addl=0),
ev(ID = 6801:7200, amt = 2.5*1, ii= 24, ss= 0, addl=10),
#Missed dose + 200% dose intake simulation
ev(ID = 6801:7200, amt = 2.5*1, ii= 24, ss= 1, addl=0),
ev(ID = 6801:7200, amt = 2.5*0, ii= 24, ss= 0, addl=0),
ev(ID = 6801:7200, amt = 2.5*2, ii= 24, ss= 0, addl=0),
ev(ID = 6801:7200, amt = 2.5*1, ii= 24, ss= 0, addl=10)))) %>%
             mutate(dose_group = "2,5 mg") %>%

    bind_rows(as_tibble( seq(
  #Steady-state simulation
ev(ID = 7201:7600, amt = 3.5*1, ii= 24, ss= 1, addl=0),
ev(ID = 7201:7600, amt = 3.5*1, ii= 24, ss= 0, addl=0),
ev(ID = 7201:7600, amt = 3.5*1, ii= 24, ss= 0, addl=10),
#3h delay simulation
ev(ID = 7201:7600, amt = 3.5*1, ii= 24, ss= 1, addl=0),
ev(ID = 7201:7600, amt = 3.5*0, ii= 3, ss= 0, addl=0),
ev(ID = 7201:7600, amt = 3.5*1, ii= 21, ss= 0, addl=0),
ev(ID = 7201:7600, amt = 3.5*1, ii= 24, ss= 0, addl=10),
#6h delay simulation
ev(ID = 7201:7600, amt = 3.5*1, ii= 24, ss= 1, addl=0),
ev(ID = 7201:7600, amt = 3.5*0, ii= 6, ss= 0, addl=0),
ev(ID = 7201:7600, amt = 3.5*1, ii= 18, ss= 0, addl=0),
ev(ID = 7201:7600, amt = 3.5*1, ii= 24, ss= 0, addl=10),
#9h delay simulation
ev(ID = 7201:7600, amt = 3.5*1, ii= 24, ss= 1, addl=0),
ev(ID = 7201:7600, amt = 3.5*0, ii= 9, ss= 0, addl=0),
ev(ID = 7201:7600, amt = 3.5*1, ii= 15, ss= 0, addl=0),
ev(ID = 7201:7600, amt = 3.5*1, ii= 24, ss= 0, addl=10),
#12h delay simulation
ev(ID = 7201:7600, amt = 3.5*1, ii= 24, ss= 1, addl=0),
ev(ID = 7201:7600, amt = 3.5*0, ii= 12, ss= 0, addl=0),
ev(ID = 7201:7600, amt = 3.5*1, ii= 12, ss= 0, addl=0),
ev(ID = 7201:7600, amt = 3.5*1, ii= 24, ss= 0, addl=10),
#15h delay + 50% dose intake simulation
ev(ID = 7201:7600, amt = 3.5*1, ii= 24, ss= 1, addl=0),
ev(ID = 7201:7600, amt = 3.5*0, ii= 15, ss= 0, addl=0),
ev(ID = 7201:7600, amt = 3.5*0.5, ii= 9, ss= 0, addl=0),
ev(ID = 7201:7600, amt = 3.5*1, ii= 24, ss= 0, addl=10),
#15h delay simulation
ev(ID = 7201:7600, amt = 3.5*1, ii= 24, ss= 1, addl=0),
ev(ID = 7201:7600, amt = 3.5*0, ii= 15, ss= 0, addl=0),
ev(ID = 7201:7600, amt = 3.5*1, ii= 9, ss= 0, addl=0),
ev(ID = 7201:7600, amt = 3.5*1, ii= 24, ss= 0, addl=10),
#18h delay + 50% dose intake simulation
ev(ID = 7201:7600, amt = 3.5*1, ii= 24, ss= 1, addl=0),
ev(ID = 7201:7600, amt = 3.5*0, ii= 18, ss= 0, addl=0),
ev(ID = 7201:7600, amt = 3.5*0.5, ii= 6, ss= 0, addl=0),
ev(ID = 7201:7600, amt = 3.5*1, ii= 24, ss= 0, addl=10),
#18h delay simulation
ev(ID = 7201:7600, amt = 3.5*1, ii= 24, ss= 1, addl=0),
ev(ID = 7201:7600, amt = 3.5*0, ii= 18, ss= 0, addl=0),
ev(ID = 7201:7600, amt = 3.5*1, ii= 6, ss= 0, addl=0),
ev(ID = 7201:7600, amt = 3.5*1, ii= 24, ss= 0, addl=10),
#21h delay + 50% dose intake simulation
ev(ID = 7201:7600, amt = 3.5*1, ii= 24, ss= 1, addl=0),
ev(ID = 7201:7600, amt = 3.5*0, ii= 21, ss= 0, addl=0),
ev(ID = 7201:7600, amt = 3.5*0.5, ii= 3, ss= 0, addl=0),
ev(ID = 7201:7600, amt = 3.5*1, ii= 24, ss= 0, addl=10),
#21h delay simulation
ev(ID = 7201:7600, amt = 3.5*1, ii= 24, ss= 1, addl=0),
ev(ID = 7201:7600, amt = 3.5*0, ii= 21, ss= 0, addl=0),
ev(ID = 7201:7600, amt = 3.5*1, ii= 3, ss= 0, addl=0),
ev(ID = 7201:7600, amt = 3.5*1, ii= 24, ss= 0, addl=10),
#Missed dose simulation
ev(ID = 7201:7600, amt = 3.5*1, ii= 24, ss= 1, addl=0),
ev(ID = 7201:7600, amt = 3.5*0, ii= 24, ss= 0, addl=0),
ev(ID = 7201:7600, amt = 3.5*1, ii= 24, ss= 0, addl=0),
ev(ID = 7201:7600, amt = 3.5*1, ii= 24, ss= 0, addl=10),
#Missed dose + 150% dose intake simulation
ev(ID = 7201:7600, amt = 3.5*1, ii= 24, ss= 1, addl=0),
ev(ID = 7201:7600, amt = 3.5*0, ii= 24, ss= 0, addl=0),
ev(ID = 7201:7600, amt = 3.5*1.5, ii= 24, ss= 0, addl=0),
ev(ID = 7201:7600, amt = 3.5*1, ii= 24, ss= 0, addl=10),
#Missed dose + 200% dose intake simulation
ev(ID = 7201:7600, amt = 3.5*1, ii= 24, ss= 1, addl=0),
ev(ID = 7201:7600, amt = 3.5*0, ii= 24, ss= 0, addl=0),
ev(ID = 7201:7600, amt = 3.5*2, ii= 24, ss= 0, addl=0),
ev(ID = 7201:7600, amt = 3.5*1, ii= 24, ss= 0, addl=10)))) %>%
             mutate(dose_group = "3,5 mg") %>%

    bind_rows(as_tibble( seq(
  #Steady-state simulation
ev(ID = 7601:8000, amt = 4.5*1, ii= 24, ss= 1, addl=0),
ev(ID = 7601:8000, amt = 4.5*1, ii= 24, ss= 0, addl=0),
ev(ID = 7601:8000, amt = 4.5*1, ii= 24, ss= 0, addl=10),
#3h delay simulation
ev(ID = 7601:8000, amt = 4.5*1, ii= 24, ss= 1, addl=0),
ev(ID = 7601:8000, amt = 4.5*0, ii= 3, ss= 0, addl=0),
ev(ID = 7601:8000, amt = 4.5*1, ii= 21, ss= 0, addl=0),
ev(ID = 7601:8000, amt = 4.5*1, ii= 24, ss= 0, addl=10),
#6h delay simulation
ev(ID = 7601:8000, amt = 4.5*1, ii= 24, ss= 1, addl=0),
ev(ID = 7601:8000, amt = 4.5*0, ii= 6, ss= 0, addl=0),
ev(ID = 7601:8000, amt = 4.5*1, ii= 18, ss= 0, addl=0),
ev(ID = 7601:8000, amt = 4.5*1, ii= 24, ss= 0, addl=10),
#9h delay simulation
ev(ID = 7601:8000, amt = 4.5*1, ii= 24, ss= 1, addl=0),
ev(ID = 7601:8000, amt = 4.5*0, ii= 9, ss= 0, addl=0),
ev(ID = 7601:8000, amt = 4.5*1, ii= 15, ss= 0, addl=0),
ev(ID = 7601:8000, amt = 4.5*1, ii= 24, ss= 0, addl=10),
#12h delay simulation
ev(ID = 7601:8000, amt = 4.5*1, ii= 24, ss= 1, addl=0),
ev(ID = 7601:8000, amt = 4.5*0, ii= 12, ss= 0, addl=0),
ev(ID = 7601:8000, amt = 4.5*1, ii= 12, ss= 0, addl=0),
ev(ID = 7601:8000, amt = 4.5*1, ii= 24, ss= 0, addl=10),
#15h delay + 50% dose intake simulation
ev(ID = 7601:8000, amt = 4.5*1, ii= 24, ss= 1, addl=0),
ev(ID = 7601:8000, amt = 4.5*0, ii= 15, ss= 0, addl=0),
ev(ID = 7601:8000, amt = 4.5*0.5, ii= 9, ss= 0, addl=0),
ev(ID = 7601:8000, amt = 4.5*1, ii= 24, ss= 0, addl=10),
#15h delay simulation
ev(ID = 7601:8000, amt = 4.5*1, ii= 24, ss= 1, addl=0),
ev(ID = 7601:8000, amt = 4.5*0, ii= 15, ss= 0, addl=0),
ev(ID = 7601:8000, amt = 4.5*1, ii= 9, ss= 0, addl=0),
ev(ID = 7601:8000, amt = 4.5*1, ii= 24, ss= 0, addl=10),
#18h delay + 50% dose intake simulation
ev(ID = 7601:8000, amt = 4.5*1, ii= 24, ss= 1, addl=0),
ev(ID = 7601:8000, amt = 4.5*0, ii= 18, ss= 0, addl=0),
ev(ID = 7601:8000, amt = 4.5*0.5, ii= 6, ss= 0, addl=0),
ev(ID = 7601:8000, amt = 4.5*1, ii= 24, ss= 0, addl=10),
#18h delay simulation
ev(ID = 7601:8000, amt = 4.5*1, ii= 24, ss= 1, addl=0),
ev(ID = 7601:8000, amt = 4.5*0, ii= 18, ss= 0, addl=0),
ev(ID = 7601:8000, amt = 4.5*1, ii= 6, ss= 0, addl=0),
ev(ID = 7601:8000, amt = 4.5*1, ii= 24, ss= 0, addl=10),
#21h delay + 50% dose intake simulation
ev(ID = 7601:8000, amt = 4.5*1, ii= 24, ss= 1, addl=0),
ev(ID = 7601:8000, amt = 4.5*0, ii= 21, ss= 0, addl=0),
ev(ID = 7601:8000, amt = 4.5*0.5, ii= 3, ss= 0, addl=0),
ev(ID = 7601:8000, amt = 4.5*1, ii= 24, ss= 0, addl=10),
#21h delay simulation
ev(ID = 7601:8000, amt = 4.5*1, ii= 24, ss= 1, addl=0),
ev(ID = 7601:8000, amt = 4.5*0, ii= 21, ss= 0, addl=0),
ev(ID = 7601:8000, amt = 4.5*1, ii= 3, ss= 0, addl=0),
ev(ID = 7601:8000, amt = 4.5*1, ii= 24, ss= 0, addl=10),
#Missed dose simulation
ev(ID = 7601:8000, amt = 4.5*1, ii= 24, ss= 1, addl=0),
ev(ID = 7601:8000, amt = 4.5*0, ii= 24, ss= 0, addl=0),
ev(ID = 7601:8000, amt = 4.5*1, ii= 24, ss= 0, addl=0),
ev(ID = 7601:8000, amt = 4.5*1, ii= 24, ss= 0, addl=10),
#Missed dose + 150% dose intake simulation
ev(ID = 7601:8000, amt = 4.5*1, ii= 24, ss= 1, addl=0),
ev(ID = 7601:8000, amt = 4.5*0, ii= 24, ss= 0, addl=0),
ev(ID = 7601:8000, amt = 4.5*1.5, ii= 24, ss= 0, addl=0),
ev(ID = 7601:8000, amt = 4.5*1, ii= 24, ss= 0, addl=10),
#Missed dose + 200% dose intake simulation
ev(ID = 7601:8000, amt = 4.5*1, ii= 24, ss= 1, addl=0),
ev(ID = 7601:8000, amt = 4.5*0, ii= 24, ss= 0, addl=0),
ev(ID = 7601:8000, amt = 4.5*2, ii= 24, ss= 0, addl=0),
ev(ID = 7601:8000, amt = 4.5*1, ii= 24, ss= 0, addl=10)))) %>%
             mutate(dose_group = "4,5 mg") %>%

    bind_rows(as_tibble( seq(
  #Steady-state simulation
ev(ID = 8001:8400, amt = 5.5*1, ii= 24, ss= 1, addl=0),
ev(ID = 8001:8400, amt = 5.5*1, ii= 24, ss= 0, addl=0),
ev(ID = 8001:8400, amt = 5.5*1, ii= 24, ss= 0, addl=10),
#3h delay simulation
ev(ID = 8001:8400, amt = 5.5*1, ii= 24, ss= 1, addl=0),
ev(ID = 8001:8400, amt = 5.5*0, ii= 3, ss= 0, addl=0),
ev(ID = 8001:8400, amt = 5.5*1, ii= 21, ss= 0, addl=0),
ev(ID = 8001:8400, amt = 5.5*1, ii= 24, ss= 0, addl=10),
#6h delay simulation
ev(ID = 8001:8400, amt = 5.5*1, ii= 24, ss= 1, addl=0),
ev(ID = 8001:8400, amt = 5.5*0, ii= 6, ss= 0, addl=0),
ev(ID = 8001:8400, amt = 5.5*1, ii= 18, ss= 0, addl=0),
ev(ID = 8001:8400, amt = 5.5*1, ii= 24, ss= 0, addl=10),
#9h delay simulation
ev(ID = 8001:8400, amt = 5.5*1, ii= 24, ss= 1, addl=0),
ev(ID = 8001:8400, amt = 5.5*0, ii= 9, ss= 0, addl=0),
ev(ID = 8001:8400, amt = 5.5*1, ii= 15, ss= 0, addl=0),
ev(ID = 8001:8400, amt = 5.5*1, ii= 24, ss= 0, addl=10),
#12h delay simulation
ev(ID = 8001:8400, amt = 5.5*1, ii= 24, ss= 1, addl=0),
ev(ID = 8001:8400, amt = 5.5*0, ii= 12, ss= 0, addl=0),
ev(ID = 8001:8400, amt = 5.5*1, ii= 12, ss= 0, addl=0),
ev(ID = 8001:8400, amt = 5.5*1, ii= 24, ss= 0, addl=10),
#15h delay + 50% dose intake simulation
ev(ID = 8001:8400, amt = 5.5*1, ii= 24, ss= 1, addl=0),
ev(ID = 8001:8400, amt = 5.5*0, ii= 15, ss= 0, addl=0),
ev(ID = 8001:8400, amt = 5.5*0.5, ii= 9, ss= 0, addl=0),
ev(ID = 8001:8400, amt = 5.5*1, ii= 24, ss= 0, addl=10),
#15h delay simulation
ev(ID = 8001:8400, amt = 5.5*1, ii= 24, ss= 1, addl=0),
ev(ID = 8001:8400, amt = 5.5*0, ii= 15, ss= 0, addl=0),
ev(ID = 8001:8400, amt = 5.5*1, ii= 9, ss= 0, addl=0),
ev(ID = 8001:8400, amt = 5.5*1, ii= 24, ss= 0, addl=10),
#18h delay + 50% dose intake simulation
ev(ID = 8001:8400, amt = 5.5*1, ii= 24, ss= 1, addl=0),
ev(ID = 8001:8400, amt = 5.5*0, ii= 18, ss= 0, addl=0),
ev(ID = 8001:8400, amt = 5.5*0.5, ii= 6, ss= 0, addl=0),
ev(ID = 8001:8400, amt = 5.5*1, ii= 24, ss= 0, addl=10),
#18h delay simulation
ev(ID = 8001:8400, amt = 5.5*1, ii= 24, ss= 1, addl=0),
ev(ID = 8001:8400, amt = 5.5*0, ii= 18, ss= 0, addl=0),
ev(ID = 8001:8400, amt = 5.5*1, ii= 6, ss= 0, addl=0),
ev(ID = 8001:8400, amt = 5.5*1, ii= 24, ss= 0, addl=10),
#21h delay + 50% dose intake simulation
ev(ID = 8001:8400, amt = 5.5*1, ii= 24, ss= 1, addl=0),
ev(ID = 8001:8400, amt = 5.5*0, ii= 21, ss= 0, addl=0),
ev(ID = 8001:8400, amt = 5.5*0.5, ii= 3, ss= 0, addl=0),
ev(ID = 8001:8400, amt = 5.5*1, ii= 24, ss= 0, addl=10),
#21h delay simulation
ev(ID = 8001:8400, amt = 5.5*1, ii= 24, ss= 1, addl=0),
ev(ID = 8001:8400, amt = 5.5*0, ii= 21, ss= 0, addl=0),
ev(ID = 8001:8400, amt = 5.5*1, ii= 3, ss= 0, addl=0),
ev(ID = 8001:8400, amt = 5.5*1, ii= 24, ss= 0, addl=10),
#Missed dose simulation
ev(ID = 8001:8400, amt = 5.5*1, ii= 24, ss= 1, addl=0),
ev(ID = 8001:8400, amt = 5.5*0, ii= 24, ss= 0, addl=0),
ev(ID = 8001:8400, amt = 5.5*1, ii= 24, ss= 0, addl=0),
ev(ID = 8001:8400, amt = 5.5*1, ii= 24, ss= 0, addl=10),
#Missed dose + 150% dose intake simulation
ev(ID = 8001:8400, amt = 5.5*1, ii= 24, ss= 1, addl=0),
ev(ID = 8001:8400, amt = 5.5*0, ii= 24, ss= 0, addl=0),
ev(ID = 8001:8400, amt = 5.5*1.5, ii= 24, ss= 0, addl=0),
ev(ID = 8001:8400, amt = 5.5*1, ii= 24, ss= 0, addl=10),
#Missed dose + 200% dose intake simulation
ev(ID = 8001:8400, amt = 5.5*1, ii= 24, ss= 1, addl=0),
ev(ID = 8001:8400, amt = 5.5*0, ii= 24, ss= 0, addl=0),
ev(ID = 8001:8400, amt = 5.5*2, ii= 24, ss= 0, addl=0),
ev(ID = 8001:8400, amt = 5.5*1, ii= 24, ss= 0, addl=10)))) %>%
             mutate(dose_group = "5,5 mg") %>%

    bind_rows(as_tibble( seq(
  #Steady-state simulation
ev(ID = 8401:8800, amt = 6.5*1, ii= 24, ss= 1, addl=0),
ev(ID = 8401:8800, amt = 6.5*1, ii= 24, ss= 0, addl=0),
ev(ID = 8401:8800, amt = 6.5*1, ii= 24, ss= 0, addl=10),
#3h delay simulation
ev(ID = 8401:8800, amt = 6.5*1, ii= 24, ss= 1, addl=0),
ev(ID = 8401:8800, amt = 6.5*0, ii= 3, ss= 0, addl=0),
ev(ID = 8401:8800, amt = 6.5*1, ii= 21, ss= 0, addl=0),
ev(ID = 8401:8800, amt = 6.5*1, ii= 24, ss= 0, addl=10),
#6h delay simulation
ev(ID = 8401:8800, amt = 6.5*1, ii= 24, ss= 1, addl=0),
ev(ID = 8401:8800, amt = 6.5*0, ii= 6, ss= 0, addl=0),
ev(ID = 8401:8800, amt = 6.5*1, ii= 18, ss= 0, addl=0),
ev(ID = 8401:8800, amt = 6.5*1, ii= 24, ss= 0, addl=10),
#9h delay simulation
ev(ID = 8401:8800, amt = 6.5*1, ii= 24, ss= 1, addl=0),
ev(ID = 8401:8800, amt = 6.5*0, ii= 9, ss= 0, addl=0),
ev(ID = 8401:8800, amt = 6.5*1, ii= 15, ss= 0, addl=0),
ev(ID = 8401:8800, amt = 6.5*1, ii= 24, ss= 0, addl=10),
#12h delay simulation
ev(ID = 8401:8800, amt = 6.5*1, ii= 24, ss= 1, addl=0),
ev(ID = 8401:8800, amt = 6.5*0, ii= 12, ss= 0, addl=0),
ev(ID = 8401:8800, amt = 6.5*1, ii= 12, ss= 0, addl=0),
ev(ID = 8401:8800, amt = 6.5*1, ii= 24, ss= 0, addl=10),
#15h delay + 50% dose intake simulation
ev(ID = 8401:8800, amt = 6.5*1, ii= 24, ss= 1, addl=0),
ev(ID = 8401:8800, amt = 6.5*0, ii= 15, ss= 0, addl=0),
ev(ID = 8401:8800, amt = 6.5*0.5, ii= 9, ss= 0, addl=0),
ev(ID = 8401:8800, amt = 6.5*1, ii= 24, ss= 0, addl=10),
#15h delay simulation
ev(ID = 8401:8800, amt = 6.5*1, ii= 24, ss= 1, addl=0),
ev(ID = 8401:8800, amt = 6.5*0, ii= 15, ss= 0, addl=0),
ev(ID = 8401:8800, amt = 6.5*1, ii= 9, ss= 0, addl=0),
ev(ID = 8401:8800, amt = 6.5*1, ii= 24, ss= 0, addl=10),
#18h delay + 50% dose intake simulation
ev(ID = 8401:8800, amt = 6.5*1, ii= 24, ss= 1, addl=0),
ev(ID = 8401:8800, amt = 6.5*0, ii= 18, ss= 0, addl=0),
ev(ID = 8401:8800, amt = 6.5*0.5, ii= 6, ss= 0, addl=0),
ev(ID = 8401:8800, amt = 6.5*1, ii= 24, ss= 0, addl=10),
#18h delay simulation
ev(ID = 8401:8800, amt = 6.5*1, ii= 24, ss= 1, addl=0),
ev(ID = 8401:8800, amt = 6.5*0, ii= 18, ss= 0, addl=0),
ev(ID = 8401:8800, amt = 6.5*1, ii= 6, ss= 0, addl=0),
ev(ID = 8401:8800, amt = 6.5*1, ii= 24, ss= 0, addl=10),
#21h delay + 50% dose intake simulation
ev(ID = 8401:8800, amt = 6.5*1, ii= 24, ss= 1, addl=0),
ev(ID = 8401:8800, amt = 6.5*0, ii= 21, ss= 0, addl=0),
ev(ID = 8401:8800, amt = 6.5*0.5, ii= 3, ss= 0, addl=0),
ev(ID = 8401:8800, amt = 6.5*1, ii= 24, ss= 0, addl=10),
#21h delay simulation
ev(ID = 8401:8800, amt = 6.5*1, ii= 24, ss= 1, addl=0),
ev(ID = 8401:8800, amt = 6.5*0, ii= 21, ss= 0, addl=0),
ev(ID = 8401:8800, amt = 6.5*1, ii= 3, ss= 0, addl=0),
ev(ID = 8401:8800, amt = 6.5*1, ii= 24, ss= 0, addl=10),
#Missed dose simulation
ev(ID = 8401:8800, amt = 6.5*1, ii= 24, ss= 1, addl=0),
ev(ID = 8401:8800, amt = 6.5*0, ii= 24, ss= 0, addl=0),
ev(ID = 8401:8800, amt = 6.5*1, ii= 24, ss= 0, addl=0),
ev(ID = 8401:8800, amt = 6.5*1, ii= 24, ss= 0, addl=10),
#Missed dose + 150% dose intake simulation
ev(ID = 8401:8800, amt = 6.5*1, ii= 24, ss= 1, addl=0),
ev(ID = 8401:8800, amt = 6.5*0, ii= 24, ss= 0, addl=0),
ev(ID = 8401:8800, amt = 6.5*1.5, ii= 24, ss= 0, addl=0),
ev(ID = 8401:8800, amt = 6.5*1, ii= 24, ss= 0, addl=10),
#Missed dose + 200% dose intake simulation
ev(ID = 8401:8800, amt = 6.5*1, ii= 24, ss= 1, addl=0),
ev(ID = 8401:8800, amt = 6.5*0, ii= 24, ss= 0, addl=0),
ev(ID = 8401:8800, amt = 6.5*2, ii= 24, ss= 0, addl=0),
ev(ID = 8401:8800, amt = 6.5*1, ii= 24, ss= 0, addl=10)))) %>%
             mutate(dose_group = "6,5 mg") %>%

    bind_rows(as_tibble( seq(
  #Steady-state simulation
ev(ID = 8801:9200, amt = 7.5*1, ii= 24, ss= 1, addl=0),
ev(ID = 8801:9200, amt = 7.5*1, ii= 24, ss= 0, addl=0),
ev(ID = 8801:9200, amt = 7.5*1, ii= 24, ss= 0, addl=10),
#3h delay simulation
ev(ID = 8801:9200, amt = 7.5*1, ii= 24, ss= 1, addl=0),
ev(ID = 8801:9200, amt = 7.5*0, ii= 3, ss= 0, addl=0),
ev(ID = 8801:9200, amt = 7.5*1, ii= 21, ss= 0, addl=0),
ev(ID = 8801:9200, amt = 7.5*1, ii= 24, ss= 0, addl=10),
#6h delay simulation
ev(ID = 8801:9200, amt = 7.5*1, ii= 24, ss= 1, addl=0),
ev(ID = 8801:9200, amt = 7.5*0, ii= 6, ss= 0, addl=0),
ev(ID = 8801:9200, amt = 7.5*1, ii= 18, ss= 0, addl=0),
ev(ID = 8801:9200, amt = 7.5*1, ii= 24, ss= 0, addl=10),
#9h delay simulation
ev(ID = 8801:9200, amt = 7.5*1, ii= 24, ss= 1, addl=0),
ev(ID = 8801:9200, amt = 7.5*0, ii= 9, ss= 0, addl=0),
ev(ID = 8801:9200, amt = 7.5*1, ii= 15, ss= 0, addl=0),
ev(ID = 8801:9200, amt = 7.5*1, ii= 24, ss= 0, addl=10),
#12h delay simulation
ev(ID = 8801:9200, amt = 7.5*1, ii= 24, ss= 1, addl=0),
ev(ID = 8801:9200, amt = 7.5*0, ii= 12, ss= 0, addl=0),
ev(ID = 8801:9200, amt = 7.5*1, ii= 12, ss= 0, addl=0),
ev(ID = 8801:9200, amt = 7.5*1, ii= 24, ss= 0, addl=10),
#15h delay + 50% dose intake simulation
ev(ID = 8801:9200, amt = 7.5*1, ii= 24, ss= 1, addl=0),
ev(ID = 8801:9200, amt = 7.5*0, ii= 15, ss= 0, addl=0),
ev(ID = 8801:9200, amt = 7.5*0.5, ii= 9, ss= 0, addl=0),
ev(ID = 8801:9200, amt = 7.5*1, ii= 24, ss= 0, addl=10),
#15h delay simulation
ev(ID = 8801:9200, amt = 7.5*1, ii= 24, ss= 1, addl=0),
ev(ID = 8801:9200, amt = 7.5*0, ii= 15, ss= 0, addl=0),
ev(ID = 8801:9200, amt = 7.5*1, ii= 9, ss= 0, addl=0),
ev(ID = 8801:9200, amt = 7.5*1, ii= 24, ss= 0, addl=10),
#18h delay + 50% dose intake simulation
ev(ID = 8801:9200, amt = 7.5*1, ii= 24, ss= 1, addl=0),
ev(ID = 8801:9200, amt = 7.5*0, ii= 18, ss= 0, addl=0),
ev(ID = 8801:9200, amt = 7.5*0.5, ii= 6, ss= 0, addl=0),
ev(ID = 8801:9200, amt = 7.5*1, ii= 24, ss= 0, addl=10),
#18h delay simulation
ev(ID = 8801:9200, amt = 7.5*1, ii= 24, ss= 1, addl=0),
ev(ID = 8801:9200, amt = 7.5*0, ii= 18, ss= 0, addl=0),
ev(ID = 8801:9200, amt = 7.5*1, ii= 6, ss= 0, addl=0),
ev(ID = 8801:9200, amt = 7.5*1, ii= 24, ss= 0, addl=10),
#21h delay + 50% dose intake simulation
ev(ID = 8801:9200, amt = 7.5*1, ii= 24, ss= 1, addl=0),
ev(ID = 8801:9200, amt = 7.5*0, ii= 21, ss= 0, addl=0),
ev(ID = 8801:9200, amt = 7.5*0.5, ii= 3, ss= 0, addl=0),
ev(ID = 8801:9200, amt = 7.5*1, ii= 24, ss= 0, addl=10),
#21h delay simulation
ev(ID = 8801:9200, amt = 7.5*1, ii= 24, ss= 1, addl=0),
ev(ID = 8801:9200, amt = 7.5*0, ii= 21, ss= 0, addl=0),
ev(ID = 8801:9200, amt = 7.5*1, ii= 3, ss= 0, addl=0),
ev(ID = 8801:9200, amt = 7.5*1, ii= 24, ss= 0, addl=10),
#Missed dose simulation
ev(ID = 8801:9200, amt = 7.5*1, ii= 24, ss= 1, addl=0),
ev(ID = 8801:9200, amt = 7.5*0, ii= 24, ss= 0, addl=0),
ev(ID = 8801:9200, amt = 7.5*1, ii= 24, ss= 0, addl=0),
ev(ID = 8801:9200, amt = 7.5*1, ii= 24, ss= 0, addl=10),
#Missed dose + 150% dose intake simulation
ev(ID = 8801:9200, amt = 7.5*1, ii= 24, ss= 1, addl=0),
ev(ID = 8801:9200, amt = 7.5*0, ii= 24, ss= 0, addl=0),
ev(ID = 8801:9200, amt = 7.5*1.5, ii= 24, ss= 0, addl=0),
ev(ID = 8801:9200, amt = 7.5*1, ii= 24, ss= 0, addl=10),
#Missed dose + 200% dose intake simulation
ev(ID = 8801:9200, amt = 7.5*1, ii= 24, ss= 1, addl=0),
ev(ID = 8801:9200, amt = 7.5*0, ii= 24, ss= 0, addl=0),
ev(ID = 8801:9200, amt = 7.5*2, ii= 24, ss= 0, addl=0),
ev(ID = 8801:9200, amt = 7.5*1, ii= 24, ss= 0, addl=10)))) %>%
             mutate(dose_group = "7,5 mg") %>%

    bind_rows(as_tibble( seq(
  #Steady-state simulation
ev(ID = 9201:9600, amt = 8.5*1, ii= 24, ss= 1, addl=0),
ev(ID = 9201:9600, amt = 8.5*1, ii= 24, ss= 0, addl=0),
ev(ID = 9201:9600, amt = 8.5*1, ii= 24, ss= 0, addl=10),
#3h delay simulation
ev(ID = 9201:9600, amt = 8.5*1, ii= 24, ss= 1, addl=0),
ev(ID = 9201:9600, amt = 8.5*0, ii= 3, ss= 0, addl=0),
ev(ID = 9201:9600, amt = 8.5*1, ii= 21, ss= 0, addl=0),
ev(ID = 9201:9600, amt = 8.5*1, ii= 24, ss= 0, addl=10),
#6h delay simulation
ev(ID = 9201:9600, amt = 8.5*1, ii= 24, ss= 1, addl=0),
ev(ID = 9201:9600, amt = 8.5*0, ii= 6, ss= 0, addl=0),
ev(ID = 9201:9600, amt = 8.5*1, ii= 18, ss= 0, addl=0),
ev(ID = 9201:9600, amt = 8.5*1, ii= 24, ss= 0, addl=10),
#9h delay simulation
ev(ID = 9201:9600, amt = 8.5*1, ii= 24, ss= 1, addl=0),
ev(ID = 9201:9600, amt = 8.5*0, ii= 9, ss= 0, addl=0),
ev(ID = 9201:9600, amt = 8.5*1, ii= 15, ss= 0, addl=0),
ev(ID = 9201:9600, amt = 8.5*1, ii= 24, ss= 0, addl=10),
#12h delay simulation
ev(ID = 9201:9600, amt = 8.5*1, ii= 24, ss= 1, addl=0),
ev(ID = 9201:9600, amt = 8.5*0, ii= 12, ss= 0, addl=0),
ev(ID = 9201:9600, amt = 8.5*1, ii= 12, ss= 0, addl=0),
ev(ID = 9201:9600, amt = 8.5*1, ii= 24, ss= 0, addl=10),
#15h delay + 50% dose intake simulation
ev(ID = 9201:9600, amt = 8.5*1, ii= 24, ss= 1, addl=0),
ev(ID = 9201:9600, amt = 8.5*0, ii= 15, ss= 0, addl=0),
ev(ID = 9201:9600, amt = 8.5*0.5, ii= 9, ss= 0, addl=0),
ev(ID = 9201:9600, amt = 8.5*1, ii= 24, ss= 0, addl=10),
#15h delay simulation
ev(ID = 9201:9600, amt = 8.5*1, ii= 24, ss= 1, addl=0),
ev(ID = 9201:9600, amt = 8.5*0, ii= 15, ss= 0, addl=0),
ev(ID = 9201:9600, amt = 8.5*1, ii= 9, ss= 0, addl=0),
ev(ID = 9201:9600, amt = 8.5*1, ii= 24, ss= 0, addl=10),
#18h delay + 50% dose intake simulation
ev(ID = 9201:9600, amt = 8.5*1, ii= 24, ss= 1, addl=0),
ev(ID = 9201:9600, amt = 8.5*0, ii= 18, ss= 0, addl=0),
ev(ID = 9201:9600, amt = 8.5*0.5, ii= 6, ss= 0, addl=0),
ev(ID = 9201:9600, amt = 8.5*1, ii= 24, ss= 0, addl=10),
#18h delay simulation
ev(ID = 9201:9600, amt = 8.5*1, ii= 24, ss= 1, addl=0),
ev(ID = 9201:9600, amt = 8.5*0, ii= 18, ss= 0, addl=0),
ev(ID = 9201:9600, amt = 8.5*1, ii= 6, ss= 0, addl=0),
ev(ID = 9201:9600, amt = 8.5*1, ii= 24, ss= 0, addl=10),
#21h delay + 50% dose intake simulation
ev(ID = 9201:9600, amt = 8.5*1, ii= 24, ss= 1, addl=0),
ev(ID = 9201:9600, amt = 8.5*0, ii= 21, ss= 0, addl=0),
ev(ID = 9201:9600, amt = 8.5*0.5, ii= 3, ss= 0, addl=0),
ev(ID = 9201:9600, amt = 8.5*1, ii= 24, ss= 0, addl=10),
#21h delay simulation
ev(ID = 9201:9600, amt = 8.5*1, ii= 24, ss= 1, addl=0),
ev(ID = 9201:9600, amt = 8.5*0, ii= 21, ss= 0, addl=0),
ev(ID = 9201:9600, amt = 8.5*1, ii= 3, ss= 0, addl=0),
ev(ID = 9201:9600, amt = 8.5*1, ii= 24, ss= 0, addl=10),
#Missed dose simulation
ev(ID = 9201:9600, amt = 8.5*1, ii= 24, ss= 1, addl=0),
ev(ID = 9201:9600, amt = 8.5*0, ii= 24, ss= 0, addl=0),
ev(ID = 9201:9600, amt = 8.5*1, ii= 24, ss= 0, addl=0),
ev(ID = 9201:9600, amt = 8.5*1, ii= 24, ss= 0, addl=10),
#Missed dose + 150% dose intake simulation
ev(ID = 9201:9600, amt = 8.5*1, ii= 24, ss= 1, addl=0),
ev(ID = 9201:9600, amt = 8.5*0, ii= 24, ss= 0, addl=0),
ev(ID = 9201:9600, amt = 8.5*1.5, ii= 24, ss= 0, addl=0),
ev(ID = 9201:9600, amt = 8.5*1, ii= 24, ss= 0, addl=10),
#Missed dose + 200% dose intake simulation
ev(ID = 9201:9600, amt = 8.5*1, ii= 24, ss= 1, addl=0),
ev(ID = 9201:9600, amt = 8.5*0, ii= 24, ss= 0, addl=0),
ev(ID = 9201:9600, amt = 8.5*2, ii= 24, ss= 0, addl=0),
ev(ID = 9201:9600, amt = 8.5*1, ii= 24, ss= 0, addl=10)))) %>%
             mutate(dose_group = "8,5 mg") %>%

    bind_rows(as_tibble( seq(
  #Steady-state simulation
ev(ID = 9601:10000, amt = 9.5*1, ii= 24, ss= 1, addl=0),
ev(ID = 9601:10000, amt = 9.5*1, ii= 24, ss= 0, addl=0),
ev(ID = 9601:10000, amt = 9.5*1, ii= 24, ss= 0, addl=10),
#3h delay simulation
ev(ID = 9601:10000, amt = 9.5*1, ii= 24, ss= 1, addl=0),
ev(ID = 9601:10000, amt = 9.5*0, ii= 3, ss= 0, addl=0),
ev(ID = 9601:10000, amt = 9.5*1, ii= 21, ss= 0, addl=0),
ev(ID = 9601:10000, amt = 9.5*1, ii= 24, ss= 0, addl=10),
#6h delay simulation
ev(ID = 9601:10000, amt = 9.5*1, ii= 24, ss= 1, addl=0),
ev(ID = 9601:10000, amt = 9.5*0, ii= 6, ss= 0, addl=0),
ev(ID = 9601:10000, amt = 9.5*1, ii= 18, ss= 0, addl=0),
ev(ID = 9601:10000, amt = 9.5*1, ii= 24, ss= 0, addl=10),
#9h delay simulation
ev(ID = 9601:10000, amt = 9.5*1, ii= 24, ss= 1, addl=0),
ev(ID = 9601:10000, amt = 9.5*0, ii= 9, ss= 0, addl=0),
ev(ID = 9601:10000, amt = 9.5*1, ii= 15, ss= 0, addl=0),
ev(ID = 9601:10000, amt = 9.5*1, ii= 24, ss= 0, addl=10),
#12h delay simulation
ev(ID = 9601:10000, amt = 9.5*1, ii= 24, ss= 1, addl=0),
ev(ID = 9601:10000, amt = 9.5*0, ii= 12, ss= 0, addl=0),
```

```r
ev(ID = 9601:10000, amt = 9.5*1, ii= 12, ss= 0, addl=0),
ev(ID = 9601:10000, amt = 9.5*1, ii= 24, ss= 0, addl=10),
#15h delay + 50% dose intake simulation
ev(ID = 9601:10000, amt = 9.5*1, ii= 24, ss= 1, addl=0),
ev(ID = 9601:10000, amt = 9.5*0, ii= 15, ss= 0, addl=0),
ev(ID = 9601:10000, amt = 9.5*0.5, ii= 9, ss= 0, addl=0),
ev(ID = 9601:10000, amt = 9.5*1, ii= 24, ss= 0, addl=10),
#15h delay simulation
ev(ID = 9601:10000, amt = 9.5*1, ii= 24, ss= 1, addl=0),
ev(ID = 9601:10000, amt = 9.5*0, ii= 15, ss= 0, addl=0),
ev(ID = 9601:10000, amt = 9.5*1, ii= 9, ss= 0, addl=0),
ev(ID = 9601:10000, amt = 9.5*1, ii= 24, ss= 0, addl=10),
#18h delay + 50% dose intake simulation
ev(ID = 9601:10000, amt = 9.5*1, ii= 24, ss= 1, addl=0),
ev(ID = 9601:10000, amt = 9.5*0, ii= 18, ss= 0, addl=0),
ev(ID = 9601:10000, amt = 9.5*0.5, ii= 6, ss= 0, addl=0),
ev(ID = 9601:10000, amt = 9.5*1, ii= 24, ss= 0, addl=10),
#18h delay simulation
ev(ID = 9601:10000, amt = 9.5*1, ii= 24, ss= 1, addl=0),
ev(ID = 9601:10000, amt = 9.5*0, ii= 18, ss= 0, addl=0),
ev(ID = 9601:10000, amt = 9.5*1, ii= 6, ss= 0, addl=0),
ev(ID = 9601:10000, amt = 9.5*1, ii= 24, ss= 0, addl=10),
#21h delay + 50% dose intake simulation
ev(ID = 9601:10000, amt = 9.5*1, ii= 24, ss= 1, addl=0),
ev(ID = 9601:10000, amt = 9.5*0, ii= 21, ss= 0, addl=0),
ev(ID = 9601:10000, amt = 9.5*0.5, ii= 3, ss= 0, addl=0),
ev(ID = 9601:10000, amt = 9.5*1, ii= 24, ss= 0, addl=10),
#21h delay simulation
ev(ID = 9601:10000, amt = 9.5*1, ii= 24, ss= 1, addl=0),
ev(ID = 9601:10000, amt = 9.5*0, ii= 21, ss= 0, addl=0),
ev(ID = 9601:10000, amt = 9.5*1, ii= 3, ss= 0, addl=0),
ev(ID = 9601:10000, amt = 9.5*1, ii= 24, ss= 0, addl=10),
#Missed dose simulation
ev(ID = 9601:10000, amt = 9.5*1, ii= 24, ss= 1, addl=0),
ev(ID = 9601:10000, amt = 9.5*0, ii= 24, ss= 0, addl=0),
ev(ID = 9601:10000, amt = 9.5*1, ii= 24, ss= 0, addl=0),
ev(ID = 9601:10000, amt = 9.5*1, ii= 24, ss= 0, addl=10),
#Missed dose + 150% dose intake simulation
ev(ID = 9601:10000, amt = 9.5*1, ii= 24, ss= 1, addl=0),
ev(ID = 9601:10000, amt = 9.5*0, ii= 24, ss= 0, addl=0),
ev(ID = 9601:10000, amt = 9.5*1.5, ii= 24, ss= 0, addl=0),
ev(ID = 9601:10000, amt = 9.5*1, ii= 24, ss= 0, addl=10),
#Missed dose + 200% dose intake simulation
ev(ID = 9601:10000, amt = 9.5*1, ii= 24, ss= 1, addl=0),
ev(ID = 9601:10000, amt = 9.5*0, ii= 24, ss= 0, addl=0),
ev(ID = 9601:10000, amt = 9.5*2, ii= 24, ss= 0, addl=0),
ev(ID = 9601:10000, amt = 9.5*1, ii= 24, ss= 0, addl=10)))) %>%
             mutate(dose_group = "9,5 mg") %>%

    bind_rows(as_tibble( seq(
  #Steady-state simulation
ev(ID = 10001:10400, amt = 10.5*1, ii= 24, ss= 1, addl=0),
ev(ID = 10001:10400, amt = 10.5*1, ii= 24, ss= 0, addl=0),
ev(ID = 10001:10400, amt = 10.5*1, ii= 24, ss= 0, addl=10),
#3h delay simulation
ev(ID = 10001:10400, amt = 10.5*1, ii= 24, ss= 1, addl=0),
ev(ID = 10001:10400, amt = 10.5*0, ii= 3, ss= 0, addl=0),
ev(ID = 10001:10400, amt = 10.5*1, ii= 21, ss= 0, addl=0),
ev(ID = 10001:10400, amt = 10.5*1, ii= 24, ss= 0, addl=10),
#6h delay simulation
ev(ID = 10001:10400, amt = 10.5*1, ii= 24, ss= 1, addl=0),
ev(ID = 10001:10400, amt = 10.5*0, ii= 6, ss= 0, addl=0),
ev(ID = 10001:10400, amt = 10.5*1, ii= 18, ss= 0, addl=0),
ev(ID = 10001:10400, amt = 10.5*1, ii= 24, ss= 0, addl=10),
#9h delay simulation
ev(ID = 10001:10400, amt = 10.5*1, ii= 24, ss= 1, addl=0),
ev(ID = 10001:10400, amt = 10.5*0, ii= 9, ss= 0, addl=0),
ev(ID = 10001:10400, amt = 10.5*1, ii= 15, ss= 0, addl=0),
ev(ID = 10001:10400, amt = 10.5*1, ii= 24, ss= 0, addl=10),
#12h delay simulation
ev(ID = 10001:10400, amt = 10.5*1, ii= 24, ss= 1, addl=0),
ev(ID = 10001:10400, amt = 10.5*0, ii= 12, ss= 0, addl=0),
ev(ID = 10001:10400, amt = 10.5*1, ii= 12, ss= 0, addl=0),
ev(ID = 10001:10400, amt = 10.5*1, ii= 24, ss= 0, addl=10),
#15h delay + 50% dose intake simulation
ev(ID = 10001:10400, amt = 10.5*1, ii= 24, ss= 1, addl=0),
ev(ID = 10001:10400, amt = 10.5*0, ii= 15, ss= 0, addl=0),
ev(ID = 10001:10400, amt = 10.5*0.5, ii= 9, ss= 0, addl=0),
ev(ID = 10001:10400, amt = 10.5*1, ii= 24, ss= 0, addl=10),
#15h delay simulation
ev(ID = 10001:10400, amt = 10.5*1, ii= 24, ss= 1, addl=0),
ev(ID = 10001:10400, amt = 10.5*0, ii= 15, ss= 0, addl=0),
ev(ID = 10001:10400, amt = 10.5*1, ii= 9, ss= 0, addl=0),
ev(ID = 10001:10400, amt = 10.5*1, ii= 24, ss= 0, addl=10),
#18h delay + 50% dose intake simulation
ev(ID = 10001:10400, amt = 10.5*1, ii= 24, ss= 1, addl=0),
ev(ID = 10001:10400, amt = 10.5*0, ii= 18, ss= 0, addl=0),
ev(ID = 10001:10400, amt = 10.5*0.5, ii= 6, ss= 0, addl=0),
ev(ID = 10001:10400, amt = 10.5*1, ii= 24, ss= 0, addl=10),
#18h delay simulation
ev(ID = 10001:10400, amt = 10.5*1, ii= 24, ss= 1, addl=0),
ev(ID = 10001:10400, amt = 10.5*0, ii= 18, ss= 0, addl=0),
ev(ID = 10001:10400, amt = 10.5*1, ii= 6, ss= 0, addl=0),
ev(ID = 10001:10400, amt = 10.5*1, ii= 24, ss= 0, addl=10),
#21h delay + 50% dose intake simulation
ev(ID = 10001:10400, amt = 10.5*1, ii= 24, ss= 1, addl=0),
ev(ID = 10001:10400, amt = 10.5*0, ii= 21, ss= 0, addl=0),
ev(ID = 10001:10400, amt = 10.5*0.5, ii= 3, ss= 0, addl=0),
ev(ID = 10001:10400, amt = 10.5*1, ii= 24, ss= 0, addl=10),
#21h delay simulation
ev(ID = 10001:10400, amt = 10.5*1, ii= 24, ss= 1, addl=0),
ev(ID = 10001:10400, amt = 10.5*0, ii= 21, ss= 0, addl=0),
ev(ID = 10001:10400, amt = 10.5*1, ii= 3, ss= 0, addl=0),
ev(ID = 10001:10400, amt = 10.5*1, ii= 24, ss= 0, addl=10),
#Missed dose simulation
ev(ID = 10001:10400, amt = 10.5*1, ii= 24, ss= 1, addl=0),
ev(ID = 10001:10400, amt = 10.5*0, ii= 24, ss= 0, addl=0),
ev(ID = 10001:10400, amt = 10.5*1, ii= 24, ss= 0, addl=0),
ev(ID = 10001:10400, amt = 10.5*1, ii= 24, ss= 0, addl=10),
#Missed dose + 150% dose intake simulation
ev(ID = 10001:10400, amt = 10.5*1, ii= 24, ss= 1, addl=0),
ev(ID = 10001:10400, amt = 10.5*0, ii= 24, ss= 0, addl=0),
ev(ID = 10001:10400, amt = 10.5*1.5, ii= 24, ss= 0, addl=0),
ev(ID = 10001:10400, amt = 10.5*1, ii= 24, ss= 0, addl=10),
#Missed dose + 200% dose intake simulation
ev(ID = 10001:10400, amt = 10.5*1, ii= 24, ss= 1, addl=0),
ev(ID = 10001:10400, amt = 10.5*0, ii= 24, ss= 0, addl=0),
ev(ID = 10001:10400, amt = 10.5*2, ii= 24, ss= 0, addl=0),
ev(ID = 10001:10400, amt = 10.5*1, ii= 24, ss= 0, addl=10)))) %>%
             mutate(dose_group = "10,5 mg") %>%

    bind_rows(as_tibble( seq(
  #Steady-state simulation
ev(ID = 10401:10800, amt = 11.5*1, ii= 24, ss= 1, addl=0),
ev(ID = 10401:10800, amt = 11.5*1, ii= 24, ss= 0, addl=0),
ev(ID = 10401:10800, amt = 11.5*1, ii= 24, ss= 0, addl=10),
#3h delay simulation
ev(ID = 10401:10800, amt = 11.5*1, ii= 24, ss= 1, addl=0),
ev(ID = 10401:10800, amt = 11.5*0, ii= 3, ss= 0, addl=0),
ev(ID = 10401:10800, amt = 11.5*1, ii= 21, ss= 0, addl=0),
ev(ID = 10401:10800, amt = 11.5*1, ii= 24, ss= 0, addl=10),
#6h delay simulation
ev(ID = 10401:10800, amt = 11.5*1, ii= 24, ss= 1, addl=0),
ev(ID = 10401:10800, amt = 11.5*0, ii= 6, ss= 0, addl=0),
ev(ID = 10401:10800, amt = 11.5*1, ii= 18, ss= 0, addl=0),
ev(ID = 10401:10800, amt = 11.5*1, ii= 24, ss= 0, addl=10),
#9h delay simulation
ev(ID = 10401:10800, amt = 11.5*1, ii= 24, ss= 1, addl=0),
ev(ID = 10401:10800, amt = 11.5*0, ii= 9, ss= 0, addl=0),
ev(ID = 10401:10800, amt = 11.5*1, ii= 15, ss= 0, addl=0),
ev(ID = 10401:10800, amt = 11.5*1, ii= 24, ss= 0, addl=10),
#12h delay simulation
ev(ID = 10401:10800, amt = 11.5*1, ii= 24, ss= 1, addl=0),
ev(ID = 10401:10800, amt = 11.5*0, ii= 12, ss= 0, addl=0),
ev(ID = 10401:10800, amt = 11.5*1, ii= 12, ss= 0, addl=0),
ev(ID = 10401:10800, amt = 11.5*1, ii= 24, ss= 0, addl=10),
#15h delay + 50% dose intake simulation
ev(ID = 10401:10800, amt = 11.5*1, ii= 24, ss= 1, addl=0),
ev(ID = 10401:10800, amt = 11.5*0, ii= 15, ss= 0, addl=0),
ev(ID = 10401:10800, amt = 11.5*0.5, ii= 9, ss= 0, addl=0),
ev(ID = 10401:10800, amt = 11.5*1, ii= 24, ss= 0, addl=10),
#15h delay simulation
ev(ID = 10401:10800, amt = 11.5*1, ii= 24, ss= 1, addl=0),
ev(ID = 10401:10800, amt = 11.5*0, ii= 15, ss= 0, addl=0),
ev(ID = 10401:10800, amt = 11.5*1, ii= 9, ss= 0, addl=0),
#18h delay + 50% dose intake simulation
ev(ID = 10401:10800, amt = 11.5*1, ii= 24, ss= 1, addl=0),
ev(ID = 10401:10800, amt = 11.5*0, ii= 18, ss= 0, addl=0),
ev(ID = 10401:10800, amt = 11.5*0.5, ii= 6, ss= 0, addl=0),
ev(ID = 10401:10800, amt = 11.5*1, ii= 24, ss= 0, addl=10),
#18h delay simulation
ev(ID = 10401:10800, amt = 11.5*1, ii= 24, ss= 1, addl=0),
ev(ID = 10401:10800, amt = 11.5*0, ii= 18, ss= 0, addl=0),
ev(ID = 10401:10800, amt = 11.5*1, ii= 6, ss= 0, addl=0),
ev(ID = 10401:10800, amt = 11.5*1, ii= 24, ss= 0, addl=10),
#21h delay + 50% dose intake simulation
ev(ID = 10401:10800, amt = 11.5*1, ii= 24, ss= 1, addl=0),
ev(ID = 10401:10800, amt = 11.5*0, ii= 21, ss= 0, addl=0),
ev(ID = 10401:10800, amt = 11.5*0.5, ii= 3, ss= 0, addl=0),
ev(ID = 10401:10800, amt = 11.5*1, ii= 24, ss= 0, addl=10),
#21h delay simulation
ev(ID = 10401:10800, amt = 11.5*1, ii= 24, ss= 1, addl=0),
ev(ID = 10401:10800, amt = 11.5*0, ii= 21, ss= 0, addl=0),
ev(ID = 10401:10800, amt = 11.5*1, ii= 3, ss= 0, addl=0),
ev(ID = 10401:10800, amt = 11.5*1, ii= 24, ss= 0, addl=10),
#Missed dose simulation
ev(ID = 10401:10800, amt = 11.5*1, ii= 24, ss= 1, addl=0),
ev(ID = 10401:10800, amt = 11.5*0, ii= 24, ss= 0, addl=0),
ev(ID = 10401:10800, amt = 11.5*1, ii= 24, ss= 0, addl=0),
ev(ID = 10401:10800, amt = 11.5*1, ii= 24, ss= 0, addl=10),
#Missed dose + 150% dose intake simulation
ev(ID = 10401:10800, amt = 11.5*1, ii= 24, ss= 1, addl=0),
ev(ID = 10401:10800, amt = 11.5*0, ii= 24, ss= 0, addl=0),
ev(ID = 10401:10800, amt = 11.5*1.5, ii= 24, ss= 0, addl=0),
ev(ID = 10401:10800, amt = 11.5*1, ii= 24, ss= 0, addl=10),
#Missed dose + 200% dose intake simulation
ev(ID = 10401:10800, amt = 11.5*1, ii= 24, ss= 1, addl=0),
ev(ID = 10401:10800, amt = 11.5*0, ii= 24, ss= 0, addl=0),
ev(ID = 10401:10800, amt = 11.5*2, ii= 24, ss= 0, addl=0),
ev(ID = 10401:10800, amt = 11.5*1, ii= 24, ss= 0, addl=10)))) %>%
             mutate(dose_group = "11,5 mg") %>%

    bind_rows(as_tibble( seq(
  #Steady-state simulation
ev(ID = 10801:11200, amt = 12.5*1, ii= 24, ss= 1, addl=0),
ev(ID = 10801:11200, amt = 12.5*1, ii= 24, ss= 0, addl=0),
ev(ID = 10801:11200, amt = 12.5*1, ii= 24, ss= 0, addl=10),
#3h delay simulation
ev(ID = 10801:11200, amt = 12.5*1, ii= 24, ss= 1, addl=0),
ev(ID = 10801:11200, amt = 12.5*0, ii= 3, ss= 0, addl=0),
ev(ID = 10801:11200, amt = 12.5*1, ii= 21, ss= 0, addl=0),
ev(ID = 10801:11200, amt = 12.5*1, ii= 24, ss= 0, addl=10),
#6h delay simulation
ev(ID = 10801:11200, amt = 12.5*1, ii= 24, ss= 1, addl=0),
ev(ID = 10801:11200, amt = 12.5*0, ii= 6, ss= 0, addl=0),
ev(ID = 10801:11200, amt = 12.5*1, ii= 18, ss= 0, addl=0),
ev(ID = 10801:11200, amt = 12.5*1, ii= 24, ss= 0, addl=10),
#9h delay simulation
ev(ID = 10801:11200, amt = 12.5*1, ii= 24, ss= 1, addl=0),
ev(ID = 10801:11200, amt = 12.5*0, ii= 9, ss= 0, addl=0),
ev(ID = 10801:11200, amt = 12.5*1, ii= 15, ss= 0, addl=0),
ev(ID = 10801:11200, amt = 12.5*1, ii= 24, ss= 0, addl=10),
#12h delay simulation
ev(ID = 10801:11200, amt = 12.5*1, ii= 24, ss= 1, addl=0),
ev(ID = 10801:11200, amt = 12.5*0, ii= 12, ss= 0, addl=0),
ev(ID = 10801:11200, amt = 12.5*1, ii= 12, ss= 0, addl=0),
ev(ID = 10801:11200, amt = 12.5*1, ii= 24, ss= 0, addl=10),
#15h delay + 50% dose intake simulation
ev(ID = 10801:11200, amt = 12.5*1, ii= 24, ss= 1, addl=0),
ev(ID = 10801:11200, amt = 12.5*0, ii= 15, ss= 0, addl=0),
ev(ID = 10801:11200, amt = 12.5*0.5, ii= 9, ss= 0, addl=0),
ev(ID = 10801:11200, amt = 12.5*1, ii= 24, ss= 0, addl=10),
#15h delay simulation
ev(ID = 10801:11200, amt = 12.5*1, ii= 24, ss= 1, addl=0),
ev(ID = 10801:11200, amt = 12.5*0, ii= 15, ss= 0, addl=0),
ev(ID = 10801:11200, amt = 12.5*1, ii= 9, ss= 0, addl=0),
ev(ID = 10801:11200, amt = 12.5*1, ii= 24, ss= 0, addl=10),
#18h delay + 50% dose intake simulation
ev(ID = 10801:11200, amt = 12.5*1, ii= 24, ss= 1, addl=0),
ev(ID = 10801:11200, amt = 12.5*0, ii= 18, ss= 0, addl=0),
ev(ID = 10801:11200, amt = 12.5*0.5, ii= 6, ss= 0, addl=0),
ev(ID = 10801:11200, amt = 12.5*1, ii= 24, ss= 0, addl=10),
#18h delay simulation
ev(ID = 10801:11200, amt = 12.5*1, ii= 24, ss= 1, addl=0),
ev(ID = 10801:11200, amt = 12.5*0, ii= 18, ss= 0, addl=0),
ev(ID = 10801:11200, amt = 12.5*1, ii= 6, ss= 0, addl=0),
ev(ID = 10801:11200, amt = 12.5*1, ii= 24, ss= 0, addl=10),
#21h delay + 50% dose intake simulation
ev(ID = 10801:11200, amt = 12.5*1, ii= 24, ss= 1, addl=0),
ev(ID = 10801:11200, amt = 12.5*0, ii= 21, ss= 0, addl=0),
ev(ID = 10801:11200, amt = 12.5*0.5, ii= 3, ss= 0, addl=0),
ev(ID = 10801:11200, amt = 12.5*1, ii= 24, ss= 0, addl=10),
#21h delay simulation
ev(ID = 10801:11200, amt = 12.5*1, ii= 24, ss= 1, addl=0),
ev(ID = 10801:11200, amt = 12.5*0, ii= 21, ss= 0, addl=0),
ev(ID = 10801:11200, amt = 12.5*1, ii= 3, ss= 0, addl=0),
ev(ID = 10801:11200, amt = 12.5*1, ii= 24, ss= 0, addl=10),
#Missed dose simulation
ev(ID = 10801:11200, amt = 12.5*1, ii= 24, ss= 1, addl=0),
ev(ID = 10801:11200, amt = 12.5*0, ii= 24, ss= 0, addl=0),
ev(ID = 10801:11200, amt = 12.5*1, ii= 24, ss= 0, addl=0),
ev(ID = 10801:11200, amt = 12.5*1, ii= 24, ss= 0, addl=10),
#Missed dose + 150% dose intake simulation
ev(ID = 10801:11200, amt = 12.5*1, ii= 24, ss= 1, addl=0),
ev(ID = 10801:11200, amt = 12.5*0, ii= 24, ss= 0, addl=0),
ev(ID = 10801:11200, amt = 12.5*1.5, ii= 24, ss= 0, addl=0),
ev(ID = 10801:11200, amt = 12.5*1, ii= 24, ss= 0, addl=10),
#Missed dose + 200% dose intake simulation
ev(ID = 10801:11200, amt = 12.5*1, ii= 24, ss= 1, addl=0),
ev(ID = 10801:11200, amt = 12.5*0, ii= 24, ss= 0, addl=0),
ev(ID = 10801:11200, amt = 12.5*2, ii= 24, ss= 0, addl=0),
ev(ID = 10801:11200, amt = 12.5*1, ii= 24, ss= 0, addl=10)))) %>%
             mutate(dose_group = "12,5 mg") %>%

    bind_rows(as_tibble( seq(
  #Steady-state simulation
ev(ID = 11201:11600, amt = 13.5*1, ii= 24, ss= 1, addl=0),
ev(ID = 11201:11600, amt = 13.5*1, ii= 24, ss= 0, addl=0),
ev(ID = 11201:11600, amt = 13.5*1, ii= 24, ss= 0, addl=10),
#3h delay simulation
ev(ID = 11201:11600, amt = 13.5*1, ii= 24, ss= 1, addl=0),
ev(ID = 11201:11600, amt = 13.5*0, ii= 3, ss= 0, addl=0),
ev(ID = 11201:11600, amt = 13.5*1, ii= 21, ss= 0, addl=0),
ev(ID = 11201:11600, amt = 13.5*1, ii= 24, ss= 0, addl=10),
#6h delay simulation
ev(ID = 11201:11600, amt = 13.5*1, ii= 24, ss= 1, addl=0),
ev(ID = 11201:11600, amt = 13.5*0, ii= 6, ss= 0, addl=0),
ev(ID = 11201:11600, amt = 13.5*1, ii= 18, ss= 0, addl=0),
ev(ID = 11201:11600, amt = 13.5*1, ii= 24, ss= 0, addl=10),
#9h delay simulation
ev(ID = 11201:11600, amt = 13.5*1, ii= 24, ss= 1, addl=0),
ev(ID = 11201:11600, amt = 13.5*0, ii= 9, ss= 0, addl=0),
ev(ID = 11201:11600, amt = 13.5*1, ii= 15, ss= 0, addl=0),
ev(ID = 11201:11600, amt = 13.5*1, ii= 24, ss= 0, addl=10),
#12h delay simulation
ev(ID = 11201:11600, amt = 13.5*1, ii= 24, ss= 1, addl=0),
ev(ID = 11201:11600, amt = 13.5*0, ii= 12, ss= 0, addl=0),
ev(ID = 11201:11600, amt = 13.5*1, ii= 12, ss= 0, addl=0),
ev(ID = 11201:11600, amt = 13.5*1, ii= 24, ss= 0, addl=10),
#15h delay + 50% dose intake simulation
ev(ID = 11201:11600, amt = 13.5*1, ii= 24, ss= 1, addl=0),
ev(ID = 11201:11600, amt = 13.5*0, ii= 15, ss= 0, addl=0),
ev(ID = 11201:11600, amt = 13.5*0.5, ii= 9, ss= 0, addl=0),
ev(ID = 11201:11600, amt = 13.5*1, ii= 24, ss= 0, addl=10),
#15h delay simulation
ev(ID = 11201:11600, amt = 13.5*1, ii= 24, ss= 1, addl=0),
ev(ID = 11201:11600, amt = 13.5*0, ii= 15, ss= 0, addl=0),
ev(ID = 11201:11600, amt = 13.5*1, ii= 9, ss= 0, addl=0),
ev(ID = 11201:11600, amt = 13.5*1, ii= 24, ss= 0, addl=10),
#18h delay + 50% dose intake simulation
ev(ID = 11201:11600, amt = 13.5*1, ii= 24, ss= 1, addl=0),
ev(ID = 11201:11600, amt = 13.5*0, ii= 18, ss= 0, addl=0),
ev(ID = 11201:11600, amt = 13.5*0.5, ii= 6, ss= 0, addl=0),
ev(ID = 11201:11600, amt = 13.5*1, ii= 24, ss= 0, addl=10),
#18h delay simulation
ev(ID = 11201:11600, amt = 13.5*1, ii= 24, ss= 1, addl=0),
ev(ID = 11201:11600, amt = 13.5*0, ii= 18, ss= 0, addl=0),
ev(ID = 11201:11600, amt = 13.5*1, ii= 6, ss= 0, addl=0),
ev(ID = 11201:11600, amt = 13.5*1, ii= 24, ss= 0, addl=10),
#21h delay + 50% dose intake simulation
ev(ID = 11201:11600, amt = 13.5*1, ii= 24, ss= 1, addl=0),
ev(ID = 11201:11600, amt = 13.5*0, ii= 21, ss= 0, addl=0),
ev(ID = 11201:11600, amt = 13.5*0.5, ii= 3, ss= 0, addl=0),
ev(ID = 11201:11600, amt = 13.5*1, ii= 24, ss= 0, addl=10),
#21h delay simulation
ev(ID = 11201:11600, amt = 13.5*1, ii= 24, ss= 1, addl=0),
ev(ID = 11201:11600, amt = 13.5*0, ii= 21, ss= 0, addl=0),
ev(ID = 11201:11600, amt = 13.5*1, ii= 3, ss= 0, addl=0),
ev(ID = 11201:11600, amt = 13.5*1, ii= 24, ss= 0, addl=10),
#Missed dose simulation
ev(ID = 11201:11600, amt = 13.5*1, ii= 24, ss= 1, addl=0),
ev(ID = 11201:11600, amt = 13.5*0, ii= 24, ss= 0, addl=0),
ev(ID = 11201:11600, amt = 13.5*1, ii= 24, ss= 0, addl=0),
ev(ID = 11201:11600, amt = 13.5*1, ii= 24, ss= 0, addl=10),
#Missed dose + 150% dose intake simulation
ev(ID = 11201:11600, amt = 13.5*1, ii= 24, ss= 1, addl=0),
ev(ID = 11201:11600, amt = 13.5*0, ii= 24, ss= 0, addl=0),
ev(ID = 11201:11600, amt = 13.5*1.5, ii= 24, ss= 0, addl=0),
ev(ID = 11201:11600, amt = 13.5*1, ii= 24, ss= 0, addl=10),
#Missed dose + 200% dose intake simulation
ev(ID = 11201:11600, amt = 13.5*1, ii= 24, ss= 1, addl=0),
ev(ID = 11201:11600, amt = 13.5*0, ii= 24, ss= 0, addl=0),
ev(ID = 11201:11600, amt = 13.5*2, ii= 24, ss= 0, addl=0),
ev(ID = 11201:11600, amt = 13.5*1, ii= 24, ss= 0, addl=10)))) %>%
             mutate(dose_group = "13,5 mg") %>%

    bind_rows(as_tibble( seq(
  #Steady-state simulation
ev(ID = 11601:12000, amt = 14.5*1, ii= 24, ss= 1, addl=0),
ev(ID = 11601:12000, amt = 14.5*1, ii= 24, ss= 0, addl=0),
ev(ID = 11601:12000, amt = 14.5*1, ii= 24, ss= 0, addl=10),
#3h delay simulation
ev(ID = 11601:12000, amt = 14.5*1, ii= 24, ss= 1, addl=0),
ev(ID = 11601:12000, amt = 14.5*0, ii= 3, ss= 0, addl=0),
ev(ID = 11601:12000, amt = 14.5*1, ii= 21, ss= 0, addl=0),
ev(ID = 11601:12000, amt = 14.5*1, ii= 24, ss= 0, addl=10),
#6h delay simulation
ev(ID = 11601:12000, amt = 14.5*1, ii= 24, ss= 1, addl=0),
ev(ID = 11601:12000, amt = 14.5*0, ii= 6, ss= 0, addl=0),
ev(ID = 11601:12000, amt = 14.5*1, ii= 18, ss= 0, addl=0),
ev(ID = 11601:12000, amt = 14.5*1, ii= 24, ss= 0, addl=10),
#9h delay simulation
ev(ID = 11601:12000, amt = 14.5*1, ii= 24, ss= 1, addl=0),
ev(ID = 11601:12000, amt = 14.5*0, ii= 9, ss= 0, addl=0),
ev(ID = 11601:12000, amt = 14.5*1, ii= 15, ss= 0, addl=0),
ev(ID = 11601:12000, amt = 14.5*1, ii= 24, ss= 0, addl=10),
#12h delay simulation
ev(ID = 11601:12000, amt = 14.5*1, ii= 24, ss= 1, addl=0),
ev(ID = 11601:12000, amt = 14.5*0, ii= 12, ss= 0, addl=0),
ev(ID = 11601:12000, amt = 14.5*1, ii= 12, ss= 0, addl=0),
ev(ID = 11601:12000, amt = 14.5*1, ii= 24, ss= 0, addl=10),
#15h delay + 50% dose intake simulation
ev(ID = 11601:12000, amt = 14.5*1, ii= 24, ss= 1, addl=0),
ev(ID = 11601:12000, amt = 14.5*0, ii= 15, ss= 0, addl=0),
ev(ID = 11601:12000, amt = 14.5*0.5, ii= 9, ss= 0, addl=0),
ev(ID = 11601:12000, amt = 14.5*1, ii= 24, ss= 0, addl=10),
#15h delay simulation
ev(ID = 11601:12000, amt = 14.5*1, ii= 24, ss= 1, addl=0),
ev(ID = 11601:12000, amt = 14.5*0, ii= 15, ss= 0, addl=0),
ev(ID = 11601:12000, amt = 14.5*1, ii= 9, ss= 0, addl=0),
ev(ID = 11601:12000, amt = 14.5*1, ii= 24, ss= 0, addl=10),
#18h delay + 50% dose intake simulation
ev(ID = 11601:12000, amt = 14.5*1, ii= 24, ss= 1, addl=0),
ev(ID = 11601:12000, amt = 14.5*0, ii= 18, ss= 0, addl=0),
ev(ID = 11601:12000, amt = 14.5*0.5, ii= 6, ss= 0, addl=0),
ev(ID = 11601:12000, amt = 14.5*1, ii= 24, ss= 0, addl=10),
#18h delay simulation
ev(ID = 11601:12000, amt = 14.5*1, ii= 24, ss= 1, addl=0),
ev(ID = 11601:12000, amt = 14.5*0, ii= 18, ss= 0, addl=0),
ev(ID = 11601:12000, amt = 14.5*1, ii= 6, ss= 0, addl=0),
ev(ID = 11601:12000, amt = 14.5*1, ii= 24, ss= 0, addl=10),
#21h delay + 50% dose intake simulation
ev(ID = 11601:12000, amt = 14.5*1, ii= 24, ss= 1, addl=0),
ev(ID = 11601:12000, amt = 14.5*0, ii= 21, ss= 0, addl=0),
ev(ID = 11601:12000, amt = 14.5*0.5, ii= 3, ss= 0, addl=0),
ev(ID = 11601:12000, amt = 14.5*1, ii= 24, ss= 0, addl=10),
#21h delay simulation
ev(ID = 11601:12000, amt = 14.5*1, ii= 24, ss= 1, addl=0),
ev(ID = 11601:12000, amt = 14.5*0, ii= 21, ss= 0, addl=0),
ev(ID = 11601:12000, amt = 14.5*1, ii= 3, ss= 0, addl=0),
ev(ID = 11601:12000, amt = 14.5*1, ii= 24, ss= 0, addl=10),
#Missed dose simulation
ev(ID = 11601:12000, amt = 14.5*1, ii= 24, ss= 1, addl=0),
ev(ID = 11601:12000, amt = 14.5*0, ii= 24, ss= 0, addl=0),
ev(ID = 11601:12000, amt = 14.5*1, ii= 24, ss= 0, addl=0),
ev(ID = 11601:12000, amt = 14.5*1, ii= 24, ss= 0, addl=10),
#Missed dose + 150% dose intake simulation
ev(ID = 11601:12000, amt = 14.5*1, ii= 24, ss= 1, addl=0),
ev(ID = 11601:12000, amt = 14.5*0, ii= 24, ss= 0, addl=0),
ev(ID = 11601:12000, amt = 14.5*1.5, ii= 24, ss= 0, addl=0),
ev(ID = 11601:12000, amt = 14.5*1, ii= 24, ss= 0, addl=10),
#Missed dose + 200% dose intake simulation
ev(ID = 11601:12000, amt = 14.5*1, ii= 24, ss= 1, addl=0),
ev(ID = 11601:12000, amt = 14.5*0, ii= 24, ss= 0, addl=0),
ev(ID = 11601:12000, amt = 14.5*2, ii= 24, ss= 0, addl=0),
ev(ID = 11601:12000, amt = 14.5*1, ii= 24, ss= 0, addl=10)))) %>%
             mutate(dose_group = "14,5 mg")


Dataset_martial_12000 <- Simulation_data_martial_12000 %>%
  arrange(ID)

set.seed(123456)
Sim_MARTIAL_by_amt_12000 <- my_model_envarsus %>%
 data_set(Dataset_martial_12000) %>%
 Req(DV) %>%
 mrgsim(end = 7000, delta = 1)
```

```
## Warning in (function (x, data, idata = no_idata_set(), carry_out = carry.out, : [steady_bolus] ID 6017 failed
to reach steady state
##   ss_n: 500, ss_rtol: 1e-08, ss_atol: 1e-08
```

```
## Warning in (function (x, data, idata = no_idata_set(), carry_out = carry.out, : [steady_bolus] ID 6208 failed
to reach steady state
##   ss_n: 500, ss_rtol: 1e-08, ss_atol: 1e-08
## Warning in (function (x, data, idata = no_idata_set(), carry_out = carry.out, : [steady_bolus] ID 6208 failed
to reach steady state
##   ss_n: 500, ss_rtol: 1e-08, ss_atol: 1e-08
## Warning in (function (x, data, idata = no_idata_set(), carry_out = carry.out, : [steady_bolus] ID 6208 failed
to reach steady state
##   ss_n: 500, ss_rtol: 1e-08, ss_atol: 1e-08
## Warning in (function (x, data, idata = no_idata_set(), carry_out = carry.out, : [steady_bolus] ID 6208 failed
to reach steady state
##   ss_n: 500, ss_rtol: 1e-08, ss_atol: 1e-08
## Warning in (function (x, data, idata = no_idata_set(), carry_out = carry.out, : [steady_bolus] ID 6208 failed
to reach steady state
##   ss_n: 500, ss_rtol: 1e-08, ss_atol: 1e-08
## Warning in (function (x, data, idata = no_idata_set(), carry_out = carry.out, : [steady_bolus] ID 6208 failed
to reach steady state
##   ss_n: 500, ss_rtol: 1e-08, ss_atol: 1e-08
```

```
## Warning in (function (x, data, idata = no_idata_set(), carry_out = carry.out, : [steady_bolus] ID 6252 failed
to reach steady state
##   ss_n: 500, ss_rtol: 1e-08, ss_atol: 1e-08
## Warning in (function (x, data, idata = no_idata_set(), carry_out = carry.out, : [steady_bolus] ID 6252 failed
to reach steady state
##   ss_n: 500, ss_rtol: 1e-08, ss_atol: 1e-08
```

```
## Warning in (function (x, data, idata = no_idata_set(), carry_out = carry.out, : [steady_bolus] ID 6323 failed
to reach steady state
##   ss_n: 500, ss_rtol: 1e-08, ss_atol: 1e-08
```

```
## Warning in (function (x, data, idata = no_idata_set(), carry_out = carry.out, : [steady_bolus] ID 6370 failed
to reach steady state
##   ss_n: 500, ss_rtol: 1e-08, ss_atol: 1e-08
## Warning in (function (x, data, idata = no_idata_set(), carry_out = carry.out, : [steady_bolus] ID 6370 failed
to reach steady state
##   ss_n: 500, ss_rtol: 1e-08, ss_atol: 1e-08
## Warning in (function (x, data, idata = no_idata_set(), carry_out = carry.out, : [steady_bolus] ID 6370 failed
to reach steady state
##   ss_n: 500, ss_rtol: 1e-08, ss_atol: 1e-08
## Warning in (function (x, data, idata = no_idata_set(), carry_out = carry.out, : [steady_bolus] ID 6370 failed
to reach steady state
##   ss_n: 500, ss_rtol: 1e-08, ss_atol: 1e-08
## Warning in (function (x, data, idata = no_idata_set(), carry_out = carry.out, : [steady_bolus] ID 6370 failed
to reach steady state
##   ss_n: 500, ss_rtol: 1e-08, ss_atol: 1e-08
## Warning in (function (x, data, idata = no_idata_set(), carry_out = carry.out, : [steady_bolus] ID 6370 failed
to reach steady state
##   ss_n: 500, ss_rtol: 1e-08, ss_atol: 1e-08
```

```
## Warning in (function (x, data, idata = no_idata_set(), carry_out = carry.out, : [steady_bolus] ID 6417 failed
to reach steady state
##   ss_n: 500, ss_rtol: 1e-08, ss_atol: 1e-08
```

```
## Warning in (function (x, data, idata = no_idata_set(), carry_out = carry.out, : [steady_bolus] ID 6541 failed
to reach steady state
##   ss_n: 500, ss_rtol: 1e-08, ss_atol: 1e-08
## Warning in (function (x, data, idata = no_idata_set(), carry_out = carry.out, : [steady_bolus] ID 6541 failed
to reach steady state
##   ss_n: 500, ss_rtol: 1e-08, ss_atol: 1e-08
```

```
## Warning in (function (x, data, idata = no_idata_set(), carry_out = carry.out, : [steady_bolus] ID 6597 failed
to reach steady state
##   ss_n: 500, ss_rtol: 1e-08, ss_atol: 1e-08
## Warning in (function (x, data, idata = no_idata_set(), carry_out = carry.out, : [steady_bolus] ID 6597 failed
to reach steady state
##   ss_n: 500, ss_rtol: 1e-08, ss_atol: 1e-08
## Warning in (function (x, data, idata = no_idata_set(), carry_out = carry.out, : [steady_bolus] ID 6597 failed
to reach steady state
##   ss_n: 500, ss_rtol: 1e-08, ss_atol: 1e-08
## Warning in (function (x, data, idata = no_idata_set(), carry_out = carry.out, : [steady_bolus] ID 6597 failed
to reach steady state
##   ss_n: 500, ss_rtol: 1e-08, ss_atol: 1e-08
## Warning in (function (x, data, idata = no_idata_set(), carry_out = carry.out, : [steady_bolus] ID 6597 failed
to reach steady state
##   ss_n: 500, ss_rtol: 1e-08, ss_atol: 1e-08
## Warning in (function (x, data, idata = no_idata_set(), carry_out = carry.out, : [steady_bolus] ID 6597 failed
to reach steady state
##   ss_n: 500, ss_rtol: 1e-08, ss_atol: 1e-08
## Warning in (function (x, data, idata = no_idata_set(), carry_out = carry.out, : [steady_bolus] ID 6597 failed
to reach steady state
##   ss_n: 500, ss_rtol: 1e-08, ss_atol: 1e-08
```

```
## Warning in (function (x, data, idata = no_idata_set(), carry_out = carry.out, : [steady_bolus] ID 6617 failed
to reach steady state
##   ss_n: 500, ss_rtol: 1e-08, ss_atol: 1e-08
## Warning in (function (x, data, idata = no_idata_set(), carry_out = carry.out, : [steady_bolus] ID 6617 failed
to reach steady state
##   ss_n: 500, ss_rtol: 1e-08, ss_atol: 1e-08
## Warning in (function (x, data, idata = no_idata_set(), carry_out = carry.out, : [steady_bolus] ID 6617 failed
to reach steady state
##   ss_n: 500, ss_rtol: 1e-08, ss_atol: 1e-08
## Warning in (function (x, data, idata = no_idata_set(), carry_out = carry.out, : [steady_bolus] ID 6617 failed
to reach steady state
##   ss_n: 500, ss_rtol: 1e-08, ss_atol: 1e-08
## Warning in (function (x, data, idata = no_idata_set(), carry_out = carry.out, : [steady_bolus] ID 6617 failed
to reach steady state
##   ss_n: 500, ss_rtol: 1e-08, ss_atol: 1e-08
## Warning in (function (x, data, idata = no_idata_set(), carry_out = carry.out, : [steady_bolus] ID 6617 failed
to reach steady state
##   ss_n: 500, ss_rtol: 1e-08, ss_atol: 1e-08
```

```
## Warning in (function (x, data, idata = no_idata_set(), carry_out = carry.out, : [steady_bolus] ID 6659 failed
to reach steady state
##   ss_n: 500, ss_rtol: 1e-08, ss_atol: 1e-08
## Warning in (function (x, data, idata = no_idata_set(), carry_out = carry.out, : [steady_bolus] ID 6659 failed
to reach steady state
##   ss_n: 500, ss_rtol: 1e-08, ss_atol: 1e-08
```

```
## Warning in (function (x, data, idata = no_idata_set(), carry_out = carry.out, : [steady_bolus] ID 6892 failed
to reach steady state
##   ss_n: 500, ss_rtol: 1e-08, ss_atol: 1e-08
```

```
## Warning in (function (x, data, idata = no_idata_set(), carry_out = carry.out, : [steady_bolus] ID 7054 failed
to reach steady state
##   ss_n: 500, ss_rtol: 1e-08, ss_atol: 1e-08
```

```
## Warning in (function (x, data, idata = no_idata_set(), carry_out = carry.out, : [steady_bolus] ID 7076 failed
to reach steady state
##   ss_n: 500, ss_rtol: 1e-08, ss_atol: 1e-08
## Warning in (function (x, data, idata = no_idata_set(), carry_out = carry.out, : [steady_bolus] ID 7076 failed
to reach steady state
##   ss_n: 500, ss_rtol: 1e-08, ss_atol: 1e-08
## Warning in (function (x, data, idata = no_idata_set(), carry_out = carry.out, : [steady_bolus] ID 7076 failed
to reach steady state
##   ss_n: 500, ss_rtol: 1e-08, ss_atol: 1e-08
## Warning in (function (x, data, idata = no_idata_set(), carry_out = carry.out, : [steady_bolus] ID 7076 failed
to reach steady state
##   ss_n: 500, ss_rtol: 1e-08, ss_atol: 1e-08
## Warning in (function (x, data, idata = no_idata_set(), carry_out = carry.out, : [steady_bolus] ID 7076 failed
to reach steady state
##   ss_n: 500, ss_rtol: 1e-08, ss_atol: 1e-08
## Warning in (function (x, data, idata = no_idata_set(), carry_out = carry.out, : [steady_bolus] ID 7076 failed
to reach steady state
##   ss_n: 500, ss_rtol: 1e-08, ss_atol: 1e-08
## Warning in (function (x, data, idata = no_idata_set(), carry_out = carry.out, : [steady_bolus] ID 7076 failed
to reach steady state
##   ss_n: 500, ss_rtol: 1e-08, ss_atol: 1e-08
```

```
## Warning in (function (x, data, idata = no_idata_set(), carry_out = carry.out, : [steady_bolus] ID 7114 failed
to reach steady state
##   ss_n: 500, ss_rtol: 1e-08, ss_atol: 1e-08
```

```
## Warning in (function (x, data, idata = no_idata_set(), carry_out = carry.out, : [steady_bolus] ID 7124 failed
to reach steady state
##   ss_n: 500, ss_rtol: 1e-08, ss_atol: 1e-08
## Warning in (function (x, data, idata = no_idata_set(), carry_out = carry.out, : [steady_bolus] ID 7124 failed
to reach steady state
##   ss_n: 500, ss_rtol: 1e-08, ss_atol: 1e-08
## Warning in (function (x, data, idata = no_idata_set(), carry_out = carry.out, : [steady_bolus] ID 7124 failed
to reach steady state
##   ss_n: 500, ss_rtol: 1e-08, ss_atol: 1e-08
## Warning in (function (x, data, idata = no_idata_set(), carry_out = carry.out, : [steady_bolus] ID 7124 failed
to reach steady state
##   ss_n: 500, ss_rtol: 1e-08, ss_atol: 1e-08
## Warning in (function (x, data, idata = no_idata_set(), carry_out = carry.out, : [steady_bolus] ID 7124 failed
to reach steady state
##   ss_n: 500, ss_rtol: 1e-08, ss_atol: 1e-08
## Warning in (function (x, data, idata = no_idata_set(), carry_out = carry.out, : [steady_bolus] ID 7124 failed
to reach steady state
##   ss_n: 500, ss_rtol: 1e-08, ss_atol: 1e-08
```

```
## Warning in (function (x, data, idata = no_idata_set(), carry_out = carry.out, : [steady_bolus] ID 7153 failed
to reach steady state
##   ss_n: 500, ss_rtol: 1e-08, ss_atol: 1e-08
## Warning in (function (x, data, idata = no_idata_set(), carry_out = carry.out, : [steady_bolus] ID 7153 failed
to reach steady state
##   ss_n: 500, ss_rtol: 1e-08, ss_atol: 1e-08
## Warning in (function (x, data, idata = no_idata_set(), carry_out = carry.out, : [steady_bolus] ID 7153 failed
to reach steady state
##   ss_n: 500, ss_rtol: 1e-08, ss_atol: 1e-08
## Warning in (function (x, data, idata = no_idata_set(), carry_out = carry.out, : [steady_bolus] ID 7153 failed
to reach steady state
##   ss_n: 500, ss_rtol: 1e-08, ss_atol: 1e-08
## Warning in (function (x, data, idata = no_idata_set(), carry_out = carry.out, : [steady_bolus] ID 7153 failed
to reach steady state
##   ss_n: 500, ss_rtol: 1e-08, ss_atol: 1e-08
## Warning in (function (x, data, idata = no_idata_set(), carry_out = carry.out, : [steady_bolus] ID 7153 failed
to reach steady state
##   ss_n: 500, ss_rtol: 1e-08, ss_atol: 1e-08
```

```
## Warning in (function (x, data, idata = no_idata_set(), carry_out = carry.out, : [steady_bolus] ID 7272 failed
to reach steady state
##   ss_n: 500, ss_rtol: 1e-08, ss_atol: 1e-08
## Warning in (function (x, data, idata = no_idata_set(), carry_out = carry.out, : [steady_bolus] ID 7272 failed
to reach steady state
##   ss_n: 500, ss_rtol: 1e-08, ss_atol: 1e-08
## Warning in (function (x, data, idata = no_idata_set(), carry_out = carry.out, : [steady_bolus] ID 7272 failed
to reach steady state
##   ss_n: 500, ss_rtol: 1e-08, ss_atol: 1e-08
## Warning in (function (x, data, idata = no_idata_set(), carry_out = carry.out, : [steady_bolus] ID 7272 failed
to reach steady state
##   ss_n: 500, ss_rtol: 1e-08, ss_atol: 1e-08
## Warning in (function (x, data, idata = no_idata_set(), carry_out = carry.out, : [steady_bolus] ID 7272 failed
to reach steady state
##   ss_n: 500, ss_rtol: 1e-08, ss_atol: 1e-08
## Warning in (function (x, data, idata = no_idata_set(), carry_out = carry.out, : [steady_bolus] ID 7272 failed
to reach steady state
##   ss_n: 500, ss_rtol: 1e-08, ss_atol: 1e-08
```

```
## Warning in (function (x, data, idata = no_idata_set(), carry_out = carry.out, : [steady_bolus] ID 7326 failed
to reach steady state
##   ss_n: 500, ss_rtol: 1e-08, ss_atol: 1e-08
## Warning in (function (x, data, idata = no_idata_set(), carry_out = carry.out, : [steady_bolus] ID 7326 failed
to reach steady state
##   ss_n: 500, ss_rtol: 1e-08, ss_atol: 1e-08
## Warning in (function (x, data, idata = no_idata_set(), carry_out = carry.out, : [steady_bolus] ID 7326 failed
to reach steady state
##   ss_n: 500, ss_rtol: 1e-08, ss_atol: 1e-08
## Warning in (function (x, data, idata = no_idata_set(), carry_out = carry.out, : [steady_bolus] ID 7326 failed
to reach steady state
##   ss_n: 500, ss_rtol: 1e-08, ss_atol: 1e-08
## Warning in (function (x, data, idata = no_idata_set(), carry_out = carry.out, : [steady_bolus] ID 7326 failed
to reach steady state
##   ss_n: 500, ss_rtol: 1e-08, ss_atol: 1e-08
## Warning in (function (x, data, idata = no_idata_set(), carry_out = carry.out, : [steady_bolus] ID 7326 failed
to reach steady state
##   ss_n: 500, ss_rtol: 1e-08, ss_atol: 1e-08
```

```
## Warning in (function (x, data, idata = no_idata_set(), carry_out = carry.out, : [steady_bolus] ID 7328 failed
to reach steady state
##   ss_n: 500, ss_rtol: 1e-08, ss_atol: 1e-08
```

```
## Warning in (function (x, data, idata = no_idata_set(), carry_out = carry.out, : [steady_bolus] ID 7329 failed
to reach steady state
##   ss_n: 500, ss_rtol: 1e-08, ss_atol: 1e-08
## Warning in (function (x, data, idata = no_idata_set(), carry_out = carry.out, : [steady_bolus] ID 7329 failed
to reach steady state
##   ss_n: 500, ss_rtol: 1e-08, ss_atol: 1e-08
## Warning in (function (x, data, idata = no_idata_set(), carry_out = carry.out, : [steady_bolus] ID 7329 failed
to reach steady state
##   ss_n: 500, ss_rtol: 1e-08, ss_atol: 1e-08
## Warning in (function (x, data, idata = no_idata_set(), carry_out = carry.out, : [steady_bolus] ID 7329 failed
to reach steady state
##   ss_n: 500, ss_rtol: 1e-08, ss_atol: 1e-08
## Warning in (function (x, data, idata = no_idata_set(), carry_out = carry.out, : [steady_bolus] ID 7329 failed
to reach steady state
##   ss_n: 500, ss_rtol: 1e-08, ss_atol: 1e-08
## Warning in (function (x, data, idata = no_idata_set(), carry_out = carry.out, : [steady_bolus] ID 7329 failed
to reach steady state
##   ss_n: 500, ss_rtol: 1e-08, ss_atol: 1e-08
## Warning in (function (x, data, idata = no_idata_set(), carry_out = carry.out, : [steady_bolus] ID 7329 failed
to reach steady state
##   ss_n: 500, ss_rtol: 1e-08, ss_atol: 1e-08
```

```
## Warning in (function (x, data, idata = no_idata_set(), carry_out = carry.out, : [steady_bolus] ID 7338 failed
to reach steady state
##   ss_n: 500, ss_rtol: 1e-08, ss_atol: 1e-08
```

```
## Warning in (function (x, data, idata = no_idata_set(), carry_out = carry.out, : [steady_bolus] ID 7374 failed
to reach steady state
##   ss_n: 500, ss_rtol: 1e-08, ss_atol: 1e-08
## Warning in (function (x, data, idata = no_idata_set(), carry_out = carry.out, : [steady_bolus] ID 7374 failed
to reach steady state
##   ss_n: 500, ss_rtol: 1e-08, ss_atol: 1e-08
## Warning in (function (x, data, idata = no_idata_set(), carry_out = carry.out, : [steady_bolus] ID 7374 failed
to reach steady state
##   ss_n: 500, ss_rtol: 1e-08, ss_atol: 1e-08
## Warning in (function (x, data, idata = no_idata_set(), carry_out = carry.out, : [steady_bolus] ID 7374 failed
to reach steady state
##   ss_n: 500, ss_rtol: 1e-08, ss_atol: 1e-08
## Warning in (function (x, data, idata = no_idata_set(), carry_out = carry.out, : [steady_bolus] ID 7374 failed
to reach steady state
##   ss_n: 500, ss_rtol: 1e-08, ss_atol: 1e-08
## Warning in (function (x, data, idata = no_idata_set(), carry_out = carry.out, : [steady_bolus] ID 7374 failed
to reach steady state
##   ss_n: 500, ss_rtol: 1e-08, ss_atol: 1e-08
```

```
## Warning in (function (x, data, idata = no_idata_set(), carry_out = carry.out, : [steady_bolus] ID 7629 failed
to reach steady state
##   ss_n: 500, ss_rtol: 1e-08, ss_atol: 1e-08
## Warning in (function (x, data, idata = no_idata_set(), carry_out = carry.out, : [steady_bolus] ID 7629 failed
to reach steady state
##   ss_n: 500, ss_rtol: 1e-08, ss_atol: 1e-08
## Warning in (function (x, data, idata = no_idata_set(), carry_out = carry.out, : [steady_bolus] ID 7629 failed
to reach steady state
##   ss_n: 500, ss_rtol: 1e-08, ss_atol: 1e-08
## Warning in (function (x, data, idata = no_idata_set(), carry_out = carry.out, : [steady_bolus] ID 7629 failed
to reach steady state
##   ss_n: 500, ss_rtol: 1e-08, ss_atol: 1e-08
## Warning in (function (x, data, idata = no_idata_set(), carry_out = carry.out, : [steady_bolus] ID 7629 failed
to reach steady state
##   ss_n: 500, ss_rtol: 1e-08, ss_atol: 1e-08
## Warning in (function (x, data, idata = no_idata_set(), carry_out = carry.out, : [steady_bolus] ID 7629 failed
to reach steady state
##   ss_n: 500, ss_rtol: 1e-08, ss_atol: 1e-08
## Warning in (function (x, data, idata = no_idata_set(), carry_out = carry.out, : [steady_bolus] ID 7629 failed
to reach steady state
##   ss_n: 500, ss_rtol: 1e-08, ss_atol: 1e-08
```

```
## Warning in (function (x, data, idata = no_idata_set(), carry_out = carry.out, : [steady_bolus] ID 7687 failed
to reach steady state
##   ss_n: 500, ss_rtol: 1e-08, ss_atol: 1e-08
```

```
## Warning in (function (x, data, idata = no_idata_set(), carry_out = carry.out, : [steady_bolus] ID 7805 failed
to reach steady state
##   ss_n: 500, ss_rtol: 1e-08, ss_atol: 1e-08
```

```
## Warning in (function (x, data, idata = no_idata_set(), carry_out = carry.out, : [steady_bolus] ID 7865 failed
to reach steady state
##   ss_n: 500, ss_rtol: 1e-08, ss_atol: 1e-08
## Warning in (function (x, data, idata = no_idata_set(), carry_out = carry.out, : [steady_bolus] ID 7865 failed
to reach steady state
##   ss_n: 500, ss_rtol: 1e-08, ss_atol: 1e-08
## Warning in (function (x, data, idata = no_idata_set(), carry_out = carry.out, : [steady_bolus] ID 7865 failed
to reach steady state
##   ss_n: 500, ss_rtol: 1e-08, ss_atol: 1e-08
## Warning in (function (x, data, idata = no_idata_set(), carry_out = carry.out, : [steady_bolus] ID 7865 failed
to reach steady state
##   ss_n: 500, ss_rtol: 1e-08, ss_atol: 1e-08
## Warning in (function (x, data, idata = no_idata_set(), carry_out = carry.out, : [steady_bolus] ID 7865 failed
to reach steady state
##   ss_n: 500, ss_rtol: 1e-08, ss_atol: 1e-08
## Warning in (function (x, data, idata = no_idata_set(), carry_out = carry.out, : [steady_bolus] ID 7865 failed
to reach steady state
##   ss_n: 500, ss_rtol: 1e-08, ss_atol: 1e-08
## Warning in (function (x, data, idata = no_idata_set(), carry_out = carry.out, : [steady_bolus] ID 7865 failed
to reach steady state
##   ss_n: 500, ss_rtol: 1e-08, ss_atol: 1e-08
## Warning in (function (x, data, idata = no_idata_set(), carry_out = carry.out, : [steady_bolus] ID 7865 failed
to reach steady state
##   ss_n: 500, ss_rtol: 1e-08, ss_atol: 1e-08
```

```
## Warning in (function (x, data, idata = no_idata_set(), carry_out = carry.out, : [steady_bolus] ID 8075 failed
to reach steady state
##   ss_n: 500, ss_rtol: 1e-08, ss_atol: 1e-08
## Warning in (function (x, data, idata = no_idata_set(), carry_out = carry.out, : [steady_bolus] ID 8075 failed
to reach steady state
##   ss_n: 500, ss_rtol: 1e-08, ss_atol: 1e-08
```

```
## Warning in (function (x, data, idata = no_idata_set(), carry_out = carry.out, : [steady_bolus] ID 8129 failed
to reach steady state
##   ss_n: 500, ss_rtol: 1e-08, ss_atol: 1e-08
## Warning in (function (x, data, idata = no_idata_set(), carry_out = carry.out, : [steady_bolus] ID 8129 failed
to reach steady state
##   ss_n: 500, ss_rtol: 1e-08, ss_atol: 1e-08
```

```
## Warning in (function (x, data, idata = no_idata_set(), carry_out = carry.out, : [steady_bolus] ID 8154 failed
to reach steady state
##   ss_n: 500, ss_rtol: 1e-08, ss_atol: 1e-08
## Warning in (function (x, data, idata = no_idata_set(), carry_out = carry.out, : [steady_bolus] ID 8154 failed
to reach steady state
##   ss_n: 500, ss_rtol: 1e-08, ss_atol: 1e-08
## Warning in (function (x, data, idata = no_idata_set(), carry_out = carry.out, : [steady_bolus] ID 8154 failed
to reach steady state
##   ss_n: 500, ss_rtol: 1e-08, ss_atol: 1e-08
## Warning in (function (x, data, idata = no_idata_set(), carry_out = carry.out, : [steady_bolus] ID 8154 failed
to reach steady state
##   ss_n: 500, ss_rtol: 1e-08, ss_atol: 1e-08
## Warning in (function (x, data, idata = no_idata_set(), carry_out = carry.out, : [steady_bolus] ID 8154 failed
to reach steady state
##   ss_n: 500, ss_rtol: 1e-08, ss_atol: 1e-08
## Warning in (function (x, data, idata = no_idata_set(), carry_out = carry.out, : [steady_bolus] ID 8154 failed
to reach steady state
##   ss_n: 500, ss_rtol: 1e-08, ss_atol: 1e-08
## Warning in (function (x, data, idata = no_idata_set(), carry_out = carry.out, : [steady_bolus] ID 8154 failed
to reach steady state
##   ss_n: 500, ss_rtol: 1e-08, ss_atol: 1e-08
```

```
## Warning in (function (x, data, idata = no_idata_set(), carry_out = carry.out, : [steady_bolus] ID 8154 failed
to reach steady state
##   ss_n: 500, ss_rtol: 1e-08, ss_atol: 1e-08
## Warning in (function (x, data, idata = no_idata_set(), carry_out = carry.out, : [steady_bolus] ID 8154 failed
to reach steady state
##   ss_n: 500, ss_rtol: 1e-08, ss_atol: 1e-08
## Warning in (function (x, data, idata = no_idata_set(), carry_out = carry.out, : [steady_bolus] ID 8154 failed
to reach steady state
##   ss_n: 500, ss_rtol: 1e-08, ss_atol: 1e-08
## Warning in (function (x, data, idata = no_idata_set(), carry_out = carry.out, : [steady_bolus] ID 8154 failed
to reach steady state
##   ss_n: 500, ss_rtol: 1e-08, ss_atol: 1e-08
## Warning in (function (x, data, idata = no_idata_set(), carry_out = carry.out, : [steady_bolus] ID 8154 failed
to reach steady state
##   ss_n: 500, ss_rtol: 1e-08, ss_atol: 1e-08
```

```
## Warning in (function (x, data, idata = no_idata_set(), carry_out = carry.out, : [steady_bolus] ID 8187 failed
to reach steady state
##   ss_n: 500, ss_rtol: 1e-08, ss_atol: 1e-08
```

```
## Warning in (function (x, data, idata = no_idata_set(), carry_out = carry.out, : [steady_bolus] ID 8217 failed
to reach steady state
##   ss_n: 500, ss_rtol: 1e-08, ss_atol: 1e-08
```

```
## Warning in (function (x, data, idata = no_idata_set(), carry_out = carry.out, : [steady_bolus] ID 8292 failed
to reach steady state
##   ss_n: 500, ss_rtol: 1e-08, ss_atol: 1e-08
```

```
## Warning in (function (x, data, idata = no_idata_set(), carry_out = carry.out, : [steady_bolus] ID 8422 failed
to reach steady state
##   ss_n: 500, ss_rtol: 1e-08, ss_atol: 1e-08
```

```
## Warning in (function (x, data, idata = no_idata_set(), carry_out = carry.out, : [steady_bolus] ID 8498 failed
to reach steady state
##   ss_n: 500, ss_rtol: 1e-08, ss_atol: 1e-08
```

```
## Warning in (function (x, data, idata = no_idata_set(), carry_out = carry.out, : [steady_bolus] ID 8517 failed
to reach steady state
##   ss_n: 500, ss_rtol: 1e-08, ss_atol: 1e-08
```

```
## Warning in (function (x, data, idata = no_idata_set(), carry_out = carry.out, : [steady_bolus] ID 8559 failed
to reach steady state
##   ss_n: 500, ss_rtol: 1e-08, ss_atol: 1e-08
## Warning in (function (x, data, idata = no_idata_set(), carry_out = carry.out, : [steady_bolus] ID 8559 failed
to reach steady state
##   ss_n: 500, ss_rtol: 1e-08, ss_atol: 1e-08
## Warning in (function (x, data, idata = no_idata_set(), carry_out = carry.out, : [steady_bolus] ID 8559 failed
to reach steady state
##   ss_n: 500, ss_rtol: 1e-08, ss_atol: 1e-08
## Warning in (function (x, data, idata = no_idata_set(), carry_out = carry.out, : [steady_bolus] ID 8559 failed
to reach steady state
##   ss_n: 500, ss_rtol: 1e-08, ss_atol: 1e-08
## Warning in (function (x, data, idata = no_idata_set(), carry_out = carry.out, : [steady_bolus] ID 8559 failed
to reach steady state
##   ss_n: 500, ss_rtol: 1e-08, ss_atol: 1e-08
## Warning in (function (x, data, idata = no_idata_set(), carry_out = carry.out, : [steady_bolus] ID 8559 failed
to reach steady state
##   ss_n: 500, ss_rtol: 1e-08, ss_atol: 1e-08
## Warning in (function (x, data, idata = no_idata_set(), carry_out = carry.out, : [steady_bolus] ID 8559 failed
to reach steady state
##   ss_n: 500, ss_rtol: 1e-08, ss_atol: 1e-08
```

```
## Warning in (function (x, data, idata = no_idata_set(), carry_out = carry.out, : [steady_bolus] ID 8565 failed
to reach steady state
##   ss_n: 500, ss_rtol: 1e-08, ss_atol: 1e-08
```

```
## Warning in (function (x, data, idata = no_idata_set(), carry_out = carry.out, : [steady_bolus] ID 8695 failed
to reach steady state
##   ss_n: 500, ss_rtol: 1e-08, ss_atol: 1e-08
## Warning in (function (x, data, idata = no_idata_set(), carry_out = carry.out, : [steady_bolus] ID 8695 failed
to reach steady state
##   ss_n: 500, ss_rtol: 1e-08, ss_atol: 1e-08
## Warning in (function (x, data, idata = no_idata_set(), carry_out = carry.out, : [steady_bolus] ID 8695 failed
to reach steady state
##   ss_n: 500, ss_rtol: 1e-08, ss_atol: 1e-08
## Warning in (function (x, data, idata = no_idata_set(), carry_out = carry.out, : [steady_bolus] ID 8695 failed
to reach steady state
##   ss_n: 500, ss_rtol: 1e-08, ss_atol: 1e-08
## Warning in (function (x, data, idata = no_idata_set(), carry_out = carry.out, : [steady_bolus] ID 8695 failed
to reach steady state
##   ss_n: 500, ss_rtol: 1e-08, ss_atol: 1e-08
## Warning in (function (x, data, idata = no_idata_set(), carry_out = carry.out, : [steady_bolus] ID 8695 failed
to reach steady state
##   ss_n: 500, ss_rtol: 1e-08, ss_atol: 1e-08
```

```
## Warning in (function (x, data, idata = no_idata_set(), carry_out = carry.out, : [steady_bolus] ID 8709 failed
to reach steady state
##   ss_n: 500, ss_rtol: 1e-08, ss_atol: 1e-08
## Warning in (function (x, data, idata = no_idata_set(), carry_out = carry.out, : [steady_bolus] ID 8709 failed
to reach steady state
##   ss_n: 500, ss_rtol: 1e-08, ss_atol: 1e-08
## Warning in (function (x, data, idata = no_idata_set(), carry_out = carry.out, : [steady_bolus] ID 8709 failed
to reach steady state
##   ss_n: 500, ss_rtol: 1e-08, ss_atol: 1e-08
## Warning in (function (x, data, idata = no_idata_set(), carry_out = carry.out, : [steady_bolus] ID 8709 failed
to reach steady state
##   ss_n: 500, ss_rtol: 1e-08, ss_atol: 1e-08
## Warning in (function (x, data, idata = no_idata_set(), carry_out = carry.out, : [steady_bolus] ID 8709 failed
to reach steady state
##   ss_n: 500, ss_rtol: 1e-08, ss_atol: 1e-08
## Warning in (function (x, data, idata = no_idata_set(), carry_out = carry.out, : [steady_bolus] ID 8709 failed
to reach steady state
##   ss_n: 500, ss_rtol: 1e-08, ss_atol: 1e-08
## Warning in (function (x, data, idata = no_idata_set(), carry_out = carry.out, : [steady_bolus] ID 8709 failed
to reach steady state
##   ss_n: 500, ss_rtol: 1e-08, ss_atol: 1e-08
## Warning in (function (x, data, idata = no_idata_set(), carry_out = carry.out, : [steady_bolus] ID 8709 failed
to reach steady state
##   ss_n: 500, ss_rtol: 1e-08, ss_atol: 1e-08
```

```
## Warning in (function (x, data, idata = no_idata_set(), carry_out = carry.out, : [steady_bolus] ID 8780 failed
to reach steady state
##   ss_n: 500, ss_rtol: 1e-08, ss_atol: 1e-08
```

```
## Warning in (function (x, data, idata = no_idata_set(), carry_out = carry.out, : [steady_bolus] ID 8815 failed
to reach steady state
##   ss_n: 500, ss_rtol: 1e-08, ss_atol: 1e-08
```

```
## Warning in (function (x, data, idata = no_idata_set(), carry_out = carry.out, : [steady_bolus] ID 8831 failed
to reach steady state
##   ss_n: 500, ss_rtol: 1e-08, ss_atol: 1e-08
## Warning in (function (x, data, idata = no_idata_set(), carry_out = carry.out, : [steady_bolus] ID 8831 failed
to reach steady state
##   ss_n: 500, ss_rtol: 1e-08, ss_atol: 1e-08
```

```
## Warning in (function (x, data, idata = no_idata_set(), carry_out = carry.out, : [steady_bolus] ID 8870 failed
to reach steady state
##   ss_n: 500, ss_rtol: 1e-08, ss_atol: 1e-08
```

```
## Warning in (function (x, data, idata = no_idata_set(), carry_out = carry.out, : [steady_bolus] ID 8906 failed
to reach steady state
##   ss_n: 500, ss_rtol: 1e-08, ss_atol: 1e-08
## Warning in (function (x, data, idata = no_idata_set(), carry_out = carry.out, : [steady_bolus] ID 8906 failed
to reach steady state
##   ss_n: 500, ss_rtol: 1e-08, ss_atol: 1e-08
## Warning in (function (x, data, idata = no_idata_set(), carry_out = carry.out, : [steady_bolus] ID 8906 failed
to reach steady state
##   ss_n: 500, ss_rtol: 1e-08, ss_atol: 1e-08
## Warning in (function (x, data, idata = no_idata_set(), carry_out = carry.out, : [steady_bolus] ID 8906 failed
to reach steady state
##   ss_n: 500, ss_rtol: 1e-08, ss_atol: 1e-08
## Warning in (function (x, data, idata = no_idata_set(), carry_out = carry.out, : [steady_bolus] ID 8906 failed
to reach steady state
##   ss_n: 500, ss_rtol: 1e-08, ss_atol: 1e-08
## Warning in (function (x, data, idata = no_idata_set(), carry_out = carry.out, : [steady_bolus] ID 8906 failed
to reach steady state
##   ss_n: 500, ss_rtol: 1e-08, ss_atol: 1e-08
## Warning in (function (x, data, idata = no_idata_set(), carry_out = carry.out, : [steady_bolus] ID 8906 failed
to reach steady state
##   ss_n: 500, ss_rtol: 1e-08, ss_atol: 1e-08
## Warning in (function (x, data, idata = no_idata_set(), carry_out = carry.out, : [steady_bolus] ID 8906 failed
to reach steady state
##   ss_n: 500, ss_rtol: 1e-08, ss_atol: 1e-08
## Warning in (function (x, data, idata = no_idata_set(), carry_out = carry.out, : [steady_bolus] ID 8906 failed
to reach steady state
##   ss_n: 500, ss_rtol: 1e-08, ss_atol: 1e-08
```

```
## Warning in (function (x, data, idata = no_idata_set(), carry_out = carry.out, : [steady_bolus] ID 8989 failed
to reach steady state
##   ss_n: 500, ss_rtol: 1e-08, ss_atol: 1e-08
## Warning in (function (x, data, idata = no_idata_set(), carry_out = carry.out, : [steady_bolus] ID 8989 failed
to reach steady state
##   ss_n: 500, ss_rtol: 1e-08, ss_atol: 1e-08
## Warning in (function (x, data, idata = no_idata_set(), carry_out = carry.out, : [steady_bolus] ID 8989 failed
to reach steady state
##   ss_n: 500, ss_rtol: 1e-08, ss_atol: 1e-08
## Warning in (function (x, data, idata = no_idata_set(), carry_out = carry.out, : [steady_bolus] ID 8989 failed
to reach steady state
##   ss_n: 500, ss_rtol: 1e-08, ss_atol: 1e-08
## Warning in (function (x, data, idata = no_idata_set(), carry_out = carry.out, : [steady_bolus] ID 8989 failed
to reach steady state
##   ss_n: 500, ss_rtol: 1e-08, ss_atol: 1e-08
## Warning in (function (x, data, idata = no_idata_set(), carry_out = carry.out, : [steady_bolus] ID 8989 failed
to reach steady state
##   ss_n: 500, ss_rtol: 1e-08, ss_atol: 1e-08
```

```
## Warning in (function (x, data, idata = no_idata_set(), carry_out = carry.out, : [steady_bolus] ID 9018 failed
to reach steady state
##   ss_n: 500, ss_rtol: 1e-08, ss_atol: 1e-08
```

```
## Warning in (function (x, data, idata = no_idata_set(), carry_out = carry.out, : [steady_bolus] ID 9027 failed
to reach steady state
##   ss_n: 500, ss_rtol: 1e-08, ss_atol: 1e-08
## Warning in (function (x, data, idata = no_idata_set(), carry_out = carry.out, : [steady_bolus] ID 9027 failed
to reach steady state
##   ss_n: 500, ss_rtol: 1e-08, ss_atol: 1e-08
## Warning in (function (x, data, idata = no_idata_set(), carry_out = carry.out, : [steady_bolus] ID 9027 failed
to reach steady state
##   ss_n: 500, ss_rtol: 1e-08, ss_atol: 1e-08
## Warning in (function (x, data, idata = no_idata_set(), carry_out = carry.out, : [steady_bolus] ID 9027 failed
to reach steady state
##   ss_n: 500, ss_rtol: 1e-08, ss_atol: 1e-08
## Warning in (function (x, data, idata = no_idata_set(), carry_out = carry.out, : [steady_bolus] ID 9027 failed
to reach steady state
##   ss_n: 500, ss_rtol: 1e-08, ss_atol: 1e-08
## Warning in (function (x, data, idata = no_idata_set(), carry_out = carry.out, : [steady_bolus] ID 9027 failed
to reach steady state
##   ss_n: 500, ss_rtol: 1e-08, ss_atol: 1e-08
## Warning in (function (x, data, idata = no_idata_set(), carry_out = carry.out, : [steady_bolus] ID 9027 failed
to reach steady state
##   ss_n: 500, ss_rtol: 1e-08, ss_atol: 1e-08
```

```
## Warning in (function (x, data, idata = no_idata_set(), carry_out = carry.out, : [steady_bolus] ID 9031 failed
to reach steady state
##   ss_n: 500, ss_rtol: 1e-08, ss_atol: 1e-08
## Warning in (function (x, data, idata = no_idata_set(), carry_out = carry.out, : [steady_bolus] ID 9031 failed
to reach steady state
##   ss_n: 500, ss_rtol: 1e-08, ss_atol: 1e-08
```

```
## Warning in (function (x, data, idata = no_idata_set(), carry_out = carry.out, : [steady_bolus] ID 9117 failed
to reach steady state
##   ss_n: 500, ss_rtol: 1e-08, ss_atol: 1e-08
## Warning in (function (x, data, idata = no_idata_set(), carry_out = carry.out, : [steady_bolus] ID 9117 failed
to reach steady state
##   ss_n: 500, ss_rtol: 1e-08, ss_atol: 1e-08
## Warning in (function (x, data, idata = no_idata_set(), carry_out = carry.out, : [steady_bolus] ID 9117 failed
to reach steady state
##   ss_n: 500, ss_rtol: 1e-08, ss_atol: 1e-08
## Warning in (function (x, data, idata = no_idata_set(), carry_out = carry.out, : [steady_bolus] ID 9117 failed
to reach steady state
##   ss_n: 500, ss_rtol: 1e-08, ss_atol: 1e-08
## Warning in (function (x, data, idata = no_idata_set(), carry_out = carry.out, : [steady_bolus] ID 9117 failed
to reach steady state
##   ss_n: 500, ss_rtol: 1e-08, ss_atol: 1e-08
## Warning in (function (x, data, idata = no_idata_set(), carry_out = carry.out, : [steady_bolus] ID 9117 failed
to reach steady state
##   ss_n: 500, ss_rtol: 1e-08, ss_atol: 1e-08
## Warning in (function (x, data, idata = no_idata_set(), carry_out = carry.out, : [steady_bolus] ID 9117 failed
to reach steady state
##   ss_n: 500, ss_rtol: 1e-08, ss_atol: 1e-08
```

```
## Warning in (function (x, data, idata = no_idata_set(), carry_out = carry.out, : [steady_bolus] ID 9127 failed
to reach steady state
##   ss_n: 500, ss_rtol: 1e-08, ss_atol: 1e-08
## Warning in (function (x, data, idata = no_idata_set(), carry_out = carry.out, : [steady_bolus] ID 9127 failed
to reach steady state
##   ss_n: 500, ss_rtol: 1e-08, ss_atol: 1e-08
## Warning in (function (x, data, idata = no_idata_set(), carry_out = carry.out, : [steady_bolus] ID 9127 failed
to reach steady state
##   ss_n: 500, ss_rtol: 1e-08, ss_atol: 1e-08
## Warning in (function (x, data, idata = no_idata_set(), carry_out = carry.out, : [steady_bolus] ID 9127 failed
to reach steady state
##   ss_n: 500, ss_rtol: 1e-08, ss_atol: 1e-08
## Warning in (function (x, data, idata = no_idata_set(), carry_out = carry.out, : [steady_bolus] ID 9127 failed
to reach steady state
##   ss_n: 500, ss_rtol: 1e-08, ss_atol: 1e-08
## Warning in (function (x, data, idata = no_idata_set(), carry_out = carry.out, : [steady_bolus] ID 9127 failed
to reach steady state
##   ss_n: 500, ss_rtol: 1e-08, ss_atol: 1e-08
## Warning in (function (x, data, idata = no_idata_set(), carry_out = carry.out, : [steady_bolus] ID 9127 failed
to reach steady state
##   ss_n: 500, ss_rtol: 1e-08, ss_atol: 1e-08
## Warning in (function (x, data, idata = no_idata_set(), carry_out = carry.out, : [steady_bolus] ID 9127 failed
to reach steady state
##   ss_n: 500, ss_rtol: 1e-08, ss_atol: 1e-08
## Warning in (function (x, data, idata = no_idata_set(), carry_out = carry.out, : [steady_bolus] ID 9127 failed
to reach steady state
##   ss_n: 500, ss_rtol: 1e-08, ss_atol: 1e-08
## Warning in (function (x, data, idata = no_idata_set(), carry_out = carry.out, : [steady_bolus] ID 9127 failed
to reach steady state
##   ss_n: 500, ss_rtol: 1e-08, ss_atol: 1e-08
## Warning in (function (x, data, idata = no_idata_set(), carry_out = carry.out, : [steady_bolus] ID 9127 failed
to reach steady state
##   ss_n: 500, ss_rtol: 1e-08, ss_atol: 1e-08
```

```
## Warning in (function (x, data, idata = no_idata_set(), carry_out = carry.out, : [steady_bolus] ID 9152 failed
to reach steady state
##   ss_n: 500, ss_rtol: 1e-08, ss_atol: 1e-08
```

```
## Warning in (function (x, data, idata = no_idata_set(), carry_out = carry.out, : [steady_bolus] ID 9264 failed
to reach steady state
##   ss_n: 500, ss_rtol: 1e-08, ss_atol: 1e-08
## Warning in (function (x, data, idata = no_idata_set(), carry_out = carry.out, : [steady_bolus] ID 9264 failed
to reach steady state
##   ss_n: 500, ss_rtol: 1e-08, ss_atol: 1e-08
## Warning in (function (x, data, idata = no_idata_set(), carry_out = carry.out, : [steady_bolus] ID 9264 failed
to reach steady state
##   ss_n: 500, ss_rtol: 1e-08, ss_atol: 1e-08
## Warning in (function (x, data, idata = no_idata_set(), carry_out = carry.out, : [steady_bolus] ID 9264 failed
to reach steady state
##   ss_n: 500, ss_rtol: 1e-08, ss_atol: 1e-08
## Warning in (function (x, data, idata = no_idata_set(), carry_out = carry.out, : [steady_bolus] ID 9264 failed
to reach steady state
##   ss_n: 500, ss_rtol: 1e-08, ss_atol: 1e-08
## Warning in (function (x, data, idata = no_idata_set(), carry_out = carry.out, : [steady_bolus] ID 9264 failed
to reach steady state
##   ss_n: 500, ss_rtol: 1e-08, ss_atol: 1e-08
```

```
## Warning in (function (x, data, idata = no_idata_set(), carry_out = carry.out, : [steady_bolus] ID 9421 failed
to reach steady state
##   ss_n: 500, ss_rtol: 1e-08, ss_atol: 1e-08
```

```
## Warning in (function (x, data, idata = no_idata_set(), carry_out = carry.out, : [steady_bolus] ID 9441 failed
to reach steady state
##   ss_n: 500, ss_rtol: 1e-08, ss_atol: 1e-08
## Warning in (function (x, data, idata = no_idata_set(), carry_out = carry.out, : [steady_bolus] ID 9441 failed
to reach steady state
##   ss_n: 500, ss_rtol: 1e-08, ss_atol: 1e-08
## Warning in (function (x, data, idata = no_idata_set(), carry_out = carry.out, : [steady_bolus] ID 9441 failed
to reach steady state
##   ss_n: 500, ss_rtol: 1e-08, ss_atol: 1e-08
## Warning in (function (x, data, idata = no_idata_set(), carry_out = carry.out, : [steady_bolus] ID 9441 failed
to reach steady state
##   ss_n: 500, ss_rtol: 1e-08, ss_atol: 1e-08
## Warning in (function (x, data, idata = no_idata_set(), carry_out = carry.out, : [steady_bolus] ID 9441 failed
to reach steady state
##   ss_n: 500, ss_rtol: 1e-08, ss_atol: 1e-08
## Warning in (function (x, data, idata = no_idata_set(), carry_out = carry.out, : [steady_bolus] ID 9441 failed
to reach steady state
##   ss_n: 500, ss_rtol: 1e-08, ss_atol: 1e-08
```

```
## Warning in (function (x, data, idata = no_idata_set(), carry_out = carry.out, : [steady_bolus] ID 9448 failed
to reach steady state
##   ss_n: 500, ss_rtol: 1e-08, ss_atol: 1e-08
```

```
## Warning in (function (x, data, idata = no_idata_set(), carry_out = carry.out, : [steady_bolus] ID 9514 failed
to reach steady state
##   ss_n: 500, ss_rtol: 1e-08, ss_atol: 1e-08
## Warning in (function (x, data, idata = no_idata_set(), carry_out = carry.out, : [steady_bolus] ID 9514 failed
to reach steady state
##   ss_n: 500, ss_rtol: 1e-08, ss_atol: 1e-08
## Warning in (function (x, data, idata = no_idata_set(), carry_out = carry.out, : [steady_bolus] ID 9514 failed
to reach steady state
##   ss_n: 500, ss_rtol: 1e-08, ss_atol: 1e-08
## Warning in (function (x, data, idata = no_idata_set(), carry_out = carry.out, : [steady_bolus] ID 9514 failed
to reach steady state
##   ss_n: 500, ss_rtol: 1e-08, ss_atol: 1e-08
## Warning in (function (x, data, idata = no_idata_set(), carry_out = carry.out, : [steady_bolus] ID 9514 failed
to reach steady state
##   ss_n: 500, ss_rtol: 1e-08, ss_atol: 1e-08
## Warning in (function (x, data, idata = no_idata_set(), carry_out = carry.out, : [steady_bolus] ID 9514 failed
to reach steady state
##   ss_n: 500, ss_rtol: 1e-08, ss_atol: 1e-08
```

```
## Warning in (function (x, data, idata = no_idata_set(), carry_out = carry.out, : [steady_bolus] ID 9579 failed
to reach steady state
##   ss_n: 500, ss_rtol: 1e-08, ss_atol: 1e-08
```

```
## Warning in (function (x, data, idata = no_idata_set(), carry_out = carry.out, : [steady_bolus] ID 9614 failed
to reach steady state
##   ss_n: 500, ss_rtol: 1e-08, ss_atol: 1e-08
```

```
## Warning in (function (x, data, idata = no_idata_set(), carry_out = carry.out, : [steady_bolus] ID 9746 failed
to reach steady state
##   ss_n: 500, ss_rtol: 1e-08, ss_atol: 1e-08
```

```
## Warning in (function (x, data, idata = no_idata_set(), carry_out = carry.out, : [steady_bolus] ID 9873 failed
to reach steady state
##   ss_n: 500, ss_rtol: 1e-08, ss_atol: 1e-08
```

```
## Warning in (function (x, data, idata = no_idata_set(), carry_out = carry.out, : [steady_bolus] ID 9875 failed
to reach steady state
##   ss_n: 500, ss_rtol: 1e-08, ss_atol: 1e-08
## Warning in (function (x, data, idata = no_idata_set(), carry_out = carry.out, : [steady_bolus] ID 9875 failed
to reach steady state
##   ss_n: 500, ss_rtol: 1e-08, ss_atol: 1e-08
## Warning in (function (x, data, idata = no_idata_set(), carry_out = carry.out, : [steady_bolus] ID 9875 failed
to reach steady state
##   ss_n: 500, ss_rtol: 1e-08, ss_atol: 1e-08
## Warning in (function (x, data, idata = no_idata_set(), carry_out = carry.out, : [steady_bolus] ID 9875 failed
to reach steady state
##   ss_n: 500, ss_rtol: 1e-08, ss_atol: 1e-08
## Warning in (function (x, data, idata = no_idata_set(), carry_out = carry.out, : [steady_bolus] ID 9875 failed
to reach steady state
##   ss_n: 500, ss_rtol: 1e-08, ss_atol: 1e-08
## Warning in (function (x, data, idata = no_idata_set(), carry_out = carry.out, : [steady_bolus] ID 9875 failed
to reach steady state
##   ss_n: 500, ss_rtol: 1e-08, ss_atol: 1e-08
```

```
## Warning in (function (x, data, idata = no_idata_set(), carry_out = carry.out, : [steady_bolus] ID 9948 failed
to reach steady state
##   ss_n: 500, ss_rtol: 1e-08, ss_atol: 1e-08
```

```
## Warning in (function (x, data, idata = no_idata_set(), carry_out = carry.out, : [steady_bolus] ID 9950 failed
to reach steady state
##   ss_n: 500, ss_rtol: 1e-08, ss_atol: 1e-08
## Warning in (function (x, data, idata = no_idata_set(), carry_out = carry.out, : [steady_bolus] ID 9950 failed
to reach steady state
##   ss_n: 500, ss_rtol: 1e-08, ss_atol: 1e-08
```

```
## Warning in (function (x, data, idata = no_idata_set(), carry_out = carry.out, : [steady_bolus] ID 10007 failed
to reach steady state
##   ss_n: 500, ss_rtol: 1e-08, ss_atol: 1e-08
```

```
## Warning in (function (x, data, idata = no_idata_set(), carry_out = carry.out, : [steady_bolus] ID 10023 failed
to reach steady state
##   ss_n: 500, ss_rtol: 1e-08, ss_atol: 1e-08
## Warning in (function (x, data, idata = no_idata_set(), carry_out = carry.out, : [steady_bolus] ID 10023 failed
to reach steady state
##   ss_n: 500, ss_rtol: 1e-08, ss_atol: 1e-08
## Warning in (function (x, data, idata = no_idata_set(), carry_out = carry.out, : [steady_bolus] ID 10023 failed
to reach steady state
##   ss_n: 500, ss_rtol: 1e-08, ss_atol: 1e-08
## Warning in (function (x, data, idata = no_idata_set(), carry_out = carry.out, : [steady_bolus] ID 10023 failed
to reach steady state
##   ss_n: 500, ss_rtol: 1e-08, ss_atol: 1e-08
## Warning in (function (x, data, idata = no_idata_set(), carry_out = carry.out, : [steady_bolus] ID 10023 failed
to reach steady state
##   ss_n: 500, ss_rtol: 1e-08, ss_atol: 1e-08
## Warning in (function (x, data, idata = no_idata_set(), carry_out = carry.out, : [steady_bolus] ID 10023 failed
to reach steady state
##   ss_n: 500, ss_rtol: 1e-08, ss_atol: 1e-08
```

```
## Warning in (function (x, data, idata = no_idata_set(), carry_out = carry.out, : [steady_bolus] ID 10053 failed
to reach steady state
##   ss_n: 500, ss_rtol: 1e-08, ss_atol: 1e-08
## Warning in (function (x, data, idata = no_idata_set(), carry_out = carry.out, : [steady_bolus] ID 10053 failed
to reach steady state
##   ss_n: 500, ss_rtol: 1e-08, ss_atol: 1e-08
## Warning in (function (x, data, idata = no_idata_set(), carry_out = carry.out, : [steady_bolus] ID 10053 failed
to reach steady state
##   ss_n: 500, ss_rtol: 1e-08, ss_atol: 1e-08
## Warning in (function (x, data, idata = no_idata_set(), carry_out = carry.out, : [steady_bolus] ID 10053 failed
to reach steady state
##   ss_n: 500, ss_rtol: 1e-08, ss_atol: 1e-08
## Warning in (function (x, data, idata = no_idata_set(), carry_out = carry.out, : [steady_bolus] ID 10053 failed
to reach steady state
##   ss_n: 500, ss_rtol: 1e-08, ss_atol: 1e-08
## Warning in (function (x, data, idata = no_idata_set(), carry_out = carry.out, : [steady_bolus] ID 10053 failed
to reach steady state
##   ss_n: 500, ss_rtol: 1e-08, ss_atol: 1e-08
```

```
## Warning in (function (x, data, idata = no_idata_set(), carry_out = carry.out, : [steady_bolus] ID 10061 failed
to reach steady state
##   ss_n: 500, ss_rtol: 1e-08, ss_atol: 1e-08
## Warning in (function (x, data, idata = no_idata_set(), carry_out = carry.out, : [steady_bolus] ID 10061 failed
to reach steady state
##   ss_n: 500, ss_rtol: 1e-08, ss_atol: 1e-08
```

```
## Warning in (function (x, data, idata = no_idata_set(), carry_out = carry.out, : [steady_bolus] ID 10078 failed
to reach steady state
##   ss_n: 500, ss_rtol: 1e-08, ss_atol: 1e-08
```

```
## Warning in (function (x, data, idata = no_idata_set(), carry_out = carry.out, : [steady_bolus] ID 10214 failed
to reach steady state
##   ss_n: 500, ss_rtol: 1e-08, ss_atol: 1e-08
## Warning in (function (x, data, idata = no_idata_set(), carry_out = carry.out, : [steady_bolus] ID 10214 failed
to reach steady state
##   ss_n: 500, ss_rtol: 1e-08, ss_atol: 1e-08
## Warning in (function (x, data, idata = no_idata_set(), carry_out = carry.out, : [steady_bolus] ID 10214 failed
to reach steady state
##   ss_n: 500, ss_rtol: 1e-08, ss_atol: 1e-08
## Warning in (function (x, data, idata = no_idata_set(), carry_out = carry.out, : [steady_bolus] ID 10214 failed
to reach steady state
##   ss_n: 500, ss_rtol: 1e-08, ss_atol: 1e-08
## Warning in (function (x, data, idata = no_idata_set(), carry_out = carry.out, : [steady_bolus] ID 10214 failed
to reach steady state
##   ss_n: 500, ss_rtol: 1e-08, ss_atol: 1e-08
## Warning in (function (x, data, idata = no_idata_set(), carry_out = carry.out, : [steady_bolus] ID 10214 failed
to reach steady state
##   ss_n: 500, ss_rtol: 1e-08, ss_atol: 1e-08
## Warning in (function (x, data, idata = no_idata_set(), carry_out = carry.out, : [steady_bolus] ID 10214 failed
to reach steady state
##   ss_n: 500, ss_rtol: 1e-08, ss_atol: 1e-08
```

```
## Warning in (function (x, data, idata = no_idata_set(), carry_out = carry.out, : [steady_bolus] ID 10217 failed
to reach steady state
##   ss_n: 500, ss_rtol: 1e-08, ss_atol: 1e-08
```

```
## Warning in (function (x, data, idata = no_idata_set(), carry_out = carry.out, : [steady_bolus] ID 10263 failed
to reach steady state
##   ss_n: 500, ss_rtol: 1e-08, ss_atol: 1e-08
## Warning in (function (x, data, idata = no_idata_set(), carry_out = carry.out, : [steady_bolus] ID 10263 failed
to reach steady state
##   ss_n: 500, ss_rtol: 1e-08, ss_atol: 1e-08
## Warning in (function (x, data, idata = no_idata_set(), carry_out = carry.out, : [steady_bolus] ID 10263 failed
to reach steady state
##   ss_n: 500, ss_rtol: 1e-08, ss_atol: 1e-08
## Warning in (function (x, data, idata = no_idata_set(), carry_out = carry.out, : [steady_bolus] ID 10263 failed
to reach steady state
##   ss_n: 500, ss_rtol: 1e-08, ss_atol: 1e-08
## Warning in (function (x, data, idata = no_idata_set(), carry_out = carry.out, : [steady_bolus] ID 10263 failed
to reach steady state
##   ss_n: 500, ss_rtol: 1e-08, ss_atol: 1e-08
## Warning in (function (x, data, idata = no_idata_set(), carry_out = carry.out, : [steady_bolus] ID 10263 failed
to reach steady state
##   ss_n: 500, ss_rtol: 1e-08, ss_atol: 1e-08
```

```
## Warning in (function (x, data, idata = no_idata_set(), carry_out = carry.out, : [steady_bolus] ID 10402 failed
to reach steady state
##   ss_n: 500, ss_rtol: 1e-08, ss_atol: 1e-08
```

```
## Warning in (function (x, data, idata = no_idata_set(), carry_out = carry.out, : [steady_bolus] ID 10412 failed
to reach steady state
##   ss_n: 500, ss_rtol: 1e-08, ss_atol: 1e-08
```

```
## Warning in (function (x, data, idata = no_idata_set(), carry_out = carry.out, : [steady_bolus] ID 10481 failed
to reach steady state
##   ss_n: 500, ss_rtol: 1e-08, ss_atol: 1e-08
```

```
## Warning in (function (x, data, idata = no_idata_set(), carry_out = carry.out, : [steady_bolus] ID 10511 failed
to reach steady state
##   ss_n: 500, ss_rtol: 1e-08, ss_atol: 1e-08
```

```
## Warning in (function (x, data, idata = no_idata_set(), carry_out = carry.out, : [steady_bolus] ID 10559 failed
to reach steady state
##   ss_n: 500, ss_rtol: 1e-08, ss_atol: 1e-08
## Warning in (function (x, data, idata = no_idata_set(), carry_out = carry.out, : [steady_bolus] ID 10559 failed
to reach steady state
##   ss_n: 500, ss_rtol: 1e-08, ss_atol: 1e-08
## Warning in (function (x, data, idata = no_idata_set(), carry_out = carry.out, : [steady_bolus] ID 10559 failed
to reach steady state
##   ss_n: 500, ss_rtol: 1e-08, ss_atol: 1e-08
## Warning in (function (x, data, idata = no_idata_set(), carry_out = carry.out, : [steady_bolus] ID 10559 failed
to reach steady state
##   ss_n: 500, ss_rtol: 1e-08, ss_atol: 1e-08
## Warning in (function (x, data, idata = no_idata_set(), carry_out = carry.out, : [steady_bolus] ID 10559 failed
to reach steady state
##   ss_n: 500, ss_rtol: 1e-08, ss_atol: 1e-08
## Warning in (function (x, data, idata = no_idata_set(), carry_out = carry.out, : [steady_bolus] ID 10559 failed
to reach steady state
##   ss_n: 500, ss_rtol: 1e-08, ss_atol: 1e-08
## Warning in (function (x, data, idata = no_idata_set(), carry_out = carry.out, : [steady_bolus] ID 10559 failed
to reach steady state
##   ss_n: 500, ss_rtol: 1e-08, ss_atol: 1e-08
```

```
## Warning in (function (x, data, idata = no_idata_set(), carry_out = carry.out, : [steady_bolus] ID 10582 failed
to reach steady state
##   ss_n: 500, ss_rtol: 1e-08, ss_atol: 1e-08
```

```
## Warning in (function (x, data, idata = no_idata_set(), carry_out = carry.out, : [steady_bolus] ID 10618 failed
to reach steady state
##   ss_n: 500, ss_rtol: 1e-08, ss_atol: 1e-08
```

```
## Warning in (function (x, data, idata = no_idata_set(), carry_out = carry.out, : [steady_bolus] ID 10728 failed
to reach steady state
##   ss_n: 500, ss_rtol: 1e-08, ss_atol: 1e-08
## Warning in (function (x, data, idata = no_idata_set(), carry_out = carry.out, : [steady_bolus] ID 10728 failed
to reach steady state
##   ss_n: 500, ss_rtol: 1e-08, ss_atol: 1e-08
## Warning in (function (x, data, idata = no_idata_set(), carry_out = carry.out, : [steady_bolus] ID 10728 failed
to reach steady state
##   ss_n: 500, ss_rtol: 1e-08, ss_atol: 1e-08
## Warning in (function (x, data, idata = no_idata_set(), carry_out = carry.out, : [steady_bolus] ID 10728 failed
to reach steady state
##   ss_n: 500, ss_rtol: 1e-08, ss_atol: 1e-08
## Warning in (function (x, data, idata = no_idata_set(), carry_out = carry.out, : [steady_bolus] ID 10728 failed
to reach steady state
##   ss_n: 500, ss_rtol: 1e-08, ss_atol: 1e-08
## Warning in (function (x, data, idata = no_idata_set(), carry_out = carry.out, : [steady_bolus] ID 10728 failed
to reach steady state
##   ss_n: 500, ss_rtol: 1e-08, ss_atol: 1e-08
```

```
## Warning in (function (x, data, idata = no_idata_set(), carry_out = carry.out, : [steady_bolus] ID 10744 failed
to reach steady state
##   ss_n: 500, ss_rtol: 1e-08, ss_atol: 1e-08
## Warning in (function (x, data, idata = no_idata_set(), carry_out = carry.out, : [steady_bolus] ID 10744 failed
to reach steady state
##   ss_n: 500, ss_rtol: 1e-08, ss_atol: 1e-08
## Warning in (function (x, data, idata = no_idata_set(), carry_out = carry.out, : [steady_bolus] ID 10744 failed
to reach steady state
##   ss_n: 500, ss_rtol: 1e-08, ss_atol: 1e-08
## Warning in (function (x, data, idata = no_idata_set(), carry_out = carry.out, : [steady_bolus] ID 10744 failed
to reach steady state
##   ss_n: 500, ss_rtol: 1e-08, ss_atol: 1e-08
## Warning in (function (x, data, idata = no_idata_set(), carry_out = carry.out, : [steady_bolus] ID 10744 failed
to reach steady state
##   ss_n: 500, ss_rtol: 1e-08, ss_atol: 1e-08
## Warning in (function (x, data, idata = no_idata_set(), carry_out = carry.out, : [steady_bolus] ID 10744 failed
to reach steady state
##   ss_n: 500, ss_rtol: 1e-08, ss_atol: 1e-08
```

```
## Warning in (function (x, data, idata = no_idata_set(), carry_out = carry.out, : [steady_bolus] ID 10746 failed
to reach steady state
##   ss_n: 500, ss_rtol: 1e-08, ss_atol: 1e-08
```

```
## Warning in (function (x, data, idata = no_idata_set(), carry_out = carry.out, : [steady_bolus] ID 10778 failed
to reach steady state
##   ss_n: 500, ss_rtol: 1e-08, ss_atol: 1e-08
## Warning in (function (x, data, idata = no_idata_set(), carry_out = carry.out, : [steady_bolus] ID 10778 failed
to reach steady state
##   ss_n: 500, ss_rtol: 1e-08, ss_atol: 1e-08
## Warning in (function (x, data, idata = no_idata_set(), carry_out = carry.out, : [steady_bolus] ID 10778 failed
to reach steady state
##   ss_n: 500, ss_rtol: 1e-08, ss_atol: 1e-08
## Warning in (function (x, data, idata = no_idata_set(), carry_out = carry.out, : [steady_bolus] ID 10778 failed
to reach steady state
##   ss_n: 500, ss_rtol: 1e-08, ss_atol: 1e-08
## Warning in (function (x, data, idata = no_idata_set(), carry_out = carry.out, : [steady_bolus] ID 10778 failed
to reach steady state
##   ss_n: 500, ss_rtol: 1e-08, ss_atol: 1e-08
## Warning in (function (x, data, idata = no_idata_set(), carry_out = carry.out, : [steady_bolus] ID 10778 failed
to reach steady state
##   ss_n: 500, ss_rtol: 1e-08, ss_atol: 1e-08
## Warning in (function (x, data, idata = no_idata_set(), carry_out = carry.out, : [steady_bolus] ID 10778 failed
to reach steady state
##   ss_n: 500, ss_rtol: 1e-08, ss_atol: 1e-08
```

```
## Warning in (function (x, data, idata = no_idata_set(), carry_out = carry.out, : [steady_bolus] ID 10887 failed
to reach steady state
##   ss_n: 500, ss_rtol: 1e-08, ss_atol: 1e-08
```

```
## Warning in (function (x, data, idata = no_idata_set(), carry_out = carry.out, : [steady_bolus] ID 10896 failed
to reach steady state
##   ss_n: 500, ss_rtol: 1e-08, ss_atol: 1e-08
## Warning in (function (x, data, idata = no_idata_set(), carry_out = carry.out, : [steady_bolus] ID 10896 failed
to reach steady state
##   ss_n: 500, ss_rtol: 1e-08, ss_atol: 1e-08
## Warning in (function (x, data, idata = no_idata_set(), carry_out = carry.out, : [steady_bolus] ID 10896 failed
to reach steady state
##   ss_n: 500, ss_rtol: 1e-08, ss_atol: 1e-08
## Warning in (function (x, data, idata = no_idata_set(), carry_out = carry.out, : [steady_bolus] ID 10896 failed
to reach steady state
##   ss_n: 500, ss_rtol: 1e-08, ss_atol: 1e-08
## Warning in (function (x, data, idata = no_idata_set(), carry_out = carry.out, : [steady_bolus] ID 10896 failed
to reach steady state
##   ss_n: 500, ss_rtol: 1e-08, ss_atol: 1e-08
## Warning in (function (x, data, idata = no_idata_set(), carry_out = carry.out, : [steady_bolus] ID 10896 failed
to reach steady state
##   ss_n: 500, ss_rtol: 1e-08, ss_atol: 1e-08
```

```
## Warning in (function (x, data, idata = no_idata_set(), carry_out = carry.out, : [steady_bolus] ID 10915 failed
to reach steady state
##   ss_n: 500, ss_rtol: 1e-08, ss_atol: 1e-08
## Warning in (function (x, data, idata = no_idata_set(), carry_out = carry.out, : [steady_bolus] ID 10915 failed
to reach steady state
##   ss_n: 500, ss_rtol: 1e-08, ss_atol: 1e-08
## Warning in (function (x, data, idata = no_idata_set(), carry_out = carry.out, : [steady_bolus] ID 10915 failed
to reach steady state
##   ss_n: 500, ss_rtol: 1e-08, ss_atol: 1e-08
## Warning in (function (x, data, idata = no_idata_set(), carry_out = carry.out, : [steady_bolus] ID 10915 failed
to reach steady state
##   ss_n: 500, ss_rtol: 1e-08, ss_atol: 1e-08
## Warning in (function (x, data, idata = no_idata_set(), carry_out = carry.out, : [steady_bolus] ID 10915 failed
to reach steady state
##   ss_n: 500, ss_rtol: 1e-08, ss_atol: 1e-08
## Warning in (function (x, data, idata = no_idata_set(), carry_out = carry.out, : [steady_bolus] ID 10915 failed
to reach steady state
##   ss_n: 500, ss_rtol: 1e-08, ss_atol: 1e-08
## Warning in (function (x, data, idata = no_idata_set(), carry_out = carry.out, : [steady_bolus] ID 10915 failed
to reach steady state
##   ss_n: 500, ss_rtol: 1e-08, ss_atol: 1e-08
```

```
## Warning in (function (x, data, idata = no_idata_set(), carry_out = carry.out, : [steady_bolus] ID 10932 failed
to reach steady state
##   ss_n: 500, ss_rtol: 1e-08, ss_atol: 1e-08
## Warning in (function (x, data, idata = no_idata_set(), carry_out = carry.out, : [steady_bolus] ID 10932 failed
to reach steady state
##   ss_n: 500, ss_rtol: 1e-08, ss_atol: 1e-08
```

```
## Warning in (function (x, data, idata = no_idata_set(), carry_out = carry.out, : [steady_bolus] ID 10942 failed
to reach steady state
##   ss_n: 500, ss_rtol: 1e-08, ss_atol: 1e-08
```

```
## Warning in (function (x, data, idata = no_idata_set(), carry_out = carry.out, : [steady_bolus] ID 10949 failed
to reach steady state
##   ss_n: 500, ss_rtol: 1e-08, ss_atol: 1e-08
## Warning in (function (x, data, idata = no_idata_set(), carry_out = carry.out, : [steady_bolus] ID 10949 failed
to reach steady state
##   ss_n: 500, ss_rtol: 1e-08, ss_atol: 1e-08
```

```
## Warning in (function (x, data, idata = no_idata_set(), carry_out = carry.out, : [steady_bolus] ID 11025 failed
to reach steady state
##   ss_n: 500, ss_rtol: 1e-08, ss_atol: 1e-08
## Warning in (function (x, data, idata = no_idata_set(), carry_out = carry.out, : [steady_bolus] ID 11025 failed
to reach steady state
##   ss_n: 500, ss_rtol: 1e-08, ss_atol: 1e-08
## Warning in (function (x, data, idata = no_idata_set(), carry_out = carry.out, : [steady_bolus] ID 11025 failed
to reach steady state
##   ss_n: 500, ss_rtol: 1e-08, ss_atol: 1e-08
## Warning in (function (x, data, idata = no_idata_set(), carry_out = carry.out, : [steady_bolus] ID 11025 failed
to reach steady state
##   ss_n: 500, ss_rtol: 1e-08, ss_atol: 1e-08
## Warning in (function (x, data, idata = no_idata_set(), carry_out = carry.out, : [steady_bolus] ID 11025 failed
to reach steady state
##   ss_n: 500, ss_rtol: 1e-08, ss_atol: 1e-08
## Warning in (function (x, data, idata = no_idata_set(), carry_out = carry.out, : [steady_bolus] ID 11025 failed
to reach steady state
##   ss_n: 500, ss_rtol: 1e-08, ss_atol: 1e-08
```

```
## Warning in (function (x, data, idata = no_idata_set(), carry_out = carry.out, : [steady_bolus] ID 11125 failed
to reach steady state
##   ss_n: 500, ss_rtol: 1e-08, ss_atol: 1e-08
```

```
## Warning in (function (x, data, idata = no_idata_set(), carry_out = carry.out, : [steady_bolus] ID 11215 failed
to reach steady state
##   ss_n: 500, ss_rtol: 1e-08, ss_atol: 1e-08
## Warning in (function (x, data, idata = no_idata_set(), carry_out = carry.out, : [steady_bolus] ID 11215 failed
to reach steady state
##   ss_n: 500, ss_rtol: 1e-08, ss_atol: 1e-08
## Warning in (function (x, data, idata = no_idata_set(), carry_out = carry.out, : [steady_bolus] ID 11215 failed
to reach steady state
##   ss_n: 500, ss_rtol: 1e-08, ss_atol: 1e-08
## Warning in (function (x, data, idata = no_idata_set(), carry_out = carry.out, : [steady_bolus] ID 11215 failed
to reach steady state
##   ss_n: 500, ss_rtol: 1e-08, ss_atol: 1e-08
## Warning in (function (x, data, idata = no_idata_set(), carry_out = carry.out, : [steady_bolus] ID 11215 failed
to reach steady state
##   ss_n: 500, ss_rtol: 1e-08, ss_atol: 1e-08
## Warning in (function (x, data, idata = no_idata_set(), carry_out = carry.out, : [steady_bolus] ID 11215 failed
to reach steady state
##   ss_n: 500, ss_rtol: 1e-08, ss_atol: 1e-08
## Warning in (function (x, data, idata = no_idata_set(), carry_out = carry.out, : [steady_bolus] ID 11215 failed
to reach steady state
##   ss_n: 500, ss_rtol: 1e-08, ss_atol: 1e-08
```

```
## Warning in (function (x, data, idata = no_idata_set(), carry_out = carry.out, : [steady_bolus] ID 11237 failed
to reach steady state
##   ss_n: 500, ss_rtol: 1e-08, ss_atol: 1e-08
```

```
## Warning in (function (x, data, idata = no_idata_set(), carry_out = carry.out, : [steady_bolus] ID 11254 failed
to reach steady state
##   ss_n: 500, ss_rtol: 1e-08, ss_atol: 1e-08
```

```
## Warning in (function (x, data, idata = no_idata_set(), carry_out = carry.out, : [steady_bolus] ID 11276 failed
to reach steady state
##   ss_n: 500, ss_rtol: 1e-08, ss_atol: 1e-08
## Warning in (function (x, data, idata = no_idata_set(), carry_out = carry.out, : [steady_bolus] ID 11276 failed
to reach steady state
##   ss_n: 500, ss_rtol: 1e-08, ss_atol: 1e-08
## Warning in (function (x, data, idata = no_idata_set(), carry_out = carry.out, : [steady_bolus] ID 11276 failed
to reach steady state
##   ss_n: 500, ss_rtol: 1e-08, ss_atol: 1e-08
## Warning in (function (x, data, idata = no_idata_set(), carry_out = carry.out, : [steady_bolus] ID 11276 failed
to reach steady state
##   ss_n: 500, ss_rtol: 1e-08, ss_atol: 1e-08
## Warning in (function (x, data, idata = no_idata_set(), carry_out = carry.out, : [steady_bolus] ID 11276 failed
to reach steady state
##   ss_n: 500, ss_rtol: 1e-08, ss_atol: 1e-08
## Warning in (function (x, data, idata = no_idata_set(), carry_out = carry.out, : [steady_bolus] ID 11276 failed
to reach steady state
##   ss_n: 500, ss_rtol: 1e-08, ss_atol: 1e-08
```

```
## Warning in (function (x, data, idata = no_idata_set(), carry_out = carry.out, : [steady_bolus] ID 11290 failed
to reach steady state
##   ss_n: 500, ss_rtol: 1e-08, ss_atol: 1e-08
```

```
## Warning in (function (x, data, idata = no_idata_set(), carry_out = carry.out, : [steady_bolus] ID 11292 failed
to reach steady state
##   ss_n: 500, ss_rtol: 1e-08, ss_atol: 1e-08
```

```
## Warning in (function (x, data, idata = no_idata_set(), carry_out = carry.out, : [steady_bolus] ID 11304 failed
to reach steady state
##   ss_n: 500, ss_rtol: 1e-08, ss_atol: 1e-08
```

```
## Warning in (function (x, data, idata = no_idata_set(), carry_out = carry.out, : [steady_bolus] ID 11313 failed
to reach steady state
##   ss_n: 500, ss_rtol: 1e-08, ss_atol: 1e-08
```

```
## Warning in (function (x, data, idata = no_idata_set(), carry_out = carry.out, : [steady_bolus] ID 11418 failed
to reach steady state
##   ss_n: 500, ss_rtol: 1e-08, ss_atol: 1e-08
```

```
## Warning in (function (x, data, idata = no_idata_set(), carry_out = carry.out, : [steady_bolus] ID 11436 failed
to reach steady state
##   ss_n: 500, ss_rtol: 1e-08, ss_atol: 1e-08
## Warning in (function (x, data, idata = no_idata_set(), carry_out = carry.out, : [steady_bolus] ID 11436 failed
to reach steady state
##   ss_n: 500, ss_rtol: 1e-08, ss_atol: 1e-08
```

```
##   ss_n: 500, ss_rtol: 1e-08, ss_atol: 1e-08
## Warning in (function (x, data, idata = no_idata_set(), carry_out = carry.out, : [steady_bolus] ID 11436 failed
to reach steady state
##   ss_n: 500, ss_rtol: 1e-08, ss_atol: 1e-08
## Warning in (function (x, data, idata = no_idata_set(), carry_out = carry.out, : [steady_bolus] ID 11436 failed
to reach steady state
##   ss_n: 500, ss_rtol: 1e-08, ss_atol: 1e-08
## Warning in (function (x, data, idata = no_idata_set(), carry_out = carry.out, : [steady_bolus] ID 11436 failed
to reach steady state
##   ss_n: 500, ss_rtol: 1e-08, ss_atol: 1e-08
## Warning in (function (x, data, idata = no_idata_set(), carry_out = carry.out, : [steady_bolus] ID 11436 failed
to reach steady state
##   ss_n: 500, ss_rtol: 1e-08, ss_atol: 1e-08
## Warning in (function (x, data, idata = no_idata_set(), carry_out = carry.out, : [steady_bolus] ID 11436 failed
to reach steady state
##   ss_n: 500, ss_rtol: 1e-08, ss_atol: 1e-08
## Warning in (function (x, data, idata = no_idata_set(), carry_out = carry.out, : [steady_bolus] ID 11436 failed
to reach steady state
##   ss_n: 500, ss_rtol: 1e-08, ss_atol: 1e-08
```

```
## Warning in (function (x, data, idata = no_idata_set(), carry_out = carry.out, : [steady_bolus] ID 11574 failed
to reach steady state
##   ss_n: 500, ss_rtol: 1e-08, ss_atol: 1e-08
```

```
## Warning in (function (x, data, idata = no_idata_set(), carry_out = carry.out, : [steady_bolus] ID 11653 failed
to reach steady state
##   ss_n: 500, ss_rtol: 1e-08, ss_atol: 1e-08
```

```
## Warning in (function (x, data, idata = no_idata_set(), carry_out = carry.out, : [steady_bolus] ID 11659 failed
to reach steady state
##   ss_n: 500, ss_rtol: 1e-08, ss_atol: 1e-08
```

```
## Warning in (function (x, data, idata = no_idata_set(), carry_out = carry.out, : [steady_bolus] ID 11695 failed
to reach steady state
##   ss_n: 500, ss_rtol: 1e-08, ss_atol: 1e-08
## Warning in (function (x, data, idata = no_idata_set(), carry_out = carry.out, : [steady_bolus] ID 11695 failed
to reach steady state
##   ss_n: 500, ss_rtol: 1e-08, ss_atol: 1e-08
## Warning in (function (x, data, idata = no_idata_set(), carry_out = carry.out, : [steady_bolus] ID 11695 failed
to reach steady state
##   ss_n: 500, ss_rtol: 1e-08, ss_atol: 1e-08
## Warning in (function (x, data, idata = no_idata_set(), carry_out = carry.out, : [steady_bolus] ID 11695 failed
to reach steady state
##   ss_n: 500, ss_rtol: 1e-08, ss_atol: 1e-08
## Warning in (function (x, data, idata = no_idata_set(), carry_out = carry.out, : [steady_bolus] ID 11695 failed
to reach steady state
##   ss_n: 500, ss_rtol: 1e-08, ss_atol: 1e-08
## Warning in (function (x, data, idata = no_idata_set(), carry_out = carry.out, : [steady_bolus] ID 11695 failed
to reach steady state
##   ss_n: 500, ss_rtol: 1e-08, ss_atol: 1e-08
## Warning in (function (x, data, idata = no_idata_set(), carry_out = carry.out, : [steady_bolus] ID 11695 failed
to reach steady state
##   ss_n: 500, ss_rtol: 1e-08, ss_atol: 1e-08
```

```
## Warning in (function (x, data, idata = no_idata_set(), carry_out = carry.out, : [steady_bolus] ID 11719 failed
to reach steady state
##   ss_n: 500, ss_rtol: 1e-08, ss_atol: 1e-08
## Warning in (function (x, data, idata = no_idata_set(), carry_out = carry.out, : [steady_bolus] ID 11719 failed
to reach steady state
##   ss_n: 500, ss_rtol: 1e-08, ss_atol: 1e-08
```

```
## Warning in (function (x, data, idata = no_idata_set(), carry_out = carry.out, : [steady_bolus] ID 11739 failed
to reach steady state
##   ss_n: 500, ss_rtol: 1e-08, ss_atol: 1e-08
## Warning in (function (x, data, idata = no_idata_set(), carry_out = carry.out, : [steady_bolus] ID 11739 failed
to reach steady state
##   ss_n: 500, ss_rtol: 1e-08, ss_atol: 1e-08
## Warning in (function (x, data, idata = no_idata_set(), carry_out = carry.out, : [steady_bolus] ID 11739 failed
to reach steady state
##   ss_n: 500, ss_rtol: 1e-08, ss_atol: 1e-08
## Warning in (function (x, data, idata = no_idata_set(), carry_out = carry.out, : [steady_bolus] ID 11739 failed
to reach steady state
##   ss_n: 500, ss_rtol: 1e-08, ss_atol: 1e-08
## Warning in (function (x, data, idata = no_idata_set(), carry_out = carry.out, : [steady_bolus] ID 11739 failed
to reach steady state
##   ss_n: 500, ss_rtol: 1e-08, ss_atol: 1e-08
## Warning in (function (x, data, idata = no_idata_set(), carry_out = carry.out, : [steady_bolus] ID 11739 failed
to reach steady state
##   ss_n: 500, ss_rtol: 1e-08, ss_atol: 1e-08
```

```
## Warning in (function (x, data, idata = no_idata_set(), carry_out = carry.out, : [steady_bolus] ID 11864 failed
to reach steady state
##   ss_n: 500, ss_rtol: 1e-08, ss_atol: 1e-08
## Warning in (function (x, data, idata = no_idata_set(), carry_out = carry.out, : [steady_bolus] ID 11864 failed
to reach steady state
##   ss_n: 500, ss_rtol: 1e-08, ss_atol: 1e-08
## Warning in (function (x, data, idata = no_idata_set(), carry_out = carry.out, : [steady_bolus] ID 11864 failed
to reach steady state
##   ss_n: 500, ss_rtol: 1e-08, ss_atol: 1e-08
## Warning in (function (x, data, idata = no_idata_set(), carry_out = carry.out, : [steady_bolus] ID 11864 failed
to reach steady state
##   ss_n: 500, ss_rtol: 1e-08, ss_atol: 1e-08
## Warning in (function (x, data, idata = no_idata_set(), carry_out = carry.out, : [steady_bolus] ID 11864 failed
to reach steady state
##   ss_n: 500, ss_rtol: 1e-08, ss_atol: 1e-08
## Warning in (function (x, data, idata = no_idata_set(), carry_out = carry.out, : [steady_bolus] ID 11864 failed
to reach steady state
##   ss_n: 500, ss_rtol: 1e-08, ss_atol: 1e-08
## Warning in (function (x, data, idata = no_idata_set(), carry_out = carry.out, : [steady_bolus] ID 11864 failed
to reach steady state
##   ss_n: 500, ss_rtol: 1e-08, ss_atol: 1e-08
```

```
## Warning in (function (x, data, idata = no_idata_set(), carry_out = carry.out, : [steady_bolus] ID 11875 failed
to reach steady state
##   ss_n: 500, ss_rtol: 1e-08, ss_atol: 1e-08
```

Fusion des dataframes

```
Sim_MARTIAL_6000_df <- as.data.frame(Sim_MARTIAL_by_amt_6000)
Sim_MARTIAL_12000_df <- as.data.frame(Sim_MARTIAL_by_amt_12000)

Data_combined_Martial <- rbind(Sim_MARTIAL_6000_df, Sim_MARTIAL_12000_df)

Data_combined_Martial
```

```
##       ID time           DV
## 1      1    0 0.000000e+00
## 2      1    0 2.252213e+00
## 3      1    1 2.410493e+00
## 4      1    2 2.618943e+00
## 5      1    3 2.739396e+00
## 6      1    4 2.786694e+00
## 7      1    5 2.791280e+00
## 8      1    6 2.764096e+00
## 9      1    7 2.729584e+00
## 10     1    8 2.691289e+00
## 11     1    9 2.655210e+00
## 12     1   10 2.614010e+00
## 13     1   11 2.579860e+00
## 14     1   12 2.538179e+00
## 15     1   13 2.506776e+00
## 16     1   14 2.479610e+00
## 17     1   15 2.449433e+00
## 18     1   16 2.427922e+00
## 19     1   17 2.401924e+00
## 20     1   18 2.375994e+00
## 21     1   19 2.349198e+00
## 22     1   20 2.332152e+00
## 23     1   21 2.309629e+00
## 24     1   22 2.288244e+00
## 25     1   23 2.270025e+00
## 26     1   24 2.249430e+00
## 27     1   24 2.248514e+00
## 28     1   25 2.408201e+00
## 29     1   26 2.626139e+00
## 30     1   27 2.745123e+00
## 31     1   28 2.789348e+00
## 32     1   29 2.784432e+00
## 33     1   30 2.760825e+00
## 34     1   31 2.736086e+00
## 35     1   32 2.692340e+00
## 36     1   33 2.654882e+00
## 37     1   34 2.611849e+00
## 38     1   35 2.578803e+00
## 39     1   36 2.541433e+00
## 40     1   37 2.512377e+00
## 41     1   38 2.478750e+00
## 42     1   39 2.448018e+00
## 43     1   40 2.422509e+00
## 44     1   41 2.395594e+00
## 45     1   42 2.371421e+00
## 46     1   43 2.350920e+00
## 47     1   44 2.326010e+00
## 48     1   45 2.307755e+00
## 49     1   46 2.289766e+00
## 50     1   47 2.265504e+00
## 51     1   48 2.250957e+00
## 52     1   48 2.249908e+00
## 53     1   49 2.410790e+00
## 54     1   50 2.617194e+00
## 55     1   51 2.746237e+00
## 56     1   52 2.788925e+00
## 57     1   53 2.783025e+00
## 58     1   54 2.759610e+00
## 59     1   55 2.725737e+00
## 60     1   56 2.691101e+00
## 61     1   57 2.651303e+00
## 62     1   58 2.613216e+00
## 63     1   59 2.582085e+00
## 64     1   60 2.540199e+00
## 65     1   61 2.511946e+00
## 66     1   62 2.482753e+00
## 67     1   63 2.450102e+00
## 68     1   64 2.424050e+00
## 69     1   65 2.396393e+00
## 70     1   66 2.368804e+00
## 71     1   67 2.347557e+00
## 72     1   68 2.325699e+00
## 73     1   69 2.306083e+00
## 74     1   70 2.291735e+00
## 75     1   71 2.265172e+00
## 76     1   72 2.251315e+00
## 77     1   73 2.407511e+00
## 78     1   74 2.622931e+00
## 79     1   75 2.736024e+00
## 80     1   76 2.783600e+00
## 81     1   77 2.789116e+00
## 82     1   78 2.758931e+00
## 83     1   79 2.727895e+00
## 84     1   80 2.692382e+00
## 85     1   81 2.651857e+00
## 86     1   82 2.615385e+00
## 87     1   83 2.571305e+00
## 88     1   84 2.540489e+00
## 89     1   85 2.509118e+00
## 90     1   86 2.480091e+00
## 91     1   87 2.449657e+00
## 92     1   88 2.426661e+00
## 93     1   89 2.397242e+00
## 94     1   90 2.370786e+00
## 95     1   91 2.348573e+00
## 96     1   92 2.329586e+00
## 97     1   93 2.308053e+00
## 98     1   94 2.289479e+00
## 99     1   95 2.267998e+00
## 100    1   96 2.246964e+00
## 101    1   97 2.409818e+00
## 102    1   98 2.617782e+00
## 103    1   99 2.743440e+00
## 104    1  100 2.785517e+00
## 105    1  101 2.786742e+00
## 106    1  102 2.763304e+00
## 107    1  103 2.730039e+00
## 108    1  104 2.689974e+00
## 109    1  105 2.654135e+00
## 110    1  106 2.614562e+00
## 111    1  107 2.581568e+00
## 112    1  108 2.544383e+00
## 113    1  109 2.507972e+00
## 114    1  110 2.480370e+00
## 115    1  111 2.450445e+00
## 116    1  112 2.424760e+00
## 117    1  113 2.397047e+00
## 118    1  114 2.371431e+00
## 119    1  115 2.350180e+00
## 120    1  116 2.331537e+00
## 121    1  117 2.305282e+00
## 122    1  118 2.282928e+00
## 123    1  119 2.269718e+00
## 124    1  120 2.248145e+00
## 125    1  121 2.410678e+00
## 126    1  122 2.618065e+00
## 127    1  123 2.738343e+00
## 128    1  124 2.785000e+00
## 129    1  125 2.787459e+00
## 130    1  126 2.767425e+00
## 131    1  127 2.730042e+00
## 132    1  128 2.692594e+00
## 133    1  129 2.657498e+00
## 134    1  130 2.616025e+00
## 135    1  131 2.574987e+00
## 136    1  132 2.539007e+00
## 137    1  133 2.510008e+00
## 138    1  134 2.481187e+00
## 139    1  135 2.449476e+00
## 140    1  136 2.424772e+00
## 141    1  137 2.396523e+00
## 142    1  138 2.374159e+00
## 143    1  139 2.353696e+00
## 144    1  140 2.328172e+00
## 145    1  141 2.304089e+00
## 146    1  142 2.286389e+00
## 147    1  143 2.269149e+00
## 148    1  144 2.248533e+00
## 149    1  145 2.407762e+00
## 150    1  146 2.621842e+00
## 151    1  147 2.742653e+00
## 152    1  148 2.790805e+00
## 153    1  149 2.789602e+00
## 154    1  150 2.760479e+00
## 155    1  151 2.736544e+00
## 156    1  152 2.691520e+00
## 157    1  153 2.654368e+00
## 158    1  154 2.616778e+00
## 159    1  155 2.577757e+00
## 160    1  156 2.545865e+00
## 161    1  157 2.513349e+00
## 162    1  158 2.480048e+00
## 163    1  159 2.451316e+00
## 164    1  160 2.421408e+00
## 165    1  161 2.398141e+00
## 166    1  162 2.372569e+00
## 167    1  163 2.346692e+00
## 168    1  164 2.325065e+00
## 169    1  165 2.305194e+00
## 170    1  166 2.290471e+00
## 171    1  167 2.269583e+00
## 172    1  168 2.245286e+00
## 173    1  169 2.407350e+00
## 174    1  170 2.619292e+00
## 175    1  171 2.737074e+00
## 176    1  172 2.786235e+00
## 177    1  173 2.790788e+00
## 178    1  174 2.766173e+00
## 179    1  175 2.726636e+00
## 180    1  176 2.692222e+00
## 181    1  177 2.649111e+00
## 182    1  178 2.619035e+00
## 183    1  179 2.576184e+00
## 184    1  180 2.543727e+00
## 185    1  181 2.513463e+00
## 186    1  182 2.477892e+00
## 187    1  183 2.450864e+00
## 188    1  184 2.422782e+00
## 189    1  185 2.397118e+00
## 190    1  186 2.370288e+00
## 191    1  187 2.354705e+00
## 192    1  188 2.323795e+00
## 193    1  189 2.304237e+00
## 194    1  190 2.289472e+00
## 195    1  191 2.267884e+00
## 196    1  192 2.245048e+00
## 197    1  193 2.407774e+00
## 198    1  194 2.619957e+00
## 199    1  195 2.740119e+00
## 200    1  196 2.785547e+00
## 201    1  197 2.788388e+00
## 202    1  198 2.763354e+00
## 203    1  199 2.724873e+00
## 204    1  200 2.688357e+00
## 205    1  201 2.656884e+00
## 206    1  202 2.614496e+00
## 207    1  203 2.582283e+00
## 208    1  204 2.544894e+00
## 209    1  205 2.508589e+00
## 210    1  206 2.479410e+00
## 211    1  207 2.450520e+00
## 212    1  208 2.421302e+00
## 213    1  209 2.397639e+00
## 214    1  210 2.371953e+00
## 215    1  211 2.349465e+00
## 216    1  212 2.323293e+00
## 217    1  213 2.309335e+00
## 218    1  214 2.290156e+00
## 219    1  215 2.271379e+00
## 220    1  216 2.249362e+00
## 221    1  217 2.406044e+00
## 222    1  218 2.618138e+00
## 223    1  219 2.740170e+00
## 224    1  220 2.783700e+00
## 225    1  221 2.786668e+00
## 226    1  222 2.761580e+00
## 227    1  223 2.729938e+00
## 228    1  224 2.689839e+00
## 229    1  225 2.654681e+00
## 230    1  226 2.618633e+00
## 231    1  227 2.579574e+00
## 232    1  228 2.546288e+00
## 233    1  229 2.511758e+00
## 234    1  230 2.483990e+00
## 235    1  231 2.451082e+00
## 236    1  232 2.425740e+00
## 237    1  233 2.396542e+00
## 238    1  234 2.369244e+00
## 239    1  235 2.350327e+00
## 240    1  236 2.327285e+00
## 241    1  237 2.307654e+00
## 242    1  238 2.290844e+00
## 243    1  239 2.269962e+00
## 244    1  240 2.254008e+00
## 245    1  241 2.411577e+00
## 246    1  242 2.618577e+00
## 247    1  243 2.742066e+00
## 248    1  244 2.786225e+00
## 249    1  245 2.783674e+00
## 250    1  246 2.764935e+00
## 251    1  247 2.729151e+00
## 252    1  248 2.689563e+00
## 253    1  249 2.655129e+00
## 254    1  250 2.613515e+00
## 255    1  251 2.576320e+00
## 256    1  252 2.543516e+00
## 257    1  253 2.504427e+00
## 258    1  254 2.478246e+00
## 259    1  255 2.449224e+00
## 260    1  256 2.426326e+00
## 261    1  257 2.397984e+00
## 262    1  258 2.372943e+00
## 263    1  259 2.349808e+00
## 264    1  260 2.331343e+00
## 265    1  261 2.307593e+00
## 266    1  262 2.286720e+00
## 267    1  263 2.266633e+00
## 268    1  264 2.251015e+00
## 269    1  265 2.410092e+00
## 270    1  266 2.620443e+00
## 271    1  267 2.741647e+00
## 272    1  268 2.791458e+00
## 273    1  269 2.787479e+00
## 274    1  270 2.769313e+00
## 275    1  271 2.733754e+00
## 276    1  272 2.689144e+00
## 277    1  273 2.652066e+00
## 278    1  274 2.615617e+00
## 279    1  275 2.576572e+00
## 280    1  276 2.538693e+00
## 281    1  277 2.512222e+00
## 282    1  278 2.484330e+00
## 283    1  279 2.453441e+00
## 284    1  280 2.425244e+00
## 285    1  281 2.401783e+00
## 286    1  282 2.370379e+00
## 287    1  283 2.347341e+00
## 288    1  284 2.330284e+00
## 289    1  285 2.307618e+00
## 290    1  286 2.286743e+00
## 291    1  287 2.268562e+00
## 292    1  288 2.247673e+00
## 293    1  289 2.413576e+00
## 294    1  290 2.617143e+00
## 295    1  291 2.735416e+00
## 296    1  292 2.790183e+00
## 297    1  293 2.786157e+00
## 298    1  294 2.763343e+00
## 299    1  295 2.731555e+00
## 300    1  296 2.689341e+00
## 301    1  297 2.650713e+00
## 302    1  298 2.617839e+00
## 303    1  299 2.578405e+00
## 304    1  300 2.541933e+00
## 305    1  301 2.512098e+00
## 306    1  302 2.478438e+00
## 307    1  303 2.450236e+00
## 308    1  304 2.419996e+00
## 309    1  305 2.395257e+00
## 310    1  306 2.372126e+00
## 311    1  307 2.349779e+00
## 312    1  308 2.324534e+00
## 313    1  309 2.306674e+00
## 314    1  310 2.287547e+00
## 315    1  311 2.268514e+00
## 316    1  312 2.252995e+00
## 317    1  312 2.251190e+00
## 318    1  313 2.405992e+00
## 319    1  314 2.617287e+00
## 320    1  315 2.737749e+00
## 321    1  316 2.789760e+00
## 322    1  317 2.788140e+00
## 323    1  318 2.761170e+00
## 324    1  319 2.732895e+00
## 325    1  320 2.697148e+00
## 326    1  321 2.654396e+00
## 327    1  322 2.612986e+00
## 328    1  323 2.576732e+00
## 329    1  324 2.546187e+00
## 330    1  325 2.515382e+00
## 331    1  326 2.482345e+00
## 332    1  327 2.454682e+00
## 333    1  328 2.421357e+00
## 334    1  329 2.397399e+00
## 335    1  330 2.374248e+00
## 336    1  331 2.348008e+00
## 337    1  332 2.329852e+00
## 338    1  333 2.303905e+00
## 339    1  334 2.285984e+00
## 340    1  335 2.272053e+00
## 341    1  336 2.252950e+00
## 342    1  336 2.251535e+00
## 343    1  337 2.231325e+00
## 344    1  338 2.213284e+00
## 345    1  339 2.200818e+00
## 346    1  339 2.201626e+00
## 347    1  340 2.358517e+00
## 348    1  341 2.572842e+00
## 349    1  342 2.698008e+00
## 350    1  343 2.746840e+00
## 351    1  344 2.748461e+00
## 352    1  345 2.718244e+00
## 353    1  346 2.699633e+00
## 354    1  347 2.655313e+00
## 355    1  348 2.615034e+00
## 356    1  349 2.581436e+00
## 357    1  350 2.547632e+00
## 358    1  351 2.512790e+00
## 359    1  352 2.481160e+00
## 360    1  353 2.449671e+00
## 361    1  354 2.420243e+00
## 362    1  355 2.389726e+00
## 363    1  356 2.369094e+00
## 364    1  357 2.344683e+00
## 365    1  358 2.321316e+00
## 366    1  359 2.299628e+00
## 367    1  360 2.279827e+00
## 368    1  360 2.281497e+00
## 369    1  361 2.439345e+00
## 370    1  362 2.644741e+00
## 371    1  363 2.763805e+00
## 372    1  364 2.804725e+00
## 373    1  365 2.808350e+00
## 374    1  366 2.786086e+00
## 375    1  367 2.746797e+00
## 376    1  368 2.710446e+00
## 377    1  369 2.668060e+00
## 378    1  370 2.629402e+00
## 379    1  371 2.589908e+00
## 380    1  372 2.558390e+00
## 381    1  373 2.525651e+00
## 382    1  374 2.492380e+00
## 383    1  375 2.460303e+00
## 384    1  376 2.435618e+00
## 385    1  377 2.403520e+00
## 386    1  378 2.384031e+00
## 387    1  379 2.356234e+00
## 388    1  380 2.336828e+00
## 389    1  381 2.312573e+00
## 390    1  382 2.292891e+00
## 391    1  383 2.272768e+00
## 392    1  384 2.256499e+00
## 393    1  385 2.419198e+00
## 394    1  386 2.619869e+00
## 395    1  387 2.751950e+00
## 396    1  388 2.792504e+00
## 397    1  389 2.791734e+00
## 398    1  390 2.767122e+00
## 399    1  391 2.735128e+00
## 400    1  392 2.698673e+00
## 401    1  393 2.655118e+00
## 402    1  394 2.616035e+00
## 403    1  395 2.580459e+00
## 404    1  396 2.548287e+00
## 405    1  397 2.516082e+00
## 406    1  398 2.487308e+00
## 407    1  399 2.453604e+00
## 408    1  400 2.428676e+00
## 409    1  401 2.402063e+00
## 410    1  402 2.373775e+00
## 411    1  403 2.350088e+00
## 412    1  404 2.328666e+00
## 413    1  405 2.308631e+00
## 414    1  406 2.288908e+00
## 415    1  407 2.270068e+00
## 416    1  408 2.250892e+00
## 417    1  409 2.409589e+00
## 418    1  410 2.618191e+00
## 419    1  411 2.741333e+00
## 420    1  412 2.789544e+00
## 421    1  413 2.787486e+00
## 422    1  414 2.765465e+00
## 423    1  415 2.730372e+00
## 424    1  416 2.691844e+00
## 425    1  417 2.654877e+00
## 426    1  418 2.615009e+00
## 427    1  419 2.580970e+00
## 428    1  420 2.542250e+00
## 429    1  421 2.513984e+00
## 430    1  422 2.476688e+00
## 431    1  423 2.452559e+00
## 432    1  424 2.424530e+00
## 433    1  425 2.402234e+00
## 434    1  426 2.377885e+00
## 435    1  427 2.350903e+00
## 436    1  428 2.329046e+00
## 437    1  429 2.310251e+00
## 438    1  430 2.290402e+00
## 439    1  431 2.270493e+00
## 440    1  432 2.251954e+00
## 441    1  433 2.411693e+00
## 442    1  434 2.621596e+00
## 443    1  435 2.741116e+00
## 444    1  436 2.790014e+00
## 445    1  437 2.789867e+00
## 446    1  438 2.767100e+00
## 447    1  439 2.728631e+00
## 448    1  440 2.696866e+00
## 449    1  441 2.655594e+00
## 450    1  442 2.617083e+00
## 451    1  443 2.577077e+00
## 452    1  444 2.542676e+00
## 453    1  445 2.513443e+00
## 454    1  446 2.483844e+00
## 455    1  447 2.450143e+00
## 456    1  448 2.426537e+00
## 457    1  449 2.400684e+00
## 458    1  450 2.370901e+00
## 459    1  451 2.351403e+00
## 460    1  452 2.331601e+00
## 461    1  453 2.307445e+00
## 462    1  454 2.282943e+00
## 463    1  455 2.267772e+00
## 464    1  456 2.248862e+00
## 465    1  457 2.413463e+00
## 466    1  458 2.626236e+00
## 467    1  459 2.743583e+00
## 468    1  460 2.791877e+00
## 469    1  461 2.792037e+00
## 470    1  462 2.771117e+00
## 471    1  463 2.734920e+00
## 472    1  464 2.693434e+00
## 473    1  465 2.655912e+00
## 474    1  466 2.616811e+00
## 475    1  467 2.580369e+00
## 476    1  468 2.543990e+00
## 477    1  469 2.514933e+00
## 478    1  470 2.488177e+00
## 479    1  471 2.448945e+00
## 480    1  472 2.422504e+00
## 481    1  473 2.397339e+00
## 482    1  474 2.371741e+00
## 483    1  475 2.348835e+00
## 484    1  476 2.328601e+00
## 485    1  477 2.308691e+00
## 486    1  478 2.289599e+00
## 487    1  479 2.273311e+00
## 488    1  480 2.246243e+00
## 489    1  481 2.410365e+00
## 490    1  482 2.621042e+00
## 491    1  483 2.740480e+00
## 492    1  484 2.790670e+00
## 493    1  485 2.793981e+00
## 494    1  486 2.765785e+00
## 495    1  487 2.735433e+00
## 496    1  488 2.697183e+00
## 497    1  489 2.653052e+00
## 498    1  490 2.617056e+00
## 499    1  491 2.583280e+00
## 500    1  492 2.540447e+00
## 501    1  493 2.510925e+00
## 502    1  494 2.480693e+00
## 503    1  495 2.451573e+00
## 504    1  496 2.421816e+00
## 505    1  497 2.398788e+00
## 506    1  498 2.369045e+00
## 507    1  499 2.347354e+00
## 508    1  500 2.327184e+00
## 509    1  501 2.307129e+00
## 510    1  502 2.291450e+00
## 511    1  503 2.270643e+00
## 512    1  504 2.256263e+00
## 513    1  505 2.411514e+00
## 514    1  506 2.619351e+00
## 515    1  507 2.743006e+00
## 516    1  508 2.786411e+00
## 517    1  509 2.788902e+00
## 518    1  510 2.764925e+00
## 519    1  511 2.732626e+00
## 520    1  512 2.687165e+00
## 521    1  513 2.654848e+00
## 522    1  514 2.614175e+00
## 523    1  515 2.580711e+00
## 524    1  516 2.545531e+00
## 525    1  517 2.516175e+00
## 526    1  518 2.482841e+00
## 527    1  519 2.457787e+00
## 528    1  520 2.416579e+00
## 529    1  521 2.400207e+00
## 530    1  522 2.376723e+00
## 531    1  523 2.350707e+00
## 532    1  524 2.328175e+00
## 533    1  525 2.307682e+00
## 534    1  526 2.287484e+00
## 535    1  527 2.270404e+00
## 536    1  528 2.251842e+00
## 537    1  529 2.408956e+00
## 538    1  530 2.621015e+00
## 539    1  531 2.741671e+00
## 540    1  532 2.790051e+00
## 541    1  533 2.786851e+00
## 542    1  534 2.766063e+00
## 543    1  535 2.730366e+00
## 544    1  536 2.690845e+00
## 545    1  537 2.649813e+00
## 546    1  538 2.616303e+00
## 547    1  539 2.580006e+00
## 548    1  540 2.539275e+00
## 549    1  541 2.511122e+00
## 550    1  542 2.481199e+00
## 551    1  543 2.450329e+00
## 552    1  544 2.424113e+00
## 553    1  545 2.398704e+00
## 554    1  546 2.376948e+00
## 555    1  547 2.352163e+00
## 556    1  548 2.329825e+00
## 557    1  549 2.305460e+00
## 558    1  550 2.289902e+00
## 559    1  551 2.269169e+00
## 560    1  552 2.246327e+00
## 561    1  553 2.412179e+00
## 562    1  554 2.616733e+00
## 563    1  555 2.744153e+00
## 564    1  556 2.787318e+00
## 565    1  557 2.789562e+00
## 566    1  558 2.764172e+00
## 567    1  559 2.730147e+00
## 568    1  560 2.690214e+00
## 569    1  561 2.650641e+00
## 570    1  562 2.614237e+00
## 571    1  563 2.582031e+00
## 572    1  564 2.542131e+00
## 573    1  565 2.510034e+00
## 574    1  566 2.482520e+00
## 575    1  567 2.447681e+00
## 576    1  568 2.425177e+00
## 577    1  569 2.395042e+00
## 578    1  570 2.374696e+00
## 579    1  571 2.347922e+00
## 580    1  572 2.323060e+00
## 581    1  573 2.308531e+00
## 582    1  574 2.288307e+00
## 583    1  575 2.267603e+00
## 584    1  576 2.250073e+00
## 585    1  577 2.411501e+00
## 586    1  578 2.622512e+00
## 587    1  579 2.745282e+00
## 588    1  580 2.787459e+00
## 589    1  581 2.789489e+00
## 590    1  582 2.768524e+00
## 591    1  583 2.730213e+00
## 592    1  584 2.692260e+00
## 593    1  585 2.656834e+00
## 594    1  586 2.613146e+00
## 595    1  587 2.582407e+00
## 596    1  588 2.548258e+00
## 597    1  589 2.512193e+00
## 598    1  590 2.482468e+00
## 599    1  591 2.450404e+00
## 600    1  592 2.425220e+00
## 601    1  593 2.398391e+00
## 602    1  594 2.374109e+00
## 603    1  595 2.349591e+00
## 604    1  596 2.333293e+00
## 605    1  597 2.307679e+00
## 606    1  598 2.286757e+00
## 607    1  599 2.268652e+00
## 608    1  600 2.249890e+00
## 609    1  601 2.411978e+00
## 610    1  602 2.622509e+00
## 611    1  603 2.746640e+00
## 612    1  604 2.787299e+00
## 613    1  605 2.788811e+00
## 614    1  606 2.763498e+00
## 615    1  607 2.729165e+00
## 616    1  608 2.688225e+00
## 617    1  609 2.651271e+00
## 618    1  610 2.611682e+00
## 619    1  611 2.577370e+00
## 620    1  612 2.541944e+00
## 621    1  613 2.509118e+00
## 622    1  614 2.483619e+00
## 623    1  615 2.445950e+00
## 624    1  616 2.419484e+00
## 625    1  617 2.400056e+00
## 626    1  618 2.377124e+00
## 627    1  619 2.348255e+00
## 628    1  620 2.331379e+00
## 629    1  621 2.307240e+00
## 630    1  622 2.286181e+00
## 631    1  623 2.267118e+00
## 632    1  624 2.250419e+00
## 633    1  624 2.247031e+00
## 634    1  625 2.408576e+00
## 635    1  626 2.614317e+00
## 636    1  627 2.742335e+00
## 637    1  628 2.782914e+00
## 638    1  629 2.789811e+00
## 639    1  630 2.760730e+00
## 640    1  631 2.725102e+00
## 641    1  632 2.687398e+00
## 642    1  633 2.653975e+00
## 643    1  634 2.617220e+00
## 644    1  635 2.572946e+00
## 645    1  636 2.541766e+00
## 646    1  637 2.514961e+00
## 647    1  638 2.484171e+00
## 648    1  639 2.450712e+00
## 649    1  640 2.418425e+00
## 650    1  641 2.393250e+00
## 651    1  642 2.369357e+00
## 652    1  643 2.351394e+00
## 653    1  644 2.326435e+00
## 654    1  645 2.305319e+00
## 655    1  646 2.286306e+00
## 656    1  647 2.263888e+00
## 657    1  648 2.253185e+00
## 658    1  648 2.247396e+00
## 659    1  649 2.237748e+00
## 660    1  650 2.211535e+00
## 661    1  651 2.198644e+00
## 662    1  652 2.185196e+00
## 663    1  653 2.168250e+00
## 664    1  654 2.161101e+00
## 665    1  654 2.160062e+00
## 666    1  655 2.318703e+00
## 667    1  656 2.530735e+00
## 668    1  657 2.655833e+00
## 669    1  658 2.707747e+00
## 670    1  659 2.714104e+00
## 671    1  660 2.687045e+00
## 672    1  661 2.654469e+00
## 673    1  662 2.621360e+00
```

```
## 674    1  663 2.585413e+00
## 675    1  664 2.552047e+00
## 676    1  665 2.518758e+00
## 677    1  666 2.483547e+00
## 678    1  667 2.451021e+00
## 679    1  668 2.424593e+00
## 680    1  669 2.392204e+00
## 681    1  670 2.364443e+00
## 682    1  671 2.342621e+00
## 683    1  672 2.318630e+00
## 684    1  672 2.315588e+00
## 685    1  673 2.470149e+00
## 686    1  674 2.677512e+00
## 687    1  675 2.796021e+00
## 688    1  676 2.837531e+00
## 689    1  677 2.835875e+00
## 690    1  678 2.808995e+00
## 691    1  679 2.771538e+00
## 692    1  680 2.723631e+00
## 693    1  681 2.691005e+00
## 694    1  682 2.647765e+00
## 695    1  683 2.608873e+00
## 696    1  684 2.570775e+00
## 697    1  685 2.535942e+00
## 698    1  686 2.503972e+00
## 699    1  687 2.473544e+00
## 700    1  688 2.445394e+00
## 701    1  689 2.421610e+00
## 702    1  690 2.390774e+00
## 703    1  691 2.366492e+00
## 704    1  692 2.341960e+00
## 705    1  693 2.320224e+00
## 706    1  694 2.302317e+00
## 707    1  695 2.277697e+00
## 708    1  696 2.262282e+00
## 709    1  697 2.422109e+00
## 710    1  698 2.631477e+00
## 711    1  699 2.756783e+00
## 712    1  700 2.800332e+00
## 713    1  701 2.800566e+00
## 714    1  702 2.771588e+00
## 715    1  703 2.739833e+00
## 716    1  704 2.698017e+00
## 717    1  705 2.660371e+00
## 718    1  706 2.624573e+00
## 719    1  707 2.583286e+00
## 720    1  708 2.553211e+00
## 721    1  709 2.514853e+00
## 722    1  710 2.482568e+00
## 723    1  711 2.456239e+00
## 724    1  712 2.431103e+00
## 725    1  713 2.404691e+00
## 726    1  714 2.378627e+00
## 727    1  715 2.354436e+00
## 728    1  716 2.330348e+00
## 729    1  717 2.311389e+00
## 730    1  718 2.294319e+00
## 731    1  719 2.269357e+00
## 732    1  720 2.255458e+00
## 733    1  721 2.414959e+00
## 734    1  722 2.620109e+00
## 735    1  723 2.746235e+00
## 736    1  724 2.791293e+00
## 737    1  725 2.794602e+00
## 738    1  726 2.769722e+00
## 739    1  727 2.736460e+00
## 740    1  728 2.699151e+00
## 741    1  729 2.655791e+00
## 742    1  730 2.618286e+00
## 743    1  731 2.579476e+00
## 744    1  732 2.550056e+00
## 745    1  733 2.517434e+00
## 746    1  734 2.483140e+00
## 747    1  735 2.456206e+00
## 748    1  736 2.427832e+00
## 749    1  737 2.402851e+00
## 750    1  738 2.375892e+00
## 751    1  739 2.348092e+00
## 752    1  740 2.330071e+00
## 753    1  741 2.309277e+00
## 754    1  742 2.288103e+00
## 755    1  743 2.268458e+00
## 756    1  744 2.252734e+00
## 757    1  745 2.416236e+00
## 758    1  746 2.623078e+00
## 759    1  747 2.741314e+00
## 760    1  748 2.798752e+00
## 761    1  749 2.794330e+00
## 762    1  750 2.766167e+00
## 763    1  751 2.729067e+00
## 764    1  752 2.695729e+00
## 765    1  753 2.655936e+00
## 766    1  754 2.615564e+00
## 767    1  755 2.579751e+00
## 768    1  756 2.543298e+00
## 769    1  757 2.512132e+00
## 770    1  758 2.479470e+00
## 771    1  759 2.449702e+00
## 772    1  760 2.430816e+00
## 773    1  761 2.399985e+00
## 774    1  762 2.379360e+00
## 775    1  763 2.353168e+00
## 776    1  764 2.330960e+00
## 777    1  765 2.310106e+00
## 778    1  766 2.288919e+00
## 779    1  767 2.275111e+00
## 780    1  768 2.254341e+00
## 781    1  769 2.410866e+00
## 782    1  770 2.624287e+00
## 783    1  771 2.744241e+00
## 784    1  772 2.792130e+00
## 785    1  773 2.795294e+00
## 786    1  774 2.769777e+00
## 787    1  775 2.732585e+00
## 788    1  776 2.695027e+00
## 789    1  777 2.653308e+00
## 790    1  778 2.618273e+00
## 791    1  779 2.583230e+00
## 792    1  780 2.550511e+00
## 793    1  781 2.516316e+00
## 794    1  782 2.483478e+00
## 795    1  783 2.457181e+00
## 796    1  784 2.424811e+00
## 797    1  785 2.404409e+00
## 798    1  786 2.374480e+00
## 799    1  787 2.354350e+00
## 800    1  788 2.327696e+00
## 801    1  789 2.307419e+00
## 802    1  790 2.293576e+00
## 803    1  791 2.269917e+00
## 804    1  792 2.254186e+00
## 805    1  793 2.413656e+00
## 806    1  794 2.623487e+00
## 807    1  795 2.743579e+00
## 808    1  796 2.792102e+00
## 809    1  797 2.789695e+00
## 810    1  798 2.771008e+00
## 811    1  799 2.735998e+00
## 812    1  800 2.690223e+00
## 813    1  801 2.659588e+00
## 814    1  802 2.615663e+00
## 815    1  803 2.582950e+00
## 816    1  804 2.550858e+00
## 817    1  805 2.512949e+00
## 818    1  806 2.485558e+00
## 819    1  807 2.454136e+00
## 820    1  808 2.428880e+00
## 821    1  809 2.399961e+00
## 822    1  810 2.378664e+00
## 823    1  811 2.350263e+00
## 824    1  812 2.331327e+00
## 825    1  813 2.307659e+00
## 826    1  814 2.287415e+00
## 827    1  815 2.269609e+00
## 828    1  816 2.252395e+00
## 829    1  817 2.411803e+00
## 830    1  818 2.618655e+00
## 831    1  819 2.742127e+00
## 832    1  820 2.787810e+00
## 833    1  821 2.791458e+00
## 834    1  822 2.766990e+00
## 835    1  823 2.737606e+00
## 836    1  824 2.695115e+00
## 837    1  825 2.649512e+00
## 838    1  826 2.616390e+00
## 839    1  827 2.576813e+00
## 840    1  828 2.544267e+00
## 841    1  829 2.519258e+00
## 842    1  830 2.482495e+00
## 843    1  831 2.452685e+00
## 844    1  832 2.428594e+00
## 845    1  833 2.401226e+00
## 846    1  834 2.373167e+00
## 847    1  835 2.351727e+00
## 848    1  836 2.327997e+00
## 849    1  837 2.309355e+00
## 850    1  838 2.292157e+00
## 851    1  839 2.267029e+00
## 852    1  840 2.251395e+00
## 853    1  841 2.415479e+00
## 854    1  842 2.621401e+00
## 855    1  843 2.747155e+00
## 856    1  844 2.786324e+00
## 857    1  845 2.789477e+00
## 858    1  846 2.769200e+00
## 859    1  847 2.730344e+00
## 860    1  848 2.694808e+00
## 861    1  849 2.657553e+00
## 862    1  850 2.613715e+00
## 863    1  851 2.581430e+00
## 864    1  852 2.545221e+00
## 865    1  853 2.514879e+00
## 866    1  854 2.485820e+00
## 867    1  855 2.456456e+00
## 868    1  856 2.423401e+00
## 869    1  857 2.403144e+00
## 870    1  858 2.374175e+00
## 871    1  859 2.354075e+00
## 872    1  860 2.333454e+00
## 873    1  861 2.306492e+00
## 874    1  862 2.284842e+00
## 875    1  863 2.269667e+00
## 876    1  864 2.252276e+00
## 877    1  865 2.418174e+00
## 878    1  866 2.619333e+00
## 879    1  867 2.742872e+00
## 880    1  868 2.787726e+00
## 881    1  869 2.784981e+00
## 882    1  870 2.770378e+00
## 883    1  871 2.730201e+00
## 884    1  872 2.693299e+00
## 885    1  873 2.651226e+00
## 886    1  874 2.615044e+00
## 887    1  875 2.579330e+00
## 888    1  876 2.547094e+00
## 889    1  877 2.514926e+00
## 890    1  878 2.488613e+00
## 891    1  879 2.449607e+00
## 892    1  880 2.421496e+00
## 893    1  881 2.395255e+00
## 894    1  882 2.374438e+00
## 895    1  883 2.351604e+00
## 896    1  884 2.331579e+00
## 897    1  885 2.309896e+00
## 898    1  886 2.291389e+00
## 899    1  887 2.271678e+00
## 900    1  888 2.251852e+00
## 901    1  889 2.412817e+00
## 902    1  890 2.620480e+00
## 903    1  891 2.742036e+00
## 904    1  892 2.789877e+00
## 905    1  893 2.787560e+00
## 906    1  894 2.764496e+00
## 907    1  895 2.724999e+00
## 908    1  896 2.690961e+00
## 909    1  897 2.652365e+00
## 910    1  898 2.623070e+00
## 911    1  899 2.579849e+00
## 912    1  900 2.546158e+00
## 913    1  901 2.513789e+00
## 914    1  902 2.479673e+00
## 915    1  903 2.448782e+00
## 916    1  904 2.425929e+00
## 917    1  905 2.398157e+00
## 918    1  906 2.375162e+00
## 919    1  907 2.350279e+00
## 920    1  908 2.323545e+00
## 921    1  909 2.304935e+00
## 922    1  910 2.285755e+00
## 923    1  911 2.266338e+00
## 924    1  912 2.252291e+00
## 925    1  913 2.411310e+00
## 926    1  914 2.617406e+00
## 927    1  915 2.746397e+00
## 928    1  916 2.790496e+00
## 929    1  917 2.790522e+00
## 930    1  918 2.768163e+00
## 931    1  919 2.734771e+00
## 932    1  920 2.695116e+00
## 933    1  921 2.654933e+00
## 934    1  922 2.614550e+00
## 935    1  923 2.574737e+00
## 936    1  924 2.547220e+00
## 937    1  925 2.515204e+00
## 938    1  926 2.479222e+00
## 939    1  927 2.448001e+00
## 940    1  928 2.423572e+00
## 941    1  929 2.394433e+00
## 942    1  930 2.370033e+00
## 943    1  931 2.356941e+00
## 944    1  932 2.327995e+00
## 945    1  933 2.306997e+00
## 946    1  934 2.292312e+00
## 947    1  935 2.267265e+00
## 948    1  936 2.248499e+00
## 949    1  936 2.249602e+00
## 950    1  937 2.409635e+00
## 951    1  938 2.618830e+00
## 952    1  939 2.739079e+00
## 953    1  940 2.784412e+00
## 954    1  941 2.786598e+00
## 955    1  942 2.764366e+00
## 956    1  943 2.730764e+00
## 957    1  944 2.690304e+00
## 958    1  945 2.650742e+00
## 959    1  946 2.610174e+00
## 960    1  947 2.575176e+00
## 961    1  948 2.543896e+00
## 962    1  949 2.509473e+00
## 963    1  950 2.481931e+00
## 964    1  951 2.458200e+00
## 965    1  952 2.424914e+00
## 966    1  953 2.396232e+00
## 967    1  954 2.370875e+00
## 968    1  955 2.351143e+00
## 969    1  956 2.326709e+00
## 970    1  957 2.309935e+00
## 971    1  958 2.289204e+00
## 972    1  959 2.268853e+00
## 973    1  960 2.250780e+00
## 974    1  960 2.251410e+00
## 975    1  961 2.229700e+00
## 976    1  962 2.217212e+00
## 977    1  963 2.200786e+00
## 978    1  964 2.186833e+00
## 979    1  965 2.168027e+00
## 980    1  966 2.155275e+00
## 981    1  967 2.142381e+00
## 982    1  968 2.131431e+00
## 983    1  969 2.117896e+00
## 984    1  969 2.117240e+00
## 985    1  970 2.279669e+00
## 986    1  971 2.500649e+00
## 987    1  972 2.621668e+00
## 988    1  973 2.674578e+00
## 989    1  974 2.677689e+00
## 990    1  975 2.656606e+00
## 991    1  976 2.627561e+00
## 992    1  977 2.589689e+00
## 993    1  978 2.555432e+00
## 994    1  979 2.518326e+00
## 995    1  980 2.481970e+00
## 996    1  981 2.455913e+00
## 997    1  982 2.422494e+00
## 998    1  983 2.393782e+00
## 999    1  984 2.362880e+00
## 1000   1  984 2.364918e+00
## 1001   1  985 2.515810e+00
## 1002   1  986 2.718800e+00
## 1003   1  987 2.833171e+00
## 1004   1  988 2.874062e+00
## 1005   1  989 2.867999e+00
## 1006   1  990 2.839857e+00
## 1007   1  991 2.790602e+00
## 1008   1  992 2.756717e+00
## 1009   1  993 2.704567e+00
## 1010   1  994 2.674104e+00
## 1011   1  995 2.629962e+00
## 1012   1  996 2.591808e+00
## 1013   1  997 2.552706e+00
## 1014   1  998 2.521893e+00
## 1015   1  999 2.484123e+00
## 1016   1 1000 2.462513e+00
## 1017   1 1001 2.432982e+00
## 1018   1 1002 2.408070e+00
## 1019   1 1003 2.381742e+00
## 1020   1 1004 2.355195e+00
## 1021   1 1005 2.335383e+00
## 1022   1 1006 2.309113e+00
## 1023   1 1007 2.293372e+00
## 1024   1 1008 2.270339e+00
## 1025   1 1009 2.431558e+00
## 1026   1 1010 2.638787e+00
## 1027   1 1011 2.760564e+00
## 1028   1 1012 2.804904e+00
## 1029   1 1013 2.804226e+00
## 1030   1 1014 2.777316e+00
## 1031   1 1015 2.751178e+00
## 1032   1 1016 2.700314e+00
## 1033   1 1017 2.667349e+00
## 1034   1 1018 2.627724e+00
## 1035   1 1019 2.590376e+00
## 1036   1 1020 2.555430e+00
## 1037   1 1021 2.530282e+00
## 1038   1 1022 2.490593e+00
## 1039   1 1023 2.457883e+00
## 1040   1 1024 2.431778e+00
## 1041   1 1025 2.407474e+00
## 1042   1 1026 2.381518e+00
## 1043   1 1027 2.353330e+00
## 1044   1 1028 2.332826e+00
## 1045   1 1029 2.311245e+00
## 1046   1 1030 2.295959e+00
## 1047   1 1031 2.276488e+00
## 1048   1 1032 2.258342e+00
## 1049   1 1033 2.418715e+00
## 1050   1 1034 2.626535e+00
## 1051   1 1035 2.750288e+00
## 1052   1 1036 2.801137e+00
## 1053   1 1037 2.795944e+00
## 1054   1 1038 2.772338e+00
## 1055   1 1039 2.734280e+00
## 1056   1 1040 2.698562e+00
## 1057   1 1041 2.658412e+00
## 1058   1 1042 2.623922e+00
## 1059   1 1043 2.587308e+00
## 1060   1 1044 2.549737e+00
## 1061   1 1045 2.516396e+00
## 1062   1 1046 2.489355e+00
## 1063   1 1047 2.459898e+00
## 1064   1 1048 2.429292e+00
## 1065   1 1049 2.404828e+00
## 1066   1 1050 2.376525e+00
## 1067   1 1051 2.356686e+00
## 1068   1 1052 2.331889e+00
## 1069   1 1053 2.312769e+00
## 1070   1 1054 2.290591e+00
## 1071   1 1055 2.273768e+00
## 1072   1 1056 2.251092e+00
## 1073   1 1057 2.414028e+00
## 1074   1 1058 2.624514e+00
## 1075   1 1059 2.743751e+00
## 1076   1 1060 2.792024e+00
## 1077   1 1061 2.792026e+00
## 1078   1 1062 2.771539e+00
## 1079   1 1063 2.736781e+00
## 1080   1 1064 2.697688e+00
## 1081   1 1065 2.658693e+00
## 1082   1 1066 2.618425e+00
## 1083   1 1067 2.582133e+00
## 1084   1 1068 2.549154e+00
## 1085   1 1069 2.515825e+00
## 1086   1 1070 2.485865e+00
## 1087   1 1071 2.457453e+00
## 1088   1 1072 2.427731e+00
## 1089   1 1073 2.404263e+00
## 1090   1 1074 2.381975e+00
## 1091   1 1075 2.353403e+00
## 1092   1 1076 2.334931e+00
## 1093   1 1077 2.311245e+00
## 1094   1 1078 2.292536e+00
## 1095   1 1079 2.273596e+00
## 1096   1 1080 2.255443e+00
## 1097   1 1081 2.415424e+00
## 1098   1 1082 2.627682e+00
## 1099   1 1083 2.741633e+00
## 1100   1 1084 2.789457e+00
## 1101   1 1085 2.793050e+00
## 1102   1 1086 2.769726e+00
## 1103   1 1087 2.737179e+00
## 1104   1 1088 2.698443e+00
## 1105   1 1089 2.654860e+00
## 1106   1 1090 2.620737e+00
## 1107   1 1091 2.581991e+00
## 1108   1 1092 2.545323e+00
## 1109   1 1093 2.518359e+00
## 1110   1 1094 2.484030e+00
## 1111   1 1095 2.453584e+00
## 1112   1 1096 2.428003e+00
## 1113   1 1097 2.401863e+00
## 1114   1 1098 2.374629e+00
## 1115   1 1099 2.351268e+00
## 1116   1 1100 2.332183e+00
## 1117   1 1101 2.308068e+00
## 1118   1 1102 2.289370e+00
## 1119   1 1103 2.270475e+00
## 1120   1 1104 2.257072e+00
## 1121   1 1105 2.412528e+00
## 1122   1 1106 2.626181e+00
## 1123   1 1107 2.743829e+00
## 1124   1 1108 2.793594e+00
## 1125   1 1109 2.795056e+00
## 1126   1 1110 2.768743e+00
## 1127   1 1111 2.732687e+00
## 1128   1 1112 2.693906e+00
## 1129   1 1113 2.656002e+00
## 1130   1 1114 2.620722e+00
## 1131   1 1115 2.584837e+00
## 1132   1 1116 2.550797e+00
## 1133   1 1117 2.516705e+00
## 1134   1 1118 2.487443e+00
## 1135   1 1119 2.451452e+00
## 1136   1 1120 2.427942e+00
## 1137   1 1121 2.402934e+00
## 1138   1 1122 2.377273e+00
## 1139   1 1123 2.355032e+00
## 1140   1 1124 2.332314e+00
## 1141   1 1125 2.311982e+00
## 1142   1 1126 2.290195e+00
## 1143   1 1127 2.266562e+00
## 1144   1 1128 2.257279e+00
## 1145   1 1129 2.415563e+00
## 1146   1 1130 2.621385e+00
## 1147   1 1131 2.741004e+00
## 1148   1 1132 2.789846e+00
## 1149   1 1133 2.793060e+00
## 1150   1 1134 2.764261e+00
## 1151   1 1135 2.736337e+00
## 1152   1 1136 2.695863e+00
## 1153   1 1137 2.656711e+00
## 1154   1 1138 2.619119e+00
## 1155   1 1139 2.576029e+00
## 1156   1 1140 2.547796e+00
## 1157   1 1141 2.517624e+00
## 1158   1 1142 2.488703e+00
## 1159   1 1143 2.455358e+00
## 1160   1 1144 2.426647e+00
## 1161   1 1145 2.402940e+00
## 1162   1 1146 2.374253e+00
## 1163   1 1147 2.351495e+00
## 1164   1 1148 2.329948e+00
## 1165   1 1149 2.310801e+00
## 1166   1 1150 2.289531e+00
## 1167   1 1151 2.267224e+00
## 1168   1 1152 2.256033e+00
## 1169   1 1153 2.417097e+00
## 1170   1 1154 2.617755e+00
## 1171   1 1155 2.744096e+00
## 1172   1 1156 2.794702e+00
## 1173   1 1157 2.788106e+00
## 1174   1 1158 2.765987e+00
## 1175   1 1159 2.735516e+00
## 1176   1 1160 2.692345e+00
## 1177   1 1161 2.656987e+00
## 1178   1 1162 2.618875e+00
## 1179   1 1163 2.581779e+00
## 1180   1 1164 2.549456e+00
## 1181   1 1165 2.515935e+00
## 1182   1 1166 2.476987e+00
## 1183   1 1167 2.452004e+00
## 1184   1 1168 2.425577e+00
## 1185   1 1169 2.402033e+00
## 1186   1 1170 2.375952e+00
## 1187   1 1171 2.356555e+00
## 1188   1 1172 2.334194e+00
## 1189   1 1173 2.305386e+00
## 1190   1 1174 2.292940e+00
## 1191   1 1175 2.268642e+00
## 1192   1 1176 2.250948e+00
## 1193   1 1177 2.412517e+00
## 1194   1 1178 2.618805e+00
## 1195   1 1179 2.745503e+00
## 1196   1 1180 2.792378e+00
## 1197   1 1181 2.787086e+00
## 1198   1 1182 2.766597e+00
## 1199   1 1183 2.730770e+00
## 1200   1 1184 2.691877e+00
## 1201   1 1185 2.659466e+00
## 1202   1 1186 2.616646e+00
## 1203   1 1187 2.580296e+00
## 1204   1 1188 2.544458e+00
## 1205   1 1189 2.516834e+00
## 1206   1 1190 2.482596e+00
## 1207   1 1191 2.455360e+00
## 1208   1 1192 2.427749e+00
## 1209   1 1193 2.401905e+00
## 1210   1 1194 2.376909e+00
## 1211   1 1195 2.352189e+00
## 1212   1 1196 2.334249e+00
## 1213   1 1197 2.309185e+00
## 1214   1 1198 2.291921e+00
## 1215   1 1199 2.270627e+00
## 1216   1 1200 2.254318e+00
## 1217   1 1201 2.412892e+00
## 1218   1 1202 2.625252e+00
## 1219   1 1203 2.748248e+00
## 1220   1 1204 2.788230e+00
## 1221   1 1205 2.789518e+00
## 1222   1 1206 2.769601e+00
## 1223   1 1207 2.730613e+00
## 1224   1 1208 2.695343e+00
## 1225   1 1209 2.652175e+00
## 1226   1 1210 2.617940e+00
## 1227   1 1211 2.582294e+00
## 1228   1 1212 2.546225e+00
## 1229   1 1213 2.518715e+00
## 1230   1 1214 2.483964e+00
## 1231   1 1215 2.453651e+00
## 1232   1 1216 2.426371e+00
## 1233   1 1217 2.407872e+00
## 1234   1 1218 2.375974e+00
## 1235   1 1219 2.353083e+00
## 1236   1 1220 2.328534e+00
## 1237   1 1221 2.306058e+00
## 1238   1 1222 2.286888e+00
## 1239   1 1223 2.270261e+00
## 1240   1 1224 2.252853e+00
## 1241   1 1225 2.414451e+00
## 1242   1 1226 2.622529e+00
## 1243   1 1227 2.742571e+00
## 1244   1 1228 2.787569e+00
## 1245   1 1229 2.785686e+00
## 1246   1 1230 2.767109e+00
## 1247   1 1231 2.731277e+00
## 1248   1 1232 2.694425e+00
## 1249   1 1233 2.655740e+00
## 1250   1 1234 2.620713e+00
## 1251   1 1235 2.580436e+00
## 1252   1 1236 2.544958e+00
## 1253   1 1237 2.514179e+00
## 1254   1 1238 2.481916e+00
## 1255   1 1239 2.454897e+00
## 1256   1 1240 2.430955e+00
## 1257   1 1241 2.400827e+00
## 1258   1 1242 2.377900e+00
## 1259   1 1243 2.352881e+00
## 1260   1 1244 2.329525e+00
## 1261   1 1245 2.306051e+00
## 1262   1 1246 2.286423e+00
## 1263   1 1247 2.270914e+00
## 1264   1 1248 2.253145e+00
## 1265   1 1248 2.251243e+00
## 1266   1 1249 2.410914e+00
## 1267   1 1250 2.616828e+00
## 1268   1 1251 2.742969e+00
## 1269   1 1252 2.790699e+00
## 1270   1 1253 2.784647e+00
## 1271   1 1254 2.770464e+00
## 1272   1 1255 2.731412e+00
## 1273   1 1256 2.689302e+00
## 1274   1 1257 2.653215e+00
## 1275   1 1258 2.613859e+00
## 1276   1 1259 2.578020e+00
## 1277   1 1260 2.542095e+00
## 1278   1 1261 2.515582e+00
## 1279   1 1262 2.477804e+00
## 1280   1 1263 2.451939e+00
## 1281   1 1264 2.424615e+00
## 1282   1 1265 2.398112e+00
## 1283   1 1266 2.368618e+00
## 1284   1 1267 2.348263e+00
## 1285   1 1268 2.324059e+00
## 1286   1 1269 2.306515e+00
## 1287   1 1270 2.284361e+00
## 1288   1 1271 2.265003e+00
## 1289   1 1272 2.248966e+00
## 1290   1 1272 2.249570e+00
## 1291   1 1273 2.231213e+00
## 1292   1 1274 2.214124e+00
## 1293   1 1275 2.200903e+00
## 1294   1 1276 2.184482e+00
## 1295   1 1277 2.168296e+00
## 1296   1 1278 2.153518e+00
## 1297   1 1279 2.139053e+00
## 1298   1 1280 2.128437e+00
## 1299   1 1281 2.116764e+00
## 1300   1 1282 2.107341e+00
## 1301   1 1283 2.096956e+00
## 1302   1 1284 2.084195e+00
## 1303   1 1284 2.080723e+00
## 1304   1 1285 2.247964e+00
## 1305   1 1286 2.461402e+00
## 1306   1 1287 2.590834e+00
## 1307   1 1288 2.644176e+00
## 1308   1 1289 2.647913e+00
## 1309   1 1290 2.628012e+00
## 1310   1 1291 2.597076e+00
## 1311   1 1292 2.566530e+00
## 1312   1 1293 2.527252e+00
## 1313   1 1294 2.489059e+00
## 1314   1 1295 2.461076e+00
## 1315   1 1296 2.424444e+00
## 1316   1 1296 2.426072e+00
## 1317   1 1297 2.571166e+00
## 1318   1 1298 2.770892e+00
## 1319   1 1299 2.880990e+00
## 1320   1 1300 2.919312e+00
## 1321   1 1301 2.910764e+00
## 1322   1 1302 2.875883e+00
## 1323   1 1303 2.835479e+00
## 1324   1 1304 2.787380e+00
## 1325   1 1305 2.742774e+00
## 1326   1 1306 2.695729e+00
## 1327   1 1307 2.651846e+00
## 1328   1 1308 2.617685e+00
## 1329   1 1309 2.578439e+00
## 1330   1 1310 2.542807e+00
## 1331   1 1311 2.509389e+00
## 1332   1 1312 2.477797e+00
## 1333   1 1313 2.445761e+00
## 1334   1 1314 2.417998e+00
## 1335   1 1315 2.391359e+00
## 1336   1 1316 2.366971e+00
## 1337   1 1317 2.346006e+00
## 1338   1 1318 2.323032e+00
## 1339   1 1319 2.306793e+00
## 1340   1 1320 2.285589e+00
## 1341   1 1321 2.438244e+00
## 1342   1 1322 2.644583e+00
## 1343   1 1323 2.772479e+00
## 1344   1 1324 2.815275e+00
## 1345   1 1325 2.812883e+00
## 1346   1 1326 2.785756e+00
## 1347   1 1327 2.754133e+00
## 1348   1 1328 2.712199e+00
## 1349   1 1329 2.673746e+00
## 1350   1 1330 2.635707e+00
## 1351   1 1331 2.594511e+00
## 1352   1 1332 2.562389e+00
## 1353   1 1333 2.525616e+00
## 1354   1 1334 2.498039e+00
## 1355   1 1335 2.464712e+00
## 1356   1 1336 2.436061e+00
## 1357   1 1337 2.411060e+00
## 1358   1 1338 2.383325e+00
## 1359   1 1339 2.362955e+00
## 1360   1 1340 2.340579e+00
## 1361   1 1341 2.318185e+00
## 1362   1 1342 2.296173e+00
## 1363   1 1343 2.276821e+00
## 1364   1 1344 2.261682e+00
## 1365   1 1345 2.420508e+00
## 1366   1 1346 2.630309e+00
## 1367   1 1347 2.752639e+00
## 1368   1 1348 2.789615e+00
## 1369   1 1349 2.795498e+00
## 1370   1 1350 2.770292e+00
## 1371   1 1351 2.738718e+00
## 1372   1 1352 2.703570e+00
## 1373   1 1353 2.663097e+00
## 1374   1 1354 2.628329e+00
## 1375   1 1355 2.588501e+00
## 1376   1 1356 2.549968e+00
## 1377   1 1357 2.519822e+00
## 1378   1 1358 2.484323e+00
## 1379   1 1359 2.461311e+00
## 1380   1 1360 2.432028e+00
## 1381   1 1361 2.404179e+00
## 1382   1 1362 2.382285e+00
## 1383   1 1363 2.358440e+00
## 1384   1 1364 2.334697e+00
## 1385   1 1365 2.313755e+00
## 1386   1 1366 2.290804e+00
## 1387   1 1367 2.273391e+00
## 1388   1 1368 2.257261e+00
## 1389   1 1369 2.418431e+00
## 1390   1 1370 2.623343e+00
## 1391   1 1371 2.747652e+00
## 1392   1 1372 2.799010e+00
## 1393   1 1373 2.794730e+00
## 1394   1 1374 2.771891e+00
## 1395   1 1375 2.734785e+00
## 1396   1 1376 2.705159e+00
## 1397   1 1377 2.659887e+00
## 1398   1 1378 2.618486e+00
## 1399   1 1379 2.582650e+00
## 1400   1 1380 2.550851e+00
## 1401   1 1381 2.521042e+00
## 1402   1 1382 2.493798e+00
## 1403   1 1383 2.455848e+00
## 1404   1 1384 2.431687e+00
## 1405   1 1385 2.401553e+00
## 1406   1 1386 2.376045e+00
## 1407   1 1387 2.350106e+00
## 1408   1 1388 2.334694e+00
## 1409   1 1389 2.312387e+00
## 1410   1 1390 2.295751e+00
## 1411   1 1391 2.274494e+00
## 1412   1 1392 2.255881e+00
## 1413   1 1393 2.412970e+00
## 1414   1 1394 2.628568e+00
## 1415   1 1395 2.742098e+00
## 1416   1 1396 2.793841e+00
## 1417   1 1397 2.795906e+00
## 1418   1 1398 2.775013e+00
## 1419   1 1399 2.735426e+00
## 1420   1 1400 2.697537e+00
## 1421   1 1401 2.664025e+00
## 1422   1 1402 2.623048e+00
## 1423   1 1403 2.587774e+00
## 1424   1 1404 2.549871e+00
## 1425   1 1405 2.511588e+00
## 1426   1 1406 2.484411e+00
## 1427   1 1407 2.455992e+00
## 1428   1 1408 2.428479e+00
## 1429   1 1409 2.405438e+00
## 1430   1 1410 2.377972e+00
## 1431   1 1411 2.354106e+00
## 1432   1 1412 2.333666e+00
## 1433   1 1413 2.311567e+00
## 1434   1 1414 2.291498e+00
## 1435   1 1415 2.272961e+00
## 1436   1 1416 2.250962e+00
## 1437   1 1417 2.415728e+00
## 1438   1 1418 2.626480e+00
## 1439   1 1419 2.752268e+00
## 1440   1 1420 2.791860e+00
## 1441   1 1421 2.797702e+00
## 1442   1 1422 2.771631e+00
## 1443   1 1423 2.732369e+00
## 1444   1 1424 2.695007e+00
## 1445   1 1425 2.654694e+00
## 1446   1 1426 2.620005e+00
## 1447   1 1427 2.580491e+00
## 1448   1 1428 2.548197e+00
## 1449   1 1429 2.513275e+00
## 1450   1 1430 2.484604e+00
## 1451   1 1431 2.456945e+00
## 1452   1 1432 2.426604e+00
## 1453   1 1433 2.404651e+00
## 1454   1 1434 2.379119e+00
## 1455   1 1435 2.356498e+00
## 1456   1 1436 2.337294e+00
## 1457   1 1437 2.310428e+00
## 1458   1 1438 2.294399e+00
## 1459   1 1439 2.273714e+00
## 1460   1 1440 2.252607e+00
## 1461   1 1441 2.416916e+00
## 1462   1 1442 2.618845e+00
## 1463   1 1443 2.747808e+00
## 1464   1 1444 2.786970e+00
## 1465   1 1445 2.791161e+00
## 1466   1 1446 2.766268e+00
## 1467   1 1447 2.738119e+00
## 1468   1 1448 2.697851e+00
## 1469   1 1449 2.657495e+00
## 1470   1 1450 2.621300e+00
## 1471   1 1451 2.580716e+00
## 1472   1 1452 2.545988e+00
## 1473   1 1453 2.516255e+00
```

```
## 1474   1 1454 2.487293e+00
## 1475   1 1455 2.454989e+00
## 1476   1 1456 2.428113e+00
## 1477   1 1457 2.399888e+00
## 1478   1 1458 2.380970e+00
## 1479   1 1459 2.353626e+00
## 1480   1 1460 2.332785e+00
## 1481   1 1461 2.310073e+00
## 1482   1 1462 2.289162e+00
## 1483   1 1463 2.273885e+00
## 1484   1 1464 2.252280e+00
## 1485   1 1465 2.412918e+00
## 1486   1 1466 2.627050e+00
## 1487   1 1467 2.741525e+00
## 1488   1 1468 2.792409e+00
## 1489   1 1469 2.792141e+00
## 1490   1 1470 2.766857e+00
## 1491   1 1471 2.733591e+00
## 1492   1 1472 2.694558e+00
## 1493   1 1473 2.659241e+00
## 1494   1 1474 2.621384e+00
## 1495   1 1475 2.581037e+00
## 1496   1 1476 2.547599e+00
## 1497   1 1477 2.515164e+00
## 1498   1 1478 2.483314e+00
## 1499   1 1479 2.455373e+00
## 1500   1 1480 2.422200e+00
## 1501   1 1481 2.406640e+00
## 1502   1 1482 2.369840e+00
## 1503   1 1483 2.352408e+00
## 1504   1 1484 2.333331e+00
## 1505   1 1485 2.310254e+00
## 1506   1 1486 2.288964e+00
## 1507   1 1487 2.271326e+00
## 1508   1 1488 2.252611e+00
## 1509   1 1489 2.418654e+00
## 1510   1 1490 2.621562e+00
## 1511   1 1491 2.744439e+00
## 1512   1 1492 2.787320e+00
## 1513   1 1493 2.789159e+00
## 1514   1 1494 2.769279e+00
## 1515   1 1495 2.732746e+00
## 1516   1 1496 2.694059e+00
## 1517   1 1497 2.660995e+00
## 1518   1 1498 2.619693e+00
## 1519   1 1499 2.585423e+00
## 1520   1 1500 2.544898e+00
## 1521   1 1501 2.522560e+00
## 1522   1 1502 2.487251e+00
## 1523   1 1503 2.451756e+00
## 1524   1 1504 2.422683e+00
## 1525   1 1505 2.400097e+00
## 1526   1 1506 2.380538e+00
## 1527   1 1507 2.347136e+00
## 1528   1 1508 2.331460e+00
## 1529   1 1509 2.309450e+00
## 1530   1 1510 2.293017e+00
## 1531   1 1511 2.267212e+00
## 1532   1 1512 2.255651e+00
## 1533   1 1513 2.412675e+00
## 1534   1 1514 2.619230e+00
## 1535   1 1515 2.743095e+00
## 1536   1 1516 2.791764e+00
## 1537   1 1517 2.791428e+00
## 1538   1 1518 2.765453e+00
## 1539   1 1519 2.736093e+00
## 1540   1 1520 2.691684e+00
## 1541   1 1521 2.655552e+00
## 1542   1 1522 2.615017e+00
## 1543   1 1523 2.583303e+00
## 1544   1 1524 2.545492e+00
## 1545   1 1525 2.514917e+00
## 1546   1 1526 2.485126e+00
## 1547   1 1527 2.455716e+00
## 1548   1 1528 2.429613e+00
## 1549   1 1529 2.397773e+00
## 1550   1 1530 2.376771e+00
## 1551   1 1531 2.352045e+00
## 1552   1 1532 2.329041e+00
## 1553   1 1533 2.312229e+00
## 1554   1 1534 2.288032e+00
## 1555   1 1535 2.270459e+00
## 1556   1 1536 2.251162e+00
## 1557   1 1537 2.415805e+00
## 1558   1 1538 2.623670e+00
## 1559   1 1539 2.740696e+00
## 1560   1 1540 2.791802e+00
## 1561   1 1541 2.789288e+00
## 1562   1 1542 2.762881e+00
## 1563   1 1543 2.725832e+00
## 1564   1 1544 2.692087e+00
## 1565   1 1545 2.655175e+00
## 1566   1 1546 2.616850e+00
## 1567   1 1547 2.583534e+00
## 1568   1 1548 2.548348e+00
## 1569   1 1549 2.514322e+00
## 1570   1 1550 2.485474e+00
## 1571   1 1551 2.456592e+00
## 1572   1 1552 2.429241e+00
## 1573   1 1553 2.400140e+00
## 1574   1 1554 2.374215e+00
## 1575   1 1555 2.351756e+00
## 1576   1 1556 2.331640e+00
## 1577   1 1557 2.312882e+00
## 1578   1 1558 2.292275e+00
## 1579   1 1559 2.269276e+00
## 1580   1 1560 2.253910e+00
## 1581   1 1560 2.248990e+00
## 1582   1 1561 2.405341e+00
## 1583   1 1562 2.619928e+00
## 1584   1 1563 2.744535e+00
## 1585   1 1564 2.788214e+00
## 1586   1 1565 2.787664e+00
## 1587   1 1566 2.768333e+00
## 1588   1 1567 2.730843e+00
## 1589   1 1568 2.695206e+00
## 1590   1 1569 2.653955e+00
## 1591   1 1570 2.613244e+00
## 1592   1 1571 2.579853e+00
## 1593   1 1572 2.543887e+00
## 1594   1 1573 2.508771e+00
## 1595   1 1574 2.482455e+00
## 1596   1 1575 2.451928e+00
## 1597   1 1576 2.423701e+00
## 1598   1 1577 2.396654e+00
## 1599   1 1578 2.372693e+00
## 1600   1 1579 2.350472e+00
## 1601   1 1580 2.330666e+00
## 1602   1 1581 2.306640e+00
## 1603   1 1582 2.288152e+00
## 1604   1 1583 2.268812e+00
## 1605   1 1584 2.251635e+00
## 1606   1 1584 2.249965e+00
## 1607   1 1585 2.232052e+00
## 1608   1 1586 2.217438e+00
## 1609   1 1587 2.199753e+00
## 1610   1 1588 2.186912e+00
## 1611   1 1589 2.166609e+00
## 1612   1 1590 2.157469e+00
## 1613   1 1591 2.141391e+00
## 1614   1 1592 2.130623e+00
## 1615   1 1593 2.120529e+00
## 1616   1 1594 2.104655e+00
## 1617   1 1595 2.095766e+00
## 1618   1 1596 2.080108e+00
## 1619   1 1597 2.070893e+00
## 1620   1 1598 2.058708e+00
## 1621   1 1599 2.050160e+00
## 1622   1 1599 2.050944e+00
## 1623   1 1600 2.125967e+00
## 1624   1 1601 2.232371e+00
## 1625   1 1602 2.292593e+00
## 1626   1 1603 2.310724e+00
## 1627   1 1604 2.310002e+00
## 1628   1 1605 2.294217e+00
## 1629   1 1606 2.274623e+00
## 1630   1 1607 2.255268e+00
## 1631   1 1608 2.235482e+00
## 1632   1 1608 2.230944e+00
## 1633   1 1609 2.389066e+00
## 1634   1 1610 2.590554e+00
## 1635   1 1611 2.709756e+00
## 1636   1 1612 2.751625e+00
## 1637   1 1613 2.748552e+00
## 1638   1 1614 2.723813e+00
## 1639   1 1615 2.691256e+00
## 1640   1 1616 2.643284e+00
## 1641   1 1617 2.605575e+00
## 1642   1 1618 2.562808e+00
## 1643   1 1619 2.526338e+00
## 1644   1 1620 2.484701e+00
## 1645   1 1621 2.451556e+00
## 1646   1 1622 2.421236e+00
## 1647   1 1623 2.387671e+00
## 1648   1 1624 2.362987e+00
## 1649   1 1625 2.334786e+00
## 1650   1 1626 2.303269e+00
## 1651   1 1627 2.280523e+00
## 1652   1 1628 2.264617e+00
## 1653   1 1629 2.239788e+00
## 1654   1 1630 2.219370e+00
## 1655   1 1631 2.199488e+00
## 1656   1 1632 2.179759e+00
## 1657   1 1633 2.338778e+00
## 1658   1 1634 2.547538e+00
## 1659   1 1635 2.672573e+00
## 1660   1 1636 2.719175e+00
## 1661   1 1637 2.715949e+00
## 1662   1 1638 2.694631e+00
## 1663   1 1639 2.656769e+00
## 1664   1 1640 2.623968e+00
## 1665   1 1641 2.580525e+00
## 1666   1 1642 2.538308e+00
## 1667   1 1643 2.503767e+00
## 1668   1 1644 2.470555e+00
## 1669   1 1645 2.439963e+00
## 1670   1 1646 2.410717e+00
## 1671   1 1647 2.378096e+00
## 1672   1 1648 2.354183e+00
## 1673   1 1649 2.326776e+00
## 1674   1 1650 2.297150e+00
## 1675   1 1651 2.278443e+00
## 1676   1 1652 2.258524e+00
## 1677   1 1653 2.233149e+00
## 1678   1 1654 2.216842e+00
## 1679   1 1655 2.198591e+00
## 1680   1 1656 2.178623e+00
## 1681   1 1657 2.339352e+00
## 1682   1 1658 2.553658e+00
## 1683   1 1659 2.668122e+00
## 1684   1 1660 2.718811e+00
## 1685   1 1661 2.721361e+00
## 1686   1 1662 2.698037e+00
## 1687   1 1663 2.659288e+00
## 1688   1 1664 2.622167e+00
## 1689   1 1665 2.584526e+00
## 1690   1 1666 2.540240e+00
## 1691   1 1667 2.507296e+00
## 1692   1 1668 2.473929e+00
## 1693   1 1669 2.441701e+00
## 1694   1 1670 2.410301e+00
## 1695   1 1671 2.383648e+00
## 1696   1 1672 2.354822e+00
## 1697   1 1673 2.332873e+00
## 1698   1 1674 2.303162e+00
## 1699   1 1675 2.286345e+00
## 1700   1 1676 2.261360e+00
## 1701   1 1677 2.242491e+00
## 1702   1 1678 2.223310e+00
## 1703   1 1679 2.201859e+00
## 1704   1 1680 2.187147e+00
## 1705   1 1681 2.345388e+00
## 1706   1 1682 2.551050e+00
## 1707   1 1683 2.675453e+00
## 1708   1 1684 2.719022e+00
## 1709   1 1685 2.728120e+00
## 1710   1 1686 2.704052e+00
## 1711   1 1687 2.668356e+00
## 1712   1 1688 2.625692e+00
## 1713   1 1689 2.586872e+00
## 1714   1 1690 2.552819e+00
## 1715   1 1691 2.515263e+00
## 1716   1 1692 2.479870e+00
## 1717   1 1693 2.443090e+00
## 1718   1 1694 2.420377e+00
## 1719   1 1695 2.388160e+00
## 1720   1 1696 2.359634e+00
## 1721   1 1697 2.337087e+00
## 1722   1 1698 2.310540e+00
## 1723   1 1699 2.288895e+00
## 1724   1 1700 2.265418e+00
## 1725   1 1701 2.247845e+00
## 1726   1 1702 2.224301e+00
## 1727   1 1703 2.210136e+00
## 1728   1 1704 2.190096e+00
## 1729   1 1705 2.354124e+00
## 1730   1 1706 2.561368e+00
## 1731   1 1707 2.684474e+00
## 1732   1 1708 2.730923e+00
## 1733   1 1709 2.732958e+00
## 1734   1 1710 2.703046e+00
## 1735   1 1711 2.673275e+00
## 1736   1 1712 2.630243e+00
## 1737   1 1713 2.594944e+00
## 1738   1 1714 2.552742e+00
## 1739   1 1715 2.517434e+00
## 1740   1 1716 2.484920e+00
## 1741   1 1717 2.452840e+00
## 1742   1 1718 2.423070e+00
## 1743   1 1719 2.399375e+00
## 1744   1 1720 2.366173e+00
## 1745   1 1721 2.338549e+00
## 1746   1 1722 2.317185e+00
## 1747   1 1723 2.294186e+00
## 1748   1 1724 2.273322e+00
## 1749   1 1725 2.249574e+00
## 1750   1 1726 2.229525e+00
## 1751   1 1727 2.215286e+00
## 1752   1 1728 2.193820e+00
## 1753   1 1729 2.355593e+00
## 1754   1 1730 2.564402e+00
## 1755   1 1731 2.689489e+00
## 1756   1 1732 2.733230e+00
## 1757   1 1733 2.737010e+00
## 1758   1 1734 2.709183e+00
## 1759   1 1735 2.679615e+00
## 1760   1 1736 2.638644e+00
## 1761   1 1737 2.594149e+00
## 1762   1 1738 2.558554e+00
## 1763   1 1739 2.533211e+00
## 1764   1 1740 2.491582e+00
## 1765   1 1741 2.459138e+00
## 1766   1 1742 2.423214e+00
## 1767   1 1743 2.401739e+00
## 1768   1 1744 2.373237e+00
## 1769   1 1745 2.345601e+00
## 1770   1 1746 2.322111e+00
## 1771   1 1747 2.299866e+00
## 1772   1 1748 2.280972e+00
## 1773   1 1749 2.255107e+00
## 1774   1 1750 2.238953e+00
## 1775   1 1751 2.216483e+00
## 1776   1 1752 2.199710e+00
## 1777   1 1753 2.362401e+00
## 1778   1 1754 2.569157e+00
## 1779   1 1755 2.694457e+00
## 1780   1 1756 2.734775e+00
## 1781   1 1757 2.741326e+00
## 1782   1 1758 2.713532e+00
## 1783   1 1759 2.681750e+00
## 1784   1 1760 2.645294e+00
## 1785   1 1761 2.609647e+00
## 1786   1 1762 2.568725e+00
## 1787   1 1763 2.528249e+00
## 1788   1 1764 2.492068e+00
## 1789   1 1765 2.467268e+00
## 1790   1 1766 2.436969e+00
## 1791   1 1767 2.403251e+00
## 1792   1 1768 2.376931e+00
## 1793   1 1769 2.352801e+00
## 1794   1 1770 2.323838e+00
## 1795   1 1771 2.305015e+00
## 1796   1 1772 2.282028e+00
## 1797   1 1773 2.261560e+00
## 1798   1 1774 2.240405e+00
## 1799   1 1775 2.221707e+00
## 1800   1 1776 2.203967e+00
## 1801   1 1777 2.361502e+00
## 1802   1 1778 2.573305e+00
## 1803   1 1779 2.693209e+00
## 1804   1 1780 2.744133e+00
## 1805   1 1781 2.743045e+00
## 1806   1 1782 2.725973e+00
## 1807   1 1783 2.687575e+00
## 1808   1 1784 2.647104e+00
## 1809   1 1785 2.608458e+00
## 1810   1 1786 2.569308e+00
## 1811   1 1787 2.531398e+00
## 1812   1 1788 2.500907e+00
## 1813   1 1789 2.465277e+00
## 1814   1 1790 2.436636e+00
## 1815   1 1791 2.410780e+00
## 1816   1 1792 2.377399e+00
## 1817   1 1793 2.352855e+00
## 1818   1 1794 2.330739e+00
## 1819   1 1795 2.304329e+00
## 1820   1 1796 2.281449e+00
## 1821   1 1797 2.262834e+00
## 1822   1 1798 2.248741e+00
## 1823   1 1799 2.225483e+00
## 1824   1 1800 2.209062e+00
## 1825   1 1801 2.370379e+00
## 1826   1 1802 2.581828e+00
## 1827   1 1803 2.702973e+00
## 1828   1 1804 2.745621e+00
## 1829   1 1805 2.745495e+00
## 1830   1 1806 2.721338e+00
## 1831   1 1807 2.686984e+00
## 1832   1 1808 2.645296e+00
## 1833   1 1809 2.605624e+00
## 1834   1 1810 2.572214e+00
## 1835   1 1811 2.538363e+00
## 1836   1 1812 2.505986e+00
## 1837   1 1813 2.474000e+00
## 1838   1 1814 2.443827e+00
## 1839   1 1815 2.406027e+00
## 1840   1 1816 2.385919e+00
## 1841   1 1817 2.358617e+00
## 1842   1 1818 2.329842e+00
## 1843   1 1819 2.308480e+00
## 1844   1 1820 2.290389e+00
## 1845   1 1821 2.270442e+00
## 1846   1 1822 2.250507e+00
## 1847   1 1823 2.231926e+00
## 1848   1 1824 2.209826e+00
## 1849   1 1825 2.369099e+00
## 1850   1 1826 2.575387e+00
## 1851   1 1827 2.702410e+00
## 1852   1 1828 2.752989e+00
## 1853   1 1829 2.749717e+00
## 1854   1 1830 2.723702e+00
## 1855   1 1831 2.691333e+00
## 1856   1 1832 2.653499e+00
## 1857   1 1833 2.614611e+00
## 1858   1 1834 2.576509e+00
## 1859   1 1835 2.541832e+00
## 1860   1 1836 2.500981e+00
## 1861   1 1837 2.469963e+00
## 1862   1 1838 2.444761e+00
## 1863   1 1839 2.414137e+00
## 1864   1 1840 2.384866e+00
## 1865   1 1841 2.362192e+00
## 1866   1 1842 2.340446e+00
## 1867   1 1843 2.314161e+00
## 1868   1 1844 2.294514e+00
## 1869   1 1845 2.270108e+00
## 1870   1 1846 2.253188e+00
## 1871   1 1847 2.229587e+00
## 1872   1 1848 2.214749e+00
## 1873   1 1849 2.374123e+00
## 1874   1 1850 2.587341e+00
## 1875   1 1851 2.705803e+00
## 1876   1 1852 2.759206e+00
## 1877   1 1853 2.756072e+00
## 1878   1 1854 2.735083e+00
## 1879   1 1855 2.692749e+00
## 1880   1 1856 2.660508e+00
## 1881   1 1857 2.621873e+00
## 1882   1 1858 2.576486e+00
## 1883   1 1859 2.543617e+00
## 1884   1 1860 2.509849e+00
## 1885   1 1861 2.479691e+00
## 1886   1 1862 2.446487e+00
## 1887   1 1863 2.417222e+00
## 1888   1 1864 2.386806e+00
## 1889   1 1865 2.367646e+00
## 1890   1 1866 2.340574e+00
## 1891   1 1867 2.314908e+00
## 1892   1 1868 2.296882e+00
## 1893   1 1869 2.272209e+00
## 1894   1 1870 2.251258e+00
## 1895   1 1871 2.236388e+00
## 1896   1 1872 2.222019e+00
## 1897   1 1872 2.247891e+00
## 1898   1 1873 2.412002e+00
## 1899   1 1874 2.622925e+00
## 1900   1 1875 2.742223e+00
## 1901   1 1876 2.791531e+00
## 1902   1 1877 2.780628e+00
## 1903   1 1878 2.765416e+00
## 1904   1 1879 2.727950e+00
## 1905   1 1880 2.689595e+00
## 1906   1 1881 2.652694e+00
## 1907   1 1882 2.612884e+00
## 1908   1 1883 2.577166e+00
## 1909   1 1884 2.542274e+00
## 1910   1 1885 2.512258e+00
## 1911   1 1886 2.481821e+00
## 1912   1 1887 2.447337e+00
## 1913   1 1888 2.426710e+00
## 1914   1 1889 2.394809e+00
## 1915   1 1890 2.375590e+00
## 1916   1 1891 2.348507e+00
## 1917   1 1892 2.328713e+00
## 1918   1 1893 2.312489e+00
## 1919   1 1894 2.288528e+00
## 1920   1 1895 2.266718e+00
## 1921   1 1896 2.251042e+00
## 1922   1 1896 2.255314e+00
## 1923   1 1897 2.227669e+00
## 1924   1 1898 2.217960e+00
## 1925   1 1899 2.199073e+00
## 1926   1 1900 2.185804e+00
## 1927   1 1901 2.168638e+00
## 1928   1 1902 2.155100e+00
## 1929   1 1903 2.144137e+00
## 1930   1 1904 2.126000e+00
## 1931   1 1905 2.118051e+00
## 1932   1 1906 2.106173e+00
## 1933   1 1907 2.092063e+00
## 1934   1 1908 2.083740e+00
## 1935   1 1909 2.074930e+00
## 1936   1 1910 2.060890e+00
## 1937   1 1911 2.050091e+00
## 1938   1 1911 2.046457e+00
## 1939   1 1912 2.217976e+00
## 1940   1 1913 2.430474e+00
## 1941   1 1914 2.561058e+00
## 1942   1 1915 2.614665e+00
## 1943   1 1916 2.616254e+00
## 1944   1 1917 2.594513e+00
## 1945   1 1918 2.571324e+00
## 1946   1 1919 2.530649e+00
## 1947   1 1920 2.497779e+00
## 1948   1 1920 2.496380e+00
## 1949   1 1921 2.643279e+00
## 1950   1 1922 2.839238e+00
## 1951   1 1923 2.938341e+00
## 1952   1 1924 2.976208e+00
## 1953   1 1925 2.961373e+00
## 1954   1 1926 2.926382e+00
## 1955   1 1927 2.873206e+00
## 1956   1 1928 2.830881e+00
## 1957   1 1929 2.782302e+00
## 1958   1 1930 2.730554e+00
## 1959   1 1931 2.687674e+00
## 1960   1 1932 2.648949e+00
## 1961   1 1933 2.607704e+00
## 1962   1 1934 2.569660e+00
## 1963   1 1935 2.532382e+00
## 1964   1 1936 2.502315e+00
## 1965   1 1937 2.468922e+00
## 1966   1 1938 2.439527e+00
## 1967   1 1939 2.412584e+00
## 1968   1 1940 2.388018e+00
## 1969   1 1941 2.360602e+00
## 1970   1 1942 2.341344e+00
## 1971   1 1943 2.314130e+00
## 1972   1 1944 2.294780e+00
## 1973   1 1945 2.453517e+00
## 1974   1 1946 2.661259e+00
## 1975   1 1947 2.779850e+00
## 1976   1 1948 2.823331e+00
## 1977   1 1949 2.822528e+00
## 1978   1 1950 2.795192e+00
## 1979   1 1951 2.757624e+00
## 1980   1 1952 2.720522e+00
## 1981   1 1953 2.677428e+00
## 1982   1 1954 2.639867e+00
## 1983   1 1955 2.601803e+00
## 1984   1 1956 2.566924e+00
## 1985   1 1957 2.530014e+00
## 1986   1 1958 2.500248e+00
## 1987   1 1959 2.473125e+00
## 1988   1 1960 2.438712e+00
## 1989   1 1961 2.412919e+00
## 1990   1 1962 2.389116e+00
## 1991   1 1963 2.365699e+00
## 1992   1 1964 2.342272e+00
## 1993   1 1965 2.319300e+00
## 1994   1 1966 2.300285e+00
## 1995   1 1967 2.279643e+00
## 1996   1 1968 2.262975e+00
## 1997   1 1969 2.422146e+00
## 1998   1 1970 2.633162e+00
## 1999   1 1971 2.756201e+00
## 2000   1 1972 2.800885e+00
## 2001   1 1973 2.802278e+00
## 2002   1 1974 2.777432e+00
## 2003   1 1975 2.742629e+00
## 2004   1 1976 2.706027e+00
## 2005   1 1977 2.664157e+00
## 2006   1 1978 2.622751e+00
## 2007   1 1979 2.590410e+00
## 2008   1 1980 2.551103e+00
## 2009   1 1981 2.520281e+00
## 2010   1 1982 2.487749e+00
## 2011   1 1983 2.459960e+00
## 2012   1 1984 2.432619e+00
## 2013   1 1985 2.405841e+00
## 2014   1 1986 2.378987e+00
## 2015   1 1987 2.355631e+00
## 2016   1 1988 2.337024e+00
## 2017   1 1989 2.315244e+00
## 2018   1 1990 2.293729e+00
## 2019   1 1991 2.272363e+00
## 2020   1 1992 2.260674e+00
## 2021   1 1993 2.415895e+00
## 2022   1 1994 2.627031e+00
## 2023   1 1995 2.745473e+00
## 2024   1 1996 2.801819e+00
## 2025   1 1997 2.794314e+00
## 2026   1 1998 2.771018e+00
## 2027   1 1999 2.738999e+00
## 2028   1 2000 2.701921e+00
## 2029   1 2001 2.658556e+00
## 2030   1 2002 2.624450e+00
## 2031   1 2003 2.581127e+00
## 2032   1 2004 2.554150e+00
## 2033   1 2005 2.516429e+00
## 2034   1 2006 2.486050e+00
## 2035   1 2007 2.456266e+00
## 2036   1 2008 2.434726e+00
## 2037   1 2009 2.407436e+00
## 2038   1 2010 2.378901e+00
## 2039   1 2011 2.358957e+00
## 2040   1 2012 2.331872e+00
## 2041   1 2013 2.314896e+00
## 2042   1 2014 2.294312e+00
## 2043   1 2015 2.275049e+00
## 2044   1 2016 2.262212e+00
## 2045   1 2017 2.413461e+00
## 2046   1 2018 2.629613e+00
## 2047   1 2019 2.750587e+00
## 2048   1 2020 2.791932e+00
## 2049   1 2021 2.796169e+00
## 2050   1 2022 2.767441e+00
## 2051   1 2023 2.740002e+00
## 2052   1 2024 2.693974e+00
## 2053   1 2025 2.659382e+00
## 2054   1 2026 2.625386e+00
## 2055   1 2027 2.584642e+00
## 2056   1 2028 2.549238e+00
## 2057   1 2029 2.516991e+00
## 2058   1 2030 2.488112e+00
## 2059   1 2031 2.462780e+00
## 2060   1 2032 2.426531e+00
## 2061   1 2033 2.403137e+00
## 2062   1 2034 2.376066e+00
## 2063   1 2035 2.359073e+00
## 2064   1 2036 2.333251e+00
## 2065   1 2037 2.314349e+00
## 2066   1 2038 2.289352e+00
## 2067   1 2039 2.272213e+00
## 2068   1 2040 2.254116e+00
## 2069   1 2041 2.419803e+00
## 2070   1 2042 2.625736e+00
## 2071   1 2043 2.752804e+00
## 2072   1 2044 2.790301e+00
## 2073   1 2045 2.792348e+00
## 2074   1 2046 2.774180e+00
## 2075   1 2047 2.738293e+00
## 2076   1 2048 2.698339e+00
## 2077   1 2049 2.661697e+00
## 2078   1 2050 2.623429e+00
## 2079   1 2051 2.579726e+00
## 2080   1 2052 2.549820e+00
## 2081   1 2053 2.518968e+00
## 2082   1 2054 2.484903e+00
## 2083   1 2055 2.462421e+00
## 2084   1 2056 2.427924e+00
## 2085   1 2057 2.403879e+00
## 2086   1 2058 2.375940e+00
## 2087   1 2059 2.352813e+00
## 2088   1 2060 2.337195e+00
## 2089   1 2061 2.309888e+00
## 2090   1 2062 2.291705e+00
## 2091   1 2063 2.273375e+00
## 2092   1 2064 2.254408e+00
## 2093   1 2065 2.416398e+00
## 2094   1 2066 2.627097e+00
## 2095   1 2067 2.744382e+00
## 2096   1 2068 2.792680e+00
## 2097   1 2069 2.791555e+00
## 2098   1 2070 2.770734e+00
## 2099   1 2071 2.735221e+00
## 2100   1 2072 2.699667e+00
## 2101   1 2073 2.661177e+00
## 2102   1 2074 2.621748e+00
## 2103   1 2075 2.580620e+00
## 2104   1 2076 2.548901e+00
## 2105   1 2077 2.516239e+00
## 2106   1 2078 2.486166e+00
## 2107   1 2079 2.455699e+00
## 2108   1 2080 2.432748e+00
## 2109   1 2081 2.406911e+00
## 2110   1 2082 2.380750e+00
## 2111   1 2083 2.356437e+00
## 2112   1 2084 2.333708e+00
## 2113   1 2085 2.314060e+00
## 2114   1 2086 2.291498e+00
## 2115   1 2087 2.274017e+00
## 2116   1 2088 2.252209e+00
## 2117   1 2089 2.417441e+00
## 2118   1 2090 2.619273e+00
## 2119   1 2091 2.752757e+00
## 2120   1 2092 2.792204e+00
## 2121   1 2093 2.786243e+00
## 2122   1 2094 2.769168e+00
## 2123   1 2095 2.734452e+00
## 2124   1 2096 2.699911e+00
## 2125   1 2097 2.658320e+00
## 2126   1 2098 2.619176e+00
## 2127   1 2099 2.581758e+00
## 2128   1 2100 2.546916e+00
## 2129   1 2101 2.517863e+00
## 2130   1 2102 2.481652e+00
## 2131   1 2103 2.456350e+00
## 2132   1 2104 2.426149e+00
## 2133   1 2105 2.403941e+00
## 2134   1 2106 2.378149e+00
## 2135   1 2107 2.354809e+00
## 2136   1 2108 2.330912e+00
## 2137   1 2109 2.314764e+00
## 2138   1 2110 2.293233e+00
## 2139   1 2111 2.274162e+00
## 2140   1 2112 2.255601e+00
## 2141   1 2113 2.416938e+00
## 2142   1 2114 2.626354e+00
## 2143   1 2115 2.749310e+00
## 2144   1 2116 2.788053e+00
## 2145   1 2117 2.793506e+00
## 2146   1 2118 2.769230e+00
## 2147   1 2119 2.736995e+00
## 2148   1 2120 2.695066e+00
## 2149   1 2121 2.659689e+00
## 2150   1 2122 2.620517e+00
## 2151   1 2123 2.590104e+00
## 2152   1 2124 2.549299e+00
## 2153   1 2125 2.515644e+00
## 2154   1 2126 2.486469e+00
## 2155   1 2127 2.452551e+00
## 2156   1 2128 2.428905e+00
## 2157   1 2129 2.398458e+00
## 2158   1 2130 2.374185e+00
## 2159   1 2131 2.352426e+00
## 2160   1 2132 2.335113e+00
## 2161   1 2133 2.316093e+00
## 2162   1 2134 2.293701e+00
## 2163   1 2135 2.272624e+00
## 2164   1 2136 2.254102e+00
## 2165   1 2137 2.416915e+00
## 2166   1 2138 2.622042e+00
## 2167   1 2139 2.746058e+00
## 2168   1 2140 2.794726e+00
## 2169   1 2141 2.794768e+00
## 2170   1 2142 2.764403e+00
## 2171   1 2143 2.730390e+00
## 2172   1 2144 2.694346e+00
## 2173   1 2145 2.655373e+00
## 2174   1 2146 2.616882e+00
## 2175   1 2147 2.585414e+00
## 2176   1 2148 2.552542e+00
## 2177   1 2149 2.517240e+00
## 2178   1 2150 2.482653e+00
## 2179   1 2151 2.454527e+00
## 2180   1 2152 2.427652e+00
## 2181   1 2153 2.406195e+00
## 2182   1 2154 2.374792e+00
## 2183   1 2155 2.353200e+00
## 2184   1 2156 2.330023e+00
## 2185   1 2157 2.315110e+00
## 2186   1 2158 2.294316e+00
## 2187   1 2159 2.270733e+00
## 2188   1 2160 2.253803e+00
## 2189   1 2161 2.416400e+00
## 2190   1 2162 2.624785e+00
## 2191   1 2163 2.747693e+00
## 2192   1 2164 2.794016e+00
## 2193   1 2165 2.794391e+00
## 2194   1 2166 2.772710e+00
## 2195   1 2167 2.733990e+00
## 2196   1 2168 2.692420e+00
## 2197   1 2169 2.654365e+00
## 2198   1 2170 2.614869e+00
## 2199   1 2171 2.579522e+00
## 2200   1 2172 2.548572e+00
## 2201   1 2173 2.513607e+00
## 2202   1 2174 2.482444e+00
## 2203   1 2175 2.454014e+00
## 2204   1 2176 2.428486e+00
## 2205   1 2177 2.400428e+00
## 2206   1 2178 2.377403e+00
## 2207   1 2179 2.352939e+00
## 2208   1 2180 2.331665e+00
## 2209   1 2181 2.315372e+00
## 2210   1 2182 2.295203e+00
## 2211   1 2183 2.270836e+00
## 2212   1 2184 2.250852e+00
## 2213   1 2184 2.251040e+00
## 2214   1 2185 2.410091e+00
## 2215   1 2186 2.621208e+00
## 2216   1 2187 2.742593e+00
## 2217   1 2188 2.787021e+00
## 2218   1 2189 2.789053e+00
## 2219   1 2190 2.766088e+00
## 2220   1 2191 2.731308e+00
## 2221   1 2192 2.695061e+00
## 2222   1 2193 2.652326e+00
## 2223   1 2194 2.614471e+00
## 2224   1 2195 2.582014e+00
## 2225   1 2196 2.540220e+00
## 2226   1 2197 2.516582e+00
## 2227   1 2198 2.478740e+00
## 2228   1 2199 2.448700e+00
## 2229   1 2200 2.423342e+00
## 2230   1 2201 2.402626e+00
## 2231   1 2202 2.372676e+00
## 2232   1 2203 2.347052e+00
## 2233   1 2204 2.326294e+00
## 2234   1 2205 2.307186e+00
## 2235   1 2206 2.286325e+00
## 2236   1 2207 2.265361e+00
## 2237   1 2208 2.250402e+00
## 2238   1 2208 2.246887e+00
## 2239   1 2209 2.230928e+00
## 2240   1 2210 2.215736e+00
## 2241   1 2211 2.200170e+00
## 2242   1 2212 2.185586e+00
## 2243   1 2213 2.174472e+00
## 2244   1 2214 2.154733e+00
## 2245   1 2215 2.142826e+00
## 2246   1 2216 2.125625e+00
## 2247   1 2217 2.118216e+00
## 2248   1 2218 2.105104e+00
## 2249   1 2219 2.095365e+00
## 2250   1 2220 2.084711e+00
## 2251   1 2221 2.072398e+00
## 2252   1 2222 2.060597e+00
## 2253   1 2223 2.051774e+00
## 2254   1 2224 2.036373e+00
## 2255   1 2225 2.028656e+00
## 2256   1 2226 2.018095e+00
## 2257   1 2226 2.020674e+00
## 2258   1 2227 2.095718e+00
## 2259   1 2228 2.199965e+00
## 2260   1 2229 2.259865e+00
## 2261   1 2230 2.283721e+00
## 2262   1 2231 2.282553e+00
## 2263   1 2232 2.271272e+00
## 2264   1 2232 2.269753e+00
## 2265   1 2233 2.427526e+00
## 2266   1 2234 2.626955e+00
## 2267   1 2235 2.749369e+00
## 2268   1 2236 2.787129e+00
## 2269   1 2237 2.788183e+00
## 2270   1 2238 2.751950e+00
## 2271   1 2239 2.714962e+00
## 2272   1 2240 2.669026e+00
## 2273   1 2241 2.630281e+00
```

```
## 2274   1 2242 2.586781e+00
## 2275   1 2243 2.545995e+00
## 2276   1 2244 2.506384e+00
## 2277   1 2245 2.468083e+00
## 2278   1 2246 2.436650e+00
## 2279   1 2247 2.406789e+00
## 2280   1 2248 2.375004e+00
## 2281   1 2249 2.345267e+00
## 2282   1 2250 2.319590e+00
## 2283   1 2251 2.292372e+00
## 2284   1 2252 2.275666e+00
## 2285   1 2253 2.250676e+00
## 2286   1 2254 2.225447e+00
## 2287   1 2255 2.206072e+00
## 2288   1 2256 2.186897e+00
## 2289   1 2257 2.345088e+00
## 2290   1 2258 2.554893e+00
## 2291   1 2259 2.677357e+00
## 2292   1 2260 2.723406e+00
## 2293   1 2261 2.724141e+00
## 2294   1 2262 2.695509e+00
## 2295   1 2263 2.667238e+00
## 2296   1 2264 2.626613e+00
## 2297   1 2265 2.585562e+00
## 2298   1 2266 2.545066e+00
## 2299   1 2267 2.513691e+00
## 2300   1 2268 2.472987e+00
## 2301   1 2269 2.441670e+00
## 2302   1 2270 2.410391e+00
## 2303   1 2271 2.387437e+00
## 2304   1 2272 2.355603e+00
## 2305   1 2273 2.326511e+00
## 2306   1 2274 2.306230e+00
## 2307   1 2275 2.279527e+00
## 2308   1 2276 2.255159e+00
## 2309   1 2277 2.234235e+00
## 2310   1 2278 2.217896e+00
## 2311   1 2279 2.197737e+00
## 2312   1 2280 2.182927e+00
## 2313   1 2281 2.341788e+00
## 2314   1 2282 2.547613e+00
## 2315   1 2283 2.677155e+00
## 2316   1 2284 2.718324e+00
## 2317   1 2285 2.716325e+00
## 2318   1 2286 2.694061e+00
## 2319   1 2287 2.659932e+00
## 2320   1 2288 2.630560e+00
## 2321   1 2289 2.581975e+00
## 2322   1 2290 2.543928e+00
## 2323   1 2291 2.512267e+00
## 2324   1 2292 2.476687e+00
## 2325   1 2293 2.440305e+00
## 2326   1 2294 2.414805e+00
## 2327   1 2295 2.384700e+00
## 2328   1 2296 2.357543e+00
## 2329   1 2297 2.329321e+00
## 2330   1 2298 2.304315e+00
## 2331   1 2299 2.284933e+00
## 2332   1 2300 2.259523e+00
## 2333   1 2301 2.238653e+00
## 2334   1 2302 2.223727e+00
## 2335   1 2303 2.201782e+00
## 2336   1 2304 2.185244e+00
## 2337   1 2305 2.347802e+00
## 2338   1 2306 2.551667e+00
## 2339   1 2307 2.680051e+00
## 2340   1 2308 2.725883e+00
## 2341   1 2309 2.721505e+00
## 2342   1 2310 2.702605e+00
## 2343   1 2311 2.662036e+00
## 2344   1 2312 2.633060e+00
## 2345   1 2313 2.590495e+00
## 2346   1 2314 2.550424e+00
## 2347   1 2315 2.519918e+00
## 2348   1 2316 2.479764e+00
## 2349   1 2317 2.448674e+00
## 2350   1 2318 2.418342e+00
## 2351   1 2319 2.390777e+00
## 2352   1 2320 2.364019e+00
## 2353   1 2321 2.338855e+00
## 2354   1 2322 2.312918e+00
## 2355   1 2323 2.287803e+00
## 2356   1 2324 2.270285e+00
## 2357   1 2325 2.244861e+00
## 2358   1 2326 2.226598e+00
## 2359   1 2327 2.206898e+00
## 2360   1 2328 2.188265e+00
## 2361   1 2329 2.352076e+00
## 2362   1 2330 2.559115e+00
## 2363   1 2331 2.678297e+00
## 2364   1 2332 2.732316e+00
## 2365   1 2333 2.734169e+00
## 2366   1 2334 2.700221e+00
## 2367   1 2335 2.673746e+00
## 2368   1 2336 2.635091e+00
## 2369   1 2337 2.592406e+00
## 2370   1 2338 2.557699e+00
## 2371   1 2339 2.516234e+00
## 2372   1 2340 2.486625e+00
## 2373   1 2341 2.456022e+00
## 2374   1 2342 2.426458e+00
## 2375   1 2343 2.389743e+00
## 2376   1 2344 2.365582e+00
## 2377   1 2345 2.338435e+00
## 2378   1 2346 2.318742e+00
## 2379   1 2347 2.298737e+00
## 2380   1 2348 2.271820e+00
## 2381   1 2349 2.252637e+00
## 2382   1 2350 2.231430e+00
## 2383   1 2351 2.217050e+00
## 2384   1 2352 2.197946e+00
## 2385   1 2353 2.354104e+00
## 2386   1 2354 2.564649e+00
## 2387   1 2355 2.685738e+00
## 2388   1 2356 2.732797e+00
## 2389   1 2357 2.733315e+00
## 2390   1 2358 2.712930e+00
## 2391   1 2359 2.675401e+00
## 2392   1 2360 2.637815e+00
## 2393   1 2361 2.599888e+00
## 2394   1 2362 2.560403e+00
## 2395   1 2363 2.525989e+00
## 2396   1 2364 2.499324e+00
## 2397   1 2365 2.455338e+00
## 2398   1 2366 2.430671e+00
## 2399   1 2367 2.397587e+00
## 2400   1 2368 2.372086e+00
## 2401   1 2369 2.347678e+00
## 2402   1 2370 2.319666e+00
## 2403   1 2371 2.299066e+00
## 2404   1 2372 2.277419e+00
## 2405   1 2373 2.257372e+00
## 2406   1 2374 2.236594e+00
## 2407   1 2375 2.221422e+00
## 2408   1 2376 2.199436e+00
## 2409   1 2377 2.359072e+00
## 2410   1 2378 2.570164e+00
## 2411   1 2379 2.693369e+00
## 2412   1 2380 2.738168e+00
## 2413   1 2381 2.736297e+00
## 2414   1 2382 2.711585e+00
## 2415   1 2383 2.684138e+00
## 2416   1 2384 2.645733e+00
## 2417   1 2385 2.601251e+00
## 2418   1 2386 2.562674e+00
## 2419   1 2387 2.531540e+00
## 2420   1 2388 2.494790e+00
## 2421   1 2389 2.463932e+00
## 2422   1 2390 2.432106e+00
## 2423   1 2391 2.399109e+00
## 2424   1 2392 2.374887e+00
## 2425   1 2393 2.353522e+00
## 2426   1 2394 2.327749e+00
## 2427   1 2395 2.303133e+00
## 2428   1 2396 2.283477e+00
## 2429   1 2397 2.260693e+00
## 2430   1 2398 2.238598e+00
## 2431   1 2399 2.224460e+00
## 2432   1 2400 2.199467e+00
## 2433   1 2401 2.364127e+00
## 2434   1 2402 2.575702e+00
## 2435   1 2403 2.696412e+00
## 2436   1 2404 2.744813e+00
## 2437   1 2405 2.745792e+00
## 2438   1 2406 2.718218e+00
## 2439   1 2407 2.689985e+00
## 2440   1 2408 2.646590e+00
## 2441   1 2409 2.609297e+00
## 2442   1 2410 2.572176e+00
## 2443   1 2411 2.532678e+00
## 2444   1 2412 2.500122e+00
## 2445   1 2413 2.471904e+00
## 2446   1 2414 2.436274e+00
## 2447   1 2415 2.411397e+00
## 2448   1 2416 2.378007e+00
## 2449   1 2417 2.348370e+00
## 2450   1 2418 2.327928e+00
## 2451   1 2419 2.307582e+00
## 2452   1 2420 2.284597e+00
## 2453   1 2421 2.269818e+00
## 2454   1 2422 2.242998e+00
## 2455   1 2423 2.224496e+00
## 2456   1 2424 2.205696e+00
## 2457   1 2425 2.368598e+00
## 2458   1 2426 2.579181e+00
## 2459   1 2427 2.697985e+00
## 2460   1 2428 2.748245e+00
## 2461   1 2429 2.752119e+00
## 2462   1 2430 2.722864e+00
## 2463   1 2431 2.694052e+00
## 2464   1 2432 2.648414e+00
## 2465   1 2433 2.616116e+00
## 2466   1 2434 2.573205e+00
## 2467   1 2435 2.539294e+00
## 2468   1 2436 2.501380e+00
## 2469   1 2437 2.470825e+00
## 2470   1 2438 2.439338e+00
## 2471   1 2439 2.412996e+00
## 2472   1 2440 2.389625e+00
## 2473   1 2441 2.358084e+00
## 2474   1 2442 2.330755e+00
## 2475   1 2443 2.313659e+00
## 2476   1 2444 2.291066e+00
## 2477   1 2445 2.268836e+00
## 2478   1 2446 2.250589e+00
## 2479   1 2447 2.228764e+00
## 2480   1 2448 2.211757e+00
## 2481   1 2449 2.373211e+00
## 2482   1 2450 2.585390e+00
## 2483   1 2451 2.703627e+00
## 2484   1 2452 2.750902e+00
## 2485   1 2453 2.746549e+00
## 2486   1 2454 2.727907e+00
## 2487   1 2455 2.695027e+00
## 2488   1 2456 2.656716e+00
## 2489   1 2457 2.620953e+00
## 2490   1 2458 2.575608e+00
## 2491   1 2459 2.543840e+00
## 2492   1 2460 2.508466e+00
## 2493   1 2461 2.475128e+00
## 2494   1 2462 2.444700e+00
## 2495   1 2463 2.414295e+00
## 2496   1 2464 2.389151e+00
## 2497   1 2465 2.361194e+00
## 2498   1 2466 2.336843e+00
## 2499   1 2467 2.317421e+00
## 2500   1 2468 2.293096e+00
## 2501   1 2469 2.270181e+00
## 2502   1 2470 2.250024e+00
## 2503   1 2471 2.238851e+00
## 2504   1 2472 2.214345e+00
## 2505   1 2473 2.373770e+00
## 2506   1 2474 2.583024e+00
## 2507   1 2475 2.707068e+00
## 2508   1 2476 2.749529e+00
## 2509   1 2477 2.757656e+00
## 2510   1 2478 2.735178e+00
## 2511   1 2479 2.701675e+00
## 2512   1 2480 2.658919e+00
## 2513   1 2481 2.620269e+00
## 2514   1 2482 2.582125e+00
## 2515   1 2483 2.541825e+00
## 2516   1 2484 2.510775e+00
## 2517   1 2485 2.476452e+00
## 2518   1 2486 2.446184e+00
## 2519   1 2487 2.420094e+00
## 2520   1 2488 2.391891e+00
## 2521   1 2489 2.364280e+00
## 2522   1 2490 2.338677e+00
## 2523   1 2491 2.311779e+00
## 2524   1 2492 2.299583e+00
## 2525   1 2493 2.276988e+00
## 2526   1 2494 2.254634e+00
## 2527   1 2495 2.235894e+00
## 2528   1 2496 2.217919e+00
## 2529   1 2496 2.252354e+00
## 2530   1 2497 2.411609e+00
## 2531   1 2498 2.615602e+00
## 2532   1 2499 2.740824e+00
## 2533   1 2500 2.791887e+00
## 2534   1 2501 2.790085e+00
## 2535   1 2502 2.763661e+00
## 2536   1 2503 2.734382e+00
## 2537   1 2504 2.690529e+00
## 2538   1 2505 2.652348e+00
## 2539   1 2506 2.610267e+00
## 2540   1 2507 2.579803e+00
## 2541   1 2508 2.546452e+00
## 2542   1 2509 2.506899e+00
## 2543   1 2510 2.483197e+00
## 2544   1 2511 2.448929e+00
## 2545   1 2512 2.424138e+00
## 2546   1 2513 2.397017e+00
## 2547   1 2514 2.366700e+00
## 2548   1 2515 2.346866e+00
## 2549   1 2516 2.328474e+00
## 2550   1 2517 2.307077e+00
## 2551   1 2518 2.282955e+00
## 2552   1 2519 2.266495e+00
## 2553   1 2520 2.249123e+00
## 2554   1 2520 2.250340e+00
## 2555   1 2521 2.233022e+00
## 2556   1 2522 2.211355e+00
## 2557   1 2523 2.199387e+00
## 2558   1 2524 2.183961e+00
## 2559   1 2525 2.169982e+00
## 2560   1 2526 2.160545e+00
## 2561   1 2527 2.143388e+00
## 2562   1 2528 2.130276e+00
## 2563   1 2529 2.119897e+00
## 2564   1 2530 2.104569e+00
## 2565   1 2531 2.092613e+00
## 2566   1 2532 2.079533e+00
## 2567   1 2533 2.072438e+00
## 2568   1 2534 2.057838e+00
## 2569   1 2535 2.050023e+00
## 2570   1 2536 2.038917e+00
## 2571   1 2537 2.030384e+00
## 2572   1 2538 2.017867e+00
## 2573   1 2538 2.019515e+00
## 2574   1 2539 2.188415e+00
## 2575   1 2540 2.403317e+00
## 2576   1 2541 2.534248e+00
## 2577   1 2542 2.583006e+00
## 2578   1 2543 2.589413e+00
## 2579   1 2544 2.574380e+00
## 2580   1 2544 2.574933e+00
## 2581   1 2545 2.719981e+00
## 2582   1 2546 2.915556e+00
## 2583   1 2547 3.011676e+00
## 2584   1 2548 3.041156e+00
## 2585   1 2549 3.023449e+00
## 2586   1 2550 2.981660e+00
## 2587   1 2551 2.933387e+00
## 2588   1 2552 2.887735e+00
## 2589   1 2553 2.834357e+00
## 2590   1 2554 2.775511e+00
## 2591   1 2555 2.724949e+00
## 2592   1 2556 2.683972e+00
## 2593   1 2557 2.640080e+00
## 2594   1 2558 2.604500e+00
## 2595   1 2559 2.559958e+00
## 2596   1 2560 2.527340e+00
## 2597   1 2561 2.495651e+00
## 2598   1 2562 2.464171e+00
## 2599   1 2563 2.438134e+00
## 2600   1 2564 2.409761e+00
## 2601   1 2565 2.377461e+00
## 2602   1 2566 2.356203e+00
## 2603   1 2567 2.333088e+00
## 2604   1 2568 2.305878e+00
## 2605   1 2569 2.465413e+00
## 2606   1 2570 2.669376e+00
## 2607   1 2571 2.787875e+00
## 2608   1 2572 2.836491e+00
## 2609   1 2573 2.826747e+00
## 2610   1 2574 2.807485e+00
## 2611   1 2575 2.772236e+00
## 2612   1 2576 2.730458e+00
## 2613   1 2577 2.687509e+00
## 2614   1 2578 2.652840e+00
## 2615   1 2579 2.607925e+00
## 2616   1 2580 2.573997e+00
## 2617   1 2581 2.538303e+00
## 2618   1 2582 2.506507e+00
## 2619   1 2583 2.474521e+00
## 2620   1 2584 2.451931e+00
## 2621   1 2585 2.417409e+00
## 2622   1 2586 2.396502e+00
## 2623   1 2587 2.372214e+00
## 2624   1 2588 2.348333e+00
## 2625   1 2589 2.324740e+00
## 2626   1 2590 2.307179e+00
## 2627   1 2591 2.284060e+00
## 2628   1 2592 2.268228e+00
## 2629   1 2593 2.429177e+00
## 2630   1 2594 2.634941e+00
## 2631   1 2595 2.757226e+00
## 2632   1 2596 2.809058e+00
## 2633   1 2597 2.800360e+00
## 2634   1 2598 2.778698e+00
## 2635   1 2599 2.742005e+00
## 2636   1 2600 2.702892e+00
## 2637   1 2601 2.668406e+00
## 2638   1 2602 2.632538e+00
## 2639   1 2603 2.590533e+00
## 2640   1 2604 2.556673e+00
## 2641   1 2605 2.523135e+00
## 2642   1 2606 2.489003e+00
## 2643   1 2607 2.462278e+00
## 2644   1 2608 2.433212e+00
## 2645   1 2609 2.407191e+00
## 2646   1 2610 2.385613e+00
## 2647   1 2611 2.363225e+00
## 2648   1 2612 2.343487e+00
## 2649   1 2613 2.320590e+00
## 2650   1 2614 2.297044e+00
## 2651   1 2615 2.280170e+00
## 2652   1 2616 2.256474e+00
## 2653   1 2617 2.418428e+00
## 2654   1 2618 2.632135e+00
## 2655   1 2619 2.749075e+00
## 2656   1 2620 2.793675e+00
## 2657   1 2621 2.795243e+00
## 2658   1 2622 2.778070e+00
## 2659   1 2623 2.742244e+00
## 2660   1 2624 2.702564e+00
## 2661   1 2625 2.660305e+00
## 2662   1 2626 2.619850e+00
## 2663   1 2627 2.586483e+00
## 2664   1 2628 2.554022e+00
## 2665   1 2629 2.522999e+00
## 2666   1 2630 2.489951e+00
## 2667   1 2631 2.462372e+00
## 2668   1 2632 2.436240e+00
## 2669   1 2633 2.408740e+00
## 2670   1 2634 2.383713e+00
## 2671   1 2635 2.359141e+00
## 2672   1 2636 2.342693e+00
## 2673   1 2637 2.318517e+00
## 2674   1 2638 2.294937e+00
## 2675   1 2639 2.282370e+00
## 2676   1 2640 2.254821e+00
## 2677   1 2641 2.414542e+00
## 2678   1 2642 2.624845e+00
## 2679   1 2643 2.751772e+00
## 2680   1 2644 2.793425e+00
## 2681   1 2645 2.800169e+00
## 2682   1 2646 2.771408e+00
## 2683   1 2647 2.736544e+00
## 2684   1 2648 2.699558e+00
## 2685   1 2649 2.660689e+00
## 2686   1 2650 2.623814e+00
## 2687   1 2651 2.584289e+00
## 2688   1 2652 2.555695e+00
## 2689   1 2653 2.516952e+00
## 2690   1 2654 2.483776e+00
## 2691   1 2655 2.460470e+00
## 2692   1 2656 2.432645e+00
## 2693   1 2657 2.403291e+00
## 2694   1 2658 2.385982e+00
## 2695   1 2659 2.359761e+00
## 2696   1 2660 2.336503e+00
## 2697   1 2661 2.314093e+00
## 2698   1 2662 2.292127e+00
## 2699   1 2663 2.276693e+00
## 2700   1 2664 2.254985e+00
## 2701   1 2665 2.416374e+00
## 2702   1 2666 2.628384e+00
## 2703   1 2667 2.751948e+00
## 2704   1 2668 2.793912e+00
## 2705   1 2669 2.799041e+00
## 2706   1 2670 2.770613e+00
## 2707   1 2671 2.738763e+00
## 2708   1 2672 2.696995e+00
## 2709   1 2673 2.662732e+00
## 2710   1 2674 2.623813e+00
## 2711   1 2675 2.587846e+00
## 2712   1 2676 2.546193e+00
## 2713   1 2677 2.521065e+00
## 2714   1 2678 2.482498e+00
## 2715   1 2679 2.459434e+00
## 2716   1 2680 2.433955e+00
## 2717   1 2681 2.404487e+00
## 2718   1 2682 2.376518e+00
## 2719   1 2683 2.354121e+00
## 2720   1 2684 2.329771e+00
## 2721   1 2685 2.313619e+00
## 2722   1 2686 2.293459e+00
## 2723   1 2687 2.272808e+00
## 2724   1 2688 2.258497e+00
## 2725   1 2689 2.421158e+00
## 2726   1 2690 2.627963e+00
## 2727   1 2691 2.743337e+00
## 2728   1 2692 2.795575e+00
## 2729   1 2693 2.796484e+00
## 2730   1 2694 2.768576e+00
## 2731   1 2695 2.737967e+00
## 2732   1 2696 2.700732e+00
## 2733   1 2697 2.656325e+00
## 2734   1 2698 2.621666e+00
## 2735   1 2699 2.590124e+00
## 2736   1 2700 2.550535e+00
## 2737   1 2701 2.520294e+00
## 2738   1 2702 2.484245e+00
## 2739   1 2703 2.461157e+00
## 2740   1 2704 2.431786e+00
## 2741   1 2705 2.398601e+00
## 2742   1 2706 2.382941e+00
## 2743   1 2707 2.358839e+00
## 2744   1 2708 2.333659e+00
## 2745   1 2709 2.314207e+00
## 2746   1 2710 2.292423e+00
## 2747   1 2711 2.277336e+00
## 2748   1 2712 2.259300e+00
## 2749   1 2713 2.412662e+00
## 2750   1 2714 2.623172e+00
## 2751   1 2715 2.747773e+00
## 2752   1 2716 2.788072e+00
## 2753   1 2717 2.794349e+00
## 2754   1 2718 2.768111e+00
## 2755   1 2719 2.734293e+00
## 2756   1 2720 2.699725e+00
## 2757   1 2721 2.658148e+00
## 2758   1 2722 2.624042e+00
## 2759   1 2723 2.582591e+00
## 2760   1 2724 2.549467e+00
## 2761   1 2725 2.520449e+00
## 2762   1 2726 2.485827e+00
## 2763   1 2727 2.459684e+00
## 2764   1 2728 2.428277e+00
## 2765   1 2729 2.402573e+00
## 2766   1 2730 2.381153e+00
## 2767   1 2731 2.358675e+00
## 2768   1 2732 2.340390e+00
## 2769   1 2733 2.309858e+00
## 2770   1 2734 2.294600e+00
## 2771   1 2735 2.273789e+00
## 2772   1 2736 2.260632e+00
## 2773   1 2737 2.420939e+00
## 2774   1 2738 2.627162e+00
## 2775   1 2739 2.749768e+00
## 2776   1 2740 2.795476e+00
## 2777   1 2741 2.795082e+00
## 2778   1 2742 2.767192e+00
## 2779   1 2743 2.738693e+00
## 2780   1 2744 2.697323e+00
## 2781   1 2745 2.657395e+00
## 2782   1 2746 2.619585e+00
## 2783   1 2747 2.582951e+00
## 2784   1 2748 2.546557e+00
## 2785   1 2749 2.513394e+00
## 2786   1 2750 2.483234e+00
## 2787   1 2751 2.458348e+00
## 2788   1 2752 2.428443e+00
## 2789   1 2753 2.404804e+00
## 2790   1 2754 2.383272e+00
## 2791   1 2755 2.355869e+00
## 2792   1 2756 2.332498e+00
## 2793   1 2757 2.306699e+00
## 2794   1 2758 2.293791e+00
## 2795   1 2759 2.275916e+00
## 2796   1 2760 2.254764e+00
## 2797   1 2761 2.414181e+00
## 2798   1 2762 2.624877e+00
## 2799   1 2763 2.747863e+00
## 2800   1 2764 2.790646e+00
## 2801   1 2765 2.793855e+00
## 2802   1 2766 2.772245e+00
## 2803   1 2767 2.736414e+00
## 2804   1 2768 2.699279e+00
## 2805   1 2769 2.656450e+00
## 2806   1 2770 2.621142e+00
## 2807   1 2771 2.581743e+00
## 2808   1 2772 2.545490e+00
## 2809   1 2773 2.511266e+00
## 2810   1 2774 2.482714e+00
## 2811   1 2775 2.459776e+00
## 2812   1 2776 2.430763e+00
## 2813   1 2777 2.403554e+00
## 2814   1 2778 2.381460e+00
## 2815   1 2779 2.356262e+00
## 2816   1 2780 2.335187e+00
## 2817   1 2781 2.308134e+00
## 2818   1 2782 2.292677e+00
## 2819   1 2783 2.271839e+00
## 2820   1 2784 2.255160e+00
## 2821   1 2785 2.417105e+00
## 2822   1 2786 2.623250e+00
## 2823   1 2787 2.745430e+00
## 2824   1 2788 2.798264e+00
## 2825   1 2789 2.787623e+00
## 2826   1 2790 2.766719e+00
## 2827   1 2791 2.734500e+00
## 2828   1 2792 2.698088e+00
## 2829   1 2793 2.658125e+00
## 2830   1 2794 2.619752e+00
## 2831   1 2795 2.578753e+00
## 2832   1 2796 2.548834e+00
## 2833   1 2797 2.516068e+00
## 2834   1 2798 2.482207e+00
## 2835   1 2799 2.454453e+00
## 2836   1 2800 2.433503e+00
## 2837   1 2801 2.405732e+00
## 2838   1 2802 2.380026e+00
## 2839   1 2803 2.351588e+00
## 2840   1 2804 2.334280e+00
## 2841   1 2805 2.312036e+00
## 2842   1 2806 2.290903e+00
## 2843   1 2807 2.274611e+00
## 2844   1 2808 2.252884e+00
## 2845   1 2808 2.248894e+00
## 2846   1 2809 2.405265e+00
## 2847   1 2810 2.615946e+00
## 2848   1 2811 2.742991e+00
## 2849   1 2812 2.786152e+00
## 2850   1 2813 2.788231e+00
## 2851   1 2814 2.766429e+00
## 2852   1 2815 2.727653e+00
## 2853   1 2816 2.692575e+00
## 2854   1 2817 2.652487e+00
## 2855   1 2818 2.616183e+00
## 2856   1 2819 2.580104e+00
## 2857   1 2820 2.544946e+00
## 2858   1 2821 2.513000e+00
## 2859   1 2822 2.480528e+00
## 2860   1 2823 2.448379e+00
## 2861   1 2824 2.421151e+00
## 2862   1 2825 2.395499e+00
## 2863   1 2826 2.375918e+00
## 2864   1 2827 2.348692e+00
## 2865   1 2828 2.325445e+00
## 2866   1 2829 2.307537e+00
## 2867   1 2830 2.287688e+00
## 2868   1 2831 2.267100e+00
## 2869   1 2832 2.253942e+00
## 2870   1 2832 2.249796e+00
## 2871   1 2833 2.233037e+00
## 2872   1 2834 2.217248e+00
## 2873   1 2835 2.202823e+00
## 2874   1 2836 2.185337e+00
## 2875   1 2837 2.169572e+00
## 2876   1 2838 2.158227e+00
## 2877   1 2839 2.145088e+00
## 2878   1 2840 2.131801e+00
## 2879   1 2841 2.116777e+00
## 2880   1 2842 2.101332e+00
## 2881   1 2843 2.095465e+00
## 2882   1 2844 2.082753e+00
## 2883   1 2845 2.068632e+00
## 2884   1 2846 2.060758e+00
## 2885   1 2847 2.050826e+00
## 2886   1 2848 2.041733e+00
## 2887   1 2849 2.030184e+00
## 2888   1 2850 2.020385e+00
## 2889   1 2851 2.009323e+00
## 2890   1 2852 1.996658e+00
## 2891   1 2853 1.991930e+00
## 2892   1 2853 1.993751e+00
## 2893   1 2854 2.071550e+00
## 2894   1 2855 2.173904e+00
## 2895   1 2856 2.232392e+00
## 2896   1 2856 2.237793e+00
## 2897   1 2857 2.432823e+00
## 2898   1 2858 2.658521e+00
## 2899   1 2859 2.781438e+00
## 2900   1 2860 2.823706e+00
## 2901   1 2861 2.822962e+00
## 2902   1 2862 2.786909e+00
## 2903   1 2863 2.748247e+00
## 2904   1 2864 2.705612e+00
## 2905   1 2865 2.659014e+00
## 2906   1 2866 2.615592e+00
## 2907   1 2867 2.567165e+00
## 2908   1 2868 2.530505e+00
## 2909   1 2869 2.496360e+00
## 2910   1 2870 2.459949e+00
## 2911   1 2871 2.424992e+00
## 2912   1 2872 2.392252e+00
## 2913   1 2873 2.366100e+00
## 2914   1 2874 2.337236e+00
## 2915   1 2875 2.309568e+00
## 2916   1 2876 2.280789e+00
## 2917   1 2877 2.259622e+00
## 2918   1 2878 2.241791e+00
## 2919   1 2879 2.223189e+00
## 2920   1 2880 2.198644e+00
## 2921   1 2881 2.357459e+00
## 2922   1 2882 2.565768e+00
## 2923   1 2883 2.686287e+00
## 2924   1 2884 2.727254e+00
## 2925   1 2885 2.729027e+00
## 2926   1 2886 2.712006e+00
## 2927   1 2887 2.670028e+00
## 2928   1 2888 2.627437e+00
## 2929   1 2889 2.589830e+00
## 2930   1 2890 2.550488e+00
## 2931   1 2891 2.522480e+00
## 2932   1 2892 2.482585e+00
## 2933   1 2893 2.451252e+00
## 2934   1 2894 2.416590e+00
## 2935   1 2895 2.390444e+00
## 2936   1 2896 2.358202e+00
## 2937   1 2897 2.331245e+00
## 2938   1 2898 2.306331e+00
## 2939   1 2899 2.286775e+00
## 2940   1 2900 2.259611e+00
## 2941   1 2901 2.239849e+00
## 2942   1 2902 2.222727e+00
## 2943   1 2903 2.199708e+00
## 2944   1 2904 2.185847e+00
## 2945   1 2905 2.344080e+00
## 2946   1 2906 2.551017e+00
## 2947   1 2907 2.675986e+00
## 2948   1 2908 2.721507e+00
## 2949   1 2909 2.724330e+00
## 2950   1 2910 2.693404e+00
## 2951   1 2911 2.664256e+00
## 2952   1 2912 2.620525e+00
## 2953   1 2913 2.585759e+00
## 2954   1 2914 2.551933e+00
## 2955   1 2915 2.508122e+00
## 2956   1 2916 2.478290e+00
## 2957   1 2917 2.446082e+00
## 2958   1 2918 2.414412e+00
## 2959   1 2919 2.381134e+00
## 2960   1 2920 2.357835e+00
## 2961   1 2921 2.331006e+00
## 2962   1 2922 2.307695e+00
## 2963   1 2923 2.284774e+00
## 2964   1 2924 2.260530e+00
## 2965   1 2925 2.241164e+00
## 2966   1 2926 2.219256e+00
## 2967   1 2927 2.202206e+00
## 2968   1 2928 2.185488e+00
## 2969   1 2929 2.348554e+00
## 2970   1 2930 2.556643e+00
## 2971   1 2931 2.678294e+00
## 2972   1 2932 2.725386e+00
## 2973   1 2933 2.722756e+00
## 2974   1 2934 2.701723e+00
## 2975   1 2935 2.669460e+00
## 2976   1 2936 2.630718e+00
## 2977   1 2937 2.592345e+00
## 2978   1 2938 2.556066e+00
## 2979   1 2939 2.512650e+00
## 2980   1 2940 2.480208e+00
## 2981   1 2941 2.449786e+00
## 2982   1 2942 2.418201e+00
## 2983   1 2943 2.387895e+00
## 2984   1 2944 2.365772e+00
## 2985   1 2945 2.337727e+00
## 2986   1 2946 2.307180e+00
## 2987   1 2947 2.290816e+00
## 2988   1 2948 2.269604e+00
## 2989   1 2949 2.248001e+00
## 2990   1 2950 2.225093e+00
## 2991   1 2951 2.203938e+00
## 2992   1 2952 2.189572e+00
## 2993   1 2953 2.351895e+00
## 2994   1 2954 2.560323e+00
## 2995   1 2955 2.681993e+00
## 2996   1 2956 2.732562e+00
## 2997   1 2957 2.728258e+00
## 2998   1 2958 2.704530e+00
## 2999   1 2959 2.665453e+00
## 3000   1 2960 2.639532e+00
## 3001   1 2961 2.598708e+00
## 3002   1 2962 2.551353e+00
## 3003   1 2963 2.522982e+00
## 3004   1 2964 2.487367e+00
## 3005   1 2965 2.451037e+00
## 3006   1 2966 2.419950e+00
## 3007   1 2967 2.397172e+00
## 3008   1 2968 2.361201e+00
## 3009   1 2969 2.341133e+00
## 3010   1 2970 2.318017e+00
## 3011   1 2971 2.299724e+00
## 3012   1 2972 2.272468e+00
## 3013   1 2973 2.252886e+00
## 3014   1 2974 2.234563e+00
## 3015   1 2975 2.211939e+00
## 3016   1 2976 2.197109e+00
## 3017   1 2977 2.357585e+00
## 3018   1 2978 2.566104e+00
## 3019   1 2979 2.686764e+00
## 3020   1 2980 2.735827e+00
## 3021   1 2981 2.737481e+00
## 3022   1 2982 2.707137e+00
## 3023   1 2983 2.675152e+00
## 3024   1 2984 2.640532e+00
## 3025   1 2985 2.596593e+00
## 3026   1 2986 2.561389e+00
## 3027   1 2987 2.527283e+00
## 3028   1 2988 2.489815e+00
## 3029   1 2989 2.464897e+00
## 3030   1 2990 2.427514e+00
## 3031   1 2991 2.399098e+00
## 3032   1 2992 2.373803e+00
## 3033   1 2993 2.349268e+00
## 3034   1 2994 2.320069e+00
## 3035   1 2995 2.298565e+00
## 3036   1 2996 2.277380e+00
## 3037   1 2997 2.256954e+00
## 3038   1 2998 2.234171e+00
## 3039   1 2999 2.224502e+00
## 3040   1 3000 2.199857e+00
## 3041   1 3001 2.362929e+00
## 3042   1 3002 2.568191e+00
## 3043   1 3003 2.693837e+00
## 3044   1 3004 2.737663e+00
## 3045   1 3005 2.740738e+00
## 3046   1 3006 2.721616e+00
## 3047   1 3007 2.685164e+00
## 3048   1 3008 2.642294e+00
## 3049   1 3009 2.606623e+00
## 3050   1 3010 2.563449e+00
## 3051   1 3011 2.530096e+00
## 3052   1 3012 2.497747e+00
## 3053   1 3013 2.464853e+00
## 3054   1 3014 2.434339e+00
## 3055   1 3015 2.403332e+00
## 3056   1 3016 2.380432e+00
## 3057   1 3017 2.352503e+00
## 3058   1 3018 2.322487e+00
## 3059   1 3019 2.300853e+00
## 3060   1 3020 2.280542e+00
## 3061   1 3021 2.260056e+00
## 3062   1 3022 2.239469e+00
## 3063   1 3023 2.223368e+00
## 3064   1 3024 2.203265e+00
## 3065   1 3025 2.363503e+00
## 3066   1 3026 2.574614e+00
## 3067   1 3027 2.694970e+00
## 3068   1 3028 2.745805e+00
## 3069   1 3029 2.746564e+00
## 3070   1 3030 2.721206e+00
## 3071   1 3031 2.686250e+00
## 3072   1 3032 2.646497e+00
## 3073   1 3033 2.608674e+00
```

```
## 3074    1 3034 2.566352e+00
## 3075    1 3035 2.531389e+00
## 3076    1 3036 2.499641e+00
## 3077    1 3037 2.467942e+00
## 3078    1 3038 2.436212e+00
## 3079    1 3039 2.410270e+00
## 3080    1 3040 2.382021e+00
## 3081    1 3041 2.358498e+00
## 3082    1 3042 2.331153e+00
## 3083    1 3043 2.308950e+00
## 3084    1 3044 2.290149e+00
## 3085    1 3045 2.268437e+00
## 3086    1 3046 2.242703e+00
## 3087    1 3047 2.224976e+00
## 3088    1 3048 2.209647e+00
## 3089    1 3049 2.367783e+00
## 3090    1 3050 2.574392e+00
## 3091    1 3051 2.704214e+00
## 3092    1 3052 2.747740e+00
## 3093    1 3053 2.745783e+00
## 3094    1 3054 2.723747e+00
## 3095    1 3055 2.692565e+00
## 3096    1 3056 2.651454e+00
## 3097    1 3057 2.613956e+00
## 3098    1 3058 2.569852e+00
## 3099    1 3059 2.538452e+00
## 3100    1 3060 2.504255e+00
## 3101    1 3061 2.473356e+00
## 3102    1 3062 2.440338e+00
## 3103    1 3063 2.413257e+00
## 3104    1 3064 2.381835e+00
## 3105    1 3065 2.356472e+00
## 3106    1 3066 2.334210e+00
## 3107    1 3067 2.309521e+00
## 3108    1 3068 2.289324e+00
## 3109    1 3069 2.268245e+00
## 3110    1 3070 2.250949e+00
## 3111    1 3071 2.229272e+00
## 3112    1 3072 2.210995e+00
## 3113    1 3073 2.374976e+00
## 3114    1 3074 2.583888e+00
## 3115    1 3075 2.705705e+00
## 3116    1 3076 2.751438e+00
## 3117    1 3077 2.749752e+00
## 3118    1 3078 2.726189e+00
## 3119    1 3079 2.689968e+00
## 3120    1 3080 2.660408e+00
## 3121    1 3081 2.616358e+00
## 3122    1 3082 2.573152e+00
## 3123    1 3083 2.538991e+00
## 3124    1 3084 2.508088e+00
## 3125    1 3085 2.473054e+00
## 3126    1 3086 2.439886e+00
## 3127    1 3087 2.415161e+00
## 3128    1 3088 2.385274e+00
## 3129    1 3089 2.360456e+00
## 3130    1 3090 2.332522e+00
## 3131    1 3091 2.315110e+00
## 3132    1 3092 2.292622e+00
## 3133    1 3093 2.271072e+00
## 3134    1 3094 2.251869e+00
## 3135    1 3095 2.232419e+00
## 3136    1 3096 2.215759e+00
## 3137    1 3097 2.375891e+00
## 3138    1 3098 2.585951e+00
## 3139    1 3099 2.706470e+00
## 3140    1 3100 2.749232e+00
## 3141    1 3101 2.758191e+00
## 3142    1 3102 2.729135e+00
## 3143    1 3103 2.700287e+00
## 3144    1 3104 2.660239e+00
## 3145    1 3105 2.615579e+00
## 3146    1 3106 2.580605e+00
## 3147    1 3107 2.550046e+00
## 3148    1 3108 2.505747e+00
## 3149    1 3109 2.479239e+00
## 3150    1 3110 2.440709e+00
## 3151    1 3111 2.415776e+00
## 3152    1 3112 2.390733e+00
## 3153    1 3113 2.363944e+00
## 3154    1 3114 2.341715e+00
## 3155    1 3115 2.317759e+00
## 3156    1 3116 2.297531e+00
## 3157    1 3117 2.271650e+00
## 3158    1 3118 2.253242e+00
## 3159    1 3119 2.234488e+00
## 3160    1 3120 2.216173e+00
## 3161    1 3120 2.248679e+00
## 3162    1 3121 2.415441e+00
## 3163    1 3122 2.621245e+00
## 3164    1 3123 2.735475e+00
## 3165    1 3124 2.786711e+00
## 3166    1 3125 2.789373e+00
## 3167    1 3126 2.766755e+00
## 3168    1 3127 2.729400e+00
## 3169    1 3128 2.690027e+00
## 3170    1 3129 2.653076e+00
## 3171    1 3130 2.611643e+00
## 3172    1 3131 2.578769e+00
## 3173    1 3132 2.542692e+00
## 3174    1 3133 2.513195e+00
## 3175    1 3134 2.481590e+00
## 3176    1 3135 2.456401e+00
## 3177    1 3136 2.421482e+00
## 3178    1 3137 2.399012e+00
## 3179    1 3138 2.378581e+00
## 3180    1 3139 2.350208e+00
## 3181    1 3140 2.325742e+00
## 3182    1 3141 2.303901e+00
## 3183    1 3142 2.282647e+00
## 3184    1 3143 2.266740e+00
## 3185    1 3144 2.247817e+00
## 3186    1 3144 2.251813e+00
## 3187    1 3145 2.231823e+00
## 3188    1 3146 2.214385e+00
## 3189    1 3147 2.202551e+00
## 3190    1 3148 2.183803e+00
## 3191    1 3149 2.171757e+00
## 3192    1 3150 2.156492e+00
## 3193    1 3151 2.144520e+00
## 3194    1 3152 2.130127e+00
## 3195    1 3153 2.120952e+00
## 3196    1 3154 2.103381e+00
## 3197    1 3155 2.096764e+00
## 3198    1 3156 2.082285e+00
## 3199    1 3157 2.072174e+00
## 3200    1 3158 2.060271e+00
## 3201    1 3159 2.048342e+00
## 3202    1 3160 2.038921e+00
## 3203    1 3161 2.027812e+00
## 3204    1 3162 2.019212e+00
## 3205    1 3163 2.013029e+00
## 3206    1 3164 1.997381e+00
## 3207    1 3165 1.993946e+00
## 3208    1 3165 1.987693e+00
## 3209    1 3166 2.161405e+00
## 3210    1 3167 2.375882e+00
## 3211    1 3168 2.506188e+00
## 3212    1 3168 2.503985e+00
## 3213    1 3169 2.732075e+00
## 3214    1 3170 2.965114e+00
## 3215    1 3171 3.089406e+00
## 3216    1 3172 3.118589e+00
## 3217    1 3173 3.101827e+00
## 3218    1 3174 3.057017e+00
## 3219    1 3175 3.002239e+00
## 3220    1 3176 2.944733e+00
## 3221    1 3177 2.884654e+00
## 3222    1 3178 2.833978e+00
## 3223    1 3179 2.778154e+00
## 3224    1 3180 2.730242e+00
## 3225    1 3181 2.683326e+00
## 3226    1 3182 2.638676e+00
## 3227    1 3183 2.601080e+00
## 3228    1 3184 2.560635e+00
## 3229    1 3185 2.525874e+00
## 3230    1 3186 2.491295e+00
## 3231    1 3187 2.461965e+00
## 3232    1 3188 2.434020e+00
## 3233    1 3189 2.403567e+00
## 3234    1 3190 2.379286e+00
## 3235    1 3191 2.355625e+00
## 3236    1 3192 2.325678e+00
## 3237    1 3193 2.482416e+00
## 3238    1 3194 2.684476e+00
## 3239    1 3195 2.809084e+00
## 3240    1 3196 2.850232e+00
## 3241    1 3197 2.849553e+00
## 3242    1 3198 2.819096e+00
## 3243    1 3199 2.783619e+00
## 3244    1 3200 2.737818e+00
## 3245    1 3201 2.694103e+00
## 3246    1 3202 2.653791e+00
## 3247    1 3203 2.619414e+00
## 3248    1 3204 2.585502e+00
## 3249    1 3205 2.547251e+00
## 3250    1 3206 2.514986e+00
## 3251    1 3207 2.480084e+00
## 3252    1 3208 2.457764e+00
## 3253    1 3209 2.425503e+00
## 3254    1 3210 2.398599e+00
## 3255    1 3211 2.376974e+00
## 3256    1 3212 2.352613e+00
## 3257    1 3213 2.333675e+00
## 3258    1 3214 2.311009e+00
## 3259    1 3215 2.287994e+00
## 3260    1 3216 2.272198e+00
## 3261    1 3217 2.432260e+00
## 3262    1 3218 2.641512e+00
## 3263    1 3219 2.761306e+00
## 3264    1 3220 2.810739e+00
## 3265    1 3221 2.807098e+00
## 3266    1 3222 2.783158e+00
## 3267    1 3223 2.747405e+00
## 3268    1 3224 2.707348e+00
## 3269    1 3225 2.670543e+00
## 3270    1 3226 2.632851e+00
## 3271    1 3227 2.594938e+00
## 3272    1 3228 2.556969e+00
## 3273    1 3229 2.530573e+00
## 3274    1 3230 2.496076e+00
## 3275    1 3231 2.463590e+00
## 3276    1 3232 2.434178e+00
## 3277    1 3233 2.411179e+00
## 3278    1 3234 2.384363e+00
## 3279    1 3235 2.363012e+00
## 3280    1 3236 2.341044e+00
## 3281    1 3237 2.318999e+00
## 3282    1 3238 2.296986e+00
## 3283    1 3239 2.279404e+00
## 3284    1 3240 2.264634e+00
## 3285    1 3241 2.422632e+00
## 3286    1 3242 2.629431e+00
## 3287    1 3243 2.750767e+00
## 3288    1 3244 2.797563e+00
## 3289    1 3245 2.807309e+00
## 3290    1 3246 2.778307e+00
## 3291    1 3247 2.739151e+00
## 3292    1 3248 2.703269e+00
## 3293    1 3249 2.660899e+00
## 3294    1 3250 2.624255e+00
## 3295    1 3251 2.587939e+00
## 3296    1 3252 2.556619e+00
## 3297    1 3253 2.527124e+00
## 3298    1 3254 2.489032e+00
## 3299    1 3255 2.464088e+00
## 3300    1 3256 2.431844e+00
## 3301    1 3257 2.412261e+00
## 3302    1 3258 2.383387e+00
## 3303    1 3259 2.356613e+00
## 3304    1 3260 2.340064e+00
## 3305    1 3261 2.318891e+00
## 3306    1 3262 2.294277e+00
## 3307    1 3263 2.278457e+00
## 3308    1 3264 2.262563e+00
## 3309    1 3265 2.426276e+00
## 3310    1 3266 2.630089e+00
## 3311    1 3267 2.745065e+00
## 3312    1 3268 2.797329e+00
## 3313    1 3269 2.800574e+00
## 3314    1 3270 2.776708e+00
## 3315    1 3271 2.741848e+00
## 3316    1 3272 2.699926e+00
## 3317    1 3273 2.663053e+00
## 3318    1 3274 2.630497e+00
## 3319    1 3275 2.587610e+00
## 3320    1 3276 2.554500e+00
## 3321    1 3277 2.522265e+00
## 3322    1 3278 2.490339e+00
## 3323    1 3279 2.464887e+00
## 3324    1 3280 2.433272e+00
## 3325    1 3281 2.406700e+00
## 3326    1 3282 2.381283e+00
## 3327    1 3283 2.353587e+00
## 3328    1 3284 2.333569e+00
## 3329    1 3285 2.313986e+00
## 3330    1 3286 2.293046e+00
## 3331    1 3287 2.277285e+00
## 3332    1 3288 2.260583e+00
## 3333    1 3289 2.420446e+00
## 3334    1 3290 2.626380e+00
## 3335    1 3291 2.751620e+00
## 3336    1 3292 2.794884e+00
## 3337    1 3293 2.797736e+00
## 3338    1 3294 2.772036e+00
## 3339    1 3295 2.738480e+00
## 3340    1 3296 2.699595e+00
## 3341    1 3297 2.656068e+00
## 3342    1 3298 2.621602e+00
## 3343    1 3299 2.592145e+00
## 3344    1 3300 2.551967e+00
## 3345    1 3301 2.517725e+00
## 3346    1 3302 2.490270e+00
## 3347    1 3303 2.455853e+00
## 3348    1 3304 2.433596e+00
## 3349    1 3305 2.407712e+00
## 3350    1 3306 2.382826e+00
## 3351    1 3307 2.357050e+00
## 3352    1 3308 2.334784e+00
## 3353    1 3309 2.316335e+00
## 3354    1 3310 2.294394e+00
## 3355    1 3311 2.277273e+00
## 3356    1 3312 2.260017e+00
## 3357    1 3313 2.419897e+00
## 3358    1 3314 2.628357e+00
## 3359    1 3315 2.746287e+00
## 3360    1 3316 2.797149e+00
## 3361    1 3317 2.798826e+00
## 3362    1 3318 2.772255e+00
## 3363    1 3319 2.738429e+00
## 3364    1 3320 2.700324e+00
## 3365    1 3321 2.658815e+00
## 3366    1 3322 2.623681e+00
## 3367    1 3323 2.588754e+00
## 3368    1 3324 2.551013e+00
## 3369    1 3325 2.520091e+00
## 3370    1 3326 2.489428e+00
## 3371    1 3327 2.453480e+00
## 3372    1 3328 2.439294e+00
## 3373    1 3329 2.409039e+00
## 3374    1 3330 2.375621e+00
## 3375    1 3331 2.356109e+00
## 3376    1 3332 2.335808e+00
## 3377    1 3333 2.311962e+00
## 3378    1 3334 2.296489e+00
## 3379    1 3335 2.275938e+00
## 3380    1 3336 2.256968e+00
## 3381    1 3337 2.418335e+00
## 3382    1 3338 2.625994e+00
## 3383    1 3339 2.750724e+00
## 3384    1 3340 2.800476e+00
## 3385    1 3341 2.794677e+00
## 3386    1 3342 2.774726e+00
## 3387    1 3343 2.741224e+00
## 3388    1 3344 2.701474e+00
## 3389    1 3345 2.658091e+00
## 3390    1 3346 2.623114e+00
## 3391    1 3347 2.584746e+00
## 3392    1 3348 2.549196e+00
## 3393    1 3349 2.519319e+00
## 3394    1 3350 2.487329e+00
## 3395    1 3351 2.458580e+00
## 3396    1 3352 2.429636e+00
## 3397    1 3353 2.405531e+00
## 3398    1 3354 2.378095e+00
## 3399    1 3355 2.356987e+00
## 3400    1 3356 2.335949e+00
## 3401    1 3357 2.316058e+00
## 3402    1 3358 2.295123e+00
## 3403    1 3359 2.276216e+00
## 3404    1 3360 2.257233e+00
## 3405    1 3361 2.417612e+00
## 3406    1 3362 2.629528e+00
## 3407    1 3363 2.750497e+00
## 3408    1 3364 2.790158e+00
## 3409    1 3365 2.796497e+00
## 3410    1 3366 2.772102e+00
## 3411    1 3367 2.736110e+00
## 3412    1 3368 2.698708e+00
## 3413    1 3369 2.660501e+00
## 3414    1 3370 2.625207e+00
## 3415    1 3371 2.587006e+00
## 3416    1 3372 2.556931e+00
## 3417    1 3373 2.515680e+00
## 3418    1 3374 2.485698e+00
## 3419    1 3375 2.457603e+00
## 3420    1 3376 2.429788e+00
## 3421    1 3377 2.405256e+00
## 3422    1 3378 2.376580e+00
## 3423    1 3379 2.356720e+00
## 3424    1 3380 2.329286e+00
## 3425    1 3381 2.310731e+00
## 3426    1 3382 2.293596e+00
## 3427    1 3383 2.271707e+00
## 3428    1 3384 2.254303e+00
## 3429    1 3385 2.415417e+00
## 3430    1 3386 2.626279e+00
## 3431    1 3387 2.748261e+00
## 3432    1 3388 2.799103e+00
## 3433    1 3389 2.792503e+00
## 3434    1 3390 2.766225e+00
## 3435    1 3391 2.740149e+00
## 3436    1 3392 2.697049e+00
## 3437    1 3393 2.659442e+00
## 3438    1 3394 2.621285e+00
## 3439    1 3395 2.581401e+00
## 3440    1 3396 2.547266e+00
## 3441    1 3397 2.517725e+00
## 3442    1 3398 2.486159e+00
## 3443    1 3399 2.459777e+00
## 3444    1 3400 2.430091e+00
## 3445    1 3401 2.404698e+00
## 3446    1 3402 2.384159e+00
## 3447    1 3403 2.355393e+00
## 3448    1 3404 2.334433e+00
## 3449    1 3405 2.309350e+00
## 3450    1 3406 2.289567e+00
## 3451    1 3407 2.277704e+00
## 3452    1 3408 2.257091e+00
## 3453    1 3409 2.415508e+00
## 3454    1 3410 2.622338e+00
## 3455    1 3411 2.744258e+00
## 3456    1 3412 2.789762e+00
## 3457    1 3413 2.796234e+00
## 3458    1 3414 2.771457e+00
## 3459    1 3415 2.737746e+00
## 3460    1 3416 2.695701e+00
## 3461    1 3417 2.658362e+00
## 3462    1 3418 2.624729e+00
## 3463    1 3419 2.584981e+00
## 3464    1 3420 2.548887e+00
## 3465    1 3421 2.517701e+00
## 3466    1 3422 2.484522e+00
## 3467    1 3423 2.456087e+00
## 3468    1 3424 2.427057e+00
## 3469    1 3425 2.403395e+00
## 3470    1 3426 2.378891e+00
## 3471    1 3427 2.353475e+00
## 3472    1 3428 2.331815e+00
## 3473    1 3429 2.308823e+00
## 3474    1 3430 2.288653e+00
## 3475    1 3431 2.270340e+00
## 3476    1 3432 2.251592e+00
## 3477    1 3432 2.249991e+00
## 3478    1 3433 2.408212e+00
## 3479    1 3434 2.619881e+00
## 3480    1 3435 2.737053e+00
## 3481    1 3436 2.783278e+00
## 3482    1 3437 2.788952e+00
## 3483    1 3438 2.763411e+00
## 3484    1 3439 2.730591e+00
## 3485    1 3440 2.688892e+00
## 3486    1 3441 2.650783e+00
## 3487    1 3442 2.608205e+00
## 3488    1 3443 2.577429e+00
## 3489    1 3444 2.541570e+00
## 3490    1 3445 2.509417e+00
## 3491    1 3446 2.478998e+00
## 3492    1 3447 2.448514e+00
## 3493    1 3448 2.422371e+00
## 3494    1 3449 2.396613e+00
## 3495    1 3450 2.373518e+00
## 3496    1 3451 2.350761e+00
## 3497    1 3452 2.326580e+00
## 3498    1 3453 2.301850e+00
## 3499    1 3454 2.287719e+00
## 3500    1 3455 2.266939e+00
## 3501    1 3456 2.248327e+00
## 3502    1 3456 2.255305e+00
## 3503    1 3457 2.232273e+00
## 3504    1 3458 2.211401e+00
## 3505    1 3459 2.199785e+00
## 3506    1 3460 2.186928e+00
## 3507    1 3461 2.167151e+00
## 3508    1 3462 2.157495e+00
## 3509    1 3463 2.144220e+00
## 3510    1 3464 2.129058e+00
## 3511    1 3465 2.116917e+00
## 3512    1 3466 2.101082e+00
## 3513    1 3467 2.092063e+00
## 3514    1 3468 2.080652e+00
## 3515    1 3469 2.068646e+00
## 3516    1 3470 2.062179e+00
## 3517    1 3471 2.049366e+00
## 3518    1 3472 2.041242e+00
## 3519    1 3473 2.031738e+00
## 3520    1 3474 2.019591e+00
## 3521    1 3475 2.006702e+00
## 3522    1 3476 1.999199e+00
## 3523    1 3477 1.988817e+00
## 3524    1 3478 1.987022e+00
## 3525    1 3479 1.973265e+00
## 3526    1 3480 1.964645e+00
## 3527    1 3480 1.962995e+00
## 3528    1 3481 2.133144e+00
## 3529    1 3482 2.351284e+00
## 3530    1 3483 2.480029e+00
## 3531    1 3484 2.531556e+00
## 3532    1 3485 2.536286e+00
## 3533    1 3486 2.517614e+00
## 3534    1 3487 2.494656e+00
## 3535    1 3488 2.461648e+00
## 3536    1 3489 2.427419e+00
## 3537    1 3490 2.392541e+00
## 3538    1 3491 2.361012e+00
## 3539    1 3492 2.330490e+00
## 3540    1 3493 2.298827e+00
## 3541    1 3494 2.273951e+00
## 3542    1 3495 2.249585e+00
## 3543    1 3496 2.230725e+00
## 3544    1 3497 2.201794e+00
## 3545    1 3498 2.173970e+00
## 3546    1 3499 2.156293e+00
## 3547    1 3500 2.132582e+00
## 3548    1 3501 2.121257e+00
## 3549    1 3502 2.102731e+00
## 3550    1 3503 2.085052e+00
## 3551    1 3504 2.067688e+00
## 3552    1 3504 2.067507e+00
## 3553    1 3505 2.232732e+00
## 3554    1 3506 2.442409e+00
## 3555    1 3507 2.563699e+00
## 3556    1 3508 2.613027e+00
## 3557    1 3509 2.615747e+00
## 3558    1 3510 2.591149e+00
## 3559    1 3511 2.555141e+00
## 3560    1 3512 2.526249e+00
## 3561    1 3513 2.487064e+00
## 3562    1 3514 2.446229e+00
## 3563    1 3515 2.410524e+00
## 3564    1 3516 2.378529e+00
## 3565    1 3517 2.350682e+00
## 3566    1 3518 2.316032e+00
## 3567    1 3519 2.288316e+00
## 3568    1 3520 2.257607e+00
## 3569    1 3521 2.233747e+00
## 3570    1 3522 2.212240e+00
## 3571    1 3523 2.190380e+00
## 3572    1 3524 2.171597e+00
## 3573    1 3525 2.149583e+00
## 3574    1 3526 2.131631e+00
## 3575    1 3527 2.111076e+00
## 3576    1 3528 2.096791e+00
## 3577    1 3529 2.257797e+00
## 3578    1 3530 2.468090e+00
## 3579    1 3531 2.587549e+00
## 3580    1 3532 2.639002e+00
## 3581    1 3533 2.633618e+00
## 3582    1 3534 2.608367e+00
## 3583    1 3535 2.583436e+00
## 3584    1 3536 2.547621e+00
## 3585    1 3537 2.501681e+00
## 3586    1 3538 2.465222e+00
## 3587    1 3539 2.431091e+00
## 3588    1 3540 2.396511e+00
## 3589    1 3541 2.362538e+00
## 3590    1 3542 2.335436e+00
## 3591    1 3543 2.307716e+00
## 3592    1 3544 2.274533e+00
## 3593    1 3545 2.252245e+00
## 3594    1 3546 2.234285e+00
## 3595    1 3547 2.208880e+00
## 3596    1 3548 2.185408e+00
## 3597    1 3549 2.169169e+00
## 3598    1 3550 2.147362e+00
## 3599    1 3551 2.126057e+00
## 3600    1 3552 2.108597e+00
## 3601    1 3553 2.272399e+00
## 3602    1 3554 2.481897e+00
## 3603    1 3555 2.601598e+00
## 3604    1 3556 2.649630e+00
## 3605    1 3557 2.649871e+00
## 3606    1 3558 2.635913e+00
## 3607    1 3559 2.593996e+00
## 3608    1 3560 2.560871e+00
## 3609    1 3561 2.520047e+00
## 3610    1 3562 2.478094e+00
## 3611    1 3563 2.443969e+00
## 3612    1 3564 2.408274e+00
## 3613    1 3565 2.375415e+00
## 3614    1 3566 2.346697e+00
## 3615    1 3567 2.319921e+00
## 3616    1 3568 2.291831e+00
## 3617    1 3569 2.264969e+00
## 3618    1 3570 2.242947e+00
## 3619    1 3571 2.219004e+00
## 3620    1 3572 2.196013e+00
## 3621    1 3573 2.179263e+00
## 3622    1 3574 2.158336e+00
## 3623    1 3575 2.140558e+00
## 3624    1 3576 2.123324e+00
## 3625    1 3577 2.283989e+00
## 3626    1 3578 2.494689e+00
## 3627    1 3579 2.613714e+00
## 3628    1 3580 2.661453e+00
## 3629    1 3581 2.666805e+00
## 3630    1 3582 2.638795e+00
## 3631    1 3583 2.604789e+00
## 3632    1 3584 2.567777e+00
## 3633    1 3585 2.528381e+00
## 3634    1 3586 2.495335e+00
## 3635    1 3587 2.452850e+00
## 3636    1 3588 2.419892e+00
## 3637    1 3589 2.387508e+00
## 3638    1 3590 2.359484e+00
## 3639    1 3591 2.331846e+00
## 3640    1 3592 2.306027e+00
## 3641    1 3593 2.276704e+00
## 3642    1 3594 2.253811e+00
## 3643    1 3595 2.231178e+00
## 3644    1 3596 2.211042e+00
## 3645    1 3597 2.188454e+00
## 3646    1 3598 2.170605e+00
## 3647    1 3599 2.154731e+00
## 3648    1 3600 2.136411e+00
## 3649    1 3601 2.296907e+00
## 3650    1 3602 2.505910e+00
## 3651    1 3603 2.626503e+00
## 3652    1 3604 2.668814e+00
## 3653    1 3605 2.678188e+00
## 3654    1 3606 2.651319e+00
## 3655    1 3607 2.612276e+00
## 3656    1 3608 2.579053e+00
## 3657    1 3609 2.540670e+00
## 3658    1 3610 2.503652e+00
## 3659    1 3611 2.464157e+00
## 3660    1 3612 2.436106e+00
## 3661    1 3613 2.398956e+00
## 3662    1 3614 2.368825e+00
## 3663    1 3615 2.340673e+00
## 3664    1 3616 2.316807e+00
## 3665    1 3617 2.291071e+00
## 3666    1 3618 2.265594e+00
## 3667    1 3619 2.241266e+00
## 3668    1 3620 2.217287e+00
## 3669    1 3621 2.198526e+00
## 3670    1 3622 2.180586e+00
## 3671    1 3623 2.162410e+00
## 3672    1 3624 2.144627e+00
## 3673    1 3625 2.305403e+00
## 3674    1 3626 2.511982e+00
## 3675    1 3627 2.632302e+00
## 3676    1 3628 2.683168e+00
## 3677    1 3629 2.682072e+00
## 3678    1 3630 2.663761e+00
## 3679    1 3631 2.624150e+00
## 3680    1 3632 2.590529e+00
## 3681    1 3633 2.548129e+00
## 3682    1 3634 2.509112e+00
## 3683    1 3635 2.477696e+00
## 3684    1 3636 2.441900e+00
## 3685    1 3637 2.408218e+00
## 3686    1 3638 2.381129e+00
## 3687    1 3639 2.349918e+00
## 3688    1 3640 2.321463e+00
## 3689    1 3641 2.296464e+00
## 3690    1 3642 2.271231e+00
## 3691    1 3643 2.250781e+00
## 3692    1 3644 2.228280e+00
## 3693    1 3645 2.208566e+00
## 3694    1 3646 2.189155e+00
## 3695    1 3647 2.172671e+00
## 3696    1 3648 2.149539e+00
## 3697    1 3649 2.313152e+00
## 3698    1 3650 2.523204e+00
## 3699    1 3651 2.641562e+00
## 3700    1 3652 2.694701e+00
## 3701    1 3653 2.697141e+00
## 3702    1 3654 2.667760e+00
## 3703    1 3655 2.634489e+00
## 3704    1 3656 2.597012e+00
## 3705    1 3657 2.558779e+00
## 3706    1 3658 2.521193e+00
## 3707    1 3659 2.487823e+00
## 3708    1 3660 2.449921e+00
## 3709    1 3661 2.415597e+00
## 3710    1 3662 2.382822e+00
## 3711    1 3663 2.358985e+00
## 3712    1 3664 2.335529e+00
## 3713    1 3665 2.311768e+00
## 3714    1 3666 2.283669e+00
## 3715    1 3667 2.252280e+00
## 3716    1 3668 2.236967e+00
## 3717    1 3669 2.217623e+00
## 3718    1 3670 2.196001e+00
## 3719    1 3671 2.178034e+00
## 3720    1 3672 2.160428e+00
## 3721    1 3673 2.318376e+00
## 3722    1 3674 2.527195e+00
## 3723    1 3675 2.652257e+00
## 3724    1 3676 2.702182e+00
## 3725    1 3677 2.700040e+00
## 3726    1 3678 2.679644e+00
## 3727    1 3679 2.640494e+00
## 3728    1 3680 2.603187e+00
## 3729    1 3681 2.562168e+00
## 3730    1 3682 2.529832e+00
## 3731    1 3683 2.492600e+00
## 3732    1 3684 2.459832e+00
## 3733    1 3685 2.425727e+00
## 3734    1 3686 2.396004e+00
## 3735    1 3687 2.365182e+00
## 3736    1 3688 2.336409e+00
## 3737    1 3689 2.316043e+00
## 3738    1 3690 2.288224e+00
## 3739    1 3691 2.262699e+00
## 3740    1 3692 2.247044e+00
## 3741    1 3693 2.222354e+00
## 3742    1 3694 2.203510e+00
## 3743    1 3695 2.186169e+00
## 3744    1 3696 2.164068e+00
## 3745    1 3697 2.329793e+00
## 3746    1 3698 2.539170e+00
## 3747    1 3699 2.665081e+00
## 3748    1 3700 2.709049e+00
## 3749    1 3701 2.710570e+00
## 3750    1 3702 2.686029e+00
## 3751    1 3703 2.651826e+00
## 3752    1 3704 2.606436e+00
## 3753    1 3705 2.574150e+00
## 3754    1 3706 2.530553e+00
## 3755    1 3707 2.495989e+00
## 3756    1 3708 2.469000e+00
## 3757    1 3709 2.432998e+00
## 3758    1 3710 2.400086e+00
## 3759    1 3711 2.370314e+00
## 3760    1 3712 2.348269e+00
## 3761    1 3713 2.320013e+00
## 3762    1 3714 2.297057e+00
## 3763    1 3715 2.270171e+00
## 3764    1 3716 2.252466e+00
## 3765    1 3717 2.230154e+00
## 3766    1 3718 2.211207e+00
## 3767    1 3719 2.194059e+00
## 3768    1 3720 2.174717e+00
## 3769    1 3721 2.335400e+00
## 3770    1 3722 2.544974e+00
## 3771    1 3723 2.670879e+00
## 3772    1 3724 2.713980e+00
## 3773    1 3725 2.716561e+00
## 3774    1 3726 2.692690e+00
## 3775    1 3727 2.656397e+00
## 3776    1 3728 2.619519e+00
## 3777    1 3729 2.582139e+00
## 3778    1 3730 2.547288e+00
## 3779    1 3731 2.504534e+00
## 3780    1 3732 2.473046e+00
## 3781    1 3733 2.436846e+00
## 3782    1 3734 2.408193e+00
## 3783    1 3735 2.380685e+00
## 3784    1 3736 2.354740e+00
## 3785    1 3737 2.327421e+00
## 3786    1 3738 2.302346e+00
## 3787    1 3739 2.279518e+00
## 3788    1 3740 2.258770e+00
## 3789    1 3741 2.237046e+00
## 3790    1 3742 2.217852e+00
## 3791    1 3743 2.202965e+00
## 3792    1 3744 2.178503e+00
## 3793    1 3745 2.341263e+00
## 3794    1 3746 2.552539e+00
## 3795    1 3747 2.669075e+00
## 3796    1 3748 2.719447e+00
## 3797    1 3749 2.722572e+00
## 3798    1 3750 2.693391e+00
## 3799    1 3751 2.666824e+00
## 3800    1 3752 2.624200e+00
## 3801    1 3753 2.583477e+00
## 3802    1 3754 2.546398e+00
## 3803    1 3755 2.514734e+00
## 3804    1 3756 2.476342e+00
## 3805    1 3757 2.443781e+00
## 3806    1 3758 2.411233e+00
## 3807    1 3759 2.387001e+00
## 3808    1 3760 2.357517e+00
## 3809    1 3761 2.335366e+00
## 3810    1 3762 2.304667e+00
## 3811    1 3763 2.285763e+00
## 3812    1 3764 2.262000e+00
## 3813    1 3765 2.243635e+00
## 3814    1 3766 2.225072e+00
## 3815    1 3767 2.203731e+00
## 3816    1 3768 2.188272e+00
## 3817    1 3768 2.249182e+00
## 3818    1 3769 2.402109e+00
## 3819    1 3770 2.615031e+00
## 3820    1 3771 2.746955e+00
## 3821    1 3772 2.792315e+00
## 3822    1 3773 2.789347e+00
## 3823    1 3774 2.767794e+00
## 3824    1 3775 2.728993e+00
## 3825    1 3776 2.690327e+00
## 3826    1 3777 2.654048e+00
## 3827    1 3778 2.613314e+00
## 3828    1 3779 2.579405e+00
## 3829    1 3780 2.542969e+00
## 3830    1 3781 2.513388e+00
## 3831    1 3782 2.482829e+00
## 3832    1 3783 2.446817e+00
## 3833    1 3784 2.421762e+00
## 3834    1 3785 2.397564e+00
## 3835    1 3786 2.372105e+00
## 3836    1 3787 2.348840e+00
## 3837    1 3788 2.329118e+00
## 3838    1 3789 2.303846e+00
## 3839    1 3790 2.283564e+00
## 3840    1 3791 2.267544e+00
## 3841    1 3792 2.251189e+00
## 3842    1 3792 2.247762e+00
## 3843    1 3793 2.230799e+00
## 3844    1 3794 2.217322e+00
## 3845    1 3795 2.200332e+00
## 3846    1 3796 2.187167e+00
## 3847    1 3797 2.171555e+00
## 3848    1 3798 2.157818e+00
## 3849    1 3799 2.145476e+00
## 3850    1 3800 2.127628e+00
## 3851    1 3801 2.115953e+00
## 3852    1 3802 2.106617e+00
## 3853    1 3803 2.094332e+00
## 3854    1 3804 2.082758e+00
## 3855    1 3805 2.072022e+00
## 3856    1 3806 2.055635e+00
## 3857    1 3807 2.054193e+00
## 3858    1 3808 2.039364e+00
## 3859    1 3809 2.024534e+00
## 3860    1 3810 2.022279e+00
## 3861    1 3811 2.010419e+00
## 3862    1 3812 1.999807e+00
## 3863    1 3813 1.987750e+00
## 3864    1 3814 1.985539e+00
## 3865    1 3815 1.970926e+00
## 3866    1 3816 1.965492e+00
## 3867    1 3816 1.962525e+00
## 3868    1 3817 2.224433e+00
## 3869    1 3818 2.548147e+00
## 3870    1 3819 2.749397e+00
## 3871    1 3820 2.836146e+00
## 3872    1 3821 2.847075e+00
## 3873    1 3822 2.827719e+00
```

```
## 3874   1 3823 2.789807e+00
## 3875   1 3824 2.744461e+00
## 3876   1 3825 2.687768e+00
## 3877   1 3826 2.642719e+00
## 3878   1 3827 2.603837e+00
## 3879   1 3828 2.558156e+00
## 3880   1 3829 2.521967e+00
## 3881   1 3830 2.484910e+00
## 3882   1 3831 2.446064e+00
## 3883   1 3832 2.417400e+00
## 3884   1 3833 2.382676e+00
## 3885   1 3834 2.354225e+00
## 3886   1 3835 2.330784e+00
## 3887   1 3836 2.301970e+00
## 3888   1 3837 2.277880e+00
## 3889   1 3838 2.251361e+00
## 3890   1 3839 2.232144e+00
## 3891   1 3840 2.210700e+00
## 3892   1 3840 2.213436e+00
## 3893   1 3841 2.366590e+00
## 3894   1 3842 2.573195e+00
## 3895   1 3843 2.693523e+00
## 3896   1 3844 2.745660e+00
## 3897   1 3845 2.739240e+00
## 3898   1 3846 2.713364e+00
## 3899   1 3847 2.672544e+00
## 3900   1 3848 2.637991e+00
## 3901   1 3849 2.596818e+00
## 3902   1 3850 2.560953e+00
## 3903   1 3851 2.518134e+00
## 3904   1 3852 2.486930e+00
## 3905   1 3853 2.451248e+00
## 3906   1 3854 2.420137e+00
## 3907   1 3855 2.388995e+00
## 3908   1 3856 2.362994e+00
## 3909   1 3857 2.337291e+00
## 3910   1 3858 2.311446e+00
## 3911   1 3859 2.288364e+00
## 3912   1 3860 2.262483e+00
## 3913   1 3861 2.239032e+00
## 3914   1 3862 2.222742e+00
## 3915   1 3863 2.201670e+00
## 3916   1 3864 2.182401e+00
## 3917   1 3865 2.344633e+00
## 3918   1 3866 2.559439e+00
## 3919   1 3867 2.678316e+00
## 3920   1 3868 2.721072e+00
## 3921   1 3869 2.721446e+00
## 3922   1 3870 2.706104e+00
## 3923   1 3871 2.668882e+00
## 3924   1 3872 2.627190e+00
## 3925   1 3873 2.588463e+00
## 3926   1 3874 2.548482e+00
## 3927   1 3875 2.512642e+00
## 3928   1 3876 2.480580e+00
## 3929   1 3877 2.450128e+00
## 3930   1 3878 2.414801e+00
## 3931   1 3879 2.383369e+00
## 3932   1 3880 2.359504e+00
## 3933   1 3881 2.332899e+00
## 3934   1 3882 2.309807e+00
## 3935   1 3883 2.286227e+00
## 3936   1 3884 2.265656e+00
## 3937   1 3885 2.245468e+00
## 3938   1 3886 2.221643e+00
## 3939   1 3887 2.209428e+00
## 3940   1 3888 2.186713e+00
## 3941   1 3889 2.346535e+00
## 3942   1 3890 2.557772e+00
## 3943   1 3891 2.680980e+00
## 3944   1 3892 2.727218e+00
## 3945   1 3893 2.726808e+00
## 3946   1 3894 2.708968e+00
## 3947   1 3895 2.671075e+00
## 3948   1 3896 2.631190e+00
## 3949   1 3897 2.590969e+00
## 3950   1 3898 2.553069e+00
## 3951   1 3899 2.516357e+00
## 3952   1 3900 2.483073e+00
## 3953   1 3901 2.451852e+00
## 3954   1 3902 2.419144e+00
## 3955   1 3903 2.388542e+00
## 3956   1 3904 2.364801e+00
## 3957   1 3905 2.335975e+00
## 3958   1 3906 2.310074e+00
## 3959   1 3907 2.291250e+00
## 3960   1 3908 2.270094e+00
## 3961   1 3909 2.244503e+00
## 3962   1 3910 2.225696e+00
## 3963   1 3911 2.208090e+00
## 3964   1 3912 2.191458e+00
## 3965   1 3913 2.348866e+00
## 3966   1 3914 2.565778e+00
## 3967   1 3915 2.683906e+00
## 3968   1 3916 2.728866e+00
## 3969   1 3917 2.727604e+00
## 3970   1 3918 2.709741e+00
## 3971   1 3919 2.673335e+00
## 3972   1 3920 2.637044e+00
## 3973   1 3921 2.594937e+00
## 3974   1 3922 2.556214e+00
## 3975   1 3923 2.521701e+00
## 3976   1 3924 2.489700e+00
## 3977   1 3925 2.454649e+00
## 3978   1 3926 2.426665e+00
## 3979   1 3927 2.395334e+00
## 3980   1 3928 2.371610e+00
## 3981   1 3929 2.342150e+00
## 3982   1 3930 2.316568e+00
## 3983   1 3931 2.296142e+00
## 3984   1 3932 2.272221e+00
## 3985   1 3933 2.252210e+00
## 3986   1 3934 2.233372e+00
## 3987   1 3935 2.213844e+00
## 3988   1 3936 2.199739e+00
## 3989   1 3937 2.355678e+00
## 3990   1 3938 2.564904e+00
## 3991   1 3939 2.690716e+00
## 3992   1 3940 2.733596e+00
## 3993   1 3941 2.734745e+00
## 3994   1 3942 2.713565e+00
## 3995   1 3943 2.681765e+00
## 3996   1 3944 2.644049e+00
## 3997   1 3945 2.600587e+00
## 3998   1 3946 2.569193e+00
## 3999   1 3947 2.530118e+00
## 4000   1 3948 2.493263e+00
## 4001   1 3949 2.460017e+00
## 4002   1 3950 2.432830e+00
## 4003   1 3951 2.396468e+00
## 4004   1 3952 2.374255e+00
## 4005   1 3953 2.347368e+00
## 4006   1 3954 2.325396e+00
## 4007   1 3955 2.304681e+00
## 4008   1 3956 2.277461e+00
## 4009   1 3957 2.257079e+00
## 4010   1 3958 2.238816e+00
## 4011   1 3959 2.220515e+00
## 4012   1 3960 2.201314e+00
## 4013   1 3961 2.361827e+00
## 4014   1 3962 2.569745e+00
## 4015   1 3963 2.686650e+00
## 4016   1 3964 2.745909e+00
## 4017   1 3965 2.739941e+00
## 4018   1 3966 2.716968e+00
## 4019   1 3967 2.681717e+00
## 4020   1 3968 2.640556e+00
## 4021   1 3969 2.607380e+00
## 4022   1 3970 2.562769e+00
## 4023   1 3971 2.530062e+00
## 4024   1 3972 2.492167e+00
## 4025   1 3973 2.462179e+00
## 4026   1 3974 2.433267e+00
## 4027   1 3975 2.408655e+00
## 4028   1 3976 2.380103e+00
## 4029   1 3977 2.349376e+00
## 4030   1 3978 2.327291e+00
## 4031   1 3979 2.301182e+00
## 4032   1 3980 2.284140e+00
## 4033   1 3981 2.262539e+00
## 4034   1 3982 2.247286e+00
## 4035   1 3983 2.221384e+00
## 4036   1 3984 2.207813e+00
## 4037   1 3985 2.368614e+00
## 4038   1 3986 2.573801e+00
## 4039   1 3987 2.697210e+00
## 4040   1 3988 2.745790e+00
## 4041   1 3989 2.738789e+00
## 4042   1 3990 2.724899e+00
## 4043   1 3991 2.685559e+00
## 4044   1 3992 2.645190e+00
## 4045   1 3993 2.609602e+00
## 4046   1 3994 2.566152e+00
## 4047   1 3995 2.537899e+00
## 4048   1 3996 2.501137e+00
## 4049   1 3997 2.468071e+00
## 4050   1 3998 2.435299e+00
## 4051   1 3999 2.411114e+00
## 4052   1 4000 2.379315e+00
## 4053   1 4001 2.354947e+00
## 4054   1 4002 2.333838e+00
## 4055   1 4003 2.307259e+00
## 4056   1 4004 2.286353e+00
## 4057   1 4005 2.259603e+00
## 4058   1 4006 2.247374e+00
## 4059   1 4007 2.226556e+00
## 4060   1 4008 2.206617e+00
## 4061   1 4009 2.370036e+00
## 4062   1 4010 2.582020e+00
## 4063   1 4011 2.701892e+00
## 4064   1 4012 2.748649e+00
## 4065   1 4013 2.745842e+00
## 4066   1 4014 2.725137e+00
## 4067   1 4015 2.692384e+00
## 4068   1 4016 2.651687e+00
## 4069   1 4017 2.617722e+00
## 4070   1 4018 2.577772e+00
## 4071   1 4019 2.539808e+00
## 4072   1 4020 2.498442e+00
## 4073   1 4021 2.473042e+00
## 4074   1 4022 2.440962e+00
## 4075   1 4023 2.415571e+00
## 4076   1 4024 2.386476e+00
## 4077   1 4025 2.359368e+00
## 4078   1 4026 2.337863e+00
## 4079   1 4027 2.315975e+00
## 4080   1 4028 2.289707e+00
## 4081   1 4029 2.269765e+00
## 4082   1 4030 2.250853e+00
## 4083   1 4031 2.235434e+00
## 4084   1 4032 2.214812e+00
## 4085   1 4033 2.370335e+00
## 4086   1 4034 2.582739e+00
## 4087   1 4035 2.707891e+00
## 4088   1 4036 2.749317e+00
## 4089   1 4037 2.753380e+00
## 4090   1 4038 2.726258e+00
## 4091   1 4039 2.696355e+00
## 4092   1 4040 2.658609e+00
## 4093   1 4041 2.619616e+00
## 4094   1 4042 2.582989e+00
## 4095   1 4043 2.546509e+00
## 4096   1 4044 2.510854e+00
## 4097   1 4045 2.472032e+00
## 4098   1 4046 2.442549e+00
## 4099   1 4047 2.418832e+00
## 4100   1 4048 2.383379e+00
## 4101   1 4049 2.359835e+00
## 4102   1 4050 2.338326e+00
## 4103   1 4051 2.317705e+00
## 4104   1 4052 2.294331e+00
## 4105   1 4053 2.270864e+00
## 4106   1 4054 2.245530e+00
## 4107   1 4055 2.234274e+00
## 4108   1 4056 2.216709e+00
## 4109   1 4057 2.376740e+00
## 4110   1 4058 2.588017e+00
## 4111   1 4059 2.706364e+00
## 4112   1 4060 2.757830e+00
## 4113   1 4061 2.755240e+00
## 4114   1 4062 2.732003e+00
## 4115   1 4063 2.695833e+00
## 4116   1 4064 2.658913e+00
## 4117   1 4065 2.617298e+00
## 4118   1 4066 2.582187e+00
## 4119   1 4067 2.543456e+00
## 4120   1 4068 2.513611e+00
## 4121   1 4069 2.480545e+00
## 4122   1 4070 2.445967e+00
## 4123   1 4071 2.422712e+00
## 4124   1 4072 2.390975e+00
## 4125   1 4073 2.364763e+00
## 4126   1 4074 2.342515e+00
## 4127   1 4075 2.318749e+00
## 4128   1 4076 2.298757e+00
## 4129   1 4077 2.273876e+00
## 4130   1 4078 2.252288e+00
## 4131   1 4079 2.235535e+00
## 4132   1 4080 2.221060e+00
## 4133   1 4081 2.377554e+00
## 4134   1 4082 2.587197e+00
## 4135   1 4083 2.711868e+00
## 4136   1 4084 2.756200e+00
## 4137   1 4085 2.759262e+00
## 4138   1 4086 2.737146e+00
## 4139   1 4087 2.694800e+00
## 4140   1 4088 2.662825e+00
## 4141   1 4089 2.626222e+00
## 4142   1 4090 2.584046e+00
## 4143   1 4091 2.548233e+00
## 4144   1 4092 2.516057e+00
## 4145   1 4093 2.474494e+00
## 4146   1 4094 2.452526e+00
## 4147   1 4095 2.423416e+00
## 4148   1 4096 2.395643e+00
## 4149   1 4097 2.367291e+00
## 4150   1 4098 2.343939e+00
## 4151   1 4099 2.317506e+00
## 4152   1 4100 2.298771e+00
## 4153   1 4101 2.278292e+00
## 4154   1 4102 2.257669e+00
## 4155   1 4103 2.239019e+00
## 4156   1 4104 2.224828e+00
## 4157   1 4104 2.248183e+00
## 4158   1 4105 2.412674e+00
## 4159   1 4106 2.616597e+00
## 4160   1 4107 2.739841e+00
## 4161   1 4108 2.787371e+00
## 4162   1 4109 2.788503e+00
## 4163   1 4110 2.763224e+00
## 4164   1 4111 2.730386e+00
## 4165   1 4112 2.689015e+00
## 4166   1 4113 2.654673e+00
## 4167   1 4114 2.612611e+00
## 4168   1 4115 2.580054e+00
## 4169   1 4116 2.541345e+00
## 4170   1 4117 2.511068e+00
## 4171   1 4118 2.480910e+00
## 4172   1 4119 2.450499e+00
## 4173   1 4120 2.423529e+00
## 4174   1 4121 2.397278e+00
## 4175   1 4122 2.373210e+00
## 4176   1 4123 2.347393e+00
## 4177   1 4124 2.329401e+00
## 4178   1 4125 2.304420e+00
## 4179   1 4126 2.291104e+00
## 4180   1 4127 2.269199e+00
## 4181   1 4128 2.251204e+00
## 4182   1 4128 2.248599e+00
## 4183   1 4129 2.230539e+00
## 4184   1 4130 2.211780e+00
## 4185   1 4131 2.200154e+00
## 4186   1 4132 2.187999e+00
## 4187   1 4133 2.170307e+00
## 4188   1 4134 2.159463e+00
## 4189   1 4135 2.144176e+00
## 4190   1 4136 2.130117e+00
## 4191   1 4137 2.118903e+00
## 4192   1 4138 2.105492e+00
## 4193   1 4139 2.095502e+00
## 4194   1 4140 2.080848e+00
## 4195   1 4141 2.072815e+00
## 4196   1 4142 2.059264e+00
## 4197   1 4143 2.045667e+00
## 4198   1 4144 2.039059e+00
## 4199   1 4145 2.025934e+00
## 4200   1 4146 2.018687e+00
## 4201   1 4147 2.009698e+00
## 4202   1 4148 2.000509e+00
## 4203   1 4149 1.990155e+00
## 4204   1 4150 1.980402e+00
## 4205   1 4151 1.970663e+00
## 4206   1 4152 1.964368e+00
## 4207   1 4152 1.963830e+00
## 4208   1 4153 2.314126e+00
## 4209   1 4154 2.751869e+00
## 4210   1 4155 3.023571e+00
## 4211   1 4156 3.138682e+00
## 4212   1 4157 3.159265e+00
## 4213   1 4158 3.128410e+00
## 4214   1 4159 3.090894e+00
## 4215   1 4160 3.017340e+00
## 4216   1 4161 2.957434e+00
## 4217   1 4162 2.907907e+00
## 4218   1 4163 2.842186e+00
## 4219   1 4164 2.791785e+00
## 4220   1 4165 2.736934e+00
## 4221   1 4166 2.692969e+00
## 4222   1 4167 2.651110e+00
## 4223   1 4168 2.606829e+00
## 4224   1 4169 2.568410e+00
## 4225   1 4170 2.533350e+00
## 4226   1 4171 2.497106e+00
## 4227   1 4172 2.470476e+00
## 4228   1 4173 2.433015e+00
## 4229   1 4174 2.403519e+00
## 4230   1 4175 2.377047e+00
## 4231   1 4176 2.353774e+00
## 4232   1 4176 2.352948e+00
## 4233   1 4177 2.508220e+00
## 4234   1 4178 2.707651e+00
## 4235   1 4179 2.827196e+00
## 4236   1 4180 2.868401e+00
## 4237   1 4181 2.862389e+00
## 4238   1 4182 2.839271e+00
## 4239   1 4183 2.796476e+00
## 4240   1 4184 2.751534e+00
## 4241   1 4185 2.709012e+00
## 4242   1 4186 2.668045e+00
## 4243   1 4187 2.627461e+00
## 4244   1 4188 2.594334e+00
## 4245   1 4189 2.559253e+00
## 4246   1 4190 2.527078e+00
## 4247   1 4191 2.486607e+00
## 4248   1 4192 2.461809e+00
## 4249   1 4193 2.434903e+00
## 4250   1 4194 2.406890e+00
## 4251   1 4195 2.383064e+00
## 4252   1 4196 2.359236e+00
## 4253   1 4197 2.336155e+00
## 4254   1 4198 2.316916e+00
## 4255   1 4199 2.293771e+00
## 4256   1 4200 2.277962e+00
## 4257   1 4201 2.435792e+00
## 4258   1 4202 2.645404e+00
## 4259   1 4203 2.767672e+00
## 4260   1 4204 2.809746e+00
## 4261   1 4205 2.811301e+00
## 4262   1 4206 2.786002e+00
## 4263   1 4207 2.747635e+00
## 4264   1 4208 2.710017e+00
## 4265   1 4209 2.671556e+00
## 4266   1 4210 2.634178e+00
## 4267   1 4211 2.600850e+00
## 4268   1 4212 2.560800e+00
## 4269   1 4213 2.532436e+00
## 4270   1 4214 2.498956e+00
## 4271   1 4215 2.464787e+00
## 4272   1 4216 2.439201e+00
## 4273   1 4217 2.416889e+00
## 4274   1 4218 2.390176e+00
## 4275   1 4219 2.366891e+00
## 4276   1 4220 2.340680e+00
## 4277   1 4221 2.317281e+00
## 4278   1 4222 2.300862e+00
## 4279   1 4223 2.286748e+00
## 4280   1 4224 2.266353e+00
## 4281   1 4225 2.430584e+00
## 4282   1 4226 2.629873e+00
## 4283   1 4227 2.756526e+00
## 4284   1 4228 2.802167e+00
## 4285   1 4229 2.803571e+00
## 4286   1 4230 2.777548e+00
## 4287   1 4231 2.742630e+00
## 4288   1 4232 2.700643e+00
## 4289   1 4233 2.664193e+00
## 4290   1 4234 2.626209e+00
## 4291   1 4235 2.588735e+00
## 4292   1 4236 2.558638e+00
## 4293   1 4237 2.523467e+00
## 4294   1 4238 2.492763e+00
## 4295   1 4239 2.464574e+00
## 4296   1 4240 2.433725e+00
## 4297   1 4241 2.407548e+00
## 4298   1 4242 2.388144e+00
## 4299   1 4243 2.356626e+00
## 4300   1 4244 2.338886e+00
## 4301   1 4245 2.315867e+00
## 4302   1 4246 2.296179e+00
## 4303   1 4247 2.277671e+00
## 4304   1 4248 2.261469e+00
## 4305   1 4249 2.423201e+00
## 4306   1 4250 2.628836e+00
## 4307   1 4251 2.751390e+00
## 4308   1 4252 2.800220e+00
## 4309   1 4253 2.803021e+00
## 4310   1 4254 2.775702e+00
## 4311   1 4255 2.742592e+00
## 4312   1 4256 2.704779e+00
## 4313   1 4257 2.664397e+00
## 4314   1 4258 2.624133e+00
## 4315   1 4259 2.591476e+00
## 4316   1 4260 2.555040e+00
## 4317   1 4261 2.522176e+00
## 4318   1 4262 2.487528e+00
## 4319   1 4263 2.464638e+00
## 4320   1 4264 2.432224e+00
## 4321   1 4265 2.410008e+00
## 4322   1 4266 2.385052e+00
## 4323   1 4267 2.357865e+00
## 4324   1 4268 2.341247e+00
## 4325   1 4269 2.317355e+00
## 4326   1 4270 2.296633e+00
## 4327   1 4271 2.277753e+00
## 4328   1 4272 2.259231e+00
## 4329   1 4273 2.425334e+00
## 4330   1 4274 2.625971e+00
## 4331   1 4275 2.751201e+00
## 4332   1 4276 2.797217e+00
## 4333   1 4277 2.800866e+00
## 4334   1 4278 2.771393e+00
## 4335   1 4279 2.744567e+00
## 4336   1 4280 2.702191e+00
## 4337   1 4281 2.667759e+00
## 4338   1 4282 2.629957e+00
## 4339   1 4283 2.586588e+00
## 4340   1 4284 2.556095e+00
## 4341   1 4285 2.518309e+00
## 4342   1 4286 2.492596e+00
## 4343   1 4287 2.464400e+00
## 4344   1 4288 2.433379e+00
## 4345   1 4289 2.407101e+00
## 4346   1 4290 2.381667e+00
## 4347   1 4291 2.363033e+00
## 4348   1 4292 2.337838e+00
## 4349   1 4293 2.316557e+00
## 4350   1 4294 2.299453e+00
## 4351   1 4295 2.279082e+00
## 4352   1 4296 2.259617e+00
## 4353   1 4297 2.417582e+00
## 4354   1 4298 2.628709e+00
## 4355   1 4299 2.757422e+00
## 4356   1 4300 2.795438e+00
## 4357   1 4301 2.799433e+00
## 4358   1 4302 2.772945e+00
## 4359   1 4303 2.736943e+00
## 4360   1 4304 2.704287e+00
## 4361   1 4305 2.661381e+00
## 4362   1 4306 2.625046e+00
## 4363   1 4307 2.591122e+00
## 4364   1 4308 2.549983e+00
## 4365   1 4309 2.524096e+00
## 4366   1 4310 2.490024e+00
## 4367   1 4311 2.461107e+00
## 4368   1 4312 2.435115e+00
## 4369   1 4313 2.408125e+00
## 4370   1 4314 2.376345e+00
## 4371   1 4315 2.353496e+00
## 4372   1 4316 2.337921e+00
## 4373   1 4317 2.317033e+00
## 4374   1 4318 2.295946e+00
## 4375   1 4319 2.279969e+00
## 4376   1 4320 2.257086e+00
## 4377   1 4321 2.416214e+00
## 4378   1 4322 2.629779e+00
## 4379   1 4323 2.754965e+00
## 4380   1 4324 2.798934e+00
## 4381   1 4325 2.797398e+00
## 4382   1 4326 2.769147e+00
## 4383   1 4327 2.740249e+00
## 4384   1 4328 2.699174e+00
## 4385   1 4329 2.658515e+00
## 4386   1 4330 2.621258e+00
## 4387   1 4331 2.592220e+00
## 4388   1 4332 2.556830e+00
## 4389   1 4333 2.523295e+00
## 4390   1 4334 2.494236e+00
## 4391   1 4335 2.459744e+00
## 4392   1 4336 2.426737e+00
## 4393   1 4337 2.404468e+00
## 4394   1 4338 2.381682e+00
## 4395   1 4339 2.357116e+00
## 4396   1 4340 2.337697e+00
## 4397   1 4341 2.313673e+00
## 4398   1 4342 2.293761e+00
## 4399   1 4343 2.281053e+00
## 4400   1 4344 2.254886e+00
## 4401   1 4345 2.417215e+00
## 4402   1 4346 2.629662e+00
## 4403   1 4347 2.749152e+00
## 4404   1 4348 2.798774e+00
## 4405   1 4349 2.795924e+00
## 4406   1 4350 2.771638e+00
## 4407   1 4351 2.735746e+00
## 4408   1 4352 2.698059e+00
## 4409   1 4353 2.658922e+00
## 4410   1 4354 2.623865e+00
## 4411   1 4355 2.585328e+00
## 4412   1 4356 2.551049e+00
## 4413   1 4357 2.521719e+00
## 4414   1 4358 2.483874e+00
## 4415   1 4359 2.457148e+00
## 4416   1 4360 2.431152e+00
## 4417   1 4361 2.401018e+00
## 4418   1 4362 2.379793e+00
## 4419   1 4363 2.354248e+00
## 4420   1 4364 2.333874e+00
## 4421   1 4365 2.315527e+00
## 4422   1 4366 2.295217e+00
## 4423   1 4367 2.277033e+00
## 4424   1 4368 2.259135e+00
## 4425   1 4369 2.417183e+00
## 4426   1 4370 2.628963e+00
## 4427   1 4371 2.750331e+00
## 4428   1 4372 2.790080e+00
## 4429   1 4373 2.794299e+00
## 4430   1 4374 2.772926e+00
## 4431   1 4375 2.742508e+00
## 4432   1 4376 2.697625e+00
## 4433   1 4377 2.661166e+00
## 4434   1 4378 2.622410e+00
## 4435   1 4379 2.584653e+00
## 4436   1 4380 2.550195e+00
## 4437   1 4381 2.515504e+00
## 4438   1 4382 2.488279e+00
## 4439   1 4383 2.458713e+00
## 4440   1 4384 2.429246e+00
## 4441   1 4385 2.404490e+00
## 4442   1 4386 2.381852e+00
## 4443   1 4387 2.355305e+00
## 4444   1 4388 2.333021e+00
## 4445   1 4389 2.320567e+00
## 4446   1 4390 2.295577e+00
## 4447   1 4391 2.278082e+00
## 4448   1 4392 2.257536e+00
## 4449   1 4393 2.413262e+00
## 4450   1 4394 2.619526e+00
## 4451   1 4395 2.748751e+00
## 4452   1 4396 2.791970e+00
## 4453   1 4397 2.796747e+00
## 4454   1 4398 2.770884e+00
## 4455   1 4399 2.736871e+00
## 4456   1 4400 2.698362e+00
## 4457   1 4401 2.654983e+00
## 4458   1 4402 2.618980e+00
## 4459   1 4403 2.582705e+00
## 4460   1 4404 2.549429e+00
## 4461   1 4405 2.517832e+00
## 4462   1 4406 2.490516e+00
## 4463   1 4407 2.457924e+00
## 4464   1 4408 2.427133e+00
## 4465   1 4409 2.405122e+00
## 4466   1 4410 2.384279e+00
## 4467   1 4411 2.353194e+00
## 4468   1 4412 2.338553e+00
## 4469   1 4413 2.317511e+00
## 4470   1 4414 2.290584e+00
## 4471   1 4415 2.273574e+00
## 4472   1 4416 2.261334e+00
## 4473   1 4417 2.411317e+00
## 4474   1 4418 2.622756e+00
## 4475   1 4419 2.748985e+00
## 4476   1 4420 2.797704e+00
## 4477   1 4421 2.793416e+00
## 4478   1 4422 2.773195e+00
## 4479   1 4423 2.734374e+00
## 4480   1 4424 2.698823e+00
## 4481   1 4425 2.665283e+00
## 4482   1 4426 2.621204e+00
## 4483   1 4427 2.582674e+00
## 4484   1 4428 2.549956e+00
## 4485   1 4429 2.519399e+00
## 4486   1 4430 2.488209e+00
## 4487   1 4431 2.463508e+00
## 4488   1 4432 2.427180e+00
## 4489   1 4433 2.404810e+00
## 4490   1 4434 2.374150e+00
## 4491   1 4435 2.354730e+00
## 4492   1 4436 2.338105e+00
## 4493   1 4437 2.313103e+00
## 4494   1 4438 2.292579e+00
## 4495   1 4439 2.268888e+00
## 4496   1 4440 2.255630e+00
## 4497   1 4441 2.236211e+00
## 4498   1 4442 2.223134e+00
## 4499   1 4443 2.204147e+00
## 4500   1 4444 2.194311e+00
## 4501   1 4445 2.174822e+00
## 4502   1 4446 2.167796e+00
## 4503   1 4447 2.150516e+00
## 4504   1 4448 2.138440e+00
## 4505   1 4449 2.121603e+00
## 4506   1 4450 2.109244e+00
## 4507   1 4451 2.100031e+00
## 4508   1 4452 2.086576e+00
## 4509   1 4453 2.076931e+00
## 4510   1 4454 2.066739e+00
## 4511   1 4455 2.055845e+00
## 4512   1 4456 2.045966e+00
## 4513   1 4457 2.031513e+00
## 4514   1 4458 2.026199e+00
## 4515   1 4459 2.011432e+00
## 4516   1 4460 2.004374e+00
## 4517   1 4461 1.993954e+00
## 4518   1 4462 1.985323e+00
## 4519   1 4463 1.981431e+00
## 4520   1 4464 1.971565e+00
## 4521   1 4465 1.963491e+00
## 4522   1 4466 1.950503e+00
## 4523   1 4467 1.944061e+00
## 4524   1 4468 1.932968e+00
## 4525   1 4469 1.928673e+00
## 4526   1 4470 1.917925e+00
## 4527   1 4471 1.912167e+00
## 4528   1 4472 1.902440e+00
## 4529   1 4473 1.896180e+00
## 4530   1 4474 1.888019e+00
## 4531   1 4475 1.879387e+00
## 4532   1 4476 1.874208e+00
## 4533   1 4477 1.865436e+00
## 4534   1 4478 1.857127e+00
## 4535   1 4479 1.848229e+00
## 4536   1 4480 1.844823e+00
## 4537   1 4481 1.839138e+00
## 4538   1 4482 1.831587e+00
## 4539   1 4483 1.820381e+00
## 4540   1 4484 1.813416e+00
## 4541   1 4485 1.809606e+00
## 4542   1 4486 1.798554e+00
## 4543   1 4487 1.794455e+00
## 4544   1 4488 1.789871e+00
## 4545   1 4489 1.781933e+00
## 4546   1 4490 1.774375e+00
## 4547   1 4491 1.768204e+00
## 4548   1 4492 1.757094e+00
## 4549   1 4493 1.754528e+00
## 4550   1 4494 1.744050e+00
## 4551   1 4495 1.740595e+00
## 4552   1 4496 1.735943e+00
## 4553   1 4497 1.725992e+00
## 4554   1 4498 1.720065e+00
## 4555   1 4499 1.714899e+00
## 4556   1 4500 1.711577e+00
## 4557   1 4501 1.700169e+00
## 4558   1 4502 1.694539e+00
## 4559   1 4503 1.688807e+00
## 4560   1 4504 1.679679e+00
## 4561   1 4505 1.676879e+00
## 4562   1 4506 1.668472e+00
## 4563   1 4507 1.662107e+00
## 4564   1 4508 1.656583e+00
## 4565   1 4509 1.649420e+00
## 4566   1 4510 1.646582e+00
## 4567   1 4511 1.640612e+00
## 4568   1 4512 1.635219e+00
## 4569   1 4513 1.625452e+00
## 4570   1 4514 1.619391e+00
## 4571   1 4515 1.610283e+00
## 4572   1 4516 1.602767e+00
## 4573   1 4517 1.600340e+00
## 4574   1 4518 1.597479e+00
## 4575   1 4519 1.591071e+00
## 4576   1 4520 1.582645e+00
## 4577   1 4521 1.577957e+00
## 4578   1 4522 1.570666e+00
## 4579   1 4523 1.562457e+00
## 4580   1 4524 1.559269e+00
## 4581   1 4525 1.553232e+00
## 4582   1 4526 1.548746e+00
## 4583   1 4527 1.541636e+00
## 4584   1 4528 1.534151e+00
## 4585   1 4529 1.530099e+00
## 4586   1 4530 1.526712e+00
## 4587   1 4531 1.522211e+00
## 4588   1 4532 1.514095e+00
## 4589   1 4533 1.507574e+00
## 4590   1 4534 1.504287e+00
## 4591   1 4535 1.497815e+00
## 4592   1 4536 1.492042e+00
## 4593   1 4537 1.485349e+00
## 4594   1 4538 1.481696e+00
## 4595   1 4539 1.474840e+00
## 4596   1 4540 1.470134e+00
## 4597   1 4541 1.463698e+00
## 4598   1 4542 1.457962e+00
## 4599   1 4543 1.452797e+00
## 4600   1 4544 1.446852e+00
## 4601   1 4545 1.443983e+00
## 4602   1 4546 1.436658e+00
## 4603   1 4547 1.430788e+00
## 4604   1 4548 1.426608e+00
## 4605   1 4549 1.420818e+00
## 4606   1 4550 1.415083e+00
## 4607   1 4551 1.410647e+00
## 4608   1 4552 1.404951e+00
## 4609   1 4553 1.402616e+00
## 4610   1 4554 1.394523e+00
## 4611   1 4555 1.389272e+00
## 4612   1 4556 1.384678e+00
## 4613   1 4557 1.377807e+00
## 4614   1 4558 1.372333e+00
## 4615   1 4559 1.367386e+00
## 4616   1 4560 1.363971e+00
## 4617   1 4561 1.356533e+00
## 4618   1 4562 1.350692e+00
## 4619   1 4563 1.347441e+00
## 4620   1 4564 1.344979e+00
## 4621   1 4565 1.339442e+00
## 4622   1 4566 1.333861e+00
## 4623   1 4567 1.327709e+00
## 4624   1 4568 1.322623e+00
## 4625   1 4569 1.316665e+00
## 4626   1 4570 1.313695e+00
## 4627   1 4571 1.307281e+00
## 4628   1 4572 1.302978e+00
## 4629   1 4573 1.298929e+00
## 4630   1 4574 1.293953e+00
## 4631   1 4575 1.291262e+00
## 4632   1 4576 1.282720e+00
## 4633   1 4577 1.281284e+00
## 4634   1 4578 1.274017e+00
## 4635   1 4579 1.270728e+00
## 4636   1 4580 1.264281e+00
## 4637   1 4581 1.261435e+00
## 4638   1 4582 1.254866e+00
## 4639   1 4583 1.251901e+00
## 4640   1 4584 1.247892e+00
## 4641   1 4585 1.241842e+00
## 4642   1 4586 1.236327e+00
## 4643   1 4587 1.231458e+00
## 4644   1 4588 1.227908e+00
## 4645   1 4589 1.222909e+00
## 4646   1 4590 1.219120e+00
## 4647   1 4591 1.214207e+00
## 4648   1 4592 1.210811e+00
## 4649   1 4593 1.204433e+00
## 4650   1 4594 1.199840e+00
## 4651   1 4595 1.197099e+00
## 4652   1 4596 1.192207e+00
## 4653   1 4597 1.186829e+00
## 4654   1 4598 1.183326e+00
## 4655   1 4599 1.178164e+00
## 4656   1 4600 1.176307e+00
## 4657   1 4601 1.170011e+00
## 4658   1 4602 1.165092e+00
## 4659   1 4603 1.160759e+00
## 4660   1 4604 1.154104e+00
## 4661   1 4605 1.150839e+00
## 4662   1 4606 1.147617e+00
## 4663   1 4607 1.145280e+00
## 4664   1 4608 1.136984e+00
## 4665   1 4609 1.135552e+00
## 4666   1 4610 1.130300e+00
## 4667   1 4611 1.127031e+00
## 4668   1 4612 1.122917e+00
## 4669   1 4613 1.118772e+00
## 4670   1 4614 1.114326e+00
## 4671   1 4615 1.111858e+00
## 4672   1 4616 1.106053e+00
## 4673   1 4617 1.102500e+00
```

```
## 4674   1 4618 1.098083e+00
## 4675   1 4619 1.093659e+00
## 4676   1 4620 1.089172e+00
## 4677   1 4621 1.084159e+00
## 4678   1 4622 1.081620e+00
## 4679   1 4623 1.076879e+00
## 4680   1 4624 1.073917e+00
## 4681   1 4625 1.069767e+00
## 4682   1 4626 1.064990e+00
## 4683   1 4627 1.061157e+00
## 4684   1 4628 1.058078e+00
## 4685   1 4629 1.054262e+00
## 4686   1 4630 1.049732e+00
## 4687   1 4631 1.046154e+00
## 4688   1 4632 1.043083e+00
## 4689   1 4633 1.037938e+00
## 4690   1 4634 1.034156e+00
## 4691   1 4635 1.030696e+00
## 4692   1 4636 1.026156e+00
## 4693   1 4637 1.022539e+00
## 4694   1 4638 1.017543e+00
## 4695   1 4639 1.014833e+00
## 4696   1 4640 1.011530e+00
## 4697   1 4641 1.007707e+00
## 4698   1 4642 1.004555e+00
## 4699   1 4643 1.000289e+00
## 4700   1 4644 9.954970e-01
## 4701   1 4645 9.921155e-01
## 4702   1 4646 9.899121e-01
## 4703   1 4647 9.861855e-01
## 4704   1 4648 9.795646e-01
## 4705   1 4649 9.768631e-01
## 4706   1 4650 9.753202e-01
## 4707   1 4651 9.718832e-01
## 4708   1 4652 9.672334e-01
## 4709   1 4653 9.634313e-01
## 4710   1 4654 9.598923e-01
## 4711   1 4655 9.558231e-01
## 4712   1 4656 9.515148e-01
## 4713   1 4657 9.499006e-01
## 4714   1 4658 9.450996e-01
## 4715   1 4659 9.410974e-01
## 4716   1 4660 9.385272e-01
## 4717   1 4661 9.335546e-01
## 4718   1 4662 9.303357e-01
## 4719   1 4663 9.279901e-01
## 4720   1 4664 9.259652e-01
## 4721   1 4665 9.200129e-01
## 4722   1 4666 9.176813e-01
## 4723   1 4667 9.167016e-01
## 4724   1 4668 9.104434e-01
## 4725   1 4669 9.060828e-01
## 4726   1 4670 9.037678e-01
## 4727   1 4671 9.025622e-01
## 4728   1 4672 8.971945e-01
## 4729   1 4673 8.944888e-01
## 4730   1 4674 8.892875e-01
## 4731   1 4675 8.861008e-01
## 4732   1 4676 8.823320e-01
## 4733   1 4677 8.796494e-01
## 4734   1 4678 8.786162e-01
## 4735   1 4679 8.737109e-01
## 4736   1 4680 8.703080e-01
## 4737   1 4681 8.677359e-01
## 4738   1 4682 8.648852e-01
## 4739   1 4683 8.595869e-01
## 4740   1 4684 8.584023e-01
## 4741   1 4685 8.560166e-01
## 4742   1 4686 8.515345e-01
## 4743   1 4687 8.489292e-01
## 4744   1 4688 8.468318e-01
## 4745   1 4689 8.420315e-01
## 4746   1 4690 8.381695e-01
## 4747   1 4691 8.350128e-01
## 4748   1 4692 8.328996e-01
## 4749   1 4693 8.295313e-01
## 4750   1 4694 8.271207e-01
## 4751   1 4695 8.227236e-01
## 4752   1 4696 8.213399e-01
## 4753   1 4697 8.188891e-01
## 4754   1 4698 8.148213e-01
## 4755   1 4699 8.112570e-01
## 4756   1 4700 8.078147e-01
## 4757   1 4701 8.053962e-01
## 4758   1 4702 8.014497e-01
## 4759   1 4703 7.988731e-01
## 4760   1 4704 7.950044e-01
## 4761   1 4705 7.927603e-01
## 4762   1 4706 7.900650e-01
## 4763   1 4707 7.875197e-01
## 4764   1 4708 7.837745e-01
## 4765   1 4709 7.796686e-01
## 4766   1 4710 7.782380e-01
## 4767   1 4711 7.767414e-01
## 4768   1 4712 7.726383e-01
## 4769   1 4713 7.697008e-01
## 4770   1 4714 7.660843e-01
## 4771   1 4715 7.642791e-01
## 4772   1 4716 7.617377e-01
## 4773   1 4717 7.589454e-01
## 4774   1 4718 7.558265e-01
## 4775   1 4719 7.529537e-01
## 4776   1 4720 7.508000e-01
## 4777   1 4721 7.476432e-01
## 4778   1 4722 7.453463e-01
## 4779   1 4723 7.412838e-01
## 4780   1 4724 7.374067e-01
## 4781   1 4725 7.369811e-01
## 4782   1 4726 7.329934e-01
## 4783   1 4727 7.303570e-01
## 4784   1 4728 7.268984e-01
## 4785   1 4729 7.247613e-01
## 4786   1 4730 7.226351e-01
## 4787   1 4731 7.200487e-01
## 4788   1 4732 7.168391e-01
## 4789   1 4733 7.140986e-01
## 4790   1 4734 7.114019e-01
## 4791   1 4735 7.092541e-01
## 4792   1 4736 7.057237e-01
## 4793   1 4737 7.037189e-01
## 4794   1 4738 7.020821e-01
## 4795   1 4739 6.988621e-01
## 4796   1 4740 6.964955e-01
## 4797   1 4741 6.931485e-01
## 4798   1 4742 6.916613e-01
## 4799   1 4743 6.869498e-01
## 4800   1 4744 6.860071e-01
## 4801   1 4745 6.831983e-01
## 4802   1 4746 6.801455e-01
## 4803   1 4747 6.779556e-01
## 4804   1 4748 6.760013e-01
## 4805   1 4749 6.736644e-01
## 4806   1 4750 6.693990e-01
## 4807   1 4751 6.673264e-01
## 4808   1 4752 6.656204e-01
## 4809   1 4753 6.634510e-01
## 4810   1 4754 6.611368e-01
## 4811   1 4755 6.582054e-01
## 4812   1 4756 6.560589e-01
## 4813   1 4757 6.530219e-01
## 4814   1 4758 6.500925e-01
## 4815   1 4759 6.487496e-01
## 4816   1 4760 6.460789e-01
## 4817   1 4761 6.427707e-01
## 4818   1 4762 6.406323e-01
## 4819   1 4763 6.386482e-01
## 4820   1 4764 6.359671e-01
## 4821   1 4765 6.329638e-01
## 4822   1 4766 6.299485e-01
## 4823   1 4767 6.293778e-01
## 4824   1 4768 6.276876e-01
## 4825   1 4769 6.254520e-01
## 4826   1 4770 6.214349e-01
## 4827   1 4771 6.198841e-01
## 4828   1 4772 6.166654e-01
## 4829   1 4773 6.151399e-01
## 4830   1 4774 6.118406e-01
## 4831   1 4775 6.107062e-01
## 4832   1 4776 6.076866e-01
## 4833   1 4777 6.059225e-01
## 4834   1 4778 6.041663e-01
## 4835   1 4779 6.017727e-01
## 4836   1 4780 6.003966e-01
## 4837   1 4781 5.969709e-01
## 4838   1 4782 5.958734e-01
## 4839   1 4783 5.923777e-01
## 4840   1 4784 5.903772e-01
## 4841   1 4785 5.881171e-01
## 4842   1 4786 5.872738e-01
## 4843   1 4787 5.836213e-01
## 4844   1 4788 5.812480e-01
## 4845   1 4789 5.783638e-01
## 4846   1 4790 5.775998e-01
## 4847   1 4791 5.751583e-01
## 4848   1 4792 5.723214e-01
## 4849   1 4793 5.708933e-01
## 4850   1 4794 5.702150e-01
## 4851   1 4795 5.671677e-01
## 4852   1 4796 5.643663e-01
## 4853   1 4797 5.617921e-01
## 4854   1 4798 5.598959e-01
## 4855   1 4799 5.586146e-01
## 4856   1 4800 5.559587e-01
## 4857   1 4801 5.545433e-01
## 4858   1 4802 5.520196e-01
## 4859   1 4803 5.496039e-01
## 4860   1 4804 5.474973e-01
## 4861   1 4805 5.468622e-01
## 4862   1 4806 5.446195e-01
## 4863   1 4807 5.419738e-01
## 4864   1 4808 5.400303e-01
## 4865   1 4809 5.372236e-01
## 4866   1 4810 5.354194e-01
## 4867   1 4811 5.338393e-01
## 4868   1 4812 5.316400e-01
## 4869   1 4813 5.289216e-01
## 4870   1 4814 5.275385e-01
## 4871   1 4815 5.258486e-01
## 4872   1 4816 5.232730e-01
## 4873   1 4817 5.222932e-01
## 4874   1 4818 5.189104e-01
## 4875   1 4819 5.187221e-01
## 4876   1 4820 5.165016e-01
## 4877   1 4821 5.133271e-01
## 4878   1 4822 5.126139e-01
## 4879   1 4823 5.105448e-01
## 4880   1 4824 5.080452e-01
## 4881   1 4825 5.073832e-01
## 4882   1 4826 5.047612e-01
## 4883   1 4827 5.028018e-01
## 4884   1 4828 5.019852e-01
## 4885   1 4829 4.990247e-01
## 4886   1 4830 4.969244e-01
## 4887   1 4831 4.950986e-01
## 4888   1 4832 4.936109e-01
## 4889   1 4833 4.908282e-01
## 4890   1 4834 4.897649e-01
## 4891   1 4835 4.871609e-01
## 4892   1 4836 4.858242e-01
## 4893   1 4837 4.849601e-01
## 4894   1 4838 4.825185e-01
## 4895   1 4839 4.805722e-01
## 4896   1 4840 4.795424e-01
## 4897   1 4841 4.768460e-01
## 4898   1 4842 4.755840e-01
## 4899   1 4843 4.731572e-01
## 4900   1 4844 4.724572e-01
## 4901   1 4845 4.706343e-01
## 4902   1 4846 4.683976e-01
## 4903   1 4847 4.666540e-01
## 4904   1 4848 4.646586e-01
## 4905   1 4849 4.630368e-01
## 4906   1 4850 4.613577e-01
## 4907   1 4851 4.595651e-01
## 4908   1 4852 4.577647e-01
## 4909   1 4853 4.566692e-01
## 4910   1 4854 4.550973e-01
## 4911   1 4855 4.534006e-01
## 4912   1 4856 4.509942e-01
## 4913   1 4857 4.487946e-01
## 4914   1 4858 4.481524e-01
## 4915   1 4859 4.458210e-01
## 4916   1 4860 4.447624e-01
## 4917   1 4861 4.423634e-01
## 4918   1 4862 4.411441e-01
## 4919   1 4863 4.395279e-01
## 4920   1 4864 4.377481e-01
## 4921   1 4865 4.373108e-01
## 4922   1 4866 4.352343e-01
## 4923   1 4867 4.327418e-01
## 4924   1 4868 4.314138e-01
## 4925   1 4869 4.294319e-01
## 4926   1 4870 4.282298e-01
## 4927   1 4871 4.268962e-01
## 4928   1 4872 4.252005e-01
## 4929   1 4873 4.223522e-01
## 4930   1 4874 4.218095e-01
## 4931   1 4875 4.203603e-01
## 4932   1 4876 4.187043e-01
## 4933   1 4877 4.170365e-01
## 4934   1 4878 4.151426e-01
## 4935   1 4879 4.132690e-01
## 4936   1 4880 4.126010e-01
## 4937   1 4881 4.113351e-01
## 4938   1 4882 4.088219e-01
## 4939   1 4883 4.080830e-01
## 4940   1 4884 4.065721e-01
## 4941   1 4885 4.047966e-01
## 4942   1 4886 4.031666e-01
## 4943   1 4887 4.018885e-01
## 4944   1 4888 4.005196e-01
## 4945   1 4889 3.991734e-01
## 4946   1 4890 3.980055e-01
## 4947   1 4891 3.962799e-01
## 4948   1 4892 3.938864e-01
## 4949   1 4893 3.930439e-01
## 4950   1 4894 3.918981e-01
## 4951   1 4895 3.899975e-01
## 4952   1 4896 3.882743e-01
## 4953   1 4897 3.874361e-01
## 4954   1 4898 3.850747e-01
## 4955   1 4899 3.844820e-01
## 4956   1 4900 3.825911e-01
## 4957   1 4901 3.814452e-01
## 4958   1 4902 3.798526e-01
## 4959   1 4903 3.785324e-01
## 4960   1 4904 3.777074e-01
## 4961   1 4905 3.758438e-01
## 4962   1 4906 3.745305e-01
## 4963   1 4907 3.725164e-01
## 4964   1 4908 3.721541e-01
## 4965   1 4909 3.703554e-01
## 4966   1 4910 3.686660e-01
## 4967   1 4911 3.674680e-01
## 4968   1 4912 3.654840e-01
## 4969   1 4913 3.648880e-01
## 4970   1 4914 3.631743e-01
## 4971   1 4915 3.626738e-01
## 4972   1 4916 3.607479e-01
## 4973   1 4917 3.592149e-01
## 4974   1 4918 3.575021e-01
## 4975   1 4919 3.559221e-01
## 4976   1 4920 3.552007e-01
## 4977   1 4921 3.542881e-01
## 4978   1 4922 3.529157e-01
## 4979   1 4923 3.511011e-01
## 4980   1 4924 3.495877e-01
## 4981   1 4925 3.499646e-01
## 4982   1 4926 3.476828e-01
## 4983   1 4927 3.463635e-01
## 4984   1 4928 3.442324e-01
## 4985   1 4929 3.438472e-01
## 4986   1 4930 3.417583e-01
## 4987   1 4931 3.413223e-01
## 4988   1 4932 3.399431e-01
## 4989   1 4933 3.382022e-01
## 4990   1 4934 3.370122e-01
## 4991   1 4935 3.357433e-01
## 4992   1 4936 3.348471e-01
## 4993   1 4937 3.334099e-01
## 4994   1 4938 3.319824e-01
## 4995   1 4939 3.311801e-01
## 4996   1 4940 3.305036e-01
## 4997   1 4941 3.286323e-01
## 4998   1 4942 3.269839e-01
## 4999   1 4943 3.264432e-01
## 5000   1 4944 3.249967e-01
## 5001   1 4945 3.241783e-01
## 5002   1 4946 3.227246e-01
## 5003   1 4947 3.213597e-01
## 5004   1 4948 3.203348e-01
## 5005   1 4949 3.186940e-01
## 5006   1 4950 3.174781e-01
## 5007   1 4951 3.162977e-01
## 5008   1 4952 3.154946e-01
## 5009   1 4953 3.142667e-01
## 5010   1 4954 3.127313e-01
## 5011   1 4955 3.117450e-01
## 5012   1 4956 3.103668e-01
## 5013   1 4957 3.091853e-01
## 5014   1 4958 3.076890e-01
## 5015   1 4959 3.071792e-01
## 5016   1 4960 3.060859e-01
## 5017   1 4961 3.049261e-01
## 5018   1 4962 3.035595e-01
## 5019   1 4963 3.023934e-01
## 5020   1 4964 3.022258e-01
## 5021   1 4965 3.003958e-01
## 5022   1 4966 2.991507e-01
## 5023   1 4967 2.984421e-01
## 5024   1 4968 2.974328e-01
## 5025   1 4969 2.954737e-01
## 5026   1 4970 2.945190e-01
## 5027   1 4971 2.937378e-01
## 5028   1 4972 2.933051e-01
## 5029   1 4973 2.912491e-01
## 5030   1 4974 2.903804e-01
## 5031   1 4975 2.892975e-01
## 5032   1 4976 2.878608e-01
## 5033   1 4977 2.873424e-01
## 5034   1 4978 2.861236e-01
## 5035   1 4979 2.849844e-01
## 5036   1 4980 2.840884e-01
## 5037   1 4981 2.831342e-01
## 5038   1 4982 2.818041e-01
## 5039   1 4983 2.805900e-01
## 5040   1 4984 2.796650e-01
## 5041   1 4985 2.789179e-01
## 5042   1 4986 2.779232e-01
## 5043   1 4987 2.761394e-01
## 5044   1 4988 2.757969e-01
## 5045   1 4989 2.749591e-01
## 5046   1 4990 2.740481e-01
## 5047   1 4991 2.722255e-01
## 5048   1 4992 2.711574e-01
## 5049   1 4993 2.705484e-01
## 5050   1 4994 2.696193e-01
## 5051   1 4995 2.687841e-01
## 5052   1 4996 2.670891e-01
## 5053   1 4997 2.663672e-01
## 5054   1 4998 2.652063e-01
## 5055   1 4999 2.638836e-01
## 5056   1 5000 2.634225e-01
## 5057   1 5001 2.626291e-01
## 5058   1 5002 2.617018e-01
## 5059   1 5003 2.607894e-01
## 5060   1 5004 2.593981e-01
## 5061   1 5005 2.588636e-01
## 5062   1 5006 2.577186e-01
## 5063   1 5007 2.570337e-01
## 5064   1 5008 2.555654e-01
## 5065   1 5009 2.545194e-01
## 5066   1 5010 2.540918e-01
## 5067   1 5011 2.526840e-01
## 5068   1 5012 2.519273e-01
## 5069   1 5013 2.505637e-01
## 5070   1 5014 2.498096e-01
## 5071   1 5015 2.492552e-01
## 5072   1 5016 2.478404e-01
## 5073   1 5017 2.474892e-01
## 5074   1 5018 2.464502e-01
## 5075   1 5019 2.451632e-01
## 5076   1 5020 2.441601e-01
## 5077   1 5021 2.434713e-01
## 5078   1 5022 2.425749e-01
## 5079   1 5023 2.420663e-01
## 5080   1 5024 2.405406e-01
## 5081   1 5025 2.403100e-01
## 5082   1 5026 2.388972e-01
## 5083   1 5027 2.379475e-01
## 5084   1 5028 2.371361e-01
## 5085   1 5029 2.365468e-01
## 5086   1 5030 2.357860e-01
## 5087   1 5031 2.343733e-01
## 5088   1 5032 2.338914e-01
## 5089   1 5033 2.327600e-01
## 5090   1 5034 2.321293e-01
## 5091   1 5035 2.310409e-01
## 5092   1 5036 2.304867e-01
## 5093   1 5037 2.292615e-01
## 5094   1 5038 2.285266e-01
## 5095   1 5039 2.279571e-01
## 5096   1 5040 2.269379e-01
## 5097   1 5041 2.258157e-01
## 5098   1 5042 2.255820e-01
## 5099   1 5043 2.246051e-01
## 5100   1 5044 2.234295e-01
## 5101   1 5045 2.227932e-01
## 5102   1 5046 2.215215e-01
## 5103   1 5047 2.209651e-01
## 5104   1 5048 2.201911e-01
## 5105   1 5049 2.192526e-01
## 5106   1 5050 2.186628e-01
## 5107   1 5051 2.176178e-01
## 5108   1 5052 2.168987e-01
## 5109   1 5053 2.157161e-01
## 5110   1 5054 2.154973e-01
## 5111   1 5055 2.142934e-01
## 5112   1 5056 2.133758e-01
## 5113   1 5057 2.128914e-01
## 5114   1 5058 2.119860e-01
## 5115   1 5059 2.111467e-01
## 5116   1 5060 2.105469e-01
## 5117   1 5061 2.097676e-01
## 5118   1 5062 2.086207e-01
## 5119   1 5063 2.080740e-01
## 5120   1 5064 2.072853e-01
## 5121   1 5065 2.062831e-01
## 5122   1 5066 2.057248e-01
## 5123   1 5067 2.050390e-01
## 5124   1 5068 2.042642e-01
## 5125   1 5069 2.036873e-01
## 5126   1 5070 2.027537e-01
## 5127   1 5071 2.021446e-01
## 5128   1 5072 2.011577e-01
## 5129   1 5073 2.003644e-01
## 5130   1 5074 1.999145e-01
## 5131   1 5075 1.986873e-01
## 5132   1 5076 1.981350e-01
## 5133   1 5077 1.970895e-01
## 5134   1 5078 1.968360e-01
## 5135   1 5079 1.958056e-01
## 5136   1 5080 1.957432e-01
## 5137   1 5081 1.945460e-01
## 5138   1 5082 1.937736e-01
## 5139   1 5083 1.930500e-01
## 5140   1 5084 1.926641e-01
## 5141   1 5085 1.917205e-01
## 5142   1 5086 1.910463e-01
## 5143   1 5087 1.905037e-01
## 5144   1 5088 1.896175e-01
## 5145   1 5089 1.891873e-01
## 5146   1 5090 1.878856e-01
## 5147   1 5091 1.873733e-01
## 5148   1 5092 1.868072e-01
## 5149   1 5093 1.862878e-01
## 5150   1 5094 1.852729e-01
## 5151   1 5095 1.844966e-01
## 5152   1 5096 1.838736e-01
## 5153   1 5097 1.830900e-01
## 5154   1 5098 1.826449e-01
## 5155   1 5099 1.818658e-01
## 5156   1 5100 1.813171e-01
## 5157   1 5101 1.806176e-01
## 5158   1 5102 1.799658e-01
## 5159   1 5103 1.792369e-01
## 5160   1 5104 1.784906e-01
## 5161   1 5105 1.779333e-01
## 5162   1 5106 1.769502e-01
## 5163   1 5107 1.763999e-01
## 5164   1 5108 1.759710e-01
## 5165   1 5109 1.756349e-01
## 5166   1 5110 1.745731e-01
## 5167   1 5111 1.739858e-01
## 5168   1 5112 1.734988e-01
## 5169   1 5113 1.727071e-01
## 5170   1 5114 1.720092e-01
## 5171   1 5115 1.713752e-01
## 5172   1 5116 1.707251e-01
## 5173   1 5117 1.700560e-01
## 5174   1 5118 1.695590e-01
## 5175   1 5119 1.690685e-01
## 5176   1 5120 1.685212e-01
## 5177   1 5121 1.675416e-01
## 5178   1 5122 1.668671e-01
## 5179   1 5123 1.665864e-01
## 5180   1 5124 1.657790e-01
## 5181   1 5125 1.650263e-01
## 5182   1 5126 1.644753e-01
## 5183   1 5127 1.641095e-01
## 5184   1 5128 1.633828e-01
## 5185   1 5129 1.625666e-01
## 5186   1 5130 1.621687e-01
## 5187   1 5131 1.616724e-01
## 5188   1 5132 1.608169e-01
## 5189   1 5133 1.603159e-01
## 5190   1 5134 1.595543e-01
## 5191   1 5135 1.591521e-01
## 5192   1 5136 1.584037e-01
## 5193   1 5137 1.578786e-01
## 5194   1 5138 1.571296e-01
## 5195   1 5139 1.566372e-01
## 5196   1 5140 1.557670e-01
## 5197   1 5141 1.553888e-01
## 5198   1 5142 1.551059e-01
## 5199   1 5143 1.545708e-01
## 5200   1 5144 1.539736e-01
## 5201   1 5145 1.532530e-01
## 5202   1 5146 1.526283e-01
## 5203   1 5147 1.521559e-01
## 5204   1 5148 1.514753e-01
## 5205   1 5149 1.508785e-01
## 5206   1 5150 1.506807e-01
## 5207   1 5151 1.497784e-01
## 5208   1 5152 1.492444e-01
## 5209   1 5153 1.488243e-01
## 5210   1 5154 1.483211e-01
## 5211   1 5155 1.474729e-01
## 5212   1 5156 1.473651e-01
## 5213   1 5157 1.464859e-01
## 5214   1 5158 1.460401e-01
## 5215   1 5159 1.454896e-01
## 5216   1 5160 1.447014e-01
## 5217   1 5161 1.444094e-01
## 5218   1 5162 1.437698e-01
## 5219   1 5163 1.433334e-01
## 5220   1 5164 1.426502e-01
## 5221   1 5165 1.421202e-01
## 5222   1 5166 1.418069e-01
## 5223   1 5167 1.413001e-01
## 5224   1 5168 1.405739e-01
## 5225   1 5169 1.402384e-01
## 5226   1 5170 1.394340e-01
## 5227   1 5171 1.390403e-01
## 5228   1 5172 1.384499e-01
## 5229   1 5173 1.380131e-01
## 5230   1 5174 1.374436e-01
## 5231   1 5175 1.371627e-01
## 5232   1 5176 1.364039e-01
## 5233   1 5177 1.361365e-01
## 5234   1 5178 1.353375e-01
## 5235   1 5179 1.349692e-01
## 5236   1 5180 1.344617e-01
## 5237   1 5181 1.339659e-01
## 5238   1 5182 1.333798e-01
## 5239   1 5183 1.329800e-01
## 5240   1 5184 1.324680e-01
## 5241   1 5185 1.321838e-01
## 5242   1 5186 1.314736e-01
## 5243   1 5187 1.309546e-01
## 5244   1 5188 1.306739e-01
## 5245   1 5189 1.299513e-01
## 5246   1 5190 1.294283e-01
## 5247   1 5191 1.291232e-01
## 5248   1 5192 1.283627e-01
## 5249   1 5193 1.280306e-01
## 5250   1 5194 1.275261e-01
## 5251   1 5195 1.270055e-01
## 5252   1 5196 1.266393e-01
## 5253   1 5197 1.261720e-01
## 5254   1 5198 1.255867e-01
## 5255   1 5199 1.251862e-01
## 5256   1 5200 1.249224e-01
## 5257   1 5201 1.242249e-01
## 5258   1 5202 1.236868e-01
## 5259   1 5203 1.232571e-01
## 5260   1 5204 1.231126e-01
## 5261   1 5205 1.223565e-01
## 5262   1 5206 1.218687e-01
## 5263   1 5207 1.214123e-01
## 5264   1 5208 1.211708e-01
## 5265   1 5209 1.206424e-01
## 5266   1 5210 1.200862e-01
## 5267   1 5211 1.197723e-01
## 5268   1 5212 1.193474e-01
## 5269   1 5213 1.187013e-01
## 5270   1 5214 1.182153e-01
## 5271   1 5215 1.179370e-01
## 5272   1 5216 1.173104e-01
## 5273   1 5217 1.171288e-01
## 5274   1 5218 1.169680e-01
## 5275   1 5219 1.160806e-01
## 5276   1 5220 1.156533e-01
## 5277   1 5221 1.154302e-01
## 5278   1 5222 1.147984e-01
## 5279   1 5223 1.145674e-01
## 5280   1 5224 1.141562e-01
## 5281   1 5225 1.136636e-01
## 5282   1 5226 1.130873e-01
## 5283   1 5227 1.126719e-01
## 5284   1 5228 1.123105e-01
## 5285   1 5229 1.119711e-01
## 5286   1 5230 1.117348e-01
## 5287   1 5231 1.112003e-01
## 5288   1 5232 1.107209e-01
## 5289   1 5233 1.105393e-01
## 5290   1 5234 1.098184e-01
## 5291   1 5235 1.094262e-01
## 5292   1 5236 1.089779e-01
## 5293   1 5237 1.085105e-01
## 5294   1 5238 1.082549e-01
## 5295   1 5239 1.079318e-01
## 5296   1 5240 1.073436e-01
## 5297   1 5241 1.070255e-01
## 5298   1 5242 1.066830e-01
## 5299   1 5243 1.061605e-01
## 5300   1 5244 1.060376e-01
## 5301   1 5245 1.053336e-01
## 5302   1 5246 1.050363e-01
## 5303   1 5247 1.048724e-01
## 5304   1 5248 1.042559e-01
## 5305   1 5249 1.039715e-01
## 5306   1 5250 1.036000e-01
## 5307   1 5251 1.031902e-01
## 5308   1 5252 1.027367e-01
## 5309   1 5253 1.024792e-01
## 5310   1 5254 1.020625e-01
## 5311   1 5255 1.015549e-01
## 5312   1 5256 1.012321e-01
## 5313   1 5257 1.008602e-01
## 5314   1 5258 1.005606e-01
## 5315   1 5259 1.002002e-01
## 5316   1 5260 9.982066e-02
## 5317   1 5261 9.952956e-02
## 5318   1 5262 9.888256e-02
## 5319   1 5263 9.844036e-02
## 5320   1 5264 9.824191e-02
## 5321   1 5265 9.784532e-02
## 5322   1 5266 9.752580e-02
## 5323   1 5267 9.699402e-02
## 5324   1 5268 9.668009e-02
## 5325   1 5269 9.658264e-02
## 5326   1 5270 9.610075e-02
## 5327   1 5271 9.545702e-02
## 5328   1 5272 9.543311e-02
## 5329   1 5273 9.489676e-02
## 5330   1 5274 9.483466e-02
## 5331   1 5275 9.411368e-02
## 5332   1 5276 9.382186e-02
## 5333   1 5277 9.365982e-02
## 5334   1 5278 9.332935e-02
## 5335   1 5279 9.285291e-02
## 5336   1 5280 9.259110e-02
## 5337   1 5281 9.212213e-02
## 5338   1 5282 9.192536e-02
## 5339   1 5283 9.134890e-02
## 5340   1 5284 9.126619e-02
## 5341   1 5285 9.077426e-02
## 5342   1 5286 9.048304e-02
## 5343   1 5287 9.012472e-02
## 5344   1 5288 8.990276e-02
## 5345   1 5289 8.946114e-02
## 5346   1 5290 8.921427e-02
## 5347   1 5291 8.866868e-02
## 5348   1 5292 8.840560e-02
## 5349   1 5293 8.823329e-02
## 5350   1 5294 8.798708e-02
## 5351   1 5295 8.741282e-02
## 5352   1 5296 8.724730e-02
## 5353   1 5297 8.679872e-02
## 5354   1 5298 8.654337e-02
## 5355   1 5299 8.608403e-02
## 5356   1 5300 8.593049e-02
## 5357   1 5301 8.545232e-02
## 5358   1 5302 8.523505e-02
## 5359   1 5303 8.495428e-02
## 5360   1 5304 8.457840e-02
## 5361   1 5305 8.433564e-02
## 5362   1 5306 8.389480e-02
## 5363   1 5307 8.372180e-02
## 5364   1 5308 8.336515e-02
## 5365   1 5309 8.309717e-02
## 5366   1 5310 8.275818e-02
## 5367   1 5311 8.243078e-02
## 5368   1 5312 8.208290e-02
## 5369   1 5313 8.180745e-02
## 5370   1 5314 8.162960e-02
## 5371   1 5315 8.120296e-02
## 5372   1 5316 8.090213e-02
## 5373   1 5317 8.053167e-02
## 5374   1 5318 8.018765e-02
## 5375   1 5319 7.997737e-02
## 5376   1 5320 7.970586e-02
## 5377   1 5321 7.919963e-02
## 5378   1 5322 7.912181e-02
## 5379   1 5323 7.886210e-02
## 5380   1 5324 7.861788e-02
## 5381   1 5325 7.814578e-02
## 5382   1 5326 7.795151e-02
## 5383   1 5327 7.745720e-02
## 5384   1 5328 7.735321e-02
## 5385   1 5329 7.703899e-02
## 5386   1 5330 7.670680e-02
## 5387   1 5331 7.639656e-02
## 5388   1 5332 7.622744e-02
## 5389   1 5333 7.596470e-02
## 5390   1 5334 7.558412e-02
## 5391   1 5335 7.530766e-02
## 5392   1 5336 7.507905e-02
## 5393   1 5337 7.489839e-02
## 5394   1 5338 7.450992e-02
## 5395   1 5339 7.428679e-02
## 5396   1 5340 7.380538e-02
## 5397   1 5341 7.371741e-02
## 5398   1 5342 7.337602e-02
## 5399   1 5343 7.311736e-02
## 5400   1 5344 7.271315e-02
## 5401   1 5345 7.256014e-02
## 5402   1 5346 7.220460e-02
## 5403   1 5347 7.201363e-02
## 5404   1 5348 7.184567e-02
## 5405   1 5349 7.144439e-02
## 5406   1 5350 7.112295e-02
## 5407   1 5351 7.110530e-02
## 5408   1 5352 7.075103e-02
## 5409   1 5353 7.042812e-02
## 5410   1 5354 7.011742e-02
## 5411   1 5355 6.998333e-02
## 5412   1 5356 6.964385e-02
## 5413   1 5357 6.935794e-02
## 5414   1 5358 6.916995e-02
## 5415   1 5359 6.886677e-02
## 5416   1 5360 6.849736e-02
## 5417   1 5361 6.814098e-02
## 5418   1 5362 6.815216e-02
## 5419   1 5363 6.793339e-02
## 5420   1 5364 6.753482e-02
## 5421   1 5365 6.737728e-02
## 5422   1 5366 6.706711e-02
## 5423   1 5367 6.690745e-02
## 5424   1 5368 6.660817e-02
## 5425   1 5369 6.639515e-02
## 5426   1 5370 6.621982e-02
## 5427   1 5371 6.601275e-02
## 5428   1 5372 6.574447e-02
## 5429   1 5373 6.538795e-02
## 5430   1 5374 6.516413e-02
## 5431   1 5375 6.498033e-02
## 5432   1 5376 6.465638e-02
## 5433   1 5377 6.435003e-02
## 5434   1 5378 6.409925e-02
## 5435   1 5379 6.389429e-02
## 5436   1 5380 6.386739e-02
## 5437   1 5381 6.346052e-02
## 5438   1 5382 6.338485e-02
## 5439   1 5383 6.286962e-02
## 5440   1 5384 6.264197e-02
## 5441   1 5385 6.237053e-02
## 5442   1 5386 6.229532e-02
## 5443   1 5387 6.200548e-02
## 5444   1 5388 6.176838e-02
## 5445   1 5389 6.145400e-02
## 5446   1 5390 6.130366e-02
## 5447   1 5391 6.108458e-02
## 5448   1 5392 6.084592e-02
## 5449   1 5393 6.064912e-02
## 5450   1 5394 6.039902e-02
## 5451   1 5395 6.013756e-02
## 5452   1 5396 5.997665e-02
## 5453   1 5397 5.978237e-02
## 5454   1 5398 5.947022e-02
## 5455   1 5399 5.928829e-02
## 5456   1 5400 5.914442e-02
## 5457   1 5401 5.880826e-02
## 5458   1 5402 5.863788e-02
## 5459   1 5403 5.852202e-02
## 5460   1 5404 5.817865e-02
## 5461   1 5405 5.805539e-02
## 5462   1 5406 5.775197e-02
## 5463   1 5407 5.752026e-02
## 5464   1 5408 5.739012e-02
## 5465   1 5409 5.727560e-02
## 5466   1 5410 5.693731e-02
## 5467   1 5411 5.672378e-02
## 5468   1 5412 5.659360e-02
## 5469   1 5413 5.635923e-02
## 5470   1 5414 5.609517e-02
## 5471   1 5415 5.578186e-02
## 5472   1 5416 5.568987e-02
## 5473   1 5417 5.536890e-02
```

```
## 5474   1 5418 5.536792e-02
## 5475   1 5419 5.502488e-02
## 5476   1 5420 5.483184e-02
## 5477   1 5421 5.461518e-02
## 5478   1 5422 5.437569e-02
## 5479   1 5423 5.427553e-02
## 5480   1 5424 5.401834e-02
## 5481   1 5425 5.375232e-02
## 5482   1 5426 5.357974e-02
## 5483   1 5427 5.345335e-02
## 5484   1 5428 5.317935e-02
## 5485   1 5429 5.295791e-02
## 5486   1 5430 5.280180e-02
## 5487   1 5431 5.260188e-02
## 5488   1 5432 5.246444e-02
## 5489   1 5433 5.235314e-02
## 5490   1 5434 5.194116e-02
## 5491   1 5435 5.185070e-02
## 5492   1 5436 5.160853e-02
## 5493   1 5437 5.153122e-02
## 5494   1 5438 5.127404e-02
## 5495   1 5439 5.096778e-02
## 5496   1 5440 5.093039e-02
## 5497   1 5441 5.066746e-02
## 5498   1 5442 5.063199e-02
## 5499   1 5443 5.042638e-02
## 5500   1 5444 5.014179e-02
## 5501   1 5445 4.993191e-02
## 5502   1 5446 4.978138e-02
## 5503   1 5447 4.955817e-02
## 5504   1 5448 4.943570e-02
## 5505   1 5449 4.918781e-02
## 5506   1 5450 4.908301e-02
## 5507   1 5451 4.888741e-02
## 5508   1 5452 4.869750e-02
## 5509   1 5453 4.849364e-02
## 5510   1 5454 4.827186e-02
## 5511   1 5455 4.815070e-02
## 5512   1 5456 4.783035e-02
## 5513   1 5457 4.782862e-02
## 5514   1 5458 4.758203e-02
## 5515   1 5459 4.745305e-02
## 5516   1 5460 4.723203e-02
## 5517   1 5461 4.706128e-02
## 5518   1 5462 4.683930e-02
## 5519   1 5463 4.668140e-02
## 5520   1 5464 4.646496e-02
## 5521   1 5465 4.636962e-02
## 5522   1 5466 4.617222e-02
## 5523   1 5467 4.603438e-02
## 5524   1 5468 4.576201e-02
## 5525   1 5469 4.569394e-02
## 5526   1 5470 4.541940e-02
## 5527   1 5471 4.541091e-02
## 5528   1 5472 4.512883e-02
## 5529   1 5473 4.499652e-02
## 5530   1 5474 4.475171e-02
## 5531   1 5475 4.455115e-02
## 5532   1 5476 4.436754e-02
## 5533   1 5477 4.428616e-02
## 5534   1 5478 4.417220e-02
## 5535   1 5479 4.402326e-02
## 5536   1 5480 4.379233e-02
## 5537   1 5481 4.372601e-02
## 5538   1 5482 4.350920e-02
## 5539   1 5483 4.334952e-02
## 5540   1 5484 4.317501e-02
## 5541   1 5485 4.308673e-02
## 5542   1 5486 4.287138e-02
## 5543   1 5487 4.266728e-02
## 5544   1 5488 4.254365e-02
## 5545   1 5489 4.244382e-02
## 5546   1 5490 4.230068e-02
## 5547   1 5491 4.206487e-02
## 5548   1 5492 4.192569e-02
## 5549   1 5493 4.174179e-02
## 5550   1 5494 4.159088e-02
## 5551   1 5495 4.142975e-02
## 5552   1 5496 4.127441e-02
## 5553   1 5497 4.104289e-02
## 5554   1 5498 4.105081e-02
## 5555   1 5499 4.082763e-02
## 5556   1 5500 4.064289e-02
## 5557   1 5501 4.053419e-02
## 5558   1 5502 4.039403e-02
## 5559   1 5503 4.020510e-02
## 5560   1 5504 4.004820e-02
## 5561   1 5505 3.989637e-02
## 5562   1 5506 3.973339e-02
## 5563   1 5507 3.960725e-02
## 5564   1 5508 3.943038e-02
## 5565   1 5509 3.937307e-02
## 5566   1 5510 3.915191e-02
## 5567   1 5511 3.904746e-02
## 5568   1 5512 3.892196e-02
## 5569   1 5513 3.871308e-02
## 5570   1 5514 3.858954e-02
## 5571   1 5515 3.848070e-02
## 5572   1 5516 3.831466e-02
## 5573   1 5517 3.816652e-02
## 5574   1 5518 3.806479e-02
## 5575   1 5519 3.784140e-02
## 5576   1 5520 3.776096e-02
## 5577   1 5521 3.758118e-02
## 5578   1 5522 3.745268e-02
## 5579   1 5523 3.734716e-02
## 5580   1 5524 3.717620e-02
## 5581   1 5525 3.705077e-02
## 5582   1 5526 3.692540e-02
## 5583   1 5527 3.680297e-02
## 5584   1 5528 3.656916e-02
## 5585   1 5529 3.646270e-02
## 5586   1 5530 3.637869e-02
## 5587   1 5531 3.624098e-02
## 5588   1 5532 3.608624e-02
## 5589   1 5533 3.589303e-02
## 5590   1 5534 3.578928e-02
## 5591   1 5535 3.567578e-02
## 5592   1 5536 3.556291e-02
## 5593   1 5537 3.541493e-02
## 5594   1 5538 3.530878e-02
## 5595   1 5539 3.516690e-02
## 5596   1 5540 3.501349e-02
## 5597   1 5541 3.488752e-02
## 5598   1 5542 3.467903e-02
## 5599   1 5543 3.463517e-02
## 5600   1 5544 3.451885e-02
## 5601   1 5545 3.433905e-02
## 5602   1 5546 3.426500e-02
## 5603   1 5547 3.414567e-02
## 5604   1 5548 3.392530e-02
## 5605   1 5549 3.387461e-02
## 5606   1 5550 3.374899e-02
## 5607   1 5551 3.368532e-02
## 5608   1 5552 3.349238e-02
## 5609   1 5553 3.328984e-02
## 5610   1 5554 3.318033e-02
## 5611   1 5555 3.315883e-02
## 5612   1 5556 3.297410e-02
## 5613   1 5557 3.289395e-02
## 5614   1 5558 3.274638e-02
## 5615   1 5559 3.265824e-02
## 5616   1 5560 3.247005e-02
## 5617   1 5561 3.234282e-02
## 5618   1 5562 3.230378e-02
## 5619   1 5563 3.210255e-02
## 5620   1 5564 3.200836e-02
## 5621   1 5565 3.189562e-02
## 5622   1 5566 3.178776e-02
## 5623   1 5567 3.171573e-02
## 5624   1 5568 3.156209e-02
## 5625   1 5569 3.139918e-02
## 5626   1 5570 3.130545e-02
## 5627   1 5571 3.115554e-02
## 5628   1 5572 3.109878e-02
## 5629   1 5573 3.094242e-02
## 5630   1 5574 3.082831e-02
## 5631   1 5575 3.069609e-02
## 5632   1 5576 3.064918e-02
## 5633   1 5577 3.057478e-02
## 5634   1 5578 3.037474e-02
## 5635   1 5579 3.032821e-02
## 5636   1 5580 3.017256e-02
## 5637   1 5581 3.007138e-02
## 5638   1 5582 2.990584e-02
## 5639   1 5583 2.979750e-02
## 5640   1 5584 2.977175e-02
## 5641   1 5585 2.961290e-02
## 5642   1 5586 2.946212e-02
## 5643   1 5587 2.936753e-02
## 5644   1 5588 2.926040e-02
## 5645   1 5589 2.914536e-02
## 5646   1 5590 2.905811e-02
## 5647   1 5591 2.899047e-02
## 5648   1 5592 2.880801e-02
## 5649   1 5593 2.874355e-02
## 5650   1 5594 2.863371e-02
## 5651   1 5595 2.851268e-02
## 5652   1 5596 2.841048e-02
## 5653   1 5597 2.829368e-02
## 5654   1 5598 2.821467e-02
## 5655   1 5599 2.806535e-02
## 5656   1 5600 2.798041e-02
## 5657   1 5601 2.790845e-02
## 5658   1 5602 2.778050e-02
## 5659   1 5603 2.767229e-02
## 5660   1 5604 2.759990e-02
## 5661   1 5605 2.748138e-02
## 5662   1 5606 2.734630e-02
## 5663   1 5607 2.723995e-02
## 5664   1 5608 2.711097e-02
## 5665   1 5609 2.704992e-02
## 5666   1 5610 2.696090e-02
## 5667   1 5611 2.689195e-02
## 5668   1 5612 2.676454e-02
## 5669   1 5613 2.667878e-02
## 5670   1 5614 2.658541e-02
## 5671   1 5615 2.644609e-02
## 5672   1 5616 2.642829e-02
## 5673   1 5617 2.623034e-02
## 5674   1 5618 2.618559e-02
## 5675   1 5619 2.610456e-02
## 5676   1 5620 2.593934e-02
## 5677   1 5621 2.588517e-02
## 5678   1 5622 2.577294e-02
## 5679   1 5623 2.570461e-02
## 5680   1 5624 2.561535e-02
## 5681   1 5625 2.550882e-02
## 5682   1 5626 2.538761e-02
## 5683   1 5627 2.527598e-02
## 5684   1 5628 2.527013e-02
## 5685   1 5629 2.512345e-02
## 5686   1 5630 2.500814e-02
## 5687   1 5631 2.493068e-02
## 5688   1 5632 2.483872e-02
## 5689   1 5633 2.473503e-02
## 5690   1 5634 2.468217e-02
## 5691   1 5635 2.452843e-02
## 5692   1 5636 2.443384e-02
## 5693   1 5637 2.438254e-02
## 5694   1 5638 2.426631e-02
## 5695   1 5639 2.421596e-02
## 5696   1 5640 2.409519e-02
## 5697   1 5641 2.401896e-02
## 5698   1 5642 2.391641e-02
## 5699   1 5643 2.382839e-02
## 5700   1 5644 2.374044e-02
## 5701   1 5645 2.367508e-02
## 5702   1 5646 2.354277e-02
## 5703   1 5647 2.353543e-02
## 5704   1 5648 2.339420e-02
## 5705   1 5649 2.329156e-02
## 5706   1 5650 2.318330e-02
## 5707   1 5651 2.308394e-02
## 5708   1 5652 2.302620e-02
## 5709   1 5653 2.295397e-02
## 5710   1 5654 2.287803e-02
## 5711   1 5655 2.280374e-02
## 5712   1 5656 2.266219e-02
## 5713   1 5657 2.262830e-02
## 5714   1 5658 2.253441e-02
## 5715   1 5659 2.247150e-02
## 5716   1 5660 2.237653e-02
## 5717   1 5661 2.230362e-02
## 5718   1 5662 2.223090e-02
## 5719   1 5663 2.213146e-02
## 5720   1 5664 2.203628e-02
## 5721   1 5665 2.195454e-02
## 5722   1 5666 2.184706e-02
## 5723   1 5667 2.176954e-02
## 5724   1 5668 2.171855e-02
## 5725   1 5669 2.162622e-02
## 5726   1 5670 2.154467e-02
## 5727   1 5671 2.144114e-02
## 5728   1 5672 2.140301e-02
## 5729   1 5673 2.131674e-02
## 5730   1 5674 2.123158e-02
## 5731   1 5675 2.111377e-02
## 5732   1 5676 2.105326e-02
## 5733   1 5677 2.092874e-02
## 5734   1 5678 2.093389e-02
## 5735   1 5679 2.084210e-02
## 5736   1 5680 2.077022e-02
## 5737   1 5681 2.065063e-02
## 5738   1 5682 2.060203e-02
## 5739   1 5683 2.052958e-02
## 5740   1 5684 2.048244e-02
## 5741   1 5685 2.037175e-02
## 5742   1 5686 2.029591e-02
## 5743   1 5687 2.020069e-02
## 5744   1 5688 2.018528e-02
## 5745   1 5689 2.007044e-02
## 5746   1 5690 1.998605e-02
## 5747   1 5691 1.993071e-02
## 5748   1 5692 1.985850e-02
## 5749   1 5693 1.973959e-02
## 5750   1 5694 1.969943e-02
## 5751   1 5695 1.962088e-02
## 5752   1 5696 1.957564e-02
## 5753   1 5697 1.947885e-02
## 5754   1 5698 1.939064e-02
## 5755   1 5699 1.936304e-02
## 5756   1 5700 1.924392e-02
## 5757   1 5701 1.921106e-02
## 5758   1 5702 1.912618e-02
## 5759   1 5703 1.905087e-02
## 5760   1 5704 1.899766e-02
## 5761   1 5705 1.888717e-02
## 5762   1 5706 1.883168e-02
## 5763   1 5707 1.875158e-02
## 5764   1 5708 1.869088e-02
## 5765   1 5709 1.862076e-02
## 5766   1 5710 1.857584e-02
## 5767   1 5711 1.847487e-02
## 5768   1 5712 1.841695e-02
## 5769   1 5713 1.835701e-02
## 5770   1 5714 1.829817e-02
## 5771   1 5715 1.822030e-02
## 5772   1 5716 1.817282e-02
## 5773   1 5717 1.808544e-02
## 5774   1 5718 1.804597e-02
## 5775   1 5719 1.793788e-02
## 5776   1 5720 1.787965e-02
## 5777   1 5721 1.781337e-02
## 5778   1 5722 1.774842e-02
## 5779   1 5723 1.768223e-02
## 5780   1 5724 1.759199e-02
## 5781   1 5725 1.758694e-02
## 5782   1 5726 1.748991e-02
## 5783   1 5727 1.743125e-02
## 5784   1 5728 1.736055e-02
## 5785   1 5729 1.728915e-02
## 5786   1 5730 1.720331e-02
## 5787   1 5731 1.715576e-02
## 5788   1 5732 1.710108e-02
## 5789   1 5733 1.703554e-02
## 5790   1 5734 1.696613e-02
## 5791   1 5735 1.692083e-02
## 5792   1 5736 1.684926e-02
## 5793   1 5737 1.676871e-02
## 5794   1 5738 1.671347e-02
## 5795   1 5739 1.666878e-02
## 5796   1 5740 1.659411e-02
## 5797   1 5741 1.652803e-02
## 5798   1 5742 1.646092e-02
## 5799   1 5743 1.639302e-02
## 5800   1 5744 1.631749e-02
## 5801   1 5745 1.629573e-02
## 5802   1 5746 1.620168e-02
## 5803   1 5747 1.615002e-02
## 5804   1 5748 1.609829e-02
## 5805   1 5749 1.605136e-02
## 5806   1 5750 1.600360e-02
## 5807   1 5751 1.592931e-02
## 5808   1 5752 1.587286e-02
## 5809   1 5753 1.580265e-02
## 5810   1 5754 1.577793e-02
## 5811   1 5755 1.569024e-02
## 5812   1 5756 1.563272e-02
## 5813   1 5757 1.557082e-02
## 5814   1 5758 1.551353e-02
## 5815   1 5759 1.542782e-02
## 5816   1 5760 1.539213e-02
## 5817   1 5761 1.535313e-02
## 5818   1 5762 1.526883e-02
## 5819   1 5763 1.521548e-02
## 5820   1 5764 1.518281e-02
## 5821   1 5765 1.511480e-02
## 5822   1 5766 1.503784e-02
## 5823   1 5767 1.499925e-02
## 5824   1 5768 1.492616e-02
## 5825   1 5769 1.487019e-02
## 5826   1 5770 1.480963e-02
## 5827   1 5771 1.473323e-02
## 5828   1 5772 1.473558e-02
## 5829   1 5773 1.468220e-02
## 5830   1 5774 1.463512e-02
## 5831   1 5775 1.455530e-02
## 5832   1 5776 1.452048e-02
## 5833   1 5777 1.443560e-02
## 5834   1 5778 1.439678e-02
## 5835   1 5779 1.433441e-02
## 5836   1 5780 1.429069e-02
## 5837   1 5781 1.421874e-02
## 5838   1 5782 1.416554e-02
## 5839   1 5783 1.414795e-02
## 5840   1 5784 1.407651e-02
## 5841   1 5785 1.400653e-02
## 5842   1 5786 1.394285e-02
## 5843   1 5787 1.390390e-02
## 5844   1 5788 1.386121e-02
## 5845   1 5789 1.381624e-02
## 5846   1 5790 1.374712e-02
## 5847   1 5791 1.369946e-02
## 5848   1 5792 1.364829e-02
## 5849   1 5793 1.360060e-02
## 5850   1 5794 1.354710e-02
## 5851   1 5795 1.350858e-02
## 5852   1 5796 1.346584e-02
## 5853   1 5797 1.340177e-02
## 5854   1 5798 1.335341e-02
## 5855   1 5799 1.332691e-02
## 5856   1 5800 1.324860e-02
## 5857   1 5801 1.320148e-02
## 5858   1 5802 1.315186e-02
## 5859   1 5803 1.310621e-02
## 5860   1 5804 1.305458e-02
## 5861   1 5805 1.300935e-02
## 5862   1 5806 1.297337e-02
## 5863   1 5807 1.290615e-02
## 5864   1 5808 1.287755e-02
## 5865   1 5809 1.283828e-02
## 5866   1 5810 1.277411e-02
## 5867   1 5811 1.271346e-02
## 5868   1 5812 1.267299e-02
## 5869   1 5813 1.262506e-02
## 5870   1 5814 1.257881e-02
## 5871   1 5815 1.252324e-02
## 5872   1 5816 1.248458e-02
## 5873   1 5817 1.242573e-02
## 5874   1 5818 1.240117e-02
## 5875   1 5819 1.233400e-02
## 5876   1 5820 1.228603e-02
## 5877   1 5821 1.223311e-02
## 5878   1 5822 1.220908e-02
## 5879   1 5823 1.216128e-02
## 5880   1 5824 1.211689e-02
## 5881   1 5825 1.205951e-02
## 5882   1 5826 1.203187e-02
## 5883   1 5827 1.201616e-02
## 5884   1 5828 1.193948e-02
## 5885   1 5829 1.191781e-02
## 5886   1 5830 1.185598e-02
## 5887   1 5831 1.180691e-02
## 5888   1 5832 1.177162e-02
## 5889   1 5833 1.169729e-02
## 5890   1 5834 1.168610e-02
## 5891   1 5835 1.162643e-02
## 5892   1 5836 1.159240e-02
## 5893   1 5837 1.152030e-02
## 5894   1 5838 1.150384e-02
## 5895   1 5839 1.145247e-02
## 5896   1 5840 1.140284e-02
## 5897   1 5841 1.134819e-02
## 5898   1 5842 1.132125e-02
## 5899   1 5843 1.129644e-02
## 5900   1 5844 1.124821e-02
## 5901   1 5845 1.120163e-02
## 5902   1 5846 1.115621e-02
## 5903   1 5847 1.111121e-02
## 5904   1 5848 1.105738e-02
## 5905   1 5849 1.104702e-02
## 5906   1 5850 1.098293e-02
## 5907   1 5851 1.096317e-02
## 5908   1 5852 1.092569e-02
## 5909   1 5853 1.087627e-02
## 5910   1 5854 1.083267e-02
## 5911   1 5855 1.079374e-02
## 5912   1 5856 1.076702e-02
## 5913   1 5857 1.071831e-02
## 5914   1 5858 1.066340e-02
## 5915   1 5859 1.063364e-02
## 5916   1 5860 1.060142e-02
## 5917   1 5861 1.055785e-02
## 5918   1 5862 1.052903e-02
## 5919   1 5863 1.046205e-02
## 5920   1 5864 1.043988e-02
## 5921   1 5865 1.041503e-02
## 5922   1 5866 1.035850e-02
## 5923   1 5867 1.033275e-02
## 5924   1 5868 1.028162e-02
## 5925   1 5869 1.024921e-02
## 5926   1 5870 1.021921e-02
## 5927   1 5871 1.016280e-02
## 5928   1 5872 1.012549e-02
## 5929   1 5873 1.006886e-02
## 5930   1 5874 1.004980e-02
## 5931   1 5875 1.001058e-02
## 5932   1 5876 9.967573e-03
## 5933   1 5877 9.944661e-03
## 5934   1 5878 9.932036e-03
## 5935   1 5879 9.883690e-03
## 5936   1 5880 9.837629e-03
## 5937   1 5881 9.795294e-03
## 5938   1 5882 9.762773e-03
## 5939   1 5883 9.715198e-03
## 5940   1 5884 9.687516e-03
## 5941   1 5885 9.649775e-03
## 5942   1 5886 9.602709e-03
## 5943   1 5887 9.580592e-03
## 5944   1 5888 9.538930e-03
## 5945   1 5889 9.519187e-03
## 5946   1 5890 9.472920e-03
## 5947   1 5891 9.428265e-03
## 5948   1 5892 9.395861e-03
## 5949   1 5893 9.357315e-03
## 5950   1 5894 9.323612e-03
## 5951   1 5895 9.306715e-03
## 5952   1 5896 9.236216e-03
## 5953   1 5897 9.241092e-03
## 5954   1 5898 9.194759e-03
## 5955   1 5899 9.139866e-03
## 5956   1 5900 9.122186e-03
## 5957   1 5901 9.070751e-03
## 5958   1 5902 9.066043e-03
## 5959   1 5903 9.018440e-03
## 5960   1 5904 8.991889e-03
## 5961   1 5905 8.953054e-03
## 5962   1 5906 8.921350e-03
## 5963   1 5907 8.894015e-03
## 5964   1 5908 8.844598e-03
## 5965   1 5909 8.817328e-03
## 5966   1 5910 8.797930e-03
## 5967   1 5911 8.763578e-03
## 5968   1 5912 8.713718e-03
## 5969   1 5913 8.703934e-03
## 5970   1 5914 8.663142e-03
## 5971   1 5915 8.623092e-03
## 5972   1 5916 8.585958e-03
## 5973   1 5917 8.563849e-03
## 5974   1 5918 8.520148e-03
## 5975   1 5919 8.506958e-03
## 5976   1 5920 8.470532e-03
## 5977   1 5921 8.428737e-03
## 5978   1 5922 8.395318e-03
## 5979   1 5923 8.367930e-03
## 5980   1 5924 8.334362e-03
## 5981   1 5925 8.303505e-03
## 5982   1 5926 8.264633e-03
## 5983   1 5927 8.237398e-03
## 5984   1 5928 8.201791e-03
## 5985   1 5929 8.188181e-03
## 5986   1 5930 8.161517e-03
## 5987   1 5931 8.142020e-03
## 5988   1 5932 8.083401e-03
## 5989   1 5933 8.070061e-03
## 5990   1 5934 8.029365e-03
## 5991   1 5935 8.007142e-03
## 5992   1 5936 7.979080e-03
## 5993   1 5937 7.943124e-03
## 5994   1 5938 7.901588e-03
## 5995   1 5939 7.882462e-03
## 5996   1 5940 7.842371e-03
## 5997   1 5941 7.823427e-03
## 5998   1 5942 7.793816e-03
## 5999   1 5943 7.761065e-03
## 6000   1 5944 7.736076e-03
## 6001   1 5945 7.709330e-03
## 6002   1 5946 7.692251e-03
## 6003   1 5947 7.648355e-03
## 6004   1 5948 7.621537e-03
## 6005   1 5949 7.593276e-03
## 6006   1 5950 7.561191e-03
## 6007   1 5951 7.539518e-03
## 6008   1 5952 7.503510e-03
## 6009   1 5953 7.478199e-03
## 6010   1 5954 7.472783e-03
## 6011   1 5955 7.424272e-03
## 6012   1 5956 7.390220e-03
## 6013   1 5957 7.378255e-03
## 6014   1 5958 7.340814e-03
## 6015   1 5959 7.306606e-03
## 6016   1 5960 7.289630e-03
## 6017   1 5961 7.250864e-03
## 6018   1 5962 7.242090e-03
## 6019   1 5963 7.210359e-03
## 6020   1 5964 7.176640e-03
## 6021   1 5965 7.152117e-03
## 6022   1 5966 7.126424e-03
## 6023   1 5967 7.098187e-03
## 6024   1 5968 7.075224e-03
## 6025   1 5969 7.051774e-03
## 6026   1 5970 7.022485e-03
## 6027   1 5971 6.990175e-03
## 6028   1 5972 6.975667e-03
## 6029   1 5973 6.939468e-03
## 6030   1 5974 6.926707e-03
## 6031   1 5975 6.886304e-03
## 6032   1 5976 6.884255e-03
## 6033   1 5977 6.844995e-03
## 6034   1 5978 6.818688e-03
## 6035   1 5979 6.794620e-03
## 6036   1 5980 6.762887e-03
## 6037   1 5981 6.745840e-03
## 6038   1 5982 6.710126e-03
## 6039   1 5983 6.685052e-03
## 6040   1 5984 6.664203e-03
## 6041   1 5985 6.633546e-03
## 6042   1 5986 6.625026e-03
## 6043   1 5987 6.602592e-03
## 6044   1 5988 6.560312e-03
## 6045   1 5989 6.530614e-03
## 6046   1 5990 6.516991e-03
## 6047   1 5991 6.495390e-03
## 6048   1 5992 6.460182e-03
## 6049   1 5993 6.444500e-03
## 6050   1 5994 6.425092e-03
## 6051   1 5995 6.389159e-03
## 6052   1 5996 6.374793e-03
## 6053   1 5997 6.351117e-03
## 6054   1 5998 6.334472e-03
## 6055   1 5999 6.311403e-03
## 6056   1 6000 6.282412e-03
## 6057   1 6001 6.255577e-03
## 6058   1 6002 6.229728e-03
## 6059   1 6003 6.204758e-03
## 6060   1 6004 6.195309e-03
## 6061   1 6005 6.157884e-03
## 6062   1 6006 6.131783e-03
## 6063   1 6007 6.114860e-03
## 6064   1 6008 6.093919e-03
## 6065   1 6009 6.081757e-03
## 6066   1 6010 6.053300e-03
## 6067   1 6011 6.030197e-03
## 6068   1 6012 5.998221e-03
## 6069   1 6013 5.982601e-03
## 6070   1 6014 5.954083e-03
## 6071   1 6015 5.941394e-03
## 6072   1 6016 5.919314e-03
## 6073   1 6017 5.884704e-03
## 6074   1 6018 5.868854e-03
## 6075   1 6019 5.842495e-03
## 6076   1 6020 5.823732e-03
## 6077   1 6021 5.796115e-03
## 6078   1 6022 5.790163e-03
## 6079   1 6023 5.756802e-03
## 6080   1 6024 5.739953e-03
## 6081   1 6025 5.717050e-03
## 6082   1 6026 5.690192e-03
## 6083   1 6027 5.676529e-03
## 6084   1 6028 5.653966e-03
## 6085   1 6029 5.636283e-03
## 6086   1 6030 5.605497e-03
## 6087   1 6031 5.591553e-03
## 6088   1 6032 5.570870e-03
## 6089   1 6033 5.543919e-03
## 6090   1 6034 5.525826e-03
## 6091   1 6035 5.518696e-03
## 6092   1 6036 5.491437e-03
## 6093   1 6037 5.481024e-03
## 6094   1 6038 5.448655e-03
## 6095   1 6039 5.427467e-03
## 6096   1 6040 5.400305e-03
## 6097   1 6041 5.380497e-03
## 6098   1 6042 5.367423e-03
## 6099   1 6043 5.344758e-03
## 6100   1 6044 5.315862e-03
## 6101   1 6045 5.304402e-03
## 6102   1 6046 5.291816e-03
## 6103   1 6047 5.259456e-03
## 6104   1 6048 5.256161e-03
## 6105   1 6049 5.228496e-03
## 6106   1 6050 5.207257e-03
## 6107   1 6051 5.186012e-03
## 6108   1 6052 5.171580e-03
## 6109   1 6053 5.147615e-03
## 6110   1 6054 5.130312e-03
## 6111   1 6055 5.103886e-03
## 6112   1 6056 5.093450e-03
## 6113   1 6057 5.069017e-03
## 6114   1 6058 5.057497e-03
## 6115   1 6059 5.033597e-03
## 6116   1 6060 5.012019e-03
## 6117   1 6061 5.002797e-03
## 6118   1 6062 4.978007e-03
## 6119   1 6063 4.953091e-03
## 6120   1 6064 4.938865e-03
## 6121   1 6065 4.930158e-03
## 6122   1 6066 4.909109e-03
## 6123   1 6067 4.886011e-03
## 6124   1 6068 4.863788e-03
## 6125   1 6069 4.843355e-03
## 6126   1 6070 4.828886e-03
## 6127   1 6071 4.812945e-03
## 6128   1 6072 4.789576e-03
## 6129   1 6073 4.772231e-03
## 6130   1 6074 4.765001e-03
## 6131   1 6075 4.747224e-03
## 6132   1 6076 4.728768e-03
## 6133   1 6077 4.710850e-03
## 6134   1 6078 4.693067e-03
## 6135   1 6079 4.676220e-03
## 6136   1 6080 4.646669e-03
## 6137   1 6081 4.636615e-03
## 6138   1 6082 4.630229e-03
## 6139   1 6083 4.604977e-03
## 6140   1 6084 4.590380e-03
## 6141   1 6085 4.570501e-03
## 6142   1 6086 4.557141e-03
## 6143   1 6087 4.536411e-03
## 6144   1 6088 4.512925e-03
## 6145   1 6089 4.500556e-03
## 6146   1 6090 4.485270e-03
## 6147   1 6091 4.471542e-03
## 6148   1 6092 4.461327e-03
## 6149   1 6093 4.436587e-03
## 6150   1 6094 4.422083e-03
## 6151   1 6095 4.411184e-03
## 6152   1 6096 4.384766e-03
## 6153   1 6097 4.365220e-03
## 6154   1 6098 4.359876e-03
## 6155   1 6099 4.338932e-03
## 6156   1 6100 4.320113e-03
## 6157   1 6101 4.309929e-03
## 6158   1 6102 4.293566e-03
## 6159   1 6103 4.278525e-03
## 6160   1 6104 4.250685e-03
## 6161   1 6105 4.240709e-03
## 6162   1 6106 4.226638e-03
## 6163   1 6107 4.202785e-03
## 6164   1 6108 4.197336e-03
## 6165   1 6109 4.179516e-03
## 6166   1 6110 4.159841e-03
## 6167   1 6111 4.144246e-03
## 6168   1 6112 4.134187e-03
## 6169   1 6113 4.110280e-03
## 6170   1 6114 4.099013e-03
## 6171   1 6115 4.088010e-03
## 6172   1 6116 4.075489e-03
## 6173   1 6117 4.053150e-03
## 6174   1 6118 4.043344e-03
## 6175   1 6119 4.023339e-03
## 6176   1 6120 4.007697e-03
## 6177   1 6121 3.996662e-03
## 6178   1 6122 3.974419e-03
## 6179   1 6123 3.964109e-03
## 6180   1 6124 3.949406e-03
## 6181   1 6125 3.937804e-03
## 6182   1 6126 3.922209e-03
## 6183   1 6127 3.905498e-03
## 6184   1 6128 3.893160e-03
## 6185   1 6129 3.873379e-03
## 6186   1 6130 3.858561e-03
## 6187   1 6131 3.854393e-03
## 6188   1 6132 3.829767e-03
## 6189   1 6133 3.810052e-03
## 6190   1 6134 3.812258e-03
## 6191   1 6135 3.790390e-03
## 6192   1 6136 3.770504e-03
## 6193   1 6137 3.764170e-03
## 6194   1 6138 3.756845e-03
## 6195   1 6139 3.735276e-03
## 6196   1 6140 3.722296e-03
## 6197   1 6141 3.705926e-03
## 6198   1 6142 3.692182e-03
## 6199   1 6143 3.681449e-03
## 6200   1 6144 3.663045e-03
## 6201   1 6145 3.645715e-03
## 6202   1 6146 3.638474e-03
## 6203   1 6147 3.626902e-03
## 6204   1 6148 3.605549e-03
## 6205   1 6149 3.598676e-03
## 6206   1 6150 3.584821e-03
## 6207   1 6151 3.572245e-03
## 6208   1 6152 3.562700e-03
## 6209   1 6153 3.545379e-03
## 6210   1 6154 3.534581e-03
## 6211   1 6155 3.521228e-03
## 6212   1 6156 3.509163e-03
## 6213   1 6157 3.494693e-03
## 6214   1 6158 3.476933e-03
## 6215   1 6159 3.473214e-03
## 6216   1 6160 3.454285e-03
## 6217   1 6161 3.440897e-03
## 6218   1 6162 3.425396e-03
## 6219   1 6163 3.415760e-03
## 6220   1 6164 3.402601e-03
## 6221   1 6165 3.392818e-03
## 6222   1 6166 3.380094e-03
## 6223   1 6167 3.361246e-03
## 6224   1 6168 3.357790e-03
## 6225   1 6169 3.342931e-03
## 6226   1 6170 3.322968e-03
## 6227   1 6171 3.319408e-03
## 6228   1 6172 3.302336e-03
## 6229   1 6173 3.290828e-03
## 6230   1 6174 3.277533e-03
## 6231   1 6175 3.264256e-03
## 6232   1 6176 3.251998e-03
## 6233   1 6177 3.239264e-03
## 6234   1 6178 3.224334e-03
## 6235   1 6179 3.221318e-03
## 6236   1 6180 3.205533e-03
## 6237   1 6181 3.193489e-03
## 6238   1 6182 3.176919e-03
## 6239   1 6183 3.166763e-03
## 6240   1 6184 3.158616e-03
## 6241   1 6185 3.143888e-03
## 6242   1 6186 3.134006e-03
## 6243   1 6187 3.125703e-03
## 6244   1 6188 3.109387e-03
## 6245   1 6189 3.097690e-03
## 6246   1 6190 3.084516e-03
## 6247   1 6191 3.076001e-03
## 6248   1 6192 3.068270e-03
## 6249   1 6193 3.048611e-03
## 6250   1 6194 3.042021e-03
## 6251   1 6195 3.033923e-03
## 6252   1 6196 3.012253e-03
## 6253   1 6197 3.007746e-03
## 6254   1 6198 2.999197e-03
## 6255   1 6199 2.980465e-03
## 6256   1 6200 2.972868e-03
## 6257   1 6201 2.963577e-03
## 6258   1 6202 2.949696e-03
## 6259   1 6203 2.935689e-03
## 6260   1 6204 2.929971e-03
## 6261   1 6205 2.915641e-03
## 6262   1 6206 2.905771e-03
## 6263   1 6207 2.895898e-03
## 6264   1 6208 2.880385e-03
## 6265   1 6209 2.876699e-03
## 6266   1 6210 2.868140e-03
## 6267   1 6211 2.852651e-03
## 6268   1 6212 2.841998e-03
## 6269   1 6213 2.839380e-03
## 6270   1 6214 2.821848e-03
## 6271   1 6215 2.813162e-03
## 6272   1 6216 2.803397e-03
## 6273   1 6217 2.795698e-03
```

```
## 6274   1 6218 2.782098e-03
## 6275   1 6219 2.769928e-03
## 6276   1 6220 2.759505e-03
## 6277   1 6221 2.745703e-03
## 6278   1 6222 2.737337e-03
## 6279   1 6223 2.725342e-03
## 6280   1 6224 2.718958e-03
## 6281   1 6225 2.709059e-03
## 6282   1 6226 2.702102e-03
## 6283   1 6227 2.689538e-03
## 6284   1 6228 2.679154e-03
## 6285   1 6229 2.665393e-03
## 6286   1 6230 2.656528e-03
## 6287   1 6231 2.651382e-03
## 6288   1 6232 2.637336e-03
## 6289   1 6233 2.628147e-03
## 6290   1 6234 2.616676e-03
## 6291   1 6235 2.605236e-03
## 6292   1 6236 2.601945e-03
## 6293   1 6237 2.588799e-03
## 6294   1 6238 2.578263e-03
## 6295   1 6239 2.572233e-03
## 6296   1 6240 2.558823e-03
## 6297   1 6241 2.551270e-03
## 6298   1 6242 2.543888e-03
## 6299   1 6243 2.528845e-03
## 6300   1 6244 2.523427e-03
## 6301   1 6245 2.510638e-03
## 6302   1 6246 2.503498e-03
## 6303   1 6247 2.496379e-03
## 6304   1 6248 2.482012e-03
## 6305   1 6249 2.477209e-03
## 6306   1 6250 2.464680e-03
## 6307   1 6251 2.457936e-03
## 6308   1 6252 2.449524e-03
## 6309   1 6253 2.439408e-03
## 6310   1 6254 2.428826e-03
## 6311   1 6255 2.419963e-03
## 6312   1 6256 2.412231e-03
## 6313   1 6257 2.402169e-03
## 6314   1 6258 2.398238e-03
## 6315   1 6259 2.387446e-03
## 6316   1 6260 2.383067e-03
## 6317   1 6261 2.365647e-03
## 6318   1 6262 2.356867e-03
## 6319   1 6263 2.348913e-03
## 6320   1 6264 2.341659e-03
## 6321   1 6265 2.334864e-03
## 6322   1 6266 2.325286e-03
## 6323   1 6267 2.317140e-03
## 6324   1 6268 2.305754e-03
## 6325   1 6269 2.300308e-03
## 6326   1 6270 2.287046e-03
## 6327   1 6271 2.278066e-03
## 6328   1 6272 2.272846e-03
## 6329   1 6273 2.260335e-03
## 6330   1 6274 2.256249e-03
## 6331   1 6275 2.244903e-03
## 6332   1 6276 2.239127e-03
## 6333   1 6277 2.232826e-03
## 6334   1 6278 2.221019e-03
## 6335   1 6279 2.213316e-03
## 6336   1 6280 2.208235e-03
## 6337   1 6281 2.199777e-03
## 6338   1 6282 2.189496e-03
## 6339   1 6283 2.183365e-03
## 6340   1 6284 2.174429e-03
## 6341   1 6285 2.161366e-03
## 6342   1 6286 2.156113e-03
## 6343   1 6287 2.147319e-03
## 6344   1 6288 2.143759e-03
## 6345   1 6289 2.130479e-03
## 6346   1 6290 2.123696e-03
## 6347   1 6291 2.121053e-03
## 6348   1 6292 2.107630e-03
## 6349   1 6293 2.097230e-03
## 6350   1 6294 2.093036e-03
## 6351   1 6295 2.084195e-03
## 6352   1 6296 2.076741e-03
## 6353   1 6297 2.067620e-03
## 6354   1 6298 2.060631e-03
## 6355   1 6299 2.055958e-03
## 6356   1 6300 2.052510e-03
## 6357   1 6301 2.037792e-03
## 6358   1 6302 2.032540e-03
## 6359   1 6303 2.025061e-03
## 6360   1 6304 2.013493e-03
## 6361   1 6305 2.004253e-03
## 6362   1 6306 2.000730e-03
## 6363   1 6307 1.992497e-03
## 6364   1 6308 1.988738e-03
## 6365   1 6309 1.979827e-03
## 6366   1 6310 1.970637e-03
## 6367   1 6311 1.969011e-03
## 6368   1 6312 1.957114e-03
## 6369   1 6313 1.951411e-03
## 6370   1 6314 1.944535e-03
## 6371   1 6315 1.932515e-03
## 6372   1 6316 1.927972e-03
## 6373   1 6317 1.920744e-03
## 6374   1 6318 1.912551e-03
## 6375   1 6319 1.910029e-03
## 6376   1 6320 1.900210e-03
## 6377   1 6321 1.892075e-03
## 6378   1 6322 1.884970e-03
## 6379   1 6323 1.877167e-03
## 6380   1 6324 1.873694e-03
## 6381   1 6325 1.863237e-03
## 6382   1 6326 1.857748e-03
## 6383   1 6327 1.849224e-03
## 6384   1 6328 1.846574e-03
## 6385   1 6329 1.836518e-03
## 6386   1 6330 1.831752e-03
## 6387   1 6331 1.820182e-03
## 6388   1 6332 1.815804e-03
## 6389   1 6333 1.809601e-03
## 6390   1 6334 1.803895e-03
## 6391   1 6335 1.792875e-03
## 6392   1 6336 1.789970e-03
## 6393   1 6337 1.781802e-03
## 6394   1 6338 1.777458e-03
## 6395   1 6339 1.769023e-03
## 6396   1 6340 1.765595e-03
## 6397   1 6341 1.757668e-03
## 6398   1 6342 1.749183e-03
## 6399   1 6343 1.741994e-03
## 6400   1 6344 1.737200e-03
## 6401   1 6345 1.730758e-03
## 6402   1 6346 1.724345e-03
## 6403   1 6347 1.716285e-03
## 6404   1 6348 1.710741e-03
## 6405   1 6349 1.704937e-03
## 6406   1 6350 1.699224e-03
## 6407   1 6351 1.691504e-03
## 6408   1 6352 1.685865e-03
## 6409   1 6353 1.679400e-03
## 6410   1 6354 1.673380e-03
## 6411   1 6355 1.662720e-03
## 6412   1 6356 1.659529e-03
## 6413   1 6357 1.653332e-03
## 6414   1 6358 1.647228e-03
## 6415   1 6359 1.641898e-03
## 6416   1 6360 1.636466e-03
## 6417   1 6361 1.629307e-03
## 6418   1 6362 1.626011e-03
## 6419   1 6363 1.617515e-03
## 6420   1 6364 1.612023e-03
## 6421   1 6365 1.604626e-03
## 6422   1 6366 1.600090e-03
## 6423   1 6367 1.594661e-03
## 6424   1 6368 1.588848e-03
## 6425   1 6369 1.581879e-03
## 6426   1 6370 1.575718e-03
## 6427   1 6371 1.567256e-03
## 6428   1 6372 1.563695e-03
## 6429   1 6373 1.558666e-03
## 6430   1 6374 1.550137e-03
## 6431   1 6375 1.548863e-03
## 6432   1 6376 1.541672e-03
## 6433   1 6377 1.534937e-03
## 6434   1 6378 1.530483e-03
## 6435   1 6379 1.525371e-03
## 6436   1 6380 1.516512e-03
## 6437   1 6381 1.515903e-03
## 6438   1 6382 1.506100e-03
## 6439   1 6383 1.501477e-03
## 6440   1 6384 1.494773e-03
## 6441   1 6385 1.490080e-03
## 6442   1 6386 1.482641e-03
## 6443   1 6387 1.479452e-03
## 6444   1 6388 1.472326e-03
## 6445   1 6389 1.468748e-03
## 6446   1 6390 1.463461e-03
## 6447   1 6391 1.459931e-03
## 6448   1 6392 1.452029e-03
## 6449   1 6393 1.446959e-03
## 6450   1 6394 1.440426e-03
## 6451   1 6395 1.434491e-03
## 6452   1 6396 1.429152e-03
## 6453   1 6397 1.422844e-03
## 6454   1 6398 1.418268e-03
## 6455   1 6399 1.415012e-03
## 6456   1 6400 1.409219e-03
## 6457   1 6401 1.403099e-03
## 6458   1 6402 1.399971e-03
## 6459   1 6403 1.392749e-03
## 6460   1 6404 1.389141e-03
## 6461   1 6405 1.380767e-03
## 6462   1 6406 1.379646e-03
## 6463   1 6407 1.374067e-03
## 6464   1 6408 1.365900e-03
## 6465   1 6409 1.360760e-03
## 6466   1 6410 1.354133e-03
## 6467   1 6411 1.353679e-03
## 6468   1 6412 1.345999e-03
## 6469   1 6413 1.342752e-03
## 6470   1 6414 1.338325e-03
## 6471   1 6415 1.331439e-03
## 6472   1 6416 1.329344e-03
## 6473   1 6417 1.318365e-03
## 6474   1 6418 1.317211e-03
## 6475   1 6419 1.309946e-03
## 6476   1 6420 1.305949e-03
## 6477   1 6421 1.301107e-03
## 6478   1 6422 1.295962e-03
## 6479   1 6423 1.293911e-03
## 6480   1 6424 1.286467e-03
## 6481   1 6425 1.283033e-03
## 6482   1 6426 1.279329e-03
## 6483   1 6427 1.272785e-03
## 6484   1 6428 1.267491e-03
## 6485   1 6429 1.265662e-03
## 6486   1 6430 1.258794e-03
## 6487   1 6431 1.254505e-03
## 6488   1 6432 1.249332e-03
## 6489   1 6433 1.245947e-03
## 6490   1 6434 1.242726e-03
## 6491   1 6435 1.235533e-03
## 6492   1 6436 1.233238e-03
## 6493   1 6437 1.228114e-03
## 6494   1 6438 1.220794e-03
## 6495   1 6439 1.217755e-03
## 6496   1 6440 1.212773e-03
## 6497   1 6441 1.208809e-03
## 6498   1 6442 1.202085e-03
## 6499   1 6443 1.200398e-03
## 6500   1 6444 1.193764e-03
## 6501   1 6445 1.190133e-03
## 6502   1 6446 1.186079e-03
## 6503   1 6447 1.181492e-03
## 6504   1 6448 1.179135e-03
## 6505   1 6449 1.171355e-03
## 6506   1 6450 1.169085e-03
## 6507   1 6451 1.164139e-03
## 6508   1 6452 1.158878e-03
## 6509   1 6453 1.157875e-03
## 6510   1 6454 1.150276e-03
## 6511   1 6455 1.147111e-03
## 6512   1 6456 1.143911e-03
## 6513   1 6457 1.139512e-03
## 6514   1 6458 1.134585e-03
## 6515   1 6459 1.128355e-03
## 6516   1 6460 1.124687e-03
## 6517   1 6461 1.121445e-03
## 6518   1 6462 1.118233e-03
## 6519   1 6463 1.113439e-03
## 6520   1 6464 1.109834e-03
## 6521   1 6465 1.104048e-03
## 6522   1 6466 1.098318e-03
## 6523   1 6467 1.096688e-03
## 6524   1 6468 1.093029e-03
## 6525   1 6469 1.088334e-03
## 6526   1 6470 1.082394e-03
## 6527   1 6471 1.079793e-03
## 6528   1 6472 1.076447e-03
## 6529   1 6473 1.072364e-03
## 6530   1 6474 1.068771e-03
## 6531   1 6475 1.065924e-03
## 6532   1 6476 1.060317e-03
## 6533   1 6477 1.055858e-03
## 6534   1 6478 1.050899e-03
## 6535   1 6479 1.047436e-03
## 6536   1 6480 1.044463e-03
## 6537   1 6481 1.039061e-03
## 6538   1 6482 1.036672e-03
## 6539   1 6483 1.032453e-03
## 6540   1 6484 1.028100e-03
## 6541   1 6485 1.024144e-03
## 6542   1 6486 1.021867e-03
## 6543   1 6487 1.018442e-03
## 6544   1 6488 1.011849e-03
## 6545   1 6489 1.009076e-03
## 6546   1 6490 1.005780e-03
## 6547   1 6491 1.002530e-03
## 6548   1 6492 9.977899e-04
## 6549   1 6493 9.961669e-04
## 6550   1 6494 9.911435e-04
## 6551   1 6495 9.882303e-04
## 6552   1 6496 9.850613e-04
## 6553   1 6497 9.784325e-04
## 6554   1 6498 9.765197e-04
## 6555   1 6499 9.750919e-04
## 6556   1 6500 9.702166e-04
## 6557   1 6501 9.657736e-04
## 6558   1 6502 9.612767e-04
## 6559   1 6503 9.579103e-04
## 6560   1 6504 9.555469e-04
## 6561   1 6505 9.526369e-04
## 6562   1 6506 9.477636e-04
## 6563   1 6507 9.450637e-04
## 6564   1 6508 9.423948e-04
## 6565   1 6509 9.358745e-04
## 6566   1 6510 9.321716e-04
## 6567   1 6511 9.318728e-04
## 6568   1 6512 9.271645e-04
## 6569   1 6513 9.244899e-04
## 6570   1 6514 9.180786e-04
## 6571   1 6515 9.165124e-04
## 6572   1 6516 9.131977e-04
## 6573   1 6517 9.090721e-04
## 6574   1 6518 9.062094e-04
## 6575   1 6519 9.010865e-04
## 6576   1 6520 8.996016e-04
## 6577   1 6521 8.946308e-04
## 6578   1 6522 8.926167e-04
## 6579   1 6523 8.901573e-04
## 6580   1 6524 8.857310e-04
## 6581   1 6525 8.831327e-04
## 6582   1 6526 8.794219e-04
## 6583   1 6527 8.770986e-04
## 6584   1 6528 8.727218e-04
## 6585   1 6529 8.686712e-04
## 6586   1 6530 8.659718e-04
## 6587   1 6531 8.629465e-04
## 6588   1 6532 8.594296e-04
## 6589   1 6533 8.562875e-04
## 6590   1 6534 8.547687e-04
## 6591   1 6535 8.510482e-04
## 6592   1 6536 8.467040e-04
## 6593   1 6537 8.434389e-04
## 6594   1 6538 8.403822e-04
## 6595   1 6539 8.385399e-04
## 6596   1 6540 8.341192e-04
## 6597   1 6541 8.323137e-04
## 6598   1 6542 8.289465e-04
## 6599   1 6543 8.254484e-04
## 6600   1 6544 8.215349e-04
## 6601   1 6545 8.187819e-04
## 6602   1 6546 8.156188e-04
## 6603   1 6547 8.141386e-04
## 6604   1 6548 8.097066e-04
## 6605   1 6549 8.072408e-04
## 6606   1 6550 8.047738e-04
## 6607   1 6551 8.007923e-04
## 6608   1 6552 7.973937e-04
## 6609   1 6553 7.958880e-04
## 6610   1 6554 7.924045e-04
## 6611   1 6555 7.886223e-04
## 6612   1 6556 7.865534e-04
## 6613   1 6557 7.826823e-04
## 6614   1 6558 7.801573e-04
## 6615   1 6559 7.776627e-04
## 6616   1 6560 7.738268e-04
## 6617   1 6561 7.722311e-04
## 6618   1 6562 7.692515e-04
## 6619   1 6563 7.661645e-04
## 6620   1 6564 7.623178e-04
## 6621   1 6565 7.606674e-04
## 6622   1 6566 7.575656e-04
## 6623   1 6567 7.554897e-04
## 6624   1 6568 7.523367e-04
## 6625   1 6569 7.482629e-04
## 6626   1 6570 7.458938e-04
## 6627   1 6571 7.441054e-04
## 6628   1 6572 7.409352e-04
## 6629   1 6573 7.381421e-04
## 6630   1 6574 7.361628e-04
## 6631   1 6575 7.328701e-04
## 6632   1 6576 7.299526e-04
## 6633   1 6577 7.265113e-04
## 6634   1 6578 7.245483e-04
## 6635   1 6579 7.215412e-04
## 6636   1 6580 7.183562e-04
## 6637   1 6581 7.165848e-04
## 6638   1 6582 7.140699e-04
## 6639   1 6583 7.098982e-04
## 6640   1 6584 7.079087e-04
## 6641   1 6585 7.043908e-04
## 6642   1 6586 7.038834e-04
## 6643   1 6587 7.010931e-04
## 6644   1 6588 6.987235e-04
## 6645   1 6589 6.943176e-04
## 6646   1 6590 6.919694e-04
## 6647   1 6591 6.900077e-04
## 6648   1 6592 6.875065e-04
## 6649   1 6593 6.845497e-04
## 6650   1 6594 6.814947e-04
## 6651   1 6595 6.806924e-04
## 6652   1 6596 6.763805e-04
## 6653   1 6597 6.742253e-04
## 6654   1 6598 6.721137e-04
## 6655   1 6599 6.701620e-04
## 6656   1 6600 6.665134e-04
## 6657   1 6601 6.644555e-04
## 6658   1 6602 6.612525e-04
## 6659   1 6603 6.598747e-04
## 6660   1 6604 6.570686e-04
## 6661   1 6605 6.547461e-04
## 6662   1 6606 6.525097e-04
## 6663   1 6607 6.498347e-04
## 6664   1 6608 6.468736e-04
## 6665   1 6609 6.454981e-04
## 6666   1 6610 6.421543e-04
## 6667   1 6611 6.406657e-04
## 6668   1 6612 6.377186e-04
## 6669   1 6613 6.354715e-04
## 6670   1 6614 6.329882e-04
## 6671   1 6615 6.308672e-04
## 6672   1 6616 6.285557e-04
## 6673   1 6617 6.259655e-04
## 6674   1 6618 6.233763e-04
## 6675   1 6619 6.197172e-04
## 6676   1 6620 6.193359e-04
## 6677   1 6621 6.173207e-04
## 6678   1 6622 6.135518e-04
## 6679   1 6623 6.114237e-04
## 6680   1 6624 6.096878e-04
## 6681   1 6625 6.085674e-04
## 6682   1 6626 6.058858e-04
## 6683   1 6627 6.029062e-04
## 6684   1 6628 6.004814e-04
## 6685   1 6629 5.985935e-04
## 6686   1 6630 5.963661e-04
## 6687   1 6631 5.938260e-04
## 6688   1 6632 5.916867e-04
## 6689   1 6633 5.895493e-04
## 6690   1 6634 5.863958e-04
## 6691   1 6635 5.859923e-04
## 6692   1 6636 5.824436e-04
## 6693   1 6637 5.815652e-04
## 6694   1 6638 5.796105e-04
## 6695   1 6639 5.765903e-04
## 6696   1 6640 5.749297e-04
## 6697   1 6641 5.722857e-04
## 6698   1 6642 5.710611e-04
## 6699   1 6643 5.676815e-04
## 6700   1 6644 5.676196e-04
## 6701   1 6645 5.641264e-04
## 6702   1 6646 5.619330e-04
## 6703   1 6647 5.597290e-04
## 6704   1 6648 5.576324e-04
## 6705   1 6649 5.553400e-04
## 6706   1 6650 5.528338e-04
## 6707   1 6651 5.519551e-04
## 6708   1 6652 5.510579e-04
## 6709   1 6653 5.470005e-04
## 6710   1 6654 5.454681e-04
## 6711   1 6655 5.429467e-04
## 6712   1 6656 5.411706e-04
## 6713   1 6657 5.389742e-04
## 6714   1 6658 5.371511e-04
## 6715   1 6659 5.352909e-04
## 6716   1 6660 5.330712e-04
## 6717   1 6661 5.309549e-04
## 6718   1 6662 5.286513e-04
## 6719   1 6663 5.274615e-04
## 6720   1 6664 5.251250e-04
## 6721   1 6665 5.227504e-04
## 6722   1 6666 5.213641e-04
## 6723   1 6667 5.185269e-04
## 6724   1 6668 5.172177e-04
## 6725   1 6669 5.153968e-04
## 6726   1 6670 5.137176e-04
## 6727   1 6671 5.115238e-04
## 6728   1 6672 5.102582e-04
## 6729   1 6673 5.084139e-04
## 6730   1 6674 5.069404e-04
## 6731   1 6675 5.040332e-04
## 6732   1 6676 5.015348e-04
## 6733   1 6677 5.007260e-04
## 6734   1 6678 4.987297e-04
## 6735   1 6679 4.962084e-04
## 6736   1 6680 4.938976e-04
## 6737   1 6681 4.933538e-04
## 6738   1 6682 4.910872e-04
## 6739   1 6683 4.894630e-04
## 6740   1 6684 4.869549e-04
## 6741   1 6685 4.861062e-04
## 6742   1 6686 4.841139e-04
## 6743   1 6687 4.818023e-04
## 6744   1 6688 4.810240e-04
## 6745   1 6689 4.781492e-04
## 6746   1 6690 4.771476e-04
## 6747   1 6691 4.749279e-04
## 6748   1 6692 4.730318e-04
## 6749   1 6693 4.711925e-04
## 6750   1 6694 4.691438e-04
## 6751   1 6695 4.668294e-04
## 6752   1 6696 4.654800e-04
## 6753   1 6697 4.636417e-04
## 6754   1 6698 4.626176e-04
## 6755   1 6699 4.610083e-04
## 6756   1 6700 4.597741e-04
## 6757   1 6701 4.567228e-04
## 6758   1 6702 4.550275e-04
## 6759   1 6703 4.548073e-04
## 6760   1 6704 4.517545e-04
## 6761   1 6705 4.510079e-04
## 6762   1 6706 4.485831e-04
## 6763   1 6707 4.479767e-04
## 6764   1 6708 4.453647e-04
## 6765   1 6709 4.438321e-04
## 6766   1 6710 4.420020e-04
## 6767   1 6711 4.400048e-04
## 6768   1 6712 4.390013e-04
## 6769   1 6713 4.369184e-04
## 6770   1 6714 4.355423e-04
## 6771   1 6715 4.342909e-04
## 6772   1 6716 4.327489e-04
## 6773   1 6717 4.312406e-04
## 6774   1 6718 4.287964e-04
## 6775   1 6719 4.279175e-04
## 6776   1 6720 4.257675e-04
## 6777   1 6721 4.246696e-04
## 6778   1 6722 4.230250e-04
## 6779   1 6723 4.215988e-04
## 6780   1 6724 4.201204e-04
## 6781   1 6725 4.180660e-04
## 6782   1 6726 4.172176e-04
## 6783   1 6727 4.141973e-04
## 6784   1 6728 4.140183e-04
## 6785   1 6729 4.124293e-04
## 6786   1 6730 4.102324e-04
## 6787   1 6731 4.088301e-04
## 6788   1 6732 4.063922e-04
## 6789   1 6733 4.061612e-04
## 6790   1 6734 4.038846e-04
## 6791   1 6735 4.027626e-04
## 6792   1 6736 4.016542e-04
## 6793   1 6737 3.999858e-04
## 6794   1 6738 3.987772e-04
## 6795   1 6739 3.965923e-04
## 6796   1 6740 3.954736e-04
## 6797   1 6741 3.944546e-04
## 6798   1 6742 3.924265e-04
## 6799   1 6743 3.908432e-04
## 6800   1 6744 3.900151e-04
## 6801   1 6745 3.874868e-04
## 6802   1 6746 3.860777e-04
## 6803   1 6747 3.848941e-04
## 6804   1 6748 3.837527e-04
## 6805   1 6749 3.823095e-04
## 6806   1 6750 3.812798e-04
## 6807   1 6751 3.791261e-04
## 6808   1 6752 3.788108e-04
## 6809   1 6753 3.763733e-04
## 6810   1 6754 3.750743e-04
## 6811   1 6755 3.735147e-04
## 6812   1 6756 3.726188e-04
## 6813   1 6757 3.711630e-04
## 6814   1 6758 3.694307e-04
## 6815   1 6759 3.676119e-04
## 6816   1 6760 3.668616e-04
## 6817   1 6761 3.656504e-04
## 6818   1 6762 3.638732e-04
## 6819   1 6763 3.628489e-04
## 6820   1 6764 3.614400e-04
## 6821   1 6765 3.595879e-04
## 6822   1 6766 3.582313e-04
## 6823   1 6767 3.570531e-04
## 6824   1 6768 3.558814e-04
## 6825   1 6769 3.546967e-04
## 6826   1 6770 3.531193e-04
## 6827   1 6771 3.516490e-04
## 6828   1 6772 3.507695e-04
## 6829   1 6773 3.497472e-04
## 6830   1 6774 3.482881e-04
## 6831   1 6775 3.468515e-04
## 6832   1 6776 3.460949e-04
## 6833   1 6777 3.442074e-04
## 6834   1 6778 3.434043e-04
## 6835   1 6779 3.417323e-04
## 6836   1 6780 3.404156e-04
## 6837   1 6781 3.391093e-04
## 6838   1 6782 3.383097e-04
## 6839   1 6783 3.360633e-04
## 6840   1 6784 3.363007e-04
## 6841   1 6785 3.345029e-04
## 6842   1 6786 3.334420e-04
## 6843   1 6787 3.311745e-04
## 6844   1 6788 3.307514e-04
## 6845   1 6789 3.293599e-04
## 6846   1 6790 3.280954e-04
## 6847   1 6791 3.265255e-04
## 6848   1 6792 3.258079e-04
## 6849   1 6793 3.236664e-04
## 6850   1 6794 3.232701e-04
## 6851   1 6795 3.221259e-04
## 6852   1 6796 3.210640e-04
## 6853   1 6797 3.194978e-04
## 6854   1 6798 3.182499e-04
## 6855   1 6799 3.171448e-04
## 6856   1 6800 3.153763e-04
## 6857   1 6801 3.148138e-04
## 6858   1 6802 3.134689e-04
## 6859   1 6803 3.126729e-04
## 6860   1 6804 3.113773e-04
## 6861   1 6805 3.098749e-04
## 6862   1 6806 3.093587e-04
## 6863   1 6807 3.081717e-04
## 6864   1 6808 3.063492e-04
## 6865   1 6809 3.057116e-04
## 6866   1 6810 3.046964e-04
## 6867   1 6811 3.038342e-04
## 6868   1 6812 3.018765e-04
## 6869   1 6813 3.010684e-04
## 6870   1 6814 3.000193e-04
## 6871   1 6815 2.981957e-04
## 6872   1 6816 2.982750e-04
## 6873   1 6817 2.961825e-04
## 6874   1 6818 2.953992e-04
## 6875   1 6819 2.946265e-04
## 6876   1 6820 2.932679e-04
## 6877   1 6821 2.917871e-04
## 6878   1 6822 2.915891e-04
## 6879   1 6823 2.899435e-04
## 6880   1 6824 2.885398e-04
## 6881   1 6825 2.876705e-04
## 6882   1 6826 2.870817e-04
## 6883   1 6827 2.859253e-04
## 6884   1 6828 2.844299e-04
## 6885   1 6829 2.837866e-04
## 6886   1 6830 2.828372e-04
## 6887   1 6831 2.813256e-04
## 6888   1 6832 2.804754e-04
## 6889   1 6833 2.794253e-04
## 6890   1 6834 2.781226e-04
## 6891   1 6835 2.771691e-04
## 6892   1 6836 2.767678e-04
## 6893   1 6837 2.751829e-04
## 6894   1 6838 2.742247e-04
## 6895   1 6839 2.732427e-04
## 6896   1 6840 2.719983e-04
## 6897   1 6841 2.713893e-04
## 6898   1 6842 2.704096e-04
## 6899   1 6843 2.690798e-04
## 6900   1 6844 2.680296e-04
## 6901   1 6845 2.671285e-04
## 6902   1 6846 2.655049e-04
## 6903   1 6847 2.652674e-04
## 6904   1 6848 2.640668e-04
## 6905   1 6849 2.630600e-04
## 6906   1 6850 2.626086e-04
## 6907   1 6851 2.608961e-04
## 6908   1 6852 2.600789e-04
## 6909   1 6853 2.591777e-04
## 6910   1 6854 2.574493e-04
## 6911   1 6855 2.571488e-04
## 6912   1 6856 2.558565e-04
## 6913   1 6857 2.555053e-04
## 6914   1 6858 2.544898e-04
## 6915   1 6859 2.535461e-04
## 6916   1 6860 2.529613e-04
## 6917   1 6861 2.517771e-04
## 6918   1 6862 2.506962e-04
## 6919   1 6863 2.497360e-04
## 6920   1 6864 2.488334e-04
## 6921   1 6865 2.480609e-04
## 6922   1 6866 2.472028e-04
## 6923   1 6867 2.458467e-04
## 6924   1 6868 2.449649e-04
## 6925   1 6869 2.439944e-04
## 6926   1 6870 2.434488e-04
## 6927   1 6871 2.423107e-04
## 6928   1 6872 2.413308e-04
## 6929   1 6873 2.402728e-04
## 6930   1 6874 2.400494e-04
## 6931   1 6875 2.389751e-04
## 6932   1 6876 2.377927e-04
## 6933   1 6877 2.373326e-04
## 6934   1 6878 2.359139e-04
## 6935   1 6879 2.353944e-04
## 6936   1 6880 2.348619e-04
## 6937   1 6881 2.336270e-04
## 6938   1 6882 2.326293e-04
## 6939   1 6883 2.321913e-04
## 6940   1 6884 2.306191e-04
## 6941   1 6885 2.297406e-04
## 6942   1 6886 2.290269e-04
## 6943   1 6887 2.280414e-04
## 6944   1 6888 2.278026e-04
## 6945   1 6889 2.268101e-04
## 6946   1 6890 2.257087e-04
## 6947   1 6891 2.250977e-04
## 6948   1 6892 2.241283e-04
## 6949   1 6893 2.235422e-04
## 6950   1 6894 2.222836e-04
## 6951   1 6895 2.218625e-04
## 6952   1 6896 2.206787e-04
## 6953   1 6897 2.200979e-04
## 6954   1 6898 2.190566e-04
## 6955   1 6899 2.181548e-04
## 6956   1 6900 2.173662e-04
## 6957   1 6901 2.167411e-04
## 6958   1 6902 2.156936e-04
## 6959   1 6903 2.153646e-04
## 6960   1 6904 2.143780e-04
## 6961   1 6905 2.136603e-04
## 6962   1 6906 2.124316e-04
## 6963   1 6907 2.122906e-04
## 6964   1 6908 2.109267e-04
## 6965   1 6909 2.102749e-04
## 6966   1 6910 2.090783e-04
## 6967   1 6911 2.087973e-04
## 6968   1 6912 2.083047e-04
## 6969   1 6913 2.073431e-04
## 6970   1 6914 2.064827e-04
## 6971   1 6915 2.054948e-04
## 6972   1 6916 2.053503e-04
## 6973   1 6917 2.041359e-04
## 6974   1 6918 2.031244e-04
## 6975   1 6919 2.026618e-04
## 6976   1 6920 2.019385e-04
## 6977   1 6921 2.006839e-04
## 6978   1 6922 2.002008e-04
## 6979   1 6923 1.996054e-04
## 6980   1 6924 1.987576e-04
## 6981   1 6925 1.982087e-04
## 6982   1 6926 1.970434e-04
## 6983   1 6927 1.966880e-04
## 6984   1 6928 1.956557e-04
## 6985   1 6929 1.951109e-04
## 6986   1 6930 1.945763e-04
## 6987   1 6931 1.937403e-04
## 6988   1 6932 1.928803e-04
## 6989   1 6933 1.921343e-04
## 6990   1 6934 1.916860e-04
## 6991   1 6935 1.907349e-04
## 6992   1 6936 1.903248e-04
## 6993   1 6937 1.893788e-04
## 6994   1 6938 1.888239e-04
## 6995   1 6939 1.880508e-04
## 6996   1 6940 1.874271e-04
## 6997   1 6941 1.867012e-04
## 6998   1 6942 1.860992e-04
## 6999   1 6943 1.852092e-04
## 7000   1 6944 1.844207e-04
## 7001   1 6945 1.837717e-04
## 7002   1 6946 1.831565e-04
## 7003   1 6947 1.824560e-04
## 7004   1 6948 1.816943e-04
## 7005   1 6949 1.809964e-04
## 7006   1 6950 1.804913e-04
## 7007   1 6951 1.795642e-04
## 7008   1 6952 1.788055e-04
## 7009   1 6953 1.785314e-04
## 7010   1 6954 1.777012e-04
## 7011   1 6955 1.770660e-04
## 7012   1 6956 1.762450e-04
## 7013   1 6957 1.757683e-04
## 7014   1 6958 1.748902e-04
## 7015   1 6959 1.744317e-04
## 7016   1 6960 1.736873e-04
## 7017   1 6961 1.733100e-04
## 7018   1 6962 1.723658e-04
## 7019   1 6963 1.720896e-04
## 7020   1 6964 1.713468e-04
## 7021   1 6965 1.706127e-04
## 7022   1 6966 1.700873e-04
## 7023   1 6967 1.692512e-04
## 7024   1 6968 1.685172e-04
## 7025   1 6969 1.682674e-04
## 7026   1 6970 1.676125e-04
## 7027   1 6971 1.667910e-04
## 7028   1 6972 1.660832e-04
## 7029   1 6973 1.658327e-04
## 7030   1 6974 1.651350e-04
## 7031   1 6975 1.645767e-04
## 7032   1 6976 1.635165e-04
## 7033   1 6977 1.629103e-04
## 7034   1 6978 1.623467e-04
## 7035   1 6979 1.620666e-04
## 7036   1 6980 1.611967e-04
## 7037   1 6981 1.604739e-04
## 7038   1 6982 1.602052e-04
## 7039   1 6983 1.595903e-04
## 7040   1 6984 1.586636e-04
## 7041   1 6985 1.581379e-04
## 7042   1 6986 1.576374e-04
## 7043   1 6987 1.572026e-04
## 7044   1 6988 1.566137e-04
## 7045   1 6989 1.558700e-04
## 7046   1 6990 1.554057e-04
## 7047   1 6991 1.547100e-04
## 7048   1 6992 1.541994e-04
## 7049   1 6993 1.535049e-04
## 7050   1 6994 1.528483e-04
## 7051   1 6995 1.527171e-04
## 7052   1 6996 1.519457e-04
## 7053   1 6997 1.514805e-04
## 7054   1 6998 1.507521e-04
## 7055   1 6999 1.499836e-04
## 7056   1 7000 1.497015e-04
## 7057   2    0 0.000000e+00
## 7058   2    0 2.221706e+00
## 7059   2    1 3.441184e+00
## 7060   2    2 3.849982e+00
## 7061   2    3 3.924055e+00
## 7062   2    4 3.829602e+00
## 7063   2    5 3.680076e+00
## 7064   2    6 3.527507e+00
## 7065   2    7 3.369178e+00
## 7066   2    8 3.231069e+00
## 7067   2    9 3.097047e+00
## 7068   2   10 2.985420e+00
## 7069   2   11 2.886538e+00
## 7070   2   12 2.797006e+00
## 7071   2   13 2.710950e+00
## 7072   2   14 2.646016e+00
## 7073   2   15 2.572339e+00
```

```
## 7074   2   16 2.517588e+00
## 7075   2   17 2.466121e+00
## 7076   2   18 2.424176e+00
## 7077   2   19 2.376693e+00
## 7078   2   20 2.344801e+00
## 7079   2   21 2.311268e+00
## 7080   2   22 2.276735e+00
## 7081   2   23 2.247160e+00
## 7082   2   24 2.220904e+00
## 7083   2   24 2.224093e+00
## 7084   2   25 3.438034e+00
## 7085   2   26 3.857417e+00
## 7086   2   27 3.915349e+00
## 7087   2   28 3.826271e+00
## 7088   2   29 3.682983e+00
## 7089   2   30 3.521998e+00
## 7090   2   31 3.366687e+00
## 7091   2   32 3.223678e+00
## 7092   2   33 3.098631e+00
## 7093   2   34 2.988722e+00
## 7094   2   35 2.883385e+00
## 7095   2   36 2.790027e+00
## 7096   2   37 2.713604e+00
## 7097   2   38 2.635261e+00
## 7098   2   39 2.572669e+00
## 7099   2   40 2.520437e+00
## 7100   2   41 2.473804e+00
## 7101   2   42 2.417680e+00
## 7102   2   43 2.378604e+00
## 7103   2   44 2.347583e+00
## 7104   2   45 2.309536e+00
## 7105   2   46 2.274737e+00
## 7106   2   47 2.249553e+00
## 7107   2   48 2.220504e+00
## 7108   2   48 2.222469e+00
## 7109   2   49 3.430850e+00
## 7110   2   50 3.856597e+00
## 7111   2   51 3.925114e+00
## 7112   2   52 3.839112e+00
## 7113   2   53 3.682312e+00
## 7114   2   54 3.529636e+00
## 7115   2   55 3.366453e+00
## 7116   2   56 3.227490e+00
## 7117   2   57 3.105532e+00
## 7118   2   58 2.989236e+00
## 7119   2   59 2.875492e+00
## 7120   2   60 2.792376e+00
## 7121   2   61 2.709514e+00
## 7122   2   62 2.642618e+00
## 7123   2   63 2.579354e+00
## 7124   2   64 2.519129e+00
## 7125   2   65 2.467744e+00
## 7126   2   66 2.418822e+00
## 7127   2   67 2.377573e+00
## 7128   2   68 2.341058e+00
## 7129   2   69 2.311901e+00
## 7130   2   70 2.273702e+00
## 7131   2   71 2.246822e+00
## 7132   2   72 2.222256e+00
## 7133   2   73 3.428136e+00
## 7134   2   74 3.851115e+00
## 7135   2   75 3.921683e+00
## 7136   2   76 3.829911e+00
## 7137   2   77 3.678822e+00
## 7138   2   78 3.523167e+00
## 7139   2   79 3.371209e+00
## 7140   2   80 3.225915e+00
## 7141   2   81 3.101704e+00
## 7142   2   82 2.982080e+00
## 7143   2   83 2.880695e+00
## 7144   2   84 2.793351e+00
## 7145   2   85 2.712939e+00
## 7146   2   86 2.639900e+00
## 7147   2   87 2.573870e+00
## 7148   2   88 2.522834e+00
## 7149   2   89 2.468541e+00
## 7150   2   90 2.418186e+00
## 7151   2   91 2.382742e+00
## 7152   2   92 2.346028e+00
## 7153   2   93 2.305883e+00
## 7154   2   94 2.273443e+00
## 7155   2   95 2.247940e+00
## 7156   2   96 2.221386e+00
## 7157   2   97 3.439982e+00
## 7158   2   98 3.863672e+00
## 7159   2   99 3.919667e+00
## 7160   2  100 3.831984e+00
## 7161   2  101 3.687084e+00
## 7162   2  102 3.521477e+00
## 7163   2  103 3.369951e+00
## 7164   2  104 3.227786e+00
## 7165   2  105 3.094952e+00
## 7166   2  106 2.982062e+00
## 7167   2  107 2.882770e+00
## 7168   2  108 2.792641e+00
## 7169   2  109 2.714335e+00
## 7170   2  110 2.640878e+00
## 7171   2  111 2.577188e+00
## 7172   2  112 2.521932e+00
## 7173   2  113 2.466593e+00
## 7174   2  114 2.423037e+00
## 7175   2  115 2.384943e+00
## 7176   2  116 2.342802e+00
## 7177   2  117 2.314661e+00
## 7178   2  118 2.277152e+00
## 7179   2  119 2.249967e+00
## 7180   2  120 2.217828e+00
## 7181   2  121 3.433844e+00
## 7182   2  122 3.859675e+00
## 7183   2  123 3.913665e+00
## 7184   2  124 3.827142e+00
## 7185   2  125 3.684984e+00
## 7186   2  126 3.523584e+00
## 7187   2  127 3.367790e+00
## 7188   2  128 3.226213e+00
## 7189   2  129 3.097697e+00
## 7190   2  130 2.980554e+00
## 7191   2  131 2.884561e+00
## 7192   2  132 2.787514e+00
## 7193   2  133 2.712525e+00
## 7194   2  134 2.642385e+00
## 7195   2  135 2.575130e+00
## 7196   2  136 2.520207e+00
## 7197   2  137 2.466719e+00
## 7198   2  138 2.416893e+00
## 7199   2  139 2.380644e+00
## 7200   2  140 2.341573e+00
## 7201   2  141 2.306554e+00
## 7202   2  142 2.278075e+00
## 7203   2  143 2.249679e+00
## 7204   2  144 2.223285e+00
## 7205   2  145 3.434331e+00
## 7206   2  146 3.857346e+00
## 7207   2  147 3.915191e+00
## 7208   2  148 3.832542e+00
## 7209   2  149 3.682548e+00
## 7210   2  150 3.527463e+00
## 7211   2  151 3.370332e+00
## 7212   2  152 3.231863e+00
## 7213   2  153 3.099862e+00
## 7214   2  154 2.984039e+00
## 7215   2  155 2.884320e+00
## 7216   2  156 2.789273e+00
## 7217   2  157 2.714401e+00
## 7218   2  158 2.638471e+00
## 7219   2  159 2.575188e+00
## 7220   2  160 2.519450e+00
## 7221   2  161 2.468174e+00
## 7222   2  162 2.423131e+00
## 7223   2  163 2.378651e+00
## 7224   2  164 2.342722e+00
## 7225   2  165 2.306928e+00
## 7226   2  166 2.277224e+00
## 7227   2  167 2.247281e+00
## 7228   2  168 2.224128e+00
## 7229   2  169 3.432098e+00
## 7230   2  170 3.851239e+00
## 7231   2  171 3.916629e+00
## 7232   2  172 3.823242e+00
## 7233   2  173 3.681557e+00
## 7234   2  174 3.525112e+00
## 7235   2  175 3.367759e+00
## 7236   2  176 3.227335e+00
## 7237   2  177 3.101508e+00
## 7238   2  178 2.981043e+00
## 7239   2  179 2.877236e+00
## 7240   2  180 2.794987e+00
## 7241   2  181 2.712348e+00
## 7242   2  182 2.641689e+00
## 7243   2  183 2.573704e+00
## 7244   2  184 2.521659e+00
## 7245   2  185 2.474772e+00
## 7246   2  186 2.422390e+00
## 7247   2  187 2.381673e+00
## 7248   2  188 2.346151e+00
## 7249   2  189 2.305217e+00
## 7250   2  190 2.279259e+00
## 7251   2  191 2.250606e+00
## 7252   2  192 2.224492e+00
## 7253   2  193 3.434011e+00
## 7254   2  194 3.858037e+00
## 7255   2  195 3.916326e+00
## 7256   2  196 3.833022e+00
## 7257   2  197 3.683194e+00
## 7258   2  198 3.528606e+00
## 7259   2  199 3.373362e+00
## 7260   2  200 3.227863e+00
## 7261   2  201 3.093461e+00
## 7262   2  202 2.988243e+00
## 7263   2  203 2.881416e+00
## 7264   2  204 2.794281e+00
## 7265   2  205 2.708951e+00
## 7266   2  206 2.641123e+00
## 7267   2  207 2.578013e+00
## 7268   2  208 2.519883e+00
## 7269   2  209 2.470566e+00
## 7270   2  210 2.416420e+00
## 7271   2  211 2.384610e+00
## 7272   2  212 2.336294e+00
## 7273   2  213 2.304440e+00
## 7274   2  214 2.280369e+00
## 7275   2  215 2.249810e+00
## 7276   2  216 2.223066e+00
## 7277   2  217 3.428357e+00
## 7278   2  218 3.861152e+00
## 7279   2  219 3.918967e+00
## 7280   2  220 3.821032e+00
## 7281   2  221 3.682296e+00
## 7282   2  222 3.522726e+00
## 7283   2  223 3.366100e+00
## 7284   2  224 3.221339e+00
## 7285   2  225 3.099854e+00
## 7286   2  226 2.984845e+00
## 7287   2  227 2.886419e+00
## 7288   2  228 2.791465e+00
## 7289   2  229 2.714568e+00
## 7290   2  230 2.640136e+00
## 7291   2  231 2.576846e+00
## 7292   2  232 2.520311e+00
## 7293   2  233 2.468584e+00
## 7294   2  234 2.422490e+00
## 7295   2  235 2.379176e+00
## 7296   2  236 2.344648e+00
## 7297   2  237 2.305651e+00
## 7298   2  238 2.283307e+00
## 7299   2  239 2.245651e+00
## 7300   2  240 2.219342e+00
## 7301   2  241 3.433118e+00
## 7302   2  242 3.857637e+00
## 7303   2  243 3.921085e+00
## 7304   2  244 3.827441e+00
## 7305   2  245 3.684640e+00
## 7306   2  246 3.524626e+00
## 7307   2  247 3.366905e+00
## 7308   2  248 3.227411e+00
## 7309   2  249 3.095545e+00
## 7310   2  250 2.984829e+00
## 7311   2  251 2.884059e+00
## 7312   2  252 2.792514e+00
## 7313   2  253 2.712067e+00
## 7314   2  254 2.637133e+00
## 7315   2  255 2.577926e+00
## 7316   2  256 2.518820e+00
## 7317   2  257 2.469623e+00
## 7318   2  258 2.422792e+00
## 7319   2  259 2.380562e+00
## 7320   2  260 2.343449e+00
## 7321   2  261 2.308151e+00
## 7322   2  262 2.279302e+00
## 7323   2  263 2.248162e+00
## 7324   2  264 2.228654e+00
## 7325   2  265 3.424990e+00
## 7326   2  266 3.859731e+00
## 7327   2  267 3.923389e+00
## 7328   2  268 3.830086e+00
## 7329   2  269 3.679990e+00
## 7330   2  270 3.524759e+00
## 7331   2  271 3.374015e+00
## 7332   2  272 3.224197e+00
## 7333   2  273 3.097922e+00
## 7334   2  274 2.980787e+00
## 7335   2  275 2.881573e+00
## 7336   2  276 2.789871e+00
## 7337   2  277 2.717136e+00
## 7338   2  278 2.641187e+00
## 7339   2  279 2.577175e+00
## 7340   2  280 2.519332e+00
## 7341   2  281 2.468279e+00
## 7342   2  282 2.424246e+00
## 7343   2  283 2.377543e+00
## 7344   2  284 2.341306e+00
## 7345   2  285 2.312070e+00
## 7346   2  286 2.275879e+00
## 7347   2  287 2.250018e+00
## 7348   2  288 2.222811e+00
## 7349   2  289 3.439421e+00
## 7350   2  290 3.853722e+00
## 7351   2  291 3.918633e+00
## 7352   2  292 3.830456e+00
## 7353   2  293 3.683842e+00
## 7354   2  294 3.529146e+00
## 7355   2  295 3.363442e+00
## 7356   2  296 3.227814e+00
## 7357   2  297 3.097635e+00
## 7358   2  298 2.987958e+00
## 7359   2  299 2.884020e+00
## 7360   2  300 2.792844e+00
## 7361   2  301 2.711993e+00
## 7362   2  302 2.641170e+00
## 7363   2  303 2.575259e+00
## 7364   2  304 2.520844e+00
## 7365   2  305 2.466274e+00
## 7366   2  306 2.423935e+00
## 7367   2  307 2.376705e+00
## 7368   2  308 2.341132e+00
## 7369   2  309 2.309818e+00
## 7370   2  310 2.278293e+00
## 7371   2  311 2.245777e+00
## 7372   2  312 2.224490e+00
## 7373   2  312 2.223510e+00
## 7374   2  313 3.436437e+00
## 7375   2  314 3.858707e+00
## 7376   2  315 3.913995e+00
## 7377   2  316 3.832709e+00
## 7378   2  317 3.683871e+00
## 7379   2  318 3.517050e+00
## 7380   2  319 3.371118e+00
## 7381   2  320 3.225805e+00
## 7382   2  321 3.100792e+00
## 7383   2  322 2.986596e+00
## 7384   2  323 2.883627e+00
## 7385   2  324 2.790690e+00
## 7386   2  325 2.710927e+00
## 7387   2  326 2.644869e+00
## 7388   2  327 2.574857e+00
## 7389   2  328 2.518503e+00
## 7390   2  329 2.464143e+00
## 7391   2  330 2.420526e+00
## 7392   2  331 2.378095e+00
## 7393   2  332 2.345326e+00
## 7394   2  333 2.303868e+00
## 7395   2  334 2.280640e+00
## 7396   2  335 2.251743e+00
## 7397   2  336 2.221202e+00
## 7398   2  336 2.218642e+00
## 7399   2  337 2.197169e+00
## 7400   2  338 2.174443e+00
## 7401   2  339 2.152196e+00
## 7402   2  339 2.153819e+00
## 7403   2  340 3.370170e+00
## 7404   2  341 3.793716e+00
## 7405   2  342 3.861186e+00
## 7406   2  343 3.773017e+00
## 7407   2  344 3.632344e+00
## 7408   2  345 3.473340e+00
## 7409   2  346 3.320479e+00
## 7410   2  347 3.182673e+00
## 7411   2  348 3.052802e+00
## 7412   2  349 2.946813e+00
## 7413   2  350 2.846261e+00
## 7414   2  351 2.745720e+00
## 7415   2  352 2.677163e+00
## 7416   2  353 2.599614e+00
## 7417   2  354 2.538978e+00
## 7418   2  355 2.483860e+00
## 7419   2  356 2.432652e+00
## 7420   2  357 2.385009e+00
## 7421   2  358 2.347313e+00
## 7422   2  359 2.307856e+00
## 7423   2  360 2.276543e+00
## 7424   2  360 2.274797e+00
## 7425   2  361 3.484292e+00
## 7426   2  362 3.891050e+00
## 7427   2  363 3.954916e+00
## 7428   2  364 3.855784e+00
## 7429   2  365 3.716209e+00
## 7430   2  366 3.550950e+00
## 7431   2  367 3.393380e+00
## 7432   2  368 3.248854e+00
## 7433   2  369 3.118106e+00
## 7434   2  370 3.001973e+00
## 7435   2  371 2.893093e+00
## 7436   2  372 2.805004e+00
## 7437   2  373 2.722926e+00
## 7438   2  374 2.648147e+00
## 7439   2  375 2.586830e+00
## 7440   2  376 2.534286e+00
## 7441   2  377 2.479232e+00
## 7442   2  378 2.431599e+00
## 7443   2  379 2.388225e+00
## 7444   2  380 2.349808e+00
## 7445   2  381 2.317584e+00
## 7446   2  382 2.278488e+00
## 7447   2  383 2.253252e+00
## 7448   2  384 2.228943e+00
## 7449   2  385 3.437053e+00
## 7450   2  386 3.861021e+00
## 7451   2  387 3.922499e+00
## 7452   2  388 3.835500e+00
## 7453   2  389 3.686242e+00
## 7454   2  390 3.529766e+00
## 7455   2  391 3.373687e+00
## 7456   2  392 3.232307e+00
## 7457   2  393 3.108909e+00
## 7458   2  394 2.992347e+00
## 7459   2  395 2.888689e+00
## 7460   2  396 2.792595e+00
## 7461   2  397 2.709700e+00
## 7462   2  398 2.642852e+00
## 7463   2  399 2.577750e+00
## 7464   2  400 2.526860e+00
## 7465   2  401 2.473454e+00
## 7466   2  402 2.431084e+00
## 7467   2  403 2.382778e+00
## 7468   2  404 2.355456e+00
## 7469   2  405 2.307652e+00
## 7470   2  406 2.282015e+00
## 7471   2  407 2.250454e+00
## 7472   2  408 2.226286e+00
## 7473   2  409 3.431163e+00
## 7474   2  410 3.857878e+00
## 7475   2  411 3.920202e+00
## 7476   2  412 3.826373e+00
## 7477   2  413 3.683067e+00
## 7478   2  414 3.526347e+00
## 7479   2  415 3.379722e+00
## 7480   2  416 3.233988e+00
## 7481   2  417 3.101584e+00
## 7482   2  418 2.986837e+00
## 7483   2  419 2.882846e+00
## 7484   2  420 2.794394e+00
## 7485   2  421 2.715541e+00
## 7486   2  422 2.645305e+00
## 7487   2  423 2.582903e+00
## 7488   2  424 2.522257e+00
## 7489   2  425 2.471737e+00
## 7490   2  426 2.428418e+00
## 7491   2  427 2.386681e+00
## 7492   2  428 2.346137e+00
## 7493   2  429 2.312386e+00
## 7494   2  430 2.282501e+00
## 7495   2  431 2.250583e+00
## 7496   2  432 2.225443e+00
## 7497   2  433 3.434579e+00
## 7498   2  434 3.856699e+00
## 7499   2  435 3.926619e+00
## 7500   2  436 3.829203e+00
## 7501   2  437 3.694255e+00
## 7502   2  438 3.529115e+00
## 7503   2  439 3.370862e+00
## 7504   2  440 3.231514e+00
## 7505   2  441 3.102403e+00
## 7506   2  442 2.987357e+00
## 7507   2  443 2.887018e+00
## 7508   2  444 2.795919e+00
## 7509   2  445 2.715144e+00
## 7510   2  446 2.642750e+00
## 7511   2  447 2.583406e+00
## 7512   2  448 2.522775e+00
## 7513   2  449 2.476410e+00
## 7514   2  450 2.424048e+00
## 7515   2  451 2.384110e+00
## 7516   2  452 2.344903e+00
## 7517   2  453 2.309960e+00
## 7518   2  454 2.279401e+00
## 7519   2  455 2.255579e+00
## 7520   2  456 2.225821e+00
## 7521   2  457 3.431702e+00
## 7522   2  458 3.861784e+00
## 7523   2  459 3.920294e+00
## 7524   2  460 3.826703e+00
## 7525   2  461 3.682379e+00
## 7526   2  462 3.522373e+00
## 7527   2  463 3.374233e+00
## 7528   2  464 3.225690e+00
## 7529   2  465 3.094676e+00
## 7530   2  466 2.988269e+00
## 7531   2  467 2.889137e+00
## 7532   2  468 2.794367e+00
## 7533   2  469 2.714094e+00
## 7534   2  470 2.645599e+00
## 7535   2  471 2.581595e+00
## 7536   2  472 2.520884e+00
## 7537   2  473 2.466500e+00
## 7538   2  474 2.425537e+00
## 7539   2  475 2.381298e+00
## 7540   2  476 2.346837e+00
## 7541   2  477 2.312309e+00
## 7542   2  478 2.277595e+00
## 7543   2  479 2.251846e+00
## 7544   2  480 2.225757e+00
## 7545   2  481 3.434813e+00
## 7546   2  482 3.861224e+00
## 7547   2  483 3.928880e+00
## 7548   2  484 3.830586e+00
## 7549   2  485 3.683498e+00
## 7550   2  486 3.525688e+00
## 7551   2  487 3.367903e+00
## 7552   2  488 3.237413e+00
## 7553   2  489 3.103365e+00
## 7554   2  490 2.992988e+00
## 7555   2  491 2.884106e+00
## 7556   2  492 2.794023e+00
## 7557   2  493 2.714143e+00
## 7558   2  494 2.644313e+00
## 7559   2  495 2.581164e+00
## 7560   2  496 2.522675e+00
## 7561   2  497 2.471521e+00
## 7562   2  498 2.423397e+00
## 7563   2  499 2.384650e+00
## 7564   2  500 2.351868e+00
## 7565   2  501 2.311092e+00
## 7566   2  502 2.279798e+00
## 7567   2  503 2.249727e+00
## 7568   2  504 2.222669e+00
## 7569   2  505 3.436866e+00
## 7570   2  506 3.859533e+00
## 7571   2  507 3.917964e+00
## 7572   2  508 3.838276e+00
## 7573   2  509 3.682845e+00
## 7574   2  510 3.527803e+00
## 7575   2  511 3.371911e+00
## 7576   2  512 3.240011e+00
## 7577   2  513 3.100499e+00
## 7578   2  514 2.981671e+00
## 7579   2  515 2.885414e+00
## 7580   2  516 2.794014e+00
## 7581   2  517 2.715065e+00
## 7582   2  518 2.643472e+00
## 7583   2  519 2.575278e+00
## 7584   2  520 2.523442e+00
## 7585   2  521 2.474019e+00
## 7586   2  522 2.426764e+00
## 7587   2  523 2.383657e+00
## 7588   2  524 2.342225e+00
## 7589   2  525 2.310687e+00
## 7590   2  526 2.280628e+00
## 7591   2  527 2.252133e+00
## 7592   2  528 2.226842e+00
## 7593   2  529 3.435093e+00
## 7594   2  530 3.863597e+00
## 7595   2  531 3.922065e+00
## 7596   2  532 3.818719e+00
## 7597   2  533 3.689489e+00
## 7598   2  534 3.526029e+00
## 7599   2  535 3.376129e+00
## 7600   2  536 3.236465e+00
## 7601   2  537 3.100052e+00
## 7602   2  538 2.989576e+00
## 7603   2  539 2.878214e+00
## 7604   2  540 2.794271e+00
## 7605   2  541 2.718799e+00
## 7606   2  542 2.641487e+00
## 7607   2  543 2.581252e+00
## 7608   2  544 2.522663e+00
## 7609   2  545 2.465901e+00
## 7610   2  546 2.423617e+00
## 7611   2  547 2.380484e+00
## 7612   2  548 2.348349e+00
## 7613   2  549 2.308827e+00
## 7614   2  550 2.280413e+00
## 7615   2  551 2.250738e+00
## 7616   2  552 2.223587e+00
## 7617   2  553 3.436351e+00
## 7618   2  554 3.854603e+00
## 7619   2  555 3.916348e+00
## 7620   2  556 3.827350e+00
## 7621   2  557 3.683616e+00
## 7622   2  558 3.526370e+00
## 7623   2  559 3.375645e+00
## 7624   2  560 3.229769e+00
## 7625   2  561 3.103120e+00
## 7626   2  562 2.987978e+00
## 7627   2  563 2.888046e+00
## 7628   2  564 2.791162e+00
## 7629   2  565 2.716856e+00
## 7630   2  566 2.638364e+00
## 7631   2  567 2.579687e+00
## 7632   2  568 2.520048e+00
## 7633   2  569 2.472890e+00
## 7634   2  570 2.422868e+00
## 7635   2  571 2.378970e+00
## 7636   2  572 2.347019e+00
## 7637   2  573 2.309119e+00
## 7638   2  574 2.279401e+00
## 7639   2  575 2.252505e+00
## 7640   2  576 2.217468e+00
## 7641   2  577 3.437298e+00
## 7642   2  578 3.856218e+00
## 7643   2  579 3.919095e+00
## 7644   2  580 3.830142e+00
## 7645   2  581 3.682135e+00
## 7646   2  582 3.518404e+00
## 7647   2  583 3.374994e+00
## 7648   2  584 3.233224e+00
## 7649   2  585 3.096177e+00
## 7650   2  586 2.986261e+00
## 7651   2  587 2.886138e+00
## 7652   2  588 2.785846e+00
## 7653   2  589 2.714695e+00
## 7654   2  590 2.642681e+00
## 7655   2  591 2.577346e+00
## 7656   2  592 2.524326e+00
## 7657   2  593 2.473069e+00
## 7658   2  594 2.421927e+00
## 7659   2  595 2.381188e+00
## 7660   2  596 2.346141e+00
## 7661   2  597 2.310669e+00
## 7662   2  598 2.276912e+00
## 7663   2  599 2.249681e+00
## 7664   2  600 2.222611e+00
## 7665   2  601 3.436903e+00
## 7666   2  602 3.857773e+00
## 7667   2  603 3.920599e+00
## 7668   2  604 3.828782e+00
## 7669   2  605 3.688263e+00
## 7670   2  606 3.530798e+00
## 7671   2  607 3.375291e+00
## 7672   2  608 3.226314e+00
## 7673   2  609 3.101152e+00
## 7674   2  610 2.983516e+00
## 7675   2  611 2.889756e+00
## 7676   2  612 2.800570e+00
## 7677   2  613 2.715624e+00
## 7678   2  614 2.638460e+00
## 7679   2  615 2.580412e+00
## 7680   2  616 2.519325e+00
## 7681   2  617 2.471691e+00
## 7682   2  618 2.420720e+00
## 7683   2  619 2.376821e+00
## 7684   2  620 2.342388e+00
## 7685   2  621 2.311245e+00
## 7686   2  622 2.274334e+00
## 7687   2  623 2.249353e+00
## 7688   2  624 2.222680e+00
## 7689   2  624 2.224323e+00
## 7690   2  625 3.434695e+00
## 7691   2  626 3.852984e+00
## 7692   2  627 3.914642e+00
## 7693   2  628 3.822479e+00
## 7694   2  629 3.680296e+00
## 7695   2  630 3.521305e+00
## 7696   2  631 3.373298e+00
## 7697   2  632 3.229577e+00
## 7698   2  633 3.098965e+00
## 7699   2  634 2.982877e+00
## 7700   2  635 2.884824e+00
## 7701   2  636 2.790729e+00
## 7702   2  637 2.710806e+00
## 7703   2  638 2.636795e+00
## 7704   2  639 2.574969e+00
## 7705   2  640 2.518036e+00
## 7706   2  641 2.469034e+00
## 7707   2  642 2.421500e+00
## 7708   2  643 2.380134e+00
## 7709   2  644 2.339627e+00
## 7710   2  645 2.312442e+00
## 7711   2  646 2.277070e+00
## 7712   2  647 2.248178e+00
## 7713   2  648 2.221369e+00
## 7714   2  648 2.222749e+00
## 7715   2  649 2.196157e+00
## 7716   2  650 2.171063e+00
## 7717   2  651 2.159337e+00
## 7718   2  652 2.133586e+00
## 7719   2  653 2.116464e+00
## 7720   2  654 2.095686e+00
## 7721   2  654 2.092762e+00
## 7722   2  655 3.315440e+00
## 7723   2  656 3.747607e+00
## 7724   2  657 3.811651e+00
## 7725   2  658 3.731619e+00
## 7726   2  659 3.586008e+00
## 7727   2  660 3.430474e+00
## 7728   2  661 3.283944e+00
## 7729   2  662 3.139295e+00
## 7730   2  663 3.009867e+00
## 7731   2  664 2.903318e+00
## 7732   2  665 2.803380e+00
## 7733   2  666 2.717694e+00
## 7734   2  667 2.640234e+00
## 7735   2  668 2.570288e+00
## 7736   2  669 2.500922e+00
## 7737   2  670 2.449358e+00
## 7738   2  671 2.398041e+00
## 7739   2  672 2.354052e+00
## 7740   2  672 2.355987e+00
## 7741   2  673 3.549629e+00
## 7742   2  674 3.954274e+00
## 7743   2  675 4.004103e+00
## 7744   2  676 3.906940e+00
## 7745   2  677 3.746740e+00
## 7746   2  678 3.580383e+00
## 7747   2  679 3.424385e+00
## 7748   2  680 3.277419e+00
## 7749   2  681 3.146406e+00
## 7750   2  682 3.026572e+00
## 7751   2  683 2.911430e+00
## 7752   2  684 2.824881e+00
## 7753   2  685 2.738666e+00
## 7754   2  686 2.666309e+00
## 7755   2  687 2.596697e+00
## 7756   2  688 2.538787e+00
## 7757   2  689 2.482120e+00
## 7758   2  690 2.444250e+00
## 7759   2  691 2.403533e+00
## 7760   2  692 2.356637e+00
## 7761   2  693 2.322450e+00
## 7762   2  694 2.294943e+00
## 7763   2  695 2.260914e+00
## 7764   2  696 2.234159e+00
## 7765   2  697 3.450758e+00
## 7766   2  698 3.861424e+00
## 7767   2  699 3.926922e+00
## 7768   2  700 3.839453e+00
## 7769   2  701 3.688228e+00
## 7770   2  702 3.534274e+00
## 7771   2  703 3.377412e+00
## 7772   2  704 3.239446e+00
## 7773   2  705 3.112620e+00
## 7774   2  706 2.999138e+00
## 7775   2  707 2.889114e+00
## 7776   2  708 2.800067e+00
## 7777   2  709 2.721783e+00
## 7778   2  710 2.647003e+00
## 7779   2  711 2.583141e+00
## 7780   2  712 2.532162e+00
## 7781   2  713 2.473706e+00
## 7782   2  714 2.432459e+00
## 7783   2  715 2.388715e+00
## 7784   2  716 2.352711e+00
## 7785   2  717 2.316642e+00
## 7786   2  718 2.284091e+00
## 7787   2  719 2.256611e+00
## 7788   2  720 2.225689e+00
## 7789   2  721 3.443823e+00
## 7790   2  722 3.862725e+00
## 7791   2  723 3.927667e+00
## 7792   2  724 3.839388e+00
## 7793   2  725 3.691681e+00
## 7794   2  726 3.532714e+00
## 7795   2  727 3.379545e+00
## 7796   2  728 3.237419e+00
## 7797   2  729 3.100378e+00
## 7798   2  730 2.994592e+00
## 7799   2  731 2.892862e+00
## 7800   2  732 2.795620e+00
## 7801   2  733 2.717745e+00
## 7802   2  734 2.646008e+00
## 7803   2  735 2.585877e+00
## 7804   2  736 2.528153e+00
## 7805   2  737 2.479699e+00
## 7806   2  738 2.431125e+00
## 7807   2  739 2.388583e+00
## 7808   2  740 2.349263e+00
## 7809   2  741 2.317431e+00
## 7810   2  742 2.286470e+00
## 7811   2  743 2.255331e+00
## 7812   2  744 2.228892e+00
## 7813   2  745 3.435348e+00
## 7814   2  746 3.858658e+00
## 7815   2  747 3.934545e+00
## 7816   2  748 3.833212e+00
## 7817   2  749 3.691880e+00
## 7818   2  750 3.532122e+00
## 7819   2  751 3.376630e+00
## 7820   2  752 3.233132e+00
## 7821   2  753 3.103514e+00
## 7822   2  754 2.996128e+00
## 7823   2  755 2.892107e+00
## 7824   2  756 2.798151e+00
## 7825   2  757 2.717982e+00
## 7826   2  758 2.645763e+00
## 7827   2  759 2.584243e+00
## 7828   2  760 2.524417e+00
## 7829   2  761 2.472751e+00
## 7830   2  762 2.426372e+00
## 7831   2  763 2.386284e+00
## 7832   2  764 2.347898e+00
## 7833   2  765 2.312887e+00
## 7834   2  766 2.288639e+00
## 7835   2  767 2.254739e+00
## 7836   2  768 2.230519e+00
## 7837   2  769 3.432585e+00
## 7838   2  770 3.868413e+00
## 7839   2  771 3.928583e+00
## 7840   2  772 3.837225e+00
## 7841   2  773 3.683554e+00
## 7842   2  774 3.527419e+00
## 7843   2  775 3.373962e+00
## 7844   2  776 3.234346e+00
## 7845   2  777 3.100382e+00
## 7846   2  778 2.991120e+00
## 7847   2  779 2.886537e+00
## 7848   2  780 2.794205e+00
## 7849   2  781 2.717931e+00
## 7850   2  782 2.647110e+00
## 7851   2  783 2.578712e+00
## 7852   2  784 2.526204e+00
## 7853   2  785 2.476897e+00
## 7854   2  786 2.425953e+00
## 7855   2  787 2.386168e+00
## 7856   2  788 2.349747e+00
## 7857   2  789 2.312760e+00
## 7858   2  790 2.284261e+00
## 7859   2  791 2.253625e+00
## 7860   2  792 2.224709e+00
## 7861   2  793 3.441230e+00
## 7862   2  794 3.856215e+00
## 7863   2  795 3.922208e+00
## 7864   2  796 3.833337e+00
## 7865   2  797 3.690249e+00
## 7866   2  798 3.522573e+00
## 7867   2  799 3.376296e+00
## 7868   2  800 3.232481e+00
## 7869   2  801 3.108189e+00
## 7870   2  802 2.991688e+00
## 7871   2  803 2.889787e+00
## 7872   2  804 2.795852e+00
## 7873   2  805 2.713963e+00
```

```
## 7874   2  806 2.643077e+00
## 7875   2  807 2.580053e+00
## 7876   2  808 2.526810e+00
## 7877   2  809 2.476819e+00
## 7878   2  810 2.426299e+00
## 7879   2  811 2.386005e+00
## 7880   2  812 2.345419e+00
## 7881   2  813 2.314661e+00
## 7882   2  814 2.282914e+00
## 7883   2  815 2.256879e+00
## 7884   2  816 2.224632e+00
## 7885   2  817 3.434571e+00
## 7886   2  818 3.856976e+00
## 7887   2  819 3.924208e+00
## 7888   2  820 3.829007e+00
## 7889   2  821 3.690833e+00
## 7890   2  822 3.529455e+00
## 7891   2  823 3.372740e+00
## 7892   2  824 3.232534e+00
## 7893   2  825 3.102568e+00
## 7894   2  826 2.988803e+00
## 7895   2  827 2.891727e+00
## 7896   2  828 2.795124e+00
## 7897   2  829 2.717654e+00
## 7898   2  830 2.640507e+00
## 7899   2  831 2.581392e+00
## 7900   2  832 2.519705e+00
## 7901   2  833 2.467946e+00
## 7902   2  834 2.427547e+00
## 7903   2  835 2.381304e+00
## 7904   2  836 2.350533e+00
## 7905   2  837 2.313037e+00
## 7906   2  838 2.281159e+00
## 7907   2  839 2.255983e+00
## 7908   2  840 2.232382e+00
## 7909   2  841 3.439754e+00
## 7910   2  842 3.858219e+00
## 7911   2  843 3.923429e+00
## 7912   2  844 3.828646e+00
## 7913   2  845 3.686607e+00
## 7914   2  846 3.523972e+00
## 7915   2  847 3.370477e+00
## 7916   2  848 3.229807e+00
## 7917   2  849 3.102829e+00
## 7918   2  850 2.985654e+00
## 7919   2  851 2.889464e+00
## 7920   2  852 2.791490e+00
## 7921   2  853 2.716204e+00
## 7922   2  854 2.643925e+00
## 7923   2  855 2.585349e+00
## 7924   2  856 2.521570e+00
## 7925   2  857 2.472316e+00
## 7926   2  858 2.428461e+00
## 7927   2  859 2.384440e+00
## 7928   2  860 2.344766e+00
## 7929   2  861 2.313885e+00
## 7930   2  862 2.281317e+00
## 7931   2  863 2.250603e+00
## 7932   2  864 2.224867e+00
## 7933   2  865 3.431934e+00
## 7934   2  866 3.858403e+00
## 7935   2  867 3.917281e+00
## 7936   2  868 3.832885e+00
## 7937   2  869 3.690156e+00
## 7938   2  870 3.529076e+00
## 7939   2  871 3.376385e+00
## 7940   2  872 3.232902e+00
## 7941   2  873 3.094739e+00
## 7942   2  874 2.984586e+00
## 7943   2  875 2.886658e+00
## 7944   2  876 2.794295e+00
## 7945   2  877 2.712985e+00
## 7946   2  878 2.641854e+00
## 7947   2  879 2.578915e+00
## 7948   2  880 2.524109e+00
## 7949   2  881 2.473732e+00
## 7950   2  882 2.425352e+00
## 7951   2  883 2.384081e+00
## 7952   2  884 2.347209e+00
## 7953   2  885 2.311433e+00
## 7954   2  886 2.283335e+00
## 7955   2  887 2.253827e+00
## 7956   2  888 2.225627e+00
## 7957   2  889 3.435417e+00
## 7958   2  890 3.862617e+00
## 7959   2  891 3.921621e+00
## 7960   2  892 3.828883e+00
## 7961   2  893 3.684238e+00
## 7962   2  894 3.528299e+00
## 7963   2  895 3.372865e+00
## 7964   2  896 3.227099e+00
## 7965   2  897 3.099956e+00
## 7966   2  898 2.985320e+00
## 7967   2  899 2.886624e+00
## 7968   2  900 2.793859e+00
## 7969   2  901 2.715816e+00
## 7970   2  902 2.639766e+00
## 7971   2  903 2.580280e+00
## 7972   2  904 2.523082e+00
## 7973   2  905 2.472901e+00
## 7974   2  906 2.422301e+00
## 7975   2  907 2.385778e+00
## 7976   2  908 2.349394e+00
## 7977   2  909 2.314221e+00
## 7978   2  910 2.276465e+00
## 7979   2  911 2.251806e+00
## 7980   2  912 2.223226e+00
## 7981   2  913 3.435143e+00
## 7982   2  914 3.854615e+00
## 7983   2  915 3.920374e+00
## 7984   2  916 3.829286e+00
## 7985   2  917 3.684540e+00
## 7986   2  918 3.531176e+00
## 7987   2  919 3.370332e+00
## 7988   2  920 3.230399e+00
## 7989   2  921 3.104690e+00
## 7990   2  922 2.986377e+00
## 7991   2  923 2.886664e+00
## 7992   2  924 2.793273e+00
## 7993   2  925 2.712430e+00
## 7994   2  926 2.642180e+00
## 7995   2  927 2.579634e+00
## 7996   2  928 2.521054e+00
## 7997   2  929 2.473460e+00
## 7998   2  930 2.428295e+00
## 7999   2  931 2.378158e+00
## 8000   2  932 2.345487e+00
## 8001   2  933 2.310131e+00
## 8002   2  934 2.279194e+00
## 8003   2  935 2.253256e+00
## 8004   2  936 2.227771e+00
## 8005   2  936 2.221638e+00
## 8006   2  937 3.435234e+00
## 8007   2  938 3.858983e+00
## 8008   2  939 3.922072e+00
## 8009   2  940 3.824505e+00
## 8010   2  941 3.682536e+00
## 8011   2  942 3.525253e+00
## 8012   2  943 3.372317e+00
## 8013   2  944 3.231913e+00
## 8014   2  945 3.100669e+00
## 8015   2  946 2.984130e+00
## 8016   2  947 2.881484e+00
## 8017   2  948 2.789026e+00
## 8018   2  949 2.713362e+00
## 8019   2  950 2.641355e+00
## 8020   2  951 2.577942e+00
## 8021   2  952 2.516179e+00
## 8022   2  953 2.467624e+00
## 8023   2  954 2.424145e+00
## 8024   2  955 2.377664e+00
## 8025   2  956 2.344185e+00
## 8026   2  957 2.307458e+00
## 8027   2  958 2.277614e+00
## 8028   2  959 2.246876e+00
## 8029   2  960 2.223981e+00
## 8030   2  960 2.225971e+00
## 8031   2  961 2.200296e+00
## 8032   2  962 2.175853e+00
## 8033   2  963 2.153336e+00
## 8034   2  964 2.130551e+00
## 8035   2  965 2.112168e+00
## 8036   2  966 2.098252e+00
## 8037   2  967 2.078640e+00
## 8038   2  968 2.061540e+00
## 8039   2  969 2.047364e+00
## 8040   2  969 2.046528e+00
## 8041   2  970 3.265362e+00
## 8042   2  971 3.694734e+00
## 8043   2  972 3.761390e+00
## 8044   2  973 3.690762e+00
## 8045   2  974 3.543081e+00
## 8046   2  975 3.389329e+00
## 8047   2  976 3.247138e+00
## 8048   2  977 3.102643e+00
## 8049   2  978 2.979881e+00
## 8050   2  979 2.865560e+00
## 8051   2  980 2.771665e+00
## 8052   2  981 2.683698e+00
## 8053   2  982 2.605146e+00
## 8054   2  983 2.536753e+00
## 8055   2  984 2.470939e+00
## 8056   2  984 2.469838e+00
## 8057   2  985 3.651597e+00
## 8058   2  986 4.044494e+00
## 8059   2  987 4.083238e+00
## 8060   2  988 3.977504e+00
## 8061   2  989 3.815931e+00
## 8062   2  990 3.635421e+00
## 8063   2  991 3.468727e+00
## 8064   2  992 3.320099e+00
## 8065   2  993 3.175910e+00
## 8066   2  994 3.054999e+00
## 8067   2  995 2.954335e+00
## 8068   2  996 2.850298e+00
## 8069   2  997 2.765867e+00
## 8070   2  998 2.684679e+00
## 8071   2  999 2.619734e+00
## 8072   2 1000 2.556678e+00
## 8073   2 1001 2.502555e+00
## 8074   2 1002 2.456118e+00
## 8075   2 1003 2.409119e+00
## 8076   2 1004 2.367106e+00
## 8077   2 1005 2.333260e+00
## 8078   2 1006 2.301128e+00
## 8079   2 1007 2.267116e+00
## 8080   2 1008 2.245750e+00
## 8081   2 1009 3.450513e+00
## 8082   2 1010 3.873210e+00
## 8083   2 1011 3.938289e+00
## 8084   2 1012 3.842187e+00
## 8085   2 1013 3.698792e+00
## 8086   2 1014 3.537255e+00
## 8087   2 1015 3.381234e+00
## 8088   2 1016 3.242704e+00
## 8089   2 1017 3.108992e+00
## 8090   2 1018 2.998817e+00
## 8091   2 1019 2.893928e+00
## 8092   2 1020 2.807182e+00
## 8093   2 1021 2.722792e+00
## 8094   2 1022 2.651968e+00
## 8095   2 1023 2.592307e+00
## 8096   2 1024 2.530800e+00
## 8097   2 1025 2.482211e+00
## 8098   2 1026 2.437342e+00
## 8099   2 1027 2.395035e+00
## 8100   2 1028 2.358396e+00
## 8101   2 1029 2.316680e+00
## 8102   2 1030 2.293173e+00
## 8103   2 1031 2.257544e+00
## 8104   2 1032 2.235371e+00
## 8105   2 1033 3.441684e+00
## 8106   2 1034 3.862559e+00
## 8107   2 1035 3.928969e+00
## 8108   2 1036 3.838975e+00
## 8109   2 1037 3.690942e+00
## 8110   2 1038 3.533381e+00
## 8111   2 1039 3.381904e+00
## 8112   2 1040 3.235909e+00
## 8113   2 1041 3.111532e+00
## 8114   2 1042 2.993512e+00
## 8115   2 1043 2.895027e+00
## 8116   2 1044 2.802220e+00
## 8117   2 1045 2.726418e+00
## 8118   2 1046 2.654072e+00
## 8119   2 1047 2.586610e+00
## 8120   2 1048 2.526928e+00
## 8121   2 1049 2.477098e+00
## 8122   2 1050 2.432580e+00
## 8123   2 1051 2.390291e+00
## 8124   2 1052 2.354097e+00
## 8125   2 1053 2.317475e+00
## 8126   2 1054 2.285236e+00
## 8127   2 1055 2.259063e+00
## 8128   2 1056 2.231423e+00
## 8129   2 1057 3.437063e+00
## 8130   2 1058 3.864351e+00
## 8131   2 1059 3.930823e+00
## 8132   2 1060 3.843046e+00
## 8133   2 1061 3.689816e+00
## 8134   2 1062 3.535790e+00
## 8135   2 1063 3.385071e+00
## 8136   2 1064 3.238881e+00
## 8137   2 1065 3.110719e+00
## 8138   2 1066 2.989416e+00
## 8139   2 1067 2.892095e+00
## 8140   2 1068 2.807836e+00
## 8141   2 1069 2.729274e+00
## 8142   2 1070 2.651141e+00
## 8143   2 1071 2.583973e+00
## 8144   2 1072 2.527091e+00
## 8145   2 1073 2.477643e+00
## 8146   2 1074 2.430287e+00
## 8147   2 1075 2.390154e+00
## 8148   2 1076 2.349049e+00
## 8149   2 1077 2.316103e+00
## 8150   2 1078 2.285745e+00
## 8151   2 1079 2.255472e+00
## 8152   2 1080 2.231987e+00
## 8153   2 1081 3.439050e+00
## 8154   2 1082 3.874076e+00
## 8155   2 1083 3.923315e+00
## 8156   2 1084 3.839439e+00
## 8157   2 1085 3.691539e+00
## 8158   2 1086 3.529903e+00
## 8159   2 1087 3.374479e+00
## 8160   2 1088 3.234230e+00
## 8161   2 1089 3.108287e+00
## 8162   2 1090 2.994634e+00
## 8163   2 1091 2.891521e+00
## 8164   2 1092 2.798817e+00
## 8165   2 1093 2.719871e+00
## 8166   2 1094 2.648659e+00
## 8167   2 1095 2.583752e+00
## 8168   2 1096 2.527757e+00
## 8169   2 1097 2.476006e+00
## 8170   2 1098 2.430669e+00
## 8171   2 1099 2.389040e+00
## 8172   2 1100 2.348686e+00
## 8173   2 1101 2.316461e+00
## 8174   2 1102 2.284576e+00
## 8175   2 1103 2.255694e+00
## 8176   2 1104 2.230927e+00
## 8177   2 1105 3.442862e+00
## 8178   2 1106 3.867716e+00
## 8179   2 1107 3.924942e+00
## 8180   2 1108 3.833902e+00
## 8181   2 1109 3.685751e+00
## 8182   2 1110 3.534159e+00
## 8183   2 1111 3.375569e+00
## 8184   2 1112 3.234800e+00
## 8185   2 1113 3.105941e+00
## 8186   2 1114 2.981892e+00
## 8187   2 1115 2.888181e+00
## 8188   2 1116 2.808902e+00
## 8189   2 1117 2.720255e+00
## 8190   2 1118 2.646537e+00
## 8191   2 1119 2.580287e+00
## 8192   2 1120 2.525716e+00
## 8193   2 1121 2.474637e+00
## 8194   2 1122 2.426322e+00
## 8195   2 1123 2.387574e+00
## 8196   2 1124 2.350194e+00
## 8197   2 1125 2.315285e+00
## 8198   2 1126 2.283998e+00
## 8199   2 1127 2.249389e+00
## 8200   2 1128 2.227143e+00
## 8201   2 1129 3.446851e+00
## 8202   2 1130 3.861779e+00
## 8203   2 1131 3.926547e+00
## 8204   2 1132 3.836378e+00
## 8205   2 1133 3.696005e+00
## 8206   2 1134 3.528699e+00
## 8207   2 1135 3.379182e+00
## 8208   2 1136 3.230422e+00
## 8209   2 1137 3.105956e+00
## 8210   2 1138 2.996051e+00
## 8211   2 1139 2.887743e+00
## 8212   2 1140 2.798889e+00
## 8213   2 1141 2.713211e+00
## 8214   2 1142 2.650057e+00
## 8215   2 1143 2.579785e+00
## 8216   2 1144 2.521231e+00
## 8217   2 1145 2.469508e+00
## 8218   2 1146 2.432148e+00
## 8219   2 1147 2.385169e+00
## 8220   2 1148 2.351898e+00
## 8221   2 1149 2.311961e+00
## 8222   2 1150 2.285378e+00
## 8223   2 1151 2.257370e+00
## 8224   2 1152 2.228405e+00
## 8225   2 1153 3.442136e+00
## 8226   2 1154 3.859353e+00
## 8227   2 1155 3.922734e+00
## 8228   2 1156 3.829639e+00
## 8229   2 1157 3.691219e+00
## 8230   2 1158 3.531668e+00
## 8231   2 1159 3.370566e+00
## 8232   2 1160 3.234372e+00
## 8233   2 1161 3.099561e+00
## 8234   2 1162 2.990113e+00
## 8235   2 1163 2.887475e+00
## 8236   2 1164 2.798627e+00
## 8237   2 1165 2.712492e+00
## 8238   2 1166 2.645582e+00
## 8239   2 1167 2.584641e+00
## 8240   2 1168 2.527094e+00
## 8241   2 1169 2.472751e+00
## 8242   2 1170 2.423762e+00
## 8243   2 1171 2.387089e+00
## 8244   2 1172 2.353048e+00
## 8245   2 1173 2.315690e+00
## 8246   2 1174 2.283122e+00
## 8247   2 1175 2.250485e+00
## 8248   2 1176 2.225524e+00
## 8249   2 1177 3.437644e+00
## 8250   2 1178 3.863125e+00
## 8251   2 1179 3.924855e+00
## 8252   2 1180 3.832829e+00
## 8253   2 1181 3.685872e+00
## 8254   2 1182 3.526337e+00
## 8255   2 1183 3.368988e+00
## 8256   2 1184 3.235017e+00
## 8257   2 1185 3.102700e+00
## 8258   2 1186 2.989242e+00
## 8259   2 1187 2.891117e+00
## 8260   2 1188 2.795283e+00
## 8261   2 1189 2.717844e+00
## 8262   2 1190 2.645998e+00
## 8263   2 1191 2.580992e+00
## 8264   2 1192 2.524020e+00
## 8265   2 1193 2.471814e+00
## 8266   2 1194 2.425680e+00
## 8267   2 1195 2.386624e+00
## 8268   2 1196 2.348131e+00
## 8269   2 1197 2.312722e+00
## 8270   2 1198 2.281965e+00
## 8271   2 1199 2.253958e+00
## 8272   2 1200 2.229122e+00
## 8273   2 1201 3.441796e+00
## 8274   2 1202 3.860005e+00
## 8275   2 1203 3.920171e+00
## 8276   2 1204 3.833449e+00
## 8277   2 1205 3.685080e+00
## 8278   2 1206 3.525860e+00
## 8279   2 1207 3.370664e+00
## 8280   2 1208 3.234110e+00
## 8281   2 1209 3.105636e+00
## 8282   2 1210 2.982506e+00
## 8283   2 1211 2.884092e+00
## 8284   2 1212 2.797742e+00
## 8285   2 1213 2.712121e+00
## 8286   2 1214 2.647921e+00
## 8287   2 1215 2.580938e+00
## 8288   2 1216 2.526340e+00
## 8289   2 1217 2.479232e+00
## 8290   2 1218 2.426450e+00
## 8291   2 1219 2.383234e+00
## 8292   2 1220 2.347684e+00
## 8293   2 1221 2.313986e+00
## 8294   2 1222 2.279550e+00
## 8295   2 1223 2.251330e+00
## 8296   2 1224 2.229850e+00
## 8297   2 1225 3.435952e+00
## 8298   2 1226 3.854900e+00
## 8299   2 1227 3.928458e+00
## 8300   2 1228 3.828928e+00
## 8301   2 1229 3.697570e+00
## 8302   2 1230 3.525187e+00
## 8303   2 1231 3.370217e+00
## 8304   2 1232 3.233615e+00
## 8305   2 1233 3.104591e+00
## 8306   2 1234 2.986256e+00
## 8307   2 1235 2.888277e+00
## 8308   2 1236 2.791692e+00
## 8309   2 1237 2.717507e+00
## 8310   2 1238 2.639997e+00
## 8311   2 1239 2.581102e+00
## 8312   2 1240 2.519230e+00
## 8313   2 1241 2.472091e+00
## 8314   2 1242 2.424454e+00
## 8315   2 1243 2.384732e+00
## 8316   2 1244 2.344511e+00
## 8317   2 1245 2.309579e+00
## 8318   2 1246 2.282811e+00
## 8319   2 1247 2.249965e+00
## 8320   2 1248 2.227648e+00
## 8321   2 1248 2.221176e+00
## 8322   2 1249 3.432452e+00
## 8323   2 1250 3.851985e+00
## 8324   2 1251 3.920112e+00
## 8325   2 1252 3.827599e+00
## 8326   2 1253 3.682416e+00
## 8327   2 1254 3.523151e+00
## 8328   2 1255 3.371858e+00
## 8329   2 1256 3.227421e+00
## 8330   2 1257 3.105003e+00
## 8331   2 1258 2.983901e+00
## 8332   2 1259 2.886452e+00
## 8333   2 1260 2.791590e+00
## 8334   2 1261 2.710741e+00
## 8335   2 1262 2.644544e+00
## 8336   2 1263 2.576529e+00
## 8337   2 1264 2.522929e+00
## 8338   2 1265 2.467703e+00
## 8339   2 1266 2.422909e+00
## 8340   2 1267 2.383956e+00
## 8341   2 1268 2.344931e+00
## 8342   2 1269 2.307503e+00
## 8343   2 1270 2.275828e+00
## 8344   2 1271 2.244763e+00
## 8345   2 1272 2.224563e+00
## 8346   2 1272 2.224287e+00
## 8347   2 1273 2.194932e+00
## 8348   2 1274 2.176182e+00
## 8349   2 1275 2.155846e+00
## 8350   2 1276 2.129588e+00
## 8351   2 1277 2.117064e+00
## 8352   2 1278 2.096942e+00
## 8353   2 1279 2.082258e+00
## 8354   2 1280 2.061012e+00
## 8355   2 1281 2.045711e+00
## 8356   2 1282 2.032898e+00
## 8357   2 1283 2.020204e+00
## 8358   2 1284 2.002391e+00
## 8359   2 1284 2.004399e+00
## 8360   2 1285 3.227343e+00
## 8361   2 1286 3.660286e+00
## 8362   2 1287 3.726471e+00
## 8363   2 1288 3.651353e+00
## 8364   2 1289 3.506123e+00
## 8365   2 1290 3.355112e+00
## 8366   2 1291 3.208109e+00
## 8367   2 1292 3.066199e+00
## 8368   2 1293 2.949095e+00
## 8369   2 1294 2.835739e+00
## 8370   2 1295 2.736901e+00
## 8371   2 1296 2.649242e+00
## 8372   2 1296 2.642561e+00
## 8373   2 1297 3.804271e+00
## 8374   2 1298 4.189209e+00
## 8375   2 1299 4.210379e+00
## 8376   2 1300 4.078478e+00
## 8377   2 1301 3.900839e+00
## 8378   2 1302 3.718178e+00
## 8379   2 1303 3.537720e+00
## 8380   2 1304 3.377303e+00
## 8381   2 1305 3.230563e+00
## 8382   2 1306 3.108121e+00
## 8383   2 1307 2.981897e+00
## 8384   2 1308 2.884384e+00
## 8385   2 1309 2.796182e+00
## 8386   2 1310 2.711448e+00
## 8387   2 1311 2.645333e+00
## 8388   2 1312 2.580012e+00
## 8389   2 1313 2.525826e+00
## 8390   2 1314 2.468840e+00
## 8391   2 1315 2.425512e+00
## 8392   2 1316 2.386076e+00
## 8393   2 1317 2.343838e+00
## 8394   2 1318 2.314662e+00
## 8395   2 1319 2.279035e+00
## 8396   2 1320 2.254639e+00
## 8397   2 1321 3.463952e+00
## 8398   2 1322 3.879511e+00
## 8399   2 1323 3.946304e+00
## 8400   2 1324 3.858331e+00
## 8401   2 1325 3.707045e+00
## 8402   2 1326 3.549370e+00
## 8403   2 1327 3.388146e+00
## 8404   2 1328 3.247465e+00
## 8405   2 1329 3.125182e+00
## 8406   2 1330 3.002427e+00
## 8407   2 1331 2.904099e+00
## 8408   2 1332 2.811208e+00
## 8409   2 1333 2.728345e+00
## 8410   2 1334 2.662506e+00
## 8411   2 1335 2.593071e+00
## 8412   2 1336 2.538629e+00
## 8413   2 1337 2.487939e+00
## 8414   2 1338 2.437063e+00
## 8415   2 1339 2.394394e+00
## 8416   2 1340 2.360440e+00
## 8417   2 1341 2.328241e+00
## 8418   2 1342 2.291249e+00
## 8419   2 1343 2.265467e+00
## 8420   2 1344 2.235198e+00
## 8421   2 1345 3.451215e+00
## 8422   2 1346 3.863093e+00
## 8423   2 1347 3.933077e+00
## 8424   2 1348 3.841311e+00
## 8425   2 1349 3.700241e+00
## 8426   2 1350 3.534940e+00
## 8427   2 1351 3.383454e+00
## 8428   2 1352 3.238077e+00
## 8429   2 1353 3.112513e+00
## 8430   2 1354 2.997247e+00
## 8431   2 1355 2.898094e+00
## 8432   2 1356 2.804182e+00
## 8433   2 1357 2.723475e+00
## 8434   2 1358 2.660170e+00
## 8435   2 1359 2.590503e+00
## 8436   2 1360 2.533529e+00
## 8437   2 1361 2.484316e+00
## 8438   2 1362 2.433201e+00
## 8439   2 1363 2.392619e+00
## 8440   2 1364 2.357833e+00
## 8441   2 1365 2.315984e+00
## 8442   2 1366 2.287605e+00
## 8443   2 1367 2.256048e+00
## 8444   2 1368 2.234274e+00
## 8445   2 1369 3.450971e+00
## 8446   2 1370 3.860008e+00
## 8447   2 1371 3.931019e+00
## 8448   2 1372 3.833466e+00
## 8449   2 1373 3.692765e+00
## 8450   2 1374 3.541599e+00
## 8451   2 1375 3.381827e+00
## 8452   2 1376 3.245206e+00
## 8453   2 1377 3.104965e+00
## 8454   2 1378 2.999698e+00
## 8455   2 1379 2.901145e+00
## 8456   2 1380 2.801391e+00
## 8457   2 1381 2.726433e+00
## 8458   2 1382 2.655891e+00
## 8459   2 1383 2.585589e+00
## 8460   2 1384 2.530474e+00
## 8461   2 1385 2.479784e+00
## 8462   2 1386 2.435303e+00
## 8463   2 1387 2.390785e+00
## 8464   2 1388 2.359366e+00
## 8465   2 1389 2.321835e+00
## 8466   2 1390 2.287389e+00
## 8467   2 1391 2.260460e+00
## 8468   2 1392 2.235706e+00
## 8469   2 1393 3.446622e+00
## 8470   2 1394 3.868381e+00
## 8471   2 1395 3.924772e+00
## 8472   2 1396 3.839342e+00
## 8473   2 1397 3.694041e+00
## 8474   2 1398 3.534563e+00
## 8475   2 1399 3.381841e+00
## 8476   2 1400 3.235356e+00
## 8477   2 1401 3.109901e+00
## 8478   2 1402 2.998952e+00
## 8479   2 1403 2.895219e+00
## 8480   2 1404 2.803861e+00
## 8481   2 1405 2.722473e+00
## 8482   2 1406 2.655506e+00
## 8483   2 1407 2.588909e+00
## 8484   2 1408 2.529867e+00
## 8485   2 1409 2.476018e+00
## 8486   2 1410 2.428027e+00
## 8487   2 1411 2.391862e+00
## 8488   2 1412 2.355220e+00
## 8489   2 1413 2.317351e+00
## 8490   2 1414 2.283595e+00
## 8491   2 1415 2.257544e+00
## 8492   2 1416 2.230253e+00
## 8493   2 1417 3.439503e+00
## 8494   2 1418 3.871206e+00
## 8495   2 1419 3.922964e+00
## 8496   2 1420 3.837389e+00
## 8497   2 1421 3.691482e+00
## 8498   2 1422 3.541185e+00
## 8499   2 1423 3.379888e+00
## 8500   2 1424 3.234994e+00
## 8501   2 1425 3.107162e+00
## 8502   2 1426 2.996671e+00
## 8503   2 1427 2.895193e+00
## 8504   2 1428 2.800493e+00
## 8505   2 1429 2.718096e+00
## 8506   2 1430 2.652598e+00
## 8507   2 1431 2.582370e+00
## 8508   2 1432 2.527314e+00
## 8509   2 1433 2.476824e+00
## 8510   2 1434 2.427801e+00
## 8511   2 1435 2.388514e+00
## 8512   2 1436 2.355706e+00
## 8513   2 1437 2.321170e+00
## 8514   2 1438 2.284184e+00
## 8515   2 1439 2.257225e+00
## 8516   2 1440 2.232160e+00
## 8517   2 1441 3.444857e+00
## 8518   2 1442 3.863878e+00
## 8519   2 1443 3.933775e+00
## 8520   2 1444 3.842260e+00
## 8521   2 1445 3.689882e+00
## 8522   2 1446 3.528241e+00
## 8523   2 1447 3.377971e+00
## 8524   2 1448 3.237555e+00
## 8525   2 1449 3.110563e+00
## 8526   2 1450 2.989082e+00
## 8527   2 1451 2.889271e+00
## 8528   2 1452 2.799326e+00
## 8529   2 1453 2.726718e+00
## 8530   2 1454 2.646165e+00
## 8531   2 1455 2.580239e+00
## 8532   2 1456 2.527361e+00
## 8533   2 1457 2.477666e+00
## 8534   2 1458 2.432240e+00
## 8535   2 1459 2.390346e+00
## 8536   2 1460 2.351205e+00
## 8537   2 1461 2.316921e+00
## 8538   2 1462 2.286490e+00
## 8539   2 1463 2.258535e+00
## 8540   2 1464 2.229728e+00
## 8541   2 1465 3.441253e+00
## 8542   2 1466 3.855385e+00
## 8543   2 1467 3.921751e+00
## 8544   2 1468 3.845624e+00
## 8545   2 1469 3.687388e+00
## 8546   2 1470 3.534445e+00
## 8547   2 1471 3.375917e+00
## 8548   2 1472 3.233853e+00
## 8549   2 1473 3.111845e+00
## 8550   2 1474 2.993813e+00
## 8551   2 1475 2.888787e+00
## 8552   2 1476 2.804553e+00
## 8553   2 1477 2.717528e+00
## 8554   2 1478 2.650762e+00
## 8555   2 1479 2.583618e+00
## 8556   2 1480 2.525872e+00
## 8557   2 1481 2.472370e+00
## 8558   2 1482 2.430325e+00
## 8559   2 1483 2.383789e+00
## 8560   2 1484 2.350284e+00
## 8561   2 1485 2.314740e+00
## 8562   2 1486 2.282549e+00
## 8563   2 1487 2.257805e+00
## 8564   2 1488 2.228771e+00
## 8565   2 1489 3.440638e+00
## 8566   2 1490 3.859727e+00
## 8567   2 1491 3.919928e+00
## 8568   2 1492 3.835936e+00
## 8569   2 1493 3.687698e+00
## 8570   2 1494 3.529242e+00
## 8571   2 1495 3.374195e+00
## 8572   2 1496 3.237848e+00
## 8573   2 1497 3.103886e+00
## 8574   2 1498 2.989547e+00
## 8575   2 1499 2.890010e+00
## 8576   2 1500 2.798049e+00
## 8577   2 1501 2.722312e+00
## 8578   2 1502 2.646641e+00
## 8579   2 1503 2.587073e+00
## 8580   2 1504 2.525611e+00
## 8581   2 1505 2.476255e+00
## 8582   2 1506 2.429770e+00
## 8583   2 1507 2.389273e+00
## 8584   2 1508 2.349021e+00
## 8585   2 1509 2.313313e+00
## 8586   2 1510 2.280929e+00
## 8587   2 1511 2.253463e+00
## 8588   2 1512 2.228763e+00
## 8589   2 1513 3.439437e+00
## 8590   2 1514 3.855757e+00
## 8591   2 1515 3.922418e+00
## 8592   2 1516 3.835053e+00
## 8593   2 1517 3.682467e+00
## 8594   2 1518 3.534716e+00
## 8595   2 1519 3.383259e+00
## 8596   2 1520 3.236353e+00
## 8597   2 1521 3.103098e+00
## 8598   2 1522 2.991035e+00
## 8599   2 1523 2.886347e+00
## 8600   2 1524 2.798034e+00
## 8601   2 1525 2.720935e+00
## 8602   2 1526 2.639582e+00
## 8603   2 1527 2.581099e+00
## 8604   2 1528 2.525351e+00
## 8605   2 1529 2.474591e+00
## 8606   2 1530 2.426219e+00
## 8607   2 1531 2.384976e+00
## 8608   2 1532 2.349644e+00
## 8609   2 1533 2.310615e+00
## 8610   2 1534 2.284113e+00
## 8611   2 1535 2.253133e+00
## 8612   2 1536 2.228342e+00
## 8613   2 1537 3.443625e+00
## 8614   2 1538 3.862179e+00
## 8615   2 1539 3.920782e+00
## 8616   2 1540 3.832350e+00
## 8617   2 1541 3.679372e+00
## 8618   2 1542 3.526866e+00
## 8619   2 1543 3.374255e+00
## 8620   2 1544 3.228532e+00
## 8621   2 1545 3.100950e+00
## 8622   2 1546 2.985910e+00
## 8623   2 1547 2.889253e+00
## 8624   2 1548 2.797309e+00
## 8625   2 1549 2.715101e+00
## 8626   2 1550 2.645512e+00
## 8627   2 1551 2.583526e+00
## 8628   2 1552 2.523783e+00
## 8629   2 1553 2.471495e+00
## 8630   2 1554 2.423772e+00
## 8631   2 1555 2.385561e+00
## 8632   2 1556 2.352712e+00
## 8633   2 1557 2.314135e+00
## 8634   2 1558 2.281109e+00
## 8635   2 1559 2.252560e+00
## 8636   2 1560 2.224759e+00
## 8637   2 1560 2.223426e+00
## 8638   2 1561 3.431772e+00
## 8639   2 1562 3.854724e+00
## 8640   2 1563 3.921173e+00
## 8641   2 1564 3.825005e+00
## 8642   2 1565 3.691625e+00
## 8643   2 1566 3.533848e+00
## 8644   2 1567 3.367316e+00
## 8645   2 1568 3.227224e+00
## 8646   2 1569 3.099676e+00
## 8647   2 1570 2.980358e+00
## 8648   2 1571 2.881895e+00
## 8649   2 1572 2.788771e+00
## 8650   2 1573 2.715974e+00
## 8651   2 1574 2.638697e+00
## 8652   2 1575 2.575029e+00
## 8653   2 1576 2.517864e+00
## 8654   2 1577 2.467754e+00
## 8655   2 1578 2.422783e+00
## 8656   2 1579 2.378437e+00
## 8657   2 1580 2.340424e+00
## 8658   2 1581 2.308298e+00
## 8659   2 1582 2.278180e+00
## 8660   2 1583 2.247178e+00
## 8661   2 1584 2.218047e+00
## 8662   2 1584 2.221069e+00
## 8663   2 1585 2.196832e+00
## 8664   2 1586 2.174461e+00
## 8665   2 1587 2.156150e+00
## 8666   2 1588 2.133864e+00
## 8667   2 1589 2.114708e+00
## 8668   2 1590 2.096954e+00
## 8669   2 1591 2.080184e+00
## 8670   2 1592 2.060847e+00
## 8671   2 1593 2.048975e+00
## 8672   2 1594 2.034407e+00
## 8673   2 1595 2.019180e+00
```

```
## 8674   2 1596 2.008070e+00
## 8675   2 1597 1.995025e+00
## 8676   2 1598 1.980651e+00
## 8677   2 1599 1.964646e+00
## 8678   2 1599 1.963465e+00
## 8679   2 1600 2.570673e+00
## 8680   2 1601 2.778471e+00
## 8681   2 1602 2.807728e+00
## 8682   2 1603 2.758349e+00
## 8683   2 1604 2.690465e+00
## 8684   2 1605 2.609919e+00
## 8685   2 1606 2.525603e+00
## 8686   2 1607 2.453086e+00
## 8687   2 1608 2.384178e+00
## 8688   2 1608 2.384446e+00
## 8689   2 1609 3.554406e+00
## 8690   2 1610 3.948216e+00
## 8691   2 1611 3.990117e+00
## 8692   2 1612 3.880099e+00
## 8693   2 1613 3.704412e+00
## 8694   2 1614 3.524078e+00
## 8695   2 1615 3.357288e+00
## 8696   2 1616 3.202659e+00
## 8697   2 1617 3.054992e+00
## 8698   2 1618 2.940151e+00
## 8699   2 1619 2.828907e+00
## 8700   2 1620 2.726606e+00
## 8701   2 1621 2.643065e+00
## 8702   2 1622 2.565540e+00
## 8703   2 1623 2.498472e+00
## 8704   2 1624 2.433364e+00
## 8705   2 1625 2.380302e+00
## 8706   2 1626 2.334849e+00
## 8707   2 1627 2.291040e+00
## 8708   2 1628 2.249154e+00
## 8709   2 1629 2.210660e+00
## 8710   2 1630 2.180255e+00
## 8711   2 1631 2.149647e+00
## 8712   2 1632 2.123677e+00
## 8713   2 1633 3.333309e+00
## 8714   2 1634 3.761626e+00
## 8715   2 1635 3.818434e+00
## 8716   2 1636 3.725955e+00
## 8717   2 1637 3.585756e+00
## 8718   2 1638 3.417877e+00
## 8719   2 1639 3.268662e+00
## 8720   2 1640 3.133571e+00
## 8721   2 1641 2.994946e+00
## 8722   2 1642 2.889304e+00
## 8723   2 1643 2.784291e+00
## 8724   2 1644 2.692030e+00
## 8725   2 1645 2.613739e+00
## 8726   2 1646 2.544184e+00
## 8727   2 1647 2.476958e+00
## 8728   2 1648 2.421895e+00
## 8729   2 1649 2.372145e+00
## 8730   2 1650 2.325402e+00
## 8731   2 1651 2.284779e+00
## 8732   2 1652 2.251445e+00
## 8733   2 1653 2.209653e+00
## 8734   2 1654 2.182926e+00
## 8735   2 1655 2.152395e+00
## 8736   2 1656 2.127119e+00
## 8737   2 1657 3.340057e+00
## 8738   2 1658 3.762593e+00
## 8739   2 1659 3.824643e+00
## 8740   2 1660 3.742586e+00
## 8741   2 1661 3.589205e+00
## 8742   2 1662 3.434910e+00
## 8743   2 1663 3.275865e+00
## 8744   2 1664 3.139436e+00
## 8745   2 1665 3.008236e+00
## 8746   2 1666 2.894516e+00
## 8747   2 1667 2.796006e+00
## 8748   2 1668 2.702784e+00
## 8749   2 1669 2.624789e+00
## 8750   2 1670 2.549718e+00
## 8751   2 1671 2.488799e+00
## 8752   2 1672 2.434851e+00
## 8753   2 1673 2.387823e+00
## 8754   2 1674 2.336559e+00
## 8755   2 1675 2.298120e+00
## 8756   2 1676 2.259182e+00
## 8757   2 1677 2.225414e+00
## 8758   2 1678 2.194549e+00
## 8759   2 1679 2.165038e+00
## 8760   2 1680 2.138346e+00
## 8761   2 1681 3.352308e+00
## 8762   2 1682 3.772598e+00
## 8763   2 1683 3.839256e+00
## 8764   2 1684 3.746358e+00
## 8765   2 1685 3.603552e+00
## 8766   2 1686 3.445705e+00
## 8767   2 1687 3.295953e+00
## 8768   2 1688 3.151747e+00
## 8769   2 1689 3.018676e+00
## 8770   2 1690 2.907185e+00
## 8771   2 1691 2.805851e+00
## 8772   2 1692 2.714831e+00
## 8773   2 1693 2.635708e+00
## 8774   2 1694 2.561197e+00
## 8775   2 1695 2.502631e+00
## 8776   2 1696 2.445673e+00
## 8777   2 1697 2.392416e+00
## 8778   2 1698 2.347413e+00
## 8779   2 1699 2.309278e+00
## 8780   2 1700 2.270209e+00
## 8781   2 1701 2.235319e+00
## 8782   2 1702 2.203537e+00
## 8783   2 1703 2.178755e+00
## 8784   2 1704 2.149511e+00
## 8785   2 1705 3.366775e+00
## 8786   2 1706 3.791649e+00
## 8787   2 1707 3.848300e+00
## 8788   2 1708 3.760448e+00
## 8789   2 1709 3.609210e+00
## 8790   2 1710 3.456223e+00
## 8791   2 1711 3.303571e+00
## 8792   2 1712 3.160018e+00
## 8793   2 1713 3.032321e+00
## 8794   2 1714 2.916857e+00
## 8795   2 1715 2.811085e+00
## 8796   2 1716 2.721723e+00
## 8797   2 1717 2.648640e+00
## 8798   2 1718 2.572825e+00
## 8799   2 1719 2.513331e+00
## 8800   2 1720 2.456142e+00
## 8801   2 1721 2.401506e+00
## 8802   2 1722 2.357406e+00
## 8803   2 1723 2.320391e+00
## 8804   2 1724 2.277918e+00
## 8805   2 1725 2.246297e+00
## 8806   2 1726 2.211820e+00
## 8807   2 1727 2.188927e+00
## 8808   2 1728 2.156945e+00
## 8809   2 1729 3.372459e+00
## 8810   2 1730 3.794555e+00
## 8811   2 1731 3.861841e+00
## 8812   2 1732 3.769785e+00
## 8813   2 1733 3.619112e+00
## 8814   2 1734 3.460480e+00
## 8815   2 1735 3.311610e+00
## 8816   2 1736 3.164773e+00
## 8817   2 1737 3.041464e+00
## 8818   2 1738 2.925030e+00
## 8819   2 1739 2.826440e+00
## 8820   2 1740 2.736752e+00
## 8821   2 1741 2.651831e+00
## 8822   2 1742 2.578845e+00
## 8823   2 1743 2.516942e+00
## 8824   2 1744 2.464209e+00
## 8825   2 1745 2.413420e+00
## 8826   2 1746 2.367844e+00
## 8827   2 1747 2.327507e+00
## 8828   2 1748 2.285880e+00
## 8829   2 1749 2.257499e+00
## 8830   2 1750 2.224034e+00
## 8831   2 1751 2.196108e+00
## 8832   2 1752 2.167443e+00
## 8833   2 1753 3.376549e+00
## 8834   2 1754 3.803461e+00
## 8835   2 1755 3.868206e+00
## 8836   2 1756 3.772187e+00
## 8837   2 1757 3.629918e+00
## 8838   2 1758 3.471522e+00
## 8839   2 1759 3.320401e+00
## 8840   2 1760 3.175361e+00
## 8841   2 1761 3.044563e+00
## 8842   2 1762 2.931297e+00
## 8843   2 1763 2.830809e+00
## 8844   2 1764 2.743633e+00
## 8845   2 1765 2.665749e+00
## 8846   2 1766 2.594865e+00
## 8847   2 1767 2.528752e+00
## 8848   2 1768 2.467365e+00
## 8849   2 1769 2.421177e+00
## 8850   2 1770 2.376478e+00
## 8851   2 1771 2.330099e+00
## 8852   2 1772 2.291627e+00
## 8853   2 1773 2.261023e+00
## 8854   2 1774 2.231448e+00
## 8855   2 1775 2.203536e+00
## 8856   2 1776 2.169296e+00
## 8857   2 1777 3.387211e+00
## 8858   2 1778 3.807352e+00
## 8859   2 1779 3.868890e+00
## 8860   2 1780 3.785958e+00
## 8861   2 1781 3.640762e+00
## 8862   2 1782 3.481383e+00
## 8863   2 1783 3.328408e+00
## 8864   2 1784 3.181130e+00
## 8865   2 1785 3.058338e+00
## 8866   2 1786 2.938301e+00
## 8867   2 1787 2.840974e+00
## 8868   2 1788 2.748030e+00
## 8869   2 1789 2.665618e+00
## 8870   2 1790 2.596891e+00
## 8871   2 1791 2.533634e+00
## 8872   2 1792 2.481379e+00
## 8873   2 1793 2.425850e+00
## 8874   2 1794 2.378294e+00
## 8875   2 1795 2.341814e+00
## 8876   2 1796 2.303354e+00
## 8877   2 1797 2.267301e+00
## 8878   2 1798 2.236941e+00
## 8879   2 1799 2.206902e+00
## 8880   2 1800 2.181582e+00
## 8881   2 1801 3.388986e+00
## 8882   2 1802 3.814199e+00
## 8883   2 1803 3.871530e+00
## 8884   2 1804 3.790768e+00
## 8885   2 1805 3.637121e+00
## 8886   2 1806 3.486006e+00
## 8887   2 1807 3.334451e+00
## 8888   2 1808 3.187793e+00
## 8889   2 1809 3.060816e+00
## 8890   2 1810 2.948279e+00
## 8891   2 1811 2.842266e+00
## 8892   2 1812 2.753918e+00
## 8893   2 1813 2.672343e+00
## 8894   2 1814 2.602824e+00
## 8895   2 1815 2.544376e+00
## 8896   2 1816 2.481026e+00
## 8897   2 1817 2.432312e+00
## 8898   2 1818 2.384308e+00
## 8899   2 1819 2.344882e+00
## 8900   2 1820 2.306905e+00
## 8901   2 1821 2.270263e+00
## 8902   2 1822 2.241471e+00
## 8903   2 1823 2.212882e+00
## 8904   2 1824 2.182266e+00
## 8905   2 1825 3.403009e+00
## 8906   2 1826 3.817972e+00
## 8907   2 1827 3.879875e+00
## 8908   2 1828 3.797424e+00
## 8909   2 1829 3.651453e+00
## 8910   2 1830 3.486843e+00
## 8911   2 1831 3.340254e+00
## 8912   2 1832 3.189719e+00
## 8913   2 1833 3.065617e+00
## 8914   2 1834 2.952841e+00
## 8915   2 1835 2.848572e+00
## 8916   2 1836 2.755108e+00
## 8917   2 1837 2.677537e+00
## 8918   2 1838 2.607761e+00
## 8919   2 1839 2.544066e+00
## 8920   2 1840 2.485861e+00
## 8921   2 1841 2.430285e+00
## 8922   2 1842 2.388005e+00
## 8923   2 1843 2.350610e+00
## 8924   2 1844 2.313769e+00
## 8925   2 1845 2.273432e+00
## 8926   2 1846 2.245730e+00
## 8927   2 1847 2.215611e+00
## 8928   2 1848 2.188885e+00
## 8929   2 1849 3.405405e+00
## 8930   2 1850 3.818340e+00
## 8931   2 1851 3.885435e+00
## 8932   2 1852 3.799125e+00
## 8933   2 1853 3.651825e+00
## 8934   2 1854 3.492048e+00
## 8935   2 1855 3.340318e+00
## 8936   2 1856 3.195526e+00
## 8937   2 1857 3.069681e+00
## 8938   2 1858 2.955342e+00
## 8939   2 1859 2.855423e+00
## 8940   2 1860 2.759070e+00
## 8941   2 1861 2.684014e+00
## 8942   2 1862 2.612123e+00
## 8943   2 1863 2.547692e+00
## 8944   2 1864 2.489794e+00
## 8945   2 1865 2.441170e+00
## 8946   2 1866 2.393050e+00
## 8947   2 1867 2.347613e+00
## 8948   2 1868 2.315424e+00
## 8949   2 1869 2.276047e+00
## 8950   2 1870 2.248097e+00
## 8951   2 1871 2.221905e+00
## 8952   2 1872 2.198535e+00
## 8953   2 1872 2.219108e+00
## 8954   2 1873 3.439927e+00
## 8955   2 1874 3.850811e+00
## 8956   2 1875 3.924188e+00
## 8957   2 1876 3.828658e+00
## 8958   2 1877 3.685972e+00
## 8959   2 1878 3.523581e+00
## 8960   2 1879 3.370773e+00
## 8961   2 1880 3.230321e+00
## 8962   2 1881 3.099682e+00
## 8963   2 1882 2.987075e+00
## 8964   2 1883 2.882799e+00
## 8965   2 1884 2.792085e+00
## 8966   2 1885 2.713018e+00
## 8967   2 1886 2.637311e+00
## 8968   2 1887 2.577490e+00
## 8969   2 1888 2.519339e+00
## 8970   2 1889 2.472764e+00
## 8971   2 1890 2.427594e+00
## 8972   2 1891 2.379971e+00
## 8973   2 1892 2.344938e+00
## 8974   2 1893 2.309841e+00
## 8975   2 1894 2.275547e+00
## 8976   2 1895 2.248379e+00
## 8977   2 1896 2.222464e+00
## 8978   2 1896 2.219485e+00
## 8979   2 1897 2.196521e+00
## 8980   2 1898 2.174435e+00
## 8981   2 1899 2.154694e+00
## 8982   2 1900 2.135024e+00
## 8983   2 1901 2.114925e+00
## 8984   2 1902 2.094489e+00
## 8985   2 1903 2.078041e+00
## 8986   2 1904 2.066890e+00
## 8987   2 1905 2.046015e+00
## 8988   2 1906 2.036374e+00
## 8989   2 1907 2.018839e+00
## 8990   2 1908 2.002709e+00
## 8991   2 1909 1.989519e+00
## 8992   2 1910 1.978591e+00
## 8993   2 1911 1.962459e+00
## 8994   2 1911 1.966823e+00
## 8995   2 1912 3.187892e+00
## 8996   2 1913 3.625030e+00
## 8997   2 1914 3.691101e+00
## 8998   2 1915 3.608733e+00
## 8999   2 1916 3.470055e+00
## 9000   2 1917 3.320453e+00
## 9001   2 1918 3.172236e+00
## 9002   2 1919 3.035041e+00
## 9003   2 1920 2.907960e+00
## 9004   2 1920 2.909533e+00
## 9005   2 1921 4.036915e+00
## 9006   2 1922 4.384284e+00
## 9007   2 1923 4.385863e+00
## 9008   2 1924 4.230295e+00
## 9009   2 1925 4.025562e+00
## 9010   2 1926 3.838512e+00
## 9011   2 1927 3.648307e+00
## 9012   2 1928 3.472516e+00
## 9013   2 1929 3.305012e+00
## 9014   2 1930 3.171386e+00
## 9015   2 1931 3.047954e+00
## 9016   2 1932 2.938446e+00
## 9017   2 1933 2.840709e+00
## 9018   2 1934 2.755798e+00
## 9019   2 1935 2.679411e+00
## 9020   2 1936 2.612526e+00
## 9021   2 1937 2.546872e+00
## 9022   2 1938 2.495260e+00
## 9023   2 1939 2.452026e+00
## 9024   2 1940 2.406505e+00
## 9025   2 1941 2.368000e+00
## 9026   2 1942 2.329424e+00
## 9027   2 1943 2.293923e+00
## 9028   2 1944 2.263691e+00
## 9029   2 1945 3.470869e+00
## 9030   2 1946 3.892370e+00
## 9031   2 1947 3.960333e+00
## 9032   2 1948 3.859789e+00
## 9033   2 1949 3.709841e+00
## 9034   2 1950 3.554953e+00
## 9035   2 1951 3.403959e+00
## 9036   2 1952 3.259527e+00
## 9037   2 1953 3.124862e+00
## 9038   2 1954 3.014521e+00
## 9039   2 1955 2.907991e+00
## 9040   2 1956 2.812792e+00
## 9041   2 1957 2.736642e+00
## 9042   2 1958 2.662349e+00
## 9043   2 1959 2.597118e+00
## 9044   2 1960 2.539125e+00
## 9045   2 1961 2.494561e+00
## 9046   2 1962 2.443485e+00
## 9047   2 1963 2.399092e+00
## 9048   2 1964 2.363712e+00
## 9049   2 1965 2.324216e+00
## 9050   2 1966 2.298232e+00
## 9051   2 1967 2.266459e+00
## 9052   2 1968 2.240095e+00
## 9053   2 1969 3.453143e+00
## 9054   2 1970 3.867585e+00
## 9055   2 1971 3.934922e+00
## 9056   2 1972 3.854491e+00
## 9057   2 1973 3.702985e+00
## 9058   2 1974 3.537843e+00
## 9059   2 1975 3.385579e+00
## 9060   2 1976 3.242983e+00
## 9061   2 1977 3.115077e+00
## 9062   2 1978 3.002461e+00
## 9063   2 1979 2.898105e+00
## 9064   2 1980 2.808199e+00
## 9065   2 1981 2.726863e+00
## 9066   2 1982 2.659721e+00
## 9067   2 1983 2.594687e+00
## 9068   2 1984 2.537160e+00
## 9069   2 1985 2.483657e+00
## 9070   2 1986 2.437712e+00
## 9071   2 1987 2.400509e+00
## 9072   2 1988 2.358163e+00
## 9073   2 1989 2.322609e+00
## 9074   2 1990 2.296426e+00
## 9075   2 1991 2.267442e+00
## 9076   2 1992 2.236999e+00
## 9077   2 1993 3.446221e+00
## 9078   2 1994 3.880074e+00
## 9079   2 1995 3.936066e+00
## 9080   2 1996 3.853147e+00
## 9081   2 1997 3.706637e+00
## 9082   2 1998 3.546490e+00
## 9083   2 1999 3.385950e+00
## 9084   2 2000 3.247160e+00
## 9085   2 2001 3.116235e+00
## 9086   2 2002 2.999890e+00
## 9087   2 2003 2.896002e+00
## 9088   2 2004 2.810442e+00
## 9089   2 2005 2.730155e+00
## 9090   2 2006 2.650745e+00
## 9091   2 2007 2.589243e+00
## 9092   2 2008 2.535731e+00
## 9093   2 2009 2.484968e+00
## 9094   2 2010 2.438079e+00
## 9095   2 2011 2.393601e+00
## 9096   2 2012 2.355256e+00
## 9097   2 2013 2.323862e+00
## 9098   2 2014 2.293273e+00
## 9099   2 2015 2.264815e+00
## 9100   2 2016 2.236064e+00
## 9101   2 2017 3.451751e+00
## 9102   2 2018 3.868237e+00
## 9103   2 2019 3.928858e+00
## 9104   2 2020 3.846674e+00
## 9105   2 2021 3.694588e+00
## 9106   2 2022 3.532354e+00
## 9107   2 2023 3.382447e+00
## 9108   2 2024 3.241583e+00
## 9109   2 2025 3.111631e+00
## 9110   2 2026 3.000117e+00
## 9111   2 2027 2.890278e+00
## 9112   2 2028 2.804921e+00
## 9113   2 2029 2.729358e+00
## 9114   2 2030 2.656582e+00
## 9115   2 2031 2.589149e+00
## 9116   2 2032 2.525336e+00
## 9117   2 2033 2.480373e+00
## 9118   2 2034 2.435845e+00
## 9119   2 2035 2.393822e+00
## 9120   2 2036 2.357685e+00
## 9121   2 2037 2.317228e+00
## 9122   2 2038 2.293189e+00
## 9123   2 2039 2.260491e+00
## 9124   2 2040 2.232830e+00
## 9125   2 2041 3.449021e+00
## 9126   2 2042 3.869040e+00
## 9127   2 2043 3.926885e+00
## 9128   2 2044 3.841621e+00
## 9129   2 2045 3.694237e+00
## 9130   2 2046 3.537680e+00
## 9131   2 2047 3.382280e+00
## 9132   2 2048 3.238166e+00
## 9133   2 2049 3.106166e+00
## 9134   2 2050 2.998121e+00
## 9135   2 2051 2.895885e+00
## 9136   2 2052 2.803582e+00
## 9137   2 2053 2.720566e+00
## 9138   2 2054 2.649821e+00
## 9139   2 2055 2.590530e+00
## 9140   2 2056 2.530037e+00
## 9141   2 2057 2.477866e+00
## 9142   2 2058 2.434412e+00
## 9143   2 2059 2.388669e+00
## 9144   2 2060 2.352232e+00
## 9145   2 2061 2.317487e+00
## 9146   2 2062 2.282962e+00
## 9147   2 2063 2.256112e+00
## 9148   2 2064 2.233014e+00
## 9149   2 2065 3.440220e+00
## 9150   2 2066 3.863853e+00
## 9151   2 2067 3.923034e+00
## 9152   2 2068 3.843065e+00
## 9153   2 2069 3.702341e+00
## 9154   2 2070 3.540905e+00
## 9155   2 2071 3.376423e+00
## 9156   2 2072 3.243314e+00
## 9157   2 2073 3.113815e+00
## 9158   2 2074 2.992054e+00
## 9159   2 2075 2.892070e+00
## 9160   2 2076 2.804786e+00
## 9161   2 2077 2.729226e+00
## 9162   2 2078 2.651987e+00
## 9163   2 2079 2.581800e+00
## 9164   2 2080 2.529225e+00
## 9165   2 2081 2.475972e+00
## 9166   2 2082 2.433056e+00
## 9167   2 2083 2.392302e+00
## 9168   2 2084 2.352962e+00
## 9169   2 2085 2.319166e+00
## 9170   2 2086 2.285527e+00
## 9171   2 2087 2.257157e+00
## 9172   2 2088 2.229372e+00
## 9173   2 2089 3.439964e+00
## 9174   2 2090 3.866614e+00
## 9175   2 2091 3.926708e+00
## 9176   2 2092 3.839128e+00
## 9177   2 2093 3.691923e+00
## 9178   2 2094 3.525640e+00
## 9179   2 2095 3.387325e+00
## 9180   2 2096 3.234400e+00
## 9181   2 2097 3.104815e+00
## 9182   2 2098 2.993731e+00
## 9183   2 2099 2.902207e+00
## 9184   2 2100 2.805867e+00
## 9185   2 2101 2.719467e+00
## 9186   2 2102 2.648740e+00
## 9187   2 2103 2.583713e+00
## 9188   2 2104 2.526763e+00
## 9189   2 2105 2.475553e+00
## 9190   2 2106 2.430786e+00
## 9191   2 2107 2.383792e+00
## 9192   2 2108 2.353100e+00
## 9193   2 2109 2.314272e+00
## 9194   2 2110 2.285794e+00
## 9195   2 2111 2.254100e+00
## 9196   2 2112 2.232870e+00
## 9197   2 2113 3.436895e+00
## 9198   2 2114 3.868160e+00
## 9199   2 2115 3.925236e+00
## 9200   2 2116 3.827695e+00
## 9201   2 2117 3.690398e+00
## 9202   2 2118 3.537349e+00
## 9203   2 2119 3.380128e+00
## 9204   2 2120 3.237122e+00
## 9205   2 2121 3.109349e+00
## 9206   2 2122 2.997524e+00
## 9207   2 2123 2.889246e+00
## 9208   2 2124 2.800120e+00
## 9209   2 2125 2.716863e+00
## 9210   2 2126 2.647876e+00
## 9211   2 2127 2.587473e+00
## 9212   2 2128 2.524954e+00
## 9213   2 2129 2.478501e+00
## 9214   2 2130 2.430028e+00
## 9215   2 2131 2.385753e+00
## 9216   2 2132 2.351777e+00
## 9217   2 2133 2.313927e+00
## 9218   2 2134 2.280585e+00
## 9219   2 2135 2.253033e+00
## 9220   2 2136 2.230079e+00
## 9221   2 2137 3.439110e+00
## 9222   2 2138 3.862289e+00
## 9223   2 2139 3.925228e+00
## 9224   2 2140 3.831023e+00
## 9225   2 2141 3.693626e+00
## 9226   2 2142 3.525954e+00
## 9227   2 2143 3.375055e+00
## 9228   2 2144 3.237666e+00
## 9229   2 2145 3.107412e+00
## 9230   2 2146 2.989256e+00
## 9231   2 2147 2.892992e+00
## 9232   2 2148 2.800471e+00
## 9233   2 2149 2.717294e+00
## 9234   2 2150 2.650886e+00
## 9235   2 2151 2.582832e+00
## 9236   2 2152 2.529196e+00
## 9237   2 2153 2.474952e+00
## 9238   2 2154 2.429907e+00
## 9239   2 2155 2.387942e+00
## 9240   2 2156 2.353511e+00
## 9241   2 2157 2.312961e+00
## 9242   2 2158 2.287057e+00
## 9243   2 2159 2.249808e+00
## 9244   2 2160 2.226063e+00
## 9245   2 2161 3.435612e+00
## 9246   2 2162 3.859950e+00
## 9247   2 2163 3.927378e+00
## 9248   2 2164 3.841009e+00
## 9249   2 2165 3.686687e+00
## 9250   2 2166 3.529289e+00
## 9251   2 2167 3.376150e+00
## 9252   2 2168 3.232639e+00
## 9253   2 2169 3.109178e+00
## 9254   2 2170 2.987897e+00
## 9255   2 2171 2.885806e+00
## 9256   2 2172 2.798311e+00
## 9257   2 2173 2.717577e+00
## 9258   2 2174 2.645899e+00
## 9259   2 2175 2.575092e+00
## 9260   2 2176 2.523782e+00
## 9261   2 2177 2.475557e+00
## 9262   2 2178 2.433134e+00
## 9263   2 2179 2.389658e+00
## 9264   2 2180 2.348774e+00
## 9265   2 2181 2.313190e+00
## 9266   2 2182 2.282179e+00
## 9267   2 2183 2.253926e+00
## 9268   2 2184 2.227134e+00
## 9269   2 2184 2.220123e+00
## 9270   2 2185 3.433824e+00
## 9271   2 2186 3.858036e+00
## 9272   2 2187 3.919928e+00
## 9273   2 2188 3.829570e+00
## 9274   2 2189 3.683850e+00
## 9275   2 2190 3.520237e+00
## 9276   2 2191 3.374435e+00
## 9277   2 2192 3.226261e+00
## 9278   2 2193 3.097611e+00
## 9279   2 2194 2.988978e+00
## 9280   2 2195 2.881507e+00
## 9281   2 2196 2.792767e+00
## 9282   2 2197 2.715669e+00
## 9283   2 2198 2.641140e+00
## 9284   2 2199 2.580839e+00
## 9285   2 2200 2.522124e+00
## 9286   2 2201 2.472273e+00
## 9287   2 2202 2.425971e+00
## 9288   2 2203 2.379517e+00
## 9289   2 2204 2.342669e+00
## 9290   2 2205 2.306003e+00
## 9291   2 2206 2.278746e+00
## 9292   2 2207 2.248046e+00
## 9293   2 2208 2.218564e+00
## 9294   2 2208 2.221157e+00
## 9295   2 2209 2.194723e+00
## 9296   2 2210 2.173762e+00
## 9297   2 2211 2.151287e+00
## 9298   2 2212 2.131615e+00
## 9299   2 2213 2.115668e+00
## 9300   2 2214 2.099734e+00
## 9301   2 2215 2.079964e+00
## 9302   2 2216 2.065939e+00
## 9303   2 2217 2.047619e+00
## 9304   2 2218 2.031126e+00
## 9305   2 2219 2.017629e+00
## 9306   2 2220 2.006427e+00
## 9307   2 2221 1.988869e+00
## 9308   2 2222 1.978234e+00
## 9309   2 2223 1.963171e+00
## 9310   2 2224 1.948877e+00
## 9311   2 2225 1.941340e+00
## 9312   2 2226 1.927263e+00
## 9313   2 2226 1.930996e+00
## 9314   2 2227 2.532282e+00
## 9315   2 2228 2.749508e+00
## 9316   2 2229 2.772314e+00
## 9317   2 2230 2.731926e+00
## 9318   2 2231 2.655307e+00
## 9319   2 2232 2.570437e+00
## 9320   2 2232 2.567893e+00
## 9321   2 2233 3.728623e+00
## 9322   2 2234 4.100962e+00
## 9323   2 2235 4.122120e+00
## 9324   2 2236 3.986638e+00
## 9325   2 2237 3.816725e+00
## 9326   2 2238 3.617736e+00
## 9327   2 2239 3.432271e+00
## 9328   2 2240 3.266040e+00
## 9329   2 2241 3.124953e+00
## 9330   2 2242 2.989307e+00
## 9331   2 2243 2.867109e+00
## 9332   2 2244 2.766533e+00
## 9333   2 2245 2.672559e+00
## 9334   2 2246 2.592275e+00
## 9335   2 2247 2.525112e+00
## 9336   2 2248 2.457326e+00
## 9337   2 2249 2.410180e+00
## 9338   2 2250 2.352516e+00
## 9339   2 2251 2.304693e+00
## 9340   2 2252 2.264303e+00
## 9341   2 2253 2.224468e+00
## 9342   2 2254 2.191984e+00
## 9343   2 2255 2.159617e+00
## 9344   2 2256 2.129086e+00
## 9345   2 2257 3.346130e+00
## 9346   2 2258 3.766598e+00
## 9347   2 2259 3.823587e+00
## 9348   2 2260 3.739973e+00
## 9349   2 2261 3.589892e+00
## 9350   2 2262 3.428556e+00
## 9351   2 2263 3.279045e+00
## 9352   2 2264 3.130510e+00
## 9353   2 2265 3.003714e+00
## 9354   2 2266 2.891774e+00
## 9355   2 2267 2.787442e+00
## 9356   2 2268 2.697687e+00
## 9357   2 2269 2.612084e+00
## 9358   2 2270 2.543450e+00
## 9359   2 2271 2.485658e+00
## 9360   2 2272 2.431851e+00
## 9361   2 2273 2.370243e+00
## 9362   2 2274 2.329050e+00
## 9363   2 2275 2.288610e+00
## 9364   2 2276 2.248876e+00
## 9365   2 2277 2.213918e+00
## 9366   2 2278 2.185535e+00
## 9367   2 2279 2.155269e+00
## 9368   2 2280 2.127980e+00
## 9369   2 2281 3.340049e+00
## 9370   2 2282 3.760906e+00
## 9371   2 2283 3.825298e+00
## 9372   2 2284 3.745036e+00
## 9373   2 2285 3.597199e+00
## 9374   2 2286 3.434784e+00
## 9375   2 2287 3.283726e+00
## 9376   2 2288 3.137897e+00
## 9377   2 2289 3.012214e+00
## 9378   2 2290 2.895285e+00
## 9379   2 2291 2.797358e+00
## 9380   2 2292 2.705872e+00
## 9381   2 2293 2.628792e+00
## 9382   2 2294 2.554102e+00
## 9383   2 2295 2.492413e+00
## 9384   2 2296 2.432283e+00
## 9385   2 2297 2.385299e+00
## 9386   2 2298 2.339930e+00
## 9387   2 2299 2.295637e+00
## 9388   2 2300 2.259310e+00
## 9389   2 2301 2.224059e+00
## 9390   2 2302 2.197983e+00
## 9391   2 2303 2.166982e+00
## 9392   2 2304 2.141587e+00
## 9393   2 2305 3.352499e+00
## 9394   2 2306 3.772142e+00
## 9395   2 2307 3.837506e+00
## 9396   2 2308 3.747606e+00
## 9397   2 2309 3.600587e+00
## 9398   2 2310 3.451549e+00
## 9399   2 2311 3.291030e+00
## 9400   2 2312 3.147848e+00
## 9401   2 2313 3.018585e+00
## 9402   2 2314 2.910320e+00
## 9403   2 2315 2.805430e+00
## 9404   2 2316 2.717793e+00
## 9405   2 2317 2.637988e+00
## 9406   2 2318 2.565686e+00
## 9407   2 2319 2.505211e+00
## 9408   2 2320 2.443303e+00
## 9409   2 2321 2.393160e+00
## 9410   2 2322 2.351322e+00
## 9411   2 2323 2.308427e+00
## 9412   2 2324 2.269573e+00
## 9413   2 2325 2.241069e+00
## 9414   2 2326 2.208735e+00
## 9415   2 2327 2.177629e+00
## 9416   2 2328 2.157649e+00
## 9417   2 2329 3.366559e+00
## 9418   2 2330 3.790559e+00
## 9419   2 2331 3.848191e+00
## 9420   2 2332 3.768072e+00
## 9421   2 2333 3.611473e+00
## 9422   2 2334 3.450241e+00
## 9423   2 2335 3.300739e+00
## 9424   2 2336 3.157384e+00
## 9425   2 2337 3.033537e+00
## 9426   2 2338 2.918552e+00
## 9427   2 2339 2.817121e+00
## 9428   2 2340 2.731258e+00
## 9429   2 2341 2.649398e+00
## 9430   2 2342 2.569183e+00
## 9431   2 2343 2.511581e+00
## 9432   2 2344 2.454529e+00
## 9433   2 2345 2.407489e+00
## 9434   2 2346 2.357798e+00
## 9435   2 2347 2.320001e+00
## 9436   2 2348 2.280626e+00
## 9437   2 2349 2.244808e+00
## 9438   2 2350 2.215310e+00
## 9439   2 2351 2.186461e+00
## 9440   2 2352 2.159692e+00
## 9441   2 2353 3.373770e+00
## 9442   2 2354 3.799444e+00
## 9443   2 2355 3.856764e+00
## 9444   2 2356 3.765572e+00
## 9445   2 2357 3.614204e+00
## 9446   2 2358 3.466445e+00
## 9447   2 2359 3.316277e+00
## 9448   2 2360 3.169466e+00
## 9449   2 2361 3.040625e+00
## 9450   2 2362 2.924042e+00
## 9451   2 2363 2.826366e+00
## 9452   2 2364 2.734423e+00
## 9453   2 2365 2.657352e+00
## 9454   2 2366 2.584863e+00
## 9455   2 2367 2.518723e+00
## 9456   2 2368 2.465644e+00
## 9457   2 2369 2.411708e+00
## 9458   2 2370 2.363232e+00
## 9459   2 2371 2.326797e+00
## 9460   2 2372 2.287259e+00
## 9461   2 2373 2.255510e+00
## 9462   2 2374 2.221206e+00
## 9463   2 2375 2.195028e+00
## 9464   2 2376 2.166449e+00
## 9465   2 2377 3.381682e+00
## 9466   2 2378 3.804878e+00
## 9467   2 2379 3.871414e+00
## 9468   2 2380 3.777577e+00
## 9469   2 2381 3.640853e+00
## 9470   2 2382 3.472088e+00
## 9471   2 2383 3.317436e+00
## 9472   2 2384 3.175625e+00
## 9473   2 2385 3.052961e+00
```

```
## 9474   2 2386 2.928761e+00
## 9475   2 2387 2.833805e+00
## 9476   2 2388 2.745477e+00
## 9477   2 2389 2.664499e+00
## 9478   2 2390 2.592030e+00
## 9479   2 2391 2.532893e+00
## 9480   2 2392 2.469869e+00
## 9481   2 2393 2.415628e+00
## 9482   2 2394 2.374192e+00
## 9483   2 2395 2.332699e+00
## 9484   2 2396 2.294390e+00
## 9485   2 2397 2.261878e+00
## 9486   2 2398 2.233963e+00
## 9487   2 2399 2.205165e+00
## 9488   2 2400 2.173206e+00
## 9489   2 2401 3.386444e+00
## 9490   2 2402 3.814025e+00
## 9491   2 2403 3.872896e+00
## 9492   2 2404 3.782153e+00
## 9493   2 2405 3.637183e+00
## 9494   2 2406 3.478307e+00
## 9495   2 2407 3.322185e+00
## 9496   2 2408 3.180363e+00
## 9497   2 2409 3.060304e+00
## 9498   2 2410 2.944406e+00
## 9499   2 2411 2.836788e+00
## 9500   2 2412 2.747896e+00
## 9501   2 2413 2.671287e+00
## 9502   2 2414 2.594796e+00
## 9503   2 2415 2.533552e+00
## 9504   2 2416 2.475434e+00
## 9505   2 2417 2.430232e+00
## 9506   2 2418 2.383019e+00
## 9507   2 2419 2.342141e+00
## 9508   2 2420 2.307424e+00
## 9509   2 2421 2.265848e+00
## 9510   2 2422 2.236822e+00
## 9511   2 2423 2.213057e+00
## 9512   2 2424 2.180954e+00
## 9513   2 2425 3.392083e+00
## 9514   2 2426 3.812823e+00
## 9515   2 2427 3.878855e+00
## 9516   2 2428 3.787400e+00
## 9517   2 2429 3.643788e+00
## 9518   2 2430 3.483046e+00
## 9519   2 2431 3.334040e+00
## 9520   2 2432 3.185938e+00
## 9521   2 2433 3.058011e+00
## 9522   2 2434 2.949086e+00
## 9523   2 2435 2.846680e+00
## 9524   2 2436 2.758664e+00
## 9525   2 2437 2.672066e+00
## 9526   2 2438 2.602859e+00
## 9527   2 2439 2.538779e+00
## 9528   2 2440 2.477992e+00
## 9529   2 2441 2.428458e+00
## 9530   2 2442 2.385932e+00
## 9531   2 2443 2.345183e+00
## 9532   2 2444 2.308412e+00
## 9533   2 2445 2.272227e+00
## 9534   2 2446 2.242262e+00
## 9535   2 2447 2.211520e+00
## 9536   2 2448 2.183914e+00
## 9537   2 2449 3.395202e+00
## 9538   2 2450 3.823416e+00
## 9539   2 2451 3.883280e+00
## 9540   2 2452 3.790957e+00
## 9541   2 2453 3.651416e+00
## 9542   2 2454 3.493311e+00
## 9543   2 2455 3.339898e+00
## 9544   2 2456 3.191186e+00
## 9545   2 2457 3.066799e+00
## 9546   2 2458 2.952914e+00
## 9547   2 2459 2.849953e+00
## 9548   2 2460 2.756726e+00
## 9549   2 2461 2.681938e+00
## 9550   2 2462 2.608200e+00
## 9551   2 2463 2.540787e+00
## 9552   2 2464 2.491175e+00
## 9553   2 2465 2.437822e+00
## 9554   2 2466 2.390298e+00
## 9555   2 2467 2.350013e+00
## 9556   2 2468 2.311248e+00
## 9557   2 2469 2.279440e+00
## 9558   2 2470 2.250753e+00
## 9559   2 2471 2.217044e+00
## 9560   2 2472 2.187501e+00
## 9561   2 2473 3.405229e+00
## 9562   2 2474 3.825614e+00
## 9563   2 2475 3.893527e+00
## 9564   2 2476 3.800893e+00
## 9565   2 2477 3.649646e+00
## 9566   2 2478 3.491565e+00
## 9567   2 2479 3.337593e+00
## 9568   2 2480 3.198846e+00
## 9569   2 2481 3.068918e+00
## 9570   2 2482 2.957113e+00
## 9571   2 2483 2.857992e+00
## 9572   2 2484 2.773213e+00
## 9573   2 2485 2.689684e+00
## 9574   2 2486 2.613386e+00
## 9575   2 2487 2.552108e+00
## 9576   2 2488 2.492209e+00
## 9577   2 2489 2.435963e+00
## 9578   2 2490 2.397132e+00
## 9579   2 2491 2.349590e+00
## 9580   2 2492 2.315086e+00
## 9581   2 2493 2.283388e+00
## 9582   2 2494 2.251555e+00
## 9583   2 2495 2.222026e+00
## 9584   2 2496 2.196706e+00
## 9585   2 2496 2.223947e+00
## 9586   2 2497 3.435471e+00
## 9587   2 2498 3.849751e+00
## 9588   2 2499 3.919431e+00
## 9589   2 2500 3.828821e+00
## 9590   2 2501 3.684278e+00
## 9591   2 2502 3.526532e+00
## 9592   2 2503 3.362307e+00
## 9593   2 2504 3.227331e+00
## 9594   2 2505 3.098675e+00
## 9595   2 2506 2.987168e+00
## 9596   2 2507 2.882547e+00
## 9597   2 2508 2.792687e+00
## 9598   2 2509 2.714499e+00
## 9599   2 2510 2.641065e+00
## 9600   2 2511 2.575849e+00
## 9601   2 2512 2.518154e+00
## 9602   2 2513 2.465393e+00
## 9603   2 2514 2.419528e+00
## 9604   2 2515 2.383774e+00
## 9605   2 2516 2.341928e+00
## 9606   2 2517 2.305991e+00
## 9607   2 2518 2.276877e+00
## 9608   2 2519 2.246445e+00
## 9609   2 2520 2.217156e+00
## 9610   2 2520 2.222054e+00
## 9611   2 2521 2.197587e+00
## 9612   2 2522 2.174631e+00
## 9613   2 2523 2.155654e+00
## 9614   2 2524 2.134634e+00
## 9615   2 2525 2.117451e+00
## 9616   2 2526 2.099881e+00
## 9617   2 2527 2.082654e+00
## 9618   2 2528 2.064079e+00
## 9619   2 2529 2.051886e+00
## 9620   2 2530 2.030940e+00
## 9621   2 2531 2.019873e+00
## 9622   2 2532 2.003034e+00
## 9623   2 2533 1.990310e+00
## 9624   2 2534 1.977785e+00
## 9625   2 2535 1.965703e+00
## 9626   2 2536 1.950505e+00
## 9627   2 2537 1.938724e+00
## 9628   2 2538 1.928688e+00
## 9629   2 2538 1.926971e+00
## 9630   2 2539 3.149481e+00
## 9631   2 2540 3.587376e+00
## 9632   2 2541 3.653876e+00
## 9633   2 2542 3.575437e+00
## 9634   2 2543 3.437545e+00
## 9635   2 2544 3.289517e+00
## 9636   2 2544 3.288599e+00
## 9637   2 2545 4.371506e+00
## 9638   2 2546 4.680880e+00
## 9639   2 2547 4.642665e+00
## 9640   2 2548 4.466115e+00
## 9641   2 2549 4.230833e+00
## 9642   2 2550 4.013187e+00
## 9643   2 2551 3.795871e+00
## 9644   2 2552 3.597342e+00
## 9645   2 2553 3.431323e+00
## 9646   2 2554 3.277051e+00
## 9647   2 2555 3.135474e+00
## 9648   2 2556 3.017825e+00
## 9649   2 2557 2.914191e+00
## 9650   2 2558 2.815427e+00
## 9651   2 2559 2.733484e+00
## 9652   2 2560 2.659850e+00
## 9653   2 2561 2.593165e+00
## 9654   2 2562 2.532146e+00
## 9655   2 2563 2.477969e+00
## 9656   2 2564 2.434197e+00
## 9657   2 2565 2.391096e+00
## 9658   2 2566 2.353115e+00
## 9659   2 2567 2.316890e+00
## 9660   2 2568 2.284963e+00
## 9661   2 2569 3.492344e+00
## 9662   2 2570 3.910442e+00
## 9663   2 2571 3.963430e+00
## 9664   2 2572 3.875154e+00
## 9665   2 2573 3.726697e+00
## 9666   2 2574 3.559085e+00
## 9667   2 2575 3.410024e+00
## 9668   2 2576 3.263099e+00
## 9669   2 2577 3.131073e+00
## 9670   2 2578 3.017926e+00
## 9671   2 2579 2.911868e+00
## 9672   2 2580 2.821714e+00
## 9673   2 2581 2.744253e+00
## 9674   2 2582 2.666789e+00
## 9675   2 2583 2.606108e+00
## 9676   2 2584 2.547133e+00
## 9677   2 2585 2.492800e+00
## 9678   2 2586 2.449106e+00
## 9679   2 2587 2.405341e+00
## 9680   2 2588 2.367650e+00
## 9681   2 2589 2.330748e+00
## 9682   2 2590 2.303354e+00
## 9683   2 2591 2.275102e+00
## 9684   2 2592 2.246249e+00
## 9685   2 2593 3.462958e+00
## 9686   2 2594 3.882704e+00
## 9687   2 2595 3.934284e+00
## 9688   2 2596 3.849949e+00
## 9689   2 2597 3.701479e+00
## 9690   2 2598 3.547168e+00
## 9691   2 2599 3.388421e+00
## 9692   2 2600 3.243655e+00
## 9693   2 2601 3.119518e+00
## 9694   2 2602 3.000270e+00
## 9695   2 2603 2.907602e+00
## 9696   2 2604 2.816578e+00
## 9697   2 2605 2.733792e+00
## 9698   2 2606 2.662813e+00
## 9699   2 2607 2.594450e+00
## 9700   2 2608 2.536443e+00
## 9701   2 2609 2.490004e+00
## 9702   2 2610 2.445235e+00
## 9703   2 2611 2.399983e+00
## 9704   2 2612 2.365005e+00
## 9705   2 2613 2.330860e+00
## 9706   2 2614 2.292786e+00
## 9707   2 2615 2.270087e+00
## 9708   2 2616 2.247326e+00
## 9709   2 2617 3.457989e+00
## 9710   2 2618 3.868393e+00
## 9711   2 2619 3.934628e+00
## 9712   2 2620 3.845761e+00
## 9713   2 2621 3.707239e+00
## 9714   2 2622 3.541177e+00
## 9715   2 2623 3.392836e+00
## 9716   2 2624 3.246737e+00
## 9717   2 2625 3.120162e+00
## 9718   2 2626 3.005923e+00
## 9719   2 2627 2.898256e+00
## 9720   2 2628 2.814534e+00
## 9721   2 2629 2.728238e+00
## 9722   2 2630 2.659227e+00
## 9723   2 2631 2.595342e+00
## 9724   2 2632 2.538732e+00
## 9725   2 2633 2.483239e+00
## 9726   2 2634 2.439972e+00
## 9727   2 2635 2.394500e+00
## 9728   2 2636 2.362239e+00
## 9729   2 2637 2.324522e+00
## 9730   2 2638 2.293483e+00
## 9731   2 2639 2.268647e+00
## 9732   2 2640 2.241256e+00
## 9733   2 2641 3.448807e+00
## 9734   2 2642 3.872470e+00
## 9735   2 2643 3.934618e+00
## 9736   2 2644 3.848985e+00
## 9737   2 2645 3.703821e+00
## 9738   2 2646 3.537890e+00
## 9739   2 2647 3.382832e+00
## 9740   2 2648 3.247448e+00
## 9741   2 2649 3.119327e+00
## 9742   2 2650 2.999448e+00
## 9743   2 2651 2.900485e+00
## 9744   2 2652 2.811094e+00
## 9745   2 2653 2.726497e+00
## 9746   2 2654 2.658680e+00
## 9747   2 2655 2.588374e+00
## 9748   2 2656 2.541718e+00
## 9749   2 2657 2.480325e+00
## 9750   2 2658 2.434301e+00
## 9751   2 2659 2.393528e+00
## 9752   2 2660 2.360317e+00
## 9753   2 2661 2.322880e+00
## 9754   2 2662 2.294906e+00
## 9755   2 2663 2.263704e+00
## 9756   2 2664 2.239487e+00
## 9757   2 2665 3.447443e+00
## 9758   2 2666 3.872721e+00
## 9759   2 2667 3.933646e+00
## 9760   2 2668 3.836109e+00
## 9761   2 2669 3.698014e+00
## 9762   2 2670 3.536927e+00
## 9763   2 2671 3.380042e+00
## 9764   2 2672 3.242605e+00
## 9765   2 2673 3.116158e+00
## 9766   2 2674 3.000223e+00
## 9767   2 2675 2.901711e+00
## 9768   2 2676 2.803638e+00
## 9769   2 2677 2.727343e+00
## 9770   2 2678 2.659858e+00
## 9771   2 2679 2.593881e+00
## 9772   2 2680 2.533752e+00
## 9773   2 2681 2.481295e+00
## 9774   2 2682 2.439098e+00
## 9775   2 2683 2.392276e+00
## 9776   2 2684 2.360031e+00
## 9777   2 2685 2.324236e+00
## 9778   2 2686 2.296411e+00
## 9779   2 2687 2.259066e+00
## 9780   2 2688 2.237174e+00
## 9781   2 2689 3.444941e+00
## 9782   2 2690 3.867467e+00
## 9783   2 2691 3.926051e+00
## 9784   2 2692 3.835735e+00
## 9785   2 2693 3.701460e+00
## 9786   2 2694 3.531648e+00
## 9787   2 2695 3.389232e+00
## 9788   2 2696 3.238129e+00
## 9789   2 2697 3.114698e+00
## 9790   2 2698 2.999411e+00
## 9791   2 2699 2.895099e+00
## 9792   2 2700 2.800447e+00
## 9793   2 2701 2.731844e+00
## 9794   2 2702 2.652049e+00
## 9795   2 2703 2.587708e+00
## 9796   2 2704 2.527183e+00
## 9797   2 2705 2.484088e+00
## 9798   2 2706 2.433930e+00
## 9799   2 2707 2.393108e+00
## 9800   2 2708 2.358549e+00
## 9801   2 2709 2.321134e+00
## 9802   2 2710 2.282662e+00
## 9803   2 2711 2.256814e+00
## 9804   2 2712 2.237095e+00
## 9805   2 2713 3.439315e+00
## 9806   2 2714 3.872670e+00
## 9807   2 2715 3.928406e+00
## 9808   2 2716 3.841579e+00
## 9809   2 2717 3.702345e+00
## 9810   2 2718 3.537926e+00
## 9811   2 2719 3.377252e+00
## 9812   2 2720 3.242793e+00
## 9813   2 2721 3.111296e+00
## 9814   2 2722 2.988414e+00
## 9815   2 2723 2.894561e+00
## 9816   2 2724 2.802758e+00
## 9817   2 2725 2.722564e+00
## 9818   2 2726 2.655449e+00
## 9819   2 2727 2.589010e+00
## 9820   2 2728 2.529256e+00
## 9821   2 2729 2.478763e+00
## 9822   2 2730 2.431617e+00
## 9823   2 2731 2.390451e+00
## 9824   2 2732 2.355669e+00
## 9825   2 2733 2.318168e+00
## 9826   2 2734 2.287153e+00
## 9827   2 2735 2.257406e+00
## 9828   2 2736 2.235347e+00
## 9829   2 2737 3.440433e+00
## 9830   2 2738 3.866201e+00
## 9831   2 2739 3.929566e+00
## 9832   2 2740 3.830003e+00
## 9833   2 2741 3.694387e+00
## 9834   2 2742 3.534772e+00
## 9835   2 2743 3.380967e+00
## 9836   2 2744 3.235574e+00
## 9837   2 2745 3.106560e+00
## 9838   2 2746 2.992956e+00
## 9839   2 2747 2.891254e+00
## 9840   2 2748 2.802265e+00
## 9841   2 2749 2.722122e+00
## 9842   2 2750 2.648354e+00
## 9843   2 2751 2.589304e+00
## 9844   2 2752 2.530238e+00
## 9845   2 2753 2.476281e+00
## 9846   2 2754 2.434202e+00
## 9847   2 2755 2.385713e+00
## 9848   2 2756 2.348967e+00
## 9849   2 2757 2.321417e+00
## 9850   2 2758 2.283934e+00
## 9851   2 2759 2.255963e+00
## 9852   2 2760 2.232322e+00
## 9853   2 2761 3.438925e+00
## 9854   2 2762 3.867421e+00
## 9855   2 2763 3.925242e+00
## 9856   2 2764 3.847607e+00
## 9857   2 2765 3.692272e+00
## 9858   2 2766 3.529788e+00
## 9859   2 2767 3.380025e+00
## 9860   2 2768 3.240040e+00
## 9861   2 2769 3.101205e+00
## 9862   2 2770 2.989581e+00
## 9863   2 2771 2.890808e+00
## 9864   2 2772 2.802794e+00
## 9865   2 2773 2.719142e+00
## 9866   2 2774 2.647354e+00
## 9867   2 2775 2.583387e+00
## 9868   2 2776 2.531895e+00
## 9869   2 2777 2.471529e+00
## 9870   2 2778 2.432831e+00
## 9871   2 2779 2.385532e+00
## 9872   2 2780 2.348753e+00
## 9873   2 2781 2.317647e+00
## 9874   2 2782 2.287640e+00
## 9875   2 2783 2.255528e+00
## 9876   2 2784 2.232239e+00
## 9877   2 2785 3.439101e+00
## 9878   2 2786 3.865487e+00
## 9879   2 2787 3.930222e+00
## 9880   2 2788 3.837954e+00
## 9881   2 2789 3.697019e+00
## 9882   2 2790 3.531184e+00
## 9883   2 2791 3.373099e+00
## 9884   2 2792 3.234425e+00
## 9885   2 2793 3.107799e+00
## 9886   2 2794 2.991256e+00
## 9887   2 2795 2.884806e+00
## 9888   2 2796 2.801601e+00
## 9889   2 2797 2.720358e+00
## 9890   2 2798 2.650599e+00
## 9891   2 2799 2.587324e+00
## 9892   2 2800 2.529472e+00
## 9893   2 2801 2.474936e+00
## 9894   2 2802 2.425880e+00
## 9895   2 2803 2.384311e+00
## 9896   2 2804 2.349283e+00
## 9897   2 2805 2.315267e+00
## 9898   2 2806 2.282926e+00
## 9899   2 2807 2.256125e+00
## 9900   2 2808 2.231908e+00
## 9901   2 2808 2.223750e+00
## 9902   2 2809 3.435536e+00
## 9903   2 2810 3.853579e+00
## 9904   2 2811 3.919980e+00
## 9905   2 2812 3.824269e+00
## 9906   2 2813 3.688011e+00
## 9907   2 2814 3.524912e+00
## 9908   2 2815 3.372498e+00
## 9909   2 2816 3.229210e+00
## 9910   2 2817 3.100101e+00
## 9911   2 2818 2.982042e+00
## 9912   2 2819 2.886411e+00
## 9913   2 2820 2.791965e+00
## 9914   2 2821 2.717243e+00
## 9915   2 2822 2.642672e+00
## 9916   2 2823 2.575501e+00
## 9917   2 2824 2.517541e+00
## 9918   2 2825 2.470894e+00
## 9919   2 2826 2.417881e+00
## 9920   2 2827 2.376191e+00
## 9921   2 2828 2.337673e+00
## 9922   2 2829 2.307507e+00
## 9923   2 2830 2.276511e+00
## 9924   2 2831 2.244594e+00
## 9925   2 2832 2.222090e+00
## 9926   2 2832 2.223050e+00
## 9927   2 2833 2.200786e+00
## 9928   2 2834 2.174793e+00
## 9929   2 2835 2.155991e+00
## 9930   2 2836 2.131491e+00
## 9931   2 2837 2.117576e+00
## 9932   2 2838 2.097618e+00
## 9933   2 2839 2.077679e+00
## 9934   2 2840 2.065660e+00
## 9935   2 2841 2.050148e+00
## 9936   2 2842 2.031508e+00
## 9937   2 2843 2.019160e+00
## 9938   2 2844 2.001838e+00
## 9939   2 2845 1.987318e+00
## 9940   2 2846 1.978124e+00
## 9941   2 2847 1.963754e+00
## 9942   2 2848 1.953455e+00
## 9943   2 2849 1.940548e+00
## 9944   2 2850 1.928568e+00
## 9945   2 2851 1.916621e+00
## 9946   2 2852 1.905799e+00
## 9947   2 2853 1.891723e+00
## 9948   2 2853 1.894903e+00
## 9949   2 2854 2.499746e+00
## 9950   2 2855 2.708913e+00
## 9951   2 2856 2.739433e+00
## 9952   2 2856 2.741361e+00
## 9953   2 2857 3.934470e+00
## 9954   2 2858 4.304324e+00
## 9955   2 2859 4.299267e+00
## 9956   2 2860 4.162968e+00
## 9957   2 2861 3.956789e+00
## 9958   2 2862 3.747300e+00
## 9959   2 2863 3.556613e+00
## 9960   2 2864 3.369228e+00
## 9961   2 2865 3.207624e+00
## 9962   2 2866 3.064421e+00
## 9963   2 2867 2.942435e+00
## 9964   2 2868 2.826989e+00
## 9965   2 2869 2.730025e+00
## 9966   2 2870 2.641444e+00
## 9967   2 2871 2.567201e+00
## 9968   2 2872 2.495430e+00
## 9969   2 2873 2.429966e+00
## 9970   2 2874 2.378130e+00
## 9971   2 2875 2.329163e+00
## 9972   2 2876 2.287623e+00
## 9973   2 2877 2.241348e+00
## 9974   2 2878 2.208936e+00
## 9975   2 2879 2.177060e+00
## 9976   2 2880 2.149872e+00
## 9977   2 2881 3.360334e+00
## 9978   2 2882 3.772389e+00
## 9979   2 2883 3.833627e+00
## 9980   2 2884 3.740963e+00
## 9981   2 2885 3.595610e+00
## 9982   2 2886 3.439166e+00
## 9983   2 2887 3.280196e+00
## 9984   2 2888 3.139550e+00
## 9985   2 2889 3.003021e+00
## 9986   2 2890 2.900080e+00
## 9987   2 2891 2.786993e+00
## 9988   2 2892 2.703542e+00
## 9989   2 2893 2.621812e+00
## 9990   2 2894 2.544048e+00
## 9991   2 2895 2.482216e+00
## 9992   2 2896 2.428969e+00
## 9993   2 2897 2.375753e+00
## 9994   2 2898 2.332950e+00
## 9995   2 2899 2.289660e+00
## 9996   2 2900 2.250670e+00
## 9997   2 2901 2.218703e+00
## 9998   2 2902 2.186809e+00
## 9999   2 2903 2.160370e+00
## 10000  2 2904 2.130488e+00
## 10001  2 2905 3.344528e+00
## 10002  2 2906 3.775047e+00
## 10003  2 2907 3.827954e+00
## 10004  2 2908 3.741664e+00
## 10005  2 2909 3.592691e+00
## 10006  2 2910 3.433379e+00
## 10007  2 2911 3.284772e+00
## 10008  2 2912 3.141053e+00
## 10009  2 2913 3.013155e+00
## 10010  2 2914 2.896199e+00
## 10011  2 2915 2.800782e+00
## 10012  2 2916 2.706662e+00
## 10013  2 2917 2.629734e+00
## 10014  2 2918 2.559558e+00
## 10015  2 2919 2.491827e+00
## 10016  2 2920 2.437586e+00
## 10017  2 2921 2.384970e+00
## 10018  2 2922 2.337504e+00
## 10019  2 2923 2.303504e+00
## 10020  2 2924 2.266190e+00
## 10021  2 2925 2.230223e+00
## 10022  2 2926 2.197767e+00
## 10023  2 2927 2.168758e+00
## 10024  2 2928 2.144476e+00
## 10025  2 2929 3.355857e+00
## 10026  2 2930 3.777626e+00
## 10027  2 2931 3.833099e+00
## 10028  2 2932 3.756722e+00
## 10029  2 2933 3.611645e+00
## 10030  2 2934 3.448619e+00
## 10031  2 2935 3.296137e+00
## 10032  2 2936 3.155434e+00
## 10033  2 2937 3.025635e+00
## 10034  2 2938 2.916438e+00
## 10035  2 2939 2.806719e+00
## 10036  2 2940 2.714004e+00
## 10037  2 2941 2.635416e+00
## 10038  2 2942 2.567628e+00
## 10039  2 2943 2.503115e+00
## 10040  2 2944 2.451940e+00
## 10041  2 2945 2.396510e+00
## 10042  2 2946 2.350908e+00
## 10043  2 2947 2.308735e+00
## 10044  2 2948 2.274092e+00
## 10045  2 2949 2.240352e+00
## 10046  2 2950 2.208629e+00
## 10047  2 2951 2.177407e+00
## 10048  2 2952 2.153212e+00
## 10049  2 2953 3.366497e+00
## 10050  2 2954 3.787442e+00
## 10051  2 2955 3.846854e+00
## 10052  2 2956 3.764994e+00
## 10053  2 2957 3.615256e+00
## 10054  2 2958 3.458318e+00
## 10055  2 2959 3.310468e+00
## 10056  2 2960 3.167396e+00
## 10057  2 2961 3.036411e+00
## 10058  2 2962 2.919608e+00
## 10059  2 2963 2.820672e+00
## 10060  2 2964 2.725921e+00
## 10061  2 2965 2.644019e+00
## 10062  2 2966 2.575605e+00
## 10063  2 2967 2.512956e+00
## 10064  2 2968 2.457742e+00
## 10065  2 2969 2.410715e+00
## 10066  2 2970 2.359329e+00
## 10067  2 2971 2.320598e+00
## 10068  2 2972 2.282239e+00
## 10069  2 2973 2.243154e+00
## 10070  2 2974 2.221167e+00
## 10071  2 2975 2.190932e+00
## 10072  2 2976 2.164050e+00
## 10073  2 2977 3.373341e+00
## 10074  2 2978 3.795896e+00
## 10075  2 2979 3.856624e+00
## 10076  2 2980 3.772964e+00
## 10077  2 2981 3.623284e+00
## 10078  2 2982 3.464115e+00
## 10079  2 2983 3.319275e+00
## 10080  2 2984 3.169164e+00
## 10081  2 2985 3.037234e+00
## 10082  2 2986 2.925523e+00
## 10083  2 2987 2.827169e+00
## 10084  2 2988 2.737573e+00
## 10085  2 2989 2.659516e+00
## 10086  2 2990 2.586069e+00
## 10087  2 2991 2.522265e+00
## 10088  2 2992 2.463012e+00
## 10089  2 2993 2.416457e+00
## 10090  2 2994 2.370290e+00
## 10091  2 2995 2.323351e+00
## 10092  2 2996 2.288199e+00
## 10093  2 2997 2.255170e+00
## 10094  2 2998 2.223698e+00
## 10095  2 2999 2.196360e+00
## 10096  2 3000 2.169316e+00
## 10097  2 3001 3.380981e+00
## 10098  2 3002 3.808418e+00
## 10099  2 3003 3.866163e+00
## 10100  2 3004 3.775537e+00
## 10101  2 3005 3.632806e+00
## 10102  2 3006 3.476300e+00
## 10103  2 3007 3.320042e+00
## 10104  2 3008 3.172977e+00
## 10105  2 3009 3.049104e+00
## 10106  2 3010 2.936991e+00
## 10107  2 3011 2.832694e+00
## 10108  2 3012 2.745614e+00
## 10109  2 3013 2.663683e+00
## 10110  2 3014 2.593944e+00
## 10111  2 3015 2.530077e+00
## 10112  2 3016 2.473062e+00
## 10113  2 3017 2.422214e+00
## 10114  2 3018 2.379230e+00
## 10115  2 3019 2.334957e+00
## 10116  2 3020 2.296965e+00
## 10117  2 3021 2.262295e+00
## 10118  2 3022 2.233976e+00
## 10119  2 3023 2.203019e+00
## 10120  2 3024 2.179708e+00
## 10121  2 3025 3.387234e+00
## 10122  2 3026 3.804770e+00
## 10123  2 3027 3.870212e+00
## 10124  2 3028 3.789781e+00
## 10125  2 3029 3.637790e+00
## 10126  2 3030 3.484756e+00
## 10127  2 3031 3.322266e+00
## 10128  2 3032 3.181901e+00
## 10129  2 3033 3.057032e+00
## 10130  2 3034 2.940478e+00
## 10131  2 3035 2.839150e+00
## 10132  2 3036 2.749565e+00
## 10133  2 3037 2.667446e+00
## 10134  2 3038 2.600765e+00
## 10135  2 3039 2.534953e+00
## 10136  2 3040 2.473829e+00
## 10137  2 3041 2.423838e+00
## 10138  2 3042 2.381732e+00
## 10139  2 3043 2.336763e+00
## 10140  2 3044 2.305372e+00
## 10141  2 3045 2.271234e+00
## 10142  2 3046 2.237096e+00
## 10143  2 3047 2.211400e+00
## 10144  2 3048 2.180549e+00
## 10145  2 3049 3.396358e+00
## 10146  2 3050 3.813275e+00
## 10147  2 3051 3.880755e+00
## 10148  2 3052 3.788909e+00
## 10149  2 3053 3.649728e+00
## 10150  2 3054 3.484452e+00
## 10151  2 3055 3.327191e+00
## 10152  2 3056 3.195358e+00
## 10153  2 3057 3.064356e+00
## 10154  2 3058 2.950865e+00
## 10155  2 3059 2.848526e+00
## 10156  2 3060 2.754476e+00
## 10157  2 3061 2.677098e+00
## 10158  2 3062 2.602112e+00
## 10159  2 3063 2.542164e+00
## 10160  2 3064 2.481440e+00
## 10161  2 3065 2.426736e+00
## 10162  2 3066 2.390365e+00
## 10163  2 3067 2.348307e+00
## 10164  2 3068 2.307312e+00
## 10165  2 3069 2.277019e+00
## 10166  2 3070 2.241889e+00
## 10167  2 3071 2.210781e+00
## 10168  2 3072 2.185829e+00
## 10169  2 3073 3.395537e+00
## 10170  2 3074 3.823632e+00
## 10171  2 3075 3.887882e+00
## 10172  2 3076 3.796063e+00
## 10173  2 3077 3.651442e+00
## 10174  2 3078 3.492301e+00
## 10175  2 3079 3.341009e+00
## 10176  2 3080 3.190556e+00
## 10177  2 3081 3.069044e+00
## 10178  2 3082 2.951517e+00
## 10179  2 3083 2.850840e+00
## 10180  2 3084 2.760293e+00
## 10181  2 3085 2.680041e+00
## 10182  2 3086 2.604938e+00
## 10183  2 3087 2.541260e+00
## 10184  2 3088 2.485934e+00
## 10185  2 3089 2.435561e+00
## 10186  2 3090 2.391475e+00
## 10187  2 3091 2.351618e+00
## 10188  2 3092 2.313644e+00
## 10189  2 3093 2.280646e+00
## 10190  2 3094 2.251424e+00
## 10191  2 3095 2.218568e+00
## 10192  2 3096 2.192933e+00
## 10193  2 3097 3.402622e+00
## 10194  2 3098 3.826889e+00
## 10195  2 3099 3.888253e+00
## 10196  2 3100 3.796691e+00
## 10197  2 3101 3.655241e+00
## 10198  2 3102 3.498671e+00
## 10199  2 3103 3.336065e+00
## 10200  2 3104 3.200642e+00
## 10201  2 3105 3.076920e+00
## 10202  2 3106 2.949552e+00
## 10203  2 3107 2.854699e+00
## 10204  2 3108 2.764791e+00
## 10205  2 3109 2.685284e+00
## 10206  2 3110 2.611742e+00
## 10207  2 3111 2.544048e+00
## 10208  2 3112 2.493959e+00
## 10209  2 3113 2.438350e+00
## 10210  2 3114 2.395471e+00
## 10211  2 3115 2.353616e+00
## 10212  2 3116 2.315893e+00
## 10213  2 3117 2.280667e+00
## 10214  2 3118 2.253623e+00
## 10215  2 3119 2.224762e+00
## 10216  2 3120 2.197829e+00
## 10217  2 3120 2.224404e+00
## 10218  2 3121 3.434732e+00
## 10219  2 3122 3.849192e+00
## 10220  2 3123 3.920687e+00
## 10221  2 3124 3.832988e+00
## 10222  2 3125 3.681486e+00
## 10223  2 3126 3.523983e+00
## 10224  2 3127 3.364762e+00
## 10225  2 3128 3.231131e+00
## 10226  2 3129 3.098176e+00
## 10227  2 3130 2.985401e+00
## 10228  2 3131 2.884917e+00
## 10229  2 3132 2.786159e+00
## 10230  2 3133 2.713326e+00
## 10231  2 3134 2.639989e+00
## 10232  2 3135 2.580298e+00
## 10233  2 3136 2.525575e+00
## 10234  2 3137 2.470996e+00
## 10235  2 3138 2.421657e+00
## 10236  2 3139 2.381979e+00
## 10237  2 3140 2.340697e+00
## 10238  2 3141 2.310664e+00
## 10239  2 3142 2.272580e+00
## 10240  2 3143 2.242585e+00
## 10241  2 3144 2.224671e+00
## 10242  2 3144 2.222581e+00
## 10243  2 3145 2.198893e+00
## 10244  2 3146 2.171594e+00
## 10245  2 3147 2.155522e+00
## 10246  2 3148 2.133975e+00
## 10247  2 3149 2.115940e+00
## 10248  2 3150 2.098578e+00
## 10249  2 3151 2.082358e+00
## 10250  2 3152 2.064451e+00
## 10251  2 3153 2.047526e+00
## 10252  2 3154 2.029708e+00
## 10253  2 3155 2.016970e+00
## 10254  2 3156 2.003220e+00
## 10255  2 3157 1.992311e+00
## 10256  2 3158 1.975251e+00
## 10257  2 3159 1.963909e+00
## 10258  2 3160 1.948633e+00
## 10259  2 3161 1.938566e+00
## 10260  2 3162 1.927772e+00
## 10261  2 3163 1.912663e+00
## 10262  2 3164 1.903070e+00
## 10263  2 3165 1.889508e+00
## 10264  2 3165 1.889633e+00
## 10265  2 3166 3.113427e+00
## 10266  2 3167 3.551501e+00
## 10267  2 3168 3.622254e+00
## 10268  2 3168 3.619802e+00
## 10269  2 3169 4.775665e+00
## 10270  2 3170 5.087808e+00
## 10271  2 3171 5.029094e+00
## 10272  2 3172 4.797656e+00
## 10273  2 3173 4.540662e+00
```

```
## 10274  2 3174 4.276889e+00
## 10275  2 3175 4.029945e+00
## 10276  2 3176 3.800725e+00
## 10277  2 3177 3.601158e+00
## 10278  2 3178 3.423188e+00
## 10279  2 3179 3.266145e+00
## 10280  2 3180 3.128036e+00
## 10281  2 3181 3.016603e+00
## 10282  2 3182 2.901258e+00
## 10283  2 3183 2.812616e+00
## 10284  2 3184 2.721346e+00
## 10285  2 3185 2.652993e+00
## 10286  2 3186 2.590565e+00
## 10287  2 3187 2.530284e+00
## 10288  2 3188 2.471115e+00
## 10289  2 3189 2.426861e+00
## 10290  2 3190 2.383914e+00
## 10291  2 3191 2.346878e+00
## 10292  2 3192 2.309000e+00
## 10293  2 3193 3.517504e+00
## 10294  2 3194 3.931441e+00
## 10295  2 3195 3.984097e+00
## 10296  2 3196 3.885319e+00
## 10297  2 3197 3.746301e+00
## 10298  2 3198 3.586981e+00
## 10299  2 3199 3.421218e+00
## 10300  2 3200 3.275982e+00
## 10301  2 3201 3.147149e+00
## 10302  2 3202 3.023566e+00
## 10303  2 3203 2.925341e+00
## 10304  2 3204 2.836068e+00
## 10305  2 3205 2.750680e+00
## 10306  2 3206 2.671471e+00
## 10307  2 3207 2.611961e+00
## 10308  2 3208 2.554789e+00
## 10309  2 3209 2.502492e+00
## 10310  2 3210 2.457569e+00
## 10311  2 3211 2.408137e+00
## 10312  2 3212 2.369673e+00
## 10313  2 3213 2.334293e+00
## 10314  2 3214 2.306152e+00
## 10315  2 3215 2.274796e+00
## 10316  2 3216 2.254148e+00
## 10317  2 3217 3.455411e+00
## 10318  2 3218 3.875443e+00
## 10319  2 3219 3.949891e+00
## 10320  2 3220 3.858385e+00
## 10321  2 3221 3.713508e+00
## 10322  2 3222 3.546229e+00
## 10323  2 3223 3.390371e+00
## 10324  2 3224 3.257572e+00
## 10325  2 3225 3.124928e+00
## 10326  2 3226 3.007228e+00
## 10327  2 3227 2.908820e+00
## 10328  2 3228 2.817872e+00
## 10329  2 3229 2.738003e+00
## 10330  2 3230 2.662334e+00
## 10331  2 3231 2.595049e+00
## 10332  2 3232 2.546592e+00
## 10333  2 3233 2.495746e+00
## 10334  2 3234 2.449676e+00
## 10335  2 3235 2.404520e+00
## 10336  2 3236 2.366400e+00
## 10337  2 3237 2.330967e+00
## 10338  2 3238 2.297686e+00
## 10339  2 3239 2.274877e+00
## 10340  2 3240 2.245917e+00
## 10341  2 3241 3.462452e+00
## 10342  2 3242 3.879817e+00
## 10343  2 3243 3.937216e+00
## 10344  2 3244 3.850488e+00
## 10345  2 3245 3.706526e+00
## 10346  2 3246 3.548732e+00
## 10347  2 3247 3.395282e+00
## 10348  2 3248 3.249128e+00
## 10349  2 3249 3.121660e+00
## 10350  2 3250 3.002364e+00
## 10351  2 3251 2.907876e+00
## 10352  2 3252 2.811872e+00
## 10353  2 3253 2.732536e+00
## 10354  2 3254 2.658320e+00
## 10355  2 3255 2.596879e+00
## 10356  2 3256 2.534891e+00
## 10357  2 3257 2.489860e+00
## 10358  2 3258 2.440675e+00
## 10359  2 3259 2.399456e+00
## 10360  2 3260 2.361358e+00
## 10361  2 3261 2.330903e+00
## 10362  2 3262 2.295405e+00
## 10363  2 3263 2.269342e+00
## 10364  2 3264 2.242348e+00
## 10365  2 3265 3.456091e+00
## 10366  2 3266 3.877579e+00
## 10367  2 3267 3.943245e+00
## 10368  2 3268 3.847944e+00
## 10369  2 3269 3.695338e+00
## 10370  2 3270 3.542308e+00
## 10371  2 3271 3.390615e+00
## 10372  2 3272 3.250500e+00
## 10373  2 3273 3.121910e+00
## 10374  2 3274 3.003065e+00
## 10375  2 3275 2.907568e+00
## 10376  2 3276 2.808495e+00
## 10377  2 3277 2.726159e+00
## 10378  2 3278 2.659473e+00
## 10379  2 3279 2.596404e+00
## 10380  2 3280 2.543928e+00
## 10381  2 3281 2.484305e+00
## 10382  2 3282 2.443143e+00
## 10383  2 3283 2.399490e+00
## 10384  2 3284 2.359321e+00
## 10385  2 3285 2.326764e+00
## 10386  2 3286 2.298701e+00
## 10387  2 3287 2.264991e+00
## 10388  2 3288 2.239765e+00
## 10389  2 3289 3.456008e+00
## 10390  2 3290 3.867851e+00
## 10391  2 3291 3.937294e+00
## 10392  2 3292 3.847747e+00
## 10393  2 3293 3.705123e+00
## 10394  2 3294 3.544226e+00
## 10395  2 3295 3.386654e+00
## 10396  2 3296 3.241600e+00
## 10397  2 3297 3.118605e+00
## 10398  2 3298 3.004786e+00
## 10399  2 3299 2.904789e+00
## 10400  2 3300 2.807624e+00
## 10401  2 3301 2.729680e+00
## 10402  2 3302 2.651941e+00
## 10403  2 3303 2.593227e+00
## 10404  2 3304 2.533248e+00
## 10405  2 3305 2.490583e+00
## 10406  2 3306 2.442026e+00
## 10407  2 3307 2.396690e+00
## 10408  2 3308 2.358175e+00
## 10409  2 3309 2.324765e+00
## 10410  2 3310 2.290392e+00
## 10411  2 3311 2.262089e+00
## 10412  2 3312 2.237892e+00
## 10413  2 3313 3.452011e+00
## 10414  2 3314 3.880034e+00
## 10415  2 3315 3.925999e+00
## 10416  2 3316 3.836446e+00
## 10417  2 3317 3.702019e+00
## 10418  2 3318 3.537060e+00
## 10419  2 3319 3.388673e+00
## 10420  2 3320 3.245288e+00
## 10421  2 3321 3.116681e+00
## 10422  2 3322 2.995908e+00
## 10423  2 3323 2.894988e+00
## 10424  2 3324 2.813089e+00
## 10425  2 3325 2.723447e+00
## 10426  2 3326 2.652804e+00
## 10427  2 3327 2.591147e+00
## 10428  2 3328 2.534709e+00
## 10429  2 3329 2.481257e+00
## 10430  2 3330 2.432189e+00
## 10431  2 3331 2.392633e+00
## 10432  2 3332 2.354312e+00
## 10433  2 3333 2.323911e+00
## 10434  2 3334 2.290321e+00
## 10435  2 3335 2.263773e+00
## 10436  2 3336 2.237514e+00
## 10437  2 3337 3.445913e+00
## 10438  2 3338 3.870177e+00
## 10439  2 3339 3.930744e+00
## 10440  2 3340 3.841345e+00
## 10441  2 3341 3.695819e+00
## 10442  2 3342 3.536993e+00
## 10443  2 3343 3.381982e+00
## 10444  2 3344 3.234974e+00
## 10445  2 3345 3.118277e+00
## 10446  2 3346 2.999972e+00
## 10447  2 3347 2.894658e+00
## 10448  2 3348 2.803735e+00
## 10449  2 3349 2.728712e+00
## 10450  2 3350 2.656774e+00
## 10451  2 3351 2.585109e+00
## 10452  2 3352 2.532030e+00
## 10453  2 3353 2.481217e+00
## 10454  2 3354 2.434015e+00
## 10455  2 3355 2.392196e+00
## 10456  2 3356 2.354233e+00
## 10457  2 3357 2.320962e+00
## 10458  2 3358 2.289581e+00
## 10459  2 3359 2.263240e+00
## 10460  2 3360 2.232954e+00
## 10461  2 3361 3.450786e+00
## 10462  2 3362 3.866616e+00
## 10463  2 3363 3.921997e+00
## 10464  2 3364 3.835441e+00
## 10465  2 3365 3.694578e+00
## 10466  2 3366 3.529771e+00
## 10467  2 3367 3.382662e+00
## 10468  2 3368 3.236047e+00
## 10469  2 3369 3.109763e+00
## 10470  2 3370 2.994173e+00
## 10471  2 3371 2.896722e+00
## 10472  2 3372 2.803764e+00
## 10473  2 3373 2.718462e+00
## 10474  2 3374 2.651287e+00
## 10475  2 3375 2.587433e+00
## 10476  2 3376 2.530094e+00
## 10477  2 3377 2.480742e+00
## 10478  2 3378 2.433255e+00
## 10479  2 3379 2.391836e+00
## 10480  2 3380 2.352142e+00
## 10481  2 3381 2.316533e+00
## 10482  2 3382 2.286184e+00
## 10483  2 3383 2.257077e+00
## 10484  2 3384 2.234725e+00
## 10485  2 3385 3.442445e+00
## 10486  2 3386 3.874007e+00
## 10487  2 3387 3.927841e+00
## 10488  2 3388 3.843663e+00
## 10489  2 3389 3.692994e+00
## 10490  2 3390 3.537733e+00
## 10491  2 3391 3.377826e+00
## 10492  2 3392 3.231855e+00
## 10493  2 3393 3.111344e+00
## 10494  2 3394 2.990176e+00
## 10495  2 3395 2.889947e+00
## 10496  2 3396 2.800561e+00
## 10497  2 3397 2.722711e+00
## 10498  2 3398 2.648685e+00
## 10499  2 3399 2.584897e+00
## 10500  2 3400 2.528379e+00
## 10501  2 3401 2.473785e+00
## 10502  2 3402 2.429786e+00
## 10503  2 3403 2.388950e+00
## 10504  2 3404 2.351908e+00
## 10505  2 3405 2.311259e+00
## 10506  2 3406 2.287463e+00
## 10507  2 3407 2.261877e+00
## 10508  2 3408 2.229673e+00
## 10509  2 3409 3.439935e+00
## 10510  2 3410 3.863902e+00
## 10511  2 3411 3.923014e+00
## 10512  2 3412 3.837106e+00
## 10513  2 3413 3.689912e+00
## 10514  2 3414 3.532480e+00
## 10515  2 3415 3.379035e+00
## 10516  2 3416 3.232393e+00
## 10517  2 3417 3.106524e+00
## 10518  2 3418 2.994966e+00
## 10519  2 3419 2.892346e+00
## 10520  2 3420 2.798743e+00
## 10521  2 3421 2.719610e+00
## 10522  2 3422 2.648738e+00
## 10523  2 3423 2.588331e+00
## 10524  2 3424 2.528536e+00
## 10525  2 3425 2.471909e+00
## 10526  2 3426 2.429447e+00
## 10527  2 3427 2.389965e+00
## 10528  2 3428 2.350294e+00
## 10529  2 3429 2.317606e+00
## 10530  2 3430 2.287048e+00
## 10531  2 3431 2.252817e+00
## 10532  2 3432 2.231260e+00
## 10533  2 3432 2.223180e+00
## 10534  2 3433 3.435101e+00
## 10535  2 3434 3.858584e+00
## 10536  2 3435 3.923547e+00
## 10537  2 3436 3.821605e+00
## 10538  2 3437 3.679032e+00
## 10539  2 3438 3.526411e+00
## 10540  2 3439 3.364943e+00
## 10541  2 3440 3.223758e+00
## 10542  2 3441 3.101033e+00
## 10543  2 3442 2.987167e+00
## 10544  2 3443 2.886508e+00
## 10545  2 3444 2.792462e+00
## 10546  2 3445 2.713739e+00
## 10547  2 3446 2.639092e+00
## 10548  2 3447 2.577988e+00
## 10549  2 3448 2.525444e+00
## 10550  2 3449 2.467151e+00
## 10551  2 3450 2.419837e+00
## 10552  2 3451 2.381155e+00
## 10553  2 3452 2.349040e+00
## 10554  2 3453 2.305241e+00
## 10555  2 3454 2.282352e+00
## 10556  2 3455 2.250996e+00
## 10557  2 3456 2.220119e+00
## 10558  2 3456 2.223965e+00
## 10559  2 3457 2.195706e+00
## 10560  2 3458 2.171693e+00
## 10561  2 3459 2.153895e+00
## 10562  2 3460 2.133777e+00
## 10563  2 3461 2.113283e+00
## 10564  2 3462 2.095079e+00
## 10565  2 3463 2.079998e+00
## 10566  2 3464 2.060666e+00
## 10567  2 3465 2.049949e+00
## 10568  2 3466 2.032513e+00
## 10569  2 3467 2.021897e+00
## 10570  2 3468 2.006128e+00
## 10571  2 3469 1.989997e+00
## 10572  2 3470 1.977491e+00
## 10573  2 3471 1.964647e+00
## 10574  2 3472 1.948831e+00
## 10575  2 3473 1.939843e+00
## 10576  2 3474 1.927682e+00
## 10577  2 3475 1.915614e+00
## 10578  2 3476 1.905382e+00
## 10579  2 3477 1.892804e+00
## 10580  2 3478 1.878087e+00
## 10581  2 3479 1.871111e+00
## 10582  2 3480 1.862271e+00
## 10583  2 3480 1.857738e+00
## 10584  2 3481 3.081966e+00
## 10585  2 3482 3.522133e+00
## 10586  2 3483 3.586078e+00
## 10587  2 3484 3.512396e+00
## 10588  2 3485 3.373330e+00
## 10589  2 3486 3.220871e+00
## 10590  2 3487 3.071761e+00
## 10591  2 3488 2.936521e+00
## 10592  2 3489 2.811044e+00
## 10593  2 3490 2.702471e+00
## 10594  2 3491 2.611438e+00
## 10595  2 3492 2.518009e+00
## 10596  2 3493 2.445924e+00
## 10597  2 3494 2.375493e+00
## 10598  2 3495 2.311802e+00
## 10599  2 3496 2.264303e+00
## 10600  2 3497 2.211397e+00
## 10601  2 3498 2.168446e+00
## 10602  2 3499 2.130738e+00
## 10603  2 3500 2.093538e+00
## 10604  2 3501 2.060826e+00
## 10605  2 3502 2.031589e+00
## 10606  2 3503 2.010984e+00
## 10607  2 3504 1.982602e+00
## 10608  2 3504 1.982594e+00
## 10609  2 3505 3.196966e+00
## 10610  2 3506 3.614377e+00
## 10611  2 3507 3.677715e+00
## 10612  2 3508 3.590299e+00
## 10613  2 3509 3.451959e+00
## 10614  2 3510 3.290078e+00
## 10615  2 3511 3.142040e+00
## 10616  2 3512 2.999666e+00
## 10617  2 3513 2.875171e+00
## 10618  2 3514 2.756501e+00
## 10619  2 3515 2.662761e+00
## 10620  2 3516 2.571091e+00
## 10621  2 3517 2.491724e+00
## 10622  2 3518 2.418360e+00
## 10623  2 3519 2.357661e+00
## 10624  2 3520 2.303173e+00
## 10625  2 3521 2.250852e+00
## 10626  2 3522 2.208423e+00
## 10627  2 3523 2.167874e+00
## 10628  2 3524 2.132928e+00
## 10629  2 3525 2.093946e+00
## 10630  2 3526 2.068066e+00
## 10631  2 3527 2.039229e+00
## 10632  2 3528 2.015393e+00
## 10633  2 3529 3.230463e+00
## 10634  2 3530 3.650161e+00
## 10635  2 3531 3.717251e+00
## 10636  2 3532 3.625835e+00
## 10637  2 3533 3.480107e+00
## 10638  2 3534 3.320789e+00
## 10639  2 3535 3.167905e+00
## 10640  2 3536 3.030522e+00
## 10641  2 3537 2.897933e+00
## 10642  2 3538 2.791424e+00
## 10643  2 3539 2.691151e+00
## 10644  2 3540 2.601792e+00
## 10645  2 3541 2.519203e+00
## 10646  2 3542 2.452919e+00
## 10647  2 3543 2.387367e+00
## 10648  2 3544 2.333752e+00
## 10649  2 3545 2.282179e+00
## 10650  2 3546 2.231316e+00
## 10651  2 3547 2.192977e+00
## 10652  2 3548 2.160943e+00
## 10653  2 3549 2.126191e+00
## 10654  2 3550 2.093524e+00
## 10655  2 3551 2.068704e+00
## 10656  2 3552 2.045471e+00
## 10657  2 3553 3.252058e+00
## 10658  2 3554 3.679242e+00
## 10659  2 3555 3.740308e+00
## 10660  2 3556 3.652563e+00
## 10661  2 3557 3.503995e+00
## 10662  2 3558 3.349743e+00
## 10663  2 3559 3.201054e+00
## 10664  2 3560 3.057111e+00
## 10665  2 3561 2.923619e+00
## 10666  2 3562 2.818409e+00
## 10667  2 3563 2.719321e+00
## 10668  2 3564 2.625491e+00
## 10669  2 3565 2.546670e+00
## 10670  2 3566 2.473620e+00
## 10671  2 3567 2.406752e+00
## 10672  2 3568 2.354770e+00
## 10673  2 3569 2.309027e+00
## 10674  2 3570 2.257599e+00
## 10675  2 3571 2.218378e+00
## 10676  2 3572 2.182873e+00
## 10677  2 3573 2.147921e+00
## 10678  2 3574 2.116477e+00
## 10679  2 3575 2.089090e+00
## 10680  2 3576 2.066661e+00
## 10681  2 3577 3.274855e+00
## 10682  2 3578 3.702661e+00
## 10683  2 3579 3.764745e+00
## 10684  2 3580 3.680331e+00
## 10685  2 3581 3.525780e+00
## 10686  2 3582 3.375122e+00
## 10687  2 3583 3.222778e+00
## 10688  2 3584 3.078382e+00
## 10689  2 3585 2.945985e+00
## 10690  2 3586 2.840378e+00
## 10691  2 3587 2.734767e+00
## 10692  2 3588 2.643235e+00
## 10693  2 3589 2.566815e+00
## 10694  2 3590 2.496843e+00
## 10695  2 3591 2.431336e+00
## 10696  2 3592 2.379952e+00
## 10697  2 3593 2.320482e+00
## 10698  2 3594 2.277751e+00
## 10699  2 3595 2.243025e+00
## 10700  2 3596 2.207826e+00
## 10701  2 3597 2.168103e+00
## 10702  2 3598 2.137382e+00
## 10703  2 3599 2.112335e+00
## 10704  2 3600 2.086543e+00
## 10705  2 3601 3.299029e+00
## 10706  2 3602 3.721338e+00
## 10707  2 3603 3.776334e+00
## 10708  2 3604 3.694024e+00
## 10709  2 3605 3.543812e+00
## 10710  2 3606 3.390189e+00
## 10711  2 3607 3.235699e+00
## 10712  2 3608 3.098551e+00
## 10713  2 3609 2.972640e+00
## 10714  2 3610 2.854754e+00
## 10715  2 3611 2.755567e+00
## 10716  2 3612 2.664418e+00
## 10717  2 3613 2.589840e+00
## 10718  2 3614 2.509106e+00
## 10719  2 3615 2.448461e+00
## 10720  2 3616 2.397281e+00
## 10721  2 3617 2.342510e+00
## 10722  2 3618 2.302115e+00
## 10723  2 3619 2.255132e+00
## 10724  2 3620 2.219701e+00
## 10725  2 3621 2.186219e+00
## 10726  2 3622 2.161806e+00
## 10727  2 3623 2.127629e+00
## 10728  2 3624 2.104180e+00
## 10729  2 3625 3.317732e+00
## 10730  2 3626 3.736906e+00
## 10731  2 3627 3.801986e+00
## 10732  2 3628 3.710999e+00
## 10733  2 3629 3.566747e+00
## 10734  2 3630 3.407778e+00
## 10735  2 3631 3.258067e+00
## 10736  2 3632 3.107118e+00
## 10737  2 3633 2.981597e+00
## 10738  2 3634 2.873588e+00
## 10739  2 3635 2.771062e+00
## 10740  2 3636 2.675875e+00
## 10741  2 3637 2.601640e+00
## 10742  2 3638 2.530971e+00
## 10743  2 3639 2.467447e+00
## 10744  2 3640 2.412903e+00
## 10745  2 3641 2.359346e+00
## 10746  2 3642 2.313949e+00
## 10747  2 3643 2.273531e+00
## 10748  2 3644 2.235897e+00
## 10749  2 3645 2.203147e+00
## 10750  2 3646 2.170191e+00
## 10751  2 3647 2.141193e+00
## 10752  2 3648 2.118825e+00
## 10753  2 3649 3.328428e+00
## 10754  2 3650 3.746720e+00
## 10755  2 3651 3.823371e+00
## 10756  2 3652 3.721398e+00
## 10757  2 3653 3.586109e+00
## 10758  2 3654 3.424373e+00
## 10759  2 3655 3.273402e+00
## 10760  2 3656 3.127189e+00
## 10761  2 3657 2.999319e+00
## 10762  2 3658 2.888157e+00
## 10763  2 3659 2.782766e+00
## 10764  2 3660 2.692469e+00
## 10765  2 3661 2.614831e+00
## 10766  2 3662 2.545808e+00
## 10767  2 3663 2.477853e+00
## 10768  2 3664 2.427873e+00
## 10769  2 3665 2.370943e+00
## 10770  2 3666 2.328766e+00
## 10771  2 3667 2.287097e+00
## 10772  2 3668 2.251094e+00
## 10773  2 3669 2.216121e+00
## 10774  2 3670 2.183200e+00
## 10775  2 3671 2.153370e+00
## 10776  2 3672 2.136237e+00
## 10777  2 3673 3.345975e+00
## 10778  2 3674 3.766649e+00
## 10779  2 3675 3.833153e+00
## 10780  2 3676 3.737955e+00
## 10781  2 3677 3.594791e+00
## 10782  2 3678 3.436644e+00
## 10783  2 3679 3.283604e+00
## 10784  2 3680 3.142440e+00
## 10785  2 3681 3.010692e+00
## 10786  2 3682 2.898707e+00
## 10787  2 3683 2.794467e+00
## 10788  2 3684 2.704836e+00
## 10789  2 3685 2.626058e+00
## 10790  2 3686 2.555140e+00
## 10791  2 3687 2.493711e+00
## 10792  2 3688 2.433968e+00
## 10793  2 3689 2.380196e+00
## 10794  2 3690 2.340229e+00
## 10795  2 3691 2.299890e+00
## 10796  2 3692 2.258524e+00
## 10797  2 3693 2.225941e+00
## 10798  2 3694 2.198549e+00
## 10799  2 3695 2.170071e+00
## 10800  2 3696 2.144702e+00
## 10801  2 3697 3.360750e+00
## 10802  2 3698 3.776404e+00
## 10803  2 3699 3.840591e+00
## 10804  2 3700 3.750610e+00
## 10805  2 3701 3.612523e+00
## 10806  2 3702 3.448821e+00
## 10807  2 3703 3.295972e+00
## 10808  2 3704 3.151289e+00
## 10809  2 3705 3.019798e+00
## 10810  2 3706 2.909518e+00
## 10811  2 3707 2.808288e+00
## 10812  2 3708 2.716101e+00
## 10813  2 3709 2.638418e+00
## 10814  2 3710 2.565163e+00
## 10815  2 3711 2.506763e+00
## 10816  2 3712 2.445637e+00
## 10817  2 3713 2.398850e+00
## 10818  2 3714 2.354372e+00
## 10819  2 3715 2.311435e+00
## 10820  2 3716 2.273040e+00
## 10821  2 3717 2.239656e+00
## 10822  2 3718 2.208074e+00
## 10823  2 3719 2.178708e+00
## 10824  2 3720 2.152054e+00
## 10825  2 3721 3.369840e+00
## 10826  2 3722 3.787971e+00
## 10827  2 3723 3.849098e+00
## 10828  2 3724 3.755334e+00
## 10829  2 3725 3.608649e+00
## 10830  2 3726 3.453660e+00
## 10831  2 3727 3.302629e+00
## 10832  2 3728 3.156715e+00
## 10833  2 3729 3.033703e+00
## 10834  2 3730 2.919180e+00
## 10835  2 3731 2.820549e+00
## 10836  2 3732 2.729647e+00
## 10837  2 3733 2.648546e+00
## 10838  2 3734 2.577400e+00
## 10839  2 3735 2.512956e+00
## 10840  2 3736 2.453800e+00
## 10841  2 3737 2.404443e+00
## 10842  2 3738 2.358713e+00
## 10843  2 3739 2.319211e+00
## 10844  2 3740 2.280587e+00
## 10845  2 3741 2.249942e+00
## 10846  2 3742 2.216623e+00
## 10847  2 3743 2.187358e+00
## 10848  2 3744 2.166200e+00
## 10849  2 3745 3.378338e+00
## 10850  2 3746 3.795503e+00
## 10851  2 3747 3.862150e+00
## 10852  2 3748 3.766274e+00
## 10853  2 3749 3.632559e+00
## 10854  2 3750 3.467098e+00
## 10855  2 3751 3.309440e+00
## 10856  2 3752 3.172588e+00
## 10857  2 3753 3.043739e+00
## 10858  2 3754 2.927423e+00
## 10859  2 3755 2.826691e+00
## 10860  2 3756 2.732012e+00
## 10861  2 3757 2.654368e+00
## 10862  2 3758 2.579428e+00
## 10863  2 3759 2.520638e+00
## 10864  2 3760 2.464609e+00
## 10865  2 3761 2.414493e+00
## 10866  2 3762 2.367690e+00
## 10867  2 3763 2.329324e+00
## 10868  2 3764 2.287889e+00
## 10869  2 3765 2.254369e+00
## 10870  2 3766 2.226408e+00
## 10871  2 3767 2.192491e+00
## 10872  2 3768 2.171778e+00
## 10873  2 3768 2.223765e+00
## 10874  2 3769 3.433941e+00
## 10875  2 3770 3.854221e+00
## 10876  2 3771 3.913627e+00
## 10877  2 3772 3.825199e+00
## 10878  2 3773 3.687372e+00
## 10879  2 3774 3.525043e+00
## 10880  2 3775 3.374609e+00
## 10881  2 3776 3.231510e+00
## 10882  2 3777 3.102587e+00
## 10883  2 3778 2.985177e+00
## 10884  2 3779 2.883761e+00
## 10885  2 3780 2.798506e+00
## 10886  2 3781 2.712208e+00
## 10887  2 3782 2.645327e+00
## 10888  2 3783 2.575557e+00
## 10889  2 3784 2.517093e+00
## 10890  2 3785 2.467280e+00
## 10891  2 3786 2.422008e+00
## 10892  2 3787 2.380238e+00
## 10893  2 3788 2.342947e+00
## 10894  2 3789 2.306161e+00
## 10895  2 3790 2.278868e+00
## 10896  2 3791 2.249013e+00
## 10897  2 3792 2.222823e+00
## 10898  2 3792 2.223357e+00
## 10899  2 3793 2.197911e+00
## 10900  2 3794 2.176294e+00
## 10901  2 3795 2.154018e+00
## 10902  2 3796 2.133622e+00
## 10903  2 3797 2.116062e+00
## 10904  2 3798 2.094442e+00
## 10905  2 3799 2.081312e+00
## 10906  2 3800 2.064446e+00
## 10907  2 3801 2.045781e+00
## 10908  2 3802 2.033679e+00
## 10909  2 3803 2.015368e+00
## 10910  2 3804 2.003715e+00
## 10911  2 3805 1.991203e+00
## 10912  2 3806 1.973310e+00
## 10913  2 3807 1.966022e+00
## 10914  2 3808 1.951248e+00
## 10915  2 3809 1.941318e+00
## 10916  2 3810 1.928589e+00
## 10917  2 3811 1.913841e+00
## 10918  2 3812 1.904610e+00
## 10919  2 3813 1.890613e+00
## 10920  2 3814 1.883886e+00
## 10921  2 3815 1.869524e+00
## 10922  2 3816 1.859867e+00
## 10923  2 3816 1.857706e+00
## 10924  2 3817 3.695413e+00
## 10925  2 3818 4.363680e+00
## 10926  2 3819 4.468864e+00
## 10927  2 3820 4.359520e+00
## 10928  2 3821 4.159163e+00
## 10929  2 3822 3.928357e+00
## 10930  2 3823 3.721141e+00
## 10931  2 3824 3.521471e+00
## 10932  2 3825 3.342787e+00
## 10933  2 3826 3.181326e+00
## 10934  2 3827 3.034440e+00
## 10935  2 3828 2.915880e+00
## 10936  2 3829 2.803507e+00
## 10937  2 3830 2.710122e+00
## 10938  2 3831 2.622263e+00
## 10939  2 3832 2.548829e+00
## 10940  2 3833 2.475720e+00
## 10941  2 3834 2.419402e+00
## 10942  2 3835 2.361730e+00
## 10943  2 3836 2.315507e+00
## 10944  2 3837 2.268733e+00
## 10945  2 3838 2.232813e+00
## 10946  2 3839 2.196962e+00
## 10947  2 3840 2.164352e+00
## 10948  2 3840 2.164865e+00
## 10949  2 3841 3.371013e+00
## 10950  2 3842 3.786836e+00
## 10951  2 3843 3.845946e+00
## 10952  2 3844 3.751972e+00
## 10953  2 3845 3.602089e+00
## 10954  2 3846 3.454648e+00
## 10955  2 3847 3.289285e+00
## 10956  2 3848 3.140014e+00
## 10957  2 3849 3.012547e+00
## 10958  2 3850 2.901654e+00
## 10959  2 3851 2.796135e+00
## 10960  2 3852 2.708411e+00
## 10961  2 3853 2.623101e+00
## 10962  2 3854 2.550486e+00
## 10963  2 3855 2.493448e+00
## 10964  2 3856 2.428768e+00
## 10965  2 3857 2.377491e+00
## 10966  2 3858 2.333276e+00
## 10967  2 3859 2.294651e+00
## 10968  2 3860 2.252424e+00
## 10969  2 3861 2.219130e+00
## 10970  2 3862 2.190550e+00
## 10971  2 3863 2.160595e+00
## 10972  2 3864 2.136318e+00
## 10973  2 3865 3.348091e+00
## 10974  2 3866 3.767804e+00
## 10975  2 3867 3.832775e+00
## 10976  2 3868 3.742846e+00
## 10977  2 3869 3.594908e+00
## 10978  2 3870 3.436924e+00
## 10979  2 3871 3.281649e+00
## 10980  2 3872 3.149118e+00
## 10981  2 3873 3.013319e+00
## 10982  2 3874 2.899383e+00
## 10983  2 3875 2.797420e+00
## 10984  2 3876 2.709460e+00
## 10985  2 3877 2.630657e+00
## 10986  2 3878 2.560353e+00
## 10987  2 3879 2.495932e+00
## 10988  2 3880 2.438885e+00
## 10989  2 3881 2.386141e+00
## 10990  2 3882 2.341790e+00
## 10991  2 3883 2.297622e+00
## 10992  2 3884 2.268482e+00
## 10993  2 3885 2.230505e+00
## 10994  2 3886 2.199730e+00
## 10995  2 3887 2.169728e+00
## 10996  2 3888 2.144902e+00
## 10997  2 3889 3.356287e+00
## 10998  2 3890 3.778943e+00
## 10999  2 3891 3.837773e+00
## 11000  2 3892 3.761081e+00
## 11001  2 3893 3.613416e+00
## 11002  2 3894 3.457666e+00
## 11003  2 3895 3.294710e+00
## 11004  2 3896 3.149673e+00
## 11005  2 3897 3.022999e+00
## 11006  2 3898 2.912091e+00
## 11007  2 3899 2.808395e+00
## 11008  2 3900 2.723824e+00
## 11009  2 3901 2.637781e+00
## 11010  2 3902 2.570721e+00
## 11011  2 3903 2.500242e+00
## 11012  2 3904 2.450883e+00
## 11013  2 3905 2.401422e+00
## 11014  2 3906 2.352180e+00
## 11015  2 3907 2.312288e+00
## 11016  2 3908 2.273301e+00
## 11017  2 3909 2.244595e+00
## 11018  2 3910 2.205612e+00
## 11019  2 3911 2.182115e+00
## 11020  2 3912 2.156822e+00
## 11021  2 3913 3.369113e+00
## 11022  2 3914 3.787733e+00
## 11023  2 3915 3.849317e+00
## 11024  2 3916 3.766108e+00
## 11025  2 3917 3.620854e+00
## 11026  2 3918 3.457423e+00
## 11027  2 3919 3.308264e+00
## 11028  2 3920 3.168599e+00
## 11029  2 3921 3.035545e+00
## 11030  2 3922 2.919618e+00
## 11031  2 3923 2.821021e+00
## 11032  2 3924 2.728558e+00
## 11033  2 3925 2.647645e+00
## 11034  2 3926 2.581015e+00
## 11035  2 3927 2.515465e+00
## 11036  2 3928 2.460058e+00
## 11037  2 3929 2.406835e+00
## 11038  2 3930 2.359535e+00
## 11039  2 3931 2.320818e+00
## 11040  2 3932 2.285062e+00
## 11041  2 3933 2.247734e+00
## 11042  2 3934 2.213498e+00
## 11043  2 3935 2.189855e+00
## 11044  2 3936 2.166585e+00
## 11045  2 3937 3.376269e+00
## 11046  2 3938 3.794574e+00
## 11047  2 3939 3.857754e+00
## 11048  2 3940 3.766264e+00
## 11049  2 3941 3.629904e+00
## 11050  2 3942 3.466516e+00
## 11051  2 3943 3.318324e+00
## 11052  2 3944 3.173790e+00
## 11053  2 3945 3.046645e+00
## 11054  2 3946 2.932103e+00
## 11055  2 3947 2.832977e+00
## 11056  2 3948 2.737514e+00
## 11057  2 3949 2.659902e+00
## 11058  2 3950 2.583223e+00
## 11059  2 3951 2.521826e+00
## 11060  2 3952 2.467829e+00
## 11061  2 3953 2.414384e+00
## 11062  2 3954 2.372779e+00
## 11063  2 3955 2.326134e+00
## 11064  2 3956 2.292925e+00
## 11065  2 3957 2.254516e+00
## 11066  2 3958 2.225256e+00
## 11067  2 3959 2.197919e+00
## 11068  2 3960 2.171319e+00
## 11069  2 3961 3.388782e+00
## 11070  2 3962 3.804108e+00
## 11071  2 3963 3.869745e+00
## 11072  2 3964 3.780006e+00
## 11073  2 3965 3.632231e+00
```

```
## 11074  2 3966 3.479668e+00
## 11075  2 3967 3.318112e+00
## 11076  2 3968 3.178368e+00
## 11077  2 3969 3.052395e+00
## 11078  2 3970 2.940438e+00
## 11079  2 3971 2.837485e+00
## 11080  2 3972 2.740499e+00
## 11081  2 3973 2.659889e+00
## 11082  2 3974 2.597122e+00
## 11083  2 3975 2.522394e+00
## 11084  2 3976 2.474324e+00
## 11085  2 3977 2.421303e+00
## 11086  2 3978 2.372949e+00
## 11087  2 3979 2.334747e+00
## 11088  2 3980 2.297010e+00
## 11089  2 3981 2.261726e+00
## 11090  2 3982 2.231835e+00
## 11091  2 3983 2.203029e+00
## 11092  2 3984 2.177607e+00
## 11093  2 3985 3.389894e+00
## 11094  2 3986 3.815906e+00
## 11095  2 3987 3.875064e+00
## 11096  2 3988 3.786244e+00
## 11097  2 3989 3.635908e+00
## 11098  2 3990 3.480479e+00
## 11099  2 3991 3.327717e+00
## 11100  2 3992 3.188495e+00
## 11101  2 3993 3.057835e+00
## 11102  2 3994 2.938574e+00
## 11103  2 3995 2.839669e+00
## 11104  2 3996 2.748076e+00
## 11105  2 3997 2.673466e+00
## 11106  2 3998 2.596178e+00
## 11107  2 3999 2.535242e+00
## 11108  2 4000 2.480803e+00
## 11109  2 4001 2.427671e+00
## 11110  2 4002 2.384055e+00
## 11111  2 4003 2.338817e+00
## 11112  2 4004 2.307277e+00
## 11113  2 4005 2.269118e+00
## 11114  2 4006 2.240868e+00
## 11115  2 4007 2.212662e+00
## 11116  2 4008 2.186689e+00
## 11117  2 4009 3.396248e+00
## 11118  2 4010 3.815180e+00
## 11119  2 4011 3.880050e+00
## 11120  2 4012 3.796692e+00
## 11121  2 4013 3.641196e+00
## 11122  2 4014 3.481059e+00
## 11123  2 4015 3.330738e+00
## 11124  2 4016 3.191096e+00
## 11125  2 4017 3.066754e+00
## 11126  2 4018 2.947367e+00
## 11127  2 4019 2.845343e+00
## 11128  2 4020 2.754384e+00
## 11129  2 4021 2.679620e+00
## 11130  2 4022 2.609422e+00
## 11131  2 4023 2.545850e+00
## 11132  2 4024 2.485315e+00
## 11133  2 4025 2.435612e+00
## 11134  2 4026 2.385672e+00
## 11135  2 4027 2.342625e+00
## 11136  2 4028 2.307093e+00
## 11137  2 4029 2.273162e+00
## 11138  2 4030 2.245799e+00
## 11139  2 4031 2.220804e+00
## 11140  2 4032 2.191072e+00
## 11141  2 4033 3.395583e+00
## 11142  2 4034 3.822633e+00
## 11143  2 4035 3.877282e+00
## 11144  2 4036 3.794021e+00
## 11145  2 4037 3.646201e+00
## 11146  2 4038 3.492440e+00
## 11147  2 4039 3.333582e+00
## 11148  2 4040 3.196755e+00
## 11149  2 4041 3.065266e+00
## 11150  2 4042 2.955443e+00
## 11151  2 4043 2.848323e+00
## 11152  2 4044 2.766090e+00
## 11153  2 4045 2.681847e+00
## 11154  2 4046 2.610668e+00
## 11155  2 4047 2.546098e+00
## 11156  2 4048 2.488136e+00
## 11157  2 4049 2.440169e+00
## 11158  2 4050 2.395628e+00
## 11159  2 4051 2.352662e+00
## 11160  2 4052 2.309923e+00
## 11161  2 4053 2.279006e+00
## 11162  2 4054 2.247652e+00
## 11163  2 4055 2.220967e+00
## 11164  2 4056 2.193154e+00
## 11165  2 4057 3.404117e+00
## 11166  2 4058 3.833210e+00
## 11167  2 4059 3.888086e+00
## 11168  2 4060 3.802001e+00
## 11169  2 4061 3.654131e+00
## 11170  2 4062 3.498144e+00
## 11171  2 4063 3.338459e+00
## 11172  2 4064 3.204213e+00
## 11173  2 4065 3.069395e+00
## 11174  2 4066 2.955305e+00
## 11175  2 4067 2.857417e+00
## 11176  2 4068 2.768326e+00
## 11177  2 4069 2.682573e+00
## 11178  2 4070 2.615063e+00
## 11179  2 4071 2.552783e+00
## 11180  2 4072 2.496138e+00
## 11181  2 4073 2.442041e+00
## 11182  2 4074 2.392077e+00
## 11183  2 4075 2.354001e+00
## 11184  2 4076 2.314365e+00
## 11185  2 4077 2.282268e+00
## 11186  2 4078 2.253530e+00
## 11187  2 4079 2.224895e+00
## 11188  2 4080 2.195702e+00
## 11189  2 4081 3.407734e+00
## 11190  2 4082 3.825674e+00
## 11191  2 4083 3.892632e+00
## 11192  2 4084 3.805215e+00
## 11193  2 4085 3.657648e+00
## 11194  2 4086 3.497256e+00
## 11195  2 4087 3.345396e+00
## 11196  2 4088 3.201572e+00
## 11197  2 4089 3.077109e+00
## 11198  2 4090 2.957021e+00
## 11199  2 4091 2.861331e+00
## 11200  2 4092 2.770329e+00
## 11201  2 4093 2.690647e+00
## 11202  2 4094 2.614451e+00
## 11203  2 4095 2.553980e+00
## 11204  2 4096 2.494188e+00
## 11205  2 4097 2.443171e+00
## 11206  2 4098 2.400214e+00
## 11207  2 4099 2.356528e+00
## 11208  2 4100 2.321088e+00
## 11209  2 4101 2.289042e+00
## 11210  2 4102 2.255446e+00
## 11211  2 4103 2.231408e+00
## 11212  2 4104 2.201072e+00
## 11213  2 4104 2.224177e+00
## 11214  2 4105 3.435522e+00
## 11215  2 4106 3.856932e+00
## 11216  2 4107 3.922867e+00
## 11217  2 4108 3.821322e+00
## 11218  2 4109 3.682973e+00
## 11219  2 4110 3.516842e+00
## 11220  2 4111 3.362924e+00
## 11221  2 4112 3.222825e+00
## 11222  2 4113 3.102271e+00
## 11223  2 4114 2.982574e+00
## 11224  2 4115 2.884086e+00
## 11225  2 4116 2.789605e+00
## 11226  2 4117 2.706593e+00
## 11227  2 4118 2.642208e+00
## 11228  2 4119 2.576215e+00
## 11229  2 4120 2.517711e+00
## 11230  2 4121 2.469495e+00
## 11231  2 4122 2.422721e+00
## 11232  2 4123 2.381023e+00
## 11233  2 4124 2.344190e+00
## 11234  2 4125 2.309683e+00
## 11235  2 4126 2.279014e+00
## 11236  2 4127 2.250305e+00
## 11237  2 4128 2.220598e+00
## 11238  2 4128 2.223205e+00
## 11239  2 4129 2.195561e+00
## 11240  2 4130 2.176635e+00
## 11241  2 4131 2.157916e+00
## 11242  2 4132 2.134822e+00
## 11243  2 4133 2.112992e+00
## 11244  2 4134 2.095633e+00
## 11245  2 4135 2.079406e+00
## 11246  2 4136 2.063531e+00
## 11247  2 4137 2.048828e+00
## 11248  2 4138 2.031797e+00
## 11249  2 4139 2.018294e+00
## 11250  2 4140 2.004829e+00
## 11251  2 4141 1.989972e+00
## 11252  2 4142 1.977727e+00
## 11253  2 4143 1.965602e+00
## 11254  2 4144 1.956146e+00
## 11255  2 4145 1.937934e+00
## 11256  2 4146 1.930244e+00
## 11257  2 4147 1.917044e+00
## 11258  2 4148 1.903840e+00
## 11259  2 4149 1.891690e+00
## 11260  2 4150 1.883596e+00
## 11261  2 4151 1.867750e+00
## 11262  2 4152 1.855521e+00
## 11263  2 4152 1.858923e+00
## 11264  2 4153 4.318904e+00
## 11265  2 4154 5.195583e+00
## 11266  2 4155 5.358287e+00
## 11267  2 4156 5.204263e+00
## 11268  2 4157 4.937452e+00
## 11269  2 4158 4.649609e+00
## 11270  2 4159 4.365421e+00
## 11271  2 4160 4.106242e+00
## 11272  2 4161 3.861598e+00
## 11273  2 4162 3.666167e+00
## 11274  2 4163 3.470143e+00
## 11275  2 4164 3.312423e+00
## 11276  2 4165 3.157240e+00
## 11277  2 4166 3.041537e+00
## 11278  2 4167 2.926565e+00
## 11279  2 4168 2.825203e+00
## 11280  2 4169 2.739606e+00
## 11281  2 4170 2.655946e+00
## 11282  2 4171 2.599721e+00
## 11283  2 4172 2.540184e+00
## 11284  2 4173 2.476465e+00
## 11285  2 4174 2.430540e+00
## 11286  2 4175 2.386826e+00
## 11287  2 4176 2.344029e+00
## 11288  2 4176 2.348645e+00
## 11289  2 4177 3.544723e+00
## 11290  2 4178 3.964573e+00
## 11291  2 4179 4.017096e+00
## 11292  2 4180 3.907048e+00
## 11293  2 4181 3.763126e+00
## 11294  2 4182 3.591980e+00
## 11295  2 4183 3.439525e+00
## 11296  2 4184 3.295029e+00
## 11297  2 4185 3.157819e+00
## 11298  2 4186 3.037349e+00
## 11299  2 4187 2.938211e+00
## 11300  2 4188 2.841980e+00
## 11301  2 4189 2.755359e+00
## 11302  2 4190 2.686664e+00
## 11303  2 4191 2.616840e+00
## 11304  2 4192 2.558328e+00
## 11305  2 4193 2.504880e+00
## 11306  2 4194 2.461220e+00
## 11307  2 4195 2.422274e+00
## 11308  2 4196 2.379980e+00
## 11309  2 4197 2.346558e+00
## 11310  2 4198 2.313767e+00
## 11311  2 4199 2.286365e+00
## 11312  2 4200 2.260017e+00
## 11313  2 4201 3.459777e+00
## 11314  2 4202 3.889169e+00
## 11315  2 4203 3.950887e+00
## 11316  2 4204 3.863228e+00
## 11317  2 4205 3.717438e+00
## 11318  2 4206 3.553250e+00
## 11319  2 4207 3.399832e+00
## 11320  2 4208 3.251307e+00
## 11321  2 4209 3.125956e+00
## 11322  2 4210 3.013765e+00
## 11323  2 4211 2.909835e+00
## 11324  2 4212 2.826504e+00
## 11325  2 4213 2.742750e+00
## 11326  2 4214 2.669810e+00
## 11327  2 4215 2.600114e+00
## 11328  2 4216 2.547420e+00
## 11329  2 4217 2.494758e+00
## 11330  2 4218 2.449872e+00
## 11331  2 4219 2.408401e+00
## 11332  2 4220 2.371936e+00
## 11333  2 4221 2.338719e+00
## 11334  2 4222 2.302651e+00
## 11335  2 4223 2.276107e+00
## 11336  2 4224 2.246394e+00
## 11337  2 4225 3.459179e+00
## 11338  2 4226 3.883855e+00
## 11339  2 4227 3.943608e+00
## 11340  2 4228 3.858695e+00
## 11341  2 4229 3.710755e+00
## 11342  2 4230 3.550360e+00
## 11343  2 4231 3.399406e+00
## 11344  2 4232 3.262806e+00
## 11345  2 4233 3.127456e+00
## 11346  2 4234 3.004756e+00
## 11347  2 4235 2.906104e+00
## 11348  2 4236 2.813943e+00
## 11349  2 4237 2.735244e+00
## 11350  2 4238 2.662067e+00
## 11351  2 4239 2.600494e+00
## 11352  2 4240 2.536494e+00
## 11353  2 4241 2.497782e+00
## 11354  2 4242 2.447446e+00
## 11355  2 4243 2.404957e+00
## 11356  2 4244 2.367465e+00
## 11357  2 4245 2.329276e+00
## 11358  2 4246 2.301371e+00
## 11359  2 4247 2.272918e+00
## 11360  2 4248 2.248550e+00
## 11361  2 4249 3.457143e+00
## 11362  2 4250 3.881015e+00
## 11363  2 4251 3.940259e+00
## 11364  2 4252 3.855035e+00
## 11365  2 4253 3.703903e+00
## 11366  2 4254 3.543275e+00
## 11367  2 4255 3.392066e+00
## 11368  2 4256 3.254057e+00
## 11369  2 4257 3.123243e+00
## 11370  2 4258 3.006143e+00
## 11371  2 4259 2.905444e+00
## 11372  2 4260 2.812440e+00
## 11373  2 4261 2.735558e+00
## 11374  2 4262 2.663495e+00
## 11375  2 4263 2.595757e+00
## 11376  2 4264 2.536672e+00
## 11377  2 4265 2.492411e+00
## 11378  2 4266 2.444854e+00
## 11379  2 4267 2.401632e+00
## 11380  2 4268 2.362427e+00
## 11381  2 4269 2.328905e+00
## 11382  2 4270 2.301848e+00
## 11383  2 4271 2.270197e+00
## 11384  2 4272 2.241490e+00
## 11385  2 4273 3.449444e+00
## 11386  2 4274 3.874984e+00
## 11387  2 4275 3.943706e+00
## 11388  2 4276 3.845987e+00
## 11389  2 4277 3.702052e+00
## 11390  2 4278 3.537776e+00
## 11391  2 4279 3.391057e+00
## 11392  2 4280 3.245735e+00
## 11393  2 4281 3.119920e+00
## 11394  2 4282 3.007332e+00
## 11395  2 4283 2.898092e+00
## 11396  2 4284 2.813613e+00
## 11397  2 4285 2.732713e+00
## 11398  2 4286 2.662725e+00
## 11399  2 4287 2.596555e+00
## 11400  2 4288 2.537711e+00
## 11401  2 4289 2.490650e+00
## 11402  2 4290 2.444558e+00
## 11403  2 4291 2.394602e+00
## 11404  2 4292 2.358866e+00
## 11405  2 4293 2.329463e+00
## 11406  2 4294 2.296508e+00
## 11407  2 4295 2.264683e+00
## 11408  2 4296 2.238968e+00
## 11409  2 4297 3.445960e+00
## 11410  2 4298 3.869105e+00
## 11411  2 4299 3.934390e+00
## 11412  2 4300 3.841384e+00
## 11413  2 4301 3.702667e+00
## 11414  2 4302 3.547945e+00
## 11415  2 4303 3.385946e+00
## 11416  2 4304 3.243023e+00
## 11417  2 4305 3.113385e+00
## 11418  2 4306 2.999725e+00
## 11419  2 4307 2.895595e+00
## 11420  2 4308 2.807809e+00
## 11421  2 4309 2.730999e+00
## 11422  2 4310 2.652431e+00
## 11423  2 4311 2.593309e+00
## 11424  2 4312 2.534643e+00
## 11425  2 4313 2.482214e+00
## 11426  2 4314 2.440630e+00
## 11427  2 4315 2.396528e+00
## 11428  2 4316 2.360264e+00
## 11429  2 4317 2.328134e+00
## 11430  2 4318 2.293228e+00
## 11431  2 4319 2.260120e+00
## 11432  2 4320 2.236320e+00
## 11433  2 4321 3.448461e+00
## 11434  2 4322 3.868000e+00
## 11435  2 4323 3.935942e+00
## 11436  2 4324 3.842340e+00
## 11437  2 4325 3.700692e+00
## 11438  2 4326 3.542924e+00
## 11439  2 4327 3.380554e+00
## 11440  2 4328 3.248442e+00
## 11441  2 4329 3.117633e+00
## 11442  2 4330 2.995029e+00
## 11443  2 4331 2.896687e+00
## 11444  2 4332 2.805877e+00
## 11445  2 4333 2.722443e+00
## 11446  2 4334 2.653288e+00
## 11447  2 4335 2.592326e+00
## 11448  2 4336 2.534280e+00
## 11449  2 4337 2.483222e+00
## 11450  2 4338 2.440096e+00
## 11451  2 4339 2.389436e+00
## 11452  2 4340 2.356917e+00
## 11453  2 4341 2.322338e+00
## 11454  2 4342 2.288453e+00
## 11455  2 4343 2.260247e+00
## 11456  2 4344 2.233547e+00
## 11457  2 4345 3.437210e+00
## 11458  2 4346 3.874123e+00
## 11459  2 4347 3.930683e+00
## 11460  2 4348 3.844184e+00
## 11461  2 4349 3.702468e+00
## 11462  2 4350 3.534747e+00
## 11463  2 4351 3.383721e+00
## 11464  2 4352 3.243316e+00
## 11465  2 4353 3.114347e+00
## 11466  2 4354 2.999110e+00
## 11467  2 4355 2.894518e+00
## 11468  2 4356 2.801567e+00
## 11469  2 4357 2.723855e+00
## 11470  2 4358 2.655892e+00
## 11471  2 4359 2.591544e+00
## 11472  2 4360 2.533767e+00
## 11473  2 4361 2.479594e+00
## 11474  2 4362 2.433728e+00
## 11475  2 4363 2.394509e+00
## 11476  2 4364 2.355376e+00
## 11477  2 4365 2.324339e+00
## 11478  2 4366 2.290144e+00
## 11479  2 4367 2.260382e+00
## 11480  2 4368 2.235270e+00
## 11481  2 4369 3.445028e+00
## 11482  2 4370 3.865643e+00
## 11483  2 4371 3.930260e+00
## 11484  2 4372 3.845912e+00
## 11485  2 4373 3.691648e+00
## 11486  2 4374 3.540761e+00
## 11487  2 4375 3.375095e+00
## 11488  2 4376 3.235804e+00
## 11489  2 4377 3.111913e+00
## 11490  2 4378 2.992730e+00
## 11491  2 4379 2.890361e+00
## 11492  2 4380 2.801255e+00
## 11493  2 4381 2.726897e+00
## 11494  2 4382 2.650596e+00
## 11495  2 4383 2.585226e+00
## 11496  2 4384 2.532991e+00
## 11497  2 4385 2.479699e+00
## 11498  2 4386 2.435263e+00
## 11499  2 4387 2.389726e+00
## 11500  2 4388 2.352375e+00
## 11501  2 4389 2.319532e+00
## 11502  2 4390 2.283570e+00
## 11503  2 4391 2.257306e+00
## 11504  2 4392 2.235255e+00
## 11505  2 4393 3.445186e+00
## 11506  2 4394 3.866443e+00
## 11507  2 4395 3.927550e+00
## 11508  2 4396 3.833765e+00
## 11509  2 4397 3.690074e+00
## 11510  2 4398 3.533607e+00
## 11511  2 4399 3.380967e+00
## 11512  2 4400 3.233748e+00
## 11513  2 4401 3.105761e+00
## 11514  2 4402 2.997598e+00
## 11515  2 4403 2.885305e+00
## 11516  2 4404 2.800569e+00
## 11517  2 4405 2.719720e+00
## 11518  2 4406 2.652675e+00
## 11519  2 4407 2.590025e+00
## 11520  2 4408 2.529549e+00
## 11521  2 4409 2.477009e+00
## 11522  2 4410 2.434204e+00
## 11523  2 4411 2.387900e+00
## 11524  2 4412 2.349340e+00
## 11525  2 4413 2.319547e+00
## 11526  2 4414 2.289853e+00
## 11527  2 4415 2.257681e+00
## 11528  2 4416 2.232325e+00
## 11529  2 4417 3.442810e+00
## 11530  2 4418 3.865646e+00
## 11531  2 4419 3.923643e+00
## 11532  2 4420 3.839238e+00
## 11533  2 4421 3.697890e+00
## 11534  2 4422 3.536495e+00
## 11535  2 4423 3.377524e+00
## 11536  2 4424 3.232438e+00
## 11537  2 4425 3.106201e+00
## 11538  2 4426 2.992583e+00
## 11539  2 4427 2.895458e+00
## 11540  2 4428 2.803105e+00
## 11541  2 4429 2.720077e+00
## 11542  2 4430 2.651008e+00
## 11543  2 4431 2.582958e+00
## 11544  2 4432 2.530166e+00
## 11545  2 4433 2.477303e+00
## 11546  2 4434 2.431098e+00
## 11547  2 4435 2.390020e+00
## 11548  2 4436 2.351047e+00
## 11549  2 4437 2.316863e+00
## 11550  2 4438 2.285910e+00
## 11551  2 4439 2.258649e+00
## 11552  2 4440 2.230317e+00
## 11553  2 4441 2.203649e+00
## 11554  2 4442 2.183301e+00
## 11555  2 4443 2.164789e+00
## 11556  2 4444 2.139450e+00
## 11557  2 4445 2.119229e+00
## 11558  2 4446 2.105581e+00
## 11559  2 4447 2.086863e+00
## 11560  2 4448 2.071803e+00
## 11561  2 4449 2.056178e+00
## 11562  2 4450 2.037334e+00
## 11563  2 4451 2.029570e+00
## 11564  2 4452 2.014499e+00
## 11565  2 4453 1.997742e+00
## 11566  2 4454 1.986438e+00
## 11567  2 4455 1.969699e+00
## 11568  2 4456 1.961385e+00
## 11569  2 4457 1.947321e+00
## 11570  2 4458 1.934837e+00
## 11571  2 4459 1.923529e+00
## 11572  2 4460 1.909239e+00
## 11573  2 4461 1.900024e+00
## 11574  2 4462 1.886799e+00
## 11575  2 4463 1.873255e+00
## 11576  2 4464 1.868005e+00
## 11577  2 4465 1.855000e+00
## 11578  2 4466 1.845684e+00
## 11579  2 4467 1.833172e+00
## 11580  2 4468 1.822019e+00
## 11581  2 4469 1.813502e+00
## 11582  2 4470 1.800256e+00
## 11583  2 4471 1.791143e+00
## 11584  2 4472 1.780683e+00
## 11585  2 4473 1.767387e+00
## 11586  2 4474 1.756112e+00
## 11587  2 4475 1.749229e+00
## 11588  2 4476 1.738770e+00
## 11589  2 4477 1.729681e+00
## 11590  2 4478 1.718004e+00
## 11591  2 4479 1.709529e+00
## 11592  2 4480 1.701816e+00
## 11593  2 4481 1.690747e+00
## 11594  2 4482 1.683243e+00
## 11595  2 4483 1.671339e+00
## 11596  2 4484 1.662705e+00
## 11597  2 4485 1.652448e+00
## 11598  2 4486 1.638327e+00
## 11599  2 4487 1.632717e+00
## 11600  2 4488 1.621519e+00
## 11601  2 4489 1.615259e+00
## 11602  2 4490 1.603079e+00
## 11603  2 4491 1.597366e+00
## 11604  2 4492 1.586032e+00
## 11605  2 4493 1.578907e+00
## 11606  2 4494 1.570282e+00
## 11607  2 4495 1.558868e+00
## 11608  2 4496 1.549242e+00
## 11609  2 4497 1.541044e+00
## 11610  2 4498 1.533531e+00
## 11611  2 4499 1.525475e+00
## 11612  2 4500 1.516627e+00
## 11613  2 4501 1.508255e+00
## 11614  2 4502 1.498545e+00
## 11615  2 4503 1.488808e+00
## 11616  2 4504 1.481242e+00
## 11617  2 4505 1.471696e+00
## 11618  2 4506 1.463988e+00
## 11619  2 4507 1.457383e+00
## 11620  2 4508 1.447861e+00
## 11621  2 4509 1.441047e+00
## 11622  2 4510 1.430923e+00
## 11623  2 4511 1.424482e+00
## 11624  2 4512 1.415752e+00
## 11625  2 4513 1.405622e+00
## 11626  2 4514 1.402039e+00
## 11627  2 4515 1.393230e+00
## 11628  2 4516 1.383443e+00
## 11629  2 4517 1.377031e+00
## 11630  2 4518 1.368366e+00
## 11631  2 4519 1.361701e+00
## 11632  2 4520 1.351726e+00
## 11633  2 4521 1.344060e+00
## 11634  2 4522 1.338522e+00
## 11635  2 4523 1.330333e+00
## 11636  2 4524 1.320452e+00
## 11637  2 4525 1.314286e+00
## 11638  2 4526 1.306797e+00
## 11639  2 4527 1.299192e+00
## 11640  2 4528 1.290779e+00
## 11641  2 4529 1.285402e+00
## 11642  2 4530 1.277302e+00
## 11643  2 4531 1.270668e+00
## 11644  2 4532 1.263429e+00
## 11645  2 4533 1.256081e+00
## 11646  2 4534 1.250146e+00
## 11647  2 4535 1.241964e+00
## 11648  2 4536 1.232390e+00
## 11649  2 4537 1.226217e+00
## 11650  2 4538 1.220586e+00
## 11651  2 4539 1.213587e+00
## 11652  2 4540 1.207897e+00
## 11653  2 4541 1.200754e+00
## 11654  2 4542 1.192905e+00
## 11655  2 4543 1.186899e+00
## 11656  2 4544 1.179806e+00
## 11657  2 4545 1.174365e+00
## 11658  2 4546 1.165909e+00
## 11659  2 4547 1.160770e+00
## 11660  2 4548 1.152211e+00
## 11661  2 4549 1.148434e+00
## 11662  2 4550 1.141841e+00
## 11663  2 4551 1.135176e+00
## 11664  2 4552 1.125838e+00
## 11665  2 4553 1.121459e+00
## 11666  2 4554 1.113690e+00
## 11667  2 4555 1.108732e+00
## 11668  2 4556 1.102581e+00
## 11669  2 4557 1.094235e+00
## 11670  2 4558 1.087788e+00
## 11671  2 4559 1.082608e+00
## 11672  2 4560 1.078192e+00
## 11673  2 4561 1.069225e+00
## 11674  2 4562 1.066330e+00
## 11675  2 4563 1.060013e+00
## 11676  2 4564 1.052864e+00
## 11677  2 4565 1.048149e+00
## 11678  2 4566 1.040811e+00
## 11679  2 4567 1.033847e+00
## 11680  2 4568 1.025552e+00
## 11681  2 4569 1.022346e+00
## 11682  2 4570 1.018735e+00
## 11683  2 4571 1.011090e+00
## 11684  2 4572 1.005049e+00
## 11685  2 4573 1.001012e+00
## 11686  2 4574 9.930046e-01
## 11687  2 4575 9.881187e-01
## 11688  2 4576 9.818345e-01
## 11689  2 4577 9.762979e-01
## 11690  2 4578 9.702807e-01
## 11691  2 4579 9.672017e-01
## 11692  2 4580 9.602470e-01
## 11693  2 4581 9.550798e-01
## 11694  2 4582 9.494786e-01
## 11695  2 4583 9.448542e-01
## 11696  2 4584 9.398294e-01
## 11697  2 4585 9.339650e-01
## 11698  2 4586 9.290949e-01
## 11699  2 4587 9.242185e-01
## 11700  2 4588 9.176736e-01
## 11701  2 4589 9.123992e-01
## 11702  2 4590 9.076711e-01
## 11703  2 4591 9.025183e-01
## 11704  2 4592 8.977876e-01
## 11705  2 4593 8.921288e-01
## 11706  2 4594 8.854540e-01
## 11707  2 4595 8.822758e-01
## 11708  2 4596 8.769242e-01
## 11709  2 4597 8.731678e-01
## 11710  2 4598 8.653962e-01
## 11711  2 4599 8.597134e-01
## 11712  2 4600 8.584997e-01
## 11713  2 4601 8.524333e-01
## 11714  2 4602 8.467032e-01
## 11715  2 4603 8.418044e-01
## 11716  2 4604 8.370752e-01
## 11717  2 4605 8.326220e-01
## 11718  2 4606 8.275504e-01
## 11719  2 4607 8.232769e-01
## 11720  2 4608 8.189886e-01
## 11721  2 4609 8.162853e-01
## 11722  2 4610 8.088959e-01
## 11723  2 4611 8.046835e-01
## 11724  2 4612 8.004186e-01
## 11725  2 4613 7.950641e-01
## 11726  2 4614 7.925952e-01
## 11727  2 4615 7.871793e-01
## 11728  2 4616 7.807317e-01
## 11729  2 4617 7.774279e-01
## 11730  2 4618 7.741759e-01
## 11731  2 4619 7.682870e-01
## 11732  2 4620 7.655113e-01
## 11733  2 4621 7.600648e-01
## 11734  2 4622 7.555118e-01
## 11735  2 4623 7.523258e-01
## 11736  2 4624 7.465228e-01
## 11737  2 4625 7.430218e-01
## 11738  2 4626 7.382227e-01
## 11739  2 4627 7.341997e-01
## 11740  2 4628 7.307143e-01
## 11741  2 4629 7.257385e-01
## 11742  2 4630 7.222596e-01
## 11743  2 4631 7.179684e-01
## 11744  2 4632 7.131031e-01
## 11745  2 4633 7.089600e-01
## 11746  2 4634 7.056374e-01
## 11747  2 4635 7.031658e-01
## 11748  2 4636 6.970656e-01
## 11749  2 4637 6.930930e-01
## 11750  2 4638 6.900434e-01
## 11751  2 4639 6.852444e-01
## 11752  2 4640 6.823529e-01
## 11753  2 4641 6.785490e-01
## 11754  2 4642 6.739256e-01
## 11755  2 4643 6.698594e-01
## 11756  2 4644 6.668374e-01
## 11757  2 4645 6.631079e-01
## 11758  2 4646 6.591620e-01
## 11759  2 4647 6.571491e-01
## 11760  2 4648 6.513764e-01
## 11761  2 4649 6.481188e-01
## 11762  2 4650 6.427847e-01
## 11763  2 4651 6.405795e-01
## 11764  2 4652 6.375958e-01
## 11765  2 4653 6.333353e-01
## 11766  2 4654 6.301093e-01
## 11767  2 4655 6.268707e-01
## 11768  2 4656 6.210186e-01
## 11769  2 4657 6.183429e-01
## 11770  2 4658 6.154889e-01
## 11771  2 4659 6.120170e-01
## 11772  2 4660 6.069145e-01
## 11773  2 4661 6.040652e-01
## 11774  2 4662 6.005149e-01
## 11775  2 4663 5.982737e-01
## 11776  2 4664 5.948371e-01
## 11777  2 4665 5.921584e-01
## 11778  2 4666 5.884121e-01
## 11779  2 4667 5.859758e-01
## 11780  2 4668 5.812085e-01
## 11781  2 4669 5.785891e-01
## 11782  2 4670 5.747970e-01
## 11783  2 4671 5.710742e-01
## 11784  2 4672 5.671064e-01
## 11785  2 4673 5.649772e-01
## 11786  2 4674 5.626458e-01
## 11787  2 4675 5.590080e-01
## 11788  2 4676 5.554680e-01
## 11789  2 4677 5.523009e-01
## 11790  2 4678 5.482074e-01
## 11791  2 4679 5.457627e-01
## 11792  2 4680 5.424526e-01
## 11793  2 4681 5.401178e-01
## 11794  2 4682 5.381919e-01
## 11795  2 4683 5.343432e-01
## 11796  2 4684 5.295240e-01
## 11797  2 4685 5.276761e-01
## 11798  2 4686 5.248294e-01
## 11799  2 4687 5.217564e-01
## 11800  2 4688 5.188436e-01
## 11801  2 4689 5.152562e-01
## 11802  2 4690 5.125678e-01
## 11803  2 4691 5.101268e-01
## 11804  2 4692 5.067571e-01
## 11805  2 4693 5.037592e-01
## 11806  2 4694 5.006249e-01
## 11807  2 4695 4.975483e-01
## 11808  2 4696 4.955372e-01
## 11809  2 4697 4.929018e-01
## 11810  2 4698 4.910286e-01
## 11811  2 4699 4.871235e-01
## 11812  2 4700 4.837116e-01
## 11813  2 4701 4.814976e-01
## 11814  2 4702 4.787586e-01
## 11815  2 4703 4.761763e-01
## 11816  2 4704 4.730693e-01
## 11817  2 4705 4.712269e-01
## 11818  2 4706 4.681386e-01
## 11819  2 4707 4.655232e-01
## 11820  2 4708 4.627225e-01
## 11821  2 4709 4.598460e-01
## 11822  2 4710 4.574506e-01
## 11823  2 4711 4.544922e-01
## 11824  2 4712 4.523110e-01
## 11825  2 4713 4.489277e-01
## 11826  2 4714 4.475921e-01
## 11827  2 4715 4.449518e-01
## 11828  2 4716 4.415892e-01
## 11829  2 4717 4.390694e-01
## 11830  2 4718 4.363866e-01
## 11831  2 4719 4.349112e-01
## 11832  2 4720 4.321315e-01
## 11833  2 4721 4.294657e-01
## 11834  2 4722 4.270204e-01
## 11835  2 4723 4.242896e-01
## 11836  2 4724 4.222724e-01
## 11837  2 4725 4.201367e-01
## 11838  2 4726 4.179140e-01
## 11839  2 4727 4.156607e-01
## 11840  2 4728 4.125081e-01
## 11841  2 4729 4.101999e-01
## 11842  2 4730 4.082003e-01
## 11843  2 4731 4.054528e-01
## 11844  2 4732 4.037853e-01
## 11845  2 4733 4.009993e-01
## 11846  2 4734 3.986083e-01
## 11847  2 4735 3.964787e-01
## 11848  2 4736 3.946797e-01
## 11849  2 4737 3.924076e-01
## 11850  2 4738 3.893319e-01
## 11851  2 4739 3.879166e-01
## 11852  2 4740 3.854663e-01
## 11853  2 4741 3.830203e-01
## 11854  2 4742 3.808626e-01
## 11855  2 4743 3.788065e-01
## 11856  2 4744 3.761490e-01
## 11857  2 4745 3.747633e-01
## 11858  2 4746 3.723200e-01
## 11859  2 4747 3.703620e-01
## 11860  2 4748 3.687517e-01
## 11861  2 4749 3.658348e-01
## 11862  2 4750 3.644054e-01
## 11863  2 4751 3.618112e-01
## 11864  2 4752 3.594910e-01
## 11865  2 4753 3.578043e-01
## 11866  2 4754 3.567847e-01
## 11867  2 4755 3.532874e-01
## 11868  2 4756 3.523853e-01
## 11869  2 4757 3.499716e-01
## 11870  2 4758 3.474883e-01
## 11871  2 4759 3.463063e-01
## 11872  2 4760 3.437418e-01
## 11873  2 4761 3.418303e-01
```

```
## 11874  2 4762 3.401640e-01
## 11875  2 4763 3.383803e-01
## 11876  2 4764 3.360487e-01
## 11877  2 4765 3.341290e-01
## 11878  2 4766 3.319496e-01
## 11879  2 4767 3.303324e-01
## 11880  2 4768 3.291207e-01
## 11881  2 4769 3.266520e-01
## 11882  2 4770 3.255177e-01
## 11883  2 4771 3.226708e-01
## 11884  2 4772 3.204436e-01
## 11885  2 4773 3.191773e-01
## 11886  2 4774 3.172181e-01
## 11887  2 4775 3.156291e-01
## 11888  2 4776 3.137361e-01
## 11889  2 4777 3.118352e-01
## 11890  2 4778 3.095197e-01
## 11891  2 4779 3.081953e-01
## 11892  2 4780 3.071417e-01
## 11893  2 4781 3.051730e-01
## 11894  2 4782 3.031653e-01
## 11895  2 4783 3.013247e-01
## 11896  2 4784 3.002184e-01
## 11897  2 4785 2.974899e-01
## 11898  2 4786 2.964032e-01
## 11899  2 4787 2.947315e-01
## 11900  2 4788 2.929868e-01
## 11901  2 4789 2.917763e-01
## 11902  2 4790 2.894180e-01
## 11903  2 4791 2.879139e-01
## 11904  2 4792 2.863580e-01
## 11905  2 4793 2.848014e-01
## 11906  2 4794 2.833651e-01
## 11907  2 4795 2.812840e-01
## 11908  2 4796 2.802375e-01
## 11909  2 4797 2.782759e-01
## 11910  2 4798 2.769498e-01
## 11911  2 4799 2.751071e-01
## 11912  2 4800 2.736089e-01
## 11913  2 4801 2.717649e-01
## 11914  2 4802 2.705003e-01
## 11915  2 4803 2.689142e-01
## 11916  2 4804 2.672127e-01
## 11917  2 4805 2.656303e-01
## 11918  2 4806 2.647691e-01
## 11919  2 4807 2.632431e-01
## 11920  2 4808 2.613512e-01
## 11921  2 4809 2.599081e-01
## 11922  2 4810 2.585247e-01
## 11923  2 4811 2.571094e-01
## 11924  2 4812 2.557147e-01
## 11925  2 4813 2.543187e-01
## 11926  2 4814 2.525194e-01
## 11927  2 4815 2.511874e-01
## 11928  2 4816 2.497856e-01
## 11929  2 4817 2.478864e-01
## 11930  2 4818 2.470395e-01
## 11931  2 4819 2.457811e-01
## 11932  2 4820 2.442615e-01
## 11933  2 4821 2.426821e-01
## 11934  2 4822 2.411573e-01
## 11935  2 4823 2.404504e-01
## 11936  2 4824 2.387636e-01
## 11937  2 4825 2.375186e-01
## 11938  2 4826 2.361434e-01
## 11939  2 4827 2.341057e-01
## 11940  2 4828 2.332032e-01
## 11941  2 4829 2.321093e-01
## 11942  2 4830 2.307643e-01
## 11943  2 4831 2.293872e-01
## 11944  2 4832 2.284323e-01
## 11945  2 4833 2.265756e-01
## 11946  2 4834 2.257080e-01
## 11947  2 4835 2.242012e-01
## 11948  2 4836 2.227670e-01
## 11949  2 4837 2.216484e-01
## 11950  2 4838 2.208466e-01
## 11951  2 4839 2.191904e-01
## 11952  2 4840 2.179617e-01
## 11953  2 4841 2.161565e-01
## 11954  2 4842 2.152203e-01
## 11955  2 4843 2.146029e-01
## 11956  2 4844 2.128024e-01
## 11957  2 4845 2.118345e-01
## 11958  2 4846 2.105260e-01
## 11959  2 4847 2.093777e-01
## 11960  2 4848 2.080238e-01
## 11961  2 4849 2.067337e-01
## 11962  2 4850 2.060433e-01
## 11963  2 4851 2.043885e-01
## 11964  2 4852 2.035634e-01
## 11965  2 4853 2.021636e-01
## 11966  2 4854 2.013689e-01
## 11967  2 4855 1.996753e-01
## 11968  2 4856 1.987567e-01
## 11969  2 4857 1.977755e-01
## 11970  2 4858 1.964439e-01
## 11971  2 4859 1.951454e-01
## 11972  2 4860 1.940486e-01
## 11973  2 4861 1.931745e-01
## 11974  2 4862 1.920045e-01
## 11975  2 4863 1.911808e-01
## 11976  2 4864 1.897520e-01
## 11977  2 4865 1.886185e-01
## 11978  2 4866 1.881426e-01
## 11979  2 4867 1.865773e-01
## 11980  2 4868 1.853981e-01
## 11981  2 4869 1.849105e-01
## 11982  2 4870 1.833178e-01
## 11983  2 4871 1.826562e-01
## 11984  2 4872 1.815973e-01
## 11985  2 4873 1.803383e-01
## 11986  2 4874 1.793571e-01
## 11987  2 4875 1.784134e-01
## 11988  2 4876 1.774587e-01
## 11989  2 4877 1.763574e-01
## 11990  2 4878 1.754172e-01
## 11991  2 4879 1.743282e-01
## 11992  2 4880 1.733433e-01
## 11993  2 4881 1.722103e-01
## 11994  2 4882 1.714760e-01
## 11995  2 4883 1.705224e-01
## 11996  2 4884 1.695338e-01
## 11997  2 4885 1.685163e-01
## 11998  2 4886 1.674855e-01
## 11999  2 4887 1.667208e-01
## 12000  2 4888 1.656268e-01
## 12001  2 4889 1.647954e-01
## 12002  2 4890 1.637019e-01
## 12003  2 4891 1.627706e-01
## 12004  2 4892 1.618969e-01
## 12005  2 4893 1.612417e-01
## 12006  2 4894 1.601538e-01
## 12007  2 4895 1.591439e-01
## 12008  2 4896 1.581730e-01
## 12009  2 4897 1.572476e-01
## 12010  2 4898 1.562809e-01
## 12011  2 4899 1.557101e-01
## 12012  2 4900 1.547908e-01
## 12013  2 4901 1.537179e-01
## 12014  2 4902 1.528811e-01
## 12015  2 4903 1.521926e-01
## 12016  2 4904 1.510981e-01
## 12017  2 4905 1.504336e-01
## 12018  2 4906 1.496989e-01
## 12019  2 4907 1.487860e-01
## 12020  2 4908 1.481729e-01
## 12021  2 4909 1.471653e-01
## 12022  2 4910 1.459627e-01
## 12023  2 4911 1.452088e-01
## 12024  2 4912 1.446303e-01
## 12025  2 4913 1.437565e-01
## 12026  2 4914 1.426004e-01
## 12027  2 4915 1.420671e-01
## 12028  2 4916 1.413025e-01
## 12029  2 4917 1.404437e-01
## 12030  2 4918 1.395994e-01
## 12031  2 4919 1.386813e-01
## 12032  2 4920 1.379668e-01
## 12033  2 4921 1.371650e-01
## 12034  2 4922 1.363394e-01
## 12035  2 4923 1.357015e-01
## 12036  2 4924 1.350932e-01
## 12037  2 4925 1.341255e-01
## 12038  2 4926 1.333386e-01
## 12039  2 4927 1.325459e-01
## 12040  2 4928 1.319406e-01
## 12041  2 4929 1.315076e-01
## 12042  2 4930 1.306036e-01
## 12043  2 4931 1.295759e-01
## 12044  2 4932 1.291557e-01
## 12045  2 4933 1.280129e-01
## 12046  2 4934 1.274576e-01
## 12047  2 4935 1.266276e-01
## 12048  2 4936 1.259993e-01
## 12049  2 4937 1.253162e-01
## 12050  2 4938 1.244337e-01
## 12051  2 4939 1.239061e-01
## 12052  2 4940 1.231426e-01
## 12053  2 4941 1.225734e-01
## 12054  2 4942 1.216840e-01
## 12055  2 4943 1.209072e-01
## 12056  2 4944 1.202873e-01
## 12057  2 4945 1.194993e-01
## 12058  2 4946 1.190758e-01
## 12059  2 4947 1.183179e-01
## 12060  2 4948 1.177306e-01
## 12061  2 4949 1.170244e-01
## 12062  2 4950 1.163506e-01
## 12063  2 4951 1.156837e-01
## 12064  2 4952 1.148419e-01
## 12065  2 4953 1.141535e-01
## 12066  2 4954 1.136038e-01
## 12067  2 4955 1.130094e-01
## 12068  2 4956 1.123092e-01
## 12069  2 4957 1.116818e-01
## 12070  2 4958 1.111930e-01
## 12071  2 4959 1.104541e-01
## 12072  2 4960 1.098458e-01
## 12073  2 4961 1.090652e-01
## 12074  2 4962 1.086259e-01
## 12075  2 4963 1.079044e-01
## 12076  2 4964 1.073092e-01
## 12077  2 4965 1.065598e-01
## 12078  2 4966 1.061294e-01
## 12079  2 4967 1.058460e-01
## 12080  2 4968 1.050328e-01
## 12081  2 4969 1.042981e-01
## 12082  2 4970 1.038003e-01
## 12083  2 4971 1.030435e-01
## 12084  2 4972 1.025346e-01
## 12085  2 4973 1.019602e-01
## 12086  2 4974 1.012838e-01
## 12087  2 4975 1.007788e-01
## 12088  2 4976 1.004736e-01
## 12089  2 4977 9.964496e-02
## 12090  2 4978 9.909616e-02
## 12091  2 4979 9.837000e-02
## 12092  2 4980 9.802062e-02
## 12093  2 4981 9.733608e-02
## 12094  2 4982 9.675736e-02
## 12095  2 4983 9.637043e-02
## 12096  2 4984 9.587717e-02
## 12097  2 4985 9.519266e-02
## 12098  2 4986 9.469140e-02
## 12099  2 4987 9.396618e-02
## 12100  2 4988 9.358045e-02
## 12101  2 4989 9.299184e-02
## 12102  2 4990 9.256623e-02
## 12103  2 4991 9.203366e-02
## 12104  2 4992 9.137579e-02
## 12105  2 4993 9.084825e-02
## 12106  2 4994 9.044832e-02
## 12107  2 4995 9.017934e-02
## 12108  2 4996 8.963688e-02
## 12109  2 4997 8.889906e-02
## 12110  2 4998 8.850573e-02
## 12111  2 4999 8.799612e-02
## 12112  2 5000 8.740335e-02
## 12113  2 5001 8.682376e-02
## 12114  2 5002 8.651654e-02
## 12115  2 5003 8.577841e-02
## 12116  2 5004 8.559316e-02
## 12117  2 5005 8.483500e-02
## 12118  2 5006 8.452056e-02
## 12119  2 5007 8.413039e-02
## 12120  2 5008 8.363401e-02
## 12121  2 5009 8.285533e-02
## 12122  2 5010 8.250245e-02
## 12123  2 5011 8.214240e-02
## 12124  2 5012 8.155531e-02
## 12125  2 5013 8.115534e-02
## 12126  2 5014 8.060842e-02
## 12127  2 5015 8.033754e-02
## 12128  2 5016 7.976889e-02
## 12129  2 5017 7.927762e-02
## 12130  2 5018 7.893994e-02
## 12131  2 5019 7.838387e-02
## 12132  2 5020 7.802786e-02
## 12133  2 5021 7.763320e-02
## 12134  2 5022 7.703701e-02
## 12135  2 5023 7.668945e-02
## 12136  2 5024 7.622530e-02
## 12137  2 5025 7.585598e-02
## 12138  2 5026 7.533561e-02
## 12139  2 5027 7.486876e-02
## 12140  2 5028 7.456810e-02
## 12141  2 5029 7.413917e-02
## 12142  2 5030 7.359707e-02
## 12143  2 5031 7.309764e-02
## 12144  2 5032 7.287586e-02
## 12145  2 5033 7.237586e-02
## 12146  2 5034 7.192725e-02
## 12147  2 5035 7.171985e-02
## 12148  2 5036 7.117941e-02
## 12149  2 5037 7.078331e-02
## 12150  2 5038 7.042404e-02
## 12151  2 5039 7.003574e-02
## 12152  2 5040 6.954447e-02
## 12153  2 5041 6.923770e-02
## 12154  2 5042 6.885381e-02
## 12155  2 5043 6.845682e-02
## 12156  2 5044 6.783194e-02
## 12157  2 5045 6.762383e-02
## 12158  2 5046 6.717387e-02
## 12159  2 5047 6.688213e-02
## 12160  2 5048 6.635462e-02
## 12161  2 5049 6.612425e-02
## 12162  2 5050 6.569392e-02
## 12163  2 5051 6.535790e-02
## 12164  2 5052 6.499217e-02
## 12165  2 5053 6.458786e-02
## 12166  2 5054 6.406351e-02
## 12167  2 5055 6.380889e-02
## 12168  2 5056 6.352177e-02
## 12169  2 5057 6.313976e-02
## 12170  2 5058 6.276927e-02
## 12171  2 5059 6.242910e-02
## 12172  2 5060 6.205259e-02
## 12173  2 5061 6.172649e-02
## 12174  2 5062 6.132326e-02
## 12175  2 5063 6.092848e-02
## 12176  2 5064 6.065502e-02
## 12177  2 5065 6.038487e-02
## 12178  2 5066 6.001142e-02
## 12179  2 5067 5.960542e-02
## 12180  2 5068 5.934147e-02
## 12181  2 5069 5.889574e-02
## 12182  2 5070 5.861818e-02
## 12183  2 5071 5.823630e-02
## 12184  2 5072 5.800899e-02
## 12185  2 5073 5.763413e-02
## 12186  2 5074 5.735350e-02
## 12187  2 5075 5.699850e-02
## 12188  2 5076 5.673288e-02
## 12189  2 5077 5.632744e-02
## 12190  2 5078 5.599915e-02
## 12191  2 5079 5.562495e-02
## 12192  2 5080 5.538632e-02
## 12193  2 5081 5.500388e-02
## 12194  2 5082 5.478011e-02
## 12195  2 5083 5.441275e-02
## 12196  2 5084 5.423337e-02
## 12197  2 5085 5.392350e-02
## 12198  2 5086 5.349784e-02
## 12199  2 5087 5.324198e-02
## 12200  2 5088 5.290549e-02
## 12201  2 5089 5.262565e-02
## 12202  2 5090 5.226872e-02
## 12203  2 5091 5.207090e-02
## 12204  2 5092 5.170580e-02
## 12205  2 5093 5.137556e-02
## 12206  2 5094 5.103620e-02
## 12207  2 5095 5.083325e-02
## 12208  2 5096 5.058266e-02
## 12209  2 5097 5.028359e-02
## 12210  2 5098 5.000254e-02
## 12211  2 5099 4.977353e-02
## 12212  2 5100 4.936823e-02
## 12213  2 5101 4.916070e-02
## 12214  2 5102 4.887041e-02
## 12215  2 5103 4.858476e-02
## 12216  2 5104 4.827868e-02
## 12217  2 5105 4.803996e-02
## 12218  2 5106 4.766369e-02
## 12219  2 5107 4.748806e-02
## 12220  2 5108 4.721146e-02
## 12221  2 5109 4.698194e-02
## 12222  2 5110 4.670805e-02
## 12223  2 5111 4.641967e-02
## 12224  2 5112 4.616829e-02
## 12225  2 5113 4.585075e-02
## 12226  2 5114 4.562647e-02
## 12227  2 5115 4.537224e-02
## 12228  2 5116 4.510938e-02
## 12229  2 5117 4.489193e-02
## 12230  2 5118 4.460438e-02
## 12231  2 5119 4.443072e-02
## 12232  2 5120 4.410577e-02
## 12233  2 5121 4.379087e-02
## 12234  2 5122 4.365774e-02
## 12235  2 5123 4.331627e-02
## 12236  2 5124 4.311939e-02
## 12237  2 5125 4.283689e-02
## 12238  2 5126 4.258383e-02
## 12239  2 5127 4.236873e-02
## 12240  2 5128 4.209685e-02
## 12241  2 5129 4.191200e-02
## 12242  2 5130 4.172037e-02
## 12243  2 5131 4.128426e-02
## 12244  2 5132 4.116818e-02
## 12245  2 5133 4.089646e-02
## 12246  2 5134 4.066599e-02
## 12247  2 5135 4.046224e-02
## 12248  2 5136 4.021057e-02
## 12249  2 5137 4.005856e-02
## 12250  2 5138 3.982416e-02
## 12251  2 5139 3.954678e-02
## 12252  2 5140 3.934663e-02
## 12253  2 5141 3.907855e-02
## 12254  2 5142 3.892477e-02
## 12255  2 5143 3.866413e-02
## 12256  2 5144 3.840227e-02
## 12257  2 5145 3.818615e-02
## 12258  2 5146 3.801577e-02
## 12259  2 5147 3.779228e-02
## 12260  2 5148 3.761742e-02
## 12261  2 5149 3.736246e-02
## 12262  2 5150 3.713113e-02
## 12263  2 5151 3.691682e-02
## 12264  2 5152 3.669927e-02
## 12265  2 5153 3.652556e-02
## 12266  2 5154 3.634887e-02
## 12267  2 5155 3.612628e-02
## 12268  2 5156 3.590255e-02
## 12269  2 5157 3.574196e-02
## 12270  2 5158 3.554218e-02
## 12271  2 5159 3.525224e-02
## 12272  2 5160 3.507165e-02
## 12273  2 5161 3.486356e-02
## 12274  2 5162 3.469081e-02
## 12275  2 5163 3.456781e-02
## 12276  2 5164 3.429088e-02
## 12277  2 5165 3.412448e-02
## 12278  2 5166 3.388714e-02
## 12279  2 5167 3.369256e-02
## 12280  2 5168 3.348923e-02
## 12281  2 5169 3.333194e-02
## 12282  2 5170 3.310548e-02
## 12283  2 5171 3.290480e-02
## 12284  2 5172 3.274115e-02
## 12285  2 5173 3.257053e-02
## 12286  2 5174 3.237311e-02
## 12287  2 5175 3.215568e-02
## 12288  2 5176 3.203592e-02
## 12289  2 5177 3.181002e-02
## 12290  2 5178 3.158454e-02
## 12291  2 5179 3.149856e-02
## 12292  2 5180 3.129483e-02
## 12293  2 5181 3.111741e-02
## 12294  2 5182 3.095236e-02
## 12295  2 5183 3.071946e-02
## 12296  2 5184 3.058703e-02
## 12297  2 5185 3.040948e-02
## 12298  2 5186 3.022956e-02
## 12299  2 5187 3.009287e-02
## 12300  2 5188 2.987409e-02
## 12301  2 5189 2.974269e-02
## 12302  2 5190 2.953682e-02
## 12303  2 5191 2.940671e-02
## 12304  2 5192 2.924698e-02
## 12305  2 5193 2.904602e-02
## 12306  2 5194 2.888552e-02
## 12307  2 5195 2.878762e-02
## 12308  2 5196 2.859374e-02
## 12309  2 5197 2.835602e-02
## 12310  2 5198 2.827767e-02
## 12311  2 5199 2.803472e-02
## 12312  2 5200 2.791697e-02
## 12313  2 5201 2.778557e-02
## 12314  2 5202 2.755862e-02
## 12315  2 5203 2.748302e-02
## 12316  2 5204 2.729169e-02
## 12317  2 5205 2.716370e-02
## 12318  2 5206 2.701646e-02
## 12319  2 5207 2.683200e-02
## 12320  2 5208 2.670793e-02
## 12321  2 5209 2.656916e-02
## 12322  2 5210 2.635385e-02
## 12323  2 5211 2.620861e-02
## 12324  2 5212 2.604932e-02
## 12325  2 5213 2.596392e-02
## 12326  2 5214 2.576888e-02
## 12327  2 5215 2.563236e-02
## 12328  2 5216 2.544641e-02
## 12329  2 5217 2.532205e-02
## 12330  2 5218 2.525758e-02
## 12331  2 5219 2.506819e-02
## 12332  2 5220 2.488437e-02
## 12333  2 5221 2.474215e-02
## 12334  2 5222 2.465588e-02
## 12335  2 5223 2.447679e-02
## 12336  2 5224 2.432501e-02
## 12337  2 5225 2.421084e-02
## 12338  2 5226 2.406462e-02
## 12339  2 5227 2.392451e-02
## 12340  2 5228 2.377727e-02
## 12341  2 5229 2.367494e-02
## 12342  2 5230 2.350419e-02
## 12343  2 5231 2.340288e-02
## 12344  2 5232 2.325819e-02
## 12345  2 5233 2.315639e-02
## 12346  2 5234 2.306776e-02
## 12347  2 5235 2.286233e-02
## 12348  2 5236 2.276441e-02
## 12349  2 5237 2.259217e-02
## 12350  2 5238 2.246424e-02
## 12351  2 5239 2.236757e-02
## 12352  2 5240 2.222033e-02
## 12353  2 5241 2.209536e-02
## 12354  2 5242 2.196218e-02
## 12355  2 5243 2.180765e-02
## 12356  2 5244 2.169714e-02
## 12357  2 5245 2.157397e-02
## 12358  2 5246 2.146737e-02
## 12359  2 5247 2.137901e-02
## 12360  2 5248 2.120358e-02
## 12361  2 5249 2.112294e-02
## 12362  2 5250 2.101926e-02
## 12363  2 5251 2.089956e-02
## 12364  2 5252 2.076546e-02
## 12365  2 5253 2.064696e-02
## 12366  2 5254 2.051966e-02
## 12367  2 5255 2.040419e-02
## 12368  2 5256 2.027381e-02
## 12369  2 5257 2.016528e-02
## 12370  2 5258 2.003707e-02
## 12371  2 5259 1.993047e-02
## 12372  2 5260 1.982683e-02
## 12373  2 5261 1.976092e-02
## 12374  2 5262 1.959579e-02
## 12375  2 5263 1.948119e-02
## 12376  2 5264 1.937081e-02
## 12377  2 5265 1.925021e-02
## 12378  2 5266 1.915027e-02
## 12379  2 5267 1.905985e-02
## 12380  2 5268 1.893622e-02
## 12381  2 5269 1.882142e-02
## 12382  2 5270 1.873924e-02
## 12383  2 5271 1.861418e-02
## 12384  2 5272 1.849358e-02
## 12385  2 5273 1.842962e-02
## 12386  2 5274 1.832254e-02
## 12387  2 5275 1.821804e-02
## 12388  2 5276 1.808517e-02
## 12389  2 5277 1.798039e-02
## 12390  2 5278 1.790389e-02
## 12391  2 5279 1.778468e-02
## 12392  2 5280 1.769247e-02
## 12393  2 5281 1.760999e-02
## 12394  2 5282 1.749062e-02
## 12395  2 5283 1.738575e-02
## 12396  2 5284 1.732345e-02
## 12397  2 5285 1.720416e-02
## 12398  2 5286 1.708269e-02
## 12399  2 5287 1.701853e-02
## 12400  2 5288 1.688714e-02
## 12401  2 5289 1.678466e-02
## 12402  2 5290 1.670088e-02
## 12403  2 5291 1.663072e-02
## 12404  2 5292 1.653203e-02
## 12405  2 5293 1.642275e-02
## 12406  2 5294 1.631688e-02
## 12407  2 5295 1.622340e-02
## 12408  2 5296 1.614074e-02
## 12409  2 5297 1.604883e-02
## 12410  2 5298 1.595723e-02
## 12411  2 5299 1.589210e-02
## 12412  2 5300 1.578553e-02
## 12413  2 5301 1.566455e-02
## 12414  2 5302 1.558906e-02
## 12415  2 5303 1.549996e-02
## 12416  2 5304 1.542660e-02
## 12417  2 5305 1.529666e-02
## 12418  2 5306 1.526872e-02
## 12419  2 5307 1.518089e-02
## 12420  2 5308 1.504875e-02
## 12421  2 5309 1.498009e-02
## 12422  2 5310 1.490833e-02
## 12423  2 5311 1.483784e-02
## 12424  2 5312 1.472824e-02
## 12425  2 5313 1.464013e-02
## 12426  2 5314 1.454156e-02
## 12427  2 5315 1.449474e-02
## 12428  2 5316 1.439907e-02
## 12429  2 5317 1.435138e-02
## 12430  2 5318 1.424504e-02
## 12431  2 5319 1.416807e-02
## 12432  2 5320 1.409604e-02
## 12433  2 5321 1.399962e-02
## 12434  2 5322 1.393033e-02
## 12435  2 5323 1.383032e-02
## 12436  2 5324 1.377064e-02
## 12437  2 5325 1.368262e-02
## 12438  2 5326 1.360498e-02
## 12439  2 5327 1.354067e-02
## 12440  2 5328 1.345559e-02
## 12441  2 5329 1.335573e-02
## 12442  2 5330 1.329772e-02
## 12443  2 5331 1.321266e-02
## 12444  2 5332 1.316608e-02
## 12445  2 5333 1.306249e-02
## 12446  2 5334 1.302091e-02
## 12447  2 5335 1.292284e-02
## 12448  2 5336 1.283500e-02
## 12449  2 5337 1.277149e-02
## 12450  2 5338 1.265435e-02
## 12451  2 5339 1.263468e-02
## 12452  2 5340 1.255391e-02
## 12453  2 5341 1.249899e-02
## 12454  2 5342 1.241411e-02
## 12455  2 5343 1.234388e-02
## 12456  2 5344 1.227593e-02
## 12457  2 5345 1.218543e-02
## 12458  2 5346 1.214303e-02
## 12459  2 5347 1.208367e-02
## 12460  2 5348 1.199159e-02
## 12461  2 5349 1.194587e-02
## 12462  2 5350 1.186719e-02
## 12463  2 5351 1.180674e-02
## 12464  2 5352 1.171840e-02
## 12465  2 5353 1.167518e-02
## 12466  2 5354 1.159390e-02
## 12467  2 5355 1.152649e-02
## 12468  2 5356 1.144428e-02
## 12469  2 5357 1.139122e-02
## 12470  2 5358 1.133621e-02
## 12471  2 5359 1.125302e-02
## 12472  2 5360 1.119834e-02
## 12473  2 5361 1.114122e-02
## 12474  2 5362 1.109409e-02
## 12475  2 5363 1.101484e-02
## 12476  2 5364 1.097313e-02
## 12477  2 5365 1.089223e-02
## 12478  2 5366 1.082969e-02
## 12479  2 5367 1.076900e-02
## 12480  2 5368 1.071255e-02
## 12481  2 5369 1.063469e-02
## 12482  2 5370 1.058532e-02
## 12483  2 5371 1.050990e-02
## 12484  2 5372 1.046564e-02
## 12485  2 5373 1.040769e-02
## 12486  2 5374 1.035032e-02
## 12487  2 5375 1.028977e-02
## 12488  2 5376 1.022490e-02
## 12489  2 5377 1.016503e-02
## 12490  2 5378 1.011528e-02
## 12491  2 5379 1.005704e-02
## 12492  2 5380 1.001721e-02
## 12493  2 5381 9.943857e-03
## 12494  2 5382 9.869699e-03
## 12495  2 5383 9.825673e-03
## 12496  2 5384 9.783353e-03
## 12497  2 5385 9.709095e-03
## 12498  2 5386 9.658347e-03
## 12499  2 5387 9.595929e-03
## 12500  2 5388 9.555807e-03
## 12501  2 5389 9.490698e-03
## 12502  2 5390 9.439643e-03
## 12503  2 5391 9.392363e-03
## 12504  2 5392 9.340684e-03
## 12505  2 5393 9.277721e-03
## 12506  2 5394 9.236003e-03
## 12507  2 5395 9.163167e-03
## 12508  2 5396 9.124686e-03
## 12509  2 5397 9.068232e-03
## 12510  2 5398 9.018654e-03
## 12511  2 5399 8.977229e-03
## 12512  2 5400 8.916454e-03
## 12513  2 5401 8.868928e-03
## 12514  2 5402 8.810121e-03
## 12515  2 5403 8.775965e-03
## 12516  2 5404 8.711846e-03
## 12517  2 5405 8.649696e-03
## 12518  2 5406 8.600408e-03
## 12519  2 5407 8.570825e-03
## 12520  2 5408 8.508230e-03
## 12521  2 5409 8.470962e-03
## 12522  2 5410 8.421590e-03
## 12523  2 5411 8.377919e-03
## 12524  2 5412 8.345145e-03
## 12525  2 5413 8.286847e-03
## 12526  2 5414 8.235632e-03
## 12527  2 5415 8.187321e-03
## 12528  2 5416 8.136347e-03
## 12529  2 5417 8.097850e-03
## 12530  2 5418 8.037731e-03
## 12531  2 5419 8.011279e-03
## 12532  2 5420 7.968663e-03
## 12533  2 5421 7.924514e-03
## 12534  2 5422 7.871546e-03
## 12535  2 5423 7.817240e-03
## 12536  2 5424 7.765208e-03
## 12537  2 5425 7.725216e-03
## 12538  2 5426 7.688141e-03
## 12539  2 5427 7.653891e-03
## 12540  2 5428 7.610157e-03
## 12541  2 5429 7.564421e-03
## 12542  2 5430 7.515733e-03
## 12543  2 5431 7.454277e-03
## 12544  2 5432 7.426098e-03
## 12545  2 5433 7.381890e-03
## 12546  2 5434 7.350771e-03
## 12547  2 5435 7.309096e-03
## 12548  2 5436 7.276872e-03
## 12549  2 5437 7.220297e-03
## 12550  2 5438 7.169476e-03
## 12551  2 5439 7.137207e-03
## 12552  2 5440 7.084689e-03
## 12553  2 5441 7.060833e-03
## 12554  2 5442 7.024155e-03
## 12555  2 5443 6.976832e-03
## 12556  2 5444 6.933518e-03
## 12557  2 5445 6.898969e-03
## 12558  2 5446 6.867840e-03
## 12559  2 5447 6.818163e-03
## 12560  2 5448 6.792113e-03
## 12561  2 5449 6.735394e-03
## 12562  2 5450 6.703117e-03
## 12563  2 5451 6.666459e-03
## 12564  2 5452 6.624335e-03
## 12565  2 5453 6.595588e-03
## 12566  2 5454 6.545307e-03
## 12567  2 5455 6.512635e-03
## 12568  2 5456 6.486125e-03
## 12569  2 5457 6.440595e-03
## 12570  2 5458 6.392255e-03
## 12571  2 5459 6.361797e-03
## 12572  2 5460 6.340536e-03
## 12573  2 5461 6.303438e-03
## 12574  2 5462 6.254226e-03
## 12575  2 5463 6.223451e-03
## 12576  2 5464 6.191367e-03
## 12577  2 5465 6.152779e-03
## 12578  2 5466 6.120816e-03
## 12579  2 5467 6.084745e-03
## 12580  2 5468 6.051124e-03
## 12581  2 5469 6.014706e-03
## 12582  2 5470 5.983576e-03
## 12583  2 5471 5.942141e-03
## 12584  2 5472 5.912833e-03
## 12585  2 5473 5.880965e-03
## 12586  2 5474 5.847016e-03
## 12587  2 5475 5.806037e-03
## 12588  2 5476 5.789200e-03
## 12589  2 5477 5.740800e-03
## 12590  2 5478 5.715004e-03
## 12591  2 5479 5.682829e-03
## 12592  2 5480 5.646826e-03
## 12593  2 5481 5.621699e-03
## 12594  2 5482 5.584891e-03
## 12595  2 5483 5.560564e-03
## 12596  2 5484 5.521490e-03
## 12597  2 5485 5.488334e-03
## 12598  2 5486 5.458902e-03
## 12599  2 5487 5.416742e-03
## 12600  2 5488 5.401375e-03
## 12601  2 5489 5.367292e-03
## 12602  2 5490 5.336189e-03
## 12603  2 5491 5.302379e-03
## 12604  2 5492 5.281038e-03
## 12605  2 5493 5.250525e-03
## 12606  2 5494 5.213901e-03
## 12607  2 5495 5.182739e-03
## 12608  2 5496 5.162874e-03
## 12609  2 5497 5.121274e-03
## 12610  2 5498 5.098131e-03
## 12611  2 5499 5.069275e-03
## 12612  2 5500 5.043855e-03
## 12613  2 5501 5.016737e-03
## 12614  2 5502 4.989185e-03
## 12615  2 5503 4.958530e-03
## 12616  2 5504 4.927993e-03
## 12617  2 5505 4.894258e-03
## 12618  2 5506 4.868781e-03
## 12619  2 5507 4.849325e-03
## 12620  2 5508 4.809306e-03
## 12621  2 5509 4.781998e-03
## 12622  2 5510 4.756815e-03
## 12623  2 5511 4.735160e-03
## 12624  2 5512 4.705273e-03
## 12625  2 5513 4.680151e-03
## 12626  2 5514 4.660418e-03
## 12627  2 5515 4.628024e-03
## 12628  2 5516 4.606075e-03
## 12629  2 5517 4.566453e-03
## 12630  2 5518 4.539953e-03
## 12631  2 5519 4.519856e-03
## 12632  2 5520 4.504901e-03
## 12633  2 5521 4.467156e-03
## 12634  2 5522 4.451268e-03
## 12635  2 5523 4.421122e-03
## 12636  2 5524 4.400530e-03
## 12637  2 5525 4.372811e-03
## 12638  2 5526 4.352154e-03
## 12639  2 5527 4.320790e-03
## 12640  2 5528 4.293665e-03
## 12641  2 5529 4.267083e-03
## 12642  2 5530 4.250455e-03
## 12643  2 5531 4.225736e-03
## 12644  2 5532 4.200733e-03
## 12645  2 5533 4.169367e-03
## 12646  2 5534 4.154702e-03
## 12647  2 5535 4.129040e-03
## 12648  2 5536 4.100719e-03
## 12649  2 5537 4.079614e-03
## 12650  2 5538 4.057214e-03
## 12651  2 5539 4.036455e-03
## 12652  2 5540 4.006447e-03
## 12653  2 5541 3.986008e-03
## 12654  2 5542 3.969553e-03
## 12655  2 5543 3.941489e-03
## 12656  2 5544 3.924973e-03
## 12657  2 5545 3.900569e-03
## 12658  2 5546 3.872656e-03
## 12659  2 5547 3.858431e-03
## 12660  2 5548 3.834369e-03
## 12661  2 5549 3.809596e-03
## 12662  2 5550 3.783111e-03
## 12663  2 5551 3.767480e-03
## 12664  2 5552 3.747724e-03
## 12665  2 5553 3.717916e-03
## 12666  2 5554 3.700759e-03
## 12667  2 5555 3.683622e-03
## 12668  2 5556 3.664693e-03
## 12669  2 5557 3.636521e-03
## 12670  2 5558 3.618061e-03
## 12671  2 5559 3.596293e-03
## 12672  2 5560 3.581546e-03
## 12673  2 5561 3.556737e-03
```

```
## 12674  2 5562 3.532860e-03
## 12675  2 5563 3.513827e-03
## 12676  2 5564 3.493508e-03
## 12677  2 5565 3.481295e-03
## 12678  2 5566 3.454689e-03
## 12679  2 5567 3.436605e-03
## 12680  2 5568 3.414719e-03
## 12681  2 5569 3.396959e-03
## 12682  2 5570 3.379168e-03
## 12683  2 5571 3.362272e-03
## 12684  2 5572 3.340066e-03
## 12685  2 5573 3.322710e-03
## 12686  2 5574 3.304478e-03
## 12687  2 5575 3.282720e-03
## 12688  2 5576 3.263186e-03
## 12689  2 5577 3.245121e-03
## 12690  2 5578 3.227608e-03
## 12691  2 5579 3.211449e-03
## 12692  2 5580 3.192805e-03
## 12693  2 5581 3.174449e-03
## 12694  2 5582 3.157227e-03
## 12695  2 5583 3.143842e-03
## 12696  2 5584 3.120953e-03
## 12697  2 5585 3.104860e-03
## 12698  2 5586 3.081752e-03
## 12699  2 5587 3.067166e-03
## 12700  2 5588 3.050854e-03
## 12701  2 5589 3.032593e-03
## 12702  2 5590 3.015359e-03
## 12703  2 5591 2.999302e-03
## 12704  2 5592 2.977090e-03
## 12705  2 5593 2.958486e-03
## 12706  2 5594 2.949215e-03
## 12707  2 5595 2.933743e-03
## 12708  2 5596 2.914243e-03
## 12709  2 5597 2.896691e-03
## 12710  2 5598 2.881626e-03
## 12711  2 5599 2.864730e-03
## 12712  2 5600 2.850770e-03
## 12713  2 5601 2.837862e-03
## 12714  2 5602 2.814866e-03
## 12715  2 5603 2.801239e-03
## 12716  2 5604 2.782473e-03
## 12717  2 5605 2.764335e-03
## 12718  2 5606 2.749311e-03
## 12719  2 5607 2.738346e-03
## 12720  2 5608 2.718132e-03
## 12721  2 5609 2.705589e-03
## 12722  2 5610 2.690731e-03
## 12723  2 5611 2.676710e-03
## 12724  2 5612 2.657072e-03
## 12725  2 5613 2.643661e-03
## 12726  2 5614 2.630090e-03
## 12727  2 5615 2.613510e-03
## 12728  2 5616 2.596169e-03
## 12729  2 5617 2.580605e-03
## 12730  2 5618 2.567636e-03
## 12731  2 5619 2.559844e-03
## 12732  2 5620 2.540321e-03
## 12733  2 5621 2.528487e-03
## 12734  2 5622 2.512587e-03
## 12735  2 5623 2.498231e-03
## 12736  2 5624 2.484039e-03
## 12737  2 5625 2.469082e-03
## 12738  2 5626 2.458796e-03
## 12739  2 5627 2.438625e-03
## 12740  2 5628 2.426131e-03
## 12741  2 5629 2.412430e-03
## 12742  2 5630 2.400810e-03
## 12743  2 5631 2.384543e-03
## 12744  2 5632 2.374387e-03
## 12745  2 5633 2.360207e-03
## 12746  2 5634 2.344394e-03
## 12747  2 5635 2.330761e-03
## 12748  2 5636 2.322766e-03
## 12749  2 5637 2.304091e-03
## 12750  2 5638 2.296523e-03
## 12751  2 5639 2.281136e-03
## 12752  2 5640 2.265862e-03
## 12753  2 5641 2.258794e-03
## 12754  2 5642 2.239879e-03
## 12755  2 5643 2.230724e-03
## 12756  2 5644 2.214298e-03
## 12757  2 5645 2.202235e-03
## 12758  2 5646 2.188690e-03
## 12759  2 5647 2.178363e-03
## 12760  2 5648 2.165580e-03
## 12761  2 5649 2.153270e-03
## 12762  2 5650 2.141538e-03
## 12763  2 5651 2.125895e-03
## 12764  2 5652 2.118735e-03
## 12765  2 5653 2.106954e-03
## 12766  2 5654 2.093301e-03
## 12767  2 5655 2.080256e-03
## 12768  2 5656 2.066155e-03
## 12769  2 5657 2.057621e-03
## 12770  2 5658 2.047722e-03
## 12771  2 5659 2.034990e-03
## 12772  2 5660 2.023230e-03
## 12773  2 5661 2.009301e-03
## 12774  2 5662 2.002947e-03
## 12775  2 5663 1.987085e-03
## 12776  2 5664 1.975828e-03
## 12777  2 5665 1.965200e-03
## 12778  2 5666 1.954884e-03
## 12779  2 5667 1.943201e-03
## 12780  2 5668 1.932580e-03
## 12781  2 5669 1.922469e-03
## 12782  2 5670 1.910325e-03
## 12783  2 5671 1.903229e-03
## 12784  2 5672 1.887656e-03
## 12785  2 5673 1.877514e-03
## 12786  2 5674 1.866262e-03
## 12787  2 5675 1.855350e-03
## 12788  2 5676 1.844326e-03
## 12789  2 5677 1.835542e-03
## 12790  2 5678 1.825194e-03
## 12791  2 5679 1.813564e-03
## 12792  2 5680 1.802796e-03
## 12793  2 5681 1.791675e-03
## 12794  2 5682 1.783365e-03
## 12795  2 5683 1.772813e-03
## 12796  2 5684 1.760979e-03
## 12797  2 5685 1.754508e-03
## 12798  2 5686 1.745133e-03
## 12799  2 5687 1.733348e-03
## 12800  2 5688 1.722947e-03
## 12801  2 5689 1.714794e-03
## 12802  2 5690 1.703005e-03
## 12803  2 5691 1.690900e-03
## 12804  2 5692 1.687073e-03
## 12805  2 5693 1.676243e-03
## 12806  2 5694 1.665915e-03
## 12807  2 5695 1.655176e-03
## 12808  2 5696 1.647978e-03
## 12809  2 5697 1.636461e-03
## 12810  2 5698 1.627131e-03
## 12811  2 5699 1.619446e-03
## 12812  2 5700 1.610615e-03
## 12813  2 5701 1.600897e-03
## 12814  2 5702 1.595103e-03
## 12815  2 5703 1.583438e-03
## 12816  2 5704 1.573669e-03
## 12817  2 5705 1.564142e-03
## 12818  2 5706 1.555878e-03
## 12819  2 5707 1.545244e-03
## 12820  2 5708 1.537828e-03
## 12821  2 5709 1.527939e-03
## 12822  2 5710 1.519834e-03
## 12823  2 5711 1.512355e-03
## 12824  2 5712 1.502372e-03
## 12825  2 5713 1.495475e-03
## 12826  2 5714 1.488806e-03
## 12827  2 5715 1.478701e-03
## 12828  2 5716 1.470339e-03
## 12829  2 5717 1.458329e-03
## 12830  2 5718 1.454096e-03
## 12831  2 5719 1.442384e-03
## 12832  2 5720 1.437606e-03
## 12833  2 5721 1.427390e-03
## 12834  2 5722 1.423843e-03
## 12835  2 5723 1.410956e-03
## 12836  2 5724 1.404468e-03
## 12837  2 5725 1.394299e-03
## 12838  2 5726 1.386581e-03
## 12839  2 5727 1.380070e-03
## 12840  2 5728 1.373984e-03
## 12841  2 5729 1.362804e-03
## 12842  2 5730 1.356624e-03
## 12843  2 5731 1.349259e-03
## 12844  2 5732 1.342286e-03
## 12845  2 5733 1.334247e-03
## 12846  2 5734 1.326469e-03
## 12847  2 5735 1.319664e-03
## 12848  2 5736 1.311382e-03
## 12849  2 5737 1.302909e-03
## 12850  2 5738 1.295578e-03
## 12851  2 5739 1.289182e-03
## 12852  2 5740 1.281215e-03
## 12853  2 5741 1.273452e-03
## 12854  2 5742 1.267346e-03
## 12855  2 5743 1.260379e-03
## 12856  2 5744 1.251395e-03
## 12857  2 5745 1.245500e-03
## 12858  2 5746 1.239296e-03
## 12859  2 5747 1.230507e-03
## 12860  2 5748 1.224891e-03
## 12861  2 5749 1.218836e-03
## 12862  2 5750 1.210109e-03
## 12863  2 5751 1.202136e-03
## 12864  2 5752 1.196866e-03
## 12865  2 5753 1.188389e-03
## 12866  2 5754 1.183531e-03
## 12867  2 5755 1.176940e-03
## 12868  2 5756 1.169725e-03
## 12869  2 5757 1.162695e-03
## 12870  2 5758 1.154498e-03
## 12871  2 5759 1.150272e-03
## 12872  2 5760 1.143831e-03
## 12873  2 5761 1.137435e-03
## 12874  2 5762 1.130696e-03
## 12875  2 5763 1.123441e-03
## 12876  2 5764 1.118628e-03
## 12877  2 5765 1.113324e-03
## 12878  2 5766 1.104556e-03
## 12879  2 5767 1.098947e-03
## 12880  2 5768 1.091653e-03
## 12881  2 5769 1.085852e-03
## 12882  2 5770 1.080146e-03
## 12883  2 5771 1.073389e-03
## 12884  2 5772 1.068974e-03
## 12885  2 5773 1.061808e-03
## 12886  2 5774 1.053546e-03
## 12887  2 5775 1.049288e-03
## 12888  2 5776 1.044995e-03
## 12889  2 5777 1.038693e-03
## 12890  2 5778 1.032611e-03
## 12891  2 5779 1.027489e-03
## 12892  2 5780 1.019070e-03
## 12893  2 5781 1.014072e-03
## 12894  2 5782 1.009289e-03
## 12895  2 5783 1.001653e-03
## 12896  2 5784 9.982846e-04
## 12897  2 5785 9.915313e-04
## 12898  2 5786 9.844057e-04
## 12899  2 5787 9.798627e-04
## 12900  2 5788 9.741788e-04
## 12901  2 5789 9.693693e-04
## 12902  2 5790 9.648025e-04
## 12903  2 5791 9.566198e-04
## 12904  2 5792 9.524081e-04
## 12905  2 5793 9.464218e-04
## 12906  2 5794 9.416272e-04
## 12907  2 5795 9.370592e-04
## 12908  2 5796 9.294897e-04
## 12909  2 5797 9.251634e-04
## 12910  2 5798 9.206622e-04
## 12911  2 5799 9.137373e-04
## 12912  2 5800 9.096359e-04
## 12913  2 5801 9.052437e-04
## 12914  2 5802 8.995635e-04
## 12915  2 5803 8.946321e-04
## 12916  2 5804 8.895101e-04
## 12917  2 5805 8.845677e-04
## 12918  2 5806 8.782960e-04
## 12919  2 5807 8.724631e-04
## 12920  2 5808 8.705749e-04
## 12921  2 5809 8.642604e-04
## 12922  2 5810 8.582768e-04
## 12923  2 5811 8.544884e-04
## 12924  2 5812 8.486279e-04
## 12925  2 5813 8.461238e-04
## 12926  2 5814 8.392522e-04
## 12927  2 5815 8.350885e-04
## 12928  2 5816 8.306373e-04
## 12929  2 5817 8.235193e-04
## 12930  2 5818 8.222439e-04
## 12931  2 5819 8.170993e-04
## 12932  2 5820 8.128884e-04
## 12933  2 5821 8.075552e-04
## 12934  2 5822 8.013052e-04
## 12935  2 5823 7.972244e-04
## 12936  2 5824 7.944166e-04
## 12937  2 5825 7.891887e-04
## 12938  2 5826 7.857380e-04
## 12939  2 5827 7.808738e-04
## 12940  2 5828 7.749889e-04
## 12941  2 5829 7.701902e-04
## 12942  2 5830 7.669556e-04
## 12943  2 5831 7.615638e-04
## 12944  2 5832 7.587157e-04
## 12945  2 5833 7.534675e-04
## 12946  2 5834 7.510692e-04
## 12947  2 5835 7.453006e-04
## 12948  2 5836 7.403613e-04
## 12949  2 5837 7.382854e-04
## 12950  2 5838 7.319020e-04
## 12951  2 5839 7.284815e-04
## 12952  2 5840 7.234340e-04
## 12953  2 5841 7.198950e-04
## 12954  2 5842 7.164541e-04
## 12955  2 5843 7.129244e-04
## 12956  2 5844 7.068331e-04
## 12957  2 5845 7.028845e-04
## 12958  2 5846 7.002378e-04
## 12959  2 5847 6.962618e-04
## 12960  2 5848 6.920787e-04
## 12961  2 5849 6.886249e-04
## 12962  2 5850 6.831245e-04
## 12963  2 5851 6.810739e-04
## 12964  2 5852 6.767354e-04
## 12965  2 5853 6.727461e-04
## 12966  2 5854 6.691670e-04
## 12967  2 5855 6.643679e-04
## 12968  2 5856 6.601467e-04
## 12969  2 5857 6.566956e-04
## 12970  2 5858 6.521570e-04
## 12971  2 5859 6.482370e-04
## 12972  2 5860 6.460382e-04
## 12973  2 5861 6.432455e-04
## 12974  2 5862 6.388290e-04
## 12975  2 5863 6.354931e-04
## 12976  2 5864 6.322665e-04
## 12977  2 5865 6.280435e-04
## 12978  2 5866 6.234878e-04
## 12979  2 5867 6.211730e-04
## 12980  2 5868 6.163596e-04
## 12981  2 5869 6.132988e-04
## 12982  2 5870 6.105524e-04
## 12983  2 5871 6.075213e-04
## 12984  2 5872 6.034544e-04
## 12985  2 5873 6.002194e-04
## 12986  2 5874 5.963979e-04
## 12987  2 5875 5.927579e-04
## 12988  2 5876 5.900336e-04
## 12989  2 5877 5.873698e-04
## 12990  2 5878 5.836234e-04
## 12991  2 5879 5.802444e-04
## 12992  2 5880 5.763935e-04
## 12993  2 5881 5.737125e-04
## 12994  2 5882 5.708364e-04
## 12995  2 5883 5.675593e-04
## 12996  2 5884 5.623002e-04
## 12997  2 5885 5.601228e-04
## 12998  2 5886 5.565129e-04
## 12999  2 5887 5.542188e-04
## 13000  2 5888 5.501575e-04
## 13001  2 5889 5.478740e-04
## 13002  2 5890 5.446327e-04
## 13003  2 5891 5.414243e-04
## 13004  2 5892 5.381770e-04
## 13005  2 5893 5.357817e-04
## 13006  2 5894 5.324142e-04
## 13007  2 5895 5.291506e-04
## 13008  2 5896 5.257636e-04
## 13009  2 5897 5.239647e-04
## 13010  2 5898 5.208829e-04
## 13011  2 5899 5.186763e-04
## 13012  2 5900 5.140399e-04
## 13013  2 5901 5.110831e-04
## 13014  2 5902 5.080546e-04
## 13015  2 5903 5.056467e-04
## 13016  2 5904 5.023319e-04
## 13017  2 5905 4.996616e-04
## 13018  2 5906 4.966246e-04
## 13019  2 5907 4.947194e-04
## 13020  2 5908 4.921518e-04
## 13021  2 5909 4.884417e-04
## 13022  2 5910 4.860972e-04
## 13023  2 5911 4.833028e-04
## 13024  2 5912 4.802047e-04
## 13025  2 5913 4.776684e-04
## 13026  2 5914 4.747081e-04
## 13027  2 5915 4.710633e-04
## 13028  2 5916 4.703615e-04
## 13029  2 5917 4.669535e-04
## 13030  2 5918 4.641366e-04
## 13031  2 5919 4.616318e-04
## 13032  2 5920 4.584465e-04
## 13033  2 5921 4.558004e-04
## 13034  2 5922 4.527402e-04
## 13035  2 5923 4.514783e-04
## 13036  2 5924 4.489332e-04
## 13037  2 5925 4.454969e-04
## 13038  2 5926 4.427535e-04
## 13039  2 5927 4.409947e-04
## 13040  2 5928 4.390123e-04
## 13041  2 5929 4.359988e-04
## 13042  2 5930 4.326767e-04
## 13043  2 5931 4.308968e-04
## 13044  2 5932 4.282676e-04
## 13045  2 5933 4.259756e-04
## 13046  2 5934 4.236368e-04
## 13047  2 5935 4.210660e-04
## 13048  2 5936 4.184257e-04
## 13049  2 5937 4.164398e-04
## 13050  2 5938 4.137120e-04
## 13051  2 5939 4.114532e-04
## 13052  2 5940 4.094125e-04
## 13053  2 5941 4.064541e-04
## 13054  2 5942 4.051143e-04
## 13055  2 5943 4.020727e-04
## 13056  2 5944 4.000915e-04
## 13057  2 5945 3.980653e-04
## 13058  2 5946 3.950412e-04
## 13059  2 5947 3.935169e-04
## 13060  2 5948 3.907382e-04
## 13061  2 5949 3.891609e-04
## 13062  2 5950 3.865043e-04
## 13063  2 5951 3.845897e-04
## 13064  2 5952 3.821875e-04
## 13065  2 5953 3.799481e-04
## 13066  2 5954 3.778515e-04
## 13067  2 5955 3.763505e-04
## 13068  2 5956 3.734928e-04
## 13069  2 5957 3.717020e-04
## 13070  2 5958 3.697530e-04
## 13071  2 5959 3.667188e-04
## 13072  2 5960 3.647166e-04
## 13073  2 5961 3.634077e-04
## 13074  2 5962 3.612683e-04
## 13075  2 5963 3.586576e-04
## 13076  2 5964 3.567490e-04
## 13077  2 5965 3.550499e-04
## 13078  2 5966 3.532012e-04
## 13079  2 5967 3.507319e-04
## 13080  2 5968 3.493015e-04
## 13081  2 5969 3.456348e-04
## 13082  2 5970 3.452165e-04
## 13083  2 5971 3.429632e-04
## 13084  2 5972 3.412761e-04
## 13085  2 5973 3.393861e-04
## 13086  2 5974 3.377676e-04
## 13087  2 5975 3.355813e-04
## 13088  2 5976 3.331618e-04
## 13089  2 5977 3.315861e-04
## 13090  2 5978 3.293129e-04
## 13091  2 5979 3.275600e-04
## 13092  2 5980 3.259775e-04
## 13093  2 5981 3.239006e-04
## 13094  2 5982 3.219801e-04
## 13095  2 5983 3.197719e-04
## 13096  2 5984 3.185575e-04
## 13097  2 5985 3.165306e-04
## 13098  2 5986 3.147955e-04
## 13099  2 5987 3.129027e-04
## 13100  2 5988 3.110597e-04
## 13101  2 5989 3.090331e-04
## 13102  2 5990 3.074946e-04
## 13103  2 5991 3.063415e-04
## 13104  2 5992 3.042979e-04
## 13105  2 5993 3.022954e-04
## 13106  2 5994 3.003844e-04
## 13107  2 5995 2.986889e-04
## 13108  2 5996 2.974440e-04
## 13109  2 5997 2.954114e-04
## 13110  2 5998 2.939673e-04
## 13111  2 5999 2.923706e-04
## 13112  2 6000 2.908871e-04
## 13113  2 6001 2.888873e-04
## 13114  2 6002 2.873394e-04
## 13115  2 6003 2.853937e-04
## 13116  2 6004 2.840406e-04
## 13117  2 6005 2.826260e-04
## 13118  2 6006 2.802039e-04
## 13119  2 6007 2.790767e-04
## 13120  2 6008 2.775126e-04
## 13121  2 6009 2.762229e-04
## 13122  2 6010 2.747615e-04
## 13123  2 6011 2.731913e-04
## 13124  2 6012 2.716270e-04
## 13125  2 6013 2.700984e-04
## 13126  2 6014 2.683420e-04
## 13127  2 6015 2.664187e-04
## 13128  2 6016 2.657352e-04
## 13129  2 6017 2.639670e-04
## 13130  2 6018 2.625947e-04
## 13131  2 6019 2.610338e-04
## 13132  2 6020 2.590157e-04
## 13133  2 6021 2.578622e-04
## 13134  2 6022 2.556397e-04
## 13135  2 6023 2.549021e-04
## 13136  2 6024 2.537661e-04
## 13137  2 6025 2.514991e-04
## 13138  2 6026 2.506144e-04
## 13139  2 6027 2.490767e-04
## 13140  2 6028 2.472766e-04
## 13141  2 6029 2.467166e-04
## 13142  2 6030 2.446023e-04
## 13143  2 6031 2.433394e-04
## 13144  2 6032 2.423286e-04
## 13145  2 6033 2.400957e-04
## 13146  2 6034 2.397261e-04
## 13147  2 6035 2.377487e-04
## 13148  2 6036 2.365773e-04
## 13149  2 6037 2.354918e-04
## 13150  2 6038 2.340941e-04
## 13151  2 6039 2.328882e-04
## 13152  2 6040 2.316401e-04
## 13153  2 6041 2.296003e-04
## 13154  2 6042 2.288873e-04
## 13155  2 6043 2.271849e-04
## 13156  2 6044 2.260604e-04
## 13157  2 6045 2.251657e-04
## 13158  2 6046 2.231266e-04
## 13159  2 6047 2.224048e-04
## 13160  2 6048 2.206916e-04
## 13161  2 6049 2.197810e-04
## 13162  2 6050 2.188604e-04
## 13163  2 6051 2.168006e-04
## 13164  2 6052 2.162373e-04
## 13165  2 6053 2.150034e-04
## 13166  2 6054 2.134483e-04
## 13167  2 6055 2.123069e-04
## 13168  2 6056 2.112315e-04
## 13169  2 6057 2.097750e-04
## 13170  2 6058 2.087734e-04
## 13171  2 6059 2.075719e-04
## 13172  2 6060 2.062562e-04
## 13173  2 6061 2.051155e-04
## 13174  2 6062 2.039627e-04
## 13175  2 6063 2.028234e-04
## 13176  2 6064 2.014422e-04
## 13177  2 6065 2.004250e-04
## 13178  2 6066 1.995467e-04
## 13179  2 6067 1.982206e-04
## 13180  2 6068 1.973595e-04
## 13181  2 6069 1.964166e-04
## 13182  2 6070 1.952513e-04
## 13183  2 6071 1.939165e-04
## 13184  2 6072 1.925527e-04
## 13185  2 6073 1.914545e-04
## 13186  2 6074 1.905207e-04
## 13187  2 6075 1.894611e-04
## 13188  2 6076 1.885334e-04
## 13189  2 6077 1.869006e-04
## 13190  2 6078 1.863084e-04
## 13191  2 6079 1.850155e-04
## 13192  2 6080 1.843235e-04
## 13193  2 6081 1.833958e-04
## 13194  2 6082 1.818259e-04
## 13195  2 6083 1.807514e-04
## 13196  2 6084 1.799966e-04
## 13197  2 6085 1.791373e-04
## 13198  2 6086 1.780382e-04
## 13199  2 6087 1.769701e-04
## 13200  2 6088 1.761109e-04
## 13201  2 6089 1.751691e-04
## 13202  2 6090 1.738718e-04
## 13203  2 6091 1.729473e-04
## 13204  2 6092 1.719649e-04
## 13205  2 6093 1.710343e-04
## 13206  2 6094 1.698282e-04
## 13207  2 6095 1.692000e-04
## 13208  2 6096 1.683160e-04
## 13209  2 6097 1.673028e-04
## 13210  2 6098 1.658530e-04
## 13211  2 6099 1.650818e-04
## 13212  2 6100 1.642832e-04
## 13213  2 6101 1.635124e-04
## 13214  2 6102 1.624271e-04
## 13215  2 6103 1.613346e-04
## 13216  2 6104 1.604698e-04
## 13217  2 6105 1.596767e-04
## 13218  2 6106 1.588468e-04
## 13219  2 6107 1.578402e-04
## 13220  2 6108 1.567560e-04
## 13221  2 6109 1.560809e-04
## 13222  2 6110 1.550029e-04
## 13223  2 6111 1.543909e-04
## 13224  2 6112 1.531826e-04
## 13225  2 6113 1.524554e-04
## 13226  2 6114 1.515877e-04
## 13227  2 6115 1.508110e-04
## 13228  2 6116 1.500246e-04
## 13229  2 6117 1.491550e-04
## 13230  2 6118 1.481177e-04
## 13231  2 6119 1.473962e-04
## 13232  2 6120 1.463972e-04
## 13233  2 6121 1.458943e-04
## 13234  2 6122 1.448022e-04
## 13235  2 6123 1.443414e-04
## 13236  2 6124 1.431495e-04
## 13237  2 6125 1.423790e-04
## 13238  2 6126 1.416145e-04
## 13239  2 6127 1.407309e-04
## 13240  2 6128 1.401679e-04
## 13241  2 6129 1.390993e-04
## 13242  2 6130 1.381902e-04
## 13243  2 6131 1.377633e-04
## 13244  2 6132 1.369350e-04
## 13245  2 6133 1.359259e-04
## 13246  2 6134 1.355578e-04
## 13247  2 6135 1.346298e-04
## 13248  2 6136 1.337796e-04
## 13249  2 6137 1.330035e-04
## 13250  2 6138 1.323367e-04
## 13251  2 6139 1.313935e-04
## 13252  2 6140 1.308647e-04
## 13253  2 6141 1.300618e-04
## 13254  2 6142 1.292155e-04
## 13255  2 6143 1.285712e-04
## 13256  2 6144 1.278286e-04
## 13257  2 6145 1.271944e-04
## 13258  2 6146 1.263538e-04
## 13259  2 6147 1.256918e-04
## 13260  2 6148 1.250167e-04
## 13261  2 6149 1.244430e-04
## 13262  2 6150 1.233413e-04
## 13263  2 6151 1.226143e-04
## 13264  2 6152 1.222517e-04
## 13265  2 6153 1.213739e-04
## 13266  2 6154 1.206150e-04
## 13267  2 6155 1.201692e-04
## 13268  2 6156 1.193362e-04
## 13269  2 6157 1.186598e-04
## 13270  2 6158 1.179196e-04
## 13271  2 6159 1.174756e-04
## 13272  2 6160 1.166328e-04
## 13273  2 6161 1.161381e-04
## 13274  2 6162 1.152331e-04
## 13275  2 6163 1.147156e-04
## 13276  2 6164 1.139440e-04
## 13277  2 6165 1.132966e-04
## 13278  2 6166 1.128121e-04
## 13279  2 6167 1.118674e-04
## 13280  2 6168 1.114251e-04
## 13281  2 6169 1.107818e-04
## 13282  2 6170 1.102190e-04
## 13283  2 6171 1.095495e-04
## 13284  2 6172 1.090257e-04
## 13285  2 6173 1.084808e-04
## 13286  2 6174 1.076579e-04
## 13287  2 6175 1.071929e-04
## 13288  2 6176 1.064244e-04
## 13289  2 6177 1.060376e-04
## 13290  2 6178 1.052866e-04
## 13291  2 6179 1.047978e-04
## 13292  2 6180 1.041055e-04
## 13293  2 6181 1.035073e-04
## 13294  2 6182 1.029213e-04
## 13295  2 6183 1.021087e-04
## 13296  2 6184 1.016672e-04
## 13297  2 6185 1.011627e-04
## 13298  2 6186 1.005642e-04
## 13299  2 6187 1.001246e-04
## 13300  2 6188 9.951488e-05
## 13301  2 6189 9.854951e-05
## 13302  2 6190 9.821798e-05
## 13303  2 6191 9.766296e-05
## 13304  2 6192 9.706991e-05
## 13305  2 6193 9.658537e-05
## 13306  2 6194 9.616151e-05
## 13307  2 6195 9.559402e-05
## 13308  2 6196 9.498031e-05
## 13309  2 6197 9.445593e-05
## 13310  2 6198 9.382526e-05
## 13311  2 6199 9.346851e-05
## 13312  2 6200 9.285132e-05
## 13313  2 6201 9.227109e-05
## 13314  2 6202 9.178281e-05
## 13315  2 6203 9.125976e-05
## 13316  2 6204 9.062837e-05
## 13317  2 6205 9.030170e-05
## 13318  2 6206 8.976549e-05
## 13319  2 6207 8.930304e-05
## 13320  2 6208 8.853660e-05
## 13321  2 6209 8.820282e-05
## 13322  2 6210 8.759420e-05
## 13323  2 6211 8.704436e-05
## 13324  2 6212 8.659425e-05
## 13325  2 6213 8.617315e-05
## 13326  2 6214 8.568938e-05
## 13327  2 6215 8.519329e-05
## 13328  2 6216 8.478983e-05
## 13329  2 6217 8.420062e-05
## 13330  2 6218 8.370758e-05
## 13331  2 6219 8.317285e-05
## 13332  2 6220 8.280323e-05
## 13333  2 6221 8.240516e-05
## 13334  2 6222 8.183516e-05
## 13335  2 6223 8.141960e-05
## 13336  2 6224 8.100051e-05
## 13337  2 6225 8.050086e-05
## 13338  2 6226 7.995422e-05
## 13339  2 6227 7.962230e-05
## 13340  2 6228 7.913482e-05
## 13341  2 6229 7.861317e-05
## 13342  2 6230 7.834150e-05
## 13343  2 6231 7.771882e-05
## 13344  2 6232 7.725120e-05
## 13345  2 6233 7.680883e-05
## 13346  2 6234 7.641723e-05
## 13347  2 6235 7.604867e-05
## 13348  2 6236 7.558721e-05
## 13349  2 6237 7.509878e-05
## 13350  2 6238 7.478362e-05
## 13351  2 6239 7.439473e-05
## 13352  2 6240 7.389700e-05
## 13353  2 6241 7.351304e-05
## 13354  2 6242 7.302012e-05
## 13355  2 6243 7.256604e-05
## 13356  2 6244 7.220773e-05
## 13357  2 6245 7.186253e-05
## 13358  2 6246 7.131568e-05
## 13359  2 6247 7.101984e-05
## 13360  2 6248 7.054780e-05
## 13361  2 6249 7.033756e-05
## 13362  2 6250 6.981269e-05
## 13363  2 6251 6.948696e-05
## 13364  2 6252 6.905714e-05
## 13365  2 6253 6.857285e-05
## 13366  2 6254 6.827880e-05
## 13367  2 6255 6.776053e-05
## 13368  2 6256 6.741811e-05
## 13369  2 6257 6.697182e-05
## 13370  2 6258 6.674408e-05
## 13371  2 6259 6.640111e-05
## 13372  2 6260 6.594086e-05
## 13373  2 6261 6.559580e-05
## 13374  2 6262 6.522747e-05
## 13375  2 6263 6.472505e-05
## 13376  2 6264 6.440573e-05
## 13377  2 6265 6.405432e-05
## 13378  2 6266 6.380649e-05
## 13379  2 6267 6.333524e-05
## 13380  2 6268 6.305643e-05
## 13381  2 6269 6.265397e-05
## 13382  2 6270 6.230559e-05
## 13383  2 6271 6.191277e-05
## 13384  2 6272 6.143858e-05
## 13385  2 6273 6.112628e-05
## 13386  2 6274 6.090318e-05
## 13387  2 6275 6.041265e-05
## 13388  2 6276 6.019738e-05
## 13389  2 6277 5.982665e-05
## 13390  2 6278 5.956171e-05
## 13391  2 6279 5.915106e-05
## 13392  2 6280 5.872292e-05
## 13393  2 6281 5.841048e-05
## 13394  2 6282 5.819438e-05
## 13395  2 6283 5.783170e-05
## 13396  2 6284 5.749311e-05
## 13397  2 6285 5.708920e-05
## 13398  2 6286 5.689174e-05
## 13399  2 6287 5.654410e-05
## 13400  2 6288 5.617185e-05
## 13401  2 6289 5.591695e-05
## 13402  2 6290 5.556667e-05
## 13403  2 6291 5.528552e-05
## 13404  2 6292 5.484333e-05
## 13405  2 6293 5.465446e-05
## 13406  2 6294 5.442392e-05
## 13407  2 6295 5.392934e-05
## 13408  2 6296 5.375665e-05
## 13409  2 6297 5.331681e-05
## 13410  2 6298 5.296034e-05
## 13411  2 6299 5.280732e-05
## 13412  2 6300 5.255881e-05
## 13413  2 6301 5.216296e-05
## 13414  2 6302 5.190672e-05
## 13415  2 6303 5.156642e-05
## 13416  2 6304 5.128859e-05
## 13417  2 6305 5.100341e-05
## 13418  2 6306 5.075127e-05
## 13419  2 6307 5.043376e-05
## 13420  2 6308 5.006183e-05
## 13421  2 6309 4.994481e-05
## 13422  2 6310 4.958519e-05
## 13423  2 6311 4.919454e-05
## 13424  2 6312 4.893625e-05
## 13425  2 6313 4.871537e-05
## 13426  2 6314 4.846670e-05
## 13427  2 6315 4.814415e-05
## 13428  2 6316 4.790273e-05
## 13429  2 6317 4.764934e-05
## 13430  2 6318 4.729196e-05
## 13431  2 6319 4.704576e-05
## 13432  2 6320 4.675504e-05
## 13433  2 6321 4.651761e-05
## 13434  2 6322 4.621858e-05
## 13435  2 6323 4.593007e-05
## 13436  2 6324 4.578036e-05
## 13437  2 6325 4.554563e-05
## 13438  2 6326 4.530121e-05
## 13439  2 6327 4.503283e-05
## 13440  2 6328 4.470599e-05
## 13441  2 6329 4.449087e-05
## 13442  2 6330 4.421620e-05
## 13443  2 6331 4.395972e-05
## 13444  2 6332 4.367898e-05
## 13445  2 6333 4.352121e-05
## 13446  2 6334 4.313451e-05
## 13447  2 6335 4.299115e-05
## 13448  2 6336 4.274444e-05
## 13449  2 6337 4.256835e-05
## 13450  2 6338 4.227133e-05
## 13451  2 6339 4.197813e-05
## 13452  2 6340 4.177912e-05
## 13453  2 6341 4.148322e-05
## 13454  2 6342 4.127299e-05
## 13455  2 6343 4.099758e-05
## 13456  2 6344 4.083231e-05
## 13457  2 6345 4.055869e-05
## 13458  2 6346 4.033540e-05
## 13459  2 6347 4.018225e-05
## 13460  2 6348 3.992444e-05
## 13461  2 6349 3.968143e-05
## 13462  2 6350 3.950390e-05
## 13463  2 6351 3.917093e-05
## 13464  2 6352 3.897237e-05
## 13465  2 6353 3.870221e-05
## 13466  2 6354 3.855115e-05
## 13467  2 6355 3.844672e-05
## 13468  2 6356 3.809665e-05
## 13469  2 6357 3.785216e-05
## 13470  2 6358 3.765786e-05
## 13471  2 6359 3.745675e-05
## 13472  2 6360 3.721566e-05
## 13473  2 6361 3.704115e-05
```

```
## 13474  2 6362 3.682167e-05
## 13475  2 6363 3.660509e-05
## 13476  2 6364 3.641041e-05
## 13477  2 6365 3.626966e-05
## 13478  2 6366 3.596763e-05
## 13479  2 6367 3.585484e-05
## 13480  2 6368 3.554988e-05
## 13481  2 6369 3.540472e-05
## 13482  2 6370 3.520755e-05
## 13483  2 6371 3.498596e-05
## 13484  2 6372 3.473874e-05
## 13485  2 6373 3.462013e-05
## 13486  2 6374 3.447386e-05
## 13487  2 6375 3.421299e-05
## 13488  2 6376 3.404189e-05
## 13489  2 6377 3.386405e-05
## 13490  2 6378 3.355537e-05
## 13491  2 6379 3.339700e-05
## 13492  2 6380 3.327103e-05
## 13493  2 6381 3.305112e-05
## 13494  2 6382 3.282826e-05
## 13495  2 6383 3.267763e-05
## 13496  2 6384 3.253378e-05
## 13497  2 6385 3.232500e-05
## 13498  2 6386 3.213771e-05
## 13499  2 6387 3.195701e-05
## 13500  2 6388 3.181309e-05
## 13501  2 6389 3.150628e-05
## 13502  2 6390 3.139724e-05
## 13503  2 6391 3.124152e-05
## 13504  2 6392 3.108163e-05
## 13505  2 6393 3.083356e-05
## 13506  2 6394 3.065868e-05
## 13507  2 6395 3.055855e-05
## 13508  2 6396 3.032498e-05
## 13509  2 6397 3.022173e-05
## 13510  2 6398 3.002560e-05
## 13511  2 6399 2.983976e-05
## 13512  2 6400 2.962441e-05
## 13513  2 6401 2.949235e-05
## 13514  2 6402 2.937872e-05
## 13515  2 6403 2.910954e-05
## 13516  2 6404 2.900313e-05
## 13517  2 6405 2.881503e-05
## 13518  2 6406 2.868658e-05
## 13519  2 6407 2.851241e-05
## 13520  2 6408 2.835929e-05
## 13521  2 6409 2.819836e-05
## 13522  2 6410 2.801866e-05
## 13523  2 6411 2.791435e-05
## 13524  2 6412 2.772139e-05
## 13525  2 6413 2.754852e-05
## 13526  2 6414 2.743610e-05
## 13527  2 6415 2.721021e-05
## 13528  2 6416 2.708387e-05
## 13529  2 6417 2.695479e-05
## 13530  2 6418 2.677999e-05
## 13531  2 6419 2.660707e-05
## 13532  2 6420 2.647687e-05
## 13533  2 6421 2.629174e-05
## 13534  2 6422 2.616275e-05
## 13535  2 6423 2.599258e-05
## 13536  2 6424 2.588519e-05
## 13537  2 6425 2.572946e-05
## 13538  2 6426 2.555640e-05
## 13539  2 6427 2.546160e-05
## 13540  2 6428 2.531720e-05
## 13541  2 6429 2.516484e-05
## 13542  2 6430 2.497156e-05
## 13543  2 6431 2.484196e-05
## 13544  2 6432 2.467232e-05
## 13545  2 6433 2.455427e-05
## 13546  2 6434 2.439760e-05
## 13547  2 6435 2.431547e-05
## 13548  2 6436 2.415502e-05
## 13549  2 6437 2.399471e-05
## 13550  2 6438 2.385911e-05
## 13551  2 6439 2.370987e-05
## 13552  2 6440 2.357454e-05
## 13553  2 6441 2.347733e-05
## 13554  2 6442 2.335670e-05
## 13555  2 6443 2.320108e-05
## 13556  2 6444 2.307950e-05
## 13557  2 6445 2.291056e-05
## 13558  2 6446 2.283423e-05
## 13559  2 6447 2.271375e-05
## 13560  2 6448 2.254777e-05
## 13561  2 6449 2.240312e-05
## 13562  2 6450 2.230798e-05
## 13563  2 6451 2.217406e-05
## 13564  2 6452 2.203785e-05
## 13565  2 6453 2.193116e-05
## 13566  2 6454 2.179151e-05
## 13567  2 6455 2.165909e-05
## 13568  2 6456 2.155066e-05
## 13569  2 6457 2.145331e-05
## 13570  2 6458 2.133992e-05
## 13571  2 6459 2.118582e-05
## 13572  2 6460 2.105439e-05
## 13573  2 6461 2.094478e-05
## 13574  2 6462 2.083744e-05
## 13575  2 6463 2.072497e-05
## 13576  2 6464 2.060117e-05
## 13577  2 6465 2.046109e-05
## 13578  2 6466 2.037618e-05
## 13579  2 6467 2.021247e-05
## 13580  2 6468 2.011634e-05
## 13581  2 6469 2.002569e-05
## 13582  2 6470 1.987399e-05
## 13583  2 6471 1.978987e-05
## 13584  2 6472 1.966858e-05
## 13585  2 6473 1.956180e-05
## 13586  2 6474 1.946321e-05
## 13587  2 6475 1.932422e-05
## 13588  2 6476 1.922022e-05
## 13589  2 6477 1.914125e-05
## 13590  2 6478 1.899130e-05
## 13591  2 6479 1.888058e-05
## 13592  2 6480 1.880094e-05
## 13593  2 6481 1.867064e-05
## 13594  2 6482 1.857242e-05
## 13595  2 6483 1.846718e-05
## 13596  2 6484 1.835455e-05
## 13597  2 6485 1.827822e-05
## 13598  2 6486 1.815837e-05
## 13599  2 6487 1.802457e-05
## 13600  2 6488 1.794659e-05
## 13601  2 6489 1.786915e-05
## 13602  2 6490 1.772084e-05
## 13603  2 6491 1.765314e-05
## 13604  2 6492 1.755515e-05
## 13605  2 6493 1.743785e-05
## 13606  2 6494 1.738783e-05
## 13607  2 6495 1.728116e-05
## 13608  2 6496 1.713165e-05
## 13609  2 6497 1.707046e-05
## 13610  2 6498 1.698228e-05
## 13611  2 6499 1.684923e-05
## 13612  2 6500 1.677504e-05
## 13613  2 6501 1.667548e-05
## 13614  2 6502 1.656405e-05
## 13615  2 6503 1.649876e-05
## 13616  2 6504 1.642487e-05
## 13617  2 6505 1.631198e-05
## 13618  2 6506 1.621559e-05
## 13619  2 6507 1.611781e-05
## 13620  2 6508 1.600986e-05
## 13621  2 6509 1.593164e-05
## 13622  2 6510 1.583789e-05
## 13623  2 6511 1.573169e-05
## 13624  2 6512 1.566563e-05
## 13625  2 6513 1.557295e-05
## 13626  2 6514 1.550427e-05
## 13627  2 6515 1.540112e-05
## 13628  2 6516 1.531605e-05
## 13629  2 6517 1.520435e-05
## 13630  2 6518 1.512041e-05
## 13631  2 6519 1.504211e-05
## 13632  2 6520 1.498408e-05
## 13633  2 6521 1.487330e-05
## 13634  2 6522 1.478477e-05
## 13635  2 6523 1.473938e-05
## 13636  2 6524 1.464042e-05
## 13637  2 6525 1.452860e-05
## 13638  2 6526 1.444242e-05
## 13639  2 6527 1.438673e-05
## 13640  2 6528 1.430238e-05
## 13641  2 6529 1.421692e-05
## 13642  2 6530 1.411594e-05
## 13643  2 6531 1.403731e-05
## 13644  2 6532 1.395779e-05
## 13645  2 6533 1.388186e-05
## 13646  2 6534 1.381281e-05
## 13647  2 6535 1.371044e-05
## 13648  2 6536 1.363711e-05
## 13649  2 6537 1.359606e-05
## 13650  2 6538 1.349180e-05
## 13651  2 6539 1.342930e-05
## 13652  2 6540 1.334768e-05
## 13653  2 6541 1.327310e-05
## 13654  2 6542 1.320283e-05
## 13655  2 6543 1.311880e-05
## 13656  2 6544 1.302580e-05
## 13657  2 6545 1.296823e-05
## 13658  2 6546 1.289631e-05
## 13659  2 6547 1.283994e-05
## 13660  2 6548 1.274572e-05
## 13661  2 6549 1.266303e-05
## 13662  2 6550 1.263803e-05
## 13663  2 6551 1.255113e-05
## 13664  2 6552 1.247737e-05
## 13665  2 6553 1.239400e-05
## 13666  2 6554 1.232775e-05
## 13667  2 6555 1.225299e-05
## 13668  2 6556 1.220066e-05
## 13669  2 6557 1.211995e-05
## 13670  2 6558 1.205438e-05
## 13671  2 6559 1.198213e-05
## 13672  2 6560 1.189559e-05
## 13673  2 6561 1.181607e-05
## 13674  2 6562 1.176909e-05
## 13675  2 6563 1.170523e-05
## 13676  2 6564 1.161999e-05
## 13677  2 6565 1.157360e-05
## 13678  2 6566 1.150277e-05
## 13679  2 6567 1.144984e-05
## 13680  2 6568 1.139786e-05
## 13681  2 6569 1.133290e-05
## 13682  2 6570 1.125218e-05
## 13683  2 6571 1.119087e-05
## 13684  2 6572 1.113031e-05
## 13685  2 6573 1.106719e-05
## 13686  2 6574 1.101061e-05
## 13687  2 6575 1.092906e-05
## 13688  2 6576 1.087019e-05
## 13689  2 6577 1.082217e-05
## 13690  2 6578 1.075887e-05
## 13691  2 6579 1.068783e-05
## 13692  2 6580 1.061263e-05
## 13693  2 6581 1.055841e-05
## 13694  2 6582 1.049501e-05
## 13695  2 6583 1.046582e-05
## 13696  2 6584 1.039599e-05
## 13697  2 6585 1.031944e-05
## 13698  2 6586 1.027665e-05
## 13699  2 6587 1.022124e-05
## 13700  2 6588 1.014296e-05
## 13701  2 6589 1.009270e-05
## 13702  2 6590 1.004441e-05
## 13703  2 6591 9.974708e-06
## 13704  2 6592 9.912295e-06
## 13705  2 6593 9.869941e-06
## 13706  2 6594 9.810859e-06
## 13707  2 6595 9.746854e-06
## 13708  2 6596 9.700422e-06
## 13709  2 6597 9.637812e-06
## 13710  2 6598 9.596210e-06
## 13711  2 6599 9.544763e-06
## 13712  2 6600 9.484796e-06
## 13713  2 6601 9.418689e-06
## 13714  2 6602 9.367856e-06
## 13715  2 6603 9.317254e-06
## 13716  2 6604 9.280350e-06
## 13717  2 6605 9.220750e-06
## 13718  2 6606 9.171005e-06
## 13719  2 6607 9.109897e-06
## 13720  2 6608 9.061212e-06
## 13721  2 6609 9.012438e-06
## 13722  2 6610 8.934922e-06
## 13723  2 6611 8.930108e-06
## 13724  2 6612 8.852720e-06
## 13725  2 6613 8.811442e-06
## 13726  2 6614 8.752591e-06
## 13727  2 6615 8.707479e-06
## 13728  2 6616 8.647380e-06
## 13729  2 6617 8.600171e-06
## 13730  2 6618 8.539213e-06
## 13731  2 6619 8.525180e-06
## 13732  2 6620 8.453458e-06
## 13733  2 6621 8.404101e-06
## 13734  2 6622 8.372627e-06
## 13735  2 6623 8.297545e-06
## 13736  2 6624 8.265199e-06
## 13737  2 6625 8.225690e-06
## 13738  2 6626 8.172797e-06
## 13739  2 6627 8.133008e-06
## 13740  2 6628 8.085992e-06
## 13741  2 6629 8.049274e-06
## 13742  2 6630 7.993162e-06
## 13743  2 6631 7.942815e-06
## 13744  2 6632 7.891516e-06
## 13745  2 6633 7.864790e-06
## 13746  2 6634 7.818645e-06
## 13747  2 6635 7.758139e-06
## 13748  2 6636 7.719748e-06
## 13749  2 6637 7.678050e-06
## 13750  2 6638 7.630226e-06
## 13751  2 6639 7.586637e-06
## 13752  2 6640 7.555584e-06
## 13753  2 6641 7.499493e-06
## 13754  2 6642 7.473650e-06
## 13755  2 6643 7.419176e-06
## 13756  2 6644 7.379133e-06
## 13757  2 6645 7.349373e-06
## 13758  2 6646 7.294258e-06
## 13759  2 6647 7.250989e-06
## 13760  2 6648 7.213124e-06
## 13761  2 6649 7.165225e-06
## 13762  2 6650 7.147044e-06
## 13763  2 6651 7.091979e-06
## 13764  2 6652 7.051347e-06
## 13765  2 6653 7.010300e-06
## 13766  2 6654 6.957032e-06
## 13767  2 6655 6.939572e-06
## 13768  2 6656 6.894392e-06
## 13769  2 6657 6.854677e-06
## 13770  2 6658 6.812025e-06
## 13771  2 6659 6.767004e-06
## 13772  2 6660 6.745171e-06
## 13773  2 6661 6.693953e-06
## 13774  2 6662 6.656101e-06
## 13775  2 6663 6.622758e-06
## 13776  2 6664 6.576172e-06
## 13777  2 6665 6.547640e-06
## 13778  2 6666 6.521020e-06
## 13779  2 6667 6.475458e-06
## 13780  2 6668 6.437348e-06
## 13781  2 6669 6.403176e-06
## 13782  2 6670 6.356536e-06
## 13783  2 6671 6.319950e-06
## 13784  2 6672 6.285640e-06
## 13785  2 6673 6.253074e-06
## 13786  2 6674 6.228668e-06
## 13787  2 6675 6.185317e-06
## 13788  2 6676 6.155865e-06
## 13789  2 6677 6.107469e-06
## 13790  2 6678 6.072236e-06
## 13791  2 6679 6.040717e-06
## 13792  2 6680 6.007102e-06
## 13793  2 6681 5.978863e-06
## 13794  2 6682 5.941387e-06
## 13795  2 6683 5.907121e-06
## 13796  2 6684 5.877298e-06
## 13797  2 6685 5.847734e-06
## 13798  2 6686 5.810387e-06
## 13799  2 6687 5.782000e-06
## 13800  2 6688 5.735390e-06
## 13801  2 6689 5.715528e-06
## 13802  2 6690 5.680183e-06
## 13803  2 6691 5.645560e-06
## 13804  2 6692 5.613505e-06
## 13805  2 6693 5.577537e-06
## 13806  2 6694 5.540621e-06
## 13807  2 6695 5.525133e-06
## 13808  2 6696 5.489998e-06
## 13809  2 6697 5.454021e-06
## 13810  2 6698 5.425607e-06
## 13811  2 6699 5.397606e-06
## 13812  2 6700 5.366917e-06
## 13813  2 6701 5.335754e-06
## 13814  2 6702 5.295569e-06
## 13815  2 6703 5.269514e-06
## 13816  2 6704 5.243108e-06
## 13817  2 6705 5.216260e-06
## 13818  2 6706 5.182844e-06
## 13819  2 6707 5.155138e-06
## 13820  2 6708 5.122315e-06
## 13821  2 6709 5.094954e-06
## 13822  2 6710 5.062876e-06
## 13823  2 6711 5.028727e-06
## 13824  2 6712 5.004933e-06
## 13825  2 6713 4.980467e-06
## 13826  2 6714 4.951648e-06
## 13827  2 6715 4.932227e-06
## 13828  2 6716 4.892355e-06
## 13829  2 6717 4.858269e-06
## 13830  2 6718 4.835345e-06
## 13831  2 6719 4.799157e-06
## 13832  2 6720 4.782580e-06
## 13833  2 6721 4.752223e-06
## 13834  2 6722 4.734425e-06
## 13835  2 6723 4.706391e-06
## 13836  2 6724 4.682543e-06
## 13837  2 6725 4.651283e-06
## 13838  2 6726 4.635266e-06
## 13839  2 6727 4.600864e-06
## 13840  2 6728 4.573902e-06
## 13841  2 6729 4.541367e-06
## 13842  2 6730 4.519586e-06
## 13843  2 6731 4.491519e-06
## 13844  2 6732 4.470121e-06
## 13845  2 6733 4.450859e-06
## 13846  2 6734 4.420159e-06
## 13847  2 6735 4.392298e-06
## 13848  2 6736 4.369499e-06
## 13849  2 6737 4.343788e-06
## 13850  2 6738 4.321930e-06
## 13851  2 6739 4.301175e-06
## 13852  2 6740 4.269524e-06
## 13853  2 6741 4.248407e-06
## 13854  2 6742 4.215213e-06
## 13855  2 6743 4.193184e-06
## 13856  2 6744 4.170455e-06
## 13857  2 6745 4.150843e-06
## 13858  2 6746 4.124753e-06
## 13859  2 6747 4.099939e-06
## 13860  2 6748 4.088128e-06
## 13861  2 6749 4.054288e-06
## 13862  2 6750 4.030003e-06
## 13863  2 6751 4.019060e-06
## 13864  2 6752 3.992043e-06
## 13865  2 6753 3.977218e-06
## 13866  2 6754 3.946266e-06
## 13867  2 6755 3.921415e-06
## 13868  2 6756 3.898845e-06
## 13869  2 6757 3.882843e-06
## 13870  2 6758 3.847586e-06
## 13871  2 6759 3.839683e-06
## 13872  2 6760 3.811559e-06
## 13873  2 6761 3.788338e-06
## 13874  2 6762 3.765537e-06
## 13875  2 6763 3.746421e-06
## 13876  2 6764 3.729402e-06
## 13877  2 6765 3.706118e-06
## 13878  2 6766 3.685676e-06
## 13879  2 6767 3.661163e-06
## 13880  2 6768 3.639440e-06
## 13881  2 6769 3.612159e-06
## 13882  2 6770 3.593875e-06
## 13883  2 6771 3.575695e-06
## 13884  2 6772 3.561191e-06
## 13885  2 6773 3.539882e-06
## 13886  2 6774 3.516168e-06
## 13887  2 6775 3.493364e-06
## 13888  2 6776 3.485689e-06
## 13889  2 6777 3.461008e-06
## 13890  2 6778 3.438392e-06
## 13891  2 6779 3.417783e-06
## 13892  2 6780 3.402077e-06
## 13893  2 6781 3.379360e-06
## 13894  2 6782 3.368362e-06
## 13895  2 6783 3.345506e-06
## 13896  2 6784 3.319550e-06
## 13897  2 6785 3.298575e-06
## 13898  2 6786 3.285511e-06
## 13899  2 6787 3.266732e-06
## 13900  2 6788 3.249537e-06
## 13901  2 6789 3.234625e-06
## 13902  2 6790 3.209033e-06
## 13903  2 6791 3.193919e-06
## 13904  2 6792 3.173403e-06
## 13905  2 6793 3.158460e-06
## 13906  2 6794 3.138371e-06
## 13907  2 6795 3.121021e-06
## 13908  2 6796 3.103782e-06
## 13909  2 6797 3.084364e-06
## 13910  2 6798 3.066772e-06
## 13911  2 6799 3.049318e-06
## 13912  2 6800 3.039133e-06
## 13913  2 6801 3.016285e-06
## 13914  2 6802 2.996374e-06
## 13915  2 6803 2.986579e-06
## 13916  2 6804 2.966166e-06
## 13917  2 6805 2.950445e-06
## 13918  2 6806 2.933613e-06
## 13919  2 6807 2.917959e-06
## 13920  2 6808 2.899733e-06
## 13921  2 6809 2.879476e-06
## 13922  2 6810 2.863053e-06
## 13923  2 6811 2.850656e-06
## 13924  2 6812 2.830589e-06
## 13925  2 6813 2.817100e-06
## 13926  2 6814 2.801559e-06
## 13927  2 6815 2.791891e-06
## 13928  2 6816 2.771276e-06
## 13929  2 6817 2.749261e-06
## 13930  2 6818 2.735124e-06
## 13931  2 6819 2.727352e-06
## 13932  2 6820 2.705867e-06
## 13933  2 6821 2.691533e-06
## 13934  2 6822 2.677555e-06
## 13935  2 6823 2.656326e-06
## 13936  2 6824 2.648165e-06
## 13937  2 6825 2.629284e-06
## 13938  2 6826 2.619672e-06
## 13939  2 6827 2.604372e-06
## 13940  2 6828 2.582413e-06
## 13941  2 6829 2.570133e-06
## 13942  2 6830 2.560370e-06
## 13943  2 6831 2.543630e-06
## 13944  2 6832 2.526189e-06
## 13945  2 6833 2.511180e-06
## 13946  2 6834 2.503706e-06
## 13947  2 6835 2.485441e-06
## 13948  2 6836 2.469224e-06
## 13949  2 6837 2.458201e-06
## 13950  2 6838 2.440078e-06
## 13951  2 6839 2.426208e-06
## 13952  2 6840 2.415236e-06
## 13953  2 6841 2.398978e-06
## 13954  2 6842 2.391189e-06
## 13955  2 6843 2.375778e-06
## 13956  2 6844 2.360001e-06
## 13957  2 6845 2.348796e-06
## 13958  2 6846 2.339416e-06
## 13959  2 6847 2.320046e-06
## 13960  2 6848 2.305244e-06
## 13961  2 6849 2.295564e-06
## 13962  2 6850 2.282085e-06
## 13963  2 6851 2.264944e-06
## 13964  2 6852 2.259654e-06
## 13965  2 6853 2.241081e-06
## 13966  2 6854 2.230191e-06
## 13967  2 6855 2.214963e-06
## 13968  2 6856 2.204582e-06
## 13969  2 6857 2.193654e-06
## 13970  2 6858 2.183449e-06
## 13971  2 6859 2.170820e-06
## 13972  2 6860 2.157182e-06
## 13973  2 6861 2.146422e-06
## 13974  2 6862 2.130930e-06
## 13975  2 6863 2.120024e-06
## 13976  2 6864 2.106111e-06
## 13977  2 6865 2.093208e-06
## 13978  2 6866 2.080809e-06
## 13979  2 6867 2.072098e-06
## 13980  2 6868 2.059876e-06
## 13981  2 6869 2.050717e-06
## 13982  2 6870 2.035812e-06
## 13983  2 6871 2.024529e-06
## 13984  2 6872 2.018003e-06
## 13985  2 6873 2.001699e-06
## 13986  2 6874 1.987522e-06
## 13987  2 6875 1.981578e-06
## 13988  2 6876 1.968343e-06
## 13989  2 6877 1.957434e-06
## 13990  2 6878 1.942324e-06
## 13991  2 6879 1.937596e-06
## 13992  2 6880 1.919897e-06
## 13993  2 6881 1.912215e-06
## 13994  2 6882 1.903060e-06
## 13995  2 6883 1.890573e-06
## 13996  2 6884 1.881881e-06
## 13997  2 6885 1.870897e-06
## 13998  2 6886 1.859215e-06
## 13999  2 6887 1.848544e-06
## 14000  2 6888 1.837718e-06
## 14001  2 6889 1.829308e-06
## 14002  2 6890 1.819579e-06
## 14003  2 6891 1.808244e-06
## 14004  2 6892 1.795854e-06
## 14005  2 6893 1.786394e-06
## 14006  2 6894 1.777168e-06
## 14007  2 6895 1.767755e-06
## 14008  2 6896 1.757660e-06
## 14009  2 6897 1.747062e-06
## 14010  2 6898 1.740162e-06
## 14011  2 6899 1.725429e-06
## 14012  2 6900 1.720895e-06
## 14013  2 6901 1.705429e-06
## 14014  2 6902 1.697618e-06
## 14015  2 6903 1.685565e-06
## 14016  2 6904 1.678874e-06
## 14017  2 6905 1.671837e-06
## 14018  2 6906 1.656431e-06
## 14019  2 6907 1.647388e-06
## 14020  2 6908 1.638198e-06
## 14021  2 6909 1.629529e-06
## 14022  2 6910 1.621913e-06
## 14023  2 6911 1.615836e-06
## 14024  2 6912 1.602140e-06
## 14025  2 6913 1.592559e-06
## 14026  2 6914 1.586922e-06
## 14027  2 6915 1.577592e-06
## 14028  2 6916 1.567014e-06
## 14029  2 6917 1.558364e-06
## 14030  2 6918 1.550406e-06
## 14031  2 6919 1.542516e-06
## 14032  2 6920 1.532179e-06
## 14033  2 6921 1.524996e-06
## 14034  2 6922 1.516798e-06
## 14035  2 6923 1.507491e-06
## 14036  2 6924 1.497424e-06
## 14037  2 6925 1.489752e-06
## 14038  2 6926 1.481418e-06
## 14039  2 6927 1.471490e-06
## 14040  2 6928 1.465435e-06
## 14041  2 6929 1.456682e-06
## 14042  2 6930 1.450295e-06
## 14043  2 6931 1.439555e-06
## 14044  2 6932 1.433249e-06
## 14045  2 6933 1.425937e-06
## 14046  2 6934 1.418392e-06
## 14047  2 6935 1.408955e-06
## 14048  2 6936 1.400193e-06
## 14049  2 6937 1.390093e-06
## 14050  2 6938 1.383164e-06
## 14051  2 6939 1.375947e-06
## 14052  2 6940 1.365322e-06
## 14053  2 6941 1.359967e-06
## 14054  2 6942 1.352701e-06
## 14055  2 6943 1.344470e-06
## 14056  2 6944 1.336411e-06
## 14057  2 6945 1.331333e-06
## 14058  2 6946 1.322551e-06
## 14059  2 6947 1.314681e-06
## 14060  2 6948 1.306007e-06
## 14061  2 6949 1.299163e-06
## 14062  2 6950 1.291479e-06
## 14063  2 6951 1.284846e-06
## 14064  2 6952 1.279356e-06
## 14065  2 6953 1.271766e-06
## 14066  2 6954 1.265141e-06
## 14067  2 6955 1.258416e-06
## 14068  2 6956 1.250305e-06
## 14069  2 6957 1.242929e-06
## 14070  2 6958 1.235670e-06
## 14071  2 6959 1.228193e-06
## 14072  2 6960 1.221363e-06
## 14073  2 6961 1.215934e-06
## 14074  2 6962 1.207741e-06
## 14075  2 6963 1.202026e-06
## 14076  2 6964 1.194260e-06
## 14077  2 6965 1.187299e-06
## 14078  2 6966 1.180130e-06
## 14079  2 6967 1.174876e-06
## 14080  2 6968 1.167493e-06
## 14081  2 6969 1.161254e-06
## 14082  2 6970 1.153948e-06
## 14083  2 6971 1.149931e-06
## 14084  2 6972 1.141207e-06
## 14085  2 6973 1.136050e-06
## 14086  2 6974 1.128351e-06
## 14087  2 6975 1.122431e-06
## 14088  2 6976 1.115766e-06
## 14089  2 6977 1.111223e-06
## 14090  2 6978 1.103282e-06
## 14091  2 6979 1.097336e-06
## 14092  2 6980 1.091475e-06
## 14093  2 6981 1.085353e-06
## 14094  2 6982 1.079457e-06
## 14095  2 6983 1.073727e-06
## 14096  2 6984 1.064090e-06
## 14097  2 6985 1.060976e-06
## 14098  2 6986 1.053388e-06
## 14099  2 6987 1.049285e-06
## 14100  2 6988 1.041147e-06
## 14101  2 6989 1.039536e-06
## 14102  2 6990 1.030643e-06
## 14103  2 6991 1.026638e-06
## 14104  2 6992 1.017301e-06
## 14105  2 6993 1.015038e-06
## 14106  2 6994 1.010102e-06
## 14107  2 6995 1.003225e-06
## 14108  2 6996 9.983973e-07
## 14109  2 6997 9.919265e-07
## 14110  2 6998 9.853127e-07
## 14111  2 6999 9.799108e-07
## 14112  2 7000 9.736741e-07
## 14113  3    0 0.000000e+00
## 14114  3    0 1.872035e+00
## 14115  3    1 2.016441e+00
## 14116  3    2 2.114862e+00
## 14117  3    3 2.145251e+00
## 14118  3    4 2.155962e+00
## 14119  3    5 2.149894e+00
## 14120  3    6 2.139170e+00
## 14121  3    7 2.118145e+00
## 14122  3    8 2.102801e+00
## 14123  3    9 2.083650e+00
## 14124  3   10 2.075376e+00
## 14125  3   11 2.052709e+00
## 14126  3   12 2.039162e+00
## 14127  3   13 2.024228e+00
## 14128  3   14 2.006833e+00
## 14129  3   15 1.992684e+00
## 14130  3   16 1.976948e+00
## 14131  3   17 1.963962e+00
## 14132  3   18 1.951740e+00
## 14133  3   19 1.937999e+00
## 14134  3   20 1.926308e+00
## 14135  3   21 1.909845e+00
## 14136  3   22 1.900002e+00
## 14137  3   23 1.885191e+00
## 14138  3   24 1.873592e+00
## 14139  3   24 1.873028e+00
## 14140  3   25 2.021459e+00
## 14141  3   26 2.115682e+00
## 14142  3   27 2.146657e+00
## 14143  3   28 2.159658e+00
## 14144  3   29 2.148832e+00
## 14145  3   30 2.138834e+00
## 14146  3   31 2.118000e+00
## 14147  3   32 2.101432e+00
## 14148  3   33 2.089102e+00
## 14149  3   34 2.070327e+00
## 14150  3   35 2.052770e+00
## 14151  3   36 2.039160e+00
## 14152  3   37 2.023312e+00
## 14153  3   38 2.006438e+00
## 14154  3   39 1.994052e+00
## 14155  3   40 1.975582e+00
## 14156  3   41 1.965110e+00
## 14157  3   42 1.951256e+00
## 14158  3   43 1.937623e+00
## 14159  3   44 1.920464e+00
## 14160  3   45 1.910358e+00
## 14161  3   46 1.899185e+00
## 14162  3   47 1.884195e+00
## 14163  3   48 1.869223e+00
## 14164  3   48 1.877746e+00
## 14165  3   49 2.018043e+00
## 14166  3   50 2.114506e+00
## 14167  3   51 2.149925e+00
## 14168  3   52 2.158748e+00
## 14169  3   53 2.147664e+00
## 14170  3   54 2.134396e+00
## 14171  3   55 2.117268e+00
## 14172  3   56 2.104160e+00
## 14173  3   57 2.089912e+00
## 14174  3   58 2.067649e+00
## 14175  3   59 2.055498e+00
## 14176  3   60 2.039604e+00
## 14177  3   61 2.026990e+00
## 14178  3   62 2.007436e+00
## 14179  3   63 1.994199e+00
## 14180  3   64 1.980381e+00
## 14181  3   65 1.962649e+00
## 14182  3   66 1.945872e+00
## 14183  3   67 1.939609e+00
## 14184  3   68 1.923648e+00
## 14185  3   69 1.909207e+00
## 14186  3   70 1.898416e+00
## 14187  3   71 1.883572e+00
## 14188  3   72 1.872993e+00
## 14189  3   73 2.023893e+00
## 14190  3   74 2.112880e+00
## 14191  3   75 2.146846e+00
## 14192  3   76 2.155552e+00
## 14193  3   77 2.147188e+00
## 14194  3   78 2.134946e+00
## 14195  3   79 2.123367e+00
## 14196  3   80 2.099796e+00
## 14197  3   81 2.087837e+00
## 14198  3   82 2.068111e+00
## 14199  3   83 2.056748e+00
## 14200  3   84 2.037329e+00
## 14201  3   85 2.021955e+00
## 14202  3   86 2.008065e+00
## 14203  3   87 1.995716e+00
## 14204  3   88 1.978062e+00
## 14205  3   89 1.961038e+00
## 14206  3   90 1.948308e+00
## 14207  3   91 1.939544e+00
## 14208  3   92 1.924734e+00
## 14209  3   93 1.906913e+00
## 14210  3   94 1.895428e+00
## 14211  3   95 1.885199e+00
## 14212  3   96 1.875560e+00
## 14213  3   97 2.020685e+00
## 14214  3   98 2.117023e+00
## 14215  3   99 2.149128e+00
## 14216  3  100 2.157003e+00
## 14217  3  101 2.150006e+00
## 14218  3  102 2.136519e+00
## 14219  3  103 2.123050e+00
## 14220  3  104 2.106643e+00
## 14221  3  105 2.087954e+00
## 14222  3  106 2.065890e+00
## 14223  3  107 2.051805e+00
## 14224  3  108 2.036383e+00
## 14225  3  109 2.019747e+00
## 14226  3  110 2.010202e+00
## 14227  3  111 1.992976e+00
## 14228  3  112 1.980424e+00
## 14229  3  113 1.963498e+00
## 14230  3  114 1.949452e+00
## 14231  3  115 1.935887e+00
## 14232  3  116 1.925402e+00
## 14233  3  117 1.914789e+00
## 14234  3  118 1.901290e+00
## 14235  3  119 1.886616e+00
## 14236  3  120 1.875345e+00
## 14237  3  121 2.021894e+00
## 14238  3  122 2.114750e+00
## 14239  3  123 2.145414e+00
## 14240  3  124 2.153555e+00
## 14241  3  125 2.149592e+00
## 14242  3  126 2.135922e+00
## 14243  3  127 2.115456e+00
## 14244  3  128 2.104032e+00
## 14245  3  129 2.086612e+00
## 14246  3  130 2.068834e+00
## 14247  3  131 2.054470e+00
## 14248  3  132 2.034247e+00
## 14249  3  133 2.021717e+00
## 14250  3  134 2.006348e+00
## 14251  3  135 1.995777e+00
## 14252  3  136 1.981510e+00
## 14253  3  137 1.964206e+00
## 14254  3  138 1.953528e+00
## 14255  3  139 1.934503e+00
## 14256  3  140 1.927100e+00
## 14257  3  141 1.910699e+00
## 14258  3  142 1.895323e+00
## 14259  3  143 1.883346e+00
## 14260  3  144 1.874289e+00
## 14261  3  145 2.017419e+00
## 14262  3  146 2.114951e+00
## 14263  3  147 2.151529e+00
## 14264  3  148 2.154762e+00
## 14265  3  149 2.152464e+00
## 14266  3  150 2.138197e+00
## 14267  3  151 2.120166e+00
## 14268  3  152 2.104370e+00
## 14269  3  153 2.087700e+00
## 14270  3  154 2.072285e+00
## 14271  3  155 2.054398e+00
## 14272  3  156 2.037112e+00
## 14273  3  157 2.020170e+00
```

```
## 14274  3  158 2.007053e+00
## 14275  3  159 1.993141e+00
## 14276  3  160 1.983404e+00
## 14277  3  161 1.965088e+00
## 14278  3  162 1.952044e+00
## 14279  3  163 1.935368e+00
## 14280  3  164 1.922159e+00
## 14281  3  165 1.909265e+00
## 14282  3  166 1.900471e+00
## 14283  3  167 1.889314e+00
## 14284  3  168 1.875015e+00
## 14285  3  169 2.022615e+00
## 14286  3  170 2.117789e+00
## 14287  3  171 2.146640e+00
## 14288  3  172 2.154424e+00
## 14289  3  173 2.149689e+00
## 14290  3  174 2.137599e+00
## 14291  3  175 2.120105e+00
## 14292  3  176 2.101279e+00
## 14293  3  177 2.084532e+00
## 14294  3  178 2.070399e+00
## 14295  3  179 2.052796e+00
## 14296  3  180 2.039266e+00
## 14297  3  181 2.024797e+00
## 14298  3  182 2.005623e+00
## 14299  3  183 1.993760e+00
## 14300  3  184 1.975725e+00
## 14301  3  185 1.964361e+00
## 14302  3  186 1.950413e+00
## 14303  3  187 1.935399e+00
## 14304  3  188 1.922066e+00
## 14305  3  189 1.908358e+00
## 14306  3  190 1.898568e+00
## 14307  3  191 1.881151e+00
## 14308  3  192 1.872728e+00
## 14309  3  193 2.021448e+00
## 14310  3  194 2.113350e+00
## 14311  3  195 2.150857e+00
## 14312  3  196 2.156991e+00
## 14313  3  197 2.148352e+00
## 14314  3  198 2.138438e+00
## 14315  3  199 2.118073e+00
## 14316  3  200 2.103090e+00
## 14317  3  201 2.081625e+00
## 14318  3  202 2.073675e+00
## 14319  3  203 2.055254e+00
## 14320  3  204 2.042214e+00
## 14321  3  205 2.020438e+00
## 14322  3  206 2.009581e+00
## 14323  3  207 1.995184e+00
## 14324  3  208 1.978925e+00
## 14325  3  209 1.962464e+00
## 14326  3  210 1.949827e+00
## 14327  3  211 1.934428e+00
## 14328  3  212 1.925148e+00
## 14329  3  213 1.909244e+00
## 14330  3  214 1.900703e+00
## 14331  3  215 1.885598e+00
## 14332  3  216 1.873198e+00
## 14333  3  217 2.016761e+00
## 14334  3  218 2.114201e+00
## 14335  3  219 2.148550e+00
## 14336  3  220 2.155090e+00
## 14337  3  221 2.149077e+00
## 14338  3  222 2.136411e+00
## 14339  3  223 2.121987e+00
## 14340  3  224 2.104627e+00
## 14341  3  225 2.083699e+00
## 14342  3  226 2.072725e+00
## 14343  3  227 2.052468e+00
## 14344  3  228 2.034611e+00
## 14345  3  229 2.022075e+00
## 14346  3  230 2.008521e+00
## 14347  3  231 1.990424e+00
## 14348  3  232 1.975066e+00
## 14349  3  233 1.963404e+00
## 14350  3  234 1.947572e+00
## 14351  3  235 1.938243e+00
## 14352  3  236 1.923358e+00
## 14353  3  237 1.910131e+00
## 14354  3  238 1.901103e+00
## 14355  3  239 1.889158e+00
## 14356  3  240 1.874266e+00
## 14357  3  241 2.023442e+00
## 14358  3  242 2.114798e+00
## 14359  3  243 2.152172e+00
## 14360  3  244 2.158549e+00
## 14361  3  245 2.149477e+00
## 14362  3  246 2.135832e+00
## 14363  3  247 2.118963e+00
## 14364  3  248 2.105261e+00
## 14365  3  249 2.088620e+00
## 14366  3  250 2.068966e+00
## 14367  3  251 2.054821e+00
## 14368  3  252 2.037083e+00
## 14369  3  253 2.021183e+00
## 14370  3  254 2.005706e+00
## 14371  3  255 1.993018e+00
## 14372  3  256 1.978586e+00
## 14373  3  257 1.963773e+00
## 14374  3  258 1.951740e+00
## 14375  3  259 1.933976e+00
## 14376  3  260 1.925214e+00
## 14377  3  261 1.910423e+00
## 14378  3  262 1.899121e+00
## 14379  3  263 1.886026e+00
## 14380  3  264 1.873126e+00
## 14381  3  265 2.017143e+00
## 14382  3  266 2.114830e+00
## 14383  3  267 2.145367e+00
## 14384  3  268 2.152982e+00
## 14385  3  269 2.147268e+00
## 14386  3  270 2.133878e+00
## 14387  3  271 2.121047e+00
## 14388  3  272 2.104977e+00
## 14389  3  273 2.087884e+00
## 14390  3  274 2.068507e+00
## 14391  3  275 2.053329e+00
## 14392  3  276 2.042306e+00
## 14393  3  277 2.022873e+00
## 14394  3  278 2.009310e+00
## 14395  3  279 1.989352e+00
## 14396  3  280 1.979631e+00
## 14397  3  281 1.965007e+00
## 14398  3  282 1.951224e+00
## 14399  3  283 1.935226e+00
## 14400  3  284 1.924150e+00
## 14401  3  285 1.910788e+00
## 14402  3  286 1.897203e+00
## 14403  3  287 1.886954e+00
## 14404  3  288 1.870312e+00
## 14405  3  289 2.020774e+00
## 14406  3  290 2.116391e+00
## 14407  3  291 2.148534e+00
## 14408  3  292 2.158282e+00
## 14409  3  293 2.148398e+00
## 14410  3  294 2.136546e+00
## 14411  3  295 2.116183e+00
## 14412  3  296 2.104624e+00
## 14413  3  297 2.088829e+00
## 14414  3  298 2.070862e+00
## 14415  3  299 2.053334e+00
## 14416  3  300 2.042855e+00
## 14417  3  301 2.023930e+00
## 14418  3  302 2.009242e+00
## 14419  3  303 1.990173e+00
## 14420  3  304 1.975636e+00
## 14421  3  305 1.965968e+00
## 14422  3  306 1.952793e+00
## 14423  3  307 1.936532e+00
## 14424  3  308 1.925044e+00
## 14425  3  309 1.914649e+00
## 14426  3  310 1.898262e+00
## 14427  3  311 1.888389e+00
## 14428  3  312 1.876552e+00
## 14429  3  312 1.872580e+00
## 14430  3  313 2.022238e+00
## 14431  3  314 2.113816e+00
## 14432  3  315 2.146888e+00
## 14433  3  316 2.158067e+00
## 14434  3  317 2.149629e+00
## 14435  3  318 2.134598e+00
## 14436  3  319 2.122842e+00
## 14437  3  320 2.103804e+00
## 14438  3  321 2.083310e+00
## 14439  3  322 2.071061e+00
## 14440  3  323 2.056529e+00
## 14441  3  324 2.036540e+00
## 14442  3  325 2.024382e+00
## 14443  3  326 2.010984e+00
## 14444  3  327 1.992289e+00
## 14445  3  328 1.979427e+00
## 14446  3  329 1.964494e+00
## 14447  3  330 1.947503e+00
## 14448  3  331 1.936811e+00
## 14449  3  332 1.925227e+00
## 14450  3  333 1.911662e+00
## 14451  3  334 1.901748e+00
## 14452  3  335 1.885021e+00
## 14453  3  336 1.870943e+00
## 14454  3  336 1.879841e+00
## 14455  3  337 1.861468e+00
## 14456  3  338 1.852058e+00
## 14457  3  339 1.841795e+00
## 14458  3  339 1.837677e+00
## 14459  3  340 1.987618e+00
## 14460  3  341 2.082317e+00
## 14461  3  342 2.113127e+00
## 14462  3  343 2.122730e+00
## 14463  3  344 2.119154e+00
## 14464  3  345 2.105504e+00
## 14465  3  346 2.091557e+00
## 14466  3  347 2.074665e+00
## 14467  3  348 2.057988e+00
## 14468  3  349 2.043852e+00
## 14469  3  350 2.026386e+00
## 14470  3  351 2.010660e+00
## 14471  3  352 1.997102e+00
## 14472  3  353 1.981716e+00
## 14473  3  354 1.969118e+00
## 14474  3  355 1.953724e+00
## 14475  3  356 1.934867e+00
## 14476  3  357 1.919816e+00
## 14477  3  358 1.911895e+00
## 14478  3  359 1.898556e+00
## 14479  3  360 1.885060e+00
## 14480  3  360 1.884607e+00
## 14481  3  361 2.034261e+00
## 14482  3  362 2.125311e+00
## 14483  3  363 2.163520e+00
## 14484  3  364 2.169738e+00
## 14485  3  365 2.160410e+00
## 14486  3  366 2.147021e+00
## 14487  3  367 2.129301e+00
## 14488  3  368 2.113575e+00
## 14489  3  369 2.098269e+00
## 14490  3  370 2.081626e+00
## 14491  3  371 2.063423e+00
## 14492  3  372 2.048186e+00
## 14493  3  373 2.028183e+00
## 14494  3  374 2.013899e+00
## 14495  3  375 2.004431e+00
## 14496  3  376 1.986665e+00
## 14497  3  377 1.972712e+00
## 14498  3  378 1.959366e+00
## 14499  3  379 1.945703e+00
## 14500  3  380 1.930601e+00
## 14501  3  381 1.921024e+00
## 14502  3  382 1.905495e+00
## 14503  3  383 1.890504e+00
## 14504  3  384 1.884138e+00
## 14505  3  385 2.025159e+00
## 14506  3  386 2.119892e+00
## 14507  3  387 2.152804e+00
## 14508  3  388 2.164585e+00
## 14509  3  389 2.150156e+00
## 14510  3  390 2.138617e+00
## 14511  3  391 2.125075e+00
## 14512  3  392 2.107378e+00
## 14513  3  393 2.092176e+00
## 14514  3  394 2.070356e+00
## 14515  3  395 2.056736e+00
## 14516  3  396 2.042052e+00
## 14517  3  397 2.024835e+00
## 14518  3  398 2.013665e+00
## 14519  3  399 1.999750e+00
## 14520  3  400 1.987069e+00
## 14521  3  401 1.969475e+00
## 14522  3  402 1.952405e+00
## 14523  3  403 1.939675e+00
## 14524  3  404 1.929340e+00
## 14525  3  405 1.915205e+00
## 14526  3  406 1.904014e+00
## 14527  3  407 1.890600e+00
## 14528  3  408 1.879195e+00
## 14529  3  409 2.020003e+00
## 14530  3  410 2.122389e+00
## 14531  3  411 2.154046e+00
## 14532  3  412 2.162760e+00
## 14533  3  413 2.151762e+00
## 14534  3  414 2.140769e+00
## 14535  3  415 2.118916e+00
## 14536  3  416 2.105646e+00
## 14537  3  417 2.087753e+00
## 14538  3  418 2.071050e+00
## 14539  3  419 2.055940e+00
## 14540  3  420 2.041266e+00
## 14541  3  421 2.023399e+00
## 14542  3  422 2.010219e+00
## 14543  3  423 1.994108e+00
## 14544  3  424 1.980798e+00
## 14545  3  425 1.967143e+00
## 14546  3  426 1.955595e+00
## 14547  3  427 1.939261e+00
## 14548  3  428 1.923966e+00
## 14549  3  429 1.912215e+00
## 14550  3  430 1.901185e+00
## 14551  3  431 1.886676e+00
## 14552  3  432 1.877496e+00
## 14553  3  433 2.024488e+00
## 14554  3  434 2.114584e+00
## 14555  3  435 2.150533e+00
## 14556  3  436 2.159027e+00
## 14557  3  437 2.151838e+00
## 14558  3  438 2.134708e+00
## 14559  3  439 2.119173e+00
## 14560  3  440 2.104488e+00
## 14561  3  441 2.090477e+00
## 14562  3  442 2.075093e+00
## 14563  3  443 2.055420e+00
## 14564  3  444 2.038169e+00
## 14565  3  445 2.025898e+00
## 14566  3  446 2.011489e+00
## 14567  3  447 1.996711e+00
## 14568  3  448 1.978929e+00
## 14569  3  449 1.963513e+00
## 14570  3  450 1.950445e+00
## 14571  3  451 1.934857e+00
## 14572  3  452 1.925679e+00
## 14573  3  453 1.910726e+00
## 14574  3  454 1.900796e+00
## 14575  3  455 1.887845e+00
## 14576  3  456 1.874484e+00
## 14577  3  457 2.021360e+00
## 14578  3  458 2.113571e+00
## 14579  3  459 2.152244e+00
## 14580  3  460 2.160194e+00
## 14581  3  461 2.148319e+00
## 14582  3  462 2.136651e+00
## 14583  3  463 2.121057e+00
## 14584  3  464 2.108133e+00
## 14585  3  465 2.084302e+00
## 14586  3  466 2.074033e+00
## 14587  3  467 2.052659e+00
## 14588  3  468 2.037769e+00
## 14589  3  469 2.024800e+00
## 14590  3  470 2.012899e+00
## 14591  3  471 1.995085e+00
## 14592  3  472 1.982577e+00
## 14593  3  473 1.965530e+00
## 14594  3  474 1.951545e+00
## 14595  3  475 1.938934e+00
## 14596  3  476 1.924765e+00
## 14597  3  477 1.911713e+00
## 14598  3  478 1.901100e+00
## 14599  3  479 1.886937e+00
## 14600  3  480 1.875392e+00
## 14601  3  481 2.019871e+00
## 14602  3  482 2.116242e+00
## 14603  3  483 2.150710e+00
## 14604  3  484 2.154698e+00
## 14605  3  485 2.153856e+00
## 14606  3  486 2.136035e+00
## 14607  3  487 2.122423e+00
## 14608  3  488 2.104325e+00
## 14609  3  489 2.086061e+00
## 14610  3  490 2.072831e+00
## 14611  3  491 2.054554e+00
## 14612  3  492 2.040720e+00
## 14613  3  493 2.025867e+00
## 14614  3  494 2.008836e+00
## 14615  3  495 1.992336e+00
## 14616  3  496 1.980237e+00
## 14617  3  497 1.967285e+00
## 14618  3  498 1.949804e+00
## 14619  3  499 1.936731e+00
## 14620  3  500 1.924424e+00
## 14621  3  501 1.915579e+00
## 14622  3  502 1.898892e+00
## 14623  3  503 1.889302e+00
## 14624  3  504 1.875686e+00
## 14625  3  505 2.025020e+00
## 14626  3  506 2.113881e+00
## 14627  3  507 2.147360e+00
## 14628  3  508 2.157171e+00
## 14629  3  509 2.152007e+00
## 14630  3  510 2.135258e+00
## 14631  3  511 2.119869e+00
## 14632  3  512 2.103312e+00
## 14633  3  513 2.089224e+00
## 14634  3  514 2.071766e+00
## 14635  3  515 2.058940e+00
## 14636  3  516 2.035789e+00
## 14637  3  517 2.024516e+00
## 14638  3  518 2.008041e+00
## 14639  3  519 1.996336e+00
## 14640  3  520 1.978396e+00
## 14641  3  521 1.963117e+00
## 14642  3  522 1.951856e+00
## 14643  3  523 1.935602e+00
## 14644  3  524 1.927395e+00
## 14645  3  525 1.913849e+00
## 14646  3  526 1.899050e+00
## 14647  3  527 1.885304e+00
## 14648  3  528 1.875638e+00
## 14649  3  529 2.020736e+00
## 14650  3  530 2.112912e+00
## 14651  3  531 2.151715e+00
## 14652  3  532 2.155004e+00
## 14653  3  533 2.149343e+00
## 14654  3  534 2.137647e+00
## 14655  3  535 2.121804e+00
## 14656  3  536 2.105559e+00
## 14657  3  537 2.089222e+00
## 14658  3  538 2.073351e+00
## 14659  3  539 2.054513e+00
## 14660  3  540 2.040834e+00
## 14661  3  541 2.025003e+00
## 14662  3  542 2.007168e+00
## 14663  3  543 1.996734e+00
## 14664  3  544 1.979633e+00
## 14665  3  545 1.962829e+00
## 14666  3  546 1.953522e+00
## 14667  3  547 1.935533e+00
## 14668  3  548 1.925922e+00
## 14669  3  549 1.914748e+00
## 14670  3  550 1.899405e+00
## 14671  3  551 1.886909e+00
## 14672  3  552 1.871167e+00
## 14673  3  553 2.021439e+00
## 14674  3  554 2.117036e+00
## 14675  3  555 2.147734e+00
## 14676  3  556 2.158460e+00
## 14677  3  557 2.145910e+00
## 14678  3  558 2.138985e+00
## 14679  3  559 2.118358e+00
## 14680  3  560 2.108460e+00
## 14681  3  561 2.085667e+00
## 14682  3  562 2.068374e+00
## 14683  3  563 2.054916e+00
## 14684  3  564 2.038495e+00
## 14685  3  565 2.024547e+00
## 14686  3  566 2.011064e+00
## 14687  3  567 1.990541e+00
## 14688  3  568 1.980356e+00
## 14689  3  569 1.967215e+00
## 14690  3  570 1.952698e+00
## 14691  3  571 1.936887e+00
## 14692  3  572 1.926677e+00
## 14693  3  573 1.911438e+00
## 14694  3  574 1.898195e+00
## 14695  3  575 1.886401e+00
## 14696  3  576 1.875408e+00
## 14697  3  577 2.022238e+00
## 14698  3  578 2.114682e+00
## 14699  3  579 2.152892e+00
## 14700  3  580 2.156089e+00
## 14701  3  581 2.149637e+00
## 14702  3  582 2.135768e+00
## 14703  3  583 2.122965e+00
## 14704  3  584 2.104743e+00
## 14705  3  585 2.088298e+00
## 14706  3  586 2.070054e+00
## 14707  3  587 2.055950e+00
## 14708  3  588 2.038472e+00
## 14709  3  589 2.024108e+00
## 14710  3  590 2.006742e+00
## 14711  3  591 1.994958e+00
## 14712  3  592 1.977517e+00
## 14713  3  593 1.962299e+00
## 14714  3  594 1.955016e+00
## 14715  3  595 1.937372e+00
## 14716  3  596 1.925607e+00
## 14717  3  597 1.907706e+00
## 14718  3  598 1.899498e+00
## 14719  3  599 1.886282e+00
## 14720  3  600 1.873805e+00
## 14721  3  601 2.023029e+00
## 14722  3  602 2.114611e+00
## 14723  3  603 2.147617e+00
## 14724  3  604 2.155877e+00
## 14725  3  605 2.149727e+00
## 14726  3  606 2.138944e+00
## 14727  3  607 2.120048e+00
## 14728  3  608 2.106570e+00
## 14729  3  609 2.089708e+00
## 14730  3  610 2.073018e+00
## 14731  3  611 2.050787e+00
## 14732  3  612 2.036553e+00
## 14733  3  613 2.022376e+00
## 14734  3  614 2.011180e+00
## 14735  3  615 1.994165e+00
## 14736  3  616 1.978248e+00
## 14737  3  617 1.964273e+00
## 14738  3  618 1.949630e+00
## 14739  3  619 1.939138e+00
## 14740  3  620 1.925224e+00
## 14741  3  621 1.913430e+00
## 14742  3  622 1.901097e+00
## 14743  3  623 1.886136e+00
## 14744  3  624 1.875448e+00
## 14745  3  624 1.875777e+00
## 14746  3  625 2.019351e+00
## 14747  3  626 2.112337e+00
## 14748  3  627 2.145837e+00
## 14749  3  628 2.153528e+00
## 14750  3  629 2.150397e+00
## 14751  3  630 2.136423e+00
## 14752  3  631 2.118313e+00
## 14753  3  632 2.106328e+00
## 14754  3  633 2.081910e+00
## 14755  3  634 2.071322e+00
## 14756  3  635 2.053016e+00
## 14757  3  636 2.037205e+00
## 14758  3  637 2.022537e+00
## 14759  3  638 2.010240e+00
## 14760  3  639 1.993504e+00
## 14761  3  640 1.979782e+00
## 14762  3  641 1.963295e+00
## 14763  3  642 1.949609e+00
## 14764  3  643 1.935534e+00
## 14765  3  644 1.921617e+00
## 14766  3  645 1.910469e+00
## 14767  3  646 1.900446e+00
## 14768  3  647 1.881994e+00
## 14769  3  648 1.874870e+00
## 14770  3  648 1.876333e+00
## 14771  3  649 1.860955e+00
## 14772  3  650 1.850825e+00
## 14773  3  651 1.841831e+00
## 14774  3  652 1.826875e+00
## 14775  3  653 1.814508e+00
## 14776  3  654 1.805381e+00
## 14777  3  654 1.807267e+00
## 14778  3  655 1.956308e+00
## 14779  3  656 2.047533e+00
## 14780  3  657 2.089471e+00
## 14781  3  658 2.091722e+00
## 14782  3  659 2.084595e+00
## 14783  3  660 2.073520e+00
## 14784  3  661 2.059313e+00
## 14785  3  662 2.047377e+00
## 14786  3  663 2.031120e+00
## 14787  3  664 2.015180e+00
## 14788  3  665 1.996989e+00
## 14789  3  666 1.988424e+00
## 14790  3  667 1.970623e+00
## 14791  3  668 1.952308e+00
## 14792  3  669 1.941956e+00
## 14793  3  670 1.925421e+00
## 14794  3  671 1.914805e+00
## 14795  3  672 1.905418e+00
## 14796  3  672 1.902946e+00
## 14797  3  673 2.046953e+00
## 14798  3  674 2.138024e+00
## 14799  3  675 2.171385e+00
## 14800  3  676 2.181263e+00
## 14801  3  677 2.176094e+00
## 14802  3  678 2.154765e+00
## 14803  3  679 2.144141e+00
## 14804  3  680 2.126993e+00
## 14805  3  681 2.108507e+00
## 14806  3  682 2.090293e+00
## 14807  3  683 2.075326e+00
## 14808  3  684 2.058787e+00
## 14809  3  685 2.039313e+00
## 14810  3  686 2.025161e+00
## 14811  3  687 2.010255e+00
## 14812  3  688 1.997499e+00
## 14813  3  689 1.980464e+00
## 14814  3  690 1.965108e+00
## 14815  3  691 1.949989e+00
## 14816  3  692 1.937140e+00
## 14817  3  693 1.926999e+00
## 14818  3  694 1.912672e+00
## 14819  3  695 1.897178e+00
## 14820  3  696 1.886806e+00
## 14821  3  697 2.031743e+00
## 14822  3  698 2.125289e+00
## 14823  3  699 2.160758e+00
## 14824  3  700 2.169411e+00
## 14825  3  701 2.162585e+00
## 14826  3  702 2.144851e+00
## 14827  3  703 2.129878e+00
## 14828  3  704 2.111699e+00
## 14829  3  705 2.100334e+00
## 14830  3  706 2.080791e+00
## 14831  3  707 2.064322e+00
## 14832  3  708 2.047810e+00
## 14833  3  709 2.035041e+00
## 14834  3  710 2.015555e+00
## 14835  3  711 2.003427e+00
## 14836  3  712 1.987403e+00
## 14837  3  713 1.970005e+00
## 14838  3  714 1.956427e+00
## 14839  3  715 1.946383e+00
## 14840  3  716 1.929507e+00
## 14841  3  717 1.914640e+00
## 14842  3  718 1.901820e+00
## 14843  3  719 1.892271e+00
## 14844  3  720 1.880473e+00
## 14845  3  721 2.026938e+00
## 14846  3  722 2.123498e+00
## 14847  3  723 2.156129e+00
## 14848  3  724 2.157933e+00
## 14849  3  725 2.155418e+00
## 14850  3  726 2.138099e+00
## 14851  3  727 2.126560e+00
## 14852  3  728 2.108525e+00
## 14853  3  729 2.091584e+00
## 14854  3  730 2.073636e+00
## 14855  3  731 2.061815e+00
## 14856  3  732 2.046522e+00
## 14857  3  733 2.029368e+00
## 14858  3  734 2.013793e+00
## 14859  3  735 1.997737e+00
## 14860  3  736 1.984762e+00
## 14861  3  737 1.965728e+00
## 14862  3  738 1.955996e+00
## 14863  3  739 1.938917e+00
## 14864  3  740 1.929753e+00
## 14865  3  741 1.915146e+00
## 14866  3  742 1.903921e+00
## 14867  3  743 1.889206e+00
## 14868  3  744 1.877202e+00
## 14869  3  745 2.020807e+00
## 14870  3  746 2.119044e+00
## 14871  3  747 2.150968e+00
## 14872  3  748 2.159690e+00
## 14873  3  749 2.155260e+00
## 14874  3  750 2.138945e+00
## 14875  3  751 2.124578e+00
## 14876  3  752 2.105013e+00
## 14877  3  753 2.090441e+00
## 14878  3  754 2.074594e+00
## 14879  3  755 2.056805e+00
## 14880  3  756 2.042138e+00
## 14881  3  757 2.024606e+00
## 14882  3  758 2.010605e+00
## 14883  3  759 2.000125e+00
## 14884  3  760 1.978901e+00
## 14885  3  761 1.965026e+00
## 14886  3  762 1.955278e+00
## 14887  3  763 1.941718e+00
## 14888  3  764 1.926933e+00
## 14889  3  765 1.916390e+00
## 14890  3  766 1.900345e+00
## 14891  3  767 1.889813e+00
## 14892  3  768 1.874306e+00
## 14893  3  769 2.023357e+00
## 14894  3  770 2.116052e+00
## 14895  3  771 2.152496e+00
## 14896  3  772 2.158881e+00
## 14897  3  773 2.150251e+00
## 14898  3  774 2.139220e+00
## 14899  3  775 2.118332e+00
## 14900  3  776 2.107944e+00
## 14901  3  777 2.089951e+00
## 14902  3  778 2.071970e+00
## 14903  3  779 2.059692e+00
## 14904  3  780 2.042069e+00
## 14905  3  781 2.025868e+00
## 14906  3  782 2.012845e+00
## 14907  3  783 1.993675e+00
## 14908  3  784 1.979959e+00
## 14909  3  785 1.967367e+00
## 14910  3  786 1.955728e+00
## 14911  3  787 1.941545e+00
## 14912  3  788 1.928121e+00
## 14913  3  789 1.913021e+00
## 14914  3  790 1.898903e+00
## 14915  3  791 1.886986e+00
## 14916  3  792 1.880883e+00
## 14917  3  793 2.022160e+00
## 14918  3  794 2.116712e+00
## 14919  3  795 2.152253e+00
## 14920  3  796 2.155217e+00
## 14921  3  797 2.154122e+00
## 14922  3  798 2.135137e+00
## 14923  3  799 2.119565e+00
## 14924  3  800 2.107335e+00
## 14925  3  801 2.087807e+00
## 14926  3  802 2.069615e+00
## 14927  3  803 2.051769e+00
## 14928  3  804 2.040080e+00
## 14929  3  805 2.026963e+00
## 14930  3  806 2.006975e+00
## 14931  3  807 1.995462e+00
## 14932  3  808 1.978062e+00
## 14933  3  809 1.964547e+00
## 14934  3  810 1.951162e+00
## 14935  3  811 1.937386e+00
## 14936  3  812 1.928538e+00
## 14937  3  813 1.912864e+00
## 14938  3  814 1.899825e+00
## 14939  3  815 1.890325e+00
## 14940  3  816 1.876306e+00
## 14941  3  817 2.020773e+00
## 14942  3  818 2.117149e+00
## 14943  3  819 2.151955e+00
## 14944  3  820 2.154177e+00
## 14945  3  821 2.150022e+00
## 14946  3  822 2.141122e+00
## 14947  3  823 2.118697e+00
## 14948  3  824 2.103681e+00
## 14949  3  825 2.090164e+00
## 14950  3  826 2.073700e+00
## 14951  3  827 2.055250e+00
## 14952  3  828 2.041102e+00
## 14953  3  829 2.024653e+00
## 14954  3  830 2.011827e+00
## 14955  3  831 1.997967e+00
## 14956  3  832 1.980438e+00
## 14957  3  833 1.967891e+00
## 14958  3  834 1.950807e+00
## 14959  3  835 1.934699e+00
## 14960  3  836 1.925305e+00
## 14961  3  837 1.913275e+00
## 14962  3  838 1.898039e+00
## 14963  3  839 1.888602e+00
## 14964  3  840 1.874510e+00
## 14965  3  841 2.022805e+00
## 14966  3  842 2.114160e+00
## 14967  3  843 2.152854e+00
## 14968  3  844 2.154845e+00
## 14969  3  845 2.150290e+00
## 14970  3  846 2.136304e+00
## 14971  3  847 2.125606e+00
## 14972  3  848 2.105061e+00
## 14973  3  849 2.093410e+00
## 14974  3  850 2.067543e+00
## 14975  3  851 2.052219e+00
## 14976  3  852 2.042964e+00
## 14977  3  853 2.023157e+00
## 14978  3  854 2.007370e+00
## 14979  3  855 1.994041e+00
## 14980  3  856 1.983313e+00
## 14981  3  857 1.966667e+00
## 14982  3  858 1.952272e+00
## 14983  3  859 1.936178e+00
## 14984  3  860 1.925877e+00
## 14985  3  861 1.912712e+00
## 14986  3  862 1.905175e+00
## 14987  3  863 1.886965e+00
## 14988  3  864 1.875021e+00
## 14989  3  865 2.025750e+00
## 14990  3  866 2.116019e+00
## 14991  3  867 2.150049e+00
## 14992  3  868 2.155375e+00
## 14993  3  869 2.151669e+00
## 14994  3  870 2.138080e+00
## 14995  3  871 2.122058e+00
## 14996  3  872 2.103116e+00
## 14997  3  873 2.087393e+00
## 14998  3  874 2.069874e+00
## 14999  3  875 2.057366e+00
## 15000  3  876 2.041514e+00
## 15001  3  877 2.026390e+00
## 15002  3  878 2.004944e+00
## 15003  3  879 1.994088e+00
## 15004  3  880 1.979150e+00
## 15005  3  881 1.965371e+00
## 15006  3  882 1.954408e+00
## 15007  3  883 1.935314e+00
## 15008  3  884 1.928416e+00
## 15009  3  885 1.911869e+00
## 15010  3  886 1.898326e+00
## 15011  3  887 1.886288e+00
## 15012  3  888 1.878148e+00
## 15013  3  889 2.020446e+00
## 15014  3  890 2.114839e+00
## 15015  3  891 2.151848e+00
## 15016  3  892 2.156691e+00
## 15017  3  893 2.149922e+00
## 15018  3  894 2.140748e+00
## 15019  3  895 2.121888e+00
## 15020  3  896 2.100502e+00
## 15021  3  897 2.087659e+00
## 15022  3  898 2.069447e+00
## 15023  3  899 2.054702e+00
## 15024  3  900 2.040138e+00
## 15025  3  901 2.024090e+00
## 15026  3  902 2.007209e+00
## 15027  3  903 1.993237e+00
## 15028  3  904 1.980124e+00
## 15029  3  905 1.963958e+00
## 15030  3  906 1.951605e+00
## 15031  3  907 1.937290e+00
## 15032  3  908 1.926835e+00
## 15033  3  909 1.912639e+00
## 15034  3  910 1.902568e+00
## 15035  3  911 1.886368e+00
## 15036  3  912 1.875019e+00
## 15037  3  913 2.017739e+00
## 15038  3  914 2.116273e+00
## 15039  3  915 2.150946e+00
## 15040  3  916 2.155375e+00
## 15041  3  917 2.148388e+00
## 15042  3  918 2.137297e+00
## 15043  3  919 2.119667e+00
## 15044  3  920 2.105243e+00
## 15045  3  921 2.087087e+00
## 15046  3  922 2.072653e+00
## 15047  3  923 2.055525e+00
## 15048  3  924 2.039602e+00
## 15049  3  925 2.023939e+00
## 15050  3  926 2.010486e+00
## 15051  3  927 1.993204e+00
## 15052  3  928 1.980492e+00
## 15053  3  929 1.967562e+00
## 15054  3  930 1.951926e+00
## 15055  3  931 1.938131e+00
## 15056  3  932 1.924932e+00
## 15057  3  933 1.911430e+00
## 15058  3  934 1.898552e+00
## 15059  3  935 1.884210e+00
## 15060  3  936 1.876112e+00
## 15061  3  936 1.877164e+00
## 15062  3  937 2.023080e+00
## 15063  3  938 2.114340e+00
## 15064  3  939 2.150611e+00
## 15065  3  940 2.156841e+00
## 15066  3  941 2.148442e+00
## 15067  3  942 2.134226e+00
## 15068  3  943 2.120034e+00
## 15069  3  944 2.101945e+00
## 15070  3  945 2.088918e+00
## 15071  3  946 2.072169e+00
## 15072  3  947 2.053379e+00
## 15073  3  948 2.037693e+00
```

```
## 15074  3  949 2.025008e+00
## 15075  3  950 2.006294e+00
## 15076  3  951 1.990842e+00
## 15077  3  952 1.979512e+00
## 15078  3  953 1.962472e+00
## 15079  3  954 1.949042e+00
## 15080  3  955 1.934784e+00
## 15081  3  956 1.927635e+00
## 15082  3  957 1.910546e+00
## 15083  3  958 1.900500e+00
## 15084  3  959 1.885327e+00
## 15085  3  960 1.870500e+00
## 15086  3  960 1.876065e+00
## 15087  3  961 1.862185e+00
## 15088  3  962 1.849762e+00
## 15089  3  963 1.840865e+00
## 15090  3  964 1.829343e+00
## 15091  3  965 1.816819e+00
## 15092  3  966 1.805351e+00
## 15093  3  967 1.796720e+00
## 15094  3  968 1.785521e+00
## 15095  3  969 1.776353e+00
## 15096  3  969 1.773063e+00
## 15097  3  970 1.925010e+00
## 15098  3  971 2.017323e+00
## 15099  3  972 2.058330e+00
## 15100  3  973 2.067592e+00
## 15101  3  974 2.062236e+00
## 15102  3  975 2.050951e+00
## 15103  3  976 2.034412e+00
## 15104  3  977 2.020950e+00
## 15105  3  978 2.008055e+00
## 15106  3  979 1.987144e+00
## 15107  3  980 1.971584e+00
## 15108  3  981 1.958432e+00
## 15109  3  982 1.943734e+00
## 15110  3  983 1.931268e+00
## 15111  3  984 1.918706e+00
## 15112  3  984 1.918548e+00
## 15113  3  985 2.064587e+00
## 15114  3  986 2.159099e+00
## 15115  3  987 2.191447e+00
## 15116  3  988 2.194574e+00
## 15117  3  989 2.188419e+00
## 15118  3  990 2.175269e+00
## 15119  3  991 2.153401e+00
## 15120  3  992 2.136311e+00
## 15121  3  993 2.121295e+00
## 15122  3  994 2.100785e+00
## 15123  3  995 2.079655e+00
## 15124  3  996 2.067756e+00
## 15125  3  997 2.048003e+00
## 15126  3  998 2.034466e+00
## 15127  3  999 2.019874e+00
## 15128  3 1000 2.003638e+00
## 15129  3 1001 1.991944e+00
## 15130  3 1002 1.978023e+00
## 15131  3 1003 1.962567e+00
## 15132  3 1004 1.945170e+00
## 15133  3 1005 1.934551e+00
## 15134  3 1006 1.922516e+00
## 15135  3 1007 1.909668e+00
## 15136  3 1008 1.893697e+00
## 15137  3 1009 2.042466e+00
## 15138  3 1010 2.131385e+00
## 15139  3 1011 2.167113e+00
## 15140  3 1012 2.168294e+00
## 15141  3 1013 2.166718e+00
## 15142  3 1014 2.149628e+00
## 15143  3 1015 2.138243e+00
## 15144  3 1016 2.116428e+00
## 15145  3 1017 2.105304e+00
## 15146  3 1018 2.086589e+00
## 15147  3 1019 2.066106e+00
## 15148  3 1020 2.049466e+00
## 15149  3 1021 2.037872e+00
## 15150  3 1022 2.019773e+00
## 15151  3 1023 2.004942e+00
## 15152  3 1024 1.992545e+00
## 15153  3 1025 1.978662e+00
## 15154  3 1026 1.963546e+00
## 15155  3 1027 1.949110e+00
## 15156  3 1028 1.931603e+00
## 15157  3 1029 1.919719e+00
## 15158  3 1030 1.913324e+00
## 15159  3 1031 1.897493e+00
## 15160  3 1032 1.885087e+00
## 15161  3 1033 2.032117e+00
## 15162  3 1034 2.125611e+00
## 15163  3 1035 2.156337e+00
## 15164  3 1036 2.165122e+00
## 15165  3 1037 2.158649e+00
## 15166  3 1038 2.148069e+00
## 15167  3 1039 2.128651e+00
## 15168  3 1040 2.111292e+00
## 15169  3 1041 2.097348e+00
## 15170  3 1042 2.076234e+00
## 15171  3 1043 2.065525e+00
## 15172  3 1044 2.046479e+00
## 15173  3 1045 2.033034e+00
## 15174  3 1046 2.015390e+00
## 15175  3 1047 2.001992e+00
## 15176  3 1048 1.986452e+00
## 15177  3 1049 1.971347e+00
## 15178  3 1050 1.954215e+00
## 15179  3 1051 1.940915e+00
## 15180  3 1052 1.933483e+00
## 15181  3 1053 1.915373e+00
## 15182  3 1054 1.905261e+00
## 15183  3 1055 1.892647e+00
## 15184  3 1056 1.880969e+00
## 15185  3 1057 2.027980e+00
## 15186  3 1058 2.120754e+00
## 15187  3 1059 2.156879e+00
## 15188  3 1060 2.158798e+00
## 15189  3 1061 2.153160e+00
## 15190  3 1062 2.136140e+00
## 15191  3 1063 2.124190e+00
## 15192  3 1064 2.107143e+00
## 15193  3 1065 2.094142e+00
## 15194  3 1066 2.075291e+00
## 15195  3 1067 2.059049e+00
## 15196  3 1068 2.042002e+00
## 15197  3 1069 2.028986e+00
## 15198  3 1070 2.012228e+00
## 15199  3 1071 1.994017e+00
## 15200  3 1072 1.982038e+00
## 15201  3 1073 1.965972e+00
## 15202  3 1074 1.952775e+00
## 15203  3 1075 1.939708e+00
## 15204  3 1076 1.925696e+00
## 15205  3 1077 1.912560e+00
## 15206  3 1078 1.902684e+00
## 15207  3 1079 1.891536e+00
## 15208  3 1080 1.878335e+00
## 15209  3 1081 2.024508e+00
## 15210  3 1082 2.116410e+00
## 15211  3 1083 2.155806e+00
## 15212  3 1084 2.158437e+00
## 15213  3 1085 2.154334e+00
## 15214  3 1086 2.137905e+00
## 15215  3 1087 2.123419e+00
## 15216  3 1088 2.105763e+00
## 15217  3 1089 2.088531e+00
## 15218  3 1090 2.075675e+00
## 15219  3 1091 2.059924e+00
## 15220  3 1092 2.041442e+00
## 15221  3 1093 2.026383e+00
## 15222  3 1094 2.008486e+00
## 15223  3 1095 1.994259e+00
## 15224  3 1096 1.985973e+00
## 15225  3 1097 1.969123e+00
## 15226  3 1098 1.953164e+00
## 15227  3 1099 1.939599e+00
## 15228  3 1100 1.925981e+00
## 15229  3 1101 1.918807e+00
## 15230  3 1102 1.899043e+00
## 15231  3 1103 1.891390e+00
## 15232  3 1104 1.877804e+00
## 15233  3 1105 2.024669e+00
## 15234  3 1106 2.125685e+00
## 15235  3 1107 2.155543e+00
## 15236  3 1108 2.159759e+00
## 15237  3 1109 2.153261e+00
## 15238  3 1110 2.141871e+00
## 15239  3 1111 2.123349e+00
## 15240  3 1112 2.108164e+00
## 15241  3 1113 2.086912e+00
## 15242  3 1114 2.075101e+00
## 15243  3 1115 2.057902e+00
## 15244  3 1116 2.041782e+00
## 15245  3 1117 2.026579e+00
## 15246  3 1118 2.009302e+00
## 15247  3 1119 1.991960e+00
## 15248  3 1120 1.979594e+00
## 15249  3 1121 1.966455e+00
## 15250  3 1122 1.953753e+00
## 15251  3 1123 1.939748e+00
## 15252  3 1124 1.924734e+00
## 15253  3 1125 1.911849e+00
## 15254  3 1126 1.900437e+00
## 15255  3 1127 1.886510e+00
## 15256  3 1128 1.875939e+00
## 15257  3 1129 2.020802e+00
## 15258  3 1130 2.117227e+00
## 15259  3 1131 2.150605e+00
## 15260  3 1132 2.155981e+00
## 15261  3 1133 2.153197e+00
## 15262  3 1134 2.138069e+00
## 15263  3 1135 2.119718e+00
## 15264  3 1136 2.107373e+00
## 15265  3 1137 2.091301e+00
## 15266  3 1138 2.067848e+00
## 15267  3 1139 2.058317e+00
## 15268  3 1140 2.041041e+00
## 15269  3 1141 2.024784e+00
## 15270  3 1142 2.012372e+00
## 15271  3 1143 1.994682e+00
## 15272  3 1144 1.979590e+00
## 15273  3 1145 1.968089e+00
## 15274  3 1146 1.953753e+00
## 15275  3 1147 1.938490e+00
## 15276  3 1148 1.927160e+00
## 15277  3 1149 1.908873e+00
## 15278  3 1150 1.899837e+00
## 15279  3 1151 1.885791e+00
## 15280  3 1152 1.876386e+00
## 15281  3 1153 2.020935e+00
## 15282  3 1154 2.119222e+00
## 15283  3 1155 2.151573e+00
## 15284  3 1156 2.161397e+00
## 15285  3 1157 2.148630e+00
## 15286  3 1158 2.139538e+00
## 15287  3 1159 2.121285e+00
## 15288  3 1160 2.107852e+00
## 15289  3 1161 2.089877e+00
## 15290  3 1162 2.070675e+00
## 15291  3 1163 2.056304e+00
## 15292  3 1164 2.039437e+00
## 15293  3 1165 2.020216e+00
## 15294  3 1166 2.006974e+00
## 15295  3 1167 1.998305e+00
## 15296  3 1168 1.983902e+00
## 15297  3 1169 1.967845e+00
## 15298  3 1170 1.952035e+00
## 15299  3 1171 1.940240e+00
## 15300  3 1172 1.923965e+00
## 15301  3 1173 1.914051e+00
## 15302  3 1174 1.902569e+00
## 15303  3 1175 1.887067e+00
## 15304  3 1176 1.875156e+00
## 15305  3 1177 2.021020e+00
## 15306  3 1178 2.118835e+00
## 15307  3 1179 2.153541e+00
## 15308  3 1180 2.156459e+00
## 15309  3 1181 2.150394e+00
## 15310  3 1182 2.135876e+00
## 15311  3 1183 2.124566e+00
## 15312  3 1184 2.104434e+00
## 15313  3 1185 2.089389e+00
## 15314  3 1186 2.069218e+00
## 15315  3 1187 2.062202e+00
## 15316  3 1188 2.040644e+00
## 15317  3 1189 2.022710e+00
## 15318  3 1190 2.010140e+00
## 15319  3 1191 1.996775e+00
## 15320  3 1192 1.980231e+00
## 15321  3 1193 1.963199e+00
## 15322  3 1194 1.954912e+00
## 15323  3 1195 1.934534e+00
## 15324  3 1196 1.925687e+00
## 15325  3 1197 1.912157e+00
## 15326  3 1198 1.902122e+00
## 15327  3 1199 1.890020e+00
## 15328  3 1200 1.873532e+00
## 15329  3 1201 2.022488e+00
## 15330  3 1202 2.120160e+00
## 15331  3 1203 2.153697e+00
## 15332  3 1204 2.154862e+00
## 15333  3 1205 2.146721e+00
## 15334  3 1206 2.134131e+00
## 15335  3 1207 2.120417e+00
## 15336  3 1208 2.101911e+00
## 15337  3 1209 2.092228e+00
## 15338  3 1210 2.069846e+00
## 15339  3 1211 2.055629e+00
## 15340  3 1212 2.039985e+00
## 15341  3 1213 2.023109e+00
## 15342  3 1214 2.009096e+00
## 15343  3 1215 1.993838e+00
## 15344  3 1216 1.982760e+00
## 15345  3 1217 1.969436e+00
## 15346  3 1218 1.950339e+00
## 15347  3 1219 1.937348e+00
## 15348  3 1220 1.926139e+00
## 15349  3 1221 1.911703e+00
## 15350  3 1222 1.898767e+00
## 15351  3 1223 1.887077e+00
## 15352  3 1224 1.876893e+00
## 15353  3 1225 2.022410e+00
## 15354  3 1226 2.118314e+00
## 15355  3 1227 2.153262e+00
## 15356  3 1228 2.158842e+00
## 15357  3 1229 2.149440e+00
## 15358  3 1230 2.135886e+00
## 15359  3 1231 2.121992e+00
## 15360  3 1232 2.105146e+00
## 15361  3 1233 2.089325e+00
## 15362  3 1234 2.073160e+00
## 15363  3 1235 2.054966e+00
## 15364  3 1236 2.036896e+00
## 15365  3 1237 2.025461e+00
## 15366  3 1238 2.009142e+00
## 15367  3 1239 1.993656e+00
## 15368  3 1240 1.978714e+00
## 15369  3 1241 1.967073e+00
## 15370  3 1242 1.951247e+00
## 15371  3 1243 1.935710e+00
## 15372  3 1244 1.923151e+00
## 15373  3 1245 1.911671e+00
## 15374  3 1246 1.900262e+00
## 15375  3 1247 1.885190e+00
## 15376  3 1248 1.873906e+00
## 15377  3 1248 1.872705e+00
## 15378  3 1249 2.017334e+00
## 15379  3 1250 2.112200e+00
## 15380  3 1251 2.150704e+00
## 15381  3 1252 2.156143e+00
## 15382  3 1253 2.144412e+00
## 15383  3 1254 2.135841e+00
## 15384  3 1255 2.121196e+00
## 15385  3 1256 2.104320e+00
## 15386  3 1257 2.081933e+00
## 15387  3 1258 2.069258e+00
## 15388  3 1259 2.055422e+00
## 15389  3 1260 2.037820e+00
## 15390  3 1261 2.024551e+00
## 15391  3 1262 2.006649e+00
## 15392  3 1263 1.993352e+00
## 15393  3 1264 1.976691e+00
## 15394  3 1265 1.963298e+00
## 15395  3 1266 1.953680e+00
## 15396  3 1267 1.938995e+00
## 15397  3 1268 1.926445e+00
## 15398  3 1269 1.911178e+00
## 15399  3 1270 1.896714e+00
## 15400  3 1271 1.885194e+00
## 15401  3 1272 1.874308e+00
## 15402  3 1272 1.875037e+00
## 15403  3 1273 1.860570e+00
## 15404  3 1274 1.847918e+00
## 15405  3 1275 1.843428e+00
## 15406  3 1276 1.826330e+00
## 15407  3 1277 1.815467e+00
## 15408  3 1278 1.807329e+00
## 15409  3 1279 1.792710e+00
## 15410  3 1280 1.784242e+00
## 15411  3 1281 1.774371e+00
## 15412  3 1282 1.767513e+00
## 15413  3 1283 1.758541e+00
## 15414  3 1284 1.747196e+00
## 15415  3 1284 1.744962e+00
## 15416  3 1285 1.893297e+00
## 15417  3 1286 1.994476e+00
## 15418  3 1287 2.029674e+00
## 15419  3 1288 2.040771e+00
## 15420  3 1289 2.031589e+00
## 15421  3 1290 2.022577e+00
## 15422  3 1291 2.009662e+00
## 15423  3 1292 1.992840e+00
## 15424  3 1293 1.979216e+00
## 15425  3 1294 1.964771e+00
## 15426  3 1295 1.948196e+00
## 15427  3 1296 1.934010e+00
## 15428  3 1296 1.936516e+00
## 15429  3 1297 2.082983e+00
## 15430  3 1298 2.172940e+00
## 15431  3 1299 2.204824e+00
## 15432  3 1300 2.211531e+00
## 15433  3 1301 2.200071e+00
## 15434  3 1302 2.182999e+00
## 15435  3 1303 2.169028e+00
## 15436  3 1304 2.151332e+00
## 15437  3 1305 2.132276e+00
## 15438  3 1306 2.115872e+00
## 15439  3 1307 2.100246e+00
## 15440  3 1308 2.080552e+00
## 15441  3 1309 2.061873e+00
## 15442  3 1310 2.049715e+00
## 15443  3 1311 2.028360e+00
## 15444  3 1312 2.012224e+00
## 15445  3 1313 1.999109e+00
## 15446  3 1314 1.983829e+00
## 15447  3 1315 1.971673e+00
## 15448  3 1316 1.955077e+00
## 15449  3 1317 1.946287e+00
## 15450  3 1318 1.928522e+00
## 15451  3 1319 1.915418e+00
## 15452  3 1320 1.901585e+00
## 15453  3 1321 2.046896e+00
## 15454  3 1322 2.142667e+00
## 15455  3 1323 2.172933e+00
## 15456  3 1324 2.179533e+00
## 15457  3 1325 2.172839e+00
## 15458  3 1326 2.160137e+00
## 15459  3 1327 2.141375e+00
## 15460  3 1328 2.126523e+00
## 15461  3 1329 2.109534e+00
## 15462  3 1330 2.091699e+00
## 15463  3 1331 2.075256e+00
## 15464  3 1332 2.061447e+00
## 15465  3 1333 2.045748e+00
## 15466  3 1334 2.026087e+00
## 15467  3 1335 2.010882e+00
## 15468  3 1336 1.996734e+00
## 15469  3 1337 1.981274e+00
## 15470  3 1338 1.971124e+00
## 15471  3 1339 1.953676e+00
## 15472  3 1340 1.939191e+00
## 15473  3 1341 1.925403e+00
## 15474  3 1342 1.913467e+00
## 15475  3 1343 1.901159e+00
## 15476  3 1344 1.890714e+00
## 15477  3 1345 2.033755e+00
## 15478  3 1346 2.129922e+00
## 15479  3 1347 2.164304e+00
## 15480  3 1348 2.171944e+00
## 15481  3 1349 2.158469e+00
## 15482  3 1350 2.147991e+00
## 15483  3 1351 2.131956e+00
## 15484  3 1352 2.117743e+00
## 15485  3 1353 2.098278e+00
## 15486  3 1354 2.082009e+00
## 15487  3 1355 2.065937e+00
## 15488  3 1356 2.046586e+00
## 15489  3 1357 2.028299e+00
## 15490  3 1358 2.015994e+00
## 15491  3 1359 2.002654e+00
## 15492  3 1360 1.986964e+00
## 15493  3 1361 1.972427e+00
## 15494  3 1362 1.963724e+00
## 15495  3 1363 1.947196e+00
## 15496  3 1364 1.931416e+00
## 15497  3 1365 1.918484e+00
## 15498  3 1366 1.910379e+00
## 15499  3 1367 1.893796e+00
## 15500  3 1368 1.883766e+00
## 15501  3 1369 2.029084e+00
## 15502  3 1370 2.123225e+00
## 15503  3 1371 2.158086e+00
## 15504  3 1372 2.159233e+00
## 15505  3 1373 2.159864e+00
## 15506  3 1374 2.146642e+00
## 15507  3 1375 2.131489e+00
## 15508  3 1376 2.111680e+00
## 15509  3 1377 2.090138e+00
## 15510  3 1378 2.079799e+00
## 15511  3 1379 2.062598e+00
## 15512  3 1380 2.046160e+00
## 15513  3 1381 2.026975e+00
## 15514  3 1382 2.014894e+00
## 15515  3 1383 1.997779e+00
## 15516  3 1384 1.985402e+00
## 15517  3 1385 1.974548e+00
## 15518  3 1386 1.957806e+00
## 15519  3 1387 1.942556e+00
## 15520  3 1388 1.927079e+00
## 15521  3 1389 1.915604e+00
## 15522  3 1390 1.903364e+00
## 15523  3 1391 1.891392e+00
## 15524  3 1392 1.873687e+00
## 15525  3 1393 2.027588e+00
## 15526  3 1394 2.120970e+00
## 15527  3 1395 2.156729e+00
## 15528  3 1396 2.158912e+00
## 15529  3 1397 2.152763e+00
## 15530  3 1398 2.140337e+00
## 15531  3 1399 2.123690e+00
## 15532  3 1400 2.105023e+00
## 15533  3 1401 2.090250e+00
## 15534  3 1402 2.076417e+00
## 15535  3 1403 2.059254e+00
## 15536  3 1404 2.040051e+00
## 15537  3 1405 2.025828e+00
## 15538  3 1406 2.012671e+00
## 15539  3 1407 2.000238e+00
## 15540  3 1408 1.985365e+00
## 15541  3 1409 1.970197e+00
## 15542  3 1410 1.953836e+00
## 15543  3 1411 1.943378e+00
## 15544  3 1412 1.925400e+00
## 15545  3 1413 1.911004e+00
## 15546  3 1414 1.905547e+00
## 15547  3 1415 1.887041e+00
## 15548  3 1416 1.876611e+00
## 15549  3 1417 2.025420e+00
## 15550  3 1418 2.119264e+00
## 15551  3 1419 2.156316e+00
## 15552  3 1420 2.159277e+00
## 15553  3 1421 2.155475e+00
## 15554  3 1422 2.139561e+00
## 15555  3 1423 2.119222e+00
## 15556  3 1424 2.106928e+00
## 15557  3 1425 2.089564e+00
## 15558  3 1426 2.074521e+00
## 15559  3 1427 2.056651e+00
## 15560  3 1428 2.043201e+00
## 15561  3 1429 2.032358e+00
## 15562  3 1430 2.012631e+00
## 15563  3 1431 1.994512e+00
## 15564  3 1432 1.980890e+00
## 15565  3 1433 1.966219e+00
## 15566  3 1434 1.950846e+00
## 15567  3 1435 1.942467e+00
## 15568  3 1436 1.924854e+00
## 15569  3 1437 1.916100e+00
## 15570  3 1438 1.898028e+00
## 15571  3 1439 1.888213e+00
## 15572  3 1440 1.876287e+00
## 15573  3 1441 2.025954e+00
## 15574  3 1442 2.117474e+00
## 15575  3 1443 2.153788e+00
## 15576  3 1444 2.158339e+00
## 15577  3 1445 2.148490e+00
## 15578  3 1446 2.136789e+00
## 15579  3 1447 2.125925e+00
## 15580  3 1448 2.110390e+00
## 15581  3 1449 2.088259e+00
## 15582  3 1450 2.072461e+00
## 15583  3 1451 2.060083e+00
## 15584  3 1452 2.043679e+00
## 15585  3 1453 2.028867e+00
## 15586  3 1454 2.008120e+00
## 15587  3 1455 1.998934e+00
## 15588  3 1456 1.980181e+00
## 15589  3 1457 1.969247e+00
## 15590  3 1458 1.954040e+00
## 15591  3 1459 1.940944e+00
## 15592  3 1460 1.923828e+00
## 15593  3 1461 1.913680e+00
## 15594  3 1462 1.897684e+00
## 15595  3 1463 1.888046e+00
## 15596  3 1464 1.874810e+00
## 15597  3 1465 2.027321e+00
## 15598  3 1466 2.121579e+00
## 15599  3 1467 2.150002e+00
## 15600  3 1468 2.155898e+00
## 15601  3 1469 2.148301e+00
## 15602  3 1470 2.138420e+00
## 15603  3 1471 2.123030e+00
## 15604  3 1472 2.109014e+00
## 15605  3 1473 2.090766e+00
## 15606  3 1474 2.072239e+00
## 15607  3 1475 2.058431e+00
## 15608  3 1476 2.038836e+00
## 15609  3 1477 2.027536e+00
## 15610  3 1478 2.013210e+00
## 15611  3 1479 1.993400e+00
## 15612  3 1480 1.980087e+00
## 15613  3 1481 1.964682e+00
## 15614  3 1482 1.952002e+00
## 15615  3 1483 1.937173e+00
## 15616  3 1484 1.924996e+00
## 15617  3 1485 1.913100e+00
## 15618  3 1486 1.898665e+00
## 15619  3 1487 1.890041e+00
## 15620  3 1488 1.874584e+00
## 15621  3 1489 2.023569e+00
## 15622  3 1490 2.117849e+00
## 15623  3 1491 2.153422e+00
## 15624  3 1492 2.156209e+00
## 15625  3 1493 2.151361e+00
## 15626  3 1494 2.139598e+00
## 15627  3 1495 2.119013e+00
## 15628  3 1496 2.105481e+00
## 15629  3 1497 2.087639e+00
## 15630  3 1498 2.073711e+00
## 15631  3 1499 2.059640e+00
## 15632  3 1500 2.037226e+00
## 15633  3 1501 2.029451e+00
## 15634  3 1502 2.009444e+00
## 15635  3 1503 1.997190e+00
## 15636  3 1504 1.981067e+00
## 15637  3 1505 1.968086e+00
## 15638  3 1506 1.954437e+00
## 15639  3 1507 1.941577e+00
## 15640  3 1508 1.927358e+00
## 15641  3 1509 1.914957e+00
## 15642  3 1510 1.898659e+00
## 15643  3 1511 1.889810e+00
## 15644  3 1512 1.878353e+00
## 15645  3 1513 2.021021e+00
## 15646  3 1514 2.117935e+00
## 15647  3 1515 2.151604e+00
## 15648  3 1516 2.156183e+00
## 15649  3 1517 2.150995e+00
## 15650  3 1518 2.139998e+00
## 15651  3 1519 2.117151e+00
## 15652  3 1520 2.106062e+00
## 15653  3 1521 2.086279e+00
## 15654  3 1522 2.070565e+00
## 15655  3 1523 2.059349e+00
## 15656  3 1524 2.039839e+00
## 15657  3 1525 2.026309e+00
## 15658  3 1526 2.014057e+00
## 15659  3 1527 1.991801e+00
## 15660  3 1528 1.981900e+00
## 15661  3 1529 1.965210e+00
## 15662  3 1530 1.953511e+00
## 15663  3 1531 1.937759e+00
## 15664  3 1532 1.927477e+00
## 15665  3 1533 1.913745e+00
## 15666  3 1534 1.899942e+00
## 15667  3 1535 1.891188e+00
## 15668  3 1536 1.877685e+00
## 15669  3 1537 2.025700e+00
## 15670  3 1538 2.116400e+00
## 15671  3 1539 2.153685e+00
## 15672  3 1540 2.155628e+00
## 15673  3 1541 2.148263e+00
## 15674  3 1542 2.138587e+00
## 15675  3 1543 2.122315e+00
## 15676  3 1544 2.109319e+00
## 15677  3 1545 2.090420e+00
## 15678  3 1546 2.072142e+00
## 15679  3 1547 2.055993e+00
## 15680  3 1548 2.037833e+00
## 15681  3 1549 2.022545e+00
## 15682  3 1550 2.009561e+00
## 15683  3 1551 1.995050e+00
## 15684  3 1552 1.979005e+00
## 15685  3 1553 1.968341e+00
## 15686  3 1554 1.952906e+00
## 15687  3 1555 1.935789e+00
## 15688  3 1556 1.926297e+00
## 15689  3 1557 1.913795e+00
## 15690  3 1558 1.897387e+00
## 15691  3 1559 1.891809e+00
## 15692  3 1560 1.875821e+00
## 15693  3 1560 1.872678e+00
## 15694  3 1561 2.020977e+00
## 15695  3 1562 2.118027e+00
## 15696  3 1563 2.150477e+00
## 15697  3 1564 2.153487e+00
## 15698  3 1565 2.148912e+00
## 15699  3 1566 2.133815e+00
## 15700  3 1567 2.118868e+00
## 15701  3 1568 2.103452e+00
## 15702  3 1569 2.087158e+00
## 15703  3 1570 2.066750e+00
## 15704  3 1571 2.054163e+00
## 15705  3 1572 2.041356e+00
## 15706  3 1573 2.019058e+00
## 15707  3 1574 2.007537e+00
## 15708  3 1575 1.992788e+00
## 15709  3 1576 1.979021e+00
## 15710  3 1577 1.965204e+00
## 15711  3 1578 1.951507e+00
## 15712  3 1579 1.938163e+00
## 15713  3 1580 1.924456e+00
## 15714  3 1581 1.910833e+00
## 15715  3 1582 1.897889e+00
## 15716  3 1583 1.884039e+00
## 15717  3 1584 1.873396e+00
## 15718  3 1584 1.873841e+00
## 15719  3 1585 1.863102e+00
## 15720  3 1586 1.849355e+00
## 15721  3 1587 1.839657e+00
## 15722  3 1588 1.825126e+00
## 15723  3 1589 1.818457e+00
## 15724  3 1590 1.807834e+00
## 15725  3 1591 1.795081e+00
## 15726  3 1592 1.785101e+00
## 15727  3 1593 1.776036e+00
## 15728  3 1594 1.767290e+00
## 15729  3 1595 1.758291e+00
## 15730  3 1596 1.747402e+00
## 15731  3 1597 1.735375e+00
## 15732  3 1598 1.728844e+00
## 15733  3 1599 1.719373e+00
## 15734  3 1599 1.723423e+00
## 15735  3 1600 1.787446e+00
## 15736  3 1601 1.836903e+00
## 15737  3 1602 1.846416e+00
## 15738  3 1603 1.849540e+00
## 15739  3 1604 1.840341e+00
## 15740  3 1605 1.830694e+00
## 15741  3 1606 1.818563e+00
## 15742  3 1607 1.809975e+00
## 15743  3 1608 1.796777e+00
## 15744  3 1608 1.800834e+00
## 15745  3 1609 1.949761e+00
## 15746  3 1610 2.041022e+00
## 15747  3 1611 2.074226e+00
## 15748  3 1612 2.082967e+00
## 15749  3 1613 2.080712e+00
## 15750  3 1614 2.066435e+00
## 15751  3 1615 2.050149e+00
## 15752  3 1616 2.032872e+00
## 15753  3 1617 2.019823e+00
## 15754  3 1618 2.005616e+00
## 15755  3 1619 1.985140e+00
## 15756  3 1620 1.970619e+00
## 15757  3 1621 1.958240e+00
## 15758  3 1622 1.943113e+00
## 15759  3 1623 1.931915e+00
## 15760  3 1624 1.916080e+00
## 15761  3 1625 1.903159e+00
## 15762  3 1626 1.892890e+00
## 15763  3 1627 1.877029e+00
## 15764  3 1628 1.863395e+00
## 15765  3 1629 1.846218e+00
## 15766  3 1630 1.836367e+00
## 15767  3 1631 1.828006e+00
## 15768  3 1632 1.813892e+00
## 15769  3 1633 1.964035e+00
## 15770  3 1634 2.055898e+00
## 15771  3 1635 2.090526e+00
## 15772  3 1636 2.096591e+00
## 15773  3 1637 2.089619e+00
## 15774  3 1638 2.081818e+00
## 15775  3 1639 2.057980e+00
## 15776  3 1640 2.046940e+00
## 15777  3 1641 2.031419e+00
## 15778  3 1642 2.012833e+00
## 15779  3 1643 1.999428e+00
## 15780  3 1644 1.982698e+00
## 15781  3 1645 1.970592e+00
## 15782  3 1646 1.952509e+00
## 15783  3 1647 1.939139e+00
## 15784  3 1648 1.922504e+00
## 15785  3 1649 1.910200e+00
## 15786  3 1650 1.898922e+00
## 15787  3 1651 1.881522e+00
## 15788  3 1652 1.870389e+00
## 15789  3 1653 1.857281e+00
## 15790  3 1654 1.845961e+00
## 15791  3 1655 1.835232e+00
## 15792  3 1656 1.819964e+00
## 15793  3 1657 1.968281e+00
## 15794  3 1658 2.062079e+00
## 15795  3 1659 2.099209e+00
## 15796  3 1660 2.103386e+00
## 15797  3 1661 2.097123e+00
## 15798  3 1662 2.082372e+00
## 15799  3 1663 2.069779e+00
## 15800  3 1664 2.054089e+00
## 15801  3 1665 2.037688e+00
## 15802  3 1666 2.019964e+00
## 15803  3 1667 2.006156e+00
## 15804  3 1668 1.987106e+00
## 15805  3 1669 1.976826e+00
## 15806  3 1670 1.956986e+00
## 15807  3 1671 1.941334e+00
## 15808  3 1672 1.930030e+00
## 15809  3 1673 1.912894e+00
## 15810  3 1674 1.901597e+00
## 15811  3 1675 1.895480e+00
## 15812  3 1676 1.876271e+00
## 15813  3 1677 1.864920e+00
## 15814  3 1678 1.854112e+00
## 15815  3 1679 1.841720e+00
## 15816  3 1680 1.828437e+00
## 15817  3 1681 1.974682e+00
## 15818  3 1682 2.067788e+00
## 15819  3 1683 2.106104e+00
## 15820  3 1684 2.106818e+00
## 15821  3 1685 2.102337e+00
## 15822  3 1686 2.089880e+00
## 15823  3 1687 2.073276e+00
## 15824  3 1688 2.056328e+00
## 15825  3 1689 2.038929e+00
## 15826  3 1690 2.025008e+00
## 15827  3 1691 2.009929e+00
## 15828  3 1692 1.989887e+00
## 15829  3 1693 1.977202e+00
## 15830  3 1694 1.965033e+00
## 15831  3 1695 1.947267e+00
## 15832  3 1696 1.931951e+00
## 15833  3 1697 1.920997e+00
## 15834  3 1698 1.902022e+00
## 15835  3 1699 1.894484e+00
## 15836  3 1700 1.881140e+00
## 15837  3 1701 1.865819e+00
## 15838  3 1702 1.855868e+00
## 15839  3 1703 1.842240e+00
## 15840  3 1704 1.831901e+00
## 15841  3 1705 1.977224e+00
## 15842  3 1706 2.071147e+00
## 15843  3 1707 2.104717e+00
## 15844  3 1708 2.112638e+00
## 15845  3 1709 2.106138e+00
## 15846  3 1710 2.093197e+00
## 15847  3 1711 2.080683e+00
## 15848  3 1712 2.061825e+00
## 15849  3 1713 2.046150e+00
## 15850  3 1714 2.027319e+00
## 15851  3 1715 2.013263e+00
## 15852  3 1716 1.999730e+00
## 15853  3 1717 1.982092e+00
## 15854  3 1718 1.967654e+00
## 15855  3 1719 1.950830e+00
## 15856  3 1720 1.936060e+00
## 15857  3 1721 1.922120e+00
## 15858  3 1722 1.906924e+00
## 15859  3 1723 1.895276e+00
## 15860  3 1724 1.881995e+00
## 15861  3 1725 1.869651e+00
## 15862  3 1726 1.857424e+00
## 15863  3 1727 1.847195e+00
## 15864  3 1728 1.829990e+00
## 15865  3 1729 1.975364e+00
## 15866  3 1730 2.077545e+00
## 15867  3 1731 2.113119e+00
## 15868  3 1732 2.120047e+00
## 15869  3 1733 2.112173e+00
## 15870  3 1734 2.098643e+00
## 15871  3 1735 2.081835e+00
## 15872  3 1736 2.059542e+00
## 15873  3 1737 2.046854e+00
```

```
## 15874  3 1738 2.032044e+00
## 15875  3 1739 2.013709e+00
## 15876  3 1740 2.001399e+00
## 15877  3 1741 1.984195e+00
## 15878  3 1742 1.968008e+00
## 15879  3 1743 1.955064e+00
## 15880  3 1744 1.939568e+00
## 15881  3 1745 1.925861e+00
## 15882  3 1746 1.913129e+00
## 15883  3 1747 1.898756e+00
## 15884  3 1748 1.884681e+00
## 15885  3 1749 1.872860e+00
## 15886  3 1750 1.859684e+00
## 15887  3 1751 1.847190e+00
## 15888  3 1752 1.836546e+00
## 15889  3 1753 1.983257e+00
## 15890  3 1754 2.077368e+00
## 15891  3 1755 2.111898e+00
## 15892  3 1756 2.120625e+00
## 15893  3 1757 2.112712e+00
## 15894  3 1758 2.100174e+00
## 15895  3 1759 2.083066e+00
## 15896  3 1760 2.067231e+00
## 15897  3 1761 2.052595e+00
## 15898  3 1762 2.036078e+00
## 15899  3 1763 2.020365e+00
## 15900  3 1764 1.998964e+00
## 15901  3 1765 1.984742e+00
## 15902  3 1766 1.972090e+00
## 15903  3 1767 1.953221e+00
## 15904  3 1768 1.943477e+00
## 15905  3 1769 1.927596e+00
## 15906  3 1770 1.914459e+00
## 15907  3 1771 1.900367e+00
## 15908  3 1772 1.890693e+00
## 15909  3 1773 1.877744e+00
## 15910  3 1774 1.863597e+00
## 15911  3 1775 1.850721e+00
## 15912  3 1776 1.838903e+00
## 15913  3 1777 1.985375e+00
## 15914  3 1778 2.082761e+00
## 15915  3 1779 2.115958e+00
## 15916  3 1780 2.122846e+00
## 15917  3 1781 2.115260e+00
## 15918  3 1782 2.099653e+00
## 15919  3 1783 2.086347e+00
## 15920  3 1784 2.069204e+00
## 15921  3 1785 2.050271e+00
## 15922  3 1786 2.037120e+00
## 15923  3 1787 2.019797e+00
## 15924  3 1788 2.008860e+00
## 15925  3 1789 1.992731e+00
## 15926  3 1790 1.975447e+00
## 15927  3 1791 1.960488e+00
## 15928  3 1792 1.946914e+00
## 15929  3 1793 1.930721e+00
## 15930  3 1794 1.919470e+00
## 15931  3 1795 1.903207e+00
## 15932  3 1796 1.890343e+00
## 15933  3 1797 1.875230e+00
## 15934  3 1798 1.868095e+00
## 15935  3 1799 1.854218e+00
## 15936  3 1800 1.841558e+00
## 15937  3 1801 1.986353e+00
## 15938  3 1802 2.082362e+00
## 15939  3 1803 2.120526e+00
## 15940  3 1804 2.123195e+00
## 15941  3 1805 2.116975e+00
## 15942  3 1806 2.106229e+00
## 15943  3 1807 2.090491e+00
## 15944  3 1808 2.073064e+00
## 15945  3 1809 2.058084e+00
## 15946  3 1810 2.042334e+00
## 15947  3 1811 2.024458e+00
## 15948  3 1812 2.011200e+00
## 15949  3 1813 1.989660e+00
## 15950  3 1814 1.978257e+00
## 15951  3 1815 1.963564e+00
## 15952  3 1816 1.944155e+00
## 15953  3 1817 1.936562e+00
## 15954  3 1818 1.920244e+00
## 15955  3 1819 1.908998e+00
## 15956  3 1820 1.894797e+00
## 15957  3 1821 1.879396e+00
## 15958  3 1822 1.867622e+00
## 15959  3 1823 1.854747e+00
## 15960  3 1824 1.843376e+00
## 15961  3 1825 1.990897e+00
## 15962  3 1826 2.088902e+00
## 15963  3 1827 2.118235e+00
## 15964  3 1828 2.128064e+00
## 15965  3 1829 2.118919e+00
## 15966  3 1830 2.107689e+00
## 15967  3 1831 2.089306e+00
## 15968  3 1832 2.074158e+00
## 15969  3 1833 2.060500e+00
## 15970  3 1834 2.040677e+00
## 15971  3 1835 2.025280e+00
## 15972  3 1836 2.006283e+00
## 15973  3 1837 1.992892e+00
## 15974  3 1838 1.977670e+00
## 15975  3 1839 1.966145e+00
## 15976  3 1840 1.953051e+00
## 15977  3 1841 1.937654e+00
## 15978  3 1842 1.924068e+00
## 15979  3 1843 1.907343e+00
## 15980  3 1844 1.900537e+00
## 15981  3 1845 1.880935e+00
## 15982  3 1846 1.872463e+00
## 15983  3 1847 1.860758e+00
## 15984  3 1848 1.848128e+00
## 15985  3 1849 1.992317e+00
## 15986  3 1850 2.087708e+00
## 15987  3 1851 2.118619e+00
## 15988  3 1852 2.127155e+00
## 15989  3 1853 2.122791e+00
## 15990  3 1854 2.107906e+00
## 15991  3 1855 2.095330e+00
## 15992  3 1856 2.077999e+00
## 15993  3 1857 2.065962e+00
## 15994  3 1858 2.043143e+00
## 15995  3 1859 2.026039e+00
## 15996  3 1860 2.013363e+00
## 15997  3 1861 1.998656e+00
## 15998  3 1862 1.980256e+00
## 15999  3 1863 1.970175e+00
## 16000  3 1864 1.952937e+00
## 16001  3 1865 1.934842e+00
## 16002  3 1866 1.923347e+00
## 16003  3 1867 1.911622e+00
## 16004  3 1868 1.897807e+00
## 16005  3 1869 1.885145e+00
## 16006  3 1870 1.872326e+00
## 16007  3 1871 1.859813e+00
## 16008  3 1872 1.848228e+00
## 16009  3 1872 1.874900e+00
## 16010  3 1873 2.020807e+00
## 16011  3 1874 2.116307e+00
## 16012  3 1875 2.148282e+00
## 16013  3 1876 2.158147e+00
## 16014  3 1877 2.143648e+00
## 16015  3 1878 2.138400e+00
## 16016  3 1879 2.117738e+00
## 16017  3 1880 2.101047e+00
## 16018  3 1881 2.088184e+00
## 16019  3 1882 2.069922e+00
## 16020  3 1883 2.058039e+00
## 16021  3 1884 2.036874e+00
## 16022  3 1885 2.023609e+00
## 16023  3 1886 2.008083e+00
## 16024  3 1887 1.992663e+00
## 16025  3 1888 1.977748e+00
## 16026  3 1889 1.966169e+00
## 16027  3 1890 1.952037e+00
## 16028  3 1891 1.936783e+00
## 16029  3 1892 1.923619e+00
## 16030  3 1893 1.912547e+00
## 16031  3 1894 1.895612e+00
## 16032  3 1895 1.883918e+00
## 16033  3 1896 1.873610e+00
## 16034  3 1896 1.871322e+00
## 16035  3 1897 1.861054e+00
## 16036  3 1898 1.849200e+00
## 16037  3 1899 1.842430e+00
## 16038  3 1900 1.828567e+00
## 16039  3 1901 1.817833e+00
## 16040  3 1902 1.806625e+00
## 16041  3 1903 1.797010e+00
## 16042  3 1904 1.785008e+00
## 16043  3 1905 1.774936e+00
## 16044  3 1906 1.768467e+00
## 16045  3 1907 1.756498e+00
## 16046  3 1908 1.743680e+00
## 16047  3 1909 1.737506e+00
## 16048  3 1910 1.730761e+00
## 16049  3 1911 1.718745e+00
## 16050  3 1911 1.720307e+00
## 16051  3 1912 1.867891e+00
## 16052  3 1913 1.963880e+00
## 16053  3 1914 2.001521e+00
## 16054  3 1915 2.009946e+00
## 16055  3 1916 2.006179e+00
## 16056  3 1917 1.997589e+00
## 16057  3 1918 1.982866e+00
## 16058  3 1919 1.968337e+00
## 16059  3 1920 1.953685e+00
## 16060  3 1920 1.954185e+00
## 16061  3 1921 2.105042e+00
## 16062  3 1922 2.192370e+00
## 16063  3 1923 2.223615e+00
## 16064  3 1924 2.230148e+00
## 16065  3 1925 2.218146e+00
## 16066  3 1926 2.203259e+00
## 16067  3 1927 2.181487e+00
## 16068  3 1928 2.169632e+00
## 16069  3 1929 2.143045e+00
## 16070  3 1930 2.133068e+00
## 16071  3 1931 2.110358e+00
## 16072  3 1932 2.093513e+00
## 16073  3 1933 2.079350e+00
## 16074  3 1934 2.060564e+00
## 16075  3 1935 2.045373e+00
## 16076  3 1936 2.025880e+00
## 16077  3 1937 2.009447e+00
## 16078  3 1938 1.995453e+00
## 16079  3 1939 1.979470e+00
## 16080  3 1940 1.962239e+00
## 16081  3 1941 1.953788e+00
## 16082  3 1942 1.939552e+00
## 16083  3 1943 1.925410e+00
## 16084  3 1944 1.913236e+00
## 16085  3 1945 2.059793e+00
## 16086  3 1946 2.154752e+00
## 16087  3 1947 2.183629e+00
## 16088  3 1948 2.186376e+00
## 16089  3 1949 2.183730e+00
## 16090  3 1950 2.164596e+00
## 16091  3 1951 2.151505e+00
## 16092  3 1952 2.127591e+00
## 16093  3 1953 2.116153e+00
## 16094  3 1954 2.098643e+00
## 16095  3 1955 2.082860e+00
## 16096  3 1956 2.065277e+00
## 16097  3 1957 2.046296e+00
## 16098  3 1958 2.035038e+00
## 16099  3 1959 2.017027e+00
## 16100  3 1960 2.000324e+00
## 16101  3 1961 1.985933e+00
## 16102  3 1962 1.975103e+00
## 16103  3 1963 1.955776e+00
## 16104  3 1964 1.944546e+00
## 16105  3 1965 1.933648e+00
## 16106  3 1966 1.918740e+00
## 16107  3 1967 1.904550e+00
## 16108  3 1968 1.892359e+00
## 16109  3 1969 2.038466e+00
## 16110  3 1970 2.129772e+00
## 16111  3 1971 2.163902e+00
## 16112  3 1972 2.170952e+00
## 16113  3 1973 2.167455e+00
## 16114  3 1974 2.152250e+00
## 16115  3 1975 2.138000e+00
## 16116  3 1976 2.114751e+00
## 16117  3 1977 2.100600e+00
## 16118  3 1978 2.084828e+00
## 16119  3 1979 2.068011e+00
## 16120  3 1980 2.054569e+00
## 16121  3 1981 2.035494e+00
## 16122  3 1982 2.018901e+00
## 16123  3 1983 2.004151e+00
## 16124  3 1984 1.991392e+00
## 16125  3 1985 1.975344e+00
## 16126  3 1986 1.962834e+00
## 16127  3 1987 1.951024e+00
## 16128  3 1988 1.934411e+00
## 16129  3 1989 1.922577e+00
## 16130  3 1990 1.908612e+00
## 16131  3 1991 1.897826e+00
## 16132  3 1992 1.882061e+00
## 16133  3 1993 2.030323e+00
## 16134  3 1994 2.126224e+00
## 16135  3 1995 2.159782e+00
## 16136  3 1996 2.165856e+00
## 16137  3 1997 2.159390e+00
## 16138  3 1998 2.144833e+00
## 16139  3 1999 2.126677e+00
## 16140  3 2000 2.115718e+00
## 16141  3 2001 2.094463e+00
## 16142  3 2002 2.078225e+00
## 16143  3 2003 2.065323e+00
## 16144  3 2004 2.048909e+00
## 16145  3 2005 2.030421e+00
## 16146  3 2006 2.015145e+00
## 16147  3 2007 2.002474e+00
## 16148  3 2008 1.984570e+00
## 16149  3 2009 1.972810e+00
## 16150  3 2010 1.960580e+00
## 16151  3 2011 1.944826e+00
## 16152  3 2012 1.930823e+00
## 16153  3 2013 1.918691e+00
## 16154  3 2014 1.904463e+00
## 16155  3 2015 1.894663e+00
## 16156  3 2016 1.880287e+00
## 16157  3 2017 2.029682e+00
## 16158  3 2018 2.120958e+00
## 16159  3 2019 2.157231e+00
## 16160  3 2020 2.159981e+00
## 16161  3 2021 2.152599e+00
## 16162  3 2022 2.143255e+00
## 16163  3 2023 2.127788e+00
## 16164  3 2024 2.112700e+00
## 16165  3 2025 2.093044e+00
## 16166  3 2026 2.074585e+00
## 16167  3 2027 2.064763e+00
## 16168  3 2028 2.043047e+00
## 16169  3 2029 2.028522e+00
## 16170  3 2030 2.011188e+00
## 16171  3 2031 1.996640e+00
## 16172  3 2032 1.983199e+00
## 16173  3 2033 1.966258e+00
## 16174  3 2034 1.957666e+00
## 16175  3 2035 1.944363e+00
## 16176  3 2036 1.927565e+00
## 16177  3 2037 1.920067e+00
## 16178  3 2038 1.901978e+00
## 16179  3 2039 1.890729e+00
## 16180  3 2040 1.877735e+00
## 16181  3 2041 2.024772e+00
## 16182  3 2042 2.120790e+00
## 16183  3 2043 2.155112e+00
## 16184  3 2044 2.163272e+00
## 16185  3 2045 2.152785e+00
## 16186  3 2046 2.139942e+00
## 16187  3 2047 2.125668e+00
## 16188  3 2048 2.109828e+00
## 16189  3 2049 2.093988e+00
## 16190  3 2050 2.072591e+00
## 16191  3 2051 2.058051e+00
## 16192  3 2052 2.042015e+00
## 16193  3 2053 2.025128e+00
## 16194  3 2054 2.012437e+00
## 16195  3 2055 1.997733e+00
## 16196  3 2056 1.985562e+00
## 16197  3 2057 1.969772e+00
## 16198  3 2058 1.952342e+00
## 16199  3 2059 1.942348e+00
## 16200  3 2060 1.929612e+00
## 16201  3 2061 1.914851e+00
## 16202  3 2062 1.900985e+00
## 16203  3 2063 1.887976e+00
## 16204  3 2064 1.876631e+00
## 16205  3 2065 2.021992e+00
## 16206  3 2066 2.115864e+00
## 16207  3 2067 2.156237e+00
## 16208  3 2068 2.157475e+00
## 16209  3 2069 2.155078e+00
## 16210  3 2070 2.140587e+00
## 16211  3 2071 2.126716e+00
## 16212  3 2072 2.107453e+00
## 16213  3 2073 2.088639e+00
## 16214  3 2074 2.073235e+00
## 16215  3 2075 2.059070e+00
## 16216  3 2076 2.044702e+00
## 16217  3 2077 2.032534e+00
## 16218  3 2078 2.014929e+00
## 16219  3 2079 2.001257e+00
## 16220  3 2080 1.981852e+00
## 16221  3 2081 1.964009e+00
## 16222  3 2082 1.955872e+00
## 16223  3 2083 1.941398e+00
## 16224  3 2084 1.928606e+00
## 16225  3 2085 1.916949e+00
## 16226  3 2086 1.896383e+00
## 16227  3 2087 1.889170e+00
## 16228  3 2088 1.879500e+00
## 16229  3 2089 2.025502e+00
## 16230  3 2090 2.120921e+00
## 16231  3 2091 2.155946e+00
## 16232  3 2092 2.160089e+00
## 16233  3 2093 2.153237e+00
## 16234  3 2094 2.136202e+00
## 16235  3 2095 2.120521e+00
## 16236  3 2096 2.104011e+00
## 16237  3 2097 2.094177e+00
## 16238  3 2098 2.077097e+00
## 16239  3 2099 2.056684e+00
## 16240  3 2100 2.044922e+00
## 16241  3 2101 2.024007e+00
## 16242  3 2102 2.010322e+00
## 16243  3 2103 1.997146e+00
## 16244  3 2104 1.983602e+00
## 16245  3 2105 1.969589e+00
## 16246  3 2106 1.952700e+00
## 16247  3 2107 1.940185e+00
## 16248  3 2108 1.925296e+00
## 16249  3 2109 1.914567e+00
## 16250  3 2110 1.896992e+00
## 16251  3 2111 1.888809e+00
## 16252  3 2112 1.877940e+00
## 16253  3 2113 2.022318e+00
## 16254  3 2114 2.118194e+00
## 16255  3 2115 2.151193e+00
## 16256  3 2116 2.158368e+00
## 16257  3 2117 2.151380e+00
## 16258  3 2118 2.136829e+00
## 16259  3 2119 2.121472e+00
## 16260  3 2120 2.110454e+00
## 16261  3 2121 2.091822e+00
## 16262  3 2122 2.071927e+00
## 16263  3 2123 2.058500e+00
## 16264  3 2124 2.042458e+00
## 16265  3 2125 2.027648e+00
## 16266  3 2126 2.007709e+00
## 16267  3 2127 1.998042e+00
## 16268  3 2128 1.980106e+00
## 16269  3 2129 1.963778e+00
## 16270  3 2130 1.951603e+00
## 16271  3 2131 1.943241e+00
## 16272  3 2132 1.929397e+00
## 16273  3 2133 1.917645e+00
## 16274  3 2134 1.902140e+00
## 16275  3 2135 1.889679e+00
## 16276  3 2136 1.875427e+00
## 16277  3 2137 2.023513e+00
## 16278  3 2138 2.112937e+00
## 16279  3 2139 2.148108e+00
## 16280  3 2140 2.157972e+00
## 16281  3 2141 2.151665e+00
## 16282  3 2142 2.137385e+00
## 16283  3 2143 2.123662e+00
## 16284  3 2144 2.107891e+00
## 16285  3 2145 2.093447e+00
## 16286  3 2146 2.073639e+00
## 16287  3 2147 2.056997e+00
## 16288  3 2148 2.042377e+00
## 16289  3 2149 2.024731e+00
## 16290  3 2150 2.009394e+00
## 16291  3 2151 1.994234e+00
## 16292  3 2152 1.982038e+00
## 16293  3 2153 1.969434e+00
## 16294  3 2154 1.957813e+00
## 16295  3 2155 1.939143e+00
## 16296  3 2156 1.927552e+00
## 16297  3 2157 1.912362e+00
## 16298  3 2158 1.902732e+00
## 16299  3 2159 1.887085e+00
## 16300  3 2160 1.876662e+00
## 16301  3 2161 2.020016e+00
## 16302  3 2162 2.117911e+00
## 16303  3 2163 2.150775e+00
## 16304  3 2164 2.161769e+00
## 16305  3 2165 2.151474e+00
## 16306  3 2166 2.137180e+00
## 16307  3 2167 2.121713e+00
## 16308  3 2168 2.111170e+00
## 16309  3 2169 2.088989e+00
## 16310  3 2170 2.071389e+00
## 16311  3 2171 2.060023e+00
## 16312  3 2172 2.042871e+00
## 16313  3 2173 2.025099e+00
## 16314  3 2174 2.011042e+00
## 16315  3 2175 1.994539e+00
## 16316  3 2176 1.981277e+00
## 16317  3 2177 1.964790e+00
## 16318  3 2178 1.952423e+00
## 16319  3 2179 1.938621e+00
## 16320  3 2180 1.925550e+00
## 16321  3 2181 1.910346e+00
## 16322  3 2182 1.897132e+00
## 16323  3 2183 1.886553e+00
## 16324  3 2184 1.877277e+00
## 16325  3 2184 1.874240e+00
## 16326  3 2185 2.019400e+00
## 16327  3 2186 2.112187e+00
## 16328  3 2187 2.149120e+00
## 16329  3 2188 2.151481e+00
## 16330  3 2189 2.149626e+00
## 16331  3 2190 2.139744e+00
## 16332  3 2191 2.120938e+00
## 16333  3 2192 2.107408e+00
## 16334  3 2193 2.085354e+00
## 16335  3 2194 2.069990e+00
## 16336  3 2195 2.053065e+00
## 16337  3 2196 2.042229e+00
## 16338  3 2197 2.019613e+00
## 16339  3 2198 2.007224e+00
## 16340  3 2199 1.994330e+00
## 16341  3 2200 1.980211e+00
## 16342  3 2201 1.964703e+00
## 16343  3 2202 1.951388e+00
## 16344  3 2203 1.938277e+00
## 16345  3 2204 1.926698e+00
## 16346  3 2205 1.910943e+00
## 16347  3 2206 1.898156e+00
## 16348  3 2207 1.891000e+00
## 16349  3 2208 1.873726e+00
## 16350  3 2208 1.872572e+00
## 16351  3 2209 1.863716e+00
## 16352  3 2210 1.851608e+00
## 16353  3 2211 1.837679e+00
## 16354  3 2212 1.830524e+00
## 16355  3 2213 1.817446e+00
## 16356  3 2214 1.807578e+00
## 16357  3 2215 1.794443e+00
## 16358  3 2216 1.787499e+00
## 16359  3 2217 1.774837e+00
## 16360  3 2218 1.766070e+00
## 16361  3 2219 1.756062e+00
## 16362  3 2220 1.746777e+00
## 16363  3 2221 1.737615e+00
## 16364  3 2222 1.729370e+00
## 16365  3 2223 1.718089e+00
## 16366  3 2224 1.711385e+00
## 16367  3 2225 1.702529e+00
## 16368  3 2226 1.693044e+00
## 16369  3 2226 1.689062e+00
## 16370  3 2227 1.763010e+00
## 16371  3 2228 1.805492e+00
## 16372  3 2229 1.824883e+00
## 16373  3 2230 1.825580e+00
## 16374  3 2231 1.817432e+00
## 16375  3 2232 1.808654e+00
## 16376  3 2232 1.809094e+00
## 16377  3 2233 1.957038e+00
## 16378  3 2234 2.056726e+00
## 16379  3 2235 2.088065e+00
## 16380  3 2236 2.091536e+00
## 16381  3 2237 2.089908e+00
## 16382  3 2238 2.072206e+00
## 16383  3 2239 2.062003e+00
## 16384  3 2240 2.041338e+00
## 16385  3 2241 2.027827e+00
## 16386  3 2242 2.013233e+00
## 16387  3 2243 1.995919e+00
## 16388  3 2244 1.980676e+00
## 16389  3 2245 1.965267e+00
## 16390  3 2246 1.949990e+00
## 16391  3 2247 1.936818e+00
## 16392  3 2248 1.920288e+00
## 16393  3 2249 1.907809e+00
## 16394  3 2250 1.897406e+00
## 16395  3 2251 1.882331e+00
## 16396  3 2252 1.868561e+00
## 16397  3 2253 1.855028e+00
## 16398  3 2254 1.839033e+00
## 16399  3 2255 1.828917e+00
## 16400  3 2256 1.819215e+00
## 16401  3 2257 1.966981e+00
## 16402  3 2258 2.061611e+00
## 16403  3 2259 2.098236e+00
## 16404  3 2260 2.100600e+00
## 16405  3 2261 2.090137e+00
## 16406  3 2262 2.078187e+00
## 16407  3 2263 2.064038e+00
## 16408  3 2264 2.054646e+00
## 16409  3 2265 2.031060e+00
## 16410  3 2266 2.017675e+00
## 16411  3 2267 1.998718e+00
## 16412  3 2268 1.985647e+00
## 16413  3 2269 1.970301e+00
## 16414  3 2270 1.957652e+00
## 16415  3 2271 1.942026e+00
## 16416  3 2272 1.927308e+00
## 16417  3 2273 1.917280e+00
## 16418  3 2274 1.895957e+00
## 16419  3 2275 1.885451e+00
## 16420  3 2276 1.873918e+00
## 16421  3 2277 1.859360e+00
## 16422  3 2278 1.842932e+00
## 16423  3 2279 1.835707e+00
## 16424  3 2280 1.822430e+00
## 16425  3 2281 1.968720e+00
## 16426  3 2282 2.066658e+00
## 16427  3 2283 2.100268e+00
## 16428  3 2284 2.106417e+00
## 16429  3 2285 2.099826e+00
## 16430  3 2286 2.086231e+00
## 16431  3 2287 2.071167e+00
## 16432  3 2288 2.051472e+00
## 16433  3 2289 2.038855e+00
## 16434  3 2290 2.023437e+00
## 16435  3 2291 2.006152e+00
## 16436  3 2292 1.991188e+00
## 16437  3 2293 1.975515e+00
## 16438  3 2294 1.957315e+00
## 16439  3 2295 1.947576e+00
## 16440  3 2296 1.929648e+00
## 16441  3 2297 1.912092e+00
## 16442  3 2298 1.904693e+00
## 16443  3 2299 1.890412e+00
## 16444  3 2300 1.877363e+00
## 16445  3 2301 1.863039e+00
## 16446  3 2302 1.849799e+00
## 16447  3 2303 1.836596e+00
## 16448  3 2304 1.829534e+00
## 16449  3 2305 1.975759e+00
## 16450  3 2306 2.072826e+00
## 16451  3 2307 2.104564e+00
## 16452  3 2308 2.107224e+00
## 16453  3 2309 2.106166e+00
## 16454  3 2310 2.090032e+00
## 16455  3 2311 2.075464e+00
## 16456  3 2312 2.056999e+00
## 16457  3 2313 2.045185e+00
## 16458  3 2314 2.026456e+00
## 16459  3 2315 2.009175e+00
## 16460  3 2316 1.992123e+00
## 16461  3 2317 1.974450e+00
## 16462  3 2318 1.962281e+00
## 16463  3 2319 1.950758e+00
## 16464  3 2320 1.933313e+00
## 16465  3 2321 1.921926e+00
## 16466  3 2322 1.904262e+00
## 16467  3 2323 1.894208e+00
## 16468  3 2324 1.878707e+00
## 16469  3 2325 1.865171e+00
## 16470  3 2326 1.857206e+00
## 16471  3 2327 1.843839e+00
## 16472  3 2328 1.830301e+00
## 16473  3 2329 1.979588e+00
## 16474  3 2330 2.072913e+00
## 16475  3 2331 2.104562e+00
## 16476  3 2332 2.115826e+00
## 16477  3 2333 2.105063e+00
## 16478  3 2334 2.093035e+00
## 16479  3 2335 2.079711e+00
## 16480  3 2336 2.063792e+00
## 16481  3 2337 2.044779e+00
## 16482  3 2338 2.026461e+00
## 16483  3 2339 2.014376e+00
## 16484  3 2340 1.994268e+00
## 16485  3 2341 1.979510e+00
## 16486  3 2342 1.967299e+00
## 16487  3 2343 1.948253e+00
## 16488  3 2344 1.939704e+00
## 16489  3 2345 1.921828e+00
## 16490  3 2346 1.910392e+00
## 16491  3 2347 1.895672e+00
## 16492  3 2348 1.884758e+00
## 16493  3 2349 1.869685e+00
## 16494  3 2350 1.857354e+00
## 16495  3 2351 1.842448e+00
## 16496  3 2352 1.832892e+00
## 16497  3 2353 1.982007e+00
## 16498  3 2354 2.076315e+00
## 16499  3 2355 2.108713e+00
## 16500  3 2356 2.117701e+00
## 16501  3 2357 2.110268e+00
## 16502  3 2358 2.096935e+00
## 16503  3 2359 2.079767e+00
## 16504  3 2360 2.061265e+00
## 16505  3 2361 2.049786e+00
## 16506  3 2362 2.032949e+00
## 16507  3 2363 2.010554e+00
## 16508  3 2364 2.000610e+00
## 16509  3 2365 1.981295e+00
## 16510  3 2366 1.971037e+00
## 16511  3 2367 1.955554e+00
## 16512  3 2368 1.938333e+00
## 16513  3 2369 1.927581e+00
## 16514  3 2370 1.911518e+00
## 16515  3 2371 1.901912e+00
## 16516  3 2372 1.888408e+00
## 16517  3 2373 1.876685e+00
## 16518  3 2374 1.861682e+00
## 16519  3 2375 1.850656e+00
## 16520  3 2376 1.834106e+00
## 16521  3 2377 1.983207e+00
## 16522  3 2378 2.076909e+00
## 16523  3 2379 2.110487e+00
## 16524  3 2380 2.121451e+00
## 16525  3 2381 2.113669e+00
## 16526  3 2382 2.096034e+00
## 16527  3 2383 2.086496e+00
## 16528  3 2384 2.068045e+00
## 16529  3 2385 2.053437e+00
## 16530  3 2386 2.035623e+00
## 16531  3 2387 2.021331e+00
## 16532  3 2388 2.005359e+00
## 16533  3 2389 1.989084e+00
## 16534  3 2390 1.974178e+00
## 16535  3 2391 1.961812e+00
## 16536  3 2392 1.938283e+00
## 16537  3 2393 1.932374e+00
## 16538  3 2394 1.914865e+00
## 16539  3 2395 1.904823e+00
## 16540  3 2396 1.889164e+00
## 16541  3 2397 1.877920e+00
## 16542  3 2398 1.865185e+00
## 16543  3 2399 1.851890e+00
## 16544  3 2400 1.840099e+00
## 16545  3 2401 1.984366e+00
## 16546  3 2402 2.085633e+00
## 16547  3 2403 2.114895e+00
## 16548  3 2404 2.117668e+00
## 16549  3 2405 2.115850e+00
## 16550  3 2406 2.100941e+00
## 16551  3 2407 2.090802e+00
## 16552  3 2408 2.072573e+00
## 16553  3 2409 2.051619e+00
## 16554  3 2410 2.034462e+00
## 16555  3 2411 2.022159e+00
## 16556  3 2412 2.003795e+00
## 16557  3 2413 1.991876e+00
## 16558  3 2414 1.973102e+00
## 16559  3 2415 1.957637e+00
## 16560  3 2416 1.950225e+00
## 16561  3 2417 1.931246e+00
## 16562  3 2418 1.915202e+00
## 16563  3 2419 1.903148e+00
## 16564  3 2420 1.891124e+00
## 16565  3 2421 1.880144e+00
## 16566  3 2422 1.869155e+00
## 16567  3 2423 1.853603e+00
## 16568  3 2424 1.843010e+00
## 16569  3 2425 1.991588e+00
## 16570  3 2426 2.083838e+00
## 16571  3 2427 2.119875e+00
## 16572  3 2428 2.119221e+00
## 16573  3 2429 2.119478e+00
## 16574  3 2430 2.104826e+00
## 16575  3 2431 2.089045e+00
## 16576  3 2432 2.072742e+00
## 16577  3 2433 2.056606e+00
## 16578  3 2434 2.041632e+00
## 16579  3 2435 2.023725e+00
## 16580  3 2436 2.010489e+00
## 16581  3 2437 1.992501e+00
## 16582  3 2438 1.975336e+00
## 16583  3 2439 1.960114e+00
## 16584  3 2440 1.950936e+00
## 16585  3 2441 1.934489e+00
## 16586  3 2442 1.921045e+00
## 16587  3 2443 1.908446e+00
## 16588  3 2444 1.893081e+00
## 16589  3 2445 1.882076e+00
## 16590  3 2446 1.866274e+00
## 16591  3 2447 1.854686e+00
## 16592  3 2448 1.847911e+00
## 16593  3 2449 1.986160e+00
## 16594  3 2450 2.088089e+00
## 16595  3 2451 2.122925e+00
## 16596  3 2452 2.128374e+00
## 16597  3 2453 2.123914e+00
## 16598  3 2454 2.104357e+00
## 16599  3 2455 2.090242e+00
## 16600  3 2456 2.076107e+00
## 16601  3 2457 2.060765e+00
## 16602  3 2458 2.042984e+00
## 16603  3 2459 2.023674e+00
## 16604  3 2460 2.011300e+00
## 16605  3 2461 1.995402e+00
## 16606  3 2462 1.976352e+00
## 16607  3 2463 1.966143e+00
## 16608  3 2464 1.952289e+00
## 16609  3 2465 1.937495e+00
## 16610  3 2466 1.922415e+00
## 16611  3 2467 1.908779e+00
## 16612  3 2468 1.898152e+00
## 16613  3 2469 1.883047e+00
## 16614  3 2470 1.873530e+00
## 16615  3 2471 1.858582e+00
## 16616  3 2472 1.847966e+00
## 16617  3 2473 1.991278e+00
## 16618  3 2474 2.086042e+00
## 16619  3 2475 2.124945e+00
## 16620  3 2476 2.127793e+00
## 16621  3 2477 2.120527e+00
## 16622  3 2478 2.108206e+00
## 16623  3 2479 2.091890e+00
## 16624  3 2480 2.073110e+00
## 16625  3 2481 2.059386e+00
## 16626  3 2482 2.044563e+00
## 16627  3 2483 2.028273e+00
## 16628  3 2484 2.015687e+00
## 16629  3 2485 1.999807e+00
## 16630  3 2486 1.981873e+00
## 16631  3 2487 1.967330e+00
## 16632  3 2488 1.955009e+00
## 16633  3 2489 1.937681e+00
## 16634  3 2490 1.923135e+00
## 16635  3 2491 1.911340e+00
## 16636  3 2492 1.897715e+00
## 16637  3 2493 1.886573e+00
## 16638  3 2494 1.875696e+00
## 16639  3 2495 1.860052e+00
## 16640  3 2496 1.848824e+00
## 16641  3 2496 1.875213e+00
## 16642  3 2497 2.019485e+00
## 16643  3 2498 2.116353e+00
## 16644  3 2499 2.148395e+00
## 16645  3 2500 2.156967e+00
## 16646  3 2501 2.150071e+00
## 16647  3 2502 2.135399e+00
## 16648  3 2503 2.120394e+00
## 16649  3 2504 2.101283e+00
## 16650  3 2505 2.086415e+00
## 16651  3 2506 2.070250e+00
## 16652  3 2507 2.055318e+00
## 16653  3 2508 2.037589e+00
## 16654  3 2509 2.024040e+00
## 16655  3 2510 2.006784e+00
## 16656  3 2511 1.995200e+00
## 16657  3 2512 1.979415e+00
## 16658  3 2513 1.966067e+00
## 16659  3 2514 1.950928e+00
## 16660  3 2515 1.936928e+00
## 16661  3 2516 1.926223e+00
## 16662  3 2517 1.909328e+00
## 16663  3 2518 1.896127e+00
## 16664  3 2519 1.883514e+00
## 16665  3 2520 1.872584e+00
## 16666  3 2520 1.874880e+00
## 16667  3 2521 1.862872e+00
## 16668  3 2522 1.852866e+00
## 16669  3 2523 1.836785e+00
## 16670  3 2524 1.827739e+00
## 16671  3 2525 1.818651e+00
## 16672  3 2526 1.806626e+00
## 16673  3 2527 1.795416e+00
```

```
## 16674  3 2528 1.785148e+00
## 16675  3 2529 1.776781e+00
## 16676  3 2530 1.763023e+00
## 16677  3 2531 1.757886e+00
## 16678  3 2532 1.749162e+00
## 16679  3 2533 1.738825e+00
## 16680  3 2534 1.730343e+00
## 16681  3 2535 1.717009e+00
## 16682  3 2536 1.709970e+00
## 16683  3 2537 1.700759e+00
## 16684  3 2538 1.692908e+00
## 16685  3 2538 1.691938e+00
## 16686  3 2539 1.841751e+00
## 16687  3 2540 1.939124e+00
## 16688  3 2541 1.974487e+00
## 16689  3 2542 1.986748e+00
## 16690  3 2543 1.982460e+00
## 16691  3 2544 1.970297e+00
## 16692  3 2544 1.974014e+00
## 16693  3 2545 2.119697e+00
## 16694  3 2546 2.212536e+00
## 16695  3 2547 2.242097e+00
## 16696  3 2548 2.246884e+00
## 16697  3 2549 2.240199e+00
## 16698  3 2550 2.224379e+00
## 16699  3 2551 2.203619e+00
## 16700  3 2552 2.185528e+00
## 16701  3 2553 2.164396e+00
## 16702  3 2554 2.143654e+00
## 16703  3 2555 2.130620e+00
## 16704  3 2556 2.106625e+00
## 16705  3 2557 2.093314e+00
## 16706  3 2558 2.072679e+00
## 16707  3 2559 2.059646e+00
## 16708  3 2560 2.039980e+00
## 16709  3 2561 2.023988e+00
## 16710  3 2562 2.007831e+00
## 16711  3 2563 1.995029e+00
## 16712  3 2564 1.981109e+00
## 16713  3 2565 1.961901e+00
## 16714  3 2566 1.947694e+00
## 16715  3 2567 1.936041e+00
## 16716  3 2568 1.924020e+00
## 16717  3 2569 2.066017e+00
## 16718  3 2570 2.161021e+00
## 16719  3 2571 2.192027e+00
## 16720  3 2572 2.199025e+00
## 16721  3 2573 2.188446e+00
## 16722  3 2574 2.175639e+00
## 16723  3 2575 2.157731e+00
## 16724  3 2576 2.142121e+00
## 16725  3 2577 2.126405e+00
## 16726  3 2578 2.103423e+00
## 16727  3 2579 2.089226e+00
## 16728  3 2580 2.071645e+00
## 16729  3 2581 2.056890e+00
## 16730  3 2582 2.038546e+00
## 16731  3 2583 2.024028e+00
## 16732  3 2584 2.006465e+00
## 16733  3 2585 1.993506e+00
## 16734  3 2586 1.980658e+00
## 16735  3 2587 1.961215e+00
## 16736  3 2588 1.950209e+00
## 16737  3 2589 1.936486e+00
## 16738  3 2590 1.922445e+00
## 16739  3 2591 1.908415e+00
## 16740  3 2592 1.897611e+00
## 16741  3 2593 2.044717e+00
## 16742  3 2594 2.135138e+00
## 16743  3 2595 2.166420e+00
## 16744  3 2596 2.175526e+00
## 16745  3 2597 2.167129e+00
## 16746  3 2598 2.154050e+00
## 16747  3 2599 2.138600e+00
## 16748  3 2600 2.123078e+00
## 16749  3 2601 2.103381e+00
## 16750  3 2602 2.088415e+00
## 16751  3 2603 2.072095e+00
## 16752  3 2604 2.057477e+00
## 16753  3 2605 2.040447e+00
## 16754  3 2606 2.024008e+00
## 16755  3 2607 2.011588e+00
## 16756  3 2608 1.992478e+00
## 16757  3 2609 1.978329e+00
## 16758  3 2610 1.964083e+00
## 16759  3 2611 1.949678e+00
## 16760  3 2612 1.937236e+00
## 16761  3 2613 1.923320e+00
## 16762  3 2614 1.910324e+00
## 16763  3 2615 1.898234e+00
## 16764  3 2616 1.889318e+00
## 16765  3 2617 2.035994e+00
## 16766  3 2618 2.125519e+00
## 16767  3 2619 2.159399e+00
## 16768  3 2620 2.166262e+00
## 16769  3 2621 2.163253e+00
## 16770  3 2622 2.148276e+00
## 16771  3 2623 2.131742e+00
## 16772  3 2624 2.113095e+00
## 16773  3 2625 2.097976e+00
## 16774  3 2626 2.079472e+00
## 16775  3 2627 2.067340e+00
## 16776  3 2628 2.049002e+00
## 16777  3 2629 2.030377e+00
## 16778  3 2630 2.018026e+00
## 16779  3 2631 1.999981e+00
## 16780  3 2632 1.987266e+00
## 16781  3 2633 1.973764e+00
## 16782  3 2634 1.958791e+00
## 16783  3 2635 1.948082e+00
## 16784  3 2636 1.931616e+00
## 16785  3 2637 1.919010e+00
## 16786  3 2638 1.913145e+00
## 16787  3 2639 1.894910e+00
## 16788  3 2640 1.884955e+00
## 16789  3 2641 2.030202e+00
## 16790  3 2642 2.127495e+00
## 16791  3 2643 2.154685e+00
## 16792  3 2644 2.165064e+00
## 16793  3 2645 2.151351e+00
## 16794  3 2646 2.145621e+00
## 16795  3 2647 2.125730e+00
## 16796  3 2648 2.112080e+00
## 16797  3 2649 2.091398e+00
## 16798  3 2650 2.074914e+00
## 16799  3 2651 2.061992e+00
## 16800  3 2652 2.046916e+00
## 16801  3 2653 2.030391e+00
## 16802  3 2654 2.015600e+00
## 16803  3 2655 2.002337e+00
## 16804  3 2656 1.982106e+00
## 16805  3 2657 1.967719e+00
## 16806  3 2658 1.953608e+00
## 16807  3 2659 1.944279e+00
## 16808  3 2660 1.928642e+00
## 16809  3 2661 1.915504e+00
## 16810  3 2662 1.906426e+00
## 16811  3 2663 1.892256e+00
## 16812  3 2664 1.878820e+00
## 16813  3 2665 2.027323e+00
## 16814  3 2666 2.120692e+00
## 16815  3 2667 2.152403e+00
## 16816  3 2668 2.160017e+00
## 16817  3 2669 2.155585e+00
## 16818  3 2670 2.143106e+00
## 16819  3 2671 2.126003e+00
## 16820  3 2672 2.110682e+00
## 16821  3 2673 2.091812e+00
## 16822  3 2674 2.075312e+00
## 16823  3 2675 2.061490e+00
## 16824  3 2676 2.044164e+00
## 16825  3 2677 2.025841e+00
## 16826  3 2678 2.008701e+00
## 16827  3 2679 1.997757e+00
## 16828  3 2680 1.983520e+00
## 16829  3 2681 1.969902e+00
## 16830  3 2682 1.955644e+00
## 16831  3 2683 1.942780e+00
## 16832  3 2684 1.928238e+00
## 16833  3 2685 1.915385e+00
## 16834  3 2686 1.905312e+00
## 16835  3 2687 1.890235e+00
## 16836  3 2688 1.878361e+00
## 16837  3 2689 2.027158e+00
## 16838  3 2690 2.122507e+00
## 16839  3 2691 2.155933e+00
## 16840  3 2692 2.158316e+00
## 16841  3 2693 2.152587e+00
## 16842  3 2694 2.139630e+00
## 16843  3 2695 2.126752e+00
## 16844  3 2696 2.110073e+00
## 16845  3 2697 2.095070e+00
## 16846  3 2698 2.074051e+00
## 16847  3 2699 2.059325e+00
## 16848  3 2700 2.042572e+00
## 16849  3 2701 2.025431e+00
## 16850  3 2702 2.015170e+00
## 16851  3 2703 1.996320e+00
## 16852  3 2704 1.982362e+00
## 16853  3 2705 1.970876e+00
## 16854  3 2706 1.954863e+00
## 16855  3 2707 1.940655e+00
## 16856  3 2708 1.930194e+00
## 16857  3 2709 1.915414e+00
## 16858  3 2710 1.903972e+00
## 16859  3 2711 1.891128e+00
## 16860  3 2712 1.875889e+00
## 16861  3 2713 2.025022e+00
## 16862  3 2714 2.115826e+00
## 16863  3 2715 2.149980e+00
## 16864  3 2716 2.160899e+00
## 16865  3 2717 2.150120e+00
## 16866  3 2718 2.136255e+00
## 16867  3 2719 2.121688e+00
## 16868  3 2720 2.108495e+00
## 16869  3 2721 2.089558e+00
## 16870  3 2722 2.071650e+00
## 16871  3 2723 2.059646e+00
## 16872  3 2724 2.044752e+00
## 16873  3 2725 2.026172e+00
## 16874  3 2726 2.012595e+00
## 16875  3 2727 1.996018e+00
## 16876  3 2728 1.982200e+00
## 16877  3 2729 1.968562e+00
## 16878  3 2730 1.956523e+00
## 16879  3 2731 1.940392e+00
## 16880  3 2732 1.926107e+00
## 16881  3 2733 1.910777e+00
## 16882  3 2734 1.898953e+00
## 16883  3 2735 1.889406e+00
## 16884  3 2736 1.875387e+00
## 16885  3 2737 2.023339e+00
## 16886  3 2738 2.120729e+00
## 16887  3 2739 2.153773e+00
## 16888  3 2740 2.159268e+00
## 16889  3 2741 2.152923e+00
## 16890  3 2742 2.137590e+00
## 16891  3 2743 2.121544e+00
## 16892  3 2744 2.108570e+00
## 16893  3 2745 2.092903e+00
## 16894  3 2746 2.070438e+00
## 16895  3 2747 2.058446e+00
## 16896  3 2748 2.042524e+00
## 16897  3 2749 2.021346e+00
## 16898  3 2750 2.009099e+00
## 16899  3 2751 1.998416e+00
## 16900  3 2752 1.980677e+00
## 16901  3 2753 1.970213e+00
## 16902  3 2754 1.955529e+00
## 16903  3 2755 1.940764e+00
## 16904  3 2756 1.932296e+00
## 16905  3 2757 1.913718e+00
## 16906  3 2758 1.902655e+00
## 16907  3 2759 1.894016e+00
## 16908  3 2760 1.876257e+00
## 16909  3 2761 2.026171e+00
## 16910  3 2762 2.116673e+00
## 16911  3 2763 2.155110e+00
## 16912  3 2764 2.159272e+00
## 16913  3 2765 2.151143e+00
## 16914  3 2766 2.141101e+00
## 16915  3 2767 2.125769e+00
## 16916  3 2768 2.106639e+00
## 16917  3 2769 2.093912e+00
## 16918  3 2770 2.074215e+00
## 16919  3 2771 2.058601e+00
## 16920  3 2772 2.037804e+00
## 16921  3 2773 2.026959e+00
## 16922  3 2774 2.012219e+00
## 16923  3 2775 1.997249e+00
## 16924  3 2776 1.980761e+00
## 16925  3 2777 1.966417e+00
## 16926  3 2778 1.955149e+00
## 16927  3 2779 1.938315e+00
## 16928  3 2780 1.926013e+00
## 16929  3 2781 1.914211e+00
## 16930  3 2782 1.905537e+00
## 16931  3 2783 1.886481e+00
## 16932  3 2784 1.877269e+00
## 16933  3 2785 2.025823e+00
## 16934  3 2786 2.119383e+00
## 16935  3 2787 2.154716e+00
## 16936  3 2788 2.157667e+00
## 16937  3 2789 2.151336e+00
## 16938  3 2790 2.136952e+00
## 16939  3 2791 2.126188e+00
## 16940  3 2792 2.110862e+00
## 16941  3 2793 2.090181e+00
## 16942  3 2794 2.072512e+00
## 16943  3 2795 2.056453e+00
## 16944  3 2796 2.040487e+00
## 16945  3 2797 2.026592e+00
## 16946  3 2798 2.010295e+00
## 16947  3 2799 1.995621e+00
## 16948  3 2800 1.980226e+00
## 16949  3 2801 1.968931e+00
## 16950  3 2802 1.951455e+00
## 16951  3 2803 1.940982e+00
## 16952  3 2804 1.928261e+00
## 16953  3 2805 1.911870e+00
## 16954  3 2806 1.899280e+00
## 16955  3 2807 1.890881e+00
## 16956  3 2808 1.880184e+00
## 16957  3 2808 1.873443e+00
## 16958  3 2809 2.019392e+00
## 16959  3 2810 2.114546e+00
## 16960  3 2811 2.149793e+00
## 16961  3 2812 2.156611e+00
## 16962  3 2813 2.148635e+00
## 16963  3 2814 2.133317e+00
## 16964  3 2815 2.120092e+00
## 16965  3 2816 2.102619e+00
## 16966  3 2817 2.089290e+00
## 16967  3 2818 2.072519e+00
## 16968  3 2819 2.054492e+00
## 16969  3 2820 2.042318e+00
## 16970  3 2821 2.022297e+00
## 16971  3 2822 2.006235e+00
## 16972  3 2823 1.995118e+00
## 16973  3 2824 1.983399e+00
## 16974  3 2825 1.966333e+00
## 16975  3 2826 1.950658e+00
## 16976  3 2827 1.937664e+00
## 16977  3 2828 1.925246e+00
## 16978  3 2829 1.905746e+00
## 16979  3 2830 1.897247e+00
## 16980  3 2831 1.883386e+00
## 16981  3 2832 1.875552e+00
## 16982  3 2832 1.875240e+00
## 16983  3 2833 1.859067e+00
## 16984  3 2834 1.848354e+00
## 16985  3 2835 1.841428e+00
## 16986  3 2836 1.828026e+00
## 16987  3 2837 1.815447e+00
## 16988  3 2838 1.807346e+00
## 16989  3 2839 1.796520e+00
## 16990  3 2840 1.786152e+00
## 16991  3 2841 1.777144e+00
## 16992  3 2842 1.767497e+00
## 16993  3 2843 1.756269e+00
## 16994  3 2844 1.743413e+00
## 16995  3 2845 1.736557e+00
## 16996  3 2846 1.726756e+00
## 16997  3 2847 1.718701e+00
## 16998  3 2848 1.712035e+00
## 16999  3 2849 1.702012e+00
## 17000  3 2850 1.694406e+00
## 17001  3 2851 1.685638e+00
## 17002  3 2852 1.678911e+00
## 17003  3 2853 1.667642e+00
## 17004  3 2853 1.668915e+00
## 17005  3 2854 1.739958e+00
## 17006  3 2855 1.782295e+00
## 17007  3 2856 1.800367e+00
## 17008  3 2856 1.798466e+00
## 17009  3 2857 1.959017e+00
## 17010  3 2858 2.059982e+00
## 17011  3 2859 2.097651e+00
## 17012  3 2860 2.103437e+00
## 17013  3 2861 2.097007e+00
## 17014  3 2862 2.083619e+00
## 17015  3 2863 2.071469e+00
## 17016  3 2864 2.054690e+00
## 17017  3 2865 2.036026e+00
## 17018  3 2866 2.016522e+00
## 17019  3 2867 2.004110e+00
## 17020  3 2868 1.988117e+00
## 17021  3 2869 1.975846e+00
## 17022  3 2870 1.952788e+00
## 17023  3 2871 1.943389e+00
## 17024  3 2872 1.931249e+00
## 17025  3 2873 1.913816e+00
## 17026  3 2874 1.896562e+00
## 17027  3 2875 1.888960e+00
## 17028  3 2876 1.872222e+00
## 17029  3 2877 1.862663e+00
## 17030  3 2878 1.846369e+00
## 17031  3 2879 1.833884e+00
## 17032  3 2880 1.825266e+00
## 17033  3 2881 1.968639e+00
## 17034  3 2882 2.062569e+00
## 17035  3 2883 2.101398e+00
## 17036  3 2884 2.104910e+00
## 17037  3 2885 2.098212e+00
## 17038  3 2886 2.083020e+00
## 17039  3 2887 2.071389e+00
## 17040  3 2888 2.055252e+00
## 17041  3 2889 2.037270e+00
## 17042  3 2890 2.021570e+00
## 17043  3 2891 2.007131e+00
## 17044  3 2892 1.986556e+00
## 17045  3 2893 1.970368e+00
## 17046  3 2894 1.960951e+00
## 17047  3 2895 1.944842e+00
## 17048  3 2896 1.927985e+00
## 17049  3 2897 1.914688e+00
## 17050  3 2898 1.903163e+00
## 17051  3 2899 1.887746e+00
## 17052  3 2900 1.877731e+00
## 17053  3 2901 1.861180e+00
## 17054  3 2902 1.850610e+00
## 17055  3 2903 1.842910e+00
## 17056  3 2904 1.826698e+00
## 17057  3 2905 1.971588e+00
## 17058  3 2906 2.067241e+00
## 17059  3 2907 2.100406e+00
## 17060  3 2908 2.108442e+00
## 17061  3 2909 2.104806e+00
## 17062  3 2910 2.086183e+00
## 17063  3 2911 2.074144e+00
## 17064  3 2912 2.056542e+00
## 17065  3 2913 2.039090e+00
## 17066  3 2914 2.023789e+00
## 17067  3 2915 2.009382e+00
## 17068  3 2916 1.993655e+00
## 17069  3 2917 1.974808e+00
## 17070  3 2918 1.959605e+00
## 17071  3 2919 1.948380e+00
## 17072  3 2920 1.931973e+00
## 17073  3 2921 1.920064e+00
## 17074  3 2922 1.906517e+00
## 17075  3 2923 1.890116e+00
## 17076  3 2924 1.876307e+00
## 17077  3 2925 1.865561e+00
## 17078  3 2926 1.853801e+00
## 17079  3 2927 1.842316e+00
## 17080  3 2928 1.830314e+00
## 17081  3 2929 1.977062e+00
## 17082  3 2930 2.072629e+00
## 17083  3 2931 2.103935e+00
## 17084  3 2932 2.108432e+00
## 17085  3 2933 2.103524e+00
## 17086  3 2934 2.093433e+00
## 17087  3 2935 2.075843e+00
## 17088  3 2936 2.059773e+00
## 17089  3 2937 2.043559e+00
## 17090  3 2938 2.026245e+00
## 17091  3 2939 2.012414e+00
## 17092  3 2940 1.995326e+00
## 17093  3 2941 1.979021e+00
## 17094  3 2942 1.964564e+00
## 17095  3 2943 1.949939e+00
## 17096  3 2944 1.936575e+00
## 17097  3 2945 1.916839e+00
## 17098  3 2946 1.910570e+00
## 17099  3 2947 1.891936e+00
## 17100  3 2948 1.881492e+00
## 17101  3 2949 1.871420e+00
## 17102  3 2950 1.856912e+00
## 17103  3 2951 1.842510e+00
## 17104  3 2952 1.831413e+00
## 17105  3 2953 1.980702e+00
## 17106  3 2954 2.070832e+00
## 17107  3 2955 2.107102e+00
## 17108  3 2956 2.117616e+00
## 17109  3 2957 2.104556e+00
## 17110  3 2958 2.093681e+00
## 17111  3 2959 2.077005e+00
## 17112  3 2960 2.061641e+00
## 17113  3 2961 2.047569e+00
## 17114  3 2962 2.032635e+00
## 17115  3 2963 2.015285e+00
## 17116  3 2964 1.997558e+00
## 17117  3 2965 1.980794e+00
## 17118  3 2966 1.968371e+00
## 17119  3 2967 1.950372e+00
## 17120  3 2968 1.939234e+00
## 17121  3 2969 1.923429e+00
## 17122  3 2970 1.910251e+00
## 17123  3 2971 1.897931e+00
## 17124  3 2972 1.887488e+00
## 17125  3 2973 1.871032e+00
## 17126  3 2974 1.856917e+00
## 17127  3 2975 1.849245e+00
## 17128  3 2976 1.834739e+00
## 17129  3 2977 1.976425e+00
## 17130  3 2978 2.073068e+00
## 17131  3 2979 2.107169e+00
## 17132  3 2980 2.117739e+00
## 17133  3 2981 2.109101e+00
## 17134  3 2982 2.101671e+00
## 17135  3 2983 2.082015e+00
## 17136  3 2984 2.065777e+00
## 17137  3 2985 2.050886e+00
## 17138  3 2986 2.031992e+00
## 17139  3 2987 2.013304e+00
## 17140  3 2988 1.999614e+00
## 17141  3 2989 1.984855e+00
## 17142  3 2990 1.971054e+00
## 17143  3 2991 1.952665e+00
## 17144  3 2992 1.938074e+00
## 17145  3 2993 1.926664e+00
## 17146  3 2994 1.914106e+00
## 17147  3 2995 1.902448e+00
## 17148  3 2996 1.887661e+00
## 17149  3 2997 1.874364e+00
## 17150  3 2998 1.863361e+00
## 17151  3 2999 1.849505e+00
## 17152  3 3000 1.840085e+00
## 17153  3 3001 1.987181e+00
## 17154  3 3002 2.080858e+00
## 17155  3 3003 2.113636e+00
## 17156  3 3004 2.119046e+00
## 17157  3 3005 2.110442e+00
## 17158  3 3006 2.100150e+00
## 17159  3 3007 2.083940e+00
## 17160  3 3008 2.068761e+00
## 17161  3 3009 2.052599e+00
## 17162  3 3010 2.036721e+00
## 17163  3 3011 2.017356e+00
## 17164  3 3012 2.001652e+00
## 17165  3 3013 1.990177e+00
## 17166  3 3014 1.973873e+00
## 17167  3 3015 1.959612e+00
## 17168  3 3016 1.942815e+00
## 17169  3 3017 1.930014e+00
## 17170  3 3018 1.916661e+00
## 17171  3 3019 1.905423e+00
## 17172  3 3020 1.889425e+00
## 17173  3 3021 1.874282e+00
## 17174  3 3022 1.861282e+00
## 17175  3 3023 1.853348e+00
## 17176  3 3024 1.842540e+00
## 17177  3 3025 1.986607e+00
## 17178  3 3026 2.084186e+00
## 17179  3 3027 2.119025e+00
## 17180  3 3028 2.122468e+00
## 17181  3 3029 2.119138e+00
## 17182  3 3030 2.102231e+00
## 17183  3 3031 2.088409e+00
## 17184  3 3032 2.069833e+00
## 17185  3 3033 2.052134e+00
## 17186  3 3034 2.035174e+00
## 17187  3 3035 2.028154e+00
## 17188  3 3036 2.004258e+00
## 17189  3 3037 1.990385e+00
## 17190  3 3038 1.973486e+00
## 17191  3 3039 1.961797e+00
## 17192  3 3040 1.950130e+00
## 17193  3 3041 1.934589e+00
## 17194  3 3042 1.917053e+00
## 17195  3 3043 1.905813e+00
## 17196  3 3044 1.893410e+00
## 17197  3 3045 1.880191e+00
## 17198  3 3046 1.867512e+00
## 17199  3 3047 1.857191e+00
## 17200  3 3048 1.842595e+00
## 17201  3 3049 1.987222e+00
## 17202  3 3050 2.082221e+00
## 17203  3 3051 2.117625e+00
## 17204  3 3052 2.124874e+00
## 17205  3 3053 2.120618e+00
## 17206  3 3054 2.104471e+00
## 17207  3 3055 2.087620e+00
## 17208  3 3056 2.073310e+00
## 17209  3 3057 2.054231e+00
## 17210  3 3058 2.039029e+00
## 17211  3 3059 2.023168e+00
## 17212  3 3060 2.008802e+00
## 17213  3 3061 1.993280e+00
## 17214  3 3062 1.974830e+00
## 17215  3 3063 1.965838e+00
## 17216  3 3064 1.949378e+00
## 17217  3 3065 1.932144e+00
## 17218  3 3066 1.919558e+00
## 17219  3 3067 1.908522e+00
## 17220  3 3068 1.893713e+00
## 17221  3 3069 1.877871e+00
## 17222  3 3070 1.867763e+00
## 17223  3 3071 1.853741e+00
## 17224  3 3072 1.844765e+00
## 17225  3 3073 1.988885e+00
## 17226  3 3074 2.085618e+00
## 17227  3 3075 2.119412e+00
## 17228  3 3076 2.126943e+00
## 17229  3 3077 2.120077e+00
## 17230  3 3078 2.104768e+00
## 17231  3 3079 2.092083e+00
## 17232  3 3080 2.078339e+00
## 17233  3 3081 2.058603e+00
## 17234  3 3082 2.038003e+00
## 17235  3 3083 2.026763e+00
## 17236  3 3084 2.010017e+00
## 17237  3 3085 1.998468e+00
## 17238  3 3086 1.980087e+00
## 17239  3 3087 1.966097e+00
## 17240  3 3088 1.951669e+00
## 17241  3 3089 1.935631e+00
## 17242  3 3090 1.920681e+00
## 17243  3 3091 1.908736e+00
## 17244  3 3092 1.893084e+00
## 17245  3 3093 1.883041e+00
## 17246  3 3094 1.872741e+00
## 17247  3 3095 1.860049e+00
## 17248  3 3096 1.846983e+00
## 17249  3 3097 1.992899e+00
## 17250  3 3098 2.087227e+00
## 17251  3 3099 2.123275e+00
## 17252  3 3100 2.128874e+00
## 17253  3 3101 2.120874e+00
## 17254  3 3102 2.107091e+00
## 17255  3 3103 2.091929e+00
## 17256  3 3104 2.074480e+00
## 17257  3 3105 2.063750e+00
## 17258  3 3106 2.047035e+00
## 17259  3 3107 2.031362e+00
## 17260  3 3108 2.012288e+00
## 17261  3 3109 1.998258e+00
## 17262  3 3110 1.977196e+00
## 17263  3 3111 1.969038e+00
## 17264  3 3112 1.951293e+00
## 17265  3 3113 1.936857e+00
## 17266  3 3114 1.923262e+00
## 17267  3 3115 1.909095e+00
## 17268  3 3116 1.894086e+00
## 17269  3 3117 1.883405e+00
## 17270  3 3118 1.872764e+00
## 17271  3 3119 1.860357e+00
## 17272  3 3120 1.851559e+00
## 17273  3 3120 1.875405e+00
## 17274  3 3121 2.021388e+00
## 17275  3 3122 2.113870e+00
## 17276  3 3123 2.150721e+00
## 17277  3 3124 2.153759e+00
## 17278  3 3125 2.146934e+00
## 17279  3 3126 2.135508e+00
## 17280  3 3127 2.120209e+00
## 17281  3 3128 2.106177e+00
## 17282  3 3129 2.084952e+00
## 17283  3 3130 2.073796e+00
## 17284  3 3131 2.053453e+00
## 17285  3 3132 2.038216e+00
## 17286  3 3133 2.024573e+00
## 17287  3 3134 2.008657e+00
## 17288  3 3135 1.992694e+00
## 17289  3 3136 1.981415e+00
## 17290  3 3137 1.962578e+00
## 17291  3 3138 1.949917e+00
## 17292  3 3139 1.937263e+00
## 17293  3 3140 1.922980e+00
## 17294  3 3141 1.915162e+00
## 17295  3 3142 1.898646e+00
## 17296  3 3143 1.886225e+00
## 17297  3 3144 1.877584e+00
## 17298  3 3144 1.874934e+00
## 17299  3 3145 1.862851e+00
## 17300  3 3146 1.850034e+00
## 17301  3 3147 1.840403e+00
## 17302  3 3148 1.827346e+00
## 17303  3 3149 1.816558e+00
## 17304  3 3150 1.808838e+00
## 17305  3 3151 1.792710e+00
## 17306  3 3152 1.784034e+00
## 17307  3 3153 1.775128e+00
## 17308  3 3154 1.766675e+00
## 17309  3 3155 1.754873e+00
## 17310  3 3156 1.745299e+00
## 17311  3 3157 1.735020e+00
## 17312  3 3158 1.728061e+00
## 17313  3 3159 1.721359e+00
## 17314  3 3160 1.710789e+00
## 17315  3 3161 1.703670e+00
## 17316  3 3162 1.693717e+00
## 17317  3 3163 1.684012e+00
## 17318  3 3164 1.678512e+00
## 17319  3 3165 1.667201e+00
## 17320  3 3165 1.668188e+00
## 17321  3 3166 1.817830e+00
## 17322  3 3167 1.921934e+00
## 17323  3 3168 1.956658e+00
## 17324  3 3168 1.957765e+00
## 17325  3 3169 2.125696e+00
## 17326  3 3170 2.224885e+00
## 17327  3 3171 2.259323e+00
## 17328  3 3172 2.263290e+00
## 17329  3 3173 2.258142e+00
## 17330  3 3174 2.241004e+00
## 17331  3 3175 2.222225e+00
## 17332  3 3176 2.202054e+00
## 17333  3 3177 2.183467e+00
## 17334  3 3178 2.164939e+00
## 17335  3 3179 2.147840e+00
## 17336  3 3180 2.122871e+00
## 17337  3 3181 2.107917e+00
## 17338  3 3182 2.088002e+00
## 17339  3 3183 2.070935e+00
## 17340  3 3184 2.057253e+00
## 17341  3 3185 2.038899e+00
## 17342  3 3186 2.021744e+00
## 17343  3 3187 2.003119e+00
## 17344  3 3188 1.991186e+00
## 17345  3 3189 1.977248e+00
## 17346  3 3190 1.961964e+00
## 17347  3 3191 1.948033e+00
## 17348  3 3192 1.933763e+00
## 17349  3 3193 2.077251e+00
## 17350  3 3194 2.168616e+00
## 17351  3 3195 2.203519e+00
## 17352  3 3196 2.205274e+00
## 17353  3 3197 2.200365e+00
## 17354  3 3198 2.185770e+00
## 17355  3 3199 2.168532e+00
## 17356  3 3200 2.150910e+00
## 17357  3 3201 2.131003e+00
## 17358  3 3202 2.113680e+00
## 17359  3 3203 2.098135e+00
## 17360  3 3204 2.079446e+00
## 17361  3 3205 2.063850e+00
## 17362  3 3206 2.047827e+00
## 17363  3 3207 2.030937e+00
## 17364  3 3208 2.015477e+00
## 17365  3 3209 1.999834e+00
## 17366  3 3210 1.983445e+00
## 17367  3 3211 1.971712e+00
## 17368  3 3212 1.955172e+00
## 17369  3 3213 1.943312e+00
## 17370  3 3214 1.927991e+00
## 17371  3 3215 1.915646e+00
## 17372  3 3216 1.900593e+00
## 17373  3 3217 2.045913e+00
## 17374  3 3218 2.143365e+00
## 17375  3 3219 2.174901e+00
## 17376  3 3220 2.182807e+00
## 17377  3 3221 2.176011e+00
## 17378  3 3222 2.157079e+00
## 17379  3 3223 2.148903e+00
## 17380  3 3224 2.124490e+00
## 17381  3 3225 2.107378e+00
## 17382  3 3226 2.093238e+00
## 17383  3 3227 2.070720e+00
## 17384  3 3228 2.058076e+00
## 17385  3 3229 2.044799e+00
## 17386  3 3230 2.024953e+00
## 17387  3 3231 2.015072e+00
## 17388  3 3232 1.997183e+00
## 17389  3 3233 1.978155e+00
## 17390  3 3234 1.968262e+00
## 17391  3 3235 1.953500e+00
## 17392  3 3236 1.941050e+00
## 17393  3 3237 1.928972e+00
## 17394  3 3238 1.914381e+00
## 17395  3 3239 1.902564e+00
## 17396  3 3240 1.888166e+00
## 17397  3 3241 2.032814e+00
## 17398  3 3242 2.130109e+00
## 17399  3 3243 2.163156e+00
## 17400  3 3244 2.171070e+00
## 17401  3 3245 2.162401e+00
## 17402  3 3246 2.149524e+00
## 17403  3 3247 2.135605e+00
## 17404  3 3248 2.116744e+00
## 17405  3 3249 2.100786e+00
## 17406  3 3250 2.082071e+00
## 17407  3 3251 2.066190e+00
## 17408  3 3252 2.050986e+00
## 17409  3 3253 2.033795e+00
## 17410  3 3254 2.018080e+00
## 17411  3 3255 2.005072e+00
## 17412  3 3256 1.989392e+00
## 17413  3 3257 1.977388e+00
## 17414  3 3258 1.961475e+00
## 17415  3 3259 1.948083e+00
## 17416  3 3260 1.932882e+00
## 17417  3 3261 1.920485e+00
## 17418  3 3262 1.911717e+00
## 17419  3 3263 1.895619e+00
## 17420  3 3264 1.884512e+00
## 17421  3 3265 2.027440e+00
## 17422  3 3266 2.119734e+00
## 17423  3 3267 2.160386e+00
## 17424  3 3268 2.165904e+00
## 17425  3 3269 2.157565e+00
## 17426  3 3270 2.144105e+00
## 17427  3 3271 2.128489e+00
## 17428  3 3272 2.110233e+00
## 17429  3 3273 2.093740e+00
## 17430  3 3274 2.080267e+00
## 17431  3 3275 2.063711e+00
## 17432  3 3276 2.047195e+00
## 17433  3 3277 2.031680e+00
## 17434  3 3278 2.015157e+00
## 17435  3 3279 2.000760e+00
## 17436  3 3280 1.988658e+00
## 17437  3 3281 1.971617e+00
## 17438  3 3282 1.959151e+00
## 17439  3 3283 1.944775e+00
## 17440  3 3284 1.931930e+00
## 17441  3 3285 1.920305e+00
## 17442  3 3286 1.908560e+00
## 17443  3 3287 1.895640e+00
## 17444  3 3288 1.879347e+00
## 17445  3 3289 2.028125e+00
## 17446  3 3290 2.123495e+00
## 17447  3 3291 2.157877e+00
## 17448  3 3292 2.163307e+00
## 17449  3 3293 2.156825e+00
## 17450  3 3294 2.137846e+00
## 17451  3 3295 2.127477e+00
## 17452  3 3296 2.112395e+00
## 17453  3 3297 2.090461e+00
## 17454  3 3298 2.081683e+00
## 17455  3 3299 2.061814e+00
## 17456  3 3300 2.044110e+00
## 17457  3 3301 2.032624e+00
## 17458  3 3302 2.013847e+00
## 17459  3 3303 1.999661e+00
## 17460  3 3304 1.982346e+00
## 17461  3 3305 1.972565e+00
## 17462  3 3306 1.956648e+00
## 17463  3 3307 1.945369e+00
## 17464  3 3308 1.929694e+00
## 17465  3 3309 1.913197e+00
## 17466  3 3310 1.907349e+00
## 17467  3 3311 1.889247e+00
## 17468  3 3312 1.877885e+00
## 17469  3 3313 2.025601e+00
## 17470  3 3314 2.116365e+00
## 17471  3 3315 2.160160e+00
## 17472  3 3316 2.163847e+00
## 17473  3 3317 2.154555e+00
```

```
## 17474  3 3318 2.139068e+00
## 17475  3 3319 2.122607e+00
## 17476  3 3320 2.109212e+00
## 17477  3 3321 2.091019e+00
## 17478  3 3322 2.074356e+00
## 17479  3 3323 2.056003e+00
## 17480  3 3324 2.046599e+00
## 17481  3 3325 2.031729e+00
## 17482  3 3326 2.011653e+00
## 17483  3 3327 2.000504e+00
## 17484  3 3328 1.981539e+00
## 17485  3 3329 1.970537e+00
## 17486  3 3330 1.956944e+00
## 17487  3 3331 1.946274e+00
## 17488  3 3332 1.927704e+00
## 17489  3 3333 1.917115e+00
## 17490  3 3334 1.903987e+00
## 17491  3 3335 1.892334e+00
## 17492  3 3336 1.878427e+00
## 17493  3 3337 2.028588e+00
## 17494  3 3338 2.118833e+00
## 17495  3 3339 2.156051e+00
## 17496  3 3340 2.157551e+00
## 17497  3 3341 2.151722e+00
## 17498  3 3342 2.139683e+00
## 17499  3 3343 2.121293e+00
## 17500  3 3344 2.111631e+00
## 17501  3 3345 2.093183e+00
## 17502  3 3346 2.075802e+00
## 17503  3 3347 2.058587e+00
## 17504  3 3348 2.041957e+00
## 17505  3 3349 2.025820e+00
## 17506  3 3350 2.012691e+00
## 17507  3 3351 1.999800e+00
## 17508  3 3352 1.981005e+00
## 17509  3 3353 1.969291e+00
## 17510  3 3354 1.954682e+00
## 17511  3 3355 1.941412e+00
## 17512  3 3356 1.928076e+00
## 17513  3 3357 1.915581e+00
## 17514  3 3358 1.904916e+00
## 17515  3 3359 1.893092e+00
## 17516  3 3360 1.876079e+00
## 17517  3 3361 2.025291e+00
## 17518  3 3362 2.115983e+00
## 17519  3 3363 2.153356e+00
## 17520  3 3364 2.156359e+00
## 17521  3 3365 2.148087e+00
## 17522  3 3366 2.139604e+00
## 17523  3 3367 2.125461e+00
## 17524  3 3368 2.104568e+00
## 17525  3 3369 2.091951e+00
## 17526  3 3370 2.071537e+00
## 17527  3 3371 2.059484e+00
## 17528  3 3372 2.043564e+00
## 17529  3 3373 2.024002e+00
## 17530  3 3374 2.011693e+00
## 17531  3 3375 1.997864e+00
## 17532  3 3376 1.984155e+00
## 17533  3 3377 1.968897e+00
## 17534  3 3378 1.956168e+00
## 17535  3 3379 1.942645e+00
## 17536  3 3380 1.928640e+00
## 17537  3 3381 1.913831e+00
## 17538  3 3382 1.899544e+00
## 17539  3 3383 1.889924e+00
## 17540  3 3384 1.876192e+00
## 17541  3 3385 2.024627e+00
## 17542  3 3386 2.117585e+00
## 17543  3 3387 2.154177e+00
## 17544  3 3388 2.157814e+00
## 17545  3 3389 2.153479e+00
## 17546  3 3390 2.142524e+00
## 17547  3 3391 2.127778e+00
## 17548  3 3392 2.106771e+00
## 17549  3 3393 2.091045e+00
## 17550  3 3394 2.075666e+00
## 17551  3 3395 2.057850e+00
## 17552  3 3396 2.041515e+00
## 17553  3 3397 2.026415e+00
## 17554  3 3398 2.013880e+00
## 17555  3 3399 1.996479e+00
## 17556  3 3400 1.980714e+00
## 17557  3 3401 1.969082e+00
## 17558  3 3402 1.956571e+00
## 17559  3 3403 1.942570e+00
## 17560  3 3404 1.925326e+00
## 17561  3 3405 1.916908e+00
## 17562  3 3406 1.903212e+00
## 17563  3 3407 1.888279e+00
## 17564  3 3408 1.876796e+00
## 17565  3 3409 2.023516e+00
## 17566  3 3410 2.116304e+00
## 17567  3 3411 2.153516e+00
## 17568  3 3412 2.160505e+00
## 17569  3 3413 2.153438e+00
## 17570  3 3414 2.138358e+00
## 17571  3 3415 2.126541e+00
## 17572  3 3416 2.108954e+00
## 17573  3 3417 2.092306e+00
## 17574  3 3418 2.075518e+00
## 17575  3 3419 2.057252e+00
## 17576  3 3420 2.040063e+00
## 17577  3 3421 2.022980e+00
## 17578  3 3422 2.011724e+00
## 17579  3 3423 1.997007e+00
## 17580  3 3424 1.978249e+00
## 17581  3 3425 1.967220e+00
## 17582  3 3426 1.952956e+00
## 17583  3 3427 1.942718e+00
## 17584  3 3428 1.927685e+00
## 17585  3 3429 1.916713e+00
## 17586  3 3430 1.899579e+00
## 17587  3 3431 1.888516e+00
## 17588  3 3432 1.874746e+00
## 17589  3 3432 1.872961e+00
## 17590  3 3433 2.015841e+00
## 17591  3 3434 2.119802e+00
## 17592  3 3435 2.148036e+00
## 17593  3 3436 2.157055e+00
## 17594  3 3437 2.150247e+00
## 17595  3 3438 2.134787e+00
## 17596  3 3439 2.117487e+00
## 17597  3 3440 2.104779e+00
## 17598  3 3441 2.086654e+00
## 17599  3 3442 2.066416e+00
## 17600  3 3443 2.050428e+00
## 17601  3 3444 2.037393e+00
## 17602  3 3445 2.023101e+00
## 17603  3 3446 2.007274e+00
## 17604  3 3447 1.991351e+00
## 17605  3 3448 1.979009e+00
## 17606  3 3449 1.963610e+00
## 17607  3 3450 1.949321e+00
## 17608  3 3451 1.931829e+00
## 17609  3 3452 1.922691e+00
## 17610  3 3453 1.910183e+00
## 17611  3 3454 1.898665e+00
## 17612  3 3455 1.887552e+00
## 17613  3 3456 1.871801e+00
## 17614  3 3456 1.872167e+00
## 17615  3 3457 1.864770e+00
## 17616  3 3458 1.846102e+00
## 17617  3 3459 1.840101e+00
## 17618  3 3460 1.824788e+00
## 17619  3 3461 1.820423e+00
## 17620  3 3462 1.805822e+00
## 17621  3 3463 1.797749e+00
## 17622  3 3464 1.787961e+00
## 17623  3 3465 1.775225e+00
## 17624  3 3466 1.764255e+00
## 17625  3 3467 1.756440e+00
## 17626  3 3468 1.748306e+00
## 17627  3 3469 1.739035e+00
## 17628  3 3470 1.728165e+00
## 17629  3 3471 1.719798e+00
## 17630  3 3472 1.712237e+00
## 17631  3 3473 1.699692e+00
## 17632  3 3474 1.696252e+00
## 17633  3 3475 1.686682e+00
## 17634  3 3476 1.680717e+00
## 17635  3 3477 1.668458e+00
## 17636  3 3478 1.658298e+00
## 17637  3 3479 1.653493e+00
## 17638  3 3480 1.648071e+00
## 17639  3 3480 1.640406e+00
## 17640  3 3481 1.793241e+00
## 17641  3 3482 1.893064e+00
## 17642  3 3483 1.931255e+00
## 17643  3 3484 1.940219e+00
## 17644  3 3485 1.936119e+00
## 17645  3 3486 1.925374e+00
## 17646  3 3487 1.918277e+00
## 17647  3 3488 1.902117e+00
## 17648  3 3489 1.889364e+00
## 17649  3 3490 1.879934e+00
## 17650  3 3491 1.863673e+00
## 17651  3 3492 1.851070e+00
## 17652  3 3493 1.833992e+00
## 17653  3 3494 1.824645e+00
## 17654  3 3495 1.815186e+00
## 17655  3 3496 1.802891e+00
## 17656  3 3497 1.787525e+00
## 17657  3 3498 1.779088e+00
## 17658  3 3499 1.765807e+00
## 17659  3 3500 1.756835e+00
## 17660  3 3501 1.739676e+00
## 17661  3 3502 1.735453e+00
## 17662  3 3503 1.728103e+00
## 17663  3 3504 1.714575e+00
## 17664  3 3504 1.716067e+00
## 17665  3 3505 1.864063e+00
## 17666  3 3506 1.959461e+00
## 17667  3 3507 1.997775e+00
## 17668  3 3508 2.005271e+00
## 17669  3 3509 2.002348e+00
## 17670  3 3510 1.987830e+00
## 17671  3 3511 1.972236e+00
## 17672  3 3512 1.958341e+00
## 17673  3 3513 1.944381e+00
## 17674  3 3514 1.927532e+00
## 17675  3 3515 1.916156e+00
## 17676  3 3516 1.900333e+00
## 17677  3 3517 1.885769e+00
## 17678  3 3518 1.873870e+00
## 17679  3 3519 1.861443e+00
## 17680  3 3520 1.842775e+00
## 17681  3 3521 1.831957e+00
## 17682  3 3522 1.819753e+00
## 17683  3 3523 1.805381e+00
## 17684  3 3524 1.793542e+00
## 17685  3 3525 1.786021e+00
## 17686  3 3526 1.771569e+00
## 17687  3 3527 1.762221e+00
## 17688  3 3528 1.751999e+00
## 17689  3 3529 1.896037e+00
## 17690  3 3530 1.993709e+00
## 17691  3 3531 2.024461e+00
## 17692  3 3532 2.034901e+00
## 17693  3 3533 2.032499e+00
## 17694  3 3534 2.013189e+00
## 17695  3 3535 1.999494e+00
## 17696  3 3536 1.988830e+00
## 17697  3 3537 1.969862e+00
## 17698  3 3538 1.957276e+00
## 17699  3 3539 1.938829e+00
## 17700  3 3540 1.926220e+00
## 17701  3 3541 1.908457e+00
## 17702  3 3542 1.893682e+00
## 17703  3 3543 1.881974e+00
## 17704  3 3544 1.869506e+00
## 17705  3 3545 1.853200e+00
## 17706  3 3546 1.842901e+00
## 17707  3 3547 1.829083e+00
## 17708  3 3548 1.814863e+00
## 17709  3 3549 1.803834e+00
## 17710  3 3550 1.793037e+00
## 17711  3 3551 1.782172e+00
## 17712  3 3552 1.766787e+00
## 17713  3 3553 1.915612e+00
## 17714  3 3554 2.008026e+00
## 17715  3 3555 2.043910e+00
## 17716  3 3556 2.052034e+00
## 17717  3 3557 2.046023e+00
## 17718  3 3558 2.032622e+00
## 17719  3 3559 2.012681e+00
## 17720  3 3560 2.003381e+00
## 17721  3 3561 1.986491e+00
## 17722  3 3562 1.968445e+00
## 17723  3 3563 1.957706e+00
## 17724  3 3564 1.941184e+00
## 17725  3 3565 1.921176e+00
## 17726  3 3566 1.912955e+00
## 17727  3 3567 1.896982e+00
## 17728  3 3568 1.879608e+00
## 17729  3 3569 1.866967e+00
## 17730  3 3570 1.854536e+00
## 17731  3 3571 1.841940e+00
## 17732  3 3572 1.828271e+00
## 17733  3 3573 1.814817e+00
## 17734  3 3574 1.804232e+00
## 17735  3 3575 1.793029e+00
## 17736  3 3576 1.779521e+00
## 17737  3 3577 1.928714e+00
## 17738  3 3578 2.017621e+00
## 17739  3 3579 2.056558e+00
## 17740  3 3580 2.061642e+00
## 17741  3 3581 2.054328e+00
## 17742  3 3582 2.042164e+00
## 17743  3 3583 2.026655e+00
## 17744  3 3584 2.012657e+00
## 17745  3 3585 1.996521e+00
## 17746  3 3586 1.983165e+00
## 17747  3 3587 1.962538e+00
## 17748  3 3588 1.948380e+00
## 17749  3 3589 1.931519e+00
## 17750  3 3590 1.916432e+00
## 17751  3 3591 1.903021e+00
## 17752  3 3592 1.890509e+00
## 17753  3 3593 1.877805e+00
## 17754  3 3594 1.863216e+00
## 17755  3 3595 1.848770e+00
## 17756  3 3596 1.836682e+00
## 17757  3 3597 1.823600e+00
## 17758  3 3598 1.815001e+00
## 17759  3 3599 1.801699e+00
## 17760  3 3600 1.786861e+00
## 17761  3 3601 1.935646e+00
## 17762  3 3602 2.027299e+00
## 17763  3 3603 2.065669e+00
## 17764  3 3604 2.072835e+00
## 17765  3 3605 2.064989e+00
## 17766  3 3606 2.055459e+00
## 17767  3 3607 2.033886e+00
## 17768  3 3608 2.020044e+00
## 17769  3 3609 2.006162e+00
## 17770  3 3610 1.989547e+00
## 17771  3 3611 1.973447e+00
## 17772  3 3612 1.956504e+00
## 17773  3 3613 1.942403e+00
## 17774  3 3614 1.926495e+00
## 17775  3 3615 1.911621e+00
## 17776  3 3616 1.899851e+00
## 17777  3 3617 1.884343e+00
## 17778  3 3618 1.870016e+00
## 17779  3 3619 1.854886e+00
## 17780  3 3620 1.846264e+00
## 17781  3 3621 1.831462e+00
## 17782  3 3622 1.816899e+00
## 17783  3 3623 1.804191e+00
## 17784  3 3624 1.793608e+00
## 17785  3 3625 1.940835e+00
## 17786  3 3626 2.036946e+00
## 17787  3 3627 2.070863e+00
## 17788  3 3628 2.080613e+00
## 17789  3 3629 2.071508e+00
## 17790  3 3630 2.055741e+00
## 17791  3 3631 2.043582e+00
## 17792  3 3632 2.027622e+00
## 17793  3 3633 2.009318e+00
## 17794  3 3634 1.992284e+00
## 17795  3 3635 1.978117e+00
## 17796  3 3636 1.965965e+00
## 17797  3 3637 1.949485e+00
## 17798  3 3638 1.934130e+00
## 17799  3 3639 1.918649e+00
## 17800  3 3640 1.905407e+00
## 17801  3 3641 1.889643e+00
## 17802  3 3642 1.877775e+00
## 17803  3 3643 1.863089e+00
## 17804  3 3644 1.850615e+00
## 17805  3 3645 1.840510e+00
## 17806  3 3646 1.822961e+00
## 17807  3 3647 1.811643e+00
## 17808  3 3648 1.802225e+00
## 17809  3 3649 1.948591e+00
## 17810  3 3650 2.040895e+00
## 17811  3 3651 2.077532e+00
## 17812  3 3652 2.082296e+00
## 17813  3 3653 2.077767e+00
## 17814  3 3654 2.057611e+00
## 17815  3 3655 2.045945e+00
## 17816  3 3656 2.032734e+00
## 17817  3 3657 2.016859e+00
## 17818  3 3658 2.001461e+00
## 17819  3 3659 1.984503e+00
## 17820  3 3660 1.967176e+00
## 17821  3 3661 1.956577e+00
## 17822  3 3662 1.938588e+00
## 17823  3 3663 1.924662e+00
## 17824  3 3664 1.905645e+00
## 17825  3 3665 1.894383e+00
## 17826  3 3666 1.882855e+00
## 17827  3 3667 1.866850e+00
## 17828  3 3668 1.855154e+00
## 17829  3 3669 1.844587e+00
## 17830  3 3670 1.830474e+00
## 17831  3 3671 1.818471e+00
## 17832  3 3672 1.808220e+00
## 17833  3 3673 1.955378e+00
## 17834  3 3674 2.047718e+00
## 17835  3 3675 2.085969e+00
## 17836  3 3676 2.088676e+00
## 17837  3 3677 2.080292e+00
## 17838  3 3678 2.066824e+00
## 17839  3 3679 2.051469e+00
## 17840  3 3680 2.039079e+00
## 17841  3 3681 2.021614e+00
## 17842  3 3682 2.003943e+00
## 17843  3 3683 1.991253e+00
## 17844  3 3684 1.974332e+00
## 17845  3 3685 1.957684e+00
## 17846  3 3686 1.939770e+00
## 17847  3 3687 1.930279e+00
## 17848  3 3688 1.916352e+00
## 17849  3 3689 1.898715e+00
## 17850  3 3690 1.889123e+00
## 17851  3 3691 1.873128e+00
## 17852  3 3692 1.863571e+00
## 17853  3 3693 1.847441e+00
## 17854  3 3694 1.837820e+00
## 17855  3 3695 1.824178e+00
## 17856  3 3696 1.812144e+00
## 17857  3 3697 1.956220e+00
## 17858  3 3698 2.052711e+00
## 17859  3 3699 2.088157e+00
## 17860  3 3700 2.092237e+00
## 17861  3 3701 2.089661e+00
## 17862  3 3702 2.078214e+00
## 17863  3 3703 2.058036e+00
## 17864  3 3704 2.042946e+00
## 17865  3 3705 2.027620e+00
## 17866  3 3706 2.009692e+00
## 17867  3 3707 1.995163e+00
## 17868  3 3708 1.978911e+00
## 17869  3 3709 1.962332e+00
## 17870  3 3710 1.947769e+00
## 17871  3 3711 1.934734e+00
## 17872  3 3712 1.917698e+00
## 17873  3 3713 1.903036e+00
## 17874  3 3714 1.889568e+00
## 17875  3 3715 1.881043e+00
## 17876  3 3716 1.864224e+00
## 17877  3 3717 1.850434e+00
## 17878  3 3718 1.840710e+00
## 17879  3 3719 1.830295e+00
## 17880  3 3720 1.817418e+00
## 17881  3 3721 1.966351e+00
## 17882  3 3722 2.061451e+00
## 17883  3 3723 2.091278e+00
## 17884  3 3724 2.096929e+00
## 17885  3 3725 2.092756e+00
## 17886  3 3726 2.080834e+00
## 17887  3 3727 2.066111e+00
## 17888  3 3728 2.044816e+00
## 17889  3 3729 2.028991e+00
## 17890  3 3730 2.012904e+00
## 17891  3 3731 1.998959e+00
## 17892  3 3732 1.985159e+00
## 17893  3 3733 1.965369e+00
## 17894  3 3734 1.951419e+00
## 17895  3 3735 1.938200e+00
## 17896  3 3736 1.924530e+00
## 17897  3 3737 1.910635e+00
## 17898  3 3738 1.894627e+00
## 17899  3 3739 1.882200e+00
## 17900  3 3740 1.873278e+00
## 17901  3 3741 1.855840e+00
## 17902  3 3742 1.844322e+00
## 17903  3 3743 1.834782e+00
## 17904  3 3744 1.819273e+00
## 17905  3 3745 1.967722e+00
## 17906  3 3746 2.060025e+00
## 17907  3 3747 2.098057e+00
## 17908  3 3748 2.107007e+00
## 17909  3 3749 2.094009e+00
## 17910  3 3750 2.080686e+00
## 17911  3 3751 2.068265e+00
## 17912  3 3752 2.051811e+00
## 17913  3 3753 2.032814e+00
## 17914  3 3754 2.018851e+00
## 17915  3 3755 2.003561e+00
## 17916  3 3756 1.990194e+00
## 17917  3 3757 1.973533e+00
## 17918  3 3758 1.960216e+00
## 17919  3 3759 1.942125e+00
## 17920  3 3760 1.927636e+00
## 17921  3 3761 1.916315e+00
## 17922  3 3762 1.900871e+00
## 17923  3 3763 1.888922e+00
## 17924  3 3764 1.875220e+00
## 17925  3 3765 1.860045e+00
## 17926  3 3766 1.846189e+00
## 17927  3 3767 1.833708e+00
## 17928  3 3768 1.823814e+00
## 17929  3 3768 1.872051e+00
## 17930  3 3769 2.020239e+00
## 17931  3 3770 2.117035e+00
## 17932  3 3771 2.154912e+00
## 17933  3 3772 2.154705e+00
## 17934  3 3773 2.145988e+00
## 17935  3 3774 2.134711e+00
## 17936  3 3775 2.125895e+00
## 17937  3 3776 2.101903e+00
## 17938  3 3777 2.086417e+00
## 17939  3 3778 2.065279e+00
## 17940  3 3779 2.053102e+00
## 17941  3 3780 2.036059e+00
## 17942  3 3781 2.025132e+00
## 17943  3 3782 2.006704e+00
## 17944  3 3783 1.991099e+00
## 17945  3 3784 1.977084e+00
## 17946  3 3785 1.960957e+00
## 17947  3 3786 1.952376e+00
## 17948  3 3787 1.938543e+00
## 17949  3 3788 1.920567e+00
## 17950  3 3789 1.908978e+00
## 17951  3 3790 1.901429e+00
## 17952  3 3791 1.884071e+00
## 17953  3 3792 1.876744e+00
## 17954  3 3792 1.875952e+00
## 17955  3 3793 1.860752e+00
## 17956  3 3794 1.851290e+00
## 17957  3 3795 1.839027e+00
## 17958  3 3796 1.826589e+00
## 17959  3 3797 1.819614e+00
## 17960  3 3798 1.807614e+00
## 17961  3 3799 1.795986e+00
## 17962  3 3800 1.785925e+00
## 17963  3 3801 1.775021e+00
## 17964  3 3802 1.765211e+00
## 17965  3 3803 1.753555e+00
## 17966  3 3804 1.749689e+00
## 17967  3 3805 1.735542e+00
## 17968  3 3806 1.728305e+00
## 17969  3 3807 1.717252e+00
## 17970  3 3808 1.710619e+00
## 17971  3 3809 1.701192e+00
## 17972  3 3810 1.694331e+00
## 17973  3 3811 1.683982e+00
## 17974  3 3812 1.678369e+00
## 17975  3 3813 1.665832e+00
## 17976  3 3814 1.659297e+00
## 17977  3 3815 1.652843e+00
## 17978  3 3816 1.644282e+00
## 17979  3 3816 1.644056e+00
## 17980  3 3817 1.872842e+00
## 17981  3 3818 2.023645e+00
## 17982  3 3819 2.089647e+00
## 17983  3 3820 2.104933e+00
## 17984  3 3821 2.105824e+00
## 17985  3 3822 2.097216e+00
## 17986  3 3823 2.082793e+00
## 17987  3 3824 2.060541e+00
## 17988  3 3825 2.050103e+00
## 17989  3 3826 2.031339e+00
## 17990  3 3827 2.011943e+00
## 17991  3 3828 1.995815e+00
## 17992  3 3829 1.983288e+00
## 17993  3 3830 1.964868e+00
## 17994  3 3831 1.950542e+00
## 17995  3 3832 1.935990e+00
## 17996  3 3833 1.922238e+00
## 17997  3 3834 1.911139e+00
## 17998  3 3835 1.895407e+00
## 17999  3 3836 1.882202e+00
## 18000  3 3837 1.868290e+00
## 18001  3 3838 1.852007e+00
## 18002  3 3839 1.844508e+00
## 18003  3 3840 1.828830e+00
## 18004  3 3840 1.830179e+00
## 18005  3 3841 1.976544e+00
## 18006  3 3842 2.066266e+00
## 18007  3 3843 2.108195e+00
## 18008  3 3844 2.109534e+00
## 18009  3 3845 2.103227e+00
## 18010  3 3846 2.094135e+00
## 18011  3 3847 2.074878e+00
## 18012  3 3848 2.058514e+00
## 18013  3 3849 2.044910e+00
## 18014  3 3850 2.027763e+00
## 18015  3 3851 2.009099e+00
## 18016  3 3852 1.992396e+00
## 18017  3 3853 1.978595e+00
## 18018  3 3854 1.965764e+00
## 18019  3 3855 1.948124e+00
## 18020  3 3856 1.934404e+00
## 18021  3 3857 1.916344e+00
## 18022  3 3858 1.904271e+00
## 18023  3 3859 1.894828e+00
## 18024  3 3860 1.877319e+00
## 18025  3 3861 1.863874e+00
## 18026  3 3862 1.854750e+00
## 18027  3 3863 1.843444e+00
## 18028  3 3864 1.830741e+00
## 18029  3 3865 1.976208e+00
## 18030  3 3866 2.071248e+00
## 18031  3 3867 2.103041e+00
## 18032  3 3868 2.114456e+00
## 18033  3 3869 2.100755e+00
## 18034  3 3870 2.091680e+00
## 18035  3 3871 2.071990e+00
## 18036  3 3872 2.059888e+00
## 18037  3 3873 2.042820e+00
## 18038  3 3874 2.027050e+00
## 18039  3 3875 2.011446e+00
## 18040  3 3876 1.993883e+00
## 18041  3 3877 1.981923e+00
## 18042  3 3878 1.964290e+00
## 18043  3 3879 1.945787e+00
## 18044  3 3880 1.935687e+00
## 18045  3 3881 1.922297e+00
## 18046  3 3882 1.904768e+00
## 18047  3 3883 1.894432e+00
## 18048  3 3884 1.880743e+00
## 18049  3 3885 1.868561e+00
## 18050  3 3886 1.855055e+00
## 18051  3 3887 1.842123e+00
## 18052  3 3888 1.831960e+00
## 18053  3 3889 1.975043e+00
## 18054  3 3890 2.070561e+00
## 18055  3 3891 2.105547e+00
## 18056  3 3892 2.112952e+00
## 18057  3 3893 2.106169e+00
## 18058  3 3894 2.094443e+00
## 18059  3 3895 2.073438e+00
## 18060  3 3896 2.059917e+00
## 18061  3 3897 2.043589e+00
## 18062  3 3898 2.029827e+00
## 18063  3 3899 2.015442e+00
## 18064  3 3900 1.994977e+00
## 18065  3 3901 1.979774e+00
## 18066  3 3902 1.963216e+00
## 18067  3 3903 1.951319e+00
## 18068  3 3904 1.933647e+00
## 18069  3 3905 1.922473e+00
## 18070  3 3906 1.910837e+00
## 18071  3 3907 1.897150e+00
## 18072  3 3908 1.883387e+00
## 18073  3 3909 1.870574e+00
## 18074  3 3910 1.860918e+00
## 18075  3 3911 1.842565e+00
## 18076  3 3912 1.835648e+00
## 18077  3 3913 1.980341e+00
## 18078  3 3914 2.072728e+00
## 18079  3 3915 2.105606e+00
## 18080  3 3916 2.112818e+00
## 18081  3 3917 2.106645e+00
## 18082  3 3918 2.096012e+00
## 18083  3 3919 2.078083e+00
## 18084  3 3920 2.067667e+00
## 18085  3 3921 2.048228e+00
## 18086  3 3922 2.030868e+00
## 18087  3 3923 2.014267e+00
## 18088  3 3924 1.993632e+00
## 18089  3 3925 1.980706e+00
## 18090  3 3926 1.967696e+00
## 18091  3 3927 1.955089e+00
## 18092  3 3928 1.940134e+00
## 18093  3 3929 1.926426e+00
## 18094  3 3930 1.912909e+00
## 18095  3 3931 1.902736e+00
## 18096  3 3932 1.887263e+00
## 18097  3 3933 1.871682e+00
## 18098  3 3934 1.856839e+00
## 18099  3 3935 1.847527e+00
## 18100  3 3936 1.833132e+00
## 18101  3 3937 1.980246e+00
## 18102  3 3938 2.079480e+00
## 18103  3 3939 2.110119e+00
## 18104  3 3940 2.117151e+00
## 18105  3 3941 2.110833e+00
## 18106  3 3942 2.094874e+00
## 18107  3 3943 2.082035e+00
## 18108  3 3944 2.064904e+00
## 18109  3 3945 2.048275e+00
## 18110  3 3946 2.033316e+00
## 18111  3 3947 2.019639e+00
## 18112  3 3948 2.001030e+00
## 18113  3 3949 1.984714e+00
## 18114  3 3950 1.971625e+00
## 18115  3 3951 1.956912e+00
## 18116  3 3952 1.938606e+00
## 18117  3 3953 1.927636e+00
## 18118  3 3954 1.916722e+00
## 18119  3 3955 1.903000e+00
## 18120  3 3956 1.888749e+00
## 18121  3 3957 1.878187e+00
## 18122  3 3958 1.863879e+00
## 18123  3 3959 1.848204e+00
## 18124  3 3960 1.838044e+00
## 18125  3 3961 1.980454e+00
## 18126  3 3962 2.079180e+00
## 18127  3 3963 2.116333e+00
## 18128  3 3964 2.118658e+00
## 18129  3 3965 2.114630e+00
## 18130  3 3966 2.099880e+00
## 18131  3 3967 2.083382e+00
## 18132  3 3968 2.069433e+00
## 18133  3 3969 2.050804e+00
## 18134  3 3970 2.033444e+00
## 18135  3 3971 2.018545e+00
## 18136  3 3972 2.004784e+00
## 18137  3 3973 1.987045e+00
## 18138  3 3974 1.975499e+00
## 18139  3 3975 1.960963e+00
## 18140  3 3976 1.945319e+00
## 18141  3 3977 1.929503e+00
## 18142  3 3978 1.915979e+00
## 18143  3 3979 1.905075e+00
## 18144  3 3980 1.887746e+00
## 18145  3 3981 1.876815e+00
## 18146  3 3982 1.865686e+00
## 18147  3 3983 1.855424e+00
## 18148  3 3984 1.843678e+00
## 18149  3 3985 1.988273e+00
## 18150  3 3986 2.081900e+00
## 18151  3 3987 2.121986e+00
## 18152  3 3988 2.126035e+00
## 18153  3 3989 2.115761e+00
## 18154  3 3990 2.102942e+00
## 18155  3 3991 2.083524e+00
## 18156  3 3992 2.072734e+00
## 18157  3 3993 2.058484e+00
## 18158  3 3994 2.040302e+00
## 18159  3 3995 2.021999e+00
## 18160  3 3996 2.005362e+00
## 18161  3 3997 1.991869e+00
## 18162  3 3998 1.977890e+00
## 18163  3 3999 1.958263e+00
## 18164  3 4000 1.945662e+00
## 18165  3 4001 1.933243e+00
## 18166  3 4002 1.921943e+00
## 18167  3 4003 1.908425e+00
## 18168  3 4004 1.887202e+00
## 18169  3 4005 1.882675e+00
## 18170  3 4006 1.866745e+00
## 18171  3 4007 1.852074e+00
## 18172  3 4008 1.843831e+00
## 18173  3 4009 1.989936e+00
## 18174  3 4010 2.087365e+00
## 18175  3 4011 2.121749e+00
## 18176  3 4012 2.124986e+00
## 18177  3 4013 2.118237e+00
## 18178  3 4014 2.103665e+00
## 18179  3 4015 2.090630e+00
## 18180  3 4016 2.073586e+00
## 18181  3 4017 2.058737e+00
## 18182  3 4018 2.045217e+00
## 18183  3 4019 2.023308e+00
## 18184  3 4020 2.005184e+00
## 18185  3 4021 1.992275e+00
## 18186  3 4022 1.976007e+00
## 18187  3 4023 1.962012e+00
## 18188  3 4024 1.948649e+00
## 18189  3 4025 1.931590e+00
## 18190  3 4026 1.922441e+00
## 18191  3 4027 1.911089e+00
## 18192  3 4028 1.895903e+00
## 18193  3 4029 1.881229e+00
## 18194  3 4030 1.867697e+00
## 18195  3 4031 1.858913e+00
## 18196  3 4032 1.843727e+00
## 18197  3 4033 1.992667e+00
## 18198  3 4034 2.087667e+00
## 18199  3 4035 2.121777e+00
## 18200  3 4036 2.128398e+00
## 18201  3 4037 2.122156e+00
## 18202  3 4038 2.107239e+00
## 18203  3 4039 2.094501e+00
## 18204  3 4040 2.077981e+00
## 18205  3 4041 2.055600e+00
## 18206  3 4042 2.040929e+00
## 18207  3 4043 2.026406e+00
## 18208  3 4044 2.012713e+00
## 18209  3 4045 1.998634e+00
## 18210  3 4046 1.981202e+00
## 18211  3 4047 1.964027e+00
## 18212  3 4048 1.952317e+00
## 18213  3 4049 1.939309e+00
## 18214  3 4050 1.922514e+00
## 18215  3 4051 1.908989e+00
## 18216  3 4052 1.895973e+00
## 18217  3 4053 1.880374e+00
## 18218  3 4054 1.868482e+00
## 18219  3 4055 1.859529e+00
## 18220  3 4056 1.845706e+00
## 18221  3 4057 1.992221e+00
## 18222  3 4058 2.089200e+00
## 18223  3 4059 2.124274e+00
## 18224  3 4060 2.125560e+00
## 18225  3 4061 2.123547e+00
## 18226  3 4062 2.112602e+00
## 18227  3 4063 2.095133e+00
## 18228  3 4064 2.083662e+00
## 18229  3 4065 2.064277e+00
## 18230  3 4066 2.043714e+00
## 18231  3 4067 2.027559e+00
## 18232  3 4068 2.010111e+00
## 18233  3 4069 2.002111e+00
## 18234  3 4070 1.978698e+00
## 18235  3 4071 1.964318e+00
## 18236  3 4072 1.949785e+00
## 18237  3 4073 1.940717e+00
## 18238  3 4074 1.924728e+00
## 18239  3 4075 1.911188e+00
## 18240  3 4076 1.900543e+00
## 18241  3 4077 1.886424e+00
## 18242  3 4078 1.875094e+00
## 18243  3 4079 1.859597e+00
## 18244  3 4080 1.849261e+00
## 18245  3 4081 1.994681e+00
## 18246  3 4082 2.091955e+00
## 18247  3 4083 2.121111e+00
## 18248  3 4084 2.131553e+00
## 18249  3 4085 2.125690e+00
## 18250  3 4086 2.111276e+00
## 18251  3 4087 2.096741e+00
## 18252  3 4088 2.079601e+00
## 18253  3 4089 2.060014e+00
## 18254  3 4090 2.047720e+00
## 18255  3 4091 2.032844e+00
## 18256  3 4092 2.015981e+00
## 18257  3 4093 1.997689e+00
## 18258  3 4094 1.985083e+00
## 18259  3 4095 1.968103e+00
## 18260  3 4096 1.955292e+00
## 18261  3 4097 1.939973e+00
## 18262  3 4098 1.926325e+00
## 18263  3 4099 1.913354e+00
## 18264  3 4100 1.901669e+00
## 18265  3 4101 1.886805e+00
## 18266  3 4102 1.876680e+00
## 18267  3 4103 1.863565e+00
## 18268  3 4104 1.850867e+00
## 18269  3 4104 1.875454e+00
## 18270  3 4105 2.022490e+00
## 18271  3 4106 2.114376e+00
## 18272  3 4107 2.152732e+00
## 18273  3 4108 2.157448e+00
```

```
## 18274  3 4109 2.151975e+00
## 18275  3 4110 2.132995e+00
## 18276  3 4111 2.116073e+00
## 18277  3 4112 2.100930e+00
## 18278  3 4113 2.085538e+00
## 18279  3 4114 2.068336e+00
## 18280  3 4115 2.052736e+00
## 18281  3 4116 2.037541e+00
## 18282  3 4117 2.021559e+00
## 18283  3 4118 2.010555e+00
## 18284  3 4119 1.995413e+00
## 18285  3 4120 1.979845e+00
## 18286  3 4121 1.961044e+00
## 18287  3 4122 1.949848e+00
## 18288  3 4123 1.936629e+00
## 18289  3 4124 1.923184e+00
## 18290  3 4125 1.910579e+00
## 18291  3 4126 1.897380e+00
## 18292  3 4127 1.885536e+00
## 18293  3 4128 1.874481e+00
## 18294  3 4128 1.870516e+00
## 18295  3 4129 1.862830e+00
## 18296  3 4130 1.851540e+00
## 18297  3 4131 1.835609e+00
## 18298  3 4132 1.828370e+00
## 18299  3 4133 1.819613e+00
## 18300  3 4134 1.805951e+00
## 18301  3 4135 1.795823e+00
## 18302  3 4136 1.783703e+00
## 18303  3 4137 1.775259e+00
## 18304  3 4138 1.763442e+00
## 18305  3 4139 1.756280e+00
## 18306  3 4140 1.743977e+00
## 18307  3 4141 1.734516e+00
## 18308  3 4142 1.728942e+00
## 18309  3 4143 1.720619e+00
## 18310  3 4144 1.710401e+00
## 18311  3 4145 1.702955e+00
## 18312  3 4146 1.692328e+00
## 18313  3 4147 1.683456e+00
## 18314  3 4148 1.674896e+00
## 18315  3 4149 1.667547e+00
## 18316  3 4150 1.661290e+00
## 18317  3 4151 1.649878e+00
## 18318  3 4152 1.641367e+00
## 18319  3 4152 1.642075e+00
## 18320  3 4153 1.954673e+00
## 18321  3 4154 2.158672e+00
## 18322  3 4155 2.246595e+00
## 18323  3 4156 2.266453e+00
## 18324  3 4157 2.270742e+00
## 18325  3 4158 2.260290e+00
## 18326  3 4159 2.241204e+00
## 18327  3 4160 2.220792e+00
## 18328  3 4161 2.203511e+00
## 18329  3 4162 2.180292e+00
## 18330  3 4163 2.161851e+00
## 18331  3 4164 2.141250e+00
## 18332  3 4165 2.127122e+00
## 18333  3 4166 2.105438e+00
## 18334  3 4167 2.088740e+00
## 18335  3 4168 2.069605e+00
## 18336  3 4169 2.054268e+00
## 18337  3 4170 2.038132e+00
## 18338  3 4171 2.018693e+00
## 18339  3 4172 2.005643e+00
## 18340  3 4173 1.988194e+00
## 18341  3 4174 1.971406e+00
## 18342  3 4175 1.955837e+00
## 18343  3 4176 1.948536e+00
## 18344  3 4176 1.941880e+00
## 18345  3 4177 2.090626e+00
## 18346  3 4178 2.183946e+00
## 18347  3 4179 2.215375e+00
## 18348  3 4180 2.216915e+00
## 18349  3 4181 2.206716e+00
## 18350  3 4182 2.193559e+00
## 18351  3 4183 2.175118e+00
## 18352  3 4184 2.157029e+00
## 18353  3 4185 2.143654e+00
## 18354  3 4186 2.124326e+00
## 18355  3 4187 2.105008e+00
## 18356  3 4188 2.082319e+00
## 18357  3 4189 2.069788e+00
## 18358  3 4190 2.051706e+00
## 18359  3 4191 2.038312e+00
## 18360  3 4192 2.017304e+00
## 18361  3 4193 2.008498e+00
## 18362  3 4194 1.989939e+00
## 18363  3 4195 1.973462e+00
## 18364  3 4196 1.962497e+00
## 18365  3 4197 1.947287e+00
## 18366  3 4198 1.935240e+00
## 18367  3 4199 1.919381e+00
## 18368  3 4200 1.908382e+00
## 18369  3 4201 2.057120e+00
## 18370  3 4202 2.149506e+00
## 18371  3 4203 2.181642e+00
## 18372  3 4204 2.184261e+00
## 18373  3 4205 2.175528e+00
## 18374  3 4206 2.164718e+00
## 18375  3 4207 2.147271e+00
## 18376  3 4208 2.128880e+00
## 18377  3 4209 2.114320e+00
## 18378  3 4210 2.096985e+00
## 18379  3 4211 2.078078e+00
## 18380  3 4212 2.063992e+00
## 18381  3 4213 2.044313e+00
## 18382  3 4214 2.031089e+00
## 18383  3 4215 2.017105e+00
## 18384  3 4216 2.002264e+00
## 18385  3 4217 1.987843e+00
## 18386  3 4218 1.972737e+00
## 18387  3 4219 1.956777e+00
## 18388  3 4220 1.943218e+00
## 18389  3 4221 1.929998e+00
## 18390  3 4222 1.919166e+00
## 18391  3 4223 1.902146e+00
## 18392  3 4224 1.889845e+00
## 18393  3 4225 2.037454e+00
## 18394  3 4226 2.129968e+00
## 18395  3 4227 2.169375e+00
## 18396  3 4228 2.173802e+00
## 18397  3 4229 2.166543e+00
## 18398  3 4230 2.152477e+00
## 18399  3 4231 2.136526e+00
## 18400  3 4232 2.119034e+00
## 18401  3 4233 2.103886e+00
## 18402  3 4234 2.082206e+00
## 18403  3 4235 2.071449e+00
## 18404  3 4236 2.050959e+00
## 18405  3 4237 2.037634e+00
## 18406  3 4238 2.021142e+00
## 18407  3 4239 2.005691e+00
## 18408  3 4240 1.994830e+00
## 18409  3 4241 1.976858e+00
## 18410  3 4242 1.960867e+00
## 18411  3 4243 1.948069e+00
## 18412  3 4244 1.937716e+00
## 18413  3 4245 1.922268e+00
## 18414  3 4246 1.910328e+00
## 18415  3 4247 1.897537e+00
## 18416  3 4248 1.882101e+00
## 18417  3 4249 2.030282e+00
## 18418  3 4250 2.124502e+00
## 18419  3 4251 2.160955e+00
## 18420  3 4252 2.167700e+00
## 18421  3 4253 2.157964e+00
## 18422  3 4254 2.145742e+00
## 18423  3 4255 2.131843e+00
## 18424  3 4256 2.112366e+00
## 18425  3 4257 2.099468e+00
## 18426  3 4258 2.078511e+00
## 18427  3 4259 2.061555e+00
## 18428  3 4260 2.049445e+00
## 18429  3 4261 2.032783e+00
## 18430  3 4262 2.016840e+00
## 18431  3 4263 2.001760e+00
## 18432  3 4264 1.986384e+00
## 18433  3 4265 1.970034e+00
## 18434  3 4266 1.958734e+00
## 18435  3 4267 1.945481e+00
## 18436  3 4268 1.934284e+00
## 18437  3 4269 1.922749e+00
## 18438  3 4270 1.907559e+00
## 18439  3 4271 1.896536e+00
## 18440  3 4272 1.882095e+00
## 18441  3 4273 2.029424e+00
## 18442  3 4274 2.123346e+00
## 18443  3 4275 2.157167e+00
## 18444  3 4276 2.162629e+00
## 18445  3 4277 2.157296e+00
## 18446  3 4278 2.142755e+00
## 18447  3 4279 2.129984e+00
## 18448  3 4280 2.112840e+00
## 18449  3 4281 2.094157e+00
## 18450  3 4282 2.076942e+00
## 18451  3 4283 2.061995e+00
## 18452  3 4284 2.046965e+00
## 18453  3 4285 2.029313e+00
## 18454  3 4286 2.017046e+00
## 18455  3 4287 2.000046e+00
## 18456  3 4288 1.985723e+00
## 18457  3 4289 1.971891e+00
## 18458  3 4290 1.961136e+00
## 18459  3 4291 1.942458e+00
## 18460  3 4292 1.930543e+00
## 18461  3 4293 1.917180e+00
## 18462  3 4294 1.906721e+00
## 18463  3 4295 1.890704e+00
## 18464  3 4296 1.880455e+00
## 18465  3 4297 2.027677e+00
## 18466  3 4298 2.117676e+00
## 18467  3 4299 2.159406e+00
## 18468  3 4300 2.162511e+00
## 18469  3 4301 2.155983e+00
## 18470  3 4302 2.143341e+00
## 18471  3 4303 2.128521e+00
## 18472  3 4304 2.108723e+00
## 18473  3 4305 2.094362e+00
## 18474  3 4306 2.073745e+00
## 18475  3 4307 2.062560e+00
## 18476  3 4308 2.043748e+00
## 18477  3 4309 2.029336e+00
## 18478  3 4310 2.017784e+00
## 18479  3 4311 1.999417e+00
## 18480  3 4312 1.983132e+00
## 18481  3 4313 1.969718e+00
## 18482  3 4314 1.954454e+00
## 18483  3 4315 1.945855e+00
## 18484  3 4316 1.930551e+00
## 18485  3 4317 1.918552e+00
## 18486  3 4318 1.904111e+00
## 18487  3 4319 1.890901e+00
## 18488  3 4320 1.880941e+00
## 18489  3 4321 2.027573e+00
## 18490  3 4322 2.120210e+00
## 18491  3 4323 2.154495e+00
## 18492  3 4324 2.159592e+00
## 18493  3 4325 2.154006e+00
## 18494  3 4326 2.142885e+00
## 18495  3 4327 2.125123e+00
## 18496  3 4328 2.112416e+00
## 18497  3 4329 2.093617e+00
## 18498  3 4330 2.078479e+00
## 18499  3 4331 2.057895e+00
## 18500  3 4332 2.041420e+00
## 18501  3 4333 2.031784e+00
## 18502  3 4334 2.015812e+00
## 18503  3 4335 1.998602e+00
## 18504  3 4336 1.981973e+00
## 18505  3 4337 1.971626e+00
## 18506  3 4338 1.954385e+00
## 18507  3 4339 1.944105e+00
## 18508  3 4340 1.930369e+00
## 18509  3 4341 1.918907e+00
## 18510  3 4342 1.904223e+00
## 18511  3 4343 1.887755e+00
## 18512  3 4344 1.880176e+00
## 18513  3 4345 2.029461e+00
## 18514  3 4346 2.118759e+00
## 18515  3 4347 2.157897e+00
## 18516  3 4348 2.165596e+00
## 18517  3 4349 2.156873e+00
## 18518  3 4350 2.139335e+00
## 18519  3 4351 2.124304e+00
## 18520  3 4352 2.112017e+00
## 18521  3 4353 2.090990e+00
## 18522  3 4354 2.074801e+00
## 18523  3 4355 2.060166e+00
## 18524  3 4356 2.047207e+00
## 18525  3 4357 2.027003e+00
## 18526  3 4358 2.009812e+00
## 18527  3 4359 1.998415e+00
## 18528  3 4360 1.982511e+00
## 18529  3 4361 1.968262e+00
## 18530  3 4362 1.955251e+00
## 18531  3 4363 1.938792e+00
## 18532  3 4364 1.926928e+00
## 18533  3 4365 1.918784e+00
## 18534  3 4366 1.901094e+00
## 18535  3 4367 1.890606e+00
## 18536  3 4368 1.876282e+00
## 18537  3 4369 2.024381e+00
## 18538  3 4370 2.119340e+00
## 18539  3 4371 2.157640e+00
## 18540  3 4372 2.159482e+00
## 18541  3 4373 2.149025e+00
## 18542  3 4374 2.142856e+00
## 18543  3 4375 2.122422e+00
## 18544  3 4376 2.108591e+00
## 18545  3 4377 2.094927e+00
## 18546  3 4378 2.072725e+00
## 18547  3 4379 2.054706e+00
## 18548  3 4380 2.042490e+00
## 18549  3 4381 2.025319e+00
## 18550  3 4382 2.013800e+00
## 18551  3 4383 1.996872e+00
## 18552  3 4384 1.982495e+00
## 18553  3 4385 1.968691e+00
## 18554  3 4386 1.951819e+00
## 18555  3 4387 1.947189e+00
## 18556  3 4388 1.929875e+00
## 18557  3 4389 1.915356e+00
## 18558  3 4390 1.901173e+00
## 18559  3 4391 1.892872e+00
## 18560  3 4392 1.878120e+00
## 18561  3 4393 2.028028e+00
## 18562  3 4394 2.121743e+00
## 18563  3 4395 2.151603e+00
## 18564  3 4396 2.158807e+00
## 18565  3 4397 2.154141e+00
## 18566  3 4398 2.141163e+00
## 18567  3 4399 2.126146e+00
## 18568  3 4400 2.106879e+00
## 18569  3 4401 2.091995e+00
## 18570  3 4402 2.071410e+00
## 18571  3 4403 2.059825e+00
## 18572  3 4404 2.040046e+00
## 18573  3 4405 2.024527e+00
## 18574  3 4406 2.012866e+00
## 18575  3 4407 1.997417e+00
## 18576  3 4408 1.985124e+00
## 18577  3 4409 1.965751e+00
## 18578  3 4410 1.954945e+00
## 18579  3 4411 1.942984e+00
## 18580  3 4412 1.925480e+00
## 18581  3 4413 1.915455e+00
## 18582  3 4414 1.902669e+00
## 18583  3 4415 1.890070e+00
## 18584  3 4416 1.877262e+00
## 18585  3 4417 2.025044e+00
## 18586  3 4418 2.122436e+00
## 18587  3 4419 2.153743e+00
## 18588  3 4420 2.158358e+00
## 18589  3 4421 2.152279e+00
## 18590  3 4422 2.141963e+00
## 18591  3 4423 2.119412e+00
## 18592  3 4424 2.111223e+00
## 18593  3 4425 2.089917e+00
## 18594  3 4426 2.079382e+00
## 18595  3 4427 2.056891e+00
## 18596  3 4428 2.043957e+00
## 18597  3 4429 2.023998e+00
## 18598  3 4430 2.013545e+00
## 18599  3 4431 1.998130e+00
## 18600  3 4432 1.985360e+00
## 18601  3 4433 1.967397e+00
## 18602  3 4434 1.953979e+00
## 18603  3 4435 1.942566e+00
## 18604  3 4436 1.929823e+00
## 18605  3 4437 1.914003e+00
## 18606  3 4438 1.903920e+00
## 18607  3 4439 1.890005e+00
## 18608  3 4440 1.876108e+00
## 18609  3 4441 1.862696e+00
## 18610  3 4442 1.853964e+00
## 18611  3 4443 1.843460e+00
## 18612  3 4444 1.833841e+00
## 18613  3 4445 1.820887e+00
## 18614  3 4446 1.810727e+00
## 18615  3 4447 1.802433e+00
## 18616  3 4448 1.790987e+00
## 18617  3 4449 1.782482e+00
## 18618  3 4450 1.771655e+00
## 18619  3 4451 1.757738e+00
## 18620  3 4452 1.750365e+00
## 18621  3 4453 1.743728e+00
## 18622  3 4454 1.730553e+00
## 18623  3 4455 1.723605e+00
## 18624  3 4456 1.716533e+00
## 18625  3 4457 1.706274e+00
## 18626  3 4458 1.695056e+00
## 18627  3 4459 1.690209e+00
## 18628  3 4460 1.676960e+00
## 18629  3 4461 1.671849e+00
## 18630  3 4462 1.661938e+00
## 18631  3 4463 1.656884e+00
## 18632  3 4464 1.649313e+00
## 18633  3 4465 1.641945e+00
## 18634  3 4466 1.632547e+00
## 18635  3 4467 1.627206e+00
## 18636  3 4468 1.618240e+00
## 18637  3 4469 1.608914e+00
## 18638  3 4470 1.601595e+00
## 18639  3 4471 1.598502e+00
## 18640  3 4472 1.589549e+00
## 18641  3 4473 1.581265e+00
## 18642  3 4474 1.577100e+00
## 18643  3 4475 1.569812e+00
## 18644  3 4476 1.560822e+00
## 18645  3 4477 1.558839e+00
## 18646  3 4478 1.548200e+00
## 18647  3 4479 1.544434e+00
## 18648  3 4480 1.536577e+00
## 18649  3 4481 1.529039e+00
## 18650  3 4482 1.525473e+00
## 18651  3 4483 1.518088e+00
## 18652  3 4484 1.513120e+00
## 18653  3 4485 1.508292e+00
## 18654  3 4486 1.500560e+00
## 18655  3 4487 1.498619e+00
## 18656  3 4488 1.489797e+00
## 18657  3 4489 1.485502e+00
## 18658  3 4490 1.477014e+00
## 18659  3 4491 1.474183e+00
## 18660  3 4492 1.466582e+00
## 18661  3 4493 1.459609e+00
## 18662  3 4494 1.453210e+00
## 18663  3 4495 1.448961e+00
## 18664  3 4496 1.443741e+00
## 18665  3 4497 1.442334e+00
## 18666  3 4498 1.434103e+00
## 18667  3 4499 1.428715e+00
## 18668  3 4500 1.423365e+00
## 18669  3 4501 1.417958e+00
## 18670  3 4502 1.416664e+00
## 18671  3 4503 1.410319e+00
## 18672  3 4504 1.403584e+00
## 18673  3 4505 1.401191e+00
## 18674  3 4506 1.394249e+00
## 18675  3 4507 1.388916e+00
## 18676  3 4508 1.384774e+00
## 18677  3 4509 1.379679e+00
## 18678  3 4510 1.373798e+00
## 18679  3 4511 1.371632e+00
## 18680  3 4512 1.366538e+00
## 18681  3 4513 1.361203e+00
## 18682  3 4514 1.356034e+00
## 18683  3 4515 1.350530e+00
## 18684  3 4516 1.345465e+00
## 18685  3 4517 1.341946e+00
## 18686  3 4518 1.337081e+00
## 18687  3 4519 1.331906e+00
## 18688  3 4520 1.329179e+00
## 18689  3 4521 1.320862e+00
## 18690  3 4522 1.319569e+00
## 18691  3 4523 1.313513e+00
## 18692  3 4524 1.310237e+00
## 18693  3 4525 1.305603e+00
## 18694  3 4526 1.300113e+00
## 18695  3 4527 1.297829e+00
## 18696  3 4528 1.294880e+00
## 18697  3 4529 1.288061e+00
## 18698  3 4530 1.284583e+00
## 18699  3 4531 1.278314e+00
## 18700  3 4532 1.274880e+00
## 18701  3 4533 1.271486e+00
## 18702  3 4534 1.266502e+00
## 18703  3 4535 1.264050e+00
## 18704  3 4536 1.259356e+00
## 18705  3 4537 1.253091e+00
## 18706  3 4538 1.250622e+00
## 18707  3 4539 1.246909e+00
## 18708  3 4540 1.241438e+00
## 18709  3 4541 1.237759e+00
## 18710  3 4542 1.235564e+00
## 18711  3 4543 1.232555e+00
## 18712  3 4544 1.224512e+00
## 18713  3 4545 1.222142e+00
## 18714  3 4546 1.215545e+00
## 18715  3 4547 1.214220e+00
## 18716  3 4548 1.210441e+00
## 18717  3 4549 1.208157e+00
## 18718  3 4550 1.201075e+00
## 18719  3 4551 1.198156e+00
## 18720  3 4552 1.196369e+00
## 18721  3 4553 1.190551e+00
## 18722  3 4554 1.187466e+00
## 18723  3 4555 1.181317e+00
## 18724  3 4556 1.177742e+00
## 18725  3 4557 1.176561e+00
## 18726  3 4558 1.171505e+00
## 18727  3 4559 1.168663e+00
## 18728  3 4560 1.163855e+00
## 18729  3 4561 1.161673e+00
## 18730  3 4562 1.154852e+00
## 18731  3 4563 1.152369e+00
## 18732  3 4564 1.149710e+00
## 18733  3 4565 1.146724e+00
## 18734  3 4566 1.144285e+00
## 18735  3 4567 1.138928e+00
## 18736  3 4568 1.135969e+00
## 18737  3 4569 1.131462e+00
## 18738  3 4570 1.126094e+00
## 18739  3 4571 1.123385e+00
## 18740  3 4572 1.120184e+00
## 18741  3 4573 1.119564e+00
## 18742  3 4574 1.114538e+00
## 18743  3 4575 1.111809e+00
## 18744  3 4576 1.105707e+00
## 18745  3 4577 1.104214e+00
## 18746  3 4578 1.099311e+00
## 18747  3 4579 1.095251e+00
## 18748  3 4580 1.094145e+00
## 18749  3 4581 1.086600e+00
## 18750  3 4582 1.084706e+00
## 18751  3 4583 1.082269e+00
## 18752  3 4584 1.079801e+00
## 18753  3 4585 1.075885e+00
## 18754  3 4586 1.072405e+00
## 18755  3 4587 1.069747e+00
## 18756  3 4588 1.064068e+00
## 18757  3 4589 1.063163e+00
## 18758  3 4590 1.059923e+00
## 18759  3 4591 1.055837e+00
## 18760  3 4592 1.050945e+00
## 18761  3 4593 1.048515e+00
## 18762  3 4594 1.046758e+00
## 18763  3 4595 1.042637e+00
## 18764  3 4596 1.037643e+00
## 18765  3 4597 1.035046e+00
## 18766  3 4598 1.032134e+00
## 18767  3 4599 1.029033e+00
## 18768  3 4600 1.026791e+00
## 18769  3 4601 1.022594e+00
## 18770  3 4602 1.020212e+00
## 18771  3 4603 1.015115e+00
## 18772  3 4604 1.013588e+00
## 18773  3 4605 1.008399e+00
## 18774  3 4606 1.006282e+00
## 18775  3 4607 1.003696e+00
## 18776  3 4608 1.000790e+00
## 18777  3 4609 9.941751e-01
## 18778  3 4610 9.934956e-01
## 18779  3 4611 9.922751e-01
## 18780  3 4612 9.880324e-01
## 18781  3 4613 9.853598e-01
## 18782  3 4614 9.816854e-01
## 18783  3 4615 9.774829e-01
## 18784  3 4616 9.760128e-01
## 18785  3 4617 9.732440e-01
## 18786  3 4618 9.683654e-01
## 18787  3 4619 9.670001e-01
## 18788  3 4620 9.627487e-01
## 18789  3 4621 9.597230e-01
## 18790  3 4622 9.569124e-01
## 18791  3 4623 9.530807e-01
## 18792  3 4624 9.502919e-01
## 18793  3 4625 9.489975e-01
## 18794  3 4626 9.454630e-01
## 18795  3 4627 9.427429e-01
## 18796  3 4628 9.395054e-01
## 18797  3 4629 9.372753e-01
## 18798  3 4630 9.332069e-01
## 18799  3 4631 9.286240e-01
## 18800  3 4632 9.275077e-01
## 18801  3 4633 9.251396e-01
## 18802  3 4634 9.219451e-01
## 18803  3 4635 9.189692e-01
## 18804  3 4636 9.171595e-01
## 18805  3 4637 9.138946e-01
## 18806  3 4638 9.110486e-01
## 18807  3 4639 9.064569e-01
## 18808  3 4640 9.029802e-01
## 18809  3 4641 9.012878e-01
## 18810  3 4642 9.000743e-01
## 18811  3 4643 8.953761e-01
## 18812  3 4644 8.927522e-01
## 18813  3 4645 8.910271e-01
## 18814  3 4646 8.872726e-01
## 18815  3 4647 8.860629e-01
## 18816  3 4648 8.811959e-01
## 18817  3 4649 8.790089e-01
## 18818  3 4650 8.767399e-01
## 18819  3 4651 8.746027e-01
## 18820  3 4652 8.714292e-01
## 18821  3 4653 8.692001e-01
## 18822  3 4654 8.664254e-01
## 18823  3 4655 8.626335e-01
## 18824  3 4656 8.588262e-01
## 18825  3 4657 8.570165e-01
## 18826  3 4658 8.552608e-01
## 18827  3 4659 8.514044e-01
## 18828  3 4660 8.502669e-01
## 18829  3 4661 8.470786e-01
## 18830  3 4662 8.433099e-01
## 18831  3 4663 8.412322e-01
## 18832  3 4664 8.394321e-01
## 18833  3 4665 8.359466e-01
## 18834  3 4666 8.331073e-01
## 18835  3 4667 8.309989e-01
## 18836  3 4668 8.286033e-01
## 18837  3 4669 8.257344e-01
## 18838  3 4670 8.231700e-01
## 18839  3 4671 8.208046e-01
## 18840  3 4672 8.181232e-01
## 18841  3 4673 8.160403e-01
## 18842  3 4674 8.118200e-01
## 18843  3 4675 8.094726e-01
## 18844  3 4676 8.086614e-01
## 18845  3 4677 8.051130e-01
## 18846  3 4678 8.038312e-01
## 18847  3 4679 7.989925e-01
## 18848  3 4680 7.964325e-01
## 18849  3 4681 7.955322e-01
## 18850  3 4682 7.934402e-01
## 18851  3 4683 7.919229e-01
## 18852  3 4684 7.874702e-01
## 18853  3 4685 7.863360e-01
## 18854  3 4686 7.824929e-01
## 18855  3 4687 7.817377e-01
## 18856  3 4688 7.782880e-01
## 18857  3 4689 7.768336e-01
## 18858  3 4690 7.726167e-01
## 18859  3 4691 7.712299e-01
## 18860  3 4692 7.673977e-01
## 18861  3 4693 7.661703e-01
## 18862  3 4694 7.642135e-01
## 18863  3 4695 7.596711e-01
## 18864  3 4696 7.597033e-01
## 18865  3 4697 7.562804e-01
## 18866  3 4698 7.533733e-01
## 18867  3 4699 7.523391e-01
## 18868  3 4700 7.496270e-01
## 18869  3 4701 7.481160e-01
## 18870  3 4702 7.445385e-01
## 18871  3 4703 7.414045e-01
## 18872  3 4704 7.402646e-01
## 18873  3 4705 7.377225e-01
## 18874  3 4706 7.352921e-01
## 18875  3 4707 7.332254e-01
## 18876  3 4708 7.315008e-01
## 18877  3 4709 7.274438e-01
## 18878  3 4710 7.255846e-01
## 18879  3 4711 7.242727e-01
## 18880  3 4712 7.206598e-01
## 18881  3 4713 7.186821e-01
## 18882  3 4714 7.169962e-01
## 18883  3 4715 7.144916e-01
## 18884  3 4716 7.135616e-01
## 18885  3 4717 7.104746e-01
## 18886  3 4718 7.080942e-01
## 18887  3 4719 7.049544e-01
## 18888  3 4720 7.038401e-01
## 18889  3 4721 7.022561e-01
## 18890  3 4722 6.989843e-01
## 18891  3 4723 6.978860e-01
## 18892  3 4724 6.936775e-01
## 18893  3 4725 6.932053e-01
## 18894  3 4726 6.905999e-01
## 18895  3 4727 6.888681e-01
## 18896  3 4728 6.861230e-01
## 18897  3 4729 6.853780e-01
## 18898  3 4730 6.825303e-01
## 18899  3 4731 6.802563e-01
## 18900  3 4732 6.787238e-01
## 18901  3 4733 6.762861e-01
## 18902  3 4734 6.733036e-01
## 18903  3 4735 6.714524e-01
## 18904  3 4736 6.694072e-01
## 18905  3 4737 6.673542e-01
## 18906  3 4738 6.654408e-01
## 18907  3 4739 6.637227e-01
## 18908  3 4740 6.600899e-01
## 18909  3 4741 6.587048e-01
## 18910  3 4742 6.566425e-01
## 18911  3 4743 6.539745e-01
## 18912  3 4744 6.528427e-01
## 18913  3 4745 6.499848e-01
## 18914  3 4746 6.490747e-01
## 18915  3 4747 6.468521e-01
## 18916  3 4748 6.440085e-01
## 18917  3 4749 6.417497e-01
## 18918  3 4750 6.406993e-01
## 18919  3 4751 6.382778e-01
## 18920  3 4752 6.376537e-01
## 18921  3 4753 6.355310e-01
## 18922  3 4754 6.326584e-01
## 18923  3 4755 6.305011e-01
## 18924  3 4756 6.281004e-01
## 18925  3 4757 6.265234e-01
## 18926  3 4758 6.251260e-01
## 18927  3 4759 6.224246e-01
## 18928  3 4760 6.214271e-01
## 18929  3 4761 6.185163e-01
## 18930  3 4762 6.168609e-01
## 18931  3 4763 6.148696e-01
## 18932  3 4764 6.122685e-01
## 18933  3 4765 6.108169e-01
## 18934  3 4766 6.091077e-01
## 18935  3 4767 6.076036e-01
## 18936  3 4768 6.055524e-01
## 18937  3 4769 6.026642e-01
## 18938  3 4770 6.020275e-01
## 18939  3 4771 5.995769e-01
## 18940  3 4772 5.989885e-01
## 18941  3 4773 5.962092e-01
## 18942  3 4774 5.942478e-01
## 18943  3 4775 5.922059e-01
## 18944  3 4776 5.921408e-01
## 18945  3 4777 5.891158e-01
## 18946  3 4778 5.876594e-01
## 18947  3 4779 5.844530e-01
## 18948  3 4780 5.829505e-01
## 18949  3 4781 5.819687e-01
## 18950  3 4782 5.795824e-01
## 18951  3 4783 5.777030e-01
## 18952  3 4784 5.753028e-01
## 18953  3 4785 5.724471e-01
## 18954  3 4786 5.711671e-01
## 18955  3 4787 5.709600e-01
## 18956  3 4788 5.681759e-01
## 18957  3 4789 5.670578e-01
## 18958  3 4790 5.648645e-01
## 18959  3 4791 5.633520e-01
## 18960  3 4792 5.627066e-01
## 18961  3 4793 5.603748e-01
## 18962  3 4794 5.581032e-01
## 18963  3 4795 5.568502e-01
## 18964  3 4796 5.550026e-01
## 18965  3 4797 5.528749e-01
## 18966  3 4798 5.506207e-01
## 18967  3 4799 5.484564e-01
## 18968  3 4800 5.475090e-01
## 18969  3 4801 5.456879e-01
## 18970  3 4802 5.437474e-01
## 18971  3 4803 5.424188e-01
## 18972  3 4804 5.412318e-01
## 18973  3 4805 5.395261e-01
## 18974  3 4806 5.371484e-01
## 18975  3 4807 5.354516e-01
## 18976  3 4808 5.342177e-01
## 18977  3 4809 5.329118e-01
## 18978  3 4810 5.317874e-01
## 18979  3 4811 5.297836e-01
## 18980  3 4812 5.274274e-01
## 18981  3 4813 5.253354e-01
## 18982  3 4814 5.240075e-01
## 18983  3 4815 5.230522e-01
## 18984  3 4816 5.204695e-01
## 18985  3 4817 5.187687e-01
## 18986  3 4818 5.184592e-01
## 18987  3 4819 5.156694e-01
## 18988  3 4820 5.151506e-01
## 18989  3 4821 5.125615e-01
## 18990  3 4822 5.115872e-01
## 18991  3 4823 5.087942e-01
## 18992  3 4824 5.079894e-01
## 18993  3 4825 5.065845e-01
## 18994  3 4826 5.052495e-01
## 18995  3 4827 5.038267e-01
## 18996  3 4828 5.022713e-01
## 18997  3 4829 5.003609e-01
## 18998  3 4830 4.986357e-01
## 18999  3 4831 4.969577e-01
## 19000  3 4832 4.962961e-01
## 19001  3 4833 4.939067e-01
## 19002  3 4834 4.931242e-01
## 19003  3 4835 4.914860e-01
## 19004  3 4836 4.891328e-01
## 19005  3 4837 4.876433e-01
## 19006  3 4838 4.862082e-01
## 19007  3 4839 4.849392e-01
## 19008  3 4840 4.828679e-01
## 19009  3 4841 4.813997e-01
## 19010  3 4842 4.807517e-01
## 19011  3 4843 4.778834e-01
## 19012  3 4844 4.776022e-01
## 19013  3 4845 4.751894e-01
## 19014  3 4846 4.749974e-01
## 19015  3 4847 4.733422e-01
## 19016  3 4848 4.717605e-01
## 19017  3 4849 4.689390e-01
## 19018  3 4850 4.681941e-01
## 19019  3 4851 4.666216e-01
## 19020  3 4852 4.655012e-01
## 19021  3 4853 4.632239e-01
## 19022  3 4854 4.622198e-01
## 19023  3 4855 4.615063e-01
## 19024  3 4856 4.596464e-01
## 19025  3 4857 4.580888e-01
## 19026  3 4858 4.573690e-01
## 19027  3 4859 4.556199e-01
## 19028  3 4860 4.536262e-01
## 19029  3 4861 4.523046e-01
## 19030  3 4862 4.514383e-01
## 19031  3 4863 4.493337e-01
## 19032  3 4864 4.479132e-01
## 19033  3 4865 4.461275e-01
## 19034  3 4866 4.461478e-01
## 19035  3 4867 4.439742e-01
## 19036  3 4868 4.425264e-01
## 19037  3 4869 4.412804e-01
## 19038  3 4870 4.393706e-01
## 19039  3 4871 4.380880e-01
## 19040  3 4872 4.360760e-01
## 19041  3 4873 4.363764e-01
## 19042  3 4874 4.343118e-01
## 19043  3 4875 4.331643e-01
## 19044  3 4876 4.323851e-01
## 19045  3 4877 4.308395e-01
## 19046  3 4878 4.284806e-01
## 19047  3 4879 4.277166e-01
## 19048  3 4880 4.262925e-01
## 19049  3 4881 4.246708e-01
## 19050  3 4882 4.231336e-01
## 19051  3 4883 4.222454e-01
## 19052  3 4884 4.213067e-01
## 19053  3 4885 4.205667e-01
## 19054  3 4886 4.179789e-01
## 19055  3 4887 4.163472e-01
## 19056  3 4888 4.154769e-01
## 19057  3 4889 4.139244e-01
## 19058  3 4890 4.132978e-01
## 19059  3 4891 4.120551e-01
## 19060  3 4892 4.104595e-01
## 19061  3 4893 4.092806e-01
## 19062  3 4894 4.090186e-01
## 19063  3 4895 4.065316e-01
## 19064  3 4896 4.059960e-01
## 19065  3 4897 4.038919e-01
## 19066  3 4898 4.018381e-01
## 19067  3 4899 4.023487e-01
## 19068  3 4900 4.009100e-01
## 19069  3 4901 3.990733e-01
## 19070  3 4902 3.984332e-01
## 19071  3 4903 3.972159e-01
## 19072  3 4904 3.946215e-01
## 19073  3 4905 3.940152e-01
```

```
## 19074  3 4906 3.928620e-01
## 19075  3 4907 3.916004e-01
## 19076  3 4908 3.902120e-01
## 19077  3 4909 3.885502e-01
## 19078  3 4910 3.884878e-01
## 19079  3 4911 3.869501e-01
## 19080  3 4912 3.857372e-01
## 19081  3 4913 3.844004e-01
## 19082  3 4914 3.831188e-01
## 19083  3 4915 3.815719e-01
## 19084  3 4916 3.811445e-01
## 19085  3 4917 3.794228e-01
## 19086  3 4918 3.787342e-01
## 19087  3 4919 3.766808e-01
## 19088  3 4920 3.763288e-01
## 19089  3 4921 3.748007e-01
## 19090  3 4922 3.735766e-01
## 19091  3 4923 3.726804e-01
## 19092  3 4924 3.714782e-01
## 19093  3 4925 3.698144e-01
## 19094  3 4926 3.693930e-01
## 19095  3 4927 3.679729e-01
## 19096  3 4928 3.672889e-01
## 19097  3 4929 3.656739e-01
## 19098  3 4930 3.643868e-01
## 19099  3 4931 3.637240e-01
## 19100  3 4932 3.625025e-01
## 19101  3 4933 3.606290e-01
## 19102  3 4934 3.601191e-01
## 19103  3 4935 3.589208e-01
## 19104  3 4936 3.575772e-01
## 19105  3 4937 3.565958e-01
## 19106  3 4938 3.552500e-01
## 19107  3 4939 3.546602e-01
## 19108  3 4940 3.529185e-01
## 19109  3 4941 3.517661e-01
## 19110  3 4942 3.506836e-01
## 19111  3 4943 3.496920e-01
## 19112  3 4944 3.485407e-01
## 19113  3 4945 3.477215e-01
## 19114  3 4946 3.471843e-01
## 19115  3 4947 3.460477e-01
## 19116  3 4948 3.446631e-01
## 19117  3 4949 3.434269e-01
## 19118  3 4950 3.422526e-01
## 19119  3 4951 3.410656e-01
## 19120  3 4952 3.397235e-01
## 19121  3 4953 3.387848e-01
## 19122  3 4954 3.374240e-01
## 19123  3 4955 3.369091e-01
## 19124  3 4956 3.356544e-01
## 19125  3 4957 3.349749e-01
## 19126  3 4958 3.343258e-01
## 19127  3 4959 3.328346e-01
## 19128  3 4960 3.320837e-01
## 19129  3 4961 3.305431e-01
## 19130  3 4962 3.301600e-01
## 19131  3 4963 3.289072e-01
## 19132  3 4964 3.278213e-01
## 19133  3 4965 3.266011e-01
## 19134  3 4966 3.255289e-01
## 19135  3 4967 3.250505e-01
## 19136  3 4968 3.235809e-01
## 19137  3 4969 3.230096e-01
## 19138  3 4970 3.210456e-01
## 19139  3 4971 3.203721e-01
## 19140  3 4972 3.191346e-01
## 19141  3 4973 3.181212e-01
## 19142  3 4974 3.177729e-01
## 19143  3 4975 3.165889e-01
## 19144  3 4976 3.154094e-01
## 19145  3 4977 3.135946e-01
## 19146  3 4978 3.137860e-01
## 19147  3 4979 3.126439e-01
## 19148  3 4980 3.112879e-01
## 19149  3 4981 3.108358e-01
## 19150  3 4982 3.095452e-01
## 19151  3 4983 3.084942e-01
## 19152  3 4984 3.073167e-01
## 19153  3 4985 3.070352e-01
## 19154  3 4986 3.060158e-01
## 19155  3 4987 3.043458e-01
## 19156  3 4988 3.039348e-01
## 19157  3 4989 3.031190e-01
## 19158  3 4990 3.021930e-01
## 19159  3 4991 3.013198e-01
## 19160  3 4992 3.001832e-01
## 19161  3 4993 2.988987e-01
## 19162  3 4994 2.979545e-01
## 19163  3 4995 2.971413e-01
## 19164  3 4996 2.964142e-01
## 19165  3 4997 2.953575e-01
## 19166  3 4998 2.943753e-01
## 19167  3 4999 2.933135e-01
## 19168  3 5000 2.923512e-01
## 19169  3 5001 2.917871e-01
## 19170  3 5002 2.908517e-01
## 19171  3 5003 2.897413e-01
## 19172  3 5004 2.885049e-01
## 19173  3 5005 2.883631e-01
## 19174  3 5006 2.870016e-01
## 19175  3 5007 2.863360e-01
## 19176  3 5008 2.857415e-01
## 19177  3 5009 2.845005e-01
## 19178  3 5010 2.838413e-01
## 19179  3 5011 2.822484e-01
## 19180  3 5012 2.819219e-01
## 19181  3 5013 2.809385e-01
## 19182  3 5014 2.802666e-01
## 19183  3 5015 2.792086e-01
## 19184  3 5016 2.780610e-01
## 19185  3 5017 2.773636e-01
## 19186  3 5018 2.762674e-01
## 19187  3 5019 2.757215e-01
## 19188  3 5020 2.753877e-01
## 19189  3 5021 2.744837e-01
## 19190  3 5022 2.725618e-01
## 19191  3 5023 2.717885e-01
## 19192  3 5024 2.712273e-01
## 19193  3 5025 2.705014e-01
## 19194  3 5026 2.697234e-01
## 19195  3 5027 2.680785e-01
## 19196  3 5028 2.678948e-01
## 19197  3 5029 2.670401e-01
## 19198  3 5030 2.663604e-01
## 19199  3 5031 2.658537e-01
## 19200  3 5032 2.646234e-01
## 19201  3 5033 2.637462e-01
## 19202  3 5034 2.632034e-01
## 19203  3 5035 2.624560e-01
## 19204  3 5036 2.611862e-01
## 19205  3 5037 2.606419e-01
## 19206  3 5038 2.598806e-01
## 19207  3 5039 2.584875e-01
## 19208  3 5040 2.579178e-01
## 19209  3 5041 2.575780e-01
## 19210  3 5042 2.569189e-01
## 19211  3 5043 2.557562e-01
## 19212  3 5044 2.550097e-01
## 19213  3 5045 2.542690e-01
## 19214  3 5046 2.532639e-01
## 19215  3 5047 2.526947e-01
## 19216  3 5048 2.519442e-01
## 19217  3 5049 2.511637e-01
## 19218  3 5050 2.501185e-01
## 19219  3 5051 2.489930e-01
## 19220  3 5052 2.489926e-01
## 19221  3 5053 2.478578e-01
## 19222  3 5054 2.472353e-01
## 19223  3 5055 2.466419e-01
## 19224  3 5056 2.459887e-01
## 19225  3 5057 2.449802e-01
## 19226  3 5058 2.442124e-01
## 19227  3 5059 2.433151e-01
## 19228  3 5060 2.422560e-01
## 19229  3 5061 2.416002e-01
## 19230  3 5062 2.411786e-01
## 19231  3 5063 2.403790e-01
## 19232  3 5064 2.395647e-01
## 19233  3 5065 2.385479e-01
## 19234  3 5066 2.378325e-01
## 19235  3 5067 2.374824e-01
## 19236  3 5068 2.364299e-01
## 19237  3 5069 2.356264e-01
## 19238  3 5070 2.346828e-01
## 19239  3 5071 2.341715e-01
## 19240  3 5072 2.333536e-01
## 19241  3 5073 2.327625e-01
## 19242  3 5074 2.321040e-01
## 19243  3 5075 2.315196e-01
## 19244  3 5076 2.302896e-01
## 19245  3 5077 2.294008e-01
## 19246  3 5078 2.294287e-01
## 19247  3 5079 2.286618e-01
## 19248  3 5080 2.275948e-01
## 19249  3 5081 2.273857e-01
## 19250  3 5082 2.262689e-01
## 19251  3 5083 2.253930e-01
## 19252  3 5084 2.249445e-01
## 19253  3 5085 2.240517e-01
## 19254  3 5086 2.236723e-01
## 19255  3 5087 2.228635e-01
## 19256  3 5088 2.217311e-01
## 19257  3 5089 2.214667e-01
## 19258  3 5090 2.208615e-01
## 19259  3 5091 2.202232e-01
## 19260  3 5092 2.198001e-01
## 19261  3 5093 2.187451e-01
## 19262  3 5094 2.180639e-01
## 19263  3 5095 2.173188e-01
## 19264  3 5096 2.171280e-01
## 19265  3 5097 2.158419e-01
## 19266  3 5098 2.149532e-01
## 19267  3 5099 2.147557e-01
## 19268  3 5100 2.137917e-01
## 19269  3 5101 2.132028e-01
## 19270  3 5102 2.123575e-01
## 19271  3 5103 2.119450e-01
## 19272  3 5104 2.115386e-01
## 19273  3 5105 2.108849e-01
## 19274  3 5106 2.100698e-01
## 19275  3 5107 2.091955e-01
## 19276  3 5108 2.084611e-01
## 19277  3 5109 2.077202e-01
## 19278  3 5110 2.077141e-01
## 19279  3 5111 2.067963e-01
## 19280  3 5112 2.059895e-01
## 19281  3 5113 2.051735e-01
## 19282  3 5114 2.046555e-01
## 19283  3 5115 2.038968e-01
## 19284  3 5116 2.035943e-01
## 19285  3 5117 2.028587e-01
## 19286  3 5118 2.021188e-01
## 19287  3 5119 2.010960e-01
## 19288  3 5120 2.011991e-01
## 19289  3 5121 2.001718e-01
## 19290  3 5122 1.994358e-01
## 19291  3 5123 1.993824e-01
## 19292  3 5124 1.982549e-01
## 19293  3 5125 1.978473e-01
## 19294  3 5126 1.968735e-01
## 19295  3 5127 1.965837e-01
## 19296  3 5128 1.957720e-01
## 19297  3 5129 1.958569e-01
## 19298  3 5130 1.951803e-01
## 19299  3 5131 1.940971e-01
## 19300  3 5132 1.937923e-01
## 19301  3 5133 1.933289e-01
## 19302  3 5134 1.923750e-01
## 19303  3 5135 1.918135e-01
## 19304  3 5136 1.909047e-01
## 19305  3 5137 1.906204e-01
## 19306  3 5138 1.900035e-01
## 19307  3 5139 1.891107e-01
## 19308  3 5140 1.885178e-01
## 19309  3 5141 1.878239e-01
## 19310  3 5142 1.873623e-01
## 19311  3 5143 1.868212e-01
## 19312  3 5144 1.862879e-01
## 19313  3 5145 1.859145e-01
## 19314  3 5146 1.853952e-01
## 19315  3 5147 1.846516e-01
## 19316  3 5148 1.840997e-01
## 19317  3 5149 1.834564e-01
## 19318  3 5150 1.828663e-01
## 19319  3 5151 1.821771e-01
## 19320  3 5152 1.816808e-01
## 19321  3 5153 1.807947e-01
## 19322  3 5154 1.806205e-01
## 19323  3 5155 1.800286e-01
## 19324  3 5156 1.796786e-01
## 19325  3 5157 1.786921e-01
## 19326  3 5158 1.784812e-01
## 19327  3 5159 1.776935e-01
## 19328  3 5160 1.772755e-01
## 19329  3 5161 1.768794e-01
## 19330  3 5162 1.762760e-01
## 19331  3 5163 1.755505e-01
## 19332  3 5164 1.749070e-01
## 19333  3 5165 1.743721e-01
## 19334  3 5166 1.740205e-01
## 19335  3 5167 1.733669e-01
## 19336  3 5168 1.727652e-01
## 19337  3 5169 1.724007e-01
## 19338  3 5170 1.718204e-01
## 19339  3 5171 1.714367e-01
## 19340  3 5172 1.705772e-01
## 19341  3 5173 1.702496e-01
## 19342  3 5174 1.697855e-01
## 19343  3 5175 1.692897e-01
## 19344  3 5176 1.686462e-01
## 19345  3 5177 1.682452e-01
## 19346  3 5178 1.675949e-01
## 19347  3 5179 1.671952e-01
## 19348  3 5180 1.665441e-01
## 19349  3 5181 1.659361e-01
## 19350  3 5182 1.656503e-01
## 19351  3 5183 1.648771e-01
## 19352  3 5184 1.644236e-01
## 19353  3 5185 1.640826e-01
## 19354  3 5186 1.634964e-01
## 19355  3 5187 1.631646e-01
## 19356  3 5188 1.623425e-01
## 19357  3 5189 1.617105e-01
## 19358  3 5190 1.615600e-01
## 19359  3 5191 1.606017e-01
## 19360  3 5192 1.606614e-01
## 19361  3 5193 1.599882e-01
## 19362  3 5194 1.593945e-01
## 19363  3 5195 1.589953e-01
## 19364  3 5196 1.582520e-01
## 19365  3 5197 1.580752e-01
## 19366  3 5198 1.571858e-01
## 19367  3 5199 1.568043e-01
## 19368  3 5200 1.564077e-01
## 19369  3 5201 1.560694e-01
## 19370  3 5202 1.553555e-01
## 19371  3 5203 1.553081e-01
## 19372  3 5204 1.543573e-01
## 19373  3 5205 1.537636e-01
## 19374  3 5206 1.537345e-01
## 19375  3 5207 1.527879e-01
## 19376  3 5208 1.527788e-01
## 19377  3 5209 1.520987e-01
## 19378  3 5210 1.516337e-01
## 19379  3 5211 1.512770e-01
## 19380  3 5212 1.506781e-01
## 19381  3 5213 1.502311e-01
## 19382  3 5214 1.499157e-01
## 19383  3 5215 1.491339e-01
## 19384  3 5216 1.487266e-01
## 19385  3 5217 1.482508e-01
## 19386  3 5218 1.477584e-01
## 19387  3 5219 1.475002e-01
## 19388  3 5220 1.467143e-01
## 19389  3 5221 1.462497e-01
## 19390  3 5222 1.460509e-01
## 19391  3 5223 1.451929e-01
## 19392  3 5224 1.448847e-01
## 19393  3 5225 1.446397e-01
## 19394  3 5226 1.442791e-01
## 19395  3 5227 1.435497e-01
## 19396  3 5228 1.430728e-01
## 19397  3 5229 1.430343e-01
## 19398  3 5230 1.423203e-01
## 19399  3 5231 1.418113e-01
## 19400  3 5232 1.415641e-01
## 19401  3 5233 1.411292e-01
## 19402  3 5234 1.405243e-01
## 19403  3 5235 1.401257e-01
## 19404  3 5236 1.398342e-01
## 19405  3 5237 1.392177e-01
## 19406  3 5238 1.387880e-01
## 19407  3 5239 1.385549e-01
## 19408  3 5240 1.377046e-01
## 19409  3 5241 1.375033e-01
## 19410  3 5242 1.371204e-01
## 19411  3 5243 1.364369e-01
## 19412  3 5244 1.361926e-01
## 19413  3 5245 1.360239e-01
## 19414  3 5246 1.352990e-01
## 19415  3 5247 1.350928e-01
## 19416  3 5248 1.346422e-01
## 19417  3 5249 1.339676e-01
## 19418  3 5250 1.337110e-01
## 19419  3 5251 1.332176e-01
## 19420  3 5252 1.328894e-01
## 19421  3 5253 1.325054e-01
## 19422  3 5254 1.322076e-01
## 19423  3 5255 1.316580e-01
## 19424  3 5256 1.311832e-01
## 19425  3 5257 1.305953e-01
## 19426  3 5258 1.303765e-01
## 19427  3 5259 1.298969e-01
## 19428  3 5260 1.295675e-01
## 19429  3 5261 1.290333e-01
## 19430  3 5262 1.288614e-01
## 19431  3 5263 1.282139e-01
## 19432  3 5264 1.278767e-01
## 19433  3 5265 1.276052e-01
## 19434  3 5266 1.273305e-01
## 19435  3 5267 1.267420e-01
## 19436  3 5268 1.262425e-01
## 19437  3 5269 1.262137e-01
## 19438  3 5270 1.255574e-01
## 19439  3 5271 1.251504e-01
## 19440  3 5272 1.248363e-01
## 19441  3 5273 1.242716e-01
## 19442  3 5274 1.242037e-01
## 19443  3 5275 1.234980e-01
## 19444  3 5276 1.233880e-01
## 19445  3 5277 1.228486e-01
## 19446  3 5278 1.226445e-01
## 19447  3 5279 1.222102e-01
## 19448  3 5280 1.217297e-01
## 19449  3 5281 1.213047e-01
## 19450  3 5282 1.207210e-01
## 19451  3 5283 1.205425e-01
## 19452  3 5284 1.201610e-01
## 19453  3 5285 1.198783e-01
## 19454  3 5286 1.194594e-01
## 19455  3 5287 1.190396e-01
## 19456  3 5288 1.185858e-01
## 19457  3 5289 1.184442e-01
## 19458  3 5290 1.179364e-01
## 19459  3 5291 1.178674e-01
## 19460  3 5292 1.171979e-01
## 19461  3 5293 1.167412e-01
## 19462  3 5294 1.165277e-01
## 19463  3 5295 1.160882e-01
## 19464  3 5296 1.156180e-01
## 19465  3 5297 1.154556e-01
## 19466  3 5298 1.150122e-01
## 19467  3 5299 1.145821e-01
## 19468  3 5300 1.141849e-01
## 19469  3 5301 1.137847e-01
## 19470  3 5302 1.135978e-01
## 19471  3 5303 1.131190e-01
## 19472  3 5304 1.129352e-01
## 19473  3 5305 1.126835e-01
## 19474  3 5306 1.121371e-01
## 19475  3 5307 1.117182e-01
## 19476  3 5308 1.115519e-01
## 19477  3 5309 1.112139e-01
## 19478  3 5310 1.105596e-01
## 19479  3 5311 1.103356e-01
## 19480  3 5312 1.101848e-01
## 19481  3 5313 1.097725e-01
## 19482  3 5314 1.092930e-01
## 19483  3 5315 1.092122e-01
## 19484  3 5316 1.087088e-01
## 19485  3 5317 1.083324e-01
## 19486  3 5318 1.080619e-01
## 19487  3 5319 1.077356e-01
## 19488  3 5320 1.074299e-01
## 19489  3 5321 1.070262e-01
## 19490  3 5322 1.065700e-01
## 19491  3 5323 1.064708e-01
## 19492  3 5324 1.058061e-01
## 19493  3 5325 1.056884e-01
## 19494  3 5326 1.052853e-01
## 19495  3 5327 1.048626e-01
## 19496  3 5328 1.046840e-01
## 19497  3 5329 1.043477e-01
## 19498  3 5330 1.039033e-01
## 19499  3 5331 1.037353e-01
## 19500  3 5332 1.034282e-01
## 19501  3 5333 1.030909e-01
## 19502  3 5334 1.026474e-01
## 19503  3 5335 1.024253e-01
## 19504  3 5336 1.021298e-01
## 19505  3 5337 1.018001e-01
## 19506  3 5338 1.013522e-01
## 19507  3 5339 1.011440e-01
## 19508  3 5340 1.008623e-01
## 19509  3 5341 1.005809e-01
## 19510  3 5342 9.997906e-02
## 19511  3 5343 9.988884e-02
## 19512  3 5344 9.959994e-02
## 19513  3 5345 9.913568e-02
## 19514  3 5346 9.899241e-02
## 19515  3 5347 9.846282e-02
## 19516  3 5348 9.853838e-02
## 19517  3 5349 9.792516e-02
## 19518  3 5350 9.771804e-02
## 19519  3 5351 9.746519e-02
## 19520  3 5352 9.714921e-02
## 19521  3 5353 9.669818e-02
## 19522  3 5354 9.654201e-02
## 19523  3 5355 9.618982e-02
## 19524  3 5356 9.584753e-02
## 19525  3 5357 9.561356e-02
## 19526  3 5358 9.543720e-02
## 19527  3 5359 9.502823e-02
## 19528  3 5360 9.468016e-02
## 19529  3 5361 9.442447e-02
## 19530  3 5362 9.428704e-02
## 19531  3 5363 9.382332e-02
## 19532  3 5364 9.361609e-02
## 19533  3 5365 9.323218e-02
## 19534  3 5366 9.291059e-02
## 19535  3 5367 9.271773e-02
## 19536  3 5368 9.221396e-02
## 19537  3 5369 9.210133e-02
## 19538  3 5370 9.174083e-02
## 19539  3 5371 9.145141e-02
## 19540  3 5372 9.142549e-02
## 19541  3 5373 9.103387e-02
## 19542  3 5374 9.072190e-02
## 19543  3 5375 9.044531e-02
## 19544  3 5376 9.005006e-02
## 19545  3 5377 8.976305e-02
## 19546  3 5378 8.941932e-02
## 19547  3 5379 8.929300e-02
## 19548  3 5380 8.888513e-02
## 19549  3 5381 8.859454e-02
## 19550  3 5382 8.853912e-02
## 19551  3 5383 8.825684e-02
## 19552  3 5384 8.772342e-02
## 19553  3 5385 8.751331e-02
## 19554  3 5386 8.727512e-02
## 19555  3 5387 8.694660e-02
## 19556  3 5388 8.677715e-02
## 19557  3 5389 8.654984e-02
## 19558  3 5390 8.619436e-02
## 19559  3 5391 8.592592e-02
## 19560  3 5392 8.569167e-02
## 19561  3 5393 8.527380e-02
## 19562  3 5394 8.513574e-02
## 19563  3 5395 8.500592e-02
## 19564  3 5396 8.447827e-02
## 19565  3 5397 8.435922e-02
## 19566  3 5398 8.400290e-02
## 19567  3 5399 8.382355e-02
## 19568  3 5400 8.368141e-02
## 19569  3 5401 8.322288e-02
## 19570  3 5402 8.297356e-02
## 19571  3 5403 8.268666e-02
## 19572  3 5404 8.256614e-02
## 19573  3 5405 8.230229e-02
## 19574  3 5406 8.202602e-02
## 19575  3 5407 8.192845e-02
## 19576  3 5408 8.162048e-02
## 19577  3 5409 8.128349e-02
## 19578  3 5410 8.108355e-02
## 19579  3 5411 8.069765e-02
## 19580  3 5412 8.049518e-02
## 19581  3 5413 8.020825e-02
## 19582  3 5414 7.994075e-02
## 19583  3 5415 7.973667e-02
## 19584  3 5416 7.935165e-02
## 19585  3 5417 7.915224e-02
## 19586  3 5418 7.911283e-02
## 19587  3 5419 7.881737e-02
## 19588  3 5420 7.849347e-02
## 19589  3 5421 7.827610e-02
## 19590  3 5422 7.797857e-02
## 19591  3 5423 7.776622e-02
## 19592  3 5424 7.756230e-02
## 19593  3 5425 7.727499e-02
## 19594  3 5426 7.710762e-02
## 19595  3 5427 7.685980e-02
## 19596  3 5428 7.656964e-02
## 19597  3 5429 7.621837e-02
## 19598  3 5430 7.605143e-02
## 19599  3 5431 7.579942e-02
## 19600  3 5432 7.552947e-02
## 19601  3 5433 7.532768e-02
## 19602  3 5434 7.499210e-02
## 19603  3 5435 7.486278e-02
## 19604  3 5436 7.460937e-02
## 19605  3 5437 7.435369e-02
## 19606  3 5438 7.406161e-02
## 19607  3 5439 7.379089e-02
## 19608  3 5440 7.379495e-02
## 19609  3 5441 7.346587e-02
## 19610  3 5442 7.322437e-02
## 19611  3 5443 7.300423e-02
## 19612  3 5444 7.277786e-02
## 19613  3 5445 7.244998e-02
## 19614  3 5446 7.222746e-02
## 19615  3 5447 7.217968e-02
## 19616  3 5448 7.190445e-02
## 19617  3 5449 7.180515e-02
## 19618  3 5450 7.132649e-02
## 19619  3 5451 7.120813e-02
## 19620  3 5452 7.108184e-02
## 19621  3 5453 7.077297e-02
## 19622  3 5454 7.058688e-02
## 19623  3 5455 7.038349e-02
## 19624  3 5456 7.011497e-02
## 19625  3 5457 6.977118e-02
## 19626  3 5458 6.956180e-02
## 19627  3 5459 6.954834e-02
## 19628  3 5460 6.912032e-02
## 19629  3 5461 6.917647e-02
## 19630  3 5462 6.886501e-02
## 19631  3 5463 6.851089e-02
## 19632  3 5464 6.832938e-02
## 19633  3 5465 6.807875e-02
## 19634  3 5466 6.797216e-02
## 19635  3 5467 6.780978e-02
## 19636  3 5468 6.749072e-02
## 19637  3 5469 6.742376e-02
## 19638  3 5470 6.721816e-02
## 19639  3 5471 6.693564e-02
## 19640  3 5472 6.666770e-02
## 19641  3 5473 6.664840e-02
## 19642  3 5474 6.633436e-02
## 19643  3 5475 6.603574e-02
## 19644  3 5476 6.586007e-02
## 19645  3 5477 6.564941e-02
## 19646  3 5478 6.545127e-02
## 19647  3 5479 6.518805e-02
## 19648  3 5480 6.489500e-02
## 19649  3 5481 6.477753e-02
## 19650  3 5482 6.454356e-02
## 19651  3 5483 6.440394e-02
## 19652  3 5484 6.415136e-02
## 19653  3 5485 6.394068e-02
## 19654  3 5486 6.382994e-02
## 19655  3 5487 6.364856e-02
## 19656  3 5488 6.344911e-02
## 19657  3 5489 6.326872e-02
## 19658  3 5490 6.308190e-02
## 19659  3 5491 6.289769e-02
## 19660  3 5492 6.257180e-02
## 19661  3 5493 6.240980e-02
## 19662  3 5494 6.234343e-02
## 19663  3 5495 6.198789e-02
## 19664  3 5496 6.188147e-02
## 19665  3 5497 6.163712e-02
## 19666  3 5498 6.138079e-02
## 19667  3 5499 6.127515e-02
## 19668  3 5500 6.119100e-02
## 19669  3 5501 6.084204e-02
## 19670  3 5502 6.072824e-02
## 19671  3 5503 6.047909e-02
## 19672  3 5504 6.025841e-02
## 19673  3 5505 6.016581e-02
## 19674  3 5506 5.997420e-02
## 19675  3 5507 5.980392e-02
## 19676  3 5508 5.952534e-02
## 19677  3 5509 5.936357e-02
## 19678  3 5510 5.919229e-02
## 19679  3 5511 5.897095e-02
## 19680  3 5512 5.871788e-02
## 19681  3 5513 5.868704e-02
## 19682  3 5514 5.851561e-02
## 19683  3 5515 5.827144e-02
## 19684  3 5516 5.812769e-02
## 19685  3 5517 5.798318e-02
## 19686  3 5518 5.775537e-02
## 19687  3 5519 5.756461e-02
## 19688  3 5520 5.741755e-02
## 19689  3 5521 5.715731e-02
## 19690  3 5522 5.704873e-02
## 19691  3 5523 5.679367e-02
## 19692  3 5524 5.663371e-02
## 19693  3 5525 5.640638e-02
## 19694  3 5526 5.629469e-02
## 19695  3 5527 5.602490e-02
## 19696  3 5528 5.581956e-02
## 19697  3 5529 5.564388e-02
## 19698  3 5530 5.551192e-02
## 19699  3 5531 5.542721e-02
## 19700  3 5532 5.523756e-02
## 19701  3 5533 5.506713e-02
## 19702  3 5534 5.487578e-02
## 19703  3 5535 5.468331e-02
## 19704  3 5536 5.463055e-02
## 19705  3 5537 5.438015e-02
## 19706  3 5538 5.416387e-02
## 19707  3 5539 5.401562e-02
## 19708  3 5540 5.385178e-02
## 19709  3 5541 5.367386e-02
## 19710  3 5542 5.352468e-02
## 19711  3 5543 5.339809e-02
## 19712  3 5544 5.320747e-02
## 19713  3 5545 5.306470e-02
## 19714  3 5546 5.281057e-02
## 19715  3 5547 5.271773e-02
## 19716  3 5548 5.263634e-02
## 19717  3 5549 5.241346e-02
## 19718  3 5550 5.226127e-02
## 19719  3 5551 5.192345e-02
## 19720  3 5552 5.187357e-02
## 19721  3 5553 5.173196e-02
## 19722  3 5554 5.159390e-02
## 19723  3 5555 5.141688e-02
## 19724  3 5556 5.131430e-02
## 19725  3 5557 5.113204e-02
## 19726  3 5558 5.092819e-02
## 19727  3 5559 5.071578e-02
## 19728  3 5560 5.058612e-02
## 19729  3 5561 5.046630e-02
## 19730  3 5562 5.014438e-02
## 19731  3 5563 5.008581e-02
## 19732  3 5564 4.994085e-02
## 19733  3 5565 4.983110e-02
## 19734  3 5566 4.970034e-02
## 19735  3 5567 4.955075e-02
## 19736  3 5568 4.928644e-02
## 19737  3 5569 4.918564e-02
## 19738  3 5570 4.908835e-02
## 19739  3 5571 4.886899e-02
## 19740  3 5572 4.866750e-02
## 19741  3 5573 4.846535e-02
## 19742  3 5574 4.846920e-02
## 19743  3 5575 4.825351e-02
## 19744  3 5576 4.816873e-02
## 19745  3 5577 4.800844e-02
## 19746  3 5578 4.781827e-02
## 19747  3 5579 4.771163e-02
## 19748  3 5580 4.749312e-02
## 19749  3 5581 4.734282e-02
## 19750  3 5582 4.725130e-02
## 19751  3 5583 4.705782e-02
## 19752  3 5584 4.695374e-02
## 19753  3 5585 4.681266e-02
## 19754  3 5586 4.669292e-02
## 19755  3 5587 4.652833e-02
## 19756  3 5588 4.633497e-02
## 19757  3 5589 4.616940e-02
## 19758  3 5590 4.608101e-02
## 19759  3 5591 4.592153e-02
## 19760  3 5592 4.573018e-02
## 19761  3 5593 4.565628e-02
## 19762  3 5594 4.549661e-02
## 19763  3 5595 4.526011e-02
## 19764  3 5596 4.517880e-02
## 19765  3 5597 4.509578e-02
## 19766  3 5598 4.496161e-02
## 19767  3 5599 4.471868e-02
## 19768  3 5600 4.467434e-02
## 19769  3 5601 4.445969e-02
## 19770  3 5602 4.430519e-02
## 19771  3 5603 4.417063e-02
## 19772  3 5604 4.401429e-02
## 19773  3 5605 4.391684e-02
## 19774  3 5606 4.387146e-02
## 19775  3 5607 4.363166e-02
## 19776  3 5608 4.358602e-02
## 19777  3 5609 4.334827e-02
## 19778  3 5610 4.326039e-02
## 19779  3 5611 4.310039e-02
## 19780  3 5612 4.298234e-02
## 19781  3 5613 4.282010e-02
## 19782  3 5614 4.270117e-02
## 19783  3 5615 4.257061e-02
## 19784  3 5616 4.251901e-02
## 19785  3 5617 4.225897e-02
## 19786  3 5618 4.217822e-02
## 19787  3 5619 4.203673e-02
## 19788  3 5620 4.192352e-02
## 19789  3 5621 4.176158e-02
## 19790  3 5622 4.166526e-02
## 19791  3 5623 4.150440e-02
## 19792  3 5624 4.144615e-02
## 19793  3 5625 4.129159e-02
## 19794  3 5626 4.115383e-02
## 19795  3 5627 4.095085e-02
## 19796  3 5628 4.086727e-02
## 19797  3 5629 4.074209e-02
## 19798  3 5630 4.061580e-02
## 19799  3 5631 4.051302e-02
## 19800  3 5632 4.041234e-02
## 19801  3 5633 4.028885e-02
## 19802  3 5634 4.007311e-02
## 19803  3 5635 4.007030e-02
## 19804  3 5636 3.989587e-02
## 19805  3 5637 3.972231e-02
## 19806  3 5638 3.966091e-02
## 19807  3 5639 3.954782e-02
## 19808  3 5640 3.941339e-02
## 19809  3 5641 3.928379e-02
## 19810  3 5642 3.915016e-02
## 19811  3 5643 3.902355e-02
## 19812  3 5644 3.888003e-02
## 19813  3 5645 3.873330e-02
## 19814  3 5646 3.865122e-02
## 19815  3 5647 3.851330e-02
## 19816  3 5648 3.838997e-02
## 19817  3 5649 3.829970e-02
## 19818  3 5650 3.817129e-02
## 19819  3 5651 3.806097e-02
## 19820  3 5652 3.790414e-02
## 19821  3 5653 3.779425e-02
## 19822  3 5654 3.765137e-02
## 19823  3 5655 3.758134e-02
## 19824  3 5656 3.744605e-02
## 19825  3 5657 3.735273e-02
## 19826  3 5658 3.714916e-02
## 19827  3 5659 3.713925e-02
## 19828  3 5660 3.695592e-02
## 19829  3 5661 3.690141e-02
## 19830  3 5662 3.676960e-02
## 19831  3 5663 3.663003e-02
## 19832  3 5664 3.657249e-02
## 19833  3 5665 3.641955e-02
## 19834  3 5666 3.629572e-02
## 19835  3 5667 3.622283e-02
## 19836  3 5668 3.604192e-02
## 19837  3 5669 3.591065e-02
## 19838  3 5670 3.579055e-02
## 19839  3 5671 3.578967e-02
## 19840  3 5672 3.564605e-02
## 19841  3 5673 3.549133e-02
## 19842  3 5674 3.543368e-02
## 19843  3 5675 3.525438e-02
## 19844  3 5676 3.519085e-02
## 19845  3 5677 3.507151e-02
## 19846  3 5678 3.494774e-02
## 19847  3 5679 3.485699e-02
## 19848  3 5680 3.476982e-02
## 19849  3 5681 3.466843e-02
## 19850  3 5682 3.457622e-02
## 19851  3 5683 3.442005e-02
## 19852  3 5684 3.429428e-02
## 19853  3 5685 3.414536e-02
## 19854  3 5686 3.407931e-02
## 19855  3 5687 3.397781e-02
## 19856  3 5688 3.380245e-02
## 19857  3 5689 3.382883e-02
## 19858  3 5690 3.367156e-02
## 19859  3 5691 3.354592e-02
## 19860  3 5692 3.343073e-02
## 19861  3 5693 3.333593e-02
## 19862  3 5694 3.329734e-02
## 19863  3 5695 3.317325e-02
## 19864  3 5696 3.303173e-02
## 19865  3 5697 3.294606e-02
## 19866  3 5698 3.282367e-02
## 19867  3 5699 3.265756e-02
## 19868  3 5700 3.265323e-02
## 19869  3 5701 3.256746e-02
## 19870  3 5702 3.241873e-02
## 19871  3 5703 3.235618e-02
## 19872  3 5704 3.218687e-02
## 19873  3 5705 3.214791e-02
```

```
## 19874  3 5706 3.200501e-02
## 19875  3 5707 3.190626e-02
## 19876  3 5708 3.185464e-02
## 19877  3 5709 3.171488e-02
## 19878  3 5710 3.161810e-02
## 19879  3 5711 3.151433e-02
## 19880  3 5712 3.142494e-02
## 19881  3 5713 3.131988e-02
## 19882  3 5714 3.120858e-02
## 19883  3 5715 3.112931e-02
## 19884  3 5716 3.100583e-02
## 19885  3 5717 3.088700e-02
## 19886  3 5718 3.080385e-02
## 19887  3 5719 3.065516e-02
## 19888  3 5720 3.064133e-02
## 19889  3 5721 3.060301e-02
## 19890  3 5722 3.042258e-02
## 19891  3 5723 3.029921e-02
## 19892  3 5724 3.023695e-02
## 19893  3 5725 3.016308e-02
## 19894  3 5726 3.011019e-02
## 19895  3 5727 3.001969e-02
## 19896  3 5728 2.988879e-02
## 19897  3 5729 2.974999e-02
## 19898  3 5730 2.967918e-02
## 19899  3 5731 2.955978e-02
## 19900  3 5732 2.954312e-02
## 19901  3 5733 2.944925e-02
## 19902  3 5734 2.931180e-02
## 19903  3 5735 2.927845e-02
## 19904  3 5736 2.911509e-02
## 19905  3 5737 2.905078e-02
## 19906  3 5738 2.896739e-02
## 19907  3 5739 2.885870e-02
## 19908  3 5740 2.878372e-02
## 19909  3 5741 2.869656e-02
## 19910  3 5742 2.865403e-02
## 19911  3 5743 2.852028e-02
## 19912  3 5744 2.837836e-02
## 19913  3 5745 2.829434e-02
## 19914  3 5746 2.827813e-02
## 19915  3 5747 2.813828e-02
## 19916  3 5748 2.809993e-02
## 19917  3 5749 2.797430e-02
## 19918  3 5750 2.793265e-02
## 19919  3 5751 2.780561e-02
## 19920  3 5752 2.772512e-02
## 19921  3 5753 2.763567e-02
## 19922  3 5754 2.751814e-02
## 19923  3 5755 2.747100e-02
## 19924  3 5756 2.734604e-02
## 19925  3 5757 2.729586e-02
## 19926  3 5758 2.725152e-02
## 19927  3 5759 2.716328e-02
## 19928  3 5760 2.701297e-02
## 19929  3 5761 2.691819e-02
## 19930  3 5762 2.684307e-02
## 19931  3 5763 2.680110e-02
## 19932  3 5764 2.670515e-02
## 19933  3 5765 2.663002e-02
## 19934  3 5766 2.653383e-02
## 19935  3 5767 2.644355e-02
## 19936  3 5768 2.634061e-02
## 19937  3 5769 2.631192e-02
## 19938  3 5770 2.621541e-02
## 19939  3 5771 2.617806e-02
## 19940  3 5772 2.603325e-02
## 19941  3 5773 2.592147e-02
## 19942  3 5774 2.586924e-02
## 19943  3 5775 2.577156e-02
## 19944  3 5776 2.572605e-02
## 19945  3 5777 2.562313e-02
## 19946  3 5778 2.552516e-02
## 19947  3 5779 2.548757e-02
## 19948  3 5780 2.535815e-02
## 19949  3 5781 2.535604e-02
## 19950  3 5782 2.523074e-02
## 19951  3 5783 2.510833e-02
## 19952  3 5784 2.511278e-02
## 19953  3 5785 2.498596e-02
## 19954  3 5786 2.488791e-02
## 19955  3 5787 2.481252e-02
## 19956  3 5788 2.475042e-02
## 19957  3 5789 2.472660e-02
## 19958  3 5790 2.460389e-02
## 19959  3 5791 2.452357e-02
## 19960  3 5792 2.444039e-02
## 19961  3 5793 2.438744e-02
## 19962  3 5794 2.429492e-02
## 19963  3 5795 2.419350e-02
## 19964  3 5796 2.410965e-02
## 19965  3 5797 2.405021e-02
## 19966  3 5798 2.402028e-02
## 19967  3 5799 2.393373e-02
## 19968  3 5800 2.385290e-02
## 19969  3 5801 2.382136e-02
## 19970  3 5802 2.372373e-02
## 19971  3 5803 2.361434e-02
## 19972  3 5804 2.354691e-02
## 19973  3 5805 2.343655e-02
## 19974  3 5806 2.340390e-02
## 19975  3 5807 2.332552e-02
## 19976  3 5808 2.324656e-02
## 19977  3 5809 2.321530e-02
## 19978  3 5810 2.310471e-02
## 19979  3 5811 2.302771e-02
## 19980  3 5812 2.299133e-02
## 19981  3 5813 2.292101e-02
## 19982  3 5814 2.284228e-02
## 19983  3 5815 2.278459e-02
## 19984  3 5816 2.268615e-02
## 19985  3 5817 2.257284e-02
## 19986  3 5818 2.256961e-02
## 19987  3 5819 2.246637e-02
## 19988  3 5820 2.240471e-02
## 19989  3 5821 2.236224e-02
## 19990  3 5822 2.226840e-02
## 19991  3 5823 2.221881e-02
## 19992  3 5824 2.212958e-02
## 19993  3 5825 2.207586e-02
## 19994  3 5826 2.196626e-02
## 19995  3 5827 2.191370e-02
## 19996  3 5828 2.187344e-02
## 19997  3 5829 2.176469e-02
## 19998  3 5830 2.170108e-02
## 19999  3 5831 2.163117e-02
## 20000  3 5832 2.153508e-02
## 20001  3 5833 2.147369e-02
## 20002  3 5834 2.142396e-02
## 20003  3 5835 2.136082e-02
## 20004  3 5836 2.129972e-02
## 20005  3 5837 2.124334e-02
## 20006  3 5838 2.117217e-02
## 20007  3 5839 2.111332e-02
## 20008  3 5840 2.106567e-02
## 20009  3 5841 2.094090e-02
## 20010  3 5842 2.091174e-02
## 20011  3 5843 2.085357e-02
## 20012  3 5844 2.075610e-02
## 20013  3 5845 2.072708e-02
## 20014  3 5846 2.064513e-02
## 20015  3 5847 2.057231e-02
## 20016  3 5848 2.052195e-02
## 20017  3 5849 2.047656e-02
## 20018  3 5850 2.039757e-02
## 20019  3 5851 2.034597e-02
## 20020  3 5852 2.026999e-02
## 20021  3 5853 2.020421e-02
## 20022  3 5854 2.014091e-02
## 20023  3 5855 2.007283e-02
## 20024  3 5856 2.005265e-02
## 20025  3 5857 1.997403e-02
## 20026  3 5858 1.988321e-02
## 20027  3 5859 1.982339e-02
## 20028  3 5860 1.974789e-02
## 20029  3 5861 1.970268e-02
## 20030  3 5862 1.966665e-02
## 20031  3 5863 1.963159e-02
## 20032  3 5864 1.953646e-02
## 20033  3 5865 1.947748e-02
## 20034  3 5866 1.940011e-02
## 20035  3 5867 1.933077e-02
## 20036  3 5868 1.928808e-02
## 20037  3 5869 1.920542e-02
## 20038  3 5870 1.916714e-02
## 20039  3 5871 1.908762e-02
## 20040  3 5872 1.904254e-02
## 20041  3 5873 1.897409e-02
## 20042  3 5874 1.889547e-02
## 20043  3 5875 1.886401e-02
## 20044  3 5876 1.881066e-02
## 20045  3 5877 1.871018e-02
## 20046  3 5878 1.865237e-02
## 20047  3 5879 1.860301e-02
## 20048  3 5880 1.856963e-02
## 20049  3 5881 1.848683e-02
## 20050  3 5882 1.842617e-02
## 20051  3 5883 1.835395e-02
## 20052  3 5884 1.833377e-02
## 20053  3 5885 1.828015e-02
## 20054  3 5886 1.822450e-02
## 20055  3 5887 1.814609e-02
## 20056  3 5888 1.807750e-02
## 20057  3 5889 1.803461e-02
## 20058  3 5890 1.801753e-02
## 20059  3 5891 1.793633e-02
## 20060  3 5892 1.787412e-02
## 20061  3 5893 1.781295e-02
## 20062  3 5894 1.776335e-02
## 20063  3 5895 1.772044e-02
## 20064  3 5896 1.767265e-02
## 20065  3 5897 1.758875e-02
## 20066  3 5898 1.753304e-02
## 20067  3 5899 1.747632e-02
## 20068  3 5900 1.743111e-02
## 20069  3 5901 1.737427e-02
## 20070  3 5902 1.734497e-02
## 20071  3 5903 1.723976e-02
## 20072  3 5904 1.721477e-02
## 20073  3 5905 1.714109e-02
## 20074  3 5906 1.705237e-02
## 20075  3 5907 1.705968e-02
## 20076  3 5908 1.699491e-02
## 20077  3 5909 1.692385e-02
## 20078  3 5910 1.687905e-02
## 20079  3 5911 1.683057e-02
## 20080  3 5912 1.680719e-02
## 20081  3 5913 1.675642e-02
## 20082  3 5914 1.668787e-02
## 20083  3 5915 1.665058e-02
## 20084  3 5916 1.660136e-02
## 20085  3 5917 1.651922e-02
## 20086  3 5918 1.643059e-02
## 20087  3 5919 1.641314e-02
## 20088  3 5920 1.638730e-02
## 20089  3 5921 1.632270e-02
## 20090  3 5922 1.626890e-02
## 20091  3 5923 1.620928e-02
## 20092  3 5924 1.613063e-02
## 20093  3 5925 1.610553e-02
## 20094  3 5926 1.606674e-02
## 20095  3 5927 1.601744e-02
## 20096  3 5928 1.596146e-02
## 20097  3 5929 1.591662e-02
## 20098  3 5930 1.587250e-02
## 20099  3 5931 1.580505e-02
## 20100  3 5932 1.577700e-02
## 20101  3 5933 1.573270e-02
## 20102  3 5934 1.570916e-02
## 20103  3 5935 1.562262e-02
## 20104  3 5936 1.557322e-02
## 20105  3 5937 1.551446e-02
## 20106  3 5938 1.546552e-02
## 20107  3 5939 1.541771e-02
## 20108  3 5940 1.541130e-02
## 20109  3 5941 1.538347e-02
## 20110  3 5942 1.529569e-02
## 20111  3 5943 1.521713e-02
## 20112  3 5944 1.517110e-02
## 20113  3 5945 1.510705e-02
## 20114  3 5946 1.509904e-02
## 20115  3 5947 1.504613e-02
## 20116  3 5948 1.498615e-02
## 20117  3 5949 1.495510e-02
## 20118  3 5950 1.488403e-02
## 20119  3 5951 1.489292e-02
## 20120  3 5952 1.481496e-02
## 20121  3 5953 1.474992e-02
## 20122  3 5954 1.471716e-02
## 20123  3 5955 1.466821e-02
## 20124  3 5956 1.463094e-02
## 20125  3 5957 1.458912e-02
## 20126  3 5958 1.455618e-02
## 20127  3 5959 1.450721e-02
## 20128  3 5960 1.445259e-02
## 20129  3 5961 1.437225e-02
## 20130  3 5962 1.431997e-02
## 20131  3 5963 1.430382e-02
## 20132  3 5964 1.425391e-02
## 20133  3 5965 1.422423e-02
## 20134  3 5966 1.416888e-02
## 20135  3 5967 1.413426e-02
## 20136  3 5968 1.411740e-02
## 20137  3 5969 1.403730e-02
## 20138  3 5970 1.398076e-02
## 20139  3 5971 1.394363e-02
## 20140  3 5972 1.390656e-02
## 20141  3 5973 1.385438e-02
## 20142  3 5974 1.379856e-02
## 20143  3 5975 1.378873e-02
## 20144  3 5976 1.374941e-02
## 20145  3 5977 1.369583e-02
## 20146  3 5978 1.367522e-02
## 20147  3 5979 1.359753e-02
## 20148  3 5980 1.355872e-02
## 20149  3 5981 1.352547e-02
## 20150  3 5982 1.349487e-02
## 20151  3 5983 1.344242e-02
## 20152  3 5984 1.339486e-02
## 20153  3 5985 1.336367e-02
## 20154  3 5986 1.330648e-02
## 20155  3 5987 1.326381e-02
## 20156  3 5988 1.320842e-02
## 20157  3 5989 1.319189e-02
## 20158  3 5990 1.319168e-02
## 20159  3 5991 1.312274e-02
## 20160  3 5992 1.309164e-02
## 20161  3 5993 1.303691e-02
## 20162  3 5994 1.297832e-02
## 20163  3 5995 1.294638e-02
## 20164  3 5996 1.291659e-02
## 20165  3 5997 1.285187e-02
## 20166  3 5998 1.281932e-02
## 20167  3 5999 1.278327e-02
## 20168  3 6000 1.275223e-02
## 20169  3 6001 1.268677e-02
## 20170  3 6002 1.265288e-02
## 20171  3 6003 1.264435e-02
## 20172  3 6004 1.258183e-02
## 20173  3 6005 1.254077e-02
## 20174  3 6006 1.249198e-02
## 20175  3 6007 1.247124e-02
## 20176  3 6008 1.243748e-02
## 20177  3 6009 1.236471e-02
## 20178  3 6010 1.235399e-02
## 20179  3 6011 1.229111e-02
## 20180  3 6012 1.228427e-02
## 20181  3 6013 1.224557e-02
## 20182  3 6014 1.218431e-02
## 20183  3 6015 1.215905e-02
## 20184  3 6016 1.212832e-02
## 20185  3 6017 1.208781e-02
## 20186  3 6018 1.204540e-02
## 20187  3 6019 1.201144e-02
## 20188  3 6020 1.197688e-02
## 20189  3 6021 1.193287e-02
## 20190  3 6022 1.188482e-02
## 20191  3 6023 1.186185e-02
## 20192  3 6024 1.183480e-02
## 20193  3 6025 1.176142e-02
## 20194  3 6026 1.174953e-02
## 20195  3 6027 1.170638e-02
## 20196  3 6028 1.165700e-02
## 20197  3 6029 1.161708e-02
## 20198  3 6030 1.160936e-02
## 20199  3 6031 1.156846e-02
## 20200  3 6032 1.151652e-02
## 20201  3 6033 1.150725e-02
## 20202  3 6034 1.146240e-02
## 20203  3 6035 1.142930e-02
## 20204  3 6036 1.139442e-02
## 20205  3 6037 1.135558e-02
## 20206  3 6038 1.132799e-02
## 20207  3 6039 1.128286e-02
## 20208  3 6040 1.124756e-02
## 20209  3 6041 1.119997e-02
## 20210  3 6042 1.116406e-02
## 20211  3 6043 1.114897e-02
## 20212  3 6044 1.111971e-02
## 20213  3 6045 1.106829e-02
## 20214  3 6046 1.103947e-02
## 20215  3 6047 1.099381e-02
## 20216  3 6048 1.096692e-02
## 20217  3 6049 1.090085e-02
## 20218  3 6050 1.089907e-02
## 20219  3 6051 1.087330e-02
## 20220  3 6052 1.082908e-02
## 20221  3 6053 1.079100e-02
## 20222  3 6054 1.076353e-02
## 20223  3 6055 1.073713e-02
## 20224  3 6056 1.066630e-02
## 20225  3 6057 1.065608e-02
## 20226  3 6058 1.061611e-02
## 20227  3 6059 1.059429e-02
## 20228  3 6060 1.054826e-02
## 20229  3 6061 1.052355e-02
## 20230  3 6062 1.049484e-02
## 20231  3 6063 1.046260e-02
## 20232  3 6064 1.042399e-02
## 20233  3 6065 1.038962e-02
## 20234  3 6066 1.035295e-02
## 20235  3 6067 1.033647e-02
## 20236  3 6068 1.029403e-02
## 20237  3 6069 1.027709e-02
## 20238  3 6070 1.024986e-02
## 20239  3 6071 1.021136e-02
## 20240  3 6072 1.016432e-02
## 20241  3 6073 1.012776e-02
## 20242  3 6074 1.009208e-02
## 20243  3 6075 1.008807e-02
## 20244  3 6076 1.005787e-02
## 20245  3 6077 9.990508e-03
## 20246  3 6078 9.979887e-03
## 20247  3 6079 9.955662e-03
## 20248  3 6080 9.919284e-03
## 20249  3 6081 9.894043e-03
## 20250  3 6082 9.840720e-03
## 20251  3 6083 9.839816e-03
## 20252  3 6084 9.797548e-03
## 20253  3 6085 9.747278e-03
## 20254  3 6086 9.734358e-03
## 20255  3 6087 9.688127e-03
## 20256  3 6088 9.674823e-03
## 20257  3 6089 9.620122e-03
## 20258  3 6090 9.621047e-03
## 20259  3 6091 9.570518e-03
## 20260  3 6092 9.565442e-03
## 20261  3 6093 9.525303e-03
## 20262  3 6094 9.499397e-03
## 20263  3 6095 9.476898e-03
## 20264  3 6096 9.434853e-03
## 20265  3 6097 9.406597e-03
## 20266  3 6098 9.377647e-03
## 20267  3 6099 9.326605e-03
## 20268  3 6100 9.314201e-03
## 20269  3 6101 9.306951e-03
## 20270  3 6102 9.265054e-03
## 20271  3 6103 9.212575e-03
## 20272  3 6104 9.182108e-03
## 20273  3 6105 9.160908e-03
## 20274  3 6106 9.153101e-03
## 20275  3 6107 9.117891e-03
## 20276  3 6108 9.091088e-03
## 20277  3 6109 9.065725e-03
## 20278  3 6110 9.021066e-03
## 20279  3 6111 8.988120e-03
## 20280  3 6112 8.970272e-03
## 20281  3 6113 8.931208e-03
## 20282  3 6114 8.910432e-03
## 20283  3 6115 8.885298e-03
## 20284  3 6116 8.869018e-03
## 20285  3 6117 8.813723e-03
## 20286  3 6118 8.798889e-03
## 20287  3 6119 8.774776e-03
## 20288  3 6120 8.754569e-03
## 20289  3 6121 8.726086e-03
## 20290  3 6122 8.700363e-03
## 20291  3 6123 8.654273e-03
## 20292  3 6124 8.638396e-03
## 20293  3 6125 8.625291e-03
## 20294  3 6126 8.591507e-03
## 20295  3 6127 8.565269e-03
## 20296  3 6128 8.538778e-03
## 20297  3 6129 8.502866e-03
## 20298  3 6130 8.488230e-03
## 20299  3 6131 8.457943e-03
## 20300  3 6132 8.412981e-03
## 20301  3 6133 8.413447e-03
## 20302  3 6134 8.373929e-03
## 20303  3 6135 8.357468e-03
## 20304  3 6136 8.305181e-03
## 20305  3 6137 8.294038e-03
## 20306  3 6138 8.279097e-03
## 20307  3 6139 8.248578e-03
## 20308  3 6140 8.223033e-03
## 20309  3 6141 8.177774e-03
## 20310  3 6142 8.178877e-03
## 20311  3 6143 8.140116e-03
## 20312  3 6144 8.109941e-03
## 20313  3 6145 8.084928e-03
## 20314  3 6146 8.068485e-03
## 20315  3 6147 8.035491e-03
## 20316  3 6148 8.033290e-03
## 20317  3 6149 7.989173e-03
## 20318  3 6150 7.965427e-03
## 20319  3 6151 7.935705e-03
## 20320  3 6152 7.916978e-03
## 20321  3 6153 7.890913e-03
## 20322  3 6154 7.841732e-03
## 20323  3 6155 7.833334e-03
## 20324  3 6156 7.814391e-03
## 20325  3 6157 7.787478e-03
## 20326  3 6158 7.751416e-03
## 20327  3 6159 7.748290e-03
## 20328  3 6160 7.730318e-03
## 20329  3 6161 7.700138e-03
## 20330  3 6162 7.674289e-03
## 20331  3 6163 7.666940e-03
## 20332  3 6164 7.613890e-03
## 20333  3 6165 7.609737e-03
## 20334  3 6166 7.569704e-03
## 20335  3 6167 7.545873e-03
## 20336  3 6168 7.522188e-03
## 20337  3 6169 7.498254e-03
## 20338  3 6170 7.475785e-03
## 20339  3 6171 7.460500e-03
## 20340  3 6172 7.437554e-03
## 20341  3 6173 7.396080e-03
## 20342  3 6174 7.384251e-03
## 20343  3 6175 7.356852e-03
## 20344  3 6176 7.343761e-03
## 20345  3 6177 7.317944e-03
## 20346  3 6178 7.293913e-03
## 20347  3 6179 7.267605e-03
## 20348  3 6180 7.256465e-03
## 20349  3 6181 7.223811e-03
## 20350  3 6182 7.195149e-03
## 20351  3 6183 7.193594e-03
## 20352  3 6184 7.155237e-03
## 20353  3 6185 7.137603e-03
## 20354  3 6186 7.112351e-03
## 20355  3 6187 7.097876e-03
## 20356  3 6188 7.078897e-03
## 20357  3 6189 7.044003e-03
## 20358  3 6190 7.035165e-03
## 20359  3 6191 7.006660e-03
## 20360  3 6192 6.981707e-03
## 20361  3 6193 6.947093e-03
## 20362  3 6194 6.939787e-03
## 20363  3 6195 6.922478e-03
## 20364  3 6196 6.911657e-03
## 20365  3 6197 6.878215e-03
## 20366  3 6198 6.838905e-03
## 20367  3 6199 6.835041e-03
## 20368  3 6200 6.799239e-03
## 20369  3 6201 6.778689e-03
## 20370  3 6202 6.769800e-03
## 20371  3 6203 6.749487e-03
## 20372  3 6204 6.723341e-03
## 20373  3 6205 6.694913e-03
## 20374  3 6206 6.682694e-03
## 20375  3 6207 6.661009e-03
## 20376  3 6208 6.636915e-03
## 20377  3 6209 6.623780e-03
## 20378  3 6210 6.594074e-03
## 20379  3 6211 6.582890e-03
## 20380  3 6212 6.555902e-03
## 20381  3 6213 6.533608e-03
## 20382  3 6214 6.525214e-03
## 20383  3 6215 6.496722e-03
## 20384  3 6216 6.475321e-03
## 20385  3 6217 6.471316e-03
## 20386  3 6218 6.436043e-03
## 20387  3 6219 6.413883e-03
## 20388  3 6220 6.390678e-03
## 20389  3 6221 6.368461e-03
## 20390  3 6222 6.345097e-03
## 20391  3 6223 6.336005e-03
## 20392  3 6224 6.318516e-03
## 20393  3 6225 6.302605e-03
## 20394  3 6226 6.276124e-03
## 20395  3 6227 6.260525e-03
## 20396  3 6228 6.236663e-03
## 20397  3 6229 6.210827e-03
## 20398  3 6230 6.188808e-03
## 20399  3 6231 6.194082e-03
## 20400  3 6232 6.148316e-03
## 20401  3 6233 6.147923e-03
## 20402  3 6234 6.107576e-03
## 20403  3 6235 6.104340e-03
## 20404  3 6236 6.088573e-03
## 20405  3 6237 6.070091e-03
## 20406  3 6238 6.042731e-03
## 20407  3 6239 6.034276e-03
## 20408  3 6240 6.016606e-03
## 20409  3 6241 5.981147e-03
## 20410  3 6242 5.965138e-03
## 20411  3 6243 5.957829e-03
## 20412  3 6244 5.927067e-03
## 20413  3 6245 5.909209e-03
## 20414  3 6246 5.895838e-03
## 20415  3 6247 5.877781e-03
## 20416  3 6248 5.855747e-03
## 20417  3 6249 5.849820e-03
## 20418  3 6250 5.829648e-03
## 20419  3 6251 5.797126e-03
## 20420  3 6252 5.789911e-03
## 20421  3 6253 5.763866e-03
## 20422  3 6254 5.749627e-03
## 20423  3 6255 5.723780e-03
## 20424  3 6256 5.703989e-03
## 20425  3 6257 5.692361e-03
## 20426  3 6258 5.677699e-03
## 20427  3 6259 5.667838e-03
## 20428  3 6260 5.637300e-03
## 20429  3 6261 5.623605e-03
## 20430  3 6262 5.610406e-03
## 20431  3 6263 5.587047e-03
## 20432  3 6264 5.568149e-03
## 20433  3 6265 5.557061e-03
## 20434  3 6266 5.541372e-03
## 20435  3 6267 5.528759e-03
## 20436  3 6268 5.510099e-03
## 20437  3 6269 5.499429e-03
## 20438  3 6270 5.466727e-03
## 20439  3 6271 5.447263e-03
## 20440  3 6272 5.431131e-03
## 20441  3 6273 5.410821e-03
## 20442  3 6274 5.402409e-03
## 20443  3 6275 5.380751e-03
## 20444  3 6276 5.374536e-03
## 20445  3 6277 5.355991e-03
## 20446  3 6278 5.337742e-03
## 20447  3 6279 5.311425e-03
## 20448  3 6280 5.298924e-03
## 20449  3 6281 5.280237e-03
## 20450  3 6282 5.262444e-03
## 20451  3 6283 5.257231e-03
## 20452  3 6284 5.237265e-03
## 20453  3 6285 5.213908e-03
## 20454  3 6286 5.204685e-03
## 20455  3 6287 5.176980e-03
## 20456  3 6288 5.174915e-03
## 20457  3 6289 5.152089e-03
## 20458  3 6290 5.140626e-03
## 20459  3 6291 5.112482e-03
## 20460  3 6292 5.097798e-03
## 20461  3 6293 5.084452e-03
## 20462  3 6294 5.071819e-03
## 20463  3 6295 5.058969e-03
## 20464  3 6296 5.038415e-03
## 20465  3 6297 5.030265e-03
## 20466  3 6298 5.006124e-03
## 20467  3 6299 4.998133e-03
## 20468  3 6300 4.976635e-03
## 20469  3 6301 4.956205e-03
## 20470  3 6302 4.935553e-03
## 20471  3 6303 4.931165e-03
## 20472  3 6304 4.915187e-03
## 20473  3 6305 4.899240e-03
## 20474  3 6306 4.873921e-03
## 20475  3 6307 4.860002e-03
## 20476  3 6308 4.853886e-03
## 20477  3 6309 4.842238e-03
## 20478  3 6310 4.826003e-03
## 20479  3 6311 4.814032e-03
## 20480  3 6312 4.788054e-03
## 20481  3 6313 4.777548e-03
## 20482  3 6314 4.768295e-03
## 20483  3 6315 4.758022e-03
## 20484  3 6316 4.734544e-03
## 20485  3 6317 4.726748e-03
## 20486  3 6318 4.707971e-03
## 20487  3 6319 4.684697e-03
## 20488  3 6320 4.669887e-03
## 20489  3 6321 4.656560e-03
## 20490  3 6322 4.651145e-03
## 20491  3 6323 4.637141e-03
## 20492  3 6324 4.620731e-03
## 20493  3 6325 4.602030e-03
## 20494  3 6326 4.594213e-03
## 20495  3 6327 4.572076e-03
## 20496  3 6328 4.564940e-03
## 20497  3 6329 4.544661e-03
## 20498  3 6330 4.537550e-03
## 20499  3 6331 4.518291e-03
## 20500  3 6332 4.495915e-03
## 20501  3 6333 4.486150e-03
## 20502  3 6334 4.465687e-03
## 20503  3 6335 4.460460e-03
## 20504  3 6336 4.443004e-03
## 20505  3 6337 4.433612e-03
## 20506  3 6338 4.428342e-03
## 20507  3 6339 4.399996e-03
## 20508  3 6340 4.392311e-03
## 20509  3 6341 4.376394e-03
## 20510  3 6342 4.362582e-03
## 20511  3 6343 4.360046e-03
## 20512  3 6344 4.343643e-03
## 20513  3 6345 4.322835e-03
## 20514  3 6346 4.306163e-03
## 20515  3 6347 4.301305e-03
## 20516  3 6348 4.287133e-03
## 20517  3 6349 4.272557e-03
## 20518  3 6350 4.260410e-03
## 20519  3 6351 4.239216e-03
## 20520  3 6352 4.220954e-03
## 20521  3 6353 4.219740e-03
## 20522  3 6354 4.200937e-03
## 20523  3 6355 4.199215e-03
## 20524  3 6356 4.171406e-03
## 20525  3 6357 4.166407e-03
## 20526  3 6358 4.153777e-03
## 20527  3 6359 4.142521e-03
## 20528  3 6360 4.124614e-03
## 20529  3 6361 4.114331e-03
## 20530  3 6362 4.105506e-03
## 20531  3 6363 4.085463e-03
## 20532  3 6364 4.076751e-03
## 20533  3 6365 4.063101e-03
## 20534  3 6366 4.049799e-03
## 20535  3 6367 4.036460e-03
## 20536  3 6368 4.012247e-03
## 20537  3 6369 4.008223e-03
## 20538  3 6370 4.003116e-03
## 20539  3 6371 3.985935e-03
## 20540  3 6372 3.967257e-03
## 20541  3 6373 3.956068e-03
## 20542  3 6374 3.953008e-03
## 20543  3 6375 3.939568e-03
## 20544  3 6376 3.924297e-03
## 20545  3 6377 3.911117e-03
## 20546  3 6378 3.898176e-03
## 20547  3 6379 3.889159e-03
## 20548  3 6380 3.873546e-03
## 20549  3 6381 3.867682e-03
## 20550  3 6382 3.853430e-03
## 20551  3 6383 3.842666e-03
## 20552  3 6384 3.825993e-03
## 20553  3 6385 3.818513e-03
## 20554  3 6386 3.802856e-03
## 20555  3 6387 3.790753e-03
## 20556  3 6388 3.779432e-03
## 20557  3 6389 3.760391e-03
## 20558  3 6390 3.753282e-03
## 20559  3 6391 3.741938e-03
## 20560  3 6392 3.731850e-03
## 20561  3 6393 3.718674e-03
## 20562  3 6394 3.702161e-03
## 20563  3 6395 3.700344e-03
## 20564  3 6396 3.687731e-03
## 20565  3 6397 3.670464e-03
## 20566  3 6398 3.668013e-03
## 20567  3 6399 3.647582e-03
## 20568  3 6400 3.635454e-03
## 20569  3 6401 3.630416e-03
## 20570  3 6402 3.618495e-03
## 20571  3 6403 3.597494e-03
## 20572  3 6404 3.594830e-03
## 20573  3 6405 3.577231e-03
## 20574  3 6406 3.567032e-03
## 20575  3 6407 3.558068e-03
## 20576  3 6408 3.544808e-03
## 20577  3 6409 3.532662e-03
## 20578  3 6410 3.526266e-03
## 20579  3 6411 3.514924e-03
## 20580  3 6412 3.505313e-03
## 20581  3 6413 3.500361e-03
## 20582  3 6414 3.484420e-03
## 20583  3 6415 3.466478e-03
## 20584  3 6416 3.461372e-03
## 20585  3 6417 3.450676e-03
## 20586  3 6418 3.437069e-03
## 20587  3 6419 3.432249e-03
## 20588  3 6420 3.420670e-03
## 20589  3 6421 3.408197e-03
## 20590  3 6422 3.398795e-03
## 20591  3 6423 3.387475e-03
## 20592  3 6424 3.376755e-03
## 20593  3 6425 3.362194e-03
## 20594  3 6426 3.356590e-03
## 20595  3 6427 3.346823e-03
## 20596  3 6428 3.334348e-03
## 20597  3 6429 3.324499e-03
## 20598  3 6430 3.312604e-03
## 20599  3 6431 3.294165e-03
## 20600  3 6432 3.291471e-03
## 20601  3 6433 3.283362e-03
## 20602  3 6434 3.268648e-03
## 20603  3 6435 3.258417e-03
## 20604  3 6436 3.246570e-03
## 20605  3 6437 3.236360e-03
## 20606  3 6438 3.228884e-03
## 20607  3 6439 3.220180e-03
## 20608  3 6440 3.204375e-03
## 20609  3 6441 3.200967e-03
## 20610  3 6442 3.186731e-03
## 20611  3 6443 3.176608e-03
## 20612  3 6444 3.169958e-03
## 20613  3 6445 3.161174e-03
## 20614  3 6446 3.147522e-03
## 20615  3 6447 3.139359e-03
## 20616  3 6448 3.131294e-03
## 20617  3 6449 3.116937e-03
## 20618  3 6450 3.110413e-03
## 20619  3 6451 3.098343e-03
## 20620  3 6452 3.087628e-03
## 20621  3 6453 3.082516e-03
## 20622  3 6454 3.070413e-03
## 20623  3 6455 3.065212e-03
## 20624  3 6456 3.054136e-03
## 20625  3 6457 3.035813e-03
## 20626  3 6458 3.032299e-03
## 20627  3 6459 3.024377e-03
## 20628  3 6460 3.016608e-03
## 20629  3 6461 3.003258e-03
## 20630  3 6462 2.996248e-03
## 20631  3 6463 2.985323e-03
## 20632  3 6464 2.983024e-03
## 20633  3 6465 2.967204e-03
## 20634  3 6466 2.957415e-03
## 20635  3 6467 2.945013e-03
## 20636  3 6468 2.940611e-03
## 20637  3 6469 2.932123e-03
## 20638  3 6470 2.923039e-03
## 20639  3 6471 2.909829e-03
## 20640  3 6472 2.902014e-03
## 20641  3 6473 2.895138e-03
## 20642  3 6474 2.885490e-03
## 20643  3 6475 2.878802e-03
## 20644  3 6476 2.868193e-03
## 20645  3 6477 2.856554e-03
## 20646  3 6478 2.846662e-03
## 20647  3 6479 2.840185e-03
## 20648  3 6480 2.827013e-03
## 20649  3 6481 2.821036e-03
## 20650  3 6482 2.819434e-03
## 20651  3 6483 2.808449e-03
## 20652  3 6484 2.798085e-03
## 20653  3 6485 2.787711e-03
## 20654  3 6486 2.781780e-03
## 20655  3 6487 2.763946e-03
## 20656  3 6488 2.762238e-03
## 20657  3 6489 2.751756e-03
## 20658  3 6490 2.744210e-03
## 20659  3 6491 2.733900e-03
## 20660  3 6492 2.730406e-03
## 20661  3 6493 2.719196e-03
## 20662  3 6494 2.708338e-03
## 20663  3 6495 2.700857e-03
## 20664  3 6496 2.697168e-03
## 20665  3 6497 2.687395e-03
## 20666  3 6498 2.679678e-03
## 20667  3 6499 2.666363e-03
## 20668  3 6500 2.654577e-03
## 20669  3 6501 2.646869e-03
## 20670  3 6502 2.643254e-03
## 20671  3 6503 2.633377e-03
## 20672  3 6504 2.621412e-03
## 20673  3 6505 2.613350e-03
```

```
## 20674  3 6506 2.604654e-03
## 20675  3 6507 2.598189e-03
## 20676  3 6508 2.588983e-03
## 20677  3 6509 2.591792e-03
## 20678  3 6510 2.582707e-03
## 20679  3 6511 2.571145e-03
## 20680  3 6512 2.560324e-03
## 20681  3 6513 2.553659e-03
## 20682  3 6514 2.546752e-03
## 20683  3 6515 2.537031e-03
## 20684  3 6516 2.530970e-03
## 20685  3 6517 2.520975e-03
## 20686  3 6518 2.512092e-03
## 20687  3 6519 2.508436e-03
## 20688  3 6520 2.502190e-03
## 20689  3 6521 2.489549e-03
## 20690  3 6522 2.478369e-03
## 20691  3 6523 2.480477e-03
## 20692  3 6524 2.465740e-03
## 20693  3 6525 2.457226e-03
## 20694  3 6526 2.451824e-03
## 20695  3 6527 2.443943e-03
## 20696  3 6528 2.436385e-03
## 20697  3 6529 2.429294e-03
## 20698  3 6530 2.421372e-03
## 20699  3 6531 2.415510e-03
## 20700  3 6532 2.407356e-03
## 20701  3 6533 2.396299e-03
## 20702  3 6534 2.385108e-03
## 20703  3 6535 2.382536e-03
## 20704  3 6536 2.370020e-03
## 20705  3 6537 2.362984e-03
## 20706  3 6538 2.360145e-03
## 20707  3 6539 2.355316e-03
## 20708  3 6540 2.345184e-03
## 20709  3 6541 2.339753e-03
## 20710  3 6542 2.335327e-03
## 20711  3 6543 2.322025e-03
## 20712  3 6544 2.317195e-03
## 20713  3 6545 2.310979e-03
## 20714  3 6546 2.305577e-03
## 20715  3 6547 2.297524e-03
## 20716  3 6548 2.284716e-03
## 20717  3 6549 2.278261e-03
## 20718  3 6550 2.271413e-03
## 20719  3 6551 2.267200e-03
## 20720  3 6552 2.263550e-03
## 20721  3 6553 2.250324e-03
## 20722  3 6554 2.248346e-03
## 20723  3 6555 2.237984e-03
## 20724  3 6556 2.230380e-03
## 20725  3 6557 2.226221e-03
## 20726  3 6558 2.219291e-03
## 20727  3 6559 2.213400e-03
## 20728  3 6560 2.204644e-03
## 20729  3 6561 2.195823e-03
## 20730  3 6562 2.190188e-03
## 20731  3 6563 2.186235e-03
## 20732  3 6564 2.176712e-03
## 20733  3 6565 2.169344e-03
## 20734  3 6566 2.160405e-03
## 20735  3 6567 2.156703e-03
## 20736  3 6568 2.151963e-03
## 20737  3 6569 2.139755e-03
## 20738  3 6570 2.134091e-03
## 20739  3 6571 2.128183e-03
## 20740  3 6572 2.118747e-03
## 20741  3 6573 2.115762e-03
## 20742  3 6574 2.109862e-03
## 20743  3 6575 2.098886e-03
## 20744  3 6576 2.093285e-03
## 20745  3 6577 2.089047e-03
## 20746  3 6578 2.084907e-03
## 20747  3 6579 2.073588e-03
## 20748  3 6580 2.067602e-03
## 20749  3 6581 2.061408e-03
## 20750  3 6582 2.053225e-03
## 20751  3 6583 2.054962e-03
## 20752  3 6584 2.044726e-03
## 20753  3 6585 2.037280e-03
## 20754  3 6586 2.030071e-03
## 20755  3 6587 2.024977e-03
## 20756  3 6588 2.017417e-03
## 20757  3 6589 2.012103e-03
## 20758  3 6590 2.008898e-03
## 20759  3 6591 1.996746e-03
## 20760  3 6592 1.993167e-03
## 20761  3 6593 1.989555e-03
## 20762  3 6594 1.983944e-03
## 20763  3 6595 1.975257e-03
## 20764  3 6596 1.970430e-03
## 20765  3 6597 1.962007e-03
## 20766  3 6598 1.958564e-03
## 20767  3 6599 1.950313e-03
## 20768  3 6600 1.943037e-03
## 20769  3 6601 1.941272e-03
## 20770  3 6602 1.931551e-03
## 20771  3 6603 1.925868e-03
## 20772  3 6604 1.918947e-03
## 20773  3 6605 1.909692e-03
## 20774  3 6606 1.904388e-03
## 20775  3 6607 1.900579e-03
## 20776  3 6608 1.898848e-03
## 20777  3 6609 1.886187e-03
## 20778  3 6610 1.884064e-03
## 20779  3 6611 1.875173e-03
## 20780  3 6612 1.872635e-03
## 20781  3 6613 1.868113e-03
## 20782  3 6614 1.860315e-03
## 20783  3 6615 1.853721e-03
## 20784  3 6616 1.850990e-03
## 20785  3 6617 1.840308e-03
## 20786  3 6618 1.836626e-03
## 20787  3 6619 1.831719e-03
## 20788  3 6620 1.824361e-03
## 20789  3 6621 1.815668e-03
## 20790  3 6622 1.817057e-03
## 20791  3 6623 1.807689e-03
## 20792  3 6624 1.801593e-03
## 20793  3 6625 1.797127e-03
## 20794  3 6626 1.790050e-03
## 20795  3 6627 1.785399e-03
## 20796  3 6628 1.781571e-03
## 20797  3 6629 1.773426e-03
## 20798  3 6630 1.767645e-03
## 20799  3 6631 1.764741e-03
## 20800  3 6632 1.755986e-03
## 20801  3 6633 1.753361e-03
## 20802  3 6634 1.748684e-03
## 20803  3 6635 1.745150e-03
## 20804  3 6636 1.735765e-03
## 20805  3 6637 1.730936e-03
## 20806  3 6638 1.722483e-03
## 20807  3 6639 1.720459e-03
## 20808  3 6640 1.714795e-03
## 20809  3 6641 1.711927e-03
## 20810  3 6642 1.705295e-03
## 20811  3 6643 1.700483e-03
## 20812  3 6644 1.694579e-03
## 20813  3 6645 1.685314e-03
## 20814  3 6646 1.682638e-03
## 20815  3 6647 1.679625e-03
## 20816  3 6648 1.671808e-03
## 20817  3 6649 1.668229e-03
## 20818  3 6650 1.662777e-03
## 20819  3 6651 1.656132e-03
## 20820  3 6652 1.650981e-03
## 20821  3 6653 1.645115e-03
## 20822  3 6654 1.639096e-03
## 20823  3 6655 1.634964e-03
## 20824  3 6656 1.630676e-03
## 20825  3 6657 1.623690e-03
## 20826  3 6658 1.622955e-03
## 20827  3 6659 1.615763e-03
## 20828  3 6660 1.607510e-03
## 20829  3 6661 1.607128e-03
## 20830  3 6662 1.599673e-03
## 20831  3 6663 1.598655e-03
## 20832  3 6664 1.592433e-03
## 20833  3 6665 1.589616e-03
## 20834  3 6666 1.578427e-03
## 20835  3 6667 1.575855e-03
## 20836  3 6668 1.570552e-03
## 20837  3 6669 1.563867e-03
## 20838  3 6670 1.559688e-03
## 20839  3 6671 1.555075e-03
## 20840  3 6672 1.551571e-03
## 20841  3 6673 1.549537e-03
## 20842  3 6674 1.540634e-03
## 20843  3 6675 1.535056e-03
## 20844  3 6676 1.531950e-03
## 20845  3 6677 1.526239e-03
## 20846  3 6678 1.523473e-03
## 20847  3 6679 1.517562e-03
## 20848  3 6680 1.512053e-03
## 20849  3 6681 1.508550e-03
## 20850  3 6682 1.502675e-03
## 20851  3 6683 1.497419e-03
## 20852  3 6684 1.493227e-03
## 20853  3 6685 1.489087e-03
## 20854  3 6686 1.484686e-03
## 20855  3 6687 1.477475e-03
## 20856  3 6688 1.477398e-03
## 20857  3 6689 1.469981e-03
## 20858  3 6690 1.465482e-03
## 20859  3 6691 1.463024e-03
## 20860  3 6692 1.458125e-03
## 20861  3 6693 1.447950e-03
## 20862  3 6694 1.449661e-03
## 20863  3 6695 1.444441e-03
## 20864  3 6696 1.439926e-03
## 20865  3 6697 1.431999e-03
## 20866  3 6698 1.428336e-03
## 20867  3 6699 1.423983e-03
## 20868  3 6700 1.420444e-03
## 20869  3 6701 1.415767e-03
## 20870  3 6702 1.410662e-03
## 20871  3 6703 1.409683e-03
## 20872  3 6704 1.403433e-03
## 20873  3 6705 1.398215e-03
## 20874  3 6706 1.394414e-03
## 20875  3 6707 1.392457e-03
## 20876  3 6708 1.388173e-03
## 20877  3 6709 1.381247e-03
## 20878  3 6710 1.377275e-03
## 20879  3 6711 1.372797e-03
## 20880  3 6712 1.370245e-03
## 20881  3 6713 1.366136e-03
## 20882  3 6714 1.359792e-03
## 20883  3 6715 1.356040e-03
## 20884  3 6716 1.349549e-03
## 20885  3 6717 1.346638e-03
## 20886  3 6718 1.341635e-03
## 20887  3 6719 1.340846e-03
## 20888  3 6720 1.333081e-03
## 20889  3 6721 1.329805e-03
## 20890  3 6722 1.329149e-03
## 20891  3 6723 1.324742e-03
## 20892  3 6724 1.317019e-03
## 20893  3 6725 1.312603e-03
## 20894  3 6726 1.308508e-03
## 20895  3 6727 1.306828e-03
## 20896  3 6728 1.300519e-03
## 20897  3 6729 1.297782e-03
## 20898  3 6730 1.291827e-03
## 20899  3 6731 1.288954e-03
## 20900  3 6732 1.284829e-03
## 20901  3 6733 1.280558e-03
## 20902  3 6734 1.279184e-03
## 20903  3 6735 1.273108e-03
## 20904  3 6736 1.269702e-03
## 20905  3 6737 1.264791e-03
## 20906  3 6738 1.259960e-03
## 20907  3 6739 1.257308e-03
## 20908  3 6740 1.252515e-03
## 20909  3 6741 1.248168e-03
## 20910  3 6742 1.247441e-03
## 20911  3 6743 1.240857e-03
## 20912  3 6744 1.235671e-03
## 20913  3 6745 1.233243e-03
## 20914  3 6746 1.229945e-03
## 20915  3 6747 1.224822e-03
## 20916  3 6748 1.221460e-03
## 20917  3 6749 1.219476e-03
## 20918  3 6750 1.214338e-03
## 20919  3 6751 1.211531e-03
## 20920  3 6752 1.209909e-03
## 20921  3 6753 1.203834e-03
## 20922  3 6754 1.199401e-03
## 20923  3 6755 1.195034e-03
## 20924  3 6756 1.192203e-03
## 20925  3 6757 1.187900e-03
## 20926  3 6758 1.184675e-03
## 20927  3 6759 1.182411e-03
## 20928  3 6760 1.175406e-03
## 20929  3 6761 1.174317e-03
## 20930  3 6762 1.170345e-03
## 20931  3 6763 1.167017e-03
## 20932  3 6764 1.163675e-03
## 20933  3 6765 1.158228e-03
## 20934  3 6766 1.154916e-03
## 20935  3 6767 1.150357e-03
## 20936  3 6768 1.148282e-03
## 20937  3 6769 1.143070e-03
## 20938  3 6770 1.140965e-03
## 20939  3 6771 1.139283e-03
## 20940  3 6772 1.133790e-03
## 20941  3 6773 1.132469e-03
## 20942  3 6774 1.126897e-03
## 20943  3 6775 1.122773e-03
## 20944  3 6776 1.118923e-03
## 20945  3 6777 1.115560e-03
## 20946  3 6778 1.113187e-03
## 20947  3 6779 1.109743e-03
## 20948  3 6780 1.105095e-03
## 20949  3 6781 1.102178e-03
## 20950  3 6782 1.098764e-03
## 20951  3 6783 1.095426e-03
## 20952  3 6784 1.091446e-03
## 20953  3 6785 1.089016e-03
## 20954  3 6786 1.086089e-03
## 20955  3 6787 1.083117e-03
## 20956  3 6788 1.078818e-03
## 20957  3 6789 1.075163e-03
## 20958  3 6790 1.071566e-03
## 20959  3 6791 1.067798e-03
## 20960  3 6792 1.064336e-03
## 20961  3 6793 1.061434e-03
## 20962  3 6794 1.058916e-03
## 20963  3 6795 1.054392e-03
## 20964  3 6796 1.053714e-03
## 20965  3 6797 1.048418e-03
## 20966  3 6798 1.044245e-03
## 20967  3 6799 1.041688e-03
## 20968  3 6800 1.038942e-03
## 20969  3 6801 1.035424e-03
## 20970  3 6802 1.030877e-03
## 20971  3 6803 1.029115e-03
## 20972  3 6804 1.025343e-03
## 20973  3 6805 1.021246e-03
## 20974  3 6806 1.018938e-03
## 20975  3 6807 1.017027e-03
## 20976  3 6808 1.013815e-03
## 20977  3 6809 1.010314e-03
## 20978  3 6810 1.005804e-03
## 20979  3 6811 1.002651e-03
## 20980  3 6812 1.001980e-03
## 20981  3 6813 9.962031e-04
## 20982  3 6814 9.919081e-04
## 20983  3 6815 9.913329e-04
## 20984  3 6816 9.879613e-04
## 20985  3 6817 9.846376e-04
## 20986  3 6818 9.813061e-04
## 20987  3 6819 9.778975e-04
## 20988  3 6820 9.761326e-04
## 20989  3 6821 9.720462e-04
## 20990  3 6822 9.698855e-04
## 20991  3 6823 9.660265e-04
## 20992  3 6824 9.634011e-04
## 20993  3 6825 9.601242e-04
## 20994  3 6826 9.569189e-04
## 20995  3 6827 9.547647e-04
## 20996  3 6828 9.513233e-04
## 20997  3 6829 9.485965e-04
## 20998  3 6830 9.449710e-04
## 20999  3 6831 9.435466e-04
## 21000  3 6832 9.390878e-04
## 21001  3 6833 9.378013e-04
## 21002  3 6834 9.346509e-04
## 21003  3 6835 9.299554e-04
## 21004  3 6836 9.280427e-04
## 21005  3 6837 9.249667e-04
## 21006  3 6838 9.236898e-04
## 21007  3 6839 9.186069e-04
## 21008  3 6840 9.148895e-04
## 21009  3 6841 9.139342e-04
## 21010  3 6842 9.092509e-04
## 21011  3 6843 9.064137e-04
## 21012  3 6844 9.035639e-04
## 21013  3 6845 9.027431e-04
## 21014  3 6846 8.990679e-04
## 21015  3 6847 8.969447e-04
## 21016  3 6848 8.927032e-04
## 21017  3 6849 8.923004e-04
## 21018  3 6850 8.875453e-04
## 21019  3 6851 8.845719e-04
## 21020  3 6852 8.811065e-04
## 21021  3 6853 8.799456e-04
## 21022  3 6854 8.770164e-04
## 21023  3 6855 8.744186e-04
## 21024  3 6856 8.703791e-04
## 21025  3 6857 8.682037e-04
## 21026  3 6858 8.674305e-04
## 21027  3 6859 8.632647e-04
## 21028  3 6860 8.604669e-04
## 21029  3 6861 8.578075e-04
## 21030  3 6862 8.561174e-04
## 21031  3 6863 8.535124e-04
## 21032  3 6864 8.508666e-04
## 21033  3 6865 8.470185e-04
## 21034  3 6866 8.431772e-04
## 21035  3 6867 8.417537e-04
## 21036  3 6868 8.390405e-04
## 21037  3 6869 8.379048e-04
## 21038  3 6870 8.331175e-04
## 21039  3 6871 8.310603e-04
## 21040  3 6872 8.277425e-04
## 21041  3 6873 8.254027e-04
## 21042  3 6874 8.236689e-04
## 21043  3 6875 8.196122e-04
## 21044  3 6876 8.183670e-04
## 21045  3 6877 8.178009e-04
## 21046  3 6878 8.143888e-04
## 21047  3 6879 8.106811e-04
## 21048  3 6880 8.066490e-04
## 21049  3 6881 8.063284e-04
## 21050  3 6882 8.028478e-04
## 21051  3 6883 8.013000e-04
## 21052  3 6884 7.971413e-04
## 21053  3 6885 7.961056e-04
## 21054  3 6886 7.937140e-04
## 21055  3 6887 7.902927e-04
## 21056  3 6888 7.882596e-04
## 21057  3 6889 7.858751e-04
## 21058  3 6890 7.834958e-04
## 21059  3 6891 7.807100e-04
## 21060  3 6892 7.783932e-04
## 21061  3 6893 7.758225e-04
## 21062  3 6894 7.735795e-04
## 21063  3 6895 7.694060e-04
## 21064  3 6896 7.681943e-04
## 21065  3 6897 7.670114e-04
## 21066  3 6898 7.637637e-04
## 21067  3 6899 7.613833e-04
## 21068  3 6900 7.592002e-04
## 21069  3 6901 7.564645e-04
## 21070  3 6902 7.548546e-04
## 21071  3 6903 7.526091e-04
## 21072  3 6904 7.483025e-04
## 21073  3 6905 7.485573e-04
## 21074  3 6906 7.444198e-04
## 21075  3 6907 7.416347e-04
## 21076  3 6908 7.396721e-04
## 21077  3 6909 7.368760e-04
## 21078  3 6910 7.348441e-04
## 21079  3 6911 7.328242e-04
## 21080  3 6912 7.313982e-04
## 21081  3 6913 7.278059e-04
## 21082  3 6914 7.266807e-04
## 21083  3 6915 7.246333e-04
## 21084  3 6916 7.214408e-04
## 21085  3 6917 7.192828e-04
## 21086  3 6918 7.174929e-04
## 21087  3 6919 7.149313e-04
## 21088  3 6920 7.130483e-04
## 21089  3 6921 7.114715e-04
## 21090  3 6922 7.085918e-04
## 21091  3 6923 7.060566e-04
## 21092  3 6924 7.036279e-04
## 21093  3 6925 7.021211e-04
## 21094  3 6926 6.989354e-04
## 21095  3 6927 6.971421e-04
## 21096  3 6928 6.964275e-04
## 21097  3 6929 6.936858e-04
## 21098  3 6930 6.903744e-04
## 21099  3 6931 6.882660e-04
## 21100  3 6932 6.875369e-04
## 21101  3 6933 6.844984e-04
## 21102  3 6934 6.827246e-04
## 21103  3 6935 6.799302e-04
## 21104  3 6936 6.785422e-04
## 21105  3 6937 6.748865e-04
## 21106  3 6938 6.751032e-04
## 21107  3 6939 6.722750e-04
## 21108  3 6940 6.709428e-04
## 21109  3 6941 6.686708e-04
## 21110  3 6942 6.657405e-04
## 21111  3 6943 6.651723e-04
## 21112  3 6944 6.610921e-04
## 21113  3 6945 6.590135e-04
## 21114  3 6946 6.584989e-04
## 21115  3 6947 6.557253e-04
## 21116  3 6948 6.537332e-04
## 21117  3 6949 6.513314e-04
## 21118  3 6950 6.490164e-04
## 21119  3 6951 6.471777e-04
## 21120  3 6952 6.452031e-04
## 21121  3 6953 6.430604e-04
## 21122  3 6954 6.404642e-04
## 21123  3 6955 6.393742e-04
## 21124  3 6956 6.359354e-04
## 21125  3 6957 6.356242e-04
## 21126  3 6958 6.332205e-04
## 21127  3 6959 6.306527e-04
## 21128  3 6960 6.284009e-04
## 21129  3 6961 6.287582e-04
## 21130  3 6962 6.262641e-04
## 21131  3 6963 6.232935e-04
## 21132  3 6964 6.218710e-04
## 21133  3 6965 6.190399e-04
## 21134  3 6966 6.166304e-04
## 21135  3 6967 6.157317e-04
## 21136  3 6968 6.123300e-04
## 21137  3 6969 6.117725e-04
## 21138  3 6970 6.096551e-04
## 21139  3 6971 6.091025e-04
## 21140  3 6972 6.055444e-04
## 21141  3 6973 6.039016e-04
## 21142  3 6974 6.022111e-04
## 21143  3 6975 6.004671e-04
## 21144  3 6976 5.983901e-04
## 21145  3 6977 5.958889e-04
## 21146  3 6978 5.958932e-04
## 21147  3 6979 5.929804e-04
## 21148  3 6980 5.910986e-04
## 21149  3 6981 5.887140e-04
## 21150  3 6982 5.863180e-04
## 21151  3 6983 5.849140e-04
## 21152  3 6984 5.832977e-04
## 21153  3 6985 5.810483e-04
## 21154  3 6986 5.804731e-04
## 21155  3 6987 5.784571e-04
## 21156  3 6988 5.746279e-04
## 21157  3 6989 5.745425e-04
## 21158  3 6990 5.721783e-04
## 21159  3 6991 5.706143e-04
## 21160  3 6992 5.692945e-04
## 21161  3 6993 5.670541e-04
## 21162  3 6994 5.655144e-04
## 21163  3 6995 5.635188e-04
## 21164  3 6996 5.616151e-04
## 21165  3 6997 5.606593e-04
## 21166  3 6998 5.585665e-04
## 21167  3 6999 5.571032e-04
## 21168  3 7000 5.561103e-04
## 21169  4    0 0.000000e+00
## 21170  4    0 1.807071e+00
## 21171  4    1 2.253667e+00
## 21172  4    2 2.542594e+00
## 21173  4    3 2.633229e+00
## 21174  4    4 2.638232e+00
## 21175  4    5 2.604025e+00
## 21176  4    6 2.557629e+00
## 21177  4    7 2.499052e+00
## 21178  4    8 2.438770e+00
## 21179  4    9 2.389456e+00
## 21180  4   10 2.333316e+00
## 21181  4   11 2.283720e+00
## 21182  4   12 2.235274e+00
## 21183  4   13 2.187540e+00
## 21184  4   14 2.143717e+00
## 21185  4   15 2.100680e+00
## 21186  4   16 2.060711e+00
## 21187  4   17 2.024503e+00
## 21188  4   18 1.988443e+00
## 21189  4   19 1.956704e+00
## 21190  4   20 1.922414e+00
## 21191  4   21 1.889998e+00
## 21192  4   22 1.864453e+00
## 21193  4   23 1.832545e+00
## 21194  4   24 1.807964e+00
## 21195  4   24 1.807441e+00
## 21196  4   25 2.252516e+00
## 21197  4   26 2.538578e+00
## 21198  4   27 2.631653e+00
## 21199  4   28 2.630426e+00
## 21200  4   29 2.605378e+00
## 21201  4   30 2.555304e+00
## 21202  4   31 2.502439e+00
## 21203  4   32 2.441447e+00
## 21204  4   33 2.389845e+00
## 21205  4   34 2.335350e+00
## 21206  4   35 2.282458e+00
## 21207  4   36 2.235421e+00
## 21208  4   37 2.187031e+00
## 21209  4   38 2.145826e+00
## 21210  4   39 2.100441e+00
## 21211  4   40 2.059598e+00
## 21212  4   41 2.025902e+00
## 21213  4   42 1.992616e+00
## 21214  4   43 1.955664e+00
## 21215  4   44 1.920584e+00
## 21216  4   45 1.890826e+00
## 21217  4   46 1.861777e+00
## 21218  4   47 1.833203e+00
## 21219  4   48 1.805596e+00
## 21220  4   48 1.807215e+00
## 21221  4   49 2.254108e+00
## 21222  4   50 2.534062e+00
## 21223  4   51 2.630024e+00
## 21224  4   52 2.639241e+00
## 21225  4   53 2.603358e+00
## 21226  4   54 2.554573e+00
## 21227  4   55 2.503076e+00
## 21228  4   56 2.442104e+00
## 21229  4   57 2.387825e+00
## 21230  4   58 2.335085e+00
## 21231  4   59 2.282515e+00
## 21232  4   60 2.232221e+00
## 21233  4   61 2.187119e+00
## 21234  4   62 2.144677e+00
## 21235  4   63 2.104191e+00
## 21236  4   64 2.060554e+00
## 21237  4   65 2.021956e+00
## 21238  4   66 1.989795e+00
## 21239  4   67 1.953862e+00
## 21240  4   68 1.922196e+00
## 21241  4   69 1.894855e+00
## 21242  4   70 1.861090e+00
## 21243  4   71 1.835354e+00
## 21244  4   72 1.803514e+00
## 21245  4   73 2.258881e+00
## 21246  4   74 2.544440e+00
## 21247  4   75 2.627340e+00
## 21248  4   76 2.638534e+00
## 21249  4   77 2.606733e+00
## 21250  4   78 2.557726e+00
## 21251  4   79 2.501284e+00
## 21252  4   80 2.445474e+00
## 21253  4   81 2.392475e+00
## 21254  4   82 2.330544e+00
## 21255  4   83 2.282247e+00
## 21256  4   84 2.239335e+00
## 21257  4   85 2.185696e+00
## 21258  4   86 2.146598e+00
## 21259  4   87 2.100532e+00
## 21260  4   88 2.060683e+00
## 21261  4   89 2.026272e+00
## 21262  4   90 1.988192e+00
## 21263  4   91 1.953730e+00
## 21264  4   92 1.923047e+00
## 21265  4   93 1.889925e+00
## 21266  4   94 1.861613e+00
## 21267  4   95 1.833242e+00
## 21268  4   96 1.806480e+00
## 21269  4   97 2.249430e+00
## 21270  4   98 2.534669e+00
## 21271  4   99 2.629206e+00
## 21272  4  100 2.638706e+00
## 21273  4  101 2.603386e+00
## 21274  4  102 2.552096e+00
## 21275  4  103 2.498957e+00
## 21276  4  104 2.444470e+00
## 21277  4  105 2.387134e+00
## 21278  4  106 2.331358e+00
## 21279  4  107 2.286128e+00
## 21280  4  108 2.234749e+00
## 21281  4  109 2.190431e+00
## 21282  4  110 2.145275e+00
## 21283  4  111 2.105353e+00
## 21284  4  112 2.060243e+00
## 21285  4  113 2.027062e+00
## 21286  4  114 1.991802e+00
## 21287  4  115 1.958854e+00
## 21288  4  116 1.922170e+00
## 21289  4  117 1.894317e+00
## 21290  4  118 1.863483e+00
## 21291  4  119 1.836826e+00
## 21292  4  120 1.806468e+00
## 21293  4  121 2.256204e+00
## 21294  4  122 2.540658e+00
## 21295  4  123 2.624877e+00
## 21296  4  124 2.635307e+00
## 21297  4  125 2.605681e+00
## 21298  4  126 2.554761e+00
## 21299  4  127 2.501328e+00
## 21300  4  128 2.444512e+00
## 21301  4  129 2.386884e+00
## 21302  4  130 2.332919e+00
## 21303  4  131 2.282286e+00
## 21304  4  132 2.235848e+00
## 21305  4  133 2.188765e+00
## 21306  4  134 2.138171e+00
## 21307  4  135 2.099508e+00
## 21308  4  136 2.061499e+00
## 21309  4  137 2.028574e+00
## 21310  4  138 1.990905e+00
## 21311  4  139 1.955136e+00
## 21312  4  140 1.923134e+00
## 21313  4  141 1.887527e+00
## 21314  4  142 1.860409e+00
## 21315  4  143 1.831757e+00
## 21316  4  144 1.809433e+00
## 21317  4  145 2.254272e+00
## 21318  4  146 2.535822e+00
## 21319  4  147 2.629023e+00
## 21320  4  148 2.634659e+00
## 21321  4  149 2.600456e+00
## 21322  4  150 2.553403e+00
## 21323  4  151 2.499130e+00
## 21324  4  152 2.442267e+00
## 21325  4  153 2.388485e+00
## 21326  4  154 2.335137e+00
## 21327  4  155 2.286096e+00
## 21328  4  156 2.235355e+00
## 21329  4  157 2.190189e+00
## 21330  4  158 2.143757e+00
## 21331  4  159 2.105887e+00
## 21332  4  160 2.063485e+00
## 21333  4  161 2.024809e+00
## 21334  4  162 1.990186e+00
## 21335  4  163 1.953854e+00
## 21336  4  164 1.916407e+00
## 21337  4  165 1.891891e+00
## 21338  4  166 1.863364e+00
## 21339  4  167 1.833275e+00
## 21340  4  168 1.807896e+00
## 21341  4  169 2.257760e+00
## 21342  4  170 2.542685e+00
## 21343  4  171 2.626699e+00
## 21344  4  172 2.637409e+00
## 21345  4  173 2.610735e+00
## 21346  4  174 2.553874e+00
## 21347  4  175 2.496241e+00
## 21348  4  176 2.444298e+00
## 21349  4  177 2.384171e+00
## 21350  4  178 2.335935e+00
## 21351  4  179 2.283331e+00
## 21352  4  180 2.236785e+00
## 21353  4  181 2.187306e+00
## 21354  4  182 2.147364e+00
## 21355  4  183 2.102096e+00
## 21356  4  184 2.060908e+00
## 21357  4  185 2.024738e+00
## 21358  4  186 1.989622e+00
## 21359  4  187 1.955827e+00
## 21360  4  188 1.921338e+00
## 21361  4  189 1.890955e+00
## 21362  4  190 1.863350e+00
## 21363  4  191 1.831253e+00
## 21364  4  192 1.807454e+00
## 21365  4  193 2.253025e+00
## 21366  4  194 2.537148e+00
## 21367  4  195 2.632043e+00
## 21368  4  196 2.638403e+00
## 21369  4  197 2.603040e+00
## 21370  4  198 2.556838e+00
## 21371  4  199 2.499980e+00
## 21372  4  200 2.442240e+00
## 21373  4  201 2.386055e+00
## 21374  4  202 2.333841e+00
## 21375  4  203 2.281265e+00
## 21376  4  204 2.237688e+00
## 21377  4  205 2.189626e+00
## 21378  4  206 2.144525e+00
## 21379  4  207 2.102307e+00
## 21380  4  208 2.066393e+00
## 21381  4  209 2.025484e+00
## 21382  4  210 1.989877e+00
## 21383  4  211 1.952970e+00
## 21384  4  212 1.921522e+00
## 21385  4  213 1.894024e+00
## 21386  4  214 1.858497e+00
## 21387  4  215 1.835978e+00
## 21388  4  216 1.805104e+00
## 21389  4  217 2.251202e+00
## 21390  4  218 2.534676e+00
## 21391  4  219 2.631418e+00
## 21392  4  220 2.641655e+00
## 21393  4  221 2.604878e+00
## 21394  4  222 2.551782e+00
## 21395  4  223 2.492171e+00
## 21396  4  224 2.445840e+00
## 21397  4  225 2.384326e+00
## 21398  4  226 2.331164e+00
## 21399  4  227 2.280340e+00
## 21400  4  228 2.230160e+00
## 21401  4  229 2.185360e+00
## 21402  4  230 2.141902e+00
## 21403  4  231 2.101470e+00
## 21404  4  232 2.063077e+00
## 21405  4  233 2.027845e+00
## 21406  4  234 1.991789e+00
## 21407  4  235 1.953676e+00
## 21408  4  236 1.923009e+00
## 21409  4  237 1.891548e+00
## 21410  4  238 1.861847e+00
## 21411  4  239 1.833629e+00
## 21412  4  240 1.806567e+00
## 21413  4  241 2.256314e+00
## 21414  4  242 2.538531e+00
## 21415  4  243 2.630699e+00
## 21416  4  244 2.638646e+00
## 21417  4  245 2.611210e+00
## 21418  4  246 2.553951e+00
## 21419  4  247 2.499120e+00
## 21420  4  248 2.441467e+00
## 21421  4  249 2.389053e+00
## 21422  4  250 2.330575e+00
## 21423  4  251 2.285045e+00
## 21424  4  252 2.232927e+00
## 21425  4  253 2.184317e+00
## 21426  4  254 2.142490e+00
## 21427  4  255 2.096774e+00
## 21428  4  256 2.062721e+00
## 21429  4  257 2.026858e+00
## 21430  4  258 1.988074e+00
## 21431  4  259 1.956006e+00
## 21432  4  260 1.925447e+00
## 21433  4  261 1.891373e+00
## 21434  4  262 1.861451e+00
## 21435  4  263 1.834839e+00
## 21436  4  264 1.809448e+00
## 21437  4  265 2.252050e+00
## 21438  4  266 2.535928e+00
## 21439  4  267 2.634323e+00
## 21440  4  268 2.637261e+00
## 21441  4  269 2.610715e+00
## 21442  4  270 2.553185e+00
## 21443  4  271 2.501522e+00
## 21444  4  272 2.441267e+00
## 21445  4  273 2.390405e+00
## 21446  4  274 2.331201e+00
## 21447  4  275 2.279903e+00
## 21448  4  276 2.238481e+00
## 21449  4  277 2.188503e+00
## 21450  4  278 2.144921e+00
## 21451  4  279 2.100911e+00
## 21452  4  280 2.063662e+00
## 21453  4  281 2.028484e+00
## 21454  4  282 1.984928e+00
## 21455  4  283 1.956222e+00
## 21456  4  284 1.920321e+00
## 21457  4  285 1.889819e+00
## 21458  4  286 1.864148e+00
## 21459  4  287 1.830884e+00
## 21460  4  288 1.806371e+00
## 21461  4  289 2.255592e+00
## 21462  4  290 2.537702e+00
## 21463  4  291 2.631585e+00
## 21464  4  292 2.636552e+00
## 21465  4  293 2.606181e+00
## 21466  4  294 2.550429e+00
## 21467  4  295 2.497371e+00
## 21468  4  296 2.440546e+00
## 21469  4  297 2.388563e+00
## 21470  4  298 2.336340e+00
## 21471  4  299 2.283873e+00
## 21472  4  300 2.236793e+00
## 21473  4  301 2.184447e+00
```

```
## 21474  4  302 2.143456e+00
## 21475  4  303 2.107621e+00
## 21476  4  304 2.065223e+00
## 21477  4  305 2.023332e+00
## 21478  4  306 1.991621e+00
## 21479  4  307 1.957811e+00
## 21480  4  308 1.920451e+00
## 21481  4  309 1.890615e+00
## 21482  4  310 1.861363e+00
## 21483  4  311 1.831112e+00
## 21484  4  312 1.806196e+00
## 21485  4  312 1.804895e+00
## 21486  4  313 2.254029e+00
## 21487  4  314 2.537415e+00
## 21488  4  315 2.630821e+00
## 21489  4  316 2.638634e+00
## 21490  4  317 2.606192e+00
## 21491  4  318 2.549579e+00
## 21492  4  319 2.499681e+00
## 21493  4  320 2.441395e+00
## 21494  4  321 2.387813e+00
## 21495  4  322 2.333796e+00
## 21496  4  323 2.281341e+00
## 21497  4  324 2.237226e+00
## 21498  4  325 2.182008e+00
## 21499  4  326 2.143904e+00
## 21500  4  327 2.103889e+00
## 21501  4  328 2.063936e+00
## 21502  4  329 2.025055e+00
## 21503  4  330 1.990756e+00
## 21504  4  331 1.956859e+00
## 21505  4  332 1.922392e+00
## 21506  4  333 1.891506e+00
## 21507  4  334 1.860273e+00
## 21508  4  335 1.835305e+00
## 21509  4  336 1.806955e+00
## 21510  4  336 1.804915e+00
## 21511  4  337 1.781746e+00
## 21512  4  338 1.758473e+00
## 21513  4  339 1.733503e+00
## 21514  4  339 1.733492e+00
## 21515  4  340 2.184553e+00
## 21516  4  341 2.467405e+00
## 21517  4  342 2.567352e+00
## 21518  4  343 2.574306e+00
## 21519  4  344 2.546710e+00
## 21520  4  345 2.496937e+00
## 21521  4  346 2.442783e+00
## 21522  4  347 2.392133e+00
## 21523  4  348 2.337306e+00
## 21524  4  349 2.284513e+00
## 21525  4  350 2.238340e+00
## 21526  4  351 2.187053e+00
## 21527  4  352 2.146815e+00
## 21528  4  353 2.097961e+00
## 21529  4  354 2.061171e+00
## 21530  4  355 2.022396e+00
## 21531  4  356 1.985538e+00
## 21532  4  357 1.953533e+00
## 21533  4  358 1.918633e+00
## 21534  4  359 1.886785e+00
## 21535  4  360 1.858094e+00
## 21536  4  360 1.855314e+00
## 21537  4  361 2.302230e+00
## 21538  4  362 2.582119e+00
## 21539  4  363 2.675751e+00
## 21540  4  364 2.680547e+00
## 21541  4  365 2.647992e+00
## 21542  4  366 2.591841e+00
## 21543  4  367 2.534771e+00
## 21544  4  368 2.478556e+00
## 21545  4  369 2.420373e+00
## 21546  4  370 2.360307e+00
## 21547  4  371 2.310833e+00
## 21548  4  372 2.261229e+00
## 21549  4  373 2.212621e+00
## 21550  4  374 2.167543e+00
## 21551  4  375 2.122079e+00
## 21552  4  376 2.078390e+00
## 21553  4  377 2.044639e+00
## 21554  4  378 2.005572e+00
## 21555  4  379 1.971968e+00
## 21556  4  380 1.938869e+00
## 21557  4  381 1.906148e+00
## 21558  4  382 1.879062e+00
## 21559  4  383 1.848576e+00
## 21560  4  384 1.819880e+00
## 21561  4  385 2.264804e+00
## 21562  4  386 2.554440e+00
## 21563  4  387 2.643757e+00
## 21564  4  388 2.650879e+00
## 21565  4  389 2.612584e+00
## 21566  4  390 2.564414e+00
## 21567  4  391 2.510802e+00
## 21568  4  392 2.454682e+00
## 21569  4  393 2.393530e+00
## 21570  4  394 2.340605e+00
## 21571  4  395 2.294412e+00
## 21572  4  396 2.243605e+00
## 21573  4  397 2.198062e+00
## 21574  4  398 2.149618e+00
## 21575  4  399 2.111644e+00
## 21576  4  400 2.070797e+00
## 21577  4  401 2.031129e+00
## 21578  4  402 1.995299e+00
## 21579  4  403 1.961971e+00
## 21580  4  404 1.927065e+00
## 21581  4  405 1.893301e+00
## 21582  4  406 1.865556e+00
## 21583  4  407 1.837604e+00
## 21584  4  408 1.813230e+00
## 21585  4  409 2.258740e+00
## 21586  4  410 2.540582e+00
## 21587  4  411 2.637480e+00
## 21588  4  412 2.645616e+00
## 21589  4  413 2.610467e+00
## 21590  4  414 2.556637e+00
## 21591  4  415 2.506232e+00
## 21592  4  416 2.445814e+00
## 21593  4  417 2.389736e+00
## 21594  4  418 2.337172e+00
## 21595  4  419 2.287707e+00
## 21596  4  420 2.237005e+00
## 21597  4  421 2.189531e+00
## 21598  4  422 2.149473e+00
## 21599  4  423 2.104096e+00
## 21600  4  424 2.064433e+00
## 21601  4  425 2.029490e+00
## 21602  4  426 1.987342e+00
## 21603  4  427 1.956950e+00
## 21604  4  428 1.927354e+00
## 21605  4  429 1.894801e+00
## 21606  4  430 1.863725e+00
## 21607  4  431 1.837601e+00
## 21608  4  432 1.811480e+00
## 21609  4  433 2.254904e+00
## 21610  4  434 2.535376e+00
## 21611  4  435 2.633864e+00
## 21612  4  436 2.639336e+00
## 21613  4  437 2.606023e+00
## 21614  4  438 2.556643e+00
## 21615  4  439 2.502199e+00
## 21616  4  440 2.447446e+00
## 21617  4  441 2.389802e+00
## 21618  4  442 2.334701e+00
## 21619  4  443 2.285482e+00
## 21620  4  444 2.236531e+00
## 21621  4  445 2.185826e+00
## 21622  4  446 2.144804e+00
## 21623  4  447 2.098967e+00
## 21624  4  448 2.065951e+00
## 21625  4  449 2.029309e+00
## 21626  4  450 1.995455e+00
## 21627  4  451 1.956513e+00
## 21628  4  452 1.921502e+00
## 21629  4  453 1.894315e+00
## 21630  4  454 1.864230e+00
## 21631  4  455 1.834586e+00
## 21632  4  456 1.806811e+00
## 21633  4  457 2.260345e+00
## 21634  4  458 2.536604e+00
## 21635  4  459 2.632597e+00
## 21636  4  460 2.641080e+00
## 21637  4  461 2.606558e+00
## 21638  4  462 2.559643e+00
## 21639  4  463 2.499254e+00
## 21640  4  464 2.443446e+00
## 21641  4  465 2.389151e+00
## 21642  4  466 2.335275e+00
## 21643  4  467 2.280805e+00
## 21644  4  468 2.241011e+00
## 21645  4  469 2.190779e+00
## 21646  4  470 2.147273e+00
## 21647  4  471 2.105567e+00
## 21648  4  472 2.067023e+00
## 21649  4  473 2.024736e+00
## 21650  4  474 1.989404e+00
## 21651  4  475 1.955859e+00
## 21652  4  476 1.928065e+00
## 21653  4  477 1.892258e+00
## 21654  4  478 1.865347e+00
## 21655  4  479 1.839010e+00
## 21656  4  480 1.805470e+00
## 21657  4  481 2.257602e+00
## 21658  4  482 2.538594e+00
## 21659  4  483 2.630755e+00
## 21660  4  484 2.636378e+00
## 21661  4  485 2.608546e+00
## 21662  4  486 2.554976e+00
## 21663  4  487 2.498995e+00
## 21664  4  488 2.443286e+00
## 21665  4  489 2.390633e+00
## 21666  4  490 2.329454e+00
## 21667  4  491 2.281336e+00
## 21668  4  492 2.233687e+00
## 21669  4  493 2.189737e+00
## 21670  4  494 2.146193e+00
## 21671  4  495 2.103644e+00
## 21672  4  496 2.064919e+00
## 21673  4  497 2.029662e+00
## 21674  4  498 1.991522e+00
## 21675  4  499 1.955329e+00
## 21676  4  500 1.926444e+00
## 21677  4  501 1.894264e+00
## 21678  4  502 1.862690e+00
## 21679  4  503 1.834380e+00
## 21680  4  504 1.808750e+00
## 21681  4  505 2.259313e+00
## 21682  4  506 2.537806e+00
## 21683  4  507 2.632647e+00
## 21684  4  508 2.639233e+00
## 21685  4  509 2.602953e+00
## 21686  4  510 2.556456e+00
## 21687  4  511 2.499513e+00
## 21688  4  512 2.446008e+00
## 21689  4  513 2.389114e+00
## 21690  4  514 2.332723e+00
## 21691  4  515 2.283281e+00
## 21692  4  516 2.237887e+00
## 21693  4  517 2.192671e+00
## 21694  4  518 2.144421e+00
## 21695  4  519 2.104059e+00
## 21696  4  520 2.064833e+00
## 21697  4  521 2.023400e+00
## 21698  4  522 1.989462e+00
## 21699  4  523 1.957810e+00
## 21700  4  524 1.920918e+00
## 21701  4  525 1.890251e+00
## 21702  4  526 1.862038e+00
## 21703  4  527 1.833674e+00
## 21704  4  528 1.809105e+00
## 21705  4  529 2.257877e+00
## 21706  4  530 2.537127e+00
## 21707  4  531 2.633985e+00
## 21708  4  532 2.640238e+00
## 21709  4  533 2.602150e+00
## 21710  4  534 2.552836e+00
## 21711  4  535 2.496782e+00
## 21712  4  536 2.442645e+00
## 21713  4  537 2.386266e+00
## 21714  4  538 2.335190e+00
## 21715  4  539 2.283780e+00
## 21716  4  540 2.234561e+00
## 21717  4  541 2.189581e+00
## 21718  4  542 2.144689e+00
## 21719  4  543 2.101299e+00
## 21720  4  544 2.063180e+00
## 21721  4  545 2.025245e+00
## 21722  4  546 1.989659e+00
## 21723  4  547 1.957897e+00
## 21724  4  548 1.922497e+00
## 21725  4  549 1.892055e+00
## 21726  4  550 1.860261e+00
## 21727  4  551 1.836634e+00
## 21728  4  552 1.807485e+00
## 21729  4  553 2.255948e+00
## 21730  4  554 2.539688e+00
## 21731  4  555 2.633832e+00
## 21732  4  556 2.642576e+00
## 21733  4  557 2.605875e+00
## 21734  4  558 2.552400e+00
## 21735  4  559 2.496843e+00
## 21736  4  560 2.446827e+00
## 21737  4  561 2.388153e+00
## 21738  4  562 2.335600e+00
## 21739  4  563 2.287634e+00
## 21740  4  564 2.235819e+00
## 21741  4  565 2.186100e+00
## 21742  4  566 2.143028e+00
## 21743  4  567 2.104029e+00
## 21744  4  568 2.062457e+00
## 21745  4  569 2.022584e+00
## 21746  4  570 1.989600e+00
## 21747  4  571 1.955832e+00
## 21748  4  572 1.923587e+00
## 21749  4  573 1.888341e+00
## 21750  4  574 1.859173e+00
## 21751  4  575 1.834142e+00
## 21752  4  576 1.807446e+00
## 21753  4  577 2.253116e+00
## 21754  4  578 2.541994e+00
## 21755  4  579 2.630066e+00
## 21756  4  580 2.639754e+00
## 21757  4  581 2.606085e+00
## 21758  4  582 2.556645e+00
## 21759  4  583 2.502378e+00
## 21760  4  584 2.440135e+00
## 21761  4  585 2.386804e+00
## 21762  4  586 2.333483e+00
## 21763  4  587 2.283025e+00
## 21764  4  588 2.235166e+00
## 21765  4  589 2.185379e+00
## 21766  4  590 2.143367e+00
## 21767  4  591 2.102289e+00
## 21768  4  592 2.063035e+00
## 21769  4  593 2.021650e+00
## 21770  4  594 1.990583e+00
## 21771  4  595 1.954981e+00
## 21772  4  596 1.923512e+00
## 21773  4  597 1.887270e+00
## 21774  4  598 1.861581e+00
## 21775  4  599 1.835986e+00
## 21776  4  600 1.807764e+00
## 21777  4  601 2.254441e+00
## 21778  4  602 2.539874e+00
## 21779  4  603 2.623162e+00
## 21780  4  604 2.639008e+00
## 21781  4  605 2.603594e+00
## 21782  4  606 2.554257e+00
## 21783  4  607 2.502045e+00
## 21784  4  608 2.442586e+00
## 21785  4  609 2.387389e+00
## 21786  4  610 2.329780e+00
## 21787  4  611 2.286186e+00
## 21788  4  612 2.234476e+00
## 21789  4  613 2.191961e+00
## 21790  4  614 2.145074e+00
## 21791  4  615 2.105873e+00
## 21792  4  616 2.066477e+00
## 21793  4  617 2.026224e+00
## 21794  4  618 1.989880e+00
## 21795  4  619 1.958736e+00
## 21796  4  620 1.925519e+00
## 21797  4  621 1.893144e+00
## 21798  4  622 1.860729e+00
## 21799  4  623 1.835703e+00
## 21800  4  624 1.807184e+00
## 21801  4  624 1.808878e+00
## 21802  4  625 2.252783e+00
## 21803  4  626 2.537811e+00
## 21804  4  627 2.630053e+00
## 21805  4  628 2.634466e+00
## 21806  4  629 2.606618e+00
## 21807  4  630 2.557106e+00
## 21808  4  631 2.497206e+00
## 21809  4  632 2.443485e+00
## 21810  4  633 2.391009e+00
## 21811  4  634 2.335977e+00
## 21812  4  635 2.282790e+00
## 21813  4  636 2.231791e+00
## 21814  4  637 2.190333e+00
## 21815  4  638 2.143648e+00
## 21816  4  639 2.102611e+00
## 21817  4  640 2.061206e+00
## 21818  4  641 2.023496e+00
## 21819  4  642 1.987437e+00
## 21820  4  643 1.957052e+00
## 21821  4  644 1.922952e+00
## 21822  4  645 1.890706e+00
## 21823  4  646 1.862710e+00
## 21824  4  647 1.837080e+00
## 21825  4  648 1.805729e+00
## 21826  4  648 1.806437e+00
## 21827  4  649 1.778947e+00
## 21828  4  650 1.760110e+00
## 21829  4  651 1.732297e+00
## 21830  4  652 1.707493e+00
## 21831  4  653 1.689027e+00
## 21832  4  654 1.669114e+00
## 21833  4  654 1.668345e+00
## 21834  4  655 2.122694e+00
## 21835  4  656 2.411372e+00
## 21836  4  657 2.508140e+00
## 21837  4  658 2.520158e+00
## 21838  4  659 2.488473e+00
## 21839  4  660 2.450398e+00
## 21840  4  661 2.396758e+00
## 21841  4  662 2.343902e+00
## 21842  4  663 2.292653e+00
## 21843  4  664 2.240703e+00
## 21844  4  665 2.192967e+00
## 21845  4  666 2.149121e+00
## 21846  4  667 2.104841e+00
## 21847  4  668 2.064902e+00
## 21848  4  669 2.025592e+00
## 21849  4  670 1.989452e+00
## 21850  4  671 1.950373e+00
## 21851  4  672 1.918076e+00
## 21852  4  672 1.918910e+00
## 21853  4  673 2.363367e+00
## 21854  4  674 2.634925e+00
## 21855  4  675 2.728019e+00
## 21856  4  676 2.721068e+00
## 21857  4  677 2.687036e+00
## 21858  4  678 2.631366e+00
## 21859  4  679 2.571862e+00
## 21860  4  680 2.516076e+00
## 21861  4  681 2.455541e+00
## 21862  4  682 2.393799e+00
## 21863  4  683 2.341085e+00
## 21864  4  684 2.290893e+00
## 21865  4  685 2.240949e+00
## 21866  4  686 2.197091e+00
## 21867  4  687 2.148649e+00
## 21868  4  688 2.107964e+00
## 21869  4  689 2.068254e+00
## 21870  4  690 2.025846e+00
## 21871  4  691 1.995842e+00
## 21872  4  692 1.960237e+00
## 21873  4  693 1.926518e+00
## 21874  4  694 1.893112e+00
## 21875  4  695 1.867336e+00
## 21876  4  696 1.833586e+00
## 21877  4  697 2.279411e+00
## 21878  4  698 2.566407e+00
## 21879  4  699 2.662532e+00
## 21880  4  700 2.661323e+00
## 21881  4  701 2.630348e+00
## 21882  4  702 2.579731e+00
## 21883  4  703 2.521635e+00
## 21884  4  704 2.464416e+00
## 21885  4  705 2.401580e+00
## 21886  4  706 2.351039e+00
## 21887  4  707 2.302601e+00
## 21888  4  708 2.248206e+00
## 21889  4  709 2.205578e+00
## 21890  4  710 2.159578e+00
## 21891  4  711 2.120280e+00
## 21892  4  712 2.077704e+00
## 21893  4  713 2.039506e+00
## 21894  4  714 1.997558e+00
## 21895  4  715 1.963202e+00
## 21896  4  716 1.934686e+00
## 21897  4  717 1.901846e+00
## 21898  4  718 1.870432e+00
## 21899  4  719 1.842971e+00
## 21900  4  720 1.815533e+00
## 21901  4  721 2.264158e+00
## 21902  4  722 2.547183e+00
## 21903  4  723 2.641068e+00
## 21904  4  724 2.646628e+00
## 21905  4  725 2.611791e+00
## 21906  4  726 2.561989e+00
## 21907  4  727 2.504900e+00
## 21908  4  728 2.446375e+00
## 21909  4  729 2.393907e+00
## 21910  4  730 2.337148e+00
## 21911  4  731 2.286873e+00
## 21912  4  732 2.240982e+00
## 21913  4  733 2.193754e+00
## 21914  4  734 2.146805e+00
## 21915  4  735 2.109440e+00
## 21916  4  736 2.066940e+00
## 21917  4  737 2.031929e+00
## 21918  4  738 1.996552e+00
## 21919  4  739 1.962939e+00
## 21920  4  740 1.927342e+00
## 21921  4  741 1.897328e+00
## 21922  4  742 1.861958e+00
## 21923  4  743 1.839530e+00
## 21924  4  744 1.812496e+00
## 21925  4  745 2.259288e+00
## 21926  4  746 2.544611e+00
## 21927  4  747 2.632882e+00
## 21928  4  748 2.641343e+00
## 21929  4  749 2.605392e+00
## 21930  4  750 2.557681e+00
## 21931  4  751 2.503101e+00
## 21932  4  752 2.446285e+00
## 21933  4  753 2.390649e+00
## 21934  4  754 2.335664e+00
## 21935  4  755 2.286495e+00
## 21936  4  756 2.238482e+00
## 21937  4  757 2.191758e+00
## 21938  4  758 2.147945e+00
## 21939  4  759 2.105070e+00
## 21940  4  760 2.066195e+00
## 21941  4  761 2.028703e+00
## 21942  4  762 1.992738e+00
## 21943  4  763 1.958662e+00
## 21944  4  764 1.924881e+00
## 21945  4  765 1.895771e+00
## 21946  4  766 1.865665e+00
## 21947  4  767 1.835225e+00
## 21948  4  768 1.810322e+00
## 21949  4  769 2.259662e+00
## 21950  4  770 2.545591e+00
## 21951  4  771 2.636877e+00
## 21952  4  772 2.641128e+00
## 21953  4  773 2.604598e+00
## 21954  4  774 2.555129e+00
## 21955  4  775 2.502222e+00
## 21956  4  776 2.447433e+00
## 21957  4  777 2.391340e+00
## 21958  4  778 2.339263e+00
## 21959  4  779 2.282343e+00
## 21960  4  780 2.236681e+00
## 21961  4  781 2.193212e+00
## 21962  4  782 2.147694e+00
## 21963  4  783 2.106094e+00
## 21964  4  784 2.064851e+00
## 21965  4  785 2.029145e+00
## 21966  4  786 1.987066e+00
## 21967  4  787 1.959293e+00
## 21968  4  788 1.925141e+00
## 21969  4  789 1.895832e+00
## 21970  4  790 1.863931e+00
## 21971  4  791 1.835994e+00
## 21972  4  792 1.807857e+00
## 21973  4  793 2.261428e+00
## 21974  4  794 2.538281e+00
## 21975  4  795 2.639062e+00
## 21976  4  796 2.639206e+00
## 21977  4  797 2.603120e+00
## 21978  4  798 2.562187e+00
## 21979  4  799 2.496407e+00
## 21980  4  800 2.447378e+00
## 21981  4  801 2.388916e+00
## 21982  4  802 2.338475e+00
## 21983  4  803 2.284403e+00
## 21984  4  804 2.240153e+00
## 21985  4  805 2.190505e+00
## 21986  4  806 2.149580e+00
## 21987  4  807 2.104496e+00
## 21988  4  808 2.066484e+00
## 21989  4  809 2.027065e+00
## 21990  4  810 1.992696e+00
## 21991  4  811 1.958081e+00
## 21992  4  812 1.924113e+00
## 21993  4  813 1.890426e+00
## 21994  4  814 1.864217e+00
## 21995  4  815 1.836785e+00
## 21996  4  816 1.805025e+00
## 21997  4  817 2.259949e+00
## 21998  4  818 2.538611e+00
## 21999  4  819 2.632023e+00
## 22000  4  820 2.640031e+00
## 22001  4  821 2.609636e+00
## 22002  4  822 2.558832e+00
## 22003  4  823 2.500448e+00
## 22004  4  824 2.448226e+00
## 22005  4  825 2.389105e+00
## 22006  4  826 2.335018e+00
## 22007  4  827 2.285599e+00
## 22008  4  828 2.238517e+00
## 22009  4  829 2.189003e+00
## 22010  4  830 2.144987e+00
## 22011  4  831 2.101609e+00
## 22012  4  832 2.062770e+00
## 22013  4  833 2.026594e+00
## 22014  4  834 1.990720e+00
## 22015  4  835 1.958632e+00
## 22016  4  836 1.927153e+00
## 22017  4  837 1.894825e+00
## 22018  4  838 1.862614e+00
## 22019  4  839 1.835234e+00
## 22020  4  840 1.808740e+00
## 22021  4  841 2.256463e+00
## 22022  4  842 2.540854e+00
## 22023  4  843 2.630809e+00
## 22024  4  844 2.639822e+00
## 22025  4  845 2.604480e+00
## 22026  4  846 2.555126e+00
## 22027  4  847 2.498412e+00
## 22028  4  848 2.442169e+00
## 22029  4  849 2.387836e+00
## 22030  4  850 2.333471e+00
## 22031  4  851 2.283554e+00
## 22032  4  852 2.235050e+00
## 22033  4  853 2.191025e+00
## 22034  4  854 2.144418e+00
## 22035  4  855 2.102990e+00
## 22036  4  856 2.061891e+00
## 22037  4  857 2.026037e+00
## 22038  4  858 1.989293e+00
## 22039  4  859 1.953906e+00
## 22040  4  860 1.925584e+00
## 22041  4  861 1.893723e+00
## 22042  4  862 1.864783e+00
## 22043  4  863 1.834155e+00
## 22044  4  864 1.810307e+00
## 22045  4  865 2.260117e+00
## 22046  4  866 2.539878e+00
## 22047  4  867 2.631099e+00
## 22048  4  868 2.640179e+00
## 22049  4  869 2.604096e+00
## 22050  4  870 2.555242e+00
## 22051  4  871 2.503689e+00
## 22052  4  872 2.445113e+00
## 22053  4  873 2.388495e+00
## 22054  4  874 2.334486e+00
## 22055  4  875 2.282101e+00
## 22056  4  876 2.239041e+00
## 22057  4  877 2.193964e+00
## 22058  4  878 2.148880e+00
## 22059  4  879 2.104983e+00
## 22060  4  880 2.065026e+00
## 22061  4  881 2.030879e+00
## 22062  4  882 1.991889e+00
## 22063  4  883 1.957977e+00
## 22064  4  884 1.924786e+00
## 22065  4  885 1.897574e+00
## 22066  4  886 1.861194e+00
## 22067  4  887 1.833103e+00
## 22068  4  888 1.805026e+00
## 22069  4  889 2.254530e+00
## 22070  4  890 2.543036e+00
## 22071  4  891 2.631547e+00
## 22072  4  892 2.640844e+00
## 22073  4  893 2.602266e+00
## 22074  4  894 2.552761e+00
## 22075  4  895 2.498371e+00
## 22076  4  896 2.442319e+00
## 22077  4  897 2.387861e+00
## 22078  4  898 2.336808e+00
## 22079  4  899 2.285735e+00
## 22080  4  900 2.236640e+00
## 22081  4  901 2.190748e+00
## 22082  4  902 2.146131e+00
## 22083  4  903 2.105844e+00
## 22084  4  904 2.063394e+00
## 22085  4  905 2.025238e+00
## 22086  4  906 1.989617e+00
## 22087  4  907 1.955723e+00
## 22088  4  908 1.923928e+00
## 22089  4  909 1.892235e+00
## 22090  4  910 1.863367e+00
## 22091  4  911 1.837325e+00
## 22092  4  912 1.809208e+00
## 22093  4  913 2.255245e+00
## 22094  4  914 2.539581e+00
## 22095  4  915 2.633714e+00
## 22096  4  916 2.637743e+00
## 22097  4  917 2.601839e+00
## 22098  4  918 2.555715e+00
## 22099  4  919 2.501833e+00
## 22100  4  920 2.443270e+00
## 22101  4  921 2.391712e+00
## 22102  4  922 2.336017e+00
## 22103  4  923 2.288293e+00
## 22104  4  924 2.240329e+00
## 22105  4  925 2.190606e+00
## 22106  4  926 2.146457e+00
## 22107  4  927 2.102374e+00
## 22108  4  928 2.065686e+00
## 22109  4  929 2.026278e+00
## 22110  4  930 1.988642e+00
## 22111  4  931 1.954174e+00
## 22112  4  932 1.922037e+00
## 22113  4  933 1.892326e+00
## 22114  4  934 1.861660e+00
## 22115  4  935 1.835155e+00
## 22116  4  936 1.807979e+00
## 22117  4  936 1.809195e+00
## 22118  4  937 2.254536e+00
## 22119  4  938 2.538511e+00
## 22120  4  939 2.636853e+00
## 22121  4  940 2.638358e+00
## 22122  4  941 2.607279e+00
## 22123  4  942 2.550812e+00
## 22124  4  943 2.499851e+00
## 22125  4  944 2.437702e+00
## 22126  4  945 2.388186e+00
## 22127  4  946 2.332830e+00
## 22128  4  947 2.281060e+00
## 22129  4  948 2.238227e+00
## 22130  4  949 2.187942e+00
## 22131  4  950 2.144326e+00
## 22132  4  951 2.101639e+00
## 22133  4  952 2.064681e+00
## 22134  4  953 2.025143e+00
## 22135  4  954 1.987987e+00
## 22136  4  955 1.957497e+00
## 22137  4  956 1.922039e+00
## 22138  4  957 1.895258e+00
## 22139  4  958 1.864320e+00
## 22140  4  959 1.836716e+00
## 22141  4  960 1.808715e+00
## 22142  4  960 1.807144e+00
## 22143  4  961 1.780824e+00
## 22144  4  962 1.756458e+00
## 22145  4  963 1.733195e+00
## 22146  4  964 1.709962e+00
## 22147  4  965 1.690516e+00
## 22148  4  966 1.668417e+00
## 22149  4  967 1.648923e+00
## 22150  4  968 1.629192e+00
## 22151  4  969 1.612823e+00
## 22152  4  969 1.610975e+00
## 22153  4  970 2.068896e+00
## 22154  4  971 2.357839e+00
## 22155  4  972 2.455847e+00
## 22156  4  973 2.473067e+00
## 22157  4  974 2.442478e+00
## 22158  4  975 2.400980e+00
## 22159  4  976 2.351653e+00
## 22160  4  977 2.298654e+00
## 22161  4  978 2.247266e+00
## 22162  4  979 2.205085e+00
## 22163  4  980 2.157730e+00
## 22164  4  981 2.109051e+00
## 22165  4  982 2.073045e+00
## 22166  4  983 2.027817e+00
## 22167  4  984 1.990763e+00
## 22168  4  984 1.992371e+00
## 22169  4  985 2.432783e+00
## 22170  4  986 2.700958e+00
## 22171  4  987 2.786662e+00
## 22172  4  988 2.787575e+00
## 22173  4  989 2.741404e+00
## 22174  4  990 2.685253e+00
## 22175  4  991 2.616797e+00
## 22176  4  992 2.558665e+00
## 22177  4  993 2.495612e+00
## 22178  4  994 2.437772e+00
## 22179  4  995 2.384540e+00
## 22180  4  996 2.325685e+00
## 22181  4  997 2.277611e+00
## 22182  4  998 2.226135e+00
## 22183  4  999 2.184243e+00
## 22184  4 1000 2.136189e+00
## 22185  4 1001 2.093931e+00
## 22186  4 1002 2.054883e+00
## 22187  4 1003 2.019186e+00
## 22188  4 1004 1.978726e+00
## 22189  4 1005 1.948961e+00
## 22190  4 1006 1.914042e+00
## 22191  4 1007 1.883132e+00
## 22192  4 1008 1.851822e+00
## 22193  4 1009 2.299585e+00
## 22194  4 1010 2.578922e+00
## 22195  4 1011 2.670709e+00
## 22196  4 1012 2.674146e+00
## 22197  4 1013 2.640193e+00
## 22198  4 1014 2.589571e+00
## 22199  4 1015 2.526910e+00
## 22200  4 1016 2.472928e+00
## 22201  4 1017 2.417252e+00
## 22202  4 1018 2.361221e+00
## 22203  4 1019 2.312220e+00
## 22204  4 1020 2.260638e+00
## 22205  4 1021 2.215458e+00
## 22206  4 1022 2.170681e+00
## 22207  4 1023 2.124114e+00
## 22208  4 1024 2.085518e+00
## 22209  4 1025 2.044612e+00
## 22210  4 1026 2.007788e+00
## 22211  4 1027 1.971250e+00
## 22212  4 1028 1.943547e+00
## 22213  4 1029 1.909152e+00
## 22214  4 1030 1.877628e+00
## 22215  4 1031 1.846984e+00
## 22216  4 1032 1.818882e+00
## 22217  4 1033 2.273344e+00
## 22218  4 1034 2.552110e+00
## 22219  4 1035 2.644237e+00
## 22220  4 1036 2.650065e+00
## 22221  4 1037 2.623332e+00
## 22222  4 1038 2.569624e+00
## 22223  4 1039 2.505043e+00
## 22224  4 1040 2.458421e+00
## 22225  4 1041 2.401183e+00
## 22226  4 1042 2.346608e+00
## 22227  4 1043 2.295514e+00
## 22228  4 1044 2.242878e+00
## 22229  4 1045 2.192888e+00
## 22230  4 1046 2.154960e+00
## 22231  4 1047 2.109841e+00
## 22232  4 1048 2.064707e+00
## 22233  4 1049 2.032953e+00
## 22234  4 1050 1.995885e+00
## 22235  4 1051 1.959354e+00
## 22236  4 1052 1.934645e+00
## 22237  4 1053 1.898049e+00
## 22238  4 1054 1.871795e+00
## 22239  4 1055 1.842997e+00
## 22240  4 1056 1.810821e+00
## 22241  4 1057 2.261832e+00
## 22242  4 1058 2.547410e+00
## 22243  4 1059 2.640475e+00
## 22244  4 1060 2.642679e+00
## 22245  4 1061 2.611578e+00
## 22246  4 1062 2.562799e+00
## 22247  4 1063 2.506154e+00
## 22248  4 1064 2.449077e+00
## 22249  4 1065 2.397303e+00
## 22250  4 1066 2.338557e+00
## 22251  4 1067 2.289252e+00
## 22252  4 1068 2.235004e+00
## 22253  4 1069 2.192036e+00
## 22254  4 1070 2.148634e+00
## 22255  4 1071 2.106023e+00
## 22256  4 1072 2.070951e+00
## 22257  4 1073 2.030950e+00
## 22258  4 1074 1.990137e+00
## 22259  4 1075 1.960178e+00
## 22260  4 1076 1.927013e+00
## 22261  4 1077 1.897028e+00
## 22262  4 1078 1.865909e+00
## 22263  4 1079 1.838396e+00
## 22264  4 1080 1.810696e+00
## 22265  4 1081 2.264658e+00
## 22266  4 1082 2.538422e+00
## 22267  4 1083 2.631309e+00
## 22268  4 1084 2.638285e+00
## 22269  4 1085 2.609052e+00
## 22270  4 1086 2.561905e+00
## 22271  4 1087 2.504837e+00
## 22272  4 1088 2.446218e+00
## 22273  4 1089 2.391056e+00
```

```
## 22274  4 1090 2.336956e+00
## 22275  4 1091 2.289342e+00
## 22276  4 1092 2.235831e+00
## 22277  4 1093 2.191919e+00
## 22278  4 1094 2.148997e+00
## 22279  4 1095 2.103348e+00
## 22280  4 1096 2.066612e+00
## 22281  4 1097 2.028110e+00
## 22282  4 1098 1.993355e+00
## 22283  4 1099 1.959506e+00
## 22284  4 1100 1.925621e+00
## 22285  4 1101 1.895929e+00
## 22286  4 1102 1.866804e+00
## 22287  4 1103 1.838732e+00
## 22288  4 1104 1.811359e+00
## 22289  4 1105 2.258953e+00
## 22290  4 1106 2.535530e+00
## 22291  4 1107 2.632348e+00
## 22292  4 1108 2.639463e+00
## 22293  4 1109 2.607706e+00
## 22294  4 1110 2.558779e+00
## 22295  4 1111 2.504067e+00
## 22296  4 1112 2.451501e+00
## 22297  4 1113 2.391726e+00
## 22298  4 1114 2.339533e+00
## 22299  4 1115 2.285942e+00
## 22300  4 1116 2.240114e+00
## 22301  4 1117 2.190298e+00
## 22302  4 1118 2.151554e+00
## 22303  4 1119 2.104261e+00
## 22304  4 1120 2.065229e+00
## 22305  4 1121 2.027114e+00
## 22306  4 1122 1.992301e+00
## 22307  4 1123 1.958640e+00
## 22308  4 1124 1.928514e+00
## 22309  4 1125 1.893758e+00
## 22310  4 1126 1.863855e+00
## 22311  4 1127 1.839240e+00
## 22312  4 1128 1.809889e+00
## 22313  4 1129 2.262635e+00
## 22314  4 1130 2.543452e+00
## 22315  4 1131 2.633840e+00
## 22316  4 1132 2.639327e+00
## 22317  4 1133 2.606706e+00
## 22318  4 1134 2.560520e+00
## 22319  4 1135 2.499344e+00
## 22320  4 1136 2.447625e+00
## 22321  4 1137 2.393195e+00
## 22322  4 1138 2.333880e+00
## 22323  4 1139 2.288423e+00
## 22324  4 1140 2.234882e+00
## 22325  4 1141 2.188756e+00
## 22326  4 1142 2.147616e+00
## 22327  4 1143 2.105625e+00
## 22328  4 1144 2.063762e+00
## 22329  4 1145 2.026928e+00
## 22330  4 1146 1.994265e+00
## 22331  4 1147 1.960144e+00
## 22332  4 1148 1.925182e+00
## 22333  4 1149 1.888586e+00
## 22334  4 1150 1.869255e+00
## 22335  4 1151 1.838540e+00
## 22336  4 1152 1.811530e+00
## 22337  4 1153 2.258792e+00
## 22338  4 1154 2.543223e+00
## 22339  4 1155 2.634353e+00
## 22340  4 1156 2.641037e+00
## 22341  4 1157 2.608519e+00
## 22342  4 1158 2.558423e+00
## 22343  4 1159 2.504200e+00
## 22344  4 1160 2.447375e+00
## 22345  4 1161 2.386008e+00
## 22346  4 1162 2.336338e+00
## 22347  4 1163 2.285316e+00
## 22348  4 1164 2.234796e+00
## 22349  4 1165 2.193171e+00
## 22350  4 1166 2.146949e+00
## 22351  4 1167 2.103571e+00
## 22352  4 1168 2.064606e+00
## 22353  4 1169 2.029051e+00
## 22354  4 1170 1.994449e+00
## 22355  4 1171 1.955617e+00
## 22356  4 1172 1.927635e+00
## 22357  4 1173 1.889848e+00
## 22358  4 1174 1.860801e+00
## 22359  4 1175 1.837946e+00
## 22360  4 1176 1.808804e+00
## 22361  4 1177 2.260632e+00
## 22362  4 1178 2.539072e+00
## 22363  4 1179 2.635267e+00
## 22364  4 1180 2.643016e+00
## 22365  4 1181 2.608652e+00
## 22366  4 1182 2.558930e+00
## 22367  4 1183 2.501338e+00
## 22368  4 1184 2.448951e+00
## 22369  4 1185 2.385493e+00
## 22370  4 1186 2.333976e+00
## 22371  4 1187 2.283018e+00
## 22372  4 1188 2.235688e+00
## 22373  4 1189 2.189921e+00
## 22374  4 1190 2.144122e+00
## 22375  4 1191 2.109232e+00
## 22376  4 1192 2.066253e+00
## 22377  4 1193 2.025755e+00
## 22378  4 1194 1.991938e+00
## 22379  4 1195 1.955972e+00
## 22380  4 1196 1.926648e+00
## 22381  4 1197 1.894108e+00
## 22382  4 1198 1.868253e+00
## 22383  4 1199 1.835919e+00
## 22384  4 1200 1.810751e+00
## 22385  4 1201 2.259461e+00
## 22386  4 1202 2.534001e+00
## 22387  4 1203 2.635893e+00
## 22388  4 1204 2.642276e+00
## 22389  4 1205 2.602694e+00
## 22390  4 1206 2.554016e+00
## 22391  4 1207 2.508844e+00
## 22392  4 1208 2.444022e+00
## 22393  4 1209 2.390247e+00
## 22394  4 1210 2.339125e+00
## 22395  4 1211 2.285474e+00
## 22396  4 1212 2.239191e+00
## 22397  4 1213 2.188821e+00
## 22398  4 1214 2.145734e+00
## 22399  4 1215 2.104051e+00
## 22400  4 1216 2.063492e+00
## 22401  4 1217 2.028455e+00
## 22402  4 1218 1.988090e+00
## 22403  4 1219 1.957094e+00
## 22404  4 1220 1.929038e+00
## 22405  4 1221 1.891835e+00
## 22406  4 1222 1.864604e+00
## 22407  4 1223 1.836036e+00
## 22408  4 1224 1.807789e+00
## 22409  4 1225 2.258025e+00
## 22410  4 1226 2.537771e+00
## 22411  4 1227 2.628667e+00
## 22412  4 1228 2.636640e+00
## 22413  4 1229 2.608888e+00
## 22414  4 1230 2.556175e+00
## 22415  4 1231 2.497769e+00
## 22416  4 1232 2.446221e+00
## 22417  4 1233 2.384357e+00
## 22418  4 1234 2.334658e+00
## 22419  4 1235 2.287480e+00
## 22420  4 1236 2.236380e+00
## 22421  4 1237 2.188805e+00
## 22422  4 1238 2.148890e+00
## 22423  4 1239 2.100113e+00
## 22424  4 1240 2.066152e+00
## 22425  4 1241 2.031460e+00
## 22426  4 1242 1.989938e+00
## 22427  4 1243 1.958963e+00
## 22428  4 1244 1.924292e+00
## 22429  4 1245 1.893502e+00
## 22430  4 1246 1.861870e+00
## 22431  4 1247 1.833477e+00
## 22432  4 1248 1.808699e+00
## 22433  4 1248 1.805641e+00
## 22434  4 1249 2.256415e+00
## 22435  4 1250 2.541430e+00
## 22436  4 1251 2.634422e+00
## 22437  4 1252 2.635763e+00
## 22438  4 1253 2.603284e+00
## 22439  4 1254 2.554811e+00
## 22440  4 1255 2.501067e+00
## 22441  4 1256 2.443395e+00
## 22442  4 1257 2.392123e+00
## 22443  4 1258 2.332787e+00
## 22444  4 1259 2.285632e+00
## 22445  4 1260 2.235445e+00
## 22446  4 1261 2.189759e+00
## 22447  4 1262 2.144319e+00
## 22448  4 1263 2.102984e+00
## 22449  4 1264 2.067507e+00
## 22450  4 1265 2.025854e+00
## 22451  4 1266 1.987122e+00
## 22452  4 1267 1.952946e+00
## 22453  4 1268 1.922245e+00
## 22454  4 1269 1.890383e+00
## 22455  4 1270 1.862201e+00
## 22456  4 1271 1.832189e+00
## 22457  4 1272 1.807541e+00
## 22458  4 1272 1.806866e+00
## 22459  4 1273 1.778770e+00
## 22460  4 1274 1.754239e+00
## 22461  4 1275 1.735189e+00
## 22462  4 1276 1.707005e+00
## 22463  4 1277 1.689906e+00
## 22464  4 1278 1.667729e+00
## 22465  4 1279 1.645706e+00
## 22466  4 1280 1.631388e+00
## 22467  4 1281 1.612080e+00
## 22468  4 1282 1.591130e+00
## 22469  4 1283 1.575737e+00
## 22470  4 1284 1.560864e+00
## 22471  4 1284 1.561626e+00
## 22472  4 1285 2.020991e+00
## 22473  4 1286 2.308399e+00
## 22474  4 1287 2.414838e+00
## 22475  4 1288 2.428586e+00
## 22476  4 1289 2.405407e+00
## 22477  4 1290 2.357905e+00
## 22478  4 1291 2.312512e+00
## 22479  4 1292 2.263704e+00
## 22480  4 1293 2.219599e+00
## 22481  4 1294 2.169490e+00
## 22482  4 1295 2.118647e+00
## 22483  4 1296 2.078189e+00
## 22484  4 1296 2.078556e+00
## 22485  4 1297 2.514914e+00
## 22486  4 1298 2.777807e+00
## 22487  4 1299 2.858861e+00
## 22488  4 1300 2.854845e+00
## 22489  4 1301 2.808190e+00
## 22490  4 1302 2.744935e+00
## 22491  4 1303 2.678522e+00
## 22492  4 1304 2.610323e+00
## 22493  4 1305 2.544979e+00
## 22494  4 1306 2.487542e+00
## 22495  4 1307 2.428457e+00
## 22496  4 1308 2.367794e+00
## 22497  4 1309 2.316483e+00
## 22498  4 1310 2.267836e+00
## 22499  4 1311 2.215182e+00
## 22500  4 1312 2.172442e+00
## 22501  4 1313 2.125877e+00
## 22502  4 1314 2.087559e+00
## 22503  4 1315 2.045796e+00
## 22504  4 1316 2.008421e+00
## 22505  4 1317 1.972979e+00
## 22506  4 1318 1.938539e+00
## 22507  4 1319 1.909912e+00
## 22508  4 1320 1.874190e+00
## 22509  4 1321 2.321789e+00
## 22510  4 1322 2.598685e+00
## 22511  4 1323 2.692354e+00
## 22512  4 1324 2.690827e+00
## 22513  4 1325 2.649270e+00
## 22514  4 1326 2.604694e+00
## 22515  4 1327 2.548934e+00
## 22516  4 1328 2.487682e+00
## 22517  4 1329 2.431265e+00
## 22518  4 1330 2.372994e+00
## 22519  4 1331 2.317804e+00
## 22520  4 1332 2.270191e+00
## 22521  4 1333 2.220425e+00
## 22522  4 1334 2.180750e+00
## 22523  4 1335 2.132363e+00
## 22524  4 1336 2.092878e+00
## 22525  4 1337 2.052979e+00
## 22526  4 1338 2.013237e+00
## 22527  4 1339 1.982299e+00
## 22528  4 1340 1.949317e+00
## 22529  4 1341 1.917569e+00
## 22530  4 1342 1.883390e+00
## 22531  4 1343 1.856391e+00
## 22532  4 1344 1.826227e+00
## 22533  4 1345 2.275410e+00
## 22534  4 1346 2.553533e+00
## 22535  4 1347 2.654277e+00
## 22536  4 1348 2.660752e+00
## 22537  4 1349 2.619531e+00
## 22538  4 1350 2.571202e+00
## 22539  4 1351 2.512030e+00
## 22540  4 1352 2.458049e+00
## 22541  4 1353 2.402431e+00
## 22542  4 1354 2.353263e+00
## 22543  4 1355 2.297233e+00
## 22544  4 1356 2.248651e+00
## 22545  4 1357 2.202522e+00
## 22546  4 1358 2.155171e+00
## 22547  4 1359 2.114851e+00
## 22548  4 1360 2.074576e+00
## 22549  4 1361 2.032977e+00
## 22550  4 1362 1.999475e+00
## 22551  4 1363 1.965450e+00
## 22552  4 1364 1.932734e+00
## 22553  4 1365 1.900861e+00
## 22554  4 1366 1.872796e+00
## 22555  4 1367 1.843468e+00
## 22556  4 1368 1.817744e+00
## 22557  4 1369 2.260414e+00
## 22558  4 1370 2.546291e+00
## 22559  4 1371 2.643522e+00
## 22560  4 1372 2.647451e+00
## 22561  4 1373 2.616065e+00
## 22562  4 1374 2.566025e+00
## 22563  4 1375 2.510207e+00
## 22564  4 1376 2.449162e+00
## 22565  4 1377 2.396040e+00
## 22566  4 1378 2.339009e+00
## 22567  4 1379 2.290152e+00
## 22568  4 1380 2.244556e+00
## 22569  4 1381 2.189181e+00
## 22570  4 1382 2.152695e+00
## 22571  4 1383 2.111980e+00
## 22572  4 1384 2.070079e+00
## 22573  4 1385 2.031683e+00
## 22574  4 1386 1.999899e+00
## 22575  4 1387 1.960988e+00
## 22576  4 1388 1.927902e+00
## 22577  4 1389 1.899478e+00
## 22578  4 1390 1.868939e+00
## 22579  4 1391 1.841893e+00
## 22580  4 1392 1.814328e+00
## 22581  4 1393 2.265400e+00
## 22582  4 1394 2.543905e+00
## 22583  4 1395 2.638477e+00
## 22584  4 1396 2.644649e+00
## 22585  4 1397 2.609326e+00
## 22586  4 1398 2.566174e+00
## 22587  4 1399 2.504696e+00
## 22588  4 1400 2.450943e+00
## 22589  4 1401 2.397425e+00
## 22590  4 1402 2.338226e+00
## 22591  4 1403 2.288379e+00
## 22592  4 1404 2.241911e+00
## 22593  4 1405 2.190406e+00
## 22594  4 1406 2.152654e+00
## 22595  4 1407 2.109289e+00
## 22596  4 1408 2.067594e+00
## 22597  4 1409 2.029376e+00
## 22598  4 1410 1.995867e+00
## 22599  4 1411 1.962320e+00
## 22600  4 1412 1.931381e+00
## 22601  4 1413 1.895354e+00
## 22602  4 1414 1.868125e+00
## 22603  4 1415 1.839424e+00
## 22604  4 1416 1.810418e+00
## 22605  4 1417 2.258666e+00
## 22606  4 1418 2.539120e+00
## 22607  4 1419 2.634446e+00
## 22608  4 1420 2.643893e+00
## 22609  4 1421 2.610566e+00
## 22610  4 1422 2.562307e+00
## 22611  4 1423 2.506165e+00
## 22612  4 1424 2.445952e+00
## 22613  4 1425 2.389548e+00
## 22614  4 1426 2.337698e+00
## 22615  4 1427 2.286207e+00
## 22616  4 1428 2.238387e+00
## 22617  4 1429 2.193321e+00
## 22618  4 1430 2.152286e+00
## 22619  4 1431 2.106797e+00
## 22620  4 1432 2.068754e+00
## 22621  4 1433 2.033126e+00
## 22622  4 1434 1.991427e+00
## 22623  4 1435 1.959617e+00
## 22624  4 1436 1.925261e+00
## 22625  4 1437 1.895373e+00
## 22626  4 1438 1.865230e+00
## 22627  4 1439 1.837191e+00
## 22628  4 1440 1.807979e+00
## 22629  4 1441 2.258380e+00
## 22630  4 1442 2.542876e+00
## 22631  4 1443 2.639811e+00
## 22632  4 1444 2.646730e+00
## 22633  4 1445 2.610612e+00
## 22634  4 1446 2.561681e+00
## 22635  4 1447 2.505129e+00
## 22636  4 1448 2.446668e+00
## 22637  4 1449 2.393592e+00
## 22638  4 1450 2.336275e+00
## 22639  4 1451 2.286131e+00
## 22640  4 1452 2.237938e+00
## 22641  4 1453 2.191274e+00
## 22642  4 1454 2.149197e+00
## 22643  4 1455 2.105581e+00
## 22644  4 1456 2.067233e+00
## 22645  4 1457 2.029633e+00
## 22646  4 1458 1.993503e+00
## 22647  4 1459 1.961878e+00
## 22648  4 1460 1.925355e+00
## 22649  4 1461 1.896367e+00
## 22650  4 1462 1.864457e+00
## 22651  4 1463 1.840588e+00
## 22652  4 1464 1.810194e+00
## 22653  4 1465 2.259471e+00
## 22654  4 1466 2.533571e+00
## 22655  4 1467 2.636172e+00
## 22656  4 1468 2.642786e+00
## 22657  4 1469 2.607166e+00
## 22658  4 1470 2.558615e+00
## 22659  4 1471 2.503654e+00
## 22660  4 1472 2.450557e+00
## 22661  4 1473 2.392277e+00
## 22662  4 1474 2.337946e+00
## 22663  4 1475 2.288412e+00
## 22664  4 1476 2.239377e+00
## 22665  4 1477 2.191101e+00
## 22666  4 1478 2.149841e+00
## 22667  4 1479 2.106169e+00
## 22668  4 1480 2.066762e+00
## 22669  4 1481 2.029852e+00
## 22670  4 1482 1.994462e+00
## 22671  4 1483 1.961636e+00
## 22672  4 1484 1.927249e+00
## 22673  4 1485 1.893446e+00
## 22674  4 1486 1.867089e+00
## 22675  4 1487 1.836412e+00
## 22676  4 1488 1.811644e+00
## 22677  4 1489 2.257714e+00
## 22678  4 1490 2.547046e+00
## 22679  4 1491 2.636777e+00
## 22680  4 1492 2.637048e+00
## 22681  4 1493 2.609622e+00
## 22682  4 1494 2.560317e+00
## 22683  4 1495 2.501415e+00
## 22684  4 1496 2.445402e+00
## 22685  4 1497 2.391211e+00
## 22686  4 1498 2.337689e+00
## 22687  4 1499 2.287073e+00
## 22688  4 1500 2.240133e+00
## 22689  4 1501 2.195146e+00
## 22690  4 1502 2.146780e+00
## 22691  4 1503 2.106554e+00
## 22692  4 1504 2.065784e+00
## 22693  4 1505 2.026592e+00
## 22694  4 1506 1.993305e+00
## 22695  4 1507 1.956351e+00
## 22696  4 1508 1.924091e+00
## 22697  4 1509 1.888965e+00
## 22698  4 1510 1.863671e+00
## 22699  4 1511 1.837920e+00
## 22700  4 1512 1.810710e+00
## 22701  4 1513 2.258213e+00
## 22702  4 1514 2.539706e+00
## 22703  4 1515 2.637424e+00
## 22704  4 1516 2.641190e+00
## 22705  4 1517 2.608926e+00
## 22706  4 1518 2.557132e+00
## 22707  4 1519 2.499167e+00
## 22708  4 1520 2.446551e+00
## 22709  4 1521 2.394844e+00
## 22710  4 1522 2.333311e+00
## 22711  4 1523 2.287677e+00
## 22712  4 1524 2.238418e+00
## 22713  4 1525 2.191578e+00
## 22714  4 1526 2.146806e+00
## 22715  4 1527 2.106170e+00
## 22716  4 1528 2.064501e+00
## 22717  4 1529 2.028484e+00
## 22718  4 1530 1.991661e+00
## 22719  4 1531 1.956978e+00
## 22720  4 1532 1.923809e+00
## 22721  4 1533 1.896258e+00
## 22722  4 1534 1.866015e+00
## 22723  4 1535 1.837055e+00
## 22724  4 1536 1.812466e+00
## 22725  4 1537 2.258632e+00
## 22726  4 1538 2.542971e+00
## 22727  4 1539 2.635453e+00
## 22728  4 1540 2.642440e+00
## 22729  4 1541 2.602692e+00
## 22730  4 1542 2.555003e+00
## 22731  4 1543 2.499885e+00
## 22732  4 1544 2.445950e+00
## 22733  4 1545 2.388819e+00
## 22734  4 1546 2.342754e+00
## 22735  4 1547 2.285982e+00
## 22736  4 1548 2.237165e+00
## 22737  4 1549 2.194317e+00
## 22738  4 1550 2.145834e+00
## 22739  4 1551 2.103348e+00
## 22740  4 1552 2.065584e+00
## 22741  4 1553 2.025689e+00
## 22742  4 1554 1.992327e+00
## 22743  4 1555 1.958221e+00
## 22744  4 1556 1.925141e+00
## 22745  4 1557 1.895693e+00
## 22746  4 1558 1.865499e+00
## 22747  4 1559 1.834272e+00
## 22748  4 1560 1.810314e+00
## 22749  4 1560 1.807854e+00
## 22750  4 1561 2.252307e+00
## 22751  4 1562 2.536771e+00
## 22752  4 1563 2.632760e+00
## 22753  4 1564 2.640243e+00
## 22754  4 1565 2.609767e+00
## 22755  4 1566 2.553433e+00
## 22756  4 1567 2.499475e+00
## 22757  4 1568 2.442820e+00
## 22758  4 1569 2.387493e+00
## 22759  4 1570 2.336944e+00
## 22760  4 1571 2.280081e+00
## 22761  4 1572 2.237044e+00
## 22762  4 1573 2.187627e+00
## 22763  4 1574 2.141174e+00
## 22764  4 1575 2.102959e+00
## 22765  4 1576 2.063482e+00
## 22766  4 1577 2.026381e+00
## 22767  4 1578 1.992332e+00
## 22768  4 1579 1.954041e+00
## 22769  4 1580 1.921714e+00
## 22770  4 1581 1.891681e+00
## 22771  4 1582 1.866483e+00
## 22772  4 1583 1.834084e+00
## 22773  4 1584 1.809921e+00
## 22774  4 1584 1.807828e+00
## 22775  4 1585 1.782090e+00
## 22776  4 1586 1.756656e+00
## 22777  4 1587 1.732003e+00
## 22778  4 1588 1.709756e+00
## 22779  4 1589 1.690366e+00
## 22780  4 1590 1.669878e+00
## 22781  4 1591 1.647665e+00
## 22782  4 1592 1.630158e+00
## 22783  4 1593 1.609893e+00
## 22784  4 1594 1.597117e+00
## 22785  4 1595 1.576847e+00
## 22786  4 1596 1.560689e+00
## 22787  4 1597 1.543639e+00
## 22788  4 1598 1.531992e+00
## 22789  4 1599 1.514673e+00
## 22790  4 1599 1.514760e+00
## 22791  4 1600 1.739644e+00
## 22792  4 1601 1.879633e+00
## 22793  4 1602 1.925879e+00
## 22794  4 1603 1.928411e+00
## 22795  4 1604 1.909996e+00
## 22796  4 1605 1.882756e+00
## 22797  4 1606 1.854822e+00
## 22798  4 1607 1.822486e+00
## 22799  4 1608 1.793927e+00
## 22800  4 1608 1.794451e+00
## 22801  4 1609 2.242170e+00
## 22802  4 1610 2.518048e+00
## 22803  4 1611 2.610426e+00
## 22804  4 1612 2.612597e+00
## 22805  4 1613 2.573181e+00
## 22806  4 1614 2.527450e+00
## 22807  4 1615 2.474279e+00
## 22808  4 1616 2.408402e+00
## 22809  4 1617 2.355865e+00
## 22810  4 1618 2.300742e+00
## 22811  4 1619 2.244998e+00
## 22812  4 1620 2.194812e+00
## 22813  4 1621 2.148066e+00
## 22814  4 1622 2.101501e+00
## 22815  4 1623 2.059432e+00
## 22816  4 1624 2.017151e+00
## 22817  4 1625 1.979987e+00
## 22818  4 1626 1.943332e+00
## 22819  4 1627 1.907037e+00
## 22820  4 1628 1.875750e+00
## 22821  4 1629 1.840415e+00
## 22822  4 1630 1.811895e+00
## 22823  4 1631 1.781165e+00
## 22824  4 1632 1.758482e+00
## 22825  4 1633 2.202687e+00
## 22826  4 1634 2.485871e+00
## 22827  4 1635 2.579666e+00
## 22828  4 1636 2.586812e+00
## 22829  4 1637 2.552380e+00
## 22830  4 1638 2.504140e+00
## 22831  4 1639 2.442095e+00
## 22832  4 1640 2.386536e+00
## 22833  4 1641 2.331475e+00
## 22834  4 1642 2.277260e+00
## 22835  4 1643 2.230787e+00
## 22836  4 1644 2.185935e+00
## 22837  4 1645 2.136917e+00
## 22838  4 1646 2.087002e+00
## 22839  4 1647 2.050671e+00
## 22840  4 1648 2.009897e+00
## 22841  4 1649 1.972206e+00
## 22842  4 1650 1.937018e+00
## 22843  4 1651 1.898904e+00
## 22844  4 1652 1.870257e+00
## 22845  4 1653 1.834235e+00
## 22846  4 1654 1.808127e+00
## 22847  4 1655 1.778631e+00
## 22848  4 1656 1.751552e+00
## 22849  4 1657 2.200424e+00
## 22850  4 1658 2.482579e+00
## 22851  4 1659 2.576153e+00
## 22852  4 1660 2.583353e+00
## 22853  4 1661 2.545234e+00
## 22854  4 1662 2.499824e+00
## 22855  4 1663 2.447529e+00
## 22856  4 1664 2.386106e+00
## 22857  4 1665 2.331326e+00
## 22858  4 1666 2.282169e+00
## 22859  4 1667 2.229091e+00
## 22860  4 1668 2.181723e+00
## 22861  4 1669 2.133580e+00
## 22862  4 1670 2.091465e+00
## 22863  4 1671 2.053106e+00
## 22864  4 1672 2.010726e+00
## 22865  4 1673 1.969148e+00
## 22866  4 1674 1.938953e+00
## 22867  4 1675 1.906295e+00
## 22868  4 1676 1.870190e+00
## 22869  4 1677 1.838819e+00
## 22870  4 1678 1.809426e+00
## 22871  4 1679 1.784392e+00
## 22872  4 1680 1.757412e+00
## 22873  4 1681 2.203421e+00
## 22874  4 1682 2.489339e+00
## 22875  4 1683 2.586240e+00
## 22876  4 1684 2.588180e+00
## 22877  4 1685 2.550714e+00
## 22878  4 1686 2.506272e+00
## 22879  4 1687 2.446116e+00
## 22880  4 1688 2.392932e+00
## 22881  4 1689 2.335913e+00
## 22882  4 1690 2.279752e+00
## 22883  4 1691 2.236561e+00
## 22884  4 1692 2.184626e+00
## 22885  4 1693 2.139704e+00
## 22886  4 1694 2.099070e+00
## 22887  4 1695 2.053102e+00
## 22888  4 1696 2.016408e+00
## 22889  4 1697 1.974127e+00
## 22890  4 1698 1.942335e+00
## 22891  4 1699 1.908919e+00
## 22892  4 1700 1.875573e+00
## 22893  4 1701 1.848360e+00
## 22894  4 1702 1.817203e+00
## 22895  4 1703 1.789924e+00
## 22896  4 1704 1.760615e+00
## 22897  4 1705 2.209646e+00
## 22898  4 1706 2.492005e+00
## 22899  4 1707 2.585344e+00
## 22900  4 1708 2.589792e+00
## 22901  4 1709 2.560101e+00
## 22902  4 1710 2.507057e+00
## 22903  4 1711 2.454459e+00
## 22904  4 1712 2.400395e+00
## 22905  4 1713 2.342088e+00
## 22906  4 1714 2.285303e+00
## 22907  4 1715 2.237793e+00
## 22908  4 1716 2.192380e+00
## 22909  4 1717 2.146570e+00
## 22910  4 1718 2.096965e+00
## 22911  4 1719 2.061860e+00
## 22912  4 1720 2.021529e+00
## 22913  4 1721 1.981526e+00
## 22914  4 1722 1.949141e+00
## 22915  4 1723 1.913186e+00
## 22916  4 1724 1.882367e+00
## 22917  4 1725 1.851593e+00
## 22918  4 1726 1.818482e+00
## 22919  4 1727 1.796440e+00
## 22920  4 1728 1.766373e+00
## 22921  4 1729 2.214082e+00
## 22922  4 1730 2.492982e+00
## 22923  4 1731 2.588422e+00
## 22924  4 1732 2.598369e+00
## 22925  4 1733 2.560212e+00
## 22926  4 1734 2.509711e+00
## 22927  4 1735 2.456516e+00
## 22928  4 1736 2.405139e+00
## 22929  4 1737 2.347881e+00
## 22930  4 1738 2.295017e+00
## 22931  4 1739 2.243160e+00
## 22932  4 1740 2.195379e+00
## 22933  4 1741 2.148202e+00
## 22934  4 1742 2.102347e+00
## 22935  4 1743 2.061698e+00
## 22936  4 1744 2.022157e+00
## 22937  4 1745 1.985259e+00
## 22938  4 1746 1.954490e+00
## 22939  4 1747 1.918934e+00
## 22940  4 1748 1.882100e+00
## 22941  4 1749 1.855433e+00
## 22942  4 1750 1.823523e+00
## 22943  4 1751 1.794467e+00
## 22944  4 1752 1.769943e+00
## 22945  4 1753 2.219533e+00
## 22946  4 1754 2.494829e+00
## 22947  4 1755 2.594728e+00
## 22948  4 1756 2.599013e+00
## 22949  4 1757 2.569746e+00
## 22950  4 1758 2.514633e+00
## 22951  4 1759 2.463555e+00
## 22952  4 1760 2.405186e+00
## 22953  4 1761 2.353186e+00
## 22954  4 1762 2.298159e+00
## 22955  4 1763 2.248654e+00
## 22956  4 1764 2.199187e+00
## 22957  4 1765 2.151186e+00
## 22958  4 1766 2.109035e+00
## 22959  4 1767 2.069106e+00
## 22960  4 1768 2.025005e+00
## 22961  4 1769 1.990676e+00
## 22962  4 1770 1.950830e+00
## 22963  4 1771 1.920688e+00
## 22964  4 1772 1.884480e+00
## 22965  4 1773 1.859783e+00
## 22966  4 1774 1.826755e+00
## 22967  4 1775 1.802100e+00
## 22968  4 1776 1.772910e+00
## 22969  4 1777 2.219594e+00
## 22970  4 1778 2.505110e+00
## 22971  4 1779 2.599485e+00
## 22972  4 1780 2.604397e+00
## 22973  4 1781 2.572021e+00
## 22974  4 1782 2.520718e+00
## 22975  4 1783 2.465766e+00
## 22976  4 1784 2.410733e+00
## 22977  4 1785 2.354002e+00
## 22978  4 1786 2.304108e+00
## 22979  4 1787 2.249961e+00
## 22980  4 1788 2.204021e+00
## 22981  4 1789 2.156879e+00
## 22982  4 1790 2.112310e+00
## 22983  4 1791 2.070662e+00
## 22984  4 1792 2.031766e+00
## 22985  4 1793 1.993618e+00
## 22986  4 1794 1.957786e+00
## 22987  4 1795 1.923609e+00
## 22988  4 1796 1.894282e+00
## 22989  4 1797 1.860290e+00
## 22990  4 1798 1.832492e+00
## 22991  4 1799 1.806281e+00
## 22992  4 1800 1.775150e+00
## 22993  4 1801 2.221529e+00
## 22994  4 1802 2.508487e+00
## 22995  4 1803 2.604731e+00
## 22996  4 1804 2.607977e+00
## 22997  4 1805 2.574324e+00
## 22998  4 1806 2.523281e+00
## 22999  4 1807 2.470147e+00
## 23000  4 1808 2.413738e+00
## 23001  4 1809 2.357127e+00
## 23002  4 1810 2.306774e+00
## 23003  4 1811 2.250793e+00
## 23004  4 1812 2.205742e+00
## 23005  4 1813 2.160829e+00
## 23006  4 1814 2.116812e+00
## 23007  4 1815 2.073555e+00
## 23008  4 1816 2.033443e+00
## 23009  4 1817 1.998414e+00
## 23010  4 1818 1.958279e+00
## 23011  4 1819 1.925755e+00
## 23012  4 1820 1.894806e+00
## 23013  4 1821 1.864044e+00
## 23014  4 1822 1.836307e+00
## 23015  4 1823 1.806203e+00
## 23016  4 1824 1.781041e+00
## 23017  4 1825 2.226684e+00
## 23018  4 1826 2.512600e+00
## 23019  4 1827 2.608035e+00
## 23020  4 1828 2.615686e+00
## 23021  4 1829 2.581357e+00
## 23022  4 1830 2.532145e+00
## 23023  4 1831 2.472976e+00
## 23024  4 1832 2.416068e+00
## 23025  4 1833 2.363510e+00
## 23026  4 1834 2.311348e+00
## 23027  4 1835 2.257769e+00
## 23028  4 1836 2.204967e+00
## 23029  4 1837 2.163196e+00
## 23030  4 1838 2.117373e+00
## 23031  4 1839 2.077370e+00
## 23032  4 1840 2.037670e+00
## 23033  4 1841 1.996969e+00
## 23034  4 1842 1.965648e+00
## 23035  4 1843 1.931022e+00
## 23036  4 1844 1.901436e+00
## 23037  4 1845 1.868989e+00
## 23038  4 1846 1.835974e+00
## 23039  4 1847 1.813771e+00
## 23040  4 1848 1.782186e+00
## 23041  4 1849 2.230198e+00
## 23042  4 1850 2.516204e+00
## 23043  4 1851 2.605456e+00
## 23044  4 1852 2.613045e+00
## 23045  4 1853 2.578624e+00
## 23046  4 1854 2.535345e+00
## 23047  4 1855 2.475580e+00
## 23048  4 1856 2.422457e+00
## 23049  4 1857 2.364655e+00
## 23050  4 1858 2.313654e+00
## 23051  4 1859 2.259491e+00
## 23052  4 1860 2.210873e+00
## 23053  4 1861 2.161861e+00
## 23054  4 1862 2.119309e+00
## 23055  4 1863 2.081823e+00
## 23056  4 1864 2.039797e+00
## 23057  4 1865 2.003591e+00
## 23058  4 1866 1.971716e+00
## 23059  4 1867 1.932743e+00
## 23060  4 1868 1.901939e+00
## 23061  4 1869 1.872179e+00
## 23062  4 1870 1.842645e+00
## 23063  4 1871 1.812339e+00
## 23064  4 1872 1.782478e+00
## 23065  4 1872 1.809834e+00
## 23066  4 1873 2.251436e+00
## 23067  4 1874 2.537663e+00
## 23068  4 1875 2.629935e+00
## 23069  4 1876 2.637004e+00
## 23070  4 1877 2.605079e+00
## 23071  4 1878 2.560471e+00
## 23072  4 1879 2.499514e+00
## 23073  4 1880 2.438992e+00
```

```
## 23074  4 1881 2.385914e+00
## 23075  4 1882 2.333950e+00
## 23076  4 1883 2.282602e+00
## 23077  4 1884 2.234114e+00
## 23078  4 1885 2.184399e+00
## 23079  4 1886 2.145382e+00
## 23080  4 1887 2.103504e+00
## 23081  4 1888 2.065239e+00
## 23082  4 1889 2.027476e+00
## 23083  4 1890 1.989059e+00
## 23084  4 1891 1.956530e+00
## 23085  4 1892 1.920496e+00
## 23086  4 1893 1.890442e+00
## 23087  4 1894 1.860645e+00
## 23088  4 1895 1.831123e+00
## 23089  4 1896 1.807779e+00
## 23090  4 1896 1.806800e+00
## 23091  4 1897 1.783556e+00
## 23092  4 1898 1.757114e+00
## 23093  4 1899 1.731997e+00
## 23094  4 1900 1.709890e+00
## 23095  4 1901 1.691197e+00
## 23096  4 1902 1.670423e+00
## 23097  4 1903 1.649979e+00
## 23098  4 1904 1.629404e+00
## 23099  4 1905 1.609861e+00
## 23100  4 1906 1.592297e+00
## 23101  4 1907 1.578843e+00
## 23102  4 1908 1.561358e+00
## 23103  4 1909 1.546216e+00
## 23104  4 1910 1.530520e+00
## 23105  4 1911 1.515086e+00
## 23106  4 1911 1.517025e+00
## 23107  4 1912 1.974931e+00
## 23108  4 1913 2.271595e+00
## 23109  4 1914 2.371956e+00
## 23110  4 1915 2.391676e+00
## 23111  4 1916 2.366107e+00
## 23112  4 1917 2.327424e+00
## 23113  4 1918 2.275299e+00
## 23114  4 1919 2.230147e+00
## 23115  4 1920 2.181631e+00
## 23116  4 1920 2.182917e+00
## 23117  4 1921 2.608092e+00
## 23118  4 1922 2.870572e+00
## 23119  4 1923 2.947324e+00
## 23120  4 1924 2.937325e+00
## 23121  4 1925 2.883448e+00
## 23122  4 1926 2.820671e+00
## 23123  4 1927 2.750234e+00
## 23124  4 1928 2.676323e+00
## 23125  4 1929 2.607977e+00
## 23126  4 1930 2.546722e+00
## 23127  4 1931 2.482268e+00
## 23128  4 1932 2.417410e+00
## 23129  4 1933 2.362630e+00
## 23130  4 1934 2.309853e+00
## 23131  4 1935 2.260202e+00
## 23132  4 1936 2.210911e+00
## 23133  4 1937 2.168254e+00
## 23134  4 1938 2.118712e+00
## 23135  4 1939 2.080122e+00
## 23136  4 1940 2.040493e+00
## 23137  4 1941 2.000895e+00
## 23138  4 1942 1.968695e+00
## 23139  4 1943 1.936737e+00
## 23140  4 1944 1.899501e+00
## 23141  4 1945 2.346048e+00
## 23142  4 1946 2.620328e+00
## 23143  4 1947 2.713310e+00
## 23144  4 1948 2.714706e+00
## 23145  4 1949 2.678682e+00
## 23146  4 1950 2.628777e+00
## 23147  4 1951 2.566647e+00
## 23148  4 1952 2.503458e+00
## 23149  4 1953 2.447044e+00
## 23150  4 1954 2.392297e+00
## 23151  4 1955 2.335130e+00
## 23152  4 1956 2.286655e+00
## 23153  4 1957 2.237403e+00
## 23154  4 1958 2.191050e+00
## 23155  4 1959 2.145719e+00
## 23156  4 1960 2.104849e+00
## 23157  4 1961 2.066265e+00
## 23158  4 1962 2.026176e+00
## 23159  4 1963 1.992515e+00
## 23160  4 1964 1.956181e+00
## 23161  4 1965 1.927437e+00
## 23162  4 1966 1.891154e+00
## 23163  4 1967 1.859797e+00
## 23164  4 1968 1.836768e+00
## 23165  4 1969 2.285752e+00
## 23166  4 1970 2.560596e+00
## 23167  4 1971 2.652905e+00
## 23168  4 1972 2.660254e+00
## 23169  4 1973 2.625965e+00
## 23170  4 1974 2.576362e+00
## 23171  4 1975 2.519409e+00
## 23172  4 1976 2.461650e+00
## 23173  4 1977 2.406587e+00
## 23174  4 1978 2.349403e+00
## 23175  4 1979 2.298216e+00
## 23176  4 1980 2.254218e+00
## 23177  4 1981 2.207041e+00
## 23178  4 1982 2.162907e+00
## 23179  4 1983 2.117306e+00
## 23180  4 1984 2.078534e+00
## 23181  4 1985 2.038714e+00
## 23182  4 1986 2.003473e+00
## 23183  4 1987 1.968235e+00
## 23184  4 1988 1.934386e+00
## 23185  4 1989 1.906245e+00
## 23186  4 1990 1.873276e+00
## 23187  4 1991 1.843753e+00
## 23188  4 1992 1.819000e+00
## 23189  4 1993 2.270667e+00
## 23190  4 1994 2.546313e+00
## 23191  4 1995 2.642903e+00
## 23192  4 1996 2.648490e+00
## 23193  4 1997 2.614067e+00
## 23194  4 1998 2.560291e+00
## 23195  4 1999 2.507754e+00
## 23196  4 2000 2.443391e+00
## 23197  4 2001 2.399368e+00
## 23198  4 2002 2.344691e+00
## 23199  4 2003 2.295571e+00
## 23200  4 2004 2.242163e+00
## 23201  4 2005 2.198481e+00
## 23202  4 2006 2.154863e+00
## 23203  4 2007 2.111692e+00
## 23204  4 2008 2.074274e+00
## 23205  4 2009 2.033147e+00
## 23206  4 2010 1.997877e+00
## 23207  4 2011 1.964605e+00
## 23208  4 2012 1.930222e+00
## 23209  4 2013 1.898873e+00
## 23210  4 2014 1.869622e+00
## 23211  4 2015 1.841810e+00
## 23212  4 2016 1.816379e+00
## 23213  4 2017 2.259829e+00
## 23214  4 2018 2.544070e+00
## 23215  4 2019 2.637789e+00
## 23216  4 2020 2.649482e+00
## 23217  4 2021 2.612915e+00
## 23218  4 2022 2.559520e+00
## 23219  4 2023 2.508301e+00
## 23220  4 2024 2.452620e+00
## 23221  4 2025 2.390202e+00
## 23222  4 2026 2.338368e+00
## 23223  4 2027 2.292298e+00
## 23224  4 2028 2.243382e+00
## 23225  4 2029 2.197814e+00
## 23226  4 2030 2.149115e+00
## 23227  4 2031 2.108454e+00
## 23228  4 2032 2.070184e+00
## 23229  4 2033 2.032627e+00
## 23230  4 2034 1.993731e+00
## 23231  4 2035 1.962785e+00
## 23232  4 2036 1.931137e+00
## 23233  4 2037 1.896440e+00
## 23234  4 2038 1.869486e+00
## 23235  4 2039 1.838756e+00
## 23236  4 2040 1.811418e+00
## 23237  4 2041 2.262813e+00
## 23238  4 2042 2.547240e+00
## 23239  4 2043 2.640925e+00
## 23240  4 2044 2.644428e+00
## 23241  4 2045 2.613591e+00
## 23242  4 2046 2.560764e+00
## 23243  4 2047 2.506786e+00
## 23244  4 2048 2.449389e+00
## 23245  4 2049 2.388200e+00
## 23246  4 2050 2.340833e+00
## 23247  4 2051 2.290598e+00
## 23248  4 2052 2.243185e+00
## 23249  4 2053 2.197402e+00
## 23250  4 2054 2.154097e+00
## 23251  4 2055 2.111135e+00
## 23252  4 2056 2.071132e+00
## 23253  4 2057 2.033322e+00
## 23254  4 2058 1.996931e+00
## 23255  4 2059 1.958571e+00
## 23256  4 2060 1.930866e+00
## 23257  4 2061 1.896798e+00
## 23258  4 2062 1.871321e+00
## 23259  4 2063 1.841999e+00
## 23260  4 2064 1.812582e+00
## 23261  4 2065 2.265960e+00
## 23262  4 2066 2.545815e+00
## 23263  4 2067 2.637430e+00
## 23264  4 2068 2.643610e+00
## 23265  4 2069 2.609071e+00
## 23266  4 2070 2.562401e+00
## 23267  4 2071 2.502262e+00
## 23268  4 2072 2.448323e+00
## 23269  4 2073 2.390465e+00
## 23270  4 2074 2.339456e+00
## 23271  4 2075 2.286215e+00
## 23272  4 2076 2.239210e+00
## 23273  4 2077 2.196199e+00
## 23274  4 2078 2.149717e+00
## 23275  4 2079 2.105584e+00
## 23276  4 2080 2.067242e+00
## 23277  4 2081 2.029319e+00
## 23278  4 2082 1.994084e+00
## 23279  4 2083 1.958230e+00
## 23280  4 2084 1.928554e+00
## 23281  4 2085 1.898248e+00
## 23282  4 2086 1.866053e+00
## 23283  4 2087 1.838270e+00
## 23284  4 2088 1.813199e+00
## 23285  4 2089 2.258364e+00
## 23286  4 2090 2.545444e+00
## 23287  4 2091 2.638145e+00
## 23288  4 2092 2.648216e+00
## 23289  4 2093 2.606981e+00
## 23290  4 2094 2.560276e+00
## 23291  4 2095 2.503174e+00
## 23292  4 2096 2.448304e+00
## 23293  4 2097 2.389392e+00
## 23294  4 2098 2.339344e+00
## 23295  4 2099 2.289209e+00
## 23296  4 2100 2.242836e+00
## 23297  4 2101 2.190840e+00
## 23298  4 2102 2.148719e+00
## 23299  4 2103 2.111092e+00
## 23300  4 2104 2.068597e+00
## 23301  4 2105 2.031875e+00
## 23302  4 2106 1.992241e+00
## 23303  4 2107 1.958746e+00
## 23304  4 2108 1.928032e+00
## 23305  4 2109 1.896545e+00
## 23306  4 2110 1.865833e+00
## 23307  4 2111 1.837744e+00
## 23308  4 2112 1.811033e+00
## 23309  4 2113 2.258574e+00
## 23310  4 2114 2.539581e+00
## 23311  4 2115 2.641254e+00
## 23312  4 2116 2.639793e+00
## 23313  4 2117 2.605493e+00
## 23314  4 2118 2.561066e+00
## 23315  4 2119 2.503186e+00
## 23316  4 2120 2.446302e+00
## 23317  4 2121 2.394272e+00
## 23318  4 2122 2.339682e+00
## 23319  4 2123 2.290409e+00
## 23320  4 2124 2.238502e+00
## 23321  4 2125 2.194704e+00
## 23322  4 2126 2.146753e+00
## 23323  4 2127 2.106134e+00
## 23324  4 2128 2.068126e+00
## 23325  4 2129 2.029121e+00
## 23326  4 2130 1.994266e+00
## 23327  4 2131 1.958124e+00
## 23328  4 2132 1.923138e+00
## 23329  4 2133 1.892717e+00
## 23330  4 2134 1.863684e+00
## 23331  4 2135 1.839328e+00
## 23332  4 2136 1.809783e+00
## 23333  4 2137 2.255268e+00
## 23334  4 2138 2.537524e+00
## 23335  4 2139 2.637825e+00
## 23336  4 2140 2.639499e+00
## 23337  4 2141 2.605955e+00
## 23338  4 2142 2.553652e+00
## 23339  4 2143 2.502235e+00
## 23340  4 2144 2.450131e+00
## 23341  4 2145 2.390500e+00
## 23342  4 2146 2.338213e+00
## 23343  4 2147 2.286318e+00
## 23344  4 2148 2.239508e+00
## 23345  4 2149 2.191336e+00
## 23346  4 2150 2.147232e+00
## 23347  4 2151 2.108345e+00
## 23348  4 2152 2.065316e+00
## 23349  4 2153 2.028300e+00
## 23350  4 2154 1.995801e+00
## 23351  4 2155 1.960637e+00
## 23352  4 2156 1.926406e+00
## 23353  4 2157 1.896983e+00
## 23354  4 2158 1.865418e+00
## 23355  4 2159 1.836658e+00
## 23356  4 2160 1.812407e+00
## 23357  4 2161 2.259556e+00
## 23358  4 2162 2.543530e+00
## 23359  4 2163 2.637148e+00
## 23360  4 2164 2.638228e+00
## 23361  4 2165 2.604135e+00
## 23362  4 2166 2.553199e+00
## 23363  4 2167 2.503471e+00
## 23364  4 2168 2.443024e+00
## 23365  4 2169 2.386006e+00
## 23366  4 2170 2.337899e+00
## 23367  4 2171 2.283152e+00
## 23368  4 2172 2.237359e+00
## 23369  4 2173 2.187662e+00
## 23370  4 2174 2.145228e+00
## 23371  4 2175 2.102788e+00
## 23372  4 2176 2.064952e+00
## 23373  4 2177 2.031481e+00
## 23374  4 2178 1.993697e+00
## 23375  4 2179 1.958300e+00
## 23376  4 2180 1.927061e+00
## 23377  4 2181 1.893660e+00
## 23378  4 2182 1.867899e+00
## 23379  4 2183 1.838264e+00
## 23380  4 2184 1.807451e+00
## 23381  4 2184 1.807606e+00
## 23382  4 2185 2.258323e+00
## 23383  4 2186 2.540001e+00
## 23384  4 2187 2.633317e+00
## 23385  4 2188 2.635211e+00
## 23386  4 2189 2.603410e+00
## 23387  4 2190 2.553470e+00
## 23388  4 2191 2.496908e+00
## 23389  4 2192 2.440929e+00
## 23390  4 2193 2.383072e+00
## 23391  4 2194 2.332637e+00
## 23392  4 2195 2.278565e+00
## 23393  4 2196 2.229215e+00
## 23394  4 2197 2.184685e+00
## 23395  4 2198 2.142289e+00
## 23396  4 2199 2.100352e+00
## 23397  4 2200 2.063390e+00
## 23398  4 2201 2.025437e+00
## 23399  4 2202 1.986442e+00
## 23400  4 2203 1.953394e+00
## 23401  4 2204 1.922608e+00
## 23402  4 2205 1.891284e+00
## 23403  4 2206 1.863755e+00
## 23404  4 2207 1.833589e+00
## 23405  4 2208 1.808389e+00
## 23406  4 2208 1.806608e+00
## 23407  4 2209 1.780338e+00
## 23408  4 2210 1.758827e+00
## 23409  4 2211 1.733677e+00
## 23410  4 2212 1.712181e+00
## 23411  4 2213 1.687512e+00
## 23412  4 2214 1.669701e+00
## 23413  4 2215 1.649657e+00
## 23414  4 2216 1.629509e+00
## 23415  4 2217 1.613696e+00
## 23416  4 2218 1.596189e+00
## 23417  4 2219 1.576858e+00
## 23418  4 2220 1.562776e+00
## 23419  4 2221 1.544499e+00
## 23420  4 2222 1.529924e+00
## 23421  4 2223 1.518461e+00
## 23422  4 2224 1.504870e+00
## 23423  4 2225 1.490628e+00
## 23424  4 2226 1.475839e+00
## 23425  4 2226 1.476887e+00
## 23426  4 2227 1.699672e+00
## 23427  4 2228 1.840565e+00
## 23428  4 2229 1.889619e+00
## 23429  4 2230 1.893136e+00
## 23430  4 2231 1.878746e+00
## 23431  4 2232 1.851024e+00
## 23432  4 2232 1.849696e+00
## 23433  4 2233 2.294535e+00
## 23434  4 2234 2.575289e+00
## 23435  4 2235 2.665381e+00
## 23436  4 2236 2.665811e+00
## 23437  4 2237 2.621092e+00
## 23438  4 2238 2.564571e+00
## 23439  4 2239 2.513187e+00
## 23440  4 2240 2.447372e+00
## 23441  4 2241 2.387541e+00
## 23442  4 2242 2.331001e+00
## 23443  4 2243 2.277503e+00
## 23444  4 2244 2.225587e+00
## 23445  4 2245 2.174004e+00
## 23446  4 2246 2.127571e+00
## 23447  4 2247 2.081094e+00
## 23448  4 2248 2.042949e+00
## 23449  4 2249 2.003444e+00
## 23450  4 2250 1.965093e+00
## 23451  4 2251 1.928420e+00
## 23452  4 2252 1.892562e+00
## 23453  4 2253 1.860235e+00
## 23454  4 2254 1.832064e+00
## 23455  4 2255 1.799161e+00
## 23456  4 2256 1.770323e+00
## 23457  4 2257 2.223578e+00
## 23458  4 2258 2.498996e+00
## 23459  4 2259 2.591273e+00
## 23460  4 2260 2.594849e+00
## 23461  4 2261 2.564451e+00
## 23462  4 2262 2.515546e+00
## 23463  4 2263 2.457322e+00
## 23464  4 2264 2.400200e+00
## 23465  4 2265 2.344025e+00
## 23466  4 2266 2.289021e+00
## 23467  4 2267 2.236591e+00
## 23468  4 2268 2.185244e+00
## 23469  4 2269 2.142779e+00
## 23470  4 2270 2.095741e+00
## 23471  4 2271 2.053745e+00
## 23472  4 2272 2.012770e+00
## 23473  4 2273 1.974991e+00
## 23474  4 2274 1.943343e+00
## 23475  4 2275 1.905438e+00
## 23476  4 2276 1.876080e+00
## 23477  4 2277 1.841125e+00
## 23478  4 2278 1.813596e+00
## 23479  4 2279 1.781906e+00
## 23480  4 2280 1.755779e+00
## 23481  4 2281 2.202453e+00
## 23482  4 2282 2.488098e+00
## 23483  4 2283 2.582299e+00
## 23484  4 2284 2.583835e+00
## 23485  4 2285 2.549331e+00
## 23486  4 2286 2.502413e+00
## 23487  4 2287 2.446514e+00
## 23488  4 2288 2.393181e+00
## 23489  4 2289 2.336561e+00
## 23490  4 2290 2.287651e+00
## 23491  4 2291 2.232813e+00
## 23492  4 2292 2.182288e+00
## 23493  4 2293 2.138440e+00
## 23494  4 2294 2.092779e+00
## 23495  4 2295 2.053795e+00
## 23496  4 2296 2.012544e+00
## 23497  4 2297 1.976707e+00
## 23498  4 2298 1.937546e+00
## 23499  4 2299 1.906798e+00
## 23500  4 2300 1.872278e+00
## 23501  4 2301 1.840478e+00
## 23502  4 2302 1.813548e+00
## 23503  4 2303 1.781910e+00
## 23504  4 2304 1.756495e+00
## 23505  4 2305 2.204021e+00
## 23506  4 2306 2.488080e+00
## 23507  4 2307 2.584575e+00
## 23508  4 2308 2.596763e+00
## 23509  4 2309 2.555190e+00
## 23510  4 2310 2.502908e+00
## 23511  4 2311 2.449587e+00
## 23512  4 2312 2.391587e+00
## 23513  4 2313 2.338927e+00
## 23514  4 2314 2.287901e+00
## 23515  4 2315 2.238270e+00
## 23516  4 2316 2.188415e+00
## 23517  4 2317 2.143352e+00
## 23518  4 2318 2.094883e+00
## 23519  4 2319 2.055492e+00
## 23520  4 2320 2.015270e+00
## 23521  4 2321 1.981371e+00
## 23522  4 2322 1.941429e+00
## 23523  4 2323 1.911525e+00
## 23524  4 2324 1.877406e+00
## 23525  4 2325 1.845873e+00
## 23526  4 2326 1.815863e+00
## 23527  4 2327 1.786072e+00
## 23528  4 2328 1.763380e+00
## 23529  4 2329 2.208510e+00
## 23530  4 2330 2.492068e+00
## 23531  4 2331 2.585142e+00
## 23532  4 2332 2.591581e+00
## 23533  4 2333 2.563282e+00
## 23534  4 2334 2.514407e+00
## 23535  4 2335 2.453472e+00
## 23536  4 2336 2.398593e+00
## 23537  4 2337 2.345821e+00
## 23538  4 2338 2.293210e+00
## 23539  4 2339 2.240113e+00
## 23540  4 2340 2.190304e+00
## 23541  4 2341 2.146677e+00
## 23542  4 2342 2.103295e+00
## 23543  4 2343 2.062419e+00
## 23544  4 2344 2.021883e+00
## 23545  4 2345 1.980120e+00
## 23546  4 2346 1.950266e+00
## 23547  4 2347 1.913445e+00
## 23548  4 2348 1.879504e+00
## 23549  4 2349 1.848814e+00
## 23550  4 2350 1.821421e+00
## 23551  4 2351 1.793049e+00
## 23552  4 2352 1.769597e+00
## 23553  4 2353 2.218132e+00
## 23554  4 2354 2.493899e+00
## 23555  4 2355 2.590314e+00
## 23556  4 2356 2.596816e+00
## 23557  4 2357 2.565591e+00
## 23558  4 2358 2.511450e+00
## 23559  4 2359 2.459001e+00
## 23560  4 2360 2.403015e+00
## 23561  4 2361 2.350987e+00
## 23562  4 2362 2.292640e+00
## 23563  4 2363 2.248033e+00
## 23564  4 2364 2.192064e+00
## 23565  4 2365 2.150320e+00
## 23566  4 2366 2.109026e+00
## 23567  4 2367 2.062805e+00
## 23568  4 2368 2.022790e+00
## 23569  4 2369 1.992589e+00
## 23570  4 2370 1.949405e+00
## 23571  4 2371 1.919647e+00
## 23572  4 2372 1.883826e+00
## 23573  4 2373 1.853245e+00
## 23574  4 2374 1.825113e+00
## 23575  4 2375 1.797928e+00
## 23576  4 2376 1.773468e+00
## 23577  4 2377 2.219872e+00
## 23578  4 2378 2.498825e+00
## 23579  4 2379 2.593426e+00
## 23580  4 2380 2.602287e+00
## 23581  4 2381 2.569788e+00
## 23582  4 2382 2.515871e+00
## 23583  4 2383 2.467847e+00
## 23584  4 2384 2.411504e+00
## 23585  4 2385 2.353479e+00
## 23586  4 2386 2.298235e+00
## 23587  4 2387 2.248587e+00
## 23588  4 2388 2.201304e+00
## 23589  4 2389 2.149497e+00
## 23590  4 2390 2.105211e+00
## 23591  4 2391 2.066985e+00
## 23592  4 2392 2.029126e+00
## 23593  4 2393 1.992002e+00
## 23594  4 2394 1.957053e+00
## 23595  4 2395 1.920529e+00
## 23596  4 2396 1.889477e+00
## 23597  4 2397 1.855758e+00
## 23598  4 2398 1.830715e+00
## 23599  4 2399 1.799929e+00
## 23600  4 2400 1.770666e+00
## 23601  4 2401 2.223728e+00
## 23602  4 2402 2.509175e+00
## 23603  4 2403 2.594192e+00
## 23604  4 2404 2.600950e+00
## 23605  4 2405 2.564465e+00
## 23606  4 2406 2.518959e+00
## 23607  4 2407 2.468403e+00
## 23608  4 2408 2.410685e+00
## 23609  4 2409 2.354003e+00
## 23610  4 2410 2.305869e+00
## 23611  4 2411 2.246834e+00
## 23612  4 2412 2.202641e+00
## 23613  4 2413 2.159474e+00
## 23614  4 2414 2.114185e+00
## 23615  4 2415 2.069033e+00
## 23616  4 2416 2.033824e+00
## 23617  4 2417 1.993095e+00
## 23618  4 2418 1.959997e+00
## 23619  4 2419 1.926603e+00
## 23620  4 2420 1.890583e+00
## 23621  4 2421 1.862296e+00
## 23622  4 2422 1.829254e+00
## 23623  4 2423 1.802711e+00
## 23624  4 2424 1.777678e+00
## 23625  4 2425 2.224243e+00
## 23626  4 2426 2.511747e+00
## 23627  4 2427 2.600017e+00
## 23628  4 2428 2.608933e+00
## 23629  4 2429 2.572400e+00
## 23630  4 2430 2.525914e+00
## 23631  4 2431 2.469353e+00
## 23632  4 2432 2.412105e+00
## 23633  4 2433 2.360376e+00
## 23634  4 2434 2.306098e+00
## 23635  4 2435 2.257925e+00
## 23636  4 2436 2.208464e+00
## 23637  4 2437 2.161075e+00
## 23638  4 2438 2.117998e+00
## 23639  4 2439 2.072030e+00
## 23640  4 2440 2.033918e+00
## 23641  4 2441 1.998013e+00
## 23642  4 2442 1.961241e+00
## 23643  4 2443 1.925607e+00
## 23644  4 2444 1.896521e+00
## 23645  4 2445 1.864511e+00
## 23646  4 2446 1.834217e+00
## 23647  4 2447 1.805443e+00
## 23648  4 2448 1.782889e+00
## 23649  4 2449 2.229255e+00
## 23650  4 2450 2.510194e+00
## 23651  4 2451 2.603565e+00
## 23652  4 2452 2.613208e+00
## 23653  4 2453 2.576513e+00
## 23654  4 2454 2.533248e+00
## 23655  4 2455 2.469810e+00
## 23656  4 2456 2.415648e+00
## 23657  4 2457 2.366458e+00
## 23658  4 2458 2.312342e+00
## 23659  4 2459 2.257682e+00
## 23660  4 2460 2.207958e+00
## 23661  4 2461 2.162647e+00
## 23662  4 2462 2.119520e+00
## 23663  4 2463 2.078121e+00
## 23664  4 2464 2.037775e+00
## 23665  4 2465 2.000556e+00
## 23666  4 2466 1.965978e+00
## 23667  4 2467 1.928989e+00
## 23668  4 2468 1.894509e+00
## 23669  4 2469 1.866668e+00
## 23670  4 2470 1.836301e+00
## 23671  4 2471 1.809045e+00
## 23672  4 2472 1.782072e+00
## 23673  4 2473 2.234088e+00
## 23674  4 2474 2.513197e+00
## 23675  4 2475 2.607412e+00
## 23676  4 2476 2.614381e+00
## 23677  4 2477 2.582017e+00
## 23678  4 2478 2.531899e+00
## 23679  4 2479 2.476819e+00
## 23680  4 2480 2.420945e+00
## 23681  4 2481 2.361280e+00
## 23682  4 2482 2.312680e+00
## 23683  4 2483 2.261034e+00
## 23684  4 2484 2.214377e+00
## 23685  4 2485 2.168548e+00
## 23686  4 2486 2.119454e+00
## 23687  4 2487 2.079049e+00
## 23688  4 2488 2.041503e+00
## 23689  4 2489 2.007765e+00
## 23690  4 2490 1.967075e+00
## 23691  4 2491 1.935902e+00
## 23692  4 2492 1.902124e+00
## 23693  4 2493 1.870506e+00
## 23694  4 2494 1.838565e+00
## 23695  4 2495 1.813256e+00
## 23696  4 2496 1.786171e+00
## 23697  4 2496 1.806246e+00
## 23698  4 2497 2.254378e+00
## 23699  4 2498 2.538647e+00
## 23700  4 2499 2.630360e+00
## 23701  4 2500 2.639074e+00
## 23702  4 2501 2.606018e+00
## 23703  4 2502 2.554341e+00
## 23704  4 2503 2.499011e+00
## 23705  4 2504 2.441808e+00
## 23706  4 2505 2.386670e+00
## 23707  4 2506 2.337206e+00
## 23708  4 2507 2.284323e+00
## 23709  4 2508 2.232360e+00
## 23710  4 2509 2.186104e+00
## 23711  4 2510 2.147003e+00
## 23712  4 2511 2.104811e+00
## 23713  4 2512 2.061633e+00
## 23714  4 2513 2.024206e+00
## 23715  4 2514 1.988996e+00
## 23716  4 2515 1.952007e+00
## 23717  4 2516 1.923349e+00
## 23718  4 2517 1.889921e+00
## 23719  4 2518 1.861066e+00
## 23720  4 2519 1.834230e+00
## 23721  4 2520 1.809754e+00
## 23722  4 2520 1.807899e+00
## 23723  4 2521 1.778425e+00
## 23724  4 2522 1.755755e+00
## 23725  4 2523 1.732049e+00
## 23726  4 2524 1.711977e+00
## 23727  4 2525 1.688289e+00
## 23728  4 2526 1.671523e+00
## 23729  4 2527 1.649709e+00
## 23730  4 2528 1.629866e+00
## 23731  4 2529 1.613653e+00
## 23732  4 2530 1.594149e+00
## 23733  4 2531 1.578215e+00
## 23734  4 2532 1.562660e+00
## 23735  4 2533 1.545484e+00
## 23736  4 2534 1.531097e+00
## 23737  4 2535 1.518218e+00
## 23738  4 2536 1.501224e+00
## 23739  4 2537 1.486449e+00
## 23740  4 2538 1.476831e+00
## 23741  4 2538 1.477192e+00
## 23742  4 2539 1.933158e+00
## 23743  4 2540 2.233277e+00
## 23744  4 2541 2.334832e+00
## 23745  4 2542 2.355213e+00
## 23746  4 2543 2.334390e+00
## 23747  4 2544 2.293285e+00
## 23748  4 2544 2.291528e+00
## 23749  4 2545 2.719078e+00
## 23750  4 2546 2.980717e+00
## 23751  4 2547 3.051747e+00
## 23752  4 2548 3.032126e+00
## 23753  4 2549 2.983188e+00
## 23754  4 2550 2.903182e+00
## 23755  4 2551 2.829505e+00
## 23756  4 2552 2.749857e+00
## 23757  4 2553 2.677597e+00
## 23758  4 2554 2.613495e+00
## 23759  4 2555 2.545878e+00
## 23760  4 2556 2.482083e+00
## 23761  4 2557 2.417303e+00
## 23762  4 2558 2.364546e+00
## 23763  4 2559 2.310777e+00
## 23764  4 2560 2.264037e+00
## 23765  4 2561 2.212318e+00
## 23766  4 2562 2.161948e+00
## 23767  4 2563 2.119350e+00
## 23768  4 2564 2.076476e+00
## 23769  4 2565 2.038375e+00
## 23770  4 2566 2.003504e+00
## 23771  4 2567 1.967474e+00
## 23772  4 2568 1.931864e+00
## 23773  4 2569 2.371511e+00
## 23774  4 2570 2.653940e+00
## 23775  4 2571 2.746618e+00
## 23776  4 2572 2.736333e+00
## 23777  4 2573 2.699025e+00
## 23778  4 2574 2.646826e+00
## 23779  4 2575 2.590843e+00
## 23780  4 2576 2.524511e+00
## 23781  4 2577 2.461634e+00
## 23782  4 2578 2.409755e+00
## 23783  4 2579 2.352785e+00
## 23784  4 2580 2.298016e+00
## 23785  4 2581 2.255336e+00
## 23786  4 2582 2.201914e+00
## 23787  4 2583 2.158450e+00
## 23788  4 2584 2.111907e+00
## 23789  4 2585 2.077325e+00
## 23790  4 2586 2.036771e+00
## 23791  4 2587 2.001877e+00
## 23792  4 2588 1.967451e+00
## 23793  4 2589 1.932998e+00
## 23794  4 2590 1.903694e+00
## 23795  4 2591 1.873428e+00
## 23796  4 2592 1.844942e+00
## 23797  4 2593 2.289787e+00
## 23798  4 2594 2.573881e+00
## 23799  4 2595 2.663336e+00
## 23800  4 2596 2.666529e+00
## 23801  4 2597 2.630788e+00
## 23802  4 2598 2.582255e+00
## 23803  4 2599 2.531554e+00
## 23804  4 2600 2.467135e+00
## 23805  4 2601 2.415927e+00
## 23806  4 2602 2.358490e+00
## 23807  4 2603 2.304943e+00
## 23808  4 2604 2.257571e+00
## 23809  4 2605 2.208186e+00
## 23810  4 2606 2.166128e+00
## 23811  4 2607 2.123669e+00
## 23812  4 2608 2.080926e+00
## 23813  4 2609 2.046071e+00
## 23814  4 2610 2.009593e+00
## 23815  4 2611 1.976309e+00
## 23816  4 2612 1.941529e+00
## 23817  4 2613 1.905284e+00
## 23818  4 2614 1.878995e+00
## 23819  4 2615 1.847709e+00
## 23820  4 2616 1.821230e+00
## 23821  4 2617 2.269673e+00
## 23822  4 2618 2.552681e+00
## 23823  4 2619 2.640514e+00
## 23824  4 2620 2.655402e+00
## 23825  4 2621 2.616982e+00
## 23826  4 2622 2.569257e+00
## 23827  4 2623 2.509017e+00
## 23828  4 2624 2.453449e+00
## 23829  4 2625 2.399611e+00
## 23830  4 2626 2.345835e+00
## 23831  4 2627 2.293909e+00
## 23832  4 2628 2.246846e+00
## 23833  4 2629 2.197763e+00
## 23834  4 2630 2.150797e+00
## 23835  4 2631 2.116284e+00
## 23836  4 2632 2.074469e+00
## 23837  4 2633 2.037681e+00
## 23838  4 2634 1.997014e+00
## 23839  4 2635 1.968271e+00
## 23840  4 2636 1.932232e+00
## 23841  4 2637 1.902323e+00
## 23842  4 2638 1.871322e+00
## 23843  4 2639 1.842806e+00
## 23844  4 2640 1.818735e+00
## 23845  4 2641 2.264625e+00
## 23846  4 2642 2.547036e+00
## 23847  4 2643 2.641124e+00
## 23848  4 2644 2.646292e+00
## 23849  4 2645 2.612097e+00
## 23850  4 2646 2.561990e+00
## 23851  4 2647 2.507387e+00
## 23852  4 2648 2.452679e+00
## 23853  4 2649 2.392022e+00
## 23854  4 2650 2.341894e+00
## 23855  4 2651 2.288124e+00
## 23856  4 2652 2.239172e+00
## 23857  4 2653 2.191883e+00
## 23858  4 2654 2.148613e+00
## 23859  4 2655 2.109324e+00
## 23860  4 2656 2.070414e+00
## 23861  4 2657 2.036146e+00
## 23862  4 2658 1.996524e+00
## 23863  4 2659 1.962519e+00
## 23864  4 2660 1.929796e+00
## 23865  4 2661 1.900583e+00
## 23866  4 2662 1.872483e+00
## 23867  4 2663 1.845438e+00
## 23868  4 2664 1.814787e+00
## 23869  4 2665 2.259521e+00
## 23870  4 2666 2.546994e+00
## 23871  4 2667 2.636453e+00
## 23872  4 2668 2.641983e+00
## 23873  4 2669 2.609387e+00
```

```
## 23874  4 2670 2.560589e+00
## 23875  4 2671 2.509296e+00
## 23876  4 2672 2.448760e+00
## 23877  4 2673 2.397118e+00
## 23878  4 2674 2.344734e+00
## 23879  4 2675 2.284722e+00
## 23880  4 2676 2.241902e+00
## 23881  4 2677 2.195528e+00
## 23882  4 2678 2.152418e+00
## 23883  4 2679 2.108938e+00
## 23884  4 2680 2.070270e+00
## 23885  4 2681 2.033767e+00
## 23886  4 2682 1.992478e+00
## 23887  4 2683 1.962618e+00
## 23888  4 2684 1.927813e+00
## 23889  4 2685 1.897241e+00
## 23890  4 2686 1.870709e+00
## 23891  4 2687 1.843410e+00
## 23892  4 2688 1.813594e+00
## 23893  4 2689 2.260738e+00
## 23894  4 2690 2.546218e+00
## 23895  4 2691 2.634504e+00
## 23896  4 2692 2.643772e+00
## 23897  4 2693 2.611116e+00
## 23898  4 2694 2.557009e+00
## 23899  4 2695 2.505791e+00
## 23900  4 2696 2.454103e+00
## 23901  4 2697 2.392219e+00
## 23902  4 2698 2.338794e+00
## 23903  4 2699 2.284261e+00
## 23904  4 2700 2.243277e+00
## 23905  4 2701 2.196791e+00
## 23906  4 2702 2.150184e+00
## 23907  4 2703 2.106510e+00
## 23908  4 2704 2.069053e+00
## 23909  4 2705 2.033224e+00
## 23910  4 2706 1.993837e+00
## 23911  4 2707 1.963156e+00
## 23912  4 2708 1.933683e+00
## 23913  4 2709 1.900033e+00
## 23914  4 2710 1.867398e+00
## 23915  4 2711 1.837709e+00
## 23916  4 2712 1.816676e+00
## 23917  4 2713 2.260663e+00
## 23918  4 2714 2.540732e+00
## 23919  4 2715 2.639146e+00
## 23920  4 2716 2.636873e+00
## 23921  4 2717 2.609545e+00
## 23922  4 2718 2.556205e+00
## 23923  4 2719 2.505215e+00
## 23924  4 2720 2.445781e+00
## 23925  4 2721 2.392774e+00
## 23926  4 2722 2.341346e+00
## 23927  4 2723 2.287889e+00
## 23928  4 2724 2.239192e+00
## 23929  4 2725 2.191333e+00
## 23930  4 2726 2.148204e+00
## 23931  4 2727 2.107967e+00
## 23932  4 2728 2.069457e+00
## 23933  4 2729 2.028167e+00
## 23934  4 2730 1.998060e+00
## 23935  4 2731 1.960743e+00
## 23936  4 2732 1.925287e+00
## 23937  4 2733 1.894052e+00
## 23938  4 2734 1.866608e+00
## 23939  4 2735 1.840218e+00
## 23940  4 2736 1.813389e+00
## 23941  4 2737 2.263745e+00
## 23942  4 2738 2.544651e+00
## 23943  4 2739 2.635300e+00
## 23944  4 2740 2.648414e+00
## 23945  4 2741 2.613054e+00
## 23946  4 2742 2.557158e+00
## 23947  4 2743 2.504052e+00
## 23948  4 2744 2.452376e+00
## 23949  4 2745 2.388198e+00
## 23950  4 2746 2.341706e+00
## 23951  4 2747 2.285313e+00
## 23952  4 2748 2.239749e+00
## 23953  4 2749 2.191034e+00
## 23954  4 2750 2.148237e+00
## 23955  4 2751 2.110636e+00
## 23956  4 2752 2.068852e+00
## 23957  4 2753 2.027552e+00
## 23958  4 2754 1.993548e+00
## 23959  4 2755 1.960667e+00
## 23960  4 2756 1.929317e+00
## 23961  4 2757 1.897136e+00
## 23962  4 2758 1.869241e+00
## 23963  4 2759 1.836780e+00
## 23964  4 2760 1.813647e+00
## 23965  4 2761 2.263151e+00
## 23966  4 2762 2.538201e+00
## 23967  4 2763 2.639595e+00
## 23968  4 2764 2.645839e+00
## 23969  4 2765 2.610480e+00
## 23970  4 2766 2.556104e+00
## 23971  4 2767 2.506451e+00
## 23972  4 2768 2.446059e+00
## 23973  4 2769 2.390437e+00
## 23974  4 2770 2.342584e+00
## 23975  4 2771 2.290328e+00
## 23976  4 2772 2.239105e+00
## 23977  4 2773 2.194967e+00
## 23978  4 2774 2.149131e+00
## 23979  4 2775 2.106050e+00
## 23980  4 2776 2.067440e+00
## 23981  4 2777 2.028219e+00
## 23982  4 2778 1.990859e+00
## 23983  4 2779 1.957732e+00
## 23984  4 2780 1.924965e+00
## 23985  4 2781 1.899390e+00
## 23986  4 2782 1.867178e+00
## 23987  4 2783 1.838297e+00
## 23988  4 2784 1.807413e+00
## 23989  4 2785 2.259155e+00
## 23990  4 2786 2.541175e+00
## 23991  4 2787 2.636402e+00
## 23992  4 2788 2.640027e+00
## 23993  4 2789 2.611225e+00
## 23994  4 2790 2.560535e+00
## 23995  4 2791 2.498140e+00
## 23996  4 2792 2.443095e+00
## 23997  4 2793 2.390305e+00
## 23998  4 2794 2.337875e+00
## 23999  4 2795 2.283680e+00
## 24000  4 2796 2.236786e+00
## 24001  4 2797 2.186857e+00
## 24002  4 2798 2.147885e+00
## 24003  4 2799 2.106182e+00
## 24004  4 2800 2.067761e+00
## 24005  4 2801 2.028709e+00
## 24006  4 2802 1.991144e+00
## 24007  4 2803 1.960309e+00
## 24008  4 2804 1.925281e+00
## 24009  4 2805 1.898454e+00
## 24010  4 2806 1.866374e+00
## 24011  4 2807 1.841181e+00
## 24012  4 2808 1.815041e+00
## 24013  4 2808 1.807118e+00
## 24014  4 2809 2.256889e+00
## 24015  4 2810 2.539558e+00
## 24016  4 2811 2.628842e+00
## 24017  4 2812 2.640278e+00
## 24018  4 2813 2.603509e+00
## 24019  4 2814 2.556550e+00
## 24020  4 2815 2.497098e+00
## 24021  4 2816 2.442003e+00
## 24022  4 2817 2.387690e+00
## 24023  4 2818 2.334991e+00
## 24024  4 2819 2.280788e+00
## 24025  4 2820 2.232426e+00
## 24026  4 2821 2.188968e+00
## 24027  4 2822 2.146291e+00
## 24028  4 2823 2.103307e+00
## 24029  4 2824 2.063759e+00
## 24030  4 2825 2.023368e+00
## 24031  4 2826 1.990680e+00
## 24032  4 2827 1.954307e+00
## 24033  4 2828 1.922298e+00
## 24034  4 2829 1.889978e+00
## 24035  4 2830 1.861513e+00
## 24036  4 2831 1.834403e+00
## 24037  4 2832 1.807559e+00
## 24038  4 2832 1.807186e+00
## 24039  4 2833 1.780713e+00
## 24040  4 2834 1.755160e+00
## 24041  4 2835 1.730778e+00
## 24042  4 2836 1.711724e+00
## 24043  4 2837 1.688298e+00
## 24044  4 2838 1.671003e+00
## 24045  4 2839 1.650552e+00
## 24046  4 2840 1.628735e+00
## 24047  4 2841 1.612527e+00
## 24048  4 2842 1.595395e+00
## 24049  4 2843 1.574914e+00
## 24050  4 2844 1.562544e+00
## 24051  4 2845 1.546040e+00
## 24052  4 2846 1.531945e+00
## 24053  4 2847 1.519584e+00
## 24054  4 2848 1.500735e+00
## 24055  4 2849 1.490523e+00
## 24056  4 2850 1.475617e+00
## 24057  4 2851 1.466990e+00
## 24058  4 2852 1.450224e+00
## 24059  4 2853 1.440254e+00
## 24060  4 2853 1.438680e+00
## 24061  4 2854 1.665706e+00
## 24062  4 2855 1.806353e+00
## 24063  4 2856 1.855206e+00
## 24064  4 2856 1.853756e+00
## 24065  4 2857 2.336316e+00
## 24066  4 2858 2.624501e+00
## 24067  4 2859 2.719083e+00
## 24068  4 2860 2.717287e+00
## 24069  4 2861 2.679214e+00
## 24070  4 2862 2.616117e+00
## 24071  4 2863 2.559177e+00
## 24072  4 2864 2.497761e+00
## 24073  4 2865 2.434511e+00
## 24074  4 2866 2.370987e+00
## 24075  4 2867 2.315202e+00
## 24076  4 2868 2.266708e+00
## 24077  4 2869 2.215798e+00
## 24078  4 2870 2.162926e+00
## 24079  4 2871 2.113544e+00
## 24080  4 2872 2.068829e+00
## 24081  4 2873 2.025301e+00
## 24082  4 2874 1.986967e+00
## 24083  4 2875 1.954415e+00
## 24084  4 2876 1.920770e+00
## 24085  4 2877 1.880021e+00
## 24086  4 2878 1.852272e+00
## 24087  4 2879 1.819639e+00
## 24088  4 2880 1.789074e+00
## 24089  4 2881 2.233666e+00
## 24090  4 2882 2.514168e+00
## 24091  4 2883 2.608832e+00
## 24092  4 2884 2.610972e+00
## 24093  4 2885 2.578292e+00
## 24094  4 2886 2.527472e+00
## 24095  4 2887 2.469092e+00
## 24096  4 2888 2.408015e+00
## 24097  4 2889 2.351652e+00
## 24098  4 2890 2.302665e+00
## 24099  4 2891 2.242402e+00
## 24100  4 2892 2.195515e+00
## 24101  4 2893 2.151439e+00
## 24102  4 2894 2.105210e+00
## 24103  4 2895 2.061705e+00
## 24104  4 2896 2.022982e+00
## 24105  4 2897 1.982586e+00
## 24106  4 2898 1.946869e+00
## 24107  4 2899 1.912157e+00
## 24108  4 2900 1.882780e+00
## 24109  4 2901 1.849862e+00
## 24110  4 2902 1.816762e+00
## 24111  4 2903 1.788344e+00
## 24112  4 2904 1.763270e+00
## 24113  4 2905 2.210538e+00
## 24114  4 2906 2.492844e+00
## 24115  4 2907 2.589208e+00
## 24116  4 2908 2.590822e+00
## 24117  4 2909 2.556681e+00
## 24118  4 2910 2.511106e+00
## 24119  4 2911 2.456501e+00
## 24120  4 2912 2.392491e+00
## 24121  4 2913 2.342091e+00
## 24122  4 2914 2.284119e+00
## 24123  4 2915 2.230278e+00
## 24124  4 2916 2.185274e+00
## 24125  4 2917 2.137971e+00
## 24126  4 2918 2.094245e+00
## 24127  4 2919 2.057077e+00
## 24128  4 2920 2.014648e+00
## 24129  4 2921 1.975725e+00
## 24130  4 2922 1.939415e+00
## 24131  4 2923 1.907945e+00
## 24132  4 2924 1.880273e+00
## 24133  4 2925 1.846200e+00
## 24134  4 2926 1.815695e+00
## 24135  4 2927 1.787132e+00
## 24136  4 2928 1.759583e+00
## 24137  4 2929 2.203123e+00
## 24138  4 2930 2.489308e+00
## 24139  4 2931 2.583635e+00
## 24140  4 2932 2.591177e+00
## 24141  4 2933 2.554105e+00
## 24142  4 2934 2.506504e+00
## 24143  4 2935 2.453311e+00
## 24144  4 2936 2.398359e+00
## 24145  4 2937 2.335233e+00
## 24146  4 2938 2.290825e+00
## 24147  4 2939 2.238594e+00
## 24148  4 2940 2.187086e+00
## 24149  4 2941 2.142023e+00
## 24150  4 2942 2.097696e+00
## 24151  4 2943 2.055401e+00
## 24152  4 2944 2.018269e+00
## 24153  4 2945 1.978297e+00
## 24154  4 2946 1.943937e+00
## 24155  4 2947 1.907227e+00
## 24156  4 2948 1.877672e+00
## 24157  4 2949 1.848916e+00
## 24158  4 2950 1.814111e+00
## 24159  4 2951 1.791706e+00
## 24160  4 2952 1.760727e+00
## 24161  4 2953 2.209072e+00
## 24162  4 2954 2.496081e+00
## 24163  4 2955 2.585556e+00
## 24164  4 2956 2.595059e+00
## 24165  4 2957 2.558379e+00
## 24166  4 2958 2.511495e+00
## 24167  4 2959 2.459513e+00
## 24168  4 2960 2.397373e+00
## 24169  4 2961 2.343992e+00
## 24170  4 2962 2.290052e+00
## 24171  4 2963 2.241597e+00
## 24172  4 2964 2.192687e+00
## 24173  4 2965 2.150120e+00
## 24174  4 2966 2.102981e+00
## 24175  4 2967 2.060352e+00
## 24176  4 2968 2.021498e+00
## 24177  4 2969 1.983305e+00
## 24178  4 2970 1.947448e+00
## 24179  4 2971 1.914711e+00
## 24180  4 2972 1.878718e+00
## 24181  4 2973 1.850561e+00
## 24182  4 2974 1.822146e+00
## 24183  4 2975 1.793461e+00
## 24184  4 2976 1.767456e+00
## 24185  4 2977 2.214354e+00
## 24186  4 2978 2.500249e+00
## 24187  4 2979 2.596094e+00
## 24188  4 2980 2.601900e+00
## 24189  4 2981 2.567945e+00
## 24190  4 2982 2.515055e+00
## 24191  4 2983 2.458960e+00
## 24192  4 2984 2.406543e+00
## 24193  4 2985 2.350124e+00
## 24194  4 2986 2.294733e+00
## 24195  4 2987 2.244929e+00
## 24196  4 2988 2.193735e+00
## 24197  4 2989 2.146867e+00
## 24198  4 2990 2.107057e+00
## 24199  4 2991 2.065899e+00
## 24200  4 2992 2.027467e+00
## 24201  4 2993 1.989163e+00
## 24202  4 2994 1.949244e+00
## 24203  4 2995 1.921177e+00
## 24204  4 2996 1.884539e+00
## 24205  4 2997 1.856486e+00
## 24206  4 2998 1.825853e+00
## 24207  4 2999 1.796821e+00
## 24208  4 3000 1.768857e+00
## 24209  4 3001 2.222196e+00
## 24210  4 3002 2.499213e+00
## 24211  4 3003 2.595938e+00
## 24212  4 3004 2.602648e+00
## 24213  4 3005 2.568661e+00
## 24214  4 3006 2.519277e+00
## 24215  4 3007 2.461061e+00
## 24216  4 3008 2.408853e+00
## 24217  4 3009 2.349367e+00
## 24218  4 3010 2.298735e+00
## 24219  4 3011 2.245853e+00
## 24220  4 3012 2.200932e+00
## 24221  4 3013 2.149486e+00
## 24222  4 3014 2.107834e+00
## 24223  4 3015 2.069680e+00
## 24224  4 3016 2.030310e+00
## 24225  4 3017 1.993417e+00
## 24226  4 3018 1.955222e+00
## 24227  4 3019 1.920545e+00
## 24228  4 3020 1.889921e+00
## 24229  4 3021 1.859721e+00
## 24230  4 3022 1.826963e+00
## 24231  4 3023 1.803183e+00
## 24232  4 3024 1.773671e+00
## 24233  4 3025 2.228670e+00
## 24234  4 3026 2.505070e+00
## 24235  4 3027 2.601216e+00
## 24236  4 3028 2.607577e+00
## 24237  4 3029 2.573493e+00
## 24238  4 3030 2.524003e+00
## 24239  4 3031 2.468510e+00
## 24240  4 3032 2.411132e+00
## 24241  4 3033 2.354965e+00
## 24242  4 3034 2.305652e+00
## 24243  4 3035 2.254907e+00
## 24244  4 3036 2.201809e+00
## 24245  4 3037 2.161332e+00
## 24246  4 3038 2.117104e+00
## 24247  4 3039 2.072239e+00
## 24248  4 3040 2.032720e+00
## 24249  4 3041 1.995366e+00
## 24250  4 3042 1.959690e+00
## 24251  4 3043 1.925704e+00
## 24252  4 3044 1.898782e+00
## 24253  4 3045 1.863711e+00
## 24254  4 3046 1.830725e+00
## 24255  4 3047 1.806821e+00
## 24256  4 3048 1.773914e+00
## 24257  4 3049 2.224528e+00
## 24258  4 3050 2.506272e+00
## 24259  4 3051 2.606558e+00
## 24260  4 3052 2.604245e+00
## 24261  4 3053 2.578832e+00
## 24262  4 3054 2.526673e+00
## 24263  4 3055 2.474234e+00
## 24264  4 3056 2.417974e+00
## 24265  4 3057 2.363560e+00
## 24266  4 3058 2.308173e+00
## 24267  4 3059 2.257421e+00
## 24268  4 3060 2.209717e+00
## 24269  4 3061 2.161987e+00
## 24270  4 3062 2.116869e+00
## 24271  4 3063 2.073571e+00
## 24272  4 3064 2.035559e+00
## 24273  4 3065 2.000360e+00
## 24274  4 3066 1.962033e+00
## 24275  4 3067 1.928706e+00
## 24276  4 3068 1.897332e+00
## 24277  4 3069 1.865648e+00
## 24278  4 3070 1.835170e+00
## 24279  4 3071 1.806788e+00
## 24280  4 3072 1.777697e+00
## 24281  4 3073 2.230210e+00
## 24282  4 3074 2.513758e+00
## 24283  4 3075 2.609664e+00
## 24284  4 3076 2.614926e+00
## 24285  4 3077 2.578616e+00
## 24286  4 3078 2.525865e+00
## 24287  4 3079 2.475663e+00
## 24288  4 3080 2.418864e+00
## 24289  4 3081 2.365851e+00
## 24290  4 3082 2.307157e+00
## 24291  4 3083 2.261424e+00
## 24292  4 3084 2.208597e+00
## 24293  4 3085 2.166903e+00
## 24294  4 3086 2.118990e+00
## 24295  4 3087 2.078358e+00
## 24296  4 3088 2.039598e+00
## 24297  4 3089 1.999265e+00
## 24298  4 3090 1.965059e+00
## 24299  4 3091 1.929961e+00
## 24300  4 3092 1.900205e+00
## 24301  4 3093 1.865772e+00
## 24302  4 3094 1.838105e+00
## 24303  4 3095 1.809754e+00
## 24304  4 3096 1.781568e+00
## 24305  4 3097 2.229129e+00
## 24306  4 3098 2.513762e+00
## 24307  4 3099 2.607453e+00
## 24308  4 3100 2.614182e+00
## 24309  4 3101 2.577786e+00
## 24310  4 3102 2.533419e+00
## 24311  4 3103 2.479178e+00
## 24312  4 3104 2.418051e+00
## 24313  4 3105 2.364714e+00
## 24314  4 3106 2.308245e+00
## 24315  4 3107 2.257785e+00
## 24316  4 3108 2.212316e+00
## 24317  4 3109 2.167553e+00
## 24318  4 3110 2.123126e+00
## 24319  4 3111 2.078606e+00
## 24320  4 3112 2.043228e+00
## 24321  4 3113 2.006169e+00
## 24322  4 3114 1.969438e+00
## 24323  4 3115 1.931009e+00
## 24324  4 3116 1.901336e+00
## 24325  4 3117 1.871149e+00
## 24326  4 3118 1.841480e+00
## 24327  4 3119 1.811946e+00
## 24328  4 3120 1.783871e+00
## 24329  4 3120 1.806363e+00
## 24330  4 3121 2.253121e+00
## 24331  4 3122 2.535382e+00
## 24332  4 3123 2.633991e+00
## 24333  4 3124 2.636428e+00
## 24334  4 3125 2.602544e+00
## 24335  4 3126 2.553484e+00
## 24336  4 3127 2.497737e+00
## 24337  4 3128 2.443451e+00
## 24338  4 3129 2.388663e+00
## 24339  4 3130 2.337076e+00
## 24340  4 3131 2.282309e+00
## 24341  4 3132 2.235744e+00
## 24342  4 3133 2.187798e+00
## 24343  4 3134 2.141998e+00
## 24344  4 3135 2.103494e+00
## 24345  4 3136 2.062644e+00
## 24346  4 3137 2.028428e+00
## 24347  4 3138 1.987199e+00
## 24348  4 3139 1.952784e+00
## 24349  4 3140 1.923539e+00
## 24350  4 3141 1.891565e+00
## 24351  4 3142 1.861234e+00
## 24352  4 3143 1.831600e+00
## 24353  4 3144 1.808222e+00
## 24354  4 3144 1.806464e+00
## 24355  4 3145 1.779693e+00
## 24356  4 3146 1.756201e+00
## 24357  4 3147 1.734461e+00
## 24358  4 3148 1.707104e+00
## 24359  4 3149 1.689355e+00
## 24360  4 3150 1.668524e+00
## 24361  4 3151 1.650416e+00
## 24362  4 3152 1.629373e+00
## 24363  4 3153 1.612263e+00
## 24364  4 3154 1.594867e+00
## 24365  4 3155 1.578216e+00
## 24366  4 3156 1.561231e+00
## 24367  4 3157 1.544968e+00
## 24368  4 3158 1.531547e+00
## 24369  4 3159 1.516564e+00
## 24370  4 3160 1.502627e+00
## 24371  4 3161 1.489178e+00
## 24372  4 3162 1.474563e+00
## 24373  4 3163 1.462835e+00
## 24374  4 3164 1.451737e+00
## 24375  4 3165 1.441362e+00
## 24376  4 3165 1.439720e+00
## 24377  4 3166 1.899714e+00
## 24378  4 3167 2.199609e+00
## 24379  4 3168 2.304943e+00
## 24380  4 3168 2.302435e+00
## 24381  4 3169 2.793835e+00
## 24382  4 3170 3.085105e+00
## 24383  4 3171 3.159994e+00
## 24384  4 3172 3.143032e+00
## 24385  4 3173 3.083751e+00
## 24386  4 3174 3.008442e+00
## 24387  4 3175 2.928979e+00
## 24388  4 3176 2.849605e+00
## 24389  4 3177 2.769488e+00
## 24390  4 3178 2.693922e+00
## 24391  4 3179 2.623205e+00
## 24392  4 3180 2.554637e+00
## 24393  4 3181 2.490239e+00
## 24394  4 3182 2.430474e+00
## 24395  4 3183 2.372992e+00
## 24396  4 3184 2.317255e+00
## 24397  4 3185 2.261634e+00
## 24398  4 3186 2.219105e+00
## 24399  4 3187 2.170572e+00
## 24400  4 3188 2.125642e+00
## 24401  4 3189 2.084104e+00
## 24402  4 3190 2.037369e+00
## 24403  4 3191 2.004583e+00
## 24404  4 3192 1.970432e+00
## 24405  4 3193 2.410511e+00
## 24406  4 3194 2.680209e+00
## 24407  4 3195 2.773742e+00
## 24408  4 3196 2.767979e+00
## 24409  4 3197 2.727319e+00
## 24410  4 3198 2.668562e+00
## 24411  4 3199 2.611163e+00
## 24412  4 3200 2.549026e+00
## 24413  4 3201 2.484562e+00
## 24414  4 3202 2.432529e+00
## 24415  4 3203 2.369914e+00
## 24416  4 3204 2.320385e+00
## 24417  4 3205 2.270170e+00
## 24418  4 3206 2.226419e+00
## 24419  4 3207 2.178588e+00
## 24420  4 3208 2.130539e+00
## 24421  4 3209 2.093573e+00
## 24422  4 3210 2.054394e+00
## 24423  4 3211 2.012476e+00
## 24424  4 3212 1.979637e+00
## 24425  4 3213 1.941597e+00
## 24426  4 3214 1.915287e+00
## 24427  4 3215 1.883791e+00
## 24428  4 3216 1.855839e+00
## 24429  4 3217 2.300857e+00
## 24430  4 3218 2.582748e+00
## 24431  4 3219 2.667979e+00
## 24432  4 3220 2.677376e+00
## 24433  4 3221 2.640991e+00
## 24434  4 3222 2.595335e+00
## 24435  4 3223 2.533504e+00
## 24436  4 3224 2.472799e+00
## 24437  4 3225 2.418072e+00
## 24438  4 3226 2.367246e+00
## 24439  4 3227 2.310140e+00
## 24440  4 3228 2.262084e+00
## 24441  4 3229 2.214009e+00
## 24442  4 3230 2.168820e+00
## 24443  4 3231 2.124330e+00
## 24444  4 3232 2.088600e+00
## 24445  4 3233 2.047446e+00
## 24446  4 3234 2.009864e+00
## 24447  4 3235 1.977675e+00
## 24448  4 3236 1.945360e+00
## 24449  4 3237 1.912082e+00
## 24450  4 3238 1.883801e+00
## 24451  4 3239 1.852861e+00
## 24452  4 3240 1.824458e+00
## 24453  4 3241 2.274598e+00
## 24454  4 3242 2.552017e+00
## 24455  4 3243 2.646480e+00
## 24456  4 3244 2.655606e+00
## 24457  4 3245 2.616093e+00
## 24458  4 3246 2.570588e+00
## 24459  4 3247 2.515514e+00
## 24460  4 3248 2.458935e+00
## 24461  4 3249 2.401191e+00
## 24462  4 3250 2.343879e+00
## 24463  4 3251 2.296867e+00
## 24464  4 3252 2.251024e+00
## 24465  4 3253 2.197398e+00
## 24466  4 3254 2.158040e+00
## 24467  4 3255 2.116654e+00
## 24468  4 3256 2.076529e+00
## 24469  4 3257 2.038811e+00
## 24470  4 3258 1.999790e+00
## 24471  4 3259 1.969508e+00
## 24472  4 3260 1.934321e+00
## 24473  4 3261 1.900196e+00
## 24474  4 3262 1.874215e+00
## 24475  4 3263 1.849346e+00
## 24476  4 3264 1.822367e+00
## 24477  4 3265 2.267513e+00
## 24478  4 3266 2.552528e+00
## 24479  4 3267 2.644459e+00
## 24480  4 3268 2.651246e+00
## 24481  4 3269 2.615018e+00
## 24482  4 3270 2.564692e+00
## 24483  4 3271 2.510715e+00
## 24484  4 3272 2.452123e+00
## 24485  4 3273 2.397950e+00
## 24486  4 3274 2.347842e+00
## 24487  4 3275 2.283027e+00
## 24488  4 3276 2.245399e+00
## 24489  4 3277 2.198508e+00
## 24490  4 3278 2.153492e+00
## 24491  4 3279 2.117171e+00
## 24492  4 3280 2.073664e+00
## 24493  4 3281 2.037634e+00
## 24494  4 3282 1.998687e+00
## 24495  4 3283 1.963064e+00
## 24496  4 3284 1.930833e+00
## 24497  4 3285 1.902295e+00
## 24498  4 3286 1.872773e+00
## 24499  4 3287 1.844950e+00
## 24500  4 3288 1.818362e+00
## 24501  4 3289 2.265309e+00
## 24502  4 3290 2.548357e+00
## 24503  4 3291 2.642014e+00
## 24504  4 3292 2.648923e+00
## 24505  4 3293 2.610420e+00
## 24506  4 3294 2.561470e+00
## 24507  4 3295 2.508641e+00
## 24508  4 3296 2.451887e+00
## 24509  4 3297 2.394345e+00
## 24510  4 3298 2.343225e+00
## 24511  4 3299 2.291889e+00
## 24512  4 3300 2.242298e+00
## 24513  4 3301 2.194653e+00
## 24514  4 3302 2.155854e+00
## 24515  4 3303 2.111300e+00
## 24516  4 3304 2.069055e+00
## 24517  4 3305 2.038752e+00
## 24518  4 3306 1.997734e+00
## 24519  4 3307 1.962069e+00
## 24520  4 3308 1.928050e+00
## 24521  4 3309 1.899633e+00
## 24522  4 3310 1.870524e+00
## 24523  4 3311 1.842304e+00
## 24524  4 3312 1.815309e+00
## 24525  4 3313 2.264149e+00
## 24526  4 3314 2.542255e+00
## 24527  4 3315 2.640792e+00
## 24528  4 3316 2.639551e+00
## 24529  4 3317 2.607504e+00
## 24530  4 3318 2.560605e+00
## 24531  4 3319 2.504184e+00
## 24532  4 3320 2.449400e+00
## 24533  4 3321 2.393660e+00
## 24534  4 3322 2.344363e+00
## 24535  4 3323 2.288088e+00
## 24536  4 3324 2.240640e+00
## 24537  4 3325 2.196805e+00
## 24538  4 3326 2.149755e+00
## 24539  4 3327 2.108568e+00
## 24540  4 3328 2.070422e+00
## 24541  4 3329 2.033367e+00
## 24542  4 3330 1.995938e+00
## 24543  4 3331 1.960181e+00
## 24544  4 3332 1.929490e+00
## 24545  4 3333 1.897779e+00
## 24546  4 3334 1.868708e+00
## 24547  4 3335 1.842150e+00
## 24548  4 3336 1.813801e+00
## 24549  4 3337 2.263234e+00
## 24550  4 3338 2.548776e+00
## 24551  4 3339 2.637136e+00
## 24552  4 3340 2.640782e+00
## 24553  4 3341 2.607533e+00
## 24554  4 3342 2.565424e+00
## 24555  4 3343 2.507090e+00
## 24556  4 3344 2.447791e+00
## 24557  4 3345 2.393290e+00
## 24558  4 3346 2.340487e+00
## 24559  4 3347 2.287145e+00
## 24560  4 3348 2.239534e+00
## 24561  4 3349 2.190053e+00
## 24562  4 3350 2.149096e+00
## 24563  4 3351 2.111380e+00
## 24564  4 3352 2.071794e+00
## 24565  4 3353 2.032433e+00
## 24566  4 3354 1.995859e+00
## 24567  4 3355 1.962360e+00
## 24568  4 3356 1.930756e+00
## 24569  4 3357 1.899895e+00
## 24570  4 3358 1.870536e+00
## 24571  4 3359 1.835217e+00
## 24572  4 3360 1.808688e+00
## 24573  4 3361 2.265519e+00
## 24574  4 3362 2.539910e+00
## 24575  4 3363 2.637533e+00
## 24576  4 3364 2.642272e+00
## 24577  4 3365 2.614394e+00
## 24578  4 3366 2.554185e+00
## 24579  4 3367 2.502388e+00
## 24580  4 3368 2.447912e+00
## 24581  4 3369 2.390501e+00
## 24582  4 3370 2.333242e+00
## 24583  4 3371 2.288489e+00
## 24584  4 3372 2.238919e+00
## 24585  4 3373 2.197146e+00
## 24586  4 3374 2.150226e+00
## 24587  4 3375 2.104933e+00
## 24588  4 3376 2.068072e+00
## 24589  4 3377 2.029788e+00
## 24590  4 3378 1.994451e+00
## 24591  4 3379 1.962379e+00
## 24592  4 3380 1.930615e+00
## 24593  4 3381 1.895136e+00
## 24594  4 3382 1.869538e+00
## 24595  4 3383 1.838509e+00
## 24596  4 3384 1.811219e+00
## 24597  4 3385 2.258815e+00
## 24598  4 3386 2.542415e+00
## 24599  4 3387 2.640517e+00
## 24600  4 3388 2.642578e+00
## 24601  4 3389 2.610640e+00
## 24602  4 3390 2.560072e+00
## 24603  4 3391 2.505069e+00
## 24604  4 3392 2.449111e+00
## 24605  4 3393 2.393115e+00
## 24606  4 3394 2.341567e+00
## 24607  4 3395 2.284732e+00
## 24608  4 3396 2.239360e+00
## 24609  4 3397 2.197113e+00
## 24610  4 3398 2.147276e+00
## 24611  4 3399 2.106279e+00
## 24612  4 3400 2.067193e+00
## 24613  4 3401 2.031335e+00
## 24614  4 3402 1.992291e+00
## 24615  4 3403 1.960113e+00
## 24616  4 3404 1.926339e+00
## 24617  4 3405 1.895122e+00
## 24618  4 3406 1.866194e+00
## 24619  4 3407 1.837150e+00
## 24620  4 3408 1.812238e+00
## 24621  4 3409 2.261888e+00
## 24622  4 3410 2.547406e+00
## 24623  4 3411 2.637225e+00
## 24624  4 3412 2.645085e+00
## 24625  4 3413 2.606090e+00
## 24626  4 3414 2.561323e+00
## 24627  4 3415 2.502860e+00
## 24628  4 3416 2.445611e+00
## 24629  4 3417 2.389345e+00
## 24630  4 3418 2.337565e+00
## 24631  4 3419 2.285928e+00
## 24632  4 3420 2.238545e+00
## 24633  4 3421 2.191609e+00
## 24634  4 3422 2.147853e+00
## 24635  4 3423 2.109309e+00
## 24636  4 3424 2.066883e+00
## 24637  4 3425 2.027819e+00
## 24638  4 3426 1.994434e+00
## 24639  4 3427 1.958579e+00
## 24640  4 3428 1.926755e+00
## 24641  4 3429 1.900388e+00
## 24642  4 3430 1.864209e+00
## 24643  4 3431 1.839190e+00
## 24644  4 3432 1.809874e+00
## 24645  4 3432 1.804378e+00
## 24646  4 3433 2.252961e+00
## 24647  4 3434 2.537291e+00
## 24648  4 3435 2.628983e+00
## 24649  4 3436 2.636150e+00
## 24650  4 3437 2.607752e+00
## 24651  4 3438 2.555368e+00
## 24652  4 3439 2.499922e+00
## 24653  4 3440 2.444458e+00
## 24654  4 3441 2.388121e+00
## 24655  4 3442 2.334040e+00
## 24656  4 3443 2.282260e+00
## 24657  4 3444 2.234516e+00
## 24658  4 3445 2.190383e+00
## 24659  4 3446 2.139332e+00
## 24660  4 3447 2.102980e+00
## 24661  4 3448 2.061587e+00
## 24662  4 3449 2.025036e+00
## 24663  4 3450 1.990109e+00
## 24664  4 3451 1.954134e+00
## 24665  4 3452 1.918027e+00
## 24666  4 3453 1.892405e+00
## 24667  4 3454 1.859915e+00
## 24668  4 3455 1.831921e+00
## 24669  4 3456 1.807227e+00
## 24670  4 3456 1.805849e+00
## 24671  4 3457 1.780287e+00
## 24672  4 3458 1.757774e+00
## 24673  4 3459 1.733240e+00
```

```
## 24674  4 3460 1.708233e+00
## 24675  4 3461 1.688170e+00
## 24676  4 3462 1.670446e+00
## 24677  4 3463 1.644102e+00
## 24678  4 3464 1.626670e+00
## 24679  4 3465 1.611512e+00
## 24680  4 3466 1.593375e+00
## 24681  4 3467 1.575737e+00
## 24682  4 3468 1.560593e+00
## 24683  4 3469 1.545959e+00
## 24684  4 3470 1.530428e+00
## 24685  4 3471 1.514921e+00
## 24686  4 3472 1.500656e+00
## 24687  4 3473 1.489037e+00
## 24688  4 3474 1.475982e+00
## 24689  4 3475 1.461559e+00
## 24690  4 3476 1.452166e+00
## 24691  4 3477 1.438650e+00
## 24692  4 3478 1.426265e+00
## 24693  4 3479 1.417608e+00
## 24694  4 3480 1.407215e+00
## 24695  4 3480 1.403420e+00
## 24696  4 3481 1.867048e+00
## 24697  4 3482 2.164456e+00
## 24698  4 3483 2.276211e+00
## 24699  4 3484 2.293531e+00
## 24700  4 3485 2.271593e+00
## 24701  4 3486 2.232920e+00
## 24702  4 3487 2.191042e+00
## 24703  4 3488 2.140361e+00
## 24704  4 3489 2.094248e+00
## 24705  4 3490 2.053183e+00
## 24706  4 3491 2.014101e+00
## 24707  4 3492 1.967595e+00
## 24708  4 3493 1.930029e+00
## 24709  4 3494 1.898646e+00
## 24710  4 3495 1.858832e+00
## 24711  4 3496 1.826799e+00
## 24712  4 3497 1.793907e+00
## 24713  4 3498 1.764943e+00
## 24714  4 3499 1.738381e+00
## 24715  4 3500 1.710386e+00
## 24716  4 3501 1.684138e+00
## 24717  4 3502 1.657758e+00
## 24718  4 3503 1.633516e+00
## 24719  4 3504 1.610839e+00
## 24720  4 3504 1.610917e+00
## 24721  4 3505 2.061684e+00
## 24722  4 3506 2.347669e+00
## 24723  4 3507 2.446441e+00
## 24724  4 3508 2.454341e+00
## 24725  4 3509 2.427304e+00
## 24726  4 3510 2.381917e+00
## 24727  4 3511 2.326474e+00
## 24728  4 3512 2.274144e+00
## 24729  4 3513 2.222491e+00
## 24730  4 3514 2.168489e+00
## 24731  4 3515 2.118531e+00
## 24732  4 3516 2.076140e+00
## 24733  4 3517 2.032423e+00
## 24734  4 3518 1.989117e+00
## 24735  4 3519 1.948968e+00
## 24736  4 3520 1.913379e+00
## 24737  4 3521 1.875776e+00
## 24738  4 3522 1.841706e+00
## 24739  4 3523 1.810812e+00
## 24740  4 3524 1.777947e+00
## 24741  4 3525 1.749385e+00
## 24742  4 3526 1.720548e+00
## 24743  4 3527 1.696591e+00
## 24744  4 3528 1.672468e+00
## 24745  4 3529 2.115298e+00
## 24746  4 3530 2.407257e+00
## 24747  4 3531 2.502115e+00
## 24748  4 3532 2.505191e+00
## 24749  4 3533 2.471713e+00
## 24750  4 3534 2.422342e+00
## 24751  4 3535 2.367318e+00
## 24752  4 3536 2.314691e+00
## 24753  4 3537 2.264357e+00
## 24754  4 3538 2.207945e+00
## 24755  4 3539 2.158041e+00
## 24756  4 3540 2.109277e+00
## 24757  4 3541 2.059083e+00
## 24758  4 3542 2.019870e+00
## 24759  4 3543 1.982823e+00
## 24760  4 3544 1.946707e+00
## 24761  4 3545 1.902793e+00
## 24762  4 3546 1.868613e+00
## 24763  4 3547 1.835755e+00
## 24764  4 3548 1.806541e+00
## 24765  4 3549 1.776064e+00
## 24766  4 3550 1.743888e+00
## 24767  4 3551 1.718113e+00
## 24768  4 3552 1.694497e+00
## 24769  4 3553 2.141722e+00
## 24770  4 3554 2.423901e+00
## 24771  4 3555 2.516426e+00
## 24772  4 3556 2.525230e+00
## 24773  4 3557 2.490284e+00
## 24774  4 3558 2.444128e+00
## 24775  4 3559 2.391837e+00
## 24776  4 3560 2.331919e+00
## 24777  4 3561 2.281009e+00
## 24778  4 3562 2.231289e+00
## 24779  4 3563 2.177475e+00
## 24780  4 3564 2.127214e+00
## 24781  4 3565 2.081748e+00
## 24782  4 3566 2.040016e+00
## 24783  4 3567 1.997923e+00
## 24784  4 3568 1.959441e+00
## 24785  4 3569 1.921405e+00
## 24786  4 3570 1.890782e+00
## 24787  4 3571 1.850410e+00
## 24788  4 3572 1.817923e+00
## 24789  4 3573 1.789522e+00
## 24790  4 3574 1.760325e+00
## 24791  4 3575 1.737030e+00
## 24792  4 3576 1.704600e+00
## 24793  4 3577 2.156364e+00
## 24794  4 3578 2.439855e+00
## 24795  4 3579 2.535494e+00
## 24796  4 3580 2.539066e+00
## 24797  4 3581 2.507605e+00
## 24798  4 3582 2.461071e+00
## 24799  4 3583 2.401335e+00
## 24800  4 3584 2.348093e+00
## 24801  4 3585 2.291037e+00
## 24802  4 3586 2.240254e+00
## 24803  4 3587 2.189204e+00
## 24804  4 3588 2.139581e+00
## 24805  4 3589 2.095612e+00
## 24806  4 3590 2.045741e+00
## 24807  4 3591 2.010406e+00
## 24808  4 3592 1.969826e+00
## 24809  4 3593 1.932983e+00
## 24810  4 3594 1.898666e+00
## 24811  4 3595 1.862110e+00
## 24812  4 3596 1.832601e+00
## 24813  4 3597 1.802017e+00
## 24814  4 3598 1.772135e+00
## 24815  4 3599 1.743638e+00
## 24816  4 3600 1.719620e+00
## 24817  4 3601 2.167680e+00
## 24818  4 3602 2.449812e+00
## 24819  4 3603 2.546133e+00
## 24820  4 3604 2.551226e+00
## 24821  4 3605 2.516254e+00
## 24822  4 3606 2.465786e+00
## 24823  4 3607 2.414835e+00
## 24824  4 3608 2.358337e+00
## 24825  4 3609 2.299617e+00
## 24826  4 3610 2.250740e+00
## 24827  4 3611 2.194586e+00
## 24828  4 3612 2.151734e+00
## 24829  4 3613 2.103781e+00
## 24830  4 3614 2.062575e+00
## 24831  4 3615 2.017821e+00
## 24832  4 3616 1.980674e+00
## 24833  4 3617 1.943202e+00
## 24834  4 3618 1.905183e+00
## 24835  4 3619 1.873956e+00
## 24836  4 3620 1.840179e+00
## 24837  4 3621 1.812074e+00
## 24838  4 3622 1.780244e+00
## 24839  4 3623 1.755187e+00
## 24840  4 3624 1.729487e+00
## 24841  4 3625 2.170398e+00
## 24842  4 3626 2.458945e+00
## 24843  4 3627 2.551644e+00
## 24844  4 3628 2.555998e+00
## 24845  4 3629 2.526713e+00
## 24846  4 3630 2.476871e+00
## 24847  4 3631 2.425647e+00
## 24848  4 3632 2.362360e+00
## 24849  4 3633 2.311114e+00
## 24850  4 3634 2.255694e+00
## 24851  4 3635 2.208709e+00
## 24852  4 3636 2.155777e+00
## 24853  4 3637 2.109772e+00
## 24854  4 3638 2.070166e+00
## 24855  4 3639 2.025657e+00
## 24856  4 3640 1.987599e+00
## 24857  4 3641 1.953373e+00
## 24858  4 3642 1.914310e+00
## 24859  4 3643 1.880790e+00
## 24860  4 3644 1.848933e+00
## 24861  4 3645 1.819995e+00
## 24862  4 3646 1.789167e+00
## 24863  4 3647 1.762649e+00
## 24864  4 3648 1.734948e+00
## 24865  4 3649 2.183794e+00
## 24866  4 3650 2.467727e+00
## 24867  4 3651 2.563423e+00
## 24868  4 3652 2.567485e+00
## 24869  4 3653 2.527237e+00
## 24870  4 3654 2.484804e+00
## 24871  4 3655 2.430709e+00
## 24872  4 3656 2.379212e+00
## 24873  4 3657 2.317010e+00
## 24874  4 3658 2.263835e+00
## 24875  4 3659 2.216937e+00
## 24876  4 3660 2.166848e+00
## 24877  4 3661 2.119611e+00
## 24878  4 3662 2.076642e+00
## 24879  4 3663 2.034953e+00
## 24880  4 3664 2.002534e+00
## 24881  4 3665 1.957122e+00
## 24882  4 3666 1.923850e+00
## 24883  4 3667 1.889373e+00
## 24884  4 3668 1.858157e+00
## 24885  4 3669 1.823987e+00
## 24886  4 3670 1.797854e+00
## 24887  4 3671 1.769682e+00
## 24888  4 3672 1.742652e+00
## 24889  4 3673 2.189305e+00
## 24890  4 3674 2.471042e+00
## 24891  4 3675 2.570288e+00
## 24892  4 3676 2.579622e+00
## 24893  4 3677 2.539010e+00
## 24894  4 3678 2.492862e+00
## 24895  4 3679 2.434905e+00
## 24896  4 3680 2.379138e+00
## 24897  4 3681 2.325293e+00
## 24898  4 3682 2.272833e+00
## 24899  4 3683 2.223270e+00
## 24900  4 3684 2.175673e+00
## 24901  4 3685 2.123888e+00
## 24902  4 3686 2.084324e+00
## 24903  4 3687 2.047095e+00
## 24904  4 3688 2.005368e+00
## 24905  4 3689 1.968253e+00
## 24906  4 3690 1.930284e+00
## 24907  4 3691 1.894890e+00
## 24908  4 3692 1.864260e+00
## 24909  4 3693 1.831541e+00
## 24910  4 3694 1.801414e+00
## 24911  4 3695 1.777179e+00
## 24912  4 3696 1.746830e+00
## 24913  4 3697 2.197681e+00
## 24914  4 3698 2.478126e+00
## 24915  4 3699 2.570688e+00
## 24916  4 3700 2.578576e+00
## 24917  4 3701 2.542368e+00
## 24918  4 3702 2.496523e+00
## 24919  4 3703 2.442335e+00
## 24920  4 3704 2.385457e+00
## 24921  4 3705 2.334519e+00
## 24922  4 3706 2.279112e+00
## 24923  4 3707 2.228993e+00
## 24924  4 3708 2.182225e+00
## 24925  4 3709 2.133176e+00
## 24926  4 3710 2.088703e+00
## 24927  4 3711 2.049498e+00
## 24928  4 3712 2.005246e+00
## 24929  4 3713 1.970302e+00
## 24930  4 3714 1.934747e+00
## 24931  4 3715 1.902748e+00
## 24932  4 3716 1.870300e+00
## 24933  4 3717 1.837762e+00
## 24934  4 3718 1.812346e+00
## 24935  4 3719 1.781464e+00
## 24936  4 3720 1.757125e+00
## 24937  4 3721 2.200121e+00
## 24938  4 3722 2.487329e+00
## 24939  4 3723 2.580170e+00
## 24940  4 3724 2.582516e+00
## 24941  4 3725 2.548617e+00
## 24942  4 3726 2.503314e+00
## 24943  4 3727 2.445798e+00
## 24944  4 3728 2.391128e+00
## 24945  4 3729 2.333816e+00
## 24946  4 3730 2.281202e+00
## 24947  4 3731 2.233492e+00
## 24948  4 3732 2.182492e+00
## 24949  4 3733 2.138701e+00
## 24950  4 3734 2.093880e+00
## 24951  4 3735 2.053758e+00
## 24952  4 3736 2.016044e+00
## 24953  4 3737 1.980188e+00
## 24954  4 3738 1.944267e+00
## 24955  4 3739 1.906871e+00
## 24956  4 3740 1.876537e+00
## 24957  4 3741 1.847493e+00
## 24958  4 3742 1.816576e+00
## 24959  4 3743 1.786238e+00
## 24960  4 3744 1.760371e+00
## 24961  4 3745 2.210947e+00
## 24962  4 3746 2.497516e+00
## 24963  4 3747 2.585316e+00
## 24964  4 3748 2.590032e+00
## 24965  4 3749 2.553880e+00
## 24966  4 3750 2.511131e+00
## 24967  4 3751 2.456030e+00
## 24968  4 3752 2.397236e+00
## 24969  4 3753 2.342242e+00
## 24970  4 3754 2.289728e+00
## 24971  4 3755 2.239420e+00
## 24972  4 3756 2.188853e+00
## 24973  4 3757 2.141220e+00
## 24974  4 3758 2.105425e+00
## 24975  4 3759 2.061050e+00
## 24976  4 3760 2.020637e+00
## 24977  4 3761 1.983541e+00
## 24978  4 3762 1.946124e+00
## 24979  4 3763 1.913184e+00
## 24980  4 3764 1.882807e+00
## 24981  4 3765 1.849142e+00
## 24982  4 3766 1.819289e+00
## 24983  4 3767 1.792608e+00
## 24984  4 3768 1.768200e+00
## 24985  4 3768 1.809157e+00
## 24986  4 3769 2.253743e+00
## 24987  4 3770 2.539063e+00
## 24988  4 3771 2.631601e+00
## 24989  4 3772 2.637024e+00
## 24990  4 3773 2.606235e+00
## 24991  4 3774 2.561387e+00
## 24992  4 3775 2.499026e+00
## 24993  4 3776 2.444844e+00
## 24994  4 3777 2.387649e+00
## 24995  4 3778 2.331387e+00
## 24996  4 3779 2.281243e+00
## 24997  4 3780 2.232885e+00
## 24998  4 3781 2.189839e+00
## 24999  4 3782 2.143980e+00
## 25000  4 3783 2.100387e+00
## 25001  4 3784 2.060806e+00
## 25002  4 3785 2.022367e+00
## 25003  4 3786 1.983718e+00
## 25004  4 3787 1.954266e+00
## 25005  4 3788 1.922121e+00
## 25006  4 3789 1.892929e+00
## 25007  4 3790 1.863034e+00
## 25008  4 3791 1.830144e+00
## 25009  4 3792 1.806504e+00
## 25010  4 3792 1.808182e+00
## 25011  4 3793 1.782985e+00
## 25012  4 3794 1.753931e+00
## 25013  4 3795 1.732565e+00
## 25014  4 3796 1.712540e+00
## 25015  4 3797 1.687896e+00
## 25016  4 3798 1.668629e+00
## 25017  4 3799 1.648340e+00
## 25018  4 3800 1.630654e+00
## 25019  4 3801 1.609164e+00
## 25020  4 3802 1.594485e+00
## 25021  4 3803 1.579529e+00
## 25022  4 3804 1.561472e+00
## 25023  4 3805 1.547003e+00
## 25024  4 3806 1.536879e+00
## 25025  4 3807 1.516601e+00
## 25026  4 3808 1.502059e+00
## 25027  4 3809 1.488812e+00
## 25028  4 3810 1.475898e+00
## 25029  4 3811 1.462035e+00
## 25030  4 3812 1.454211e+00
## 25031  4 3813 1.438138e+00
## 25032  4 3814 1.429653e+00
## 25033  4 3815 1.418777e+00
## 25034  4 3816 1.405983e+00
## 25035  4 3816 1.406538e+00
## 25036  4 3817 2.106583e+00
## 25037  4 3818 2.562751e+00
## 25038  4 3819 2.725868e+00
## 25039  4 3820 2.756328e+00
## 25040  4 3821 2.723610e+00
## 25041  4 3822 2.674918e+00
## 25042  4 3823 2.612520e+00
## 25043  4 3824 2.551493e+00
## 25044  4 3825 2.481619e+00
## 25045  4 3826 2.422060e+00
## 25046  4 3827 2.360606e+00
## 25047  4 3828 2.302721e+00
## 25048  4 3829 2.252481e+00
## 25049  4 3830 2.202538e+00
## 25050  4 3831 2.153669e+00
## 25051  4 3832 2.107467e+00
## 25052  4 3833 2.062045e+00
## 25053  4 3834 2.021254e+00
## 25054  4 3835 1.979623e+00
## 25055  4 3836 1.945855e+00
## 25056  4 3837 1.909208e+00
## 25057  4 3838 1.877253e+00
## 25058  4 3839 1.840832e+00
## 25059  4 3840 1.815223e+00
## 25060  4 3840 1.811276e+00
## 25061  4 3841 2.257733e+00
## 25062  4 3842 2.541219e+00
## 25063  4 3843 2.628746e+00
## 25064  4 3844 2.629458e+00
## 25065  4 3845 2.590456e+00
## 25066  4 3846 2.542196e+00
## 25067  4 3847 2.481346e+00
## 25068  4 3848 2.422822e+00
## 25069  4 3849 2.366353e+00
## 25070  4 3850 2.312704e+00
## 25071  4 3851 2.260406e+00
## 25072  4 3852 2.212173e+00
## 25073  4 3853 2.164046e+00
## 25074  4 3854 2.115016e+00
## 25075  4 3855 2.071914e+00
## 25076  4 3856 2.030045e+00
## 25077  4 3857 1.990652e+00
## 25078  4 3858 1.958986e+00
## 25079  4 3859 1.923572e+00
## 25080  4 3860 1.884307e+00
## 25081  4 3861 1.855006e+00
## 25082  4 3862 1.819520e+00
## 25083  4 3863 1.793641e+00
## 25084  4 3864 1.765966e+00
## 25085  4 3865 2.218803e+00
## 25086  4 3866 2.495851e+00
## 25087  4 3867 2.590351e+00
## 25088  4 3868 2.592871e+00
## 25089  4 3869 2.560868e+00
## 25090  4 3870 2.509870e+00
## 25091  4 3871 2.458462e+00
## 25092  4 3872 2.397368e+00
## 25093  4 3873 2.345205e+00
## 25094  4 3874 2.289884e+00
## 25095  4 3875 2.237313e+00
## 25096  4 3876 2.190514e+00
## 25097  4 3877 2.143060e+00
## 25098  4 3878 2.098928e+00
## 25099  4 3879 2.056163e+00
## 25100  4 3880 2.018689e+00
## 25101  4 3881 1.976644e+00
## 25102  4 3882 1.944044e+00
## 25103  4 3883 1.908328e+00
## 25104  4 3884 1.876536e+00
## 25105  4 3885 1.848591e+00
## 25106  4 3886 1.815614e+00
## 25107  4 3887 1.788114e+00
## 25108  4 3888 1.761770e+00
## 25109  4 3889 2.208858e+00
## 25110  4 3890 2.491531e+00
## 25111  4 3891 2.586161e+00
## 25112  4 3892 2.594859e+00
## 25113  4 3893 2.559638e+00
## 25114  4 3894 2.512514e+00
## 25115  4 3895 2.453101e+00
## 25116  4 3896 2.398146e+00
## 25117  4 3897 2.342111e+00
## 25118  4 3898 2.291084e+00
## 25119  4 3899 2.241233e+00
## 25120  4 3900 2.183864e+00
## 25121  4 3901 2.143153e+00
## 25122  4 3902 2.103489e+00
## 25123  4 3903 2.060927e+00
## 25124  4 3904 2.017459e+00
## 25125  4 3905 1.979745e+00
## 25126  4 3906 1.942735e+00
## 25127  4 3907 1.907955e+00
## 25128  4 3908 1.879939e+00
## 25129  4 3909 1.846755e+00
## 25130  4 3910 1.818437e+00
## 25131  4 3911 1.787632e+00
## 25132  4 3912 1.764375e+00
## 25133  4 3913 2.211077e+00
## 25134  4 3914 2.499554e+00
## 25135  4 3915 2.587893e+00
## 25136  4 3916 2.595660e+00
## 25137  4 3917 2.564048e+00
## 25138  4 3918 2.515401e+00
## 25139  4 3919 2.457879e+00
## 25140  4 3920 2.400936e+00
## 25141  4 3921 2.343575e+00
## 25142  4 3922 2.293300e+00
## 25143  4 3923 2.243118e+00
## 25144  4 3924 2.194310e+00
## 25145  4 3925 2.144666e+00
## 25146  4 3926 2.102489e+00
## 25147  4 3927 2.063694e+00
## 25148  4 3928 2.026259e+00
## 25149  4 3929 1.985464e+00
## 25150  4 3930 1.949271e+00
## 25151  4 3931 1.911250e+00
## 25152  4 3932 1.879931e+00
## 25153  4 3933 1.850663e+00
## 25154  4 3934 1.824248e+00
## 25155  4 3935 1.792034e+00
## 25156  4 3936 1.767285e+00
## 25157  4 3937 2.216190e+00
## 25158  4 3938 2.499253e+00
## 25159  4 3939 2.590071e+00
## 25160  4 3940 2.594909e+00
## 25161  4 3941 2.564660e+00
## 25162  4 3942 2.513879e+00
## 25163  4 3943 2.464022e+00
## 25164  4 3944 2.405681e+00
## 25165  4 3945 2.349888e+00
## 25166  4 3946 2.298970e+00
## 25167  4 3947 2.239525e+00
## 25168  4 3948 2.202544e+00
## 25169  4 3949 2.148749e+00
## 25170  4 3950 2.108130e+00
## 25171  4 3951 2.065272e+00
## 25172  4 3952 2.026085e+00
## 25173  4 3953 1.988479e+00
## 25174  4 3954 1.950027e+00
## 25175  4 3955 1.919978e+00
## 25176  4 3956 1.887245e+00
## 25177  4 3957 1.853542e+00
## 25178  4 3958 1.825423e+00
## 25179  4 3959 1.797834e+00
## 25180  4 3960 1.771611e+00
## 25181  4 3961 2.222402e+00
## 25182  4 3962 2.502621e+00
## 25183  4 3963 2.593834e+00
## 25184  4 3964 2.605158e+00
## 25185  4 3965 2.574015e+00
## 25186  4 3966 2.522510e+00
## 25187  4 3967 2.464995e+00
## 25188  4 3968 2.409250e+00
## 25189  4 3969 2.346198e+00
## 25190  4 3970 2.300640e+00
## 25191  4 3971 2.251735e+00
## 25192  4 3972 2.201701e+00
## 25193  4 3973 2.156685e+00
## 25194  4 3974 2.110548e+00
## 25195  4 3975 2.070960e+00
## 25196  4 3976 2.028078e+00
## 25197  4 3977 1.992462e+00
## 25198  4 3978 1.954078e+00
## 25199  4 3979 1.923834e+00
## 25200  4 3980 1.889256e+00
## 25201  4 3981 1.859222e+00
## 25202  4 3982 1.829468e+00
## 25203  4 3983 1.800736e+00
## 25204  4 3984 1.773407e+00
## 25205  4 3985 2.221710e+00
## 25206  4 3986 2.506952e+00
## 25207  4 3987 2.602015e+00
## 25208  4 3988 2.607950e+00
## 25209  4 3989 2.574315e+00
## 25210  4 3990 2.523598e+00
## 25211  4 3991 2.471289e+00
## 25212  4 3992 2.410942e+00
## 25213  4 3993 2.357381e+00
## 25214  4 3994 2.307915e+00
## 25215  4 3995 2.254717e+00
## 25216  4 3996 2.204827e+00
## 25217  4 3997 2.160504e+00
## 25218  4 3998 2.114216e+00
## 25219  4 3999 2.074310e+00
## 25220  4 4000 2.032076e+00
## 25221  4 4001 1.992450e+00
## 25222  4 4002 1.959885e+00
## 25223  4 4003 1.925225e+00
## 25224  4 4004 1.890921e+00
## 25225  4 4005 1.865316e+00
## 25226  4 4006 1.832875e+00
## 25227  4 4007 1.807924e+00
## 25228  4 4008 1.779201e+00
## 25229  4 4009 2.227277e+00
## 25230  4 4010 2.510495e+00
## 25231  4 4011 2.606689e+00
## 25232  4 4012 2.609674e+00
## 25233  4 4013 2.577997e+00
## 25234  4 4014 2.526163e+00
## 25235  4 4015 2.473785e+00
## 25236  4 4016 2.416712e+00
## 25237  4 4017 2.362264e+00
## 25238  4 4018 2.309043e+00
## 25239  4 4019 2.255260e+00
## 25240  4 4020 2.206176e+00
## 25241  4 4021 2.159207e+00
## 25242  4 4022 2.117672e+00
## 25243  4 4023 2.077214e+00
## 25244  4 4024 2.036250e+00
## 25245  4 4025 1.996214e+00
## 25246  4 4026 1.965937e+00
## 25247  4 4027 1.927979e+00
## 25248  4 4028 1.892806e+00
## 25249  4 4029 1.866334e+00
## 25250  4 4030 1.837337e+00
## 25251  4 4031 1.808416e+00
## 25252  4 4032 1.782173e+00
## 25253  4 4033 2.228765e+00
## 25254  4 4034 2.510945e+00
## 25255  4 4035 2.607578e+00
## 25256  4 4036 2.612679e+00
## 25257  4 4037 2.581612e+00
## 25258  4 4038 2.528773e+00
## 25259  4 4039 2.471279e+00
## 25260  4 4040 2.417843e+00
## 25261  4 4041 2.361665e+00
## 25262  4 4042 2.311419e+00
## 25263  4 4043 2.253850e+00
## 25264  4 4044 2.206522e+00
## 25265  4 4045 2.162485e+00
## 25266  4 4046 2.118957e+00
## 25267  4 4047 2.081016e+00
## 25268  4 4048 2.037028e+00
## 25269  4 4049 2.004304e+00
## 25270  4 4050 1.964086e+00
## 25271  4 4051 1.930453e+00
## 25272  4 4052 1.899965e+00
## 25273  4 4053 1.868359e+00
## 25274  4 4054 1.838901e+00
## 25275  4 4055 1.809935e+00
## 25276  4 4056 1.780150e+00
## 25277  4 4057 2.228218e+00
## 25278  4 4058 2.512030e+00
## 25279  4 4059 2.606967e+00
## 25280  4 4060 2.609599e+00
## 25281  4 4061 2.578149e+00
## 25282  4 4062 2.530376e+00
## 25283  4 4063 2.476467e+00
## 25284  4 4064 2.416591e+00
## 25285  4 4065 2.364214e+00
## 25286  4 4066 2.315413e+00
## 25287  4 4067 2.261401e+00
## 25288  4 4068 2.214132e+00
## 25289  4 4069 2.167814e+00
## 25290  4 4070 2.123644e+00
## 25291  4 4071 2.083187e+00
## 25292  4 4072 2.041183e+00
## 25293  4 4073 2.004192e+00
## 25294  4 4074 1.966241e+00
## 25295  4 4075 1.935338e+00
## 25296  4 4076 1.899335e+00
## 25297  4 4077 1.869703e+00
## 25298  4 4078 1.843311e+00
## 25299  4 4079 1.814611e+00
## 25300  4 4080 1.790136e+00
## 25301  4 4081 2.235653e+00
## 25302  4 4082 2.514980e+00
## 25303  4 4083 2.612363e+00
## 25304  4 4084 2.611594e+00
## 25305  4 4085 2.586682e+00
## 25306  4 4086 2.537393e+00
## 25307  4 4087 2.477062e+00
## 25308  4 4088 2.424501e+00
## 25309  4 4089 2.364890e+00
## 25310  4 4090 2.316462e+00
## 25311  4 4091 2.262769e+00
## 25312  4 4092 2.218691e+00
## 25313  4 4093 2.167198e+00
## 25314  4 4094 2.128296e+00
## 25315  4 4095 2.081451e+00
## 25316  4 4096 2.043891e+00
## 25317  4 4097 2.004927e+00
## 25318  4 4098 1.972215e+00
## 25319  4 4099 1.935340e+00
## 25320  4 4100 1.902996e+00
## 25321  4 4101 1.870777e+00
## 25322  4 4102 1.847139e+00
## 25323  4 4103 1.817320e+00
## 25324  4 4104 1.789647e+00
## 25325  4 4104 1.806336e+00
## 25326  4 4105 2.257293e+00
## 25327  4 4106 2.533023e+00
## 25328  4 4107 2.629769e+00
## 25329  4 4108 2.638921e+00
## 25330  4 4109 2.604144e+00
## 25331  4 4110 2.556889e+00
## 25332  4 4111 2.496513e+00
## 25333  4 4112 2.439294e+00
## 25334  4 4113 2.389303e+00
## 25335  4 4114 2.333933e+00
## 25336  4 4115 2.278417e+00
## 25337  4 4116 2.238683e+00
## 25338  4 4117 2.189111e+00
## 25339  4 4118 2.144972e+00
## 25340  4 4119 2.100166e+00
## 25341  4 4120 2.064861e+00
## 25342  4 4121 2.024074e+00
## 25343  4 4122 1.989439e+00
## 25344  4 4123 1.957143e+00
## 25345  4 4124 1.921234e+00
## 25346  4 4125 1.892033e+00
## 25347  4 4126 1.863492e+00
## 25348  4 4127 1.832543e+00
## 25349  4 4128 1.806439e+00
## 25350  4 4128 1.810140e+00
## 25351  4 4129 1.780121e+00
## 25352  4 4130 1.758168e+00
## 25353  4 4131 1.731831e+00
## 25354  4 4132 1.709533e+00
## 25355  4 4133 1.688248e+00
## 25356  4 4134 1.670137e+00
## 25357  4 4135 1.647586e+00
## 25358  4 4136 1.630986e+00
## 25359  4 4137 1.611166e+00
## 25360  4 4138 1.593698e+00
## 25361  4 4139 1.578356e+00
## 25362  4 4140 1.561165e+00
## 25363  4 4141 1.547375e+00
## 25364  4 4142 1.530454e+00
## 25365  4 4143 1.517739e+00
## 25366  4 4144 1.501050e+00
## 25367  4 4145 1.489876e+00
## 25368  4 4146 1.477759e+00
## 25369  4 4147 1.466950e+00
## 25370  4 4148 1.451631e+00
## 25371  4 4149 1.439018e+00
## 25372  4 4150 1.427199e+00
## 25373  4 4151 1.418071e+00
## 25374  4 4152 1.405340e+00
## 25375  4 4152 1.404743e+00
## 25376  4 4153 2.348110e+00
## 25377  4 4154 2.950025e+00
## 25378  4 4155 3.178005e+00
## 25379  4 4156 3.217872e+00
## 25380  4 4157 3.194048e+00
## 25381  4 4158 3.117814e+00
## 25382  4 4159 3.039970e+00
## 25383  4 4160 2.952246e+00
## 25384  4 4161 2.870743e+00
## 25385  4 4162 2.796330e+00
## 25386  4 4163 2.712715e+00
## 25387  4 4164 2.643165e+00
## 25388  4 4165 2.573240e+00
## 25389  4 4166 2.510676e+00
## 25390  4 4167 2.443986e+00
## 25391  4 4168 2.386112e+00
## 25392  4 4169 2.325862e+00
## 25393  4 4170 2.276084e+00
## 25394  4 4171 2.229740e+00
## 25395  4 4172 2.179406e+00
## 25396  4 4173 2.132472e+00
## 25397  4 4174 2.087100e+00
## 25398  4 4175 2.058750e+00
## 25399  4 4176 2.009643e+00
## 25400  4 4176 2.007517e+00
## 25401  4 4177 2.443771e+00
## 25402  4 4178 2.722991e+00
## 25403  4 4179 2.805995e+00
## 25404  4 4180 2.798478e+00
## 25405  4 4181 2.755602e+00
## 25406  4 4182 2.704403e+00
## 25407  4 4183 2.636147e+00
## 25408  4 4184 2.575366e+00
## 25409  4 4185 2.511699e+00
## 25410  4 4186 2.453981e+00
## 25411  4 4187 2.391099e+00
## 25412  4 4188 2.342298e+00
## 25413  4 4189 2.290241e+00
## 25414  4 4190 2.240980e+00
## 25415  4 4191 2.190871e+00
## 25416  4 4192 2.149952e+00
## 25417  4 4193 2.104646e+00
## 25418  4 4194 2.069674e+00
## 25419  4 4195 2.028799e+00
## 25420  4 4196 1.991956e+00
## 25421  4 4197 1.959850e+00
## 25422  4 4198 1.922094e+00
## 25423  4 4199 1.894427e+00
## 25424  4 4200 1.868512e+00
## 25425  4 4201 2.306638e+00
## 25426  4 4202 2.589719e+00
## 25427  4 4203 2.688083e+00
## 25428  4 4204 2.692768e+00
## 25429  4 4205 2.647362e+00
## 25430  4 4206 2.601325e+00
## 25431  4 4207 2.541512e+00
## 25432  4 4208 2.476368e+00
## 25433  4 4209 2.426741e+00
## 25434  4 4210 2.370105e+00
## 25435  4 4211 2.322827e+00
## 25436  4 4212 2.270091e+00
## 25437  4 4213 2.224587e+00
## 25438  4 4214 2.176093e+00
## 25439  4 4215 2.132766e+00
## 25440  4 4216 2.092312e+00
## 25441  4 4217 2.051194e+00
## 25442  4 4218 2.017884e+00
## 25443  4 4219 1.979494e+00
## 25444  4 4220 1.950030e+00
## 25445  4 4221 1.912159e+00
## 25446  4 4222 1.886317e+00
## 25447  4 4223 1.857261e+00
## 25448  4 4224 1.827355e+00
## 25449  4 4225 2.281593e+00
## 25450  4 4226 2.559642e+00
## 25451  4 4227 2.654466e+00
## 25452  4 4228 2.660271e+00
## 25453  4 4229 2.624781e+00
## 25454  4 4230 2.570749e+00
## 25455  4 4231 2.523353e+00
## 25456  4 4232 2.463464e+00
## 25457  4 4233 2.403273e+00
## 25458  4 4234 2.348807e+00
## 25459  4 4235 2.301669e+00
## 25460  4 4236 2.252398e+00
## 25461  4 4237 2.205486e+00
## 25462  4 4238 2.159222e+00
## 25463  4 4239 2.119254e+00
## 25464  4 4240 2.077617e+00
## 25465  4 4241 2.039119e+00
## 25466  4 4242 2.002186e+00
## 25467  4 4243 1.972661e+00
## 25468  4 4244 1.935713e+00
## 25469  4 4245 1.908273e+00
## 25470  4 4246 1.876194e+00
## 25471  4 4247 1.849098e+00
## 25472  4 4248 1.820778e+00
## 25473  4 4249 2.264256e+00
```

```
## 25474  4 4250 2.548818e+00
## 25475  4 4251 2.646773e+00
## 25476  4 4252 2.654128e+00
## 25477  4 4253 2.615778e+00
## 25478  4 4254 2.563909e+00
## 25479  4 4255 2.510300e+00
## 25480  4 4256 2.457273e+00
## 25481  4 4257 2.399805e+00
## 25482  4 4258 2.347005e+00
## 25483  4 4259 2.294702e+00
## 25484  4 4260 2.247952e+00
## 25485  4 4261 2.197786e+00
## 25486  4 4262 2.156266e+00
## 25487  4 4263 2.111640e+00
## 25488  4 4264 2.073651e+00
## 25489  4 4265 2.035380e+00
## 25490  4 4266 2.003056e+00
## 25491  4 4267 1.968469e+00
## 25492  4 4268 1.930814e+00
## 25493  4 4269 1.901901e+00
## 25494  4 4270 1.874695e+00
## 25495  4 4271 1.846405e+00
## 25496  4 4272 1.816226e+00
## 25497  4 4273 2.266495e+00
## 25498  4 4274 2.548223e+00
## 25499  4 4275 2.646922e+00
## 25500  4 4276 2.644029e+00
## 25501  4 4277 2.616060e+00
## 25502  4 4278 2.562692e+00
## 25503  4 4279 2.510143e+00
## 25504  4 4280 2.452133e+00
## 25505  4 4281 2.398194e+00
## 25506  4 4282 2.346759e+00
## 25507  4 4283 2.288457e+00
## 25508  4 4284 2.244402e+00
## 25509  4 4285 2.200260e+00
## 25510  4 4286 2.155168e+00
## 25511  4 4287 2.111007e+00
## 25512  4 4288 2.075741e+00
## 25513  4 4289 2.032470e+00
## 25514  4 4290 1.999932e+00
## 25515  4 4291 1.964635e+00
## 25516  4 4292 1.932738e+00
## 25517  4 4293 1.898038e+00
## 25518  4 4294 1.871209e+00
## 25519  4 4295 1.840858e+00
## 25520  4 4296 1.815010e+00
## 25521  4 4297 2.265456e+00
## 25522  4 4298 2.548209e+00
## 25523  4 4299 2.639846e+00
## 25524  4 4300 2.648530e+00
## 25525  4 4301 2.618735e+00
## 25526  4 4302 2.558014e+00
## 25527  4 4303 2.511089e+00
## 25528  4 4304 2.456486e+00
## 25529  4 4305 2.394261e+00
## 25530  4 4306 2.346152e+00
## 25531  4 4307 2.288125e+00
## 25532  4 4308 2.243017e+00
## 25533  4 4309 2.196921e+00
## 25534  4 4310 2.155978e+00
## 25535  4 4311 2.112784e+00
## 25536  4 4312 2.071276e+00
## 25537  4 4313 2.031664e+00
## 25538  4 4314 1.994200e+00
## 25539  4 4315 1.966250e+00
## 25540  4 4316 1.929277e+00
## 25541  4 4317 1.896207e+00
## 25542  4 4318 1.868839e+00
## 25543  4 4319 1.843758e+00
## 25544  4 4320 1.813956e+00
## 25545  4 4321 2.264924e+00
## 25546  4 4322 2.546249e+00
## 25547  4 4323 2.640450e+00
## 25548  4 4324 2.650059e+00
## 25549  4 4325 2.611116e+00
## 25550  4 4326 2.561758e+00
## 25551  4 4327 2.506024e+00
## 25552  4 4328 2.453811e+00
## 25553  4 4329 2.397847e+00
## 25554  4 4330 2.337863e+00
## 25555  4 4331 2.292545e+00
## 25556  4 4332 2.239338e+00
## 25557  4 4333 2.193086e+00
## 25558  4 4334 2.151070e+00
## 25559  4 4335 2.110683e+00
## 25560  4 4336 2.065732e+00
## 25561  4 4337 2.033767e+00
## 25562  4 4338 1.995065e+00
## 25563  4 4339 1.962974e+00
## 25564  4 4340 1.927733e+00
## 25565  4 4341 1.899133e+00
## 25566  4 4342 1.869801e+00
## 25567  4 4343 1.842597e+00
## 25568  4 4344 1.814984e+00
## 25569  4 4345 2.262539e+00
## 25570  4 4346 2.538689e+00
## 25571  4 4347 2.640969e+00
## 25572  4 4348 2.647229e+00
## 25573  4 4349 2.615400e+00
## 25574  4 4350 2.567573e+00
## 25575  4 4351 2.505513e+00
## 25576  4 4352 2.445646e+00
## 25577  4 4353 2.395630e+00
## 25578  4 4354 2.343744e+00
## 25579  4 4355 2.291240e+00
## 25580  4 4356 2.237255e+00
## 25581  4 4357 2.197385e+00
## 25582  4 4358 2.151278e+00
## 25583  4 4359 2.112375e+00
## 25584  4 4360 2.073101e+00
## 25585  4 4361 2.030583e+00
## 25586  4 4362 1.994581e+00
## 25587  4 4363 1.963964e+00
## 25588  4 4364 1.931598e+00
## 25589  4 4365 1.896152e+00
## 25590  4 4366 1.868106e+00
## 25591  4 4367 1.842025e+00
## 25592  4 4368 1.811911e+00
## 25593  4 4369 2.262359e+00
## 25594  4 4370 2.544550e+00
## 25595  4 4371 2.634080e+00
## 25596  4 4372 2.646080e+00
## 25597  4 4373 2.612066e+00
## 25598  4 4374 2.557595e+00
## 25599  4 4375 2.508161e+00
## 25600  4 4376 2.449538e+00
## 25601  4 4377 2.390662e+00
## 25602  4 4378 2.341946e+00
## 25603  4 4379 2.294109e+00
## 25604  4 4380 2.242703e+00
## 25605  4 4381 2.195997e+00
## 25606  4 4382 2.154572e+00
## 25607  4 4383 2.109144e+00
## 25608  4 4384 2.070888e+00
## 25609  4 4385 2.029677e+00
## 25610  4 4386 1.996885e+00
## 25611  4 4387 1.960895e+00
## 25612  4 4388 1.930909e+00
## 25613  4 4389 1.897830e+00
## 25614  4 4390 1.866285e+00
## 25615  4 4391 1.839060e+00
## 25616  4 4392 1.810929e+00
## 25617  4 4393 2.260874e+00
## 25618  4 4394 2.548491e+00
## 25619  4 4395 2.639314e+00
## 25620  4 4396 2.641530e+00
## 25621  4 4397 2.610511e+00
## 25622  4 4398 2.559789e+00
## 25623  4 4399 2.505372e+00
## 25624  4 4400 2.447292e+00
## 25625  4 4401 2.390939e+00
## 25626  4 4402 2.339941e+00
## 25627  4 4403 2.288895e+00
## 25628  4 4404 2.243725e+00
## 25629  4 4405 2.192396e+00
## 25630  4 4406 2.147729e+00
## 25631  4 4407 2.107603e+00
## 25632  4 4408 2.072271e+00
## 25633  4 4409 2.029292e+00
## 25634  4 4410 1.995092e+00
## 25635  4 4411 1.963431e+00
## 25636  4 4412 1.929072e+00
## 25637  4 4413 1.896558e+00
## 25638  4 4414 1.868713e+00
## 25639  4 4415 1.837549e+00
## 25640  4 4416 1.813875e+00
## 25641  4 4417 2.258901e+00
## 25642  4 4418 2.537679e+00
## 25643  4 4419 2.633166e+00
## 25644  4 4420 2.644298e+00
## 25645  4 4421 2.604232e+00
## 25646  4 4422 2.564919e+00
## 25647  4 4423 2.505298e+00
## 25648  4 4424 2.447961e+00
## 25649  4 4425 2.389676e+00
## 25650  4 4426 2.336264e+00
## 25651  4 4427 2.287838e+00
## 25652  4 4428 2.239049e+00
## 25653  4 4429 2.196421e+00
## 25654  4 4430 2.150544e+00
## 25655  4 4431 2.108917e+00
## 25656  4 4432 2.066204e+00
## 25657  4 4433 2.032282e+00
## 25658  4 4434 1.996062e+00
## 25659  4 4435 1.957623e+00
## 25660  4 4436 1.928626e+00
## 25661  4 4437 1.898664e+00
## 25662  4 4438 1.869343e+00
## 25663  4 4439 1.836386e+00
## 25664  4 4440 1.810101e+00
## 25665  4 4441 1.785184e+00
## 25666  4 4442 1.759462e+00
## 25667  4 4443 1.738163e+00
## 25668  4 4444 1.715218e+00
## 25669  4 4445 1.693633e+00
## 25670  4 4446 1.671262e+00
## 25671  4 4447 1.653537e+00
## 25672  4 4448 1.633246e+00
## 25673  4 4449 1.615111e+00
## 25674  4 4450 1.600886e+00
## 25675  4 4451 1.583848e+00
## 25676  4 4452 1.567567e+00
## 25677  4 4453 1.547058e+00
## 25678  4 4454 1.538760e+00
## 25679  4 4455 1.522026e+00
## 25680  4 4456 1.506940e+00
## 25681  4 4457 1.493537e+00
## 25682  4 4458 1.480471e+00
## 25683  4 4459 1.466525e+00
## 25684  4 4460 1.454021e+00
## 25685  4 4461 1.443379e+00
## 25686  4 4462 1.434347e+00
## 25687  4 4463 1.421414e+00
## 25688  4 4464 1.411524e+00
## 25689  4 4465 1.403105e+00
## 25690  4 4466 1.388235e+00
## 25691  4 4467 1.382673e+00
## 25692  4 4468 1.368955e+00
## 25693  4 4469 1.360789e+00
## 25694  4 4470 1.351956e+00
## 25695  4 4471 1.345766e+00
## 25696  4 4472 1.332644e+00
## 25697  4 4473 1.322504e+00
## 25698  4 4474 1.316644e+00
## 25699  4 4475 1.309797e+00
## 25700  4 4476 1.300402e+00
## 25701  4 4477 1.292871e+00
## 25702  4 4478 1.285121e+00
## 25703  4 4479 1.278398e+00
## 25704  4 4480 1.271308e+00
## 25705  4 4481 1.260707e+00
## 25706  4 4482 1.257409e+00
## 25707  4 4483 1.249977e+00
## 25708  4 4484 1.241851e+00
## 25709  4 4485 1.233225e+00
## 25710  4 4486 1.230266e+00
## 25711  4 4487 1.221105e+00
## 25712  4 4488 1.212963e+00
## 25713  4 4489 1.207727e+00
## 25714  4 4490 1.198793e+00
## 25715  4 4491 1.195951e+00
## 25716  4 4492 1.188854e+00
## 25717  4 4493 1.183602e+00
## 25718  4 4494 1.176722e+00
## 25719  4 4495 1.171665e+00
## 25720  4 4496 1.166490e+00
## 25721  4 4497 1.160978e+00
## 25722  4 4498 1.154321e+00
## 25723  4 4499 1.146368e+00
## 25724  4 4500 1.141060e+00
## 25725  4 4501 1.136335e+00
## 25726  4 4502 1.131317e+00
## 25727  4 4503 1.124927e+00
## 25728  4 4504 1.120680e+00
## 25729  4 4505 1.114657e+00
## 25730  4 4506 1.110103e+00
## 25731  4 4507 1.103113e+00
## 25732  4 4508 1.098977e+00
## 25733  4 4509 1.093678e+00
## 25734  4 4510 1.088674e+00
## 25735  4 4511 1.082028e+00
## 25736  4 4512 1.077052e+00
## 25737  4 4513 1.071126e+00
## 25738  4 4514 1.064394e+00
## 25739  4 4515 1.060608e+00
## 25740  4 4516 1.057316e+00
## 25741  4 4517 1.051454e+00
## 25742  4 4518 1.045379e+00
## 25743  4 4519 1.042786e+00
## 25744  4 4520 1.037086e+00
## 25745  4 4521 1.032852e+00
## 25746  4 4522 1.027304e+00
## 25747  4 4523 1.020945e+00
## 25748  4 4524 1.019539e+00
## 25749  4 4525 1.011269e+00
## 25750  4 4526 1.008030e+00
## 25751  4 4527 1.002971e+00
## 25752  4 4528 9.991082e-01
## 25753  4 4529 9.935484e-01
## 25754  4 4530 9.884719e-01
## 25755  4 4531 9.846212e-01
## 25756  4 4532 9.803127e-01
## 25757  4 4533 9.779856e-01
## 25758  4 4534 9.699007e-01
## 25759  4 4535 9.696629e-01
## 25760  4 4536 9.621840e-01
## 25761  4 4537 9.578335e-01
## 25762  4 4538 9.531222e-01
## 25763  4 4539 9.498491e-01
## 25764  4 4540 9.446323e-01
## 25765  4 4541 9.388020e-01
## 25766  4 4542 9.352195e-01
## 25767  4 4543 9.312813e-01
## 25768  4 4544 9.275844e-01
## 25769  4 4545 9.246598e-01
## 25770  4 4546 9.179289e-01
## 25771  4 4547 9.148856e-01
## 25772  4 4548 9.105924e-01
## 25773  4 4549 9.046957e-01
## 25774  4 4550 9.023697e-01
## 25775  4 4551 8.979587e-01
## 25776  4 4552 8.931726e-01
## 25777  4 4553 8.894115e-01
## 25778  4 4554 8.854583e-01
## 25779  4 4555 8.816937e-01
## 25780  4 4556 8.783569e-01
## 25781  4 4557 8.740352e-01
## 25782  4 4558 8.698738e-01
## 25783  4 4559 8.664947e-01
## 25784  4 4560 8.620834e-01
## 25785  4 4561 8.585377e-01
## 25786  4 4562 8.548577e-01
## 25787  4 4563 8.498316e-01
## 25788  4 4564 8.463873e-01
## 25789  4 4565 8.428815e-01
## 25790  4 4566 8.380670e-01
## 25791  4 4567 8.341790e-01
## 25792  4 4568 8.304708e-01
## 25793  4 4569 8.283279e-01
## 25794  4 4570 8.240732e-01
## 25795  4 4571 8.207166e-01
## 25796  4 4572 8.164768e-01
## 25797  4 4573 8.125577e-01
## 25798  4 4574 8.084095e-01
## 25799  4 4575 8.040261e-01
## 25800  4 4576 8.025623e-01
## 25801  4 4577 7.978101e-01
## 25802  4 4578 7.938891e-01
## 25803  4 4579 7.895095e-01
## 25804  4 4580 7.855759e-01
## 25805  4 4581 7.830796e-01
## 25806  4 4582 7.790610e-01
## 25807  4 4583 7.757608e-01
## 25808  4 4584 7.722397e-01
## 25809  4 4585 7.676774e-01
## 25810  4 4586 7.659219e-01
## 25811  4 4587 7.595746e-01
## 25812  4 4588 7.586043e-01
## 25813  4 4589 7.529516e-01
## 25814  4 4590 7.508406e-01
## 25815  4 4591 7.477103e-01
## 25816  4 4592 7.452109e-01
## 25817  4 4593 7.407355e-01
## 25818  4 4594 7.393133e-01
## 25819  4 4595 7.347691e-01
## 25820  4 4596 7.304095e-01
## 25821  4 4597 7.283186e-01
## 25822  4 4598 7.240768e-01
## 25823  4 4599 7.211987e-01
## 25824  4 4600 7.184486e-01
## 25825  4 4601 7.152651e-01
## 25826  4 4602 7.111691e-01
## 25827  4 4603 7.079100e-01
## 25828  4 4604 7.054315e-01
## 25829  4 4605 7.013742e-01
## 25830  4 4606 6.982948e-01
## 25831  4 4607 6.952606e-01
## 25832  4 4608 6.930382e-01
## 25833  4 4609 6.891465e-01
## 25834  4 4610 6.854377e-01
## 25835  4 4611 6.819487e-01
## 25836  4 4612 6.786390e-01
## 25837  4 4613 6.767597e-01
## 25838  4 4614 6.728960e-01
## 25839  4 4615 6.714329e-01
## 25840  4 4616 6.677004e-01
## 25841  4 4617 6.640538e-01
## 25842  4 4618 6.610679e-01
## 25843  4 4619 6.566662e-01
## 25844  4 4620 6.553843e-01
## 25845  4 4621 6.511012e-01
## 25846  4 4622 6.498205e-01
## 25847  4 4623 6.461385e-01
## 25848  4 4624 6.438044e-01
## 25849  4 4625 6.399593e-01
## 25850  4 4626 6.376741e-01
## 25851  4 4627 6.343869e-01
## 25852  4 4628 6.311842e-01
## 25853  4 4629 6.274465e-01
## 25854  4 4630 6.258469e-01
## 25855  4 4631 6.228476e-01
## 25856  4 4632 6.211566e-01
## 25857  4 4633 6.174844e-01
## 25858  4 4634 6.157866e-01
## 25859  4 4635 6.110836e-01
## 25860  4 4636 6.093345e-01
## 25861  4 4637 6.070822e-01
## 25862  4 4638 6.046483e-01
## 25863  4 4639 6.007259e-01
## 25864  4 4640 5.975100e-01
## 25865  4 4641 5.959190e-01
## 25866  4 4642 5.926874e-01
## 25867  4 4643 5.892158e-01
## 25868  4 4644 5.878069e-01
## 25869  4 4645 5.842955e-01
## 25870  4 4646 5.825497e-01
## 25871  4 4647 5.793439e-01
## 25872  4 4648 5.765431e-01
## 25873  4 4649 5.745153e-01
## 25874  4 4650 5.711471e-01
## 25875  4 4651 5.677182e-01
## 25876  4 4652 5.665164e-01
## 25877  4 4653 5.632318e-01
## 25878  4 4654 5.613654e-01
## 25879  4 4655 5.578230e-01
## 25880  4 4656 5.565509e-01
## 25881  4 4657 5.538056e-01
## 25882  4 4658 5.511889e-01
## 25883  4 4659 5.478380e-01
## 25884  4 4660 5.456770e-01
## 25885  4 4661 5.425656e-01
## 25886  4 4662 5.411244e-01
## 25887  4 4663 5.379236e-01
## 25888  4 4664 5.359867e-01
## 25889  4 4665 5.330742e-01
## 25890  4 4666 5.315005e-01
## 25891  4 4667 5.292515e-01
## 25892  4 4668 5.267925e-01
## 25893  4 4669 5.231650e-01
## 25894  4 4670 5.207049e-01
## 25895  4 4671 5.195520e-01
## 25896  4 4672 5.170073e-01
## 25897  4 4673 5.142696e-01
## 25898  4 4674 5.115460e-01
## 25899  4 4675 5.098263e-01
## 25900  4 4676 5.073263e-01
## 25901  4 4677 5.046989e-01
## 25902  4 4678 5.023183e-01
## 25903  4 4679 5.004787e-01
## 25904  4 4680 4.978491e-01
## 25905  4 4681 4.954330e-01
## 25906  4 4682 4.942494e-01
## 25907  4 4683 4.914763e-01
## 25908  4 4684 4.887698e-01
## 25909  4 4685 4.863040e-01
## 25910  4 4686 4.841187e-01
## 25911  4 4687 4.826675e-01
## 25912  4 4688 4.795643e-01
## 25913  4 4689 4.782348e-01
## 25914  4 4690 4.755380e-01
## 25915  4 4691 4.736565e-01
## 25916  4 4692 4.715810e-01
## 25917  4 4693 4.690936e-01
## 25918  4 4694 4.671026e-01
## 25919  4 4695 4.647235e-01
## 25920  4 4696 4.632144e-01
## 25921  4 4697 4.609132e-01
## 25922  4 4698 4.583462e-01
## 25923  4 4699 4.579177e-01
## 25924  4 4700 4.547280e-01
## 25925  4 4701 4.520201e-01
## 25926  4 4702 4.503045e-01
## 25927  4 4703 4.486866e-01
## 25928  4 4704 4.459031e-01
## 25929  4 4705 4.447271e-01
## 25930  4 4706 4.416945e-01
## 25931  4 4707 4.405545e-01
## 25932  4 4708 4.387746e-01
## 25933  4 4709 4.372783e-01
## 25934  4 4710 4.331334e-01
## 25935  4 4711 4.323424e-01
## 25936  4 4712 4.299120e-01
## 25937  4 4713 4.280035e-01
## 25938  4 4714 4.263131e-01
## 25939  4 4715 4.255632e-01
## 25940  4 4716 4.220012e-01
## 25941  4 4717 4.203285e-01
## 25942  4 4718 4.183935e-01
## 25943  4 4719 4.167632e-01
## 25944  4 4720 4.154754e-01
## 25945  4 4721 4.133470e-01
## 25946  4 4722 4.113295e-01
## 25947  4 4723 4.094573e-01
## 25948  4 4724 4.074512e-01
## 25949  4 4725 4.057935e-01
## 25950  4 4726 4.032911e-01
## 25951  4 4727 4.021518e-01
## 25952  4 4728 4.000702e-01
## 25953  4 4729 3.981666e-01
## 25954  4 4730 3.969472e-01
## 25955  4 4731 3.944250e-01
## 25956  4 4732 3.932144e-01
## 25957  4 4733 3.911435e-01
## 25958  4 4734 3.893813e-01
## 25959  4 4735 3.872929e-01
## 25960  4 4736 3.856680e-01
## 25961  4 4737 3.838799e-01
## 25962  4 4738 3.824580e-01
## 25963  4 4739 3.798943e-01
## 25964  4 4740 3.796651e-01
## 25965  4 4741 3.768774e-01
## 25966  4 4742 3.748626e-01
## 25967  4 4743 3.732197e-01
## 25968  4 4744 3.718540e-01
## 25969  4 4745 3.702262e-01
## 25970  4 4746 3.680569e-01
## 25971  4 4747 3.668076e-01
## 25972  4 4748 3.651121e-01
## 25973  4 4749 3.642373e-01
## 25974  4 4750 3.611533e-01
## 25975  4 4751 3.606439e-01
## 25976  4 4752 3.584688e-01
## 25977  4 4753 3.572174e-01
## 25978  4 4754 3.554559e-01
## 25979  4 4755 3.536489e-01
## 25980  4 4756 3.512929e-01
## 25981  4 4757 3.501071e-01
## 25982  4 4758 3.490916e-01
## 25983  4 4759 3.474042e-01
## 25984  4 4760 3.450892e-01
## 25985  4 4761 3.442283e-01
## 25986  4 4762 3.423357e-01
## 25987  4 4763 3.409206e-01
## 25988  4 4764 3.392051e-01
## 25989  4 4765 3.384685e-01
## 25990  4 4766 3.364562e-01
## 25991  4 4767 3.345143e-01
## 25992  4 4768 3.335415e-01
## 25993  4 4769 3.315933e-01
## 25994  4 4770 3.297925e-01
## 25995  4 4771 3.287924e-01
## 25996  4 4772 3.270864e-01
## 25997  4 4773 3.258994e-01
## 25998  4 4774 3.241908e-01
## 25999  4 4775 3.233456e-01
## 26000  4 4776 3.212880e-01
## 26001  4 4777 3.202011e-01
## 26002  4 4778 3.180928e-01
## 26003  4 4779 3.175937e-01
## 26004  4 4780 3.160738e-01
## 26005  4 4781 3.137572e-01
## 26006  4 4782 3.127985e-01
## 26007  4 4783 3.111923e-01
## 26008  4 4784 3.099392e-01
## 26009  4 4785 3.088185e-01
## 26010  4 4786 3.066035e-01
## 26011  4 4787 3.058708e-01
## 26012  4 4788 3.042519e-01
## 26013  4 4789 3.024174e-01
## 26014  4 4790 3.013892e-01
## 26015  4 4791 2.996964e-01
## 26016  4 4792 2.983090e-01
## 26017  4 4793 2.974208e-01
## 26018  4 4794 2.962819e-01
## 26019  4 4795 2.947239e-01
## 26020  4 4796 2.936827e-01
## 26021  4 4797 2.922261e-01
## 26022  4 4798 2.904134e-01
## 26023  4 4799 2.893327e-01
## 26024  4 4800 2.875311e-01
## 26025  4 4801 2.869593e-01
## 26026  4 4802 2.855259e-01
## 26027  4 4803 2.841217e-01
## 26028  4 4804 2.822148e-01
## 26029  4 4805 2.814924e-01
## 26030  4 4806 2.803807e-01
## 26031  4 4807 2.789446e-01
## 26032  4 4808 2.776725e-01
## 26033  4 4809 2.769448e-01
## 26034  4 4810 2.750247e-01
## 26035  4 4811 2.742822e-01
## 26036  4 4812 2.729586e-01
## 26037  4 4813 2.714726e-01
## 26038  4 4814 2.694998e-01
## 26039  4 4815 2.688594e-01
## 26040  4 4816 2.677609e-01
## 26041  4 4817 2.662907e-01
## 26042  4 4818 2.649780e-01
## 26043  4 4819 2.637760e-01
## 26044  4 4820 2.625388e-01
## 26045  4 4821 2.613600e-01
## 26046  4 4822 2.601051e-01
## 26047  4 4823 2.590665e-01
## 26048  4 4824 2.580029e-01
## 26049  4 4825 2.566521e-01
## 26050  4 4826 2.557723e-01
## 26051  4 4827 2.543092e-01
## 26052  4 4828 2.530610e-01
## 26053  4 4829 2.521720e-01
## 26054  4 4830 2.511613e-01
## 26055  4 4831 2.498108e-01
## 26056  4 4832 2.489470e-01
## 26057  4 4833 2.476238e-01
## 26058  4 4834 2.459748e-01
## 26059  4 4835 2.454024e-01
## 26060  4 4836 2.442033e-01
## 26061  4 4837 2.431308e-01
## 26062  4 4838 2.419567e-01
## 26063  4 4839 2.408263e-01
## 26064  4 4840 2.399066e-01
## 26065  4 4841 2.386952e-01
## 26066  4 4842 2.376261e-01
## 26067  4 4843 2.364183e-01
## 26068  4 4844 2.353073e-01
## 26069  4 4845 2.345421e-01
## 26070  4 4846 2.332297e-01
## 26071  4 4847 2.320169e-01
## 26072  4 4848 2.311378e-01
## 26073  4 4849 2.301908e-01
## 26074  4 4850 2.290486e-01
## 26075  4 4851 2.281313e-01
## 26076  4 4852 2.271428e-01
## 26077  4 4853 2.261074e-01
## 26078  4 4854 2.249171e-01
## 26079  4 4855 2.237674e-01
## 26080  4 4856 2.228668e-01
## 26081  4 4857 2.217185e-01
## 26082  4 4858 2.208573e-01
## 26083  4 4859 2.196177e-01
## 26084  4 4860 2.189438e-01
## 26085  4 4861 2.176805e-01
## 26086  4 4862 2.170332e-01
## 26087  4 4863 2.160414e-01
## 26088  4 4864 2.149083e-01
## 26089  4 4865 2.141176e-01
## 26090  4 4866 2.133122e-01
## 26091  4 4867 2.118881e-01
## 26092  4 4868 2.110663e-01
## 26093  4 4869 2.103265e-01
## 26094  4 4870 2.092543e-01
## 26095  4 4871 2.081963e-01
## 26096  4 4872 2.075452e-01
## 26097  4 4873 2.063250e-01
## 26098  4 4874 2.051219e-01
## 26099  4 4875 2.046706e-01
## 26100  4 4876 2.033236e-01
## 26101  4 4877 2.024626e-01
## 26102  4 4878 2.016857e-01
## 26103  4 4879 2.006905e-01
## 26104  4 4880 1.998638e-01
## 26105  4 4881 1.987719e-01
## 26106  4 4882 1.980544e-01
## 26107  4 4883 1.970470e-01
## 26108  4 4884 1.961792e-01
## 26109  4 4885 1.953906e-01
## 26110  4 4886 1.945553e-01
## 26111  4 4887 1.932916e-01
## 26112  4 4888 1.923566e-01
## 26113  4 4889 1.916110e-01
## 26114  4 4890 1.907243e-01
## 26115  4 4891 1.901302e-01
## 26116  4 4892 1.890313e-01
## 26117  4 4893 1.882266e-01
## 26118  4 4894 1.873000e-01
## 26119  4 4895 1.866434e-01
## 26120  4 4896 1.857372e-01
## 26121  4 4897 1.847171e-01
## 26122  4 4898 1.842397e-01
## 26123  4 4899 1.829489e-01
## 26124  4 4900 1.823591e-01
## 26125  4 4901 1.817858e-01
## 26126  4 4902 1.809533e-01
## 26127  4 4903 1.799120e-01
## 26128  4 4904 1.791253e-01
## 26129  4 4905 1.785177e-01
## 26130  4 4906 1.774880e-01
## 26131  4 4907 1.763005e-01
## 26132  4 4908 1.756689e-01
## 26133  4 4909 1.749250e-01
## 26134  4 4910 1.745291e-01
## 26135  4 4911 1.737008e-01
## 26136  4 4912 1.726185e-01
## 26137  4 4913 1.717693e-01
## 26138  4 4914 1.710468e-01
## 26139  4 4915 1.704473e-01
## 26140  4 4916 1.695026e-01
## 26141  4 4917 1.689549e-01
## 26142  4 4918 1.680784e-01
## 26143  4 4919 1.675676e-01
## 26144  4 4920 1.662453e-01
## 26145  4 4921 1.656169e-01
## 26146  4 4922 1.648992e-01
## 26147  4 4923 1.640765e-01
## 26148  4 4924 1.635653e-01
## 26149  4 4925 1.627009e-01
## 26150  4 4926 1.618651e-01
## 26151  4 4927 1.609877e-01
## 26152  4 4928 1.602626e-01
## 26153  4 4929 1.599745e-01
## 26154  4 4930 1.591487e-01
## 26155  4 4931 1.581675e-01
## 26156  4 4932 1.575455e-01
## 26157  4 4933 1.569885e-01
## 26158  4 4934 1.561795e-01
## 26159  4 4935 1.554773e-01
## 26160  4 4936 1.548265e-01
## 26161  4 4937 1.539121e-01
## 26162  4 4938 1.531957e-01
## 26163  4 4939 1.524677e-01
## 26164  4 4940 1.516568e-01
## 26165  4 4941 1.511734e-01
## 26166  4 4942 1.504488e-01
## 26167  4 4943 1.496514e-01
## 26168  4 4944 1.492857e-01
## 26169  4 4945 1.486518e-01
## 26170  4 4946 1.477209e-01
## 26171  4 4947 1.471417e-01
## 26172  4 4948 1.464896e-01
## 26173  4 4949 1.457586e-01
## 26174  4 4950 1.451595e-01
## 26175  4 4951 1.446989e-01
## 26176  4 4952 1.436938e-01
## 26177  4 4953 1.431085e-01
## 26178  4 4954 1.424282e-01
## 26179  4 4955 1.417703e-01
## 26180  4 4956 1.413459e-01
## 26181  4 4957 1.406444e-01
## 26182  4 4958 1.399116e-01
## 26183  4 4959 1.391931e-01
## 26184  4 4960 1.387168e-01
## 26185  4 4961 1.377786e-01
## 26186  4 4962 1.373368e-01
## 26187  4 4963 1.369592e-01
## 26188  4 4964 1.360585e-01
## 26189  4 4965 1.352176e-01
## 26190  4 4966 1.347172e-01
## 26191  4 4967 1.342327e-01
## 26192  4 4968 1.335966e-01
## 26193  4 4969 1.331677e-01
## 26194  4 4970 1.325252e-01
## 26195  4 4971 1.318470e-01
## 26196  4 4972 1.312842e-01
## 26197  4 4973 1.306081e-01
## 26198  4 4974 1.299732e-01
## 26199  4 4975 1.293087e-01
## 26200  4 4976 1.287789e-01
## 26201  4 4977 1.281748e-01
## 26202  4 4978 1.279229e-01
## 26203  4 4979 1.272403e-01
## 26204  4 4980 1.265434e-01
## 26205  4 4981 1.259621e-01
## 26206  4 4982 1.255199e-01
## 26207  4 4983 1.250006e-01
## 26208  4 4984 1.242310e-01
## 26209  4 4985 1.235318e-01
## 26210  4 4986 1.230608e-01
## 26211  4 4987 1.224000e-01
## 26212  4 4988 1.221803e-01
## 26213  4 4989 1.214613e-01
## 26214  4 4990 1.207676e-01
## 26215  4 4991 1.203571e-01
## 26216  4 4992 1.198386e-01
## 26217  4 4993 1.194267e-01
## 26218  4 4994 1.187639e-01
## 26219  4 4995 1.181849e-01
## 26220  4 4996 1.177176e-01
## 26221  4 4997 1.171976e-01
## 26222  4 4998 1.165698e-01
## 26223  4 4999 1.159232e-01
## 26224  4 5000 1.154812e-01
## 26225  4 5001 1.150157e-01
## 26226  4 5002 1.146002e-01
## 26227  4 5003 1.141613e-01
## 26228  4 5004 1.134691e-01
## 26229  4 5005 1.128198e-01
## 26230  4 5006 1.123098e-01
## 26231  4 5007 1.116342e-01
## 26232  4 5008 1.113969e-01
## 26233  4 5009 1.110116e-01
## 26234  4 5010 1.103982e-01
## 26235  4 5011 1.096243e-01
## 26236  4 5012 1.091932e-01
## 26237  4 5013 1.088597e-01
## 26238  4 5014 1.083411e-01
## 26239  4 5015 1.079199e-01
## 26240  4 5016 1.074046e-01
## 26241  4 5017 1.069255e-01
## 26242  4 5018 1.066176e-01
## 26243  4 5019 1.059225e-01
## 26244  4 5020 1.054623e-01
## 26245  4 5021 1.050195e-01
## 26246  4 5022 1.045204e-01
## 26247  4 5023 1.039061e-01
## 26248  4 5024 1.035448e-01
## 26249  4 5025 1.030086e-01
## 26250  4 5026 1.026894e-01
## 26251  4 5027 1.019175e-01
## 26252  4 5028 1.015730e-01
## 26253  4 5029 1.011181e-01
## 26254  4 5030 1.007439e-01
## 26255  4 5031 1.002217e-01
## 26256  4 5032 9.959943e-02
## 26257  4 5033 9.935800e-02
## 26258  4 5034 9.881157e-02
## 26259  4 5035 9.863588e-02
## 26260  4 5036 9.788663e-02
## 26261  4 5037 9.750583e-02
## 26262  4 5038 9.712181e-02
## 26263  4 5039 9.652185e-02
## 26264  4 5040 9.629778e-02
## 26265  4 5041 9.575368e-02
## 26266  4 5042 9.540977e-02
## 26267  4 5043 9.488535e-02
## 26268  4 5044 9.460202e-02
## 26269  4 5045 9.395945e-02
## 26270  4 5046 9.348262e-02
## 26271  4 5047 9.309365e-02
## 26272  4 5048 9.285091e-02
## 26273  4 5049 9.224907e-02
```

```
## 26274  4 5050 9.181033e-02
## 26275  4 5051 9.167295e-02
## 26276  4 5052 9.126500e-02
## 26277  4 5053 9.060363e-02
## 26278  4 5054 9.021108e-02
## 26279  4 5055 8.995474e-02
## 26280  4 5056 8.956464e-02
## 26281  4 5057 8.895502e-02
## 26282  4 5058 8.872654e-02
## 26283  4 5059 8.830072e-02
## 26284  4 5060 8.791391e-02
## 26285  4 5061 8.744686e-02
## 26286  4 5062 8.690661e-02
## 26287  4 5063 8.676754e-02
## 26288  4 5064 8.611814e-02
## 26289  4 5065 8.585959e-02
## 26290  4 5066 8.538905e-02
## 26291  4 5067 8.512163e-02
## 26292  4 5068 8.471088e-02
## 26293  4 5069 8.428973e-02
## 26294  4 5070 8.393257e-02
## 26295  4 5071 8.358557e-02
## 26296  4 5072 8.316163e-02
## 26297  4 5073 8.279096e-02
## 26298  4 5074 8.224124e-02
## 26299  4 5075 8.196347e-02
## 26300  4 5076 8.164947e-02
## 26301  4 5077 8.122778e-02
## 26302  4 5078 8.091065e-02
## 26303  4 5079 8.045132e-02
## 26304  4 5080 8.017287e-02
## 26305  4 5081 7.978514e-02
## 26306  4 5082 7.946562e-02
## 26307  4 5083 7.912826e-02
## 26308  4 5084 7.871357e-02
## 26309  4 5085 7.843271e-02
## 26310  4 5086 7.796300e-02
## 26311  4 5087 7.753412e-02
## 26312  4 5088 7.719557e-02
## 26313  4 5089 7.688650e-02
## 26314  4 5090 7.644585e-02
## 26315  4 5091 7.619898e-02
## 26316  4 5092 7.589187e-02
## 26317  4 5093 7.546514e-02
## 26318  4 5094 7.516123e-02
## 26319  4 5095 7.480878e-02
## 26320  4 5096 7.456165e-02
## 26321  4 5097 7.423838e-02
## 26322  4 5098 7.379339e-02
## 26323  4 5099 7.347004e-02
## 26324  4 5100 7.316459e-02
## 26325  4 5101 7.296934e-02
## 26326  4 5102 7.240743e-02
## 26327  4 5103 7.216949e-02
## 26328  4 5104 7.180864e-02
## 26329  4 5105 7.143132e-02
## 26330  4 5106 7.118198e-02
## 26331  4 5107 7.088434e-02
## 26332  4 5108 7.051455e-02
## 26333  4 5109 7.007160e-02
## 26334  4 5110 6.982417e-02
## 26335  4 5111 6.953709e-02
## 26336  4 5112 6.939704e-02
## 26337  4 5113 6.900324e-02
## 26338  4 5114 6.866573e-02
## 26339  4 5115 6.830621e-02
## 26340  4 5116 6.809949e-02
## 26341  4 5117 6.756567e-02
## 26342  4 5118 6.750100e-02
## 26343  4 5119 6.698740e-02
## 26344  4 5120 6.684845e-02
## 26345  4 5121 6.653723e-02
## 26346  4 5122 6.612493e-02
## 26347  4 5123 6.579771e-02
## 26348  4 5124 6.552408e-02
## 26349  4 5125 6.529495e-02
## 26350  4 5126 6.477408e-02
## 26351  4 5127 6.468257e-02
## 26352  4 5128 6.432945e-02
## 26353  4 5129 6.392086e-02
## 26354  4 5130 6.376504e-02
## 26355  4 5131 6.349915e-02
## 26356  4 5132 6.318760e-02
## 26357  4 5133 6.289105e-02
## 26358  4 5134 6.261255e-02
## 26359  4 5135 6.223524e-02
## 26360  4 5136 6.195810e-02
## 26361  4 5137 6.169833e-02
## 26362  4 5138 6.140392e-02
## 26363  4 5139 6.111669e-02
## 26364  4 5140 6.096193e-02
## 26365  4 5141 6.052610e-02
## 26366  4 5142 6.033094e-02
## 26367  4 5143 6.007116e-02
## 26368  4 5144 5.978887e-02
## 26369  4 5145 5.946282e-02
## 26370  4 5146 5.937732e-02
## 26371  4 5147 5.899518e-02
## 26372  4 5148 5.869791e-02
## 26373  4 5149 5.855421e-02
## 26374  4 5150 5.813644e-02
## 26375  4 5151 5.799326e-02
## 26376  4 5152 5.758855e-02
## 26377  4 5153 5.731298e-02
## 26378  4 5154 5.712652e-02
## 26379  4 5155 5.692826e-02
## 26380  4 5156 5.670900e-02
## 26381  4 5157 5.641749e-02
## 26382  4 5158 5.609295e-02
## 26383  4 5159 5.592388e-02
## 26384  4 5160 5.558606e-02
## 26385  4 5161 5.528765e-02
## 26386  4 5162 5.507469e-02
## 26387  4 5163 5.485739e-02
## 26388  4 5164 5.469369e-02
## 26389  4 5165 5.431052e-02
## 26390  4 5166 5.410523e-02
## 26391  4 5167 5.379036e-02
## 26392  4 5168 5.358437e-02
## 26393  4 5169 5.334044e-02
## 26394  4 5170 5.318952e-02
## 26395  4 5171 5.283687e-02
## 26396  4 5172 5.264956e-02
## 26397  4 5173 5.246755e-02
## 26398  4 5174 5.223722e-02
## 26399  4 5175 5.196127e-02
## 26400  4 5176 5.163908e-02
## 26401  4 5177 5.151963e-02
## 26402  4 5178 5.128472e-02
## 26403  4 5179 5.092658e-02
## 26404  4 5180 5.080255e-02
## 26405  4 5181 5.051511e-02
## 26406  4 5182 5.025875e-02
## 26407  4 5183 5.010739e-02
## 26408  4 5184 4.988215e-02
## 26409  4 5185 4.960663e-02
## 26410  4 5186 4.939391e-02
## 26411  4 5187 4.913037e-02
## 26412  4 5188 4.887594e-02
## 26413  4 5189 4.871234e-02
## 26414  4 5190 4.850227e-02
## 26415  4 5191 4.826019e-02
## 26416  4 5192 4.802059e-02
## 26417  4 5193 4.794172e-02
## 26418  4 5194 4.755327e-02
## 26419  4 5195 4.738664e-02
## 26420  4 5196 4.713121e-02
## 26421  4 5197 4.698645e-02
## 26422  4 5198 4.674285e-02
## 26423  4 5199 4.651285e-02
## 26424  4 5200 4.635395e-02
## 26425  4 5201 4.611557e-02
## 26426  4 5202 4.597762e-02
## 26427  4 5203 4.572422e-02
## 26428  4 5204 4.539628e-02
## 26429  4 5205 4.524382e-02
## 26430  4 5206 4.508096e-02
## 26431  4 5207 4.483986e-02
## 26432  4 5208 4.467232e-02
## 26433  4 5209 4.438251e-02
## 26434  4 5210 4.425095e-02
## 26435  4 5211 4.415910e-02
## 26436  4 5212 4.381768e-02
## 26437  4 5213 4.365561e-02
## 26438  4 5214 4.348247e-02
## 26439  4 5215 4.326379e-02
## 26440  4 5216 4.303442e-02
## 26441  4 5217 4.280559e-02
## 26442  4 5218 4.268210e-02
## 26443  4 5219 4.249971e-02
## 26444  4 5220 4.225578e-02
## 26445  4 5221 4.210221e-02
## 26446  4 5222 4.190806e-02
## 26447  4 5223 4.164132e-02
## 26448  4 5224 4.152439e-02
## 26449  4 5225 4.129237e-02
## 26450  4 5226 4.108649e-02
## 26451  4 5227 4.098576e-02
## 26452  4 5228 4.075296e-02
## 26453  4 5229 4.065128e-02
## 26454  4 5230 4.035941e-02
## 26455  4 5231 4.022713e-02
## 26456  4 5232 4.003523e-02
## 26457  4 5233 3.992919e-02
## 26458  4 5234 3.968846e-02
## 26459  4 5235 3.949251e-02
## 26460  4 5236 3.929899e-02
## 26461  4 5237 3.907930e-02
## 26462  4 5238 3.898118e-02
## 26463  4 5239 3.877417e-02
## 26464  4 5240 3.861458e-02
## 26465  4 5241 3.840056e-02
## 26466  4 5242 3.827080e-02
## 26467  4 5243 3.808127e-02
## 26468  4 5244 3.788971e-02
## 26469  4 5245 3.771790e-02
## 26470  4 5246 3.750689e-02
## 26471  4 5247 3.735896e-02
## 26472  4 5248 3.723009e-02
## 26473  4 5249 3.705606e-02
## 26474  4 5250 3.692341e-02
## 26475  4 5251 3.678163e-02
## 26476  4 5252 3.657752e-02
## 26477  4 5253 3.641567e-02
## 26478  4 5254 3.623730e-02
## 26479  4 5255 3.603177e-02
## 26480  4 5256 3.580525e-02
## 26481  4 5257 3.573872e-02
## 26482  4 5258 3.560829e-02
## 26483  4 5259 3.535413e-02
## 26484  4 5260 3.519316e-02
## 26485  4 5261 3.501222e-02
## 26486  4 5262 3.489323e-02
## 26487  4 5263 3.478689e-02
## 26488  4 5264 3.462855e-02
## 26489  4 5265 3.440534e-02
## 26490  4 5266 3.427621e-02
## 26491  4 5267 3.419141e-02
## 26492  4 5268 3.388787e-02
## 26493  4 5269 3.378266e-02
## 26494  4 5270 3.363161e-02
## 26495  4 5271 3.346405e-02
## 26496  4 5272 3.338491e-02
## 26497  4 5273 3.318709e-02
## 26498  4 5274 3.307166e-02
## 26499  4 5275 3.292897e-02
## 26500  4 5276 3.275859e-02
## 26501  4 5277 3.256428e-02
## 26502  4 5278 3.247539e-02
## 26503  4 5279 3.227650e-02
## 26504  4 5280 3.210862e-02
## 26505  4 5281 3.204663e-02
## 26506  4 5282 3.186055e-02
## 26507  4 5283 3.169857e-02
## 26508  4 5284 3.155653e-02
## 26509  4 5285 3.137195e-02
## 26510  4 5286 3.129569e-02
## 26511  4 5287 3.112353e-02
## 26512  4 5288 3.099605e-02
## 26513  4 5289 3.087926e-02
## 26514  4 5290 3.074419e-02
## 26515  4 5291 3.055833e-02
## 26516  4 5292 3.043292e-02
## 26517  4 5293 3.033383e-02
## 26518  4 5294 3.018161e-02
## 26519  4 5295 3.005341e-02
## 26520  4 5296 2.987010e-02
## 26521  4 5297 2.979385e-02
## 26522  4 5298 2.961152e-02
## 26523  4 5299 2.946713e-02
## 26524  4 5300 2.934165e-02
## 26525  4 5301 2.924658e-02
## 26526  4 5302 2.905042e-02
## 26527  4 5303 2.890688e-02
## 26528  4 5304 2.879524e-02
## 26529  4 5305 2.867617e-02
## 26530  4 5306 2.858242e-02
## 26531  4 5307 2.842180e-02
## 26532  4 5308 2.830933e-02
## 26533  4 5309 2.815958e-02
## 26534  4 5310 2.798968e-02
## 26535  4 5311 2.790873e-02
## 26536  4 5312 2.781101e-02
## 26537  4 5313 2.763809e-02
## 26538  4 5314 2.753399e-02
## 26539  4 5315 2.736489e-02
## 26540  4 5316 2.723998e-02
## 26541  4 5317 2.713839e-02
## 26542  4 5318 2.702206e-02
## 26543  4 5319 2.691635e-02
## 26544  4 5320 2.679106e-02
## 26545  4 5321 2.664375e-02
## 26546  4 5322 2.657702e-02
## 26547  4 5323 2.646227e-02
## 26548  4 5324 2.630640e-02
## 26549  4 5325 2.620120e-02
## 26550  4 5326 2.597430e-02
## 26551  4 5327 2.594166e-02
## 26552  4 5328 2.581368e-02
## 26553  4 5329 2.567908e-02
## 26554  4 5330 2.560186e-02
## 26555  4 5331 2.543273e-02
## 26556  4 5332 2.534483e-02
## 26557  4 5333 2.525359e-02
## 26558  4 5334 2.510187e-02
## 26559  4 5335 2.501063e-02
## 26560  4 5336 2.491173e-02
## 26561  4 5337 2.479903e-02
## 26562  4 5338 2.465290e-02
## 26563  4 5339 2.454599e-02
## 26564  4 5340 2.446840e-02
## 26565  4 5341 2.439651e-02
## 26566  4 5342 2.428851e-02
## 26567  4 5343 2.410566e-02
## 26568  4 5344 2.401916e-02
## 26569  4 5345 2.390960e-02
## 26570  4 5346 2.379635e-02
## 26571  4 5347 2.369992e-02
## 26572  4 5348 2.362846e-02
## 26573  4 5349 2.344965e-02
## 26574  4 5350 2.337608e-02
## 26575  4 5351 2.329865e-02
## 26576  4 5352 2.315223e-02
## 26577  4 5353 2.304488e-02
## 26578  4 5354 2.294831e-02
## 26579  4 5355 2.279083e-02
## 26580  4 5356 2.273449e-02
## 26581  4 5357 2.262467e-02
## 26582  4 5358 2.254229e-02
## 26583  4 5359 2.244002e-02
## 26584  4 5360 2.228453e-02
## 26585  4 5361 2.220054e-02
## 26586  4 5362 2.210940e-02
## 26587  4 5363 2.200505e-02
## 26588  4 5364 2.195511e-02
## 26589  4 5365 2.177887e-02
## 26590  4 5366 2.171578e-02
## 26591  4 5367 2.163251e-02
## 26592  4 5368 2.149218e-02
## 26593  4 5369 2.141263e-02
## 26594  4 5370 2.132065e-02
## 26595  4 5371 2.118622e-02
## 26596  4 5372 2.113772e-02
## 26597  4 5373 2.104052e-02
## 26598  4 5374 2.091563e-02
## 26599  4 5375 2.085076e-02
## 26600  4 5376 2.076564e-02
## 26601  4 5377 2.067700e-02
## 26602  4 5378 2.054886e-02
## 26603  4 5379 2.043999e-02
## 26604  4 5380 2.035042e-02
## 26605  4 5381 2.025239e-02
## 26606  4 5382 2.015872e-02
## 26607  4 5383 2.012360e-02
## 26608  4 5384 1.999326e-02
## 26609  4 5385 1.992321e-02
## 26610  4 5386 1.979121e-02
## 26611  4 5387 1.971532e-02
## 26612  4 5388 1.962939e-02
## 26613  4 5389 1.957886e-02
## 26614  4 5390 1.946542e-02
## 26615  4 5391 1.936640e-02
## 26616  4 5392 1.927332e-02
## 26617  4 5393 1.920119e-02
## 26618  4 5394 1.909298e-02
## 26619  4 5395 1.898939e-02
## 26620  4 5396 1.892911e-02
## 26621  4 5397 1.883462e-02
## 26622  4 5398 1.873812e-02
## 26623  4 5399 1.866003e-02
## 26624  4 5400 1.860670e-02
## 26625  4 5401 1.852030e-02
## 26626  4 5402 1.842644e-02
## 26627  4 5403 1.834678e-02
## 26628  4 5404 1.826941e-02
## 26629  4 5405 1.814811e-02
## 26630  4 5406 1.814481e-02
## 26631  4 5407 1.801467e-02
## 26632  4 5408 1.790577e-02
## 26633  4 5409 1.782760e-02
## 26634  4 5410 1.775854e-02
## 26635  4 5411 1.767624e-02
## 26636  4 5412 1.756888e-02
## 26637  4 5413 1.749945e-02
## 26638  4 5414 1.743134e-02
## 26639  4 5415 1.733851e-02
## 26640  4 5416 1.731424e-02
## 26641  4 5417 1.719851e-02
## 26642  4 5418 1.712391e-02
## 26643  4 5419 1.706263e-02
## 26644  4 5420 1.694529e-02
## 26645  4 5421 1.692184e-02
## 26646  4 5422 1.681060e-02
## 26647  4 5423 1.674177e-02
## 26648  4 5424 1.664181e-02
## 26649  4 5425 1.658628e-02
## 26650  4 5426 1.651048e-02
## 26651  4 5427 1.642160e-02
## 26652  4 5428 1.634516e-02
## 26653  4 5429 1.627614e-02
## 26654  4 5430 1.621916e-02
## 26655  4 5431 1.611811e-02
## 26656  4 5432 1.602930e-02
## 26657  4 5433 1.596532e-02
## 26658  4 5434 1.591007e-02
## 26659  4 5435 1.584323e-02
## 26660  4 5436 1.573889e-02
## 26661  4 5437 1.569743e-02
## 26662  4 5438 1.560899e-02
## 26663  4 5439 1.556292e-02
## 26664  4 5440 1.546149e-02
## 26665  4 5441 1.540080e-02
## 26666  4 5442 1.532456e-02
## 26667  4 5443 1.526598e-02
## 26668  4 5444 1.521390e-02
## 26669  4 5445 1.514096e-02
## 26670  4 5446 1.505881e-02
## 26671  4 5447 1.500542e-02
## 26672  4 5448 1.495329e-02
## 26673  4 5449 1.483443e-02
## 26674  4 5450 1.481214e-02
## 26675  4 5451 1.471673e-02
## 26676  4 5452 1.464003e-02
## 26677  4 5453 1.457182e-02
## 26678  4 5454 1.453205e-02
## 26679  4 5455 1.444605e-02
## 26680  4 5456 1.439334e-02
## 26681  4 5457 1.434028e-02
## 26682  4 5458 1.427059e-02
## 26683  4 5459 1.420630e-02
## 26684  4 5460 1.411662e-02
## 26685  4 5461 1.407667e-02
## 26686  4 5462 1.402407e-02
## 26687  4 5463 1.393336e-02
## 26688  4 5464 1.387437e-02
## 26689  4 5465 1.384071e-02
## 26690  4 5466 1.374298e-02
## 26691  4 5467 1.370453e-02
## 26692  4 5468 1.362280e-02
## 26693  4 5469 1.355939e-02
## 26694  4 5470 1.351005e-02
## 26695  4 5471 1.345078e-02
## 26696  4 5472 1.336106e-02
## 26697  4 5473 1.330911e-02
## 26698  4 5474 1.324114e-02
## 26699  4 5475 1.319130e-02
## 26700  4 5476 1.311732e-02
## 26701  4 5477 1.306959e-02
## 26702  4 5478 1.303121e-02
## 26703  4 5479 1.295089e-02
## 26704  4 5480 1.289322e-02
## 26705  4 5481 1.284208e-02
## 26706  4 5482 1.276050e-02
## 26707  4 5483 1.271446e-02
## 26708  4 5484 1.268667e-02
## 26709  4 5485 1.260956e-02
## 26710  4 5486 1.255366e-02
## 26711  4 5487 1.247769e-02
## 26712  4 5488 1.242948e-02
## 26713  4 5489 1.238448e-02
## 26714  4 5490 1.233696e-02
## 26715  4 5491 1.225171e-02
## 26716  4 5492 1.221928e-02
## 26717  4 5493 1.215255e-02
## 26718  4 5494 1.209281e-02
## 26719  4 5495 1.204461e-02
## 26720  4 5496 1.198193e-02
## 26721  4 5497 1.193109e-02
## 26722  4 5498 1.186381e-02
## 26723  4 5499 1.181799e-02
## 26724  4 5500 1.177003e-02
## 26725  4 5501 1.174533e-02
## 26726  4 5502 1.165956e-02
## 26727  4 5503 1.160059e-02
## 26728  4 5504 1.155934e-02
## 26729  4 5505 1.149118e-02
## 26730  4 5506 1.144853e-02
## 26731  4 5507 1.139489e-02
## 26732  4 5508 1.133550e-02
## 26733  4 5509 1.130486e-02
## 26734  4 5510 1.125475e-02
## 26735  4 5511 1.117219e-02
## 26736  4 5512 1.115663e-02
## 26737  4 5513 1.109465e-02
## 26738  4 5514 1.104548e-02
## 26739  4 5515 1.101460e-02
## 26740  4 5516 1.093183e-02
## 26741  4 5517 1.088927e-02
## 26742  4 5518 1.082374e-02
## 26743  4 5519 1.079879e-02
## 26744  4 5520 1.074004e-02
## 26745  4 5521 1.070305e-02
## 26746  4 5522 1.066123e-02
## 26747  4 5523 1.060468e-02
## 26748  4 5524 1.056723e-02
## 26749  4 5525 1.050261e-02
## 26750  4 5526 1.046527e-02
## 26751  4 5527 1.039547e-02
## 26752  4 5528 1.036746e-02
## 26753  4 5529 1.029811e-02
## 26754  4 5530 1.024659e-02
## 26755  4 5531 1.024233e-02
## 26756  4 5532 1.015770e-02
## 26757  4 5533 1.013752e-02
## 26758  4 5534 1.007661e-02
## 26759  4 5535 1.003018e-02
## 26760  4 5536 9.981097e-03
## 26761  4 5537 9.935638e-03
## 26762  4 5538 9.880135e-03
## 26763  4 5539 9.839937e-03
## 26764  4 5540 9.815503e-03
## 26765  4 5541 9.754482e-03
## 26766  4 5542 9.717946e-03
## 26767  4 5543 9.674318e-03
## 26768  4 5544 9.610067e-03
## 26769  4 5545 9.593133e-03
## 26770  4 5546 9.536044e-03
## 26771  4 5547 9.481117e-03
## 26772  4 5548 9.455436e-03
## 26773  4 5549 9.432126e-03
## 26774  4 5550 9.371318e-03
## 26775  4 5551 9.333119e-03
## 26776  4 5552 9.276447e-03
## 26777  4 5553 9.249856e-03
## 26778  4 5554 9.196980e-03
## 26779  4 5555 9.170799e-03
## 26780  4 5556 9.112753e-03
## 26781  4 5557 9.079968e-03
## 26782  4 5558 9.035633e-03
## 26783  4 5559 8.988098e-03
## 26784  4 5560 8.960965e-03
## 26785  4 5561 8.906125e-03
## 26786  4 5562 8.874715e-03
## 26787  4 5563 8.817186e-03
## 26788  4 5564 8.786490e-03
## 26789  4 5565 8.736109e-03
## 26790  4 5566 8.710022e-03
## 26791  4 5567 8.693513e-03
## 26792  4 5568 8.629243e-03
## 26793  4 5569 8.584876e-03
## 26794  4 5570 8.543049e-03
## 26795  4 5571 8.511976e-03
## 26796  4 5572 8.464058e-03
## 26797  4 5573 8.431428e-03
## 26798  4 5574 8.385606e-03
## 26799  4 5575 8.348471e-03
## 26800  4 5576 8.328167e-03
## 26801  4 5577 8.269258e-03
## 26802  4 5578 8.251376e-03
## 26803  4 5579 8.202046e-03
## 26804  4 5580 8.175715e-03
## 26805  4 5581 8.134113e-03
## 26806  4 5582 8.083945e-03
## 26807  4 5583 8.049826e-03
## 26808  4 5584 8.019945e-03
## 26809  4 5585 7.973615e-03
## 26810  4 5586 7.942625e-03
## 26811  4 5587 7.918261e-03
## 26812  4 5588 7.891859e-03
## 26813  4 5589 7.839690e-03
## 26814  4 5590 7.807403e-03
## 26815  4 5591 7.767807e-03
## 26816  4 5592 7.734691e-03
## 26817  4 5593 7.706295e-03
## 26818  4 5594 7.661879e-03
## 26819  4 5595 7.621778e-03
## 26820  4 5596 7.576635e-03
## 26821  4 5597 7.555756e-03
## 26822  4 5598 7.521522e-03
## 26823  4 5599 7.486964e-03
## 26824  4 5600 7.464678e-03
## 26825  4 5601 7.426331e-03
## 26826  4 5602 7.372293e-03
## 26827  4 5603 7.340647e-03
## 26828  4 5604 7.316392e-03
## 26829  4 5605 7.292817e-03
## 26830  4 5606 7.254822e-03
## 26831  4 5607 7.217497e-03
## 26832  4 5608 7.189672e-03
## 26833  4 5609 7.163372e-03
## 26834  4 5610 7.115639e-03
## 26835  4 5611 7.098841e-03
## 26836  4 5612 7.044634e-03
## 26837  4 5613 7.031983e-03
## 26838  4 5614 6.990856e-03
## 26839  4 5615 6.966214e-03
## 26840  4 5616 6.938461e-03
## 26841  4 5617 6.899488e-03
## 26842  4 5618 6.873205e-03
## 26843  4 5619 6.827617e-03
## 26844  4 5620 6.801343e-03
## 26845  4 5621 6.774073e-03
## 26846  4 5622 6.733366e-03
## 26847  4 5623 6.710980e-03
## 26848  4 5624 6.689011e-03
## 26849  4 5625 6.641356e-03
## 26850  4 5626 6.617032e-03
## 26851  4 5627 6.592735e-03
## 26852  4 5628 6.555937e-03
## 26853  4 5629 6.525571e-03
## 26854  4 5630 6.491635e-03
## 26855  4 5631 6.469965e-03
## 26856  4 5632 6.432128e-03
## 26857  4 5633 6.401495e-03
## 26858  4 5634 6.374116e-03
## 26859  4 5635 6.355281e-03
## 26860  4 5636 6.321870e-03
## 26861  4 5637 6.296511e-03
## 26862  4 5638 6.269841e-03
## 26863  4 5639 6.233353e-03
## 26864  4 5640 6.204666e-03
## 26865  4 5641 6.178794e-03
## 26866  4 5642 6.150843e-03
## 26867  4 5643 6.119052e-03
## 26868  4 5644 6.097578e-03
## 26869  4 5645 6.082017e-03
## 26870  4 5646 6.051456e-03
## 26871  4 5647 6.020954e-03
## 26872  4 5648 5.983202e-03
## 26873  4 5649 5.960819e-03
## 26874  4 5650 5.928455e-03
## 26875  4 5651 5.905699e-03
## 26876  4 5652 5.882680e-03
## 26877  4 5653 5.858158e-03
## 26878  4 5654 5.820600e-03
## 26879  4 5655 5.805434e-03
## 26880  4 5656 5.774007e-03
## 26881  4 5657 5.758595e-03
## 26882  4 5658 5.716698e-03
## 26883  4 5659 5.703288e-03
## 26884  4 5660 5.675216e-03
## 26885  4 5661 5.647940e-03
## 26886  4 5662 5.614079e-03
## 26887  4 5663 5.599083e-03
## 26888  4 5664 5.569254e-03
## 26889  4 5665 5.548777e-03
## 26890  4 5666 5.522993e-03
## 26891  4 5667 5.485417e-03
## 26892  4 5668 5.473597e-03
## 26893  4 5669 5.445037e-03
## 26894  4 5670 5.426701e-03
## 26895  4 5671 5.395043e-03
## 26896  4 5672 5.359816e-03
## 26897  4 5673 5.338065e-03
## 26898  4 5674 5.323107e-03
## 26899  4 5675 5.293584e-03
## 26900  4 5676 5.274741e-03
## 26901  4 5677 5.238055e-03
## 26902  4 5678 5.211953e-03
## 26903  4 5679 5.199253e-03
## 26904  4 5680 5.171645e-03
## 26905  4 5681 5.145226e-03
## 26906  4 5682 5.122335e-03
## 26907  4 5683 5.107469e-03
## 26908  4 5684 5.080656e-03
## 26909  4 5685 5.056417e-03
## 26910  4 5686 5.029308e-03
## 26911  4 5687 5.013530e-03
## 26912  4 5688 4.988178e-03
## 26913  4 5689 4.962576e-03
## 26914  4 5690 4.937305e-03
## 26915  4 5691 4.916892e-03
## 26916  4 5692 4.904662e-03
## 26917  4 5693 4.881099e-03
## 26918  4 5694 4.846599e-03
## 26919  4 5695 4.835787e-03
## 26920  4 5696 4.812268e-03
## 26921  4 5697 4.786008e-03
## 26922  4 5698 4.766818e-03
## 26923  4 5699 4.738741e-03
## 26924  4 5700 4.723402e-03
## 26925  4 5701 4.701751e-03
## 26926  4 5702 4.678190e-03
## 26927  4 5703 4.652424e-03
## 26928  4 5704 4.638405e-03
## 26929  4 5705 4.610540e-03
## 26930  4 5706 4.592864e-03
## 26931  4 5707 4.572943e-03
## 26932  4 5708 4.559350e-03
## 26933  4 5709 4.532569e-03
## 26934  4 5710 4.506672e-03
## 26935  4 5711 4.485899e-03
## 26936  4 5712 4.464864e-03
## 26937  4 5713 4.458340e-03
## 26938  4 5714 4.435982e-03
## 26939  4 5715 4.416692e-03
## 26940  4 5716 4.393080e-03
## 26941  4 5717 4.368113e-03
## 26942  4 5718 4.349485e-03
## 26943  4 5719 4.324124e-03
## 26944  4 5720 4.305347e-03
## 26945  4 5721 4.288997e-03
## 26946  4 5722 4.264171e-03
## 26947  4 5723 4.250379e-03
## 26948  4 5724 4.232206e-03
## 26949  4 5725 4.215181e-03
## 26950  4 5726 4.195004e-03
## 26951  4 5727 4.175483e-03
## 26952  4 5728 4.162979e-03
## 26953  4 5729 4.138740e-03
## 26954  4 5730 4.113732e-03
## 26955  4 5731 4.097143e-03
## 26956  4 5732 4.079112e-03
## 26957  4 5733 4.062580e-03
## 26958  4 5734 4.045492e-03
## 26959  4 5735 4.021150e-03
## 26960  4 5736 4.006623e-03
## 26961  4 5737 3.989682e-03
## 26962  4 5738 3.971921e-03
## 26963  4 5739 3.949263e-03
## 26964  4 5740 3.939818e-03
## 26965  4 5741 3.913075e-03
## 26966  4 5742 3.901614e-03
## 26967  4 5743 3.883853e-03
## 26968  4 5744 3.858045e-03
## 26969  4 5745 3.841298e-03
## 26970  4 5746 3.834523e-03
## 26971  4 5747 3.807432e-03
## 26972  4 5748 3.790785e-03
## 26973  4 5749 3.776762e-03
## 26974  4 5750 3.755793e-03
## 26975  4 5751 3.744518e-03
## 26976  4 5752 3.729341e-03
## 26977  4 5753 3.704367e-03
## 26978  4 5754 3.698392e-03
## 26979  4 5755 3.668416e-03
## 26980  4 5756 3.654242e-03
## 26981  4 5757 3.638119e-03
## 26982  4 5758 3.628306e-03
## 26983  4 5759 3.606840e-03
## 26984  4 5760 3.590543e-03
## 26985  4 5761 3.571672e-03
## 26986  4 5762 3.559328e-03
## 26987  4 5763 3.542102e-03
## 26988  4 5764 3.531025e-03
## 26989  4 5765 3.509727e-03
## 26990  4 5766 3.497365e-03
## 26991  4 5767 3.476501e-03
## 26992  4 5768 3.461618e-03
## 26993  4 5769 3.445273e-03
## 26994  4 5770 3.428145e-03
## 26995  4 5771 3.408227e-03
## 26996  4 5772 3.401629e-03
## 26997  4 5773 3.378478e-03
## 26998  4 5774 3.370191e-03
## 26999  4 5775 3.351674e-03
## 27000  4 5776 3.337145e-03
## 27001  4 5777 3.324667e-03
## 27002  4 5778 3.307401e-03
## 27003  4 5779 3.291315e-03
## 27004  4 5780 3.271921e-03
## 27005  4 5781 3.261791e-03
## 27006  4 5782 3.244332e-03
## 27007  4 5783 3.232014e-03
## 27008  4 5784 3.218403e-03
## 27009  4 5785 3.202866e-03
## 27010  4 5786 3.192266e-03
## 27011  4 5787 3.173189e-03
## 27012  4 5788 3.158487e-03
## 27013  4 5789 3.150072e-03
## 27014  4 5790 3.128575e-03
## 27015  4 5791 3.115525e-03
## 27016  4 5792 3.100878e-03
## 27017  4 5793 3.092743e-03
## 27018  4 5794 3.080518e-03
## 27019  4 5795 3.058393e-03
## 27020  4 5796 3.049346e-03
## 27021  4 5797 3.033749e-03
## 27022  4 5798 3.016763e-03
## 27023  4 5799 3.010385e-03
## 27024  4 5800 2.990582e-03
## 27025  4 5801 2.978562e-03
## 27026  4 5802 2.967559e-03
## 27027  4 5803 2.952670e-03
## 27028  4 5804 2.937560e-03
## 27029  4 5805 2.921522e-03
## 27030  4 5806 2.912503e-03
## 27031  4 5807 2.893275e-03
## 27032  4 5808 2.883097e-03
## 27033  4 5809 2.870998e-03
## 27034  4 5810 2.855647e-03
## 27035  4 5811 2.840845e-03
## 27036  4 5812 2.834979e-03
## 27037  4 5813 2.823493e-03
## 27038  4 5814 2.809220e-03
## 27039  4 5815 2.789727e-03
## 27040  4 5816 2.780166e-03
## 27041  4 5817 2.769748e-03
## 27042  4 5818 2.757914e-03
## 27043  4 5819 2.743691e-03
## 27044  4 5820 2.732223e-03
## 27045  4 5821 2.719851e-03
## 27046  4 5822 2.700946e-03
## 27047  4 5823 2.689649e-03
## 27048  4 5824 2.682345e-03
## 27049  4 5825 2.670753e-03
## 27050  4 5826 2.655390e-03
## 27051  4 5827 2.644774e-03
## 27052  4 5828 2.632508e-03
## 27053  4 5829 2.621640e-03
## 27054  4 5830 2.606934e-03
## 27055  4 5831 2.594968e-03
## 27056  4 5832 2.589063e-03
## 27057  4 5833 2.568182e-03
## 27058  4 5834 2.557919e-03
## 27059  4 5835 2.549482e-03
## 27060  4 5836 2.538922e-03
## 27061  4 5837 2.529112e-03
## 27062  4 5838 2.514548e-03
## 27063  4 5839 2.501622e-03
## 27064  4 5840 2.490729e-03
## 27065  4 5841 2.479483e-03
## 27066  4 5842 2.466473e-03
## 27067  4 5843 2.455685e-03
## 27068  4 5844 2.445205e-03
## 27069  4 5845 2.437954e-03
## 27070  4 5846 2.426430e-03
## 27071  4 5847 2.416232e-03
## 27072  4 5848 2.401845e-03
## 27073  4 5849 2.390907e-03
```

```
## 27074  4 5850 2.376791e-03
## 27075  4 5851 2.366034e-03
## 27076  4 5852 2.361290e-03
## 27077  4 5853 2.352258e-03
## 27078  4 5854 2.335321e-03
## 27079  4 5855 2.323566e-03
## 27080  4 5856 2.313594e-03
## 27081  4 5857 2.305608e-03
## 27082  4 5858 2.298642e-03
## 27083  4 5859 2.285552e-03
## 27084  4 5860 2.275170e-03
## 27085  4 5861 2.264001e-03
## 27086  4 5862 2.249548e-03
## 27087  4 5863 2.243548e-03
## 27088  4 5864 2.233305e-03
## 27089  4 5865 2.223604e-03
## 27090  4 5866 2.207677e-03
## 27091  4 5867 2.203865e-03
## 27092  4 5868 2.192168e-03
## 27093  4 5869 2.181961e-03
## 27094  4 5870 2.168959e-03
## 27095  4 5871 2.161236e-03
## 27096  4 5872 2.155585e-03
## 27097  4 5873 2.139978e-03
## 27098  4 5874 2.132877e-03
## 27099  4 5875 2.118793e-03
## 27100  4 5876 2.111618e-03
## 27101  4 5877 2.106105e-03
## 27102  4 5878 2.093459e-03
## 27103  4 5879 2.083389e-03
## 27104  4 5880 2.076039e-03
## 27105  4 5881 2.067444e-03
## 27106  4 5882 2.061262e-03
## 27107  4 5883 2.043842e-03
## 27108  4 5884 2.039835e-03
## 27109  4 5885 2.027437e-03
## 27110  4 5886 2.020206e-03
## 27111  4 5887 2.010817e-03
## 27112  4 5888 2.001384e-03
## 27113  4 5889 1.993453e-03
## 27114  4 5890 1.987111e-03
## 27115  4 5891 1.975733e-03
## 27116  4 5892 1.967092e-03
## 27117  4 5893 1.956079e-03
## 27118  4 5894 1.946801e-03
## 27119  4 5895 1.937763e-03
## 27120  4 5896 1.930883e-03
## 27121  4 5897 1.919879e-03
## 27122  4 5898 1.910224e-03
## 27123  4 5899 1.902369e-03
## 27124  4 5900 1.895849e-03
## 27125  4 5901 1.883695e-03
## 27126  4 5902 1.879581e-03
## 27127  4 5903 1.865497e-03
## 27128  4 5904 1.858791e-03
## 27129  4 5905 1.849654e-03
## 27130  4 5906 1.842223e-03
## 27131  4 5907 1.836578e-03
## 27132  4 5908 1.826096e-03
## 27133  4 5909 1.820205e-03
## 27134  4 5910 1.806483e-03
## 27135  4 5911 1.800814e-03
## 27136  4 5912 1.792551e-03
## 27137  4 5913 1.785849e-03
## 27138  4 5914 1.780478e-03
## 27139  4 5915 1.768467e-03
## 27140  4 5916 1.759273e-03
## 27141  4 5917 1.750318e-03
## 27142  4 5918 1.745433e-03
## 27143  4 5919 1.735719e-03
## 27144  4 5920 1.728133e-03
## 27145  4 5921 1.719830e-03
## 27146  4 5922 1.713520e-03
## 27147  4 5923 1.703029e-03
## 27148  4 5924 1.699107e-03
## 27149  4 5925 1.688855e-03
## 27150  4 5926 1.681886e-03
## 27151  4 5927 1.673773e-03
## 27152  4 5928 1.666936e-03
## 27153  4 5929 1.661057e-03
## 27154  4 5930 1.651193e-03
## 27155  4 5931 1.647275e-03
## 27156  4 5932 1.638331e-03
## 27157  4 5933 1.627549e-03
## 27158  4 5934 1.623047e-03
## 27159  4 5935 1.611995e-03
## 27160  4 5936 1.604860e-03
## 27161  4 5937 1.601418e-03
## 27162  4 5938 1.589090e-03
## 27163  4 5939 1.583567e-03
## 27164  4 5940 1.578056e-03
## 27165  4 5941 1.568543e-03
## 27166  4 5942 1.562516e-03
## 27167  4 5943 1.556924e-03
## 27168  4 5944 1.549261e-03
## 27169  4 5945 1.542766e-03
## 27170  4 5946 1.532640e-03
## 27171  4 5947 1.532112e-03
## 27172  4 5948 1.522588e-03
## 27173  4 5949 1.514551e-03
## 27174  4 5950 1.507394e-03
## 27175  4 5951 1.500848e-03
## 27176  4 5952 1.493431e-03
## 27177  4 5953 1.487188e-03
## 27178  4 5954 1.480376e-03
## 27179  4 5955 1.471587e-03
## 27180  4 5956 1.466517e-03
## 27181  4 5957 1.461568e-03
## 27182  4 5958 1.455779e-03
## 27183  4 5959 1.447545e-03
## 27184  4 5960 1.442195e-03
## 27185  4 5961 1.433910e-03
## 27186  4 5962 1.424870e-03
## 27187  4 5963 1.421982e-03
## 27188  4 5964 1.414008e-03
## 27189  4 5965 1.405255e-03
## 27190  4 5966 1.404266e-03
## 27191  4 5967 1.392818e-03
## 27192  4 5968 1.388673e-03
## 27193  4 5969 1.382152e-03
## 27194  4 5970 1.375667e-03
## 27195  4 5971 1.369241e-03
## 27196  4 5972 1.362782e-03
## 27197  4 5973 1.354185e-03
## 27198  4 5974 1.351912e-03
## 27199  4 5975 1.345243e-03
## 27200  4 5976 1.338637e-03
## 27201  4 5977 1.333539e-03
## 27202  4 5978 1.326787e-03
## 27203  4 5979 1.320781e-03
## 27204  4 5980 1.313503e-03
## 27205  4 5981 1.307796e-03
## 27206  4 5982 1.302428e-03
## 27207  4 5983 1.298662e-03
## 27208  4 5984 1.289545e-03
## 27209  4 5985 1.284259e-03
## 27210  4 5986 1.279096e-03
## 27211  4 5987 1.272105e-03
## 27212  4 5988 1.266685e-03
## 27213  4 5989 1.262651e-03
## 27214  4 5990 1.253320e-03
## 27215  4 5991 1.250826e-03
## 27216  4 5992 1.246139e-03
## 27217  4 5993 1.238347e-03
## 27218  4 5994 1.234620e-03
## 27219  4 5995 1.228302e-03
## 27220  4 5996 1.221613e-03
## 27221  4 5997 1.217998e-03
## 27222  4 5998 1.210356e-03
## 27223  4 5999 1.206101e-03
## 27224  4 6000 1.199795e-03
## 27225  4 6001 1.193821e-03
## 27226  4 6002 1.188134e-03
## 27227  4 6003 1.182015e-03
## 27228  4 6004 1.177275e-03
## 27229  4 6005 1.172818e-03
## 27230  4 6006 1.167455e-03
## 27231  4 6007 1.161972e-03
## 27232  4 6008 1.155241e-03
## 27233  4 6009 1.152787e-03
## 27234  4 6010 1.146149e-03
## 27235  4 6011 1.141467e-03
## 27236  4 6012 1.135166e-03
## 27237  4 6013 1.129654e-03
## 27238  4 6014 1.125719e-03
## 27239  4 6015 1.120489e-03
## 27240  4 6016 1.115263e-03
## 27241  4 6017 1.110158e-03
## 27242  4 6018 1.106268e-03
## 27243  4 6019 1.103245e-03
## 27244  4 6020 1.094960e-03
## 27245  4 6021 1.089571e-03
## 27246  4 6022 1.083067e-03
## 27247  4 6023 1.080615e-03
## 27248  4 6024 1.076572e-03
## 27249  4 6025 1.069767e-03
## 27250  4 6026 1.064676e-03
## 27251  4 6027 1.062882e-03
## 27252  4 6028 1.057080e-03
## 27253  4 6029 1.048820e-03
## 27254  4 6030 1.046038e-03
## 27255  4 6031 1.041440e-03
## 27256  4 6032 1.036430e-03
## 27257  4 6033 1.032348e-03
## 27258  4 6034 1.028259e-03
## 27259  4 6035 1.020694e-03
## 27260  4 6036 1.017471e-03
## 27261  4 6037 1.014102e-03
## 27262  4 6038 1.008999e-03
## 27263  4 6039 1.003580e-03
## 27264  4 6040 9.991313e-04
## 27265  4 6041 9.932870e-04
## 27266  4 6042 9.892594e-04
## 27267  4 6043 9.862260e-04
## 27268  4 6044 9.825897e-04
## 27269  4 6045 9.787709e-04
## 27270  4 6046 9.731767e-04
## 27271  4 6047 9.673885e-04
## 27272  4 6048 9.621649e-04
## 27273  4 6049 9.592051e-04
## 27274  4 6050 9.541829e-04
## 27275  4 6051 9.507800e-04
## 27276  4 6052 9.458573e-04
## 27277  4 6053 9.425105e-04
## 27278  4 6054 9.394988e-04
## 27279  4 6055 9.337538e-04
## 27280  4 6056 9.290024e-04
## 27281  4 6057 9.248669e-04
## 27282  4 6058 9.193691e-04
## 27283  4 6059 9.165981e-04
## 27284  4 6060 9.121249e-04
## 27285  4 6061 9.090137e-04
## 27286  4 6062 9.033430e-04
## 27287  4 6063 8.998627e-04
## 27288  4 6064 8.952732e-04
## 27289  4 6065 8.930289e-04
## 27290  4 6066 8.883338e-04
## 27291  4 6067 8.820195e-04
## 27292  4 6068 8.789530e-04
## 27293  4 6069 8.753985e-04
## 27294  4 6070 8.710725e-04
## 27295  4 6071 8.670103e-04
## 27296  4 6072 8.638531e-04
## 27297  4 6073 8.593673e-04
## 27298  4 6074 8.553764e-04
## 27299  4 6075 8.529435e-04
## 27300  4 6076 8.482054e-04
## 27301  4 6077 8.434230e-04
## 27302  4 6078 8.392007e-04
## 27303  4 6079 8.352369e-04
## 27304  4 6080 8.319368e-04
## 27305  4 6081 8.298029e-04
## 27306  4 6082 8.244436e-04
## 27307  4 6083 8.203505e-04
## 27308  4 6084 8.165748e-04
## 27309  4 6085 8.123659e-04
## 27310  4 6086 8.107163e-04
## 27311  4 6087 8.062723e-04
## 27312  4 6088 8.030740e-04
## 27313  4 6089 7.988401e-04
## 27314  4 6090 7.958440e-04
## 27315  4 6091 7.910600e-04
## 27316  4 6092 7.886720e-04
## 27317  4 6093 7.848354e-04
## 27318  4 6094 7.831852e-04
## 27319  4 6095 7.764128e-04
## 27320  4 6096 7.740202e-04
## 27321  4 6097 7.718158e-04
## 27322  4 6098 7.663349e-04
## 27323  4 6099 7.628594e-04
## 27324  4 6100 7.606214e-04
## 27325  4 6101 7.553589e-04
## 27326  4 6102 7.533748e-04
## 27327  4 6103 7.495146e-04
## 27328  4 6104 7.459861e-04
## 27329  4 6105 7.429602e-04
## 27330  4 6106 7.399164e-04
## 27331  4 6107 7.372576e-04
## 27332  4 6108 7.321284e-04
## 27333  4 6109 7.291999e-04
## 27334  4 6110 7.261403e-04
## 27335  4 6111 7.224577e-04
## 27336  4 6112 7.194350e-04
## 27337  4 6113 7.166201e-04
## 27338  4 6114 7.130129e-04
## 27339  4 6115 7.092605e-04
## 27340  4 6116 7.060297e-04
## 27341  4 6117 7.034262e-04
## 27342  4 6118 7.003301e-04
## 27343  4 6119 6.970708e-04
## 27344  4 6120 6.936599e-04
## 27345  4 6121 6.919007e-04
## 27346  4 6122 6.869372e-04
## 27347  4 6123 6.842373e-04
## 27348  4 6124 6.806368e-04
## 27349  4 6125 6.766219e-04
## 27350  4 6126 6.755397e-04
## 27351  4 6127 6.705797e-04
## 27352  4 6128 6.682712e-04
## 27353  4 6129 6.656970e-04
## 27354  4 6130 6.625222e-04
## 27355  4 6131 6.582796e-04
## 27356  4 6132 6.559768e-04
## 27357  4 6133 6.542228e-04
## 27358  4 6134 6.506798e-04
## 27359  4 6135 6.470486e-04
## 27360  4 6136 6.453460e-04
## 27361  4 6137 6.412911e-04
## 27362  4 6138 6.387652e-04
## 27363  4 6139 6.350972e-04
## 27364  4 6140 6.327038e-04
## 27365  4 6141 6.311787e-04
## 27366  4 6142 6.279468e-04
## 27367  4 6143 6.241403e-04
## 27368  4 6144 6.217610e-04
## 27369  4 6145 6.181013e-04
## 27370  4 6146 6.156055e-04
## 27371  4 6147 6.133434e-04
## 27372  4 6148 6.110238e-04
## 27373  4 6149 6.079000e-04
## 27374  4 6150 6.044770e-04
## 27375  4 6151 6.021729e-04
## 27376  4 6152 5.988037e-04
## 27377  4 6153 5.965179e-04
## 27378  4 6154 5.933744e-04
## 27379  4 6155 5.911524e-04
## 27380  4 6156 5.877285e-04
## 27381  4 6157 5.850590e-04
## 27382  4 6158 5.830976e-04
## 27383  4 6159 5.804125e-04
## 27384  4 6160 5.780409e-04
## 27385  4 6161 5.754271e-04
## 27386  4 6162 5.725655e-04
## 27387  4 6163 5.705390e-04
## 27388  4 6164 5.666176e-04
## 27389  4 6165 5.648711e-04
## 27390  4 6166 5.610628e-04
## 27391  4 6167 5.595608e-04
## 27392  4 6168 5.566835e-04
## 27393  4 6169 5.540396e-04
## 27394  4 6170 5.531916e-04
## 27395  4 6171 5.495713e-04
## 27396  4 6172 5.464170e-04
## 27397  4 6173 5.449390e-04
## 27398  4 6174 5.414423e-04
## 27399  4 6175 5.391145e-04
## 27400  4 6176 5.372079e-04
## 27401  4 6177 5.342829e-04
## 27402  4 6178 5.316564e-04
## 27403  4 6179 5.292919e-04
## 27404  4 6180 5.269050e-04
## 27405  4 6181 5.254804e-04
## 27406  4 6182 5.212952e-04
## 27407  4 6183 5.194877e-04
## 27408  4 6184 5.169510e-04
## 27409  4 6185 5.151220e-04
## 27410  4 6186 5.128489e-04
## 27411  4 6187 5.108330e-04
## 27412  4 6188 5.085451e-04
## 27413  4 6189 5.054794e-04
## 27414  4 6190 5.033944e-04
## 27415  4 6191 5.020252e-04
## 27416  4 6192 4.990575e-04
## 27417  4 6193 4.968948e-04
## 27418  4 6194 4.944914e-04
## 27419  4 6195 4.921459e-04
## 27420  4 6196 4.906823e-04
## 27421  4 6197 4.873596e-04
## 27422  4 6198 4.854006e-04
## 27423  4 6199 4.837373e-04
## 27424  4 6200 4.813972e-04
## 27425  4 6201 4.795916e-04
## 27426  4 6202 4.772608e-04
## 27427  4 6203 4.750662e-04
## 27428  4 6204 4.722580e-04
## 27429  4 6205 4.700330e-04
## 27430  4 6206 4.687235e-04
## 27431  4 6207 4.655392e-04
## 27432  4 6208 4.639091e-04
## 27433  4 6209 4.618869e-04
## 27434  4 6210 4.599924e-04
## 27435  4 6211 4.574290e-04
## 27436  4 6212 4.557143e-04
## 27437  4 6213 4.534822e-04
## 27438  4 6214 4.519456e-04
## 27439  4 6215 4.478481e-04
## 27440  4 6216 4.471765e-04
## 27441  4 6217 4.451765e-04
## 27442  4 6218 4.433472e-04
## 27443  4 6219 4.415797e-04
## 27444  4 6220 4.380560e-04
## 27445  4 6221 4.378512e-04
## 27446  4 6222 4.345741e-04
## 27447  4 6223 4.342564e-04
## 27448  4 6224 4.314604e-04
## 27449  4 6225 4.297275e-04
## 27450  4 6226 4.269159e-04
## 27451  4 6227 4.252410e-04
## 27452  4 6228 4.242888e-04
## 27453  4 6229 4.216837e-04
## 27454  4 6230 4.192877e-04
## 27455  4 6231 4.179492e-04
## 27456  4 6232 4.166761e-04
## 27457  4 6233 4.141927e-04
## 27458  4 6234 4.119541e-04
## 27459  4 6235 4.098578e-04
## 27460  4 6236 4.079628e-04
## 27461  4 6237 4.064156e-04
## 27462  4 6238 4.037356e-04
## 27463  4 6239 4.030517e-04
## 27464  4 6240 4.002718e-04
## 27465  4 6241 3.994466e-04
## 27466  4 6242 3.970421e-04
## 27467  4 6243 3.958466e-04
## 27468  4 6244 3.934420e-04
## 27469  4 6245 3.917926e-04
## 27470  4 6246 3.898512e-04
## 27471  4 6247 3.877490e-04
## 27472  4 6248 3.865839e-04
## 27473  4 6249 3.850537e-04
## 27474  4 6250 3.828073e-04
## 27475  4 6251 3.812761e-04
## 27476  4 6252 3.793289e-04
## 27477  4 6253 3.777888e-04
## 27478  4 6254 3.761470e-04
## 27479  4 6255 3.751081e-04
## 27480  4 6256 3.730696e-04
## 27481  4 6257 3.701848e-04
## 27482  4 6258 3.692143e-04
## 27483  4 6259 3.679088e-04
## 27484  4 6260 3.661352e-04
## 27485  4 6261 3.648184e-04
## 27486  4 6262 3.625452e-04
## 27487  4 6263 3.607413e-04
## 27488  4 6264 3.593498e-04
## 27489  4 6265 3.575239e-04
## 27490  4 6266 3.562901e-04
## 27491  4 6267 3.545018e-04
## 27492  4 6268 3.530603e-04
## 27493  4 6269 3.516849e-04
## 27494  4 6270 3.500432e-04
## 27495  4 6271 3.481187e-04
## 27496  4 6272 3.463092e-04
## 27497  4 6273 3.453956e-04
## 27498  4 6274 3.428887e-04
## 27499  4 6275 3.419906e-04
## 27500  4 6276 3.403352e-04
## 27501  4 6277 3.387717e-04
## 27502  4 6278 3.373488e-04
## 27503  4 6279 3.350539e-04
## 27504  4 6280 3.335483e-04
## 27505  4 6281 3.323255e-04
## 27506  4 6282 3.310893e-04
## 27507  4 6283 3.295400e-04
## 27508  4 6284 3.276943e-04
## 27509  4 6285 3.255450e-04
## 27510  4 6286 3.251486e-04
## 27511  4 6287 3.232825e-04
## 27512  4 6288 3.221420e-04
## 27513  4 6289 3.206411e-04
## 27514  4 6290 3.190038e-04
## 27515  4 6291 3.178462e-04
## 27516  4 6292 3.162786e-04
## 27517  4 6293 3.143510e-04
## 27518  4 6294 3.136399e-04
## 27519  4 6295 3.121705e-04
## 27520  4 6296 3.102657e-04
## 27521  4 6297 3.095857e-04
## 27522  4 6298 3.075005e-04
## 27523  4 6299 3.065435e-04
## 27524  4 6300 3.050107e-04
## 27525  4 6301 3.029648e-04
## 27526  4 6302 3.018986e-04
## 27527  4 6303 3.006953e-04
## 27528  4 6304 2.998594e-04
## 27529  4 6305 2.977557e-04
## 27530  4 6306 2.964852e-04
## 27531  4 6307 2.948978e-04
## 27532  4 6308 2.936989e-04
## 27533  4 6309 2.923933e-04
## 27534  4 6310 2.910170e-04
## 27535  4 6311 2.895508e-04
## 27536  4 6312 2.879597e-04
## 27537  4 6313 2.874110e-04
## 27538  4 6314 2.857581e-04
## 27539  4 6315 2.848601e-04
## 27540  4 6316 2.832779e-04
## 27541  4 6317 2.816757e-04
## 27542  4 6318 2.805637e-04
## 27543  4 6319 2.797570e-04
## 27544  4 6320 2.781960e-04
## 27545  4 6321 2.768110e-04
## 27546  4 6322 2.755436e-04
## 27547  4 6323 2.744172e-04
## 27548  4 6324 2.730481e-04
## 27549  4 6325 2.720931e-04
## 27550  4 6326 2.705290e-04
## 27551  4 6327 2.693146e-04
## 27552  4 6328 2.686252e-04
## 27553  4 6329 2.674022e-04
## 27554  4 6330 2.657460e-04
## 27555  4 6331 2.642880e-04
## 27556  4 6332 2.635583e-04
## 27557  4 6333 2.618949e-04
## 27558  4 6334 2.610375e-04
## 27559  4 6335 2.597591e-04
## 27560  4 6336 2.581195e-04
## 27561  4 6337 2.571918e-04
## 27562  4 6338 2.563155e-04
## 27563  4 6339 2.547859e-04
## 27564  4 6340 2.535688e-04
## 27565  4 6341 2.525206e-04
## 27566  4 6342 2.511857e-04
## 27567  4 6343 2.504411e-04
## 27568  4 6344 2.494419e-04
## 27569  4 6345 2.479967e-04
## 27570  4 6346 2.467671e-04
## 27571  4 6347 2.457932e-04
## 27572  4 6348 2.449272e-04
## 27573  4 6349 2.438539e-04
## 27574  4 6350 2.428279e-04
## 27575  4 6351 2.415406e-04
## 27576  4 6352 2.402804e-04
## 27577  4 6353 2.390729e-04
## 27578  4 6354 2.381824e-04
## 27579  4 6355 2.372239e-04
## 27580  4 6356 2.360167e-04
## 27581  4 6357 2.353293e-04
## 27582  4 6358 2.336419e-04
## 27583  4 6359 2.329004e-04
## 27584  4 6360 2.312535e-04
## 27585  4 6361 2.311140e-04
## 27586  4 6362 2.295039e-04
## 27587  4 6363 2.282275e-04
## 27588  4 6364 2.274093e-04
## 27589  4 6365 2.267400e-04
## 27590  4 6366 2.253765e-04
## 27591  4 6367 2.243803e-04
## 27592  4 6368 2.228479e-04
## 27593  4 6369 2.225223e-04
## 27594  4 6370 2.214037e-04
## 27595  4 6371 2.200322e-04
## 27596  4 6372 2.196376e-04
## 27597  4 6373 2.186482e-04
## 27598  4 6374 2.170343e-04
## 27599  4 6375 2.163467e-04
## 27600  4 6376 2.157494e-04
## 27601  4 6377 2.145358e-04
## 27602  4 6378 2.134088e-04
## 27603  4 6379 2.126015e-04
## 27604  4 6380 2.114550e-04
## 27605  4 6381 2.104315e-04
## 27606  4 6382 2.097542e-04
## 27607  4 6383 2.087045e-04
## 27608  4 6384 2.076185e-04
## 27609  4 6385 2.068660e-04
## 27610  4 6386 2.057671e-04
## 27611  4 6387 2.050859e-04
## 27612  4 6388 2.039001e-04
## 27613  4 6389 2.029389e-04
## 27614  4 6390 2.023065e-04
## 27615  4 6391 2.009329e-04
## 27616  4 6392 2.002289e-04
## 27617  4 6393 1.994653e-04
## 27618  4 6394 1.986225e-04
## 27619  4 6395 1.976320e-04
## 27620  4 6396 1.965164e-04
## 27621  4 6397 1.959454e-04
## 27622  4 6398 1.951898e-04
## 27623  4 6399 1.941744e-04
## 27624  4 6400 1.927954e-04
## 27625  4 6401 1.923077e-04
## 27626  4 6402 1.910661e-04
## 27627  4 6403 1.904435e-04
## 27628  4 6404 1.898006e-04
## 27629  4 6405 1.885531e-04
## 27630  4 6406 1.879231e-04
## 27631  4 6407 1.868555e-04
## 27632  4 6408 1.861446e-04
## 27633  4 6409 1.854934e-04
## 27634  4 6410 1.843263e-04
## 27635  4 6411 1.836122e-04
## 27636  4 6412 1.825959e-04
## 27637  4 6413 1.818794e-04
## 27638  4 6414 1.812953e-04
## 27639  4 6415 1.804020e-04
## 27640  4 6416 1.791056e-04
## 27641  4 6417 1.787710e-04
## 27642  4 6418 1.778110e-04
## 27643  4 6419 1.771510e-04
## 27644  4 6420 1.761753e-04
## 27645  4 6421 1.751198e-04
## 27646  4 6422 1.744035e-04
## 27647  4 6423 1.739850e-04
## 27648  4 6424 1.731378e-04
## 27649  4 6425 1.721086e-04
## 27650  4 6426 1.714074e-04
## 27651  4 6427 1.705785e-04
## 27652  4 6428 1.697072e-04
## 27653  4 6429 1.690806e-04
## 27654  4 6430 1.682923e-04
## 27655  4 6431 1.674456e-04
## 27656  4 6432 1.669009e-04
## 27657  4 6433 1.662485e-04
## 27658  4 6434 1.651787e-04
## 27659  4 6435 1.646773e-04
## 27660  4 6436 1.638278e-04
## 27661  4 6437 1.630182e-04
## 27662  4 6438 1.623293e-04
## 27663  4 6439 1.616811e-04
## 27664  4 6440 1.608918e-04
## 27665  4 6441 1.603516e-04
## 27666  4 6442 1.594955e-04
## 27667  4 6443 1.587142e-04
## 27668  4 6444 1.577455e-04
## 27669  4 6445 1.572956e-04
## 27670  4 6446 1.563091e-04
## 27671  4 6447 1.557967e-04
## 27672  4 6448 1.550901e-04
## 27673  4 6449 1.542397e-04
## 27674  4 6450 1.535437e-04
## 27675  4 6451 1.528214e-04
## 27676  4 6452 1.521558e-04
## 27677  4 6453 1.517298e-04
## 27678  4 6454 1.506586e-04
## 27679  4 6455 1.499725e-04
## 27680  4 6456 1.497267e-04
## 27681  4 6457 1.489660e-04
## 27682  4 6458 1.481427e-04
## 27683  4 6459 1.475101e-04
## 27684  4 6460 1.467542e-04
## 27685  4 6461 1.458182e-04
## 27686  4 6462 1.454900e-04
## 27687  4 6463 1.447543e-04
## 27688  4 6464 1.441990e-04
## 27689  4 6465 1.434093e-04
## 27690  4 6466 1.427573e-04
## 27691  4 6467 1.421750e-04
## 27692  4 6468 1.416425e-04
## 27693  4 6469 1.410263e-04
## 27694  4 6470 1.401953e-04
## 27695  4 6471 1.393952e-04
## 27696  4 6472 1.391041e-04
## 27697  4 6473 1.380657e-04
## 27698  4 6474 1.374745e-04
## 27699  4 6475 1.372051e-04
## 27700  4 6476 1.365037e-04
## 27701  4 6477 1.361298e-04
## 27702  4 6478 1.352801e-04
## 27703  4 6479 1.347130e-04
## 27704  4 6480 1.338646e-04
## 27705  4 6481 1.335419e-04
## 27706  4 6482 1.326251e-04
## 27707  4 6483 1.319483e-04
## 27708  4 6484 1.312431e-04
## 27709  4 6485 1.309114e-04
## 27710  4 6486 1.302684e-04
## 27711  4 6487 1.298187e-04
## 27712  4 6488 1.290571e-04
## 27713  4 6489 1.285399e-04
## 27714  4 6490 1.279354e-04
## 27715  4 6491 1.272664e-04
## 27716  4 6492 1.264867e-04
## 27717  4 6493 1.263397e-04
## 27718  4 6494 1.259204e-04
## 27719  4 6495 1.251709e-04
## 27720  4 6496 1.245732e-04
## 27721  4 6497 1.237249e-04
## 27722  4 6498 1.231412e-04
## 27723  4 6499 1.226911e-04
## 27724  4 6500 1.220451e-04
## 27725  4 6501 1.216779e-04
## 27726  4 6502 1.211300e-04
## 27727  4 6503 1.205718e-04
## 27728  4 6504 1.200705e-04
## 27729  4 6505 1.197271e-04
## 27730  4 6506 1.191140e-04
## 27731  4 6507 1.182919e-04
## 27732  4 6508 1.177744e-04
## 27733  4 6509 1.172612e-04
## 27734  4 6510 1.169482e-04
## 27735  4 6511 1.163169e-04
## 27736  4 6512 1.157339e-04
## 27737  4 6513 1.150206e-04
## 27738  4 6514 1.144669e-04
## 27739  4 6515 1.143705e-04
## 27740  4 6516 1.135225e-04
## 27741  4 6517 1.131293e-04
## 27742  4 6518 1.126875e-04
## 27743  4 6519 1.119914e-04
## 27744  4 6520 1.116448e-04
## 27745  4 6521 1.111202e-04
## 27746  4 6522 1.106533e-04
## 27747  4 6523 1.101993e-04
## 27748  4 6524 1.094751e-04
## 27749  4 6525 1.090724e-04
## 27750  4 6526 1.086845e-04
## 27751  4 6527 1.080300e-04
## 27752  4 6528 1.075230e-04
## 27753  4 6529 1.072757e-04
## 27754  4 6530 1.067614e-04
## 27755  4 6531 1.060711e-04
## 27756  4 6532 1.058759e-04
## 27757  4 6533 1.050530e-04
## 27758  4 6534 1.044314e-04
## 27759  4 6535 1.040351e-04
## 27760  4 6536 1.036926e-04
## 27761  4 6537 1.032889e-04
## 27762  4 6538 1.029653e-04
## 27763  4 6539 1.021526e-04
## 27764  4 6540 1.018955e-04
## 27765  4 6541 1.012884e-04
## 27766  4 6542 1.009091e-04
## 27767  4 6543 1.005201e-04
## 27768  4 6544 1.001561e-04
## 27769  4 6545 9.955494e-05
## 27770  4 6546 9.908750e-05
## 27771  4 6547 9.854009e-05
## 27772  4 6548 9.806230e-05
## 27773  4 6549 9.768223e-05
## 27774  4 6550 9.723732e-05
## 27775  4 6551 9.682547e-05
## 27776  4 6552 9.632766e-05
## 27777  4 6553 9.604077e-05
## 27778  4 6554 9.562438e-05
## 27779  4 6555 9.495000e-05
## 27780  4 6556 9.459312e-05
## 27781  4 6557 9.439960e-05
## 27782  4 6558 9.365749e-05
## 27783  4 6559 9.319807e-05
## 27784  4 6560 9.293108e-05
## 27785  4 6561 9.264104e-05
## 27786  4 6562 9.194405e-05
## 27787  4 6563 9.155916e-05
## 27788  4 6564 9.117545e-05
## 27789  4 6565 9.093229e-05
## 27790  4 6566 9.054350e-05
## 27791  4 6567 9.003414e-05
## 27792  4 6568 8.971120e-05
## 27793  4 6569 8.922921e-05
## 27794  4 6570 8.878418e-05
## 27795  4 6571 8.829605e-05
## 27796  4 6572 8.806082e-05
## 27797  4 6573 8.766659e-05
## 27798  4 6574 8.718113e-05
## 27799  4 6575 8.669651e-05
## 27800  4 6576 8.632589e-05
## 27801  4 6577 8.607961e-05
## 27802  4 6578 8.553925e-05
## 27803  4 6579 8.524064e-05
## 27804  4 6580 8.486288e-05
## 27805  4 6581 8.422571e-05
## 27806  4 6582 8.416567e-05
## 27807  4 6583 8.374847e-05
## 27808  4 6584 8.327864e-05
## 27809  4 6585 8.293096e-05
## 27810  4 6586 8.249063e-05
## 27811  4 6587 8.204357e-05
## 27812  4 6588 8.181661e-05
## 27813  4 6589 8.152620e-05
## 27814  4 6590 8.109142e-05
## 27815  4 6591 8.071834e-05
## 27816  4 6592 8.046934e-05
## 27817  4 6593 7.993869e-05
## 27818  4 6594 7.968967e-05
## 27819  4 6595 7.920134e-05
## 27820  4 6596 7.894034e-05
## 27821  4 6597 7.834302e-05
## 27822  4 6598 7.818387e-05
## 27823  4 6599 7.781731e-05
## 27824  4 6600 7.717515e-05
## 27825  4 6601 7.700477e-05
## 27826  4 6602 7.670062e-05
## 27827  4 6603 7.631600e-05
## 27828  4 6604 7.603560e-05
## 27829  4 6605 7.573825e-05
## 27830  4 6606 7.532943e-05
## 27831  4 6607 7.489572e-05
## 27832  4 6608 7.459031e-05
## 27833  4 6609 7.412289e-05
## 27834  4 6610 7.405393e-05
## 27835  4 6611 7.362477e-05
## 27836  4 6612 7.318727e-05
## 27837  4 6613 7.301951e-05
## 27838  4 6614 7.278681e-05
## 27839  4 6615 7.221186e-05
## 27840  4 6616 7.178254e-05
## 27841  4 6617 7.161585e-05
## 27842  4 6618 7.111212e-05
## 27843  4 6619 7.089306e-05
## 27844  4 6620 7.067026e-05
## 27845  4 6621 7.034974e-05
## 27846  4 6622 7.009455e-05
## 27847  4 6623 6.964296e-05
## 27848  4 6624 6.933181e-05
## 27849  4 6625 6.904343e-05
## 27850  4 6626 6.881776e-05
## 27851  4 6627 6.842641e-05
## 27852  4 6628 6.808221e-05
## 27853  4 6629 6.785786e-05
## 27854  4 6630 6.757107e-05
## 27855  4 6631 6.728064e-05
## 27856  4 6632 6.691924e-05
## 27857  4 6633 6.660579e-05
## 27858  4 6634 6.621755e-05
## 27859  4 6635 6.603654e-05
## 27860  4 6636 6.574658e-05
## 27861  4 6637 6.537154e-05
## 27862  4 6638 6.506642e-05
## 27863  4 6639 6.475839e-05
## 27864  4 6640 6.466125e-05
## 27865  4 6641 6.422265e-05
## 27866  4 6642 6.397707e-05
## 27867  4 6643 6.366363e-05
## 27868  4 6644 6.336614e-05
## 27869  4 6645 6.306660e-05
## 27870  4 6646 6.278399e-05
## 27871  4 6647 6.236516e-05
## 27872  4 6648 6.221667e-05
## 27873  4 6649 6.191136e-05
```

```
## 27874  4 6650 6.166598e-05
## 27875  4 6651 6.129245e-05
## 27876  4 6652 6.110138e-05
## 27877  4 6653 6.079654e-05
## 27878  4 6654 6.053134e-05
## 27879  4 6655 6.025344e-05
## 27880  4 6656 6.002597e-05
## 27881  4 6657 5.966587e-05
## 27882  4 6658 5.945043e-05
## 27883  4 6659 5.923059e-05
## 27884  4 6660 5.896505e-05
## 27885  4 6661 5.864986e-05
## 27886  4 6662 5.836748e-05
## 27887  4 6663 5.795134e-05
## 27888  4 6664 5.777657e-05
## 27889  4 6665 5.754387e-05
## 27890  4 6666 5.736886e-05
## 27891  4 6667 5.712424e-05
## 27892  4 6668 5.671222e-05
## 27893  4 6669 5.643178e-05
## 27894  4 6670 5.626792e-05
## 27895  4 6671 5.595402e-05
## 27896  4 6672 5.576973e-05
## 27897  4 6673 5.542018e-05
## 27898  4 6674 5.518932e-05
## 27899  4 6675 5.489768e-05
## 27900  4 6676 5.479921e-05
## 27901  4 6677 5.453240e-05
## 27902  4 6678 5.424920e-05
## 27903  4 6679 5.388401e-05
## 27904  4 6680 5.377363e-05
## 27905  4 6681 5.346628e-05
## 27906  4 6682 5.324979e-05
## 27907  4 6683 5.303082e-05
## 27908  4 6684 5.273914e-05
## 27909  4 6685 5.250100e-05
## 27910  4 6686 5.235357e-05
## 27911  4 6687 5.209876e-05
## 27912  4 6688 5.175694e-05
## 27913  4 6689 5.152760e-05
## 27914  4 6690 5.134359e-05
## 27915  4 6691 5.106867e-05
## 27916  4 6692 5.089451e-05
## 27917  4 6693 5.061828e-05
## 27918  4 6694 5.045887e-05
## 27919  4 6695 5.012640e-05
## 27920  4 6696 4.983861e-05
## 27921  4 6697 4.963006e-05
## 27922  4 6698 4.941922e-05
## 27923  4 6699 4.920902e-05
## 27924  4 6700 4.903706e-05
## 27925  4 6701 4.876842e-05
## 27926  4 6702 4.867660e-05
## 27927  4 6703 4.846500e-05
## 27928  4 6704 4.807993e-05
## 27929  4 6705 4.785225e-05
## 27930  4 6706 4.773606e-05
## 27931  4 6707 4.734992e-05
## 27932  4 6708 4.725653e-05
## 27933  4 6709 4.704188e-05
## 27934  4 6710 4.681958e-05
## 27935  4 6711 4.658355e-05
## 27936  4 6712 4.640298e-05
## 27937  4 6713 4.618084e-05
## 27938  4 6714 4.606093e-05
## 27939  4 6715 4.584379e-05
## 27940  4 6716 4.569347e-05
## 27941  4 6717 4.540454e-05
## 27942  4 6718 4.519255e-05
## 27943  4 6719 4.497901e-05
## 27944  4 6720 4.485704e-05
## 27945  4 6721 4.456173e-05
## 27946  4 6722 4.432524e-05
## 27947  4 6723 4.423660e-05
## 27948  4 6724 4.392839e-05
## 27949  4 6725 4.369515e-05
## 27950  4 6726 4.352527e-05
## 27951  4 6727 4.345771e-05
## 27952  4 6728 4.312184e-05
## 27953  4 6729 4.306943e-05
## 27954  4 6730 4.283943e-05
## 27955  4 6731 4.259657e-05
## 27956  4 6732 4.233394e-05
## 27957  4 6733 4.210603e-05
## 27958  4 6734 4.200704e-05
## 27959  4 6735 4.180454e-05
## 27960  4 6736 4.163895e-05
## 27961  4 6737 4.141797e-05
## 27962  4 6738 4.122915e-05
## 27963  4 6739 4.104143e-05
## 27964  4 6740 4.084559e-05
## 27965  4 6741 4.074012e-05
## 27966  4 6742 4.046627e-05
## 27967  4 6743 4.034190e-05
## 27968  4 6744 4.008660e-05
## 27969  4 6745 3.989651e-05
## 27970  4 6746 3.971718e-05
## 27971  4 6747 3.956757e-05
## 27972  4 6748 3.937532e-05
## 27973  4 6749 3.917907e-05
## 27974  4 6750 3.898127e-05
## 27975  4 6751 3.886692e-05
## 27976  4 6752 3.871487e-05
## 27977  4 6753 3.853911e-05
## 27978  4 6754 3.833344e-05
## 27979  4 6755 3.812794e-05
## 27980  4 6756 3.804203e-05
## 27981  4 6757 3.782086e-05
## 27982  4 6758 3.762479e-05
## 27983  4 6759 3.740203e-05
## 27984  4 6760 3.726652e-05
## 27985  4 6761 3.713126e-05
## 27986  4 6762 3.696980e-05
## 27987  4 6763 3.676532e-05
## 27988  4 6764 3.657860e-05
## 27989  4 6765 3.641886e-05
## 27990  4 6766 3.627927e-05
## 27991  4 6767 3.615889e-05
## 27992  4 6768 3.595517e-05
## 27993  4 6769 3.572562e-05
## 27994  4 6770 3.563944e-05
## 27995  4 6771 3.550528e-05
## 27996  4 6772 3.538349e-05
## 27997  4 6773 3.510549e-05
## 27998  4 6774 3.495518e-05
## 27999  4 6775 3.480644e-05
## 28000  4 6776 3.460383e-05
## 28001  4 6777 3.452750e-05
## 28002  4 6778 3.436995e-05
## 28003  4 6779 3.424710e-05
## 28004  4 6780 3.401134e-05
## 28005  4 6781 3.381671e-05
## 28006  4 6782 3.380149e-05
## 28007  4 6783 3.359999e-05
## 28008  4 6784 3.342966e-05
## 28009  4 6785 3.327788e-05
## 28010  4 6786 3.307811e-05
## 28011  4 6787 3.292254e-05
## 28012  4 6788 3.277679e-05
## 28013  4 6789 3.257927e-05
## 28014  4 6790 3.248624e-05
## 28015  4 6791 3.233347e-05
## 28016  4 6792 3.221515e-05
## 28017  4 6793 3.210796e-05
## 28018  4 6794 3.192378e-05
## 28019  4 6795 3.179083e-05
## 28020  4 6796 3.169709e-05
## 28021  4 6797 3.144498e-05
## 28022  4 6798 3.140241e-05
## 28023  4 6799 3.119831e-05
## 28024  4 6800 3.109540e-05
## 28025  4 6801 3.094228e-05
## 28026  4 6802 3.073534e-05
## 28027  4 6803 3.057744e-05
## 28028  4 6804 3.047205e-05
## 28029  4 6805 3.039556e-05
## 28030  4 6806 3.023203e-05
## 28031  4 6807 3.013277e-05
## 28032  4 6808 2.998325e-05
## 28033  4 6809 2.981653e-05
## 28034  4 6810 2.965316e-05
## 28035  4 6811 2.956496e-05
## 28036  4 6812 2.939604e-05
## 28037  4 6813 2.933247e-05
## 28038  4 6814 2.908723e-05
## 28039  4 6815 2.898613e-05
## 28040  4 6816 2.886382e-05
## 28041  4 6817 2.864298e-05
## 28042  4 6818 2.861501e-05
## 28043  4 6819 2.847480e-05
## 28044  4 6820 2.831411e-05
## 28045  4 6821 2.822472e-05
## 28046  4 6822 2.807811e-05
## 28047  4 6823 2.794262e-05
## 28048  4 6824 2.784312e-05
## 28049  4 6825 2.769581e-05
## 28050  4 6826 2.756670e-05
## 28051  4 6827 2.746562e-05
## 28052  4 6828 2.734960e-05
## 28053  4 6829 2.723832e-05
## 28054  4 6830 2.703434e-05
## 28055  4 6831 2.701078e-05
## 28056  4 6832 2.682125e-05
## 28057  4 6833 2.673245e-05
## 28058  4 6834 2.654538e-05
## 28059  4 6835 2.646065e-05
## 28060  4 6836 2.636913e-05
## 28061  4 6837 2.620661e-05
## 28062  4 6838 2.609034e-05
## 28063  4 6839 2.602532e-05
## 28064  4 6840 2.586396e-05
## 28065  4 6841 2.574614e-05
## 28066  4 6842 2.564657e-05
## 28067  4 6843 2.555522e-05
## 28068  4 6844 2.536449e-05
## 28069  4 6845 2.528838e-05
## 28070  4 6846 2.522191e-05
## 28071  4 6847 2.504167e-05
## 28072  4 6848 2.499863e-05
## 28073  4 6849 2.482746e-05
## 28074  4 6850 2.471346e-05
## 28075  4 6851 2.465328e-05
## 28076  4 6852 2.451282e-05
## 28077  4 6853 2.443418e-05
## 28078  4 6854 2.425811e-05
## 28079  4 6855 2.419336e-05
## 28080  4 6856 2.402791e-05
## 28081  4 6857 2.393704e-05
## 28082  4 6858 2.386010e-05
## 28083  4 6859 2.369186e-05
## 28084  4 6860 2.362420e-05
## 28085  4 6861 2.348067e-05
## 28086  4 6862 2.338752e-05
## 28087  4 6863 2.332285e-05
## 28088  4 6864 2.323263e-05
## 28089  4 6865 2.308413e-05
## 28090  4 6866 2.299744e-05
## 28091  4 6867 2.288533e-05
## 28092  4 6868 2.277094e-05
## 28093  4 6869 2.263534e-05
## 28094  4 6870 2.254799e-05
## 28095  4 6871 2.247467e-05
## 28096  4 6872 2.235801e-05
## 28097  4 6873 2.226121e-05
## 28098  4 6874 2.213970e-05
## 28099  4 6875 2.202388e-05
## 28100  4 6876 2.193588e-05
## 28101  4 6877 2.184774e-05
## 28102  4 6878 2.177809e-05
## 28103  4 6879 2.164128e-05
## 28104  4 6880 2.152304e-05
## 28105  4 6881 2.146011e-05
## 28106  4 6882 2.133520e-05
## 28107  4 6883 2.125795e-05
## 28108  4 6884 2.119057e-05
## 28109  4 6885 2.107310e-05
## 28110  4 6886 2.095297e-05
## 28111  4 6887 2.087225e-05
## 28112  4 6888 2.075919e-05
## 28113  4 6889 2.069467e-05
## 28114  4 6890 2.059138e-05
## 28115  4 6891 2.052279e-05
## 28116  4 6892 2.043347e-05
## 28117  4 6893 2.036779e-05
## 28118  4 6894 2.022092e-05
## 28119  4 6895 2.012689e-05
## 28120  4 6896 2.004716e-05
## 28121  4 6897 1.997126e-05
## 28122  4 6898 1.987066e-05
## 28123  4 6899 1.976895e-05
## 28124  4 6900 1.965625e-05
## 28125  4 6901 1.956058e-05
## 28126  4 6902 1.949629e-05
## 28127  4 6903 1.941969e-05
## 28128  4 6904 1.929784e-05
## 28129  4 6905 1.922086e-05
## 28130  4 6906 1.914913e-05
## 28131  4 6907 1.905127e-05
## 28132  4 6908 1.894551e-05
## 28133  4 6909 1.887612e-05
## 28134  4 6910 1.878761e-05
## 28135  4 6911 1.868783e-05
## 28136  4 6912 1.856292e-05
## 28137  4 6913 1.855175e-05
## 28138  4 6914 1.849544e-05
## 28139  4 6915 1.836809e-05
## 28140  4 6916 1.826575e-05
## 28141  4 6917 1.820956e-05
## 28142  4 6918 1.812302e-05
## 28143  4 6919 1.802196e-05
## 28144  4 6920 1.796538e-05
## 28145  4 6921 1.784267e-05
## 28146  4 6922 1.779061e-05
## 28147  4 6923 1.770882e-05
## 28148  4 6924 1.763711e-05
## 28149  4 6925 1.755553e-05
## 28150  4 6926 1.748543e-05
## 28151  4 6927 1.739423e-05
## 28152  4 6928 1.732201e-05
## 28153  4 6929 1.723698e-05
## 28154  4 6930 1.713886e-05
## 28155  4 6931 1.704794e-05
## 28156  4 6932 1.699677e-05
## 28157  4 6933 1.693877e-05
## 28158  4 6934 1.686386e-05
## 28159  4 6935 1.674740e-05
## 28160  4 6936 1.668362e-05
## 28161  4 6937 1.659372e-05
## 28162  4 6938 1.654538e-05
## 28163  4 6939 1.648735e-05
## 28164  4 6940 1.639237e-05
## 28165  4 6941 1.630189e-05
## 28166  4 6942 1.624578e-05
## 28167  4 6943 1.613261e-05
## 28168  4 6944 1.610589e-05
## 28169  4 6945 1.598563e-05
## 28170  4 6946 1.596200e-05
## 28171  4 6947 1.586031e-05
## 28172  4 6948 1.580028e-05
## 28173  4 6949 1.572131e-05
## 28174  4 6950 1.567282e-05
## 28175  4 6951 1.561039e-05
## 28176  4 6952 1.549485e-05
## 28177  4 6953 1.543523e-05
## 28178  4 6954 1.538110e-05
## 28179  4 6955 1.530213e-05
## 28180  4 6956 1.525220e-05
## 28181  4 6957 1.514819e-05
## 28182  4 6958 1.510198e-05
## 28183  4 6959 1.503710e-05
## 28184  4 6960 1.495273e-05
## 28185  4 6961 1.487343e-05
## 28186  4 6962 1.483108e-05
## 28187  4 6963 1.474439e-05
## 28188  4 6964 1.467660e-05
## 28189  4 6965 1.458735e-05
## 28190  4 6966 1.456805e-05
## 28191  4 6967 1.447852e-05
## 28192  4 6968 1.441159e-05
## 28193  4 6969 1.435639e-05
## 28194  4 6970 1.427850e-05
## 28195  4 6971 1.422667e-05
## 28196  4 6972 1.416193e-05
## 28197  4 6973 1.409037e-05
## 28198  4 6974 1.403193e-05
## 28199  4 6975 1.397024e-05
## 28200  4 6976 1.389983e-05
## 28201  4 6977 1.384110e-05
## 28202  4 6978 1.375469e-05
## 28203  4 6979 1.371342e-05
## 28204  4 6980 1.365626e-05
## 28205  4 6981 1.359297e-05
## 28206  4 6982 1.352521e-05
## 28207  4 6983 1.344575e-05
## 28208  4 6984 1.340434e-05
## 28209  4 6985 1.332911e-05
## 28210  4 6986 1.327814e-05
## 28211  4 6987 1.322983e-05
## 28212  4 6988 1.314331e-05
## 28213  4 6989 1.310757e-05
## 28214  4 6990 1.304361e-05
## 28215  4 6991 1.297311e-05
## 28216  4 6992 1.293337e-05
## 28217  4 6993 1.286480e-05
## 28218  4 6994 1.280278e-05
## 28219  4 6995 1.274925e-05
## 28220  4 6996 1.268690e-05
## 28221  4 6997 1.263971e-05
## 28222  4 6998 1.255797e-05
## 28223  4 6999 1.250380e-05
## 28224  4 7000 1.245494e-05
## 28225  5    0 0.000000e+00
## 28226  5    0 6.587558e+00
## 28227  5    1 6.785303e+00
## 28228  5    2 6.882898e+00
## 28229  5    3 6.916211e+00
## 28230  5    4 6.905572e+00
## 28231  5    5 6.909190e+00
## 28232  5    6 6.897266e+00
## 28233  5    7 6.890426e+00
## 28234  5    8 6.854902e+00
## 28235  5    9 6.841741e+00
## 28236  5   10 6.835700e+00
## 28237  5   11 6.805127e+00
## 28238  5   12 6.798343e+00
## 28239  5   13 6.772644e+00
## 28240  5   14 6.753740e+00
## 28241  5   15 6.732777e+00
## 28242  5   16 6.713478e+00
## 28243  5   17 6.703664e+00
## 28244  5   18 6.703417e+00
## 28245  5   19 6.669443e+00
## 28246  5   20 6.660967e+00
## 28247  5   21 6.646264e+00
## 28248  5   22 6.618894e+00
## 28249  5   23 6.613726e+00
## 28250  5   24 6.591872e+00
## 28251  5   24 6.594042e+00
## 28252  5   25 6.791354e+00
## 28253  5   26 6.892027e+00
## 28254  5   27 6.912167e+00
## 28255  5   28 6.910751e+00
## 28256  5   29 6.920507e+00
## 28257  5   30 6.911306e+00
## 28258  5   31 6.878898e+00
## 28259  5   32 6.866317e+00
## 28260  5   33 6.845033e+00
## 28261  5   34 6.818043e+00
## 28262  5   35 6.798717e+00
## 28263  5   36 6.784102e+00
## 28264  5   37 6.776226e+00
## 28265  5   38 6.745813e+00
## 28266  5   39 6.742129e+00
## 28267  5   40 6.719292e+00
## 28268  5   41 6.708462e+00
## 28269  5   42 6.685680e+00
## 28270  5   43 6.673138e+00
## 28271  5   44 6.648451e+00
## 28272  5   45 6.630544e+00
## 28273  5   46 6.621868e+00
## 28274  5   47 6.593023e+00
## 28275  5   48 6.590989e+00
## 28276  5   48 6.591834e+00
## 28277  5   49 6.790370e+00
## 28278  5   50 6.876557e+00
## 28279  5   51 6.909704e+00
## 28280  5   52 6.912688e+00
## 28281  5   53 6.903666e+00
## 28282  5   54 6.914155e+00
## 28283  5   55 6.879204e+00
## 28284  5   56 6.871001e+00
## 28285  5   57 6.843618e+00
## 28286  5   58 6.827107e+00
## 28287  5   59 6.807114e+00
## 28288  5   60 6.787741e+00
## 28289  5   61 6.777785e+00
## 28290  5   62 6.752633e+00
## 28291  5   63 6.729827e+00
## 28292  5   64 6.721553e+00
## 28293  5   65 6.715912e+00
## 28294  5   66 6.693068e+00
## 28295  5   67 6.670511e+00
## 28296  5   68 6.649936e+00
## 28297  5   69 6.632436e+00
## 28298  5   70 6.613286e+00
## 28299  5   71 6.607563e+00
## 28300  5   72 6.595961e+00
## 28301  5   73 6.801003e+00
## 28302  5   74 6.869334e+00
## 28303  5   75 6.916115e+00
## 28304  5   76 6.919971e+00
## 28305  5   77 6.921851e+00
## 28306  5   78 6.895995e+00
## 28307  5   79 6.882105e+00
## 28308  5   80 6.868700e+00
## 28309  5   81 6.844001e+00
## 28310  5   82 6.828871e+00
## 28311  5   83 6.799366e+00
## 28312  5   84 6.793968e+00
## 28313  5   85 6.781420e+00
## 28314  5   86 6.756717e+00
## 28315  5   87 6.733064e+00
## 28316  5   88 6.713554e+00
## 28317  5   89 6.700388e+00
## 28318  5   90 6.694360e+00
## 28319  5   91 6.669120e+00
## 28320  5   92 6.662193e+00
## 28321  5   93 6.653413e+00
## 28322  5   94 6.623243e+00
## 28323  5   95 6.617694e+00
## 28324  5   96 6.589303e+00
## 28325  5   97 6.791662e+00
## 28326  5   98 6.880366e+00
## 28327  5   99 6.924093e+00
## 28328  5  100 6.914163e+00
## 28329  5  101 6.913700e+00
## 28330  5  102 6.901330e+00
## 28331  5  103 6.882022e+00
## 28332  5  104 6.868040e+00
## 28333  5  105 6.845187e+00
## 28334  5  106 6.814861e+00
## 28335  5  107 6.804721e+00
## 28336  5  108 6.790930e+00
## 28337  5  109 6.765864e+00
## 28338  5  110 6.747678e+00
## 28339  5  111 6.731437e+00
## 28340  5  112 6.716721e+00
## 28341  5  113 6.698148e+00
## 28342  5  114 6.695329e+00
## 28343  5  115 6.667424e+00
## 28344  5  116 6.645404e+00
## 28345  5  117 6.633568e+00
## 28346  5  118 6.627257e+00
## 28347  5  119 6.616934e+00
## 28348  5  120 6.596451e+00
## 28349  5  121 6.788344e+00
## 28350  5  122 6.883225e+00
## 28351  5  123 6.918121e+00
## 28352  5  124 6.922281e+00
## 28353  5  125 6.904959e+00
## 28354  5  126 6.889704e+00
## 28355  5  127 6.886271e+00
## 28356  5  128 6.859794e+00
## 28357  5  129 6.846684e+00
## 28358  5  130 6.829095e+00
## 28359  5  131 6.802458e+00
## 28360  5  132 6.797463e+00
## 28361  5  133 6.770675e+00
## 28362  5  134 6.764987e+00
## 28363  5  135 6.739633e+00
## 28364  5  136 6.710733e+00
## 28365  5  137 6.710050e+00
## 28366  5  138 6.704783e+00
## 28367  5  139 6.672044e+00
## 28368  5  140 6.634231e+00
## 28369  5  141 6.639208e+00
## 28370  5  142 6.622358e+00
## 28371  5  143 6.610512e+00
## 28372  5  144 6.598363e+00
## 28373  5  145 6.789581e+00
## 28374  5  146 6.882045e+00
## 28375  5  147 6.911828e+00
## 28376  5  148 6.915420e+00
## 28377  5  149 6.904613e+00
## 28378  5  150 6.891193e+00
## 28379  5  151 6.886410e+00
## 28380  5  152 6.869645e+00
## 28381  5  153 6.833925e+00
## 28382  5  154 6.841267e+00
## 28383  5  155 6.809788e+00
## 28384  5  156 6.784926e+00
## 28385  5  157 6.778179e+00
## 28386  5  158 6.747141e+00
## 28387  5  159 6.736536e+00
## 28388  5  160 6.728468e+00
## 28389  5  161 6.705328e+00
## 28390  5  162 6.689366e+00
## 28391  5  163 6.687415e+00
## 28392  5  164 6.648970e+00
## 28393  5  165 6.636308e+00
## 28394  5  166 6.630153e+00
## 28395  5  167 6.612301e+00
## 28396  5  168 6.598641e+00
## 28397  5  169 6.797110e+00
## 28398  5  170 6.874331e+00
## 28399  5  171 6.927168e+00
## 28400  5  172 6.918420e+00
## 28401  5  173 6.908910e+00
## 28402  5  174 6.892684e+00
## 28403  5  175 6.875669e+00
## 28404  5  176 6.858457e+00
## 28405  5  177 6.860849e+00
## 28406  5  178 6.826691e+00
## 28407  5  179 6.812572e+00
## 28408  5  180 6.771531e+00
## 28409  5  181 6.776559e+00
## 28410  5  182 6.759464e+00
## 28411  5  183 6.731749e+00
## 28412  5  184 6.729734e+00
## 28413  5  185 6.704917e+00
## 28414  5  186 6.687757e+00
## 28415  5  187 6.667812e+00
## 28416  5  188 6.650424e+00
## 28417  5  189 6.649118e+00
## 28418  5  190 6.623590e+00
## 28419  5  191 6.600810e+00
## 28420  5  192 6.584575e+00
## 28421  5  193 6.777586e+00
## 28422  5  194 6.889635e+00
## 28423  5  195 6.902512e+00
## 28424  5  196 6.917366e+00
## 28425  5  197 6.899848e+00
## 28426  5  198 6.895499e+00
## 28427  5  199 6.873433e+00
## 28428  5  200 6.854907e+00
## 28429  5  201 6.855316e+00
## 28430  5  202 6.829558e+00
## 28431  5  203 6.801779e+00
## 28432  5  204 6.793224e+00
## 28433  5  205 6.777993e+00
## 28434  5  206 6.763550e+00
## 28435  5  207 6.737877e+00
## 28436  5  208 6.718595e+00
## 28437  5  209 6.709865e+00
## 28438  5  210 6.694767e+00
## 28439  5  211 6.666502e+00
## 28440  5  212 6.652480e+00
## 28441  5  213 6.648295e+00
## 28442  5  214 6.626478e+00
## 28443  5  215 6.611361e+00
## 28444  5  216 6.588582e+00
## 28445  5  217 6.784142e+00
## 28446  5  218 6.878663e+00
## 28447  5  219 6.913350e+00
## 28448  5  220 6.920611e+00
## 28449  5  221 6.902287e+00
## 28450  5  222 6.903416e+00
## 28451  5  223 6.891818e+00
## 28452  5  224 6.872207e+00
## 28453  5  225 6.842399e+00
## 28454  5  226 6.835925e+00
## 28455  5  227 6.813325e+00
## 28456  5  228 6.785120e+00
## 28457  5  229 6.759318e+00
## 28458  5  230 6.743528e+00
## 28459  5  231 6.745273e+00
## 28460  5  232 6.723229e+00
## 28461  5  233 6.707088e+00
## 28462  5  234 6.683007e+00
## 28463  5  235 6.668281e+00
## 28464  5  236 6.658320e+00
## 28465  5  237 6.650385e+00
## 28466  5  238 6.634029e+00
## 28467  5  239 6.609906e+00
## 28468  5  240 6.590233e+00
## 28469  5  241 6.786560e+00
## 28470  5  242 6.880711e+00
## 28471  5  243 6.916632e+00
## 28472  5  244 6.925509e+00
## 28473  5  245 6.907168e+00
## 28474  5  246 6.889003e+00
## 28475  5  247 6.880355e+00
## 28476  5  248 6.858589e+00
## 28477  5  249 6.839968e+00
## 28478  5  250 6.838208e+00
## 28479  5  251 6.802685e+00
## 28480  5  252 6.780665e+00
## 28481  5  253 6.775447e+00
## 28482  5  254 6.752080e+00
## 28483  5  255 6.736056e+00
## 28484  5  256 6.717599e+00
## 28485  5  257 6.700723e+00
## 28486  5  258 6.683371e+00
## 28487  5  259 6.660760e+00
## 28488  5  260 6.641159e+00
## 28489  5  261 6.645346e+00
## 28490  5  262 6.630701e+00
## 28491  5  263 6.609031e+00
## 28492  5  264 6.595896e+00
## 28493  5  265 6.789772e+00
## 28494  5  266 6.885324e+00
## 28495  5  267 6.910330e+00
## 28496  5  268 6.925667e+00
## 28497  5  269 6.920063e+00
## 28498  5  270 6.896423e+00
## 28499  5  271 6.890029e+00
## 28500  5  272 6.869309e+00
## 28501  5  273 6.841157e+00
## 28502  5  274 6.819450e+00
## 28503  5  275 6.817008e+00
## 28504  5  276 6.790390e+00
## 28505  5  277 6.783000e+00
## 28506  5  278 6.746471e+00
## 28507  5  279 6.741916e+00
## 28508  5  280 6.722693e+00
## 28509  5  281 6.707360e+00
## 28510  5  282 6.686365e+00
## 28511  5  283 6.670713e+00
## 28512  5  284 6.653490e+00
## 28513  5  285 6.643557e+00
## 28514  5  286 6.625386e+00
## 28515  5  287 6.602538e+00
## 28516  5  288 6.597947e+00
## 28517  5  289 6.787931e+00
## 28518  5  290 6.880251e+00
## 28519  5  291 6.906650e+00
## 28520  5  292 6.922980e+00
## 28521  5  293 6.902177e+00
## 28522  5  294 6.899668e+00
## 28523  5  295 6.881185e+00
## 28524  5  296 6.853440e+00
## 28525  5  297 6.855983e+00
## 28526  5  298 6.826545e+00
## 28527  5  299 6.802699e+00
## 28528  5  300 6.786081e+00
## 28529  5  301 6.779942e+00
## 28530  5  302 6.758339e+00
## 28531  5  303 6.736988e+00
## 28532  5  304 6.723770e+00
## 28533  5  305 6.700552e+00
## 28534  5  306 6.708135e+00
## 28535  5  307 6.674903e+00
## 28536  5  308 6.658631e+00
## 28537  5  309 6.645956e+00
## 28538  5  310 6.622123e+00
## 28539  5  311 6.611840e+00
## 28540  5  312 6.597210e+00
## 28541  5  312 6.587973e+00
## 28542  5  313 6.785809e+00
## 28543  5  314 6.884673e+00
## 28544  5  315 6.916809e+00
## 28545  5  316 6.907830e+00
## 28546  5  317 6.919604e+00
## 28547  5  318 6.908375e+00
## 28548  5  319 6.887631e+00
## 28549  5  320 6.867491e+00
## 28550  5  321 6.827506e+00
## 28551  5  322 6.813348e+00
## 28552  5  323 6.803516e+00
## 28553  5  324 6.791661e+00
## 28554  5  325 6.777853e+00
## 28555  5  326 6.761320e+00
## 28556  5  327 6.737566e+00
## 28557  5  328 6.716880e+00
## 28558  5  329 6.699905e+00
## 28559  5  330 6.698108e+00
## 28560  5  331 6.670931e+00
## 28561  5  332 6.660355e+00
## 28562  5  333 6.638233e+00
## 28563  5  334 6.626137e+00
## 28564  5  335 6.606107e+00
## 28565  5  336 6.600821e+00
## 28566  5  336 6.588806e+00
## 28567  5  337 6.570525e+00
## 28568  5  338 6.567653e+00
## 28569  5  339 6.540311e+00
## 28570  5  339 6.541011e+00
## 28571  5  340 6.751981e+00
## 28572  5  341 6.839467e+00
## 28573  5  342 6.871493e+00
## 28574  5  343 6.868864e+00
## 28575  5  344 6.876846e+00
## 28576  5  345 6.860364e+00
## 28577  5  346 6.844349e+00
## 28578  5  347 6.832739e+00
## 28579  5  348 6.791978e+00
## 28580  5  349 6.784616e+00
## 28581  5  350 6.765484e+00
## 28582  5  351 6.751618e+00
## 28583  5  352 6.723398e+00
## 28584  5  353 6.714568e+00
## 28585  5  354 6.700611e+00
## 28586  5  355 6.671575e+00
## 28587  5  356 6.667604e+00
## 28588  5  357 6.649580e+00
## 28589  5  358 6.624903e+00
## 28590  5  359 6.616507e+00
## 28591  5  360 6.603213e+00
## 28592  5  360 6.595007e+00
## 28593  5  361 6.795150e+00
## 28594  5  362 6.890618e+00
## 28595  5  363 6.913713e+00
## 28596  5  364 6.919062e+00
## 28597  5  365 6.931719e+00
## 28598  5  366 6.898461e+00
## 28599  5  367 6.898260e+00
## 28600  5  368 6.867543e+00
## 28601  5  369 6.848366e+00
## 28602  5  370 6.827325e+00
## 28603  5  371 6.814524e+00
## 28604  5  372 6.790065e+00
## 28605  5  373 6.780751e+00
## 28606  5  374 6.774926e+00
## 28607  5  375 6.743948e+00
## 28608  5  376 6.738162e+00
## 28609  5  377 6.716044e+00
## 28610  5  378 6.695913e+00
## 28611  5  379 6.670129e+00
## 28612  5  380 6.659212e+00
## 28613  5  381 6.662209e+00
## 28614  5  382 6.624006e+00
## 28615  5  383 6.613011e+00
## 28616  5  384 6.600440e+00
## 28617  5  385 6.787773e+00
## 28618  5  386 6.899880e+00
## 28619  5  387 6.905934e+00
## 28620  5  388 6.930175e+00
## 28621  5  389 6.917932e+00
## 28622  5  390 6.906405e+00
## 28623  5  391 6.879048e+00
## 28624  5  392 6.876524e+00
## 28625  5  393 6.845166e+00
## 28626  5  394 6.819529e+00
## 28627  5  395 6.818950e+00
## 28628  5  396 6.795060e+00
## 28629  5  397 6.770477e+00
## 28630  5  398 6.757200e+00
## 28631  5  399 6.739157e+00
## 28632  5  400 6.727589e+00
## 28633  5  401 6.695091e+00
## 28634  5  402 6.689406e+00
## 28635  5  403 6.661231e+00
## 28636  5  404 6.659032e+00
## 28637  5  405 6.650779e+00
## 28638  5  406 6.623113e+00
## 28639  5  407 6.628974e+00
## 28640  5  408 6.590774e+00
## 28641  5  409 6.790411e+00
## 28642  5  410 6.878996e+00
## 28643  5  411 6.918381e+00
## 28644  5  412 6.927896e+00
## 28645  5  413 6.919220e+00
## 28646  5  414 6.909456e+00
## 28647  5  415 6.875966e+00
## 28648  5  416 6.867570e+00
## 28649  5  417 6.835469e+00
## 28650  5  418 6.821135e+00
## 28651  5  419 6.820712e+00
## 28652  5  420 6.784668e+00
## 28653  5  421 6.776281e+00
## 28654  5  422 6.767097e+00
## 28655  5  423 6.733190e+00
## 28656  5  424 6.727581e+00
## 28657  5  425 6.713693e+00
## 28658  5  426 6.692046e+00
## 28659  5  427 6.670924e+00
## 28660  5  428 6.657955e+00
## 28661  5  429 6.633455e+00
## 28662  5  430 6.624157e+00
## 28663  5  431 6.609556e+00
## 28664  5  432 6.597967e+00
## 28665  5  433 6.785334e+00
## 28666  5  434 6.882859e+00
## 28667  5  435 6.926619e+00
## 28668  5  436 6.923791e+00
## 28669  5  437 6.916057e+00
## 28670  5  438 6.897014e+00
## 28671  5  439 6.873102e+00
## 28672  5  440 6.870762e+00
## 28673  5  441 6.852378e+00
```

```
## 28674  5  442 6.826756e+00
## 28675  5  443 6.798778e+00
## 28676  5  444 6.781925e+00
## 28677  5  445 6.792097e+00
## 28678  5  446 6.756169e+00
## 28679  5  447 6.742306e+00
## 28680  5  448 6.723067e+00
## 28681  5  449 6.709772e+00
## 28682  5  450 6.685724e+00
## 28683  5  451 6.677298e+00
## 28684  5  452 6.657301e+00
## 28685  5  453 6.636962e+00
## 28686  5  454 6.633317e+00
## 28687  5  455 6.602139e+00
## 28688  5  456 6.592491e+00
## 28689  5  457 6.790974e+00
## 28690  5  458 6.886287e+00
## 28691  5  459 6.914037e+00
## 28692  5  460 6.916890e+00
## 28693  5  461 6.901060e+00
## 28694  5  462 6.894579e+00
## 28695  5  463 6.891682e+00
## 28696  5  464 6.868179e+00
## 28697  5  465 6.852282e+00
## 28698  5  466 6.830322e+00
## 28699  5  467 6.813893e+00
## 28700  5  468 6.786958e+00
## 28701  5  469 6.779821e+00
## 28702  5  470 6.763349e+00
## 28703  5  471 6.741779e+00
## 28704  5  472 6.721232e+00
## 28705  5  473 6.713267e+00
## 28706  5  474 6.692113e+00
## 28707  5  475 6.674412e+00
## 28708  5  476 6.649252e+00
## 28709  5  477 6.635103e+00
## 28710  5  478 6.615659e+00
## 28711  5  479 6.620901e+00
## 28712  5  480 6.590207e+00
## 28713  5  481 6.790705e+00
## 28714  5  482 6.882517e+00
## 28715  5  483 6.922228e+00
## 28716  5  484 6.919605e+00
## 28717  5  485 6.898265e+00
## 28718  5  486 6.905258e+00
## 28719  5  487 6.883248e+00
## 28720  5  488 6.863795e+00
## 28721  5  489 6.854110e+00
## 28722  5  490 6.831115e+00
## 28723  5  491 6.817548e+00
## 28724  5  492 6.793722e+00
## 28725  5  493 6.783914e+00
## 28726  5  494 6.756967e+00
## 28727  5  495 6.736878e+00
## 28728  5  496 6.718721e+00
## 28729  5  497 6.705719e+00
## 28730  5  498 6.690993e+00
## 28731  5  499 6.661703e+00
## 28732  5  500 6.663610e+00
## 28733  5  501 6.643562e+00
## 28734  5  502 6.623721e+00
## 28735  5  503 6.619659e+00
## 28736  5  504 6.588706e+00
## 28737  5  505 6.791782e+00
## 28738  5  506 6.885577e+00
## 28739  5  507 6.921088e+00
## 28740  5  508 6.930565e+00
## 28741  5  509 6.909358e+00
## 28742  5  510 6.901861e+00
## 28743  5  511 6.885473e+00
## 28744  5  512 6.875288e+00
## 28745  5  513 6.839028e+00
## 28746  5  514 6.827793e+00
## 28747  5  515 6.808057e+00
## 28748  5  516 6.798675e+00
## 28749  5  517 6.777858e+00
## 28750  5  518 6.746511e+00
## 28751  5  519 6.750139e+00
## 28752  5  520 6.717269e+00
## 28753  5  521 6.700369e+00
## 28754  5  522 6.686383e+00
## 28755  5  523 6.665864e+00
## 28756  5  524 6.657113e+00
## 28757  5  525 6.622032e+00
## 28758  5  526 6.626506e+00
## 28759  5  527 6.609517e+00
## 28760  5  528 6.585233e+00
## 28761  5  529 6.796687e+00
## 28762  5  530 6.867601e+00
## 28763  5  531 6.905965e+00
## 28764  5  532 6.919960e+00
## 28765  5  533 6.906340e+00
## 28766  5  534 6.898026e+00
## 28767  5  535 6.879902e+00
## 28768  5  536 6.861564e+00
## 28769  5  537 6.838348e+00
## 28770  5  538 6.825759e+00
## 28771  5  539 6.810232e+00
## 28772  5  540 6.783549e+00
## 28773  5  541 6.783474e+00
## 28774  5  542 6.755075e+00
## 28775  5  543 6.733537e+00
## 28776  5  544 6.707493e+00
## 28777  5  545 6.704842e+00
## 28778  5  546 6.692006e+00
## 28779  5  547 6.674299e+00
## 28780  5  548 6.657392e+00
## 28781  5  549 6.640339e+00
## 28782  5  550 6.621665e+00
## 28783  5  551 6.600928e+00
## 28784  5  552 6.592683e+00
## 28785  5  553 6.796343e+00
## 28786  5  554 6.871830e+00
## 28787  5  555 6.911369e+00
## 28788  5  556 6.935217e+00
## 28789  5  557 6.919839e+00
## 28790  5  558 6.892920e+00
## 28791  5  559 6.876567e+00
## 28792  5  560 6.868989e+00
## 28793  5  561 6.847110e+00
## 28794  5  562 6.824588e+00
## 28795  5  563 6.804709e+00
## 28796  5  564 6.782027e+00
## 28797  5  565 6.770185e+00
## 28798  5  566 6.749643e+00
## 28799  5  567 6.742847e+00
## 28800  5  568 6.727101e+00
## 28801  5  569 6.709699e+00
## 28802  5  570 6.694656e+00
## 28803  5  571 6.670437e+00
## 28804  5  572 6.659933e+00
## 28805  5  573 6.655709e+00
## 28806  5  574 6.614050e+00
## 28807  5  575 6.607994e+00
## 28808  5  576 6.587290e+00
## 28809  5  577 6.788757e+00
## 28810  5  578 6.869526e+00
## 28811  5  579 6.919569e+00
## 28812  5  580 6.917856e+00
## 28813  5  581 6.919665e+00
## 28814  5  582 6.901511e+00
## 28815  5  583 6.877188e+00
## 28816  5  584 6.868889e+00
## 28817  5  585 6.849000e+00
## 28818  5  586 6.822567e+00
## 28819  5  587 6.818569e+00
## 28820  5  588 6.798503e+00
## 28821  5  589 6.774706e+00
## 28822  5  590 6.742801e+00
## 28823  5  591 6.741959e+00
## 28824  5  592 6.717080e+00
## 28825  5  593 6.708077e+00
## 28826  5  594 6.691123e+00
## 28827  5  595 6.664076e+00
## 28828  5  596 6.654668e+00
## 28829  5  597 6.640697e+00
## 28830  5  598 6.627889e+00
## 28831  5  599 6.604191e+00
## 28832  5  600 6.596065e+00
## 28833  5  601 6.789842e+00
## 28834  5  602 6.887436e+00
## 28835  5  603 6.919151e+00
## 28836  5  604 6.923035e+00
## 28837  5  605 6.917334e+00
## 28838  5  606 6.888346e+00
## 28839  5  607 6.880392e+00
## 28840  5  608 6.856739e+00
## 28841  5  609 6.843039e+00
## 28842  5  610 6.833644e+00
## 28843  5  611 6.797989e+00
## 28844  5  612 6.797970e+00
## 28845  5  613 6.764004e+00
## 28846  5  614 6.755542e+00
## 28847  5  615 6.742043e+00
## 28848  5  616 6.711991e+00
## 28849  5  617 6.706985e+00
## 28850  5  618 6.696357e+00
## 28851  5  619 6.674381e+00
## 28852  5  620 6.660387e+00
## 28853  5  621 6.639089e+00
## 28854  5  622 6.622059e+00
## 28855  5  623 6.616511e+00
## 28856  5  624 6.598516e+00
## 28857  5  624 6.598714e+00
## 28858  5  625 6.789779e+00
## 28859  5  626 6.870994e+00
## 28860  5  627 6.922819e+00
## 28861  5  628 6.925443e+00
## 28862  5  629 6.916181e+00
## 28863  5  630 6.910986e+00
## 28864  5  631 6.882620e+00
## 28865  5  632 6.860808e+00
## 28866  5  633 6.839424e+00
## 28867  5  634 6.823615e+00
## 28868  5  635 6.818116e+00
## 28869  5  636 6.793350e+00
## 28870  5  637 6.770893e+00
## 28871  5  638 6.751833e+00
## 28872  5  639 6.743860e+00
## 28873  5  640 6.716877e+00
## 28874  5  641 6.698337e+00
## 28875  5  642 6.689820e+00
## 28876  5  643 6.664333e+00
## 28877  5  644 6.669924e+00
## 28878  5  645 6.631586e+00
## 28879  5  646 6.616871e+00
## 28880  5  647 6.613614e+00
## 28881  5  648 6.599767e+00
## 28882  5  648 6.580418e+00
## 28883  5  649 6.580772e+00
## 28884  5  650 6.561622e+00
## 28885  5  651 6.551210e+00
## 28886  5  652 6.538152e+00
## 28887  5  653 6.522507e+00
## 28888  5  654 6.505639e+00
## 28889  5  654 6.511215e+00
## 28890  5  655 6.699529e+00
## 28891  5  656 6.803802e+00
## 28892  5  657 6.845102e+00
## 28893  5  658 6.850256e+00
## 28894  5  659 6.830313e+00
## 28895  5  660 6.826819e+00
## 28896  5  661 6.800251e+00
## 28897  5  662 6.779825e+00
## 28898  5  663 6.767116e+00
## 28899  5  664 6.757932e+00
## 28900  5  665 6.738544e+00
## 28901  5  666 6.728035e+00
## 28902  5  667 6.706036e+00
## 28903  5  668 6.675602e+00
## 28904  5  669 6.659609e+00
## 28905  5  670 6.642765e+00
## 28906  5  671 6.637623e+00
## 28907  5  672 6.612916e+00
## 28908  5  672 6.611388e+00
## 28909  5  673 6.818745e+00
## 28910  5  674 6.911128e+00
## 28911  5  675 6.940028e+00
## 28912  5  676 6.943030e+00
## 28913  5  677 6.929562e+00
## 28914  5  678 6.929782e+00
## 28915  5  679 6.887409e+00
## 28916  5  680 6.896581e+00
## 28917  5  681 6.869776e+00
## 28918  5  682 6.824764e+00
## 28919  5  683 6.822502e+00
## 28920  5  684 6.808339e+00
## 28921  5  685 6.787107e+00
## 28922  5  686 6.780780e+00
## 28923  5  687 6.753412e+00
## 28924  5  688 6.733392e+00
## 28925  5  689 6.730413e+00
## 28926  5  690 6.703925e+00
## 28927  5  691 6.698849e+00
## 28928  5  692 6.674013e+00
## 28929  5  693 6.647140e+00
## 28930  5  694 6.628932e+00
## 28931  5  695 6.617693e+00
## 28932  5  696 6.609355e+00
## 28933  5  697 6.805336e+00
## 28934  5  698 6.873848e+00
## 28935  5  699 6.923629e+00
## 28936  5  700 6.929318e+00
## 28937  5  701 6.913503e+00
## 28938  5  702 6.911919e+00
## 28939  5  703 6.894013e+00
## 28940  5  704 6.866967e+00
## 28941  5  705 6.859958e+00
## 28942  5  706 6.828331e+00
## 28943  5  707 6.826854e+00
## 28944  5  708 6.800405e+00
## 28945  5  709 6.784426e+00
## 28946  5  710 6.762646e+00
## 28947  5  711 6.753219e+00
## 28948  5  712 6.735590e+00
## 28949  5  713 6.709906e+00
## 28950  5  714 6.693472e+00
## 28951  5  715 6.684821e+00
## 28952  5  716 6.659253e+00
## 28953  5  717 6.655873e+00
## 28954  5  718 6.626911e+00
## 28955  5  719 6.621388e+00
## 28956  5  720 6.608556e+00
## 28957  5  721 6.780295e+00
## 28958  5  722 6.883442e+00
## 28959  5  723 6.932754e+00
## 28960  5  724 6.921259e+00
## 28961  5  725 6.910920e+00
## 28962  5  726 6.906074e+00
## 28963  5  727 6.880816e+00
## 28964  5  728 6.863938e+00
## 28965  5  729 6.859070e+00
## 28966  5  730 6.829263e+00
## 28967  5  731 6.818481e+00
## 28968  5  732 6.805765e+00
## 28969  5  733 6.775740e+00
## 28970  5  734 6.761994e+00
## 28971  5  735 6.751547e+00
## 28972  5  736 6.725534e+00
## 28973  5  737 6.706763e+00
## 28974  5  738 6.694778e+00
## 28975  5  739 6.678704e+00
## 28976  5  740 6.648864e+00
## 28977  5  741 6.642904e+00
## 28978  5  742 6.623366e+00
## 28979  5  743 6.617386e+00
## 28980  5  744 6.593400e+00
## 28981  5  745 6.800292e+00
## 28982  5  746 6.895588e+00
## 28983  5  747 6.919534e+00
## 28984  5  748 6.928213e+00
## 28985  5  749 6.920870e+00
## 28986  5  750 6.908136e+00
## 28987  5  751 6.876473e+00
## 28988  5  752 6.867529e+00
## 28989  5  753 6.848717e+00
## 28990  5  754 6.835044e+00
## 28991  5  755 6.811048e+00
## 28992  5  756 6.795235e+00
## 28993  5  757 6.773369e+00
## 28994  5  758 6.753677e+00
## 28995  5  759 6.747561e+00
## 28996  5  760 6.721363e+00
## 28997  5  761 6.702794e+00
## 28998  5  762 6.681964e+00
## 28999  5  763 6.681414e+00
## 29000  5  764 6.649645e+00
## 29001  5  765 6.659002e+00
## 29002  5  766 6.627737e+00
## 29003  5  767 6.607579e+00
## 29004  5  768 6.598467e+00
## 29005  5  769 6.784690e+00
## 29006  5  770 6.880813e+00
## 29007  5  771 6.918921e+00
## 29008  5  772 6.915128e+00
## 29009  5  773 6.899654e+00
## 29010  5  774 6.900235e+00
## 29011  5  775 6.890122e+00
## 29012  5  776 6.874506e+00
## 29013  5  777 6.842697e+00
## 29014  5  778 6.840604e+00
## 29015  5  779 6.815015e+00
## 29016  5  780 6.789393e+00
## 29017  5  781 6.772025e+00
## 29018  5  782 6.744893e+00
## 29019  5  783 6.742462e+00
## 29020  5  784 6.728117e+00
## 29021  5  785 6.701599e+00
## 29022  5  786 6.675594e+00
## 29023  5  787 6.683727e+00
## 29024  5  788 6.661824e+00
## 29025  5  789 6.643937e+00
## 29026  5  790 6.621664e+00
## 29027  5  791 6.619097e+00
## 29028  5  792 6.597929e+00
## 29029  5  793 6.791644e+00
## 29030  5  794 6.887677e+00
## 29031  5  795 6.907140e+00
## 29032  5  796 6.917753e+00
## 29033  5  797 6.915113e+00
## 29034  5  798 6.903845e+00
## 29035  5  799 6.883189e+00
## 29036  5  800 6.863669e+00
## 29037  5  801 6.852835e+00
## 29038  5  802 6.823466e+00
## 29039  5  803 6.812238e+00
## 29040  5  804 6.789041e+00
## 29041  5  805 6.781640e+00
## 29042  5  806 6.760529e+00
## 29043  5  807 6.752003e+00
## 29044  5  808 6.723553e+00
## 29045  5  809 6.721175e+00
## 29046  5  810 6.693532e+00
## 29047  5  811 6.665685e+00
## 29048  5  812 6.665483e+00
## 29049  5  813 6.645504e+00
## 29050  5  814 6.629183e+00
## 29051  5  815 6.600779e+00
## 29052  5  816 6.589313e+00
## 29053  5  817 6.798582e+00
## 29054  5  818 6.888048e+00
## 29055  5  819 6.917086e+00
## 29056  5  820 6.909301e+00
## 29057  5  821 6.917440e+00
## 29058  5  822 6.898115e+00
## 29059  5  823 6.876899e+00
## 29060  5  824 6.874776e+00
## 29061  5  825 6.850689e+00
## 29062  5  826 6.835727e+00
## 29063  5  827 6.802725e+00
## 29064  5  828 6.783983e+00
## 29065  5  829 6.769906e+00
## 29066  5  830 6.758277e+00
## 29067  5  831 6.743959e+00
## 29068  5  832 6.715579e+00
## 29069  5  833 6.696606e+00
## 29070  5  834 6.687185e+00
## 29071  5  835 6.680978e+00
## 29072  5  836 6.651311e+00
## 29073  5  837 6.638678e+00
## 29074  5  838 6.630205e+00
## 29075  5  839 6.611318e+00
## 29076  5  840 6.583190e+00
## 29077  5  841 6.791582e+00
## 29078  5  842 6.877722e+00
## 29079  5  843 6.906720e+00
## 29080  5  844 6.931058e+00
## 29081  5  845 6.910209e+00
## 29082  5  846 6.903589e+00
## 29083  5  847 6.891143e+00
## 29084  5  848 6.858706e+00
## 29085  5  849 6.852291e+00
## 29086  5  850 6.834906e+00
## 29087  5  851 6.799942e+00
## 29088  5  852 6.796337e+00
## 29089  5  853 6.770583e+00
## 29090  5  854 6.758209e+00
## 29091  5  855 6.733017e+00
## 29092  5  856 6.717454e+00
## 29093  5  857 6.700239e+00
## 29094  5  858 6.686397e+00
## 29095  5  859 6.670574e+00
## 29096  5  860 6.659374e+00
## 29097  5  861 6.644349e+00
## 29098  5  862 6.625472e+00
## 29099  5  863 6.610069e+00
## 29100  5  864 6.595265e+00
## 29101  5  865 6.798052e+00
## 29102  5  866 6.884618e+00
## 29103  5  867 6.915115e+00
## 29104  5  868 6.924363e+00
## 29105  5  869 6.906070e+00
## 29106  5  870 6.899055e+00
## 29107  5  871 6.875858e+00
## 29108  5  872 6.872400e+00
## 29109  5  873 6.861248e+00
## 29110  5  874 6.828851e+00
## 29111  5  875 6.810117e+00
## 29112  5  876 6.790622e+00
## 29113  5  877 6.764279e+00
## 29114  5  878 6.758843e+00
## 29115  5  879 6.744667e+00
## 29116  5  880 6.719478e+00
## 29117  5  881 6.696450e+00
## 29118  5  882 6.686692e+00
## 29119  5  883 6.676022e+00
## 29120  5  884 6.652151e+00
## 29121  5  885 6.629858e+00
## 29122  5  886 6.626053e+00
## 29123  5  887 6.624562e+00
## 29124  5  888 6.589673e+00
## 29125  5  889 6.787464e+00
## 29126  5  890 6.891492e+00
## 29127  5  891 6.922010e+00
## 29128  5  892 6.922589e+00
## 29129  5  893 6.918913e+00
## 29130  5  894 6.901912e+00
## 29131  5  895 6.881947e+00
## 29132  5  896 6.879052e+00
## 29133  5  897 6.844763e+00
## 29134  5  898 6.828171e+00
## 29135  5  899 6.811470e+00
## 29136  5  900 6.792338e+00
## 29137  5  901 6.767857e+00
## 29138  5  902 6.752529e+00
## 29139  5  903 6.735146e+00
## 29140  5  904 6.719713e+00
## 29141  5  905 6.706054e+00
## 29142  5  906 6.689001e+00
## 29143  5  907 6.654265e+00
## 29144  5  908 6.656923e+00
## 29145  5  909 6.640599e+00
## 29146  5  910 6.626858e+00
## 29147  5  911 6.603533e+00
## 29148  5  912 6.593086e+00
## 29149  5  913 6.789168e+00
## 29150  5  914 6.880415e+00
## 29151  5  915 6.914190e+00
## 29152  5  916 6.926463e+00
## 29153  5  917 6.925281e+00
## 29154  5  918 6.902599e+00
## 29155  5  919 6.879287e+00
## 29156  5  920 6.863101e+00
## 29157  5  921 6.851048e+00
## 29158  5  922 6.821042e+00
## 29159  5  923 6.808607e+00
## 29160  5  924 6.787929e+00
## 29161  5  925 6.771259e+00
## 29162  5  926 6.753613e+00
## 29163  5  927 6.734957e+00
## 29164  5  928 6.712674e+00
## 29165  5  929 6.706442e+00
## 29166  5  930 6.685679e+00
## 29167  5  931 6.681816e+00
## 29168  5  932 6.657311e+00
## 29169  5  933 6.647409e+00
## 29170  5  934 6.629262e+00
## 29171  5  935 6.601558e+00
## 29172  5  936 6.599945e+00
## 29173  5  936 6.587440e+00
## 29174  5  937 6.782503e+00
## 29175  5  938 6.883717e+00
## 29176  5  939 6.911509e+00
## 29177  5  940 6.920783e+00
## 29178  5  941 6.916215e+00
## 29179  5  942 6.887217e+00
## 29180  5  943 6.884925e+00
## 29181  5  944 6.863307e+00
## 29182  5  945 6.849156e+00
## 29183  5  946 6.817174e+00
## 29184  5  947 6.810038e+00
## 29185  5  948 6.792341e+00
## 29186  5  949 6.776821e+00
## 29187  5  950 6.750890e+00
## 29188  5  951 6.749019e+00
## 29189  5  952 6.726307e+00
## 29190  5  953 6.708461e+00
## 29191  5  954 6.686006e+00
## 29192  5  955 6.680131e+00
## 29193  5  956 6.671697e+00
## 29194  5  957 6.636081e+00
## 29195  5  958 6.629366e+00
## 29196  5  959 6.606016e+00
## 29197  5  960 6.599153e+00
## 29198  5  960 6.595676e+00
## 29199  5  961 6.580681e+00
## 29200  5  962 6.563562e+00
## 29201  5  963 6.552869e+00
## 29202  5  964 6.524194e+00
## 29203  5  965 6.522997e+00
## 29204  5  966 6.504068e+00
## 29205  5  967 6.499298e+00
## 29206  5  968 6.470258e+00
## 29207  5  969 6.463375e+00
## 29208  5  969 6.464063e+00
## 29209  5  970 6.659501e+00
## 29210  5  971 6.751518e+00
## 29211  5  972 6.795122e+00
## 29212  5  973 6.794534e+00
## 29213  5  974 6.792834e+00
## 29214  5  975 6.780011e+00
## 29215  5  976 6.761760e+00
## 29216  5  977 6.754453e+00
## 29217  5  978 6.732449e+00
## 29218  5  979 6.718798e+00
## 29219  5  980 6.686626e+00
## 29220  5  981 6.664991e+00
## 29221  5  982 6.672831e+00
## 29222  5  983 6.639233e+00
## 29223  5  984 6.626579e+00
## 29224  5  984 6.620782e+00
## 29225  5  985 6.829586e+00
## 29226  5  986 6.909021e+00
## 29227  5  987 6.946048e+00
## 29228  5  988 6.940582e+00
## 29229  5  989 6.937059e+00
## 29230  5  990 6.935395e+00
## 29231  5  991 6.911377e+00
## 29232  5  992 6.882638e+00
## 29233  5  993 6.886126e+00
## 29234  5  994 6.840775e+00
## 29235  5  995 6.836998e+00
## 29236  5  996 6.813143e+00
## 29237  5  997 6.792212e+00
## 29238  5  998 6.777308e+00
## 29239  5  999 6.761153e+00
## 29240  5 1000 6.747580e+00
## 29241  5 1001 6.734802e+00
## 29242  5 1002 6.717246e+00
## 29243  5 1003 6.697395e+00
## 29244  5 1004 6.677538e+00
## 29245  5 1005 6.658766e+00
## 29246  5 1006 6.632668e+00
## 29247  5 1007 6.624097e+00
## 29248  5 1008 6.605606e+00
## 29249  5 1009 6.804337e+00
## 29250  5 1010 6.898531e+00
## 29251  5 1011 6.923017e+00
## 29252  5 1012 6.938959e+00
## 29253  5 1013 6.919180e+00
## 29254  5 1014 6.903285e+00
## 29255  5 1015 6.891224e+00
## 29256  5 1016 6.880078e+00
## 29257  5 1017 6.859800e+00
## 29258  5 1018 6.853753e+00
## 29259  5 1019 6.833744e+00
## 29260  5 1020 6.804259e+00
## 29261  5 1021 6.785056e+00
## 29262  5 1022 6.768004e+00
## 29263  5 1023 6.756557e+00
## 29264  5 1024 6.730743e+00
## 29265  5 1025 6.719286e+00
## 29266  5 1026 6.706990e+00
## 29267  5 1027 6.683311e+00
## 29268  5 1028 6.669319e+00
## 29269  5 1029 6.649407e+00
## 29270  5 1030 6.631130e+00
## 29271  5 1031 6.621565e+00
## 29272  5 1032 6.594822e+00
## 29273  5 1033 6.789095e+00
## 29274  5 1034 6.890345e+00
## 29275  5 1035 6.938285e+00
## 29276  5 1036 6.929581e+00
## 29277  5 1037 6.921000e+00
## 29278  5 1038 6.895183e+00
## 29279  5 1039 6.899974e+00
## 29280  5 1040 6.888248e+00
## 29281  5 1041 6.851629e+00
## 29282  5 1042 6.831051e+00
## 29283  5 1043 6.822682e+00
## 29284  5 1044 6.800430e+00
## 29285  5 1045 6.767696e+00
## 29286  5 1046 6.756459e+00
## 29287  5 1047 6.750916e+00
## 29288  5 1048 6.720701e+00
## 29289  5 1049 6.698617e+00
## 29290  5 1050 6.703878e+00
## 29291  5 1051 6.683842e+00
## 29292  5 1052 6.665801e+00
## 29293  5 1053 6.645471e+00
## 29294  5 1054 6.632426e+00
## 29295  5 1055 6.611441e+00
## 29296  5 1056 6.603792e+00
## 29297  5 1057 6.793653e+00
## 29298  5 1058 6.888567e+00
## 29299  5 1059 6.921258e+00
## 29300  5 1060 6.922358e+00
## 29301  5 1061 6.912562e+00
## 29302  5 1062 6.900918e+00
## 29303  5 1063 6.884961e+00
## 29304  5 1064 6.867841e+00
## 29305  5 1065 6.851399e+00
## 29306  5 1066 6.827544e+00
## 29307  5 1067 6.811026e+00
## 29308  5 1068 6.799149e+00
## 29309  5 1069 6.771563e+00
## 29310  5 1070 6.757446e+00
## 29311  5 1071 6.750405e+00
## 29312  5 1072 6.734895e+00
## 29313  5 1073 6.702537e+00
## 29314  5 1074 6.694914e+00
## 29315  5 1075 6.678462e+00
## 29316  5 1076 6.650122e+00
## 29317  5 1077 6.661390e+00
## 29318  5 1078 6.623652e+00
## 29319  5 1079 6.609562e+00
## 29320  5 1080 6.603805e+00
## 29321  5 1081 6.784153e+00
## 29322  5 1082 6.884834e+00
## 29323  5 1083 6.915958e+00
## 29324  5 1084 6.923821e+00
## 29325  5 1085 6.927133e+00
## 29326  5 1086 6.902347e+00
## 29327  5 1087 6.897032e+00
## 29328  5 1088 6.871517e+00
## 29329  5 1089 6.853572e+00
## 29330  5 1090 6.818776e+00
## 29331  5 1091 6.802952e+00
## 29332  5 1092 6.799221e+00
## 29333  5 1093 6.778422e+00
## 29334  5 1094 6.766316e+00
## 29335  5 1095 6.735434e+00
## 29336  5 1096 6.726179e+00
## 29337  5 1097 6.708140e+00
## 29338  5 1098 6.689370e+00
## 29339  5 1099 6.671415e+00
## 29340  5 1100 6.660080e+00
## 29341  5 1101 6.645730e+00
## 29342  5 1102 6.617994e+00
## 29343  5 1103 6.619528e+00
## 29344  5 1104 6.581817e+00
## 29345  5 1105 6.805816e+00
## 29346  5 1106 6.884526e+00
## 29347  5 1107 6.916097e+00
## 29348  5 1108 6.929451e+00
## 29349  5 1109 6.919932e+00
## 29350  5 1110 6.897066e+00
## 29351  5 1111 6.887550e+00
## 29352  5 1112 6.866053e+00
## 29353  5 1113 6.851336e+00
## 29354  5 1114 6.832963e+00
## 29355  5 1115 6.815005e+00
## 29356  5 1116 6.800035e+00
## 29357  5 1117 6.770378e+00
## 29358  5 1118 6.761074e+00
## 29359  5 1119 6.745950e+00
## 29360  5 1120 6.722079e+00
## 29361  5 1121 6.702090e+00
## 29362  5 1122 6.690157e+00
## 29363  5 1123 6.680339e+00
## 29364  5 1124 6.664059e+00
## 29365  5 1125 6.642245e+00
## 29366  5 1126 6.622921e+00
## 29367  5 1127 6.607421e+00
## 29368  5 1128 6.594623e+00
## 29369  5 1129 6.784529e+00
## 29370  5 1130 6.874647e+00
## 29371  5 1131 6.923387e+00
## 29372  5 1132 6.899285e+00
## 29373  5 1133 6.926350e+00
## 29374  5 1134 6.901126e+00
## 29375  5 1135 6.888936e+00
## 29376  5 1136 6.873240e+00
## 29377  5 1137 6.843124e+00
## 29378  5 1138 6.829023e+00
## 29379  5 1139 6.813572e+00
## 29380  5 1140 6.798112e+00
## 29381  5 1141 6.782481e+00
## 29382  5 1142 6.757467e+00
## 29383  5 1143 6.740236e+00
## 29384  5 1144 6.733373e+00
## 29385  5 1145 6.712711e+00
## 29386  5 1146 6.686560e+00
## 29387  5 1147 6.683768e+00
## 29388  5 1148 6.651532e+00
## 29389  5 1149 6.637290e+00
## 29390  5 1150 6.622976e+00
## 29391  5 1151 6.609557e+00
## 29392  5 1152 6.585174e+00
## 29393  5 1153 6.790381e+00
## 29394  5 1154 6.883118e+00
## 29395  5 1155 6.912430e+00
## 29396  5 1156 6.922023e+00
## 29397  5 1157 6.914172e+00
## 29398  5 1158 6.900148e+00
## 29399  5 1159 6.889724e+00
## 29400  5 1160 6.857946e+00
## 29401  5 1161 6.847671e+00
## 29402  5 1162 6.836548e+00
## 29403  5 1163 6.815240e+00
## 29404  5 1164 6.787110e+00
## 29405  5 1165 6.770159e+00
## 29406  5 1166 6.752230e+00
## 29407  5 1167 6.729850e+00
## 29408  5 1168 6.722370e+00
## 29409  5 1169 6.709961e+00
## 29410  5 1170 6.689003e+00
## 29411  5 1171 6.673535e+00
## 29412  5 1172 6.641502e+00
## 29413  5 1173 6.646165e+00
## 29414  5 1174 6.623916e+00
## 29415  5 1175 6.606544e+00
## 29416  5 1176 6.593311e+00
## 29417  5 1177 6.790551e+00
## 29418  5 1178 6.891022e+00
## 29419  5 1179 6.921216e+00
## 29420  5 1180 6.916162e+00
## 29421  5 1181 6.906121e+00
## 29422  5 1182 6.907423e+00
## 29423  5 1183 6.889209e+00
## 29424  5 1184 6.855229e+00
## 29425  5 1185 6.843611e+00
## 29426  5 1186 6.831350e+00
## 29427  5 1187 6.806463e+00
## 29428  5 1188 6.795173e+00
## 29429  5 1189 6.769450e+00
## 29430  5 1190 6.762554e+00
## 29431  5 1191 6.739341e+00
## 29432  5 1192 6.727012e+00
## 29433  5 1193 6.709626e+00
## 29434  5 1194 6.685247e+00
## 29435  5 1195 6.677315e+00
## 29436  5 1196 6.660437e+00
## 29437  5 1197 6.642756e+00
## 29438  5 1198 6.617440e+00
## 29439  5 1199 6.602899e+00
## 29440  5 1200 6.594135e+00
## 29441  5 1201 6.774868e+00
## 29442  5 1202 6.868183e+00
## 29443  5 1203 6.923900e+00
## 29444  5 1204 6.913968e+00
## 29445  5 1205 6.919651e+00
## 29446  5 1206 6.900748e+00
## 29447  5 1207 6.884717e+00
## 29448  5 1208 6.850950e+00
## 29449  5 1209 6.854470e+00
## 29450  5 1210 6.837989e+00
## 29451  5 1211 6.819619e+00
## 29452  5 1212 6.786206e+00
## 29453  5 1213 6.768288e+00
## 29454  5 1214 6.748459e+00
## 29455  5 1215 6.742647e+00
## 29456  5 1216 6.722121e+00
## 29457  5 1217 6.703072e+00
## 29458  5 1218 6.684764e+00
## 29459  5 1219 6.677013e+00
## 29460  5 1220 6.658334e+00
## 29461  5 1221 6.648901e+00
## 29462  5 1222 6.630270e+00
## 29463  5 1223 6.611061e+00
## 29464  5 1224 6.599907e+00
## 29465  5 1225 6.808079e+00
## 29466  5 1226 6.895582e+00
## 29467  5 1227 6.909665e+00
## 29468  5 1228 6.924641e+00
## 29469  5 1229 6.913425e+00
## 29470  5 1230 6.901281e+00
## 29471  5 1231 6.893113e+00
## 29472  5 1232 6.852942e+00
## 29473  5 1233 6.853913e+00
```

```
## 29474  5 1234 6.827064e+00
## 29475  5 1235 6.802791e+00
## 29476  5 1236 6.798357e+00
## 29477  5 1237 6.764744e+00
## 29478  5 1238 6.755758e+00
## 29479  5 1239 6.744097e+00
## 29480  5 1240 6.726983e+00
## 29481  5 1241 6.709676e+00
## 29482  5 1242 6.692687e+00
## 29483  5 1243 6.668133e+00
## 29484  5 1244 6.653437e+00
## 29485  5 1245 6.647463e+00
## 29486  5 1246 6.635764e+00
## 29487  5 1247 6.598168e+00
## 29488  5 1248 6.595811e+00
## 29489  5 1248 6.582435e+00
## 29490  5 1249 6.785230e+00
## 29491  5 1250 6.875648e+00
## 29492  5 1251 6.921342e+00
## 29493  5 1252 6.924239e+00
## 29494  5 1253 6.910864e+00
## 29495  5 1254 6.905929e+00
## 29496  5 1255 6.895441e+00
## 29497  5 1256 6.862395e+00
## 29498  5 1257 6.846944e+00
## 29499  5 1258 6.820791e+00
## 29500  5 1259 6.817883e+00
## 29501  5 1260 6.792865e+00
## 29502  5 1261 6.772680e+00
## 29503  5 1262 6.754321e+00
## 29504  5 1263 6.732943e+00
## 29505  5 1264 6.711550e+00
## 29506  5 1265 6.707578e+00
## 29507  5 1266 6.691370e+00
## 29508  5 1267 6.667018e+00
## 29509  5 1268 6.643193e+00
## 29510  5 1269 6.645098e+00
## 29511  5 1270 6.615454e+00
## 29512  5 1271 6.613513e+00
## 29513  5 1272 6.599903e+00
## 29514  5 1272 6.591926e+00
## 29515  5 1273 6.578078e+00
## 29516  5 1274 6.561584e+00
## 29517  5 1275 6.540093e+00
## 29518  5 1276 6.536800e+00
## 29519  5 1277 6.520639e+00
## 29520  5 1278 6.515225e+00
## 29521  5 1279 6.488743e+00
## 29522  5 1280 6.462833e+00
## 29523  5 1281 6.470761e+00
## 29524  5 1282 6.450960e+00
## 29525  5 1283 6.443014e+00
## 29526  5 1284 6.428895e+00
## 29527  5 1284 6.427779e+00
## 29528  5 1285 6.623225e+00
## 29529  5 1286 6.717067e+00
## 29530  5 1287 6.745544e+00
## 29531  5 1288 6.764966e+00
## 29532  5 1289 6.755464e+00
## 29533  5 1290 6.741586e+00
## 29534  5 1291 6.705158e+00
## 29535  5 1292 6.704016e+00
## 29536  5 1293 6.688860e+00
## 29537  5 1294 6.676332e+00
## 29538  5 1295 6.639378e+00
## 29539  5 1296 6.633659e+00
## 29540  5 1296 6.641879e+00
## 29541  5 1297 6.840689e+00
## 29542  5 1298 6.935759e+00
## 29543  5 1299 6.956188e+00
## 29544  5 1300 6.953102e+00
## 29545  5 1301 6.950910e+00
## 29546  5 1302 6.930772e+00
## 29547  5 1303 6.916732e+00
## 29548  5 1304 6.910100e+00
## 29549  5 1305 6.877129e+00
## 29550  5 1306 6.865565e+00
## 29551  5 1307 6.839813e+00
## 29552  5 1308 6.828643e+00
## 29553  5 1309 6.798848e+00
## 29554  5 1310 6.791998e+00
## 29555  5 1311 6.773858e+00
## 29556  5 1312 6.756126e+00
## 29557  5 1313 6.729023e+00
## 29558  5 1314 6.717031e+00
## 29559  5 1315 6.693543e+00
## 29560  5 1316 6.678396e+00
## 29561  5 1317 6.655847e+00
## 29562  5 1318 6.658910e+00
## 29563  5 1319 6.635533e+00
## 29564  5 1320 6.603144e+00
## 29565  5 1321 6.805001e+00
## 29566  5 1322 6.911252e+00
## 29567  5 1323 6.935910e+00
## 29568  5 1324 6.948567e+00
## 29569  5 1325 6.935033e+00
## 29570  5 1326 6.925935e+00
## 29571  5 1327 6.902364e+00
## 29572  5 1328 6.876785e+00
## 29573  5 1329 6.863946e+00
## 29574  5 1330 6.846085e+00
## 29575  5 1331 6.830674e+00
## 29576  5 1332 6.811386e+00
## 29577  5 1333 6.795366e+00
## 29578  5 1334 6.779116e+00
## 29579  5 1335 6.761138e+00
## 29580  5 1336 6.749400e+00
## 29581  5 1337 6.709165e+00
## 29582  5 1338 6.704950e+00
## 29583  5 1339 6.688680e+00
## 29584  5 1340 6.671720e+00
## 29585  5 1341 6.650570e+00
## 29586  5 1342 6.633176e+00
## 29587  5 1343 6.616224e+00
## 29588  5 1344 6.598246e+00
## 29589  5 1345 6.808083e+00
## 29590  5 1346 6.881013e+00
## 29591  5 1347 6.922801e+00
## 29592  5 1348 6.924809e+00
## 29593  5 1349 6.925740e+00
## 29594  5 1350 6.899752e+00
## 29595  5 1351 6.886449e+00
## 29596  5 1352 6.863459e+00
## 29597  5 1353 6.853030e+00
## 29598  5 1354 6.839270e+00
## 29599  5 1355 6.820085e+00
## 29600  5 1356 6.789652e+00
## 29601  5 1357 6.779304e+00
## 29602  5 1358 6.767962e+00
## 29603  5 1359 6.752183e+00
## 29604  5 1360 6.736753e+00
## 29605  5 1361 6.722216e+00
## 29606  5 1362 6.698415e+00
## 29607  5 1363 6.682758e+00
## 29608  5 1364 6.662647e+00
## 29609  5 1365 6.652807e+00
## 29610  5 1366 6.633081e+00
## 29611  5 1367 6.618583e+00
## 29612  5 1368 6.597942e+00
## 29613  5 1369 6.796981e+00
## 29614  5 1370 6.894280e+00
## 29615  5 1371 6.935158e+00
## 29616  5 1372 6.920297e+00
## 29617  5 1373 6.913839e+00
## 29618  5 1374 6.896111e+00
## 29619  5 1375 6.900259e+00
## 29620  5 1376 6.880320e+00
## 29621  5 1377 6.850030e+00
## 29622  5 1378 6.835731e+00
## 29623  5 1379 6.816377e+00
## 29624  5 1380 6.788691e+00
## 29625  5 1381 6.778887e+00
## 29626  5 1382 6.765203e+00
## 29627  5 1383 6.742393e+00
## 29628  5 1384 6.730174e+00
## 29629  5 1385 6.715487e+00
## 29630  5 1386 6.698924e+00
## 29631  5 1387 6.687300e+00
## 29632  5 1388 6.666764e+00
## 29633  5 1389 6.653271e+00
## 29634  5 1390 6.638420e+00
## 29635  5 1391 6.611456e+00
## 29636  5 1392 6.592979e+00
## 29637  5 1393 6.804806e+00
## 29638  5 1394 6.882211e+00
## 29639  5 1395 6.923220e+00
## 29640  5 1396 6.931341e+00
## 29641  5 1397 6.925259e+00
## 29642  5 1398 6.905132e+00
## 29643  5 1399 6.882667e+00
## 29644  5 1400 6.868685e+00
## 29645  5 1401 6.853120e+00
## 29646  5 1402 6.830759e+00
## 29647  5 1403 6.813360e+00
## 29648  5 1404 6.791582e+00
## 29649  5 1405 6.781699e+00
## 29650  5 1406 6.758283e+00
## 29651  5 1407 6.743752e+00
## 29652  5 1408 6.718131e+00
## 29653  5 1409 6.700516e+00
## 29654  5 1410 6.698341e+00
## 29655  5 1411 6.682585e+00
## 29656  5 1412 6.660922e+00
## 29657  5 1413 6.649212e+00
## 29658  5 1414 6.627794e+00
## 29659  5 1415 6.605327e+00
## 29660  5 1416 6.593499e+00
## 29661  5 1417 6.796232e+00
## 29662  5 1418 6.896047e+00
## 29663  5 1419 6.924548e+00
## 29664  5 1420 6.923735e+00
## 29665  5 1421 6.921374e+00
## 29666  5 1422 6.903851e+00
## 29667  5 1423 6.884058e+00
## 29668  5 1424 6.873277e+00
## 29669  5 1425 6.852153e+00
## 29670  5 1426 6.831132e+00
## 29671  5 1427 6.823159e+00
## 29672  5 1428 6.794290e+00
## 29673  5 1429 6.784328e+00
## 29674  5 1430 6.759676e+00
## 29675  5 1431 6.746777e+00
## 29676  5 1432 6.728736e+00
## 29677  5 1433 6.702187e+00
## 29678  5 1434 6.689369e+00
## 29679  5 1435 6.675176e+00
## 29680  5 1436 6.665561e+00
## 29681  5 1437 6.649290e+00
## 29682  5 1438 6.639324e+00
## 29683  5 1439 6.611593e+00
## 29684  5 1440 6.587474e+00
## 29685  5 1441 6.797953e+00
## 29686  5 1442 6.885871e+00
## 29687  5 1443 6.912308e+00
## 29688  5 1444 6.912383e+00
## 29689  5 1445 6.922005e+00
## 29690  5 1446 6.902317e+00
## 29691  5 1447 6.881033e+00
## 29692  5 1448 6.877413e+00
## 29693  5 1449 6.841818e+00
## 29694  5 1450 6.831548e+00
## 29695  5 1451 6.813683e+00
## 29696  5 1452 6.783519e+00
## 29697  5 1453 6.778330e+00
## 29698  5 1454 6.765981e+00
## 29699  5 1455 6.756090e+00
## 29700  5 1456 6.735303e+00
## 29701  5 1457 6.708361e+00
## 29702  5 1458 6.695207e+00
## 29703  5 1459 6.683078e+00
## 29704  5 1460 6.651285e+00
## 29705  5 1461 6.646881e+00
## 29706  5 1462 6.638070e+00
## 29707  5 1463 6.613029e+00
## 29708  5 1464 6.597414e+00
## 29709  5 1465 6.781664e+00
## 29710  5 1466 6.884892e+00
## 29711  5 1467 6.906210e+00
## 29712  5 1468 6.922666e+00
## 29713  5 1469 6.910865e+00
## 29714  5 1470 6.910534e+00
## 29715  5 1471 6.886880e+00
## 29716  5 1472 6.869606e+00
## 29717  5 1473 6.842347e+00
## 29718  5 1474 6.832763e+00
## 29719  5 1475 6.804769e+00
## 29720  5 1476 6.797382e+00
## 29721  5 1477 6.777906e+00
## 29722  5 1478 6.757573e+00
## 29723  5 1479 6.750303e+00
## 29724  5 1480 6.731836e+00
## 29725  5 1481 6.704601e+00
## 29726  5 1482 6.688637e+00
## 29727  5 1483 6.670625e+00
## 29728  5 1484 6.660155e+00
## 29729  5 1485 6.649902e+00
## 29730  5 1486 6.620724e+00
## 29731  5 1487 6.607783e+00
## 29732  5 1488 6.583924e+00
## 29733  5 1489 6.793991e+00
## 29734  5 1490 6.879186e+00
## 29735  5 1491 6.917813e+00
## 29736  5 1492 6.933342e+00
## 29737  5 1493 6.907405e+00
## 29738  5 1494 6.899721e+00
## 29739  5 1495 6.875577e+00
## 29740  5 1496 6.853404e+00
## 29741  5 1497 6.848944e+00
## 29742  5 1498 6.848693e+00
## 29743  5 1499 6.826046e+00
## 29744  5 1500 6.780704e+00
## 29745  5 1501 6.783537e+00
## 29746  5 1502 6.759146e+00
## 29747  5 1503 6.747871e+00
## 29748  5 1504 6.716392e+00
## 29749  5 1505 6.690618e+00
## 29750  5 1506 6.689660e+00
## 29751  5 1507 6.671357e+00
## 29752  5 1508 6.658953e+00
## 29753  5 1509 6.638472e+00
## 29754  5 1510 6.628277e+00
## 29755  5 1511 6.612855e+00
## 29756  5 1512 6.610441e+00
## 29757  5 1513 6.777068e+00
## 29758  5 1514 6.884228e+00
## 29759  5 1515 6.921993e+00
## 29760  5 1516 6.907357e+00
## 29761  5 1517 6.925034e+00
## 29762  5 1518 6.891768e+00
## 29763  5 1519 6.879749e+00
## 29764  5 1520 6.863810e+00
## 29765  5 1521 6.843038e+00
## 29766  5 1522 6.840104e+00
## 29767  5 1523 6.823371e+00
## 29768  5 1524 6.789047e+00
## 29769  5 1525 6.775759e+00
## 29770  5 1526 6.759294e+00
## 29771  5 1527 6.729746e+00
## 29772  5 1528 6.723261e+00
## 29773  5 1529 6.710771e+00
## 29774  5 1530 6.693650e+00
## 29775  5 1531 6.665689e+00
## 29776  5 1532 6.658141e+00
## 29777  5 1533 6.647336e+00
## 29778  5 1534 6.630859e+00
## 29779  5 1535 6.610840e+00
## 29780  5 1536 6.587879e+00
## 29781  5 1537 6.795450e+00
## 29782  5 1538 6.881204e+00
## 29783  5 1539 6.923067e+00
## 29784  5 1540 6.923233e+00
## 29785  5 1541 6.906118e+00
## 29786  5 1542 6.908410e+00
## 29787  5 1543 6.885424e+00
## 29788  5 1544 6.863903e+00
## 29789  5 1545 6.844263e+00
## 29790  5 1546 6.823264e+00
## 29791  5 1547 6.809261e+00
## 29792  5 1548 6.804850e+00
## 29793  5 1549 6.772745e+00
## 29794  5 1550 6.772553e+00
## 29795  5 1551 6.756690e+00
## 29796  5 1552 6.741874e+00
## 29797  5 1553 6.711623e+00
## 29798  5 1554 6.688546e+00
## 29799  5 1555 6.661890e+00
## 29800  5 1556 6.653855e+00
## 29801  5 1557 6.646125e+00
## 29802  5 1558 6.627234e+00
## 29803  5 1559 6.614305e+00
## 29804  5 1560 6.598141e+00
## 29805  5 1560 6.591401e+00
## 29806  5 1561 6.797683e+00
## 29807  5 1562 6.880030e+00
## 29808  5 1563 6.910094e+00
## 29809  5 1564 6.921550e+00
## 29810  5 1565 6.900168e+00
## 29811  5 1566 6.895599e+00
## 29812  5 1567 6.881588e+00
## 29813  5 1568 6.870044e+00
## 29814  5 1569 6.845249e+00
## 29815  5 1570 6.820661e+00
## 29816  5 1571 6.812030e+00
## 29817  5 1572 6.789730e+00
## 29818  5 1573 6.768048e+00
## 29819  5 1574 6.762694e+00
## 29820  5 1575 6.743237e+00
## 29821  5 1576 6.724545e+00
## 29822  5 1577 6.699689e+00
## 29823  5 1578 6.695256e+00
## 29824  5 1579 6.666455e+00
## 29825  5 1580 6.649488e+00
## 29826  5 1581 6.633085e+00
## 29827  5 1582 6.625652e+00
## 29828  5 1583 6.603512e+00
## 29829  5 1584 6.609193e+00
## 29830  5 1584 6.596046e+00
## 29831  5 1585 6.578442e+00
## 29832  5 1586 6.561150e+00
## 29833  5 1587 6.551344e+00
## 29834  5 1588 6.535892e+00
## 29835  5 1589 6.523575e+00
## 29836  5 1590 6.498381e+00
## 29837  5 1591 6.489593e+00
## 29838  5 1592 6.482018e+00
## 29839  5 1593 6.468394e+00
## 29840  5 1594 6.453584e+00
## 29841  5 1595 6.442826e+00
## 29842  5 1596 6.427255e+00
## 29843  5 1597 6.400873e+00
## 29844  5 1598 6.403179e+00
## 29845  5 1599 6.380848e+00
## 29846  5 1599 6.388374e+00
## 29847  5 1600 6.485942e+00
## 29848  5 1601 6.517076e+00
## 29849  5 1602 6.525188e+00
## 29850  5 1603 6.520326e+00
## 29851  5 1604 6.513910e+00
## 29852  5 1605 6.505755e+00
## 29853  5 1606 6.487761e+00
## 29854  5 1607 6.481391e+00
## 29855  5 1608 6.461739e+00
## 29856  5 1608 6.466321e+00
## 29857  5 1609 6.676158e+00
## 29858  5 1610 6.759986e+00
## 29859  5 1611 6.791663e+00
## 29860  5 1612 6.800572e+00
## 29861  5 1613 6.782452e+00
## 29862  5 1614 6.762416e+00
## 29863  5 1615 6.758412e+00
## 29864  5 1616 6.744872e+00
## 29865  5 1617 6.720731e+00
## 29866  5 1618 6.702876e+00
## 29867  5 1619 6.695268e+00
## 29868  5 1620 6.659163e+00
## 29869  5 1621 6.646633e+00
## 29870  5 1622 6.643255e+00
## 29871  5 1623 6.623458e+00
## 29872  5 1624 6.604967e+00
## 29873  5 1625 6.595462e+00
## 29874  5 1626 6.559156e+00
## 29875  5 1627 6.555559e+00
## 29876  5 1628 6.552425e+00
## 29877  5 1629 6.519782e+00
## 29878  5 1630 6.507474e+00
## 29879  5 1631 6.497440e+00
## 29880  5 1632 6.486872e+00
## 29881  5 1633 6.669684e+00
## 29882  5 1634 6.776219e+00
## 29883  5 1635 6.806289e+00
## 29884  5 1636 6.808422e+00
## 29885  5 1637 6.801374e+00
## 29886  5 1638 6.795425e+00
## 29887  5 1639 6.762431e+00
## 29888  5 1640 6.749576e+00
## 29889  5 1641 6.741510e+00
## 29890  5 1642 6.712728e+00
## 29891  5 1643 6.687886e+00
## 29892  5 1644 6.684849e+00
## 29893  5 1645 6.666383e+00
## 29894  5 1646 6.639243e+00
## 29895  5 1647 6.624747e+00
## 29896  5 1648 6.618530e+00
## 29897  5 1649 6.606203e+00
## 29898  5 1650 6.588042e+00
## 29899  5 1651 6.566911e+00
## 29900  5 1652 6.541689e+00
## 29901  5 1653 6.541413e+00
## 29902  5 1654 6.516525e+00
## 29903  5 1655 6.494133e+00
## 29904  5 1656 6.487334e+00
## 29905  5 1657 6.685514e+00
## 29906  5 1658 6.772243e+00
## 29907  5 1659 6.809065e+00
## 29908  5 1660 6.825325e+00
## 29909  5 1661 6.824567e+00
## 29910  5 1662 6.799154e+00
## 29911  5 1663 6.789022e+00
## 29912  5 1664 6.771496e+00
## 29913  5 1665 6.737265e+00
## 29914  5 1666 6.722948e+00
## 29915  5 1667 6.713881e+00
## 29916  5 1668 6.686709e+00
## 29917  5 1669 6.665712e+00
## 29918  5 1670 6.648039e+00
## 29919  5 1671 6.634960e+00
## 29920  5 1672 6.621166e+00
## 29921  5 1673 6.608462e+00
## 29922  5 1674 6.573938e+00
## 29923  5 1675 6.576175e+00
## 29924  5 1676 6.547624e+00
## 29925  5 1677 6.542149e+00
## 29926  5 1678 6.525123e+00
## 29927  5 1679 6.513729e+00
## 29928  5 1680 6.495621e+00
## 29929  5 1681 6.694036e+00
## 29930  5 1682 6.781453e+00
## 29931  5 1683 6.821098e+00
## 29932  5 1684 6.830058e+00
## 29933  5 1685 6.821307e+00
## 29934  5 1686 6.804626e+00
## 29935  5 1687 6.794490e+00
## 29936  5 1688 6.759668e+00
## 29937  5 1689 6.741601e+00
## 29938  5 1690 6.731057e+00
## 29939  5 1691 6.703496e+00
## 29940  5 1692 6.687495e+00
## 29941  5 1693 6.677894e+00
## 29942  5 1694 6.652875e+00
## 29943  5 1695 6.636356e+00
## 29944  5 1696 6.624363e+00
## 29945  5 1697 6.616998e+00
## 29946  5 1698 6.588037e+00
## 29947  5 1699 6.570802e+00
## 29948  5 1700 6.568504e+00
## 29949  5 1701 6.530761e+00
## 29950  5 1702 6.528261e+00
## 29951  5 1703 6.506639e+00
## 29952  5 1704 6.505510e+00
## 29953  5 1705 6.700011e+00
## 29954  5 1706 6.798274e+00
## 29955  5 1707 6.819357e+00
## 29956  5 1708 6.835554e+00
## 29957  5 1709 6.818938e+00
## 29958  5 1710 6.790412e+00
## 29959  5 1711 6.779055e+00
## 29960  5 1712 6.777179e+00
## 29961  5 1713 6.747307e+00
## 29962  5 1714 6.738937e+00
## 29963  5 1715 6.720130e+00
## 29964  5 1716 6.699196e+00
## 29965  5 1717 6.676443e+00
## 29966  5 1718 6.655802e+00
## 29967  5 1719 6.642650e+00
## 29968  5 1720 6.642933e+00
## 29969  5 1721 6.619323e+00
## 29970  5 1722 6.606938e+00
## 29971  5 1723 6.580495e+00
## 29972  5 1724 6.568619e+00
## 29973  5 1725 6.553984e+00
## 29974  5 1726 6.516139e+00
## 29975  5 1727 6.511296e+00
## 29976  5 1728 6.506465e+00
## 29977  5 1729 6.705569e+00
## 29978  5 1730 6.795735e+00
## 29979  5 1731 6.818296e+00
## 29980  5 1732 6.833817e+00
## 29981  5 1733 6.815897e+00
## 29982  5 1734 6.818646e+00
## 29983  5 1735 6.797991e+00
## 29984  5 1736 6.769448e+00
## 29985  5 1737 6.751693e+00
## 29986  5 1738 6.730999e+00
## 29987  5 1739 6.715509e+00
## 29988  5 1740 6.707417e+00
## 29989  5 1741 6.691966e+00
## 29990  5 1742 6.656402e+00
## 29991  5 1743 6.644683e+00
## 29992  5 1744 6.630944e+00
## 29993  5 1745 6.621648e+00
## 29994  5 1746 6.610936e+00
## 29995  5 1747 6.588255e+00
## 29996  5 1748 6.568849e+00
## 29997  5 1749 6.553281e+00
## 29998  5 1750 6.540021e+00
## 29999  5 1751 6.521175e+00
## 30000  5 1752 6.510451e+00
## 30001  5 1753 6.701788e+00
## 30002  5 1754 6.789854e+00
## 30003  5 1755 6.823981e+00
## 30004  5 1756 6.837083e+00
## 30005  5 1757 6.834517e+00
## 30006  5 1758 6.813000e+00
## 30007  5 1759 6.787136e+00
## 30008  5 1760 6.772741e+00
## 30009  5 1761 6.760053e+00
## 30010  5 1762 6.744495e+00
## 30011  5 1763 6.718961e+00
## 30012  5 1764 6.693264e+00
## 30013  5 1765 6.679881e+00
## 30014  5 1766 6.679419e+00
## 30015  5 1767 6.656788e+00
## 30016  5 1768 6.636544e+00
## 30017  5 1769 6.604594e+00
## 30018  5 1770 6.583527e+00
## 30019  5 1771 6.580814e+00
## 30020  5 1772 6.567178e+00
## 30021  5 1773 6.563043e+00
## 30022  5 1774 6.532678e+00
## 30023  5 1775 6.513006e+00
## 30024  5 1776 6.503679e+00
## 30025  5 1777 6.710909e+00
## 30026  5 1778 6.798374e+00
## 30027  5 1779 6.829915e+00
## 30028  5 1780 6.834700e+00
## 30029  5 1781 6.823984e+00
## 30030  5 1782 6.820302e+00
## 30031  5 1783 6.784308e+00
## 30032  5 1784 6.775019e+00
## 30033  5 1785 6.767478e+00
## 30034  5 1786 6.740396e+00
## 30035  5 1787 6.724138e+00
## 30036  5 1788 6.699842e+00
## 30037  5 1789 6.679177e+00
## 30038  5 1790 6.670808e+00
## 30039  5 1791 6.659091e+00
## 30040  5 1792 6.639639e+00
## 30041  5 1793 6.610957e+00
## 30042  5 1794 6.599007e+00
## 30043  5 1795 6.593096e+00
## 30044  5 1796 6.576032e+00
## 30045  5 1797 6.556436e+00
## 30046  5 1798 6.541991e+00
## 30047  5 1799 6.530963e+00
## 30048  5 1800 6.515823e+00
## 30049  5 1801 6.698755e+00
## 30050  5 1802 6.798526e+00
## 30051  5 1803 6.833110e+00
## 30052  5 1804 6.837904e+00
## 30053  5 1805 6.831445e+00
## 30054  5 1806 6.819639e+00
## 30055  5 1807 6.793275e+00
## 30056  5 1808 6.783662e+00
## 30057  5 1809 6.770452e+00
## 30058  5 1810 6.747453e+00
## 30059  5 1811 6.730852e+00
## 30060  5 1812 6.708899e+00
## 30061  5 1813 6.689213e+00
## 30062  5 1814 6.676439e+00
## 30063  5 1815 6.661889e+00
## 30064  5 1816 6.638795e+00
## 30065  5 1817 6.618907e+00
## 30066  5 1818 6.597445e+00
## 30067  5 1819 6.592704e+00
## 30068  5 1820 6.573794e+00
## 30069  5 1821 6.562270e+00
## 30070  5 1822 6.540348e+00
## 30071  5 1823 6.529263e+00
## 30072  5 1824 6.514698e+00
## 30073  5 1825 6.713860e+00
## 30074  5 1826 6.806715e+00
## 30075  5 1827 6.839441e+00
## 30076  5 1828 6.845844e+00
## 30077  5 1829 6.838023e+00
## 30078  5 1830 6.823180e+00
## 30079  5 1831 6.815578e+00
## 30080  5 1832 6.796259e+00
## 30081  5 1833 6.764584e+00
## 30082  5 1834 6.749611e+00
## 30083  5 1835 6.716103e+00
## 30084  5 1836 6.723230e+00
## 30085  5 1837 6.694825e+00
## 30086  5 1838 6.668980e+00
## 30087  5 1839 6.663035e+00
## 30088  5 1840 6.652608e+00
## 30089  5 1841 6.631463e+00
## 30090  5 1842 6.619154e+00
## 30091  5 1843 6.598963e+00
## 30092  5 1844 6.578035e+00
## 30093  5 1845 6.579906e+00
## 30094  5 1846 6.560115e+00
## 30095  5 1847 6.523907e+00
## 30096  5 1848 6.514548e+00
## 30097  5 1849 6.700239e+00
## 30098  5 1850 6.812277e+00
## 30099  5 1851 6.838162e+00
## 30100  5 1852 6.842189e+00
## 30101  5 1853 6.842361e+00
## 30102  5 1854 6.821664e+00
## 30103  5 1855 6.805625e+00
## 30104  5 1856 6.783107e+00
## 30105  5 1857 6.762693e+00
## 30106  5 1858 6.759671e+00
## 30107  5 1859 6.730792e+00
## 30108  5 1860 6.706074e+00
## 30109  5 1861 6.690448e+00
## 30110  5 1862 6.677849e+00
## 30111  5 1863 6.652974e+00
## 30112  5 1864 6.653318e+00
## 30113  5 1865 6.638575e+00
## 30114  5 1866 6.619091e+00
## 30115  5 1867 6.610039e+00
## 30116  5 1868 6.582609e+00
## 30117  5 1869 6.563370e+00
## 30118  5 1870 6.541189e+00
## 30119  5 1871 6.535171e+00
## 30120  5 1872 6.519593e+00
## 30121  5 1872 6.581319e+00
## 30122  5 1873 6.785324e+00
## 30123  5 1874 6.888788e+00
## 30124  5 1875 6.920506e+00
## 30125  5 1876 6.924314e+00
## 30126  5 1877 6.913299e+00
## 30127  5 1878 6.903088e+00
## 30128  5 1879 6.875352e+00
## 30129  5 1880 6.861561e+00
## 30130  5 1881 6.841415e+00
## 30131  5 1882 6.815617e+00
## 30132  5 1883 6.807396e+00
## 30133  5 1884 6.792420e+00
## 30134  5 1885 6.764457e+00
## 30135  5 1886 6.753881e+00
## 30136  5 1887 6.730687e+00
## 30137  5 1888 6.730087e+00
## 30138  5 1889 6.711300e+00
## 30139  5 1890 6.700926e+00
## 30140  5 1891 6.667351e+00
## 30141  5 1892 6.657725e+00
## 30142  5 1893 6.649664e+00
## 30143  5 1894 6.623792e+00
## 30144  5 1895 6.609873e+00
## 30145  5 1896 6.596919e+00
## 30146  5 1896 6.599700e+00
## 30147  5 1897 6.584279e+00
## 30148  5 1898 6.564734e+00
## 30149  5 1899 6.549919e+00
## 30150  5 1900 6.533051e+00
## 30151  5 1901 6.521629e+00
## 30152  5 1902 6.502415e+00
## 30153  5 1903 6.494035e+00
## 30154  5 1904 6.478474e+00
## 30155  5 1905 6.465927e+00
## 30156  5 1906 6.454008e+00
## 30157  5 1907 6.425331e+00
## 30158  5 1908 6.427942e+00
## 30159  5 1909 6.411681e+00
## 30160  5 1910 6.392414e+00
## 30161  5 1911 6.391961e+00
## 30162  5 1911 6.389447e+00
## 30163  5 1912 6.588753e+00
## 30164  5 1913 6.675552e+00
## 30165  5 1914 6.710205e+00
## 30166  5 1915 6.724526e+00
## 30167  5 1916 6.724821e+00
## 30168  5 1917 6.699457e+00
## 30169  5 1918 6.676396e+00
## 30170  5 1919 6.667908e+00
## 30171  5 1920 6.646152e+00
## 30172  5 1920 6.654311e+00
## 30173  5 1921 6.850827e+00
## 30174  5 1922 6.937169e+00
## 30175  5 1923 6.965104e+00
## 30176  5 1924 6.978922e+00
## 30177  5 1925 6.954887e+00
## 30178  5 1926 6.944309e+00
## 30179  5 1927 6.932662e+00
## 30180  5 1928 6.924537e+00
## 30181  5 1929 6.899532e+00
## 30182  5 1930 6.866545e+00
## 30183  5 1931 6.849384e+00
## 30184  5 1932 6.841663e+00
## 30185  5 1933 6.831606e+00
## 30186  5 1934 6.788308e+00
## 30187  5 1935 6.778911e+00
## 30188  5 1936 6.761810e+00
## 30189  5 1937 6.752263e+00
## 30190  5 1938 6.715041e+00
## 30191  5 1939 6.713263e+00
## 30192  5 1940 6.684265e+00
## 30193  5 1941 6.668218e+00
## 30194  5 1942 6.658168e+00
## 30195  5 1943 6.637052e+00
## 30196  5 1944 6.631971e+00
## 30197  5 1945 6.821735e+00
## 30198  5 1946 6.909926e+00
## 30199  5 1947 6.936522e+00
## 30200  5 1948 6.951030e+00
## 30201  5 1949 6.936626e+00
## 30202  5 1950 6.930753e+00
## 30203  5 1951 6.897260e+00
## 30204  5 1952 6.886793e+00
## 30205  5 1953 6.864704e+00
## 30206  5 1954 6.863112e+00
## 30207  5 1955 6.838419e+00
## 30208  5 1956 6.810249e+00
## 30209  5 1957 6.795188e+00
## 30210  5 1958 6.772858e+00
## 30211  5 1959 6.774231e+00
## 30212  5 1960 6.746091e+00
## 30213  5 1961 6.739944e+00
## 30214  5 1962 6.710621e+00
## 30215  5 1963 6.687471e+00
## 30216  5 1964 6.679735e+00
## 30217  5 1965 6.665246e+00
## 30218  5 1966 6.636327e+00
## 30219  5 1967 6.620150e+00
## 30220  5 1968 6.609677e+00
## 30221  5 1969 6.802919e+00
## 30222  5 1970 6.897259e+00
## 30223  5 1971 6.939453e+00
## 30224  5 1972 6.939179e+00
## 30225  5 1973 6.933090e+00
## 30226  5 1974 6.910995e+00
## 30227  5 1975 6.911992e+00
## 30228  5 1976 6.885159e+00
## 30229  5 1977 6.845801e+00
## 30230  5 1978 6.838630e+00
## 30231  5 1979 6.807095e+00
## 30232  5 1980 6.806444e+00
## 30233  5 1981 6.788681e+00
## 30234  5 1982 6.770165e+00
## 30235  5 1983 6.739441e+00
## 30236  5 1984 6.739878e+00
## 30237  5 1985 6.718770e+00
## 30238  5 1986 6.697695e+00
## 30239  5 1987 6.685958e+00
## 30240  5 1988 6.668694e+00
## 30241  5 1989 6.663847e+00
## 30242  5 1990 6.643981e+00
## 30243  5 1991 6.631958e+00
## 30244  5 1992 6.599011e+00
## 30245  5 1993 6.794416e+00
## 30246  5 1994 6.878062e+00
## 30247  5 1995 6.947649e+00
## 30248  5 1996 6.939991e+00
## 30249  5 1997 6.910773e+00
## 30250  5 1998 6.906755e+00
## 30251  5 1999 6.895369e+00
## 30252  5 2000 6.881249e+00
## 30253  5 2001 6.854895e+00
## 30254  5 2002 6.829353e+00
## 30255  5 2003 6.820007e+00
## 30256  5 2004 6.785079e+00
## 30257  5 2005 6.786826e+00
## 30258  5 2006 6.764233e+00
## 30259  5 2007 6.756044e+00
## 30260  5 2008 6.729553e+00
## 30261  5 2009 6.708372e+00
## 30262  5 2010 6.687468e+00
## 30263  5 2011 6.686479e+00
## 30264  5 2012 6.664318e+00
## 30265  5 2013 6.648060e+00
## 30266  5 2014 6.627855e+00
## 30267  5 2015 6.616173e+00
## 30268  5 2016 6.609002e+00
## 30269  5 2017 6.785520e+00
## 30270  5 2018 6.892941e+00
## 30271  5 2019 6.923671e+00
## 30272  5 2020 6.931125e+00
## 30273  5 2021 6.929931e+00
```

```
## 30274  5 2022 6.902788e+00
## 30275  5 2023 6.882362e+00
## 30276  5 2024 6.863436e+00
## 30277  5 2025 6.857608e+00
## 30278  5 2026 6.832941e+00
## 30279  5 2027 6.832364e+00
## 30280  5 2028 6.799465e+00
## 30281  5 2029 6.776734e+00
## 30282  5 2030 6.763542e+00
## 30283  5 2031 6.743909e+00
## 30284  5 2032 6.734210e+00
## 30285  5 2033 6.719405e+00
## 30286  5 2034 6.704151e+00
## 30287  5 2035 6.667489e+00
## 30288  5 2036 6.663020e+00
## 30289  5 2037 6.658874e+00
## 30290  5 2038 6.635661e+00
## 30291  5 2039 6.610134e+00
## 30292  5 2040 6.608642e+00
## 30293  5 2041 6.799918e+00
## 30294  5 2042 6.886749e+00
## 30295  5 2043 6.925272e+00
## 30296  5 2044 6.924226e+00
## 30297  5 2045 6.904417e+00
## 30298  5 2046 6.903802e+00
## 30299  5 2047 6.885006e+00
## 30300  5 2048 6.867162e+00
## 30301  5 2049 6.854869e+00
## 30302  5 2050 6.833376e+00
## 30303  5 2051 6.821757e+00
## 30304  5 2052 6.805944e+00
## 30305  5 2053 6.770991e+00
## 30306  5 2054 6.756200e+00
## 30307  5 2055 6.745613e+00
## 30308  5 2056 6.726277e+00
## 30309  5 2057 6.714405e+00
## 30310  5 2058 6.695597e+00
## 30311  5 2059 6.670377e+00
## 30312  5 2060 6.675561e+00
## 30313  5 2061 6.638410e+00
## 30314  5 2062 6.619069e+00
## 30315  5 2063 6.597293e+00
## 30316  5 2064 6.600436e+00
## 30317  5 2065 6.790648e+00
## 30318  5 2066 6.877764e+00
## 30319  5 2067 6.926545e+00
## 30320  5 2068 6.923410e+00
## 30321  5 2069 6.915498e+00
## 30322  5 2070 6.910723e+00
## 30323  5 2071 6.895868e+00
## 30324  5 2072 6.865637e+00
## 30325  5 2073 6.844980e+00
## 30326  5 2074 6.829366e+00
## 30327  5 2075 6.809597e+00
## 30328  5 2076 6.805649e+00
## 30329  5 2077 6.762905e+00
## 30330  5 2078 6.750126e+00
## 30331  5 2079 6.737502e+00
## 30332  5 2080 6.716277e+00
## 30333  5 2081 6.693002e+00
## 30334  5 2082 6.685871e+00
## 30335  5 2083 6.682854e+00
## 30336  5 2084 6.648694e+00
## 30337  5 2085 6.644284e+00
## 30338  5 2086 6.627000e+00
## 30339  5 2087 6.621728e+00
## 30340  5 2088 6.594140e+00
## 30341  5 2089 6.797071e+00
## 30342  5 2090 6.880399e+00
## 30343  5 2091 6.923071e+00
## 30344  5 2092 6.928429e+00
## 30345  5 2093 6.922361e+00
## 30346  5 2094 6.908532e+00
## 30347  5 2095 6.869787e+00
## 30348  5 2096 6.866786e+00
## 30349  5 2097 6.834539e+00
## 30350  5 2098 6.843391e+00
## 30351  5 2099 6.808353e+00
## 30352  5 2100 6.786460e+00
## 30353  5 2101 6.771815e+00
## 30354  5 2102 6.751871e+00
## 30355  5 2103 6.738997e+00
## 30356  5 2104 6.724754e+00
## 30357  5 2105 6.705170e+00
## 30358  5 2106 6.699767e+00
## 30359  5 2107 6.676212e+00
## 30360  5 2108 6.658272e+00
## 30361  5 2109 6.639135e+00
## 30362  5 2110 6.627566e+00
## 30363  5 2111 6.621035e+00
## 30364  5 2112 6.595488e+00
## 30365  5 2113 6.798952e+00
## 30366  5 2114 6.885293e+00
## 30367  5 2115 6.918676e+00
## 30368  5 2116 6.920337e+00
## 30369  5 2117 6.917873e+00
## 30370  5 2118 6.906641e+00
## 30371  5 2119 6.879299e+00
## 30372  5 2120 6.869582e+00
## 30373  5 2121 6.849071e+00
## 30374  5 2122 6.826415e+00
## 30375  5 2123 6.806045e+00
## 30376  5 2124 6.782300e+00
## 30377  5 2125 6.764407e+00
## 30378  5 2126 6.771622e+00
## 30379  5 2127 6.731035e+00
## 30380  5 2128 6.737050e+00
## 30381  5 2129 6.712206e+00
## 30382  5 2130 6.698577e+00
## 30383  5 2131 6.673546e+00
## 30384  5 2132 6.658428e+00
## 30385  5 2133 6.657064e+00
## 30386  5 2134 6.635768e+00
## 30387  5 2135 6.602932e+00
## 30388  5 2136 6.597055e+00
## 30389  5 2137 6.790570e+00
## 30390  5 2138 6.894036e+00
## 30391  5 2139 6.920829e+00
## 30392  5 2140 6.931930e+00
## 30393  5 2141 6.906049e+00
## 30394  5 2142 6.900944e+00
## 30395  5 2143 6.881824e+00
## 30396  5 2144 6.860567e+00
## 30397  5 2145 6.844519e+00
## 30398  5 2146 6.817762e+00
## 30399  5 2147 6.816454e+00
## 30400  5 2148 6.796981e+00
## 30401  5 2149 6.783596e+00
## 30402  5 2150 6.763850e+00
## 30403  5 2151 6.748941e+00
## 30404  5 2152 6.716612e+00
## 30405  5 2153 6.696568e+00
## 30406  5 2154 6.694615e+00
## 30407  5 2155 6.674128e+00
## 30408  5 2156 6.659277e+00
## 30409  5 2157 6.660348e+00
## 30410  5 2158 6.621609e+00
## 30411  5 2159 6.604936e+00
## 30412  5 2160 6.592595e+00
## 30413  5 2161 6.800637e+00
## 30414  5 2162 6.883544e+00
## 30415  5 2163 6.926858e+00
## 30416  5 2164 6.923755e+00
## 30417  5 2165 6.914638e+00
## 30418  5 2166 6.903232e+00
## 30419  5 2167 6.896410e+00
## 30420  5 2168 6.866341e+00
## 30421  5 2169 6.837280e+00
## 30422  5 2170 6.833432e+00
## 30423  5 2171 6.816820e+00
## 30424  5 2172 6.800612e+00
## 30425  5 2173 6.775434e+00
## 30426  5 2174 6.762515e+00
## 30427  5 2175 6.737431e+00
## 30428  5 2176 6.717229e+00
## 30429  5 2177 6.713537e+00
## 30430  5 2178 6.691665e+00
## 30431  5 2179 6.676398e+00
## 30432  5 2180 6.647178e+00
## 30433  5 2181 6.648132e+00
## 30434  5 2182 6.627391e+00
## 30435  5 2183 6.615761e+00
## 30436  5 2184 6.599129e+00
## 30437  5 2184 6.587441e+00
## 30438  5 2185 6.802590e+00
## 30439  5 2186 6.878296e+00
## 30440  5 2187 6.922891e+00
## 30441  5 2188 6.923009e+00
## 30442  5 2189 6.901539e+00
## 30443  5 2190 6.892824e+00
## 30444  5 2191 6.881830e+00
## 30445  5 2192 6.858238e+00
## 30446  5 2193 6.844999e+00
## 30447  5 2194 6.817187e+00
## 30448  5 2195 6.800716e+00
## 30449  5 2196 6.793789e+00
## 30450  5 2197 6.767961e+00
## 30451  5 2198 6.765563e+00
## 30452  5 2199 6.737985e+00
## 30453  5 2200 6.716828e+00
## 30454  5 2201 6.705612e+00
## 30455  5 2202 6.678587e+00
## 30456  5 2203 6.673906e+00
## 30457  5 2204 6.651898e+00
## 30458  5 2205 6.642694e+00
## 30459  5 2206 6.639520e+00
## 30460  5 2207 6.609160e+00
## 30461  5 2208 6.599492e+00
## 30462  5 2208 6.603417e+00
## 30463  5 2209 6.585712e+00
## 30464  5 2210 6.555518e+00
## 30465  5 2211 6.550969e+00
## 30466  5 2212 6.541868e+00
## 30467  5 2213 6.512923e+00
## 30468  5 2214 6.500870e+00
## 30469  5 2215 6.491643e+00
## 30470  5 2216 6.478850e+00
## 30471  5 2217 6.465224e+00
## 30472  5 2218 6.451812e+00
## 30473  5 2219 6.429625e+00
## 30474  5 2220 6.431623e+00
## 30475  5 2221 6.401449e+00
## 30476  5 2222 6.407981e+00
## 30477  5 2223 6.381512e+00
## 30478  5 2224 6.360025e+00
## 30479  5 2225 6.357794e+00
## 30480  5 2226 6.349782e+00
## 30481  5 2226 6.349813e+00
## 30482  5 2227 6.449999e+00
## 30483  5 2228 6.482948e+00
## 30484  5 2229 6.496847e+00
## 30485  5 2230 6.487150e+00
## 30486  5 2231 6.491350e+00
## 30487  5 2232 6.470159e+00
## 30488  5 2232 6.461901e+00
## 30489  5 2233 6.675643e+00
## 30490  5 2234 6.775431e+00
## 30491  5 2235 6.798750e+00
## 30492  5 2236 6.795441e+00
## 30493  5 2237 6.781949e+00
## 30494  5 2238 6.788290e+00
## 30495  5 2239 6.753155e+00
## 30496  5 2240 6.745464e+00
## 30497  5 2241 6.734208e+00
## 30498  5 2242 6.717760e+00
## 30499  5 2243 6.687223e+00
## 30500  5 2244 6.671518e+00
## 30501  5 2245 6.647045e+00
## 30502  5 2246 6.644740e+00
## 30503  5 2247 6.622438e+00
## 30504  5 2248 6.601487e+00
## 30505  5 2249 6.585230e+00
## 30506  5 2250 6.567484e+00
## 30507  5 2251 6.564530e+00
## 30508  5 2252 6.548266e+00
## 30509  5 2253 6.528977e+00
## 30510  5 2254 6.520386e+00
## 30511  5 2255 6.493434e+00
## 30512  5 2256 6.483108e+00
## 30513  5 2257 6.673387e+00
## 30514  5 2258 6.775266e+00
## 30515  5 2259 6.811652e+00
## 30516  5 2260 6.814962e+00
## 30517  5 2261 6.809675e+00
## 30518  5 2262 6.803101e+00
## 30519  5 2263 6.778763e+00
## 30520  5 2264 6.747888e+00
## 30521  5 2265 6.733606e+00
## 30522  5 2266 6.718571e+00
## 30523  5 2267 6.699807e+00
## 30524  5 2268 6.683595e+00
## 30525  5 2269 6.658092e+00
## 30526  5 2270 6.651929e+00
## 30527  5 2271 6.636630e+00
## 30528  5 2272 6.618143e+00
## 30529  5 2273 6.606818e+00
## 30530  5 2274 6.591362e+00
## 30531  5 2275 6.575340e+00
## 30532  5 2276 6.546531e+00
## 30533  5 2277 6.530477e+00
## 30534  5 2278 6.514219e+00
## 30535  5 2279 6.494204e+00
## 30536  5 2280 6.482853e+00
## 30537  5 2281 6.689268e+00
## 30538  5 2282 6.780925e+00
## 30539  5 2283 6.808298e+00
## 30540  5 2284 6.818585e+00
## 30541  5 2285 6.817470e+00
## 30542  5 2286 6.800704e+00
## 30543  5 2287 6.775975e+00
## 30544  5 2288 6.754901e+00
## 30545  5 2289 6.746516e+00
## 30546  5 2290 6.736226e+00
## 30547  5 2291 6.710651e+00
## 30548  5 2292 6.683133e+00
## 30549  5 2293 6.673788e+00
## 30550  5 2294 6.661882e+00
## 30551  5 2295 6.640773e+00
## 30552  5 2296 6.611506e+00
## 30553  5 2297 6.601490e+00
## 30554  5 2298 6.580791e+00
## 30555  5 2299 6.558622e+00
## 30556  5 2300 6.551161e+00
## 30557  5 2301 6.539440e+00
## 30558  5 2302 6.518138e+00
## 30559  5 2303 6.516630e+00
## 30560  5 2304 6.504316e+00
## 30561  5 2305 6.694411e+00
## 30562  5 2306 6.795803e+00
## 30563  5 2307 6.817635e+00
## 30564  5 2308 6.821306e+00
## 30565  5 2309 6.812274e+00
## 30566  5 2310 6.789653e+00
## 30567  5 2311 6.784969e+00
## 30568  5 2312 6.761544e+00
## 30569  5 2313 6.732806e+00
## 30570  5 2314 6.738684e+00
## 30571  5 2315 6.712327e+00
## 30572  5 2316 6.690167e+00
## 30573  5 2317 6.673328e+00
## 30574  5 2318 6.665045e+00
## 30575  5 2319 6.639654e+00
## 30576  5 2320 6.622734e+00
## 30577  5 2321 6.597943e+00
## 30578  5 2322 6.592444e+00
## 30579  5 2323 6.580879e+00
## 30580  5 2324 6.566627e+00
## 30581  5 2325 6.540314e+00
## 30582  5 2326 6.530232e+00
## 30583  5 2327 6.513309e+00
## 30584  5 2328 6.499886e+00
## 30585  5 2329 6.674487e+00
## 30586  5 2330 6.782200e+00
## 30587  5 2331 6.813756e+00
## 30588  5 2332 6.819798e+00
## 30589  5 2333 6.816450e+00
## 30590  5 2334 6.802716e+00
## 30591  5 2335 6.788808e+00
## 30592  5 2336 6.771082e+00
## 30593  5 2337 6.745963e+00
## 30594  5 2338 6.740772e+00
## 30595  5 2339 6.714476e+00
## 30596  5 2340 6.691492e+00
## 30597  5 2341 6.685169e+00
## 30598  5 2342 6.662876e+00
## 30599  5 2343 6.633930e+00
## 30600  5 2344 6.626401e+00
## 30601  5 2345 6.618308e+00
## 30602  5 2346 6.594106e+00
## 30603  5 2347 6.570770e+00
## 30604  5 2348 6.563630e+00
## 30605  5 2349 6.536727e+00
## 30606  5 2350 6.538489e+00
## 30607  5 2351 6.524461e+00
## 30608  5 2352 6.492268e+00
## 30609  5 2353 6.692385e+00
## 30610  5 2354 6.791844e+00
## 30611  5 2355 6.825571e+00
## 30612  5 2356 6.830001e+00
## 30613  5 2357 6.839685e+00
## 30614  5 2358 6.798556e+00
## 30615  5 2359 6.797215e+00
## 30616  5 2360 6.772815e+00
## 30617  5 2361 6.757155e+00
## 30618  5 2362 6.734728e+00
## 30619  5 2363 6.716741e+00
## 30620  5 2364 6.712716e+00
## 30621  5 2365 6.677368e+00
## 30622  5 2366 6.674706e+00
## 30623  5 2367 6.648662e+00
## 30624  5 2368 6.632037e+00
## 30625  5 2369 6.616019e+00
## 30626  5 2370 6.587152e+00
## 30627  5 2371 6.593812e+00
## 30628  5 2372 6.572531e+00
## 30629  5 2373 6.547554e+00
## 30630  5 2374 6.531294e+00
## 30631  5 2375 6.520765e+00
## 30632  5 2376 6.511653e+00
## 30633  5 2377 6.699969e+00
## 30634  5 2378 6.794757e+00
## 30635  5 2379 6.826159e+00
## 30636  5 2380 6.836012e+00
## 30637  5 2381 6.834422e+00
## 30638  5 2382 6.799237e+00
## 30639  5 2383 6.798661e+00
## 30640  5 2384 6.769662e+00
## 30641  5 2385 6.764540e+00
## 30642  5 2386 6.745148e+00
## 30643  5 2387 6.723359e+00
## 30644  5 2388 6.708864e+00
## 30645  5 2389 6.682507e+00
## 30646  5 2390 6.671021e+00
## 30647  5 2391 6.661623e+00
## 30648  5 2392 6.635696e+00
## 30649  5 2393 6.622340e+00
## 30650  5 2394 6.600086e+00
## 30651  5 2395 6.605049e+00
## 30652  5 2396 6.560933e+00
## 30653  5 2397 6.557498e+00
## 30654  5 2398 6.543629e+00
## 30655  5 2399 6.515010e+00
## 30656  5 2400 6.502321e+00
## 30657  5 2401 6.693661e+00
## 30658  5 2402 6.787937e+00
## 30659  5 2403 6.843517e+00
## 30660  5 2404 6.835509e+00
## 30661  5 2405 6.820614e+00
## 30662  5 2406 6.823641e+00
## 30663  5 2407 6.804190e+00
## 30664  5 2408 6.780523e+00
## 30665  5 2409 6.769214e+00
## 30666  5 2410 6.737283e+00
## 30667  5 2411 6.708975e+00
## 30668  5 2412 6.705259e+00
## 30669  5 2413 6.691291e+00
## 30670  5 2414 6.671018e+00
## 30671  5 2415 6.656673e+00
## 30672  5 2416 6.641584e+00
## 30673  5 2417 6.613532e+00
## 30674  5 2418 6.612900e+00
## 30675  5 2419 6.595502e+00
## 30676  5 2420 6.563866e+00
## 30677  5 2421 6.555836e+00
## 30678  5 2422 6.545979e+00
## 30679  5 2423 6.524222e+00
## 30680  5 2424 6.504078e+00
## 30681  5 2425 6.704680e+00
## 30682  5 2426 6.809378e+00
## 30683  5 2427 6.836283e+00
## 30684  5 2428 6.846655e+00
## 30685  5 2429 6.825586e+00
## 30686  5 2430 6.810864e+00
## 30687  5 2431 6.807813e+00
## 30688  5 2432 6.782179e+00
## 30689  5 2433 6.755457e+00
## 30690  5 2434 6.747094e+00
## 30691  5 2435 6.738536e+00
## 30692  5 2436 6.701824e+00
## 30693  5 2437 6.700947e+00
## 30694  5 2438 6.672910e+00
## 30695  5 2439 6.644539e+00
## 30696  5 2440 6.638711e+00
## 30697  5 2441 6.625535e+00
## 30698  5 2442 6.606661e+00
## 30699  5 2443 6.594139e+00
## 30700  5 2444 6.578311e+00
## 30701  5 2445 6.567159e+00
## 30702  5 2446 6.553387e+00
## 30703  5 2447 6.526513e+00
## 30704  5 2448 6.518551e+00
## 30705  5 2449 6.702029e+00
## 30706  5 2450 6.795443e+00
## 30707  5 2451 6.841581e+00
## 30708  5 2452 6.851231e+00
## 30709  5 2453 6.820201e+00
## 30710  5 2454 6.812867e+00
## 30711  5 2455 6.804843e+00
## 30712  5 2456 6.787124e+00
## 30713  5 2457 6.754587e+00
## 30714  5 2458 6.753767e+00
## 30715  5 2459 6.727535e+00
## 30716  5 2460 6.720199e+00
## 30717  5 2461 6.685834e+00
## 30718  5 2462 6.667915e+00
## 30719  5 2463 6.655860e+00
## 30720  5 2464 6.649945e+00
## 30721  5 2465 6.639212e+00
## 30722  5 2466 6.619307e+00
## 30723  5 2467 6.593559e+00
## 30724  5 2468 6.581765e+00
## 30725  5 2469 6.564143e+00
## 30726  5 2470 6.555825e+00
## 30727  5 2471 6.524033e+00
## 30728  5 2472 6.510722e+00
## 30729  5 2473 6.717312e+00
## 30730  5 2474 6.803383e+00
## 30731  5 2475 6.830207e+00
## 30732  5 2476 6.845523e+00
## 30733  5 2477 6.832111e+00
## 30734  5 2478 6.813269e+00
## 30735  5 2479 6.811751e+00
## 30736  5 2480 6.782914e+00
## 30737  5 2481 6.769390e+00
## 30738  5 2482 6.757545e+00
## 30739  5 2483 6.729446e+00
## 30740  5 2484 6.720811e+00
## 30741  5 2485 6.694951e+00
## 30742  5 2486 6.679379e+00
## 30743  5 2487 6.657146e+00
## 30744  5 2488 6.643191e+00
## 30745  5 2489 6.625455e+00
## 30746  5 2490 6.612238e+00
## 30747  5 2491 6.585929e+00
## 30748  5 2492 6.578077e+00
## 30749  5 2493 6.574716e+00
## 30750  5 2494 6.550394e+00
## 30751  5 2495 6.546753e+00
## 30752  5 2496 6.521789e+00
## 30753  5 2496 6.581519e+00
## 30754  5 2497 6.792580e+00
## 30755  5 2498 6.894248e+00
## 30756  5 2499 6.906820e+00
## 30757  5 2500 6.927097e+00
## 30758  5 2501 6.907536e+00
## 30759  5 2502 6.893123e+00
## 30760  5 2503 6.881953e+00
## 30761  5 2504 6.858805e+00
## 30762  5 2505 6.849809e+00
## 30763  5 2506 6.830856e+00
## 30764  5 2507 6.814524e+00
## 30765  5 2508 6.790739e+00
## 30766  5 2509 6.762935e+00
## 30767  5 2510 6.753557e+00
## 30768  5 2511 6.736866e+00
## 30769  5 2512 6.724317e+00
## 30770  5 2513 6.694954e+00
## 30771  5 2514 6.684623e+00
## 30772  5 2515 6.665230e+00
## 30773  5 2516 6.664876e+00
## 30774  5 2517 6.656998e+00
## 30775  5 2518 6.623431e+00
## 30776  5 2519 6.609381e+00
## 30777  5 2520 6.595313e+00
## 30778  5 2520 6.586392e+00
## 30779  5 2521 6.579992e+00
## 30780  5 2522 6.567009e+00
## 30781  5 2523 6.554196e+00
## 30782  5 2524 6.538848e+00
## 30783  5 2525 6.515153e+00
## 30784  5 2526 6.501667e+00
## 30785  5 2527 6.487403e+00
## 30786  5 2528 6.481776e+00
## 30787  5 2529 6.454267e+00
## 30788  5 2530 6.455400e+00
## 30789  5 2531 6.432547e+00
## 30790  5 2532 6.424418e+00
## 30791  5 2533 6.418976e+00
## 30792  5 2534 6.407348e+00
## 30793  5 2535 6.368971e+00
## 30794  5 2536 6.370545e+00
## 30795  5 2537 6.367204e+00
## 30796  5 2538 6.347768e+00
## 30797  5 2538 6.337067e+00
## 30798  5 2539 6.550670e+00
## 30799  5 2540 6.656661e+00
## 30800  5 2541 6.679406e+00
## 30801  5 2542 6.686741e+00
## 30802  5 2543 6.669315e+00
## 30803  5 2544 6.657947e+00
## 30804  5 2544 6.660961e+00
## 30805  5 2545 6.861293e+00
## 30806  5 2546 6.951526e+00
## 30807  5 2547 7.003025e+00
## 30808  5 2548 6.986986e+00
## 30809  5 2549 6.982063e+00
## 30810  5 2550 6.957232e+00
## 30811  5 2551 6.950433e+00
## 30812  5 2552 6.929496e+00
## 30813  5 2553 6.906984e+00
## 30814  5 2554 6.888359e+00
## 30815  5 2555 6.874051e+00
## 30816  5 2556 6.836416e+00
## 30817  5 2557 6.814615e+00
## 30818  5 2558 6.805132e+00
## 30819  5 2559 6.792088e+00
## 30820  5 2560 6.778586e+00
## 30821  5 2561 6.752976e+00
## 30822  5 2562 6.728697e+00
## 30823  5 2563 6.712523e+00
## 30824  5 2564 6.701682e+00
## 30825  5 2565 6.688790e+00
## 30826  5 2566 6.668595e+00
## 30827  5 2567 6.654247e+00
## 30828  5 2568 6.637684e+00
## 30829  5 2569 6.824956e+00
## 30830  5 2570 6.915089e+00
## 30831  5 2571 6.951990e+00
## 30832  5 2572 6.961313e+00
## 30833  5 2573 6.954202e+00
## 30834  5 2574 6.932479e+00
## 30835  5 2575 6.924447e+00
## 30836  5 2576 6.889356e+00
## 30837  5 2577 6.878910e+00
## 30838  5 2578 6.860943e+00
## 30839  5 2579 6.836923e+00
## 30840  5 2580 6.822955e+00
## 30841  5 2581 6.808191e+00
## 30842  5 2582 6.792222e+00
## 30843  5 2583 6.765623e+00
## 30844  5 2584 6.747606e+00
## 30845  5 2585 6.729455e+00
## 30846  5 2586 6.724195e+00
## 30847  5 2587 6.702820e+00
## 30848  5 2588 6.683286e+00
## 30849  5 2589 6.662698e+00
## 30850  5 2590 6.654814e+00
## 30851  5 2591 6.630169e+00
## 30852  5 2592 6.609040e+00
## 30853  5 2593 6.823645e+00
## 30854  5 2594 6.908585e+00
## 30855  5 2595 6.935724e+00
## 30856  5 2596 6.938165e+00
## 30857  5 2597 6.938478e+00
## 30858  5 2598 6.921581e+00
## 30859  5 2599 6.905357e+00
## 30860  5 2600 6.888909e+00
## 30861  5 2601 6.869722e+00
## 30862  5 2602 6.848136e+00
## 30863  5 2603 6.811491e+00
## 30864  5 2604 6.804250e+00
## 30865  5 2605 6.788608e+00
## 30866  5 2606 6.760792e+00
## 30867  5 2607 6.744412e+00
## 30868  5 2608 6.734033e+00
## 30869  5 2609 6.722819e+00
## 30870  5 2610 6.710213e+00
## 30871  5 2611 6.689490e+00
## 30872  5 2612 6.676689e+00
## 30873  5 2613 6.649067e+00
## 30874  5 2614 6.644253e+00
## 30875  5 2615 6.631736e+00
## 30876  5 2616 6.600248e+00
## 30877  5 2617 6.804476e+00
## 30878  5 2618 6.893119e+00
## 30879  5 2619 6.928891e+00
## 30880  5 2620 6.933291e+00
## 30881  5 2621 6.933745e+00
## 30882  5 2622 6.910066e+00
## 30883  5 2623 6.898623e+00
## 30884  5 2624 6.866559e+00
## 30885  5 2625 6.868898e+00
## 30886  5 2626 6.835393e+00
## 30887  5 2627 6.818958e+00
## 30888  5 2628 6.803905e+00
## 30889  5 2629 6.771042e+00
## 30890  5 2630 6.770889e+00
## 30891  5 2631 6.755775e+00
## 30892  5 2632 6.733995e+00
## 30893  5 2633 6.698358e+00
## 30894  5 2634 6.687968e+00
## 30895  5 2635 6.688518e+00
## 30896  5 2636 6.664704e+00
## 30897  5 2637 6.648631e+00
## 30898  5 2638 6.632070e+00
## 30899  5 2639 6.627973e+00
## 30900  5 2640 6.605016e+00
## 30901  5 2641 6.789148e+00
## 30902  5 2642 6.887172e+00
## 30903  5 2643 6.930708e+00
## 30904  5 2644 6.935680e+00
## 30905  5 2645 6.920957e+00
## 30906  5 2646 6.903679e+00
## 30907  5 2647 6.883597e+00
## 30908  5 2648 6.866767e+00
## 30909  5 2649 6.836737e+00
## 30910  5 2650 6.836139e+00
## 30911  5 2651 6.809815e+00
## 30912  5 2652 6.811747e+00
## 30913  5 2653 6.784266e+00
## 30914  5 2654 6.769662e+00
## 30915  5 2655 6.744423e+00
## 30916  5 2656 6.736502e+00
## 30917  5 2657 6.713085e+00
## 30918  5 2658 6.693314e+00
## 30919  5 2659 6.677838e+00
## 30920  5 2660 6.660901e+00
## 30921  5 2661 6.642540e+00
## 30922  5 2662 6.638824e+00
## 30923  5 2663 6.607441e+00
## 30924  5 2664 6.605842e+00
## 30925  5 2665 6.794391e+00
## 30926  5 2666 6.884394e+00
## 30927  5 2667 6.911457e+00
## 30928  5 2668 6.931523e+00
## 30929  5 2669 6.925528e+00
## 30930  5 2670 6.891827e+00
## 30931  5 2671 6.888436e+00
## 30932  5 2672 6.875386e+00
## 30933  5 2673 6.853025e+00
## 30934  5 2674 6.835751e+00
## 30935  5 2675 6.808327e+00
## 30936  5 2676 6.813840e+00
## 30937  5 2677 6.784936e+00
## 30938  5 2678 6.765992e+00
## 30939  5 2679 6.729383e+00
## 30940  5 2680 6.734210e+00
## 30941  5 2681 6.705398e+00
## 30942  5 2682 6.695142e+00
## 30943  5 2683 6.684103e+00
## 30944  5 2684 6.658690e+00
## 30945  5 2685 6.640801e+00
## 30946  5 2686 6.625553e+00
## 30947  5 2687 6.607937e+00
## 30948  5 2688 6.615741e+00
## 30949  5 2689 6.789306e+00
## 30950  5 2690 6.880391e+00
## 30951  5 2691 6.919626e+00
## 30952  5 2692 6.939832e+00
## 30953  5 2693 6.921200e+00
## 30954  5 2694 6.910151e+00
## 30955  5 2695 6.884555e+00
## 30956  5 2696 6.861031e+00
## 30957  5 2697 6.842061e+00
## 30958  5 2698 6.835287e+00
## 30959  5 2699 6.819049e+00
## 30960  5 2700 6.796673e+00
## 30961  5 2701 6.762848e+00
## 30962  5 2702 6.752682e+00
## 30963  5 2703 6.744680e+00
## 30964  5 2704 6.728403e+00
## 30965  5 2705 6.714527e+00
## 30966  5 2706 6.686822e+00
## 30967  5 2707 6.680178e+00
## 30968  5 2708 6.653211e+00
## 30969  5 2709 6.637994e+00
## 30970  5 2710 6.635709e+00
## 30971  5 2711 6.606497e+00
## 30972  5 2712 6.599183e+00
## 30973  5 2713 6.793128e+00
## 30974  5 2714 6.874357e+00
## 30975  5 2715 6.925306e+00
## 30976  5 2716 6.927771e+00
## 30977  5 2717 6.922524e+00
## 30978  5 2718 6.905024e+00
## 30979  5 2719 6.868071e+00
## 30980  5 2720 6.868289e+00
## 30981  5 2721 6.841554e+00
## 30982  5 2722 6.835937e+00
## 30983  5 2723 6.815878e+00
## 30984  5 2724 6.782791e+00
## 30985  5 2725 6.780144e+00
## 30986  5 2726 6.758375e+00
## 30987  5 2727 6.732912e+00
## 30988  5 2728 6.740760e+00
## 30989  5 2729 6.711838e+00
## 30990  5 2730 6.689452e+00
## 30991  5 2731 6.677441e+00
## 30992  5 2732 6.658337e+00
## 30993  5 2733 6.639983e+00
## 30994  5 2734 6.629387e+00
## 30995  5 2735 6.617222e+00
## 30996  5 2736 6.584482e+00
## 30997  5 2737 6.805696e+00
## 30998  5 2738 6.880580e+00
## 30999  5 2739 6.927673e+00
## 31000  5 2740 6.933864e+00
## 31001  5 2741 6.909860e+00
## 31002  5 2742 6.895928e+00
## 31003  5 2743 6.884773e+00
## 31004  5 2744 6.856590e+00
## 31005  5 2745 6.848168e+00
## 31006  5 2746 6.833221e+00
## 31007  5 2747 6.821752e+00
## 31008  5 2748 6.798966e+00
## 31009  5 2749 6.761786e+00
## 31010  5 2750 6.756418e+00
## 31011  5 2751 6.744928e+00
## 31012  5 2752 6.731698e+00
## 31013  5 2753 6.710272e+00
## 31014  5 2754 6.692688e+00
## 31015  5 2755 6.684066e+00
## 31016  5 2756 6.667327e+00
## 31017  5 2757 6.641872e+00
## 31018  5 2758 6.624153e+00
## 31019  5 2759 6.603434e+00
## 31020  5 2760 6.592811e+00
## 31021  5 2761 6.799731e+00
## 31022  5 2762 6.882797e+00
## 31023  5 2763 6.917922e+00
## 31024  5 2764 6.924103e+00
## 31025  5 2765 6.912858e+00
## 31026  5 2766 6.910507e+00
## 31027  5 2767 6.904049e+00
## 31028  5 2768 6.868256e+00
## 31029  5 2769 6.843448e+00
## 31030  5 2770 6.825169e+00
## 31031  5 2771 6.812443e+00
## 31032  5 2772 6.784822e+00
## 31033  5 2773 6.779274e+00
## 31034  5 2774 6.769132e+00
## 31035  5 2775 6.743870e+00
## 31036  5 2776 6.726043e+00
## 31037  5 2777 6.711133e+00
## 31038  5 2778 6.697444e+00
## 31039  5 2779 6.665759e+00
## 31040  5 2780 6.662933e+00
## 31041  5 2781 6.653752e+00
## 31042  5 2782 6.642389e+00
## 31043  5 2783 6.617341e+00
## 31044  5 2784 6.589063e+00
## 31045  5 2785 6.800184e+00
## 31046  5 2786 6.883386e+00
## 31047  5 2787 6.917153e+00
## 31048  5 2788 6.925244e+00
## 31049  5 2789 6.903694e+00
## 31050  5 2790 6.908945e+00
## 31051  5 2791 6.901549e+00
## 31052  5 2792 6.863235e+00
## 31053  5 2793 6.853386e+00
## 31054  5 2794 6.830568e+00
## 31055  5 2795 6.808675e+00
## 31056  5 2796 6.790097e+00
## 31057  5 2797 6.787069e+00
## 31058  5 2798 6.759616e+00
## 31059  5 2799 6.754175e+00
## 31060  5 2800 6.729072e+00
## 31061  5 2801 6.715784e+00
## 31062  5 2802 6.687474e+00
## 31063  5 2803 6.678116e+00
## 31064  5 2804 6.662538e+00
## 31065  5 2805 6.643936e+00
## 31066  5 2806 6.642904e+00
## 31067  5 2807 6.610266e+00
## 31068  5 2808 6.593002e+00
## 31069  5 2808 6.592571e+00
## 31070  5 2809 6.786621e+00
## 31071  5 2810 6.885283e+00
## 31072  5 2811 6.906665e+00
## 31073  5 2812 6.922878e+00
```

```
## 31074  5 2813 6.913990e+00
## 31075  5 2814 6.899590e+00
## 31076  5 2815 6.888590e+00
## 31077  5 2816 6.866203e+00
## 31078  5 2817 6.852196e+00
## 31079  5 2818 6.830566e+00
## 31080  5 2819 6.796933e+00
## 31081  5 2820 6.791223e+00
## 31082  5 2821 6.767941e+00
## 31083  5 2822 6.747907e+00
## 31084  5 2823 6.744435e+00
## 31085  5 2824 6.723594e+00
## 31086  5 2825 6.696181e+00
## 31087  5 2826 6.680708e+00
## 31088  5 2827 6.667206e+00
## 31089  5 2828 6.650976e+00
## 31090  5 2829 6.636231e+00
## 31091  5 2830 6.631467e+00
## 31092  5 2831 6.609257e+00
## 31093  5 2832 6.585806e+00
## 31094  5 2832 6.597418e+00
## 31095  5 2833 6.570710e+00
## 31096  5 2834 6.565086e+00
## 31097  5 2835 6.548571e+00
## 31098  5 2836 6.532835e+00
## 31099  5 2837 6.506091e+00
## 31100  5 2838 6.505138e+00
## 31101  5 2839 6.497161e+00
## 31102  5 2840 6.467940e+00
## 31103  5 2841 6.457490e+00
## 31104  5 2842 6.450742e+00
## 31105  5 2843 6.435874e+00
## 31106  5 2844 6.427010e+00
## 31107  5 2845 6.406129e+00
## 31108  5 2846 6.402576e+00
## 31109  5 2847 6.394793e+00
## 31110  5 2848 6.371320e+00
## 31111  5 2849 6.361303e+00
## 31112  5 2850 6.346399e+00
## 31113  5 2851 6.324557e+00
## 31114  5 2852 6.325213e+00
## 31115  5 2853 6.306787e+00
## 31116  5 2853 6.300103e+00
## 31117  5 2854 6.405843e+00
## 31118  5 2855 6.440905e+00
## 31119  5 2856 6.449388e+00
## 31120  5 2856 6.457042e+00
## 31121  5 2857 6.673360e+00
## 31122  5 2858 6.765947e+00
## 31123  5 2859 6.808237e+00
## 31124  5 2860 6.811711e+00
## 31125  5 2861 6.801736e+00
## 31126  5 2862 6.772844e+00
## 31127  5 2863 6.757459e+00
## 31128  5 2864 6.758234e+00
## 31129  5 2865 6.722437e+00
## 31130  5 2866 6.708718e+00
## 31131  5 2867 6.709834e+00
## 31132  5 2868 6.673135e+00
## 31133  5 2869 6.666983e+00
## 31134  5 2870 6.641945e+00
## 31135  5 2871 6.628804e+00
## 31136  5 2872 6.619962e+00
## 31137  5 2873 6.587549e+00
## 31138  5 2874 6.572794e+00
## 31139  5 2875 6.574240e+00
## 31140  5 2876 6.548989e+00
## 31141  5 2877 6.533335e+00
## 31142  5 2878 6.500479e+00
## 31143  5 2879 6.499364e+00
## 31144  5 2880 6.483208e+00
## 31145  5 2881 6.687559e+00
## 31146  5 2882 6.774557e+00
## 31147  5 2883 6.804548e+00
## 31148  5 2884 6.813417e+00
## 31149  5 2885 6.805696e+00
## 31150  5 2886 6.784150e+00
## 31151  5 2887 6.777474e+00
## 31152  5 2888 6.770996e+00
## 31153  5 2889 6.729044e+00
## 31154  5 2890 6.718210e+00
## 31155  5 2891 6.699511e+00
## 31156  5 2892 6.682132e+00
## 31157  5 2893 6.673636e+00
## 31158  5 2894 6.666252e+00
## 31159  5 2895 6.627383e+00
## 31160  5 2896 6.617704e+00
## 31161  5 2897 6.602595e+00
## 31162  5 2898 6.593576e+00
## 31163  5 2899 6.565278e+00
## 31164  5 2900 6.561852e+00
## 31165  5 2901 6.543931e+00
## 31166  5 2902 6.532403e+00
## 31167  5 2903 6.506552e+00
## 31168  5 2904 6.492261e+00
## 31169  5 2905 6.694969e+00
## 31170  5 2906 6.788487e+00
## 31171  5 2907 6.807439e+00
## 31172  5 2908 6.809322e+00
## 31173  5 2909 6.812429e+00
## 31174  5 2910 6.806200e+00
## 31175  5 2911 6.790913e+00
## 31176  5 2912 6.768412e+00
## 31177  5 2913 6.740856e+00
## 31178  5 2914 6.720536e+00
## 31179  5 2915 6.708845e+00
## 31180  5 2916 6.702227e+00
## 31181  5 2917 6.673527e+00
## 31182  5 2918 6.657336e+00
## 31183  5 2919 6.634459e+00
## 31184  5 2920 6.624590e+00
## 31185  5 2921 6.603650e+00
## 31186  5 2922 6.583237e+00
## 31187  5 2923 6.569435e+00
## 31188  5 2924 6.553414e+00
## 31189  5 2925 6.528598e+00
## 31190  5 2926 6.519836e+00
## 31191  5 2927 6.509724e+00
## 31192  5 2928 6.492632e+00
## 31193  5 2929 6.695528e+00
## 31194  5 2930 6.780646e+00
## 31195  5 2931 6.831224e+00
## 31196  5 2932 6.821277e+00
## 31197  5 2933 6.820896e+00
## 31198  5 2934 6.797178e+00
## 31199  5 2935 6.767592e+00
## 31200  5 2936 6.762487e+00
## 31201  5 2937 6.741147e+00
## 31202  5 2938 6.735008e+00
## 31203  5 2939 6.726704e+00
## 31204  5 2940 6.704328e+00
## 31205  5 2941 6.675819e+00
## 31206  5 2942 6.672896e+00
## 31207  5 2943 6.633421e+00
## 31208  5 2944 6.628248e+00
## 31209  5 2945 6.607667e+00
## 31210  5 2946 6.601785e+00
## 31211  5 2947 6.579370e+00
## 31212  5 2948 6.573032e+00
## 31213  5 2949 6.548099e+00
## 31214  5 2950 6.526469e+00
## 31215  5 2951 6.517275e+00
## 31216  5 2952 6.503618e+00
## 31217  5 2953 6.696893e+00
## 31218  5 2954 6.789075e+00
## 31219  5 2955 6.811434e+00
## 31220  5 2956 6.824641e+00
## 31221  5 2957 6.819170e+00
## 31222  5 2958 6.804621e+00
## 31223  5 2959 6.785134e+00
## 31224  5 2960 6.778590e+00
## 31225  5 2961 6.744351e+00
## 31226  5 2962 6.742335e+00
## 31227  5 2963 6.706819e+00
## 31228  5 2964 6.707567e+00
## 31229  5 2965 6.691318e+00
## 31230  5 2966 6.660270e+00
## 31231  5 2967 6.660252e+00
## 31232  5 2968 6.634617e+00
## 31233  5 2969 6.615747e+00
## 31234  5 2970 6.582678e+00
## 31235  5 2971 6.585357e+00
## 31236  5 2972 6.567912e+00
## 31237  5 2973 6.553566e+00
## 31238  5 2974 6.540537e+00
## 31239  5 2975 6.516201e+00
## 31240  5 2976 6.499010e+00
## 31241  5 2977 6.703983e+00
## 31242  5 2978 6.794166e+00
## 31243  5 2979 6.828104e+00
## 31244  5 2980 6.825128e+00
## 31245  5 2981 6.825500e+00
## 31246  5 2982 6.805750e+00
## 31247  5 2983 6.788430e+00
## 31248  5 2984 6.773778e+00
## 31249  5 2985 6.752456e+00
## 31250  5 2986 6.748608e+00
## 31251  5 2987 6.724468e+00
## 31252  5 2988 6.719047e+00
## 31253  5 2989 6.681160e+00
## 31254  5 2990 6.667277e+00
## 31255  5 2991 6.631168e+00
## 31256  5 2992 6.632412e+00
## 31257  5 2993 6.616075e+00
## 31258  5 2994 6.599511e+00
## 31259  5 2995 6.583693e+00
## 31260  5 2996 6.561274e+00
## 31261  5 2997 6.546811e+00
## 31262  5 2998 6.530645e+00
## 31263  5 2999 6.514129e+00
## 31264  5 3000 6.515081e+00
## 31265  5 3001 6.691930e+00
## 31266  5 3002 6.791657e+00
## 31267  5 3003 6.832388e+00
## 31268  5 3004 6.829045e+00
## 31269  5 3005 6.816967e+00
## 31270  5 3006 6.816512e+00
## 31271  5 3007 6.801218e+00
## 31272  5 3008 6.775030e+00
## 31273  5 3009 6.756431e+00
## 31274  5 3010 6.739751e+00
## 31275  5 3011 6.730056e+00
## 31276  5 3012 6.698305e+00
## 31277  5 3013 6.692691e+00
## 31278  5 3014 6.676747e+00
## 31279  5 3015 6.655852e+00
## 31280  5 3016 6.634969e+00
## 31281  5 3017 6.623290e+00
## 31282  5 3018 6.606364e+00
## 31283  5 3019 6.587783e+00
## 31284  5 3020 6.565866e+00
## 31285  5 3021 6.562132e+00
## 31286  5 3022 6.534971e+00
## 31287  5 3023 6.526739e+00
## 31288  5 3024 6.510956e+00
## 31289  5 3025 6.702847e+00
## 31290  5 3026 6.801894e+00
## 31291  5 3027 6.810149e+00
## 31292  5 3028 6.836039e+00
## 31293  5 3029 6.842283e+00
## 31294  5 3030 6.819052e+00
## 31295  5 3031 6.799598e+00
## 31296  5 3032 6.779220e+00
## 31297  5 3033 6.756795e+00
## 31298  5 3034 6.741614e+00
## 31299  5 3035 6.727430e+00
## 31300  5 3036 6.699618e+00
## 31301  5 3037 6.682392e+00
## 31302  5 3038 6.667476e+00
## 31303  5 3039 6.672299e+00
## 31304  5 3040 6.647966e+00
## 31305  5 3041 6.628356e+00
## 31306  5 3042 6.606516e+00
## 31307  5 3043 6.582794e+00
## 31308  5 3044 6.563679e+00
## 31309  5 3045 6.550676e+00
## 31310  5 3046 6.546195e+00
## 31311  5 3047 6.527417e+00
## 31312  5 3048 6.511447e+00
## 31313  5 3049 6.717417e+00
## 31314  5 3050 6.804259e+00
## 31315  5 3051 6.817412e+00
## 31316  5 3052 6.844050e+00
## 31317  5 3053 6.827714e+00
## 31318  5 3054 6.827791e+00
## 31319  5 3055 6.798523e+00
## 31320  5 3056 6.771660e+00
## 31321  5 3057 6.753952e+00
## 31322  5 3058 6.753773e+00
## 31323  5 3059 6.724828e+00
## 31324  5 3060 6.705202e+00
## 31325  5 3061 6.696231e+00
## 31326  5 3062 6.669444e+00
## 31327  5 3063 6.658254e+00
## 31328  5 3064 6.628734e+00
## 31329  5 3065 6.625411e+00
## 31330  5 3066 6.616140e+00
## 31331  5 3067 6.592348e+00
## 31332  5 3068 6.581056e+00
## 31333  5 3069 6.550445e+00
## 31334  5 3070 6.542947e+00
## 31335  5 3071 6.524778e+00
## 31336  5 3072 6.519794e+00
## 31337  5 3073 6.712950e+00
## 31338  5 3074 6.817353e+00
## 31339  5 3075 6.840043e+00
## 31340  5 3076 6.842831e+00
## 31341  5 3077 6.839175e+00
## 31342  5 3078 6.818905e+00
## 31343  5 3079 6.799885e+00
## 31344  5 3080 6.787909e+00
## 31345  5 3081 6.770892e+00
## 31346  5 3082 6.744661e+00
## 31347  5 3083 6.728085e+00
## 31348  5 3084 6.720411e+00
## 31349  5 3085 6.689376e+00
## 31350  5 3086 6.680209e+00
## 31351  5 3087 6.652734e+00
## 31352  5 3088 6.647228e+00
## 31353  5 3089 6.628423e+00
## 31354  5 3090 6.607565e+00
## 31355  5 3091 6.602918e+00
## 31356  5 3092 6.575357e+00
## 31357  5 3093 6.578018e+00
## 31358  5 3094 6.557543e+00
## 31359  5 3095 6.533807e+00
## 31360  5 3096 6.513214e+00
## 31361  5 3097 6.712991e+00
## 31362  5 3098 6.813164e+00
## 31363  5 3099 6.847931e+00
## 31364  5 3100 6.853090e+00
## 31365  5 3101 6.831791e+00
## 31366  5 3102 6.810894e+00
## 31367  5 3103 6.809863e+00
## 31368  5 3104 6.776944e+00
## 31369  5 3105 6.769966e+00
## 31370  5 3106 6.747577e+00
## 31371  5 3107 6.729824e+00
## 31372  5 3108 6.714520e+00
## 31373  5 3109 6.695824e+00
## 31374  5 3110 6.672443e+00
## 31375  5 3111 6.672818e+00
## 31376  5 3112 6.653116e+00
## 31377  5 3113 6.639523e+00
## 31378  5 3114 6.617125e+00
## 31379  5 3115 6.590743e+00
## 31380  5 3116 6.577482e+00
## 31381  5 3117 6.571714e+00
## 31382  5 3118 6.552401e+00
## 31383  5 3119 6.533710e+00
## 31384  5 3120 6.516857e+00
## 31385  5 3120 6.597745e+00
## 31386  5 3121 6.795382e+00
## 31387  5 3122 6.892694e+00
## 31388  5 3123 6.917318e+00
## 31389  5 3124 6.922194e+00
## 31390  5 3125 6.924999e+00
## 31391  5 3126 6.898381e+00
## 31392  5 3127 6.886957e+00
## 31393  5 3128 6.851479e+00
## 31394  5 3129 6.826078e+00
## 31395  5 3130 6.826504e+00
## 31396  5 3131 6.799733e+00
## 31397  5 3132 6.786313e+00
## 31398  5 3133 6.758942e+00
## 31399  5 3134 6.746609e+00
## 31400  5 3135 6.738596e+00
## 31401  5 3136 6.730067e+00
## 31402  5 3137 6.703885e+00
## 31403  5 3138 6.692507e+00
## 31404  5 3139 6.674573e+00
## 31405  5 3140 6.651967e+00
## 31406  5 3141 6.642067e+00
## 31407  5 3142 6.632950e+00
## 31408  5 3143 6.598115e+00
## 31409  5 3144 6.590937e+00
## 31410  5 3144 6.586996e+00
## 31411  5 3145 6.577875e+00
## 31412  5 3146 6.568775e+00
## 31413  5 3147 6.547509e+00
## 31414  5 3148 6.527922e+00
## 31415  5 3149 6.515489e+00
## 31416  5 3150 6.494773e+00
## 31417  5 3151 6.483390e+00
## 31418  5 3152 6.478977e+00
## 31419  5 3153 6.452937e+00
## 31420  5 3154 6.447727e+00
## 31421  5 3155 6.447334e+00
## 31422  5 3156 6.415396e+00
## 31423  5 3157 6.410425e+00
## 31424  5 3158 6.381646e+00
## 31425  5 3159 6.388002e+00
## 31426  5 3160 6.366718e+00
## 31427  5 3161 6.353361e+00
## 31428  5 3162 6.344408e+00
## 31429  5 3163 6.344792e+00
## 31430  5 3164 6.323007e+00
## 31431  5 3165 6.315984e+00
## 31432  5 3165 6.303264e+00
## 31433  5 3166 6.505144e+00
## 31434  5 3167 6.601400e+00
## 31435  5 3168 6.640464e+00
## 31436  5 3168 6.654834e+00
## 31437  5 3169 6.874463e+00
## 31438  5 3170 6.966350e+00
## 31439  5 3171 7.003116e+00
## 31440  5 3172 6.997235e+00
## 31441  5 3173 6.992690e+00
## 31442  5 3174 6.976018e+00
## 31443  5 3175 6.958362e+00
## 31444  5 3176 6.923813e+00
## 31445  5 3177 6.916274e+00
## 31446  5 3178 6.897260e+00
## 31447  5 3179 6.881959e+00
## 31448  5 3180 6.856870e+00
## 31449  5 3181 6.830035e+00
## 31450  5 3182 6.815985e+00
## 31451  5 3183 6.803314e+00
## 31452  5 3184 6.787373e+00
## 31453  5 3185 6.760686e+00
## 31454  5 3186 6.748033e+00
## 31455  5 3187 6.706256e+00
## 31456  5 3188 6.705950e+00
## 31457  5 3189 6.704215e+00
## 31458  5 3190 6.669983e+00
## 31459  5 3191 6.654051e+00
## 31460  5 3192 6.650221e+00
## 31461  5 3193 6.838988e+00
## 31462  5 3194 6.936399e+00
## 31463  5 3195 6.949139e+00
## 31464  5 3196 6.956230e+00
## 31465  5 3197 6.947820e+00
## 31466  5 3198 6.929121e+00
## 31467  5 3199 6.917428e+00
## 31468  5 3200 6.898053e+00
## 31469  5 3201 6.882507e+00
## 31470  5 3202 6.855757e+00
## 31471  5 3203 6.843442e+00
## 31472  5 3204 6.830223e+00
## 31473  5 3205 6.817307e+00
## 31474  5 3206 6.798262e+00
## 31475  5 3207 6.775879e+00
## 31476  5 3208 6.761175e+00
## 31477  5 3209 6.734995e+00
## 31478  5 3210 6.717300e+00
## 31479  5 3211 6.695968e+00
## 31480  5 3212 6.688017e+00
## 31481  5 3213 6.670917e+00
## 31482  5 3214 6.650866e+00
## 31483  5 3215 6.642196e+00
## 31484  5 3216 6.613867e+00
## 31485  5 3217 6.812150e+00
## 31486  5 3218 6.901254e+00
## 31487  5 3219 6.938423e+00
## 31488  5 3220 6.948100e+00
## 31489  5 3221 6.933450e+00
## 31490  5 3222 6.918850e+00
## 31491  5 3223 6.899198e+00
## 31492  5 3224 6.884779e+00
## 31493  5 3225 6.860318e+00
## 31494  5 3226 6.834054e+00
## 31495  5 3227 6.825602e+00
## 31496  5 3228 6.814222e+00
## 31497  5 3229 6.777699e+00
## 31498  5 3230 6.783629e+00
## 31499  5 3231 6.759127e+00
## 31500  5 3232 6.727129e+00
## 31501  5 3233 6.726473e+00
## 31502  5 3234 6.707484e+00
## 31503  5 3235 6.693128e+00
## 31504  5 3236 6.668573e+00
## 31505  5 3237 6.657816e+00
## 31506  5 3238 6.647356e+00
## 31507  5 3239 6.630772e+00
## 31508  5 3240 6.626459e+00
## 31509  5 3241 6.808334e+00
## 31510  5 3242 6.901326e+00
## 31511  5 3243 6.936767e+00
## 31512  5 3244 6.937357e+00
## 31513  5 3245 6.928745e+00
## 31514  5 3246 6.906289e+00
## 31515  5 3247 6.893950e+00
## 31516  5 3248 6.879777e+00
## 31517  5 3249 6.853880e+00
## 31518  5 3250 6.846405e+00
## 31519  5 3251 6.819354e+00
## 31520  5 3252 6.803062e+00
## 31521  5 3253 6.776663e+00
## 31522  5 3254 6.765947e+00
## 31523  5 3255 6.756023e+00
## 31524  5 3256 6.739817e+00
## 31525  5 3257 6.718230e+00
## 31526  5 3258 6.700520e+00
## 31527  5 3259 6.690042e+00
## 31528  5 3260 6.660021e+00
## 31529  5 3261 6.647791e+00
## 31530  5 3262 6.632780e+00
## 31531  5 3263 6.620167e+00
## 31532  5 3264 6.592500e+00
## 31533  5 3265 6.797678e+00
## 31534  5 3266 6.881697e+00
## 31535  5 3267 6.937385e+00
## 31536  5 3268 6.943262e+00
## 31537  5 3269 6.924310e+00
## 31538  5 3270 6.899844e+00
## 31539  5 3271 6.909081e+00
## 31540  5 3272 6.874343e+00
## 31541  5 3273 6.850451e+00
## 31542  5 3274 6.842060e+00
## 31543  5 3275 6.813434e+00
## 31544  5 3276 6.799037e+00
## 31545  5 3277 6.779114e+00
## 31546  5 3278 6.778177e+00
## 31547  5 3279 6.747549e+00
## 31548  5 3280 6.730581e+00
## 31549  5 3281 6.726133e+00
## 31550  5 3282 6.685248e+00
## 31551  5 3283 6.675690e+00
## 31552  5 3284 6.673310e+00
## 31553  5 3285 6.655815e+00
## 31554  5 3286 6.631637e+00
## 31555  5 3287 6.611866e+00
## 31556  5 3288 6.611633e+00
## 31557  5 3289 6.801754e+00
## 31558  5 3290 6.900810e+00
## 31559  5 3291 6.925777e+00
## 31560  5 3292 6.921555e+00
## 31561  5 3293 6.923922e+00
## 31562  5 3294 6.906908e+00
## 31563  5 3295 6.893021e+00
## 31564  5 3296 6.868204e+00
## 31565  5 3297 6.859624e+00
## 31566  5 3298 6.842798e+00
## 31567  5 3299 6.801002e+00
## 31568  5 3300 6.803754e+00
## 31569  5 3301 6.776866e+00
## 31570  5 3302 6.763388e+00
## 31571  5 3303 6.736929e+00
## 31572  5 3304 6.734751e+00
## 31573  5 3305 6.712547e+00
## 31574  5 3306 6.695492e+00
## 31575  5 3307 6.679676e+00
## 31576  5 3308 6.662394e+00
## 31577  5 3309 6.644782e+00
## 31578  5 3310 6.618533e+00
## 31579  5 3311 6.604816e+00
## 31580  5 3312 6.600303e+00
## 31581  5 3313 6.789123e+00
## 31582  5 3314 6.882231e+00
## 31583  5 3315 6.914677e+00
## 31584  5 3316 6.921404e+00
## 31585  5 3317 6.914554e+00
## 31586  5 3318 6.904158e+00
## 31587  5 3319 6.880299e+00
## 31588  5 3320 6.862919e+00
## 31589  5 3321 6.842411e+00
## 31590  5 3322 6.817696e+00
## 31591  5 3323 6.823715e+00
## 31592  5 3324 6.793711e+00
## 31593  5 3325 6.776201e+00
## 31594  5 3326 6.772832e+00
## 31595  5 3327 6.738580e+00
## 31596  5 3328 6.738263e+00
## 31597  5 3329 6.709421e+00
## 31598  5 3330 6.703274e+00
## 31599  5 3331 6.678359e+00
## 31600  5 3332 6.662665e+00
## 31601  5 3333 6.633804e+00
## 31602  5 3334 6.632030e+00
## 31603  5 3335 6.606334e+00
## 31604  5 3336 6.604902e+00
## 31605  5 3337 6.793488e+00
## 31606  5 3338 6.876632e+00
## 31607  5 3339 6.921674e+00
## 31608  5 3340 6.925658e+00
## 31609  5 3341 6.924947e+00
## 31610  5 3342 6.906608e+00
## 31611  5 3343 6.880616e+00
## 31612  5 3344 6.868761e+00
## 31613  5 3345 6.851697e+00
## 31614  5 3346 6.834006e+00
## 31615  5 3347 6.819095e+00
## 31616  5 3348 6.790693e+00
## 31617  5 3349 6.768635e+00
## 31618  5 3350 6.771630e+00
## 31619  5 3351 6.752482e+00
## 31620  5 3352 6.722324e+00
## 31621  5 3353 6.706893e+00
## 31622  5 3354 6.683585e+00
## 31623  5 3355 6.683263e+00
## 31624  5 3356 6.647175e+00
## 31625  5 3357 6.649724e+00
## 31626  5 3358 6.621885e+00
## 31627  5 3359 6.600835e+00
## 31628  5 3360 6.601462e+00
## 31629  5 3361 6.797476e+00
## 31630  5 3362 6.895588e+00
## 31631  5 3363 6.914301e+00
## 31632  5 3364 6.918050e+00
## 31633  5 3365 6.911509e+00
## 31634  5 3366 6.901445e+00
## 31635  5 3367 6.875782e+00
## 31636  5 3368 6.866114e+00
## 31637  5 3369 6.849603e+00
## 31638  5 3370 6.840935e+00
## 31639  5 3371 6.808985e+00
## 31640  5 3372 6.791411e+00
## 31641  5 3373 6.771936e+00
## 31642  5 3374 6.757274e+00
## 31643  5 3375 6.746675e+00
## 31644  5 3376 6.727521e+00
## 31645  5 3377 6.712000e+00
## 31646  5 3378 6.690991e+00
## 31647  5 3379 6.668200e+00
## 31648  5 3380 6.650615e+00
## 31649  5 3381 6.647560e+00
## 31650  5 3382 6.624841e+00
## 31651  5 3383 6.610760e+00
## 31652  5 3384 6.593857e+00
## 31653  5 3385 6.782296e+00
## 31654  5 3386 6.897782e+00
## 31655  5 3387 6.921916e+00
## 31656  5 3388 6.935074e+00
## 31657  5 3389 6.921607e+00
## 31658  5 3390 6.899472e+00
## 31659  5 3391 6.883022e+00
## 31660  5 3392 6.857220e+00
## 31661  5 3393 6.864187e+00
## 31662  5 3394 6.824012e+00
## 31663  5 3395 6.818382e+00
## 31664  5 3396 6.781758e+00
## 31665  5 3397 6.780140e+00
## 31666  5 3398 6.768374e+00
## 31667  5 3399 6.758424e+00
## 31668  5 3400 6.722384e+00
## 31669  5 3401 6.709800e+00
## 31670  5 3402 6.697748e+00
## 31671  5 3403 6.685802e+00
## 31672  5 3404 6.669233e+00
## 31673  5 3405 6.646619e+00
## 31674  5 3406 6.628375e+00
## 31675  5 3407 6.610368e+00
## 31676  5 3408 6.601681e+00
## 31677  5 3409 6.786306e+00
## 31678  5 3410 6.881361e+00
## 31679  5 3411 6.926804e+00
## 31680  5 3412 6.915750e+00
## 31681  5 3413 6.912501e+00
## 31682  5 3414 6.896231e+00
## 31683  5 3415 6.882191e+00
## 31684  5 3416 6.868466e+00
## 31685  5 3417 6.850465e+00
## 31686  5 3418 6.831924e+00
## 31687  5 3419 6.810566e+00
## 31688  5 3420 6.797302e+00
## 31689  5 3421 6.776646e+00
## 31690  5 3422 6.760906e+00
## 31691  5 3423 6.751249e+00
## 31692  5 3424 6.723913e+00
## 31693  5 3425 6.717856e+00
## 31694  5 3426 6.683199e+00
## 31695  5 3427 6.670330e+00
## 31696  5 3428 6.661589e+00
## 31697  5 3429 6.649074e+00
## 31698  5 3430 6.632138e+00
## 31699  5 3431 6.614587e+00
## 31700  5 3432 6.593246e+00
## 31701  5 3432 6.580203e+00
## 31702  5 3433 6.787527e+00
## 31703  5 3434 6.878488e+00
## 31704  5 3435 6.917170e+00
## 31705  5 3436 6.924237e+00
## 31706  5 3437 6.923719e+00
## 31707  5 3438 6.903036e+00
## 31708  5 3439 6.876668e+00
## 31709  5 3440 6.862128e+00
## 31710  5 3441 6.847146e+00
## 31711  5 3442 6.821447e+00
## 31712  5 3443 6.815642e+00
## 31713  5 3444 6.770933e+00
## 31714  5 3445 6.756115e+00
## 31715  5 3446 6.758434e+00
## 31716  5 3447 6.745259e+00
## 31717  5 3448 6.736088e+00
## 31718  5 3449 6.711238e+00
## 31719  5 3450 6.693765e+00
## 31720  5 3451 6.678759e+00
## 31721  5 3452 6.650199e+00
## 31722  5 3453 6.627163e+00
## 31723  5 3454 6.628015e+00
## 31724  5 3455 6.594649e+00
## 31725  5 3456 6.596452e+00
## 31726  5 3456 6.598035e+00
## 31727  5 3457 6.574492e+00
## 31728  5 3458 6.571328e+00
## 31729  5 3459 6.550718e+00
## 31730  5 3460 6.533026e+00
## 31731  5 3461 6.512053e+00
## 31732  5 3462 6.506181e+00
## 31733  5 3463 6.497974e+00
## 31734  5 3464 6.474150e+00
## 31735  5 3465 6.462135e+00
## 31736  5 3466 6.459345e+00
## 31737  5 3467 6.431449e+00
## 31738  5 3468 6.422353e+00
## 31739  5 3469 6.410379e+00
## 31740  5 3470 6.390291e+00
## 31741  5 3471 6.387610e+00
## 31742  5 3472 6.378364e+00
## 31743  5 3473 6.365929e+00
## 31744  5 3474 6.345867e+00
## 31745  5 3475 6.327439e+00
## 31746  5 3476 6.321459e+00
## 31747  5 3477 6.300851e+00
## 31748  5 3478 6.302201e+00
## 31749  5 3479 6.273514e+00
## 31750  5 3480 6.266670e+00
## 31751  5 3480 6.277303e+00
## 31752  5 3481 6.471057e+00
## 31753  5 3482 6.572609e+00
## 31754  5 3483 6.609496e+00
## 31755  5 3484 6.615268e+00
## 31756  5 3485 6.605346e+00
## 31757  5 3486 6.588322e+00
## 31758  5 3487 6.586417e+00
## 31759  5 3488 6.565261e+00
## 31760  5 3489 6.557953e+00
## 31761  5 3490 6.528577e+00
## 31762  5 3491 6.517185e+00
## 31763  5 3492 6.505685e+00
## 31764  5 3493 6.495612e+00
## 31765  5 3494 6.480983e+00
## 31766  5 3495 6.464542e+00
## 31767  5 3496 6.452767e+00
## 31768  5 3497 6.431410e+00
## 31769  5 3498 6.407180e+00
## 31770  5 3499 6.401317e+00
## 31771  5 3500 6.386464e+00
## 31772  5 3501 6.375175e+00
## 31773  5 3502 6.358164e+00
## 31774  5 3503 6.339096e+00
## 31775  5 3504 6.339632e+00
## 31776  5 3504 6.328259e+00
## 31777  5 3505 6.530001e+00
## 31778  5 3506 6.624618e+00
## 31779  5 3507 6.654527e+00
## 31780  5 3508 6.673090e+00
## 31781  5 3509 6.649391e+00
## 31782  5 3510 6.646545e+00
## 31783  5 3511 6.642326e+00
## 31784  5 3512 6.604369e+00
## 31785  5 3513 6.592923e+00
## 31786  5 3514 6.583548e+00
## 31787  5 3515 6.558575e+00
## 31788  5 3516 6.550726e+00
## 31789  5 3517 6.529705e+00
## 31790  5 3518 6.513111e+00
## 31791  5 3519 6.495552e+00
## 31792  5 3520 6.477914e+00
## 31793  5 3521 6.476602e+00
## 31794  5 3522 6.441640e+00
## 31795  5 3523 6.432607e+00
## 31796  5 3524 6.413292e+00
## 31797  5 3525 6.409551e+00
## 31798  5 3526 6.387371e+00
## 31799  5 3527 6.381045e+00
## 31800  5 3528 6.357573e+00
## 31801  5 3529 6.569862e+00
## 31802  5 3530 6.640206e+00
## 31803  5 3531 6.701232e+00
## 31804  5 3532 6.698995e+00
## 31805  5 3533 6.696563e+00
## 31806  5 3534 6.672695e+00
## 31807  5 3535 6.655248e+00
## 31808  5 3536 6.643266e+00
## 31809  5 3537 6.617863e+00
## 31810  5 3538 6.609437e+00
## 31811  5 3539 6.588278e+00
## 31812  5 3540 6.579988e+00
## 31813  5 3541 6.547576e+00
## 31814  5 3542 6.526937e+00
## 31815  5 3543 6.520213e+00
## 31816  5 3544 6.499367e+00
## 31817  5 3545 6.490445e+00
## 31818  5 3546 6.471406e+00
## 31819  5 3547 6.458000e+00
## 31820  5 3548 6.437321e+00
## 31821  5 3549 6.424842e+00
## 31822  5 3550 6.415561e+00
## 31823  5 3551 6.398816e+00
## 31824  5 3552 6.378527e+00
## 31825  5 3553 6.577171e+00
## 31826  5 3554 6.672328e+00
## 31827  5 3555 6.704389e+00
## 31828  5 3556 6.711704e+00
## 31829  5 3557 6.701105e+00
## 31830  5 3558 6.696482e+00
## 31831  5 3559 6.678194e+00
## 31832  5 3560 6.651484e+00
## 31833  5 3561 6.643431e+00
## 31834  5 3562 6.621603e+00
## 31835  5 3563 6.604529e+00
## 31836  5 3564 6.591339e+00
## 31837  5 3565 6.567505e+00
## 31838  5 3566 6.556545e+00
## 31839  5 3567 6.539907e+00
## 31840  5 3568 6.514057e+00
## 31841  5 3569 6.503876e+00
## 31842  5 3570 6.484198e+00
## 31843  5 3571 6.477264e+00
## 31844  5 3572 6.451100e+00
## 31845  5 3573 6.442899e+00
## 31846  5 3574 6.422163e+00
## 31847  5 3575 6.423301e+00
## 31848  5 3576 6.406057e+00
## 31849  5 3577 6.596011e+00
## 31850  5 3578 6.683961e+00
## 31851  5 3579 6.722246e+00
## 31852  5 3580 6.716968e+00
## 31853  5 3581 6.724971e+00
## 31854  5 3582 6.701716e+00
## 31855  5 3583 6.683886e+00
## 31856  5 3584 6.669464e+00
## 31857  5 3585 6.659328e+00
## 31858  5 3586 6.641384e+00
## 31859  5 3587 6.612883e+00
## 31860  5 3588 6.605645e+00
## 31861  5 3589 6.576486e+00
## 31862  5 3590 6.555129e+00
## 31863  5 3591 6.535406e+00
## 31864  5 3592 6.541242e+00
## 31865  5 3593 6.509293e+00
## 31866  5 3594 6.516241e+00
## 31867  5 3595 6.472403e+00
## 31868  5 3596 6.462416e+00
## 31869  5 3597 6.437921e+00
## 31870  5 3598 6.446644e+00
## 31871  5 3599 6.415781e+00
## 31872  5 3600 6.402307e+00
## 31873  5 3601 6.601459e+00
```

```
## 31874  5 3602 6.706116e+00
## 31875  5 3603 6.734344e+00
## 31876  5 3604 6.737760e+00
## 31877  5 3605 6.745355e+00
## 31878  5 3606 6.726067e+00
## 31879  5 3607 6.689768e+00
## 31880  5 3608 6.677871e+00
## 31881  5 3609 6.662418e+00
## 31882  5 3610 6.650190e+00
## 31883  5 3611 6.618767e+00
## 31884  5 3612 6.599501e+00
## 31885  5 3613 6.594860e+00
## 31886  5 3614 6.575298e+00
## 31887  5 3615 6.547666e+00
## 31888  5 3616 6.536750e+00
## 31889  5 3617 6.512516e+00
## 31890  5 3618 6.499922e+00
## 31891  5 3619 6.486674e+00
## 31892  5 3620 6.480233e+00
## 31893  5 3621 6.463631e+00
## 31894  5 3622 6.445055e+00
## 31895  5 3623 6.424452e+00
## 31896  5 3624 6.420578e+00
## 31897  5 3625 6.614258e+00
## 31898  5 3626 6.700459e+00
## 31899  5 3627 6.729954e+00
## 31900  5 3628 6.733312e+00
## 31901  5 3629 6.733750e+00
## 31902  5 3630 6.725712e+00
## 31903  5 3631 6.701761e+00
## 31904  5 3632 6.705247e+00
## 31905  5 3633 6.680186e+00
## 31906  5 3634 6.654430e+00
## 31907  5 3635 6.627596e+00
## 31908  5 3636 6.613340e+00
## 31909  5 3637 6.602537e+00
## 31910  5 3638 6.585790e+00
## 31911  5 3639 6.572396e+00
## 31912  5 3640 6.542734e+00
## 31913  5 3641 6.535162e+00
## 31914  5 3642 6.508759e+00
## 31915  5 3643 6.499806e+00
## 31916  5 3644 6.486217e+00
## 31917  5 3645 6.460144e+00
## 31918  5 3646 6.442260e+00
## 31919  5 3647 6.453239e+00
## 31920  5 3648 6.419937e+00
## 31921  5 3649 6.613464e+00
## 31922  5 3650 6.706942e+00
## 31923  5 3651 6.743713e+00
## 31924  5 3652 6.739238e+00
## 31925  5 3653 6.739219e+00
## 31926  5 3654 6.723384e+00
## 31927  5 3655 6.714765e+00
## 31928  5 3656 6.688597e+00
## 31929  5 3657 6.677231e+00
## 31930  5 3658 6.658253e+00
## 31931  5 3659 6.646296e+00
## 31932  5 3660 6.632227e+00
## 31933  5 3661 6.614507e+00
## 31934  5 3662 6.589946e+00
## 31935  5 3663 6.575194e+00
## 31936  5 3664 6.563518e+00
## 31937  5 3665 6.538144e+00
## 31938  5 3666 6.521766e+00
## 31939  5 3667 6.507727e+00
## 31940  5 3668 6.489687e+00
## 31941  5 3669 6.470285e+00
## 31942  5 3670 6.465080e+00
## 31943  5 3671 6.441306e+00
## 31944  5 3672 6.427088e+00
## 31945  5 3673 6.617919e+00
## 31946  5 3674 6.726767e+00
## 31947  5 3675 6.755954e+00
## 31948  5 3676 6.752153e+00
## 31949  5 3677 6.746154e+00
## 31950  5 3678 6.721635e+00
## 31951  5 3679 6.711642e+00
## 31952  5 3680 6.704893e+00
## 31953  5 3681 6.682118e+00
## 31954  5 3682 6.651156e+00
## 31955  5 3683 6.637808e+00
## 31956  5 3684 6.625506e+00
## 31957  5 3685 6.608448e+00
## 31958  5 3686 6.594264e+00
## 31959  5 3687 6.581103e+00
## 31960  5 3688 6.556249e+00
## 31961  5 3689 6.553419e+00
## 31962  5 3690 6.534452e+00
## 31963  5 3691 6.513824e+00
## 31964  5 3692 6.494006e+00
## 31965  5 3693 6.475521e+00
## 31966  5 3694 6.456706e+00
## 31967  5 3695 6.445546e+00
## 31968  5 3696 6.438873e+00
## 31969  5 3697 6.638874e+00
## 31970  5 3698 6.719677e+00
## 31971  5 3699 6.758420e+00
## 31972  5 3700 6.744488e+00
## 31973  5 3701 6.751084e+00
## 31974  5 3702 6.734824e+00
## 31975  5 3703 6.725191e+00
## 31976  5 3704 6.723647e+00
## 31977  5 3705 6.680468e+00
## 31978  5 3706 6.665973e+00
## 31979  5 3707 6.653819e+00
## 31980  5 3708 6.639190e+00
## 31981  5 3709 6.606054e+00
## 31982  5 3710 6.595725e+00
## 31983  5 3711 6.567185e+00
## 31984  5 3712 6.562535e+00
## 31985  5 3713 6.542798e+00
## 31986  5 3714 6.531831e+00
## 31987  5 3715 6.508763e+00
## 31988  5 3716 6.505911e+00
## 31989  5 3717 6.489219e+00
## 31990  5 3718 6.462395e+00
## 31991  5 3719 6.454460e+00
## 31992  5 3720 6.435971e+00
## 31993  5 3721 6.629004e+00
## 31994  5 3722 6.737867e+00
## 31995  5 3723 6.762925e+00
## 31996  5 3724 6.771503e+00
## 31997  5 3725 6.763148e+00
## 31998  5 3726 6.734523e+00
## 31999  5 3727 6.730857e+00
## 32000  5 3728 6.710129e+00
## 32001  5 3729 6.683493e+00
## 32002  5 3730 6.682324e+00
## 32003  5 3731 6.646603e+00
## 32004  5 3732 6.638262e+00
## 32005  5 3733 6.608816e+00
## 32006  5 3734 6.592900e+00
## 32007  5 3735 6.578143e+00
## 32008  5 3736 6.565119e+00
## 32009  5 3737 6.547585e+00
## 32010  5 3738 6.521864e+00
## 32011  5 3739 6.530318e+00
## 32012  5 3740 6.507605e+00
## 32013  5 3741 6.495110e+00
## 32014  5 3742 6.474269e+00
## 32015  5 3743 6.447641e+00
## 32016  5 3744 6.450587e+00
## 32017  5 3745 6.629226e+00
## 32018  5 3746 6.735493e+00
## 32019  5 3747 6.767219e+00
## 32020  5 3748 6.770044e+00
## 32021  5 3749 6.769196e+00
## 32022  5 3750 6.749103e+00
## 32023  5 3751 6.729902e+00
## 32024  5 3752 6.712469e+00
## 32025  5 3753 6.685793e+00
## 32026  5 3754 6.677473e+00
## 32027  5 3755 6.648964e+00
## 32028  5 3756 6.638420e+00
## 32029  5 3757 6.636582e+00
## 32030  5 3758 6.600013e+00
## 32031  5 3759 6.594277e+00
## 32032  5 3760 6.568391e+00
## 32033  5 3761 6.556102e+00
## 32034  5 3762 6.534712e+00
## 32035  5 3763 6.527700e+00
## 32036  5 3764 6.517291e+00
## 32037  5 3765 6.495627e+00
## 32038  5 3766 6.477969e+00
## 32039  5 3767 6.471483e+00
## 32040  5 3768 6.450138e+00
## 32041  5 3768 6.603047e+00
## 32042  5 3769 6.783211e+00
## 32043  5 3770 6.872519e+00
## 32044  5 3771 6.907565e+00
## 32045  5 3772 6.923871e+00
## 32046  5 3773 6.910055e+00
## 32047  5 3774 6.898387e+00
## 32048  5 3775 6.878811e+00
## 32049  5 3776 6.848136e+00
## 32050  5 3777 6.849633e+00
## 32051  5 3778 6.829923e+00
## 32052  5 3779 6.810070e+00
## 32053  5 3780 6.798687e+00
## 32054  5 3781 6.757243e+00
## 32055  5 3782 6.757573e+00
## 32056  5 3783 6.739367e+00
## 32057  5 3784 6.720528e+00
## 32058  5 3785 6.701478e+00
## 32059  5 3786 6.697400e+00
## 32060  5 3787 6.667402e+00
## 32061  5 3788 6.662899e+00
## 32062  5 3789 6.640345e+00
## 32063  5 3790 6.619590e+00
## 32064  5 3791 6.607217e+00
## 32065  5 3792 6.590710e+00
## 32066  5 3792 6.597887e+00
## 32067  5 3793 6.577053e+00
## 32068  5 3794 6.560809e+00
## 32069  5 3795 6.545440e+00
## 32070  5 3796 6.542712e+00
## 32071  5 3797 6.514649e+00
## 32072  5 3798 6.500921e+00
## 32073  5 3799 6.498213e+00
## 32074  5 3800 6.484395e+00
## 32075  5 3801 6.473210e+00
## 32076  5 3802 6.453098e+00
## 32077  5 3803 6.434113e+00
## 32078  5 3804 6.432507e+00
## 32079  5 3805 6.409820e+00
## 32080  5 3806 6.398757e+00
## 32081  5 3807 6.393687e+00
## 32082  5 3808 6.372016e+00
## 32083  5 3809 6.361911e+00
## 32084  5 3810 6.352579e+00
## 32085  5 3811 6.337059e+00
## 32086  5 3812 6.322419e+00
## 32087  5 3813 6.301555e+00
## 32088  5 3814 6.302564e+00
## 32089  5 3815 6.289310e+00
## 32090  5 3816 6.274022e+00
## 32091  5 3816 6.281043e+00
## 32092  5 3817 6.564656e+00
## 32093  5 3818 6.723804e+00
## 32094  5 3819 6.807154e+00
## 32095  5 3820 6.802540e+00
## 32096  5 3821 6.799444e+00
## 32097  5 3822 6.796834e+00
## 32098  5 3823 6.785034e+00
## 32099  5 3824 6.761871e+00
## 32100  5 3825 6.736720e+00
## 32101  5 3826 6.716227e+00
## 32102  5 3827 6.710559e+00
## 32103  5 3828 6.685898e+00
## 32104  5 3829 6.682554e+00
## 32105  5 3830 6.646613e+00
## 32106  5 3831 6.635041e+00
## 32107  5 3832 6.619628e+00
## 32108  5 3833 6.600808e+00
## 32109  5 3834 6.579991e+00
## 32110  5 3835 6.573524e+00
## 32111  5 3836 6.551093e+00
## 32112  5 3837 6.545429e+00
## 32113  5 3838 6.509843e+00
## 32114  5 3839 6.507978e+00
## 32115  5 3840 6.486690e+00
## 32116  5 3840 6.486546e+00
## 32117  5 3841 6.696482e+00
## 32118  5 3842 6.778265e+00
## 32119  5 3843 6.819142e+00
## 32120  5 3844 6.821657e+00
## 32121  5 3845 6.805243e+00
## 32122  5 3846 6.791521e+00
## 32123  5 3847 6.782168e+00
## 32124  5 3848 6.757423e+00
## 32125  5 3849 6.740228e+00
## 32126  5 3850 6.734317e+00
## 32127  5 3851 6.697771e+00
## 32128  5 3852 6.694542e+00
## 32129  5 3853 6.673595e+00
## 32130  5 3854 6.669725e+00
## 32131  5 3855 6.637854e+00
## 32132  5 3856 6.618814e+00
## 32133  5 3857 6.606883e+00
## 32134  5 3858 6.584573e+00
## 32135  5 3859 6.580331e+00
## 32136  5 3860 6.551379e+00
## 32137  5 3861 6.543958e+00
## 32138  5 3862 6.525967e+00
## 32139  5 3863 6.509250e+00
## 32140  5 3864 6.494074e+00
## 32141  5 3865 6.689902e+00
## 32142  5 3866 6.778566e+00
## 32143  5 3867 6.822084e+00
## 32144  5 3868 6.821233e+00
## 32145  5 3869 6.815367e+00
## 32146  5 3870 6.793898e+00
## 32147  5 3871 6.788474e+00
## 32148  5 3872 6.760292e+00
## 32149  5 3873 6.759782e+00
## 32150  5 3874 6.731772e+00
## 32151  5 3875 6.716751e+00
## 32152  5 3876 6.690723e+00
## 32153  5 3877 6.679080e+00
## 32154  5 3878 6.662539e+00
## 32155  5 3879 6.632051e+00
## 32156  5 3880 6.626459e+00
## 32157  5 3881 6.613263e+00
## 32158  5 3882 6.598399e+00
## 32159  5 3883 6.573981e+00
## 32160  5 3884 6.563632e+00
## 32161  5 3885 6.557128e+00
## 32162  5 3886 6.539573e+00
## 32163  5 3887 6.513810e+00
## 32164  5 3888 6.510475e+00
## 32165  5 3889 6.693001e+00
## 32166  5 3890 6.790763e+00
## 32167  5 3891 6.815099e+00
## 32168  5 3892 6.822995e+00
## 32169  5 3893 6.822170e+00
## 32170  5 3894 6.803069e+00
## 32171  5 3895 6.783676e+00
## 32172  5 3896 6.762090e+00
## 32173  5 3897 6.746511e+00
## 32174  5 3898 6.732563e+00
## 32175  5 3899 6.723638e+00
## 32176  5 3900 6.698016e+00
## 32177  5 3901 6.672265e+00
## 32178  5 3902 6.657056e+00
## 32179  5 3903 6.644199e+00
## 32180  5 3904 6.626911e+00
## 32181  5 3905 6.608504e+00
## 32182  5 3906 6.615167e+00
## 32183  5 3907 6.569313e+00
## 32184  5 3908 6.575622e+00
## 32185  5 3909 6.554521e+00
## 32186  5 3910 6.534763e+00
## 32187  5 3911 6.523427e+00
## 32188  5 3912 6.501235e+00
## 32189  5 3913 6.700481e+00
## 32190  5 3914 6.793184e+00
## 32191  5 3915 6.816750e+00
## 32192  5 3916 6.818789e+00
## 32193  5 3917 6.833544e+00
## 32194  5 3918 6.808287e+00
## 32195  5 3919 6.781790e+00
## 32196  5 3920 6.761893e+00
## 32197  5 3921 6.757566e+00
## 32198  5 3922 6.730853e+00
## 32199  5 3923 6.717157e+00
## 32200  5 3924 6.700994e+00
## 32201  5 3925 6.689938e+00
## 32202  5 3926 6.650442e+00
## 32203  5 3927 6.647581e+00
## 32204  5 3928 6.628873e+00
## 32205  5 3929 6.629197e+00
## 32206  5 3930 6.601947e+00
## 32207  5 3931 6.581321e+00
## 32208  5 3932 6.561039e+00
## 32209  5 3933 6.542547e+00
## 32210  5 3934 6.521218e+00
## 32211  5 3935 6.534267e+00
## 32212  5 3936 6.510110e+00
## 32213  5 3937 6.707445e+00
## 32214  5 3938 6.791921e+00
## 32215  5 3939 6.816118e+00
## 32216  5 3940 6.836551e+00
## 32217  5 3941 6.826879e+00
## 32218  5 3942 6.814751e+00
## 32219  5 3943 6.788171e+00
## 32220  5 3944 6.780281e+00
## 32221  5 3945 6.746826e+00
## 32222  5 3946 6.748253e+00
## 32223  5 3947 6.719652e+00
## 32224  5 3948 6.704275e+00
## 32225  5 3949 6.684210e+00
## 32226  5 3950 6.665958e+00
## 32227  5 3951 6.658934e+00
## 32228  5 3952 6.634342e+00
## 32229  5 3953 6.622142e+00
## 32230  5 3954 6.608545e+00
## 32231  5 3955 6.594442e+00
## 32232  5 3956 6.562498e+00
## 32233  5 3957 6.547939e+00
## 32234  5 3958 6.544979e+00
## 32235  5 3959 6.532396e+00
## 32236  5 3960 6.503990e+00
## 32237  5 3961 6.708200e+00
## 32238  5 3962 6.784264e+00
## 32239  5 3963 6.833709e+00
## 32240  5 3964 6.825905e+00
## 32241  5 3965 6.825644e+00
## 32242  5 3966 6.808333e+00
## 32243  5 3967 6.807983e+00
## 32244  5 3968 6.777500e+00
## 32245  5 3969 6.760193e+00
## 32246  5 3970 6.734403e+00
## 32247  5 3971 6.728994e+00
## 32248  5 3972 6.709495e+00
## 32249  5 3973 6.690774e+00
## 32250  5 3974 6.665167e+00
## 32251  5 3975 6.658396e+00
## 32252  5 3976 6.631677e+00
## 32253  5 3977 6.625075e+00
## 32254  5 3978 6.611110e+00
## 32255  5 3979 6.579459e+00
## 32256  5 3980 6.557703e+00
## 32257  5 3981 6.550416e+00
## 32258  5 3982 6.548437e+00
## 32259  5 3983 6.517907e+00
## 32260  5 3984 6.504270e+00
## 32261  5 3985 6.711154e+00
## 32262  5 3986 6.794081e+00
## 32263  5 3987 6.842032e+00
## 32264  5 3988 6.838976e+00
## 32265  5 3989 6.816059e+00
## 32266  5 3990 6.812912e+00
## 32267  5 3991 6.816805e+00
## 32268  5 3992 6.787929e+00
## 32269  5 3993 6.765060e+00
## 32270  5 3994 6.740216e+00
## 32271  5 3995 6.727404e+00
## 32272  5 3996 6.708385e+00
## 32273  5 3997 6.682139e+00
## 32274  5 3998 6.674210e+00
## 32275  5 3999 6.653270e+00
## 32276  5 4000 6.636283e+00
## 32277  5 4001 6.631160e+00
## 32278  5 4002 6.605787e+00
## 32279  5 4003 6.587929e+00
## 32280  5 4004 6.582251e+00
## 32281  5 4005 6.548874e+00
## 32282  5 4006 6.558283e+00
## 32283  5 4007 6.520424e+00
## 32284  5 4008 6.518051e+00
## 32285  5 4009 6.714300e+00
## 32286  5 4010 6.805110e+00
## 32287  5 4011 6.839725e+00
## 32288  5 4012 6.835144e+00
## 32289  5 4013 6.838006e+00
## 32290  5 4014 6.820007e+00
## 32291  5 4015 6.805567e+00
## 32292  5 4016 6.788773e+00
## 32293  5 4017 6.772352e+00
## 32294  5 4018 6.744501e+00
## 32295  5 4019 6.726862e+00
## 32296  5 4020 6.704201e+00
## 32297  5 4021 6.686379e+00
## 32298  5 4022 6.681615e+00
## 32299  5 4023 6.664108e+00
## 32300  5 4024 6.649760e+00
## 32301  5 4025 6.625212e+00
## 32302  5 4026 6.605335e+00
## 32303  5 4027 6.601062e+00
## 32304  5 4028 6.573959e+00
## 32305  5 4029 6.561122e+00
## 32306  5 4030 6.551280e+00
## 32307  5 4031 6.514039e+00
## 32308  5 4032 6.529999e+00
## 32309  5 4033 6.720318e+00
## 32310  5 4034 6.802477e+00
## 32311  5 4035 6.839826e+00
## 32312  5 4036 6.832724e+00
## 32313  5 4037 6.837193e+00
## 32314  5 4038 6.811659e+00
## 32315  5 4039 6.812637e+00
## 32316  5 4040 6.777762e+00
## 32317  5 4041 6.775002e+00
## 32318  5 4042 6.747491e+00
## 32319  5 4043 6.731939e+00
## 32320  5 4044 6.717685e+00
## 32321  5 4045 6.717072e+00
## 32322  5 4046 6.681897e+00
## 32323  5 4047 6.670812e+00
## 32324  5 4048 6.642057e+00
## 32325  5 4049 6.635352e+00
## 32326  5 4050 6.611002e+00
## 32327  5 4051 6.594128e+00
## 32328  5 4052 6.584816e+00
## 32329  5 4053 6.570605e+00
## 32330  5 4054 6.562225e+00
## 32331  5 4055 6.531651e+00
## 32332  5 4056 6.509991e+00
## 32333  5 4057 6.717569e+00
## 32334  5 4058 6.812165e+00
## 32335  5 4059 6.847004e+00
## 32336  5 4060 6.852637e+00
## 32337  5 4061 6.832706e+00
## 32338  5 4062 6.822521e+00
## 32339  5 4063 6.801580e+00
## 32340  5 4064 6.783814e+00
## 32341  5 4065 6.780113e+00
## 32342  5 4066 6.757897e+00
## 32343  5 4067 6.743370e+00
## 32344  5 4068 6.721296e+00
## 32345  5 4069 6.709837e+00
## 32346  5 4070 6.671900e+00
## 32347  5 4071 6.661900e+00
## 32348  5 4072 6.647777e+00
## 32349  5 4073 6.630405e+00
## 32350  5 4074 6.611820e+00
## 32351  5 4075 6.592938e+00
## 32352  5 4076 6.580098e+00
## 32353  5 4077 6.563545e+00
## 32354  5 4078 6.554970e+00
## 32355  5 4079 6.528544e+00
## 32356  5 4080 6.517178e+00
## 32357  5 4081 6.719196e+00
## 32358  5 4082 6.807303e+00
## 32359  5 4083 6.846408e+00
## 32360  5 4084 6.859974e+00
## 32361  5 4085 6.839234e+00
## 32362  5 4086 6.839285e+00
## 32363  5 4087 6.813920e+00
## 32364  5 4088 6.788033e+00
## 32365  5 4089 6.768235e+00
## 32366  5 4090 6.755669e+00
## 32367  5 4091 6.739468e+00
## 32368  5 4092 6.712137e+00
## 32369  5 4093 6.696058e+00
## 32370  5 4094 6.692436e+00
## 32371  5 4095 6.666050e+00
## 32372  5 4096 6.654567e+00
## 32373  5 4097 6.635800e+00
## 32374  5 4098 6.623519e+00
## 32375  5 4099 6.598360e+00
## 32376  5 4100 6.590627e+00
## 32377  5 4101 6.567871e+00
## 32378  5 4102 6.553756e+00
## 32379  5 4103 6.531744e+00
## 32380  5 4104 6.523224e+00
## 32381  5 4104 6.587382e+00
## 32382  5 4105 6.787142e+00
## 32383  5 4106 6.886442e+00
## 32384  5 4107 6.916656e+00
## 32385  5 4108 6.919222e+00
## 32386  5 4109 6.911458e+00
## 32387  5 4110 6.892643e+00
## 32388  5 4111 6.882630e+00
## 32389  5 4112 6.865015e+00
## 32390  5 4113 6.842990e+00
## 32391  5 4114 6.826264e+00
## 32392  5 4115 6.807310e+00
## 32393  5 4116 6.786498e+00
## 32394  5 4117 6.773382e+00
## 32395  5 4118 6.745581e+00
## 32396  5 4119 6.723690e+00
## 32397  5 4120 6.715776e+00
## 32398  5 4121 6.713595e+00
## 32399  5 4122 6.691252e+00
## 32400  5 4123 6.681216e+00
## 32401  5 4124 6.653884e+00
## 32402  5 4125 6.637615e+00
## 32403  5 4126 6.629052e+00
## 32404  5 4127 6.609979e+00
## 32405  5 4128 6.589979e+00
## 32406  5 4128 6.599024e+00
## 32407  5 4129 6.562297e+00
## 32408  5 4130 6.567837e+00
## 32409  5 4131 6.541049e+00
## 32410  5 4132 6.541197e+00
## 32411  5 4133 6.525583e+00
## 32412  5 4134 6.508852e+00
## 32413  5 4135 6.481886e+00
## 32414  5 4136 6.479168e+00
## 32415  5 4137 6.473505e+00
## 32416  5 4138 6.447142e+00
## 32417  5 4139 6.448551e+00
## 32418  5 4140 6.429795e+00
## 32419  5 4141 6.410491e+00
## 32420  5 4142 6.399105e+00
## 32421  5 4143 6.391569e+00
## 32422  5 4144 6.363386e+00
## 32423  5 4145 6.357197e+00
## 32424  5 4146 6.348956e+00
## 32425  5 4147 6.344447e+00
## 32426  5 4148 6.320776e+00
## 32427  5 4149 6.299242e+00
## 32428  5 4150 6.305164e+00
## 32429  5 4151 6.279336e+00
## 32430  5 4152 6.279744e+00
## 32431  5 4152 6.274059e+00
## 32432  5 4153 6.681009e+00
## 32433  5 4154 6.880369e+00
## 32434  5 4155 6.973391e+00
## 32435  5 4156 7.000507e+00
## 32436  5 4157 7.010124e+00
## 32437  5 4158 6.994872e+00
## 32438  5 4159 6.982044e+00
## 32439  5 4160 6.956219e+00
## 32440  5 4161 6.946848e+00
## 32441  5 4162 6.917823e+00
## 32442  5 4163 6.889671e+00
## 32443  5 4164 6.878106e+00
## 32444  5 4165 6.850310e+00
## 32445  5 4166 6.836731e+00
## 32446  5 4167 6.812912e+00
## 32447  5 4168 6.791305e+00
## 32448  5 4169 6.777419e+00
## 32449  5 4170 6.752384e+00
## 32450  5 4171 6.741420e+00
## 32451  5 4172 6.727698e+00
## 32452  5 4173 6.725780e+00
## 32453  5 4174 6.694860e+00
## 32454  5 4175 6.670797e+00
## 32455  5 4176 6.657857e+00
## 32456  5 4176 6.664803e+00
## 32457  5 4177 6.847434e+00
## 32458  5 4178 6.938168e+00
## 32459  5 4179 6.964617e+00
## 32460  5 4180 6.984361e+00
## 32461  5 4181 6.957955e+00
## 32462  5 4182 6.943012e+00
## 32463  5 4183 6.937386e+00
## 32464  5 4184 6.918861e+00
## 32465  5 4185 6.892790e+00
## 32466  5 4186 6.869892e+00
## 32467  5 4187 6.847317e+00
## 32468  5 4188 6.828065e+00
## 32469  5 4189 6.824086e+00
## 32470  5 4190 6.792097e+00
## 32471  5 4191 6.771734e+00
## 32472  5 4192 6.757585e+00
## 32473  5 4193 6.739500e+00
## 32474  5 4194 6.721987e+00
## 32475  5 4195 6.701053e+00
## 32476  5 4196 6.690052e+00
## 32477  5 4197 6.669538e+00
## 32478  5 4198 6.663364e+00
## 32479  5 4199 6.647546e+00
## 32480  5 4200 6.626114e+00
## 32481  5 4201 6.829772e+00
## 32482  5 4202 6.897214e+00
## 32483  5 4203 6.944883e+00
## 32484  5 4204 6.952748e+00
## 32485  5 4205 6.940327e+00
## 32486  5 4206 6.933604e+00
## 32487  5 4207 6.908064e+00
## 32488  5 4208 6.887660e+00
## 32489  5 4209 6.875562e+00
## 32490  5 4210 6.857208e+00
## 32491  5 4211 6.832579e+00
## 32492  5 4212 6.821738e+00
## 32493  5 4213 6.803569e+00
## 32494  5 4214 6.781181e+00
## 32495  5 4215 6.772549e+00
## 32496  5 4216 6.748741e+00
## 32497  5 4217 6.726925e+00
## 32498  5 4218 6.722488e+00
## 32499  5 4219 6.702697e+00
## 32500  5 4220 6.676208e+00
## 32501  5 4221 6.671913e+00
## 32502  5 4222 6.634977e+00
## 32503  5 4223 6.631259e+00
## 32504  5 4224 6.609114e+00
## 32505  5 4225 6.799412e+00
## 32506  5 4226 6.896973e+00
## 32507  5 4227 6.937301e+00
## 32508  5 4228 6.947757e+00
## 32509  5 4229 6.933714e+00
## 32510  5 4230 6.909244e+00
## 32511  5 4231 6.884806e+00
## 32512  5 4232 6.888853e+00
## 32513  5 4233 6.851448e+00
## 32514  5 4234 6.831565e+00
## 32515  5 4235 6.827238e+00
## 32516  5 4236 6.805153e+00
## 32517  5 4237 6.773813e+00
## 32518  5 4238 6.766195e+00
## 32519  5 4239 6.746439e+00
## 32520  5 4240 6.729926e+00
## 32521  5 4241 6.725428e+00
## 32522  5 4242 6.700611e+00
## 32523  5 4243 6.683686e+00
## 32524  5 4244 6.670152e+00
## 32525  5 4245 6.665955e+00
## 32526  5 4246 6.634492e+00
## 32527  5 4247 6.629657e+00
## 32528  5 4248 6.606255e+00
## 32529  5 4249 6.808658e+00
## 32530  5 4250 6.890983e+00
## 32531  5 4251 6.923678e+00
## 32532  5 4252 6.937177e+00
## 32533  5 4253 6.924915e+00
## 32534  5 4254 6.908452e+00
## 32535  5 4255 6.894227e+00
## 32536  5 4256 6.866185e+00
## 32537  5 4257 6.844841e+00
## 32538  5 4258 6.832496e+00
## 32539  5 4259 6.815237e+00
## 32540  5 4260 6.805285e+00
## 32541  5 4261 6.766579e+00
## 32542  5 4262 6.774528e+00
## 32543  5 4263 6.751239e+00
## 32544  5 4264 6.729795e+00
## 32545  5 4265 6.717987e+00
## 32546  5 4266 6.697398e+00
## 32547  5 4267 6.683277e+00
## 32548  5 4268 6.671242e+00
## 32549  5 4269 6.630698e+00
## 32550  5 4270 6.640142e+00
## 32551  5 4271 6.631440e+00
## 32552  5 4272 6.613197e+00
## 32553  5 4273 6.810140e+00
## 32554  5 4274 6.887998e+00
## 32555  5 4275 6.918890e+00
## 32556  5 4276 6.925382e+00
## 32557  5 4277 6.912767e+00
## 32558  5 4278 6.903828e+00
## 32559  5 4279 6.899809e+00
## 32560  5 4280 6.875139e+00
## 32561  5 4281 6.857434e+00
## 32562  5 4282 6.838638e+00
## 32563  5 4283 6.823728e+00
## 32564  5 4284 6.796812e+00
## 32565  5 4285 6.780034e+00
## 32566  5 4286 6.765920e+00
## 32567  5 4287 6.737209e+00
## 32568  5 4288 6.725005e+00
## 32569  5 4289 6.724010e+00
## 32570  5 4290 6.694405e+00
## 32571  5 4291 6.670376e+00
## 32572  5 4292 6.646715e+00
## 32573  5 4293 6.652777e+00
## 32574  5 4294 6.615054e+00
## 32575  5 4295 6.620804e+00
## 32576  5 4296 6.602127e+00
## 32577  5 4297 6.802598e+00
## 32578  5 4298 6.882348e+00
## 32579  5 4299 6.930374e+00
## 32580  5 4300 6.927058e+00
## 32581  5 4301 6.947331e+00
## 32582  5 4302 6.899900e+00
## 32583  5 4303 6.885624e+00
## 32584  5 4304 6.868796e+00
## 32585  5 4305 6.857667e+00
## 32586  5 4306 6.830263e+00
## 32587  5 4307 6.815988e+00
## 32588  5 4308 6.795711e+00
## 32589  5 4309 6.776641e+00
## 32590  5 4310 6.761844e+00
## 32591  5 4311 6.749835e+00
## 32592  5 4312 6.728715e+00
## 32593  5 4313 6.717267e+00
## 32594  5 4314 6.688414e+00
## 32595  5 4315 6.678566e+00
## 32596  5 4316 6.653911e+00
## 32597  5 4317 6.638256e+00
## 32598  5 4318 6.643412e+00
## 32599  5 4319 6.623519e+00
## 32600  5 4320 6.603385e+00
## 32601  5 4321 6.803361e+00
## 32602  5 4322 6.876978e+00
## 32603  5 4323 6.932131e+00
## 32604  5 4324 6.918537e+00
## 32605  5 4325 6.921613e+00
## 32606  5 4326 6.915010e+00
## 32607  5 4327 6.873421e+00
## 32608  5 4328 6.867535e+00
## 32609  5 4329 6.856462e+00
## 32610  5 4330 6.832271e+00
## 32611  5 4331 6.814571e+00
## 32612  5 4332 6.794220e+00
## 32613  5 4333 6.774494e+00
## 32614  5 4334 6.765633e+00
## 32615  5 4335 6.752898e+00
## 32616  5 4336 6.731925e+00
## 32617  5 4337 6.715724e+00
## 32618  5 4338 6.691797e+00
## 32619  5 4339 6.685238e+00
## 32620  5 4340 6.665042e+00
## 32621  5 4341 6.642569e+00
## 32622  5 4342 6.638022e+00
## 32623  5 4343 6.607820e+00
## 32624  5 4344 6.604165e+00
## 32625  5 4345 6.800348e+00
## 32626  5 4346 6.879106e+00
## 32627  5 4347 6.921929e+00
## 32628  5 4348 6.925784e+00
## 32629  5 4349 6.917449e+00
## 32630  5 4350 6.905327e+00
## 32631  5 4351 6.883217e+00
## 32632  5 4352 6.860577e+00
## 32633  5 4353 6.851237e+00
## 32634  5 4354 6.832175e+00
## 32635  5 4355 6.815632e+00
## 32636  5 4356 6.804313e+00
## 32637  5 4357 6.781149e+00
## 32638  5 4358 6.768001e+00
## 32639  5 4359 6.743743e+00
## 32640  5 4360 6.745418e+00
## 32641  5 4361 6.712413e+00
## 32642  5 4362 6.699076e+00
## 32643  5 4363 6.684240e+00
## 32644  5 4364 6.653964e+00
## 32645  5 4365 6.646881e+00
## 32646  5 4366 6.628984e+00
## 32647  5 4367 6.615876e+00
## 32648  5 4368 6.613120e+00
## 32649  5 4369 6.793970e+00
## 32650  5 4370 6.888014e+00
## 32651  5 4371 6.931039e+00
## 32652  5 4372 6.918214e+00
## 32653  5 4373 6.910421e+00
## 32654  5 4374 6.897039e+00
## 32655  5 4375 6.894285e+00
## 32656  5 4376 6.854819e+00
## 32657  5 4377 6.856053e+00
## 32658  5 4378 6.816051e+00
## 32659  5 4379 6.806046e+00
## 32660  5 4380 6.795240e+00
## 32661  5 4381 6.784970e+00
## 32662  5 4382 6.750937e+00
## 32663  5 4383 6.733890e+00
## 32664  5 4384 6.731342e+00
## 32665  5 4385 6.705546e+00
## 32666  5 4386 6.703261e+00
## 32667  5 4387 6.677427e+00
## 32668  5 4388 6.662076e+00
## 32669  5 4389 6.650064e+00
## 32670  5 4390 6.625913e+00
## 32671  5 4391 6.612717e+00
## 32672  5 4392 6.604722e+00
## 32673  5 4393 6.793952e+00
```

```
## 32674  5 4394 6.891463e+00
## 32675  5 4395 6.928423e+00
## 32676  5 4396 6.918929e+00
## 32677  5 4397 6.915223e+00
## 32678  5 4398 6.902650e+00
## 32679  5 4399 6.891048e+00
## 32680  5 4400 6.871784e+00
## 32681  5 4401 6.860229e+00
## 32682  5 4402 6.822394e+00
## 32683  5 4403 6.807217e+00
## 32684  5 4404 6.801399e+00
## 32685  5 4405 6.773810e+00
## 32686  5 4406 6.762336e+00
## 32687  5 4407 6.735307e+00
## 32688  5 4408 6.733038e+00
## 32689  5 4409 6.703826e+00
## 32690  5 4410 6.696668e+00
## 32691  5 4411 6.667725e+00
## 32692  5 4412 6.658910e+00
## 32693  5 4413 6.645154e+00
## 32694  5 4414 6.634398e+00
## 32695  5 4415 6.619365e+00
## 32696  5 4416 6.606328e+00
## 32697  5 4417 6.795740e+00
## 32698  5 4418 6.888041e+00
## 32699  5 4419 6.921459e+00
## 32700  5 4420 6.921941e+00
## 32701  5 4421 6.915030e+00
## 32702  5 4422 6.906242e+00
## 32703  5 4423 6.882387e+00
## 32704  5 4424 6.874945e+00
## 32705  5 4425 6.856131e+00
## 32706  5 4426 6.835547e+00
## 32707  5 4427 6.804504e+00
## 32708  5 4428 6.801398e+00
## 32709  5 4429 6.770161e+00
## 32710  5 4430 6.754343e+00
## 32711  5 4431 6.737642e+00
## 32712  5 4432 6.730975e+00
## 32713  5 4433 6.709860e+00
## 32714  5 4434 6.694117e+00
## 32715  5 4435 6.671839e+00
## 32716  5 4436 6.656260e+00
## 32717  5 4437 6.649119e+00
## 32718  5 4438 6.637070e+00
## 32719  5 4439 6.612045e+00
## 32720  5 4440 6.600458e+00
## 32721  5 4441 6.572599e+00
## 32722  5 4442 6.576110e+00
## 32723  5 4443 6.539339e+00
## 32724  5 4444 6.545121e+00
## 32725  5 4445 6.531283e+00
## 32726  5 4446 6.505186e+00
## 32727  5 4447 6.506823e+00
## 32728  5 4448 6.474330e+00
## 32729  5 4449 6.470425e+00
## 32730  5 4450 6.457533e+00
## 32731  5 4451 6.446771e+00
## 32732  5 4452 6.419521e+00
## 32733  5 4453 6.410856e+00
## 32734  5 4454 6.408215e+00
## 32735  5 4455 6.384530e+00
## 32736  5 4456 6.379814e+00
## 32737  5 4457 6.363872e+00
## 32738  5 4458 6.358371e+00
## 32739  5 4459 6.343981e+00
## 32740  5 4460 6.318991e+00
## 32741  5 4461 6.319522e+00
## 32742  5 4462 6.305396e+00
## 32743  5 4463 6.287652e+00
## 32744  5 4464 6.275292e+00
## 32745  5 4465 6.257946e+00
## 32746  5 4466 6.255839e+00
## 32747  5 4467 6.246491e+00
## 32748  5 4468 6.230081e+00
## 32749  5 4469 6.224395e+00
## 32750  5 4470 6.209265e+00
## 32751  5 4471 6.195743e+00
## 32752  5 4472 6.182163e+00
## 32753  5 4473 6.179775e+00
## 32754  5 4474 6.159533e+00
## 32755  5 4475 6.154638e+00
## 32756  5 4476 6.135996e+00
## 32757  5 4477 6.130478e+00
## 32758  5 4478 6.122078e+00
## 32759  5 4479 6.106555e+00
## 32760  5 4480 6.087821e+00
## 32761  5 4481 6.084929e+00
## 32762  5 4482 6.081452e+00
## 32763  5 4483 6.076043e+00
## 32764  5 4484 6.053353e+00
## 32765  5 4485 6.048177e+00
## 32766  5 4486 6.037664e+00
## 32767  5 4487 6.029447e+00
## 32768  5 4488 6.012009e+00
## 32769  5 4489 6.000590e+00
## 32770  5 4490 5.998192e+00
## 32771  5 4491 5.980941e+00
## 32772  5 4492 5.989230e+00
## 32773  5 4493 5.970269e+00
## 32774  5 4494 5.953468e+00
## 32775  5 4495 5.946862e+00
## 32776  5 4496 5.928735e+00
## 32777  5 4497 5.932129e+00
## 32778  5 4498 5.926349e+00
## 32779  5 4499 5.910015e+00
## 32780  5 4500 5.899810e+00
## 32781  5 4501 5.885010e+00
## 32782  5 4502 5.875407e+00
## 32783  5 4503 5.876579e+00
## 32784  5 4504 5.866354e+00
## 32785  5 4505 5.850388e+00
## 32786  5 4506 5.846843e+00
## 32787  5 4507 5.831273e+00
## 32788  5 4508 5.820707e+00
## 32789  5 4509 5.810122e+00
## 32790  5 4510 5.804279e+00
## 32791  5 4511 5.795649e+00
## 32792  5 4512 5.784302e+00
## 32793  5 4513 5.776329e+00
## 32794  5 4514 5.775392e+00
## 32795  5 4515 5.749230e+00
## 32796  5 4516 5.754437e+00
## 32797  5 4517 5.748723e+00
## 32798  5 4518 5.734864e+00
## 32799  5 4519 5.733966e+00
## 32800  5 4520 5.720938e+00
## 32801  5 4521 5.710809e+00
## 32802  5 4522 5.697273e+00
## 32803  5 4523 5.688803e+00
## 32804  5 4524 5.681247e+00
## 32805  5 4525 5.680719e+00
## 32806  5 4526 5.676287e+00
## 32807  5 4527 5.653088e+00
## 32808  5 4528 5.650148e+00
## 32809  5 4529 5.629914e+00
## 32810  5 4530 5.624024e+00
## 32811  5 4531 5.613083e+00
## 32812  5 4532 5.612759e+00
## 32813  5 4533 5.596986e+00
## 32814  5 4534 5.597589e+00
## 32815  5 4535 5.582561e+00
## 32816  5 4536 5.581207e+00
## 32817  5 4537 5.575622e+00
## 32818  5 4538 5.567298e+00
## 32819  5 4539 5.544498e+00
## 32820  5 4540 5.541940e+00
## 32821  5 4541 5.529285e+00
## 32822  5 4542 5.529630e+00
## 32823  5 4543 5.520889e+00
## 32824  5 4544 5.507008e+00
## 32825  5 4545 5.507224e+00
## 32826  5 4546 5.497058e+00
## 32827  5 4547 5.487522e+00
## 32828  5 4548 5.475053e+00
## 32829  5 4549 5.470265e+00
## 32830  5 4550 5.465097e+00
## 32831  5 4551 5.459016e+00
## 32832  5 4552 5.438782e+00
## 32833  5 4553 5.434381e+00
## 32834  5 4554 5.424411e+00
## 32835  5 4555 5.422377e+00
## 32836  5 4556 5.408535e+00
## 32837  5 4557 5.406379e+00
## 32838  5 4558 5.408426e+00
## 32839  5 4559 5.381777e+00
## 32840  5 4560 5.381049e+00
## 32841  5 4561 5.368268e+00
## 32842  5 4562 5.359686e+00
## 32843  5 4563 5.354723e+00
## 32844  5 4564 5.348562e+00
## 32845  5 4565 5.343246e+00
## 32846  5 4566 5.332765e+00
## 32847  5 4567 5.323153e+00
## 32848  5 4568 5.326979e+00
## 32849  5 4569 5.298072e+00
## 32850  5 4570 5.298459e+00
## 32851  5 4571 5.301944e+00
## 32852  5 4572 5.291910e+00
## 32853  5 4573 5.271450e+00
## 32854  5 4574 5.274310e+00
## 32855  5 4575 5.271724e+00
## 32856  5 4576 5.254070e+00
## 32857  5 4577 5.245220e+00
## 32858  5 4578 5.236085e+00
## 32859  5 4579 5.227293e+00
## 32860  5 4580 5.213530e+00
## 32861  5 4581 5.212410e+00
## 32862  5 4582 5.215749e+00
## 32863  5 4583 5.199100e+00
## 32864  5 4584 5.188127e+00
## 32865  5 4585 5.190409e+00
## 32866  5 4586 5.175577e+00
## 32867  5 4587 5.173817e+00
## 32868  5 4588 5.152814e+00
## 32869  5 4589 5.145475e+00
## 32870  5 4590 5.151390e+00
## 32871  5 4591 5.146458e+00
## 32872  5 4592 5.125869e+00
## 32873  5 4593 5.122140e+00
## 32874  5 4594 5.106848e+00
## 32875  5 4595 5.111539e+00
## 32876  5 4596 5.099694e+00
## 32877  5 4597 5.092771e+00
## 32878  5 4598 5.085802e+00
## 32879  5 4599 5.079283e+00
## 32880  5 4600 5.068281e+00
## 32881  5 4601 5.066100e+00
## 32882  5 4602 5.057967e+00
## 32883  5 4603 5.050308e+00
## 32884  5 4604 5.048317e+00
## 32885  5 4605 5.035886e+00
## 32886  5 4606 5.023962e+00
## 32887  5 4607 5.026273e+00
## 32888  5 4608 5.010527e+00
## 32889  5 4609 4.998296e+00
## 32890  5 4610 4.990223e+00
## 32891  5 4611 4.994551e+00
## 32892  5 4612 4.989292e+00
## 32893  5 4613 4.979133e+00
## 32894  5 4614 4.968523e+00
## 32895  5 4615 4.961394e+00
## 32896  5 4616 4.962802e+00
## 32897  5 4617 4.944328e+00
## 32898  5 4618 4.939521e+00
## 32899  5 4619 4.937931e+00
## 32900  5 4620 4.923841e+00
## 32901  5 4621 4.915620e+00
## 32902  5 4622 4.910893e+00
## 32903  5 4623 4.906232e+00
## 32904  5 4624 4.894228e+00
## 32905  5 4625 4.894027e+00
## 32906  5 4626 4.883337e+00
## 32907  5 4627 4.864131e+00
## 32908  5 4628 4.860273e+00
## 32909  5 4629 4.858201e+00
## 32910  5 4630 4.856183e+00
## 32911  5 4631 4.841453e+00
## 32912  5 4632 4.831351e+00
## 32913  5 4633 4.837224e+00
## 32914  5 4634 4.821886e+00
## 32915  5 4635 4.824308e+00
## 32916  5 4636 4.812097e+00
## 32917  5 4637 4.807886e+00
## 32918  5 4638 4.795175e+00
## 32919  5 4639 4.785765e+00
## 32920  5 4640 4.785661e+00
## 32921  5 4641 4.784833e+00
## 32922  5 4642 4.762531e+00
## 32923  5 4643 4.756723e+00
## 32924  5 4644 4.753834e+00
## 32925  5 4645 4.754755e+00
## 32926  5 4646 4.742753e+00
## 32927  5 4647 4.731092e+00
## 32928  5 4648 4.732911e+00
## 32929  5 4649 4.725896e+00
## 32930  5 4650 4.715757e+00
## 32931  5 4651 4.705064e+00
## 32932  5 4652 4.694828e+00
## 32933  5 4653 4.690429e+00
## 32934  5 4654 4.691540e+00
## 32935  5 4655 4.673791e+00
## 32936  5 4656 4.674510e+00
## 32937  5 4657 4.664133e+00
## 32938  5 4658 4.657217e+00
## 32939  5 4659 4.654352e+00
## 32940  5 4660 4.650119e+00
## 32941  5 4661 4.639197e+00
## 32942  5 4662 4.629704e+00
## 32943  5 4663 4.631025e+00
## 32944  5 4664 4.615839e+00
## 32945  5 4665 4.609034e+00
## 32946  5 4666 4.604037e+00
## 32947  5 4667 4.595586e+00
## 32948  5 4668 4.594846e+00
## 32949  5 4669 4.582471e+00
[illegible]
[illegible]
[illegible]
[illegible]
## 32954  5 4674 4.547393e+00
## 32955  5 4675 4.540910e+00
## 32956  5 4676 4.532696e+00
## 32957  5 4677 4.529048e+00
## 32958  5 4678 4.511307e+00
## 32959  5 4679 4.513244e+00
## 32960  5 4680 4.518992e+00
## 32961  5 4681 4.501014e+00
## 32962  5 4682 4.501190e+00
## 32963  5 4683 4.491730e+00
## 32964  5 4684 4.476031e+00
## 32965  5 4685 4.481073e+00
## 32966  5 4686 4.471950e+00
## 32967  5 4687 4.456600e+00
## 32968  5 4688 4.463270e+00
## 32969  5 4689 4.442805e+00
## 32970  5 4690 4.451499e+00
## 32971  5 4691 4.441211e+00
## 32972  5 4692 4.428338e+00
## 32973  5 4693 4.427609e+00
## 32974  5 4694 4.420842e+00
## 32975  5 4695 4.409125e+00
## 32976  5 4696 4.409986e+00
## 32977  5 4697 4.400403e+00
## 32978  5 4698 4.394445e+00
## 32979  5 4699 4.391931e+00
## 32980  5 4700 4.381539e+00
## 32981  5 4701 4.369542e+00
## 32982  5 4702 4.368111e+00
## 32983  5 4703 4.352678e+00
## 32984  5 4704 4.358131e+00
## 32985  5 4705 4.354561e+00
## 32986  5 4706 4.341082e+00
## 32987  5 4707 4.335753e+00
## 32988  5 4708 4.331217e+00
## 32989  5 4709 4.320091e+00
## 32990  5 4710 4.316886e+00
## 32991  5 4711 4.314112e+00
## 32992  5 4712 4.298597e+00
## 32993  5 4713 4.296870e+00
## 32994  5 4714 4.294207e+00
## 32995  5 4715 4.280610e+00
## 32996  5 4716 4.280708e+00
## 32997  5 4717 4.269087e+00
## 32998  5 4718 4.266652e+00
## 32999  5 4719 4.258793e+00
## 33000  5 4720 4.252225e+00
## 33001  5 4721 4.250732e+00
## 33002  5 4722 4.242251e+00
## 33003  5 4723 4.228024e+00
## 33004  5 4724 4.229859e+00
## 33005  5 4725 4.221590e+00
## 33006  5 4726 4.210033e+00
## 33007  5 4727 4.205689e+00
## 33008  5 4728 4.207965e+00
## 33009  5 4729 4.196949e+00
## 33010  5 4730 4.190972e+00
## 33011  5 4731 4.191411e+00
## 33012  5 4732 4.184119e+00
## 33013  5 4733 4.166147e+00
## 33014  5 4734 4.169078e+00
## 33015  5 4735 4.163132e+00
## 33016  5 4736 4.161464e+00
## 33017  5 4737 4.157237e+00
## 33018  5 4738 4.145407e+00
## 33019  5 4739 4.136401e+00
## 33020  5 4740 4.139337e+00
## 33021  5 4741 4.121549e+00
## 33022  5 4742 4.115255e+00
## 33023  5 4743 4.109041e+00
## 33024  5 4744 4.111261e+00
## 33025  5 4745 4.097899e+00
## 33026  5 4746 4.098972e+00
## 33027  5 4747 4.088375e+00
## 33028  5 4748 4.083928e+00
## 33029  5 4749 4.075875e+00
## 33030  5 4750 4.064192e+00
## 33031  5 4751 4.072911e+00
## 33032  5 4752 4.047967e+00
## 33033  5 4753 4.047967e+00
## 33034  5 4754 4.042797e+00
## 33035  5 4755 4.045628e+00
## 33036  5 4756 4.037238e+00
## 33037  5 4757 4.036138e+00
## 33038  5 4758 4.026140e+00
## 33039  5 4759 4.019999e+00
## 33040  5 4760 4.011380e+00
## 33041  5 4761 4.001938e+00
## 33042  5 4762 4.003944e+00
## 33043  5 4763 3.997985e+00
## 33044  5 4764 3.989232e+00
## 33045  5 4765 3.979575e+00
## 33046  5 4766 3.977693e+00
## 33047  5 4767 3.975718e+00
## 33048  5 4768 3.966353e+00
## 33049  5 4769 3.962232e+00
## 33050  5 4770 3.953068e+00
## 33051  5 4771 3.947433e+00
## 33052  5 4772 3.938825e+00
## 33053  5 4773 3.939087e+00
## 33054  5 4774 3.935618e+00
## 33055  5 4775 3.928632e+00
## 33056  5 4776 3.922943e+00
## 33057  5 4777 3.911201e+00
## 33058  5 4778 3.912795e+00
## 33059  5 4779 3.906361e+00
## 33060  5 4780 3.898090e+00
## 33061  5 4781 3.892234e+00
## 33062  5 4782 3.888731e+00
## 33063  5 4783 3.883974e+00
## 33064  5 4784 3.873994e+00
## 33065  5 4785 3.871129e+00
## 33066  5 4786 3.859216e+00
## 33067  5 4787 3.858671e+00
## 33068  5 4788 3.852831e+00
## 33069  5 4789 3.852147e+00
## 33070  5 4790 3.842970e+00
## 33071  5 4791 3.843408e+00
## 33072  5 4792 3.832291e+00
## 33073  5 4793 3.821106e+00
## 33074  5 4794 3.819585e+00
## 33075  5 4795 3.809242e+00
## 33076  5 4796 3.802776e+00
## 33077  5 4797 3.802310e+00
## 33078  5 4798 3.797467e+00
## 33079  5 4799 3.794353e+00
## 33080  5 4800 3.790122e+00
## 33081  5 4801 3.780287e+00
## 33082  5 4802 3.778669e+00
## 33083  5 4803 3.766487e+00
## 33084  5 4804 3.754439e+00
## 33085  5 4805 3.758968e+00
## 33086  5 4806 3.753032e+00
## 33087  5 4807 3.736235e+00
## 33088  5 4808 3.741710e+00
## 33089  5 4809 3.737366e+00
## 33090  5 4810 3.730320e+00
## 33091  5 4811 3.725613e+00
## 33092  5 4812 3.718269e+00
## 33093  5 4813 3.715718e+00
## 33094  5 4814 3.707002e+00
## 33095  5 4815 3.700084e+00
## 33096  5 4816 3.696176e+00
## 33097  5 4817 3.697199e+00
## 33098  5 4818 3.687807e+00
## 33099  5 4819 3.678306e+00
## 33100  5 4820 3.671273e+00
## 33101  5 4821 3.669238e+00
## 33102  5 4822 3.669904e+00
## 33103  5 4823 3.660061e+00
## 33104  5 4824 3.661902e+00
## 33105  5 4825 3.646912e+00
## 33106  5 4826 3.649548e+00
## 33107  5 4827 3.635997e+00
## 33108  5 4828 3.631062e+00
## 33109  5 4829 3.632042e+00
## 33110  5 4830 3.623268e+00
## 33111  5 4831 3.620240e+00
## 33112  5 4832 3.611641e+00
## 33113  5 4833 3.608402e+00
## 33114  5 4834 3.600638e+00
## 33115  5 4835 3.592147e+00
## 33116  5 4836 3.586393e+00
## 33117  5 4837 3.583076e+00
## 33118  5 4838 3.577589e+00
## 33119  5 4839 3.579272e+00
## 33120  5 4840 3.572788e+00
## 33121  5 4841 3.560873e+00
## 33122  5 4842 3.559002e+00
## 33123  5 4843 3.554616e+00
## 33124  5 4844 3.549307e+00
## 33125  5 4845 3.545054e+00
## 33126  5 4846 3.536274e+00
## 33127  5 4847 3.539413e+00
## 33128  5 4848 3.529291e+00
## 33129  5 4849 3.525707e+00
## 33130  5 4850 3.516550e+00
## 33131  5 4851 3.510783e+00
## 33132  5 4852 3.507410e+00
## 33133  5 4853 3.499660e+00
## 33134  5 4854 3.498396e+00
## 33135  5 4855 3.493793e+00
## 33136  5 4856 3.493962e+00
## 33137  5 4857 3.487824e+00
## 33138  5 4858 3.475039e+00
## 33139  5 4859 3.472216e+00
## 33140  5 4860 3.463336e+00
## 33141  5 4861 3.458643e+00
## 33142  5 4862 3.450334e+00
## 33143  5 4863 3.448875e+00
## 33144  5 4864 3.446167e+00
## 33145  5 4865 3.443750e+00
## 33146  5 4866 3.436612e+00
## 33147  5 4867 3.431675e+00
## 33148  5 4868 3.426329e+00
## 33149  5 4869 3.425608e+00
## 33150  5 4870 3.418630e+00
## 33151  5 4871 3.418739e+00
## 33152  5 4872 3.411927e+00
## 33153  5 4873 3.400774e+00
## 33154  5 4874 3.393861e+00
## 33155  5 4875 3.391782e+00
## 33156  5 4876 3.385818e+00
## 33157  5 4877 3.384776e+00
## 33158  5 4878 3.378030e+00
## 33159  5 4879 3.372974e+00
## 33160  5 4880 3.364504e+00
## 33161  5 4881 3.358102e+00
## 33162  5 4882 3.364373e+00
## 33163  5 4883 3.352473e+00
## 33164  5 4884 3.348788e+00
## 33165  5 4885 3.336078e+00
## 33166  5 4886 3.337311e+00
## 33167  5 4887 3.335546e+00
## 33168  5 4888 3.331294e+00
## 33169  5 4889 3.327773e+00
## 33170  5 4890 3.322440e+00
## 33171  5 4891 3.314868e+00
## 33172  5 4892 3.314913e+00
## 33173  5 4893 3.300306e+00
## 33174  5 4894 3.298095e+00
## 33175  5 4895 3.290827e+00
## 33176  5 4896 3.288566e+00
## 33177  5 4897 3.287311e+00
## 33178  5 4898 3.284313e+00
## 33179  5 4899 3.269871e+00
## 33180  5 4900 3.274306e+00
## 33181  5 4901 3.259492e+00
## 33182  5 4902 3.255420e+00
## 33183  5 4903 3.256859e+00
## 33184  5 4904 3.252961e+00
## 33185  5 4905 3.247933e+00
## 33186  5 4906 3.236500e+00
## 33187  5 4907 3.238455e+00
## 33188  5 4908 3.234022e+00
## 33189  5 4909 3.226698e+00
## 33190  5 4910 3.221185e+00
## 33191  5 4911 3.224519e+00
## 33192  5 4912 3.215100e+00
## 33193  5 4913 3.211434e+00
## 33194  5 4914 3.201309e+00
## 33195  5 4915 3.198068e+00
## 33196  5 4916 3.195122e+00
## 33197  5 4917 3.194730e+00
## 33198  5 4918 3.183798e+00
## 33199  5 4919 3.179605e+00
## 33200  5 4920 3.182346e+00
## 33201  5 4921 3.176295e+00
## 33202  5 4922 3.162231e+00
## 33203  5 4923 3.155382e+00
## 33204  5 4924 3.155555e+00
## 33205  5 4925 3.157161e+00
## 33206  5 4926 3.145690e+00
## 33207  5 4927 3.145108e+00
## 33208  5 4928 3.137450e+00
## 33209  5 4929 3.131415e+00
## 33210  5 4930 3.131109e+00
## 33211  5 4931 3.123608e+00
## 33212  5 4932 3.122092e+00
## 33213  5 4933 3.109706e+00
## 33214  5 4934 3.111944e+00
## 33215  5 4935 3.100609e+00
## 33216  5 4936 3.103135e+00
## 33217  5 4937 3.095887e+00
## 33218  5 4938 3.100136e+00
## 33219  5 4939 3.084169e+00
## 33220  5 4940 3.090146e+00
## 33221  5 4941 3.078305e+00
## 33222  5 4942 3.072538e+00
## 33223  5 4943 3.074794e+00
## 33224  5 4944 3.066869e+00
## 33225  5 4945 3.058186e+00
## 33226  5 4946 3.057670e+00
## 33227  5 4947 3.053885e+00
## 33228  5 4948 3.051066e+00
## 33229  5 4949 3.042495e+00
## 33230  5 4950 3.039503e+00
## 33231  5 4951 3.038286e+00
## 33232  5 4952 3.029112e+00
## 33233  5 4953 3.029148e+00
## 33234  5 4954 3.024197e+00
## 33235  5 4955 3.016168e+00
## 33236  5 4956 3.010803e+00
## 33237  5 4957 3.008718e+00
## 33238  5 4958 2.999482e+00
## 33239  5 4959 2.997374e+00
## 33240  5 4960 2.995702e+00
## 33241  5 4961 2.989160e+00
## 33242  5 4962 2.985976e+00
## 33243  5 4963 2.975518e+00
## 33244  5 4964 2.979788e+00
## 33245  5 4965 2.971050e+00
## 33246  5 4966 2.967599e+00
## 33247  5 4967 2.963404e+00
## 33248  5 4968 2.959821e+00
## 33249  5 4969 2.959708e+00
## 33250  5 4970 2.953766e+00
## 33251  5 4971 2.947480e+00
## 33252  5 4972 2.945449e+00
## 33253  5 4973 2.938474e+00
## 33254  5 4974 2.936531e+00
## 33255  5 4975 2.928448e+00
## 33256  5 4976 2.929251e+00
## 33257  5 4977 2.920954e+00
## 33258  5 4978 2.917407e+00
## 33259  5 4979 2.917363e+00
## 33260  5 4980 2.910659e+00
## 33261  5 4981 2.901846e+00
## 33262  5 4982 2.900463e+00
## 33263  5 4983 2.896323e+00
## 33264  5 4984 2.891936e+00
## 33265  5 4985 2.886501e+00
## 33266  5 4986 2.882654e+00
## 33267  5 4987 2.879354e+00
## 33268  5 4988 2.875282e+00
## 33269  5 4989 2.875843e+00
## 33270  5 4990 2.870052e+00
## 33271  5 4991 2.861035e+00
## 33272  5 4992 2.858037e+00
## 33273  5 4993 2.855183e+00
## 33274  5 4994 2.852241e+00
## 33275  5 4995 2.844632e+00
## 33276  5 4996 2.841514e+00
## 33277  5 4997 2.836308e+00
## 33278  5 4998 2.832129e+00
## 33279  5 4999 2.830304e+00
## 33280  5 5000 2.826175e+00
## 33281  5 5001 2.824705e+00
## 33282  5 5002 2.811348e+00
## 33283  5 5003 2.812603e+00
## 33284  5 5004 2.807793e+00
## 33285  5 5005 2.802250e+00
## 33286  5 5006 2.801449e+00
## 33287  5 5007 2.793493e+00
## 33288  5 5008 2.792037e+00
## 33289  5 5009 2.789987e+00
## 33290  5 5010 2.785187e+00
## 33291  5 5011 2.775053e+00
## 33292  5 5012 2.778610e+00
## 33293  5 5013 2.778140e+00
## 33294  5 5014 2.766967e+00
## 33295  5 5015 2.766058e+00
## 33296  5 5016 2.761502e+00
## 33297  5 5017 2.752880e+00
## 33298  5 5018 2.753602e+00
## 33299  5 5019 2.745942e+00
## 33300  5 5020 2.744699e+00
## 33301  5 5021 2.737744e+00
## 33302  5 5022 2.734358e+00
## 33303  5 5023 2.733248e+00
## 33304  5 5024 2.731732e+00
## 33305  5 5025 2.724347e+00
## 33306  5 5026 2.716157e+00
## 33307  5 5027 2.716919e+00
## 33308  5 5028 2.710020e+00
## 33309  5 5029 2.709111e+00
## 33310  5 5030 2.705189e+00
## 33311  5 5031 2.698556e+00
## 33312  5 5032 2.696611e+00
## 33313  5 5033 2.694509e+00
## 33314  5 5034 2.693561e+00
## 33315  5 5035 2.683153e+00
## 33316  5 5036 2.679425e+00
## 33317  5 5037 2.675002e+00
## 33318  5 5038 2.669750e+00
## 33319  5 5039 2.661380e+00
## 33320  5 5040 2.671405e+00
## 33321  5 5041 2.664412e+00
## 33322  5 5042 2.656024e+00
## 33323  5 5043 2.656843e+00
## 33324  5 5044 2.652144e+00
## 33325  5 5045 2.645363e+00
## 33326  5 5046 2.645737e+00
## 33327  5 5047 2.642213e+00
## 33328  5 5048 2.629843e+00
## 33329  5 5049 2.629448e+00
## 33330  5 5050 2.620601e+00
## 33331  5 5051 2.623726e+00
## 33332  5 5052 2.615285e+00
## 33333  5 5053 2.617391e+00
##  [ reached 'max' / getOption("max.print") -- omitted 84638267 rows ]
```

Visualisation of C0 profiles at steady-state: <5, >8 or between 5 and 8 ug/L

```
ID_C0_Martial <- as_tibble(Data_combined_Martial) %>%
  dplyr::filter(time == 24) %>%
  select(ID, DV, time) %>%
  group_by(ID, time) %>%
  dplyr::filter(DV == max(DV)) %>%
  ungroup() %>%
  mutate(Group = case_when(
    DV < 5  ~ "Below 5",
    DV > 8  ~ "Above 8",
    TRUE    ~ "Between 5 and 8"
  ))

print(ID_C0_Martial)
```

```
## # A tibble: 12,000 × 4
##       ID    DV  time Group
##    <dbl> <dbl> <dbl> <chr>
##  1     1  2.25    24 Below 5
##  2     2  2.22    24 Below 5
##  3     3  1.87    24 Below 5
##  4     4  1.81    24 Below 5
##  5     5  6.59    24 Between 5 and 8
##  6     6  3.79    24 Below 5
##  7     7  1.60    24 Below 5
##  8     8  2.83    24 Below 5
##  9     9  1.38    24 Below 5
## 10    10  2.37    24 Below 5
## # ℹ 11,990 more rows
```

Profiles counting

```
count_Below_5_M <- nrow(ID_C0_Martial %>% dplyr::filter(Group == "Below 5"))
count_Above_8_M <- nrow(ID_C0_Martial %>% dplyr::filter(Group == "Above 8"))
count_Between_5_8_M <- nrow(ID_C0_Martial %>% dplyr::filter(Group == "Between 5 and 8"))

print(count_Below_5_M)
```

```
## [1] 1773
```

```
print(count_Above_8_M)
```

```
## [1] 9044
```

```
print(count_Between_5_8_M)
```

```
## [1] 1183
```

#Selection of profiles with a C0 in the therapeutic target: 5-8 ug/L

```
selection_5_8_M <- ID_C0_Martial %>%
  dplyr::filter(Group == "Between 5 and 8") %>%
  select(ID, DV, time)

Results_test_12000_M <- Data_combined_Martial %>%
  dplyr::filter(ID %in%selection_5_8_M$ID)

sim_data_filtered_12000_M <- Results_test_12000_M %>%
  group_by(ID, time) %>%
  dplyr::filter(DV == max(DV)) %>%
  ungroup()

sim_data_filtered_12000_M
```

```
## # A tibble: 8,282,183 × 3
##       ID  time    DV
##    <dbl> <dbl> <dbl>
##  1     5     0  6.59
##  2     5     1  6.79
##  3     5     2  6.88
##  4     5     3  6.92
##  5     5     4  6.91
##  6     5     5  6.91
##  7     5     6  6.90
##  8     5     7  6.89
##  9     5     8  6.85
## 10     5     9  6.84
## # ℹ 8,282,173 more rows
```

Dose distribution of selected profiles

```
# For IDs 1 to 6000
ids_to_keep_6000_M <- sim_data_filtered_12000_M$ID

filtered_data_with_info_6000_M <- Dataset_martial_6000 %>%
  dplyr::filter(ID %in% ids_to_keep_6000_M) %>%
  select(ID) %>%
  distinct(ID, .keep_all = TRUE) %>%
  mutate(
    Group_of_dose = case_when(
      ID >= 1 & ID <= 400 ~ "1 mg",
      ID >= 401 & ID <= 800 ~ "2 mg",
      ID >= 801 & ID <= 1200 ~ "3 mg",
      ID >= 1201 & ID <= 1600 ~ "4 mg",
      ID >= 1601 & ID <= 2000 ~ "5 mg",
      ID >= 2001 & ID <= 2400 ~ "6 mg",
      ID >= 2401 & ID <= 2800 ~ "7 mg",
      ID >= 2801 & ID <= 3200 ~ "8 mg",
      ID >= 3201 & ID <= 3600 ~ "9 mg",
      ID >= 3601 & ID <= 4000 ~ "10 mg",
      ID >= 4001 & ID <= 4400 ~ "11 mg",
      ID >= 4401 & ID <= 4800 ~ "12 mg",
      ID >= 4801 & ID <= 5200 ~ "13 mg",
      ID >= 5201 & ID <= 5600 ~ "14 mg",
      ID >= 5601 & ID <= 6000 ~ "15 mg",
      TRUE ~ NA_character_
    )
  )

# IDs de 6001 à 12000
ids_to_keep_12000_M <- sim_data_filtered_12000_M$ID

filtered_data_with_info_12000_M <- Dataset_martial_12000 %>%
  dplyr::filter(ID %in% ids_to_keep_12000_M) %>%
  select(ID) %>%
  distinct(ID, .keep_all = TRUE) %>%
  mutate(
    Group_of_dose = case_when(
      ID >= 6001 & ID <= 6400 ~ "0,5 mg",
      ID >= 6401 & ID <= 6800 ~ "1,5 mg",
      ID >= 6801 & ID <= 7200 ~ "2,5 mg",
      ID >= 7201 & ID <= 7600 ~ "3,5 mg",
      ID >= 7601 & ID <= 8000 ~ "4,5 mg",
      ID >= 8001 & ID <= 8400 ~ "5,5 mg",
```

```
      ID >= 8401 & ID <= 8800 ~ "6,5 mg",
      ID >= 8801 & ID <= 9200 ~ "7,5 mg",
      ID >= 9201 & ID <= 9600 ~ "8,5 mg",
      ID >= 9601 & ID <= 10000 ~ "9,5 mg",
      ID >= 10001 & ID <= 10400 ~ "10,5 mg",
      ID >= 10401 & ID <= 10800 ~ "11,5 mg",
      ID >= 10801 & ID <= 11200 ~ "12,5 mg",
      ID >= 11201 & ID <= 11600 ~ "13,5 mg",
      ID >= 11601 & ID <= 12000 ~ "14,5 mg",
      TRUE ~ NA_character_
    )
  )

# Combinaison des deux data frames
filtered_data_with_info_M <- bind_rows(filtered_data_with_info_6000_M, filtered_data_with_info_12000_M)

filtered_data_with_info_M
```

```
## # A tibble: 1,183 × 2
##       ID Group_of_dose
##    <int> <chr>
##  1     5 1 mg
##  2    28 1 mg
##  3    48 1 mg
##  4    72 1 mg
##  5    74 1 mg
##  6   101 1 mg
##  7   103 1 mg
##  8   112 1 mg
##  9   133 1 mg
## 10   142 1 mg
## # ℹ 1,173 more rows
```

#Dose distribution of selected profiles

```
filtered_data_with_info_histo_M <- filtered_data_with_info_M %>%
  mutate(
    Group_of_dose = factor(
      Group_of_dose,
      levels = c(
        "0,5 mg", "1 mg", "1,5 mg", "2 mg", "2,5 mg", "3 mg",
        "3,5 mg", "4 mg", "4,5 mg", "5 mg", "5,5 mg", "6 mg",
        "6,5 mg", "7 mg", "7,5 mg", "8 mg", "8,5 mg", "9 mg",
        "9,5 mg", "10 mg", "10,5 mg", "11 mg", "11,5 mg", "12 mg",
        "12,5 mg", "13 mg", "13,5 mg", "14 mg", "14,5 mg", "15 mg"))) %>%
  mutate(
    Group_of_dose = gsub(",", ".", Group_of_dose) %>% factor(),
    numeric_dose = as.numeric(gsub(" mg", "", Group_of_dose)),
    Group_of_dose = factor(Group_of_dose, levels = unique(Group_of_dose[order(numeric_dose)]))
  )

#histogram
ggplot(filtered_data_with_info_histo_M, aes(x = Group_of_dose)) +
  geom_bar(fill = "grey", color = "black") +
  geom_text(
    stat = "count",
    aes(label = ..count..),
    vjust = -0.5,
    size = 3
  ) +
  labs(
    title = "                                               Martial model",
    x = "Dose group",
    y = "Number of pharmacokinetic profiles"
  ) +
  theme_minimal() +
  theme(
    axis.text.x = element_text(angle = 45, hjust = 1))
```

```
## Warning: The dot-dot notation (`..count..`) was deprecated in ggplot2 3.4.0.
## ℹ Please use `after_stat(count)` instead.
## This warning is displayed once every 8 hours.
## Call `lifecycle::last_lifecycle_warnings()` to see where this warning was
## generated.
```

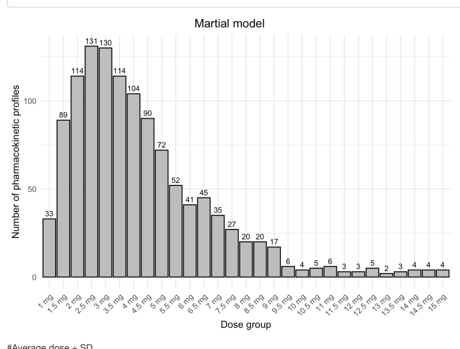

#Average dose + SD

```
filtered_data_combined_M <- filtered_data_with_info_M %>%
  mutate(
    Dose_numeric = as.numeric(sub(" mg", "", gsub(",", ".", Group_of_dose)))  # Convertir en numérique
  )

dose_stats_M <- filtered_data_combined_M %>%
  summarize(
    Mean_dose = mean(Dose_numeric, na.rm = TRUE),
    SD_dose = sd(Dose_numeric, na.rm = TRUE)
  )

dose_stats_M
```

```
## # A tibble: 1 × 2
##   Mean_dose SD_dose
##       <dbl>   <dbl>
## 1      4.26    2.51
```

Time separation by simulated event

```
# Définir les intervalles et les durées d'oubli
sim_data_by_scenario_M <- sim_data_filtered_12000_M %>%
  mutate(
    forget_event = case_when(
      time >= 0 & time < 312 ~ "no_forget",
            time >= 312 & time < 624 ~ "forget_3h_retake_x1dose",
      time >= 624 & time < 936 ~ "forget_6h_retake_x1dose",
            time >= 936 & time < 1248 ~ "forget_9h_retake_x1dose",
      time >= 1248 & time < 1560 ~ "forget_12h_retake_x1dose",
            time >= 1560 & time < 1872 ~ "forget_15h_retake_x0.5dose",
      time >= 1872 & time < 2184 ~ "forget_15h_retake_x1dose",
            time >= 2184 & time < 2496 ~ "forget_18h_retake_x0.5dose",
      time >= 2496 & time < 2808 ~ "forget_18h_retake_x1dose",
      time >= 2808 & time < 3120 ~ "forget_21h_retake_x0.5dose",
            time >= 3120 & time < 3432 ~ "forget_21h_retake_x1dose",
      time >= 3456 & time < 3768 ~ "forget_24h_retake_x1dose",
      time >= 3788 & time < 4104 ~ "forget_24h_retake_x1.5dose",
            time >= 4128 & time < 4440 ~ "forget_24h_retake_x2dose"))

sim_data_by_scenario_M
```

```
## # A tibble: 8,282,183 × 4
##       ID  time    DV forget_event
##    <dbl> <dbl> <dbl> <chr>
##  1     5     0  6.59 no_forget
##  2     5     1  6.79 no_forget
##  3     5     2  6.88 no_forget
##  4     5     3  6.92 no_forget
##  5     5     4  6.91 no_forget
##  6     5     5  6.91 no_forget
##  7     5     6  6.90 no_forget
##  8     5     7  6.89 no_forget
##  9     5     8  6.85 no_forget
## 10     5     9  6.84 no_forget
## # ℹ 8,282,173 more rows
```

#C0 measurements for each profile every 24 hours

```
time_intervals <- list(
  c(24, 312), c(336, 624), c(648, 936), c(960, 1248),
  c(1272, 1560), c(1584, 1872), c(1896, 2184), c(2208, 2496),
  c(2520, 2808), c(2832, 3120), c(3144, 3432), c(3456, 3768),
  c(3772, 4104), c(4128, 4440)
)

filter_by_interval <- function(forget_event_group, interval) {
  sim_data_by_scenario_M %>%
    dplyr::filter(forget_event == forget_event_group,
                  time >= interval[1],
                  time < interval[2],
                  time %% 24 == 0)
}

forget_events <- unique(sim_data_by_scenario_M$forget_event)

# C0 calculation by forget_event and interval of time
results_final_C0_scenario_M <- map_dfr(forget_events, function(event) {
  map_dfr(time_intervals, function(interval) {
    filter_by_interval(event, interval)
  })
})

results_final_C0_scenario_M <- results_final_C0_scenario_M %>%
  arrange(forget_event, time)

print(results_final_C0_scenario_M)
```

```
## # A tibble: 202,293 × 4
##       ID  time    DV forget_event
##    <dbl> <dbl> <dbl> <chr>
##  1     5  1272  6.60 forget_12h_retake_x1dose
##  2    28  1272  7.80 forget_12h_retake_x1dose
##  3    48  1272  6.55 forget_12h_retake_x1dose
##  4    72  1272  5.22 forget_12h_retake_x1dose
##  5    74  1272  5.43 forget_12h_retake_x1dose
##  6   101  1272  6.72 forget_12h_retake_x1dose
##  7   103  1272  7.32 forget_12h_retake_x1dose
##  8   112  1272  5.77 forget_12h_retake_x1dose
##  9   133  1272  5.06 forget_12h_retake_x1dose
## 10   142  1272  5.46 forget_12h_retake_x1dose
## # ℹ 202,283 more rows
```

Calculation of relative differences in C0 for each profile

```
C0_basale_by_scenario_M <- results_final_C0_scenario_M %>%
  group_by(ID, forget_event) %>%
  arrange(time) %>%
  mutate(C0_basale = first(DV)) %>%
  ungroup()

Relative_C0_change_by_scenario_M <- C0_basale_by_scenario_M %>%
  group_by(forget_event) %>%
  mutate(relative_C0 = (DV - C0_basale) / C0_basale * 100) %>%
  ungroup()

Relative_C0_change_by_scenario_M
```

```
## # A tibble: 202,293 × 6
##       ID  time    DV forget_event C0_basale relative_C0
##    <dbl> <dbl> <dbl> <chr>            <dbl>       <dbl>
##  1     5    24  6.59 no_forget         6.59           0
##  2    28    24  7.79 no_forget         7.79           0
##  3    48    24  6.56 no_forget         6.56           0
##  4    72    24  5.23 no_forget         5.23           0
##  5    74    24  5.45 no_forget         5.45           0
##  6   101    24  6.73 no_forget         6.73           0
##  7   103    24  7.33 no_forget         7.33           0
##  8   112    24  5.77 no_forget         5.77           0
##  9   133    24  5.05 no_forget         5.05           0
## 10   142    24  5.47 no_forget         5.47           0
## # ℹ 202,283 more rows
```

Rename time for each group of forget_event

```
Data_with_time_renamed_M <- Relative_C0_change_by_scenario_M %>%
  mutate(time_adjusted = case_when(
    time >= 24 & time < 312 ~ paste0((time - 24) / 24 * 24 + 24, "h"),
    time >= 336 & time < 624 ~ paste0((time - 336) / 24 * 24 + 24, "h"),
    time >= 648 & time < 936 ~ paste0((time - 648) / 24 * 24 + 24, "h"),
    time >= 960 & time < 1248 ~ paste0((time - 960) / 24 * 24 + 24, "h"),
    time >= 1272 & time < 1560 ~ paste0((time - 1272) / 24 * 24 + 24, "h"),
    time >= 1584 & time < 1872 ~ paste0((time - 1584) / 24 * 24 + 24, "h"),
    time >= 1896 & time < 2184 ~ paste0((time - 1896) / 24 * 24 + 24, "h"),
    time >= 2208 & time < 2496 ~ paste0((time - 2208) / 24 * 24 + 24, "h"),
    time >= 2520 & time < 2808 ~ paste0((time - 2520) / 24 * 24 + 24, "h"),
    time >= 2832 & time < 3120 ~ paste0((time - 2832) / 24 * 24 + 24, "h"),
    time >= 3144 & time < 3432 ~ paste0((time - 3144) / 24 * 24 + 24, "h"),

      time >= 3456 & time < 3768 ~ paste0((time - 3456) / 24 * 24 + 24, "h"),
    time >= 3792 & time < 4104 ~ paste0((time - 3792) / 24 * 24 + 24, "h"),
    time >= 4128 & time < 4440 ~ paste0((time - 4128) / 24 * 24 + 24, "h"),

    TRUE ~ paste0(time, "h")
  ))

print(Data_with_time_renamed_M)
```

```
## # A tibble: 202,293 × 7
##       ID  time    DV forget_event C0_basale relative_C0 time_adjusted
##    <dbl> <dbl> <dbl> <chr>            <dbl>       <dbl> <chr>
##  1     5    24  6.59 no_forget         6.59           0 24h
##  2    28    24  7.79 no_forget         7.79           0 24h
##  3    48    24  6.56 no_forget         6.56           0 24h
##  4    72    24  5.23 no_forget         5.23           0 24h
##  5    74    24  5.45 no_forget         5.45           0 24h
##  6   101    24  6.73 no_forget         6.73           0 24h
##  7   103    24  7.33 no_forget         7.33           0 24h
##  8   112    24  5.77 no_forget         5.77           0 24h
##  9   133    24  5.05 no_forget         5.05           0 24h
## 10   142    24  5.47 no_forget         5.47           0 24h
## # ℹ 202,283 more rows
```

#Average statistics of C0 and RD: all profiles

```
summary_stats_expanded_M <- Data_with_time_renamed_M %>%
  group_by(forget_event, time_adjusted) %>%
  summarize(
    min_DV = fivenum(DV)[1],        # Minimum
    Q1_DV = fivenum(DV)[2],         # First quartile (Q1)
    median_DV = fivenum(DV)[3],     # Median (Q2)
    Q3_DV = fivenum(DV)[4],         # Third quartile (Q3)
    max_DV = fivenum(DV)[5],        # Maximum
    mean_DV = mean(DV, na.rm = TRUE),  # Mean
    sd_DV = sd(DV, na.rm = TRUE),      # SD

    min_relative_C0 = fivenum(relative_C0)[1],        # Minimum
    Q1_relative_C0 = fivenum(relative_C0)[2],         # First quartile (Q1)
    median_relative_C0 = fivenum(relative_C0)[3],     # Median (Q2)
    Q3_relative_C0 = fivenum(relative_C0)[4],         # Third quartile (Q3)
    max_relative_C0 = fivenum(relative_C0)[5],        # Maximum
    mean_relative_C0 = mean(relative_C0, na.rm = TRUE),  # Mean
    sd_relative_C0 = sd(relative_C0, na.rm = TRUE)       # SD
  ) %>%
  ungroup() %>%
  arrange(forget_event, time_adjusted)
```

```
## `summarise()` has grouped output by 'forget_event'. You can override using the
## `.groups` argument.
```

```
summary_stats_expanded_M
```

```
## # A tibble: 171 × 16
##    forget_event  time_adjusted min_DV Q1_DV median_DV Q3_DV max_DV mean_DV sd_DV
##    <chr>         <chr>          <dbl> <dbl>     <dbl> <dbl>  <dbl>   <dbl> <dbl>
##  1 forget_12h_r… 120h            5.02  5.78      6.49  7.24   8.05    6.50 0.867
##  2 forget_12h_r… 144h            5.01  5.78      6.49  7.23   8.02    6.50 0.866
##  3 forget_12h_r… 168h            5.01  5.77      6.48  7.23   8.02    6.49 0.866
##  4 forget_12h_r… 192h            5.01  5.77      6.48  7.23   8.02    6.49 0.865
##  5 forget_12h_r… 216h            5.00  5.76      6.47  7.23   8.02    6.49 0.865
##  6 forget_12h_r… 240h            5.00  5.76      6.47  7.23   8.00    6.49 0.865
##  7 forget_12h_r… 24h             5.00  5.75      6.45  7.21   7.99    6.48 0.863
##  8 forget_12h_r… 264h            5.01  5.76      6.47  7.23   8.01    6.48 0.864
##  9 forget_12h_r… 288h            5.01  5.76      6.47  7.22   8.00    6.48 0.864
## 10 forget_12h_r… 48h             5.00  6.35      7.20  8.04  11.7     7.26 1.18
## # ℹ 161 more rows
## # ℹ 7 more variables: min_relative_C0 <dbl>, Q1_relative_C0 <dbl>,
## #   median_relative_C0 <dbl>, Q3_relative_C0 <dbl>, max_relative_C0 <dbl>,
## #   mean_relative_C0 <dbl>, sd_relative_C0 <dbl>
```

#Histograms of mean C0 and mean RD by simulated event

```
desired_order <- c("no_forget",
                   "forget_3h_retake_x1dose",
                   "forget_6h_retake_x1dose",
                   "forget_9h_retake_x1dose",
                   "forget_12h_retake_x1dose",
                   "forget_15h_retake_x0.5dose",
                   "forget_15h_retake_x1dose",
                   "forget_18h_retake_x0.5dose",
                   "forget_18h_retake_x1dose",
                   "forget_21h_retake_x0.5dose",
                   "forget_21h_retake_x1dose",
                   "forget_24h_retake_x1dose",
                   "forget_24h_retake_x1.5dose",
                   "forget_24h_retake_x2dose")

summary_stats_expanded_M$forget_event <- factor(summary_stats_expanded_M$forget_event, levels = desired_order)

time_adjusted_order <- c("24h", "48h", "72h", "96h", "120h", "144h", "168h", "192h",
                         "216h", "240h", "264h", "288h", "312h")

summary_stats_expanded_M <- summary_stats_expanded_M %>%
  mutate(time_adjusted = factor(time_adjusted, levels = time_adjusted_order))

forget_events_M_C0 <- levels(summary_stats_expanded_M$forget_event)

for (event in forget_events_M_C0) {
  data_subset_M_C0 <- summary_stats_expanded_M %>%
    dplyr::filter(forget_event == event)

  p9 <- ggplot(data_subset_M_C0, aes(x = time_adjusted, y = mean_DV)) +
    geom_bar(stat = "identity", fill = "grey", color = "black") +
    geom_text(aes(label = round(mean_DV, 1)), vjust = -0.4, color = "black") +
    labs(title = paste("Mean residual concentration for :", event),
         x = "Time (h)", y = "Mean Residual Concentration") +
    theme_bw() +
    theme(plot.title = element_text(hjust = 0.5),
          axis.text.x = element_text(angle = 45, hjust = 1))

  print(p9)

  p10 <- ggplot(data_subset_M_C0, aes(x = time_adjusted, y = mean_relative_C0)) +
    geom_bar(stat = "identity", fill = "steelblue", color = "black") +
    geom_text(aes(label = paste0(round(mean_relative_C0, 1), "%")), vjust = -0.4, color = "black") +
    labs(title = paste("Relative difference of mean through concentration for:", event),
         x = "Time (h)", y = "Mean relative difference of through concentration") +
    theme_bw() +
    theme(plot.title = element_text(hjust = 0.5),
          axis.text.x = element_text(angle = 45, hjust = 1)) +
    ylim(-100, 170) +
    geom_hline(yintercept = c(-5, 5), linetype = "dashed", color = "red")

  print(p10)
}
```

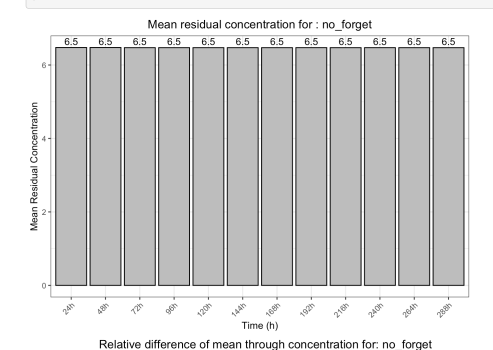

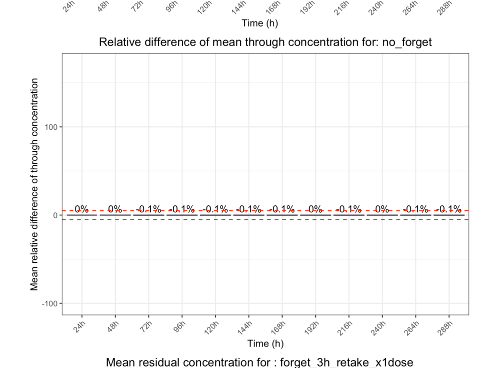

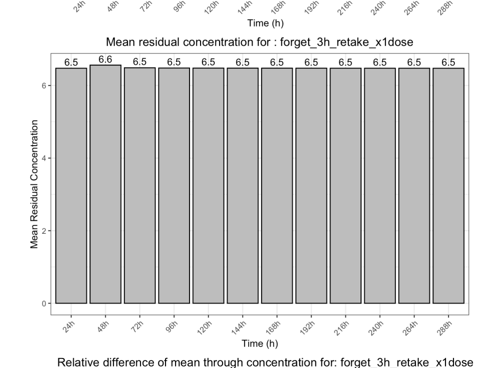

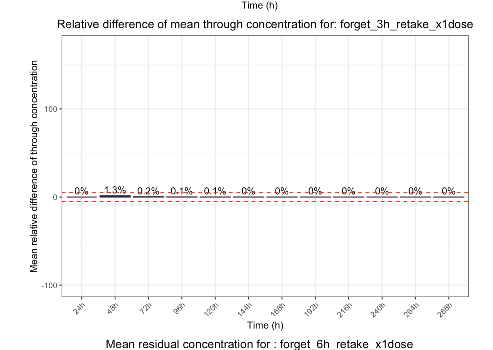

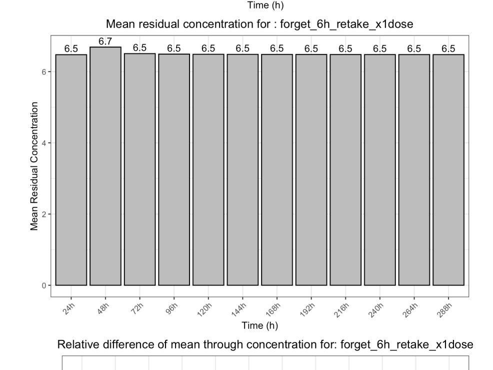

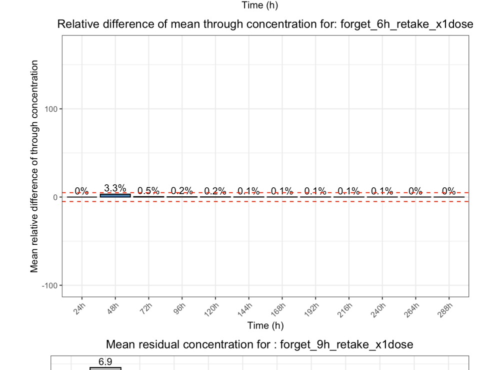

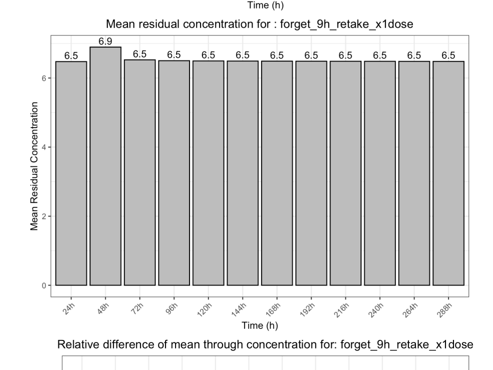

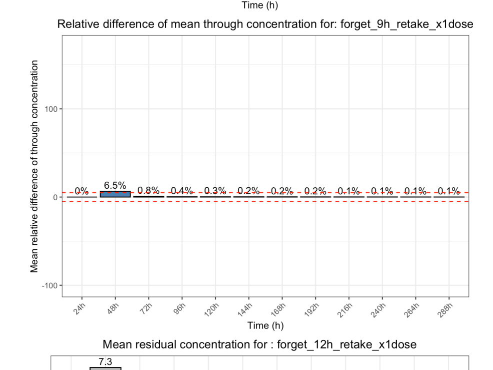

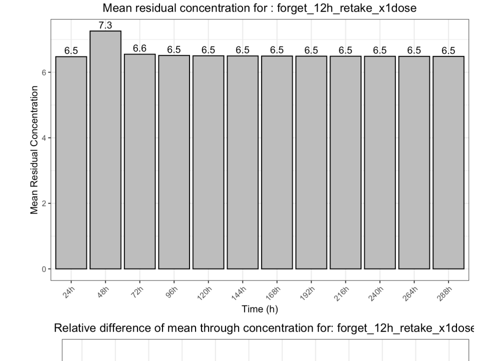

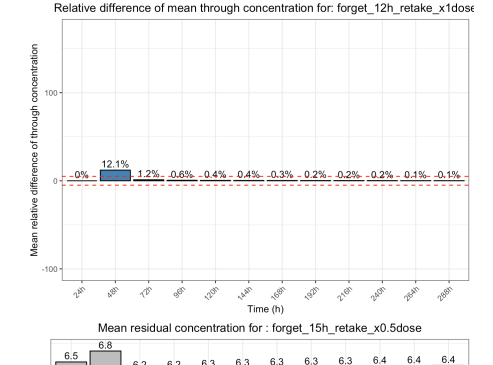

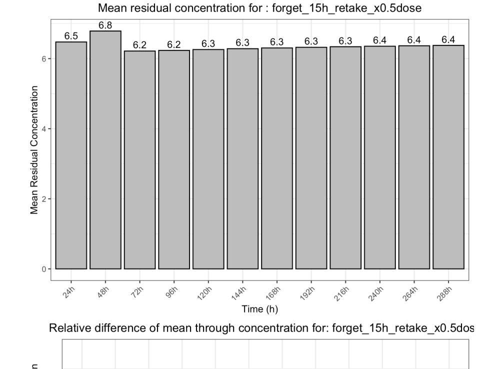

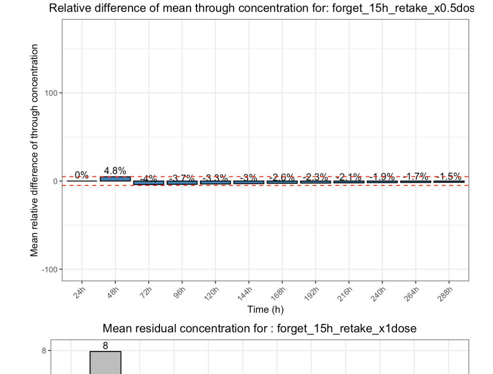

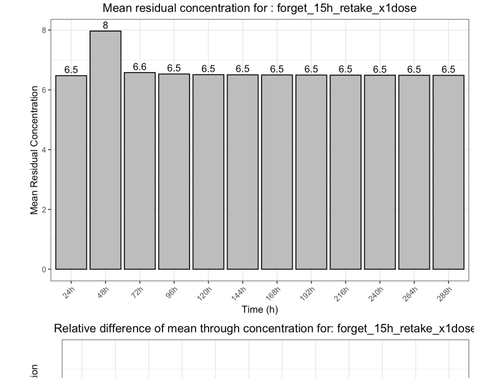

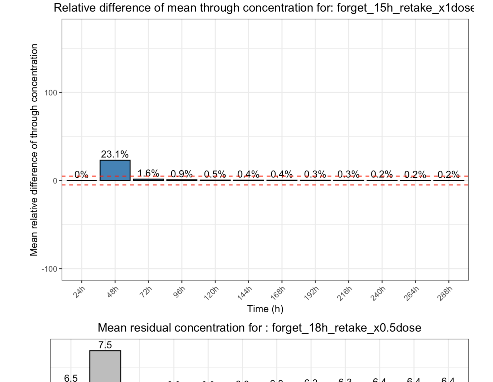

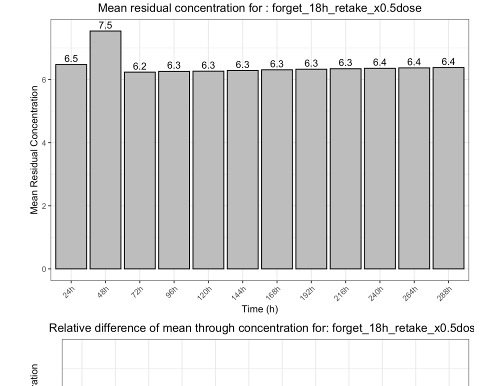

Relative difference of mean through concentration for: forget_18h_retake_x0.5dose

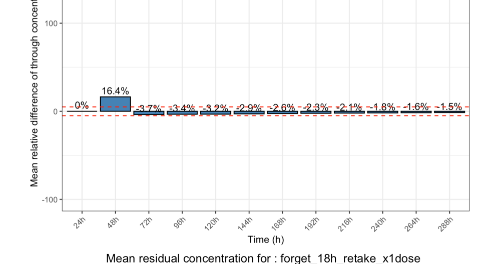

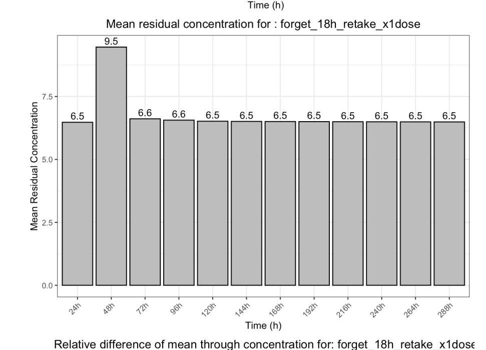

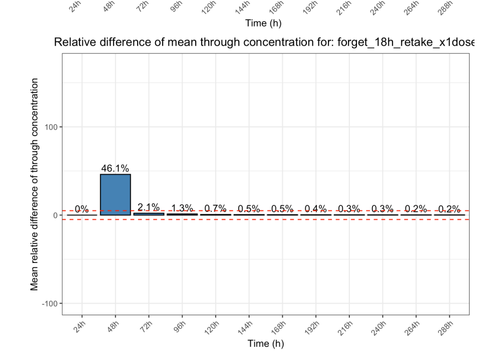

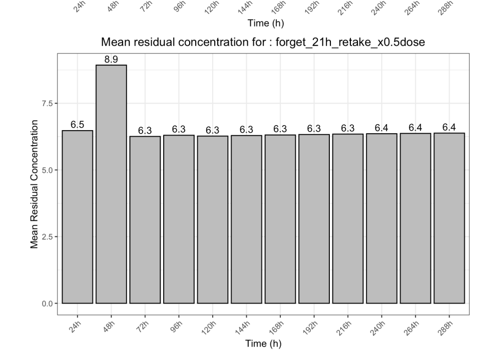

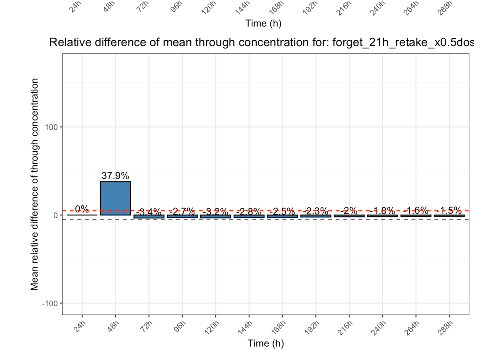

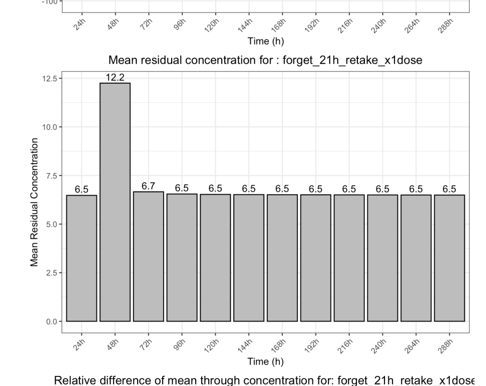

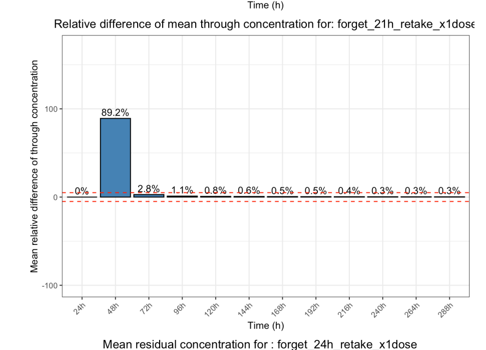

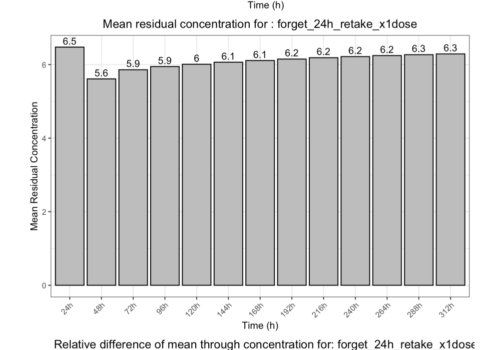

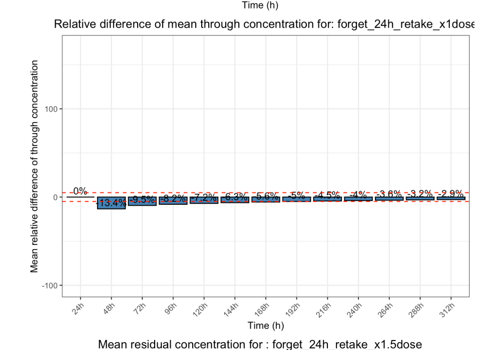

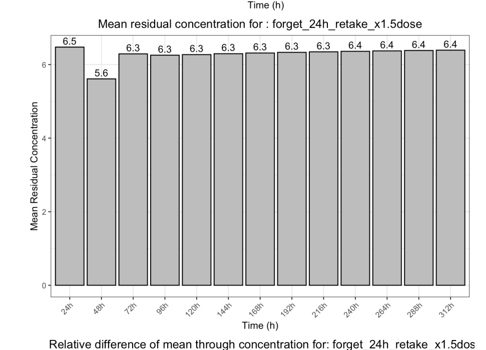

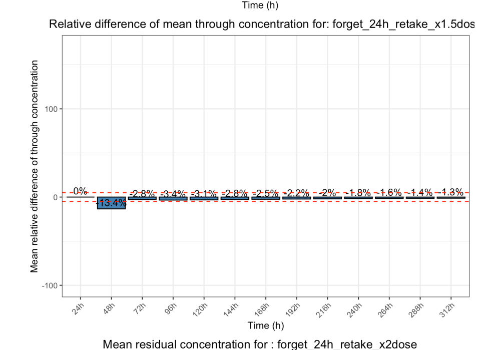

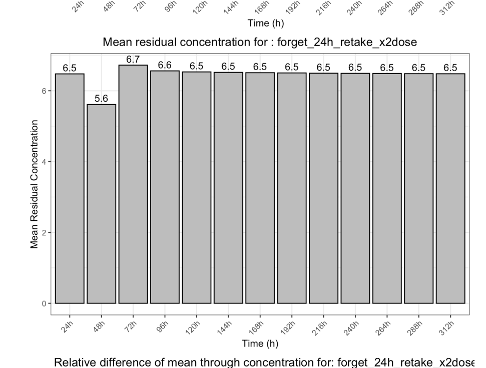

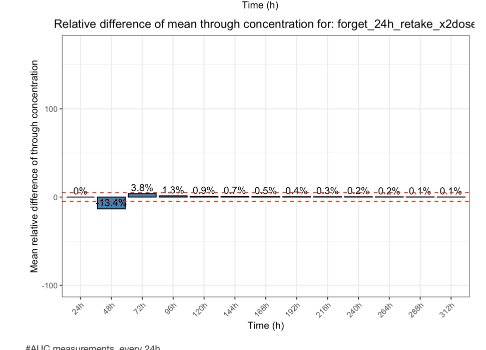

#AUC measurements, every 24h

```r
start <- seq(0, 4416, by = 24)
end <- seq(24, 4440, by = 24)

iterate_auc <- function(start, end) {
  sim_data_by_scenario_M %>%
    group_by(ID) %>%
    rename(id = ID) %>%
    makeAUC(DV ~ time, start = start, end = end) %>%
    mutate(
      auc = round(tau, 0),
      intervalle = paste0(start, "_", end)
    ) %>%
    select(id, auc, intervalle)
}

auc_test_TEST_M <- map2_df(start, end, iterate_auc) %>%
  arrange(id, intervalle)


assign_forget_event <- function(start, end) {
  case_when(
    start >= 0 & end <= 312 ~ "no_forget",
    start >= 312 & end <= 624 ~ "forget_3h_retake_x1dose",
    start >= 624 & end <= 936 ~ "forget_6h_retake_x1dose",
    start >= 936 & end <= 1248 ~ "forget_9h_retake_x1dose",
    start >= 1248 & end <= 1560 ~ "forget_12h_retake_x1dose",
    start >= 1560 & end <= 1872 ~ "forget_15h_retake_x0.5dose",
    start >= 1872 & end <= 2184 ~ "forget_15h_retake_x1dose",
    start >= 2184 & end <= 2496 ~ "forget_18h_retake_x0.5dose",
    start >= 2496 & end <= 2808 ~ "forget_18h_retake_x1dose",
    start >= 2808 & end <= 3120 ~ "forget_21h_retake_x0.5dose",
    start >= 3120 & end <= 3432 ~ "forget_21h_retake_x1dose",
    start >= 3432 & end <= 3768 ~ "forget_24h_retake_x1dose",
    start >= 3768 & end <= 4104 ~ "forget_24h_retake_x1.5dose",
    start >= 4104 & end <= 4440 ~ "forget_24h_retake_x2dose",
    TRUE ~ NA_character_
  )
}

auc_test_TEST_by_scenario_M <- auc_test_TEST_M %>%
  mutate(
    start = as.numeric(str_extract(intervalle, "^[0-9]+")),
    end = as.numeric(str_extract(intervalle, "(?<=_)[0-9]+")),
    forget_event = assign_forget_event(start, end)
  )

auc_test_TEST_by_scenario_M <- auc_test_TEST_by_scenario_M %>%
  select(-start, -end)

print(auc_test_TEST_by_scenario_M)
```

```
## # A tibble: 218,855 × 4
## # Groups:   id [1,183]
##       id   auc intervalle forget_event
##    <dbl> <dbl> <chr>      <chr>
##  1     5   163 0_24       no_forget
##  2     5   163 1008_1032  forget_9h_retake_x1dose
##  3     5   163 1032_1056  forget_9h_retake_x1dose
##  4     5   163 1056_1080  forget_9h_retake_x1dose
##  5     5   163 1080_1104  forget_9h_retake_x1dose
##  6     5   163 1104_1128  forget_9h_retake_x1dose
##  7     5   163 1128_1152  forget_9h_retake_x1dose
##  8     5   163 1152_1176  forget_9h_retake_x1dose
##  9     5   163 1176_1200  forget_9h_retake_x1dose
## 10     5   163 1200_1224  forget_9h_retake_x1dose
## # ℹ 218,845 more rows
```

Calculation of relative differences in AUC for each profile

```r
AUC_basale_by_scenario_M <- auc_test_TEST_by_scenario_M %>%
  group_by(id, forget_event) %>%
  arrange(intervalle) %>%
  mutate(AUC_basale = first(auc)) %>%
  ungroup()

Relative_AUC_change_by_scenario_M <- AUC_basale_by_scenario_M %>%
  group_by(forget_event) %>%
  mutate(relative_AUC_by_scenario = (auc - AUC_basale) / AUC_basale * 100) %>%
  ungroup()

Relative_AUC_change_by_scenario_M
```

```
## # A tibble: 218,855 × 6
##       id   auc intervalle forget_event AUC_basale relative_AUC_by_scenario
##    <dbl> <dbl> <chr>      <chr>             <dbl>                    <dbl>
##  1     5   163 0_24       no_forget           163                        0
##  2    28   204 0_24       no_forget           204                        0
##  3    48   193 0_24       no_forget           193                        0
##  4    72   140 0_24       no_forget           140                        0
##  5    74   151 0_24       no_forget           151                        0
##  6   101   186 0_24       no_forget           186                        0
##  7   103   192 0_24       no_forget           192                        0
##  8   112   157 0_24       no_forget           157                        0
##  9   133   148 0_24       no_forget           148                        0
## 10   142   141 0_24       no_forget           141                        0
## # ℹ 218,845 more rows
```

#Renaming intervals

```r
rename_interval <- function(intervalle) {
  start <- as.numeric(sub("_.*", "", intervalle))
  end <- as.numeric(sub(".*_", "", intervalle))

 if ((start >= 3432 && end <= 3768) || (start >= 3768 && end <= 4104) || (start >= 4104 && end <= 4440)) {
    interval_index <- ((start - 3432) %% 336) %/% 24
    new_start <- interval_index * 24
    new_end <- new_start + 24
  } else {
    interval_index <- ((start - 312) %% 312) %/% 24
    new_start <- interval_index * 24
    new_end <- new_start + 24
  }

  return(paste0(new_start, "_", new_end))
}

Renamed_AUC_M <- Relative_AUC_change_by_scenario_M %>%
  mutate(intervalle_renamed = sapply(intervalle, rename_interval))

print(Renamed_AUC_M)
```

```
## # A tibble: 218,855 × 7
##       id   auc intervalle forget_event AUC_basale relative_AUC_by_scenario
##    <dbl> <dbl> <chr>      <chr>             <dbl>                    <dbl>
##  1     5   163 0_24       no_forget           163                        0
##  2    28   204 0_24       no_forget           204                        0
##  3    48   193 0_24       no_forget           193                        0
##  4    72   140 0_24       no_forget           140                        0
##  5    74   151 0_24       no_forget           151                        0
##  6   101   186 0_24       no_forget           186                        0
##  7   103   192 0_24       no_forget           192                        0
##  8   112   157 0_24       no_forget           157                        0
##  9   133   148 0_24       no_forget           148                        0
## 10   142   141 0_24       no_forget           141                        0
## # ℹ 218,845 more rows
## # ℹ 1 more variable: intervalle_renamed <chr>
```

#AUC and RD average statistics: all profiles

```r
summary_stats_auc_M <- Renamed_AUC_M %>%
  group_by(forget_event, intervalle_renamed) %>%
  summarize(
    min_auc = fivenum(auc)[1],        # Minimum
    Q1_auc = fivenum(auc)[2],         # First quartile (Q1)
    median_auc = fivenum(auc)[3],     # Median (Q2)
    Q3_auc = fivenum(auc)[4],         # Third quartile (Q3)
    max_auc = fivenum(auc)[5],        # Maximum
    mean_auc = mean(auc, na.rm = TRUE),  # Mean
    sd_auc = sd(auc, na.rm = TRUE),      # SD

    min_relative_auc = fivenum(relative_AUC_by_scenario)[1],        # Minimum
    Q1_relative_auc = fivenum(relative_AUC_by_scenario)[2],         # First quartile (Q1)
    median_relative_auc = fivenum(relative_AUC_by_scenario)[3],     # Median (Q2)
    Q3_relative_auc = fivenum(relative_AUC_by_scenario)[4],         # Third quartile (Q3)
    max_relative_auc = fivenum(relative_AUC_by_scenario)[5],        # Maximum
    mean_relative_auc = mean(relative_AUC_by_scenario, na.rm = TRUE),  # Mean
    sd_relative_auc = sd(relative_AUC_by_scenario, na.rm = TRUE)       # SD
  ) %>%
  ungroup() %>%
  arrange(intervalle_renamed)
```

```
## `summarise()` has grouped output by 'forget_event'. You can override using the
## `.groups` argument.
```

```r
summary_stats_auc_M
```

```
## # A tibble: 185 × 16
##    forget_event      intervalle_renamed min_auc Q1_auc median_auc Q3_auc max_auc
##    <chr>             <chr>                <dbl>  <dbl>      <dbl>  <dbl>   <dbl>
##  1 forget_12h_retak… 0_24                   125   172         199    231     377
##  2 forget_15h_retak… 0_24                   125   172         199    231     377
##  3 forget_15h_retak… 0_24                   125   172         199    231     377
##  4 forget_18h_retak… 0_24                   125   172         199    231     377
##  5 forget_18h_retak… 0_24                   125   172.        199    231     377
##  6 forget_21h_retak… 0_24                   125   172         199    231     377
##  7 forget_21h_retak… 0_24                   125   172         199    231     377
##  8 forget_24h_retak… 0_24                   125   172         199    231     377
##  9 forget_24h_retak… 0_24                   125   172         199    231     377
## 10 forget_24h_retak… 0_24                   125   172         199    231     377
## # ℹ 175 more rows
## # ℹ 9 more variables: mean_auc <dbl>, sd_auc <dbl>, min_relative_auc <dbl>,
## #   Q1_relative_auc <dbl>, median_relative_auc <dbl>, Q3_relative_auc <dbl>,
## #   max_relative_auc <dbl>, mean_relative_auc <dbl>, sd_relative_auc <dbl>
```

#AUC and RD histograms: all profiles

```r
desired_order <- c("no_forget",
                   "forget_3h_retake_x1dose",
                   "forget_6h_retake_x1dose",
                   "forget_9h_retake_x1dose",
                   "forget_12h_retake_x1dose",
                   "forget_15h_retake_x0.5dose",
                   "forget_15h_retake_x1dose",
                   "forget_18h_retake_x0.5dose",
                   "forget_18h_retake_x1dose",
                   "forget_21h_retake_x0.5dose",
                   "forget_21h_retake_x1dose",
                   "forget_24h_retake_x1dose",
                   "forget_24h_retake_x1.5dose",
                   "forget_24h_retake_x2dose")

summary_stats_auc_M <- summary_stats_auc_M %>%
  mutate(
    interval_start = as.numeric(str_extract(intervalle_renamed, "^[0-9]+"))
  )

summary_stats_auc_M <- summary_stats_auc_M %>%
  arrange(forget_event, interval_start)

summary_stats_auc_M$intervalle_renamed <- factor(summary_stats_auc_M$intervalle_renamed, levels = unique(summary_
stats_auc_M$intervalle_renamed))

summary_stats_auc_M$forget_event <- factor(summary_stats_auc_M$forget_event, levels = desired_order)

forget_events_M <- levels(summary_stats_auc_M$forget_event)

for (event in forget_events_M) {
  data_subset_M <- summary_stats_auc_M %>%
    dplyr::filter(forget_event == event)

  p11 <- ggplot(data_subset_M, aes(x = intervalle_renamed, y = mean_auc)) +
    geom_bar(stat = "identity", fill = "grey", color = "black") +
    geom_text(aes(label = round(mean_auc, 1)), vjust = -0.5, color = "black") +
    labs(title = paste("Mean AUC for Forget Event:", event),
         x = "Interval of time (h)", y = "Mean AUC") +
    theme_bw() +
    theme(plot.title = element_text(hjust = 0.5)) +
  theme(axis.text.x = element_text(angle = 45, hjust = 1))

  print(p11)

p12 <- ggplot(data_subset_M, aes(x = intervalle_renamed, y = mean_relative_auc)) +
  geom_bar(stat = "identity", fill = "steelblue", color = "black") +
  geom_text(aes(label = round(mean_relative_auc, 1)), vjust = -0.5, color = "black") +
  labs(title = paste("Mean Relative Difference of AUC by Forget Event:", event),
       x = "Interval of time (h)", y = "Mean RD of AUC") +
  theme_bw() +
  theme(plot.title = element_text(hjust = 0.5)) +
  ylim(-100, 100) +
  geom_hline(yintercept = c(-5, 5), linetype = "dashed", color = "red") +
  theme(plot.title = element_text(hjust = 0.5)) +
  theme(axis.text.x = element_text(angle = 45, hjust = 1))

print(p12)
}
```

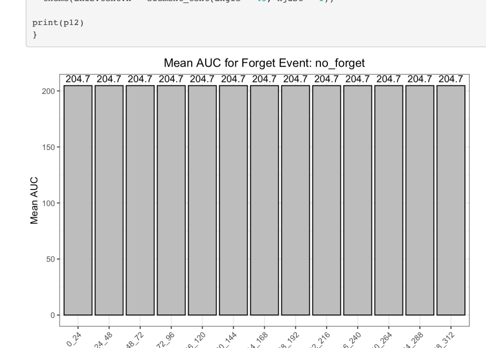

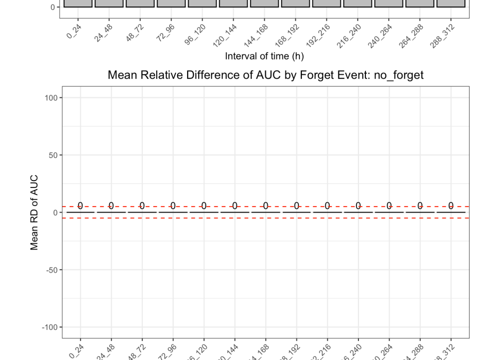

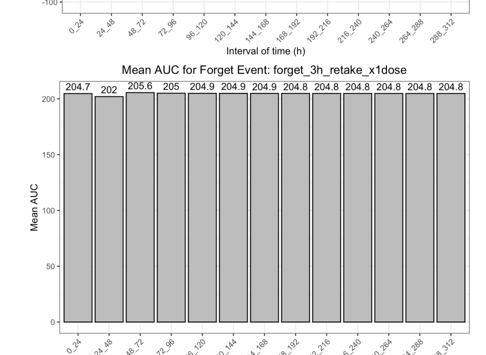

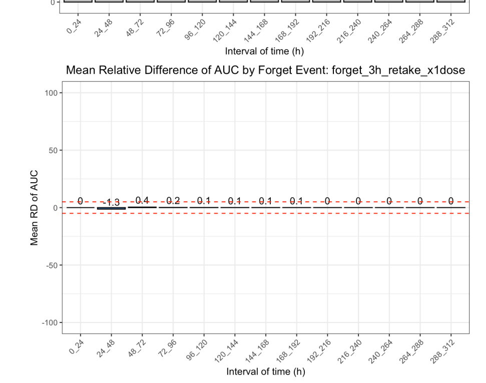

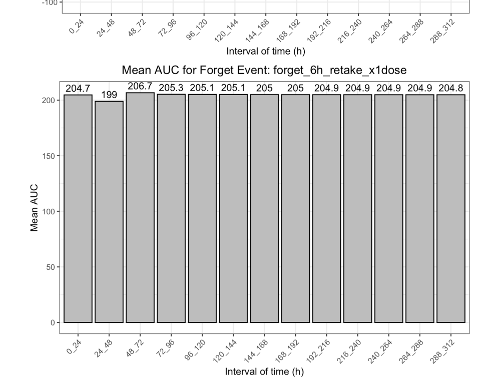

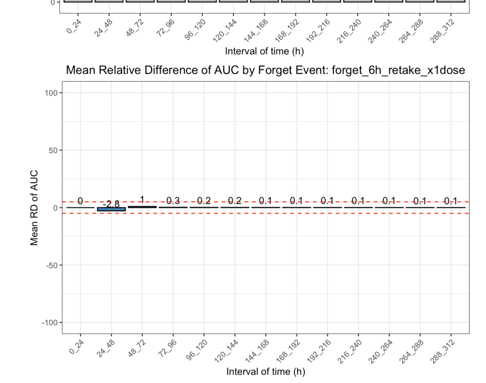

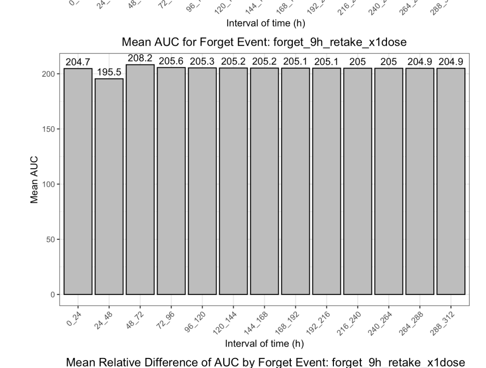

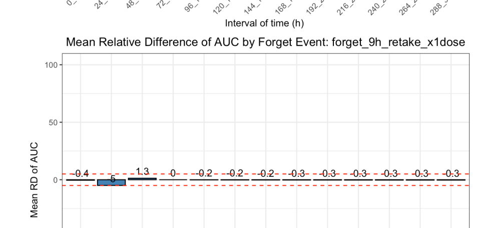

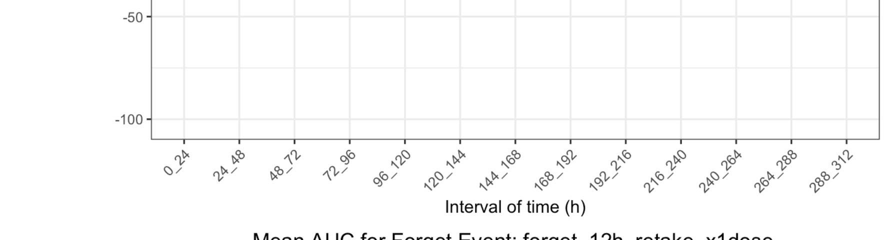

Mean AUC for Forget Event: forget_12h_retake_x1dose

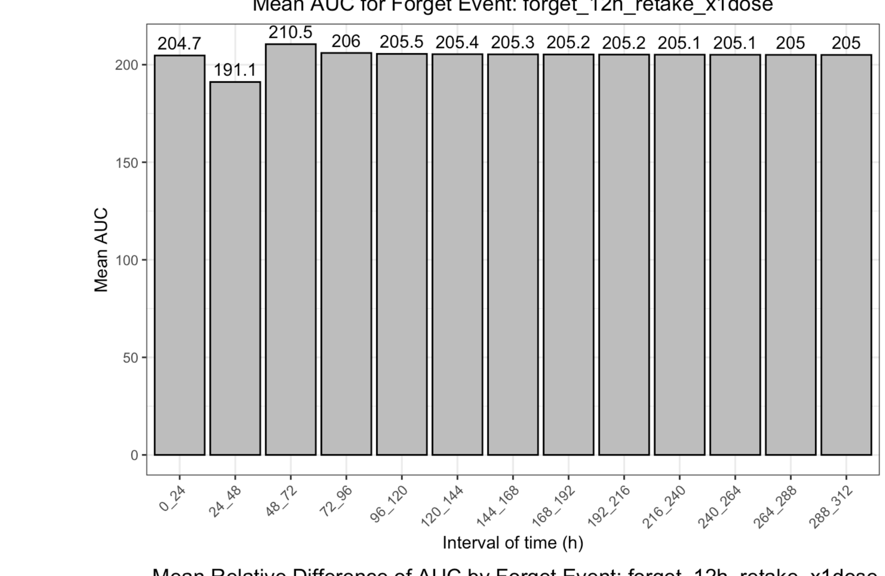

Mean Relative Difference of AUC by Forget Event: forget_12h_retake_x1dose

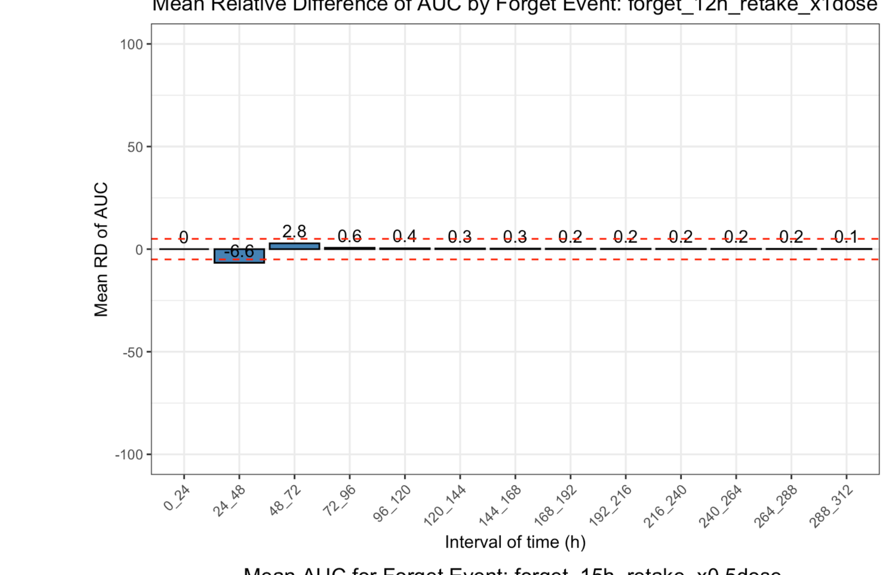

Mean AUC for Forget Event: forget_15h_retake_x0.5dose

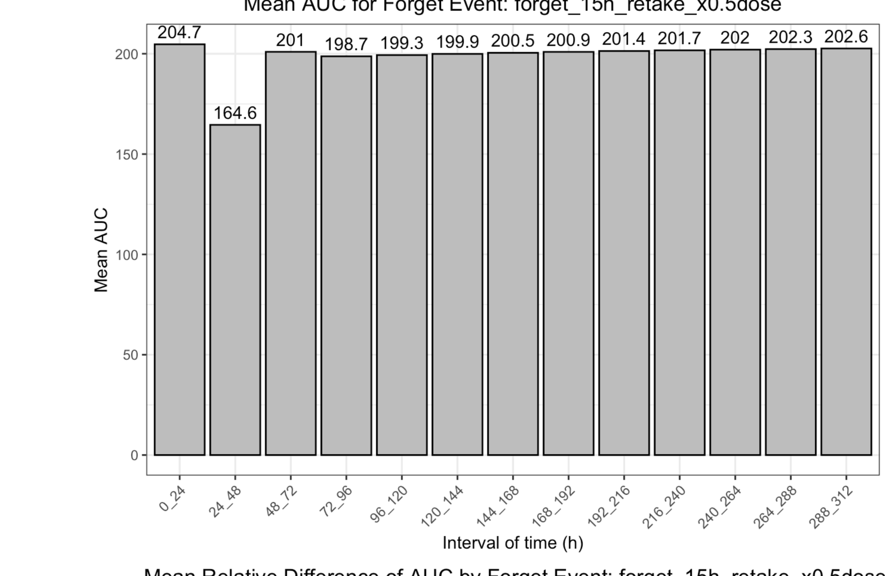

Mean Relative Difference of AUC by Forget Event: forget_15h_retake_x0.5dose

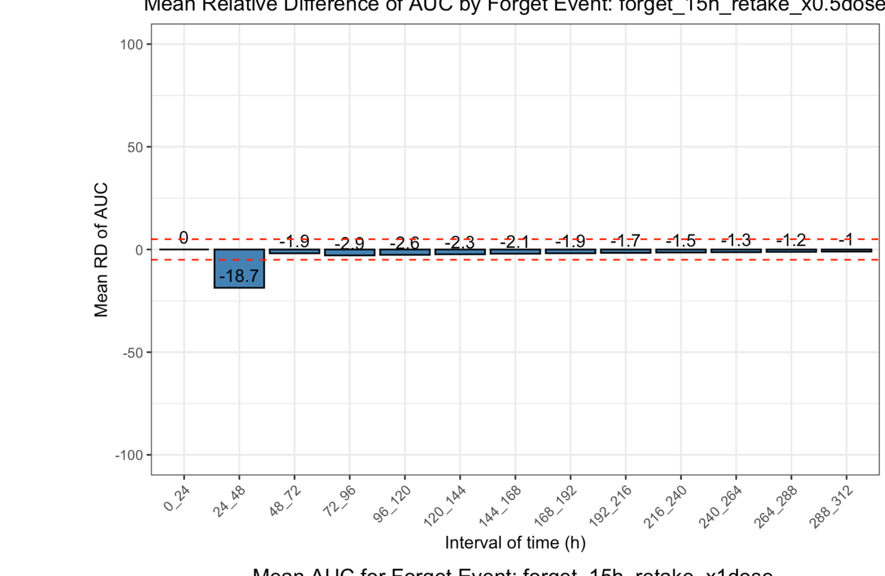

Mean AUC for Forget Event: forget_15h_retake_x1dose

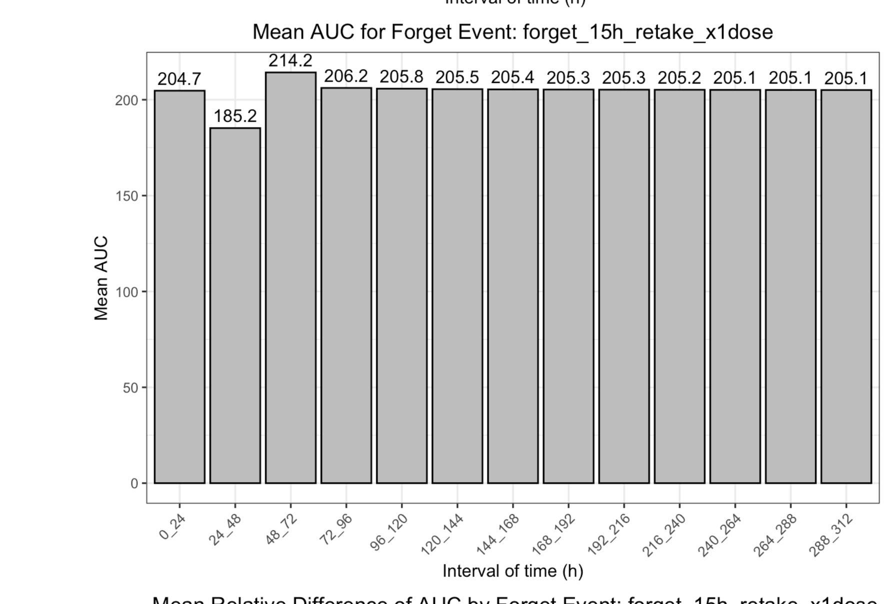

Mean Relative Difference of AUC by Forget Event: forget_15h_retake_x1dose

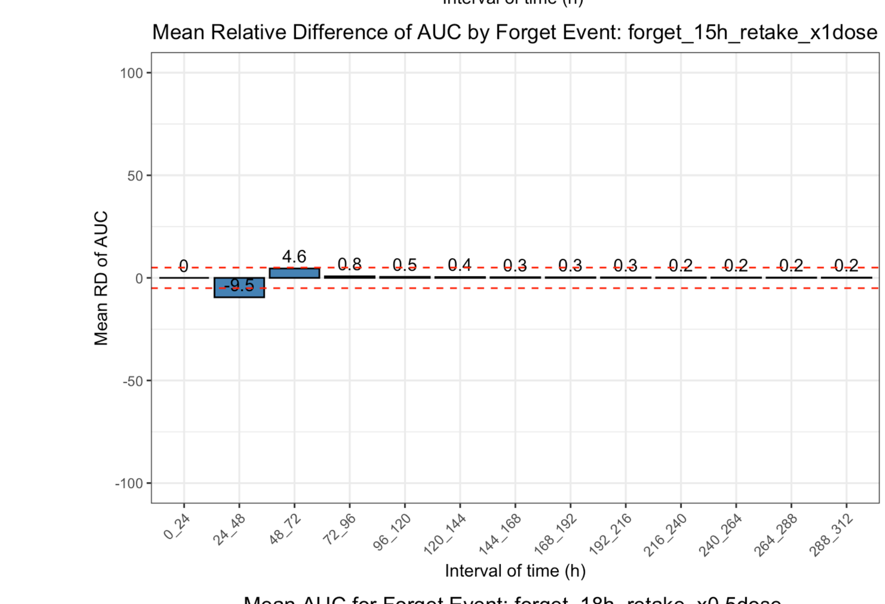

Mean AUC for Forget Event: forget_18h_retake_x0.5dose

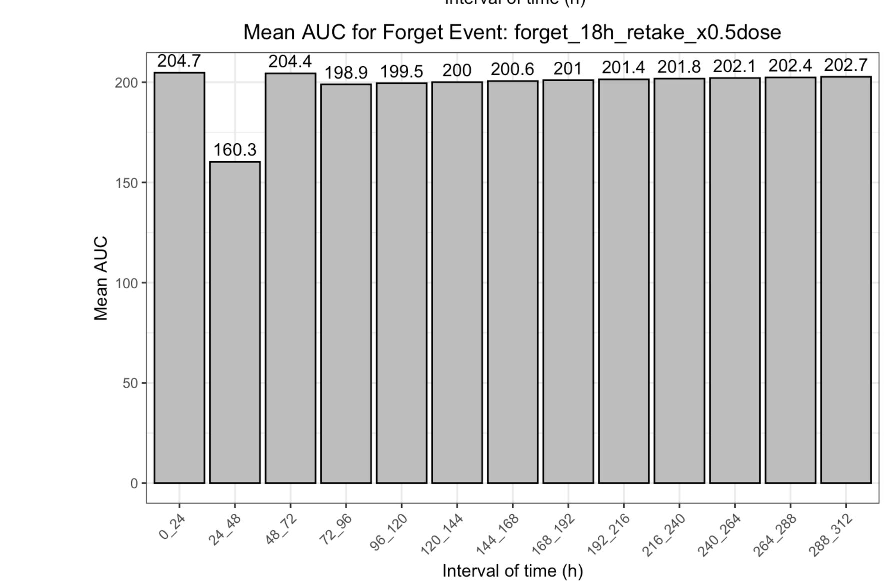

Mean Relative Difference of AUC by Forget Event: forget_18h_retake_x0.5dose

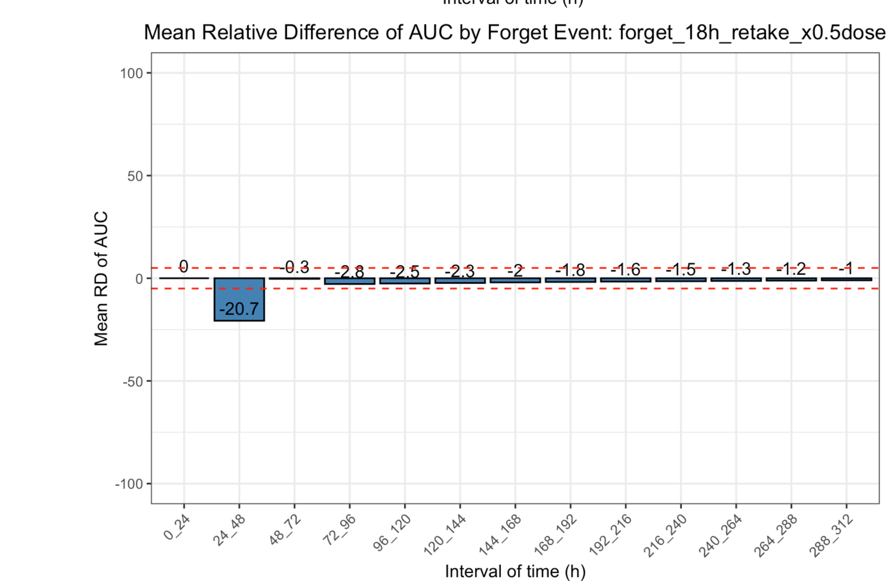

Mean AUC for Forget Event: forget_18h_retake_x1dose

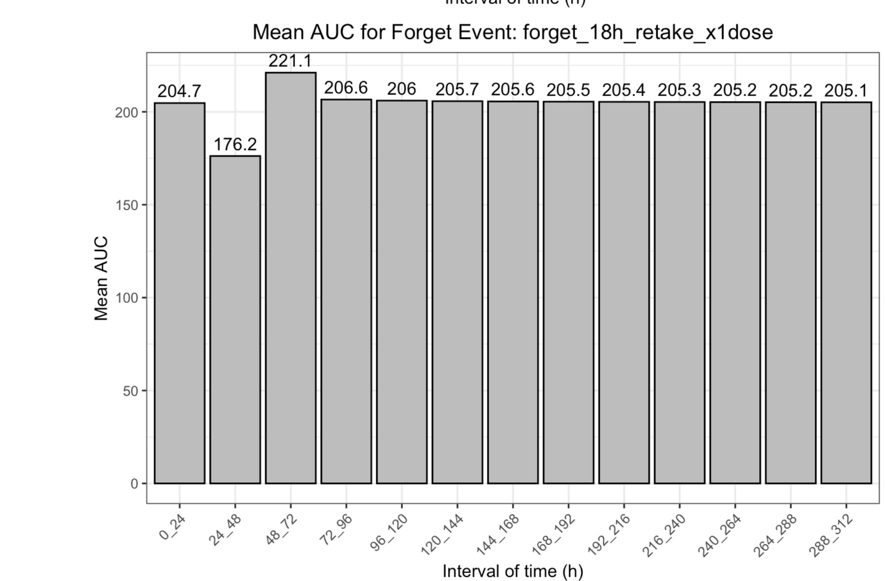

Mean Relative Difference of AUC by Forget Event: forget_18h_retake_x1dose

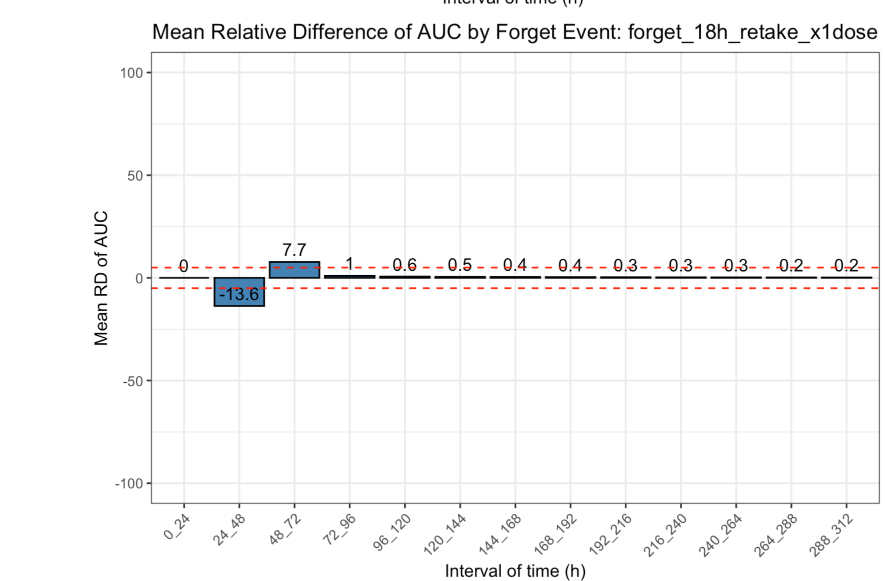

Mean AUC for Forget Event: forget_21h_retake_x0.5dose

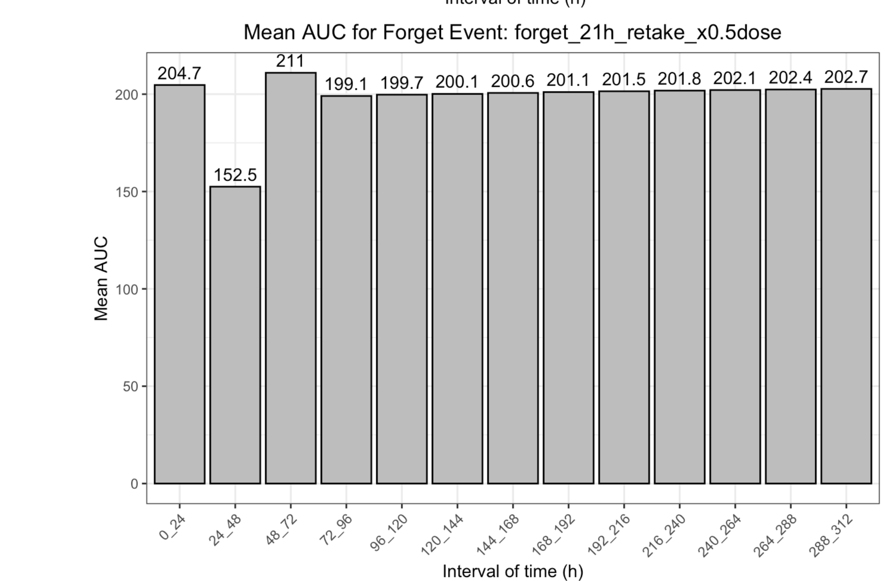

Mean Relative Difference of AUC by Forget Event: forget_21h_retake_x0.5dose

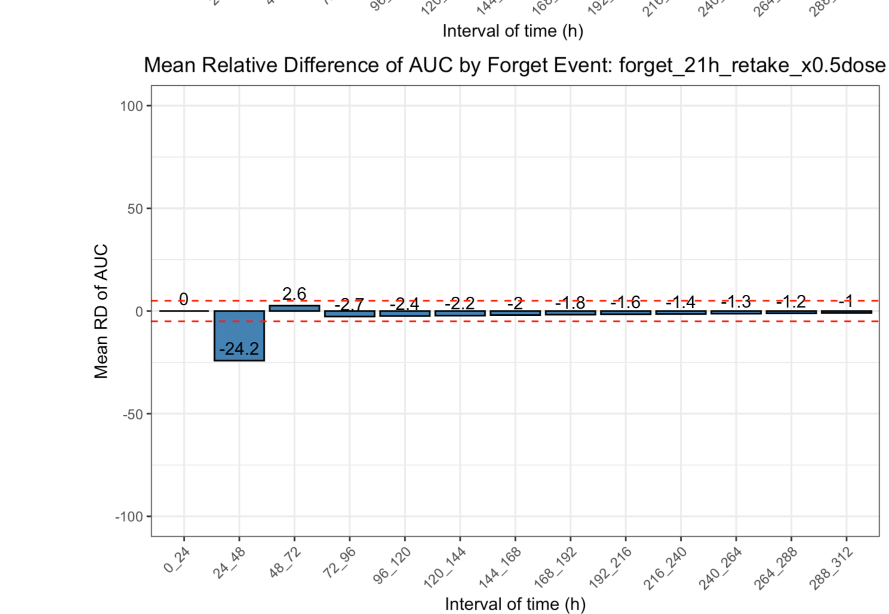

Mean AUC for Forget Event: forget_21h_retake_x1dose

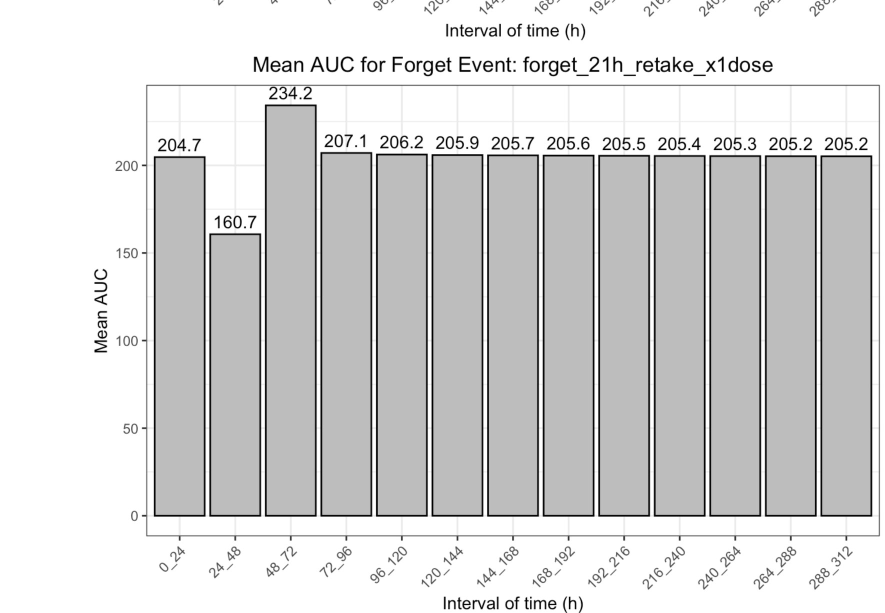

Mean Relative Difference of AUC by Forget Event: forget_21h_retake_x1dose

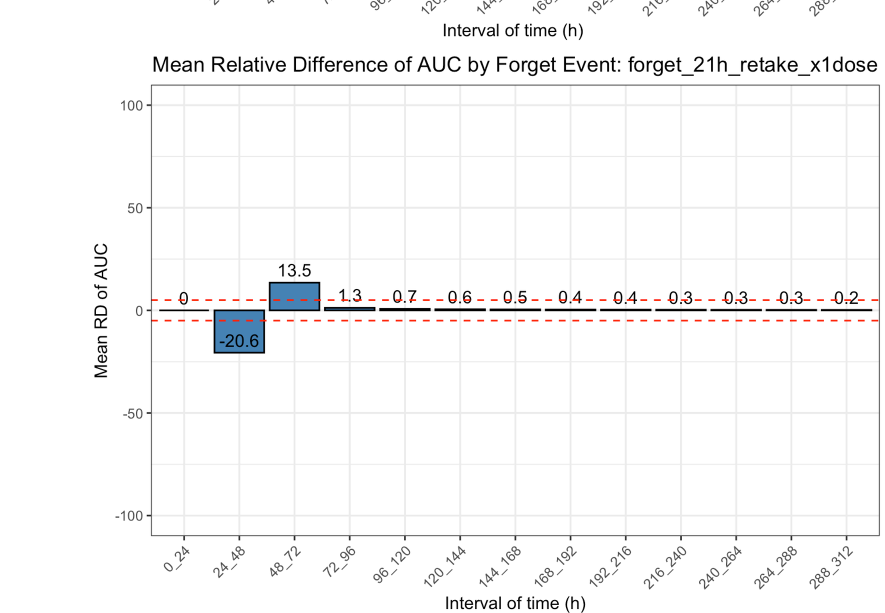

Mean AUC for Forget Event: forget_24h_retake_x1dose

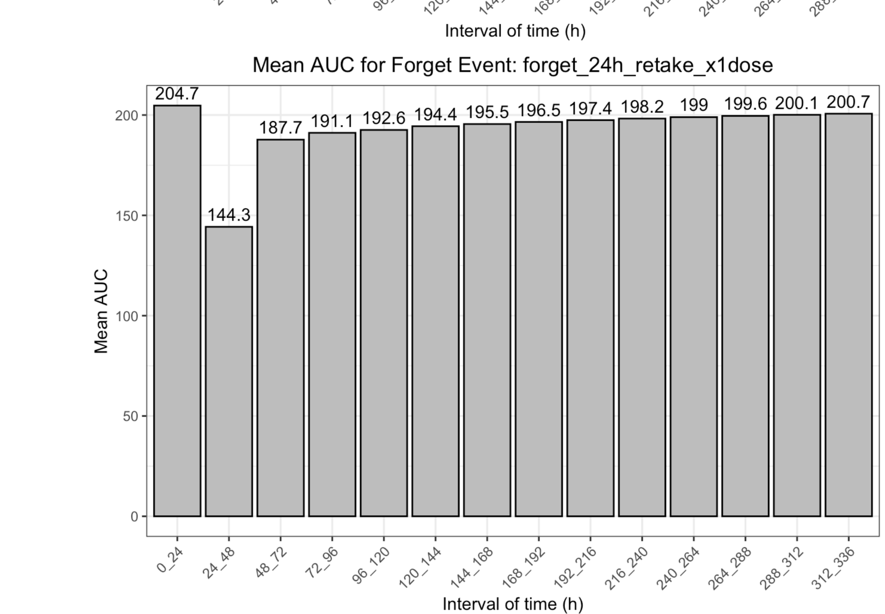

Mean Relative Difference of AUC by Forget Event: forget_24h_retake_x1dose

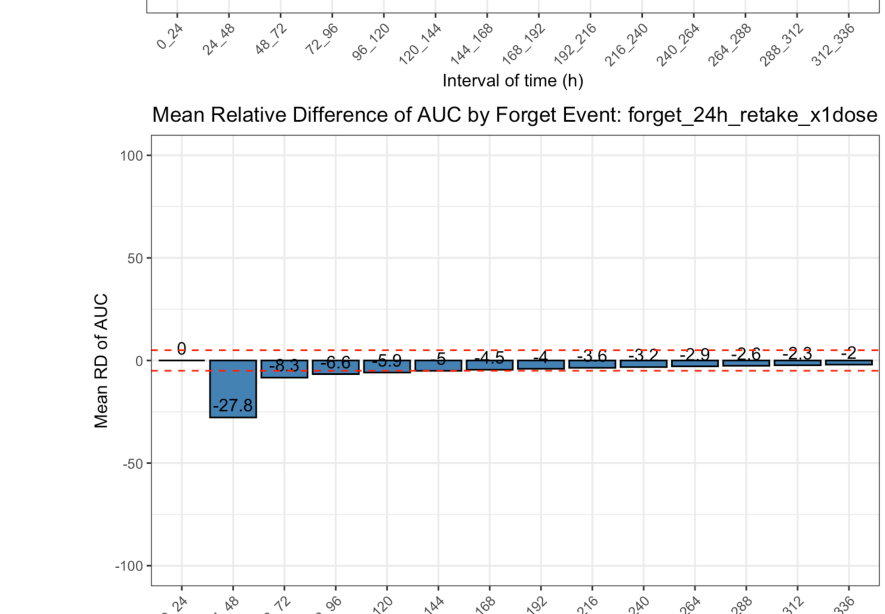

Mean AUC for Forget Event: forget_24h_retake_x1.5dose

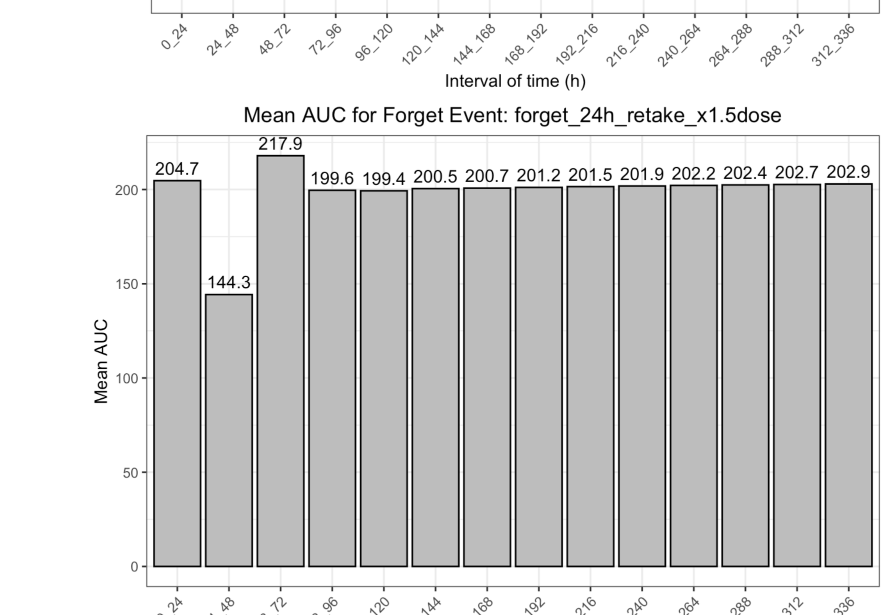

Mean Relative Difference of AUC by Forget Event: forget_24h_retake_x1.5dose

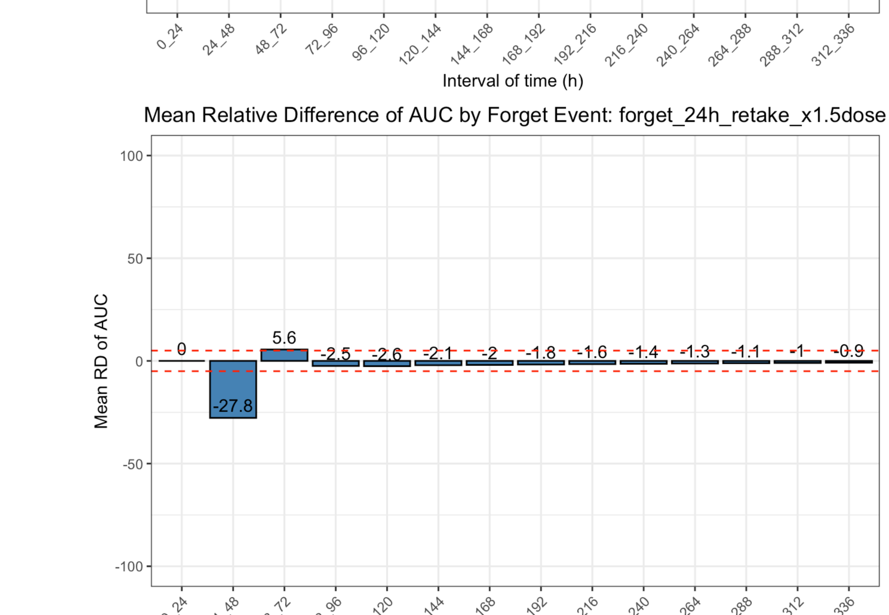

Mean AUC for Forget Event: forget_24h_retake_x2dose

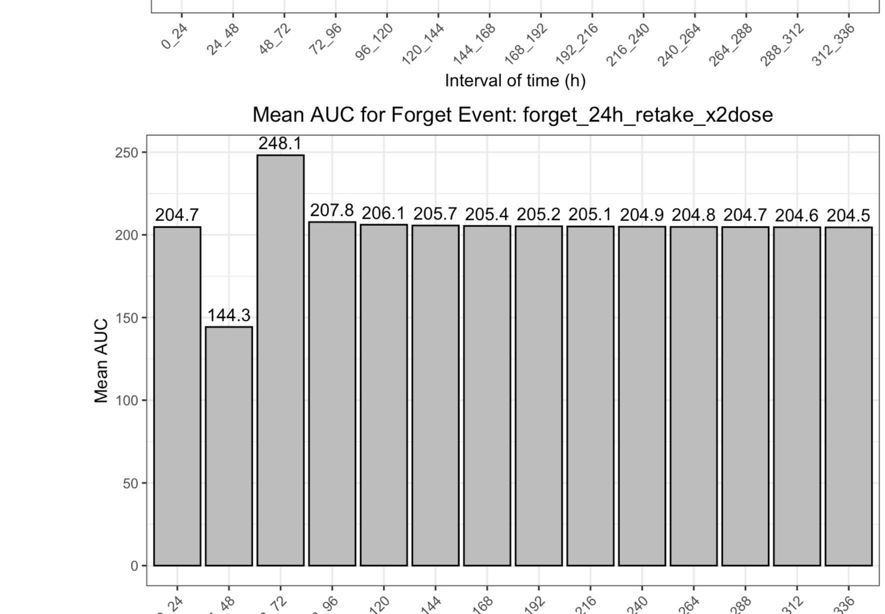

Mean Relative Difference of AUC by Forget Event: forget_24h_retake_x2dose

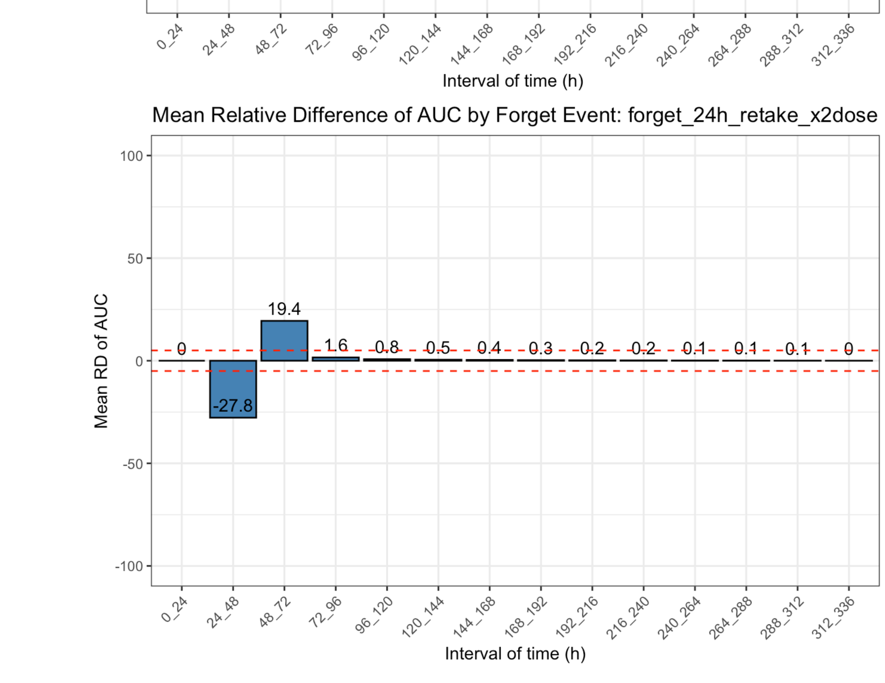

Supplement: Supplementary file 2 — Data S2: Supporting Information. [file BCPT-138-0-s002.pdf]
